# Supplementary figures and images for: Mycn regulates intestinal development through ribosomal biogenesis in a zebrafish model of Feingold syndrome 1
Source: PLoS Biol. 2022 Nov 1;20(11):e3001856. doi: 10.1371/journal.pbio.3001856 (PMC9624419; doi:10.1371/journal.pbio.3001856)

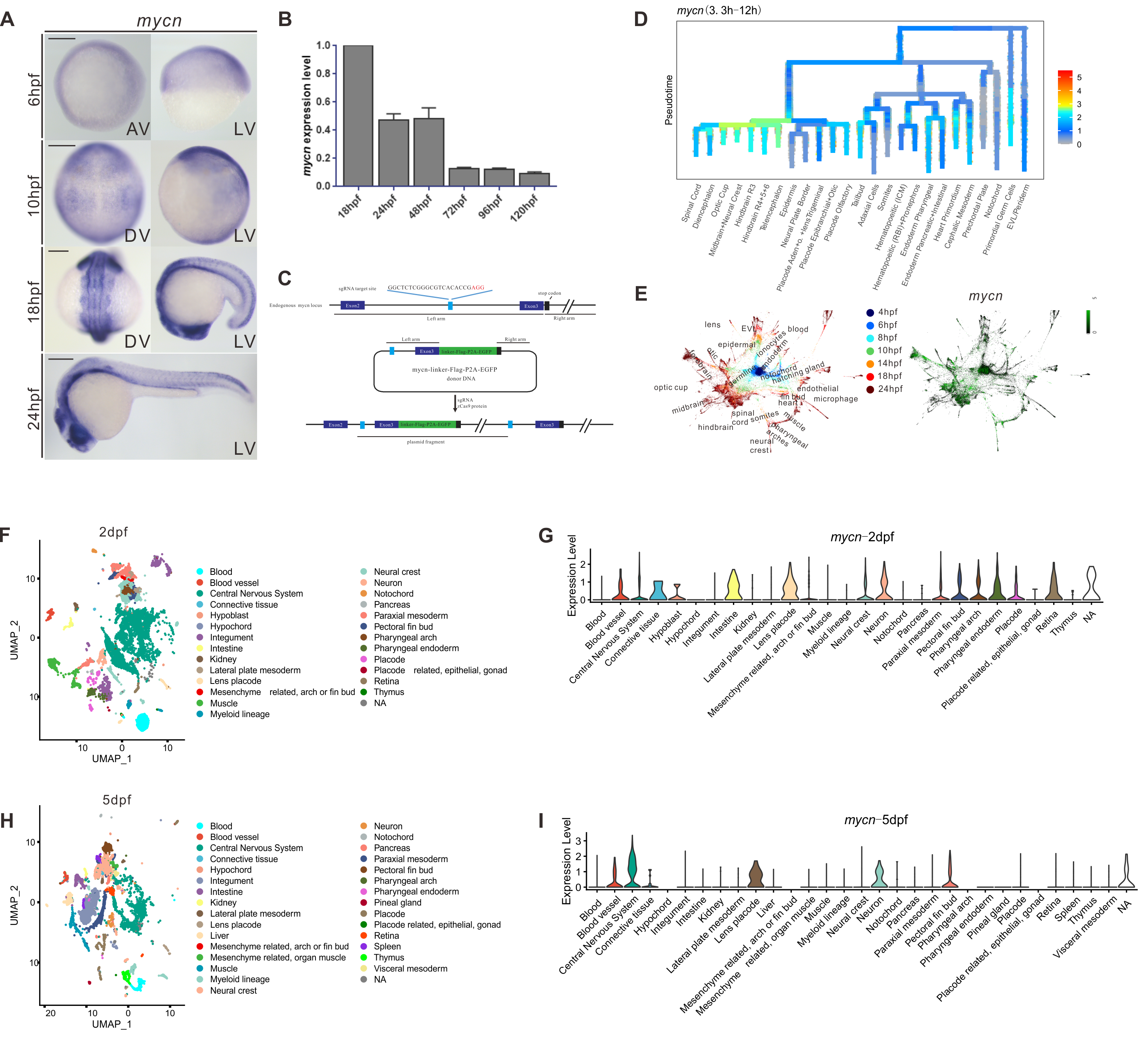

Supplement: S1 Fig — (A) Expression of mycn shown by WISH in embryos at 6, 10, 18, and 24 hpf; AV, LV, and DV. (B) mycn mRNA expression levels in the zebrafish embryos were assessed via qPCR at different stages. (C) Schematic representing the EGFP knock-in strategy using CRISPR/Cas9 in zebrafish. (D, E). Analysis of expression dynamics of mycn during early development of zebrafish embryos using published single-cell RNA-seq datasets. mycn expression dynamics are shown in the URD tree (D) [27] and the zebrafish developmental landscape graph (E) [26]. (F–I). UMAP plot showing cell types of zebrafish embryonic scRNA-seq datasets at 2 dpf (F) and 5 dpf (H) [25]. Violin plots showing mycn expression levels in different cell types of WT embryonic scRNA-seq datasets at 2 dpf (G) and 5 dpf (I). Scale bars: 200 μm. AV, animal view; DV, dorsal view; hpf, hours postfertilization; LV, lateral view; WISH, whole mount in situ hybridization; WT, wild-type. (TIF) [file pbio.3001856.s001.tif]

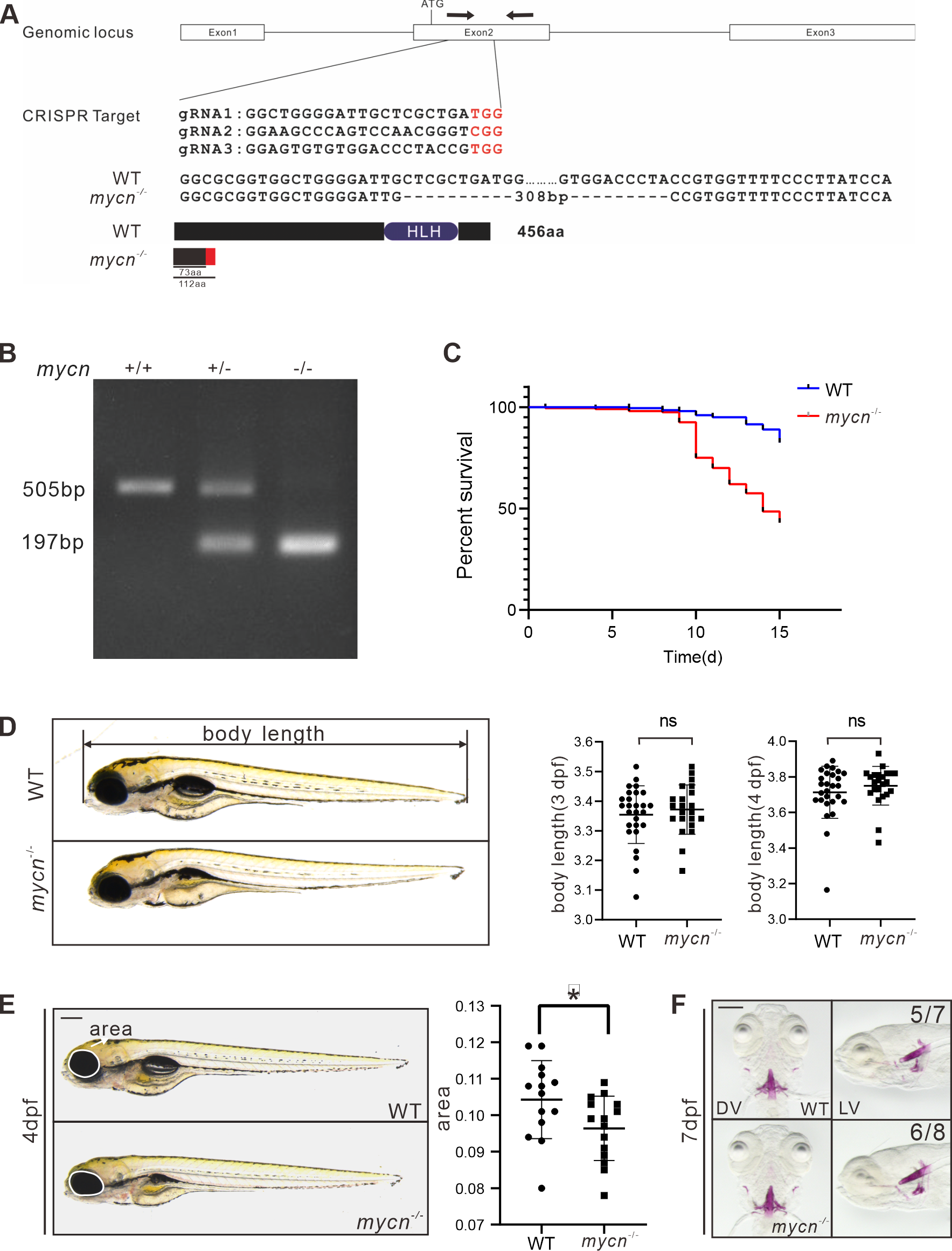

Supplement: S2 Fig — (A) Schematic representation of the mycn gene editing strategy and the resulted mutation. Arrows indicate the PCR primers for mutation detection. (B) Verification of mycn mutants by PCR. The 505-bp strip represents the WT mycn fragment; the 197-bp strip represents mutated mycn fragment; and the lane of 2 strips (505 and 197 bp) represents the heterozygotes. (C) Survival curve of the WT and mycn mutants during the first 15 dpf development. (D) Representative photos of body length measurement of the WT and mycn mutant embryos at 72 hpf and 96 hpf. Statistics in the right panel. (E) Representative photos of eye area measurement of the WT and mycn mutant embryos at 96 hpf. Statistics in the right panel. (F) Alizarin red staining show the skull development in WT and mycn mutant embryos at 7 dpf, DV and LV. The data underlying this figure can be found in S1 Data. Raw images of this figure are provided in S1 Raw Images. dpf, days postfertilization; DV, dorsal view; hpf, hours postfertilization; LV, lateral view; WT, wild-type. (TIF) [file pbio.3001856.s002.tif]

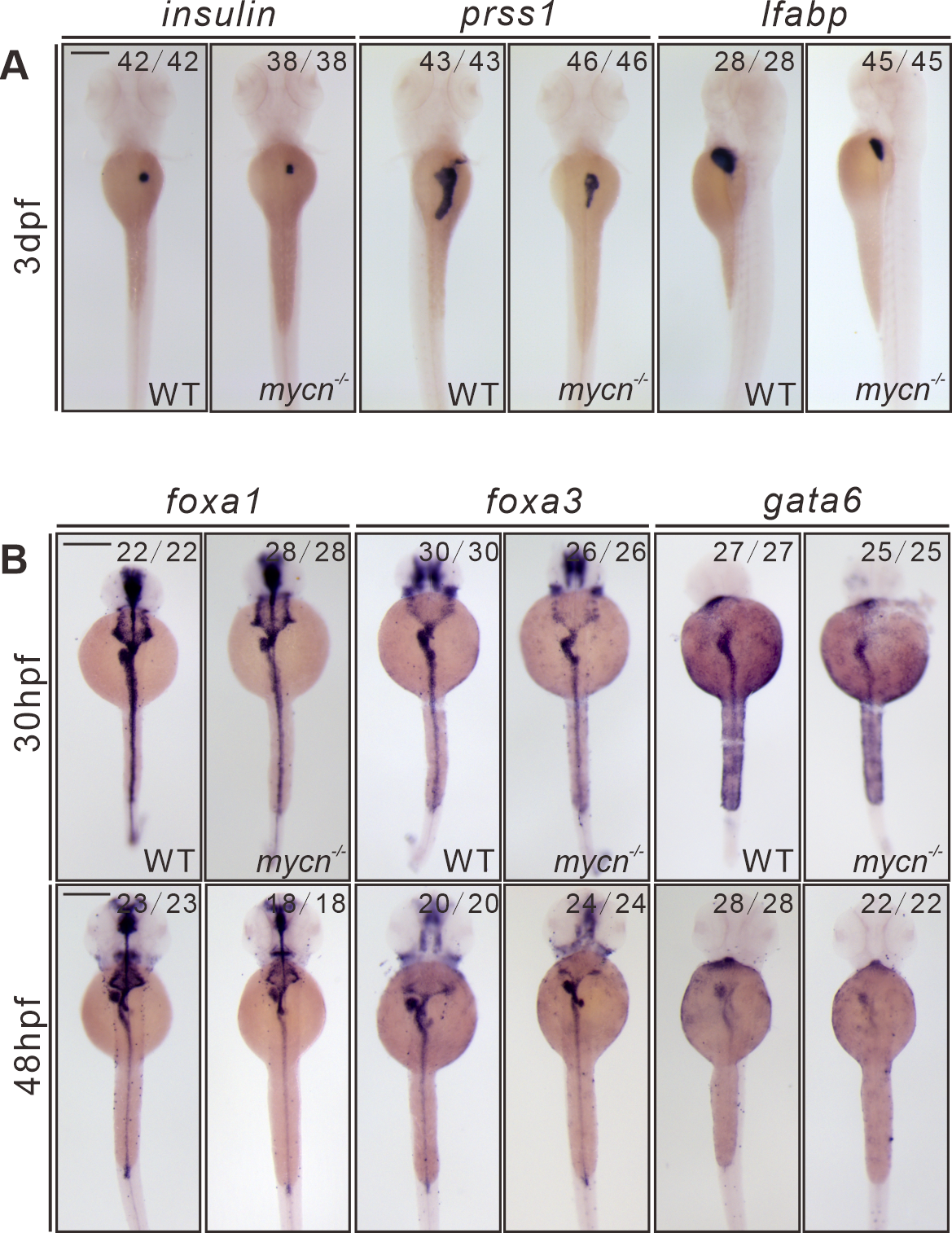

Supplement: S3 Fig — (A) Expression of markers of digestive organs: insulin (islet), prss1 (pancreas), and lfabp (liver) by WISH at 3 dpf in the mycn mutant and WT embryos. (B) Analysis of endoderm development by expression of pan-endoderm markers foxa1, foxa3, and gata6 at 30 and 48 hpf. All embryos are in dorsal view. Scale bars: 200 μm. dpf, days postfertilization; hpf, hours postfertilization; WISH, whole mount in situ hybridization; WT, wild-type. (TIF) [file pbio.3001856.s003.tif]

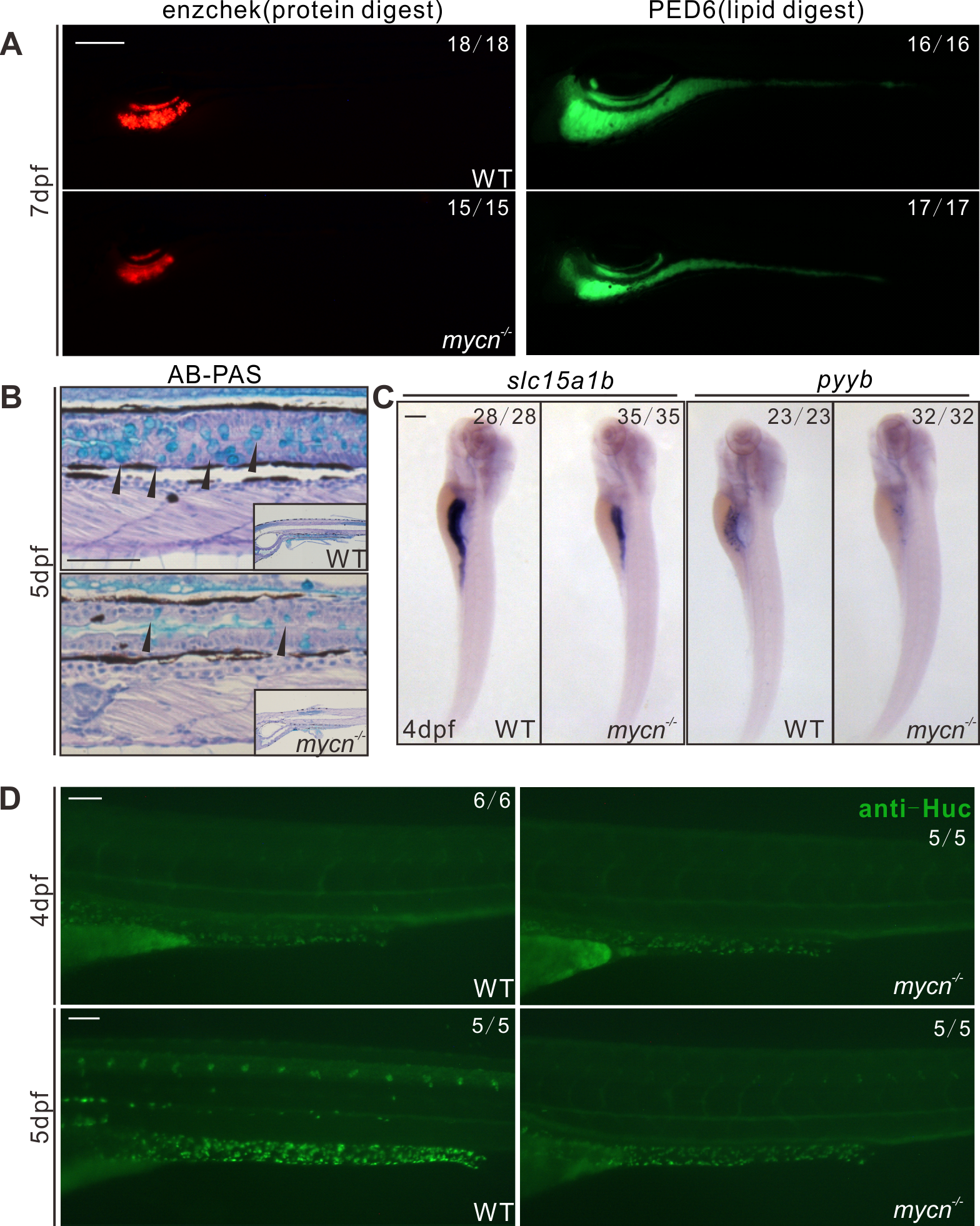

Supplement: S4 Fig — (A) Assessment of the effectiveness of protein and lipid digestion in the intestines of WT and mycn mutant embryos by EnzChek and PED6 treatment. The quenched fluorescent reporter PED6 or Enzchek is activated only after cleavage by intestinal phospholipase or protease. (B, C). Goblet cells (AB-PAS), absorptive cells (slc15a1b), and enteroendocrine cells (pyyb) in the intestines were analyzed by AB-PAS staining (left) and WISH (right) in WT and mycn mutant embryos at indicated developmental stages. Sections were cut along the sagittal plane. Arrow heads indicate the goblet cell. (D) Enteric neurons in WT and mycn mutant were shown by immunofluorescence with anti-Huc antibody at 4 and 5 dpf. All embryos are in lateral view. Scale bars: 200 μm (A, C, D), 50 μm (B). dpf, days postfertilization; WISH, whole mount in situ hybridization; WT, wild-type. (TIF) [file pbio.3001856.s004.tif]

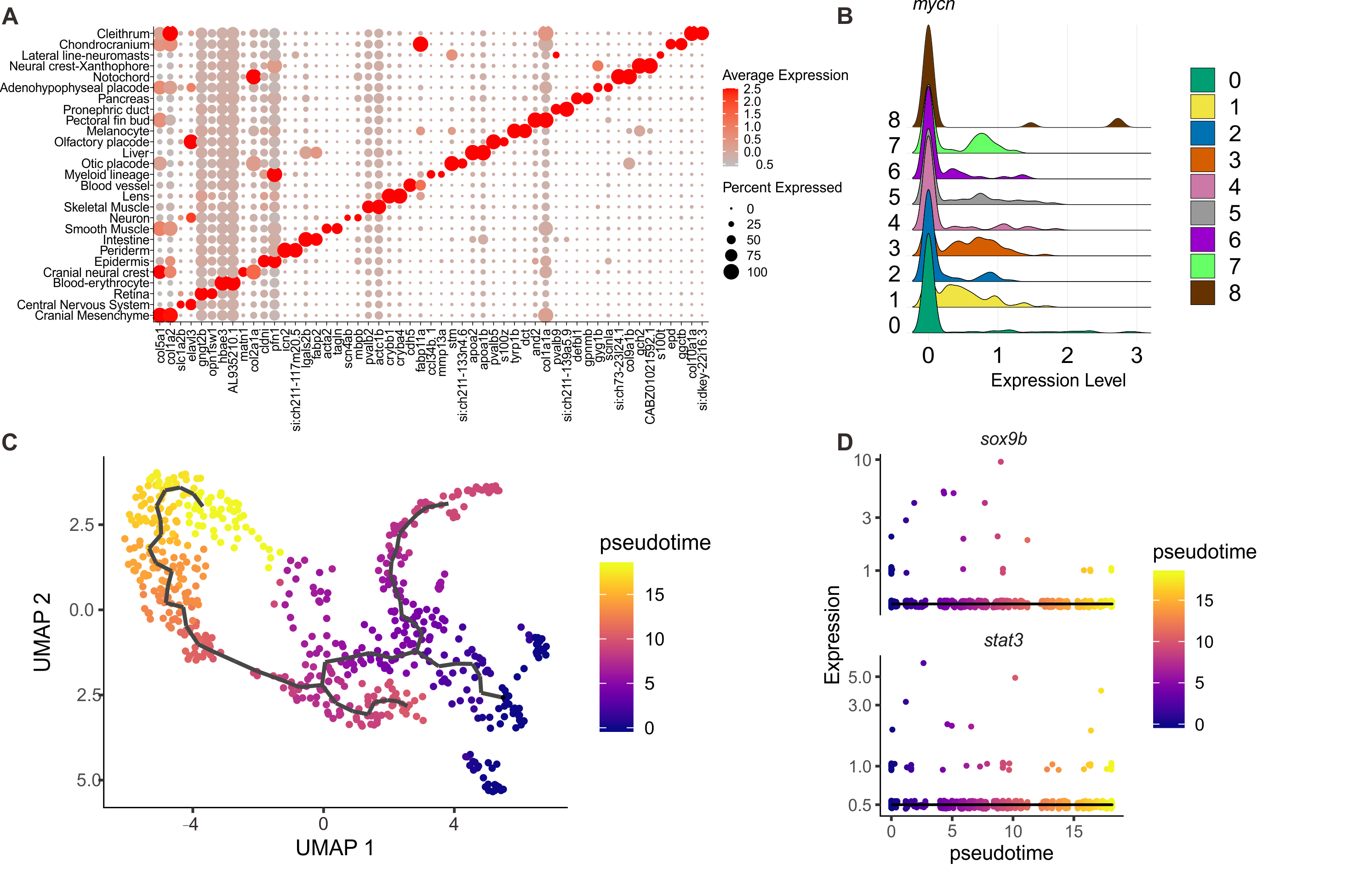

Supplement: S5 Fig — (A) Dot plot showing expressions of 2 selected marker genes in each cell type. Dot size indicates the percentage of cells expressing the indicated genes; dot color indicates the average expression level of indicated genes. (B) Ridge plot showing the expression distributions of mycn in each subcluster of WT intestinal cells. (C) Pseudotime differentiation trajectory analysis for intestinal cells. (D) Spline plot representing changes in expression over pseudotime for the intestinal stem cell markers, sox9b and stat3. dpf, days postfertilization; scRNA-seq, single-cell RNA-seq; WT, wild-type. (TIF) [file pbio.3001856.s005.tif]

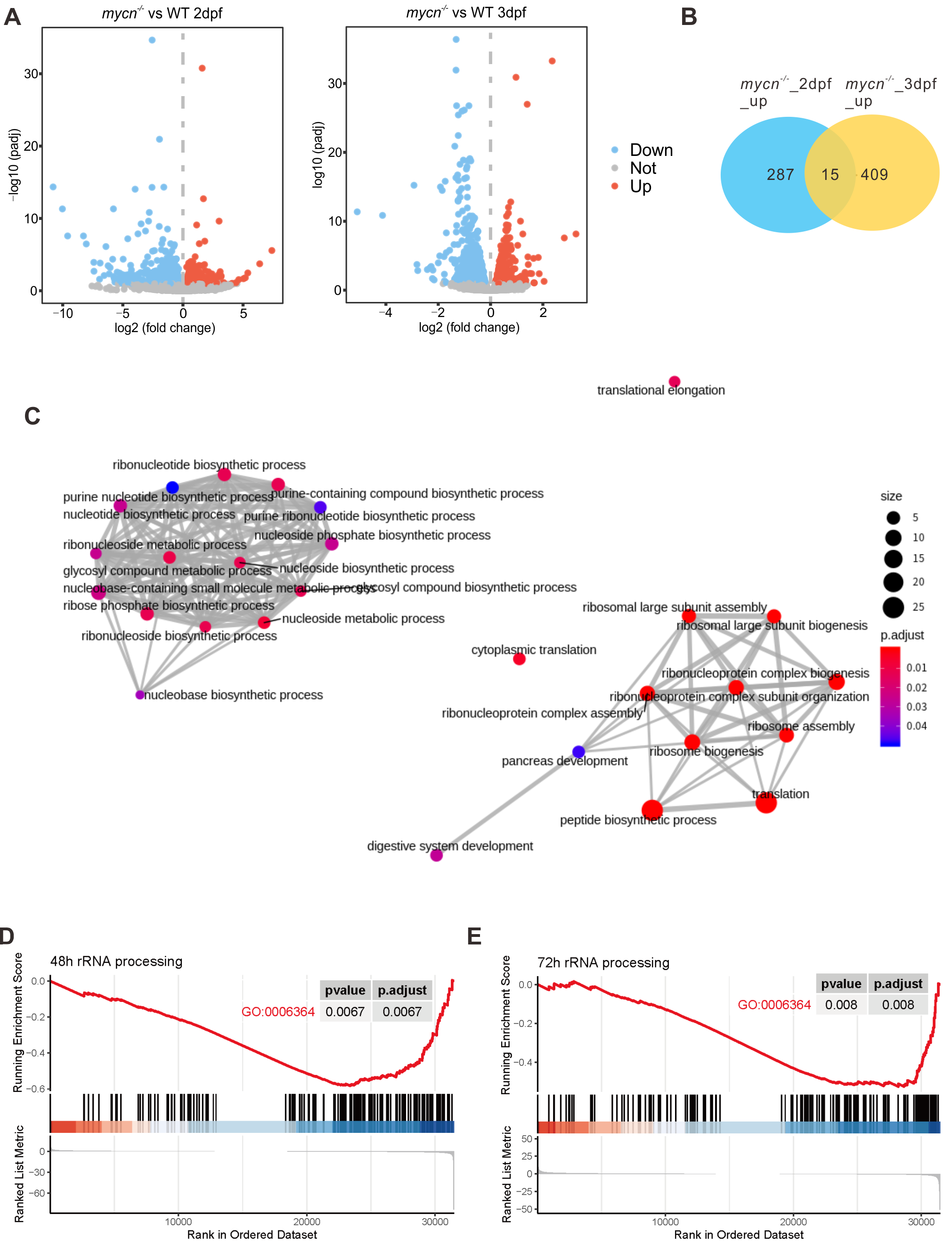

Supplement: S6 Fig — (A) Volcano plot showing DEGs between mycn mutant and WT embryos at 2 (left) and 3 dpf (right). DEGs were identified by |log2 (fold change) | > 0 and an adjusted p < 0.1. (B) Venn plot showing up-regulated genes in mycn mutants compared with WT at 2 and 3 dpf. The number of congruently up-regulated genes is indicated in the middle. (C) Emapplot showing interaction networks between enriched GO terms analyzed by 85 overlapped down-regulated genes in mycn mutants. The color scales indicate different thresholds of adjusted p-values, and the dot sizes represent the gene counts of each GO term. (D, E) GSEA of bulk RNA-seq datasets of mycn and WT at 48 hpf (D) and 72 hpf (E) using RNA processing signature. The data underlying this figure can be found in S2 Data. DEG, differentially expressed gene; dpf, days postfertilization; GO, gene ontology; GSEA, gene set enrichment analysis; hpf, hours postfertilization; WT, wild-type. (TIF) [file pbio.3001856.s006.tif]

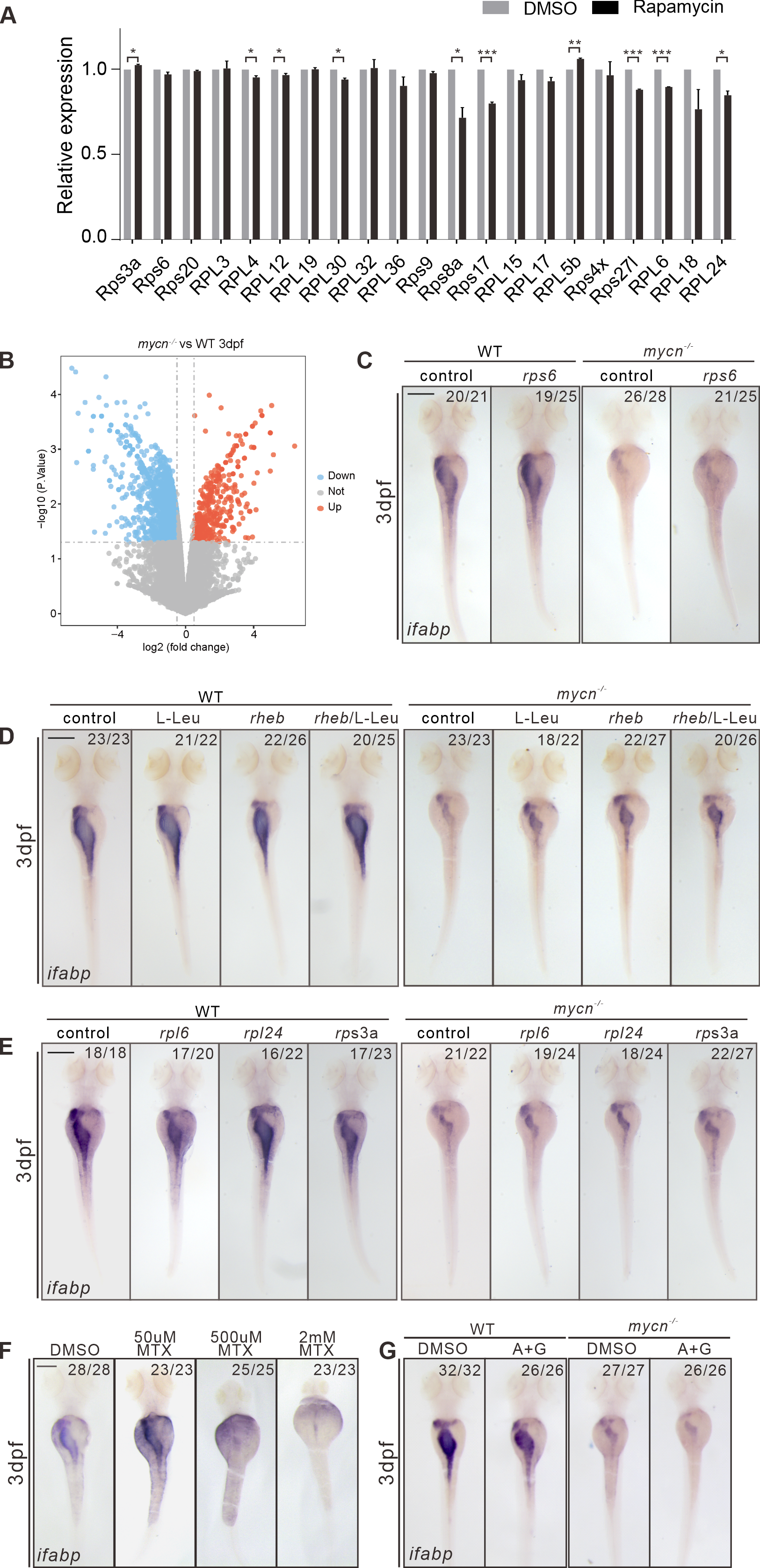

Supplement: S7 Fig — (A) qPCR of rpl and rps genes at 3 dpf in WT and embryos treated with rapamycin. Asterisks indicate that the significant difference by Student t test. ***p < 0.0001; **p < 0.001; *p < 0.05. (B) Volcano plot shows genes that have differential translation efficiency between mycn mutant and WT embryos at 72 hpf. Blue dots and red dots indicate down-regulated genes and up-regulated genes in mycn mutants, respectively (p-value < 0.05, |log2 fold change| > 0.5). (C) Rescue experiment of the intestinal defects in the mycn mutants by injecting rps6 mRNA. (D) Rescue experiment of the intestinal defects in the mycn mutants by activating the mTOR pathway: injecting rheb mRNA, or L-Leu and rheb/L-leu treatment. (E) Rescue experiment of the intestinal defects in the mycn mutants by injecting rpl6, rpl24, or rps3a mRNA. (F) Inhibition of purine de novo synthesis by methotrexate induces intestinal defects in zebrafish embryos. (G) Adenine (A) and guanine (G) supplementation cannot rescue the intestinal defects of mycn mutant. All embryos are in dorsal view. Scale bar: 200 μm. The data underlying this figure can be found in S1 and S2 Data. dpf, days postfertilization; hpf, hours postfertilization; WT, wild-type. (TIF) [file pbio.3001856.s007.tif]

Figure 6A

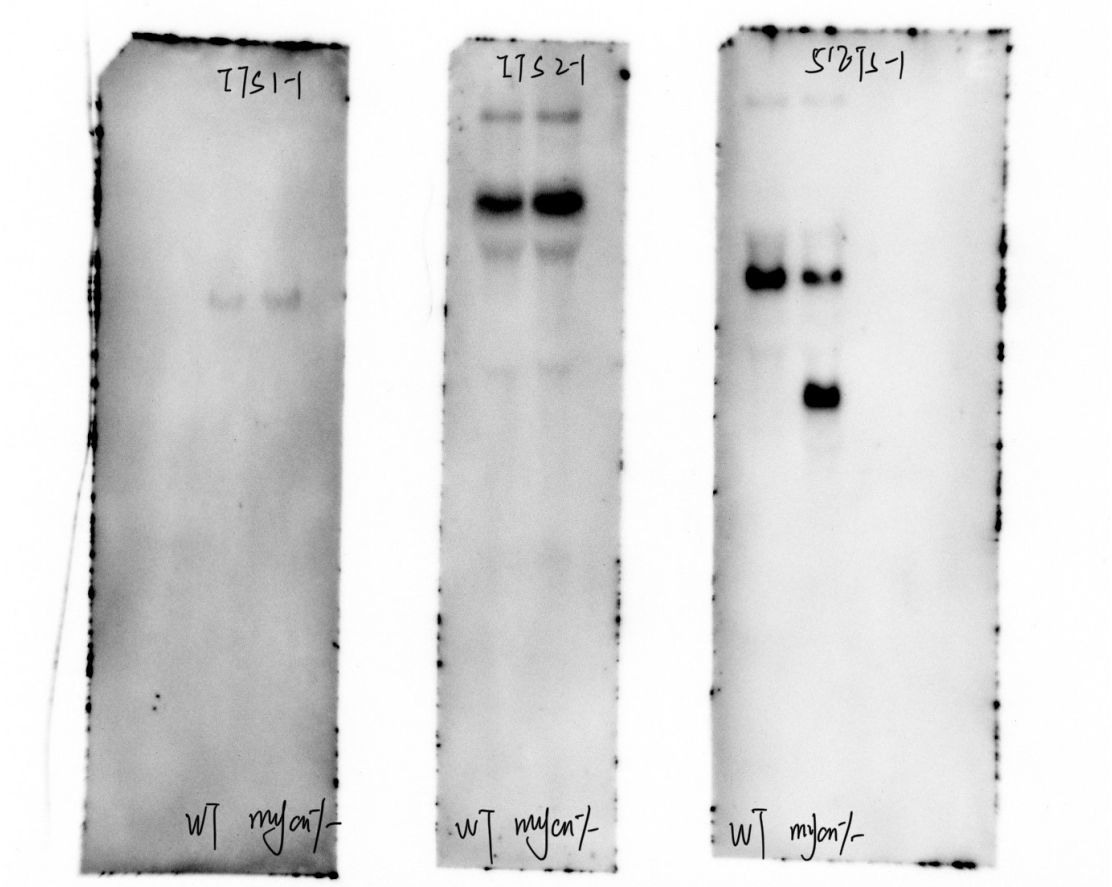

Figure 6C

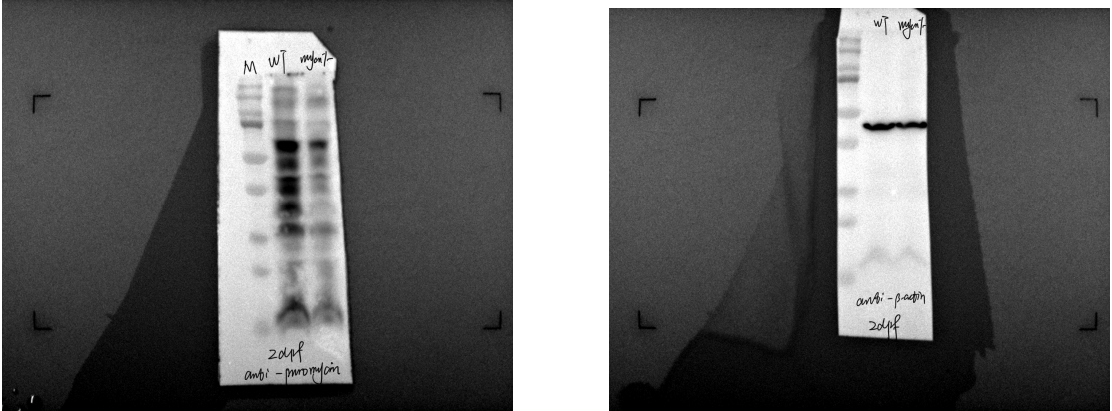

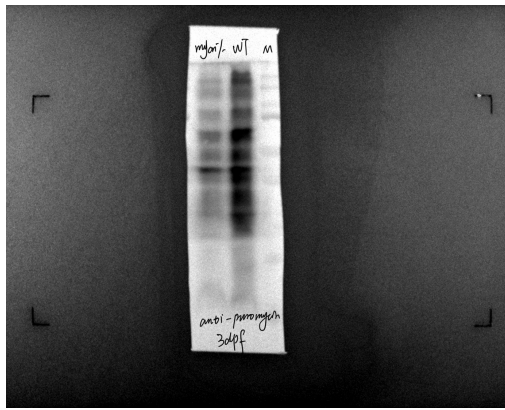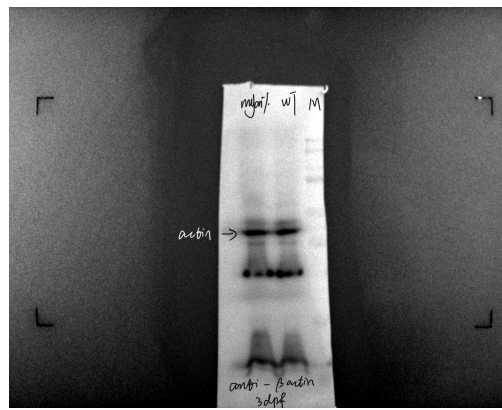

Figure 7C

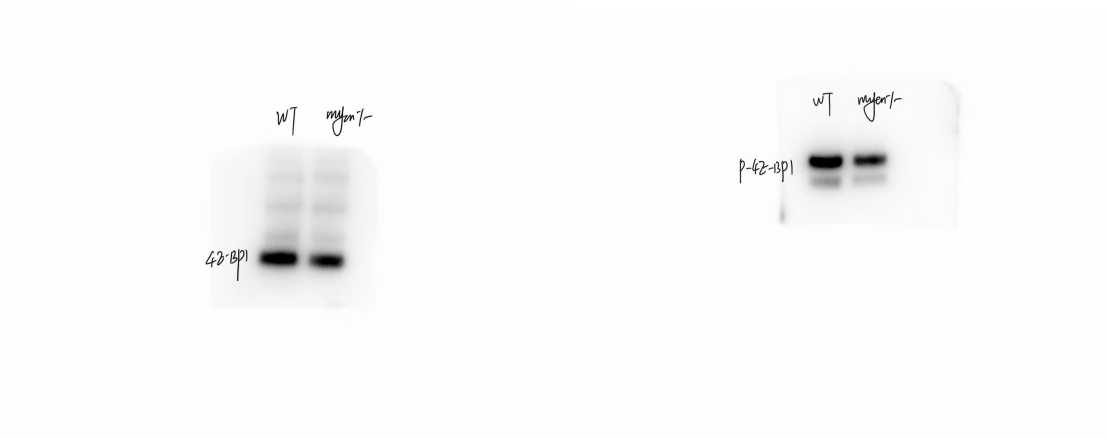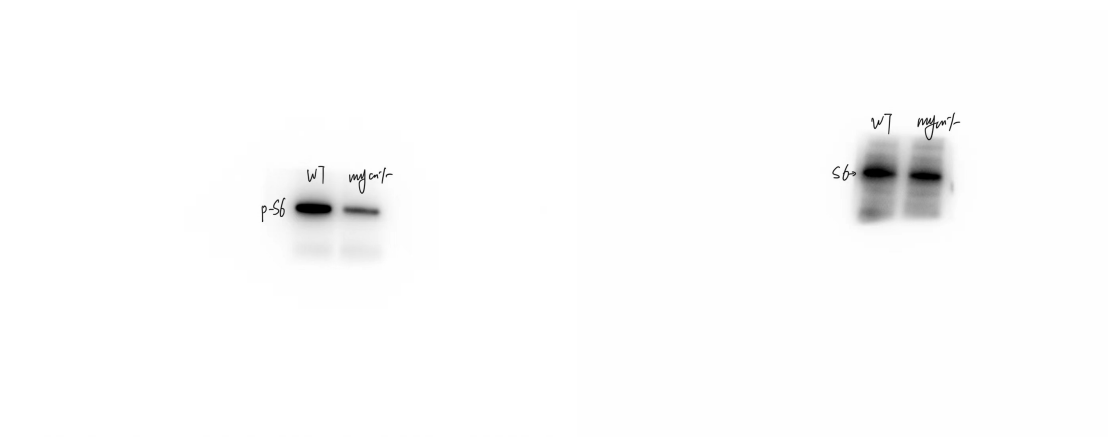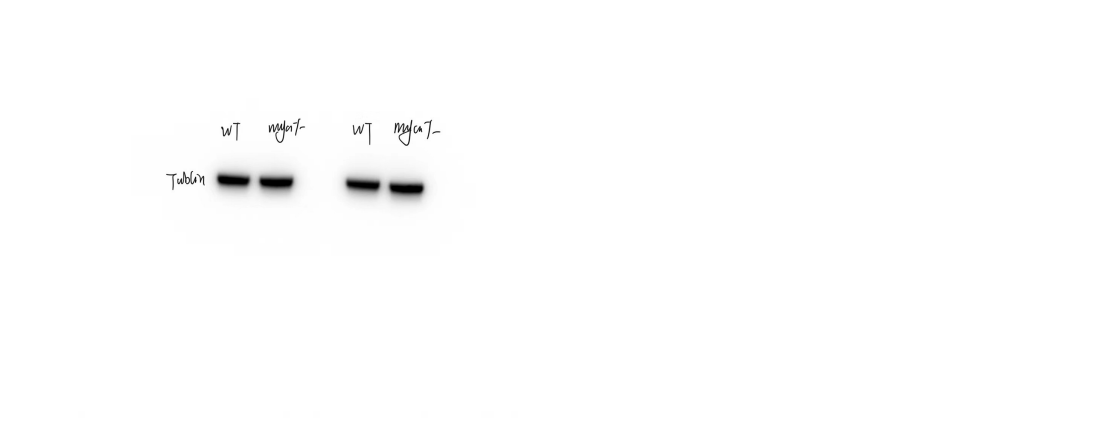

Figure S2B

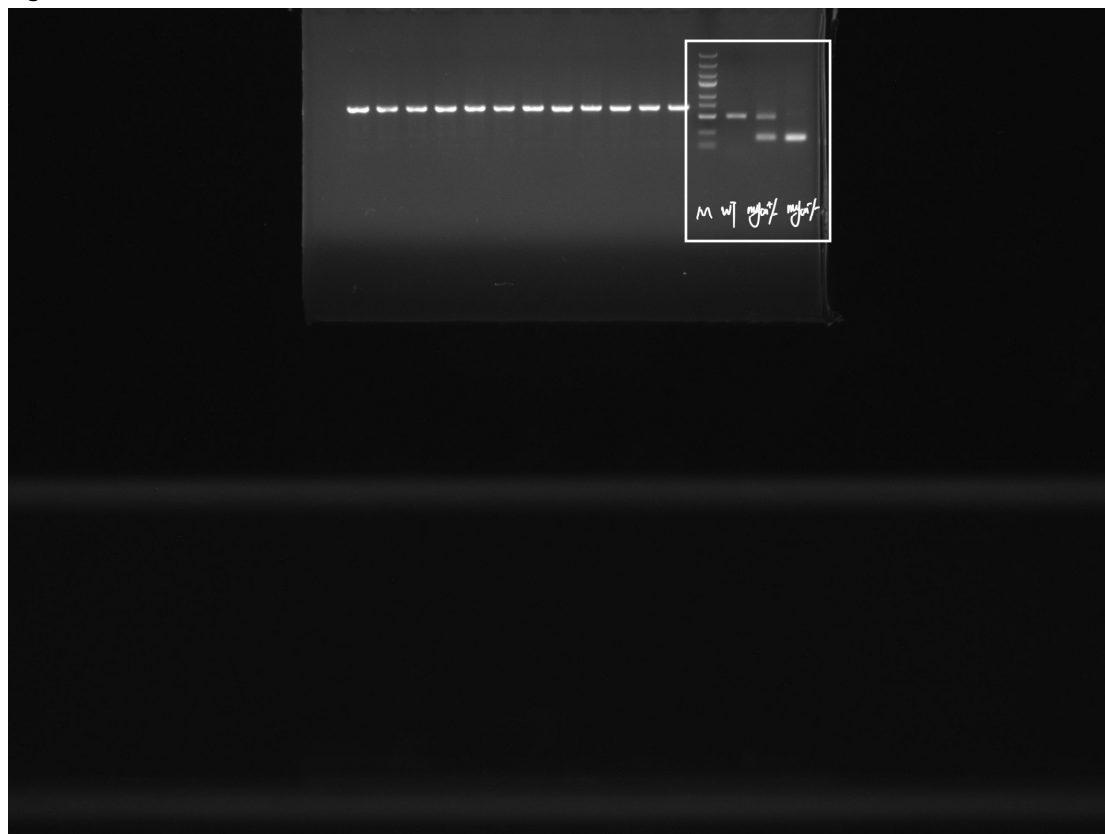

Supplement: S1 Raw Images — (PDF) [file pbio.3001856.s014.pdf]
