# Supplementary material for: Mycn regulates intestinal development through ribosomal biogenesis in a zebrafish model of Feingold syndrome 1
Source: PLoS Biol. 2022 Nov 1;20(11):e3001856. doi: 10.1371/journal.pbio.3001856 (PMC9624419; doi:10.1371/journal.pbio.3001856)
Supplement: S1 Table — (PDF) [file pbio.3001856.s008.pdf]

NAMES	PRIMERS
GATA6 F	ATGGACCTGGGTGATAACAGCTG
GATA6 R	TTAGGCCAAGGCCATGGGGCACC
foxa3 F	ATGTTGAGCTCCGTGAAGATGG
foxa3 R	CCTTAAAGCATTTCTTTTTCTCAG
prss1 F	ATGAAGGCTTTCATTCTTCTG
prss1 R	CCATGATAACGACCTCAAAAC
FOXA1 F	CTCACACACACGCACGCTGTGAG
FOXA1 R	GGGGATCACAACGAGTATTTTTAC
lfabp F	TCTCCAGAAAGCATGGCCTTCAG
lfabp R	GTCAGTCAGAGCTTTATTTAAATAG
ifabp F	GTCATCATCATGACCTTCAACG
ifabp R	CTCACAGGTGCAAATGACACG
MYCN KI F	GGGTCATCTCTCTTTACAGGGTG
MYCN KI R	CTGACAAAAGCAGTTCTACTGTC
Grna1（MYCN KI）	TAATACGACTCACTATAGGCTCTCGGGCGTCACACCGGTTTTAGAGCTAGAAATAGC
Grna2（MYCN KI）	TAATACGACTCACTATAGGAGACTAAAGTGCCCCGTTGTTTTAGAGCTAGAAATAGC
Grna3（MYCN KI）	TAATACGACTCACTATAGGCGTGGTCCAATTATTCGCGTTTTAGAGCTAGAAATAGC
PUC19 F	GACGAAAGGGCCTCGTGATACG
PUC19 R	GCCTGGGGTGCCTAATGAGTGAG
Left Arm fusion F	TTAGGCACCCCAGGCGAGAATAAGGGCTGGCACTGGGC
Left Arm R	CGAACCACCTCCCGAGCGAGTCCTGGCCTGCTCAAGTC
LINKER F	CAGGCCAGGACTCGCTCGGGAGGTGGTTCGGGTGGC
LINKER R	AATCAGACTCACCATAGGACCAGGATTTTCTTCAAC
EGFP fusion F	GAAAATCCTGGTCCTATGGTGAGCAAGGGCGAGGAG
EGFP fusion R	GAGCGTAAAGGGTTACTTGTACAGCTCGTCCATGCC
RIGHT ARM F	GACAACGTCAAGTAACCCTTTACGCTCATTGGCTGAATG
RIGHT Arm fushion R	CGAGGCCCTTTCGTCGTCTCTCTCTCTCTAGCTGTGTG
Mycn KO F	CTCTGTCCCCGAGCCGGGCA
Mycn KO R	GCTGCTGCTGCTCCCGTTTC
RPL3 F	CGAACGTGCTTCCAGCTTTATAAG
T7-RPL3 R	GTAATACGACTCACTATAGGGTTAGGCCATCTCTTCCTTGGCCAG
RPL12 F	GGTGAAGGTGCAACAGTTTTCG
T7-RPL12 R	GTAATACGACTCACTATAGGGTTACTCAGTTGGGCACTCAACTG
RPL15 F	ATGGGAGCGTACAAGTATATGC
T7-RPL15 R	GTAATACGACTCACTATAGGGATGTACAGTTTAAGCACACTTTG
RPL19 F	GCTTTCCGCTGACCACCAGCCATG
T7-RPL19 R	GTAATACGACTCACTATAGGGTTATTTCTTGGTCTCATCCTCTTTG
RPL5 F	GCAGCGGAGTGTAAACTCCCCAG
T7-RPL5 R	GTAATACGACTCACTATAGGGGCAGGGTCGAGCTCTAGTCCTCC
RPL18 F	GTCCAAGATGGGAGTTGACATC
T7-RPL18 R	GTAATACGACTCACTATAGGGCCTTAGTGTCTGCGACCACGG
RPL30 F	ACGAGGCCATCTTGGTGTGATCAG
T7-RPL30 R	GTAATACGACTCACTATAGGGGTGGTGGAAAAGGCTCTACTTCTC
RPL24D1 F	ATGCGCATTGAGAAGTGTTATTTC
T7-RPL24D1 R	GTAATACGACTCACTATAGGGTGATCAGCTGAATGCAGAGATG
RPL4 F	ATCGAGTTAGGAGCGTGGTTCCTG
T7-RPL4 R	GTAATACGACTCACTATAGGGCTAAGCTTGAGCTGGAGTAGCTTC
RPL32 F	GCGGCCGCGGATCCGACGTCTC
T7-RPL32 R	GTAATACGACTCACTATAGGGGATTATTCGTTCTCCTCGCTGC
RPL17 F	ATGGTCCGCTACTCTCTCGACCC

T7-RPL17 R	GTAATACGACTCACTATAGGGTTACTCCCGAGCCATAAGCTTCTG
RPL6 F	TCCACACACAATCTGCATTAATC
T7-RPL6 R	GTAATACGACTCACTATAGGGGCATTTTAGAAAACCAGTTTGTG
RPL36 F	GGACGCCATTAGCAAAGAGCAG
T7-RPL36 R	GTAATACGACTCACTATAGGGCACAAACAGTTTTATTTTTGATG
Rps9 F	TCTCTTTAGTGTTCGAGCCTGAC
Rps9 R	GTAATACGACTCACTATAGGGTTAATCTTCCTCTTCATCATCACC
Rps4x F	ATGGCCCGAGGACCGAAGAAGCACC
Rps4x R	GTAATACGACTCACTATAGGGGAAACCAAGGTGTGCTAGTCCTG
Rps6 F	CTCCAAGCGAGAAAGTCCTCCATC
Rps6 R	GTAATACGACTCACTATAGGGTCACTTCTGGCTTGACTCTGAC
Rps3A F	ATGGCAGTCGGCAAAAATAAGAGG
Rps3A R	GTAATACGACTCACTATAGGGCAGCACACAAAGGCTCAAATTTAC
Rps20 F	CACCGCTGTTCGCCTTTCAGCTG
Rps20 R	GTAATACGACTCACTATAGGGTTAAGCATCTGCAATTGTGACC
Rps8A F	CTCTTTCTAGCCGGCGCCTGAG
Rps8A R	GTAATACGACTCACTATAGGGCTATTTGCCTTTCTTGGCTTTAATC
Rps27l F	ATGCCTTTGTCCAGAGATTTGATC
Rps27l R	GTAATACGACTCACTATAGGGGTGAAGGTCATTCATTGTAATC
Rps17 F	ATGGGACGCGTGAGGACCAAGACGG
Rps17 R	GTAATACGACTCACTATAGGGTTTTGCAGTCTTTATTGACATCTG
Mycn qF	TACCAAGTCAGCGCTTGAGG
Mycn qR	TTTGCTGCCTTGTCGTTGTG
RPL3 qF	AAGGGACATGGATGCAAGGG
RPL3 qR	AGGCTCCAATACAAGCCACC
RPL12 qF	GATGACATCGCAAAGGCCAC
RPL12 qR	CAGAGGCTGATGGCACTACC
RPL15 qF	CTGTGCATCATGGCGTCAAC
RPL15 qR	TAGGAGTTCAGGACCCGGAG
RPL19 qF	TCTTGCACGAAGGAAGGGTC
RPL19 qR	ACGTTACCCTTTGCCCTCAG
RPL5b qF	ATTTTGCCAGAAAGCGCCTG
RPL5b qR	CCCACACTGATGCCGTACTT
RPL18 qF	CAACAAGGACCGCAAGGTTC
RPL18 qR	GGGAGCATCAGAACGACGAG
RPL30 qF	GTTGGCAAAAACCGGTGTCC
RPL30 qR	CTGCTGGTCTGGCATACTCC
RPL24d1 qF	CGCAACCCAAGAAAGACCAG
RPL24d1 qR	ATGATGAAGCGAGCCTGACG
RPL4 qF	CGTATCCAGCGTAAGGGACC
RPL4 qR	AGAGGTCATCCAGTTTGCGG
RPL32 qF	AGGGTCAGATGTTGATGCCC
RPL32 qR	TTGAGGACACGTTGTGAGCG
RPL17 qF	CGGCCTGAGAACATCTCTGAA
RPL17 qR	AACACACTGGTGCTTGACGA
RPL6 qF	TCATCATCGCGTTATGGCTGAG
RPL6 qR	CACCTTCGTCACTGGAGCTTT
RPL36 qF	ACGCCATTAGCAAAGAGCAG
RPL36 qR	TCTCTCGTAGGGTGCGAAAC
RPs9 qF	GCTCATTCGTCAGAGGCACA
RPs9 qR	CCACCATATGGAGAGCGGAG

RPs4x qF	GGACCGAAGAAGCACCTGAA
RPs4x qR	CTCCCTCAGTTTGTGGGGAC
RPs6 qF	TTCAGCGTCTGGTTACACCC
RPs6 qR	TGACGTTTCTCCTTGGCCTC
RPs3A qF	AAGAGGCTGACCAAAGGTGG
RPs3A qR	ACTGACCTCGAACACACGTC
RPs20 qF	AGGTGGCCATTCACCGTATC
RPs20 qR	TTGGTAGGCATACGCACAGG
RPs8A qF	CGCCGCATCCACACAATAAG
RPs8A qR	CCAGGGTCTTGGTTCTCACC
RPs27l qF	TGTTCAACAGTGCTCTGCCA
RPs27l qR	GGCATCAACAAGTCACGACAG
RPs17 qF	CCACATTAACAAGCGGGTGTG
RPs17 qR	TGCAGTTTGATGGAGATGCCT
Hsp70 P F	CCAAGTTTGTACAAAAAAGCAGGC
Hsp70 P R	AGCAACTTTTCTATACAAAGTTG
Sox17 promoter F	CTTTGTATAGAAAAGTTGCTTACACTCTCTGACAAAAGGTACAC
Sox17 promoter R	CTTTTTTGTACAAACTTGGTCCACAGTGAAAGTTTCAGGCAG
ZMYCN F	GTACAAAAAAGCAGGCTTCGGATCCATGCCAGCTAAAACCATGAGC
ZMYCN R	CAGAGAGAAGTTTGTAGCGGATCCGCGAGTCCTGGCCTGCTCAAGTC
Grna1（mycn）	TAATACGACTCACTATAGGCTGGGGATTGCTCGCTGAGTTTTAGAGCTAGAAATAGC
Grna2（mycn）	TAATACGACTCACTATAGGAAGCCCAGTCCAACGGGTGTTTTAGAGCTAGAAATAGC
Grna3（mycn）	TAATACGACTCACTATAGGAGTGTGTGGACCCTACCGGTTTTAGAGCTAGAAATAGC
Constant GRNA	AAAAGCACCGACTCGGTGCCACTTTTTCAAGTTGATAACGGACTAGCCTTATTTTAACTTGC TATTTCTAGCTCTAAAAC
T7 PROMOTER F	TAATACGACTCACTATAGG
Grna scaffold R	AAAAGCACCGACTCGGTGC
