# Supplementary material for: Mycn regulates intestinal development through ribosomal biogenesis in a zebrafish model of Feingold syndrome 1
Source: PLoS Biol. 2022 Nov 1;20(11):e3001856. doi: 10.1371/journal.pbio.3001856 (PMC9624419; doi:10.1371/journal.pbio.3001856)
Supplement: S2 Table — (PDF) [file pbio.3001856.s009.pdf]

gene	p_val	avg_log2FC	pct.1	pct.2	p_val_adj	cluster
nolc1	3.27E-11	0.30805719	0.119	0.508	7.80E-07	0
prdx6	5.79E-11	0.259476337	0.19	0.623	1.38E-06	0
gngt2b	6.35E-11	2.168844209	0.69	0.65	1.51E-06	0
si:ch211-212k18.7	1.45E-10	0.318990809	0.167	0.555	3.46E-06	0
hsdl2	1.51E-10	0.320937422	0.151	0.551	3.61E-06	0
rbm8a	4.31E-10	0.334788642	0.27	0.713	1.03E-05	0
sf3b6	6.23E-10	0.321428513	0.167	0.559	1.48E-05	0
krtt1c19e	6.62E-10	1.845516034	0.698	0.699	1.58E-05	0
rpn1	6.93E-10	0.255528527	0.167	0.541	1.65E-05	0
farsa	8.05E-10	0.33564754	0.095	0.426	1.92E-05	0
wdr83os	9.44E-10	0.325985776	0.143	0.518	2.25E-05	0
eif4a3	1.16E-09	0.266542649	0.119	0.475	2.77E-05	0
fam32a	1.38E-09	0.256280611	0.198	0.594	3.29E-05	0
pdlim2	1.83E-09	0.319247275	0.111	0.449	4.35E-05	0
ssrp1a	2.33E-09	0.314372127	0.079	0.377	5.55E-05	0
leo1	3.40E-09	0.258583353	0.111	0.428	8.10E-05	0
ndufa9a	3.52E-09	0.344074883	0.175	0.543	8.40E-05	0
mphosph10	6.77E-09	0.277822541	0.111	0.439	0.0001614	0
ndufv2	7.12E-09	0.299265234	0.183	0.551	0.0001698	0
skp1	8.15E-09	0.263842581	0.23	0.625	0.0001944	0
prelid1a	8.45E-09	0.298437038	0.246	0.631	0.0002016	0
cpsf6	1.14E-08	0.377883085	0.103	0.404	0.0002713	0
aifm2	1.14E-08	0.297625645	0.103	0.408	0.0002715	0
timm23a	1.15E-08	0.354491767	0.119	0.445	0.0002744	0
cct6a	1.44E-08	0.256575131	0.23	0.631	0.0003435	0
eif3ja	1.55E-08	0.252528191	0.246	0.648	0.0003698	0
bcas2	1.64E-08	0.312527324	0.111	0.422	0.0003911	0
psma2	2.14E-08	0.282985597	0.214	0.592	0.0005103	0
si:dkey-17e16.10	2.23E-08	0.271372305	0.159	0.492	0.0005324	0
srsf10b	2.25E-08	0.293336404	0.111	0.416	0.0005356	0
zgc:85777	2.38E-08	0.326910274	0.095	0.402	0.0005674	0
eif3d	2.39E-08	0.261558328	0.262	0.666	0.0005692	0
zgc:86598	2.51E-08	0.286706352	0.175	0.531	0.0005975	0
cct8	2.61E-08	0.253023674	0.19	0.549	0.0006212	0
mrpl40	3.03E-08	0.254562464	0.143	0.463	0.0007229	0
anp32b	3.11E-08	0.303849548	0.143	0.467	0.0007404	0
myef2	3.26E-08	0.252089397	0.127	0.457	0.0007773	0
cstf2	3.27E-08	0.250078726	0.111	0.42	0.0007794	0
ifrd1	3.40E-08	0.3149818	0.222	0.617	0.00081	0
cab39l	4.18E-08	0.519072575	0.119	0.418	0.0009971	0
ubxn1	4.56E-08	0.300576962	0.111	0.412	0.0010884	0
smim20	4.81E-08	0.370056679	0.071	0.346	0.0011477	0
npepps	5.04E-08	0.37013343	0.079	0.344	0.0012011	0
pdhb	5.42E-08	0.426748359	0.119	0.43	0.0012925	0
sept7a	5.80E-08	0.302306963	0.111	0.408	0.0013833	0
nutf2l	6.64E-08	0.294276258	0.198	0.547	0.0015824	0
spcs1	7.42E-08	0.440956639	0.238	0.621	0.0017686	0
chmp4bb	7.53E-08	0.253996608	0.167	0.486	0.0017967	0
calm2b	7.72E-08	0.334141131	0.294	0.711	0.0018405	0
cope	9.41E-08	0.37976789	0.198	0.561	0.0022443	0
epn1	1.12E-07	0.302667402	0.175	0.496	0.0026779	0
cdkn2aipnl	1.17E-07	0.321340757	0.19	0.535	0.0027895	0
pet100	1.28E-07	0.280330146	0.127	0.428	0.0030414	0

si:dkey-74k8.3	1.28E-07	0.438964283	0.111	0.404	0.0030468	0
tomm22	1.29E-07	0.266712539	0.19	0.535	0.0030739	0
smc1a1	1.29E-07	0.327821988	0.143	0.455	0.0030757	0
cisd1	1.30E-07	0.266001378	0.111	0.4	0.003092	0
emc2	1.30E-07	0.28728188	0.175	0.51	0.003105	0
pdcd10a	1.46E-07	0.287107677	0.111	0.398	0.003476	0
tomm40	1.74E-07	0.282055595	0.159	0.482	0.0041555	0
CABZ01079011.1	1.75E-07	0.314469768	0.222	0.586	0.0041675	0
capza1a	1.81E-07	0.54663943	0.143	0.453	0.0043151	0
acsbg2	1.82E-07	0.259214308	0.095	0.361	0.0043439	0
higd2a	1.85E-07	0.353936103	0.103	0.383	0.0044174	0
wdr82	2.09E-07	0.305270497	0.071	0.314	0.0049908	0
nip7	2.20E-07	0.324463186	0.143	0.439	0.0052468	0
grsf1	2.56E-07	0.290254059	0.071	0.328	0.0060946	0
acadl	2.83E-07	0.313973993	0.103	0.373	0.0067422	0
coa5	2.88E-07	0.302822534	0.111	0.393	0.0068713	0
acadm	2.89E-07	0.390200414	0.175	0.504	0.0068916	0
fabp1b.1	3.43E-07	0.644399333	0.754	0.531	0.0081725	0
rbb4l	3.88E-07	0.321413873	0.087	0.342	0.0092451	0
ccdc47	4.10E-07	0.254174874	0.198	0.529	0.0097691	0
noc2l	4.10E-07	0.269679474	0.119	0.402	0.0097809	0
srm	4.19E-07	0.391321025	0.056	0.291	0.0099996	0
metap1	4.95E-07	0.297281722	0.127	0.408	0.0117947	0
mrpl54	5.21E-07	0.338192156	0.222	0.582	0.0124342	0
hp1bp3	5.56E-07	0.416601775	0.222	0.574	0.0132674	0
vamp3	5.73E-07	0.300093045	0.151	0.443	0.0136611	0
mif	5.92E-07	0.332389155	0.262	0.643	0.014121	0
pwp2h	6.04E-07	0.379675491	0.087	0.334	0.0144009	0
snrpd3l	6.27E-07	0.29020599	0.214	0.572	0.0149572	0
apex1	6.48E-07	0.28751008	0.119	0.377	0.0154516	0
exosc2	6.94E-07	0.276662613	0.079	0.314	0.0165436	0
psmd12	7.33E-07	0.427631009	0.167	0.465	0.0174811	0
ddx18	7.70E-07	0.43954668	0.238	0.605	0.0183506	0
fam210b	8.00E-07	0.29547618	0.103	0.365	0.0190752	0
rbbp4	8.12E-07	0.46979565	0.111	0.385	0.0193737	0
adh5	8.42E-07	0.325721681	0.262	0.625	0.0200711	0
etfb	8.97E-07	0.655142048	0.222	0.576	0.0213863	0
vbp1	9.18E-07	0.29831409	0.151	0.432	0.0218921	0
bola3	9.49E-07	0.269132419	0.079	0.322	0.0226218	0
mpc1	9.75E-07	0.337343663	0.302	0.656	0.023245	0
stl3	9.90E-07	0.485068566	0.143	0.439	0.0236165	0
pycard	1.04E-06	0.290348838	0.119	0.377	0.0248501	0
dus3l	1.09E-06	0.322585282	0.056	0.275	0.0259963	0
sigmar1	1.11E-06	0.282837166	0.175	0.482	0.0264868	0
mrpl32	1.13E-06	0.304212956	0.206	0.539	0.0268892	0
txndc17	1.14E-06	0.343716754	0.079	0.316	0.0272231	0
zmat2	1.14E-06	0.363052316	0.151	0.436	0.0272804	0
mrps35	1.17E-06	0.47957786	0.167	0.471	0.0278984	0
znf593	1.25E-06	0.41524159	0.198	0.531	0.0297245	0
psmb2	1.31E-06	0.430383116	0.246	0.617	0.0313384	0
si:dkey-69o16.5	1.33E-06	0.29696337	0.095	0.34	0.0317825	0
ccdc124	1.35E-06	0.27855164	0.167	0.467	0.0322452	0
ranbp3b	1.37E-06	0.432000054	0.087	0.326	0.0327237	0
dync1li2	1.40E-06	0.269195377	0.056	0.264	0.0333659	0

npepl1	1.45E-06	0.376129947	0.19	0.494	0.0345967	0
ddx24	1.49E-06	0.380892909	0.183	0.488	0.0354432	0
mrpl30	1.49E-06	0.517077324	0.214	0.559	0.035569	0
chchd4a	1.49E-06	0.285143397	0.095	0.342	0.035591	0
naa38	1.56E-06	0.25355228	0.111	0.367	0.0371649	0
tnk1	1.58E-06	0.447550844	0.19	0.496	0.0375684	0
triap1	1.60E-06	0.330741551	0.103	0.355	0.0381165	0
h3f3b.1.2	1.60E-06	0.430690206	0.278	0.652	0.0382326	0
blcap	1.61E-06	0.556446573	0.119	0.391	0.0382851	0
park7	1.82E-06	0.457404063	0.111	0.373	0.043492	0
nap1l4a	1.85E-06	0.410897061	0.079	0.311	0.0441288	0
ciarta	1.89E-06	0.340160427	0.079	0.309	0.0451765	0
dhx9	1.93E-06	0.278069938	0.079	0.303	0.0459958	0
opa1	2.01E-06	0.491998572	0.095	0.342	0.0478906	0
nucks1b	2.19E-06	0.486029352	0.159	0.443	0.0522852	0
prdx4	2.35E-06	0.406113773	0.206	0.527	0.056078	0
zgc:91910	2.40E-06	0.386862279	0.198	0.504	0.057283	0
nifk	2.49E-06	0.251458449	0.159	0.441	0.0593642	0
aifm1	2.61E-06	0.635958667	0.119	0.379	0.0622059	0
ier3ip1	2.72E-06	0.47276095	0.127	0.395	0.0647534	0
zgc:77486.1	2.89E-06	0.374539927	0.23	0.574	0.0689178	0
mt-atp8	3.04E-06	0.284217928	0.27	0.639	0.0725277	0
spcs3	3.05E-06	0.351390634	0.23	0.568	0.0726125	0
mrps10	3.06E-06	0.428216414	0.222	0.545	0.0729845	0
ccng1	3.13E-06	0.358003098	0.254	0.59	0.0746508	0
acaa1	3.13E-06	0.271427548	0.079	0.299	0.0747017	0
ube2nb	3.15E-06	0.333791211	0.095	0.332	0.0751041	0
arpc4	3.38E-06	0.582260111	0.19	0.496	0.0805764	0
iars	3.66E-06	0.268920337	0.127	0.383	0.0873125	0
psma4	3.75E-06	0.262465264	0.31	0.701	0.0893792	0
ddx39ab	3.77E-06	0.262991711	0.31	0.668	0.0898756	0
gtf2f2a	3.78E-06	0.393840746	0.127	0.375	0.0900477	0
fahd1	3.80E-06	0.345118377	0.175	0.461	0.0905609	0
lonrf1l	3.88E-06	0.264086734	0.183	0.469	0.0925742	0
rnaseka	4.00E-06	0.414664007	0.151	0.434	0.0954319	0
ppp4cb	4.36E-06	0.346742877	0.103	0.34	0.1040438	0
npm3	4.49E-06	0.500309027	0.19	0.494	0.1071201	0
fabp2	4.55E-06	0.80483243	0.865	0.766	0.1085693	0
thoc5	4.88E-06	0.251319841	0.056	0.254	0.1164177	0
ptrhd1	4.91E-06	0.531603144	0.135	0.398	0.1170624	0
lpgat1	4.92E-06	0.352231916	0.127	0.381	0.1172957	0
cited4b	4.94E-06	0.365539348	0.135	0.391	0.1178001	0
rbm34	5.02E-06	0.250895132	0.056	0.256	0.1197543	0
timm8a	5.06E-06	0.394950964	0.206	0.496	0.120675	0
polr2h	5.26E-06	0.311079674	0.087	0.314	0.1254729	0
akr7a3	5.28E-06	0.306821415	0.079	0.299	0.125942	0
tpi1b	5.32E-06	0.455821244	0.333	0.705	0.1268902	0
txndc9	5.37E-06	0.661258544	0.135	0.387	0.1281204	0
glrx5	5.47E-06	0.337026416	0.214	0.525	0.1305264	0
timm10	6.55E-06	0.486727862	0.19	0.471	0.1561316	0
polr1d	6.56E-06	0.547070366	0.135	0.393	0.156456	0
hadhaa	6.99E-06	0.342975989	0.079	0.289	0.1666277	0
lig1	7.45E-06	0.295773098	0.079	0.287	0.17766	0
ckba	8.01E-06	0.329553506	0.095	0.322	0.190982	0

zgc:112255	8.05E-06	0.421468161	0.111	0.355	0.1919057	0
cdk11b	8.22E-06	0.626737675	0.127	0.379	0.1960935	0
rbx1	8.65E-06	0.279828021	0.349	0.744	0.2062537	0
ppifb	8.94E-06	0.443964795	0.167	0.445	0.2131304	0
abcc2	9.11E-06	0.295235719	0.103	0.33	0.2172681	0
sgk1	9.41E-06	0.375734009	0.127	0.371	0.2242921	0
lsm7	9.79E-06	0.300562429	0.27	0.598	0.2333592	0
matr3l1.1.1	1.03E-05	0.275647662	0.103	0.328	0.2445981	0
impdh2	1.03E-05	0.299715155	0.151	0.414	0.2462937	0
cox17	1.04E-05	0.329723329	0.317	0.719	0.2479471	0
krt5	1.06E-05	1.882331159	0.563	0.559	0.2531461	0
mrps18a	1.09E-05	0.340521907	0.167	0.451	0.259069	0
gadd45gip1	1.13E-05	0.376772868	0.206	0.52	0.2693923	0
trmt112	1.17E-05	0.538667466	0.159	0.418	0.2778746	0
mtap	1.19E-05	0.443135169	0.167	0.436	0.2833832	0
srd5a2a	1.20E-05	0.320095745	0.103	0.332	0.2857048	0
hsd11b1la	1.21E-05	0.345216306	0.119	0.355	0.2897039	0
si:ch211-288g17.3	1.23E-05	0.25865011	0.302	0.639	0.2941484	0
sae1	1.29E-05	0.359658327	0.079	0.283	0.3080441	0
si:ch211-248a14.8	1.30E-05	0.316351353	0.119	0.359	0.3097377	0
cherp	1.37E-05	0.340943735	0.063	0.26	0.3278164	0
timmdc1	1.41E-05	0.265290002	0.095	0.303	0.3352429	0
ktn1	1.47E-05	0.253594963	0.31	0.68	0.3499379	0
hmgn3	1.48E-05	0.300434362	0.111	0.328	0.3523044	0
emg1	1.61E-05	0.262379311	0.071	0.264	0.3830932	0
smim4	1.71E-05	0.341609236	0.159	0.412	0.4072872	0
ccdc58	1.71E-05	0.262066135	0.151	0.393	0.4074454	0
ube2f	1.86E-05	0.343513977	0.071	0.266	0.4428809	0
tusc2b	2.04E-05	0.253082174	0.103	0.314	0.4856458	0
ndufv3	2.05E-05	0.322664838	0.238	0.547	0.4890009	0
mrpl43	2.08E-05	0.384921352	0.111	0.332	0.4953296	0
snrpd2	2.23E-05	0.428622676	0.333	0.707	0.5317798	0
timm13	2.30E-05	0.341164818	0.198	0.473	0.5475598	0
sdhdb	2.45E-05	0.351744193	0.278	0.598	0.5847383	0
ctr9	2.50E-05	0.36841232	0.079	0.275	0.5956189	0
ablim1b	2.58E-05	0.323134018	0.087	0.287	0.6147718	0
lta4h	2.63E-05	0.31640275	0.119	0.34	0.6270928	0
vdac1	2.69E-05	0.463155732	0.167	0.43	0.6413355	0
med28	2.76E-05	0.450465593	0.103	0.316	0.6578296	0
mrps25	3.04E-05	0.275205569	0.23	0.523	0.7256374	0
zgc:136564	3.17E-05	0.276143292	0.167	0.408	0.7564976	0
mbd3a	3.24E-05	0.400961754	0.071	0.26	0.771654	0
lama3	3.29E-05	0.250578763	0.119	0.34	0.784787	0
mrpl24	3.30E-05	0.598661551	0.206	0.494	0.7875338	0
dnaja2	3.38E-05	0.492722601	0.286	0.623	0.8062528	0
CHCHD5	3.55E-05	0.389153304	0.103	0.309	0.8456958	0
mrps18c	3.61E-05	0.355358478	0.238	0.535	0.86124	0
crebzf	3.66E-05	0.559809874	0.222	0.52	0.873252	0
sec22ba	3.72E-05	0.282531563	0.095	0.297	0.8868415	0
rrm2.1	3.79E-05	0.424940111	0.111	0.318	0.9037777	0
rho	4.27E-05	1.46043159	0.54	0.498	1	0
aqp8a.1	4.59E-05	0.407439405	0.135	0.371	1	0
ddx42	4.63E-05	0.276234334	0.079	0.268	1	0
arhgdig	4.73E-05	0.292440269	0.111	0.328	1	0

txndc12	4.90E-05	0.337129925	0.087	0.277	1	0
nr1h4	5.21E-05	0.311183095	0.079	0.268	1	0
nop16	5.24E-05	0.471280377	0.19	0.455	1	0
zdhhc4	5.57E-05	0.312348223	0.087	0.273	1	0
cdkn1a	5.58E-05	0.291803095	0.119	0.328	1	0
myo15b	5.60E-05	0.373103937	0.079	0.27	1	0
hexb	5.64E-05	0.408692058	0.095	0.289	1	0
snrnp40	5.80E-05	0.300034924	0.087	0.279	1	0
rsl1d1	5.91E-05	0.378243681	0.278	0.607	1	0
eif1axa	6.08E-05	0.651407261	0.151	0.385	1	0
acsl4a	6.10E-05	0.455266824	0.135	0.35	1	0
fam120a	6.12E-05	0.388502825	0.087	0.275	1	0
slirp	6.29E-05	0.300520197	0.325	0.672	1	0
mpv17	6.61E-05	0.398599981	0.071	0.252	1	0
bcap31	6.61E-05	0.3775084	0.095	0.293	1	0
si:ch211-161h7.5	6.62E-05	0.52546813	0.119	0.326	1	0
bhlhe40	6.81E-05	0.265227975	0.071	0.252	1	0
tsc22d3	7.16E-05	0.324663887	0.167	0.404	1	0
rnf19a	7.32E-05	0.426408447	0.095	0.285	1	0
cnih1	7.37E-05	0.460683181	0.079	0.266	1	0
pin4	7.56E-05	0.309657089	0.206	0.469	1	0
ube2c	8.07E-05	0.282745825	0.111	0.314	1	0
ifrd2	8.20E-05	0.544022914	0.087	0.275	1	0
ctsba	8.28E-05	0.547427119	0.079	0.262	1	0
mrpl57	8.66E-05	0.374277764	0.31	0.629	1	0
lhfpl2a	8.71E-05	0.294562043	0.111	0.311	1	0
tufm	9.09E-05	0.68994341	0.238	0.51	1	0
dhrs1	9.30E-05	0.404271625	0.262	0.557	1	0
btf3l4	9.32E-05	0.369491286	0.278	0.588	1	0
aldh9a1a.1	9.49E-05	0.334147493	0.31	0.65	1	0
supt4h1	9.85E-05	0.383495143	0.222	0.498	1	0
chchd10	0.0001005	0.253154922	0.302	0.6	1	0
mycbp	0.0001008	0.377831321	0.135	0.348	1	0
id2a	0.0001111	0.770711207	0.31	0.631	1	0
glulb	0.0001112	0.458867797	0.167	0.402	1	0
mrpl22	0.0001128	0.319430653	0.119	0.314	1	0
ndufaf4	0.0001177	0.272140555	0.087	0.266	1	0
tmsb4x	0.0001228	0.366371782	1	0.992	1	0
lsm12b	0.0001252	0.63409874	0.31	0.631	1	0
xpo1b	0.0001305	0.319669969	0.135	0.344	1	0
tuba8l2	0.0001336	0.338103793	0.333	0.664	1	0
slc39a7	0.0001366	0.462753057	0.087	0.268	1	0
mrps9	0.0001454	0.49952601	0.151	0.373	1	0
msmo1	0.0001471	0.256434254	0.222	0.465	1	0
rgcc	0.0001539	0.64495038	0.214	0.482	1	0
zgc:162025	0.0001621	0.454663234	0.087	0.256	1	0
gstr	0.0001624	0.279932253	0.357	0.664	1	0
cox7a3	0.0001698	0.504292188	0.325	0.65	1	0
pfdn4	0.0001802	0.461761199	0.31	0.639	1	0
thoc7	0.000185	0.327545594	0.111	0.295	1	0
eif4bb	0.000191	0.373116599	0.349	0.672	1	0
lgals1l1	0.0002549	1.616998504	0.294	0.186	1	0
mrpl52	0.000256	0.37145546	0.246	0.527	1	0
si:ch211-68a17.7	0.0002692	0.387860292	0.238	0.498	1	0

cicb	0.0002854	0.268035659	0.135	0.328	1	0
gsta.1.1	0.0003242	0.493780899	0.889	0.828	1	0
sik1	0.0003275	0.375521851	0.151	0.361	1	0
rbm19	0.000334	0.564725631	0.135	0.34	1	0
rtca	0.0003373	0.302264363	0.095	0.268	1	0
mrpl35	0.0003573	0.562348245	0.214	0.463	1	0
jdp2b	0.0003591	0.482276905	0.373	0.734	1	0
angptl4	0.00036	0.445578901	0.357	0.711	1	0
cct5	0.000369	0.33883792	0.333	0.656	1	0
bin2a	0.0003762	0.322328742	0.294	0.559	1	0
mt-nd2	0.0003784	0.276701025	0.532	0.926	1	0
ndufa6	0.0003964	0.330798265	0.397	0.775	1	0
nuak1b	0.0003973	0.393512127	0.135	0.32	1	0
ndufb8	0.0004144	0.410427721	0.437	0.773	1	0
mcm2	0.0004202	0.387060215	0.103	0.275	1	0
hbae1.1	0.0004443	0.475867149	0.095	0.26	1	0
NDUFAF8	0.0004694	0.277339472	0.111	0.287	1	0
mgst1.2	0.0004758	0.318322505	0.143	0.328	1	0
atp5mea	0.0004902	0.449480489	0.325	0.676	1	0
rpl35	0.0004989	0.541325319	0.968	0.98	1	0
coq10b	0.0005177	0.258142596	0.095	0.258	1	0
si:ch211-156b7.4	0.0005306	0.746299588	0.135	0.328	1	0
mt-nd3	0.0005368	0.284295502	0.437	0.855	1	0
srp19	0.0005798	0.479639696	0.238	0.498	1	0
rgn	0.0005981	0.486014722	0.111	0.287	1	0
ndufa11	0.0005995	0.486362385	0.286	0.6	1	0
NPC1L1	0.0006012	0.312842543	0.095	0.254	1	0
si:ch211-198m17.1	0.0006227	0.291180166	0.175	0.389	1	0
cops5	0.0006247	0.606288155	0.111	0.287	1	0
hmgn7	0.0006582	0.347955741	0.357	0.689	1	0
psmb3	0.0006599	0.422491151	0.365	0.699	1	0
fosab	0.0006758	0.394988135	0.397	0.738	1	0
id1	0.0006864	0.403105464	0.175	0.371	1	0
mesd	0.0006955	0.311988861	0.167	0.375	1	0
slc35b1	0.0007262	0.348947088	0.159	0.355	1	0
mrps14	0.0007835	0.427940583	0.238	0.494	1	0
tgif1	0.0008506	0.285397592	0.119	0.291	1	0
EIF1B	0.001153	0.496789738	0.333	0.67	1	0
mrpl46	0.0012042	0.389646022	0.159	0.342	1	0
lsm4	0.0012462	0.520202609	0.31	0.613	1	0
gnpda1	0.0012622	0.362983425	0.262	0.512	1	0
coa3a	0.0012934	0.788447941	0.31	0.609	1	0
cbr1l	0.0015107	0.60501805	0.238	0.463	1	0
hsp70.3	0.0015727	0.696517259	0.183	0.373	1	0
psmb6	0.0015809	0.446604549	0.381	0.709	1	0
pptc7a	0.0016035	0.262667171	0.111	0.266	1	0
si:ch211-107o10.3	0.0016743	0.630348403	0.23	0.467	1	0
dhrs13l1	0.0016889	0.368203565	0.111	0.266	1	0
mgst3b	0.0017156	0.438037254	0.365	0.668	1	0
pam16	0.0017515	0.682248188	0.246	0.488	1	0
si:dkey-16p21.8	0.0018541	0.356415251	0.27	0.533	1	0
cyr61	0.0018587	0.573865061	0.103	0.254	1	0
prdx1	0.001911	0.769503405	0.294	0.561	1	0
tomm6	0.0019369	0.259945739	0.373	0.701	1	0

pcna	0.0020387	0.589742516	0.262	0.5	1	0
rpl29	0.002067	0.499332083	0.841	0.957	1	0
ndufa3	0.0021342	0.3471055	0.397	0.709	1	0
si:dkey-183i3.5	0.0022011	1.731440375	0.5	0.523	1	0
tomm7	0.0022875	0.591412813	0.373	0.707	1	0
hnf4a	0.0030069	0.363489486	0.119	0.27	1	0
zanl	0.0034201	0.250739402	0.27	0.48	1	0
mrps33	0.0034685	0.620581206	0.23	0.455	1	0
mt-nd4	0.0037578	0.290581455	0.563	0.908	1	0
imp3	0.0039276	0.326335098	0.175	0.359	1	0
cnbpb	0.0039865	0.396722919	0.46	0.775	1	0
zgc:175088	0.0042357	0.311145845	0.119	0.262	1	0
pvalb2	0.0042568	0.643861266	0.23	0.445	1	0
zgc:172079.2	0.0042945	0.354684604	0.31	0.541	1	0
ndufb9	0.0044239	0.467129516	0.429	0.752	1	0
hmgb1b	0.0049055	0.604830224	0.143	0.309	1	0
mbl2	0.0049073	0.262318998	0.143	0.295	1	0
rbp2a	0.0057	0.550435334	0.69	0.539	1	0
gstm.1	0.0057232	0.653622735	0.23	0.439	1	0
scp2a	0.0058892	0.34612856	0.31	0.535	1	0
mt-co2	0.0066055	0.352534188	0.802	0.973	1	0
mt-cyb	0.0067524	0.417711956	0.706	0.955	1	0
dnajb1b	0.0075025	0.506738158	0.135	0.279	1	0
rbp5	0.0081179	0.333039776	0.167	0.322	1	0
hspe1	0.0083887	0.669195409	0.706	0.826	1	0
tuba8l	0.0087945	0.296249521	0.444	0.701	1	0
mt-nd1	0.0092948	0.384662862	0.587	0.918	1	0
rltgr	2.25E-52	1.101401839	0.938	0.167	5.37E-48	1
acsl5	5.19E-52	0.631704012	0.7	0.062	1.24E-47	1
slc13a3	7.71E-51	0.780805217	0.825	0.105	1.84E-46	1
pck2	8.28E-51	0.694626222	0.812	0.103	1.98E-46	1
stra6	4.19E-50	0.666061886	0.825	0.103	9.99E-46	1
faah2a	4.23E-49	0.530965924	0.8	0.096	1.01E-44	1
cyp4v8	2.45E-48	0.539078767	0.788	0.097	5.84E-44	1
ugt1b5	2.90E-48	0.622628768	0.9	0.142	6.92E-44	1
gyg1a	1.46E-47	0.485330977	0.788	0.097	3.48E-43	1
chpt1	2.03E-46	1.228636146	0.975	0.245	4.83E-42	1
mat1a	6.66E-44	0.737277947	0.862	0.148	1.59E-39	1
slc37a4a	1.83E-43	0.601046702	0.888	0.15	4.36E-39	1
lta4h1	1.14E-42	1.036413892	0.912	0.202	2.71E-38	1
acad11	1.37E-42	0.441127226	0.75	0.097	3.26E-38	1
cyp2x9	1.54E-42	0.646522151	0.875	0.159	3.68E-38	1
mogat2	1.18E-41	0.500749118	0.738	0.101	2.82E-37	1
trap1	1.38E-41	0.41231872	0.762	0.105	3.30E-37	1
slco1d1	5.24E-40	1.128334461	0.938	0.236	1.25E-35	1
si:ch211-127i16.2	6.19E-40	0.357765252	0.6	0.058	1.48E-35	1
enpep	6.64E-40	0.6336689	0.862	0.155	1.58E-35	1
gpt2l	7.23E-40	0.72554152	0.875	0.185	1.72E-35	1
mttp	1.36E-39	0.927878098	0.95	0.24	3.25E-35	1
slc7a8a	3.06E-39	0.942405062	0.975	0.255	7.30E-35	1
got2a	3.63E-39	0.40475649	0.65	0.077	8.64E-35	1
si:ch211-161h7.8	6.79E-39	0.72859383	0.888	0.176	1.62E-34	1
cyp2k19	8.15E-39	0.507628924	0.662	0.082	1.94E-34	1
apobb.1	1.16E-38	0.995981711	0.888	0.2	2.76E-34	1

acsf2	2.55E-38	0.631434079	0.788	0.137	6.09E-34	1
srd5a2a1	2.68E-38	0.638151712	0.912	0.191	6.38E-34	1
slc7a7	2.79E-38	0.619859845	0.8	0.133	6.66E-34	1
tbrg4	3.67E-38	0.462032808	0.688	0.096	8.74E-34	1
lap3	4.23E-38	0.379295966	0.725	0.105	1.01E-33	1
bco1	2.26E-37	0.535038997	0.588	0.064	5.39E-33	1
nt5c2a	2.77E-37	0.311427796	0.812	0.137	6.61E-33	1
zgc:152830	3.17E-37	0.768832154	0.862	0.195	7.56E-33	1
mxra5a	4.58E-37	0.410967483	0.625	0.071	1.09E-32	1
ilvbl	4.69E-37	0.649730176	0.85	0.184	1.12E-32	1
slc5a1	5.68E-37	0.74308537	0.8	0.152	1.35E-32	1
pla2g12b	5.86E-37	0.842929916	0.812	0.167	1.40E-32	1
lin28a	5.99E-37	0.431960474	0.812	0.14	1.43E-32	1
aifm11	7.67E-37	0.708299995	0.925	0.236	1.83E-32	1
cd36	8.27E-37	0.726782019	0.875	0.189	1.97E-32	1
lpcat3	1.60E-36	0.7000491	0.888	0.215	3.81E-32	1
gapdh	3.76E-36	1.624916371	1	0.62	8.97E-32	1
dpydb	4.22E-36	0.754386354	0.862	0.193	1.01E-31	1
zgc:103681	4.45E-36	0.323149724	0.762	0.125	1.06E-31	1
cpox	4.75E-36	0.466911793	0.75	0.125	1.13E-31	1
heatr3	4.89E-36	0.327210039	0.65	0.082	1.17E-31	1
pipox	5.07E-36	0.43474126	0.788	0.131	1.21E-31	1
sccpdha	5.27E-36	0.306045838	0.638	0.081	1.26E-31	1
pcxb	6.85E-36	0.950822317	0.975	0.29	1.63E-31	1
gatm	8.69E-36	1.103902462	0.938	0.243	2.07E-31	1
aqp8a.11	9.54E-36	0.91697002	0.95	0.228	2.28E-31	1
akt2l	1.00E-35	0.408175165	0.888	0.178	2.39E-31	1
mthfd1b	1.08E-35	0.572778097	0.762	0.135	2.57E-31	1
pnkp	1.26E-35	0.325100697	0.575	0.062	3.00E-31	1
atp1a1a.4	1.49E-35	1.238456646	1	0.433	3.55E-31	1
ada	1.54E-35	0.844596229	0.888	0.228	3.66E-31	1
amt	1.70E-35	0.30130771	0.662	0.088	4.05E-31	1
zgc:110843	3.47E-35	0.368349288	0.788	0.14	8.28E-31	1
dgat1a	7.04E-35	0.260448774	0.575	0.064	1.68E-30	1
retsat	1.10E-34	0.591744198	0.862	0.193	2.63E-30	1
dnpep	1.46E-34	0.480334639	0.8	0.161	3.49E-30	1
glud1b	1.72E-34	1.487743317	1	0.446	4.11E-30	1
rdh1	1.73E-34	0.688711153	0.85	0.189	4.13E-30	1
atp8a2	2.37E-34	0.63727295	0.85	0.193	5.65E-30	1
mylk5	2.67E-34	0.477043945	0.7	0.11	6.38E-30	1
ace	2.73E-34	0.812824402	0.938	0.23	6.51E-30	1
zgc:153031	3.73E-34	0.301803678	0.488	0.043	8.89E-30	1
adsl	5.35E-34	0.500669804	0.762	0.146	1.28E-29	1
CYP2C9	6.63E-34	0.691224546	0.875	0.197	1.58E-29	1
cpt2	6.73E-34	0.494790684	0.788	0.15	1.61E-29	1
gstt2	8.51E-34	0.33253385	0.725	0.122	2.03E-29	1
zgc:85843	1.10E-33	0.461071609	0.812	0.155	2.63E-29	1
a1cf	1.19E-33	0.461660953	0.888	0.185	2.85E-29	1
got1	1.23E-33	0.58673009	0.812	0.17	2.94E-29	1
aldh1a2	1.48E-33	0.684652074	0.75	0.14	3.53E-29	1
oxa1l	1.73E-33	0.561764572	0.838	0.185	4.12E-29	1
slc2a2	2.02E-33	0.3543662	0.6	0.079	4.81E-29	1
ak3	2.68E-33	0.689834051	0.938	0.272	6.40E-29	1
dhcr7	2.98E-33	0.771603956	0.938	0.268	7.11E-29	1

st8sia7.1	5.40E-33	0.440532922	0.662	0.103	1.29E-28	1
sult2st2	5.66E-33	1.28435573	1	0.41	1.35E-28	1
abcb11a	6.15E-33	0.479067078	0.712	0.129	1.47E-28	1
dpp3	6.20E-33	0.40212259	0.7	0.118	1.48E-28	1
slc25a16	6.48E-33	0.255771087	0.6	0.079	1.55E-28	1
l2hgdh	7.03E-33	0.322638061	0.638	0.09	1.68E-28	1
hprt1	9.00E-33	0.348120288	0.75	0.135	2.15E-28	1
ugt5b2	9.47E-33	0.483169203	0.75	0.144	2.26E-28	1
cat	9.85E-33	0.874087144	0.962	0.333	2.35E-28	1
si:ch211-161h7.51	1.55E-32	0.627751891	0.875	0.195	3.70E-28	1
abat	1.56E-32	0.600561012	0.85	0.2	3.72E-28	1
sdha	2.53E-32	0.976788059	0.962	0.369	6.03E-28	1
fbp1b	2.98E-32	1.058522811	0.975	0.345	7.11E-28	1
slc22a6l	3.49E-32	0.456543	0.688	0.114	8.33E-28	1
slc25a44b	3.73E-32	0.416839996	0.688	0.118	8.90E-28	1
msrb2	3.77E-32	0.703093531	0.888	0.232	8.98E-28	1
slc16a10	3.96E-32	0.521598526	0.8	0.174	9.44E-28	1
pa2g4a	4.70E-32	1.06590659	0.988	0.438	1.12E-27	1
aldob	5.28E-32	1.463227374	1	0.721	1.26E-27	1
fmo5	5.31E-32	0.259365427	0.612	0.082	1.27E-27	1
CU856520.1	6.46E-32	0.590890319	0.862	0.208	1.54E-27	1
lgals9l3	6.69E-32	0.295661323	0.712	0.118	1.59E-27	1
zgc:112056	6.69E-32	0.360692877	0.65	0.101	1.60E-27	1
mrpl37	7.82E-32	0.710828098	0.925	0.268	1.87E-27	1
creb3l3a	8.02E-32	0.416728816	0.775	0.146	1.91E-27	1
ehhadh	1.61E-31	0.361442649	0.75	0.144	3.84E-27	1
g6pca.2	1.70E-31	0.483920869	0.588	0.079	4.05E-27	1
abhd14b	1.74E-31	0.450316807	0.788	0.163	4.14E-27	1
nap1l4a1	1.88E-31	0.402009671	0.812	0.182	4.47E-27	1
msmo1l	2.06E-31	1.0565242	0.975	0.331	4.91E-27	1
paics	2.13E-31	0.865241444	0.962	0.33	5.07E-27	1
tha1	2.84E-31	0.352797462	0.625	0.094	6.76E-27	1
ptcd3	3.00E-31	0.527808008	0.8	0.17	7.16E-27	1
npr1b	3.39E-31	0.300074647	0.438	0.037	8.09E-27	1
suclg2	4.29E-31	0.715744908	0.95	0.294	1.02E-26	1
slc25a10	4.93E-31	0.484194317	0.738	0.148	1.18E-26	1
aco2	5.56E-31	0.786113701	0.938	0.32	1.32E-26	1
ddx10	6.17E-31	0.380432834	0.65	0.103	1.47E-26	1
cyp2p6	1.14E-30	0.28088406	0.475	0.047	2.72E-26	1
cyp2p9	1.19E-30	0.482622769	0.538	0.069	2.85E-26	1
mrps27	1.22E-30	0.387854131	0.7	0.125	2.91E-26	1
hsd11b1la1	1.35E-30	0.876511995	0.85	0.225	3.22E-26	1
dpp4	1.61E-30	0.340453779	0.7	0.122	3.83E-26	1
mrpl44	2.11E-30	0.535510558	0.85	0.228	5.02E-26	1
baiap2l2a	2.40E-30	0.282470971	0.475	0.047	5.72E-26	1
hsd3b7	2.61E-30	0.401942907	0.625	0.099	6.22E-26	1
pdss1	2.62E-30	0.437102908	0.875	0.223	6.24E-26	1
si:ch211-201h21.5	3.31E-30	0.559358905	0.975	0.268	7.90E-26	1
tecrb	3.52E-30	0.655940382	0.912	0.288	8.40E-26	1
IZUMO1R	3.64E-30	0.469301884	0.775	0.159	8.67E-26	1
dlat	4.72E-30	0.498839201	0.9	0.253	1.13E-25	1
mrps2	4.88E-30	0.481625954	0.825	0.197	1.16E-25	1
rgn1	4.99E-30	0.323799856	0.8	0.169	1.19E-25	1
mrps22	6.05E-30	0.358575832	0.725	0.14	1.44E-25	1

tsr1	6.32E-30	0.550602597	0.775	0.176	1.51E-25	1
si:ch211-221j21.3	6.41E-30	0.471269021	0.738	0.148	1.53E-25	1
si:dkey-69o16.51	7.72E-30	0.441997428	0.875	0.202	1.84E-25	1
atp5fa1	8.95E-30	1.164258811	1	0.745	2.13E-25	1
papss2a	8.95E-30	0.798233413	0.912	0.29	2.13E-25	1
pla2g15	9.27E-30	0.257426374	0.575	0.082	2.21E-25	1
zgc:56235	9.27E-30	0.270705709	0.512	0.062	2.21E-25	1
cryz	9.29E-30	0.287574275	0.712	0.133	2.22E-25	1
pwp2h1	9.83E-30	0.393392544	0.838	0.2	2.35E-25	1
slc27a4	1.02E-29	0.557286084	0.8	0.191	2.42E-25	1
acaa2	1.03E-29	0.729355955	0.912	0.3	2.47E-25	1
uqcrc1	1.12E-29	0.949700616	1	0.468	2.68E-25	1
tktb	1.18E-29	0.77489017	0.988	0.384	2.82E-25	1
hadhab	1.26E-29	0.750691902	0.95	0.352	3.00E-25	1
aldh1l1	1.28E-29	0.627280662	0.925	0.272	3.04E-25	1
immt	1.38E-29	0.55273726	0.85	0.223	3.30E-25	1
hmgcra	1.47E-29	0.972834556	0.838	0.238	3.50E-25	1
aldh7a1	1.58E-29	0.898449807	0.962	0.343	3.77E-25	1
ociad1	1.71E-29	0.435650426	0.838	0.215	4.08E-25	1
them4	1.83E-29	0.268530089	0.538	0.071	4.37E-25	1
atp5f1c	1.88E-29	1.207639235	0.988	0.56	4.49E-25	1
noc4l	1.93E-29	0.3167841	0.7	0.127	4.61E-25	1
cyp8b1	1.97E-29	0.733472649	0.775	0.191	4.69E-25	1
aldh9a1b	2.02E-29	0.28009499	0.675	0.114	4.82E-25	1
crls1	2.13E-29	0.264294531	0.65	0.107	5.08E-25	1
acss2	2.40E-29	0.291765698	0.738	0.142	5.71E-25	1
ace2	2.65E-29	0.771794553	0.812	0.215	6.31E-25	1
zgc:113054	2.85E-29	0.283527458	0.55	0.075	6.80E-25	1
hibadhb	3.05E-29	0.464164066	0.825	0.187	7.28E-25	1
sh3gl3b	3.15E-29	0.41417221	0.812	0.184	7.52E-25	1
dap3	3.21E-29	0.492698168	0.888	0.266	7.66E-25	1
si:ch73-269m23.5	3.24E-29	0.319394981	0.688	0.124	7.73E-25	1
slc1a2	3.84E-29	0.394370819	0.8	0.178	9.15E-25	1
cmc2	4.60E-29	0.373493474	0.8	0.169	1.10E-24	1
ecsit	4.73E-29	0.365315577	0.688	0.135	1.13E-24	1
rpia	5.17E-29	0.325069806	0.688	0.127	1.23E-24	1
decr1	5.28E-29	0.293985985	0.75	0.155	1.26E-24	1
oplah	6.01E-29	0.266610384	0.662	0.112	1.43E-24	1
zgc:136858	6.25E-29	0.28747103	0.55	0.073	1.49E-24	1
clpp	6.84E-29	0.531730362	0.925	0.266	1.63E-24	1
asrgl1	6.95E-29	0.251001157	0.525	0.064	1.66E-24	1
sc5d	7.35E-29	0.486627547	0.662	0.124	1.75E-24	1
chs1	9.06E-29	0.481170921	0.638	0.107	2.16E-24	1
atp5f1b	9.40E-29	1.138436855	1	0.807	2.24E-24	1
cox10	1.00E-28	0.311778466	0.712	0.142	2.39E-24	1
ech1	1.09E-28	0.532720704	0.862	0.219	2.61E-24	1
mmaa	1.15E-28	0.2665586	0.625	0.105	2.74E-24	1
lclat1	1.27E-28	0.298255384	0.712	0.146	3.03E-24	1
lipf	1.46E-28	0.539640996	0.838	0.217	3.48E-24	1
mgst1.2l	1.64E-28	0.597593383	0.838	0.208	3.91E-24	1
hspd1	1.75E-28	1.262931471	1	0.625	4.18E-24	1
hadhaa1	1.79E-28	0.283257075	0.775	0.167	4.28E-24	1
agpat3	1.80E-28	0.550946302	0.838	0.221	4.28E-24	1
slc25a11	1.95E-28	0.379152085	0.8	0.197	4.64E-24	1

sec14l8	2.02E-28	0.312720447	0.8	0.172	4.81E-24	1
ebp	2.14E-28	0.633282959	0.85	0.217	5.11E-24	1
prpf19	2.20E-28	0.584605578	0.838	0.227	5.25E-24	1
tomm40l	2.31E-28	0.74728949	0.95	0.331	5.50E-24	1
sucla2	2.42E-28	0.279681696	0.712	0.14	5.78E-24	1
faah2b	2.66E-28	0.531812371	0.8	0.187	6.34E-24	1
phb	2.76E-28	0.968563275	1	0.485	6.57E-24	1
dachc	2.80E-28	0.313941914	0.6	0.097	6.67E-24	1
polr1a	3.64E-28	0.461843795	0.788	0.189	8.67E-24	1
bcl2l13	3.79E-28	0.256411969	0.612	0.099	9.04E-24	1
cers1	4.04E-28	0.357013774	0.362	0.026	9.64E-24	1
rhoub	4.06E-28	0.397800228	0.725	0.152	9.68E-24	1
samm50	4.11E-28	0.348770177	0.875	0.223	9.80E-24	1
mccc2	4.21E-28	0.45021818	0.825	0.221	1.00E-23	1
aldh2.2	4.31E-28	0.636488223	0.912	0.296	1.03E-23	1
porb	5.04E-28	0.625393061	0.9	0.279	1.20E-23	1
cyb5r2	5.13E-28	0.651197106	0.925	0.272	1.22E-23	1
slc43a2b	5.14E-28	0.350172577	0.662	0.124	1.22E-23	1
si:ch211-235e9.8	5.37E-28	0.866597591	0.988	0.395	1.28E-23	1
mrpl39	5.49E-28	0.60127634	0.912	0.294	1.31E-23	1
aldh9a1a.1l	6.71E-28	1.184611984	0.988	0.519	1.60E-23	1
HADHB	7.12E-28	0.799375542	0.975	0.403	1.70E-23	1
slc6a19a.1	7.16E-28	0.432558576	0.562	0.084	1.71E-23	1
slc3a2a	9.59E-28	0.761886654	0.988	0.388	2.29E-23	1
mybbp1a	9.88E-28	0.530802692	0.888	0.242	2.35E-23	1
ndufa9a1	1.02E-27	0.753043464	0.95	0.395	2.43E-23	1
adka	1.09E-27	0.477692641	0.875	0.255	2.60E-23	1
ppp5c	1.10E-27	0.297228581	0.788	0.184	2.61E-23	1
mrps15	1.11E-27	0.445975477	0.888	0.249	2.64E-23	1
rbb4l1	1.12E-27	0.370812666	0.825	0.21	2.67E-23	1
gmps	1.14E-27	0.437254719	0.762	0.17	2.73E-23	1
gstk1	1.25E-27	0.588737322	0.9	0.262	2.99E-23	1
pgam1a	1.28E-27	0.827069165	0.95	0.401	3.05E-23	1
eno3	1.31E-27	1.149378156	1	0.721	3.11E-23	1
acadl1	1.48E-27	0.442834844	0.862	0.236	3.53E-23	1
dnajc11a	1.53E-27	0.428648765	0.8	0.202	3.64E-23	1
zgc:1365641	1.63E-27	0.481253162	0.938	0.272	3.90E-23	1
mdh1aa	1.84E-27	1.332611843	0.988	0.592	4.39E-23	1
si:ch211-117n7.7	1.84E-27	0.538695623	0.85	0.223	4.39E-23	1
pdhx	1.95E-27	0.426514385	0.812	0.206	4.65E-23	1
abcb7	2.08E-27	0.250856753	0.75	0.161	4.95E-23	1
ncln	2.17E-27	0.370473715	0.838	0.228	5.18E-23	1
pex5	2.45E-27	0.308145736	0.55	0.082	5.84E-23	1
cluha	3.04E-27	0.666603534	0.85	0.257	7.25E-23	1
zgc:77748	3.29E-27	0.967584878	1	0.453	7.83E-23	1
aamp	3.41E-27	0.502003824	0.875	0.257	8.13E-23	1
ccl25a	3.64E-27	0.359412141	0.8	0.185	8.68E-23	1
adh8b	3.78E-27	1.335692387	0.975	0.476	9.01E-23	1
slc25a26	3.86E-27	0.308069408	0.612	0.105	9.20E-23	1
ndufa10	3.89E-27	0.753947033	0.962	0.397	9.28E-23	1
h6pd	3.91E-27	0.260064079	0.55	0.084	9.33E-23	1
itpkca	3.95E-27	0.369930974	0.738	0.157	9.41E-23	1
tbce1a	4.17E-27	0.253048298	0.788	0.18	9.94E-23	1
uqcrc2b	6.08E-27	0.887406535	0.95	0.391	1.45E-22	1

acsl1b	6.53E-27	0.924738661	0.95	0.397	1.56E-22	1
ca4b	6.74E-27	0.857501589	0.662	0.139	1.61E-22	1
zbtb16a	6.91E-27	0.758749626	0.925	0.27	1.65E-22	1
pdzk1	6.95E-27	0.524406529	0.912	0.262	1.66E-22	1
ACSF3	7.35E-27	0.252358484	0.55	0.081	1.75E-22	1
naprt	8.45E-27	0.506656244	0.9	0.273	2.01E-22	1
mrps30	9.03E-27	0.472515589	0.85	0.234	2.15E-22	1
ugt1ab	9.75E-27	1.07947381	1	0.414	2.32E-22	1
si:ch211-248a14.81	9.92E-27	0.519042196	0.838	0.23	2.37E-22	1
hao2	1.10E-26	0.269150226	0.575	0.088	2.63E-22	1
cs	1.16E-26	0.598616418	0.95	0.324	2.77E-22	1
aldh8a1	1.22E-26	0.564692955	0.838	0.225	2.91E-22	1
khk	1.31E-26	0.438409694	0.762	0.182	3.13E-22	1
rpe	1.38E-26	0.383243033	0.825	0.208	3.28E-22	1
ppp2r1ba	1.39E-26	0.481210052	0.9	0.272	3.32E-22	1
eci1	1.40E-26	0.456963183	0.95	0.294	3.35E-22	1
kyat1	1.49E-26	0.303455737	0.588	0.094	3.56E-22	1
eaf2	1.60E-26	0.372518426	0.775	0.18	3.80E-22	1
igf2bp3	1.88E-26	0.879604765	0.988	0.451	4.48E-22	1
dlst	1.88E-26	0.826787773	0.975	0.453	4.48E-22	1
cyc1	1.93E-26	0.98271691	1	0.536	4.61E-22	1
hnf4a1	2.00E-26	0.327929925	0.738	0.165	4.77E-22	1
nedd4a	2.03E-26	0.841409962	0.962	0.369	4.85E-22	1
dhrs9	2.14E-26	0.454448121	0.8	0.195	5.09E-22	1
slc35d1b	2.27E-26	0.546695465	0.762	0.184	5.42E-22	1
nomo	2.32E-26	0.375776008	0.838	0.21	5.53E-22	1
si:ch73-21k16.5	2.38E-26	0.29346918	0.538	0.079	5.67E-22	1
acot18	2.54E-26	0.321494844	0.65	0.129	6.05E-22	1
acadm1	2.54E-26	0.599779705	0.962	0.358	6.06E-22	1
mdh2	2.70E-26	1.031819125	0.975	0.566	6.43E-22	1
anpepa	2.79E-26	0.30049532	0.712	0.146	6.64E-22	1
slc25a3b	2.95E-26	1.014409674	1	0.757	7.04E-22	1
polr2b	3.21E-26	0.405485656	0.738	0.167	7.66E-22	1
si:ch211-121j5.4	3.24E-26	0.327342568	0.575	0.092	7.72E-22	1
lrrc20	3.33E-26	0.312869395	0.75	0.17	7.94E-22	1
si:ch73-308m11.1	3.40E-26	0.371809898	0.7	0.15	8.11E-22	1
parm1	3.61E-26	0.275049323	0.612	0.109	8.62E-22	1
cpt1ab	3.73E-26	0.697577387	0.925	0.341	8.88E-22	1
ppip5k1b	4.04E-26	0.440327887	0.6	0.114	9.64E-22	1
myo15b1	4.49E-26	0.346264496	0.725	0.157	1.07E-21	1
slc25a1b	4.59E-26	0.302297777	0.638	0.118	1.09E-21	1
igfbp2a	5.35E-26	0.251444478	0.488	0.064	1.28E-21	1
hmgcs1	5.81E-26	1.076734366	0.975	0.386	1.38E-21	1
ptges1	5.84E-26	0.380753421	0.812	0.2	1.39E-21	1
mrpl19	6.17E-26	0.387333221	0.825	0.212	1.47E-21	1
mrpl51	6.22E-26	0.372681642	0.825	0.212	1.48E-21	1
gmpr2	8.02E-26	0.265987834	0.75	0.169	1.91E-21	1
map2k2a	9.35E-26	0.295726319	0.7	0.148	2.23E-21	1
zgc:136472	9.38E-26	0.320086318	0.812	0.204	2.24E-21	1
eif2a	9.41E-26	0.408410735	0.825	0.225	2.24E-21	1
nle1	9.62E-26	0.264578823	0.625	0.114	2.29E-21	1
hadh	1.03E-25	0.720044541	0.975	0.38	2.45E-21	1
coq9	1.12E-25	0.417488402	0.712	0.159	2.68E-21	1
mtch2	1.16E-25	0.586433057	0.95	0.335	2.77E-21	1

ugt2a4	1.32E-25	0.694387057	0.962	0.369	3.14E-21	1
sult3st1	1.35E-25	0.330811821	0.612	0.114	3.21E-21	1
ndrg2	1.39E-25	0.515207296	0.925	0.279	3.31E-21	1
abcc12	1.49E-25	0.442163404	0.8	0.202	3.55E-21	1
ivd	1.53E-25	0.266580062	0.725	0.159	3.64E-21	1
acox1	1.54E-25	0.325811612	0.775	0.185	3.68E-21	1
clic5b	1.79E-25	0.469526737	0.85	0.247	4.28E-21	1
slc25a20	2.06E-25	0.725129656	0.962	0.397	4.92E-21	1
fkbp8	2.32E-25	0.32437447	0.575	0.103	5.53E-21	1
cracr2b	2.48E-25	0.409937785	0.688	0.146	5.92E-21	1
got2b	2.55E-25	0.759831609	0.925	0.352	6.08E-21	1
ahcy	2.55E-25	1.114805449	1	0.597	6.09E-21	1
mrpl38	2.74E-25	0.536852607	0.888	0.29	6.52E-21	1
lonrf1	2.83E-25	0.276356904	0.638	0.122	6.75E-21	1
prmt1	2.91E-25	0.778721201	0.912	0.343	6.94E-21	1
mrpl36	2.95E-25	0.657772066	0.962	0.363	7.04E-21	1
osr2	3.25E-25	0.3171123	0.538	0.086	7.74E-21	1
atic	3.26E-25	0.3344086	0.8	0.195	7.78E-21	1
timm50	3.45E-25	0.266757259	0.662	0.139	8.22E-21	1
chchd4a1	4.74E-25	0.334564961	0.825	0.212	1.13E-20	1
dcaf13	4.84E-25	0.364253531	0.7	0.165	1.15E-20	1
cyp2aa1	5.32E-25	0.320123354	0.7	0.146	1.27E-20	1
akap1b	5.34E-25	0.350308295	0.7	0.163	1.27E-20	1
ethe1	5.39E-25	0.346749479	0.712	0.163	1.29E-20	1
fbxl8	5.57E-25	0.382081624	0.612	0.116	1.33E-20	1
dohh	5.62E-25	0.3552036	0.788	0.204	1.34E-20	1
gstr1	5.71E-25	0.982594487	1	0.541	1.36E-20	1
mvk	5.79E-25	0.349671388	0.488	0.071	1.38E-20	1
emc8	5.91E-25	0.353231162	0.738	0.169	1.41E-20	1
cdh17	6.09E-25	0.908792316	1	0.485	1.45E-20	1
eci2	6.74E-25	0.548117409	0.925	0.316	1.61E-20	1
atp5pb	7.07E-25	0.94317104	1	0.697	1.69E-20	1
fntb	7.56E-25	0.319303691	0.638	0.125	1.80E-20	1
mrps91	7.87E-25	0.346145533	0.85	0.249	1.88E-20	1
sdhaf3	8.05E-25	0.282832447	0.7	0.155	1.92E-20	1
sfxn4	8.15E-25	0.259590766	0.538	0.092	1.94E-20	1
rrp12	8.45E-25	0.604787994	0.812	0.251	2.01E-20	1
uqcrc2a	9.48E-25	0.623429686	0.938	0.404	2.26E-20	1
sco1	1.06E-24	0.3330125	0.7	0.165	2.53E-20	1
phyhd1	1.08E-24	0.411643525	0.838	0.232	2.57E-20	1
acox3	1.12E-24	0.333294201	0.6	0.112	2.66E-20	1
lrpprc	1.12E-24	0.506396074	0.825	0.242	2.67E-20	1
hccsb	1.14E-24	0.398796198	0.712	0.172	2.71E-20	1
sf3b3	1.22E-24	0.412177173	0.812	0.217	2.91E-20	1
PEPD	1.41E-24	0.437332944	0.788	0.212	3.35E-20	1
tfb2m	1.44E-24	0.553889744	0.85	0.272	3.43E-20	1
iars1	1.54E-24	0.323240387	0.875	0.249	3.67E-20	1
ndrg3b	1.60E-24	0.257161704	0.612	0.116	3.81E-20	1
tmem86b	1.61E-24	0.27487829	0.538	0.088	3.83E-20	1
ugt5b3.1	1.63E-24	0.312959532	0.638	0.131	3.89E-20	1
acaa11	1.82E-24	0.322490758	0.75	0.18	4.34E-20	1
apoa4b.1	1.83E-24	1.185490716	0.988	0.421	4.35E-20	1
ndufv1	1.83E-24	0.510397338	0.912	0.313	4.37E-20	1
shmt1	1.92E-24	0.328425052	0.75	0.189	4.58E-20	1

pgm1	1.94E-24	0.276083555	0.712	0.163	4.64E-20	1
ppargc1b	2.32E-24	0.486713628	0.875	0.255	5.54E-20	1
rtn4b	2.35E-24	0.676599507	0.938	0.36	5.61E-20	1
rpf2	2.36E-24	0.520860158	0.862	0.273	5.64E-20	1
upb1	2.50E-24	0.403415482	0.638	0.135	5.97E-20	1
myo1b	2.64E-24	0.604004018	0.825	0.247	6.29E-20	1
bin2a1	2.75E-24	0.789704485	1	0.431	6.55E-20	1
smfn	2.79E-24	0.341691817	0.65	0.139	6.64E-20	1
tmem147	3.42E-24	0.390679927	0.912	0.298	8.15E-20	1
ap1s3a	3.60E-24	0.385334529	0.675	0.15	8.59E-20	1
fh	3.71E-24	0.525466796	0.9	0.322	8.85E-20	1
scp2a1	3.86E-24	0.940622579	1	0.412	9.22E-20	1
uqcc1	3.98E-24	0.294132576	0.712	0.172	9.48E-20	1
kmo	3.99E-24	0.254589819	0.638	0.127	9.51E-20	1
idh1	3.99E-24	0.782835597	0.95	0.404	9.51E-20	1
fdps	4.25E-24	0.694515227	0.925	0.348	1.01E-19	1
pus7	4.43E-24	0.317096898	0.75	0.18	1.06E-19	1
dldh	4.56E-24	0.725491715	0.912	0.399	1.09E-19	1
gamt	4.70E-24	1.068160367	1	0.476	1.12E-19	1
atp23	4.94E-24	0.289941251	0.562	0.101	1.18E-19	1
wdr43	5.10E-24	0.46489339	0.925	0.303	1.22E-19	1
slc13a2	5.22E-24	0.393762888	0.675	0.152	1.24E-19	1
pdcd11	5.53E-24	0.523814368	0.9	0.303	1.32E-19	1
idh3g	6.48E-24	0.297030984	0.738	0.172	1.54E-19	1
mrpl23	6.79E-24	0.490038883	0.775	0.204	1.62E-19	1
dhdh1	7.65E-24	0.731505984	0.988	0.434	1.82E-19	1
cyp46a1.1	8.06E-24	0.439838927	0.838	0.243	1.92E-19	1
pdhb1	8.28E-24	0.362176641	0.888	0.288	1.97E-19	1
etfa	9.00E-24	0.573701544	0.962	0.375	2.15E-19	1
prpf8	9.38E-24	0.473427374	0.875	0.279	2.24E-19	1
mat2al	1.04E-23	0.608419796	0.725	0.189	2.47E-19	1
sult3st4	1.09E-23	0.458400882	0.65	0.14	2.59E-19	1
si:zfos-943e10.1	1.16E-23	0.502885918	0.875	0.275	2.77E-19	1
eftud2	1.18E-23	0.309917416	0.638	0.135	2.81E-19	1
mrpl4	1.34E-23	0.417506379	0.888	0.294	3.19E-19	1
mrpl12	1.37E-23	0.739069831	0.988	0.463	3.27E-19	1
tufm1	1.40E-23	0.586000416	0.938	0.382	3.33E-19	1
aldh6a1	1.60E-23	0.615383419	0.938	0.375	3.82E-19	1
zgc:56095	1.61E-23	0.376650163	0.875	0.266	3.85E-19	1
ptk6b	1.63E-23	0.272651747	0.612	0.122	3.88E-19	1
stt3b	1.92E-23	0.341915059	0.888	0.272	4.57E-19	1
idh3b	1.93E-23	0.48788853	0.888	0.3	4.59E-19	1
ndufs2	2.12E-23	0.695069904	0.962	0.427	5.05E-19	1
ldha	2.14E-23	0.970146426	0.95	0.442	5.11E-19	1
pter	2.30E-23	0.276439903	0.675	0.142	5.49E-19	1
pdha1a	2.33E-23	0.559387211	0.862	0.292	5.56E-19	1
idi1	2.34E-23	0.541468108	0.812	0.232	5.57E-19	1
bms1	2.53E-23	0.405382166	0.9	0.273	6.03E-19	1
mrpl2	2.77E-23	0.464922664	0.912	0.318	6.60E-19	1
suclg1	3.05E-23	1.015739397	1	0.601	7.28E-19	1
pgm3	3.71E-23	0.255326339	0.575	0.109	8.85E-19	1
vmp1	3.73E-23	0.31274474	0.812	0.221	8.90E-19	1
etfdh	3.90E-23	0.348431937	0.788	0.221	9.29E-19	1
cyp2k16	4.13E-23	0.754676499	0.975	0.38	9.85E-19	1

rrp9	4.14E-23	0.253280817	0.7	0.163	9.88E-19	1
stl31	4.20E-23	0.368560153	0.9	0.3	1.00E-18	1
atp5mc3b	4.20E-23	0.950372789	1	0.837	1.00E-18	1
decr2	4.24E-23	0.276363179	0.75	0.182	1.01E-18	1
taz	5.29E-23	0.255512469	0.538	0.097	1.26E-18	1
larp1b	5.83E-23	0.29766024	0.875	0.255	1.39E-18	1
gfm1	6.22E-23	0.364625466	0.638	0.15	1.48E-18	1
cad	7.34E-23	0.393945442	0.775	0.21	1.75E-18	1
sf3b4	7.42E-23	0.390862967	0.775	0.206	1.77E-18	1
sdhb	7.94E-23	0.800384197	0.962	0.509	1.89E-18	1
wu:fb72h05	8.19E-23	0.398215033	0.762	0.197	1.95E-18	1
mrps11	8.48E-23	0.469881699	0.912	0.33	2.02E-18	1
stard7	8.75E-23	0.290452029	0.562	0.105	2.09E-18	1
drg1	8.98E-23	0.386190627	0.788	0.217	2.14E-18	1
cpsf3	9.31E-23	0.298050961	0.65	0.148	2.22E-18	1
strap	9.91E-23	0.372685106	0.738	0.189	2.36E-18	1
si:ch211-225b11.1	1.00E-22	0.42443931	0.775	0.204	2.38E-18	1
heatr1	1.01E-22	0.255858037	0.575	0.116	2.42E-18	1
ddx19	1.16E-22	0.264704677	0.612	0.124	2.76E-18	1
nr2f6b	1.16E-22	0.466782333	0.862	0.27	2.77E-18	1
ckmt1	1.17E-22	0.761922587	0.988	0.444	2.79E-18	1
cndp2	1.17E-22	0.269387433	0.762	0.2	2.80E-18	1
khsrp	1.26E-22	0.432691824	0.875	0.273	3.02E-18	1
fth1a	1.29E-22	0.946704846	1	0.781	3.07E-18	1
usp5	1.31E-22	0.422243756	0.8	0.238	3.12E-18	1
cers3a	1.31E-22	0.399702272	0.8	0.232	3.12E-18	1
gstt1a	1.35E-22	1.063281149	1	0.551	3.21E-18	1
btr05	1.39E-22	0.251012325	0.45	0.066	3.31E-18	1
agk	1.39E-22	0.254190387	0.7	0.167	3.32E-18	1
atp5po	1.43E-22	0.923256378	0.988	0.687	3.40E-18	1
pecr	1.46E-22	0.25749868	0.65	0.146	3.49E-18	1
npepl1l	1.52E-22	0.378202635	0.95	0.354	3.62E-18	1
ube2nb1	1.56E-22	0.280036748	0.788	0.208	3.72E-18	1
dnaja3a	1.63E-22	0.328658828	0.662	0.155	3.90E-18	1
sod2	1.64E-22	0.625742436	1	0.39	3.91E-18	1
hnf1ba	1.76E-22	0.298993204	0.688	0.161	4.19E-18	1
acmsd	1.78E-22	0.361578624	0.738	0.189	4.25E-18	1
grhprb	1.83E-22	0.354501934	0.662	0.15	4.36E-18	1
utp6	1.93E-22	0.290707973	0.65	0.148	4.59E-18	1
cct4	2.03E-22	0.89466295	0.975	0.532	4.83E-18	1
zgc:77739	2.04E-22	0.397105906	0.725	0.184	4.86E-18	1
tmx2b	2.15E-22	0.328323942	0.712	0.18	5.14E-18	1
si:dkey-28n18.9	2.19E-22	0.305802904	0.575	0.109	5.23E-18	1
acadvl	2.22E-22	0.63764887	0.962	0.367	5.30E-18	1
cse1l	2.37E-22	0.282580687	0.688	0.165	5.66E-18	1
pdia5	2.55E-22	0.353619605	0.825	0.247	6.08E-18	1
ppifb1	2.58E-22	0.505118506	0.888	0.313	6.15E-18	1
ppp4r3b	2.60E-22	0.252915505	0.588	0.12	6.19E-18	1
slc9a3r1a	2.93E-22	0.507202334	0.95	0.358	6.98E-18	1
txn2	3.00E-22	0.326046354	0.862	0.264	7.16E-18	1
hmbsa	3.24E-22	0.331615627	0.612	0.133	7.73E-18	1
ftcd	3.68E-22	0.558121	0.562	0.118	8.77E-18	1
mrpl48	3.90E-22	0.51971127	0.962	0.333	9.31E-18	1
mtx2	4.22E-22	0.435332394	0.862	0.281	1.01E-17	1

pa2g4b	4.31E-22	0.759378312	0.962	0.457	1.03E-17	1
nid2a	4.48E-22	0.26415411	0.562	0.11	1.07E-17	1
gar1	4.49E-22	0.567182911	0.938	0.352	1.07E-17	1
eef2b	4.51E-22	0.812236468	1	0.897	1.08E-17	1
sgta	4.54E-22	0.387202573	0.962	0.339	1.08E-17	1
nipsnap1	4.78E-22	0.260618183	0.562	0.109	1.14E-17	1
mrps18b	4.95E-22	0.426772705	0.85	0.266	1.18E-17	1
vars	4.96E-22	0.360845296	0.875	0.283	1.18E-17	1
azin1b	5.00E-22	0.538316817	0.962	0.371	1.19E-17	1
c3a.1	5.26E-22	0.308591827	0.675	0.152	1.25E-17	1
mrpl431	5.66E-22	0.264003601	0.788	0.212	1.35E-17	1
bud23	5.83E-22	0.33394223	0.688	0.17	1.39E-17	1
aqp8a.2	6.49E-22	0.806440236	0.9	0.3	1.55E-17	1
eif3s6ip	6.59E-22	0.623985804	0.938	0.386	1.57E-17	1
gpd1b	6.65E-22	0.342810838	0.612	0.137	1.59E-17	1
rangap1a	6.65E-22	0.291057093	0.712	0.178	1.59E-17	1
zgc:112175	7.08E-22	0.323481248	0.8	0.242	1.69E-17	1
fip1l1b	7.82E-22	0.338517959	0.762	0.2	1.86E-17	1
sec13	7.85E-22	0.37777009	0.9	0.294	1.87E-17	1
bpnt1	8.26E-22	0.376671333	0.8	0.227	1.97E-17	1
rpn11	9.09E-22	0.437430434	0.962	0.39	2.17E-17	1
eif3i	9.80E-22	0.646730715	0.988	0.425	2.34E-17	1
grsf1l	9.93E-22	0.285133273	0.75	0.204	2.37E-17	1
ssfa2	1.01E-21	0.259404878	0.612	0.135	2.41E-17	1
fam210b1	1.06E-21	0.356982947	0.788	0.24	2.52E-17	1
actr2a	1.09E-21	0.494403089	0.9	0.33	2.59E-17	1
rtn4a	1.17E-21	0.61391084	0.962	0.453	2.78E-17	1
nit2	1.36E-21	0.340697326	0.65	0.15	3.25E-17	1
ttll4	1.47E-21	0.268019497	0.538	0.101	3.50E-17	1
myo7bb	1.57E-21	0.337832735	0.638	0.146	3.73E-17	1
mrps31	1.67E-21	0.284778446	0.95	0.324	3.99E-17	1
sgk2a	1.98E-21	0.290002609	0.675	0.157	4.71E-17	1
ndrg1a	2.02E-21	0.827200719	1	0.537	4.81E-17	1
gnpda11	2.06E-21	0.617888393	0.988	0.382	4.91E-17	1
alad	2.08E-21	0.304831265	0.775	0.217	4.96E-17	1
mpv17l2	2.11E-21	0.377169717	0.775	0.219	5.03E-17	1
mmadhc	2.15E-21	0.361962629	0.712	0.191	5.12E-17	1
slc22a18	2.15E-21	0.287686854	0.638	0.15	5.13E-17	1
ptgr1	2.19E-21	0.574929754	0.925	0.373	5.22E-17	1
zgc:77938	2.21E-21	0.459362634	0.85	0.26	5.26E-17	1
nup50	2.31E-21	0.433149724	0.825	0.255	5.51E-17	1
rtraf	2.36E-21	0.280073753	0.7	0.17	5.64E-17	1
metap11	2.43E-21	0.28155035	0.85	0.275	5.79E-17	1
thop1	2.51E-21	0.34812719	0.85	0.268	5.98E-17	1
slc25a5	2.57E-21	0.783776616	1	0.966	6.12E-17	1
gapvd1	3.03E-21	0.252651728	0.488	0.084	7.23E-17	1
ppid	3.11E-21	0.46097154	0.938	0.33	7.41E-17	1
ddx56	3.11E-21	0.295758415	0.688	0.169	7.42E-17	1
abcd3a	3.22E-21	0.438542387	0.85	0.288	7.67E-17	1
selenop	3.28E-21	0.595204846	0.875	0.277	7.81E-17	1
clic4	3.43E-21	0.292127593	0.712	0.182	8.17E-17	1
si:ch211-107o10.31	3.51E-21	0.557170324	0.938	0.341	8.37E-17	1
agpat9l	3.72E-21	0.26938333	0.6	0.129	8.88E-17	1
timm44	4.19E-21	0.267140782	0.8	0.227	1.00E-16	1

ndufs8a	4.26E-21	0.568913212	0.912	0.376	1.02E-16	1
timm29	4.30E-21	0.397329194	0.688	0.176	1.03E-16	1
ppm1g	4.79E-21	0.378632197	0.8	0.23	1.14E-16	1
copg2	5.19E-21	0.310154951	0.8	0.223	1.24E-16	1
ldhba	5.55E-21	0.909370952	1	0.551	1.32E-16	1
stard4	5.75E-21	0.298930691	0.525	0.103	1.37E-16	1
chia.1	5.86E-21	0.493864334	0.425	0.066	1.40E-16	1
cyp51	5.87E-21	0.91634341	0.812	0.288	1.40E-16	1
slc4a4a	6.10E-21	0.325054705	0.475	0.081	1.45E-16	1
ccdc47l	6.27E-21	0.444504205	0.95	0.388	1.50E-16	1
tomm40l	6.86E-21	0.261130792	0.925	0.339	1.63E-16	1
hnrnpul1	6.90E-21	0.389293379	0.85	0.281	1.64E-16	1
pes	7.13E-21	0.471141421	0.95	0.348	1.70E-16	1
zgc:158846	7.17E-21	1.012122044	1	0.743	1.71E-16	1
wdr3	7.51E-21	0.305929917	0.662	0.172	1.79E-16	1
selenot1b	7.86E-21	0.325585878	0.838	0.262	1.87E-16	1
mrpl15	8.92E-21	0.326290284	0.838	0.27	2.13E-16	1
nup93	9.05E-21	0.327859023	0.688	0.18	2.16E-16	1
abce1	9.94E-21	0.729305602	0.912	0.386	2.37E-16	1
gpia	1.02E-20	0.463504037	0.938	0.365	2.43E-16	1
zgc:56304	1.04E-20	0.314408846	0.812	0.242	2.48E-16	1
cyp26a1	1.09E-20	0.403497931	0.288	0.024	2.61E-16	1
tmem97	1.20E-20	0.344319481	0.8	0.242	2.85E-16	1
twf1b	1.36E-20	0.271907693	0.712	0.193	3.25E-16	1
hsd17b7	1.41E-20	0.565677071	0.825	0.27	3.36E-16	1
AL831745.1	1.43E-20	0.348403	0.912	0.275	3.42E-16	1
adssl	1.45E-20	0.331708915	0.788	0.227	3.45E-16	1
esd	1.46E-20	0.474138144	0.838	0.296	3.49E-16	1
acsbg2l	1.48E-20	0.449632361	0.775	0.236	3.53E-16	1
chia.2	1.51E-20	0.830870339	0.6	0.148	3.59E-16	1
acy3.2	1.53E-20	0.298109284	0.7	0.185	3.66E-16	1
micu2	1.56E-20	0.408054263	0.95	0.35	3.71E-16	1
PXMP4	1.56E-20	0.260252676	0.525	0.103	3.71E-16	1
pgk1	1.57E-20	0.597740445	0.962	0.446	3.74E-16	1
rmdn1	1.66E-20	0.474053116	0.95	0.363	3.96E-16	1
krt92	1.75E-20	0.980256019	1	0.792	4.18E-16	1
cct7	1.89E-20	0.674876675	0.988	0.461	4.50E-16	1
echs1	1.91E-20	0.456233207	0.888	0.326	4.55E-16	1
usp14	1.93E-20	0.254954647	0.688	0.172	4.59E-16	1
si:dkey-283b1.6	2.00E-20	0.454951243	0.912	0.305	4.78E-16	1
tjap1	2.01E-20	0.28139474	0.688	0.167	4.79E-16	1
tmem126a	2.02E-20	0.343504961	0.725	0.21	4.81E-16	1
insig1	2.06E-20	0.933168342	0.925	0.448	4.90E-16	1
pak1ip1	2.59E-20	0.310261358	0.838	0.27	6.17E-16	1
pvalb9	2.61E-20	2.098443416	0.638	0.154	6.21E-16	1
dhrs11	2.63E-20	0.69353705	0.938	0.431	6.27E-16	1
zgc:153968	2.67E-20	0.684290537	0.675	0.182	6.37E-16	1
slc31a1	2.93E-20	0.513006998	0.912	0.354	7.00E-16	1
hnrnpl	3.07E-20	0.419389057	0.875	0.279	7.33E-16	1
coq5	3.17E-20	0.293586571	0.638	0.157	7.56E-16	1
wdr75	3.17E-20	0.306236513	0.675	0.174	7.56E-16	1
mrps35l	3.54E-20	0.336698156	0.912	0.333	8.45E-16	1
gpatch4	3.64E-20	0.345095945	0.838	0.247	8.68E-16	1
phb2b	3.68E-20	0.642817262	0.975	0.434	8.77E-16	1

agmat	3.81E-20	0.253599644	0.525	0.105	9.09E-16	1
ndrg3a	4.73E-20	0.320263092	0.875	0.301	1.13E-15	1
etfb1	4.74E-20	0.392764946	0.962	0.434	1.13E-15	1
erlin1	5.02E-20	0.355088602	0.838	0.285	1.20E-15	1
znf622	5.12E-20	0.283446085	0.8	0.228	1.22E-15	1
rhebl1	5.44E-20	0.252440738	0.675	0.17	1.30E-15	1
psmd1	5.47E-20	0.557936713	0.988	0.414	1.30E-15	1
selenot1a	5.48E-20	0.57229149	0.988	0.463	1.31E-15	1
kalrnb	5.54E-20	0.422886564	0.95	0.341	1.32E-15	1
mrpl45	6.26E-20	0.397006012	0.825	0.268	1.49E-15	1
pmpcb	6.99E-20	0.344246457	0.675	0.187	1.67E-15	1
pdzk1ip1	7.90E-20	0.500527012	0.6	0.137	1.88E-15	1
si:dkey-4e7.3	8.00E-20	0.29455414	0.5	0.097	1.91E-15	1
uqcrfs1	8.62E-20	0.829520909	0.962	0.556	2.05E-15	1
cebpz	9.75E-20	0.29076576	0.725	0.197	2.32E-15	1
si:ch211-212k18.71	1.05E-19	0.399996501	0.95	0.404	2.51E-15	1
rpl3	1.09E-19	0.712150737	1	0.931	2.59E-15	1
ndufs7	1.11E-19	0.597507758	0.975	0.506	2.64E-15	1
mvda	1.13E-19	0.431200103	0.588	0.137	2.69E-15	1
gcshb	1.17E-19	0.774245018	0.988	0.431	2.79E-15	1
elovl2	1.21E-19	0.674013696	0.55	0.125	2.87E-15	1
sehl1	1.21E-19	0.25616185	0.688	0.182	2.88E-15	1
fdx1	1.27E-19	0.738309362	1	0.453	3.03E-15	1
psma5	1.28E-19	0.496404491	0.938	0.395	3.06E-15	1
blmh	1.29E-19	0.406273633	0.875	0.313	3.07E-15	1
eed	1.32E-19	0.263212511	0.662	0.17	3.14E-15	1
lman2	1.42E-19	0.346046227	0.725	0.2	3.39E-15	1
rhbdl2	1.45E-19	0.273782247	0.4	0.06	3.45E-15	1
phb2a	1.46E-19	0.340120777	0.7	0.199	3.47E-15	1
etv5b	1.59E-19	0.576885206	0.812	0.285	3.78E-15	1
mpc2	1.59E-19	0.492915605	0.912	0.386	3.79E-15	1
atp5f1d	1.74E-19	0.786073485	1	0.708	4.14E-15	1
eef1a1l1	2.03E-19	0.696514279	1	0.983	4.83E-15	1
gnl2	2.03E-19	0.352861099	0.788	0.257	4.84E-15	1
pprc1	2.05E-19	0.287163096	0.85	0.272	4.88E-15	1
prdx2	2.09E-19	0.869890688	1	0.824	4.98E-15	1
sypl2a	2.30E-19	0.452951679	0.925	0.38	5.49E-15	1
tsfm	2.31E-19	0.258374907	0.5	0.101	5.50E-15	1
anks4b	2.55E-19	0.494217774	0.95	0.354	6.07E-15	1
gpr39	2.59E-19	0.280735173	0.662	0.172	6.16E-15	1
pfdn2	2.75E-19	0.340493879	0.762	0.225	6.57E-15	1
rnf121	2.82E-19	0.255062987	0.575	0.135	6.71E-15	1
fkbp4	2.93E-19	0.360018427	0.725	0.208	6.99E-15	1
qars	2.99E-19	0.28890681	0.775	0.223	7.14E-15	1
adh5l	3.16E-19	0.547760241	0.975	0.487	7.54E-15	1
pfdn5	3.19E-19	0.49487953	0.85	0.322	7.60E-15	1
fdx1l	3.31E-19	0.333771958	0.812	0.249	7.90E-15	1
cct8l	3.39E-19	0.548475615	0.938	0.406	8.08E-15	1
zgc:158258	3.51E-19	0.284154273	0.775	0.225	8.37E-15	1
prelid1a1	3.67E-19	0.516043783	0.975	0.489	8.76E-15	1
mrpl42	4.06E-19	0.27627501	0.862	0.273	9.68E-15	1
mpc1l	4.69E-19	0.597275346	1	0.521	1.12E-14	1
kpnb3	4.74E-19	0.43854766	0.825	0.277	1.13E-14	1
sdad1	4.92E-19	0.346794229	0.762	0.227	1.17E-14	1

cyb5a	5.00E-19	0.528280718	0.95	0.44	1.19E-14	1
mrpl34	5.26E-19	0.404042979	0.938	0.354	1.25E-14	1
pbld	5.59E-19	0.354022295	0.838	0.262	1.33E-14	1
krt97	5.64E-19	0.950748787	1	0.575	1.34E-14	1
nipsnap3a	5.84E-19	0.298418143	0.85	0.29	1.39E-14	1
prkar2aa	5.89E-19	0.450565418	0.912	0.361	1.41E-14	1
coro2a	6.17E-19	0.290471934	0.65	0.167	1.47E-14	1
ndufa8	6.32E-19	0.563383496	0.988	0.552	1.51E-14	1
eif4ebp2	6.40E-19	0.393096877	0.838	0.301	1.53E-14	1
mrpl16	6.44E-19	0.270690609	0.712	0.21	1.54E-14	1
mcm4	6.48E-19	0.282055387	0.6	0.146	1.54E-14	1
ndufs3	6.49E-19	0.560632578	0.95	0.414	1.55E-14	1
gcsha	6.89E-19	0.347962396	0.925	0.316	1.64E-14	1
nolc1l	7.02E-19	0.297162174	0.938	0.352	1.67E-14	1
srp72	7.36E-19	0.296709621	0.762	0.234	1.76E-14	1
g3bp1	7.50E-19	0.348616723	0.9	0.324	1.79E-14	1
dnajc3a	7.66E-19	0.463321112	0.862	0.294	1.83E-14	1
prdx3	7.96E-19	0.554828179	0.925	0.416	1.90E-14	1
hspa4b	8.71E-19	0.525036249	0.838	0.33	2.08E-14	1
rars	9.20E-19	0.428040139	0.825	0.275	2.19E-14	1
sdhc	9.23E-19	0.634065502	1	0.494	2.20E-14	1
hsdl2l	9.77E-19	0.420170014	0.95	0.397	2.33E-14	1
si:dkey-44g23.5	9.94E-19	0.451428536	0.862	0.298	2.37E-14	1
ddx54	1.04E-18	0.267311362	0.762	0.232	2.49E-14	1
vdac2	1.05E-18	0.84110055	1	0.646	2.51E-14	1
ptges3a	1.07E-18	0.345289493	0.838	0.298	2.55E-14	1
cldn15a	1.08E-18	0.799293753	0.975	0.438	2.58E-14	1
igf2bp1	1.14E-18	0.674882679	0.988	0.481	2.72E-14	1
afp4.1	1.29E-18	0.664376753	0.988	0.376	3.09E-14	1
pabpc1a	1.30E-18	0.706475855	1	0.831	3.09E-14	1
eif5a2	1.30E-18	0.770335391	1	0.59	3.10E-14	1
CR388364.1	1.35E-18	0.250843987	0.562	0.125	3.22E-14	1
timm13l	1.42E-18	0.350740795	0.912	0.343	3.38E-14	1
acadsb	1.48E-18	0.353315946	0.725	0.21	3.54E-14	1
pgd	1.51E-18	0.496818015	0.888	0.35	3.59E-14	1
gspt1l	1.57E-18	0.492420439	0.988	0.472	3.74E-14	1
mphosph10l	1.58E-18	0.283066449	0.875	0.296	3.76E-14	1
maip1	1.60E-18	0.286480389	0.588	0.152	3.83E-14	1
grpel1	1.77E-18	0.31649463	0.825	0.285	4.23E-14	1
tfam	2.11E-18	0.278386581	0.838	0.281	5.02E-14	1
aimp2	2.13E-18	0.338124745	0.85	0.296	5.07E-14	1
tomm22l	2.18E-18	0.441161092	0.912	0.397	5.19E-14	1
nop58	2.25E-18	0.649220043	1	0.56	5.36E-14	1
chchd4b	2.47E-18	0.261588537	0.45	0.084	5.90E-14	1
zan1l	2.48E-18	0.54471429	0.938	0.361	5.91E-14	1
ide	2.69E-18	0.283469586	0.7	0.2	6.41E-14	1
klf11b	2.79E-18	0.41424104	0.925	0.345	6.66E-14	1
ezh2	2.83E-18	0.314834432	0.7	0.212	6.75E-14	1
mrpl3	2.94E-18	0.323716695	0.725	0.219	7.00E-14	1
gata6	2.96E-18	0.266597878	0.838	0.275	7.06E-14	1
eif4ebp1	3.22E-18	0.46944419	0.975	0.419	7.68E-14	1
mrpl20	3.23E-18	0.458388941	0.95	0.371	7.70E-14	1
psmc1b	3.46E-18	0.346263077	0.8	0.275	8.24E-14	1
adrm1	3.72E-18	0.429954962	0.862	0.324	8.86E-14	1

nip71	3.79E-18	0.284820533	0.862	0.305	9.04E-14	1
abcc21	3.85E-18	0.352921205	0.738	0.215	9.18E-14	1
c23h20orf24	4.03E-18	0.598129544	0.962	0.448	9.62E-14	1
mrps18a1	4.31E-18	0.296680417	0.925	0.313	1.03E-13	1
hspa14	4.50E-18	0.271730188	0.662	0.187	1.07E-13	1
apoa1b	4.79E-18	0.966298935	0.962	0.532	1.14E-13	1
bckdhb	5.03E-18	0.310034636	0.75	0.24	1.20E-13	1
pllp	5.04E-18	0.256962109	0.812	0.247	1.20E-13	1
nansa	5.33E-18	0.335976771	0.862	0.301	1.27E-13	1
tspan13a	5.34E-18	0.430974284	0.95	0.346	1.27E-13	1
dnmt1	5.58E-18	0.33293625	0.538	0.122	1.33E-13	1
atpaf1	6.25E-18	0.289206438	0.738	0.23	1.49E-13	1
gstm.11	6.47E-18	0.343481237	0.862	0.326	1.54E-13	1
ndufa4	7.08E-18	0.685291691	0.988	0.459	1.69E-13	1
hacd2	7.21E-18	0.582174268	0.912	0.393	1.72E-13	1
bop1	9.06E-18	0.294883671	0.838	0.27	2.16E-13	1
fads2	9.17E-18	0.255876091	0.325	0.043	2.19E-13	1
atp2a2a.1	9.58E-18	0.483608663	0.95	0.419	2.28E-13	1
cox16	1.02E-17	0.256556815	0.688	0.195	2.43E-13	1
si:dkey-183n20.15	1.03E-17	0.363760257	0.688	0.195	2.45E-13	1
mrps24	1.03E-17	0.444330303	0.95	0.397	2.45E-13	1
bxdc2	1.04E-17	0.508616148	0.975	0.41	2.47E-13	1
dhrs4	1.04E-17	0.329162335	0.862	0.298	2.49E-13	1
tegt	1.08E-17	0.297224848	0.65	0.178	2.57E-13	1
srsf1a	1.18E-17	0.545319666	0.962	0.474	2.81E-13	1
mrps5	1.22E-17	0.278691411	0.638	0.174	2.91E-13	1
eif4a1b	1.25E-17	0.462156078	0.975	0.472	2.97E-13	1
nmt1a	1.33E-17	0.335553495	0.825	0.288	3.17E-13	1
gtpbp4	1.34E-17	0.470492867	0.95	0.412	3.19E-13	1
apoa1a	1.49E-17	0.696650422	0.988	0.436	3.55E-13	1
gpx4a	1.58E-17	0.862332616	1	0.541	3.76E-13	1
vil1	1.70E-17	0.80040717	0.988	0.541	4.06E-13	1
zgc:101569	1.78E-17	0.271858046	0.725	0.213	4.24E-13	1
dhx15	1.78E-17	0.344575492	0.825	0.303	4.25E-13	1
slc25a36a	1.82E-17	0.264399753	0.8	0.26	4.33E-13	1
idh3a	1.84E-17	0.435302262	0.938	0.431	4.38E-13	1
acat1	1.98E-17	0.262143441	0.562	0.14	4.73E-13	1
pklr	2.06E-17	0.552114306	0.962	0.433	4.90E-13	1
tmem11	2.06E-17	0.312717531	0.838	0.328	4.92E-13	1
ddx39aa	2.17E-17	0.342172437	0.925	0.369	5.17E-13	1
lonrf1l1	2.17E-17	0.46687708	0.85	0.345	5.17E-13	1
u2af1	2.28E-17	0.383102629	0.988	0.404	5.44E-13	1
si:ch211-250c4.4	2.43E-17	0.328572834	0.762	0.227	5.79E-13	1
nop2	2.63E-17	0.41893697	0.938	0.39	6.28E-13	1
larp4ab	2.70E-17	0.261918904	0.762	0.257	6.45E-13	1
ndufv21	2.76E-17	0.35249045	0.938	0.406	6.58E-13	1
tnpo2	3.02E-17	0.309865018	0.788	0.251	7.21E-13	1
hspa9	3.22E-17	0.631308519	1	0.575	7.67E-13	1
si:ch73-194h10.2	3.36E-17	0.301265895	0.925	0.341	8.01E-13	1
hspa8	3.94E-17	0.713924238	1	0.929	9.39E-13	1
itpk1a	4.20E-17	0.439929987	0.95	0.39	1.00E-12	1
cd3eap	4.72E-17	0.276338495	0.875	0.311	1.13E-12	1
ndufb5	4.96E-17	0.592009068	0.975	0.496	1.18E-12	1
sdr16c5b	4.99E-17	0.399231039	0.912	0.363	1.19E-12	1

tspo	5.02E-17	0.346949365	0.662	0.206	1.20E-12	1
mcee	5.27E-17	0.279078079	0.762	0.264	1.26E-12	1
ckba1	5.39E-17	0.2696824	0.7	0.212	1.29E-12	1
rnaseh2a	5.68E-17	0.253292914	0.588	0.154	1.35E-12	1
ndufab1a	6.40E-17	0.557365192	0.975	0.483	1.53E-12	1
uchl3	6.67E-17	0.282908554	0.788	0.264	1.59E-12	1
usp10	6.85E-17	0.479413803	0.938	0.395	1.63E-12	1
pgrmc1	6.91E-17	0.574685718	0.988	0.522	1.65E-12	1
mrps101	7.57E-17	0.376540922	0.925	0.412	1.81E-12	1
cryl1	8.28E-17	0.341990091	0.762	0.249	1.97E-12	1
dnttip2	8.40E-17	0.258334206	0.65	0.189	2.00E-12	1
emc6	8.42E-17	0.335076141	0.938	0.365	2.01E-12	1
esf1	8.47E-17	0.447513843	0.8	0.29	2.02E-12	1
chchd1	9.79E-17	0.267050952	0.8	0.279	2.33E-12	1
cdx1b	1.05E-16	0.525929423	0.988	0.466	2.50E-12	1
cct3	1.05E-16	0.616843619	0.975	0.511	2.50E-12	1
naa15a	1.11E-16	0.273769972	0.762	0.255	2.66E-12	1
fam207a	1.16E-16	0.293022729	0.8	0.268	2.76E-12	1
txnrd3	1.25E-16	0.317521941	0.812	0.29	2.98E-12	1
cstf3	1.28E-16	0.254467353	0.638	0.18	3.05E-12	1
gclc	1.32E-16	0.336757214	0.838	0.287	3.14E-12	1
ddx39ab1	1.33E-16	0.546429092	0.988	0.536	3.16E-12	1
sdhdb1	1.36E-16	0.49433019	0.938	0.472	3.24E-12	1
jpt2	1.39E-16	0.272401546	0.888	0.322	3.31E-12	1
tuba8l21	1.45E-16	0.648641949	0.975	0.539	3.45E-12	1
CU462878.1	1.59E-16	0.374644718	0.675	0.21	3.80E-12	1
myg1	1.72E-16	0.333945577	0.875	0.346	4.11E-12	1
acp1	1.79E-16	0.283960831	0.875	0.322	4.27E-12	1
ngdn	1.81E-16	0.260450023	0.825	0.283	4.31E-12	1
eif4h	1.96E-16	0.453966787	0.95	0.442	4.67E-12	1
kars	2.02E-16	0.583765502	0.938	0.479	4.81E-12	1
chchd6b	2.08E-16	0.28174287	0.8	0.279	4.96E-12	1
ivns1abpa	2.08E-16	0.371936386	0.9	0.326	4.97E-12	1
hnf4g	2.19E-16	0.328111797	0.862	0.326	5.23E-12	1
ppa1b	2.26E-16	0.354544504	0.95	0.401	5.39E-12	1
mrpl17	2.38E-16	0.253022058	0.775	0.251	5.68E-12	1
mcm21	2.80E-16	0.3806978	0.612	0.184	6.67E-12	1
cbr1l1	3.10E-16	0.349404143	0.888	0.346	7.38E-12	1
auh	3.14E-16	0.329783058	0.762	0.255	7.48E-12	1
prdx1l	3.57E-16	0.397673776	0.95	0.44	8.52E-12	1
nfe2l2a	3.57E-16	0.395911255	0.95	0.38	8.52E-12	1
prdx41	3.70E-16	0.347730475	0.912	0.393	8.83E-12	1
herpud1	3.73E-16	0.685646779	0.988	0.543	8.90E-12	1
stoml3b	4.09E-16	0.869843534	0.95	0.421	9.76E-12	1
gpx1a	4.12E-16	0.682001937	0.988	0.509	9.82E-12	1
rap1aa	4.48E-16	0.417928455	0.812	0.313	1.07E-11	1
cyp24a1	4.62E-16	0.661134486	0.562	0.154	1.10E-11	1
sf3a1	4.75E-16	0.267799368	0.738	0.249	1.13E-11	1
sephs2	4.89E-16	0.285814343	0.75	0.257	1.17E-11	1
timm9	5.14E-16	0.268542628	0.912	0.354	1.23E-11	1
tsr2	5.42E-16	0.313354753	0.788	0.277	1.29E-11	1
mrpl13	5.48E-16	0.365686036	0.938	0.361	1.31E-11	1
mrpl47	5.55E-16	0.253473151	0.862	0.318	1.32E-11	1
p4hb	6.08E-16	0.409446515	0.925	0.429	1.45E-11	1

farsb	6.13E-16	0.272937145	0.712	0.212	1.46E-11	1
mrpl27	6.44E-16	0.282542418	0.862	0.311	1.54E-11	1
tpi1b1	6.48E-16	0.542904855	0.975	0.577	1.54E-11	1
ldlra	6.89E-16	0.554923944	0.962	0.479	1.64E-11	1
eif2s1a	6.94E-16	0.386009297	0.888	0.354	1.65E-11	1
cant1b	6.98E-16	0.272321062	0.738	0.242	1.66E-11	1
esama	8.22E-16	0.25163016	0.45	0.096	1.96E-11	1
thrap3b	8.56E-16	0.280703519	0.888	0.343	2.04E-11	1
mrps7	8.58E-16	0.360519712	0.812	0.292	2.05E-11	1
bzw1a	9.34E-16	0.349538727	0.925	0.367	2.23E-11	1
dnajc15	1.00E-15	0.261402966	0.6	0.169	2.39E-11	1
gdi2	1.08E-15	0.519850357	0.962	0.509	2.58E-11	1
vdac3	1.14E-15	0.291063646	0.862	0.361	2.71E-11	1
eif4bb1	1.34E-15	0.469847955	0.975	0.551	3.21E-11	1
eif3c	1.37E-15	0.54863926	0.975	0.545	3.28E-11	1
col14a1a	1.47E-15	0.269410425	0.85	0.301	3.51E-11	1
sub1b	1.57E-15	0.306438177	0.838	0.298	3.74E-11	1
eva1a	1.61E-15	0.286553097	0.588	0.169	3.84E-11	1
sar1b	1.63E-15	0.299212398	0.938	0.395	3.89E-11	1
mrpl11	1.74E-15	0.497436344	0.988	0.476	4.15E-11	1
akirin2	1.81E-15	0.287488579	0.8	0.273	4.31E-11	1
eif5a	1.85E-15	0.702651779	0.988	0.607	4.41E-11	1
hnrnpl2	2.25E-15	0.349758637	0.862	0.322	5.37E-11	1
rpl4	2.40E-15	0.57889612	1	0.884	5.72E-11	1
serpinb1l3	2.40E-15	0.64659496	0.988	0.601	5.73E-11	1
apoc2	2.53E-15	0.559539904	1	0.455	6.04E-11	1
ciapin1	2.73E-15	0.302339164	0.775	0.287	6.51E-11	1
mrpl18	2.85E-15	0.3180944	0.975	0.408	6.79E-11	1
mrps36	2.90E-15	0.397514924	0.95	0.498	6.93E-11	1
cnbpa	3.15E-15	0.5553515	0.962	0.496	7.52E-11	1
glrx5l	3.20E-15	0.329111667	0.9	0.395	7.63E-11	1
tuft1a	3.24E-15	0.430371195	0.812	0.298	7.73E-11	1
sult3st3	3.37E-15	0.416933764	0.912	0.361	8.04E-11	1
tomm5	3.67E-15	0.468898319	1	0.494	8.74E-11	1
stip1	3.79E-15	0.252503603	0.838	0.324	9.04E-11	1
CU694197.1	4.22E-15	0.361913525	0.962	0.44	1.01E-10	1
atp1b1a	4.22E-15	0.582200465	1	0.816	1.01E-10	1
pitpnaa	4.32E-15	0.325291317	0.838	0.315	1.03E-10	1
atp5mc1	4.53E-15	0.660279163	1	0.779	1.08E-10	1
si:dkey-251i10.3	4.60E-15	0.301742411	0.75	0.26	1.10E-10	1
mrpl9	4.71E-15	0.298716784	0.725	0.243	1.12E-10	1
ak2	5.17E-15	0.537805964	1	0.552	1.23E-10	1
si:dkey-17e16.101	5.21E-15	0.251349072	0.9	0.352	1.24E-10	1
kpnb1	5.52E-15	0.430939013	0.925	0.414	1.32E-10	1
eif3s10	6.96E-15	0.564798721	1	0.577	1.66E-10	1
ipo7	7.10E-15	0.445861363	0.938	0.401	1.69E-10	1
mrps16	7.16E-15	0.32562472	0.825	0.311	1.71E-10	1
cast	7.37E-15	0.408581414	0.938	0.419	1.76E-10	1
rpl7l1	7.55E-15	0.511309218	0.962	0.453	1.80E-10	1
slc25a33	7.59E-15	0.272520098	0.588	0.169	1.81E-10	1
nsdhl	7.63E-15	0.330293197	0.662	0.212	1.82E-10	1
arhgdia	7.96E-15	0.453926929	0.95	0.47	1.90E-10	1
ponzr1	8.32E-15	0.393459855	0.862	0.337	1.98E-10	1
ftsj3	8.72E-15	0.251717227	0.662	0.208	2.08E-10	1

gstp1	8.86E-15	0.731783754	1	0.785	2.11E-10	1
rbp2a1	9.62E-15	0.810420909	0.988	0.507	2.29E-10	1
prpf39	9.92E-15	0.276829115	0.912	0.367	2.37E-10	1
ddost	1.02E-14	0.282686342	0.962	0.446	2.43E-10	1
hars	1.04E-14	0.26347248	0.925	0.386	2.48E-10	1
CCT2	1.20E-14	0.528810783	0.962	0.564	2.85E-10	1
dnajc7	1.30E-14	0.320381824	0.912	0.361	3.10E-10	1
zgc:77486.11	1.36E-14	0.373300591	0.962	0.434	3.24E-10	1
srsf7a	1.38E-14	0.286474393	0.9	0.38	3.30E-10	1
mbl21	1.41E-14	0.280690786	0.662	0.204	3.36E-10	1
llph	1.46E-14	0.269584776	0.938	0.373	3.48E-10	1
sec61a1	1.51E-14	0.329735572	0.95	0.431	3.60E-10	1
eps8l3b	1.54E-14	0.420049994	0.9	0.44	3.67E-10	1
atp5pd	1.54E-14	0.605260084	1	0.757	3.68E-10	1
nop56	1.55E-14	0.459187282	0.988	0.575	3.69E-10	1
acad9	1.66E-14	0.255171555	0.675	0.221	3.96E-10	1
dbi	1.88E-14	0.69259561	1	0.764	4.48E-10	1
lsm12a	2.03E-14	0.251960794	0.738	0.251	4.84E-10	1
uso1	2.26E-14	0.262873773	0.75	0.258	5.38E-10	1
tcp1	2.81E-14	0.573575484	0.962	0.537	6.70E-10	1
rpl5b	2.85E-14	0.539609383	1	0.837	6.80E-10	1
CU682777.2	2.88E-14	0.503286478	0.962	0.489	6.87E-10	1
prkab1b	3.18E-14	0.278604979	0.825	0.326	7.58E-10	1
aurkaip1	3.26E-14	0.338385411	0.85	0.35	7.77E-10	1
mrpl28	3.46E-14	0.280617208	0.862	0.339	8.25E-10	1
uba1	3.53E-14	0.360657236	0.912	0.399	8.41E-10	1
hdlbpa	3.82E-14	0.628663655	1	0.654	9.10E-10	1
ilf2	3.93E-14	0.339749219	0.912	0.436	9.37E-10	1
atp5mc3a	4.08E-14	0.556107785	0.988	0.648	9.73E-10	1
si:dkey-16p21.81	4.53E-14	0.723265637	0.9	0.416	1.08E-09	1
timm8b	4.61E-14	0.428285765	0.975	0.449	1.10E-09	1
csnk2b	4.79E-14	0.260006957	0.975	0.442	1.14E-09	1
pfn2l	5.07E-14	0.432842028	0.975	0.528	1.21E-09	1
eif4g1a	5.48E-14	0.463595884	1	0.597	1.31E-09	1
cldn15lb	5.84E-14	0.594235328	1	0.549	1.39E-09	1
eif3m	6.03E-14	0.617641009	0.95	0.541	1.44E-09	1
rbm47	6.09E-14	0.313343724	0.938	0.438	1.45E-09	1
mibp2	6.65E-14	0.62063712	1	0.678	1.59E-09	1
rpsa	7.50E-14	0.540939308	1	0.944	1.79E-09	1
pfn2	8.11E-14	0.563438128	1	0.667	1.93E-09	1
cct5l	9.05E-14	0.468779814	0.975	0.532	2.16E-09	1
c1qbp	9.46E-14	0.567210748	1	0.672	2.26E-09	1
mcm6	9.84E-14	0.296591191	0.538	0.157	2.35E-09	1
snd1	1.01E-13	0.341495874	0.888	0.41	2.40E-09	1
muc13b	1.01E-13	0.360176736	0.988	0.449	2.40E-09	1
degs2	1.04E-13	0.324097827	0.938	0.451	2.47E-09	1
psmc3	1.06E-13	0.278609317	0.862	0.35	2.52E-09	1
psmd3	1.14E-13	0.340178054	0.912	0.425	2.71E-09	1
eif2s1b	1.19E-13	0.484259373	1	0.543	2.84E-09	1
acot19	1.19E-13	0.37192643	0.688	0.232	2.84E-09	1
mrpl301	1.21E-13	0.266302687	0.925	0.423	2.89E-09	1
ccdc137	1.24E-13	0.331331773	0.562	0.17	2.96E-09	1
rps2	1.27E-13	0.582807682	1	0.948	3.03E-09	1
ccnb1	1.32E-13	0.391591955	0.35	0.075	3.15E-09	1

gsta.1.11	1.45E-13	0.748164182	1	0.816	3.45E-09	1
si:ch211-71m22.1	1.55E-13	0.568080699	0.95	0.468	3.71E-09	1
rsl1d11	1.93E-13	0.415192137	0.95	0.478	4.60E-09	1
ogdha	1.99E-13	0.300349174	0.95	0.468	4.74E-09	1
ndufa111	2.06E-13	0.353232245	0.95	0.474	4.90E-09	1
cct6a1	2.10E-13	0.360946285	0.975	0.485	5.00E-09	1
fam213b	2.56E-13	0.330230654	0.988	0.478	6.10E-09	1
ptbp1a	2.62E-13	0.250357706	0.862	0.358	6.26E-09	1
snrpb	2.73E-13	0.613592725	0.988	0.657	6.51E-09	1
psmb4	2.79E-13	0.358457693	0.95	0.464	6.65E-09	1
vcp	2.86E-13	0.403926567	0.962	0.526	6.82E-09	1
gstm.3	3.37E-13	0.259070667	0.912	0.427	8.03E-09	1
ccng11	3.47E-13	0.422265468	0.912	0.463	8.28E-09	1
ola1	3.83E-13	0.369351113	0.938	0.494	9.13E-09	1
hnrnpa1b	4.24E-13	0.442956344	1	0.592	1.01E-08	1
eef1g	4.56E-13	0.583701195	1	0.854	1.09E-08	1
trpm4a	4.66E-13	0.30913806	0.862	0.35	1.11E-08	1
npm1a	5.36E-13	0.546831104	1	0.738	1.28E-08	1
si:ch211-214j24.10	5.96E-13	0.254437856	0.738	0.266	1.42E-08	1
bub3	6.49E-13	0.25277548	0.65	0.227	1.55E-08	1
mphosph8	6.61E-13	0.33687615	0.975	0.498	1.58E-08	1
eif3ea	7.16E-13	0.495301935	0.975	0.554	1.71E-08	1
denr	7.51E-13	0.293534112	0.938	0.455	1.79E-08	1
eif2s3	8.47E-13	0.433819204	0.988	0.536	2.02E-08	1
eif3ha	9.34E-13	0.501147784	0.938	0.511	2.23E-08	1
eif6	1.01E-12	0.355741943	0.938	0.427	2.41E-08	1
fbl	1.10E-12	0.422984003	0.975	0.528	2.61E-08	1
romo1	1.15E-12	0.364139794	0.962	0.547	2.74E-08	1
ndufab1b	1.27E-12	0.50942768	0.988	0.573	3.02E-08	1
eef2a.1	1.29E-12	0.258961806	0.75	0.305	3.07E-08	1
efhd2	1.97E-12	0.256157176	0.925	0.434	4.70E-08	1
psmb5	1.97E-12	0.276131148	0.888	0.395	4.71E-08	1
prdx61	2.04E-12	0.323775157	0.912	0.478	4.86E-08	1
rack1	2.25E-12	0.584307461	0.988	0.891	5.37E-08	1
psma3	2.39E-12	0.368776057	0.962	0.536	5.69E-08	1
bsg	2.57E-12	0.303394365	0.988	0.571	6.13E-08	1
cox8b	2.62E-12	0.609092078	0.988	0.573	6.25E-08	1
COX5B	3.44E-12	0.563657132	1	0.801	8.21E-08	1
bysl	3.69E-12	0.269676331	0.9	0.418	8.79E-08	1
rps6	3.75E-12	0.486592963	1	0.921	8.94E-08	1
gnl3	3.98E-12	0.359163394	0.938	0.457	9.50E-08	1
perp	4.03E-12	0.271665136	0.95	0.459	9.61E-08	1
psmc6	4.06E-12	0.302705824	0.85	0.376	9.68E-08	1
bccip	4.23E-12	0.29388877	0.962	0.429	1.01E-07	1
sb:cb1058	4.51E-12	0.484073758	1	0.558	1.08E-07	1
pfdn6	4.59E-12	0.286186009	0.925	0.431	1.09E-07	1
chchd101	5.88E-12	0.380078173	0.975	0.474	1.40E-07	1
cltca	6.88E-12	0.308343988	0.938	0.423	1.64E-07	1
coro1ca	7.03E-12	0.314489396	0.875	0.414	1.68E-07	1
gsta.2	7.28E-12	0.316389704	0.6	0.21	1.74E-07	1
tuba811	8.18E-12	0.855130083	1	0.596	1.95E-07	1
dkc1	8.41E-12	0.461403233	0.938	0.481	2.01E-07	1
ranbp1	8.77E-12	0.295801712	0.962	0.534	2.09E-07	1
hmga1a	9.19E-12	0.451142396	1	0.839	2.19E-07	1

si:dkey-239h2.3	9.35E-12	0.305818233	0.912	0.41	2.23E-07	1
pfdn1	1.08E-11	0.258974708	0.875	0.406	2.57E-07	1
rbm4.3	1.11E-11	0.490437173	0.975	0.545	2.65E-07	1
mgst3b1	1.18E-11	0.451470863	0.975	0.551	2.82E-07	1
rplp0	1.24E-11	0.557552012	1	0.925	2.96E-07	1
tob1a	1.30E-11	0.254115643	1	0.485	3.10E-07	1
eif3ba	1.30E-11	0.397136176	0.938	0.522	3.11E-07	1
actn4	1.33E-11	0.286325167	0.962	0.427	3.17E-07	1
eif4a1a	1.36E-11	0.345443323	0.975	0.577	3.24E-07	1
tpt1	1.99E-11	0.578483426	0.988	0.839	4.75E-07	1
eppk1	2.02E-11	0.495604688	1	0.519	4.82E-07	1
zgc:92630	2.05E-11	0.252468015	0.862	0.369	4.89E-07	1
psma8	2.06E-11	0.39420031	0.95	0.504	4.90E-07	1
cox14	2.10E-11	0.348407448	0.925	0.423	5.01E-07	1
dlgap5	2.19E-11	0.296360024	0.488	0.154	5.23E-07	1
eif3k	2.81E-11	0.401757191	1	0.552	6.70E-07	1
eif3d1	2.88E-11	0.33779931	0.95	0.528	6.86E-07	1
nap1l1	3.13E-11	0.272708725	0.938	0.438	7.47E-07	1
si:ch211-69g19.2	3.51E-11	0.44392649	0.362	0.094	8.38E-07	1
eif3g	3.96E-11	0.479933895	0.938	0.562	9.44E-07	1
hsp90b1	4.98E-11	0.473780267	1	0.77	1.19E-06	1
pcna1	5.69E-11	0.420333463	0.85	0.391	1.36E-06	1
cox5aa	5.75E-11	0.470131151	0.988	0.734	1.37E-06	1
ndufs5	6.13E-11	0.493577287	0.988	0.582	1.46E-06	1
aldoaa	6.74E-11	0.258633074	0.988	0.474	1.61E-06	1
naa10	7.16E-11	0.269112438	0.925	0.453	1.71E-06	1
sod1	7.34E-11	0.495887654	0.988	0.684	1.75E-06	1
cox6a1	7.65E-11	0.490370828	1	0.824	1.82E-06	1
nnt	7.67E-11	0.269260449	0.65	0.253	1.83E-06	1
aurka	9.73E-11	0.280674436	0.325	0.079	2.32E-06	1
rpl10	1.06E-10	0.518438843	1	0.91	2.53E-06	1
gars	1.13E-10	0.302885712	0.888	0.419	2.69E-06	1
banf1	1.14E-10	0.334422675	0.938	0.513	2.73E-06	1
rpl8	1.19E-10	0.443341942	1	0.953	2.83E-06	1
rida	1.43E-10	0.454277053	0.862	0.444	3.40E-06	1
cox5ab	1.67E-10	0.47584261	1	0.695	3.99E-06	1
rps3a	1.84E-10	0.520734354	1	0.933	4.38E-06	1
minos1	1.85E-10	0.364773754	1	0.556	4.40E-06	1
pdia3	2.00E-10	0.384926473	1	0.661	4.78E-06	1
nme2b.1	2.03E-10	0.532861753	1	0.865	4.84E-06	1
lxn	2.22E-10	0.270828865	0.912	0.406	5.29E-06	1
cirbpa	2.61E-10	0.47691573	1	0.719	6.22E-06	1
ndufa12	2.93E-10	0.3885213	1	0.584	6.98E-06	1
serbp1a	2.94E-10	0.411446339	1	0.873	7.01E-06	1
tacc3	3.05E-10	0.414360843	0.325	0.088	7.28E-06	1
khdrbs1a	4.27E-10	0.458266312	1	0.715	1.02E-05	1
rpl6	4.32E-10	0.378401137	1	0.942	1.03E-05	1
rps3	4.92E-10	0.481226674	1	0.91	1.17E-05	1
cenpf	5.36E-10	0.377296356	0.388	0.112	1.28E-05	1
pdap1a	6.00E-10	0.29922591	0.962	0.526	1.43E-05	1
snu13b	6.33E-10	0.386818749	0.975	0.56	1.51E-05	1
btf3	6.55E-10	0.483406638	1	0.749	1.56E-05	1
ezra	6.64E-10	0.43141014	1	0.699	1.58E-05	1
cox7a2a	7.28E-10	0.43404867	1	0.717	1.74E-05	1

dap	7.53E-10	0.438905916	1	0.629	1.80E-05	1
ywhaba	8.61E-10	0.353589545	0.975	0.607	2.05E-05	1
ndufs4	8.70E-10	0.320221848	0.988	0.541	2.08E-05	1
btg2	9.05E-10	0.409206651	0.988	0.64	2.16E-05	1
s100a10a	1.04E-09	0.561969931	1	0.798	2.47E-05	1
ran	1.04E-09	0.454370818	1	0.745	2.49E-05	1
ebna1bp2	1.32E-09	0.273596581	0.95	0.483	3.14E-05	1
rps4x	2.08E-09	0.389320716	1	0.961	4.95E-05	1
eif5	2.20E-09	0.348938206	0.988	0.639	5.25E-05	1
rps9	2.83E-09	0.404085358	1	0.949	6.75E-05	1
serpinb1	2.88E-09	0.322637409	0.975	0.592	6.86E-05	1
rpl5a	3.17E-09	0.469749226	1	0.813	7.56E-05	1
anp32a	3.19E-09	0.268242875	0.95	0.519	7.61E-05	1
rpl7	3.33E-09	0.454765909	1	0.921	7.94E-05	1
abracl	3.41E-09	0.30945478	0.95	0.496	8.13E-05	1
ftr82	3.52E-09	0.418278313	0.962	0.622	8.40E-05	1
ndufb6	3.91E-09	0.250482224	0.962	0.558	9.32E-05	1
ppl	4.87E-09	0.358928345	0.95	0.5	0.0001162	1
rpl7a	4.98E-09	0.42220082	1	0.949	0.0001187	1
psma1	5.03E-09	0.290185521	0.925	0.493	0.00012	1
ndufb81	5.38E-09	0.29907497	1	0.659	0.0001283	1
pdia4	6.37E-09	0.388653689	0.975	0.61	0.0001518	1
zgc:92027	6.38E-09	0.250836268	0.938	0.493	0.0001522	1
rpl12	6.49E-09	0.439532428	1	0.934	0.0001548	1
ATP5MD	6.69E-09	0.432190231	1	0.7	0.0001596	1
pdia6	7.31E-09	0.257747361	0.988	0.532	0.0001742	1
spdl1	1.06E-08	0.261118218	0.325	0.096	0.0002539	1
rpl10a	1.40E-08	0.47191904	1	0.921	0.0003336	1
tpx2	2.20E-08	0.365990201	0.45	0.17	0.0005244	1
atp5l	2.64E-08	0.417741532	1	0.82	0.0006301	1
setb	2.94E-08	0.307242395	0.988	0.625	0.000701	1
ociad2	3.23E-08	0.251142549	0.938	0.466	0.0007713	1
tubb2b	4.95E-08	0.442122206	0.975	0.625	0.0011793	1
kpna2	5.15E-08	0.886639254	0.425	0.17	0.0012291	1
calr3a	6.03E-08	0.389561171	0.988	0.607	0.0014369	1
cox6c	9.02E-08	0.345157557	1	0.734	0.0021498	1
rpl18	1.08E-07	0.343957281	1	0.934	0.0025639	1
plk1	1.09E-07	0.27988517	0.338	0.116	0.0025992	1
si:dkeyp-73b11.8	1.15E-07	0.432970431	1	0.753	0.0027424	1
rpl18a	1.49E-07	0.369611255	1	0.936	0.0035617	1
eef1b2	1.59E-07	0.4091697	1	0.831	0.0037969	1
hnrnpabb	1.61E-07	0.273521495	0.988	0.642	0.0038428	1
fabp21	2.00E-07	0.341995284	1	0.755	0.0047571	1
si:dkey-36i7.3	4.15E-07	0.32128358	0.988	0.605	0.0099028	1
anxa2b	4.30E-07	0.458417779	1	0.742	0.0102485	1
cox7b	4.63E-07	0.325714985	1	0.798	0.0110488	1
uqcrq	5.28E-07	0.340737788	1	0.725	0.0125974	1
mki67	5.62E-07	0.54203886	0.538	0.253	0.013411	1
cirbpb	6.75E-07	0.313526398	1	0.858	0.0161017	1
fa2h	7.48E-07	0.264099632	0.988	0.622	0.0178384	1
nusap1	8.05E-07	0.286813375	0.288	0.092	0.0192002	1
mgst3a	8.95E-07	0.280107631	1	0.676	0.0213366	1
ncl	9.15E-07	0.31784098	1	0.785	0.0218102	1
eif3f	1.32E-06	0.310436225	0.975	0.64	0.0315229	1

hspe11	1.53E-06	0.275904677	1	0.772	0.0364447	1
rpl19	1.62E-06	0.365201958	1	0.949	0.0386825	1
CABZ01058261.1	2.38E-06	0.256364835	0.388	0.159	0.0567402	1
uqcrh	2.72E-06	0.289800625	0.988	0.749	0.0647441	1
rpl13a	2.90E-06	0.30145715	1	0.938	0.0692631	1
gpx4b	3.43E-06	0.341263708	0.988	0.687	0.0818383	1
nupr1	8.57E-06	0.351104408	0.988	0.74	0.2043395	1
cox6b2	9.82E-06	0.263988573	1	0.742	0.2341528	1
rplp2l	1.19E-05	0.263639679	1	0.976	0.2833326	1
cldn15la	3.04E-05	0.302189927	1	0.783	0.7260013	1
rps8a	0.0001153	0.277255129	1	0.972	1	1
ppiaa	0.0003561	0.264162532	1	0.882	1	1
rps11	0.0010708	0.25923957	1	0.919	1	1
anxa4	4.24E-33	3.883420773	0.892	0.435	1.01E-28	2
bsnb	1.55E-27	0.911272926	0.338	0.022	3.70E-23	2
shroom4	2.58E-27	1.174379216	0.365	0.03	6.14E-23	2
met	1.27E-24	1.074504176	0.432	0.057	3.02E-20	2
neurl1aa	1.78E-24	1.438792455	0.446	0.063	4.24E-20	2
fhl1b	6.71E-24	1.715448176	0.473	0.076	1.60E-19	2
map4l	5.48E-23	0.80032408	0.324	0.028	1.31E-18	2
hepacam2	7.74E-23	1.221242423	0.257	0.013	1.85E-18	2
si:ch211-222k6.3	9.01E-23	1.011589537	0.311	0.026	2.15E-18	2
arrdc3a	1.63E-21	1.295031925	0.608	0.165	3.88E-17	2
krt18	2.39E-21	2.543506192	0.878	0.52	5.70E-17	2
ret	7.13E-21	0.857298433	0.257	0.017	1.70E-16	2
si:ch73-359m17.9	1.58E-20	2.012608727	0.27	0.02	3.78E-16	2
gdi1	4.00E-20	0.68192466	0.324	0.037	9.54E-16	2
soga1	1.14E-19	0.948618202	0.365	0.054	2.71E-15	2
FADS6	4.53E-19	0.998307731	0.392	0.065	1.08E-14	2
lygl1	1.34E-18	0.897128024	0.297	0.033	3.19E-14	2
si:dkey-28b4.8	4.22E-18	0.811680711	0.351	0.054	1.01E-13	2
mgll	7.16E-18	0.875478251	0.257	0.024	1.71E-13	2
b4galt6	1.38E-17	0.884725076	0.554	0.169	3.29E-13	2
calm1b	2.26E-17	1.67410922	0.581	0.178	5.39E-13	2
dnaja2l	2.55E-17	0.923210683	0.527	0.144	6.07E-13	2
h1f0	4.78E-17	2.387178861	0.73	0.363	1.14E-12	2
jpt1b	1.53E-16	1.178675081	0.608	0.207	3.65E-12	2
vamp2	1.75E-16	0.750147676	0.311	0.044	4.18E-12	2
zgc:113142	2.20E-16	1.665632762	0.257	0.028	5.26E-12	2
ptmaa	2.78E-16	1.669799403	0.851	0.609	6.63E-12	2
adgrg1	6.25E-16	1.066383872	0.311	0.048	1.49E-11	2
tspan7b	9.59E-16	0.822829686	0.257	0.03	2.29E-11	2
sypa	1.10E-15	1.022855012	0.257	0.03	2.63E-11	2
CR383676.1	1.33E-15	0.968770205	1	0.978	3.18E-11	2
syt1a	1.59E-15	1.062218488	0.324	0.054	3.78E-11	2
dixdc1a	2.04E-15	0.661176671	0.297	0.044	4.87E-11	2
lysmd2	2.72E-15	0.620840317	0.311	0.05	6.49E-11	2
tmeff1b	5.80E-15	0.621996742	0.284	0.041	1.38E-10	2
igfbp5b	7.24E-15	1.599459558	0.324	0.059	1.73E-10	2
arrdc3b	9.42E-15	1.004422824	0.405	0.098	2.25E-10	2
prkar1aa	1.52E-14	1.026850051	0.527	0.17	3.63E-10	2
meis1b	1.59E-14	1.342912584	0.486	0.146	3.80E-10	2
tm4sf4	1.59E-14	1.823556033	0.892	0.733	3.80E-10	2
foxp4	1.87E-14	1.77167054	0.716	0.376	4.45E-10	2

slc1a4	2.02E-14	1.597561422	0.392	0.094	4.83E-10	2
relb	2.86E-14	1.446880488	0.514	0.178	6.81E-10	2
BX901920.1	3.03E-14	1.065463734	0.27	0.039	7.22E-10	2
rtn1a	3.53E-14	1.314314045	0.446	0.122	8.42E-10	2
gnao1a	5.30E-14	0.601895425	0.257	0.035	1.26E-09	2
frmd6	6.64E-14	0.644805699	0.27	0.041	1.58E-09	2
sptlc2b	8.40E-14	1.198170983	0.743	0.43	2.00E-09	2
tox	9.17E-14	0.760870925	0.392	0.087	2.19E-09	2
nfkbib	1.02E-13	1.413819893	0.568	0.233	2.42E-09	2
h3f3b.1.21	1.06E-13	1.151263018	0.797	0.544	2.53E-09	2
h3f3c	1.30E-13	1.076139337	0.851	0.637	3.11E-09	2
ywhabl	1.39E-13	0.905540376	0.77	0.487	3.32E-09	2
histh1l	1.65E-13	2.431656442	0.716	0.431	3.93E-09	2
gabarapb	2.05E-13	0.88147961	0.662	0.307	4.88E-09	2
stx1b	2.11E-13	1.262478436	0.311	0.059	5.03E-09	2
ptbp1b	3.02E-13	1.277825455	0.635	0.304	7.20E-09	2
si:ch211-147g22.5	3.82E-13	0.937301535	0.311	0.061	9.11E-09	2
scg3	4.71E-13	2.060280648	0.297	0.056	1.12E-08	2
irf1b	5.36E-13	1.255845669	0.365	0.089	1.28E-08	2
phgdh	6.02E-13	0.755983861	0.392	0.096	1.44E-08	2
tenm3	9.33E-13	0.746638339	0.311	0.063	2.23E-08	2
plpp5	1.01E-12	0.408219596	0.338	0.074	2.40E-08	2
hsbp1a	1.08E-12	0.675417759	0.365	0.087	2.59E-08	2
cyfip2	1.22E-12	0.642624328	0.257	0.041	2.91E-08	2
gnb1a	1.26E-12	1.146592569	0.77	0.494	3.01E-08	2
sat1a.2	1.31E-12	1.176700359	0.824	0.663	3.12E-08	2
tox2	1.87E-12	0.582947223	0.27	0.046	4.46E-08	2
atrx	2.78E-12	1.061508425	0.73	0.42	6.63E-08	2
ap3s2	3.22E-12	1.02325681	0.392	0.107	7.67E-08	2
atf4a	3.91E-12	1.018421068	0.851	0.652	9.33E-08	2
bik	4.04E-12	1.003470361	0.27	0.05	9.64E-08	2
slc7a10a	4.88E-12	0.654913344	0.324	0.07	1.16E-07	2
odc1	6.10E-12	1.448130828	0.608	0.3	1.46E-07	2
golgb1	6.72E-12	1.182920335	0.676	0.374	1.60E-07	2
sdc4	7.35E-12	1.435451094	0.824	0.63	1.75E-07	2
gse1	7.88E-12	0.887816221	0.581	0.252	1.88E-07	2
elovl1a	9.95E-12	0.938729905	0.324	0.078	2.37E-07	2
tmsb	1.10E-11	1.076022669	0.419	0.126	2.63E-07	2
myl12.1	1.28E-11	0.88475198	0.865	0.724	3.06E-07	2
pbxip1a	1.39E-11	1.34090013	0.351	0.089	3.31E-07	2
notch2	2.17E-11	0.976043787	0.432	0.146	5.18E-07	2
ptp4a1	2.19E-11	1.086573443	0.689	0.422	5.22E-07	2
ankrd12	2.55E-11	1.037236948	0.635	0.33	6.07E-07	2
spint2	3.01E-11	1.374377911	0.622	0.326	7.17E-07	2
tsc22d1	3.80E-11	0.86916597	0.716	0.404	9.06E-07	2
pard6a	4.23E-11	0.496231432	0.284	0.059	1.01E-06	2
atp6v0cb	4.24E-11	1.011809451	0.351	0.093	1.01E-06	2
tmem56a	5.17E-11	0.57194994	0.378	0.111	1.23E-06	2
nfkb2	5.47E-11	1.161948656	0.446	0.163	1.30E-06	2
ywhaz	5.64E-11	1.063406233	0.703	0.42	1.34E-06	2
reep5	7.39E-11	1.025385422	0.703	0.407	1.76E-06	2
rassf1	8.76E-11	0.704957818	0.419	0.137	2.09E-06	2
mgea5	8.76E-11	0.631284649	0.419	0.137	2.09E-06	2
celf3a	9.24E-11	0.648576447	0.311	0.076	2.20E-06	2

baz2a	1.07E-10	0.577287293	0.473	0.178	2.55E-06	2
ctsf	1.37E-10	0.713516246	0.419	0.141	3.27E-06	2
jmjd1cb	1.58E-10	1.026737252	0.622	0.33	3.77E-06	2
smim29	1.66E-10	0.523461655	0.257	0.052	3.96E-06	2
mkrn1	1.83E-10	0.572744761	0.324	0.085	4.36E-06	2
klf7a	1.92E-10	0.538713644	0.257	0.052	4.59E-06	2
smim7	2.06E-10	0.471602955	0.257	0.052	4.92E-06	2
sytl3.1	2.26E-10	1.082918953	0.662	0.365	5.40E-06	2
serinc1	2.44E-10	0.985104939	0.568	0.269	5.82E-06	2
cebpg	2.49E-10	1.191282522	0.662	0.417	5.93E-06	2
hmgb3a	3.50E-10	1.365255797	0.527	0.241	8.35E-06	2
klf7b	3.72E-10	1.125489779	0.649	0.415	8.88E-06	2
cnn3a	3.97E-10	1.287536685	0.5	0.23	9.48E-06	2
xirp2a	4.01E-10	0.699408718	0.284	0.069	9.56E-06	2
zgc:86896	4.20E-10	0.711483688	0.257	0.054	1.00E-05	2
phf20a	4.48E-10	0.338235578	0.27	0.059	1.07E-05	2
nlk1	5.00E-10	0.660002585	0.446	0.161	1.19E-05	2
zfhx4	5.04E-10	1.453350195	0.338	0.096	1.20E-05	2
tm9sf2	5.74E-10	0.793180387	0.595	0.294	1.37E-05	2
h3f3d	5.88E-10	0.681009704	0.865	0.811	1.40E-05	2
si:ch73-335l21.4	6.80E-10	1.142418803	0.878	0.709	1.62E-05	2
slc12a2	8.18E-10	0.366272624	0.284	0.067	1.95E-05	2
cdkn1bb	8.52E-10	0.856996277	0.689	0.424	2.03E-05	2
actb2	8.64E-10	0.916906973	0.959	0.963	2.06E-05	2
cpe	8.77E-10	0.932283311	0.311	0.085	2.09E-05	2
rnf10	9.51E-10	0.865262007	0.689	0.43	2.27E-05	2
marcksl1b	1.00E-09	1.720428506	0.581	0.296	2.39E-05	2
creb3l3l	1.02E-09	0.992098642	0.622	0.369	2.43E-05	2
gapdhs	1.07E-09	1.323552053	0.622	0.322	2.55E-05	2
fam177a1	1.08E-09	0.576610829	0.446	0.165	2.59E-05	2
txnipa	1.12E-09	1.046640301	0.432	0.157	2.66E-05	2
syncripl	1.22E-09	0.589004906	0.378	0.124	2.91E-05	2
tspan14	1.24E-09	0.311694295	0.257	0.056	2.95E-05	2
gnb1b	1.31E-09	0.829537153	0.77	0.556	3.11E-05	2
atp1b2a	1.58E-09	0.826121036	0.365	0.117	3.76E-05	2
sntb2	1.73E-09	0.644525701	0.297	0.08	4.12E-05	2
stard10	1.91E-09	0.690930337	0.365	0.115	4.56E-05	2
asap3	2.03E-09	0.751625832	0.338	0.102	4.83E-05	2
si:busm1-57f23.1	2.66E-09	0.824991331	0.365	0.119	6.35E-05	2
si:dkey-42i9.4	2.90E-09	0.899554696	0.635	0.383	6.92E-05	2
cblb	3.36E-09	0.405512427	0.27	0.065	8.02E-05	2
ero1b	3.50E-09	0.534943007	0.446	0.174	8.36E-05	2
rimkla	3.52E-09	0.423027315	0.297	0.081	8.39E-05	2
ppdpfb	3.57E-09	1.086880277	0.743	0.53	8.52E-05	2
ash1l	3.83E-09	0.757478874	0.541	0.263	9.14E-05	2
slc38a10	3.90E-09	0.711628695	0.392	0.135	9.31E-05	2
fam168a	4.16E-09	0.642362103	0.365	0.119	9.93E-05	2
clstn1	4.39E-09	0.942501855	0.378	0.13	0.0001047	2
purab	4.92E-09	0.609443654	0.257	0.063	0.0001174	2
cldnh	4.98E-09	1.295958744	0.514	0.217	0.0001188	2
CR762483.1	5.00E-09	2.727720585	0.459	0.196	0.0001193	2
appb	5.32E-09	0.515410288	0.338	0.102	0.0001269	2
mef2d	5.44E-09	0.821407586	0.459	0.193	0.0001297	2
ywhaqb	5.81E-09	0.630040814	0.838	0.637	0.0001384	2

znf395b	6.24E-09	0.367539991	0.257	0.061	0.0001487	2
klf6a	7.18E-09	0.981474766	0.851	0.752	0.0001711	2
cc2d1b	7.56E-09	0.419117308	0.365	0.12	0.0001803	2
zgc:92606	8.52E-09	0.656951052	0.527	0.263	0.0002032	2
tfe3a	8.53E-09	1.326054692	0.595	0.363	0.0002033	2
rab2a	9.49E-09	0.967870542	0.703	0.537	0.0002264	2
oaz1b	1.01E-08	0.761267865	0.73	0.563	0.0002413	2
hs3st1l2	1.22E-08	0.804346595	0.297	0.089	0.0002916	2
cdh1	1.34E-08	1.14637743	0.77	0.646	0.0003184	2
sec63	1.40E-08	0.80165915	0.568	0.317	0.0003331	2
pip4p1a	1.57E-08	0.596292154	0.297	0.089	0.000375	2
ddx5	1.61E-08	0.758041919	0.865	0.663	0.0003849	2
rnf44	1.69E-08	0.549049954	0.297	0.087	0.0004031	2
zgc:174906	1.70E-08	0.412573342	0.27	0.07	0.0004051	2
gosr1	1.85E-08	0.731511927	0.432	0.178	0.0004417	2
dgcr2	1.88E-08	0.392591268	0.284	0.08	0.000449	2
clk2a	1.89E-08	0.415099966	0.284	0.078	0.0004511	2
arl8a	1.90E-08	0.47568111	0.338	0.115	0.0004534	2
pam	1.93E-08	1.001390301	0.473	0.231	0.0004592	2
arf2a	2.08E-08	0.830956586	0.662	0.413	0.000497	2
marcksb	2.12E-08	0.882106217	0.351	0.122	0.0005059	2
slc38a2	2.25E-08	0.940274363	0.635	0.376	0.0005358	2
rab11a	2.34E-08	0.827703082	0.689	0.448	0.0005572	2
stk26	2.74E-08	0.448653673	0.311	0.096	0.000653	2
arg2	2.76E-08	0.275853022	0.297	0.083	0.0006577	2
hmgn3l	3.01E-08	0.574054318	0.514	0.252	0.0007184	2
zgc:92066	3.16E-08	0.637627513	0.959	0.87	0.0007534	2
hk1	3.30E-08	0.3637909	0.297	0.085	0.000786	2
map3k14a	3.48E-08	0.781066743	0.378	0.144	0.0008305	2
atf6	3.76E-08	0.995331654	0.635	0.393	0.0008974	2
si:dkey-28b4.7	3.89E-08	0.713823714	0.581	0.309	0.0009273	2
mthfd2	3.91E-08	0.817121635	0.595	0.348	0.0009327	2
myh10	4.05E-08	0.542089796	0.284	0.083	0.0009654	2
kdm6bb	4.10E-08	0.799101984	0.541	0.27	0.000977	2
tmem54a	4.13E-08	0.783687233	0.324	0.106	0.0009837	2
ep300b	4.14E-08	0.845714084	0.419	0.17	0.000988	2
kdm4b	4.21E-08	0.632594013	0.514	0.261	0.0010034	2
tpp2	4.31E-08	0.49256692	0.419	0.172	0.0010276	2
pkma	4.47E-08	0.596370567	0.554	0.281	0.0010648	2
ranbp9	4.72E-08	0.509015946	0.392	0.159	0.0011254	2
smarca4a	4.73E-08	0.793141102	0.649	0.424	0.001128	2
nuak2	5.47E-08	0.443164355	0.338	0.119	0.0013032	2
nfkbiaa	5.79E-08	1.284334089	0.635	0.389	0.0013809	2
elovl1b	5.99E-08	0.859236198	0.676	0.467	0.001428	2
stag2b	6.01E-08	0.620452051	0.595	0.337	0.0014323	2
sox4a.1	6.46E-08	1.054255481	0.378	0.143	0.0015397	2
mbnl3	7.20E-08	0.668520036	0.284	0.083	0.0017174	2
actn1	7.58E-08	0.491348217	0.378	0.135	0.0018066	2
cfl1l	8.37E-08	1.148069345	0.716	0.481	0.0019969	2
stxbp2	8.48E-08	0.589029609	0.378	0.143	0.0020214	2
fstl1b	8.63E-08	0.586536802	0.365	0.139	0.0020572	2
si:ch211-137a8.4	8.98E-08	0.341119847	0.284	0.081	0.0021418	2
ing4	9.12E-08	0.431276231	0.338	0.12	0.0021757	2
clip2	9.26E-08	0.310787151	0.257	0.069	0.0022079	2

map1lc3b	9.40E-08	0.394754608	0.351	0.128	0.0022405	2
zswim8	1.04E-07	0.281047895	0.365	0.133	0.002487	2
cica	1.06E-07	0.672600389	0.392	0.156	0.0025275	2
tuba1c	1.17E-07	1.468500408	0.405	0.159	0.0027854	2
chd4b	1.21E-07	0.524885688	0.716	0.489	0.0028765	2
marcksl1a	1.26E-07	0.837404136	0.581	0.339	0.0030064	2
hmgn6	1.30E-07	0.748672923	0.824	0.685	0.0030903	2
ptprfb	1.30E-07	0.880238074	0.622	0.376	0.0030954	2
calm1a	1.31E-07	1.040223117	0.797	0.624	0.0031167	2
tcima	1.35E-07	0.792612675	0.5	0.226	0.0032117	2
kdm5c	1.36E-07	0.884066878	0.568	0.311	0.0032414	2
tspan3a	1.37E-07	0.508228966	0.338	0.122	0.0032661	2
pdcl	1.38E-07	0.694245584	0.554	0.313	0.0032855	2
cldn7b	1.52E-07	1.145510759	0.554	0.307	0.0036357	2
rnasekb	1.74E-07	0.573656374	0.297	0.096	0.0041412	2
ptenb	1.77E-07	0.530352909	0.446	0.204	0.0042122	2
atp6ap2	1.82E-07	0.773037843	0.446	0.211	0.0043467	2
sox9b	1.86E-07	0.362544096	0.27	0.08	0.0044326	2
atf7ip	1.92E-07	0.673826555	0.595	0.37	0.0045664	2
trim33	1.99E-07	0.484738306	0.405	0.176	0.0047468	2
plekha1a	2.08E-07	0.531427966	0.284	0.091	0.0049643	2
fndc3a	2.27E-07	0.379718407	0.284	0.089	0.0054091	2
zgc:158564	2.29E-07	0.781074066	0.595	0.369	0.0054706	2
irf2bpl	2.32E-07	0.517473309	0.351	0.133	0.0055324	2
ppp3r1b	2.38E-07	0.381862806	0.459	0.217	0.0056736	2
rad21a	2.38E-07	0.720076097	0.676	0.448	0.0056796	2
ppp2r1bb	2.50E-07	0.48115669	0.459	0.22	0.0059595	2
arglu1a	2.56E-07	0.633692821	0.432	0.2	0.0061155	2
sgsm3	2.57E-07	0.451859964	0.284	0.091	0.0061253	2
rab3ip	2.65E-07	0.357435333	0.257	0.076	0.0063074	2
ifi30	2.69E-07	0.633386805	0.473	0.239	0.0064169	2
arhgef1b	2.75E-07	1.006058767	0.459	0.233	0.0065486	2
ginm1	2.78E-07	0.538824134	0.419	0.185	0.0066211	2
ppp1cb	2.92E-07	0.52353509	0.459	0.23	0.0069523	2
ppiab	2.93E-07	0.484696084	0.905	0.874	0.0069909	2
rel	3.10E-07	0.275634756	0.324	0.111	0.0073934	2
alcama	3.40E-07	0.764331223	0.257	0.076	0.008114	2
trip11	3.50E-07	0.448103185	0.378	0.152	0.0083565	2
yif1a	3.61E-07	0.383932876	0.365	0.139	0.0086151	2
rapgef2	3.96E-07	0.426163625	0.257	0.074	0.0094502	2
calr	4.14E-07	1.066032389	0.77	0.593	0.0098807	2
ammecr1	4.15E-07	0.559170898	0.554	0.313	0.0098923	2
homeza	4.20E-07	0.467624253	0.27	0.083	0.0100173	2
krt94	4.22E-07	1.290626148	0.284	0.091	0.0100546	2
atf3	4.28E-07	1.030271881	0.919	0.839	0.0102049	2
schip1	4.32E-07	0.843303906	0.473	0.239	0.0103085	2
rhoaa	4.37E-07	0.688491357	0.649	0.435	0.0104172	2
lin7c	4.41E-07	0.602139636	0.392	0.172	0.0105079	2
si:ch211-79l17.1	4.63E-07	0.318393339	0.27	0.085	0.0110444	2
dstyk	4.94E-07	0.542467814	0.297	0.106	0.011788	2
kdm5bb	5.32E-07	0.37069221	0.446	0.206	0.0126819	2
ubald1b	5.59E-07	0.25037464	0.365	0.143	0.0133188	2
dcaf7	5.71E-07	0.629404039	0.432	0.206	0.0136237	2
tmed2	5.79E-07	0.928312258	0.743	0.548	0.0137956	2

rc3h1b	5.86E-07	0.527399263	0.27	0.087	0.0139704	2
mknk2b	6.03E-07	1.007771767	0.743	0.619	0.0143764	2
tmem30aa	6.58E-07	0.337468778	0.27	0.085	0.0156885	2
atf1	6.61E-07	0.572490946	0.351	0.144	0.0157682	2
acsl3b	6.83E-07	0.587110224	0.324	0.124	0.0162758	2
praf2	7.31E-07	0.502644358	0.284	0.094	0.0174417	2
atg13	8.60E-07	0.502336882	0.338	0.137	0.0205059	2
birc2	8.94E-07	0.811680086	0.473	0.257	0.0213282	2
srsf6b	8.96E-07	0.527421277	0.581	0.363	0.0213669	2
si:ch211-160o17.4	9.13E-07	0.517101384	0.541	0.302	0.0217743	2
ubc	9.17E-07	0.488815832	0.865	0.8	0.0218772	2
zgc:162939	9.37E-07	0.525362007	0.297	0.107	0.0223409	2
otud5a	9.54E-07	0.55709633	0.432	0.2	0.0227475	2
btaf1	9.76E-07	0.738416606	0.284	0.1	0.0232723	2
arfgap3	9.80E-07	0.543630313	0.338	0.137	0.0233703	2
stm	9.91E-07	0.401687579	0.338	0.124	0.0236402	2
tnksb	1.03E-06	0.37802623	0.297	0.106	0.0244436	2
si:ch211-238n5.4	1.13E-06	0.413680872	0.27	0.087	0.0268817	2
kctd5a	1.19E-06	0.639948707	0.554	0.337	0.0282802	2
appa	1.21E-06	0.816063187	0.649	0.481	0.0287733	2
fxyd1	1.21E-06	1.089197026	0.554	0.374	0.0288527	2
gpt2	1.22E-06	0.402782319	0.311	0.111	0.0289743	2
arid1aa	1.23E-06	0.530178778	0.378	0.172	0.0292758	2
aldocb	1.27E-06	0.776944745	0.257	0.083	0.0301905	2
zfr	1.28E-06	0.83121738	0.527	0.296	0.0305806	2
baz2ba	1.35E-06	0.674441179	0.568	0.346	0.0322066	2
smarce1	1.41E-06	0.783684923	0.568	0.389	0.0335604	2
tnip1	1.46E-06	0.941465207	0.514	0.313	0.0348767	2
arl6ip1	1.53E-06	0.647304897	0.486	0.263	0.0365575	2
hipk3a	1.55E-06	0.667904252	0.459	0.235	0.037026	2
sorl1	1.58E-06	0.493320037	0.311	0.113	0.0376828	2
cdc42	1.59E-06	0.579007707	0.527	0.304	0.037977	2
ogt.1.1	1.62E-06	0.702375712	0.405	0.193	0.0386656	2
pdcd6ip	1.67E-06	0.802452774	0.514	0.309	0.0397283	2
ago3b	1.72E-06	0.409996423	0.284	0.1	0.0410994	2
pnrc2	1.77E-06	0.542748422	0.905	0.791	0.0422265	2
eif2s31	1.78E-06	0.578139725	0.757	0.572	0.0423972	2
MYO9B	1.82E-06	0.383594651	0.338	0.137	0.0434379	2
atf4b	1.99E-06	0.606819412	0.838	0.717	0.0475465	2
rnf38	2.39E-06	0.558751015	0.324	0.13	0.0570381	2
gnas	2.40E-06	0.551516604	0.297	0.109	0.0571785	2
ctnnb2	2.40E-06	0.716113161	0.595	0.398	0.0571832	2
map7d3	2.61E-06	0.488143561	0.338	0.139	0.062126	2
golga7	2.63E-06	0.56492783	0.514	0.289	0.0627175	2
rnf11b	2.67E-06	0.6805991	0.297	0.113	0.0637519	2
mid1ip1b	2.80E-06	0.621408401	0.595	0.376	0.0668184	2
chd7	2.85E-06	0.789924215	0.689	0.496	0.0679335	2
rab13	2.90E-06	0.556012924	0.324	0.128	0.0691214	2
TCIM (1 of many)	2.97E-06	1.04578256	0.419	0.219	0.0707778	2
rab1ab	3.01E-06	0.626416387	0.703	0.541	0.0717266	2
chd4a	3.04E-06	0.920603403	0.635	0.428	0.0724925	2
btbd10a	3.06E-06	0.532292618	0.338	0.144	0.0729278	2
plxna3	3.08E-06	0.379680487	0.257	0.085	0.0733276	2
krt8	3.08E-06	0.855223713	0.73	0.626	0.0735237	2

slc7a6os	3.09E-06	0.434071957	0.378	0.169	0.0737814	2
tmem50a	3.10E-06	0.567650278	0.419	0.204	0.0739711	2
hnrnpa01.1	3.22E-06	0.387109567	0.932	0.854	0.0767507	2
atp2b2.1	3.31E-06	0.939636403	0.595	0.417	0.0788308	2
zgc:111986	3.52E-06	0.650891915	0.649	0.469	0.0840518	2
socs3a	3.61E-06	1.024680425	0.446	0.219	0.0861233	2
dock7	3.62E-06	0.378746359	0.432	0.213	0.0863544	2
tmem59	3.68E-06	0.544634317	0.311	0.128	0.0876739	2
gadd45ba	3.73E-06	1.601270043	0.716	0.565	0.0889526	2
kdm2ba	3.76E-06	0.544583679	0.405	0.193	0.0895426	2
fam120c	3.79E-06	0.557161987	0.459	0.246	0.0902563	2
ldb1a	4.05E-06	0.466790479	0.338	0.143	0.096678	2
mycbp2	4.10E-06	0.586244697	0.662	0.437	0.0978087	2
15-Sep	4.10E-06	0.452922054	0.284	0.111	0.0978254	2
ip6k2a	4.12E-06	0.421408487	0.378	0.178	0.0982318	2
tmf1	4.14E-06	0.316367624	0.284	0.106	0.0988182	2
gnai1	4.21E-06	0.435735148	0.324	0.137	0.1004383	2
sec61a11	4.22E-06	0.832404691	0.649	0.478	0.1007186	2
myl12.2	4.23E-06	0.446205326	0.473	0.259	0.1008047	2
rbpms2a	4.30E-06	0.57692928	0.27	0.094	0.1025018	2
slc3a2b	4.42E-06	0.496767681	0.338	0.148	0.1053189	2
b2ml	4.47E-06	0.531484722	0.581	0.367	0.1065452	2
usp36	4.47E-06	0.589762224	0.608	0.407	0.106634	2
mef2aa	4.54E-06	0.591959069	0.297	0.113	0.1082151	2
ugt8	4.63E-06	0.921579008	0.581	0.415	0.1103998	2
sinhcaf	4.67E-06	0.643428795	0.446	0.233	0.1113494	2
canx	4.68E-06	0.583072393	0.824	0.643	0.1115669	2
actb1	4.90E-06	0.530581574	0.959	0.97	0.1168321	2
pim1	4.91E-06	0.943087212	0.676	0.483	0.1170952	2
ubr3	4.97E-06	0.47627299	0.338	0.144	0.1185779	2
ctnnd1	5.16E-06	0.599331701	0.676	0.504	0.1229415	2
acin1a	5.16E-06	0.707488026	0.743	0.613	0.12298	2
cdh2	5.41E-06	0.463096058	0.27	0.1	0.1289529	2
kansl1b	5.41E-06	0.518181653	0.5	0.294	0.1289849	2
rhoab	5.64E-06	0.711860936	0.689	0.58	0.1345676	2
casc4	5.83E-06	0.719209059	0.378	0.178	0.1389838	2
hdac1	5.91E-06	0.699991497	0.662	0.454	0.1408371	2
sox6	6.03E-06	0.543317316	0.297	0.119	0.1438934	2
slc1a5	6.25E-06	0.705817811	0.554	0.383	0.1489354	2
si:ch211-276i12.9	6.31E-06	0.272859388	0.27	0.098	0.1504066	2
ube2e1	6.60E-06	0.657770782	0.554	0.367	0.1574918	2
rtf1	7.05E-06	0.606177107	0.595	0.402	0.1681922	2
sec23b	7.14E-06	0.451007487	0.541	0.328	0.1703319	2
zmiz1a	7.31E-06	0.76209406	0.365	0.181	0.1742766	2
csnk2a2a	7.37E-06	0.268794679	0.257	0.093	0.1758169	2
usp9	7.80E-06	0.460208821	0.514	0.302	0.1860955	2
tax1bp1b	8.38E-06	0.481491865	0.541	0.317	0.1999281	2
akap12b	8.46E-06	0.873421477	0.311	0.128	0.2016389	2
mtss1	8.48E-06	0.589237869	0.297	0.124	0.2021038	2
med19b	8.51E-06	0.364466255	0.257	0.091	0.2028563	2
gpm6aa	8.52E-06	1.606703217	0.27	0.104	0.2031442	2
slc7a3a	8.63E-06	0.694597063	0.662	0.515	0.2056782	2
synrg	8.72E-06	0.564420761	0.297	0.122	0.207939	2
gtpbp1	8.93E-06	0.906179868	0.486	0.285	0.2129275	2

copb1	9.31E-06	0.614066325	0.568	0.357	0.2219942	2
tmem184ba	9.33E-06	0.254197959	0.257	0.087	0.2225711	2
plk3	9.44E-06	0.642399434	0.365	0.176	0.2250009	2
pdpk1b	9.47E-06	0.46250799	0.392	0.189	0.2258724	2
prrc2a	9.52E-06	0.597107477	0.392	0.194	0.2270924	2
atp6v0ca	9.67E-06	0.553714638	0.77	0.552	0.2304978	2
brd3b	9.73E-06	0.699621834	0.446	0.248	0.2319867	2
AKAP13	9.93E-06	0.887943606	0.432	0.239	0.2368799	2
tanc1b	1.05E-05	0.281315856	0.257	0.093	0.2494307	2
arf1	1.06E-05	0.590669754	0.581	0.394	0.2527958	2
zswim5	1.12E-05	0.452494391	0.5	0.298	0.2668714	2
agap1	1.12E-05	0.382116354	0.338	0.148	0.2670716	2
cltca1	1.14E-05	0.540763741	0.662	0.467	0.2721393	2
ptp4a2a	1.15E-05	0.336622159	0.365	0.176	0.2732968	2
mbpb	1.17E-05	0.683028861	0.473	0.259	0.2788756	2
kdm6ba	1.22E-05	0.816621032	0.581	0.404	0.2918592	2
si:ch211-212k18.4	1.24E-05	0.569480467	0.527	0.328	0.2946823	2
lima1a	1.33E-05	1.255419494	0.419	0.231	0.3181228	2
atf5b	1.36E-05	0.762175976	0.541	0.35	0.3235168	2
brd3a	1.39E-05	0.419842448	0.5	0.283	0.3324867	2
scrib	1.41E-05	0.677007964	0.378	0.185	0.3369346	2
tspan7	1.43E-05	0.592893331	0.419	0.217	0.3404422	2
cwc25	1.55E-05	0.341356967	0.351	0.163	0.3693095	2
pafah1b1b	1.55E-05	0.450436548	0.446	0.228	0.3694637	2
tmed7	1.56E-05	0.645936002	0.635	0.467	0.3710276	2
slc38a5a	1.57E-05	0.358496841	0.297	0.117	0.3744911	2
kdm4ab	1.57E-05	0.383625315	0.257	0.093	0.3750602	2
smarca5	1.58E-05	0.684996245	0.446	0.256	0.3760761	2
gna11b	1.66E-05	0.401418606	0.351	0.161	0.3964804	2
add3a	1.66E-05	1.161296774	0.527	0.339	0.3965111	2
si:ch73-46j18.5	1.70E-05	0.923531695	0.527	0.357	0.404614	2
nova2	1.73E-05	1.229953103	0.284	0.117	0.4113435	2
dctn2	1.74E-05	0.481137419	0.419	0.241	0.4151506	2
actr1	1.78E-05	0.380786213	0.473	0.269	0.4254147	2
golga5	1.80E-05	0.54531482	0.297	0.122	0.4290673	2
midn	1.81E-05	0.811953655	0.743	0.615	0.4310932	2
ddx3a	1.82E-05	0.59969635	0.581	0.391	0.4348067	2
ccni	1.85E-05	0.630527887	0.581	0.424	0.440522	2
ptena	1.86E-05	0.572537786	0.473	0.274	0.4434512	2
gdi2l	1.87E-05	0.536707176	0.716	0.548	0.4450598	2
btg1	1.88E-05	0.611221267	0.865	0.791	0.4481748	2
vangl2	1.91E-05	0.441199521	0.324	0.144	0.4559257	2
sp4	1.93E-05	0.305796779	0.27	0.106	0.4590204	2
rhoac	1.96E-05	0.399481272	0.581	0.393	0.4668121	2
kmt2cb	1.98E-05	0.657112008	0.541	0.352	0.4719141	2
fam210aa	1.99E-05	0.515930665	0.405	0.211	0.4737333	2
ssbp4	2.03E-05	0.474399411	0.257	0.096	0.4838647	2
bcam	2.04E-05	0.715501875	0.459	0.256	0.4867153	2
stat3	2.04E-05	0.412359056	0.324	0.141	0.4873331	2
vat1	2.05E-05	1.067873399	0.324	0.156	0.4892722	2
ikbkg	2.09E-05	0.443158237	0.378	0.185	0.4979919	2
zgc:165555.12	2.09E-05	0.627807424	0.851	0.717	0.4983072	2
si:ch211-222l21.1	2.11E-05	0.66826896	0.959	0.856	0.5026905	2
nfkbiab	2.12E-05	1.596690461	0.608	0.485	0.5058769	2

wipf2a	2.27E-05	0.456252861	0.432	0.244	0.542148	2
arf5	2.31E-05	0.657129101	0.649	0.493	0.5516019	2
irf2bp2b	2.33E-05	0.566443572	0.473	0.294	0.556234	2
chmp4ba	2.53E-05	0.478556036	0.527	0.331	0.6038054	2
slc35e1	2.56E-05	0.493070197	0.324	0.15	0.610618	2
tnrc6c1	2.59E-05	0.685739613	0.378	0.183	0.617022	2
hnrnpua	2.62E-05	0.581728646	0.568	0.383	0.6243707	2
laptm4a	2.65E-05	0.476077467	0.419	0.233	0.6316563	2
sh3bgrl	2.71E-05	0.467555279	0.405	0.211	0.6470109	2
spry4	2.77E-05	0.842902457	0.486	0.298	0.6615143	2
h1fx	2.78E-05	1.795120068	0.405	0.222	0.6632153	2
tp53inp1	2.79E-05	0.505149107	0.365	0.174	0.6649579	2
grinab	2.86E-05	0.510435127	0.405	0.222	0.6824408	2
tial1	2.90E-05	0.391306557	0.378	0.191	0.6916855	2
tmem181	2.91E-05	0.307767845	0.257	0.1	0.6946465	2
abca3b	2.94E-05	0.486067451	0.297	0.122	0.7002004	2
amd1	2.96E-05	0.660852549	0.486	0.306	0.7048468	2
slc30a5	2.97E-05	0.318033024	0.27	0.107	0.70786	2
si:dkey-276j7.1	2.98E-05	0.635356928	0.338	0.163	0.7116974	2
dtnba	3.05E-05	0.346142025	0.27	0.109	0.7273121	2
cd81a	3.10E-05	0.618174183	0.514	0.319	0.7395928	2
ube2e3	3.20E-05	0.641910009	0.514	0.343	0.7636001	2
macf1a	3.27E-05	0.608119807	0.527	0.331	0.7793542	2
si:ch211-137i24.10	3.32E-05	1.396503688	0.797	0.774	0.7911994	2
csnk1a1	3.42E-05	0.810790289	0.662	0.502	0.8147581	2
abca1a	3.42E-05	0.42958764	0.297	0.13	0.816527	2
ncoa3	3.48E-05	0.310938936	0.297	0.128	0.8289676	2
ppp2r5eb	3.53E-05	0.540038296	0.419	0.228	0.8418449	2
glipr2l	3.61E-05	0.507271673	0.27	0.113	0.8618841	2
cebpd	3.62E-05	0.972652436	0.595	0.469	0.8624089	2
tmem184a	3.66E-05	0.729700386	0.392	0.215	0.8718184	2
meaf6	3.66E-05	0.423503654	0.5	0.307	0.8734175	2
ss18	3.69E-05	0.320455457	0.432	0.237	0.8798336	2
dyrk1ab	3.74E-05	0.385075666	0.257	0.1	0.8918708	2
ing3	3.76E-05	0.381059411	0.392	0.196	0.8974302	2
ywhae1	3.91E-05	0.408437705	0.689	0.515	0.93248	2
mlf2	3.94E-05	0.493004117	0.378	0.202	0.9395582	2
hmga2	4.08E-05	0.638065182	0.284	0.122	0.9722384	2
trim8b	4.08E-05	0.538510731	0.324	0.152	0.9729595	2
ywhag2	4.13E-05	0.840219204	0.257	0.104	0.9838182	2
rtca1	4.15E-05	0.386515045	0.405	0.209	0.9885212	2
hsp90ab1	4.17E-05	0.342759257	0.986	0.98	0.993665	2
fundc1	4.22E-05	0.390171506	0.284	0.124	1	2
hax1	4.35E-05	0.611467134	0.459	0.281	1	2
ctdspl3	4.35E-05	0.5409546	0.257	0.102	1	2
lsm14ab	4.36E-05	0.42708405	0.541	0.361	1	2
alcamb	4.47E-05	0.707319942	0.351	0.174	1	2
adgrg1.1	4.48E-05	0.32911596	0.392	0.202	1	2
ubtf	4.54E-05	0.474589423	0.5	0.311	1	2
ptmab	4.59E-05	0.513586544	0.973	0.894	1	2
pbx4	4.69E-05	0.528605082	0.473	0.302	1	2
tcf3b	4.70E-05	0.604881938	0.514	0.324	1	2
daam1a	4.70E-05	0.681198341	0.446	0.27	1	2
rab14	4.77E-05	0.398699035	0.514	0.319	1	2

tollip	4.78E-05	0.404147056	0.365	0.178	1	2
camk2g2	4.80E-05	0.437358068	0.311	0.146	1	2
ywhah	4.85E-05	0.758741775	0.635	0.491	1	2
dip2ba	5.16E-05	0.435221136	0.351	0.17	1	2
elf3	5.21E-05	1.130212354	0.676	0.552	1	2
h3f3b.1.1	5.27E-05	0.543034761	0.649	0.526	1	2
leng8	5.28E-05	0.350208144	0.405	0.224	1	2
fxr2	5.36E-05	0.33165763	0.365	0.181	1	2
zbtb17	5.39E-05	0.254315473	0.284	0.117	1	2
nrbf2b	5.41E-05	0.40502256	0.324	0.152	1	2
stau1	5.48E-05	0.342698038	0.365	0.185	1	2
n4bp1	5.55E-05	0.652815524	0.284	0.12	1	2
letm2	5.71E-05	0.561458443	0.527	0.359	1	2
eif1b	5.77E-05	0.437959389	0.824	0.763	1	2
sumo1	5.79E-05	0.342297169	0.419	0.241	1	2
rbm25b	5.90E-05	0.590550223	0.595	0.419	1	2
jund	5.91E-05	0.745631032	0.716	0.578	1	2
ube2e2	5.96E-05	0.397945482	0.324	0.154	1	2
kdm7ab	6.03E-05	0.285524118	0.338	0.161	1	2
rbm14b	6.06E-05	0.444143252	0.365	0.193	1	2
sox11a	6.10E-05	0.599749137	0.432	0.257	1	2
crb3b	6.11E-05	0.554978556	0.257	0.109	1	2
hyou1	6.14E-05	0.967696445	0.77	0.683	1	2
si:ch73-269m14.2	6.22E-05	0.53087524	0.459	0.265	1	2
znf318	6.36E-05	0.480760474	0.392	0.207	1	2
cdkn1ba	6.52E-05	0.515818702	0.27	0.113	1	2
arpp19a	6.61E-05	0.323153993	0.351	0.172	1	2
sesn2	6.71E-05	0.441937289	0.432	0.254	1	2
tmed10	6.88E-05	0.670001452	0.662	0.526	1	2
hivep1	7.36E-05	0.776177737	0.459	0.28	1	2
csnk1da	7.38E-05	0.395898677	0.514	0.33	1	2
fbxw11b	7.47E-05	0.695169652	0.514	0.346	1	2
susd6	7.58E-05	0.593693907	0.419	0.239	1	2
ctnnbip1	7.65E-05	0.62072859	0.257	0.107	1	2
rsrc2	7.65E-05	0.505688601	0.446	0.27	1	2
golt1bb	7.71E-05	0.321682142	0.378	0.194	1	2
jak1	7.76E-05	0.553947692	0.568	0.411	1	2
lpin2	7.82E-05	0.534248568	0.297	0.133	1	2
ets2	7.84E-05	0.792513962	0.703	0.55	1	2
si:dkeyp-44a8.2	8.00E-05	0.334994769	0.338	0.17	1	2
rhbdf1a	8.12E-05	0.577470416	0.392	0.222	1	2
ucp2	8.17E-05	0.650815789	0.568	0.404	1	2
uck2a	8.49E-05	0.272344439	0.284	0.126	1	2
pfn1	8.51E-05	0.490746057	0.905	0.8	1	2
kif1b	8.56E-05	0.372882933	0.284	0.12	1	2
si:dkey-253i9.4	8.93E-05	0.394419814	0.297	0.135	1	2
si:ch211-196l7.4	8.98E-05	0.903187162	0.554	0.396	1	2
stau2	9.18E-05	0.555258307	0.5	0.335	1	2
syncrip	9.19E-05	0.578381838	0.77	0.646	1	2
hmgb1b1	9.57E-05	0.453995203	0.446	0.252	1	2
calua	9.58E-05	0.451756672	0.378	0.202	1	2
il6st	9.61E-05	0.607174972	0.446	0.274	1	2
dnase1l4.1	9.91E-05	0.948285899	0.257	0.106	1	2
macc1	0.0001025	0.46933979	0.338	0.167	1	2

selenow1	0.0001065	0.40498301	0.77	0.656	1	2
bsdc1	0.0001071	0.384983948	0.297	0.139	1	2
pdzd11	0.0001073	0.436876245	0.297	0.135	1	2
zgc:162698	0.0001097	0.422786834	0.311	0.148	1	2
wasla	0.0001098	0.653337923	0.27	0.119	1	2
nucb2b	0.0001116	1.083403901	0.432	0.276	1	2
anp32e	0.000113	0.973910775	0.514	0.35	1	2
SERP1	0.0001168	0.448362703	0.622	0.452	1	2
bcl2l10	0.0001237	0.377866259	0.351	0.185	1	2
crebrf	0.0001252	0.355316263	0.284	0.122	1	2
hcfc1b	0.0001298	0.376945868	0.419	0.248	1	2
hcfc1a	0.000132	0.506067317	0.419	0.244	1	2
eif3ea1	0.0001325	0.394197475	0.716	0.594	1	2
ubald1a	0.0001349	0.273916246	0.338	0.169	1	2
gnai2a	0.0001354	0.362702147	0.541	0.367	1	2
top2b	0.0001358	0.510712235	0.446	0.285	1	2
pdia6l	0.0001363	0.990250471	0.73	0.572	1	2
rasal2	0.0001373	0.901241897	0.297	0.143	1	2
trib3	0.0001384	0.400103529	0.676	0.539	1	2
psma1l	0.000147	0.546800658	0.676	0.531	1	2
dusp2	0.0001486	1.102428102	0.324	0.17	1	2
med12	0.0001498	0.572248463	0.351	0.187	1	2
cep350	0.0001508	0.662348346	0.473	0.294	1	2
fosl1a	0.0001568	0.705935566	0.703	0.598	1	2
ap1s1	0.0001587	0.430884477	0.419	0.265	1	2
tnfb	0.0001588	1.610480319	0.297	0.148	1	2
snx12	0.0001598	0.255262975	0.459	0.281	1	2
kmt5b	0.0001605	0.361601791	0.324	0.157	1	2
dnajb11	0.000162	0.511923738	0.568	0.383	1	2
per1b	0.0001632	0.568613264	0.27	0.126	1	2
arpc4l	0.0001633	0.368883148	0.73	0.557	1	2
flna	0.0001648	0.950938676	0.324	0.174	1	2
rnf25	0.0001683	0.479857623	0.324	0.159	1	2
ywhaqa	0.0001707	0.802700814	0.716	0.557	1	2
ppp3r1a	0.0001748	0.37196886	0.324	0.167	1	2
eef1g1	0.0001758	0.369182039	0.878	0.872	1	2
smurf2	0.00018	0.351208316	0.257	0.117	1	2
myl6	0.0001811	0.338022028	0.338	0.176	1	2
ist1	0.0001844	0.457487752	0.554	0.389	1	2
aip	0.0001846	0.500376841	0.405	0.23	1	2
oxsr1a	0.0001859	0.312702959	0.446	0.259	1	2
sumo2b	0.0001887	0.425481118	0.581	0.433	1	2
fryl	0.0001891	0.261667467	0.311	0.152	1	2
rnf146	0.0001894	0.450404074	0.338	0.174	1	2
tubb4b	0.0001911	0.720160413	0.824	0.719	1	2
vps28	0.0001915	0.370412285	0.446	0.285	1	2
cirbpa1	0.0001933	0.497229204	0.824	0.746	1	2
crebbpb	0.0001933	0.460432693	0.514	0.352	1	2
erf	0.0001938	0.263770704	0.284	0.128	1	2
bud31	0.0001953	0.342486198	0.716	0.556	1	2
emc7	0.0001966	0.441329754	0.527	0.354	1	2
hnrnpa0a	0.0002003	0.490397306	0.581	0.426	1	2
capza1b	0.0002025	0.69514615	0.676	0.544	1	2
adam10a	0.0002041	0.577271911	0.541	0.378	1	2

gch1	0.0002042	0.668943323	0.257	0.115	1	2
tcf7l2	0.0002044	0.924515173	0.473	0.307	1	2
otub1a	0.0002046	0.40268586	0.338	0.172	1	2
qkia	0.0002069	0.758153652	0.378	0.224	1	2
dazap2	0.0002071	0.26579578	0.486	0.307	1	2
junba	0.0002079	0.637741896	0.784	0.715	1	2
clint1a	0.0002108	0.666581466	0.568	0.398	1	2
arid1ab	0.0002123	0.380137694	0.392	0.222	1	2
gars1	0.0002144	0.629174031	0.608	0.463	1	2
edem1	0.0002167	0.50546533	0.432	0.261	1	2
slc35b11	0.0002183	0.432814728	0.459	0.294	1	2
si:dkeyp-27c8.2	0.0002243	0.575115223	0.378	0.209	1	2
zgc:100918	0.0002274	0.306579574	0.459	0.289	1	2
perp1	0.0002288	0.707667793	0.649	0.506	1	2
prkar2aa1	0.0002293	0.509888123	0.568	0.415	1	2
hagh	0.0002314	0.417847811	0.459	0.294	1	2
hnrnpaba	0.000232	0.405631878	0.811	0.715	1	2
ppp2cb	0.00024	0.31610311	0.662	0.535	1	2
ctnnb1	0.0002441	0.484523289	0.595	0.463	1	2
rab1ba	0.0002451	0.379854049	0.514	0.354	1	2
si:ch211-195b15.8	0.0002515	0.403987402	0.311	0.15	1	2
acbd7	0.0002601	0.566458167	0.311	0.161	1	2
spen	0.000261	0.72333797	0.432	0.27	1	2
maff	0.000262	0.722446056	0.608	0.507	1	2
ube2v1	0.0002638	0.334220241	0.662	0.511	1	2
BX936337.1	0.000265	0.593859921	0.608	0.461	1	2
znf865	0.0002677	0.651767767	0.378	0.233	1	2
DYRK3	0.0002685	0.38608926	0.257	0.117	1	2
hivep2a	0.0002698	0.541527921	0.351	0.196	1	2
prr12b	0.0002711	0.360427077	0.257	0.109	1	2
si:dkey-250d21.1	0.0002727	0.437777904	0.311	0.159	1	2
kctd10	0.0002732	0.35967328	0.338	0.18	1	2
gpr108	0.0002767	0.489836443	0.338	0.185	1	2
cpda	0.0002778	0.363743635	0.27	0.124	1	2
gabarapa	0.0002962	0.359774288	0.5	0.333	1	2
kdm1a	0.0002968	0.312846089	0.554	0.374	1	2
si:ch211-262e15.1	0.0003008	0.566527926	0.432	0.27	1	2
nap1l4b	0.0003037	0.534056051	0.311	0.165	1	2
smc1al1	0.0003105	0.483660815	0.541	0.37	1	2
vdac1l	0.0003131	0.308187165	0.527	0.356	1	2
prkcsh	0.0003148	0.5317266	0.608	0.454	1	2
tardbp	0.0003156	0.538455244	0.554	0.411	1	2
eprs	0.0003166	0.532437527	0.486	0.313	1	2
nfyba	0.0003281	0.421929719	0.297	0.15	1	2
ptbp3	0.0003464	0.51684592	0.311	0.163	1	2
pcdh1b	0.0003483	0.500582827	0.405	0.233	1	2
igfbp1a	0.0003496	1.044961805	0.608	0.465	1	2
zfhx3	0.0003528	0.90830698	0.432	0.254	1	2
sf1	0.0003535	0.409528653	0.635	0.519	1	2
fam199x	0.0003537	0.288857251	0.338	0.176	1	2
fam107b	0.0003543	0.405851392	0.473	0.354	1	2
cbx7a	0.00036	1.027177311	0.311	0.157	1	2
kdm5ba	0.0003642	0.396806326	0.284	0.141	1	2
clk4a	0.0003712	0.313988442	0.5	0.32	1	2

ctif	0.0003871	0.361524205	0.27	0.126	1	2
erlec1	0.0003934	0.806019792	0.378	0.237	1	2
ythdf1	0.0003994	0.360010558	0.338	0.183	1	2
zgc:153867	0.0004039	0.513418505	0.811	0.757	1	2
rnf40	0.0004069	0.483902292	0.378	0.228	1	2
calm2b1	0.0004157	0.524093307	0.757	0.607	1	2
drap1	0.0004159	0.405021315	0.5	0.344	1	2
ubl3b	0.000438	0.447884434	0.297	0.156	1	2
nsd1a	0.0004414	0.336826354	0.338	0.185	1	2
ctbp1	0.0004551	0.426077579	0.432	0.272	1	2
lpp	0.000464	0.267470931	0.324	0.17	1	2
cxcl18b	0.0004738	0.9488239	0.405	0.252	1	2
uba1l	0.0004755	0.291481914	0.622	0.444	1	2
pdgfba	0.0004763	1.013263476	0.365	0.222	1	2
EPB41L2	0.0004764	0.289327681	0.338	0.183	1	2
furina	0.0004771	0.518108808	0.365	0.213	1	2
nfkbie	0.0004813	0.410525167	0.284	0.144	1	2
dusp6	0.0004837	0.5659858	0.378	0.222	1	2
tfg	0.0004997	0.317736946	0.581	0.415	1	2
ap3d1	0.0005064	0.427710864	0.392	0.235	1	2
tet3	0.0005066	0.485246767	0.5	0.343	1	2
FAM83G	0.0005128	0.265758271	0.297	0.15	1	2
rab5aa	0.0005172	0.315903128	0.5	0.356	1	2
epc1b	0.0005174	0.432723956	0.257	0.119	1	2
nucb2a	0.0005208	0.369656852	0.392	0.228	1	2
uhrf1bp1l	0.0005213	0.530148527	0.311	0.163	1	2
rtf2	0.0005342	0.504102217	0.351	0.202	1	2
tpt1l	0.0005345	0.437894966	0.892	0.854	1	2
si:dkey-177p2.6	0.0005346	0.569018675	0.473	0.339	1	2
pkp2	0.0005369	0.406069413	0.311	0.159	1	2
uso1l	0.000541	0.585498798	0.459	0.304	1	2
vmp1l	0.0005457	0.772214294	0.419	0.281	1	2
arid4a	0.0005501	0.474291462	0.527	0.359	1	2
st3gal7	0.0005653	0.49316364	0.568	0.406	1	2
pbrm1l	0.000569	0.328586613	0.351	0.198	1	2
hbegfa	0.0005713	1.051272646	0.784	0.796	1	2
anxa11a	0.0005718	0.360568246	0.554	0.363	1	2
cap1	0.0005804	0.792288531	0.419	0.263	1	2
cnot3a	0.0005813	0.35464531	0.338	0.176	1	2
taok2a	0.0005843	0.483798649	0.27	0.131	1	2
si:dkey-112a7.4	0.0005973	0.514783741	0.5	0.376	1	2
ube2d2	0.0006034	0.309823464	0.662	0.517	1	2
chac1	0.000604	0.560295482	0.838	0.783	1	2
cib1	0.000607	0.809363609	0.527	0.396	1	2
rps6ka1	0.000609	0.304795158	0.338	0.176	1	2
pde7a	0.0006177	0.470564741	0.284	0.139	1	2
kif5ba	0.0006177	0.3922444	0.473	0.311	1	2
cgnb	0.0006211	0.435976071	0.311	0.165	1	2
atl3	0.0006274	0.820242275	0.378	0.213	1	2
slc43a1a	0.0006283	0.40672596	0.297	0.157	1	2
rsf1b.1	0.0006392	0.297871849	0.311	0.159	1	2
magi1b	0.0006631	0.275186264	0.311	0.161	1	2
snx1a	0.0006693	0.376062134	0.311	0.163	1	2
pnisr	0.0006743	0.588790052	0.459	0.304	1	2

fgfr2	0.0006859	0.502658457	0.405	0.25	1	2
mta2	0.0007	0.302093319	0.392	0.243	1	2
eif5b	0.0007019	0.766164218	0.703	0.615	1	2
mycb	0.0007035	0.362661145	0.324	0.178	1	2
srebf2	0.0007152	0.59956077	0.432	0.294	1	2
rab5c	0.000717	0.280330025	0.514	0.361	1	2
KIF2A	0.0007325	0.368328212	0.351	0.198	1	2
cct6a2	0.0007328	0.480579972	0.662	0.533	1	2
pcbd1	0.0007405	0.419102906	0.446	0.306	1	2
pak2b	0.0007491	0.38115711	0.432	0.272	1	2
vdac31	0.000751	0.535243802	0.554	0.409	1	2
celsr1a	0.0007518	0.684045409	0.419	0.27	1	2
smg7	0.0007553	0.341039524	0.432	0.276	1	2
dnajc5ab	0.0007625	0.379335674	0.324	0.176	1	2
wasf2	0.0007677	0.496916079	0.432	0.289	1	2
tet2	0.0008026	0.349136123	0.284	0.139	1	2
zfand3	0.0008054	0.330884827	0.432	0.272	1	2
rsl24d1	0.000808	0.326316441	0.757	0.659	1	2
eif4g2b	0.0008101	0.584768903	0.581	0.457	1	2
wdr26b	0.0008121	0.326847272	0.297	0.154	1	2
nars	0.0008157	0.504894795	0.622	0.531	1	2
hgs	0.0008482	0.300854893	0.284	0.148	1	2
bnip3lb	0.000867	0.506458781	0.257	0.119	1	2
sec24b	0.0008708	0.331287098	0.297	0.157	1	2
enc1	0.0008785	0.359553055	0.324	0.189	1	2
hnrnpub	0.0008823	0.542676491	0.689	0.602	1	2
per3	0.000886	0.460941603	0.432	0.276	1	2
mbnl2	0.0009055	0.346917138	0.392	0.235	1	2
rabgap1l	0.0009079	0.407806713	0.257	0.122	1	2
ppp1r12a	0.0009346	0.496123383	0.473	0.335	1	2
tln1	0.0009406	0.636854035	0.473	0.337	1	2
srpr	0.0009439	0.671365962	0.608	0.467	1	2
lims1	0.0009939	0.318297509	0.432	0.28	1	2
arrb2b	0.0009963	0.280339089	0.338	0.193	1	2
ralab	0.0010016	0.307274304	0.378	0.228	1	2
chmp1b	0.0010149	0.443298386	0.554	0.422	1	2
prkci	0.0010248	0.354140145	0.378	0.22	1	2
chmp5b	0.0010311	0.697184244	0.662	0.546	1	2
hspa81	0.0010364	0.319804483	0.986	0.931	1	2
gadd45ga	0.0010474	0.498839612	0.27	0.135	1	2
sptan1	0.0010538	0.461182813	0.527	0.407	1	2
brd1b	0.0010584	0.398818093	0.432	0.302	1	2
rbms1a	0.0010627	0.480389716	0.324	0.183	1	2
fubp1	0.0010629	0.445969026	0.554	0.426	1	2
hip1rb	0.0010632	0.397504307	0.297	0.157	1	2
sart1	0.0010643	0.490780519	0.527	0.374	1	2
plecb	0.0010721	1.19152699	0.581	0.5	1	2
srgap3	0.001095	0.794367265	0.257	0.128	1	2
slc23a2	0.0011339	0.364535575	0.27	0.139	1	2
jupa	0.0011353	0.463251636	0.649	0.55	1	2
atp2c1	0.0011412	0.347751617	0.257	0.12	1	2
cyth1a.1	0.0011544	0.641729611	0.419	0.281	1	2
cst3	0.0011577	0.459999713	0.432	0.272	1	2
nfyc	0.0011585	0.608776176	0.419	0.267	1	2

syvn1	0.001197	0.419717008	0.405	0.252	1	2
ankrd11	0.0012024	0.531373769	0.622	0.535	1	2
cnppd1	0.0012147	0.289729304	0.311	0.178	1	2
hmga1a1	0.0012183	0.616858746	0.919	0.852	1	2
atp2a2a.11	0.0012238	0.421987198	0.622	0.47	1	2
trappc3	0.0012469	0.400761913	0.297	0.165	1	2
CU462878.11	0.0012663	0.295659585	0.405	0.252	1	2
psat1	0.0012679	0.593866014	0.432	0.293	1	2
sf3b1	0.0012705	0.480540073	0.5	0.369	1	2
tpm3	0.0012936	0.569885195	0.811	0.759	1	2
ppiaa1	0.0013041	0.281133303	0.932	0.893	1	2
nipblb	0.0013055	0.327037322	0.432	0.289	1	2
bptf	0.0013206	0.484410178	0.365	0.228	1	2
prkcdb	0.0013302	0.776115849	0.432	0.309	1	2
tdh	0.00134	0.443940798	0.486	0.341	1	2
shc1	0.0013468	0.484008634	0.297	0.17	1	2
otud5b	0.0013499	0.450644728	0.419	0.276	1	2
aars	0.0013571	0.550194806	0.527	0.428	1	2
syne2b	0.001362	0.387233375	0.27	0.143	1	2
rhocb	0.0013743	0.323881378	0.473	0.315	1	2
sptbn2	0.0013894	0.367386597	0.257	0.133	1	2
yipf4	0.00139	0.48255792	0.378	0.233	1	2
ppp1caa	0.0014135	0.463418853	0.595	0.476	1	2
atp6v1e1b	0.0014337	0.680673559	0.459	0.328	1	2
ctdsp2	0.0014383	0.284975251	0.419	0.272	1	2
smarcd1	0.0014546	0.403043221	0.486	0.354	1	2
zgc:136930	0.0014638	0.655116429	0.541	0.381	1	2
paip2b	0.0014822	0.517307985	0.514	0.404	1	2
eif4a1b1	0.0015231	0.514377966	0.676	0.519	1	2
hs6st2	0.0015291	0.333497222	0.27	0.137	1	2
nckap1	0.0015373	0.573136074	0.446	0.296	1	2
tgif11	0.0015444	0.459363036	0.378	0.239	1	2
cldn12	0.0015466	0.36192164	0.257	0.131	1	2
tm9sf1	0.0015499	0.314495887	0.257	0.133	1	2
gtpbp41	0.0015729	0.425455718	0.595	0.467	1	2
wap1b	0.0015746	0.31933593	0.324	0.181	1	2
ap2s1	0.0015842	0.366020046	0.432	0.294	1	2
pdzk1ip11	0.0015843	0.526371661	0.324	0.18	1	2
rbms2a	0.001591	0.275526761	0.284	0.15	1	2
hm13	0.0016036	0.301114313	0.446	0.287	1	2
rbm39a	0.0016738	0.431650046	0.568	0.463	1	2
eno1a	0.0016743	0.331808342	0.392	0.252	1	2
smarcc1a	0.0017167	0.294931233	0.446	0.317	1	2
ostf1	0.0017176	0.416020181	0.365	0.222	1	2
khdrbs1a1	0.0017236	0.283452011	0.851	0.739	1	2
ipo71	0.0017269	0.317717694	0.608	0.452	1	2
mycbp1	0.0017406	0.484965928	0.432	0.287	1	2
dedd1	0.0017431	0.573878115	0.635	0.507	1	2
mst1	0.0017496	0.751144096	0.284	0.161	1	2
erp44	0.0017727	0.434137238	0.459	0.328	1	2
basp1	0.0017753	0.454046985	0.446	0.291	1	2
syngr2a	0.0017957	0.386830548	0.338	0.2	1	2
cxcl8a	0.0018145	0.523181722	0.473	0.317	1	2
CR589944.1	0.0018618	0.343561849	0.27	0.141	1	2

stt3b1	0.0019232	0.289322993	0.486	0.333	1	2
phc2a	0.0019465	0.469788545	0.297	0.169	1	2
tm9sf3	0.0019912	0.384464551	0.486	0.337	1	2
ddx39aa1	0.0019995	0.345681523	0.554	0.426	1	2
yipf5	0.0020219	0.300588191	0.27	0.143	1	2
psmd11b	0.0020338	0.294070542	0.419	0.276	1	2
pcbp2	0.0020601	0.424044435	0.486	0.385	1	2
cdc42l	0.0020648	0.388553681	0.689	0.578	1	2
purba	0.0021009	0.474340223	0.392	0.269	1	2
scfd1	0.0021201	0.322163271	0.338	0.194	1	2
rnpc3	0.0021292	0.403221472	0.27	0.143	1	2
ccnl1a	0.0021413	0.613604761	0.446	0.333	1	2
sec62	0.0021878	0.561062168	0.527	0.363	1	2
uck2b	0.0022151	0.303924261	0.365	0.224	1	2
mapre1b	0.0022264	0.333932141	0.419	0.287	1	2
snw1	0.0022429	0.362420055	0.419	0.298	1	2
ckbb	0.0022573	0.734367637	0.419	0.274	1	2
FP017217.1	0.0022915	0.464417724	0.459	0.304	1	2
ier2a	0.0023028	0.490165502	0.662	0.572	1	2
dab2ipb	0.0023092	0.455837323	0.351	0.215	1	2
psip1b	0.0023335	0.379656946	0.459	0.333	1	2
pdxdc1	0.0023728	0.712719559	0.324	0.196	1	2
ccnd1	0.0023914	0.39017815	0.297	0.167	1	2
tuba1a	0.002399	1.312534596	0.27	0.146	1	2
cry1aa	0.0023991	0.312636158	0.338	0.2	1	2
ppp6r3	0.0024015	0.278152933	0.459	0.319	1	2
afdna	0.0024194	0.797709999	0.514	0.387	1	2
copb2	0.0024443	0.361876461	0.527	0.372	1	2
pcf11	0.0024928	0.340011563	0.608	0.483	1	2
yars	0.0024988	0.592886324	0.432	0.32	1	2
ptp4a2b	0.0025173	0.377419821	0.649	0.511	1	2
optn	0.0025416	0.445870149	0.27	0.146	1	2
ddost1	0.0025566	0.593316849	0.635	0.496	1	2
elf1	0.0026154	0.470936431	0.527	0.419	1	2
ccnt1	0.002618	0.512155499	0.297	0.169	1	2
copz2	0.0026921	0.556516863	0.338	0.2	1	2
eif3ba1	0.0027127	0.367397991	0.703	0.559	1	2
chmp2a	0.0027375	0.308457538	0.568	0.457	1	2
si:dkey-172h23.2	0.0027678	0.267619889	0.351	0.215	1	2
rack1l	0.0027993	0.261880494	0.959	0.896	1	2
rbm5	0.0028116	0.459870059	0.351	0.224	1	2
her9	0.0028137	0.627557007	0.527	0.357	1	2
llgl2	0.0028359	0.573032521	0.432	0.337	1	2
srrm2	0.0030462	0.480465382	0.568	0.433	1	2
gpatch8	0.0031	0.621363746	0.405	0.285	1	2
eif3m1	0.0031922	0.341806756	0.676	0.583	1	2
wnk1b	0.0031965	0.260071612	0.27	0.148	1	2
cnot1	0.0032599	0.366724095	0.473	0.335	1	2
yy1a	0.0032627	0.479871435	0.432	0.307	1	2
ube2d2l	0.003286	0.271937022	0.311	0.187	1	2
sept7a1	0.0033981	0.382986718	0.446	0.333	1	2
elmsan1b	0.0034281	0.552263204	0.486	0.361	1	2
pafah1b1a	0.0034344	0.303533851	0.419	0.281	1	2
znf609b	0.0035278	0.635991531	0.392	0.28	1	2

tp53	0.0035506	0.320574551	0.405	0.283	1	2
ypel5	0.0035876	0.38425556	0.27	0.148	1	2
zzz3	0.0036002	0.363440195	0.27	0.152	1	2
kdm3b	0.0036437	0.334197796	0.257	0.141	1	2
kpna4	0.0036473	0.353680429	0.392	0.263	1	2
jun	0.0037287	0.279532855	0.878	0.813	1	2
mark2b	0.0037764	0.391257963	0.5	0.352	1	2
luc7l	0.0038182	0.393225191	0.514	0.407	1	2
tmem115	0.0038392	0.310282477	0.405	0.272	1	2
galnt2	0.0038566	0.601521265	0.27	0.152	1	2
ppm1k	0.0038712	0.298237377	0.297	0.17	1	2
clasp2	0.0038872	0.35036242	0.324	0.193	1	2
fat1a	0.0039216	0.594408474	0.473	0.344	1	2
cct82	0.0039264	0.255282625	0.581	0.461	1	2
zgc:56525	0.0039637	0.250924673	0.297	0.176	1	2
trioa	0.0040365	0.36493496	0.297	0.172	1	2
hif1ab	0.0040461	0.360913143	0.527	0.394	1	2
mbd2	0.0040526	0.282352853	0.432	0.309	1	2
fam91a1	0.0040805	0.473546724	0.257	0.139	1	2
prrc2c	0.0041275	0.340287567	0.405	0.261	1	2
waca	0.0041354	0.363581447	0.365	0.235	1	2
cebpb	0.0042045	0.548281019	0.77	0.757	1	2
ocrl	0.0042278	0.361392198	0.338	0.207	1	2
rpn12	0.0042504	0.275026207	0.595	0.446	1	2
gnaia	0.0043271	0.277482936	0.378	0.252	1	2
trim3b	0.004431	0.306430359	0.284	0.167	1	2
foxo4	0.0044658	0.406647149	0.378	0.248	1	2
mfn2	0.0045729	0.265117961	0.297	0.174	1	2
si:ch1073-392o20.2	0.0046239	0.288812389	0.378	0.256	1	2
chd9	0.0046684	0.793645459	0.459	0.352	1	2
arpc3	0.004669	0.455489776	0.649	0.554	1	2
tspan15	0.0046889	0.390571472	0.284	0.169	1	2
copa	0.004784	0.399631509	0.473	0.354	1	2
h2afx1	0.004904	0.455624594	0.676	0.607	1	2
ralgapa1	0.0049727	0.347539711	0.324	0.194	1	2
papola	0.0050255	0.403065851	0.5	0.37	1	2
hnrnpk	0.0051084	0.440508866	0.473	0.341	1	2
csnk1g2b	0.0051964	0.512124172	0.473	0.339	1	2
ccdc187	0.0052849	0.359755501	0.284	0.163	1	2
gtf2a1	0.0053945	0.324322056	0.284	0.165	1	2
txndc91	0.0055083	0.292813568	0.446	0.32	1	2
cyp3c1	0.0055386	0.372952848	0.27	0.154	1	2
atp2b1a	0.0057435	0.803110132	0.649	0.528	1	2
mgaa	0.0057528	0.42270359	0.257	0.143	1	2
si:ch73-238c9.1	0.0057581	0.295841328	0.284	0.169	1	2
si:ch211-11k18.4	0.0058249	0.286074328	0.27	0.163	1	2
tpm4a	0.0059461	1.175072577	0.405	0.311	1	2
si:dkey-222f8.3	0.0060323	0.292698083	0.27	0.148	1	2
irf2	0.0060595	0.290870751	0.338	0.213	1	2
pcyt2	0.0061338	0.367635834	0.473	0.372	1	2
rab35b	0.0061464	0.259381819	0.419	0.293	1	2
bloc1s6	0.0061783	0.264246668	0.297	0.178	1	2
tp53bp2a	0.0061816	0.258053125	0.27	0.15	1	2
aldoaa1	0.0062039	0.369789664	0.608	0.531	1	2

tnrc6a	0.0062064	0.599488519	0.473	0.359	1	2
ccnl1b	0.006252	0.262156274	0.514	0.393	1	2
atp6v0a1a	0.0062554	0.442338843	0.324	0.204	1	2
kmt2ba	0.0062747	0.28572425	0.27	0.161	1	2
celf1	0.0063445	0.527962735	0.392	0.274	1	2
ankrd54	0.0063656	0.492193122	0.297	0.185	1	2
cand1	0.006457	0.309832811	0.338	0.213	1	2
arcn1a	0.0064835	0.369360657	0.351	0.233	1	2
zgc:110425	0.00651	0.72577203	0.378	0.257	1	2
setd2	0.0065208	0.387944297	0.257	0.146	1	2
tardbpl	0.0065701	0.548096191	0.541	0.461	1	2
fam32a1	0.0066369	0.377942746	0.608	0.5	1	2
epc2	0.0066505	0.279247873	0.27	0.159	1	2
kif13ba	0.006663	0.596762919	0.405	0.289	1	2
creb1a	0.0066754	0.394785058	0.365	0.244	1	2
cldnb	0.0068658	0.666469523	0.811	0.774	1	2
tsc22d3l	0.0069033	0.583185408	0.446	0.343	1	2
sdf2l1	0.0069124	0.501450442	0.459	0.374	1	2
si:dkey-42i9.6	0.0069153	0.283873306	0.27	0.156	1	2
rock2a	0.007096	0.500047909	0.311	0.198	1	2
derl1	0.0071568	0.266905951	0.284	0.174	1	2
flii	0.0071638	0.339348175	0.311	0.202	1	2
sh3bp5la	0.0071807	0.410885206	0.324	0.217	1	2
sec61b	0.0072072	0.457108452	0.784	0.698	1	2
pik3r1	0.0072113	0.25097622	0.27	0.163	1	2
cltcb	0.007243	0.57963194	0.432	0.32	1	2
helz	0.0073877	0.387961084	0.338	0.217	1	2
pdap1b	0.0074166	0.50764066	0.514	0.413	1	2
ap1ar	0.0075095	0.425837993	0.405	0.287	1	2
taok1a	0.007536	0.396773627	0.27	0.163	1	2
hspa5	0.0075614	0.647400785	0.932	0.906	1	2
tmem33	0.0077279	0.395344733	0.351	0.241	1	2
kmt2ca	0.0077505	0.4912031	0.486	0.376	1	2
manf	0.0078002	0.361576741	0.703	0.609	1	2
selenos	0.0078834	0.412540209	0.378	0.263	1	2
kmt2e	0.0079139	0.46204137	0.311	0.196	1	2
pdia3l	0.0079475	0.460697044	0.77	0.696	1	2
sec31a	0.0081459	0.556191424	0.351	0.237	1	2
gigyf1b	0.0085304	0.284853438	0.405	0.287	1	2
chd6	0.0085772	0.361831724	0.311	0.193	1	2
cars	0.0086037	0.362475294	0.405	0.283	1	2
ctgfa	0.0087461	0.292241043	0.284	0.167	1	2
rab11ba	0.0087675	0.413771806	0.365	0.248	1	2
si:ch211-114c12.2	0.0090014	0.379542967	0.365	0.241	1	2
si:ch211-286b5.5	0.0090074	0.320270276	0.338	0.226	1	2
mych	0.00904	0.579269283	0.554	0.494	1	2
ganab	0.0091832	0.473817412	0.27	0.169	1	2
si:ch1073-174d20.2	0.0091932	0.305288379	0.27	0.163	1	2
ssr3	0.0093253	0.509076523	0.824	0.72	1	2
arhgap1	0.0093265	0.305847671	0.324	0.209	1	2
sf3b2	0.0093366	0.576001748	0.541	0.444	1	2
zgc:162730	0.0094292	0.552281114	0.784	0.789	1	2
ap2m1b	0.00949	0.392343203	0.595	0.498	1	2
ubap2a	0.0095675	0.458455735	0.446	0.339	1	2

yipf6	0.0097359	0.270601582	0.311	0.196	1	2
gadd45ab	0.0098259	0.375264821	0.378	0.269	1	2
arhgap5	0.0099704	0.286766976	0.703	0.619	1	2
nme2b.11	0.0099941	0.2525184	0.932	0.876	1	2
aqp8a.21	5.78E-22	1.003440138	0.892	0.307	1.38E-17	3
rbp2a2	1.11E-20	1.211620814	1	0.511	2.64E-16	3
mat2al1	1.92E-20	0.677226935	0.703	0.198	4.58E-16	3
fabp1b.11	5.74E-20	1.35129316	0.986	0.52	1.37E-15	3
ugt1ab1	9.54E-19	0.822051399	0.973	0.424	2.27E-14	3
cyp8b11	9.94E-19	0.465452272	0.743	0.202	2.37E-14	3
park71	1.68E-18	0.411252137	0.757	0.259	4.01E-14	3
sod21	1.83E-18	0.789966101	0.905	0.409	4.35E-14	3
apoc21	2.45E-18	0.774803402	0.986	0.463	5.84E-14	3
stoml3b1	6.30E-18	1.010716445	0.946	0.428	1.50E-13	3
gatm1	9.69E-18	0.573273111	0.811	0.269	2.31E-13	3
apoa4b.11	1.65E-17	0.968501576	0.959	0.431	3.93E-13	3
ssbp1	1.75E-17	0.501266692	0.784	0.287	4.16E-13	3
apoa1a1	2.13E-17	0.898019421	0.986	0.443	5.09E-13	3
mrps241	3.76E-17	0.533783606	0.919	0.407	8.97E-13	3
gcshb1	5.67E-17	0.875694625	0.946	0.443	1.35E-12	3
afp4.11	1.48E-16	0.936656587	0.905	0.394	3.53E-12	3
srd5a2a2	1.81E-16	0.389017692	0.73	0.224	4.31E-12	3
ada1	1.87E-16	0.386466747	0.784	0.25	4.46E-12	3
fdx11	1.95E-16	0.884617701	0.946	0.467	4.65E-12	3
pla2g12b1	2.70E-16	0.411874762	0.662	0.194	6.43E-12	3
si:ch211-117n7.71	3.11E-16	0.329496234	0.757	0.243	7.42E-12	3
gstt1a1	3.12E-16	0.944859402	0.973	0.559	7.43E-12	3
gstk11	4.73E-16	0.513651443	0.784	0.285	1.13E-11	3
ndufa41	5.23E-16	0.747919465	0.973	0.467	1.25E-11	3
msrb21	6.44E-16	0.441481207	0.77	0.256	1.53E-11	3
sult2st21	7.63E-16	0.727130658	0.986	0.419	1.82E-11	3
si:dkey-69o16.52	7.66E-16	0.424788675	0.689	0.235	1.83E-11	3
sult3st2	1.02E-15	0.338915065	0.622	0.189	2.43E-11	3
COX5B1	1.21E-15	0.725362369	1	0.804	2.88E-11	3
gpx4a1	1.49E-15	0.959196742	0.959	0.552	3.54E-11	3
mrpl91	1.63E-15	0.362593096	0.73	0.248	3.88E-11	3
rida1	1.88E-15	1.042120656	0.892	0.444	4.48E-11	3
ociad21	2.17E-15	0.791980009	0.946	0.47	5.18E-11	3
mbl22	2.46E-15	0.512880056	0.662	0.209	5.87E-11	3
prdx12	2.75E-15	0.438636025	0.932	0.448	6.57E-11	3
eif4e2rs1	2.83E-15	0.29640853	0.554	0.169	6.75E-11	3
cryl11	3.14E-15	0.444932383	0.716	0.261	7.48E-11	3
eci21	3.60E-15	0.453493806	0.824	0.337	8.59E-11	3
ndufab1a1	4.34E-15	0.635057037	0.946	0.493	1.04E-10	3
cox8b1	4.64E-15	0.880729276	0.986	0.578	1.11E-10	3
atp5l1	5.55E-15	0.681408538	1	0.822	1.32E-10	3
si:ch211-201h21.51	7.14E-15	0.381753001	0.824	0.296	1.70E-10	3
mttp1	7.55E-15	0.27796076	0.797	0.269	1.80E-10	3
ldha1	7.68E-15	0.675222519	0.919	0.452	1.83E-10	3
slc25a101	8.96E-15	0.279821786	0.595	0.174	2.14E-10	3
cox141	1.03E-14	0.692658042	0.865	0.437	2.45E-10	3
hsd11b1la2	1.03E-14	0.435401731	0.73	0.248	2.45E-10	3
ndufa81	1.14E-14	0.597873062	0.959	0.561	2.73E-10	3
zgc:112146	1.17E-14	0.562470239	0.568	0.17	2.78E-10	3

chia.21	1.38E-14	0.342875335	0.568	0.157	3.30E-10	3
mrpl321	1.56E-14	0.468583464	0.892	0.413	3.71E-10	3
ivns1abpa1	1.64E-14	0.607536103	0.797	0.346	3.91E-10	3
mrpl391	2.02E-14	0.45102715	0.797	0.317	4.82E-10	3
mgst3b2	2.13E-14	0.711472026	0.959	0.557	5.07E-10	3
zgc:926301	2.70E-14	0.444648074	0.865	0.374	6.45E-10	3
ndufb3	2.74E-14	0.678409942	0.973	0.604	6.54E-10	3
zgc:56231	2.83E-14	0.252069233	0.297	0.046	6.76E-10	3
mrpl181	2.93E-14	0.515630537	0.878	0.428	7.00E-10	3
chchd102	3.05E-14	0.649503941	0.932	0.485	7.28E-10	3
mrpl421	3.36E-14	0.45754656	0.716	0.3	8.01E-10	3
timm101	3.49E-14	0.368816037	0.851	0.354	8.33E-10	3
cbr1l2	4.13E-14	0.563746937	0.811	0.363	9.84E-10	3
ca4b1	4.20E-14	0.440273368	0.554	0.159	1.00E-09	3
fabp22	6.53E-14	0.990397958	1	0.757	1.56E-09	3
mrpl471	7.20E-14	0.411661019	0.797	0.333	1.72E-09	3
mrpl341	8.83E-14	0.431478738	0.838	0.374	2.11E-09	3
AL831745.11	9.51E-14	0.257523092	0.797	0.298	2.27E-09	3
gamt1	9.83E-14	0.763595868	0.959	0.487	2.34E-09	3
rrp7a	1.06E-13	0.291582725	0.649	0.23	2.53E-09	3
hint1	1.13E-13	0.555438977	0.946	0.541	2.69E-09	3
zgc:777391	1.38E-13	0.434933661	0.608	0.206	3.28E-09	3
suclg21	1.58E-13	0.359461613	0.811	0.32	3.78E-09	3
timm8a1	1.90E-13	0.442493382	0.824	0.383	4.54E-09	3
ace21	2.08E-13	0.282512673	0.716	0.235	4.96E-09	3
cox7a1	2.21E-13	0.423178984	0.649	0.233	5.26E-09	3
mrpl14	2.76E-13	0.478717325	0.851	0.404	6.57E-09	3
phb2b1	2.95E-13	0.563682473	0.905	0.45	7.03E-09	3
mrpl481	3.21E-13	0.507272707	0.824	0.359	7.66E-09	3
ndufa121	3.22E-13	0.659131471	0.959	0.594	7.67E-09	3
cd361	3.34E-13	0.395380515	0.662	0.226	7.96E-09	3
cldn15a1	3.51E-13	0.64715661	0.946	0.448	8.37E-09	3
si:ch211-161h7.81	3.60E-13	0.397286162	0.635	0.219	8.58E-09	3
lyrm7	4.04E-13	0.353702192	0.581	0.191	9.63E-09	3
pgrmc11	4.27E-13	0.562678496	0.959	0.531	1.02E-08	3
bckdhb1	4.62E-13	0.301668374	0.689	0.254	1.10E-08	3
mrpl161	4.64E-13	0.321948233	0.608	0.23	1.11E-08	3
ap1s3a1	4.75E-13	0.358397538	0.541	0.174	1.13E-08	3
tmem242	4.93E-13	0.365550627	0.568	0.189	1.18E-08	3
naa20	5.83E-13	0.45340112	0.649	0.25	1.39E-08	3
cox6a11	6.33E-13	0.580415011	1	0.826	1.51E-08	3
cers3a1	6.40E-13	0.402968034	0.689	0.254	1.53E-08	3
nop161	6.43E-13	0.312865399	0.797	0.346	1.53E-08	3
ndufa2	6.47E-13	0.527606662	0.959	0.541	1.54E-08	3
echs11	6.78E-13	0.4194083	0.811	0.343	1.62E-08	3
cox6b1	6.84E-13	0.646120222	0.973	0.73	1.63E-08	3
CU682777.21	7.45E-13	0.578184814	0.959	0.494	1.78E-08	3
mrpl201	8.03E-13	0.508847551	0.824	0.394	1.91E-08	3
smim41	8.90E-13	0.441681071	0.716	0.311	2.12E-08	3
scp2a2	9.44E-13	0.580103759	0.919	0.43	2.25E-08	3
gnpda12	1.00E-12	0.540145996	0.878	0.404	2.40E-08	3
NPC1L11	1.07E-12	0.251630711	0.541	0.178	2.55E-08	3
aqp8a.12	1.12E-12	0.399140145	0.73	0.267	2.67E-08	3
tomm51	1.13E-12	0.522888362	0.946	0.507	2.70E-08	3

mrps34	1.19E-12	0.290308206	0.716	0.291	2.84E-08	3
nol7	1.21E-12	0.441834167	0.662	0.27	2.88E-08	3
ccdc25	1.46E-12	0.355796757	0.757	0.317	3.47E-08	3
zgc:193742	1.53E-12	0.334670188	0.595	0.207	3.64E-08	3
mrpl271	1.63E-12	0.441233203	0.743	0.333	3.89E-08	3
mrpl21	1.63E-12	0.379843898	0.757	0.324	3.89E-08	3
zfp36l2	1.69E-12	0.470083374	0.797	0.359	4.03E-08	3
sdr16c5b1	1.81E-12	0.402064035	0.838	0.38	4.32E-08	3
ndufa101	2.26E-12	0.461508858	0.892	0.413	5.40E-08	3
prdx31	2.52E-12	0.429390671	0.865	0.43	6.01E-08	3
hsd17b10	2.78E-12	0.287625719	0.662	0.248	6.63E-08	3
ugt5b21	2.82E-12	0.346208727	0.554	0.178	6.72E-08	3
snrnp401	2.85E-12	0.250861338	0.568	0.194	6.79E-08	3
gcsha1	3.05E-12	0.409879616	0.757	0.346	7.28E-08	3
grsf12	3.49E-12	0.270930642	0.608	0.23	8.31E-08	3
aldh7a11	3.56E-12	0.401057294	0.851	0.365	8.48E-08	3
paics1	3.74E-12	0.36451124	0.824	0.356	8.92E-08	3
mpc12	3.89E-12	0.518368435	0.946	0.533	9.27E-08	3
si:dkey-183n20.151	4.40E-12	0.317083622	0.608	0.211	1.05E-07	3
hadh1	4.52E-12	0.47021516	0.878	0.4	1.08E-07	3
bin2a2	4.61E-12	0.502009802	0.932	0.446	1.10E-07	3
mrps251	5.41E-12	0.362922917	0.851	0.409	1.29E-07	3
suclg11	5.68E-12	0.706915419	0.973	0.609	1.35E-07	3
cmc21	6.18E-12	0.284860565	0.581	0.206	1.47E-07	3
timm132	6.22E-12	0.422733974	0.797	0.365	1.48E-07	3
tspan13a1	6.37E-12	0.485036109	0.824	0.37	1.52E-07	3
ak31	6.71E-12	0.305423245	0.757	0.304	1.60E-07	3
cyp2k161	6.71E-12	0.507448462	0.865	0.402	1.60E-07	3
ndufab1b1	6.75E-12	0.628946606	0.973	0.58	1.61E-07	3
zgc:172079.21	6.76E-12	0.58435525	0.865	0.443	1.61E-07	3
sdhb1	6.96E-12	0.543624322	0.919	0.52	1.66E-07	3
mrpl241	7.00E-12	0.251450454	0.824	0.381	1.67E-07	3
zanl2	7.05E-12	0.490672434	0.865	0.378	1.68E-07	3
uqcrq1	7.15E-12	0.70176566	0.973	0.731	1.70E-07	3
chia.11	7.23E-12	0.253364567	0.351	0.08	1.72E-07	3
ndufb82	7.27E-12	0.500077453	0.986	0.665	1.73E-07	3
uqcrfs11	7.50E-12	0.595603738	0.973	0.559	1.79E-07	3
zgc:1365642	8.02E-12	0.336134552	0.73	0.307	1.91E-07	3
adh8b1	8.67E-12	0.620600409	0.946	0.485	2.07E-07	3
si:ch211-235e9.81	8.86E-12	0.527965577	0.878	0.417	2.11E-07	3
mrpl41	9.65E-12	0.252645482	0.703	0.287	2.30E-07	3
si:ch211-161h7.52	1.05E-11	0.410906845	0.635	0.235	2.50E-07	3
llph1	1.07E-11	0.428657405	0.824	0.394	2.56E-07	3
timm91	1.10E-11	0.520809966	0.77	0.38	2.61E-07	3
dnph1	1.17E-11	0.292355802	0.554	0.196	2.78E-07	3
rdh11	1.17E-11	0.349477662	0.622	0.228	2.80E-07	3
slc35d1b1	1.32E-11	0.329377822	0.581	0.215	3.16E-07	3
sdhdb2	1.38E-11	0.403655363	0.932	0.478	3.29E-07	3
krt971	1.44E-11	0.760099888	0.959	0.585	3.42E-07	3
si:ch211-107o10.32	1.47E-11	0.348321845	0.824	0.363	3.51E-07	3
mrps18b1	1.49E-11	0.353990695	0.703	0.293	3.55E-07	3
dldh1	1.63E-11	0.364764683	0.865	0.411	3.88E-07	3
mrpl541	1.64E-11	0.317555559	0.905	0.454	3.90E-07	3
ckmt11	1.66E-11	0.414511734	0.919	0.459	3.95E-07	3

mrpl281	1.66E-11	0.392202681	0.77	0.357	3.96E-07	3
nipsnap3a1	1.74E-11	0.287330246	0.743	0.311	4.14E-07	3
fdps1	1.89E-11	0.372733817	0.838	0.367	4.52E-07	3
gapdh1	1.92E-11	0.732550441	0.973	0.628	4.58E-07	3
fbl1	2.20E-11	0.489543865	0.959	0.535	5.24E-07	3
got2b1	2.24E-11	0.41902681	0.824	0.372	5.35E-07	3
uqcr10	2.25E-11	0.627633796	0.986	0.731	5.37E-07	3
mrpl33	2.53E-11	0.367324221	0.824	0.413	6.04E-07	3
mrps71	2.71E-11	0.38243578	0.703	0.313	6.46E-07	3
si:ch211-68a17.71	2.79E-11	0.285778124	0.824	0.393	6.66E-07	3
dhrs12	2.83E-11	0.350801358	0.905	0.441	6.74E-07	3
ebna1bp21	2.87E-11	0.495685267	0.919	0.493	6.84E-07	3
mrps26	2.95E-11	0.310221204	0.797	0.374	7.04E-07	3
ndufa5	3.03E-11	0.578771301	0.946	0.611	7.23E-07	3
mrpl381	3.11E-11	0.281038245	0.743	0.317	7.41E-07	3
entpd8	3.26E-11	0.282171133	0.514	0.161	7.78E-07	3
gsta.1.12	3.34E-11	0.716086873	1	0.819	7.95E-07	3
mrpl111	3.46E-11	0.507908815	0.905	0.493	8.26E-07	3
dtd2	4.02E-11	0.264495306	0.446	0.135	9.58E-07	3
ndufs31	4.02E-11	0.413391273	0.865	0.431	9.59E-07	3
cyp2x91	4.02E-11	0.340965234	0.581	0.207	9.59E-07	3
si:dkey-36i7.31	4.08E-11	0.608015613	0.986	0.609	9.74E-07	3
bxdc21	4.10E-11	0.481805658	0.865	0.431	9.78E-07	3
sult3st31	4.40E-11	0.273225726	0.851	0.376	1.05E-06	3
slirp1	4.59E-11	0.472716134	0.946	0.554	1.09E-06	3
CYP2C91	4.65E-11	0.335955382	0.649	0.235	1.11E-06	3
mrpl231	4.86E-11	0.342606879	0.608	0.233	1.16E-06	3
ndufb61	5.28E-11	0.55222566	0.946	0.565	1.26E-06	3
mrpl171	5.41E-11	0.303469896	0.662	0.272	1.29E-06	3
mrps361	5.53E-11	0.417485696	0.905	0.509	1.32E-06	3
ccdc581	5.56E-11	0.361364804	0.689	0.296	1.33E-06	3
atp5pf	6.03E-11	0.536970088	1	0.75	1.44E-06	3
spink4	6.29E-11	0.512033689	0.797	0.356	1.50E-06	3
pbld1	6.86E-11	0.328391874	0.703	0.287	1.64E-06	3
atp231	7.05E-11	0.265828937	0.419	0.126	1.68E-06	3
rtn4b1	7.07E-11	0.315211095	0.865	0.376	1.69E-06	3
cox7b1	7.22E-11	0.676143881	1	0.8	1.72E-06	3
uqcrc2b1	7.22E-11	0.456081701	0.851	0.411	1.72E-06	3
timm8b1	7.89E-11	0.453614482	0.878	0.469	1.88E-06	3
fh1	8.26E-11	0.251685688	0.797	0.343	1.97E-06	3
cyp2k191	8.43E-11	0.488307472	0.405	0.124	2.01E-06	3
bccip1	8.68E-11	0.453635444	0.878	0.446	2.07E-06	3
idh11	9.50E-11	0.395203174	0.892	0.419	2.27E-06	3
ndufa112	9.91E-11	0.424130985	0.878	0.489	2.36E-06	3
aurkaip11	1.00E-10	0.354669729	0.784	0.365	2.39E-06	3
psma21	1.07E-10	0.364125788	0.892	0.463	2.54E-06	3
prdx62	1.09E-10	0.278978143	0.946	0.478	2.60E-06	3
CU694197.11	1.14E-10	0.292589437	0.905	0.454	2.72E-06	3
etfb2	1.16E-10	0.288612515	0.878	0.452	2.76E-06	3
atp5f1c1	1.17E-10	0.576111291	0.946	0.57	2.79E-06	3
cyb5r21	1.19E-10	0.344934651	0.73	0.306	2.83E-06	3
clpp1	1.19E-10	0.362024157	0.689	0.306	2.84E-06	3
ndufv31	1.40E-10	0.270040185	0.878	0.43	3.34E-06	3
ndufs41	1.41E-10	0.573790269	0.878	0.561	3.36E-06	3

mapre1a	1.56E-10	0.277876863	0.568	0.226	3.73E-06	3
ecil1	1.63E-10	0.397616608	0.743	0.33	3.88E-06	3
hmgcs11	1.69E-10	0.274622531	0.865	0.407	4.02E-06	3
si:ch211-71m22.11	1.87E-10	0.506272688	0.919	0.478	4.46E-06	3
gsta.21	1.96E-10	0.47992338	0.568	0.219	4.68E-06	3
ndufs6	2.09E-10	0.455996362	0.932	0.589	4.97E-06	3
ndufv22	2.16E-10	0.377500385	0.838	0.426	5.15E-06	3
ftcd1	2.18E-10	0.289924727	0.446	0.139	5.20E-06	3
mrpl432	2.24E-10	0.308612169	0.595	0.244	5.35E-06	3
tomm40l1	2.30E-10	0.308434194	0.797	0.359	5.48E-06	3
fahd11	2.32E-10	0.257290021	0.757	0.354	5.52E-06	3
atp5po1	2.53E-10	0.587922364	0.973	0.693	6.03E-06	3
sf3b5	2.60E-10	0.38969667	0.892	0.543	6.20E-06	3
ndufb4	2.64E-10	0.530832432	0.946	0.622	6.30E-06	3
si:dkey-44g23.51	2.91E-10	0.406825147	0.716	0.324	6.95E-06	3
pbdc1	2.93E-10	0.421836228	0.838	0.443	6.99E-06	3
ndrg3a1	3.01E-10	0.292674287	0.757	0.324	7.18E-06	3
cnbpa1	3.47E-10	0.500619437	0.932	0.506	8.27E-06	3
zgc:777481	3.59E-10	0.465182065	0.946	0.467	8.55E-06	3
mrpl1	3.90E-10	0.27859565	0.419	0.137	9.31E-06	3
atp5mc3a1	3.94E-10	0.517647083	0.973	0.654	9.41E-06	3
dbi1	4.09E-10	0.661961299	0.986	0.769	9.76E-06	3
ppil1	4.22E-10	0.272506962	0.743	0.357	1.01E-05	3
ndufb10	4.52E-10	0.479774144	0.959	0.631	1.08E-05	3
tpi1b2	4.54E-10	0.399976715	0.959	0.583	1.08E-05	3
chchd11	4.62E-10	0.289053549	0.689	0.3	1.10E-05	3
eppk11	4.75E-10	0.643275434	0.959	0.53	1.13E-05	3
mrpl571	4.96E-10	0.32643071	0.892	0.519	1.18E-05	3
uqcrh1	5.09E-10	0.540900554	0.973	0.754	1.21E-05	3
gstp11	5.19E-10	0.633423382	0.986	0.789	1.24E-05	3
bpnt11	5.58E-10	0.367094484	0.608	0.259	1.33E-05	3
mrto4	5.58E-10	0.258052964	0.595	0.243	1.33E-05	3
coa7	5.63E-10	0.298337629	0.608	0.256	1.34E-05	3
ndufb51	5.89E-10	0.359988578	0.932	0.507	1.40E-05	3
ponzr11	6.12E-10	0.284015332	0.797	0.352	1.46E-05	3
slc31a11	6.13E-10	0.387486151	0.811	0.374	1.46E-05	3
tbca	6.15E-10	0.278752843	0.905	0.494	1.47E-05	3
cox7a2a1	6.25E-10	0.529622835	1	0.72	1.49E-05	3
fkbp3	6.69E-10	0.350733544	0.824	0.402	1.60E-05	3
zgc:92907	6.86E-10	0.267163511	0.297	0.072	1.64E-05	3
sod11	6.90E-10	0.557541703	0.986	0.687	1.65E-05	3
fbp1b1	6.96E-10	0.400753318	0.811	0.374	1.66E-05	3
ndufs71	7.07E-10	0.366476926	0.919	0.519	1.69E-05	3
sssca1	7.11E-10	0.329319646	0.446	0.161	1.69E-05	3
tomm71	7.44E-10	0.3145708	0.973	0.593	1.77E-05	3
mrps18c1	7.59E-10	0.27077266	0.838	0.424	1.81E-05	3
aimp1	7.81E-10	0.276053238	0.919	0.485	1.86E-05	3
ndufs51	7.84E-10	0.659814851	0.932	0.594	1.87E-05	3
hsdl22	8.06E-10	0.273150732	0.838	0.419	1.92E-05	3
cox4i1	8.25E-10	0.400741591	1	0.844	1.97E-05	3
mgst1.22	8.44E-10	0.259884235	0.608	0.246	2.01E-05	3
mrpl121	8.91E-10	0.436523557	0.865	0.485	2.12E-05	3
cyb5a1	9.12E-10	0.417597979	0.865	0.457	2.17E-05	3
pwp1	9.59E-10	0.340577308	0.824	0.409	2.29E-05	3

atp5mf	1.02E-09	0.54012045	1	0.822	2.43E-05	3
abcc22	1.07E-09	0.255458311	0.595	0.241	2.56E-05	3
elovl21	1.18E-09	0.291828117	0.446	0.144	2.82E-05	3
prdx21	1.22E-09	0.601061998	1	0.826	2.90E-05	3
apoa1b1	1.36E-09	0.469532983	0.932	0.541	3.25E-05	3
anks4b1	1.41E-09	0.316185132	0.824	0.378	3.37E-05	3
phb1	1.47E-09	0.481434232	0.919	0.502	3.50E-05	3
apobb.11	1.52E-09	0.327382754	0.635	0.243	3.62E-05	3
mrps18a2	1.54E-09	0.28405214	0.716	0.348	3.66E-05	3
cyc11	1.70E-09	0.384417694	0.946	0.548	4.06E-05	3
ola11	1.84E-09	0.376221985	0.892	0.506	4.40E-05	3
papss2a1	1.86E-09	0.294488345	0.743	0.32	4.43E-05	3
sdhc1	1.89E-09	0.430634412	0.878	0.517	4.50E-05	3
dhdh11	1.94E-09	0.39900362	0.851	0.459	4.63E-05	3
c23h20orf241	2.00E-09	0.369138968	0.892	0.463	4.77E-05	3
mrps6	2.08E-09	0.339959567	0.838	0.433	4.96E-05	3
tomm222	2.09E-09	0.250024369	0.811	0.417	4.97E-05	3
dhrs41	2.17E-09	0.301389386	0.703	0.326	5.17E-05	3
sord	2.20E-09	0.293211238	0.378	0.119	5.25E-05	3
idh3b1	2.24E-09	0.295853609	0.73	0.328	5.34E-05	3
aifm4	2.24E-09	0.316172958	0.459	0.167	5.34E-05	3
lrp1ab	2.30E-09	0.313195791	0.541	0.215	5.49E-05	3
fam207a1	2.43E-09	0.381321774	0.635	0.296	5.80E-05	3
rpl23	2.48E-09	0.405836504	1	0.981	5.90E-05	3
mrps21	2.49E-09	0.261844788	0.568	0.239	5.93E-05	3
minos11	2.49E-09	0.445042296	0.905	0.574	5.93E-05	3
ATP5MD1	2.53E-09	0.645888451	0.973	0.707	6.02E-05	3
coq51	2.58E-09	0.275015466	0.486	0.183	6.15E-05	3
rwdd1	2.60E-09	0.277742152	0.892	0.467	6.20E-05	3
mrpl131	2.70E-09	0.423746024	0.77	0.391	6.44E-05	3
HADHB1	2.80E-09	0.371661199	0.838	0.428	6.68E-05	3
rpl7l11	2.83E-09	0.405150621	0.865	0.472	6.76E-05	3
mgst3a1	2.86E-09	0.461800292	0.986	0.681	6.82E-05	3
aldh1l11	3.15E-09	0.265643942	0.689	0.311	7.51E-05	3
ptgr11	3.19E-09	0.346264425	0.797	0.396	7.60E-05	3
snrpb1	3.28E-09	0.520952286	0.986	0.661	7.81E-05	3
DUT	3.33E-09	0.536280732	0.784	0.411	7.94E-05	3
gstr2	3.33E-09	0.42505306	0.986	0.548	7.95E-05	3
acad91	3.34E-09	0.262388202	0.581	0.239	7.97E-05	3
rfc3	3.48E-09	0.257612999	0.446	0.161	8.29E-05	3
dap1	3.57E-09	0.501127571	0.959	0.639	8.52E-05	3
mrps331	3.60E-09	0.253283334	0.73	0.365	8.59E-05	3
ddt	3.72E-09	0.320086064	0.905	0.526	8.87E-05	3
atp5pb1	3.74E-09	0.539534865	0.986	0.702	8.91E-05	3
atp5f1d1	3.77E-09	0.531113414	0.973	0.715	9.00E-05	3
ech11	3.92E-09	0.258155232	0.622	0.259	9.35E-05	3
mrpl361	4.08E-09	0.34246891	0.77	0.396	9.72E-05	3
slco1d11	4.15E-09	0.290858906	0.703	0.276	9.90E-05	3
plac8.1	4.44E-09	0.428240338	0.959	0.652	0.0001059	3
aldh8a11	4.52E-09	0.290407288	0.622	0.261	0.0001077	3
si:dkey-88p24.11	4.65E-09	0.279521121	0.757	0.369	0.0001108	3
atp5meb	4.76E-09	0.425904132	1	0.772	0.0001136	3
cox6c1	4.79E-09	0.48800735	0.986	0.739	0.0001142	3
cox161	4.93E-09	0.31398841	0.527	0.222	0.0001176	3

zgc:153911	4.96E-09	0.269322737	0.608	0.257	0.0001183	3
ndufb2	5.06E-09	0.391470548	0.959	0.641	0.0001207	3
si:ch211-133l5.7	5.23E-09	0.288921806	0.757	0.363	0.0001248	3
mrpl451	5.29E-09	0.266156625	0.662	0.296	0.000126	3
si:dkey-13i19.8	5.35E-09	0.279376251	0.77	0.369	0.0001277	3
lpcat4	5.68E-09	0.29702179	0.432	0.15	0.0001355	3
rpl27	5.75E-09	0.371821093	1	0.957	0.0001372	3
aspdh	6.10E-09	0.261449404	0.432	0.156	0.0001454	3
eif3k1	6.20E-09	0.454851418	0.905	0.57	0.0001477	3
glrx3	6.37E-09	0.2572606	0.703	0.344	0.0001519	3
cenpx	6.43E-09	0.271913146	0.635	0.283	0.0001534	3
cat1	6.95E-09	0.373769187	0.77	0.367	0.0001656	3
slc7a71	7.05E-09	0.325212963	0.5	0.181	0.0001682	3
drg11	7.15E-09	0.285530518	0.581	0.252	0.0001704	3
pdcd5	7.23E-09	0.311518467	0.649	0.307	0.0001724	3
eno31	7.25E-09	0.514565504	0.986	0.726	0.0001728	3
atp5mc11	7.38E-09	0.482780531	1	0.781	0.000176	3
ndufb7	7.43E-09	0.36523136	0.973	0.646	0.0001771	3
atp5mc3b1	7.48E-09	0.537954977	1	0.839	0.0001783	3
eif2s1b1	7.54E-09	0.455172391	0.919	0.559	0.0001799	3
atp5if1b	7.59E-09	0.471095667	0.973	0.693	0.0001811	3
zgc:77929	7.84E-09	0.297768185	0.392	0.135	0.0001871	3
banf11	8.34E-09	0.279066568	0.946	0.517	0.000199	3
acsl1b1	8.84E-09	0.291708526	0.878	0.413	0.0002108	3
tsr21	9.12E-09	0.280905482	0.662	0.3	0.0002174	3
slc9a3r1a1	9.80E-09	0.326425588	0.797	0.385	0.0002337	3
mtch21	1.02E-08	0.275655607	0.77	0.367	0.0002443	3
sub1b1	1.03E-08	0.299610821	0.689	0.324	0.0002456	3
zgc:1588461	1.06E-08	0.599275423	0.986	0.748	0.0002525	3
ndufa61	1.08E-08	0.391871217	0.959	0.661	0.0002584	3
ccl25a1	1.09E-08	0.34835492	0.554	0.226	0.0002603	3
chchd2	1.12E-08	0.299004054	0.946	0.585	0.0002659	3
snu13b1	1.20E-08	0.450395631	0.892	0.576	0.0002851	3
smim8	1.35E-08	0.265148051	0.608	0.27	0.0003211	3
slc25a331	1.35E-08	0.288256592	0.486	0.187	0.000322	3
apoa4b.2	1.45E-08	0.289538344	0.554	0.224	0.0003454	3
atp5f1e	1.47E-08	0.504594772	1	0.874	0.0003498	3
rbm8a1	1.53E-08	0.268258693	0.946	0.578	0.0003653	3
nupr11	1.60E-08	0.621293778	0.986	0.743	0.000381	3
gpx1a1	1.68E-08	0.416493554	0.905	0.526	0.0004005	3
ppa1b1	1.71E-08	0.305555384	0.824	0.424	0.0004067	3
s100a10a1	1.73E-08	0.586594267	1	0.8	0.0004133	3
cmc1	1.85E-08	0.261988064	0.622	0.283	0.0004403	3
sub1a	1.91E-08	0.446301611	0.946	0.628	0.0004554	3
si:dkey-16p21.82	1.94E-08	0.471393749	0.797	0.435	0.0004626	3
acox31	2.12E-08	0.27546216	0.405	0.144	0.0005057	3
si:ch211-225b11.11	2.12E-08	0.356987726	0.554	0.241	0.0005063	3
glud1b1	2.30E-08	0.352980448	0.878	0.469	0.0005478	3
mrpl521	2.31E-08	0.370989725	0.77	0.428	0.0005504	3
ugt2a41	2.57E-08	0.323219364	0.77	0.402	0.0006124	3
zgc:194392	2.58E-08	0.255584544	0.473	0.181	0.0006152	3
mpv17l21	2.60E-08	0.293425031	0.568	0.254	0.0006203	3
ndufb91	2.73E-08	0.332163237	0.973	0.646	0.0006517	3
gstp2	2.80E-08	0.353500367	0.581	0.241	0.0006676	3

si:ch211-282b22.1	2.82E-08	0.322403697	0.554	0.25	0.0006726	3
vil1l	2.85E-08	0.349813621	0.973	0.548	0.0006805	3
oxa1l1	2.87E-08	0.267198246	0.554	0.231	0.0006851	3
dlst1	2.97E-08	0.362224754	0.865	0.474	0.0007085	3
gnl31	3.16E-08	0.340097113	0.851	0.474	0.0007526	3
tmcc3	3.42E-08	0.302871738	0.541	0.233	0.0008148	3
polr2k	3.50E-08	0.289400685	0.797	0.413	0.000834	3
aldh1a21	3.55E-08	0.380747131	0.473	0.185	0.0008463	3
nop53	3.62E-08	0.338487599	0.919	0.543	0.0008622	3
mcm5	3.81E-08	0.266838983	0.473	0.185	0.0009087	3
pa2g4a1	3.95E-08	0.384585879	0.905	0.456	0.0009414	3
ptges3b	4.00E-08	0.369893672	0.919	0.57	0.0009539	3
eif3g1	4.07E-08	0.402688782	0.946	0.565	0.0009716	3
coa6	4.16E-08	0.304589493	0.784	0.43	0.0009915	3
mdh1aa1	4.32E-08	0.468429025	0.946	0.602	0.0010297	3
nedd4a1	4.37E-08	0.260991776	0.838	0.393	0.0010409	3
tomm61	4.38E-08	0.381499258	0.946	0.591	0.0010449	3
rps5	4.52E-08	0.366719878	1	0.959	0.0010784	3
ndufa7	4.90E-08	0.40834955	0.878	0.502	0.0011695	3
zgc:1539681	4.94E-08	0.427160421	0.514	0.209	0.0011772	3
cox5ab1	5.07E-08	0.444256519	0.959	0.704	0.0012083	3
mrps141	5.36E-08	0.28979706	0.757	0.398	0.0012784	3
mrpl371	5.59E-08	0.2903232	0.649	0.313	0.0013337	3
eif2s2	6.49E-08	0.404172722	0.973	0.706	0.0015476	3
gsto2	7.10E-08	0.300143775	0.662	0.328	0.0016922	3
nop10	7.36E-08	0.292664289	0.932	0.567	0.001756	3
RPL41	7.47E-08	0.393214899	1	0.998	0.0017805	3
IZUMO1R1	7.57E-08	0.287329364	0.486	0.206	0.0018044	3
cox7a31	8.00E-08	0.253991227	0.878	0.543	0.0019087	3
hypk	8.27E-08	0.279399166	0.932	0.611	0.001973	3
rsl1d12	8.70E-08	0.303326569	0.878	0.493	0.0020737	3
tomm20a	8.93E-08	0.270816354	0.851	0.481	0.0021293	3
c1qbp1	9.02E-08	0.457085462	0.946	0.683	0.0021516	3
dhfr	9.15E-08	0.271454175	0.541	0.241	0.0021818	3
zgc:110182	1.00E-07	0.266345549	0.743	0.367	0.0023927	3
psmc61	1.02E-07	0.327869188	0.757	0.394	0.002428	3
mrps28	1.04E-07	0.280871562	0.581	0.274	0.0024834	3
zc3h13	1.12E-07	0.278554159	0.595	0.281	0.0026801	3
cox5aa1	1.15E-07	0.427145696	0.986	0.737	0.0027464	3
ahcy1	1.24E-07	0.365624459	0.973	0.606	0.0029573	3
rps10	1.35E-07	0.373266607	1	0.944	0.0032256	3
atp5pd1	1.42E-07	0.437509314	0.986	0.761	0.0033778	3
prkab1b1	1.44E-07	0.307207952	0.703	0.348	0.0034337	3
sb:cb10581	1.64E-07	0.350993285	0.946	0.57	0.0039084	3
psmb1	1.79E-07	0.334084918	0.932	0.57	0.0042612	3
aldob1	1.93E-07	0.450654947	0.986	0.726	0.0046046	3
gpx1b	1.94E-07	0.310162057	0.459	0.193	0.004627	3
nop581	1.94E-07	0.338627829	0.946	0.572	0.0046327	3
smim15	2.22E-07	0.262894331	0.77	0.426	0.0053045	3
idh3g1	2.28E-07	0.254279529	0.486	0.213	0.0054265	3
si:ch211-121j5.41	2.66E-07	0.457710217	0.351	0.128	0.0063471	3
rpl121	2.99E-07	0.440995466	1	0.935	0.0071221	3
gpx4b1	2.99E-07	0.421801565	0.946	0.696	0.0071403	3
etfa1	3.01E-07	0.283525993	0.784	0.406	0.007182	3

NDUFC1	3.22E-07	0.261446151	0.905	0.58	0.0076666	3
si:ch211-217k17.7	3.25E-07	0.251189264	0.838	0.469	0.0077444	3
ndufc2	3.44E-07	0.330689861	0.959	0.665	0.0082077	3
slc25a201	3.51E-07	0.275143627	0.797	0.426	0.008364	3
decr21	3.52E-07	0.331241065	0.486	0.224	0.0083953	3
cldn15lb1	3.61E-07	0.334666914	0.959	0.559	0.0086018	3
serpinb1l31	4.10E-07	0.362941259	0.986	0.606	0.0097841	3
hnrnpa1b1	4.13E-07	0.333510232	0.986	0.598	0.0098592	3
slc25a3b1	4.29E-07	0.458127512	1	0.759	0.0102298	3
eif3ha1	4.30E-07	0.401367832	0.878	0.524	0.0102513	3
hspe12	4.61E-07	0.418485031	0.973	0.778	0.0109884	3
zgc:195170	4.79E-07	0.25352739	0.473	0.206	0.011418	3
hibadhb1	5.50E-07	0.264704535	0.514	0.237	0.0131186	3
snrpg	5.60E-07	0.266898338	0.919	0.594	0.0133469	3
anxa2b1	5.74E-07	0.438637934	1	0.744	0.0136752	3
pfdn51	5.88E-07	0.259570611	0.689	0.35	0.0140161	3
mdh21	6.10E-07	0.382565601	0.919	0.578	0.0145358	3
magoh	6.17E-07	0.277486779	0.811	0.485	0.0147073	3
ndufa31	6.53E-07	0.337689435	0.919	0.607	0.0155816	3
rtcb	6.59E-07	0.361780149	0.554	0.278	0.0157127	3
ybey	6.82E-07	0.257483622	0.432	0.187	0.0162523	3
zgc:193541	7.26E-07	0.416573587	0.973	0.748	0.0173137	3
ssuh2rs1	7.50E-07	0.274576286	0.676	0.376	0.01788	3
hikeshi	8.35E-07	0.304393711	0.608	0.302	0.0198993	3
rps15	8.39E-07	0.313225933	1	0.959	0.0199966	3
cycsb	8.94E-07	0.406621955	0.973	0.819	0.0213206	3
pklr1	9.24E-07	0.289801046	0.851	0.454	0.0220332	3
cox7c	9.35E-07	0.436734577	1	0.809	0.022301	3
creb3l3a1	9.45E-07	0.261201033	0.459	0.196	0.0225282	3
ndnl2	1.17E-06	0.278962526	0.5	0.233	0.027787	3
dkc11	1.28E-06	0.270905092	0.865	0.496	0.0304824	3
dtymk	1.36E-06	0.308695125	0.405	0.169	0.032396	3
hnrnpm	1.43E-06	0.35564525	0.716	0.376	0.0340171	3
rps23	1.48E-06	0.332069114	0.986	0.974	0.0353503	3
ccz1	1.57E-06	0.265781352	0.392	0.161	0.037328	3
zgc:56493	1.66E-06	0.364162802	0.973	0.826	0.0395192	3
rars1	1.76E-06	0.272098882	0.608	0.311	0.0420796	3
rps111	1.82E-06	0.373551719	1	0.92	0.0434003	3
krt921	1.86E-06	0.405399708	1	0.794	0.0444654	3
hspd11	1.87E-06	0.360798263	0.946	0.637	0.0445047	3
ak21	1.90E-06	0.281734661	0.932	0.567	0.0454184	3
si:dkeyp-73b11.81	2.04E-06	0.407770103	1	0.756	0.0485401	3
fth1a1	2.04E-06	0.371991541	1	0.783	0.0485902	3
glrx	2.04E-06	0.258470347	0.919	0.611	0.0487534	3
march51	2.08E-06	0.272796478	0.446	0.211	0.0494925	3
pa2g4b1	2.22E-06	0.262012965	0.838	0.48	0.0529719	3
rpl22l1	2.57E-06	0.368467534	0.986	0.852	0.061356	3
uqcrb	2.80E-06	0.268170792	0.986	0.813	0.0667929	3
uqcrc11	2.88E-06	0.253242988	0.824	0.498	0.0687457	3
rpl35a	2.95E-06	0.314163148	1	0.957	0.0702265	3
rps8a1	2.95E-06	0.364420586	0.986	0.974	0.0702569	3
hccsb1	3.19E-06	0.258349721	0.459	0.213	0.0760847	3
rpl15	3.52E-06	0.329219905	1	0.95	0.0838864	3
btf31	3.61E-06	0.39177414	0.986	0.754	0.0859623	3

cyp24a11	3.69E-06	0.353496738	0.419	0.178	0.0880117	3
tmem86b1	3.81E-06	0.253370323	0.324	0.122	0.0908553	3
rplp2l1	4.55E-06	0.287052598	0.986	0.978	0.1085817	3
sdha1	4.71E-06	0.262228058	0.73	0.407	0.1122917	3
slbp	4.83E-06	0.262264856	0.432	0.198	0.1152644	3
tmem258	6.34E-06	0.315068905	0.986	0.663	0.1510587	3
porb1	6.82E-06	0.259091953	0.622	0.324	0.1625914	3
snrpe	7.04E-06	0.273638867	0.932	0.646	0.167927	3
timm291	7.44E-06	0.256745468	0.459	0.213	0.1774223	3
atp5fa11	7.59E-06	0.358089022	1	0.748	0.1810861	3
nup621	8.08E-06	0.273280138	0.446	0.206	0.192678	3
rpl30	8.29E-06	0.28964036	1	0.943	0.1977681	3
insig11	9.42E-06	0.31596635	0.824	0.467	0.2246838	3
naca	9.70E-06	0.279686288	1	0.93	0.231248	3
rps12	1.05E-05	0.344938836	1	0.957	0.2499587	3
setb1	1.06E-05	0.304803134	0.959	0.633	0.2516509	3
rps15a	1.06E-05	0.263972334	1	0.961	0.2524204	3
hmgb2b	1.12E-05	0.290024997	0.973	0.743	0.2674373	3
rps31	1.16E-05	0.354081938	1	0.911	0.2763243	3
eif3f1	1.21E-05	0.306722784	0.946	0.648	0.2885035	3
cldn15la1	1.29E-05	0.306910929	1	0.785	0.3080305	3
atp5f1b1	1.34E-05	0.39716943	0.986	0.811	0.3183814	3
rpl39	1.34E-05	0.286780598	1	0.961	0.3184817	3
rps7	1.36E-05	0.278631578	1	0.981	0.3252653	3
rpl13	1.80E-05	0.265077816	1	0.978	0.4283978	3
rps16	2.20E-05	0.288124045	1	0.93	0.5255668	3
si:ch211-161h7.4	2.32E-05	0.335258644	0.311	0.131	0.5537461	3
rpl37.1	2.76E-05	0.328787838	1	0.978	0.6583267	3
rps3a1	3.08E-05	0.3516406	1	0.933	0.7340275	3
tma7	5.05E-05	0.274441769	0.973	0.767	1	3
ndufa1	5.62E-05	0.275130157	0.946	0.737	1	3
rps91	6.53E-05	0.289958394	0.986	0.952	1	3
rpl36	6.63E-05	0.270177665	1	0.948	1	3
rps24	6.93E-05	0.266966351	1	0.963	1	3
ran1	6.97E-05	0.296940869	0.986	0.75	1	3
atp5if1a	0.0001023	0.273226727	0.973	0.791	1	3
rpl23a	0.0001062	0.262377333	0.986	0.956	1	3
rpl17	0.0001418	0.27048257	1	0.946	1	3
nme2b.12	0.0001582	0.352929848	1	0.867	1	3
npm1a1	0.0002787	0.26267042	0.959	0.746	1	3
ndufa41	0.0002957	0.294773027	0.959	0.767	1	3
rplp01	0.0004233	0.30787902	0.986	0.928	1	3
htatip2	0.0004263	0.331874572	0.297	0.141	1	3
rpl5a1	0.0014235	0.255004545	1	0.815	1	3
rpl191	0.001653	0.251140626	1	0.95	1	3
rpl71	0.0018994	0.250591333	1	0.922	1	3
zgc:165555.15	0.0032476	0.398920061	0.473	0.3	1	3
rps29	6.29E-10	0.481721215	1	0.998	1.50E-05	4
CT027638.1	3.83E-09	2.466249404	0.944	0.954	9.14E-05	4
fabp6	1.17E-08	1.330944148	0.292	0.079	0.0002789	4
zgc:158463	1.28E-08	1.279291113	0.958	0.972	0.0003064	4
rpl38	1.73E-08	0.739388059	1	0.98	0.0004124	4
sptbn5	6.82E-08	1.431436646	0.347	0.137	0.0016273	4
CR383676.11	1.01E-07	0.627223761	0.972	0.982	0.0024084	4

si:ch211-153b23.5	4.48E-07	1.332278818	0.444	0.212	0.0106887	4
si:ch73-335l21.41	1.22E-06	0.751862256	0.819	0.718	0.0292065	4
atf3l	1.59E-06	0.777792711	0.889	0.843	0.03802	4
cldnbl	1.67E-06	0.860782698	0.861	0.768	0.0397188	4
si:ch211-139a5.9	2.78E-06	1.632439377	0.403	0.205	0.0662658	4
ier2b	3.67E-06	0.987523141	0.764	0.71	0.0874466	4
mt-co1	4.12E-06	0.844421568	0.875	0.9	0.0981532	4
1-Jun	7.05E-06	0.69801305	0.819	0.821	0.1681469	4
epcam	7.34E-06	0.600607032	0.958	0.908	0.1751227	4
mt-nd2l	7.35E-06	0.801227878	0.861	0.843	0.1752896	4
psmd6	9.24E-06	0.337345745	0.125	0.411	0.2202721	4
rps25	1.38E-05	0.402139489	0.986	0.969	0.3290665	4
rps28	1.43E-05	0.435499903	0.986	0.983	0.3410016	4
tnks1bp1	1.83E-05	1.635892308	0.542	0.435	0.4371881	4
hbegfal	1.95E-05	0.830945292	0.847	0.788	0.4646441	4
mt-cybl	6.62E-05	0.576590779	0.917	0.902	1	4
krt18l	6.88E-05	0.683995456	0.694	0.546	1	4
mt-co2l	7.08E-05	0.635966005	0.931	0.939	1	4
rpl29l	7.49E-05	0.455323329	0.972	0.928	1	4
mt-co3	0.0001017	0.584726314	0.917	0.937	1	4
wu:fb18f06	0.0001765	0.997601334	0.542	0.421	1	4
selenowl1	0.0002012	0.724916054	0.694	0.666	1	4
mt-nd1l	0.0002573	0.624819914	0.833	0.852	1	4
mt-nd5	0.0003401	0.653879828	0.778	0.76	1	4
krtt1c19e1	0.0004143	0.421038484	0.736	0.694	1	4
mt-nd3l	0.0004722	0.56127752	0.806	0.764	1	4
zgc:64022	0.0006474	1.033155061	0.444	0.308	1	4
mt-nd4l	0.0008308	0.485053147	0.861	0.834	1	4
dnmbp	0.000867	0.886740726	0.653	0.622	1	4
mmgt1	0.0008792	0.39001299	0.167	0.424	1	4
fosab1	0.0009856	0.62861685	0.694	0.664	1	4
bahcc1a	0.0011046	0.613983923	0.278	0.149	1	4
ier5	0.0012381	0.866145661	0.667	0.666	1	4
zgc:1627301	0.0019015	0.785433725	0.75	0.793	1	4
sec23b1	0.0023139	0.326341699	0.167	0.378	1	4
phlda2	0.0026319	0.364896219	0.889	0.902	1	4
fosb	0.0029285	0.863256116	0.361	0.247	1	4
mt-atp6	0.0031062	0.356910011	0.903	0.915	1	4
tcf3b1	0.0034444	0.443477887	0.153	0.373	1	4
decr1l	0.0038606	0.250001192	0.083	0.253	1	4
pnrc2l	0.0046315	0.45826687	0.833	0.801	1	4
tagln2	0.0048288	0.533000002	0.917	0.847	1	4
hnrnph1l	0.0060981	0.316349201	0.222	0.448	1	4
lmo7a	0.0074819	0.673287742	0.528	0.485	1	4
gmfb	0.0076746	0.468504272	0.111	0.286	1	4
mcfd2	0.0078458	0.276558105	0.111	0.282	1	4
igfbp1a1	0.0078802	0.495388057	0.569	0.47	1	4
arhgap5l	0.0081964	0.906950195	0.611	0.631	1	4
ccnl1b1	0.00823	0.260023951	0.208	0.434	1	4
foxa3	0.0082878	0.309208402	0.222	0.448	1	4
mpp1	0.0083048	0.250515658	0.111	0.269	1	4
stard14	0.0085994	0.588015654	0.597	0.585	1	4
tjp2a	0.0090074	0.288854728	0.097	0.26	1	4
fosl1a1	0.0092604	0.637566109	0.611	0.611	1	4

tmem184a1	0.0093251	0.321091308	0.097	0.255	1	4
myzap	0.0093901	0.398142873	0.111	0.277	1	4
zfhx31	0.0094108	0.709335159	0.361	0.264	1	4
rpl37.11	0.009613	0.318063893	0.986	0.98	1	4
apoc22	1.97E-12	0.975999366	0.915	0.475	4.69E-08	5
ndufb101	6.83E-12	0.82800113	0.944	0.635	1.63E-07	5
fdx12	1.48E-11	0.743610666	0.873	0.479	3.52E-07	5
timm8b2	2.24E-11	0.724985423	0.817	0.479	5.34E-07	5
fabp1b.12	3.33E-11	1.30080086	0.901	0.534	7.93E-07	5
rbp2a3	6.20E-11	0.815828885	0.958	0.519	1.48E-06	5
apoa4b.12	1.16E-10	0.812834391	0.901	0.442	2.77E-06	5
ndufa32	3.82E-10	0.62172228	0.901	0.611	9.10E-06	5
uqcrq2	5.07E-10	0.768246712	0.915	0.74	1.21E-05	5
afp4.12	5.33E-10	0.779864754	0.817	0.409	1.27E-05	5
uqcrh2	9.22E-10	0.604690518	0.958	0.757	2.20E-05	5
atp5pf1	1.10E-09	0.626858734	0.915	0.762	2.62E-05	5
ndufb92	1.38E-09	0.500834257	0.901	0.657	3.28E-05	5
atp5meb1	1.60E-09	0.594538444	0.958	0.779	3.81E-05	5
cox7b2	3.27E-09	0.578679501	0.986	0.803	7.79E-05	5
mgst3b3	3.82E-09	0.780866218	0.887	0.569	9.10E-05	5
atp5l2	4.68E-09	0.582659304	0.958	0.829	0.0001115	5
atp5f1e1	6.36E-09	0.654891312	0.958	0.88	0.0001517	5
aqp8a.22	7.58E-09	0.620189376	0.732	0.331	0.0001808	5
tmem14ca	7.97E-09	0.668538296	0.69	0.405	0.00019	5
plac8.11	9.00E-09	0.735594842	0.901	0.661	0.0002145	5
apoa1a2	1.18E-08	0.600369341	0.887	0.459	0.0002811	5
ociad22	1.22E-08	0.933473851	0.775	0.495	0.0002912	5
ndufa13	1.27E-08	0.597233028	0.944	0.738	0.0003032	5
fabp23	1.43E-08	0.716241515	1	0.759	0.0003408	5
mrpl182	1.44E-08	0.542756707	0.746	0.448	0.0003439	5
timm8a2	1.88E-08	0.483410391	0.704	0.401	0.0004484	5
atp5if1a1	2.24E-08	0.585253464	0.944	0.796	0.0005331	5
atp5if1b1	2.33E-08	0.605399933	0.901	0.703	0.0005566	5
gsta.1.13	2.41E-08	0.80273597	0.958	0.825	0.0005736	5
stoml3b2	3.05E-08	0.690835168	0.789	0.451	0.0007281	5
ndufb31	3.18E-08	0.607523567	0.873	0.619	0.0007593	5
gpx4a2	7.46E-08	0.669014385	0.901	0.562	0.0017776	5
tspan13a2	8.46E-08	0.632532158	0.676	0.392	0.002017	5
mgst3a2	8.99E-08	0.577713397	0.944	0.689	0.0021447	5
uqcrb1	1.01E-07	0.512349221	0.944	0.82	0.0023968	5
ndufs52	1.19E-07	0.653647555	0.845	0.608	0.0028261	5
si:ch211-133l5.71	1.27E-07	0.611697511	0.676	0.376	0.0030174	5
mrpl401	1.28E-07	0.53466712	0.634	0.366	0.0030628	5
aurkaip12	1.31E-07	0.66840266	0.662	0.383	0.003131	5
ndufb71	1.47E-07	0.604332676	0.859	0.663	0.0035167	5
s100a10a2	1.58E-07	0.599475034	0.972	0.805	0.0037777	5
ndufa122	1.67E-07	0.555160741	0.887	0.606	0.0039738	5
pgrmc12	1.97E-07	0.572629594	0.859	0.547	0.0046862	5
atp5mf1	2.06E-07	0.510341312	0.944	0.831	0.0049071	5
uqcr101	2.30E-07	0.630167291	0.887	0.746	0.0054902	5
cycsb1	2.65E-07	0.52392994	0.958	0.821	0.0063166	5
snu13b2	3.29E-07	0.430788298	0.901	0.576	0.00784	5
ndufb83	3.30E-07	0.395374885	0.915	0.676	0.0078657	5
polr2l	4.08E-07	0.401583301	0.718	0.457	0.0097189	5

si:dkey-33i11.9	4.39E-07	0.340714702	0.859	0.558	0.0104641	5
ndufa71	5.06E-07	0.50067213	0.761	0.519	0.0120555	5
hspe13	5.29E-07	0.548310011	0.972	0.779	0.0126042	5
si:ch211-71m22.12	5.66E-07	0.622810198	0.789	0.497	0.0134963	5
mrpl202	6.12E-07	0.607652371	0.676	0.416	0.0146025	5
ndufab1b2	6.83E-07	0.575421356	0.817	0.602	0.016295	5
sf3b51	6.99E-07	0.479493578	0.789	0.558	0.0166599	5
cox6c2	7.04E-07	0.512197839	0.944	0.746	0.0167937	5
chchd103	7.35E-07	0.454046479	0.831	0.501	0.0175288	5
cox5ab2	8.38E-07	0.607043672	0.93	0.709	0.0199864	5
ndufa21	9.56E-07	0.541336167	0.761	0.569	0.0227874	5
CABZ01079011.11	9.81E-07	0.777086708	0.704	0.486	0.0233882	5
zgc:1935411	9.87E-07	0.583141679	0.887	0.761	0.0235237	5
uqcc2	1.18E-06	0.405074718	0.634	0.374	0.0282155	5
dbi2	1.33E-06	0.556478117	0.93	0.777	0.0316201	5
ugt1ab2	1.42E-06	0.613990736	0.803	0.449	0.0337776	5
nupr12	1.49E-06	0.544549834	0.972	0.746	0.0356004	5
cldn15a2	1.50E-06	0.49438083	0.831	0.466	0.0358077	5
si:dkeyp-73b11.82	1.56E-06	0.567933319	0.986	0.759	0.0370978	5
ATP5MD2	1.59E-06	0.571679159	0.915	0.716	0.037989	5
si:dkey-36i7.32	1.84E-06	0.481175912	0.944	0.617	0.0438477	5
cox6b11	1.89E-06	0.464553967	0.915	0.738	0.0450864	5
adh8b2	2.10E-06	0.521693846	0.901	0.494	0.0499902	5
gcshb2	2.82E-06	0.361117742	0.831	0.46	0.0671705	5
RPL411	2.84E-06	0.390121976	1	0.998	0.0677965	5
dap2	3.00E-06	0.585182657	0.873	0.652	0.0715346	5
pam161	3.46E-06	0.460231007	0.634	0.413	0.082595	5
ndufs61	3.76E-06	0.43456763	0.845	0.602	0.0895523	5
zgc:172079.22	4.03E-06	0.760536373	0.732	0.462	0.0961384	5
rpl381	4.45E-06	0.424862785	0.986	0.982	0.1060157	5
ndufa51	5.56E-06	0.469656207	0.831	0.628	0.1325656	5
chchd21	5.64E-06	0.558831504	0.845	0.6	0.1344212	5
ndufs42	5.97E-06	0.408076459	0.803	0.573	0.1423296	5
mrpl331	6.00E-06	0.445987453	0.69	0.433	0.1429604	5
llph2	6.04E-06	0.401103937	0.648	0.42	0.1439843	5
rps15a1	6.34E-06	0.33863012	1	0.961	0.1511642	5
cyp2k162	6.43E-06	0.480341447	0.718	0.424	0.1533773	5
mrpl141	6.66E-06	0.378031751	0.676	0.429	0.1587987	5
sod12	6.72E-06	0.498168125	0.901	0.7	0.1603564	5
cox7a2a2	6.82E-06	0.460758966	0.93	0.731	0.1626874	5
si:dkey-183n20.152	7.39E-06	0.392981482	0.451	0.234	0.1761314	5
mrpl112	7.51E-06	0.467517027	0.761	0.514	0.1791653	5
timm92	8.64E-06	0.35714159	0.648	0.398	0.2059739	5
cox6a12	9.46E-06	0.466925487	0.972	0.831	0.2256895	5
sult3st32	9.52E-06	0.62254406	0.648	0.405	0.2269138	5
btf3141	1.12E-05	0.324425268	0.746	0.495	0.2670203	5
rplp1	1.14E-05	0.297501859	1	0.983	0.271416	5
anxa2b2	1.29E-05	0.506082914	0.958	0.751	0.3073359	5
ndufa62	1.31E-05	0.311804471	0.873	0.674	0.3125289	5
mrpl282	1.39E-05	0.379501266	0.62	0.379	0.3304787	5
ost4	1.46E-05	0.364684417	0.887	0.718	0.3484719	5
ndufb21	1.89E-05	0.369871583	0.873	0.654	0.4499845	5
cox171	1.89E-05	0.29744271	0.845	0.61	0.450472	5
ndufb62	1.89E-05	0.308830546	0.845	0.58	0.4516509	5

scp2a3	1.96E-05	0.372180176	0.761	0.453	0.4682334	5
cox8b2	2.01E-05	0.507714593	0.845	0.599	0.4790113	5
mt-atp81	2.11E-05	0.478540019	0.732	0.541	0.5033805	5
mrps18c2	2.39E-05	0.392526599	0.69	0.446	0.5689249	5
mrpl572	2.43E-05	0.379579312	0.746	0.54	0.5803174	5
rps241	2.55E-05	0.324676364	0.986	0.965	0.6082955	5
cox142	3.08E-05	0.465847538	0.662	0.466	0.7348968	5
c1qbp2	3.13E-05	0.404736995	0.944	0.685	0.7455803	5
mrpl92	3.21E-05	0.286580083	0.507	0.28	0.7665226	5
rida2	3.33E-05	0.515583429	0.732	0.468	0.7937271	5
apoa4b.21	3.35E-05	0.402630256	0.451	0.239	0.7992986	5
minos12	3.46E-05	0.391390502	0.817	0.587	0.824364	5
tomm72	3.86E-05	0.294897342	0.817	0.615	0.9214259	5
rpl361	3.91E-05	0.358105729	0.986	0.95	0.9316941	5
mrps161	4.07E-05	0.344650775	0.563	0.354	0.9696441	5
rpl37.12	4.41E-05	0.435895272	0.986	0.98	1	5
lsm6	4.52E-05	0.389268453	0.746	0.565	1	5
gamt2	4.75E-05	0.492937311	0.789	0.512	1	5
rpl231	4.85E-05	0.304612654	0.986	0.983	1	5
hsd17b101	4.88E-05	0.59387249	0.465	0.276	1	5
rps231	5.18E-05	0.333012904	1	0.972	1	5
mrpl322	5.33E-05	0.410704062	0.634	0.449	1	5
mpc13	5.68E-05	0.3439426	0.775	0.558	1	5
sub1a1	5.83E-05	0.327511573	0.859	0.641	1	5
naa381	6.28E-05	0.261621506	0.493	0.291	1	5
tomm52	6.38E-05	0.403984589	0.746	0.536	1	5
snrpd21	7.02E-05	0.295084279	0.789	0.61	1	5
COX5B2	7.30E-05	0.392729	0.944	0.812	1	5
si:dkey-88p24.111	7.57E-05	0.396389238	0.606	0.39	1	5
supt4h11	7.63E-05	0.264546396	0.62	0.418	1	5
hint11	7.66E-05	0.350706416	0.775	0.565	1	5
mrpl411	8.19E-05	0.441898642	0.507	0.315	1	5
rpl36a	8.54E-05	0.305565531	1	0.982	1	5
ndufb41	8.68E-05	0.333118304	0.845	0.637	1	5
gadd45gip11	8.76E-05	0.274340303	0.648	0.431	1	5
mrps17	8.95E-05	0.390539396	0.521	0.33	1	5
NDUFC11	9.13E-05	0.294871375	0.789	0.597	1	5
naca1	9.21E-05	0.297568346	0.972	0.934	1	5
ndufb52	9.95E-05	0.458168559	0.732	0.536	1	5
mt-nd32	0.0001017	0.472015051	0.831	0.761	1	5
zgc:564931	0.0001022	0.328442556	0.958	0.829	1	5
cox20	0.0001075	0.370774213	0.521	0.33	1	5
tmem2581	0.0001106	0.374000889	0.873	0.68	1	5
lsm3	0.0001124	0.433235749	0.676	0.49	1	5
cbr113	0.0001177	0.381761845	0.606	0.392	1	5
zgc:1588462	0.000128	0.435952026	1	0.748	1	5
tmem971	0.0001376	0.408969283	0.493	0.291	1	5
mt-nd41	0.0001386	0.34924405	0.662	0.49	1	5
atp5mc12	0.0001388	0.440300933	0.986	0.785	1	5
mrps242	0.0001405	0.37416804	0.648	0.446	1	5
tomm70a	0.0001459	0.491131016	0.704	0.516	1	5
tomm62	0.0001474	0.371031305	0.803	0.611	1	5
ndufa82	0.0001495	0.266010763	0.831	0.58	1	5
snrpd1	0.0001518	0.333337138	0.859	0.692	1	5

gstp21	0.0001555	0.655389057	0.465	0.258	1	5
smdt1b	0.0001639	0.405579285	0.761	0.567	1	5
gstt1b	0.0001815	0.463997877	0.676	0.459	1	5
gstp12	0.0001818	0.398325568	0.986	0.79	1	5
gsta.22	0.0001828	0.42701099	0.423	0.239	1	5
aimp11	0.0001892	0.428711284	0.69	0.517	1	5
nop101	0.0001949	0.318301346	0.775	0.589	1	5
atp5f1d2	0.0002105	0.462640251	0.887	0.727	1	5
mrpl272	0.0002128	0.359119495	0.549	0.361	1	5
dhdhl2	0.0002133	0.373887368	0.746	0.475	1	5
prdx63	0.0002221	0.271262222	0.718	0.51	1	5
tmsb1	0.0002352	0.287713688	0.972	0.783	1	5
rps261	0.0002369	0.290138357	1	0.958	1	5
ndufc21	0.0002494	0.417536426	0.831	0.683	1	5
mt-atp61	0.0002512	0.411242553	0.901	0.915	1	5
si:ch211-162e15.3	0.0002516	0.306802123	0.507	0.326	1	5
mt-nd51	0.0002583	0.506705277	0.845	0.751	1	5
chchd12	0.0002699	0.284308071	0.521	0.324	1	5
rps27.2	0.000273	0.306827487	0.944	0.849	1	5
ptges3b1	0.0002876	0.25693829	0.803	0.587	1	5
uqcrfs12	0.0002877	0.365565425	0.789	0.586	1	5
tfam1	0.0003068	0.522296333	0.493	0.335	1	5
dnph11	0.0003111	0.50599322	0.38	0.221	1	5
denr1	0.0003145	0.301812215	0.704	0.494	1	5
mrps332	0.0003214	0.263868375	0.606	0.383	1	5
dhrs42	0.0003257	0.385940667	0.535	0.35	1	5
ckmt12	0.0003592	0.332215098	0.761	0.483	1	5
timm441	0.0003684	0.367531964	0.465	0.28	1	5
bxdc22	0.000372	0.323732064	0.676	0.459	1	5
ppifb2	0.0004013	0.35574149	0.563	0.365	1	5
aifm41	0.0004116	0.2666552	0.352	0.182	1	5
prdx22	0.0004117	0.361244578	0.986	0.829	1	5
slc31a12	0.0004207	0.864461315	0.606	0.403	1	5
zgc:162780	0.0004222	0.373033199	0.352	0.188	1	5
psma6b	0.0004274	0.300845131	0.62	0.446	1	5
ndufa42	0.0004303	0.25763187	0.746	0.499	1	5
mt-nd42	0.0004329	0.414711343	0.831	0.838	1	5
snrpe1	0.0004336	0.27826735	0.845	0.659	1	5
gpx1a2	0.0004382	0.350225068	0.775	0.545	1	5
AL831745.12	0.000442	0.59461589	0.507	0.339	1	5
cox7c1	0.0004429	0.323495503	0.93	0.82	1	5
gstt1a2	0.0004546	0.268231198	0.915	0.569	1	5
chchd7	0.0004811	0.434830366	0.521	0.344	1	5
mt-cyb2	0.000485	0.356187222	0.901	0.904	1	5
ndufa411	0.0004927	0.262245306	0.93	0.772	1	5
mrpl522	0.0005018	0.364678992	0.62	0.449	1	5
rpl14	0.0005089	0.254809719	0.986	0.967	1	5
mt-co11	0.0005095	0.387614786	0.901	0.897	1	5
rpl26	0.0005166	0.334418005	0.859	0.724	1	5
mt-nd12	0.0005357	0.297782942	0.831	0.853	1	5
mt-co31	0.0005566	0.342413215	0.972	0.93	1	5
tma71	0.0006099	0.36658024	0.887	0.779	1	5
fkbp1aa	0.0006148	0.306010445	0.93	0.742	1	5
mt-co22	0.0006585	0.286550248	0.958	0.936	1	5

pdzk11	0.0006912	0.528590448	0.521	0.324	1	5
cldn15la2	0.0007072	0.358344347	1	0.786	1	5
rpsl21	0.000721	0.331722656	0.986	0.959	1	5
pbld2	0.0007679	0.296254943	0.493	0.317	1	5
aifm21	0.000825	0.327209961	0.493	0.326	1	5
rwdd11	0.000847	0.366647408	0.662	0.499	1	5
triap11	0.0009087	0.443839088	0.437	0.285	1	5
pdx1	0.0010403	0.298732259	0.38	0.206	1	5
EIF1B1	0.0011111	0.261272453	0.718	0.586	1	5
mt-nd22	0.0011139	0.291562999	0.831	0.847	1	5
selenoh	0.0012223	0.384054035	0.535	0.398	1	5
pfdn61	0.0013442	0.275001802	0.606	0.481	1	5
znf5931	0.0014048	0.369217844	0.577	0.448	1	5
atp5pd2	0.0014954	0.312692928	0.915	0.772	1	5
cox5aa2	0.0015194	0.318427155	0.944	0.744	1	5
mrps252	0.0015261	0.277779425	0.634	0.44	1	5
rps21	0.0015671	0.272708452	0.986	0.963	1	5
si:ch211-217k17.71	0.0016381	0.327795303	0.662	0.494	1	5
mrpl31	0.0016476	0.319622497	0.423	0.267	1	5
mrpl53	0.001673	0.298916138	0.493	0.337	1	5
CU682777.22	0.0017686	0.270068165	0.761	0.523	1	5
mbl23	0.0017921	0.478893236	0.408	0.245	1	5
mrps211	0.0018815	0.445694425	0.577	0.409	1	5
pagr1	0.0020044	0.292537218	0.324	0.192	1	5
cbx3a	0.0020305	0.385262053	0.662	0.516	1	5
idh3a1	0.0020546	0.384041898	0.648	0.477	1	5
mrpl362	0.0021614	0.386652692	0.592	0.422	1	5
mrpl461	0.0022202	0.341126288	0.437	0.287	1	5
fdps2	0.0022234	0.309305374	0.592	0.401	1	5
smim81	0.0023906	0.281229261	0.437	0.295	1	5
eppk12	0.0023979	0.364038864	0.761	0.558	1	5
DUT1	0.0024192	0.389595069	0.592	0.438	1	5
anks4b2	0.0024437	0.324782486	0.62	0.407	1	5
mpc21	0.002445	0.300078881	0.606	0.435	1	5
magoh1	0.0025106	0.333968888	0.662	0.506	1	5
rtn4b2	0.0026	0.325753443	0.592	0.414	1	5
dhrs13l11	0.0027216	0.42335318	0.352	0.219	1	5
spink41	0.0028527	0.43479349	0.535	0.392	1	5
abcf1	0.0030119	0.300296391	0.845	0.659	1	5
rps51	0.003063	0.276662031	0.986	0.961	1	5
ndufs72	0.003121	0.385707677	0.775	0.54	1	5
atp5f1c2	0.0031493	0.304376501	0.831	0.587	1	5
rsrp1	0.0032431	0.291467331	0.859	0.659	1	5
mrpl49	0.0036529	0.529394608	0.507	0.354	1	5
rdh12	0.0039407	0.356549733	0.437	0.254	1	5
mrpl452	0.0040341	0.36703693	0.465	0.324	1	5
emg11	0.0040385	0.258017627	0.338	0.21	1	5
ndufs32	0.0041683	0.373955244	0.634	0.464	1	5
eif3ja1	0.0041902	0.313427034	0.718	0.545	1	5
mrps72	0.0045574	0.275683808	0.493	0.343	1	5
hirip3	0.0045793	0.378487167	0.338	0.21	1	5
nop162	0.0053501	0.355650266	0.521	0.385	1	5
rrs1	0.0054858	0.323411366	0.535	0.405	1	5
sult1st6	0.005595	0.303223097	0.296	0.18	1	5

dtymk1	0.0060094	0.261280745	0.296	0.184	1	5
auh1	0.0065777	0.338032741	0.437	0.306	1	5
nr0b2a	0.0073763	0.304784261	0.324	0.193	1	5
hccsa.1	0.0075006	0.349266614	0.31	0.199	1	5
sub1b2	0.007682	0.385890919	0.465	0.355	1	5
polr2k1	0.0079365	0.324342108	0.577	0.444	1	5
nedd8	0.0083938	0.253357128	0.493	0.372	1	5
eif3g2	0.0085142	0.362669922	0.789	0.587	1	5
cyp8b12	0.0086687	0.497012042	0.38	0.252	1	5
zgc:777392	0.0086785	0.331784876	0.366	0.239	1	5
zgc:1121461	0.0089051	0.287908939	0.324	0.204	1	5
cdkn1a1	0.0091337	0.267113571	0.394	0.271	1	5
mrps18a3	0.0092577	0.275539668	0.493	0.379	1	5
ceacam1	7.21E-94	3.585484322	0.788	0.009	1.72E-89	6
mnx1	2.46E-73	1.893766122	0.558	0	5.86E-69	6
myof	9.05E-72	1.715400854	0.654	0.011	2.16E-67	6
zgc:92380	3.20E-68	3.135976864	0.904	0.069	7.63E-64	6
wnt11r	5.32E-67	0.926439663	0.596	0.009	1.27E-62	6
anxa5b	2.02E-63	2.34770854	0.846	0.06	4.81E-59	6
cnfn	2.55E-59	3.721767609	0.654	0.027	6.07E-55	6
sftpba	4.08E-56	1.901785443	0.481	0.005	9.74E-52	6
ctrl	1.27E-55	3.696817072	0.462	0.004	3.03E-51	6
si:cabz01007794.1	1.06E-53	1.54886133	0.635	0.032	2.53E-49	6
tspan35	7.65E-53	1.20434227	0.673	0.039	1.83E-48	6
zgc:162879	1.46E-52	0.898587505	0.538	0.016	3.47E-48	6
gna15.1	7.56E-49	0.807119883	0.577	0.027	1.80E-44	6
abca12	1.03E-47	3.05621649	0.558	0.027	2.46E-43	6
s100w	2.97E-47	1.184340675	0.462	0.011	7.07E-43	6
fer1l4	1.43E-46	1.460539724	0.692	0.053	3.41E-42	6
capgb	1.36E-45	1.38551893	0.654	0.048	3.25E-41	6
si:ch73-288o11.5	2.13E-45	2.374071025	0.385	0.004	5.08E-41	6
BX908782.2	1.01E-44	4.443056323	0.462	0.014	2.41E-40	6
anxa3a	5.76E-44	0.608815828	0.5	0.02	1.37E-39	6
vwa1	6.12E-43	1.754835876	0.692	0.064	1.46E-38	6
glud1a	1.18E-41	1.079787315	0.673	0.062	2.81E-37	6
tmem136a	1.23E-41	0.673527746	0.442	0.014	2.94E-37	6
opcml	1.53E-41	0.565078009	0.404	0.009	3.66E-37	6
agr1	2.12E-41	1.204133585	0.538	0.03	5.06E-37	6
oclnb	2.63E-41	0.521774146	0.442	0.014	6.28E-37	6
zgc:91860	1.80E-40	0.559061422	0.442	0.016	4.29E-36	6
lgals3b	1.91E-40	1.970088926	0.904	0.178	4.57E-36	6
atp10b	2.00E-40	0.424991394	0.308	0	4.77E-36	6
zgc:158403	4.54E-40	0.905237994	0.615	0.052	1.08E-35	6
myl9a	6.11E-40	1.025295139	0.596	0.046	1.46E-35	6
cx35.4	2.14E-38	0.590345789	0.462	0.021	5.11E-34	6
mal	4.71E-38	2.335333404	0.673	0.071	1.12E-33	6
itpka	7.92E-38	0.838563722	0.538	0.037	1.89E-33	6
pard3bb	9.64E-38	0.609557112	0.577	0.046	2.30E-33	6
sox2	4.03E-37	0.731482649	0.462	0.023	9.60E-33	6
si:dkey-30c15.2	4.56E-37	0.659492476	0.365	0.009	1.09E-32	6
ahnak	4.93E-37	2.947520331	0.942	0.254	1.17E-32	6
capn2b	1.16E-36	0.982067348	0.481	0.028	2.77E-32	6
si:dkey-222n6.2	2.47E-36	0.850177395	0.346	0.007	5.88E-32	6
si:dkey-19b23.12	3.42E-36	0.580041539	0.385	0.012	8.16E-32	6

cav1	6.06E-36	1.896255387	0.654	0.078	1.45E-31	6
cal5a	1.79E-35	0.611893037	0.308	0.004	4.26E-31	6
agr2	1.97E-34	2.343181789	0.942	0.26	4.70E-30	6
s100v2	4.26E-34	1.38493646	0.673	0.085	1.02E-29	6
sox19a	6.78E-34	0.460606993	0.327	0.007	1.62E-29	6
myh9a	9.03E-34	2.078173301	0.808	0.149	2.15E-29	6
si:dkey-262k9.2	1.23E-33	2.075068633	0.788	0.157	2.94E-29	6
si:ch211-264f5.6	2.94E-33	0.455300936	0.423	0.023	7.02E-29	6
si:dkey-208c12.2	7.10E-33	0.475230186	0.269	0.002	1.69E-28	6
cabp2b	1.21E-32	1.717474133	0.538	0.053	2.88E-28	6
zgc:174164	2.01E-32	0.50524053	0.365	0.014	4.79E-28	6
stox2a	2.85E-32	1.025633744	0.615	0.073	6.79E-28	6
si:dkeyp-69e1.8	6.74E-32	0.531415644	0.5	0.041	1.61E-27	6
ptgs2a	3.29E-31	2.167940122	0.712	0.117	7.84E-27	6
kbtbd11	3.44E-31	0.368024549	0.288	0.005	8.21E-27	6
clic2	4.62E-31	1.021887799	0.577	0.059	1.10E-26	6
mid1ip1a	4.99E-31	1.196188324	0.558	0.059	1.19E-26	6
rnf183	8.84E-31	0.725459976	0.519	0.05	2.11E-26	6
bcam1	2.82E-30	2.356239922	0.865	0.226	6.73E-26	6
enosf1	4.25E-30	0.426041182	0.423	0.027	1.01E-25	6
tmem120a	5.36E-30	0.861590938	0.596	0.077	1.28E-25	6
rin2	5.61E-30	0.528414343	0.519	0.052	1.34E-25	6
smox	6.87E-29	0.639542634	0.615	0.087	1.64E-24	6
anxa3b	7.11E-29	1.969992268	0.712	0.141	1.69E-24	6
aqp1a.1	8.04E-29	3.532088282	0.442	0.037	1.92E-24	6
cd151	1.30E-28	0.419953869	0.346	0.016	3.10E-24	6
anxa2a	1.61E-28	2.195689696	0.712	0.13	3.85E-24	6
s100a10b	2.76E-28	2.526751943	0.962	0.384	6.58E-24	6
rpz5	3.05E-28	1.083817712	0.404	0.028	7.28E-24	6
padi2	3.44E-28	0.613950671	0.481	0.046	8.20E-24	6
cav2	6.41E-28	0.703194092	0.442	0.037	1.53E-23	6
cobl11b	8.10E-28	0.465423533	0.558	0.066	1.93E-23	6
icn	9.25E-28	4.527781932	1	0.696	2.20E-23	6
si:ch211-157p22.10	2.60E-27	0.451377488	0.519	0.057	6.20E-23	6
si:dkey-19b23.8	6.48E-27	0.434602202	0.423	0.034	1.55E-22	6
sox13	7.55E-27	0.450956765	0.269	0.007	1.80E-22	6
wif1	1.58E-26	1.455804671	0.442	0.043	3.77E-22	6
nipal4	1.76E-26	0.37137944	0.327	0.016	4.21E-22	6
rhogb	1.79E-26	0.747471944	0.577	0.082	4.27E-22	6
muc5.3	2.26E-26	0.743160708	0.288	0.011	5.38E-22	6
zgc:92818	2.87E-26	0.802776526	0.635	0.101	6.83E-22	6
zyx	3.86E-26	1.100949984	0.692	0.132	9.21E-22	6
si:ch211-202f5.3	4.11E-26	0.708773171	0.577	0.08	9.80E-22	6
cwc15	5.14E-26	1.089090708	0.769	0.162	1.23E-21	6
palm2.1	5.47E-26	0.877209046	0.423	0.039	1.30E-21	6
basp11	8.94E-26	1.297799079	0.885	0.256	2.13E-21	6
abca3b1	1.95E-25	0.942689801	0.615	0.1	4.65E-21	6
zgc:174917	2.10E-25	2.389015456	0.346	0.021	5.02E-21	6
hk2	2.40E-25	0.660786655	0.481	0.053	5.73E-21	6
spock3	2.49E-25	2.273829123	0.423	0.041	5.94E-21	6
serpinb14	3.44E-25	0.912491184	0.692	0.141	8.20E-21	6
cldn7b1	3.98E-25	1.357508088	0.923	0.283	9.49E-21	6
zgc:92480	1.16E-24	0.258412095	0.288	0.012	2.77E-20	6
calm1b1	1.96E-24	1.059249178	0.788	0.174	4.68E-20	6

rab32a	5.21E-24	1.265445785	0.481	0.062	1.24E-19	6
foxp41	7.10E-24	1.677479856	0.942	0.368	1.69E-19	6
frem2b	9.91E-24	0.70418418	0.327	0.021	2.36E-19	6
pnp5a	1.06E-23	1.109551515	0.769	0.176	2.53E-19	6
nr4a3	1.10E-23	0.568632414	0.596	0.096	2.62E-19	6
cxcr3.3	1.32E-23	0.454200893	0.462	0.052	3.15E-19	6
vamp8	1.91E-23	0.428894862	0.423	0.043	4.55E-19	6
cavin2b	3.15E-23	1.618574611	0.788	0.24	7.51E-19	6
ical1	4.14E-23	0.396404362	0.404	0.039	9.87E-19	6
sytl3.11	4.63E-23	1.496546267	0.904	0.354	1.10E-18	6
slc38a5a1	8.28E-23	0.672341329	0.577	0.098	1.97E-18	6
col4a3bpa	8.80E-23	0.389296134	0.5	0.068	2.10E-18	6
pgm2l1	9.83E-23	0.498383055	0.365	0.032	2.34E-18	6
si:dkey-222f2.1	1.92E-22	0.351762911	0.269	0.012	4.57E-18	6
qkia1	2.85E-22	1.1907679	0.75	0.196	6.78E-18	6
tp53bp2b	2.87E-22	0.583147018	0.5	0.069	6.83E-18	6
ltb4r2a	2.93E-22	0.439791372	0.346	0.028	6.99E-18	6
stx3a	3.33E-22	0.735804546	0.673	0.144	7.94E-18	6
ajuba	5.48E-22	0.484453922	0.462	0.06	1.31E-17	6
fam83fa	6.12E-22	0.363400484	0.365	0.032	1.46E-17	6
CLDN23	6.41E-22	0.693493149	0.538	0.087	1.53E-17	6
sh3d21	6.51E-22	0.757598708	0.635	0.132	1.55E-17	6
abhd17b	6.54E-22	0.819415692	0.692	0.158	1.56E-17	6
plxnb1a	1.50E-21	0.486424304	0.423	0.05	3.57E-17	6
tcnl	1.53E-21	4.546210849	0.577	0.114	3.65E-17	6
grnb	1.58E-21	0.386384318	0.385	0.039	3.77E-17	6
litaf	1.97E-21	0.526083535	0.538	0.084	4.69E-17	6
scel	2.17E-21	0.715077341	0.327	0.025	5.17E-17	6
gdpd3a	2.18E-21	0.537111564	0.462	0.062	5.19E-17	6
cldnb2	2.31E-21	1.861406582	1	0.758	5.50E-17	6
b4galnt3b	2.65E-21	0.424759687	0.442	0.053	6.31E-17	6
syngr2a1	5.64E-21	0.93442373	0.692	0.173	1.34E-16	6
tprg1	5.86E-21	1.684769063	0.731	0.217	1.40E-16	6
ftr83	9.15E-21	1.651010133	0.673	0.164	2.18E-16	6
tmem238a	9.75E-21	0.425887881	0.481	0.069	2.32E-16	6
rbpms2a1	1.21E-20	0.37946972	0.519	0.078	2.89E-16	6
zgc:194930	1.24E-20	0.533867532	0.577	0.105	2.95E-16	6
FP102018.1	1.29E-20	0.868756854	0.288	0.02	3.07E-16	6
mroh1	1.55E-20	0.489091746	0.481	0.069	3.70E-16	6
cd99	1.64E-20	0.355570949	0.462	0.062	3.91E-16	6
capn2a	2.03E-20	1.074120399	0.558	0.109	4.84E-16	6
slc38a5b	2.45E-20	1.007872758	0.827	0.27	5.85E-16	6
arl4cb	2.64E-20	0.634247937	0.423	0.055	6.30E-16	6
rabac1	4.21E-20	0.500688832	0.635	0.133	1.00E-15	6
igf1rb	4.36E-20	0.424323224	0.481	0.068	1.04E-15	6
si:dkey-222f8.31	4.68E-20	0.800603055	0.596	0.123	1.12E-15	6
iqgap2	8.73E-20	1.04878776	0.75	0.217	2.08E-15	6
sytl4	1.09E-19	0.778060641	0.442	0.064	2.61E-15	6
hk11	1.33E-19	0.851808685	0.481	0.077	3.18E-15	6
si:ch211-132p1.3	1.50E-19	1.000256526	0.346	0.034	3.57E-15	6
sgsm31	1.56E-19	0.319101522	0.5	0.078	3.71E-15	6
raraa	1.67E-19	1.321329699	0.692	0.203	3.99E-15	6
ccdc125	2.22E-19	0.917008471	0.462	0.069	5.29E-15	6
sh3bp4a	2.24E-19	0.550233185	0.442	0.06	5.34E-15	6

sept9a	3.49E-19	0.678813931	0.615	0.141	8.32E-15	6
cavin2a	4.29E-19	0.838709357	0.615	0.148	1.02E-14	6
fn1b	4.70E-19	2.993483206	0.462	0.075	1.12E-14	6
rock2b.1	6.31E-19	0.456708453	0.635	0.144	1.50E-14	6
si:ch211-153b23.51	9.71E-19	2.44204505	0.731	0.194	2.31E-14	6
tspan3a1	1.11E-18	0.506964342	0.558	0.11	2.66E-14	6
penkb	1.42E-18	0.3524988	0.288	0.023	3.39E-14	6
paqr5b	1.44E-18	0.377393978	0.327	0.032	3.43E-14	6
nxn	1.46E-18	0.371392624	0.385	0.046	3.49E-14	6
itm2bb	1.83E-18	0.960343731	0.769	0.269	4.36E-14	6
tgfbr2b	1.99E-18	0.260524386	0.327	0.032	4.75E-14	6
fgfr2l	2.21E-18	1.271210175	0.731	0.226	5.27E-14	6
cd9b	2.45E-18	1.579061773	0.942	0.587	5.83E-14	6
col4a6	2.83E-18	1.647331531	0.481	0.087	6.75E-14	6
anxa1a	2.87E-18	1.026300771	0.538	0.101	6.85E-14	6
flot1b	2.92E-18	0.885901449	0.827	0.292	6.96E-14	6
rab9a	4.04E-18	0.304841184	0.404	0.055	9.63E-14	6
prr15lb	4.77E-18	0.926207815	0.423	0.066	1.14E-13	6
lamb2l	4.92E-18	1.102938749	0.712	0.21	1.17E-13	6
itgb6	5.26E-18	0.531100212	0.596	0.13	1.25E-13	6
atp6v1e1a	6.44E-18	0.254045607	0.423	0.064	1.54E-13	6
cgnb1	6.59E-18	0.705319401	0.615	0.142	1.57E-13	6
ralab1	7.12E-18	0.727836189	0.731	0.201	1.70E-13	6
actb1l	9.74E-18	1.323346024	0.981	0.968	2.32E-13	6
parp4	1.16E-17	0.525538561	0.558	0.125	2.77E-13	6
f3a	1.28E-17	1.151290562	0.558	0.126	3.04E-13	6
capns1a	1.28E-17	1.202550062	0.865	0.383	3.05E-13	6
cadps2	1.47E-17	0.63817395	0.538	0.11	3.50E-13	6
btbd10b	1.87E-17	0.762351973	0.827	0.31	4.46E-13	6
si:ch211-266g18.6	2.53E-17	1.567900952	0.769	0.278	6.02E-13	6
st5	3.02E-17	0.353631406	0.404	0.057	7.20E-13	6
id1l	3.65E-17	0.997974795	0.827	0.285	8.71E-13	6
slc17a9b	3.80E-17	0.440768123	0.462	0.08	9.07E-13	6
msna	4.04E-17	2.42184889	0.577	0.141	9.64E-13	6
pkma1	5.07E-17	1.141809652	0.75	0.274	1.21E-12	6
arhgap29b	5.08E-17	0.503632389	0.346	0.043	1.21E-12	6
pip5k1ab	5.22E-17	0.290953935	0.462	0.08	1.25E-12	6
zgc:77650	6.86E-17	0.716421598	0.692	0.206	1.64E-12	6
krt94l	7.32E-17	1.655132012	0.462	0.082	1.74E-12	6
rab27a	7.38E-17	0.356731565	0.327	0.037	1.76E-12	6
npc1	9.94E-17	0.707327371	0.731	0.244	2.37E-12	6
baiap2l1b	1.09E-16	0.623005534	0.538	0.119	2.59E-12	6
slc35c2	1.12E-16	0.799512675	0.538	0.121	2.68E-12	6
map3k21	1.19E-16	0.550318252	0.596	0.144	2.84E-12	6
rnf38l	1.37E-16	0.471471228	0.538	0.117	3.26E-12	6
cx43.4	1.72E-16	0.324101058	0.442	0.071	4.10E-12	6
flna1	1.98E-16	0.539809558	0.635	0.151	4.73E-12	6
ptprfb1	2.00E-16	1.165260095	0.808	0.368	4.76E-12	6
zdhhc13	2.15E-16	0.493238532	0.673	0.198	5.13E-12	6
alox12	2.36E-16	0.722177501	0.558	0.133	5.62E-12	6
itgav	2.60E-16	0.356772086	0.404	0.062	6.20E-12	6
CR753886.1	2.72E-16	0.39718984	0.423	0.069	6.48E-12	6
stm1	2.85E-16	3.834214493	0.5	0.117	6.79E-12	6
stxbp2l	3.46E-16	0.451440763	0.577	0.133	8.26E-12	6

bnip3lb1	3.77E-16	0.68240062	0.5	0.101	8.99E-12	6
agrn	4.00E-16	1.575187591	0.808	0.335	9.55E-12	6
selenow2b	4.19E-16	0.40655856	0.365	0.052	1.00E-11	6
zgc:100829	5.55E-16	0.280443622	0.423	0.068	1.32E-11	6
trib1	6.04E-16	0.325337751	0.5	0.098	1.44E-11	6
sgms1	6.05E-16	0.634264269	0.346	0.048	1.44E-11	6
clip2l	7.76E-16	0.420907489	0.404	0.062	1.85E-11	6
tmem189	8.92E-16	0.56815864	0.654	0.192	2.13E-11	6
tmem54a1	8.99E-16	0.351965876	0.5	0.098	2.14E-11	6
si:ch211-153b23.4	9.02E-16	0.963040763	0.346	0.046	2.15E-11	6
pkig	9.12E-16	0.357975963	0.385	0.059	2.18E-11	6
ctgfa1	9.40E-16	1.278628965	0.577	0.144	2.24E-11	6
arl8	9.76E-16	0.311208227	0.385	0.06	2.33E-11	6
ppdpfb1	1.01E-15	1.350457784	0.923	0.521	2.41E-11	6
foxa2	1.10E-15	0.441902797	0.327	0.041	2.62E-11	6
rrbp1b	1.16E-15	0.883663572	0.577	0.157	2.77E-11	6
cpda1	1.21E-15	0.657836495	0.5	0.109	2.88E-11	6
slc4a10a	1.33E-15	0.355715658	0.308	0.036	3.16E-11	6
alcama1	1.44E-15	1.032741126	0.404	0.069	3.43E-11	6
zmp:0000001114	1.49E-15	0.3281354	0.404	0.066	3.55E-11	6
slc7a10a1	1.63E-15	0.486901871	0.423	0.071	3.89E-11	6
timp2b	1.65E-15	0.408685521	0.5	0.105	3.94E-11	6
actb21	1.70E-15	1.216578467	1	0.959	4.06E-11	6
spint21	1.76E-15	0.77205686	0.788	0.322	4.19E-11	6
plscr3b	1.77E-15	0.483200487	0.5	0.107	4.22E-11	6
hip1rb1	1.81E-15	0.542369525	0.558	0.139	4.32E-11	6
zfhx32	1.85E-15	0.692709937	0.731	0.233	4.41E-11	6
s100v1	1.97E-15	1.175961423	0.865	0.443	4.69E-11	6
si:ch211-105c13.3	2.03E-15	0.955264586	0.538	0.123	4.83E-11	6
mvp	2.13E-15	1.560104586	0.923	0.598	5.08E-11	6
palm3	2.51E-15	0.347179462	0.5	0.107	5.98E-11	6
fam46bb	2.57E-15	0.650278547	0.346	0.05	6.14E-11	6
rbms2a1	3.08E-15	0.427844315	0.558	0.13	7.35E-11	6
atl31	3.17E-15	0.497326827	0.654	0.194	7.56E-11	6
serpine1	3.71E-15	1.383226315	0.673	0.215	8.85E-11	6
nckap11	4.46E-15	0.521283691	0.788	0.27	1.06E-10	6
serinc5	4.49E-15	0.378807363	0.423	0.075	1.07E-10	6
mxra8b	5.14E-15	0.688533363	0.269	0.028	1.23E-10	6
si:dkey-229e3.2	5.33E-15	0.283660805	0.288	0.032	1.27E-10	6
asap2b	5.39E-15	0.582290525	0.385	0.066	1.28E-10	6
prkci1	6.52E-15	0.45908914	0.673	0.199	1.55E-10	6
si:ch211-210c8.7	6.94E-15	0.254787991	0.365	0.055	1.66E-10	6
TSTA3 (1 of many)	7.19E-15	0.431552109	0.346	0.05	1.71E-10	6
chpfa	7.31E-15	0.333035529	0.327	0.043	1.74E-10	6
skia	8.65E-15	0.879548057	0.692	0.244	2.06E-10	6
ahcyl2	1.05E-14	0.590877649	0.673	0.219	2.50E-10	6
snx18a	1.10E-14	0.273215372	0.423	0.078	2.61E-10	6
sult6b1	1.22E-14	1.27366094	0.788	0.347	2.91E-10	6
hspg2	1.25E-14	0.5765169	0.538	0.137	2.97E-10	6
map3k5	1.35E-14	0.519472712	0.327	0.046	3.22E-10	6
si:ch211-207j7.2	1.39E-14	0.633292663	0.654	0.194	3.31E-10	6
rnd3b	1.40E-14	0.329146103	0.288	0.034	3.35E-10	6
tpd52l2a	1.43E-14	0.619206841	0.404	0.073	3.41E-10	6
rhoca	1.50E-14	0.552519471	0.712	0.249	3.57E-10	6

tfa	2.00E-14	4.546825118	0.462	0.112	4.77E-10	6
chac11	2.16E-14	1.289266846	0.981	0.772	5.15E-10	6
madd	2.27E-14	0.368911748	0.404	0.073	5.41E-10	6
si:busm1-57f23.11	2.67E-14	0.426344383	0.519	0.114	6.37E-10	6
cebpd1	2.76E-14	1.326826069	0.904	0.445	6.59E-10	6
s100u	3.10E-14	0.775879176	0.769	0.302	7.38E-10	6
ica1	3.18E-14	0.270805764	0.269	0.03	7.59E-10	6
chmp2a1	3.24E-14	0.808291462	0.885	0.432	7.73E-10	6
si:ch73-70c5.1	3.40E-14	0.834961094	0.769	0.315	8.10E-10	6
selenom	3.41E-14	0.395533519	0.442	0.089	8.13E-10	6
fgfr3	3.67E-14	0.424728655	0.288	0.036	8.75E-10	6
fam3c	3.85E-14	0.595757193	0.596	0.173	9.19E-10	6
mxi1	4.24E-14	0.393412079	0.385	0.068	1.01E-09	6
rbms1a1	4.33E-14	0.51822465	0.596	0.164	1.03E-09	6
kctd5a1	4.69E-14	0.529201593	0.827	0.32	1.12E-09	6
rhocb1	4.89E-14	0.825380045	0.731	0.297	1.17E-09	6
pacsin3	5.42E-14	0.379496746	0.423	0.085	1.29E-09	6
si:ch1073-392o20.21	5.54E-14	0.38700428	0.731	0.228	1.32E-09	6
yap1	5.62E-14	0.883296431	0.788	0.306	1.34E-09	6
epcam1	5.95E-14	0.971898914	1	0.906	1.42E-09	6
ptpn3	6.05E-14	0.59432497	0.654	0.21	1.44E-09	6
rap1b	6.21E-14	1.000148473	0.827	0.393	1.48E-09	6
taok1b	6.39E-14	0.487429848	0.442	0.093	1.52E-09	6
b3gnt2b	6.74E-14	0.270425519	0.288	0.036	1.61E-09	6
fundc2	6.80E-14	0.457826618	0.673	0.217	1.62E-09	6
rras	7.31E-14	0.431529997	0.692	0.242	1.74E-09	6
actn11	7.74E-14	0.958346144	0.519	0.132	1.84E-09	6
si:ch211-202a12.4	7.80E-14	1.14709646	0.923	0.646	1.86E-09	6
postna	8.56E-14	0.67279496	0.288	0.037	2.04E-09	6
cyfip1	1.07E-13	0.581415692	0.635	0.206	2.55E-09	6
cldnh1	1.08E-13	2.426261751	0.615	0.219	2.59E-09	6
her91	1.14E-13	1.188295188	0.808	0.338	2.73E-09	6
crip1	1.28E-13	1.441604808	0.846	0.454	3.06E-09	6
elovl1b1	1.45E-13	1.218628975	0.846	0.459	3.46E-09	6
hs6st21	1.48E-13	0.464879485	0.5	0.121	3.53E-09	6
chmp5b1	1.55E-13	0.798495373	0.923	0.527	3.70E-09	6
sdcbp2	1.56E-13	1.009906558	0.846	0.491	3.73E-09	6
spred2a	1.58E-13	0.294442853	0.558	0.144	3.77E-09	6
papss2b	1.59E-13	0.66067585	0.558	0.151	3.79E-09	6
chmp1b1	1.59E-13	0.724688641	0.846	0.4	3.80E-09	6
hpn	1.66E-13	0.286474825	0.346	0.055	3.96E-09	6
scarb2a	1.90E-13	1.082079773	0.827	0.434	4.52E-09	6
spaca41	2.03E-13	0.922354775	0.538	0.141	4.85E-09	6
csrp1a	2.06E-13	0.552035382	0.731	0.269	4.92E-09	6
ucp21	2.15E-13	0.730386418	0.846	0.384	5.12E-09	6
tmem30c	2.22E-13	0.638008119	0.692	0.249	5.29E-09	6
si:dkeyp-94b4.1	2.50E-13	0.547897578	0.712	0.269	5.95E-09	6
noxo1a	2.72E-13	1.155679167	0.346	0.055	6.48E-09	6
tjp1b	3.22E-13	0.467783253	0.596	0.165	7.67E-09	6
map1lc3a	3.48E-13	0.667763553	0.673	0.244	8.30E-09	6
lrch4	3.58E-13	0.338083539	0.346	0.059	8.54E-09	6
si:ch211-286b5.51	4.42E-13	0.583514818	0.635	0.203	1.05E-08	6
sdc41	4.52E-13	0.939234157	0.923	0.628	1.08E-08	6
fxyd11	4.57E-13	0.570224646	0.846	0.354	1.09E-08	6

zgc:1750881	5.04E-13	0.384710216	0.635	0.196	1.20E-08	6
lipia	5.38E-13	0.400089668	0.558	0.158	1.28E-08	6
pacsin2	5.51E-13	0.372425628	0.462	0.107	1.31E-08	6
gadd45ab1	5.61E-13	0.816893501	0.673	0.246	1.34E-08	6
cnn3a1	7.70E-13	0.55593675	0.673	0.224	1.84E-08	6
aqp3a	1.02E-12	0.751689746	0.5	0.13	2.44E-08	6
anxa11b	1.10E-12	0.839649748	0.827	0.422	2.63E-08	6
adgrg1.11	1.29E-12	0.509435491	0.596	0.19	3.07E-08	6
maccl1	1.39E-12	0.477715194	0.558	0.153	3.31E-08	6
tpm31	1.41E-12	0.951270903	0.962	0.747	3.36E-08	6
sulf2b	1.44E-12	0.290926685	0.308	0.048	3.43E-08	6
epb41l5	1.48E-12	0.277008999	0.462	0.11	3.53E-08	6
klhl24b	1.51E-12	0.482013088	0.404	0.084	3.61E-08	6
cers2a	1.77E-12	0.330177362	0.5	0.119	4.23E-08	6
scamp2l	1.85E-12	0.445989207	0.308	0.048	4.42E-08	6
aplp2	1.86E-12	0.730798789	0.75	0.345	4.43E-08	6
plekhg4	1.89E-12	0.491567079	0.654	0.224	4.50E-08	6
si:cabz01102082.1	1.96E-12	0.411773823	0.442	0.101	4.66E-08	6
slc12a7a	2.04E-12	0.432082465	0.462	0.114	4.87E-08	6
sash1a	2.06E-12	0.318776126	0.308	0.048	4.91E-08	6
si:ch211-152p11.4	2.20E-12	0.345395629	0.308	0.048	5.24E-08	6
ddah2	2.39E-12	0.307366929	0.288	0.043	5.69E-08	6
rap2b	2.43E-12	0.276507344	0.577	0.169	5.80E-08	6
serpinh1a	2.44E-12	0.551044222	0.269	0.037	5.81E-08	6
jpt1b1	2.80E-12	0.575729329	0.673	0.217	6.67E-08	6
f3b	3.29E-12	0.774104127	0.519	0.142	7.85E-08	6
lman21	3.39E-12	0.564318365	0.654	0.233	8.08E-08	6
camk2g1	3.63E-12	0.35746345	0.538	0.16	8.65E-08	6
ntan1	3.66E-12	0.485665398	0.462	0.116	8.72E-08	6
dapk3	4.04E-12	0.355144151	0.365	0.069	9.63E-08	6
si:dkey-240h12.3	4.20E-12	0.373741308	0.346	0.064	1.00E-07	6
cldnc	4.44E-12	1.075337402	0.981	0.785	1.06E-07	6
fras1	4.73E-12	0.27265301	0.385	0.077	1.13E-07	6
btr02	5.25E-12	0.697918831	0.731	0.301	1.25E-07	6
mgat1a	5.33E-12	0.612898827	0.635	0.233	1.27E-07	6
arpc2	5.37E-12	0.733903082	0.904	0.482	1.28E-07	6
col4a5	6.36E-12	1.833769028	0.462	0.128	1.52E-07	6
ppfibp1b	6.36E-12	0.408684117	0.558	0.171	1.52E-07	6
nrg1	6.47E-12	0.443619813	0.577	0.176	1.54E-07	6
zgc:565251	6.55E-12	0.295459091	0.558	0.157	1.56E-07	6
arg21	6.63E-12	0.991167924	0.385	0.084	1.58E-07	6
zmynd11	6.69E-12	0.415075622	0.538	0.16	1.59E-07	6
pdcd6ip1	6.69E-12	0.606119706	0.712	0.299	1.60E-07	6
gapdhs1	6.88E-12	1.137987378	0.712	0.326	1.64E-07	6
frmd8	7.66E-12	0.282488701	0.346	0.068	1.83E-07	6
zgc:92242	8.49E-12	0.335776228	0.635	0.206	2.02E-07	6
arhgef1a	8.77E-12	0.411756165	0.423	0.096	2.09E-07	6
arf11	9.14E-12	0.509527568	0.827	0.379	2.18E-07	6
sh3bgrl1	9.77E-12	0.635320951	0.596	0.201	2.33E-07	6
nos1apa	9.81E-12	0.405669512	0.442	0.109	2.34E-07	6
rest	1.03E-11	0.280924991	0.481	0.125	2.44E-07	6
ctnnb11	1.03E-11	0.838232374	0.846	0.445	2.45E-07	6
cygb1	1.04E-11	2.853460614	0.673	0.29	2.47E-07	6
tes	1.04E-11	0.320152022	0.481	0.123	2.47E-07	6

hopx	1.16E-11	0.467590021	0.385	0.08	2.76E-07	6
tdh1	1.18E-11	0.848033786	0.731	0.324	2.83E-07	6
selenow12	1.21E-11	1.473383231	0.981	0.641	2.88E-07	6
rab5aa1	1.39E-11	0.54886255	0.769	0.336	3.30E-07	6
cd63	1.39E-11	0.867705942	0.904	0.628	3.32E-07	6
gsna	1.42E-11	0.343286946	0.577	0.183	3.39E-07	6
vaspb	1.47E-11	0.65591289	0.808	0.463	3.51E-07	6
mkrn11	1.50E-11	0.27331448	0.404	0.087	3.58E-07	6
slc25a43	1.51E-11	0.307220324	0.423	0.101	3.61E-07	6
rhpn2	1.61E-11	0.573480341	0.673	0.262	3.83E-07	6
trip111	1.68E-11	0.443769991	0.519	0.148	4.02E-07	6
cicb1	1.77E-11	0.377120794	0.692	0.251	4.21E-07	6
tax1bp1b1	1.81E-11	0.523499108	0.75	0.306	4.32E-07	6
ywhaz1	1.87E-11	0.751110669	0.846	0.418	4.46E-07	6
capns1b	1.88E-11	0.764560189	0.75	0.37	4.49E-07	6
shroom2a	2.03E-11	0.432311595	0.481	0.139	4.84E-07	6
cmpk	2.04E-11	0.610593917	0.827	0.377	4.88E-07	6
pef1	2.20E-11	0.560464155	0.673	0.254	5.24E-07	6
psat11	2.25E-11	0.693452967	0.673	0.276	5.36E-07	6
glb11	2.40E-11	0.374852272	0.288	0.048	5.73E-07	6
ctnnb21	2.47E-11	0.554033744	0.788	0.388	5.88E-07	6
lama5	2.68E-11	1.857832313	0.75	0.365	6.40E-07	6
pik3c2a	2.71E-11	0.355490074	0.346	0.073	6.47E-07	6
gale	2.77E-11	0.630188446	0.673	0.272	6.61E-07	6
uck2b1	2.77E-11	0.574679622	0.596	0.208	6.61E-07	6
nfil3	2.85E-11	0.435888051	0.519	0.151	6.79E-07	6
si:ch211-250c4.41	3.52E-11	0.490072706	0.692	0.26	8.38E-07	6
stau21	3.67E-11	0.498653856	0.75	0.319	8.75E-07	6
BX908782.3	3.67E-11	0.387206333	0.288	0.05	8.76E-07	6
scin1b	3.71E-11	0.45133579	0.385	0.091	8.84E-07	6
socs3a1	3.74E-11	0.387386925	0.635	0.21	8.93E-07	6
mid1ip1b1	3.78E-11	0.51115718	0.827	0.363	9.02E-07	6
si:ch211-191a24.4	3.91E-11	0.377936804	0.423	0.105	9.32E-07	6
jarid2b	3.94E-11	0.270195841	0.423	0.098	9.40E-07	6
zgc:85932	4.01E-11	0.277877833	0.423	0.103	9.55E-07	6
sh3rf2	4.11E-11	0.322960215	0.308	0.055	9.81E-07	6
ppp1cb1	4.22E-11	0.598296528	0.615	0.224	1.01E-06	6
ctsla	4.26E-11	1.392338016	0.923	0.632	1.01E-06	6
gsr	4.44E-11	0.400505538	0.577	0.187	1.06E-06	6
creb3l3l1	4.45E-11	0.631624904	0.769	0.365	1.06E-06	6
nfkbiab1	4.49E-11	1.457881069	0.846	0.468	1.07E-06	6
fasn	5.60E-11	0.296870302	0.288	0.048	1.33E-06	6
sdc2	5.77E-11	0.371850399	0.538	0.173	1.38E-06	6
zgc:123010	5.82E-11	0.659333892	0.519	0.169	1.39E-06	6
fam199x1	6.38E-11	0.371348061	0.519	0.165	1.52E-06	6
zswim51	6.42E-11	0.406640797	0.712	0.286	1.53E-06	6
zgc:92140	6.77E-11	0.568013198	0.365	0.085	1.61E-06	6
foxp1b	6.98E-11	0.263997785	0.269	0.041	1.67E-06	6
capza1a1	7.35E-11	0.518545696	0.75	0.356	1.75E-06	6
sh3gl1a	7.52E-11	0.485433864	0.635	0.221	1.79E-06	6
mcl1a	7.55E-11	0.976676403	0.923	0.749	1.80E-06	6
glsb	8.45E-11	0.484769855	0.654	0.235	2.02E-06	6
chmp4bb1	8.77E-11	0.511310425	0.788	0.386	2.09E-06	6
atp1a1a.1	8.79E-11	0.842646284	0.923	0.582	2.10E-06	6

shcl1	9.98E-11	0.392791634	0.519	0.155	2.38E-06	6
cpne1	1.02E-10	0.761271878	0.808	0.404	2.44E-06	6
rnf19a1	1.11E-10	0.455174499	0.596	0.214	2.66E-06	6
tagln21	1.13E-10	0.837819983	1	0.842	2.68E-06	6
DYRK31	1.16E-10	0.499405514	0.423	0.107	2.77E-06	6
BX936337.11	1.19E-10	0.77574761	0.846	0.445	2.83E-06	6
cfl1l1	1.27E-10	1.027439361	0.846	0.479	3.02E-06	6
uacab	1.30E-10	0.492790598	0.654	0.242	3.11E-06	6
slc37a2	1.30E-10	0.500669127	0.692	0.288	3.11E-06	6
CU928117.1	1.39E-10	0.329386214	0.596	0.185	3.31E-06	6
myo9aa	1.43E-10	0.419967702	0.442	0.119	3.40E-06	6
letm1	1.46E-10	0.287908542	0.519	0.16	3.48E-06	6
tbc1d10aa	1.47E-10	0.324338621	0.481	0.139	3.50E-06	6
irf2bp1	1.50E-10	0.331081774	0.462	0.13	3.58E-06	6
schip11	1.50E-10	0.691630526	0.635	0.233	3.58E-06	6
tead1b	1.52E-10	0.331457294	0.481	0.137	3.61E-06	6
pbxip1a1	1.55E-10	0.328863017	0.404	0.094	3.70E-06	6
itih5	1.57E-10	0.347612284	0.346	0.077	3.74E-06	6
adam17a	1.58E-10	0.250647156	0.404	0.1	3.77E-06	6
tjp1a	1.65E-10	0.658202884	0.692	0.304	3.93E-06	6
lamc1	1.68E-10	0.600184602	0.558	0.19	4.00E-06	6
btg3	1.77E-10	0.549219514	0.481	0.141	4.21E-06	6
spag1a	1.85E-10	0.591869883	0.519	0.18	4.40E-06	6
ap3d11	1.85E-10	0.59034798	0.615	0.221	4.41E-06	6
ccni1	1.88E-10	0.59716209	0.808	0.409	4.47E-06	6
krt91	2.10E-10	1.670078104	0.865	0.609	5.02E-06	6
gata5	2.14E-10	1.236513772	0.5	0.167	5.10E-06	6
tjp2a1	2.20E-10	0.331296244	0.596	0.208	5.25E-06	6
fam177a11	2.21E-10	0.368370505	0.538	0.167	5.27E-06	6
smad3a	2.42E-10	0.68069074	0.635	0.254	5.76E-06	6
csnk1da1	2.64E-10	0.695867858	0.731	0.317	6.28E-06	6
cebpb1	2.72E-10	1.167266338	0.962	0.74	6.50E-06	6
pls3	2.80E-10	0.410622041	0.596	0.21	6.67E-06	6
cobl	2.92E-10	0.475110383	0.308	0.06	6.96E-06	6
leng9	2.95E-10	0.402157209	0.596	0.221	7.03E-06	6
cab39l1	3.14E-10	0.603764841	0.635	0.247	7.48E-06	6
ncoa4	3.17E-10	0.387165191	0.635	0.237	7.56E-06	6
tmem79b	3.40E-10	0.348101544	0.442	0.121	8.11E-06	6
tax1bp3	3.43E-10	0.47988459	0.827	0.454	8.19E-06	6
itsn2a	4.20E-10	0.397533227	0.423	0.11	1.00E-05	6
arl1	4.23E-10	0.413335321	0.75	0.338	1.01E-05	6
cnn2	4.42E-10	0.674957216	0.962	0.669	1.05E-05	6
cyb5r1	4.49E-10	0.561352117	0.673	0.263	1.07E-05	6
itsn2b	4.65E-10	0.407915562	0.673	0.267	1.11E-05	6
atp2c11	4.66E-10	0.260198257	0.423	0.11	1.11E-05	6
pbxip1b	4.68E-10	0.383130888	0.577	0.203	1.12E-05	6
CU929259.1	4.68E-10	0.57604035	0.769	0.375	1.12E-05	6
pdgfba1	4.77E-10	0.410697998	0.596	0.206	1.14E-05	6
limk2	4.80E-10	0.600437263	0.5	0.16	1.14E-05	6
mef2aa1	5.03E-10	0.3266465	0.423	0.109	1.20E-05	6
fbxo45	5.15E-10	0.318163185	0.346	0.077	1.23E-05	6
zgc:153115	6.34E-10	0.26297624	0.615	0.222	1.51E-05	6
ppp1r9a1a	6.45E-10	0.30653355	0.365	0.089	1.54E-05	6
tm9sf31	6.53E-10	0.451169077	0.731	0.32	1.56E-05	6

wipf2a1	6.57E-10	0.339670255	0.615	0.235	1.57E-05	6
si:dkey-172h23.21	6.58E-10	0.263485071	0.577	0.199	1.57E-05	6
rilpl2	6.80E-10	0.379084168	0.423	0.117	1.62E-05	6
wnk1b1	7.41E-10	0.327421191	0.462	0.135	1.77E-05	6
mpzl2b	7.71E-10	0.499849666	0.596	0.23	1.84E-05	6
uap1	8.07E-10	0.297969299	0.635	0.244	1.92E-05	6
tln11	8.27E-10	0.778285947	0.692	0.322	1.97E-05	6
rab11fip1a	8.62E-10	0.378672949	0.462	0.137	2.06E-05	6
zgc:158343	8.84E-10	1.178559919	0.885	0.6	2.11E-05	6
anxa11a1	9.00E-10	0.678459713	0.692	0.358	2.15E-05	6
sdf2l11	1.12E-09	0.612213214	0.731	0.352	2.67E-05	6
fosl1a2	1.19E-09	0.878649566	0.885	0.585	2.83E-05	6
tp53inp11	1.27E-09	0.622553241	0.519	0.167	3.02E-05	6
fermt1	1.27E-09	0.477803103	0.673	0.31	3.04E-05	6
baiap2a	1.33E-09	0.300258482	0.346	0.085	3.18E-05	6
10-Sep	1.40E-09	0.570046942	0.558	0.212	3.33E-05	6
si:dkey-177p2.61	1.44E-09	0.437983014	0.712	0.322	3.44E-05	6
mapk3	1.48E-09	0.364555078	0.519	0.176	3.52E-05	6
cldn121	1.50E-09	0.26566351	0.423	0.121	3.58E-05	6
gprc5c	1.51E-09	0.436446693	0.481	0.148	3.60E-05	6
nfil3-5	1.61E-09	0.872422531	0.865	0.544	3.83E-05	6
ssh2a	1.62E-09	0.392052461	0.442	0.126	3.87E-05	6
arhgap42a	1.63E-09	0.279217024	0.5	0.16	3.90E-05	6
lims11	1.66E-09	0.53805555	0.635	0.267	3.96E-05	6
pcdh19	1.70E-09	0.54558929	0.288	0.057	4.06E-05	6
si:dkeyp-110e4.6	1.70E-09	0.387807044	0.269	0.052	4.06E-05	6
fosl2	1.73E-09	0.709785327	0.865	0.448	4.12E-05	6
si:ch1073-165f9.2	1.78E-09	0.380412411	0.365	0.089	4.25E-05	6
ptp4a11	1.82E-09	0.509066278	0.827	0.42	4.35E-05	6
derl11	1.85E-09	0.3520274	0.5	0.158	4.41E-05	6
itm2ba	1.87E-09	0.463305665	0.769	0.381	4.47E-05	6
gabpa	1.88E-09	0.44249665	0.577	0.212	4.49E-05	6
pim3	1.90E-09	0.31947781	0.423	0.119	4.53E-05	6
glulb1	1.93E-09	0.543054918	0.692	0.322	4.59E-05	6
stim1a	1.98E-09	0.453562392	0.519	0.169	4.72E-05	6
mst1ra	2.03E-09	0.254078788	0.462	0.135	4.83E-05	6
rac1a	2.08E-09	0.582904493	0.692	0.306	4.95E-05	6
rab10	2.11E-09	0.617053284	0.885	0.525	5.03E-05	6
relb1	2.23E-09	0.742520868	0.558	0.187	5.31E-05	6
nipal3	2.32E-09	0.314821033	0.423	0.123	5.53E-05	6
plecb1	2.40E-09	0.892973603	0.827	0.48	5.71E-05	6
sptlc2b1	2.43E-09	0.642573638	0.808	0.436	5.79E-05	6
tnfb1	2.57E-09	0.795198951	0.462	0.139	6.13E-05	6
DST	2.61E-09	0.47232574	0.442	0.133	6.22E-05	6
cnpy1	2.62E-09	0.357238661	0.75	0.352	6.25E-05	6
zgc:92313	2.67E-09	0.526679168	0.769	0.381	6.36E-05	6
abracl1	2.71E-09	0.70217875	0.904	0.523	6.47E-05	6
large2	2.79E-09	0.402515098	0.519	0.189	6.65E-05	6
spint1b	2.87E-09	0.334704438	0.269	0.052	6.84E-05	6
si:ch1073-443f11.2	2.97E-09	0.523080113	0.615	0.253	7.08E-05	6
rab2a1	3.01E-09	0.583424433	0.865	0.528	7.17E-05	6
dlg5a	3.17E-09	0.417695596	0.385	0.103	7.56E-05	6
srebf21	3.30E-09	0.479419797	0.635	0.281	7.87E-05	6
gabarapb1	3.31E-09	0.473407219	0.712	0.317	7.90E-05	6

arpc31	3.39E-09	0.679670603	0.865	0.537	8.10E-05	6
scfd11	3.52E-09	0.358641697	0.519	0.183	8.40E-05	6
si:dkey-42i9.61	3.65E-09	0.300939051	0.462	0.142	8.69E-05	6
ctnnd11	3.97E-09	0.539200779	0.846	0.495	9.46E-05	6
cmtm6	3.97E-09	0.467934301	0.481	0.158	9.47E-05	6
irf6	4.01E-09	0.483772895	0.846	0.422	9.56E-05	6
nfkbiaa1	4.21E-09	1.548912946	0.731	0.39	0.0001004	6
CABZ01005876.1	4.26E-09	0.371559376	0.404	0.112	0.0001016	6
stx5a	4.40E-09	0.402239739	0.538	0.196	0.0001049	6
net1	4.43E-09	0.796093084	0.596	0.276	0.0001057	6
rnf13	4.58E-09	0.352463667	0.327	0.077	0.0001092	6
vapb	4.94E-09	0.688015106	0.788	0.413	0.0001178	6
gak	4.95E-09	0.333130631	0.5	0.173	0.000118	6
c1galt1b	5.05E-09	0.341397227	0.558	0.212	0.0001204	6
arf6b	5.08E-09	0.405373106	0.712	0.31	0.0001212	6
abi1a	5.26E-09	0.313156713	0.712	0.297	0.0001254	6
iqgap1	5.26E-09	0.296295584	0.538	0.194	0.0001255	6
marcksl1a1	5.29E-09	1.941107896	0.692	0.338	0.0001262	6
myo1eb	5.34E-09	0.310593122	0.596	0.235	0.0001274	6
arrdc3b1	5.53E-09	0.335469655	0.404	0.11	0.0001319	6
spon1b	5.62E-09	0.534723137	0.269	0.055	0.0001339	6
ggcx	5.97E-09	0.289395599	0.327	0.078	0.0001423	6
pnpla8	6.23E-09	0.33797982	0.365	0.094	0.0001485	6
jupa1	6.42E-09	0.564439855	0.885	0.532	0.0001531	6
trib31	6.55E-09	0.643973909	0.885	0.525	0.0001561	6
xiap	6.73E-09	0.417344856	0.365	0.098	0.0001604	6
maff1	6.96E-09	0.602449481	0.827	0.491	0.000166	6
clint1a1	7.04E-09	0.62947886	0.75	0.388	0.0001678	6
CT030188.1	7.07E-09	0.288806611	0.327	0.078	0.0001686	6
arhgef1b1	7.34E-09	0.378684339	0.615	0.228	0.0001751	6
pde7a1	7.86E-09	0.496402159	0.423	0.132	0.0001875	6
ildr1a	7.95E-09	0.587350642	0.654	0.283	0.0001895	6
cd1511	8.61E-09	0.444637323	0.596	0.242	0.0002053	6
csnk1a11	8.72E-09	0.433821905	0.827	0.493	0.0002079	6
lect21	9.30E-09	1.222069057	0.385	0.114	0.0002217	6
lsp1	9.55E-09	1.203792258	0.75	0.432	0.0002276	6
rnf169	9.57E-09	0.330244407	0.481	0.16	0.0002282	6
slc25a55a	1.02E-08	0.603111701	0.885	0.525	0.0002428	6
rcan1a	1.04E-08	0.345347367	0.481	0.158	0.0002482	6
arnt2	1.10E-08	0.27998135	0.346	0.089	0.0002614	6
zgc:77849	1.10E-08	0.569485436	0.827	0.509	0.0002625	6
ell	1.11E-08	0.400940759	0.5	0.174	0.0002654	6
wasf21	1.22E-08	0.321234962	0.654	0.274	0.0002915	6
ctsz	1.22E-08	0.415055385	0.538	0.208	0.0002916	6
foxo41	1.27E-08	0.413037159	0.577	0.235	0.0003033	6
tnip11	1.31E-08	0.433077447	0.692	0.304	0.0003114	6
ctdsp21	1.37E-08	0.461411096	0.635	0.258	0.0003259	6
ywhae2	1.37E-08	0.383339968	0.692	0.319	0.000326	6
si:ch211-195b11.3	1.38E-08	0.821820585	0.269	0.055	0.0003291	6
kdelr2a	1.41E-08	0.528029491	0.769	0.406	0.0003371	6
ppiaa2	1.43E-08	0.570602849	0.981	0.89	0.0003418	6
irs2a	1.45E-08	0.263446758	0.365	0.098	0.0003455	6
lpp1	1.47E-08	0.338047137	0.481	0.162	0.0003505	6
llgl21	1.48E-08	0.375990362	0.712	0.315	0.0003518	6

vps281	1.51E-08	0.343611585	0.635	0.274	0.0003598	6
myo9b	1.59E-08	0.269772104	0.365	0.098	0.000379	6
kdm2ba1	1.60E-08	0.45123249	0.5	0.192	0.000381	6
fndc3ba	1.74E-08	0.271283542	0.308	0.071	0.0004159	6
tpd52l2b	1.81E-08	0.359268989	0.519	0.205	0.0004323	6
golt1bb1	1.85E-08	0.315612058	0.538	0.187	0.0004423	6
MYO1D	1.87E-08	0.954693775	0.635	0.308	0.0004463	6
mknk2b1	1.91E-08	0.982732319	0.865	0.612	0.0004553	6
tubb4b1	2.12E-08	0.780495451	0.962	0.71	0.0005052	6
frmd4ba	2.29E-08	0.617520954	0.731	0.356	0.0005472	6
serpinb11	2.38E-08	0.8275654	0.923	0.616	0.0005666	6
f11r.1	2.40E-08	0.492568084	0.923	0.617	0.0005726	6
sec23b2	2.70E-08	0.384059515	0.673	0.324	0.0006431	6
bzw1b	2.70E-08	0.573344542	0.904	0.562	0.0006445	6
CU984600.1	2.74E-08	0.3141982	0.346	0.094	0.0006535	6
egr2a	2.79E-08	1.036606622	0.635	0.285	0.000665	6
cast1	2.91E-08	0.58106394	0.827	0.456	0.0006933	6
jak11	3.03E-08	0.427710299	0.769	0.399	0.0007228	6
pcbp21	3.06E-08	0.459506289	0.75	0.365	0.0007304	6
zswim81	3.19E-08	0.355314844	0.423	0.137	0.0007608	6
jmjd1cb1	3.26E-08	0.383088669	0.712	0.333	0.0007781	6
lipg	3.27E-08	0.291349755	0.385	0.112	0.0007789	6
rel1	3.32E-08	0.294654675	0.404	0.112	0.0007923	6
atp6v1e1b1	3.33E-08	0.434857126	0.692	0.311	0.000794	6
kif5ba1	3.33E-08	0.514920237	0.635	0.302	0.0007942	6
ccdc186	3.43E-08	0.339298474	0.481	0.162	0.000818	6
pnrc22	3.75E-08	0.769933089	0.923	0.794	0.0008944	6
smc6	3.79E-08	0.27179073	0.269	0.057	0.0009038	6
tmsb11	3.82E-08	0.732048661	1	0.786	0.0009112	6
gnai2a1	4.13E-08	0.627883491	0.731	0.356	0.0009842	6
ddx3b	4.25E-08	0.486969196	0.788	0.397	0.0010123	6
spartb	4.55E-08	0.270848992	0.288	0.068	0.001085	6
tmf11	4.65E-08	0.324981666	0.365	0.105	0.0011089	6
wu:fc21g02	4.72E-08	0.261839405	0.346	0.094	0.0011252	6
vkorc1l1	4.76E-08	0.38956215	0.481	0.178	0.0011355	6
cyth1a.11	4.95E-08	0.276036582	0.615	0.269	0.00118	6
klf51	4.98E-08	0.652584685	0.788	0.52	0.0011872	6
zgc:1538671	5.63E-08	0.658676145	0.923	0.749	0.0013433	6
rbm14b1	5.64E-08	0.490568578	0.5	0.187	0.0013451	6
rb1cc1	5.66E-08	0.255443709	0.462	0.157	0.0013486	6
vsig10	5.70E-08	0.31053877	0.481	0.164	0.0013583	6
b3gnt5a	5.92E-08	0.656743091	0.288	0.071	0.0014112	6
palld	6.07E-08	0.499241872	0.462	0.164	0.0014468	6
ist11	6.07E-08	0.490434633	0.712	0.381	0.001448	6
arpc5a	6.67E-08	0.622628199	0.712	0.375	0.0015903	6
chmp4ba1	6.68E-08	0.390764717	0.692	0.324	0.0015939	6
zgc:92360	6.81E-08	0.260470248	0.442	0.153	0.0016239	6
nrarpa	6.90E-08	0.368561222	0.673	0.294	0.0016443	6
trim16	6.93E-08	0.345367875	0.731	0.37	0.0016527	6
zdhhc5a	7.09E-08	0.379215962	0.596	0.251	0.0016902	6
mknk1	7.57E-08	0.284807917	0.404	0.13	0.0018054	6
ubb	7.61E-08	0.759867509	0.923	0.6	0.0018144	6
notch21	7.62E-08	0.318409121	0.462	0.155	0.0018162	6
ppp1r12a1	7.71E-08	0.327296362	0.673	0.322	0.0018375	6

gadd45ba1	7.81E-08	1.149069042	0.827	0.56	0.0018622	6
csnk1db	7.98E-08	0.402849564	0.712	0.335	0.0019033	6
mob4	8.15E-08	0.317565927	0.635	0.279	0.0019424	6
CR762483.11	8.50E-08	0.373150576	0.538	0.199	0.0020278	6
cdca4	8.53E-08	0.308157325	0.423	0.144	0.0020348	6
CU694481.1	8.64E-08	0.435395137	0.346	0.098	0.0020603	6
CABZ01020840.1	8.74E-08	0.373483099	0.481	0.173	0.0020848	6
afdna1	9.20E-08	0.386860375	0.712	0.374	0.0021945	6
gmppb	9.29E-08	0.259163165	0.654	0.294	0.0022147	6
tmed101	9.41E-08	0.476313655	0.846	0.514	0.0022437	6
mapk6	9.58E-08	0.314956105	0.558	0.233	0.0022837	6
mbpb1	9.62E-08	0.303026038	0.596	0.256	0.0022946	6
stam2	9.84E-08	0.27889852	0.308	0.078	0.0023463	6
CR383676.12	1.01E-07	0.614239825	1	0.979	0.0024105	6
si:dkey-112a7.41	1.02E-07	0.523705003	0.692	0.363	0.0024298	6
nedd9	1.02E-07	0.3522778	0.442	0.157	0.0024419	6
adam10a1	1.05E-07	0.313223731	0.75	0.365	0.0024924	6
cgnl1	1.15E-07	0.48963082	0.538	0.231	0.0027332	6
igf2bp2b	1.16E-07	0.268741247	0.481	0.181	0.0027552	6
akap12b1	1.18E-07	1.163099358	0.404	0.126	0.0028081	6
dsc2l	1.21E-07	0.536686366	0.596	0.262	0.0028851	6
appbp2	1.22E-07	0.277730238	0.308	0.08	0.0029128	6
PPP2CA	1.29E-07	0.331271856	0.577	0.254	0.0030847	6
myo18ab	1.31E-07	0.259970156	0.615	0.262	0.003126	6
gipc2	1.31E-07	0.423604185	0.769	0.413	0.0031332	6
tpm4a1	1.32E-07	0.373635881	0.673	0.29	0.0031407	6
foxa31	1.33E-07	0.277975228	0.769	0.39	0.0031627	6
ppm1bb	1.33E-07	0.307333988	0.423	0.144	0.0031635	6
zmiz1a1	1.34E-07	0.268539877	0.481	0.178	0.0032009	6
wls	1.45E-07	0.390347002	0.519	0.203	0.003462	6
idh2	1.46E-07	1.219878095	0.865	0.571	0.003492	6
eif4g2b1	1.57E-07	0.456919309	0.808	0.441	0.0037465	6
phgdh1	1.62E-07	0.288650893	0.365	0.11	0.0038526	6
tmem131	1.63E-07	0.367291004	0.635	0.295	0.003893	6
gsel1	1.75E-07	0.284836919	0.615	0.262	0.0041668	6
optn1	1.75E-07	0.290317544	0.423	0.137	0.0041674	6
ubc1	1.77E-07	0.537239558	0.981	0.792	0.0042216	6
cntf	1.99E-07	0.441222626	0.327	0.089	0.0047468	6
slc37a1	2.08E-07	0.31980861	0.327	0.096	0.0049528	6
si:dkey-204f11.64	2.09E-07	0.443765657	0.423	0.149	0.0049867	6
tmtc3	2.11E-07	0.286269345	0.269	0.068	0.0050371	6
cdh11	2.12E-07	0.436393925	0.885	0.641	0.0050466	6
psap	2.24E-07	0.529118489	0.635	0.308	0.0053411	6
wipi1	2.36E-07	0.264338252	0.269	0.062	0.0056179	6
rnf20	2.44E-07	0.276646232	0.481	0.181	0.0058297	6
rab141	2.50E-07	0.427503498	0.654	0.313	0.0059724	6
wbp2nl	2.62E-07	0.530448345	0.519	0.228	0.0062363	6
arl5c	2.84E-07	0.565203226	0.654	0.34	0.0067703	6
ctnna1	2.94E-07	0.37780218	0.692	0.338	0.0070163	6
klf7b1	3.05E-07	0.426813324	0.769	0.413	0.0072639	6
mid1ip11	3.05E-07	0.258747972	0.385	0.123	0.007281	6
hmgn32	3.17E-07	0.320973847	0.577	0.256	0.0075611	6
si:ch211-15b10.6	3.23E-07	0.650593636	0.769	0.415	0.0076979	6
smad2	3.23E-07	0.291099362	0.538	0.233	0.0077041	6

ihha	3.27E-07	0.566811696	0.615	0.297	0.0077962	6
lepr	3.59E-07	0.341033891	0.269	0.068	0.0085686	6
gmds	3.62E-07	0.263456989	0.654	0.313	0.0086384	6
pim2	3.63E-07	0.406005206	0.269	0.068	0.0086454	6
aim1b	3.74E-07	1.154532852	0.404	0.151	0.0089132	6
uggt1	3.75E-07	0.304473639	0.538	0.222	0.008946	6
rac2	3.79E-07	0.339212289	0.615	0.281	0.0090468	6
ivns1abpb	3.80E-07	0.284264359	0.404	0.142	0.0090605	6
rrbp1a	3.84E-07	0.370960622	0.577	0.242	0.0091657	6
prrc1	4.02E-07	0.290479762	0.346	0.109	0.0095747	6
zgc:91908	4.12E-07	0.252939973	0.327	0.098	0.0098295	6
si:dkey-16l2.20	4.16E-07	0.688021839	0.769	0.457	0.0099126	6
tbc1d17	4.18E-07	0.33329861	0.385	0.132	0.0099556	6
si:ch211-284f22.3	4.19E-07	0.301409004	0.288	0.078	0.0099917	6
tcf7l21	4.33E-07	0.272778578	0.635	0.299	0.0103357	6
copa1	4.37E-07	0.364805796	0.673	0.34	0.010427	6
kdf1a	4.38E-07	0.505955873	0.288	0.075	0.0104347	6
arf51	4.42E-07	0.403773816	0.808	0.484	0.010543	6
rhoaa1	4.54E-07	0.423537166	0.75	0.434	0.0108358	6
copg21	4.58E-07	0.577812247	0.596	0.27	0.0109301	6
stat1a	4.66E-07	0.281591544	0.615	0.272	0.0111188	6
her6	4.69E-07	1.178299669	0.769	0.464	0.0111765	6
si:dkey-229b18.3	4.77E-07	0.304891733	0.346	0.105	0.0113678	6
fosb1	4.78E-07	0.50112132	0.558	0.233	0.0113961	6
golgb11	4.84E-07	0.536118524	0.692	0.384	0.0115513	6
yif1a1	4.88E-07	0.293330503	0.404	0.144	0.0116342	6
bri3	5.14E-07	0.317898477	0.423	0.153	0.0122522	6
nos1apa.1	5.26E-07	0.521329387	0.577	0.274	0.0125421	6
ano9b	5.34E-07	0.741235536	0.635	0.322	0.0127402	6
txn	5.53E-07	0.974667687	0.865	0.614	0.0131851	6
myl12.11	5.59E-07	0.503816446	0.942	0.722	0.013337	6
ppp4r2b	5.62E-07	0.278672063	0.462	0.176	0.0134119	6
oaz1b1	5.67E-07	0.476213389	0.923	0.552	0.0135308	6
copb11	5.76E-07	0.308361448	0.692	0.354	0.013728	6
phactr4a	5.77E-07	0.452520301	0.808	0.473	0.0137553	6
fat1a1	6.34E-07	0.437433858	0.654	0.333	0.0151172	6
mark2b1	6.75E-07	0.250656272	0.692	0.34	0.0160899	6
pfn11	6.81E-07	0.324271018	0.942	0.801	0.0162276	6
atic1	7.24E-07	1.203085307	0.519	0.251	0.017268	6
tcima1	7.25E-07	0.514986233	0.538	0.233	0.0172878	6
nav3	7.39E-07	0.380271518	0.308	0.087	0.0176149	6
hif1a1	7.57E-07	0.275190908	0.558	0.242	0.018061	6
zgc:162964	7.78E-07	0.27188921	0.385	0.13	0.0185533	6
st14a	8.04E-07	0.376335012	0.712	0.37	0.0191603	6
rab1ab1	8.32E-07	0.386396139	0.846	0.534	0.0198411	6
ap2s11	8.40E-07	0.35719433	0.615	0.283	0.0200323	6
ilk	8.40E-07	0.322759486	0.519	0.219	0.0200329	6
kctd13	8.54E-07	0.25608823	0.288	0.078	0.0203562	6
si:dkey-56m19.5	9.78E-07	0.57180196	0.308	0.089	0.0233087	6
arhgap23a	9.79E-07	0.309506602	0.442	0.165	0.0233502	6
zfp36l1a	9.84E-07	0.436710919	0.538	0.228	0.0234742	6
h1fx1	9.92E-07	0.791446898	0.519	0.219	0.0236453	6
spint1a	1.00E-06	0.367760579	0.788	0.512	0.0239234	6
arhgdia1	1.02E-06	0.565067564	0.808	0.507	0.0243257	6

rab1ba1	1.03E-06	0.297507443	0.654	0.347	0.0245281	6
ptena1	1.10E-06	0.371771576	0.596	0.27	0.0261791	6
il6st1	1.14E-06	0.30280149	0.615	0.265	0.0270854	6
yipf61	1.15E-06	0.328045786	0.462	0.187	0.0275303	6
appa1	1.28E-06	0.255866668	0.846	0.47	0.0304309	6
fkbp5	1.30E-06	0.298304324	0.365	0.126	0.0309219	6
si:dkey-42i9.41	1.32E-06	0.281269681	0.75	0.383	0.0313585	6
ywhae11	1.32E-06	0.439018511	0.846	0.507	0.0314737	6
si:dkey-79d12.5	1.32E-06	0.440227146	0.808	0.504	0.0315643	6
gabarapa1	1.33E-06	0.30896636	0.635	0.327	0.0316029	6
zgc:1009181	1.42E-06	0.266024261	0.596	0.283	0.0338738	6
si:ch211-166a6.5	1.43E-06	0.283899102	0.615	0.292	0.0341951	6
aldoaa2	1.44E-06	0.47591225	0.788	0.518	0.0343472	6
hist2h21	1.50E-06	0.31082548	0.635	0.308	0.0358467	6
snx121	1.54E-06	0.331184082	0.596	0.276	0.0367102	6
mtpn	1.55E-06	0.387355877	0.673	0.354	0.036967	6
si:ch73-138n13.1	1.56E-06	0.436248835	0.712	0.39	0.0371514	6
ube2d1b	1.60E-06	0.424816442	0.846	0.534	0.0382435	6
sri	1.64E-06	0.495972695	0.846	0.541	0.0391348	6
fam107b1	1.74E-06	0.561492692	0.673	0.34	0.0414477	6
h2afy2	1.75E-06	0.422889627	0.75	0.406	0.0418261	6
col18a1a	1.87E-06	1.869562428	0.731	0.441	0.0446632	6
ywhab11	1.88E-06	0.436002486	0.788	0.496	0.0448565	6
cdc42ep5	2.07E-06	0.272813001	0.538	0.262	0.0492438	6
crema	2.10E-06	0.553980602	0.615	0.306	0.050155	6
myl9b	2.11E-06	0.748931514	0.596	0.288	0.0503991	6
srpr1	2.21E-06	0.274925871	0.808	0.454	0.0526427	6
bcar1	2.31E-06	0.381903914	0.635	0.319	0.0549797	6
ythdc1	2.33E-06	0.389303169	0.596	0.272	0.0555179	6
dock71	2.39E-06	0.295908966	0.519	0.214	0.0569076	6
si:rp71-62i8.1	2.44E-06	0.274993293	0.481	0.19	0.0582599	6
nfkbib1	2.53E-06	0.680674526	0.538	0.249	0.0603653	6
prkcdb1	2.54E-06	0.410877525	0.615	0.297	0.0604646	6
prelid3b	2.56E-06	0.414478788	0.885	0.6	0.0610737	6
dpf2	2.60E-06	0.492138844	0.346	0.112	0.0621074	6
lasp1	2.62E-06	0.330252394	0.846	0.575	0.0625685	6
itgb4	2.71E-06	0.265969037	0.712	0.384	0.0645175	6
serpinh1b	2.73E-06	0.45812602	0.404	0.153	0.0650407	6
trak1a	2.75E-06	0.370076475	0.308	0.096	0.0655265	6
nabp1a	2.77E-06	0.252535251	0.577	0.269	0.0659394	6
pawr	2.92E-06	0.456970313	0.712	0.372	0.0695193	6
nucb2a1	2.93E-06	0.263707616	0.519	0.222	0.0699514	6
cxcl8a1	3.00E-06	2.090316008	0.596	0.311	0.0715366	6
oclna	3.04E-06	0.707832903	0.692	0.413	0.0725137	6
pcxa	3.05E-06	0.287454787	0.365	0.126	0.0726757	6
erbb3a	3.07E-06	0.344596557	0.423	0.162	0.0730993	6
neo1a	3.07E-06	0.381979091	0.365	0.126	0.0731468	6
arpc1a	3.10E-06	0.355578514	0.846	0.495	0.0738533	6
cry1aa1	3.13E-06	0.252516095	0.462	0.194	0.0745631	6
kras	3.14E-06	0.382364877	0.615	0.34	0.074787	6
epha2a	3.14E-06	0.473446708	0.712	0.391	0.0748551	6
cst14a.2	3.23E-06	0.54664263	0.904	0.573	0.0770754	6
shha	3.27E-06	0.357043872	0.423	0.162	0.0779782	6
ERC1	3.31E-06	0.281701553	0.442	0.173	0.0788613	6

prkd2	3.37E-06	0.295246813	0.404	0.148	0.080284	6
cpeb4b	3.49E-06	0.363502158	0.769	0.445	0.0831708	6
atf4b1	3.52E-06	0.401010923	0.942	0.712	0.0839605	6
yy1a1	3.62E-06	0.293470273	0.615	0.295	0.0862932	6
pof1b	3.67E-06	0.306882373	0.538	0.246	0.0874775	6
efnb2a	3.70E-06	0.336271136	0.288	0.085	0.088111	6
erf13	3.71E-06	0.270782679	0.365	0.133	0.0883961	6
tfg1	3.72E-06	0.414216622	0.692	0.411	0.0887115	6
ahr2	3.86E-06	0.40876978	0.558	0.274	0.0921085	6
ppp1caa1	3.88E-06	0.308762799	0.769	0.464	0.0925679	6
atf5b1	3.95E-06	0.323064465	0.673	0.345	0.0941736	6
znf185	3.95E-06	0.398084903	0.423	0.167	0.0941833	6
sav1	3.97E-06	0.296750813	0.288	0.089	0.094765	6
aclya	4.11E-06	0.31685971	0.462	0.196	0.0979027	6
gnb1a1	4.19E-06	0.27707517	0.808	0.502	0.1000092	6
smarca4a1	4.24E-06	0.415283051	0.75	0.423	0.1010235	6
wu:fj16a03	4.28E-06	0.464070177	0.308	0.101	0.1020562	6
tep1	4.35E-06	0.350314861	0.673	0.354	0.1036075	6
ccdc167	4.43E-06	0.314981944	0.404	0.164	0.1055888	6
si:dkey-156n14.5	4.44E-06	0.26797279	0.615	0.299	0.1058244	6
si:ch73-335l21.42	4.59E-06	0.923467904	0.904	0.714	0.1095374	6
nme3	4.64E-06	0.460689009	0.365	0.132	0.1105937	6
tnrc18	4.92E-06	0.392792297	0.519	0.23	0.117225	6
mthfd21	5.15E-06	0.375642965	0.635	0.354	0.1227701	6
nfkb21	5.15E-06	0.332561387	0.442	0.174	0.1228798	6
si:ch211-114c12.21	5.18E-06	0.307412638	0.519	0.231	0.1235851	6
sqstm1	5.25E-06	0.550201643	0.654	0.359	0.1251966	6
letm21	5.28E-06	0.354042166	0.673	0.352	0.1258363	6
mydgf	5.36E-06	0.281896677	0.519	0.235	0.1277458	6
eif3jb	5.42E-06	0.467124108	0.827	0.486	0.1291979	6
naa35	5.55E-06	0.37261121	0.327	0.11	0.1322837	6
atf7ip1	5.59E-06	0.292494127	0.712	0.368	0.133397	6
clk4a1	5.67E-06	0.267930284	0.635	0.315	0.135115	6
btg11	5.69E-06	0.652476695	0.962	0.785	0.1355838	6
rhoab1	5.77E-06	0.463703391	0.827	0.571	0.1375903	6
lxn1	5.85E-06	0.596777462	0.731	0.448	0.1394288	6
tfpia	6.03E-06	0.500109794	0.577	0.286	0.1437322	6
dap1b	6.03E-06	0.402560772	0.712	0.39	0.1437973	6
rab11a1	6.15E-06	0.466684172	0.769	0.45	0.146669	6
WDR1	6.19E-06	0.375098831	0.308	0.098	0.1475875	6
ptgs2b	6.48E-06	0.791230959	0.308	0.11	0.1544505	6
cyr611	6.91E-06	0.707633545	0.481	0.199	0.1646812	6
vps35	6.92E-06	0.477055982	0.481	0.21	0.1650476	6
zgc:165573	6.94E-06	0.374440154	0.673	0.352	0.1655928	6
c1galt1c1	7.08E-06	0.361413489	0.481	0.201	0.1687045	6
alas1	7.12E-06	0.403359582	0.827	0.489	0.1698168	6
gsto21	7.18E-06	0.258361888	0.692	0.338	0.1712126	6
si:ch73-375g18.1	7.22E-06	0.313331106	0.404	0.162	0.1721175	6
slc38a21	7.30E-06	0.280973175	0.692	0.381	0.1739827	6
irf2bp2b1	7.94E-06	0.348795967	0.577	0.292	0.1894205	6
purba1	8.57E-06	0.406535036	0.558	0.258	0.2042332	6
cdc42l1	8.90E-06	0.374095516	0.846	0.568	0.2122963	6
wsb1	9.08E-06	0.343932195	0.635	0.327	0.2165256	6
cnot4b	9.10E-06	0.419472739	0.327	0.121	0.2170639	6

h1f01	9.16E-06	0.591097063	0.654	0.384	0.2183469	6
eif1b1	9.30E-06	0.456220918	0.981	0.751	0.2217929	6
myh9b	9.58E-06	0.321252071	0.788	0.491	0.2283487	6
lrrfip1a	9.72E-06	0.564124734	0.596	0.32	0.2318424	6
elf31	9.88E-06	1.004093834	0.769	0.548	0.2355414	6
pgd1	1.02E-05	0.382941146	0.692	0.395	0.2434752	6
atf32	1.06E-05	0.708025697	0.981	0.836	0.2522831	6
txndc92	1.06E-05	0.287133898	0.615	0.31	0.2522965	6
si:dkey-244a7.1	1.06E-05	0.260446573	0.538	0.242	0.252558	6
nr3c1	1.08E-05	0.268484927	0.481	0.219	0.2578368	6
jund1	1.10E-05	0.444835458	0.846	0.571	0.2618043	6
hectd1	1.12E-05	0.293542162	0.654	0.365	0.2663587	6
chp1	1.12E-05	0.319148568	0.5	0.233	0.2664594	6
foxa1	1.13E-05	0.298910891	0.519	0.253	0.2696352	6
prkag1	1.19E-05	0.255529614	0.519	0.256	0.2846504	6
dag1	1.21E-05	0.625417139	0.692	0.425	0.2876536	6
rab5ab	1.27E-05	0.408133628	0.615	0.319	0.303969	6
elf11	1.31E-05	0.250901393	0.731	0.404	0.313452	6
ctsf1	1.32E-05	0.402392132	0.385	0.155	0.3150076	6
atp6v0ca1	1.34E-05	0.36605923	0.846	0.553	0.3189141	6
ppiab1	1.42E-05	0.4048696	0.981	0.868	0.3391531	6
psmd4b	1.42E-05	0.298795413	0.577	0.278	0.3392833	6
rab5c1	1.49E-05	0.345434095	0.635	0.356	0.3546912	6
puf60b	1.53E-05	0.427536587	0.558	0.276	0.3649087	6
zfand5a	1.55E-05	0.360444301	0.731	0.416	0.3689231	6
lmna	1.56E-05	0.553131145	0.596	0.322	0.3721149	6
nfe2l2a1	1.59E-05	0.361852799	0.75	0.427	0.3781904	6
hdac4	1.59E-05	0.52621114	0.308	0.109	0.3799085	6
lsm14aa	1.60E-05	0.257040136	0.404	0.16	0.3821919	6
zgc:65873	1.75E-05	0.265469665	0.462	0.203	0.4161975	6
smap1	1.79E-05	0.357633416	0.577	0.295	0.4263427	6
pitpnaa1	1.85E-05	0.389630087	0.654	0.358	0.4405687	6
cltca2	1.86E-05	0.447748474	0.769	0.464	0.4442071	6
ube2e31	1.99E-05	0.271418225	0.635	0.338	0.4744264	6
lrba	2.06E-05	0.365970205	0.673	0.379	0.4905393	6
cul1a	2.13E-05	0.307697748	0.404	0.167	0.5082896	6
tnfrsf1a	2.25E-05	0.394611123	0.385	0.16	0.5357633	6
ubr5	2.28E-05	0.365236078	0.462	0.205	0.5437483	6
cebpa	2.31E-05	0.56458322	0.75	0.466	0.5511516	6
vdac12	2.44E-05	0.288030898	0.654	0.351	0.5820034	6
eif4ebp11	2.61E-05	0.422536861	0.769	0.466	0.6229929	6
pdia32	2.79E-05	0.553863938	0.942	0.683	0.665634	6
CABZ01030862.1	2.89E-05	0.284018347	0.442	0.187	0.6881357	6
pleca	3.02E-05	0.683912052	0.692	0.393	0.7200936	6
ddx3a1	3.05E-05	0.402867084	0.673	0.39	0.726613	6
ywhaba1	3.17E-05	0.493290016	0.865	0.635	0.7566516	6
waslb	3.24E-05	0.268175609	0.596	0.288	0.7735902	6
lnx2b	3.41E-05	0.279688952	0.481	0.219	0.8128104	6
rbm51	3.42E-05	0.344064911	0.481	0.217	0.8165134	6
rtn3	3.44E-05	0.272877727	0.596	0.294	0.8202725	6
zgc:920271	3.54E-05	0.366615526	0.827	0.525	0.8438695	6
zgc:91976	3.60E-05	0.331212449	0.538	0.267	0.8578407	6
psmd11	3.72E-05	0.326762326	0.788	0.461	0.8858556	6
atf4a1	3.74E-05	0.408443317	0.923	0.653	0.8906582	6

degs2l		3.91E-05	0.305262778	0.788	0.489	0.9316251	6
aff4		4.24E-05	0.335033602	0.635	0.367	1	6
bzw1a1		4.52E-05	0.253166377	0.712	0.415	1	6
tfe3a1		4.64E-05	0.450645364	0.654	0.367	1	6
mboat1		4.71E-05	0.291236167	0.442	0.203	1	6
ip6k2b		4.74E-05	0.331013538	0.596	0.326	1	6
amd11		4.76E-05	0.259247196	0.577	0.304	1	6
ggps1		4.88E-05	0.383262609	0.577	0.304	1	6
syne2b1		4.96E-05	0.323224562	0.346	0.141	1	6
arrdc3a1		5.09E-05	0.449576605	0.442	0.198	1	6
klf3		5.55E-05	0.594275391	0.788	0.523	1	6
lsr		5.95E-05	0.32550881	0.75	0.475	1	6
	2-Jun	6.36E-05	0.514619345	0.981	0.806	1	6
prrg2		6.41E-05	0.271928998	0.385	0.173	1	6
ywhah1		6.49E-05	0.355464739	0.788	0.482	1	6
ubfd1		6.69E-05	0.266096132	0.462	0.208	1	6
mapkapk2a		7.15E-05	0.268786082	0.731	0.466	1	6
pdap1b1		7.50E-05	0.446892045	0.654	0.404	1	6
usp361		7.50E-05	0.373904335	0.673	0.409	1	6
wu:fb18f061		7.51E-05	0.74630212	0.635	0.416	1	6
slc13a21		7.52E-05	1.453609749	0.404	0.203	1	6
ppl1		7.60E-05	0.589837522	0.846	0.532	1	6
prkaa1		7.85E-05	0.318331347	0.423	0.198	1	6
krt81		8.16E-05	0.584751925	0.846	0.619	1	6
rnf101		9.12E-05	0.286728899	0.712	0.438	1	6
cdkn1bb1		9.39E-05	0.281932177	0.731	0.431	1	6
scamp2		0.0001026	0.27501973	0.635	0.363	1	6
pmp22b		0.0001036	1.89272686	0.481	0.295	1	6
fam53b		0.0001059	0.346564081	0.5	0.254	1	6
elmsan1b1		0.0001076	0.366002449	0.635	0.352	1	6
sptssa		0.00011	0.251941493	0.731	0.48	1	6
macf1a1		0.0001109	0.282125269	0.615	0.331	1	6
sec61a12		0.0001137	0.278327728	0.75	0.475	1	6
ube2v11		0.0001143	0.533708173	0.769	0.507	1	6
nccrp1		0.000115	0.481146895	0.692	0.399	1	6
prdm1a		0.0001205	0.705960582	0.538	0.315	1	6
irf1b1		0.0001212	0.503348288	0.288	0.107	1	6
si:dkey-87o1.2		0.0001216	0.267516239	0.481	0.233	1	6
tmem263		0.0001287	0.286212365	0.462	0.221	1	6
nnt1		0.0001351	0.334821367	0.538	0.283	1	6
egr1		0.0001386	0.603015022	0.423	0.198	1	6
pllp1		0.0001496	0.469198206	0.538	0.301	1	6
slc1a51		0.000154	0.332080068	0.635	0.383	1	6
si:dkey-95p16.2		0.0001613	0.264129224	0.558	0.313	1	6
fnbp4		0.0001667	0.31809398	0.519	0.274	1	6
ptp4a2b1		0.0001672	0.32634375	0.788	0.504	1	6
ano10a		0.0001693	0.333295976	0.288	0.112	1	6
tmsb4x1		0.0001753	0.371839136	1	0.993	1	6
snx5		0.0001767	0.283568299	0.288	0.11	1	6
calm2a.1		0.0001789	0.448170515	0.827	0.587	1	6
ndel1b		0.0001875	0.281490982	0.442	0.224	1	6
ube2e21		0.0001888	0.323481659	0.365	0.157	1	6
mapk14b		0.0001909	0.269702971	0.346	0.148	1	6
vcp1		0.0002009	0.314780078	0.827	0.56	1	6

krt4	0.0002094	0.70615784	0.981	0.929	1	6
tmem1151	0.0002408	0.442961205	0.481	0.27	1	6
col14a1a1	0.0002427	0.321721326	0.615	0.351	1	6
eef1a1l11	0.0002576	0.382454674	1	0.984	1	6
sept8a	0.0002634	0.276969779	0.327	0.144	1	6
cox8a	0.0002771	0.382401156	0.981	0.74	1	6
arrdc1a	0.0003058	0.513604046	0.635	0.406	1	6
ap1s3b	0.0003109	0.281734823	0.731	0.452	1	6
junba1	0.0003144	0.449596557	0.885	0.708	1	6
dab2ipb1	0.0003184	0.257177244	0.423	0.214	1	6
gadd45aa	0.0003263	0.342397313	0.519	0.286	1	6
ddx51	0.0003412	0.331927672	0.904	0.667	1	6
dnmbp1	0.0003415	0.333806475	0.885	0.601	1	6
egr3	0.0003815	0.484929634	0.346	0.164	1	6
rhbdf1a1	0.0003868	0.276646294	0.442	0.224	1	6
gstt1b1	0.0003905	0.880051338	0.712	0.463	1	6
fundc11	0.0004008	0.353107919	0.308	0.128	1	6
npc2	0.000451	0.280691682	0.75	0.466	1	6
pdgfab	0.0004687	0.294912178	0.519	0.285	1	6
ap2b1	0.0004689	0.286851838	0.462	0.244	1	6
ier2a1	0.0004738	0.768842977	0.788	0.564	1	6
gnpnat1	0.0004897	0.283131104	0.712	0.443	1	6
pcyt21	0.0005166	0.375231646	0.615	0.363	1	6
ginm11	0.0005494	0.284854911	0.404	0.196	1	6
zgc:920661	0.0005859	0.433088579	1	0.87	1	6
zgc:123068	0.0005879	0.828725324	0.288	0.125	1	6
fkbp11	0.0006263	0.431101211	0.365	0.187	1	6
slc7a6os1	0.0006547	0.262907223	0.365	0.178	1	6
slc5a81	0.0006694	0.39241975	0.346	0.173	1	6
pdlim1	0.0006783	0.2536707	0.885	0.635	1	6
rgcc1	0.0007083	0.581860311	0.635	0.407	1	6
hspa51	0.0007098	0.325618203	0.942	0.906	1	6
AL935186.9	0.0007598	0.287589436	0.654	0.404	1	6
gstp22	0.0008333	0.787031437	0.462	0.265	1	6
actn41	0.0008475	0.285192394	0.692	0.479	1	6
dnajc3b	0.0008505	0.251784986	0.423	0.219	1	6
abhd16a	0.0009085	0.376592301	0.404	0.196	1	6
si:ch211-207i1.2	0.0009664	0.396889991	0.346	0.169	1	6
tuba8l4	0.0009901	0.459349158	0.904	0.678	1	6
syvn11	0.0009989	0.276048992	0.462	0.253	1	6
rpl192	0.0010024	0.310893664	1	0.952	1	6
fkbp1aa1	0.00101	0.40338247	0.962	0.746	1	6
abhd2a	0.0010641	0.362749961	0.288	0.121	1	6
kin	0.0011209	0.290683682	0.269	0.117	1	6
histh111	0.0011263	0.327123769	0.654	0.448	1	6
rbm4.31	0.0011765	0.258496632	0.846	0.578	1	6
FP017217.11	0.0012216	0.31156478	0.519	0.304	1	6
si:dkey-248g15.3	0.0013935	0.830382973	0.269	0.116	1	6
h3f3d1	0.0014171	0.264324585	0.962	0.804	1	6
krtcap2	0.0014314	0.356383291	0.731	0.472	1	6
tmem2582	0.001441	0.263086727	0.942	0.68	1	6
sparc	0.0014926	0.644757174	0.615	0.415	1	6
psmd4a	0.0015403	0.38037741	0.596	0.351	1	6
rbmx	0.0015586	0.327632406	0.654	0.391	1	6

sap30l	0.0015754	0.256789714	0.731	0.48	1	6
drap1l	0.0016792	0.341278132	0.558	0.345	1	6
smdt1b1	0.0017194	0.266276135	0.846	0.566	1	6
eef2b1	0.0022348	0.292512324	1	0.902	1	6
ubqln4	0.0022712	0.25611174	0.731	0.463	1	6
gspt1l1	0.0022734	0.264558086	0.788	0.516	1	6
rpl101	0.0023245	0.341946231	0.942	0.92	1	6
tpt12	0.0023345	0.437039198	0.942	0.851	1	6
apmap	0.0024724	0.272414291	0.346	0.189	1	6
coro2a1	0.0026176	0.263987945	0.404	0.214	1	6
nup153	0.002693	0.421949348	0.423	0.246	1	6
ywhaqb1	0.0028195	0.262443399	0.808	0.648	1	6
brk1	0.0029805	0.257883677	0.538	0.342	1	6
btg21	0.0030459	0.584309497	0.865	0.669	1	6
glipr2l1	0.0031163	0.286002196	0.269	0.119	1	6
tet31	0.0031359	0.297603349	0.538	0.345	1	6
xbp1	0.0033453	0.276823257	0.962	0.767	1	6
eef1da	0.0036388	0.274285723	0.942	0.696	1	6
g3bp11	0.003681	0.312833889	0.596	0.381	1	6
ppp1cab	0.0040419	0.25766437	0.462	0.26	1	6
gk5	0.0042562	0.273460584	0.385	0.228	1	6
gstm.31	0.0044712	0.589381234	0.692	0.472	1	6
ddt1	0.0045936	0.775788876	0.769	0.553	1	6
hspa82	0.0048055	0.322635942	0.962	0.936	1	6
nme2b.13	0.0050131	0.289131847	0.962	0.875	1	6
rack12	0.0053302	0.291710499	0.981	0.897	1	6
amotl2b	0.0053426	0.592606299	0.577	0.393	1	6
cyp3a65	0.0055287	0.364013443	0.423	0.278	1	6
hnrnpub1	0.0063217	0.26968108	0.865	0.589	1	6
glod5	0.0063218	0.360046577	0.615	0.404	1	6
notch3	0.0075617	0.681648913	0.327	0.192	1	6
rps22	0.007875	0.275718243	0.981	0.952	1	6
fgd4a	0.0088354	0.298491293	0.327	0.185	1	6
gpx1a3	0.0097431	0.525241944	0.769	0.553	1	6
thbs1b	8.51E-15	1.769245167	0.659	0.19	2.03E-10	7
si:ch211-153b23.52	1.67E-10	0.866519	0.659	0.209	3.99E-06	7
anpepb	1.37E-09	1.004060542	0.561	0.178	3.26E-05	7
sfrp5	2.42E-09	0.736927126	0.463	0.14	5.76E-05	7
baiap2l1a	2.76E-09	0.979239392	0.829	0.447	6.57E-05	7
muc13b1	3.04E-09	1.122242284	0.927	0.49	7.24E-05	7
fabp61	7.28E-09	2.594233697	0.366	0.086	0.0001735	7
anxa2b3	2.08E-07	0.868608242	0.951	0.763	0.004964	7
si:ch211-153b23.41	4.65E-07	0.868361514	0.268	0.058	0.0110865	7
shroom1	1.23E-06	0.557678774	0.439	0.161	0.0292858	7
cdaa	1.38E-06	0.757854281	0.341	0.105	0.0329063	7
si:ch211-105c13.31	1.86E-06	0.610546894	0.415	0.14	0.044469	7
wu:fb59d01	2.53E-06	0.732548703	0.951	0.749	0.0602261	7
zgc:640221	3.27E-06	0.850206423	0.585	0.305	0.0779345	7
traf4b	4.05E-06	0.584720162	0.463	0.194	0.0966758	7
abracl2	4.66E-06	0.513776995	0.829	0.536	0.1111682	7
cldn15la3	7.07E-06	0.636495338	0.951	0.801	0.1684765	7
si:ch73-194h10.21	7.21E-06	0.765487083	0.707	0.396	0.17191	7
ponzr12	8.66E-06	0.915751033	0.659	0.387	0.2065226	7
si:ch211-139a5.91	1.51E-05	1.122894309	0.463	0.211	0.3599153	7

tjp3	1.60E-05	0.677351452	0.854	0.565	0.3822345	7
zgc:1583431	1.64E-05	0.840877036	0.854	0.607	0.3903078	7
txnl	2.06E-05	0.439787996	0.854	0.62	0.4905315	7
trpm4a1	2.34E-05	0.769602555	0.659	0.4	0.5574615	7
ppp1r9a1b	2.47E-05	0.75864205	0.659	0.435	0.5894729	7
chmp4c	2.49E-05	0.466597492	0.488	0.236	0.5927389	7
ddit3	3.59E-05	0.562899472	0.902	0.688	0.8558847	7
cyp3c3	3.82E-05	0.830285513	0.683	0.429	0.9098406	7
krt82	4.37E-05	0.51509234	0.902	0.62	1	7
tmem176l.2	4.92E-05	0.805548302	0.805	0.532	1	7
snrpd11	7.40E-05	0.458790194	0.878	0.7	1	7
mcl1b	7.66E-05	0.501963693	0.707	0.471	1	7
si:dkey-191g9.7	8.50E-05	0.681326197	0.439	0.211	1	7
cldnb3	8.68E-05	0.436445257	0.927	0.768	1	7
tuft1a1	8.80E-05	0.635972876	0.61	0.347	1	7
amotl2b1	9.89E-05	0.769244252	0.634	0.393	1	7
trim161	0.0001071	0.452802545	0.634	0.384	1	7
pisd	0.0001092	0.398613515	0.366	0.155	1	7
cdh171	0.0001124	0.638570588	0.878	0.529	1	7
ftr831	0.0001189	0.417103384	0.439	0.19	1	7
cgna	0.0001204	0.70436359	0.61	0.391	1	7
pdlim11	0.0001219	0.481081814	0.902	0.639	1	7
si:dkey-33i11.91	0.0001242	0.266491915	0.878	0.572	1	7
epcam2	0.0001517	0.593597039	0.976	0.909	1	7
slc10a3	0.0001858	0.718272239	0.463	0.248	1	7
s100a10a3	0.0001871	0.521835542	1	0.812	1	7
hdgfl2	0.0001909	0.353866925	0.585	0.351	1	7
tagln22	0.0002019	0.46813095	0.927	0.85	1	7
hoxc3a	0.0002395	0.300297306	0.341	0.138	1	7
kars1	0.0002671	0.952882864	0.732	0.525	1	7
eif1axb	0.0002708	0.275126333	0.854	0.583	1	7
nav31	0.0002948	0.382763979	0.268	0.094	1	7
hbegfa2	0.0003106	0.332428358	0.951	0.784	1	7
ppp2r5ea	0.0003301	0.516321881	0.537	0.318	1	7
arhgap52	0.0003307	0.586140935	0.805	0.616	1	7
zgc:922421	0.0003673	0.382637997	0.463	0.227	1	7
si:ch211-191j22.3	0.0004123	0.442538238	0.512	0.286	1	7
itpk1a1	0.0004331	0.530927026	0.659	0.449	1	7
wu:fb18f062	0.0004761	0.52990021	0.659	0.419	1	7
col18a1a1	0.000493	0.54969111	0.683	0.45	1	7
serpinb12	0.0005141	0.580246866	0.805	0.63	1	7
pllp2	0.0005678	0.590209518	0.512	0.307	1	7
zgc:923131	0.0005836	0.367261403	0.634	0.398	1	7
tpm32	0.0006399	0.487558345	0.902	0.756	1	7
nhp2	0.0006434	0.381247032	0.659	0.442	1	7
dnmbp2	0.0006701	0.479074266	0.829	0.611	1	7
igf2bp31	0.000703	0.512587102	0.756	0.504	1	7
glod51	0.0007199	0.380884556	0.61	0.408	1	7
max	0.000733	0.328312824	0.732	0.51	1	7
rab20	0.0007428	0.367511915	0.268	0.106	1	7
pum1	0.0007792	0.345612903	0.561	0.354	1	7
lxn2	0.0007829	0.412373049	0.659	0.459	1	7
kynu	0.0007967	0.422605278	0.366	0.173	1	7
pdap1a1	0.0008409	0.463819657	0.78	0.569	1	7

si:zfos-1714f5.3	0.0008447	0.449712367	0.317	0.138	1	7
si:ch73-22o12.1	0.0008519	0.460612447	0.732	0.529	1	7
cldn15lb2	0.0008575	0.295234458	0.878	0.588	1	7
higd1a	0.0009466	0.465438363	0.829	0.64	1	7
dag11	0.0010427	0.364160879	0.659	0.433	1	7
ctgfa2	0.0010782	0.428181449	0.366	0.168	1	7
lsp11	0.0011149	0.388874229	0.659	0.445	1	7
pklr2	0.0011507	0.482244016	0.707	0.487	1	7
bcl2l1	0.001162	0.629390204	0.463	0.267	1	7
elf32	0.0011857	0.284636619	0.756	0.553	1	7
zgc:165555.9	0.0012215	0.677691173	0.463	0.25	1	7
nrarpa1	0.0012905	0.418537177	0.537	0.311	1	7
eps8l3b1	0.0013331	0.514606852	0.707	0.485	1	7
si:ch73-86n18.1	0.0014044	0.356919655	0.512	0.293	1	7
ctnnb12	0.0015002	0.504152634	0.683	0.464	1	7
epdl2	0.0015364	0.994935472	0.415	0.22	1	7
rbm471	0.0015409	0.505847762	0.707	0.489	1	7
ptmab1	0.0015428	0.271781524	1	0.897	1	7
angptl41	0.0015876	0.539578681	0.902	0.62	1	7
si:dkey-283b1.61	0.0016236	0.304880853	0.61	0.368	1	7
naa101	0.001787	0.361655014	0.683	0.503	1	7
anks4b3	0.0018474	0.377044701	0.634	0.417	1	7
u2surp	0.0019302	0.785476313	0.659	0.473	1	7
ndufa4l2	0.0019337	0.341561225	0.927	0.78	1	7
rpl37.13	0.0019444	0.346320573	1	0.979	1	7
rnf128a	0.0019947	0.351826115	0.634	0.454	1	7
lmna1	0.0019994	0.513164569	0.512	0.333	1	7
acsf21	0.002007	0.684612167	0.39	0.209	1	7
myh9b1	0.0020111	0.343908354	0.683	0.504	1	7
cx28.9	0.0020582	0.380497943	0.463	0.258	1	7
dap1b1	0.0020642	0.370771955	0.61	0.403	1	7
nagk	0.0020649	0.660659486	0.268	0.122	1	7
s100u1	0.0020812	0.333540755	0.537	0.328	1	7
tmsb4x2	0.0021513	0.257706226	1	0.993	1	7
hspa91	0.0021535	0.360250484	0.854	0.614	1	7
impdh21	0.0021902	0.374304048	0.561	0.346	1	7
cfd	0.0022891	0.491167465	0.341	0.166	1	7
zc3h11a	0.0022972	0.652622316	0.585	0.387	1	7
pdzk1ip12	0.0024678	0.41776605	0.366	0.185	1	7
taf15	0.0024833	0.321092103	0.756	0.574	1	7
zbtb8os	0.0024963	0.40565716	0.634	0.469	1	7
prpf81	0.0026323	0.450986162	0.537	0.344	1	7
slc26a5	0.0026774	0.464402647	0.341	0.182	1	7
cry1ba	0.0027319	0.350987271	0.78	0.555	1	7
lmo7a1	0.0028881	0.283273468	0.659	0.478	1	7
ncf1	0.0029971	0.683217614	0.341	0.173	1	7
sptbn51	0.003199	0.40265662	0.317	0.15	1	7
mt-nd52	0.003299	0.47042273	0.927	0.75	1	7
serpinb1l32	0.003495	0.309488551	0.854	0.637	1	7
ano9b1	0.003553	0.430883867	0.512	0.337	1	7
ugt81	0.0037034	0.256937772	0.634	0.421	1	7
elf12	0.0038179	0.54850184	0.585	0.421	1	7
srrm1	0.0039062	0.260425771	0.659	0.496	1	7
nop102	0.0039715	0.273575682	0.829	0.595	1	7

si:ch73-138n13.11	0.0039919	0.485800075	0.61	0.403	1	7
isoc2	0.0041692	0.433194731	0.341	0.188	1	7
sub1a2	0.0042149	0.27057126	0.829	0.654	1	7
pttg1ipb	0.0044436	0.347229973	0.341	0.182	1	7
lgals2b	0.0046416	0.591362946	1	0.939	1	7
rnf213a	0.0047154	0.470517069	0.317	0.161	1	7
smdt1b2	0.0048086	0.462292102	0.78	0.576	1	7
dspa	0.0048919	0.382912083	0.78	0.606	1	7
epha2a1	0.0049738	0.322716874	0.585	0.407	1	7
seta	0.0050701	0.335542831	0.78	0.551	1	7
myo7bb1	0.0053368	0.570445439	0.366	0.199	1	7
sptan11	0.0053991	0.358880963	0.561	0.412	1	7
zgc:113314	0.0054165	0.315367597	0.341	0.176	1	7
rassf7b	0.0055204	0.407488277	0.659	0.476	1	7
ubl3a	0.005651	0.46925216	0.585	0.426	1	7
pdk2a	0.0058068	0.386066945	0.415	0.265	1	7
MYO1D1	0.00619	0.278762793	0.512	0.323	1	7
mt-nd4l1	0.0062331	0.382149719	0.659	0.499	1	7
jupa2	0.0062399	0.344683864	0.756	0.548	1	7
csde1	0.0064923	0.279457521	0.805	0.565	1	7
smim151	0.0065077	0.324573461	0.61	0.457	1	7
actn42	0.0065457	0.475818972	0.659	0.485	1	7
gstt1a3	0.0065953	0.301744317	0.927	0.586	1	7
CU929259.11	0.0066792	0.259931254	0.561	0.398	1	7
aldh8a12	0.006739	0.538325235	0.488	0.291	1	7
nsa2	0.0070019	0.316899384	0.78	0.599	1	7
zgc:1584631	0.0070605	0.321707943	1	0.969	1	7
chp11	0.0071134	0.343699233	0.39	0.246	1	7
ezra1	0.0072233	0.373687219	0.878	0.728	1	7
grn1	0.0072544	0.252463564	0.341	0.18	1	7
dnajc2	0.0072648	0.332347865	0.366	0.222	1	7
foxa11	0.0073933	0.43160981	0.439	0.264	1	7
sdcbp21	0.0074603	0.301609163	0.707	0.508	1	7
gstr3	0.0075456	0.317738775	0.78	0.588	1	7
safb	0.0075583	0.320258017	0.634	0.48	1	7
phlda21	0.0076221	0.257864803	1	0.894	1	7
ddx52	0.0079066	0.274468643	0.878	0.674	1	7
si:dkey-202l16.5	0.0079591	0.595217901	0.463	0.314	1	7
rpsl22	0.0086597	0.329400513	1	0.96	1	7
chd4a1	0.0091268	0.378387257	0.61	0.442	1	7
nr0b2a1	0.0095712	0.531356799	0.341	0.199	1	7
ush1c	0.0096893	0.309474515	0.537	0.365	1	7
cnn21	0.0098813	0.325519135	0.805	0.686	1	7
upf1	0.0099518	0.355406183	0.488	0.326	1	7
rho1	2.35E-06	1.738331026	0.792	0.495	0.0560771	8
mt-co23	0.0003103	1.483410616	0.958	0.937	1	8
mt-nd43	0.0003407	1.290985086	0.875	0.836	1	8
mt-co32	0.0006826	1.438994341	0.875	0.937	1	8
prelid3b1	0.0009703	0.299389951	0.208	0.641	1	8
copz1	0.000976	0.301236769	0.042	0.408	1	8
mt-cyb3	0.0010058	1.255590172	0.917	0.903	1	8
tmed102	0.0010628	0.297023033	0.125	0.559	1	8
llph3	0.0012582	0.265145469	0.083	0.461	1	8
stard141	0.0021189	0.521362609	0.167	0.603	1	8

atp2b2.11	0.0023689	0.793198795	0.083	0.453	1	8
mt-nd23	0.0047559	1.274654705	0.875	0.844	1	8
sfpq	0.0060795	0.352781632	0.125	0.49	1	8
ehd1b	0.0063867	0.548364897	0.042	0.325	1	8
klf31	0.0066569	0.335521881	0.167	0.561	1	8
mt-co12	0.0075267	1.022886629	0.875	0.898	1	8
emc10	0.007629	0.327908071	0.125	0.476	1	8
tmem45b	0.0081802	0.4283919	0.042	0.314	1	8
mt-atp62	0.0089673	1.349976674	0.875	0.915	1	8
klf7b2	0.0092853	0.473813455	0.125	0.456	1	8
