# Supplementary material for: Mycn regulates intestinal development through ribosomal biogenesis in a zebrafish model of Feingold syndrome 1
Source: PLoS Biol. 2022 Nov 1;20(11):e3001856. doi: 10.1371/journal.pbio.3001856 (PMC9624419; doi:10.1371/journal.pbio.3001856)
Supplement: S3 Table — (PDF) [file pbio.3001856.s010.pdf]

external_gene_name	baseMean	log2FoldChange	lfcSE	stat	pvalue	padj
slc35a5	118.0957	0.138332419	0.216139	0.640015	0.522163	0.887769
ccdc80	204.5786	0.667121999	0.196714	3.391322	0.000696	0.034884
nrf1	645.5006	0.179803727	0.116936	1.537621	0.124141	0.571369
ube2h	1977.436	-0.056246	0.071018	-0.792	0.428363	0.846133
slc9a3r1a	328.7106	0.044235208	0.163773	0.2701	0.787083	0.963065
dap	1337.363	0.167933466	0.130751	1.28438	0.199009	0.679593
itsn1	566.1092	-0.094719369	0.148573	-0.63753	0.523782	0.888642
myh10	2374.121	0.090600692	0.157815	0.574094	0.565904	0.901726
tmem267	73.85192	-0.223448521	0.244177	-0.91511	0.360135	0.811057
thraa	79.31861	-0.33157794	0.22693	-1.46115	0.143975	0.603144
itsn2a	181.5203	0.187071665	0.186403	1.003586	0.315578	0.77795
hoxb2a	126.2727	-0.186821278	0.218519	-0.85494	0.392583	0.827902
ptpn4b	320.0827	0.272050438	0.189022	1.439255	0.150078	0.613391
sema6e	414.4191	0.068626535	0.170777	0.401848	0.687796	0.938835
krt97	10017.04	-0.32325501	0.131935	-2.4501	0.014282	0.209217
usp49	244.7783	-0.161041055	0.17196	-0.9365	0.349015	0.801306
slc40a1	1322.731	-0.121807615	0.204745	-0.59492	0.551895	0.8974
dcaf8	305.9647	-0.10517891	0.17171	-0.61254	0.540183	0.893049
cfap300	20.81778	0.01514882	0.385248	0.039322	0.968633	1
prkag3b	199.0066	0.244593155	0.211413	1.156947	0.247294	0.727871
triob	466.5137	0.305852562	0.146243	2.091401	0.036492	0.335935
pde6a	132.7582	-0.474910063	0.308647	-1.53868	0.123882	0.571162
matr3l1.1	964.0491	0.087886534	0.104344	0.84228	0.399631	0.831196
si:ch73-314g15.3	82.88596	0.006933536	0.220986	0.031375	0.97497	1
slc39a13	346.1549	0.347299768	0.163181	2.128312	0.033311	0.323127
nitr2b	4.536112	1.402299391	1.090216	1.286258	0.198353	0.679109
cntn2	131.597	0.092688797	0.221997	0.417523	0.676296	0.93557
tnni1a	5.786459	1.055428444	1.777568	0.593748	0.55268	0.8974
pms1	88.98435	0.14563694	0.271281	0.536849	0.591372	0.909437
upf3b	605.5693	-0.092745394	0.120052	-0.77254	0.439792	0.852079
stx1b	2744.315	0.28021458	0.194118	1.443529	0.148871	0.610726
ankar	8.781245	-0.738601733	0.530253	-1.39292	0.163643	0.631915
ofd1	128.2947	0.075828432	0.181092	0.418728	0.675415	0.935422
asdurf	297.8941	-0.081497962	0.143207	-0.56909	0.569293	0.902303
gtf2e1	251.9598	-0.094273307	0.122736	-0.7681	0.442428	0.853789
slc1a4	821.3637	-0.195471144	0.136713	-1.42979	0.152778	0.617015
ttn.1	5496.635	-0.016464703	0.262568	-0.06271	0.95	0.99801
znf281a	415.6062	0.117140376	0.111839	1.047405	0.294913	0.763708
ell	503.7507	-0.059260509	0.120175	-0.49312	0.621929	0.918461
grasp	113.9098	-0.303424775	0.227414	-1.33424	0.182126	0.658775
dnah6	40.405	0.323344095	0.327526	0.987233	0.323528	0.783161
ptpn4a	561.6631	0.034414846	0.12739	0.270153	0.787042	0.963065
opn1mw4	0.128836	0	5.267649	0	1	NA
osgepl1	29.25713	-0.065452238	0.367856	-0.17793	0.858779	0.979951
psmb9a	4.131826	2.293483148	1.513929	1.514921	0.129792	0.579886
creg2	634.3608	-0.426468675	0.199447	-2.13826	0.032496	0.319445
sypl2b	204.6097	-0.393646479	0.130244	-3.02237	0.002508	0.08071
daxx	255.2643	-0.123712701	0.144216	-0.85783	0.390988	0.827902
slc6a22.1	201.3817	0.051833218	0.190289	0.272392	0.785321	0.962605
lcmt2	38.14294	-0.243434905	0.359533	-0.67709	0.498352	0.879132
znf511	81.96516	-0.748802888	0.227024	-3.29834	0.000973	0.043444
spi1b	73.18201	0.148530427	0.24201	0.613736	0.53939	0.893049
scfd1	683.7883	0.127237992	0.103951	1.224013	0.220947	0.702242

nr4a1	497.5112	-0.357396467	0.599656	-0.596	0.551174	0.8974
rassf6	173.4035	0.301666795	0.156641	1.925852	0.054123	0.402505
telo2	150.1353	0.071266001	0.170902	0.416998	0.67668	0.935656
snx9a	281.2678	0.115556174	0.150473	0.767952	0.442516	0.853789
dstyk	1254.436	-0.090185724	0.124464	-0.72459	0.468702	0.866951
mapk14a	517.4562	-0.068113069	0.125146	-0.54427	0.586258	0.906711
scrib	1554.842	0.093783318	0.133826	0.700785	0.483437	0.872099
zgc:56699	108.6732	-0.336251936	0.213115	-1.57779	0.114613	0.554279
skap2	79.39406	0.338860117	0.26392	1.283951	0.199159	0.679605
unk	106.3742	0.009571021	0.224451	0.042642	0.965987	1
dzip1	350.9059	0.048373899	0.1575	0.307135	0.75874	0.957882
ncor2	1509.007	0.048182865	0.122003	0.394932	0.692893	0.940444
myh9b	2318.49	0.046641659	0.199072	0.234295	0.814756	0.970686
znf384l	440.9022	-0.107923446	0.165386	-0.65255	0.514045	0.88631
bysl	1455.774	-0.13090452	0.172949	-0.7569	0.449112	0.856796
slc17a6a	81.66448	0.465811805	0.26007	1.7911	0.073277	0.460742
dicer1	668.062	0.000823473	0.151654	0.00543	0.995668	1
rimbp2	83.94345	0.021260874	0.351149	0.060547	0.95172	0.99801
matr3l1.1	618.32	-0.093510362	0.127695	-0.73229	0.46399	0.864593
hsd17b8	129.0817	0.029264277	0.154335	0.189616	0.84961	0.978209
arl14ep	346.4709	0.019793201	0.214075	0.092459	0.926333	0.994815
mycbp2	1643.958	0.110642258	0.159467	0.693826	0.487791	0.873291
map4k2	158.7571	0.115670496	0.168294	0.687312	0.491886	0.876086
puf60b	1618.343	0.037821719	0.094256	0.401267	0.688224	0.93909
srrm1	1564.129	-0.089906221	0.164866	-0.54533	0.585527	0.906655
pcgf1	76.76687	-0.314161947	0.277971	-1.1302	0.258393	0.735636
gas2a	31.68654	-0.380059647	0.382557	-0.99347	0.320479	0.780821
psmb8a	4.397652	-1.378650788	1.658115	-0.83146	0.405716	0.834645
g2e3	438.9288	0.270497304	0.234501	1.153503	0.248704	0.728758
slc6a22.2	158.1514	-0.668236035	0.202702	-3.29664	0.000978	0.043444
mctp2a	107.9434	-0.33448633	0.248594	-1.34551	0.178461	0.652712
actn3b	8925.677	0.1212822	0.210479	0.576219	0.564467	0.901433
slc2a1a	56.91533	-0.775586592	0.456456	-1.69915	0.089291	0.501424
inppl1b	182.6042	-0.040786814	0.220438	-0.18503	0.853208	0.978839
mier1b	233.7677	0.019896822	0.176891	0.11248	0.910442	0.992208
adam8a	134.1958	0.079380367	0.231955	0.342223	0.732183	0.950617
tdh2	508.9021	0.302044395	0.261786	1.153785	0.248588	0.728723
mhc1zea	131.2977	0.007991898	0.190438	0.041966	0.966526	1
skap1	12.14574	-1.20034116	0.570622	-2.10357	0.035416	0.330833
sp3a	105.0466	0.266968184	0.175764	1.518901	0.128787	0.578173
tomm34	250.6532	0.585637647	0.188364	3.109079	0.001877	0.066898
kifc1	659.3963	0.29341253	0.220307	1.331836	0.182914	0.659278
csmd2	52.37148	-0.188720783	0.335485	-0.56253	0.573754	0.904296
prf1.3	0.441618	-0.868132876	3.17633	-0.27331	0.784613	NA
echs1	1329.517	-0.112093739	0.107537	-1.04237	0.297239	0.764844
nfya1	213.8527	0.094710846	0.164756	0.574856	0.565388	0.901538
kirrel1a	546.8383	0.215769112	0.154565	1.395977	0.162721	0.630455
psmb13a	2.086309	-0.868149099	2.165098	-0.40097	0.688439	NA
gpm6bb	552.5294	-0.010684048	0.152926	-0.06986	0.944302	0.997371
uckl1a	31.37656	0.251725994	0.427183	0.58927	0.55568	0.898723
flot1a	1566.41	0.062717748	0.10293	0.609322	0.542311	0.894395
si:ch211-247n2.1	76.38959	0.236053282	0.346298	0.681648	0.495461	0.87806
oc90	18.34908	0.285940656	0.444349	0.643505	0.519897	0.887148
strn3	1219.245	0.163888372	0.0865	1.894666	0.058137	0.416521

gulp1a	415.5726	-0.040203145	0.130469	-0.30814	0.757973	0.957487
fkbp8	432.9672	0.028645039	0.144323	0.198478	0.842671	0.9766
noc2l	625.2574	-0.007448286	0.16661	-0.0447	0.964343	0.999925
tnxba	375.85	0.534731355	0.370159	1.444601	0.14857	0.61024
slc29a2	195.37	0.985529407	0.330652	2.980564	0.002877	0.088391
ptpra	382.3258	0.110928781	0.158359	0.700491	0.483621	0.872148
otol1a	9.409358	-0.59311211	0.912783	-0.64978	0.515832	0.886634
stim2b	46.2779	0.242797707	0.250639	0.968714	0.332688	0.789807
nup155	530.3451	0.203169939	0.12152	1.67191	0.094542	0.513368
tspan11	51.56158	-0.466227206	0.265138	-1.75843	0.078674	0.474455
prkacba	120.5776	-0.298707465	0.253355	-1.17901	0.238395	0.719474
irx2a	282.5125	0.278962039	0.152724	1.82658	0.067763	0.443773
atp5po	8457.899	4.57E-05	0.139444	0.000328	0.999738	1
kcnh6a	70.65874	-0.090379256	0.258562	-0.34955	0.72668	0.949095
zgc:112148	105.2907	0.847553312	0.233861	3.62417	0.00029	0.019028
tnfrsf21	291.969	-0.201048275	0.159461	-1.2608	0.207382	0.689456
zmp:0000000521	12.23245	-0.995957369	0.506217	-1.96745	0.049131	0.386041
c3b.2	383.7958	-0.539516733	0.49893	-1.08135	0.279542	0.752477
cfap43	19.07791	-0.287068856	0.396871	-0.72333	0.469477	0.867224
zgc:112982	979.5078	0.031739524	0.141769	0.223881	0.82285	0.971966
nalcn	299.0616	0.112658378	0.274532	0.410365	0.681538	0.937418
aff4	292.6174	0.005053852	0.178412	0.028327	0.977401	1
dbx1b	95.60424	0.273406789	0.222255	1.230148	0.218642	0.699445
atp1a1a.4	3162.927	0.163955084	0.242148	0.677087	0.498351	0.879132
phgdh	1327.774	-0.072760123	0.132392	-0.54958	0.582606	0.90571
nav2b	431.1402	-0.029228429	0.141162	-0.20706	0.835967	0.976007
stx3a	282.9642	0.055197704	0.159785	0.345451	0.729755	0.950056
cacnb3a	8.704609	0.354652275	0.753162	0.470884	0.637723	0.924779
kbtbd12	945.9557	0.112085158	0.173955	0.644332	0.51936	0.887148
ppm1ba	175.9017	0.217153978	0.145744	1.489973	0.136231	0.591544
tuba1a	4693.417	0.164257031	0.352194	0.466382	0.640942	0.925364
stt3b	1062.604	0.15286006	0.094821	1.612097	0.106941	0.537
syt14b	64.69812	0.951577538	0.268934	3.538334	0.000403	0.023889
man2b1	477.8451	0.050522013	0.154962	0.326029	0.744403	0.95491
manea	161.5879	0.233920175	0.194045	1.205493	0.228013	0.709324
si:dkey-247m21.3	12.98714	-0.974535582	0.708759	-1.37499	0.169135	0.640442
vill	131.0346	0.237634122	0.180518	1.3164	0.18804	0.667313
rorab	1306.542	-0.13080753	0.207552	-0.63024	0.528537	0.890002
drd4-rs	78.49216	-0.058550363	0.231598	-0.25281	0.800415	0.967364
palmda	185.598	-0.007618098	0.248298	-0.03068	0.975524	1
pink1	354.5998	0.04322298	0.151084	0.286086	0.774812	0.960899
klhl15	293.1312	-0.17051177	0.147608	-1.15517	0.248021	0.728489
prkag2b	80.44624	0.092642324	0.31533	0.293795	0.768915	0.960395
LO018422.1	38.24182	-0.02269034	0.427582	-0.05307	0.957679	0.999109
b3gat3	247.2921	-0.246008078	0.140029	-1.75683	0.078946	0.475183
dnajc1	823.0803	0.061756892	0.100791	0.612721	0.540061	0.893049
ak1	20964.84	-0.017160838	0.131719	-0.13028	0.896342	0.98939
pfkfb3	747.4668	-0.302669314	0.39563	-0.76503	0.444252	0.854431
dock5	61.96294	0.081447157	0.330467	0.246461	0.805325	0.969348
txlng	288.1748	-0.300072404	0.124375	-2.41265	0.015837	0.222022
cthrc1b	26.96573	-1.137014197	1.065043	-1.06758	0.285712	0.757084
hsd11b2	395.6046	-0.332090582	0.222273	-1.49407	0.135158	0.590536
fkbp16	124.8048	-0.333596248	0.24849	-1.34249	0.179436	0.654677
myhb	1278.662	-0.351310937	0.310017	-1.1332	0.257131	0.734679

stxbp1a	3333.55	0.273432423	0.211955	1.290052	0.197033	0.677235
adamts18	203.1237	-0.13757466	0.211471	-0.65056	0.515331	0.886401
lmcd1	246.3644	-0.210185708	0.225516	-0.93202	0.351326	0.802305
rxrbb	413.8676	-0.062451799	0.130447	-0.47875	0.632114	0.923176
grb10a	136.2354	-0.620902584	0.222082	-2.79582	0.005177	0.12067
ppil2	376.8841	-0.09714156	0.128099	-0.75833	0.448251	0.856741
rictorb	222.6867	0.00744525	0.169823	0.043841	0.965031	1
pygb	650.7976	0.141268904	0.113814	1.241225	0.214523	0.69658
qkib	82.69519	0.367251345	0.218146	1.68351	0.092277	0.508314
tox4a	252.0672	0.185116716	0.144823	1.278227	0.201169	0.681045
pfkfb2b	86.07643	0.406985952	0.283428	1.435944	0.151018	0.615085
brf2	18.70627	-0.226366138	0.4273	-0.52976	0.596279	0.911626
adss	402.5414	0.179047028	0.160724	1.114001	0.265279	0.742063
mnat1	101.3866	0.020839319	0.216874	0.09609	0.923449	0.994525
csnk2a1	2031.881	-0.089333777	0.075517	-1.18296	0.236827	0.718136
lamb2	924.9409	0.154493218	0.178225	0.866843	0.386028	0.826948
si:dkeyp-52c3.7	3.180791	-0.178447871	1.328468	-0.13433	0.893145	0.988566
stam	943.6197	0.090039292	0.098748	0.911809	0.361869	0.811787
cwf19l1	258.7856	-0.023824052	0.125812	-0.18936	0.849809	0.97824
celf2	554.7982	0.10831608	0.13913	0.778525	0.436259	0.850864
psme1	84.72572	0.065201704	0.345984	0.188453	0.850522	0.978251
cacnb1	175.0829	0.100620972	0.185432	0.542631	0.587384	0.907373
tra2b	530.6197	0.023528182	0.125658	0.18724	0.851472	0.978548
aplnra	246.0822	-0.323377188	0.145615	-2.22077	0.026367	0.287876
itfg2	126.8951	-0.17796717	0.207143	-0.85915	0.390256	0.827902
igbp1	463.9022	-0.254057825	0.129742	-1.95817	0.05021	0.390261
chmp2bb	1147.422	-0.258240031	0.154721	-1.66907	0.095104	0.51461
aspn	334.8077	0.308209489	0.202974	1.518465	0.128897	0.578173
rho	5841.953	-0.470414229	0.441166	-1.0663	0.286289	0.757624
rhd	102.8181	0.439270275	0.248739	1.765986	0.077398	0.470172
bach1b	518.4567	0.154270394	0.170815	0.903144	0.366449	0.81589
pygl	1556.32	-0.728965228	0.245643	-2.96758	0.003002	0.090435
hspb11	353.3604	-0.330903923	0.291222	-1.13626	0.255848	0.733642
stk25a	180.7702	0.698108654	0.173942	4.013465	5.98E-05	0.006302
invs	71.35987	0.086918584	0.212676	0.40869	0.682767	0.938006
rtca	487.5255	-0.074500166	0.117376	-0.63472	0.525614	0.888928
tbx3a	394.9098	-0.02336721	0.12958	-0.18033	0.856893	0.979558
scocb	726.4548	0.031098898	0.150457	0.206696	0.836247	0.976007
si:dkey-103j14.5	21.64345	-0.195254924	0.665942	-0.2932	0.769368	0.960484
zgc:103692	255.0908	-0.061970831	0.148165	-0.41826	0.675761	0.935422
sypb	1610.634	0.319584737	0.185973	1.718444	0.085716	0.491277
plpp2a	45.80102	-0.602520581	0.291168	-2.06932	0.038516	0.344223
mmp14a	1284.808	0.043695307	0.123079	0.355018	0.722576	0.947632
psmb6	2496.286	0.004064204	0.186057	0.021844	0.982572	1
kcna2a	4.97011	-0.397178824	1.042788	-0.38088	0.703291	0.943622
tbxas1	326.3066	-0.453517811	0.21461	-2.11322	0.034582	0.328029
epb41l3a	490.6019	0.089435138	0.156944	0.569853	0.568778	0.902234
ca15c	78.36669	0.262743708	0.768454	0.341912	0.732417	0.950617
mrpl41	362.9043	0.147933147	0.134346	1.101133	0.270839	0.746845
dcaf13	406.6236	0.021612325	0.136809	0.157975	0.874477	0.984052
zfand5b	2202.736	0.06123515	0.09501	0.644513	0.519243	0.887148
mcoln1a	333.3673	-0.311322724	0.119734	-2.60012	0.009319	0.165999
si:ch211-197g15.6	42.71992	0.732253881	0.454404	1.61146	0.107079	0.537019
si:dkey-21p1.3	9.377898	0.725521469	0.645216	1.124463	0.260817	0.738407

ankrd22	88.71217	0.085845027	0.361986	0.23715	0.81254	0.970291
arsh	61.02675	-0.526243622	0.239028	-2.2016	0.027694	0.29556
p2rx2	35.26727	-1.177812487	0.583491	-2.01856	0.043533	0.365455
gins2	106.7632	-0.157507539	0.344117	-0.45771	0.647157	0.926968
me3	1514.009	0.040619041	0.080723	0.503189	0.614831	0.915998
fabp11b	94.66091	-0.407838266	0.296735	-1.37442	0.169311	0.640794
lhx8a	92.94381	0.679071628	0.204607	3.318908	0.000904	0.041218
frmd6	368.0598	0.009511226	0.140247	0.067817	0.945931	0.997371
fam102bb	108.4628	-0.855764107	0.205002	-4.17442	2.99E-05	0.003783
dlc	578.3038	0.103168538	0.147544	0.699237	0.484404	0.872824
sgf29	242.4792	-0.070361633	0.187472	-0.37532	0.707424	0.944981
tubb4b	20454.61	-0.097693713	0.141558	-0.69013	0.490112	0.875079
cyp11a1	5.04448	0.026305098	0.755261	0.034829	0.972216	1
tagapa	23.1074	-1.258039748	0.548641	-2.29301	0.021847	0.261875
shpk	15.86331	0.081103171	0.43376	0.186977	0.851678	0.978548
ubald1a	600.2824	-0.060756371	0.109071	-0.55704	0.577503	0.904842
cers5	455.6742	-0.214672605	0.131397	-1.63378	0.102306	0.528996
UBB	4361.995	0.195095677	0.195534	0.997756	0.318398	0.779923
mief2	215.7825	-0.025924569	0.20577	-0.12599	0.899741	0.99045
atm	178.2869	0.192623248	0.157656	1.221791	0.221787	0.703656
tlcd1	114.3437	0.482340435	0.24684	1.954058	0.050694	0.39166
ugt5d1	38.90915	-0.618192568	0.488924	-1.26439	0.206089	0.688448
cry-dash	557.2135	-0.00306869	0.200705	-0.01529	0.987801	1
gale	406.2545	-0.02852684	0.154572	-0.18455	0.853579	0.978887
ercc3	423.2416	0.220984434	0.138197	1.599058	0.109808	0.543642
nusap1	600.6257	0.221580173	0.278255	0.79632	0.425846	0.844714
si:ch211-225b11.1	279.7349	-0.122491355	0.208974	-0.58616	0.557771	0.899184
zmynd10	26.40377	1.260113681	0.3435	3.668449	0.000244	0.016928
pde4cb	52.9926	0.122589991	0.319506	0.383686	0.701211	0.943062
prdm1a	349.0113	-0.010236317	0.191568	-0.05343	0.957386	0.999109
arhgef37	99.00199	0.292086451	0.260092	1.123013	0.261432	0.738975
rdh14b	275.7679	0.145800856	0.136746	1.066215	0.286327	0.757624
ercc6l	223.0333	0.446500424	0.180814	2.469386	0.013535	0.202995
wnt7ab	18.55678	0.090745353	0.447742	0.202673	0.83939	0.976417
noc3l	371.5145	0.05039061	0.154397	0.326371	0.744143	0.95491
itgb6	172.9777	0.32561134	0.167575	1.943073	0.052007	0.396487
zgc:92818	511.1272	0.081270415	0.156093	0.520652	0.602609	0.914072
itga10	196.9689	0.474707366	0.187261	2.535002	0.011245	0.183909
ankrd33ab	6.619336	1.985822438	1.504831	1.319632	0.186958	0.665301
zgc:153911	27.12598	-1.266113698	0.551467	-2.2959	0.021682	0.261221
dmbx1b	33.29555	0.583448838	0.352643	1.654505	0.098025	0.521071
hsdl2	1617.6	0.099906758	0.140729	0.709922	0.477752	0.869292
adora2b	18.68528	-0.439416171	0.505397	-0.86945	0.384603	0.826588
tm9sf4	646.8804	0.011315988	0.090526	0.125003	0.900521	0.99045
smu1a	614.6052	-0.096883508	0.106591	-0.90893	0.363387	0.813662
rundc3ab	424.3467	0.396562122	0.148697	2.666918	0.007655	0.147606
eif3eb	272.6039	-0.599706039	0.168133	-3.56686	0.000361	0.022086
mapkapk2a	800.636	-0.147204689	0.114459	-1.2861	0.19841	0.679148
syngr1a	392.2764	-0.181984909	0.153988	-1.18181	0.23728	0.718568
tmod2	111.7828	-0.816553165	0.158998	-5.13563	2.81E-07	0.00011
cabp5a	34.52936	-3.119050349	1.33283	-2.34017	0.019275	0.244673
tbx15	371.0793	-0.102920515	0.168608	-0.61041	0.541588	0.893902
dpysl3	2257.168	-0.030609133	0.118537	-0.25822	0.796234	0.966357
mylpfb	31222.61	0.187309741	0.144522	1.296067	0.194953	0.67483

ruvbl1	676.9414	0.303795076	0.124886	2.432574	0.014992	0.214289
slc45a2	771.4674	-0.329400387	0.16371	-2.01209	0.04421	0.368106
pabpc1l	1.416545	0.339784479	1.570491	0.216356	0.828711	NA
pcsk1	41.53892	-0.209156186	0.470314	-0.44472	0.656525	0.929753
irx7	73.01267	-0.241274509	0.227314	-1.06142	0.2885	0.759522
unm_sa1614	98.85729	-0.052362968	0.174167	-0.30065	0.763682	0.959055
rnf145a	198.6577	0.099324436	0.213006	0.466299	0.641001	0.925364
rpa3	325.4319	-0.149370725	0.235962	-0.63303	0.526714	0.889699
anxa11b	2793.67	-0.138623115	0.084966	-1.63151	0.102782	0.529448
b4galt1	124.6592	-0.371775443	0.240938	-1.54303	0.122823	0.569468
zgc:85722	284.4956	0.129477899	0.282592	0.45818	0.646823	0.926728
sh3gl1b	367.4738	0.044503564	0.17579	0.253164	0.800142	0.967256
rgs5a	735.1663	0.073711485	0.180617	0.408109	0.683194	0.938212
stxbp5a	215.9537	-0.235414144	0.298895	-0.78762	0.430921	0.84772
mapre1b	2294.462	0.226996139	0.125859	1.803573	0.071298	0.455342
optn	547.9435	-0.382027492	0.137337	-2.78167	0.005408	0.123724
kcnj1b	27.5751	0.198897966	0.317162	0.627118	0.530582	0.890545
ATG14	765.7664	-0.148206442	0.169517	-0.87429	0.381962	0.824957
tbce1a	305.6459	-0.247529866	0.137144	-1.80489	0.071093	0.455158
fam113	0.758103	-0.85269986	2.885151	-0.29555	0.767575	NA
rabif	218.1311	0.177147575	0.188628	0.939137	0.347661	0.80026
gnb3b	4011.64	1.181072103	0.484358	2.438427	0.014751	0.213272
rasl12	36.16556	-0.3613457	0.305932	-1.18113	0.237551	0.718568
her11	3.057268	1.676162255	1.353633	1.23827	0.215616	0.697157
ncl	8180.211	-0.229090874	0.122478	-1.87046	0.06142	0.425187
utp15	394.3881	-0.080709952	0.197526	-0.4086	0.682831	0.938006
ankrd2	0.319876	0.093659364	3.460444	0.027066	0.978407	NA
kiss1ra	2.234783	0	1.959022	0	1	1
sdc2	1193.896	-0.122153687	0.11617	-1.05151	0.293024	0.762508
spon2b	309.4512	-0.184958966	0.292621	-0.63208	0.527336	0.889948
tdh	6705.084	-0.285462975	0.217235	-1.31407	0.188822	0.668515
sema6d	184.4104	-0.197386864	0.188049	-1.04965	0.293877	0.763068
phf20a	179.1573	0.13326754	0.163804	0.813579	0.415886	0.839284
pdcd2l	124.3066	-0.003099829	0.258053	-0.01201	0.990416	1
dedd1	898.1648	-0.254331102	0.194427	-1.30811	0.190837	0.670774
gdf5	50.71716	0.487008165	0.324453	1.501013	0.133352	0.586883
miox	540.7889	-0.590319445	0.19267	-3.06388	0.002185	0.073902
pvalb2	232553	0.163097803	0.13216	1.234097	0.217167	0.69815
si:ch211-48m9.1	68.93091	-2.994101113	1.09748	-2.72816	0.006369	0.134763
pdx1	48.30226	-0.595157355	0.31205	-1.90725	0.056488	0.410418
tle3a	888.1563	0.09889533	0.145213	0.681034	0.49585	0.87806
ap2m1a	4413.946	0.151112095	0.117468	1.286406	0.198302	0.679109
atp1a1a.1	4682.075	-0.029027024	0.154106	-0.18836	0.850596	0.978251
arcn1a	919.5911	0.043213734	0.106561	0.405529	0.685089	0.938486
meis3	402.469	-0.262373573	0.12831	-2.04485	0.04087	0.353604
BX908804.1	0.342035	1.055411721	3.412846	0.309247	0.757134	NA
cog8	141.6059	-0.094253869	0.159921	-0.58938	0.555608	0.898723
rasgrf2b	11.15162	0.832793207	0.720532	1.155803	0.247762	0.728489
cep57l1	203.3209	0.556839379	0.154468	3.604893	0.000312	0.020043
trmt2a	125.3226	-0.3042596	0.184106	-1.65263	0.098406	0.521076
col4a4	86.17774	0.014127511	0.286618	0.04929	0.960688	0.999243
si:dkey-28b4.8	754.198	0.136028546	0.141408	0.961958	0.336071	0.792304
fndc1	81.22225	0.232920202	0.305449	0.762551	0.445731	0.855403
zgc:113210	190.3821	-0.078592748	0.150672	-0.52162	0.601938	0.914001

reps1	577.5444	0.079800077	0.140785	0.566823	0.570834	0.902501
vps50	206.2058	0.232671451	0.158674	1.466349	0.142553	0.601741
aven	200.4474	-0.294160528	0.162041	-1.81534	0.069471	0.45
si:ch211-160o17.2	229.5246	0.334041488	0.239	1.397664	0.162214	0.629748
tjp3	528.5198	-0.072275453	0.167666	-0.43107	0.666418	0.932664
adipor1a	843.2996	0.146226334	0.118047	1.23871	0.215453	0.697157
sec31b	223.5352	-0.251740923	0.199726	-1.26043	0.207514	0.68962
gls2b	166.1797	-0.831005599	0.611931	-1.35801	0.174462	0.647905
gbx2	201.9394	-0.286271948	0.193052	-1.48287	0.138108	0.594663
meis1a	799.8332	-0.249160477	0.10184	-2.44659	0.014422	0.210484
bgnb	158.7836	-0.108207522	0.225648	-0.47954	0.631554	0.923003
abcd3b	139.0558	0.114616961	0.206602	0.554772	0.579051	0.905417
ppp6c	1358.4	-0.135013521	0.11871	-1.13734	0.255395	0.733542
smo	605.2573	-0.06758563	0.175839	-0.38436	0.700712	0.942918
si:dkey-32n7.4	53.72641	0.089763343	0.269499	0.333074	0.739078	0.95338
gpr34b	0.564074	-0.868132643	3.005784	-0.28882	0.772719	NA
pdap1b	550.2837	0.229847658	0.162237	1.416744	0.156558	0.622452
a1cf	374.6921	-0.196172329	0.206899	-0.94816	0.34305	0.797639
ss18	1014.039	0.042544244	0.073389	0.579711	0.562109	0.900715
phc1	235.879	0.11009594	0.166607	0.660813	0.508732	0.883333
gda	110.444	2.52850643	0.782313	3.232092	0.001229	0.050845
tnnt2d	2964.208	-0.059372064	0.134808	-0.44042	0.659633	0.930581
cep135	153.9401	-0.127505257	0.287072	-0.44416	0.656929	0.929939
itpkca	391.1138	-0.113663878	0.142342	-0.79853	0.424564	0.844535
prkcea	474.0037	-0.039877938	0.138678	-0.28756	0.773685	0.960624
ube2a1	1015.852	0.033853843	0.152203	0.222426	0.823982	0.972032
lrrc4.2	90.07559	0.103638252	0.351367	0.294958	0.768026	0.960296
hdac8	217.7882	0.006850885	0.179273	0.038215	0.969516	1
limk1b	159.9413	-0.1315325	0.157723	-0.83395	0.404312	0.833888
scnm1	84.56774	-0.172057503	0.19474	-0.88353	0.376952	0.822324
eif4a1b	12087.52	-0.277957493	0.115494	-2.40669	0.016098	0.223897
hspa9	3206.891	0.191707685	0.156536	1.22469	0.220692	0.701913
CABZ01111496.1	51.82245	-0.277007831	0.356422	-0.77719	0.437047	0.851125
sorbs2a	87.00131	0.35947115	0.213979	1.679933	0.09297	0.510456
tacr3l	20.43313	1.44518485	0.64319	2.246903	0.024646	0.278415
fgfr1op	360.7454	0.057808055	0.123469	0.468198	0.639643	0.924779
cd276	557.2463	-0.032159644	0.141197	-0.22777	0.819829	0.971785
gorasp1a	546.5174	-0.101036375	0.135767	-0.74419	0.45676	0.860698
btbd7	79.78951	-0.044710844	0.302954	-0.14758	0.882672	0.986908
gpx9	143.9312	0.308458652	0.221065	1.395329	0.162917	0.630534
mybphb	23806.54	-0.003383559	0.213755	-0.01583	0.987371	1
spire2	43.2843	1.008310811	0.385183	2.617744	0.008851	0.16123
stambpl1	119.6287	0.281330839	0.23935	1.175396	0.239836	0.720392
ubxn6	492.8107	-0.384339722	0.137662	-2.7919	0.00524	0.121252
or103-4	0	NA	NA	NA	NA	NA
oclnb	416.1781	-0.07918421	0.138159	-0.57314	0.56655	0.901739
kdm5bb	2744.425	-0.037181135	0.088141	-0.42184	0.673143	0.93414
rcan1a	862.3737	0.131093712	0.093427	1.403169	0.160567	0.627313
ada	1905.057	-0.044820909	0.210458	-0.21297	0.831352	0.974908
zgc:123105	1015.884	0.042410171	0.126284	0.335833	0.736997	0.952276
apip	706.7722	0.472693104	0.471393	1.002759	0.315977	0.778545
dachc	207.495	-0.031738924	0.17289	-0.18358	0.854344	0.979086
pxmp2	153.9444	0.236824169	0.31701	0.747055	0.455031	0.860008
si:dkey-27m7.4	28.42293	0.406882069	0.512607	0.793751	0.42734	0.845774

skp1	3799.985	0.006664725	0.125167	0.053247	0.957535	0.999109
nudt21	654.8711	-0.009881495	0.134111	-0.07368	0.941264	0.997371
nfkbil1	108.5723	-0.287022278	0.188947	-1.51907	0.128746	0.578173
nr2f6b	850.7244	-0.252439374	0.103963	-2.42816	0.015175	0.216246
mlst8	223.6668	-0.100203872	0.166138	-0.60313	0.546419	0.896107
magi1b	1180.527	0.13340918	0.102661	1.299514	0.193768	0.674122
stx5al	605.6733	-0.142177779	0.144055	-0.98697	0.323659	0.783161
sult1st4	207.2295	-0.644589241	0.157153	-4.10166	4.10E-05	0.004825
psmd1	2557.582	0.322475144	0.166759	1.933782	0.05314	0.400175
rassf7b	182.9394	-0.041825115	0.152222	-0.27476	0.783497	0.96245
wdr21	61.81059	-1.423773894	0.280492	-5.07598	3.85E-07	0.000137
foxm1	146.7308	0.22155381	0.175023	1.265856	0.205565	0.68784
rhcga	582.0108	0.126833284	0.714243	0.177577	0.859055	0.979975
ngly1	373.5281	0.040560299	0.156838	0.258613	0.795934	0.966247
chchd6a	278.0568	-0.101416785	0.26168	-0.38756	0.698342	0.942433
mterf3	156.5762	-0.185922092	0.232767	-0.79875	0.424436	0.844412
pomk	196.6102	-0.26301953	0.150262	-1.7504	0.080049	0.478179
ano2a	66.41621	0.156168972	0.390474	0.399948	0.689195	0.939277
naa15a	1081.089	-0.128232107	0.128154	-1.00061	0.317015	0.778879
anxa2a	1785.999	-0.051271398	0.137834	-0.37198	0.709907	0.945821
urb2	211.4805	-0.052599516	0.246781	-0.21314	0.831216	0.974908
bin2a	309.4234	-0.755367985	0.17986	-4.19976	2.67E-05	0.003515
gdf9	0.821926	0	2.495039	0	1	NA
nup88	391.071	-0.053818795	0.113797	-0.47294	0.636259	0.924779
gbp3	81.42048	-1.394534012	0.330718	-4.21669	2.48E-05	0.00332
pik3cd	63.48738	-0.545034329	0.314237	-1.73447	0.082835	0.483014
zbtb22b	101.3275	-0.001066383	0.227612	-0.00469	0.996262	1
zgc:101559	157.7189	-0.15677973	0.140673	-1.1145	0.265066	0.742063
loxa	109.0763	-0.464059011	0.256423	-1.80974	0.070336	0.452395
clcn3	732.6587	-0.007198808	0.092407	-0.0779	0.937905	0.997305
dhps	271.6869	-0.250220703	0.175134	-1.42874	0.153078	0.617699
pik3ip1	942.7671	-0.514050229	0.165106	-3.11346	0.001849	0.066115
wdr12	345.181	-0.114451064	0.169433	-0.67549	0.499362	0.879603
dab1b	22.07148	0.763752377	0.410533	1.860393	0.06283	0.428925
sox9a	564.0924	0.151722434	0.186385	0.814026	0.41563	0.839167
stc1	2.304225	-0.799506556	1.506076	-0.53085	0.59552	0.911376
stx16	409.2113	-0.117282975	0.105603	-1.1106	0.26674	0.743188
emc2	1354.202	0.080228899	0.11758	0.682334	0.495028	0.87806
pank2	428.6177	-0.512292811	0.125012	-4.09796	4.17E-05	0.004878
eps8l1b	128.1389	-0.037155078	0.186365	-0.19937	0.841975	0.97643
fam91a1	414.6095	0.136705211	0.134131	1.019192	0.308112	0.773116
cacng5a	54.79495	0.686759592	0.539787	1.272278	0.203274	0.684076
slf2	158.2472	-0.28136392	0.160072	-1.75773	0.078793	0.474808
trpc2b	10.81898	1.078522975	0.578931	1.862955	0.062469	0.427718
commd2	367.8343	-0.094097033	0.167807	-0.56075	0.57497	0.904516
gng12a	359.9294	-0.216217245	0.110091	-1.96399	0.049531	0.387714
si:dkey-275b16.2	0	NA	NA	NA	NA	NA
si:ch211-266g18.6	191.2988	0.035873671	0.175745	0.204124	0.838257	0.976395
cwc25	529.6015	-0.23566289	0.136117	-1.73132	0.083394	0.484513
asb8	454.3382	0.024645344	0.114653	0.214956	0.829802	0.974816
col4a3	74.73765	0.190888965	0.349042	0.546893	0.584452	0.90647
rbpja	337.9084	-0.027600291	0.117536	-0.23482	0.814345	0.970503
fgf8a	69.29872	-0.284683129	0.245048	-1.16174	0.24534	0.726909
tenm1	318.1697	-0.659404676	0.253888	-2.59723	0.009398	0.166683

foxa2	138.1685	-0.114067734	0.255468	-0.4465	0.655233	0.929606
capslb	24.28086	0.683175671	0.399664	1.709373	0.087382	0.496224
chrna5	96.72511	-0.249526018	0.260688	-0.95718	0.338476	0.795226
igf2bp2a	137.8999	0.053562587	0.241133	0.222129	0.824214	0.972107
hnrnpdl	1846.608	0.081582775	0.092147	0.885356	0.375965	0.822258
dhrs7	416.1756	0.027915282	0.1714	0.162866	0.870624	0.983365
ippk	155.9548	-0.253188889	0.196544	-1.2882	0.197675	0.678337
pde10a	47.56877	-0.572107078	0.345562	-1.65559	0.097806	0.521071
fech	86.93404	0.380334483	0.261415	1.454908	0.145695	0.604973
neurod4	720.2728	0.192784721	0.148425	1.298871	0.193988	0.674122
sult1st5	45.16548	-0.35321986	0.331269	-1.06626	0.286304	0.757624
ppp1caa	2605.082	-0.057928391	0.125097	-0.46307	0.643316	0.92593
nup93	802.8228	0.258478747	0.115447	2.238941	0.02516	0.281483
madd	854.392	0.017706898	0.122201	0.1449	0.88479	0.987266
anks1b	541.9882	0.311536983	0.134679	2.313178	0.020713	0.255657
impa1	333.4016	-0.046963117	0.184296	-0.25482	0.798859	0.96696
aprt	138.981	-0.902774464	0.224374	-4.02353	5.73E-05	0.006122
tbc1d14	192.0444	0.205912869	0.174017	1.183295	0.236692	0.71801
itln3	53.75981	-1.760370295	1.330086	-1.3235	0.185669	0.663476
psma5	1712.257	-0.089133597	0.19257	-0.46286	0.643462	0.925997
mak16	424.2813	-0.277669512	0.179999	-1.54262	0.122923	0.569468
tcea1	945.3188	-0.011507278	0.095023	-0.1211	0.903612	0.991366
col8a1b	84.47871	-0.301744274	0.32986	-0.91477	0.360315	0.811057
adarb1b	63.41458	0.318253469	0.279666	1.137975	0.255131	0.733542
gnrhr2	1.268943	1.055435795	2.034206	0.518844	0.60387	NA
dohh	443.4762	-0.048001717	0.150561	-0.31882	0.749864	0.955074
hsp90b1	3197.093	0.301525746	0.132639	2.273281	0.023009	0.268036
dctn5	386.9836	0.138102898	0.13236	1.043387	0.296769	0.764495
klhl32	213.456	-0.216956061	0.195469	-1.10993	0.267031	0.743418
pla2g7	444.9304	0.187811632	0.144453	1.300154	0.193548	0.673755
surf1	278.62	0.023228983	0.1815	0.127983	0.898162	0.990171
rpl3	63504.03	-0.424434713	0.11478	-3.69783	0.000217	0.015696
nr2f6a	543.3084	-0.109483304	0.11052	-0.99062	0.321873	0.782075
slc26a3.2	68.83113	0.67509869	0.948011	0.712121	0.47639	0.869292
arih1	434.7855	-0.149504028	0.110803	-1.34928	0.177246	0.650801
clockb	315.6914	0.131656749	0.191227	0.688484	0.491148	0.875595
zgc:172270	54.52571	0.726920689	0.267734	2.715086	0.006626	0.136957
mogat3b	144.6167	-0.276717743	0.19932	-1.38831	0.165043	0.634185
tfg	1278.305	0.071284941	0.125126	0.569704	0.568879	0.902234
zfyve19	328.4882	0.082535173	0.142197	0.580429	0.561625	0.900673
tmem59l	1511.467	0.27518793	0.155659	1.767885	0.07708	0.469501
cryl1	268.3928	0.184202657	0.167716	1.098302	0.272073	0.748056
runx1t1	792.6405	0.021875427	0.10949	0.199794	0.841642	0.97643
eif5	3694.174	-0.009773282	0.115311	-0.08476	0.932455	0.996175
obsl1a	133.8328	-0.003587864	0.189785	-0.0189	0.984917	1
hars	1529.819	0.088175344	0.124221	0.709829	0.47781	0.869292
vdac3	9767.536	-0.087057939	0.128794	-0.67595	0.499073	0.879596
slc5a8l	53.99399	0.034408156	0.340144	0.101158	0.919425	0.993892
atp7a	367.7477	-0.056977496	0.166727	-0.34174	0.732546	0.950617
cldng	16.98953	-0.080510602	0.470097	-0.17126	0.864016	0.981867
prnprs3	1223.417	0.170527224	0.108209	1.575904	0.115048	0.554651
ryr2b	29.03678	-0.368303429	0.572081	-0.6438	0.519708	0.887148
mitfa	71.50847	-0.553881325	0.250798	-2.20848	0.027211	0.293137
polb	470.5122	0.024710633	0.16462	0.150107	0.88068	0.986624

pfas	807.5559	-0.07439485	0.119948	-0.62022	0.53511	0.891993
lats1	296.5451	-0.211394727	0.179254	-1.1793	0.238278	0.719242
tspan2a	634.4503	0.627787958	0.165118	3.80205	0.000144	0.01196
cnpy1	299.8556	-0.016227763	0.145238	-0.11173	0.911036	0.992208
tada2b	115.7132	-0.285048827	0.207957	-1.37071	0.170466	0.64208
pip4k2aa	454.2579	-0.069394549	0.123586	-0.56151	0.57445	0.904516
ctnnd2b	508.1698	0.383382925	0.140821	2.722494	0.006479	0.136134
dennd5b	931.0058	-0.035831075	0.150914	-0.23743	0.812325	0.970134
idh2	10877.66	-0.023618123	0.093958	-0.25137	0.80153	0.967776
asb2a.1	308.7665	-0.092926164	0.232866	-0.39905	0.689853	0.939679
LHX3	47.46458	0.191639132	0.29139	0.657671	0.510749	0.884139
aqp3a	3879.032	-0.041860554	0.114301	-0.36623	0.714192	0.945821
plxnb2a	674.526	0.030502847	0.128126	0.23807	0.811827	0.97012
prkag1	495.7735	-0.117130974	0.109747	-1.06729	0.285843	0.757258
nr1d2a	1137.498	-0.457313649	0.437092	-1.04626	0.295439	0.763988
ankrd16	80.28849	0.030572872	0.243368	0.125624	0.900029	0.99045
snapc1b	145.199	-0.677475648	0.16205	-4.18065	2.91E-05	0.003705
galnt2	546.9139	-0.24679627	0.155395	-1.58819	0.112243	0.549012
stom	1648.425	-0.116595741	0.112813	-1.03353	0.301356	0.767552
usb1	144.717	0.561480632	0.239198	2.347349	0.018908	0.242305
chm	399.3301	0.070116812	0.13347	0.525337	0.599349	0.913192
mrpl21	278.8046	-0.07522539	0.184711	-0.40726	0.683817	0.938486
acsl1b	752.5296	-0.030940098	0.094009	-0.32912	0.742066	0.954329
cbx3a	1532.583	0.053451066	0.112674	0.474386	0.635225	0.924272
pard6b	32.01021	-0.36945765	0.281691	-1.31157	0.189664	0.669289
tm9sf2	1601.534	0.054919266	0.074752	0.734683	0.462533	0.863917
cdk20	23.25722	-0.212237176	0.389249	-0.54525	0.585584	0.906655
decr1	368.248	-0.054374058	0.244924	-0.222	0.824311	0.972171
ccdc102a	516.0425	0.228026276	0.099125	2.300382	0.021427	0.25974
pbrm1	237.6497	-0.083284234	0.168018	-0.49569	0.620116	0.917678
zdhhc23b	50.14706	-0.157209796	0.275587	-0.57045	0.568369	0.902146
gpr61l	6.729386	1.221744546	0.835509	1.462276	0.143666	0.602773
ctsl.1	691.9105	0.015610523	0.263058	0.059343	0.952679	0.998395
hapln2	15.82259	0.929276211	0.579944	1.602354	0.109077	0.541879
sycp1	53.55793	-0.080274653	0.261127	-0.30742	0.758527	0.957865
ftr01	20.22813	2.297483995	1.398873	1.642382	0.100511	0.525223
med13b	598.0814	0.036104755	0.177583	0.203312	0.838891	0.976417
setb	3852.408	0.028685629	0.082305	0.348529	0.727443	0.949141
cx28.6	21.00278	-0.427842772	0.41519	-1.03047	0.302787	0.768916
cldn12	464.0354	0.040795159	0.111489	0.365912	0.714431	0.945821
cndp2	951.8227	-0.05279344	0.13948	-0.3785	0.705058	0.944016
nxnl2	114.5374	-0.063662736	0.230991	-0.27561	0.78285	0.962328
rpa1	1159.962	0.171219659	0.125865	1.360344	0.173721	0.646897
mtfr1l	582.6359	0.223463018	0.128554	1.738282	0.082161	0.482431
rrs1	323.1664	-0.118163928	0.160998	-0.73395	0.46298	0.864191
nmur1b	1.574059	0	2.315837	0	1	NA
pfn2	7963.925	-0.044698898	0.116182	-0.38473	0.700437	0.942841
parp3	182.3054	0.325086533	0.164211	1.979692	0.047738	0.380493
traf4a	468.7306	0.03028877	0.100722	0.300715	0.763632	0.959055
tlx1	29.57079	-0.119882389	0.416176	-0.28806	0.773303	0.960624
larp6b	3.156791	-1.425161365	1.953448	-0.72956	0.465658	0.864997
trim35-13	62.60499	0.216400882	0.246523	0.877811	0.380046	0.823607
isl2a	321.9822	0.059581905	0.133829	0.445209	0.656169	0.929684
chordc1a	90.4627	0.219860928	0.186779	1.177121	0.239147	0.719781

spop1b	446.1165	-0.138955723	0.145906	-0.95237	0.340911	0.795603
zgc:153896	52.80378	0.014033183	0.370733	0.037852	0.969805	1
LTN1	315.4447	-0.004632654	0.171676	-0.02698	0.978472	1
crhr1	33.65474	0.325137152	0.367843	0.883902	0.376749	0.822258
fhl2b	259.1468	-1.102398125	0.243909	-4.51971	6.19E-06	0.00127
syt9a	235.7282	0.145779287	0.168735	0.863954	0.387613	0.827592
phyhip1a	50.92516	0.7850077	0.305476	2.569786	0.010176	0.175289
adcyap1a	86.1541	-0.186773555	0.33624	-0.55548	0.578568	0.905417
spag1a	295.9941	0.176387882	0.136597	1.291301	0.196599	0.676952
CHST13	73.64908	0.187674839	0.230154	0.815433	0.414824	0.838698
isl1	781.4178	0.070325191	0.152685	0.46059	0.645093	0.926728
arhgdig	1690.636	0.072756318	0.129366	0.562406	0.57384	0.904296
cog2	328.576	0.049363884	0.1294	0.381483	0.702845	0.943622
phf6	304.455	-0.05187325	0.118388	-0.43816	0.661268	0.930581
marcksa	401.2216	0.155726082	0.163131	0.954606	0.339777	0.795226
CR354540.1	21.58185	-0.58215748	0.379486	-1.53407	0.125013	0.572594
uhrf1bp1l	277.7404	0.152379616	0.162031	0.940433	0.346996	0.79983
galk2	57.77718	-1.248697366	0.528005	-2.36493	0.018033	0.237135
bhlhe40	1976.054	-0.050733088	0.268929	-0.18865	0.850368	0.978251
bach2b	219.7976	0.010903717	0.180506	0.060406	0.951832	0.99801
acsl4a	705.1702	-0.198632291	0.195583	-1.01559	0.309824	0.774621
cx39.9	282.3959	-0.140430686	0.241701	-0.58101	0.561234	0.90039
acsbg2	1054.082	0.256325036	0.116493	2.200347	0.027782	0.29637
tie1	413.8308	-0.063590493	0.161967	-0.39261	0.694605	0.941093
esr1	10.62673	0.891475177	0.66488	1.340805	0.179984	0.655675
tti1	78.73449	-0.114206792	0.233924	-0.48822	0.625393	0.920625
mgat4b	511.6075	0.074251687	0.147167	0.504541	0.613881	0.915998
mvk	60.1912	-0.271555979	0.280082	-0.96956	0.332267	0.789602
rttn	69.09113	0.289872432	0.264368	1.096472	0.272872	0.748807
usp19	801.0554	-0.041681669	0.133689	-0.31178	0.755206	0.956367
DHRS11 (1 of many)	289.8405	-0.415128314	0.325495	-1.27537	0.202177	0.682294
grm2a	137.1699	0.958345307	0.252588	3.794103	0.000148	0.012175
rab23	70.64192	-0.297769307	0.228297	-1.30431	0.192128	0.672283
orai1b	50.21748	-0.380401302	0.322948	-1.1779	0.238835	0.719781
tmem63c	84.23586	0.635148564	0.249428	2.546423	0.010883	0.181938
reep3b	618.007	-0.193175233	0.111564	-1.73152	0.083359	0.484434
ik	1086.705	-0.121921775	0.0944	-1.29154	0.196516	0.676952
zgc:110366	114.0501	-0.397539634	0.230026	-1.72824	0.083945	0.486647
stmn1a	1682.542	-0.173217699	0.211528	-0.81889	0.412851	0.838123
rabac1	771.5432	-0.004154469	0.111195	-0.03736	0.970196	1
copa	2772.909	0.027841541	0.064385	0.432423	0.665434	0.932595
cnot1	2384.098	0.001037103	0.132815	0.007809	0.99377	1
ulk4	46.12373	-0.007713007	0.303271	-0.02543	0.97971	1
fam169ab	226.9766	-0.058199017	0.173847	-0.33477	0.737797	0.95259
nf1b	123.3257	0.076560641	0.197406	0.387834	0.698139	0.942433
zgc:122979	73.57118	-0.40100426	0.447359	-0.89638	0.370049	0.818446
cbx1a	1206.976	0.098120045	0.129125	0.759883	0.447325	0.856073
tnfsf10l	478.7702	0.061077317	0.128725	0.474481	0.635157	0.924272
spam1	144.3329	-0.036571636	0.580979	-0.06295	0.949808	0.99801
efhc2	27.22923	0.068366877	0.365512	0.187044	0.851626	0.978548
ccdc106a	653.3053	-0.034874146	0.144528	-0.2413	0.809325	0.969828
rnd1b	255.1291	-0.157367368	0.205375	-0.76624	0.443531	0.854255
slc6a3	63.97224	1.01877879	0.504844	2.018008	0.04359	0.365542
pde3a	109.0658	0.010116369	0.22146	0.04568	0.963565	0.999842

dlb	489.3287	0.074972189	0.280174	0.267591	0.789014	0.963602
asic4b	9.442133	1.067896081	0.738128	1.446763	0.147963	0.608936
slit2	648.1384	0.342419397	0.144696	2.366473	0.017958	0.2367
dhfr	234.4508	-0.146194486	0.170524	-0.85733	0.391265	0.827902
wnt11	44.02984	-0.574291621	0.267413	-2.14758	0.031747	0.316921
cul5b	28.96509	0.144980291	0.448365	0.323353	0.746428	0.955074
tmed9	1766.856	0.100944642	0.109516	0.921736	0.356666	0.807342
cyp46a1.2	32.60075	-0.212913732	0.362313	-0.58765	0.556766	0.899063
atl3	745.4753	-0.005528993	0.115303	-0.04795	0.961755	0.999451
zgc:112496	73.96407	0.294211952	0.266069	1.105772	0.268825	0.744925
seh1l	647.2762	-0.014897837	0.137469	-0.10837	0.9137	0.992796
zgc:77375	258.8546	-0.673536936	0.215106	-3.13119	0.001741	0.064499
hecw1b	72.20321	0.263863832	0.260863	1.011503	0.311776	0.776164
ambp	2317.271	-0.398351162	0.212901	-1.87107	0.061336	0.424867
irf2bpl	1304.829	0.057557896	0.122123	0.47131	0.637419	0.924779
rhogb	235.6658	0.031824405	0.150871	0.210938	0.832936	0.975379
slc45a1	105.6725	0.22245683	0.348655	0.638043	0.523446	0.888516
vangl1	115.5937	0.074588268	0.201945	0.369349	0.711868	0.945821
klhl18	119.9598	0.002896779	0.20871	0.013879	0.988926	1
lypd6	141.534	-0.139292135	0.205049	-0.67931	0.49694	0.878572
fam8a1b	204.7419	-0.068489755	0.159759	-0.42871	0.668136	0.933086
ldlrap1a	302.2208	-0.087010604	0.174606	-0.49833	0.618254	0.916585
cbwd	220.8179	-0.002964137	0.171841	-0.01725	0.986238	1
ush1ga	49.04125	0.252519266	0.35569	0.709943	0.47774	0.869292
casp9	270.2221	-0.11629677	0.173317	-0.67101	0.502217	0.881277
LIN28A	16.89683	-0.348459098	0.390964	-0.89128	0.372777	0.819953
exoc4	276.7812	0.12887221	0.157102	0.820308	0.41204	0.838123
nelfe	438.8991	0.046361	0.160552	0.28876	0.772765	0.960624
gnb3a	603.8697	-0.23658963	0.538715	-0.43917	0.660536	0.930581
lhfpl6	392.8461	0.193837718	0.124309	1.559326	0.118919	0.562318
fmnl3	359.8835	-0.106678853	0.161197	-0.66179	0.508105	0.883056
ppp1r13ba	629.1558	-0.000169109	0.143541	-0.00118	0.99906	1
yrk	349.0601	0.0433796	0.128569	0.337402	0.735814	0.951543
si:dkey-153k10.9	498.4	-0.15524091	0.181165	-0.8569	0.391498	0.827902
hdr	177.433	0.262784295	0.210536	1.248169	0.211969	0.693592
b3gnt5b	54.33686	-0.081396874	0.303798	-0.26793	0.788753	0.963568
elovl6	408.1048	0.104900697	0.18725	0.560216	0.575332	0.904739
snx10a	76.00641	-0.61741351	0.40525	-1.52354	0.127624	0.576985
spra	219.7409	0.269061163	0.1592	1.690086	0.091012	0.50549
tcf7l2	1462.15	0.108416714	0.12435	0.871869	0.38328	0.825809
dpy30	528.2937	-0.329465758	0.170267	-1.93499	0.052991	0.399808
btk	54.44593	0.096720611	0.239898	0.403174	0.68682	0.93875
fam92a1	133.5042	-0.174718408	0.177753	-0.98293	0.325643	0.784564
grm5a	72.61776	-0.072494368	0.462392	-0.15678	0.875417	0.984383
aplnr2	33.05708	-0.053707071	0.461313	-0.11642	0.907318	0.991745
tnfrsfa	293.8863	-0.043446696	0.143564	-0.30263	0.762172	0.95835
tardbpl	2848.323	0.099554842	0.088236	1.128281	0.259201	0.736127
p2rx5	119.3471	0.326160078	0.184602	1.76683	0.077257	0.46958
unc119.2	22.73711	0.184246147	0.525119	0.350866	0.725689	0.948653
desi2	252.5677	0.085479278	0.168746	0.506555	0.612467	0.915727
prkcsh	1031.29	0.002382793	0.091249	0.026113	0.979167	1
atat1	253.2767	-0.001508101	0.185168	-0.00814	0.993502	1
tbx21	42.95063	0.462368516	0.460083	1.004968	0.314912	0.777649
pcyt1ab	195.41	-0.034765405	0.170359	-0.20407	0.838298	0.976395

tspan33a	307.2963	0.01259734	0.207236	0.060787	0.951529	0.99801
xkr8.1	14.29258	0.092535558	0.450951	0.205201	0.837415	0.9761
rcor3	70.54437	0.080835161	0.257918	0.313415	0.753966	0.955912
ppat	445.1331	-0.717251771	0.165759	-4.32708	1.51E-05	0.002448
zgc:63882	157.5429	-0.000140187	0.192596	-0.00073	0.999419	1
pin4	304.0139	-0.209457282	0.206834	-1.01268	0.311211	0.775936
zgc:173742	15.79968	-0.122040964	0.544636	-0.22408	0.822697	0.971966
kmt2a	613.77	0.034482824	0.204311	0.168777	0.865972	0.98245
ptgs2a	576.3543	0.151998316	0.145511	1.044582	0.296216	0.764246
prkcg	7.161905	0.660095838	1.123435	0.587569	0.556822	0.899063
gtf3c2	81.06669	-0.102109055	0.313869	-0.32532	0.744936	0.954956
plk4	195.4002	0.357692976	0.192603	1.857156	0.063289	0.429493
zgc:194665	147.0434	0.001535745	0.212885	0.007214	0.994244	1
nmnat2	426.8391	0.179757872	0.169272	1.061946	0.28826	0.759235
sell1	590.9603	0.085831194	0.102783	0.835075	0.403676	0.833888
ntrk1	14.88456	0.663692662	0.734216	0.903947	0.366023	0.815714
kat5a	508.7323	-0.235062214	0.149155	-1.57596	0.115035	0.554651
sox4a	3446.009	-0.036109746	0.108866	-0.33169	0.740123	0.953709
gpr22a	130.2592	0.035643015	0.239123	0.149058	0.881508	0.986908
znf800a	184.917	0.006137036	0.13969	0.043933	0.964958	1
lrrc4ba	31.75913	0.202898811	0.38351	0.529057	0.596766	0.911757
or102-2	1.3164	1.936587051	2.030803	0.953606	0.340283	NA
mtmr2	319.7028	-0.037894851	0.128432	-0.29506	0.767949	0.960296
stx2a	90.7598	0.155193626	0.256824	0.604281	0.545657	0.895515
gpm6ab	3112.558	-0.075578109	0.124714	-0.60601	0.544507	0.895206
panx3	35.11145	-0.478928858	0.469605	-1.01986	0.307797	0.772996
egln1b	340.1481	-0.148058945	0.189558	-0.78108	0.434758	0.850002
epn3b	11.51722	0.533434883	0.644277	0.827959	0.407694	0.835802
osbp	247.3266	-0.172003207	0.167015	-1.02987	0.303072	0.769218
epha7	273.3547	0.457262949	0.163608	2.794863	0.005192	0.120907
ccdc9	390.8884	0.261412198	0.161697	1.616682	0.105947	0.534956
cdhr1a	130.2686	0.335276644	0.509579	0.657948	0.510571	0.884085
spice1	96.36751	0.035279447	0.353721	0.099738	0.920552	0.994085
kctd16a	5.2297	0.06906946	0.833639	0.082853	0.933968	0.996638
zgc:101810	1240.176	-0.084677062	0.110254	-0.76802	0.442477	0.853789
ptk2ab	497.3969	-0.025230543	0.136689	-0.18458	0.853556	0.978887
dnajb6a	946.0172	-0.031076457	0.105563	-0.29439	0.768462	0.960395
acaa1	314.0824	0.068015096	0.259409	0.262192	0.793173	0.965312
nabp1a	259.391	0.100962308	0.151928	0.664541	0.506344	0.883007
six4a	404.0857	0.066473186	0.200038	0.332303	0.739661	0.953588
eefsec	165.8408	-0.14361135	0.151885	-0.94553	0.34439	0.798147
rxrgb	216.5516	-0.200624015	0.182842	-1.09725	0.272531	0.748416
irf2bp2a	861.6776	0.059554941	0.130261	0.457199	0.647528	0.927091
syf2	914.1608	-0.129576656	0.129888	-0.9976	0.318473	0.779923
tbr1b	225.108	0.37790935	0.240304	1.572629	0.115805	0.555893
mad2l1	376.5833	0.125315362	0.27351	0.458175	0.646826	0.926728
tcf12	1484.974	-0.096960705	0.144241	-0.67221	0.501447	0.880897
mpp5b	23.08154	0.374353133	0.569226	0.657653	0.510761	0.884139
tcea3	339.1796	-0.225276856	0.201556	-1.11769	0.263701	0.741708
trappc11	666.1324	-0.052957542	0.097747	-0.54178	0.587969	0.907732
hnrnpub	2833.219	0.118706692	0.091611	1.29577	0.195055	0.67483
lmbr1l	242.6139	-0.12052363	0.150298	-0.8019	0.422612	0.843493
zgc:100868	899.4162	-0.057619183	0.155841	-0.36973	0.711583	0.945821
mylkb	54.40983	-0.423040349	0.299492	-1.41253	0.157795	0.623766

hspa4a	769.6385	0.293347742	0.225578	1.300427	0.193455	0.673662
ybx1	40864.27	-0.109096748	0.088433	-1.23366	0.21733	0.69815
hhla2a.2	7.558471	-1.85806024	0.905949	-2.05095	0.040271	0.350654
ankrd10b	305.3185	0.044309	0.13777	0.321615	0.747744	0.955074
wdr36	364.5249	-0.376714293	0.157809	-2.38715	0.01698	0.230743
fgfr3	1115.162	-0.086605947	0.124529	-0.69547	0.48676	0.873245
cops2	1089.021	-0.126000657	0.119758	-1.05213	0.29274	0.762197
lrp1ba	28.7948	-0.36593169	0.512502	-0.71401	0.475221	0.869292
ccdc146	16.41372	1.209932424	0.426592	2.836277	0.004564	0.111673
mgrn1b	574.4046	-0.023456907	0.144782	-0.16202	0.871294	0.983365
grwd1	274.3999	-0.117737787	0.138003	-0.85315	0.393574	0.828374
asz1	4.435088	0.09817038	0.877362	0.111893	0.910909	0.992208
flot2a	3407.397	-0.064769349	0.10786	-0.6005	0.548175	0.896644
dnajc5ab	1125.704	0.034091509	0.116595	0.292392	0.769987	0.960484
rassf1	463.5301	-0.23253359	0.142417	-1.63277	0.102518	0.529149
foxp1a	819.0953	-0.140246673	0.130961	-1.07091	0.284212	0.75523
hinfp	95.02741	-0.017119512	0.212611	-0.08052	0.935823	0.996829
esrrga	190.8754	0.103090357	0.192627	0.535181	0.592524	0.909996
zgc:171734	0.139092	1.055370259	5.267649	0.200349	0.841207	NA
mindy1	525.5155	-0.15584555	0.138907	-1.12194	0.261889	0.739623
penka	91.02751	0.182309146	0.437202	0.416991	0.676685	0.935656
psen1	632.0794	0.218616689	0.116552	1.875702	0.060696	0.423211
mst1rb	5.69334	0.329749578	0.822541	0.400891	0.6885	0.939118
spata61	26.53083	-0.277581439	0.384235	-0.72243	0.470032	0.867349
tmc6b	645.4742	-0.293152471	0.11781	-2.48835	0.012834	0.19771
rock2b	414.3467	0.129646596	0.16653	0.778516	0.436265	0.850864
acsm3	126.5168	-0.337132573	0.243223	-1.3861	0.165715	0.635372
aga	166.9158	0.023361149	0.23898	0.097754	0.922128	0.994346
pex19	743.623	-0.081306133	0.10532	-0.77199	0.44012	0.852079
zp2l2	0.732041	-3.146754107	2.971264	-1.05906	0.289571	NA
gys2	600.8784	-0.132440075	0.238385	-0.55557	0.578503	0.905417
stip1	1220.66	0.073102603	0.108697	0.672538	0.501241	0.880764
immp2l	106.8273	-0.118955594	0.232705	-0.51119	0.609221	0.915204
lmo7a	1092.393	-0.278517336	0.166134	-1.67646	0.093649	0.511703
slc8a3	38.84587	0.548795082	0.543248	1.01021	0.312395	0.776164
anos1b	288.3274	-0.273240159	0.195539	-1.39737	0.162303	0.629802
skp2	177.3616	0.091451898	0.175138	0.52217	0.601552	0.913965
mtdhb	384.5509	0.4353009	0.160215	2.716974	0.006588	0.136957
bccip	1567.627	-0.024262543	0.146848	-0.16522	0.868769	0.982801
rsad2	23.19401	-0.620762264	0.938142	-0.66169	0.508168	0.883089
mxb	4.570826	0.239116332	1.15182	0.207599	0.835542	0.976007
grna	70.23142	-0.392540176	0.359467	-1.09201	0.27483	0.750181
zmat2	722.3791	-0.108787249	0.119078	-0.91358	0.360936	0.811253
cyp4t8	164.8997	0.237793479	0.394211	0.603214	0.546367	0.896107
elovl5	134.8215	0.246326945	0.232909	1.057611	0.290233	0.76078
ppp3ca	742.3557	0.13710407	0.230141	0.595739	0.55135	0.8974
brf1b	121.4809	-0.125646637	0.162662	-0.77244	0.439854	0.852079
slc5a12	69.22141	1.349148893	0.586081	2.301983	0.021336	0.258949
si:dkeyp-2e4.8	50.23758	0.001375273	0.270928	0.005076	0.99595	1
tspy	694.8223	-0.065670389	0.108816	-0.6035	0.546176	0.89597
fkbp9	556.141	-0.236435842	0.28124	-0.84069	0.40052	0.831725
tshz1	538.7325	0.003531151	0.114415	0.030863	0.975379	1
crppa	289.0116	0.098641482	0.154979	0.636482	0.524462	0.888808
rab20	171.9867	-0.149425015	0.155819	-0.95896	0.337578	0.794562

wnt2ba	24.91737	0.665279442	0.416863	1.595919	0.110507	0.545461
dimt1l	212.3089	0.127389124	0.293486	0.434056	0.664248	0.93177
ncapd2	705.7747	0.253469206	0.242529	1.045111	0.295972	0.764246
eif4e3	871.5869	0.058340513	0.142897	0.408268	0.683077	0.938167
ggctb	741.4346	0.047271935	0.264838	0.178494	0.858335	0.979774
cep89	55.99451	0.479229474	0.264739	1.810196	0.070265	0.452111
glcea	152.8116	0.298911208	0.205549	1.454209	0.145888	0.605234
zgc:86764	279.7885	0.335643987	0.205275	1.635094	0.102029	0.528864
limk2	622.6652	-0.479285151	0.120615	-3.97369	7.08E-05	0.007021
oclna	818.1572	-0.236674433	0.12376	-1.91237	0.055828	0.408826
cdh17	829.9105	-0.178975648	0.186983	-0.95717	0.338479	0.795226
calub	759.1045	0.328914339	0.155728	2.112112	0.034677	0.328212
atp2a2b	1106.633	0.19626589	0.124317	1.578752	0.114393	0.553523
large1	57.44411	0.232376659	0.326013	0.712784	0.475979	0.869292
rnaseh2b	239.5628	0.007408958	0.131993	0.056132	0.955237	0.998611
psmd11b	1200.923	0.226875332	0.166089	1.36599	0.171942	0.644123
hhatlb	824.2248	0.031011661	0.133223	0.23278	0.815932	0.971207
camkvb	150.1296	-0.796876071	0.39738	-2.00532	0.044928	0.371348
tbx20	42.86981	0.023256724	0.246667	0.094284	0.924884	0.994815
aspa	55.08511	0.223513841	0.339366	0.658621	0.510139	0.883733
cyth1a	203.1093	-0.149092496	0.186922	-0.79762	0.425092	0.844714
tpm3	13092.62	0.129125897	0.081805	1.578467	0.114458	0.553653
efr3a	102.2911	0.086138277	0.214943	0.40075	0.688604	0.939118
dynll2b	287.1695	-0.256884446	0.187119	-1.37284	0.169802	0.641498
zgc:101040	43.80475	-0.304043513	0.322683	-0.94224	0.346071	0.799369
dglucy	360.4585	-1.440864679	0.246023	-5.85662	4.72E-09	3.13E-06
cpn1	11.99542	-0.342399417	0.586268	-0.58403	0.559199	0.899517
sp4	462.5229	0.108589192	0.131815	0.8238	0.410053	0.836939
ahcy	19111.88	-0.366539412	0.144609	-2.53469	0.011255	0.183909
coro2a	283.9401	-0.112394197	0.139228	-0.80727	0.419511	0.842383
zgc:158328	73.99391	-0.04119912	0.28126	-0.14648	0.883542	0.987014
wrnip1	66.89706	-0.17154923	0.241418	-0.71059	0.477338	0.869292
pdcd6	495.3017	-0.039860738	0.141183	-0.28233	0.777687	0.961073
desmb	705.316	-0.852992442	0.380404	-2.24233	0.02494	0.279833
ssr2	2651.251	0.02508183	0.133717	0.187573	0.851211	0.978548
ccdc85ca	108.3577	0.146990238	0.215795	0.681158	0.495771	0.87806
srcap	824.4647	0.15350227	0.169251	0.90695	0.364433	0.814648
asb3	87.92926	0.278331886	0.23641	1.177328	0.239065	0.719781
rpe	791.0685	0.028632271	0.124465	0.230043	0.818058	0.971518
kdelr2a	1554.63	-0.081052238	0.119349	-0.67912	0.497063	0.878572
zc3h12a	22.80348	0.211076567	0.387302	0.544993	0.585759	0.906655
rad52	59.00618	0.397609741	0.214073	1.857355	0.063261	0.429493
fbxl15	57.81314	0.027469707	0.262372	0.104698	0.916616	0.993451
CALHM1 (1 of many)	3.520198	0	1.901218	0	1	1
celf1	1050.753	0.036919192	0.104283	0.35403	0.723317	0.947898
stam2	607.4058	-0.054483506	0.10351	-0.52636	0.598638	0.912889
nipsnap1	89.84315	0.093791673	0.235527	0.39822	0.690468	0.939906
ftr66	2.300343	-1.815818051	1.675863	-1.08351	0.278581	0.752152
lipib	17.9443	0.095989312	0.970026	0.098955	0.921174	0.994085
slc22a4	12.93992	0.678006286	1.391395	0.487285	0.626056	0.920625
ccdc85al	144.3562	0.564192554	0.233989	2.41119	0.015901	0.222371
dip2ba	722.0653	-0.038529169	0.123409	-0.31221	0.754882	0.956283
larp6a	675.7026	-0.04258609	0.133725	-0.31846	0.750135	0.955074
tyro3	49.54453	0.086979555	0.295015	0.294831	0.768123	0.960296

sucla2	3977.906	-0.080523309	0.093474	-0.86145	0.38899	0.827725
grin3ba	28.54455	1.388398472	0.367628	3.776642	0.000159	0.012833
mcamb	571.864	0.05687452	0.121228	0.469153	0.638961	0.924779
gprc6a	27.10585	-0.469903097	0.504129	-0.93211	0.351281	0.802305
camk4	203.1931	-0.119169675	0.253801	-0.46954	0.638684	0.924779
tubgcp4	293.0508	0.142314355	0.139395	1.020943	0.307281	0.772541
prdm9	203.6486	-0.29153496	0.147886	-1.97135	0.048684	0.384066
slc5a5	9.663694	-1.880319753	1.288722	-1.45906	0.144549	0.603539
dnmt3aa	77.13677	0.096680348	0.242662	0.398416	0.690324	0.93988
trim3b	705.498	0.078657637	0.125447	0.627018	0.530648	0.890545
grapa	63.85896	0.186032301	0.28885	0.644044	0.519547	0.887148
map3k5	488.3307	0.131810919	0.196491	0.670825	0.502332	0.881277
pgam1a	1437.157	-0.600219641	0.200181	-2.99838	0.002714	0.085055
wdfy1	128.4402	-0.146076198	0.162049	-0.90143	0.36736	0.816473
mtfmt	113.8717	0.139876009	0.173567	0.805891	0.420306	0.842383
gdi2	4066.982	0.000196647	0.089585	0.002195	0.998249	1
foxp2	256.0952	0.357134566	0.173732	2.055659	0.039815	0.348912
tacc3	627.3827	0.199613349	0.228967	0.871801	0.383317	0.825809
katnb1	547.0209	-0.278713518	0.127119	-2.19254	0.028341	0.299296
csnk1g2a	671.9297	0.076810646	0.081547	0.941914	0.346237	0.799463
kif4	469.8626	0.410108025	0.231633	1.77051	0.076642	0.468636
slc30a1a	325.1319	-0.213174943	0.127028	-1.67817	0.093313	0.511128
dnase1l1	182.1027	-0.49910455	0.178097	-2.80243	0.005072	0.119186
irf2bp1	600.4145	-0.153420072	0.152824	-1.0039	0.315428	0.777834
nav3	230.3675	-0.048537094	0.168642	-0.28781	0.773492	0.960624
tenm3	1172.628	0.040239668	0.134608	0.29894	0.764986	0.959622
nfkbiaa	384.8695	-0.303058157	0.328696	-0.922	0.356527	0.807342
rapgef2	1063.445	-0.068813126	0.133236	-0.51648	0.605522	0.915016
ucmab	402.9476	0.444234103	0.207449	2.141417	0.03224	0.318747
dgcr6	200.1671	-0.352659744	0.177894	-1.98242	0.047433	0.379101
gdf6b	16.34529	0.017887899	0.384373	0.046538	0.962882	0.99973
naca	16291.54	-0.347082367	0.094067	-3.68973	0.000224	0.015984
slc5a9	41.29073	1.118086916	0.311327	3.591353	0.000329	0.0206
galr1a	6.70121	-0.251854916	0.745055	-0.33804	0.735337	0.951543
acy3.2	281.1548	0.051254319	0.212061	0.241697	0.809015	0.969828
igfn1.1	632.5109	0.040337901	0.308032	0.130954	0.895812	0.98926
ubr7	603.1055	0.008335579	0.125899	0.066208	0.947212	0.997371
xpo7	807.0617	0.115123966	0.116431	0.988771	0.322775	0.782618
wif1	529.4537	-0.133003832	0.175973	-0.75582	0.449757	0.856979
btbd3a	58.68406	-0.15539014	0.279343	-0.55627	0.578026	0.90529
rint1	201.0905	-0.06689738	0.154706	-0.43242	0.665439	0.932595
znf710b	641.6661	0.063606774	0.096514	0.659039	0.509871	0.883733
hnrnph1l	1629.746	0.141720816	0.148593	0.95375	0.34021	0.795345
pou4f1	206.4241	0.153500105	0.148276	1.035235	0.300559	0.766629
ywhah	2804.612	0.209268772	0.116651	1.793972	0.072818	0.458474
gfm2	178.5381	-0.011290625	0.171527	-0.06582	0.947518	0.997371
entpd8	75.41467	-0.137194119	0.393772	-0.34841	0.727533	0.949205
vsx2	216.5089	0.32220931	0.152466	2.113315	0.034574	0.328029
mphosph8	1133.033	-0.057392546	0.089748	-0.63949	0.522507	0.887826
ugp2a	394.7212	-0.498204936	0.334883	-1.4877	0.13683	0.592353
zbtb20	7.366371	0.407208327	0.649009	0.627431	0.530377	0.890545
mtf2	279.1176	0.220412847	0.126485	1.742603	0.081403	0.480419
rxrga	129.8189	-0.348009373	0.169807	-2.04945	0.040419	0.351287
adgb	39.17644	0.646229659	0.515679	1.253162	0.210147	0.692011

paxip1	345.4773	0.211956943	0.100404	2.11104	0.034769	0.328533
aph1b	588.6136	0.00522687	0.1435	0.036424	0.970944	1
bfb	91.91779	-0.528684173	0.347264	-1.52243	0.127902	0.577117
nek2	86.43755	0.124146221	0.299578	0.414404	0.678578	0.936026
maco1a	224.3441	-0.085721342	0.168758	-0.50796	0.611485	0.915331
RHOU (1 of many)	37.58724	-0.161271246	0.450857	-0.3577	0.720568	0.947263
zmp:0000000527	5.712151	0.779422262	0.924866	0.842741	0.399373	0.831196
smyd2b	512.3435	-0.155488009	0.399725	-0.38899	0.697286	0.942027
adat3	62.3259	0.18562334	0.252325	0.735651	0.461943	0.863402
gcat	671.7521	0.053074749	0.177561	0.298911	0.765008	0.959622
robo3	795.1172	-0.07983336	0.09323	-0.85631	0.391828	0.827902
olfml2a	132.9423	-0.151481948	0.2007	-0.75477	0.450388	0.857151
hrasb	533.3975	-0.07115369	0.119585	-0.59501	0.551839	0.8974
man1a1	525.0251	0.031615029	0.115978	0.272594	0.785165	0.962605
ttbk2a	41.91398	-0.11798437	0.328772	-0.35886	0.719697	0.94694
f3b	128.3704	-0.197742666	0.264667	-0.74714	0.454981	0.860002
sec61a1l	426.6191	-0.139642478	0.123733	-1.12857	0.259077	0.736127
trip10a	401.1077	0.070625403	0.128376	0.550144	0.582221	0.90571
slc25a23a	311.6066	-0.136879537	0.159819	-0.85647	0.39174	0.827902
ddx19	354.5737	0.116885132	0.110199	1.060677	0.288837	0.760057
eps8l3b	144.502	-0.486165679	0.323801	-1.50143	0.133243	0.586706
ethe1	409.1154	-0.223685109	0.203423	-1.09961	0.271504	0.74783
olfml2ba	83.31228	0.119289538	0.271672	0.439094	0.660593	0.930581
rpf1	434.8084	-0.161185399	0.188735	-0.85403	0.393088	0.828061
gpm6ba	549.8915	0.222948688	0.240017	0.928888	0.352947	0.803792
cand2	546.6581	-0.872861327	0.404215	-2.1594	0.030819	0.312711
ptprfb	1624.27	-0.040850867	0.131872	-0.30978	0.756731	0.95667
col14a1a	251.999	-0.202214841	0.147403	-1.37185	0.170111	0.641806
tsr2	399.4448	-0.08812631	0.206295	-0.42719	0.669243	0.933115
ddx3xb	3397.995	0.046325957	0.109255	0.424018	0.671553	0.933953
scn8aa	292.6837	0.57022122	0.228242	2.498319	0.012478	0.194346
guk1b	273.5026	-0.958808822	0.33626	-2.85139	0.004353	0.108751
npy8br	10.9527	1.297868162	0.529576	2.450769	0.014255	0.209045
ncanb	50.72313	0.377238331	0.310835	1.213628	0.22489	0.70685
ppm1f	25.4167	0.810557339	0.345338	2.34714	0.018918	0.242307
enpp1	576.7241	0.100835806	0.099621	1.012198	0.311444	0.77613
rpl28	21750.58	-0.345143789	0.119433	-2.88986	0.003854	0.103374
smim14	224.2868	-0.635667661	0.232436	-2.73481	0.006242	0.133784
ampd3a	136.9324	0.082464852	0.279895	0.294627	0.768279	0.960296
klhl10a	0.715078	-1.782789235	2.212689	-0.80571	0.420409	NA
ncf2	35.83132	0.276979927	0.506953	0.546362	0.584817	0.906655
slc39a10	503.7511	-0.061246598	0.11259	-0.54398	0.586456	0.906711
eif2s1a	659.0523	0.038478736	0.108367	0.355077	0.722532	0.947632
faima	58.53188	0.058916263	0.32666	0.18036	0.85687	0.979558
gatad2b	120.874	0.073666514	0.22341	0.329736	0.741599	0.954276
got2b	2461.267	-0.06676327	0.128658	-0.51892	0.603817	0.914603
tnni2a.2	306.844	0.510932864	0.267798	1.907901	0.056404	0.410324
cd9a	491.8381	-0.489717245	0.149414	-3.27759	0.001047	0.045343
SP5	5.444699	0.372807427	0.748702	0.497938	0.618528	0.916674
slc25a6	82.55693	0.849483325	0.286651	2.963481	0.003042	0.09094
ralgapa2	590.7983	-0.010547919	0.106638	-0.09891	0.921207	0.994085
or131-1	0.793022	0.093655071	2.301846	0.040687	0.967545	NA
gon4l	428.7905	0.034326204	0.172315	0.199206	0.842101	0.97643
mmadhc	689.2852	-0.054132414	0.151001	-0.35849	0.719977	0.947004

rfx1a	174.861	0.511594948	0.181522	2.818362	0.004827	0.115344
cyb5r3	588.4913	0.05333078	0.175906	0.303178	0.761755	0.958242
slc7a9	34.55646	-0.135651702	0.346635	-0.39134	0.695547	0.9414
dera	195.1343	0.24391322	0.218282	1.117425	0.263813	0.741813
clk4b	455.9252	-0.067859065	0.143237	-0.47375	0.635675	0.924524
tgm1l3	29.65252	-1.126146329	1.103646	-1.02039	0.307545	0.772879
tcf3a	119.6374	-0.072776739	0.286914	-0.25365	0.799764	0.967206
srpk3	322.6096	0.121829364	0.144725	0.841798	0.399901	0.831277
serpina10a	1360.264	-0.247059986	0.107813	-2.29157	0.021931	0.262532
ak2	3104.849	-0.547827283	0.137157	-3.99417	6.49E-05	0.006631
tbc1d17	576.7796	-0.134278439	0.121859	-1.10191	0.270499	0.746683
clul1	934.4621	-0.02143995	0.085295	-0.25136	0.801533	0.967776
v2rl1	13.65328	1.368785076	0.683082	2.003838	0.045087	0.372035
htra4	47.15841	-0.204357699	0.325597	-0.62764	0.53024	0.890545
sart1	1103.426	0.164797728	0.081145	2.030894	0.042266	0.359771
ankmy2a	157.8644	-0.220108972	0.193995	-1.13461	0.256539	0.73401
ric8b	395.6318	-0.285851202	0.141676	-2.01764	0.043629	0.3656
sfswap	866.184	-0.044290509	0.123483	-0.35868	0.719837	0.94694
rgl1	719.3283	-0.253738661	0.146949	-1.72671	0.08422	0.487221
prc1b	221.2514	0.411913002	0.270581	1.522327	0.127927	0.577117
mycla	290.6024	0.091930959	0.190955	0.481428	0.630212	0.922869
dct	1499.049	-0.343103777	0.177287	-1.9353	0.052954	0.399666
bmi1a	273.3435	-0.310324415	0.159598	-1.94441	0.051846	0.395809
klhl42	50.35134	-0.22381048	0.306043	-0.7313	0.464594	0.8647
tktb	2969.806	-0.216771353	0.108753	-1.99324	0.046235	0.375744
tp53i11b	329.2917	-0.064523924	0.157103	-0.41071	0.681284	0.937331
lta4h	664.7027	-0.054145962	0.191247	-0.28312	0.777085	0.961073
abat	2262.322	-0.269768424	0.205386	-1.31347	0.189024	0.668516
aebp2	390.6226	-0.034319877	0.105017	-0.3268	0.743817	0.95491
cyp20a1	347.4484	-0.097859034	0.170416	-0.57424	0.565807	0.901634
usta	121.8937	-0.236825118	0.204682	-1.15704	0.247257	0.727871
malt2	60.6938	-0.253138604	0.270737	-0.935	0.34979	0.801408
crybg1b	289.203	-0.065244676	0.25127	-0.25966	0.795126	0.965909
akap1b	765.5783	-0.152126847	0.129055	-1.17877	0.238489	0.719595
znf385b	80.61455	-0.608690017	0.248443	-2.45002	0.014285	0.209217
uck2a	399.3587	0.053376146	0.121704	0.438572	0.660971	0.930581
gpr37l1a	44.37671	0.119138966	0.35906	0.331808	0.740035	0.953684
rad21a	4757.936	-0.026367455	0.083458	-0.31594	0.75205	0.955423
cdk15	80.37736	-0.350858443	0.20359	-1.72336	0.084824	0.488883
igf2r	184.2938	0.021696841	0.235468	0.092143	0.926584	0.994815
pimr185	2.532612	-0.259936576	1.242663	-0.20918	0.83431	0.975731
chrd	43.45968	0.087030116	0.385627	0.225685	0.821446	0.971788
myof	348.0128	-0.31977065	0.179448	-1.78197	0.074755	0.464131
tbx2b	702.0674	0.099265665	0.125736	0.789477	0.429833	0.847418
kdm5c	2189.429	0.010210633	0.09917	0.102961	0.917994	0.993875
csnk1db	1807.046	-0.097797779	0.115611	-0.84592	0.397597	0.830253
cep170aa	690.4736	0.074565487	0.157807	0.47251	0.636562	0.924779
star	151.363	-0.108739837	0.268016	-0.40572	0.684947	0.938486
pde11al	25.78619	0.012123883	0.372485	0.032549	0.974035	1
lrrk2	337.4989	0.241584727	0.200185	1.206806	0.227507	0.70878
lrrc23	23.14854	0.287580097	0.371637	0.773819	0.439038	0.852079
hif1aa	98.18223	0.302214384	0.266425	1.13433	0.256656	0.734072
gatad2ab	461.4803	0.146817963	0.16374	0.896651	0.369905	0.818439
sav1	529.5004	-0.121289152	0.140274	-0.86466	0.387228	0.827592

eif4g1a	2484.257	-0.043381321	0.150717	-0.28783	0.773475	0.960624
erbb3a	405.3764	0.034300827	0.206362	0.166217	0.867986	0.982801
pou4f3	45.32673	-0.166057648	0.380901	-0.43596	0.662866	0.931106
gpx1b	314.2225	-0.215421405	0.294675	-0.73105	0.464751	0.864823
akr1b1.1	1045.08	-0.539230103	0.21969	-2.4545	0.014108	0.208027
gnl3	714.4212	-0.098078411	0.177719	-0.55187	0.581035	0.905618
ugt1ab	717.4505	-0.13194497	0.255172	-0.51708	0.605099	0.914985
ddx39aa	1841.361	0.057427709	0.107601	0.533711	0.593541	0.910427
brms1la	903.5467	-0.317870538	0.150541	-2.11153	0.034727	0.328285
slc27a1a	535.0889	0.088569677	0.145936	0.606909	0.543911	0.895043
ptp4a1	3304.554	-0.051210234	0.128572	-0.3983	0.690408	0.93988
ell2	641.1851	0.016238738	0.090849	0.178744	0.858139	0.979774
mier3a	341.7334	-0.048874727	0.110884	-0.44078	0.659376	0.930581
vldlr	690.161	0.160180876	0.117236	1.366312	0.171841	0.643953
tuba8l4	8413.713	-0.020071947	0.152309	-0.13178	0.895155	0.989187
stat1a	637.3306	-0.314999797	0.131854	-2.38901	0.016894	0.230481
mpp5a	199.2654	-0.103501258	0.210438	-0.49184	0.622834	0.918894
rragd	54.57007	-0.219984161	0.348712	-0.63085	0.52814	0.889948
waslb	469.9975	0.119907861	0.129755	0.924109	0.35543	0.806546
prdm5	77.07328	0.41557954	0.196686	2.11291	0.034609	0.328035
ndufs5	2076.897	0.084529044	0.168843	0.500636	0.616627	0.916365
maats1	26.85449	0.632059192	0.427138	1.479753	0.138939	0.596373
arhgef7a	507.641	0.232726567	0.13119	1.773971	0.076068	0.467233
psmd9	246.9922	0.343022252	0.158689	2.161606	0.030649	0.312139
RAPH1 (1 of many)	453.4378	0.152279154	0.191674	0.794471	0.426922	0.845491
shisa4	200.5963	0.239625369	0.20463	1.171019	0.241591	0.723306
ptpn18	17.22226	-0.010577563	0.446962	-0.02367	0.981119	1
itgav	1685.043	0.059116651	0.114484	0.516376	0.605592	0.915049
rpl23a	30115.11	-0.265756675	0.109416	-2.42886	0.015146	0.215962
snx14	623.7074	-0.015143569	0.09991	-0.15157	0.879525	0.986187
tas1r3	1.407239	-0.868145009	2.311282	-0.37561	0.707205	NA
med17	270.4827	-0.214804246	0.128468	-1.67205	0.094515	0.513368
nkx1.2la	8.451475	-0.350910201	0.698334	-0.5025	0.615318	0.916365
itga5	227.759	-0.273191756	0.14179	-1.92674	0.054012	0.402269
slc18a3a	290.6148	-0.259455758	0.294134	-0.8821	0.377722	0.822478
crlf1b	55.67763	0.22356673	0.269509	0.829535	0.406802	0.835226
atp6v1h	1686.04	-0.003224722	0.116769	-0.02762	0.977968	1
ugt5c2	4.795863	-2.067944967	1.261874	-1.63879	0.101257	0.526828
stxbp5l	134.2732	0.318442675	0.256706	1.240498	0.214791	0.696864
triobpb	438.5375	-0.072142761	0.134618	-0.53591	0.592022	0.909686
smad2	552.7639	0.18507438	0.120317	1.53822	0.123995	0.57117
exosc9	183.0661	-0.122828634	0.190514	-0.64472	0.519106	0.887148
rbm25b	876.5028	0.042376396	0.115231	0.367752	0.713058	0.945821
nrcama	99.68725	-0.000161674	0.289308	-0.00056	0.999554	1
laptm4a	1576.365	0.079579886	0.091148	0.873082	0.382618	0.825435
ywhae1	3677.577	0.147878898	0.095592	1.546984	0.121867	0.56863
hmgb3b	932.2544	-0.399350396	0.163907	-2.43644	0.014833	0.213592
mapk12b	38.27731	0.198182714	0.335251	0.591148	0.554421	0.898444
rpl38	6117.221	-0.273205268	0.14359	-1.90268	0.057082	0.412539
atrnl1b	713.9731	0.05826673	0.134263	0.433974	0.664308	0.93177
esyt3	164.5324	0.237795387	0.19249	1.235368	0.216694	0.69815
fabp2	3625.954	0.03950149	0.219962	0.179583	0.85748	0.979741
polr1e	252.9329	-0.186397538	0.177719	-1.04883	0.294255	0.763323
slc19a3a	14.87267	-0.678477977	0.693827	-0.97788	0.328135	0.786542

pdgfrl	466.937	-0.077627553	0.225808	-0.34378	0.731015	0.950056
pcdh8	118.028	-0.114665849	0.230913	-0.49658	0.619488	0.917435
grap2a	55.42816	0.160340379	0.281283	0.570033	0.568656	0.902234
saxo2	11.62423	0.597838246	0.681091	0.877766	0.380071	0.823607
ddi2	798.2594	-0.234547867	0.134096	-1.7491	0.080274	0.478505
anp32a	6771.264	0.001759099	0.097583	0.018027	0.985618	1
agpat9l	207.4922	0.17645752	0.194587	0.906832	0.364496	0.814648
asb7	165.4438	0.161376031	0.210504	0.766618	0.443309	0.854063
rtn1a	5790.372	0.000619519	0.114166	0.005426	0.99567	1
cyp2x10.2	5.689354	-3.203790167	1.038793	-3.08415	0.002041	0.070215
pip5k1bb	219.732	0.07286336	0.157326	0.463136	0.643267	0.92593
her6	773.7849	0.12477803	0.153094	0.815043	0.415048	0.838732
otop2	16.18949	-0.493890592	1.061486	-0.46528	0.641729	0.925586
supt6h	1401.411	0.036481403	0.130065	0.280486	0.779105	0.961073
fn1b	3596.062	-0.274078127	0.220578	-1.24254	0.214036	0.696474
brd3a	779.8675	0.005731097	0.106013	0.05406	0.956887	0.999109
c1qtnf6b	61.29189	0.272220543	0.33121	0.821896	0.411136	0.837797
rgp1	246.2166	-0.044699264	0.151048	-0.29593	0.767285	0.960296
ak4	647.7623	-0.107082343	0.182409	-0.58705	0.557173	0.899165
rras	579.8898	-0.062195216	0.166231	-0.37415	0.708294	0.945344
irf4a	7.940095	-1.404720514	0.669636	-2.09774	0.035928	0.332937
nlk1	868.2468	0.031657318	0.115385	0.274363	0.783805	0.96245
dus4l	94.26026	0.394664663	0.238517	1.65466	0.097993	0.521071
kcnmb2	73.62457	0.162109397	0.27715	0.584916	0.558604	0.899409
nadka	40.28187	-0.187752679	0.340287	-0.55175	0.581121	0.905618
cldnd	3.313379	-1.587871954	1.028108	-1.54446	0.122477	0.569468
phf8	628.7697	0.09428713	0.136788	0.689293	0.490639	0.875539
DMWD	50.72786	-0.009868218	0.265217	-0.03721	0.970319	1
zgc:111983	2652.958	0.982914242	0.285995	3.436823	0.000589	0.031278
sgk2b	230.9487	-0.37193452	0.363825	-1.02229	0.306644	0.771918
lsm14aa	566.3405	-0.004088586	0.153653	-0.02661	0.978771	1
kaznb	177.5964	0.065175648	0.170249	0.382826	0.701849	0.943292
chrna2a	18.22292	-0.943030301	0.618098	-1.5257	0.127085	0.576323
csrp1a	576.6249	0.351275849	0.12679	2.770528	0.005597	0.126154
nectin3b	102.9938	0.140850251	0.19009	0.740967	0.458713	0.861537
gpr27	171.6313	0.015381177	0.203683	0.075515	0.939805	0.997371
map2k2a	776.7763	0.126213923	0.097127	1.299468	0.193783	0.674122
hnf1ba	205.7022	-0.426855224	0.183946	-2.32055	0.020311	0.252973
napga	353.1337	0.160060212	0.117252	1.365099	0.172222	0.644849
pspc1	489.717	0.057182403	0.11745	0.486868	0.626352	0.920665
ppp2r2ab	637.1835	0.222757079	0.120037	1.855744	0.06349	0.430136
slitrk2	109.6508	0.590535257	0.261324	2.259781	0.023835	0.273663
lnpk	221.487	-0.108299084	0.150987	-0.71728	0.473204	0.869064
eomesa	271.8708	0.283189662	0.17339	1.633252	0.102416	0.529087
drg2	565.9471	0.084151496	0.12706	0.662296	0.507781	0.883056
bud13	203.9796	0.008666405	0.162862	0.053213	0.957562	0.999109
fam49a	399.4797	-0.211709272	0.148481	-1.42583	0.153917	0.618475
ripk1l	62.57617	-0.185189973	0.223702	-0.82784	0.407761	0.835802
pth2r	11.77155	-0.047619899	0.585405	-0.08135	0.935167	0.996829
arhgef6	360.505	0.036501984	0.116372	0.313666	0.753774	0.955912
rpl12	29588.57	-0.480070167	0.132027	-3.63614	0.000277	0.018426
wipf2b	336.9636	0.127840892	0.132714	0.963278	0.335408	0.792031
specc1la	562.3337	0.218166193	0.127283	1.71402	0.086525	0.493048
tmem178b	236.8231	0.084082687	0.246498	0.341109	0.733022	0.950736

mllt3	186.9279	-0.014602101	0.183091	-0.07975	0.936434	0.997044
klhl41b	3630.517	-0.068492025	0.193098	-0.3547	0.722814	0.947691
fam234b	305.3987	0.254992857	0.158676	1.607002	0.108054	0.53992
slc24a3	62.07144	0.305636553	0.277705	1.10058	0.271079	0.747282
snd1	3350.775	0.07161952	0.136174	0.525941	0.598929	0.913109
elovl4a	127.1354	-0.146825343	0.176867	-0.83015	0.406457	0.834893
cass4	42.64195	-0.104720159	0.367234	-0.28516	0.775522	0.961073
rb1	310.8118	0.190620172	0.129671	1.470024	0.141555	0.600284
copz2	544.3872	0.003541465	0.098784	0.035851	0.971401	1
arntl1a	559.8183	0.062698246	0.218005	0.287601	0.773653	0.960624
nt5dc1	74.90804	0.069615431	0.267218	0.260519	0.794464	0.965431
sult1st6	137.8256	0.021357709	0.281777	0.075796	0.939581	0.997371
wdr26b	1052.443	-0.090399105	0.099964	-0.90432	0.365826	0.815714
gtf2f2b	0.21156	1.055363231	5.117974	0.206207	0.836629	NA
urod	294.9575	-0.194224782	0.169789	-1.14392	0.252657	0.732759
galnt9	275.1292	0.317092711	0.234332	1.353176	0.176	0.649415
mycn	1698.964	1.601186634	0.128649	12.44617	1.47E-35	1.70E-31
eef1a2	23.23422	0.405415927	0.795348	0.509734	0.610238	0.915331
gnmt	1509.859	0.009504173	0.151153	0.062878	0.949864	0.99801
parp9	157.7745	0.124259572	0.200772	0.61891	0.535975	0.892102
asic2	44.13574	0.563269027	0.434057	1.297685	0.194396	0.674677
nkain1	536.4932	-0.079526526	0.122253	-0.65051	0.515365	0.886401
kifap3b	455.0577	-0.071397031	0.149859	-0.47643	0.63377	0.923974
si:ch1073-349o24.2	48.25298	0.738348143	0.371003	1.990138	0.046576	0.376263
glra4a	212.4425	0.175764025	0.185891	0.945522	0.344392	0.798147
trh	86.46793	-0.27082842	0.380768	-0.71127	0.476918	0.869292
stx17	98.07893	0.083290508	0.194262	0.428753	0.668103	0.933086
enpp4	12.62089	-0.24400914	0.551307	-0.4426	0.658054	0.930581
phf21aa	297.6011	0.207455571	0.173738	1.194074	0.232449	0.713923
mpped2a	424.908	0.052868038	0.114861	0.460278	0.645317	0.926728
kcns3a	47.56164	0.350634729	0.406418	0.862744	0.388278	0.827592
myo3b	70.71892	-0.406586506	0.328123	-1.23913	0.215297	0.697157
oprk1	6.521084	-0.252721163	0.970654	-0.26036	0.794585	0.965469
lhx6	157.8013	-0.011398399	0.16219	-0.07028	0.943972	0.997371
far1	155.9128	-0.15266195	0.196623	-0.77642	0.437501	0.851283
impdh2	808.9467	-0.149430339	0.140524	-1.06338	0.28761	0.758397
si:ch1073-459j12.1	868.8361	-0.081113343	0.146949	-0.55198	0.580959	0.905618
wnt8b	32.25386	0.190318592	0.324906	0.585765	0.558033	0.899301
arl8ba	657.0122	-0.301577082	0.140508	-2.14633	0.031847	0.317164
lrp5	853.9836	0.072026432	0.168792	0.426716	0.669586	0.933416
cacna1ab	51.09494	0.791113465	0.334869	2.36246	0.018154	0.237981
fbxo38	305.133	-0.208979034	0.155054	-1.34778	0.177729	0.65136
tbx16l	43.88057	0.687991375	0.25629	2.684426	0.007265	0.144498
gdpd3b	19.9291	1.263637378	0.627886	2.012525	0.044165	0.368091
csell	848.8597	0.277204863	0.138493	2.001587	0.045329	0.372707
zak	34.7873	0.124256659	0.442787	0.280624	0.778999	0.961073
ccdc113	19.49149	-0.290928764	0.404746	-0.71879	0.472269	0.868726
celf3b	287.9959	-0.070726997	0.205133	-0.34479	0.730255	0.950056
creld1b	146.0776	0.063664366	0.205283	0.31013	0.756462	0.956532
hoxa11b	168.0156	-0.140824063	0.154229	-0.91308	0.361199	0.81142
ms4a17a.6	7.718372	0.994177649	0.767577	1.295215	0.195246	0.67483
uox	1278.879	-1.412363208	0.335526	-4.2094	2.56E-05	0.00341
ttc9c	107.216	-0.067806259	0.197131	-0.34397	0.730872	0.950056
hnrpkl	983.5617	-0.291858826	0.159184	-1.83347	0.066732	0.441264

tmigd1	19.64088	0.492736739	0.596013	0.826722	0.408395	0.83628
cnot4a	311.6939	0.022664916	0.14678	0.154414	0.877283	0.985341
mrps18b	331.2951	-0.265826653	0.141932	-1.87292	0.061079	0.424477
si:dkey-19a16.16	0	NA	NA	NA	NA	NA
ankrd50l	338.2831	-0.116123088	0.150498	-0.77159	0.440357	0.852245
rhcgl1	402.7912	1.302231658	0.662101	1.966816	0.049204	0.386201
pias4a	741.8415	0.115210159	0.096893	1.189041	0.234424	0.715154
aqp10a	59.34	-0.261817767	0.670566	-0.39044	0.696209	0.941657
xab2	362.6802	-0.099957988	0.13745	-0.72723	0.467084	0.866065
her13	113.0583	-0.133489002	0.256769	-0.51988	0.603147	0.914226
itm2ba	2940.28	-0.07166326	0.076642	-0.93504	0.349769	0.801408
cx43.4	1308.171	-0.053227233	0.139452	-0.38169	0.702693	0.943622
lipia	180.3414	-0.248189899	0.148595	-1.67025	0.094871	0.514067
asb6	84.38049	0.278888526	0.268026	1.040527	0.298095	0.765416
acat2	301.4098	-0.076821524	0.241834	-0.31766	0.750741	0.955074
slc6a16a	132.8657	0.492094689	0.231932	2.121717	0.033862	0.325143
mrto4	137.943	0.066938556	0.207538	0.322536	0.747047	0.955074
cnot3a	747.5036	0.025899887	0.105052	0.246543	0.805262	0.969348
gtf2h4	98.61611	0.158102995	0.255756	0.618178	0.536458	0.892397
psmc3	2284.834	0.141950709	0.123125	1.152903	0.24895	0.728918
l1cama	186.4934	-0.007150535	0.199881	-0.03577	0.971463	1
kcnk2b	9.494645	0.925068887	0.562032	1.645937	0.099777	0.524166
itgb1bp1	396.45	0.100021644	0.131294	0.761814	0.446171	0.855403
aig1	90.72244	-0.665161917	0.259276	-2.56545	0.010304	0.176586
plxna3	348.9137	0.228875894	0.164824	1.388606	0.164953	0.634185
recql	205.2928	0.074090739	0.18199	0.407113	0.683925	0.938486
sash1a	567.5131	-0.050643266	0.114849	-0.44096	0.659245	0.930581
slc30a4	823.4601	-0.039548338	0.151245	-0.26149	0.793718	0.965431
nadk2	240.8634	0.107318024	0.143511	0.747804	0.454578	0.85968
zbtb16a	436.5955	-0.059572101	0.165496	-0.35996	0.718877	0.94694
ppp1r8a	46.69991	0.08163584	0.25069	0.325645	0.744693	0.95491
grm2b	381.1665	0.293487121	0.151401	1.938481	0.052565	0.398274
rae1	596.2368	0.132157965	0.121437	1.088286	0.276469	0.751059
mta1	305.0729	0.158141539	0.138888	1.138625	0.254859	0.733542
CR846090.1	0.174693	0	5.267649	0	1	NA
cnpy2	194.141	-0.096613594	0.15197	-0.63574	0.524944	0.888928
abce1	2016.211	0.069362236	0.172475	0.402159	0.687567	0.938835
wdr55	134.8935	0.141245589	0.194405	0.726553	0.4675	0.866174
actn1	1118.84	0.02431916	0.106382	0.228603	0.819178	0.971785
ncam1b	226.3789	0.094598737	0.26657	0.354874	0.722684	0.947632
pbk	347.2239	0.231160786	0.278238	0.830802	0.406085	0.834645
ryk	545.3897	-0.155088347	0.127202	-1.21923	0.222758	0.704616
mycb	577.6052	0.043403863	0.293408	0.14793	0.882398	0.986908
acp2	638.2256	-0.382109627	0.177654	-2.15086	0.031487	0.315416
rundc3aa	34.2578	0.547062428	0.355664	1.538145	0.124013	0.57117
ric8a	238.8079	0.439813525	0.17549	2.506196	0.012204	0.191703
use1	258.9921	0.160513876	0.133155	1.205462	0.228025	0.709324
rab5ab	467.7101	0.055665361	0.174113	0.319708	0.74919	0.955074
nipsnap2	823.6097	-0.03239553	0.115907	-0.2795	0.779864	0.961089
mynn	367.2306	0.196306274	0.123742	1.586419	0.112644	0.549814
si:ch211-251b21.1	11860.48	-0.237576555	0.145578	-1.63196	0.102688	0.529448
ela3l	21225.64	0.673370417	0.958923	0.702215	0.482545	0.871721
myf5	114.9143	0.046442989	0.202367	0.229499	0.818481	0.971656
ect2	239.6823	0.376133963	0.211936	1.774749	0.075939	0.46659

yipf5	402.6034	-0.103352497	0.146128	-0.70728	0.479395	0.86985
mrpl57	335.0254	0.129517285	0.15519	0.834571	0.40396	0.833888
metrnlb	37.80571	0.120512241	0.269216	0.447641	0.654412	0.929488
aco2	5625.931	0.02580289	0.12939	0.199419	0.841935	0.97643
sh3gl3b	198.3601	-0.389735902	0.196558	-1.98281	0.047389	0.379101
tmpoa	475.356	0.072407605	0.134089	0.539997	0.589199	0.9083
rpl7	45390.14	-0.330418167	0.138718	-2.38195	0.017221	0.231824
chmp4bb	1221.887	-0.145863283	0.112888	-1.2921	0.196321	0.67688
tbx16	4.64111	-0.31388142	0.796038	-0.3943	0.693356	0.940668
tcap	275.7176	0.02091399	0.489185	0.042753	0.965899	1
degs1	793.5978	0.206232885	0.143929	1.432877	0.151893	0.615406
dmrt1	3.289139	0.509158551	1.383263	0.368085	0.71281	0.945821
fgf20a	50.55994	0.257011353	0.322562	0.796781	0.425578	0.844714
cxcr3.1	6.629385	0.874752795	0.855999	1.021908	0.306824	0.772019
UBL4A	332.7751	-0.044828338	0.155677	-0.28796	0.773379	0.960624
sft2d1	587.5439	0.036171409	0.11007	0.328621	0.742442	0.954362
AMDHD1	60.48252	-0.106395889	0.50123	-0.21227	0.831897	0.974925
tcf7l1b	411.7629	-0.105161077	0.196144	-0.53614	0.59186	0.909603
dysf	482.9072	0.115489759	0.197172	0.585732	0.558055	0.899301
slc26a3.1	2.816426	-2.369116215	1.82846	-1.29569	0.195083	0.67483
odc1	1922.092	0.104271575	0.170683	0.610908	0.541261	0.893744
ubtd1a	116.483	0.730459491	0.285218	2.561055	0.010435	0.178049
kcnk6	271.2268	-0.366028728	0.192016	-1.90624	0.056619	0.410984
cct7	7197.275	-0.111995766	0.14592	-0.76752	0.442775	0.853929
irf2a	357.763	-0.284185996	0.152917	-1.85843	0.063108	0.429313
rnd3b	283.6946	-0.101254605	0.145357	-0.69659	0.486058	0.873244
lrrk1	27.30441	-0.516457058	0.460619	-1.12122	0.262193	0.739695
strap	981.2329	-0.196146236	0.10421	-1.88222	0.059807	0.421883
phox2a	203.9492	-0.273839082	0.16389	-1.67087	0.094748	0.513797
barx1	204.1423	-0.244887837	0.146125	-1.67588	0.093761	0.511703
ppifa	190.2428	-0.176204914	0.238075	-0.74012	0.459226	0.861818
slc2a1b	196.6908	-0.133698135	0.242573	-0.55117	0.581519	0.905618
arid2	694.3363	0.181866234	0.174839	1.040194	0.29825	0.765416
chmp4c	131.4271	-0.112783973	0.196893	-0.57282	0.566768	0.901739
ftcd	1266.778	-0.464761219	0.136962	-3.39337	0.00069	0.034774
apol1	203.7191	-0.426508328	0.177617	-2.40128	0.016338	0.225785
ndor1	176.1865	0.083178602	0.152852	0.544177	0.58632	0.906711
prdm12b	212.535	0.194382228	0.172897	1.124268	0.260899	0.738467
avpr2aa	24.00382	0.701654769	0.423765	1.655763	0.09777	0.521071
ube2ib	1742.69	0.007460472	0.12601	0.059205	0.952789	0.998406
fbxl18	108.8291	0.04299486	0.212275	0.202544	0.839492	0.976417
ift81	79.90874	0.45703553	0.218691	2.089868	0.03663	0.336389
slc25a39	494.5867	0.013479952	0.133186	0.101211	0.919383	0.993892
srgap1a	161.5014	0.107061826	0.20584	0.520122	0.602979	0.914226
tdrd1	31.78503	0.409134755	0.502269	0.814573	0.415317	0.838754
ifit10	4.49618	-0.487895834	1.116318	-0.43706	0.662069	0.930742
fbxo8	295.7106	0.263893089	0.188196	1.402224	0.160848	0.627788
rpe65a	995.5239	0.060050223	0.24782	0.242314	0.808537	0.969828
epc2	534.961	0.009894548	0.150125	0.065909	0.94745	0.997371
adrb1	25.82984	-0.179154104	0.437041	-0.40993	0.681861	0.937498
st3gal8	234.8924	-0.511827442	0.226765	-2.25709	0.024003	0.274503
ufl1	552.0971	-0.130022792	0.137579	-0.94508	0.344618	0.798147
kmt2e	970.654	0.014879376	0.179895	0.082711	0.934081	0.996669
ndufs2	3408.308	0.001540124	0.11148	0.013815	0.988977	1

zcchc9	259.8387	0.355043766	0.178882	1.984797	0.047167	0.378022
itln1	19.12916	-0.772433967	0.701172	-1.10163	0.270621	0.746683
opn4.1	186.8842	-0.252690034	0.267065	-0.94617	0.344061	0.798147
sema3bl	179.7205	0.252944037	0.211677	1.19495	0.232106	0.71325
cdh23	12.67762	0.096736328	0.770424	0.125562	0.900078	0.99045
appbp2	567.4126	-0.21574011	0.133751	-1.613	0.106744	0.536615
crybb1l1	10676.56	0.542680767	0.360863	1.503843	0.132622	0.585592
si:ch1073-155h21.1	2.968294	-1.7611445	1.803886	-0.97631	0.328913	0.787203
bloc1s4	215.1171	-0.486200667	0.154635	-3.14418	0.001666	0.062852
zmynd8	153.8957	0.338993137	0.180817	1.874788	0.060822	0.423578
stxbp2	511.4723	-0.072211627	0.099882	-0.72297	0.4697	0.867305
wwox	98.1924	0.162420745	0.205957	0.788615	0.430337	0.847541
gins1	117.9843	0.063797693	0.165351	0.385832	0.699621	0.942499
mkrn2	270.8931	0.106882923	0.132964	0.803846	0.421486	0.842754
cnot4b	554.6368	-0.196169619	0.125253	-1.56618	0.117305	0.558519
nsfa	1472.33	0.299837064	0.21638	1.385695	0.16584	0.635405
crybb1l3	46.57412	0.520594143	0.370069	1.406748	0.159502	0.6257
ccnh	153.3784	-0.201987088	0.157297	-1.28411	0.199103	0.679605
amph	950.8317	0.313722056	0.194429	1.613552	0.106625	0.536345
ghrb	771.2758	-0.0562621	0.238228	-0.23617	0.813301	0.970328
ttyh3b	1587.606	-0.055645024	0.128672	-0.43246	0.66541	0.932595
ppdpfa	2286.591	-0.044775739	0.232138	-0.19288	0.84705	0.9773
cul1b	748.3674	-0.274911631	0.137492	-1.99948	0.045557	0.373513
nfkbiab	1175.332	-0.231453386	0.293371	-0.78895	0.430144	0.847541
fabp7a	5730.665	0.279987445	0.225326	1.242586	0.214021	0.696474
nsa2	7956.402	-0.375027157	0.15016	-2.49752	0.012507	0.194482
adamts8a	70.83172	0.355859716	0.278434	1.278076	0.201223	0.681045
rab3il1	249.7556	-0.025401929	0.147443	-0.17228	0.863215	0.981725
lgsn	608.0624	0.766325913	0.275345	2.783153	0.005383	0.123514
sub1b	549.2419	0.02292307	0.255521	0.089711	0.928517	0.995052
nudt8	237.7697	-0.055745161	0.153821	-0.3624	0.71705	0.94627
efnb1	141.4452	-0.00784948	0.178708	-0.04392	0.964965	1
rgl2	110.1169	0.01413455	0.204426	0.069143	0.944876	0.997371
stn1	90.25551	-0.029796242	0.260236	-0.1145	0.908844	0.992033
zyg11	580.427	-0.279054486	0.174703	-1.5973	0.110198	0.544875
atp1a1a.2	1788.705	0.052643465	0.219055	0.240321	0.810081	0.969828
il15l	42.19135	-0.459227829	0.279241	-1.64455	0.100062	0.524334
tsr1	772.0928	0.069269275	0.140644	0.492516	0.622354	0.918644
uqcrfs1	4049.31	0.016517323	0.105454	0.156631	0.875536	0.984383
lmnl3	50.94409	0.252658885	0.254333	0.993417	0.320507	0.780821
cpne2	264.7036	0.219836228	0.208324	1.055263	0.291305	0.761454
wdr61	339.4409	0.173900321	0.139412	1.247388	0.212255	0.69394
spg21	387.2214	-0.148277035	0.145256	-1.0208	0.307349	0.772541
klhdc10	320.4634	-0.204307267	0.114666	-1.78176	0.074789	0.464151
sult5a1	22.70525	-0.882784077	0.490067	-1.80135	0.071647	0.4564
vash2	469.1895	-0.32164381	0.18894	-1.70236	0.088688	0.5001
blk	14.54875	-0.366341664	0.515382	-0.71082	0.477198	0.869292
tmx2b	637.2486	0.32023669	0.130055	2.462317	0.013804	0.205712
zgc:112965	2.204354	-0.723780486	1.4348	-0.50445	0.613947	0.915998
atp2b1b	414.5193	0.498880145	0.21721	2.296767	0.021632	0.261049
ppp2r1ba	2771.707	0.002768811	0.092669	0.029879	0.976164	1
cst3	4499.03	-0.489221061	0.168143	-2.90956	0.003619	0.099519
gas6	474.8481	0.020815957	0.15078	0.138055	0.890197	0.98835
cyth3a	198.75	-0.161130098	0.154458	-1.0432	0.296856	0.764495

zdhhc16a	211.3252	-0.053964065	0.198601	-0.27172	0.785836	0.962605
sp3b	637.6727	0.07116742	0.10345	0.68794	0.49149	0.87565
rnpepl1	246.9002	-0.102429056	0.13661	-0.74979	0.453379	0.858656
palm1b	1190.812	0.092853952	0.107528	0.863532	0.387845	0.827592
atf3	305.5615	-0.08870023	0.290607	-0.30522	0.760195	0.957886
camta1b	599.9487	0.302025934	0.219568	1.375548	0.168962	0.640442
map2k1	496.9415	0.157484889	0.129364	1.217379	0.22346	0.70531
ctsla	12772.46	-0.111145163	0.139724	-0.79546	0.426345	0.845031
MCHR2 (1 of many)	4.606556	-2.120760331	1.537749	-1.37913	0.167854	0.639036
rtn4rl1a	10.12286	-0.452538096	0.667888	-0.67757	0.498047	0.878951
tra2a	1439.665	-0.042517799	0.104727	-0.40599	0.684753	0.938486
pimr195	0.217027	0	5.267649	0	1	NA
dazap2	1918.932	-0.154354121	0.088457	-1.74496	0.080992	0.479801
ehd3	86.55049	0.564903884	0.229004	2.46679	0.013633	0.203924
fancl	13.02394	-0.191719371	0.421818	-0.45451	0.649464	0.927756
slc35b2	206.2623	0.072218345	0.14449	0.499816	0.617204	0.916365
meox1	352.6728	-0.164868808	0.171132	-0.9634	0.335346	0.79203
pdcd2	37.97154	0.154568653	0.315552	0.489836	0.62425	0.919537
crnkl1	340.7367	-0.342389719	0.130038	-2.63299	0.008464	0.157114
lrp2b	28.16815	0.908711856	0.918395	0.989456	0.32244	0.78246
si:ch211-196i2.1	324.5857	0.052555871	0.172082	0.305412	0.760052	0.957886
xpnpep3	252.8907	-0.04713302	0.162269	-0.29046	0.771462	0.960597
ttc27	366.1707	-0.135375833	0.159884	-0.84671	0.397154	0.830253
ptpn1	536.6465	-0.128162979	0.110957	-1.15507	0.248061	0.728489
hmx4	335.9358	0.015626921	0.162462	0.096188	0.923371	0.994525
gabpb2a	326.2521	0.306049151	0.176411	1.734862	0.082765	0.483014
lhx1b	74.89986	0.158619837	0.258836	0.612819	0.539996	0.893049
itga11b	47.66189	0.383232118	0.304782	1.257398	0.208609	0.690728
iars	1420.642	0.210624686	0.122405	1.720722	0.085301	0.490552
hibadhb	1203.257	0.011363735	0.128754	0.088259	0.929671	0.995576
hnrnpaba	10488.35	0.216796759	0.087558	2.47605	0.013285	0.200523
cks1b	285.5015	0.021735513	0.282189	0.077025	0.938604	0.997371
fth1b	621.0976	0.37620646	0.181487	2.072917	0.03818	0.342672
si:ch211-220f16.2	220.5402	-0.266368129	0.16656	-1.59923	0.10977	0.543595
onecut1	71.6348	0.132532695	0.215373	0.615364	0.538314	0.892568
masp2	176.7079	0.312710947	0.257862	1.212705	0.225243	0.70685
nedd8	571.4461	0.090535022	0.168757	0.536483	0.591625	0.909437
wt1b	38.88738	-0.591510468	0.262536	-2.25306	0.024255	0.275475
pou2f1b	254.3893	0.15082695	0.157682	0.956527	0.338806	0.795226
zgc:194189	9.901347	-0.121089709	0.621382	-0.19487	0.845494	0.976867
nudt17	258.199	0.237694547	0.175838	1.351778	0.176446	0.649859
mxg	1.323149	-3.118530476	2.082889	-1.49721	0.134338	NA
med31	296.298	0.110843961	0.148256	0.747651	0.454671	0.859767
kif18a	146.022	0.31410722	0.210453	1.492527	0.135561	0.591012
fam129bb	339.3236	0.007831893	0.173421	0.045161	0.963979	0.999842
mep1a.2	30.97056	2.278899022	0.691795	3.294185	0.000987	0.043563
myl9b	1869.316	-0.369157427	0.221452	-1.66698	0.095518	0.516124
sart3	644.4903	0.263434245	0.124657	2.113265	0.034578	0.328029
skib	521.3197	0.064098678	0.114317	0.560711	0.574995	0.904516
si:dkey-42i9.4	1739.26	-0.29140287	0.187329	-1.55556	0.119812	0.564602
scara3	119.4553	0.163737623	0.223563	0.7324	0.463924	0.864593
tma16	212.659	0.112655356	0.161237	0.698694	0.484743	0.872911
il1r4	3.27947	-0.059167078	1.029921	-0.05745	0.954188	0.998611
olfcd1	1.238642	0.234441558	2.186227	0.107236	0.914602	NA

srsf9	861.2088	0.013351494	0.111747	0.119479	0.904896	0.991631
ifit9	1.984495	3.061626684	1.46004	2.096947	0.035998	NA
slc7a10a	285.0658	0.374567987	0.199022	1.882038	0.059831	0.421883
mettl2a	262.0449	-0.164215445	0.154418	-1.06345	0.287578	0.758397
src	130.2346	0.155280283	0.210439	0.737888	0.460583	0.862369
si:ch211-195d17.2	299.9046	-0.131951113	0.125546	-1.05102	0.29325	0.762581
pcdh15b	82.96089	-0.345822769	0.288088	-1.20041	0.229981	0.711763
sox1b	266.9322	0.308158487	0.196095	1.571475	0.116072	0.556131
foxl1	127.8324	-0.043246453	0.218573	-0.19786	0.843156	0.976791
kcnab1a	29.02733	0.590802052	0.365215	1.617684	0.105731	0.534827
rbl1	246.0962	-0.262604802	0.150593	-1.74381	0.081193	0.479923
stxbp3	596.4242	0.014022258	0.131671	0.106495	0.91519	0.993066
serinc5	593.2511	-0.006875098	0.090526	-0.07595	0.939462	0.997371
sms	643.518	-0.015078093	0.14308	-0.10538	0.916073	0.993234
btr33	18.87259	0.147854388	0.527718	0.280177	0.779342	0.961073
caspa	136.8055	0.146633091	0.168362	0.870939	0.383788	0.826299
dbn1a	197.504	-0.017231974	0.196772	-0.08757	0.930216	0.995707
hoxb1a	11.23353	-0.207145755	0.502757	-0.41202	0.680325	0.936798
si:ch73-111e15.1	4.487196	-1.48012092	1.042076	-1.42036	0.155503	0.620161
mia3	729.4284	-0.146435904	0.12372	-1.18361	0.236568	0.717725
syk	114.7041	-0.289612909	0.239873	-1.20736	0.227294	0.708598
sf1	1559.624	-0.00239179	0.123622	-0.01935	0.984564	1
tmeff1a	14.74217	-0.484806482	0.580807	-0.83471	0.40388	0.833888
pank1a	176.4822	-0.088012458	0.157935	-0.55727	0.577343	0.904832
ugp2b	735.7421	-0.067598454	0.131677	-0.51337	0.607696	0.915204
pax1a	60.54773	0.251785845	0.266383	0.945202	0.344556	0.798147
myt1la	368.7052	0.439449965	0.270393	1.625225	0.104115	0.532183
kcnk13a	5.317573	-0.723884328	1.098592	-0.65892	0.509947	0.883733
znf410	1063.935	-0.071289072	0.157646	-0.45221	0.651119	0.928395
crygm4	0.893208	1.771652394	2.100681	0.84337	0.399021	NA
vps35	1216.94	0.068569838	0.078292	0.875823	0.381126	0.824296
cog5	248.8219	-0.009671627	0.139375	-0.06939	0.944677	0.997371
sars	2126.775	0.022561497	0.123145	0.183211	0.854633	0.979161
manba	88.12115	-0.029095821	0.192006	-0.15154	0.879553	0.986187
mocs3	79.0621	-0.356551145	0.240025	-1.48547	0.137418	0.593264
cct8	5173.078	-0.126048992	0.109458	-1.15157	0.249496	0.729721
vezf1a	109.0304	0.116695445	0.223274	0.522656	0.601214	0.913756
ptchd4	13.90798	1.181013746	0.548917	2.151533	0.031434	0.315297
cnot6a	946.4349	0.102800304	0.115012	0.893824	0.371416	0.818945
mfsd4ab	93.67955	-0.882182167	0.762013	-1.1577	0.246987	0.727706
klhl24b	1262.704	-0.26554148	0.1668	-1.59198	0.11139	0.547032
rcor2	517.2414	-0.060423367	0.141405	-0.42731	0.669155	0.933115
map2k7	48.9696	0.110511482	0.280395	0.394128	0.693487	0.940668
fam114a1	336.9074	0.139515425	0.106889	1.305241	0.191811	0.672149
eif2s3	7277.433	-0.47410516	0.11034	-4.29675	1.73E-05	0.002695
hand2	299.1999	-0.338271146	0.143196	-2.36229	0.018162	0.237981
rdh5	241.2217	-0.205941067	0.216248	-0.95234	0.340927	0.795603
ip6k2a	1914.721	-0.065881916	0.155234	-0.4244	0.671271	0.933953
ambra1a	159.3863	0.158385052	0.223533	0.708552	0.478602	0.869292
asic1a	47.31772	0.183676864	0.337207	0.5447	0.58596	0.906655
znfl2a	157.56	0.328641572	0.194759	1.687427	0.091521	0.506944
mcl1b	1490.885	-0.237807806	0.20062	-1.18536	0.235874	0.717225
csnk1da	2462.926	-0.06943387	0.107799	-0.6441	0.519509	0.887148
spred2b	287.5964	-0.144995584	0.133433	-1.08666	0.277188	0.751426

epn2	1200.782	0.144871411	0.116867	1.239628	0.215113	0.697086
brd7	728.4505	-0.028603776	0.123442	-0.23172	0.816757	0.971207
arpc1a	2656.648	0.175040358	0.110838	1.579238	0.114281	0.553312
hdac6	250.6444	0.267041174	0.151356	1.76433	0.077676	0.471265
mmp14b	1310.877	0.10849077	0.130885	0.828904	0.407159	0.835299
snupn	277.2698	0.180157553	0.143184	1.258222	0.208311	0.690398
ftr24	114.1927	-0.218497031	0.191468	-1.14116	0.253801	0.732873
cacna1c	59.9502	0.490292626	0.369149	1.328169	0.184122	0.661004
phospho1	170.3612	-0.368027555	0.582865	-0.63141	0.527772	0.889948
tspan7b	1370.34	0.068445652	0.159832	0.428235	0.66848	0.933086
atp1la	580.396	-0.146677597	0.128876	-1.13813	0.255067	0.733542
exoc3l2a	103.8293	-0.3319257	0.191344	-1.7347	0.082793	0.483014
unc45b	380.8973	0.296163524	0.235464	1.257789	0.208468	0.69072
bcl2l1	468.819	-0.026629345	0.14424	-0.18462	0.853528	0.978887
atxn10	58.87863	-0.145334216	0.257039	-0.56542	0.57179	0.903234
fkbp4	1130.298	0.059642448	0.10965	0.543937	0.586485	0.906711
pcolce2a	51.3206	0.317477616	0.256683	1.236845	0.216145	0.69815
cpeb1b	13.2116	-0.148444558	0.561856	-0.2642	0.791623	0.964817
faah2a	118.7574	0.398148542	0.275686	1.44421	0.14868	0.610476
anapc5	180.4777	0.253823113	0.159235	1.594017	0.110932	0.54583
apex2	105.3621	0.149389552	0.208925	0.715041	0.474584	0.869292
asb14a	0.18149	-0.868115394	5.267649	-0.1648	0.8691	NA
trmt61a	150.7113	-0.360916217	0.208072	-1.73457	0.082816	0.483014
lrp3	74.15044	0.154795179	0.217329	0.712263	0.476302	0.869292
dmd	917.1727	-0.084934235	0.220228	-0.38567	0.699744	0.942499
ltv1	570.5593	-0.189285076	0.146509	-1.29197	0.196369	0.67688
si:ch211-107o10.3	142.5086	-0.603928317	0.328985	-1.83573	0.066398	0.440056
myl6	875.6318	0.438210599	0.177447	2.469525	0.013529	0.202995
pno1	2525.828	-0.599265915	0.131259	-4.56553	4.98E-06	0.001059
glcci1a	99.57664	0.401494522	0.177988	2.255736	0.024087	0.27487
sox21b	188.0708	0.111079154	0.237407	0.467886	0.639866	0.924779
chia.4	10.90296	-0.881841184	1.128898	-0.78115	0.434713	0.850002
yod1	619.4066	-0.098646621	0.113087	-0.8723	0.383043	0.825809
arhgap12a	263.3015	0.063618951	0.132231	0.481118	0.630433	0.92291
pole3	385.3639	0.23515076	0.154311	1.523872	0.127541	0.576964
zgc:136872	82.53541	-0.043159523	0.345409	-0.12495	0.900562	0.99045
brap	214.8341	-0.128521552	0.139192	-0.92334	0.355829	0.806879
slc25a24	476.0426	-0.389717433	0.140276	-2.77823	0.005466	0.12457
fam20b	364.145	-0.017829225	0.147167	-0.12115	0.903573	0.991366
lyst	206.9679	-0.313625327	0.263987	-1.18804	0.23482	0.715533
mtmr8	230.2483	-0.198345673	0.203856	-0.97297	0.330569	0.788126
nbas	143.8293	-0.300095964	0.184607	-1.62559	0.104036	0.532096
cops3	757.8817	-0.112026237	0.125701	-0.89121	0.372815	0.819953
mttp	463.7181	0.318548985	0.194884	1.634556	0.102142	0.528963
kifap3a	476.7622	0.12977191	0.105127	1.234433	0.217041	0.69815
coro1b	755.7463	-0.153500244	0.129995	-1.18082	0.237675	0.718568
fign	47.35651	0.370634864	0.317286	1.16814	0.24275	0.724345
snx3	802.111	0.049473138	0.133117	0.371652	0.710152	0.945821
epas1a	1092.204	-0.316397754	0.134497	-2.35244	0.01865	0.2416
apeh	432.6679	-0.008133161	0.144446	-0.05631	0.955098	0.998611
paip2b	2426.665	-0.088124857	0.111045	-0.7936	0.427429	0.845877
lmo3	880.6789	0.22909791	0.129612	1.767568	0.077133	0.469501
prkcba	305.3236	0.081702554	0.136702	0.597671	0.55006	0.8974
ranbp3a	57.09978	-0.338780697	0.304676	-1.11194	0.266165	0.742912

zgc:66479	749.3797	-0.076994721	0.141121	-0.54559	0.585346	0.906655
atp6ap2	1366.992	-0.062646368	0.105632	-0.59306	0.553139	0.897809
esf1	271.2263	0.127684861	0.210107	0.607713	0.543378	0.894925
ube2na	1763.732	0.071477831	0.112466	0.635548	0.525071	0.888928
entpd6	532.7945	0.093327297	0.087633	1.064979	0.286886	0.757915
tmed5	430.4183	0.154098039	0.123836	1.244373	0.213362	0.695695
tle2b	87.88222	-0.385342992	0.246521	-1.56312	0.118024	0.560168
cacng1a	686.0548	0.349016145	0.157615	2.214363	0.026804	0.290512
prpf4ba	207.7326	-0.138375212	0.187105	-0.73956	0.459567	0.862129
dldh	4356.992	-0.09487143	0.075608	-1.25478	0.209558	0.691743
camk1gb	300.9092	0.490471197	0.272346	1.800912	0.071717	0.4564
actr3b	97.60071	-0.511427802	0.356895	-1.43299	0.15186	0.615406
her5	2.490717	-1.427888344	1.14231	-1.25	0.211299	0.692983
marcksb	5186.77	0.13844286	0.132208	1.047156	0.295028	0.763708
orc3	208.2779	-0.176212097	0.202022	-0.87224	0.383077	0.825809
bokb	12.9993	-1.444632417	0.671798	-2.1504	0.031524	0.315646
eml2	839.1796	-0.083359442	0.128983	-0.64628	0.518098	0.887148
cmtm3	152.9754	-0.149349722	0.177237	-0.84266	0.399421	0.831196
glud1a	1935.984	-0.295243129	0.115121	-2.56463	0.010329	0.176586
hsf1	229.705	-0.2783734	0.159446	-1.74588	0.080831	0.479801
chga	364.4445	0.438690079	0.184202	2.381566	0.017239	0.231824
neu1	260.9348	0.138876062	0.120613	1.151416	0.249561	0.729721
si:dkey-46g23.5	2.856776	-1.565911192	1.052823	-1.48734	0.136924	0.5925
hmbsa	295.5628	0.199948043	0.151145	1.322885	0.185874	0.663692
ptprq	16.8909	-0.920528404	0.651698	-1.41251	0.1578	0.623766
elp4	117.4454	0.11073197	0.197396	0.560965	0.574821	0.904516
cyp7b1	73.2099	0.232976998	0.293434	0.793968	0.427214	0.845741
mylipa	490.5638	-0.272969847	0.174233	-1.56669	0.117187	0.558341
tfap2e	272.5205	-0.191827509	0.14581	-1.3156	0.188308	0.667547
cabp4	12.28234	-1.805993548	1.445263	-1.2496	0.211448	0.692983
rap1b	1349.478	0.121640744	0.123232	0.987087	0.3236	0.783161
usp28	1632.73	0.105409215	0.18278	0.5767	0.564142	0.901398
hprt1	600.2007	0.060225584	0.153017	0.393588	0.693885	0.940854
msx1b	535.9018	0.010886336	0.134961	0.080663	0.93571	0.996829
ctns	119.0205	-0.059401075	0.160849	-0.3693	0.711906	0.945821
smarca2	548.2926	-0.026029425	0.161984	-0.16069	0.872336	0.983447
si:ch211-241e1.5	121.2041	-0.164146313	0.21463	-0.76479	0.444399	0.85448
slc8a2a	28.70837	-0.134954428	0.434498	-0.3106	0.756106	0.956532
mrpl53	175.3393	0.130659586	0.163223	0.800496	0.423424	0.843967
renbp	282.5119	-0.410368268	0.228589	-1.79522	0.072618	0.457864
sec11a	527.3868	-0.070036987	0.186814	-0.3749	0.707733	0.945194
ccdc127b	68.83678	-0.103667917	0.225652	-0.45942	0.645936	0.926728
rtf1	1555.243	0.079812585	0.082227	0.970633	0.331731	0.789531
pla2g3	2.443856	-1.383334241	1.388679	-0.99615	0.319177	0.779923
pofut1	213.6576	0.170252408	0.139414	1.221198	0.222011	0.703829
tbl1xr1b	450.6037	-0.100478015	0.14117	-0.71175	0.476617	0.869292
fgb	3787.575	-0.079185915	0.216875	-0.36512	0.71502	0.945821
mdn1	476.2948	-0.011367231	0.218439	-0.05204	0.958498	0.999109
golga1	348.8745	-0.11017242	0.133307	-0.82645	0.408547	0.836443
casq2	2371.06	0.039159324	0.184076	0.212735	0.831534	0.974908
sh3glb2a	22.07234	-0.157219061	0.454061	-0.34625	0.729154	0.949983
e2f7	117.4898	0.429288869	0.242321	1.771568	0.076466	0.468417
pdia6	1205.527	0.54811129	0.148315	3.695578	0.000219	0.015758
col11a1b	2936.579	0.166274374	0.185801	0.894906	0.370837	0.818484

rhbg	2608.019	0.258528926	0.156791	1.648879	0.099172	0.522589
cnr1	25.25057	0.302605165	0.45146	0.670282	0.502678	0.881453
chrna1	641.9811	0.046918801	0.174753	0.268487	0.788324	0.963471
ankrd28b	617.5089	0.018042226	0.114995	0.156896	0.875327	0.984383
ank2a	288.9563	0.490388179	0.24997	1.961787	0.049787	0.38821
tnikb	525.9605	-0.049567309	0.127259	-0.3895	0.696906	0.94198
si:ch73-141c7.1	306.5875	-0.433489413	0.460847	-0.94064	0.346891	0.799748
nphp1	51.16157	0.768732768	0.269043	2.857284	0.004273	0.10808
cbr4	71.94591	-0.113473381	0.274218	-0.41381	0.679015	0.936141
BX296551.1	19.52515	-0.321367391	0.427564	-0.75162	0.452277	0.857994
cd74a	32.07317	-1.076443961	0.842842	-1.27716	0.201546	0.681423
gata2b	14.12466	0.599169338	0.625271	0.958256	0.337934	0.794808
serinc1	4070.258	0.003433484	0.120441	0.028508	0.977257	1
pigc	122.175	0.150870221	0.167188	0.902401	0.366844	0.816111
sele	111.1151	-0.253942532	0.317143	-0.80072	0.423294	0.843894
slc10a4	45.56489	0.376291217	0.370943	1.014419	0.310383	0.775121
myo1eb	452.338	-0.047592717	0.128072	-0.37161	0.710185	0.945821
hlx1	263.3663	-0.20075473	0.189109	-1.06158	0.288426	0.759522
tp53bp2a	1210.651	-0.030883561	0.125682	-0.24573	0.805893	0.969625
ppp1r13bb	821.7609	-0.0248742	0.144761	-0.17183	0.863572	0.981794
pla2g1b	1271.935	1.947156993	1.576879	1.234817	0.216899	0.69815
kmo	151.8095	-0.56624797	0.270804	-2.09099	0.036529	0.335935
ftr55	2.584579	1.072732025	1.579002	0.679373	0.496901	0.878572
mob1ba	549.6625	-0.109173227	0.120038	-0.90949	0.363092	0.813385
ctr9	896.034	-0.06647207	0.099552	-0.66771	0.504318	0.881824
zfand1	200.6759	-0.074932322	0.179147	-0.41827	0.675747	0.935422
col16a1	1165.08	0.209419257	0.168132	1.245567	0.212923	0.695242
anxa3a	365.311	-0.09128582	0.117714	-0.77549	0.438051	0.851454
prkcda	326.815	-0.037591449	0.131552	-0.28575	0.775068	0.960899
slc9a6a	227.9647	-0.137126163	0.142128	-0.9648	0.334643	0.791583
ppiaa	31149.2	-0.241079963	0.146652	-1.64389	0.100198	0.524654
mfsd13a	418.8222	-0.037836209	0.107765	-0.3511	0.725513	0.948653
zgc:112437	116.6648	0.2652829	0.25335	1.0471	0.295054	0.763708
toml12	131.6503	-0.138334479	0.197005	-0.70219	0.482562	0.871721
mars2	58.76573	0.195318823	0.244358	0.799315	0.424108	0.844355
fev	60.66798	0.03465952	0.278354	0.124516	0.900907	0.99045
etfb	3899.431	-0.101347384	0.134487	-0.75359	0.451097	0.857606
napepld	137.4532	-0.49363293	0.228661	-2.15879	0.030866	0.312739
rtraf	548.0397	-0.059778501	0.139065	-0.42986	0.667298	0.933049
FAR2	52.35927	-0.365408871	0.310468	-1.17696	0.23921	0.719781
uevld	126.6872	0.192896243	0.176625	1.092122	0.274779	0.750181
zgc:136439	0	NA	NA	NA	NA	NA
ppm1da	497.5742	0.015552745	0.100125	0.155333	0.876559	0.985007
smyd1a	6997.757	-0.053606616	0.174349	-0.30747	0.758488	0.957865
dnm1b	211.4163	0.368834001	0.246438	1.49666	0.134482	0.588914
rpl15	29034.91	-0.377323036	0.124963	-3.01947	0.002532	0.08126
dusp8a	356.3234	0.172123741	0.164546	1.046054	0.295536	0.764153
pvalb6	440.9452	0.221178744	0.288844	0.765737	0.443833	0.854255
napsa	192.2656	-0.332303383	0.223524	-1.48666	0.137106	0.592785
clgn	984.3454	0.242032207	0.084469	2.865333	0.004166	0.106651
aif1l	1127.202	-0.006666642	0.15683	-0.04251	0.966093	1
nrap	844.1847	0.041998308	0.164119	0.255902	0.798027	0.966458
txndc5	1571.281	0.123995197	0.125535	0.987733	0.323283	0.783113
caprin1a	645.2212	-0.039002149	0.116409	-0.33504	0.737591	0.952496

atg3	688.4276	-0.3144701	0.143354	-2.19367	0.028259	0.298982
fgf6a	161.1787	0.197812939	0.323013	0.612398	0.540274	0.893049
diras1b	5.777499	-0.201252256	0.686896	-0.29299	0.769531	0.960484
mpp4l	46.82438	-0.893659142	0.652144	-1.37034	0.170581	0.642139
robo4	449.9186	0.06138745	0.161483	0.380148	0.703836	0.943735
npl	377.227	-0.116243565	0.188656	-0.61617	0.537785	0.892498
vcanb	1863.33	0.267559871	0.170248	1.571585	0.116047	0.556131
si:ch211-210b2.2	48.1335	-0.876554137	0.372873	-2.35081	0.018733	0.241927
mef2cb	741.9066	0.0034322	0.16813	0.020414	0.983713	1
tmem30c	73.75946	-0.325319822	0.271311	-1.19907	0.230502	0.712099
ubxn7	401.4995	0.107583553	0.161477	0.666248	0.505253	0.882188
myog	199.5306	-0.105550539	0.273714	-0.38562	0.699776	0.942499
zgc:92137	6245.375	-1.762299637	1.654754	-1.06499	0.28688	0.757915
atp5mc3b	21670.17	-0.103333007	0.154391	-0.66929	0.503308	0.881759
slc35e4	45.17824	-0.286674756	0.419943	-0.68265	0.494827	0.878016
rgs9bp	30.12305	-0.990097714	0.5603	-1.76709	0.077214	0.469565
hnf1a	71.59845	-0.450652317	0.226063	-1.99348	0.046209	0.375668
nid2b	1.77896	-1.781178268	1.912814	-0.93118	0.351759	NA
med19b	156.8605	-0.027182034	0.169088	-0.16076	0.872285	0.983447
ppardb	498.4474	-0.145293636	0.127082	-1.1433	0.252912	0.732759
prkar2ab	66.22129	0.920482807	0.383428	2.400664	0.016365	0.225867
pdcl	1313.874	-0.465629879	0.112552	-4.13703	3.52E-05	0.004291
slc38a8a	22.07652	0.395884024	0.391718	1.010635	0.312191	0.776164
arf1	2145.179	0.135721679	0.08569	1.583873	0.113223	0.551017
gipc2	290.4341	0.066148061	0.136455	0.484759	0.627847	0.921643
MAP3K13	25.63885	0.269253844	0.372356	0.723108	0.469614	0.867224
xrcc1	414.6295	0.012155455	0.128273	0.094762	0.924504	0.994815
syne1a	1145.541	0.162391545	0.161275	1.006925	0.313971	0.777479
prelid3b	1578.796	0.14429491	0.377329	0.382411	0.702156	0.943292
tnfa	2.137033	0	2.037611	0	1	NA
rnf150b	212.4755	-0.582869821	0.140102	-4.16034	3.18E-05	0.00398
tial1	1278.272	-0.06897759	0.091407	-0.75462	0.450477	0.857151
wls	1791.372	0.252010144	0.122335	2.06	0.039399	0.347538
cldnb	1315.765	-0.08289341	0.262649	-0.31561	0.752302	0.955481
ubr4	1865.02	-0.02852197	0.223954	-0.12736	0.898658	0.990199
foxi3b	210.5693	-0.145204268	0.209671	-0.69253	0.488602	0.873964
gng3	1458.476	0.164662404	0.194049	0.84856	0.396126	0.8295
wrap73	169.1463	0.012475441	0.165499	0.075381	0.939912	0.997371
spegb	490.8696	0.176740516	0.220727	0.800718	0.423295	0.843894
ca10b	53.12444	-0.990678991	0.421981	-2.34768	0.018891	0.242305
papola	2043.677	0.157070413	0.079706	1.970633	0.048766	0.384322
nr1d2b	1409.33	-0.145089604	0.695697	-0.20855	NA	NA
ccnc	556.0545	-0.088849527	0.125262	-0.70931	0.478132	0.869292
rgs12a	248.0655	0.220623057	0.151314	1.458052	0.144826	0.603718
chia.3	94.73276	-1.036054128	0.861674	-1.20237	0.229219	0.711317
CABZ01027646.1	31.62904	-0.475103717	0.499571	-0.95102	0.341592	0.796327
nrbp2b	80.041	-0.386083911	0.237269	-1.6272	0.103695	0.531435
unc119.1	388.2513	-0.168558581	0.105225	-1.60189	0.109179	0.542034
rcvrn3	3546.366	0.192450423	0.159764	1.204592	0.228361	0.709551
psmb1	2335.052	0.029441252	0.134897	0.21825	0.827235	0.973459
lhfpl2a	215.2185	-0.334993852	0.208021	-1.61039	0.107314	0.537961
fgfr1op2	484.4306	-0.15855447	0.15	-1.05703	0.290499	0.76078
si:ch211-197g15.6	5.644859	-0.474811365	0.741544	-0.6403	0.521977	0.88764
dlg1	539.6414	-0.057993435	0.123928	-0.46796	0.639813	0.924779

med27	216.3612	0.00112667	0.13047	0.008636	0.99311	1
ppa2	154.678	-0.166520235	0.180745	-0.9213	0.356895	0.807545
daam1b	782.8227	-0.009975594	0.103868	-0.09604	0.923488	0.994525
llgl1	503.7927	0.203062707	0.144676	1.403569	0.160447	0.627013
ascl1b	30.2221	-0.251922015	0.516074	-0.48815	0.625443	0.920625
rpn2	2309.657	0.012174994	0.111543	0.109151	0.913083	0.99273
twf2b	60.45436	-0.115098316	0.302856	-0.38004	0.703913	0.943735
kif21b	65.5755	-0.206005897	0.233941	-0.88059	0.378539	0.822927
gigyf2	822.5146	0.201462688	0.107321	1.877201	0.06049	0.422849
cacnb4a	11.43734	-0.160813164	0.585674	-0.27458	0.78364	0.96245
ppp1r7	645.8312	0.21727861	0.123479	1.759637	0.078469	0.474337
efhc1	48.71316	-0.617093406	0.320146	-1.92754	0.053913	0.402007
fbxw2	437.1237	-0.085923889	0.137326	-0.62569	0.531518	0.890835
dffb	122.7405	-0.177472332	0.201677	-0.87998	0.37887	0.823136
sf3b6	781.5244	-0.178060286	0.160821	-1.1072	0.268209	0.744507
zc3h11a	1044.116	-0.099835201	0.13329	-0.74901	0.453852	0.858859
srek1	155.1527	0.187245345	0.156972	1.192855	0.232926	0.714492
ppme1	1018.65	-0.06754024	0.122444	-0.5516	0.58122	0.905618
mcl1a	3712.295	0.05586347	0.181779	0.307316	0.758603	0.957865
myh11a	429.0585	0.46130653	0.220342	2.09359	0.036297	0.335171
slc49a4	441.7422	0.011912113	0.165695	0.071892	0.942688	0.997371
klc1a	399.7306	0.196704171	0.130392	1.508561	0.131411	0.583069
smfn	139.3371	-0.10319049	0.205141	-0.50302	0.614948	0.915998
her4.4	156.7294	0.347814561	0.29375	1.184049	0.236394	0.717674
pou3f1	931.2643	0.150510676	0.114264	1.317222	0.187764	0.666964
mmp23bb	162.0812	-0.060445313	0.25543	-0.23664	0.812935	0.970328
hdlbpa	17692.18	-0.121012588	0.065189	-1.85634	0.063405	0.429783
agk	357.3323	-0.09583713	0.15006	-0.63866	0.523045	0.88829
fam117bb	113.9037	0.115564885	0.236285	0.489092	0.624777	0.920109
dusp23a	139.7146	-0.279127113	0.299714	-0.93131	0.351692	0.802513
wdr41	402.5474	0.112295067	0.131814	0.851919	0.394259	0.828374
dcps	221.4887	-0.083826811	0.169592	-0.49429	0.621105	0.918461
ddx55	196.5365	-0.078207031	0.16872	-0.46353	0.642983	0.925854
dcaf11	724.5279	-0.27178851	0.105283	-2.5815	0.009837	0.171403
mapk8b	108.9201	0.01975945	0.184402	0.107154	0.914667	0.992972
snrpc	664.5321	-0.040379559	0.139775	-0.28889	0.772665	0.960624
cyp2k6	0.368977	-3.616250748	5.162302	-0.70051	0.483608	NA
ier5	1654.411	0.025392056	0.149502	0.169844	0.865133	0.982142
sult3st1	12.09989	0.155742014	0.485292	0.320924	0.748268	0.955074
rnf114	229.7511	-0.268847099	0.14584	-1.84344	0.065265	0.435359
znf385a	72.54003	0.029502708	0.236802	0.124588	0.90085	0.99045
slc38a5a	300.9411	-0.523533253	0.160229	-3.2674	0.001085	0.046486
dmbx1a	294.4061	0.478011307	0.177342	2.695414	0.00703	0.141889
cadm2a	28.28553	0.261288492	0.37945	0.688598	0.491076	0.875595
micu2	514.4928	-0.036515803	0.149793	-0.24378	0.807405	0.969736
erc1a	274.0728	0.311685923	0.192975	1.615164	0.106275	0.535537
cdc6	213.09	0.273720845	0.196958	1.389745	0.164606	0.634007
fndc4b	265.1267	0.349949673	0.252455	1.386186	0.16569	0.635372
edf1	2424.913	-0.125513268	0.124231	-1.01032	0.312341	0.776164
med14	534.8351	0.010965651	0.145569	0.075329	0.939953	0.997371
cbl	174.7346	0.07828971	0.18253	0.428913	0.667986	0.933086
rundc3b	14.18438	-0.135564684	0.570712	-0.23754	0.812241	0.970129
icn	12173.96	-0.096177938	0.224887	-0.42767	0.66889	0.933115
fam199x	797.992	0.217755403	0.106396	2.046645	0.040693	0.352601

gna11a	151.106	0.063736469	0.159035	0.400769	0.68859	0.939118
vim	1686.139	-0.090761456	0.164707	-0.55105	0.581602	0.905618
trim13	366.5187	-0.008737261	0.177904	-0.04911	0.96083	0.999249
qki2	342.2831	0.123727411	0.145544	0.850104	0.395268	0.829083
grpel1	517.6971	0.221337458	0.121708	1.818592	0.068974	0.448201
CU856539.1	154.8949	0.498964973	0.288682	1.728426	0.083912	0.486647
spon1a	15.77457	0.280981221	0.587581	0.4782	0.632508	0.923396
ssh1b	206.8907	-0.10457146	0.164877	-0.63424	0.525925	0.889076
cmtm7	281.2787	-0.309479859	0.12919	-2.39555	0.016595	0.2282
engase	83.22414	-0.119197606	0.210874	-0.56525	0.571901	0.90333
dnm1a	129.9223	0.54799486	0.292355	1.874416	0.060873	0.423588
neu3.2	241.0417	0.522554515	0.274511	1.903582	0.056965	0.412306
ufd1l	1082.937	0.206131509	0.166369	1.239	0.215345	0.697157
ndrg3b	972.407	0.148953528	0.123888	1.202329	0.229236	0.711317
itpkb	95.80026	0.246849482	0.255436	0.966385	0.333851	0.790692
adam9	280.7241	-0.333108483	0.15366	-2.16783	0.030172	0.309482
CR792422.1	7.297995	-0.705190968	0.971085	-0.72619	0.467723	0.866174
nup133	424.639	0.222375605	0.112974	1.968384	0.049024	0.385568
rbfox3a	442.6705	0.165939509	0.146757	1.130709	0.258178	0.735569
p4ha2	353.1574	0.030592114	0.219945	0.13909	0.889379	0.988318
slc1a6	29.45055	0.943460947	0.415139	2.272637	0.023048	0.26812
f9a	12.34456	0.674072143	0.70706	0.953344	0.340416	0.795496
pank1b	455.8911	0.079153375	0.106833	0.740908	0.458749	0.861537
bri3bp	148.3132	0.100748802	0.228203	0.441488	0.65886	0.930581
ndufb8	3351.616	-0.097464025	0.133485	-0.73015	0.465299	0.864997
smpdl3b	126.4446	0.649013572	0.283856	2.286417	0.02223	0.263658
dacha	412.1258	-0.062021056	0.108155	-0.57345	0.566341	0.901739
ldb1a	1890.631	-0.046956922	0.078279	-0.59987	0.548595	0.896847
plppr3a	503.7154	-0.069874282	0.151441	-0.4614	0.644514	0.926639
tor4aa	196.8623	0.14858393	0.164501	0.90324	0.366398	0.81589
cpa2	32.76177	1.98462201	0.611782	3.244005	0.001179	0.049383
atp5fa1	24971.97	0.038243392	0.118591	0.322481	0.747088	0.955074
vstm4a	202.3441	-0.219329598	0.215773	-1.01648	0.309399	0.774075
abi1a	632.0011	0.085685058	0.099201	0.863749	0.387726	0.827592
rps15a	16989.26	-0.351901276	0.128234	-2.74422	0.006066	0.131345
myd88	210.4606	-0.306015896	0.178243	-1.71685	0.086006	0.491972
asap2a	207.8061	0.129266904	0.136863	0.944497	0.344916	0.79837
si:ch73-265h17.1	10.63095	-0.810530486	0.643301	-1.25996	0.207685	0.689888
myo3a	83.81779	-0.21877448	0.253681	-0.8624	0.388467	0.827613
pax3a	48.59031	-0.156791293	0.318751	-0.49189	0.622795	0.918894
eif4bb	1932.397	0.014069802	0.097632	0.14411	0.885414	0.987533
smad3b	166.4469	0.036628012	0.174787	0.209558	0.834012	0.975618
ppm1na	54.76904	-0.830261244	0.625569	-1.32721	0.184439	0.661629
rbm22	445.1766	-0.032279357	0.120104	-0.26876	0.788113	0.963467
rpl22l1	4067.142	-0.357159864	0.147991	-2.41339	0.015805	0.221904
prmt1	2468.979	-0.180989809	0.137204	-1.31913	0.187127	0.665603
wdr54	156.7677	0.12251597	0.169513	0.722752	0.469832	0.867343
nqo1	917.781	-0.992018076	0.370258	-2.67926	0.007379	0.145623
jak3	35.43653	-0.120933742	0.380446	-0.31787	0.750581	0.955074
bin2b	100.1132	0.140887073	0.252289	0.558435	0.576548	0.90475
igf2bp3	944.3413	-0.073791106	0.137072	-0.53834	0.590344	0.908901
dpydb	763.6326	-1.001152729	0.233564	-4.28642	1.82E-05	0.002746
fezf1	92.88106	0.134391134	0.23461	0.572828	0.566761	0.901739
ptgs2b	130.0151	0.037098278	0.407548	0.091028	0.92747	0.994973

scamp2	531.9134	0.043267667	0.119378	0.362442	0.717022	0.94627
clasp1a	587.4322	-0.042978151	0.103635	-0.41471	0.678357	0.936026
xpo4	400.925	-0.01306283	0.144138	-0.09063	0.927789	0.99504
fmodb	172.2534	0.216900281	0.188527	1.1505	0.249938	0.729721
kcnh6b	8.591672	-1.397360944	1.594542	-0.87634	0.380845	0.824073
traf3ip1	63.14024	-0.081581613	0.250179	-0.32609	0.744354	0.95491
b4galt6	443.8635	0.000831954	0.156783	0.005306	0.995766	1
cp	713.9458	0.623052921	0.378075	1.647962	0.09936	0.523342
qars	689.5999	0.250175764	0.119257	2.097794	0.035923	0.332937
gpr183a	27.06505	-0.156898801	0.404762	-0.38763	0.698288	0.942433
srpx	409.6207	0.09334199	0.15807	0.590511	0.554848	0.898569
radx	31.67149	0.234729267	0.344219	0.681918	0.495291	0.87806
zgc:56231	1.935869	-0.782289405	1.870374	-0.41825	0.675762	NA
acer1	53.31403	-0.040404778	0.380376	-0.10622	0.915405	0.993068
c1ql3a	14.53726	0.210237397	0.52929	0.397206	0.691215	0.940173
slc5a3a	0.045161	0	5.267649	0	1	NA
rnf2	1323.251	-0.049847114	0.104696	-0.47611	0.633993	0.924049
lpar5a	17.09714	-0.371404371	0.46115	-0.80539	0.420596	0.842383
sept4a	134.0574	-0.142924411	0.229523	-0.6227	0.533481	0.891181
lrrfip2	493.2349	0.093501495	0.109264	0.855739	0.392142	0.827902
ppp4r4	64.30641	0.618625954	0.409202	1.511787	0.130588	0.581871
igsf9b	209.5652	0.037056914	0.174923	0.211847	0.832226	0.975013
epn1	853.9205	0.096439189	0.13367	0.72147	0.470621	0.867752
sirt4	106.4694	0.954145925	0.588044	1.622575	0.10468	0.53308
ndrg1b	771.8504	-0.20379138	0.268473	-0.75908	0.447806	0.856379
npsn	493.4067	0.04817919	0.199643	0.241327	0.809302	0.969828
scara5	109.4323	-0.068401071	0.262299	-0.26078	0.794266	0.965431
eaf2	418.7583	0.06573809	0.130581	0.503429	0.614663	0.915998
clu	856.4905	1.142100419	0.158194	7.219606	5.21E-13	8.05E-10
tent5c	147.5875	0.394941174	0.290429	1.359853	0.173876	0.646897
rnf11a	491.6062	0.023280985	0.184758	0.126008	0.899726	0.99045
trabd	263.315	0.269894883	0.14337	1.882508	0.059767	0.42181
guca1a	10.96434	0.906978745	0.713076	1.271924	0.2034	0.684359
atp1a2a	1070.459	0.263007602	0.245292	1.072224	0.283619	0.755095
p2rx3a	19.18187	0.034531507	0.429696	0.080363	0.935949	0.996901
hsp90aa1.1	1160.556	0.916438802	0.215269	4.257186	2.07E-05	0.002979
bzw1a	2297.519	-0.09654072	0.127507	-0.75714	0.448967	0.856796
prg4a	23.66232	0.394222527	0.373677	1.054981	0.291434	0.761454
sae1	661.1614	0.252675877	0.149905	1.685576	0.091878	0.507669
cxcr5	2.509465	-1.291016595	1.592637	-0.81062	0.417586	0.841149
dvl1a	65.74384	0.413248482	0.304701	1.356241	0.175022	0.648933
rpl21	18579.29	-0.176472577	0.115933	-1.52219	0.127961	0.577144
per3	812.163	0.234470719	0.637855	0.367592	0.713177	0.945821
si:ch211-282j22.3	115.7262	0.077064097	0.230751	0.333971	0.738402	0.952912
scamp4	619.76	0.112002431	0.094672	1.183053	0.236788	0.718119
mmgt1	539.8558	0.184074006	0.147319	1.249496	0.211484	0.692983
pdha1b	46.05905	-1.261268848	0.63666	-1.98107	0.047583	0.379782
mmp25b	30.9203	-0.468306424	0.335079	-1.3976	0.162234	0.629748
spop1a	343.821	-0.105143928	0.123673	-0.85017	0.395228	0.829083
aqp4	14.16125	-0.218998253	0.642806	-0.34069	0.733336	0.95079
ezh2	344.271	0.133166097	0.184904	0.720191	0.471407	0.86809
slc25a25a	643.7649	-0.248562836	0.483997	-0.51356	0.607558	0.915204
pard3ab	333.6098	0.157028122	0.152855	1.027302	0.304278	0.769725
tex9	67.61168	0.542362244	0.248949	2.178607	0.029361	0.304886

foxn4	215.7729	0.207931505	0.175354	1.18578	0.235709	0.716905
slc7a6os	550.2208	0.076821844	0.112262	0.684308	0.493781	0.877235
mtmr10	223.4612	-0.409101833	0.198082	-2.06532	0.038893	0.345399
zgc:112294	26.69937	0.28842995	0.503331	0.573042	0.566616	0.901739
kctd9b	172.7519	-0.045183204	0.190864	-0.23673	0.812867	0.970328
sarm1	179.6916	0.273392638	0.183204	1.492287	0.135624	0.591012
themis	12.5089	-2.25861217	0.683124	-3.3063	0.000945	0.042765
clic2	260.6331	-0.263527383	0.162582	-1.62089	0.105042	0.533616
hoxb6a	141.1216	0.118169812	0.173169	0.682394	0.49499	0.87806
slc20a1b	2135.729	0.07994958	0.159269	0.501977	0.615684	0.916365
arhgap42b	27.13104	-0.137484957	0.432245	-0.31807	0.75043	0.955074
ppm1k	408.6697	-0.515344831	0.122987	-4.19023	2.79E-05	0.00362
insig1	674.2671	-0.184498782	0.181306	-1.01761	0.308863	0.77371
fbxo36a	10.25466	-0.526417278	0.614383	-0.85682	0.391543	0.827902
trim16	189.9237	0.193709083	0.148622	1.30337	0.192448	0.672283
gngt2a	505.4423	0.21686282	0.276179	0.785225	0.432322	0.848419
tjap1	340.0578	0.370259441	0.167861	2.205755	0.027401	0.293857
il17d	3.697368	0.339130193	0.814907	0.416158	0.677294	0.935952
crygs4	0.408343	1.992516752	2.940751	0.677554	0.498055	NA
msi1	715.8484	0.254020426	0.138661	1.831947	0.066959	0.441882
npffr1l3	9.481469	-0.319040149	0.659421	-0.48382	0.628514	0.921864
chchd10	671.9195	0.105645304	0.180484	0.585345	0.558316	0.899409
6-Sep	1866.606	0.02009985	0.094151	0.213484	0.830949	0.974908
ttyh2l	289.4682	-0.388165001	0.141297	-2.74716	0.006011	0.131072
scin	223.7472	0.126580548	0.196479	0.644246	0.519416	0.887148
CABZ01073795.1	8.216573	0	1.871067	0	1	1
xrcc4	94.49552	-0.476984922	0.204516	-2.33226	0.019687	0.247431
zgc:101716	61.53856	0.269391168	0.254324	1.059244	0.289489	0.760499
dnaja2b	1277.017	-0.194025325	0.112961	-1.71763	0.085865	0.491473
acsl4b	274.6999	0.112868345	0.152146	0.741843	0.458183	0.861001
nod2	62.02668	0.253908011	0.236716	1.072629	0.283438	0.755095
capn12	427.236	0.57968535	0.152581	3.799203	0.000145	0.012055
flj13639	162.4294	1.187086858	0.482575	2.4599	0.013898	0.206547
epha4l	161.6123	0.064021117	0.161389	0.396688	0.691598	0.940173
sox19a	784.6786	0.075824898	0.167269	0.45331	0.650326	0.927835
pnpla6	469.0158	0.400824931	0.222443	1.801923	0.071558	0.456244
tlk2	481.867	0.141575516	0.115527	1.225475	0.220396	0.701707
zgc:55621	0	NA	NA	NA	NA	NA
ppp1r12a	518.7031	0.048427655	0.138878	0.348707	0.72731	0.949095
thbs1b	2834.676	-0.041206388	0.133636	-0.30835	0.757818	0.957397
homer3b	508.8943	-0.183657444	0.104655	-1.75489	0.079279	0.476619
dla	477.3152	0.191273855	0.195551	0.978128	0.328011	0.786438
cdc25b	269.1083	0.073913357	0.138872	0.532242	0.594558	0.910865
tfeb	164.8256	0.439522723	0.193862	2.267196	0.023378	0.270393
slc7a14a	271.0391	0.386562429	0.187656	2.059947	0.039404	0.347538
memo1	505.7109	0.078997475	0.149112	0.529785	0.596261	0.911626
churc1	229.3856	-0.142094373	0.196053	-0.72477	0.46859	0.866951
fshb	0.516575	0	4.042926	0	1	NA
kras	171.0728	0.090426998	0.196095	0.461139	0.644699	0.926639
amt	312.4704	-0.583858074	0.166264	-3.51164	0.000445	0.025478
DDX17	1884.953	-0.555543	0.181809	-3.05564	0.002246	0.075413
cdkn1ca	678.7527	-0.07923084	0.128567	-0.61626	0.537721	0.892498
mettl13	135.8303	0.129967959	0.171548	0.757619	0.448679	0.856741
nprl3	233.5291	0.244901723	0.18217	1.34436	0.178832	0.653658

HTRA3	93.83895	-0.23658751	0.270451	-0.87479	0.381689	0.824957
cacna1fa	16.047	-0.110726202	1.038314	-0.10664	0.915074	0.993066
syt14a	10.42371	0.603490741	0.597322	1.010328	0.312338	0.776164
abcb4	262.6713	-0.189971951	0.298807	-0.63577	0.524927	0.888928
BX957278.1	69.06814	-0.882052627	0.318221	-2.77182	0.005574	0.126154
pomgnt2	56.96735	0.078291382	0.27731	0.282324	0.777695	0.961073
rapgef6	296.5565	-0.355511884	0.165039	-2.1541	0.031232	0.314357
cbsb	842.6436	-0.077816769	0.275177	-0.28279	0.777339	0.961073
kif11	1042.426	0.364932134	0.260084	1.40313	0.160578	0.627313
ndel1a	759.044	0.017519246	0.129523	0.13526	0.892406	0.988566
sh3glb1b	713.3941	-0.310829684	0.136781	-2.27247	0.023058	0.26812
mpp2b	372.6132	0.400549532	0.16376	2.445956	0.014447	0.210484
glmnb	81.49951	0.154572999	0.381997	0.404645	0.685739	0.938486
fkbp7	508.6725	0.052670494	0.213645	0.246533	0.80527	0.969348
psma8	2802.326	-0.039303369	0.134305	-0.29264	0.769795	0.960484
rab34b	96.2029	-0.103919587	0.169846	-0.61185	0.54064	0.893355
trmt1	120.6195	0.325820854	0.192919	1.688903	0.091238	0.505618
zdhhc3a	135.7049	0.398883383	0.274814	1.451465	0.14665	0.606437
gtf2a1	749.7141	-0.005125848	0.089423	-0.05732	0.954289	0.998611
hnrnpa1a	4138.397	0.354858279	0.127065	2.792739	0.005226	0.121217
pdlim3a	22.65631	1.420858151	0.914457	1.553773	0.120238	0.565555
zgc:92907	139.3729	-0.198485308	0.177027	-1.12121	0.262197	0.739695
fgfr1a	670.9575	0.139488199	0.165409	0.843293	0.399064	0.831196
pgbd5	23.542	0.048871478	0.364534	0.134066	0.893351	0.988566
slc17a9b	123.5313	-0.028589691	0.16333	-0.17504	0.861047	0.980388
fbxo9	1617.603	-0.142557159	0.113967	-1.25086	0.210984	0.69272
camk2b1	1472.666	0.153812856	0.098747	1.55765	0.119316	0.562753
glra3	129.0645	0.185315527	0.367098	0.504812	0.613691	0.915998
ddx11	116.8391	0.160312761	0.285756	0.561014	0.574788	0.904516
zgc:91968	558.9961	-0.313154776	0.200201	-1.5642	0.11777	0.559739
wdr3	888.4362	-0.242802378	0.152752	-1.58952	0.111942	0.548295
si:dkey-42l23.2	5.137893	1.073601201	1.07313	1.000439	0.317098	0.778879
si:ch211-288d18.1	324.728	0.628521817	0.180895	3.474506	0.000512	0.027902
drd2b	27.04104	0.584978614	0.44872	1.30366	0.192349	0.672283
ccna2	667.5791	0.264328276	0.288012	0.917767	0.358741	0.80995
chrna10a	16.31031	0	1.698795	0	1	1
pptc7a	584.5002	-0.176300863	0.134904	-1.30686	0.19126	0.671109
snrpb	3480.547	-0.104283312	0.147271	-0.70811	0.47888	0.869292
dpysl5a	932.9826	0.083748433	0.16978	0.493276	0.621817	0.918461
uqcrb	8.310464	0.109563435	0.899619	0.121789	0.903066	0.991243
rpp14	145.8007	0.217671902	0.195532	1.113227	0.265611	0.742247
arhgef18a	363.4023	-0.027136413	0.128836	-0.21063	0.833177	0.975379
sema3fa	483.7238	-0.115193769	0.123823	-0.93031	0.352209	0.802776
cahz	2537.107	0.007652336	0.159098	0.048098	0.961638	0.999451
ndrg2	2865.493	-0.04043971	0.154867	-0.26113	0.793995	0.965431
si:ch211-12m10.1	460.4561	0.073024347	0.184071	0.396717	0.691576	0.940173
atp6v1d	1881.276	-0.097869671	0.124743	-0.78457	0.432705	0.84885
acvr2aa	856.742	0.21103603	0.130552	1.616485	0.10599	0.534956
fgfr1b	189.3798	0.037918658	0.235652	0.160909	0.872165	0.983447
dnajc11a	866.1132	0.005367399	0.113327	0.047362	0.962225	0.999451
rplp2l	16546.35	-0.277345493	0.139427	-1.98918	0.046681	0.376436
akt2	1431.651	-0.065874444	0.081714	-0.80615	0.420154	0.842383
ccdc28b	88.13748	0.515744277	0.211946	2.433378	0.014959	0.214257
pcyt1aa	157.9487	-0.570260909	0.166448	-3.42606	0.000612	0.032103

otx2b	624.7296	0.070119796	0.104855	0.668734	0.503665	0.881759
ppp1r14aa	147.6644	-0.246293971	0.204032	-1.20713	0.227381	0.708598
ift46	324.6569	0.210414689	0.184279	1.141826	0.253526	0.732873
esrp1	573.4105	-0.23184714	0.130182	-1.78095	0.074921	0.464197
krr1	223.9377	-0.390869333	0.210355	-1.85814	0.063149	0.429313
rnpc3	261.9167	-0.277197285	0.126023	-2.19958	0.027836	0.296561
enpp2	174.6937	0.154608302	0.209419	0.738272	0.460349	0.862369
trpm1a	90.78976	-0.485967219	0.30816	-1.577	0.114796	0.554602
traip	112.5264	0.116816113	0.250843	0.465694	0.641435	0.925586
uqcc2	437.3322	-0.008955479	0.144143	-0.06213	0.95046	0.99801
tlx2	151.6204	0.246199928	0.21774	1.130709	0.258178	0.735569
si:dkey-110k5.10	7.671304	0.578853714	0.734375	0.788226	0.430565	0.847542
atf7a	183.667	0.000209499	0.216036	0.00097	0.999226	1
blzf1	677.4203	0.042882742	0.116823	0.367074	0.713564	0.945821
nae1	534.3667	0.09589335	0.141732	0.67658	0.498672	0.879251
stk3	520.8766	0.025036571	0.134198	0.186564	0.852003	0.978558
adra1ba	19.58216	-0.072044643	0.391937	-0.18382	0.854157	0.979016
ankrd45	15.91602	-0.032559288	0.432664	-0.07525	0.940013	0.997371
arhgap33	135.6136	0.292863161	0.271146	1.080094	0.2801	0.752951
ncaldb	164.957	0.245756507	0.256787	0.957044	0.338545	0.795226
nfatc2ip	262.2353	0.071831797	0.189673	0.378713	0.704901	0.944015
fyna	671.5385	0.218919965	0.135456	1.616167	0.106058	0.534956
zgc:64106	237.1522	-0.387675211	0.167072	-2.32041	0.020319	0.252973
mknk2a	217.7084	-0.393338319	0.247812	-1.58724	0.112458	0.549251
dnase1l4.2	175.3901	-0.461064857	0.157421	-2.92887	0.003402	0.095894
tnnc1a	102.4009	-0.025724976	0.352277	-0.07302	0.941786	0.997371
fen1	469.2528	-0.133739965	0.149377	-0.89532	0.370618	0.818484
rps9	41697.14	-0.432033771	0.148374	-2.9118	0.003594	0.09929
col2a1b	6578.965	0.036762828	0.167856	0.219014	0.826639	0.973383
aadat	40.77186	0.07649681	0.373311	0.204914	0.837639	0.976213
sigmar1	264.7948	0.123466074	0.143119	0.86268	0.388313	0.827592
ryr1a	699.9198	0.204045864	0.256403	0.795802	0.426147	0.844714
ptger2a	55.47507	-0.086404752	0.275211	-0.31396	0.753552	0.955864
elmsan1a	217.4993	0.038170401	0.170838	0.223431	0.8232	0.972005
fes	62.77696	-0.102014548	0.24773	-0.4118	0.680488	0.936945
gsna	687.8773	-0.051162551	0.138806	-0.36859	0.712434	0.945821
otub1b	508.4254	-0.079389762	0.134398	-0.59071	0.554717	0.89855
CABZ01044048.1	21.13878	0.078982159	0.528437	0.149464	0.881188	0.986892
afg1la	140.6701	0.1688249	0.18055	0.935059	0.349758	0.801408
calcrla	75.79815	0.007251223	0.225413	0.032169	0.974338	1
gnaq	113.5929	-0.297826801	0.283769	-1.04954	0.29393	0.763068
sirt2	737.4385	-0.239418979	0.145475	-1.64577	0.09981	0.524166
scube3	57.32038	0.319591353	0.253234	1.262037	0.206935	0.689276
dcun1d3	253.8031	-0.021509733	0.134057	-0.16045	0.872525	0.983447
ppm1bb	1449.898	0.136620876	0.085551	1.596944	0.110278	0.544923
si:ch211-212o1.2	188.1389	-0.00735414	0.138213	-0.05321	0.957566	0.999109
lpcat1	126.62	0.049976508	0.173334	0.288326	0.773098	0.960624
rcc2	2205.561	0.048391803	0.125076	0.386899	0.698831	0.942456
orai1a	130.2766	0.01338688	0.17921	0.074699	0.940454	0.997371
myl4	644.6286	-0.87413545	0.191006	-4.57649	4.73E-06	0.001024
upb1	251.4401	0.071997305	0.22171	0.324737	0.74538	0.955013
sema6dl	436.313	-0.028223944	0.158525	-0.17804	0.858691	0.979934
ugt2a5	125.2485	0.318134658	0.194476	1.635859	0.101869	0.528631
arrb2b	1484.986	-0.161047155	0.097101	-1.65856	0.097205	0.519839

c1ql4a	14.56169	1.417864875	0.666459	2.127461	0.033382	0.323127
atp5pb	7790.503	0.066432507	0.108159	0.614213	0.539074	0.892985
spag7	1018.111	0.212671286	0.133598	1.591873	0.111413	0.547032
sfpq	2886.975	0.145347508	0.092677	1.568316	0.116807	0.557831
pou6f1	322.7765	-0.110502949	0.115321	-0.95822	0.337952	0.794808
calcr1b	55.80883	0.165903693	0.243759	0.680606	0.496121	0.878093
abcb11a	69.7222	-0.768443464	0.431538	-1.78071	0.07496	0.464266
hoxb10a	159.3807	-0.039398799	0.185796	-0.21205	0.832065	0.975013
si:dkey-23k10.5	41.99289	-0.214760891	0.435066	-0.49363	0.621569	0.918461
sox5	967.2398	-0.015943643	0.132601	-0.12024	0.904295	0.991581
cry1b	323.5611	-0.459745907	0.155386	-2.95873	0.003089	0.091315
smarcb1b	522.4535	0.035607903	0.162103	0.219663	0.826134	0.973084
atxn2l	608.381	0.059027769	0.140093	0.421346	0.673503	0.934323
epha4b	77.67859	0.341205983	0.269987	1.263787	0.206307	0.688779
si:dkeyp-117h8.2	334.5288	-0.085144964	0.142612	-0.59704	0.55048	0.8974
dennd6b	216.4653	-0.117021731	0.145296	-0.8054	0.420588	0.842383
FOXK2	734.3739	-0.119486624	0.10768	-1.10964	0.267153	0.743418
actr1	1592.987	0.102720463	0.083723	1.226907	0.219858	0.700895
rbm39a	2229.159	-0.083947616	0.124558	-0.67397	0.500333	0.880145
mybpc3	2434.078	-0.098555085	0.097381	-1.01206	0.31151	0.77613
slc26a2	257	0.056309659	0.187703	0.299993	0.764183	0.959228
scxb	20.05977	-0.647667761	0.398512	-1.62521	0.104117	0.532183
syt5b	967.1186	-0.106782305	0.393347	-0.27147	0.786029	0.962605
snrpd1	874.9374	-0.129450693	0.119732	-1.08117	0.279621	0.752477
ftr52p	44.58458	-0.0630383	0.298327	-0.21131	0.832648	0.975239
twf1b	325.8859	0.207483154	0.162955	1.273253	0.202928	0.683508
chuk	941.7516	-0.161913037	0.109514	-1.47846	0.139284	0.597457
aldoaa	8864.725	0.030742966	0.180849	0.169992	0.865016	0.982136
pde6b	113.9827	-0.510136153	0.448813	-1.13663	0.255692	0.733642
sema3b	204.6789	0.299772571	0.194426	1.541837	0.123113	0.569695
si:ch211-246m6.5	108.0302	0.380072614	0.177556	2.140578	0.032308	0.318811
prtfdc1	192.6569	-0.3157933	0.203628	-1.55083	0.120942	0.566816
ttc36	456.0448	-0.734954932	0.244981	-3.00005	0.002699	0.084979
esrrgb	11.59066	0.40035592	0.542498	0.737986	0.460523	0.862369
ctsll	152.6544	0.783331938	0.364488	2.149129	0.031624	0.316333
clocka	128.4724	0.021957329	0.304353	0.072144	0.942487	0.997371
zgc:56556	71.69348	0.218036012	0.220329	0.989594	0.322373	0.78246
marveld1	152.1577	-0.17161943	0.173974	-0.98647	0.323904	0.783161
b9d1	273.7835	-0.085683192	0.173713	-0.49325	0.621839	0.918461
u2af2b	1622.538	0.038381902	0.07888	0.486586	0.626552	0.920665
pigo	274.965	0.220254789	0.160415	1.373029	0.169743	0.641486
ints13	284.9402	-0.005319565	0.158744	-0.03351	0.973268	1
cpm	338.0869	-0.037492997	0.147807	-0.25366	0.799756	0.967206
dhrs12	439.9962	0.059423044	0.205443	0.289243	0.772396	0.960624
cttn	1026.194	0.066380317	0.100285	0.661917	0.508024	0.883056
stag2a	698.2878	-0.129741148	0.13386	-0.96923	0.332431	0.789726
tbx6	17.9339	0.840633397	0.604458	1.390723	0.16431	0.633351
tent5bb	224.2337	-0.075818328	0.166093	-0.45648	0.648043	0.927245
wars2	65.27005	-0.092286667	0.226138	-0.4081	0.683201	0.938212
mical1	59.79898	-0.253286895	0.30851	-0.821	0.411647	0.837966
batf	2.699619	-1.765174955	1.84739	-0.9555	0.339327	0.795226
pcnx1	532.8029	-0.055296052	0.149457	-0.36998	0.711397	0.945821
plod2	332.8643	-0.129681671	0.232344	-0.55815	0.576745	0.904769
pbxip1b	413.526	0.048382062	0.163683	0.295583	0.767548	0.960296

cetn3	187.5072	0.405158824	0.223166	1.815508	0.069446	0.45
zgc:100846	138.2953	0.116356962	0.162994	0.713874	0.475305	0.869292
atp5l	4960.521	0.137480821	0.123205	1.115871	0.264477	0.742063
chn2	33.56031	-0.073077132	0.348221	-0.20986	0.833778	0.975482
map2k5	146.2216	0.035472784	0.186238	0.19047	0.848941	0.978021
aak1a	40.0064	0.402023101	0.278691	1.442538	0.149151	0.610893
inaa	161.8782	0.485437969	0.237576	2.043293	0.041023	0.353808
ptk7a	589.7106	0.084137913	0.158309	0.53148	0.595087	0.910894
galnt8a.2	5.48048	1.096897037	1.095336	1.001425	0.316621	0.778879
sp8a	445.7986	0.197848496	0.282162	0.701188	0.483186	0.871988
ednraa	406.0865	-0.205447659	0.184788	-1.1118	0.266223	0.742912
foxn1	19.84854	0.443853766	0.429992	1.032238	0.301961	0.767995
MFN1	18.55811	1.939702096	1.618611	1.198375	0.230771	0.712454
mrpl19	460.1101	0.097906926	0.127104	0.770287	0.44113	0.852823
pdca	71.95176	2.310742674	0.568321	4.065908	4.78E-05	0.005408
cry4	739.0691	0.005146787	0.200176	0.025711	0.979488	1
tsg101b	305.0337	0.202093982	0.190618	1.060202	0.289053	0.76024
itpr2	164.7559	0.02571415	0.170566	0.150758	0.880167	0.986408
txnl1	1363.596	0.310849255	0.159622	1.947407	0.051486	0.394488
spns1	1227.729	-0.057270007	0.144268	-0.39697	0.691391	0.940173
tpgs2	81.73723	0.42114078	0.221555	1.900837	0.057323	0.413377
plp1b	286.1212	0.176186673	0.394003	0.447171	0.654752	0.929527
prkcbp1l	874.0932	-0.039382535	0.106149	-0.37101	0.710628	0.945821
gyg1a	1293.326	-0.24929483	0.164666	-1.51394	0.130041	0.580771
bmpr2a	332.8668	0.171878282	0.159403	1.078263	0.280917	0.75325
slc35e1	431.0317	0.108574439	0.119649	0.90744	0.364174	0.814549
insra	773.7378	0.040728298	0.114256	0.356466	0.721492	0.947343
dlx4a	177.3143	-0.213978737	0.208866	-1.02448	0.305609	0.770852
ctnnbl1	497.3976	0.007999446	0.162751	0.049152	0.960798	0.999249
csrp2	701.3364	-0.04541841	0.188111	-0.24144	0.809211	0.969828
slc35d1a	155.2902	0.065326111	0.138692	0.471015	0.63763	0.924779
fmn1	17.81518	-0.077516129	0.490595	-0.158	0.874453	0.984052
zgc:136908	1163.422	0.01555602	0.157995	0.098459	0.921568	0.994085
crx	896.6241	-0.013615852	0.286169	-0.04758	0.962051	0.999451
bfsp2	1309.014	0.60503576	0.246654	2.452978	0.014168	0.208294
ybey	0	NA	NA	NA	NA	NA
slc38a7	276.0845	0.113015918	0.212176	0.532652	0.594275	0.910731
mob2b	476.9225	-0.076836538	0.130644	-0.58813	0.556442	0.899063
cpa6	118.7335	0.113876763	0.17048	0.667976	0.504149	0.881759
hpgd	260.0281	0.044752138	0.173161	0.258443	0.796065	0.966305
glra1	291.7047	0.376154785	0.254512	1.477943	0.139423	0.597663
kdsr	263.8445	0.041969003	0.12952	0.324035	0.745912	0.955074
hs6st2	610.0496	-0.06327938	0.094463	-0.66988	0.502931	0.881461
dnaaf1	14.57142	-0.1245174	0.423092	-0.2943	0.768526	0.960395
zgc:100832	128.9949	-0.349531365	0.220741	-1.58345	0.11332	0.551138
ttc26	154.1329	0.208186431	0.175704	1.184871	0.236068	0.717364
rnf19a	792.4301	0.059580722	0.130896	0.455176	0.648983	0.927649
polr3gla	99.75952	-0.674669796	0.283953	-2.37599	0.017502	0.233637
mettl21a	41.71987	-0.215454283	0.336318	-0.64063	0.521766	0.887551
mtnr1al	3.586654	0.112530897	1.694634	0.066404	0.947056	0.997371
thbs3b	415.5538	-0.000775646	0.198312	-0.00391	0.996879	1
dcn	2441.908	0.078454744	0.10898	0.719902	0.471585	0.86809
angptl1a	244.9333	0.13994679	0.2429	0.576149	0.564514	0.901433
dmac2l	212.3601	0.053236276	0.190955	0.278789	0.780407	0.961411

kif15	415.3543	0.084688329	0.208544	0.406094	0.684674	0.938486
apoa1a	53390.78	-0.302438743	0.232957	-1.29826	0.194198	0.674395
meis1b	2623.295	0.026766397	0.115271	0.232204	0.81638	0.971207
c19h1orf109	116.2874	-0.486619928	0.247837	-1.96347	0.049591	0.387751
cnga1b	38.79442	-0.398038849	0.894497	-0.44499	0.65633	0.929684
zgc:109965	173.5872	0.054928818	0.371409	0.147893	0.882427	0.986908
fbxl2	195.9814	-0.298204824	0.1682	-1.77292	0.076242	0.467585
cyp46a1.1	70.70013	-0.715206233	0.296447	-2.41259	0.01584	0.222022
sgce	654.5478	-0.008740642	0.122357	-0.07144	0.943051	0.997371
ccnl1b	672.8741	-0.135253125	0.144946	-0.93313	0.350753	0.802215
papolg	1225.515	0.062255508	0.079504	0.783054	0.433596	0.849735
emc7	777.0822	0.029823553	0.097625	0.305491	0.759993	0.957886
rab4b	76.98871	0.147931452	0.253566	0.583405	0.559621	0.899571
zgc:162872	81.81509	-0.099252537	0.203395	-0.48798	0.625565	0.920625
cant1a	401.2215	-0.04999842	0.119865	-0.41712	0.676589	0.935635
scp2a	2065.927	-0.056630147	0.162607	-0.34826	0.727642	0.949241
fer	88.38026	0.080578295	0.233856	0.344563	0.730423	0.950056
gpt2	352.3205	-0.149592929	0.170715	-0.87627	0.380881	0.824073
CDK18	66.07578	-0.047722267	0.274047	-0.17414	0.861756	0.980588
umps	267.4186	0.05623718	0.194663	0.288896	0.772661	0.960624
dpf2	215.0472	-0.006797735	0.164353	-0.04136	0.967008	1
nup35	220.9674	0.013346924	0.129065	0.103413	0.917635	0.993777
psme3	519.4908	0.018508622	0.124155	0.149076	0.881493	0.986908
ube2e3	1826.547	-0.086965258	0.112815	-0.77086	0.440787	0.852579
def6a	19.63898	-0.337374198	0.456984	-0.73826	0.460354	0.862369
rgma	1327.684	0.049051121	0.113041	0.433924	0.664344	0.93177
eftud2	869.7097	0.237092819	0.109144	2.172295	0.029833	0.307628
clcn1b	56.50117	0.096890515	0.369675	0.262096	0.793247	0.965312
tmem206	130.1722	0.174060354	0.198611	0.876388	0.380819	0.824073
eif4e1c	726.7409	0.356618707	0.177691	2.006957	0.044754	0.370491
katnal2	8.145987	0.177464681	0.676263	0.26242	0.792998	0.965312
zgc:65851	136.0202	1.057148225	0.284642	3.713958	0.000204	0.015177
bcs1l	188.9829	0.346615984	0.164765	2.103694	0.035405	0.330833
cnga3b	15.38567	2.528223955	1.210871	2.087939	0.036803	0.336916
syt13	196.6824	0.122925273	0.270486	0.454461	0.649497	0.927756
myoz2a	601.9614	-0.160348111	0.164849	-0.9727	0.330703	0.788149
usp25	1043.742	-0.230750619	0.151435	-1.52376	0.12757	0.576964
zgc:110269	175.1679	-0.685928544	0.235395	-2.91395	0.003569	0.099143
ptpn11b	132.6917	-0.01235089	0.242704	-0.05089	0.959414	0.999164
capn9	571.1678	0.15869724	0.133589	1.187949	0.234854	0.715533
nacad	218.0082	0.24262878	0.202586	1.197658	0.23105	0.712561
vipr2	49.26775	-0.016628909	0.319115	-0.05211	0.958441	0.999109
galnt8a.1	6.094091	0	1.752786	0	1	1
fbp2	721.842	0.008245292	0.296346	0.027823	0.977803	1
trim46a	62.83831	0.300092268	0.311351	0.96384	0.335126	0.79185
ttc21b	183.9203	-0.204880724	0.166322	-1.23183	0.218011	0.699144
rdh10b	167.5743	-0.068103019	0.172135	-0.39564	0.692373	0.940414
naa35	662.2197	-0.120657679	0.133481	-0.90393	0.366033	0.815714
hsc70	4514.699	0.395876799	0.13481	2.936557	0.003319	0.0947
pdha1a	4246.671	0.090490145	0.084759	1.067611	0.285696	0.757084
cox4i1l	129.5018	-0.97647739	0.534228	-1.82783	0.067575	0.443563
adar	375.0712	0.136761047	0.151271	0.904078	0.365954	0.815714
kcnk5b	98.11593	-0.142737694	0.235049	-0.60727	0.543674	0.894925
mmp13a	59.49417	-0.311660946	0.400836	-0.77753	0.436848	0.851125

eya4	510.8646	-0.032399481	0.127447	-0.25422	0.799326	0.967111
adssl	798.6349	0.124720409	0.111424	1.119331	0.262999	0.740604
ercc6l2	113.5022	-0.241911021	0.195682	-1.23625	0.216366	0.69815
col1a1a	37394.46	0.098126471	0.15082	0.650618	0.515293	0.886401
mgat1a	260.773	-0.209161317	0.136223	-1.53544	0.124676	0.57237
rnf207b	138.9548	-0.207001967	0.187155	-1.10604	0.268708	0.744925
col11a2	5100.199	0.328019051	0.231362	1.417774	0.156257	0.621949
neflb	375.8563	0.656829139	0.276681	2.373955	0.017599	0.234077
fam76b	672.1855	0.048479076	0.12245	0.395908	0.692173	0.940333
spic	2.954327	0	1.990245	0	1	1
zgc:113057	2.266465	-1.595580454	1.429265	-1.11636	0.264266	0.742063
si:ch211-169p10.1	38.0324	-0.198819798	0.360535	-0.55146	0.58132	0.905618
vmp1	493.3976	0.053137545	0.110684	0.480084	0.631167	0.923003
rnls	259.7119	0.159203523	0.13995	1.137578	0.255297	0.733542
zgc:77849	2904.287	-0.134806994	0.088537	-1.52261	0.127857	0.577117
rassf4	54.56855	0.17503741	0.246382	0.710432	0.477436	0.869292
spint1b	408.4784	0.176952896	0.14508	1.219696	0.22258	0.704536
aacs	60.79435	-0.078322957	0.279502	-0.28022	0.779306	0.961073
aurka	283.4161	0.219592755	0.255998	0.857791	0.391008	0.827902
mphosph10	320.0184	-0.107411334	0.16311	-0.65852	0.510205	0.883733
GPR62 (1 of many)	7.492189	-1.779806101	1.752956	-1.01532	0.309955	0.774671
per1b	855.2987	-0.366219789	0.776565	-0.47159	NA	NA
rlbp1a	449.6661	0.510785066	0.173461	2.944667	0.003233	0.093414
u2af2a	789.736	0.189707748	0.091871	2.064944	0.038928	0.345405
rnpep	224.7433	0.430849124	0.212421	2.028278	0.042532	0.361124
sdcbp2	1955.699	-0.083292158	0.097302	-0.85602	0.391987	0.827902
hcfc1b	1044.157	0.193992294	0.180989	1.071844	0.28379	0.755095
slc6a6a	26.97763	0.14785996	0.411839	0.359024	0.719577	0.94694
epx	12.11588	-1.158288696	1.144913	-1.01168	0.31169	0.776164
dnase1	129.7173	0.307647462	0.847607	0.36296	0.716635	0.94627
rap1aa	1058.843	0.160893496	0.113157	1.421867	0.155065	0.619776
ak5	135.4074	0.808355955	0.315743	2.560173	0.010462	0.178239
pdss2	174.9241	0.384782866	0.18184	2.116046	0.034341	0.327305
polr3f	189.7608	-0.539396947	0.200594	-2.689	0.007167	0.143346
slkb	419.8444	0.230694441	0.152155	1.51618	0.129474	0.579133
waca	945.2782	0.134127096	0.10917	1.228613	0.219217	0.700266
AL590149.1	127.6837	0.161253452	0.237135	0.680007	0.4965	0.878161
fmnl2a	109.2439	0.327338602	0.295007	1.109594	0.267174	0.743418
ptdss1a	258.8417	-0.117759886	0.175135	-0.6724	0.501332	0.880791
pdcd10b	423.0292	-0.193903339	0.140314	-1.38192	0.166995	0.637452
col5a1	5025.9	0.170875578	0.164679	1.037629	0.299443	0.766128
si:dkey-97a13.6	36.62263	0.172881539	0.402197	0.429843	0.66731	0.933049
eif3ja	522.8614	-0.089465935	0.140037	-0.63887	0.522905	0.888222
hpxa	49.05744	-1.715186602	1.074023	-1.59697	0.110271	0.544923
saga	362.1608	0.534950992	0.359875	1.486492	0.137149	0.592789
prkag2a	316.691	-0.229234715	0.139503	-1.64322	0.100338	0.524753
cdc34b	710.8433	0.112866414	0.146547	0.770172	0.441198	0.852874
zgc:92749	39.90691	0.496043274	0.309519	1.602627	0.109017	0.541807
cideb	78.14667	0.050608229	0.266873	0.189634	0.849596	0.978209
si:ch211-266d19.4	160.2075	-0.094620549	0.146734	-0.64485	0.519027	0.887148
smad4b	122.4355	0.135746254	0.174654	0.77723	0.437023	0.851125
atf6	648.5651	0.496031971	0.10054	4.933689	8.07E-07	0.000253
tfap2b	672.3012	0.009403928	0.209947	0.044792	0.964273	0.999925
inhbaa	92.13136	-0.313326262	0.212973	-1.4712	0.141236	0.599899

gtf2e2	533.8694	0.027308794	0.13261	0.205933	0.836843	0.976007
rpusd4	99.0762	-0.203350509	0.214855	-0.94645	0.343917	0.798147
pfn2l	3371.108	0.10795616	0.090362	1.194702	0.232203	0.713454
atp2b1a	867.9536	0.068944087	0.124589	0.553371	0.58001	0.905618
eif1b	8907.004	0.100632284	0.110209	0.913105	0.361188	0.81142
c3a.1	3229.306	0.155755044	0.24436	0.6374	0.523864	0.888642
bbx	43.75368	-0.225201634	0.345814	-0.65122	0.514903	0.886401
wee2	3.169467	0.573674361	1.341462	0.427649	0.668907	0.933115
rbm14a	716.8231	0.059996829	0.118617	0.505803	0.612995	0.915998
calhm5.2	35.83775	-0.38700963	0.279462	-1.38484	0.166101	0.635707
hcls1	365.1904	-0.005926117	0.130087	-0.04556	0.963665	0.999842
maco1b	533.3319	0.145480163	0.113498	1.281786	0.199918	0.6804
poc1bl	133.3567	0.187192772	0.189925	0.985612	0.324324	0.783308
rer1	779.5226	0.093192016	0.133483	0.698159	0.485078	0.872911
arl13b	265.961	-0.270761779	0.127814	-2.1184	0.034141	0.326054
smarca1	315.6997	-0.17869114	0.143509	-1.24516	0.213074	0.695467
nucks1b	1312.832	0.174219434	0.10843	1.606741	0.108111	0.54009
foxa3	217.182	-0.334475321	0.143283	-2.33436	0.019577	0.246694
plek2	84.13485	0.036159957	0.382932	0.094429	0.924768	0.994815
tmem161a	383.9786	0.142909603	0.135352	1.055838	0.291042	0.761197
hnmt	97.41132	-0.750862163	0.243399	-3.08491	0.002036	0.070205
pfkpb	225.4512	0.424369783	0.207788	2.042318	0.04112	0.353918
dipk2aa	88.19934	0.492677328	0.230078	2.141346	0.032246	0.318747
csnk2a2a	617.2878	-0.117909475	0.114262	-1.03192	0.302107	0.76803
nop56	2525.263	-0.267480611	0.133554	-2.00279	0.0452	0.372359
dlgap4b	42.15691	-0.020169235	0.297842	-0.06772	0.94601	0.997371
itga3b	311.1347	0.279726693	0.159463	1.754178	0.0794	0.477151
mettl3	167.7377	0.116091125	0.173246	0.670094	0.502798	0.881453
asah2	85.74405	-0.255858309	0.442197	-0.57861	0.562854	0.900772
foxn3	72.52193	0.259285276	0.247454	1.047812	0.294725	0.763708
alg9	299.0122	0.230955014	0.142895	1.616255	0.106039	0.534956
arih2	1368.754	-0.054354937	0.171341	-0.31723	0.751067	0.955222
tmprss4b	22.20746	-0.510757207	0.482963	-1.05755	0.290261	0.76078
picalma	764.6193	-0.074018804	0.118276	-0.62581	0.531437	0.890835
npepl1	1644.761	-0.132824084	0.092977	-1.42857	0.153129	0.617769
snap23.1	905.5268	-0.041806091	0.103982	-0.40205	0.687646	0.938835
slc4a1a	3627.445	0.315586499	0.223547	1.411724	0.158031	0.623766
znf143a	419.4431	0.045749191	0.120923	0.378332	0.705184	0.944028
ammecr1	322.594	-0.019257204	0.117281	-0.1642	0.869576	0.983265
anos1a	399.6266	-0.134541165	0.16579	-0.81152	0.417069	0.84067
ddx49	190.105	0.08571933	0.18398	0.465916	0.641275	0.925523
slc34a2a	67.77596	0.085279842	1.491351	0.057183	0.954399	0.998611
arhgef4	1146.668	0.122756953	0.103919	1.181271	0.237495	0.718568
ech1	461.4014	-0.087676885	0.185315	-0.47312	0.636126	0.924779
tcea2	197.0784	0.171244771	0.161624	1.059525	0.289361	0.760405
eif2d	206.9749	0.410463605	0.147048	2.791352	0.005249	0.121252
mat2b	395.5113	-0.074359552	0.16013	-0.46437	0.642383	0.92567
itgb5	432.1381	0.156154805	0.101253	1.542229	0.123018	0.569606
myhz2	58247.93	-0.01159401	0.373612	-0.03103	0.975244	1
tnfsf13b	10.80939	0.593177527	0.525673	1.128414	0.259145	0.736127
syap1	720.3605	0.019894591	0.121432	0.163833	0.869862	0.983365
scml2	542.5267	-0.10983548	0.130269	-0.84314	0.399148	0.831196
mob1bb	245.918	-0.363744257	0.171123	-2.12563	0.033534	0.323214
rhoub	831.304	-0.028843225	0.132419	-0.21782	0.82757	0.973588

cfl1l	8125.61	-0.0286533	0.138982	-0.20617	0.836662	0.976007
osbpl7	573.2386	-0.086501159	0.140672	-0.61491	0.538611	0.892777
nf1a	457.7511	-0.090088278	0.125137	-0.71992	0.471576	0.86809
adal	27.71349	0.045427196	0.349662	0.129918	0.896632	0.98939
gpia	3424.841	-0.222089502	0.202699	-1.09566	0.273228	0.749148
ppfia2	234.634	-0.185131559	0.240473	-0.76986	0.441381	0.852874
tdg.1	702.5065	0.138375625	0.109253	1.266562	0.205312	0.68744
opcml	29.63839	0.487061246	0.54419	0.89502	0.370776	0.818484
pibf1	0.179863	2.667699955	5.195716	0.513442	0.607642	NA
ubiad1	467.2442	-0.108975297	0.149177	-0.73051	0.465079	0.864892
or101-1	5.453898	0.022218398	0.956388	0.023232	0.981466	1
pole4	97.91044	0.046159414	0.240833	0.191666	0.848004	0.977829
dtnbb	23.943	0.370467696	0.458693	0.807659	0.419287	0.842286
si:ch211-59h6.1	37.3533	-0.273760446	0.320453	-0.85429	0.392944	0.827902
nsun3	50.46745	-0.051547712	0.274854	-0.18755	0.851233	0.978548
erap1a	73.62288	0.063331149	0.278694	0.227243	0.820235	0.971788
ints6	172.6932	0.141750983	0.166867	0.849487	0.39561	0.829389
mta2	1867.586	0.070252968	0.109276	0.642893	0.520293	0.887149
ndufv2	2229.677	0.205440146	0.123468	1.663917	0.096129	0.517378
abcc12	154.2776	-1.338709853	0.384152	-3.48484	0.000492	0.027296
zgc:158412	14.03666	1.078563724	1.298577	0.830574	0.406215	0.834645
itpk1a	272.4492	-0.188847661	0.201232	-0.93846	0.348009	0.800392
hoxb5a	355.8939	-0.22285082	0.126939	-1.75558	0.07916	0.47627
ascc2	222.6101	-0.24504936	0.158779	-1.54334	0.122749	0.569468
oxct1a	918.5822	0.158817122	0.159636	0.99487	0.3198	0.780475
mmp15b	131.9502	0.031167095	0.203184	0.153393	0.878088	0.98573
mrpl11	426.9408	0.13076323	0.136066	0.961027	0.336539	0.793245
bmi1b	321.5951	-0.005808821	0.158582	-0.03663	0.97078	1
ywhaba	8630.655	0.156549719	0.079105	1.978999	0.047816	0.380801
tubgcp2	290.9695	-0.085445644	0.136862	-0.62432	0.532419	0.890978
uap1l1	305.2001	-0.051880343	0.129682	-0.40006	0.689114	0.93924
ndrg3a	1956.71	0.064701769	0.153256	0.422181	0.672893	0.93414
gclc	563.8204	-0.129694035	0.162175	-0.79971	0.423876	0.844281
dmtn	264.5505	0.528280597	0.148158	3.565648	0.000363	0.022086
hiat1a	423.0673	0.013716799	0.13898	0.098696	0.921379	0.994085
maf1	765.1141	2.47E-05	0.127544	0.000194	0.999845	1
dlx1a	165.1993	0.028891749	0.143482	0.201361	0.840416	0.976417
pdk1	166.2983	-0.794821895	0.283831	-2.80034	0.005105	0.119235
prph2la	30.19882	-0.114930384	0.448004	-0.25654	0.797535	0.966447
atp1b1a	3871.475	0.097477094	0.122154	0.797983	0.42488	0.844714
dhx16	325.0226	-0.064503989	0.126968	-0.50803	0.611431	0.915331
dennd3b	13.10804	-0.375174773	0.530777	-0.70684	0.479665	0.869938
zgc:55558	847.5363	0.047602018	0.105763	0.450084	0.65265	0.929035
jag1b	412.5073	-0.014984539	0.19235	-0.0779	0.937906	0.997305
nkd3	137.8832	-0.416819636	0.210208	-1.98289	0.04738	0.379101
si:ch211-1f22.1	1.61926	-2.812508223	1.551512	-1.81275	0.06987	NA
zeb1b	628.4707	-0.108556846	0.100751	-1.07748	0.281266	0.753577
htr2cl2	3.358693	-0.217505235	1.404758	-0.15483	0.876952	0.985067
pde4ca	109.5249	0.178038126	0.286837	0.620694	0.534801	0.891993
slc35a3a	169.1907	0.097893353	0.152484	0.641989	0.52088	0.887411
phc2b	384.7556	0.237138509	0.121958	1.944424	0.051844	0.395809
glulc	23.06374	-0.355751414	0.470158	-0.75666	0.449252	0.856862
dhcr24	98.36573	0.443046053	0.201972	2.193604	0.028264	0.298982
zgc:172271	136.0471	-0.627365923	0.439948	-1.426	0.153868	0.618475

wbp11a	105.6978	0.035273045	0.180683	0.195221	0.84522	0.976791
tars	1107.201	0.222166144	0.099905	2.223774	0.026164	0.286896
hsf4	76.11099	0.300808392	0.388193	0.774895	0.438402	0.851685
tmc5	70.34356	-0.022510876	0.303388	-0.0742	0.940853	0.997371
colec11	82.17285	-0.877651987	0.419748	-2.0909	0.036537	0.335935
eif4eb	268.7647	0.296808287	0.12398	2.393999	0.016666	0.228896
znf76	139.8091	-0.139979957	0.196293	-0.71312	0.475774	0.869292
tmem179aa	149.131	0.52515317	0.230814	2.275222	0.022893	0.267028
crispld1b	182.1796	0.389601907	0.202441	1.924525	0.054289	0.402961
slc2a3a	782.3283	-0.773914051	0.144596	-5.35223	8.69E-08	4.38E-05
gpx8	232.7288	-0.220420486	0.183424	-1.2017	0.22948	0.711596
rpl19	28479.32	-0.407411723	0.114951	-3.54422	0.000394	0.023515
map3k15	246.2137	0.058966529	0.192423	0.306442	0.759268	0.957886
cadps2	128.5051	0.093776707	0.201474	0.465453	0.641607	0.925586
pygmb	2020.455	-0.070553007	0.191987	-0.36749	0.713254	0.945821
ttc5	252.9809	-0.047814197	0.135975	-0.35164	0.725109	0.948653
ndufa10	2928.681	0.001202446	0.118869	0.010116	0.991929	1
anxa6	1166.8	0.690074095	0.146706	4.703798	2.55E-06	0.00061
kif26ba	453.7617	0.239500598	0.180657	1.325721	0.184932	0.662268
tasorb	783.7742	0.122669526	0.142733	0.859433	0.390102	0.827902
cirbpb	31819.57	-0.090047809	0.112455	-0.80074	0.423279	0.843894
sh3gl3a	263.7507	0.368596529	0.13996	2.63359	0.008449	0.156983
isoc2	280.1038	-0.153308278	0.198401	-0.77272	0.439688	0.852079
ppp6r3	1350.461	0.04373186	0.114356	0.382419	0.702151	0.943292
cwc15	80.74883	0.530071869	0.221685	2.391105	0.016798	0.230026
gabra4	9.800422	0.604807261	0.817086	0.7402	0.459179	0.861818
CABZ01088367.1	10.97346	1.649886454	0.685209	2.407858	0.016046	0.223838
guca1b	26.45703	1.055418542	1.683642	0.626866	0.530747	0.890545
bnip2	340.2526	-0.048645185	0.135159	-0.35991	0.718915	0.94694
bmp2a	116.7779	0.037733143	0.165451	0.228062	0.819598	0.971785
lin7a	605.1903	-0.155803982	0.157561	-0.98885	0.322738	0.782609
lmna	392.7807	0.092275691	0.126768	0.727907	0.46667	0.865643
slc8a1a	28.23022	0.116261219	0.374898	0.310115	0.756474	0.956532
bhmt	68375.44	-0.305574389	0.183377	-1.66637	0.095639	0.516418
hira	495.9396	0.076213628	0.139489	0.546376	0.584807	0.906655
sycp3	3.484658	2.98002908	1.368921	2.176919	0.029487	0.305685
hey2	92.08665	0.230595869	0.207705	1.11021	0.266908	0.743418
atp6v1ba	2373.127	0.030600869	0.068229	0.448504	0.653789	0.929269
tdrd9	26.39562	-0.46069148	0.419234	-1.09889	0.271817	0.748006
mtrex	632.7202	0.043320797	0.084641	0.511819	0.608778	0.915204
wfikkn2a	131.9475	0.142836812	0.216638	0.659333	0.509682	0.883733
supt7l	65.10795	-0.409382006	0.28023	-1.46088	0.144048	0.603144
nedd1	289.2233	-0.05651349	0.14839	-0.38084	0.703319	0.943622
cct4	7459.11	-0.118040198	0.132675	-0.8897	0.373628	0.820566
arhgef39	63.49233	-0.146213911	0.386222	-0.37857	0.705004	0.944016
gata1a	48.82347	-0.023292402	0.330517	-0.07047	0.943817	0.997371
rrp15	252.5285	-0.147755553	0.175096	-0.84385	0.398751	0.830925
map3k9	25.23859	0.582056005	0.450557	1.291858	0.196406	0.67688
zbtb34	26.09579	-0.220140189	0.352604	-0.62433	0.532413	0.890978
abca2	239.1189	0.18732273	0.191381	0.978793	0.327682	0.786438
ube2kb	773.1961	-0.062400551	0.125087	-0.49886	0.617881	0.916478
pck1	3815.738	-0.959026587	0.359541	-2.66737	0.007645	0.147606
cyp2ae1	26.47329	0.606986546	0.335695	1.808151	0.070583	0.453373
svep1	367.7897	0.115826729	0.170699	0.678542	0.497428	0.878744

mcm9	44.7202	0.314772144	0.369977	0.850789	0.394887	0.828825
hoxb4a	189.259	-0.19877553	0.151883	-1.30874	0.190622	0.670716
ikzf1	51.63603	0.053460136	0.263184	0.203128	0.839035	0.976417
lpgat1	396.349	0.15401256	0.141576	1.087843	0.276665	0.751239
pgm1	2710.104	-0.133144632	0.114327	-1.16459	0.244183	0.724792
rfx2	332.0062	0.161158108	0.169598	0.950235	0.341993	0.79678
gadd45bb	168.2934	-0.457141265	0.300361	-1.52197	0.128016	0.577144
csnk2a2b	554.4462	0.068559914	0.12193	0.562291	0.573917	0.904296
brd1a	329.3713	-0.015986924	0.149397	-0.10701	0.914782	0.992996
tnfb	37.21623	-0.226603103	0.525534	-0.43119	0.666333	0.932664
sema3gb	253.3451	0.135617886	0.139986	0.968795	0.332647	0.789807
anxa13l	218.5559	0.390820784	0.140117	2.789244	0.005283	0.121922
pbx3b	1268.25	0.178094903	0.157796	1.128639	0.25905	0.736127
vdac2	22497.81	-0.188069934	0.101222	-1.85799	0.063171	0.429313
cd164	1580.166	-0.343666456	0.096674	-3.55491	0.000378	0.022825
cntn3a.2	4.613495	1.020955675	0.905007	1.128119	0.25927	0.736127
casr	0.857277	2.577022989	2.3233	1.109208	0.26734	NA
adgrl4	66.51369	-0.219711967	0.218882	-1.00379	0.31548	0.777872
tpd52l2b	1679.779	0.040380844	0.107035	0.377268	0.705974	0.944512
brox	325.9011	-0.230795448	0.137134	-1.683	0.092376	0.508314
tbck	213.8637	0.161476505	0.170369	0.947807	0.343228	0.797757
napba	227.129	0.437145293	0.211961	2.062382	0.039171	0.346149
hyou1	662.2223	0.337387987	0.125742	2.683174	0.007293	0.144668
si:dkey-42i9.6	1279.448	-0.174967835	0.106197	-1.64758	0.09944	0.523641
vsig8b	42.5866	-0.079331642	0.299321	-0.26504	0.79098	0.964766
cilp2	114.7619	0.35796776	0.279973	1.278582	0.201044	0.681045
stimate	209.3492	-0.057391905	0.14855	-0.38635	0.69924	0.942499
cers2a	69.18299	0.233142099	0.268169	0.869385	0.384636	0.826588
ccm2	176.5287	-0.667900655	0.148334	-4.50267	6.71E-06	0.001322
usp9	2303.51	0.083528874	0.142283	0.58706	0.557163	0.899165
zgc:77486	293.657	0.294382853	0.201868	1.458291	0.14476	0.603718
gtse1	167.8755	0.390039491	0.175376	2.22402	0.026147	0.286896
g6pca.2	1785.853	-0.088451485	0.239762	-0.36891	0.712192	0.945821
ap4b1	204.8013	0.033863704	0.164024	0.206456	0.836435	0.976007
srsf6a	1384.585	0.030037223	0.103236	0.290956	0.771085	0.960484
slc4a4a	987.3089	-0.333609727	0.158541	-2.10424	0.035357	0.330833
vta1	597.7856	-0.04414592	0.119591	-0.36914	0.712023	0.945821
polr3glb	226.848	-0.179467008	0.150057	-1.19599	0.231699	0.713202
lancl1	325.0836	0.064116535	0.135452	0.473351	0.635963	0.92471
slc25a55b	767.0385	-0.264102101	0.223542	-1.18144	0.237427	0.718568
eno1b	506.4194	0.675802294	0.215678	3.133388	0.001728	0.064499
tnni2a.3	67.29855	0.645159416	0.49342	1.307526	0.191034	0.67094
actn3a	25711.07	0.108643395	0.197477	0.550157	0.582211	0.90571
rrbp1a	747.1441	0.276662157	0.100608	2.749911	0.005961	0.13073
ctss2.2	164.7182	-0.618641094	0.207502	-2.98137	0.00287	0.088299
si:dkey-246g23.4	28.45672	-1.58715324	0.450138	-3.52593	0.000422	0.024691
thop1	707.6768	0.195286299	0.10852	1.799541	0.071933	0.4564
ppp1r13l	579.137	0.085396309	0.111344	0.766958	0.443106	0.854063
zgc:77158	177.2571	-0.121697968	0.203452	-0.59817	0.54973	0.897373
gucy1a1	146.5586	-0.397509835	0.263901	-1.50628	0.131995	0.584317
sim1b	11.12832	0.096204461	0.686633	0.14011	0.888573	0.988188
klf11b	439.0974	-0.093894926	0.250661	-0.37459	0.707966	0.945335
hps4	127.8967	-0.202969493	0.166724	-1.2174	0.223453	0.70531
snrpd3	0.176868	0	5.267649	0	1	NA

pcid2	325.8824	0.129718097	0.166493	0.779122	0.435908	0.850596
capns1b	2100.017	0.058101027	0.113928	0.509982	0.610064	0.915331
fam219ab	167.4125	0.169499658	0.279958	0.605447	0.544882	0.895206
anapc16	779.8393	-0.143677843	0.182674	-0.78653	0.431559	0.848419
snx13	87.50935	-0.051184981	0.205475	-0.24911	0.803279	0.968443
osbpl2b	343.5658	0.102658364	0.138925	0.738949	0.459938	0.862369
arhgef3	29.41926	0.015360016	0.325363	0.047209	0.962347	0.999461
spo11	2.943551	-0.8546605	1.092224	-0.7825	0.433923	0.850002
sulf2b	1345.43	-0.126716517	0.120252	-1.05376	0.291995	0.761655
aldh3b1	168.6412	-0.180704877	0.181762	-0.99418	0.320134	0.780709
abl2	299.6726	-0.0797843	0.137136	-0.58179	0.560709	0.899982
tbc1d19	71.85352	-0.202970529	0.246755	-0.82256	0.410759	0.837567
sept5a	534.3476	0.1895134	0.154442	1.227087	0.21979	0.700776
ttc14	378.7089	0.321807422	0.236578	1.360258	0.173748	0.646897
egfra	291.0422	-0.147322658	0.182648	-0.80659	0.4199	0.842383
lmo4a	420.6685	-0.114817081	0.135079	-0.85	0.395325	0.829083
slc12a3	111.9195	0.917986401	0.672969	1.364084	0.172541	0.645319
amy2a	28514.23	-2.23426862	1.142119	-1.95625	0.050436	0.39074
cdc14aa	22.96467	0.232653734	0.495846	0.469205	0.638923	0.924779
ptpmt1	167.0181	-0.131381446	0.170213	-0.77186	0.440194	0.852079
armc1	321.2393	-0.0177303	0.127234	-0.13935	0.889172	0.988253
fam133b	940.0866	0.00104014	0.114554	0.00908	0.992755	1
slc5a1	208.7493	-2.36319945	0.828769	-2.85146	0.004352	0.108751
spata20	258.5271	-0.936517682	0.208022	-4.50202	6.73E-06	0.001322
pi4k2b	263.2883	-0.195116008	0.121455	-1.60649	0.108166	0.54011
sorl1	533.84	-0.118010161	0.158875	-0.74279	0.457612	0.860993
si:ch211-253p14.2	1.507818	-0.106655893	1.40189	-0.07608	0.939355	NA
frya	774.0436	-0.092068625	0.202868	-0.45383	0.649948	0.927756
slc16a9a	211.5221	-0.091289251	0.259829	-0.35134	0.72533	0.948653
plekhb2	375.7213	0.037763247	0.140537	0.268707	0.788155	0.963467
eif3m	6538.906	-0.356489726	0.143742	-2.48007	0.013135	0.199185
XRCC2	10.98196	0.627236473	0.633405	0.990262	0.322046	0.782237
psmb3	1554.609	0.15740471	0.154603	1.018124	0.308619	0.773546
ivns1abpb	1068.275	-0.211097303	0.176124	-1.19857	0.230694	0.712405
pdf	98.11386	-0.010723082	0.211981	-0.05059	0.959656	0.999164
slc31a1	475.3114	-0.099553035	0.114774	-0.86738	0.385731	0.826939
mipb	1182.256	0.153761042	0.412447	0.372802	0.709296	0.945546
cul2	587.6968	-0.109729325	0.107858	-1.01735	0.308986	0.773791
psma6b	1146.406	-0.014694881	0.160889	-0.09134	0.927226	0.994973
psap	6036.288	-0.274573063	0.135063	-2.03293	0.04206	0.358807
pex3	162.533	0.551755	0.177003	3.117213	0.001826	0.065787
anxa13	1425.939	0.166845604	0.10555	1.580726	0.113941	0.552686
ndfip1	1432.807	0.070913335	0.114543	0.619096	0.535853	0.892102
ube2q2	922.1142	0.064862405	0.100658	0.644382	0.519327	0.887148
ern1	215.3496	0.136819604	0.253771	0.539145	0.589787	0.908601
stard8	316.0319	0.08391737	0.110142	0.761899	0.44612	0.855403
eif2b4	981.9909	-0.23389638	0.145943	-1.60265	0.109012	0.541807
lrrc45	161.837	0.289105092	0.169723	1.70339	0.088495	0.499597
cwc22	447.9111	0.052681016	0.107934	0.488085	0.625489	0.920625
lbr	868.5713	0.219354786	0.128917	1.701514	0.088846	0.500363
pdia7	838.6801	0.082952451	0.128799	0.644048	0.519545	0.887148
rrm1	1716.634	0.335980551	0.207228	1.621311	0.104951	0.533388
lhx1a	536.4798	-0.073006984	0.203168	-0.35934	0.719339	0.94694
ms4a17a.4	3.657338	2.508036449	1.599806	1.567713	0.116948	0.557831

abcc2	816.2611	0.220176823	0.264423	0.832668	0.405032	0.834284
si:dkeyp-93d12.1	13.56997	-0.11788821	0.605559	-0.19468	0.845646	0.976945
smarcad1a	337.2591	0.268995702	0.323487	0.831549	0.405663	0.834645
cldn7b	1660.862	-0.094393509	0.14723	-0.64113	0.521437	0.887551
ngfb	78.65056	0.05012679	0.208282	0.240667	0.809813	0.969828
olfm1b	818.0634	0.039602288	0.111946	0.353761	0.723518	0.947917
gabrr2b	23.82547	-0.438023132	0.412442	-1.06202	0.288225	0.759229
rab36	27.99913	0.602295822	0.407811	1.476901	0.139702	0.598033
cldn5b	369.7551	-0.438171082	0.225526	-1.94288	0.052031	0.396487
pgam1b	1405.572	0.142602727	0.165228	0.863068	0.3881	0.827592
fam184a	224.7456	0.239158576	0.222487	1.074932	0.282405	0.754744
wdr82	864.1373	0.314732872	0.126496	2.488095	0.012843	0.197722
atp6v1e1a	329.7562	-0.373209181	0.162138	-2.3018	0.021346	0.258949
osr1	250.7587	-0.168805718	0.16235	-1.03977	0.298448	0.765446
pkd2	177.979	0.274436434	0.234088	1.172364	0.241051	0.722623
pyroxd2	357.6373	0.27963033	0.122369	2.285144	0.022304	0.264074
dkk1a	60.49338	-0.09589933	0.239292	-0.40076	0.688595	0.939118
cfl2	2381.672	0.259900721	0.134843	1.927435	0.053925	0.402007
wasla	503.8697	-0.001340847	0.120091	-0.01117	0.991092	1
picalmb	897.8248	0.07060387	0.096443	0.732076	0.464122	0.864593
acot8	68.74612	-0.111205516	0.289584	-0.38402	0.700965	0.942918
srp72	1129.421	0.001852437	0.130783	0.014164	0.988699	1
med22	241.2049	-0.117894131	0.165454	-0.71255	0.476125	0.869292
mtdha	1555.831	-0.033815186	0.100015	-0.3381	0.735286	0.951543
ercc4	164.7413	0.333801147	0.169154	1.973353	0.048455	0.383271
ssr3	2272.408	-0.208489535	0.141243	-1.47611	0.139915	0.598455
CABZ01041610.1	12.13014	0.604742572	0.593143	1.019557	0.307939	0.772996
bloc1s1	231.6925	-0.179665205	0.180838	-0.99351	0.320461	0.780821
pfkma	1724.558	0.072558449	0.247779	0.292835	0.769648	0.960484
ildr2	199.8714	-0.148692432	0.176659	-0.84169	0.39996	0.831277
foxp1b	264.0244	0.138976895	0.17164	0.809702	0.418112	0.84138
sst2	49.83154	-0.358702395	0.385239	-0.93112	0.351794	0.802513
myl1	34783.98	0.536946667	0.182415	2.943541	0.003245	0.093414
otpa	561.795	0.186648573	0.244643	0.762942	0.445498	0.855403
zgc:55413	12.76448	-0.090122387	0.487164	-0.18499	0.853234	0.978839
odf3l2a	27.51742	0.03270077	0.402271	0.08129	0.935211	0.996829
cdh13	486.6756	0.884756422	0.205164	4.312428	1.61E-05	0.002576
mecp2	34.67485	0.449901984	0.400672	1.122869	0.261493	0.738975
zgc:86598	1575.374	-0.009583611	0.100301	-0.09555	0.923879	0.994713
dlst	5186.481	0.054549932	0.133761	0.407815	0.683409	0.938291
si:dkey-121j17.5	26.48827	0.928289706	0.633812	1.464614	0.143026	0.602002
sept8b	194.233	-0.044135324	0.236245	-0.18682	0.851801	0.978558
esyt1b	160.278	-0.575759049	0.276131	-2.0851	0.037061	0.337668
rbmx	1559.712	0.007958049	0.084231	0.094479	0.924728	0.994815
jag2a	36.209	0.146221237	0.264944	0.551895	0.581021	0.905618
pdlim3b	123.1624	-0.360302963	0.185269	-1.94476	0.051804	0.395809
eya1	749.2579	0.151965655	0.114574	1.326355	0.184722	0.661987
camk2d2	662.9439	0.141068649	0.177387	0.795259	0.426463	0.84512
rfc2	241.9332	0.111626136	0.171558	0.650663	0.515264	0.886401
dlgap1a	15.20303	1.29861982	0.604794	2.147211	0.031776	0.317081
eno2	822.1619	0.527820412	0.245349	2.151304	0.031452	0.315339
med15	245.863	-0.287338365	0.154276	-1.8625	0.062533	0.427724
brinp2	67.24614	0.311333666	0.339063	0.918217	0.358506	0.80995
vac14	271.0606	0.315968804	0.152794	2.067941	0.038646	0.344438

spaw	1.92711	0.670789936	1.185675	0.565745	0.571567	NA
pgghg	151.4767	0.190235891	0.30558	0.622541	0.533586	0.891181
atp5pf	2906.137	0.013461859	0.125152	0.107564	0.914342	0.992972
gucy2c	122.7811	-0.574448147	0.301956	-1.90242	0.057116	0.41265
en1a	108.8267	-0.00186491	0.218195	-0.00855	0.993181	1
sh2d5	61.83035	0.594569607	0.269586	2.205488	0.02742	0.293857
npm1a	1972.757	-0.270564459	0.162745	-1.6625	0.096412	0.518297
ormdl1	635.2498	-0.36721853	0.159726	-2.29905	0.021502	0.260159
rab11al	157.271	0.159154009	0.173304	0.918354	0.358434	0.809919
stk17b	200.0698	0.071129473	0.144242	0.493127	0.621923	0.918461
krt95	1.135056	0	2.762817	0	1	NA
amh	12.38091	0.88689199	0.472145	1.878431	0.060322	0.422591
optc	25.97377	0.101963322	0.358015	0.284801	0.775796	0.961073
tmbim4	876.0928	0.033109828	0.112145	0.295242	0.767809	0.960296
luc7l3	779.0108	-0.260367065	0.145054	-1.79496	0.07266	0.457864
ddx4	71.40374	0.529996388	0.387709	1.366996	0.171627	0.643878
slc12a4	386.4425	-0.160736156	0.135436	-1.18681	0.235304	0.716329
galnt6	252.3084	0.314290542	0.176425	1.781437	0.074841	0.464151
selenoo2	12.4867	-0.629297024	0.510196	-1.23344	0.217411	0.69815
smc1b	6.465414	0.766888199	1.042733	0.73546	0.46206	0.863402
elavl3	1256.574	0.227048535	0.137324	1.653379	0.098254	0.521076
mxe	4.704868	-1.485552216	1.404547	-1.05767	0.290204	0.76078
ppp2r5d	624.7228	-0.024294884	0.10777	-0.22543	0.821642	0.971794
rabgef1	468.2805	0.12856124	0.103017	1.247963	0.212045	0.693739
dgkza	324.4294	0.173514226	0.134404	1.290994	0.196706	0.677117
rab3c	214.0973	0.092643052	0.153901	0.601964	0.547198	0.896121
usf1l	572.5397	-0.124568696	0.133644	-0.93209	0.351289	0.802305
arhgef25b	233.5528	0.068279039	0.151175	0.451657	0.651516	0.928562
yipf1	248.349	-0.031457685	0.140398	-0.22406	0.82271	0.971966
sstr3	6.35842	0.495688072	0.968632	0.51174	0.608833	0.915204
ptf1a	65.35783	-0.088928062	0.30514	-0.29143	0.77072	0.960484
nr6a1b	27.62005	0.032490656	0.451691	0.071931	0.942657	0.997371
ca2	608.8914	0.046227113	0.151154	0.305827	0.759736	0.957886
tac1	142.5963	-0.006687087	0.245346	-0.02726	0.978256	1
trpv6	314.1473	-0.017811297	0.202031	-0.08816	0.929749	0.995576
ythdf2	447.4564	-0.01240857	0.134138	-0.09251	0.926296	0.994815
nutf2l	535.5963	-0.113730752	0.131933	-0.86203	0.38867	0.827613
tmem126a	374.2989	-0.30447836	0.182271	-1.67047	0.094827	0.513949
usp5	1700.029	0.16592589	0.115899	1.43164	0.152247	0.616018
cdh6	742.448	0.025095836	0.148099	0.169453	0.86544	0.982279
pdk3a	61.00967	0.292149813	0.256585	1.138609	0.254866	0.733542
atg4a	253.9146	-0.294159444	0.136241	-2.15911	0.030842	0.312738
aida	185.3175	0.0928934	0.175672	0.52879	0.596951	0.91176
pfdn1	552.8471	0.115647599	0.119802	0.96532	0.334384	0.791508
golim4b	85.28593	0.205816556	0.223136	0.922381	0.35633	0.807306
rfx3	123.7267	0.390097294	0.206191	1.891921	0.058501	0.418172
ppp1r3cb	673.3223	-0.326636624	0.294833	-1.10787	0.267917	0.74434
serpinb1l3	919.0361	0.037459619	0.225348	0.16623	0.867976	0.982801
eif4e1b	1.410696	-1.390573248	1.783159	-0.77984	0.435487	NA
hnrnpr	1582.69	0.139692037	0.098334	1.420587	0.155437	0.620161
ctnnb1	7387.907	-0.017100633	0.084916	-0.20138	0.840399	0.976417
chpt1	1115.369	0.054061036	0.103449	0.522587	0.601262	0.913756
rhpn2	413.8454	-0.074062182	0.153121	-0.48368	0.62861	0.921946
exoc3	577.843	0.010881215	0.119562	0.091009	0.927486	0.994973

slc38a5b	2566.586	-0.153610515	0.158295	-0.97041	0.331843	0.789531
adcy2b	213.2279	-0.250725129	0.169233	-1.48154	0.138463	0.595018
ilf2	2500.276	0.129410837	0.116752	1.108426	0.267678	0.744014
dennd1a	95.65748	0.121827722	0.263294	0.462707	0.643575	0.925997
slc5a6b	51.72556	0.370995444	0.283426	1.308969	0.190545	0.670655
si:ch211-212d10.1	15.19717	1.083985308	1.305217	0.830502	0.406255	0.834645
si:dkeyp-94g1.1	4.948111	-3.175442349	1.377034	-2.306	0.021111	0.25794
dlx3b	607.4528	-0.122889287	0.090718	-1.35463	0.175535	0.649391
mbtps1	851.6365	0.036208229	0.101012	0.358454	0.720003	0.947004
aoc2	1888.28	-0.143740529	0.170537	-0.84287	0.399301	0.831196
svilb	72.79627	-0.31666361	0.289413	-1.09416	0.273886	0.749234
ankrd13b	83.89304	0.181974439	0.245855	0.740169	0.459197	0.861818
itpr1a	163.1808	-0.112521704	0.163345	-0.68886	0.490911	0.875595
zgc:171731	3.276591	-4.197385362	1.610918	-2.60559	0.009172	0.164481
colgalt2	268.884	0.054470008	0.203484	0.267687	0.78894	0.963568
fzd9b	318.2812	0.00387238	0.122521	0.031606	0.974786	1
acsl3b	753.1963	-0.044976468	0.116267	-0.38684	0.698877	0.942456
efna3a	116.4206	0.090167781	0.227101	0.397038	0.691339	0.940173
bckdhb	1142.895	-0.034191269	0.12151	-0.28139	0.778414	0.961073
znf710a	286.0994	0.106813389	0.179505	0.595043	0.551815	0.8974
tmem26b	9.09188	-0.494459956	1.097285	-0.45062	0.652262	0.928768
esco2	368.1371	0.236063793	0.228442	1.033364	0.301434	0.767562
rps4x	30131.49	-0.372196026	0.111132	-3.34913	0.000811	0.038177
znf622	590.7441	-0.062087467	0.1355	-0.45821	0.646802	0.926728
pgm2l1	101.4278	-0.191510224	0.20125	-0.9516	0.341299	0.796175
dync1h1	725.1532	0.111167071	0.26317	0.422416	0.672722	0.93414
her1	0.757682	2.01026229	2.724716	0.737788	0.460643	NA
acox1	574.3843	0.251073991	0.153303	1.637761	0.101471	0.527632
cacybp	348.6617	-0.058326117	0.138192	-0.42207	0.672976	0.93414
rbfox1	1603.351	0.125303133	0.119888	1.045172	0.295944	0.764246
mfsd6l	34.42666	-0.009729583	0.339215	-0.02868	0.977118	1
arf2a	4759.758	0.096737612	0.09559	1.012002	0.311537	0.77613
usp4	990.3348	0.137110241	0.147126	0.931925	0.351376	0.802305
zgc:113220	106.6634	0.059694296	0.185511	0.321783	0.747617	0.955074
g3bp2	640.0707	-0.109637815	0.113644	-0.96475	0.334672	0.791583
lrrc4bb	205.5473	0.071960796	0.238542	0.301669	0.762904	0.958842
ehd1b	909.9228	0.228028018	0.154837	1.472699	0.140832	0.599658
uqcrc2a	4548.32	-0.060876491	0.126978	-0.47942	0.631637	0.923003
wnt11r	1024.226	0.042047462	0.130653	0.321825	0.747585	0.955074
cryba1l2	175.8517	-0.541052782	0.293131	-1.84577	0.064925	0.434811
cacna2d1a	286.2823	-0.083423812	0.22155	-0.37655	0.706511	0.944683
fhl5	78.7057	0.829689204	1.072697	0.773461	0.43925	0.852079
hacd2	957.8281	-0.248729249	0.161199	-1.54299	0.122832	0.569468
ranbp1	2190.347	0.038063612	0.119511	0.318495	0.750109	0.955074
si:dkeyp-69b9.6	720.5011	0.05738629	0.104814	0.547504	0.584032	0.906376
l3mbtl1a	123.6957	0.449103211	0.283828	1.582305	0.11358	0.551824
prph2b	1353.219	1.08966324	0.741799	1.468947	0.141847	0.600644
pih1d2	11.26951	0.193907855	0.571622	0.339224	0.734441	0.951134
reep1	19.96384	-0.194899375	0.434612	-0.44844	0.653832	0.929269
drd2l	2.299947	-0.942856816	1.419511	-0.66421	0.506554	0.883007
hprt1l	238.9898	0.051416783	0.176704	0.290977	0.771069	0.960484
rpl8	38059.64	-0.318236173	0.123453	-2.5778	0.009943	0.172469
syngr3a	517.7726	-0.134009531	0.178079	-0.75253	0.451733	0.857676
abi2b	784.8771	0.183930831	0.11673	1.575698	0.115095	0.554651

robo2	579.436	0.086592767	0.176694	0.490072	0.624083	0.919497
ncbp2	321.1423	-0.007771062	0.141883	-0.05477	0.956321	0.99899
htra1b	324.7731	0.221453589	0.231215	0.957781	0.338173	0.795076
panx1b	115.0638	-0.088341509	0.20027	-0.44111	0.659132	0.930581
lamp2	683.8213	0.178694185	0.132031	1.353426	0.17592	0.649415
ndufab1b	1305.644	0.05988463	0.125874	0.475752	0.634251	0.924077
slc10a2	39.55385	-0.836706772	0.620372	-1.34872	0.177428	0.651026
uts1	35.01508	-0.091277172	0.462986	-0.19715	0.843711	0.976791
p2ry11	11.31301	-0.018640395	0.562426	-0.03314	0.973561	1
KCNN2	16.19863	0.27627632	0.50723	0.544676	0.585976	0.906655
tprkb	52.31105	-0.297673777	0.317958	-0.9362	0.349168	0.801408
kif23	527.4979	0.353900402	0.229217	1.543957	0.122599	0.569468
znf598	561.6211	0.160895348	0.1368	1.176139	0.239539	0.720048
angpt2a	21.79114	0.234437426	0.560602	0.418188	0.675809	0.935422
svopb	2.391439	-0.370137341	1.517351	-0.24394	0.80728	0.969736
cnih3	66.2901	-0.280083144	0.36286	-0.77188	0.440188	0.852079
snx33	84.87223	0.195397846	0.237919	0.821279	0.411487	0.837797
diablob	375.982	-0.069897487	0.173167	-0.40364	0.686476	0.938732
ppp3cca	316.5806	0.084459778	0.15621	0.540682	0.588727	0.908018
triap1	131.2762	0.060951727	0.190663	0.319683	0.749208	0.955074
ccdc82	294.5371	0.01300659	0.155725	0.083523	0.933436	0.996472
g6pcb	3.37831	0.826816025	1.306972	0.63262	0.526982	0.889861
ankhb	311.1029	0.033315135	0.148724	0.224007	0.822752	0.971966
ntng1a	104.5745	0.323867413	0.384862	0.841516	0.400059	0.831408
irge1	5.983475	0	1.831379	0	1	1
lims2	699.1119	0.078529622	0.17364	0.452255	0.651085	0.928395
acvr1l	983.8573	-0.006499617	0.087888	-0.07395	0.941047	0.997371
tbp	276.6742	-0.043772829	0.131549	-0.33275	0.739324	0.953422
jagn1b	366.3792	0.014659579	0.136313	0.107543	0.914358	0.992972
cdh4	345.2851	0.388973398	0.259917	1.496528	0.134516	0.588953
arhgap4b	382.502	0.177709933	0.148041	1.200412	0.22998	0.711763
dnm1l	946.2759	0.184028934	0.095626	1.924466	0.054296	0.402961
ccdc114	50.6078	0.309905347	0.30687	1.009893	0.312547	0.776164
tspan18b	602.7269	-0.120659845	0.099708	-1.21014	0.226227	0.707597
kif26ab	529.0824	-0.034730565	0.17156	-0.20244	0.839573	0.976417
l1camb	128.6998	-0.026691757	0.217486	-0.12273	0.902322	0.990938
slc23a1	49.9069	-0.041368128	0.351372	-0.11773	0.906279	0.991631
calm2b	8306.664	0.058037187	0.142909	0.406112	0.684661	0.938486
grip1	374.5813	0.125015933	0.139172	0.898284	0.369034	0.817763
ube2d4	40.55627	0.251989384	0.287918	0.875213	0.381458	0.82463
daam1a	712.1689	-0.279125357	0.115665	-2.41323	0.015812	0.221904
esrrd	8.880395	-1.077448169	0.657329	-1.63913	0.101186	0.526828
c4	24.25468	-3.233624263	0.76505	-4.22669	2.37E-05	0.003251
txk	1.832644	0	2.605311	0	1	NA
pola2	258.0031	-0.002282269	0.177391	-0.01287	0.989735	1
dmrt2a	242.7441	0.115559746	0.143743	0.803933	0.421436	0.842754
gja8b	84.82148	0.033479224	0.364201	0.091925	0.926757	0.994815
dnajb11	601.7387	0.201672903	0.170531	1.182616	0.236962	0.718451
slc18a2	12.312	-0.562164326	0.461013	-1.21941	0.222689	0.704588
ddx39ab	3935.201	0.12087662	0.121044	0.998614	0.317982	0.779809
dnase1l4.1	724.7296	-0.767689354	0.210245	-3.65141	0.000261	0.017773
gorasp2	877.9903	0.157370502	0.127634	1.232987	0.217581	0.69815
rpl27	19189.88	-0.276010821	0.132366	-2.0852	0.037051	0.337668
camkk1b	103.4408	0.086553991	0.597844	0.144777	0.884887	0.987266

sh2d4bb	10.72155	-0.204060866	0.634908	-0.3214	0.747906	0.955074
wasa	22.36213	-0.041345592	0.401415	-0.103	0.917963	0.993875
wdr91	179.253	0.006666561	0.195251	0.034144	0.972763	1
fbln2	576.1534	-0.218358939	0.145201	-1.50383	0.132624	0.585592
slc50a1	46.03827	-0.221242644	0.270019	-0.81936	0.412581	0.838123
zgc:92664	300.2461	0.119374209	0.153145	0.779487	0.435693	0.850467
mknk2b	4147.5	-0.010332147	0.203042	-0.05089	0.959416	0.999164
atp6v0a1b	616.7778	0.096402683	0.228353	0.422165	0.672904	0.93414
ror1	174.6943	0.260173216	0.166397	1.563573	0.117918	0.559998
mpp3a	260.5629	-0.003737553	0.164702	-0.02269	0.981895	1
cblb	280.8912	-0.049472496	0.162458	-0.30452	0.760728	0.957886
pcmt	862.9612	0.031288172	0.106585	0.293552	0.7691	0.960484
hnrnpua	1064.095	0.235719042	0.116801	2.018127	0.043578	0.365542
rbb4l	927.7905	0.055229388	0.12714	0.434397	0.664	0.93174
nsrp1	189.2118	0.015413259	0.167328	0.092114	0.926608	0.994815
cbll1	140.6708	0.064848984	0.175939	0.368587	0.712435	0.945821
cd2ap	630.9397	-0.052035832	0.120468	-0.43195	0.66578	0.932664
ero1a	37.18038	-1.002874675	0.401727	-2.49641	0.012546	0.194767
pycr3	91.96014	-0.040946621	0.319806	-0.12804	0.898121	0.990171
prpf19	1176.752	0.084738958	0.112947	0.750257	0.4531	0.858335
cdkl5	162.7526	0.535432058	0.262209	2.042004	0.041151	0.353924
st3gal3b	103.2701	-0.092007261	0.194601	-0.4728	0.636357	0.924779
fzr1a	1036.046	-0.133295432	0.144785	-0.92065	0.357235	0.808077
adma	217.9124	-0.05243763	0.161421	-0.32485	0.745295	0.954956
mab21l2	1700.59	0.028132372	0.097831	0.287562	0.773682	0.960624
alpi.1	274.4383	0.231756637	0.124724	1.858159	0.063146	0.429313
plxnc1	97.32418	0.097597925	0.242139	0.403066	0.6869	0.93875
sacm1la	230.5147	0.054117338	0.163692	0.330605	0.740943	0.954103
ube2g1a	466.632	0.115347024	0.166198	0.694034	0.487661	0.873291
fam110a	65.7667	-0.215775285	0.257764	-0.8371	0.402534	0.833401
pitpnbl	1168.887	0.058796691	0.104764	0.561232	0.57464	0.904516
klf12a	251.2394	0.165665761	0.164283	1.008416	0.313255	0.776663
zc4h2	1591.654	0.143275754	0.133519	1.073075	0.283238	0.755028
psmc5	2857.052	0.052903532	0.145326	0.364033	0.715834	0.946196
u2af1	1498.156	-0.033671133	0.128581	-0.26187	0.793424	0.965366
comta	177.6348	0.481328578	0.181831	2.647126	0.008118	0.152796
pgd	1030.582	-0.086650049	0.189131	-0.45815	0.646846	0.926728
mfge8a	477.7927	0.004509561	0.209429	0.021533	0.982821	1
lsg1	541.919	-0.22567817	0.138776	-1.6262	0.103907	0.532096
fosl1a	279.1917	-1.045932528	0.571204	-1.8311	0.067085	0.442084
fcho1	130.3758	-0.521921737	0.20794	-2.50996	0.012074	0.190742
st3gal3a	308.1064	0.308324318	0.129067	2.388863	0.016901	0.230481
ndufs3	1697.089	0.019035425	0.103554	0.183821	0.854154	0.979016
dhx15	1517.68	-0.034374741	0.089544	-0.38388	0.701064	0.942918
thoc7	347.9495	-0.284848471	0.195361	-1.45806	0.144824	0.603718
foxf1	197.3304	-0.192178	0.169952	-1.13078	0.258147	0.735569
arl3l2	100.1218	-0.75530673	0.454916	-1.66032	0.09685	0.519447
ppil1	669.7791	-0.197363146	0.178241	-1.10728	0.268173	0.744507
slc24a4a	21.99865	0.136857535	0.378707	0.361381	0.717815	0.946466
hdac1	4217.982	0.166820159	0.118309	1.410036	0.158529	0.623937
lim2.4	1120.49	0.406024108	0.376282	1.079043	0.280568	0.753147
fut8a	707.685	0.281230895	0.105634	2.662316	0.00776	0.14885
racgap1	317.5108	0.25639055	0.249583	1.027274	0.304291	0.769725
chd1l	212.9921	0.179121805	0.147846	1.211545	0.225687	0.707068

gpc4	1253.927	0.251348438	0.104295	2.409966	0.015954	0.222818
ppp2r5ea	656.1144	0.09659065	0.113782	0.84891	0.395932	0.829389
iqch	25.59025	0.104745513	0.417416	0.250938	0.801862	0.967776
zgc:113518	143.1634	-0.028677743	0.184151	-0.15573	0.876246	0.984895
klf3	559.1289	-0.311465611	0.202918	-1.53493	0.124801	0.572486
adam10b	490.2973	0.156146693	0.186673	0.836471	0.40289	0.833479
pibf1	427.6254	0.238089366	0.148531	1.602961	0.108943	0.541793
mc4r	10.42331	-0.207144899	0.905307	-0.22881	0.819015	0.971785
prps1a	2651.481	-0.158934581	0.152208	-1.04419	0.296397	0.764246
CU929145.1	1.594151	-0.868144599	2.123316	-0.40886	0.682641	NA
slc4a8	218.2408	-0.008763622	0.304579	-0.02877	0.977046	1
sox6	654.6781	0.069617317	0.089937	0.77407	0.438889	0.851966
gad2	1046.474	0.347014586	0.273497	1.268808	0.20451	0.68614
znf207a	664.1183	-0.055500665	0.109598	-0.5064	0.612575	0.91573
psen2	420.9366	0.008153165	0.170236	0.047893	0.961801	0.999451
s100a1	16.08655	-0.584010101	0.544913	-1.07175	0.283833	0.755095
alpl	307.2333	-0.019969051	0.141172	-0.14145	0.887513	0.987974
fth1a	15721.29	-0.370110426	0.156622	-2.36308	0.018124	0.237918
phactr4a	456.3629	0.017435073	0.143601	0.121413	0.903364	0.99133
zic2a	894.6509	0.123736651	0.115587	1.070505	0.284392	0.755402
zgc:100829	687.241	-0.105076629	0.161208	-0.65181	0.514526	0.88631
fstl1a	678.4869	-0.091027508	0.153272	-0.59389	0.552583	0.8974
zgc:123305	224.3292	-0.171651125	0.191784	-0.89502	0.370776	0.818484
dnmt3ab	834.5114	0.064761058	0.129075	0.501732	0.615856	0.916365
zic1	870.7447	0.139760239	0.119885	1.165787	0.2437	0.724437
cyp3c1	857.5666	-0.048595641	0.125942	-0.38586	0.699603	0.942499
itprip	49.29164	-0.286617311	0.278503	-1.02914	0.303415	0.769363
ankrd33bb	6.07794	-1.376392774	1.542782	-0.89215	0.372313	0.819778
c1ql2	1.636289	-0.656025744	1.505687	-0.4357	0.663055	NA
si:ch211-220f21.2	10.10953	0.628028155	0.695328	0.903211	0.366414	0.81589
tubg1	401.4953	0.192871545	0.141138	1.366544	0.171768	0.643953
rasl11b	230.7677	-0.034565295	0.269828	-0.1281	0.898069	0.990171
ada2b	20.45225	-0.684305317	0.462075	-1.48094	0.138622	0.595394
rgs6	55.17765	0.096141635	0.25505	0.376953	0.706209	0.944606
psmd10	202.7188	-0.157131921	0.225065	-0.69816	0.485075	0.872911
gemin2	309.4513	-0.158081319	0.191012	-0.8276	0.407897	0.835879
rap2c	922.8794	0.127247201	0.108658	1.171076	0.241568	0.723306
tnip1	1561.239	-0.033404749	0.099098	-0.33709	0.73605	0.95169
ca15a	350.7618	-0.825324139	0.215279	-3.83374	0.000126	0.011039
zgc:77112	55.42281	-0.142669019	0.421412	-0.33855	0.734949	0.951434
pla2g12b	4083.831	-0.259741515	0.197405	-1.31578	0.188247	0.667434
msx3	591.6138	-0.102369408	0.147541	-0.69384	0.487783	0.873291
nmd3	776.9245	-0.24825764	0.200007	-1.24125	0.214514	0.69658
si:dkeyp-75b4.9	0	NA	NA	NA	NA	NA
gsk3ab	1329.565	-0.004881235	0.085153	-0.05732	0.954288	0.998611
bmp6	103.6881	-0.222850233	0.211325	-1.05454	0.291636	0.761454
dvl3a	415.5585	0.071552079	0.148456	0.481974	0.629824	0.92285
hsd17b12a	544.8865	-0.030784221	0.21392	-0.14391	0.885575	0.987533
kctd5a	357.3822	0.227240455	0.145552	1.561231	0.118469	0.561566
ano5a	68.90167	0.037991587	0.274208	0.13855	0.889806	0.98835
cax2	116.0416	-0.697250551	0.257132	-2.71164	0.006695	0.137643
aicda	0.046941	0	5.267649	0	1	NA
aarsd1	133.0635	0.268394554	0.208124	1.289591	0.197193	0.67737
hps3	222.3965	-0.055834652	0.136141	-0.41012	0.681716	0.937427

zap70	9.573242	-1.149816504	0.704049	-1.63315	0.102438	0.529087
tmem50a	1385.794	-0.271979045	0.115309	-2.3587	0.018339	0.239117
tspan7	1640.627	0.155945147	0.168971	0.922908	0.356055	0.807063
iah1	102.8837	-0.174202389	0.228804	-0.76136	0.446441	0.855403
ankrd46b	350.4453	0.091107589	0.152449	0.597627	0.550089	0.8974
atp1b3a	1749.338	-0.332498518	0.113695	-2.92447	0.00345	0.096904
creb3l1	225.6849	0.023130901	0.163984	0.141056	0.887826	0.988077
smcr8b	90.68058	-0.532482883	0.327826	-1.62428	0.104315	0.532541
cgnl1	596.5144	0.079546379	0.139186	0.571512	0.567652	0.901809
rab22a	316.0913	-0.349693628	0.111835	-3.12686	0.001767	0.064775
sesn3	902.982	-0.334827364	0.207718	-1.61193	0.106977	0.537
vps26b	505.9144	-0.143851827	0.14333	-1.00364	0.315553	0.77795
lemd3	480.4365	0.112513125	0.137008	0.821218	0.411522	0.837797
mov10a	363.2296	0.069803899	0.123198	0.566599	0.570987	0.902501
eif4e2rs1	363.8261	-0.067435977	0.109148	-0.61784	0.53668	0.892421
pnn	1158.384	-0.12578778	0.115523	-1.08886	0.276216	0.750674
chata	252.5712	0.01221946	0.253266	0.048248	0.961519	0.999434
slc25a40	139.3693	-0.010969919	0.178106	-0.06159	0.950888	0.99801
mtmr9	181.1755	0.281595794	0.159254	1.768216	0.077025	0.469486
rpl5b	25488.35	-0.444537912	0.135151	-3.2892	0.001005	0.044091
socs6b	255.8388	0.024504859	0.180277	0.135929	0.891878	0.988566
apoa2	74025.29	-0.178917062	0.234108	-0.76425	0.444719	0.854903
slc22a16	247.0121	-0.247610355	0.185641	-1.33381	0.182265	0.658775
npas1	78.44025	-0.06911672	0.245874	-0.28111	0.778629	0.961073
pax8	98.15023	-0.222858938	0.226825	-0.98252	0.325846	0.784773
b2ml	2029.621	-0.1527658	0.097598	-1.56526	0.117522	0.559019
zc3h15	693.038	0.000521499	0.117135	0.004452	0.996448	1
mafa	259.7259	0.066215995	0.139568	0.474437	0.635188	0.924272
ptprea	220.5178	0.290168994	0.132923	2.182978	0.029037	0.303472
ptbp2b	885.1849	0.147486534	0.092903	1.587531	0.112393	0.549251
stat6	285.7941	0.12586514	0.164727	0.764083	0.444818	0.85495
lztr1	614.8849	-0.08389147	0.135219	-0.62041	0.534988	0.891993
mxtx2	0.97443	-0.804745957	2.170229	-0.37081	0.710778	NA
dcbld1	209.6963	-0.066132928	0.140617	-0.4703	0.638138	0.924779
slc25a36b	495.833	-0.338803868	0.147966	-2.28974	0.022036	0.262782
rnft1	401.6773	-0.181085303	0.151377	-1.19626	0.231597	0.713198
dlat	1941.341	-0.048061979	0.106931	-0.44947	0.653093	0.929197
pwp1	371.1135	-0.087919838	0.167193	-0.52586	0.598987	0.913109
stk17al	207.3011	-0.21870649	0.197466	-1.10756	0.26805	0.744423
rwdd1	733.6057	0.189075085	0.131884	1.433648	0.151673	0.615406
snap91b	1006.89	0.365227951	0.226051	1.615691	0.106161	0.535194
kptn	288.3168	-0.380019303	0.179402	-2.11826	0.034153	0.326054
slc44a1a	224.0859	-0.141801178	0.171054	-0.82899	0.407112	0.835278
matn4	6243.48	0.220362937	0.146542	1.503757	0.132644	0.585592
cldnc	368.4546	-0.299209918	0.269609	-1.10979	0.267089	0.743418
zgc:153311	24.44479	-0.864221771	0.368757	-2.34361	0.019098	0.243268
yaf2	433.6031	0.118344249	0.107107	1.104914	0.269197	0.745285
rabgef1l	60.29621	-0.210755325	0.280472	-0.75143	0.452393	0.857994
phip	155.1827	0.085051635	0.184712	0.460456	0.645189	0.926728
rapgef1b	218.0371	-0.04989588	0.158427	-0.31494	0.752804	0.955756
COX5B	6969.017	-0.011968294	0.117483	-0.10187	0.918858	0.993892
abcc9	177.2256	-0.13112396	0.228991	-0.57262	0.566904	0.901747
zdhhc16b	185.2263	0.299741937	0.148182	2.022798	0.043094	0.36374
hcfc1a	1079.292	0.353423186	0.12372	2.856648	0.004281	0.108179

mfsd5	131.3168	-0.045784942	0.194856	-0.23497	0.814233	0.970483
wdhd1	185.4765	0.173558683	0.203741	0.851861	0.394291	0.828374
nup107	788.9083	0.124819971	0.095807	1.302826	0.192634	0.672683
gpcpd1	750.3615	0.157397043	0.143409	1.097541	0.272405	0.748416
dbn1b	748.3135	-0.089095454	0.105311	-0.84602	0.397539	0.830253
zic5	392.9148	0.124688705	0.122001	1.022028	0.306767	0.772
hacd3	362.4023	0.251210772	0.16639	1.50977	0.131102	0.582816
gins4	185.4537	-0.060552929	0.257293	-0.23535	0.81394	0.970483
gpat3	631.1643	0.047419724	0.107309	0.441898	0.658563	0.930581
mhc1laa	99.98055	0.120112087	0.215569	0.557186	0.5774	0.904842
fibpa	473.3707	-0.013856881	0.150751	-0.09192	0.926763	0.994815
rab5b	243.3281	0.188163435	0.181352	1.037557	0.299476	0.766128
tlr3	12.96499	-1.218322666	0.651115	-1.87113	0.061327	0.424867
cryz	217.4916	-0.298464974	0.159182	-1.87499	0.060794	0.423559
nob1	438.3212	-0.131115336	0.139483	-0.94001	0.347211	0.799992
cldn15la	376.5223	-0.351461969	0.189453	-1.85514	0.063577	0.430344
spsb4a	279.117	-0.113652703	0.128654	-0.8834	0.37702	0.822324
mbnl3	380.8458	0.194500088	0.14643	1.328276	0.184087	0.660978
smurf1	365.6537	0.073447082	0.230913	0.318072	0.75043	0.955074
rtn2a	167.7153	-0.339875565	0.280157	-1.21316	0.225068	0.70685
bicral	171.6669	0.146424072	0.149587	0.978854	0.327652	0.786438
qtrt2	97.89467	-0.077505602	0.229239	-0.3381	0.735288	0.951543
htr6	11.8023	-1.279120864	0.612377	-2.08878	0.036728	0.33671
bbs12	63.30777	-0.344368789	0.301683	-1.14149	0.253665	0.732873
exd2	202.6406	0.118891479	0.157854	0.753176	0.451344	0.857606
ptrhd1	125.1105	0.025272941	0.197469	0.127984	0.898162	0.990171
ap3m2	502.8918	0.079252899	0.108805	0.728394	0.466373	0.865508
keap1a	307.0967	-0.091028685	0.175154	-0.51971	0.603267	0.914226
nme6	67.60192	0.029158432	0.248614	0.117284	0.906635	0.991644
mkks	20.66672	0.032743122	0.379827	0.086205	0.931303	0.995809
slc6a2	143.1569	-0.058791074	0.258473	-0.22745	0.82007	0.971788
xiap	407.4002	-0.263544361	0.169112	-1.5584	0.119138	0.562753
dag1	1121.752	0.240258027	0.15492	1.550853	0.120937	0.566816
zfp36l1a	1709.081	0.084767305	0.134044	0.632386	0.527135	0.889948
fam53b	965.3155	-0.008611711	0.142513	-0.06043	0.951815	0.99801
gmpr	1405.695	-0.006115573	0.180599	-0.03386	0.972987	1
ccdc170	19.12514	0.644464235	0.407816	1.580283	0.114042	0.55294
usp33	650.1203	0.023321964	0.113364	0.205727	0.837004	0.976007
cct3	4744.792	-0.059004614	0.109902	-0.53689	0.591347	0.909437
eif4enif1	290.326	-0.270158459	0.18997	-1.42211	0.154993	0.619776
trim33	591.9096	-0.02926951	0.117248	-0.24964	0.802868	0.968323
SBNO2	326.6276	0.001369359	0.173891	0.007875	0.993717	1
trib3	564.6116	-0.188575371	0.188188	-1.00206	0.316315	0.778879
zgc:112408	12.32439	-0.643179716	0.44795	-1.43583	0.151051	0.615085
nup214	287.7732	0.057272103	0.204411	0.280181	0.779339	0.961073
zmp:0000001048	42.87014	-1.29603384	0.479355	-2.70371	0.006857	0.139692
mhc1lba	6.626517	0.674540518	0.842874	0.800286	0.423545	0.843967
bcap29	145.9989	0.232147627	0.159447	1.455959	0.145404	0.604523
sdr16c5a	533.6983	-0.662482031	0.126177	-5.25044	1.52E-07	6.51E-05
arl6ip5b	602.9854	-0.2967709	0.204683	-1.44991	0.147084	0.607283
psmd13	1512.189	0.027656187	0.146155	0.189224	0.849917	0.978251
ralab	567.3004	-0.167242852	0.133454	-1.25318	0.210138	0.692011
chmp4ba	557.2157	-0.357092718	0.132068	-2.70386	0.006854	0.139692
nudt3a	342.2857	-0.162620948	0.153576	-1.05889	0.289648	0.760643

fxr2	1602.425	0.146845021	0.211194	0.695309	0.486862	0.873246
zdhhc5a	453.504	0.163122499	0.131725	1.238356	0.215584	0.697157
eif1ad	334.3418	-0.399039827	0.182173	-2.19044	0.028492	0.300486
clca1	19.78857	-0.868151365	1.701398	-0.51026	0.609871	0.915331
zgc:65894	1396.958	0.358601606	0.162092	2.212338	0.026943	0.291718
upf1	1130.572	-0.103628822	0.13634	-0.76008	0.447209	0.856073
tfdp1b	344.469	-0.137277887	0.14585	-0.94123	0.346589	0.79947
mrpl46	236.9296	0.046556342	0.148577	0.313348	0.754016	0.955912
dock9b	331.4561	0.05412822	0.122404	0.442209	0.658338	0.930581
med7	322.6124	-0.3128636	0.122919	-2.54528	0.010919	0.182142
c9	2333.16	-0.607596784	0.212195	-2.86339	0.004191	0.10707
tmco6	111.7471	-0.588702896	0.208024	-2.82997	0.004655	0.11318
lctlb	212.0272	0.458745411	0.288896	1.587926	0.112303	0.549075
zgc:92518	253.8932	0.217365928	0.250214	0.86872	0.385	0.826939
ncoa5	899.8666	-0.07004344	0.115143	-0.60832	0.542976	0.894917
dip2bb	78.13856	0.259250464	0.272513	0.951333	0.341435	0.796175
cap1	274.0059	-0.026141458	0.162604	-0.16077	0.872277	0.983447
fmo5	322.8596	-0.649024898	0.157158	-4.12975	3.63E-05	0.004382
kpna1	666.8361	-0.044212784	0.144448	-0.30608	0.759543	0.957886
her8a	199.2414	0.296368878	0.215109	1.377764	0.168276	0.639712
gna15.1	209.4262	0.089702091	0.159936	0.560864	0.57489	0.904516
zbtb8os	311.8199	-0.379857604	0.139772	-2.71769	0.006574	0.136957
asns	252.2643	0.41820355	0.176719	2.36649	0.017958	0.2367
calcoco1b	1103.02	0.175416211	0.39895	0.439694	0.660159	0.930581
arf2b	2.079528	0.044120698	1.183305	0.037286	0.970257	NA
fam219aa	34.10182	-0.281925102	0.302375	-0.93237	0.351146	0.80227
riok1	296.921	-0.512750241	0.18104	-2.83225	0.004622	0.112688
ptch1	575.9672	0.072275798	0.235227	0.30726	0.758646	0.957867
agt	712.4604	-2.048322786	0.478145	-4.2839	1.84E-05	0.002746
dhtkd1	1377.991	-0.04770304	0.147939	-0.32245	0.747111	0.955074
crygm1	1.182559	0	4.560512	0	1	NA
fignl1	108.9384	0.274467569	0.181702	1.510534	0.130907	0.582503
farp2	515.4167	0.351575534	0.174574	2.013903	0.04402	0.367392
slc7a1	60.70397	0.182829507	0.393915	0.464134	0.642552	0.92567
eif3c	7200.1	-0.232290915	0.112293	-2.06862	0.038582	0.344335
ythdf1	690.3206	-0.043529072	0.126375	-0.34444	0.730512	0.950056
vtg3	1.725038	1.130689033	1.451485	0.778988	0.435987	NA
esr2a	6.918692	0.306945597	0.772208	0.397491	0.691006	0.940173
irf9	123.0716	0.387422188	0.204963	1.890208	0.05873	0.418427
fut9a	184.2042	0.228850631	0.274287	0.834347	0.404086	0.833888
trappc6b	206.219	-0.225120035	0.214392	-1.05004	0.2937	0.76281
cdc42bpab	257.5499	-0.15219342	0.145573	-1.04548	0.295803	0.764246
anxa5b	1084.883	-0.000920592	0.250408	-0.00368	0.997067	1
galcb	270.0654	0.111594332	0.143616	0.777032	0.43714	0.851125
eif4a2	1114.228	-0.39992963	0.12082	-3.31013	0.000933	0.042367
ugt5a1	10.88879	2.547223098	1.49356	1.705471	0.088107	0.498396
slc17a7a	100.8384	-0.091004881	0.316408	-0.28762	0.773638	0.960624
ptpn2a	174.5924	-0.29884296	0.186641	-1.60116	0.109341	0.542487
baiap2l2a	40.13671	-0.207530921	0.596786	-0.34775	0.72803	0.949325
dkc1	928.8551	-0.137392496	0.188315	-0.72959	0.465641	0.864997
rab11fip3	180.3986	0.264655774	0.17506	1.511797	0.130585	0.581871
ddc	92.06944	-0.262543395	0.337003	-0.77905	0.435949	0.850604
cdk8	337.0142	0.21422781	0.138902	1.542299	0.123001	0.569606
apbb1ip	157.3819	0.21876671	0.161725	1.352706	0.17615	0.649415

setd3	469.663	0.040640633	0.135758	0.29936	0.764665	0.959403
gtf2h2	213.2207	0.140791687	0.141147	0.997482	0.318531	0.779923
rbm8a	1649.053	-0.132270116	0.115366	-1.14653	0.251576	0.731828
ctdnep1a	199.9728	-0.089991099	0.15463	-0.58198	0.560581	0.899928
gata3	715.9423	-0.025364872	0.109614	-0.2314	0.817003	0.971207
helz2	9.733562	-1.259433122	0.909627	-1.38456	0.166187	0.63593
wdcp	93.3569	0.271629345	0.227981	1.191456	0.233475	0.714569
klhl4	64.912	-0.380424224	0.216271	-1.75902	0.078574	0.474455
suco	964.6469	-0.062844142	0.11926	-0.52695	0.598229	0.912715
npas2	67.8761	-0.34101528	0.40808	-0.83566	0.403347	0.833803
zgc:55888	1.516671	0.794076749	1.651239	0.480898	0.630589	NA
sash3	31.02585	0.781683693	0.380668	2.053453	0.040029	0.349722
mrm3a	52.46819	-0.136819395	0.27459	-0.49827	0.618296	0.916585
zgc:154046	447.1859	0.517713929	0.12934	4.002735	6.26E-05	0.006506
eif5b	976.7722	0.070093315	0.135674	0.516629	0.605415	0.914985
iqsec1b	668.8543	0.215518751	0.129493	1.66433	0.096047	0.517378
prlra	142.608	0.410728542	0.199896	2.054712	0.039907	0.349185
mroh1	499.5473	-0.340327069	0.129541	-2.62718	0.008609	0.158949
rgs7a	207.1024	0.179026742	0.229747	0.779236	0.435841	0.850596
haus4	105.9693	0.523503278	0.250594	2.089052	0.036703	0.336694
ckmt1	3665.403	0.598545608	0.247544	2.417932	0.015609	0.220659
pimr110	0.329315	-0.868116841	5.251677	-0.1653	0.868706	NA
echdc2	189.9169	-0.127446943	0.184083	-0.69233	0.488728	0.873964
scrn3	200.2401	0.157359102	0.235287	0.668797	0.503625	0.881759
mcat	30.44791	-0.328076276	0.371161	-0.88392	0.376741	0.822258
si:ch211-195b13.1	593.6584	-0.373751899	0.212474	-1.75905	0.078569	0.474455
tprb	771.1711	0.061087119	0.143823	0.424738	0.671027	0.933953
akr7a3	400.248	0.078590409	0.165674	0.474366	0.635239	0.924272
znf106a	1106.662	0.114662054	0.188781	0.607382	0.543597	0.894925
mgme1	108.6538	-0.465908125	0.238832	-1.95078	0.051084	0.393482
rnf20	538.8641	0.06871361	0.102801	0.668414	0.503869	0.881759
gabbr1b	58.47321	0.563014586	0.359241	1.567233	0.11706	0.557969
si:ch73-166o21.1	4.477723	1.132601428	0.962153	1.177153	0.239135	0.719781
gnao1a	1372.676	0.312764097	0.105813	2.955818	0.003118	0.091566
acadvl	4039.784	-0.024453794	0.11146	-0.2194	0.826342	0.973229
cd9b	1178.499	-0.178830848	0.130047	-1.37513	0.169091	0.640442
p2rx1	68.72299	-0.312046504	0.253244	-1.2322	0.217876	0.698904
trpa1a	220.565	-0.434208667	0.151447	-2.86706	0.004143	0.106542
pbld	91.16873	0.283323383	0.247034	1.1469	0.251423	0.731717
atic	2521.978	0.086042292	0.124439	0.691439	0.48929	0.874352
rchy1	225.7424	0.068076973	0.179104	0.380097	0.703873	0.943735
mmp11b	188.7981	0.229772397	0.227589	1.009593	0.31269	0.776189
sdha	4905.432	0.019595854	0.143203	0.13684	0.891157	0.988566
zgc:162339	115.8764	-0.285279537	0.22613	-1.26157	0.207103	0.689352
gadd45gb.1	599.3605	0.146319704	0.156215	0.936655	0.348936	0.801306
rfxank	20.52325	-0.628240783	0.465553	-1.34945	0.177192	0.65076
psat1	977.0298	-0.047415462	0.183936	-0.25778	0.796574	0.966447
lrrc4cb	105.0339	0.348925161	0.328154	1.063296	0.287648	0.758397
rgs7bpb	124.0661	0.569668206	0.24058	2.3679	0.017889	0.236508
angel2	393.4396	0.067271707	0.122503	0.549144	0.582907	0.90571
slc35f6	700.8098	-0.121961035	0.130762	-0.93269	0.350978	0.802224
slco1c1	109.4791	-0.241863831	0.193229	-1.25169	0.210681	0.692347
abcc6a	56.26569	0.510406358	0.485913	1.050407	0.293531	0.76277
ipo9	479.1461	0.200240456	0.165153	1.212454	0.225339	0.70685

tbca	823.5431	-0.069758482	0.188032	-0.37099	0.710642	0.945821
znf292a	364.3082	0.143126365	0.141532	1.011265	0.311889	0.776164
scamp3	453.9622	-0.204377701	0.09988	-2.04623	0.040734	0.352816
sgtb	127.6321	-0.035702358	0.201553	-0.17714	0.859401	0.979975
tfa	21336.46	-0.33830269	0.25042	-1.35094	0.176714	0.650047
cishb	92.4645	-0.427803939	0.349535	-1.22392	0.220982	0.702255
plekho2	234.6443	-0.154656938	0.144628	-1.06934	0.284914	0.755935
aqr	477.3314	0.072152958	0.132097	0.54621	0.584921	0.906655
huwe1	2796.099	0.079756055	0.18258	0.436828	0.662236	0.930742
srsf6b	2267.856	0.142294344	0.090639	1.569902	0.116438	0.557474
zeb1a	96.15913	0.10908333	0.229622	0.475057	0.634747	0.924272
zgc:152891	48.68142	-0.625024074	0.415073	-1.50582	0.132114	0.584623
crybb1l2	16682.73	0.401872098	0.239553	1.677591	0.093427	0.511267
mtmr6	401.4247	0.065056502	0.123816	0.525429	0.599285	0.913192
prkacab	551.2252	0.231911693	0.159318	1.455651	0.145489	0.604661
ints3	404.5259	0.083062273	0.105089	0.790397	0.429296	0.846956
casc1	21.15681	0.372220761	0.373437	0.996743	0.318889	0.779923
abcg2d	71.67625	0.198430031	0.304166	0.652375	0.51416	0.88631
vtg6	0.229641	-0.868117144	4.983452	-0.1742	0.861708	NA
rimk1a	3042.842	0.03949059	0.130032	0.303698	0.761358	0.958159
tcirg1a	256.9773	-1.280404239	0.182349	-7.02171	2.19E-12	2.99E-09
glipr2l	89.65152	-0.06807942	0.209566	-0.32486	0.745288	0.954956
neurod2	83.48669	0.164526386	0.294224	0.559187	0.576034	0.904739
sf3b5	632.3602	-0.100759218	0.15435	-0.6528	0.513888	0.88631
pon2	315.7257	-0.426693177	0.200171	-2.13164	0.033036	0.321751
smad7	315.3032	-0.052709737	0.116857	-0.45106	0.651945	0.928716
farsb	765.963	0.276136703	0.139174	1.984106	0.047244	0.378508
fam102ab	135.7131	-0.032008075	0.202815	-0.15782	0.8746	0.984095
rnf128a	664.5933	-0.120955229	0.147942	-0.81759	0.413594	0.83842
rhobtb4	92.75761	-0.154564773	0.239639	-0.64499	0.518934	0.887148
smarce1	3483.486	-0.056330061	0.114005	-0.4941	0.621235	0.918461
gys1	1785.065	0.071194401	0.191512	0.37175	0.710079	0.945821
dnajb9b	22.8632	0.644451063	0.370989	1.737119	0.082366	0.482779
eif3g	4103.883	-0.314057524	0.124261	-2.52741	0.011491	0.186054
fbxo15	8.253833	-0.905843637	0.669164	-1.35369	0.175834	0.649413
wdr5	947.0228	-0.012140474	0.104246	-0.11646	0.907288	0.991745
bckdk	1268.105	-0.174051654	0.130689	-1.3318	0.182925	0.659278
zp3e	6.072162	-1.37265468	0.951307	-1.44291	0.149045	0.610726
ace2	647.3582	0.097366618	1.476046	0.065964	0.947406	0.997371
si:dkey-94f20.4	15.7976	-0.352933249	1.213165	-0.29092	0.771113	0.960484
ttyh2	53.21811	0.257654971	0.23848	1.080407	0.279961	0.752866
hmcn1	478.5763	-0.064583919	0.173883	-0.37142	0.710324	0.945821
itgb2	119.1902	-0.047239557	0.214144	-0.2206	0.825406	0.972721
neurog3	2.96068	-0.698957686	1.156626	-0.60431	0.545639	0.895515
slc16a1a	96.74275	-0.160824687	0.248564	-0.64702	0.517621	0.887148
MCUR1	207.0538	0.172994811	0.198256	0.872585	0.382889	0.825721
tmem214	910.7449	-0.091621365	0.117003	-0.78307	0.433588	0.849735
snx27b	169.6094	0.034188988	0.174452	0.19598	0.844626	0.976791
pmpcb	487.124	-0.035627347	0.178678	-0.19939	0.841955	0.97643
bcar3	261.6683	-0.056926957	0.132337	-0.43017	0.667073	0.933049
ssrp1b	178.5175	0.210311369	0.160729	1.308486	0.190709	0.670725
lin28a	85.59518	0.073626595	0.261674	0.281368	0.778429	0.961073
myo10	627.9405	-0.053530376	0.200566	-0.2669	0.789549	0.9639
dennd5a	249.0269	0.154086133	0.138284	1.114273	0.265162	0.742063

nr4a2a	231.0858	-0.103344534	0.242695	-0.42582	0.670239	0.933809
scamp2l	150.2827	0.336963305	0.147537	2.283929	0.022376	0.264783
ywhae2	1544.535	-0.042604619	0.14503	-0.29376	0.768938	0.960395
ak7b	32.12194	0.071620655	0.342907	0.208863	0.834555	0.97576
sqor	608.4026	-0.184816653	0.220459	-0.83833	0.401847	0.832807
svilc	1045.782	-0.125526148	0.206476	-0.60795	0.543223	0.894925
ikbkg	393.1806	0.08697109	0.1178	0.738296	0.460334	0.862369
zgc:152670	4.629114	-4.656565775	1.312558	-3.5477	0.000389	0.023306
slc27a4	38.3513	0.38438882	0.27901	1.377688	0.1683	0.639712
adsl	2311.863	0.111075267	0.1301	0.853767	0.393234	0.828219
zmynd12	8.201767	-0.924914878	0.614439	-1.5053	0.132247	0.584912
large2	417.377	-0.142588659	0.201298	-0.70835	0.47873	0.869292
chordc1b	332.1976	-0.068705388	0.113942	-0.60299	0.546518	0.896107
mespaa	0.045161	0	5.267649	0	1	NA
bud31	873.9091	-0.235975812	0.177528	-1.32923	0.183771	0.66056
asb12a	376.7201	0.013815364	0.141404	0.097701	0.92217	0.994346
emc8	436.2534	0.001343067	0.136763	0.00982	0.992165	1
vaspb	1594.979	0.023151322	0.085249	0.271572	0.785951	0.962605
nr2e1	159.2713	0.200714485	0.272934	0.735397	0.462098	0.863402
kcna1b	4.554494	1.634245078	1.11295	1.46839	0.141998	0.601041
fam8a1a	17.00135	-0.660213852	0.489834	-1.34783	0.177713	0.65136
kctd10	551.5251	0.07616651	0.128748	0.591592	0.554124	0.898275
vps37a	552.575	-0.119363314	0.144553	-0.82574	0.408952	0.836637
mafba	483.1079	-0.141067154	0.110376	-1.27806	0.201227	0.681045
ilvbl	343.323	-0.022277517	0.132229	-0.16848	0.866208	0.982573
pdzk1ip1	83.78017	0.14362803	0.318672	0.450709	0.6522	0.928768
myof1	188.7601	-0.167433355	0.163985	-1.02103	0.307242	0.772541
zgc:63587	707.6718	0.00471544	0.115037	0.040991	0.967303	1
pla2g4ab	7.324402	-0.168750311	1.052008	-0.16041	0.87256	0.983447
brd9	430.4595	0.106838578	0.1024	1.043347	0.296788	0.764495
cables2b	32.36067	-0.29636834	0.313431	-0.94556	0.344372	0.798147
wnt10a	21.58724	-0.345952343	0.419275	-0.82512	0.409303	0.83666
kif3ca	88.34041	0.469285271	0.289873	1.618935	0.105461	0.534737
sinup	1.667147	-0.36617708	1.624003	-0.22548	0.821607	NA
slc3a1	51.32873	-0.008232933	0.339522	-0.02425	0.980654	1
nr2f1b	259.8428	-0.121015374	0.177704	-0.68099	0.495875	0.87806
si:dkey-29p10.4	113.3056	0.094526225	0.18851	0.501438	0.616063	0.916365
dlx2b	127.6486	0.206916881	0.201626	1.02624	0.304779	0.770005
npc1	1196.9	-0.162622168	0.12205	-1.33242	0.182721	0.659278
zpax2	1.603838	0.093650384	1.894577	0.049431	0.960576	NA
cnn1a	13.95468	-1.087739635	1.238654	-0.87816	0.379856	0.823607
foxf2a	278.8112	-0.21154262	0.140731	-1.50317	0.132796	0.585961
elp2	200.4549	-0.167080532	0.159006	-1.05078	0.29336	0.762713
plxnb1b	486.7559	0.26488586	0.150035	1.765493	0.077481	0.470449
pabpc1a	21065.94	-0.21797662	0.106064	-2.05514	0.039866	0.348958
otud7b	99.05864	-0.007782712	0.232587	-0.03346	0.973307	1
megf10	144.0331	0.026314947	0.18737	0.140444	0.888309	0.988188
fbxw11b	990.7537	-0.032303431	0.115457	-0.27979	0.77964	0.961073
npy8ar	2.482412	-0.10105541	1.080152	-0.09356	0.925461	0.994815
eif5a	15954.79	-0.127573843	0.11098	-1.14952	0.25034	0.730066
traf5	13.77013	-0.296294272	0.529983	-0.55906	0.576119	0.904749
garem	273.2507	-0.198350877	0.140738	-1.40936	0.158729	0.624456
chd6	463.1098	0.1775465	0.184534	0.962132	0.335983	0.792177
prx	1252.165	-0.083108523	0.260027	-0.31961	0.74926	0.955074

smtlb	4.562067	1.22703311	0.935438	1.31172	0.189615	0.669289
kcnk1b	15.05577	-0.080964799	0.594506	-0.13619	0.891672	0.988566
tmed1b	258.7706	-0.012311765	0.180532	-0.0682	0.945629	0.997371
gdpd1	565.9578	0.069859746	0.195513	0.357316	0.720855	0.947343
scml4	26.40981	-0.166911689	0.398169	-0.4192	0.675072	0.935189
eapp	155.8946	-0.059995472	0.176257	-0.34039	0.733565	0.950808
opn1sw2	3526.165	-0.740171296	0.459225	-1.61178	0.107009	0.537
gnai2b	468.9983	0.228929778	0.131128	1.745847	0.080838	0.479801
fabp11a	1481.699	0.227594111	0.187621	1.213055	0.225109	0.70685
gpsm2	367.7359	0.163715792	0.194769	0.840562	0.400593	0.831725
lmbrd2b	452.838	0.399867396	0.179747	2.224611	0.026107	0.286686
cela1.6	29116.08	0.424007929	0.694503	0.61052	0.541517	0.893858
larp7	256.1053	0.008921175	0.202163	0.044129	0.964802	1
bag5	115.7407	-0.118037851	0.232076	-0.50862	0.61102	0.915331
f11r.1	1032.395	-0.151121138	0.12399	-1.21882	0.222912	0.704786
kdr	126.4293	-0.09690664	0.274064	-0.35359	0.723645	0.947923
cdkl1	143.5417	0.061574765	0.193292	0.318559	0.750061	0.955074
zgc:77929	276.7943	0.017437742	0.159327	0.109446	0.912849	0.99273
kidins220b	2988.65	0.219503057	0.129001	1.701566	0.088837	0.500363
uts2b	5.620385	0	1.775766	0	1	1
epha2a	329.7768	0.137865192	0.192171	0.71741	0.473121	0.868983
rsph9	25.97456	0.199188968	0.389792	0.511014	0.609341	0.915204
mettl8	29.16956	-0.37138342	0.345968	-1.07346	0.283064	0.755028
slc23a2	31.13088	0.103127382	0.323736	0.318554	0.750065	0.955074
prdm4	259.4585	-0.216392745	0.161813	-1.3373	0.181124	0.657265
rhbdf1b	129.3144	0.264246105	0.272826	0.968552	0.332769	0.789823
sema3d	464.5376	-0.151463973	0.185542	-0.81633	0.41431	0.838631
b9d2	57.17955	-0.028092229	0.256217	-0.10964	0.912693	0.992635
sobpb	250.2603	0.022323855	0.212891	0.10486	0.916487	0.99343
gstt1b	1086.243	0.221805216	0.203293	1.091062	0.275245	0.750424
unc13ba	28.35715	0.014681905	0.482326	0.03044	0.975716	1
smarcc1a	1642.843	0.095292792	0.16093	0.592139	0.553758	0.89821
slc44a5a	6.115444	1.751149309	0.832513	2.103449	0.035426	0.330833
klf1	68.96095	0.172700001	0.386652	0.446654	0.655125	0.929606
apmap	756.1772	0.038185166	0.154495	0.247162	0.804783	0.96927
patl1	1485.07	-0.055548005	0.0947	-0.58657	0.557494	0.899184
crocc2	34.50057	0.442753233	0.294084	1.505535	0.132186	0.584831
phf20l1	347.987	-0.038036911	0.148553	-0.25605	0.797913	0.966447
sphkap	219.7847	-0.056963824	0.24549	-0.23204	0.816506	0.971207
mindy2	105.7778	-0.069331525	0.220579	-0.31432	0.753281	0.955861
adcy3b	13.80082	1.222449851	0.560586	2.180665	0.029208	0.304273
eif4ba	503.1303	0.046203263	0.097445	0.474146	0.635396	0.924292
mylz3	160396.5	0.115440574	0.147311	0.78365	0.433245	0.849392
hal	469.5088	-1.233925286	0.354307	-3.48265	0.000496	0.027455
eif3i	5902.359	-0.235491412	0.132317	-1.77975	0.075117	0.464618
camk1db	208.3018	0.796555945	0.232432	3.427045	0.00061	0.032059
nup50	687.7569	0.228119302	0.139575	1.634388	0.102177	0.528963
ncam2	357.2466	-0.052137681	0.290612	-0.17941	0.857618	0.979756
zgc:110699	40.17066	-0.790375945	0.425047	-1.8595	0.062956	0.429313
ifi45	103.4908	-2.354787716	0.543358	-4.33377	1.47E-05	0.002392
cel.1	9582.765	-0.900328774	0.954655	-0.94309	0.345633	0.798835
tgfbr1a	39.68073	-0.132542653	0.334517	-0.39622	0.691942	0.940274
rock2a	615.958	0.034780776	0.168071	0.206941	0.836056	0.976007
si:ch211-1o7.3	31.71069	-0.734028538	0.396838	-1.84969	0.064358	0.43348

ctdp1	167.531	0.219042964	0.173276	1.264125	0.206185	0.688671
ccnt1	77.54491	0.430000816	0.343264	1.252683	0.210321	0.692011
kif25	51.16378	-0.298529004	0.376561	-0.79278	0.427908	0.845893
prkag3a	58.47982	0.214338431	0.229166	0.9353	0.349634	0.801408
fgf1a	30.70659	0.060907397	0.363843	0.1674	0.867055	0.982582
banp	171.0169	0.144615701	0.148087	0.976562	0.328786	0.787144
itk	4.19627	-1.199131703	1.13259	-1.05875	0.289713	0.760705
reep3a	353.897	-0.139238134	0.172189	-0.80864	0.418725	0.84177
mccc2	1622.792	0.223246479	0.130798	1.706802	0.087859	0.497853
si:ch211-195b15.7	19.36237	-0.484112301	0.649123	-0.74579	0.455792	0.860272
nit2	270.2602	0.005521206	0.214154	0.025781	0.979432	1
fat1a	1589.143	0.054763155	0.197359	0.27748	0.781411	0.962024
ccdc6b	214.9647	0.034323063	0.153096	0.224193	0.822607	0.971966
ccng2	1294.148	-0.123982874	0.167521	-0.7401	0.459236	0.861818
rpp40	78.58054	0.242659318	0.197289	1.229969	0.218709	0.699445
sys1	239.0236	-0.111670829	0.15898	-0.70242	0.482417	0.871721
pnpo	271.4938	-0.208214257	0.140836	-1.47842	0.139295	0.597457
tlx3b	46.86261	0.412503963	0.295628	1.39535	0.16291	0.630534
alg6	221.3393	0.134074327	0.161369	0.830853	0.406057	0.834645
krt4	124194.1	-0.093901664	0.133951	-0.70101	0.483295	0.871988
pdcb	790.0256	-0.134389628	0.2767	-0.48569	0.627189	0.921095
rnf167	176.6112	0.077406941	0.181893	0.425562	0.670427	0.933883
cdh7b	4.754628	-0.85761914	1.104712	-0.77633	0.437555	0.851283
rgs13	29.06264	-0.072988847	0.670255	-0.1089	0.913284	0.99273
zgc:112163	37.71083	-0.712851509	0.314577	-2.26606	0.023448	0.270635
gmppb	890.0462	0.041772447	0.146149	0.285821	0.775015	0.960899
sephs2	968.7803	0.148346794	0.121917	1.216784	0.223686	0.705624
braf	204.8866	-0.016278966	0.161373	-0.10088	0.919647	0.993892
snrkb	243.4162	-0.307298711	0.164376	-1.86949	0.061555	0.425614
nova2	2080.503	0.131354296	0.099024	1.326486	0.184679	0.661987
utp4	564.5654	-0.121414018	0.174398	-0.69619	0.48631	0.873244
mmp2	2786.439	0.246236232	0.173796	1.416811	0.156538	0.622452
dnajc5gb	245.6012	-0.10487842	0.150696	-0.69596	0.486454	0.873244
utp25	323.0812	0.068934714	0.174116	0.395912	0.69217	0.940333
paqr3a	223.9077	-0.205138323	0.169646	-1.20921	0.226581	0.707798
lrrc40	165.8542	0.305357288	0.157028	1.944598	0.051823	0.395809
ppp1r14ab	167.3938	-0.039744353	0.226896	-0.17517	0.860949	0.980388
si:ch211-106h11.1	37.83033	-0.025342728	0.454718	-0.05573	0.955555	0.998611
si:ch73-151m17.5	3.189954	-2.138514112	1.65065	-1.29556	0.195128	0.67483
phb2b	741.9625	0.250011647	0.132399	1.888316	0.058984	0.418576
ak5l	77.91101	-0.210346764	0.277587	-0.75777	0.448589	0.856741
sec63	560.7275	0.056507921	0.107355	0.526363	0.598636	0.912889
g3bp1	1500.977	0.071718885	0.131322	0.546128	0.584978	0.906655
grm6a	4.917907	-0.205025947	1.12319	-0.18254	0.85516	0.979197
smc2	758.7507	0.348115305	0.252276	1.379897	0.167619	0.638828
arhgap29b	468.0781	0.121176538	0.139242	0.870261	0.384158	0.826565
pik3cg	137.0154	-0.477106982	0.165869	-2.87641	0.004022	0.105636
srrt	925.6944	0.213376644	0.148535	1.436542	0.150848	0.615085
mdh1aa	8192.616	-0.425974393	0.14628	-2.91205	0.003591	0.09929
slc16a12a	103.2125	-0.261476971	0.386263	-0.67694	0.498443	0.879132
atp5mc3a	1930.324	0.231256964	0.129201	1.789907	0.073469	0.461196
rorcb	591.5918	-0.092052546	0.323809	-0.28428	0.776195	0.961073
hsd17b10	692.9721	-0.016777414	0.160334	-0.10464	0.916661	0.993453
zgc:158689	90.72843	0.114694142	0.245144	0.467864	0.639882	0.924779

chrnb2	185.7328	0.127214971	0.234563	0.54235	0.587578	0.907552
sdr16c5b	629.9844	-0.261553663	0.134636	-1.94268	0.052055	0.396487
bcor	970.0754	0.097231331	0.148519	0.654673	0.512678	0.885483
tgm1	91.88315	-0.818083777	0.283187	-2.88885	0.003867	0.103403
gsk3ba	885.1758	-0.028725119	0.110221	-0.26061	0.79439	0.965431
stard3	292.9739	-0.309600488	0.144445	-2.14338	0.032082	0.318495
si:ch211-210c8.6	176.9996	0.023148125	0.248568	0.093126	0.925804	0.994815
pigs	189.3202	0.188599648	0.155268	1.214672	0.224491	0.706406
gata5	256.2149	-0.137644357	0.158911	-0.86617	0.386396	0.827278
micall2b	90.07505	-0.025935626	0.289483	-0.08959	0.928611	0.995093
brf1a	83.3393	0.12361807	0.255737	0.483379	0.628827	0.922088
cand1	1001.702	0.045829557	0.107901	0.424737	0.671029	0.933953
syvn1	427.9477	0.229278101	0.110363	2.077484	0.037757	0.340665
srsf1b	1277.538	-0.000446311	0.108672	-0.00411	0.996723	1
copz1	1141.21	-0.075255097	0.188522	-0.39918	0.689758	0.939657
prepl	62.99579	-0.224681266	0.327795	-0.68543	0.493072	0.876715
rgs5b	48.88147	0.18912239	0.420713	0.449528	0.653051	0.929197
dnajc3b	343.2105	0.319529783	0.156038	2.047773	0.040582	0.351905
kcnip3b	60.77659	0.270840754	0.339281	0.798279	0.424709	0.844714
rdh1	297.201	-0.096039764	0.309132	-0.31068	0.756047	0.956532
bgna	54.29769	-1.422512262	0.526314	-2.70278	0.006876	0.139692
zbtb11	497.579	-0.113187958	0.121255	-0.93347	0.350576	0.802215
tcp1	5806.968	-0.089345515	0.106915	-0.83567	0.403342	0.833803
tln2a	366.552	-0.032092789	0.199097	-0.16119	0.871942	0.983447
casp3a	1233.905	0.01527487	0.107611	0.141945	0.887123	0.987739
pdss1	451.7784	-0.021488851	0.129733	-0.16564	0.868441	0.982801
her7	2.819159	0.226402029	1.123818	0.201458	0.840341	0.976417
cx36.7	3.072483	-0.476482892	1.025059	-0.46483	0.64205	0.925586
xrcc3	101.4798	-0.093164841	0.235286	-0.39597	0.692131	0.940333
ncoa2	934.4681	0.119987886	0.138825	0.864312	0.387416	0.827592
ints6l	120.9277	0.255682391	0.181959	1.405164	0.159972	0.626214
ptk6a	51.74941	0.354436692	0.300984	1.177593	0.238959	0.719781
tp73	31.84491	0.459591719	0.35091	1.309713	0.190293	0.670176
sfxn2	1396.689	-0.089255562	0.166328	-0.53662	0.591528	0.909437
cyp39a1	17.6771	-0.129827323	0.635495	-0.20429	0.838124	0.976386
rsph14	19.7919	0.900814128	0.391901	2.298578	0.021529	0.260177
zgc:172106	2.237954	-0.391542118	1.281607	-0.30551	0.759979	0.957886
zgc:77739	110.0107	-0.141612071	0.221001	-0.64078	0.521668	0.887551
vps13d	368.6027	-0.10858032	0.172778	-0.62844	0.529717	0.890484
dse	473.4135	0.032672732	0.118583	0.275527	0.782912	0.962328
tssk6	0	NA	NA	NA	NA	NA
ubp1	182.0341	-0.059547007	0.305303	-0.19504	0.84536	0.976791
eci1	679.4899	-0.037623525	0.227803	-0.16516	0.86882	0.982801
nkx2.5	20.49139	0.061472338	0.420795	0.146086	0.883853	0.987014
vrk1	292.0547	-0.24724325	0.20874	-1.18446	0.236232	0.717364
mdh1b	12.38334	0.153083806	0.53169	0.287919	0.773409	0.960624
crtap	697.5163	0.110921921	0.123986	0.894635	0.370982	0.818556
msh2	359.0865	0.012825053	0.159861	0.080226	0.936057	0.996941
tbx2a	358.091	0.037781671	0.151189	0.249897	0.802667	0.968181
scn8ab	49.94981	0.853380014	0.321553	2.653933	0.007956	0.150851
msantd4	25.14004	0.211289437	0.375234	0.563088	0.573375	0.904056
brms1lb	144.0694	0.417611513	0.207701	2.01064	0.044363	0.368519
zgc:171704	9.620208	0.016302546	0.615913	0.026469	0.978883	1
sf3b2	1543.669	0.044064316	0.103917	0.424033	0.671542	0.933953

pik3r2	618.5163	-0.124331163	0.104233	-1.19281	0.232942	0.714492
neil1	325.4897	0.37978601	0.131557	2.886866	0.003891	0.103477
ntm	16.623	-0.443739767	0.446094	-0.99472	0.319871	0.780475
ptchd1	62.76137	-0.097924919	0.31156	-0.31431	0.753289	0.955861
rdh12	178.5431	-0.269330869	0.213999	-1.25856	0.208188	0.69024
mrps22	390.1234	0.262519019	0.126751	2.071135	0.038346	0.343352
rbp1.1	5.40095	-2.265499633	0.7789	-2.90859	0.003631	0.099671
scap	126.6294	0.049973344	0.168439	0.296685	0.766707	0.960296
casq1b	1976.807	-0.016670097	0.126692	-0.13158	0.895317	0.989223
eif2b3	329.483	-0.22790813	0.130026	-1.75279	0.079639	0.477652
rab3da	86.58756	-0.038865845	0.240472	-0.16162	0.871603	0.983365
mafga	5.708759	-0.539708178	0.81368	-0.66329	0.507143	0.883007
pak4	429.458	-0.111199572	0.12609	-0.88191	0.377828	0.822615
kdm7aa	270.7853	-0.043331341	0.156497	-0.27688	0.78187	0.96218
CR392347.1	2.88922	1.994118482	1.677586	1.188683	0.234564	0.715489
cenpj	61.29568	0.627980569	0.227764	2.757151	0.005831	0.129157
psmd3	2383.837	-0.083366086	0.149951	-0.55596	0.578242	0.905417
midlipl1	529.0958	0.104726017	0.189719	0.552007	0.580944	0.905618
gpx1a	1220.95	0.035608679	0.159178	0.223703	0.822988	0.971966
slc38a4	1637.973	-0.150719616	0.15123	-0.99663	0.318946	0.779923
nt5c1ba	75.04979	0.679376162	0.361205	1.880862	0.059991	0.421883
ano10b	163.2279	-0.262667363	0.206066	-1.27468	0.202424	0.682855
ctnnal1	156.6391	-0.247009747	0.155482	-1.58867	0.112136	0.548603
depdc1a	174.4346	0.128154007	0.213314	0.600776	0.547989	0.896611
gnai2a	1453.428	-0.031409526	0.094763	-0.33145	0.740302	0.953888
pgm2	542.4104	-0.519925429	0.155437	-3.34493	0.000823	0.038681
acvrl1	416.3437	-0.107087173	0.152764	-0.701	0.483305	0.871988
dnajc28	134.2152	-0.130858368	0.206012	-0.6352	0.5253	0.888928
asna1	1086.819	-0.085104761	0.136823	-0.622	0.533939	0.891181
ubr5	2000.442	0.009731411	0.172114	0.05654	0.954911	0.998611
nck2a	245.9892	-0.049410905	0.135456	-0.36477	0.71528	0.945917
htr2cl1	87.26363	0.283351266	0.267697	1.058476	0.289838	0.760705
mfap1	623.8107	0.066801768	0.139163	0.480026	0.631209	0.923003
ncoa1	187.593	-0.102693562	0.247737	-0.41453	0.678489	0.936026
atp1a3a	3215.306	0.262807857	0.20681	1.270769	0.203811	0.684589
bmp7a	10.01869	-0.343251621	0.576105	-0.59581	0.551299	0.8974
pdia2	1526.697	-0.660407425	0.329519	-2.00415	0.045054	0.371889
trim101	913.0645	0.099215951	0.123205	0.805292	0.420651	0.842383
mthfd1a	857.6216	-0.122867817	0.303449	-0.4049	0.685547	0.938486
olfm1a	228.1448	0.290649595	0.152969	1.900056	0.057426	0.413602
wdr33	1006.02	0.036811321	0.095881	0.383928	0.701032	0.942918
cfap161	8.647936	0.305284814	0.5574	0.547694	0.583902	0.906376
cyba	213.2712	0.430932426	0.181921	2.368788	0.017846	0.236422
pdpk1b	1018.315	0.007002692	0.094226	0.074318	0.940757	0.997371
rev1	237.1883	-0.038972689	0.149236	-0.26115	0.793979	0.965431
si:ch73-186j5.2	1.305286	1.042755207	1.733963	0.601371	0.547593	NA
etv4	490.0271	0.085352391	0.150496	0.567139	0.57062	0.902481
rell1	255.8749	0.113347989	0.160538	0.70605	0.480157	0.870001
arglu1b	689.5951	-0.027238473	0.136087	-0.20015	0.841359	0.97643
lbx1a	66.65062	0.213756238	0.30187	0.708107	0.478879	0.869292
taf5	341.0465	0.125290007	0.107852	1.161681	0.245365	0.726909
il1r3	0.380278	0	5.117832	0	1	NA
rhocb	1142.394	0.055297601	0.098864	0.559329	0.575938	0.904739
gc2	11.15104	0.135567706	0.649915	0.208593	0.834766	0.975908

rpl35	20257.35	-0.216562835	0.13286	-1.63	0.103101	0.530217
ggt1b	32.09814	0.007658255	0.589959	0.012981	0.989643	1
hpda	1836.989	-0.894930833	0.231443	-3.86674	0.00011	0.009944
sult1st3	89.24409	-0.916244405	0.944222	-0.97037	0.331862	0.789531
prkcha	27.67886	1.024397312	0.442899	2.312936	0.020726	0.255657
frzb	258.215	-0.178303928	0.226078	-0.78868	0.430297	0.847541
kank2	154.4176	-0.463378472	0.338478	-1.36901	0.170997	0.643066
hpca	1043.463	0.03871022	0.162906	0.237624	0.812173	0.970129
rps6kb1a	380.064	-0.052187793	0.12578	-0.41491	0.678206	0.936026
krt18a.1	7729.624	-0.264584622	0.138403	-1.9117	0.055915	0.409076
mknk1	309.2699	-0.057132846	0.155121	-0.36831	0.712641	0.945821
pth1rb	67.83949	-0.539098137	0.308942	-1.74498	0.080989	0.479801
sulf2a	503.3421	0.07984142	0.160047	0.498861	0.617877	0.916478
ddx61	1466.843	0.106615574	0.102499	1.04016	0.298266	0.765416
aldh7a1	2068.003	0.09228401	0.116473	0.79232	0.428174	0.846133
m6pr	345.1838	-0.248685963	0.137193	-1.81267	0.069882	0.450896
heg1	123.802	0.090609843	0.217007	0.417544	0.67628	0.93557
msrb2	605.3794	-0.998841342	0.277741	-3.59631	0.000323	0.020489
mbnl2	555.1896	-0.022107794	0.130611	-0.16926	0.865589	0.982364
zgc:56095	908.2945	-0.047491101	0.174166	-0.27268	0.785101	0.962605
pak2b	1076.927	0.055788851	0.08186	0.681515	0.495546	0.87806
agxtb	3395.644	-0.368631798	0.30983	-1.18979	0.23413	0.714938
cyp2v1	353.1269	-0.391714382	0.170756	-2.294	0.02179	0.261665
pdia4	922.5098	0.185272534	0.166305	1.114052	0.265257	0.742063
znf503	1048.149	-0.013439369	0.108562	-0.12379	0.901478	0.990643
smn1	247.2894	-0.121733533	0.217982	-0.55846	0.576532	0.90475
ftr06	0.753543	0.88883378	2.418357	0.367536	0.713219	NA
zdhhc8b	223.9049	0.023855628	0.153866	0.155041	0.876789	0.985052
stk38b	158.9942	-0.088307439	0.191371	-0.46145	0.644479	0.926639
or115-1	5.870648	-0.634955397	1.264973	-0.50195	0.615701	0.916365
midn	1283.089	-0.141652507	0.174918	-0.80982	0.418042	0.841312
lipf	1357.225	-0.150990906	0.167954	-0.899	0.368651	0.81746
mapkapk2b	20.73591	-2.493394706	0.833609	-2.99108	0.00278	0.086456
slc6a9	991.4709	0.015521172	0.145668	0.106551	0.915145	0.993066
hapln4	24.92231	-1.029705722	0.631547	-1.63045	0.103007	0.530217
ifih1	13.69559	-1.26601098	0.669405	-1.89125	0.058591	0.418278
kdm7ab	317.9891	-0.023438144	0.133128	-0.17606	0.860249	0.980264
cope	1532.675	-0.049437191	0.137297	-0.36007	0.718791	0.94694
flnca	303.4977	-0.064137024	0.192526	-0.33314	0.739032	0.95338
tnfrsf1a	306.7687	-0.059778765	0.170314	-0.35099	0.725595	0.948653
sf3b4	388.9342	-0.082861343	0.142326	-0.58219	0.560436	0.899928
zgc:152658	2.191264	-0.868195539	4.968163	-0.17475	0.861275	NA
parp16	366.9373	-0.094577341	0.143848	-0.65748	0.51087	0.884206
retsat	390.5333	0.196070623	0.133057	1.473578	0.140595	0.599658
rab5aa	1314.211	-0.007439347	0.085956	-0.08655	0.931031	0.995809
lbx1b	43.56072	0.597046273	0.341433	1.748649	0.080352	0.478844
agtr1a	8.611582	-0.843066653	0.554826	-1.51952	0.128633	0.578162
cpsf6	1487.195	-0.009448559	0.101476	-0.09311	0.925815	0.994815
cers4b	49.03873	-0.20705477	0.358035	-0.57831	0.563055	0.90078
slc6a19a.1	60.61924	-0.931913089	0.785469	-1.18644	0.235448	0.716581
rad54l	172.3296	0.209695874	0.201322	1.041593	0.297601	0.765407
arl5a	292.2844	0.068616463	0.1541	0.445272	0.656123	0.929684
sec61g	2318.351	-0.256466052	0.175964	-1.45749	0.14498	0.604004
igf2a	344.26	-0.057811176	0.158225	-0.36537	0.714834	0.945821

gtpbp3	37.52112	0.717266295	0.292163	2.455018	0.014088	0.208027
acot7	169.0319	0.443068431	0.197651	2.24167	0.024983	0.280179
aar2	244.5523	-0.476329942	0.188745	-2.52367	0.011614	0.187517
si:ch211-262i1.4	0.574772	3.33904249	3.176816	1.051066	0.293228	NA
saal1	127.9497	-0.365514534	0.269722	-1.35515	0.175369	0.649279
itga2b	7.248333	1.226671062	0.960332	1.277341	0.201482	0.68141
elk3	354.9329	-0.06128097	0.169316	-0.36193	0.717403	0.946272
phf2	535.0679	0.12877885	0.112855	1.141104	0.253827	0.732873
cdh2	2198.284	0.006580146	0.106265	0.061922	0.950625	0.99801
carm1	1003.39	0.198372176	0.118406	1.675362	0.093863	0.511703
dgkh	86.03195	-0.081000202	0.221247	-0.36611	0.714285	0.945821
npnta	256.5284	0.367024507	0.163522	2.244502	0.0248	0.279131
slco3a1	73.84176	-0.166175079	0.254098	-0.65398	0.513124	0.886055
zw10	453.0245	0.026412356	0.108301	0.243879	0.807324	0.969736
psme4b	898.6161	0.128524204	0.144267	0.890879	0.372994	0.820208
scamp5a	106.147	0.182048488	0.251726	0.723201	0.469556	0.867224
prl2	9.737671	2.862912915	0.791048	3.619141	0.000296	0.019346
npr1b	97.58243	-0.392107761	0.249807	-1.56965	0.116498	0.557474
klf5l	250.8174	-0.272795477	0.158289	-1.7234	0.084816	0.488883
pknox1.1	379.8097	-0.060122428	0.123377	-0.48731	0.626042	0.920625
hivep2b	35.27892	-0.229086338	0.40512	-0.56548	0.571749	0.903234
kdm4aa	370.5621	0.086089477	0.199155	0.432273	0.665543	0.932606
efna1b	503.0449	-0.092262555	0.122915	-0.75062	0.452881	0.858256
st8sia2	17.67498	0.255811787	0.599334	0.426826	0.669506	0.93336
crygmxl1	61.43522	-0.794864088	0.435907	-1.82347	0.068232	0.445546
abhd3	96.37231	1.089452898	0.302693	3.599203	0.000319	0.020374
esrp2	604.3683	-0.042001242	0.141738	-0.29633	0.766978	0.960296
bdnf	173.3133	0.519799598	0.221511	2.346608	0.018945	0.242378
flncb	660.9403	-0.036963343	0.266319	-0.13879	0.889613	0.988318
tmem248	107.6467	-0.08430344	0.177005	-0.47628	0.633877	0.923974
trpc4apa	764.6348	0.010617743	0.079066	0.134289	0.893174	0.988566
dgat2	322.4493	0.212370356	0.289711	0.733042	0.463533	0.864593
si:dkey-199f5.8	609.6864	0.131741578	0.116814	1.127792	0.259408	0.736127
scaf4b	413.2328	0.102101725	0.11885	0.859078	0.390298	0.827902
dclk1a	280.7589	0.208310383	0.230555	0.903517	0.366252	0.81589
henmt1	18.9112	-0.491116153	0.457863	-1.07263	0.283438	0.755095
apobec2a	477.7813	0.081697839	0.207233	0.394232	0.69341	0.940668
jak2b	682.1233	-0.135123016	0.109853	-1.23003	0.218686	0.699445
snrpa	904.5109	-0.131795038	0.09724	-1.35536	0.175303	0.649279
rnaseh2a	315.1167	-0.015422686	0.17656	-0.08735	0.930393	0.995707
mmp24	66.87942	0.315983225	0.256232	1.23319	0.217505	0.69815
zfand5a	1474.841	-0.050435323	0.146439	-0.34441	0.730537	0.950056
pes	698.6872	-0.091262607	0.151651	-0.60179	0.547313	0.896137
aimp2	2001.12	-0.289705763	0.149605	-1.93647	0.05281	0.399532
cstf3	1229.6	-0.10372605	0.099728	-1.04009	0.2983	0.765416
kat6a	1043.917	0.233558595	0.146782	1.591195	0.111566	0.547433
hnrnpk	1750.464	-0.07252555	0.099461	-0.72919	0.465886	0.86502
rad17	144.3477	0.038058429	0.157517	0.241615	0.809078	0.969828
lratd1	279.5609	-0.214759659	0.135144	-1.58912	0.112034	0.548404
fat2	266.9664	0.252718739	0.225576	1.120326	0.262575	0.740129
zcchc17	201.6623	0.132769121	0.127823	1.038695	0.298947	0.765877
hoga1	366.7811	0.225855697	0.161862	1.395359	0.162908	0.630534
gclm	375.2698	-0.115225345	0.15264	-0.75488	0.450318	0.857151
tmem39b	221.4647	-0.416409575	0.160912	-2.58781	0.009659	0.16916

nrarpb	221.9063	-0.037021667	0.187982	-0.19694	0.843873	0.976791
gtpbp4	7468.104	-0.479809268	0.143631	-3.34058	0.000836	0.039213
cyb5r1	340.7898	-0.162232155	0.159113	-1.0196	0.307918	0.772996
gabbr1a	24.47927	0.177871895	0.322873	0.550904	0.581699	0.905618
acvr1ba	1171.332	-0.073286353	0.114919	-0.63772	0.523653	0.888628
b3gnt5a	610.1656	-0.059524805	0.147505	-0.40354	0.686548	0.938732
clptm1	910.2607	0.06980009	0.084642	0.824646	0.409573	0.836787
sptlc2a	298.1327	-0.237651707	0.160411	-1.48151	0.138469	0.595018
zgc:91890	126.1881	-0.009785615	0.195367	-0.05009	0.960052	0.999164
eya2	548.729	-0.011011734	0.117047	-0.09408	0.925046	0.994815
castor2	1422.874	-0.173192676	0.143573	-1.2063	0.2277	0.708829
hspa4b	1894.168	0.044151383	0.099963	0.441678	0.658722	0.930581
cplx2l	996.0052	0.364461692	0.18617	1.957683	0.050267	0.390574
smc3	1914.906	0.139486041	0.102939	1.355042	0.175404	0.649279
ak7a	69.96043	0.23888768	0.24336	0.981622	0.326286	0.785215
smarcd1	1413.206	-0.051834666	0.099136	-0.52286	0.601069	0.913756
erp44	1192.196	0.168595969	0.106303	1.586001	0.112739	0.549875
barhl1b	181.1312	0.350002375	0.163586	2.139556	0.032391	0.319256
podn	9.421329	0.359561351	1.078143	0.333501	0.738756	0.953168
tmem59	1140.235	-0.139412947	0.113495	-1.22836	0.219311	0.700266
sgsm3	468.1321	-0.074327259	0.134718	-0.55173	0.581136	0.905618
snx12	1515.542	0.027363215	0.087232	0.313682	0.753762	0.955912
arpc5b	760.4945	-0.058572223	0.127815	-0.45826	0.646768	0.926728
fat1b	113.4733	-0.388197742	0.264151	-1.46961	0.141668	0.600284
DHDH	844.7556	-0.319404957	0.141579	-2.25602	0.024069	0.274857
si:ch73-160i9.2	4.166079	-0.893038955	1.133529	-0.78784	0.43079	0.847629
myl7	820.9849	-0.014803947	0.183121	-0.08084	0.935567	0.996829
cd82a	1897.722	0.086322382	0.19825	0.435422	0.663256	0.93126
kdm4ab	472.6599	-0.136593606	0.14371	-0.95048	0.341869	0.796651
mob3a	462.1981	-0.152231386	0.135819	-1.12084	0.262358	0.73998
ing1	234.3674	0.012921184	0.14347	0.090062	0.928238	0.995052
parvb	853.4713	-0.102400677	0.124756	-0.82081	0.411757	0.83798
he1.2	1764.76	-0.989115281	0.452802	-2.18443	0.02893	0.3029
klhl40b	97.31541	-0.047897524	0.33495	-0.143	0.886291	0.987533
tpm4b	877.6936	-0.490493349	0.112696	-4.35237	1.35E-05	0.002278
plk2b	402.694	-0.229886978	0.157772	-1.45708	0.145094	0.604004
ankef1a	18.09942	0.102961995	0.375807	0.273976	0.784103	0.962605
tram1	748.1281	0.111731044	0.120584	0.926583	0.354143	0.80525
fam185a	108.9754	-0.181136379	0.220541	-0.82133	0.411459	0.837797
rab4a	184.6028	-0.005311593	0.151655	-0.03502	0.97206	1
haus5	111.3321	0.51975959	0.271769	1.912507	0.055811	0.408826
sh3bp5la	212.3461	0.080777306	0.12748	0.633647	0.526311	0.88947
lrit1a	112.6749	1.33914943	0.627751	2.133251	0.032904	0.321141
rpsa	54981.12	-0.263470053	0.123076	-2.14071	0.032298	0.318811
slain1a	107.1167	0.521546781	0.206094	2.530625	0.011386	0.185262
12-Sep	629.6072	0.131544049	0.13167	0.999045	0.317773	0.779627
vps9d1	237.1982	0.127579711	0.160529	0.794744	0.426762	0.845352
fam120c	815.8741	-0.002435745	0.171577	-0.0142	0.988673	1
abo	5.798164	1.709931327	1.13622	1.50493	0.132342	0.585048
camsap1a	250.2273	0.211105613	0.221018	0.95515	0.339502	0.795226
CTBP1	717.203	0.19091314	0.111862	1.706679	0.087882	0.497853
mrpl24	422.5661	0.232643325	0.132817	1.751602	0.079842	0.478112
arglu1a	1500.995	0.049525614	0.159887	0.309754	0.756748	0.95667
mogat2	94.64698	-0.866914972	0.469686	-1.84573	0.064931	0.434811

rpl7a	60163.15	-0.352698818	0.129164	-2.73064	0.006321	0.134501
sptan1	4481.106	0.022067936	0.135217	0.163203	0.870358	0.983365
nln	162.1989	-0.276740118	0.22276	-1.24233	0.214116	0.696474
sema3aa	996.0365	0.232266993	0.118729	1.956275	0.050433	0.39074
gsr	487.6955	0.149198403	0.146384	1.019227	0.308095	0.773116
cul1a	877.0396	-0.005265056	0.090195	-0.05837	0.953451	0.998436
fancf	46.25018	0.369827024	0.296115	1.248931	0.21169	0.693267
rhag	5313.873	-0.365726014	0.143238	-2.55327	0.010672	0.179908
dhrs9	207.4717	0.215182893	0.235441	0.913958	0.360739	0.811253
zbtb39	125.0795	-0.329836001	0.223735	-1.47423	0.140421	0.599512
rasd1	504.8871	-0.203925893	0.2057	-0.99138	0.321502	0.781986
GNB4	135.9644	0.003539123	0.179995	0.019662	0.984313	1
larsb	558.2914	0.078649215	0.134563	0.584477	0.558899	0.899409
tfdp1a	655.3903	0.023644096	0.117843	0.200641	0.840979	0.97643
cbln8	247.4698	-3.084090902	1.39705	-2.20757	0.027274	0.293515
znf687b	506.8099	-0.1415929	0.112164	-1.26237	0.206816	0.689189
ints7	191.9567	0.196080895	0.154942	1.265513	0.205688	0.687948
mid1ip1b	1333.234	0.263057838	0.138976	1.892824	0.058381	0.417756
phactr3b	249.8323	0.302350447	0.179851	1.681116	0.09274	0.509444
dusp5	135.6192	0.068442588	0.287409	0.238137	0.811775	0.97012
fbxo30b	82.12962	-0.203159206	0.238014	-0.85356	0.393349	0.828374
rab39ba	130.3877	0.288472382	0.218859	1.318074	0.187479	0.666343
fer1l6	42.06673	0.51562999	0.332787	1.549429	0.121279	0.567338
prpsap2	602.9462	-0.085490324	0.133632	-0.63974	0.52234	0.887826
plxna4	658.6098	0.239247077	0.121822	1.963915	0.04954	0.387714
ndufb4	0.703826	-0.796210895	2.556718	-0.31142	0.755482	NA
hes6	461.4575	0.122144564	0.20622	0.592303	0.553648	0.898195
adprhl2	248.6415	-0.006098612	0.220165	-0.0277	0.977901	1
uxt	142.5969	-0.380429527	0.237074	-1.60469	0.108563	0.541057
gpc1a	110.0657	0.168956979	0.209539	0.806328	0.420054	0.842383
chrnd	330.8024	0.054961755	0.173091	0.317531	0.750841	0.955074
mark3a	730.1916	0.138153579	0.11268	1.226069	0.220173	0.701416
sparc	18608.24	0.027563753	0.157558	0.174944	0.861124	0.980406
coa7	173.7108	-0.203410121	0.196318	-1.03613	0.300144	0.766629
fabp1a	0	NA	NA	NA	NA	NA
sec23b	1131.385	-0.047661588	0.248772	-0.19159	0.848065	0.977839
ptbp1a	1095.597	-0.04013152	0.115895	-0.34627	0.729137	0.949983
mbip	159.3937	-0.041336389	0.192225	-0.21504	0.829735	0.974816
krt99	159.5943	-0.514259653	0.218441	-2.35422	0.018562	0.241071
tgfb3	770.7343	0.028637661	0.137441	0.208363	0.834946	0.975994
flt1	113.5947	0.033286737	0.218422	0.152396	0.878875	0.985841
cdc42bpb	1273.607	-0.015007782	0.132076	-0.11363	0.909531	0.99213
stat5a	108.5518	0.110928557	0.287342	0.38605	0.69946	0.942499
rergla	98.69066	-1.289434464	0.413908	-3.11527	0.001838	0.065916
psma6a	1184.105	0.137641309	0.153537	0.896471	0.370001	0.818446
atp5f1d	4347.154	0.062025072	0.111763	0.554969	0.578916	0.905417
zgc:63470	363.4966	0.159429794	0.137345	1.160799	0.245724	0.727177
gadd45ga	445.0377	-0.047509799	0.179495	-0.26469	0.791252	0.964766
kcnj2a	68.42594	0.582377475	0.25467	2.286789	0.022208	0.263608
etnk1	961.8611	-0.658525815	0.278257	-2.36661	0.017952	0.2367
trioa	409.1494	0.086387453	0.203219	0.425096	0.670767	0.933939
rnf13	749.3248	-0.263491139	0.153607	-1.71535	0.08628	0.493048
CABZ01086041.1	51.4192	0.090616085	0.371957	0.24362	0.807525	0.969736
ssr4	3748.321	0.143320624	0.139688	1.026004	0.30489	0.770035

pcsk2	88.51426	0.496461812	0.282089	1.759948	0.078417	0.474337
tfe3b	61.88525	0.444433477	0.343824	1.292621	0.196142	0.676562
elf2a	366.251	-0.103656194	0.145862	-0.71064	0.477305	0.869292
zdhhc7	62.82959	0.084515329	0.236086	0.357986	0.720354	0.947199
tmx1	214.245	0.037474137	0.176827	0.211925	0.832166	0.975013
shbg	229.1751	-2.091538599	0.489482	-4.27297	1.93E-05	0.002811
esd	1190.678	-0.041191886	0.140925	-0.2923	0.770061	0.960484
cry5	399.9955	-0.070655772	0.292393	-0.24165	0.809054	0.969828
pcnx3	60.46444	-0.642590893	0.263853	-2.43541	0.014875	0.213725
zgc:103759	24.09135	0.369550645	0.410934	0.899295	0.368495	0.817272
mcm5	849.519	-0.061724154	0.219903	-0.28069	0.77895	0.961073
sp7	112.9397	0.316792178	0.234653	1.350046	0.177001	0.650558
mpx	307.2436	0.035478372	0.234936	0.151013	0.879966	0.986348
tbcelb	175.0784	0.098318533	0.184151	0.533903	0.593409	0.910427
agfg1b	249.302	0.027523082	0.152139	0.180907	0.85644	0.979558
parp1	773.563	0.029954867	0.140822	0.212715	0.83155	0.974908
hdgfl2	552.4303	0.100264677	0.126594	0.79202	0.428349	0.846133
fads2	215.4971	0.919081157	0.290141	3.167707	0.001536	0.05933
gpt2l	1600.004	-0.309632518	0.155835	-1.98693	0.046931	0.377563
cds1	140.2291	0.038780719	0.199518	0.194372	0.845884	0.977039
clcn7	337.3256	-0.215150527	0.125564	-1.71348	0.086625	0.493145
asap2b	531.1498	0.048205701	0.120899	0.398726	0.690095	0.939785
neurod1	1343.945	0.033914447	0.191199	0.177378	0.859211	0.979975
taf7	730.0909	-0.100020297	0.122586	-0.81592	0.414547	0.838683
ldb2a	451.723	-0.02022712	0.232724	-0.08691	0.930739	0.995809
uso1	953.1234	-0.219960359	0.112409	-1.95679	0.050372	0.390726
gstk2	1.245207	1.60801865	1.987502	0.809065	0.418478	NA
chico	77.89072	-0.095038247	0.229531	-0.41405	0.678834	0.936051
igl4v9	0	NA	NA	NA	NA	NA
senp8	36.49455	-0.60796505	0.293139	-2.07398	0.038081	0.342345
nrd1a	808.9234	0.234862389	0.112907	2.080142	0.037512	0.340049
col12a1b	1255.031	0.184732446	0.222429	0.830523	0.406243	0.834645
ip6k2b	1222.255	-0.034769554	0.164937	-0.21081	0.833039	0.975379
jade3	292.1599	-0.069204189	0.168637	-0.41037	0.681533	0.937418
yif1a	447.891	-0.12479629	0.141051	-0.88476	0.376285	0.822258
ldhba	20218.17	0.038627624	0.15162	0.254766	0.798903	0.966962
twist3	239.2741	-0.12885297	0.157507	-0.81808	0.413313	0.838317
zgc:162239	2.045405	-0.281911321	1.192495	-0.2364	0.813119	NA
pou2f2a	120.9215	0.075641419	0.17924	0.422011	0.673017	0.93414
rxfp2b	5.925421	1.706136246	1.104976	1.544048	0.122577	0.569468
fgl2b	70.60414	-1.801326203	1.39887	-1.2877	0.19785	0.678554
colq	102.1962	0.497626188	0.232491	2.140413	0.032321	0.318811
clcn5a	630.2315	-0.189945723	0.131729	-1.44194	0.149319	0.611148
aldocb	4406.35	0.219764842	0.242975	0.904477	0.365743	0.815695
zar1	9.269098	-1.883282874	0.723631	-2.60255	0.009253	0.165407
spout1	129.0025	-0.142933448	0.178414	-0.80113	0.423055	0.843894
rhoua	114.0154	-0.112568387	0.198537	-0.56699	0.570721	0.902481
oatx	131.4919	0.016859926	0.243675	0.06919	0.944838	0.997371
metap1d	120.1789	-0.51788245	0.203419	-2.54589	0.0109	0.181956
pbx2	1061.367	0.000235549	0.09663	0.002438	0.998055	1
celsr2	351.608	0.250671461	0.203729	1.230414	0.218542	0.699445
bmpr1aa	934.8876	0.130947274	0.111031	1.179375	0.238249	0.719242
mtch2	1123.834	0.013771295	0.116847	0.117858	0.90618	0.991631
ccndx	37.36563	-0.163205397	0.272638	-0.59862	0.549429	0.897192

tlr4ba	3.992041	-0.543819133	0.957129	-0.56818	0.569915	0.902322
dctn1a	181.2854	0.373246646	0.190966	1.954523	0.050639	0.391589
rfng	200.6957	-0.303191396	0.190063	-1.59521	0.110665	0.545461
hsd3b2	12.03726	-0.701324126	0.533795	-1.31385	0.188898	0.668515
rom1a	298.6521	0.090434315	0.66336	0.136328	0.891562	0.988566
kcnn3	218.3835	-0.002101064	0.211865	-0.00992	0.992088	1
acp5a	274.618	-1.173500214	0.44914	-2.61277	0.008981	0.162446
zgc:158803	777.9843	-0.182819116	0.180242	-1.0143	0.310439	0.775146
si:ch1073-280e3.1	55.49763	-0.301404165	0.467842	-0.64424	0.519417	0.887148
zhx3	352.6445	-0.238388924	0.181088	-1.31643	0.18803	0.667313
rps6	38397.33	-0.431713055	0.114108	-3.78338	0.000155	0.012534
impg2a	30.68766	-0.183939629	0.981613	-0.18739	0.851359	0.978548
prmt3	324.2656	-0.068364197	0.194202	-0.35203	0.724818	0.948548
qrich1	244.8141	0.206402152	0.170635	1.209615	0.226427	0.707798
evpla	636.1788	-0.169747889	0.145467	-1.16691	0.243245	0.72442
nfe2l3	422.7119	0.138536122	0.143712	0.963986	0.335053	0.79185
fn1a	1106.031	-0.080399593	0.217721	-0.36928	0.71192	0.945821
edrf1	199.7634	0.034424362	0.167241	0.205837	0.836918	0.976007
nkx2.1	74.50862	0.557538755	0.26064	2.139115	0.032426	0.319303
ugdh	626.7063	0.036622421	0.157398	0.232673	0.816015	0.971207
cnot3b	399.0537	0.13735931	0.130155	1.055355	0.291263	0.761431
pdlim1	934.5564	-0.151396985	0.108319	-1.39769	0.162206	0.629748
atp1a1b	2973.22	0.265413157	0.176375	1.504827	0.132369	0.585048
fgl2a	104.7322	-0.551026539	0.297357	-1.85308	0.063871	0.431455
hsph1	41.04326	0.207585496	0.325768	0.637219	0.523982	0.888694
rbks	87.64406	-0.215307515	0.197769	-1.08868	0.276295	0.750674
bsg	8091.311	-0.103302128	0.1095	-0.9434	0.345478	0.79876
gpat4	555.1359	0.082074474	0.127614	0.643145	0.52013	0.887148
rcvrn2	2224.955	-0.183193869	0.22696	-0.80717	0.419571	0.842383
dscc1	84.24868	0.186553717	0.263272	0.708596	0.478575	0.869292
epb41l3b	2481.099	-0.35897837	0.158286	-2.26791	0.023335	0.270061
lft1	0.653229	1.882458892	2.544082	0.739936	0.459339	NA
cmpk	1133.252	0.045576018	0.12097	0.376754	0.706356	0.944623
tal1	351.6681	0.007600537	0.117761	0.064542	0.948539	0.997738
slc5a11	27.25576	1.079887574	1.305205	0.82737	0.408027	0.835879
sgta	1178.699	0.056926898	0.116901	0.486966	0.626282	0.92063
ptprdb	88.83751	0.503154094	0.24738	2.033932	0.041958	0.35834
serpinh1b	2037.988	0.442129059	0.530528	0.833376	0.404633	0.834253
carf	220.3587	0.137241173	0.177326	0.773949	0.438961	0.852034
sec62	829.1163	-0.223419421	0.09002	-2.48189	0.013069	0.198789
znf513a	265.5749	-0.086478927	0.122486	-0.70603	0.480169	0.870001
dop1a	373.3076	-0.059077818	0.137772	-0.42881	0.668062	0.933086
sfxn1	63.82657	0.261815149	0.254642	1.028169	0.30387	0.769473
smarcd3b	526.1681	0.018916263	0.135252	0.139859	0.888771	0.988188
stk38a	133.649	0.413903901	0.2073	1.996637	0.045865	0.374848
idi1	1154.491	-0.219399382	0.144823	-1.51494	0.129786	0.579886
grhprb	2729.976	-0.294632027	0.216466	-1.3611	0.173483	0.64634
cabp1a	48.65807	0.438425662	0.339261	1.292294	0.196255	0.676773
bmp4	455.1228	-0.030733455	0.120346	-0.25537	0.798434	0.966698
slc30a7	457.0511	-0.16026853	0.13013	-1.2316	0.218098	0.69923
sh3bp4a	355.7997	0.110729912	0.128106	0.864363	0.387389	0.827592
rad23aa	421.8455	-0.310647095	0.143814	-2.16006	0.030768	0.312673
col1a2	12510.75	0.124130474	0.155014	0.800769	0.423265	0.843894
vcp	5462.036	0.235029288	0.112056	2.097434	0.035955	0.332966

bmp2k	344.5129	0.048471307	0.151899	0.319101	0.74965	0.955074
mrps33	303.4308	0.229784206	0.162012	1.418314	0.156099	0.621751
fam151b	86.68512	0.173494799	0.252781	0.686343	0.492497	0.876385
cps1	65.42921	0.069698002	0.354175	0.19679	0.843992	0.976791
cldn11a	571.4148	0.262412313	0.186617	1.406157	0.159678	0.625998
vps35l	528.2315	-0.018022809	0.111209	-0.16206	0.871257	0.983365
cnot8	494.7199	-0.007557768	0.116986	-0.0646	0.948489	0.997738
ahr1a	4.213779	2.022887374	1.574688	1.284627	0.198923	0.679593
nkd1	721.8261	-0.070732668	0.118673	-0.59603	0.551154	0.8974
aox6	152.5815	-0.245043011	0.195734	-1.25192	0.2106	0.692339
bmpr2b	147.9576	0.028391192	0.233792	0.121438	0.903344	0.99133
thbs4b	3381.117	-0.271902405	0.130423	-2.08478	0.03709	0.337668
nol10	415.2923	0.011853302	0.162106	0.07312	0.94171	0.997371
neil3	62.04067	-0.246140077	0.425436	-0.57856	0.562886	0.900772
ek1	128.8092	0.287417179	0.206256	1.393494	0.16347	0.631479
tg	223.2911	0.048431369	0.390031	0.124173	0.901178	0.990512
slc33a1	181.012	-0.103899243	0.167181	-0.62148	0.534285	0.891573
nuak1a	235.481	-0.089027589	0.258149	-0.34487	0.730193	0.950056
psmc2	2043.627	0.210175351	0.138045	1.522518	0.127879	0.577117
kctd16b	21.4814	1.480766021	0.621238	2.383574	0.017145	0.231774
usp2a	1911.34	0.022475528	0.140611	0.159841	0.873006	0.983537
slc20a1a	243.8535	0.007535715	0.194579	0.038728	0.969107	1
endou2	213.9412	0.691097845	0.240929	2.868467	0.004125	0.106542
coq8aa	1412.383	-0.578409891	0.126018	-4.5899	4.43E-06	0.000979
fnbp1l	481.1418	-0.037523109	0.108306	-0.34645	0.729001	0.949983
jdp2b	119.2256	-0.006191651	0.583434	-0.01061	0.991533	1
sipa1l1	382.9245	-0.065362819	0.131181	-0.49826	0.618299	0.916585
ptges	114.7949	-0.431581832	0.22145	-1.94889	0.051309	0.394059
pah	2376.736	-0.23354688	0.176946	-1.31988	0.186876	0.665301
acox1	27.16341	0.411024984	0.342952	1.198491	0.230726	0.71241
spag6	51.97965	0.157196676	0.267949	0.586666	0.557428	0.899184
efnb2a	856.463	0.33873766	0.13472	2.51439	0.011924	0.190141
zbtb22a	100.6992	-0.151482177	0.243455	-0.62222	0.533798	0.891181
nova1	308.8199	0.209010251	0.202591	1.031686	0.302219	0.768145
GCA	1082	0.032915584	0.160089	0.205608	0.837097	0.976007
ppef1	16.1054	-0.321114176	0.659684	-0.48677	0.626422	0.920665
rpl5a	24772.73	-0.271205363	0.124774	-2.17358	0.029737	0.307042
cdc73	504.5195	-0.244694947	0.115238	-2.12339	0.033721	0.324467
nf2a	73.48176	-0.074000894	0.245468	-0.30147	0.763057	0.958891
slc1a1	254.3949	-0.118763794	0.215395	-0.55138	0.581375	0.905618
cpsf4	422.7189	-0.031605961	0.140099	-0.2256	0.821515	0.971788
amfra	681.9021	-0.070465865	0.109967	-0.64079	0.521657	0.887551
dld	271.4778	-0.043468846	0.166711	-0.26074	0.79429	0.965431
si:ch211-225b10.3	51.06476	-0.433808437	0.275304	-1.57574	0.115085	0.554651
usf2	955.4549	0.004192043	0.105956	0.039564	0.968441	1
mapre3a	66.06373	0.572880443	0.295738	1.937123	0.05273	0.399138
eif6	974.3704	-0.149143348	0.176768	-0.84372	0.398825	0.831005
sept9a	1054.566	-0.078602139	0.097874	-0.8031	0.421918	0.843181
mc1r	70.25455	-0.405122557	0.276962	-1.46274	0.143539	0.602607
lpin1	1749.065	-0.55921328	0.26549	-2.10634	0.035174	0.330358
icmt	256.0835	-0.102880822	0.179772	-0.57229	0.567129	0.901759
fermt2	197.2349	-0.084176123	0.240986	-0.3493	0.726865	0.949095
rhoj	79.93434	0.071867163	0.275049	0.261288	0.79387	0.965431
btbd10b	805.2121	0.035315815	0.102506	0.344526	0.730451	0.950056

ewsr1a	3627.172	0.245809553	0.108199	2.27182	0.023097	0.26812
rab2a	3844.427	0.093089118	0.102657	0.906796	0.364515	0.814648
angptl6	124.5656	0.196078776	0.212597	0.922302	0.356371	0.807306
si:dkeyp-120h9.1	274.6388	0.145184488	0.152646	0.951119	0.341544	0.796294
efcab7	171.7584	0.182181409	0.171827	1.060261	0.289026	0.76024
si:ch211-278j3.3	175.1976	0.397502347	0.181294	2.19259	0.028337	0.299296
pif1	151.4933	0.202025718	0.261142	0.773625	0.439153	0.852079
TAFA3	120.5245	-0.239189254	0.287978	-0.83058	0.40621	0.834645
btg2	1367.076	-0.285957988	0.633686	-0.45126	0.651801	0.928625
os9	455.7813	-0.164095809	0.113604	-1.44446	0.148609	0.610295
cnih1	871.8005	0.119484753	0.110489	1.081419	0.279511	0.752477
tyk2	117.5363	-0.288419486	0.212949	-1.35441	0.175606	0.649413
plekha6	820.8046	-0.1730502	0.13349	-1.29636	0.194853	0.67483
nkx2.9	4.482112	0.539403979	0.882791	0.611021	0.541186	0.893683
ptpn11a	699.9581	0.053056217	0.091222	0.581619	0.560823	0.900005
clasp2	1885.066	0.091274968	0.102361	0.891692	0.372558	0.819953
lmx1a	7.724465	0.796763448	0.705945	1.128648	0.259046	0.736127
snrnp25	126.7959	0.114348825	0.221331	0.516641	0.605407	0.914985
fbp1b	3494.564	-0.346674398	0.218613	-1.58579	0.112787	0.549875
g6pc3	72.96282	-0.274448275	0.214905	-1.27707	0.201579	0.681423
eif4g2a	2664.231	0.131678739	0.091944	1.432159	0.152098	0.615705
nipsnap3a	205.3421	-0.417794683	0.193405	-2.16021	0.030757	0.312673
mical2b	369.2299	-0.217696015	0.192489	-1.13095	0.258075	0.735569
snx15	172.453	-0.119044313	0.164769	-0.72249	0.469993	0.867349
napab	910.5394	-0.051976984	0.148928	-0.34901	0.727083	0.949095
emx3	152.9305	0.036665482	0.151894	0.241389	0.809254	0.969828
nceh1a	1.51882	-0.149096834	1.308264	-0.11397	0.909265	NA
snx5	701.8195	0.038609917	0.120839	0.319517	0.749335	0.955074
xkr6a	3.970672	-0.542804497	1.204553	-0.45063	0.652258	0.928768
tmtc4	257.5143	0.172635369	0.148302	1.164082	0.244391	0.725223
adi1	430.5592	-0.32994148	0.167109	-1.97441	0.048335	0.382747
yipf3	724.3256	-0.089374107	0.146133	-0.6116	0.540806	0.893438
cacng8a	169.6117	0.275429103	0.25595	1.076107	0.28188	0.754378
psmd8	2452.179	0.249822981	0.176362	1.416534	0.156619	0.622452
si:ch211-171h4.3	23.94455	0.028913075	0.39104	0.073939	0.941059	0.997371
ewsr1b	2367.984	-0.002968983	0.097539	-0.03044	0.975717	1
safb	1402.239	0.112291612	0.150419	0.746525	0.45535	0.860008
map3k7	569.4908	-0.059568437	0.104941	-0.56764	0.570282	0.902481
hddc3	197.9962	0.177760226	0.171372	1.037277	0.299607	0.766128
znrf1	303.6773	0.184472988	0.170385	1.082682	0.27895	0.752417
nono	1920.991	0.170890876	0.095834	1.783201	0.074554	0.463807
lta	1.002965	0.548281047	1.691616	0.324117	0.74585	NA
arhgap17a	128.3284	0.003885355	0.205188	0.018936	0.984892	1
lgi1a	493.5894	0.002837178	0.154579	0.018354	0.985356	1
znf330	409.2122	-0.235927753	0.143553	-1.64349	0.100281	0.524748
rab7a	1933.634	-0.13598888	0.11338	-1.19941	0.230369	0.712099
h3f3a	10478.49	-0.054860883	0.167522	-0.32749	0.743301	0.954856
srd5a1	16.62946	0.413182763	0.503101	0.821272	0.411491	0.837797
exoc6	346.8052	0.031133272	0.113431	0.274469	0.783724	0.96245
nup62l	892.1185	-0.13181521	0.153807	-0.85702	0.391436	0.827902
hacl1	177.5954	-0.143697802	0.20522	-0.70021	0.483794	0.87219
ism1	558.3669	-0.026239489	0.107457	-0.24419	0.807088	0.969726
rem1	171.5974	-0.026951456	0.284153	-0.09485	0.924435	0.994815
si:dkey-77f5.10	0.599257	-0.868134652	2.479506	-0.35012	0.726246	NA

ddx3xa	1873.639	0.004732899	0.116671	0.040566	0.967642	1
atp2a1	146592.7	-0.072674195	0.209591	-0.34674	0.728785	0.94987
otofb	362.3984	0.159941936	0.191435	0.835488	0.403443	0.833803
txlnba	1842.437	0.246928134	0.227539	1.085214	0.277827	0.751752
gnl3l	483.0291	-0.211359781	0.160353	-1.31809	0.187472	0.666343
miga1	316.7264	0.061218674	0.162181	0.377471	0.705823	0.944413
rnf144aa	29.21088	-0.125070969	0.378655	-0.3303	0.741171	0.954103
grk7a	828.0702	1.049681999	0.390738	2.686408	0.007222	0.144046
nfe2	43.18525	-0.315606194	0.331171	-0.953	0.34059	0.795538
emc3	861.0082	0.133524294	0.086126	1.550328	0.121063	0.566931
snap25a	3180.905	0.208731627	0.20046	1.041265	0.297753	0.765407
tnnt2a	262.6653	0.039687577	0.221723	0.178996	0.857941	0.979774
si:ch211-160d20.3	94.9849	0.359613303	0.19306	1.862706	0.062504	0.427724
gatd3a	326.2931	-0.126179941	0.126784	-0.99524	0.319622	0.780289
ap1b1	866.7137	0.111213691	0.094185	1.180799	0.237683	0.718568
baxa	545.759	0.08386377	0.130266	0.643788	0.519713	0.887148
jak1	1144.05	-0.193110204	0.097248	-1.98575	0.047061	0.378022
gtdc1	63.87237	0.014056491	0.225058	0.062457	0.950199	0.99801
slc7a3a	1388.029	-0.015023356	0.165867	-0.09057	0.92783	0.99504
trpc4apb	325.0556	-0.980795572	0.237067	-4.1372	3.52E-05	0.004291
INSM2	67.53889	0.011299712	0.309725	0.036483	0.970897	1
prkg1a	87.24977	0.309031376	0.209934	1.47204	0.14101	0.599658
dpp3	648.2361	-0.030004774	0.148897	-0.20151	0.840297	0.976417
clpp	712.9754	0.372282355	0.110113	3.380919	0.000722	0.035767
sesn1	2746.378	-0.126800803	0.176153	-0.71983	0.471627	0.868097
slc9a8	180.1448	0.156983543	0.149195	1.052206	0.292705	0.762191
odf2b	90.51898	0.025251846	0.185316	0.136263	0.891613	0.988566
mdkb	3665.383	0.180993865	0.126915	1.426107	0.153838	0.618475
rrm2	719.0262	0.00032783	0.31924	0.001027	0.999181	1
slc25a22a	52.26269	0.351231893	0.272616	1.288378	0.197614	0.678229
smpd4	185.2857	-0.024210702	0.192453	-0.1258	0.89989	0.99045
efcab6	26.77394	-0.113835011	0.320814	-0.35483	0.722716	0.947632
zgc:153921	8.007864	0.387518373	0.608221	0.637135	0.524037	0.888694
fga	3269.561	0.053042694	0.221307	0.239679	0.810579	0.969828
bdkrb1	18.05502	0.577850663	0.556184	1.038956	0.298825	0.765735
gfi1aa	40.06702	-0.428985255	0.354669	-1.20954	0.226456	0.707798
caprin2	22.65963	0.503010687	0.43336	1.160722	0.245755	0.727177
tmem178	171.5788	0.5144944	0.201441	2.554066	0.010647	0.179908
elf1	550.8149	0.026495793	0.12618	0.209984	0.83368	0.975482
arrdc2	1989.831	-0.17465632	0.20781	-0.84046	0.40065	0.831725
slmapb	246.1825	0.026355121	0.25296	0.104187	0.917021	0.993657
tnr	79.64894	0.279001072	0.371645	0.75072	0.452821	0.858256
cep85l	389.1896	0.128539576	0.145235	0.885043	0.376133	0.822258
lama4	1531.323	0.076873319	0.150277	0.511545	0.60897	0.915204
sla2	10.22571	-1.482881062	0.83397	-1.7781	0.075388	0.465238
neurod6b	438.2242	0.193315901	0.192472	1.004383	0.315194	0.777834
rac3b	974.7581	0.277028098	0.139702	1.982993	0.047368	0.379101
necap1	516.5401	0.399607393	0.204862	1.950617	0.051103	0.393482
efemp2b	158.5478	-0.235163847	0.222447	-1.05717	0.290436	0.76078
si:ch73-177h5.2	0.755383	-0.818428908	2.227156	-0.36748	0.713263	NA
faimb	64.43913	0.034808585	0.550793	0.063197	0.949609	0.99801
srp68	1084.604	-0.07458754	0.097359	-0.76611	0.44361	0.854255
ift22	67.844	-0.194359421	0.296812	-0.65482	0.512581	0.885483
si:dkey-230p4.1	82.97314	0.193782488	0.218154	0.888281	0.37439	0.820992

mcrs1	554.4202	-0.084024733	0.109578	-0.7668	0.443198	0.854063
rbm7	274.0574	-0.265548566	0.135716	-1.95666	0.050388	0.390726
tns1b	275.7751	0.047633035	0.2373	0.200729	0.84091	0.97643
atp6v0a1a	915.5267	0.001184851	0.123085	0.009626	0.992319	1
eef1a1l1	182429.7	-0.563956341	0.105496	-5.34574	9.00E-08	4.44E-05
galr2a	0.978379	-0.868144414	2.47147	-0.35127	0.725389	NA
ccdc149b	185.7777	0.120146853	0.203686	0.589862	0.555283	0.898723
trim25l	39.06138	-0.42134132	0.436995	-0.96418	0.334956	0.79181
apoa4b.2	4247.562	0.704594047	0.353189	1.994951	0.046048	0.375021
ptprz1b	651.9763	-0.037975963	0.126098	-0.30116	0.76329	0.958924
nfat5a	11.80741	-0.028163056	0.567778	-0.0496	0.960439	0.999164
pdk2a	2633.923	-0.309719288	0.139486	-2.22043	0.02639	0.287876
gli2b	172.3146	-0.002901661	0.178251	-0.01628	0.987012	1
armc1l	236.6851	0.122368029	0.17738	0.689862	0.490281	0.875178
tmod4	5410.526	0.130694373	0.112867	1.157955	0.246882	0.727706
LO018197.1	130.5706	0.073710006	0.2311	0.318952	0.749763	0.955074
slc25a55a	2600.578	-0.220511263	0.197941	-1.11403	0.265268	0.742063
bag1	297.7108	0.080561659	0.140406	0.573775	0.56612	0.901739
cyb5r4	128.0052	-0.141892295	0.171898	-0.82545	0.409119	0.836637
gabrp	156.8055	0.290922492	0.21406	1.359069	0.174125	0.647379
ddx56	288.1027	-0.077752951	0.180101	-0.43172	0.665946	0.932664
myo1ca	286.65	-0.029804424	0.154261	-0.19321	0.846796	0.977263
creb3l3l	1228.674	-0.152086555	0.125704	-1.20987	0.226327	0.707597
fam49ba	403.7585	-0.124196487	0.134409	-0.92402	0.355475	0.806546
ezra	458.8048	0.391125971	0.143868	2.718651	0.006555	0.136957
auts2b	268.2109	0.021728026	0.195328	0.111238	0.911427	0.992236
gmcl1	424.6386	-0.074887288	0.114212	-0.65568	0.512027	0.885217
si:ch211-214j8.1	56.70315	-1.67723513	0.777431	-2.15741	0.030974	0.313376
dnajb6b	295.1238	-0.18204508	0.133818	-1.36039	0.173705	0.646897
pck2	2131.592	0.551155633	0.133448	4.130107	3.63E-05	0.004382
pth1ra	10.6983	-0.786237116	0.549931	-1.4297	0.152802	0.617015
hgs	566.2737	-0.214195236	0.149371	-1.43398	0.151577	0.615372
thap12b	150.5387	0.197814152	0.160003	1.236315	0.216341	0.69815
ubald1b	541.8272	-0.391731521	0.159438	-2.45696	0.014012	0.207448
ripor3	209.04	0.045159456	0.144219	0.313132	0.754181	0.955996
slc35a3b	21.56468	-0.084129787	0.468523	-0.17956	0.857495	0.979741
prickle2a	183.6937	0.347501906	0.248451	1.398673	0.161911	0.629443
slc16a10	977.7216	-0.14537554	0.1169	-1.24359	0.213649	0.696139
ctu1	77.37135	-0.016721111	0.275029	-0.0608	0.95152	0.99801
c5	599.54	-0.165118848	0.244814	-0.67447	0.500016	0.879879
trim35-28	317.7767	-0.148426216	0.179419	-0.82726	0.408091	0.835879
ift57	108.5893	-0.013327176	0.171825	-0.07756	0.938176	0.997364
foxd3	299.4751	-0.104472451	0.244356	-0.42754	0.668985	0.933115
herpud2	550.8245	-0.001503497	0.129689	-0.01159	0.99075	1
rhbdd1	90.09884	-0.012142126	0.283252	-0.04287	0.965808	1
clptm1l	349.6982	0.00124063	0.149457	0.008301	0.993377	1
isl1l	2.659212	-0.491879919	1.639335	-0.30005	0.76414	0.959228
alas1	1806.452	-0.534918948	0.140359	-3.81107	0.000138	0.01183
sec31a	556.6871	-0.152374156	0.127894	-1.19141	0.233492	0.714569
znf367	115.4472	0.254067361	0.256163	0.991819	0.321286	0.781707
nelfcd	397.559	-0.23227202	0.145118	-1.60057	0.109472	0.542678
sf3a2	1072.318	-0.010040389	0.118313	-0.08486	0.93237	0.996174
poc1b	102.598	0.040841158	0.208023	0.19633	0.844352	0.976791
c1d	330.1669	-0.226629821	0.198135	-1.14381	0.252701	0.732759

ptmaa	8711.538	0.017116537	0.177828	0.096253	0.92332	0.994525
spata6	39.01701	-0.420246789	0.299772	-1.40189	0.160949	0.627788
ndc1	406.6521	-0.056327897	0.138596	-0.40642	0.684435	0.938486
cfl1	9565.434	0.402828298	0.14297	2.817575	0.004839	0.115344
dhrs4	384.8679	-0.048450646	0.267307	-0.18125	0.856168	0.979558
adgrv1	365.9787	0.019536582	0.133729	0.146091	0.88385	0.987014
pabpc1b	2086.116	0.64174083	0.182969	3.507378	0.000453	0.025763
plekhf2	271.9687	-0.10943419	0.121244	-0.90259	0.366741	0.816111
rtn1b	1328.389	0.021212534	0.163533	0.129714	0.896792	0.98939
nipal3	402.7537	-0.210776095	0.133912	-1.57399	0.115491	0.555747
cbr1l	636.4696	-0.280841994	0.226977	-1.23731	0.215971	0.697913
valopa	12.38733	0.531424709	0.649852	0.817762	0.413493	0.838369
ptpreb	294.9588	0.208762942	0.170914	1.221449	0.221916	0.703829
zgc:92040	722.7175	0.098264733	0.165206	0.5948	0.551977	0.8974
thrb	294.3822	0.18500327	0.151167	1.223836	0.221014	0.702262
cyp2ad2	273.4132	0.084270579	0.619451	0.136041	0.891789	0.988566
cept1b	144.1596	0.057223913	0.180479	0.317068	0.751192	0.955226
rbfox1l	1586.288	0.162704326	0.146659	1.109407	0.267255	0.743462
si:dkeyp-4h4.1	3.985662	0.080587837	1.674292	0.048132	0.961611	0.999451
coro1cb	1447.813	0.195469522	0.111534	1.752561	0.079677	0.477652
st8sia6	595.6232	0.121768671	0.120001	1.014731	0.310234	0.775062
nebl	23.773	-1.198930142	0.490443	-2.44459	0.014502	0.210663
noto	8.751621	0.104185285	0.583473	0.178561	0.858283	0.979774
serpind1	129.5182	-0.49961782	0.246138	-2.02983	0.042374	0.360425
kctd9a	25.25741	0.250380316	0.391581	0.639408	0.522557	0.887826
hmgcl	582.6408	-0.086868417	0.108475	-0.80082	0.423237	0.843894
prkci	1031.316	-0.21123421	0.08236	-2.56477	0.010324	0.176586
nkx2.7	24.73685	-0.284466028	0.331526	-0.85805	0.390865	0.827902
pgls	134.3266	0.469357149	0.204299	2.2974	0.021596	0.26075
apaf1	397.8574	0.290403338	0.13183	2.20287	0.027604	0.295283
zgc:165604	58.92593	-0.393365782	0.37975	-1.03585	0.30027	0.766629
mvp	1401.462	0.019254897	0.166348	0.11575	0.90785	0.991745
slc25a48	483.3031	-0.252448778	0.193623	-1.30382	0.192296	0.672283
cct6a	5850.086	-0.301883228	0.12022	-2.51109	0.012036	0.190742
arhgap22	28.23558	0.242174212	0.372554	0.650037	0.515668	0.88655
eif3d	5866.93	-0.330983215	0.118771	-2.78673	0.005324	0.122593
mybpc2b	2671.437	-0.058072119	0.261299	-0.22224	0.824124	0.97205
rab7b	276.1616	0.088394165	0.153506	0.575835	0.564726	0.901461
mier3b	124.3147	0.040218618	0.204318	0.196843	0.84395	0.976791
slc30a2	5.871313	-1.519492219	0.83252	-1.82517	0.067975	0.44461
rhoca	834.4139	0.060316834	0.09399	0.641736	0.521045	0.887466
nsun4	54.97314	-0.280353059	0.219628	-1.27649	0.201781	0.681726
pax4	2.934756	3.889488365	1.616013	2.406843	0.016091	0.223897
cpa5	31471.09	-0.039017116	0.219059	-0.17811	0.858635	0.979931
wsb1	2636.836	-0.107434841	0.203277	-0.52851	0.597143	0.91193
prph2lb	42.20537	0.297225935	0.515794	0.576249	0.564447	0.901433
pdhb	4057.787	0.129563976	0.095961	1.350179	0.176958	0.650504
nefma	623.9723	0.771776332	0.274615	2.810391	0.004948	0.117227
gria1a	292.6068	0.105496574	0.220899	0.477578	0.632951	0.923885
fbp1a	816.0422	-0.162210587	0.18551	-0.8744	0.381899	0.824957
mc3r	3.28164	1.550197613	1.088912	1.423621	0.154556	0.619425
actr6	290.317	0.205242725	0.138322	1.483804	0.137861	0.593945
tob1b	2833.587	-0.173966143	0.167125	-1.04093	0.297907	0.765407
cab39l1	752.9278	-0.070321998	0.140325	-0.50114	0.616276	0.916365

phf21ab	135.4592	0.139811893	0.21695	0.644442	0.519289	0.887148
ppm1db	171.7364	0.052514342	0.199141	0.263705	0.792007	0.964867
naalad2	175.0127	0.100092656	0.139779	0.716079	0.473942	0.869292
jag2b	403.3488	0.032217322	0.135367	0.238	0.811881	0.97012
chrnb5b	16.44684	0.791542022	0.51665	1.532066	0.125506	0.57334
mul1a	279.5781	0.166910534	0.138417	1.20585	0.227875	0.709278
yipf2	504.2114	-0.153096729	0.140039	-1.09325	0.274286	0.749612
mcm3ap	454.8267	-0.066445974	0.175189	-0.37928	0.704479	0.943785
zgc:110319	309.6451	0.225163989	0.135	1.667881	0.095339	0.515402
chd3	290.1528	0.80425929	0.186547	4.311287	1.62E-05	0.002576
poglut3	195.0714	0.075639114	0.160509	0.471245	0.637466	0.924779
odf3l2b	4.548047	-0.656049005	0.739897	-0.88668	0.375253	0.822186
asmtl	475.0163	0.289186294	0.165118	1.751395	0.079878	0.478112
ncoa4	1697.825	0.164592915	0.099987	1.646139	0.099735	0.524166
cdh11	1467.379	0.18833261	0.138342	1.361351	0.173403	0.64634
zfp36l1b	1399.672	0.039943192	0.119119	0.335322	0.737382	0.952375
prf1.2	0	NA	NA	NA	NA	NA
strbp	469.1674	0.053923588	0.104436	0.516332	0.605623	0.915049
daw1	48.55626	-0.297866703	0.274423	-1.08543	0.277731	0.751752
ireb2	601.6881	-0.041104311	0.141748	-0.28998	0.771831	0.960624
olfml2bb	91.60308	-0.142850406	0.262075	-0.54507	0.585703	0.906655
cdc14b	913.9754	0.081953627	0.126336	0.648695	0.516535	0.886933
cyth1b	286.296	-0.053488382	0.116973	-0.45727	0.647477	0.927082
hnf4a	207.979	-0.412566343	0.200983	-2.05274	0.040097	0.350191
gpr132a	0.129399	0	5.267649	0	1	NA
tab2	408.4508	-0.016679905	0.121288	-0.13752	0.890618	0.988446
rgs14a	14.81134	0.012157081	0.449268	0.02706	0.978412	1
ephb2b	443.6029	0.163009683	0.15863	1.027611	0.304133	0.769716
vrk2	42.04516	-0.701747252	0.274811	-2.55356	0.010663	0.179908
rad23b	2828.94	0.07333229	0.118632	0.618147	0.536478	0.892397
prkn	102.1101	-0.657747345	0.29033	-2.26552	0.023481	0.270809
ubl3b	720.658	-0.005423526	0.106929	-0.05072	0.959548	0.999164
wdr45b	1142.755	-0.196522787	0.176804	-1.11153	0.26634	0.742912
OTOP1	93.32595	-0.672606362	0.192733	-3.48984	0.000483	0.027042
gdap2	238.6842	-0.086202512	0.16282	-0.52943	0.596504	0.911676
cgas	5.238192	-1.808126405	0.88646	-2.03972	0.041379	0.355014
slc16a7	64.44668	-1.624324308	0.645421	-2.51669	0.011846	0.189426
orc5	97.3935	-0.470583559	0.207129	-2.27194	0.02309	0.26812
dynlrb1	258.6289	-0.291060913	0.224636	-1.2957	0.195078	0.67483
cntn5	255.3328	0.048875232	0.229065	0.213368	0.83104	0.974908
tmem60	108.7144	-0.152819465	0.183606	-0.83232	0.405226	0.834483
magi2a	113.6234	-0.281103164	0.198839	-1.41372	0.157443	0.623263
lhfpl3	442.8519	0.255976944	0.240234	1.065532	0.286635	0.757624
negr1	203.9797	0.4570195	0.208112	2.196029	0.02809	0.298489
si:dkey-151g10.3	161.605	0.113770256	0.237121	0.479798	0.631371	0.923003
sh3bgr	881.0199	0.294674104	0.198828	1.482056	0.138325	0.59495
gnai1	1144.442	-0.017177167	0.11637	-0.14761	0.882652	0.986908
fzd3a	398.9144	-0.015504821	0.146148	-0.10609	0.915511	0.993078
sec61a1	5322.372	-0.465436174	0.132921	-3.5016	0.000462	0.026072
phf11	16.72325	1.083670628	0.628149	1.72518	0.084495	0.487852
asrgl1	160.3503	0.101679298	0.218084	0.466239	0.641045	0.925364
mxa	4.557484	-1.556645584	1.08736	-1.43158	0.152263	0.616018
morn3	27.46086	0.025994185	0.359093	0.072389	0.942293	0.997371
pdcd4a	1553.091	-0.403204011	0.141809	-2.8433	0.004465	0.110056

col7a1	394.6362	0.125692403	0.173728	0.723502	0.469371	0.867224
cacna1ba	64.53879	-0.207753131	0.406438	-0.51116	0.609242	0.915204
klhl24a	300.3199	-0.202671094	0.190645	-1.06308	0.287744	0.758441
ccdc25	522.8203	0.041218266	0.162801	0.253182	0.800127	0.967256
abcb5	1141.796	-0.183897634	0.237452	-0.77446	0.438657	0.851801
myoc	965.2429	-0.132365945	0.121555	-1.08893	0.276183	0.750674
hmmr	326.9007	0.297296957	0.306574	0.969739	0.332176	0.789602
tle5	88.17971	-0.020748187	0.287749	-0.07211	0.942518	0.997371
zfp36l2	1317.577	-0.109333604	0.154002	-0.70995	0.477736	0.869292
nmt2	419.8543	-0.003193475	0.118114	-0.02704	0.97843	1
calm1a	5299.82	0.118998961	0.127251	0.935151	0.34971	0.801408
cherp	644.1596	0.029626031	0.130944	0.22625	0.821007	0.971788
katna1	99.15047	0.111353951	0.224912	0.495101	0.620529	0.918076
ahr2	1191.508	0.059656417	0.130698	0.456443	0.648071	0.927245
rps23	22813.61	-0.339251807	0.123458	-2.74792	0.005997	0.131072
hid1a	40.56593	-0.070351558	0.32584	-0.21591	0.829059	0.974547
si:dkey-256h2.1	34.65742	-0.397838938	0.388994	-1.02274	0.306432	0.771574
ntpcr	84.40772	-0.530722608	0.246566	-2.15245	0.031362	0.314976
arnt	152.1383	0.452600341	0.298201	1.517771	0.129072	0.578533
erap1b	35.61115	-1.079216949	0.554497	-1.9463	0.051619	0.394983
rplp1	34204.85	-0.273554281	0.135452	-2.01956	0.043429	0.365245
upk1a	5.659523	0.298365702	1.083709	0.275319	0.783071	0.962328
rcan2	149.831	-0.321409168	0.264618	-1.21461	0.224513	0.706406
fbxl3b	272.4033	-0.271353469	0.15716	-1.72661	0.084239	0.487221
cacul1	4.509271	2.052416271	1.029384	1.993829	0.046171	0.375668
gja3	36.96783	0.980453857	0.372274	2.633689	0.008446	0.156983
zbtb7b	151.108	-0.072859782	0.176779	-0.41215	0.680229	0.936798
disc1	59.86483	-0.066457117	0.261056	-0.25457	0.799055	0.967043
asap3	305.1577	-0.126807196	0.149362	-0.84899	0.395887	0.829389
b4galt7	165.1456	0.071424413	0.177828	0.401649	0.687943	0.938937
derl1	875.6614	-0.023658545	0.138005	-0.17143	0.863884	0.981859
VSTM2A	4.472623	-2.052052831	0.962333	-2.13237	0.032976	0.321438
ak9	26.48236	-0.807458415	0.407953	-1.97929	0.047783	0.380722
vax1	98.60123	0.701528603	0.221046	3.173683	0.001505	0.058515
hsp70.3	436.2775	-0.099805824	0.609593	-0.16373	0.869947	0.983365
smad9	305.5486	-0.002834433	0.142208	-0.01993	0.984098	1
ddx23	948.9428	-0.016908261	0.09364	-0.18057	0.856708	0.979558
tnc	1913.744	0.273076661	0.228986	1.192547	0.233047	0.714569
zgc:101100	57.62481	0.430718879	0.229125	1.879842	0.06013	0.422294
stl3	1780.398	-0.187375646	0.142964	-1.31065	0.189976	0.66964
sfr1	15.11682	-0.291211617	0.452618	-0.64339	0.519969	0.887148
ginm1	996.3884	-0.439864572	0.119616	-3.67731	0.000236	0.01655
mical3a	84.60019	0.037692282	0.315095	0.119622	0.904783	0.991619
sh3bp2	13.81711	-0.461476364	0.466931	-0.98832	0.322997	0.782754
ndufa2	1011.706	0.028179093	0.157764	0.178616	0.85824	0.979774
ercc2	209.0977	-0.167475788	0.150696	-1.11135	0.266419	0.742912
plecb	2013.61	0.05622826	0.186321	0.301782	0.762818	0.958842
erlin1	1528.035	-0.048586744	0.125456	-0.38728	0.698547	0.942433
ppp2r2aa	90.96161	0.048648133	0.173078	0.281076	0.778652	0.961073
cfap206	11.54669	-0.05574858	0.516649	-0.1079	0.914072	0.992969
traf3	155.0631	-0.402825362	0.229161	-1.75783	0.078777	0.474808
zgc:66426	10.59984	0.224386068	0.476424	0.47098	0.637655	0.924779
focad	142.0984	0.385785866	0.196444	1.963847	0.049548	0.387714
map1ab	252.5911	0.169863466	0.291984	0.581756	0.560731	0.899982

tlr4bb	0.253593	0	5.267649	0	1	NA
si:dkey-34e4.1	92.6942	0.307109545	0.22203	1.38319	0.166607	0.636904
opn4a	2.875921	2.612410716	1.59656	1.636274	0.101782	0.528581
obscnb	247.1796	0.028767465	0.341926	0.084134	0.93295	0.996264
rbm4.3	2703.849	0.102548759	0.094804	1.081688	0.279391	0.752477
ocstamp	8.809735	-0.575593398	0.57989	-0.99259	0.32091	0.781284
ttc23	50.68663	-0.326799924	0.301734	-1.08307	0.278776	0.752299
mgst1.2	578.7936	0.142449984	0.168095	0.847437	0.396751	0.830253
IGLON5	161.2264	-0.134341829	0.27886	-0.48175	0.629981	0.922869
ddx59	147.8262	0.127959358	0.203037	0.630226	0.528547	0.890002
gsto1	89.65468	0.027318983	0.3178	0.085963	0.931496	0.995809
usp6nl	284.9672	0.280396156	0.130324	2.151529	0.031434	0.315297
gtf2ird1	401.9613	0.205178568	0.188009	1.091324	0.27513	0.750424
uck2b	631.5658	-0.225515355	0.102217	-2.20625	0.027367	0.293836
uck1	56.25402	-0.2771747	0.323146	-0.85774	0.391037	0.827902
ppan	299.5174	0.004637343	0.230883	0.020085	0.983975	1
prkcbb	351.0795	0.55746291	0.173545	3.212215	0.001317	0.053354
si:ch211-57i17.1	282.4931	-0.147130886	0.168566	-0.87284	0.382751	0.825577
pdzk1	487.2384	-0.131326204	0.354493	-0.37046	0.711039	0.945821
trim35-34	2.254786	-0.950472762	1.166064	-0.81511	0.415008	0.838732
brd2a	1323.997	-0.046505325	0.144826	-0.32111	0.748126	0.955074
hnf1bb	53.1122	-0.684249088	0.373464	-1.83217	0.066927	0.44184
higd1a	634.783	0.515346563	0.203213	2.535996	0.011213	0.183909
dspa	1070.418	-0.025421617	0.177181	-0.14348	0.885913	0.987533
atp6v1g1	2991.094	0.014845	0.13025	0.113973	0.909259	0.992067
or115-5	0.127238	0	5.267649	0	1	NA
si:ch211-156l18.7	0.321363	0.042929173	3.863243	0.011112	0.991134	NA
ufc1	441.2136	-0.141949324	0.196562	-0.72216	0.470196	0.86742
nek1	300.7439	0.169095264	0.126215	1.339737	0.180331	0.656129
kng1	678.7903	-0.241857765	0.247462	-0.97735	0.328394	0.786776
mtmr1b	102.9261	-0.007753631	0.198296	-0.0391	0.96881	1
ntmt1	570.9969	0.144605791	0.124567	1.16087	0.245695	0.727177
rrp12	1528.248	0.236448358	0.184475	1.281737	0.199935	0.6804
ing5a	242.9199	-0.043625153	0.178587	-0.24428	0.807014	0.969726
faf1	551.1103	0.223343024	0.159878	1.396955	0.162427	0.629825
slc26a5	700.2433	-0.313011795	0.126982	-2.465	0.013701	0.204799
ppp1r8b	524.9442	0.031305404	0.131953	0.237246	0.812466	0.970252
cd81b	239.7404	-0.13408901	0.222789	-0.60186	0.547264	0.896121
cox6a1	4489.852	0.01133626	0.118021	0.096053	0.923478	0.994525
eno1a	10503.42	-0.041773291	0.200629	-0.20821	0.835064	0.976007
clcn5b	136.7926	0.023289748	0.232135	0.100328	0.920084	0.993955
rad21l1	2.882628	-0.887544628	1.669238	-0.53171	0.594929	0.910869
pkd2l1	40.6834	0.147347002	0.376955	0.390887	0.695881	0.941649
cox4i2	659.2961	-0.143568145	0.162081	-0.88578	0.375737	0.822237
zgc:109913	9.752484	0.509273723	0.621373	0.819595	0.412447	0.838123
pex5la	18.49057	0.357426493	0.498783	0.716597	0.473623	0.869146
mchr1b	154.3387	0.88244009	0.373872	2.360276	0.018261	0.238375
ntn1b	362.8461	0.592234238	0.149272	3.967471	7.26E-05	0.007162
chrnb1l	21.8629	0.065699203	0.474567	0.13844	0.889892	0.98835
gxylt1b	75.6332	0.149381457	0.28555	0.523136	0.600879	0.913756
clcnk	41.87045	-0.25312029	0.335209	-0.75511	0.450182	0.857151
zgc:56106	220.8804	0.125560332	0.157564	0.796886	0.425517	0.844714
nkx6.1	142.5726	-0.349857782	0.259642	-1.34746	0.177831	0.651389
nmur3	12.14754	-0.50926588	1.39824	-0.36422	0.715694	0.9461

dnpep	586.0173	-0.107021567	0.174222	-0.61428	0.539029	0.892985
ddx43	36.84021	0.482642813	0.365099	1.32195	0.186185	0.664263
si:ch211-241e1.3	167.816	0.156567049	0.220706	0.709393	0.478081	0.869292
fbxo44	312.9453	-0.01090808	0.156047	-0.0699	0.944271	0.997371
cyp2p9	147.78	0.118864794	0.379779	0.312984	0.754293	0.956018
otud3	219.1289	0.00274858	0.210825	0.013037	0.989598	1
cyp2ad3	1510.364	-0.730604475	0.143117	-5.10495	3.31E-07	0.000124
psmf1	176.6803	0.078822302	0.168432	0.467976	0.639802	0.924779
snx4	654.2642	0.029742458	0.102834	0.289227	0.772408	0.960624
armc2	41.41248	0.782986635	0.310988	2.517739	0.011811	0.189124
grapb	11.63328	0.663324236	1.028235	0.64511	0.518856	0.887148
he1.3	1.65706	1.736349992	1.961027	0.885429	0.375925	NA
fkbp1aa	6965.343	0.06433746	0.134445	0.478542	0.632265	0.923215
itgb1b.2	549.7366	0.21365246	0.260833	0.819116	0.41272	0.838123
chic2	427.8138	0.021332732	0.155335	0.137334	0.890767	0.988556
lad1	152.535	-0.199107483	0.265749	-0.74923	0.453718	0.858742
stat3	331.4017	0.072444667	0.154693	0.468313	0.639561	0.924779
casp8ap2	154.484	-0.259579812	0.210401	-1.23374	0.2173	0.69815
epha2b	251.446	-0.042360451	0.222037	-0.19078	0.848697	0.977918
aasdh	45.90481	-0.679953949	0.42396	-1.60382	0.108754	0.541319
hpcal1	122.2038	-0.277713505	0.197439	-1.40658	0.159552	0.6257
apobb.1	19409.81	0.425595506	0.239637	1.776002	0.075733	0.46631
gid8a	312.3074	-0.072213757	0.141401	-0.5107	0.60956	0.915204
si:ch211-106a19.1	201.2209	0.003549311	0.164653	0.021556	0.982802	1
cops7a	602.8459	0.008470556	0.132936	0.063719	0.949194	0.99801
golt1ba	84.32129	0.028274913	0.228814	0.123571	0.901655	0.990643
pitpnc1b	204.8634	-0.07334048	0.129435	-0.56662	0.570973	0.902501
fzr1b	509.0661	0.006582659	0.138015	0.047695	0.961959	0.999451
dnd1	4.660731	1.41653146	0.7242	1.955994	0.050466	0.390807
pvalb3	544.9061	0.152646983	0.256613	0.594853	0.551942	0.8974
bnip4	367.2154	-0.575840333	0.238098	-2.4185	0.015584	0.220447
ptk2ba	22.93014	0.199758656	0.344308	0.580174	0.561797	0.900715
lias	297.8211	0.029421844	0.145944	0.201597	0.840232	0.976417
eogt	71.08649	0.095883731	0.335942	0.285417	0.775325	0.960964
wnt7ba	11.19696	-0.309814543	0.589865	-0.52523	0.599424	0.913246
zgc:175214	690.3579	-0.643768977	0.209158	-3.07791	0.002085	0.071344
mcf2a	68.72836	0.264049082	0.282142	0.935873	0.349339	0.801408
skilb	133.19	-0.149288684	0.177205	-0.84246	0.39953	0.831196
znf1163	24.08813	0.375677765	0.468404	0.802038	0.422531	0.843493
tbcc	193.7854	-0.21674602	0.153568	-1.4114	0.158128	0.623766
taar11	1.234672	4.205882136	1.85357	2.269071	0.023264	NA
ino80e	226.5192	-0.203904224	0.198859	-1.02537	0.305188	0.770369
ecd	170.1692	-0.392188039	0.172871	-2.26867	0.023288	0.269659
pth2	32.43214	0.097118195	0.571879	0.169823	0.865149	0.982142
zgc:66448	352.5511	-0.07347781	0.123714	-0.59393	0.552556	0.8974
fxr1	2789.191	-0.258598642	0.134337	-1.925	0.05423	0.402725
tm9sf1	347.3765	0.158188454	0.120193	1.316122	0.188133	0.667333
epha6	160.7279	0.535969912	0.309522	1.731605	0.083344	0.484434
wdr13	537.5085	0.097386533	0.125159	0.778105	0.436507	0.850912
tmpob	686.4921	0.23371176	0.130164	1.795512	0.072572	0.457864
ccdc28a	10.53608	-0.761048052	0.562226	-1.35363	0.175853	0.649413
clic4	1485.469	0.377931823	0.113291	3.335948	0.00085	0.039393
setx	243.2552	-0.112568658	0.176282	-0.63857	0.523101	0.888312
dtl	76.05487	0.266066199	0.244985	1.086051	0.277456	0.751426

ccdc12	166.1365	0.08667805	0.19452	0.445599	0.655887	0.929606
pkp2	101.9608	-0.501860644	0.232477	-2.15876	0.030869	0.312739
cdv3	1875.584	0.167858391	0.127124	1.320426	0.186693	0.665181
hoxa2b	141.9596	0.000652267	0.166996	0.003906	0.996884	1
si:dkey-23a23.2	98.81865	-0.091367635	0.333021	-0.27436	0.783808	0.96245
zbtb1	161.1624	0.074781055	0.180829	0.413546	0.679207	0.936209
fam129ab	34.24591	0.328229211	0.317655	1.033287	0.30147	0.767562
foxo3a	1112.425	0.069665561	0.142187	0.489958	0.624164	0.919499
ccn1	653.8815	-0.006811855	0.457042	-0.0149	0.988109	1
krt1-19d	2486.904	-0.211897948	0.166074	-1.27592	0.201983	0.68211
scaper	350.9113	0.175958362	0.125941	1.397151	0.162368	0.629825
mapk7	210.1724	0.075213745	0.147432	0.510157	0.609941	0.915331
plg	2078.653	0.290322719	0.252882	1.148056	0.250945	0.731279
gopc	167.4944	-0.092690422	0.162479	-0.57048	0.568355	0.902146
ucp1	17484.12	-0.666479923	0.213047	-3.12832	0.001758	0.064556
wdr73	82.69236	-0.11484382	0.236097	-0.48643	0.626664	0.920665
cbln10	362.8735	-0.47677131	0.244932	-1.94654	0.05159	0.394954
snrnp40	286.1137	0.063512594	0.1554	0.408703	0.682757	0.938006
v2rx4	0.307083	0	4.634512	0	1	NA
fez1	1912.381	0.176992678	0.108665	1.628795	0.103356	0.530446
tdo2b	369.7044	-3.758883311	1.309182	-2.87117	0.00409	0.106542
pcp4l1	201.4534	-0.196067963	0.251411	-0.77987	0.435467	0.850467
si:rp71-1g18.13	45.18037	-0.392402425	0.287664	-1.3641	0.172537	0.645319
lcp1	406.5022	0.007278103	0.175073	0.041572	0.96684	1
kpna4	1715.867	0.034087846	0.108729	0.313511	0.753892	0.955912
mhc1lda	5.132798	-0.276789783	1.347005	-0.20549	0.837193	0.976007
mob2a	275.9487	0.045393154	0.164381	0.276146	0.782436	0.962328
crema	228.5176	-0.103825613	0.257803	-0.40273	0.687145	0.938835
selenot2	1528.226	0.10259089	0.11435	0.897168	0.369629	0.818064
vsnl1a	1142.48	0.64948394	0.218706	2.969673	0.002981	0.090034
kcnh1b	6.879118	-0.083768294	1.064897	-0.07866	0.9373	0.997305
syne3	567.4705	-0.069384936	0.112509	-0.6167	0.53743	0.892498
atad1a	106.6736	0.010952551	0.20355	0.053808	0.957088	0.999109
tfap2d	133.8685	0.082050466	0.203234	0.403725	0.686415	0.938732
psmd4b	1057.859	0.138345936	0.119844	1.15438	0.248345	0.728649
hsd17b3	9.185037	0.364164839	0.610606	0.596399	0.550908	0.8974
fabp3	6291.354	-0.06548823	0.136294	-0.48049	0.630877	0.923003
rps27.1	25099.68	-0.261199837	0.131208	-1.99073	0.046511	0.376263
snu13b	813.2002	-0.20100468	0.174047	-1.15489	0.248136	0.728489
gpbar1	3.616249	0.211915703	0.927439	0.228496	0.819261	0.971785
zgc:66427	547.9345	-0.247757666	0.135532	-1.82803	0.067545	0.443563
spryd4	446.1295	-0.423098128	0.150381	-2.81352	0.0049	0.116571
sim1a	78.10719	0.204274609	0.261293	0.781782	0.434342	0.850002
fmn2a	188.677	-0.627922401	0.180406	-3.48061	0.0005	0.027598
ywhaqb	6246.939	-0.010085984	0.106052	-0.0951	0.924232	0.994815
anp32b	2646.898	0.018220952	0.125139	0.145605	0.884233	0.987047
nr5a1b	3.523011	1.065070906	1.005651	1.059086	0.289561	0.760499
mxd	6.744447	-0.221941392	0.856703	-0.25906	0.795585	0.966077
slc37a2	964.3469	-0.224109216	0.088084	-2.54426	0.010951	0.182182
atg5	370.5289	-0.18071216	0.116876	-1.54618	0.122061	0.569043
tjp2b	915.4651	-0.230857221	0.132877	-1.73738	0.08232	0.482739
atp2b3b	593.1466	0.639328129	0.232664	2.747854	0.005999	0.131072
galnt14	197.9138	-0.321285171	0.230265	-1.39528	0.16293	0.630534
ctnnb2	3374.154	-0.002479343	0.09779	-0.02535	0.979773	1

lrp13	1.546634	-1.404349311	1.743338	-0.80555	0.420501	NA
rflna	51.93901	-0.121808025	0.29371	-0.41472	0.678345	0.936026
ift74	374.3113	0.689148573	0.183727	3.750931	0.000176	0.013744
gmppab	265.2204	0.330113035	0.175266	1.883492	0.059634	0.421125
ankrd49	132.2061	0.154647949	0.193754	0.798165	0.424775	0.844714
BX323555.1	0.323151	1.854116729	5.241768	0.35372	0.723549	NA
ggt1a	15.80845	1.045279319	0.702654	1.487616	0.136852	0.592353
pinx1	705.9409	-0.122184068	0.192459	-0.63486	0.525522	0.888928
nnt	8168.6	0.117538026	0.133078	0.883227	0.377113	0.822451
ahr1b	101.8803	0.817814819	0.209304	3.907312	9.33E-05	0.008719
cdh7a	157.9288	0.353228629	0.252009	1.401649	0.16102	0.627788
kcnn1b	20.18739	0.302078067	0.487132	0.620115	0.535182	0.891993
lpp	385.3112	-0.064315971	0.106349	-0.60476	0.545338	0.895275
coq9	825.0915	0.126589134	0.120786	1.048048	0.294617	0.763708
cdc7	89.00056	0.301787386	0.219059	1.377654	0.16831	0.639712
kcnk5a	39.49162	-0.818344006	0.364419	-2.24561	0.024729	0.27868
nrbf2b	345.4197	-0.078635237	0.136841	-0.57465	0.56553	0.901564
sh3gl2a	622.6867	0.423743997	0.272787	1.553387	0.120331	0.565852
AL845324.1	169.7287	-0.291609308	0.331828	-0.8788	0.379512	0.823607
ggps1	437.3501	0.109575905	0.168325	0.650977	0.515061	0.886401
kyat1	303.3085	0.372853921	0.218769	1.70433	0.08832	0.499113
idh3g	1100.195	-0.181265949	0.112592	-1.60993	0.107413	0.53811
he1.1	6924.238	0.124954803	0.587467	0.212701	0.83156	0.974908
gabpb1	83.82189	0.255794122	0.261482	0.978249	0.327951	0.786438
timm8a	294.0582	0.170675906	0.207917	0.820885	0.411712	0.837966
ercc5	223.1849	-0.353898158	0.152569	-2.31959	0.020363	0.253253
cacna1fb	143.381	-0.083092123	0.372842	-0.22286	0.823643	0.972005
spon1b	975.6863	0.282115568	0.098392	2.867262	0.00414	0.106542
kctd6b	225.6895	0.339064263	0.178732	1.897051	0.057821	0.415158
mao	426.9178	-0.316426001	0.153717	-2.0585	0.039542	0.347625
aqp1a.1	2248.967	-0.265870301	0.143304	-1.85529	0.063555	0.430337
hcrtr2	1.402238	0.026776878	1.838384	0.014565	0.988379	NA
cdc42se1	403.2223	0.006894504	0.129891	0.053079	0.957669	0.999109
zgc:73226	155.0751	-1.010214344	0.420714	-2.40119	0.016342	0.225785
gnsa	431.4886	0.038325557	0.162505	0.235843	0.813555	0.970328
mfsd4aa	41.99777	-0.365450001	0.414053	-0.88262	0.377443	0.822478
gabrb3	301.7953	0.60869365	0.275494	2.209461	0.027143	0.293072
ryr1b	1512.816	-0.037275113	0.365452	-0.102	0.918759	0.993884
dyrk1ab	574.2991	0.006791132	0.132765	0.051151	0.959205	0.999164
uvrag	61.29427	-0.102590409	0.272907	-0.37592	0.706979	0.944895
faxdc2	906.3294	-0.357517676	0.231323	-1.54553	0.122218	0.569366
pfkfb2a	51.30117	0.227322631	0.264199	0.860422	0.389556	0.827843
lsm4	737.4793	0.103134419	0.13252	0.778255	0.436419	0.850912
ccdc174	290.5385	-0.139178845	0.213012	-0.65338	0.513509	0.88631
dnase1l1l	190.0811	0.60115467	0.275298	2.183648	0.028988	0.303224
si:dkey-172h23.2	220.1831	0.19815447	0.181445	1.092092	0.274793	0.750181
stk24b	665.8503	0.061837644	0.112047	0.55189	0.581024	0.905618
cacna2d4b	128.3281	0.205275818	0.594384	0.345359	0.729825	0.950056
casd1	272.8682	-0.066729284	0.147134	-0.45353	0.65017	0.927835
atf2	19.65124	0.179211607	0.426323	0.420366	0.674218	0.934868
mark4a	56.22345	0.362238043	0.279761	1.294812	0.195385	0.675078
phactr1	132.0623	0.55353615	0.249321	2.220176	0.026407	0.287927
llgl2	805.4441	-0.00822325	0.13453	-0.06113	0.951259	0.99801
dnajc24	49.54177	-0.36260649	0.253784	-1.4288	0.153062	0.617699

skila	106.6474	-0.019849293	0.212375	-0.09346	0.925535	0.994815
si:ch73-362m14.4	82.10716	0.847532449	0.341465	2.482045	0.013063	0.198789
znhit3	92.11365	-0.845256004	0.287714	-2.93783	0.003305	0.094544
fam167ab	58.38891	-0.008245092	0.247477	-0.03332	0.973422	1
rnf181	113.8651	0.008513794	0.186524	0.045645	0.963594	0.999842
tpm4a	2679.56	-0.056644124	0.158974	-0.35631	0.721608	0.947343
atp6v1c1a	1326.401	-0.103016779	0.14509	-0.71002	0.477692	0.869292
dctn4	395.3588	0.307386757	0.142083	2.163424	0.030509	0.311199
pef1	328.0018	0.031866778	0.136874	0.232818	0.815902	0.971207
heatr5a	399.3185	-0.062339342	0.128842	-0.48384	0.628496	0.921864
rprmb	40.33588	-0.055287484	0.312334	-0.17701	0.859498	0.979975
mdga2a	124.8276	0.305491149	0.242039	1.262156	0.206893	0.689246
sdf2	259.174	0.185782314	0.169728	1.094586	0.273698	0.749234
angptl2a	133.9899	-0.617470089	0.181076	-3.41	0.00065	0.033227
coch	445.4973	1.539254496	0.425904	3.614091	0.000301	0.019507
tmem38a	5949.966	-0.083581569	0.137268	-0.60889	0.542595	0.894595
dnajc4	366.9234	-0.034099468	0.137395	-0.24819	0.803991	0.968814
lmbr1	104.5081	0.281794647	0.200643	1.404459	0.160182	0.626611
adra1d	75.95899	-0.054185889	0.243383	-0.22264	0.823819	0.972005
naa40	767.5987	0.023264994	0.105771	0.219957	0.825905	0.972962
gmppaa	276.3235	-0.30203656	0.175341	-1.72256	0.084967	0.489359
vamp8	519.099	-0.038160074	0.138112	-0.2763	0.78232	0.962328
sde2	356.0207	-0.01132333	0.134612	-0.08412	0.932963	0.996264
p2rx8	8.393761	1.402318564	0.67815	2.067859	0.038653	0.344438
cav3	1490.763	0.192279673	0.124575	1.543487	0.122713	0.569468
hao1	1570.078	-0.096370296	0.188912	-0.51013	0.609958	0.915331
kcnh1a	7.932051	0.312137856	0.613932	0.508424	0.611156	0.915331
narf	513.436	-0.302527414	0.14812	-2.04244	0.041108	0.353918
pnocb	29.80122	-0.043949867	0.424543	-0.10352	0.917548	0.993777
znf395b	442.3682	-0.070727089	0.279806	-0.25277	0.800444	0.967364
mcm3	652.6423	-0.079255175	0.251869	-0.31467	0.753014	0.955756
opn7a	0.235135	0	5.267649	0	1	NA
wasf2	857.2854	-0.07692589	0.099828	-0.77058	0.440954	0.852687
ifng1	0.166044	0	5.267649	0	1	NA
cdk5rap2	321.4219	0.142294568	0.199836	0.712055	0.476431	0.869292
ubl7a	130.103	-0.526383453	0.263726	-1.99595	0.04594	0.375002
pcbp4	693.015	0.016917116	0.101422	0.166799	0.867528	0.982582
pigm	62.95988	0.288540533	0.27637	1.044038	0.296468	0.764344
adh8b	1551.127	-1.072102217	0.221787	-4.83394	1.34E-06	0.000365
slc11a2	757.1943	0.14625	0.14432	1.013376	0.310881	0.775776
c1qtnf4	500.5027	0.118676623	0.208863	0.568205	0.569896	0.902322
zbed4	371.7601	-0.053525052	0.134903	-0.39677	0.691539	0.940173
nsmce4a	125.2942	0.065243411	0.223576	0.291817	0.770426	0.960484
herpud1	552.4116	0.020678378	0.129611	0.159542	0.873242	0.983537
trap1	743.5121	0.327272394	0.157615	2.076398	0.037857	0.34119
arhgap1	1227.912	-0.055623868	0.104389	-0.53285	0.594137	0.910701
col4a3bpa	324.4466	0.136806806	0.125043	1.094076	0.273921	0.749234
crlf1a	178.2234	-0.10202699	0.145179	-0.70276	0.482202	0.871592
msto1	369.8729	-0.322153013	0.159118	-2.02462	0.042906	0.362823
get4	648.9021	0.030715586	0.113615	0.270349	0.786892	0.963061
twistnb	214.4349	-0.392123905	0.153837	-2.54895	0.010805	0.181278
ets1	78.3609	-0.087909288	0.212695	-0.41331	0.679378	0.936209
pvalb4	17100.55	-0.145226989	0.153941	-0.94339	0.345479	0.79876
gnpda2	1592.48	-0.061065607	0.186542	-0.32736	0.743398	0.954856

mrps18a	362.6924	0.087641125	0.167051	0.524636	0.599836	0.913598
nsl1	83.87229	-0.216559592	0.282454	-0.76671	0.443255	0.854063
mpc2	2486.078	0.157322188	0.178467	0.88152	0.378037	0.82265
pigk	54.56094	-0.059875111	0.262149	-0.2284	0.819335	0.971785
top2a	1554.501	0.121579546	0.269763	0.45069	0.652213	0.928768
col9a2	7684.029	0.39702525	0.182018	2.181238	0.029166	0.304273
c6ast3	809.2704	1.055406661	1.691222	0.62405	0.532595	0.890978
prf1.6	0.106927	0	5.267649	0	1	NA
emilin1a	337.876	0.415831966	0.24613	1.689478	0.091128	0.50549
tspan36	1345.377	-0.318194091	0.187818	-1.69416	0.090235	0.50416
pla2g4aa	93.08528	-0.085290403	0.224527	-0.37987	0.704044	0.943735
cryba4	8404.305	0.662333197	0.261843	2.529507	0.011422	0.185332
slc4a1b	79.3756	-1.019402167	0.294747	-3.45857	0.000543	0.029126
nolc1	686.9183	0.594211719	0.269379	2.205854	0.027394	0.293857
sccpdhb	388.0878	-0.719288029	0.146948	-4.89485	9.84E-07	0.000296
kif26bb	40.77272	0.189481583	0.331953	0.570809	0.568129	0.902108
gpc5b	92.2997	0.778085126	0.231353	3.363192	0.00077	0.03696
homezb	532.7756	0.020199649	0.140698	0.143568	0.885842	0.987533
zgc:162964	102.2548	0.193140774	0.211347	0.913854	0.360793	0.811253
foxo6b	146.4983	0.174073816	0.203048	0.857305	0.391276	0.827902
nlrc5	5.516117	0.128138807	0.78736	0.162745	0.870719	0.983365
pip5k1ab	418.2439	-0.055962885	0.103475	-0.54083	0.588622	0.908014
snrpa1	497.4159	0.08804696	0.128872	0.683211	0.494474	0.877861
pgm3	206.735	-0.001095184	0.13747	-0.00797	0.993644	1
oser1	669.4059	-0.046342853	0.146524	-0.31628	0.751789	0.955391
pane1	128.8956	0.123978561	0.243776	0.508576	0.61105	0.915331
polr3g	1.328855	3.279249409	1.903152	1.723062	0.084877	NA
lysmd3	101.2927	-0.151181466	0.208612	-0.7247	0.468636	0.866951
myo1b	1044.961	-0.146269704	0.134495	-1.08755	0.276795	0.751311
slc25a23b	23.15303	0.029599218	0.487652	0.060697	0.9516	0.99801
selenbp1	386.952	-0.182763567	0.165688	-1.10306	0.270001	0.746532
hdhd2	105.5757	0.398606152	0.210912	1.889921	0.058769	0.418455
gng13a	84.93712	-1.604359572	0.870152	-1.84377	0.065217	0.435355
dpp6b	267.7378	0.624389175	0.233282	2.676543	0.007439	0.146061
hsp90aa1.2	1099.699	0.368872569	0.427153	0.863561	0.387829	0.827592
inhbab	21.76499	0.745649316	0.614829	1.212776	0.225216	0.70685
slc24a5	94.34421	-0.301097535	0.334759	-0.89945	0.368415	0.817272
si:ch211-129c21.1	338.8276	-0.181692661	0.15387	-1.18082	0.237674	0.718568
ctnna2	612.6257	0.294953416	0.135527	2.176339	0.02953	0.30586
mxc	61.13717	-0.066510054	0.396733	-0.16764	0.866863	0.982582
ogfrl2	58.8211	-0.055663828	0.342483	-0.16253	0.870888	0.983365
rilpl2	144.9493	0.019211419	0.174243	0.110257	0.912206	0.992371
rnf150a	29.47113	-0.291266495	0.366005	-0.7958	0.426149	0.844714
tmem134	425.0556	-0.035241453	0.119589	-0.29469	0.768232	0.960296
tnn	4986.719	0.088370456	0.176441	0.500849	0.616477	0.916365
crhbp	186.4467	-0.911187805	0.253584	-3.59324	0.000327	0.020507
max	1855.308	-0.092864283	0.114881	-0.80835	0.418887	0.841915
cops8	647.3269	0.10606868	0.159372	0.665542	0.505704	0.882449
col5a2b	98.41316	-0.448707315	0.374773	-1.19728	0.231198	0.712822
dscama	510.9217	0.338482813	0.171536	1.97325	0.048467	0.383271
dock4b	342.9562	-0.137567759	0.14655	-0.93871	0.347879	0.800274
ptgr1	1129.324	0.058393568	0.114841	0.508474	0.611121	0.915331
tbx5a	42.28794	-0.200542286	0.263264	-0.76175	0.446208	0.855403
fam50a	565.6884	-0.030333436	0.161256	-0.18811	0.850792	0.978402

stil	82.94278	0.406100594	0.300484	1.351489	0.176539	0.649967
tmem136a	246.9127	0.071207276	0.137835	0.516612	0.605427	0.914985
spast	276.7965	0.1608348	0.155913	1.031569	0.302274	0.768145
rnf144b	303.9452	-0.089397011	0.150807	-0.59279	0.55332	0.89793
tmed4	252.3111	-0.039002255	0.233702	-0.16689	0.867457	0.982582
patj	235.2222	0.263869162	0.184152	1.432889	0.15189	0.615406
MARK4	638.184	-0.069471507	0.103319	-0.6724	0.50133	0.880791
fancg	63.55251	-0.039101064	0.207303	-0.18862	0.850392	0.978251
synj1	692.4333	0.146115503	0.128229	1.139489	0.254499	0.733426
tpi1a	201.0817	-0.4704027	0.197508	-2.38168	0.017234	0.231824
gna14a	9.432593	-0.88992767	1.249367	-0.7123	0.476277	0.869292
prdm8	65.23359	-0.154001802	0.24315	-0.63336	0.526498	0.889591
pnoca	70.2596	-0.237158542	0.350726	-0.67619	0.498918	0.879457
nr3c1	1031.605	-0.067271928	0.134392	-0.50057	0.616677	0.916365
stx5a	641.6274	0.036645041	0.10218	0.358632	0.719871	0.94694
syngr3b	191.854	0.046674351	0.226384	0.206173	0.836655	0.976007
trip13	106.1479	0.352033835	0.241143	1.459856	0.14433	0.603413
AL935186.1	23.65457	0.691260511	0.662779	1.042973	0.296961	0.764508
brd8	768.8669	0.233781736	0.090861	2.572955	0.010083	0.174093
rpl18a	26622.84	-0.343816199	0.12779	-2.69048	0.007135	0.143006
slc25a10	565.8901	-0.102847166	0.149597	-0.68749	0.491772	0.87595
marveld2a	243.1538	-0.057095416	0.135378	-0.42175	0.67321	0.93414
grnb	306.5739	0.205180351	0.164817	1.244897	0.213169	0.695467
loxl4	122.5322	-0.594634639	0.289824	-2.05171	0.040198	0.350542
ezrb	1732.623	0.007922972	0.103998	0.076184	0.939273	0.997371
edem1	520.4573	0.093795094	0.133494	0.702619	0.482293	0.871664
ppp3cb	859.6179	0.221119044	0.218674	1.011182	0.311929	0.776164
magixa	39.01416	0.498189951	0.303716	1.640313	0.10094	0.526541
lbx2	222.4778	-0.084763426	0.144879	-0.58506	0.558505	0.899409
cd63	1975.284	-0.074593831	0.094404	-0.79016	0.429436	0.846956
si:dkey-181c13.1	0.6279	0	4.377438	0	1	NA
asic4a	93.6793	-0.098560804	0.333264	-0.29574	0.767426	0.960296
mpp7b	1.384044	-1.74909094	2.017058	-0.86715	0.38586	NA
zgc:103482	16.30889	-0.006312698	0.541879	-0.01165	0.990705	1
six6a	53.63063	0.150370467	0.317756	0.473226	0.636052	0.924779
cpne8	9.716311	1.563786138	0.805525	1.941326	0.052219	0.396951
syt2a	42.56157	0.783857941	0.390179	2.008968	0.044541	0.369365
ints11	261.6824	-0.021801718	0.148008	-0.1473	0.882895	0.986908
myo5ab	161.0597	0.041002522	0.198767	0.206285	0.836569	0.976007
sppl3	422.9888	-0.011661464	0.164203	-0.07102	0.943383	0.997371
znf668	107.3898	0.090225239	0.172949	0.521687	0.601888	0.914001
lipt1	70.07545	-0.039981854	0.225769	-0.17709	0.859436	0.979975
s100a10b	3571.253	0.198710506	0.13467	1.47554	0.140067	0.598705
pdcd6ip	2042.548	-0.062860038	0.123044	-0.51087	0.60944	0.915204
gbgt1l1	19.50997	-0.039796245	0.431246	-0.09228	0.926474	0.994815
panx1a	528.5616	0.129815987	0.180526	0.719099	0.47208	0.868517
tspan9a	18.29373	0.682105132	0.454438	1.500985	0.133359	0.586883
gfap	2546.559	0.010749184	0.170588	0.063013	0.949756	0.99801
dixdc1a	429.4683	0.040016293	0.147529	0.271244	0.786203	0.962605
dpf3	111.9839	-0.177499696	0.180948	-0.98094	0.32662	0.785469
cuzd1.2	10.20709	-0.868149847	1.759656	-0.49336	0.621756	0.918461
fynb	115.4719	-0.002574526	0.186999	-0.01377	0.989015	1
col21a1	61.48695	0.395180645	0.30257	1.30608	0.191525	0.671557
ccsapa	143.64	-0.265262567	0.231075	-1.14795	0.250989	0.731313

myl12.2	800.8409	-0.020760185	0.135802	-0.15287	0.8785	0.985761
rbm28	473.2022	-0.096369988	0.16896	-0.57037	0.568425	0.902146
nek10	10.50057	0.450693901	0.60686	0.742666	0.457684	0.860993
hagh	762.895	-0.52870679	0.147128	-3.59351	0.000326	0.020507
tusc2b	658.3565	0.005348793	0.132611	0.040335	0.967826	1
znf277	123.4527	-0.640658276	0.198208	-3.23225	0.001228	0.050845
igfbp5b	912.3735	0.131867306	0.14452	0.912451	0.361532	0.811773
prdx2	9423.257	-0.097304846	0.167153	-0.58213	0.560478	0.899928
admp	0.484442	-3.150117619	4.050988	-0.77762	0.436795	NA
idh1	1816.464	0.140266746	0.158162	0.886856	0.375157	0.822052
arl2bp	114.4846	-0.059560594	0.218653	-0.2724	0.785316	0.962605
lsm14b	2.596946	2.776329826	1.531453	1.812873	0.069851	0.450824
pfdn2	995.7125	0.273738192	0.125662	2.178371	0.029378	0.304886
srsf3a	32.81155	-0.017110683	0.328589	-0.05207	0.95847	0.999109
ftr83	412.4289	0.010517103	0.170291	0.06176	0.950754	0.99801
ube2g2	567.6747	0.106458581	0.103226	1.031314	0.302394	0.768252
fzd5	123.9917	0.250827491	0.215405	1.164447	0.244243	0.724876
tdrd3	371.0421	0.182569941	0.141237	1.29265	0.196132	0.676562
socs3a	431.3094	0.034357208	0.724147	0.047445	0.962158	0.999451
msrb1a	734.499	-0.252064184	0.262795	-0.95917	0.337474	0.7944
spon2a	142.512	0.136868982	0.213478	0.641138	0.521433	0.887551
aatf	205.3066	-0.04442748	0.167851	-0.26468	0.791253	0.964766
bnip3la	338.1738	0.22723734	0.12777	1.77849	0.075323	0.465238
gipr	20.6594	0.698813635	0.532272	1.312888	0.189221	0.668738
nhlh2	806.1545	0.035401084	0.147908	0.239345	0.810838	0.969828
mtx1a	167.6613	0.190950162	0.164084	1.163733	0.244532	0.725549
gucy2f	52.43495	0.832304673	0.425328	1.956856	0.050364	0.390726
si:ch73-335d12.2	0.194	0	5.267649	0	1	NA
wdr24	243.1852	-0.110637695	0.167575	-0.66023	0.509107	0.883443
dip2cb	434.2089	0.266874385	0.212888	1.253589	0.209991	0.691821
zgc:101679	254.7999	0.009667344	0.173989	0.055563	0.95569	0.998707
sgk1	1375.06	-0.293074516	0.231701	-1.26488	0.205913	0.688314
clint1a	1002.795	-0.057693572	0.107539	-0.53649	0.59162	0.909437
si:ch73-34314.6	0.07132	0	5.267649	0	1	NA
ndufaf1	802.0236	0.222032618	0.148964	1.49051	0.13609	0.591153
wdr83os	630.4121	0.116105684	0.176788	0.65675	0.511341	0.884691
ormdl3	39.1653	-0.051424707	0.324718	-0.15837	0.874167	0.98385
pex11g	76.01932	0.010786288	0.286726	0.037619	0.969992	1
slc25a3b	17366.91	-0.14807328	0.113029	-1.31005	0.190179	0.669977
nf2b	387.7046	-0.087559526	0.123101	-0.71128	0.476911	0.869292
mkln1	371.6843	0.030227592	0.138595	0.2181	0.827351	0.973459
zgc:66447	192.2499	-0.118724654	0.147465	-0.80511	0.420758	0.842383
rpl10	33018.68	-0.323016775	0.11458	-2.81914	0.004815	0.115256
plekhj1	189.2028	-0.167173421	0.143739	-1.16304	0.244815	0.726138
agmo	396.2942	-0.395121209	0.195941	-2.01653	0.043744	0.3663
or102-5	0.194091	2.43638772	5.206324	0.467967	0.639808	NA
trim35-14	0.236389	2.079536233	5.22636	0.397894	0.690708	NA
nvl	438.1177	-0.097012403	0.160136	-0.60581	0.544639	0.895206
ifnphi1	2.154153	-0.934726704	1.480419	-0.63139	0.527783	NA
casp6l1	38.05507	0.073663216	0.363694	0.202542	0.839493	0.976417
prr15la	69.42806	-0.192276956	0.24375	-0.78883	0.430213	0.847541
gli2a	461.8565	-0.014136884	0.161926	-0.0873	0.930429	0.995707
or111-2	5.245874	1.557512898	1.262267	1.233901	0.21724	0.69815
adgrb2	42.60548	0.15448354	0.436475	0.353934	0.723388	0.947908

fstl3	826.1823	0.035802406	0.204261	0.175278	0.860862	0.980388
grm6b	122.8051	0.311753438	0.318208	0.979717	0.327226	0.786088
antxr1a	92.69944	-0.124063448	0.344931	-0.35968	0.719089	0.94694
comtb	214.0115	0.030943065	0.194875	0.158784	0.873839	0.98385
tmem9b	589.7991	0.077359046	0.146998	0.52626	0.598708	0.912935
mbd1b	481.3826	0.024335009	0.108983	0.223291	0.823309	0.972005
dmgdh	2153.453	0.206800392	0.142891	1.447257	0.147825	0.608687
cxxc1b	260.8945	0.229206214	0.126577	1.810806	0.070171	0.452005
grin1b	1338.978	0.923108155	0.212062	4.353014	1.34E-05	0.002278
ube2f	444.4866	0.084781178	0.151132	0.560976	0.574814	0.904516
tspan35	328.2352	-0.379284454	0.185068	-2.04943	0.04042	0.351287
zgc:66440	116.3416	-0.362756702	0.231408	-1.5676	0.116974	0.557831
si:ch211-125e6.11	0.069091	0	5.267649	0	1	NA
chp2	268.4915	-0.149095472	0.195999	-0.76069	0.44684	0.855784
chd4b	2281.218	0.129200472	0.145389	0.888655	0.374188	0.820862
abhd2a	14.39622	-0.085188624	0.585429	-0.14551	0.884304	0.987047
taf5l	109.9856	0.017930636	0.172979	0.103658	0.917441	0.993777
chek2	59.0594	0.188921216	0.329736	0.572947	0.56668	0.901739
dmtf1	249.8684	0.151237471	0.149769	1.009806	0.312588	0.776164
gna12a	535.4969	0.056452225	0.10963	0.514934	0.606599	0.915204
sox12	731.3562	-0.041777	0.107949	-0.38701	0.698752	0.942456
rps21	14259.16	-0.284039118	0.149023	-1.90601	0.056649	0.411073
ddx51	202.0109	-0.126688272	0.153328	-0.82625	0.408659	0.836526
camk2n1a	953.5967	0.003155019	0.160987	0.019598	0.984364	1
zgc:56525	990.0015	-0.014158814	0.094772	-0.1494	0.881239	0.986892
lmf2b	429.9186	0.787259391	0.174379	4.51465	6.34E-06	0.001289
ankdd1b	20.27841	-0.305172053	0.473996	-0.64383	0.519686	0.887148
kctd2	47.11209	0.010076912	0.271896	0.037062	0.970436	1
usp14	1110.432	0.197342552	0.156413	1.261678	0.207065	0.689323
shox	216.0389	0.027002917	0.145031	0.186187	0.852298	0.978626
pnx	1.223402	0.357237681	1.830993	0.195106	0.84531	NA
plekhg5a	363.2348	-0.203676295	0.163502	-1.24571	0.212872	0.695171
lgals9l1	78.59102	0.172971959	0.236709	0.730736	0.464941	0.864823
tecrb	1779.978	-0.039960426	0.120291	-0.3322	0.739741	0.953588
bscl2l	526.4255	-0.400565831	0.206873	-1.93628	0.052833	0.399532
si:dkey-190g11.3	18.0542	0.477549728	0.719389	0.663827	0.506801	0.883007
tiam1b	177.5727	-0.123436887	0.166141	-0.74296	0.457503	0.860993
crygs3	0.965961	0	2.520467	0	1	NA
mlh1	116.6162	-0.472237533	0.183904	-2.56785	0.010233	0.176021
irak1	59.97045	-0.213687702	0.254875	-0.8384	0.401806	0.832807
rhoq	259.5854	0.281416317	0.175184	1.606404	0.108185	0.54011
mcts1	567.5315	0.060690302	0.137569	0.441163	0.659095	0.930581
magi3b	26.01579	0.281555238	0.452573	0.622121	0.533862	0.891181
blmh	1070.879	0.086318205	0.164162	0.525812	0.599019	0.913109
l3mbtl3	36.05352	-0.025041061	0.301071	-0.08317	0.933714	0.996598
xpnpep2	62.74268	-0.40539882	0.395317	-1.0255	0.305126	0.770296
mospd2	309.9726	-0.35306097	0.116375	-3.03382	0.002415	0.078804
ankrd44	83.53667	0.516610561	0.258067	2.001846	0.045301	0.372707
ube2b	432.8845	0.182880802	0.146572	1.24772	0.212134	0.693739
tsc1a	705.3055	-0.181693535	0.109992	-1.65188	0.098559	0.521133
mxf	6.05314	-1.392013833	1.643876	-0.84679	0.397113	0.830253
ccdc3a	71.5809	-0.372258605	0.327674	-1.13606	0.25593	0.733716
hltf	155.6999	0.270440597	0.16206	1.668767	0.095164	0.514692
trappc5	493.4924	-0.024426143	0.120566	-0.2026	0.839451	0.976417

cd82b	1062.262	-0.036062882	0.146149	-0.24675	0.805098	0.969348
pdcd5	827.6098	-0.11533442	0.176777	-0.65243	0.514125	0.88631
lhcgr	1.569835	-2.111128191	1.696812	-1.24417	0.213436	NA
si:dkey-43k4.5	24.71159	0.588496507	0.417666	1.409011	0.158832	0.624456
zgc:162324	9.901659	0.305342761	0.656581	0.46505	0.641896	0.925586
adprm	86.1445	0.112305034	0.273486	0.410643	0.681335	0.937331
si:dkey-13a21.4	46.38672	-0.771785675	0.461593	-1.67201	0.094523	0.513368
slc48a1b	407.7914	-0.063730483	0.159165	-0.40041	0.688858	0.93924
sfxn5b	381.3207	0.070290727	0.163638	0.429551	0.667522	0.933086
slc46a1	88.14862	-0.308902603	0.24365	-1.26781	0.204864	0.686735
col11a1a	8718.516	0.304685234	0.174377	1.747274	0.08059	0.479386
emilin1b	575.8167	0.255189531	0.135319	1.885832	0.059318	0.419405
hps1	155.1585	-0.492923768	0.163782	-3.00963	0.002616	0.083393
ube3d	107.0052	0.040564842	0.18602	0.218067	0.827377	0.973459
prpf8	2439.782	0.108800338	0.155718	0.698701	0.484739	0.872911
polr2eb	765.6697	0.046544078	0.155747	0.298843	0.76506	0.959622
yju2	345.4208	0.109047528	0.128873	0.846159	0.397464	0.830253
enoph1	459.554	0.027019742	0.130407	0.207196	0.835857	0.976007
fanci	102.1516	0.452140049	0.341406	1.324349	0.185387	0.663181
nsd2	445.3436	0.311185794	0.18916	1.645096	0.09995	0.524166
prnpa	100.0696	-2.123125434	0.570594	-3.7209	0.000199	0.014933
zgc:56585	207.5336	0.059552361	0.194774	0.305751	0.759794	0.957886
CR847944.1	1.759073	-2.349187399	2.029082	-1.15776	0.246962	NA
insyn1	137.0491	0.037173033	0.296286	0.125463	0.900157	0.99045
slc1a7b	23.11555	-2.386494143	1.348599	-1.76961	0.076792	0.469341
lyrm1	40.36949	-0.461657005	0.340735	-1.35489	0.175454	0.649279
erbb2	136.71	0.189441668	0.213838	0.885914	0.375664	0.822237
zgc:112994	22.10369	-0.39102882	0.503846	-0.77609	0.437697	0.851283
adgre5a	0.137587	0	5.267649	0	1	NA
dhrs13a.1	327.5115	0.142402893	0.194338	0.732758	0.463706	0.864593
mmp11a	170.5486	-0.643141356	0.23808	-2.70137	0.006905	0.139981
arhgap29a	231.5809	0.095737677	0.151651	0.631304	0.527842	0.889948
xkr7	43.84981	0.123283691	0.440367	0.279957	0.779511	0.961073
susd4	90.93054	0.368607508	0.298429	1.235162	0.21677	0.69815
styxl1	4.713457	-0.029395444	0.732427	-0.04013	0.967986	1
csad	278.3611	-0.246436006	0.148299	-1.66175	0.096563	0.518868
wasb	54.75104	-0.401245733	0.279506	-1.43555	0.15113	0.615085
pbld2	146.6225	-0.593109068	0.227752	-2.60419	0.009209	0.165025
dbi	3618.508	-0.132824146	0.188966	-0.7029	0.482118	0.87159
aco1	158.2684	-0.156727519	0.212382	-0.73795	0.460544	0.862369
si:dkey-27j5.5	13.58413	3.266678326	1.397095	2.338194	0.019377	0.245204
pip4p1b	73.13869	-0.135953426	0.254751	-0.53367	0.593568	0.910427
rfx4	146.7212	0.256444498	0.193053	1.328361	0.184059	0.660978
rad51d	36.46731	0.525875699	0.471095	1.116283	0.264301	0.742063
hephl1b	11.17699	-1.17310881	0.639469	-1.83451	0.066579	0.440501
anxa5a	196.684	0.018356557	0.282439	0.064993	0.94818	0.997653
ccr12b.2	3.459397	-2.087338074	1.112427	-1.87638	0.060603	0.423018
apoob	197.309	0.218977558	0.185823	1.178423	0.238628	0.719642
med29	229.2752	0.262181502	0.126641	2.070268	0.038427	0.343768
zgc:66474	246.2646	-0.129890906	0.157382	-0.82532	0.409189	0.836637
mis12	145.4627	0.160555281	0.20448	0.785186	0.432344	0.848419
six1b	751.0691	0.003943505	0.152333	0.025887	0.979347	1
tial1	1897.282	0.005169444	0.097209	0.053179	0.95759	0.999109
arhgap12b	445.2208	0.04929457	0.133248	0.369946	0.711423	0.945821

rab15	193.268	0.094443537	0.1915	0.493179	0.621886	0.918461
khsrp	1328.076	0.062524863	0.161435	0.387308	0.698528	0.942433
ppm1e	61.12268	0.0753502	0.277318	0.27171	0.785845	0.962605
xkr9	19.6498	-0.484284451	0.378078	-1.28091	0.200225	0.680711
si:ch211-285c6.3	0.533138	-2.406573819	3.109496	-0.77394	0.438964	NA
hoxb6b	131.4555	0.359070323	0.20933	1.715332	0.086284	0.493048
ralgps2	367.8316	0.404504667	0.124887	3.238955	0.0012	0.049994
alcama	871.3968	0.108465987	0.134403	0.807022	0.419654	0.842383
ppp4r2a	476.7062	-0.036777088	0.095368	-0.38563	0.699768	0.942499
cyp2u1	73.10275	0.109962767	0.459722	0.239194	0.810955	0.969848
pimr196	0.087381	0	5.267649	0	1	NA
cdk2	379.2776	0.1665527	0.129653	1.284604	0.198931	0.679593
iscub	245.2582	0.274199986	0.218289	1.256133	0.209068	0.691152
pafah1b1b	1556.197	0.102495559	0.081744	1.253865	0.209891	0.691821
en2a	203.4199	-0.015872334	0.177803	-0.08927	0.928868	0.995164
socs3b	519.8421	0.179408142	0.18773	0.955671	0.339239	0.795226
zgc:113425	9.041038	0.594292471	0.71397	0.832377	0.405196	0.834483
gmds	622.1534	0.453988528	0.129158	3.514989	0.00044	0.025327
mark3b	632.31	-0.029075171	0.105432	-0.27577	0.782724	0.962328
ehmt1b	745.4766	0.022083511	0.104949	0.210421	0.833339	0.975388
asb1	22.86653	-0.120333787	0.424165	-0.2837	0.776644	0.961073
gosr1	293.5045	0.145045964	0.131245	1.105152	0.269094	0.745266
tspo	1155.302	0.157191694	0.139176	1.129446	0.25871	0.73587
tlr19	2.130795	1.951755946	1.642614	1.188201	0.234754	NA
uri1	693.4001	-0.087421203	0.109523	-0.7982	0.424755	0.844714
slc25a14	459.9919	0.25845236	0.126592	2.041614	0.04119	0.354125
zgc:101583	139.8489	-0.298114694	0.183861	-1.62142	0.104928	0.533388
gtf2h1	194.5306	0.013654114	0.139683	0.097751	0.92213	0.994346
ftr72	2.10485	-0.234832786	1.269815	-0.18493	0.85328	NA
rab5c	1914.679	0.06077415	0.098858	0.614765	0.53871	0.892777
syncripl	569.7702	0.252466837	0.11037	2.287454	0.022169	0.263608
anxa1a	4789.824	-0.414535175	0.122996	-3.3703	0.000751	0.036396
il11ra	67.78284	0.52647329	0.268535	1.960539	0.049933	0.388888
grik4	84.84726	0.046747075	0.326911	0.142996	0.886293	0.987533
ldlrb	296.9764	0.389094356	0.215711	1.803777	0.071266	0.455342
fam126a	272.0492	0.005938464	0.15357	0.038669	0.969154	1
ahsg1	222.8255	-1.02955377	0.468896	-2.1957	0.028114	0.29853
bcl2l10	626.4846	0.079273879	0.125317	0.632586	0.527004	0.889861
nol11	405.6238	-0.092053374	0.14489	-0.63533	0.525211	0.888928
tmem41ab	174.8201	0.109047028	0.277706	0.39267	0.694563	0.941093
robo1	805.6343	0.097434805	0.121861	0.799559	0.423966	0.844352
aqp7	76.87107	-0.467410526	0.271536	-1.72136	0.085186	0.490251
grm1a	221.8226	0.2109487	0.348494	0.605314	0.54497	0.895206
suv39h1a	4.033021	-0.521231752	1.174121	-0.44393	0.657091	0.929939
stau2	4799.519	0.135695241	0.083303	1.628935	0.103327	0.530446
taf8	148.6261	0.190125212	0.164939	1.152699	0.249034	0.728918
extl3	208.8014	0.608651563	0.195153	3.118841	0.001816	0.06578
gucy2d	456.1358	0.961248006	0.557893	1.722998	0.084889	0.489029
cotl1	5142.476	0.041362235	0.112879	0.366428	0.714045	0.945821
cdr2l	452.5473	0.020766692	0.150285	0.138182	0.890097	0.98835
slc25a32b	139.94	0.140621285	0.171627	0.81934	0.412592	0.838123
med21	249.8555	0.007767386	0.173458	0.04478	0.964283	0.999925
arhgap23b	34.24114	-0.031395104	0.470456	-0.06673	0.946794	0.997371
acin1b	1694.114	0.007205548	0.132404	0.054421	0.9566	0.999074

rhoaa	3192.132	0.099131872	0.098096	1.010563	0.312226	0.776164
cacna2d4a	21.01009	0.961481782	0.626697	1.534204	0.124979	0.572594
aktip	943.4682	0.056038269	0.102766	0.545301	0.585547	0.906655
si:ch211-226m16.3	1.746272	1.520679867	1.883513	0.807364	0.419457	NA
fam107b	978.5571	-0.170278797	0.154347	-1.10322	0.269933	0.746532
uchl1	318.9352	0.253517761	0.197425	1.284124	0.199098	0.679605
fam57ba	247.851	-0.080158493	0.171	-0.46876	0.63924	0.924779
palm1a	367.213	0.215430398	0.177113	1.216345	0.223853	0.705807
gpatch11	180.3986	-0.090392631	0.149326	-0.60534	0.544954	0.895206
torl13	7.45819	0.981739212	1.701854	0.576864	0.564031	0.901345
cryzl1	212.5356	0.294061027	0.232389	1.265385	0.205733	0.687948
cbln13	59.7591	-1.415860625	0.387666	-3.65227	0.00026	0.017766
slc48a1a	91.81402	-0.116818901	0.282167	-0.41401	0.678869	0.936051
tmed2	2126.089	-0.0620565	0.086844	-0.71457	0.474873	0.869292
nos2a	9.181249	-1.34654402	1.190716	-1.13087	0.25811	0.735569
rom1b	218.0192	-0.676485053	0.592764	-1.14124	0.253771	0.732873
fbxl4	287.8728	-0.189844282	0.152668	-1.24351	0.213679	0.696139
ap2s1	1674.354	0.096623342	0.127928	0.755295	0.450072	0.85711
si:ch211-119o8.6	1.59157	-0.645463857	1.351828	-0.47747	0.633024	NA
krt1-c5	223.3033	-0.601726351	0.257318	-2.33845	0.019364	0.245171
lims1	910.3975	-0.005357251	0.183272	-0.02923	0.97668	1
rexo1	188.8489	0.036746314	0.161584	0.227413	0.820103	0.971788
tbc1d22b	15.20766	0.230632579	0.419385	0.549931	0.582367	0.90571
klhl6	19.82332	-0.342944019	0.471042	-0.72805	0.466581	0.865588
zgc:85777	880.0179	-0.205305189	0.139696	-1.46965	0.141655	0.600284
gbf1	567.492	0.145876529	0.133814	1.090144	0.27565	0.750653
ppp2r5a	22.04756	0.64546321	0.462939	1.394272	0.163235	0.631199
pcdh17	791.6387	0.370686241	0.161604	2.2938	0.021802	0.261665
scyl3	481.081	0.05535977	0.140347	0.39445	0.693248	0.940668
arpc1b	1011.519	-0.028308871	0.128195	-0.22083	0.825227	0.97261
slc38a3a	769.3596	0.257809263	0.179934	1.432797	0.151916	0.615406
acbd4	55.67619	2.127003506	0.490413	4.337166	1.44E-05	0.002389
cnppd1	1325.809	0.103401766	0.100964	1.024149	0.305765	0.771068
znf804a	72.87101	0.254420868	0.287546	0.884801	0.376264	0.822258
hmbox1a	25.50112	0.014672831	0.420587	0.034887	0.97217	1
tgfb2	72.566	0.119936154	0.20308	0.590586	0.554798	0.89855
ptgdsb.1	9574.695	-0.167310841	0.186936	-0.89502	0.370778	0.818484
psmc4	2707.994	0.113304852	0.148787	0.761523	0.446345	0.855403
zfr	2196.844	0.136885586	0.068819	1.989075	0.046693	0.376436
ephb4b	409.9542	-0.107037344	0.164476	-0.65078	0.515189	0.886401
tmem218	33.77206	0.174068077	0.305727	0.569358	0.569113	0.902234
tmem169a	68.86272	-0.151100975	0.256091	-0.59003	0.555171	0.898723
nup160	688.5768	0.041129956	0.118775	0.346286	0.729128	0.949983
ube2l3b	1353.616	-0.054258076	0.089351	-0.60725	0.543686	0.894925
aptx	224.8677	0.414838504	0.174118	2.382507	0.017195	0.231824
gabrr3a	23.53412	-0.052212367	0.525187	-0.09942	0.920807	0.994085
tpd52l2a	652.0985	-0.281642053	0.158401	-1.77804	0.075398	0.465238
fbxo28	405.8348	-0.173407982	0.11651	-1.48836	0.136657	0.591964
igl3v2	0.043308	0	5.267649	0	1	NA
kpna7	3.170933	0.279941477	0.965574	0.289922	0.771876	0.960624
phactr2	203.2343	-0.057965989	0.149504	-0.38772	0.698222	0.942433
rnf25	1171.575	-0.291322626	0.147032	-1.98135	0.047552	0.379659
namptb	164.1223	-0.097617731	0.179855	-0.54276	0.587297	0.9073
kat8	456.8872	-0.070502455	0.12845	-0.54887	0.583095	0.90582

hcn3	16.88214	0.083316339	0.593217	0.140448	0.888306	0.988188
si:dkey-16l2.17	58.45141	-0.274327796	0.240988	-1.13834	0.254977	0.733542
smad1	695.592	0.054023465	0.103224	0.52336	0.600724	0.913756
gabarapl2	916.6192	-0.193221768	0.152237	-1.26922	0.204362	0.685846
adgrg1	176.6386	-0.279669986	0.160434	-1.74321	0.081297	0.480158
ift88	172.3709	0.090729087	0.152563	0.594701	0.552043	0.8974
rs1a	1153.92	0.02846639	0.429673	0.066251	0.947178	0.997371
btg1	5647.523	-0.212358919	0.089312	-2.37773	0.017419	0.232896
numb	275.8279	-0.172589846	0.200916	-0.85901	0.390333	0.827902
kcnf1b	53.61677	0.828424565	0.347691	2.382645	0.017189	0.231824
nrp1b	107.783	0.051511911	0.190554	0.270328	0.786908	0.963061
lsp1	789.5661	-0.271262194	0.123976	-2.18801	0.028669	0.301471
tcp11l2	976.2213	-0.168517892	0.128192	-1.31457	0.188654	0.668367
lin7c	1397.208	0.060392615	0.102901	0.5869	0.557271	0.899165
mpzl2b	536.5081	-0.110635711	0.146113	-0.75719	0.448934	0.856796
zmym2	792.4927	0.002705859	0.137531	0.019675	0.984303	1
slc25a4	17592.81	0.165794301	0.137815	1.203024	0.228967	0.710863
cd2bp2	348.2835	0.307335347	0.162696	1.889015	0.05889	0.418455
si:ch211-106h11.3	68.87058	-0.214465765	0.37326	-0.57458	0.565579	0.901579
ccdc171	60.60511	0.343675168	0.25381	1.354064	0.175716	0.649413
ccnd3	6.305438	-0.45826765	0.813943	-0.56302	0.57342	0.904065
si:ch211-150g13.3	102.3287	0.197916807	0.198072	0.999215	0.317691	0.77959
vangl2	833.1182	-0.010164545	0.155062	-0.06555	0.947735	0.997371
zgpat	358.0582	0.088936267	0.130372	0.682173	0.49513	0.87806
trappc4	444.8931	-0.21834552	0.182637	-1.19552	0.231885	0.713202
gad1b	1388.931	0.435552284	0.255662	1.703625	0.088451	0.499597
igf1ra	223.4797	-0.101906551	0.236398	-0.43108	0.66641	0.932664
slc25a3a	345.2723	-0.056940741	0.422809	-0.13467	0.892871	0.988566
rpp30	103.4272	-0.192983963	0.241935	-0.79767	0.425063	0.844714
mettl26	240.6462	-0.2639243	0.179591	-1.46958	0.141675	0.600284
rmnd1	157.6454	-0.059307335	0.176896	-0.33527	0.737424	0.952375
hsd17b1	0.656871	1.055416151	2.998276	0.352008	0.724833	NA
gphb5	4.353084	-0.045731148	0.903854	-0.0506	0.959648	0.999164
uros	500.9716	-0.056460558	0.140227	-0.40264	0.687216	0.938835
elovl4b	467.3567	0.117757392	0.456781	0.257798	0.796562	0.966447
mast1a	31.48125	0.6770357	0.36355	1.862293	0.062562	0.427724
oxsr1b	465.6382	-0.242347641	0.131095	-1.84864	0.064509	0.433491
usp20	274.3221	-0.028500387	0.139799	-0.20387	0.838458	0.976395
trpc7a	9.081165	0.87057405	0.752858	1.15636	0.247534	0.728384
hmox1a	564.9793	-0.1378775	0.474223	-0.29074	0.771247	0.960484
noxred1	2.64348	0.365554677	1.139572	0.320783	0.748375	0.955074
TPGS1	285.9626	-0.134587838	0.199325	-0.67522	0.499537	0.879603
mapk1	167.1352	0.286125732	0.144646	1.978108	0.047917	0.380801
pak6a	9.252965	0.645302307	0.734519	0.878537	0.379652	0.823607
as3mt	171.5418	-0.49457982	0.18934	-2.61213	0.008998	0.162625
angptl7	861.6625	0.293236058	0.135548	2.163334	0.030515	0.311199
ttpa	178.0265	0.201878706	0.174744	1.15528	0.247976	0.728489
top3b	188.2676	-0.146212106	0.185605	-0.78776	0.430839	0.847629
htr7b	2.2759	0.077735396	1.294199	0.060064	0.952104	0.998066
fzd7b	882.1485	-0.117348447	0.127859	-0.9178	0.358725	0.80995
2-Sep	1874.97	0.024240275	0.123155	0.196828	0.843962	0.976791
selenot1b	515.7439	-0.083618062	0.121416	-0.68869	0.491017	0.875595
fam221a	10.57838	-0.369193288	0.670077	-0.55097	0.581653	0.905618
pdlim5b	612.5237	-0.143437613	0.130852	-1.09618	0.273001	0.748889

lrfn1	24.57608	0.480224937	0.471707	1.018058	0.30865	0.773546
hpn	53.47312	-0.080238958	0.325849	-0.24625	0.805492	0.969368
cavin2a	752.8655	-0.134288769	0.128946	-1.04144	0.297673	0.765407
gatad1	182.7636	0.184643315	0.165277	1.117172	0.263921	0.741937
grip2b	11.34534	0.087506331	0.649809	0.134665	0.892877	0.988566
bco2l	63.28205	-0.700693087	0.368585	-1.90104	0.057297	0.413318
adra1ab	24.3487	-0.136776344	0.453829	-0.30138	0.763123	0.958905
zgc:165539	1.43367	0.317341475	1.411946	0.224755	0.82217	NA
cpsf3	748.3308	0.005369943	0.095723	0.056099	0.955263	0.998611
crhb	52.42152	-0.689216873	0.372968	-1.84793	0.064613	0.433937
irf10	14.1828	-0.342444095	0.5876	-0.58278	0.560039	0.899738
si:ch211-13315.8	33.85963	0.509216477	0.277939	1.832118	0.066934	0.44184
tmem230a	78.43783	0.016250176	0.25762	0.063078	0.949704	0.99801
pold1	478.5509	0.097535943	0.150356	0.648701	0.516532	0.886933
zgc:103510	10.02513	-0.423575393	0.563378	-0.75185	0.452141	0.857994
btbd16	3.368211	-1.175163439	0.947569	-1.24019	0.214906	0.697
dgkb	7.600851	-0.907661063	1.350444	-0.67212	0.501507	0.880897
srsf5b	2816.44	-0.082160262	0.10079	-0.81516	0.414981	0.838732
si:ch211-13c6.2	509.3867	0.138688838	0.136742	1.014238	0.310469	0.775146
adcyap1b	858.5539	0.14355118	0.151509	0.947478	0.343395	0.797961
gadd45ba	951.96	-0.414601255	0.239718	-1.72954	0.083713	0.485998
hirip3	251.6351	0.170702549	0.11527	1.480897	0.138634	0.595394
dpp7	1184.277	0.273334159	0.158967	1.719445	0.085533	0.491033
tnfaip3	55.2857	0.253724059	0.265331	0.956254	0.338944	0.795226
adcy6b	152.1047	-0.07698439	0.152031	-0.50637	0.612594	0.91573
ucmaa	87.2693	0.94752875	0.560346	1.690971	0.090842	0.505206
sbds	253.3116	-0.104350635	0.156141	-0.66831	0.503937	0.881759
frk	118.744	0.089850868	0.178169	0.504301	0.61405	0.915998
nus1	504.9597	0.221044661	0.14981	1.475505	0.140077	0.598705
tmtops2b	9.294465	-0.012819143	0.699115	-0.01834	0.985371	1
top1mt	965.5993	0.176359588	0.140134	1.25851	0.208207	0.69024
naa50	1227.538	0.08766488	0.101751	0.861565	0.388927	0.827725
pudp	102.4541	-0.636373486	0.190745	-3.33624	0.000849	0.039393
grin1a	1510.068	0.294066104	0.176461	1.666464	0.095621	0.516418
ccdc191	29.05935	8.73E-05	0.353413	0.000247	0.999803	1
paplna	249.8656	0.186704665	0.174196	1.071809	0.283806	0.755095
zfyve21	464.307	0.011199439	0.129869	0.086236	0.931279	0.995809
sem1	1664.656	-0.117660983	0.165415	-0.71131	0.476894	0.869292
adamtsl3	34.64379	0.147540487	0.332811	0.443316	0.657537	0.93039
si:ch211-216p19.6	21.29544	-0.123678883	0.41298	-0.29948	0.764574	0.959393
zc2hc1a	378.0373	0.006514284	0.127631	0.05104	0.959294	0.999164
fntb	785.0926	-0.25791351	0.153877	-1.6761	0.093718	0.511703
naprt	596.3301	-0.166211062	0.139883	-1.18821	0.234749	0.715533
glis1b	36.03387	-0.318382373	0.304201	-1.04662	0.295275	0.763896
rad18	115.6515	1.13495135	0.256976	4.416563	1.00E-05	0.001874
kcnf1a	50.8277	-0.449012443	0.280619	-1.60008	0.109581	0.543101
fgf12a	182.6994	0.832333881	0.315092	2.641562	0.008252	0.154575
camkva	78.44771	0.126359319	0.375278	0.336709	0.736336	0.951919
ap1s3b	236.6496	-0.346828922	0.166012	-2.08918	0.036692	0.336694
yy1b	1007.146	-0.110386585	0.097063	-1.13727	0.255426	0.733542
gstz1	615.2675	-0.075652245	0.226334	-0.33425	0.73819	0.952898
slc35d1b	200.8142	-0.51982766	0.194392	-2.67412	0.007493	0.146749
hao2	499.4036	-0.500923473	0.17225	-2.90812	0.003636	0.099701
pfkpa	1166.555	-0.143787149	0.191448	-0.75105	0.452622	0.857994

tpmt.2	15.92205	-0.563108272	0.579828	-0.97117	0.331466	0.789156
tp53inp1	401.5384	-0.11416804	0.180543	-0.63236	0.527153	0.889948
trim63a	4143.097	0.373401163	0.154629	2.414821	0.015743	0.221337
rdh8a	446.1075	-0.359340419	0.217322	-1.65349	0.098231	0.521076
bmp1b	62.96592	0.211405834	0.273517	0.772917	0.439572	0.852079
traf6	277.8863	-0.250775167	0.134984	-1.85781	0.063196	0.429313
dirasla	893.0274	0.162686975	0.188491	0.863102	0.388082	0.827592
bnip3lb	1493.969	-0.155048404	0.15578	-0.9953	0.319588	0.780289
bmp1a	907.4699	0.146907686	0.130531	1.125462	0.260393	0.737749
mapkapk5	165.5261	-0.051826961	0.163053	-0.31785	0.750596	0.955074
zbtb2b	441.5549	-0.104804418	0.119875	-0.87428	0.381965	0.824957
aldh2.2	1655.251	0.019808633	0.13691	0.144684	0.884961	0.987266
galk1	97.51422	-0.308478105	0.266455	-1.15771	0.246982	0.727706
cldn23a	457.4143	0.044870806	0.123017	0.364753	0.715296	0.945917
fut9d	785.8486	0.144430255	0.201097	0.71821	0.472628	0.868786
sh2d3ca	103.2233	0.16990705	0.160323	1.059776	0.289246	0.760276
glrx	452.3967	-0.592249105	0.217208	-2.72665	0.006398	0.135026
dscamb	493.2384	0.323991384	0.208912	1.550854	0.120937	0.566816
sumo3a	3020.591	0.146807527	0.123895	1.184934	0.236044	0.717364
hccsa.1	237.0624	0.058674707	0.199493	0.294119	0.768667	0.960395
pax2a	304.2695	0.112805005	0.148611	0.759062	0.447815	0.856379
tead1a	93.68982	-0.037964666	0.221361	-0.17151	0.863826	0.981842
prg4b	126.0799	-0.205383783	0.345796	-0.59395	0.552549	0.8974
cst14a.1	39.46527	-0.898201139	0.363362	-2.47192	0.013439	0.202065
slc4a2a	274.4033	-0.119143311	0.135173	-0.88142	0.378092	0.82265
cradd	30.62795	-0.140669268	0.316791	-0.44404	0.65701	0.929939
ube2v2	2083.592	-0.022825826	0.09561	-0.23874	0.811308	0.97002
commd8	287.2731	0.437537011	0.172413	2.53772	0.011158	0.183611
ttn.2	5177.861	-0.056234833	0.289533	-0.19423	0.845999	0.977039
itgb8	24.75271	-0.399838414	0.379086	-1.05474	0.291543	0.761454
zbtb18	853.3934	0.055943118	0.142184	0.393457	0.693982	0.940919
cyb561a3b	44.03279	-0.831434744	0.26682	-3.11609	0.001833	0.065916
aldh3a2a	284.1748	-0.030964809	0.157711	-0.19634	0.844345	0.976791
sult1st1	978.3467	-0.149136588	0.123084	-1.21166	0.225642	0.707068
si:ch211-196h16.12	13.92997	2.512296006	1.566993	1.60326	0.108877	0.541698
mkrn4	246.6524	-0.025661872	0.168987	-0.15186	0.879299	0.986147
prph	296.2845	0.252464818	0.192839	1.309203	0.190465	0.670633
synj2bp	277.1199	0.262112259	0.158584	1.652827	0.098366	0.521076
pex14	318.8119	0.150058848	0.151389	0.991211	0.321583	0.78202
gmfb	1873.932	-0.100186395	0.155731	-0.64333	0.520009	0.887148
nitr9	0.446561	-0.86812373	4.000989	-0.21698	0.828226	NA
hmga1a	19301.06	0.094866067	0.157781	0.601253	0.547671	0.896211
dhdhl	930.2155	0.394658825	0.193684	2.037647	0.041585	0.356072
tesca	256.6022	0.039259794	0.219076	0.179206	0.857776	0.979774
pax3b	33.09428	0.164945398	0.290127	0.568529	0.569676	0.902322
stxbp6l	82.0923	-0.014314902	0.412653	-0.03469	0.972327	1
sult2st3	142.7606	0.405074884	0.260626	1.554237	0.120128	0.565265
trub2	208.9636	0.023443361	0.179441	0.130647	0.896055	0.98926
htatip2	272.4722	-0.300818354	0.159931	-1.88093	0.059982	0.421883
rab44	14.21762	-0.06281517	0.493091	-0.12739	0.898631	0.990199
daam2	45.16453	-0.589998936	0.395022	-1.49358	0.135284	0.590585
fkbp5	1086.779	-0.09247987	0.555532	-0.16647	0.867786	0.982728
rhogc	453.9256	-0.084341565	0.120821	-0.69807	0.485132	0.872911
adnp2b	494.2468	-0.007904842	0.109871	-0.07195	0.942645	0.997371

cplane2	49.34002	0.20641951	0.25402	0.81261	0.416442	0.839843
edem2	224.4576	-0.111329132	0.201468	-0.55259	0.580544	0.905618
rps6ka2	14.61941	0.979789292	0.633188	1.54739	0.121769	0.56863
lin9	192.9606	0.210488762	0.156805	1.342363	0.179478	0.654677
si:ch211-173n18.3	66.60795	1.528662549	0.373228	4.095787	4.21E-05	0.004899
cabp5b	82.33669	0.171448876	0.528827	0.324206	0.745782	0.955074
itgb4	690.4311	0.053820945	0.22226	0.242153	0.808661	0.969828
hbp1	1030.899	-0.052962718	0.129026	-0.41048	0.681454	0.937418
c1ql3b	24.59576	0.233706416	0.425977	0.548637	0.583255	0.905947
inip	205.5047	0.133869692	0.173032	0.773671	0.439125	0.852079
macf1a	2278.152	0.097047037	0.185621	0.522825	0.601096	0.913756
morc2	813.2144	-0.049893715	0.107489	-0.46418	0.642521	0.92567
si:ch211-121a2.4	15.32735	0.095269985	0.420283	0.226681	0.820672	0.971788
si:ch211-114c12.2	1290.373	-0.105156533	0.134565	-0.78146	0.434535	0.850002
ndufs1	1.463341	-2.324639292	2.005108	-1.15936	0.24631	NA
plppr3b	242.1954	-0.040293807	0.341709	-0.11792	0.906132	0.991631
march5l	544.9518	-0.004807543	0.123349	-0.03898	0.96891	1
jkamp	221.3781	0.050667718	0.123489	0.410302	0.681584	0.937418
si:ch211-234h8.7	34.97213	0.223565296	0.351986	0.635153	0.525329	0.888928
gpr135	21.90033	-0.749824469	0.413307	-1.81421	0.069646	0.450502
mospd1	782.5199	-0.056547523	0.175675	-0.32189	0.747538	0.955074
krt18b	5236.377	-0.135040161	0.17296	-0.78076	0.434944	0.850002
jph2	3256.989	-0.42955528	0.222617	-1.92957	0.05366	0.401106
gdap1l1	29.96207	-0.024755658	0.315043	-0.07858	0.937368	0.997305
ms4a17c.2	0	NA	NA	NA	NA	NA
cntfr	684.9539	0.075824961	0.087751	0.864093	0.387537	0.827592
tek	129.8222	0.0703302	0.321654	0.218652	0.826921	0.973458
ahsa1a	181.1903	0.625473987	0.271083	2.307316	0.021037	0.257628
nuak1b	1414.087	-0.004368454	0.169642	-0.02575	0.979456	1
exosc2	262.574	-0.106486956	0.185737	-0.57332	0.566427	0.901739
crkl	988.4359	0.049631905	0.090147	0.550564	0.581932	0.90571
mindy3	324.1775	0.076888657	0.15537	0.494873	0.62069	0.918151
mapk14b	428.3484	-0.065951933	0.128614	-0.51279	0.608097	0.915204
adra1aa	6.806012	-0.350722185	0.766584	-0.45751	0.647303	0.927082
stat4	82.26548	-0.782987452	0.283144	-2.76533	0.005686	0.127671
msnb	21.74552	-0.054535249	0.385983	-0.14129	0.887641	0.988069
tm9sf3	784.3681	0.033054899	0.098001	0.33729	0.735898	0.951589
gzmk	1.699389	-0.393354276	1.882656	-0.20894	0.834498	NA
si:dkey-24p1.6	24.51279	0.214143175	0.451568	0.474222	0.635342	0.924272
chst12a	23.11561	0.381371691	0.397556	0.959289	0.337413	0.794381
nlk2	202.1239	0.114683096	0.180597	0.635024	0.525413	0.888928
CYTH2	51.67467	-0.066295595	0.267084	-0.24822	0.803964	0.968814
ankrd9	470.9275	-0.062426704	0.292924	-0.21312	0.831237	0.974908
zgc:162945	128.7454	0.032943755	0.243918	0.135061	0.892564	0.988566
tmed3	461.1727	0.124504858	0.142755	0.872155	0.383124	0.825809
slc34a1a	39.83716	0.237496507	0.560681	0.423586	0.671868	0.933953
calcr	33.99379	0.44852525	0.37002	1.212166	0.225449	0.70685
lsm12a	437.7028	0.214067248	0.167123	1.280894	0.200231	0.680711
btr16	10.34705	-0.596993796	0.71681	-0.83285	0.404931	0.834284
sgsm1a	20.50697	0.609515226	0.452704	1.346389	0.178177	0.65219
rdh20	10.65509	0.783138133	0.926784	0.845006	0.398107	0.830737
vipr1a	94.31154	0.358490369	0.230968	1.552118	0.120634	0.566496
ndufb10	2034.764	0.049857748	0.129667	0.384505	0.700604	0.942918
mrgbp	264.5195	0.134421183	0.131012	1.026023	0.304881	0.770035

plpp2b	227.2697	0.038828596	0.165862	0.234102	0.814906	0.970814
tekt4	37.09723	0.124800204	0.292188	0.427124	0.669289	0.933115
odf2a	88.50489	0.194897375	0.237003	0.822341	0.410883	0.83771
si:dkey-10h3.2	2.709589	1.638418374	1.262528	1.297728	0.194381	0.674677
taf1b	117.9645	-0.067846413	0.217885	-0.31139	0.755507	0.956532
mtss1la	389.1358	0.110265955	0.168068	0.65608	0.511773	0.885041
ZBTB26	68.34181	-0.050898383	0.308274	-0.16511	0.868859	0.982801
maff	813.619	-0.09858171	0.110275	-0.89396	0.371343	0.818945
cpsf2	557.951	0.130671022	0.119308	1.09524	0.273412	0.749148
tekt2	20.65537	0.372625482	0.408174	0.912908	0.361291	0.811547
pus3	59.77699	-0.100795319	0.231569	-0.43527	0.663365	0.931356
pcca	748.7207	-0.070844315	0.105178	-0.67357	0.500588	0.880351
eif1axa	357.9138	0.218725181	0.145004	1.508404	0.131451	0.583069
xpnpep1	773.4237	0.005369811	0.151463	0.035453	0.971719	1
cdkn1ba	580.2369	-0.14443207	0.108763	-1.32796	0.184193	0.661153
epb41b	472.7912	0.041607878	0.203596	0.204365	0.838069	0.97637
rab32a	202.7986	-0.299088191	0.208292	-1.43591	0.151028	0.615085
zdhhc23a	16.07647	0.050261575	0.461834	0.10883	0.913337	0.99273
nupl2	170.7718	-0.107677282	0.206394	-0.52171	0.601875	0.914001
peli2	284.1133	-0.159076671	0.148182	-1.07352	0.283036	0.755028
gbe1a	24.40114	-0.174545104	0.539995	-0.32323	0.746518	0.955074
tm6sf2	107.7441	-0.103048185	0.281266	-0.36637	0.714087	0.945821
rbbp4	1869.059	0.222067499	0.203859	1.089319	0.276013	0.750674
clpxa	1856.981	-0.072107182	0.101057	-0.71353	0.475516	0.869292
uqcrq	2220.251	0.075050871	0.187293	0.400714	0.688631	0.939118
tnni2a.4	70292.9	0.021818039	0.165909	0.131506	0.895375	0.989223
creld2	119.6904	0.369662087	0.228779	1.615801	0.106137	0.535191
klf6a	1929.12	-0.066554608	0.122962	-0.54126	0.588326	0.90797
pfkfb4b	871.8824	-0.456261598	0.295604	-1.54349	0.122713	0.569468
guf1	110.7326	0.366957377	0.193886	1.892645	0.058405	0.417797
chchd1	384.8241	-0.259408061	0.185075	-1.40163	0.161024	0.627788
ftr51	110.4624	-0.115085368	0.405186	-0.28403	0.776387	0.961073
ube2d1a	338.8532	0.069963378	0.167884	0.416738	0.67687	0.935664
gem	80.87777	-0.0578181	0.445784	-0.1297	0.896804	0.98939
si:dkey-40m6.8	572.3402	0.056353186	0.198121	0.284438	0.776075	0.961073
adamts15a	161.9089	-0.271530334	0.240176	-1.13055	0.258245	0.73562
tubgcp3	486.4883	0.229650045	0.10232	2.244427	0.024805	0.279131
lrp1ab	1028.954	0.130222535	0.20615	0.631688	0.527591	0.889948
hsp90ab1	54594.26	-0.361620741	0.078557	-4.60327	4.16E-06	0.000936
med23	425.9908	0.18595473	0.176588	1.053042	0.292322	0.761879
ppfibp2b	437.5998	0.083090282	0.107063	0.776091	0.437696	0.851283
si:ch211-200p22.4	979.0087	0.10006143	0.119781	0.83537	0.403509	0.833867
lnx2a	299.6103	0.12316484	0.159296	0.773183	0.439414	0.852079
foxd1	300.8269	0.150986686	0.144014	1.048417	0.294446	0.763544
tyrp1a	1046.676	-0.227749089	0.197408	-1.1537	0.248623	0.728723
ube2z	453.5869	0.015271853	0.131754	0.115911	0.907723	0.991745
avpr2ab	13.71769	0.52418765	0.454055	1.154458	0.248312	0.728647
pnp4b	787.104	0.289540051	0.321502	0.900586	0.367809	0.817078
FO704779.1	17.33875	0.346212975	0.562138	0.615886	0.53797	0.892498
stxbp5b	113.141	-0.078056499	0.205869	-0.37916	0.704572	0.943785
syt9b	224.4148	0.270301408	0.215352	1.255158	0.209421	0.691505
fbxo3	374.1026	0.01600036	0.159679	0.100203	0.920183	0.993982
fubp1	3748.898	0.087578599	0.077751	1.126394	0.259999	0.73712
poli	152.6114	-0.2803292	0.219699	-1.27597	0.201966	0.68211

ssb	972.3107	-0.129519238	0.112229	-1.15406	0.248475	0.728723
zgc:136493	67.03879	-0.179560741	0.237617	-0.75567	0.449845	0.857077
hoxb3a	612.5859	0.057174105	0.108018	0.529301	0.596596	0.911696
stx11b.1	329.324	0.162437545	0.262931	0.617796	0.53671	0.892421
ipmkb	260.3165	0.10678239	0.150739	0.708393	0.478701	0.869292
zgc:55943	571.021	0.144237497	0.134885	1.069334	0.284919	0.755935
nexmifb	208.3983	-0.31664446	0.205272	-1.54256	0.122938	0.569468
baiap2l1a	147.0621	-0.27181711	0.204492	-1.32923	0.183771	0.66056
rmc1	179.6262	0.036606482	0.153935	0.237804	0.812033	0.97012
prr15lb	31.65481	0.143912993	0.470772	0.305695	0.759837	0.957886
atxn7l3	185.9079	0.104032789	0.204891	0.507746	0.611632	0.915331
serpine2	643.125	-0.356328422	0.167494	-2.12742	0.033386	0.323127
mboat1	105.2231	-0.443052197	0.206745	-2.14299	0.032114	0.318495
ankrd6b	231.9142	0.003578904	0.195774	0.018281	0.985415	1
rasa4	170.1761	-0.356799642	0.204216	-1.74717	0.080608	0.479386
aldh3a2b	64.17918	0.087007837	0.298349	0.291631	0.770568	0.960484
si:dkey-90m5.4	148.1863	-0.358802725	0.344067	-1.04283	0.297028	0.764598
sort1a	545.4593	-0.108881365	0.115961	-0.93895	0.347758	0.800274
h2afx	371.5061	-0.311452834	0.239433	-1.30079	0.193329	0.673396
mtx2	560.1936	0.186957724	0.133428	1.401185	0.161159	0.627888
si:ch211-147k10.5	7.97966	0.835932603	0.598445	1.396841	0.162461	0.629825
tph1a	33.35586	0.04024536	0.360497	0.111639	0.91111	0.992208
atp2a2a	1416.372	0.574850573	0.191366	3.003928	0.002665	0.084161
zgc:92242	490.9461	-0.041485036	0.186598	-0.22232	0.824062	0.97205
zgc:56676	1744.381	0.011875116	0.151794	0.078232	0.937644	0.997305
cacna1sa	296.9662	0.079274687	0.242981	0.326259	0.744228	0.95491
cd209	0.094679	0	5.267649	0	1	NA
asap1a	792.3086	0.078485914	0.0982	0.799242	0.42415	0.844355
afdna	744.1412	-0.124183008	0.156333	-0.79435	0.426992	0.845491
gstm.2	157.232	-0.457085561	0.152184	-3.00351	0.002669	0.084161
camk1b	452.1542	0.217382183	0.1821	1.19375	0.232576	0.71403
ldlra	476.2792	0.507274602	0.307185	1.651366	0.098664	0.521402
zgc:86609	289.0569	0.106298001	0.134133	0.792484	0.428079	0.846079
speccl	146.4251	-0.524481245	0.175315	-2.99164	0.002775	0.086456
ush2a	37.92647	0.416351883	0.464975	0.895429	0.370558	0.818484
f9b	141.7223	-0.72378294	0.279847	-2.58635	0.0097	0.169747
tfcp2l1	59.60537	0.736512066	0.32831	2.243342	0.024875	0.279374
rpl34	19145.93	-0.325464757	0.131802	-2.46935	0.013536	0.202995
gsk3aa	229.0743	0.006403954	0.139924	0.045767	0.963496	0.999842
timm17a	1303.089	0.103084217	0.143873	0.716496	0.473685	0.869192
kcns3b	0.650507	1.003195533	2.757676	0.363783	0.71602	NA
impdh1b	1681.489	-0.673060204	0.165973	-4.05525	5.01E-05	0.005606
rpl18	24651.31	-0.398200734	0.117593	-3.38625	0.000709	0.035458
id2b	240.2179	0.110407289	0.148496	0.743504	0.457177	0.860993
grem1a	14.28811	0.399409461	0.686098	0.582147	0.560468	0.899928
kansl3	973.0638	0.092293427	0.098618	0.935871	0.349339	0.801408
snapc1a	1.411257	0.610427116	1.922934	0.317446	0.750905	NA
tbc1d7	314.6951	0.117957591	0.141217	0.835295	0.403552	0.83388
msra	550.7932	-0.032487985	0.206436	-0.15738	0.874949	0.984191
trim55a	289.1067	0.165522135	0.238641	0.693604	0.48793	0.873337
wdr32	311.727	0.039556846	0.121353	0.325966	0.74445	0.95491
macrod1	44.49917	0.194516148	0.353752	0.549866	0.582411	0.90571
gpkow	350.8538	0.20717768	0.125143	1.655522	0.097819	0.521071
zgc:77056	292.7529	-0.072632229	0.205287	-0.35381	0.723482	0.947917

sgms1	305.8649	-0.109606523	0.14769	-0.74214	0.458002	0.860993
si:dkey-47k20.3	0.566088	-1.379831724	2.806544	-0.49165	0.622968	NA
tomm70a	1222.161	0.486379382	0.202624	2.400402	0.016377	0.225867
commd5	153.4808	-0.147659546	0.154621	-0.95497	0.339591	0.795226
rab1ab	1532.923	-0.022928864	0.101698	-0.22546	0.821621	0.971794
crim1	152.4438	-0.121835481	0.266693	-0.45684	0.647787	0.927183
xpr1b	605.1856	-0.109499075	0.109974	-0.99568	0.319404	0.780208
hsp70.1	120.1731	-0.721424546	0.426614	-1.69105	0.090827	0.505206
tkta	1738.897	0.433386199	0.147622	2.935781	0.003327	0.09482
rufy3	519.1798	0.221458015	0.130953	1.691132	0.090812	0.505206
pgp	342.997	0.227356294	0.137011	1.659407	0.097034	0.519526
lrrc30a	77.76385	0.375616081	0.263896	1.423347	0.154636	0.619425
p2rx3b	61.45469	0.754046668	0.272123	2.770978	0.005589	0.126154
si:dkeyp-9d4.2	5.745247	-1.716681317	1.435853	-1.19558	0.231859	0.713202
hmgb2a	6.411549	-0.720483001	0.667197	-1.07987	0.280202	0.752951
pqbp1	259.8093	-0.189476941	0.163583	-1.1583	0.246743	0.727706
clybl	375.3677	0.050371051	0.140228	0.359208	0.719439	0.94694
mep1a.1	35.04873	1.023131822	0.697455	1.466951	0.142389	0.601598
wbp11b	176.1321	-0.247990226	0.186565	-1.32924	0.183768	0.66056
mpp6b	422.0912	0.193928198	0.153144	1.266314	0.205401	0.687537
mef2ca	992.4464	-0.03703764	0.192916	-0.19199	0.847751	0.97772
nr1i2	230.0002	-0.030841587	0.169597	-0.18185	0.855699	0.979524
wap1b	684.11	-0.021728682	0.117963	-0.1842	0.853858	0.978905
dedd	604.5351	-0.058860919	0.117894	-0.49927	0.61759	0.916398
prxl2b	526.8539	0.085325987	0.204385	0.417478	0.676329	0.93557
oxgr1b	0.260652	0	4.828052	0	1	NA
srsf5a	4354.191	-0.119748269	0.10319	-1.16047	0.245859	0.727177
cel.2	3287.775	0.865579282	0.710848	1.217672	0.223349	0.70531
myf6	177.2605	0.011921051	0.238077	0.050072	0.960065	0.999164
slc26a1	111.6703	0.085408705	0.210427	0.405882	0.684829	0.938486
si:dkey-23a13.11	1.650264	-0.493442874	1.58485	-0.31135	0.755535	NA
tmprss9	419.2032	-0.067116099	0.251253	-0.26713	0.789373	0.963786
zgc:161973	28.77056	0.961930243	0.617529	1.557708	0.119303	0.562753
cnn3b	579.0116	0.224982951	0.155542	1.446445	0.148052	0.609064
armh1	10.33005	0.244991712	0.642725	0.381177	0.703072	0.943622
rassf2a	583.0912	-0.148648432	0.098338	-1.5116	0.130635	0.581957
slc6a14	28.33469	-0.024266981	0.566147	-0.04286	0.96581	1
khk	126.707	0.022827219	0.19401	0.11766	0.906337	0.991631
kcnh2a	16.76941	0.442906919	0.596007	0.743123	0.457407	0.860993
rab41	1387.176	0.126034208	0.110699	1.138527	0.2549	0.733542
si:dkeyp-86f7.4	5.839107	0	1.902524	0	1	1
slc2a15a	285.4845	-0.382170642	0.194389	-1.96601	0.049298	0.386671
cnga1a	35.15252	1.16189089	0.630488	1.842845	0.065352	0.435359
phyhd1	665.4251	0.570213736	0.255589	2.230979	0.025682	0.284447
trim54	21.28516	-0.716932566	0.72007	-0.99564	0.319424	0.780208
casc3	693.6349	-0.148495096	0.142276	-1.04371	0.296618	0.764495
ap3m1	333.1742	0.012171349	0.127208	0.095681	0.923774	0.994713
SPDEF	14.58249	0.192775162	0.500749	0.384973	0.700257	0.942739
atp13a1	442.2393	0.049875707	0.148589	0.335662	0.737126	0.952312
grtp1a	231.2379	-0.4555693	0.198676	-2.29303	0.021846	0.261875
parpbp	142.758	0.056466968	0.223991	0.252094	0.800968	0.96768
ampd2b	309.4501	-0.002149271	0.156666	-0.01372	0.989054	1
glb1l	240.3412	0.075126532	0.167381	0.448835	0.653551	0.929269
si:dkey-42l23.9	0.184502	0	5.267649	0	1	NA

tnni2b.2	5823.328	-0.055509169	0.159628	-0.34774	0.728034	0.949325
BX511121.1	3.019233	1.055425436	1.943378	0.543088	0.587069	0.907249
slc6a7	189.4909	-0.048023663	0.139843	-0.34341	0.73129	0.950169
lrrfip1a	509.935	0.036655358	0.133154	0.275286	0.783096	0.962328
agfg1a	300.4292	0.081975263	0.162234	0.505289	0.613356	0.915998
nup188	803.6318	0.135499325	0.102273	1.324882	0.18521	0.662813
pimr135	0.09025	0	5.267649	0	1	NA
grcc10	423.1182	-0.028980064	0.128836	-0.22494	0.822027	0.971966
eef1db	3410.4	-0.47645124	0.109654	-4.34503	1.39E-05	0.002321
tbx4	47.81637	-0.314509097	0.302573	-1.03945	0.298596	0.765656
plekha1a	554.8514	-0.24068487	0.138688	-1.73544	0.082663	0.483014
glipr1b	103.1657	0.410279602	0.274472	1.494796	0.134968	0.589926
nfkbib	1153.655	-0.160329419	0.104438	-1.53517	0.124742	0.572486
tram2	335.7086	-0.058264766	0.138081	-0.42196	0.673053	0.93414
malb	151.4593	0.415953997	0.244765	1.699402	0.089243	0.501424
ccdc78	12.44043	-0.271592092	0.496461	-0.54706	0.58434	0.906412
sdcbp	68.54656	0.995962321	0.343129	2.902586	0.003701	0.100884
sh3bp4	118.4313	-0.197103439	0.176896	-1.11423	0.265179	0.742063
stmn4	972.1734	0.119545805	0.215405	0.554981	0.578908	0.905417
si:ch211-207i1.2	313.9276	0.120056598	0.177882	0.674921	0.499726	0.879636
sec22ba	227.2095	0.080780943	0.185833	0.434697	0.663782	0.931603
myod1	201.5095	-0.159947859	0.253321	-0.6314	0.527776	0.889948
vps4a	123.3238	-0.238598722	0.166161	-1.43595	0.151016	0.615085
susd6	955.6886	0.168366956	0.108306	1.554554	0.120052	0.565105
sox7	198.8874	0.108210921	0.159664	0.677743	0.497935	0.878926
si:ch211-87m7.2	45.39914	0.009975245	0.333965	0.029869	0.976171	1
fkbp6	3.980489	0.840876658	0.842775	0.997748	0.318402	0.779923
sdhdb	1233.234	0.055232923	0.125441	0.440309	0.659714	0.930581
gnptab	514.8842	-0.053730126	0.122781	-0.43761	0.661669	0.930742
pak7	296.2602	0.139904547	0.159688	0.876111	0.38097	0.824157
ssbp3b	1074.944	-0.197798461	0.134293	-1.47288	0.140783	0.599658
naglu	106.8972	0.505223874	0.200213	2.523429	0.011622	0.187517
mybpc2a	265.0922	-0.057972448	0.275939	-0.21009	0.833596	0.975482
ppp1r14bb	566.266	0.032899936	0.174064	0.189011	0.850084	0.978251
cdk21	1.604877	-0.54429958	1.570995	-0.34647	0.728991	NA
hyi	578.3336	0.047359439	0.222577	0.212778	0.8315	0.974908
traf2a	92.96035	-0.212132768	0.246129	-0.86188	0.388756	0.827664
zgc:92429	364.0873	0.230338262	0.204108	1.128509	0.259105	0.736127
uchl3	498.8506	0.122536186	0.209935	0.583686	0.559431	0.899571
sap30l	1492.662	-0.155958207	0.199767	-0.7807	0.434979	0.850002
matn1	6289.289	-0.693575046	0.493492	-1.40544	0.15989	0.626123
ppp2ca	445.6982	0.0319717	0.101601	0.314679	0.753006	0.955756
tmem30aa	495.9003	0.364280904	0.145753	2.499297	0.012444	0.194346
pgrmc2	942.313	-0.075479038	0.107084	-0.70486	0.480898	0.870179
dennd6aa	65.61707	-0.179143395	0.199524	-0.89785	0.369264	0.817763
vbp1	1155.331	-0.042674271	0.15101	-0.28259	0.777489	0.961073
dennd2db	36.60708	-0.749771491	0.448708	-1.67096	0.09473	0.513797
klhl36	70.9694	-0.011220135	0.243961	-0.04599	0.963317	0.999842
cbln9	17.89358	-1.95560895	0.822252	-2.37836	0.01739	0.232896
si:ch211-220i18.4	51.96412	0.086060889	0.320921	0.268168	0.78857	0.963567
mfsd2b	78.83869	-0.349271728	0.351825	-0.99274	0.320835	0.781266
scdb	841.078	0.094475062	0.170427	0.554345	0.579343	0.905578
gtf3aa	140.7813	0.194905517	0.222118	0.877488	0.380222	0.823648
grtp1b	34.62589	0.235648312	0.337427	0.698368	0.484947	0.872911

tnnt3a	10612.9	-0.060870888	0.178056	-0.34186	0.732453	0.950617
idh3a	1322.449	0.207667033	0.112152	1.851659	0.064075	0.432202
jag1a	150.4908	0.62420759	0.193728	3.222077	0.001273	0.052006
sox13	434.3011	0.079251403	0.142194	0.557349	0.577289	0.904809
hspa12b	178.5626	0.263967683	0.189644	1.391911	0.16395	0.632697
tmc2b	14.54647	0.349592238	0.613183	0.570127	0.568592	0.902234
efna1a	420.4977	-0.227486264	0.155317	-1.46466	0.143015	0.602002
zc3h10	226.7206	0.371845913	0.16033	2.319252	0.020381	0.253346
guk1a	818.0348	-0.047940329	0.11654	-0.41136	0.680806	0.937189
mespba	0.145288	0	5.267649	0	1	NA
cryba2a	4932.121	0.506566244	0.262319	1.931106	0.05347	0.400549
mnx2b	9.669699	0.725860891	0.699461	1.037743	0.29939	0.766128
zgc:66313	16.93117	-1.755441036	0.539866	-3.25163	0.001147	0.04834
angpt1	90.66063	0.236433781	0.3315	0.713224	0.475707	0.869292
metrn	443.7573	-0.089362209	0.129348	-0.69086	0.489651	0.874629
naa15b	715.5827	0.056513304	0.108841	0.51923	0.6036	0.914558
grin2bb	247.5856	0.42948049	0.228227	1.881816	0.059861	0.421883
prf1.1	0.383482	-0.868143408	4.388648	-0.19782	0.843189	NA
twist1a	459.6995	0.00823462	0.146055	0.05638	0.955039	0.998611
rps26l	13123.59	-0.263031141	0.136125	-1.93227	0.053326	0.400175
crygn2	8395.202	0.6837976	0.340907	2.005817	0.044876	0.371082
pkd1a	288.7332	0.698182258	0.270109	2.584817	0.009743	0.170247
rsrp1	1894.24	-0.143315924	0.250374	-0.57241	0.567046	0.901747
ppa1b	1172.814	0.067966539	0.097676	0.695837	0.486531	0.873244
sppl2	482.4481	0.153125684	0.202973	0.754414	0.4506	0.857151
crabp2b	783.5088	-0.078652156	0.136813	-0.57489	0.565368	0.901538
tppp3	151.1971	-0.159297075	0.213258	-0.74697	0.455082	0.860008
tmem263	833.7818	0.084707549	0.095666	0.885456	0.375911	0.822237
slc16a5a	46.57012	-0.131873623	0.278051	-0.47428	0.635301	0.924272
zgc:66484	152.0806	-0.07484439	0.187607	-0.39894	0.689936	0.939679
hmgb1b	4356.396	0.104800911	0.144223	0.726661	0.467434	0.86616
sptb	1013.034	0.313646072	0.215625	1.454592	0.145782	0.60501
hjv	319.7547	0.184052913	0.180875	1.017569	0.308883	0.77371
si:dkey-167k11.5	0	NA	NA	NA	NA	NA
tsnaxip1	31.6373	0.229121044	0.378967	0.604594	0.545449	0.8953
acsl1a	391.6251	-0.042899301	0.134875	-0.31807	0.750433	0.955074
slc22a2	492.8838	0.228886488	0.268241	0.853287	0.3935	0.828374
arhgef12a	464.0189	-0.071280723	0.146266	-0.48734	0.626021	0.920625
psmc1a	2071.582	0.18260973	0.134977	1.352897	0.176089	0.649415
rnd1a	678.4565	0.030141408	0.2008	0.150107	0.88068	0.986624
helz	959.3161	0.116136007	0.186154	0.623872	0.532712	0.890978
zgc:77880	107.8836	-0.096926419	0.219487	-0.44161	0.658775	0.930581
nudt1	65.12895	0.098898271	0.276824	0.357261	0.720897	0.947343
foxk2	826.7226	0.006697683	0.102427	0.06539	0.947863	0.997411
slc10a1	37.50131	-2.860752802	0.386023	-7.41083	1.26E-13	2.36E-10
lrrc42	59.0282	0.013773455	0.262831	0.052404	0.958207	0.999109
nampta	260.4869	-0.098759474	0.165151	-0.598	0.549843	0.897373
rps19	23779.77	-0.298131228	0.134964	-2.20896	0.027177	0.293072
phkg1a	152.0016	0.314867507	0.28245	1.114774	0.264947	0.742063
rhogd	126.7834	0.214128533	0.244049	0.8774	0.38027	0.823655
syt1a	2258.012	0.288826646	0.174783	1.652487	0.098435	0.521076
nfe2l1a	392.6477	-1.190823159	0.321674	-3.70195	0.000214	0.015588
th	54.0605	-0.34784333	0.368913	-0.94289	0.345738	0.798919
lrit2	22.61865	1.984020671	1.423248	1.394009	0.163315	0.631199

mfsd2aa	77.42366	0.397303346	0.261071	1.521822	0.128054	0.577144
ccdc3b	216.6063	0.409692056	0.18361	2.23132	0.02566	0.284447
plekhm3	161.2827	-0.198289864	0.192499	-1.03008	0.302972	0.76913
pimr189	2.340495	2.656325421	1.325028	2.004732	0.044992	0.37151
tex30	17.47541	-0.444835692	0.412041	-1.07959	0.280325	0.753146
letmd1	106.6213	0.108896701	0.216339	0.503361	0.614711	0.915998
gnai3	97.22658	-0.075550309	0.178879	-0.42235	0.672767	0.93414
r3hdml	24.58738	-0.485349549	0.561092	-0.86501	0.387034	0.827592
3-Sep	641.5571	0.165766103	0.158565	1.045413	0.295832	0.764246
CU929230.1	370.686	0.085010431	0.162138	0.524309	0.600064	0.913756
phka2	533.283	-0.15660953	0.185555	-0.84401	0.398666	0.830925
atp6v1e1b	2562.344	0.009802886	0.133254	0.073565	0.941356	0.997371
ctps1a	1081.258	-0.137235151	0.188475	-0.72814	0.46653	0.865588
otx2a	78.35174	0.248105034	0.204784	1.211547	0.225686	0.707068
ing4	1337.398	-0.131790593	0.116251	-1.13368	0.25693	0.734493
xirp1	171.8908	0.220359979	0.204166	1.079318	0.280446	0.753147
cldn11b	289.7277	0.157427234	0.136521	1.153135	0.248855	0.728918
b3gat2	152.9347	0.144830692	0.204001	0.709953	0.477734	0.869292
sptlc3	156.6128	-0.1515368	0.196451	-0.77137	0.440487	0.852385
gabrr3b	28.7735	-1.057557019	0.676577	-1.5631	0.118029	0.560168
prrg4	357.1336	-0.106410495	0.158783	-0.67016	0.502753	0.881453
dnmt1	959.4754	0.124313986	0.179047	0.694309	0.487488	0.873291
guca1c	41.44386	-0.332421966	1.152885	-0.28834	0.773087	0.960624
melk	178.6661	0.095690091	0.209834	0.456027	0.648371	0.927474
syt16	5.424386	0.90627583	0.872852	1.038293	0.299134	0.766018
hadh	1138.613	0.165236624	0.177462	0.93111	0.351797	0.802513
sybl1	375.855	0.023821279	0.128576	0.185269	0.853018	0.978824
BX323854.1	5.783944	0.508812578	1.33791	0.380304	0.70372	0.943735
acads	688.5957	0.055146488	0.167113	0.329994	0.741404	0.954103
exoc3l2b	110.8729	0.178748824	0.207353	0.86205	0.38866	0.827613
mpi	102.2179	0.039971928	0.197535	0.202354	0.83964	0.976417
ddx18	946.2132	0.032048794	0.192506	0.166482	0.867777	0.982728
zgc:110006	37.67173	-0.543191586	0.326135	-1.66554	0.095805	0.516953
fam120b	78.88825	0.059829661	0.245626	0.243581	0.807556	0.969736
znhit2	73.3971	-0.047596703	0.217638	-0.2187	0.826887	0.973458
esyt2a	329.5758	0.040868301	0.160448	0.254714	0.798944	0.966962
cmtr1	435.9405	-0.289032421	0.13217	-2.18683	0.028755	0.301537
otofa	193.2516	0.518918814	0.248288	2.089987	0.036619	0.336389
tescb	66.62043	-0.320328765	0.226819	-1.41226	0.157872	0.623766
klf11a	709.3005	0.257116623	0.215942	1.190675	0.233781	0.714749
opn7c	18.65608	-0.426641053	0.47805	-0.89246	0.372146	0.819641
siah1	663.3144	-0.041878677	0.115319	-0.36316	0.716488	0.94627
cetp	277.7643	-0.870610026	0.225228	-3.86546	0.000111	0.009958
baxb	83.0039	0.248377653	0.209096	1.187863	0.234887	0.715533
phf23a	211.3383	-0.241058643	0.159482	-1.51151	0.130658	0.581957
foxq1a	340.823	-0.271346857	0.281662	-0.96338	0.335357	0.79203
cited2	536.9279	-0.48248364	0.168338	-2.86617	0.004155	0.106606
cluha	873.2369	0.076612638	0.162081	0.472681	0.636441	0.924779
tmem120a	175.5101	0.056521902	0.165445	0.341635	0.732626	0.950617
cpa1	13888.08	0.222151458	0.623085	0.356535	0.72144	0.947343
fgf10a	147.3801	-0.09212363	0.171108	-0.53839	0.590305	0.908901
ksr1b	202.5144	0.01832456	0.175518	0.104403	0.91685	0.99353
fermt3b	21.75686	-1.172314518	0.481256	-2.43595	0.014853	0.213725
si:ch211-259g3.4	350.9767	-0.059285578	0.155653	-0.38088	0.70329	0.943622

srprb	802.258	-0.112678595	0.172852	-0.65188	0.514479	0.88631
ftr56	29.83343	-0.086427424	0.401023	-0.21552	0.829364	0.974609
yipf4	792.1176	-0.069364111	0.124446	-0.55738	0.577267	0.904809
ak8	24.74192	0.233496048	0.364549	0.640506	0.521843	0.887551
pde12	120.4988	-0.14589396	0.204902	-0.71202	0.476454	0.869292
si:ch211-51a6.2	179.5954	0.019099026	0.171252	0.111526	0.911199	0.992208
dnaja1	197.4989	0.405565268	0.196017	2.069027	0.038544	0.344223
ccdc80l1	168.1343	0.190950575	0.185038	1.031954	0.302094	0.76803
csrp1b	232.9497	-1.583130618	0.764817	-2.06995	0.038457	0.343904
tmem127	218.4799	-0.098456257	0.149083	-0.66041	0.508988	0.883442
mybl1	120.305	0.27222476	0.217474	1.251757	0.210658	0.692347
dtna	75.28055	0.136780212	0.206888	0.66113	0.508529	0.883333
pip4k2ca	916.5678	-0.074661754	0.100309	-0.74432	0.456683	0.860698
lipg	247.0638	0.287496934	0.357416	0.804377	0.421179	0.84265
nr1h5	70.41413	-1.876519507	0.396818	-4.72891	2.26E-06	0.000551
gspt1	701.0809	0.292213148	0.126748	2.305464	0.021141	0.25794
igsf21a	240.3612	-0.034367818	0.367446	-0.09353	0.925481	0.994815
cadm1a	158.1801	0.200568426	0.173692	1.154733	0.2482	0.728489
veph1	30.58931	-0.226510714	0.404905	-0.55942	0.575878	0.904739
timm50	324.9707	0.163308779	0.157965	1.033831	0.301215	0.767371
ivns1abpa	3287.977	-0.154744189	0.157313	-0.98367	0.325278	0.784089
lrba	661.643	0.339424942	0.134939	2.515402	0.01189	0.189858
dnal4a	44.5767	0.140965058	0.263463	0.535048	0.592617	0.909996
baiap2l1b	86.71929	-0.420481417	0.207886	-2.02265	0.043109	0.36374
notum1a	205.5344	0.339556021	0.240648	1.411009	0.158242	0.623766
moxd1	90.83347	0.237547856	0.190944	1.244072	0.213473	0.69586
irx3b	76.60941	0.164030379	0.186296	0.88048	0.378599	0.82298
gba3	12.44783	0.813570958	0.545449	1.491563	0.135814	0.591131
nr1d4a	25.2015	0.343580911	0.745226	0.461043	0.644768	0.926639
tuba8l2	5457.105	-0.174504599	0.150574	-1.15893	0.246485	0.727706
hmg20a	282.4177	0.22827218	0.147828	1.544177	0.122546	0.569468
ppp2r5cb	828.4561	0.011133029	0.093922	0.118535	0.905644	0.991631
alg11	150.2416	-0.160120561	0.174641	-0.91685	0.35922	0.810194
commd1	189.6097	-0.152147533	0.186931	-0.81392	0.415689	0.839167
arcn1b	1215.768	-0.003516993	0.096093	-0.0366	0.970804	1
ssu72	725.2036	-0.004546199	0.11518	-0.03947	0.968515	1
lhx2b	238.4919	0.050594744	0.169552	0.298403	0.765396	0.959831
podxl	1154.382	-0.180228448	0.130294	-1.38324	0.16659	0.636904
kidins220a	484.1599	-0.140224728	0.44277	-0.3167	0.751472	0.955331
snai3	18.9468	0.765791338	0.631776	1.212124	0.225465	0.70685
hbegfb	61.97676	0.329344071	0.29177	1.128778	0.258991	0.736127
enkur	82.97394	0.016558035	0.208907	0.07926	0.936826	0.997044
sap30bp	480.278	0.020250997	0.155586	0.13016	0.89644	0.98939
rnf123	119.8729	0.177408267	0.239911	0.739476	0.459618	0.862156
si:ch73-193i2.2	5.161168	1.055427222	1.805656	0.584512	0.558876	0.899409
vamp1	664.0086	0.482130064	0.223179	2.16028	0.030751	0.312673
dnajc9	331.2444	0.016986508	0.198162	0.08572	0.931689	0.995827
dhrs11b	176.3759	-0.201696382	0.179351	-1.12459	0.260762	0.738343
smap1	1142.959	0.135959078	0.080466	1.689642	0.091096	0.50549
zic4	268.6648	0.144420074	0.158739	0.909795	0.362931	0.813115
six6b	204.4337	0.11413208	0.222217	0.513606	0.607527	0.915204
ppdpfb	9974.949	-0.141222512	0.161615	-0.87382	0.382215	0.825191
tmed1a	149.8472	-0.061232291	0.204296	-0.29972	0.764388	0.959254
hsd20b2	196.5406	0.095478663	0.203622	0.468901	0.63914	0.924779

hoxa10b	209.9324	-0.119494557	0.157718	-0.75765	0.448663	0.856741
rab6bb	651.5636	0.22224734	0.173262	1.282721	0.19959	0.680373
RTKN	84.33074	-0.061386787	0.230536	-0.26628	0.790025	0.964274
rbm17	347.0145	-0.1267862	0.111709	-1.13497	0.256388	0.73401
mc5ra	60.81908	-0.43186103	0.311044	-1.38842	0.165008	0.634185
cmpk2	3.719358	-0.868148047	1.862481	-0.46612	0.641126	0.925424
rtn4ip1	224.969	0.199645401	0.171281	1.165599	0.243777	0.724437
efna2a	184.4615	0.182722823	0.176718	1.033978	0.301147	0.767371
reep2	339.6186	0.374183232	0.185318	2.01914	0.043473	0.365455
klhl38a	47.86105	-0.338885187	0.328283	-1.0323	0.301934	0.767995
cers4a	120.3361	0.164695156	0.260938	0.631165	0.527933	0.889948
dctn2	2041.188	-0.069518698	0.126593	-0.54915	0.5829	0.90571
wt1a	73.6349	0.338529586	0.2023	1.673401	0.094248	0.512734
igfbp2b	73.65798	-0.221010891	0.178444	-1.23855	0.215513	0.697157
csrnp1a	323.9443	-0.29682326	0.17564	-1.68995	0.091037	0.50549
calm2a	2465.842	-0.386353297	0.136623	-2.82788	0.004686	0.113564
rcor1	555.5136	-0.074437658	0.138844	-0.53612	0.591874	0.909603
TXN	4959.54	-0.171801554	0.137435	-1.25006	0.211279	0.692983
kcnj11l	98.1262	0.168909702	0.311121	0.542907	0.587194	0.907261
syt6b	72.2071	0.020908768	0.322216	0.064891	0.948261	0.997694
col9a1b	1788.277	0.208229055	0.441917	0.471195	0.637501	0.924779
ognb	5.638268	-0.459992902	1.150494	-0.39982	0.689288	0.939348
prrc2a	2320.875	-0.032344324	0.115101	-0.28101	0.778705	0.961073
seta	4901.381	0.15919559	0.111401	1.429036	0.152994	0.617573
ap1ar	169.5255	0.354838727	0.199743	1.776478	0.075654	0.465951
flvcr2b	140.5055	-0.229299133	0.215547	-1.0638	0.28742	0.758397
psmb2	1710.085	-0.061325777	0.138625	-0.44239	0.65821	0.930581
ap5m1	101.5492	-0.608171689	0.276954	-2.19593	0.028097	0.298489
CU571315.1	52.12913	-0.265528015	0.350414	-0.75775	0.448598	0.856741
tmem200a	35.77992	0.45445898	0.352998	1.287425	0.197946	0.678664
ephb3a	657.3512	-0.013352342	0.102932	-0.12972	0.896788	0.98939
srpk1b	332.4982	0.045793083	0.135189	0.338734	0.73481	0.951361
tada2a	229.2219	-0.286173966	0.144647	-1.97843	0.04788	0.380801
flvcr1	200.444	0.135176712	0.189759	0.712359	0.476243	0.869292
si:dkey-239b22.1	306.3138	0.477196414	0.565945	0.843185	0.399125	0.831196
calb1	134.9662	0.339410805	0.273958	1.238918	0.215376	0.697157
rd3	78.4975	-0.199516353	0.282278	-0.70681	0.479686	0.869938
g6pca.1	230.6545	-0.829796888	0.238922	-3.47309	0.000515	0.027984
unm_sa1261	54.03787	-0.194773043	0.245676	-0.7928	0.427892	0.845893
chst10	210.5193	-0.141643286	0.195018	-0.72631	0.46765	0.866174
pan3	416.6642	0.054358623	0.14042	0.387115	0.698671	0.942456
stat2	246.0176	-0.173488056	0.194667	-0.89121	0.372819	0.819953
cort	152.6955	0.094932396	0.334634	0.28369	0.776648	0.961073
fahd1	124.7618	-0.470213383	0.219451	-2.14268	0.032139	0.318495
si:ch211-207d6.2	216.7243	-0.206084304	0.181474	-1.13561	0.25612	0.73401
sox21a	112.0435	-0.180971446	0.263507	-0.68678	0.492221	0.876345
col5a2a	4333.223	0.125531271	0.189302	0.663128	0.507249	0.883007
ercc1	232.2823	-0.142255711	0.171019	-0.83181	0.405515	0.834645
atp6v0b	1177.653	0.086648969	0.093647	0.925268	0.354826	0.805933
fosab	250.8867	-0.427878531	0.95814	-0.44657	0.655184	0.929606
kif9	16.56271	-0.499008894	0.466018	-1.07079	0.284263	0.75523
mylk2	34.34824	0.363947146	0.332384	1.094959	0.273535	0.749148
prkg1b	569.5686	-0.331363373	0.304117	-1.08959	0.275893	0.750674
mllt1b	118.4824	0.093374151	0.179906	0.519015	0.60375	0.914603

grm3	33.50911	0.746026463	0.436824	1.707842	0.087666	0.496994
clstn1	2961.036	0.292919796	0.146029	2.005898	0.044867	0.371082
si:ch73-27e22.6	0.477482	0	5.267649	0	1	NA
pthlha	50.9952	0.424735359	0.296962	1.430266	0.152641	0.616684
si:busm1-266f07.2	13.15369	0.087434381	1.511706	0.057838	0.953877	0.998611
C3AR1	4.101111	0.109311981	1.03338	0.105781	0.915756	0.99311
npr1a	99.37137	0.122039876	0.172997	0.705444	0.480534	0.870179
mef2aa	152.647	0.364424161	0.200147	1.820786	0.068639	0.44686
tmc4	29.84496	0.633477046	0.653714	0.969044	0.332523	0.789726
dab2	437.0417	-0.136269881	0.154818	-0.88019	0.378754	0.823136
smad6b	610.4711	-0.010530126	0.146844	-0.07171	0.942833	0.997371
roraa	708.6779	-0.125749841	0.215667	-0.58307	0.559843	0.899668
kat7b	495.8577	0.054792461	0.119957	0.456769	0.647837	0.927196
pus7	356.6296	0.082366468	0.193925	0.424734	0.671031	0.933953
ube2s	883.548	0.190448469	0.107301	1.774897	0.075915	0.46659
fthl27	7990.596	0.235964714	0.193246	1.221061	0.222063	0.703829
pparaa	90.39109	-0.088176005	0.234147	-0.37658	0.706483	0.944683
crybg1a	905.5821	-0.036369568	0.150101	-0.2423	0.808547	0.969828
adcy8	121.0612	0.152169402	0.23966	0.634938	0.525469	0.888928
abcf1	1538.446	0.331499639	0.123889	2.675779	0.007456	0.146147
ache	872.9145	-0.013291488	0.122813	-0.10823	0.913817	0.992796
rbm24b	111.431	-0.031765272	0.32821	-0.09678	0.922898	0.994525
dhrs13b.1	113.2293	-0.567741045	0.188832	-3.00659	0.002642	0.083741
trim2a	36.04557	0.173230373	0.417129	0.415292	0.677928	0.936026
eif4ebp2	4081.329	-0.017157501	0.083354	-0.20584	0.836917	0.976007
vps37c	555.3299	0.107882777	0.104901	1.028428	0.303748	0.769473
mboat2a	30.4735	-0.799118086	0.333625	-2.39526	0.016609	0.228244
pparg	51.75425	0.099053729	0.330857	0.299385	0.764646	0.959403
trpa1b	39.74449	0.097179605	0.447036	0.217386	0.827907	0.973883
pard6ga	2.195878	1.754747863	1.633318	1.074346	0.282668	NA
psmb12	4.104277	0.119331702	1.191719	0.100134	0.920238	0.993982
ift140	177.5957	0.15927322	0.152502	1.044399	0.296301	0.764246
mapk8a	156.3469	0.203817946	0.169664	1.201304	0.229633	0.711596
tcp11l1	215.1182	0.242533514	0.176959	1.370567	0.17051	0.64208
tbx1	332.6705	-0.060049708	0.165656	-0.3625	0.716982	0.94627
lef1	554.7155	0.112599146	0.134886	0.834772	0.403846	0.833888
ptbp1b	1159.69	-0.055030577	0.120806	-0.45553	0.648729	0.927546
tns3.2	758.4066	0.158527216	0.136553	1.160919	0.245675	0.727177
hif1an	648.1436	-0.074652358	0.19174	-0.38934	0.697023	0.94198
stard14	283.5967	-0.327015044	0.23038	-1.41946	0.155766	0.620972
erc1b	0.131777	0	5.267649	0	1	NA
mb	812.995	-0.116997802	0.200694	-0.58297	0.559915	0.89967
rbck1	172.4039	-0.139637512	0.209721	-0.66582	0.505524	0.8824
tmem63a	129.8031	0.03556646	0.180262	0.197304	0.84359	0.976791
cpped1	223.4892	-0.417559971	0.218785	-1.90854	0.056321	0.410107
poglut2	163.0415	0.053471768	0.174599	0.306255	0.75941	0.957886
nos2b	21.57787	-0.228753616	0.355681	-0.64314	0.520131	0.887148
pcbd1	487.059	0.111600435	0.187283	0.595893	0.551247	0.8974
six4b	234.5614	-0.036975884	0.204125	-0.18114	0.856255	0.979558
ptpa	466.4747	-0.162543668	0.137136	-1.18527	0.235911	0.717225
ccdc65	21.79235	0.630944679	0.370451	1.703179	0.088535	0.499597
slc15a2	159.0817	0.319533808	0.171707	1.860925	0.062755	0.42872
pafah1b1a	1233.978	-0.010752868	0.095391	-0.11272	0.910249	0.992208
bet1l	135.729	-0.187748673	0.197765	-0.94935	0.342441	0.797179

mxd1	59.57359	0.035541071	0.235234	0.151088	0.879906	0.986339
ftr16	12.67151	0.90643843	0.713101	1.271121	0.203685	0.684367
enah	928.32	0.149118685	0.132314	1.127008	0.259739	0.736707
arl6	276.4122	0.17092442	0.147165	1.161444	0.245461	0.726909
cdk16	350.6688	0.03868306	0.138712	0.278874	0.780341	0.961411
BX323082.1	6.330881	-0.744185864	1.0385	-0.7166	0.473623	0.869146
acsl3a	47.02434	0.256107623	0.311166	0.823057	0.410476	0.8372
dpysl2b	744.7291	0.226972897	0.107458	2.112194	0.03467	0.328212
sgsh	207.8617	-0.18161818	0.203717	-0.89152	0.372649	0.819953
c8g	292.6132	-0.418219838	0.252841	-1.65408	0.098111	0.521071
prdx3	1666.22	-0.598891691	0.157303	-3.80725	0.000141	0.011879
mapk6	1010.909	-0.597217728	0.169662	-3.52004	0.000431	0.02512
slain2	338.051	0.036673031	0.14163	0.258935	0.795685	0.966097
cnot7	96.16417	-0.200550995	0.202937	-0.98824	0.323034	0.782754
ddx1	553.1163	0.14108802	0.127639	1.105365	0.269002	0.745101
scg5	168.2185	0.494666185	0.246978	2.002876	0.045191	0.372359
gtf2f1	506.7229	-0.065623834	0.109769	-0.59783	0.54995	0.8974
drd3	41.81035	-1.265794237	0.42829	-2.95546	0.003122	0.091566
ppm1aa	349.1642	0.082640016	0.169305	0.488113	0.62547	0.920625
zgc:171776	2.163307	-2.229408547	1.761276	-1.26579	0.205588	NA
grk6	41.79427	0.483405685	0.301114	1.605392	0.108408	0.54085
srgap2	610.9196	0.039802499	0.116529	0.341569	0.732676	0.950617
puf60a	1250.28	-0.00703865	0.094449	-0.07452	0.940594	0.997371
lrrc8aa	134.1451	-0.430837146	0.194983	-2.20962	0.027132	0.293072
klf12b	40.42474	0.101114169	0.2968	0.340681	0.733343	0.95079
gpc3	12.52542	0.442221265	0.636505	0.694765	0.487203	0.873291
cthl	77.62325	-1.555727649	0.540909	-2.87614	0.004026	0.105636
acss3	146.3814	-0.173670959	0.195397	-0.88881	0.374104	0.820835
phactr4b	344.8627	-0.012061653	0.139541	-0.08644	0.931118	0.995809
wnt2bb	43.08335	-0.023520881	0.243852	-0.09646	0.923159	0.994525
anapc4	182.6865	0.306374653	0.15715	1.949564	0.051228	0.394059
dnm3a	110.7152	-0.233362765	0.217186	-1.07449	0.282605	0.754808
tnnt2c	446.9392	0.268971697	0.168859	1.592877	0.111188	0.54662
tmtopsb	26.58778	0.22039386	0.429472	0.513174	0.607829	0.915204
txndc16	111.3761	0.056242857	0.210992	0.266564	0.789805	0.964162
rngtt	347.459	0.11541071	0.131432	0.878104	0.379887	0.823607
zgc:110224	51.26644	0.063450985	0.348461	0.182089	0.855513	0.979408
mybl2b	296.1429	0.32452648	0.174559	1.859124	0.06301	0.429313
satlb	172.6025	0.016209187	0.214003	0.075743	0.939624	0.997371
rars2	88.65513	-0.04780961	0.232007	-0.20607	0.836736	0.976007
zgc:113294	58.31399	-0.321716356	0.287035	-1.12082	0.262362	0.73998
pomp	1339.897	0.160486962	0.170819	0.939517	0.347465	0.800037
tbc1d20	134.5529	0.014025073	0.163168	0.085955	0.931503	0.995809
tox	1205.436	0.074486833	0.092039	0.809299	0.418343	0.841565
mfsd6a	94.00113	-0.144575815	0.204863	-0.70572	0.480362	0.870074
mest	135.2217	0.065355774	0.256272	0.255025	0.798704	0.966823
epb41l5	508.4006	0.005050607	0.111203	0.045418	0.963774	0.999842
mecr	173.3672	-0.189275391	0.246089	-0.76913	0.441813	0.853281
usp36	864.7824	-0.220569155	0.156281	-1.41136	0.158138	0.623766
serhl	177.3225	0.047117343	0.163557	0.288078	0.773287	0.960624
pde4a	210.6252	0.437597844	0.198757	2.201669	0.027689	0.29556
tiprl	404.5707	0.40198932	0.158194	2.541122	0.01105	0.18325
btbd6b	645.4364	0.010953687	0.093312	0.117387	0.906553	0.991636
ccdc180	12.53579	0.679320984	0.542617	1.251935	0.210594	0.692339

rnf145b	684.5316	0.114545259	0.109041	1.050483	0.293496	0.76277
ompb	52.68268	0.794967463	0.323941	2.454048	0.014126	0.208027
tspan12	180.1207	-0.014070735	0.194559	-0.07232	0.942346	0.997371
ckap4	2122.726	0.103530114	0.186897	0.553943	0.579618	0.905618
coq8ab	77.16936	0.226243182	0.278293	0.812969	0.416236	0.839647
ppp2r1bb	3300.524	0.140030982	0.127665	1.096859	0.272703	0.748552
mpv17	172.0082	-0.261098253	0.192035	-1.35964	0.173944	0.647021
itga9	108.0205	-0.291069042	0.256377	-1.13532	0.256242	0.73401
mark2b	668.4986	0.086092986	0.119982	0.717548	0.473036	0.868965
slc1a8b	16.77179	-0.487553848	1.411075	-0.34552	0.729704	0.950056
ampd3b	1143.518	0.059543256	0.152531	0.390367	0.696265	0.941657
si:dkey-40c11.2	437.183	0.014811267	0.153451	0.096521	0.923107	0.994525
pon1	246.8524	-0.7374672	0.238371	-3.09377	0.001976	0.068961
rrp36	146.445	-0.213899802	0.221773	-0.9645	0.334796	0.791753
arpp19a	1424.306	0.090337617	0.102024	0.885451	0.375914	0.822237
praf2	296.6435	0.321175742	0.120137	2.673417	0.007508	0.146931
5-Mar	169.0527	-0.145777974	0.15082	-0.96657	0.333759	0.790676
egln3	374.1827	0.026134395	0.319114	0.081897	0.934729	0.996829
cacng2a	206.3944	0.191541951	0.263088	0.728053	0.466581	0.865588
il20ra	94.17388	0.46169505	0.338665	1.36328	0.172794	0.645664
ywhaz	5186.219	0.067925277	0.107447	0.632174	0.527273	0.889948
clcn6	63.10551	-0.007483744	0.232648	-0.03217	0.974338	1
pax2b	16.98599	-0.005671605	0.534433	-0.01061	0.991533	1
thnsl2	33.74942	-0.84744561	0.523602	-1.61849	0.105557	0.534823
macrod2	366.7363	0.221786566	0.129356	1.714541	0.086429	0.493048
st7l	133.7438	0.038737065	0.219671	0.176342	0.860026	0.980167
sept8a	1157.993	-0.04537479	0.142438	-0.31856	0.750061	0.955074
msi2b	325.6976	0.096985211	0.135305	0.716787	0.473506	0.869146
fgfrl1a	623.5417	-0.140280451	0.155068	-0.90464	0.365658	0.815695
mgst1.1	201.9234	0.051442086	0.186793	0.275396	0.783012	0.962328
tob1a	1743.561	-0.126614378	0.181567	-0.69734	0.485589	0.87303
rcan3	517.4729	-0.013022691	0.163681	-0.07956	0.936586	0.997044
SCYL2	323.066	0.031777959	0.177424	0.179108	0.857853	0.979774
neb	6030.655	-0.031783569	0.275395	-0.11541	0.908119	0.991745
ltb4r	33.6524	-0.084184473	0.308589	-0.2728	0.785004	0.962605
si:dkey-261m9.12	2.766893	0.407922077	1.279236	0.318879	0.749818	0.955074
cd36	333.494	0.713740852	0.321516	2.219923	0.026424	0.287979
fuk	150.1153	-0.30602523	0.227123	-1.3474	0.177851	0.651389
ppp1r10	999.8937	0.063265362	0.101291	0.62459	0.53224	0.890978
sinhcaf	352.514	-0.376773336	0.146315	-2.57509	0.010021	0.173583
qrsl1	138.6165	0.325560452	0.193245	1.6847	0.092047	0.507669
foxg1b	511.9262	-0.116439007	0.294135	-0.39587	0.692202	0.940333
arl4cb	718.6176	-0.097210437	0.157303	-0.61798	0.536588	0.892421
gria1b	40.96004	-0.067663492	0.340796	-0.19855	0.842619	0.9766
rps27a	34946.11	-0.255027588	0.114294	-2.23133	0.025659	0.284447
gria3a	493.4999	-0.166815308	0.24706	-0.6752	0.499548	0.879603
si:dkey-11p23.7	1.809831	2.003633121	1.920798	1.043125	0.29689	NA
tnfsf10l3	44.10037	-0.894065038	0.298572	-2.99447	0.002749	0.085914
pde4d	34.50389	0.353859231	0.321245	1.101524	0.270669	0.746683
net1	1821.951	-0.216853914	0.09265	-2.34057	0.019254	0.244673
slc38a9	95.46728	0.251436955	0.225834	1.113368	0.26555	0.742247
pigf	166.3495	-0.365962536	0.199061	-1.83845	0.065997	0.438653
tmem8a	77.82847	-0.026689873	0.24258	-0.11002	0.91239	0.992398
kcnd2	167.8394	-0.076692186	0.242963	-0.31565	0.752265	0.955481

grk5	45.77587	0.546872642	0.315272	1.734605	0.082811	0.483014
ktn1	2210.492	-0.06202266	0.082526	-0.75155	0.452321	0.857994
tdrd7a	12.44108	0.895842038	0.518583	1.727479	0.084082	0.486971
tm7sf2	106.2792	0.408276145	0.233184	1.750878	0.079967	0.478179
rxfp2a	35.11905	-1.346173052	0.434527	-3.09802	0.001948	0.068449
htra1a	308.5309	-0.015977479	0.201487	-0.0793	0.936796	0.997044
pvalb5	1092.805	0.758654762	0.19526	3.885348	0.000102	0.009321
si:dkey-206f10.1	118.7366	-0.080604555	0.419265	-0.19225	0.847545	0.977676
ndrg1a	1442.834	-0.298850608	0.173406	-1.72342	0.084813	0.488883
ccdc120	106.64	0.023868456	0.208823	0.1143	0.909	0.992033
lratd2	38.65316	-0.127015923	0.309061	-0.41097	0.681092	0.937331
ugt5g1	81.13741	-0.202684771	0.240043	-0.84437	0.398463	0.830887
pacsin1a	330.8832	0.127183594	0.219534	0.579335	0.562363	0.900715
erh	1623.957	-0.107706012	0.152064	-0.7083	0.478762	0.869292
pde4ba	1300.659	-0.066644851	0.115667	-0.57618	0.564495	0.901433
trit1	178.4528	-0.231634981	0.184367	-1.25638	0.208978	0.691152
si:ch211-226m7.4	18.62661	0.51748088	0.442168	1.170326	0.24187	0.72348
xgb	5.42516	-2.461128693	1.101582	-2.23418	0.025471	0.283609
chchd3a	1069.965	-0.070846706	0.12301	-0.57594	0.564655	0.901445
cryba1l1	9885.935	0.621871866	0.272045	2.285917	0.022259	0.263807
cnksr1	142.3104	-0.013957631	0.179599	-0.07772	0.938054	0.997331
dtx2	277.2347	-0.137042094	0.126177	-1.08611	0.277429	0.751426
nrbp2a	339.4244	0.098240294	0.166146	0.591288	0.554327	0.898354
kcnc1b	64.79939	0.622226733	0.317104	1.962218	0.049737	0.388069
her12	319.4784	0.139441157	0.206688	0.674644	0.499902	0.879766
cox4i1	6775.535	0.018421433	0.107741	0.170979	0.86424	0.981867
cmlc1	404.2429	-0.091091486	0.207034	-0.43998	0.65995	0.930581
mrpl27	324.3317	0.122123351	0.190547	0.640911	0.521581	0.887551
plxnb3	89.67217	0.027762177	0.23727	0.117007	0.906855	0.991644
ccl34b.3	1.692474	2.284631727	1.852357	1.233365	0.21744	NA
rnf185	690.7372	0.025756113	0.149026	0.172829	0.862786	0.981285
h3f3c	12773.71	0.01583707	0.115115	0.137576	0.890576	0.988446
pcnt	486.4135	0.010785911	0.185068	0.058281	0.953525	0.998436
ppcdc	95.12231	-0.408655973	0.234353	-1.74376	0.081201	0.479923
pkd1b	80.32037	0.776660144	0.331637	2.341899	0.019186	0.243983
ptprja	26.4688	0.383803834	0.373345	1.028013	0.303944	0.76949
ccni2	61.22955	-0.253495219	0.364516	-0.69543	0.486786	0.873245
zgc:171927	10.48212	0.104223534	0.588333	0.177151	0.85939	0.979975
trim35-10	1.942209	1.652281937	1.31666	1.254904	0.209513	NA
CR735102.1	136.2953	0.060692376	0.221487	0.274022	0.784068	0.962605
kif7	226.4159	0.15564618	0.181685	0.85668	0.391622	0.827902
tmc2a	21.22806	-0.382632631	0.555986	-0.68821	0.491323	0.875595
prkrip1	175.4626	-0.179771162	0.155132	-1.15883	0.246527	0.727706
ndufaf7	115.858	-0.00229788	0.203906	-0.01127	0.991009	1
lyrm2	218.6204	-0.063040928	0.178033	-0.3541	0.723267	0.947898
desi1a	466.2271	0.024896619	0.240936	0.103333	0.917699	0.993777
psme2	46.71613	0.060031984	0.36244	0.165633	0.868446	0.982801
nr1d1	478.045	-0.667910266	0.787862	-0.84775	0.396577	0.830053
sst1.2	215.2409	-0.030194662	0.226832	-0.13311	0.894103	0.988762
sult2st1	520.772	-0.214675093	0.141114	-1.52129	0.128187	0.57717
trappc6bl	239.3061	-0.243978109	0.193488	-1.26095	0.207327	0.689456
nr2f5	594.0824	0.046960725	0.183234	0.256289	0.797728	0.966447
snrpe	830.5708	-0.064522965	0.204315	-0.3158	0.752153	0.955481
tmem243b	151.5809	-0.438318394	0.17392	-2.52023	0.011728	0.188354

arl4ab	283.0561	-0.367115169	0.165584	-2.21709	0.026617	0.289132
prkar2aa	1431.102	-0.219172536	0.109826	-1.99564	0.045973	0.375002
zgc:101840	400.1529	0.249258732	0.205316	1.214024	0.224739	0.706634
lect2l	472.284	-1.166819418	0.297032	-3.92826	8.56E-05	0.008158
mcm6l	5.419676	-1.579118084	0.938225	-1.68309	0.092358	0.508314
stmn2a	1911.068	0.366168863	0.187884	1.948914	0.051306	0.394059
osbpl3a	130.6597	-0.154891571	0.195985	-0.79032	0.429338	0.846956
dis3l2	206.5708	-0.006631982	0.190775	-0.03476	0.972268	1
primpol	178.4041	0.520814363	0.258445	2.015181	0.043886	0.366822
gsto2	881.2639	0.276649052	0.168617	1.640694	0.100861	0.526442
gpr37b	79.14415	0.147588045	0.25039	0.589434	0.55557	0.898723
igf2b	878.2989	0.046304201	0.123034	0.376352	0.706655	0.944735
sar1ab	812.0011	0.323260151	0.127343	2.53849	0.011133	0.183611
unc5b	209.054	-0.076699192	0.179465	-0.42738	0.669104	0.933115
cntln	65.05181	-0.053798586	0.224185	-0.23997	0.81035	0.969828
zgc:101699	4.016435	0.063251808	1.12163	0.056393	0.955029	0.998611
asl	104.8086	-0.139253231	0.19059	-0.73064	0.464996	0.864823
mgst3b	2389.514	-0.022530559	0.143008	-0.15755	0.874814	0.984191
rrm2b	661.1772	0.007166857	0.180777	0.039645	0.968376	1
ppil3	94.45279	-0.172219845	0.235988	-0.72978	0.465523	0.864997
chchd4a	189.1403	-0.027464273	0.203698	-0.13483	0.892747	0.988566
gyg2	39.56675	1.084314781	0.775385	1.398422	0.161986	0.629618
grifin	123.7128	-0.257678419	0.363167	-0.70953	0.477994	0.869292
cabp1b	12.34517	0.418765641	0.779863	0.536973	0.591286	0.909437
acot18	198.3346	0.334037574	0.170926	1.954278	0.050668	0.391589
gpatch1	380.2842	0.095030415	0.149905	0.633939	0.526121	0.889213
mboat7	280.3609	0.102790298	0.125648	0.818081	0.413311	0.838317
rps6ka1	272.7177	0.101078375	0.138586	0.729354	0.465785	0.86502
metap1	1758.662	-0.271794659	0.132954	-2.04427	0.040927	0.353808
mdm2	714.319	0.155960703	0.117085	1.332035	0.182849	0.659278
map4k6	124.6106	-0.191322542	0.271035	-0.70589	0.480254	0.870001
tap2t	67.26031	-0.585064644	0.317609	-1.84209	0.065462	0.4356
nxph1	367.7241	0.215392655	0.318079	0.677168	0.4983	0.879132
nkap	675.2387	-0.115237163	0.120241	-0.95839	0.337868	0.794808
atpsckmt	106.0113	-0.024974597	0.202081	-0.12359	0.901642	0.990643
pimr111	0	NA	NA	NA	NA	NA
tagln2	1791.68	-0.0652132	0.132179	-0.49337	0.621751	0.918461
ube2j1	330.6445	0.029195485	0.148404	0.196729	0.844039	0.976791
rorb	73.46961	0.260934636	0.249102	1.047503	0.294868	0.763708
zgc:64189	15.34326	0.292516806	0.502935	0.58162	0.560823	0.900005
efr3ba	95.01181	0.321223334	0.311163	1.032333	0.301916	0.767995
ccdc115	213.3241	-0.347649277	0.193381	-1.79774	0.072218	0.456932
p4ha1a	288.7585	0.205556006	0.116881	1.758671	0.078633	0.474455
paics	5827.924	-0.223285818	0.225528	-0.99006	0.322145	0.782237
adamts15b	146.167	0.108258657	0.211757	0.511239	0.609184	0.915204
fbxw4	332.8727	-0.214241474	0.160624	-1.3338	0.182268	0.658775
cyp17a1	140.4561	0.728544048	0.286353	2.544216	0.010952	0.182182
fkbp1ab	118.4031	0.166707918	0.186192	0.895357	0.370596	0.818484
mtrf1	72.12992	-0.192694729	0.26199	-0.73551	0.462032	0.863402
CABZ01088134.1	28.48534	0.202903801	0.397198	0.510838	0.609465	0.915204
ido1	15.41194	0.58768642	0.957823	0.613565	0.539503	0.893049
polr2c	491.7451	0.054901205	0.14969	0.366767	0.713793	0.945821
api5	1406.975	0.099232511	0.095054	1.043954	0.296507	0.764359
pdia8	205.6454	0.013796353	0.197885	0.069719	0.944417	0.997371

lrpap1	550.5291	0.030120355	0.140296	0.214692	0.830007	0.974908
map1lc3a	948.3121	-0.314371659	0.129565	-2.42636	0.015251	0.217192
morn5	7.946022	-0.413308577	0.7281	-0.56765	0.57027	0.902481
rasgef1ba	586.1835	-0.069551742	0.193375	-0.35967	0.719092	0.94694
selenon	706.0966	0.20828482	0.159903	1.302567	0.192723	0.67273
NPFFR2	10.12828	-0.654095718	0.854102	-0.76583	0.443778	0.854255
smarcb1a	543.9362	0.00661377	0.114133	0.057948	0.95379	0.998611
stmn1b	4682.839	0.105922027	0.20485	0.517072	0.605106	0.914985
scd	10.80921	0.84238334	0.709005	1.18812	0.234786	0.715533
pi4k2a	393.1838	-0.134954692	0.130292	-1.03578	0.300303	0.766629
tpma	180965.7	0.042212665	0.18092	0.233322	0.815511	0.971158
oxgr1a.1	0.88479	-3.359420186	1.99976	-1.67991	0.092975	NA
adora2aa	32.19874	0.295082059	0.292828	1.007697	0.3136	0.777207
jade1	550.0655	-0.012529385	0.112548	-0.11133	0.911358	0.992208
tshba	10.37722	2.184565562	0.694306	3.146404	0.001653	0.062485
il12ba	0.613554	-3.302237009	3.363149	-0.98189	0.326155	NA
ncf1	116.4285	-0.307135023	0.217399	-1.41277	0.157723	0.623766
zgc:153867	6505.155	0.041178839	0.110938	0.371186	0.710499	0.945821
nt5c1bb	76.00878	-0.083049123	0.263801	-0.31482	0.7529	0.955756
rbm48	289.7785	-0.227572413	0.221736	-1.02632	0.304739	0.770005
ncaph2	235.1224	0.218118197	0.195023	1.118421	0.263387	0.741418
pmelb	457.9291	0.069905975	0.172716	0.404745	0.685665	0.938486
trappc13	438.9626	-0.072589891	0.137471	-0.52804	0.597473	0.911953
hspbap1	37.17134	-0.910714589	0.319965	-2.8463	0.004423	0.109607
ndufb7	1865.757	0.049776415	0.177301	0.280745	0.778906	0.961073
cyp27a7	322.6705	-0.468948734	0.19363	-2.42187	0.015441	0.218848
snx27a	312.1483	0.022704466	0.138039	0.164479	0.869354	0.983062
ampd1	17947.56	0.398296583	0.121057	3.290161	0.001001	0.044023
fat3a	124.8152	0.119588975	0.260767	0.458605	0.646518	0.926728
igsf9ba	437.172	0.196171364	0.187435	1.04661	0.29528	0.763896
mad1l1	180.1161	0.104018941	0.173666	0.598959	0.5492	0.897092
abrab	206.0965	-0.025622397	0.258875	-0.09898	0.921157	0.994085
cnot9	572.2961	-0.040179694	0.114818	-0.34994	0.726382	0.948987
sepsecs	104.2924	-0.027021068	0.213858	-0.12635	0.899454	0.99045
derl3	291.2255	-0.569036462	0.196835	-2.89093	0.003841	0.103366
spty2d1	422.8951	-0.029075452	0.128856	-0.22564	0.821479	0.971788
ap2m1b	1966.421	0.144888199	0.098937	1.464452	0.14307	0.602002
msi2a	272.3076	0.131496421	0.168275	0.781436	0.434546	0.850002
si:ch211-243j20.2	257.0495	0.11284803	0.149583	0.754415	0.4506	0.857151
ndfip2	1105.898	0.030404558	0.120711	0.251879	0.801135	0.967743
fbxo16	36.46837	-1.147165709	0.359648	-3.18969	0.001424	0.056314
lamb2l	546.6231	0.208573833	0.174518	1.195142	0.232032	0.713202
entpd2a.2	39.2536	-0.230706441	0.363481	-0.63471	0.525615	0.888928
CABZ01088025.1	5.702952	-0.432602143	0.750014	-0.57679	0.56408	0.901361
oxtr	6.63202	-1.029826291	0.728368	-1.41388	0.157397	0.623263
trmt44	128.8523	-0.132759829	0.165147	-0.80389	0.421462	0.842754
nitr1i	0.045161	0	5.267649	0	1	NA
nup58	386.2858	-0.099480674	0.12247	-0.81229	0.416627	0.840071
trmt2b	57.99903	0.07897183	0.216163	0.365334	0.714862	0.945821
prrx1a	1050.696	-0.197859254	0.11174	-1.77071	0.07661	0.468597
mrps23	250.8236	0.13815211	0.162941	0.847864	0.396514	0.830053
nxn	450.1256	0.099373058	0.126466	0.785771	0.432001	0.848419
rsrc1	341.7344	0.217114517	0.169406	1.281622	0.199975	0.6804
slc19a1	109.6633	0.045388835	0.203155	0.22342	0.823209	0.972005

cyp26a1	270.5693	-0.014901311	0.177195	-0.0841	0.93298	0.996264
tango6	166.619	-0.361785946	0.17439	-2.07459	0.038025	0.342284
prom1b	732.5238	-0.390201994	0.211212	-1.84745	0.064683	0.434279
rgmd	1157.564	-0.056104961	0.136562	-0.41084	0.68119	0.937331
dnal1	52.49385	-0.209429897	0.314245	-0.66645	0.505121	0.882097
irx5a	440.2526	0.120684055	0.118536	1.018124	0.308619	0.773546
zgc:92275	68.55166	1.229271489	0.39397	3.120214	0.001807	0.065671
csnk1g2b	523.3204	0.105065901	0.098116	1.070829	0.284247	0.75523
elac2	231.8702	0.033130492	0.168781	0.196293	0.844381	0.976791
unm_sa911	95.71129	-0.862780439	0.357253	-2.41504	0.015733	0.221337
lrp11	97.74328	0.504251262	0.245493	2.054034	0.039972	0.349495
plcd1b	619.2531	0.1468012	0.119346	1.230045	0.21868	0.699445
zgc:65895	837.7868	0.145795057	0.215774	0.675685	0.499241	0.879603
dclk2a	295.9034	0.010478552	0.158715	0.066021	0.947361	0.997371
si:dkey-59l11.10	1.592899	1.563362099	1.776031	0.880256	0.37872	NA
mtus1b	614.9966	-0.121695081	0.09262	-1.31391	0.188876	0.668515
actl6b	20.58334	0.157603088	0.496482	0.31744	0.75091	0.955074
rarga	1068.332	-0.074044765	0.119696	-0.61861	0.536175	0.892277
shisa2a	27.0979	-0.230235252	0.443806	-0.51877	0.603918	0.914603
ccnk	200.3386	0.052945121	0.137263	0.385721	0.699703	0.942499
slc4a1ap	258.7764	0.138281221	0.141473	0.977439	0.328352	0.786776
lingo1a	28.96775	0.655624398	0.410128	1.598585	0.109913	0.54393
serpini1	579.4821	0.05407883	0.210904	0.256414	0.797631	0.966447
prkcq	76.4503	-1.089147526	0.438617	-2.48314	0.013023	0.198789
mrrf	252.8509	-0.139725514	0.207237	-0.67423	0.500165	0.880007
cpsf1	697.4344	0.056263188	0.125144	0.449587	0.653008	0.929197
esr2b	37.80922	-0.631067282	0.36376	-1.73485	0.082768	0.483014
calm3a	2119.004	0.29607159	0.149144	1.985136	0.047129	0.378022
oxsr1a	550.4696	-0.070568076	0.118253	-0.59675	0.550671	0.8974
top2b	1632.991	0.021007649	0.100487	0.209058	0.834403	0.975731
cx44.2	113.1649	0.404360147	0.213357	1.895226	0.058062	0.416246
si:dkey-240h12.4	104.0393	-0.21961234	0.21769	-1.00883	0.313057	0.776599
capn2l	818.0099	0.660958754	0.280661	2.355006	0.018522	0.240697
rab42a	137.8227	-0.039660443	0.287039	-0.13817	0.890105	0.98835
hs1bp3	61.31357	-0.025085342	0.282859	-0.08868	0.929332	0.995452
si:dkey-243i1.1	123.1856	0.39115326	0.194381	2.0123	0.044188	0.368106
kcnip3a	175.2906	0.541862117	0.303338	1.786334	0.074045	0.46206
capza1a	2801.797	0.008226377	0.093014	0.088442	0.929525	0.995576
cdk10	159.13	0.102015665	0.165662	0.615804	0.538024	0.892498
pld2	247.4974	-0.041325982	0.163666	-0.2525	0.800653	0.967515
slit3	1023.882	-0.002985824	0.144937	-0.0206	0.983564	1
ogdha	3242.752	0.154090585	0.103189	1.49328	0.135364	0.590767
rpl37	20517.86	-0.345990214	0.174599	-1.98162	0.047521	0.379549
hif1ab	1804.943	-0.001859444	0.123747	-0.01503	0.988011	1
sema3c	99.87478	-0.261828291	0.247671	-1.05716	0.290437	0.76078
chrne	431.3945	0.156550948	0.211982	0.73851	0.460205	0.862369
cox10	558.2889	0.118190223	0.117546	1.005481	0.314665	0.777518
ppp5c	981.3766	0.059696537	0.114168	0.522885	0.601054	0.913756
palmdb	24.18931	-0.204235702	0.493053	-0.41423	0.678708	0.936026
ing3	934.4467	-0.185940298	0.122616	-1.51644	0.129408	0.579015
si:dkey-88n24.7	0.183528	2.079549577	5.22636	0.397896	0.690707	NA
pcdh19	666.194	0.136247983	0.126048	1.08092	0.279733	0.75269
hexb	896.2885	0.290929964	0.127046	2.289962	0.022024	0.262762
chst11	127.1134	0.274703939	0.187587	1.464408	0.143083	0.602002

mars	629.7044	0.004135129	0.203047	0.020365	0.983752	1
zgc:173443	86.62654	-0.448466284	0.44162	-1.0155	0.309866	0.774621
pik3r3b	938.534	-0.241484215	0.129659	-1.86246	0.062539	0.427724
irx6a	70.38798	-0.413627611	0.285194	-1.45034	0.146964	0.60703
sncga	719.3224	-0.044242573	0.244663	-0.18083	0.856501	0.979558
atp1b2b	340.3288	0.119898608	0.260645	0.460007	0.645511	0.926728
jpt2	1392.892	0.119774144	0.099178	1.207672	0.227173	0.708426
zgc:172121	49.05638	-0.72343148	0.30007	-2.41088	0.015914	0.222395
igf1rb	299.3654	-0.1289656	0.140954	-0.91495	0.36022	0.811057
mpped2	241.1274	0.386064547	0.134156	2.877719	0.004006	0.105636
unc119a	81.0738	-0.578638726	0.228312	-2.53443	0.011263	0.183909
si:ch211-163121.7	24.90368	-0.155478408	0.377283	-0.4121	0.680266	0.936798
aldoab	16663.73	0.197520089	0.139121	1.419775	0.155673	0.62071
ttyh3a	471.1581	-0.037042119	0.128541	-0.28817	0.773213	0.960624
grin2aa	238.9473	0.119087401	0.351366	0.338927	0.734664	0.951332
fbxl14a	312.1154	0.024342219	0.166459	0.146235	0.883736	0.987014
per2	1551.481	0.201235763	0.156579	1.285203	0.198722	0.679447
lmo1	453.3768	-0.031885201	0.195989	-0.16269	0.870764	0.983365
trdmt1	87.53944	0.261424724	0.215098	1.215374	0.224224	0.70607
rab6ba	347.78	0.078583537	0.218865	0.35905	0.719558	0.94694
chodl	74.90043	0.256294516	0.253079	1.012706	0.311201	0.775936
atp6v1aa	2221.738	0.086665049	0.097125	0.8923	0.372232	0.819753
tgfbr2b	664.8926	-0.055601682	0.136959	-0.40597	0.684763	0.938486
pard3bb	159.3704	-0.046084512	0.16027	-0.28754	0.773697	0.960624
srpx2	19.19837	-0.216280216	0.350447	-0.61715	0.537133	0.89244
fitm2	206.0454	-0.208115024	0.165308	-1.25895	0.208048	0.690193
gpr143	201.0191	-0.541630498	0.186901	-2.89795	0.003756	0.102148
si:dkey-23o4.6	66.81116	0.36032388	0.676669	0.532496	0.594382	0.910768
scn4ab	322.4881	0.449832563	0.226079	1.989711	0.046623	0.376263
tmem165	955.8035	-0.031463547	0.119118	-0.26414	0.791675	0.964817
zgc:153169	45.91216	-0.404832077	0.350921	-1.15363	0.248653	0.728723
insb	3.780871	-2.211530381	1.03499	-2.13676	0.032617	0.319477
mlf2	1270.505	0.015151468	0.086864	0.174428	0.861529	0.980527
nuf2	254.0741	0.179740575	0.226982	0.791873	0.428435	0.846133
emc9	304.3414	0.19117053	0.162979	1.172974	0.240806	0.722178
fhl3a	592.6726	-0.389071471	0.138473	-2.80972	0.004958	0.117232
fabp7b	146.0756	0.430140637	0.278253	1.545863	0.122138	0.569287
CABZ01090041.1	58.2897	0.89124128	0.472526	1.886122	0.059278	0.419384
celf3a	871.3307	0.259761547	0.106753	2.433299	0.014962	0.214257
ube2e2	792.758	0.083584244	0.115543	0.723404	0.469432	0.867224
scel	980.3769	-0.381186031	0.173433	-2.19788	0.027958	0.297418
tmem198b	389.1399	-0.229410411	0.14423	-1.59059	0.111703	0.547596
mysm1	225.016	-0.156487464	0.152182	-1.02829	0.303812	0.769473
vegfab	212.2797	0.086988958	0.214305	0.405911	0.684808	0.938486
pvalb7	2787.712	-0.014298649	0.195285	-0.07322	0.941632	0.997371
ftr85	29.75654	0.108638925	0.371651	0.292314	0.770046	0.960484
esyt1a	87.93507	0.146052822	0.290318	0.503079	0.614909	0.915998
def6c	62.57493	0.053892265	0.278658	0.1934	0.846646	0.977214
tfpia	702.3709	0.200735033	0.110102	1.823176	0.068277	0.445546
med6	313.5118	-0.070157774	0.148832	-0.47139	0.637363	0.924779
tspan3a	1437.231	0.083879583	0.134092	0.625539	0.531618	0.890835
zdhhc2	64.77187	0.375112438	0.308719	1.215063	0.224342	0.706347
phf3	466.132	-0.037601711	0.161596	-0.23269	0.816002	0.971207
rab13	315.6699	-0.047288858	0.203478	-0.2324	0.816225	0.971207

ncapd3	343.9793	0.3294941	0.229744	1.434178	0.151521	0.615372
txn2	824.2802	-0.009600515	0.102595	-0.09358	0.925446	0.994815
dachb	197.3341	-0.204758185	0.14993	-1.36569	0.172037	0.644374
mylka	269.7071	-0.122882025	0.210163	-0.5847	0.55875	0.899409
kcnip1b	335.4987	0.204078886	0.185004	1.103104	0.269982	0.746532
asah1b	415.9371	-0.328250046	0.167904	-1.95499	0.050584	0.391331
copg2	1698.638	-0.023007509	0.117614	-0.19562	0.844909	0.976791
uspl1	316.9164	-0.184978659	0.192948	-0.9587	0.337712	0.794797
si:dkey-222n6.2	275.3905	-0.094623726	0.200379	-0.47222	0.636767	0.924779
nt5c2l1	1102.829	-0.048225271	0.176703	-0.27292	0.784916	0.962605
ccnb3	387.1887	0.1557673	0.190197	0.818978	0.412799	0.838123
f7	285.593	-0.133291865	0.213198	-0.6252	0.531838	0.890978
mid2	124.7186	0.175850672	0.197592	0.889967	0.373484	0.820566
acbd5a	400.9973	-0.002819544	0.166974	-0.01689	0.986527	1
bet1	211.3735	-0.194824205	0.164361	-1.18534	0.235882	0.717225
rarab	596.9623	0.107663892	0.117327	0.917636	0.358809	0.80995
tgfb1b	34.6904	0.09730931	0.305463	0.318564	0.750057	0.955074
ldb2b	496.3306	0.093311196	0.1268	0.735892	0.461797	0.863402
rps10	27241.5	-0.315623567	0.128776	-2.45095	0.014248	0.209045
paqr7b	27.72964	0.266977605	0.375207	0.711547	0.476746	0.869292
si:ch211-152f2.2	0.625144	1.055425623	2.564796	0.411505	0.680702	NA
hat1	801.8397	0.057498068	0.146649	0.39208	0.694999	0.941197
cx52.6	12.08017	-0.099408879	0.902365	-0.11016	0.912279	0.992371
chchd3b	239.614	0.155010692	0.252961	0.612785	0.540019	0.893049
slc1a7a	16.6681	1.455224982	0.656552	2.216466	0.02666	0.289458
si:dkey-224e22.2	102.2816	0.19261687	0.322013	0.598165	0.54973	0.897373
tm9sf5	455.8859	0.117852173	0.11016	1.069832	0.284695	0.755774
myclb	394.5068	-0.146250999	0.153105	-0.95523	0.339459	0.795226
hddc2	219.7747	0.092239549	0.151912	0.60719	0.543725	0.894925
gpr157	74.30452	0.024799358	0.283978	0.087329	0.93041	0.995707
zcchc8	334.7946	0.296192617	0.122406	2.419751	0.015531	0.219827
asb4	54.47827	0.516640273	0.307004	1.682847	0.092405	0.508314
retsatl	2.955814	-1.50170185	1.086686	-1.38191	0.166999	0.637452
bag2	167.375	0.267676292	0.145707	1.837092	0.066196	0.439276
trim35-27	53.60788	-0.144405207	0.28706	-0.50305	0.61493	0.915998
thy1	696.756	0.738397914	0.281457	2.623483	0.008704	0.1602
khdc4	651.8121	0.053363823	0.122183	0.436754	0.66229	0.930742
lpcat4	155.5484	-0.453783764	0.241196	-1.88139	0.059919	0.421883
pfdn5	1308.994	-0.347507877	0.156078	-2.2265	0.025981	0.285997
or115-10	0.190858	0	5.267649	0	1	NA
pdcd10a	979.7359	-0.026277188	0.132022	-0.19904	0.842235	0.97643
fgf13a	535.3841	0.239247115	0.136874	1.747939	0.080475	0.479085
ubtf	1537.784	-0.132780469	0.083895	-1.58269	0.113492	0.551541
rtel1	138.9565	0.109696812	0.222524	0.492966	0.622037	0.918461
hsf2bp	2.085062	-3.077531891	1.810817	-1.69953	0.08922	NA
si:ch211-254c8.3	3.110854	-1.386968651	1.696092	-0.81774	0.413504	0.838369
tdgf1	20.0791	-1.292206524	0.435182	-2.96935	0.002984	0.090034
dicp2.1	1.146082	0	2.777668	0	1	NA
gldc	833.9852	-0.096822129	0.213099	-0.45435	0.649574	0.927756
camsap1b	300.4536	0.056854463	0.140361	0.405058	0.685435	0.938486
rxrab	23.01232	-0.234492077	0.380097	-0.61693	0.537283	0.892498
zgc:101858	362.5676	-0.154605987	0.149275	-1.03571	0.300336	0.766629
surf4l	170.3876	-0.053156073	0.174395	-0.3048	0.760517	0.957886
rgs3b	260.0533	0.180016201	0.135263	1.330858	0.183236	0.659455

pho	82.20988	-0.293773281	0.317978	-0.92388	0.355549	0.806546
selenow1	5650.844	-0.000506455	0.127184	-0.00398	0.996823	1
ehd2a	2.327151	0.098253273	1.208225	0.08132	0.935187	0.996829
polr2j	293.881	0.051450026	0.148883	0.345573	0.729663	0.950056
lrwd1	84.73745	-0.183193237	0.223844	-0.8184	0.413131	0.838274
orai2	71.29677	0.309339461	0.368109	0.840347	0.400714	0.831725
pex12	71.45531	-0.533620672	0.242547	-2.20007	0.027802	0.296445
drl1.2	11.98723	0.011073939	0.651701	0.016992	0.986443	1
drl1.1	24.47626	1.470056549	0.589208	2.494972	0.012597	0.195228
ap2b1	1498.714	-0.060178795	0.080854	-0.74429	0.456701	0.860698
pkdcca	112.8081	0.064955545	0.235973	0.275267	0.783111	0.962328
tmem136b	45.13834	0.48859113	0.488272	1.000653	0.316995	0.778879
mrpl48	262.2438	-0.164525747	0.140798	-1.16852	0.242596	0.724345
layna	9.799415	0.467176089	0.565789	0.825707	0.40897	0.836637
btg4	0.247052	-0.868112681	5.267649	-0.1648	0.869101	NA
schip1	1046.19	0.210981579	0.10257	2.05695	0.039691	0.348481
gna14	222.0241	-0.06234563	0.189336	-0.32929	0.74194	0.954329
slc25a46	195.1714	0.152906812	0.159998	0.95568	0.339234	0.795226
tmem150ab	38.63451	-0.117551663	0.314915	-0.37328	0.708939	0.945546
fzd9a	38.00735	0.290064499	0.402776	0.720163	0.471425	0.86809
abl1	400.0622	-0.05164629	0.185332	-0.27867	0.780498	0.961411
rab14l	737.4479	0.17240376	0.106125	1.624531	0.104263	0.532541
fam81b	0.840859	0.062899601	2.313121	0.027193	0.978306	NA
gcnt4a	205.3543	-0.350561955	0.178876	-1.9598	0.050019	0.389217
si:dkey-47k20.8	0.435748	1.055409122	3.50244	0.301335	0.763159	NA
zgc:110249	29.90147	-0.693023321	0.377277	-1.83691	0.066223	0.439276
npr3	282.0317	0.083139936	0.171665	0.484316	0.628162	0.921864
eef2l2	13868.08	-0.254812764	0.136762	-1.86318	0.062436	0.427718
akr1a1a	195.2488	0.037129605	0.305761	0.121433	0.903348	0.99133
riok2	437.337	-0.100528881	0.198875	-0.50549	0.613217	0.915998
tmtc2b	45.03241	0.008572981	0.294822	0.029078	0.976802	1
emc6	633.2762	0.061299163	0.141235	0.434022	0.664272	0.93177
ncor1	1464.321	0.068488189	0.156845	0.436661	0.662357	0.930742
dmrt3a	33.52961	-0.542397106	0.330936	-1.63898	0.101218	0.526828
grb10b	72.50582	-0.303728129	0.280018	-1.08467	0.278066	0.751959
entpd3	346.2338	0.246142485	0.159682	1.541455	0.123206	0.569695
myh7bb	494.9234	0.000903217	0.193994	0.004656	0.996285	1
hnrnpl	3624.142	0.058471738	0.135233	0.432378	0.665466	0.932595
srsf7a	1386.979	-0.038207632	0.086746	-0.44045	0.65961	0.930581
nccrp1	851.9464	0.034688693	0.212343	0.163361	0.870234	0.983365
ckma	175954.9	0.014172593	0.186973	0.0758	0.939578	0.997371
capns1a	2435.95	-0.22796693	0.085159	-2.67697	0.007429	0.145999
taf1	740.7532	0.013165457	0.157685	0.083492	0.93346	0.996472
cfap298	141.5871	0.232938929	0.180156	1.292983	0.196017	0.676453
mis18a	83.88726	0.208459234	0.241473	0.863281	0.387983	0.827592
tppp2	196.5153	0.06943488	0.238411	0.291241	0.770867	0.960484
ftr99	7.117948	-1.526533702	0.741113	-2.05979	0.039419	0.347542
ins	312.2981	0.653842299	0.293451	2.228117	0.025873	0.285598
pafah1b2	80.413	-0.280763917	0.249507	-1.12527	0.260473	0.737886
gnb2	664.3769	0.009415851	0.143267	0.065722	0.947599	0.997371
mink1	708.9609	-0.05274609	0.161492	-0.32662	0.743957	0.95491
fgf5	11.47855	-0.173384335	0.615487	-0.2817	0.778171	0.961073
tmem174	16.50827	-0.798647572	1.619942	-0.49301	0.622006	0.918461
fcho2	520.3866	0.028793966	0.126656	0.22734	0.820159	0.971788

tnpo1	553.7852	-0.004673295	0.119	-0.03927	0.968674	1
ptcd2	62.2896	-0.184261959	0.23567	-0.78186	0.434294	0.850002
enc1	676.1798	0.134289625	0.148764	0.902702	0.366684	0.816111
ankra2	612.3593	-0.020986388	0.1592	-0.13182	0.895123	0.989187
btf3	22500.79	-0.508430646	0.191747	-2.65156	0.008012	0.151789
ttc33	193.7131	-0.194643086	0.189042	-1.02963	0.303184	0.769249
ptger4b	38.07908	-0.103804973	0.261577	-0.39684	0.691483	0.940173
ophn1	141.7442	-0.039975566	0.191181	-0.2091	0.834372	0.975731
ccn1l1	47.04717	-0.125259901	0.326891	-0.38319	0.701582	0.943254
surf6	29.63679	-0.107022912	0.415808	-0.25739	0.796881	0.966447
arpc5lb	255.0957	-0.159902389	0.178052	-0.89807	0.36915	0.817763
zmat5	234.7024	-0.485706231	0.15736	-3.08659	0.002025	0.070121
ml7	2.626302	-1.480367043	1.538265	-0.96236	0.335868	0.792139
myhc4	1484.966	1.227231169	0.391679	3.133255	0.001729	0.064499
camkk1a	39.06257	-0.336669021	0.338618	-0.99424	0.320104	0.780709
atp2a1l	52511.91	0.283919262	0.222953	1.273446	0.20286	0.683508
spns3	70.70163	0.11310386	0.409599	0.276133	0.782446	0.962328
slc25a25b	47.49523	0.44218215	0.287272	1.539243	0.123745	0.570837
sh3glb2b	501.3787	-0.094372482	0.143185	-0.6591	0.509834	0.883733
si:dkey-220k22.1	180.3607	0.190615177	0.165807	1.149621	0.2503	0.730041
nelfb	304.4454	0.225569954	0.12178	1.85227	0.063987	0.431861
entpd2a.1	48.32804	0.244348054	0.350672	0.6968	0.485928	0.873244
ddx31	131.7287	0.037397776	0.21801	0.171542	0.863798	0.981842
barhl1a	181.0733	0.540757273	0.150689	3.588557	0.000333	0.020711
adam28	127.2299	0.161529482	0.224113	0.720749	0.471064	0.868025
tm2d2	269.9673	-0.069834788	0.139313	-0.50128	0.616175	0.916365
histh1l	13761.54	-0.158896514	0.188017	-0.84512	0.398046	0.830737
sfrp1a	331.3736	-0.671746592	0.150795	-4.45471	8.40E-06	0.001609
zgc:110329	134.2744	-0.066592177	0.16636	-0.40029	0.688944	0.93924
tacr1a	21.14438	0.515348115	0.494045	1.04312	0.296893	0.764503
rasa1a	549.0493	-0.038380371	0.128852	-0.29786	0.765806	0.959951
atp6v0a2a	234.2252	0.309906554	0.153202	2.022857	0.043088	0.36374
brwd3	381.4088	0.085901664	0.145657	0.589754	0.555356	0.898723
etnppl	522.2843	0.388179618	0.281939	1.376821	0.168568	0.639966
jmjd7	117.158	-0.060295806	0.210824	-0.286	0.774877	0.960899
uprt	652.9165	-0.232604042	0.136344	-1.70601	0.088006	0.498194
cfap73	5.083099	1.704730702	0.816431	2.088028	0.036795	0.336916
iqcd	15.99613	0.143333589	0.422389	0.33934	0.734353	0.951134
agtr2	222.7189	0.010740574	0.16423	0.0654	0.947856	0.997411
cx27.5	26.17279	-0.958800981	0.557298	-1.72045	0.085351	0.490595
gbgt1l3	58.45809	-1.034825708	0.256616	-4.03258	5.52E-05	0.006008
rps6ka3a	652.6284	-0.040658232	0.118711	-0.3425	0.731977	0.950617
gabarapa	1487.167	-0.169342785	0.135652	-1.24836	0.211898	0.693489
gps2	451.0118	0.032757167	0.142003	0.23068	0.817564	0.971383
tp53	765.9156	0.091192779	0.15388	0.592622	0.553434	0.897974
capga	11.96255	0.46415317	0.576553	0.805048	0.420792	0.842383
mpdu1a	219.6783	-0.044145623	0.18028	-0.24487	0.806555	0.969726
znf703	808.185	-0.004906624	0.117151	-0.04188	0.966592	1
dgcr8	588.5203	-0.142056942	0.124463	-1.14136	0.25372	0.732873
atp6v0a2b	227.6093	-0.204952938	0.175772	-1.16602	0.243608	0.724437
cyp1d1	231.5183	0.860274194	0.17632	4.879038	1.07E-06	0.000308
zgc:101016	200.2785	-0.508055306	0.168098	-3.02238	0.002508	0.08071
abhd17b	288.5703	-0.231937507	0.141126	-1.64348	0.100284	0.524748
AK6	296.9887	-0.627705179	0.236196	-2.65757	0.007871	0.150059

cds2	808.0838	-0.168654237	0.105987	-1.59127	0.111548	0.547433
hs3st1l2	112.962	0.294662146	0.180337	1.63395	0.102269	0.528996
si:dkey-174n20.1	62.36163	-0.575608014	0.260988	-2.2055	0.027419	0.293857
ficd	113.8627	0.106418224	0.223033	0.477142	0.633261	0.923885
ISCU (1 of many)	1218.945	-0.032211297	0.141383	-0.22783	0.819778	0.971785
coro1ca	1186.813	0.042693457	0.095651	0.446344	0.655349	0.929606
dao.3	10.52018	-0.911885123	0.533134	-1.71042	0.087187	0.495407
dao.1	890.0178	-0.139083896	0.376955	-0.36897	0.712153	0.945821
dao.2	150.9993	-1.185030011	0.293338	-4.03981	5.35E-05	0.005902
tchp	172.408	0.088882747	0.196718	0.451828	0.651393	0.928562
aldh3b2	20.80334	-0.660888546	0.456194	-1.4487	0.147421	0.608001
ankle2	96.89483	-0.247067851	0.201138	-1.22835	0.219316	0.700266
pgam5	705.2975	0.058525893	0.133891	0.437117	0.662026	0.930742
mtnr1c	8.717777	1.722480963	0.832727	2.068482	0.038595	0.344335
zgc:113208	343.3446	-0.004655035	0.212765	-0.02188	0.982545	1
coq5	239.3841	0.227978084	0.158764	1.435954	0.151015	0.615085
xbp1	4043.405	-0.143828887	0.182006	-0.79024	0.429386	0.846956
snrnp27	631.0761	-0.12372245	0.145689	-0.84922	0.395758	0.829389
parvab	105.0351	-0.045672292	0.220339	-0.20728	0.83579	0.976007
ypel1	171.5642	-0.088405453	0.195	-0.45336	0.650288	0.927835
sdf2l1	521.8561	0.244977969	0.192546	1.272309	0.203263	0.684076
ccl19a.2	0.14762	0	5.267649	0	1	NA
tctn2	69.49695	0.076864183	0.253937	0.30269	0.762126	0.958345
rfc5	428.1075	0.010590935	0.153338	0.069069	0.944935	0.997371
pimr123	0.04222	0	5.267649	0	1	NA
irx4a	100.7072	0.354682571	0.270132	1.312997	0.189184	0.66871
ext1c	324.1515	0.256545968	0.162896	1.574908	0.115278	0.555298
evla	572.6631	0.020077393	0.119784	0.167613	0.866887	0.982582
sat1a.1	488.0374	-0.282438374	0.179702	-1.57171	0.116019	0.556131
rad21b	375.8859	0.47414456	0.252662	1.876599	0.060573	0.423018
BX649384.1	0.659156	2.613837829	2.781274	0.939799	0.347321	NA
si:ch211-193k19.1	210.2722	-0.092874026	0.18241	-0.50915	0.610646	0.915331
zgc:103438	27.60938	-1.086724945	0.337824	-3.21684	0.001296	0.052702
si:dkey-267n13.1	38.98105	-0.082715272	0.343542	-0.24077	0.809732	0.969828
ptp4a2b	1344.649	0.085647789	0.109092	0.785097	0.432397	0.848419
bmp8a	23.88989	0.070949425	0.424702	0.167057	0.867325	0.982582
zgc:91910	957.2045	0.179534875	0.157097	1.142825	0.253111	0.732759
si:rp71-45k5.4	1321.989	0.106407023	0.156493	0.679947	0.496538	0.878161
zgc:113424	0.405877	-0.776942606	2.968444	-0.26173	0.793527	NA
FP102018.1	375.2044	-0.001388192	0.183274	-0.00757	0.993957	1
mon1ba	24.29566	-0.199761756	0.39111	-0.51076	0.609522	0.915204
rps3a	55038.1	-0.420651305	0.138015	-3.04787	0.002305	0.076394
stm	1559.791	0.121582839	0.117611	1.033769	0.301244	0.767371
scxa	96.03984	-0.16045417	0.204382	-0.78507	0.432412	0.848419
spinb	48.24032	-0.282721841	0.403344	-0.70094	0.483338	0.871988
srp19	429.8658	-0.059571973	0.150824	-0.39498	0.692861	0.940444
zgc:101664	98.89272	-0.000316035	0.172894	-0.00183	0.998542	1
dock6	309.0046	-0.355475013	0.218671	-1.62562	0.104031	0.532096
marcksl1b	8796.423	0.037327492	0.109335	0.341404	0.732799	0.950617
cbln6	0.335261	0	4.390552	0	1	NA
arl5c	193.9663	-0.312795011	0.311225	-1.00505	0.314875	0.777649
arntl1b	310.0327	0.267207768	0.202425	1.320031	0.186825	0.665301
gsx1	111.2523	0.116840452	0.201131	0.580917	0.561296	0.90039
slc25a11	1976.074	0.170019152	0.109638	1.550738	0.120965	0.566816

ipo7	5537.163	-0.357082977	0.132803	-2.68883	0.00717	0.143346
stx18	227.4866	-0.235371931	0.137115	-1.71661	0.086051	0.492054
CU633984.1	7.784638	-0.384760949	0.721894	-0.53299	0.594042	0.910615
si:ch1073-157b13.1	442.605	-0.352301127	0.138885	-2.53664	0.011192	0.183794
ctdnep1b	143.1373	-0.060185243	0.163386	-0.36836	0.712603	0.945821
cdh31	0.710488	1.055430078	2.449813	0.430821	0.666599	NA
si:dkey-17m8.2	4.487501	-6.018071578	5.139612	-1.17092	0.241631	0.723333
gngt1	871.9677	0.172063045	0.277326	0.620436	0.534971	0.891993
clcn4	324.7307	-0.062299648	0.176317	-0.35334	0.723834	0.94801
col1a1b	14120.91	0.18949755	0.158339	1.196784	0.231391	0.712847
rgcc	1836.883	-0.247966725	0.169297	-1.46469	0.143007	0.602002
sirt3	168.6184	-0.292535035	0.190448	-1.53603	0.12453	0.572138
drd4b	10.52501	1.106244634	0.863851	1.280597	0.200335	0.680711
si:ch211-173p18.3	844.0212	-0.007583389	0.122367	-0.06197	0.950585	0.99801
zmym4	320.644	0.065748588	0.123263	0.533399	0.593758	0.910427
pyyb	45.30382	-0.220517362	0.480795	-0.45865	0.646485	0.926728
eef2k	1912.054	-0.150875911	0.210071	-0.71821	0.472626	0.868786
mosmob	153.3246	-0.553934284	0.236763	-2.33961	0.019304	0.244676
cart3	63.73839	0.007298972	0.365901	0.019948	0.984085	1
cdkn2aip	133.6393	0.006800599	0.163456	0.041605	0.966814	1
cnn2	1851.478	-0.262652056	0.154559	-1.69937	0.08925	0.501424
angptl4	1614.969	0.185341743	0.195998	0.945632	0.344336	0.798147
rps28	9237.728	-0.231677714	0.141852	-1.63324	0.102419	0.529087
si:rp71-39b20.4	40.73686	1.310370812	1.038814	1.261411	0.207161	0.689445
insl3	0.641012	-1.819808666	3.434447	-0.52987	0.596202	NA
spire1a	207.4934	-0.05151028	0.162195	-0.31758	0.750802	0.955074
azin1b	5124.876	-0.040997075	0.120803	-0.33937	0.73433	0.951134
laptm4b	546.3253	-0.063947676	0.162914	-0.39252	0.694671	0.941093
rpl30	22531.94	-0.443679783	0.131471	-3.37473	0.000739	0.036194
hsd17b12b	284.8522	-0.056244106	0.199707	-0.28163	0.778224	0.961073
fjx1	52.98207	-0.367624799	0.390263	-0.94199	0.346197	0.799463
tmem54b	6.279587	1.363763301	1.393363	0.978757	0.3277	0.786438
fuca1.2	219.1849	0.029395686	0.176237	0.166796	0.867531	0.982582
atp6v1c1b	610.6404	0.054632083	0.118994	0.459115	0.646152	0.926728
rida	1742.385	0.180818082	0.272719	0.663019	0.507318	0.883007
nt5c1aa	60.48517	0.670240525	0.333495	2.009748	0.044458	0.368943
zgc:91944	220.8834	-0.267460413	0.185974	-1.43816	0.150388	0.614113
zbtb8b	219.3341	-0.38976289	0.165777	-2.35112	0.018717	0.241927
fuca1.1	1273.157	-0.096695235	0.148564	-0.65087	0.515132	0.886401
acana	721.3061	-0.081389016	0.22328	-0.36452	0.715473	0.945988
fndc5b	62.12977	0.484463409	0.303878	1.594267	0.110876	0.54583
lingo1b	179.5393	0.182798035	0.264577	0.690906	0.489624	0.874629
crabp1b	252.4041	-0.201233541	0.178777	-1.12561	0.260331	0.737663
slc25a44b	203.6572	0.168902808	0.144035	1.17265	0.240936	0.722465
fam49al	1236.348	0.046970143	0.1803	0.260511	0.79447	0.965431
mfsd2ab	460.7673	0.030592822	0.228645	0.133801	0.89356	0.988566
znf281b	448.9535	0.011225491	0.128793	0.087159	0.930545	0.995741
yars	811.8194	0.221006152	0.158629	1.39323	0.16355	0.631682
tmem167a	646.584	-0.184995705	0.198859	-0.93029	0.352224	0.802776
bzw2	1035.007	0.1729319	0.118205	1.462985	0.143471	0.602607
dync1li2	742.2026	-0.196278099	0.110579	-1.775	0.075899	0.46659
hrh3	43.61537	-0.05642359	0.297654	-0.18956	0.849653	0.978209
sync	40.9147	-0.655917571	0.43715	-1.50044	0.1335	0.586903
tmem106bb	227.164	-0.05694559	0.142171	-0.40054	0.688757	0.939177

cdr2a	26.64386	-0.210677479	0.313991	-0.67097	0.502242	0.881277
tdp2b	108.01	0.085241761	0.228129	0.373656	0.70866	0.94551
gmnn	113.1319	-0.076155348	0.203695	-0.37387	0.708501	0.94551
tnni2b.1	2018.163	0.216488948	0.15326	1.412564	0.157784	0.623766
kcng2	34.07245	0.594566467	0.350442	1.69662	0.089769	0.502765
ccdc96	31.2158	0.936102692	0.374581	2.499069	0.012452	0.194346
pgr	4.493481	-0.240826744	0.88091	-0.27338	0.784558	0.962605
ptprn2	786.2458	0.106735325	0.157355	0.678311	0.497575	0.878787
ube3c	478.0518	0.055742915	0.126751	0.439784	0.660094	0.930581
mnx1	34.58758	-0.536868573	0.326158	-1.64604	0.099756	0.524166
fam210aa	318.8519	0.125135992	0.178669	0.700379	0.48369	0.872148
ptpn2b	421.9638	-0.000296647	0.10821	-0.00274	0.997813	1
psmg2	488.7092	-0.361504021	0.135992	-2.65827	0.007854	0.149996
cited4a	1138.61	-0.129564583	0.176997	-0.73202	0.464158	0.864593
sumo3b	1080.031	0.100702304	0.168531	0.597529	0.550154	0.8974
pitpnb	116.4504	0.01430835	0.203944	0.070158	0.944068	0.997371
caly	372.3233	0.408038953	0.199082	2.049606	0.040403	0.351287
arrdc3b	2621.497	0.268149711	0.222764	1.203738	0.228691	0.710195
sept5b	47.96348	0.282574938	0.329183	0.858412	0.390665	0.827902
mdka	6968.728	0.147137343	0.129158	1.139206	0.254617	0.733441
harbi1	200.1112	-0.207811336	0.179052	-1.16062	0.245797	0.727177
atg13	1258.532	-0.067213623	0.128539	-0.5229	0.601041	0.913756
f2	1530.325	-0.055544914	0.217694	-0.25515	0.798606	0.966807
znf408	38.68735	0.223417744	0.317638	0.703373	0.481824	0.871155
rps20	32380.8	-0.456946742	0.128817	-3.54725	0.000389	0.023306
penkb	78.98115	0.255942376	0.261017	0.980559	0.32681	0.785579
irx4b	48.24069	-0.250351926	0.271466	-0.92222	0.356414	0.807322
thap11	256.8259	-0.160955062	0.155448	-1.03543	0.300469	0.766629
vrk3	203.3047	0.101935607	0.169932	0.599863	0.548598	0.896847
tradd	177.2889	-0.179130433	0.166606	-1.07518	0.282296	0.754679
gnao1b	1143.271	0.511518653	0.187208	2.732357	0.006288	0.134409
tmppe	99.08518	-0.120404189	0.231953	-0.51909	0.603699	0.914588
exosc6	183.488	-0.107860802	0.232047	-0.46482	0.642059	0.925586
ogfod1	159.9379	-0.65504647	0.19002	-3.44725	0.000566	0.030304
ppp1r11	377.565	-0.091464117	0.134282	-0.68113	0.495788	0.87806
cnep1r1	206.5209	-0.027867052	0.14783	-0.18851	0.850479	0.978251
mgat1b	261.9117	0.001637175	0.169838	0.00964	0.992309	1
si:dkey-78a14.4	32.78284	-0.422173414	0.361042	-1.16932	0.242275	0.724106
ddx39b	347.0627	0.169636731	0.126091	1.345351	0.178512	0.652732
znrd1	42.31473	-0.435533012	0.320281	-1.35985	0.173878	0.646897
gtf2h4	0.288341	1.055360606	4.752284	0.222074	0.824256	NA
cebpg	1358.476	-0.024711594	0.101712	-0.24296	0.80804	0.969828
cebpa	748.1562	-0.405360568	0.2436	-1.66404	0.096104	0.517378
heatr3	344.5233	-0.010271601	0.176277	-0.05827	0.953534	0.998436
cd81a	11357.65	0.128822544	0.107607	1.197162	0.231243	0.712838
tph1b	43.56842	-0.28641436	0.255736	-1.11996	0.26273	0.740207
itln2	3.035344	-3.266012486	1.417936	-2.30336	0.021259	0.258701
itga11a	172.4235	0.114892021	0.186522	0.61597	0.537914	0.892498
pard6gb	216.1722	0.239221227	0.152449	1.569186	0.116605	0.557506
sdhaf3	171.3664	-0.00887409	0.171166	-0.05184	0.958652	0.999109
pias1a	154.9912	-0.053175363	0.170798	-0.31134	0.755545	0.956532
smad3a	406.5234	-0.043028872	0.123766	-0.34766	0.728093	0.949348
eya3	382.6452	-0.094867837	0.127122	-0.74627	0.455503	0.860008
ctdsp2	2632.673	-0.150219519	0.105545	-1.42328	0.154656	0.619425

myo1g	8.185108	-1.039311645	0.704179	-1.47592	0.139965	0.598517
si:dkeyp-92c9.2	12.00493	-0.204656012	0.575156	-0.35583	0.72197	0.947403
rgs18	29.25087	-0.001504603	0.427209	-0.00352	0.99719	1
txnipa	8839.492	0.330032778	0.152284	2.167217	0.030218	0.309805
mtx1b	132.4897	-0.491256527	0.16288	-3.01607	0.002561	0.082065
eif3hb	243.4704	0.35171387	0.176291	1.995071	0.046035	0.375021
mchr2	15.75472	1.07709658	0.450566	2.39054	0.016824	0.230232
nitr13	0.722495	1.055403349	3.296538	0.320155	0.748851	NA
bbox1	373.4265	0.453171185	0.256772	1.764876	0.077585	0.470832
tipin	166.1976	0.056623804	0.143455	0.394715	0.693053	0.94055
lctla	340.3294	0.609066966	0.301727	2.018603	0.043528	0.365455
crybgx	1241.709	-0.287497578	0.518272	-0.55472	0.579084	0.905417
tssc4	435.2922	-0.210619934	0.150281	-1.4015	0.161064	0.627834
ano5b	357.4576	-0.043011827	0.136745	-0.31454	0.75311	0.955777
gas2b	26.71252	-0.025816995	0.452682	-0.05703	0.95452	0.998611
gla	43.4933	-0.012935148	0.321441	-0.04024	0.967901	1
fnbp1a	23.07469	0.184772669	0.424044	0.435739	0.663026	0.931141
nudcd1	184.5631	-0.059406763	0.143215	-0.41481	0.678282	0.936026
trhrb	3.108427	-2.762954711	1.620926	-1.70455	0.088278	0.498998
hnrnpa01	8006.845	-0.026054211	0.111932	-0.23277	0.815942	0.971207
hnrnpa0b	82.15962	-0.144910435	0.251759	-0.57559	0.564891	0.901538
cars	485.6963	0.045235981	0.117981	0.383416	0.701411	0.943166
nfatc1	137.4852	-0.117973152	0.26445	-0.44611	0.655519	0.929606
rnasel3	540.6332	0.19478034	0.473447	0.411409	0.680772	0.937189
pcdh1b	442.9273	-0.102224994	0.128252	-0.79706	0.425415	0.844714
myo1ea	429.512	-0.161043594	0.132005	-1.21998	0.222471	0.704478
ccnb2	90.26869	-0.079358454	0.212047	-0.37425	0.708218	0.945344
mbpa	1022.207	0.830435456	1.062545	0.781554	0.434477	0.850002
spata4	30.54816	-0.139013206	0.341684	-0.40685	0.684121	0.938486
txnl4a	541.6904	0.113075255	0.234956	0.481262	0.63033	0.922869
pitx2	665.2612	-0.022951447	0.137164	-0.16733	0.867111	0.982582
tbc1d5	567.7903	-0.069471865	0.116331	-0.59719	0.550381	0.8974
dazl	7.467904	1.020425561	0.737928	1.382826	0.166718	0.637156
lcp2b	5.954434	0.134405529	1.076516	0.124852	0.90064	0.99045
npy	51.32363	0.68842416	0.508415	1.354059	0.175718	0.649413
npvf	36.89863	0.292740985	0.42376	0.690818	0.48968	0.874629
trpm7	542.4508	0.09710052	0.144178	0.673475	0.500645	0.880385
zbtb3	116.491	0.030100419	0.180326	0.166922	0.867431	0.982582
slc27a2a	2584.572	0.454753423	0.22487	2.022294	0.043146	0.363921
gatm	15762.04	0.008146516	0.166191	0.049019	0.960904	0.999249
sb:cb649	71.8976	0.061428166	0.280662	0.218869	0.826752	0.973458
tmem144b	1.095622	0	2.305674	0	1	NA
rras2	531.6609	-0.068623552	0.112907	-0.60779	0.543328	0.894925
hoxa13b	128.9361	-0.382651418	0.244424	-1.56552	0.117461	0.558959
rasal2	481.1602	0.10452336	0.137837	0.758309	0.448266	0.856741
gcgrb	32.18414	-1.019420006	0.369645	-2.75783	0.005819	0.129012
lamc1	2052.489	0.108135688	0.155489	0.695455	0.486771	0.873245
CC2D1A	126.5897	0.086739511	0.203574	0.426084	0.670047	0.933809
rnaset2	815.6962	-0.083898365	0.189461	-0.44283	0.657891	0.930581
nucb2b	180.0556	-0.111059467	0.172653	-0.64325	0.520061	0.887148
cdx4	5.989009	-0.134439426	0.814011	-0.16516	0.868821	0.982801
gnb1l	23.80712	-0.04436842	0.396892	-0.11179	0.91099	0.992208
golga7ba	414.9922	0.240923875	0.264126	0.912157	0.361686	0.811787
rps13	20956.08	-0.323380785	0.132522	-2.44021	0.014679	0.212568

nucb2a	1676.053	-0.303188824	0.127343	-2.38089	0.017271	0.231982
dnaaf3l	11.01673	0.674489234	0.6139	1.098695	0.271901	0.748035
phf23b	277.047	-0.08029555	0.130999	-0.61295	0.539911	0.893049
nod1	11.30793	0.348152509	0.662023	0.525892	0.598963	0.913109
rpl39	15689.49	-0.373233047	0.165549	-2.25451	0.024164	0.275116
ndufa1	1162.567	0.039242723	0.160723	0.244163	0.807104	0.969726
c1galt1c1	316.0176	0.08320228	0.123185	0.675424	0.499407	0.879603
cers3b	40.68605	0.500040741	0.28936	1.728095	0.083971	0.486647
vps11	332.8115	-0.120539959	0.136116	-0.88557	0.375849	0.822237
calb2b	1322.972	0.424245103	0.284589	1.490727	0.136033	0.591131
cmtr2	138.9257	-0.120780029	0.196002	-0.61622	0.537751	0.892498
riox2	358.0942	0.180004159	0.20738	0.867993	0.385398	0.826939
acta1a	9642.426	0.23727496	0.250024	0.94901	0.342616	0.797189
cyfip2	1243.266	0.189760407	0.135489	1.400557	0.161347	0.628218
cldn7a	451.4543	0.121554745	0.266003	0.456967	0.647695	0.927153
ponzr6	94.40173	0.185216244	0.275174	0.673088	0.500891	0.880413
slc24a6a	241.5569	-0.3231738	0.258274	-1.25128	0.210831	0.692523
rbm4.1	555.725	0.062775472	0.175214	0.35828	0.720134	0.947004
pop7	433.4175	0.037503521	0.16063	0.233478	0.81539	0.971158
si:ch211-81a5.5	15.82019	0.642790597	0.443557	1.449173	0.147289	0.607673
si:ch73-335l21.1	678.2616	0.08052723	0.146064	0.551313	0.581419	0.905618
glb1	165.6034	-0.268636684	0.207279	-1.29602	0.194969	0.67483
si:dkey-33c9.6	18.82938	0.229156617	0.489916	0.467747	0.639966	0.924843
ntn5	296.6534	-0.145420217	0.179566	-0.80984	0.418031	0.841312
pcdh20	110.6087	-0.169461701	0.205357	-0.8252	0.409255	0.836637
zgc:103586	252.3055	0.208182274	0.164729	1.263787	0.206307	0.688779
slc3a2a	1882.712	-0.178314032	0.172236	-1.03529	0.300533	0.766629
si:dkey-264d12.4	141.298	-0.513138175	0.194134	-2.64322	0.008212	0.153944
sat2a	0	NA	NA	NA	NA	NA
ndufv1	5352.949	0.300832564	0.116704	2.577747	0.009945	0.172469
atp11c	165.3634	0.117549687	0.176599	0.665629	0.505648	0.882418
serpinh2	167.8828	0.020386634	0.145728	0.139895	0.888743	0.988188
PRKAR2B	102.4713	-0.031031926	0.274377	-0.1131	0.909952	0.992208
anxa4	2071.396	-0.02305374	0.132208	-0.17437	0.861571	0.980527
cacng6a	400.6534	-0.160213687	0.200391	-0.7995	0.423998	0.844352
prlhr2b	1.986002	-0.445613642	1.605475	-0.27756	0.781351	NA
tmtops3a	6.003013	-1.812552937	0.958783	-1.89047	0.058695	0.418329
rab11fip1b	57.40221	0.249660931	0.251981	0.990794	0.321786	0.782075
dand5	1.753916	-0.944511857	1.474461	-0.64058	0.521795	NA
snapc2	108.4336	0.35793153	0.229349	1.560641	0.118608	0.561771
si:dkeyp-80c12.4	10.40975	-0.351010981	0.569466	-0.61639	0.53764	0.892498
tcn2	1070.924	-0.066455112	0.184705	-0.35979	0.719004	0.94694
hexim1	812.754	-0.064496636	0.109654	-0.58818	0.556409	0.899063
c1ql3b	23.45304	-0.026702792	0.477876	-0.05588	0.955439	0.998611
zgc:112356	1419.168	0.128703842	0.100472	1.280993	0.200196	0.680711
si:ch73-174h16.4	210.3256	-0.166142894	0.158963	-1.04517	0.295946	0.764246
gstcd	99.732	0.153997185	0.277536	0.554872	0.578982	0.905417
zgc:162171	21.32486	-0.059437762	0.417422	-0.14239	0.88677	0.987668
fam122b	348.3247	-0.216691245	0.169145	-1.2811	0.20016	0.680711
rab39bb	149.7398	0.268498233	0.211388	1.27017	0.204024	0.685206
syt4	436.1098	-0.06317534	0.157474	-0.40118	0.688288	0.93909
ccnt2b	867.8174	0.105082619	0.088973	1.181067	0.237576	0.718568
rhbdf1a	511.5196	0.068995348	0.150047	0.459826	0.645641	0.926728
pknox1.2	115.2248	0.249260048	0.180573	1.380383	0.167469	0.638828

tmprss3b	12.39748	0.614574805	0.564482	1.088741	0.276268	0.750674
pdxkb	39.71231	0.196590195	0.355672	0.552729	0.580449	0.905618
s1pr2	299.4453	0.130995804	0.168421	0.777789	0.436693	0.851093
agpat3	1354.199	-0.165067465	0.135536	-1.21789	0.223267	0.70531
col18a1a	2999.099	0.205655784	0.131899	1.559188	0.118952	0.562358
gpr185b	6.934115	0.122883351	0.827835	0.148439	0.881996	0.986908
aanat1	24.21277	1.840110752	0.514612	3.575723	0.000349	0.021465
bach2a	38.83281	-0.066908379	0.393724	-0.16994	0.865059	0.982136
sema3e	511.1675	0.10223524	0.130817	0.781512	0.434501	0.850002
si:ch73-71d17.2	67.6783	0.354648296	0.270916	1.309073	0.19051	0.670633
atp6v0cb	3095.543	0.361095407	0.142777	2.529091	0.011436	0.185422
st8sia5	150.1844	0.183382789	0.183839	0.997519	0.318513	0.779923
cbr1	284.6685	-0.237170869	0.168777	-1.40523	0.159953	0.626214
mhc1zba	749.7999	0.203051762	0.210696	0.963718	0.335187	0.791912
setd4	119.2702	-0.155513278	0.213879	-0.72711	0.467159	0.866134
kdm2ba	936.3024	-0.093080017	0.092557	-1.00566	0.314581	0.777479
tab1	623.2537	-0.341929912	0.163432	-2.09219	0.036422	0.335676
gpr182	205.1635	-0.128832414	0.214641	-0.60022	0.548358	0.896644
mgat3b	115.3133	0.034032064	0.252358	0.134857	0.892725	0.988566
polr2f	510.6913	0.117370115	0.168691	0.695768	0.486574	0.873244
sema6ba	12.16101	-0.661493804	0.503632	-1.31345	0.189033	0.668516
cd74b	50.26496	-0.009081112	0.624123	-0.01455	0.988391	1
rps14	26142.25	-0.2933197	0.140476	-2.08805	0.036794	0.336916
plp2	721.9175	-0.014205286	0.127577	-0.11135	0.911342	0.992208
ebpl	27.89254	0.523774782	0.423474	1.236852	0.216142	0.69815
arl11	104.8398	0.165577009	0.203208	0.814816	0.415178	0.838754
rcbtb1	152.1482	-0.274324318	0.164807	-1.66452	0.096009	0.517378
cdadc1	126.5069	-0.169391379	0.198243	-0.85446	0.392848	0.827902
cab39l	1101.215	-0.052727633	0.111686	-0.4721	0.636852	0.924779
aplnrb	239.9477	-0.442591135	0.135247	-3.27247	0.001066	0.045972
tnni4b.2	1214.137	-0.032702507	0.125664	-0.26024	0.79468	0.965534
hnrnpa1b	3918.549	0.165383576	0.112198	1.474037	0.140472	0.599618
trmt10a	113.9936	0.214510668	0.233809	0.917459	0.358902	0.80995
ndufa7	896.8249	-0.01288915	0.159848	-0.08063	0.935733	0.996829
ccnt2a	576.3915	-0.044938313	0.097373	-0.46151	0.644434	0.926639
bcl9	1021.059	0.206082001	0.136559	1.509104	0.131272	0.583069
calcoco1a	1022.742	-0.273316619	0.139465	-1.95975	0.050025	0.389217
znf865	897.9351	0.032012053	0.144388	0.221709	0.824541	0.972227
si:ch211-114n24.6	1133.728	1.072692114	0.475672	2.255111	0.024126	0.274967
tmem18	390.9019	-0.25734954	0.134649	-1.91126	0.055971	0.409108
tomm40l	390.3512	0.235341724	0.126029	1.867355	0.061852	0.426649
slc9a3.2	34.83836	-0.590696736	0.465952	-1.26772	0.204898	0.686746
si:dkey-211g8.1	0.140441	1.055383603	5.267649	0.200352	0.841205	NA
nitr7a	0.103749	0	5.267649	0	1	NA
nitr8	0.358284	1.702985565	4.36166	0.390444	0.696208	NA
hmgn3	1992.405	0.23241532	0.133362	1.742734	0.08138	0.480419
prmt9	88.17656	-0.326156449	0.216138	-1.50902	0.131294	0.583069
hax1	468.9132	0.324642932	0.138119	2.350459	0.01875	0.241927
urgcp	77.78914	0.948515676	0.633583	1.497067	0.134376	0.588706
pygo2	559.7939	0.077686172	0.130723	0.594281	0.552324	0.8974
s100s	498.988	0.290490891	0.218003	1.33251	0.182693	0.659278
rgs12b	563.8352	-0.03499212	0.118996	-0.29406	0.768711	0.960395
dtd2	67.96735	-0.051172144	0.270985	-0.18884	0.85022	0.978251
aldh8a1	255.1001	-0.172748236	0.212815	-0.81173	0.416946	0.840494

hbs1l	1047.864	0.009479485	0.105676	0.089703	0.928523	0.995052
si:dkey-170l10.1	54.06209	0.157382056	0.325961	0.482825	0.62922	0.922431
commd7	292.5531	-0.213948897	0.188653	-1.13409	0.256757	0.73427
si:ch211-226m16.2	439.9881	0.088454087	0.163484	0.541057	0.588468	0.90797
tap2a	1.083937	-0.868144733	2.233633	-0.38867	0.697521	NA
dnmt3bb.1	374.9193	-0.1575592	0.153596	-1.02581	0.304983	0.770102
ttll5	29.52448	0.047647538	0.32389	0.14711	0.883045	0.986908
si:ch211-106j24.1	381.7472	0.040027755	0.206902	0.193462	0.846597	0.977214
ifrd2	661.4541	0.054787382	0.120608	0.454259	0.649643	0.927756
pou2f2a	363.9219	0.085424572	0.128956	0.662434	0.507693	0.883056
hyal3	15.41191	-0.13692296	0.575126	-0.23807	0.811823	0.97012
mgll	769.7697	-0.081414772	0.124288	-0.65505	0.512435	0.885459
ankrd52a	95.69278	0.240946851	0.314561	0.765979	0.443689	0.854255
krt91	44703.41	0.038235841	0.1784	0.214326	0.830293	0.974908
rnf41	484.3406	0.004673253	0.128036	0.036499	0.970884	1
cytl1	33608.91	0.152398643	0.244157	0.624182	0.532508	0.890978
upp2	204.6057	0	1.700425	0	1	1
krt92	5712.646	-0.370244611	0.228929	-1.61729	0.105816	0.534827
krt15	7674.189	-0.146570412	0.198763	-0.73741	0.460871	0.862482
tsen54	188.2288	-0.141846417	0.184857	-0.76733	0.442885	0.853998
anks4b	132.2327	-1.326831541	0.312139	-4.25076	2.13E-05	0.003005
slc43a2a	1042.571	-0.433242592	0.14684	-2.95043	0.003173	0.09274
DXO	46.782	0.162750217	0.271504	0.599439	0.54888	0.897091
plagx	817.0645	-0.021577209	0.116409	-0.18536	0.852949	0.978794
myo1d	302.3369	0.177797667	0.198046	0.897761	0.369313	0.817763
slc34a2b	238.0441	-0.148649375	0.209538	-0.70941	0.478067	0.869292
slc2a12	173.1999	-0.179267329	0.184151	-0.97348	0.330314	0.788126
tbpl1	113.2116	0.077467844	0.20983	0.369194	0.711983	0.945821
tcf21	55.20468	-0.069291645	0.25754	-0.26905	0.787889	0.963339
arih1l	286.6963	-0.140320972	0.139492	-1.00595	0.314442	0.777479
rps12	21477.55	-0.286050233	0.121702	-2.35041	0.018753	0.241927
zgc:153284	396.8938	0.130149357	0.22272	0.584362	0.558977	0.899409
sh3bgrl2	283.4311	-0.246137474	0.156991	-1.56785	0.116916	0.557831
zgc:153935	135.5983	-0.585897278	0.328362	-1.7843	0.074374	0.463115
f13a1b	298.7459	0.416234036	0.200267	2.078397	0.037673	0.340171
aimp1b	1521.171	0.105676536	0.174748	0.604737	0.545354	0.895275
sgms2b	42.37471	-0.068887081	0.298842	-0.23051	0.817693	0.971441
irak1bp1	109.7144	-0.242708992	0.214799	-1.12993	0.258504	0.735636
scamp1	200.1546	0.194743683	0.158635	1.22762	0.21959	0.700438
edn1	71.86406	-0.043365684	0.283854	-0.15277	0.878576	0.985761
st6galnac6	9.708924	0.759599178	0.587312	1.293349	0.19589	0.676219
dolpp1	107.7937	0.230472625	0.214207	1.075934	0.281957	0.754378
tatdn1	64.11336	-0.098888778	0.268347	-0.36851	0.712493	0.945821
rnf139	388.4725	-0.294558884	0.128134	-2.29883	0.021515	0.260176
tbx18	94.33612	0.155478556	0.239993	0.647847	0.517084	0.887148
ctss1	20.28101	-0.715822684	0.469657	-1.52414	0.127474	0.576964
gpd1c	766.0615	-0.171407996	0.143215	-1.19685	0.231363	0.712847
golph3l	251.3261	-0.402475125	0.132755	-3.03171	0.002432	0.07907
ensaa	59.11125	-0.138931342	0.34652	-0.40093	0.688469	0.939118
zgc:198371	35.62657	0.778668023	0.358753	2.170484	0.02997	0.30849
asxl1	1041.752	-0.022406271	0.138537	-0.16173	0.871515	0.983365
rnf24	158.3582	-0.251147767	0.231607	-1.08437	0.2782	0.751971
hsd3b7	445.1051	-0.014761249	0.129859	-0.11367	0.909498	0.99213
smox	1504.827	-0.154315333	0.118536	-1.30184	0.19297	0.672855

si:ch1073-416d2.3	91.05765	-0.832859295	0.3454	-2.41129	0.015896	0.222371
ripply3	7.741247	-1.231722048	0.660905	-1.86369	0.062365	0.427718
plxnb2b	84.15607	-0.14975714	0.241979	-0.61888	0.535993	0.892102
has2	55.09879	0.414589502	0.24107	1.719788	0.085471	0.490796
dctd	111.8034	-0.209198861	0.248712	-0.84113	0.400275	0.831485
erbb3b	154.2542	0.037472833	0.228316	0.164127	0.869631	0.983279
tbc1d16	190.5369	0.09029201	0.134251	0.672563	0.501226	0.880764
lsm6	589.376	0.057337794	0.229387	0.249961	0.802617	0.968181
arf3b	36.47825	0.159315626	0.345838	0.460666	0.645038	0.926728
DISP3	21.62807	-0.642000785	0.467564	-1.37308	0.169729	0.641486
fkbp11	287.3697	0.004401726	0.220131	0.019996	0.984047	1
zgc:152968	2.702457	-1.189853696	1.517929	-0.78387	0.433118	0.849392
bscl2	372.3428	0.151491242	0.142284	1.064707	0.287008	0.757955
banf1	1792.016	-0.03071046	0.148086	-0.20738	0.835711	0.976007
slc3a2b	2514.907	-0.294308625	0.176145	-1.67084	0.094754	0.513797
ube4b	1046.438	0.023968151	0.110599	0.216711	0.828433	0.974137
gab1	463.2297	0.090569474	0.112557	0.804652	0.42102	0.842517
kif1b	1417.574	0.039466564	0.140439	0.281023	0.778693	0.961073
casz1	169.1012	-0.030288918	0.229008	-0.13226	0.894778	0.989112
ptger2b	12.84285	-1.466218367	0.717809	-2.04263	0.041089	0.353918
psmc6	1856.134	0.242542936	0.135769	1.786441	0.074028	0.46206
cgrrf1	47.1026	0.259301535	0.265656	0.976081	0.329024	0.787268
ddhd1b	140.7261	0.312099617	0.215927	1.445395	0.148347	0.609649
rad51b	50.99576	0.476856519	0.291965	1.633268	0.102413	0.529087
zte38	1.685868	-0.749496067	1.401024	-0.53496	0.592675	NA
gcdha	1868.369	0.386806811	0.133737	2.892284	0.003825	0.10304
slc44a2	605.6993	0.174632192	0.123107	1.418536	0.156034	0.621616
kmt2d	454.4237	0.136660739	0.209289	0.652976	0.513772	0.88631
aldh9a1b	360.9232	-0.126658301	0.193759	-0.65369	0.513311	0.886245
dhh	11.33934	-0.531734097	0.561722	-0.94661	0.343835	0.798147
galca	245.2548	-0.261969038	0.145011	-1.80655	0.070832	0.454179
lgals8a	111.6335	0.425666233	0.18953	2.245907	0.02471	0.27868
dnajc22	23.19397	0.088779304	0.965481	0.091953	0.926735	0.994815
ikzf4	46.23221	-0.104017337	0.319355	-0.32571	0.744643	0.95491
zdhhc14	51.48663	0.449217992	0.301526	1.489814	0.136273	0.591569
tmem242	194.073	0.282710405	0.211271	1.338144	0.18085	0.656785
rps26	6029.906	-0.166687744	0.122769	-1.35773	0.174549	0.648125
si:dkey-60a16.1	133.498	-0.105210751	0.184032	-0.5717	0.567526	0.901809
cfap36	469.849	0.142601385	0.136392	1.045524	0.295781	0.764246
asah1a	226.894	-0.02814744	0.18545	-0.15178	0.879361	0.986147
slc7a2	330.1306	-0.077623401	0.161101	-0.48183	0.629927	0.922869
irs2a	551.1117	-0.204991434	0.185408	-1.10562	0.268889	0.744967
ankrd10a	145.6483	0.130642105	0.233268	0.560051	0.575445	0.904739
mtmr7a	159.1068	0.273663763	0.198695	1.377305	0.168418	0.639712
pfdn6	420.9754	0.130711063	0.143079	0.913557	0.36095	0.811253
pwp2h	386.7527	-0.009031033	0.134275	-0.06726	0.946376	0.997371
cxcl12a	1349.166	-0.073089668	0.180996	-0.40382	0.686345	0.938732
mat2ab	618.2847	-0.109698952	0.457011	-0.24004	0.810303	0.969828
TENM2	409.9061	0.626223838	0.202635	3.090401	0.001999	0.06954
pfkfb1	224.1704	-0.271653191	0.217895	-1.24672	0.212501	0.694155
zgc:153146	38.97151	-0.400581344	0.286756	-1.39694	0.162431	0.629825
cpb2	204.7259	-0.046575382	0.258761	-0.17999	0.857158	0.979645
slc8a4b	91.16935	0.147281072	0.320507	0.459525	0.645857	0.926728
atp6ap1b	564.4023	-0.461178363	0.124677	-3.69898	0.000216	0.015696

hivep3b	206.3143	-0.50003828	0.222504	-2.24732	0.02462	0.278337
si:dkey-78k11.9	3.992134	0.366357696	1.325241	0.276446	0.782205	0.962328
rcc1	234.8756	0.191515091	0.18463	1.037289	0.299601	0.766128
oprd1b	20.89976	-1.564488155	0.730324	-2.14218	0.032179	0.318495
ddrgk1	105.2821	-0.467236135	0.17957	-2.60197	0.009269	0.165455
nek12	21.39767	1.158641785	0.408607	2.835593	0.004574	0.111794
ggh	107.8059	0.033516378	0.208412	0.160818	0.872237	0.983447
zc3h13	914.2651	0.096143424	0.148672	0.646681	0.517838	0.887148
zic2b	254.8273	0.274147825	0.155039	1.768247	0.07702	0.469486
tpbgb	137.4976	-0.174399503	0.184372	-0.94591	0.344195	0.798147
rpa2	520.8568	-0.209602661	0.20018	-1.04707	0.295066	0.763708
ttr	699.6755	-1.492695034	0.979582	-1.52381	0.127557	0.576964
zgc:113364	37.3381	0.658995589	0.323864	2.034791	0.041872	0.357865
tshr	2.840145	0.540370857	1.532861	0.352524	0.724445	0.948381
arid5b	386.8909	0.079891149	0.160227	0.498612	0.618053	0.916507
zgc:101851	135.1957	0.334556098	0.160616	2.082959	0.037255	0.338774
denr	768.457	0.197719398	0.168217	1.175385	0.239841	0.720392
smad5	585.896	-0.027762333	0.138244	-0.20082	0.840838	0.97643
gpr137	124.3951	-0.367472049	0.191672	-1.91719	0.055213	0.406983
trmo	34.07393	0.102403393	0.388132	0.263837	0.791906	0.964842
pimr212	0.043309	0	5.267649	0	1	NA
si:ch211-145b13.5	52.17265	0.159092748	0.373474	0.425981	0.670122	0.933809
ndufb6	1208.779	0.127260249	0.149912	0.848899	0.395938	0.829389
toporsa	108.2842	0.06345851	0.226847	0.279741	0.779676	0.961073
mtap	282.6057	-0.477235617	0.168861	-2.8262	0.00471	0.113805
cdkn2a/b	3.058274	0.093644522	1.851118	0.050588	0.959654	0.999164
myoz2b	308.5783	0.363897985	0.170396	2.135595	0.032712	0.320079
zgc:158263	251.1382	-0.315319728	0.150291	-2.09806	0.0359	0.332937
map9	66.28844	0.174641762	0.218207	0.80035	0.423508	0.843967
lrata	137.6865	-0.021724067	0.24956	-0.08705	0.930632	0.995786
fgg	3453.723	-0.176604987	0.218358	-0.80879	0.418638	0.84177
plrg1	508.5478	-0.02942912	0.103365	-0.28471	0.775865	0.961073
ptges3a	1045.906	0.036190353	0.143912	0.251476	0.801446	0.967776
mipa	975.5144	0.282638733	0.261105	1.082472	0.279043	0.752444
si:dkey-30k22.7	5.198764	-0.094118553	0.870522	-0.10812	0.913903	0.992832
dpf2l	603.024	-0.072286713	0.103796	-0.69643	0.486157	0.873244
chrm1a	1.614954	0.09364889	2.034797	0.046024	0.963291	NA
gnpda1	512.708	-0.00376352	0.206123	-0.01826	0.985433	1
gatb	196.1509	-0.344768882	0.159152	-2.16629	0.030289	0.310203
ddost	1684.133	0.276892992	0.123817	2.236303	0.025332	0.282591
fam131c	29.08507	-0.271220853	0.314917	-0.86125	0.389103	0.827725
klhl17	229.0615	0.13110659	0.18022	0.727482	0.46693	0.865918
wdr92	128.8172	-0.014169565	0.150761	-0.09399	0.92512	0.994815
cnrip1b	116.6368	0.318945274	0.349829	0.911719	0.361917	0.811787
rpl9	112.248	-0.198059712	0.246893	-0.80221	0.422432	0.843493
frs2b	274.1425	-0.001968707	0.155417	-0.01267	0.989893	1
polr3e	704.7678	-0.162425159	0.12603	-1.28878	0.197475	0.678053
si:ch1073-272o11.3	7.622979	1.032080896	0.757945	1.361683	0.173298	0.646172
kdelr2b	908.0912	0.070048453	0.094205	0.743574	0.457134	0.860993
scube2	209.3007	-0.083353934	0.202184	-0.41227	0.680144	0.936798
st5	564.4489	0.021257905	0.145419	0.146184	0.883777	0.987014
ephb2a	48.97068	0.341720119	0.300714	1.136361	0.255806	0.733642
qdprb1	175.3275	-0.08548777	0.196968	-0.43402	0.664275	0.93177
pax5	12.17369	-0.395693059	0.414336	-0.955	0.339575	0.795226

gsg1l	59.19304	-0.529952611	0.428975	-1.23539	0.216684	0.69815
med19a	579.9345	-0.189835867	0.146963	-1.29173	0.196451	0.67688
slc43a1a	303.0073	0.004979419	0.226907	0.021945	0.982492	1
ssrp1a	765.0974	0.076039967	0.167472	0.454045	0.649797	0.927756
zgc:113229	24.5487	-0.027790318	0.388878	-0.07146	0.943029	0.997371
lim2.3	281.2991	0.493414509	0.349392	1.412211	0.157888	0.623766
si:dkey-4p15.3	55.11334	0.688535261	0.319977	2.151827	0.031411	0.315297
zgc:112083	102.8151	-0.016814071	0.195991	-0.08579	0.931633	0.995827
wdr19	137.8557	0.002610868	0.205751	0.012689	0.989876	1
tbc1d10c	17.85821	-0.83624836	0.462945	-1.80637	0.070861	0.454179
tmem144a	115.989	0.0329629	0.208822	0.157852	0.874574	0.984095
med28	232.3169	0.004879098	0.140869	0.034636	0.97237	1
npy1r	4.69355	1.30455653	1.161678	1.122993	0.26144	0.738975
dlgap1b	23.4373	0.336608412	0.372584	0.903444	0.366291	0.81589
egr1	546.7751	-0.113105842	0.518487	-0.21815	0.827315	0.973459
smim19	427.8065	-0.018592518	0.167412	-0.11106	0.91157	0.992326
s100a10a	697.0757	-0.416015377	0.253805	-1.63911	0.10119	0.526828
si:ch211-39a7.1	12.25863	-1.899533076	0.625325	-3.03767	0.002384	0.078183
tll1	53.207	0.200827069	0.340724	0.589412	0.555585	0.898723
haus7	132.9369	0.230608524	0.186192	1.238551	0.215512	0.697157
fmr1	599.1498	-0.204715968	0.122725	-1.66809	0.095297	0.515296
ugt8	246.1367	-0.237666486	0.139665	-1.70169	0.088814	0.500363
cd79a	4.678934	0.710460341	1.588502	0.447252	0.654693	0.929527
foxb2	12.01143	1.695967205	0.788427	2.151077	0.03147	0.315382
sorbs3	315.7803	-0.180773309	0.150111	-1.20427	0.228487	0.70974
tmem192	169.5505	-0.141759634	0.167334	-0.84716	0.396904	0.830253
1-Mar	20.89798	0.97958673	0.612261	1.599949	0.10961	0.543127
canx	3417.834	0.130136353	0.110667	1.175929	0.239623	0.720113
bmp15	2.673184	-1.215790181	1.448755	-0.8392	0.401359	0.832371
rtn4rl2b	54.5497	0.573008163	0.492995	1.162301	0.245113	0.726529
gria4a	181.0834	0.318889519	0.317581	1.004119	0.315321	0.777834
gria3b	245.4827	-0.011020021	0.272454	-0.04045	0.967737	1
aasdhppt	72.56484	-0.131743519	0.251934	-0.52293	0.601023	0.913756
thoc2	1188.133	-0.045535526	0.104539	-0.43558	0.663138	0.931207
prps1b	328.755	0.137162452	0.136257	1.006642	0.314107	0.777479
kctd12b	17.95553	0.582514852	0.58404	0.997388	0.318576	0.779923
hdac3	559.6135	-0.022274685	0.129418	-0.17211	0.863348	0.981732
slc35c2	914.3835	0.283198993	0.124225	2.27972	0.022624	0.265959
CR749762.1	0.716385	0	2.643398	0	1	NA
si:rp71-17i16.5	3.722966	0	1.887477	0	1	1
si:ch211-191a24.4	139.1068	-0.108828407	0.212268	-0.51269	0.608165	0.915204
mcm3l	8.881113	-0.450239337	0.611415	-0.73639	0.461494	0.863229
mep1b	37.4019	-1.763478544	1.597458	-1.10393	0.269624	0.746036
ccnq	146.4956	0.069113027	0.144912	0.476932	0.633411	0.923885
tnnc1b	4378.272	0.175865629	0.123117	1.428443	0.153164	0.617769
mtnr1aa	11.27342	0.039843698	0.63938	0.062316	0.950311	0.99801
cyp21a2	31.47722	-0.439192062	0.384453	-1.14238	0.253295	0.732759
pm20d1.1	85.31808	0.249469658	0.527806	0.472654	0.63646	0.924779
il1rapl2	28.68392	0.224511727	0.698115	0.321597	0.747758	0.955074
arhgef3l	54.73637	0.248203626	0.256132	0.969047	0.332522	0.789726
atoh8	626.0467	0.046726672	0.126171	0.370344	0.711126	0.945821
st3gal5	375.845	-0.138422902	0.148277	-0.93354	0.350541	0.802215
asb12b	51.28914	0.274711761	0.270026	1.017352	0.308986	0.773791
uba1	3857.255	0.112824375	0.126023	0.895267	0.370645	0.818484

mtm1	215.61	0.062847555	0.141573	0.443924	0.657098	0.929939
polr1d	136.0167	-0.173021206	0.185899	-0.93073	0.351996	0.802591
rps6ka1	699.5269	-0.150048459	0.125814	-1.19262	0.233018	0.714569
BX530018.1	3.149523	-1.089753351	1.038971	-1.04888	0.294235	0.763323
SYNPR	192.0926	-0.437222699	0.303968	-1.43838	0.150325	0.613966
bhlhe23	219.6164	0.361761091	0.277956	1.301506	0.193085	0.67315
gid8b	475.9551	0.172152622	0.128089	1.344008	0.178946	0.653868
prickle2b	351.5775	-0.089824286	0.18916	-0.47486	0.634888	0.924272
prf1.5	47.32899	0.020271658	0.347855	0.058276	0.953529	0.998436
zgc:162144	17.99482	-0.718453396	0.438051	-1.64011	0.100982	0.526541
nol4lb	47.24288	0.082134827	0.271737	0.302258	0.762455	0.958551
lgals8b	36.1666	-0.030999154	0.425581	-0.07284	0.941934	0.997371
ddit4	608.2349	-0.169863827	0.597259	-0.28441	0.7761	0.961073
BX569798.1	7.781589	0.74554255	0.744755	1.001058	0.316799	0.778879
trpt1	209.432	0.29658107	0.194099	1.527992	0.126514	0.575111
leng1	154.637	-0.016397708	0.161554	-0.1015	0.919154	0.993892
nkx3-2	250.9986	0.295563703	0.157932	1.871457	0.061282	0.424867
aurkb	564.286	0.285544342	0.241237	1.18367	0.236544	0.717725
zgc:173570	65.60863	0.154596305	0.262717	0.588452	0.556229	0.899002
rgs11	100.1157	0.00059983	0.292297	0.002052	0.998363	1
rnf175	383.221	-0.022187483	0.16784	-0.13219	0.89483	0.989112
zgc:56409	100.7198	-0.097790765	0.19195	-0.50946	0.610431	0.915331
pmm2	233.0807	0.026582128	0.150509	0.176615	0.85981	0.980066
pls3	2425.946	0.086170281	0.113176	0.761382	0.446429	0.855403
npm1b	325.1399	0.020280738	0.132177	0.153436	0.878054	0.98573
fgf24	119.5449	-0.216607333	0.224371	-0.9654	0.334345	0.791508
zgc:103678	317.572	-0.411855465	0.199504	-2.06439	0.03898	0.345405
pcdh2ab2	0.274427	0.093651644	4.79789	0.019519	0.984427	NA
pcdh2aa1	0.251206	-2.340119894	5.201853	-0.44986	0.652809	NA
gss	67.37018	-0.230713622	0.282803	-0.81581	0.414609	0.838683
exosc10	477.3077	-0.138372948	0.115649	-1.19649	0.231504	0.713101
pcnp	587.4867	-0.074911037	0.135937	-0.55107	0.581585	0.905618
fbxl31	45.91801	-0.918535844	0.786921	-1.16725	0.243108	0.724396
zgc:112980	170.603	-0.097671175	0.16665	-0.58609	0.557817	0.899184
actb1	52277.38	0.074831708	0.126388	0.59208	0.553797	0.89821
fscn1b	171.9649	0.407855072	0.281031	1.45128	0.146702	0.606437
slc43a1b	231.9832	0.170112215	0.207706	0.819003	0.412785	0.838123
tlr2	1.524455	-0.205564011	1.579156	-0.13017	0.896429	NA
uncx4.1	312.6505	0.289800888	0.130065	2.228131	0.025872	0.285598
rybpa	434.8561	0.112632189	0.130621	0.862285	0.388531	0.827613
stk16	201.9809	-0.103729956	0.127343	-0.81457	0.415318	0.838754
acss2	715.7475	-0.03187094	0.176799	-0.18027	0.856943	0.979558
sox8b	15.18197	1.127895201	0.534737	2.109254	0.034923	0.329194
proza	1454.881	-0.14892623	0.117014	-1.27272	0.203117	0.683943
pvalb1	225411.1	0.007282365	0.174014	0.041849	0.966619	1
pvalb8	3280.171	-0.026080801	0.204646	-0.12744	0.898589	0.990199
nptx2a	12.08404	1.212142866	0.747111	1.622439	0.104709	0.53308
phlda3	158.8802	-0.286786065	0.225377	-1.27247	0.203206	0.684044
lgals3bpa	17.12375	-4.075494256	1.332494	-3.05855	0.002224	0.074795
adtrp1	100.6739	-0.530276023	0.202768	-2.61519	0.008918	0.161876
zgc:113278	31.74784	-0.044902783	0.385737	-0.11641	0.907329	0.991745
b4galt5	7.912991	0.322122788	0.68442	0.470651	0.63789	0.924779
mitfb	20.89252	0.206505695	0.450138	0.458761	0.646406	0.926728
polr3k	178.7728	-0.163284255	0.193816	-0.84247	0.399524	0.831196

igfals	13.03451	2.52961685	1.54008	1.642523	0.100482	0.525188
ogfr	459.6877	-0.270457727	0.146485	-1.84632	0.064846	0.434744
unkl	651.2721	-0.243436951	0.159454	-1.52669	0.126838	0.575696
col9a3	5835.337	0.401726492	0.185767	2.162524	0.030578	0.311697
hml3	827.3506	0.190569233	0.104183	1.829179	0.067373	0.443222
zgc:101663	280.2137	0.034218746	0.183389	0.186591	0.851981	0.978558
taf11	456.7328	0.069662724	0.135878	0.512684	0.608172	0.915204
il11a	11.83407	1.573682017	0.875291	1.797896	0.072194	0.456904
cox6b2	7145.343	0.018545541	0.129803	0.142875	0.886389	0.987533
slc2a3b	85.00757	0.600292589	0.263734	2.276126	0.022838	0.266968
cln3	306.9376	-0.19068747	0.155359	-1.2274	0.219674	0.700598
atp5mf	3395.675	-0.03219825	0.161551	-0.19931	0.842023	0.97643
actb2	77937.75	-0.127677752	0.134985	-0.94586	0.344218	0.798147
wipi2	1053.683	-0.131799056	0.115504	-1.14108	0.253835	0.732873
foxk1	550.7769	-0.17881523	0.15908	-1.12406	0.260989	0.738533
cyp3c3	103.3908	1.072494352	0.574826	1.865772	0.062073	0.42715
cyp3c3	11.01858	0.905522829	0.851865	1.062989	0.287787	0.758441
lfng	1467.864	0.059734396	0.130839	0.456549	0.647995	0.927245
prcp	184.5092	-0.501889739	0.22459	-2.23469	0.025438	0.283498
rab30	123.3727	0.066910618	0.170455	0.392541	0.694659	0.941093
wnt9b	244.6241	-0.142971039	0.198975	-0.71854	0.472426	0.868786
mlx	430.8119	0.020529377	0.122304	0.167855	0.866697	0.982582
psmc3ip	50.29808	0.119955041	0.294537	0.407266	0.683812	0.938486
ezh1	175.7386	-0.559330372	0.238177	-2.34838	0.018855	0.242305
ramp2	297.5472	0.023491433	0.140515	0.167181	0.867228	0.982582
cacna1aa	192.807	0.005486741	0.21456	0.025572	0.979599	1
alkbh7	144.2233	0.029568501	0.184134	0.160581	0.872423	0.983447
timm29	258.4224	-0.137703732	0.148155	-0.92946	0.352651	0.803513
nmrk1	23.25255	-0.310936279	0.327636	-0.94903	0.342606	0.797189
znf11	1.099459	-0.935203979	1.740356	-0.53736	0.591016	NA
cdk5r1a	50.37161	-0.515930022	0.386429	-1.33512	0.181837	0.658469
itga3a	71.56381	-0.059713675	0.31642	-0.18872	0.850315	0.978251
rbbp6	800.3659	-0.136404167	0.151949	-0.8977	0.369346	0.817763
gng13b	687.35	0.170091177	0.247851	0.686265	0.492546	0.876385
gna13b	242.0568	-0.313003227	0.157344	-1.98929	0.046669	0.376436
rgs9a	84.53267	0.313331137	0.855291	0.366344	0.714108	0.945821
ddx42	674.2384	-0.038739654	0.097705	-0.3965	0.69174	0.940256
strada	256.9044	0.078023332	0.174696	0.446625	0.655146	0.929606
rnf113a	323.9856	-0.02736627	0.144486	-0.1894	0.849776	0.97824
lin7b	508.1391	0.116939096	0.179328	0.652097	0.514338	0.88631
aldh16a1	270.2867	-0.352421514	0.148448	-2.37404	0.017595	0.234077
syt5a	261.8958	0.202181884	0.296088	0.682843	0.494706	0.878016
cpt1cb	99.35862	0.259156912	0.225192	1.150825	0.249804	0.729721
prl	41.16561	0.020544482	0.289278	0.07102	0.943382	0.997371
tnnt1	172.2982	0.71561535	0.224566	3.18666	0.001439	0.05681
pih1d1	94.68401	-0.063528147	0.198132	-0.32064	0.748487	0.955074
nosip	435.2626	-0.062461492	0.144645	-0.43183	0.665867	0.932664
lrrc17	889.7751	-0.032226683	0.089482	-0.36015	0.718738	0.94694
rcn3	964.1138	0.216436619	0.152163	1.422399	0.154911	0.619752
psmb7	2574.398	0.028207218	0.145043	0.194475	0.845804	0.977039
nek6	181.0164	-0.429159682	0.18933	-2.26673	0.023407	0.270489
lhx2a	7.318831	1.683991929	0.629323	2.67588	0.007453	0.146147
srrm2	775.1548	-0.029954572	0.131439	-0.2279	0.819726	0.971785
thoc6	134.2686	-0.131627686	0.149392	-0.88109	0.378269	0.822672

si:dkey-16l2.16	325.4625	-0.442744369	0.132881	-3.3319	0.000863	0.039733
fus	1609.374	0.078102914	0.151831	0.514407	0.606968	0.915204
elob	3456.766	0.049935859	0.12613	0.395909	0.692172	0.940333
gdf3	41.70697	-0.150086001	0.450782	-0.33295	0.739175	0.953422
tubb5	4652.927	0.253529207	0.192257	1.318701	0.187269	0.66594
flot1b	3821.655	-0.172300219	0.088105	-1.95563	0.050509	0.391012
crls1	391.4329	0.027425323	0.150738	0.18194	0.855629	0.979493
med25	364.7699	-0.026960162	0.144108	-0.18708	0.851596	0.978548
osr2	147.9192	-0.272406897	0.219606	-1.24043	0.214815	0.696864
CT573429.1	4.263338	-0.483219635	1.504977	-0.32108	0.748149	0.955074
rac2	645.95	0.17036536	0.123782	1.376329	0.16872	0.640124
cyth4a	21.47346	0.620127836	0.479596	1.29302	0.196004	0.676453
fam83fa	55.1981	0.096050462	0.273298	0.351449	0.725251	0.948653
prph2a	924.1618	-1.491262922	1.192189	-1.25086	0.210985	0.69272
flr	230.1883	-0.016808618	0.131788	-0.12754	0.898511	0.990199
cbx7a	487.1587	-0.585941168	0.275523	-2.12665	0.033449	0.323151
nol12	228.8301	-0.150653312	0.186919	-0.80598	0.420252	0.842383
ndufa6	2025.376	0.069030342	0.14656	0.471004	0.637638	0.924779
rbx1	1190.313	0.016227116	0.158419	0.102432	0.918414	0.993884
tpk1	132.6112	0.441815694	0.168628	2.620062	0.008791	0.160701
si:ch211-149k23.9	3.968863	0.217050283	1.157325	0.187545	0.851234	0.978548
fgfbp2b	748.3981	0.006627059	0.242755	0.027299	0.978221	1
grb2b	910.1324	-0.142700502	0.105298	-1.3552	0.175353	0.649279
chmp3	248.3449	-0.123321002	0.15483	-0.79649	0.425745	0.844714
kpna2	1268.487	0.095100548	0.272113	0.34949	0.726722	0.949095
smurf2	383.3474	0.306452116	0.135715	2.258055	0.023942	0.274217
ddx5	7009.949	0.101451261	0.118507	0.856079	0.391954	0.827902
mgat3a	23.89852	-1.065696985	0.476471	-2.23665	0.025309	0.282476
ptx4	2.533769	-0.47138272	1.286243	-0.36648	0.714007	0.945821
ergic3	244.4592	0.103470731	0.135225	0.765173	0.444168	0.854431
cyc1	4962.034	0.017169974	0.089086	0.192735	0.847166	0.977337
romo1	2783.887	-0.126140096	0.113307	-1.11326	0.265595	0.742247
TMEM114	7.744159	0.547011314	0.942483	0.580394	0.561649	0.900673
clec16a	461.954	-0.157095902	0.127367	-1.23341	0.217423	0.69815
socs1a	132.6684	-0.392054572	0.662449	-0.59183	0.553967	0.89821
pigq	261.7941	0.144648211	0.139666	1.03567	0.300356	0.766629
gskip	866.0562	-0.125767665	0.125759	-1.00007	0.317276	0.779151
slc37a4a	352.6836	0.184282277	0.19313	0.954188	0.339989	0.795302
sgcg	729.2147	-0.106235449	0.172429	-0.61611	0.537822	0.892498
si:dkeyp-59c12.1	15.3517	-0.14014151	0.451554	-0.31035	0.756292	0.956532
rprd1b	419.1244	-0.124358016	0.129224	-0.96234	0.335877	0.792139
xpa	70.81537	0.164434392	0.202234	0.813091	0.416166	0.839647
gnrhr4	2.046421	-1.08605802	1.765723	-0.61508	0.538503	NA
si:ch211-13k12.2	93.82966	-0.201625536	0.204581	-0.98555	0.324353	0.783308
myl9a	741.5316	0.535700996	0.152846	3.504853	0.000457	0.025945
zgc:113411	81.85041	0.1600134	0.202044	0.791972	0.428377	0.846133
pdgfbb	84.94467	-0.438981386	0.288304	-1.52263	0.12785	0.577117
atf4b	3384.71	-0.026467413	0.138399	-0.19124	0.848337	0.977912
hbbe3	5315.295	0.247542688	0.458658	0.539711	0.589397	0.908456
zgc:92360	657.2165	0.008299116	0.132333	0.062714	0.949994	0.99801
lgals2b	3036.586	-0.649925037	0.199309	-3.26089	0.001111	0.047149
isca2	403.2767	-0.106468959	0.159421	-0.66785	0.50423	0.881803
dusp11	280.808	-0.005022703	0.176361	-0.02848	0.97728	1
tcf7l1a	574.7651	0.011775486	0.136976	0.085968	0.931492	0.995809

hyal6	23.36014	2.941898542	1.411722	2.083908	0.037169	0.338153
cyb561	15.30866	0.103293549	0.57316	0.180218	0.856982	0.979558
gh1	243.5008	0.590336981	0.484374	1.218764	0.222934	0.704786
zgc:103564	29.68187	0.269658241	0.343734	0.784497	0.432748	0.848864
smtla	1.577464	-1.795861729	2.061369	-0.8712	0.383646	NA
cdab	339.6548	0.093836712	1.050227	0.089349	0.928805	0.995163
her2	109.3566	0.189560358	0.237823	0.797066	0.425413	0.844714
aldh4a1	566.2976	-0.262238765	0.138835	-1.88885	0.058912	0.418455
slc35b1	466.1118	0.06763497	0.128212	0.527524	0.59783	0.912437
tyw3	88.20334	-0.24361379	0.222325	-1.09575	0.273187	0.749148
nras	471.8839	-0.039766686	0.161236	-0.24664	0.80519	0.969348
CABZ01074994.1	0.952448	-0.54965283	1.924292	-0.28564	0.775155	NA
selenow2a	3054.785	0.030135409	0.135032	0.223173	0.823401	0.972005
pkdccb	95.98722	0.021339804	0.258443	0.082571	0.934193	0.996696
tnpo2	1493.398	0.074778007	0.111663	0.669678	0.503063	0.881598
zgc:113333	222.7812	0.088762516	0.231986	0.38262	0.702001	0.943292
e4f1	248.0884	-0.014845448	0.175686	-0.0845	0.932659	0.996197
ggact.2	397.2244	0.995788034	0.261818	3.803359	0.000143	0.01194
proca	87.35098	0.218565332	0.330341	0.661635	0.508205	0.883089
PIGG	79.66082	0.226229127	0.260562	0.868235	0.385266	0.826939
zgc:113293	2.602003	0.13677272	1.850656	0.073905	0.941086	0.997371
ptgir	19.44663	0.229748162	0.407268	0.564121	0.572672	0.903562
natd1	872.725	-0.087405518	0.1015	-0.86114	0.389163	0.827725
wnt6a	18.89081	0.08966975	0.595207	0.150653	0.880249	0.986408
cd151	320.3035	-0.19666469	0.200929	-0.97878	0.32769	0.786438
thoc5	749.2819	0.014609385	0.125193	0.116695	0.907101	0.991745
zgc:103559	52.2832	-2.813724261	0.662507	-4.24709	2.17E-05	0.003005
tmem86b	255.6098	-1.00138015	0.28493	-3.51448	0.000441	0.025327
aspdh	41.4612	-1.243955995	0.434716	-2.86154	0.004216	0.107344
rnf34a	135.3778	0.00228086	0.1678	0.013593	0.989155	1
ccar2	287.3208	0.136959766	0.148577	0.921809	0.356628	0.807342
vgll3	26.44399	0.100420039	0.395625	0.253826	0.79963	0.967206
tsc22d1	1579.027	-0.002908486	0.115225	-0.02524	0.979862	1
ykt6	298.6055	0.120568992	0.14698	0.82031	0.412039	0.838123
dnajc15	348.877	0.207020516	0.191462	1.081262	0.27958	0.752477
rcl1	414.734	-0.083367613	0.221051	-0.37714	0.706068	0.944549
zgc:172053	9.936633	-0.808552373	1.269575	-0.63687	0.524211	0.888801
pigh	309.7993	-0.260730727	0.151717	-1.71854	0.085699	0.491277
slc27a1b	0.069091	0	5.267649	0	1	NA
enosf1	397.8363	-0.50244449	0.173687	-2.89282	0.003818	0.10304
thgl1	101.5719	0.095504513	0.217846	0.438403	0.661094	0.930581
drd4a	39.39343	-0.844466755	0.637962	-1.32369	0.185605	0.663476
sagb	1334.708	-0.651278458	0.451406	-1.44278	0.149084	0.610726
th2	11.41957	0.95091324	0.829206	1.146776	0.251474	0.731717
ascl1a	287.5873	0.126758405	0.147165	0.861337	0.389053	0.827725
si:dkeyp-52c3.7	0.900838	-3.078020265	2.66545	-1.15478	0.248179	NA
rpap2	110.5526	-0.282266346	0.206308	-1.36818	0.171256	0.643325
pmm1	15.14726	-0.740329383	0.559077	-1.3242	0.185437	0.663256
zgc:153921	5.727248	-0.510904406	0.915223	-0.55823	0.576688	0.904769
b3galt6	187.5423	-0.011173341	0.199728	-0.05594	0.955387	0.998611
entpd4	226.0285	0.505385862	0.200538	2.520151	0.01173	0.188354
c4b	224.0896	-0.418290271	0.320811	-1.30385	0.192284	0.672283
sulf1	557.963	0.184736762	0.156962	1.176952	0.239215	0.719781
csrnp1b	340.9592	-0.05904959	0.206316	-0.28621	0.774717	0.96088

actr10	998.9577	0.027502975	0.118247	0.232589	0.81608	0.971207
fabp10a	5182.642	-1.91335881	0.447034	-4.28012	1.87E-05	0.002746
plcg1	1047.935	0.017283983	0.117274	0.147381	0.882831	0.986908
spire1b	21.54733	-0.619162082	0.489049	-1.26605	0.205494	0.68775
nrp2b	866.0406	0.20773637	0.128661	1.614602	0.106397	0.535773
mrps35	656.6909	-0.103624031	0.149317	-0.69398	0.487692	0.873291
stmn3	288.8731	0.388284975	0.242292	1.602547	0.109035	0.541807
igsf8	161.3873	0.211222173	0.237773	0.888334	0.374361	0.820992
ube2d3	1440.887	0.123291	0.106415	1.158589	0.246624	0.727706
acy1	919.9413	-0.47266871	0.202556	-2.33352	0.019621	0.246937
si:dkeyp-68b7.7	104.7755	-0.061671079	0.192696	-0.32004	0.748936	0.955074
b3gnt7	150.491	0.095901345	0.250672	0.382578	0.702033	0.943292
cep41	83.55557	0.521535536	0.275325	1.894256	0.058191	0.416652
polr2h	220.3782	0.119554397	0.178452	0.669951	0.502889	0.881453
chrnb3b	14.47429	0.551286482	0.63906	0.862652	0.388329	0.827592
snap29	455.2205	-0.062604684	0.118126	-0.52998	0.596125	0.911517
pik3r1	1906.265	-0.09398426	0.151671	-0.61966	0.535482	0.891993
gga1	544.7321	-0.048158306	0.109057	-0.44159	0.658788	0.930581
ccr12a	30.36512	-0.18918191	0.513577	-0.36836	0.712604	0.945821
wdr48b	221.3156	-0.089012393	0.153527	-0.57978	0.562061	0.900715
BX890608.1	1.173188	0.09365102	2.1842	0.042877	0.9658	NA
zgc:112185	67.31665	0.217856339	0.278724	0.781619	0.434438	0.850002
anks3	132.7099	0.363882809	0.226149	1.609042	0.107607	0.538826
h1f0	11860.22	-0.129929315	0.191022	-0.68018	0.49639	0.878161
klf6b	42.71511	-0.499650494	0.383246	-1.30373	0.192324	0.672283
pitrm1	581.7721	-0.011442414	0.137154	-0.08343	0.933512	0.996474
gnptg	264.7199	-0.546290639	0.19717	-2.77066	0.005594	0.126154
tsr3	275.4305	0.066081772	0.185456	0.35632	0.721601	0.947343
fzd8b	44.13947	-0.124326477	0.272501	-0.45624	0.648216	0.927337
BX548047.1	1.464219	0	4.388294	0	1	NA
scg2b	1043.294	0.330558827	0.244923	1.349646	0.17713	0.650742
ube2d1b	950.8313	0.059846194	0.14256	0.419797	0.674634	0.934877
cox6c	3514.172	0.23156779	0.158676	1.459373	0.144463	0.603539
abraa	584.161	-0.221903594	0.28082	-0.7902	0.429412	0.846956
eif4e2	240.9526	0.09127057	0.220059	0.414754	0.678322	0.936026
CU929150.1	4.957503	0.626381615	1.102139	0.568333	0.569809	0.902322
rspo4	15.5508	0.373644457	0.478347	0.781116	0.434734	0.850002
sdhc	1828.581	-0.056187332	0.158859	-0.35369	0.723569	0.947923
mpz	159.6682	-0.320494995	0.431479	-0.74278	0.457613	0.860993
pih1d3	21.78342	0.460525429	0.387173	1.189456	0.23426	0.714938
pip4p1a	242.7655	-0.151326172	0.145849	-1.03756	0.299476	0.766128
cpt2	308.9074	-0.056739226	0.196837	-0.28825	0.773152	0.960624
magoh	1003.881	-0.060664575	0.154973	-0.39145	0.695464	0.9414
elovl6l	246.8437	-0.214506403	0.287951	-0.74494	0.456307	0.860403
alas2	2219.113	-0.38054628	0.239374	-1.58975	0.11189	0.548216
ftr11	1.051771	0.09364454	2.400524	0.03901	0.968882	NA
ajap1	44.48441	-0.000258727	0.385672	-0.00067	0.999465	1
cavin4a	1454.97	0.183700131	0.169358	1.084686	0.278061	0.751959
igfbp1b	855.6002	-1.569459892	0.557707	-2.81413	0.004891	0.116469
fggy	198.7597	-0.866669535	0.300515	-2.88395	0.003927	0.104111
gbp1	89.19895	0.661529541	0.343648	1.925021	0.054227	0.402725
gbp2	84.00682	-0.725232537	0.33627	-2.1567	0.031029	0.313376
tcf7	104.7594	-0.153072548	0.192911	-0.79349	0.427495	0.845893
MFAP4 (1 of many)	6.19438	-0.910078182	0.981246	-0.92747	0.353682	0.804596

zgc:171687	6.301787	0.201405043	0.77641	0.259406	0.795322	0.965909
si:zfos-2330d3.1	19.58457	-0.191417299	0.517577	-0.36983	0.711507	0.945821
si:ch211-134m17.9	101.8137	-0.106820717	0.244061	-0.43768	0.661618	0.930742
nfkb2	571.9681	-0.06579446	0.148673	-0.44255	0.658095	0.930581
gng7	343.2901	0.017411133	0.152279	0.114337	0.90897	0.992033
zgc:101744	1140.335	-0.43108642	0.157258	-2.74127	0.00612	0.131913
elavl1a	2286.895	0.000567558	0.084173	0.006743	0.99462	1
gpr160	28.79797	-0.375689783	0.330375	-1.13716	0.255471	0.733544
hkdc1	113.5381	0.192704914	0.243838	0.790298	0.429354	0.846956
si:dkeyp-66d1.7	60.22671	-0.550681869	0.269462	-2.04363	0.04099	0.353808
tep1	43.86889	-0.014817059	0.300434	-0.04932	0.960665	0.999243
casq1a	1839.749	-0.089891398	0.233191	-0.38548	0.699879	0.942583
zp3f.1	2.225959	-0.537129359	1.370103	-0.39204	0.695032	0.941197
ch25hl2	22.63284	-0.541431222	0.317859	-1.70337	0.088499	0.499597
s100z	102.6514	0.202598954	0.22244	0.910804	0.362399	0.812303
slc25a36a	732.9815	-0.325870863	0.162637	-2.00367	0.045105	0.372051
phf20b	587.2535	0.03536165	0.133405	0.265069	0.790956	0.964766
rbp1.2	85.73134	0	1.688376	0	1	1
copb2	3063.404	0.013979828	0.110787	0.126187	0.899584	0.99045
plk3	749.0304	0.050634496	0.163313	0.310045	0.756527	0.956547
mrpl12	876.3363	0.126771467	0.145842	0.869236	0.384718	0.826588
zgc:103625	41.37768	0.352265171	0.347193	1.014609	0.310292	0.775062
ubtfl	757.7375	0.013701521	0.123712	0.110754	0.911812	0.992336
faim2a	111.0978	0.026832492	0.201112	0.13342	0.893861	0.988675
abcf2a	779.2212	0.093884624	0.168085	0.558556	0.576465	0.90475
smarcd3a	308.1284	0.197182638	0.142973	1.379163	0.167845	0.639036
tmub1	511.9601	-0.221464027	0.148551	-1.49083	0.136006	0.591131
dnai1.2	42.43491	0.269907592	0.272899	0.989038	0.322645	0.782525
tmem53	115.5424	-0.2127786	0.183856	-1.15731	0.247146	0.727871
klf17	120.861	-0.538808882	0.208448	-2.58486	0.009742	0.170247
ccdc24	11.53282	0.020977508	0.57727	0.036339	0.971012	1
zgc:113531	234.2186	0.151123422	0.254941	0.592779	0.553329	0.89793
klhl20	290.388	-0.10482266	0.13794	-0.75991	0.447306	0.856073
cenpl	74.32047	0.13603081	0.278953	0.487648	0.625799	0.920625
pigp	313.1737	1.160147746	0.301658	3.845901	0.00012	0.010622
e2f5	28.30709	-0.02566738	0.282674	-0.0908	0.92765	0.995016
myrip	270.5284	0.088834701	0.252166	0.352286	0.724623	0.9484
mrc1b	150.9977	-0.221089516	0.176582	-1.25205	0.210552	0.692339
jph1b	2891.13	-0.083745206	0.185394	-0.45171	0.651475	0.928562
etfdh	1364.425	-0.195967637	0.147607	-1.32763	0.184301	0.661379
ppid	925.5415	-0.10451928	0.125352	-0.83381	0.40439	0.833939
ldhd	162.0663	0.16939574	0.211671	0.800278	0.42355	0.843967
gins3	101.563	0.014617275	0.187363	0.078016	0.937815	0.997305
chmp5a	72.94106	0.436271212	0.338446	1.289044	0.197383	0.677836
setd7	691.9572	0.129447138	0.120313	1.07592	0.281963	0.754378
rgs20	215.636	0.119018011	0.199487	0.59662	0.550761	0.8974
kcnb2	160.0095	-0.7844057	0.255163	-3.07414	0.002111	0.072145
babam2	318.2664	-0.213203021	0.136243	-1.56488	0.117612	0.559331
acox3	223.7605	0.477071279	0.179728	2.65441	0.007945	0.150851
trappc8	388.0195	-0.043841901	0.128355	-0.34157	0.732676	0.950617
shhb	100.3492	0.186747807	0.303111	0.616104	0.537826	0.892498
en2b	129.8015	-0.009222886	0.218053	-0.0423	0.966262	1
htr5aa	2.997314	-1.298079608	1.403493	-0.92489	0.355022	0.80622
slu7	839.524	-0.207961486	0.114557	-1.81536	0.069469	0.45

zgc:101569	344.2525	-0.166264073	0.181866	-0.91421	0.360605	0.811237
il12a	1.292904	-2.306308306	2.433808	-0.94761	0.343326	NA
ift80	276.6252	-0.063266015	0.135023	-0.46856	0.639386	0.924779
acaa2	1342.038	-0.145853847	0.202603	-0.7199	0.471586	0.86809
smc4	694.866	0.240310399	0.241253	0.996091	0.319206	0.779923
AL954146.1	58.47031	-2.047566541	0.749371	-2.73238	0.006288	0.134409
tmx3a	198.3768	0.363069591	0.368236	0.985969	0.324148	0.783161
zgc:113691	9.442673	-1.014666227	0.606839	-1.67205	0.094514	0.513368
acadm	3298.616	0.146854188	0.154012	0.953522	0.340326	0.795464
tmem256	264.2087	0.268715107	0.204728	1.312545	0.189336	0.668942
hectd3	878.6617	-0.106086145	0.111845	-0.94851	0.34287	0.79738
msl2a	272.4785	0.270791216	0.157016	1.724613	0.084597	0.488321
pccb	1167.736	-0.003106663	0.137011	-0.02267	0.98191	1
rad50	133.2171	0.078732788	0.161143	0.48859	0.625132	0.920398
drd1b	14.71283	0.296897757	0.588352	0.504626	0.613822	0.915998
itprid2	305.6474	-0.03566886	0.176681	-0.20188	0.840008	0.976417
ppp1r1c	107.2062	-0.179458828	0.275941	-0.65035	0.515464	0.886401
ctnnbip1	916.0146	0.072190381	0.107702	0.670279	0.50268	0.881453
pimr68	10.78418	0.706262346	0.634903	1.112394	0.265969	0.742773
fdx1b	18.62269	0.290336036	0.375439	0.773324	0.439331	0.852079
flvcr2a	19.36057	0.713939804	0.50353	1.41787	0.156229	0.621949
traf4b	127.2684	-0.135922357	0.166865	-0.81456	0.415322	0.838754
cul3a	1134.289	0.098286155	0.095167	1.032774	0.30171	0.767693
ccr6b	5.415364	1.396281303	1.074969	1.298904	0.193977	0.674122
zgc:113142	41.79173	-0.648887782	0.560829	-1.15702	0.247266	0.727871
DPYSL2 (1 of many)	99.38582	0.572210606	0.262431	2.180423	0.029226	0.304273
pdcd7	188.453	-0.122211912	0.171169	-0.71398	0.475238	0.869292
slc25a24l	2.803945	-1.357234775	1.286871	-1.05468	0.291573	0.761454
dnajb4	151.9146	0.401137888	0.215894	1.858033	0.063164	0.429313
si:dkey-24c2.9	14.30018	-0.568448369	0.476985	-1.19175	0.233358	0.714569
txndc12	677.2656	0.090136586	0.14455	0.623568	0.532911	0.890978
zgc:153615	294.8684	0.259146699	0.226761	1.142819	0.253114	0.732759
cdyl	140.8476	-0.245167648	0.193872	-1.26458	0.206021	0.688321
si:dkey-31m5.5	0.04222	0	5.267649	0	1	NA
wnt3	47.95834	0.024555496	0.327778	0.074915	0.940282	0.997371
nsfb	335.7433	0.03747321	0.115242	0.325169	0.745053	0.954956
egln1a	45.04496	0.131287137	0.313982	0.418136	0.675848	0.935422
trappc1	494.6138	0.001929906	0.18229	0.010587	0.991553	1
eno3	19578.81	0.028033788	0.14697	0.190745	0.848725	0.977918
zgc:85858	413.7645	-0.04178043	0.146559	-0.28508	0.775587	0.961073
fbxo5	182.0054	0.446803055	0.212479	2.102814	0.035482	0.330833
stk25b	950.2497	0.132387445	0.11667	1.13472	0.256493	0.73401
marcksl1a	5824.044	0.255627846	0.137673	1.856777	0.063343	0.429516
sfrp5	248.7993	-0.197093821	0.159491	-1.23576	0.216546	0.69815
hhatla	5856.297	0.16822773	0.170009	0.989522	0.322408	0.78246
klhl40a	121.8484	0.204643884	0.268573	0.761969	0.446079	0.855403
morn4	152.6453	0.301721439	0.195055	1.546854	0.121898	0.56863
CR847936.1	55.40882	-0.441430704	0.277293	-1.59193	0.111401	0.547032
st3gal7	168.2294	0.039295418	0.21068	0.186517	0.85204	0.978558
trpc2a	2.634874	0.168023726	1.020741	0.16461	0.869251	0.982993
klhl31	5844.525	0.028038194	0.261359	0.107279	0.914568	0.992972
srd5a2b	6.37995	-0.913915667	1.370294	-0.66695	0.504805	0.88195
slx4ip	38.69649	0.457215167	0.280532	1.629814	0.103141	0.530217
im:7138239	5.334255	-1.317869519	0.805151	-1.6368	0.101673	0.528316

tyr	316.4011	-0.530328589	0.311075	-1.70482	0.088227	0.498835
zgc:123010	343.9507	0.057845934	0.131253	0.440722	0.659414	0.930581
got1	3789.14	0.186318508	0.155564	1.197696	0.231035	0.712561
nkx2.3	120.8051	-0.636602557	0.193076	-3.29716	0.000977	0.043444
si:dkey-24p1.1	0.30471	-0.868130786	3.977116	-0.21828	0.82721	NA
ca8	165.5431	-0.369768255	0.173826	-2.12724	0.0334	0.323127
aep1	3676.123	0.019462644	0.262244	0.074216	0.940839	0.997371
jac7	14.04256	0.951569782	0.941204	1.011013	0.31201	0.776164
trim35-9	23.99112	0.523684437	0.406644	1.287819	0.197809	0.678554
nr5a5	208.2666	-1.715078669	0.943472	-1.81784	0.069089	0.448527
tefa	2195.507	-0.122004773	0.264524	-0.46122	0.644638	0.926639
trim9	237.1875	0.141285225	0.247255	0.571415	0.567719	0.901809
map4k5	675.7582	-0.096924306	0.112177	-0.86403	0.38757	0.827592
cdkn3	126.3258	0.174789384	0.203143	0.860426	0.389554	0.827843
atp1a1a.3	611.1055	-0.259528657	0.248484	-1.04445	0.296278	0.764246
lamb4	268.0681	0.070673372	0.253729	0.278539	0.780599	0.961421
cox16	100.5163	0.252902388	0.179529	1.408696	0.158925	0.62448
arpc5a	999.892	0.098358439	0.143999	0.683049	0.494576	0.877975
plaub	29.07336	0.645750222	0.421635	1.531539	0.125636	0.573594
lgmn	3043.175	-0.174052053	0.148998	-1.16815	0.242745	0.724345
fbxo36b	5.505139	-0.51483212	0.783991	-0.65668	0.511386	0.884702
rhebl1	988.6344	-0.020622364	0.102096	-0.20199	0.839925	0.976417
mhc1uma	3.012953	-5.512874196	5.141837	-1.07216	0.283648	0.755095
ctslb	73.66759	1.349192355	1.27577	1.057552	0.29026	0.76078
olfm3b	10.32924	0.356495213	0.816673	0.436522	0.662458	0.93076
trpm3	47.85232	0.330017399	0.329539	1.001451	0.316609	0.778879
nfyba	1013.597	0.038847787	0.116298	0.334037	0.738351	0.952912
greb1l	136.5106	-0.08182486	0.226836	-0.36072	0.718307	0.946603
zgc:110130	193.2593	0.585572575	0.203989	2.870611	0.004097	0.106542
nasp	1101.923	0.09101124	0.106382	0.855513	0.392267	0.827902
zgc:77439	1144.791	0.005371639	0.148152	0.036258	0.971077	1
prpf38a	316.2998	0.011079566	0.128454	0.086253	0.931265	0.995809
arrb2a	317.8996	-0.052266391	0.142489	-0.36681	0.713761	0.945821
orc1	134.8722	0.065823904	0.191335	0.344025	0.730827	0.950056
gpr101	7.352857	-0.301085259	0.717034	-0.4199	0.674556	0.934877
st6galnac5a	4.325793	-0.438231826	1.05676	-0.41469	0.678366	0.936026
mpl	3.689161	-0.367736519	0.943911	-0.38959	0.696841	0.94198
vps13a	1023.407	-0.143130855	0.142332	-1.00561	0.314603	0.777479
cep78	72.43875	-0.071863066	0.241938	-0.29703	0.766443	0.960229
zgc:171579	4.675865	0.504461659	0.959693	0.525649	0.599132	0.913173
cacng5b	5.564867	-2.207026825	1.239764	-1.7802	0.075043	0.464411
zgc:152791	33.33056	-1.060638697	1.247672	-0.85009	0.395273	0.829083
klhl21	1002.707	-0.007258765	0.112029	-0.06479	0.948338	0.99773
spega	137.0588	0.202100383	0.201549	1.002737	0.315988	0.778545
zbtb48	80.60772	-0.029687458	0.209188	-0.14192	0.887145	0.987739
igfbp5a	108.9471	0.024933652	0.252409	0.098783	0.921311	0.994085
arhgap4a	36.37943	0.485691484	0.332537	1.460564	0.144135	0.603144
rbm25a	194.8045	0.787793665	0.246852	3.191364	0.001416	0.056314
emc4	163.3493	0.757730242	0.213521	3.548741	0.000387	0.023295
arg2	3416.927	-0.131261812	0.217875	-0.60246	0.546866	0.896107
vti1b	179.3284	-0.25171065	0.183681	-1.37037	0.170573	0.642139
pls1	116.6552	-0.195139473	0.324514	-0.60133	0.547621	0.896211
fbln5	197.7797	-0.084536381	0.163497	-0.51705	0.605119	0.914985
golga5	723.3485	-0.132285319	0.100474	-1.31662	0.187967	0.667259

rdh14a	223.7204	0.103590937	0.214871	0.482108	0.629729	0.92285
terfa	681.7642	-0.126596468	0.180115	-0.70287	0.48214	0.87159
six1a	318.073	0.130957674	0.179529	0.729453	0.465724	0.864997
hook2	62.78502	0.654193288	0.278633	2.347866	0.018881	0.242305
par1a	210.8379	-0.401674311	0.215501	-1.86391	0.062334	0.427718
vps37b	308.6766	-0.007733516	0.132044	-0.05857	0.953296	0.998436
pmepa1	45.05543	-0.332535204	0.340113	-0.97772	0.328214	0.786586
ccndbp1	332.8985	0.103848748	0.139082	0.746671	0.455262	0.860008
st6gal2a	896.3436	-0.140194125	0.101761	-1.37768	0.168303	0.639712
gadd45gip1	436.7978	0.093674916	0.108274	0.865169	0.386946	0.827592
tmem209	336.277	0.270878999	0.150285	1.802433	0.071477	0.456109
ahcyl2	1195.422	0.024198176	0.122382	0.197726	0.843259	0.976791
drg1	501.1606	0.022492572	0.150581	0.149372	0.88126	0.986892
ndufa5	1503.925	-0.116196899	0.156434	-0.74278	0.457613	0.860993
rps24	22384.09	-0.436244303	0.138794	-3.1431	0.001672	0.062877
qrfpra	2.919455	0.039361113	1.195561	0.032923	0.973736	1
ssbp1	221.4073	0.097778029	0.232938	0.419759	0.674661	0.934877
ccl19b	58.56623	-0.064229468	0.416413	-0.15424	0.877417	0.985398
pald1b	123.8807	-0.076705302	0.250601	-0.30609	0.75954	0.957886
ska1	70.8708	-0.197117458	0.229661	-0.8583	0.390729	0.827902
zgc:194209	179.8022	-0.500246734	0.206461	-2.42296	0.015394	0.218559
dnajb12a	446.9053	0.053244486	0.131393	0.40523	0.685309	0.938486
cuedc2	172.4136	0.216498974	0.172316	1.25641	0.208967	0.691152
epha3	66.94771	0.209404747	0.268592	0.779638	0.435604	0.850467
apool	386.3662	0.194945478	0.163844	1.189824	0.234115	0.714938
rcn2	127.3337	0.354017146	0.169087	2.093703	0.036286	0.335171
ch25hl1.1	234.657	-0.085898915	0.270272	-0.31782	0.750618	0.955074
dlg4b	148.1586	0.178074685	0.261856	0.680047	0.496474	0.878161
sdhaf4	234.944	-0.050528995	0.179106	-0.28212	0.777853	0.961073
wnk1b	1171.388	0.102424806	0.177461	0.577168	0.563826	0.901196
si:ch211-240l19.5	1444.889	2.096646924	1.165979	1.798185	0.072148	0.456773
nt5c2a	773.221	0.020378097	0.098261	0.207387	0.835708	0.976007
polr1c	170.9012	-0.012525501	0.211083	-0.05934	0.952682	0.998395
prom2	192.6899	0.352793561	0.224892	1.568726	0.116712	0.557685
cep68	81.63161	0.099646914	0.208194	0.478625	0.632206	0.923187
dmrta2	100.3435	0.262392356	0.162231	1.617402	0.105792	0.534827
fuom	11.16763	-0.160559047	0.495971	-0.32373	0.746145	0.955074
AL831745.1	107.4809	0.021683055	0.535803	0.040468	0.96772	1
snrpb2	383.354	-0.044737406	0.183132	-0.24429	0.807006	0.969726
adka	1397.646	0.131274374	0.123308	1.064605	0.287055	0.757955
msl2b	441.3873	-0.009537292	0.104761	-0.09104	0.927462	0.994973
oprm1	10.46257	0.579997968	0.826318	0.701906	0.482737	0.871787
rgs17	105.2882	0.219747378	0.193011	1.138524	0.254902	0.733542
il13ra2	171.7735	0.377818591	0.211996	1.782194	0.074718	0.46413
il22ra2	0.315272	-2.340129263	5.201853	-0.44986	0.652808	NA
zgc:110353	24.90418	0.44536794	0.349784	1.273265	0.202924	0.683508
hk1	1500.248	0.242916519	0.131788	1.84324	0.065294	0.435359
dhrs7ca	93.10129	0.102588549	0.284067	0.361142	0.717993	0.946533
tspan15	203.5536	0.210971024	0.174213	1.210996	0.225897	0.707208
acbd6	365.1498	0.070243944	0.156554	0.44869	0.653656	0.929269
lhx4	180.9522	-0.336625714	0.215162	-1.56452	0.117695	0.559498
qsox1	532.6109	0.023151975	0.170912	0.135462	0.892247	0.988566
hnrnph3	185.0082	-0.050087823	0.167963	-0.29821	0.765545	0.959831
terb2	4.933438	-0.146202075	0.929136	-0.15735	0.874967	0.984191

calhm2.1	82.63043	-0.059296175	0.244141	-0.24288	0.808101	0.969828
si:dkey-51a16.9	404.0118	-0.291917881	0.129575	-2.25289	0.024266	0.275475
bag3	200.0583	0.175749591	0.245888	0.714754	0.474761	0.869292
git1	377.502	-0.046645731	0.115332	-0.40445	0.685884	0.938574
pitpnaa	629.2566	-0.126883896	0.19111	-0.66393	0.506734	0.883007
naa20	586.1278	0.0802219	0.116209	0.690324	0.48999	0.874929
ccdc85a	52.1973	-0.238991754	0.34221	-0.69838	0.484941	0.872911
soul2	587.2693	-0.127047964	0.127568	-0.99592	0.319289	0.780043
si:cabz01090165.1	47.67963	0.602082865	0.415848	1.447843	0.147661	0.608558
ugt2a6	56.69577	1.423815487	0.400121	3.558465	0.000373	0.022626
eef1a1a	865.0734	0.227167683	0.209041	1.086712	0.277164	0.751426
ap1m3	128.6593	-0.212417912	0.203968	-1.04143	0.297676	0.765407
zgc:153119	28.89174	0.336598453	0.362762	0.927877	0.353471	0.804432
c8a	545.9982	-0.138445606	0.181711	-0.7619	0.446121	0.855403
c8b	474.946	-0.210858315	0.214244	-0.9842	0.325019	0.783955
ssx2ipa	255.1481	0.19808774	0.156547	1.26536	0.205742	0.687948
tubb2	462.859	0.103958824	0.182774	0.568783	0.569504	0.902322
rrh	26.58044	-0.911572607	0.530402	-1.71864	0.085679	0.491277
dapp1	23.9367	0.373896028	0.539898	0.692531	0.488604	0.873964
mrps6	373.6678	0.151499865	0.148176	1.022433	0.306576	0.771853
loxl3b	607.0689	0.208496966	0.110502	1.886816	0.059185	0.419186
emx1	49.32563	-0.028466727	0.23979	-0.11872	0.905501	0.991631
fstl1b	4474.195	0.198039954	0.136928	1.446308	0.148091	0.609064
ptk2bb	402.9703	-0.17009966	0.129159	-1.31698	0.187846	0.667033
pa2g4a	2830.165	-0.026991568	0.119515	-0.22584	0.821325	0.971788
cfd	2203.685	0.570006598	0.179873	3.168938	0.00153	0.059181
ier3ip1	503.3987	0.158699557	0.178501	0.889069	0.373966	0.820695
atoh1c	12.64844	-0.116493543	0.603827	-0.19293	0.847017	0.9773
mat1a	3363.297	0.556984681	0.219429	2.538337	0.011138	0.183611
poll	157.089	0.025652341	0.157105	0.163281	0.870297	0.983365
fgf8b	33.60137	0.838525693	0.365576	2.293708	0.021807	0.261665
cenpk	114.1467	-0.354847755	0.329249	-1.07775	0.281145	0.753557
nrgna	441.4898	0.113677802	0.276373	0.41132	0.680838	0.937189
slc6a1b	1516.256	0.209632477	0.222646	0.941551	0.346422	0.799463
mtif3	143.6714	0.018697178	0.152958	0.122238	0.902711	0.991036
itm2cb	280.4351	0.242134706	0.223317	1.084264	0.278248	0.751971
si:dkey-235d18.5	227.1033	0.089273029	0.2138	0.417554	0.676273	0.93557
fank1	7.048493	0.95514384	0.742492	1.286403	0.198302	0.679109
slc36a4	266.1011	0.145135606	0.134083	1.082429	0.279062	0.752444
tsta3	164.5778	-0.027736888	0.165348	-0.16775	0.866781	0.982582
mrpl38	623.6092	-0.01473106	0.116989	-0.12592	0.899797	0.99045
TSTA3 (1 of many)	890.8417	-0.110068316	0.131444	-0.83738	0.40238	0.833317
dsc2l	408.9772	-0.064126349	0.185343	-0.34599	0.729352	0.950056
samm50l	457.0568	0.383092868	0.098179	3.901964	9.54E-05	0.008792
si:ch211-121a2.2	44.14593	-3.704391489	0.976715	-3.7927	0.000149	0.012187
sirt5	236.9602	-0.144520758	0.147674	-0.97865	0.327753	0.786438
zgc:194221	75.42728	2.236611092	1.075709	2.079197	0.037599	0.340171
shtn1	69.28109	-0.220247979	0.247874	-0.88855	0.374247	0.820913
fam204a	239.8846	0.453790839	0.169218	2.681689	0.007325	0.14495
emx2	371.858	-0.087450437	0.128148	-0.68242	0.494974	0.87806
mrps26	560.8467	-0.138104755	0.138586	-0.99653	0.318994	0.779923
OSBPL8	12.39651	0.01757054	0.649708	0.027044	0.978425	1
zgc:123321	145.6002	-0.045203476	0.247097	-0.18294	0.854847	0.979161
asap1b	454.866	-0.043891009	0.152868	-0.28712	0.774022	0.960636

zgc:112160	22478.16	2.52993036	1.45694	1.736469	0.082481	0.482825
BX914200.1	4.035548	-4.008406013	1.349049	-2.97128	0.002966	0.090034
ldlrap1b	44.93621	0.233940251	0.295921	0.790549	0.429207	0.846956
si:ch73-308m11.1	101.6567	-0.383205912	0.311528	-1.23008	0.218666	0.699445
xpc	326.4008	-0.202433811	0.184167	-1.09919	0.271686	0.747976
prop1	1.509365	3.01080981	1.881851	1.599919	0.109616	NA
mcfd2	393.5431	0.162673469	0.151557	1.073348	0.283115	0.755028
dync2li1	42.95152	-0.332246363	0.244769	-1.35739	0.174658	0.648268
tbxtb	6.518374	0.570676035	0.664093	0.859331	0.390158	0.827902
mea1	917.9189	-0.128240881	0.132765	-0.96592	0.334083	0.791078
zgc:172182	7.988091	0.541014066	0.634743	0.852335	0.394028	0.828374
bbs5	84.57325	-0.232263517	0.249292	-0.93169	0.351495	0.802378
zp3b	19.47889	-0.352018788	0.398841	-0.8826	0.37745	0.822478
gng5	957.2883	0.069626565	0.14187	0.490777	0.623585	0.919171
gsta.2	186.2324	-0.209794565	0.537422	-0.39037	0.696262	0.941657
znf800b	141.3668	-0.895091136	0.212222	-4.21771	2.47E-05	0.00332
dusp22b	68.47282	-0.139266607	0.244083	-0.57057	0.568291	0.902146
dhdds	295.0896	0.072146319	0.178997	0.40306	0.686904	0.93875
ftr93	17.75168	-0.375144498	0.512589	-0.73186	0.464253	0.864593
lifrb	79.09812	-0.33659682	0.254017	-1.3251	0.18514	0.662704
rpz4	135.0427	-0.167193386	0.180178	-0.92794	0.35344	0.804432
lamtor2	351.3151	0.034404884	0.154484	0.222708	0.823763	0.972005
ambra1b	400.5108	-0.033156571	0.168796	-0.19643	0.844273	0.976791
arpp19b	625.2553	0.264811603	0.156762	1.689261	0.091169	0.505533
cemip	376.7304	0.031404344	0.13208	0.237767	0.812062	0.97012
ap3s2	478.3654	-0.025047472	0.149218	-0.16786	0.866695	0.982582
c1qbp	1581.518	-0.063972677	0.13315	-0.48046	0.630902	0.923003
CR855393.1	0.316692	0.960779704	4.530092	0.212088	0.832038	NA
BX323555.2	0.127474	-0.868089684	5.267649	-0.1648	0.869104	NA
gnpnat1	166.4973	-0.006816814	0.195731	-0.03483	0.972217	1
zbtb7a	18.70154	0.064136336	0.373796	0.171581	0.863767	0.981842
si:ch73-168d20.1	5.982248	-1.244182512	1.348373	-0.92273	0.356149	0.807117
sh3gl2b	11.683	0.270534029	0.528425	0.511963	0.608677	0.915204
rbm42	256.5188	-0.204792034	0.171859	-1.19163	0.233406	0.714569
tmem147	790.6294	0.049593109	0.139317	0.355974	0.72186	0.947403
gapdhs	10063.93	-0.033213634	0.160983	-0.20632	0.836543	0.976007
bola1	209.5777	0.204841083	0.143771	1.424769	0.154224	0.61894
ckmt2b	7743.534	0.258398514	0.122719	2.105616	0.035238	0.330683
slc25a33	832.5738	-0.160612672	0.271585	-0.59139	0.554259	0.898306
hlcs	284.1453	-0.055499127	0.175355	-0.3165	0.751626	0.955356
CABZ01064671.1	14.6597	-0.616892366	0.55366	-1.11421	0.26519	0.742063
ccdc172	6.46346	1.233232337	0.71045	1.735847	0.082591	0.483014
tent5ba	679.5455	-0.529629522	0.289388	-1.83017	0.067225	0.44275
fibina	336.2055	0.250051673	0.20888	1.197108	0.231265	0.712838
si:ch211-87j1.4	13.73332	-0.080899974	0.482155	-0.16779	0.86675	0.982582
utp11	149.9369	-0.334675174	0.205489	-1.62868	0.103381	0.530446
gdnfa	41.0226	-0.039263493	0.30411	-0.12911	0.897271	0.989719
st7	99.75799	-0.045018775	0.241412	-0.18648	0.852068	0.978558
fgfbp1b	16.72476	-0.654616012	0.548953	-1.19248	0.233073	0.714569
fgfbp2a	48.55737	-0.343127065	0.332147	-1.03306	0.301577	0.767607
prom1a	346.151	0.104205795	0.165749	0.628695	0.529549	0.890434
akirin1	728.1233	0.068580082	0.115834	0.592057	0.553812	0.89821
cnr2	4.176556	0.078330752	1.334122	0.058713	0.95318	0.998436
rai14	277.4682	0.099940189	0.169326	0.590224	0.55504	0.898692

tecrl2b	48.0323	-0.487597428	0.351512	-1.38714	0.165398	0.634653
ppt1	815.4044	-0.211747654	0.108172	-1.95751	0.050288	0.390605
hivep2a	379.8484	0.167283597	0.199126	0.84009	0.400858	0.8318
snrnp48	101.8318	0.07106853	0.217828	0.326259	0.744228	0.95491
xkr8.3	95.49696	-0.001787873	0.218661	-0.00818	0.993476	1
ptp4a3a	1211.807	0.22827092	0.141644	1.611585	0.107052	0.537
slc13a5b	14.47345	0.276603282	0.645528	0.428492	0.668293	0.933086
fryl	960.2256	-0.258387213	0.16112	-1.6037	0.108781	0.541334
pnpla7b	560.6122	-0.069792633	0.14107	-0.49474	0.620785	0.9182
si:ch211-244a23.1	18.29275	1.09474002	1.322066	0.828053	0.407641	0.83577
scfd2	111.7743	0.007579893	0.214263	0.035377	0.971779	1
neurod6a	197.7445	0.221865099	0.246694	0.899353	0.368465	0.817272
palld	1723.001	-0.083675678	0.138566	-0.60387	0.545932	0.895711
ebag9	412.5118	-0.151167144	0.128261	-1.17859	0.23856	0.719595
gpd1l	339.1073	0.161018794	0.156784	1.027007	0.304417	0.769848
osbpl10b	289.8393	-0.155016421	0.200102	-0.77469	0.438524	0.851685
tardbp	2579.812	0.12456065	0.086125	1.446277	0.1481	0.609064
trmt1l	109.1942	-0.220568684	0.248162	-0.88881	0.374105	0.820835
tcf19l	31.36806	0.735379705	0.339119	2.168502	0.030121	0.309482
pttg1ipb	432.9516	-0.124224686	0.173712	-0.71512	0.474536	0.869292
mcm4	1034.178	0.041576015	0.180811	0.229942	0.818137	0.971518
cldn1	1495.004	0.079321327	0.222392	0.356674	0.721336	0.947343
snai2	971.4694	0.028638476	0.148432	0.19294	0.847006	0.9773
GALNTL6	232.9979	-0.226924216	0.256034	-0.8863	0.375453	0.822237
gbp	387.7061	-0.010467158	0.236271	-0.0443	0.964664	1
acp6	132.9447	0.389973403	0.301972	1.291422	0.196557	0.676952
gja5a	14.26085	0.419310728	0.535962	0.782352	0.434008	0.850002
coil	111.4693	0.164942388	0.182055	0.906005	0.364933	0.814972
scpep1	436.8461	0.053225825	0.140535	0.378737	0.704883	0.944015
pycard	1009.71	-0.163572206	0.209512	-0.78073	0.434962	0.850002
fli1b	134.4864	-0.204620784	0.179798	-1.13806	0.255095	0.733542
zp3d.1	4.459777	-0.37974016	0.959695	-0.39569	0.692335	0.940414
ccsapb	328.2154	-0.057585952	0.128631	-0.44768	0.654382	0.929488
epb41l4a	255.0303	0.015748708	0.132905	0.118496	0.905674	0.991631
si:dkey-95h12.1	267.6198	0.350408821	0.24617	1.423442	0.154608	0.619425
kif20bb	183.1789	0.157479754	0.2144	0.734515	0.462635	0.864037
pi4kb	626.3247	-0.042550239	0.096898	-0.43912	0.660572	0.930581
znf366	21.1984	0.284013086	0.379028	0.74932	0.453664	0.858742
zgc:113232	638.6803	0.185127311	0.531468	0.348332	0.727591	0.949228
psma2	1111.75	0.165431948	0.148606	1.113227	0.265611	0.742247
zfpm2a	237.4606	0.40470554	0.243288	1.663485	0.096215	0.517722
si:ch211-215k15.4	1.224114	0	2.138267	0	1	NA
zfyve26	349.8684	-0.195529226	0.15734	-1.24272	0.213971	0.696474
ackr4b	111.5356	-0.303942546	0.215704	-1.40907	0.158814	0.624456
fosaa	26.05401	-0.099510344	0.509459	-0.19533	0.845138	0.976791
jdp2a	55.5001	0.300107717	0.262954	1.141293	0.253748	0.732873
si:ch73-138e16.5	10.98896	0.60932511	0.65128	0.935581	0.349489	0.801408
nek4	161.7559	-0.036355605	0.159196	-0.22837	0.819358	0.971785
grm4	222.1828	0.465034393	0.194405	2.392094	0.016753	0.229543
glt8d1	202.4052	-0.176205441	0.158508	-1.11165	0.266289	0.742912
cdc42l	1936.139	0.003278279	0.088048	0.037233	0.970299	1
wnt4b	157.3829	0.158946519	0.280432	0.566792	0.570855	0.902501
zgc:92287	418.6069	0.219493594	0.217278	1.010198	0.3124	0.776164
zgc:165461	1262.853	0.010354431	0.18341	0.056455	0.954979	0.998611

prim1	305.7839	-0.015472383	0.191126	-0.08095	0.935479	0.996829
rgs16	187.497	-0.358527939	0.281503	-1.27362	0.202798	0.683465
havcr1	87.48501	-0.534449613	0.262017	-2.03975	0.041375	0.355014
zgc:101562	97.24656	0.24043359	0.441156	0.545008	0.585748	0.906655
slc35a1	351.3945	0.176399209	0.131814	1.338244	0.180817	0.656768
syncrip	4203.308	0.0199401	0.098852	0.201718	0.840137	0.976417
fam210ab	215.243	-0.08971845	0.128562	-0.69786	0.485265	0.872911
qdpra	902.2752	-0.681557496	0.200807	-3.3941	0.000689	0.034762
nenf	327.8857	0.183148226	0.262917	0.696601	0.486053	0.873244
fstl5	1035.951	0.502855365	0.231592	2.171296	0.029909	0.307994
BX465228.1	16.76012	1.313373313	0.79285	1.656521	0.097616	0.521026
scrt1b	123.6113	-0.167166391	0.263279	-0.63494	0.525468	0.888928
krcp	374.2414	0.219158693	0.20598	1.063982	0.287337	0.758355
si:dkey-37m8.11	13.48556	-0.492738817	0.588976	-0.8366	0.402816	0.833401
mef2d	975.7509	-0.033677381	0.108533	-0.3103	0.756335	0.956532
kpnb3	2582.165	0.2365372	0.115088	2.055265	0.039853	0.348958
rap2ab	12.66037	0.205505046	0.519967	0.395227	0.692675	0.940444
oxgr1a.3	0.118261	0	5.267649	0	1	NA
crata	761.9103	-0.205228628	0.106549	-1.92615	0.054086	0.402505
tlr18	54.15009	-0.27164043	0.213676	-1.27127	0.203632	0.684359
si:ch211-132g1.1	1.311624	1.913169792	2.249329	0.850551	0.395019	NA
ctsk	718.0851	0.36559433	0.160367	2.279742	0.022623	0.265959
atp1a1a.5	96.10767	-0.6557422	0.361662	-1.81314	0.069811	0.450824
onecut1	218.0195	0.012573561	0.154715	0.081269	0.935228	0.996829
hormad1	6.883782	-0.486028394	0.659546	-0.73691	0.461175	0.862771
ephx2	461.3031	0.136072848	0.223729	0.608204	0.543052	0.894925
slc38a11	26.44331	-1.847453718	0.509274	-3.62762	0.000286	0.018882
si:ch73-340m8.2	36.60486	-0.151828326	0.320025	-0.47443	0.635196	0.924272
runx2a	3.370443	-1.431089637	1.095244	-1.30664	0.191335	0.671193
mpdu1b	390.153	0.529966767	0.145067	3.653243	0.000259	0.017751
sox19b	234.349	-0.02331809	0.182162	-0.12801	0.898143	0.990171
scamp5b	389.0988	0.065834418	0.279982	0.235138	0.814101	0.970483
fbxo32	1791.867	-0.665574847	0.179229	-3.71355	0.000204	0.015177
klhl38b	821.5339	-0.459053099	0.382525	-1.20006	0.230116	0.711949
zgc:110182	128.4083	-0.004157438	0.215138	-0.01932	0.984582	1
zgc:92590	625.6448	0.103280015	1.571776	0.065709	0.947609	0.997371
si:dkey-79d12.5	172.0381	-0.049667569	0.294119	-0.16887	0.8659	0.982416
ubl7b	976.9574	-0.124727763	0.178008	-0.70068	0.4835	0.872099
ywhab1	9311.756	0.120714523	0.092273	1.308229	0.190796	0.670774
pon3.2	262.62	-0.021130722	0.168396	-0.12548	0.900142	0.99045
cadm4	810.403	0.173829037	0.165101	1.052865	0.292403	0.761879
apoeb	18929.43	0.399288786	0.172665	2.3125	0.02075	0.255734
apoa4b.1	13763.04	-0.093353741	0.211663	-0.44105	0.659178	0.930581
gtpbp10	178.9951	-0.258500638	0.154702	-1.67095	0.094731	0.513797
osgin2	8.248034	-0.350116836	0.620859	-0.56392	0.572807	0.90359
nbn	162.7269	0.119440606	0.165833	0.720246	0.471374	0.86809
otomp	465.2569	0.20575277	0.145858	1.410634	0.158353	0.623766
pxdc1b	130.3714	0.361260227	0.200122	1.805197	0.071044	0.454971
prpf4bb	545.392	0.039012719	0.115617	0.337432	0.735791	0.951543
psph	1397.474	0.118302907	0.102337	1.15601	0.247677	0.728489
CABZ01041494.1	9.063128	0.882171508	0.95088	0.927742	0.353541	0.804513
rx2	72.23627	0.197831017	0.258758	0.76454	0.444546	0.85464
dhx29	216.9369	0.167514389	0.196847	0.850989	0.394776	0.828825
crfb2	43.20059	-0.070040797	0.340317	-0.20581	0.836939	0.976007

mat2aa	1968.435	0.170302269	0.192588	0.884282	0.376544	0.822258
hvcn1	411.1805	-0.177698297	0.138761	-1.28061	0.200331	0.680711
rpp38	116.6264	-0.04135156	0.192156	-0.2152	0.829613	0.974753
crot	535.8306	-0.116378826	0.162347	-0.71685	0.473466	0.869146
spred3	302.9752	0.405259023	0.191597	2.115164	0.034416	0.327696
ehd2b	434.3203	-0.000950482	0.160067	-0.00594	0.995262	1
tmem182a	1001.046	-0.142252795	0.149376	-0.95231	0.340938	0.795603
arhgef1a	247.2522	-0.155680858	0.156125	-0.99715	0.318691	0.779923
mmd	194.6393	0.304021655	0.191482	1.587728	0.112348	0.549177
bcl7bb	208.2146	0.12445882	0.19996	0.622418	0.533667	0.891181
slc25a20	1342.156	0.002287471	0.157106	0.01456	0.988383	1
usp22	283.9994	0.200807312	0.185728	1.081188	0.279614	0.752477
acot19	15.97367	-0.234462602	1.029417	-0.22776	0.819831	0.971785
nptxra	92.14747	0.420972114	0.42055	1.001004	0.316825	0.778879
klf2b	536.7563	-0.202428867	0.196877	-1.0282	0.303855	0.769473
cyp2k22	0.271213	0.811792156	5.168395	0.157069	0.875191	NA
rsl24d1	6353.446	-0.722323178	0.141751	-5.09571	3.47E-07	0.000126
snrpd2	1018.377	-0.110838394	0.166041	-0.66754	0.504429	0.881885
prtgb	13.99017	-0.050891007	0.498513	-0.10209	0.918689	0.993884
polr2i	353.3879	-0.278205022	0.148294	-1.87604	0.06065	0.423018
si:ch211-219a4.3	78.26867	0.028334468	0.239952	0.118084	0.906001	0.991631
fam214a	587.1033	-0.181263736	0.123665	-1.46576	0.142714	0.601981
wdfy2	436.2473	-0.031980243	0.135524	-0.23597	0.813452	0.970328
unc50	400.2151	0.023260927	0.167595	0.138793	0.889614	0.988318
slc25a51b	515.8091	0.71435351	0.264281	2.703011	0.006871	0.139692
irf2	168.6072	-0.498986682	0.183288	-2.72242	0.006481	0.136134
vil1	907.5033	-0.952695743	0.34293	-2.7781	0.005468	0.12457
enpp6	139.0023	0.406185246	0.165104	2.460174	0.013887	0.206522
snx10b	12.23302	-0.218404915	0.71758	-0.30436	0.760851	0.957937
lrp2bp	7.783401	0.024744403	0.676617	0.036571	0.970827	1
cga	32.51017	0.459804372	0.472456	0.973221	0.330443	0.788126
osbpl3b	99.73574	0.111790158	0.195023	0.573215	0.566499	0.901739
gsdmeb	134.4907	-0.02312135	0.242654	-0.09529	0.924088	0.994802
zgc:113176	54.45543	-0.076561266	0.23158	-0.3306	0.740943	0.954103
dusp19a	13.06974	-0.824244643	0.584878	-1.40926	0.158759	0.624456
mthfd1b	1172.465	-0.361798945	0.106679	-3.39149	0.000695	0.034884
BX004816.1	3.234438	-3.548472392	1.745717	-2.03267	0.042086	0.358832
sb:cb81	411.3666	0.085042318	0.183259	0.464054	0.642609	0.92567
yif1b	213.3449	-0.71280847	0.213628	-3.33668	0.000848	0.039393
ca15b	1.948483	0.346786974	1.327986	0.261138	0.793986	NA
zgc:92313	368.5953	-0.025357158	0.161335	-0.15717	0.87511	0.984191
smpd2a	218.6279	0.213853747	0.200656	1.065771	0.286527	0.757624
s1pr5a	166.1402	-0.541564177	0.27078	-2.00001	0.045499	0.373513
siae	126.9864	0.27334327	0.22419	1.21925	0.222749	0.704616
lgals3bpb	122.5118	-0.366744225	0.362772	-1.01095	0.312041	0.776164
epcam	5916.35	0.471426514	0.187978	2.507885	0.012146	0.191226
csgalnact1a	142.6327	-0.167849787	0.26131	-0.64234	0.520652	0.887165
ftr90	0.324809	1.055391818	3.949751	0.267205	0.789312	NA
spsb4b	188.6256	-0.178891317	0.169416	-1.05593	0.291	0.761178
b3gat1a	45.22808	0.656094105	0.335915	1.953153	0.050801	0.392225
tmem97	106.4281	-0.215098093	0.194525	-1.10576	0.268831	0.744925
bckdha	1101.24	0.057251983	0.097684	0.586091	0.557814	0.899184
ift20	209.9968	-0.009814674	0.143689	-0.06831	0.945543	0.997371
exosc5	175.1725	0.021115677	0.149737	0.141019	0.887855	0.988077

rad1	111.3096	0.277086148	0.210742	1.314809	0.188574	0.668287
hnrnpul1	1263.205	-0.055081207	0.104126	-0.52899	0.596814	0.911757
ckmb	150031.7	0.050710458	0.184818	0.27438	0.783792	0.96245
phykpl	174.9777	0.007213991	0.196764	0.036663	0.970754	1
pdzd3a	79.70212	-0.356011771	0.262365	-1.35693	0.174802	0.648577
ube4a	326.0999	0.002678842	0.134858	0.019864	0.984152	1
c18h3orf33	40.89472	0.102011694	0.345224	0.295494	0.767616	0.960296
eef1akmt1	192.9194	-0.086255539	0.212221	-0.40644	0.684418	0.938486
v2rh32	1.399586	0	2.151101	0	1	NA
CU570684.1	0	NA	NA	NA	NA	NA
tfap2c	771.1832	0.090272512	0.104156	0.866709	0.386102	0.82701
rtf2	728.2071	-0.318139313	0.162096	-1.96266	0.049686	0.388006
gcnt7	80.16147	0.715619705	0.341021	2.098461	0.035864	0.332937
si:ch73-352p4.8	250.1158	-0.134642716	0.14459	-0.9312	0.351749	0.802513
sestd1	820.7943	0.018936458	0.124174	0.1525	0.878793	0.985841
ilkap	100.3919	0.074807283	0.226602	0.330127	0.741304	0.954103
psmg1	430.2955	-0.150739815	0.174786	-0.86243	0.388454	0.827613
lrrc32	93.3782	0.367910139	0.278322	1.321887	0.186206	0.664263
fosl2	232.2936	0.406151501	0.203519	1.995641	0.045973	0.375002
grik1b	66.37452	-0.441636887	0.383919	-1.15034	0.250004	0.72973
zgc:110333	480.1407	-0.190742402	0.114627	-1.66402	0.096108	0.517378
v2rx1	3.588207	0.16212713	1.010979	0.160366	0.872592	0.983447
PRMT8	12.24717	0.704224213	0.59155	1.190473	0.233861	0.71479
rnf11b	134.6496	-0.1285529	0.1702	-0.75531	0.450065	0.85711
si:ch73-44m9.3	0.989657	-1.746128066	5.122181	-0.3409	0.733182	NA
ccr10	1.430538	-0.868138197	2.25869	-0.38435	0.700716	NA
pxylp1	189.1106	0.000260999	0.18866	0.001383	0.998896	1
prickle1a	228.8966	0.229402708	0.142046	1.61499	0.106313	0.53561
gps1	947.0275	-0.021918194	0.102544	-0.21375	0.830746	0.974908
wipi1	415.4606	-0.052592587	0.119605	-0.43972	0.66014	0.930581
nifk	390.2308	-0.269691557	0.215019	-1.25427	0.209745	0.691766
lrrc51	7.332865	-0.701498102	0.719949	-0.97437	0.329872	0.788084
ctdspl	830.3939	-0.017293462	0.086937	-0.19892	0.842327	0.97643
ccdc93	275.7401	-0.442867456	0.131226	-3.37485	0.000739	0.036194
MEP1B	22.77885	-0.227942575	0.804087	-0.28348	0.776809	0.961073
plcd4b	112.0892	-0.261436685	0.231553	-1.12906	0.258873	0.736046
fkbp14	443.9665	0.080344815	0.145806	0.55104	0.581606	0.905618
plekha8	202.9979	-0.032154529	0.18678	-0.17215	0.863318	0.981732
nab1b	17.82187	-0.217873227	0.485289	-0.44896	0.653464	0.929269
si:ch211-113j13.2	0.995264	0	2.689558	0	1	NA
BX936415.1	7.531361	1.358819985	0.93056	1.460217	0.14423	0.603325
glsb	439.7552	-0.019385069	0.165061	-0.11744	0.90651	0.991636
chchd4b	74.63247	0.162334617	0.317611	0.511111	0.609273	0.915204
adprh	124.7994	-0.185277806	0.242616	-0.76367	0.445066	0.855219
rbbp9	279.1463	-0.118696437	0.168152	-0.70589	0.480257	0.870001
maccl	148.8137	-0.25840529	0.183839	-1.40561	0.159841	0.626123
zgc:114130	234.9324	-0.273838375	0.247277	-1.10741	0.268115	0.744507
tfb1m	75.24615	-0.007461401	0.264599	-0.0282	0.977504	1
wdr75	477.202	-0.118400658	0.187948	-0.62997	0.528717	0.890094
elavl2	9.38395	0.358900074	0.664232	0.540323	0.588974	0.908074
crygm6	0.14264	0	5.267649	0	1	NA
crygm7	4.728638	0	1.894199	0	1	1
zgc:153846	10.05938	0	1.751229	0	1	1
kcnab1b	87.07263	-0.293168733	0.306863	-0.95537	0.339389	0.795226

tm4sf4	431.1566	-0.668382918	0.373564	-1.78921	0.073581	0.461398
id1	933.5401	-0.05461346	0.138622	-0.39398	0.693599	0.940668
drd6b	2.888765	1.676123476	1.397076	1.199736	0.230242	0.712099
inhbb	338.6903	0.097182368	0.124454	0.78087	0.434879	0.850002
ralba	207.9644	0.27990175	0.177528	1.576662	0.114873	0.554651
med16	207.8874	0.042209272	0.129721	0.325384	0.74489	0.954956
sult3st4	68.28426	-0.527104126	0.519006	-1.0156	0.309818	0.774621
sst1.1	197.4946	0.002444208	0.204615	0.011945	0.990469	1
lactb	219.9056	-0.003254376	0.149896	-0.02171	0.982679	1
ncf4	30.32259	-0.2485047	0.318535	-0.78015	0.435303	0.850347
tsku	274.6311	0.13352461	0.153203	0.871551	0.383453	0.825875
si:dkey-27n14.1	30.559	-0.219863191	0.392155	-0.56065	0.575033	0.904516
fundc1	646.304	0.041591257	0.151955	0.273708	0.784309	0.962605
dcun1d2a	1161.13	-0.057711104	0.111592	-0.51716	0.605042	0.914985
adra2a	30.34832	-0.162110668	0.348298	-0.46544	0.641619	0.925586
plekhh2	55.77344	0.632329194	0.236102	2.678199	0.007402	0.145771
shoc2	301.1716	0.179715864	0.144997	1.239442	0.215182	0.697157
tsg101a	520.1333	0.227160682	0.135318	1.678723	0.093206	0.510759
tcf25	400.3217	-0.067735978	0.130258	-0.52001	0.603055	0.914226
si:dkey-286j15.1	410.1098	-0.794210635	0.26661	-2.97892	0.002893	0.088537
upp1	13.10573	0.663146978	0.510567	1.298845	0.193997	0.674122
add3a	1087.841	0.160431853	0.105004	1.527861	0.126547	0.575146
hnrnph1	224.3225	0.178392723	0.131989	1.351577	0.176511	0.649967
mxi1	1125.568	-0.155523374	0.09587	-1.62224	0.104752	0.53308
fdps	805.4648	0.656287229	0.126696	5.180035	2.22E-07	9.02E-05
gpa33b	18.19923	-0.749526601	0.931362	-0.80476	0.420956	0.842494
si:dkey-11d18.4	0.57542	0	2.795901	0	1	NA
si:ch73-113g13.1	0.164191	0	5.267649	0	1	NA
gcgb	97.4616	0.174485304	0.2883	0.605221	0.545032	0.895206
ildr1b	145.1188	-0.302408213	0.183834	-1.645	0.099969	0.524166
meox2a	29.87577	-0.181286648	0.330054	-0.54926	0.582825	0.90571
kdelr3	308.4338	-0.105093763	0.157632	-0.6667	0.504961	0.88195
cbfb	585.6625	0.059250411	0.162157	0.365388	0.714822	0.945821
si:dkey-49n23.1	438.6674	-0.11138574	0.128771	-0.86499	0.387042	0.827592
zmp:0000000606	6.387032	-0.851854779	1.221558	-0.69735	0.485583	0.87303
wnt10b	49.58541	-0.004402206	0.249378	-0.01765	0.985916	1
nr2f2	1499.452	0.001124545	0.10131	0.0111	0.991144	1
deptor	479.5532	0.082524152	0.187873	0.439255	0.660477	0.930581
ssuh2.1	7.264535	0.338292472	0.584361	0.57891	0.56265	0.900772
pnp6	130.0618	0.39570239	0.228265	1.73352	0.083003	0.483577
ntd5	1035.71	-0.089364453	0.140783	-0.63477	0.52558	0.888928
olig2	202.5399	0.08249117	0.192745	0.427982	0.668664	0.933086
olig1	57.59282	0.518719965	0.290711	1.784318	0.074372	0.463115
dharma	0.129927	0	5.267649	0	1	NA
rabl3	108.1604	0.243374023	0.181523	1.340736	0.180006	0.655675
zgc:101731	31.34394	0.626725132	0.347316	1.804482	0.071156	0.455341
zgc:113149	217.9151	0.309244064	0.149084	2.074294	0.038052	0.342345
zgc:92606	1910.383	-0.176622044	0.166429	-1.06125	0.288577	0.759638
fgf14	60.63908	0.775883882	0.332393	2.334236	0.019583	0.246694
hspa13	147.962	0.12909556	0.173709	0.74317	0.457379	0.860993
itgbl1	563.0486	0.089839308	0.13039	0.689002	0.490822	0.87556
tpi1b	7489.242	0.053543754	0.138762	0.385868	0.699594	0.942499
usp37	310.9775	0.129374992	0.113733	1.137533	0.255316	0.733542
cttnbp2	593.8238	0.110907605	0.131628	0.842584	0.399461	0.831196

or102-4	0.474181	-0.868142428	3.638709	-0.23859	0.811427	NA
or102-1	0.046941	0	5.267649	0	1	NA
st3gal4	190.8408	0.051180603	0.154265	0.331772	0.740062	0.953684
AL845428.1	0.718241	1.925682623	3.311877	0.581447	0.560939	NA
gemin7	167.6813	0.037499448	0.216759	0.173	0.862651	0.981229
or111-11	16.77592	0.073705726	0.411499	0.179115	0.857847	0.979774
pdcd4b	744.6202	-0.180757244	0.139528	-1.29549	0.195152	0.67483
or111-6	5.063572	1.540070761	0.967442	1.5919	0.111407	0.547032
or111-1	8.777284	0.325859646	0.668772	0.48725	0.626081	0.920625
or107-1	3.640194	-0.406601769	0.846537	-0.48031	0.631005	0.923003
or119-2	8.02274	0.425865258	0.764004	0.557412	0.577246	0.904809
zgc:152857	4.59116	0.102379769	0.873803	0.117166	0.906729	0.991644
piga	104.7353	0.209669246	0.209408	1.001245	0.316708	0.778879
cxcr3.2	55.08845	0.098769253	0.322074	0.306666	0.759097	0.957886
mid1ip1a	1084.296	-0.077292006	0.245264	-0.31514	0.752657	0.955756
lgals9l3	198.3983	-0.014615667	0.187462	-0.07797	0.937855	0.997305
calb2a	1204.788	0.640170251	0.199515	3.208639	0.001334	0.053834
hspb1	531.4599	-0.424611782	0.207339	-2.04791	0.040568	0.351905
got2a	1609.463	0.059046502	0.117206	0.503784	0.614413	0.915998
jagn1a	181.7271	0.009072874	0.199497	0.045479	0.963726	0.999842
mier1a	338.1362	0.086281222	0.110438	0.781263	0.434648	0.850002
si:dkey-12e7.4	15.26443	-0.490350278	0.584341	-0.83915	0.401385	0.832371
chka	625.1957	-0.577947084	0.204035	-2.83259	0.004617	0.112688
thap3	42.92458	0.686828831	0.291821	2.353594	0.018593	0.241343
kmt5b	823.3293	-0.129791451	0.108869	-1.19218	0.233189	0.714569
anpepa	59.27439	-0.377090455	0.498726	-0.75611	0.449585	0.856979
arpin	139.8441	0.019631921	0.24305	0.080773	0.935622	0.996829
LO018380.1	10.7773	0.861531289	0.608807	1.415113	0.157035	0.622926
pgpep1l	6.423134	2.239855825	1.06517	2.102816	0.035482	0.330833
barx2	50.23706	0.188812082	0.289331	0.652582	0.514026	0.88631
cftr	53.47578	0.009322014	0.364342	0.025586	0.979588	1
ctsh	281.9306	0.011687931	0.153773	0.076008	0.939413	0.997371
dnajc3a	300.8932	0.213442767	0.199136	1.071843	0.28379	0.755095
wdr18	287.4684	0.238766629	0.126276	1.890837	0.058646	0.418329
cnfn	110.1631	-0.009511472	0.580606	-0.01638	0.98693	1
wnt2	109.1815	-0.175712139	0.175458	-1.00145	0.31661	0.778879
ceacam1	945.2016	-0.587519348	0.236175	-2.48765	0.012859	0.197838
rapsn	317.0251	0.144176771	0.208933	0.690062	0.490155	0.875082
dhrs13a.3	395.176	0.04853305	0.126288	0.384304	0.700753	0.942918
ddb2	613.2289	0.00421529	0.258168	0.016328	0.986973	1
cryba1a	39.19038	0.928831386	0.386215	2.404961	0.016174	0.224405
paqr5a	42.71912	0.090749014	0.322451	0.281435	0.778377	0.961073
slc17a6b	684.2806	0.339393348	0.195427	1.736679	0.082444	0.482825
morf4l1	882.8354	0.012850323	0.145905	0.088073	0.929818	0.995605
si:ch211-63p21.4	2.812413	0.133931452	1.814747	0.073802	0.941168	0.997371
rab32b	7.215503	-1.76644204	1.634655	-1.08062	0.279866	0.752785
aagab	255.9598	-0.103012788	0.14126	-0.72924	0.465855	0.86502
crygs2	0.547474	0.704048524	2.70292	0.260477	0.794496	NA
adra1bb	10.5748	-0.370107135	0.675177	-0.54816	0.58358	0.906087
crygm5	277.6945	-0.231322991	0.611851	-0.37807	0.705378	0.944122
rpl4	39873.38	-0.32001741	0.108252	-2.95623	0.003114	0.091566
alg12	101.5641	0.091076567	0.212379	0.42884	0.668039	0.933086
drap1	1575.559	-0.064513033	0.132356	-0.48742	0.625959	0.920625
slc6a11	47.73647	0.483815437	0.696039	0.695098	0.486994	0.873291

cetn4	319.457	-0.24386138	0.135809	-1.79562	0.072555	0.457864
xpo6	531.4217	0.037913349	0.145503	0.260568	0.794426	0.965431
ftr53	21.65195	0.258837098	0.329552	0.785421	0.432207	0.848419
zmp:0000000608	1.789234	0.965619582	1.865367	0.517657	0.604698	NA
fam173a	199.9759	0.205044838	0.166964	1.228078	0.219418	0.700359
cdc40	605.2878	-0.086237893	0.150691	-0.57228	0.567131	0.901759
rps29	9296.386	-0.139747865	0.163415	-0.85517	0.392457	0.827902
med18	113.7365	-0.121687029	0.189631	-0.6417	0.521065	0.887466
wdr20b	78.31209	-0.466837741	0.282711	-1.65129	0.098679	0.521402
setdb1a	406.8345	0.105233453	0.132839	0.792186	0.428252	0.846133
si:dkey-241l7.6	0.555069	3.203868512	3.31976	0.96509	0.3345	NA
gemin4	3.960222	1.39932042	0.998108	1.401973	0.160923	0.627788
smtnl1	140.5769	0.068714573	0.323584	0.212355	0.83183	0.974925
si:ch211-231m23.4	1.112891	0.539379867	1.733169	0.31121	0.755641	NA
noxo1a	147.0884	0.247083381	0.262489	0.94131	0.346546	0.79947
lim2.5	173.9078	1.169825784	0.302063	3.872784	0.000108	0.009739
zgc:110796	87.513	0.117029624	0.257838	0.453889	0.649909	0.927756
drc3	37.92444	-0.604075169	0.332822	-1.81501	0.069522	0.450071
crybb3	3.641594	-0.065736492	0.893688	-0.07356	0.941363	0.997371
trak1a	326.6267	0.079939346	0.142061	0.562711	0.573632	0.904248
mrps25	651.7813	-0.08609505	0.12041	-0.71502	0.474598	0.869292
ndufb9	2314.88	0.02868323	0.126744	0.226308	0.820962	0.971788
rangap1a	788.9816	-0.02242827	0.138658	-0.16175	0.871501	0.983365
csdc2a	430.0558	0.311560914	0.174087	1.789683	0.073505	0.461297
tmem33	541.5278	0.110504902	0.123325	0.896049	0.370227	0.818484
si:dkey-125e8.4	3.923142	0.109135074	1.209011	0.090268	0.928074	0.995052
zgc:92380	1690.178	-0.189759467	0.130287	-1.45647	0.145263	0.604476
mrpl51	530.488	0.281292274	0.098058	2.868624	0.004123	0.106542
cyp19a1a	1.110509	-3.534953372	2.123764	-1.66448	0.096017	NA
ankrd39	110.035	-0.39091287	0.238527	-1.63886	0.101241	0.526828
lgi3	20.49499	0.260066145	0.761207	0.34165	0.732615	0.950617
zgc:161969	405.6824	-0.099700782	0.122733	-0.81234	0.416599	0.840071
ttk	285.8896	0.10402268	0.196692	0.528859	0.596903	0.91176
chmp7	470.6316	0.036202994	0.148273	0.244164	0.807104	0.969726
dctn3	919.2897	-0.123538801	0.161298	-0.7659	0.443734	0.854255
zgc:136564	179.798	0.310407462	0.240905	1.288505	0.19757	0.678182
si:ch211-57i17.5	11.35018	0.313103875	0.606094	0.516593	0.60544	0.914985
rmi1	74.88783	0.20057609	0.268891	0.745938	0.455705	0.860177
sh2d4a	60.45148	-0.093681037	0.251171	-0.37298	0.709166	0.945546
si:ch211-245j22.3	11.62377	0.306281559	0.649832	0.471324	0.637409	0.924779
arntl2	243.6852	-0.019305398	0.352737	-0.05473	0.956353	0.99899
si:dkey-283b15.2	84.96411	0.393984507	0.506584	0.777728	0.43673	0.851093
si:dkey-81n2.1	0.441166	0.08374504	2.866273	0.029217	0.976691	NA
tmed10	2414.022	0.036135485	0.081213	0.444949	0.656356	0.929684
dnajb1b	223.3494	-0.016493922	0.213119	-0.07739	0.938311	0.997371
eif2b2	693.6532	-0.015506795	0.154255	-0.10053	0.919926	0.993892
adgre7	0.060442	0	5.267649	0	1	NA
ndufa3	1149.783	-0.060242472	0.17558	-0.34311	0.731519	0.950365
zc3h14	562.1467	-0.113721977	0.115329	-0.98607	0.324099	0.783161
rmdn3	216.882	0.364703018	0.216453	1.684908	0.092006	0.507669
rad51	259.2267	0.054205364	0.187929	0.288435	0.773014	0.960624
adgrg11	8.73261	-1.198980273	0.842321	-1.42342	0.154613	0.619425
bmf2	85.46745	-0.196813919	0.326553	-0.6027	0.546707	0.896107
atp6ap1a	1108.569	0.412836318	0.177125	2.330763	0.019766	0.248225

taz	358.2206	-0.330545055	0.21594	-1.53073	0.125837	0.573743
yrdc	164.4507	-0.011072707	0.147919	-0.07486	0.940329	0.997371
ints14	341.0514	0.058788712	0.159641	0.368256	0.712683	0.945821
bmp2b	374.1234	-0.091620283	0.143242	-0.63962	0.522421	0.887826
slc24a1	30.29146	-0.098705451	0.532117	-0.1855	0.85284	0.978794
si:dkey-7c18.24	602.5865	0.483202541	0.690685	0.699599	0.484178	0.872553
uba52	20476.07	-0.300948914	0.139105	-2.16347	0.030505	0.311199
spred1	634.3193	0.197467405	0.090784	2.175129	0.02962	0.306524
rab11a	2692.623	0.097725255	0.091198	1.071572	0.283912	0.755095
taar12b	0.177631	0	5.267649	0	1	NA
pimr183	0.621085	0.078601748	2.487875	0.031594	0.974796	NA
smg5	837.2561	0.16241286	0.114828	1.414396	0.157246	0.623263
paqr6	346.9249	0.0865755	0.194648	0.444779	0.65648	0.929745
afp4	1593.566	-0.578003304	0.576225	-1.00309	0.31582	0.778379
si:ch211-196f5.2	40.59469	1.228507352	0.599839	2.04806	0.040554	0.351905
ipo4	284.6239	0.077839833	0.234545	0.331876	0.739983	0.953684
tgfb1a	372.1218	0.049329874	0.140567	0.350934	0.725638	0.948653
med4	153.252	0.048823482	0.17049	0.286372	0.774593	0.960871
itm2bb	3236.043	-0.531354239	0.147843	-3.59405	0.000326	0.020507
pex10	39.11529	0.148658674	0.293557	0.506405	0.612573	0.91573
rasal1b	4.001531	2.947578845	1.525607	1.932069	0.053351	0.400175
ppih	295.4132	0.042092457	0.154927	0.271691	0.785859	0.962605
ccdc130	134.8578	0.309590188	0.189223	1.636117	0.101815	0.528581
mrps28	236.257	0.016779717	0.124442	0.134839	0.892739	0.988566
sult1st2	40.19246	1.320125399	0.484743	2.723351	0.006462	0.135996
gypc	216.2405	-0.013180133	0.197446	-0.06675	0.946778	0.997371
pimr138	4.152633	0.876078745	1.041207	0.841407	0.40012	0.831461
unc93a	6.106513	0.046085895	0.698352	0.065992	0.947384	0.997371
si:dkey-102f14.5	137.5692	-0.289714197	0.171135	-1.6929	0.090474	0.505009
tnfaip1	183.344	-0.258596558	0.143214	-1.80567	0.07097	0.454693
ces2	6905.376	-0.765137895	0.380423	-2.01128	0.044295	0.368387
mrpl39	665.5107	0.088007969	0.117259	0.750543	0.452928	0.858256
zfpm1	160.7841	-0.263172548	0.170183	-1.54641	0.122005	0.568897
trmt10c	190.1732	-0.176728755	0.19839	-0.89082	0.373028	0.820208
nudt18	141.9789	0.226507427	0.256297	0.883771	0.37682	0.822258
znf143b	291.941	0.274606062	0.115092	2.385971	0.017034	0.231104
dhx40	193.0792	-0.277138021	0.258837	-1.0707	0.284303	0.755251
adprhl1	49.63405	0.482409496	0.332877	1.44921	0.147279	0.607673
dcun1d2b	883.2492	0.034226522	0.144115	0.237495	0.812273	0.970129
ces3	1121.806	-1.567522283	0.508954	-3.07989	0.002071	0.071081
zgc:91860	642.1165	0.001165391	0.178249	0.006538	0.994783	1
wdr83	96.83835	-0.188777872	0.199337	-0.94703	0.343624	0.798147
zgc:92335	483.4422	-0.176246471	0.183526	-0.96033	0.336887	0.793513
eif4ebp3l	2986.281	-0.149262513	0.204016	-0.73162	0.464399	0.864593
adarb1a	232.7068	-0.084074775	0.218999	-0.38391	0.701049	0.942918
b3gnt9	38.35705	-0.489287505	0.320066	-1.52871	0.126337	0.574665
nme5	34.55096	-0.078951121	0.322978	-0.24445	0.806884	0.969726
rack1	51419.37	-0.347971576	0.129534	-2.68633	0.007224	0.144046
bbs2	304.3555	0.049363928	0.151221	0.326436	0.744095	0.95491
mt2	735.5414	-0.729345767	0.383199	-1.90331	0.057001	0.412333
fam192a	536.6966	0.040204499	0.109882	0.365888	0.714449	0.945821
si:dkey-194e6.1	85.18447	-1.320857339	0.37988	-3.47704	0.000507	0.02777
sb:cb37	720.6824	-0.224905766	0.396628	-0.56704	0.570684	0.902481
oprd1a	4.992999	1.414394508	1.073939	1.317016	0.187833	0.667033

mkrn1	2389.416	-0.33761392	0.14375	-2.34861	0.018843	0.242305
si:dkey-105h12.2	305.3614	0.092884747	0.342314	0.271343	0.786127	0.962605
usf1	562.3794	-0.066137568	0.112379	-0.58852	0.556183	0.899002
bhlhe41	360.1849	-0.606871274	0.218593	-2.77626	0.005499	0.125156
piwil1	33.88871	0.357087222	0.433002	0.824678	0.409554	0.836787
rfx6	1.168353	1.054527333	1.693355	0.622744	0.533452	NA
rrbp1b	956.3271	0.190952439	0.165962	1.15058	0.249905	0.729721
si:busm1-228j01.6	0.965541	-1.759343207	2.141448	-0.82157	0.411323	NA
vgll2a	41.20296	0.015471922	0.372344	0.041553	0.966855	1
kat14	430.4039	-0.050545652	0.115049	-0.43934	0.660414	0.930581
bco2b	20.53745	-0.185286085	0.741231	-0.24997	0.80261	0.968181
arpc5la	463.884	-0.109473174	0.125488	-0.87238	0.383001	0.825809
TUBB4B	602.9235	0.144374725	0.117576	1.227929	0.219474	0.700391
glipr2	121.8601	0.132927226	0.201832	0.658604	0.51015	0.883733
man1a2	527.2459	0.263509315	0.120776	2.181796	0.029125	0.304246
sntb1	202.4873	0.037851087	0.154572	0.244876	0.806552	0.969726
tbc1d32	76.57579	0.248356135	0.306455	0.810416	0.417701	0.841149
tmem237a	189.0244	0.635988213	0.403221	1.577268	0.114734	0.554525
hsdl1	67.09792	0.068986407	0.245989	0.280445	0.779136	0.961073
sspn	195.842	-0.281938996	0.215095	-1.31077	0.189937	0.669635
ccdc92	246.2507	0.322150477	0.2211	1.457036	0.145106	0.604004
ndst3	262.0483	-0.113439777	0.124971	-0.90773	0.364023	0.814497
trdn	345.5574	-0.113179857	0.170442	-0.66404	0.506667	0.883007
cx32.3	106.1789	-0.393814964	0.391325	-1.00636	0.314241	0.777479
mgat4c	13.82183	0.809333706	0.5933	1.364122	0.172529	0.645319
cx28.1	4.509102	-0.40293984	0.998153	-0.40369	0.686444	0.938732
cx28.9	132.7176	1.463731484	0.458752	3.190681	0.001419	0.056314
cx43	1625.516	-0.163275689	0.128761	-1.26805	0.20478	0.686713
rnf141	171.8948	-0.153820107	0.178891	-0.85985	0.38987	0.827843
rps25	18779	-0.307689753	0.149347	-2.06023	0.039377	0.347538
si:dkeyp-28d2.4	5.480509	-0.424724763	0.941018	-0.45135	0.65174	0.928625
tsn	302.3124	-0.0816347	0.174152	-0.46875	0.639245	0.924779
ccl38a.5	5.971585	-2.11565348	1.439066	-1.47016	0.141519	0.600284
tsc22d2	595.088	-0.072927785	0.146823	-0.49671	0.619396	0.917435
rh50	17.74994	0.319447437	0.874371	0.365345	0.714854	0.945821
rbm39b	1119.415	-0.048545924	0.09525	-0.50967	0.610284	0.915331
ren	12.8676	0.023804585	0.71805	0.033152	0.973554	1
capn3a	2133.534	0.705563148	0.362452	1.946638	0.051578	0.394954
tnfrsf19	469.5094	-0.083624976	0.138869	-0.60219	0.54705	0.896121
ift172	329.4015	-0.169703687	0.130875	-1.29669	0.194739	0.67483
si:dkey-221h15.4	13.67683	-1.373403069	0.576024	-2.38428	0.017113	0.231611
ube2v1	1661.469	0.004427407	0.117288	0.037748	0.969888	1
rab11ba	1086.242	0.165159525	0.107589	1.535093	0.124761	0.572486
sf3a1	1351.643	0.015156791	0.139758	0.10845	0.913639	0.992796
cad	1335.583	-0.665565048	0.156711	-4.24709	2.17E-05	0.003005
dnajc5ga	543.2727	-0.205333928	0.135127	-1.51957	0.12862	0.578162
ankzf1	148.8112	0.031827659	0.189177	0.168243	0.866392	0.982582
usp39	321.3602	0.00289209	0.122611	0.023587	0.981182	1
znf512	25.73841	-0.049574252	0.407263	-0.12173	0.903117	0.991246
si:ch211-245h14.1	18.61422	-0.342684031	0.48983	-0.6996	0.484179	0.872553
stum	34.51544	-0.48703554	0.305308	-1.59523	0.110662	0.545461
ccl38a.4	2.566926	0.943319455	2.01291	0.468635	0.639331	0.924779
ccl38.1	17.05151	-0.150463331	0.593412	-0.25356	0.799838	0.967237
hsbp1b	1002.321	-0.153512725	0.186922	-0.82127	0.411494	0.837797

ccl38.6	17.9306	1.000427224	0.703588	1.421893	0.155057	0.619776
cryba2b	7489.623	0.375962361	0.237043	1.586051	0.112728	0.549875
dlg4a	20.40935	0.94220687	0.791541	1.190345	0.233911	0.71479
adat2	62.1665	-0.723303903	0.243803	-2.96676	0.00301	0.090442
styk1b	304.347	-0.15705518	0.129878	-1.20925	0.226566	0.707798
selenoo1	440.8969	0.097797991	0.17557	0.557032	0.577505	0.904842
prox2	37.59878	0.543869459	0.45074	1.206614	0.227581	0.708829
cxcr4b	137.7099	0.028407433	0.204632	0.138822	0.889591	0.988318
si:ch211-173a9.6	112.9972	-1.440390489	0.357571	-4.02827	5.62E-05	0.006027
brk1	1913.736	0.072720215	0.158303	0.459374	0.645966	0.926728
il17a/f3	3.28789	-0.024937894	1.011525	-0.02465	0.980331	1
calhm6	13.52033	0.338560054	0.534827	0.633027	0.526716	0.889699
adamts6	32.75996	0.059255143	0.353852	0.167457	0.86701	0.982582
rrp9	227.3009	-0.297160598	0.198652	-1.49588	0.134684	0.589356
zgc:113337	152.6883	-0.077890515	0.227065	-0.34303	0.731575	0.950365
si:ch211-63o20.7	79.51769	0.441807402	0.39855	1.108537	0.26763	0.74397
nfya	957.6842	0.040680238	0.116196	0.350099	0.726265	0.948987
elp3	239.9928	-0.189147901	0.172233	-1.09821	0.272112	0.748056
pklr	3384.735	0.466616889	0.188423	2.476429	0.01327	0.200523
cyp11c1	50.25446	-0.024429979	0.307952	-0.07933	0.93677	0.997044
fhl2a	754.4922	-0.179765298	0.144988	-1.23987	0.215025	0.697086
mapk12a	430.6938	-0.090514403	0.119689	-0.75625	0.449501	0.856979
si:dkeyp-118h3.6	171.7998	0.024218166	0.162593	0.14895	0.881593	0.986908
prrx1b	711.1323	-0.055754207	0.119304	-0.46733	0.640264	0.924877
kpna5	877.7455	-0.098483414	0.101478	-0.97049	0.331804	0.789531
gorab	120.4722	-0.147419106	0.187547	-0.78604	0.431844	0.848419
pou3f3a	316.3353	0.004513546	0.147364	0.030629	0.975566	1
mettl11b	47.08942	0.253131939	0.270317	0.936425	0.349054	0.801306
tal2	205.3736	-0.00115091	0.165395	-0.00696	0.994448	1
hmg20b	611.2647	-0.003159183	0.151243	-0.02089	0.983335	1
zgc:171971	89.91013	0.136250914	0.210244	0.64806	0.516946	0.887148
fam129aa	203.9802	-0.264373155	0.224708	-1.17652	0.239389	0.719781
mau2	727.6235	-0.161959459	0.11442	-1.41548	0.156929	0.622766
trmt11	147.4767	-0.394229278	0.205374	-1.91957	0.054912	0.405196
si:ch211-145c1.1	1.075195	-0.561706611	2.113327	-0.26579	0.790399	NA
eef2a.2	14.66671	-0.922584006	0.565445	-1.63161	0.102762	0.529448
pebp1	573.5803	-0.589239321	0.188857	-3.12004	0.001808	0.065671
fbxl17	68.60808	0.088376373	0.232887	0.379482	0.70433	0.943785
dmap1	442.0157	0.324493905	0.165115	1.965258	0.049384	0.387221
si:ch73-55i23.1	15.76486	-2.158695509	0.770777	-2.80067	0.0051	0.119231
btbd2a	518.9343	0.122435768	0.107759	1.136201	0.255872	0.733642
eef2a.1	296.1152	-0.337729322	0.241535	-1.39827	0.162033	0.629618
mnx2a	16.46575	-0.597287579	0.488238	-1.22335	0.221197	0.702457
si:dkey-44k1.5	78.01526	0.775005893	0.388364	1.995567	0.045981	0.375002
dio1	99.60299	-0.285466594	0.247639	-1.15275	0.249012	0.728918
bin1a	30.89358	1.004552027	0.368194	2.72832	0.006366	0.134763
tmem198a	88.47675	0.164957819	0.235064	0.701756	0.482832	0.871821
trim35-40	168.112	0.121039343	0.152049	0.796056	0.425999	0.844714
si:dkey-97o5.1	109.2101	0.144636695	0.223274	0.647798	0.517116	0.887148
acot11b	228.8139	-0.455444679	0.183467	-2.48243	0.013049	0.198789
nudt3b	47.76841	0.242126877	0.250469	0.966693	0.333698	0.790676
si:dkey-4e7.3	151.0407	0.374628357	0.198058	1.891508	0.058557	0.418278
elmo3	155.8094	0.15309635	0.21199	0.722185	0.470181	0.86742
pacsin1b	365.5038	0.014032857	0.121547	0.115452	0.908087	0.991745

zp3a.1	1.511922	0.125154036	1.643736	0.07614	0.939308	NA
zp3a.2	12.04798	-0.691722577	0.617718	-1.1198	0.262798	0.740207
si:dkey-51e6.1	316.8114	0.50930447	0.180479	2.821952	0.004773	0.114488
selp	2.757078	-3.922908497	1.647581	-2.38101	0.017265	0.231982
fpgt	41.5416	0.012728866	0.262212	0.048544	0.961283	0.99935
myo6b	146.6574	0.083165426	0.247888	0.335496	0.737251	0.952312
lhx8b	4.650714	1.239475441	0.83094	1.491654	0.13579	0.591131
rbbp5	501.3875	-0.047124565	0.104196	-0.45227	0.651075	0.928395
skia	814.7008	0.017795879	0.104129	0.170903	0.8643	0.981867
ppil6	8.092064	-0.267874184	0.653329	-0.41001	0.681796	0.937464
RFESD (1 of many)	36.52399	0.583783292	0.326094	1.79023	0.073417	0.46112
c7a	142.3341	0.539829618	0.340365	1.586032	0.112732	0.549875
foxl2a	77.93749	0.398003651	0.247875	1.605666	0.108347	0.540687
drc1	27.14682	0.04125486	0.409393	0.100771	0.919732	0.993892
paplnb	8.625405	0.805779422	0.818774	0.984129	0.325052	0.783955
smek1	1320.584	0.058066763	0.093019	0.624246	0.532466	0.890978
ttc38	92.56585	-0.171508664	0.248896	-0.68908	0.490775	0.87556
tspan33b	14.20273	0.46054715	0.559382	0.823315	0.410329	0.837066
sema3ab	128.3597	0.340402439	0.194859	1.746914	0.080652	0.479526
si:ch211-233h19.2	27.57707	0.83664642	0.415881	2.011746	0.044247	0.368166
tmem243a	71.28722	-0.611059297	0.26565	-2.30024	0.021435	0.25974
pias4b	5.403788	-1.264301705	0.861841	-1.46698	0.142382	0.601598
mthfd1l	1737.379	-0.462617668	0.24521	-1.88662	0.059212	0.419186
stx11b.2	39.97606	-0.201678071	0.344486	-0.58545	0.558248	0.899409
specc1lb	93.27723	0.077306574	0.224012	0.3451	0.730019	0.950056
fgf16	46.38491	-0.326707497	0.274007	-1.19233	0.233131	0.714569
atrx	2335.146	0.194149846	0.130843	1.483841	0.137851	0.593945
myl13	16935.68	-0.003540319	0.114995	-0.03079	0.97544	1
FRMD4A	180.1763	-0.162310376	0.213872	-0.75891	0.447904	0.856477
eif4h	1677.051	0.071352347	0.092762	0.769196	0.441777	0.853281
tgfbr1b	127.2189	-0.030173639	0.205141	-0.14709	0.883063	0.986908
cdan1	82.18105	0.454185745	0.270066	1.681761	0.092615	0.509109
arsa	99.40485	0.305182834	0.205676	1.483807	0.13786	0.593945
tmem231	110.4887	0.006916254	0.209602	0.032997	0.973677	1
efnb3b	156.1504	0.154501279	0.178923	0.863506	0.38786	0.827592
itga6a	143.8772	0.092301414	0.21508	0.429149	0.667815	0.933086
atp8b3	331.2313	0.399710602	0.162463	2.460314	0.013882	0.206522
dlx6a	88.05148	-0.138586704	0.228212	-0.60727	0.54367	0.894925
ca4b	80.79797	0.313388648	0.874566	0.358336	0.720092	0.947004
dlx5a	411.7607	-0.024404993	0.121241	-0.20129	0.840469	0.976417
arhgef18b	81.6839	0.278032367	0.204966	1.356483	0.174945	0.648869
cdca4	970.8548	-0.017590549	0.112567	-0.15627	0.875822	0.984562
si:dkey-177p2.6	461.9284	-0.125848881	0.142205	-0.88498	0.376167	0.822258
si:dkey-91m11.5	224.6689	0.035122057	0.18389	0.190995	0.84853	0.977912
plin2	101.3105	-0.723287649	0.275785	-2.62265	0.008725	0.160441
impdh1a	238.3489	0.62300231	0.235605	2.644269	0.008187	0.153717
chrac1	357.8887	-0.174441695	0.197926	-0.88135	0.37813	0.822654
lpar2a	112.9148	-0.294741711	0.209683	-1.40565	0.159827	0.626123
tmem251	247.0898	-0.312256225	0.136616	-2.28565	0.022275	0.263857
trmt6	101.4346	0.106348111	0.201947	0.526615	0.598461	0.91286
si:dkeyp-55f12.3	160.2516	-0.138470647	0.263459	-0.52559	0.599175	0.913173
syt1b	8.880662	0.823904384	0.945415	0.871474	0.383496	0.825875
cldnk	156.46	0.343439495	0.303063	1.13323	0.257118	0.734679
kif26aa	174.3566	-0.118795185	0.186221	-0.63792	0.523523	0.888581

ptafr	8.397221	-0.662458454	0.568328	-1.16563	0.243766	0.724437
zgc:103681	87.94055	0.017740797	0.256855	0.069069	0.944934	0.997371
zbtb25	36.31639	0.208120853	0.324678	0.641007	0.521518	0.887551
zgc:77752	27.38118	0.290677811	0.817166	0.355714	0.722054	0.947403
syndig1l	25.7072	0.502199356	0.49903	1.006351	0.314247	0.777479
trim35-30	19.35143	0.31990687	0.438321	0.729847	0.465484	0.864997
derl2	270.3001	0.04117586	0.149263	0.275862	0.782654	0.962328
RALGDS	110.5957	0.109648736	0.213018	0.51474	0.606735	0.915204
rab40b	279.131	-0.204589108	0.178082	-1.14885	0.250619	0.730604
galnt17	88.84991	-0.023277443	0.293293	-0.07937	0.936742	0.997044
gstt1a	2163.609	-0.790344862	0.180813	-4.37105	1.24E-05	0.002154
p2rx7	108.0934	-0.260355441	0.289309	-0.89992	0.368162	0.817272
p2rx4b	8.316671	-0.396545519	0.617222	-0.64247	0.520569	0.887165
mad2l2	160.0939	-0.188118173	0.221156	-0.85061	0.394984	0.828825
rfc4	402.523	-0.049088765	0.141055	-0.34801	0.727831	0.949325
tlk1a	697.0694	-0.027200207	0.138364	-0.19658	0.844152	0.976791
ndufa12	1698.979	0.180019875	0.140462	1.28163	0.199972	0.6804
s1pr3a	36.73019	-0.145955085	0.339701	-0.42966	0.667445	0.933086
nr2c2	407.2088	-0.053895118	0.162348	-0.33197	0.73991	0.953684
tle2a	832.1565	0.039426989	0.09191	0.428972	0.667944	0.933086
foxd5	16.95856	0.082924665	0.500175	0.165791	0.868321	0.982801
thap12a	308.8152	0.005708406	0.156022	0.036587	0.970814	1
si:dkey-250d21.1	94.8931	-0.141838132	0.217954	-0.65077	0.515195	0.886401
parp12a	52.85923	-0.185521581	0.320119	-0.57954	0.562226	0.900715
ubr3	565.3481	-0.001339561	0.164707	-0.00813	0.993511	1
glod4	129.2134	-0.262528165	0.195058	-1.3459	0.178335	0.652458
acot20	112.3605	-0.135890313	0.200387	-0.67814	0.497683	0.878845
acot21	1.630724	2.570516871	1.759939	1.460572	0.144133	NA
gpr176	46.25427	-0.793740245	0.318381	-2.49305	0.012665	0.196026
hipk2	194.9248	0.366680813	0.243854	1.503688	0.132662	0.585592
UTP14C	297.6695	-0.201680549	0.123959	-1.62699	0.103738	0.531539
si:dkey-251i10.1	70.29014	-0.103978027	0.231689	-0.44878	0.653589	0.929269
katnbl1	201.3714	0.085855948	0.132239	0.649247	0.516179	0.88677
ebf2	245.2432	-0.027292326	0.234533	-0.11637	0.90736	0.991745
sebox	21.4981	0.092209579	1.47307	0.062597	0.950088	0.99801
unga	139.5303	-0.437110967	0.213933	-2.04321	0.041031	0.353808
gnat2	3532.467	0.096360284	0.273013	0.352952	0.724125	0.948273
nup205	878.454	0.123971368	0.107251	1.1559	0.247722	0.728489
gstm.1	944.785	-0.09345027	0.168045	-0.5561	0.578141	0.905409
rsrc2	651.039	0.019394097	0.084009	0.230858	0.817425	0.971268
actc1a	3939.635	-0.669827711	0.205414	-3.26086	0.001111	0.047149
si:dkey-48j7.3	0.197334	-1.826676282	5.23046	-0.34924	0.72691	NA
ywhaqa	2469.576	0.139894988	0.124711	1.121756	0.261966	0.739673
degs2	1.040206	-1.758849814	2.2706	-0.77462	0.438565	NA
sema3ga	81.39339	0.461926578	0.243227	1.899155	0.057544	0.4139
tpd52l1	326.4318	-0.120231965	0.140414	-0.85627	0.39185	0.827902
mboat2b	213.1976	-0.054923182	0.199127	-0.27582	0.782687	0.962328
cacna1sb	1060.084	-0.13052813	0.222962	-0.58543	0.55826	0.899409
pex7	148.2241	-0.235936684	0.184945	-1.27571	0.202057	0.682257
dym	114.9257	0.155418922	0.164622	0.944093	0.345122	0.79837
rnf144ab	243.795	0.083836796	0.142516	0.588262	0.556357	0.899063
tnni1c	933.0544	0.168202407	0.146743	1.146238	0.251697	0.732003
lpar2b	257.7976	0.072873733	0.156772	0.464838	0.642047	0.925586
allc	93.77638	0.400473304	0.443011	0.903981	0.366006	0.815714

mis18bp1	93.02402	0.271616415	0.225332	1.205408	0.228046	0.709324
rps7	28122.65	-0.341475817	0.119125	-2.86654	0.00415	0.106599
rnaseh1	244.7719	0.137978259	0.182248	0.757092	0.448995	0.856796
batf3	9.556686	-0.018122912	0.605547	-0.02993	0.976124	1
rbm19	250.6143	-0.071239566	0.19179	-0.37145	0.710305	0.945821
ica1	55.71527	0.601883631	0.332179	1.811926	0.069998	0.451265
crp3	267.1831	0.786903002	0.467312	1.683891	0.092203	0.508046
sema3h	37.48254	-0.004195076	0.363398	-0.01154	0.990789	1
gstr	1417.495	-0.248873362	0.112669	-2.20889	0.027182	0.293072
cryaba	0.171522	1.055370259	5.267649	0.200349	0.841207	NA
acbd3	413.7667	-0.223170683	0.177681	-1.25602	0.20911	0.691152
nhsl1b	523.776	0.069357384	0.163111	0.425216	0.670679	0.933939
hebp2	975.0723	0.155879824	0.240184	0.649002	0.516337	0.886895
tert	117.7621	0.12622125	0.352276	0.358302	0.720117	0.947004
cyp51	317.2555	0.658699835	0.239536	2.749898	0.005961	0.13073
wtap	1194.858	-0.053323331	0.108203	-0.49281	0.622148	0.918461
si:ch1073-440p11.2	0.2705	1.055348013	5.267649	0.200345	0.841211	NA
sod2	1227.968	0.053464381	0.168079	0.318091	0.750416	0.955074
zgc:77784	244.5601	-0.012728793	0.245123	-0.05193	0.958586	0.999109
rreb1b	403.8771	-0.049151206	0.171052	-0.28735	0.773848	0.960624
nrn1b	17.79359	-0.972578466	0.635379	-1.53071	0.125842	0.573743
acad8	383.2477	0.353715616	0.124704	2.836447	0.004562	0.111673
thyn1	96.13025	0.296458194	0.17814	1.664188	0.096075	0.517378
fam32a	1595.621	-0.270768834	0.189954	-1.42545	0.154028	0.618621
tsta3l2	0.17928	0	5.267649	0	1	NA
si:dkeyp-70f9.7	2.311166	0.093653673	1.950517	0.048015	0.961704	0.999451
klf2a	765.2396	-0.110241146	0.538191	-0.20484	0.8377	0.976219
eps15l1a	468.613	0.105207639	0.142572	0.737928	0.460558	0.862369
odr4	122.2927	0.179173055	0.172979	1.035809	0.300291	0.766629
cadm1b	17.45338	0.840761955	0.48045	1.749946	0.080128	0.478357
si:dkey-15j16.3	4.705953	0.566807337	1.031816	0.54933	0.582779	0.90571
serpinc1	1300.973	-0.168742254	0.199632	-0.84527	0.397963	0.830737
bora	65.13227	0.255008562	0.253904	1.004348	0.315211	0.777834
zbtb37	59.04523	-0.357126308	0.243497	-1.46666	0.142469	0.60163
s1pr1	863.3944	0.027730046	0.131511	0.210857	0.832999	0.975379
si:dkey-15j16.6	9.961627	-1.37200194	1.123667	-1.221	0.222084	0.703829
parp6a	123.046	0.443422185	0.301138	1.472488	0.140889	0.599658
cx30.3	552.9073	-0.703963272	0.113404	-6.20755	5.38E-10	4.30E-07
tuba8l	5428.159	0.059235076	0.160438	0.369208	0.711973	0.945821
adipor1b	355.2909	-0.139927139	0.140199	-0.99806	0.31825	0.779923
blnk	22.71896	-0.829309438	0.449546	-1.84477	0.065071	0.435355
gfra2a	22.43475	-0.073402191	0.417306	-0.1759	0.860376	0.980264
supt5h	1450.776	-0.063411509	0.087979	-0.72076	0.471057	0.868025
cebpb	1336.484	-0.269724736	0.416736	-0.64723	0.517482	0.887148
grb7	39.64311	-0.477107722	0.294714	-1.61888	0.105473	0.534737
exo5	101.998	0.383659973	0.213364	1.79815	0.072153	0.456773
plaa	434.1779	0.195554884	0.162619	1.202537	0.229156	0.711257
tmem189	517.966	-0.190145914	0.123997	-1.53347	0.125161	0.572892
slc52a3	26.69537	-0.110218554	0.45905	-0.2401	0.810252	0.969828
stx6	965.1905	0.067796439	0.120839	0.561047	0.574766	0.904516
coasy	254.764	-0.143119494	0.15934	-0.8982	0.369077	0.817763
riok3	912.5759	-0.269031958	0.14231	-1.89046	0.058696	0.418329
si:ch211-282j17.11	0.250071	-1.826677	5.23046	-0.34924	0.72691	NA
ctsl2	45.95309	0.238552938	0.526457	0.453129	0.650456	0.927861

cnstb	71.09068	-0.828809156	0.242516	-3.41754	0.000632	0.032718
ndufa11	1228.149	-0.038880148	0.171262	-0.22702	0.820407	0.971788
apoba	3764.82	0.275287011	0.272752	1.009295	0.312833	0.776304
gdf7	29.88619	0.057668165	0.325104	0.177384	0.859207	0.979975
git2b	197.078	0.100011917	0.174887	0.571865	0.567413	0.901809
tpp1	557.5434	-0.402465121	0.142305	-2.82819	0.004681	0.113564
yy1a	1239.84	0.029611365	0.140736	0.210404	0.833353	0.975388
atpaf1	496.4086	0.059473538	0.099466	0.597928	0.549888	0.897373
slc16a4	285.5366	0.027961232	0.137179	0.20383	0.838486	0.976395
or128-10	7.05058	0.679629426	0.626238	1.085257	0.277808	0.751752
fgf1b	38.53988	-0.296043818	0.3673	-0.806	0.420242	0.842383
alkal2b	35.10435	-0.510909007	0.363099	-1.40708	0.159404	0.6257
mmp9	446.4059	1.049470565	0.222762	4.711174	2.46E-06	0.000594
agps	139.4073	0.306265226	0.165862	1.846501	0.064819	0.434694
spcs2	918.1797	0.075724577	0.136561	0.554512	0.579228	0.905578
nfe2l2a	1709.649	-0.238682564	0.127298	-1.87498	0.060795	0.423559
rnf169	283.6599	0.024532748	0.174813	0.140337	0.888393	0.988188
egr2b	41.47519	0.713319314	0.520131	1.371422	0.170243	0.641806
adoa	250.931	0.045688826	0.151063	0.30245	0.762309	0.958467
si:dkey-30j22.1	53.56869	-0.435928626	0.434844	-1.00249	0.316105	0.778668
rffl	171.0264	-0.112964889	0.149973	-0.75323	0.45131	0.857606
tfdp2	551.6526	0.093653642	0.089835	1.042503	0.297178	0.764844
atp1b3b	2529.663	0.074775488	0.0929	0.804903	0.420876	0.842479
dph6	207.6235	-0.024475195	0.283576	-0.08631	0.931221	0.995809
eml1	544.5831	-0.008299456	0.122814	-0.06758	0.946122	0.997371
oxt	38.4232	0.476181782	0.582947	0.816852	0.414013	0.838631
disp2	157.0669	0.407021684	0.202807	2.006937	0.044756	0.370491
pcmtl	220.0675	0.224512054	0.186943	1.200965	0.229765	0.711763
nphs2	21.16169	-0.485263575	0.456758	-1.06241	0.28805	0.75894
srp9	373.3222	-0.044804796	0.222438	-0.20143	0.840366	0.976417
ivd	1706.693	0.073642334	0.207652	0.354643	0.722857	0.947694
ephx1	285.1243	0.15865693	0.174588	0.908748	0.363483	0.81371
itpka	263.8545	-0.483721134	0.141375	-3.42156	0.000623	0.032419
si:ch211-246m6.4	159.4152	0.216782977	0.279833	0.774688	0.438524	0.851685
slc5a6a	16.42079	-0.412506118	0.456298	-0.90403	0.365981	0.815714
ltk	145.2682	-0.3268062	0.261613	-1.2492	0.211592	0.693044
rpl7l1	823.5446	-0.33569012	0.207971	-1.61412	0.106501	0.536018
cx35.4	316.5588	0.014431598	0.210032	0.068711	0.945219	0.997371
zdhhc8a	63.64288	-0.113825329	0.40836	-0.27874	0.780446	0.961411
slc25a27	24.10069	0.827533967	0.432292	1.914293	0.055583	0.408323
phlda2	410.8418	-0.17328	0.19994	-0.86666	0.386128	0.82701
abracl	1445.795	0.2129748	0.145367	1.465088	0.142897	0.602002
heca	196.2741	-0.133323375	0.181287	-0.73543	0.46208	0.863402
si:ch211-222f23.6	7.672362	0.467530876	0.706078	0.662152	0.507874	0.883056
nthl1	116.5985	-0.255465988	0.223426	-1.1434	0.252871	0.732759
TNPO2	453.6869	0.020802579	0.140579	0.147978	0.88236	0.986908
paip1	639.9724	0.053464034	0.104439	0.511917	0.608709	0.915204
tyms	321.5928	0.154388519	0.316495	0.487807	0.625687	0.920625
si:ch211-163l21.8	163.0685	-0.389066997	0.244089	-1.59395	0.110946	0.54583
gtpbp1l	225.3064	0.18392993	0.163708	1.123522	0.261216	0.738737
afg1lb	59.60115	-0.265162192	0.246208	-1.07698	0.281487	0.753734
foxo3b	1399.042	0.081609351	0.136755	0.596756	0.550671	0.8974
rpl10a	37451.4	-0.413983804	0.149223	-2.77426	0.005533	0.125682
hsh2d	19.68429	0.46536647	0.495649	0.938902	0.347781	0.800274

sult3st3	96.80119	0.383724764	0.586076	0.654735	0.512638	0.885483
gpr173	36.11382	0.390617214	0.31291	1.248337	0.211908	0.693489
mfng	72.98253	0.058406711	0.261467	0.223381	0.823239	0.972005
mapre1a	193.0282	-0.159482457	0.169643	-0.94011	0.347162	0.799992
tmem120b	56.59226	-0.04476012	0.275072	-0.16272	0.870738	0.983365
bloc1s6	620.9123	-0.024253272	0.143915	-0.16853	0.86617	0.982573
ccn2a	1850.139	-0.154450768	0.186585	-0.82778	0.407797	0.835802
fam228a	23.70891	0.37080708	0.339764	1.091366	0.275112	0.750424
si:dkey-256e7.5	19.54943	-0.081642808	0.351076	-0.23255	0.816111	0.971207
nab1a	81.19445	-0.218118501	0.342556	-0.63674	0.524296	0.888801
hook1	270.7724	0.035316872	0.167166	0.211268	0.832678	0.975239
mstna	0.043308	0	5.267649	0	1	NA
dnajc5aa	129.1132	0.534927876	0.201445	2.65546	0.00792	0.150539
cyp2n13	484.349	0.252504397	0.354506	0.71227	0.476297	0.869292
slc17a9a	26.08526	-0.143474065	0.352367	-0.40717	0.683881	0.938486
cyp2ad6	151.5502	-0.307960799	0.400885	-0.7682	0.442367	0.853782
zgc:100920	208.0661	-0.928843046	0.255571	-3.63438	0.000279	0.018447
dgcr2	152.6867	-0.50027919	0.323967	-1.54423	0.122533	0.569468
znf1035	745.6563	0.012472989	0.157409	0.079239	0.936842	0.997044
gng8	109.4044	0.020520744	0.296499	0.06921	0.944822	0.997371
zgc:136605	1.314151	0	2.41718	0	1	NA
auh	525.6512	-0.041512831	0.149807	-0.27711	0.781696	0.962077
nfil3	745.6901	-0.231514367	0.19811	-1.16862	0.242559	0.724345
cyp2p6	218.4434	-0.558082298	0.301675	-1.84995	0.064321	0.433359
cyp2p7	142.9898	0.09508632	0.388653	0.244656	0.806723	0.969726
cyp2p8	103.8807	-0.422813688	0.434034	-0.97415	0.329983	0.788084
has1	1.32616	1.055430053	2.147002	0.491583	0.623014	NA
fpr1	57.0793	0.131035406	0.267364	0.490101	0.624062	0.919497
slc24a2	407.9087	1.007667818	0.429151	2.348049	0.018872	0.242305
si:dkeyp-84f3.5	395.8004	-0.003193445	0.126471	-0.02525	0.979855	1
cyp2p10	57.75649	0.33332715	0.446459	0.746601	0.455304	0.860008
crygs1	0.411973	1.945175611	3.168524	0.613906	0.539277	NA
prss1	67066.71	-1.583691375	0.396178	-3.99742	6.40E-05	0.006594
sptlc1	407.62	-0.160625441	0.130039	-1.23521	0.216751	0.69815
vmo1a	23.43089	0.817476761	1.046764	0.780956	0.434828	0.850002
pcyt2	699.3323	0.352678259	0.144222	2.445387	0.01447	0.210484
si:dkeyp-117h8.4	79.80729	0.113998346	0.259391	0.439484	0.660311	0.930581
gnav1	138.6579	0.046527288	0.169223	0.274946	0.783358	0.96245
fam43a	789.1756	-0.343404882	0.149533	-2.29652	0.021646	0.261086
camk2d1	446.0513	-0.018778447	0.130919	-0.14344	0.885946	0.987533
chst14	82.72437	0.085121492	0.196126	0.434014	0.664278	0.93177
napgb	247.0866	0.185452326	0.289911	0.639687	0.522376	0.887826
exoc1	1052.228	-0.097768277	0.085547	-1.14286	0.253099	0.732759
slc26a11	117.1525	0.006576712	0.175181	0.037542	0.970053	1
ttbk2b	264.2689	0.181877691	0.166072	1.09517	0.273442	0.749148
ints10	184.5412	0.1247706	0.146311	0.852775	0.393784	0.828374
capn3b	333.5639	0.214324668	0.144211	1.486187	0.13723	0.59288
fbxl5	858.8146	-0.017370346	0.148465	-0.117	0.90686	0.991644
hiat1b	464.5023	-0.070842516	0.100961	-0.70168	0.482876	0.871834
nog2	200.9457	0.117109259	0.169096	0.692563	0.488584	0.873964
grp	96.22911	-0.085188533	0.242717	-0.35098	0.725604	0.948653
nisch	428.8641	0.056783505	0.123486	0.459837	0.645633	0.926728
MMP23B	77.94077	0.060616723	0.215378	0.281443	0.778371	0.961073
ctsz	916.5382	-0.364834391	0.170295	-2.14236	0.032164	0.318495

abhd17ab	90.49592	0.438820552	0.193658	2.265953	0.023454	0.270635
alox5b.1	5.791441	1.137322244	0.767497	1.481858	0.138378	0.594956
alox5b.2	1.156389	-2.026120769	1.619186	-1.25132	0.210818	NA
mpeg1.2	140.4259	0.626990733	0.379961	1.650145	0.098913	0.522055
gnas	346.2674	0.110622173	0.124373	0.889437	0.373768	0.820644
kctd6a	99.13818	0.049489084	0.216858	0.228209	0.819484	0.971785
lxn	2285.206	-0.419634222	0.188845	-2.22211	0.026276	0.28745
qtrt1	93.306	-0.095384721	0.17162	-0.55579	0.578354	0.905417
blf	101.8085	-0.302089862	0.294412	-1.02608	0.304854	0.770035
cldne	1686.828	0.067601926	0.112299	0.601983	0.547186	0.896121
notch2	1061.54	-0.003227824	0.193173	-0.01671	0.986668	1
BX664625.1	11.04576	-1.057698223	0.763512	-1.38531	0.165959	0.635475
cldnf	242.3187	-0.01084928	0.191824	-0.05656	0.954897	0.998611
cux1b	436.2772	0.063182105	0.119165	0.530207	0.595969	0.911458
pomca	27.05157	-0.01336915	0.743979	-0.01797	0.985663	1
cdca8	388.4373	0.137997957	0.220276	0.626476	0.531002	0.890545
si:ch211-132f19.7	95.02506	-0.620630536	0.550881	-1.12661	0.259906	0.737088
or108-3	0.688014	0.742998986	2.24612	0.330792	0.740801	NA
slc1a3b	385.125	0.450991068	0.129676	3.477825	0.000505	0.027755
ucp2	5594.075	0.112826999	0.236911	0.476243	0.633901	0.923974
dnajb13	15.05226	0.598032595	0.510678	1.171056	0.241576	0.723306
cela1.5	313.7186	0	1.692399	0	1	1
cela1.4	885.3015	-0.868185887	1.682614	-0.51597	0.605872	0.915055
cela1.3	2155.044	0	1.708455	0	1	1
cela1.1	363.8368	-1.762177553	1.66742	-1.05683	0.29059	0.76078
lsm1	137.257	0.081470757	0.210222	0.387547	0.698351	0.942433
bag4	161.7326	0.043608544	0.162719	0.267999	0.7887	0.963568
gpd1b	2214.761	-0.092646566	0.172054	-0.53847	0.590251	0.908901
hhla2a.1	458.3635	-0.137720647	0.159602	-0.8629	0.388193	0.827592
mllt1a	346.3089	-0.009887521	0.139777	-0.07074	0.943606	0.997371
rnasel2	0.480281	-2.745953887	2.764868	-0.99316	0.320632	NA
si:rp71-1i20.2	13.79734	-1.597148088	0.516009	-3.0952	0.001967	0.068839
nsun5	106.2019	0.04796872	0.205429	0.233506	0.815369	0.971158
nfic	16.77163	-0.817534	0.473735	-1.72572	0.084398	0.487532
ripk4	181.1722	-0.206801785	0.166453	-1.2424	0.214088	0.696474
adam17a	575.1042	0.017135995	0.129953	0.131863	0.895092	0.989187
kcnh5a	5.440967	-0.203956109	0.756994	-0.26943	0.7876	0.963263
nfixa	11.13013	-0.65022078	0.584387	-1.11265	0.265857	0.742605
CT573139.1	3.02447	1.617424964	1.647442	0.98178	0.326208	0.785142
ccna1	2.65534	-1.107421526	1.395071	-0.79381	0.427306	0.845774
atg16l2	7.404225	0.363862771	1.062297	0.342525	0.731956	0.950617
arrb1	35.56238	0.849202708	0.365545	2.323115	0.020173	0.251701
si:dkey-222f2.1	761.9945	-0.100226134	0.168588	-0.5945	0.552175	0.8974
prkchb	77.87844	-0.134126233	0.286533	-0.4681	0.639713	0.924779
gtf3c3	206.6298	-0.266123178	0.138323	-1.92393	0.054363	0.40321
irf1b	153.953	-0.069860297	0.467324	-0.14949	0.881167	0.986892
ppm1ab	261.8349	-0.037372852	0.135711	-0.27539	0.78302	0.962328
ckbb	12256	0.039333934	0.172893	0.227504	0.820031	0.971788
xylb	86.65879	0.753987677	0.277699	2.715122	0.006625	0.136957
morc3b	10.06001	0.400857537	0.804698	0.498147	0.618381	0.916585
mgmt	89.84518	-0.50121037	0.240858	-2.08094	0.037439	0.339918
aqp12	1025.003	0.402711535	0.264261	1.523917	0.127529	0.576964
stap2b	153.1904	0.34039968	0.193302	1.760976	0.078243	0.473959
prrg1	260.6026	0.09279747	0.156134	0.594344	0.552282	0.8974

zbtb21	20.86943	-0.222530138	0.419234	-0.5308	0.595556	0.911376
atg101	421.7224	-0.085288833	0.165795	-0.51442	0.606956	0.915204
impact	245.0308	-0.089739109	0.174882	-0.51314	0.607853	0.915204
nmu	19.03461	1.499636644	1.04287	1.43799	0.150437	0.614169
gtf3c6	73.4198	0.34304475	0.260619	1.316269	0.188084	0.667333
nop2	476.2886	-0.147287132	0.189853	-0.7758	0.437869	0.851283
srd5a3	35.33144	-0.19794442	0.389469	-0.50824	0.611283	0.915331
obscna	81.40541	-0.905446251	0.221748	-4.08322	4.44E-05	0.005146
ank2b	1836.623	0.135588211	0.165366	0.819928	0.412257	0.838123
kita	142.1171	-0.047404259	0.218994	-0.21646	0.828626	0.974137
gsx2	58.06566	0.021053276	0.218342	0.096423	0.923184	0.994525
lnx1	306.5707	0.138042224	0.20822	0.662964	0.507353	0.883007
fip1l1a	29.24938	0.075474961	0.555411	0.13589	0.891908	0.988566
prkcz	50.67639	0.366736651	0.234552	1.563559	0.117921	0.559998
ccdc6a	926.3105	-0.12515479	0.109118	-1.14696	0.251397	0.731717
abraxas1	71.29784	-0.150997663	0.250575	-0.6026	0.546772	0.896107
npy4r	2.199406	-1.560794953	1.614634	-0.96666	0.333716	NA
gpx3	175.3191	-0.161496075	0.293116	-0.55096	0.581659	0.905618
tbl2	216.8103	0.005515216	0.12878	0.042827	0.96584	1
rmnd5b	829.5253	0.058452758	0.098278	0.59477	0.551997	0.8974
dph3	29.29549	0.297534801	0.355544	0.836845	0.40268	0.833401
nck2b	225.1644	0.074044165	0.155217	0.477035	0.633337	0.923885
mdh2	10402.55	0.008505345	0.092389	0.09206	0.926651	0.994815
atraid	0	NA	NA	NA	NA	NA
si:dkey-71h2.2	89.23993	-0.69436025	0.28086	-2.47226	0.013426	0.202001
crip3	111.0983	-0.069062255	0.198975	-0.34709	0.728524	0.949696
fndc4a	31.39584	0.404912949	0.696494	0.581359	0.560999	0.900224
nitr2a	1.206243	-0.857271862	1.886919	-0.45432	0.649596	NA
zgc:171480	317.4847	0.211179331	0.184562	1.144219	0.252533	0.732759
paox1	110.8359	0.245621585	0.160551	1.529866	0.12605	0.574239
rpp21	86.32033	-0.030697323	0.213632	-0.14369	0.885743	0.987533
slc8a1b	233.9164	0.541088202	0.21758	2.486845	0.012888	0.198116
xkr6b	23.40971	-0.348620249	0.43401	-0.80325	0.421828	0.843078
tnfaip2a	228.7577	-0.816049305	0.153058	-5.33162	9.73E-08	4.70E-05
eipr1	470.3837	0.011883005	0.196073	0.060605	0.951674	0.99801
b3gntl1	68.77445	0.298007227	0.228605	1.303588	0.192374	0.672283
si:dkey-5n18.1	207.3622	0.305670072	0.225197	1.357345	0.174671	0.648268
zgc:153665	416.9606	-0.79330689	0.174679	-4.5415	5.59E-06	0.001166
kcnj13	150.2239	0.024444494	0.186462	0.131096	0.895699	0.98926
si:ch211-152f2.3	1.270904	2.474449269	2.602656	0.95074	0.341736	NA
efhd1	387.5253	-0.085543627	0.201025	-0.42554	0.670445	0.933883
gpr55a	3.661158	-0.275636997	0.964508	-0.28578	0.775047	0.960899
itm2ca	894.9578	0.099442152	0.197355	0.503875	0.61435	0.915998
cab39	92.81598	0.163210733	0.188077	0.867789	0.38551	0.826939
rps5	26159.73	-0.34682368	0.124123	-2.79419	0.005203	0.121039
gapdh	80817.32	0.108760866	0.172893	0.629064	0.529307	0.890418
wu:fj39g12	88.59885	0.554380939	0.415361	1.334696	0.181976	0.658775
atp6v1b2	1338.433	0.109407153	0.184084	0.594332	0.55229	0.8974
kcnk3a	7.538602	-0.611261466	1.2443	-0.49125	0.62325	0.918971
ndufb11	1010.619	0.003117029	0.140803	0.022138	0.982338	1
sytl3	2.776657	-1.77389359	1.297936	-1.3667	0.171718	0.643953
rsph3	17.11363	0.063238908	0.396151	0.159633	0.87317	0.983537
atp2b3a	991.8175	0.352353627	0.227518	1.548682	0.121458	0.567605
tagapb	46.53151	0.075665629	0.311019	0.243283	0.807786	0.969828

opn8c	3.17426	0.593687643	1.183647	0.501575	0.615967	0.916365
rbbp8	54.59798	-0.100391761	0.242832	-0.41342	0.679298	0.936209
b3gnt3.1	98.87637	-0.33753064	0.262937	-1.2837	0.199249	0.679739
otx5	470.379	0.124897622	0.234135	0.533443	0.593727	0.910427
ube2d2	1557.395	0.334732778	0.150105	2.229995	0.025748	0.28497
si:ch211-22i13.2	444.7968	-0.011814881	0.122696	-0.09629	0.923287	0.994525
irf1a	14.08283	-0.405964124	0.532893	-0.76181	0.446172	0.855403
cltca	2982.988	0.108417051	0.125996	0.860477	0.389526	0.827843
scrn2	370.6352	0.200403088	0.211483	0.947607	0.34333	0.797888
ccdc124	1031.325	0.056712985	0.142131	0.39902	0.689879	0.939679
rpl11	29141.56	-0.374107689	0.1728	-2.16498	0.030389	0.310733
vamp4	367.9347	0.139467155	0.149509	0.932837	0.350904	0.802215
prdx6	1109.951	0.097408748	0.140316	0.69421	0.48755	0.873291
si:dkey-239i20.4	28.41629	0.204610852	0.399435	0.512251	0.608475	0.915204
si:dkey-239i20.2	38.00252	0.527712767	0.449861	1.173058	0.240773	0.722178
plpp6	27.0072	-0.151997816	0.349448	-0.43497	0.663588	0.931556
jun	1892.011	-0.002620056	0.127195	-0.0206	0.983566	1
si:dkey-86e18.1	99.99324	-0.075385318	0.232024	-0.3249	0.745254	0.954956
zpr1	277.6942	0.091531754	0.204799	0.446934	0.654922	0.929606
atg4da	199.2085	-0.48279291	0.178316	-2.70751	0.006779	0.139038
ENKD1	40.52649	0.126855028	0.403732	0.314206	0.753364	0.955861
ches1	40.26272	-0.316104591	0.320228	-0.98712	0.323582	0.783161
mrpl40	311.0107	0.0458606	0.132421	0.346325	0.729099	0.949983
tmem30ab	565.0367	-0.019104061	0.118521	-0.16119	0.871946	0.983447
kcnk13b	0.205351	1.055361142	5.167946	0.204213	0.838187	NA
gle1	263.4338	0.25249992	0.161366	1.564762	0.117639	0.559345
psmc1b	1697.976	0.16360073	0.182084	0.898493	0.368923	0.817763
zgc:65997	533.3597	0.159693475	0.20542	0.7774	0.436923	0.851125
nepro	81.07853	0.204964701	0.246133	0.832738	0.404992	0.834284
dram2a	15.52259	-0.322975174	0.462288	-0.69864	0.484774	0.872911
KIF2A	743.239	0.156270617	0.095138	1.642563	0.100473	0.525188
gadd45aa	196.1039	-0.042983468	0.220192	-0.19521	0.84523	0.976791
ifit15	11.86501	0.010579849	1.235892	0.00856	NA	NA
fas	10.53	0.247285365	0.774364	0.31934	0.749469	0.955074
srd5a2a	157.3042	-0.058872238	0.258401	-0.22783	0.819776	0.971785
ca4a	46.58432	0.073839649	0.33551	0.220082	0.825807	0.972946
rapgef1a	61.2852	-0.023379842	0.276825	-0.08446	0.932693	0.996197
eif4ebp1	1412.438	-0.075517664	0.137726	-0.54832	0.583472	0.906042
pqlc2	327.2686	-0.315896024	0.175534	-1.79963	0.071919	0.4564
tbc1d9	88.70083	-0.110588504	0.266052	-0.41566	0.677655	0.936026
bcas2	746.6198	0.034324008	0.141041	0.243362	0.807725	0.969828
cmc2	259.3266	0.158262885	0.181551	0.871726	0.383358	0.825809
cenpn	110.5966	0.02726509	0.221422	0.123136	0.901999	0.990847
ehbp1	441.4734	0.065596231	0.15346	0.427449	0.669053	0.933115
vkorc1l1	170.1629	0.17817561	0.205401	0.867453	0.385694	0.826939
slc6a8	124.7347	-0.282300896	0.386334	-0.73072	0.464952	0.864823
pnck	3.384834	-1.250925872	1.415545	-0.88371	0.376855	0.822258
cxadr	315.2876	0.049153083	0.17358	0.283173	0.777044	0.961073
cadpsa	57.61208	0.578852321	0.373399	1.550223	0.121088	0.566931
cnih2	217.1432	0.240996374	0.212959	1.131658	0.257778	0.735557
faub	262.8021	-0.123306554	0.16085	-0.76659	0.443324	0.854063
si:dkey-63b1.1	3.081858	-2.144716567	1.605315	-1.33601	0.181546	0.657892
glrx5	746.1399	-0.052321228	0.137838	-0.37959	0.704253	0.943757
abt1	108.3443	-0.059418359	0.24656	-0.24099	0.809563	0.969828

zgc:100997	44.63283	0.609659917	0.310531	1.963283	0.049613	0.387751
si:dkey-174m14.3	226.4142	0.614201281	0.257842	2.382087	0.017215	0.231824
ylpm1	520.5043	-0.112213506	0.169151	-0.66339	0.50708	0.883007
bpnt1	259.4998	-0.151620412	0.157206	-0.96447	0.334812	0.791753
nabp1b	0.739175	0.114697838	2.630453	0.043604	0.96522	NA
si:ch211-59d15.9	69.99589	-0.218165522	0.229812	-0.94932	0.342457	0.797179
nefmb	528.3983	0.630422291	0.248182	2.540159	0.01108	0.18325
gpd1a	662.177	-0.148130628	0.18533	-0.79928	0.424127	0.844355
mob3c	96.0895	-0.29462887	0.196003	-1.50318	0.132792	0.585961
parvaa	283.619	0.492246004	0.166811	2.950926	0.003168	0.09274
asf1bb	345.5461	0.21567041	0.150528	1.432762	0.151926	0.615406
cldn5a	200.9518	0.191688539	0.164211	1.167331	0.243077	0.724396
c3a.6	374.2981	-1.165275353	0.306144	-3.8063	0.000141	0.011879
cdc45	123.6836	0.236654463	0.292491	0.809099	0.418458	0.841565
cpa4	4412.511	-1.673813847	0.617067	-2.71253	0.006677	0.137398
cwc27	262.7463	0.213843817	0.165695	1.290586	0.196847	0.67714
srek1ip1	146.9759	-0.026600394	0.148089	-0.17962	0.857447	0.979741
plac8.1	481.6594	-1.320589158	0.376951	-3.50335	0.000459	0.026028
cops4	1073.346	0.150364031	0.126868	1.185201	0.235938	0.717225
trmu	85.49813	0.362034781	0.241411	1.499659	0.133703	0.587056
si:ch211-282j17.8	0.711884	3.582637119	2.3127	1.549115	0.121354	NA
efcab11	29.53202	0.130306435	0.335644	0.388228	0.697847	0.942355
nrxn3a	536.3868	-0.095991716	0.2372	-0.40469	0.685708	0.938486
ptbp3	105.6148	0.202900385	0.2626	0.772661	0.439723	0.852079
si:ch211-282j17.3	0	NA	NA	NA	NA	NA
si:ch211-282j17.2	0.368	0	5.038065	0	1	NA
si:ch211-282j17.1	0.159496	0	5.267649	0	1	NA
si:dkey-192d15.2	95.66633	-0.530563002	0.301912	-1.75734	0.078859	0.475082
psmb10	255.9512	1.112991826	0.472047	2.357797	0.018384	0.239433
si:rp71-7l19.2	2.899804	0.762291553	1.23994	0.614781	0.538699	0.892777
arhgdia	2518.467	-0.02887039	0.087148	-0.33128	0.740432	0.953939
ms4a17a.7	187.0911	-0.168149384	0.313294	-0.53671	0.591465	0.909437
cdc5l	1021.89	0.028845965	0.081486	0.353999	0.723339	0.947898
ms4a17a.1	10.58629	1.943949499	0.973955	1.995933	0.045941	0.375002
zmp:0000000619	27.15984	-0.882700441	0.406187	-2.17314	0.02977	0.307249
ms4a17a.8	43.00802	-1.207779464	0.566986	-2.13018	0.033157	0.322388
postna	585.2787	-0.039026541	0.143791	-0.27141	0.786075	0.962605
st6galnac1.1	71.18373	-0.142638393	0.228428	-0.62443	0.532342	0.890978
st6galnac1.2	3.205941	-4.027165627	1.46663	-2.74586	0.006035	0.131072
si:ch211-119c20.2	75.12688	0.294632076	0.289729	1.016923	0.30919	0.773791
nme2a	251.5864	0.581405932	0.421615	1.378996	0.167896	0.639091
klf7b	525.5956	0.369123743	0.150936	2.445565	0.014463	0.210484
rab3ab	51.4241	0.221996142	0.398736	0.55675	0.577698	0.905055
si:ch73-111k22.2	776.487	-0.089061242	0.113656	-0.7836	0.433274	0.849392
akap7	226.9191	0.091017007	0.203946	0.446281	0.655395	0.929606
tmem244	28.28906	-0.15404068	0.4413	-0.34906	0.727044	0.949095
sod1	1032.544	-0.079088059	0.184776	-0.42802	0.668635	0.933086
ftr54	3.693175	0.951599613	1.212663	0.784719	0.432619	0.848753
ppil4	256.901	-0.096248648	0.153569	-0.62675	0.530826	0.890545
amd1	2918.02	-0.129370978	0.125537	-1.03054	0.302755	0.768916
ufm1	677.7845	0.237688901	0.128674	1.847224	0.064715	0.434368
cdk19	30.45498	-0.321138494	0.367698	-0.87338	0.382458	0.825252
nptnb	468.7416	0.21216603	0.266936	0.794819	0.426719	0.845352
timm9	304.4084	-0.114047233	0.158936	-0.71757	0.473024	0.868965

arid4a	941.8772	0.197692197	0.105257	1.87819	0.060355	0.422591
mmachc	49.00237	-0.246869933	0.31017	-0.79592	0.42608	0.844714
nup43	249.6362	0.114875036	0.164069	0.700161	0.483827	0.87219
si:ch211-214k5.3	0.558105	0	3.457376	0	1	NA
timm10b	133.4956	-0.066324488	0.163609	-0.40539	0.685194	0.938486
psma6l	40.4444	0.47932374	0.485854	0.986558	0.323859	0.783161
ugt5g2	0.46481	0	3.157007	0	1	NA
gabrr1	81.83712	-0.91729122	1.219584	-0.75213	0.45197	0.857994
pnrc1	32.85935	0.270320981	0.409808	0.659628	0.509492	0.883733
rnf14	468.8243	0.268143325	0.131479	2.039438	0.041406	0.355065
fgf11b	137.058	1.312512208	0.491419	2.670862	0.007566	0.14728
mei4	3.092532	0.53926692	1.090779	0.494387	0.621033	0.918449
FO904943.1	1.913367	-1.44203601	1.369751	-1.05277	0.292445	NA
sox9b	715.9212	0.081284891	0.175195	0.463968	0.642671	0.92567
gpn1	251.9967	-0.137029222	0.125011	-1.09613	0.27302	0.748889
stmn4l	454.7167	0.402981503	0.244905	1.645458	0.099875	0.524166
il17a/f1	0.046941	0	5.267649	0	1	NA
il17a/f2	0.060442	0	5.267649	0	1	NA
lrrc57	233.92	-0.086898162	0.15044	-0.57763	0.563516	0.901075
rpf2	499.8203	-0.168538981	0.196885	-0.85603	0.391983	0.827902
fgf18b	36.63187	0.054111915	0.290468	0.186292	0.852215	0.978626
si:ch211-241j12.3	219.9674	-0.121104088	0.160929	-0.75253	0.451733	0.857676
lrpprc	1394.472	0.088672222	0.114096	0.77717	0.437058	0.851125
ppp2r3c	377.4681	-0.169534159	0.139654	-1.21396	0.224763	0.706634
znf292b	658.3589	0.175467044	0.170699	1.02793	0.303983	0.769505
etf1b	2409.694	0.051661897	0.11021	0.468761	0.639241	0.924779
tube1	0.053506	-0.86807634	5.267649	-0.16479	0.869106	NA
setd6	94.4884	-0.196873386	0.2449	-0.80389	0.421459	0.842754
cyp2x8	59.43312	-4.857738746	1.288121	-3.77118	0.000162	0.012981
lgals3b	2175.544	-0.03539155	0.090546	-0.39087	0.695894	0.941649
cyp2x7	195.0133	-0.02857238	0.217974	-0.13108	0.895711	0.98926
loxl2a	339.5348	-0.037586316	0.308174	-0.12196	0.902927	0.991179
xkrx	310.7412	0.007516958	0.15117	0.049725	0.960341	0.999164
gfra2b	43.0993	-0.016995057	0.655546	-0.02593	0.979317	1
myo6a	866.0518	-0.117180417	0.114866	-1.02015	0.307659	0.772983
nkl.4	0.632517	0	3.288974	0	1	NA
tmem86a	64.41526	-0.163866321	0.402625	-0.40699	0.684012	0.938486
zbtb2a	33.11938	-0.057757938	0.367365	-0.15722	0.87507	0.984191
stx11a	44.28504	-0.430322317	0.343293	-1.25351	0.210019	0.691821
rassf2b	15.63538	-0.948462343	0.493833	-1.92061	0.05478	0.405097
slc18b1	50.27422	-0.086085219	0.364207	-0.23636	0.81315	0.970328
prnpb	41.76505	0.419038414	0.273535	1.531935	0.125538	0.573341
vsnl1b	617.6828	0.458558275	0.273471	1.67681	0.09358	0.511703
ahi1	268.1321	-0.035465951	0.138141	-0.25674	0.797381	0.966447
lft2	3.229769	-1.376720157	1.31627	-1.04593	0.295595	0.764221
adora4a	1.364396	0.275634316	1.446646	0.190533	0.848891	NA
ctbp2a	1115.083	0.064806779	0.113323	0.571877	0.567405	0.901809
TATDN3	196.5823	-0.202761238	0.181873	-1.11485	0.264914	0.742063
fuca2	1126.821	-0.033295946	0.148326	-0.22448	0.822385	0.971966
loxl2b	647.3885	-0.184313881	0.224832	-0.81979	0.412339	0.838123
peli1b	179.8196	-0.165035731	0.150349	-1.09768	0.272343	0.748416
zmpste24	441.8649	0.136660907	0.110929	1.231969	0.21796	0.699079
pitpnab	358.6021	0.191136977	0.188627	1.013308	0.310913	0.775776
atp5if1b	1721.499	0.139749692	0.137795	1.014182	0.310496	0.775146

rpl13a	25911.47	-0.258897973	0.140877	-1.83776	0.066098	0.439076
gfpt2	190.4373	-0.142696936	0.19843	-0.71913	0.472061	0.868517
egr2a	32.57978	-0.479883664	0.555603	-0.86372	0.387743	0.827592
znf593	447.083	-0.225744159	0.177773	-1.26984	0.20414	0.685399
cyb561d2	13.60116	0.312477322	0.438412	0.712748	0.476002	0.869292
txn	1061.343	0.68537263	0.148963	4.60097	4.21E-06	0.000937
si:ch211-214p16.1	15.67729	-1.142951543	0.643965	-1.77487	0.07592	0.46659
ogna	930.9919	0.257912417	0.118264	2.180811	0.029197	0.304273
iars2	469.2213	-0.008952176	0.102032	-0.08774	0.930084	0.995706
cenpp	107.9831	-0.133884453	0.192036	-0.69719	0.485687	0.87303
rab3gap2	461.4854	0.047943493	0.113722	0.421584	0.673329	0.934249
acss1	328.4145	-0.14364242	0.17054	-0.84228	0.399631	0.831196
nol8	143.0959	0.180472043	0.237941	0.758473	0.448168	0.856741
wapla	191.8281	0.186137902	0.158931	1.171184	0.241525	0.723306
ppp1cb	3305.305	-0.057493516	0.123127	-0.46695	0.640539	0.925152
mafaa	270.3615	0.094594442	0.325605	0.290519	0.771419	0.960594
grid1b	45.50192	1.154137213	0.5585	2.066493	0.038782	0.344867
padi2	927.4865	0.140430464	0.108425	1.295189	0.195255	0.67483
prelid1b	28.45949	0.23947824	0.402074	0.595608	0.551437	0.8974
oxtrl	5.39887	-0.634580956	0.800275	-0.79295	0.427805	0.845893
rbms2a	532.7562	-0.542839037	0.203399	-2.66884	0.007611	0.147466
stau1	2515.2	0.097741963	0.093683	1.043326	0.296797	0.764495
prkab1a	521.1867	0.176802453	0.139617	1.266338	0.205392	0.687537
gxylt2	240.119	0.176474269	0.173205	1.018878	0.308261	0.773155
tcta	382.8618	-0.071747514	0.162595	-0.44127	0.65902	0.930581
gnat1	832.5882	0.087856505	0.249502	0.352127	0.724743	0.948503
sumf1	243.2599	0.003057514	0.172133	0.017762	0.985828	1
endou	388.7349	-1.773884166	0.975441	-1.81855	0.068981	0.448201
fto	113.7748	0.099164048	0.234908	0.42214	0.672923	0.93414
si:dkey-27b3.2	559.2404	-0.035307477	0.102723	-0.34371	0.731061	0.950056
BX005421.1	1.53762	-1.404547129	1.748553	-0.80326	0.421823	NA
dram2b	485.7848	-0.03585599	0.148702	-0.24113	0.809457	0.969828
snx18b	75.12314	-0.115233158	0.178579	-0.64528	0.518747	0.887148
rasgef1bb	375.9383	-0.126929644	0.160481	-0.79093	0.428984	0.846713
paqr3b	111.7379	-0.123463459	0.320627	-0.38507	0.700186	0.942723
anxa3b	800.6619	-0.045239356	0.159039	-0.28445	0.776062	0.961073
rbm18	253.5594	0.052512796	0.150981	0.347811	0.727982	0.949325
si:ch211-243g18.2	1181.714	-0.005354603	0.163648	-0.03272	0.973898	1
pimr71	1.074523	3.269356263	1.88494	1.734462	0.082836	NA
cdc37l1	101.8489	-0.030163477	0.174481	-0.17288	0.862749	0.981285
sumo1	1412.529	-0.352782992	0.164421	-2.14561	0.031904	0.317397
spp1	203.0052	-0.862607877	0.409255	-2.10775	0.035052	0.329872
synpr	43.72045	0.912874446	0.319423	2.857889	0.004265	0.107992
opn1mw3	1.054206	3.288288309	1.842001	1.785171	0.074234	NA
opn1mw2	175.1077	-2.371078468	1.434198	-1.65324	0.098281	0.521076
erbin	753.0628	0.057988084	0.130805	0.443316	0.657537	0.93039
smc5	275.2021	0.154525975	0.132591	1.16543	0.243845	0.724437
rph3al	0	NA	NA	NA	NA	NA
pip5k1ba	74.33371	0.226336724	0.224983	1.006018	0.314407	0.777479
phax	292.8791	0.054217178	0.184488	0.293879	0.76885	0.960395
lmnb1	1733.073	0.236493411	0.170686	1.385546	0.165885	0.635405
atf1	760.6262	0.007826664	0.110481	0.070842	0.943524	0.997371
prrc1	385.8492	0.159543935	0.124978	1.276573	0.201753	0.681726
itgb7	29.18473	0.325164108	0.418529	0.776922	0.437205	0.851125

fstl4	57.36115	0.021570405	0.346911	0.062179	0.950421	0.99801
zgc:193690	26.33947	-0.471729509	0.507894	-0.9288	0.352995	0.803822
BX950188.1	19.66113	-0.737119151	0.523962	-1.40682	0.159481	0.6257
tmem185	307.3003	0.069919612	0.144424	0.484127	0.628296	0.921864
ankrd46a	732.1977	0.095191693	0.111004	0.857555	0.391138	0.827902
zranb2	701.4928	-0.030169386	0.104665	-0.28825	0.773157	0.960624
si:ch211-218m3.16	2.126937	0	1.959315	0	1	NA
or115-6	1.474002	0.775593229	1.574504	0.492595	0.622299	NA
or115-7	2.428128	0.416679469	1.309528	0.318191	0.75034	0.955074
rp2	348.9511	-0.266311907	0.165997	-1.60431	0.108645	0.541123
chst7	182.4404	0.093858986	0.166641	0.563241	0.57327	0.903952
or115-13	0	NA	NA	NA	NA	NA
cyfip1	1765.1	-0.053712926	0.090512	-0.59344	0.55289	0.897594
CT030712.1	6.660184	-2.152589287	1.098092	-1.9603	0.049961	0.388976
tp63	522.6386	-0.129888993	0.164191	-0.79109	0.428894	0.846607
tbc1d23	883.0877	-0.126480236	0.096299	-1.31342	0.189043	0.668516
unc119b	519.8472	0.223005139	0.149566	1.491014	0.135958	0.591131
angptl3	241.3168	-0.056392653	0.200798	-0.28084	0.778831	0.961073
atg4c	352.9449	-0.006499268	0.172662	-0.03764	0.969973	1
zgc:158291	421.9744	0.063076974	0.189528	0.332811	0.739277	0.953422
rbmx2	100.0306	0.092689219	0.223431	0.414845	0.678255	0.936026
siah2l	343.6601	0.12153993	0.166671	0.729221	0.465867	0.86502
cldn2	97.26615	-0.549444253	0.361067	-1.52172	0.128078	0.577144
wdr78	20.58976	0.492527884	0.461997	1.066085	0.286385	0.757624
nop16	267.3708	-0.169662868	0.184642	-0.91887	0.358162	0.809542
pars2	63.06888	-0.08405461	0.234998	-0.35768	0.720581	0.947263
ttc4	210.0505	-0.103238673	0.184788	-0.55869	0.576375	0.90475
csmd1a	2.478297	0.971665872	1.754411	0.553842	0.579687	0.905618
tlr5a	25.6278	-0.623338703	0.477197	-1.30625	0.191468	0.671456
disp1	229.9953	-0.012570885	0.152465	-0.08245	0.934288	0.996752
dnajc19	295.9089	-0.746511847	0.220755	-3.38163	0.000721	0.035767
acvr2ba	73.06837	-0.462618765	0.231863	-1.99523	0.046018	0.375021
ppig	445.5796	0.151356512	0.143577	1.054186	0.291798	0.761454
sned1	90.49622	-0.266032304	0.311886	-0.85298	0.393671	0.828374
rps6ka4	94.11452	0.051690171	0.203307	0.254247	0.799304	0.967111
cdca5	205.8637	0.129266791	0.253915	0.509094	0.610686	0.915331
si:ch73-194h10.2	100.6536	0.297517662	0.3216	0.925116	0.354905	0.806034
gfi1ab	46.75149	-0.110542718	0.283051	-0.39054	0.696137	0.941657
cdc23	194.0159	0.268674231	0.166987	1.608957	0.107626	0.538826
sall4	336.635	0.011530127	0.144302	0.079902	0.936315	0.997032
pak1ip1	370.8949	-0.24092969	0.159486	-1.51067	0.130874	0.582503
tlr9	0.298346	0	5.267649	0	1	NA
kif20a	270.268	0.235456847	0.246551	0.955002	0.339577	0.795226
ublcp1	208.9221	0.416696538	0.164197	2.537779	0.011156	0.183611
viml	30.02396	-1.407245971	0.539027	-2.61072	0.009035	0.16317
etv5b	781.8936	-0.332895044	0.178667	-1.86321	0.062432	0.427718
ca4c	20.13153	-0.474693613	0.488131	-0.97247	0.330816	0.788291
tpst1l	287.3033	0.084819424	0.136832	0.619881	0.535336	0.891993
st6gal1	23.46805	0.649724104	0.519265	1.251238	0.210847	0.692523
eef1b2	9308.534	-0.483822017	0.153772	-3.14636	0.001653	0.062485
def6b	35.10481	-0.301993472	0.441784	-0.68358	0.494242	0.877583
pparda	105.1549	0.33506409	0.229661	1.458953	0.144578	0.603539
camk1ga	102.7363	-0.251308741	0.521901	-0.48153	0.630143	0.922869
slc15a1b	163.4388	2.636976722	0.927194	2.84404	0.004455	0.109917

nr4a2b	187.1912	-0.166760051	0.324777	-0.51346	0.607629	0.915204
CR631122.1	2.794746	0.958730969	1.802109	0.532005	0.594722	0.910869
fkbp2	915.633	-0.215383948	0.192308	-1.12	0.262715	0.740207
ppp1r14ba	791.1632	0.368749905	0.188414	1.957123	0.050333	0.390692
zgc:110782	31.41131	-0.058822164	0.361137	-0.16288	0.870612	0.983365
zgc:77262	923.7874	0.146591024	0.111038	1.320192	0.186771	0.665301
zgc:112416	6.070629	0.492920176	0.789555	0.624301	0.53243	0.890978
hif1al2	205.1918	-0.269303168	0.184331	-1.46097	0.144023	0.603144
atg9a	585.1502	-0.245896736	0.135237	-1.81827	0.069023	0.448349
pacs1a	239.0319	-0.007178841	0.185975	-0.0386	0.969208	1
ccr7	1.960897	-3.767512146	1.63161	-2.30908	0.020939	NA
cycsb	5246.292	0.513486575	0.117098	4.385119	1.16E-05	0.00208
ola1	4018.223	-0.276473533	0.106488	-2.59628	0.009424	0.166808
fabp6	1050.195	1.055395163	1.69478	0.622733	0.53346	0.891181
ttc1	161.9522	0.087267468	0.167512	0.520964	0.602392	0.914001
cldn19	171.7875	0.116885382	0.201975	0.578712	0.562784	0.900772
cdc42	2806.45	0.129885412	0.103054	1.260357	0.207541	0.68962
rbms3	116.6603	0.232040722	0.254442	0.91196	0.36179	0.811787
vars	791.0436	0.072203285	0.125637	0.574695	0.565497	0.901564
tex261	206.7439	0.174141755	0.149076	1.168144	0.242749	0.724345
emp2	1882.127	-0.037688103	0.149248	-0.25252	0.80064	0.967515
pgap2	88.10695	0.007300615	0.184337	0.039605	0.968408	1
ncbp3	332.1829	0.022368584	0.108823	0.205549	0.837143	0.976007
plppr2b	16.10207	0.416285921	0.659239	0.631465	0.527737	0.889948
rtn4a	4776.76	0.021601386	0.130422	0.165626	0.868451	0.982801
stx12l	253.4935	-0.039121176	0.167299	-0.23384	0.815109	0.970957
c1qb	12.96554	-1.323422372	0.791661	-1.6717	0.094583	0.51347
c1qa	14.11602	-0.0038732	0.819157	-0.00473	0.996227	1
zak	374.5089	-0.047828194	0.201393	-0.23749	0.812279	0.970129
birc2	1829.017	-0.000721148	0.114675	-0.00629	0.994982	1
polq	53.54952	0.075637944	0.249137	0.3036	0.761433	0.958159
pcf11	941.6778	0.004672133	0.125633	0.037189	0.970334	1
ccdc90b	180.8266	-0.130727895	0.24645	-0.53044	0.595804	0.911427
alg8	107.321	0.500781588	0.210882	2.374698	0.017563	0.234077
dtd1	194.7623	0.069600396	0.157503	0.4419	0.658561	0.930581
guca1d	5.352452	-0.812469663	1.678686	-0.48399	0.628392	0.921864
myo7ab	64.1601	0.286403013	0.314519	0.910607	0.362502	0.812457
sc5d	78.01762	0.558840701	0.227294	2.458666	0.013945	0.207125
st14b	203.556	-0.301359209	0.161174	-1.86978	0.061515	0.425588
hp1bp3	4756.084	0.031516484	0.098341	0.320481	0.748604	0.955074
parapinopsinb	36.83773	-0.04736572	0.401651	-0.11793	0.906125	0.991631
rbp4l	154.6088	-0.223704635	0.329413	-0.6791	0.497074	0.878572
nr0b2a	352.7276	-0.91209397	0.304155	-2.99878	0.002711	0.085055
dusp4	202.8427	-0.438229869	0.261153	-1.67806	0.093336	0.51113
ppp1r3b	205.2307	0.115982385	0.351485	0.329978	0.741416	0.954103
eri1	157.8117	0.062596889	0.198008	0.316133	0.751901	0.955391
fybb	55.33384	-0.321970626	0.258651	-1.24481	0.213202	0.695467
ccdc80l2	476.1667	0.058571919	0.163282	0.358716	0.719808	0.94694
or137-2	0.787119	0	2.6798	0	1	NA
vav2	225.0761	-0.12749026	0.149818	-0.85097	0.394788	0.828825
or115-12	11.73209	0.781974231	0.5231	1.494884	0.134945	0.589926
ddt	1694.45	0.332747666	0.175162	1.899657	0.057478	0.413721
p2rx4a	31.12134	-0.203087002	0.47671	-0.42602	0.670095	0.933809
idh3b	1569.047	0.031476039	0.114182	0.275666	0.782805	0.962328

gnrh2	18.44539	0.233261998	0.526793	0.442796	0.657913	0.930581
si:dkeyp-87d8.8	41.11631	-0.296910851	0.310437	-0.95643	0.338855	0.795226
gnaia	784.1368	0.032044375	0.154466	0.207453	0.835656	0.976007
mitd1	90.27899	-0.437897265	0.228083	-1.91991	0.05487	0.405196
tspan10	65.9684	-0.477022095	0.258498	-1.84536	0.064985	0.434945
kctd13	302.6687	-0.221600521	0.144627	-1.53222	0.125467	0.57334
pou5f3	15.63735	-0.910656939	0.51522	-1.76751	0.077143	0.469501
fut7	11.74076	0.076220394	0.4958	0.153732	0.877821	0.985613
clic3	73.81601	-0.072935385	0.252262	-0.28913	0.772485	0.960624
bace2	247.557	-0.095729729	0.152679	-0.627	0.530658	0.890545
entpd2b	4.135366	0.529465059	1.251921	0.422922	0.672352	0.934089
cd8a	0.289971	-1.378335838	5.201853	-0.26497	0.791032	NA
wdr38	6.095822	-0.298900086	0.630895	-0.47377	0.635663	0.924524
aadacl4	3.447715	-0.826987642	1.478771	-0.55924	0.575998	0.904739
dhrs3b	252.7075	-0.135391465	0.120047	-1.12782	0.259395	0.736127
dck	87.26687	0.620537159	0.396956	1.563238	0.117997	0.560168
slc4a4b	207.0943	-0.175247513	0.226201	-0.77474	0.438491	0.851685
fpgs	373.0009	-0.459287792	0.192477	-2.3862	0.017024	0.231104
cdk9	686.2742	-0.241160692	0.088796	-2.71591	0.006609	0.136957
ttc17	264.5038	0.017058362	0.164902	0.103445	0.91761	0.993777
vps33b	230.9732	-0.245864263	0.182226	-1.34923	0.177264	0.650801
serinc4	88.58724	0.133237482	0.278567	0.478296	0.632439	0.923353
cstf1	373.1688	0.020683745	0.130433	0.158578	0.874002	0.98385
wnt7aa	323.3319	0.072672851	0.168493	0.431311	0.666242	0.932664
zgc:171599	97.97174	-0.07367911	0.268222	-0.27469	0.783551	0.96245
wbp2nl	1160.1	-0.088758046	0.131233	-0.67634	0.498825	0.879386
opn1lw2	6911.024	0.194732308	0.505536	0.3852	0.700089	0.942647
opn1lw1	10.38699	-0.868155027	1.842275	-0.47124	0.637469	0.924779
zgc:92107	509.3798	-0.072872636	0.124701	-0.58438	0.558966	0.899409
crygm2e	21.95442	1.410912355	1.30468	1.081424	0.279508	0.752477
zgc:113307	255.0545	-0.009474462	0.201728	-0.04697	0.96254	0.999509
fmoda	3308.778	-0.135550277	0.145164	-0.93377	0.350421	0.802215
tmem183a	407.3025	-0.147499547	0.126267	-1.16816	0.242743	0.724345
prlhr2a	13.20102	0.930198409	0.477519	1.947981	0.051417	0.394202
atp2b4	1828.646	0.024238259	0.12052	0.201113	0.84061	0.97643
suclg2	1145.851	0.113429028	0.153593	0.738505	0.460207	0.862369
ube2w	198.1709	-0.245286223	0.180913	-1.35582	0.175155	0.649231
gdf11	19.87549	0.075427063	0.362834	0.207883	0.83532	0.976007
cdk5rap1	86.59454	-0.216516224	0.205337	-1.05444	0.29168	0.761454
hpdb	3932.138	0.003747359	0.270243	0.013867	0.988936	1
cbx2	413.0082	0.052826364	0.151019	0.3498	0.726489	0.949072
mrpl45	415.7206	0.292733939	0.157703	1.85624	0.063419	0.429783
npepps	1561.726	-0.06820026	0.115727	-0.58932	0.555647	0.898723
rnf8	368.6713	-0.102393484	0.135056	-0.75816	0.448356	0.856741
slit1a	418.502	-0.095485734	0.230951	-0.41345	0.67928	0.936209
zgc:114123	1.810116	1.418003882	1.465122	0.96784	0.333124	NA
vcla	410.4082	-0.024892709	0.199464	-0.1248	0.900683	0.99045
bcap31	710.5161	-0.120204127	0.111603	-1.07707	0.28145	0.753734
krt98	11.04389	-1.089418806	1.00516	-1.08383	0.278442	0.752101
krt94	1725.998	0.405438395	0.184703	2.195087	0.028157	0.298748
krt93	7.854018	1.055425418	1.774779	0.59468	0.552057	0.8974
kpna6	146.0408	-0.22151746	0.236217	-0.93777	0.348362	0.800896
dhrs3a	178.9171	-0.596427985	0.22416	-2.66072	0.007797	0.149185
plvapb	1079.412	-0.126542413	0.120493	-1.0502	0.293625	0.762788

ptprga	55.26144	-0.103161024	0.317415	-0.325	0.745178	0.954956
tapbp.2	27.70581	-0.693702115	0.437664	-1.58501	0.112964	0.55033
tuba2	2751.685	0.155183735	0.168315	0.921982	0.356538	0.807342
cyp27b1	43.26937	-0.225844395	0.286088	-0.78942	0.429865	0.847418
npffl	86.05124	0.939193195	0.251253	3.738042	0.000185	0.014229
pdrg1	155.1069	0.140325659	0.16824	0.834082	0.404235	0.833888
aamp	2492.071	-0.140431716	0.206111	-0.68134	0.495657	0.87806
lhfpl5a	15.2805	0.795349635	0.500387	1.58947	0.111954	0.548295
ift52	226.4407	0.071643835	0.178294	0.401829	0.68781	0.938835
rab9a	75.14006	0.12109541	0.19309	0.627145	0.530564	0.890545
cdc42ep4b	601.8836	0.003414755	0.102097	0.033446	0.973319	1
tekt3	35.76692	-0.79151552	0.435414	-1.81784	0.069088	0.448527
kcnj16	6.873371	0.612608744	0.710696	0.861984	0.388696	0.827613
slc16a3	664.8361	0.097749545	0.173153	0.564528	0.572395	0.903461
ablim1b	796.493	-0.308613489	0.17293	-1.78461	0.074324	0.463115
vti1a	138.6458	-0.185579668	0.184492	-1.00589	0.314467	0.777479
entpd1	726.2704	0.108755094	0.140611	0.773446	0.439259	0.852079
kcnk1a	28.12129	-0.181126854	0.320998	-0.56426	0.572576	0.903533
itgb3b	85.34981	0.549364931	0.217697	2.523529	0.011618	0.187517
chad	207.8904	0.132177291	0.3374	0.391752	0.695241	0.941316
arl4d	630.1017	-0.192620617	0.136741	-1.40866	0.158937	0.62448
tmem106a	384.5886	-0.206795384	0.141418	-1.4623	0.143659	0.602773
cdk5r1b	289.6289	0.1814117	0.251588	0.721065	0.470869	0.867874
si:ch211-270n8.1	187.6664	-2.342981241	0.840558	-2.78741	0.005313	0.122491
mrpl10	260.9361	0.127032468	0.154856	0.820329	0.412029	0.838123
cdk5rap3	311.0997	0.325735757	0.15083	2.159623	0.030802	0.312711
si:dkey-222b8.1	62.21995	-0.072808669	0.25356	-0.28715	0.774001	0.960636
elac1	274.4252	-0.082111123	0.165854	-0.49508	0.620544	0.918076
foxn2b	346.6374	-0.193114457	0.131869	-1.46444	0.143073	0.602002
oplah	383.9752	-0.182808819	0.150133	-1.21765	0.223359	0.70531
FAM53C	323.6276	-0.019987359	0.152349	-0.13119	0.895621	0.98926
fkbp10b	502.3393	0.116201573	0.147954	0.785388	0.432226	0.848419
klhl11	168.8729	-0.043534052	0.154197	-0.28233	0.777692	0.961073
id4	379.7853	0.074327875	0.186711	0.39809	0.690564	0.939981
vdac1	3968.596	0.087596003	0.161806	0.541363	0.588257	0.907935
CABZ01084564.1	1.819684	-0.925234173	1.703873	-0.54302	0.587117	NA
ca7	35.25653	0.685091211	0.443604	1.544374	0.122498	0.569468
lcmt1	204.111	0.100224279	0.164964	0.607551	0.543485	0.894925
aqp8a.1	680.3164	-0.419549482	0.155045	-2.70599	0.00681	0.139148
hbae5	99.69583	-0.57956753	0.271155	-2.1374	0.032565	0.319477
hbbe2	2922.457	-0.452829088	0.22811	-1.98514	0.047129	0.378022
zgc:163057	138.4674	0.194902919	0.29394	0.663071	0.507285	0.883007
shisa9a	153.7678	-0.074463889	0.276211	-0.26959	0.787475	0.963263
tomm22	883.0041	0.046946198	0.122966	0.381783	0.702622	0.943591
tmem184ba	601.889	0.034833085	0.116182	0.299814	0.764319	0.959228
csnk1e	439.541	0.04536092	0.139082	0.326145	0.744315	0.95491
slc16a8	11.53006	-0.589195841	0.878444	-0.67073	0.502395	0.881321
phf5a	293.1534	-0.187551098	0.213029	-0.8804	0.378643	0.822997
rgs9b	114.6673	0.401182524	0.354013	1.133243	0.257112	0.734679
si:ch211-147h1.4	0.854649	-0.387386303	2.066291	-0.18748	0.851285	NA
zdhhc4	222.5844	-0.168775912	0.157066	-1.07456	0.282574	0.754808
msrb1b	0	NA	NA	NA	NA	NA
nptx2b	7.861584	-1.491803271	1.262825	-1.18132	0.237475	0.718568
bhlha15	16.41184	-0.726865866	0.612796	-1.18615	0.235564	0.716683

dlgap5	442.6557	0.322970808	0.244036	1.323458	0.185683	0.663476
minpp1b	47.59594	-0.049391018	0.299304	-0.16502	0.868929	0.982801
pofut2	131.8757	0.172736741	0.216962	0.79616	0.425939	0.844714
acta2	2649.747	-0.507979436	0.218624	-2.32353	0.02015	0.25169
ch25h	11.69288	-0.491305015	1.018318	-0.48247	0.629474	0.922687
zgc:55262	430.4751	-0.346516487	0.104558	-3.31412	0.000919	0.041848
dusp19b	98.43881	-0.060161511	0.194954	-0.30859	0.757631	0.957364
dkk1b	171.6469	0.063733502	0.220413	0.289155	0.772463	0.960624
glipr1a	38.05343	-0.373670482	0.295784	-1.26332	0.206473	0.688857
stub1	596.6019	-0.021644729	0.142549	-0.15184	0.879313	0.986147
cox6b1	971.2621	-0.019326001	0.194694	-0.09926	0.920929	0.994085
h3f3b.1	9450.34	0.021491326	0.093658	0.229467	0.818506	0.971656
nif3l1	125.5378	0.226175617	0.217682	1.039021	0.298795	0.765735
zmp:0000000624	77.17071	0.30737417	0.252009	1.219694	0.222581	0.704536
decr2	221.3599	-0.006526579	0.161206	-0.04049	0.967706	1
gsnb	1366.061	0.366214364	0.177826	2.059396	0.039456	0.34755
gsg1l2b	27.48535	-0.546434338	0.501749	-1.08906	0.276127	0.750674
tectb	85.14758	-0.072838324	0.239725	-0.30384	0.761248	0.958159
pts	354.61	-0.370254119	0.188238	-1.96695	0.049189	0.386201
tmem204	288.2252	0.095221885	0.145125	0.656138	0.511735	0.885041
klhl14	90.27788	0.143360678	0.243631	0.588435	0.556241	0.899002
rdh12l	70.36128	-0.506486789	0.290769	-1.74189	0.081528	0.480662
gpr137c	104.8298	-0.193679465	0.200343	-0.96674	0.333674	0.790676
sh3glb1a	503.3948	-0.025519989	0.16223	-0.15731	0.875002	0.984191
cops9	372.3293	-0.005667719	0.210448	-0.02693	0.978514	1
phb2a	1285.142	0.151144808	0.086356	1.750251	0.080075	0.478179
wipf2a	626.4427	0.007971862	0.115241	0.069176	0.94485	0.997371
vmo1b	341.1544	0.201732363	0.29047	0.694504	0.487366	0.873291
st8sia3	5.890736	0.654470166	0.721855	0.90665	0.364592	0.814648
smpx	553.5875	-0.075535741	0.13974	-0.54055	0.588821	0.908018
mtfr1	211.5621	-0.127037338	0.144702	-0.87793	0.379984	0.823607
pde7a	666.647	-0.032812689	0.138666	-0.23663	0.812942	0.970328
slc51a	237.9738	-0.677656056	0.302369	-2.24115	0.025016	0.280283
pola1	426.448	0.202860294	0.21601	0.939127	0.347666	0.80026
map7d2b	879.5978	0.359720676	0.175283	2.052224	0.040148	0.350281
pkp4	396.9396	0.021522974	0.128962	0.166895	0.867453	0.982582
si:ch211-140b10.6	174.0947	-0.051793999	0.188871	-0.27423	0.783908	0.96249
usp46	10.06064	0.434277959	0.517769	0.838749	0.40161	0.832651
ccdc58	143.3498	0.023382391	0.188597	0.123981	0.901331	0.990553
cst14a.2	952.9404	-0.26836626	0.184795	-1.45224	0.146435	0.605984
eloca	217.2019	-0.290477818	0.162266	-1.79013	0.073433	0.46112
elocb	1427.648	0.004080142	0.192878	0.021154	0.983123	1
flj11011l	296.9098	0.081605073	0.127752	0.63878	0.522966	0.888222
trappc3	594.7003	0.009098005	0.155043	0.05868	0.953207	0.998436
tuba1b	1634.378	0.627829099	0.183825	3.41536	0.000637	0.032797
prdm14	10.4552	-0.082528166	0.694701	-0.1188	0.905436	0.991631
ngdn	264.7685	-0.393233173	0.234745	-1.67515	0.093904	0.511703
pabpn1	2827.097	0.017599424	0.08609	0.20443	0.838017	0.976359
pi15a	14.13403	0.153722876	0.464887	0.330668	0.740896	0.954103
gpr22b	73.68226	-0.451771292	0.422752	-1.06864	0.285231	0.756415
cd226	22.72777	-0.128676185	0.859286	-0.14975	0.880964	0.986846
mtrr	148.3849	0.333763661	0.176516	1.89084	0.058646	0.418329
cct5	4976.94	0.031085015	0.10974	0.283261	0.776977	0.961073
mllt10	1101.417	-0.055869599	0.112422	-0.49696	0.619216	0.917397

commd3	240.1488	0.006880683	0.221905	0.031007	0.975264	1
tagln	1232.404	-0.211883883	0.208693	-1.01529	0.309969	0.774671
acadl1	208.0153	0.217542802	0.190268	1.143348	0.252894	0.732759
gcm2	154.8201	-0.462594872	0.186443	-2.48116	0.013095	0.198965
elovl2	177.5931	0.61139544	0.273864	2.232477	0.025583	0.283893
gnal	68.16796	-0.234272011	0.300127	-0.78058	0.435051	0.85007
mppe1	145.8781	-0.091435041	0.153412	-0.59601	0.551169	0.8974
fam49bb	242.4154	0.01076113	0.154394	0.069699	0.944433	0.997371
epdr1	176.8792	-0.237645161	0.220605	-1.07724	0.281373	0.753688
stard3nl	242.4228	-0.131023018	0.143551	-0.91273	0.361384	0.811679
otulina	101.098	0.172586881	0.274163	0.629504	0.529019	0.890325
si:ch211-146l10.8	0.286387	3.343250914	5.172828	0.64631	0.518079	NA
zgc:173856	0.053262	1.055396947	5.267649	0.200354	0.841203	NA
si:dkey-96n2.3	2.004351	1.305720357	1.079927	1.209082	0.226631	NA
mcee	215.0815	0.215868719	0.161149	1.339563	0.180387	0.656134
slc35b3	163.6864	0.140358792	0.166742	0.841774	0.399914	0.831277
cpb1	15040.39	-0.111630794	0.305673	-0.3652	0.714964	0.945821
agtr1b	32.49725	-0.477468999	0.297516	-1.60485	0.108527	0.541057
fzd8a	463.609	0.017801072	0.158244	0.112491	0.910434	0.992208
slc35g2b	309.2892	0.022413274	0.234469	0.095592	0.923845	0.994713
f13a1a.1	1.633422	2.572456716	2.032609	1.265593	0.205659	NA
ralaa	396.8878	0.248958118	0.192235	1.29507	0.195296	0.674872
lancl2	654.5701	0.278268334	0.149145	1.865753	0.062076	0.42715
gsap	93.13588	-0.451064572	0.19408	-2.32412	0.020119	0.251571
stk38l	200.5358	0.379171198	0.177319	2.138352	0.032488	0.319445
lrtm2a	128.4205	0.094666141	0.222579	0.425314	0.670608	0.933939
dcp1b	173.3657	0.010403073	0.208951	0.049787	0.960292	0.999164
rassf8b	168.5538	-0.138205588	0.161307	-0.85678	0.391564	0.827902
pawr	386.45	-0.164575557	0.119443	-1.37786	0.168247	0.639712
rps16	17833.16	-0.363047023	0.135274	-2.68379	0.007279	0.144623
ndufb2	1723.001	0.052895343	0.127628	0.414449	0.678545	0.936026
slc35e3	141.2189	0.042475197	0.205742	0.206449	0.83644	0.976007
gramd4b	363.6459	0.070811855	0.134979	0.524613	0.599852	0.913598
tbc1d22a	158.0306	-0.298391894	0.171025	-1.74472	0.081033	0.479801
taf3	565.167	0.003824181	0.151226	0.025288	0.979825	1
atp5f1c	9725.858	-0.012130954	0.125535	-0.09663	0.923017	0.994525
kin	151.9822	-0.188749654	0.147081	-1.2833	0.199386	0.679878
itih2	1602.571	-0.141904265	0.229891	-0.61727	0.537059	0.89244
itih5	174.8462	-0.058763008	0.195037	-0.30129	0.763192	0.958905
tmem110l	419.422	-0.49457039	0.192105	-2.57449	0.010039	0.173583
sfmbt2	50.62255	-0.239560519	0.281077	-0.85229	0.394051	0.828374
acot15	610.9034	0.735094604	0.568301	1.293496	0.19584	0.676219
lamb1b	616.2285	0.31835824	0.188969	1.684709	0.092045	0.507669
tmcc3	610.6248	0.048879037	0.095286	0.512972	0.607971	0.915204
nr2c1	386.8451	0.082927571	0.10441	0.794249	0.427051	0.845491
fgd6	174.8266	0.261385216	0.211554	1.23555	0.216626	0.69815
si:ch211-244b2.4	62.79213	-0.111255512	0.337637	-0.32951	0.741769	0.954329
ppp6r2a	682.8495	0.041987428	0.115781	0.362645	0.71687	0.94627
gcc1	272.9746	-0.193726355	0.125949	-1.53813	0.124016	0.57117
lamtor4	714.2387	0.036895811	0.17707	0.208369	0.834941	0.975994
atp6v1f	821.7052	0.067161001	0.129893	0.517047	0.605123	0.914985
hgfa	50.86334	0.03328728	0.282313	0.117909	0.90614	0.991631
lepb	13.53633	0	1.787021	0	1	1
bik	98.65558	-0.312775056	0.271311	-1.15283	0.248981	0.728918

si:dkey-202b22.6	5.607197	0.192543482	0.905422	0.212656	0.831595	0.974908
hsd17b2	28.19615	-0.067920521	0.37087	-0.18314	0.854689	0.979161
snx20	28.68355	-0.824678631	0.442025	-1.86568	0.062085	0.42715
sco2	0	NA	NA	NA	NA	NA
OTUD7A	63.79403	0.222795611	0.308065	0.723209	0.469551	0.867224
socs2	121.9436	-0.202865107	0.268811	-0.75467	0.450444	0.857151
mybpc1	497.1119	-0.559987424	0.21294	-2.62979	0.008544	0.158366
dram1	49.42916	-0.157480602	0.354207	-0.4446	0.656609	0.929815
pus1	125.1668	-0.305168818	0.194488	-1.56909	0.116627	0.557506
washc3	275.0087	-0.190396156	0.146707	-1.2978	0.194357	0.674677
noc4l	246.5838	-0.275292712	0.194981	-1.4119	0.15798	0.623766
phpt1	202.9308	0.146143576	0.13811	1.05817	0.289978	0.76078
bcat1	292.1464	-0.228750125	0.242585	-0.94297	0.345698	0.798904
lrmp	164.652	-0.107364657	0.216082	-0.49687	0.619281	0.917397
lum	1889.764	0.044279484	0.168295	0.263107	0.792468	0.965226
zgc:172145	55.21315	0.035776602	0.234242	0.152734	0.878608	0.985761
kcnj8	18.03432	-0.64872825	0.466263	-1.39134	0.164124	0.633166
tnni2a.1	919.1254	0.534311475	0.525261	1.01723	0.309044	0.773791
shisal1a	62.57208	-0.045521462	0.320805	-0.1419	0.887161	0.987739
si:dkey-14k9.3	74.05054	-0.488755594	0.209762	-2.33005	0.019804	0.24843
rassf8a	141.5821	-0.17615616	0.216	-0.81554	0.414764	0.838698
akr1b1.2	410.5373	-0.502969601	0.217969	-2.30753	0.021025	0.257618
cax1	223.3007	-0.773868865	0.170609	-4.53592	5.74E-06	0.001186
ticrr	153.5134	0.328046314	0.197143	1.664	0.096112	0.517378
alkbh3	179.2278	0.01917796	0.181651	0.105576	0.915919	0.993113
tafa5b	93.45696	0.32850359	0.232288	1.41421	0.1573	0.623263
nfybb	336.8789	-0.41704429	0.1934	-2.15639	0.031054	0.313376
lrrc10	12.01987	-0.734286059	0.530567	-1.38397	0.166369	0.636415
clta	2666.358	0.172540817	0.132873	1.29854	0.194102	0.674365
nansa	407.9381	0.212595095	0.127602	1.666079	0.095698	0.516616
nek8	55.75697	0.00686425	0.233226	0.029432	0.97652	1
rab34a	203.9128	-0.119398725	0.210526	-0.56714	0.570617	0.902481
asb15a	193.0553	0.944940882	0.32028	2.950357	0.003174	0.09274
lmod2a	3.719466	-0.914573491	0.822991	-1.11128	0.266448	0.742912
rbl2	222.2513	0.036015476	0.168751	0.213424	0.830996	0.974908
slc13a1	82.89895	-0.260774724	0.584173	-0.4464	0.655309	0.929606
elavl4	2458.112	0.111503145	0.184041	0.60586	0.544608	0.895206
usp3	168.7316	0.16488629	0.162938	1.01196	0.311557	0.77613
cal2	8.152053	0.69506241	1.459266	0.47631	0.633854	0.923974
msrb3	761.2472	0.195232584	0.140962	1.384997	0.166053	0.635628
hic1l	236.733	-0.233141343	0.125436	-1.85864	0.063078	0.429313
irf7	72.88822	-0.17591678	0.291875	-0.60271	0.5467	0.896107
ANKRD50	218.1796	0.050758332	0.226738	0.223863	0.822864	0.971966
si:dkey-39a18.1	19.01435	-0.515489676	0.390611	-1.3197	0.186935	0.665301
ifngl1r	0	NA	NA	NA	NA	NA
il26	0.906363	1.982951675	2.315716	0.856302	0.391831	NA
il22	1.447194	-1.731926611	2.235947	-0.77458	0.438586	NA
mdm1	39.55889	-0.006297596	0.29464	-0.02137	0.982947	1
calua	1069.246	0.1235042	0.130208	0.948516	0.342867	0.79738
opn1sw1	13480.69	1.413766473	1.220469	1.158379	0.246709	0.727706
tnpo3	1043.12	-0.034190198	0.136256	-0.25093	0.801871	0.967776
irf5	105.2424	-0.187378391	0.199813	-0.93777	0.348364	0.800896
ccdc87	5.881925	-0.196907413	0.817146	-0.24097	0.809579	0.969828
cntn1b	275.5322	0.197698549	0.210062	0.941142	0.346632	0.79947

pdzrn4	188.5325	0.303064876	0.208661	1.452427	0.146383	0.605984
zcrb1	126.1147	-0.242057494	0.196745	-1.23031	0.218581	0.699445
pphln1	540.0584	-0.020979613	0.116132	-0.18065	0.856639	0.979558
prickle1b	740.2701	-0.065727328	0.115294	-0.57009	0.568619	0.902234
myca	706.66	0.002286225	0.147829	0.015465	0.987661	1
mrpl23	902.4096	-0.052062706	0.157535	-0.33048	0.741035	0.954103
dclre1c	182.7666	-0.220818276	0.150767	-1.46464	0.14302	0.602002
meig1	82.37375	-0.00573474	0.216032	-0.02655	0.978822	1
tspan9b	292.8441	0.01993882	0.177255	0.112487	0.910437	0.992208
adm2a	50.2776	0.231519584	0.314059	0.737186	0.461009	0.862601
ciapin1	716.9244	-0.017729639	0.205842	-0.08613	0.931361	0.995809
guca1g	28.56685	-0.920523803	0.632586	-1.45518	0.14562	0.604973
cped1	379.7114	-0.019837088	0.134596	-0.14738	0.88283	0.986908
stab2	287.9503	0.197130795	0.217068	0.908151	0.363799	0.814181
ppfibp1a	547.1067	-0.250080337	0.118909	-2.10313	0.035455	0.330833
syt10	71.46168	0.291779965	0.324135	0.90018	0.368025	0.817272
si:ch211-161n3.4	1.14696	3.547774334	2.081831	1.704161	0.088351	NA
tmtc2a	146.6506	0.297248092	0.179909	1.652215	0.098491	0.521131
mettl25	58.81148	-0.166206921	0.264399	-0.62862	0.529596	0.890434
ccdc59	32.04114	-0.614460969	0.351905	-1.7461	0.080793	0.479801
cracr2ab	16.99806	-0.245806244	0.480766	-0.51128	0.609154	0.915204
prmt8b	80.04634	1.158087849	0.37732	3.069247	0.002146	0.073122
asb13a.2	16.303	-1.189538104	0.523823	-2.27088	0.023154	0.268647
cry1a	1452.063	0.123100568	0.103186	1.192996	0.232871	0.714492
PYURF	150.1546	-0.177033468	0.193651	-0.91419	0.360619	0.811237
cnbpa	2564.14	-0.016716264	0.112786	-0.14821	0.882175	0.986908
zgc:103499	98.05256	-0.176058766	0.203172	-0.86655	0.386189	0.827064
avpr1ab	64.5532	-0.039000146	0.296376	-0.13159	0.895309	0.989223
srgap1b	263.9694	0.473607079	0.246235	1.923397	0.05443	0.403361
si:ch211-125a15.1	161.8586	-0.15201645	0.226379	-0.67151	0.501894	0.881148
si:dkey-106n21.1	60.36014	-0.278686607	0.319777	-0.8715	0.383479	0.825875
si:ch211-68a17.7	195.2445	0.307551834	0.208965	1.471789	0.141078	0.599658
acanb	89.98251	-1.082983956	0.364093	-2.97447	0.002935	0.089477
fbxl14b	494.1553	-0.077918408	0.149241	-0.5221	0.601603	0.913982
hapln3	367.0084	-0.07803247	0.178618	-0.43687	0.662207	0.930742
mfge8b	177.3363	0.189682574	0.274899	0.690007	0.49019	0.875082
abhd2b	111.5024	0.689174907	0.279893	2.462276	0.013806	0.205712
rlbp1b	130.6669	0.052776942	0.334677	0.157695	0.874697	0.984156
lrtm2b	30.85059	1.422969194	1.249017	1.139271	0.25459	0.733441
samm50	695.172	0.27979559	0.127951	2.186745	0.028761	0.301537
mcm10	68.10384	0.195775313	0.272595	0.718191	0.472639	0.868786
tnnt2e	3120.16	0.122541261	0.098019	1.25018	0.211234	0.692983
zgc:101783	269.1447	-0.127452735	0.151	-0.84406	0.398638	0.830925
lyrm5b	73.43132	-0.067307459	0.319718	-0.21052	0.833261	0.975379
cart2	46.71564	0.224536102	0.671835	0.334213	0.738219	0.952898
si:dkey-14d8.7	1138.487	0.093618061	1.673369	0.055946	0.955385	0.998611
si:dkey-14d8.6	8590.07	-3.232888224	1.086128	-2.97653	0.002915	0.089033
mapk11	181.4258	0.045939778	0.163834	0.280404	0.779167	0.961073
si:dkey-14d8.20	40.25374	-0.676763995	0.359957	-1.88013	0.060091	0.422231
zgc:113263	316.677	-0.008476975	0.147045	-0.05765	0.954028	0.998611
apex1	1234.523	0.11968505	0.139123	0.860285	0.389632	0.827843
osgep	259.1374	-0.370589188	0.20215	-1.83324	0.066766	0.441363
si:ch211-214j24.10	939.1774	-0.163429514	0.11543	-1.41583	0.156826	0.622675
ccdc167	223.1769	-0.310774638	0.166759	-1.86361	0.062376	0.427718

galnt8b.1	26.82952	0.403495149	0.421092	0.958211	0.337956	0.794808
rad51ap1	78.16812	0.004124256	0.244479	0.01687	0.986541	1
fgf23	22.1596	1.084271364	1.300731	0.833586	0.404514	0.834083
fgf6b	12.30849	0.172882456	0.56607	0.305408	0.760055	0.957886
cebpz	417.5287	-0.153563702	0.146215	-1.05026	0.293598	0.762788
tigarb	84.54811	0.425977853	0.264118	1.612831	0.106781	0.536686
asb15b	86.77466	0.326416636	1.004626	0.324914	0.745246	0.954956
lmod2b	78.91686	-0.408755965	0.487319	-0.83879	0.401589	0.832651
plekha5	695.042	0.003438878	0.123378	0.027873	0.977764	1
frs2a	55.24304	-0.200597781	0.273403	-0.73371	0.463127	0.864191
yeats4	302.6099	0.047273565	0.113236	0.417478	0.676329	0.93557
ube2nb	886.8625	0.000616359	0.107114	0.005754	0.995409	1
nudt4b	334.2739	-0.327187825	0.151524	-2.15932	0.030826	0.312711
gas2l3	112.2815	-0.106376767	0.197111	-0.53968	0.589418	0.908456
scaf11	973.5962	-0.195167744	0.120467	-1.62009	0.105213	0.534134
slc38a2	1144.248	0.09204572	0.143416	0.641811	0.520996	0.887466
mmp30	518.2905	0.201891567	0.329726	0.612301	0.540339	0.893049
acat1	2369.879	0.060540309	0.09851	0.614558	0.538846	0.892875
kctd15a	390.6951	-0.04313853	0.155089	-0.27815	0.780895	0.961608
si:ch211-152c2.3	544.5574	-0.043283749	0.206131	-0.20998	0.833682	0.975482
agbl5	308.0273	0.107789541	0.131548	0.819392	0.412563	0.838123
nr2e3	146.7901	0.172274444	0.250381	0.68805	0.491422	0.875595
dynlt1	47.10679	-0.090953897	0.298001	-0.30521	0.760204	0.957886
tmem181	458.788	0.144145596	0.16381	0.879954	0.378884	0.823136
tulp4a	524.8459	0.068129204	0.134369	0.507029	0.612135	0.915668
mrps10	585.3149	-0.196104063	0.186472	-1.05166	0.292957	0.762505
si:ch211-51e12.7	2249.71	0.342095908	0.127043	2.69275	0.007087	0.142662
crabp1a	441.7958	-0.397394819	0.203673	-1.95115	0.05104	0.393353
slc25a44a	57.7775	0.363118872	0.227454	1.596447	0.110389	0.545238
psma4	1852.729	0.134486879	0.124941	1.076402	0.281748	0.754257
oaz2a	4477.187	-0.074679468	0.095519	-0.78182	0.434318	0.850002
rbpms2a	1029.556	-0.170136841	0.101942	-1.66896	0.095126	0.51461
cpeb1a	17.51814	-0.149051995	0.442795	-0.33662	0.736406	0.951919
immp1l	50.43985	0.223997187	0.326368	0.686334	0.492502	0.876385
pax6b	390.3076	0.149858602	0.17984	0.833289	0.404682	0.83428
slc6a1a	202.3387	0.889156078	0.320431	2.774873	0.005522	0.125566
syn2a	985.9235	0.563020233	0.249495	2.256641	0.024031	0.27455
sec24d	791.4968	0.075540723	0.120804	0.625318	0.531762	0.890949
hrc	1706.011	-0.170591055	0.136624	-1.24862	0.211806	0.693489
kat5b	305.6044	-0.039085528	0.120964	-0.32312	0.746606	0.955074
prlrb	12.93444	0.259179814	0.451521	0.574015	0.565958	0.901739
kcnk10b	8.952901	-0.418658485	0.500777	-0.83602	0.403145	0.833709
gpr65	1.815675	-3.936384205	2.073012	-1.89887	0.057581	NA
egfl6	1592.473	0.072691835	0.103807	0.700257	0.483767	0.87219
csf3r	25.80362	0.100644024	0.52708	0.190946	0.848568	0.977912
spg11	416.0813	0.0567653	0.145392	0.390428	0.69622	0.941657
sidt2	1049.524	-0.362346364	0.17415	-2.08066	0.037465	0.339939
chchd6b	600.6746	0.138770949	0.155382	0.893097	0.371805	0.819358
zgc:153704	1156.673	-0.767865335	0.246303	-3.11757	0.001824	0.065787
si:ch73-55i23.1	2.861729	-0.236680823	1.211768	-0.19532	0.845144	0.976791
zbtb43	15.15318	-0.595337671	0.506096	-1.17633	0.239462	0.719909
arf4a	392.0303	0.022425149	0.159041	0.141002	0.887868	0.988077
saa	13.00368	0	1.810401	0	1	1
necap2	652.3328	-0.128188331	0.144665	-0.8861	0.375562	0.822237

med20	238.4487	-0.120851322	0.160901	-0.75109	0.452598	0.857994
tmcc2	182.4956	-0.059448815	0.212973	-0.27914	0.780139	0.961275
kdm2bb	564.3699	-0.280153226	0.118003	-2.37412	0.017591	0.234077
slc47a1	96.72309	-0.495602561	0.283321	-1.74926	0.080245	0.478492
rasl11a	259.0879	0.131058466	0.208941	0.62725	0.530495	0.890545
kcna6a	84.13525	0.06620405	0.289791	0.228454	0.819293	0.971785
snai1b	347.5298	-0.026988403	0.157195	-0.17169	0.863683	0.981842
CU914487.1	43.85867	1.945567207	0.512777	3.79418	0.000148	0.012175
tmed7	1290.968	0.134848345	0.11283	1.195147	0.232029	0.713202
pym1	211.8817	-0.452287374	0.177117	-2.5536	0.010662	0.179908
zgc:110339	3263.361	-0.235046706	0.129423	-1.81611	0.069354	0.449618
slc27a6	112.0041	-0.034252479	0.204314	-0.16765	0.866862	0.982582
CT027815.1	172.4447	-0.312762953	0.192559	-1.62424	0.104324	0.532541
zgc:77838	142.4092	0.056986878	0.199008	0.286355	0.774606	0.960871
snx16	276.4393	0.015295072	0.129803	0.117833	0.9062	0.991631
stk11	656.1228	0.185065438	0.114662	1.614009	0.106526	0.536018
cacng6b	81.37951	-0.13694852	0.326104	-0.41995	0.674519	0.934877
bloc1s2	222.7216	0.011428277	0.17844	0.064045	0.948934	0.997922
brd2b	86.41793	-0.014266886	0.258548	-0.05518	0.955994	0.998845
dhrs11a	55.18091	-0.635311298	0.252009	-2.52098	0.011703	0.188169
si:ch211-283g2.1	72.6879	3.007524088	0.406232	7.403468	1.33E-13	2.36E-10
si:ch211-283g2.3	0.050624	1.055396947	5.267649	0.200354	0.841203	NA
ebp	80.4502	0.500718855	0.25098	1.995052	0.046037	0.375021
rab10	1545.098	0.279201957	0.102712	2.718312	0.006562	0.136957
caskin1	587.3882	-0.149479717	0.134709	-1.10965	0.267151	0.743418
rab11fip1a	118.6224	-0.174486986	0.178568	-0.97715	0.328497	0.786794
tk2	131.3214	0.022962995	0.186698	0.122995	0.902111	0.990847
dynlt3	631.263	0.006201384	0.138014	0.044933	0.964161	0.999925
b3galnt2	103.1348	-0.034624236	0.236599	-0.14634	0.883652	0.987014
zgc:112038	11.5684	-2.153415906	0.696047	-3.09378	0.001976	0.068961
zgc:162025	72.54416	-0.520229089	0.237847	-2.18725	0.028725	0.301537
si:ch211-170d8.5	122.3433	-3.08145796	1.364286	-2.25866	0.023905	0.274193
prkab1b	862.188	-0.224054994	0.106103	-2.11167	0.034715	0.328285
tnfaip8l2b	163.1086	-0.135885316	0.187182	-0.72595	0.467868	0.866201
b4galnt4a	65.20876	0.212691819	0.26779	0.794247	0.427051	0.845491
RPS17	21498.87	-0.285743071	0.118982	-2.40156	0.016325	0.225785
CR339041.1	4.030711	0.38242752	1.228723	0.31124	0.755618	0.956532
CR339041.2	4.499347	1.397521448	1.430951	0.976638	0.328748	0.787144
mhc1lfa	8.597	0.414742406	0.995988	0.416413	0.677108	0.935906
mhc1lga	5.759857	0.413658477	1.006219	0.411102	0.680998	0.937257
si:ch211-113a14.24	1.704473	2.086293362	1.498375	1.392371	0.16381	NA
nfatc3b	183.8335	-0.207076858	0.184092	-1.12486	0.260649	0.738121
slc7a10b	73.80597	0.016849032	0.344009	0.048979	0.960936	0.999249
faap24	20.03772	0.281315338	0.465846	0.60388	0.545923	0.895711
si:ch211-113a14.18	3.838685	3.547444685	1.486913	2.385779	0.017043	0.231104
hist1h2a4	1.610592	4.852616068	2.216059	2.18975	0.028542	NA
zgc:173552	2.384778	0.109280119	1.582106	0.069073	0.944932	0.997371
ada2a	428.2864	-0.273744584	0.147757	-1.85267	0.063929	0.431598
ccnd2a	281.374	0.007155985	0.13601	0.052614	0.95804	0.999109
tigara	70.97361	0.06418577	0.277198	0.231552	0.816886	0.971207
rpgrip1l	62.12762	0.431743892	0.321428	1.343207	0.179205	0.654489
pdhx	1456.374	0.052425563	0.13689	0.382976	0.701738	0.943292
igl4v10	0.526784	1.055416085	3.087854	0.341796	0.732504	NA
trim44	167.0366	-0.074885002	0.154948	-0.48329	0.62889	0.922122

CABZ01080568.1	368.018	0.862863067	0.309983	2.783583	0.005376	0.123514
sntb2	363.733	-0.042680103	0.133162	-0.32051	0.74858	0.955074
fhod1	134.8116	0.061145085	0.180494	0.338766	0.734786	0.951361
rplp0	94091.24	-0.295389834	0.172058	-1.7168	0.086015	0.491972
dhx33	69.85421	-0.269533201	0.406354	-0.6633	0.507141	0.883007
zgc:113426	38.02623	-0.339357454	0.320183	-1.05989	0.289196	0.760276
si:dkeyp-110c12.3	0.7892	1.930172717	2.563302	0.753003	0.451448	NA
brd1b	359.7333	-0.142556781	0.183733	-0.77589	0.437814	0.851283
fnbp4	1451.938	0.176763699	0.106687	1.656843	0.097551	0.520966
BX664721.1	1.672444	-0.087389104	1.506043	-0.05803	0.953728	NA
CU469568.1	79.61709	0.0722725	0.209016	0.345774	0.729512	0.950056
btr12	120.0699	-0.021226302	0.170598	-0.12442	0.90098	0.99045
ptprz1a	484.1082	0.116934059	0.14114	0.828497	0.407389	0.835698
aass	527.9961	0.20691482	0.18837	1.098452	0.272007	0.748056
swap70a	76.37814	-0.011528845	0.24462	-0.04713	0.96241	0.999461
hyal4	207.9227	0.076716468	0.179387	0.427659	0.6689	0.933115
tmeff2a	487.453	0.225672842	0.142945	1.578739	0.114396	0.553523
si:dkeyp-19e1.3	82.02105	-0.210941189	0.256523	-0.82231	0.410901	0.83771
ctu2	109.0538	-0.392791018	0.236615	-1.66004	0.096906	0.51949
kcnc1a	254.9624	0.196212204	0.214263	0.915755	0.359795	0.810703
galns	174.8186	-0.368230239	0.178379	-2.06431	0.038988	0.345405
cdt1	124.2374	0.299334348	0.23869	1.254072	0.209816	0.691821
tes	353.7773	0.032072256	0.141807	0.226168	0.821071	0.971788
ccdc33	2.305012	-1.495238843	1.270803	-1.17661	0.239351	0.719781
pkp3a	946.4297	-0.314392031	0.143228	-2.19505	0.02816	0.298748
zgc:158222	165.7157	-0.279212105	0.192111	-1.45339	0.146115	0.605672
stra6	406.1568	0.047895233	0.227424	0.210599	0.8332	0.975379
islr2	2798.967	-0.008509385	0.16022	-0.05311	0.957644	0.999109
ush1c	109.9187	0.217646204	0.339725	0.640655	0.521747	0.887551
abcc8	277.121	0.100806608	0.182769	0.551552	0.581255	0.905618
kcnj11	160.5109	0.263565294	0.219346	1.201598	0.229519	0.711596
ankrd11	384.8051	0.045011942	0.18834	0.238993	0.811111	0.969984
ist1	1110.79	0.080070809	0.094884	0.84388	0.398736	0.830925
dhodh	311.9514	-0.084063865	0.171054	-0.49145	0.62311	0.918894
hp	4.686291	0	2.918574	0	1	1
rsph10b	21.34516	-0.139960558	0.41456	-0.33761	0.735656	0.951543
kcng4b	18.40555	-1.438873604	0.671607	-2.14243	0.032159	0.318495
fbn2a	115.7909	-0.225975895	0.213688	-1.0575	0.290282	0.76078
exoc3l1	167.1085	-0.042086482	0.171354	-0.24561	0.805984	0.969625
prmt7	223.3842	0.205932373	0.164286	1.253498	0.210025	0.691821
hpxb	230.2248	0.702776772	0.360132	1.951442	0.051004	0.393269
slc14a2	65.11493	0.357474687	0.55302	0.646405	0.518017	0.887148
pde5ab	29.74275	0.390071486	0.383003	1.018456	0.308461	0.773474
cdk7	295.4559	0.085755435	0.154452	0.555224	0.578742	0.905417
nsmaf	205.2248	-0.147221187	0.169933	-0.86635	0.386299	0.827215
slc30a5	523.2955	-0.086494026	0.121199	-0.71365	0.475443	0.869292
ccnb1	650.8086	0.198141413	0.215966	0.917466	0.358898	0.80995
cyb5r2	110.764	0.199385706	0.24558	0.811898	0.41685	0.840374
plag1	169.7324	-0.075011164	0.18516	-0.40512	0.685392	0.938486
edc3	385.4162	-0.175155925	0.114556	-1.529	0.126265	0.574654
kxd1	104.6405	0.011364398	0.18298	0.062107	0.950477	0.99801
ergic2	326.2019	-0.154145372	0.114501	-1.34624	0.178225	0.652262
pcxb	1419.939	0.175224441	0.170693	1.026547	0.304634	0.769932
ugt2a4	276.0657	-0.044317914	0.220137	-0.20132	0.840449	0.976417

ythdc1	753.2166	0.058273467	0.14563	0.400146	0.689049	0.93924
brms1	173.1994	0.133028463	0.174591	0.761942	0.446095	0.855403
isca1	1332.432	-0.16085927	0.11729	-1.37147	0.170228	0.641806
selenom	272.6686	-0.047217064	0.20097	-0.23495	0.81425	0.970483
zpd	0	NA	NA	NA	NA	NA
mmp15a	243.0675	0.068169986	0.250402	0.272242	0.785436	0.962605
btr01	64.04852	-0.126388409	0.274968	-0.45965	0.645769	0.926728
smu1b	180.9872	0.225817714	0.230276	0.980641	0.32677	0.785579
cd99	809.4692	0.25263513	0.108078	2.337536	0.019411	0.245214
STX3	74.55027	-0.076008362	0.26254	-0.28951	0.77219	0.960624
ndufs8a	3327.531	0.006644294	0.107901	0.061578	0.950899	0.99801
tmem187	43.74742	-0.537314788	0.305538	-1.75859	0.078648	0.474455
si:dkey-9i23.4	2.408564	2.291341441	1.597307	1.434503	0.151429	0.615372
cav2	405.7016	-0.443820287	0.168434	-2.63498	0.008414	0.156847
ciao2b	360.4787	-0.499457663	0.152294	-3.27956	0.00104	0.045197
rrad	115.2538	0.234625448	0.239861	0.978172	0.327989	0.786438
rtn4rl2a	47.78764	-0.345441418	0.604235	-0.5717	0.567525	0.901809
cabp2a	59.12607	0.986279093	0.376736	2.61796	0.008846	0.16123
si:ch73-109d9.1	101.307	-0.837770146	0.248018	-3.37786	0.000731	0.036014
cmtm4	69.85053	0.025874319	0.236711	0.109308	0.912958	0.99273
pomgnt1	291.9042	0.316979973	0.150792	2.102107	0.035544	0.331075
tspan1	25.23489	0.310703904	0.523025	0.594051	0.552478	0.8974
akr1a1b	338.1337	-0.558725217	0.183213	-3.0496	0.002291	0.076134
nxnl1	41.48559	0.085622942	1.21541	0.070448	0.943837	0.997371
trim35-1	36.28617	-0.060114368	0.304124	-0.19766	0.843308	0.976791
caspb	1051.267	-0.514501631	0.162278	-3.17049	0.001522	0.059063
ggt5a	210.4099	0.272997849	0.180863	1.509421	0.131191	0.583069
parietopsin	1.360712	-0.36765008	1.417345	-0.25939	0.795332	NA
pcolceb	229.1972	0.093291191	1.45716	0.064023	0.948952	0.997922
PAQR9	67.18119	1.025681885	0.985333	1.040949	0.297899	0.765407
col4a6	2060.767	0.081166042	0.158308	0.512709	0.608155	0.915204
col4a5	4563.436	0.189367649	0.157256	1.204197	0.228514	0.70974
si:dkeyp-93a5.2	1.323681	0.652046966	2.34491	0.278069	0.780959	NA
dnajb9a	36.25589	-0.083632837	0.299078	-0.27964	0.779757	0.961073
blcap	1480.934	-0.154381602	0.140509	-1.09873	0.271887	0.748035
si:ch211-122f10.4	246.4498	0.593061836	0.154699	3.833655	0.000126	0.011039
gabarapb	3544.333	-0.287505692	0.139287	-2.06412	0.039006	0.345405
cxcr1	4.420085	2.939147722	1.5751	1.866007	0.06204	0.42715
CCKAR	45.87614	-0.41028608	0.423128	-0.96965	0.332221	0.789602
rbpjb	76.47164	0.147802927	0.274664	0.538124	0.590492	0.908901
notch1b	307.0603	0.273129137	0.249418	1.095066	0.273488	0.749148
trim35-31	2.853463	0.334680558	0.911763	0.36707	0.713567	0.945821
agxta	728.9683	-0.884479174	0.246664	-3.58576	0.000336	0.020824
dtymk	109.9175	-0.370940435	0.2552	-1.45353	0.146076	0.605672
atg4b	538.4718	-0.100511482	0.16786	-0.59878	0.549319	0.897174
zgc:114041	37.7669	-0.519415305	0.421154	-1.23331	0.217459	0.69815
mboat4	3.709135	-0.798950223	1.866219	-0.42811	0.66857	0.933086
hexa	306.8209	0.648776199	0.149683	4.334336	1.46E-05	0.002392
crtc3	73.89778	0.052267947	0.221948	0.235496	0.813824	0.970423
ehf	12.81178	0.937630598	0.770306	1.217219	0.223521	0.705391
adamtsl5	86.01941	-0.00051792	0.227214	-0.00228	0.998181	1
rag2	12.16572	0.23814657	0.691401	0.344441	0.730515	0.950056
rag1	44.78114	0.525066584	0.393516	1.334295	0.182107	0.658775
accs	182.0787	0.318653805	0.158162	2.014736	0.043932	0.367079

boka	218.808	-0.236242588	0.151316	-1.56125	0.118465	0.561566
gli3	194.9362	0.110295712	0.201303	0.547909	0.583754	0.906298
slc1a2a	212.1606	3.57110658	1.288885	2.770695	0.005594	0.126154
notch3	1377.766	0.101453298	0.236397	0.429164	0.667804	0.933086
acvr1bb	96.66129	0.020275522	0.165539	0.122482	0.902517	0.990964
ptgs1	158.3961	0.096203614	0.176128	0.546215	0.584918	0.906655
pbx4	1960.252	0.121276838	0.118712	1.021605	0.306968	0.772084
lix1	307.173	-0.042082104	0.138214	-0.30447	0.760769	0.957886
lrrc8ab	224.1738	0.205645799	0.15666	1.312692	0.189287	0.668869
il12rb2	17.52531	0.190048546	0.444827	0.427242	0.669203	0.933115
il23r	9.311946	-1.758920299	1.658406	-1.06061	0.288868	0.760057
znf507	61.63205	0.031171631	0.261013	0.119426	0.904938	0.991631
si:ch211-236k19.2	0.407697	-3.322174862	3.497813	-0.94979	0.342221	NA
uap1	341.0817	-0.089661033	0.160291	-0.55936	0.575913	0.904739
zgc:171711	11.13991	-0.537831641	0.756568	-0.71088	0.477157	0.869292
eif2s1b	3187.605	9.68E-05	0.132629	0.00073	0.999418	1
rbfox2	1629.872	0.06649078	0.105372	0.631013	0.528032	0.889948
znf541	5.350312	-0.318064488	0.846465	-0.37576	0.707098	0.944945
fdx2	255.5409	0.10400277	0.196124	0.530292	0.59591	0.911428
s1pr5b	19.79021	0.046413843	0.385964	0.120254	0.904282	0.991581
c3a.3	575.1208	0.277421514	0.694209	0.399623	0.689434	0.939493
btr02	123.9015	-0.340541827	0.172164	-1.97801	0.047928	0.380801
zgc:114118	10.68056	0.118422869	1.148303	0.103129	0.917861	0.99387
rcvrna	395.5572	-0.33918681	0.260433	-1.3024	0.192781	0.672803
aff2	165.254	0.09682543	0.203107	0.476721	0.633561	0.923924
zgc:158564	1187.519	0.013140276	0.118353	0.111026	0.911595	0.992326
alkbh4	104.4092	-0.105403951	0.223091	-0.47247	0.636591	0.924779
trim35-3	2.392898	-2.320832206	1.839062	-1.26197	0.206961	0.689276
rab1aa	303.1711	0.172231208	0.150998	1.140621	0.254028	0.733071
slc22a18	295.2683	-0.181377823	0.148527	-1.22118	0.22202	0.703829
cabp2b	68.56321	0.839482032	0.33055	2.539654	0.011096	0.18325
osgn1	346.7905	0.305932011	0.144526	2.116797	0.034277	0.326832
zmp:0000000634	41.94689	0.350950473	0.431852	0.812664	0.41641	0.839843
rab33ba	203.1387	0.239850938	0.155036	1.547062	0.121848	0.56863
pimr198	0	NA	NA	NA	NA	NA
zgc:171489	23.70105	0.308820898	0.369864	0.834957	0.403742	0.833888
uqcrc1	5110.369	0.041661989	0.12689	0.328332	0.742661	0.954484
pimr196	0.137703	1.055372236	5.267649	0.20035	0.841207	NA
tlr5b	127.8744	0.050740906	0.195747	0.259217	0.795468	0.966036
slc4a2b	192.3907	0.063582518	0.198045	0.321052	0.748171	0.955074
abcf2b	121.1684	0.206329988	0.276938	0.745041	0.456247	0.860359
ftr98	3.305038	-0.367164021	1.586902	-0.23137	0.817026	0.971207
ociad1	797.3783	0.024912933	0.08015	0.310829	0.755931	0.956532
ociad2	232.4582	-0.325626302	0.182105	-1.78812	0.073757	0.461398
sgcb	380.705	-0.283498925	0.214124	-1.32399	0.185506	0.6634
spata18	45.73501	-0.013006326	0.35644	-0.03649	0.970892	1
tbl3	771.0303	-0.085336914	0.198296	-0.43035	0.666941	0.932984
smarca5	1533.696	0.055923927	0.199183	0.280766	0.77889	0.961073
si:dkey-156n14.3	484.3179	0.099874124	0.131906	0.757163	0.448952	0.856796
cacng1b	2.197347	0	1.936902	0	1	NA
il15	79.68507	-0.345436462	0.262528	-1.31581	0.188238	0.667434
cltb	148.0108	0.041668343	0.195103	0.213571	0.830882	0.974908
higd2a	779.7312	-0.246105295	0.164537	-1.49575	0.13472	0.5894
faf2	718.341	0.030899131	0.131614	0.23477	0.814387	0.970503

zgc:66455	3.418526	-1.387067185	0.822862	-1.68566	0.091861	0.507669
b3gnt2a	7.537324	-1.678650537	0.990645	-1.6945	0.09017	0.50413
psmd12	1594.498	-0.02381117	0.143301	-0.16616	0.868029	0.982801
ccdc22	443.1607	-0.098211076	0.136413	-0.71995	0.471555	0.86809
cldnd1b	253.0758	-0.09816437	0.130862	-0.75013	0.453174	0.858337
pou2f3	114.8436	0.185817492	0.223655	0.830822	0.406074	0.834645
pcgf5b	68.82431	-0.03936161	0.275111	-0.14308	0.886231	0.987533
tmed8	305.6304	0.199828861	0.13808	1.4472	0.147841	0.608687
pkz	25.09917	2.166941241	0.793611	2.730482	0.006324	0.134501
gpr6	3.202616	1.055420281	2.070051	0.509852	0.610155	0.915331
dnmt3ba	822.5128	0.099715371	0.151874	0.656567	0.51146	0.884763
pak6b	79.99464	-0.685218046	0.318854	-2.149	0.031634	0.316333
mgat2	223.3087	0.019663909	0.191393	0.102741	0.918169	0.993884
si:dkey-71l1.1	27.62068	0.521671599	0.372649	1.3999	0.161543	0.628646
crfb12	75.94658	-0.149908087	0.300067	-0.49958	0.61737	0.916365
ankrd12	736.388	0.027578367	0.122457	0.225209	0.821816	0.971852
ppp1r12c	340.9725	-0.114242178	0.128104	-0.89179	0.372504	0.819953
si:ch211-256e16.3	143.9705	0.224431448	0.208804	1.074842	0.282446	0.754744
bicdl2	159.4546	-0.439265736	0.203692	-2.15652	0.031043	0.313376
si:dkey-43k4.3	22.1282	0.170147368	0.384814	0.442154	0.658377	0.930581
syngr2a	541.4188	-0.171209729	0.155292	-1.1025	0.270244	0.746532
inha	0.759844	3.205177372	3.057942	1.048149	0.29457	NA
spred2a	326.6872	0.005556035	0.156428	0.035518	0.971667	1
mia	471.9308	-0.432095739	0.326684	-1.32267	0.185945	0.663819
actr2a	1154.68	0.193739724	0.092547	2.09342	0.036312	0.335171
hspb2	154.5731	0.095607851	0.233706	0.409095	0.68247	0.938006
rhpn1	8.457277	0.753056721	0.548953	1.371806	0.170124	0.641806
porcnl	130.9287	-0.129689611	0.186617	-0.69495	0.487085	0.873291
pisd	436.9891	0.022453596	0.116887	0.192096	0.847667	0.97772
dusp10	19.51633	-0.489175893	0.430831	-1.13542	0.256198	0.73401
gsk3bb	21.79075	0.342705843	0.412346	0.831113	0.40591	0.834645
igfbp2a	888.0257	-0.030014441	0.161133	-0.18627	0.852232	0.978626
taf1a	113.5499	-0.002708793	0.199188	-0.0136	0.98915	1
pdcd11	689.6663	-0.311567221	0.190904	-1.63206	0.102667	0.529448
arhgef11	593.4381	-0.075694562	0.114329	-0.66208	0.507921	0.883056
pcdh18b	929.2404	0.128596228	0.170879	0.752556	0.451717	0.857676
arhgap45a	48.95009	0.260803934	0.318011	0.82011	0.412154	0.838123
zgc:110789	184.375	-1.011788839	0.198371	-5.10049	3.39E-07	0.000125
lin28b	40.45866	-0.274290982	0.347449	-0.78944	0.429854	0.847418
tsga10	32.34449	0.851371184	0.477695	1.782247	0.074709	0.46413
calcoco2	359.7776	0.175972876	0.205637	0.855744	0.392139	0.827902
stx4	506.5356	-0.084712378	0.168192	-0.50367	0.614497	0.915998
sgms2a	57.69347	0.019186857	0.313735	0.061156	0.951235	0.99801
snx11	284.3574	-0.136255928	0.165347	-0.82406	0.409906	0.836939
mul1b	69.58182	0.101963547	0.222312	0.45865	0.646485	0.926728
CR762407.1	13.67457	1.179517429	0.665872	1.771388	0.076496	0.468417
ubxn10	6.561299	0.346569022	0.804138	0.430982	0.666481	0.932664
tia1	1126.385	0.016698349	0.083329	0.200391	0.841174	0.97643
cptp	156.7361	-0.065691036	0.206835	-0.3176	0.750787	0.955074
lig3	618.1028	0.06357911	0.127248	0.499645	0.617325	0.916365
fgfrl1b	409.1702	-0.107700335	0.17096	-0.62997	0.528712	0.890094
porcn	74.78549	-0.354428312	0.276788	-1.28051	0.200367	0.680719
cenpi	198.1624	0.152373735	0.211159	0.721607	0.470536	0.867743
tmem35	1685.85	0.122591965	0.125156	0.979513	0.327326	0.786249

arl13a	121.552	0.070447282	0.608582	0.115756	0.907846	0.991745
c6ast4	9177.002	0	1.713423	0	1	1
nkiras2	411.4806	-0.143841117	0.130524	-1.10203	0.270448	0.746683
cpeb2	56.66329	0.31222682	0.286683	1.089099	0.27611	0.750674
dpp9	1026.639	0.132071523	0.106957	1.234812	0.2169	0.69815
CU468164.1	0.872449	3.695529351	2.316655	1.595201	0.110667	NA
olig4	79.29441	0.033440966	0.277887	0.12034	0.904214	0.991581
tnmd	721.097	0.211423844	0.191014	1.106847	0.26836	0.744748
raver1	192.7554	0.1707599	0.192213	0.888391	0.374331	0.820992
ahrrb	4.874225	1.569091095	1.110361	1.413136	0.157616	0.623766
bsdc1	1430.159	0.033119984	0.101183	0.327326	0.743421	0.954856
spryd7a	319.1495	-0.05009374	0.12555	-0.39899	0.689898	0.939679
fkbp1b	674.9019	0.305831938	0.14877	2.055741	0.039807	0.348912
AL954655.1	6.014295	-1.789442746	1.514875	-1.18125	0.237504	0.718568
si:ch211-106n13.3	66.14176	-0.567746474	0.468168	-1.2127	0.225245	0.70685
fam210b	551.0243	0.155844706	0.208159	0.74868	0.45405	0.858944
kpna3	1042.647	0.157843626	0.105252	1.499676	0.133698	0.587056
shisa9b	3.905577	-0.220579886	1.186926	-0.18584	0.852569	0.978672
ca10a	256.0121	0.187775652	0.265094	0.708337	0.478736	0.869292
ftr69	3.575352	0.120523933	1.176036	0.102483	0.918373	0.993884
hs3st4	10.50761	0.450711802	0.62259	0.72393	0.469109	0.867181
ube2ia	976.8403	0.055024058	0.119885	0.458973	0.646254	0.926728
fermt1	398.8823	0.157663242	0.127099	1.240479	0.214798	0.696864
thrab	88.26111	0.186930325	0.221301	0.844688	0.398285	0.830737
si:ch211-193e13.5	10.22976	-0.342976277	0.593542	-0.57785	0.563368	0.901075
pptc7b	103.1314	-0.113379937	0.241703	-0.46909	0.639007	0.924779
sept7b	1358.384	-0.007039818	0.094523	-0.07448	0.94063	0.997371
csnk1a1	3039.818	0.024978519	0.08934	0.279589	0.779793	0.961073
si:ch211-138g9.2	0.28075	1.889444579	4.729616	0.399492	0.689531	NA
si:dkey-182g1.2	174.6047	0.35778869	0.248581	1.439327	0.150058	0.613391
paqr5b	99.88255	-0.499491757	0.256281	-1.949	0.051296	0.394059
arrdc3a	2368.824	-0.091303093	0.119131	-0.76641	0.443434	0.854205
si:dkey-172m14.1	284.4699	0.006677331	0.197425	0.033822	0.973019	1
nr2f1a	1206.595	0.028344482	0.091203	0.310786	0.755963	0.956532
bdh2	0.063619	0	5.267649	0	1	NA
fam172a	263.4885	-0.155875438	0.145701	-1.06983	0.284695	0.755774
SLC9B2	151.6838	0.256345622	0.327001	0.783929	0.433082	0.849392
smpd2b	90.23716	-0.346161265	0.227656	-1.52055	0.128374	0.577658
capn1a	579.4867	-0.17450629	0.112675	-1.54876	0.12144	0.567605
cisd2	169.4482	0.040578046	0.164541	0.246614	0.805207	0.969348
pkp1b	238.9432	-0.253336114	0.176293	-1.43702	0.150713	0.614793
tnni1b	226.7907	-0.098121177	0.238859	-0.41079	0.681226	0.937331
suclg1	5310.076	-0.056448031	0.115909	-0.487	0.626256	0.92063
lrrtm1	268.0107	0.135772883	0.184003	0.737883	0.460585	0.862369
prim2	334.1551	0.07753893	0.159343	0.486617	0.626529	0.920665
sltm	1745.743	0.030093283	0.085978	0.350012	0.726329	0.948987
ankrd31	2.058368	-2.776046446	1.871102	-1.48364	0.137904	NA
hmgcra	257.8943	1.336717416	0.2741	4.876753	1.08E-06	0.000308
egf	37.34567	0.090217026	0.388608	0.232154	0.816418	0.971207
card11	81.49637	0.545764445	0.22298	2.447592	0.014381	0.210099
grinab	806.7989	-0.04613948	0.120175	-0.38394	0.701026	0.942918
gpatch3	238.9946	-0.136429014	0.175317	-0.77819	0.436459	0.850912
capn1b	179.7811	0.157898284	0.170491	0.92614	0.354373	0.805403
pimr130	0.865839	-1.746148446	2.187027	-0.79841	0.424632	NA

chrnb3a	125.9243	-0.091728663	0.257661	-0.35601	0.721836	0.947403
gria2b	402.9982	0.364656299	0.204952	1.779226	0.075203	0.465024
si:ch211-239f4.1	28.78658	0.10202727	0.355439	0.287046	0.774077	0.960652
glrbb	568.8851	0.304897005	0.223694	1.363009	0.17288	0.645664
opn3	40.06444	-1.925929517	0.650609	-2.9602	0.003074	0.091315
zgc:153932	59.48137	0	1.705509	0	1	1
glrba	88.12613	-0.434098788	0.493428	-0.87976	0.378989	0.823145
cdc42ep3	318.172	0.10373646	0.184475	0.562334	0.573889	0.904296
zdhhc12b	54.08265	0.625933475	0.322189	1.942755	0.052046	0.396487
azin1a	367.3915	0.020826209	0.133057	0.15652	0.875623	0.984433
shmt1	1498.496	0.073491986	0.161806	0.454197	0.649687	0.927756
smcr8a	128.5002	-0.233503876	0.210986	-1.10673	0.268412	0.744804
runx3	143.2783	-0.293381702	0.179761	-1.63206	0.102666	0.529448
top3a	126.7379	-0.068084974	0.246173	-0.27657	0.782108	0.962319
zfand3	1584.727	-0.04947996	0.092203	-0.53664	0.591517	0.909437
ndufs4	1605.63	-0.102541984	0.151157	-0.67838	0.497529	0.878774
ppifb	884.068	-0.219435033	0.099604	-2.20308	0.027589	0.295257
fsta	653.258	-0.074981309	0.168277	-0.44558	0.6559	0.929606
golph3	961.7978	-0.16486251	0.08053	-2.04722	0.040636	0.352243
ctso	111.0744	-0.612055417	0.218081	-2.80656	0.005007	0.118028
khdrbs1a	11098.98	-0.02493712	0.095344	-0.26155	0.793669	0.965431
chp1	182.4642	-0.268822697	0.156457	-1.71819	0.085762	0.491305
htt	459.4255	0.006206083	0.152795	0.040617	0.967601	1
CR933791.1	2.703162	-1.443859865	1.542729	-0.93591	0.349318	0.801408
CR933791.2	0.587937	0	3.913617	0	1	NA
dnajb5	25.79966	-0.471395159	0.368171	-1.28037	0.200415	0.680781
rx3	89.21249	-0.099525248	0.292332	-0.34045	0.733516	0.950808
znf532	113.4901	0.123275992	0.19363	0.636656	0.524349	0.888801
htra3a	11.65436	1.540902773	0.627811	2.454407	0.014112	0.208027
atxn2	279.3954	0.194739533	0.1954	0.996618	0.31895	0.779923
kcnk3b	10.04734	0.511059944	1.29206	0.395539	0.692445	0.940414
CU550714.1	0.049519	0	5.267649	0	1	NA
zgc:153642	0.059954	1.055396947	5.267649	0.200354	0.841203	NA
rtkn2b	84.74	-0.120158655	0.205448	-0.58486	0.558642	0.899409
zgc:165423	515.0255	1.113118753	0.273385	4.071609	4.67E-05	0.005329
bcl7a	692.1391	-0.011239119	0.097736	-0.11499	0.90845	0.991812
wnt8a	0.063619	0	5.267649	0	1	NA
si:dkey-57n24.6	61.27447	0.071510774	0.279089	0.256229	0.797774	0.966447
si:ch211-202f3.3	341.8832	-0.355887296	0.153149	-2.3238	0.020136	0.251648
adrb3b	0.420014	0	4.18507	0	1	NA
zgc:113162	161.8645	-0.696549836	0.17492	-3.98211	6.83E-05	0.006851
arf6b	1065.536	-0.042931757	0.108199	-0.39679	0.691525	0.940173
aspg	720.1589	0.35492647	0.144981	2.448098	0.014361	0.209937
nppal	44.38629	0.384969119	0.456853	0.842654	0.399422	0.831196
arhgap23a	819.7106	0.010412537	0.150154	0.069346	0.944714	0.997371
rab25b	48.21511	-0.333378428	0.265592	-1.25523	0.209396	0.691505
plcd3a	369.8686	-0.000274907	0.202379	-0.00136	0.998916	1
nppb	36.92907	-0.794807058	0.535236	-1.48497	0.137553	0.59348
nppa	279.8716	-0.334507125	0.234949	-1.42374	0.154521	0.619425
nmt1a	1406.685	0.00169897	0.087699	0.019373	0.984544	1
ubqln4	1595.39	0.14951455	0.102895	1.453074	0.146203	0.605672
mbnl1	124.3943	0.530992715	0.225727	2.352366	0.018654	0.2416
gabrr2a	39.08018	-0.081273234	0.75856	-0.10714	0.914677	0.992972
xcr1b.1	0.466023	1.055376718	4.65548	0.226696	0.82066	NA

slc25a28	1805.876	-0.238113415	0.123206	-1.93265	0.05328	0.400175
sema4e	236.8025	-0.001897489	0.231712	-0.00819	0.993466	1
slc35f1	118.1789	0.281888434	0.184671	1.526435	0.126902	0.575854
cd22	8.307796	0.30770171	0.695834	0.442206	0.65834	0.930581
lpcat2	128.8427	0.564252123	0.198363	2.844538	0.004448	0.109917
rnft2	162.0883	-0.09103774	0.250954	-0.36277	0.716779	0.94627
grsf1	312.0457	0.067032384	0.137384	0.48792	0.625606	0.920625
kif19	231.1679	0.054196544	0.193468	0.280132	0.779376	0.961073
si:dkey-33i11.1	3.168089	0.435679611	1.054915	0.413	0.679607	0.936344
si:ch211-203b20.7	1.291379	0	2.111903	0	1	NA
eif2s2	3443.508	0.258994589	0.101952	2.54037	0.011074	0.18325
rps11	29011.96	-0.364620469	0.148076	-2.46239	0.013801	0.205712
CR628323.2	3.55872	0.534083333	0.848679	0.629311	0.529145	0.890363
tubb1	116.2999	0.347134389	0.312327	1.111446	0.266376	0.742912
cyp8b1	187.7053	-0.457683556	0.364395	-1.25601	0.209112	0.691152
gosr2	546.0378	-0.028752173	0.127595	-0.22534	0.821715	0.971801
gipc3	67.08354	-0.634742426	0.253691	-2.50203	0.012349	0.19319
thada	127.7663	-0.216699917	0.203184	-1.06652	0.286187	0.757624
mthfr	313.3187	0.123492118	0.235914	0.523463	0.600652	0.913756
si:ch211-204c21.1	736.8529	0.150843934	0.134529	1.121272	0.262172	0.739695
hsf2	524.2905	-0.191885676	0.28988	-0.66195	0.508004	0.883056
prpf38b	658.34	0.1727478	0.135082	1.278839	0.200954	0.681045
fam102ba	139.5949	-0.001636899	0.25147	-0.00651	0.994806	1
cep44	70.69347	0.100378122	0.214011	0.469032	0.639046	0.924779
pkib	137.1846	0.000293812	0.242406	0.001212	0.999033	1
ly75	319.831	-0.010750853	0.24565	-0.04376	0.965092	1
si:ch211-272h9.3	0.045161	0	5.267649	0	1	NA
smpdl3a	51.17126	0.225752563	0.345885	0.652681	0.513962	0.88631
clvs2	33.32096	0.613609605	0.335487	1.82901	0.067398	0.443262
si:dkey-148a17.6	13.05877	-0.543803267	0.543841	-0.99993	0.317344	0.779234
helb	110.3878	-0.15849642	0.189803	-0.83506	0.403686	0.833888
carhsp1	636.8882	-0.284760053	0.148866	-1.91286	0.055766	0.408826
pcp4a	578.3944	0.256219773	0.243488	1.052289	0.292667	0.762191
irak3	16.71715	-0.968022926	0.588058	-1.64613	0.099736	0.524166
b3gnt7l	132.5452	-0.009526903	0.228185	-0.04175	0.966697	1
tas1r2.1	0	NA	NA	NA	NA	NA
b2m	75.71233	0.250287314	0.328343	0.762274	0.445897	0.855403
lrrc47	323.6618	-0.172913909	0.151522	-1.14118	0.253796	0.732873
pcmtd1	842.9278	-0.038653176	0.153883	-0.25118	0.801671	0.967776
alg3	244.9428	0.022146871	0.15164	0.146049	0.883883	0.987014
prss56	6.293366	0.4770426	0.86827	0.549417	0.582719	0.90571
zgc:110626	320.1359	0.198492085	0.142886	1.389163	0.164783	0.634185
zgc:113377	22.43031	0.240395806	0.39375	0.610529	0.541511	0.893858
chic1	128.0197	-0.002785499	0.211945	-0.01314	0.989514	1
cttnbp2nla	99.88056	0.033519289	0.204878	0.163606	0.870041	0.983365
vstm4b	64.89187	-0.426240576	0.262925	-1.62115	0.104986	0.533448
galnt16	136.9607	0.437008645	0.273456	1.598096	0.110022	0.544236
pdzd11	376.0085	-0.086810944	0.155146	-0.55954	0.575791	0.904739
mtor	665.8974	0.04309617	0.09511	0.453118	0.650464	0.927861
dis3l	112.6952	-0.037132804	0.209779	-0.17701	0.859501	0.979975
zgc:172323	16.8869	0.162012567	0.598506	0.270695	0.786626	0.962867
snx22	65.57609	0.212175872	0.248272	0.85461	0.392767	0.827902
ppfia4	93.38195	0.145751867	0.248652	0.586168	0.557763	0.899184
smad6a	574.2428	-0.064071841	0.131783	-0.48619	0.626832	0.920795

me1	228.92	-0.073633239	0.269332	-0.27339	0.784552	0.962605
cox7a2a	2493.614	-0.151137737	0.1752	-0.86266	0.388324	0.827592
asb5b	275.8636	0.342565493	0.137815	2.485686	0.01293	0.198143
itgb1b.1	492.857	0.226388852	0.149091	1.518462	0.128898	0.578173
kif6	15.038	0.71502064	0.508522	1.406077	0.159701	0.625998
sts	51.42642	0.322597439	0.386508	0.834646	0.403917	0.833888
depdc7b	0.986046	1.983423503	2.336935	0.848728	0.396032	NA
inab	272.364	0.502442336	0.21037	2.388377	0.016923	0.230621
mylpfa	206100.3	-0.028836882	0.158268	-0.1822	0.855424	0.979354
zgc:113090	58.1842	0.187064266	0.286206	0.6536	0.513369	0.886281
atp1b4	1431.41	0.232502735	0.118568	1.960924	0.049888	0.388669
zgc:113372	109.6242	-0.339401327	0.240057	-1.41383	0.15741	0.623263
slc2a15b	429.4359	-0.675669716	0.128464	-5.2596	1.44E-07	6.43E-05
RAP1GAP	92.10862	0.089987694	0.253618	0.354816	0.722728	0.947632
apln	1133.144	-0.011516913	0.118037	-0.09757	0.922273	0.994346
pnrc2	7315.462	-0.001767396	0.102361	-0.01727	0.986224	1
ftr14	93.82603	-0.504238678	0.26068	-1.93432	0.053073	0.400175
nkx2.2a	137.442	0.435868272	0.196136	2.222274	0.026265	0.28745
insm1b	362.1152	0.269346235	0.158972	1.694301	0.090208	0.50413
tespa1	14.60571	0.465757171	0.499828	0.931835	0.351422	0.802305
tmprss3a	83.49914	-1.000413579	0.458642	-2.18125	0.029165	0.304273
lrrc6	21.99949	0.195045847	0.37308	0.5228	0.601114	0.913756
zgc:112285	460.4999	0.253637144	0.299921	0.84568	0.397731	0.830444
gna11b	91.52564	0.142494036	0.216219	0.659026	0.509879	0.883733
si:ch211-138g9.3	0.170783	-0.868103029	5.267649	-0.1648	0.869102	NA
adgrd1	34.29349	-0.927911069	0.361784	-2.56482	0.010323	0.176586
si:dkey-56i24.1	25.02371	0.182130822	0.363649	0.500842	0.616482	0.916365
basp1	3226.119	0.204216153	0.165217	1.236051	0.21644	0.69815
cilp	711.6846	0.152630351	0.23363	0.653299	0.513563	0.88631
pax10	133.2405	0.274514859	0.32212	0.852214	0.394095	0.828374
rpl31	27821.55	-0.305849134	0.143146	-2.13662	0.032629	0.319477
ftr12	5.597665	2.408024104	1.259215	1.912322	0.055835	0.408826
eif3jb	619.6875	-0.015400574	0.130419	-0.11808	0.906	0.991631
nptxrb	203.6322	0.390154121	0.186301	2.094209	0.036241	0.335095
fam189a1	111.8816	0.574610687	0.216983	2.648177	0.008093	0.152599
plpp1a	670.8884	-0.149315581	0.127824	-1.16814	0.242751	0.724345
rprma	36.73562	0.730444523	0.405933	1.79942	0.071952	0.4564
wdr27	4.441403	-0.587925743	1.067136	-0.55094	0.581676	0.905618
CU207281.1	1.70295	-3.620445623	5.162221	-0.70133	0.483094	NA
jade2	39.2675	-0.110089796	0.393979	-0.27943	0.779914	0.961101
cdkn2aipnl	418.7361	-0.184745048	0.177619	-1.04012	0.298284	0.765416
PPP2CA	1534.392	0.001385866	0.088505	0.015659	0.987507	1
sord	433.9959	-0.360835275	0.282566	-1.277	0.201603	0.681423
myl2b	122.6337	0.253228606	0.39669	0.638354	0.523243	0.888312
serinc2l	43.6192	-0.276633218	0.282661	-0.97867	0.327741	0.786438
si:ch211-257p13.3	162.1481	0.362069283	0.233644	1.54966	0.121223	0.567243
sub1a	1602.705	-0.046621078	0.203648	-0.22893	0.818923	0.971785
ppp4r2b	629.8388	0.088728427	0.093712	0.946824	0.343728	0.798147
si:ch211-251f6.6	572.9472	-0.849354683	0.22711	-3.73983	0.000184	0.014175
pyya	134.9442	0.290569373	0.301822	0.962716	0.33569	0.792139
ftr06	2.016029	-5.192375203	1.986287	-2.61411	0.008946	NA
pop5	128.4144	-0.326143694	0.207343	-1.57297	0.115726	0.555893
mpp2a	106.5382	-0.012222053	0.212785	-0.05744	0.954196	0.998611
cntn3b	20.10438	0.465282718	0.463236	1.004419	0.315177	0.777834

ccdc103	29.57656	-0.205866799	0.332883	-0.61844	0.536288	0.892371
GK3P	355.4117	0.436951284	0.185866	2.350894	0.018728	0.241927
rpl23	25803.17	-0.347026837	0.151745	-2.2869	0.022202	0.263608
rybpb	401.2187	0.150050888	0.142001	1.056687	0.290654	0.76078
si:dkey-283b1.7	10.70299	-1.939816037	0.831766	-2.33217	0.019692	0.247431
c3a.5	0.966523	-0.868139687	2.835281	-0.30619	0.759459	NA
poglut1	183.2736	0.390939336	0.202164	1.933774	0.053141	0.400175
timmdc1	347.348	-0.057557006	0.115368	-0.4989	0.617851	0.916478
gtpbp1	721.4982	0.054851845	0.118371	0.46339	0.643085	0.92593
adam10a	1706.6	-0.085778451	0.09937	-0.86322	0.388016	0.827592
tdrkh	194.8494	-0.094705649	0.153445	-0.6172	0.537106	0.89244
ngb	213.6278	0.195626193	0.280032	0.698585	0.484812	0.872911
lipca	27.25085	-1.762556743	1.621963	-1.08668	0.277178	0.751426
gdf6a	174.458	-0.044101889	0.206119	-0.21396	0.830575	0.974908
aqp9b	676.074	0.311102406	0.348169	0.89354	0.371568	0.819115
entpd5a	79.70861	-0.368373993	0.303669	-1.21308	0.225101	0.70685
zgc:113054	458.3704	-0.042701653	0.125038	-0.34151	0.73272	0.950617
syne2b	494.483	-0.272274422	0.113175	-2.40578	0.016138	0.224038
aldh6a1	8271.236	-0.174476467	0.114119	-1.5289	0.12629	0.574656
osbp2	172.4606	0.644266322	0.318074	2.025524	0.042814	0.362408
si:ch73-22a13.3	5.413245	0.203626456	0.751399	0.270996	0.786394	0.962701
aldh1a2	967.0583	-0.153064552	0.128079	-1.19508	0.232054	0.713202
gemin8	267.8742	0.040286737	0.150414	0.267838	0.788824	0.963568
ednrbb	0.581027	0	2.831403	0	1	NA
isl2b	253.3965	0.253681322	0.192082	1.320691	0.186604	0.664968
cbsa	307.2393	-0.70118267	0.348491	-2.01206	0.044214	0.368106
cryaa	363.9713	0.503611597	0.300449	1.676199	0.093699	0.511703
fbxo22	406.3776	0.228311636	0.115744	1.972562	0.048545	0.383628
kazald3	246.0145	0.212707372	0.234666	0.906426	0.36471	0.814648
crybb2	7.298954	0.335628725	0.74867	0.4483	0.653937	0.929325
si:dkey-1f12.3	260.7025	-0.438732134	0.30868	-1.42132	0.155224	0.61985
adhfe1	252.9634	-0.460853127	0.142462	-3.23493	0.001217	0.050523
LO017829.1	60.63836	-2.528207224	0.433361	-5.83395	5.41E-09	3.39E-06
enpp7.2	1.641364	0.09364295	2.758555	0.033946	0.97292	NA
enpp7.1	15.88564	0.087259792	1.486656	0.058695	0.953195	0.998436
snx24	48.45196	-0.21950586	0.308743	-0.71097	0.477106	0.869292
nog3	215.2411	-0.402252809	0.126899	-3.16986	0.001525	0.059092
cyp4f3	9.990779	0.425195291	0.705566	0.60263	0.546755	0.896107
lmo7b	131.9957	-0.110502298	0.288395	-0.38316	0.701599	0.943254
kctd12.2	263.964	-0.082518085	0.15024	-0.54924	0.58284	0.90571
hspa4l	370.8952	0.30001632	0.177879	1.686632	0.091674	0.507427
jakmip2	446.514	0.421909726	0.169076	2.495384	0.012582	0.195132
wdr76	215.4388	0.279779903	0.190026	1.472323	0.140934	0.599658
rtkn2a	103.9267	0.365386399	0.243557	1.500207	0.133561	0.586903
tspan3b	247.1278	-0.295559332	0.217426	-1.35935	0.174035	0.647252
ms4a17a.12	7.758101	2.320314428	1.461224	1.587925	0.112303	0.549075
pstpip1b	8.958138	0.556631517	0.666204	0.835528	0.403421	0.833803
sox3	706.7648	0.053414438	0.124931	0.42755	0.668979	0.933115
AL590151.1	7.797079	0.01499195	0.619642	0.024195	0.980697	1
adob	406.8791	-0.01037183	0.139442	-0.07438	0.940707	0.997371
fancd2	235.3867	-0.037681718	0.149876	-0.25142	0.80149	0.967776
gpat2	15.69205	-0.318319469	0.62566	-0.50877	0.610911	0.915331
htr1fb	0.40881	0	4.25335	0	1	NA
cers6	112.4708	0.136690663	0.234215	0.583613	0.559481	0.899571

creb1a	751.4477	0.119164304	0.118702	1.003892	0.315431	0.777834
gatd1	254.1183	0.201251621	0.18748	1.073459	0.283065	0.755028
camk2a	101.1117	-0.372793673	0.314706	-1.18458	0.236185	0.717364
sra1	273.5612	0.112074712	0.159798	0.701352	0.483083	0.871988
ebi3	37.77505	-2.911796659	0.683771	-4.25844	2.06E-05	0.002979
csf1rb	21.75753	-0.605171026	0.424628	-1.42518	0.154106	0.618826
anxa2b	473.4235	-0.70740403	0.312036	-2.26706	0.023387	0.270393
si:dkey-182o15.5	0.148387	-1.755438701	5.23528	-0.33531	0.737392	NA
cracr2b	136.2903	-0.022502702	0.253423	-0.0888	0.929245	0.995404
got1l1	20.71133	0.871199497	0.430475	2.023811	0.04299	0.363264
adrb3a	125.3862	-0.213908474	0.1867	-1.14573	0.251905	0.732418
or135-1	0.806365	2.898070707	2.381004	1.217163	0.223542	NA
foxb1b	32.45477	-0.482434449	0.403573	-1.19541	0.231928	0.713202
ndufaf6	132.9066	-0.076498195	0.211086	-0.3624	0.717051	0.94627
lypla2	1125.014	-0.305349565	0.121599	-2.51113	0.012035	0.190742
gabrg2	16.39975	-0.081838534	0.458418	-0.17852	0.858311	0.979774
myb	285.7453	-0.323673121	0.193214	-1.67521	0.093894	0.511703
stag2b	1486.263	0.166224991	0.132226	1.257126	0.208708	0.690923
aldob	26477.85	-0.2740264	0.201207	-1.36192	0.173224	0.646158
gtf2a2	216.383	0.03245693	0.165491	0.196125	0.844513	0.976791
maea	781.958	-0.105273036	0.111959	-0.94029	0.347071	0.799924
zgc:162948	88.2573	-0.164618615	0.218243	-0.75429	0.450675	0.857151
il2rgb	10.95557	-0.950487838	0.685054	-1.38746	0.165301	0.634423
ghrhrb	15.96782	-1.081211803	0.514957	-2.09961	0.035763	0.33238
si:ch73-89b15.3	67.93567	-0.915381954	0.251003	-3.6469	0.000265	0.017992
GPR68	2.52128	4.493957317	1.555948	2.888244	0.003874	0.10341
ccdc88c	462.4619	0.046963545	0.159192	0.295013	0.767984	0.960296
zgc:153990	288.3903	0.101172076	0.123676	0.818042	0.413333	0.838317
adcyap1r1a	71.47963	-0.076066786	0.249042	-0.30544	0.760033	0.957886
ttll3	16.66088	0.659981603	0.466651	1.414295	0.157275	0.623263
AL954695.1	2.439151	-1.738317098	2.017661	-0.86155	0.388935	0.827725
dcdc2b	44.00151	0.566116617	0.286617	1.975167	0.048249	0.38233
CABZ01084566.1	69.7301	0.064415666	0.469279	0.137265	0.890821	0.988566
mff	403.6063	-0.113058172	0.127975	-0.88344	0.376999	0.822324
si:dkey-187j14.4	105.0224	-0.063177025	0.381144	-0.16576	0.868349	0.982801
apbb3	33.36133	0.05863004	0.308573	0.190004	0.849306	0.978102
vgll2b	271.0062	-0.26278549	0.205675	-1.27768	0.201364	0.681208
alpi.2	25.02251	-3.274718141	0.675172	-4.8502	1.23E-06	0.000346
si:ch211-218m3.13	3.073524	-0.305654153	1.47975	-0.20656	0.836355	0.976007
si:rp71-1g18.1	189.0783	-0.281279082	0.16469	-1.70793	0.087649	0.496994
si:ch211-276c2.4	331.9318	-0.119105116	0.134017	-0.88873	0.374147	0.820849
cbln5	18.74463	0.20484032	0.634433	0.322872	0.746792	0.955074
klhl43	1320.789	0.01184361	0.265089	0.044678	0.964364	0.999925
osbpl2a	82.11724	0.053920162	0.291356	0.185066	0.853177	0.978839
trub1	92.17925	-0.126723731	0.18197	-0.6964	0.48618	0.873244
hnrnpc	1039.664	-0.02981279	0.126142	-0.23634	0.813166	0.970328
or115-11	9.875607	0.920750015	0.708293	1.299956	0.193616	0.673889
or115-2	0.060442	0	5.267649	0	1	NA
pcmtd2a	391.2537	-0.34541956	0.232623	-1.48489	0.137574	0.59348
ubxn11	19.74158	0.066488781	0.390967	0.170063	0.864961	0.982136
pax9	237.4199	-0.074024154	0.167517	-0.44189	0.658569	0.930581
vtnb	1239.934	-0.152477513	0.195949	-0.77815	0.436482	0.850912
mtnr1ba	1.721173	-0.033355284	1.574382	-0.02119	0.983097	NA
plekhh1	96.49809	0.25353409	0.25747	0.984714	0.324765	0.783588

si:ch211-284o19.8	36.27558	-0.211264162	0.378684	-0.55789	0.576919	0.904798
ei24	302.5445	-0.234040952	0.128588	-1.82009	0.068745	0.447298
cbln14	55.92336	-1.245322051	0.330688	-3.76586	0.000166	0.013152
slc13a2	799.4441	-0.165056405	0.293455	-0.56246	0.573803	0.904296
uts2a	5.130328	0.205634029	0.807927	0.25452	0.799094	0.967043
ccdc187	292.0191	-0.155564033	0.154633	-1.00602	0.314405	0.777479
crip1	1025.797	-0.086984369	0.232208	-0.3746	0.70796	0.945335
ism2b	13.7466	0.269206697	0.492147	0.547005	0.584375	0.906412
crygmx	1748.7	0.813292933	0.391251	2.0787	0.037645	0.340171
elmsan1b	556.3156	-0.050642543	0.225085	-0.22499	0.821985	0.971966
etv2	163.3658	-0.232309566	0.200567	-1.15826	0.246757	0.727706
cryba1b	7019.232	0.718344322	0.359263	1.999494	0.045555	0.373513
klhl26	43.98903	-0.20132907	0.271028	-0.74283	0.457582	0.860993
cpdb	1.216093	-0.868143358	2.094032	-0.41458	0.67845	NA
inpp5l	402.1998	0.028529256	0.126618	0.225318	0.821732	0.971801
med13a	994.712	0.162210443	0.131755	1.23115	0.218267	0.699433
tmem62	62.0948	-0.190628449	0.223063	-0.85459	0.392776	0.827902
slc30a1b	46.35061	-0.222721792	0.250799	-0.88805	0.374514	0.821189
angpt2b	45.8514	-0.273763202	0.293636	-0.93232	0.351171	0.80227
si:dkey-42l23.3	0	NA	NA	NA	NA	NA
fbl	1596.066	0.022613348	0.152744	0.148047	0.882306	0.986908
alg10	76.68866	0.224624818	0.223726	1.004017	0.31537	0.777834
srfa	57.3314	-0.251988442	0.290575	-0.86721	0.385828	0.826939
si:dkey-117a8.3	0.502582	1.055411945	3.189057	0.330948	0.740684	NA
opn4b	17.28609	0.113682677	0.450548	0.252321	0.800793	0.967609
si:dkey-42l23.5	0.07132	0	5.267649	0	1	NA
hdhd3	72.95408	0.428438693	0.25507	1.679691	0.093017	0.510593
si:ch211-137j23.8	2.304882	-1.510118292	1.195369	-1.26331	0.206479	0.688857
tgfa	53.79614	-0.714510517	0.35518	-2.01169	0.044253	0.368166
btr32	8.53356	-2.193953247	1.190766	-1.84247	0.065406	0.435359
lin37	147.2358	0.419577585	0.190101	2.207135	0.027305	0.293572
myadml2	17.41458	1.170337325	0.619573	1.888941	0.0589	0.418455
sptbn2	1093.649	-0.125089317	0.14002	-0.89337	0.37166	0.819115
cdc42ep2	48.91742	0.103300698	0.290366	0.35576	0.72202	0.947403
slc2a11a	10.51279	-2.661430363	1.037689	-2.56477	0.010325	0.176586
npm2b	9.554809	-1.44652915	0.732827	-1.9739	0.048393	0.383075
cyp17a2	13.56463	0.002460019	0.638481	0.003853	0.996926	1
fetub	21142.58	0.01047982	0.343326	0.030524	0.975649	1
chmp2a	487.3249	0.019436922	0.107875	0.18018	0.857011	0.979558
htr1b	24.70974	0.214564203	0.477425	0.44942	0.653129	0.929197
hmgb2b	5867.352	0.087328347	0.169874	0.514078	0.607197	0.915204
sfi1	108.2676	0.265254531	0.19788	1.34048	0.180089	0.655771
gpank1	197.1947	0.109528099	0.17792	0.615603	0.538156	0.892498
rargb	26.35238	0.526310566	0.35265	1.492445	0.135583	0.591012
ppp1r2	276.4527	-0.018175749	0.146043	-0.12445	0.900955	0.99045
scaf1	1305.601	0.064364101	0.138658	0.464192	0.64251	0.92567
ttll6	48.94129	-0.270686586	0.31212	-0.86725	0.385804	0.826939
hoxb8b	94.09726	-0.280076498	0.184205	-1.52046	0.128396	0.577658
mustn1a	16.67636	-1.572041347	0.761312	-2.06491	0.038931	0.345405
hoxb5b	279.6457	-0.11184948	0.153557	-0.72839	0.466373	0.865508
mxd4	270.8637	-0.296952693	0.273137	-1.08719	0.276951	0.751311
rab24	152.6579	-0.285585951	0.17041	-1.67588	0.093762	0.511703
hoxb1b	143.6055	-0.443278924	0.169858	-2.6097	0.009062	0.163401
rnf34b	8.077044	-0.216045049	0.618536	-0.34928	0.726876	0.949095

plin1	3.207272	-1.155196493	1.285395	-0.89871	0.368807	0.817729
vsig8a	107.2107	0.548867926	0.251893	2.178973	0.029334	0.304886
zgc:153219	15.7566	-1.414406455	0.561415	-2.51936	0.011757	0.188516
ostn	23.34909	0.009762853	0.53659	0.018194	0.985484	1
hlfx	3677.983	-0.394432085	0.17542	-2.2485	0.024544	0.277819
pof1b	713.3538	-0.225198759	0.126024	-1.78695	0.073946	0.461828
arpc4	1315.19	0.034040557	0.127291	0.267423	0.789143	0.963658
tatdn3	25.84652	-0.528595866	0.351974	-1.50181	0.133147	0.586514
irs1	255.9139	-0.007702912	0.176861	-0.04355	0.96526	1
rbm41	48.45906	0.004888076	0.314857	0.015525	0.987614	1
ripply1	48.07824	0.171148635	0.264347	0.64744	0.517347	0.887148
tmem30b	350.3097	-0.109474987	0.110914	-0.98703	0.32363	0.783161
htr1d	55.3798	-0.211429143	0.43444	-0.48667	0.626492	0.920665
slc26a6l	25.3039	0.10372047	0.820885	0.126352	0.899453	0.99045
im:7136021	70.15664	-0.542353073	0.260551	-2.08156	0.037383	0.339535
ccdc105	32.37	-0.928839357	0.4158	-2.23386	0.025492	0.283691
adgrg6	484.3043	0.092734819	0.145671	0.636607	0.524381	0.888801
cx23	208.1021	0.352363799	0.30677	1.148624	0.250711	0.730689
nmbr	5.105395	1.95570457	0.881056	2.219727	0.026437	0.287989
ftr50	1.085668	-1.351191996	2.084787	-0.64812	0.516907	NA
bms1	450.3669	-0.071857326	0.197768	-0.36334	0.716349	0.94627
pcna	4579.727	0.149779715	0.199913	0.749226	0.453721	0.858742
zgc:113625	14.58875	0.158252529	0.598837	0.264266	0.791575	0.964817
nfatc4	139.1014	-0.011741881	0.232067	-0.0506	0.959647	0.999164
mrps17	232.058	0.022215646	0.188831	0.117648	0.906347	0.991631
ltb4r2b	26.66228	0.35197046	0.411726	0.854865	0.392626	0.827902
pgrmc1	1104.103	-0.139510807	0.162806	-0.85692	0.391491	0.827902
adgra1a	153.5636	0.199873621	0.329386	0.606807	0.543979	0.895043
valopb	6.228118	2.109030805	1.346659	1.566121	0.11732	0.558519
btr06	11.73614	1.946046451	1.612968	1.2065	0.227625	0.708829
ncs1b	52.82041	-0.135971582	0.33757	-0.4028	0.687099	0.938835
pgk1	7975.519	-0.289850728	0.160276	-1.80845	0.070536	0.453224
slc38a8b	17.46787	0.003582359	0.432105	0.00829	0.993385	1
mthfd2l	25.33442	0.2645968	0.444577	0.595166	0.551733	0.8974
rnf40	1084.639	0.0411997	0.087854	0.468956	0.639101	0.924779
hbl4	81.93033	-0.70746183	0.273766	-2.58419	0.009761	0.170429
npffr1l1	1.283886	1.53036819	1.940948	0.788464	0.430425	NA
CU682777.1	4.7169	0.516384108	1.008525	0.512019	0.608638	0.915204
phkg2	235.8296	0.038072	0.15897	0.239492	0.810724	0.969828
st8sia7.1	28.19922	-1.777912612	0.980441	-1.81338	0.069773	0.450821
hectd1	2894.84	-0.03983003	0.16152	-0.2466	0.805222	0.969348
si:ch73-269m23.5	63.10873	0.770750282	0.313583	2.457883	0.013976	0.207312
ap4s1	169.2041	-0.310999547	0.188201	-1.65249	0.098435	0.521076
fbxl8	44.12663	0.316605702	0.270152	1.171956	0.241215	0.722739
ipo11	247.7926	0.166963775	0.187733	0.889367	0.373806	0.820644
taar16a	0.402588	0	4.965055	0	1	NA
ghrl	8.623995	-0.692251426	1.23964	-0.55843	0.576551	0.90475
crbn	212.7167	-0.072145877	0.158644	-0.45477	0.649277	0.927756
pqlc3	119.0036	-0.218671945	0.188175	-1.16207	0.245208	0.726715
taar18d	0.120883	0	5.267649	0	1	NA
sobpa	31.9528	0.062829533	0.389465	0.161323	0.871839	0.983447
taar18a	0.09025	0	5.267649	0	1	NA
tctex1d2	83.46845	-0.068558327	0.336785	-0.20357	0.838692	0.976417
nat10	460.1855	-0.033917098	0.149943	-0.2262	0.821046	0.971788

taar20c	0.094679	0	5.267649	0	1	NA
cog4	331.034	0.13508863	0.118134	1.143518	0.252824	0.732759
cdkn1bb	1589.391	-0.039397594	0.104043	-0.37867	0.704935	0.944015
caprin1b	5790.997	-0.538630299	0.117523	-4.58319	4.58E-06	0.001001
zgc:63972	56.07336	-0.524459711	0.32092	-1.63424	0.102209	0.528963
acin1a	2512.396	0.089017082	0.138675	0.641912	0.520931	0.887432
si:ch211-14a17.11	322.1978	-0.279352205	0.179656	-1.55493	0.119963	0.564831
dhrs1	609.2468	-0.114125448	0.157504	-0.72459	0.468705	0.866951
kti12	93.54636	0.079816202	0.228728	0.348956	0.727122	0.949095
ehd4	138.3676	-0.117221169	0.22475	-0.52156	0.601975	0.914001
homeza	307.0969	-0.169077404	0.16822	-1.0051	0.31485	0.777649
zgc:77151	341.7597	0.001305639	0.153777	0.00849	0.993226	1
slc38a6	126.7338	-0.226789609	0.228619	-0.992	0.321198	0.781574
zp3f.2	1.389739	-1.721923993	1.710274	-1.00681	0.314025	NA
stk33	13.67645	-0.182247974	0.54416	-0.33492	0.737688	0.952532
oxct1b	1045.755	-0.000233837	0.22776	-0.00103	0.999181	1
ap2a1	2114.572	0.177708422	0.080488	2.207901	0.027251	0.293406
ngs	1101.12	-0.093661135	0.374786	-0.24991	0.80266	0.968181
pparab	26.93388	-0.404002152	0.431346	-0.93661	0.34896	0.801306
rerglb	107.2737	0.054101401	0.225836	0.239561	0.810671	0.969828
ccn4a	7.480611	0.832475264	0.703293	1.183682	0.236539	0.717725
trim66	64.62099	0.176101994	0.3417	0.51537	0.606294	0.915204
dctpp1	0	NA	NA	NA	NA	NA
ap1g2	378.2286	-0.056388252	0.131333	-0.42935	0.667666	0.933086
sla1a	39.2384	-0.008331797	0.283036	-0.02944	0.976516	1
slc7a8b	18.41611	0.227359608	0.711282	0.319648	0.749236	0.955074
gabpb2b	93.47117	0.040068871	0.241743	0.16575	0.868354	0.982801
lrrc9	10.18298	0.279706989	0.577984	0.483936	0.628432	0.921864
dcaf7	1978.812	0.144065645	0.08732	1.649857	0.098972	0.522136
ccdc47	998.183	0.118659769	0.095812	1.23846	0.215545	0.697157
si:zfos-464b6.2	87.94741	-0.287512826	0.182647	-1.57414	0.115455	0.555689
b3gnt3.2	2.01085	-2.14910981	1.719131	-1.25011	0.211258	NA
alg13	1.983712	0.149743951	1.38798	0.107886	0.914086	NA
pcbp3	99.25774	0.355030562	0.252419	1.406515	0.159571	0.6257
rhof	66.3022	-0.163128567	0.250895	-0.65019	0.515571	0.886459
si:ch211-198n5.11	189.5408	-0.340668576	0.191051	-1.78313	0.074565	0.463807
spata17	7.16938	-0.39216909	0.579739	-0.67646	0.49875	0.879321
ssuh2.2	13.07008	-0.560301994	0.531474	-1.05424	0.291772	0.761454
rpe65c	53.04138	-0.436645833	0.256654	-1.7013	0.088886	0.500363
slc7a6	283.328	0.156836629	0.14753	1.063085	0.287744	0.758441
yipf6	419.7172	-0.274211351	0.12669	-2.16443	0.030431	0.311024
fzd2	1218.535	-0.014693852	0.138517	-0.10608	0.915519	0.993078
cbx1b	671.6107	-0.019396042	0.180641	-0.10737	0.914493	0.992972
ccdc43	242.5365	0.026237425	0.15837	0.165671	0.868416	0.982801
slc29a1b	250.4503	0.150363611	0.158956	0.945946	0.344176	0.798147
kif1c	52.27954	-0.137317569	0.300274	-0.45731	0.64745	0.927082
loxl1	545.3988	0.169884612	0.140134	1.212299	0.225398	0.70685
epha4a	668.1244	0.100122436	0.148076	0.676157	0.498941	0.879457
clip3	185.8453	0.388077437	0.25307	1.533481	0.125157	0.572892
slmapa	610.6032	0.144069456	0.160222	0.899184	0.368555	0.817325
tinf2	79.94511	0.352355249	0.208287	1.691681	0.090707	0.505206
stambpa	333.2961	-0.044638866	0.132667	-0.33647	0.736515	0.951919
adrb2b	30.75124	-0.374308203	0.37443	-0.99967	0.317468	0.779375
rars	684.5746	0.209240891	0.141885	1.474718	0.140289	0.599409

araf	541.684	-0.113056363	0.137632	-0.82144	0.411394	0.837797
ACOT12	61.37682	-0.227979609	0.365786	-0.62326	0.533114	0.890978
nhsl1a	490.692	0.263166727	0.143835	1.829643	0.067303	0.442891
lrp10	537.4192	-0.079950696	0.120659	-0.66262	0.507576	0.883055
imp4	211.7431	-0.10419299	0.165076	-0.63118	0.527921	0.889948
il12bb	4.301917	0.405798207	1.122947	0.361369	0.717824	0.946466
samsn1a	289.0547	-0.314254894	0.220088	-1.42786	0.153332	0.617847
si:ch73-285p12.4	0.218128	2.459808624	5.205172	0.47257	0.63652	NA
her15.2	149.3877	-0.110575215	0.182532	-0.60579	0.544657	0.895206
her15.1	431.9551	0.321687868	0.141057	2.280546	0.022575	0.265653
smg1	1711.041	-0.012972191	0.144455	-0.0898	0.928445	0.995052
wdr95	19.1386	-0.630030255	0.438844	-1.43566	0.151099	0.615085
znhit1	228.0105	0.098978065	0.196022	0.504934	0.613605	0.915998
waif2	7.914459	0.533191661	0.855594	0.623183	0.533164	0.890978
arl6ip1	2199.633	0.041767112	0.078209	0.534048	0.593308	0.910427
mfsd6b	98.88289	-0.220973975	0.210298	-1.05077	0.293366	0.762713
FO681288.1	63.92848	0.136687719	0.322495	0.423845	0.671679	0.933953
cox6a2	125.9877	-0.730886124	0.382681	-1.90991	0.056145	0.409466
cnot6l	798.177	-0.274264482	0.127838	-2.14541	0.03192	0.317405
mrpl1	202.3562	0.040784295	0.148032	0.275509	0.782925	0.962328
slco2b1	386.0615	-0.19690345	0.151298	-1.30143	0.193113	0.67315
coro1a	296.133	-0.273169501	0.209953	-1.3011	0.193225	0.673236
cldni	5759.827	0.088898073	0.121055	0.734362	0.462728	0.864104
fras1	1096.722	-0.014879684	0.209713	-0.07095	0.943435	0.997371
fli1a	523.1629	-0.099531592	0.162723	-0.61166	0.540762	0.893429
tent5ab	203.4591	0.114034468	0.187403	0.6085	0.542856	0.894847
si:ch211-51c14.1	530.5335	-0.125874556	0.185861	-0.67725	0.498248	0.879132
rac1l	1.246776	2.575877656	1.801666	1.42972	0.152798	NA
si:ch211-1f22.5	0.046941	0	5.267649	0	1	NA
slc34a1b	4.518955	0.087838158	1.312998	0.066899	0.946662	0.997371
pgpep1	242.8387	-0.384715501	0.186636	-2.06131	0.039273	0.346914
adra2db	6.300403	-0.95431403	0.815359	-1.17042	0.241831	0.72348
chrna9	39.10407	0.568739625	0.29183	1.94887	0.051311	0.394059
prdm8b	362.7741	-0.207234309	0.207281	-0.99978	0.317419	0.779336
si:dkey-166k12.1	68.58455	-0.544143188	0.266454	-2.04217	0.041135	0.353918
psmb4	2685.675	0.193712824	0.147401	1.31419	0.188782	0.668514
prlh2r	0.515363	0.965224017	3.034998	0.318031	0.750461	NA
dhx8	687.8534	-0.083240181	0.107776	-0.77234	0.439912	0.852079
or118-2	0.043308	0	5.267649	0	1	NA
or118-3	0	NA	NA	NA	NA	NA
or118-1	0.053262	1.055396947	5.267649	0.200354	0.841203	NA
si:ch211-242b18.1	1354.365	-0.133856544	0.127777	-1.04758	0.294831	0.763708
or116-1	2.835297	2.166075979	1.332518	1.625551	0.104045	0.532096
prkg2	11.89942	0.251026473	0.613555	0.409134	0.682441	0.938006
si:ch211-244e12.7	9.037015	2.109510078	0.97366	2.166579	0.030267	0.310167
uggt1	215.8892	0.179475796	0.177498	1.011141	0.311949	0.776164
cuedc1b	67.06615	0.006683722	0.318511	0.020984	0.983258	1
lmo4b	795.1062	-0.023865496	0.09961	-0.23959	0.810648	0.969828
col10a1a	2222.954	-0.028483891	0.271715	-0.10483	0.916511	0.99343
hs6st1a	69.90456	0.360524323	0.222331	1.621569	0.104896	0.533388
alox5ap	19.51841	-0.013977028	0.425737	-0.03283	0.97381	1
ghra	56.49208	0.079773847	0.313852	0.254177	0.799359	0.967111
si:dkey-46a10.3	482.1176	-0.066163823	0.123826	-0.53433	0.593114	0.910427
pogza	127.2392	-0.079286152	0.174552	-0.45423	0.649666	0.927756

faah2b	34.94706	-1.164931118	0.717669	-1.62322	0.104543	0.53308
lysmd1	112.9976	-0.251016084	0.170814	-1.46953	0.14169	0.600284
emc10	621.8236	0.068644094	0.112587	0.609696	0.542063	0.894241
plcxd3	182.4453	0.861134915	0.255405	3.371643	0.000747	0.036381
zgc:153441	80.50939	0.628134526	0.32323	1.943306	0.051979	0.396431
rfc1	330.3479	0.230722084	0.146819	1.571474	0.116073	0.556131
anp32e	5348.709	0.245705233	0.101346	2.424413	0.015333	0.21791
btr09	9.733697	-0.085880526	0.655906	-0.13093	0.895827	0.98926
sec13	1173.694	0.175497369	0.117818	1.489563	0.136339	0.591569
ptp4a3b	1387.844	-0.099454415	0.108979	-0.9126	0.361453	0.811711
rpl32	22901.78	-0.386268612	0.13006	-2.96993	0.002979	0.090034
nubpl	119.9532	-0.059710602	0.182989	-0.32631	0.744191	0.95491
id3	1051.679	-0.354235206	0.149296	-2.3727	0.017659	0.234598
ssuh2.4	50.66229	-0.105803385	0.370538	-0.28554	0.775231	0.960899
pik3c3	566.614	-0.353950637	0.107226	-3.30097	0.000964	0.043349
nucb1	770.3937	0.195771283	0.162485	1.204859	0.228258	0.709397
ugt5f1	46.91923	-1.359216892	0.38638	-3.51782	0.000435	0.025267
zgc:136870	24.99186	-1.150895743	0.421904	-2.72786	0.006375	0.134763
hsd17b14	198.9919	-0.067376361	0.147457	-0.45692	0.647727	0.927153
CU657980.1	13.48088	-3.731682118	0.915328	-4.07688	4.56E-05	0.005236
xcr1a.1	0.871777	0	3.067953	0	1	NA
pdk4	500.7474	-0.146551108	0.169402	-0.86511	0.38698	0.827592
bcat2	1301.9	0.102537907	0.098849	1.037314	0.29959	0.766128
CSKMT	50.38195	-0.143314698	0.374629	-0.38255	0.702053	0.943292
tp53bp2b	336.2369	-0.012314475	0.12271	-0.10035	0.920063	0.993955
aplp2	2139.35	0.147873914	0.15269	0.968456	0.332816	0.789855
hibch	376.5205	0.036678995	0.124447	0.294736	0.768195	0.960296
gpsm1b	114.5428	0.087726323	0.2013	0.435799	0.662982	0.931141
six2b	97.3655	-0.221107367	0.249485	-0.88626	0.375479	0.822237
six3b	398.2175	0.217533552	0.15897	1.368389	0.17119	0.643325
FP015808.1	0.547201	-1.784869867	2.705538	-0.65971	0.50944	NA
rgra	238.4634	-0.466212517	0.270043	-1.72644	0.084269	0.487271
ms4a17a.16	14.80573	-1.226179786	1.328092	-0.92326	0.35587	0.806879
mta3	567.9793	0.027684282	0.119506	0.231656	0.816805	0.971207
ier5l	1473.79	0.086554309	0.104338	0.829557	0.406789	0.835226
cox7a2l	1300.06	-0.00671611	0.131563	-0.05105	0.959287	0.999164
tmsb	600.584	0.168010424	0.262861	0.639161	0.522718	0.887996
dgat1b	162.5242	0.25044306	0.187715	1.334163	0.18215	0.658775
eif4ebp3	1199.86	-0.535263082	0.232533	-2.30188	0.021342	0.258949
zgc:110540	236.757	0.204605967	0.248455	0.823515	0.410215	0.837066
tpra1	223.2063	-0.545912579	0.154698	-3.52889	0.000417	0.02455
ppp2r5b	294.8136	0.363142319	0.171242	2.120634	0.033953	0.325344
zgc:101765	45.24558	0.182027532	0.363428	0.500863	0.616468	0.916365
badb	148.4022	0.01551348	0.179045	0.086646	0.930953	0.995809
ldlrad4b	37.97697	-0.265366758	0.305594	-0.86836	0.385196	0.826939
lgals2a	684.7446	-0.217480536	0.156017	-1.39396	0.16333	0.631199
mc5rb	4.929764	0.439317951	0.774057	0.567552	0.570339	0.902481
mc2r	1.033855	-3.814674904	2.231434	-1.70952	0.087355	NA
eef2kmt	49.26938	-0.219201306	0.28798	-0.76117	0.446557	0.855454
znf1015	61.75902	-0.124135913	0.29981	-0.41405	0.678839	0.936051
alg1	167.3724	-0.043894406	0.172949	-0.2538	0.79965	0.967206
cd40	25.73119	-1.042538118	0.459291	-2.26989	0.023214	0.268939
itsn2b	848.116	-0.029354267	0.122188	-0.24024	0.810145	0.969828
cxcr2	2.303871	-1.760330615	1.936571	-0.90899	0.363354	0.813662

kifc3	956.7443	0.14098888	0.106837	1.319668	0.186946	0.665301
ebna1bp2	483.8339	0.027092745	0.177669	0.15249	0.8788	0.985841
zbtb41	123.1329	-0.210167877	0.18169	-1.15674	0.247378	0.728026
col4a1	494.331	0.02761139	0.206515	0.133701	0.893639	0.988566
si:dkey-33m11.7	1.607402	0	2.029349	0	1	NA
si:dkey-33m11.8	123.5916	-0.078611474	0.321747	-0.24433	0.806977	0.969726
pds5a	1125.537	0.049862286	0.095701	0.521022	0.602351	0.914001
ptch2	645.8939	0.209255702	0.214466	0.975706	0.32921	0.787409
pomt2	274.0995	-0.02464915	0.151805	-0.16237	0.871012	0.983365
itih3a	402.1663	0.382170235	0.250329	1.526675	0.126842	0.575696
depdc7a	96.69678	-0.078520991	0.206824	-0.37965	0.704204	0.943746
casp3b	217.3026	0.703338422	0.165704	4.244549	2.19E-05	0.003021
ponzr5	477.2147	-0.345582351	0.193051	-1.79011	0.073437	0.46112
map2	566.6338	0.132196196	0.131265	1.007092	0.313891	0.777479
itih1	117.5391	-0.277430692	0.758941	-0.36555	0.714701	0.945821
bcl9l	431.0889	0.077776829	0.126033	0.617115	0.537159	0.89244
prdx5	3218.958	0.111608781	0.143951	0.775325	0.438148	0.851454
zdhhc20a	34.54536	0.017106263	0.279091	0.061293	0.951126	0.99801
borcs7	97.16277	-0.333450896	0.276651	-1.20531	0.228083	0.709324
svila	1226.78	-0.18192199	0.152794	-1.19063	0.233797	0.714749
nxf1	1206.638	0.077074989	0.128007	0.602114	0.547098	0.896121
chac2	81.25619	-0.054661662	0.231097	-0.23653	0.813021	0.970328
rbm4.2	953.2614	0.071330106	0.106361	0.670641	0.502449	0.881342
ggnbp2	531.5845	-0.153049049	0.120577	-1.2693	0.204333	0.685846
pora	960.2784	-0.010353033	0.111776	-0.09262	0.926203	0.994815
cdh27	157.5645	0.226903009	0.311792	0.727738	0.466774	0.865766
fam184b	263.2897	0.108185637	0.183168	0.590635	0.554765	0.89855
cxcl12b	637.2579	-0.333807193	0.173063	-1.92882	0.053754	0.401636
znf148	254.6866	-0.06221933	0.149706	-0.41561	0.677695	0.936026
gde1	330.9918	0.204859664	0.233179	0.878551	0.379645	0.823607
pom121	236.2709	0.03488445	0.163321	0.213595	0.830863	0.974908
dtnbp1a	424.8879	-0.180956132	0.146598	-1.23437	0.217064	0.69815
mylipb	211.1993	-0.102793827	0.24371	-0.42179	0.673181	0.93414
ctsba	3268.567	0.700416208	0.259145	2.702798	0.006876	0.139692
kcnk2a	26.19233	0.209932373	0.440031	0.477086	0.633301	0.923885
ccnl1a	1019.762	-0.15611521	0.087327	-1.78771	0.073823	0.461665
armc4	48.50762	0.782348386	0.271028	2.886594	0.003894	0.103477
pim3	653.8282	0.128419859	0.171799	0.7475	0.454762	0.859845
lrfn4a	128.0719	-0.011775279	0.228168	-0.05161	0.958841	0.999164
cenpf	496.5532	0.432138459	0.293359	1.473072	0.140732	0.599658
si:dkey-101k6.5	84.86101	-0.309060469	0.201863	-1.53104	0.12576	0.573743
zgc:110410	39.24656	0.34817593	0.382112	0.911188	0.362196	0.812085
smyd2a	658.3945	0.032317272	0.240346	0.134461	0.893038	0.988566
slc8a4a	26.00673	0.348655733	0.47871	0.728324	0.466415	0.865517
prox1a	293.3492	-0.049359211	0.181023	-0.27267	0.785108	0.962605
cyp27a1.4	20.62174	0.297414544	0.451109	0.659296	0.509706	0.883733
chad1a	20.85357	-1.242790791	0.382112	-3.25243	0.001144	0.048292
zhx2a	354.652	-0.295632631	0.127801	-2.31323	0.02071	0.255657
epoa	19.17145	-0.188833337	0.444039	-0.42526	0.670645	0.933939
si:ch211-256m1.8	657.1794	-1.028198149	0.223226	-4.60609	4.10E-06	0.000932
c1qtnf6a	33.23561	0.15281469	0.386604	0.395275	0.69264	0.940444
pxdc1a	31.31271	0.310932116	0.561364	0.553887	0.579656	0.905618
tm4sf5	129.9787	0.5147781	0.804492	0.63988	0.522251	0.887826
ccr9a	36.30005	-0.186856385	0.533448	-0.35028	0.726128	0.948987

slc17a5	674.5513	0.088530639	0.279591	0.316644	0.751514	0.955331
zgc:136930	13575.25	0.34679227	0.212699	1.63044	0.103009	0.530217
dtnbp1b	71.4171	-0.178460706	0.30349	-0.58803	0.556513	0.899063
tuba1c	6587.069	0.668236572	0.167148	3.997868	6.39E-05	0.006594
ablim2	357.8508	-0.479838222	0.283798	-1.69077	0.09088	0.505206
dock8	457.4989	-0.446458677	0.137671	-3.24295	0.001183	0.049477
slc7a7	204.6511	-0.137882639	0.245166	-0.5624	0.573841	0.904296
ncs1a	133.8668	-0.243411756	0.199949	-1.21737	0.223465	0.70531
fancc	56.21191	0.527348703	0.247243	2.132915	0.032932	0.321256
kif17	13.82233	0.373269344	0.53116	0.702743	0.482216	0.871592
xdh	625.6464	-0.064553517	0.182565	-0.35359	0.723644	0.947923
zmp:0000000650	4.185174	-1.650968101	1.140572	-1.44749	0.147759	0.608687
cntd2	3.542685	0.102046901	0.999671	0.10208	0.918693	0.993884
snap23.2	115.679	0.419805351	0.334303	1.255765	0.209201	0.691271
slc12a10.3	36.72809	-0.262739887	0.456688	-0.57532	0.565078	0.901538
pitpnm3	108.9929	-0.138053983	0.255203	-0.54096	0.588537	0.908004
si:ch1073-358c10.1	83.19719	0.379187333	0.392316	0.966535	0.333776	0.790676
nkd2a	114.2922	0.033608223	0.178089	0.188716	0.850315	0.978251
rel	161.6827	-0.036451978	0.257041	-0.14181	0.887227	0.98775
cfb	1010.653	0.018034914	0.226128	0.079755	0.936432	0.997044
b3gat1b	75.38874	-0.346807224	0.313501	-1.10624	0.268623	0.744925
id2a	2039.779	-0.161545303	0.1462	-1.10496	0.269178	0.745285
afap1	251.6829	0.273365395	0.193037	1.416127	0.156738	0.622646
prelid3a	106.3376	-0.159321028	0.337924	-0.47147	0.637305	0.924779
mpeg1.1	179.6604	-0.149197754	0.204459	-0.72972	0.465563	0.864997
rab18a	742.8293	-0.115218198	0.153214	-0.75201	0.452045	0.857994
atg9b	673.7688	-0.167674502	0.188572	-0.88918	0.373905	0.820695
atoh1a	93.4512	0.088171703	0.305723	0.288404	0.773037	0.960624
cyb561d1	27.13151	-0.309829711	0.500374	-0.6192	0.535787	0.892102
atxn7l2a	117.1502	-0.307883832	0.181036	-1.70067	0.089004	0.500695
grid2	106.6546	0.126960495	0.276062	0.459898	0.645589	0.926728
ret	304.1701	-0.12189136	0.156853	-0.7771	0.437098	0.851125
sypl2a	1574.716	-0.123818986	0.109119	-1.13472	0.256495	0.73401
slc12a10.1	20.68875	-2.109246485	1.27862	-1.64963	0.099019	0.522138
mcmbp	492.3654	-0.165736036	0.09132	-1.81489	0.069541	0.450071
c1ql4b	35.23443	0.399995816	0.435185	0.91914	0.358022	0.809315
aldh9a1a.2	96.23435	-1.135815108	0.425159	-2.67151	0.007551	0.14728
si:dkey-28d5.11	0	NA	NA	NA	NA	NA
zmp:0000000652	2.636366	0.020422464	1.103696	0.018504	0.985237	1
si:dkeyp-50d11.2	43.6859	0.099496729	0.362337	0.274597	0.783625	0.96245
si:ch73-1a9.4	626.9849	0.19473634	0.127325	1.529443	0.126155	0.574377
slc16a13	71.1894	-0.485410378	0.33201	-1.46203	0.143732	0.602871
mipep	193.5067	0.141009948	0.162183	0.86945	0.384601	0.826588
si:dkey-83f18.2	0.11861	0	5.267649	0	1	NA
pknox2	317.7766	0.203562514	0.130689	1.557605	0.119327	0.562753
sun1	847.0971	-0.007944282	0.158155	-0.05023	0.959938	0.999164
leo1	531.4189	0.231321496	0.108796	2.126199	0.033487	0.323151
sp100.1	6.972377	1.187325078	0.968067	1.22649	0.220014	0.701105
llph	488.3496	0.002756393	0.194972	0.014137	0.98872	1
si:dkey-25e12.3	41.36945	0.677141335	0.350316	1.932946	0.053243	0.400175
zgc:163121	51.34858	0.135108075	0.331998	0.406955	0.684041	0.938486
sema3fb	380.2425	-0.074197097	0.193075	-0.38429	0.700762	0.942918
irf4b	1.740526	1.055417118	2.28784	0.461316	0.644572	NA
gnb5b	446.6888	0.406637589	0.226602	1.794497	0.072734	0.458195

bambia	918.1922	-0.159879917	0.100953	-1.5837	0.113262	0.551017
zgc:66160	583.8907	-0.050159331	0.118063	-0.42485	0.670946	0.933953
vtna	650.7608	-0.434242744	0.244918	-1.77301	0.076227	0.467585
si:dkey-67c22.2	744.8264	-0.080527803	0.127222	-0.63297	0.526754	0.889699
foxq1b	235.636	-0.162542574	0.22218	-0.73158	0.464425	0.864593
foxc1b	546.3197	0.189801346	0.138945	1.366013	0.171935	0.644123
si:dkey-56f14.7	298.967	0.258713428	0.223132	1.159461	0.246268	0.727532
mylk4b	86.50315	-0.098559439	0.383844	-0.25677	0.797357	0.966447
zp2l1	0.728756	-3.345436825	2.360295	-1.41738	0.156372	NA
serpinb1	792.8856	-0.125792106	0.172549	-0.72902	0.465987	0.865069
rwdd2b	79.09314	-0.277908076	0.20311	-1.36827	0.171229	0.643325
kat6b	530.673	0.213787673	0.138	1.549185	0.121337	0.567497
BX248515.1	8.669541	0.728989921	0.701458	1.03925	0.298689	0.765669
vps26bl	69.49521	-0.07664192	0.233752	-0.32788	0.743004	0.9547
ftr97	59.96639	-0.093937431	0.322386	-0.29138	0.77076	0.960484
METTL21C	16.20383	0.091597046	1.493003	0.061351	0.95108	0.99801
adamtsl7	239.7514	-0.168899173	0.208544	-0.8099	0.417998	0.841312
zcchc7	129.3198	0.199682853	0.191219	1.044263	0.296364	0.764246
slc22a15	157.582	0.188466962	0.170125	1.107813	0.267943	0.74434
gpm6aa	9908.526	-0.002599121	0.123415	-0.02106	0.983198	1
dlgap3	62.59326	-0.08780083	0.28623	-0.30675	0.759034	0.957886
lrit3a	88.9644	0.563526867	0.7241	0.778245	0.436425	0.850912
si:dkeyp-3f10.14	7.265843	1.147712073	0.681966	1.682945	0.092386	0.508314
cdc16	207.5528	0.131924918	0.145644	0.905802	0.365041	0.815072
nubp1	215.1493	0.085038459	0.153129	0.55534	0.578662	0.905417
dexi	0	NA	NA	NA	NA	NA
rps27.2	14217.11	-0.296002823	0.145365	-2.03628	0.041723	0.356776
CR352329.1	3.732372	0.119054359	1.25354	0.094975	0.924335	0.994815
pelo	433.7442	0.085091504	0.148955	0.571258	0.567825	0.901872
mmp20b	4.072297	-2.095571017	1.193857	-1.7553	0.079209	0.476322
atf7b	559.1496	-0.087035812	0.124814	-0.69732	0.485601	0.87303
galnt12	60.43337	-0.072970792	0.241914	-0.30164	0.762927	0.958842
hic1	152.9481	0.245070166	0.206407	1.187317	0.235103	0.715906
si:ch1073-184j22.1	59.07132	0.267995209	0.306822	0.873456	0.382414	0.825252
cicb	768.4852	0.056856178	0.124322	0.457329	0.647434	0.927082
si:ch211-212k18.7	1034.314	0.144540182	0.163665	0.883148	0.377156	0.822467
si:ch211-37e10.1	1.587198	1.084429211	1.429178	0.758778	0.447985	NA
ypel3	754.0078	-0.128047638	0.26259	-0.48763	0.62581	0.920625
icn2	4734.075	-0.220303182	0.186405	-1.18185	0.237263	0.718568
pygma	2895.862	0.201287411	0.159078	1.265336	0.205751	0.687948
ftr61	10.20257	4.642059535	1.340383	3.463232	0.000534	0.028826
slc22a6l	162.2832	-0.57458594	0.229577	-2.5028	0.012321	0.193027
rnf7	1325.387	0.040169744	0.153165	0.262265	0.793117	0.965312
lgals1b	21.34013	-0.243865431	0.412799	-0.59076	0.554681	0.89855
cmn	562.9055	0.455144741	0.531397	0.856506	0.391718	0.827902
sertad2b	342.8742	0.070414306	0.144578	0.487033	0.626235	0.92063
grk7b	26.34845	0.310803916	0.523337	0.593889	0.552587	0.8974
otub1a	607.7789	0.021929346	0.123828	0.177096	0.859433	0.979975
avl9	384.8508	-0.153958086	0.140187	-1.09823	0.272103	0.748056
epdl2	880.7421	-1.49419107	0.705165	-2.11892	0.034097	0.326054
pfkfb4a	217.7649	0.106916105	0.188277	0.567865	0.570126	0.902481
appb	2856.414	0.079083021	0.083652	0.945382	0.344464	0.798147
npat	294.2476	0.169194623	0.145852	1.160042	0.246032	0.727297
FO834888.1	14.37057	0.562503049	0.681028	0.825962	0.408826	0.836637

dvl1b	62.09357	-0.030266923	0.237582	-0.1274	0.898627	0.990199
wnt1	35.1623	0.386441796	0.334208	1.15629	0.247562	0.728384
chrna6	115.5255	-0.09936136	0.300774	-0.33035	0.741134	0.954103
p2ry8	2.616207	0.349118646	1.265746	0.27582	0.782686	0.962328
c1galt1b	232.9348	0.083872487	0.135672	0.618199	0.536444	0.892397
drosha	240.7628	0.21204383	0.175405	1.208878	0.22671	0.707913
cacnb2b	54.14107	0.165684134	0.410457	0.403657	0.686465	0.938732
mastl	182.6347	0.103824638	0.168708	0.615409	0.538285	0.892568
ghdc	62.48057	0.107989798	0.373829	0.288875	0.772677	0.960624
gpr61	51.38301	-0.294779583	0.295541	-0.99742	0.318559	0.779923
FOXN2 (1 of many)	20.01671	0.012719448	0.458414	0.027747	0.977864	1
pcolce2b	180.8078	0.158737481	0.188694	0.841243	0.400212	0.831472
c1galt1a	53.19906	0.321918904	0.292586	1.100254	0.271222	0.747344
stat5b	168.3693	0.202905016	0.166843	1.216142	0.223931	0.705807
s100t	1015.101	-0.372537837	0.206899	-1.80058	0.071769	0.4564
pipox	97.54364	1.016162698	0.257861	3.940743	8.12E-05	0.007875
capn2b	518.2701	-0.565925938	0.168356	-3.36148	0.000775	0.037036
clul1	88.55798	-0.726783512	0.68176	-1.06604	0.286406	0.757624
cgna	135.2649	0.188071515	0.203591	0.923773	0.355605	0.806594
exoc2	769.2539	-0.023747815	0.099659	-0.23829	0.811656	0.97012
zgc:123238	137.4891	-0.16381914	0.192125	-0.85267	0.393842	0.828374
acta1b	28114.94	-0.068520498	0.269854	-0.25392	0.79956	0.967206
acad9	211.95	0.178464858	0.258444	0.690536	0.489857	0.874825
zgc:153345	9.318249	0.08746391	0.690293	0.126705	0.899174	0.99045
dipk2ab	171.4149	-0.139416262	0.150083	-0.92893	0.352926	0.803792
foxh1	5.183301	1.329492282	0.905695	1.467925	0.142125	0.601137
smtnl	234.3537	-0.016623868	0.229883	-0.07231	0.942352	0.997371
crk	1159.704	0.041929728	0.079807	0.525387	0.599314	0.913192
ankrd33aa	41.2917	-0.64269951	0.660426	-0.97316	0.330475	0.788126
ruvbl2	622.4833	0.540725418	0.184393	2.932459	0.003363	0.095589
si:ch211-217a12.1	304.6445	0.070594378	0.153465	0.460003	0.645514	0.926728
prss60.2	93.52224	-0.226428519	0.287061	-0.78878	0.43024	0.847541
ftr82	645.8366	-0.190328392	0.149353	-1.27435	0.202538	0.683016
cpda	400.2555	0.462932953	0.132278	3.499685	0.000466	0.026196
aclyb	94.02246	0.508432074	0.286065	1.777328	0.075514	0.46546
nubp2	28.7875	0.237360631	0.38724	0.612955	0.539906	0.893049
birc5b	4.545433	1.181363143	0.890751	1.326255	0.184755	0.661987
pdzd3b	20.55447	-0.411620739	0.570382	-0.72166	0.470505	0.867743
gpr161	97.60916	-0.028977457	0.194722	-0.14881	0.8817	0.986908
map3k10	21.26429	0.379151051	0.406812	0.932006	0.351333	0.802305
armt1	206.6973	-0.292736547	0.17682	-1.65556	0.097811	0.521071
akap12b	4072.239	0.163833516	0.115152	1.422759	0.154806	0.619752
mto1	209.7743	-0.212630186	0.13408	-1.58585	0.112773	0.549875
pimr191	5.968038	-1.508059418	0.881498	-1.71079	0.08712	0.495232
CU655961.1	37.19331	0.112121588	0.305526	0.366979	0.713635	0.945821
pcnx2	287.4514	0.290819041	0.24452	1.189345	0.234304	0.714977
f5	262.4789	-0.457079704	0.193073	-2.36739	0.017914	0.236638
timm8b	498.8904	-0.548751066	0.167053	-3.28489	0.00102	0.044601
sdhda	16.65711	0.456971096	0.488581	0.935302	0.349632	0.801408
fmnl1a	249.6742	0.073159611	0.19705	0.371275	0.710433	0.945821
capn8	374.2735	-0.60625083	0.162227	-3.73706	0.000186	0.014237
ino80c	313.878	-0.091029873	0.139412	-0.65296	0.513784	0.88631
bco2a	146.4542	-0.171801588	0.248195	-0.6922	0.488809	0.873964
hsp70l	638.6327	-0.446926956	0.527711	-0.84692	0.397042	0.830253

trim35-29	6.405821	1.022703282	0.86998	1.175548	0.239776	0.720384
si:dkeyp-69e1.8	88.35457	-0.056512137	0.166013	-0.34041	0.733549	0.950808
ube3a	1172.404	0.021301137	0.127155	0.167521	0.86696	0.982582
ribc1	10.27425	-0.401819253	0.724782	-0.5544	0.579305	0.905578
9-Mar	142.1155	0.331278642	0.225802	1.467119	0.142344	0.601598
ankrd54	410.1764	0.211614777	0.114753	1.844083	0.065171	0.435355
si:ch211-238g23.1	1.241212	0.604041338	1.548875	0.389987	0.696546	NA
foxp3a	3.463053	-0.340106516	1.141729	-0.29789	0.765789	0.959951
fosb	27.84137	-0.864427241	0.639837	-1.35101	0.176692	0.650047
npas4a	57.44394	0.243335336	0.937439	0.259575	0.795192	0.965909
suv39h1b	259.2142	0.309216882	0.183377	1.686239	0.09175	0.507483
smc1a	17.66101	-0.397139312	0.690503	-0.57514	0.565193	0.901538
tmem9	307.7574	0.11713916	0.192405	0.608816	0.542646	0.894595
efhd2	867.4154	0.145340903	0.108971	1.333752	0.182285	0.658775
srm	363.161	0.325507735	0.156523	2.079618	0.037561	0.340171
tmem115	538.7376	-0.140658324	0.122939	-1.14413	0.252568	0.732759
dffa	209.8551	-0.098715824	0.148454	-0.66496	0.506077	0.882901
ptger3	15.76894	0.702706074	0.455263	1.543517	0.122705	0.569468
ptpn3	443.7658	-0.234850782	0.149776	-1.56801	0.116879	0.557831
prss23	331.0078	0.130621175	0.151129	0.864305	0.38742	0.827592
dzank1	24.41491	0.172711922	0.465798	0.370787	0.710796	0.945821
zgc:158423	323.3474	-0.31286101	0.221121	-1.41488	0.157102	0.623076
foxo4	1122.061	-0.162916871	0.109601	-1.48646	0.137158	0.592789
cnpy4	211.0741	0.262433761	0.141955	1.848706	0.0645	0.433491
hey1	31.18186	-0.134139384	0.406264	-0.33018	0.741265	0.954103
vtg2	0.983728	0.099849506	2.083933	0.047914	0.961785	NA
si:dkey-225f5.5	27.11709	0.310148818	0.363935	0.852209	0.394098	0.828374
celsr3	146.4606	0.023630514	0.243577	0.097015	0.922715	0.994525
si:dkey-182g1.6	3.900853	-0.916462901	1.391096	-0.65881	0.51002	0.883733
lectin	0.214395	-1.826677	5.23046	-0.34924	0.72691	NA
slc26a6	39.34593	-0.659174103	0.446469	-1.47642	0.139832	0.598212
arhgef1b	906.9706	-0.005784868	0.105897	-0.05463	0.956435	0.99899
mettl16	92.0106	0.158127198	0.255303	0.61937	0.535672	0.89207
march4l	29.07706	0.866386867	0.517619	1.673791	0.094172	0.512557
CABZ01046427.1	0.169362	-1.710071829	5.238473	-0.32644	0.744088	NA
cdh10a	77.61463	0.156267199	0.267847	0.583419	0.559611	0.899571
mtnr1ab	33.58108	0.790073054	0.473287	1.66933	0.095052	0.51461
nr4a3	204.639	-0.324379215	0.615075	-0.52738	0.597928	0.912528
kcnc3a	66.997	0.234339592	0.419279	0.558911	0.576222	0.90475
tor1l1	0.858482	2.918315487	2.409079	1.211382	0.225749	NA
dop1b	628.7352	-0.162784252	0.156256	-1.04178	0.297515	0.765351
rsl1d1	2414.308	-0.527388581	0.173964	-3.03159	0.002433	0.07907
cpe	3629.781	0.263592372	0.146146	1.803619	0.071291	0.455342
pag1	560.8596	0.135808709	0.124495	1.090874	0.275328	0.750424
msmo1	238.4084	0.501471546	0.244348	2.052283	0.040142	0.350281
dmc1	0.58321	1.055409124	3.289456	0.320846	0.748327	NA
tex10	630.5446	-0.057878684	0.116423	-0.49714	0.619088	0.917329
zgc:153215	54.59867	-0.331521479	0.239899	-1.38192	0.166997	0.637452
nmnat1-rbp7a	0.174358	0	5.267649	0	1	NA
zgc:154093	257.2337	-0.195874267	0.206311	-0.94941	0.342412	0.797179
lzic	193.1278	0.091550851	0.180509	0.507182	0.612027	0.915649
zswim5	1340.688	0.066372989	0.100932	0.657602	0.510794	0.884139
steap4	258.433	-0.15343681	0.212688	-0.72142	0.470654	0.867752
luc7l	1232.285	-0.14270881	0.117665	-1.21284	0.225192	0.70685

nipa2	288.1719	-0.08859297	0.135236	-0.6551	0.512405	0.885459
nipa1	49.57088	-0.55214726	0.292993	-1.88451	0.059497	0.420414
si:dkey-47k20.9	0.063619	0	5.267649	0	1	NA
foxi3a	177.4923	-0.375512656	0.182704	-2.05531	0.039849	0.348958
zc3h7bb	759.4374	0.195898044	0.125627	1.559364	0.11891	0.562318
si:ch1073-416j23.1	7.412323	0.860835652	0.655158	1.313937	0.188867	0.668515
tspan14	684.7274	0.006807245	0.102847	0.066188	0.947228	0.997371
asph	1718.853	0.195357839	0.110629	1.765885	0.077415	0.470172
v2rc2	1.275556	-0.94868142	1.673006	-0.56705	0.570679	NA
lcp2a	28.71918	-0.43398668	0.413559	-1.04939	0.293997	0.763068
klc3	38.29357	0.386693206	0.300957	1.28488	0.198834	0.679593
cflara	137.8317	-0.729402657	0.227323	-3.20866	0.001334	0.053834
rfc3	239.6713	-0.032593458	0.160365	-0.20324	0.838944	0.976417
zgc:136971	108.0126	0.223517379	0.221312	1.009965	0.312512	0.776164
zar1l	1.266764	-2.753311621	2.09437	-1.31463	0.188636	NA
tpmt.1	193.392	-0.174985532	0.212661	-0.82284	0.410599	0.837316
pecr	220.9309	0.157513251	0.189172	0.832647	0.405044	0.834284
gzm3.2	0.21328	0	5.267649	0	1	NA
tmem161b	227.4299	0.019317875	0.150042	0.128749	0.897556	0.989832
hmbsb	368.2121	0.616343291	0.186439	3.305863	0.000947	0.042765
v2rh1	0.092827	0	5.267649	0	1	NA
rps8a	30490.42	-0.407936261	0.129298	-3.15501	0.001605	0.061411
si:ch211-59d17.3	89.94377	-0.26798682	0.307924	-0.8703	0.384136	0.826565
v2rh7	0.08847	0	5.267649	0	1	NA
fryb	914.6213	-0.063955805	0.144855	-0.44152	0.65884	0.930581
sprn	197.9542	0.225085578	0.265771	0.846917	0.397042	0.830253
dnajc18	573.4499	0.125776933	0.119285	1.054428	0.291687	0.761454
si:dkey-47k20.2	1.954318	-1.784330537	1.75736	-1.01535	0.30994	NA
cyb5d1	13.32178	0.303275123	0.488278	0.621112	0.534526	0.891711
atp5mc1	4645.955	0.318151535	0.190198	1.672743	0.094378	0.513319
v2rh9	0	NA	NA	NA	NA	NA
eve1	26.0976	0.030254129	0.331673	0.091217	0.92732	0.994973
si:dkey-47k20.7	0	NA	NA	NA	NA	NA
hoxb13a	142.628	0.122952449	0.18779	0.654733	0.51264	0.885483
itga6l	29.53745	-0.316524118	0.470167	-0.67322	0.500809	0.880413
ttbk1a	239.3374	0.038399274	0.217095	0.176878	0.859604	0.980024
sostdc1b	28.71253	-0.318346723	0.405181	-0.78569	0.432049	0.848419
hoxb9a	408.6138	-0.014897322	0.136879	-0.10884	0.913333	0.99273
eva1c	10.49767	-0.723633368	0.686295	-1.0544	0.291698	0.761454
tprg1	219.0646	-0.089899	0.169429	-0.5306	0.595697	0.911427
hoxb8a	353.7082	0.099361582	0.124752	0.79647	0.425759	0.844714
slc22a7a	740.2323	-0.305663171	0.141554	-2.15933	0.030824	0.312711
cyp26c1	175.2162	-0.240048022	0.218009	-1.10109	0.270857	0.746845
hoxb7a	174.2469	-0.059426395	0.152113	-0.39067	0.696039	0.941657
pip4p2	106.9204	-0.496442624	0.203817	-2.43573	0.014862	0.213725
stxbp1b	643.7091	0.315623949	0.177567	1.777489	0.075488	0.465421
itih6	61.7348	-0.176049735	0.665624	-0.26449	0.791404	0.964817
si:ch73-139e5.4	48.18213	-1.478465183	0.363348	-4.069	4.72E-05	0.005363
noxa1	12.41935	0.38975568	0.559936	0.696072	0.486384	0.873244
kctd17	291.5839	-0.055412143	0.123633	-0.4482	0.65401	0.929373
grem2a	16.67491	-0.353352944	0.685701	-0.51532	0.606332	0.915204
rangap1b	292.4851	-0.02620615	0.181864	-0.1441	0.885424	0.987533
adgre14	2.567332	-0.641521368	1.455357	-0.4408	0.659358	0.930581
mov10b.2	4.560079	-0.566542643	0.938829	-0.60346	0.546205	0.89597

rca2.1	715.4071	-0.166174726	0.193182	-0.8602	0.389679	0.827843
dcst1	2.188524	-3.570774497	1.587518	-2.24928	0.024495	NA
rftn2	446.8596	-0.051778721	0.183542	-0.28211	0.777861	0.961073
l3mbtl2	241.6398	0.429685158	0.173307	2.479325	0.013163	0.19947
si:dkey-191g9.5	46.91215	-0.229507846	0.445894	-0.51471	0.606753	0.915204
igsf21b	174.6325	-0.169400894	0.33741	-0.50206	0.615624	0.916365
mob4	1633.67	-0.067188804	0.12832	-0.5236	0.600554	0.913756
ecrg4a	345.3856	0.562463418	0.266639	2.109458	0.034905	0.329161
nrbp1	624.6019	0.139050846	0.13372	1.039863	0.298403	0.765416
capza1b	5397.111	0.000547808	0.115481	0.004744	0.996215	1
cttnbp2nlb	167.9792	0.033218393	0.145159	0.228841	0.818992	0.971785
si:dkey-12h9.6	244.0138	0.06662856	0.189568	0.351476	0.725231	0.948653
gtf2h5	313.3625	0.244296347	0.177309	1.377797	0.168266	0.639712
kcnd3	65.02356	-0.010874955	0.328192	-0.03314	0.973566	1
uxs1	395.1029	-0.049159694	0.109739	-0.44797	0.654174	0.929434
syt11b	124.298	0.275110388	0.194598	1.41374	0.157438	0.623263
ndufa4	2695.021	0.129705111	0.156195	0.830403	0.406311	0.834669
lmod1a	80.65146	0.482740226	0.234649	2.057284	0.039659	0.348331
pikfyve	579.5181	-0.31420502	0.164721	-1.9075	0.056456	0.410349
eef1g	36914.94	-0.447893339	0.119737	-3.74065	0.000184	0.014175
serac1	235.6679	-0.099284397	0.179753	-0.55234	0.580717	0.905618
gdi1	1860.857	0.039042202	0.13028	0.299679	0.764422	0.959254
myct1a	29.47769	0.203215099	0.322189	0.630733	0.528215	0.889948
polr2gl	1031.338	-0.118450903	0.155985	-0.75937	0.44763	0.856366
fastkd1	212.6645	0.211480375	0.180285	1.173035	0.240782	0.722178
neurog1	120.0985	0.137839189	0.193628	0.711876	0.476542	0.869292
kitb	33.74072	0.366927566	0.315906	1.161508	0.245435	0.726909
c1qtnf5	280.3445	0.166881563	0.211148	0.790355	0.42932	0.846956
sf3b1	4710.538	-0.039890472	0.140388	-0.28415	0.776299	0.961073
coq10b	1171.396	-0.038670452	0.185226	-0.20877	0.834624	0.975792
zgc:123244	17.00028	-1.257211539	0.587268	-2.14078	0.032292	0.318811
si:dkey-159a18.1	25.88337	0.291408962	0.419536	0.694598	0.487307	0.873291
rbms2b	407.2491	-0.067306538	0.165412	-0.4069	0.684079	0.938486
tyrp1b	3559.379	0.086051954	0.16106	0.534286	0.593144	0.910427
fam3c	338.1726	-0.134400678	0.135106	-0.99478	0.319844	0.780475
si:ch211-229c8.4	0.135409	0	5.267649	0	1	NA
npdc1b	433.8454	-0.078889506	0.208666	-0.37807	0.705382	0.944122
hspd1	2291.501	0.234465121	0.195166	1.201365	0.22961	0.711596
nr2c2ap	44.08759	-0.181039674	0.279809	-0.64701	0.517625	0.887148
slc25a51a	16.4963	0.288206535	0.372588	0.773525	0.439212	0.852079
si:ch211-215a10.4	198.5265	0.058001303	0.211408	0.274358	0.78381	0.96245
jac2	0.844294	-2.377293472	2.198662	-1.08125	0.279588	NA
hspe1	1645.84	-0.095650847	0.197134	-0.48521	0.627529	0.921429
scrt2	208.6547	-0.217364712	0.132374	-1.64204	0.100581	0.525351
dpt	18.69145	0.628686835	0.699683	0.898531	0.368903	0.817763
v2ra17	0.2861	-0.868128509	4.675833	-0.18566	0.852709	NA
ncam1a	436.6349	0.240161744	0.171966	1.396564	0.162545	0.630004
dvl2	483.4288	-0.089999626	0.145394	-0.61901	0.535912	0.892102
eif5a2	4850.125	-0.006087427	0.089396	-0.0681	0.94571	0.997371
si:ch211-168k14.2	15.72159	-0.220167925	0.614584	-0.35824	0.720164	0.947004
nme7	132.4395	-0.388820386	0.180618	-2.15272	0.031341	0.314904
slc2a2	343.221	0.02789488	0.212131	0.131498	0.895381	0.989223
abcb9	79.18098	0.070307833	0.268151	0.262195	0.793171	0.965312
si:ch211-201h21.5	125.0263	0.796011453	0.241602	3.294727	0.000985	0.043562

zgc:113436	183.9202	-0.097836178	0.187253	-0.52248	0.601336	0.913756
camk2g2	1617.75	0.111608642	0.08436	1.323001	0.185835	0.663655
myoz1a	1821.744	-0.019114733	0.188355	-0.10148	0.919167	0.993892
si:ch211-199o1.2	49.99089	-0.030294308	0.255075	-0.11877	0.90546	0.991631
ogfod2	28.99845	-0.201514014	0.336151	-0.59948	0.548856	0.897091
si:ch73-130a3.4	55.15527	-0.012439182	0.241886	-0.05143	0.958986	0.999164
gnrh3	70.05491	0.564323105	0.381264	1.480138	0.138836	0.596141
tnika	441.9498	-0.054099624	0.144074	-0.3755	0.707289	0.944981
creb3l3a	64.78075	-0.042883796	0.434761	-0.09864	0.921426	0.994085
pld1a	246.1476	-0.013229987	0.185554	-0.0713	0.943159	0.997371
ghsra	1.017922	0	2.488573	0	1	NA
zgc:110591	331.831	-0.400900151	0.179827	-2.22937	0.025789	0.285137
smndc1	970.9503	-0.073391331	0.116214	-0.63152	0.527701	0.889948
tmem45b	287.9047	-0.149833076	0.150917	-0.99282	0.320798	0.781257
zgc:171740	28.60699	0.646561918	0.392559	1.647043	0.099549	0.523919
zgc:162952	63.43202	-0.253419207	0.269218	-0.94132	0.346543	0.79947
bricd5	87.88018	0.262160633	0.300678	0.871899	0.383263	0.825809
si:dkey-183i3.5	11076.86	0.060022674	0.238938	0.251206	0.801655	0.967776
add3b	105.8696	-0.306575089	0.211281	-1.45103	0.146772	0.60662
sort1b	485.2406	0.174127554	0.144102	1.20836	0.226909	0.707983
cdc27	697.3474	0.093496482	0.10658	0.877246	0.380353	0.823655
tmem131	940.0304	-0.13498806	0.150002	-0.89991	0.368168	0.817272
slc35g2a	169.1781	0.745567543	0.269465	2.766845	0.00566	0.127203
dixdc1b	193.3249	0.10607536	0.170038	0.623833	0.532737	0.890978
atxn7l2b	190.3002	0.198284346	0.167952	1.180602	0.237761	0.718607
atpaf2	191.9336	-0.094434174	0.156899	-0.60188	0.547254	0.896121
or132-5	13.90638	1.13352045	0.477191	2.375401	0.01753	0.233833
ccdc157	20.69741	0.000358316	0.344907	0.001039	0.999171	1
cyb561a3a	94.88584	0.105829468	0.236921	0.446687	0.655101	0.929606
drd7	4.08516	2.292428889	1.569536	1.460577	0.144132	0.603144
vsx1	698.6085	0.014303674	0.284335	0.050306	0.959879	0.999164
si:ch211-183d21.3	53.27466	0.136029583	0.325822	0.417497	0.676315	0.93557
znf706	1589.04	-0.144780062	0.10248	-1.41277	0.157724	0.623766
a2ml	2315.694	-0.243410354	0.356985	-0.68185	0.495333	0.87806
kansl2	2.112541	-0.272972696	1.334328	-0.20458	0.837903	NA
ldb3a	2599.207	0.204875816	0.211876	0.966961	0.333563	0.790676
zgc:123295	85.33156	-0.627781983	0.256344	-2.44899	0.014326	0.209552
rhot2	182.9229	0.08775592	0.182651	0.480458	0.630902	0.923003
mhc2dbb	16.71613	-1.63141257	0.623693	-2.61573	0.008904	0.161802
ahcyl1	771.1087	0.221195471	0.126453	1.74923	0.080251	0.478492
mlh3	65.47239	-0.205881301	0.202463	-1.01688	0.30921	0.773791
szrd1	644.6314	-0.042876676	0.130717	-0.32801	0.742904	0.954655
stk31	6.24238	0.10775402	0.897588	0.120048	0.904445	0.991581
acap1	98.59471	-0.395058314	0.220832	-1.78895	0.073622	0.461398
rab3aa	327.7354	-0.232992713	0.199249	-1.16936	0.242261	0.724106
mpv17l2	124.4608	0.464403065	0.20293	2.288494	0.022109	0.263263
ufsp2	412.8242	0.036641937	0.136624	0.268195	0.788549	0.963567
noxo1b	14.47642	0.236351954	0.564157	0.418947	0.675255	0.935297
ankrd37	206.982	0.220694667	0.355405	0.620966	0.534622	0.891806
ifi30	785.3218	-0.038791562	0.222231	-0.17455	0.861429	0.9805
si:ch73-86n18.1	377.8103	-0.310798197	0.184703	-1.68269	0.092434	0.508357
cfap97	104.1371	0.136035621	0.227027	0.599206	0.549036	0.897092
TMC1	31.82111	-2.041014847	0.821295	-2.48512	0.012951	0.198329
rnf17	30.3297	0.024298365	0.45331	0.053602	0.957252	0.999109

TOM1L2	371.6038	0.00015597	0.204487	0.000763	0.999391	1
onecut3a	30.30256	0.374284205	0.285693	1.310092	0.190165	0.669977
dusp22a	162.756	-0.380703603	0.267207	-1.42475	0.154229	0.61894
helt	84.73107	0.009299564	0.185498	0.050133	0.960016	0.999164
irf8	49.91557	-0.690520139	0.324863	-2.12557	0.033539	0.323214
fdx1	649.4516	0.021884585	0.158183	0.13835	0.889964	0.98835
usp1	455.3784	0.052507747	0.102843	0.510562	0.609658	0.915204
auts2a	956.8481	0.107676124	0.162921	0.660911	0.50867	0.883333
her9	625.4546	0.145525981	0.128551	1.132052	0.257612	0.735174
4-Mar	4.12505	2.277755162	1.567843	1.452795	0.146281	0.605885
zgc:152753	0.28719	1.055395973	4.034919	0.261566	0.793656	NA
MANEAL	200.5849	0.184390051	0.213567	0.863381	0.387928	0.827592
afap1l1b	115.6874	-0.085013342	0.194589	-0.43689	0.662194	0.930742
lhfpl5b	37.30736	-0.160189589	0.36692	-0.43658	0.662416	0.930757
crp2	6.411663	-0.867241451	1.030255	-0.84177	0.399915	0.831277
fitm1l	365.2057	0.325430632	0.196139	1.659184	0.097079	0.519526
tmub2	311.7469	-0.277670158	0.143083	-1.94062	0.052304	0.397339
asb16	107.2276	0.463642772	0.21535	2.152975	0.031321	0.314904
chaf1b	222.1735	0.21371304	0.228045	0.937153	0.34868	0.801264
trnau1apb	984.7621	-0.221963857	0.157794	-1.40667	0.159525	0.6257
ccdc125	136.8785	0.047062868	0.289348	0.162651	0.870793	0.983365
si:ch211-168b3.1	132.7455	-0.078188468	0.21979	-0.35574	0.722034	0.947403
rab1bb	461.457	-0.053380045	0.176113	-0.3031	0.761813	0.958263
cpvl	604.1612	-0.161524999	0.121315	-1.33145	0.183041	0.659373
vat1	1911.277	0.039586411	0.09468	0.418106	0.67587	0.935422
ssbp4	1707.32	0.080637173	0.088838	0.907684	0.364045	0.814497
zgc:110158	824.6316	0.12057365	0.134963	0.893384	0.371652	0.819115
ikzf5	234.5838	0.207758947	0.133362	1.557855	0.119268	0.562753
crp2	1362.209	3.275235856	1.36656	2.3967	0.016543	0.22762
ca6	1299.706	-0.402470654	0.106041	-3.79544	0.000147	0.012175
si:ch211-208g24.8	0.129927	0	5.267649	0	1	NA
si:ch73-334d15.4	55.47088	-2.754310261	0.977528	-2.81763	0.004838	0.115344
frg1	292.1863	-0.062716451	0.144318	-0.43457	0.663874	0.931675
si:ch73-113g13.2	0.198634	1.055359535	5.228811	0.201835	0.840045	NA
pdik1l	139.2407	-0.023487427	0.209013	-0.11237	0.910528	0.992208
gstk1	616.0085	0.050821741	0.238506	0.213083	0.831262	0.974908
arr3a	4820.446	0.045258466	0.232775	0.19443	0.845839	0.977039
spsb1	422.1251	0.159891644	0.161909	0.98754	0.323378	0.783161
thoc3	314.3773	0.161434469	0.139374	1.158279	0.24675	0.727706
si:dkey-280e21.3	249.8173	0.488011191	0.15912	3.066937	0.002163	0.073473
bcar1	519.4609	-0.08479832	0.127941	-0.66279	0.507464	0.883007
cpamd8	550.9794	0.069175233	0.125559	0.550939	0.581676	0.905618
nutf2	1068.216	0.201898691	0.092953	2.172059	0.029851	0.307675
serinc2	637.5124	-0.018619829	0.12167	-0.15304	0.87837	0.985761
pla2g4f.1	97.65247	-0.002281369	0.212237	-0.01075	0.991424	1
nr0b1	78.44822	-0.043895117	0.38216	-0.11486	0.908556	0.99186
vps26a	775.7798	-0.140831353	0.105401	-1.33615	0.1815	0.657892
tango2	353.1769	0.16563143	0.157475	1.051792	0.292895	0.762428
slc22a13a	3.061643	1.372744513	1.51406	0.906664	0.364584	0.814648
rap1gap	94.33938	-0.350144185	0.259014	-1.35184	0.176427	0.649859
copb1	1907.554	0.028782687	0.097689	0.294635	0.768273	0.960296
ccz1	391.797	0.015273535	0.115564	0.132165	0.894854	0.989112
asb11	1.495565	0.821968941	1.694878	0.484972	0.627696	NA
necab1	74.67686	-0.930798233	0.315984	-2.94571	0.003222	0.093414

nrsn1l	70.63341	0.216005826	0.454822	0.474924	0.634841	0.924272
arf2b	106.2547	0.099820247	0.295621	0.337663	0.735617	0.951543
ndufs6	971.4599	0.024693737	0.168762	0.146323	0.883667	0.987014
cyp2r1	87.01026	-0.083031722	0.458443	-0.18112	0.856276	0.979558
calca	95.92166	0.488968542	0.328842	1.486942	0.13703	0.59257
gucy1b2	1.715683	1.470451669	1.327301	1.107851	0.267926	NA
irx1b	202.8681	0.10533725	0.15238	0.69128	0.489389	0.874462
pimr47	0.049519	0	5.267649	0	1	NA
papss2b	730.8728	0.034095498	0.10835	0.31468	0.753005	0.955756
plekha3	309.7675	-0.243073367	0.150212	-1.6182	0.10562	0.534827
mcf2b	6.147105	0.501805313	1.273803	0.393943	0.693623	0.940668
wbp2	245.5302	0.118306887	0.193477	0.611478	0.540883	0.893439
atad1b	193.9695	0.019916681	0.138905	0.143383	0.885988	0.987533
cybb	105.2101	-0.141215503	0.27605	-0.51156	0.60896	0.915204
pimr48	1.359716	0.595457543	1.798474	0.33109	0.740576	NA
rpgra	12.17113	-1.235862169	0.583373	-2.11848	0.034135	0.326054
xk	59.0966	0.310502929	0.339922	0.913454	0.361004	0.811296
arhgap42a	294.7879	0.032352771	0.126062	0.256642	0.797455	0.966447
grpr	6.763793	0.080961916	0.902381	0.08972	0.928509	0.995052
ctcf	1723.092	0.124064968	0.132445	0.936728	0.348898	0.801306
ptenb	858.9942	0.100742108	0.090617	1.111736	0.266252	0.742912
vegfd	37.61126	0.135247939	0.296796	0.455694	0.64861	0.927501
trpc6a	52.11823	-0.206917907	0.282064	-0.73358	0.463202	0.864262
cxcl14	859.6753	-0.089641782	0.162248	-0.5525	0.580606	0.905618
tmem170a	168.4506	-0.067145214	0.16421	-0.4089	0.682615	0.938006
angptl5	192.0692	-1.190952388	0.236548	-5.03471	4.79E-07	0.000161
fgf13b	345.1763	0.16444543	0.189532	0.867639	0.385592	0.826939
pir	321.023	0.23307163	0.218728	1.065578	0.286614	0.757624
carmil2	336.8561	0.310341835	0.173483	1.788886	0.073633	0.461398
rpia	980.5781	-0.086724086	0.14847	-0.58412	0.559142	0.899487
hdac9b	289.774	0.112989145	0.13997	0.807241	0.419528	0.842383
slc22a7b.1	39.50259	-0.097821568	0.514738	-0.19004	0.849277	0.978102
klhl3	196.5837	0.390852062	0.152109	2.569555	0.010183	0.175289
ext2	724.5592	-0.059851451	0.119859	-0.49935	0.617535	0.916375
htatsf1	448.992	0.136149965	0.114502	1.189063	0.234415	0.715154
zgc:113276	97.99907	0.535819284	0.255993	2.093101	0.03634	0.335171
gfra4a	55.96429	-0.520053459	0.380612	-1.36636	0.171826	0.643953
SLC22A7 (1 of many)	15.58002	-0.476129428	0.577754	-0.8241	0.409881	0.836939
fhl1b	292.0164	-0.282352971	0.158103	-1.78588	0.074118	0.462265
gna15.3	9.592043	0.688161911	1.420871	0.484324	0.628156	0.921864
tspan18a	193.4833	-0.459247412	0.14967	-3.06839	0.002152	0.073224
ttbk1b	119.5896	-0.041678391	0.187338	-0.22248	0.823943	0.972032
arfip2b	0.526814	1.055417365	2.877503	0.366782	0.713781	NA
nsun2	310.641	-0.23197087	0.121014	-1.91689	0.055252	0.406983
sp8b	7.816532	-0.002375738	0.673782	-0.00353	0.997187	1
abcb8	283.5416	-0.024177434	0.152183	-0.15887	0.873771	0.983831
BX530018.2	51.12284	0.648978676	0.28583	2.270505	0.023177	0.268774
trim47	321.3177	-0.183890727	0.134027	-1.37204	0.170051	0.641806
morc3a	162.3585	0.134190776	0.191269	0.701581	0.482941	0.871882
stc2a	236.6092	0.148234663	0.237184	0.624977	0.531986	0.890978
cdk5	104.2519	-0.699330083	0.430145	-1.6258	0.103992	0.532096
mtmr1a	401.1439	0.115430646	0.16132	0.715539	0.474276	0.869292
cpeb4a	331.1576	-0.001323218	0.161187	-0.00821	0.99345	1
phc2a	696.9045	-0.191093752	0.091098	-2.09768	0.035933	0.332937

RAMP1	236.8051	-0.210948568	0.170393	-1.23801	0.215712	0.697271
pskh1	171.4543	-0.034820577	0.206633	-0.16851	0.866179	0.982573
vars2	164.7218	-0.049586619	0.196544	-0.25229	0.800815	0.967609
slc6a19b	126.5139	-0.75887861	0.829837	-0.91449	0.360459	0.811237
utp3	253.0936	-0.513416294	0.189496	-2.70938	0.006741	0.138465
cd99l2	2302.413	0.182749621	0.124824	1.464058	0.143178	0.602183
fhad1	18.84275	0.651943031	0.53388	1.221142	0.222032	0.703829
hmgb3a	6759.972	-0.02615218	0.097843	-0.26729	0.789248	0.963735
mettl7a	63.93557	-0.70003774	0.362274	-1.93234	0.053317	0.400175
her4.2	188.6965	0.111740657	0.177872	0.628208	0.529867	0.890545
her4.1	95.21501	0.155392129	0.225602	0.688787	0.490957	0.875595
tas1r1	2.797173	-0.921436353	1.480236	-0.62249	0.533618	0.891181
tmeff1b	1572.614	0.09802397	0.12313	0.7961	0.425974	0.844714
senp3b	303.2034	0.011895616	0.128476	0.09259	0.926229	0.994815
crmp1	339.6361	0.358228525	0.245871	1.456977	0.145123	0.604004
cavin4b	1466.943	0.143591469	0.228828	0.627509	0.530326	0.890545
ela2	24261.93	0.91946549	0.359507	2.55757	0.010541	0.179446
necab2	785.6316	0.329571075	0.167132	1.971924	0.048618	0.383778
si:ch211-254p10.2	28.16459	-0.126188766	0.42294	-0.29836	0.765428	0.959831
dctn1b	364.0364	0.220979257	0.202686	1.090255	0.275601	0.750653
slc39a8	29.85511	-1.134096341	0.631774	-1.7951	0.072638	0.457864
zgc:172079	4.433713	-1.66868774	0.898627	-1.85693	0.063321	0.429493
hydin	51.56318	0.998686103	0.38098	2.621363	0.008758	0.160701
ela2l	27435.35	-0.339042888	0.296571	-1.14321	0.252952	0.732759
itgb3a	165.6282	0.151539841	0.207384	0.730722	0.464949	0.864823
rprml	49.74461	0.190262205	0.396809	0.479481	0.631597	0.923003
zgc:163079	0.285605	0.093652163	4.739054	0.019762	0.984233	NA
gpr37l1b	51.53741	0.269727501	0.277174	0.973133	0.330487	0.788126
ssh1a	20.18688	-0.429438023	0.448559	-0.95737	0.338379	0.795226
zanl	259.9914	-2.593462466	0.614054	-4.22351	2.41E-05	0.003278
raraa	413.8699	-0.010969253	0.129487	-0.08471	0.93249	0.996175
aire	2.907652	1.490057172	1.318947	1.129733	0.258589	0.735786
pde6ga	17.42863	0.288943666	0.860932	0.335617	0.737159	0.952312
jazf1b	94.52495	0.264528575	0.245274	1.078501	0.28081	0.753193
arl3a	23.24522	-0.268629429	0.426727	-0.62951	0.529015	0.890325
serpine1	183.457	-0.540981794	0.277676	-1.94825	0.051385	0.394202
ppp2r2ca	112.8936	0.16410226	0.332346	0.49377	0.621469	0.918461
zgc:123297	2.018291	-0.868138778	1.95242	-0.44465	0.656574	NA
sufu	588.1726	-0.030996198	0.124019	-0.24993	0.802641	0.968181
ap1s1	613.9738	0.255406611	0.160739	1.588952	0.112071	0.548404
si:dkey-40g16.6	26.90963	-0.04951842	0.504318	-0.09819	0.921782	0.99426
plch2b	25.02961	0.142129523	0.439851	0.323131	0.746596	0.955074
pde5aa	18.67465	-1.237928392	0.700496	-1.76722	0.077192	0.469556
hoxa9b	160.4475	-0.035069711	0.152317	-0.23024	0.817904	0.971518
zmp:0000000662	201.3714	-0.104018509	0.144745	-0.71863	0.472366	0.868767
si:ch211-107m4.1	64.01382	0.276223812	0.202891	1.361438	0.173375	0.64634
gng2	820.7113	0.165285159	0.165745	0.997223	0.318656	0.779923
exo1	55.6393	-0.042158791	0.224988	-0.18738	0.851361	0.978548
svopa	23.41968	0.127769326	0.457467	0.279297	0.780017	0.961176
laynb	44.91057	-0.069876534	0.321516	-0.21733	0.827947	0.973883
si:ch211-125o16.4	1539.256	-0.179006282	0.180392	-0.99232	0.321041	0.781417
pimr93	0.821023	-1.212208504	2.11724	-0.57254	0.566955	NA
otud5b	656.0629	0.081564331	0.100598	0.810797	0.417483	0.841149
usp30	53.52562	-0.023332574	0.271497	-0.08594	0.931514	0.995809

si:dkey-27c15.3	75.58844	-0.026371504	0.197314	-0.13365	0.893677	0.988566
gatc	223.891	-0.063989784	0.150869	-0.42414	0.671463	0.933953
tax1bp1b	2219.1	-0.062847991	0.080977	-0.77612	0.437679	0.851283
spx	16.93493	0.647962673	0.595569	1.087972	0.276608	0.751172
cep63	200.4852	-0.059978478	0.185355	-0.32359	0.746251	0.955074
ap4m1	113.8455	-0.276031065	0.233568	-1.1818	0.237285	0.718568
and3	3579.491	-0.215493399	0.146263	-1.47332	0.140664	0.599658
lygl1	228.6898	-0.169606077	0.17589	-0.96427	0.334909	0.79181
vamp2	556.8188	0.219911678	0.235637	0.933266	0.350683	0.802215
per1a	141.3436	-0.618593149	0.407263	-1.5189	0.128786	0.578173
tbccd1	120.2989	-0.178803611	0.204471	-0.87447	0.381863	0.824957
dnah5l	57.35559	0.276004559	0.370399	0.745155	0.456178	0.860359
mpp6a	300.7416	-0.169549221	0.15451	-1.09734	0.272494	0.748416
ttc12	17.94456	0.014514136	0.47558	0.030519	0.975653	1
mrpl13	421.6885	0.044513574	0.136047	0.327193	0.743522	0.954856
si:rp71-45g20.10	1.864329	1.055416891	2.281249	0.462649	0.643616	NA
LO018309.1	33.17377	-1.380053543	0.547936	-2.51864	0.011781	0.18877
CU234171.1	9.739895	-0.479772702	0.646419	-0.7422	0.457966	0.860993
or133-1	5.783663	-0.023483467	0.831939	-0.02823	0.977481	1
pcolcea	1044.072	0.163684104	0.156298	1.047257	0.294981	0.763708
emg1	164.7861	0.201625552	0.166321	1.21227	0.225409	0.70685
si:ch211-237l4.6	66.34419	1.120826975	0.320532	3.496773	0.000471	0.02642
si:rp71-45g20.4	3.904656	-0.871535285	1.175058	-0.7417	0.458272	0.861001
tmem88a	120.757	-0.181107882	0.181248	-0.99923	0.317685	0.77959
ankk1	37.6397	-0.037381377	0.319903	-0.11685	0.906977	0.991679
ltbp1	431.3673	-0.216614613	0.149761	-1.4464	0.148065	0.609064
dbr1	353.2445	-0.070277064	0.112702	-0.62356	0.532914	0.890978
sap130a	327.8625	-0.062949031	0.125062	-0.50334	0.614723	0.915998
drd2a	12.53672	0.008288602	0.666747	0.012431	0.990081	1
kdm6bb	592.2133	0.028197622	0.221626	0.12723	0.898758	0.990262
tfip11	501.4893	0.011780269	0.115001	0.102437	0.91841	0.993884
hepacama	268.0794	0.368892202	0.151619	2.433017	0.014974	0.214278
kera	434.0923	0.293165015	0.19512	1.502489	0.132971	0.58639
or125-8	6.494167	0.472531532	0.724433	0.652278	0.514222	0.88631
htr7a	4.518745	0.634975581	1.209382	0.525041	0.599554	0.913385
epyc	1967.854	0.217477661	0.239289	0.90885	0.363429	0.813669
si:dkeyp-38g8.5	22.41783	-0.573817153	0.417303	-1.37506	0.169112	0.640442
ccdc30	94.86622	0.087786742	0.273587	0.320874	0.748306	0.955074
ilk	1035.793	-0.166280619	0.110253	-1.50817	0.131511	0.583069
nek7	490.206	-0.057958304	0.116011	-0.49959	0.617363	0.916365
ifit16	26.31998	-0.572425535	0.478732	-1.19571	0.231809	0.713202
letm1	129.873	0.099051928	0.173262	0.571688	0.567534	0.901809
lhx9	485.5019	0.121267712	0.127446	0.951519	0.341341	0.796175
rad9a	42.55454	0.252759334	0.290748	0.869341	0.38466	0.826588
tpte	569.1381	-0.279038049	0.165662	-1.68438	0.092109	0.507702
dact2	146.1486	0.148798764	0.1972	0.754558	0.450514	0.857151
snai1a	359.8106	-0.003732914	0.149748	-0.02493	0.980112	1
kirrel1b	211.9955	0.215925671	0.159218	1.356165	0.175047	0.648933
camkvl	179.4397	0.146151831	0.273897	0.533601	0.593618	0.910427
si:ch211-287a12.9	104.3225	-0.393978529	0.23051	-1.70916	0.087421	0.496224
fam241a	222.0194	-0.362805923	0.174405	-2.08025	0.037503	0.340049
ctbp1	2718.221	0.06916108	0.134334	0.514846	0.606661	0.915204
si:dkey-154b15.1	35.13017	0.212493962	0.342561	0.620309	0.535054	0.891993
dpm1	318.1821	0.139214253	0.15696	0.886942	0.37511	0.822028

cadm3	217.3852	0.127701034	0.162372	0.786473	0.431591	0.848419
cdc14ab	376.6717	-0.193103488	0.118629	-1.6278	0.103567	0.531015
slc6a4b	7.473352	0.10900679	1.061518	0.10269	0.918209	0.993884
vps36	258.1261	-0.049045841	0.152602	-0.3214	0.747909	0.955074
chrna2b	57.38387	0.342010056	0.33384	1.024471	0.305613	0.770852
ran	10273.74	-0.091357041	0.113324	-0.80615	0.420154	0.842383
rtn2b	455.0627	-0.142058502	0.17716	-0.80186	0.422631	0.843493
htr2aa	14.29245	1.864671329	0.654374	2.849548	0.004378	0.108843
ppm1nb	316.2293	0.52758664	0.250685	2.104584	0.035328	0.330833
stoml3b	243.3264	-0.656891095	0.343927	-1.90997	0.056136	0.409466
kcnk12l	0.898066	-2.304171659	2.598873	-0.8866	0.375292	NA
si:ch211-191j22.8	9.164612	-0.471170592	0.730726	-0.6448	0.519058	0.887148
tm2d1	139.1975	-0.276031516	0.184771	-1.49391	0.135199	0.590585
kcnk18	31.87634	0.010267292	0.308471	0.033284	0.973448	1
zgc:91976	362.864	-0.29501758	0.135555	-2.17638	0.029527	0.30586
lrit3b	4.704249	2.306504972	0.969844	2.378223	0.017396	0.232896
ndufs8b	6.007846	-0.519956556	1.496246	-0.34751	0.72821	0.949447
minpp1a	169.4427	-0.166068313	0.216074	-0.76857	0.442148	0.853572
tvp23b	321.1757	0.133373547	0.135799	0.982137	0.326033	0.784919
si:dkey-157g16.6	289.0731	-0.001964434	0.18376	-0.01069	0.991471	1
enpep	120.073	-0.300996149	0.28374	-1.06082	0.288773	0.759979
rpgrb	119.2858	0.097396536	0.201224	0.48402	0.628372	0.921864
lamtor3	228.5503	0.023547982	0.197554	0.119198	0.905119	0.991631
ftsj1	177.4276	-0.104465696	0.176337	-0.59242	0.553569	0.89813
zmp:0000001103	1.010942	0	2.415065	0	1	NA
kdm5ba	1683.946	-0.115038431	0.154903	-0.74265	0.457696	0.860993
ndr1	1.430233	-0.742558841	1.775133	-0.41831	0.675719	NA
htr1ab	19.77901	-0.289699521	0.455467	-0.63605	0.524745	0.888928
papss1	252.6509	0.262042341	0.231427	1.132288	0.257513	0.735155
zwilch	58.54307	0.364499569	0.311831	1.1689	0.242444	0.724345
rnf180	95.24059	-0.343565898	0.197459	-1.73993	0.081871	0.481671
nle1	187.7876	-0.015391933	0.21132	-0.07284	0.941936	0.997371
si:ch73-383g2.1	14.51455	0.535191447	0.574349	0.931822	0.351428	0.802305
serpine3	63.24597	-0.475718377	0.30635	-1.55286	0.120456	0.565995
slc25a1a	255.2879	0.053699993	0.168392	0.318898	0.749804	0.955074
gpr25	0.392458	0	3.772209	0	1	NA
c6	267.0201	-0.254173677	0.231018	-1.10023	0.271231	0.747344
hpf1	379.7995	-0.065856403	0.168045	-0.3919	0.695133	0.94128
ghsrb	3.109829	1.631411935	1.443771	1.129966	0.258491	0.735636
c7b	106.8223	-1.272362385	0.476756	-2.66879	0.007612	0.147466
pld1b	189.8525	-0.162744771	0.171818	-0.94719	0.343541	0.798147
hadhaa	586.9374	0.010187273	0.239842	0.042475	0.96612	1
zgc:174164	80.96959	0.162192214	0.263685	0.615097	0.53849	0.892732
zgc:110045	166.0429	0.369675093	0.166484	2.220488	0.026386	0.287876
zgc:194252	10.71846	-0.616224611	0.820274	-0.75124	0.452507	0.857994
nradd	151.9405	-0.159931493	0.16206	-0.98687	0.323707	0.783161
mocos	222.3011	-0.485271252	0.165529	-2.93164	0.003372	0.095623
prep	185.6419	0.132070867	0.137425	0.961037	0.336534	0.793245
ankrd29	154.0431	-0.153216038	0.257498	-0.59502	0.551831	0.8974
lrmda	30.4382	-0.195051491	0.295552	-0.65996	0.509281	0.883568
eif4g2b	4140.368	-0.087892709	0.110889	-0.79262	0.428001	0.845998
abca4a	118.7002	-0.83018035	0.341757	-2.42916	0.015134	0.21592
ifit8	3.00749	-1.802049468	2.244567	-0.80285	0.422062	0.843323
il17c	0.903457	-1.811171747	2.657102	-0.68163	0.49547	NA

zgc:55781	159.6656	-0.04688255	0.140888	-0.33276	0.739312	0.953422
nmt1b	225.3222	-0.179175452	0.304057	-0.58928	0.555672	0.898723
plppr5a	30.89954	-0.576274365	0.361256	-1.5952	0.110669	0.545461
has3	19.54741	-0.269962712	0.328633	-0.82147	0.411378	0.837797
tbc1d15	610.8362	-0.292125709	0.149855	-1.94938	0.05125	0.394059
si:dkey-222b8.4	29.85255	0.554331404	0.463849	1.195069	0.23206	0.713202
rorca	392.272	0.045427863	0.175906	0.25825	0.796214	0.966357
chtopa	1536.696	0.047325343	0.083902	0.564055	0.572717	0.903571
si:dkey-30k6.5	111.967	0.016147828	0.167907	0.096171	0.923384	0.994525
tph2	59.42769	0.619238068	0.294344	2.103793	0.035397	0.330833
tnfsf10	493.4661	-0.135307434	0.148103	-0.9136	0.360926	0.811253
im:7138535	116.5981	-0.134888384	0.218138	-0.61836	0.536336	0.892371
sclt1	177.9691	0.091392402	0.155289	0.58853	0.556177	0.899002
zmynd11	1132.651	-0.014634571	0.10185	-0.14369	0.885747	0.987533
pimr141	1.7951	-3.932751193	4.785644	-0.82178	0.411202	NA
emc1	816.5188	0.106841631	0.10439	1.023485	0.306079	0.771472
si:dkey-91i10.3	115.2443	0.476910058	0.446997	1.066919	0.286008	0.757455
zgc:173729	5.778933	0.081244265	0.805279	0.10089	0.919638	0.993892
pimr52	0.211242	-2.8108188	5.183137	-0.5423	0.587611	NA
slc30a9	642.1088	-0.077049281	0.130219	-0.59169	0.554057	0.898275
alox5a	42.84926	0.236842722	0.253885	0.932873	0.350885	0.802215
iqca1	24.6629	-0.101210216	0.375795	-0.26932	0.787681	0.963263
pimr54	0	NA	NA	NA	NA	NA
swap70b	256.2377	-0.051914067	0.143223	-0.36247	0.717001	0.94627
slc25a16	371.1163	0.035259674	0.151835	0.232224	0.816364	0.971207
cbln1	407.2318	0.404461086	0.215817	1.874097	0.060917	0.423604
pbdc1	918.6683	0.000271697	0.164266	0.001654	0.99868	1
galnt7	774.4247	0.025745598	0.109417	0.235299	0.813977	0.970483
rwdd3	57.63543	-0.548603048	0.309739	-1.77118	0.076531	0.468485
nexn	781.2219	0.163773224	0.227579	0.719632	0.471751	0.868256
tut1	176.0989	0.003306358	0.165559	0.019971	0.984067	1
tmem47	823.8024	0.304901894	0.125206	2.435209	0.014883	0.213725
e2f8	222.5567	0.135709187	0.229286	0.591877	0.553933	0.89821
cth1	0.234763	-1.826688039	5.230459	-0.34924	0.726909	NA
kcnk7	3.25576	0.854068352	1.128167	0.757041	0.449025	0.856796
cmasa	530.3503	-0.334182462	0.115083	-2.90384	0.003686	0.100716
slc25a29	51.38626	0.019284247	0.38658	0.049884	0.960215	0.999164
ehbp1l1a	385.9885	-0.052220047	0.238962	-0.21853	0.827017	0.973458
elovl8b	104.8134	1.190707852	0.959391	1.241108	0.214566	0.69658
rps8b	83.2711	0.170108572	0.268696	0.633088	0.526676	0.889699
sfxn3	151.0748	0.007275139	0.19029	0.038232	0.969503	1
selenou1a	1245.461	0.319915062	0.14362	2.227509	0.025913	0.285638
lrrc31	36.88379	-0.189261138	0.367468	-0.51504	0.606524	0.915204
rab33a	122.8956	0.114820681	0.213949	0.536674	0.591493	0.909437
si:ch211-130m23.3	27.18498	0.502095017	0.415512	1.208376	0.226903	0.707983
CR847953.1	245.8759	0.097713618	0.142247	0.68693	0.492127	0.87629
CABZ01067232.1	267.0036	0.654130259	0.386439	1.692714	0.09051	0.505085
rxfp3	12.71551	0.709412146	0.764977	0.927364	0.353738	0.804644
phb	2534.098	0.156367081	0.10947	1.428403	0.153176	0.617769
nudt13	24.15787	-0.524294527	0.722132	-0.72604	0.467816	0.866174
slc44a5b	213.3263	0.084127644	0.171756	0.489808	0.624269	0.919537
oard1	37.35929	0.28496564	0.367726	0.774939	0.438375	0.851685
sv2ba	628.1271	0.133777889	0.359895	0.371714	0.710106	0.945821
arg1	13.5928	-2.390465216	1.37414	-1.73961	0.081928	0.481794

st6galnac5b	72.38353	-0.125270916	0.21875	-0.57267	0.56687	0.901747
amacr	98.25057	0.189276037	0.201979	0.937105	0.348704	0.801264
apodb	861.1024	0.068736658	0.215123	0.319523	0.74933	0.955074
phf1	4.311272	3.261528369	1.537173	2.121771	0.033857	0.325143
si:dkey-228d14.5	46.2188	-0.387867421	0.347803	-1.11519	0.264767	0.742063
ston2	647.287	-0.047124518	0.138056	-0.34134	0.732846	0.950624
ppp3ccb	431.7552	-0.096749204	0.12694	-0.76216	0.445962	0.855403
crygm2d13	8002.707	0.645134605	0.34621	1.86342	0.062403	0.427718
gfpt1	390.3878	-0.052492285	0.10918	-0.48079	0.630668	0.923003
kcnc2	38.86888	0.446221432	0.384121	1.16167	0.24537	0.726909
lrrc28	50.08031	-0.022573249	0.275043	-0.08207	0.93459	0.996829
hrh2b	2.071423	1.452775038	1.167362	1.244494	0.213318	NA
RNF157	288.5834	0.249495137	0.14666	1.701182	0.088909	0.500368
srsf2a	2136.571	0.037173042	0.09498	0.391377	0.695518	0.9414
n4bp1	221.7278	0.153009462	0.1882	0.813014	0.41621	0.839647
stk35l	466.728	0.246773847	0.141957	1.73837	0.082146	0.482431
habp2	174.9002	-0.65410587	0.307375	-2.12804	0.033334	0.323127
si:dkey-202g17.3	66.82565	-0.161978111	0.350264	-0.46245	0.643762	0.925997
mdm4	859.9875	-0.056622306	0.093647	-0.60464	0.54542	0.8953
pcdh1g9	109.8508	0.077867941	0.25801	0.301802	0.762803	0.958842
mef2ab	34.07724	0.477010668	0.409582	1.164628	0.24417	0.724792
itpa	195.7862	-0.08818122	0.197411	-0.44669	0.6551	0.929606
pgap3	28.94714	0.408598697	0.378062	1.080772	0.279798	0.752778
crygm2a	3.983145	-1.747637577	1.795489	-0.97335	0.33038	0.788126
rpl17	24590.14	-0.417925387	0.127491	-3.27807	0.001045	0.045343
polr3c	80.53959	-0.126967169	0.206076	-0.61612	0.537816	0.892498
nefla	152.7078	0.860924331	0.332582	2.588607	0.009636	0.169022
pgam2	2775.08	0.334660746	0.166031	2.015657	0.043836	0.366547
pnp4a	631.3643	-0.72808693	0.232582	-3.13045	0.001745	0.064499
mbtps2	280.4551	-0.021591888	0.148173	-0.14572	0.884142	0.987047
prrc2c	987.5163	0.187465755	0.176595	1.061559	0.288436	0.759522
sall3b	181.9981	0.220773212	0.148733	1.48436	0.137713	0.593771
si:ch1073-396h14.1	260.4908	0.219832867	0.20126	1.092285	0.274708	0.750181
s100b	205.2497	-0.138979959	0.207552	-0.66962	0.503103	0.881598
arl4ca	288.3797	-0.10189288	0.153725	-0.66282	0.507443	0.883007
cdkn2c	48.55339	0.393394846	0.250564	1.570039	0.116406	0.557474
nitr3c	2.710999	2.910339361	1.244341	2.338861	0.019343	0.245035
cops5	485.9098	0.210606381	0.160201	1.314642	0.18863	0.668367
dusp14	98.07716	-0.081933855	0.268792	-0.30482	0.760501	0.957886
josd2	260.037	0.043814026	0.147159	0.297732	0.765908	0.959974
galm	247.5517	-0.197567785	0.227143	-0.86979	0.384413	0.826588
ppp1r42	12.82406	0.168605895	0.543573	0.310181	0.756423	0.956532
cxcr4a	133.5934	-0.144117525	0.184556	-0.78089	0.434868	0.850002
wdr34	42.01536	-0.034135232	0.283187	-0.12054	0.904056	0.991581
adam8b	55.9253	-0.253350599	0.273259	-0.92714	0.353852	0.804826
dnttip2	521.0513	-0.208214294	0.183553	-1.13436	0.256645	0.734072
satb1a	92.39123	0.016432155	0.230415	0.071315	0.943147	0.997371
dbpb	210.5624	-0.624063381	0.293615	-2.12545	0.033549	0.323214
dhx57	247.4514	0.100311533	0.137402	0.730058	0.465355	0.864997
aldoca	160.9006	-0.492277508	0.965881	-0.50967	0.610285	0.915331
sprn2	104.0336	0.142026213	0.230702	0.615626	0.538141	0.892498
epas1b	411.075	-0.209074112	0.162105	-1.28974	0.19714	0.677304
plpp5	367.8643	0.127013651	0.148962	0.852656	0.39385	0.828374
golga7	1354.092	0.026776737	0.117265	0.228344	0.819379	0.971785

neu4	12.35821	0.549547413	0.554801	0.99053	0.321915	0.782075
sfrp1b	272.4015	0.744621175	0.325161	2.290005	0.022021	0.262762
sh2b1	656.9711	-0.02038046	0.138361	-0.1473	0.882896	0.986908
foxj2	103.6286	0.095212306	0.211671	0.449813	0.652846	0.929142
si:ch211-266o15.1	512.2889	-0.190995312	0.120183	-1.58921	0.112013	0.548404
mcm6	782.6914	0.013151941	0.152387	0.086306	0.931223	0.995809
sbk3	65.72696	0.183917507	0.415335	0.442817	0.657898	0.930581
ical1	219.4644	-0.244781377	0.181395	-1.34944	0.177196	0.65076
srsf1a	2800.771	0.096556455	0.085519	1.129061	0.258872	0.736046
ctsd	4035.623	-0.023394883	0.143584	-0.16294	0.870569	0.983365
styx	218.6654	-0.078399915	0.143813	-0.54515	0.58565	0.906655
si:ch211-137i24.10	232.3681	-0.452303762	0.269569	-1.67788	0.093371	0.5112
zgc:66443	106.2538	-0.099221802	0.190783	-0.52008	0.603009	0.914226
zgc:175264	473.5055	-0.041039509	0.116849	-0.35122	0.725425	0.948653
cmah	301.71	-0.189160323	0.162057	-1.16724	0.243112	0.724396
atl2	519.5132	0.039634599	0.115127	0.344268	0.730645	0.950056
slc17a8	37.68214	0.324821066	0.393888	0.824654	0.409568	0.836787
ephb6	90.82441	1.051449942	0.257801	4.078528	4.53E-05	0.005224
ano10a	456.732	0.291449544	0.109421	2.663563	0.007732	0.148545
rxraa	132.321	-0.284807027	0.184899	-1.54034	0.123478	0.570142
hells	325.0395	0.079035584	0.154351	0.512051	0.608615	0.915204
nr1h4	57.46291	-0.508594019	0.381139	-1.33441	0.182071	0.658775
si:dkey-10f21.4	67.42808	1.358873526	0.216766	6.268862	3.64E-10	3.12E-07
zbtb47a	37.09149	0.006571154	0.355382	0.01849	0.985248	1
nudt4a	498.5316	-0.209665215	0.110394	-1.89924	0.057533	0.4139
pimr55	2.197791	-0.798877568	1.87783	-0.42543	0.670526	NA
7-Mar	230.6168	-0.180015107	0.164576	-1.09381	0.274037	0.749284
baz2ba	1354.016	0.122182817	0.171441	0.712682	0.476042	0.869292
itgae.2	18.28041	0.344160172	0.471422	0.730048	0.465361	0.864997
lyz	60.02864	-0.150542954	0.308759	-0.48757	0.625852	0.920625
ankrd6a	48.01675	-1.59113544	0.437115	-3.64008	0.000273	0.018252
cx52.7	10.18916	0.080013999	1.536123	0.052088	0.958458	0.999109
mrpl28	546.4497	-0.055845281	0.118984	-0.46935	0.63882	0.924779
si:ch73-61d6.3	153.528	-0.152389797	0.200515	-0.75999	0.447259	0.856073
dnmt3bb.2	150.1681	0.235011767	0.167376	1.404096	0.16029	0.626823
gulp1b	32.80941	0.40570137	0.349864	1.159596	0.246213	0.727532
zgc:171470	8.358171	4.128230724	1.408817	2.930282	0.003387	0.095869
atp6v0ca	3480.069	0.01107284	0.079379	0.139493	0.889061	0.988224
sap18	0	NA	NA	NA	NA	NA
mrps31	426.7847	-0.176865933	0.129299	-1.36789	0.171347	0.64335
arfrp1	180.2596	0.130948365	0.212088	0.617424	0.536955	0.892421
hoxd10a	166.9043	-0.235611953	0.188984	-1.24673	0.212497	0.694155
dnmt3bb.3	96.52674	0.291174707	0.18981	1.534033	0.125022	0.572594
lasp1	2439.814	-0.060531641	0.09283	-0.65207	0.514357	0.88631
cdc42l2	53.80182	-0.351388945	0.278089	-1.26358	0.206379	0.688825
p3h3	238.7843	0.234371386	0.167619	1.398239	0.162041	0.629618
si:dkey-33c12.3	1121.645	-0.142913574	0.23564	-0.60649	0.544189	0.895206
arpc3	1681.884	0.217397159	0.143746	1.512367	0.13044	0.581523
zgc:114174	48.31174	0.525225326	0.34043	1.542827	0.122873	0.569468
BX901922.1	1.209983	-1.929686288	1.717204	-1.12374	0.261124	NA
nts	15.72899	-0.032583204	0.719082	-0.04531	0.963858	0.999842
si:dkey-33c12.4	406.2909	0.08269529	0.147713	0.559838	0.57559	0.904739
zgc:114081	46.58885	0.549169487	0.374506	1.466385	0.142543	0.601741
si:ch211-266g18.10	374.9726	0.166968392	0.273057	0.611477	0.540884	0.893439

si:ch73-181d5.4	182.4905	-0.305420097	0.185736	-1.64437	0.100099	0.524371
mrps34	593.8391	0.026707549	0.120481	0.221674	0.824568	0.972227
zgc:86709	1116.375	0.710110486	0.282741	2.511522	0.012021	0.190742
eif1axb	2497.829	-0.029138077	0.140175	-0.20787	0.83533	0.976007
syt11a	916.9132	0.475309979	0.182647	2.602337	0.009259	0.165407
tsc1b	677.5051	-0.082381102	0.11809	-0.69762	0.485418	0.873021
ccdc136b	111.7788	0.597464458	0.208009	2.872297	0.004075	0.106446
rps6ka3b	95.52946	-0.00337193	0.208633	-0.01616	0.987105	1
mzt2b	353.0788	-0.01938361	0.15536	-0.12477	0.900709	0.99045
naf1	124.1796	-0.084722373	0.187724	-0.45131	0.651764	0.928625
lhx5	310.9582	0.133312427	0.241707	0.551545	0.58126	0.905618
ythdf3	242.9936	0.088205058	0.187578	0.470231	0.63819	0.924779
dido1	998.4584	-0.046312159	0.143461	-0.32282	0.746831	0.955074
glt8d2	37.64562	-0.384662118	0.281242	-1.36773	0.171398	0.643433
slc43a3b	244.8341	-0.158698348	0.170361	-0.93154	0.351573	0.802399
dph1	186.0374	0.366117429	0.166632	2.197168	0.028008	0.297823
parp12b	184.3982	-0.055329917	0.158098	-0.34997	0.72636	0.948987
plcd4a	44.81662	-0.581244238	0.358264	-1.62239	0.104719	0.53308
svopl	326.5988	-0.28753504	0.231208	-1.24362	0.213639	0.696139
eps8l1a	282.0752	-0.251512382	0.159551	-1.57638	0.114938	0.554651
ints8	347.276	-0.009670073	0.116398	-0.08308	0.93379	0.996633
uba3	459.9348	0.079314987	0.162706	0.487475	0.625922	0.920625
fstb	415.0201	-0.193691775	0.135365	-1.43089	0.152462	0.61658
arl15b	258.0523	0.047578629	0.140654	0.338267	0.735162	0.951543
tmf1	604.8268	-0.087335967	0.09855	-0.88621	0.375506	0.822237
cep350	583.4747	0.057737818	0.15225	0.37923	0.704517	0.943785
wfdc1	408.6689	0.320285544	0.182133	1.758527	0.078658	0.474455
six2a	404.2133	0.016726839	0.137762	0.121418	0.90336	0.99133
hgd	3266.49	-0.235208939	0.232828	-1.01023	0.312387	0.776164
six3a	358.3074	0.117973221	0.159807	0.738225	0.460378	0.862369
arxa	186.6287	0.153705991	0.180639	0.850904	0.394823	0.828825
ano3	28.08065	0.746402387	0.488264	1.528688	0.126342	0.574665
fbxw9	38.34823	0.406177223	0.312348	1.300401	0.193463	0.673662
ormdl2	208.3644	-0.359516804	0.159387	-2.25562	0.024094	0.27487
si:dkey-11o15.7	19.71062	0.841477917	0.61881	1.359832	0.173883	0.646897
hspa14	379.3903	-0.079183139	0.158957	-0.49814	0.618384	0.916585
si:dkey-6n6.2	242.6043	-0.192078428	0.172393	-1.11419	0.265198	0.742063
eml3	234.0353	-0.728970204	0.213339	-3.41696	0.000633	0.032718
bhlhe22	437.5731	0.149430338	0.22958	0.650885	0.515121	0.886401
ndufa8	1430.493	0.069555784	0.095782	0.726185	0.467725	0.866174
kitlgb	19.70898	-0.271688347	0.448447	-0.60584	0.544619	0.895206
rab1ba	3005.018	0.197420661	0.092336	2.138058	0.032512	0.319448
tlr21	8.975119	0.394083686	0.682848	0.577118	0.56386	0.901196
pafah1b3	599.6779	-0.08776193	0.17132	-0.51227	0.608462	0.915204
ugt5e1	0	NA	NA	NA	NA	NA
smyd3	67.59223	0.003023587	0.239392	0.01263	0.989923	1
serping1	205.2054	-0.676999492	0.259453	-2.60934	0.009072	0.163447
igf3	0.58885	-1.806363648	2.245208	-0.80454	0.421084	NA
magt1	759.5714	-0.205877677	0.124568	-1.65273	0.098386	0.521076
cx31.7	1.410256	0	2.055366	0	1	NA
tnfsf10l4	26.92287	-0.304420127	0.386248	-0.78815	0.430611	0.847542
snf8	305.1831	0.194998478	0.185937	1.048732	0.294301	0.763345
txndc17	294.3999	0.220899684	0.179068	1.23361	0.217348	0.69815
birc7	106.357	0.565731493	0.243555	2.32281	0.020189	0.25177

aifm1	1034.387	0.338767767	0.095618	3.542929	0.000396	0.02357
rprd2a	352.0286	0.100538439	0.179109	0.561325	0.574576	0.904516
cfap57	17.99933	0.881032602	0.540828	1.629044	0.103304	0.530446
ciarta	728.4365	-0.353558129	0.171705	-2.05911	0.039484	0.34755
b3gnt3.4	83.72485	-0.267103051	0.246354	-1.08423	0.278265	0.751971
sardh	1349.208	0.135356822	0.147968	0.914774	0.36031	0.811057
glra4b	49.49408	0.192016369	0.58871	0.326164	0.7443	0.95491
rpl36a	21821.07	-0.395084244	0.152528	-2.59023	0.009591	0.168452
eps8l2	160.9	-0.312632807	0.161017	-1.94162	0.052184	0.396885
impad1	258.5013	-0.095380442	0.140026	-0.68116	0.49577	0.87806
fgfr2	2076.319	-0.000708589	0.106513	-0.00665	0.994692	1
snap25b	1699.146	0.434955281	0.200445	2.169945	0.030011	0.308772
GPR83	2.587867	-0.805848014	1.133723	-0.7108	0.47721	0.869292
hcfc2	95.65209	-0.229785735	0.202167	-1.13662	0.255699	0.733642
msna	2188.02	0.249310215	0.114436	2.178599	0.029361	0.304886
foxd2	343.0999	0.279829622	0.138955	2.013817	0.044029	0.367392
ttc25	37.22337	-0.326870578	0.329688	-0.99146	0.321463	0.781986
atp7b	21.74473	-0.172338297	0.483535	-0.35641	0.721531	0.947343
dnajc5b	45.10075	0.142172341	0.41098	0.345935	0.729392	0.950056
dnajc7	1527.016	0.080575063	0.084102	0.958065	0.33803	0.79489
znf385c	82.06425	0.041221566	0.344342	0.119711	0.904712	0.991588
trim55b	533.6736	-0.044849988	0.21302	-0.21054	0.833244	0.975379
ipo8	293.6967	-0.212670112	0.152178	-1.39751	0.162261	0.629748
tnfaip2b	86.44724	-1.456966208	0.2509	-5.80696	6.36E-09	3.88E-06
pcyt1ba	42.64114	0.30437539	0.305869	0.995116	0.31968	0.78034
htr2ab	4.90894	-2.396066473	1.454817	-1.64699	0.09956	0.523919
zdhhc20b	102.2525	-0.1064437	0.206566	-0.5153	0.606342	0.915204
ackr3b	867.4374	-0.046802935	0.122883	-0.38088	0.703296	0.943622
exoc3l4	34.52032	-0.366253868	0.351153	-1.043	0.296947	0.764508
plcxd2	13.25631	2.569504201	1.372875	1.871623	0.061259	0.424867
pex16	125.0251	-0.247503211	0.181757	-1.36173	0.173284	0.646172
smc1a1	1471.002	0.087329528	0.123776	0.705547	0.48047	0.870179
si:ch211-153b23.5	934.2341	0.111652529	0.189413	0.589467	0.555548	0.898723
abi2a	229.3956	-0.106247456	0.149712	-0.70968	0.477903	0.869292
slc25a26	312.1408	-0.099540342	0.150763	-0.66025	0.509096	0.883443
ndnl2	144.6365	-0.140128313	0.183626	-0.76312	0.445393	0.855403
fam151a	23.08967	0.502998353	0.558295	0.900955	0.367612	0.816956
tubd1	70.8767	0.79438855	0.236485	3.359156	0.000782	0.037196
tada3l	437.662	0.019510397	0.128509	0.151821	0.879328	0.986147
ugcg	160.3705	-0.059185751	0.16404	-0.3608	0.718248	0.946598
mpp4a	29.04451	-0.147186867	0.528087	-0.27872	0.780462	0.961411
arpc4l	1201.311	-0.065054	0.139823	-0.46526	0.641745	0.925586
ak3	547.2902	-0.000265085	0.131211	-0.00202	0.998388	1
acot11a	84.37738	-0.030552774	0.214735	-0.14228	0.886858	0.987671
rps6kb1b	946.2418	0.092939422	0.111596	0.832817	0.404948	0.834284
nt5c2b	537.5168	0.102280801	0.158956	0.643455	0.519929	0.887148
fbrsl1	710.1388	0.061699085	0.126089	0.489331	0.624607	0.919918
ssbp3a	134.5242	-0.161165269	0.184516	-0.87345	0.382419	0.825252
phactr3a	187.3689	0.37990336	0.229106	1.658196	0.097278	0.519937
il17ra1a	75.00679	-0.369209809	0.297747	-1.24001	0.214972	0.697086
cysltr1	22.11671	-0.363398838	0.377609	-0.96237	0.335865	0.792139
si:dkeyp-77h1.4	22.7355	-0.126895328	0.483399	-0.26251	0.792931	0.965312
adamts15b	34.50164	0.573942467	0.629269	0.912077	0.361728	0.811787
si:ch211-213d14.1	5.226074	1.918208883	1.310951	1.463219	0.143408	0.602607

draxin	195.7053	0.065134235	0.288024	0.226142	0.821091	0.971788
slc39a1	137.5326	-0.37468469	0.175868	-2.13048	0.033132	0.322362
celsr1b	365.0066	0.195181108	0.222119	0.878721	0.379552	0.823607
cip2a	120.4118	0.34654803	0.204462	1.694922	0.09009	0.50413
glmna	186.1181	-0.149388954	0.161663	-0.92408	0.355446	0.806546
COLGALT1	168.2386	0.605342376	0.205583	2.944523	0.003235	0.093414
si:dkey-208b23.5	7.838666	-0.246305068	0.537628	-0.45813	0.646857	0.926728
CT990561.1	19.21667	0.513042595	0.403165	1.272539	0.203182	0.684044
cpt1b	361.6883	0.279696092	0.291238	0.960369	0.33687	0.793513
gpalpp1	245.1701	0.09970282	0.140841	0.707913	0.478999	0.869441
sephs1	616.6087	-0.097261118	0.17695	-0.54965	0.582556	0.90571
timm13	782.5405	0.198099389	0.118287	1.674731	0.093987	0.511793
zgc:173737	6.305038	0.592448837	0.708146	0.83662	0.402806	0.833401
sh3bgrl	782.729	-0.290922791	0.176962	-1.64399	0.100179	0.524654
prpf18	407.7914	-0.075392961	0.121304	-0.62152	0.534258	0.891573
c1qtnf9	96.27261	-0.642839369	0.273996	-2.34616	0.018968	0.242406
cep76	83.37759	0.221965385	0.271419	0.817797	0.413473	0.838369
enc3	620.2359	-0.185142363	0.130708	-1.41646	0.156641	0.622452
tmbim1a	183.5463	-0.112490117	0.184471	-0.6098	0.541996	0.894241
casp8	166.7941	-0.151091474	0.143443	-1.05332	0.292195	0.761879
neu3.3	39.98526	-0.409915822	0.731524	-0.56036	0.575235	0.904718
lsm7	1104.456	-0.027628169	0.205968	-0.13414	0.893294	0.988566
btr21	3.70771	0.184947725	1.132985	0.163239	0.87033	0.983365
krt18a.2	33.14002	-3.107670118	0.736683	-4.21847	2.46E-05	0.00332
slc25a22b	9.965636	0.943768948	0.799602	1.180298	0.237882	0.718607
grhl2a	143.8103	-0.162448717	0.183827	-0.8837	0.376856	0.822258
si:dkey-5i3.5	141.8604	-0.173786166	0.210319	-0.8263	0.408634	0.836526
sass6	77.2083	0.307989724	0.264912	1.16261	0.244988	0.726435
scin1b	652.5051	0.608991689	0.142301	4.279594	1.87E-05	0.002746
polr2l	426.8368	0.247749135	0.234799	1.055153	0.291356	0.761454
tapbpl	269.7273	-0.172716348	0.225136	-0.76716	0.442984	0.854063
acot9.1	244.6131	-0.144232019	0.179027	-0.80564	0.420448	0.842383
selenot1a	961.0063	0.04501282	0.129312	0.348094	0.72777	0.9493
ankrd33ba	49.58799	-0.017791348	0.336468	-0.05288	0.95783	0.999109
krt8	26150.48	-0.297285372	0.129701	-2.29208	0.021901	0.262381
hspb8	620.7577	0.321145601	0.110526	2.905598	0.003666	0.100272
si:dkey-222f8.3	609.9028	-0.242504581	0.164231	-1.47661	0.13978	0.59821
abhd14a	50.83105	0.509154393	0.296213	1.718879	0.085636	0.491277
mex3b	1069.444	0.039704656	0.159029	0.24967	0.802843	0.968323
ropn1l	40.35943	-0.113444415	0.356072	-0.3186	0.75003	0.955074
krt5	43395.29	-0.010332354	0.176865	-0.05842	0.953414	0.998436
mcph1	31.62753	0.819039547	0.285624	2.867545	0.004137	0.106542
otpb	354.6124	0.197100881	0.191652	1.02843	0.303747	0.769473
zgc:171775	844.5616	-0.096575915	0.112779	-0.85633	0.391816	0.827902
pimr57	0.18768	0	5.267649	0	1	NA
ccl19a.1	33.98673	-0.270134647	0.432202	-0.62502	0.531958	0.890978
acot9.2	58.51654	-0.081838381	0.294181	-0.27819	0.780866	0.961608
adcy2a	66.61303	1.034822592	0.348463	2.969675	0.002981	0.090034
tagln3b	533.3404	-0.13034918	0.110784	-1.1766	0.239354	0.719781
hce2l1	3.556619	-0.180260037	1.107616	-0.16275	0.870718	0.983365
CR956623.1	13.82069	-1.489347141	0.949142	-1.56915	0.116613	0.557506
rnaset2l	21.30997	-0.332155124	0.519288	-0.63964	0.522409	0.887826
bglap	0.046941	0	5.267649	0	1	NA
si:dkey-11m19.5	3.116127	-0.620176676	1.161371	-0.534	0.593339	0.910427

gcn1	2260.164	0.000152888	0.106311	0.001438	0.998853	1
lgi1b	94.54358	0.19381929	0.18848	1.028326	0.303796	0.769473
rab35b	252.1432	0.05831763	0.174813	0.3336	0.738682	0.953124
snx6	85.33136	-0.026439193	0.268401	-0.09851	0.92153	0.994085
zgc:136472	72.03607	-0.989940941	0.510089	-1.94072	0.052292	0.397339
zrsr2	103.96	0.111075691	0.220631	0.503444	0.614652	0.915998
rpl6	44392.75	-0.373019191	0.127697	-2.92113	0.003488	0.097712
abhd17c	456.984	0.105515851	0.118544	0.8901	0.373412	0.820558
dynll1	3415.516	0.040540195	0.169194	0.239608	0.810634	0.969828
plekhg2	306.3619	0.185541217	0.209912	0.883899	0.376751	0.822258
cacng3a	1.649705	0.093645988	2.100615	0.04458	0.964442	NA
ice1	150.6648	0.038113078	0.176028	0.216518	0.828584	0.974137
zgc:158846	2162.62	0.564177521	0.284689	1.981734	0.047509	0.379549
ndufab1a	1377.091	-0.002211276	0.133506	-0.01656	0.986785	1
rasd3	29.75704	-0.373046183	0.331814	-1.12426	0.260902	0.738467
prpf40a	745.1096	0.124441953	0.103905	1.197654	0.231052	0.712561
mapk13	107.7825	-0.016561056	0.183898	-0.09006	0.928243	0.995052
plk1	742.3918	0.303320794	0.242115	1.252798	0.210279	0.692011
st6galnac3	36.12544	0.708825408	0.269656	2.628629	0.008573	0.158655
ern2	86.52373	0.17835763	0.256758	0.694652	0.487273	0.873291
stc1l	64.62062	-0.956803091	0.412733	-2.31821	0.020438	0.253747
pimr72	0.096459	0	5.267649	0	1	NA
zgc:110425	13.08792	-0.934221519	0.638043	-1.4642	0.14314	0.602132
chtf18	122.1426	0.28950866	0.199677	1.449888	0.14709	0.607283
caps2	5.179477	0.406570728	0.707343	0.574786	0.565436	0.901538
adora2ab	10.52793	-0.355408614	0.630511	-0.56368	0.57297	0.903718
si:dkey-30c15.17	34.44875	-0.823410495	0.402433	-2.04608	0.040748	0.352816
dnaja3a	1108.239	0.054642692	0.147279	0.371016	0.710626	0.945821
slc9a3.1	51.47282	-0.14185544	0.613302	-0.2313	0.817083	0.971207
olah	88.15948	0.198534128	0.367844	0.539724	0.589388	0.908456
flt3	4.790464	-0.650120123	1.276926	-0.50913	0.610662	0.915331
ap1s2	1198.364	-0.004982424	0.134051	-0.03717	0.970351	1
cfap70	19.51334	0.918552437	0.548314	1.67523	0.093889	0.511703
shq1	107.0183	-0.287413997	0.249539	-1.15178	0.249411	0.729721
cnot10	271.0047	0.044433301	0.139178	0.319255	0.749533	0.955074
pimr209	0.91501	-1.941463146	2.149931	-0.90304	0.366507	NA
pole	269.364	0.186957707	0.307992	0.607022	0.543836	0.894999
si:dkey-211g8.7	0.046941	0	5.267649	0	1	NA
si:dkey-102c8.2	2.905227	-2.991309102	1.711136	-1.74814	0.080439	0.478998
alcamb	1086.567	-0.12667185	0.119115	-1.06344	0.287583	0.758397
lama5	1519.3	0.068158431	0.155425	0.438529	0.661003	0.930581
si:dkey-30c15.12	3.384077	-0.388282625	1.197312	-0.3243	0.745715	0.955074
si:ch73-160i9.3	2.134678	2.534836885	1.147298	2.209397	0.027147	NA
bves	283.0733	0.203352534	0.201612	1.008635	0.31315	0.776599
popdc3	85.91318	0.316445201	0.304612	1.038848	0.298876	0.76578
sprb	170.5167	-0.477254484	0.239029	-1.99664	0.045864	0.374848
muc5.2	194.0057	0.297845051	0.336859	0.884183	0.376598	0.822258
il11b	2.176398	-0.398236304	1.647263	-0.24176	0.808969	NA
lzts3b	35.22415	0.605987956	0.397723	1.523644	0.127598	0.576979
adrm1	1606.848	0.081792494	0.140137	0.583662	0.559447	0.899571
fbxo30a	112.5788	-0.082713532	0.1892	-0.43717	0.661984	0.930742
znf991	104.6527	-0.157740155	0.216462	-0.72872	0.466172	0.865297
ubox5	152.3335	-0.010508521	0.244402	-0.043	0.965704	1
si:ch73-368j24.17	2.802274	2.41133426	1.457549	1.654377	0.098051	0.521071

socs6a	45.91767	-0.1083581	0.345031	-0.31405	0.75348	0.955861
si:dkey-253d23.5	36.02573	-0.021544822	0.319337	-0.06747	0.94621	0.997371
avp	16.81233	0.431308243	0.540007	0.798709	0.424459	0.844412
ccl27a	21.80649	-1.912925345	1.252997	-1.52668	0.126841	0.575696
pdp2	64.22286	-0.260987946	0.246616	-1.05828	0.28993	0.760779
abcg2c	232.4376	-0.137564709	0.164153	-0.83803	0.402014	0.832956
ccdc106b	21.63321	-0.357625768	0.438764	-0.81508	0.415029	0.838732
si:ch211-274f20.2	15.92628	-0.342876071	0.532175	-0.64429	0.519386	0.887148
chst2b	152.1353	0.234260301	0.203561	1.150809	0.249811	0.729721
terb1	3.128868	1.281504166	1.364817	0.938957	0.347753	0.800274
sri	978.608	0.143186455	0.110213	1.299181	0.193882	0.674122
nt5c3a	1781.404	-0.152247851	0.162563	-0.93655	0.348992	0.801306
sox18	157.8998	0.037901154	0.194282	0.195083	0.845328	0.976791
gdap1	138.9436	-0.218995006	0.27892	-0.78515	0.432364	0.848419
jph1a	213.4066	0.498343612	0.272759	1.827047	0.067693	0.443563
vsig10	146.1678	0.066592273	0.184286	0.361353	0.717836	0.946466
sik1	1164.512	-0.513513574	0.439995	-1.16709	0.243174	0.72442
wsb2	54.5733	0.309364294	0.299965	1.031334	0.302384	0.768252
zgc:162472	226.118	0.010897078	0.155546	0.070057	0.944148	0.997371
CABZ01030107.1	76.92775	0.18386837	0.255691	0.719105	0.472076	0.868517
si:ch211-71m22.1	186.7518	0.010852823	0.18924	0.057349	0.954267	0.998611
dnajb2	168.4963	0.426042	0.220508	1.932092	0.053348	0.400175
ptprna	578.5821	0.682740803	0.171835	3.973224	7.09E-05	0.007021
hck	63.62676	-0.183829667	0.305581	-0.60157	0.547457	0.896211
trim46b	114.7333	0.310785943	0.25585	1.214722	0.224472	0.706406
dpm3	245.2801	0.093635141	0.175527	0.533451	0.593722	0.910427
desma	6581.299	0.279392729	0.173028	1.614721	0.106371	0.535773
si:dkey-178e17.3	126.8426	0.008840901	0.191725	0.046112	0.963221	0.999842
ilf3a	1018.781	0.038069086	0.088228	0.431483	0.666117	0.932664
pimr94	0.249435	2.850002157	5.18846	0.549296	0.582802	NA
dennd2da	239.6912	-0.065503427	0.128265	-0.51069	0.609569	0.915204
nuggc.3	9.971276	0.305002087	0.828708	0.368045	0.712839	0.945821
nkl.2	0.608229	2.062659919	3.246724	0.635305	0.525229	NA
aqp10b	10.85858	0.497109758	0.876383	0.567229	0.570558	0.902481
zgc:114104	74.57458	-0.351771313	0.261168	-1.34692	0.178007	0.651772
cd8b	0.199839	-1.826676548	5.23046	-0.34924	0.72691	NA
pimr204	0.621603	0	3.962111	0	1	NA
si:dkey-204a24.11	1.37578	1.055433978	2.191537	0.481595	0.630093	NA
cgnb	185.4597	-0.194275595	0.177074	-1.09714	0.27258	0.748416
fzd3b	73.98977	-0.047419672	0.258423	-0.1835	0.854408	0.979088
cast	4204.912	-0.014479509	0.141987	-0.10198	0.918775	0.993884
ddr2b	161.0441	0.479901942	0.261442	1.835598	0.066417	0.440058
tbl1xr1a	950.225	0.065990745	0.084481	0.781131	0.434726	0.850002
tmeff2b	253.8388	-0.205879242	0.17037	-1.20842	0.226885	0.707983
si:dkey-73p2.2	1.681	0	2.106719	0	1	NA
nos1apb	12.77913	0.200378705	0.628401	0.318871	0.749825	0.955074
vax2	6.926931	0.88549978	0.622898	1.42158	0.155148	0.619776
terf1	102.0752	0.049606994	0.235591	0.210564	0.833228	0.975379
cept1a	11.96798	0.127234691	0.635163	0.200318	0.841232	0.97643
si:dkey-119f1.1	110.9633	0.315344401	0.180213	1.74984	0.080146	0.478357
or126-7	0.101247	1.854150541	5.241768	0.353726	0.723544	NA
rfesd	68.36721	0.113005374	0.35904	0.314743	0.752957	0.955756
nudt2	225.5258	0.167981729	0.171913	0.977131	0.328504	0.786794
akirin2	949.6512	-0.0097103	0.11097	-0.0875	0.930271	0.995707

rdh10a	982.2359	-0.295383303	0.155062	-1.90494	0.056788	0.411692
slc2a6	46.02371	-0.528092475	0.323779	-1.63103	0.102884	0.529859
scgn	78.6864	-0.146918344	0.222945	-0.65899	0.509903	0.883733
ihha	38.42167	-0.481316033	0.333604	-1.44278	0.149084	0.610726
prdx1	1238.081	-0.063512291	0.214316	-0.29635	0.766963	0.960296
gabra6b	9.436778	0.097574943	0.797829	0.122301	0.902661	0.991028
si:ch211-117c9.2	17.459	0.133709551	0.598386	0.22345	0.823185	0.972005
ube2r2	190.0096	-0.122944209	0.171535	-0.71673	0.47354	0.869146
ubap1	472.991	0.023286754	0.120787	0.192791	0.847122	0.977335
si:ch73-213k20.5	6.25698	-6.887444873	1.361681	-5.05805	4.24E-07	0.000149
or128-3	1.190119	1.949627913	2.465418	0.79079	0.429066	NA
or128-2	0.26002	0	4.787542	0	1	NA
nav1b	126.5153	0.118479216	0.242384	0.488807	0.624978	0.920289
CABZ01053221.1	3.77938	-0.021181653	1.148299	-0.01845	0.985283	1
2-Oct	45.17723	-0.595534975	0.396567	-1.50172	0.133168	0.586514
slc9a2	65.46532	0.299615915	0.252185	1.188081	0.234802	0.715533
si:ch73-242m19.1	16.22448	0.80701068	0.498435	1.619089	0.105428	0.534737
zgc:171887	2.446903	-0.625514431	1.376894	-0.45429	0.649617	0.927756
si:dkey-19a16.2	49.88538	-0.173729435	0.298946	-0.58114	0.561146	0.90039
mybpha	340.4327	0.099293244	0.257451	0.385678	0.699735	0.942499
rab25a	164.9928	-0.004979281	0.165748	-0.03004	0.976034	1
rev3l	440.6636	-0.038001514	0.183609	-0.20697	0.836034	0.976007
grk1a	225.3101	0.696849039	0.448002	1.555461	0.119836	0.564602
naxe	284.0858	0.209270763	0.234797	0.891284	0.372777	0.819953
ihhb	15.15524	0.004936823	0.628309	0.007857	0.993731	1
rbm38	238.3721	-0.198624492	0.135132	-1.46985	0.141601	0.600284
nog1	206.8275	-0.124433127	0.177203	-0.70221	0.48255	0.871721
bin1b	294.1824	-0.040953075	0.138035	-0.29669	0.766706	0.960296
sema5a	502.5662	-0.02873939	0.148018	-0.19416	0.84605	0.977039
wnt3a	16.67547	0.391401432	0.470021	0.832731	0.404996	0.834284
mrpl4	623.5344	0.139236697	0.157768	0.882539	0.377485	0.822478
ccdc51	168.041	0.060531306	0.19849	0.304959	0.760397	0.957886
zgc:171424	2.467787	-5.049247881	1.701757	-2.96708	0.003006	0.090442
zdhhc3b	191.9249	-0.084890783	0.155763	-0.545	0.585755	0.906655
si:ch73-212j7.3	69.02469	0.058323226	0.301172	0.193654	0.846447	0.977213
susd1	59.26414	-0.526556497	0.313578	-1.67919	0.093115	0.510759
mcoln1b	32.76714	-0.23700156	0.393322	-0.60256	0.546799	0.896107
sash1b	247.4929	-0.063160314	0.116181	-0.54364	0.586692	0.906788
zgc:112052	440.5025	0.02825213	0.133502	0.211624	0.832401	0.975062
ncln	570.485	0.204819571	0.143441	1.427897	0.153321	0.617847
endog	162.6714	0.278768253	0.16846	1.654804	0.097964	0.521071
apc	1675.099	0.199685985	0.117825	1.694762	0.090121	0.50413
CABZ01040556.1	36.88683	-0.16859604	0.358704	-0.47001	0.638345	0.924779
zgc:171857	69.10819	0.389194804	0.280879	1.38563	0.16586	0.635405
ptpdc1b	83.06203	0.313840494	0.592776	0.529442	0.596499	0.911676
zgc:113227	34.37226	-0.243437961	0.320414	-0.75976	0.447397	0.856073
mmp16b	22.58466	0.50106962	0.531157	0.943356	0.345499	0.79876
cldnj	49.32022	0.124499288	0.347115	0.358669	0.719843	0.94694
ttc29	3.104672	0.919426707	0.937231	0.981004	0.326591	0.785469
cep170ab	49.69638	0.080855894	0.310311	0.260564	0.794429	0.965431
nhej1	125.539	0.171045667	0.199378	0.857897	0.390949	0.827902
si:ch211-237c6.4	232.8408	0.033867776	0.177277	0.191044	0.848491	0.977912
si:ch211-181l5.2	6.998923	-0.752974326	0.990064	-0.76053	0.446937	0.85583
nnr	0.049021	-0.86807634	5.267649	-0.16479	0.869106	NA

pou1f1	3.657827	1.069141417	1.070107	0.999098	0.317747	0.779627
si:busm1-52i16.2	2.573776	-1.510909288	1.560961	-0.96794	0.333077	0.790126
cldn10a	3.182104	-0.078998791	1.183808	-0.06673	0.946794	0.997371
b4galnt3b	129.399	0.269558794	0.211576	1.274051	0.202645	0.683051
si:ch211-248e11.2	123.1738	-0.164920801	0.284216	-0.58027	0.561736	0.900715
cdcp1a	64.75909	-0.724684746	0.295215	-2.45477	0.014098	0.208027
si:dkey-8e10.3	75.04428	-0.002117581	0.268564	-0.00788	0.993709	1
acot22	0.500464	1.055404854	3.537012	0.298389	0.765406	NA
CU856539.2	383.7623	0.015469894	0.126034	0.122744	0.90231	0.990938
spina	107.0293	0.068484725	0.224327	0.30529	0.760145	0.957886
abcc4	388.2517	0.233714359	0.165633	1.41104	0.158233	0.623766
trim35-7	17.61656	0.025073492	0.483733	0.051833	0.958661	0.999109
otol1b	14.35891	-1.105823865	0.801661	-1.37942	0.167767	0.638989
ngfa	37.65765	-0.216220836	0.323709	-0.66795	0.504167	0.881759
txnl4b	124.8089	-0.14359118	0.194357	-0.7388	0.460027	0.862369
themis2	9.998463	0.012399653	0.651318	0.019038	0.984811	1
zgc:112332	65.6505	-0.067741979	0.316313	-0.21416	0.830421	0.974908
haus1	70.66549	-0.429739938	0.277787	-1.54701	0.12186	0.56863
cntnap2a	304.3008	-0.011975917	0.17702	-0.06765	0.946062	0.997371
zgc:113423	124.3999	-0.11728633	0.277822	-0.42216	0.672906	0.93414
rad23ab	468.5013	0.064225135	0.19723	0.325635	0.7447	0.95491
KCNJ6	53.29529	0.24339717	0.357317	0.681179	0.495758	0.87806
KCNJ15	55.71883	-0.347330076	0.310777	-1.11762	0.263731	0.741708
slc35a2	130.7378	0.200211217	0.155543	1.287173	0.198034	0.678765
cers2b	462.1416	0.214802644	0.131015	1.639529	0.101103	0.526828
rock1	623.3416	0.009757764	0.148122	0.065876	0.947476	0.997371
stx1a	8.647984	-0.710200792	1.03772	-0.68439	0.493731	0.877215
jmjd4	51.28759	0.046160008	0.249887	0.184723	0.853446	0.978887
jam2a	212.8612	-0.308682134	0.187803	-1.64364	0.10025	0.524748
pim2	581.6002	-0.237306789	0.165464	-1.43419	0.151518	0.615372
strip2	175.3502	0.347834835	0.242331	1.435369	0.151182	0.615188
otud5a	695.3412	0.028897452	0.112833	0.256107	0.797868	0.966447
lim2.2	19.38122	-0.073018507	0.456239	-0.16004	0.872846	0.983537
thap1	164.2776	-0.018667424	0.249179	-0.07492	0.940282	0.997371
zgc:123217	49.41765	-0.217160285	0.32505	-0.66808	0.50408	0.881759
srrd	50.65898	0.12041861	0.27552	0.437059	0.662068	0.930742
mmp28	128.5598	-0.144615482	0.229904	-0.62903	0.529332	0.890418
porb	919.8233	0.372047927	0.204179	1.822166	0.06843	0.445916
FO904903.1	5.785046	-6.834477554	5.137318	-1.33036	NA	NA
ntf3	16.18578	0.308266068	0.523117	0.589287	0.555669	0.898723
epm2a	44.03459	-0.558527789	0.440428	-1.26815	0.204745	0.686713
psmd5	335.0234	0.075648708	0.158485	0.477323	0.633132	0.923885
mpzl1l	276.8144	-0.133965662	0.133599	-1.00275	0.315983	0.778545
zgc:174904	257.3124	-0.449325469	0.198358	-2.26523	0.023499	0.270878
slc13a4	480.3671	-0.213925814	0.233669	-0.91551	0.359925	0.810891
pdk2b	6837.375	-1.324271438	0.251641	-5.26255	1.42E-07	6.43E-05
ft1	8.967343	-0.778498955	0.574908	-1.35413	0.175696	0.649413
atpv0e2	760.3995	-0.20912262	0.207115	-1.00969	0.312643	0.776164
vipr1b	10.18785	-0.181723724	0.612681	-0.2966	0.766769	0.960296
emsy	787.7397	0.119446997	0.125225	0.953858	0.340156	0.795345
lgals1a	76.33259	0.06438619	0.228036	0.282352	0.777674	0.961073
rassf3	133.4969	-0.197245546	0.188702	-1.04527	0.295897	0.764246
jupb	29.2217	0.860072713	0.389667	2.207198	0.0273	0.293572
gars	2354.025	0.089617145	0.117614	0.761958	0.446085	0.855403

gsc	222.2739	-0.440250205	0.172651	-2.54994	0.010774	0.181029
nip7	257.227	-0.045594552	0.228421	-0.19961	0.841788	0.97643
vps25	808.4992	0.192654737	0.138529	1.39072	0.16431	0.633351
LO018513.1	10.21143	1.423430429	0.849498	1.675614	0.093814	0.511703
ppp2r5ca	242.7795	-0.079274194	0.179435	-0.4418	0.658634	0.930581
sstr2a	70.13062	-0.048940852	0.274199	-0.17849	0.858341	0.979774
ank1a	416.917	0.067869242	0.196744	0.344963	0.730122	0.950056
nktr	587.8705	-0.262692569	0.249132	-1.05443	0.291686	0.761454
si:ch73-126o18.1	0.416155	-0.868122166	5.267649	-0.1648	0.869099	NA
si:dkeyp-113d7.1	836.1509	-0.139110552	0.127095	-1.09454	0.273717	0.749234
heatr5b	229.7075	0.166642472	0.195629	0.85183	0.394309	0.828374
EFEMP1	12.05132	-1.774840289	1.022545	-1.73571	0.082615	0.483014
plcd1a	499.1198	-0.081175803	0.119622	-0.6786	0.497391	0.878744
prkacbb	899.9197	-0.135293352	0.138946	-0.97371	0.330201	0.788126
uqcrh	2966.647	-0.013694318	0.140386	-0.09755	0.922291	0.994346
gata1b	1.421831	-1.330495511	1.547456	-0.8598	0.389902	NA
spdya	4.508425	-0.742805564	0.724675	-1.02502	0.305354	0.770528
wfikkn2b	5.555173	0.543867809	0.927654	0.586283	0.557686	0.899184
tmem107	150.2914	-0.291426091	0.158136	-1.84288	0.065347	0.435359
rbp3	1322.823	0.491953483	0.244641	2.010921	0.044334	0.368519
slc22a13b	13.3281	-0.603489625	0.713837	-0.84542	0.397878	0.830675
gdf2	7.190209	-0.384394415	1.005635	-0.38224	0.702283	0.943402
cep19	76.43631	0.42253612	0.235084	1.797381	0.072275	0.457045
tax1bp3	659.1283	0.158655792	0.155008	1.023533	0.306056	0.771472
CU467633.1	1.839139	-3.56404874	1.615674	-2.20592	0.02739	NA
tspan2b	165.606	0.09754764	0.209744	0.46508	0.641874	0.925586
abhd13	37.0638	-0.329078259	0.268619	-1.22508	0.220547	0.701837
ciao1	412.3719	0.276936202	0.1771	1.56373	0.117881	0.559998
zbtb8a	56.08008	0.243489361	0.256682	0.948605	0.342822	0.79738
fabp1b.1	6440.173	0.152262352	0.1885	0.80776	0.419229	0.842286
hephl1a	194.6894	-0.448164531	0.212676	-2.10726	0.035095	0.329877
runx2b	20.71529	0.011616075	0.395272	0.029388	0.976556	1
mrps27	491.701	0.238062068	0.14502	1.641586	0.100676	0.525729
zgc:113274	64.41555	-0.132676772	0.261873	-0.50665	0.612403	0.915727
ptger4a	11.44809	-1.944113478	0.687612	-2.82734	0.004694	0.113638
arfip2a	399.8163	0.060832841	0.150808	0.40338	0.686669	0.93875
hnrnpd	935.1953	0.167620356	0.108458	1.545489	0.122228	0.569366
tmem54a	392.9922	-0.102648511	0.145574	-0.70513	0.48073	0.870179
zpcx	2.159264	2.399767482	1.807669	1.327548	0.184327	NA
il1r1	1.396005	1.888650401	1.675085	1.127495	0.259533	NA
evx2	116.9672	0.25144733	0.178014	1.412514	0.157799	0.623766
hoxd13a	53.80439	-0.162317775	0.206021	-0.78787	0.430772	0.847629
pabpc4	10285.88	-0.066836223	0.145568	-0.45914	0.646132	0.926728
hoxd12a	84.43968	-0.141965064	0.216554	-0.65556	0.512105	0.885285
hoxd11a	125.0779	0.126892685	0.247285	0.513143	0.607852	0.915204
hoxd9a	230.2129	-0.292656481	0.160506	-1.82333	0.068253	0.445546
hoxd4a	94.71405	-0.005988774	0.200529	-0.02986	0.976175	1
tfap2a	1604.122	-0.077925556	0.121367	-0.64207	0.52083	0.887391
hoxd3a	232.3088	-0.029056293	0.157669	-0.18429	0.853788	0.978905
mak	306.0225	0.013440696	0.135159	0.099443	0.920786	0.994085
marco	105.3353	-0.282868346	0.286477	-0.9874	0.323445	0.783161
spcs1	229.531	-0.112145707	0.206272	-0.54368	0.586663	0.906788
hnrnpl2	1841.129	0.072130595	0.11626	0.620426	0.534977	0.891993
chchd2	1091.967	0.197307436	0.139238	1.417047	0.156469	0.622452

tmem37	36.49752	-1.59587034	0.958817	-1.66442	0.096029	0.517378
golt1bb	266.9666	-0.061462738	0.195731	-0.31402	0.753509	0.955861
dpysl5b	1257.163	0.220935929	0.220404	1.002413	0.316144	0.778682
ora3	11.36157	0.3791901	0.517211	0.733144	0.463471	0.864553
topbp1	206.2668	0.368728639	0.146924	2.509653	0.012085	0.190742
rpn1	1578.719	0.052926537	0.14169	0.373536	0.708749	0.945518
gata2a	289.928	-0.170593122	0.148379	-1.14971	0.250263	0.730026
si:ch211-285f17.1	471.5206	0.035192483	0.201636	0.174535	0.861445	0.9805
tgif1	877.1476	-0.093050127	0.123524	-0.75329	0.451273	0.857606
dlgap2a	19.2223	1.404894079	0.565022	2.486443	0.012903	0.198116
avil	31.69409	-0.745775456	0.865716	-0.86146	0.388987	0.827725
calml4b	1.764203	0	1.989214	0	1	NA
rxfp3.3b	24.44244	-0.601803621	0.747996	-0.80455	0.421077	0.842517
homer2	578.4726	0.04396563	0.170385	0.258037	0.796378	0.966432
hnrnpa3	166.313	0.283205089	0.206117	1.373999	0.169442	0.640975
glmp	422.5886	0.017293892	0.128253	0.134842	0.892737	0.988566
sarnp	706.4288	-0.126883766	0.15658	-0.81034	0.417743	0.841149
srsf3b	2310.177	-0.06209311	0.102766	-0.60422	0.5457	0.895522
slc39a4	6.864545	-0.905723114	1.09238	-0.82913	0.407032	0.835261
cavin1b	592.8454	0.173301166	0.155296	1.115938	0.264449	0.742063
tgfbr2a	49.28537	-0.424176855	0.301542	-1.40669	0.159518	0.6257
nipal4	169.1679	0.111421108	0.187612	0.593891	0.552585	0.8974
mfap2	1833.683	-0.120993739	0.20287	-0.59641	0.550901	0.8974
gria4b	289.9214	1.032065428	0.221541	4.658576	3.18E-06	0.000745
si:ch1073-15f12.3	3.204246	-0.650453839	1.300807	-0.50004	0.617048	0.916365
nr1d4b	40.73826	-0.885063751	0.628375	-1.4085	0.158984	0.624561
dnajc8	897.3189	0.299728441	0.122516	2.44645	0.014427	0.210484
fgf7	23.55078	0.08731615	0.452357	0.193025	0.84694	0.9773
bdh1	186.9106	-0.097560952	0.182877	-0.53348	0.593702	0.910427
gyg1b	3078.694	-0.20816308	0.142569	-1.46008	0.144267	0.603371
tas1r2.2	0.103263	1.055383603	5.267649	0.200352	0.841205	NA
myef2	1339.975	0.167454666	0.104761	1.598449	0.109943	0.543963
si:ch211-160o17.6	34.8078	-0.186372798	0.707582	-0.26339	0.792247	0.965108
tmem167b	463.0741	0.072305786	0.130181	0.555426	0.578603	0.905417
rab43	243.0461	-0.134935458	0.131356	-1.02725	0.304302	0.769725
raf1b	564.2849	-0.299613441	0.142984	-2.09543	0.036133	0.334343
abcd2	8.574027	-0.249668399	0.880405	-0.28358	0.77673	0.961073
ccr12b.1	0.502193	1.05540286	3.52685	0.299248	0.764751	NA
impa2	50.59632	0.840855538	0.338471	2.484276	0.012982	0.198536
zgc:111976	4.068582	0.083699249	0.930908	0.089911	0.928358	0.995052
prune2	392.5907	0.827542667	0.160103	5.168802	2.36E-07	9.41E-05
rnf215	41.08473	0.388552549	0.31016	1.25275	0.210297	0.692011
fsd1l	184.8798	-0.147293421	0.153329	-0.96064	0.336735	0.793455
fktn	253.171	-0.303180453	0.169913	-1.78433	0.07437	0.463115
galnt18b	147.1009	-0.238870963	0.193946	-1.23163	0.218086	0.69923
smtnb	440.4625	-0.148606956	0.141207	-1.0524	0.292615	0.762191
dqx1	143.5274	-0.226748833	0.170556	-1.32947	0.183692	0.66056
si:ch211-283h6.4	81.57202	-0.178925023	0.220732	-0.8106	0.417596	0.841149
sbf2	488.194	-0.082392955	0.110582	-0.74509	0.45622	0.860359
mepce	407.9785	-0.113306887	0.186937	-0.60612	0.544434	0.895206
wasf3a	55.63337	-0.708407946	0.393224	-1.80154	0.071618	0.4564
arhgap31	173.4305	-0.077677019	0.225226	-0.34488	0.730181	0.950056
kank4	188.5056	0.022181939	0.203381	0.109066	0.91315	0.99273
mlxip	270.5087	0.156563458	0.206014	0.759966	0.447275	0.856073

camsap3	247.3168	0.038591131	0.15827	0.243832	0.807361	0.969736
tead1b	349.887	0.079528786	0.187691	0.423722	0.671769	0.933953
emb	43.15892	0.368825475	0.37592	0.981129	0.326529	0.785469
cplx4b	0.392712	-1.756251467	3.756677	-0.4675	0.640141	NA
arl16	149.0895	-0.308353959	0.206758	-1.49138	0.135863	0.591131
ACSF3	68.41714	0.372595793	0.2408	1.547326	0.121785	0.56863
si:dkey-163m14.2	33.13053	-0.356979794	0.393388	-0.90745	0.36417	0.814549
coq2	232.2321	0.559126576	0.144846	3.860141	0.000113	0.010099
rims4	15.01629	1.13633445	0.71834	1.58189	0.113675	0.551905
KIAA0895L	58.25241	-0.184601908	0.311456	-0.59271	0.553378	0.897946
si:ch211-146m13.3	239.3378	-0.117036521	0.226742	-0.51617	0.605738	0.915055
akap10	180.8513	-0.035435315	0.166138	-0.21329	0.831102	0.974908
sympk	647.5888	0.08558571	0.112884	0.758172	0.448348	0.856741
recql5	237.3326	-0.329216079	0.155659	-2.11499	0.034431	0.327696
xylt2	35.90294	-0.29635088	0.344832	-0.85941	0.390116	0.827902
tecta	65.54677	0.219533307	0.324396	0.676744	0.498568	0.879201
si:ch73-281f12.4	132.4884	0.31835739	0.182479	1.744622	0.081051	0.479801
fscn2a	33.31171	1.089432741	1.286055	0.847112	0.396933	0.830253
uhmk1	78.0754	-0.169539771	0.22077	-0.76795	0.442518	0.853789
mbd5	215.1247	0.118822972	0.195811	0.606824	0.543967	0.895043
sowahcb	100.5942	0.244691218	0.261552	0.935534	0.349513	0.801408
rcc1l	275.4847	-0.215838643	0.152317	-1.41704	0.156472	0.622452
CU928117.1	268.0793	0.175782968	0.165865	1.059797	0.289237	0.760276
clip2	209.8383	0.256314915	0.159544	1.606548	0.108154	0.54011
rabep1	508.3029	-0.021410253	0.104259	-0.20536	0.837293	0.976007
map1aa	791.3755	0.422173703	0.185243	2.279027	0.022665	0.266308
mtss1	415.9411	-0.110854531	0.109253	-1.01466	0.310267	0.775062
ccdc84	84.66716	0.044848017	0.209755	0.213811	0.830694	0.974908
gpr146	691.4739	-0.166315483	0.096047	-1.7316	0.083345	0.484434
nprl2	206.2051	0.110576852	0.175321	0.63071	0.52823	0.889948
hs3st2	21.82348	-1.218604914	0.442941	-2.75117	0.005938	0.130541
asphd2	326.5369	-0.163645452	0.150947	-1.08413	0.278309	0.752003
mlec	162.8308	0.012944604	0.165155	0.078378	0.937527	0.997305
thumpd3	253.8163	-0.279566905	0.201354	-1.38843	0.165005	0.634185
si:ch211-127d4.3	44.65703	0.027884979	0.334014	0.083484	0.933466	0.996472
mtmr14	405.3085	-0.084922899	0.118805	-0.71481	0.474725	0.869292
nt5dc2	2267.923	-0.266592042	0.16944	-1.57337	0.115633	0.555893
cidec	53.41993	0.035528485	0.395832	0.089757	0.928481	0.995052
kdm2aa	704.3116	0.139736332	0.137067	1.019473	0.307978	0.772996
eif3ba	7974.062	-0.328586813	0.11142	-2.94908	0.003187	0.093009
mks1	103.1785	0.105274416	0.193484	0.544098	0.586374	0.906711
mrm1	25.67331	-0.302372242	0.39278	-0.76983	0.441404	0.852874
arhgap36	171.9606	0.112774848	0.177353	0.635878	0.524856	0.888928
fscn1a	2480.627	0.090858098	0.137259	0.661947	0.508005	0.883056
slc43a3a	123.1904	0.440799905	0.258276	1.706698	0.087878	0.497853
mblac2	13.6548	0.309503053	0.567665	0.545221	0.585602	0.906655
slc29a4	200.627	0.278418614	0.201788	1.379757	0.167661	0.638828
adam19a	267.2169	-0.074875405	0.167737	-0.44639	0.655319	0.929606
dusp28	12.81075	0.283989526	0.54965	0.516674	0.605384	0.914985
dnajc16	31.30091	-0.314459682	0.385144	-0.81647	0.414229	0.838631
agmat	59.62801	-0.217471253	0.299276	-0.72666	0.467435	0.86616
flii	834.042	0.231384656	0.107679	2.148841	0.031647	0.316333
vwa5b1	2.329435	-0.495463264	1.62648	-0.30462	0.760653	0.957886
si:ch211-216l23.1	754.5468	0.105197739	0.122556	0.858364	0.390691	0.827902

nol6	347.0482	-0.162581436	0.191208	-0.85029	0.395165	0.829083
slc19a2	19.05575	-0.052725977	0.439449	-0.11998	0.904497	0.991581
arsk	52.87538	-0.438816419	0.345096	-1.27158	0.203523	0.684359
fam169aa	33.16569	-0.259072165	0.477844	-0.54217	0.587702	0.907608
ubac1	544.9375	0.074599489	0.145634	0.512238	0.608484	0.915204
hypk	843.7485	-0.021916411	0.148108	-0.14798	0.882361	0.986908
sec14l8	153.535	0.120426521	0.223784	0.538138	0.590482	0.908901
ptprsa	282.0366	0.263890041	0.260079	1.014652	0.310272	0.775062
tmem98	72.35076	-0.149692075	0.231496	-0.64663	0.517872	0.887148
plod1a	835.8966	0.187815076	0.177933	1.055537	0.29118	0.761299
kcnj19a	21.5444	0.855799768	0.447203	1.913672	0.055662	0.408467
abtb2a	80.52057	-0.000418548	0.259439	-0.00161	0.998713	1
wdtc1	371.8969	-0.146340345	0.130921	-1.11778	0.263661	0.741708
gabrd	137.3297	0.883793494	0.393826	2.24412	0.024825	0.279217
gpatch8	1640.297	0.007733891	0.154997	0.049897	0.960204	0.999164
cpt1aa	395.4969	-0.131421528	0.143897	-0.9133	0.361083	0.811396
SRCIN1	102.7573	0.393901699	0.289387	1.36116	0.173463	0.64634
dub	225.6621	-0.582871774	0.244318	-2.38571	0.017046	0.231104
slc32a1	966.913	0.249542528	0.211411	1.180366	0.237855	0.718607
atcayb	87.88563	0.231632006	0.28302	0.81843	0.413112	0.838274
parp8	126.0252	0.197777276	0.168613	1.172967	0.240809	0.722178
trpm5	41.51675	-0.114351322	0.639792	-0.17873	0.858148	0.979774
kdm6al	637.8367	0.006316201	0.140689	0.044895	0.964191	0.999925
kcnq1.1	27.82288	-0.548556527	0.361959	-1.51552	0.129641	0.579651
pdzd2	220.1076	-0.065869378	0.179795	-0.36636	0.714098	0.945821
retreg3	415.5164	-0.215478896	0.128028	-1.68306	0.092364	0.508314
slc25a38a	45.9674	-0.334493377	0.431058	-0.77598	0.43776	0.851283
sacm1lb	267.5989	0.190248102	0.125733	1.513107	0.130252	0.581464
caska	659.938	-0.014367935	0.141875	-0.10127	0.919335	0.993892
smpd5	164.84	0.086031048	0.204887	0.419895	0.674562	0.934877
sin3ab	665.9382	0.072120771	0.105271	0.685099	0.493281	0.876787
oaz2b	106.2627	0.890117646	0.401393	2.217571	0.026584	0.288909
zgc:136864	0	NA	NA	NA	NA	NA
mtmr12	189.8776	-0.070733471	0.166091	-0.42587	0.670202	0.933809
kif5ab	55.98549	0.847726079	0.429015	1.975984	0.048157	0.381858
soat2	215.6107	-1.033234004	0.347142	-2.9764	0.002917	0.089033
crtac1a	1030.989	1.306811993	0.297963	4.385822	1.16E-05	0.00208
adgrb3	416.2433	0.508856002	0.226487	2.246731	0.024657	0.278415
zfyve27	246.0169	-0.37655592	0.141297	-2.66499	0.007699	0.148279
ddit3	851.2439	0.064126809	0.183699	0.349087	0.727024	0.949095
exosc1	449.5287	-0.571090427	0.178994	-3.19055	0.00142	0.056314
fam135a	507.0207	0.189406358	0.114736	1.650798	0.09878	0.521469
epg5	212.483	-0.128262631	0.213627	-0.6004	0.548237	0.896644
slc35f3b	6.600297	-1.815345946	0.849675	-2.13652	0.032637	0.319477
manbal	510.6517	0.039199606	0.156118	0.251089	0.801745	0.967776
CABZ01041604.1	27.45575	0.529939033	0.507444	1.044329	0.296333	0.764246
wdr66	18.58067	0.237202234	0.448431	0.52896	0.596833	0.911757
alkbh2	51.43103	-0.10019717	0.276249	-0.36271	0.716825	0.94627
blvra	134.0198	-0.048752857	0.192719	-0.25297	0.800289	0.967327
pappab	23.85871	0.726279363	0.573994	1.265308	0.205761	0.687948
cactin	486.261	0.014833357	0.110488	0.134253	0.893203	0.988566
clk2b	268.5377	0.059280153	0.129228	0.458724	0.646432	0.926728
shrprbck1r	701.1527	-0.151319497	0.111822	-1.35322	0.175986	0.649415
trpv1	44.8681	-0.487121519	0.345395	-1.41033	0.158442	0.623806

frmd3	20.29874	0.161493382	0.373465	0.432419	0.665437	0.932595
fam222ba	273.5778	0.123315686	0.16105	0.765699	0.443856	0.854255
eral1	200.6604	0.058277176	0.170271	0.342262	0.732154	0.950617
ago3a	56.46792	0.005674099	0.323362	0.017547	0.986	1
AL929217.1	7.130934	1.431469093	1.02687	1.394012	0.163314	0.631199
ntrk2a	147.1963	0.311767755	0.206639	1.508753	0.131362	0.583069
CR847944.2	1.742431	-0.86814315	2.021059	-0.42955	0.667524	NA
tbc1d9	96.94676	0.220615565	0.240572	0.917045	0.359119	0.810124
vps16	608.4603	-0.356240065	0.09225	-3.8617	0.000113	0.010073
hs3st3l	46.14714	0.055602528	0.336639	0.165169	0.868811	0.982801
sdc4	2557.87	0.015995698	0.143233	0.111676	0.91108	0.992208
si:ch211-217k17.7	761.9996	-0.140090906	0.215737	-0.64936	0.516106	0.88677
bbs7	175.8812	-0.707213276	0.181246	-3.90195	9.54E-05	0.008792
sdr42e2	60.83221	-0.809366396	0.368908	-2.19395	0.028239	0.298982
hyal1	54.19033	0.154616186	0.253686	0.609479	0.542207	0.89435
ccnj	21.03372	0.490795839	0.335527	1.462759	0.143533	0.602607
zgc:162613	136.6577	0.236965871	0.154178	1.536965	0.124302	0.571556
slc25a47a	362.4042	0.338902824	0.289312	1.17141	0.241434	0.723209
usp24	908.5224	0.201486997	0.180971	1.113364	0.265552	0.742247
hyal2a	78.37084	0.175899798	0.239586	0.734183	0.462837	0.864104
plpp3	1840.575	0.231324481	0.106063	2.181008	0.029183	0.304273
herc4	140.083	-0.050794567	0.194382	-0.26131	0.793851	0.965431
dab1a	35.42119	0.202726096	0.271336	0.74714	0.454979	0.860002
c1qtnf2	27.99643	-0.035927125	0.396065	-0.09071	0.927723	0.995016
sv2a	1838.901	0.108570424	0.243964	0.445026	0.656301	0.929684
si:dkey-121b10.7	13.69452	0.382855821	0.579415	0.660762	0.508765	0.883333
plxdc2	865.6309	-0.105522194	0.102281	-1.03169	0.302219	0.768145
pld6	5.646735	-1.747378065	0.968371	-1.80445	0.071161	0.455341
ptk6b	63.39808	0.442770553	0.436637	1.014047	0.31056	0.775146
plch2a	442.3669	0.218528975	0.147436	1.482199	0.138287	0.594938
chpf2	260.8685	-0.217096627	0.133961	-1.6206	0.105104	0.533762
polk	73.308	0.350791207	0.198431	1.767826	0.07709	0.469501
camsap2b	161.4294	0.139873845	0.208175	0.671904	0.501645	0.881073
raly	373.9548	0.38757682	0.149204	2.597632	0.009387	0.166663
cplx4a	496.3978	0.089782053	0.335621	0.26751	0.789076	0.963627
poc5	44.3281	0.280342338	0.306018	0.916098	0.359615	0.810668
zufsp	87.11612	-0.161409644	0.200406	-0.80542	0.42058	0.842383
dnah12	45.69819	0.53821628	0.384159	1.401025	0.161207	0.627968
trpm4a	521.2726	-0.228124244	0.150778	-1.51298	0.130285	0.581464
sv2ca	6.967904	0.136498971	0.798455	0.170954	0.86426	0.981867
mettl9	252.4743	0.188878779	0.174325	1.083489	0.278592	0.752152
ogg1	55.87884	-0.531514679	0.268708	-1.97804	0.047924	0.380801
fzd7a	1525.405	-0.014025557	0.158938	-0.08825	0.929682	0.995576
mtrf1l	70.75065	-0.058933948	0.201959	-0.29181	0.770431	0.960484
nedd4l	493.6105	-0.089352042	0.115186	-0.77572	0.437913	0.851283
n4bp3	24.68392	0.871504825	0.378201	2.304342	0.021203	0.258321
hhipl2	129.2971	-0.106793234	0.213167	-0.50098	0.616382	0.916365
iqgap2	270.7588	-0.088469046	0.171855	-0.51479	0.6067	0.915204
f2r	35.68309	0.052806595	0.291068	0.181424	0.856035	0.979558
nsd1a	461.0338	-0.260430885	0.130103	-2.00173	0.045314	0.372707
si:ch1073-44g3.1	218.6625	0.39186031	0.186311	2.103265	0.035443	0.330833
vcpkmt	117.8752	-0.016067517	0.166747	-0.09636	0.923236	0.994525
ncapg2	200.3991	0.395541759	0.269143	1.469632	0.141661	0.600284
nsmfa	10.58982	0.108514525	0.902253	0.120271	0.904269	0.991581

nom1	151.647	-0.26634606	0.189744	-1.40371	0.160404	0.626951
fam160b2	338.9688	-0.201561077	0.147384	-1.36759	0.17144	0.643433
setd1a	653.8879	0.137544754	0.183638	0.749	0.453857	0.858859
tmem151ba	39.49874	0.167586318	0.276695	0.605671	0.544733	0.895206
rnf32	19.04214	0.457518934	0.41058	1.114324	0.26514	0.742063
aimp1a	1446.769	-0.04663494	0.149403	-0.31214	0.754933	0.956283
lig1	465.5738	0.065855967	0.207338	0.317627	0.750768	0.955074
zgc:195075	2.828427	-2.616723891	1.461753	-1.79013	0.073433	0.46112
slc47a2.1	35.74213	0.734970824	0.501819	1.464613	0.143027	0.602002
epc1b	527.6065	-0.410352645	0.126684	-3.23918	0.001199	0.049994
cylda	407.3658	0.197975498	0.110139	1.797499	0.072256	0.457045
lrrc74a	4.651525	-1.482055326	1.039245	-1.42609	0.153843	0.618475
ubap2b	5608.041	-0.035096626	0.153109	-0.22923	0.818693	0.971679
fam98b	147.5455	0.14176331	0.205758	0.688981	0.490835	0.87556
isoc1	134.5988	0.16914059	0.177976	0.950357	0.341931	0.796716
adcy7	194.6537	-0.568594419	0.165096	-3.44402	0.000573	0.030598
tent4b	365.1581	-0.171554711	0.166742	-1.02886	0.303545	0.769406
abi3a	85.47587	0.167208103	0.253555	0.659455	0.509603	0.883733
crb2b	16.49529	-1.769781082	0.617648	-2.86535	0.004165	0.106651
kcnq3	23.57328	0.674202369	0.367843	1.832852	0.066825	0.441496
btaf1	640.0625	0.154858406	0.110807	1.397546	0.162249	0.629748
zgc:165481	67.19707	-0.27422829	0.292772	-0.93666	0.348932	0.801306
dapk1	388.1015	0.024827548	0.133926	0.185383	0.852929	0.978794
ptgis	110.2571	-0.336345134	0.266771	-1.2608	0.207381	0.689456
kcnb1	4.923083	3.074841779	1.227075	2.50583	0.012216	0.191771
tbc1d10ab	38.11213	-0.386912362	0.308438	-1.25442	0.209688	0.691766
trappc12	333.8651	-0.080862913	0.152439	-0.53046	0.595791	0.911427
kank1a	1022.861	-0.156883267	0.125789	-1.24719	0.212327	0.694078
cpeb3	63.02133	-0.092559191	0.35352	-0.26182	0.793459	0.965366
crb2a	52.41632	-0.755964464	0.348537	-2.16896	0.030085	0.309264
aggf1	142.5016	0.009697848	0.18876	0.051377	0.959025	0.999164
sybu	204.9862	-0.61183059	0.316385	-1.93381	0.053136	0.400175
znf395a	1652.174	-0.090382325	0.226905	-0.39833	0.69039	0.93988
lrrn1	944.3179	0.18239729	0.145193	1.256241	0.209029	0.691152
camk1a	23.8655	0.241174578	0.484154	0.498136	0.618388	0.916585
glyctk	277.9914	-0.054022186	0.149121	-0.36227	0.717149	0.94627
ca16b	312.0191	0.31302588	0.20897	1.497945	0.134148	0.588117
timm17b	39.77998	-0.937005534	0.341266	-2.74567	0.006039	0.131072
acyp1	88.66193	-0.725431966	0.402068	-1.80425	0.071192	0.455342
adamts3	66.72691	0.095332397	0.242958	0.392382	0.694776	0.941181
dusp3b	19.32709	-0.995152798	0.740687	-1.34355	0.179093	0.654301
sh3pxd2aa	167.3296	-0.415677316	0.24014	-1.73098	0.083456	0.484751
ablim1a	187.9154	0.184473327	0.140756	1.31059	0.189996	0.66964
psmd11a	961.6214	0.074442911	0.163353	0.455719	0.648592	0.927501
fam155b	138.8971	0.229119952	0.186833	1.226333	0.220073	0.701196
h6pd	313.4173	0.123177647	0.191365	0.64368	0.519783	0.887148
dnah7	21.29444	0.081296402	0.522852	0.155486	0.876438	0.985007
fam160b1	173.0832	0.099091603	0.192105	0.51582	0.60598	0.915112
mns1	75.18177	0.241375117	0.238028	1.014062	0.310553	0.775146
trmt10b	48.76627	-0.538432872	0.257108	-2.09419	0.036243	0.335095
vcpip1	115.641	-0.332349548	0.234909	-1.4148	0.157127	0.623076
pkn1a	494.0381	0.009884516	0.113949	0.086745	0.930874	0.995809
si:ch211-214c7.5	0.540527	0	3.923051	0	1	NA
rpusd1	58.89431	0.331475361	0.293963	1.127608	0.259485	0.736167

lhpp	184.7585	0.212424039	0.257405	0.825252	0.409228	0.836637
atp2c1	236.5025	0.141222038	0.185486	0.761362	0.446441	0.855403
mrpl18	522.0417	0.039115653	0.16011	0.244305	0.806994	0.969726
CU019662.1	14.34674	-0.700822567	0.750159	-0.93423	0.350184	0.802152
traf7	221.0417	0.068011348	0.155371	0.437736	0.661578	0.930742
d2hgdh	85.5895	-0.098661751	0.218709	-0.45111	0.65191	0.928716
ankef1b	1.538626	0.770880782	1.44746	0.532575	0.594328	NA
cep85	240.3774	0.058031989	0.172076	0.337246	0.735931	0.951589
scn1ba	135.8441	0.227149657	0.203127	1.118263	0.263455	0.741437
mst1ra	78.45055	-0.134572699	0.244875	-0.54956	0.582623	0.90571
dok7	168.0435	0.542124704	0.199694	2.714773	0.006632	0.136957
uacab	1241.943	-0.108796904	0.187633	-0.57984	0.562023	0.900715
enox1	138.8792	0.522934289	0.300303	1.741358	0.081621	0.480965
slc16a6b	562.584	-0.081433435	0.217641	-0.37416	0.708283	0.945344
fgd4b	41.95564	-0.114201646	0.338166	-0.33771	0.735583	0.951543
wdr44	820.6907	-0.070812936	0.108508	-0.65261	0.51401	0.88631
mogs	333.4888	0.029337761	0.12196	0.240552	0.809902	0.969828
si:ch211-216b21.2	51.80805	-0.317069569	0.322368	-0.98356	0.32533	0.784089
alkbh8	71.02092	-0.095579178	0.291068	-0.32837	0.742629	0.954484
zranb1a	971.9955	-0.084380426	0.113777	-0.74163	0.458311	0.861001
zgc:162160	17.61537	0.2381516	0.473063	0.503425	0.614666	0.915998
pecam1	363.6883	-0.175071685	0.154484	-1.13327	0.257103	0.734679
si:dkey-37g12.1	6.602766	-2.758451863	1.373893	-2.00776	0.044668	0.37016
pde9a	49.61989	0.663416368	0.340587	1.94786	0.051432	0.394202
rnf121	386.2184	0.091206027	0.124297	0.733775	0.463086	0.864191
plpbp	686.4827	0.233009819	0.166924	1.395906	0.162743	0.630455
arhgap35b	409.0033	0.156426438	0.162457	0.96288	0.335608	0.792139
nin	143.649	0.311202414	0.277523	1.121356	0.262136	0.739695
slc4a10b	31.65393	0.303248394	0.487443	0.622121	0.533863	0.891181
tfcp2	583.0113	-0.025232444	0.113057	-0.22318	0.823393	0.972005
urb1	187.5596	-0.248060518	0.218913	-1.13315	0.257153	0.734679
srgap3	137.3716	-0.21130288	0.221363	-0.95455	0.339804	0.795226
wdr62	252.9076	0.125417403	0.230494	0.544124	0.586356	0.906711
rabgap1l2	207.2149	-0.335824531	0.206029	-1.62998	0.103105	0.530217
cish	500.1844	-0.474074583	0.251977	-1.88142	0.059915	0.421883
scn4bb	23.06897	-0.530733884	0.607655	-0.87341	0.382438	0.825252
dcaf5	343.0157	-0.032924356	0.179508	-0.18341	0.854473	0.979088
zgc:153654	7.566008	-0.031677214	0.671669	-0.04716	0.962384	0.999461
exoc5	297.9554	-0.066299519	0.194453	-0.34095	0.733138	0.950758
fam20cl	0.536034	0	4.060258	0	1	NA
map1sb	157.2364	-0.17680602	0.267312	-0.66142	0.508342	0.883261
si:ch211-284e13.4	59.89955	-0.127459017	0.303059	-0.42058	0.674065	0.934789
trmt12	52.25674	-0.484941169	0.293402	-1.65282	0.098367	0.521076
si:dkey-157l19.2	38.22431	0.071984996	0.343894	0.209323	0.834196	0.975731
apoda.1	686.899	-0.900500316	0.194523	-4.62926	3.67E-06	0.000845
map3k3	199.1012	-0.097966232	0.166939	-0.58684	0.557312	0.899165
wasf1	213.2365	0.146751063	0.176622	0.830879	0.406042	0.834645
apoda.2	33430.45	-1.402701394	0.334083	-4.19866	2.69E-05	0.003515
samd7	130.0886	0.105967792	0.297451	0.356252	0.721652	0.947347
cep104	68.27404	0.162641081	0.223708	0.727024	0.467211	0.86616
nadkb	358.9658	-0.180947451	0.149131	-1.21335	0.224997	0.70685
slc12a9	185.1924	-0.26574242	0.180501	-1.47225	0.140953	0.599658
syn1	679.1231	0.105361536	0.219791	0.479371	0.631674	0.923003
plxna2	94.13897	0.17517087	0.197004	0.889174	0.373909	0.820695

ttll11	32.51965	-0.275296361	0.352103	-0.78186	0.434295	0.850002
coq6	458.3503	0.207357279	0.141172	1.468832	0.141878	0.600644
mmaa	274.9029	0.057850245	0.129339	0.447278	0.654675	0.929527
stk26	504.6338	-0.024707281	0.105269	-0.23471	0.814437	0.970506
ash2l	724.6876	0.067174872	0.100745	0.666783	0.50491	0.88195
fibcd1	32.88914	-0.4756469	0.463362	-1.02651	0.30465	0.769932
mtfr2	145.7526	0.037741502	0.216133	0.174622	0.861377	0.9805
si:dkey-57k2.7	204.5838	-0.527236776	0.176935	-2.97983	0.002884	0.088391
hhip	683.7992	0.007569181	0.16746	0.0452	0.963948	0.999842
rft1	88.99339	0.034737414	0.19714	0.176207	0.860131	0.980191
gpr185a	3.498353	0.206740874	0.875035	0.236266	0.813226	0.970328
thbs2a	112.1224	0.551354323	0.267181	2.063602	0.039055	0.345519
ddx28	149.3125	-0.557456493	0.167543	-3.32725	0.000877	0.040082
si:ch211-157b11.8	25.59424	-0.229197859	0.555316	-0.41273	0.679801	0.9365
arhgef28a	285.7447	-0.250246626	0.154669	-1.61795	0.105674	0.534827
zfyve28	72.45138	0.170809222	0.225561	0.757264	0.448892	0.856796
map1b	384.1707	0.1805941	0.222779	0.810642	0.417571	0.841149
bnipl	140.0538	0.109271085	0.192673	0.567133	0.570624	0.902481
tbl1x	210.301	-0.001289347	0.196607	-0.00656	0.994768	1
clcn2c	204.5967	-0.615396813	0.322528	-1.90804	0.056386	0.410319
tmem259	401.6487	0.054517534	0.139293	0.391386	0.695512	0.9414
elp6	113.4321	-0.240961595	0.206276	-1.16815	0.242746	0.724345
sdk2b	210.7993	-0.023385763	0.19749	-0.11841	0.905739	0.991631
pmp22b	2363.952	-0.049062069	0.141597	-0.34649	0.728973	0.949983
pik3r4	579.619	-0.253254898	0.156102	-1.62237	0.104724	0.53308
gcnt3	20.23495	0.458821337	0.464243	0.988322	0.322995	0.782754
vps8	462.2801	0.121411604	0.153076	0.793146	0.427693	0.845893
ubr1	80.14749	0.019789377	0.296414	0.066763	0.946771	0.997371
atl1	424.4081	0.240423798	0.168072	1.430484	0.152578	0.61658
mid1	25.92937	0.034491642	0.368478	0.093606	0.925422	0.994815
selenoi	106.235	0.10845771	0.206939	0.524105	0.600205	0.913756
mrpl32	615.9093	-0.373051758	0.191836	-1.94464	0.051818	0.395809
eprs	3017.961	0.056014075	0.144216	0.388405	0.697717	0.9423
cacna1ha	105.5447	-0.262187465	0.281707	-0.93071	0.352004	0.802591
tnfrsf9a	36.30608	0.224571975	0.400359	0.560927	0.574847	0.904516
l2hgdh	150.235	-0.020035783	0.193809	-0.10338	0.917662	0.993777
dpcd	82.70518	0.07040078	0.262118	0.268584	0.78825	0.963471
pfkla	99.24757	0.073451446	0.238211	0.308346	0.757819	0.957397
sos2	389.4289	-0.107179527	0.182471	-0.58738	0.556951	0.899145
cul4b	1162.447	-0.125806386	0.095716	-1.31437	0.188722	0.668504
kbtbd3	39.50088	0.704389118	0.308621	2.282379	0.022467	0.265534
zdhhc9	394.6548	-0.097954058	0.137946	-0.71009	0.477648	0.869292
pcsk5b	305.1023	0.124098038	0.155182	0.799692	0.423889	0.844281
rbm27	138.6953	-0.065691477	0.219887	-0.29875	0.76513	0.959622
rfk	53.34902	0.003733423	0.290874	0.012835	0.989759	1
bmp3	241.7491	0.022326183	0.186402	0.119774	0.904662	0.991581
nkx6.3	18.28096	-0.363414782	0.461458	-0.78754	0.430968	0.84774
adam22	115.1892	0.066157327	0.178423	0.370789	0.710794	0.945821
sgppl	62.77902	0.072947522	0.290438	0.251163	0.801688	0.967776
atad2b	306.6013	0.04008042	0.194342	0.206236	0.836606	0.976007
shank1	50.405	0.136854472	0.454994	0.300783	0.76358	0.959055
lgr4	563.0432	0.205338534	0.171429	1.197805	0.230993	0.712561
ecel1	83.98687	-0.860020461	0.293586	-2.92937	0.003397	0.095894
rps6ka5	67.46752	0.455569611	0.278232	1.637376	0.101552	0.527925

mut	1332.848	-0.392896373	0.1287	-3.0528	0.002267	0.075801
dis3	182.1949	0.150330389	0.154327	0.974103	0.330005	0.788084
slc25a21	99.72134	-0.362089435	0.202597	-1.78724	0.073899	0.461767
cluhb	34.35769	0.386363824	0.511415	0.755481	0.449961	0.85711
tmem229b	271.3903	0.564468427	0.197842	2.853122	0.004329	0.108751
si:dkey-226m8.10	40.33179	0.403305376	0.47529	0.848547	0.396134	0.8295
taspl	192.2777	0.095369018	0.168071	0.567434	0.570419	0.902481
kank1b	15.58384	0.059768558	0.556052	0.107487	0.914402	0.992972
ctdspl2b	915.3016	0.097778453	0.11354	0.861184	0.389137	0.827725
hadhab	1526.132	0.218147904	0.197867	1.102495	0.270246	0.746532
prkaa1	481.8876	-0.078085504	0.117299	-0.6657	0.505604	0.882409
zgc:158659	44.36175	-0.056243066	0.336583	-0.1671	0.867291	0.982582
capn15	161.9049	0.207324537	0.208717	0.993326	0.320551	0.780821
rgs7bpa	160.683	-0.222697908	0.207419	-1.07366	0.282975	0.755028
cd109	113.5158	0.242558971	0.264444	0.91724	0.359017	0.810051
pcdh7b	222.4306	0.235681478	0.143905	1.637755	0.101473	0.527632
ipo13	133.1043	0.551327984	0.171445	3.215776	0.001301	0.052789
lig4	105.4587	-0.142493227	0.203623	-0.69979	0.484058	0.872532
si:ch73-14h1.2	86.23115	-2.9775181	0.662584	-4.4938	7.00E-06	0.001353
dgkaa	677.2116	-0.080001963	0.109487	-0.7307	0.464964	0.864823
hip1ra	40.54146	-0.239181277	0.371184	-0.64437	0.519333	0.887148
GARNL3	136.5916	-0.026293822	0.217193	-0.12106	0.903642	0.991366
usp16	2.350317	0.66827637	1.534519	0.435496	0.663203	0.931241
clstn2	258.2693	-0.478104635	0.299814	-1.59467	0.110786	0.545801
CLSTN2 (1 of many)	39.06006	-0.880897358	0.386691	-2.27804	0.022724	0.266612
apba2b	640.9548	0.065852766	0.190296	0.346055	0.729302	0.950056
prkaa2	48.2755	0.056212485	0.32926	0.170724	0.864441	0.981867
sirt7	51.07145	0.077888011	0.31406	0.248003	0.804132	0.968814
senp5	148.9686	0.180327605	0.149043	1.209904	0.226316	0.707597
BX005421.2	0.773189	-1.742413428	2.488658	-0.70014	0.483839	NA
si:ch211-10a23.2	258.3537	0.21884784	0.196288	1.114931	0.26488	0.742063
klf8	340.1172	0.027150778	0.135044	0.201052	0.840658	0.97643
prf1.9	4.182894	0.066929099	0.980589	0.068254	0.945583	0.997371
tm4sf18	139.9246	-0.181285326	0.249594	-0.72632	0.467642	0.866174
lct	14.33582	0.842782553	1.112138	0.757804	0.448568	0.856741
ndst2b	93.52606	0.343509259	0.223625	1.536097	0.124515	0.572138
kdm1a	2298.637	-0.056004019	0.102973	-0.54387	0.586529	0.906711
si:ch73-127m5.1	70.14633	0.295449494	0.275757	1.071414	0.283983	0.755095
agr1	1319.485	-0.082007099	0.127446	-0.64347	0.519922	0.887148
lrsam1	171.0677	0.016475355	0.24075	0.068433	0.945441	0.997371
chd2	1060.736	-0.164394266	0.168068	-0.97814	0.328004	0.786438
trim69	74.22955	-0.014152606	0.274629	-0.05153	0.9589	0.999164
znf346	835.7025	0.294913139	0.136947	2.153483	0.031281	0.314709
kmt2bb	769.383	0.125720564	0.180671	0.695852	0.486521	0.873244
npffr2b	1.225814	2.090186755	2.060152	1.014579	0.310307	NA
nsd1b	73.91751	0.463815881	0.268716	1.726042	0.08434	0.48732
pex11a	53.03572	0.250624681	0.294011	0.852433	0.393974	0.828374
sv2bb	578.4464	0.190445861	0.176549	1.078713	0.280716	0.753147
prkx	157.7674	-0.674600009	0.240094	-2.80973	0.004958	0.117232
stim1a	542.5657	0.047014727	0.106521	0.441367	0.658947	0.930581
dnajc16l	389.1773	-0.104436926	0.153476	-0.68048	0.496201	0.878102
trim8b	796.9673	0.04027989	0.110177	0.365593	0.714668	0.945821
appl1	187.2268	-0.084956007	0.176988	-0.48001	0.631221	0.923003
syt17	5.566058	0.71423018	0.725732	0.984152	0.325041	0.783955

pgm5	25.43603	-0.258176318	0.398514	-0.64785	0.517083	0.887148
tsfm	230.9822	0.502584523	0.162914	3.084974	0.002036	0.070205
taok3a	254.344	-0.212345748	0.151204	-1.40437	0.160209	0.626611
pear1	196.6673	0.062240527	0.189296	0.3288	0.742307	0.954329
abhd6a	167.2171	0.50216121	0.230831	2.175453	0.029596	0.306409
nphs1	33.82304	-0.080246882	0.348691	-0.23014	0.817985	0.971518
pxnb	65.5392	-0.179369769	0.330345	-0.54298	0.587146	0.907261
smg7	1428.112	0.007444153	0.127751	0.058271	0.953533	0.998436
ankib1a	113.9424	-0.192818798	0.218037	-0.88434	0.376513	0.822258
map7d3	582.7645	-0.056298223	0.150927	-0.37302	0.709136	0.945546
acap2	259.1271	-0.129825233	0.163923	-0.79199	0.428366	0.846133
pcnx4	290.1705	-0.026846042	0.148104	-0.18126	0.85616	0.979558
lrrc14b	41.38546	0.31050038	0.484065	0.641443	0.521235	0.887473
slc20a2	790.4478	-0.133982577	0.162284	-0.8256	0.409029	0.836637
pfkmb	703.0545	-0.245498908	0.306223	-0.8017	0.422726	0.843493
map1sa	281.2657	-0.137674339	0.141956	-0.96984	0.332127	0.789602
mecom	148.0696	0.025627896	0.268363	0.095497	0.92392	0.994713
plekha7a	180.8236	-0.164876531	0.201942	-0.81646	0.414239	0.838631
car15	8.665073	0.684520187	0.847655	0.807545	0.419352	0.84234
fam83hb	324.5487	-0.165089318	0.14432	-1.14391	0.252659	0.732759
tecpr2	116.7873	-0.077834676	0.208523	-0.37327	0.70895	0.945546
trpc5b	30.00093	0.387419487	0.569156	0.680692	0.496067	0.878093
trim35-33	3.976718	1.883215862	1.085975	1.734125	0.082896	0.483073
pik3c2a	618.708	0.072879198	0.121215	0.601237	0.547682	0.896211
antxr1d	19.93489	0.211041553	0.3924	0.537823	0.5907	0.90904
cabp7b	49.73781	1.580274866	0.609771	2.591588	0.009553	0.168223
setd1ba	307.6851	-0.097980617	0.167575	-0.5847	0.558751	0.899409
dlgap4a	308.984	0.269701656	0.159457	1.691377	0.090765	0.505206
nckap1	1925.53	0.095901654	0.08602	1.114878	0.264903	0.742063
kctd3	123.9029	0.27581016	0.21686	1.271837	0.203431	0.684359
pstpip1a	8.269284	-0.569885814	0.61849	-0.92142	0.356834	0.807485
dars2	106.7878	0.313592746	0.216327	1.449623	0.147164	0.607479
atxn1b	483.6746	-0.026412973	0.113605	-0.2325	0.816151	0.971207
scai	18.43694	0.191229671	0.446576	0.428213	0.668496	0.933086
mbtd1	249.3658	0.109896203	0.153637	0.715296	0.474426	0.869292
mctp1b	122.1599	0.428251481	0.169637	2.524516	0.011586	0.18746
pde1a	47.43634	0.68381552	0.333693	2.049237	0.040439	0.35132
edil3b	9.704979	2.175385067	0.92642	2.348163	0.018866	0.242305
slc28a1	39.65678	-2.234808741	1.150998	-1.94163	0.052182	0.396885
nmur1a	1.309238	0.107712103	2.129929	0.050571	0.959668	NA
znf592	195.7759	-0.117201364	0.147137	-0.79655	0.425714	0.844714
mettl7a	0	NA	NA	NA	NA	NA
col8a2	317.5118	-0.112925189	0.179085	-0.63057	0.528323	0.889948
znf362a	475.624	0.109404413	0.148696	0.735757	0.461879	0.863402
trim62.1	35.21728	0.441522946	0.389123	1.134661	0.256518	0.73401
si:ch211-126i22.5	188.6562	-0.114375046	0.136667	-0.83689	0.402656	0.833401
fbxl16	99.50727	0.186905487	0.237596	0.786651	0.431486	0.848419
anln	555.7366	0.316173742	0.25407	1.244435	0.213339	0.695695
pla2g6	450.2807	-0.103091508	0.135205	-0.76248	0.445771	0.855403
ess2	480.1586	-0.054571728	0.143322	-0.38076	0.703379	0.943622
jarid2a	315.4844	0.006744187	0.18069	0.037325	0.970226	1
rnf19b	46.21655	-0.254456565	0.305748	-0.83224	0.405271	0.834483
si:ch211-136a13.1	230.4676	0.030174298	0.219401	0.137531	0.890611	0.988446
rnf220a	522.1274	0.064439271	0.102478	0.62881	0.529473	0.890434

baiap2l2b	21.33319	-0.536056113	0.49349	-1.08626	0.277366	0.751426
adnp2a	164.2326	0.088601519	0.177256	0.499849	0.617181	0.916365
ANKFN1	19.01521	0.190534107	0.481745	0.395508	0.692468	0.940414
rnf217	32.79878	0.043994237	0.340756	0.129108	0.897272	0.989719
pip5kl1	65.95285	0.607160206	0.287543	2.111543	0.034726	0.328285
dync1i1	344.5956	0.148467359	0.164141	0.90451	0.365725	0.815695
polg	119.6653	-0.124348296	0.215443	-0.57717	0.563822	0.901196
si:ch1073-214b20.2	2.88265	-1.17436765	1.305278	-0.89971	0.368276	0.817272
tmcc1b	464.4794	0.084634463	0.137479	0.615619	0.538146	0.892498
btr04	140.9842	-0.343887671	0.226503	-1.51825	0.128952	0.578173
zgc:153018	768.9219	-0.114313784	0.139972	-0.81669	0.414105	0.838631
atp2a3	641.3187	0.128706809	0.150956	0.852614	0.393874	0.828374
atp8b1	311.7088	-0.192005753	0.172258	-1.11464	0.265005	0.742063
smyd4	64.71265	0.390684739	0.232089	1.683343	0.092309	0.508314
zgc:154077	201.0334	-0.16436261	0.233092	-0.70514	0.480722	0.870179
mief1	290.5453	0.060711296	0.119784	0.506842	0.612266	0.915709
rpusd2	62.5959	0.160235446	0.230779	0.694323	0.48748	0.873291
fbxw7	515.2984	0.26417568	0.116772	2.262312	0.023678	0.272269
klhdc8a	15.54966	-0.007017549	0.410648	-0.01709	0.986366	1
cox11	265.2748	0.005216115	0.147658	0.035326	0.97182	1
cyb5d2	74.46274	0.314071981	0.275327	1.140721	0.253986	0.733071
hlfb	75.14855	-0.167719638	0.325798	-0.5148	0.606695	0.915204
ankfy1	664.2215	0.124314677	0.108704	1.14361	0.252785	0.732759
kcnj5	7.156322	-0.013585164	0.763007	-0.0178	0.985795	1
spns2	353.5308	-0.319156752	0.130476	-2.44609	0.014442	0.210484
ccdc186	283.9319	0.215895455	0.153017	1.410925	0.158267	0.623766
fam160a1a	97.19586	0.068380044	0.186991	0.365687	0.714599	0.945821
si:dkey-72l14.4	470.2645	-0.098185124	0.111966	-0.87692	0.380529	0.823655
rabggta	257.1224	-0.711267829	0.234852	-3.02858	0.002457	0.079624
vezf1b	97.70505	0.172950212	0.20824	0.830533	0.406237	0.834645
dner	36.72359	-0.130739746	0.408406	-0.32012	0.748876	0.955074
atp10a	44.56914	-0.457551324	0.263128	-1.73889	0.082053	0.482272
pum2	134.8111	0.060621709	0.237659	0.255078	0.798663	0.966823
gabbr2	15.02571	0.30257012	0.562992	0.537432	0.590969	0.9093
si:dkey-72l14.3	103.3988	-0.209408552	0.402998	-0.51963	0.603323	0.914226
abcg4a	17.03818	-0.123387001	0.550061	-0.22432	0.822512	0.971966
ranbp9	897.5394	-0.121481887	0.131608	-0.92306	0.355978	0.807045
rnf182	35.47504	0.812090595	0.613641	1.323397	0.185703	0.663476
brinp3a.2	104.085	-0.412459244	0.250605	-1.64585	0.099794	0.524166
nipblb	839.618	0.125681733	0.17604	0.71394	0.475264	0.869292
cyhr1	626.2323	0.06763559	0.118224	0.572096	0.567257	0.901809
stpg2	8.963868	-0.899548422	0.678188	-1.3264	0.184707	0.661987
dpagt1	185.1272	0.10080264	0.168257	0.5991	0.549106	0.897092
nemp2	158.9122	0.067306231	0.153591	0.438217	0.661229	0.930581
c2cd2l	207.7435	0.045093376	0.184547	0.244346	0.806963	0.969726
chst3a	14.57555	-0.139223723	0.423746	-0.32855	0.742492	0.954374
arpp21	301.1977	0.458147328	0.262602	1.744643	0.081047	0.479801
igl4v8	0.301241	0	4.604814	0	1	NA
gtpbp6	73.60604	-0.078630277	0.227544	-0.34556	0.729673	0.950056
phldb1a	324.5407	-0.179842993	0.175134	-1.02689	0.304474	0.769885
stk36	61.66106	0.522263567	0.340027	1.535946	0.124552	0.572138
dapk2a	342.0706	0.175188671	0.216503	0.809173	0.418416	0.841565
nfasca	443.2379	0.057785244	0.139833	0.413243	0.679428	0.936209
nars	995.1448	0.381592145	0.165321	2.308188	0.020989	0.257578

snx19a	57.10711	-0.118346348	0.866975	-0.1365	0.891422	0.988566
slc19a3b	6.13389	1.564710382	1.605496	0.974596	0.329761	0.788011
ep300b	1013.368	0.065328365	0.16553	0.394662	0.693093	0.94055
zbtb44	36.23536	-0.994006919	0.351338	-2.8292	0.004666	0.113335
parga	457.5765	0.198837316	0.113067	1.758577	0.078649	0.474455
slc43a2b	2715.547	-0.494125259	0.224803	-2.19804	0.027946	0.297418
adgrl3.1	683.9818	0.226665415	0.141443	1.602525	0.109039	0.541807
srpra	958.715	0.044684555	0.125755	0.355331	0.722342	0.947586
kif21a	276.7865	0.197105162	0.173659	1.135013	0.25637	0.73401
senp7b	224.8058	-0.060967743	0.155023	-0.39328	0.694112	0.940996
colec12	1073.323	0.075164568	0.15497	0.485026	0.627658	0.921538
tapt1b	343.2035	0.124651101	0.140083	0.889836	0.373554	0.820566
gfod2	213.361	-0.083571765	0.167602	-0.49863	0.618038	0.916507
zfp64	84.23488	-0.345377089	0.226165	-1.5271	0.126735	0.575662
nrn1lb	39.32448	-1.112004237	0.834442	-1.33263	0.182653	0.659278
bicra	486.4767	-0.005158242	0.193022	-0.02672	0.97868	1
ube2j2	37.61293	0.23725672	0.389678	0.608853	0.542622	0.894595
slc6a4a	245.9121	0.289793459	0.191593	1.512548	0.130394	0.581523
dhx34	135.2314	-0.292067119	0.190527	-1.53295	0.125289	0.573026
larp1b	1202.912	-0.071310718	0.126877	-0.56204	0.574085	0.904376
st14a	705.4314	0.268357141	0.121427	2.210031	0.027103	0.293036
znf740b	294.8196	-0.015580119	0.134416	-0.11591	0.907724	0.991745
mov10b.1	105.9691	-0.222305801	0.240708	-0.92355	0.35572	0.806777
vps45	366.9707	0.010909449	0.133511	0.081712	0.934876	0.996829
nat15	277.9073	0.015647127	0.126048	0.124136	0.901208	0.990512
cbx5	2862.762	0.128322923	0.098453	1.303398	0.192439	0.672283
plekho1a	125.406	0.638634424	0.206308	3.095537	0.001965	0.068839
hectd2	59.79413	0.357797293	0.321558	1.112699	0.265838	0.742605
cnnm2a	19.96485	0.833324027	0.501592	1.661358	0.096641	0.51893
emilin2a	180.7032	0.263973973	0.187793	1.405663	0.159824	0.626123
armc7	50.74524	-0.136342454	0.27173	-0.50176	0.615838	0.916365
acsf2	945.646	-0.231060623	0.181072	-1.27607	0.20193	0.68211
trpc1	94.93568	0.475709743	0.390858	1.217092	0.223569	0.705397
ddx20	171.2009	-0.06735831	0.161797	-0.41631	0.67718	0.93595
stag1a	450.2589	0.246077309	0.136715	1.799935	0.071871	0.4564
ttc7b	568.5607	0.291808709	0.107577	2.71256	0.006677	0.137398
rab40c	571.0358	0.002908482	0.107785	0.026984	0.978472	1
rabep2	291.1352	-0.052295677	0.114166	-0.45807	0.646904	0.926728
lpin2	913.2146	-0.030177101	0.135163	-0.22326	0.82333	0.972005
fam149b1	313.2093	0.050985101	0.174112	0.29283	0.769652	0.960484
hexdc	92.60143	-0.208474635	0.232447	-0.89687	0.369788	0.818318
si:ch211-157j23.2	3.324773	-0.560511144	1.15576	-0.48497	0.627696	0.921538
uba7	27.71409	0.999435132	0.547645	1.824968	0.068006	0.44461
ogfrl1	46.68615	0.12927784	0.297916	0.433941	0.664331	0.93177
timp2a	1532.009	0.036869093	0.121581	0.303246	0.761702	0.958242
pfdn4	646.5923	-0.162228023	0.151774	-1.06888	0.285125	0.756222
tinagl1	43.54388	-0.07696669	0.3437	-0.22394	0.822807	0.971966
abcc5	1586.866	-0.028562358	0.14965	-0.19086	0.848635	0.977912
alg5	84.55214	-0.259875564	0.303165	-0.85721	0.39133	0.827902
cbln4	34.0183	-0.340409192	0.38928	-0.87446	0.381869	0.824957
tuft1a	253.6303	-0.261873117	0.225698	-1.16028	0.245934	0.727194
ubash3ba	40.67181	0.495285691	0.294772	1.680233	0.092912	0.510257
xylt1	78.43073	0.070465615	0.238065	0.295993	0.767235	0.960296
myom1a	4591.966	-0.041166932	0.226744	-0.18156	0.855931	0.979558

dusp3a	112.4045	0.114062504	0.219305	0.520108	0.602988	0.914226
si:dkey-44g23.5	514.6015	-0.196481807	0.173492	-1.13251	0.257419	0.735125
zgc:153240	242.5656	0.122059482	0.164724	0.740995	0.458697	0.861537
sost	330.9739	-0.086984039	0.17257	-0.50405	0.614226	0.915998
si:dkey-202e22.2	236.4782	0.356231412	0.173124	2.057668	0.039622	0.348139
lmln	28.56771	0.518779284	0.475739	1.090471	0.275506	0.750643
ago2	55.90198	0.341378678	0.249299	1.369352	0.170889	0.642777
BX294434.1	1655.943	0.093868699	0.152928	0.613809	0.539341	0.893049
lss	56.93397	0.724250022	0.277903	2.606126	0.009157	0.164349
glg1a	1421.305	0.060023097	0.101852	0.589316	0.555649	0.898723
kcnc4	23.42116	-0.444969302	0.437481	-1.01712	0.309098	0.773791
pi15b	21.26309	1.669963756	1.210141	1.379974	0.167595	0.638828
arhgap5	773.3347	-0.014726326	0.156535	-0.09408	0.925048	0.994815
gmpr2	446.5363	0.05498543	0.135834	0.404798	0.685626	0.938486
oca2	99.77851	-0.004690692	0.250062	-0.01876	0.985034	1
ppp1r9a	37.44067	0.819407263	0.352533	2.324343	0.020107	0.251556
znf438	97.09142	-0.07434392	0.20554	-0.3617	0.717576	0.946393
ngrn	272.8884	-0.362073205	0.177591	-2.0388	0.04147	0.355348
cdon	1518.68	0.028576059	0.1471	0.194263	0.84597	0.977039
galnt1	539.8987	0.084460181	0.120764	0.69938	0.484315	0.872731
ddx6	317.502	-0.080533562	0.174146	-0.46245	0.643759	0.925997
wwp2	2036.749	-0.032616961	0.104624	-0.31175	0.755228	0.956367
adat1	42.35238	0.173801888	0.342985	0.506733	0.612342	0.915709
tfpi2	92.39373	0.010016306	0.25222	0.039713	0.968322	1
bcl11aa	506.9755	0.182057788	0.118674	1.534094	0.125006	0.572594
foxred2	331.5754	-0.364698053	0.17281	-2.1104	0.034824	0.328663
aoc1	75.47885	0	1.692202	0	1	1
chst6	33.98139	-0.863876226	0.429658	-2.01061	0.044367	0.368519
dipk1ab	26.94616	-0.156117245	0.498198	-0.31336	0.754004	0.955912
GFOD1	21.93189	0.581903331	0.381731	1.524381	0.127413	0.576964
hepacam2	68.19187	0.155112789	0.245865	0.630885	0.528115	0.889948
klf13	79.86473	0.007530301	0.315455	0.023871	0.980955	1
tsen34	156.8733	-0.405042284	0.151665	-2.67064	0.007571	0.14728
cdh18a	166.3963	0.060748863	0.214287	0.283493	0.776799	0.961073
cog7	382.033	-0.011020438	0.127018	-0.08676	0.93086	0.995809
znf142	150.2171	-0.099934103	0.159103	-0.62811	0.529933	0.890545
sgpl1	1081.505	-0.124477936	0.187169	-0.66506	0.506014	0.882858
efcab1	26.23964	0.493034753	0.390405	1.262881	0.206632	0.688972
smg8	363.1842	0.009708471	0.148716	0.065282	0.947949	0.997456
cmya5	197.09	0.394142429	0.328076	1.201377	0.229605	0.711596
serpinf2b	957.3899	-0.446695773	0.21254	-2.10171	0.035579	0.331205
haus3	125.5109	0.21413373	0.192168	1.114304	0.265149	0.742063
grhl1	296.4016	-0.081554571	0.156956	-0.5196	0.603342	0.914226
trip12	1922.801	-0.008961917	0.123738	-0.07243	0.942262	0.997371
epb41l4b	104.6983	-0.097244958	0.196754	-0.49425	0.621132	0.918461
csgalnact2	302.6821	-0.233129484	0.119652	-1.9484	0.051367	0.394202
eva1a	111.6481	-0.100949456	0.195665	-0.51593	0.605903	0.915055
bivm	77.65865	0.188404649	0.230348	0.817913	0.413407	0.838369
nelfa	234.1999	-0.219031292	0.149898	-1.4612	0.14396	0.603144
sec23ip	471.0454	0.085067994	0.152899	0.556367	0.57796	0.905248
slx4	170.9819	0.179461829	0.217024	0.826923	0.408281	0.83612
c2cd4a	63.12015	-0.328068359	0.562092	-0.58366	0.559452	0.899571
nub1	50.80773	0.081670077	0.397272	0.205577	0.837121	0.976007
zmat4b	16.41894	0.156545364	0.494244	0.316737	0.751443	0.955331

FQ311928.1	100.4575	0.099247661	0.339445	0.292382	0.769995	0.960484
znf646	509.4019	0.015772146	0.137931	0.114348	0.908962	0.992033
zgc:194330	19.05701	-0.272599132	0.408771	-0.66688	0.504852	0.88195
col6a2	1832.912	0.030885459	0.181301	0.170355	0.864731	0.982052
inpp5f	88.26786	0.193123177	0.207958	0.928666	0.353062	0.803896
ugt5c3	88.76033	-0.496865834	0.275844	-1.80125	0.071663	0.4564
ugt5c1	3.343335	0.084000947	1.040188	0.080756	0.935636	0.996829
adcy6a	233.9516	0.043621853	0.169135	0.257911	0.796476	0.966447
smap2	12.30891	-0.252951554	0.598132	-0.4229	0.672366	0.934089
tesk2	333.3448	-0.180065921	0.148646	-1.21137	0.225753	0.707068
n4bp2	266.5086	0.054129591	0.143411	0.377444	0.705844	0.944413
nrxn2a	555.7423	0.267127688	0.219672	1.216032	0.223973	0.705807
phf14	187.5052	0.259893722	0.13562	1.916333	0.055323	0.407317
si:dkey-253i9.4	599.5995	-0.131347321	0.131829	-0.99635	0.319081	0.779923
dsela	30.37835	0.323349692	0.360981	0.895752	0.370385	0.818484
vwa7	39.36923	0.530299177	0.274358	1.932876	0.053251	0.400175
cpne5a	13.98708	1.194372912	0.73782	1.618785	0.105493	0.534737
ncaph	312.5646	0.234547569	0.242975	0.965314	0.334388	0.791508
sema5ba	69.28186	0.18671228	0.264337	0.706342	0.479976	0.870001
gba2	321.0272	0.080227649	0.118813	0.675243	0.499522	0.879603
tbkbp1	135.212	0.40334486	0.346047	1.165579	0.243785	0.724437
igf2bp1	3786.297	0.121194595	0.135341	0.895473	0.370534	0.818484
thsd7aa	151.1021	0.397496472	0.20091	1.97848	0.047875	0.380801
si:ch211-132b12.1	4.125299	-1.723452333	1.39558	-1.23494	0.216854	0.69815
wu:fj05g07	111.2473	0.14822209	0.240691	0.615819	0.538014	0.892498
DNAH10	84.72235	-0.094570407	0.344737	-0.27433	0.783834	0.96245
u2surp	1202.802	-0.151234127	0.13555	-1.11571	0.264548	0.742063
si:ch211-250e5.2	55.13008	0.002245613	0.265821	0.008448	0.99326	1
thsd7bb	11.84669	1.800846294	0.6664	2.70235	0.006885	0.139692
dusp13a	0.963788	2.003307096	2.133656	0.938908	0.347778	NA
kdm4c	298.9524	-0.113878131	0.172447	-0.66037	0.509018	0.883442
mrps9	452.6778	0.056112313	0.10481	0.53537	0.592394	0.909996
tgfbrap1	88.58116	0.028376485	0.205021	0.138408	0.889918	0.98835
tbx3b	13.40178	-0.295723029	0.51127	-0.57841	0.562988	0.900772
tmtops2a	60.07914	0.666451088	0.321613	2.072213	0.038246	0.342672
lrp1bb	149.6125	0.113798694	0.240924	0.472343	0.636682	0.924779
b4galnt1a	42.11388	-0.488444768	0.362974	-1.34567	0.178408	0.652623
slka	440.1571	-0.050689456	0.145373	-0.34869	0.727325	0.949095
nxt2	150.9569	-0.293527077	0.211504	-1.38781	0.165196	0.634423
unc5db	23.43121	0.486027429	0.411416	1.181354	0.237462	0.718568
ccdc85b	694.4915	-0.188940076	0.105736	-1.7869	0.073954	0.461828
ano6	102.0383	0.515793595	0.297764	1.732223	0.083234	0.48419
zgc:153409	9.559503	-0.435156085	0.708599	-0.61411	0.539144	0.892985
si:ch211-145o7.3	28.18731	0.074746106	0.381268	0.196046	0.844574	0.976791
foxo1b	27.46277	0.279805847	0.328004	0.853056	0.393628	0.828374
rap1gap2a	91.65924	-0.06050614	0.285624	-0.21184	0.832233	0.975013
tmem246	193.8993	0.003792787	0.135627	0.027965	0.97769	1
stim1b	74.23602	-0.09746313	0.225384	-0.43243	0.665427	0.932595
tmem121aa	49.64737	0.667901365	0.415231	1.608504	0.107725	0.539089
slc7a3b	116.9878	-0.235613945	0.246126	-0.95729	0.338421	0.795226
gldn	20.49522	-0.816580882	0.491047	-1.66294	0.096325	0.518071
myo1cb	955.3127	-0.102001853	0.120429	-0.84698	0.397004	0.830253
kctd7	161.3605	0.300027857	0.23132	1.297024	0.194623	0.67483
cyp4v7	113.6206	-2.117495675	0.769027	-2.75347	0.005897	0.129875

ctdspl2a	595.0918	0.227713632	0.109023	2.088672	0.036737	0.33671
cachd1	707.9326	0.054556798	0.1506	0.362263	0.717156	0.94627
abcb10	145.3974	0.317706194	0.256288	1.239645	0.215107	0.697086
cemip2	452.3922	0.070789887	0.151433	0.467467	0.640166	0.924877
ssx2ipb	57.80592	1.022869367	0.347072	2.947141	0.003207	0.093414
sorbs2b	608.7891	-0.133582046	0.142856	-0.93508	0.349748	0.801408
ufsp1	32.70773	0.055778387	0.427805	0.130383	0.896264	0.98939
si:dkey-85k7.11	0.782488	0	4.341141	0	1	NA
them4	127.7313	0.486112399	0.207366	2.344227	0.019067	0.242999
kcng3	11.86927	-0.345664881	0.522323	-0.66178	0.50811	0.883056
ndufaf5	450.8176	-0.091739378	0.138617	-0.66182	0.508088	0.883056
fbxo11a	606.4739	0.255975917	0.107539	2.380299	0.017299	0.232084
aifm4	259.447	-0.204741808	0.1683	-1.21653	0.223783	0.705807
myo5aa	195.64	-0.140326307	0.237826	-0.59004	0.555165	0.898723
kcnj9	7.640209	0.254470058	0.657648	0.38694	0.698801	0.942456
dcakd	61.3137	-0.191370843	0.296491	-0.64545	0.518635	0.887148
sipa1l2	1039.245	0.11119386	0.130168	0.854231	0.392977	0.827902
ankrd34bb	8.282278	0.083061671	1.180678	0.070351	0.943914	0.997371
ensab	92.66653	-0.19886885	0.250853	-0.79277	0.427912	0.845893
nrxn1a	1070.629	0.177682466	0.180604	0.983822	0.325203	0.784075
marveld2b	136.927	-0.120710044	0.184363	-0.65474	0.512635	0.885483
wwc3	408.4136	-0.049090114	0.11493	-0.42713	0.669284	0.933115
ptpdc1a	143.8333	0.230498621	0.171359	1.345122	0.178586	0.652862
CABZ01055347.1	110.8369	0.161932225	0.279533	0.579296	0.56239	0.900715
zgc:162707	123.423	-0.156212085	0.177007	-0.88252	0.377496	0.822478
hcn2b	95.57266	-0.012183122	0.256813	-0.04744	0.962163	0.999451
si:ch73-334d15.1	167.6585	0.209623839	0.168416	1.24468	0.21325	0.695523
bicc1a	202.1382	0.149050862	0.168913	0.882414	0.377553	0.822478
slc25a17	118.9577	0.279801463	0.176931	1.581414	0.113783	0.552117
hcn4	4.550049	0.296915933	0.88568	0.335241	0.737443	0.952375
atxn1a	81.77021	0.198213511	0.279649	0.708793	0.478453	0.869292
arvcfb	806.5686	-0.014574694	0.117406	-0.12414	0.901205	0.990512
nxpe3	103.0567	0.018683476	0.197697	0.094506	0.924707	0.994815
qpctla	174.1548	-0.039227357	0.157749	-0.24867	0.803617	0.968722
yjefn3	172.901	0.076753865	0.204311	0.375672	0.707161	0.944975
ca14	30.37697	0.43624105	0.557768	0.78212	0.434144	0.850002
sipa1l3	151.0499	0.241573257	0.190516	1.267998	0.204799	0.686713
map3k8	52.79224	-0.365207977	0.34679	-1.05311	0.29229	0.761879
tpd52	276.3147	-0.301832145	0.158237	-1.90746	0.056461	0.410349
si:dkey-45k15.1	20.5578	0.165237041	0.54579	0.302748	0.762082	0.958345
cytip	10.21274	-0.524368839	0.982577	-0.53367	0.593572	0.910427
znf704	31.71216	-0.314937003	0.40051	-0.78634	0.431669	0.848419
washc4	350.5039	-0.361007256	0.139383	-2.59003	0.009597	0.168452
tmem64	45.09362	-0.255383988	0.285289	-0.89518	0.370693	0.818484
mast3a	249.1493	0.081977637	0.144949	0.565563	0.571691	0.903234
acot13	139.6475	0.623490324	0.271598	2.295637	0.021697	0.261221
raph1b	305.9418	-0.355492159	0.20484	-1.73546	0.082659	0.483014
hnrnpm	3089.386	0.096004283	0.09701	0.989636	0.322352	0.78246
ank3a	983.1457	0.119391284	0.129008	0.925455	0.354729	0.805792
ece1	361.9448	-0.107815149	0.170329	-0.63298	0.526746	0.889699
2-Mar	72.77026	-0.131661704	0.234827	-0.56067	0.575019	0.904516
itpr3	244.9367	-0.039868847	0.175553	-0.2271	0.820342	0.971788
clasrp	395.042	9.52E-05	0.12229	0.000779	0.999379	1
arhgef9a	209.4267	-0.266235339	0.307693	-0.86526	0.386895	0.827592

dipk2b	78.09697	0.154269471	0.323204	0.477313	0.633139	0.923885
htr3b	15.67449	1.081239929	0.758795	1.424943	0.154174	0.61894
ripor2	207.4271	0.453377898	0.165089	2.746269	0.006028	0.131072
rabgap1l	1007.011	-0.030940033	0.129079	-0.2397	0.810564	0.969828
sh3pxd2ab	235.4886	-0.213428986	0.181083	-1.17862	0.238549	0.719595
kdm6a	619.2985	0.143773741	0.142943	1.005809	0.314507	0.777479
opa3	130.944	0.064319705	0.180283	0.356771	0.721263	0.947343
trim108	80.35186	1.167132776	0.439091	2.658064	0.007859	0.149996
spata5l1	153.6573	-0.185548513	0.212418	-0.87351	0.382386	0.825252
ahnak	4669.768	-0.045218284	0.184437	-0.24517	0.806326	0.969726
bmp10	2.664663	0.212329334	1.170377	0.18142	0.856038	0.979558
abcd4	172.1622	-0.182415603	0.182007	-1.00224	0.316226	0.7788
neurl1ab	58.68146	0.332200966	0.272698	1.2182	0.223148	0.70527
mbd3a	895.113	-0.019777731	0.113454	-0.17432	0.861611	0.980527
fmn2b	395.3826	0.341348655	0.163562	2.086968	0.036891	0.337141
arhgap25	16.84512	-0.157307177	0.484521	-0.32467	0.745434	0.955021
klhl2	188.7209	0.054683146	0.154436	0.354084	0.723276	0.947898
gnl1	406.3297	-0.33941916	0.164892	-2.05843	0.039549	0.347625
nyx	48.02225	-0.868163361	1.690674	-0.5135	0.607601	0.915204
jam3b	967.1776	-0.114171549	0.098836	-1.15517	0.248022	0.728489
rasgrp4	201.1423	-0.074803041	0.170577	-0.43853	0.661003	0.930581
zgc:154006	25.35132	0.126714956	0.414304	0.30585	0.759719	0.957886
arhgap27l	352.5684	0.096021917	0.158639	0.605286	0.544989	0.895206
si:dkey-208k22.6	21.41657	-1.138536666	0.522602	-2.17859	0.029362	0.304886
cnot2	854.4039	0.025586774	0.08225	0.311084	0.755737	0.956532
si:ch211-194e15.5	168.2225	0.194209046	0.196918	0.986242	0.324014	0.783161
si:dkey-13n15.2	40.02249	0.588783353	0.394966	1.49072	0.136035	0.591131
mbd3b	1253.072	0.053631194	0.123747	0.433395	0.664728	0.93201
kif1aa	777.6584	0.532078809	0.180944	2.940579	0.003276	0.093825
meox2b	53.02475	0.299565874	0.251708	1.190131	0.233995	0.714881
wscd2	26.37699	-0.077032833	0.418422	-0.1841	0.853933	0.978905
UNC13A	83.70837	0.020966867	0.246499	0.085059	0.932215	0.996053
prss12	186.133	0.309949672	0.17522	1.768914	0.076908	0.469467
sntg1	3.427365	-1.2404377	1.225267	-1.01238	0.311356	0.77613
zpld1a	42.48289	0.772454551	0.402407	1.919586	0.05491	0.405196
nfixb	49.71115	0.404016141	0.3202	1.261761	0.207035	0.689322
tiparp	606.5673	-0.029980169	0.167556	-0.17893	0.857996	0.979774
si:dkey-38p12.3	367.5899	-0.087258278	0.110263	-0.79137	0.428731	0.846502
pld7	240.6127	-0.048833378	0.141719	-0.34458	0.730411	0.950056
rxfp3.2b	14.64047	-0.426727257	0.596491	-0.7154	0.474365	0.869292
col15a1b	712.2364	0.223563774	0.142202	1.572156	0.115914	0.556115
si:dkey-19b23.13	78.97226	-0.024187978	0.325899	-0.07422	0.940836	0.997371
olfml3a	137.9165	0.042426659	0.199646	0.21251	0.831709	0.974914
lman2	977.4613	0.00248089	0.124952	0.019855	0.984159	1
zgc:153968	291.4469	0	1.682229	0	1	1
myo18ab	1161.978	0.451635343	0.15203	2.970707	0.002971	0.090034
hdlbpb	446.3643	0.007521287	0.116949	0.064312	0.948721	0.99777
ddx10	344.36	-0.019119795	0.172816	-0.11064	0.911904	0.992336
v2rx3	1.292089	-3.073696822	1.829121	-1.68042	0.092875	NA
v2rh10	0	NA	NA	NA	NA	NA
satb2	55.71989	-0.071158855	0.289592	-0.24572	0.805898	0.969625
ppcs	120.5135	-0.119564131	0.196157	-0.60953	0.542171	0.89435
atp13a2	172.2664	0.201416763	0.224653	0.896569	0.369949	0.818446
usp32	469.0102	0.311932733	0.163798	1.90438	0.056861	0.411856

slco2a1	135.2316	0.363820024	0.261951	1.388884	0.164868	0.634185
iqub	18.7236	-0.015216176	0.37904	-0.04014	0.967978	1
ankrd13a	159.9667	0.059500217	0.169386	0.351271	0.725385	0.948653
v2ra18	0.922257	-0.868141227	2.69032	-0.32269	0.746929	NA
fhod3b	83.17363	0.246308606	0.211191	1.166281	0.243501	0.724437
mfsd12a	147.4631	-0.187751899	0.193408	-0.97075	0.331671	0.789531
si:ch73-233f7.1	467.6314	-0.180636453	0.260199	-0.69422	0.487542	0.873291
git2a	177.8813	0.046388483	0.152246	0.304694	0.760599	0.957886
tmem51b	96.19825	-0.193287629	0.215647	-0.89631	0.370085	0.818447
cplx2	519.701	-0.063674943	0.169789	-0.37502	0.707642	0.945128
gltpa	118.8002	-0.226513643	0.165776	-1.36638	0.171819	0.643953
amotl2a	1763.917	-0.192104713	0.119986	-1.60105	0.109365	0.542487
fbxo10	40.48197	-0.790589489	0.310025	-2.55008	0.01077	0.181029
htr4	9.882798	-0.359443959	0.702066	-0.51198	0.608665	0.915204
trpv4	102.2716	-0.134154284	0.228266	-0.58771	0.556727	0.899063
commd4	189.3636	0.137832342	0.176695	0.780059	0.435356	0.850379
amotl2b	567.9505	-0.093724948	0.17526	-0.53478	0.592803	0.910162
golgb1	643.8406	-0.077365082	0.152194	-0.50833	0.611221	0.915331
ecpas	787.0435	-0.051922402	0.203258	-0.25545	0.798375	0.966698
sytl2a	75.30797	0.522807592	0.24802	2.107927	0.035037	0.329872
ube3b	63.07511	0.33839648	0.275317	1.229116	0.219028	0.699984
vrtn	6.126122	0.778742874	0.704006	1.10616	0.268657	0.744925
myo1ha	10.61883	0.058106388	0.708488	0.082015	0.934635	0.996829
lingo3a	73.66487	-0.185052268	0.331243	-0.55866	0.576394	0.90475
grhl2b	174.8592	-0.091708792	0.199661	-0.45932	0.646003	0.926728
sema6bb	41.89924	0.12682045	0.288996	0.43883	0.660784	0.930581
ppfibp2a	466.8518	0.220759606	0.143841	1.534749	0.124846	0.572491
lap3	338.1104	0.141450825	0.130309	1.085507	0.277697	0.751752
rbm47	561.7503	-0.020397076	0.13564	-0.15038	0.880467	0.986481
tbc1d2b	97.18128	-0.12258068	0.191892	-0.6388	0.522952	0.888222
mpnd	283.4985	0.073384354	0.158638	0.46259	0.643658	0.925997
kcnh4b	30.83533	0.413424551	0.422601	0.978285	0.327933	0.786438
dot1l	146.3058	0.071815548	0.271568	0.264448	0.791435	0.964817
wdr74	182.3551	-0.240138898	0.180325	-1.3317	0.182959	0.659278
acacb	1284.326	0.030320275	0.181325	0.167215	0.867201	0.982582
abtb2b	157.6647	-0.055391218	0.146546	-0.37798	0.705446	0.944153
adnpa	0	NA	NA	NA	NA	NA
myo5b	835.4499	-0.351077971	0.148707	-2.36088	0.018232	0.238258
hs2st1b	124.5538	-0.19660778	0.209261	-0.93953	0.347457	0.800037
gpr18	2.31155	1.075504794	1.577104	0.681949	0.495271	0.87806
vps51	276.2003	0.185024099	0.144154	1.28352	0.19931	0.679819
fsd1	108.1423	0.354943477	0.226376	1.567939	0.116895	0.557831
rufy2	693.303	-0.033393876	0.109011	-0.30634	0.759349	0.957886
gse1	874.6194	0.019188195	0.133708	0.143509	0.885888	0.987533
ccdc181	40.8014	0.243636208	0.32432	0.751221	0.452519	0.857994
fndc3bb	311.667	-0.021007252	0.170121	-0.12348	0.901724	0.990656
kif1ab	208.8075	0.609929341	0.263804	2.312058	0.020774	0.255762
cpox	304.3714	-0.092862893	0.183198	-0.5069	0.612225	0.915709
gpr17	43.15448	0.125085005	0.387348	0.322927	0.746751	0.955074
atp8b5a	105.031	0.364961561	0.24036	1.518393	0.128915	0.578173
mthfsd	11.8012	0.104575758	0.591621	0.176761	0.859696	0.980066
il1rapl1a	275.7611	0.86509329	0.268934	3.216753	0.001297	0.052702
arhgap45b	77.79684	-0.22168291	0.243262	-0.91129	0.362141	0.812082
casp2	202.4866	-0.016499348	0.149101	-0.11066	0.911887	0.992336

kif1bp	323.5776	-0.133657256	0.114675	-1.16553	0.243805	0.724437
cpt1ab	1201.061	-0.000414679	0.211937	-0.00196	0.998439	1
rnf38	287.6685	0.020544455	0.186185	0.110344	0.912136	0.992371
elmod1	889.9721	0.307441201	0.21046	1.460806	0.144069	0.603144
zgc:174945	3.176723	-0.339014559	0.956245	-0.35453	0.722944	0.947755
slc12a7b	1302.626	0.183279387	0.14233	1.287703	0.197849	0.678554
si:ch211-236l14.4	155.9619	0.546747529	0.227175	2.406728	0.016096	0.223897
gmeb1	325.9675	0.041655938	0.149768	0.278137	0.780907	0.961608
tab3	259.3312	-0.010497813	0.145091	-0.07235	0.942321	0.997371
acsbg1	30.68016	0.413328801	0.654553	0.631467	0.527735	0.889948
shc2	68.97312	-0.574615306	0.255192	-2.2517	0.024342	0.276029
tbc1d1	631.5063	0.072629192	0.112703	0.644431	0.519296	0.887148
hipk3b	202.4377	0.093650969	0.208851	0.44841	0.653858	0.929269
dph7	229.0726	-0.054930947	0.144047	-0.38134	0.70295	0.943622
clcn1a	94.72817	0.631063747	0.240801	2.620689	0.008775	0.160701
tkfc	206.1826	-0.077642749	0.157535	-0.49286	0.62211	0.918461
kiaa1549lb	7.459622	-2.613130252	1.367709	-1.91059	0.056057	0.409215
nceh1b.1	39.8493	0.338422142	0.392185	0.862914	0.388185	0.827592
uggt2	382.2816	0.376338504	0.147689	2.548182	0.010829	0.181481
pm20d1.2	134.2614	0.139198734	0.208668	0.667083	0.504719	0.88195
iffo2a	29.45772	-0.133798552	0.329199	-0.40644	0.684422	0.938486
ankrd24	112.2189	-0.076113355	0.21801	-0.34913	0.726994	0.949095
klf5b	26.24691	-0.379186171	0.473961	-0.80004	0.423689	0.844048
iffo1b	45.62775	0.678500506	0.281403	2.411138	0.015903	0.222371
shdb	262.0693	-0.16932394	0.118761	-1.42576	0.153938	0.618475
rxfp3.3a2	4.261836	1.43550281	1.330258	1.079116	0.280536	0.753147
tent4a	45.89454	0.450248475	0.279548	1.610631	0.10726	0.537809
ctdspl1b	615.7743	-0.010325162	0.129784	-0.07956	0.93659	0.997044
klhl5	21.61997	0.326603625	0.483289	0.675794	0.499171	0.879596
agap1	793.2692	-0.007886921	0.139574	-0.05651	0.954938	0.998611
mxra8b	803.558	-0.131345994	0.146309	-0.89773	0.369331	0.817763
cyp4v8	310.8304	-0.737979775	0.230185	-3.20603	0.001346	0.05423
kcnab2b	42.51861	0.15904691	0.306946	0.51816	0.604347	0.914971
sytl5	18.64012	0.607593075	0.467758	1.298948	0.193962	0.674122
stag1b	727.6647	0.170550049	0.117808	1.447699	0.147701	0.608614
ranbp10	343.9207	-0.065127051	0.170587	-0.38178	0.702623	0.943591
eif2ak3	415.8286	-0.092180257	0.142745	-0.64577	0.51843	0.887148
stoml1	112.5472	-0.10221323	0.19516	-0.52374	0.60046	0.913756
larp4b	345.3769	0.1873134	0.157427	1.18984	0.234109	0.714938
otc	75.50644	0.662894107	0.377905	1.754129	0.079408	0.477151
SENP7	365.6251	0.137284661	0.126814	1.082571	0.278999	0.752444
chaf1a	924.0206	0.233100349	0.157778	1.477398	0.139569	0.597884
dip2ca	371.4167	0.216354945	0.120045	1.802284	0.071501	0.456132
abi3bpa	73.54568	-2.028682174	0.978816	-2.07259	0.038211	0.342672
atrn	375.2468	-0.026913522	0.153197	-0.17568	0.860546	0.980264
tub	195.8546	0.290088986	0.222506	1.303734	0.192324	0.672283
tmem168b	24.47512	0.404173365	0.37514	1.077393	0.281305	0.753593
olfml3b	446.0695	0.094214582	0.136344	0.691005	0.489563	0.874629
camsap2a	188.5015	0.176257932	0.168565	1.045636	0.295729	0.764246
cacnb4b	783.314	0.187506928	0.143955	1.302542	0.192731	0.67273
dcbld2	200.5105	0.06891724	0.151715	0.454254	0.649646	0.927756
ripor1	532.7353	-0.041512639	0.113394	-0.36609	0.714296	0.945821
bmt2	105.9615	-0.292482279	0.17004	-1.72008	0.085419	0.490781
slc22a7b.3	9.249189	2.590677937	1.389255	1.864797	0.06221	0.427589

kif14	182.3069	0.233006585	0.214501	1.086272	0.277358	0.751426
PDE3B	315.4297	-0.320272975	0.205273	-1.56023	0.118706	0.561771
TBC1D8B	327.8839	-0.250005091	0.140581	-1.77838	0.075342	0.465238
fbxo31	117.6798	0.181026447	0.191413	0.94574	0.344281	0.798147
pcm1	739.0971	0.065884252	0.137487	0.479205	0.631793	0.923003
si:dkey-217l24.1	64.46429	0.164131771	0.230272	0.712773	0.475986	0.869292
sigirr	67.64744	0.299586135	0.24892	1.203545	0.228765	0.710332
skiv2l	202.463	0.219158777	0.154707	1.416607	0.156598	0.622452
prrt1	73.29056	0.408356609	0.28198	1.448176	0.147568	0.608498
mrps5	547.6245	0.075067315	0.118385	0.634097	0.526018	0.889156
narfl	303.6427	0.112470575	0.119588	0.94048	0.346971	0.79983
kcnj3b	132.1233	0.846042059	0.308439	2.742983	0.006088	0.131486
plekha7b	190.693	1.016274772	0.184094	5.520404	3.38E-08	1.96E-05
arap1a	101.397	0.428521233	0.231298	1.852678	0.063929	0.431598
mxra8a	503.7694	-0.091162401	0.232947	-0.39134	0.695543	0.9414
ino80db	265.35	0.210455545	0.167358	1.25752	0.208565	0.690728
adipoqa	3.594687	0.426111464	1.103809	0.386037	0.699469	0.942499
znf362b	69.68061	0.117834171	0.291856	0.403741	0.686403	0.938732
lrrc34	8.705107	0.025608993	0.69001	0.037114	0.970394	1
nfs1	474.5376	0.156709091	0.144792	1.082305	0.279117	0.752444
si:ch211-15p9.2	0	NA	NA	NA	NA	NA
setd2	897.4251	0.072247977	0.158636	0.455432	0.648798	0.927546
ptpn21	191.9015	0.066599035	0.253323	0.262902	0.792626	0.965266
fbxl7	106.7453	-0.134599304	0.195529	-0.68838	0.491211	0.875595
armc8	332.2589	-0.210713764	0.139275	-1.51294	0.130296	0.581464
ednrab	9.268152	0.300895942	0.620665	0.484796	0.627821	0.921643
arhgap17b	289.6233	-0.213770286	0.147743	-1.4469	0.147924	0.608882
edc4	477.6746	0.105055756	0.110248	0.952905	0.340638	0.795544
kdm3b	1571.924	-0.061833855	0.153703	-0.4023	0.687467	0.938835
jarid2b	36.85615	0.157297103	0.420001	0.374516	0.70802	0.945335
fan1	158.2182	-0.096091123	0.16326	-0.58858	0.556144	0.899002
slc25a15b	98.74779	-0.012850363	0.244744	-0.05251	0.958126	0.999109
afg3l2	822.5186	0.04268066	0.10542	0.404864	0.685577	0.938486
ppp2r2cb	116.6237	0.304483756	0.256214	1.188394	0.234678	0.715533
si:dkey-202e17.1	27.43194	-1.480826699	0.443562	-3.33849	0.000842	0.039393
hace1	140.307	0.175766548	0.155937	1.127166	0.259672	0.736607
edaradd	27.09395	-0.374817624	0.36204	-1.03529	0.300532	0.766629
qpctlb	12.98281	-0.150136949	0.617305	-0.24321	0.80784	0.969828
soat1	37.88152	-0.607347307	0.415164	-1.46291	0.143493	0.602607
znf652	216.3513	0.076820436	0.172919	0.444257	0.656857	0.929939
pdpk1a	262.393	-0.027357801	0.215264	-0.12709	0.89887	0.990291
rims3	10.1302	0.887157582	0.648424	1.368176	0.171257	0.643325
ccdc61	169.7069	0.148834985	0.147562	1.008627	0.313154	0.776599
si:ch211-1f22.14	0.404638	0	4.281749	0	1	NA
wdr26a	249.8404	-0.044615364	0.189126	-0.2359	0.813508	0.970328
rasl10a	5.827901	0.049996373	0.714429	0.069981	0.944209	0.997371
sik2b	748.6104	-0.132722501	0.200007	-0.66359	0.506954	0.883007
si:dkey-103g5.3	353.2809	-0.472567209	0.205087	-2.30423	0.02121	0.258321
adam23a	629.7848	0.32741026	0.205246	1.595208	0.110666	0.545461
shank2b	120.4233	-0.180708621	0.326226	-0.55394	0.579622	0.905618
hmgn7	6811.529	0.054155706	0.130836	0.413922	0.678932	0.936081
rasgrp2	12.69689	-0.872445933	0.575516	-1.51594	0.129535	0.579296
b4galt3	48.31178	-0.082622598	0.294809	-0.28026	0.77928	0.961073
si:ch211-233a24.2	995.7147	0.068183501	0.184533	0.369491	0.711762	0.945821

nudt14	89.91787	-0.074763278	0.212724	-0.35146	0.725246	0.948653
zeb2a	1252.439	0.157394659	0.082215	1.914435	0.055565	0.408319
wfs1a	167.4846	0.363231328	0.243202	1.493537	0.135297	0.590585
cacna1ea	41.62983	0.169012004	0.375921	0.449594	0.653003	0.929197
mtus2a	91.16214	0.348414311	0.226618	1.537455	0.124182	0.571369
arhgap15	20.92698	0.041654879	0.442243	0.09419	0.924958	0.994815
synm	360.4342	-0.05237395	0.198151	-0.26431	0.791538	0.964817
sema4ab	278.7312	-0.031516185	0.142979	-0.22043	0.825539	0.972729
axdnd1	30.20923	-0.033428182	0.345945	-0.09663	0.923021	0.994525
tafa1a	70.40655	-0.104168363	0.434221	-0.2399	0.81041	0.969828
scn3b	123.7949	0.019868954	0.244062	0.08141	0.935116	0.996829
PAXBP1	657.5503	0.041480165	0.17647	0.235054	0.814166	0.970483
tpcn1	166.2547	0.109697942	0.177571	0.617771	0.536726	0.892421
phex	569.3786	0.028922454	0.194543	0.148669	0.881815	0.986908
bcl2l13	497.8243	0.256917021	0.137636	1.866647	0.061951	0.42715
nrd1b	151.3658	0.517695712	0.165671	3.124851	0.001779	0.065013
leng9	162.4867	-0.447052338	0.243221	-1.83805	0.066055	0.438914
nlgn3b	374.8047	0.063490526	0.190105	0.333976	0.738398	0.952912
CRHR2	7.514442	0.088959944	0.888072	0.100172	0.920208	0.993982
slc35a4	399.1507	0.065240922	0.104995	0.621374	0.534354	0.891616
si:ch211-238n5.4	247.0739	0.000905214	0.144971	0.006244	0.995018	1
snapin	536.0862	-0.138785482	0.161788	-0.85782	0.39099	0.827902
flcn	559.5631	-0.240036537	0.127855	-1.87741	0.060462	0.422849
cdc42ep5	317.5626	0.009943589	0.118156	0.084157	0.932932	0.996264
anks1aa	101.5621	0.089161949	0.200367	0.444994	0.656324	0.929684
ndst1a	220.3841	-0.50618763	0.196344	-2.57806	0.009936	0.172469
znf319b	186.698	-0.110310992	0.196221	-0.56218	0.573996	0.904358
frem1b	234.9972	-0.327136158	0.214564	-1.52465	0.127346	0.576964
ctnnd2a	262.5849	-0.010660255	0.174448	-0.06111	0.951273	0.99801
ttl	69.90047	-0.00981202	0.249478	-0.03933	0.968627	1
nfia	190.7819	0.209319739	0.172938	1.210375	0.226135	0.707473
pex2	37.5682	-0.055279308	0.28695	-0.19264	0.847238	0.977371
zgc:153901	67.36276	-0.319934144	0.239516	-1.33575	0.18163	0.657968
zgc:153521	148.6236	0.095975804	0.188122	0.510179	0.609926	0.915331
clcn2a	11.82289	0.653894644	0.633898	1.031545	0.302285	0.768145
trim109	189.8172	-0.487833571	0.197003	-2.47627	0.013276	0.200523
gpd2	135.9752	0.307522836	0.187989	1.635853	0.10187	0.528631
msrb1b	3.685461	2.235205602	1.366842	1.635307	0.101985	0.528846
pias1b	108.6334	0.137013958	0.190921	0.717646	0.472976	0.868965
neurl2	104.4432	0.225463806	0.217926	1.03459	0.300861	0.767015
hecw2a	178.4396	0.003267692	0.232601	0.014048	0.988791	1
skor1b	127.2764	-0.201024797	0.213576	-0.94123	0.346586	0.79947
xpr1a	359.6857	-0.039263341	0.182158	-0.21555	0.829342	0.974609
rnf115	567.6003	0.064600329	0.11604	0.556709	0.577727	0.905055
las1l	373.9391	-0.122214442	0.172463	-0.70864	0.478547	0.869292
sgk3	321.5542	-0.019865481	0.128081	-0.1551	0.876741	0.985052
shisa7a	47.88039	0.595791105	0.391446	1.522026	0.128003	0.577144
zc3h12b	41.47779	0.271625177	0.2746	0.989168	0.322581	0.782475
pgap1	40.83957	0.204922037	0.349581	0.586194	0.557745	0.899184
stra6l	47.79451	-1.057641887	0.373489	-2.83179	0.004629	0.112688
hipk3a	537.161	-0.335528259	0.147644	-2.27255	0.023054	0.26812
tafa4b	36.06078	0.10461335	0.400601	0.261141	0.793984	0.965431
sin3b	509.5666	0.19353833	0.163498	1.183736	0.236518	0.717725
snapc5	263.6438	-0.047477418	0.233689	-0.20317	0.839006	0.976417

kiaa1549la	412.7219	0.081119358	0.292644	0.277195	0.781631	0.962077
ackr3a	48.32797	-0.011208711	0.333078	-0.03365	0.973155	1
vcam1b	382.748	0.150174222	0.126437	1.187735	0.234938	0.715592
mlphb	160.9457	-0.460168606	0.167677	-2.74437	0.006063	0.131345
lyve1b	119.8329	-0.308259648	0.206052	-1.49603	0.134646	0.589298
dock11	186.4013	-0.163263838	0.186055	-0.8775	0.380214	0.823648
si:dkey-6n6.1	202.5857	-0.337906584	0.288689	-1.17048	0.241806	0.72348
8-Mar	336.1318	0.259914385	0.155732	1.668984	0.09512	0.51461
hhipl1	133.7618	0.064303207	0.253328	0.253834	0.799624	0.967206
si:ch73-19616.5	21.43421	0.14487209	0.396743	0.365154	0.714997	0.945821
zc3h18	699.4483	-0.00153778	0.108797	-0.01413	0.988723	1
cplx3a	11.9859	0.433479327	0.692514	0.625951	0.531347	0.890833
bcl11ba	707.1278	0.176113183	0.163892	1.074565	0.282569	0.754808
golga3	350.4679	-0.227874219	0.173457	-1.31372	0.18894	0.668516
tecpr1a	78.97925	-0.262081341	0.289507	-0.90527	0.365324	0.815339
si:ch211-107n13.1	78.30178	-0.186407821	0.230023	-0.81039	0.417718	0.841149
ulk1a	391.7079	-0.223586412	0.19877	-1.12485	0.260652	0.738121
abcc13	39.93368	-0.320466495	0.302777	-1.05842	0.289863	0.760705
atp9b	62.62309	0.008681304	0.236588	0.036694	0.970729	1
ASTE1	48.22043	0.379313464	0.299261	1.267501	0.204976	0.686811
mapk8ip3	880.2625	0.302989569	0.157497	1.923781	0.054382	0.40321
washc2c	625.8023	-0.307147206	0.118452	-2.593	0.009514	0.167766
si:dkey-117i10.1	13.99822	-0.223593222	0.5984	-0.37365	0.708663	0.94551
TMEM132C	44.46114	-0.145002602	0.487886	-0.29721	0.766309	0.960229
acmsd	166.6897	-0.077782554	0.275119	-0.28272	0.777389	0.961073
rc3h1a	119.2504	-0.015813255	0.25509	-0.06199	0.95057	0.99801
lpar6a	457.4794	-0.293732772	0.118298	-2.48298	0.013029	0.198789
bach1a	146.7217	0.010851409	0.18958	0.057239	0.954355	0.998611
rspry1	452.2632	-0.082573036	0.101206	-0.81589	0.414565	0.838683
adck1	108.3074	0.865047981	0.218268	3.963235	7.39E-05	0.007259
egln2	127.2111	0.238645218	0.382431	0.624021	0.532614	0.890978
kcnh4a	16.83863	-0.849562647	0.649946	-1.30713	0.191169	0.671109
mrpl2	1551.87	0.095475318	0.152178	0.627392	0.530402	0.890545
znrf3	169.9958	-0.015308742	0.201761	-0.07588	0.939518	0.997371
si:ch211-210g13.5	55.39945	0.385273361	0.284845	1.352572	0.176192	0.649415
arhgap35a	565.5632	0.480118246	0.129479	3.708074	0.000209	0.015361
kremen1	61.94565	-0.234813479	0.315774	-0.74361	0.45711	0.860993
brat1	176.9426	0.032174018	0.186855	0.172187	0.863291	0.981732
pleca	1938.874	-0.012462505	0.181685	-0.06859	0.945313	0.997371
myl10	12170.9	0.052973923	0.141158	0.375281	0.707451	0.944981
stox1	309.846	-0.267087999	0.134229	-1.9898	0.046613	0.376263
coq7	277.946	-0.415166683	0.145021	-2.8628	0.004199	0.107151
ltc4s	13.17589	2.95453671	0.735422	4.017473	5.88E-05	0.006235
piwil2	22.20713	-0.86830518	0.644173	-1.34794	0.177678	0.65136
si:dkey-26i13.8	46.14234	1.869355328	0.503691	3.711316	0.000206	0.015263
dhx37	337.3942	0.005628095	0.173816	0.03238	0.974169	1
kcnj12b	240.0894	0.169812984	0.257037	0.660656	0.508833	0.883385
satb1b	60.40575	0.167307069	0.27432	0.609898	0.541929	0.894241
dnah9l	31.94848	-0.15348413	0.448786	-0.342	0.732352	0.950617
npffr1l2	8.055064	-1.220649739	0.579223	-2.10739	0.035084	0.329877
duox	61.35464	-0.378428661	0.323922	-1.16827	0.242697	0.724345
cadm2b	72.88031	0.108247682	0.436347	0.248077	0.804075	0.968814
kat2b	66.05304	-0.056974105	0.36615	-0.1556	0.876346	0.984959
fem1b	195.678	-0.351495439	0.162137	-2.1679	0.030167	0.309482

kcnh8	8.302786	-0.173346519	0.739143	-0.23452	0.814578	0.970574
tet3	1666.089	0.190760271	0.139077	1.371617	0.170183	0.641806
rif1	553.8576	-0.02944661	0.151905	-0.19385	0.846294	0.97711
zc3hc1	366.3074	0.05126689	0.135016	0.37971	0.704161	0.943741
ydjc	103.6835	-0.346711319	0.278308	-1.24578	0.212843	0.695171
abca4b	256.5067	0.296810211	0.290085	1.023184	0.306221	0.771472
lmod3	221.9211	0.060216188	0.190747	0.315687	0.75224	0.955481
oxnad1	228.6562	0.420945292	0.154832	2.718716	0.006554	0.136957
mpp3b	70.02948	0.310621083	0.349088	0.889808	0.373569	0.820566
kcnj19b	55.05714	-0.128087405	0.306986	-0.41724	0.676502	0.93557
btbd10a	733.0615	0.209616975	0.09931	2.110726	0.034796	0.328533
megf11	90.51246	0.901246613	0.337587	2.669675	0.007592	0.147466
kcnh7	23.81253	0.339772908	0.45491	0.746901	0.455123	0.860008
gpnmb	1115.6	-1.024979996	0.163133	-6.2831	3.32E-10	3.08E-07
nrxn3b	150.8934	0.283950643	0.311403	0.911843	0.361851	0.811787
ccdc126	104.8915	0.017855816	0.193148	0.092446	0.926344	0.994815
dgkg	18.9193	-0.300374529	0.383312	-0.78363	0.433257	0.849392
nkain4	230.7733	-0.010997164	0.137966	-0.07971	0.936468	0.997044
ankmy1	21.88799	-0.030235411	0.442575	-0.06832	0.945533	0.997371
plat	63.24422	-0.061168916	0.32649	-0.18735	0.851384	0.978548
gal3st1a	96.21102	-0.286851324	0.208768	-1.37402	0.169436	0.640975
tmem264	20.49465	1.489738973	0.643947	2.313451	0.020698	0.255657
pcdh1a	184.54	0.155237304	0.258711	0.600041	0.548479	0.896779
dennd4a	246.2308	0.173310087	0.176607	0.981334	0.326428	0.785445
cep290	303.1299	0.178835231	0.194459	0.919656	0.357752	0.80901
scn1lab	94.58912	0.136402428	0.254404	0.536164	0.591845	0.909603
socs5b	122.985	-0.094570715	0.212206	-0.44566	0.655846	0.929606
pkn1b	54.92529	0.019204997	0.26033	0.073772	0.941192	0.997371
ino80b	373.6063	0.051526173	0.140299	0.367261	0.713425	0.945821
si:ch73-74h11.1	358.6267	0.214156983	0.199848	1.071599	0.2839	0.755095
mfap3l	841.2367	-0.166901697	0.113938	-1.46485	0.142961	0.602002
naif1	16.53471	-0.033482159	0.434241	-0.07711	0.93854	0.997371
pllp	147.2592	-0.58920655	0.24884	-2.36781	0.017894	0.236508
zgc:158403	237.1925	-0.614935764	0.147681	-4.16394	3.13E-05	0.003939
CR352265.1	22.58861	0.434871403	0.476639	0.912371	0.361573	0.811787
nsd3	139.2739	-0.164485	0.20745	-0.79289	0.427842	0.845893
snx21	37.74273	0.54428326	0.265697	2.048513	0.04051	0.351803
aifm3	19.89284	0.080886804	0.478705	0.16897	0.86582	0.982395
glsl	82.04374	-0.002941052	0.273667	-0.01075	0.991425	1
adpgk2	48.63584	0.908703123	0.279243	3.254163	0.001137	0.048173
irg1l	254.2433	-0.166590101	0.220352	-0.75602	0.449639	0.856979
CU639469.1	1200.997	0.052728154	0.116263	0.453524	0.650171	0.927835
trim36	295.1308	0.507008376	0.16744	3.028001	0.002462	0.079664
abcb7	619.7597	-0.014363451	0.104224	-0.13781	0.890388	0.988446
nell2b	1147.875	0.228963555	0.183929	1.244849	0.213187	0.695467
baiap2a	311.4077	0.068446847	0.133684	0.512006	0.608647	0.915204
erfl3	542.0717	-0.010098359	0.13314	-0.07585	0.93954	0.997371
ttll9	18.8054	0.189829321	0.427511	0.444034	0.657018	0.929939
CR786571.1	13.53434	0.70489458	0.455264	1.548319	0.121546	0.567899
p2ry10	7.44193	-2.254104415	0.953603	-2.36378	0.01809	0.237607
glsa	46.29043	0.314961348	0.239836	1.313238	0.189103	0.668577
crym	114.3436	0.406618776	0.255094	1.593998	0.110936	0.54583
irgf3	7.032123	-0.216617152	1.19308	-0.18156	0.855927	0.979558
slc6a15	70.28899	0.248187102	0.296579	0.836832	0.402687	0.833401

pi4kab	56.6924	-0.328730912	0.337828	-0.97307	0.330518	0.788126
alx1	121.9114	-0.376086511	0.200433	-1.87637	0.060604	0.423018
si:ch73-171o20.1	0.102175	1.055383603	5.267649	0.200352	0.841205	NA
tent5d	8.518437	0.668084665	0.963942	0.693075	0.488262	0.873728
si:ch73-22o12.1	544.8497	-0.072266123	0.136468	-0.52955	0.596427	0.911676
ecsit	307.4985	0.19786203	0.127313	1.554134	0.120152	0.565265
spata13	938.1972	-0.230867871	0.155177	-1.48777	0.136811	0.592353
tmtc3	553.0849	0.132967494	0.12367	1.075181	0.282294	0.754679
kcnk10a	11.93983	-0.330355118	0.769015	-0.42958	0.6675	0.933086
sdk1b	54.47066	0.291321311	0.311865	0.934126	0.350239	0.802197
plekhg7	132.314	0.385361127	0.250611	1.537685	0.124126	0.571369
gk5	328.4021	-0.290529075	0.168237	-1.7269	0.084185	0.487221
mras	347.9544	0.196351876	0.126313	1.554482	0.12007	0.565105
eea1	138.0841	-0.019017421	0.179326	-0.10605	0.915543	0.993078
mrs2	123.338	0.421819899	0.163821	2.574885	0.010027	0.173583
slc2a11l	8.710115	0.55118485	0.954639	0.577375	0.563686	0.901166
hcar1-3	10.36555	0.592995335	0.588836	1.007064	0.313904	0.777479
cntn3a.1	143.5659	0.41126621	0.247539	1.661417	0.09663	0.51893
map3k2	25.2918	0.178119095	0.398295	0.447204	0.654728	0.929527
steap2	51.34828	-0.017318415	0.426561	-0.0406	0.967615	1
foxe3	26.10212	0.039264388	0.324371	0.121048	0.903653	0.991366
si:dkey-103i16.6	34.22344	-0.513472088	0.318978	-1.60974	0.107455	0.538203
tnksb	984.2191	-0.037747354	0.102011	-0.37003	0.711359	0.945821
si:ch211-81a5.1	85.8191	0.349264688	0.229338	1.522927	0.127777	0.577117
si:dkey-220f10.4	58.11979	-0.317454518	0.377589	-0.84074	0.400493	0.831725
idua	58.87635	-1.450726103	0.27941	-5.19211	2.08E-07	8.60E-05
kcnv2b	15.86857	0.20959731	0.73764	0.284146	0.776299	0.961073
furina	420.8769	0.016040312	0.14071	0.113996	0.909241	0.992067
polr3b	387.2141	0.016615377	0.125819	0.132058	0.894939	0.989158
mrpl37	789.7827	0.047087691	0.129822	0.362711	0.716821	0.94627
tceanc2	76.11949	0.278108984	0.224909	1.23654	0.216258	0.69815
si:ch211-243o19.4	7.765169	0.797442667	0.728004	1.095383	0.273349	0.749148
FAM83G	343.8095	-0.045235332	0.152697	-0.29624	0.767045	0.960296
cmip	897.3403	0.091686698	0.099458	0.921868	0.356598	0.807342
gpr12	12.60127	0.657358344	0.565191	1.163072	0.2448	0.726138
ndnf	943.8835	0.089601001	0.111274	0.805229	0.420688	0.842383
kcna1a	27.77708	0.915469485	0.375459	2.438269	0.014758	0.213272
taco1	207.2094	0.261080197	0.243305	1.073058	0.283245	0.755028
amn	28.43448	0.756805596	0.519101	1.457916	0.144864	0.603718
wasf3b	939.0749	0.041154669	0.112638	0.36537	0.714835	0.945821
nt5dc3	34.77323	0.041695244	0.35772	0.116558	0.90721	0.991745
meak7	0	NA	NA	NA	NA	NA
clk2a	988.5167	0.00458778	0.129053	0.03555	0.971642	1
dipk1aa	146.8316	0.100083456	0.197149	0.507655	0.611695	0.915331
dagla	231.5488	0.641877013	0.19493	3.292865	0.000992	0.043685
zgc:158785	59.63871	0.493248516	0.402966	1.224045	0.220935	0.702242
armc10	172.9139	-0.182935234	0.146933	-1.24503	0.213121	0.695467
kmt2ba	605.7035	0.161722261	0.161925	0.99875	0.317916	0.77973
si:dkey-246g23.2	23.85301	0.181972976	0.401308	0.45345	0.650225	0.927835
fam45a	183.5883	-0.173700429	0.182193	-0.95339	0.340394	0.795496
kcng4a	42.39891	-0.128575402	0.330321	-0.38924	0.697096	0.94198
sbf1	973.9275	0.048105239	0.131407	0.366077	0.714307	0.945821
fam129ba	395.6353	0.060337699	0.126665	0.476357	0.63382	0.923974
sdhaf2	476.0876	0.313385007	0.1703	1.840189	0.06574	0.437326

hip1	650.6944	0.17380848	0.122739	1.416088	0.15675	0.622646
ttll7	238.8753	0.330840248	0.226507	1.460621	0.144119	0.603144
itga2.2	42.89196	0.217360098	0.293448	0.74071	0.458869	0.861588
iffo1a	15.78033	1.574451949	0.660703	2.382993	0.017172	0.231824
samd13	142.8323	0.088500816	0.171667	0.515539	0.606176	0.915204
ccdc151	32.75984	0.540840491	0.318666	1.697204	0.089658	0.50251
fbxo18	318.0001	0.090502214	0.129641	0.6981	0.485115	0.872911
pnpla7a	641.7212	-0.016088076	0.119981	-0.13409	0.893332	0.988566
tyw1	197.3892	-0.265791497	0.139259	-1.90861	0.056313	0.410107
abi1b	32.70999	0.940698874	0.300207	3.133504	0.001727	0.064499
si:dkey-14o18.2	5.943092	1.416572785	1.383804	1.02368	0.305986	0.771459
hyal2b	361.1441	0.061762447	0.148018	0.417263	0.676486	0.93557
pglyrp2	25.75396	0.162679071	0.52009	0.31279	0.75444	0.956153
atp8a1	19.78994	0.138455686	0.573839	0.24128	0.809338	0.969828
alkbh5	138.3466	0.156347828	0.170907	0.914811	0.360291	0.811057
anapc7	206.3766	0.334602955	0.193907	1.725588	0.084422	0.48755
cacng7a	21.32384	0.351550848	0.687768	0.511147	0.609248	0.915204
apc2	217.1032	0.694967205	0.231393	3.003404	0.00267	0.084161
si:dkeyp-27e10.3	201.8092	0.355700252	0.223418	1.592087	0.111365	0.547032
catip	48.93664	0.246198332	0.306503	0.80325	0.42183	0.843078
antxr2b	41.66502	-1.142816659	0.362368	-3.15374	0.001612	0.06152
dbpa	200.8846	0.195609121	0.397626	0.491943	0.62276	0.918894
fam78ba	26.42987	0.112342135	0.347726	0.323077	0.746637	0.955074
panx2	25.69926	0.07304325	0.60848	0.120042	0.90445	0.991581
rpain	72.49609	-0.135903211	0.24959	-0.54451	0.586094	0.906711
mlc1	113.9352	0.465702273	0.27077	1.719919	0.085447	0.490781
mesd	345.7828	0.168391595	0.114413	1.471784	0.141079	0.599658
rad54l2	718.1367	0.255560354	0.143718	1.778211	0.075369	0.465238
dok6	14.79434	0.754552201	0.564949	1.335611	0.181676	0.658034
psd2	353.8577	0.126240616	0.115598	1.092065	0.274804	0.750181
lipea	42.76359	0.188522857	0.333905	0.564601	0.572345	0.903461
btbd11b	34.50934	0.738677097	0.345541	2.137742	0.032538	0.319448
thtpa	83.77481	-0.008369712	0.195061	-0.04291	0.965775	1
rc3h2	89.40257	0.014249382	0.270818	0.052616	0.958038	0.999109
bap1	344.0315	-0.099835582	0.123979	-0.80526	0.42067	0.842383
shank3b	131.5794	-0.152115712	0.189923	-0.80093	0.423171	0.843894
tulp4b	275.3472	0.781981882	0.249926	3.128857	0.001755	0.064556
abcg8	38.83248	0.548594789	0.451209	1.215832	0.224049	0.705807
ubn2a	364.4717	-0.185930145	0.146394	-1.27006	0.204062	0.685236
syne1b	541.2273	0.00253327	0.20515	0.012348	0.990148	1
gripap1	147.1898	0.006876421	0.185924	0.036985	0.970497	1
soga1	255.2264	0.163627425	0.230304	0.710483	0.477405	0.869292
atrip	157.8734	-0.177805194	0.166045	-1.07083	0.284248	0.75523
abcg5	33.54922	0.138932024	0.576943	0.240807	0.809705	0.969828
ago3b	50.68629	0.456963117	0.225738	2.02431	0.042938	0.362962
si:dkey-211g8.6	0.772922	3.264329551	2.313173	1.411192	0.158188	NA
ccm2l	65.034	-0.382778498	0.268425	-1.42601	0.153864	0.618475
ctsf	2282.113	-0.041142546	0.090225	-0.456	0.648391	0.927474
scube1	68.4196	0.068267506	0.234899	0.290625	0.771338	0.960546
psmd14	1855.61	0.160631801	0.131849	1.218304	0.223108	0.705242
man2a2	379.8349	-0.046741607	0.138268	-0.33805	0.735324	0.951543
CABZ01059392.1	5.036057	-2.111887196	1.203035	-1.75547	0.07918	0.47627
si:ch73-160i9.1	0.320972	-1.826685199	5.230459	-0.34924	0.726909	NA
gusb	251.5555	0.290556245	0.189428	1.533864	0.125063	0.572671

si:ch211-38m6.7	12.85946	0.060475157	0.548486	0.110258	0.912204	0.992371
slc4a10a	1029.428	0.275334508	0.189393	1.453772	0.146009	0.605628
exd3	156.5427	-0.525947322	0.177871	-2.95691	0.003107	0.091566
shisa7b	507.202	0.125897993	0.191017	0.659094	0.509835	0.883733
tfam	443.3725	0.065480044	0.177331	0.369252	0.71194	0.945821
tmtc1	395.9079	-0.143912917	0.128181	-1.12273	0.261553	0.738975
nrxn2b	356.7454	0.159156743	0.21998	0.723504	0.46937	0.867224
bcl11ab	337.3243	0.145282919	0.182278	0.797041	0.425427	0.844714
dcaf12	531.2374	-0.05422963	0.103778	-0.52256	0.601283	0.913756
mapk8ip2	65.94478	0.374591402	0.307286	1.219033	0.222832	0.704655
CABZ01081780.1	13.98262	-0.194594688	0.599815	-0.32442	0.745617	0.955074
si:dkey-32e23.4	59.66302	0.291599438	0.318	0.916979	0.359154	0.810124
ppwd1	267.3528	0.158439973	0.157408	1.006559	0.314147	0.777479
cmtm8b	17.58358	0.192984026	0.461146	0.418488	0.675591	0.935422
chkb	260.693	0.040518481	0.137016	0.295721	0.767443	0.960296
ubn1	20.15514	0.715207956	0.627213	1.140295	0.254163	0.733279
manf	462.3827	0.402831485	0.18958	2.124859	0.033598	0.323555
dock3	51.04041	-0.504384416	0.319948	-1.57646	0.114921	0.554651
rc3h1b	461.9022	-0.028239299	0.142106	-0.19872	0.842482	0.976552
si:ch211-220f12.4	12.05574	0.215019612	0.704539	0.305192	0.76022	0.957886
agpat5	301.3034	-0.128675336	0.159617	-0.80615	0.420157	0.842383
zranb1b	990.0496	-0.036166513	0.105809	-0.34181	0.732494	0.950617
extl2	74.33056	-0.658692397	0.27859	-2.36438	0.01806	0.237356
lin54	344.1383	0.155640682	0.136582	1.139538	0.254479	0.733426
pxk	449.3207	-0.390276698	0.107298	-3.63731	0.000275	0.018396
golga2	499.699	0.048634245	0.138024	0.35236	0.724568	0.9484
erbb4a	8.916166	-0.203251375	0.706082	-0.28786	0.773455	0.960624
exoc8	196.4849	-0.036585698	0.169998	-0.21521	0.829602	0.974753
prmt2	42.84787	-0.277883667	0.332363	-0.83609	0.403107	0.833704
mtif2	182.7146	0.321943216	0.136057	2.366244	0.01797	0.2367
lrrc3b	7.677666	-0.015153527	0.625459	-0.02423	0.980671	1
ppm1la	688.4439	-0.16130476	0.107998	-1.49359	0.135282	0.590585
ubap2l	1470.815	-0.068272058	0.15268	-0.44716	0.654761	0.929527
arl14	91.72096	0.565892491	0.299505	1.889427	0.058835	0.418455
nxph2a	54.26554	0.531740878	0.347058	1.53214	0.125488	0.57334
xpo1b	2052.203	0.08275995	0.174779	0.473513	0.635847	0.924669
bmp7b	154.7555	-0.278234128	0.168729	-1.649	0.099148	0.522578
baz1a	191.3678	-0.09905051	0.156045	-0.63476	0.525586	0.888928
ttc13	292.3278	0.004030506	0.127817	0.031533	0.974844	1
lix1l	285.8772	-0.030114638	0.17663	-0.1705	0.864621	0.982015
mrpl9	277.563	0.024093974	0.146	0.165028	0.868922	0.982801
spata2	290.925	0.140591107	0.126689	1.109734	0.267114	0.743418
prcc	593.7052	0.322509711	0.106796	3.01988	0.002529	0.08126
hecw2b	101.706	-0.146841405	0.221341	-0.66342	0.507064	0.883007
r3hdm4	402.9849	-0.100654339	0.192477	-0.52294	0.601014	0.913756
btbd11a	187.865	0.091050675	0.14853	0.613012	0.539868	0.893049
si:ch211-194e18.2	13.3709	-0.462351607	0.574296	-0.80508	0.420776	0.842383
rfx5	191.2719	-0.104464043	0.15302	-0.68268	0.494807	0.878016
pcdh9	61.46082	0.177087847	0.297572	0.595109	0.551771	0.8974
SLC35G1	47.95885	0.84355112	0.279943	3.013293	0.002584	0.08259
retreg2	230.0821	0.10182518	0.19229	0.529539	0.596432	0.911676
msh3	70.12682	-0.057507398	0.240345	-0.23927	0.810896	0.969828
abi3b	115.4297	0.059035431	0.23755	0.248518	0.803734	0.968762
ube2t	111.7242	-0.237975174	0.21508	-1.10645	0.268533	0.744925

slc2a11b	814.4162	-0.39894065	0.172665	-2.31048	0.020861	0.256424
ssuh2rs1	1743.924	0.154821513	0.102945	1.503922	0.132601	0.585592
neto2b	11.12977	-3.083448273	1.444811	-2.13415	0.03283	0.32069
myh9a	2810.67	-0.013685526	0.126182	-0.10846	0.913632	0.992796
abcb6a	96.69036	0.730311628	0.234815	3.110164	0.00187	0.066755
pcloa	606.0052	0.123184431	0.201479	0.6114	0.540935	0.89346
CU655845.1	52.72068	-0.328891649	0.340455	-0.96603	0.334027	0.791027
sgsm2	29.05511	-0.202584902	0.37188	-0.54476	0.58592	0.906655
tjp2a	513.0295	0.081295466	0.116841	0.695778	0.486568	0.873244
oxr1b	676.3693	-0.358214631	0.197071	-1.81769	0.069112	0.448548
soga3a	0	NA	NA	NA	NA	NA
plbd1	91.79459	-0.761436008	0.221525	-3.43725	0.000588	0.031278
apba2a	130.3688	0.071948343	0.21405	0.336129	0.736773	0.952147
FP085394.1	17.85065	-1.604343582	0.970851	-1.65251	0.09843	0.521076
rabl2	151.8366	0.096401843	0.202531	0.475986	0.634084	0.924077
mgat4a	447.4566	-0.19490921	0.125996	-1.54695	0.121875	0.56863
shank3a	18.85155	-0.011181779	0.512032	-0.02184	0.982577	1
nup210	542.647	0.142664616	0.144624	0.986453	0.323911	0.783161
czib	386.2542	0.075101507	0.172168	0.43621	0.662684	0.930964
fam162a	365.4654	-0.090884313	0.191963	-0.47345	0.635895	0.924669
hrc	8.379765	0.03730813	0.646396	0.057717	0.953974	0.998611
inpp4aa	521.2206	0.176498291	0.110003	1.60448	0.108608	0.541057
abtb1	649.4844	-0.378200513	0.1445	-2.6173	0.008863	0.161314
GPR45	7.572965	0.586679193	0.708346	0.828238	0.407536	0.83577
micu1	1012.856	-0.14229502	0.118493	-1.20087	0.2298	0.711763
si:ch211-1e14.1	25.7332	0.503420401	0.419952	1.198756	0.230623	0.71228
sgk2a	145.4101	-0.152348776	0.178308	-0.85441	0.392876	0.827902
rasa3	272.1653	0.138827484	0.183777	0.755412	0.450002	0.85711
zpld1b	15.20339	-0.188166864	0.577387	-0.32589	0.744505	0.95491
pter	146.8739	-0.390230754	0.227124	-1.71814	0.085772	0.491305
cenpe	698.8568	0.177711519	0.260094	0.683258	0.494444	0.877861
sec22c	20.59894	0.151722782	0.364678	0.416046	0.677376	0.935952
pvrl2l	57.58746	-0.068859969	0.32496	-0.2119	0.832183	0.975013
LO018340.1	372.2659	0.292310241	0.194608	1.502046	0.133085	0.586514
lrrc73	101.8304	-0.422003878	0.307754	-1.37124	0.170301	0.641918
cers1	122.9005	-0.587696451	0.174653	-3.36493	0.000766	0.036804
rxylt1	64.99454	0.400760003	0.203713	1.967282	0.049151	0.386041
ptprk	643.6173	-0.000746109	0.131667	-0.00567	0.995479	1
erf	454.5653	0.070014639	0.128422	0.545194	0.58562	0.906655
DHX35	174.1878	-0.185048107	0.158771	-1.1655	0.243815	0.724437
atp2b2	508.0647	0.000225389	0.225417	0.001	0.999202	1
trpm4b.2	120.3367	-0.151165891	0.220186	-0.68654	0.492374	0.876385
rph3aa	10.70107	0.102207315	1.012758	0.10092	0.919614	0.993892
wnt9a	29.42672	-0.035788906	0.313163	-0.11428	0.909014	0.992033
srebf2	950.7381	0.357473283	0.159579	2.240106	0.025084	0.280772
si:dkey-32n7.7	1.226591	-2.101483706	1.923152	-1.09273	0.274513	NA
snap47	192.1698	0.202768562	0.243124	0.834012	0.404274	0.833888
iba57	194.2613	0.083410173	0.157563	0.529378	0.596544	0.911676
ccdc13	8.20764	0.805616991	0.609376	1.322037	0.186156	0.664263
wash1	82.89815	0.378337939	0.214631	1.762734	0.077945	0.47265
isy1	351.3213	-0.020949402	0.105163	-0.19921	0.842099	0.97643
arfgef1	561.5179	0.135200826	0.121183	1.115673	0.264562	0.742063
abcg1	60.86781	0.083026568	0.41018	0.202415	0.839592	0.976417
si:ch211-214j24.15	13.58427	-0.471576979	0.619381	-0.76137	0.446437	0.855403

lrrc58b	478.2672	-0.249507522	0.205114	-1.21643	0.223821	0.705807
si:ch211-15d5.11	176.9076	0.179590418	0.220627	0.813999	0.415646	0.839167
zgc:153913	468.2543	-0.185059579	0.253397	-0.73032	0.465197	0.864989
PLEKHB1	93.01115	0.088800173	0.705193	0.125923	0.899793	0.99045
bbs4	92.96737	0.334685069	0.322548	1.037627	0.299444	0.766128
adpgk	211.561	0.266194747	0.157399	1.69121	0.090797	0.505206
elmo2	551.0262	0.149978654	0.178879	0.838436	0.401786	0.832807
chd4a	1879.875	0.158758303	0.185711	0.854868	0.392624	0.827902
kalrnb	681.5762	-0.009973224	0.129462	-0.07704	0.938595	0.997371
slc25a15a	58.09959	0.37591084	0.285072	1.318654	0.187285	0.66594
pip4k2ab	24.71092	0.239228606	0.389879	0.613598	0.539481	0.893049
rlf	316.0904	0.278731856	0.249788	1.115872	0.264477	0.742063
si:ch211-166g5.4	93.90863	0.030250147	0.297303	0.101748	0.918956	0.993892
pigx	127.235	-0.228162869	0.223679	-1.02004	0.307707	0.772996
creb3l2	527.7417	-0.012003062	0.114591	-0.10475	0.916577	0.993451
syt7a	17.68037	0.901838201	0.594214	1.5177	0.12909	0.578533
dyrk1aa	960.8614	0.048392809	0.102572	0.471795	0.637073	0.924779
perp	2912.696	-0.022154896	0.095585	-0.23178	0.816707	0.971207
vps41	555.6247	-0.017022039	0.151103	-0.11265	0.910307	0.992208
mtg2	195.9511	0.053666177	0.155501	0.345118	0.730006	0.950056
klhl35	12.61559	-0.265828495	0.641792	-0.4142	0.67873	0.936026
DGKI	187.7685	0.025558665	0.193285	0.132233	0.8948	0.989112
ppp1r9ala	297.7869	0.142230475	0.145902	0.974839	0.32964	0.788
map2k4a	100.7363	-0.032445112	0.212377	-0.15277	0.878579	0.985761
uba5	289.1493	0.155492505	0.13513	1.150688	0.249861	0.729721
hipk1a	47.24361	0.195369878	0.31136	0.627473	0.530349	0.890545
bud23	333.5007	0.086954784	0.186498	0.466249	0.641037	0.925364
coro2bb	32.06589	-0.28424201	0.427751	-0.6645	0.506368	0.883007
antxr1c	796.2337	-0.055520365	0.091688	-0.60554	0.544823	0.895206
si:ch211-133n4.10	1.754822	-2.156384079	2.057213	-1.04821	0.294543	NA
skor2	78.76554	0.39391192	0.321969	1.223445	0.221162	0.702457
aadac	72.34697	0.035853332	0.329288	0.108881	0.913297	0.99273
gfm1	476.8925	0.236829647	0.143174	1.654143	0.098099	0.521071
ddx21	7148.091	-0.923916442	0.158125	-5.84295	5.13E-09	3.30E-06
bop1	420.7171	-0.14318132	0.162007	-0.8838	0.376805	0.822258
VIT	631.9094	-0.160382129	0.18492	-0.86731	0.385775	0.826939
hpse	140.4877	-0.469742422	0.198863	-2.36214	0.01817	0.237981
nrxn1b	131.9411	0.151789876	0.243225	0.624071	0.532581	0.890978
galnt11	694.4645	-0.022432107	0.122754	-0.18274	0.855002	0.979161
lnpa	118.1425	0.083117631	0.232312	0.357785	0.720505	0.947263
tead3b	138.3787	-0.328762984	0.266962	-1.2315	0.218137	0.699257
nuak2	243.0912	-0.47972035	0.196823	-2.43732	0.014796	0.213551
clp1	159.2674	0.023653873	0.178123	0.132795	0.894355	0.988891
mat2al	193.7858	0.490023779	0.177581	2.759442	0.00579	0.128995
gtf2a1l	14.9871	-0.315605357	0.547517	-0.57643	0.564324	0.901433
zmp:0000000711	309.3081	0.020546781	0.184438	0.111402	0.911298	0.992208
ccny	86.25611	-0.101811501	0.212272	-0.47963	0.631492	0.923003
fhod3a	493.761	0.139405209	0.214103	0.651114	0.514973	0.886401
ppm1h	191.1597	-1.590126387	0.180035	-8.83234	1.03E-18	4.75E-15
atp23	288.7193	0.085427879	0.178987	0.477284	0.63316	0.923885
nrm	157.9985	0.256376529	0.199597	1.284469	0.198978	0.679593
rreb1a	381.8987	0.012090536	0.16843	0.071784	0.942774	0.997371
pde8b	18.81694	-0.604520143	0.441934	-1.3679	0.171345	0.64335
trim3a	197.5216	0.24201404	0.16376	1.477857	0.139446	0.597663

syngap1a	140.0453	0.727674763	0.353825	2.056598	0.039725	0.348523
vps72a	419.485	0.001378093	0.109942	0.012535	0.989999	1
usp8	1499.814	0.072196327	0.130839	0.551796	0.581088	0.905618
cdk12	668.6019	-0.073343812	0.14792	-0.49583	0.620011	0.91764
osbpl6	19.13319	0.608338164	0.363321	1.674383	0.094055	0.512045
rbm45	7.869914	0.26916752	0.750076	0.358854	0.719705	0.94694
pde11a	14.21701	0.095948361	0.509349	0.188375	0.850583	0.978251
cox19	129.9502	-0.007313568	0.182439	-0.04009	0.968023	1
mt-nd1	21604.97	0.152256751	0.156933	0.970204	0.331945	0.789602
mt-nd2	14876.38	0.155111938	0.130953	1.184485	0.236221	0.717364
mt-co1	173529.8	-0.061516925	0.080337	-0.76574	0.443832	0.854255
mt-co2	96788.39	-0.304265602	0.162172	-1.87619	0.06063	0.423018
mt-atp8	1134.856	0.106237643	0.254549	0.417356	0.676418	0.93557
mt-atp6	34123.93	0.127107487	0.162688	0.781294	0.43463	0.850002
mt-co3	65725.57	-0.090547582	0.105899	-0.85504	0.39253	0.827902
mt-nd3	8998.441	0.081343689	0.16547	0.49159	0.623009	0.918894
mt-nd4l	3547.563	0.127063445	0.114145	1.113176	0.265633	0.742247
mt-nd4	14120.98	0.079866306	0.106304	0.751302	0.452471	0.857994
mt-nd5	12021.65	0.110782559	0.095304	1.162412	0.245068	0.726512
mt-nd6	3236.642	0.153960879	0.135789	1.133826	0.256867	0.734405
mt-cyb	38126.9	0.008391099	0.138243	0.060698	0.9516	0.99801
kctd8	175.7088	-0.449439113	0.240433	-1.86929	0.061583	0.425659
slc24a4b	5.459317	-1.120657187	1.136399	-0.98615	0.324061	0.783161
ccdc15	22.66814	-0.405504882	0.474289	-0.85497	0.392566	0.827902
zgc:158482	496.1979	0.631394282	0.221498	2.850558	0.004364	0.108751
fut9b	97.40082	-0.111003105	0.235865	-0.47062	0.637911	0.924779
pcsk5a	85.98976	0.484094025	0.348697	1.388296	0.165047	0.634185
tcf15	110.4724	0.027356088	0.176447	0.155039	0.876791	0.985052
adam19b	188.1215	-0.031458479	0.182932	-0.17197	0.863462	0.981766
si:ch211-193c2.2	18.8353	-0.180973729	0.50067	-0.36146	0.717753	0.946466
adamts12	139.801	0.498670874	0.263834	1.89009	0.058746	0.418427
gtf2h3	205.3055	-0.050420102	0.17955	-0.28081	0.778854	0.961073
kiss1rb	3.267496	-2.3228331	1.179565	-1.96923	0.048927	0.385066
zgc:153044	39.35503	-0.863228085	0.354223	-2.43696	0.014811	0.213551
sftpbb	71.375	-0.155059293	0.235252	-0.65912	0.509819	0.883733
SLC6A13	47.94097	0.530580378	0.41997	1.263376	0.206454	0.688857
puraa	132.1275	0.128620288	0.169731	0.757788	0.448578	0.856741
BX901974.1	1.421032	-2.37995259	2.102433	-1.132	0.257635	NA
sco1	264.123	0.179097675	0.166718	1.074258	0.282707	0.754993
fancb	24.59304	0.130721187	0.340776	0.383599	0.701276	0.943094
arfgap2	910.6452	-0.027708654	0.121044	-0.22891	0.818935	0.971785
napaa	322.1519	0.038060619	0.157428	0.241766	0.808962	0.969828
rsph4a	40.31113	-0.082749423	0.392824	-0.21065	0.833158	0.975379
srebf1	236.793	-0.06172744	0.273173	-0.22596	0.821229	0.971788
zswim7	21.59223	0.461872134	0.400962	1.151909	0.249358	0.729721
dnajc25	104.3006	-0.125126948	0.194526	-0.64324	0.520069	0.887148
ywhag1	496.3615	0.348842698	0.180754	1.929928	0.053616	0.400993
zgc:154075	211.3464	-0.375714735	0.178267	-2.1076	0.035066	0.329872
prpf4	369.561	-0.044548152	0.123683	-0.36018	0.718712	0.94694
tmem232	9.839004	0.424728085	0.700861	0.606009	0.544509	0.895206
kcmf1	403.0587	0.008583916	0.128206	0.066954	0.946618	0.997371
BX470189.1	183.0272	-0.161253013	0.169859	-0.94933	0.342451	0.797179
ftr80	0.921382	0.093654024	2.450537	0.038218	0.969514	NA
si:dkey-172m14.2	16.92261	0.356121693	0.710463	0.501253	0.616193	0.916365

fam167aa	21.59068	-1.828622908	0.725072	-2.52199	0.011669	0.187763
cep57	47.09065	-0.177160381	0.352062	-0.50321	0.614818	0.915998
zglp1	1.330176	-0.84491141	1.527605	-0.5531	0.580198	NA
pomt1	172.5539	-0.232416477	0.162307	-1.43196	0.152156	0.6158
card9	26.68829	0.373781843	0.411115	0.909191	0.363249	0.813595
snapc4	105.433	-0.236529654	0.261084	-0.90595	0.364961	0.814972
plcl1	88.63116	0.238578322	0.259943	0.917809	0.358719	0.80995
myoz3a	278.9161	-1.046779544	0.572789	-1.82751	0.067622	0.443563
shisal1b	63.66378	0.132209081	0.250276	0.528253	0.597324	0.911953
parvg	68.3501	-0.26781468	0.284706	-0.94067	0.346873	0.799748
snx18a	439.815	-0.040130949	0.133236	-0.3012	0.763261	0.958924
hspb3	51.08321	-0.149274726	0.250599	-0.59567	0.551394	0.8974
tomm7	727.9042	-0.575362811	0.157369	-3.65614	0.000256	0.017645
si:dkey-3h3.3	33.78748	0.305817766	0.335901	0.91044	0.36259	0.81253
wwtr1	194.5382	-0.015290564	0.213409	-0.07165	0.942881	0.997371
tank	70.09588	0.257801974	0.255391	1.009441	0.312763	0.776213
arid3c	262.233	0.027063028	0.15843	0.17082	0.864365	0.981867
trpc6b	6.772964	-0.395459958	0.889024	-0.44482	0.656446	0.929745
itpkcb	497.9213	-0.087480097	0.162164	-0.53946	0.589572	0.908513
si:rp71-68n21.9	76.92845	-0.27557821	0.209979	-1.31241	0.189383	0.668949
pigl	82.43853	-0.11682878	0.214269	-0.54524	0.585587	0.906655
zgc:158640	52.44242	0.091300212	0.223992	0.407604	0.683564	0.938405
urp2	18.87356	1.105766259	0.56675	1.951065	0.051049	0.393353
CABZ01088428.1	21.98264	-0.641480196	0.420959	-1.52385	0.127545	0.576964
cabz01076234.2	12.01812	-0.588884122	0.734065	-0.80222	0.422424	0.843493
ifngr2	515.8471	-0.229656412	0.144826	-1.58574	0.112799	0.549875
clpxb	762.1294	0.024312121	0.130844	0.185811	0.852593	0.978672
spi1a	68.30216	0.122366235	0.273564	0.447303	0.654656	0.929527
CABZ01064472.1	78.25381	0.598338595	0.266093	2.248608	0.024537	0.277819
ggcx	493.0395	0.071365921	0.107404	0.664464	0.506393	0.883007
zgc:158432	15.08949	0.085697782	1.270291	0.067463	0.946213	0.997371
si:dkeyp-27c8.1	0.150235	0	5.267649	0	1	NA
zgc:171844	30.23546	-0.689461329	0.371827	-1.85425	0.063703	0.430571
zmp:0000001127	0.474251	0	4.891932	0	1	NA
CABZ01060891.1	4.220285	0.677563291	1.056624	0.641253	0.521358	0.887551
CABZ01083448.1	414.2707	-0.050135503	0.187075	-0.268	0.788701	0.963568
cntnap3	185.919	0.417667611	0.311888	1.339158	0.180519	0.656202
si:ch211-229c8.13	1.870029	0.249574988	1.73432	0.143904	0.885577	NA
ppargc1a	87.17482	0.211888188	0.304658	0.695495	0.486745	0.873245
tbata	35.49868	-0.327515157	0.295556	-1.10813	0.267805	0.744189
tle2c	152.9034	0.016934804	0.200112	0.084627	0.932558	0.996175
riox1	467.9265	-0.027189197	0.203034	-0.13391	0.89347	0.988566
il21	2.478481	0.297227269	1.217101	0.244209	0.807069	0.969726
nmrk2	671.0068	0.158749828	0.40896	0.38818	0.697883	0.942355
jund	403.4408	-0.499306337	0.145333	-3.4356	0.000591	0.031348
lcn15	262.372	-0.217843661	0.192417	-1.13214	0.257574	0.735155
scospondin	27.08608	0.455939503	0.379806	1.200454	0.229963	0.711763
CU469531.1	2.266765	-0.875406845	1.542586	-0.56749	0.570379	0.902481
gltpd2	49.09526	-0.732632438	0.337345	-2.17176	0.029874	0.307772
MAN1C1	35.985	-0.338583944	0.350878	-0.96496	0.334563	0.79151
ints9	138.8389	0.084182677	0.161017	0.52282	0.6011	0.913756
lyn	53.57744	-0.224918714	0.245546	-0.91599	0.35967	0.810668
elp5	162.4084	0.085489092	0.156216	0.547248	0.584208	0.906402
rab8a	189.9346	-0.249257995	0.153913	-1.61947	0.105346	0.534575

sstr1b	28.82756	-0.045025267	0.575549	-0.07823	0.937645	0.997305
EVA1A	7.272685	-0.530292821	0.761361	-0.69651	0.486111	0.873244
zcchc2	142.8671	0.080259789	0.179153	0.447995	0.654157	0.929434
sh3gl1a	288.8919	-0.010251989	0.149768	-0.06845	0.945425	0.997371
slc6a5	277.5992	-0.197839313	0.304205	-0.65035	0.515467	0.886401
fem1a	632.6449	0.093311016	0.168192	0.554789	0.579039	0.905417
atp5if1a	948.9174	-0.117717329	0.15906	-0.74008	0.459252	0.861818
ar	57.78143	0.378035684	0.285586	1.323718	0.185597	0.663476
nitr7b	0.043309	0	5.267649	0	1	NA
gtf3c5	149.6316	-0.063124355	0.185717	-0.33989	0.733936	0.95093
zgc:103697	262.768	-0.154614766	0.172777	-0.89488	0.37085	0.818484
gas1b	352.8695	0.085063212	0.12846	0.662178	0.507857	0.883056
ftr23	51.12806	-0.850004556	0.274822	-3.09293	0.001982	0.069053
myhz1.1	199088.4	-0.22879475	0.411056	-0.5566	0.577799	0.905057
si:dkey-31e10.1	7.483442	-0.706841791	0.70718	-0.99952	0.317542	0.779441
myhz1.2	65223.91	-0.078967287	0.419046	-0.18845	0.850528	0.978251
csk	660.2727	0.095550289	0.114814	0.832221	0.405284	0.834483
myhz1.3	37427.35	0.265276735	0.416935	0.636254	0.524611	0.888928
CABZ01084793.1	108.6826	0.577567315	0.272305	2.121029	0.033919	0.325295
gck	49.87394	-0.647204529	0.506867	-1.27687	0.201647	0.68147
si:ch211-230g14.3	5.509088	0.154221328	0.653452	0.23601	0.813425	0.970328
ece2a	44.22081	-0.068622017	0.273815	-0.25061	0.802112	0.96794
si:cabz01007802.1	323.7169	0.208700875	0.256163	0.814719	0.415233	0.838754
tmem119b	468.0877	-0.300343333	0.138449	-2.16934	0.030057	0.309109
agbl4	16.84266	-1.213198146	0.528364	-2.29614	0.021668	0.26121
sirt1	720.3687	-0.285490853	0.118369	-2.41187	0.015871	0.222329
BX664721.2	0.275769	0	5.267649	0	1	NA
wbp4	90.64821	0.052077567	0.216965	0.240027	0.810309	0.969828
CT573494.1	0.043309	0	5.267649	0	1	NA
psmb11b	0.135482	0	5.267649	0	1	NA
tcnba	687.5083	0.674038629	1.337581	0.503924	0.614315	0.915998
atf5a	612.6292	0.458756431	0.178747	2.56652	0.010272	0.176568
si:ch211-198o12.4	0.437654	0	3.686175	0	1	NA
tafa5l	65.42313	0.77154208	0.456098	1.691615	0.090719	0.505206
cfap45	48.66851	0.068557759	0.319876	0.214326	0.830293	0.974908
CABZ01040999.1	4.239866	0.630668405	0.878066	0.718247	0.472605	0.868786
CABZ01079818.1	2.157287	1.627281465	1.7128	0.950071	0.342076	NA
f11r.2	189.0293	0.236004386	0.280227	0.842191	0.399681	0.831219
CFAP77	29.83197	0.294618079	0.334331	0.881216	0.378201	0.822672
gkap1	820.7768	-0.034099303	0.133161	-0.25608	0.797892	0.966447
opn7d	4.675239	-1.550293507	1.223023	-1.26759	0.204944	0.686802
nfu1	526.9476	-0.088524133	0.113428	-0.78044	0.435132	0.850085
nppc	30.63742	-0.289515722	0.450353	-0.64286	0.520313	0.887149
si:ch211-194e1.7	7.736428	0.025147393	0.640336	0.039272	0.968673	1
ppp1r15b	69.57967	0.452115308	0.260224	1.737406	0.082315	0.482739
spaca9	0	NA	NA	NA	NA	NA
ch25hl1.2	6.713944	1.632236544	0.991247	1.646649	0.09963	0.524166
si:dkey-24l11.2	187.1345	-0.02358042	0.160579	-0.14685	0.883253	0.986978
tnfsf11	14.48162	0.564769888	0.623979	0.905111	0.365407	0.815339
slc39a6	317.5168	0.010432542	0.148036	0.070473	0.943817	0.997371
nmba	12.2295	-0.324997929	0.710478	-0.45744	0.647358	0.927082
pimr180	0.137189	0	5.267649	0	1	NA
arrdc1a	199.7832	0.034925448	0.168448	0.207336	0.835747	0.976007
tprg1l	35.0388	-0.438045609	0.411	-1.0658	0.286513	0.757624

malt3	55.94412	-0.040752189	0.286098	-0.14244	0.886731	0.987668
chchd7	225.6267	0.273561594	0.192173	1.42352	0.154585	0.619425
ehmt1a	321.2074	0.145415226	0.147472	0.986056	0.324106	0.783161
si:dkey-16p6.4	1.059908	-0.887194369	1.882771	-0.47122	0.637486	NA
whrnb	36.08087	0.878750457	0.485355	1.810532	0.070213	0.452026
hes2.2	51.33585	-0.216558775	0.280203	-0.77287	0.439602	0.852079
ing5b	66.79452	0.242185415	0.211967	1.142562	0.253221	0.732759
uqcc1	874.7565	0.118311238	0.144615	0.818112	0.413293	0.838317
pak2a	1004.223	-0.029881183	0.116341	-0.25684	0.7973	0.966447
trib2	242.0293	0.016032941	0.212978	0.07528	0.939992	0.997371
dpep1	104.177	1.676052887	0.98751	1.697252	0.089649	0.50251
crb3b	138.3203	-0.388933567	0.229514	-1.6946	0.090152	0.50413
spg7	892.0329	0.014104664	0.107894	0.130727	0.895991	0.98926
pimr188	0.562612	1.928556693	2.773627	0.69532	0.486855	NA
cdh15	129.3641	-0.052910899	0.29363	-0.1802	0.856999	0.979558
taf4a	166.5342	0.03080499	0.167676	0.183717	0.854235	0.979058
tmem44	7.158736	0.498451852	0.764321	0.65215	0.514305	0.88631
klf9	842.6249	-0.621932921	0.400903	-1.55133	0.120823	0.566816
pld3	98.9513	0.198162825	0.234607	0.844657	0.398302	0.830737
def8	374.0225	-0.209404051	0.140641	-1.48893	0.136506	0.591902
fzd10	429.9258	0.097920301	0.172038	0.569178	0.569235	0.902303
ccni	5542.475	0.126185745	0.168326	0.749652	0.453464	0.858722
stx2b	143.7591	0.217167971	0.191778	1.132395	0.257468	0.735125
b3gnt2l	167.7269	-0.054155056	0.180426	-0.30015	0.764062	0.959228
mbl2	46.85024	-0.81180108	0.541713	-1.49858	0.133983	0.58783
MBL2 (1 of many)	28.39636	-0.065478534	0.37611	-0.17409	0.861791	0.980588
tmem150aa	64.72595	-0.164548021	0.246079	-0.66868	0.5037	0.881759
CRACR2A	31.21582	-0.051681019	0.324325	-0.15935	0.873394	0.983645
cbln12	27.75738	-0.273555027	0.678996	-0.40288	0.687035	0.938818
zgc:64051	46.03094	0.178897605	0.367662	0.486581	0.626555	0.920665
trim110	17.49529	-0.241332727	0.879151	-0.27451	0.783695	0.96245
cngb1a	72.53734	0.286779822	0.769386	0.372739	0.709343	0.945546
plcb3	388.8518	-0.222562087	0.162867	-1.36653	0.171774	0.643953
luzp2	234.4268	0.193251258	0.236538	0.816999	0.413929	0.838631
si:ch211-151h10.2	10.14223	-0.362878667	0.551712	-0.65773	0.51071	0.884139
nanos3	19.80448	-0.078805122	0.489111	-0.16112	0.872	0.983447
rwdd	267.6168	0.159106647	0.134754	1.180717	0.237715	0.718572
kcne4	43.95041	-1.014886732	0.443497	-2.28837	0.022116	0.263263
lrch4	285.3778	0.097009857	0.133723	0.725456	0.468172	0.866625
pros1	356.8857	-0.378597895	0.196654	-1.92519	0.054205	0.402725
vamp5	560.922	-0.169932597	0.156463	-1.08609	0.277441	0.751426
csf1b	107.1715	0.111539485	0.171152	0.651699	0.514595	0.88631
grhpra	100.4568	-0.208572884	0.246895	-0.84478	0.398232	0.830737
tomm5	1214.441	-0.160718068	0.202308	-0.79442	0.426949	0.845491
si:ch211-203b8.6	61.33931	-0.201885871	0.313561	-0.64385	0.519673	0.887148
ptx3a	1017.701	0.159775639	0.156797	1.018996	0.308205	0.773155
si:ch211-240b21.2	1.381041	-3.341239093	2.360627	-1.4154	0.15695	NA
GRB14	23.77595	0.118975134	0.394448	0.301624	0.762939	0.958842
cyp2x10.2	2.111777	-1.816622076	1.693149	-1.07292	0.283305	NA
slc7a4	28.91881	0.191763553	0.316474	0.605938	0.544556	0.895206
lamc2	40.04083	0.758435373	0.556007	1.364074	0.172544	0.645319
eif3k	1375.524	-0.288517799	0.125942	-2.29088	0.02197	0.262534
cyp2x12	30.61185	1.352669734	0.438612	3.08398	0.002043	0.070215
TMEM208	313.0494	-0.114244351	0.145406	-0.78569	0.432046	0.848419

SLCO3A1 (1 of many)	144.0033	0.282577216	0.195754	1.443535	0.14887	0.610726
zgc:171509	1.997476	0	1.993373	0	1	NA
mrps14	360.5222	0.14895965	0.164377	0.90621	0.364825	0.814824
astn1	513.563	0.217589413	0.20784	1.046909	0.295142	0.763815
si:dkey-21n10.3	0	NA	NA	NA	NA	NA
mmab	110.6682	0.054637422	0.185751	0.294144	0.768648	0.960395
si:ch211-132b12.3	0.15197	0.892446925	5.241767	0.170257	0.864808	NA
lmx1bb	222.4062	-0.041805835	0.131145	-0.31878	0.749896	0.955074
si:dkey-154p10.3	81.53801	-0.173165684	0.208418	-0.83086	0.406054	0.834645
nfkbie	211.1057	-0.086018655	0.18868	-0.4559	0.648464	0.927501
angptl2b	443.1425	-0.276268202	0.1241	-2.22617	0.026003	0.286077
ralgps1	23.84803	0.104644489	0.589795	0.177425	0.859174	0.979975
zgc:158427	0.128836	0	5.267649	0	1	NA
si:ch211-132b12.7	184.1143	0.470856913	0.778057	0.60517	0.545066	0.895206
foxg1c	20.36754	0.826343275	0.403489	2.047996	0.04056	0.351905
pglyrp5	23.93525	-0.011316262	0.380563	-0.02974	0.976278	1
slc6a18	19.65706	1.408863512	1.284004	1.097243	0.272535	0.748416
tns2b	391.3678	0.476690646	0.156234	3.051125	0.00228	0.07594
cep126	14.4711	0.499631093	0.660522	0.756418	0.449399	0.856979
znf131	415.1108	0.055362186	0.129548	0.427348	0.669126	0.933115
yap1	1552.947	0.021367758	0.095393	0.223998	0.822759	0.971966
tbcb	885.8827	0.153294048	0.090733	1.6895	0.091124	0.50549
six5	103.7453	-0.235795697	0.271971	-0.86699	0.385949	0.826939
six9	9.403354	0.417648631	0.669783	0.623558	0.532918	0.890978
vgll4l	254.0388	-0.164702158	0.196978	-0.83614	0.403074	0.833704
ptgesl	535.8643	0.164641133	0.163018	1.009959	0.312515	0.776164
foxl2b	79.31744	-0.145582606	0.239623	-0.60755	0.543488	0.894925
ttc9b	27.59676	0.587456082	0.3462	1.696869	0.089722	0.502623
si:ch211-153j24.3	12.46656	-0.233187829	0.63786	-0.36558	0.71468	0.945821
si:ch211-195h23.3	119.3343	-2.799452062	0.359712	-7.78248	7.11E-15	1.50E-11
si:dkey-56m19.5	2700.519	0.094507983	0.108638	0.869933	0.384337	0.826565
h3f3b.1	2248.738	0.040310405	0.118997	0.338752	0.734797	0.951361
si:ch1073-429i10.3	4662.603	0.009862915	0.085506	0.115348	0.90817	0.991745
gucd1	289.2619	-0.280624219	0.160502	-1.74841	0.080393	0.478844
CABZ01112732.1	6.840654	0.021513615	0.691319	0.03112	0.975174	1
tmem91	8.054635	1.242935116	0.945746	1.314238	0.188766	0.668514
tnnt3b	95907.71	0.155800725	0.143274	1.087433	0.276845	0.751311
otog	97.09914	0.426144861	0.22806	1.868567	0.061683	0.42588
aopep	163.9767	0.090035296	0.179259	0.502264	0.615482	0.916365
zgc:136254	8.01399	-0.569672922	0.894144	-0.63712	0.524049	0.888694
dnah3	12.81737	1.378243065	0.663418	2.077489	0.037756	0.340665
si:dkey-94e7.2	44.47477	-0.813816206	0.675802	-1.20422	0.228504	0.70974
gpx4a	12821.88	-0.131929876	0.187147	-0.70495	0.48084	0.870179
map7d2a	216.6409	0.130045951	0.200197	0.64959	0.515957	0.886651
neto1	34.84951	-0.899497296	0.763796	-1.17767	0.238929	0.719781
CABZ01100185.1	133.6353	0.133543279	0.18857	0.708189	0.478828	0.869292
cyp2y3	48.64565	3.741746985	1.334651	2.803539	0.005055	0.119017
gbgt1l4	407.2507	-0.076529712	0.156741	-0.48825	0.625369	0.920625
crybb1	7969.075	0.648754529	0.364781	1.778475	0.075326	0.465238
chs1	141.2278	0.451653572	0.468449	0.964146	0.334973	0.79181
hapln1b	374.9835	0.114207042	0.214409	0.532661	0.594268	0.910731
v2rh14	1.724132	-0.342408553	1.683494	-0.20339	0.838829	NA
BX530037.1	0.600148	1.055370274	3.992675	0.264327	0.791528	NA
si:dkey-47k20.4	1.315159	-0.914071679	1.624244	-0.56277	0.573593	NA

elovl8a	15.0603	0.842456969	0.726044	1.160339	0.245911	0.727194
htr5ab	22.47103	0.233558221	0.508921	0.458928	0.646286	0.926728
rbm33a	750.7568	0.171027471	0.156434	1.093291	0.274266	0.749612
olfcq19	0.04222	0	5.267649	0	1	NA
shha	239.0955	-0.023165618	0.191321	-0.12108	0.903626	0.991366
slc16a1b	415.7087	-0.080627299	0.148663	-0.54235	0.587579	0.907552
tmem51a	166.9327	-0.314000452	0.189478	-1.65719	0.097481	0.520785
olfcd3	0.918069	3.007987516	2.105657	1.428527	0.15314	NA
glis1a	14.8069	-0.518652289	0.635408	-0.81625	0.414357	0.838631
rnf44	75.38471	0.093738489	0.309526	0.302845	0.762008	0.958345
CU302253.1	81.70466	-1.235679711	1.237203	-0.99877	0.317907	0.77973
rabggtb	391.804	0.224868017	0.187184	1.201319	0.229627	0.711596
vimr1	526.1138	-0.33941913	0.463292	-0.73262	0.463787	0.864593
naa80	47.77054	-0.119631176	0.329587	-0.36297	0.716625	0.94627
CU326366.2	1.599564	-0.219832154	1.885375	-0.1166	0.907178	NA
cnot11	369.922	-0.003405143	0.17037	-0.01999	0.984054	1
atmin	39.27437	0.053349849	0.268957	0.198358	0.842765	0.9766
dalrd3	547.7754	-0.091195021	0.141038	-0.6466	0.517892	0.887148
si:ch211-181d7.3	10.78165	-5.554451477	5.141623	-1.08029	0.280012	0.752917
sptbn4a	139.0807	0.141817641	0.386082	0.367325	0.713377	0.945821
rab8b	448.6301	0.032291837	0.111287	0.290167	0.771689	0.960624
cd151l	554.7335	0.163084019	0.147217	1.107782	0.267956	0.74434
si:ch211-281l24.3	606.4269	0.021652934	0.258731	0.083689	0.933304	0.996472
lpar5b	4.934433	0.071315969	1.569149	0.045449	0.96375	0.999842
rsf1b.1	510.1795	0.221538077	0.109196	2.028811	0.042478	0.360968
taf10	681.9392	-0.024434628	0.137841	-0.17727	0.859299	0.979975
fam214b	597.9843	0.229054204	0.095938	2.387513	0.016963	0.230743
rhoga	6.302289	-0.170208865	0.638766	-0.26647	0.789881	0.964203
dnlz	110.7228	-0.054409562	0.194197	-0.28018	0.779342	0.961073
irgq2	18.63481	0.540036763	0.514037	1.05058	0.293451	0.76277
or104-2	0	NA	NA	NA	NA	NA
or105-1	0	NA	NA	NA	NA	NA
or106-1	2.831993	-0.096091078	1.3318	-0.07215	0.942482	0.997371
zgc:152951	19.14301	-0.524631274	0.486439	-1.07851	0.280804	0.753193
iqcg	8.074674	0.154456411	0.660023	0.234017	0.814972	0.970843
ctrl	14632.67	1.945129455	1.57688	1.233531	0.217378	0.69815
crfb1	65.28511	-0.260129345	0.270288	-0.96242	0.335841	0.792139
chmp2ba	19.32969	-1.198900201	0.538476	-2.22647	0.025983	0.285997
kctd4	190.6923	0.140798163	0.189168	0.744301	0.456694	0.860698
psenen	571.8403	-0.027989755	0.139364	-0.20084	0.840824	0.97643
gpr85	310.2268	0.07840225	0.24241	0.323428	0.746371	0.955074
lysmd4	189.0159	0.120055691	0.166476	0.721158	0.470812	0.867838
or108-1	0.712904	3.512983695	2.688535	1.306653	0.19133	NA
tmem168a	220.3874	-0.000796817	0.142538	-0.00559	0.99554	1
ifrd1	1526.118	0.260518103	0.160534	1.622821	0.104628	0.53308
fam174b	208.4879	-0.132019379	0.149572	-0.88265	0.377427	0.822478
nid1a	1601.702	0.278589773	0.183498	1.518216	0.12896	0.578173
crfb4	95.53371	0.157646612	0.269656	0.584621	0.558803	0.899409
si:dkey-14o1.20	0.322862	1.05539041	3.952386	0.267026	0.789449	NA
ksr1a	34.69162	0.55613716	0.344576	1.613976	0.106533	0.536018
cuedc1a	270.6257	-0.524451055	0.198595	-2.6408	0.008271	0.154797
gmnc	9.151513	0.328518211	0.489622	0.670963	0.502244	0.881277
masp1	168.1264	-0.294347071	0.167203	-1.76042	0.078337	0.47433
eif2ak2	24.23204	-0.604439855	0.395182	-1.52952	0.126135	0.574377

rxfp2l	10.10992	-0.899449558	0.816457	-1.10165	0.270614	0.746683
spry4	721.0411	-0.058282095	0.182658	-0.31908	0.749667	0.955074
cox5b2	247.0565	-0.211658935	0.268049	-0.78963	0.429745	0.847418
CR384085.1	0.659255	-1.806909736	2.608416	-0.69272	0.488483	NA
map4l	736.6369	0.13477046	0.280144	0.481076	0.630463	0.92291
si:ch211-250k18.5	3.774072	-0.014525936	1.165418	-0.01246	0.990055	1
exosc8	277.5883	-0.372195599	0.183773	-2.0253	0.042837	0.362408
si:ch211-244o22.2	360.1382	0.076587689	0.150481	0.508952	0.610786	0.915331
tnks1bp1	251.931	-0.203558479	0.2174	-0.93633	0.349102	0.801336
mespab	0.553879	-0.595450744	2.862235	-0.20804	0.8352	NA
plcg2	68.43599	-0.575669654	0.317344	-1.81402	0.069674	0.450548
sparta	42.11763	0.16911553	0.420875	0.401819	0.687817	0.938835
vsir	179.4087	-0.329004569	0.179306	-1.83488	0.066523	0.440383
slc6a17	50.11346	0.529696423	0.357237	1.48276	0.138138	0.594663
BX324132.1	0.875437	0.955145386	2.124743	0.449535	0.653046	NA
BX548044.1	0.429242	2.074396812	4.273253	0.485437	0.627366	NA
tlr7	0.981592	-0.150908506	1.919367	-0.07862	0.937332	NA
spata2l	92.20789	0.145983778	0.217695	0.670588	0.502483	0.881342
pimr149	0	NA	NA	NA	NA	NA
BX548044.2	0.221663	0	5.267649	0	1	NA
h2afva	1793.978	0.00819469	0.164849	0.04971	0.960353	0.999164
purba	1470.633	0.121420706	0.08727	1.391328	0.164126	0.633166
bloc1s3	32.27887	0.572079499	0.281475	2.032432	0.04211	0.358841
zgc:172139	45.50411	-0.029056056	0.284853	-0.102	0.918754	0.993884
dclre1b	237.5835	0.224996614	0.139948	1.607715	0.107898	0.539565
zgc:66024	10.53358	-0.162826761	0.646122	-0.25201	0.801036	0.96768
zgc:66024	2.072458	0.667208206	1.556256	0.428726	0.668122	NA
fam83ha	160.9343	0.137537956	0.187546	0.733355	0.463342	0.864408
rnf183	337.2116	-0.064295142	0.121744	-0.52812	0.597419	0.911953
si:ch211-163l21.10	17.74563	-0.203630188	0.431109	-0.47234	0.636684	0.924779
f2rl1.1	35.34236	0.164260396	0.349257	0.470314	0.638131	0.924779
gbp4	16.77619	-0.033114633	0.703394	-0.04708	0.962451	0.999461
il2rga	14.74345	0.664284396	0.673388	0.986481	0.323897	0.783161
si:dkey-98f17.5	60.29784	0.424442861	0.251939	1.684707	0.092045	0.507669
si:dkey-61p9.7	8.40287	-2.613806077	1.407593	-1.85693	0.063321	0.429493
nap1l4b	1075.916	0.037243426	0.129817	0.286893	0.774194	0.960694
si:ch211-250m6.4	0.644893	0	2.826244	0	1	NA
fance	71.64773	0.176970208	0.323747	0.546631	0.584632	0.906628
si:ch211-250m6.7	3.116562	1.055430612	1.928893	0.547169	0.584263	0.906412
zgc:153031	220.0963	-0.241257363	0.214728	-1.12355	0.261204	0.738737
tspan13a	193.3903	0.09716685	0.267937	0.362649	0.716867	0.94627
mrpl14	318.8921	0.356137923	0.172609	2.063266	0.039087	0.34567
klhl41a	118.0828	-0.320595645	0.223991	-1.43129	0.152348	0.616255
sostdc1a	210.3942	-0.526038893	0.206827	-2.54338	0.010979	0.182478
mettl5	274.8454	-0.013834312	0.139225	-0.09937	0.920847	0.994085
nrip1b	384.691	0.034774678	0.145266	0.239386	0.810806	0.969828
nos1	125.1001	0.278071693	0.282025	0.985981	0.324143	0.783161
zgc:153012	269.2219	-0.026970939	0.197519	-0.13655	0.891387	0.988566
si:ch73-42k18.1	1.877044	-1.501100135	1.332452	-1.12657	0.259924	NA
ppp1r35	26.33205	-0.213263494	0.361279	-0.5903	0.554988	0.898671
map2k2b	148.6254	0.379029548	0.189348	2.001765	0.04531	0.372707
rad51c	37.01935	-0.280598941	0.342846	-0.81844	0.413106	0.838274
umodl1	14.78391	-0.182976737	0.454849	-0.40228	0.687478	0.938835
si:ch211-137a8.2	65.79135	-0.283481776	0.247598	-1.14493	0.252239	0.732759

cyp1b1	48.0546	-0.241143757	0.383715	-0.62844	0.529713	0.890484
si:ch211-218m3.11	0.120883	0	5.267649	0	1	NA
xaf1	25.74488	0.020271405	0.621638	0.03261	0.973986	1
atp5meb	3162.352	0.093672154	0.13666	0.685438	0.493067	0.876715
zgc:113983	16.3971	0.145903805	0.578981	0.252001	0.80104	0.96768
BX897692.1	1.043795	-2.441912773	2.23664	-1.09178	0.274931	NA
si:ch211-264e16.1	118.2273	-0.358966675	0.244063	-1.4708	0.141346	0.600146
si:ch211-219a15.4	6.628281	0.784300375	0.974562	0.804773	0.420951	0.842494
frmd7	4.811906	-0.547800993	0.694274	-0.78903	0.430096	0.847541
nrip1a	309.7767	-0.257421157	0.148908	-1.72873	0.083858	0.486647
si:ch211-261n11.7	9.129477	-2.221789425	0.97095	-2.28826	0.022122	0.263263
zgc:153372	95.98677	-0.230051847	0.21433	-1.07335	0.283112	0.755028
zgc:153759	8.532844	0.390398334	0.706123	0.552876	0.580348	0.905618
pdlim4	254.7565	-0.144538989	0.17259	-0.83747	0.402328	0.833284
lim2.1	311.9336	0.372997332	0.261981	1.423756	0.154517	0.619425
bsx	40.38289	0.094209888	0.293521	0.320965	0.748237	0.955074
glo1	1638.8	-0.096120275	0.123281	-0.77968	0.435577	0.850467
glceb	435.6416	-0.010148349	0.122517	-0.08283	0.933985	0.996638
jhy	16.30241	-0.26554094	0.45882	-0.57875	0.56276	0.900772
btbd9	327.0052	0.125546832	0.139403	0.900605	0.367798	0.817078
gabra1	68.05262	0.425852394	0.270742	1.572908	0.11574	0.555893
zgc:158862	35.89328	1.871591052	1.032641	1.812431	0.06992	0.451012
hspa8	56840.57	-0.770093433	0.126142	-6.10498	1.03E-09	7.76E-07
zgc:153631	140.1711	0.22343628	0.202375	1.104072	0.269562	0.746029
pimr129	0.544138	-2.788832344	2.668452	-1.04511	0.295971	NA
h2afx1	6134.644	0.193709093	0.153928	1.258437	0.208234	0.69024
hist2h2l	1274.113	-0.105954428	0.167806	-0.63141	0.527773	0.889948
pcyox1l	252.6501	0.015027272	0.129992	0.115601	0.907969	0.991745
tle3b	862.8264	0.131955992	0.119512	1.10412	0.269541	0.746029
si:ch211-147a11.3	153.7673	-0.203418969	0.203078	-1.00168	0.316499	0.778879
eps8b	11.21169	1.608263958	0.816233	1.97035	0.048798	0.384446
ifnphi2	0	NA	NA	NA	NA	NA
prdx4	1085.27	0.15892811	0.117838	1.348702	0.177433	0.651026
nphp4	87.90402	-0.002012276	0.196814	-0.01022	0.991842	1
elnb	206.3887	-0.307351977	0.62235	-0.49386	0.621407	0.918461
cyp7a1	275.7736	-0.385807754	0.825569	-0.46732	0.640268	0.924877
adcy1a	9.111873	1.325817127	0.709148	1.869592	0.06154	0.425614
admb	140.0184	0.081360882	0.214624	0.379086	0.704624	0.943785
rxfp3.3a1	14.78053	0.266862535	0.556315	0.479696	0.631443	0.923003
acss2l	53.80883	0.886821033	0.492626	1.80019	0.071831	0.4564
skor1a	119.4788	0.20834778	0.231099	0.901553	0.367294	0.816459
plac8l1	58.77022	-0.510878445	0.286807	-1.78126	0.07487	0.464197
pimr51	0.127238	0	5.267649	0	1	NA
akip1	31.83301	-0.086976298	0.356676	-0.24385	0.807345	0.969736
si:ch211-242e8.1	48.83111	0.094803771	0.856636	0.11067	0.911878	0.992336
agpat4	145.1098	0.137183796	0.162965	0.8418	0.3999	0.831277
chtf8	122.6023	-0.15155209	0.196478	-0.77134	0.440503	0.852385
wu:fk65c09	2.313507	-0.426560299	1.45836	-0.29249	0.76991	0.960484
serpinf1	840.5323	0.206674689	0.160185	1.290229	0.196971	0.677235
ranbp3b	860.597	0.040662229	0.105535	0.385298	0.700017	0.942647
lgals9l4	0.800048	-0.868148328	2.805975	-0.30939	0.757023	NA
btg3	463.7877	0.030461449	0.132453	0.229978	0.818108	0.971518
nufip2	702.0592	0.211592508	0.14727	1.436765	0.150785	0.614977
cry3a	4509.137	0.146211944	0.152539	0.958521	0.3378	0.794808

mrm3b	88.81971	0.062298928	0.213505	0.291791	0.770447	0.960484
ints2	246.6619	-0.020383757	0.137143	-0.14863	0.881845	0.986908
agrp	58.77899	-0.000641405	0.365607	-0.00175	0.9986	1
atp6v0d1	1518.467	-2.45E-05	0.12051	-0.0002	0.999838	1
col2a1a	27122.57	0.427515449	0.200971	2.127254	0.033399	0.323127
gls2a	215.42	-0.045899429	0.300445	-0.15277	0.878579	0.985761
tmco1	538.2311	0.31201507	0.156507	1.993615	0.046194	0.375668
aldh9a1a.1	10212.7	-0.041542835	0.119012	-0.34906	0.727041	0.949095
napbb	87.89295	0.896046774	0.22967	3.901447	9.56E-05	0.008792
zgc:112962	216.8367	-0.423163128	0.146493	-2.88863	0.003869	0.103403
si:dkeyp-82a1.4	0.856564	1.055433907	2.197014	0.480395	0.630947	NA
fgfr4	1286.826	0.018935022	0.141589	0.133732	0.893615	0.988566
fam89b	150.3128	-0.471553552	0.236831	-1.9911	0.04647	0.376263
znrd2	406.1452	-0.039473934	0.143278	-0.27551	0.782928	0.962328
ikzf2	44.45132	-0.236502252	0.2709	-0.87302	0.38265	0.825435
dbn1	199.6704	-0.233867432	0.148691	-1.57284	0.115755	0.555893
unc93b1	38.91802	0.475982729	0.347044	1.371536	0.170208	0.641806
timm10	359.791	-0.039192385	0.145901	-0.26862	0.788219	0.963471
kcnh5b	18.38788	-0.70147699	0.615265	-1.14012	0.254235	0.733304
ppp2r5eb	817.3642	0.218596461	0.089776	2.434912	0.014895	0.213725
si:ch211-216l23.2	443.4618	-0.126007632	0.109816	-1.14745	0.251198	0.731476
fhdc3	141.1205	-0.147096995	0.161258	-0.91219	0.361671	0.811787
mstnb	29.60605	1.722774602	0.511699	3.366771	0.000761	0.036636
vwc2l	2.600045	-0.484041685	1.186611	-0.40792	0.683333	0.938241
ppp1r15a	1224.798	-0.053425688	0.22902	-0.23328	0.815544	0.971158
grik1a	106.2036	-0.076033588	0.381545	-0.19928	0.842045	0.97643
aars	3302.466	0.042183442	0.110782	0.380779	0.703367	0.943622
map3k7cl	39.53067	-0.259617992	0.329093	-0.78889	0.430177	0.847541
pex11b	109.6511	-0.185664227	0.215184	-0.86282	0.388238	0.827592
rit1	237.3121	0.018311889	0.150365	0.121783	0.903071	0.991243
tmem135	323.5948	0.324314969	0.191016	1.697845	0.089537	0.502318
tafa5a	112.1325	0.006176278	0.237282	0.026029	0.979234	1
si:dkey-73n10.1	202.0492	0.091433294	0.164799	0.554817	0.57902	0.905417
cerk	84.97674	-0.175298777	0.241979	-0.72444	0.468797	0.866951
gramd4a	23.93087	0.519464846	0.422277	1.230152	0.21864	0.699445
vps4b	1211.099	-0.00816714	0.082794	-0.09864	0.921421	0.994085
ubtd2	384.1845	0.024495209	0.171115	0.143151	0.886171	0.987533
celsr1a	412.1015	0.052978772	0.141981	0.373139	0.709045	0.945546
cyp27a1.2	6.639987	-2.544143114	1.064343	-2.39034	0.016833	0.230232
si:dkey-242h9.3	39.57406	1.772780891	1.266867	1.399343	0.16171	0.628908
zgc:172339	9.71127	-0.199539923	0.673849	-0.29612	0.767139	0.960296
zgc:163030	491.3819	0.093007279	0.29969	0.310344	0.756299	0.956532
mycbpap	14.30233	-0.230647481	0.507686	-0.45431	0.649605	0.927756
ebf1b	52.23196	0.48058045	0.278401	1.726217	0.084308	0.487271
zgc:172120	27.15677	0.990868309	0.390135	2.539812	0.011091	0.18325
taar15	0	NA	NA	NA	NA	NA
stx7l	99.24125	0.183783917	0.213105	0.86241	0.388462	0.827613
taar14i	0	NA	NA	NA	NA	NA
taar14l	0.97437	-1.482254003	1.751332	-0.84636	0.397353	NA
taar10	0.174762	0	5.267649	0	1	NA
c6ast1	5.501008	-1.695825857	0.855394	-1.98251	0.047422	0.379101
zgc:153738	19.05878	-0.301867126	0.39729	-0.75982	0.447365	0.856073
ttc32	147.6458	0.03471555	0.230667	0.150501	0.88037	0.986419
pdia5	484.7945	0.108276144	0.100449	1.077927	0.281066	0.753478

si:ch211-244b2.3	52.94094	-0.516269523	0.385455	-1.33938	0.180448	0.656148
matn3a	624.0326	-0.225754919	0.317231	-0.71164	0.476686	0.869292
rxfp3.3a3	6.857449	0.449707865	0.766296	0.586859	0.557298	0.899165
aftpha	462.798	0.011626492	0.113703	0.102254	0.918555	0.993884
uacaa	229.4566	-0.189285506	0.147283	-1.28519	0.198727	0.679447
zpax1	5.003262	0.361037725	0.822836	0.438773	0.660826	0.930581
mixl1	8.401208	-0.552190254	0.608125	-0.90802	0.363867	0.814255
chrm4a	92.50031	-0.015545411	0.361332	-0.04302	0.965684	1
rec8b	2.187096	-0.298859023	1.549969	-0.19282	0.847103	NA
metap2a	160.0371	0.429234512	0.228371	1.879545	0.06017	0.422294
erich3	19.49	0.610621502	0.523667	1.166049	0.243595	0.724437
si:ch211-196h16.5	120.1162	0.423821075	0.222897	1.90142	0.057247	0.413213
matn3b	148.2213	0.113436584	0.321587	0.35274	0.724283	0.948276
esrra	166.7351	0.044475423	0.22227	0.200097	0.841405	0.97643
wdr35	206.1673	0.035001075	0.172704	0.202665	0.839397	0.976417
si:dkey-182i3.11	1.482443	-3.939035572	1.965419	-2.00417	0.045052	NA
kbtbd4	129.1062	-0.009159247	0.182295	-0.05024	0.959928	0.999164
ighmbp2	98.47449	0.052233447	0.2314	0.225728	0.821413	0.971788
pimr132	0.049519	0	5.267649	0	1	NA
trmt5	133.2352	-0.521514139	0.191009	-2.73031	0.006327	0.134501
elovl7a	425.1397	0.19126309	0.139962	1.36654	0.171769	0.643953
tsen15	137.5132	-0.341186426	0.180152	-1.89388	0.058241	0.416878
pimr109	0.304838	2.072152707	3.994796	0.518713	0.603961	NA
bbc3	194.8809	-0.006382638	0.211265	-0.03021	0.975898	1
ercc8	264.9656	0.041969285	0.161441	0.259966	0.79489	0.965688
ndufaf2	204.4468	-0.027428663	0.185639	-0.14775	0.882538	0.986908
gabpa	552.5567	0.047263	0.110205	0.428865	0.668021	0.933086
bida	265.2275	0.139931936	0.189689	0.737691	0.460702	0.862374
acot16	115.7844	-1.664604167	0.359634	-4.6286	3.68E-06	0.000845
ahsg2	347.7416	0	1.693502	0	1	1
insl5b	6.29348	-2.503466349	1.311463	-1.90891	0.056274	0.410018
bcl6ab	263.7805	-0.050222704	0.174921	-0.28712	0.774023	0.960636
moxd11	88.99707	0.593762444	0.305736	1.942076	0.052128	0.396782
upf3a	317.758	0.167302307	0.181275	0.922922	0.356048	0.807063
osbpl9	626.918	0.064087728	0.093861	0.682794	0.494737	0.878016
tmem177	299.2976	0.253403866	0.135348	1.872245	0.061173	0.424852
snx9b	867.0013	-0.034196319	0.09096	-0.37595	0.706956	0.944895
si:ch211-203k16.3	14.24709	0.682015733	0.878214	0.776594	0.437398	0.851283
pomcb	6.173128	0.68926651	0.728507	0.946136	0.344079	0.798147
il15ra	2.911205	0.812986177	1.35424	0.600327	0.548289	0.896644
oxa1l	533.2221	-0.440717852	0.13507	-3.26288	0.001103	0.047146
tmem199	83.67689	-0.022003602	0.241857	-0.09098	0.92751	0.994973
efr3bb	67.36625	0.191086514	0.21726	0.87953	0.379114	0.823173
fam169b	10.26596	1.322737322	0.915532	1.444774	0.148521	0.610149
si:ch73-122g19.1	2.499079	1.99427455	1.837953	1.085052	0.277899	0.751841
vps53	312.356	-0.043957509	0.173758	-0.25298	0.800283	0.967327
mus81	125.2673	0.346954284	0.234994	1.476439	0.139826	0.598212
nkx3.3	90.58526	-0.277903037	0.264579	-1.05036	0.293553	0.76277
si:dkey-12e7.1	139.0635	-0.499052071	0.197234	-2.53025	0.011398	0.185329
drgx	50.85018	-0.240881746	0.426301	-0.56505	0.572039	0.903461
trip6	212.9863	-0.210617179	0.176717	-1.19183	0.233327	0.714569
tmem72	16.56274	0.345094351	0.578313	0.596726	0.55069	0.8974
FP102052.1	52.94468	0.600320951	0.333324	1.801014	0.071701	0.4564
zgc:114045	214.7455	-0.338391681	0.207383	-1.63172	0.102738	0.529448

bcl6b	262.4598	-0.374723485	0.188705	-1.98576	0.04706	0.378022
tbc1d24	502.9929	-0.082307285	0.152258	-0.54058	0.588799	0.908018
zgc:153115	485.4698	-0.001267503	0.247517	-0.00512	0.995914	1
adgrl2a	629.6816	0.127392028	0.143923	0.885143	0.37608	0.822258
ppp3r1b	1431.841	0.05318128	0.093122	0.571091	0.567938	0.901989
spa17	223.0471	0.114890267	0.224082	0.512716	0.60815	0.915204
CR847571.1	0.403336	1.055393839	3.764118	0.280383	0.779184	NA
zgc:153142	0	NA	NA	NA	NA	NA
nrn1la	75.96554	0.147070137	0.297418	0.494491	0.62096	0.918399
crcp	115.343	0.034520597	0.206556	0.167124	0.867272	0.982582
sh2d4ba	14.41973	-0.651787823	0.742327	-0.87803	0.379925	0.823607
zgc:162608	420.5812	-0.626761589	0.235417	-2.66234	0.00776	0.14885
tnfsf12	110.176	0.138879131	0.211061	0.658004	0.510536	0.884085
si:dkey-242g16.2	144.1482	-0.401514955	0.294573	-1.36304	0.17287	0.645664
pard6a	396.0787	0.159769601	0.124871	1.279474	0.20073	0.681045
zgc:158868	29.905	-0.073353365	0.398646	-0.18401	0.854008	0.978943
si:ch211-125e6.5	21.31128	0.291855664	0.653796	0.446402	0.655307	0.929606
mxtx1	1.292426	-1.012433727	1.919344	-0.52749	0.597854	NA
cnga4	22.72602	0.860024535	0.412201	2.086423	0.03694	0.337141
mtg1	131.1773	-0.137875455	0.187518	-0.73527	0.462178	0.863463
tmem88b	73.64934	-0.28145975	0.209605	-1.34281	0.179334	0.654677
itga2.1	9.631839	-0.412707853	0.879953	-0.46901	0.639061	0.924779
zswim8	1101.046	-0.054411001	0.108506	-0.50146	0.616049	0.916365
si:dkeyp-114g9.1	2.626052	-1.864564012	1.063197	-1.75373	0.079476	0.477246
lrrc4.1	398.1514	-0.039804949	0.222701	-0.17874	0.858144	0.979774
pln	77.58723	0.033847232	0.328395	0.103069	0.917908	0.993875
zgc:194990	39.06314	0.464454642	0.356518	1.302751	0.19266	0.672683
gkup	125.4934	-0.642969206	0.211522	-3.03973	0.002368	0.077821
cx34.5	4.245582	-0.191648933	0.860828	-0.22263	0.823821	0.972005
col17a1a	2410.852	0.321112381	0.199219	1.611858	0.106993	0.537
trim23	172.9103	-0.035483943	0.173429	-0.2046	0.837883	0.976301
grpel2	769.6534	0.103682519	0.114157	0.908248	0.363747	0.814144
nhp2	565.3395	-0.183872317	0.182649	-1.0067	0.31408	0.777479
tmie	61.66661	0.319565321	0.33268	0.960578	0.336764	0.793455
hsbp1a	552.6881	-0.055895414	0.186313	-0.30001	0.764171	0.959228
nkain2	64.44435	0.16619785	0.250172	0.664335	0.506476	0.883007
slbp2	2.240359	0.10314964	1.301323	0.079265	0.936822	0.997044
tp53i11a	130.4363	-0.11657614	0.186014	-0.6267	0.530853	0.890545
slc26a4	6.332179	-0.020473257	0.764543	-0.02678	0.978636	1
plscr3b	1870.913	-0.046462854	0.126645	-0.36687	0.713713	0.945821
tnk1	341.9235	-0.191479926	0.152982	-1.25165	0.210698	0.692347
cfap58	18.21219	-0.044137282	0.471623	-0.09359	0.925438	0.994815
neurl1aa	54.77884	0.032212438	0.284961	0.113042	0.909998	0.992208
ptgdsa	138.9537	-0.860706663	0.212488	-4.05062	5.11E-05	0.00569
dachd	487.3361	0.046003733	0.113995	0.403558	0.686538	0.938732
lpar6b	56.58069	0.154763441	0.258109	0.599604	0.54877	0.897065
pimr128	1.200191	0.974291111	2.062531	0.472377	0.636658	NA
dbh	36.2252	-0.447351782	0.386119	-1.15858	0.246626	0.727706
gja5b	11.25157	0.849791068	0.603835	1.407324	0.159331	0.625607
gja8a	34.05372	0.809717449	0.466085	1.737275	0.082339	0.482739
zgc:113314	99.99213	-0.117661391	0.225944	-0.52076	0.602537	0.914023
si:dkey-6n21.13	18.58038	0.333005944	0.452739	0.735537	0.462013	0.863402
rnaseka	602.0751	0.036028519	0.168854	0.213371	0.831037	0.974908
alox12	655.9839	-0.054378958	0.113772	-0.47797	0.632675	0.923581

cox7a1	197.2088	-2.270442227	0.603011	-3.76517	0.000166	0.013152
igsf9bb	21.63474	-1.139471372	0.349447	-3.26078	0.001111	0.047149
khdrbs2	261.3371	0.056286769	0.218647	0.257433	0.796845	0.966447
mhc1zca	203.7753	-0.201456087	0.301539	-0.66809	0.504074	0.881759
chsy3	626.2611	-0.020397076	0.136651	-0.14926	0.881345	0.986908
frem1a	713.4911	0.089093879	0.167869	0.530735	0.595603	0.91138
spint2	1741.33	-0.026164989	0.157075	-0.16658	0.867704	0.982728
slc13a3	77.22676	-0.30519807	0.274731	-1.1109	0.266613	0.743108
ghrh	1.74923	0.901060037	1.493549	0.603301	0.546308	NA
traf1	17.97726	-0.571646264	0.456263	-1.25289	0.210246	0.692011
dab2ipa	99.08004	0.087860165	0.261902	0.335469	0.737271	0.952312
spryd7b	185.3931	-0.000254556	0.212848	-0.0012	0.999046	1
tmem223	125.8165	0.08673908	0.176955	0.490176	0.624009	0.919497
tmem179b	53.24774	-0.853107979	0.334807	-2.54806	0.010832	0.181481
arl2	244.6579	0.232344543	0.202055	1.149906	0.250182	0.729991
sumf2	106.9146	0.076277905	0.185652	0.410866	0.681171	0.937331
phkg1b	169.9464	0.200340459	0.195118	1.026765	0.304531	0.769885
spag1b	38.10173	-0.639949282	0.310763	-2.05929	0.039467	0.34755
hs6st3b	4.003098	1.251850336	1.087865	1.15074	0.249839	0.729721
abhd11	119.549	0.012174349	0.184364	0.066034	0.947351	0.997371
cldnh	949.856	0.030330762	0.159192	0.190529	0.848895	0.978016
si:dkey-88e18.2	2.935886	0.065269626	1.123566	0.058091	0.953676	0.998539
zgc:136892	134.9455	0.15881237	0.207661	0.764768	0.44441	0.85448
mettl27	32.82682	0.309506437	0.31417	0.985157	0.324547	0.783308
bbs10	39.07676	0.273916504	0.293675	0.932721	0.350964	0.802224
aqp3b	31.66573	-0.054688761	0.399	-0.13706	0.89098	0.988566
vps29	775.7445	-0.060192318	0.120541	-0.49935	0.617532	0.916375
gpn3	108.5075	-0.177180431	0.200459	-0.88387	0.376764	0.822258
ube2g1b	636.3946	-0.018342403	0.126566	-0.14492	0.884771	0.987266
zgc:153981	192.0441	0.325470612	0.149416	2.17829	0.029384	0.304886
si:dkey-238c7.16	486.7168	0.021154331	0.144589	0.146307	0.883679	0.987014
gtf3c4	113.4235	-0.126272164	0.222448	-0.56765	0.570274	0.902481
si:dkey-30c15.2	40.49251	0.267510142	0.477901	0.55976	0.575643	0.904739
si:dkey-8e10.2	5.434695	-1.69223353	1.417379	-1.19392	0.23251	0.713923
galt	167.0967	0.376380932	0.147733	2.547705	0.010843	0.181533
atg12	167.7206	0.124406448	0.23209	0.536026	0.591941	0.909621
zgc:162780	69.00867	-1.743222913	0.335644	-5.19366	2.06E-07	8.60E-05
zgc:162396	271.5958	-0.194430685	0.190906	-1.01846	0.308458	0.773474
atoh7	63.39268	0.394795378	0.258128	1.529457	0.126151	0.574377
CR388165.1	1.892911	0.093648139	2.010292	0.046584	0.962845	NA
muc13a	73.07825	-0.168832449	0.292065	-0.57806	0.563221	0.90092
kcnh3	20.68689	-0.238260143	0.421856	-0.56479	0.572217	0.903461
zgc:153151	25.01065	-0.560181223	0.44615	-1.25559	0.209265	0.691286
mucms1	19.69953	-2.811389307	0.963224	-2.91873	0.003515	0.098113
zgc:114181	2251.45	0.122142125	0.182509	0.669239	0.503343	0.881759
tmem222a	233.3383	-0.131389799	0.137533	-0.95533	0.339409	0.795226
tmem150c	35.20223	0.124842136	0.351707	0.35496	0.722619	0.947632
kif13a	230.4223	-0.097879374	0.223623	-0.4377	0.661605	0.930742
si:ch211-214c7.4	410.3713	-0.089970982	0.121513	-0.74042	0.459044	0.861818
chrm5b	2.112235	0.069408224	1.386618	0.050056	0.960078	NA
zgc:109889	558.7796	0.167432583	0.183633	0.911778	0.361886	0.811787
snx30	236.8178	0.293641722	0.151653	1.936274	0.052834	0.399532
taok1a	629.4194	0.086412645	0.143651	0.601546	0.547477	0.896211
zgc:162331	47.4838	0.740420999	0.66962	1.105734	0.268842	0.744925

palm2	77.60322	-0.4643769	0.374382	-1.24038	0.214834	0.696864
ckmt2a	1671.405	0.366349509	0.519761	0.704843	0.480908	0.870179
atf7ip	1854.183	0.078446493	0.071653	1.094807	0.273601	0.749153
mrps18c	336.8994	-0.252883391	0.188698	-1.34015	0.180197	0.656017
si:ch211-255g12.8	8.860231	0.201734989	0.625068	0.322741	0.746891	0.955074
tat	3937.541	-0.676423755	0.253616	-2.66712	0.00765	0.147606
emp1	58.15448	0.705470903	0.335757	2.101132	0.035629	0.331406
vegfc	55.09565	-0.708778718	0.258159	-2.74551	0.006042	0.131072
tmem160	104.5646	0.060460741	0.218187	0.277106	0.781699	0.962077
ppp6r2b	69.58803	0.577516733	0.335131	1.723255	0.084842	0.488883
chrnb1	324.5981	-0.09534741	0.198975	-0.47919	0.631801	0.923003
si:dkey-121j17.6	49.92729	0.056727692	0.290898	0.195009	0.845386	0.976791
fgf11a	11.08736	-0.207467572	0.603623	-0.3437	0.731069	0.950056
adra2c	33.12179	-0.237949482	0.517347	-0.45994	0.645558	0.926728
maip1	262.8512	0.212699516	0.138046	1.540785	0.123369	0.570096
tyw5	159.2275	-0.173206145	0.189963	-0.91179	0.36188	0.811787
chid1	198.3635	0.003124509	0.177024	0.01765	0.985918	1
tmem230b	281.8624	-0.294086377	0.150147	-1.95866	0.050153	0.389949
her8.2	97.94925	0.495609856	0.233094	2.126223	0.033485	0.323151
nicn1	24.95039	0.81434043	0.364228	2.235796	0.025365	0.282825
pcgf6	71.86147	-0.11976425	0.22667	-0.52837	0.597246	0.911953
col7a1l	273.8803	-0.238066645	0.196901	-1.20907	0.226636	0.707798
si:ch211-261n11.5	270.4932	-0.104067033	0.134535	-0.77353	0.439207	0.852079
lrguk	12.33673	0.16009085	0.471592	0.339469	0.734256	0.951134
lgi2b	85.65321	-0.176684823	0.255234	-0.69225	0.488782	0.873964
efhb	6.161705	-0.867084173	0.920813	-0.94165	0.346372	0.799463
prmt6	136.7851	0.289982898	0.205469	1.41132	0.15815	0.623766
si:ch211-113e8.5	0.10996	0	5.267649	0	1	NA
cdc34a	761.2353	-0.027243258	0.13033	-0.20903	0.834423	0.975731
vwa10.2	5.78951	0.074198471	1.150122	0.064514	0.948561	0.997738
foxi2	28.5314	-0.21492628	0.306152	-0.70203	0.482663	0.871787
zbbx	6.399072	0.15006663	0.623332	0.240749	0.80975	0.969828
glis3	22.50717	-0.895364254	0.574026	-1.5598	0.118808	0.562134
rpz3	42.82324	0.783553964	0.311013	2.51936	0.011757	0.188516
mpg	29.25054	0.016483378	0.391841	0.042067	0.966446	1
mavs	138.9941	0.298502325	0.208189	1.433804	0.151628	0.615384
hbba2	3.212835	-0.190689721	0.971025	-0.19638	0.844313	0.976791
hbaa2	0.38778	-3.092001853	4.211471	-0.73419	0.462836	NA
pou4f2	417.263	0.10130855	0.208614	0.485626	0.627232	0.921095
fndc5a	7.772786	-1.679482589	0.723127	-2.32253	0.020204	0.251822
cul5a	191.4702	0.016578798	0.171159	0.096862	0.922836	0.994525
mos	16.61372	-0.126481339	0.5758	-0.21966	0.826134	0.973084
slc35f2	120.0691	-0.252672946	0.211989	-1.19192	0.233293	0.714569
capn5b	6.572877	1.050926402	0.814618	1.290084	0.197021	0.677235
ckba	817.3329	0.031688599	0.156525	0.202451	0.839564	0.976417
mtmr11	273.6866	0.084135426	0.142634	0.589871	0.555277	0.898723
si:dkey-189g17.2	10.97967	1.042357303	0.557656	1.869176	0.061598	0.425659
etv5a	617.1597	0.018478222	0.17316	0.106712	0.915018	0.993066
syngap1b	192.8897	-0.070583543	0.380323	-0.18559	0.852767	0.978779
caln2	9.862035	0.56296445	0.666865	0.844195	0.39856	0.830925
nectin1a	32.63621	-0.714515861	0.463814	-1.54052	0.123433	0.570142
gig2j	2.326594	-2.49170059	1.553395	-1.60404	0.108706	0.541209
nitr1b	0.805442	0	2.623392	0	1	NA
flot2b	763.4641	0.063830712	0.110835	0.575908	0.564678	0.901445

fbxo40.1	157.9937	-0.306421454	0.259445	-1.18106	0.237577	0.718568
pus7l	118.4822	-0.245521141	0.285272	-0.86066	0.389428	0.827843
rec114	0.517657	1.055406064	3.187285	0.33113	0.740546	NA
tmc6a	6.26257	5.031751169	1.408906	3.571388	0.000355	0.021766
dynll2a	786.3656	-0.203641521	0.132884	-1.53248	0.125405	0.573278
crygm2d5	1766.656	0.411256494	0.258692	1.589751	0.111891	0.548216
zgc:154058	54.43809	0.197914044	0.261131	0.757912	0.448504	0.856741
znf1124	231.1758	-0.035176356	0.277729	-0.12666	0.899212	0.99045
si:dkey-228l14.1	0.120883	0	5.267649	0	1	NA
crygm2d12	3355.815	0.215340299	0.389781	0.552465	0.58063	0.905618
si:ch211-198k9.6	16.49763	-1.190551965	0.514463	-2.31416	0.020659	0.255657
sstr2b	4.128914	1.314886895	1.181038	1.113332	0.265566	0.742247
zdhhc18a	254.1089	-0.201126681	0.165553	-1.21488	0.224411	0.706406
ostm1	282.1577	-0.161979611	0.16488	-0.98241	0.325899	0.784773
dnali1	32.27935	0.639051781	0.355625	1.79698	0.072339	0.457323
crygm2d17	10608.11	-0.041040408	0.296432	-0.13845	0.889886	0.98835
proca1	81.18512	0.475513032	0.203725	2.334096	0.019591	0.246694
crygm2d15	3359.064	0.418672661	0.359285	1.165295	0.2439	0.724507
crygm2d11	4099.516	-0.920943569	0.52547	-1.75261	0.079669	0.477652
si:ch211-117n7.7	170.5157	0.022783598	0.17857	0.127589	0.898474	0.990199
rragca	563.5059	0.124697348	0.109869	1.134965	0.25639	0.73401
sfxn4	185.4681	0.024396134	0.176714	0.138054	0.890198	0.98835
rhbdl2	228.6227	-0.003579149	0.155621	-0.023	0.981651	1
pnpla4	17.97181	-0.912133667	0.464306	-1.96451	0.049471	0.387507
CU571382.1	0.21379	2.079536253	5.22636	0.397894	0.690708	NA
kctd12.1	133.7196	0.214523612	0.302838	0.708377	0.478711	0.869292
acod1	44.0462	-0.083738705	0.393717	-0.21269	0.831571	0.974908
zgc:162944	1118.636	-0.251712276	0.168936	-1.48999	0.136228	0.591544
nitr1b	0.649459	1.055420296	3.128929	0.33731	0.735883	NA
mrpl30	656.1555	0.031010485	0.190018	0.163197	0.870363	0.983365
lipt2	36.42161	-0.197054577	0.356957	-0.55204	0.580921	0.905618
txndc9	1148.871	0.025699558	0.114482	0.224486	0.822379	0.971966
pnisr	1137.712	-0.089695538	0.1321	-0.679	0.49714	0.878609
coq3	270.7057	0.088466549	0.19493	0.453838	0.649946	0.927756
grxcr1b	13.11631	-0.245037346	0.576372	-0.42514	0.670736	0.933939
sox1a	278.7042	0.02946727	0.212043	0.138969	0.889475	0.988318
zgc:113030	70.36195	-0.157501661	0.2317	-0.67976	0.496653	0.878298
snu13a	393.6777	0.053491599	0.139089	0.384586	0.700544	0.942918
cldna	115.9779	0.445314822	0.232663	1.91399	0.055621	0.408467
sphk2	346.4439	0.198959261	0.118334	1.681342	0.092697	0.509435
CR392036.1	0.940775	2.233632971	2.060156	1.084206	0.278274	NA
pimr152	0	NA	NA	NA	NA	NA
CABZ01071903.1	145.6405	0.132271068	0.205162	0.644714	0.519112	0.887148
si:dkey-33c12.12	192.8915	0.036495299	0.163626	0.223041	0.823504	0.972005
gtf2f2a	490.73	0.126721058	0.120943	1.047772	0.294744	0.763708
hmga2	698.9226	0.116222549	0.185664	0.625985	0.531325	0.890833
ska3	181.7939	0.385266859	0.21994	1.75169	0.079827	0.478112
cox17	888.3111	0.372058796	0.174031	2.137889	0.032526	0.319448
pla1a	130.2432	0.071792799	0.256761	0.27961	0.779777	0.961073
hsd3b1	18.77013	-0.381131085	0.569852	-0.66882	0.503607	0.881759
prkceb	14.30534	0.160089688	0.496038	0.322737	0.746894	0.955074
CABZ01053976.1	136.236	0.435379457	0.221275	1.967593	0.049115	0.386041
parp4	225.5871	-0.110443363	0.202149	-0.54635	0.584827	0.906655
dnm2a	361.0401	0.211551289	0.193825	1.091454	0.275073	0.750424

ppap2d	650.7385	-0.00692776	0.10405	-0.06658	0.946915	0.997371
tdp2a	1.622628	0.511045011	1.449585	0.352546	0.724429	NA
p2ry13	11.90415	0.599123527	0.676567	0.885534	0.375869	0.822237
p2ry12	8.094556	-0.326386927	0.562307	-0.58044	0.561616	0.900673
itga6b	651.8908	0.02661298	0.199015	0.133723	0.893621	0.988566
eef1a1l2	174.3983	0.469356986	0.245294	1.91345	0.05569	0.408467
kcnq5b	56.0683	-0.174586122	0.320513	-0.54471	0.585954	0.906655
kcnq5a	27.41903	-0.315116986	0.412639	-0.76366	0.445068	0.855219
pdlim2	580.0844	-0.189016081	0.133175	-1.41931	0.15581	0.621042
zgc:162936	56.78421	-0.162965581	0.246896	-0.66006	0.509217	0.883523
sh2b3	28.76456	-0.264660588	0.34664	-0.7635	0.445164	0.855332
il21r.1	23.0721	0.238401097	0.37793	0.630808	0.528166	0.889948
alox5b.3	5.310239	0.770414611	0.772275	0.997591	0.318478	0.779923
pcsk7	376.3046	0.11379863	0.150215	0.757572	0.448707	0.856741
lingo2b	147.8515	0.575116376	0.226699	2.536921	0.011183	0.183794
myg1	945.6316	0.139431989	0.165311	0.84345	0.398977	0.831172
cd28l	198.1031	-0.394668384	0.187542	-2.10442	0.035341	0.330833
lman1	401.7688	0.245740923	0.12808	1.918657	0.055028	0.405919
cspg5a	1637.421	0.265743058	0.145611	1.825016	0.067999	0.44461
nkd3	0.99491	3.196586445	2.026697	1.57724	0.11474	NA
arid6	129.5238	-0.489278416	0.290489	-1.68433	0.092118	0.507702
bnc2	402.378	0.229577357	0.168265	1.364384	0.172447	0.645319
dpy19l1l	81.83479	0.149959432	0.29127	0.514846	0.606661	0.915204
dnajb14	132.8786	0.112608359	0.1517	0.742308	0.457901	0.860993
si:ch211-145b13.6	82.31171	-0.294200497	0.360309	-0.81652	0.414202	0.838631
txnipb	814.8612	0.010280204	0.380695	0.027004	0.978457	1
rcn1	501.6071	0.154009669	0.178075	0.864857	0.387117	0.827592
hce2l2	27.94721	-3.478896697	0.478103	-7.27646	3.43E-13	5.67E-10
sesn2	175.0656	-0.054970458	0.199594	-0.27541	0.783	0.962328
dmrt2b	33.4587	-0.762998295	0.349915	-2.18053	0.029219	0.304273
pimr173	0.266154	-0.868126796	4.123291	-0.21054	0.833245	NA
taf15	2941.437	0.151003935	0.143802	1.050079	0.293682	0.76281
cyp3c4	30.14447	-0.126583165	0.549274	-0.23046	0.817738	0.971441
dchs1a	196.2431	0.389353959	0.175187	2.222503	0.026249	0.287427
tmem41aa	203.4357	-0.270228035	0.170065	-1.58897	0.112067	0.548404
ehhadh	157.1877	-0.383525386	0.172059	-2.22903	0.025812	0.285195
rgs8	260.859	0.336874122	0.31172	1.080694	0.279833	0.752785
rbp2a	2414.579	0.230800806	0.218429	1.056638	0.290677	0.76078
AL935184.1	0.169362	-1.710071829	5.238473	-0.32644	0.744088	NA
zgc:162344	57.84586	-0.301975293	0.268987	-1.12264	0.261591	0.738975
zgc:153920	8.975473	0.786385713	0.712622	1.103511	0.269805	0.746434
dars	3382.59	-0.265186688	0.116555	-2.27521	0.022893	0.267028
celf4	134.7628	0.45731789	0.290122	1.576297	0.114957	0.554651
eny2	285.4509	0.096999881	0.171004	0.567237	0.570553	0.902481
rgs4	1075.219	-0.494193904	0.131252	-3.76523	0.000166	0.013152
zgc:173548	36.35208	0.034421282	0.321423	0.10709	0.914717	0.992972
sfrp2	429.4153	-0.055667877	0.191326	-0.29096	0.771084	0.960484
tdrd6	18.82311	-0.021989493	0.491584	-0.04473	0.964321	0.999925
foxg1d	79.56142	0.384823195	0.318516	1.208176	0.226979	0.708107
arfip1	315.5049	0.094333313	0.155783	0.605542	0.544819	0.895206
adora1a	1.182325	-1.809041922	2.538104	-0.71275	0.475998	NA
si:dkey-69o16.5	803.8383	0.602835206	0.279738	2.155001	0.031162	0.313784
si:ch211-262i1.5	78.96781	-0.076305959	0.224207	-0.34034	0.733603	0.950808
gfer	203.0962	0.328464299	0.20116	1.632852	0.1025	0.529149

si:ch211-163c2.2	2.768803	-1.129899517	1.046254	-1.07995	0.280165	0.752951
pitx3	232.1195	-0.224885977	0.160483	-1.40131	0.161122	0.627852
si:dkey-13m1.5	2.862964	2.073635721	1.453533	1.426617	0.15369	0.61823
lrp8	439.1525	0.144530043	0.184513	0.783307	0.433447	0.849658
actr2b	135.9053	0.159734171	0.194795	0.82001	0.41221	0.838123
abcb11b	98.6772	-0.637625665	0.664711	-0.95925	0.337432	0.794381
nbeaa	837.7387	0.275394079	0.131699	2.091084	0.03652	0.335935
rspo3	151.8649	0.046770187	0.192736	0.242665	0.808265	0.969828
atp5f1b	28650.66	0.060073436	0.102407	0.586616	0.557462	0.899184
phf10	254.334	-0.04061254	0.168939	-0.2404	0.810021	0.969828
mettl6	115.4901	-0.124799131	0.194808	-0.64063	0.521765	0.887551
KCNV1	4.259564	0.436937118	0.866872	0.504039	0.614234	0.915998
sytl1	77.43819	0.036376238	0.279804	0.130006	0.896562	0.98939
selenos	1249.493	-0.189726987	0.102038	-1.85938	0.062974	0.429313
lcorl	533.3994	0.394693482	0.126437	3.12165	0.001798	0.065518
si:ch211-168d1.3	25.85885	0.253893024	0.639668	0.396914	0.691431	0.940173
six7	401.378	0.298740993	0.268742	1.111629	0.266298	0.742912
dek	738.3878	0.103599541	0.10846	0.955186	0.339483	0.795226
ncapg	677.9288	0.381571819	0.1856	2.055881	0.039794	0.348912
opn5	31.80138	0.519924824	0.331677	1.567563	0.116983	0.557831
nit1	62.57324	-0.652633489	0.33261	-1.96216	0.049744	0.388069
ctc1	295.8836	-0.12969034	0.116481	-1.1134	0.265535	0.742247
rhoh	2.304343	-2.803002608	1.81355	-1.54559	0.122204	0.569366
stk11ip	370.7228	-0.465723233	0.18642	-2.49824	0.012481	0.194346
ptpn23a	471.2921	0.047141453	0.162126	0.29077	0.771227	0.960484
si:dkey-22l11.6	19.56265	1.51314058	0.583034	2.595287	0.009451	0.167036
nfxl1	221.7517	-0.074998693	0.189888	-0.39496	0.69287	0.940444
rpp25l	78.68984	-0.032422246	0.224017	-0.14473	0.884923	0.987266
aste1a	8.491215	1.670848866	1.39316	1.199323	0.230402	0.712099
si:dkey-84o3.3	1.064935	1.98531353	1.865732	1.064094	0.287286	NA
RETSAT	21.29258	0.473243715	0.457395	1.03465	0.300832	0.767015
si:ch211-191i18.2	102.1605	0.057120666	0.201583	0.28336	0.776901	0.961073
cart4	41.05027	0.189390365	0.4044	0.468325	0.639552	0.924779
magi1a	432.198	0.179787327	0.163755	1.097902	0.272247	0.748277
cygb2	130.051	0.568140157	0.261495	2.172663	0.029806	0.307479
jtb	388.4545	0.102185388	0.13237	0.771966	0.440135	0.852079
nfyc	1217.047	0.072041293	0.116837	0.616598	0.5375	0.892498
tuba8l3	1170.048	0.201077878	0.13429	1.497339	0.134305	0.588585
tgm2a	473.0261	0.705900085	0.288938	2.443088	0.014562	0.211168
vamp3	1463.211	-0.007453886	0.108618	-0.06863	0.945288	0.997371
stox2a	652.2745	0.193423184	0.12701	1.522893	0.127786	0.577117
ubr2	891.8009	-0.039509784	0.116751	-0.33841	0.735054	0.951517
tnfrsf1b	90.31561	0.588144825	0.338684	1.73656	0.082465	0.482825
zgc:153499	0.327109	-0.868131356	4.526148	-0.1918	0.847896	NA
sod3a	153.9042	0.24857008	0.26009	0.955709	0.339219	0.795226
si:ch211-225p5.8	108.7872	0.635451878	0.219922	2.889444	0.003859	0.103374
gria2a	517.7124	0.093554789	0.170317	0.549297	0.582802	0.90571
PLEKHH3	401.4195	0.167087965	0.136102	1.227672	0.21957	0.700438
zp3d.2	5.086138	-1.416936553	0.812139	-1.7447	0.081038	0.479801
dyrk1b	762.0023	0.156961173	0.175368	0.895036	0.370768	0.818484
si:dkey-13e3.1	0	NA	NA	NA	NA	NA
si:zfos-2070c2.1	0.652193	-1.936674715	2.594458	-0.74647	0.455386	NA
etnk2	17.59135	0.055481678	0.486454	0.114053	0.909196	0.992067
slc41a1	44.85124	-0.271131518	0.363775	-0.74533	0.456074	0.860359

si:ch211-120e1.1	0	NA	NA	NA	NA	NA
pou3f2a	235.503	0.099271668	0.140963	0.70424	0.481283	0.87045
fam43b	55.37121	-0.314176446	0.426291	-0.737	0.461122	0.862743
nhsa	66.09998	0.156629598	0.21737	0.720566	0.471176	0.86809
cdk6	70.30457	0.203651561	0.297317	0.684964	0.493367	0.876836
zgc:158258	45.92672	-0.612476293	0.612149	-1.00053	0.317052	0.778879
aldh1l2	289.5785	0.249743003	0.271015	0.921509	0.356785	0.807453
polh	198.7494	0.428866987	0.229079	1.872133	0.061188	0.424852
knl1	265.395	0.222433709	0.237478	0.936652	0.348938	0.801306
suz12a	303.4789	0.049282345	0.124039	0.397314	0.691136	0.940173
crlf3	603.233	-0.304317421	0.110761	-2.74751	0.006005	0.131072
spock3	1264.921	0.092034825	0.123321	0.746301	0.455486	0.860008
tut4	63.25566	-0.013857276	0.225486	-0.06146	0.950997	0.99801
casp8l2	25.58399	-0.631431171	0.417496	-1.51243	0.130426	0.581523
mettl14	134.0143	0.208554905	0.200853	1.038346	0.299109	0.766018
hist1h2a3	14.06251	0.317109442	0.527368	0.601306	0.547636	0.896211
sft2d3	279.8537	-0.143417222	0.15142	-0.94715	0.343564	0.798147
si:ch211-113a14.22	3.984606	-2.228960279	1.34933	-1.6519	0.098555	0.521133
CR354435.1	5.228048	1.227368472	1.067376	1.149893	0.250188	0.729991
BX942819.1	3.960864	1.095747737	1.047266	1.046294	0.295425	0.763988
cald1a	606.5672	0.009993032	0.137682	0.07258	0.94214	0.997371
irge4	8.625113	0.07012425	1.055076	0.066464	0.947009	0.997371
arl8bb	90.40266	-0.057747682	0.205361	-0.2812	0.778556	0.961073
si:dkey-245p14.4	84.16576	0.240432758	0.215114	1.117701	0.263695	0.741708
clrn2	46.73312	0.486731873	0.373126	1.304472	0.192073	0.672283
muc5.1	561.5688	0.183469281	0.275488	0.66598	0.505424	0.882293
hoxc1a	131.0034	0.083603721	0.226444	0.369203	0.711976	0.945821
hoxc4a	162.1513	0.095136843	0.145648	0.653195	0.51363	0.88631
hoxc3a	398.878	0.124602844	0.119225	1.045108	0.295973	0.764246
hoxc5a	144.3795	-0.124746571	0.217754	-0.57288	0.566727	0.901739
or134-1	2.522397	0.84023887	1.294873	0.648897	0.516405	0.886895
hoxc6a	170.8832	0.072258198	0.153278	0.471419	0.637342	0.924779
or137-3	3.737526	0.904975501	1.213922	0.745497	0.455971	0.86033
hoxc8a	235.2792	-0.002030558	0.143588	-0.01414	0.988717	1
hoxc10a	80.3709	-0.148560311	0.218731	-0.67919	0.497016	0.878572
hoxc11a	123.215	0.220533612	0.296893	0.742806	0.457599	0.860993
hoxc12a	179.9104	0.052934039	0.179626	0.29469	0.76823	0.960296
hoxc13a	118.1015	-0.117252062	0.168027	-0.69782	0.485292	0.872911
cx39.4	29.69795	-0.201229996	0.375996	-0.53519	0.592518	0.909996
smim12	247.6451	0.121883341	0.153156	0.79581	0.426142	0.844714
inka1a	301.7101	-0.287064096	0.194783	-1.47377	0.140545	0.599658
cx30.9	11.77697	-0.519192599	0.676297	-0.7677	0.442666	0.853879
casp6l2	0.707961	0	2.451119	0	1	NA
kat7a	269.6489	0.07746331	0.147908	0.523728	0.600468	0.913756
ccl35.2	0.410346	0	4.327352	0	1	NA
krtcap2	558.8851	0.083471563	0.153088	0.545252	0.58558	0.906655
nudt7	53.93235	0.031003591	0.307867	0.100705	0.919785	0.993892
foxf2b	67.61893	-0.687286438	0.24097	-2.85217	0.004342	0.108751
flad1	318.4007	-0.084264489	0.12284	-0.68597	0.492732	0.876558
tspan4b	404.6348	-0.334583623	0.131146	-2.55124	0.010734	0.180622
tlr19	2.27755	-0.429981783	1.333604	-0.32242	0.747134	0.955074
COQ10A	148.0198	0.409692502	0.202262	2.025556	0.04281	0.362408
uroc1	578.9023	-0.135369798	0.285122	-0.47478	0.634944	0.924272
serpinb1l2	21.29262	-0.243449675	0.443701	-0.54868	0.583225	0.905947

alg2	149.4648	-0.120689879	0.196505	-0.61418	0.539094	0.892985
mala	0	NA	NA	NA	NA	NA
wdyhv1	0	NA	NA	NA	NA	NA
inka1b	188.4225	-0.367524618	0.157074	-2.33982	0.019293	0.244673
abitram	113.0974	-0.343128021	0.235859	-1.4548	0.145724	0.604984
ccnd2b	79.49312	-0.212020753	0.236606	-0.89609	0.370203	0.818484
mtmr7b	342.0234	-0.173636791	0.180363	-0.9627	0.335696	0.792139
si:dkey-61f9.1	1.578303	-0.868149393	2.337623	-0.37138	0.710354	NA
zgc:162816	146.3184	-0.411730597	0.198837	-2.0707	0.038387	0.343542
nkd3l	0	NA	NA	NA	NA	NA
zgc:153157	45.98292	0.810833248	0.345724	2.345322	0.019011	0.242726
dll4	207.238	-0.072531448	0.167628	-0.43269	0.665238	0.932595
chac1	0	NA	NA	NA	NA	NA
s100v1	678.9396	0.046267005	0.114149	0.405321	0.685241	0.938486
si:dkey-222b8.1	113.2286	-0.118526256	0.217167	-0.54578	0.585214	0.906655
chtopb	14.57607	-0.729746065	0.435529	-1.67554	0.093828	0.511703
fip1l1b	842.3504	-0.031084362	0.111824	-0.27798	0.781031	0.961659
ino80	385.0428	-0.227957233	0.218207	-1.04468	0.29617	0.764246
vps18	358.2527	-0.124028509	0.131109	-0.946	0.34415	0.798147
rhov	232.3319	-0.314989097	0.153161	-2.05659	0.039726	0.348523
vapb	804.8048	0.150062124	0.125648	1.194305	0.232359	0.713741
rpl22	12174.58	-0.293955366	0.131146	-2.24143	0.024998	0.280216
atp6v1c2	17.56302	0.091028912	0.591171	0.153981	0.877625	0.985488
zdhhc17	146.6761	0.137875859	0.158483	0.869973	0.384315	0.826565
si:ch211-113g11.6	412.0549	0.096400135	0.296018	0.325656	0.744684	0.95491
slc39a9	343.1835	-0.036480319	0.162877	-0.22397	0.822777	0.971966
grk4	627.518	-0.000533143	0.127931	-0.00417	0.996675	1
tspan5b	237.7914	0.122544332	0.145796	0.84052	0.400617	0.831725
saraf	298.4753	-0.002393842	0.147058	-0.01628	0.987012	1
gch1	380.2936	-0.080502344	0.190458	-0.42268	0.67253	0.93414
pla2g12a	161.0421	0.387651846	0.23433	1.654299	0.098067	0.521071
tmem245	150.653	-0.510022684	0.189611	-2.68984	0.007149	0.143159
zgc:153292	117.7006	-0.031474981	0.194518	-0.16181	0.871455	0.983365
zgc:154061	39.0533	0.089289635	0.276042	0.323464	0.746344	0.955074
e2f3	250.2538	0.030418841	0.221006	0.137638	0.890526	0.988446
si:dkey-13n23.3	10.60992	0.826845553	0.632095	1.308104	0.190838	0.670774
lactbl1b	65.78517	1.233671456	0.582575	2.11762	0.034207	0.326435
sh3rf1	388.5833	0.193733905	0.126423	1.532421	0.125419	0.573278
tarbp2	315.7735	0.044394467	0.143731	0.308871	0.75742	0.957364
arf5	2128.848	0.020153746	0.125561	0.160509	0.87248	0.983447
parp6b	291.184	-0.00342199	0.179664	-0.01905	0.984804	1
khdrbs1b	3133.403	-0.024487307	0.074914	-0.32687	0.743765	0.95491
dnajc2	212.1376	0.018174609	0.179878	0.101038	0.91952	0.993892
amn1	97.9781	-0.352473406	0.288112	-1.22339	0.221182	0.702457
tspan13b	170.9079	-0.214630223	0.202617	-1.05929	0.289468	0.760499
agr2	1408.339	0.370447692	0.149271	2.481713	0.013075	0.198789
zgc:195001	2304.418	0.260368517	0.092955	2.801017	0.005094	0.119231
cenps	80.23532	0.184594794	0.229254	0.805197	0.420706	0.842383
rbp7b	63.09993	0.008823199	0.333377	0.026466	0.978886	1
zgc:110783	157.2312	0.186153092	0.213545	0.871727	0.383357	0.825809
si:dkeyp-75b4.7	0.344739	-0.863085617	4.488111	-0.1923	0.847503	NA
hpcal4	208.5117	0.333570172	0.243004	1.372697	0.169847	0.641525
pdgfra	1073.038	0.19171523	0.157457	1.217572	0.223387	0.70531
FRMD7	6.843022	-1.573093314	0.851859	-1.84666	0.064797	0.434674

bicc1b	89.83675	-0.236596315	0.259509	-0.91171	0.361923	0.811787
phyhiplb	106.1305	0.013068001	0.203077	0.06435	0.948692	0.99777
si:dkey-31e10.5	12.09448	1.007250587	0.666043	1.512291	0.13046	0.581523
trpc5a	4.588823	0.974737018	1.083075	0.899972	0.368135	0.817272
trpc4a	54.61572	0.333329068	0.275466	1.210054	0.226258	0.707597
si:dkey-183j2.10	311.7726	0.726486664	0.415805	1.747183	0.080606	0.479386
cnpy3	203.5575	0.09636665	0.146625	0.657232	0.511032	0.884394
brpf3a	214.9304	0.043748374	0.170787	0.256158	0.797829	0.966447
cacna1i	9.329382	-0.925416398	0.829665	-1.11541	0.264675	0.742063
zc2hc1c	8.388243	-0.588252804	0.582586	-1.00973	0.312627	0.776164
ripply2	0.908689	-0.9087579	2.002688	-0.45377	0.649995	NA
creb5b	28.46592	-0.585389042	0.39476	-1.4829	0.138102	0.594663
stmn2b	1361.96	0.15528116	0.16922	0.917627	0.358814	0.80995
hey1	650.8842	-0.055333001	0.122536	-0.45156	0.651583	0.9286
arf3a	186.8059	0.565554895	0.211178	2.6781	0.007404	0.145771
si:dkeyp-97e7.9	11.31323	-0.079717308	0.506136	-0.1575	0.87485	0.984191
mafbb	476.2377	-0.464206223	0.182088	-2.54936	0.010792	0.181199
grin2ab	213.4177	-0.26070812	0.310367	-0.84	0.400908	0.83183
top1l	1964.354	0.467408067	0.112624	4.150169	3.32E-05	0.004139
msgn1	1.390194	-0.66123949	1.494225	-0.44253	0.658106	NA
rnmt	494.7011	0.038365031	0.10463	0.366673	0.713863	0.945821
ldlrad4a	310.9128	-0.094734368	0.16373	-0.5786	0.562858	0.900772
si:ch211-93g23.2	37.02711	0.296237826	0.467586	0.633547	0.526377	0.889515
nap1l4a	1926.39	0.0537741	0.140392	0.38303	0.701698	0.943292
adap2	57.52342	0.294347486	0.298028	0.987651	0.323324	0.783129
cadpsb	949.1133	0.053199914	0.213912	0.2487	0.803593	0.968722
atad5a	291.8299	0.360468398	0.137335	2.624729	0.008672	0.159845
ppp4ca	381.2386	-0.088052548	0.112744	-0.78099	0.434806	0.850002
inavab	121.0805	-0.303559222	0.236478	-1.28367	0.199257	0.679739
mapk3	1291.854	0.027239917	0.080239	0.339483	0.734246	0.951134
fam131bb	31.12472	0.080072362	0.366134	0.218697	0.826886	0.973458
edn2	29.14294	-0.221332935	0.294113	-0.75254	0.451724	0.857676
ggact.3	100.7658	0.954161705	0.308015	3.097777	0.00195	0.068449
ggact.1	234.7499	0.87039073	0.272862	3.189861	0.001423	0.056314
itpk1b	194.467	-0.121507564	0.192761	-0.63035	0.528463	0.88999
clic5b	357.1396	0.356908911	0.193003	1.849242	0.064423	0.433491
mrpl35	271.4544	0.208493027	0.180566	1.154666	0.248227	0.728489
nt5c1ab	18.48829	0.621512328	0.582969	1.066116	0.286371	0.757624
prelp	256.9352	-0.88784686	0.260962	-3.40221	0.000668	0.034114
eno4	20.18958	-0.192625764	0.401737	-0.47948	0.631595	0.923003
dyrk3	1055.635	0.078663703	0.124435	0.632167	0.527278	0.889948
RASSF5	216.739	0.238171649	0.148484	1.604023	0.108709	0.541209
hspa12a	83.26658	0.315447061	0.226526	1.39254	0.163759	0.632171
zgc:162509	18.77161	0.906201225	0.518288	1.748453	0.080386	0.478844
b4galnt2.2	1.952831	0.632144465	1.544756	0.40922	0.682378	NA
ikbke	113.507	0.069666238	0.240413	0.289777	0.771987	0.960624
mrpl36	583.0186	-0.013349384	0.132609	-0.10067	0.919815	0.993892
zgc:152986	0.23516	2.53946606	4.85586	0.522969	0.600996	NA
vhl	146.5679	0.23894762	0.199268	1.199125	0.230479	0.712099
tatdn2	3.53627	1.216931994	1.083953	1.12268	0.261573	0.738975
grin2db	36.31543	0.27722367	0.355772	0.779216	0.435852	0.850596
FAM72B	226.5885	-0.041624365	0.132729	-0.3136	0.753821	0.955912
med10	584.2378	-0.020024485	0.155245	-0.12899	0.897368	0.989719
cacng7b	37.15458	0.608562143	0.763189	0.797394	0.425223	0.844714

enpp5	145.7965	-0.085298451	0.208614	-0.40888	0.682627	0.938006
cacng8b	233.4619	0.263173034	0.230649	1.141013	0.253865	0.732873
pimr60	0.049519	0	5.267649	0	1	NA
ttpal	328.2945	-0.066731062	0.141485	-0.47165	0.637178	0.924779
prkcdb	732.5628	-0.051903286	0.121901	-0.42578	0.670266	0.933809
pex5	360.008	0.000558887	0.141247	0.003957	0.996843	1
si:ch211-69g19.2	946.4455	0.116036066	0.236042	0.49159	0.623009	0.918894
pa2g4b	3117.958	0.048446878	0.148015	0.327312	0.743432	0.954856
zbtb12.2	142.763	0.163320264	0.168059	0.971801	0.331149	0.788766
si:ch211-182e10.4	0.200823	-0.748301491	5.238472	-0.14285	0.886411	NA
rhol	47.36161	2.989455533	1.31	2.282028	0.022488	0.265534
cd4-1	3.056126	-2.603838415	1.645224	-1.58266	0.113498	0.551541
cxcr3.3	191.8236	-0.133248061	0.227976	-0.58448	0.558895	0.899409
crip2	614.1789	0.095378998	0.105363	0.905238	0.365339	0.815339
slc2a9l1	1.389614	-0.868143444	2.207362	-0.39329	0.694102	NA
ptgr2	153.8384	0.048633341	0.21495	0.226254	0.821004	0.971788
psmd6	2025.621	0.170358242	0.121638	1.400539	0.161352	0.628218
fam3a	320.6619	0.098796737	0.155365	0.6359	0.524842	0.888928
ifnphi3	1.551709	0.823058949	2.11194	0.389717	0.696746	NA
fezf2	152.464	0.187744513	0.281061	0.667985	0.504143	0.881759
c1qtnf12	201.7654	-0.012514592	0.271071	-0.04617	0.963177	0.999842
sdf4	744.8272	0.048086951	0.126152	0.381183	0.703067	0.943622
dkk3b	134.457	0.844309919	0.212167	3.979461	6.91E-05	0.006898
CR759887.1	1.586114	0.077524332	1.667125	0.046502	0.96291	NA
cdipt	393.645	0.152585099	0.186414	0.818528	0.413056	0.838274
ncalda	845.9581	0.042401872	0.122221	0.346927	0.728646	0.949787
si:dkey-80c24.1	0.79395	-0.673217679	2.180243	-0.30878	0.757488	NA
nxph3	5.088033	0.104000096	0.807342	0.128818	0.897502	0.989819
kbtbd8	165.1595	-0.147199185	0.13998	-1.05157	0.292995	0.762508
s100v2	920.6602	0.060678975	0.132111	0.459302	0.646017	0.926728
thap7	181.6787	0.266190081	0.146028	1.822876	0.068322	0.445546
zgc:64022	55.54781	-0.551594519	0.297405	-1.85469	0.063641	0.430571
wu:fi42e03	2.027868	-1.531301395	1.308809	-1.17	0.242003	NA
prss60.3	30.04779	3.178760196	0.812395	3.912826	9.12E-05	0.008627
prss60.1	1.212926	-0.231101929	1.563556	-0.14781	0.882496	NA
zgc:173544	0.291188	-0.771890306	4.642984	-0.16625	0.867961	NA
slc25a18	170.456	0.004282328	0.20319	0.021076	0.983185	1
vdrb	173.5913	-0.182249507	0.293666	-0.6206	0.534862	0.891993
ndufa9a	2210.103	0.04054486	0.116457	0.348152	0.727726	0.949297
chrna4b	35.31754	-0.667086541	0.59356	-1.12387	0.261067	0.738663
cnga3a	69.20236	1.962205671	0.531868	3.689273	0.000225	0.015984
gabra5	92.30588	0.721100165	0.376331	1.916131	0.055348	0.407377
dyrk4	236.7649	0.335834382	0.185247	1.812898	0.069848	0.450824
rnd2	239.9035	0.583203929	0.298712	1.952398	0.050891	0.392525
pimr89	0	NA	NA	NA	NA	NA
tmem45a	232.8123	-0.111637593	0.124878	-0.89397	0.371337	0.818945
sft2d2a	66.86116	0.160444188	0.270498	0.593143	0.553086	0.897809
ccr11.1	2.617292	-1.522292547	1.322506	-1.15107	0.249705	0.729721
si:ch211-155i14.1	1.203083	-1.449282488	1.699765	-0.85264	0.393861	NA
foxg1a	260.5348	0.26686321	0.167427	1.593909	0.110956	0.54583
her4.3	110.9418	0.685284253	0.235168	2.914016	0.003568	0.099143
irgf1	7.462893	-0.737115462	1.061476	-0.69442	0.487416	0.873291
cyp2x9	84.49846	-0.802651224	0.557589	-1.4395	0.150008	0.613319
rln3a	27.04545	0.774326051	0.9118	0.849228	0.395754	0.829389

gjd2b	101.679	-0.075652039	0.285731	-0.26477	0.791189	0.964766
znf770	430.6269	0.051957418	0.115684	0.449133	0.653336	0.929269
jupa	2377.533	-0.141708765	0.070738	-2.00329	0.045146	0.372256
lrrc15	98.47437	0.144065513	0.177591	0.811222	0.417238	0.840937
greb1	93.06577	-0.024647092	0.217881	-0.11312	0.909934	0.992208
eaf1	271.3696	-0.049884486	0.164704	-0.30287	0.761986	0.958345
pmvk	38.61211	0.03614005	0.308504	0.117146	0.906744	0.991644
zgc:109744	114.4514	-0.101634304	0.180109	-0.56429	0.572554	0.903533
opa1	927.8601	0.057453805	0.098594	0.582731	0.560075	0.899738
znf516	484.2955	0.003543905	0.161388	0.021959	0.982481	1
ccka	64.59546	-0.755733086	0.397682	-1.90034	0.057388	0.413587
si:ch211-156l18.6	0.046941	0	5.267649	0	1	NA
pax7b	283.0316	-0.075787773	0.220309	-0.34401	0.730842	0.950056
cnp	1244.425	-0.122505222	0.1094	-1.11979	0.262803	0.740207
ndufb5	1620.801	-0.048321143	0.124597	-0.38782	0.69815	0.942433
bpgm	530.0793	0.144153206	0.132743	1.085957	0.277498	0.751426
actl6a	1080.635	0.099706794	0.120058	0.830487	0.406264	0.834645
zgc:136858	234.4459	-0.174570186	0.217751	-0.80169	0.42273	0.843493
rftn1a	17.65206	0.368887427	0.482783	0.764085	0.444816	0.85495
snrka	563.6509	0.054949932	0.142592	0.385365	0.699967	0.942646
lin52	146.5457	-0.042917248	0.181881	-0.23596	0.813461	0.970328
taf13	635.977	0.427929119	0.172331	2.483175	0.013022	0.198789
tnnc2	80343.1	0.020330994	0.136534	0.148908	0.881626	0.986908
evi5b	238.8772	-0.39887442	0.160973	-2.4779	0.013216	0.200006
arid3a	163.8357	-0.446286349	0.180854	-2.46766	0.0136	0.203563
gamt	12596.56	-0.539721125	0.189208	-2.85253	0.004337	0.108751
si:dkey-56d12.4	34.04557	-1.173240651	0.386254	-3.03748	0.002386	0.078183
dazap1	139.3306	0.142655083	0.198039	0.720339	0.471316	0.86809
trim35-20	14.97591	0.119837053	0.619773	0.193356	0.84668	0.977214
rps15	24776.32	-0.338473748	0.127372	-2.65737	0.007875	0.150059
si:ch211-282j17.10	0.477875	0	2.87599	0	1	NA
si:dkey-88n24.6	0.093881	0	5.267649	0	1	NA
zgc:195077	4.211317	0.763536038	1.276946	0.597939	0.549881	0.897373
si:dkey-32e6.6	46.29932	0.134851823	0.36876	0.36569	0.714596	0.945821
si:busm1-105l16.2	26.03761	-0.741524069	0.412929	-1.79577	0.072532	0.457864
bcl6aa	174.5416	-0.071985564	0.232029	-0.31024	0.756376	0.956532
tmem11	828.1067	0.042181194	0.106991	0.394248	0.693398	0.940668
smx5	0	NA	NA	NA	NA	NA
cfap126	34.78479	0.285636655	0.387014	0.738053	0.460482	0.862369
ccl25b	678.2949	-0.238181817	0.171003	-1.39285	0.163665	0.631915
asmt2	124.1114	0.032921766	0.209456	0.157177	0.875105	0.984191
AL953867.1	0.150467	-2.324715954	5.202574	-0.44684	0.654991	NA
BX510941.1	2.672808	-4.378505463	1.502976	-2.91322	0.003577	0.099143
si:ch211-262h13.5	44.5234	-0.868161036	1.689765	-0.51378	0.607409	0.915204
abhd6b	14.40548	-0.337085192	0.594918	-0.56661	0.570981	0.902501
si:ch211-262h13.3	185.9932	0.049471312	0.169254	0.29229	0.770065	0.960484
met	126.1963	-0.106396834	0.231117	-0.46036	0.645259	0.926728
sox2	852.9197	0.247618172	0.11315	2.188401	0.02864	0.301362
dusp6	2037.187	-0.098017306	0.143386	-0.68359	0.494235	0.877583
dnajc27	190.1734	-0.088544171	0.154306	-0.57382	0.566088	0.901739
kitlga	322.1022	-0.131156826	0.119851	-1.09433	0.273809	0.749234
si:ch211-284e20.8	368.5282	-0.31961169	0.219045	-1.45911	0.144534	0.603539
cpne5b	20.80035	0.020171998	0.498389	0.040474	0.967715	1
cnbpb	4317.965	0.183927494	0.091728	2.005131	0.044949	0.371348

zgc:158296	362.539	0.315862072	0.240947	1.310919	0.189885	0.669635
sox14	183.0683	0.12063347	0.238694	0.50539	0.613285	0.915998
cart1	6.158166	2.316538802	1.472073	1.573657	0.115567	0.555766
si:ch211-232m10.6	48.78292	0.393796614	0.60869	0.646958	0.517659	0.887148
hcrt	8.116484	1.166372231	0.778091	1.499017	0.133869	0.58766
znf740a	432.1284	-0.087590661	0.112724	-0.77704	0.437138	0.851125
igfbp6a	3.92114	0	1.906578	0	1	1
styk1a	71.41227	-0.461899979	0.2703	-1.70884	0.087481	0.49631
hmga1b	947.334	0.238673006	0.192124	1.242286	0.214131	0.696474
fam131ba	81.09019	0.618886222	0.216057	2.864452	0.004177	0.10683
pla2g4f.2	0.714527	2.262191246	2.319502	0.975292	0.329416	NA
hmx2	101.6547	0.320009443	0.205787	1.55505	0.119934	0.564812
hmx3a	137.0292	0.120271895	0.162982	0.737947	0.460547	0.862369
im:7160594	253.6021	-0.371153373	0.179529	-2.06737	0.038699	0.344605
pex6	96.92755	-0.136093055	0.204338	-0.66602	0.505398	0.882293
si:ch211-288g17.3	4908.414	0.234341677	0.144607	1.620537	0.105117	0.533762
si:ch211-288g17.4	91.67782	-0.092778039	0.515019	-0.18014	0.857039	0.979558
lepr	414.3306	0.006115725	0.169974	0.03598	0.971298	1
si:rp71-15k1.1	1.496158	0	2.242247	0	1	NA
insl5a	15.4758	0.860030062	0.588561	1.461241	0.143949	0.603144
furinb	279.4803	0.313354818	0.230814	1.35761	0.174588	0.648164
si:ch211-81a5.8	196.3075	0.172466619	0.295562	0.583521	0.559543	0.899571
si:dkey-88e12.3	1.116909	-1.396793765	1.992089	-0.70117	0.483197	NA
ash1l	1122.855	0.095556579	0.178974	0.533913	0.593402	0.910427
mrps21	322.1177	-0.17307221	0.244713	-0.70724	0.479415	0.86985
sergef	68.80316	-0.018465226	0.273006	-0.06764	0.946075	0.997371
mlpha	322.1952	-0.60348266	0.199182	-3.02981	0.002447	0.07941
inpp4ab	58.60918	-0.22256276	0.298215	-0.74632	0.455476	0.860008
nuggc.1	2.599087	-0.11743887	1.181962	-0.09936	0.920853	0.994085
abhd12	375.5623	-0.229365774	0.184892	-1.24054	0.214776	0.696864
ppp1r3ca	186.186	-0.402196898	0.243063	-1.6547	0.097985	0.521071
pcgf5a	58.79823	-0.30510937	0.387613	-0.78715	0.431195	0.848102
kif20ba	201.9791	0.309176267	0.238291	1.297475	0.194468	0.674826
tuft1b	163.0155	-0.138216154	0.242258	-0.57053	0.568317	0.902146
cdk5r2a	543.1431	0.379724106	0.297049	1.278322	0.201136	0.681045
ifit14	9.747488	0.534272434	0.851653	0.627336	0.530439	0.890545
arl6ip6	50.48735	0.679732009	0.353934	1.920507	0.054794	0.405097
s100u	891.1434	-0.120829922	0.122798	-0.98397	0.32513	0.783981
pbxip1a	576.9085	0.331344969	0.154381	2.146283	0.03185	0.317164
nt5e	275.6748	-0.181860196	0.19403	-0.93728	0.348615	0.801264
ptena	1995.111	0.070712717	0.099682	0.709381	0.478088	0.869292
si:dkey-42l16.1	0.465482	-1.713176346	4.083567	-0.41953	0.674829	NA
papss2a	467.2505	-0.471657817	0.199108	-2.36886	0.017843	0.236422
zgc:171679	374.0241	-0.328237253	0.266066	-1.23367	0.217326	0.69815
dytn	12.94985	0.898331533	0.506684	1.772962	0.076235	0.467585
mllt11	2895.486	-0.006211937	0.208917	-0.02973	0.976279	1
si:dkey-147f3.4	146.1732	0.490764872	0.216531	2.266486	0.023422	0.270528
snx1a	2697.538	-0.01993494	0.079368	-0.25117	0.801681	0.967776
pnkd	193.079	-0.201836764	0.180922	-1.1156	0.264593	0.742063
si:dkey-286j15.3	275.5913	0.44998214	0.269249	1.671247	0.094673	0.513797
pex13	297.2116	-0.148907517	0.117445	-1.26789	0.204836	0.686735
smim8	266.3746	0.237448042	0.222515	1.067109	0.285923	0.757383
cx28.8	47.05158	-0.093693134	0.295609	-0.31695	0.751282	0.955285
zgc:153722	15.05936	0.255817169	0.469673	0.544671	0.58598	0.906655

bbip1	289.1048	0.058541756	0.176693	0.331318	0.740404	0.953939
ccn6	180.189	-0.028091766	0.33023	-0.08507	0.932208	0.996053
si:dkey-119m7.4	59.57271	-0.033240328	0.277291	-0.11988	0.904582	0.991581
cbx6a	484.5101	0.097069655	0.139104	0.697819	0.485291	0.872911
si:dkey-150i13.2	33.89707	0.574544239	0.269134	2.134788	0.032778	0.32041
ZNF512B	18.243	-0.552052597	0.496999	-1.11077	0.266666	0.743163
gtpbp2b	118.2496	0.097698285	0.207752	0.470265	0.638166	0.924779
naa10	1185.637	-0.12794335	0.136159	-0.93966	0.34739	0.800035
hps5	386.4246	-0.21933826	0.144407	-1.51889	0.128791	0.578173
g6pd	499.5011	-0.157826705	0.113226	-1.39391	0.163344	0.631199
pthlhb	1.513472	0	2.260325	0	1	NA
rccd1	72.99993	-0.398636392	0.297505	-1.33993	0.180268	0.656111
ldhbb	347.4299	0.046170432	0.323532	0.142707	0.886521	0.987533
p4ha1b	683.0299	0.230583755	0.281364	0.819522	0.412489	0.838123
si:dkeyp-34c12.1	11.05137	-1.391733018	0.985374	-1.41239	0.157835	0.623766
wipf1b	366.1376	-0.144558374	0.135785	-1.06461	0.287051	0.757955
adgrg7.2	0.973199	0	2.63908	0	1	NA
gpr155b	55.5022	0.106470905	0.350892	0.30343	0.761563	0.958237
slc35b4	338.4113	0.096240933	0.110139	0.873811	0.382221	0.825191
adgrg7.1	0.350948	0	4.440764	0	1	NA
actn2b	328.1844	0.519472487	0.204025	2.546116	0.010893	0.181956
chrm3a	34.78008	0.417118114	0.52582	0.793272	0.427619	0.845893
abi3bpb	973.5346	-0.072175761	0.116415	-0.61999	0.535267	0.891993
si:ch73-367f21.4	126.0087	-0.443082385	0.209088	-2.11912	0.03408	0.326054
si:dkey-222p3.1	35.5592	-0.895993304	0.876871	-1.02181	0.306872	0.772019
wnt7bb	79.82421	-0.006211643	0.215019	-0.02889	0.976953	1
xirp2a	825.4212	0.130404387	0.188109	0.69324	0.488159	0.873611
eepd1	280.632	0.004275271	0.192958	0.022156	0.982323	1
zgc:64065	17.91204	0.199708649	0.46547	0.429047	0.667889	0.933086
si:dkey-208m12.2	39.48345	0.917767061	0.488691	1.878009	0.06038	0.422591
cica	682.6235	-0.169856538	0.193884	-0.87607	0.38099	0.824157
pick1	97.55674	-0.252768433	0.237308	-1.06515	0.286809	0.757911
sft2d2b	89.94415	-0.009012626	0.233663	-0.03857	0.969232	1
alkbh1	54.18478	0.039646717	0.254492	0.155788	0.8762	0.984891
zmp:0000001138	1.005503	0	3.058996	0	1	NA
slc12a10.2	188.1373	-0.360543486	0.358097	-1.00683	0.314016	0.777479
pimr73	0.418251	0	4.099753	0	1	NA
cx40.8	16.64199	-0.817157928	0.512783	-1.59358	0.111031	0.545965
cavin2b	1606.351	-0.209884752	0.101596	-2.06588	0.03884	0.345058
usp40	299.234	0.064218017	0.158521	0.405106	0.685399	0.938486
fopnl	60.33237	0.052874473	0.29498	0.179247	0.857743	0.979774
sptssa	297.5707	0.021848072	0.148611	0.147015	0.88312	0.986924
tmem170b	8.092134	1.234704668	0.636923	1.938547	0.052557	0.398274
wnt4a	88.80909	0.105085495	0.202233	0.519626	0.603324	0.914226
oprl1	99.38339	-0.597079131	0.325161	-1.83626	0.066319	0.439788
zgc:112255	397.4678	0.193502818	0.21148	0.914994	0.360195	0.811057
p3h1	359.0441	-0.053531957	0.146155	-0.36627	0.714165	0.945821
rgl3a	198.4793	-0.375513852	0.211773	-1.77319	0.076198	0.467585
palm3	135.2283	-0.026498861	0.159817	-0.16581	0.868309	0.982801
si:ch211-133n4.9	20.79696	0	1.700607	0	1	1
hs6st3a	8.641275	-0.027768024	0.636238	-0.04364	0.965188	1
zgc:158445	17.82329	0.489305375	0.786205	0.622364	0.533703	0.891181
si:ch211-133n4.4	1100.051	0.111735663	0.136674	0.817535	0.413623	0.83842
lrfn5a	21.33379	0.415630415	0.573965	0.724139	0.468981	0.867083

zgc:158254	126.7341	-0.159984516	0.203534	-0.78603	0.431848	0.848419
si:ch211-117c9.5	434.3315	0.103156258	0.173626	0.59413	0.552425	0.8974
ftr58	12.8671	-0.956542602	0.575833	-1.66115	0.096684	0.519038
trim35-39	90.98534	0.031465006	0.270789	0.116197	0.907496	0.991745
tmem79a	52.55792	-0.166228	0.337311	-0.4928	0.622151	0.918461
ppp1r18	126.4166	-0.561682745	0.289908	-1.93745	0.05269	0.398963
fbxo34	977.1691	-0.116686997	0.103202	-1.13067	0.258195	0.735569
zmp:0000001139	8.037595	-0.080031408	0.930905	-0.08597	0.931489	0.995809
si:zfos-452g4.1	69.44449	0.22248753	0.263013	0.845918	0.397598	0.830253
ncoa6	864.2565	0.065347837	0.161446	0.404765	0.68565	0.938486
samhd1	120.3651	-0.220903134	0.182982	-1.20724	0.227341	0.708598
tonsl	102.4348	0.324183993	0.217233	1.492336	0.135611	0.591012
chrm3b	0.2609	0	5.267649	0	1	NA
tbc1d2	111.5808	0.01497781	0.17703	0.084606	0.932575	0.996175
gip	35.04025	-2.757120498	1.42597	-1.93351	0.053174	0.400175
si:dkey-7l4.5	6.151592	-3.241472626	1.397988	-2.31867	0.020413	0.253602
cbarpb	52.71187	-0.025642448	0.362159	-0.0708	0.943553	0.997371
ryr3	3947.452	6.85E-05	0.359126	0.000191	0.999848	1
si:ch211-212d10.2	153.6678	-0.04192862	0.185652	-0.22585	0.821322	0.971788
mif	469.1927	-0.056548728	0.191714	-0.29496	0.768022	0.960296
borcs8	139.2467	-0.155469855	0.21878	-0.71062	0.477319	0.869292
abch1	99.72409	-0.259370131	0.185659	-1.39702	0.162407	0.629825
mgst2	213.2291	-2.561031747	0.194106	-13.194	9.51E-40	2.20E-35
aftphb	188.3044	-0.031728921	0.163867	-0.19363	0.846468	0.977213
si:ch211-235e9.8	2327.837	0.34157218	0.13101	2.607231	0.009128	0.163946
si:ch211-197g15.7	19.00201	-0.961708646	0.478818	-2.0085	0.04459	0.36964
map4k3b	112.1891	-0.063205027	0.219713	-0.28767	0.773598	0.960624
morn2	25.46982	-1.029335023	0.388705	-2.64811	0.008094	0.152599
si:ch211-197g15.10	0.418872	0	4.300054	0	1	NA
lrrtm2	193.7482	-0.114637025	0.238448	-0.48076	0.630685	0.923003
celf5a	27.70779	0.232753721	0.402947	0.577629	0.563514	0.901075
hsd11b1la	274.7681	0.182654652	0.170539	1.07104	0.284151	0.75523
micos13	856.7589	-0.17739916	0.188722	-0.94	0.347216	0.799992
si:dkey-7l4.11	0.133557	0	5.267649	0	1	NA
slc7a11	67.02813	0.454320459	0.301437	1.507181	0.131764	0.583645
si:dkey-21e2.3	1.550425	0	2.187724	0	1	NA
si:dkey-7l4.6	2.697509	-1.382162306	1.943568	-0.71115	0.476993	0.869292
foxq2	24.75047	0.060219744	0.399862	0.150601	0.88029	0.986408
camk2g1	2208.498	-0.129840975	0.103371	-1.25606	0.209093	0.691152
timm23a	1150.671	-4.63E-05	0.107262	-0.00043	0.999656	1
BX247870.1	0.463886	1.055420598	2.891469	0.365012	0.715102	NA
si:dkey-172o19.2	1.158471	0	2.393265	0	1	NA
oaz1a	5352.293	0.1598558	0.122327	1.306796	0.191282	0.671109
tepsin	89.29804	-0.742737987	0.26309	-2.82313	0.004756	0.114424
AL935279.1	0.04222	0	5.267649	0	1	NA
arl3b	1015.333	-0.096738528	0.136976	-0.70624	0.480037	0.870001
cks2	100.8756	0.268883882	0.205897	1.305915	0.191581	0.671651
mier2	263.5285	-0.006664224	0.134134	-0.04968	0.960375	0.999164
cybc1	134.8666	-0.070112734	0.182362	-0.38447	0.700631	0.942918
gna15.4	1.788488	0	2.013464	0	1	NA
gbx1	73.90181	-0.148000397	0.240356	-0.61576	0.538056	0.892498
ap3d1	623.0604	-0.278829971	0.125261	-2.22599	0.026015	0.286078
nat9	306.9193	0.000334114	0.136648	0.002445	0.998049	1
lrrc59	408.285	-0.045329582	0.156489	-0.28967	0.772072	0.960624

tdo2a	401.2576	-0.00059239	0.413268	-0.00143	0.998856	1
ptprc	59.09843	-0.152341483	0.282454	-0.53935	0.589645	0.908565
si:dkey-121a9.3	33.88144	-0.315691258	0.310823	-1.01566	0.309791	0.774621
myoz1b	5555.558	0.074092505	0.129674	0.571373	0.567747	0.901809
zgc:153659	0	NA	NA	NA	NA	NA
fam234a	34.11492	-0.125894799	0.356847	-0.3528	0.72424	0.948273
tdrd5	6.664282	-0.345428756	1.01178	-0.34141	0.732797	0.950617
crp1	11.26788	1.565513211	1.503634	1.041153	0.297805	0.765407
crp7	2.242775	-0.483629311	1.78098	-0.27155	0.785966	0.962605
crp6	0.644911	0.945525336	2.499597	0.378271	0.705229	NA
si:dkey-121a11.3	215.3885	-0.10607041	0.145386	-0.72958	0.46565	0.864997
si:ch211-234p6.5	113.8317	0.465427969	0.285695	1.629109	0.10329	0.530446
CU896602.1	0.130487	0	5.267649	0	1	NA
lama3	44.02247	-0.58900005	0.484507	-1.21567	0.224111	0.705812
zgc:154125	11.46487	-0.084674872	0.715531	-0.11834	0.905799	0.991631
lrrc39	278.0899	0.119144718	0.174244	0.68378	0.494114	0.877558
zbtb33	241.4057	0.115802612	0.150062	0.771699	0.440293	0.852192
mrpl54	369.331	-0.034658141	0.185268	-0.18707	0.851605	0.978548
znf618	132.8158	0.129096535	0.167918	0.768806	0.442009	0.853374
aox5	895.0284	-0.369042441	0.173825	-2.12307	0.033748	0.324589
lnx2b	329.8547	-0.514524755	0.144307	-3.56549	0.000363	0.022086
zgc:152948	83.88841	0.310599197	0.26069	1.191449	0.233477	0.714569
nrros	64.23846	-0.451499343	0.25657	-1.75975	0.07845	0.474337
fbxo45	263.8384	0.152387857	0.14241	1.070064	0.28459	0.755668
olfm3a	101.6043	0.375062182	0.356311	1.052625	0.292513	0.762033
fshr	0.175549	-1.791424827	5.232816	-0.34234	0.732092	NA
sult3st5	13.29763	0.648463692	1.460465	0.444012	0.657034	0.929939
zic6	140.4557	0.082249475	0.19811	0.41517	0.678017	0.936026
zic3	817.9263	0.199775214	0.104401	1.913531	0.05568	0.408467
fhl1a	243.5183	-0.398567565	0.22367	-1.78195	0.074758	0.464131
cxcl32b.1	2.339906	0	2.044489	0	1	1
fam207a	182.3312	-0.152423743	0.246826	-0.61754	0.536881	0.892421
hs6st1b	151.4129	0.055786344	0.178773	0.312052	0.755001	0.956283
mgat5	404.5475	0.112890858	0.116433	0.969575	0.332259	0.789602
krt222	304.0465	-0.098331728	0.189682	-0.5184	0.604177	0.91484
insrb	304.1512	0.162512284	0.20647	0.787097	0.431225	0.848102
si:dkey-42i9.8	2.663972	-1.495294447	1.097484	-1.36247	0.173048	0.645824
si:dkey-42i9.7	1.424733	0	2.702864	0	1	NA
josd1	453.5506	-0.347797638	0.127163	-2.73506	0.006237	0.133784
ecm2	378.0378	0.061796206	0.207218	0.298218	0.765537	0.959831
xrcc6	382.4775	-0.072105112	0.130974	-0.55053	0.581957	0.90571
zgc:171500	2.458197	0	5.267649	0	1	1
CU929149.1	0.45168	-0.868130581	3.142372	-0.27627	0.782344	NA
zgc:153675	420.939	0.00749403	0.163088	0.045951	0.96335	0.999842
fblim1	285.7964	0.146158368	0.190529	0.76712	0.44301	0.854063
dlx4b	73.279	-0.054226179	0.236443	-0.22934	0.818604	0.971679
mtus1a	150.7943	-0.163123729	0.250612	-0.6509	0.515111	0.886401
hnf4g	52.90953	-1.018275749	0.446099	-2.28262	0.022453	0.265534
ppp1cab	841.5019	0.242164032	0.105684	2.291405	0.02194	0.262532
TSTD1	377.6231	-0.257538568	0.221791	-1.16118	0.24557	0.727137
si:dkeyp-87e7.4	537.8022	0.109124472	0.11774	0.926828	0.354016	0.80504
lsm5	318.5423	-0.096264374	0.223452	-0.4308	0.66661	0.932664
si:ch211-222k6.3	72.54985	0.181547009	0.427006	0.425163	0.670718	0.933939
urad	80.11533	-0.205562095	0.221604	-0.92761	0.353611	0.804513

si:ch211-222k6.1	24.95587	-0.069269677	0.396927	-0.17452	0.861461	0.9805
gtf3ab	3.642692	-2.751531975	1.145182	-2.4027	0.016274	0.22539
stard15	121.8608	-0.440681735	0.212019	-2.07851	0.037663	0.340171
tgfbi	4882.505	0.224336633	0.123209	1.820786	0.068639	0.44686
BX539307.1	2.194691	-1.908281462	2.234458	-0.85402	0.393091	NA
si:dkey-253d23.2	71.96548	0.283457818	0.228713	1.23936	0.215212	0.697157
si:ch211-236g6.1	34.74856	1.257311599	1.015334	1.238323	0.215596	0.697157
aqp8a.2	120.9095	1.408229901	1.227286	1.147435	0.251202	0.731476
TIMM21	124.3821	0.107548559	0.191136	0.56268	0.573653	0.904248
netol1	83.11179	0.022587918	0.280319	0.080579	0.935777	0.996829
pvalb9	5023.499	0.149899592	0.19646	0.763002	0.445462	0.855403
si:ch211-156p11.1	15.87317	-0.919905239	0.810905	-1.13442	0.256619	0.734072
si:ch211-213a13.5	6.154342	-0.595296575	0.881751	-0.67513	0.499593	0.879603
ptgdsb.2	0	NA	NA	NA	NA	NA
BX511034.1	48.17709	-0.157991685	0.354109	-0.44617	0.655477	0.929606
BX511034.2	1.443698	-1.766016861	2.028476	-0.87061	0.383966	NA
drd1a	5.71448	0.446294177	1.133217	0.393829	0.693707	0.940668
zgc:171490	8.850645	-6.934274774	1.973392	-3.51389	0.000442	0.025327
si:dkey-19a16.7	60.60789	-9.591122769	1.43439	-6.68655	2.28E-11	2.65E-08
zgc:113298	24.89231	0.036852252	0.398185	0.092551	0.926261	0.994815
rrp8	116.1201	-0.003286161	0.246863	-0.01331	0.989379	1
si:ch73-256j6.2	0.226485	0	5.267649	0	1	NA
si:dkey-19a16.5	2.720982	-1.32918691	1.449256	-0.91715	0.359063	0.810078
si:dkey-19a16.4	34.34007	0.417252716	0.601068	0.694186	0.487566	0.873291
ywhag2	2146.271	0.422950694	0.159356	2.65412	0.007952	0.150851
si:rp71-36a1.3	4.40473	-1.365550052	1.052979	-1.29684	0.194685	0.67483
si:dkey-222f2.7	4.525597	-0.613878331	1.00949	-0.60811	0.543116	0.894925
smyd5	136.0032	0.188884563	0.234256	0.806317	0.42006	0.842383
tma7	1474.216	0.008851838	0.206763	0.042812	0.965852	1
nudt22	181.9544	-0.212438362	0.177393	-1.19756	0.231089	0.712582
ctdspla	890.2932	-0.080469749	0.142715	-0.56385	0.572857	0.903608
atcaya	410.1074	0.222954277	0.173722	1.2834	0.199352	0.679863
mydgf	439.8396	0.248269353	0.172683	1.437716	0.150515	0.6142
loxl5a	3.330523	-1.055399479	1.18243	-0.89257	0.372088	0.819592
zfr2	682.9492	-0.051486408	0.139085	-0.37018	0.71125	0.945821
rx1	331.9229	0.51716408	0.198244	2.608719	0.009088	0.163488
slco5a1a	84.38674	0.237665664	0.230113	1.032821	0.301688	0.767693
uqcrc2b	3702.546	0.151015291	0.111567	1.353581	0.17587	0.649413
prex2	265.3493	0.048976395	0.156132	0.313686	0.753759	0.955912
ndc80	441.6693	0.161710862	0.202064	0.800293	0.423541	0.843967
pimr193	0.043309	0	5.267649	0	1	NA
zgc:66433	933.8579	0.050776408	0.092578	0.548472	0.583368	0.905948
ids	97.89812	-0.130582858	0.23306	-0.5603	0.575277	0.904718
zgc:162941	0.111311	0	5.267649	0	1	NA
ppp1r9bb	109.0268	0.174261277	0.238186	0.73162	0.464401	0.864593
si:dkey-20i20.8	58.55733	0.03168516	0.266351	0.11896	0.905307	0.991631
si:dkey-20i20.2	68.0952	0.033730972	0.249151	0.135383	0.892309	0.988566
ankha	112.449	-0.227244552	0.261296	-0.86968	0.384473	0.826588
znf1167	36.20354	-0.233673437	0.276629	-0.84472	0.398268	0.830737
si:dkey-37o8.1	0	NA	NA	NA	NA	NA
si:ch211-207i20.3	81.4555	-0.211338813	0.262905	-0.80386	0.421477	0.842754
prlh2	16.54025	-0.346772432	0.705633	-0.49143	0.623119	0.918894
zgc:112977	26.72513	-0.289802342	0.327748	-0.88422	0.376576	0.822258
pimr46	0.427465	1.921877964	4.938949	0.389127	0.697182	NA

si:ch211-253p2.2	37.32636	-0.069073252	0.377931	-0.18277	0.854981	0.979161
eef1e1	395.4608	0.295500924	0.15814	1.868602	0.061678	0.42588
znf1153	42.51377	-0.051437109	0.271705	-0.18931	0.849848	0.97824
znf1180	41.03212	-0.084546238	0.324959	-0.26018	0.794729	0.965543
adarb2	20.0754	0.499691341	0.381834	1.308662	0.190649	0.670716
znf1162	31.30576	0.113843739	0.354189	0.32142	0.747892	0.955074
si:dkey-32n7.9	0	NA	NA	NA	NA	NA
nrn1a	257.9616	0.071557023	0.150372	0.475867	0.634169	0.924077
itgb1a	1569.25	0.040883403	0.141306	0.289326	0.772332	0.960624
si:ch73-134f24.1	0	NA	NA	NA	NA	NA
znf711	277.5974	0.064451124	0.121795	0.529176	0.596684	0.911709
glod5	268.9651	-0.306346729	0.234222	-1.30793	0.190895	0.670774
zdhhc15b	446.4232	-0.029881917	0.176872	-0.16895	0.865839	0.982395
rell2	117.5805	-0.158831621	0.223985	-0.70912	0.478251	0.869292
dhrs7cb	944.354	0.192371826	0.12346	1.55817	0.119193	0.562753
trmt112	276.8676	0.016783712	0.226397	0.074134	0.940904	0.997371
tgfb5	60.42827	0.319465311	0.329475	0.96962	0.332236	0.789602
pimr56	0.04222	0	5.267649	0	1	NA
arl9	300.0063	-0.208632709	0.170447	-1.22403	0.22094	0.702242
pcdh2ab1	4.474507	-1.691707302	0.94914	-1.78236	0.074691	0.46413
CR847851.1	0.616803	1.432421282	2.53834	0.564314	0.57254	NA
sec24b	675.5128	0.020036093	0.145603	0.137608	0.890551	0.988446
rasa1b	122.3448	-0.414789491	0.210165	-1.97364	0.048423	0.383181
adgra3	178.4612	-0.087301831	0.172978	-0.5047	0.61377	0.915998
tlr8b	4.443001	0.122923554	0.927545	0.132526	0.894569	0.988985
quo	670.8428	0.045794891	0.154537	0.296335	0.766974	0.960296
heatr6	146.6573	-0.060013558	0.179687	-0.33399	0.738388	0.952912
nav2a	531.431	0.222822634	0.12624	1.76507	0.077552	0.470757
mamdc2b	63.54757	-0.14367065	0.308041	-0.4664	0.640928	0.925364
frmd8	663.8987	-0.249996924	0.081205	-3.07858	0.00208	0.071289
col9a1a	5068.414	0.459375983	0.1851	2.48177	0.013073	0.198789
si:dkeyp-72g9.4	598.2337	0.273049072	0.198367	1.376486	0.168671	0.640045
rbfa	74.41068	-0.506566255	0.264494	-1.91523	0.055463	0.407706
cnksr2b	240.0196	-0.106257473	0.276676	-0.38405	0.700942	0.942918
zgc:171551	7.145447	0.291456408	0.722258	0.403535	0.686555	0.938732
fam149a	31.92792	-0.28221578	0.376932	-0.74872	0.454027	0.858944
usp7	1004.933	-0.010414555	0.15812	-0.06586	0.947486	0.997371
mmrn2b	76.96573	-0.01706188	0.232129	-0.0735	0.941407	0.997371
vav3b	96.16788	0.619314089	0.374096	1.655496	0.097824	0.521071
cpxm1a	431.9767	0.153321035	0.152372	1.006229	0.314305	0.777479
si:ch73-233k15.2	40.82155	0.244254968	0.40152	0.608326	0.542971	0.894917
si:dkey-250123.4	79.41254	0.847477315	0.366932	2.309631	0.020909	0.256868
smarcad1b	292.2388	0.126744404	0.130925	0.96807	0.333009	0.790126
zgc:194679	65.95299	0.066795346	0.247342	0.270053	0.78712	0.963065
nlrx1	257.9435	-0.059319496	0.177069	-0.33501	0.737619	0.952496
zbtb32	2.059478	-2.615756603	1.619438	-1.61523	0.106262	NA
gal3st3	64.52404	-0.12586503	0.291126	-0.43234	0.665495	0.932595
dok4	221.7188	0.08019593	0.154599	0.518735	0.603946	0.914603
myh14	493.6226	-0.026880508	0.182175	-0.14755	0.882696	0.986908
ftr95	1.361946	0	2.162922	0	1	NA
suds3	485.8341	-0.178795739	0.114415	-1.5627	0.118123	0.560498
dennd2c	194.1892	-0.094013862	0.179638	-0.52335	0.60073	0.913756
RAP1GDS1	22.93458	0.054332163	0.379723	0.143084	0.886224	0.987533
prss59.2	91496.95	-3.567687213	1.317888	-2.70712	0.006787	0.139038

slc25a37	30.67083	0.028869719	0.336409	0.085817	0.931612	0.995827
ip6k1	385.2116	0.277320332	0.15999	1.733362	0.083031	0.483619
si:dkey-1j5.4	41.3387	-0.704905493	0.54137	-1.30208	0.19289	0.672855
crygm2d18	5805.543	0.426910504	0.369899	1.154127	0.248448	0.728723
CACNA2D1	12.80087	0.964384827	0.468365	2.059045	0.03949	0.34755
taccl	220.3874	-0.254352757	0.196312	-1.29566	0.195093	0.67483
slc12a7a	255.5515	0.05011965	0.168903	0.296737	0.766668	0.960296
pdzrn3a	53.08077	-0.391986428	0.231535	-1.69299	0.090457	0.505009
myo19	47.66999	0.314220835	0.231927	1.354827	0.175473	0.649279
si:ch211-113j14.1	156.6855	0.22321779	0.250377	0.891527	0.372647	0.819953
gna13a	193.8065	0.558152196	0.170725	3.269298	0.001078	0.04626
tnni1d	549.4942	0.299909014	0.150486	1.992939	0.046268	0.375756
magi2b	16.29586	0.318559467	0.539015	0.591003	0.554518	0.898538
tbc1d12b	129.3832	-0.038161503	0.159244	-0.23964	0.810608	0.969828
tti2	84.98234	-0.148983635	0.246299	-0.60489	0.545252	0.895275
si:dkey-17e16.9	3.539225	-0.286781391	0.983082	-0.29172	0.770503	0.960484
si:dkey-288i20.2	26.77432	0.399976555	0.455283	0.878523	0.37966	0.823607
cmbl	518.3141	-0.002621576	0.196191	-0.01336	0.989339	1
si:ch73-303b9.1	0.944785	2.649256149	2.096074	1.263913	0.206261	NA
zmp:0000000735	19.40854	-0.695231763	0.452742	-1.5356	0.124636	0.572298
man1b1a	175.0659	0.148880286	0.184	0.809132	0.418439	0.841565
zgc:194210	15.57763	-0.11496719	0.497095	-0.23128	0.817099	0.971207
si:dkeyp-104h9.5	230.6939	0.119534319	0.141255	0.846228	0.397426	0.830253
cntnap5l	126.0479	-0.198178445	0.258904	-0.76545	0.444002	0.854304
WDR31	15.85602	0.35254599	0.53644	0.657195	0.511055	0.884394
thbs2b	205.691	0.152440981	0.23597	0.646019	0.518267	0.887148
or131-2	6.758704	0.534154352	0.864475	0.617894	0.536645	0.892421
scly	42.44419	-0.431130352	0.257134	-1.67668	0.093606	0.511703
pax1b	76.64987	0.523009227	0.226623	2.307836	0.021008	0.257618
zgc:174917	77.08121	1.137513792	0.519073	2.191432	0.028421	0.299922
znf1177	10.14129	-0.239239502	0.670573	-0.35677	0.721265	0.947343
LO017700.1	69.97837	-0.132510452	0.238828	-0.55484	0.579007	0.905417
RASGRF1	100.4181	0.765008625	0.310388	2.464683	0.013713	0.204799
senp2	505.2662	0.205649849	0.102158	2.013058	0.044108	0.367756
herc2	1638.478	0.033247939	0.204923	0.162246	0.871112	0.983365
myo9ab	428.5117	0.003318722	0.125174	0.026513	0.978848	1
si:dkey-85k7.10	3.109236	1.939869123	1.856256	1.045044	0.296003	0.764246
zgc:110843	583.9624	0.267967324	0.159399	1.681107	0.092742	0.509444
rbm15	387.8757	-0.247347628	0.159619	-1.54962	0.121234	0.567243
arhgef7b	506.3788	-0.005855314	0.133204	-0.04396	0.964938	1
hdac7b	22.05397	1.04427093	0.595552	1.753449	0.079525	0.477355
klf7a	41.55426	0.080399833	0.239367	0.335885	0.736958	0.952276
pex26	201.0683	0.022629935	0.154781	0.146206	0.883758	0.987014
kif13bb	135.6687	-0.109311431	0.238383	-0.45855	0.646555	0.926728
gas2l2	0.968356	0.107318227	1.607622	0.066756	0.946776	NA
tdp1	80.87717	0.03925628	0.20965	0.187246	0.851467	0.978548
rex1bd	238.5534	-0.166219236	0.184342	-0.90169	0.367222	0.816459
pdzrn3b	445.8742	-0.130020783	0.136296	-0.95396	0.340104	0.795345
gdpd2	276.5598	0.508556084	0.1462	3.478489	0.000504	0.027752
tpst1	614.6561	-0.018910955	0.134888	-0.1402	0.888504	0.988188
crygm2d6	2168.978	0.46873371	0.54663	0.857497	0.39117	0.827902
si:ch1073-165f9.2	33.80034	0.419374496	0.547025	0.766646	0.443292	0.854063
slc41a2b	279.8834	0.092368166	0.137859	0.670019	0.502846	0.881453
clstn3	951.2783	0.53370652	0.253901	2.102027	0.035551	0.331075

gdf10b	69.33085	0.40867792	0.277494	1.472743	0.14082	0.599658
secisbp2	119.9041	-0.156295064	0.19451	-0.80353	0.421667	0.842943
dnase2	130.0292	-0.038429636	0.189159	-0.20316	0.83901	0.976417
cx47.1	17.68171	-1.769087718	1.618502	-1.09304	0.274376	0.74977
nemp1	72.1134	0.075254991	0.31367	0.239918	0.810394	0.969828
inka2	38.16157	-0.068200655	0.322169	-0.21169	0.832347	0.975062
CABZ01046997.1	1.91067	0.093642174	2.166477	0.043223	0.965524	NA
ptpn9b	248.9213	0.250309898	0.125758	1.990412	0.046546	0.376263
zgc:92481	276.0021	-0.995008137	0.343979	-2.89264	0.00382	0.10304
ppip5k1b	281.8957	-0.200854944	0.162804	-1.23372	0.217307	0.69815
cinp	181.9819	-0.029555016	0.279788	-0.10563	0.915873	0.99311
tmem255a	469.8283	0.082534577	0.107912	0.764831	0.444372	0.85448
si:ch211-202h22.7	22.2096	-0.013920322	0.505963	-0.02751	0.978051	1
pimr213	4.145791	2.650453953	2.877187	0.921196	0.356948	0.807586
fam163ba	14.96098	0.084968341	0.542388	0.156656	0.875516	0.984383
znf1085	14.40389	-1.425179773	0.589621	-2.41711	0.015644	0.220889
b3glcta	151.3155	0.019205881	0.195089	0.098447	0.921578	0.994085
rnf41l	92.72134	0.01393572	0.260891	0.053416	0.957401	0.999109
cntnap5a	38.43831	0.74533064	0.34066	2.187901	0.028677	0.301471
adck2	127.9421	-0.0040304	0.188993	-0.02133	0.982986	1
synrg	413.7042	0.114077935	0.138489	0.823731	0.410092	0.836945
otulina	59.97478	-0.104282961	0.309732	-0.33669	0.736352	0.951919
gareml	19.174	-0.203794566	0.438461	-0.4648	0.642078	0.925586
wnt6b	7.22317	0.091134819	0.686605	0.132733	0.894405	0.988899
BX511021.1	23.43716	0.510159529	0.434392	1.174421	0.240226	0.721177
si:ch73-386h18.1	694.0066	0.169947059	0.176617	0.962233	0.335933	0.792139
si:dkeyp-67e1.6	0.17956	-0.868114638	5.267649	-0.1648	0.8691	NA
prok1	6.41606	0.436618294	0.797568	0.547437	0.584078	0.906376
cd4-2.2	2.179476	0.146764249	1.422239	0.103192	0.91781	NA
slc4a7	144.312	0.107598048	0.199888	0.538291	0.590376	0.908901
tox3	313.6246	-0.026651068	0.132228	-0.20155	0.840266	0.976417
pmch	26.36537	-1.232459904	1.237126	-0.99623	0.319139	0.779923
si:ch211-160j14.2	50.44349	-0.510150624	0.39127	-1.30383	0.19229	0.672283
ccdc32	369.496	0.044776893	0.137936	0.32462	0.745469	0.955021
si:ch211-258f14.2	40.93638	-0.036865735	0.376137	-0.09801	0.921923	0.994346
si:dkey-202b22.5	6.139535	0.639033263	0.724968	0.881464	0.378067	0.82265
mctp2b	83.6589	-0.141361061	0.265847	-0.53174	0.594907	0.910869
colgalt1	76.96906	0.444070244	0.284617	1.560239	0.118703	0.561771
crabp2a	1101.194	-0.059363023	0.136246	-0.4357	0.663051	0.931141
pctp	57.68182	-0.021451383	0.273777	-0.07835	0.937547	0.997305
mntb	244.9845	-0.19708161	0.138018	-1.42794	0.15331	0.617847
si:ch211-276k2.1	1.077223	-0.868172732	5.267649	-0.16481	0.869092	NA
prickle3	58.96264	-0.295280521	0.261544	-1.12899	0.258903	0.736046
srpk1a	466.8921	0.045711203	0.115681	0.395148	0.692734	0.940444
si:ch211-23l10.3	22.84965	0.403818909	0.392367	1.029186	0.303392	0.769363
taptla	120.3751	-0.071562005	0.216853	-0.33	0.741399	0.954103
crygmxl2	4506.551	0.555187031	0.260709	2.129524	0.033211	0.32264
slc6a11a	148.464	0.303897802	0.1907	1.593587	0.111029	0.545965
stac	21.34046	0.536401779	0.508813	1.054222	0.291781	0.761454
znf1059	54.62117	-0.174038616	0.269939	-0.64473	0.5191	0.887148
si:ch1073-159d7.7	7.57451	1.610590329	0.820604	1.962688	0.049682	0.388006
zgc:194275	2.899168	-1.370301807	1.221441	-1.12187	0.261916	0.739623
ptgfr	0.914385	-1.404621054	2.190197	-0.64132	0.521314	NA
usp31	108.7853	0.151795848	0.203709	0.745159	0.456176	0.860359

rbms1a	1077.158	0.084954653	0.097115	0.874786	0.38169	0.824957
CT573248.1	17.71086	-0.60239865	0.471308	-1.27814	0.2012	0.681045
myt1a	1044.619	0.141779015	0.147091	0.963886	0.335103	0.79185
fastkd2	354.1202	-0.047363928	0.134621	-0.35183	0.724964	0.948578
adamtsl2	30.48293	-0.167721353	0.381691	-0.43942	0.660359	0.930581
si:dkey-91i10.2	113.6383	-0.044140969	0.184192	-0.23965	0.810604	0.969828
ky	26.38002	0.080411098	0.458384	0.175423	0.860747	0.980388
abca5	174.4808	-0.082611282	0.198248	-0.41671	0.676893	0.935664
efnb2b	123.5288	-0.083733565	0.261485	-0.32022	0.7488	0.955074
si:ch211-281g13.5	2.673233	-5.660614794	1.800957	-3.14311	0.001672	0.062877
kctd1	113.2163	-0.078641127	0.255324	-0.30801	0.758078	0.95756
calm3b	9428.282	0.2898269	0.113403	2.555724	0.010597	0.179908
dlg5a	538.2961	0.021527573	0.150732	0.14282	0.886432	0.987533
map3k14a	257.947	0.002423653	0.142906	0.01696	0.986469	1
si:dkey-234i14.15	0	NA	NA	NA	NA	NA
rem2	22.87079	0.836289393	0.433706	1.928239	0.053825	0.401878
tas2r200.2	0.109463	-0.868089684	5.267649	-0.1648	0.869104	NA
zgc:171452	7.305063	-1.315913036	0.81375	-1.6171	0.105857	0.534827
irx5b	138.5804	0.125380207	0.233573	0.536792	0.591411	0.909437
mrnip	19.06739	0.196783475	0.365408	0.538531	0.59021	0.908901
BX511100.1	0.214816	1.055359662	5.267649	0.200347	0.841209	NA
map6b	42.30189	0.270828514	0.299947	0.902922	0.366567	0.816033
rnf26	101.6435	0.161267082	0.226628	0.711594	0.476717	0.869292
antxr1b	69.58696	-0.013532656	0.336654	-0.0402	0.967936	1
si:dkey-3d4.3	73.98213	-0.286056785	0.236835	-1.20783	0.227112	0.70833
dnai2b	9.814353	0.122022946	0.516758	0.236132	0.81333	0.970328
gpc5c	19.54143	0.700884199	0.484089	1.447841	0.147661	0.608558
strip1	613.5193	0.033530581	0.118628	0.282654	0.777442	0.961073
si:ch1073-188e1.1	0.14264	0	5.267649	0	1	NA
tecpr1b	93.02037	0.333745182	0.350101	0.953284	0.340446	0.795496
nim1k	48.71455	0.349518261	0.308032	1.134682	0.256509	0.73401
tgm2b	964.0139	0.328090588	0.206235	1.590855	0.111642	0.547577
si:ch211-86h15.1	93.88596	-0.201135947	0.192189	-1.04656	0.295305	0.763896
zgc:163014	3.700903	-0.870067777	1.047467	-0.83064	0.406177	0.834645
bcdin3d	44.746	0.435470553	0.418562	1.040396	0.298156	0.765416
si:dkey-1h24.2	19.16819	0.182272441	0.404416	0.450705	0.652202	0.928768
ftr12	96.80565	0.312570007	0.238453	1.310822	0.189918	0.669635
frmpd2	20.76845	0.506041047	0.854845	0.591969	0.553872	0.89821
usp2b	58.88068	0.047518338	0.386248	0.123026	0.902087	0.990847
dnmbp	531.4388	0.065123516	0.14115	0.461378	0.644528	0.926639
znf1161	52.64449	-0.034749831	0.299959	-0.11585	0.907773	0.991745
ttc39a	85.69601	-0.387643852	0.205148	-1.88958	0.058814	0.418455
edem3	536.0065	0.145145873	0.097372	1.490631	0.136058	0.591131
kif5ba	1263.14	-0.151056009	0.092688	-1.62972	0.10316	0.530217
scpp7	1.51681	2.521610991	2.176396	1.158618	0.246612	NA
slc47a2.2	7.922743	-7.400154334	1.683039	-4.3969	1.10E-05	0.002003
c2cd3	152.3056	-0.168188532	0.208524	-0.80657	0.419917	0.842383
myo1013	199.0958	-0.092825068	0.211259	-0.43939	0.660379	0.930581
zgc:113452	105.6177	-0.249524829	0.218037	-1.14441	0.252452	0.732759
rbpjl	15.80301	-0.494038306	1.380411	-0.35789	0.720424	0.947238
itpr1b	242.695	0.24292882	0.240297	1.01095	0.31204	0.776164
si:ch211-226h7.5	8.571528	-3.065252024	1.502578	-2.04	0.041351	0.355014
slitrk5b	8.999436	0.232480908	0.809612	0.287151	0.773997	0.960636
sgca	397.8145	0.252694614	0.171706	1.471673	0.141109	0.599658

paqr4b	23.47178	0.795105527	0.669339	1.187896	0.234874	0.715533
tnrc6b	884.4837	0.0117299	0.135032	0.086868	0.930777	0.995809
zgc:162879	58.95242	-0.182588222	0.24944	-0.73199	0.464174	0.864593
birc6	1383.988	0.067771362	0.196392	0.345082	0.730033	0.950056
tasor2	530.7342	-0.446392822	0.170702	-2.61504	0.008922	0.161876
gpam	297.6933	-0.22705546	0.181122	-1.25361	0.209985	0.691821
fbxo4	43.6819	-0.581030777	0.279262	-2.08059	0.037471	0.339939
calhm2.2	0.281472	2.436367833	5.206324	0.467963	0.639811	NA
prokr1a	2.83574	0.860851376	1.318258	0.653022	0.513742	0.88631
si:dkey-172j4.3	305.7582	-0.088995056	0.152908	-0.58202	0.560555	0.899928
arhgap32b	331.5828	0.192447507	0.190232	1.011646	0.311707	0.776164
pcsk9	21.54193	1.476280152	0.842876	1.751479	0.079863	0.478112
zgc:172253	48.04238	-0.831885779	0.484367	-1.71747	0.085893	0.491515
flna	2798.258	0.02511293	0.148495	0.169117	0.865705	0.982364
commd6	78.14385	0.146962622	0.256053	0.573954	0.565999	0.901739
si:dkey-1c7.3	0.319913	-0.868124223	5.267649	-0.1648	0.869099	NA
zgc:110286	11.55935	1.055417026	1.904803	0.554082	0.579523	0.905618
SLC5A10	10.11045	-0.203717387	0.689612	-0.29541	0.767682	0.960296
eif3bb	15.30132	0.506068816	0.620573	0.815486	0.414794	0.838698
wu:fc38h03	179.4917	-0.206664574	0.291681	-0.70853	0.478616	0.869292
si:dkey-188i13.6	41.34337	-4.13182331	1.034505	-3.99401	6.50E-05	0.006631
map3k22	59.40878	-0.243930662	0.29404	-0.82958	0.406774	0.835226
zgc:172302	234.4348	0.629669763	0.267926	2.350167	0.018765	0.24195
rap1gap2b	13.01786	0.173577494	0.607298	0.285819	0.775017	0.960899
MDFIC	184.2688	0.246853309	0.167434	1.474333	0.140392	0.599499
si:ch211-269e2.1	361.7828	0.229475851	0.12584	1.823552	0.06822	0.445546
zgc:158398	315.279	0.130343413	0.149823	0.869982	0.38431	0.826565
prr12a	1396.112	0.16205909	0.150776	1.07483	0.282451	0.754744
CU138547.1	32.68619	-0.4477179	0.362037	-1.23666	0.216212	0.69815
pde4bb	48.51871	0.401152124	0.351058	1.142694	0.253166	0.732759
CR376783.1	32.76718	-0.545680004	0.380534	-1.43398	0.151577	0.615372
brpf3b	189.8656	0.071433754	0.178112	0.401062	0.688375	0.939118
serbp1a	26335.18	-0.331995178	0.11763	-2.82237	0.004767	0.114459
arhgef10lb	161.7837	0.406464125	0.174497	2.329349	0.019841	0.248624
spen	695.6169	0.03812385	0.189695	0.200975	0.840718	0.97643
rab14	1769.83	-0.103773741	0.090745	-1.14358	0.252797	0.732759
tmem237b	234.2399	-0.214106991	0.161155	-1.32857	0.183988	0.66083
olig3	69.01804	-0.016527087	0.217179	-0.0761	0.939341	0.997371
abcb6b	30.09529	0.131721011	0.400433	0.328946	0.742196	0.954329
micu3b	818.4237	0.115565777	0.145738	0.79297	0.427795	0.845893
gramd1bb	40.48942	-0.043829027	0.389217	-0.11261	0.910341	0.992208
nck1a	482.6625	-0.146002237	0.136213	-1.07187	0.28378	0.755095
dzip1l	14.16742	1.292470971	0.629301	2.053819	0.039993	0.349544
si:ch1073-314i13.4	189.7115	-0.020855517	0.20191	-0.10329	0.917732	0.993777
si:ch211-214e3.5	228.1342	-0.205854157	0.159478	-1.2908	0.196773	0.67714
wrb	318.1319	-0.064812261	0.159369	-0.40668	0.684242	0.938486
POLR2E (1 of many)	0	NA	NA	NA	NA	NA
limch1a	183.7739	0.191162853	0.273599	0.698697	0.484741	0.872911
si:ch73-12o23.1	298.1876	0.08171735	0.173947	0.469783	0.63851	0.924779
inpp5d	72.96429	-0.231973322	0.30711	-0.75534	0.450043	0.85711
si:ch73-335m24.2	79.03257	-0.450901311	0.656726	-0.68659	0.492341	0.876385
hint3	71.6534	0.36702054	0.243629	1.506475	0.131945	0.584315
sptlc2b	531.6873	-0.010311368	0.110374	-0.09342	0.925568	0.994815
tuba4l	20.3968	-0.800725319	0.474821	-1.68637	0.091724	0.507459

adnpb	466.2787	-0.225374933	0.128036	-1.76025	0.078366	0.47433
znf1015	224.0475	-0.402169382	0.169534	-2.37221	0.017682	0.234646
cth	962.2022	0.335548332	0.221268	1.51648	0.129398	0.579015
gpr183b	3.390461	0.699405935	0.886333	0.7891	0.430053	0.847541
phf12a	360.5527	0.085683999	0.118348	0.724	0.469066	0.867171
si:ch73-257c13.2	99.08354	0.505835374	0.199138	2.54012	0.011081	0.18325
ctslb	153.5001	-0.088392379	0.301551	-0.29313	0.769426	0.960484
rapgef5b	102.1235	0.276250933	0.229835	1.201952	0.229382	0.711579
lrrc75ba	98.00564	0.695028743	0.282841	2.45731	0.013998	0.207377
cnnm4b	31.05812	0.146558629	0.383629	0.382032	0.702437	0.943506
rbfox3b	7.050264	0.526191601	0.793204	0.663375	0.50709	0.883007
mast2	467.1682	0.012802937	0.126973	0.100832	0.919684	0.993892
ttc37	211.9562	0.464532477	0.16576	2.802432	0.005072	0.119186
itga1	236.0538	-0.187817259	0.187454	-1.00194	0.316374	0.778879
fam20ca	15.20731	0.268050305	0.480484	0.557876	0.576929	0.904798
sall1a	418.8451	0.010300152	0.111938	0.092016	0.926685	0.994815
CABZ01083937.1	11.96816	0.099152518	0.739233	0.134129	0.893301	0.988566
tead3a	123.0053	0.246011331	0.235507	1.044604	0.296206	0.764246
si:ch211-194m7.3	230.0344	-0.797505071	0.256832	-3.10516	0.001902	0.067376
efcab2	11.09363	-0.388493649	0.515984	-0.75292	0.4515	0.857676
taar12m	0	NA	NA	NA	NA	NA
mcm8	58.28037	0.523667254	0.245417	2.13379	0.03286	0.320845
apba1b	466.6574	0.161997101	0.135191	1.198286	0.230806	0.712466
arvcfa	137.0856	0.377121559	0.327774	1.150553	0.249916	0.729721
eif4g3b	426.8428	0.261453922	0.175192	1.492388	0.135597	0.591012
si:ch1073-335m2.2	1101.985	0.013499504	0.165197	0.081717	0.934871	0.996829
si:dkey-1c7.1	0.598889	-0.821755525	2.535718	-0.32407	0.745883	NA
boc	1555.161	0.116473134	0.129681	0.898153	0.369104	0.817763
cbfa2t2	289.008	-0.071175325	0.197144	-0.36103	0.718075	0.946566
serf2	2866.431	-0.285213763	0.137936	-2.06773	0.038666	0.344438
hykk.2	50.69756	-1.218520097	0.369679	-3.29616	0.00098	0.043444
si:ch211-214b16.4	164.2107	1.108655619	0.317773	3.488827	0.000485	0.027042
l3mbtl1b	148.6988	-0.024243296	0.184548	-0.13137	0.895486	0.989245
fxn	106.1671	0.126945026	0.167665	0.757134	0.448969	0.856796
znf1lc	1.236545	-0.925697916	1.523853	-0.60747	0.543538	NA
ftr57	0.912935	0	2.342083	0	1	NA
zgc:165582	13.78568	-0.40827042	0.462729	-0.88231	0.377609	0.822478
TTC9	194.2148	0.136625266	0.211649	0.645528	0.518585	0.887148
zgc:171901	61.95334	0.141602426	0.263133	0.538141	0.59048	0.908901
usp12b	545.4673	0.066314281	0.121376	0.546353	0.584823	0.906655
tirap	3.789551	-0.462091107	1.102409	-0.41916	0.675096	0.935189
elfn1b	40.4122	0.735087829	0.451691	1.627413	0.103649	0.531318
mdga1	12.93697	0.413065629	0.616919	0.669562	0.503137	0.881598
junba	330.9045	0.038053474	0.283186	0.134376	0.893105	0.988566
knstrn	165.064	0.289554185	0.177443	1.631812	0.102719	0.529448
farp1	737.4361	-0.162751315	0.156333	-1.04106	0.29785	0.765407
slc12a8	18.81521	-0.051350453	0.464991	-0.11043	0.912066	0.992371
alkal1	9.187951	-0.413837932	0.707164	-0.58521	0.558408	0.899409
gpr26	2.909954	0.69575822	1.246354	0.558235	0.576684	0.904769
tmem176l.4	1075.8	-0.015129839	0.139542	-0.10842	0.913659	0.992796
sh3yl1	56.74412	-0.248347367	0.257345	-0.96504	0.334526	0.79151
fscn2b	151.9062	-0.031259746	0.218325	-0.14318	0.886148	0.987533
gnpat	78.18764	0.089998574	0.211593	0.425339	0.67059	0.933939
pex5lb	26.76772	0.753524228	0.416008	1.811321	0.070091	0.451617

brip1	85.92695	0.214772345	0.197099	1.089665	0.275861	0.750674
sema4ba	58.60447	0.038981146	0.29724	0.131144	0.895662	0.98926
zgc:171482	34.43344	0.090789587	0.409868	0.221509	0.824696	0.972272
hcn4l	13.76818	0.405417204	0.774262	0.523618	0.600544	0.913756
ibtk	520.4648	0.078107494	0.157379	0.496303	0.619681	0.91747
si:busm1-57f23.1	201.3076	0.019911002	0.192142	0.103626	0.917466	0.993777
BX571825.1	0	NA	NA	NA	NA	NA
hhat	16.94559	0.662352	0.399076	1.659713	0.096972	0.519502
ddb1	1584.796	0.066819824	0.115678	0.577635	0.56351	0.901075
ttc19	154.4695	-0.079392391	0.211889	-0.37469	0.707892	0.945335
gp1bb	68.28384	0.12536555	0.262706	0.477209	0.633214	0.923885
alx4b	78.55065	-0.420501558	0.217235	-1.9357	0.052905	0.399547
gas7a	62.91652	0.165664325	0.361218	0.458627	0.646502	0.926728
dapk3	404.1459	0.022931071	0.134532	0.17045	0.864656	0.982015
mnd1	179.7269	-0.067207654	0.185049	-0.36319	0.716464	0.94627
zfx	561.7139	-0.081693883	0.107786	-0.75792	0.448496	0.856741
setd9	35.53205	0.685420599	0.292067	2.346795	0.018936	0.242378
si:dkey-96f10.1	22.31519	-1.390647138	1.473917	-0.9435	0.345423	0.79876
vps13c	421.6685	-0.140332429	0.140081	-1.00179	0.316444	0.778879
gdpd3a	224.494	-0.107871008	0.146815	-0.73474	0.462498	0.863917
prdm11	147.44	0.158546796	0.193499	0.819367	0.412577	0.838123
amigo3	70.54121	0.046199854	0.369373	0.125077	0.900463	0.99045
vps39	525.0573	-0.13919838	0.123232	-1.12957	0.258658	0.73587
odam	68.48731	1.583240402	1.485304	1.065937	0.286452	0.757624
cnksr2a	21.86701	0.248518981	0.402903	0.616821	0.537352	0.892498
ulk1b	187.9182	0.089464321	0.212511	0.420986	0.673765	0.934519
ifngr1l	103.8295	-0.320743955	0.269308	-1.19099	0.233657	0.714749
casc4	351.5248	0.014302486	0.116643	0.122618	0.90241	0.99094
chd9	1228.676	0.271139317	0.143522	1.889189	0.058867	0.418455
tnip2	51.8592	-0.112444934	0.429294	-0.26193	0.793376	0.965366
zmiz1a	1009.253	0.110279953	0.0803	1.373346	0.169645	0.641445
mturn	49.79456	0.537806951	0.318889	1.686501	0.091699	0.507445
tmem94	365.2316	-0.42553298	0.139069	-3.05988	0.002214	0.07471
rmdn1	626.3542	0.406783469	0.211521	1.923133	0.054463	0.403426
si:dkey-28e7.3	215.7943	0.120675215	0.177878	0.678415	0.497509	0.878774
tacr2	5.248645	0.76578209	1.037999	0.737748	0.460667	0.862374
si:busm1-104n07.3	0.862178	-2.768272187	2.304358	-1.20132	0.229627	NA
BX248501.1	12.13289	-1.815687432	0.679761	-2.67107	0.007561	0.14728
map6a	89.02271	0.612547981	0.261185	2.345262	0.019014	0.242726
cntnap1	14.73781	0.099887302	0.625635	0.159658	0.873151	0.983537
zbtb16b	157.6197	-0.22472513	0.247009	-0.90978	0.362936	0.813115
chst15	39.14163	0.332966134	0.269342	1.236221	0.216376	0.69815
unm_sa821	90.76054	-0.970063829	0.285602	-3.39656	0.000682	0.034597
KCNK12	0.62777	-0.782788027	2.298653	-0.34054	0.733448	NA
spag9a	118.6973	0.209404758	0.255731	0.818848	0.412873	0.838123
slc25a38b	330.7621	0.019145564	0.16175	0.118366	0.905778	0.991631
lingo2a	162.478	0.299611088	0.241758	1.239299	0.215235	0.697157
si:dkey-179j5.5	0.149833	1.921567426	5.236909	0.366928	0.713673	NA
gpr63	115.7301	0.327036537	0.252787	1.293725	0.19576	0.676072
znf1007	171.5899	0.194861056	0.169894	1.146956	0.2514	0.731717
si:ch211-213a13.2	39.97139	-0.504932213	0.466539	-1.08229	0.279122	0.752444
si:ch211-240l19.8	138.8932	0	1.734525	0	1	1
zbtb17	335.3945	0.005045126	0.126262	0.039958	0.968127	1
ndufs7	2615.738	0.059048998	0.148391	0.397927	0.690684	0.940089

tspan31	716.1497	0.124066663	0.130518	0.950569	0.341823	0.796624
ganc	82.01454	0.378672936	0.274931	1.377338	0.168408	0.639712
cntnap2b	162.8746	0.027015162	0.186228	0.145065	0.88466	0.987263
CHST8	58.98195	0.072107624	0.281616	0.25605	0.797912	0.966447
slc47a3	33.61976	-0.907550872	0.623219	-1.45623	0.145329	0.604476
gpr37a	34.86709	0.635164655	0.30125	2.108428	0.034994	0.329598
fam57bb	31.78971	0.255501417	0.360457	0.708827	0.478432	0.869292
pusl1	26.10581	-0.06694837	0.408698	-0.16381	0.869882	0.983365
gpaa1	211.2671	-0.237752205	0.144789	-1.64206	0.100577	0.525351
zgc:56304	1411.409	-0.161075036	0.085809	-1.87714	0.060498	0.422849
ppp1r9alb	329.2595	0.045209551	0.116718	0.387339	0.698505	0.942433
add2	585.833	0.021337787	0.145217	0.146937	0.883182	0.986945
gfra4b	64.2253	-0.323351192	0.283031	-1.14246	0.253263	0.732759
grid1a	92.68072	0.357593011	0.357473	1.000335	0.317149	0.77892
METTL18	76.95057	-0.703251986	0.251084	-2.80086	0.005097	0.119231
rin2	386.4005	-0.156253508	0.126604	-1.23419	0.217132	0.69815
wdpcp	65.54452	-0.181834465	0.230691	-0.78822	0.430569	0.847542
eda	20.37101	-0.172580681	0.522453	-0.33033	0.741152	0.954103
lzts2a	154.7628	0.108696989	0.18663	0.58242	0.560284	0.899928
frmd4ba	461.5795	0.086498515	0.11012	0.785495	0.432164	0.848419
gpr78a	21.58221	-0.686939352	0.374286	-1.83533	0.066456	0.440066
cbln2a	29.63644	0.872888568	0.464288	1.880059	0.0601	0.422231
mrvi1	16.9676	-0.686301945	0.59178	-1.15973	0.24616	0.727532
phc3	5.547693	0.284707416	1.018381	0.279569	0.779809	0.961073
unm_sa1506	0.70378	-3.109803344	2.584268	-1.20336	0.228837	NA
si:ch211-113e8.6	0.792035	0	2.354959	0	1	NA
wdr37	527.9386	-0.013391885	0.126769	-0.10564	0.915868	0.99311
si:ch211-240l19.6	915.5972	-2.763117552	1.411789	-1.95717	0.050327	0.390692
wfs1b	122.2693	-0.103872265	0.216911	-0.47887	0.63203	0.923163
tbc1d31	135.9775	-0.062299261	0.237584	-0.26222	0.793152	0.965312
si:ch211-198a12.6	2013.752	0.076427205	0.078599	0.972366	0.330868	0.7883
pidd1	72.12941	0.094141872	0.258417	0.364302	0.715633	0.9461
si:dkey-10o6.2	19.98633	0.305299231	0.398958	0.765241	0.444128	0.854431
gpr35.1	21.60248	0.549843535	0.48683	1.129437	0.258714	0.73587
keap1b	1164.137	0.221910061	0.179962	1.233091	0.217542	0.69815
abca1a	1287.027	-0.689271034	0.18639	-3.698	0.000217	0.015696
cep170b	162.1604	0.106547446	0.234664	0.454042	0.649799	0.927756
loxhd1b	24.52748	-1.555850872	0.614803	-2.53065	0.011385	0.185262
si:dkeyp-51b7.3	9.271707	3.443704649	1.324473	2.600056	0.009321	0.165999
spock1	10.38317	-0.609066157	0.524773	-1.16063	0.245793	0.727177
CABZ01032476.1	153.0999	0.090020915	0.233683	0.385227	0.700069	0.942647
adamts16	23.94091	-0.13468221	0.425995	-0.31616	0.751882	0.955391
smtna	16.3502	-0.431374394	0.744357	-0.57953	0.562234	0.900715
si:ch211-233m11.1	0.394742	-0.868121413	4.966869	-0.17478	0.861251	NA
ctss2.1	162.9459	-0.130025075	0.318204	-0.40862	0.682817	0.938006
rilp	71.66949	-0.453569942	0.268399	-1.68991	0.091045	0.50549
gper1	8.129192	-0.628012394	0.766408	-0.81942	0.412545	0.838123
cntf	26.52823	0.244309215	0.343579	0.711071	0.47704	0.869292
SAMD8	178.4351	-0.402524782	0.196969	-2.0436	0.040994	0.353808
cdk14	32.99952	-0.115499171	0.403305	-0.28638	0.774586	0.960871
zgc:153760	13.85635	1.393257767	0.72348	1.925772	0.054133	0.402505
akt1s1	3356.488	-0.454997061	0.170477	-2.66897	0.007608	0.147466
cacng4b	13.62922	0.018960079	0.529624	0.035799	0.971443	1
matk	1.740052	-0.031182941	1.32764	-0.02349	0.981261	NA

nptx1l	274.888	0.391558514	0.241956	1.618304	0.105597	0.534827
pan2	326.4387	-0.030852866	0.131675	-0.23431	0.814744	0.970686
rprd2b	268.0167	0.160864005	0.130772	1.23011	0.218656	0.699445
frem3	237.8311	0.228209	0.319381	0.714536	0.474896	0.869292
rims1a	336.6488	0.136921858	0.232268	0.589499	0.555527	0.898723
mlsl	118.034	-0.220128585	0.261769	-0.84093	0.400388	0.831645
oga	1900.296	-0.287788164	0.173394	-1.65973	0.096968	0.519502
fbrs	687.5466	0.15004717	0.134928	1.112051	0.266116	0.742912
ppm1j	38.50367	0.119066004	0.347257	0.342876	0.731692	0.950457
mms22l	150.0608	-0.045897236	0.215931	-0.21256	0.831674	0.974914
mertka	168.9526	0.104888204	0.263931	0.397407	0.691067	0.940173
zgc:194621	17.27771	-0.092024325	0.512202	-0.17966	0.857416	0.979741
npdc1a	354.0057	-0.087526889	0.217737	-0.40198	0.687695	0.938835
sdsl	68.49235	0.156472675	0.363266	0.430738	0.666659	0.932664
arfgef2	621.1928	0.068561397	0.113741	0.602786	0.546651	0.896107
ccdc97	203.2236	0.340507056	0.177629	1.916959	0.055243	0.406983
armc3	32.76728	-0.576201458	0.337813	-1.70568	0.088067	0.498295
si:ch211-186e20.2	12.76596	0	2.262245	0	1	1
stk17a	444.9006	-0.080326372	0.128497	-0.62512	0.531891	0.890978
rab38c	55.65162	-0.405007144	0.280703	-1.44283	0.149069	0.610726
si:ch211-51h9.7	101.4063	0.464615332	0.233776	1.98744	0.046874	0.377236
rassf9	25.69664	0.023770468	0.330642	0.071892	0.942688	0.997371
myo10l1	47.23173	0.387141607	0.31308	1.236556	0.216252	0.69815
nrde2	130.0759	-0.237269428	0.177419	-1.33734	0.181112	0.657265
dnajc10	302.5559	0.062987996	0.136442	0.461647	0.644334	0.926639
mfsd9	18.25755	-0.715200203	0.388692	-1.84002	0.065766	0.437367
pkmyt1	79.44941	-0.177549556	0.21507	-0.82554	0.409063	0.836637
BX682234.1	6.274947	-0.378648277	0.658439	-0.57507	0.565244	0.901538
msl1a	186.7354	-0.257384742	0.183485	-1.40275	0.16069	0.627541
slitrk3b	62.47544	0.681191299	0.395258	1.723407	0.084815	0.488883
arhgap18	83.67209	0.009940365	0.377615	0.026324	0.978999	1
elmod3	19.27128	-0.002639308	0.379839	-0.00695	0.994456	1
zmp:0000000760	782.524	0.247802518	0.485352	0.510562	0.609658	0.915204
kcnd1	46.8187	0.996288255	0.403702	2.467881	0.013592	0.203563
brwd1	431.1143	0.028308531	0.158481	0.178624	0.858233	0.979774
abca12	745.8753	-0.064954535	0.208166	-0.31203	0.755016	0.956283
qser1	295.2047	-0.185227143	0.157272	-1.17775	0.238897	0.719781
hlfa	811.7078	-0.150961392	0.145522	-1.03738	0.29956	0.766128
si:dkey-188i13.9	10.49156	0.099648562	1.686297	0.059093	0.952878	0.998425
tfb2m	608.7409	0.044501584	0.14611	0.304577	0.760689	0.957886
arv1	137.915	0.018119019	0.220403	0.082209	0.934481	0.99682
csde1	8009.451	0.007630444	0.108107	0.070582	0.94373	0.997371
ccar1	457.7906	0.029034648	0.14511	0.200086	0.841413	0.97643
ttc7a	127.3157	0.126511898	0.218413	0.579233	0.562432	0.900715
bicdl1	15.92124	-0.043171562	0.438089	-0.09855	0.921499	0.994085
BX571825.2	0	NA	NA	NA	NA	NA
mrap2a	33.75369	0.014181915	0.415562	0.034127	0.972776	1
zgc:171426	14.9058	1.524750091	0.572241	2.664526	0.00771	0.148279
mhc1zja	11.55879	-0.292996469	0.668484	-0.4383	0.661169	0.930581
zgc:113295	83.30394	-0.389442667	0.270444	-1.44001	0.149864	0.613056
ifngr1	38.25284	0.051164456	0.314501	0.162685	0.870767	0.983365
ccl44	170.0504	-0.746451571	0.224104	-3.33083	0.000866	0.039806
si:dkey-110g7.8	52.09336	0.378371578	0.334565	1.130937	0.258081	0.735569
tmem154	103.8434	0.105022946	0.226511	0.463656	0.642894	0.925784

ank1b	168.339	0.632115752	0.285467	2.214319	0.026807	0.290512
alms1	287.832	0.228291613	0.171252	1.333077	0.182507	0.659083
ptprt	30.42576	0.47198161	0.330485	1.428148	0.153249	0.617847
adgrg3	13.74214	-0.264613912	0.575647	-0.45968	0.645745	0.926728
rab5if	1007.306	0.219068622	0.174657	1.254278	0.209741	0.691766
aldh3a1	102.905	-0.339497407	0.216285	-1.56968	0.116491	0.557474
necab3	97.0909	0.782177997	0.283825	2.755849	0.005854	0.129451
dbf4	117.9998	0.107423315	0.207257	0.518309	0.604243	0.914873
cflarb	15.03738	0.01258022	0.588982	0.021359	0.982959	1
atxn7	146.5947	-0.031489876	0.197293	-0.15961	0.873188	0.983537
afap1l2	370.2276	0.058750274	0.138019	0.425669	0.670349	0.933861
tbrg4	474.5129	0.228791817	0.182662	1.25254	0.210373	0.692083
megf6b	337.0994	0.209677049	0.158556	1.322419	0.186029	0.663938
zgc:194392	116.0695	0.087272074	0.285082	0.30613	0.759506	0.957886
fhdc1	55.72119	0.193319264	0.274101	0.705285	0.480633	0.870179
btr05	24.11403	-1.313734991	1.039412	-1.26392	0.206258	0.688779
mxra5a	249.7515	-0.084379331	0.222994	-0.37839	0.705139	0.944028
si:zfos-367g9.1	0	NA	NA	NA	NA	NA
si:cabz01036022.1	11.55861	0.491230691	0.576911	0.851485	0.3945	0.828625
si:dkeyp-23e4.3	84.61419	0.306484115	0.252562	1.213498	0.224939	0.70685
si:dkey-253d23.9	37.65071	-0.716064473	0.282847	-2.53163	0.011353	0.18512
si:dkey-49c17.4	1.10981	0	2.292479	0	1	NA
si:ch73-184c24.1	19.81274	-0.306696105	0.448645	-0.6836	0.494225	0.877583
rhobtb2a	13.38682	0.70408562	0.493894	1.42558	0.15399	0.618574
rasip1	111.591	-0.190762168	0.208756	-0.9138	0.36082	0.811253
setmar	25.73048	-0.690908791	0.384004	-1.79922	0.071983	0.4564
impg1b	57.65658	-0.20626555	0.386907	-0.53311	0.593955	0.910542
snx8a	116.1348	-0.361146522	0.160792	-2.24605	0.024701	0.27868
phldb2b	498.4708	0.055220962	0.140939	0.391807	0.695201	0.941316
CABZ01061495.1	191.1059	0.517945942	0.167024	3.101018	0.001929	0.068013
si:dkeyp-68b7.12	19.18244	-0.032270662	0.41766	-0.07727	0.938412	0.997371
smim7	207.4033	0.0817613	0.201158	0.406454	0.684409	0.938486
rac1a	1672.281	0.169943967	0.132753	1.280148	0.200493	0.68081
il12rb2l	110.8013	-0.317001379	0.261653	-1.21154	0.22569	0.707068
s1pr4	6.735964	-0.356739662	0.708443	-0.50355	0.614574	0.915998
myo15b	104.1383	0.529676485	0.339088	1.562064	0.118273	0.561094
sh2d1ab	1.024546	-0.498770184	2.08072	-0.23971	0.810555	NA
stard7	646.2988	0.038329077	0.106421	0.360164	0.718724	0.94694
slc5a7a	67.52801	0.101247485	0.467631	0.216511	0.828589	0.974137
si:dkey-112m2.1	100.1199	0.5787318	0.250177	2.313289	0.020707	0.255657
frmpd3	176.2168	-0.396890648	0.271649	-1.46104	0.144004	0.603144
ptpn5	243.031	-0.126102522	0.254177	-0.49612	0.619809	0.91747
stim2a	115.7555	0.072266717	0.230803	0.313111	0.754197	0.955996
AL929017.1	99.48809	0.106117465	0.256605	0.413544	0.679208	0.936209
si:ch1073-291c23.2	1154.918	-0.71810105	0.146519	-4.90107	9.53E-07	0.000291
pimr170	0.099744	-1.732891383	5.236854	-0.3309	0.740718	NA
abcd1	35.52237	-0.05000065	0.389976	-0.12821	0.897979	0.990171
CT025742.1	97.86118	-0.126807326	0.238138	-0.5325	0.594383	0.910768
pde6d	241.003	-0.086228744	0.13549	-0.63642	0.5245	0.888808
myorg	743.5901	-0.057848772	0.144105	-0.40143	0.6881	0.939
tysnd1	46.85796	-0.290266656	0.252108	-1.15136	0.249586	0.729721
hes2.1	20.91709	0.250878785	0.408158	0.614661	0.538779	0.892826
taok2a	603.1471	-0.061703579	0.112412	-0.54891	0.58307	0.90582
nol4la	29.31136	0.04942659	0.442646	0.111662	0.911092	0.992208

itcha	83.11388	0.08303442	0.267671	0.310211	0.7564	0.956532
si:ch211-253b8.5	125.5349	1.075322395	0.394044	2.728938	0.006354	0.134763
camk1da	35.95058	0.554440275	0.330559	1.677282	0.093487	0.511476
znf831	8.725645	-1.539353159	0.779512	-1.97477	0.048295	0.38256
col6a1	2891.48	0.084788669	0.160214	0.52922	0.596653	0.911709
dcaf17	38.07468	-0.317278189	0.356611	-0.8897	0.373624	0.820566
crtc2	181.4123	0.003243259	0.185647	0.01747	0.986062	1
CU929346.1	8.657237	0.367470656	0.54271	0.677104	0.49834	0.879132
etaa1	63.67367	0.234367545	0.235491	0.995227	0.319626	0.780289
BFSP1	276.2135	0.479947123	0.29355	1.634973	0.102055	0.528877
fam117ba	197.4896	0.378601223	0.185934	2.036212	0.041729	0.356776
bub1bb	222.0435	0.13783862	0.219266	0.628636	0.529588	0.890434
si:dkey-234i14.13	0.069091	0	5.267649	0	1	NA
si:ch1073-70f20.1	12.20702	-0.006560843	0.500192	-0.01312	0.989535	1
polg2	76.15176	-0.246977528	0.206197	-1.19778	0.231004	0.712561
calhm5.1	28.89544	0.289524459	0.355746	0.813852	0.41573	0.839167
c1qtnf7	4.190293	0.634975637	1.241416	0.511493	0.609006	0.915204
zgc:194114	1.25087	2.320651873	1.845004	1.257803	0.208463	NA
nup98	1148.259	-0.046924426	0.154662	-0.3034	0.761585	0.958237
or102-3	0.526316	1.055411128	3.121548	0.338105	0.735284	NA
zgc:162698	383.2536	0.023356321	0.123603	0.188963	0.850122	0.978251
polr2m	106.6795	0.103486558	0.219622	0.471203	0.637496	0.924779
larp4ab	361.2294	-0.305121034	0.166603	-1.83143	0.067036	0.441984
jac9	3.450393	2.958348224	1.907738	1.55071	0.120971	0.566816
LO017656.1	5.370188	-1.465809528	1.013668	-1.44605	0.148164	0.609115
aurkaip1	518.2779	0.246441532	0.141464	1.742077	0.081495	0.48059
sparcl1	337.5782	0.446513452	0.249818	1.787355	0.07388	0.461767
eif2b5	590.0926	0.388860415	0.124138	3.132481	0.001733	0.064499
tbrg1	88.07422	-0.215322969	0.250608	-0.8592	0.390229	0.827902
chst2a	471.5409	0.114171997	0.136446	0.836758	0.402729	0.833401
pask	78.37078	-0.089672402	0.2157	-0.41573	0.677609	0.936026
glra2	78.12756	0.538832712	0.404503	1.332086	0.182832	0.659278
usp45	145.7068	0.070332014	0.177826	0.395511	0.692466	0.940414
sqstm1	360.6404	-0.681125428	0.183588	-3.71008	0.000207	0.015289
soul5	3896.573	1.470325321	0.361893	4.062871	4.85E-05	0.005452
apobb.2	725.8758	-0.000982213	0.236316	-0.00416	0.996684	1
myzap	229.4538	-0.454942344	0.159947	-2.84433	0.00445	0.109917
fam120a	694.7942	-0.020508224	0.145509	-0.14094	0.887916	0.988077
si:ch211-93e11.8	0.772208	1.055420622	2.760339	0.382352	0.7022	NA
si:ch73-375g18.1	586.4465	-0.053368405	0.161596	-0.33026	0.741205	0.954103
cdc123	398.33	0.030924151	0.17212	0.179666	0.857415	0.979741
fgfr1bl	7.347879	-7.064379511	2.707888	-2.60881	0.009086	0.163488
stx10	67.60249	0.284430961	0.225989	1.258605	0.208173	0.69024
GTPBP8	56.27854	0.294929146	0.225506	1.307855	0.190923	0.670774
raver2	183.8911	0.391597798	0.186019	2.105151	0.035278	0.330796
si:ch211-225k7.6	0	NA	NA	NA	NA	NA
fmnl2b	602.4914	-0.150622367	0.127993	-1.1768	0.239273	0.719781
si:dkeyp-123h10.2	220.7466	0.01354456	0.224029	0.060459	0.95179	0.99801
cxcl18b	110.3723	-0.43608648	0.309319	-1.40983	0.158591	0.624074
rnf151	7.205016	2.959767805	1.526056	1.939488	0.052442	0.397997
lonrf1	1239.814	0.017783704	0.149074	0.119294	0.905042	0.991631
sccpdha.2	22.27375	0.223917831	0.482622	0.463961	0.642676	0.92567
rasgrf2a	46.58465	0.092955112	0.334819	0.277628	0.781298	0.961936
ggt1l2.1	1.036345	0	2.315188	0	1	NA

si:dkeyp-33b5.4	72.99773	-0.006044817	0.216188	-0.02796	0.977693	1
gabra6a	71.36668	-0.492009025	1.335823	-0.36832	0.712635	0.945821
aipl1	37.72393	-0.468635214	0.444539	-1.05421	0.291789	0.761454
rpap1	192.896	0.111676176	0.147564	0.756796	0.449172	0.856796
prkdc	175.6703	-0.198114433	0.186301	-1.06341	0.287595	0.758397
CU467646.1	0	NA	NA	NA	NA	NA
si:dkey-52l18.4	90.01559	0.037191222	0.286503	0.129811	0.896716	0.98939
wdr17	141.9643	0.002801131	0.227285	0.012324	0.990167	1
neo1b	297.7069	0.112891374	0.188047	0.600337	0.548282	0.896644
nkx2.4a	44.94842	0.269234227	0.316487	0.850695	0.394939	0.828825
tmco3	103.2043	-0.002956999	0.180647	-0.01637	0.98694	1
dab2ipb	159.7931	-0.067259578	0.185094	-0.36338	0.716321	0.94627
als2a	50.43497	-0.533491684	0.298795	-1.78548	0.074184	0.462553
tacr3a	1.28304	1.055432342	2.020577	0.522342	0.601432	NA
nanog	2.474086	0.138361393	1.387905	0.099691	0.92059	0.994085
sall2	148.6068	0.362702052	0.181259	2.001011	0.045391	0.37308
hbegfa	482.9496	0.124799257	0.213501	0.584538	0.558858	0.899409
trafd1	269.3273	-0.061497095	0.1187	-0.51809	0.604396	0.914972
TMEM8B	146.9464	0.153977538	0.23201	0.663669	0.506902	0.883007
or103-5	4.861021	0.837059001	0.928295	0.901717	0.367207	0.816459
mrps11	576.7114	-0.077393019	0.156907	-0.49324	0.621843	0.918461
fh	3366.669	-0.181142974	0.120886	-1.49846	0.134015	0.58783
adgrb1a	494.3373	0.004848219	0.186955	0.025933	0.979311	1
adgrf11	0	NA	NA	NA	NA	NA
hdac5	101.5385	-0.05982666	0.293243	-0.20402	0.83834	0.976395
SAT2	253.7391	0.212116324	0.191142	1.109729	0.267116	0.743418
gprc5bb	23.31309	-1.415629373	0.66819	-2.1186	0.034124	0.326054
nanp	57.20281	0.00847225	0.253546	0.033415	0.973344	1
lrrc38a	51.21713	-0.418591581	0.36294	-1.15334	0.248773	0.728797
nagpa	111.7805	0.123247113	0.217666	0.566222	0.571243	0.902817
si:dkey-188i13.10	18.77777	-0.250277447	0.677568	-0.36938	0.711847	0.945821
tbc1d4	294.5816	-0.281241782	0.156199	-1.80054	0.071775	0.4564
igdcc3	499.9632	0.081080404	0.12993	0.624033	0.532606	0.890978
meltf	94.91428	0.089985188	0.37778	0.238194	0.81173	0.97012
defbl1	947.4294	-0.895134767	0.29999	-2.98388	0.002846	0.087695
cxcl20	81.31838	0.11440838	0.38891	0.294177	0.768623	0.960395
mylk5	664.9872	-0.276975812	0.098074	-2.82414	0.004741	0.114199
zc3h6	71.34652	-0.821392287	0.256536	-3.20186	0.001365	0.054641
bbs1	72.44527	0.399785636	0.272586	1.466638	0.142475	0.60163
zdhhc22	31.41717	0.480883175	0.503906	0.954311	0.339926	0.795243
fbxo25	407.1675	0.115702142	0.2695	0.429321	0.667689	0.933086
si:ch1073-322p19.1	343.9433	0.104653965	0.117405	0.89139	0.37272	0.819953
ribc2	18.79944	0.028388276	0.449683	0.063129	0.949663	0.99801
lpcat3	320.1218	-0.023884813	0.154082	-0.15501	0.876811	0.985052
si:dkey-59p5.2	0	NA	NA	NA	NA	NA
crfb16	58.31467	0.189495828	0.282118	0.671691	0.50178	0.88111
rnf220b	78.91704	-0.387872104	0.255685	-1.51699	0.129268	0.578874
adamts10	156.6309	0.068857207	0.244949	0.281108	0.778627	0.961073
cntnap5b	234.5951	0.590766127	0.258959	2.281313	0.02253	0.265534
oacyl	281.6976	-0.185584017	0.163796	-1.13302	0.257207	0.734741
yme1l1a	438.3897	0.164110623	0.232061	0.707189	0.479449	0.86985
BX908780.1	0.060442	0	5.267649	0	1	NA
si:dkey-56e3.3	139.7663	0.347967765	0.177603	1.959243	0.050084	0.389545
inpp4b	210.177	-0.603337941	0.158366	-3.80977	0.000139	0.011849

zgc:195212	2.128416	0.475378524	1.230202	0.386423	0.699183	NA
si:dkey-100n19.2	9.184785	0.230668453	0.705107	0.32714	0.743562	0.954856
p4htm	114.6391	0.476896188	0.27242	1.75059	0.080017	0.478179
chd7	1966.781	0.12499194	0.190748	0.655273	0.512292	0.885459
11-Mar	4.593354	0.081528257	0.966943	0.084315	0.932806	0.996264
vezt	77.25757	-0.484193329	0.235216	-2.05851	0.039542	0.347625
tdrd12	4.406453	-0.316837402	0.853315	-0.3713	0.710413	0.945821
amer2	653.2561	0.077685848	0.155392	0.499936	0.61712	0.916365
si:ch211-223a10.1	55.75122	0.600012827	0.254087	2.361447	0.018204	0.238026
smad4a	461.0937	-0.278760457	0.184329	-1.5123	0.130458	0.581523
slc46a2	16.03196	0.129052021	0.477344	0.270355	0.786887	0.963061
tet1	58.64293	0.017806482	0.304838	0.058413	0.95342	0.998436
pimr74	1.281417	1.621872657	1.98483	0.817134	0.413852	NA
wdr11	218.2025	-0.43798752	0.179142	-2.44492	0.014489	0.210602
zgc:172133	2.720938	-1.760782513	1.860293	-0.94651	0.343889	0.798147
fam171a2a	541.5952	0.035152572	0.126821	0.277182	0.781641	0.962077
phrf1	650.4535	-0.030828737	0.131177	-0.23502	0.814196	0.970483
pik3cb	383.2	0.172700395	0.129664	1.331906	0.182891	0.659278
timp2b	376.3032	0.359931485	0.174332	2.064629	0.038958	0.345405
ankrd1a	61.74818	-0.37450995	0.308922	-1.21231	0.225393	0.70685
si:dkeyp-74a11.1	58.72996	-0.272774569	0.30074	-0.90701	0.3644	0.814648
plaua	9.530861	0.698945246	0.855114	0.817371	0.413716	0.838434
si:dkey-20i10.7	125.2282	-0.063304546	0.260952	-0.24259	0.808322	0.969828
rapgef5a	34.43671	0.205676703	0.34315	0.599378	0.548921	0.897091
thsd1	1.353603	-1.248789184	1.416389	-0.88167	0.377955	NA
si:dkeyp-14d3.1	71.23576	-0.105555035	0.270684	-0.38996	0.696568	0.941847
tbc1d30	239.5007	0.119210221	0.236458	0.50415	0.614156	0.915998
irs2b	680.8854	-0.006484092	0.123582	-0.05247	0.958156	0.999109
kansl1l	16.03086	-1.53364325	0.708676	-2.1641	0.030457	0.311151
MRAS	19.37644	-0.056829492	0.423183	-0.13429	0.893173	0.988566
nitr4a	0.690751	-0.868140113	2.933238	-0.29597	0.767256	NA
myo15aa	65.31275	0.676669937	0.297847	2.271873	0.023094	0.26812
tulp1a	179.9174	-0.534677323	0.222562	-2.40238	0.016289	0.225456
fam83c	27.08285	0.588456321	0.33769	1.742593	0.081405	0.480419
trnt1	274.4388	0.055131784	0.148486	0.371292	0.71042	0.945821
prxl2c	101.4901	-0.593729131	0.238147	-2.49312	0.012663	0.196026
gsdf	10.51235	-1.917807603	0.998578	-1.92054	0.05479	0.405097
kcnq2a	24.24229	1.370106131	0.613436	2.233496	0.025516	0.283691
zgc:174906	304.0481	-0.254231331	0.192158	-1.32303	0.185824	0.663655
CABZ01071972.1	4.23691	0.578284125	0.918653	0.629491	0.529028	0.890325
srfbp1	207.52	0.038426629	0.193127	0.198971	0.842286	0.97643
mrpl16	999.8007	0.128677509	0.120164	1.070848	0.284238	0.75523
dock10	254.2102	0.130724629	0.190588	0.685902	0.492775	0.876558
phox2ba	4.727782	-0.581829984	0.726513	-0.80085	0.423217	0.843894
golga4	422.6046	0.183616997	0.196921	0.932438	0.35111	0.80227
si:ch211-119d14.3	16.07934	0.10413059	0.441343	0.23594	0.813479	0.970328
tbc1d25	238.5699	0.07402838	0.140555	0.526687	0.598411	0.912844
arhgap32a	201.4621	0.283725305	0.242977	1.167703	0.242926	0.724396
lars2	116.2476	-0.271653995	0.208355	-1.3038	0.192301	0.672283
ipp	106.6686	0.2990907	0.225067	1.328898	0.183882	0.660725
med1	307.7532	-0.170886447	0.171404	-0.99698	0.318775	0.779923
csf1a	89.33142	-0.575450306	0.207075	-2.77895	0.005454	0.124491
si:ch211-76l23.7	16.33826	0.346273178	0.536145	0.645857	0.518372	0.887148
tmem129	208.031	-0.29149066	0.152869	-1.9068	0.056546	0.410638

chfr	279.1173	0.293215209	0.16501	1.776952	0.075576	0.465594
si:ch73-138n13.1	671.0294	0.026643081	0.128957	0.206605	0.836318	0.976007
brinp3b	66.58494	0.45109142	0.342927	1.315416	0.18837	0.667666
espl1	312.1829	0.340945412	0.176865	1.927715	0.053891	0.402007
ftr29	3.515074	-1.841991242	1.831762	-1.00558	0.314616	0.777479
whrna	67.01423	0.192364237	0.279471	0.688316	0.491254	0.875595
mrm2	27.19767	-0.076271365	0.376864	-0.20238	0.839616	0.976417
nck1b	619.076	-0.085360121	0.112518	-0.75863	0.448072	0.856727
zgc:171501	11.55999	0.462850901	0.571363	0.810082	0.417893	0.841312
sgsm1b	43.97977	0.268919934	0.462977	0.58085	0.561342	0.900401
kri1	256.8249	-0.100216309	0.160928	-0.62274	0.533454	0.891181
slc9a6b	181.2835	0.117375398	0.176802	0.663879	0.506768	0.883007
gramd1ba	108.7572	0.004818337	0.263635	0.018277	0.985418	1
LO017852.1	257.6647	0.237637624	0.135013	1.760109	0.078389	0.474337
si:ch211-281g13.4	4.52358	-0.12817095	1.219146	-0.10513	0.916271	0.993263
spock2	886.5594	0.12420849	0.190986	0.650355	0.515463	0.886401
dguok	107.2008	-0.143970431	0.201787	-0.71348	0.475551	0.869292
cipca	75.17668	-0.082790304	0.347969	-0.23792	0.81194	0.97012
fbxo33	201.8191	-0.00824305	0.210351	-0.03919	0.968741	1
gpha2	2.826085	1.403190306	1.032713	1.358741	0.174229	0.647454
adck5	135.1687	0.335235289	0.180693	1.855276	0.063557	0.430337
BX571825.3	0	NA	NA	NA	NA	NA
si:ch211-207k7.4	52.99312	-0.269796065	0.40006	-0.67439	0.500064	0.879896
zgc:175107	75.09074	0.005518587	0.256048	0.021553	0.982805	1
pttg1	100.4539	0.144484147	0.22156	0.652121	0.514323	0.88631
MRPL49	204.8001	-0.120668528	0.159781	-0.75521	0.450121	0.85711
BX547992.1	3.522992	-0.321519902	1.076108	-0.29878	0.765108	0.959622
myom2a	7919.484	0.08803185	0.236985	0.371465	0.710291	0.945821
akap11	416.3573	-0.133341822	0.147239	-0.90562	0.365138	0.81521
sytl2b	146.0154	-0.209354915	0.182581	-1.14664	0.25153	0.731788
shc1	622.913	0.013330295	0.094902	0.140464	0.888293	0.988188
vwa2	357.2515	0.138938625	0.141257	0.983591	0.325317	0.784089
cgref1	286.8321	0.316216312	0.442332	0.714884	0.474681	0.869292
psmb5	2428.351	0.07425292	0.151026	0.491656	0.622963	0.918894
naa25	625.1999	0.045165677	0.121505	0.37172	0.710102	0.945821
mkxa	208.5547	0.264496532	0.182181	1.451834	0.146548	0.606341
hdc	27.6973	-0.139526396	0.478235	-0.29175	0.770476	0.960484
soga3b	46.43225	0.286276481	0.317553	0.901508	0.367318	0.816459
pik3ca	155.224	-0.31421575	0.229059	-1.37177	0.170135	0.641806
ptpn20	64.76962	-0.22846338	0.286528	-0.79735	0.425248	0.844714
kmt5c	461.1304	0.13427	0.149292	0.899378	0.368451	0.817272
mss51	378.2121	0.312796901	0.188438	1.659947	0.096925	0.51949
shisa3	295.5768	0.108530446	0.170856	0.635216	0.525287	0.888928
vwa1	474.3145	0.262588907	0.144255	1.820308	0.068712	0.447208
znf989	26.40602	-0.049263878	0.364865	-0.13502	0.892597	0.988566
nlrp15	0.521121	1.530653646	2.672842	0.572669	0.566869	NA
si:dkey-63d15.12	13.76691	0.395094191	0.632475	0.62468	0.532181	0.890978
zgc:173587	8.899527	1.212516094	0.663441	1.827617	0.067607	0.443563
si:ch211-267e7.3	234.0116	-0.370042334	0.160249	-2.30917	0.020934	0.257047
si:ch211-26b3.4	359.5398	0.426833766	0.254837	1.67493	0.093948	0.511719
ccdc160	70.36184	0.165372217	0.235066	0.703514	0.481735	0.871063
smim1	54.51275	0.700920663	0.294664	2.378711	0.017373	0.232896
cdnf	380.7591	0.654980101	0.156387	4.18819	2.81E-05	0.00362
si:dkey-40c23.2	17.1261	-1.245694793	0.952479	-1.30784	0.190926	0.670774

si:ch211-132g1.7	13.63776	0.912161479	0.528045	1.727431	0.08409	0.486971
si:dkey-108k21.10	5.297352	-0.041048615	1.054485	-0.03893	0.968948	1
phf12b	530.467	-0.012183365	0.114691	-0.10623	0.915402	0.993068
ccdc136b	155.0149	0.778711679	0.507933	1.5331	0.125251	0.572966
cd37	19.68301	-0.121801925	0.525605	-0.23174	0.816743	0.971207
si:ch211-120k19.1	56.95645	0.687511013	0.37606	1.828195	0.06752	0.443563
cpz	111.676	0.251997416	0.296257	0.850605	0.394989	0.828825
cln8	41.77993	0.381020154	0.25792	1.477279	0.139601	0.597884
pwwp2a	319.402	0.062879086	0.1433	0.438794	0.660811	0.930581
zgc:174154	52.82112	-0.163916711	0.411384	-0.39845	0.690297	0.93988
lyrm9	54.11497	0.003383073	0.241722	0.013996	0.988833	1
pigu	87.02701	0.016102882	0.210179	0.076615	0.93893	0.997371
slc4a11	55.60802	0.575396506	0.268604	2.142175	0.032179	0.318495
zbtb24	30.3319	0.255092642	0.338206	0.754252	0.450698	0.857151
cpne1	1749.238	-0.238217181	0.088905	-2.67946	0.007374	0.145623
zgc:163061	27.42211	0.016040627	0.328121	0.048886	0.96101	0.999264
snphb	14.66576	-0.746869038	0.633502	-1.17895	0.238417	0.719474
BX649498.1	29.74404	-0.432324273	0.475511	-0.90918	0.363256	0.813595
si:ch73-236c18.3	0.895088	0	3.096	0	1	NA
zfhx4	1259.072	-0.087758911	0.150593	-0.58275	0.560059	0.899738
chd8	905.758	0.017282796	0.151467	0.114103	0.909156	0.992067
si:dkey-89b17.4	1286.22	-0.059147812	0.123647	-0.47836	0.632393	0.923343
cdh5	514.6009	0.091482487	0.144993	0.630943	0.528078	0.889948
aifm5	33.36701	0.292558508	0.434608	0.673155	0.500849	0.880413
adamts17	36.18079	0.485995265	0.324344	1.498393	0.134031	0.58783
luzp1	583.4847	-0.099050789	0.114339	-0.86629	0.386331	0.827215
apcdd1l	182.0612	-0.11559891	0.179766	-0.64305	0.520189	0.887148
ppm1g	840.7105	0.049490007	0.162268	0.30499	0.760374	0.957886
kmt2cb	616.4955	-0.049490723	0.150889	-0.32799	0.742917	0.954655
si:dkey-76k16.5	15.26243	-0.95618372	0.471869	-2.02637	0.042726	0.362408
si:ch211-119o8.4	1.081212	-3.608765951	2.045056	-1.76463	0.077626	NA
fam13a	736.7445	-0.070545245	0.156169	-0.45172	0.651469	0.928562
lzts1	118.4898	0.331018946	0.20181	1.640248	0.100954	0.526541
fbxl20	61.03527	-0.161485266	0.314189	-0.51398	0.607269	0.915204
gpbp1l1	868.5278	-0.027626998	0.101526	-0.27212	0.785532	0.962605
ide	1133.996	0.140656829	0.10792	1.303342	0.192458	0.672283
bicd2	127.553	0.021348688	0.244946	0.087157	0.930547	0.995741
lrch2	31.84765	-0.223489456	0.451313	-0.4952	0.62046	0.91807
itfg1	161.3104	-0.189211414	0.192891	-0.98093	0.326629	0.785469
tnfaip8l2a	52.40154	-0.018551275	0.352493	-0.05263	0.958028	0.999109
trim71	611.7277	-0.14449598	0.14265	-1.01294	0.31109	0.775936
flrt1b	185.0087	0.834409315	0.23699	3.520862	0.00043	0.025105
ambn	0.13363	0	5.267649	0	1	NA
cdc25d	25.47192	0.45598553	0.334736	1.362222	0.173128	0.645953
si:dkeyp-41f9.3	20.89987	0.011406193	0.491211	0.023221	0.981474	1
RNF5	171.2989	0.281310274	0.139924	2.010449	0.044384	0.368519
mical2a	142.8535	-0.308430857	0.315613	-0.97724	0.328448	0.786794
ercc6	344.2963	0.287006242	0.149847	1.915328	0.055451	0.407706
apoc4	7.027881	3.750968428	1.527525	2.455586	0.014066	0.208027
polr2k	174.5724	-0.14662027	0.22499	-0.65168	0.514611	0.88631
slc36a1	147.4874	-0.272319933	0.180675	-1.50724	0.131749	0.583645
cenpo	44.37354	0.357734364	0.327433	1.092543	0.274595	0.750181
birc5a	331.8388	0.161478263	0.218925	0.737597	0.46076	0.862409
zgc:174938	348.5811	-0.165303828	0.253632	-0.65175	0.514565	0.88631

bend5	24.85286	-0.349775721	0.439524	-0.79581	0.426145	0.844714
lrig1	485.709	0.229193826	0.163166	1.40467	0.160119	0.626578
zgc:172090	0.926597	-2.309247843	5.203305	-0.4438	0.657184	NA
si:dkey-197i20.6	142.7924	-1.178937891	0.269108	-4.38091	1.18E-05	0.00209
rad9b	0.791697	0.09365502	2.460949	0.038056	0.969643	NA
si:ch211-225k7.4	0.87271	3.244836075	2.87273	1.129531	0.258674	NA
si:ch211-89o9.6	170.2543	-0.11620241	0.164997	-0.70427	0.481266	0.87045
steap3	41.13352	-0.940463597	0.315099	-2.98466	0.002839	0.087586
ifi35	59.78836	0.236629069	0.365784	0.646909	0.517691	0.887148
sema5bb	61.88394	0.057184272	0.286529	0.199576	0.841813	0.97643
vps26c	512.1446	-0.093513841	0.133877	-0.6985	0.484862	0.872911
si:ch1073-429i10.1	59.0843	-0.576500211	0.309248	-1.8642	0.062294	0.427718
tsc22d3	1383.216	0.039344581	0.217828	0.180623	0.856664	0.979558
rereb	334.7827	-0.032702226	0.164843	-0.19838	0.842745	0.9766
tlr4al	0.554556	0	3.309503	0	1	NA
pms2	82.1241	0.086402098	0.178263	0.484688	0.627898	0.921659
arhgap21b	993.5017	0.036863741	0.100592	0.366469	0.714015	0.945821
fndc7b	73.13246	0.301954507	0.644248	0.468693	0.639289	0.924779
mrpl17	305.2206	0.070289587	0.206089	0.341064	0.733055	0.950736
orc6	125.4211	-0.177367277	0.197522	-0.89796	0.369206	0.817763
tlr7	84.6141	0.167684602	0.225982	0.742026	0.458072	0.860993
anapc1	247.3346	0.230841606	0.182033	1.268128	0.204752	0.686713
adora1b	18.03811	0.892417557	0.423869	2.105407	0.035256	0.33072
si:ch73-380l3.2	171.7851	-0.036683823	0.255293	-0.14369	0.885743	0.987533
nkpd1	12.11365	-0.639487046	1.125327	-0.56827	0.569853	0.902322
zswim2	7.01752	0.324190098	0.760088	0.426516	0.669732	0.933451
si:ch211-106h4.9	122.6239	-0.156657275	0.242068	-0.64716	0.517527	0.887148
nid2a	1021.991	0.08951027	0.16561	0.540489	0.58886	0.908018
ndufb3	1346.744	-0.11102277	0.117997	-0.94089	0.346759	0.799681
shox2	176.618	0.014503663	0.152926	0.094841	0.924441	0.994815
si:dkey-27p23.3	24.67762	0.322212565	0.420116	0.766961	0.443105	0.854063
TMEM236	3.47047	-0.703002777	1.08848	-0.64586	0.518372	0.887148
rpz5	664.4361	-0.24507041	0.127558	-1.92125	0.054701	0.404924
il2rb	12.40587	-0.65388499	0.51438	-1.27121	0.203654	0.684359
zdhhc6	296.749	-0.102002037	0.115788	-0.88094	0.378351	0.822672
si:dkey-242e21.3	8.046047	0.314359025	0.692645	0.453853	0.649934	0.927756
cep152	88.00894	0.271543754	0.224542	1.209324	0.226538	0.707798
dhrs7b	83.91606	-0.279002811	0.223264	-1.24965	0.211426	0.692983
map1lc3cl	99.2892	-1.159078393	0.416469	-2.78311	0.005384	0.123514
dnph1	119.5111	0.453081356	0.29804	1.520202	0.12846	0.577835
zgc:174698	2.159437	1.612077595	1.512677	1.065712	0.286554	NA
taf4b	1.401991	0.104030745	1.550862	0.067079	0.946519	NA
zyx	414.3663	-0.102538211	0.143661	-0.71375	0.475381	0.869292
cxxc5b	438.2006	-0.181302085	0.109275	-1.65913	0.097089	0.519526
zgc:165518	12.88147	-1.782656463	0.529426	-3.36715	0.000759	0.036636
mrpl47	313.3257	-0.053038274	0.139604	-0.37992	0.704006	0.943735
ovgp1	4.294667	0.072349437	1.081536	0.066895	0.946665	0.997371
zfyve16	194.5397	-0.116043408	0.191892	-0.60473	0.545357	0.895275
NCKAP1L	138.8238	-0.577340707	0.1775	-3.25262	0.001143	0.048292
myo18aa	536.9627	-0.042894673	0.169221	-0.25348	0.799895	0.967255
mri1	101.5447	0.175111045	0.268811	0.651429	0.51477	0.886401
gig2e	43.48583	1.836439719	0.41249	4.452083	8.50E-06	0.001615
ywhabb	3618.589	0.03743901	0.135165	0.276987	0.78179	0.962133
samd4a	334.398	0.130216967	0.188005	0.692625	0.488545	0.873964

si:ch1073-287p18.1	67.02523	-0.427696346	0.305491	-1.40003	0.161505	0.628602
sccpdha.1	139.1156	-0.71171959	0.347218	-2.04978	0.040386	0.351287
sdhb	3368.634	-0.049619963	0.11446	-0.43351	0.664643	0.93201
foxj3	315.3758	-0.110621678	0.162982	-0.67874	0.497305	0.878713
si:ch211-57m13.5	0.514572	3.752289759	3.38662	1.107975	0.267873	NA
hmgxb4a	115.8827	0.603184101	0.220331	2.737624	0.006188	0.133012
si:ch73-190m4.1	2.885731	-3.636071055	3.725311	-0.97604	0.329042	0.787268
si:dkey-79f11.5	0.67225	2.576963393	2.277613	1.131432	0.257873	NA
prex1	312.0562	0.327197611	0.149693	2.185785	0.028831	0.302136
nol7	396.0759	-0.176580755	0.190706	-0.92593	0.354481	0.805464
usp38	262.5153	0.033386116	0.154755	0.215736	0.829194	0.974606
calml4a	114.4852	-0.662899564	0.320801	-2.06639	0.038792	0.344867
grin3a	42.05288	0.074909333	0.39391	0.190169	0.849177	0.978098
slc41a2a	221.0196	-0.244331718	0.225821	-1.08197	0.279266	0.752477
si:dkey-219c3.2	122.7655	0.095383132	0.235647	0.404771	0.685646	0.938486
kirrel3a	115.6468	0.100362751	0.266762	0.376225	0.706749	0.944806
traj39	0	NA	NA	NA	NA	NA
kcnt2	12.44832	0.603435417	0.533546	1.13099	0.258059	0.735569
asphd1	96.66913	0.346390984	0.304837	1.136317	0.255824	0.733642
slc12a5a	25.35478	0.339180147	0.4854	0.698764	0.4847	0.872911
mettl24	111.3973	-0.440337606	0.220381	-1.99807	0.045709	0.374363
dok2	29.40335	1.796743614	0.363255	4.946229	7.57E-07	0.00024
arhgef2	294.8261	-0.090807723	0.180154	-0.50406	0.614222	0.915998
CU694442.1	6.650405	-0.202177214	1.013578	-0.19947	0.841896	0.97643
plxnb1a	457.7952	0.131668553	0.155634	0.846015	0.397544	0.830253
virma	731.4266	0.034688589	0.123782	0.28024	0.779293	0.961073
dlec1	41.86206	-0.06410914	0.368774	-0.17384	0.861988	0.980696
msh4	6.364059	-0.004643719	0.845462	-0.00549	0.995618	1
f7i	278.3881	-0.994861807	0.339675	-2.92886	0.003402	0.095894
taar14j	0	NA	NA	NA	NA	NA
kiss1	2.716109	0.833010276	1.302481	0.639556	0.522461	0.887826
syt3	111.5812	0.310370043	0.301097	1.030797	0.302636	0.7687
slc7a8a	1739.705	-0.072157814	0.144716	-0.49862	0.61805	0.916507
lyve1a	126.6819	0.088896724	0.284972	0.311949	0.755079	0.956283
si:dkey-182i3.8	61.59173	-0.44088278	0.236355	-1.86534	0.062134	0.427289
si:dkey-1d7.3	48.53547	0.246132467	0.513121	0.479677	0.631457	0.923003
pigt	22.22142	0.329256538	0.40084	0.821416	0.411409	0.837797
kcnip2	3.354795	-2.319638207	1.383863	-1.67621	0.093698	0.511703
prr12b	1021.731	0.187741322	0.172225	1.090095	0.275671	0.750653
gnal2	208.7646	-0.221177723	0.146433	-1.51044	0.130931	0.582503
oafa	161.6565	-0.096199116	0.173843	-0.55337	0.580013	0.905618
sh3kbp1	569.4235	-0.226382201	0.102393	-2.21092	0.027041	0.292604
cx34.4	50.72178	0.097802255	0.287517	0.340162	0.733734	0.950924
adgre10	7.886215	-0.977605874	0.703461	-1.38971	0.164617	0.634007
pgs1	89.39377	0.005695686	0.235582	0.024177	0.980711	1
fam155a	13.66952	0.706243664	0.618214	1.142394	0.25329	0.732759
mcf2la	508.7055	0.033382322	0.109373	0.305215	0.760202	0.957886
igsf9a	231.6261	-0.00565224	0.284933	-0.01984	0.984173	1
cdcp2	23.31277	-0.033532453	0.437123	-0.07671	0.938853	0.997371
mrtfaa	30.04	0.3469936	0.38096	0.910841	0.362379	0.812303
triobpa	114.074	0.083671619	0.22765	0.367546	0.713212	0.945821
si:ch211-39k3.2	174.7727	0.428721826	0.230828	1.857319	0.063266	0.429493
bub3	507.9663	0.238313802	0.155665	1.530942	0.125784	0.573743
shprh	124.9664	-0.013868066	0.20236	-0.06853	0.945362	0.997371

vwa5b2	12.37552	0.639717406	0.517005	1.237352	0.215956	0.697913
herc3	218.6187	0.020719953	0.149116	0.138952	0.889488	0.988318
sall1b	32.00488	-0.09229088	0.403663	-0.22863	0.819154	0.971785
bag6	911.4339	0.071672538	0.120605	0.594275	0.552328	0.8974
spata7	170.4486	-0.036969031	0.257442	-0.1436	0.885815	0.987533
si:ch1073-186i23.1	137.6108	0.173849209	0.182623	0.951955	0.34112	0.795946
chadlb	105.0579	0.678200456	0.313857	2.160861	0.030706	0.312455
rfx1b	388.0112	0.097971246	0.160668	0.609774	0.542011	0.894241
si:ch211-266g18.9	29.51943	-0.257971564	0.377069	-0.68415	0.493881	0.877345
slc35f3a	4.35316	2.548058237	1.319598	1.930935	0.053491	0.400578
kcnj14	4.690574	0.83439844	1.183664	0.704929	0.480855	0.870179
si:ch73-70k4.1	9.468402	0.356889372	0.637978	0.559407	0.575884	0.904739
zgc:66472	133.7747	-0.099762114	0.163485	-0.61022	0.541716	0.894049
cacna1db	40.49531	0.314408263	0.319515	0.984018	0.325107	0.783981
ccser1	24.7344	-0.662710504	0.38349	-1.72811	0.083969	0.486647
txnrd2.2	27.16822	0.014158536	0.431512	0.032811	0.973825	1
zgc:171558	245.9003	0.510993194	0.179264	2.8505	0.004365	0.108751
igfn1.2	6.870308	0.656857317	1.158179	0.567147	0.570615	0.902481
hivep3a	38.80863	0.012772523	0.436072	0.02929	0.976633	1
si:dkey-219c10.4	14.30086	-0.129747945	0.663155	-0.19565	0.844882	0.976791
adamts2	87.77728	0.128199627	0.254124	0.504478	0.613926	0.915998
acsl5	395.8162	0.429821292	0.419064	1.025669	0.305047	0.770181
si:ch73-211l2.3	3.014743	2.535327958	1.695217	1.495577	0.134764	0.589481
cox15	423.0604	-0.058053176	0.177535	-0.327	0.743671	0.95491
sugp1	221.2593	0.179820651	0.150094	1.198051	0.230897	0.712561
FRMD5	70.95523	-0.005515961	0.233126	-0.02366	0.981123	1
si:ch211-250c4.5	7.884964	0.194715634	0.638135	0.305132	0.760265	0.957886
slco4a1	18.67412	0.7160052	0.559994	1.278594	0.20104	0.681045
prkd1	61.51953	0.36691316	0.374353	0.980126	0.327024	0.785766
mbd2	153.0054	-0.099236961	0.230398	-0.43072	0.666672	0.932664
serpinh1a	641.7674	0.071586485	0.148671	0.481511	0.630153	0.922869
faxca	39.53241	0.063122183	0.348013	0.181379	0.85607	0.979558
si:dkey-266f7.9	301.6062	2.535937082	1.468258	1.727174	0.084136	0.487116
cdh24a	20.20373	0.196306984	0.519641	0.377774	0.705598	0.944274
vav3b	22.60355	-0.429014137	0.393973	-1.08894	0.27618	0.750674
mhc1uba	3.66832	-3.997633836	1.867686	-2.14042	0.032321	0.318811
plekhg4	981.9112	0.109726701	0.121498	0.903117	0.366464	0.81589
BX324188.1	3.781865	1.764529022	1.017223	1.734653	0.082802	0.483014
csrnp2	18.70917	-0.050465715	0.410751	-0.12286	0.902216	0.990915
si:dkey-258f14.3	29.62325	0.102898487	0.380007	0.27078	0.78656	0.962838
slc45a3	14.63575	-0.840339224	0.627603	-1.33897	0.180582	0.656326
prr5a	83.82356	0.009077252	0.285908	0.031749	0.974672	1
tmem125b	68.03805	-0.142434433	0.270522	-0.52652	0.598529	0.912889
hsbp1l1	47.74731	-0.228881142	0.300631	-0.76134	0.446456	0.855403
arpc2	3131.917	-0.051505716	0.120212	-0.42846	0.668318	0.933086
acap3a	121.3529	0.139279202	0.169037	0.823958	0.409963	0.836939
clic5a	710.4964	-0.310294969	0.186913	-1.66011	0.096893	0.51949
znf1lb	0.735729	-0.806220249	2.830378	-0.28485	0.775763	NA
pip5k1ca	80.13638	0.336078683	0.243883	1.378031	0.168194	0.639712
osbpl5	192.6215	0.021970995	0.21797	0.100798	0.919711	0.993892
ppp2r3b	166.0853	-0.000486193	0.170436	-0.00285	0.997724	1
piezo2a.2	371.7836	0.122706021	0.163044	0.752596	0.451693	0.857676
umad1	103.4791	-0.272408623	0.216849	-1.25622	0.209038	0.691152
twist1b	356.1786	-0.155117476	0.147067	-1.05474	0.291543	0.761454

si:ch211-149b19.3	719.42	0.043664499	0.195101	0.223804	0.82291	0.971966
mab21l3	20.25114	0.868115928	0.449692	1.930468	0.053549	0.400752
ERC1	66.86568	-0.629210415	0.298353	-2.10894	0.034949	0.329312
si:ch73-139e5.2	5.357964	-1.246662056	1.013002	-1.23066	0.21845	0.699445
tmem69	156.8542	-0.048311957	0.187648	-0.25746	0.796823	0.966447
ftr70	0.655223	1.055416827	2.988005	0.353218	0.723925	NA
pappa2	136.1099	0.459292124	0.251366	1.827181	0.067672	0.443563
dgkzb	108.7944	-0.110658972	0.207886	-0.53231	0.594514	0.910857
kif5c	113.6993	0.142588564	0.267387	0.533266	0.593849	0.910482
cacnb3b	18.16364	0.119550754	0.467402	0.255777	0.798123	0.966524
arl15a	31.49243	0.246086465	0.382353	0.64361	0.519828	0.887148
wwc1	89.10438	0.035381736	0.246378	0.143608	0.88581	0.987533
si:dkeyp-73d8.9	19.18808	-0.679691059	1.460698	-0.46532	0.641703	0.925586
si:dkey-283j8.1	17.49082	-0.145065831	0.578903	-0.25059	0.802133	0.96794
sez6l2	264.1071	0.163546011	0.348781	0.468908	0.639136	0.924779
rpgrip1	83.83979	0.008285322	0.40357	0.02053	0.983621	1
IYD	39.276	-0.467892615	0.536464	-0.87218	0.383111	0.825809
gba	254.8087	-0.187867423	0.165301	-1.13652	0.255739	0.733642
diaph2	563.2572	0.002179442	0.148712	0.014655	0.988307	1
6-Mar	869.2677	-0.038610149	0.095537	-0.40414	0.68611	0.938732
crtc1a	31.29525	0.101809154	0.422715	0.240846	0.809675	0.969828
dlgap2b	4.084447	1.887450953	1.328287	1.420966	0.155327	0.620076
dda1	922.118	-0.21345291	0.112582	-1.89598	0.057962	0.415784
myh7ba	92.7545	0.573689859	0.266939	2.149145	0.031623	0.316333
crtc1b	13.39723	0.764136033	0.597014	1.279929	0.20057	0.68081
ush1gb	0.266429	0	4.807834	0	1	NA
CABZ01041962.1	220.4291	-0.174470781	0.18377	-0.9494	0.342419	0.797179
jakmip1	127.7556	0.253724947	0.177807	1.426968	0.153589	0.618187
slc31a2	102.8575	0.211251309	0.214979	0.982659	0.325775	0.784719
ftr76	1.909144	-0.87715582	1.746395	-0.50227	0.61548	NA
klhl30	81.07567	0.814936562	0.329258	2.475068	0.013321	0.200944
grid2ipa	32.12968	0.022565921	0.506633	0.044541	0.964473	0.999948
sema4bb	253.1369	-0.46515542	0.169162	-2.74976	0.005964	0.13073
ftr43	3.644811	-4.363120599	1.64629	-2.65027	0.008043	0.151997
nwd1	14.58514	0.777011812	0.573387	1.355127	0.175377	0.649279
CABZ01072254.1	51.58314	-0.060136606	0.272087	-0.22102	0.825077	0.97258
stard5	5.341862	0.007585883	0.702409	0.0108	0.991383	1
cdip1	234.2864	0.009021745	0.169345	0.053274	0.957513	0.999109
emid1	617.8762	-0.124902499	0.13328	-0.93714	0.348685	0.801264
foxp4	1007.102	-0.161523909	0.180952	-0.89263	0.372055	0.819592
smpd1	331.6841	-0.273716556	0.153978	-1.77763	0.075464	0.465421
si:ch211-160b11.4	4.62052	1.994291968	1.838111	1.084968	0.277936	0.751841
actc1a	1217.953	0.928651048	0.446691	2.078956	0.037621	0.340171
gabrb1	49.85318	1.521130734	0.516821	2.943247	0.003248	0.093414
prkar1aa	2182.863	-0.029160955	0.07204	-0.40479	0.685632	0.938486
siglec15l	0.191229	2.079549577	5.22636	0.397896	0.690707	NA
zgc:162972	19.25836	-0.044254412	0.425563	-0.10399	0.917177	0.993735
mmrn2a	225.634	0.194018031	0.176957	1.096411	0.272899	0.748807
si:dkey-98f17.3	22.45432	0.005771707	0.372478	0.015495	0.987637	1
si:dkey-71p21.13	9.791518	1.452691819	0.610869	2.378075	0.017403	0.232896
clcf1	46.78928	-0.04099109	0.302746	-0.1354	0.892298	0.988566
ppip5k1a	271.3686	-0.145126018	0.211628	-0.68576	0.492865	0.876617
icam3	159.7342	-0.10131065	0.201745	-0.50217	0.615547	0.916365
zgc:172075	69.09114	-0.298531736	0.304487	-0.98044	0.326869	0.785637

si:ch211-1a19.2	0.373549	-1.410123995	3.246764	-0.43432	0.664058	NA
lca5	40.9119	0.130298325	0.354089	0.367982	0.712887	0.945821
mtpap	122.0737	0.010945791	0.182399	0.06001	0.952148	0.998066
zgc:165583	0.239853	0	4.943673	0	1	NA
si:dkey-285e18.2	0.678734	-1.811681098	2.688258	-0.67392	0.50036	NA
col19a1	162.7059	-0.08424105	0.253389	-0.33246	0.739544	0.953573
pold3	368.5031	0.176321687	0.225741	0.78108	0.434756	0.850002
pcsk1nl	643.6753	0.185093868	0.268843	0.688482	0.491149	0.875595
znf827	623.9747	-0.085456423	0.164345	-0.51998	0.603076	0.914226
trim2b	137.7914	0.177752986	0.255308	0.696231	0.486284	0.873244
ptcd1	178.0304	-0.262237119	0.164197	-1.59709	0.110247	0.544923
ngef	167.4823	-0.037655946	0.173822	-0.21664	0.828492	0.974137
stat1b	9.262675	-0.494762201	1.525745	-0.32428	0.745729	0.955074
frrs1a	2.652136	-2.383476028	1.511409	-1.57699	0.114798	0.554602
lrrc3cb	1.849471	-2.74728371	1.712288	-1.60445	0.108614	NA
ccdc88b	45.1999	-0.057699703	0.284662	-0.2027	0.839373	0.976417
fbxl13	4.293779	-0.356168862	1.167913	-0.30496	0.760395	0.957886
ankrd1b	703.7364	0.259148889	0.163946	1.580693	0.113948	0.552686
si:ch211-226h7.6	4.16859	0	1.912984	0	1	1
zdhhc11	9.274653	0.413232559	0.730025	0.566052	0.571358	0.902903
DOCK4 (1 of many)	28.91647	0.114030525	0.37474	0.304292	0.760905	0.957953
zgc:165514	0.937349	0.974203152	2.045801	0.476197	0.633934	NA
b3galt4	10.6022	0.074915329	0.675083	0.110972	0.911638	0.992327
fthl28	320.6958	-0.744566349	0.317321	-2.34641	0.018955	0.242378
SERP1	2856.448	-0.113671751	0.171331	-0.66346	0.507033	0.883007
tha1	147.0643	-0.047368518	0.160109	-0.29585	0.767343	0.960296
ror2	223.2476	-0.047086106	0.145442	-0.32375	0.746131	0.955074
kif2c	271.6732	0.234995851	0.243466	0.96521	0.33444	0.79151
mrtfab	958.7492	-0.029878877	0.122929	-0.24306	0.807961	0.969828
atp10b	85.80115	0.067570286	0.353976	0.19089	0.848612	0.977912
lrrc18b	6.012242	0.998989393	0.765804	1.304498	0.192064	0.672283
gramd1c	191.1789	-0.152838073	0.184797	-0.82706	0.408203	0.836036
si:ch211-74f19.2	479.9197	0.239175642	0.192572	1.242008	0.214234	0.696474
txlnbb	3626.51	-0.283107202	0.245125	-1.15495	0.248111	0.728489
prune	53.88353	-0.338734713	0.229135	-1.47832	0.139323	0.597467
dlk2	46.74325	-0.059832898	0.281014	-0.21292	0.831391	0.974908
ppp1r3db	101.6629	1.071893074	0.294017	3.645687	0.000267	0.017992
rasef	95.7852	-0.613740802	0.201039	-3.05284	0.002267	0.075801
irf3	35.31681	0.364323706	0.321781	1.132209	0.257547	0.735155
si:dkey-247i3.1	1.565478	-1.751781293	1.485709	-1.17909	0.238363	NA
znf1084	13.10729	-0.344798211	0.460027	-0.74952	0.453546	0.858722
si:ch211-285c6.1	12.08538	-1.471542038	0.66243	-2.22143	0.026322	0.287677
tbr1a	28.90865	0.250932778	0.439443	0.571024	0.567983	0.902
pou3f2b	641.6586	0.087325336	0.161827	0.539622	0.589458	0.908456
zgc:195170	17.73865	-1.602435795	0.869087	-1.84382	0.06521	0.435355
ano8a	51.17368	0.049136116	0.307674	0.159702	0.873116	0.983537
zgc:172131	28.17308	-0.405933833	0.563694	-0.72013	0.471444	0.86809
adamts13	79.60493	0.741016381	0.383615	1.931666	0.053401	0.400289
znf1041	87.43535	-0.28155552	0.223563	-1.2594	0.207886	0.689937
CR385050.1	3.149439	-2.651891962	1.116153	-2.37592	0.017505	0.233637
ppp1r1b	314.9254	-0.22384312	0.122005	-1.83471	0.066548	0.440425
snrpd3l	537.7311	-0.004037136	0.167797	-0.02406	0.980805	1
si:dkey-125e8.1	0.07132	0	5.267649	0	1	NA
calr	1551.901	0.176024629	0.162972	1.08009	0.280102	0.752951

tns2a	267.0498	-0.045488771	0.270727	-0.16802	0.866564	0.982582
klb	42.8389	0.007711239	0.368337	0.020935	0.983297	1
triqk	322.9592	0.182420137	0.140511	1.298267	0.194196	0.674395
nfatc3a	202.9941	-0.333987928	0.168819	-1.97838	0.047885	0.380801
FQ323119.1	250.1016	-0.168475416	0.29273	-0.57553	0.564931	0.901538
dtx4a	445.8447	-0.235442359	0.169673	-1.38762	0.165252	0.634423
usp11	135.7479	-0.053605456	0.171583	-0.31242	0.754724	0.956283
loxl3a	27.1095	-0.32010412	0.814973	-0.39278	0.694483	0.941093
si:zfos-2070c2.3	0.04222	0	5.267649	0	1	NA
pdp1	235.4414	-0.156226497	0.199792	-0.78195	0.434246	0.850002
mxra5b	104.0718	-0.684397645	0.39205	-1.74569	0.080865	0.479801
si:ch73-290k24.5	10.92024	-0.04775471	0.630089	-0.07579	0.939586	0.997371
myot	442.7864	0.132798394	0.159205	0.834133	0.404206	0.833888
si:ch211-240l19.7	35.01521	0	1.741242	0	1	1
plod3	834.2579	0.012987138	0.104707	0.124033	0.901289	0.990553
atp6v1ab	949.1639	0.033449763	0.104521	0.320029	0.748946	0.955074
ano9a	98.27384	0.071263514	0.220149	0.323706	0.74616	0.955074
col28a2a	783.8227	0.592456088	0.404911	1.463178	0.143419	0.602607
lmtk2	606.2735	0.06661492	0.131391	0.506997	0.612157	0.915668
osbpl11	48.42948	-0.148791661	0.261303	-0.56942	0.56907	0.902234
dusp12	74.40434	-1.004227777	0.260514	-3.8548	0.000116	0.010282
si:ch211-159i8.4	279.9671	0.461105816	0.260198	1.772137	0.076372	0.468131
mrpl43	189.3347	0.229740253	0.167049	1.375285	0.169043	0.640442
cln5	40.75996	-0.193448823	0.348076	-0.55577	0.578371	0.905417
si:dkeyp-13d11.1	0	NA	NA	NA	NA	NA
taar19p	0.046941	0	5.267649	0	1	NA
mylk3	97.90704	-0.476289364	0.260545	-1.82805	0.067542	0.443563
brinp3a.1	190.1317	-0.208363222	0.301041	-0.69214	0.488849	0.873964
CABZ01102039.1	139.7015	-0.59482024	0.23714	-2.5083	0.012131	0.191211
zmp:0000001161	0.492524	0	3.970805	0	1	NA
rpl37	181.194	-0.337920748	0.18744	-1.80282	0.071416	0.455969
CABZ01039859.1	162.3704	-0.406718369	0.179801	-2.26204	0.023695	0.272324
tmem260	91.19231	0.12158958	0.181926	0.668348	0.503912	0.881759
si:dkey-178k16.1	682.7725	0.242703086	0.166469	1.457951	0.144854	0.603718
CABZ01080074.1	149.9646	-0.82501288	0.223894	-3.68483	0.000229	0.016166
vopp1	68.18653	0.072493313	0.256643	0.282467	0.777585	0.961073
tmem175	56.91451	-0.12777562	0.284607	-0.44896	0.653464	0.929269
nsmce2	95.088	-0.370023746	0.225178	-1.64325	0.100331	0.524753
hnrnpa01	60.99989	0.05285447	0.248742	0.212487	0.831727	0.974914
slc25a1b	358.1262	0.015799475	0.12543	0.125962	0.899762	0.99045
epdl1	849.333	-1.758386409	0.28021	-6.27524	3.49E-10	3.11E-07
zgc:194686	1.926138	1.965953982	1.821328	1.079407	0.280406	NA
si:dkey-217f16.6	8.419036	-1.647972011	0.785276	-2.09859	0.035853	0.332937
npm2a	4.61869	-1.439231478	0.920179	-1.56408	0.117799	0.559764
tmem65	189.6383	0.449831315	0.157333	2.859112	0.004248	0.107812
si:dkey-201l21.4	0.117125	-0.868089684	5.267649	-0.1648	0.869104	NA
cacng3b	242.5434	0.116375014	0.276841	0.420368	0.674217	0.934868
nat16l	24.18059	-0.118678152	0.358384	-0.33115	0.740533	0.953939
mctp1a	66.69562	0.306603011	0.280448	1.093261	0.274279	0.749612
ggcta	6.055589	-0.620464941	0.792196	-0.78322	0.433497	0.849685
cavin1a	59.04807	-0.129924572	0.272442	-0.47689	0.633441	0.923885
espn	28.10897	-0.318641553	0.46457	-0.68589	0.492785	0.876558
shroom2a	565.88	0.096408923	0.106871	0.902109	0.366999	0.816297
si:dkeyp-117b11.2	75.36935	-0.130992565	0.304599	-0.43005	0.66716	0.933049

dkk2	31.03893	-0.469008656	0.315893	-1.48471	0.137621	0.593574
lrrc29	69.97	-0.365430654	0.321818	-1.13552	0.256158	0.73401
ARHGAP22 (1 of many	125.5819	-0.056570874	0.231723	-0.24413	0.807129	0.969726
abhd10a	240.5749	-0.222068303	0.156547	-1.41854	0.156033	0.621616
zgc:163143	45.85747	-0.135258851	0.322238	-0.41975	0.674669	0.934877
ppp4cb	949.7118	-0.018817468	0.14466	-0.13008	0.896503	0.98939
btbd6a	229.1554	-0.030995324	0.126656	-0.24472	0.806673	0.969726
zgc:174310	113.3759	0.309605965	0.23051	1.343133	0.179229	0.654489
si:dkey-183c6.8	176.1001	-0.104914605	0.179478	-0.58455	0.558848	0.899409
serpinf2a	246.5909	-1.974275866	0.75341	-2.62045	0.008781	0.160701
kbtbd13	19.13286	0.058373897	0.390333	0.149549	0.88112	0.986892
prdm2b	537.5154	0.03415394	0.212227	0.160931	0.872148	0.983447
ndnfl	253.4475	-0.053029408	0.190412	-0.2785	0.78063	0.961421
lamtor1	139.1394	0.073319031	0.160272	0.457466	0.647336	0.927082
maml1	267.438	-0.127494712	0.17672	-0.72145	0.470631	0.867752
ovol1a	179.3237	-0.023125502	0.171044	-0.1352	0.892452	0.988566
trim37	239.3267	-0.035072547	0.146547	-0.23933	0.810852	0.969828
org	4.484211	-0.768720477	0.761463	-1.00953	0.31272	0.776189
zgc:173770	2.975551	2.693682696	4.384996	0.614295	0.53902	0.892985
mcf2lb	164.9625	0.208419526	0.18405	1.132406	0.257464	0.735125
dhrs13b.2	6.471263	-1.444308039	1.079136	-1.33839	0.180768	0.656768
stab1	257.9554	-0.058578533	0.156547	-0.37419	0.708261	0.945344
acp1	574.3915	-0.023720747	0.160235	-0.14804	0.882313	0.986908
haao	228.4106	-0.099978884	0.282213	-0.35427	0.723138	0.947898
ankrd40	328.7476	0.164315201	0.132324	1.241766	0.214323	0.696474
vwc2	37.79602	0.100306888	0.419136	0.239318	0.810859	0.969828
frmd4bb	69.5944	0.178548143	0.257857	0.692431	0.488667	0.873964
usp53b	46.90232	0.122979527	0.367463	0.334671	0.737873	0.952611
chgb	157.8063	0.052168611	0.354661	0.147094	0.883058	0.986908
asxl2	87.66632	-0.35445345	0.228568	-1.55076	0.120959	0.566816
grm8b	99.92794	1.210856886	0.342241	3.538019	0.000403	0.023889
polr2d	652.9214	-0.137831038	0.194333	-0.70925	0.478168	0.869292
fgf22	13.30033	0.318047943	0.519287	0.61247	0.540227	0.893049
si:ch211-265g22.4	9.310888	-0.407497071	0.681441	-0.59799	0.549844	0.897373
mettl1	168.8928	-0.021850944	0.199826	-0.10935	0.912925	0.99273
mafb	51.99062	0.507132747	0.27355	1.853893	0.063754	0.430794
gar1	921.7689	0.117300277	0.152988	0.766729	0.443243	0.854063
mn1a	464.932	-0.099346225	0.170133	-0.58393	0.559266	0.899562
jac6	0.989719	-2.310731024	2.182221	-1.05889	0.28965	NA
si:ch211-222l21.1	23346.78	0.009279088	0.150566	0.061628	0.950859	0.99801
nfe2l1b	240.9002	0.052211109	0.153382	0.3404	0.733555	0.950808
si:ch211-14a17.10	45.02753	1.801143007	0.80268	2.243913	0.024838	0.279232
sc:d156	5.22725	0.403286625	0.809843	0.497981	0.618497	0.916674
clec3ba	39.87688	-1.606480286	0.545498	-2.94498	0.00323	0.093414
pogzb	461.8529	0.280021071	0.169395	1.653068	0.098317	0.521076
aldh5a1	281.9938	-0.115229481	0.130099	-0.88571	0.375774	0.822237
trhra	5.887189	0.143114696	0.840754	0.170222	0.864836	0.982074
si:ch211-221f10.2	334.0976	-0.040265843	0.187389	-0.21488	0.829862	0.974816
si:dkey-266m15.5	89.16048	-0.590112669	0.297299	-1.98491	0.047154	0.378022
zgc:193801	90.98866	-0.067637592	0.233379	-0.28982	0.771955	0.960624
cdkn1a	82.98965	-0.202022289	0.41472	-0.48713	0.626167	0.92063
bicc2	74.7167	0.243751523	0.238884	1.020375	0.30755	0.772879
rb1cc1	784.8227	-0.106699	0.116004	-0.91978	0.357685	0.808938
apbb1	140.0448	0.257379475	0.284389	0.905027	0.365451	0.815359

rarres3l	80.71801	-0.404724993	0.292804	-1.38224	0.166899	0.637452
hspg2	469.9491	0.111199207	0.245255	0.453403	0.650259	0.927835
kank3	456.1018	-0.076933162	0.102525	-0.75038	0.453024	0.858265
sec61b	1954.289	0.088681943	0.14054	0.631008	0.528035	0.889948
ndp	45.17431	0.233535675	0.305379	0.764741	0.444426	0.85448
sp5a	122.806	0.142104923	0.212655	0.668242	0.503979	0.881759
crygm2d7	5526.742	0.781512526	0.374106	2.089014	0.036706	0.336694
si:dkey-88j15.3	3.550723	-4.585270426	1.548472	-2.96116	0.003065	0.091315
si:ch73-160i9.3	18.01792	0.8250299	0.466344	1.769143	0.07687	0.469467
patz1	263.1913	0.382801307	0.16062	2.38328	0.017159	0.231824
si:ch211-113e8.11	406.3507	0.151259571	0.144958	1.043475	0.296728	0.764495
csf2rb	18.26165	-0.161022683	0.553678	-0.29082	0.771186	0.960484
atad2	502.397	0.181409552	0.146839	1.235435	0.216669	0.69815
man1b1b	53.29159	-0.225766012	0.287267	-0.78591	0.43192	0.848419
pcdh12	117.265	-0.060748122	0.246567	-0.24638	0.805392	0.969348
sema4ga	28.71251	-0.273038262	0.334691	-0.81579	0.414619	0.838683
prss16	369.6349	0.230575841	0.148462	1.5531	0.120399	0.565852
si:ch211-199g17.9	53.91561	-0.269371274	0.243913	-1.10437	0.269432	0.745846
fbxo21	124.6441	-0.005302293	0.216178	-0.02453	0.980432	1
ssuh2.3	2.445257	0.902850847	0.978339	0.92284	0.35609	0.807064
tm2d3	630.6009	0.051723809	0.175408	0.294877	0.768088	0.960296
abhd8b	22.99625	-0.606135845	0.576611	-1.0512	0.293165	0.762581
atp13a3	39.53077	-0.021735384	0.326881	-0.06649	0.946985	0.997371
col14a1b	598.1278	-0.044239002	0.199518	-0.22173	0.824524	0.972227
ptprb	362.5087	-0.062112495	0.148605	-0.41797	0.675969	0.935448
smg9	215.6358	0.252824831	0.153647	1.645496	0.099868	0.524166
zdhhc12a	72.03284	-0.456009295	0.208279	-2.18941	0.028567	0.300821
capn10	99.56119	-0.149295354	0.252352	-0.59162	0.554108	0.898275
FO082781.1	74.39369	0.147330064	0.231865	0.635414	0.525158	0.888928
esamb	36.03476	0.219067979	0.32154	0.681309	0.495676	0.87806
kcnv2a	24.96028	1.367052175	0.544716	2.509659	0.012085	0.190742
pde9al	12.67656	-0.449158733	0.498201	-0.90156	0.36729	0.816459
zgc:193807	0.222343	-0.868114395	5.267649	-0.1648	0.869101	NA
plagl2	103.0667	-0.252571467	0.274365	-0.92057	0.357277	0.808092
cdca7b	325.1315	0.276992121	0.167264	1.656019	0.097718	0.521071
ccngl	15980.13	0.121768803	0.109845	1.108549	0.267625	0.74397
wu:fi04e12	646.294	-0.030638558	0.165978	-0.18459	0.853547	0.978887
rai1	201.53	0.451600998	0.313909	1.438638	0.150253	0.613889
ccn4b	21.76775	0.25068848	0.50483	0.49658	0.619486	0.917435
si:rp71-84d9.1	6.288541	-0.646110426	1.003481	-0.64387	0.51966	0.887148
gramd1a	642.5511	0.002042037	0.130036	0.015704	0.987471	1
avpr2l	4.173482	-1.302018914	0.99149	-1.31319	0.189118	0.668577
NAMPT (1 of many)	0.22275	0	5.267649	0	1	NA
SIPA1	47.08673	0.290124724	0.365906	0.792893	0.42784	0.845893
ipmka	18.96537	0.115725266	0.510655	0.226621	0.820718	0.971788
b4galnt4b	82.14795	-0.03761599	0.280195	-0.13425	0.893205	0.988566
CABZ01068356.1	8.326818	1.23280836	0.764138	1.613331	0.106673	0.536372
zfand4	163.2095	0.159727697	0.185153	0.86268	0.388314	0.827592
si:dkey-42l23.7	46.21032	0.599849323	0.537228	1.116565	0.26418	0.742063
si:dkey-265c15.6	6.613301	1.190982003	0.834409	1.427335	0.153483	0.618041
palb2	33.05614	-0.245882811	0.343572	-0.71567	0.474197	0.869292
prkra	250.7565	0.388096407	0.178502	2.174181	0.029692	0.306711
zgc:171686	12.02752	-0.070751999	0.669172	-0.10573	0.915796	0.99311
pi4kaa	188.8351	0.21639933	0.231183	0.936054	0.349246	0.801408

si:ch211-247i17.1	3.795875	-0.950180497	1.108141	-0.85745	0.391194	0.827902
myo9aa	592.766	0.000456077	0.156879	0.002907	0.99768	1
syt6a	56.84075	-0.050182973	0.361265	-0.13891	0.889522	0.988318
bard1	29.47265	-0.18691998	0.372185	-0.50222	0.61551	0.916365
si:dkey-193c22.2	332.578	0.007424846	0.193365	0.038398	0.96937	1
cyth1a	846.0324	-0.101758277	0.142708	-0.71305	0.475814	0.869292
zgc:193811	7.139863	-0.453925999	0.672824	-0.67466	0.499893	0.879766
TMCC1	25.4179	-0.073282844	0.417935	-0.17535	0.860808	0.980388
ntn2	51.11408	0.421475407	0.281927	1.494983	0.134919	0.589926
tmem67	172.4971	-0.177781795	0.196096	-0.90661	0.364615	0.814648
slc9a7	349.7315	0.001838147	0.169632	0.010836	0.991354	1
ap5s1	188.5572	0.022052071	0.152305	0.144789	0.884878	0.987266
ephb1	13.81678	-0.402758731	0.488274	-0.82486	0.40945	0.836787
sp2	309.249	0.066305123	0.144243	0.459677	0.645748	0.926728
ddhd2	259.0065	0.492402438	0.254798	1.932518	0.053296	0.400175
zgc:163061	12.69341	-1.898725645	0.623144	-3.04701	0.002311	0.076505
pgfb	13.96829	0.549179473	0.436247	1.258873	0.208076	0.690193
reps2	47.45211	-0.415278783	0.33065	-1.25595	0.209135	0.691152
lrrc58a	81.47326	-0.04573216	0.337621	-0.13545	0.892253	0.988566
gucy2g	10.9087	0.027867088	0.552735	0.050417	0.95979	0.999164
si:ch73-343l4.5	0.169904	-0.868100281	5.267649	-0.1648	0.869103	NA
fam13b	7076.83	0.021641272	0.130929	0.16529	0.868716	0.982801
acadsb	811.0096	-0.101112919	0.134363	-0.75253	0.451729	0.857676
trim45	167.4772	-0.257964647	0.231647	-1.11361	0.265445	0.742247
angpt4	35.13166	0.178346668	0.381834	0.467079	0.640443	0.925072
BX571825.4	0	NA	NA	NA	NA	NA
cx32.2	29.11929	0.277212086	0.390409	0.710056	0.477669	0.869292
crygm2d16	5449.532	0.051183312	0.528972	0.09676	0.922917	0.994525
cdk13	597.4674	-0.019822129	0.130823	-0.15152	0.879567	0.986187
dlg3	780.8406	0.148586757	0.112028	1.326338	0.184728	0.661987
si:dkey-178o16.4	15.02567	-1.038843126	0.580955	-1.78816	0.07375	0.461398
si:ch211-261p9.4	54.77909	-0.072837845	0.315267	-0.23104	0.817287	0.971207
rnd3a	1326.325	-0.034405666	0.104651	-0.32876	0.742334	0.954329
tnfrsf18	3.026637	2.534672779	1.612936	1.571465	0.116075	0.556131
arap3	171.2097	0.121312702	0.175694	0.690476	0.489895	0.874826
tradv30.0.1	0	NA	NA	NA	NA	NA
ttyh1	291.2453	0.610384182	0.196764	3.102114	0.001921	0.067865
leng8	1357.98	-0.014596238	0.180644	-0.0808	0.9356	0.996829
zgc:174268	90.5364	-0.030460633	0.189693	-0.16058	0.872425	0.983447
ganab	948.1278	0.023960555	0.105154	0.227862	0.819753	0.971785
gzm3.4	4.536226	0.093368779	0.834724	0.111856	0.910938	0.992208
eif3s10	7883.97	-0.215012395	0.103626	-2.07488	0.037997	0.342167
nlrc8	9.549754	-4.051666213	2.587048	-1.56613	0.117317	0.558519
xkr8.2	42.38722	-0.014269847	0.542679	-0.0263	0.979022	1
CCKBR	4.827432	0.820206782	1.000929	0.819445	0.412532	0.838123
dpp6a	66.51243	-0.545385565	0.324833	-1.67897	0.093157	0.510759
zgc:194101	2.142011	0.093647559	1.940681	0.048255	0.961513	NA
ankib1b	477.3415	0.045205803	0.144768	0.312265	0.754839	0.956283
si:dkey-65b12.6	931.9498	0.431426385	0.225448	1.913641	0.055666	0.408467
iqck	27.5294	0.434187918	0.40514	1.071697	0.283856	0.755095
atp1b1b	1777.293	0.000537684	0.134394	0.004001	0.996808	1
LRRC75A	149.4995	0.166371174	0.187653	0.886588	0.375301	0.822213
gpx4b	3810.64	-0.1216337	0.155454	-0.78244	0.433954	0.850002
apom	2535.34	-0.68159274	0.274154	-2.48616	0.012913	0.198139

ftr86	132.0947	-0.285960481	0.287019	-0.99631	0.319098	0.779923
si:dkey-174i8.1	32.52086	0.071207194	0.531259	0.134035	0.893375	0.988566
plin6	202.2362	-0.123910588	0.282034	-0.43935	0.66041	0.930581
nitr6a	0	NA	NA	NA	NA	NA
tnrc6c1	745.7304	0.113788566	0.113696	1.000817	0.316915	0.778879
zgc:112492	107.6655	-0.434947627	0.390139	-1.11485	0.264913	0.742063
si:ch211-157b11.14	533.3883	0.027573175	0.134013	0.20575	0.836986	0.976007
areg	47.46189	0.401208551	0.325892	1.231108	0.218282	0.699433
frem2a	672.6793	-0.017567706	0.244562	-0.07183	0.942735	0.997371
her3	7.902162	0.687777436	0.60485	1.137105	0.255495	0.733544
TCIM (1 of many)	72.30865	-0.715772814	0.26067	-2.7459	0.006035	0.131072
tmem79b	236.747	0.063332438	0.197273	0.32104	0.74818	0.955074
gask1a	200.911	0.394542768	0.238533	1.654038	0.09812	0.521071
si:ch73-193c12.2	44.19834	0.146115816	0.287542	0.508154	0.611345	0.915331
mrtfbb	159.9852	-0.049890066	0.22107	-0.22568	0.821454	0.971788
piezo1	199.5824	-0.165171213	0.191041	-0.86459	0.387265	0.827592
mpp4l	88.36109	-0.344551321	0.446239	-0.77212	0.440042	0.852079
unm_hu7912	93.76703	0.356570977	0.283532	1.257604	0.208535	0.690728
zgc:171967	315.3417	0.036898531	0.175797	0.209893	0.833751	0.975482
ralgapa1	653.675	-0.079625621	0.124317	-0.64051	0.521843	0.887551
rfxap	46.72269	0.118266776	0.424024	0.278915	0.78031	0.961411
flrt3	906.6897	0.083096883	0.148154	0.560883	0.574877	0.904516
si:dkey-188g12.1	5.256165	0.093775463	0.837181	0.112013	0.910813	0.992208
slc2a13b	155.0038	-0.558071526	0.241538	-2.31049	0.020861	0.256424
prozb	100.831	-1.771376392	0.457346	-3.87316	0.000107	0.009739
CR376751.1	0.169362	-1.710071829	5.238473	-0.32644	0.744088	NA
bend3	240.1101	-0.143585337	0.142866	-1.00503	0.314881	0.777649
loxl5b	28.29156	-0.994222388	0.493534	-2.0145	0.043957	0.367156
eme1	108.5544	0.082906832	0.225889	0.367025	0.7136	0.945821
lacc1	42.35263	0.256578639	0.443584	0.578422	0.562979	0.900772
igdcc4	166.94	-0.165287384	0.248393	-0.66543	0.505778	0.882512
ZNF335	250.4423	0.075801927	0.148934	0.508964	0.610777	0.915331
kazna	145.3459	0.038080569	0.164427	0.231595	0.816852	0.971207
als2b	492.8098	-0.108455704	0.1595	-0.67997	0.496523	0.878161
tet2	656.5429	0.077193052	0.124511	0.61997	0.535278	0.891993
aldh1a3	126.9473	0.09491108	0.18761	0.505895	0.61293	0.915995
wdr60	110.5335	0.486956997	0.218377	2.229896	0.025754	0.28497
znf451	744.918	-0.1959055	0.11973	-1.63623	0.101792	0.528581
lhfpl4b	51.90561	0.4036323	0.361393	1.11688	0.264046	0.742018
dsg2.1	460.913	-0.113087536	0.196671	-0.57501	0.565286	0.901538
zgc:173837	0.059954	1.055396947	5.267649	0.200354	0.841203	NA
kel	71.40244	0.003442121	0.295761	0.011638	0.990714	1
ferl14	52.30511	-0.661595697	0.326531	-2.02613	0.042751	0.362408
jac8	1.925086	0.139666739	1.740723	0.080235	0.93605	NA
si:ch211-38m6.6	21.21702	-1.317449571	0.455272	-2.89376	0.003807	0.103034
paqr4a	17.79092	0.477281525	0.357867	1.333684	0.182308	0.658775
gdpd5b	361.3389	-0.002777144	0.115129	-0.02412	0.980755	1
cables2a	150.8058	0.166228688	0.195182	0.851661	0.394402	0.828495
cep70	80.53572	-0.263928039	0.209643	-1.25894	0.208052	0.690193
pagr1	281.252	0.160606667	0.153326	1.047487	0.294875	0.763708
ftr22	0.53932	1.055361864	4.134864	0.255235	0.798542	NA
si:ch211-165b10.3	30.71259	0.04431898	0.418249	0.105963	0.915612	0.993106
plvapa	123.9441	0.06887537	0.235704	0.292211	0.770125	0.960484
pdzd7a	7.411483	0.542018694	1.022566	0.530057	0.596072	0.911517

pmchl	77.3542	1.177092518	0.634681	1.85462	0.06365	0.430571
ppp1r16a	26.72321	-0.028944737	0.350294	-0.08263	0.934146	0.996692
zgc:198329	45.08416	-0.880059014	0.343109	-2.56495	0.010319	0.176586
rnasel4	7.816616	0	5.267649	0	1	1
znf839	508.3032	-0.200843708	0.13488	-1.48906	0.136472	0.591902
dolk	14.65232	0.209956568	0.474405	0.442568	0.658078	0.930581
adgra2	168.6313	0.086879858	0.243587	0.356668	0.72134	0.947343
sharpin	237.6828	-0.074132204	0.14551	-0.50946	0.610427	0.915331
si:ch73-182a11.2	105.0142	-0.364452783	0.256555	-1.42056	0.155444	0.620161
stxbp4	19.51629	-0.245606958	0.693555	-0.35413	0.723243	0.947898
si:ch73-92i20.1	20.00665	0.352843187	0.524188	0.673124	0.500869	0.880413
igsf3	164.2784	0.071189232	0.183442	0.388075	0.697961	0.942355
aldh1l1	1688.994	-0.262298725	0.211115	-1.24244	0.214073	0.696474
abcc8b	68.29929	-1.491086432	0.776273	-1.92083	0.054753	0.405097
wdfy4	132.722	-0.041973143	0.239524	-0.17524	0.860895	0.980388
uhrf1bp1	52.54432	-0.114671926	0.242939	-0.47202	0.636913	0.924779
eif4ea	301.7271	0.059469874	0.174515	0.340773	0.733275	0.95079
znf280d	451.7213	-0.029211988	0.12727	-0.22953	0.818458	0.971656
socs7	253.9556	0.306803084	0.153672	1.996484	0.045881	0.374848
calhm1	10.53085	-0.809500475	1.643178	-0.49264	0.622265	0.91857
fam131a	21.83836	1.188974444	0.401057	2.964605	0.003031	0.090902
pcdhb	11.133	0.633663082	0.598687	1.058422	0.289863	0.760705
si:dkey-94e7.1	8.75977	-0.581233279	1.003136	-0.57942	0.562308	0.900715
si:ch211-81n22.1	19.56468	-1.173404983	0.391888	-2.99424	0.002751	0.085914
bub1	374.6635	0.297272533	0.247898	1.199171	0.230461	0.712099
nmnat3	53.69393	0.045882797	0.267324	0.171637	0.863723	0.981842
pimr177	0.338044	0.095044446	3.852933	0.024668	0.98032	NA
BX901889.1	0.423091	2.921055457	4.185343	0.697925	0.485224	NA
txlna	544.4238	0.025752857	0.114816	0.224296	0.822527	0.971966
pbrm1l	849.6407	0.063908762	0.15359	0.416099	0.677337	0.935952
esama	108.261	-0.037940885	0.221874	-0.171	0.864222	0.981867
si:ch211-85n16.4	54.59843	0.389976426	0.239978	1.625053	0.104151	0.532242
arl6ip5a	121.2543	0.274795096	0.262068	1.048564	0.294379	0.763461
scg5	12.61265	0.146981069	1.626872	0.090346	0.928012	0.995052
ptprnb	50.09865	0.350570814	0.387281	0.905211	0.365354	0.815339
tnrc18	1010.917	-0.033615338	0.149587	-0.22472	0.822196	0.971966
ppfia3	522.5695	0.535447578	0.196076	2.73082	0.006318	0.134501
gask1b	108.9021	0.040188676	0.265721	0.151244	0.879783	0.986287
esco1	222.8755	-0.124591395	0.134502	-0.92632	0.354282	0.805403
cela1.5	146.8455	0	3.564701	0	1	1
rbm6	512.3519	0.110195641	0.114748	0.960324	0.336892	0.793513
si:ch211-11p18.6	22.09988	-0.239049997	0.643994	-0.3712	0.710489	0.945821
srgn	607.0906	-0.137303589	0.150726	-0.91095	0.362321	0.812287
si:ch73-280o22.2	64.11468	0.371338208	0.265128	1.4006	0.161334	0.628218
tanc1a	408.3185	0.025286757	0.156291	0.161793	0.871469	0.983365
fastk	481.3821	0.003347298	0.131283	0.025497	0.979659	1
zgc:173726	59.4701	-0.120785905	0.249806	-0.48352	0.628727	0.922044
gprc5ba	342.0807	0.084358633	0.179758	0.46929	0.638862	0.924779
slc38a10	492.5418	0.321628147	0.117003	2.748891	0.00598	0.130955
agtrap	169.2423	-0.380094451	0.182106	-2.08721	0.036869	0.337141
avpr1aa	45.73085	-0.295212183	0.28446	-1.0378	0.299364	0.766128
col28a1a	143.0672	0.081930016	0.329953	0.248308	0.803896	0.968812
zgc:110821	94.38355	-0.186553935	0.192716	-0.96802	0.333032	0.790126
ankrd34ba	7.764959	0.781140795	0.948993	0.823126	0.410436	0.8372

si:ch211-196g2.4	48.64434	-0.442424039	0.37899	-1.16738	0.243058	0.724396
blm	118.0653	0.223660298	0.235966	0.94785	0.343206	0.797757
si:ch211-127b11.1	14.70638	-1.135195281	1.011001	-1.12284	0.261504	0.738975
elk4	43.68938	-0.332533766	0.315573	-1.05375	0.291999	0.761655
scaf4a	890.231	0.129465171	0.110699	1.169522	0.242194	0.72408
si:ch211-160o17.4	1601.19	0.05272219	0.12757	0.41328	0.679402	0.936209
fndc7a	83.41355	0.001696051	0.343666	0.004935	0.996062	1
si:ch211-226h8.6	0	NA	NA	NA	NA	NA
trim65	31.60825	-0.252736392	0.298519	-0.84663	0.397199	0.830253
zbtb10	151.3425	-0.200758063	0.192583	-1.04245	0.297204	0.764844
sema4aa	14.38685	0.596477996	0.548632	1.08721	0.276944	0.751311
oafb	20.32439	0.316851653	0.493138	0.642521	0.520535	0.887165
si:ch211-180f4.1	64.11376	0.640260807	0.331724	1.930098	0.053595	0.400965
arhgef16	273.7016	-0.205477722	0.142092	-1.44609	0.148153	0.609115
si:ch73-44m9.1	0.74877	-3.706698546	4.89315	-0.75753	0.448734	NA
ccnjl	266.4985	-0.037185761	0.144763	-0.25687	0.797277	0.966447
naxd	634.2273	-0.141435553	0.094357	-1.49893	0.133891	0.58766
arid5a	10.4966	-0.040837426	0.669336	-0.06101	0.95135	0.99801
cyp26b1	539.7618	0.008042536	0.108027	0.074449	0.940653	0.997371
si:ch211-255p10.4	17.57726	0.166547849	0.446391	0.373099	0.709075	0.945546
snrnp70	1782.145	-0.022577167	0.109841	-0.20554	0.837147	0.976007
bcl10	148.455	0.354933953	0.196382	1.807367	0.070705	0.453582
gpr158a	23.02101	0.517547964	0.537737	0.962455	0.335821	0.792139
si:dkey-28d5.14	0.14762	0	5.267649	0	1	NA
zgc:195173	174.4656	-1.096382914	0.243466	-4.50323	6.69E-06	0.001322
col6a3	1500.852	0.244919793	0.228989	1.069572	0.284812	0.75591
znf526	144.4241	0.086465986	0.204913	0.421965	0.67305	0.93414
adcy3a	131.132	-0.003012851	0.233357	-0.01291	0.989699	1
cbln2b	283.8861	0.274082025	0.262129	1.045599	0.295746	0.764246
si:ch211-157p22.10	57.21613	-0.504715467	0.276238	-1.8271	0.067684	0.443563
synpo2b	164.825	0.163134657	0.321177	0.507927	0.611504	0.915331
ccdc153	9.124059	0.126630888	0.680219	0.186162	0.852318	0.978626
nwd2	255.3793	-0.070061625	0.195086	-0.35913	0.719496	0.94694
kcnj3a	57.6964	-0.025953551	0.321818	-0.08065	0.935723	0.996829
nmbb	28.67242	-0.795946585	0.578467	-1.37596	0.168834	0.640393
si:ch211-153b23.4	231.5249	0.21627256	0.222242	0.973138	0.330485	0.788126
qsox2	91.62298	0.293436844	0.275193	1.066294	0.286291	0.757624
zgc:174888	145.0187	-0.113691346	0.197404	-0.57593	0.564661	0.901445
map2k4b	1022.357	-0.160947864	0.118755	-1.3553	0.175322	0.649279
zgc:152977	40.18071	-0.32263613	0.404768	-0.79709	0.425399	0.844714
ccdc83	4.853412	0.862593328	0.686029	1.257371	0.208619	0.690728
slc37a4b	426.904	0.160098557	0.160861	0.995259	0.31961	0.780289
impg1a	75.49662	0.590536359	0.755738	0.781403	0.434565	0.850002
atrnl1a	40.05079	0.425587019	0.301718	1.410548	0.158378	0.623766
nags	21.80451	1.000844388	0.432079	2.316348	0.020539	0.254489
chst12b	7.65461	-1.232447075	0.695316	-1.7725	0.076312	0.467887
myo7bb	84.42548	0.977106364	0.621328	1.57261	0.115809	0.555893
mocs2	140.7094	-0.027593305	0.181005	-0.15244	0.878836	0.985841
zgc:153116	29.24019	-0.283690566	0.344022	-0.82463	0.409582	0.836787
lzts2b	40.31969	0.054514698	0.278246	0.195922	0.844671	0.976791
or112-1	7.898329	0.460535108	0.690725	0.666742	0.504937	0.88195
eif4g3a	609.7616	0.194904015	0.171092	1.139173	0.254631	0.733441
zgc:162184	71.80022	0.222983385	0.286065	0.779484	0.435694	0.850467
si:ch211-106h4.6	9.208913	-0.74633099	0.65302	-1.14289	0.253083	0.732759

si:ch211-208f21.3	5.968198	-1.497840836	1.107258	-1.35275	0.176136	0.649415
ypel2a	52.01821	-0.057580053	0.296764	-0.19403	0.846155	0.977095
smarca4a	3331.488	0.141232744	0.113746	1.241645	0.214367	0.696521
fbxl6	123.0856	-0.015970355	0.152956	-0.10441	0.916843	0.99353
ntrk3a	207.822	0.215479761	0.208225	1.034843	0.300742	0.767001
ano8b	34.59561	-0.656408956	0.322302	-2.03663	0.041688	0.356684
vwf	24.98684	0.434210601	0.626101	0.693515	0.487986	0.873369
hspb6	196.0929	-0.060076993	0.531002	-0.11314	0.90992	0.992208
rfx7a	317.3348	-0.393193891	0.178532	-2.20237	0.027639	0.295524
si:ch211-165i18.2	2.996124	2.446695251	1.431215	1.709523	0.087354	0.496199
cd247l	11.15384	-1.670484515	0.65688	-2.54306	0.010989	0.182514
setd1bb	4.872356	1.839160862	1.152941	1.595191	0.11067	0.545461
si:dkey-225n22.4	13.19582	0.471037019	0.632697	0.744491	0.456579	0.860698
zgc:171459	66.20973	0.193498249	0.201893	0.958421	0.33785	0.794808
gmip	70.59226	-0.415994751	0.321074	-1.29563	0.195102	0.67483
si:rp71-36a1.5	33.54201	1.343557898	0.387401	3.468136	0.000524	0.028371
alkbh6	145.7042	0.118228799	0.214012	0.552441	0.580646	0.905618
nat8l	1282.756	0.021435547	0.151995	0.141028	0.887848	0.988077
timd4	60.22641	-0.061444575	0.379328	-0.16198	0.87132	0.983365
si:dkey-28d5.13	0.79913	1.550931437	2.464273	0.629367	0.529109	NA
lmod1b	19.09404	-1.054311585	0.643303	-1.6389	0.101234	0.526828
si:ch73-27e22.3	8.35144	0.174020643	0.670789	0.259427	0.795306	0.965909
wdr43	1644.09	-0.30510973	0.169531	-1.79973	0.071903	0.4564
wdr89	85.03658	0.100757258	0.221984	0.453894	0.649905	0.927756
zgc:174698	5.284947	0.925848997	0.919834	1.006539	0.314156	0.777479
crispld1a	53.02708	-0.120341209	0.356622	-0.33745	0.73578	0.951543
gdpd5a	128.8232	-0.145005447	0.194349	-0.74611	0.455602	0.860124
scarf1	76.75256	0.114592912	0.23859	0.480292	0.63102	0.923003
si:ch211-188c16.1	13.03173	-0.576611418	0.721611	-0.79906	0.424255	0.844355
mmp25a	2.28116	0.833524873	0.974592	0.855256	0.39241	0.827902
rps2	119160.7	-0.392813483	0.141637	-2.77338	0.005548	0.125896
synpo2la	777.0569	0.080831395	0.257879	0.313447	0.753941	0.955912
akap6	413.7648	0.211267456	0.166506	1.268825	0.204504	0.68614
nbr1a	979.8094	-0.243358356	0.12334	-1.97307	0.048487	0.3833
gas1a	1216.365	-0.081338942	0.120844	-0.67309	0.500889	0.880413
yars2	69.67101	-0.052945512	0.238607	-0.22189	0.824397	0.972223
usp42	313.5924	0.239443297	0.130302	1.83761	0.06612	0.439094
mlf1	1330.808	0.147022669	0.233794	0.628855	0.529444	0.890434
erg	73.57147	-0.552666378	0.222843	-2.48007	0.013136	0.199185
cutc	344.5127	-0.021750021	0.161628	-0.13457	0.892953	0.988566
tmem201	167.7922	-0.084088994	0.181252	-0.46393	0.642695	0.92567
gpr84	59.00327	1.396415349	0.525841	2.655583	0.007917	0.150539
si:ch211-209f23.6	7.390351	-0.836166706	0.5657	-1.47811	0.139378	0.597593
slc46a3	39.45571	-0.049753996	0.321319	-0.15484	0.876945	0.985067
mblac1	43.4918	-2.015396285	0.466156	-4.32344	1.54E-05	0.002472
MB21D2	18.90791	-0.30883386	0.490871	-0.62915	0.529248	0.890406
snx29	221.563	0.106979887	0.170904	0.625964	0.531338	0.890833
CR376737.1	191.4667	-0.145475576	0.199156	-0.73046	0.465108	0.864892
thap4	184.4085	-0.301417978	0.181225	-1.66322	0.096268	0.517886
slc45a4	318.3618	-0.092422862	0.14215	-0.65018	0.515577	0.886459
ano11	88.49348	0.097780898	0.386245	0.253158	0.800146	0.967256
nlgn2a	283.7303	0.157948355	0.219266	0.720352	0.471308	0.86809
SH3TC1	46.65192	-0.132534029	0.331768	-0.39948	0.689541	0.939582
si:ch211-171h4.5	0.594001	0	2.752011	0	1	NA

ptrh2	263.1858	0.248381579	0.217533	1.141809	0.253533	0.732873
ppp1r14c	1204.668	-0.086625821	0.198226	-0.43701	0.662108	0.930742
sorcs3b	20.98714	0.942680464	0.501925	1.878128	0.060364	0.422591
b4galnt1b	70.50639	-0.347640668	0.249312	-1.3944	0.163197	0.631199
rerea	680.3525	-0.310424843	0.193915	-1.60083	0.109414	0.542505
sypl1	126.3941	-0.253770336	0.193942	-1.30848	0.190709	0.670725
lrrc61	19.05959	-0.004206827	0.475199	-0.00885	0.992937	1
fthl30	89.84368	0.441396926	0.328744	1.342679	0.179376	0.654677
bptf	1067.832	0.009647086	0.162036	0.059537	0.952525	0.998371
mapk9	80.17378	-0.033869226	0.212137	-0.15966	0.873151	0.983537
ntng2a	104.6502	-0.073424901	0.285257	-0.2574	0.79687	0.966447
slc30a6	221.7894	0.056700213	0.154369	0.367303	0.713393	0.945821
kcnk15	26.40084	-0.130449362	0.50894	-0.25632	0.797707	0.966447
tfr1b	443.0959	-0.460912528	0.206359	-2.23354	0.025513	0.283691
si:dkey-85n7.6	122.2846	0.391671227	0.231238	1.693798	0.090304	0.50442
hcn5	47.31138	-0.29346616	0.296941	-0.9883	0.323007	0.782754
anxa11a	1958.854	-0.045201586	0.108648	-0.41604	0.677384	0.935952
dnah11	5.171727	-1.236056045	0.886716	-1.39397	0.163327	0.631199
rgs19	45.91419	-0.226203195	0.257325	-0.87906	0.37937	0.823515
tcte1	2.447264	0.371965144	0.993268	0.374486	0.708043	0.945335
obsl1b	304.9446	-0.107427485	0.294698	-0.36453	0.715459	0.945988
tradv27.0	0.295069	1.055362043	5.267649	0.200348	0.841209	NA
si:dkey-81n2.2	0.069091	0	5.267649	0	1	NA
tlcd2	362.9474	-0.332138074	0.166006	-2.00076	0.045418	0.373167
ubash3bb	66.58133	-0.211967005	0.277879	-0.7628	0.445581	0.855403
rbm10	322.0788	0.094969897	0.182285	0.520996	0.602369	0.914001
col8a1a	252.5721	0.170188683	0.289631	0.587606	0.556797	0.899063
ncoa3	731.4341	-0.102629184	0.114957	-0.89276	0.371984	0.819592
psip1b	346.8185	0.275009371	0.181987	1.511145	0.130752	0.582151
si:dkey-184p18.2	211.6007	0.433283664	0.285735	1.516383	0.129422	0.579015
cby1	185.3479	-0.020979853	0.160313	-0.13087	0.895879	0.98926
myo9b	394.0834	-0.099587346	0.206879	-0.48138	0.630246	0.922869
ftr08	0.247147	2.079533563	5.22636	0.397893	0.690709	NA
zgc:194312	138.4982	-0.094053176	0.236778	-0.39722	0.691204	0.940173
zmp:0000001175	9.811241	0.299432501	0.833097	0.359421	0.71928	0.94694
nectin4a	126.5789	0.007307223	0.223502	0.032694	0.973918	1
gpr20	3.44696	-1.813823045	1.612397	-1.12492	0.260622	0.738121
camta1a	419.1137	0.387183564	0.207804	1.863217	0.062432	0.427718
usf3	233.4083	0.270022691	0.203531	1.32669	0.184611	0.661987
mon1bb	250.1977	0.010333324	0.163491	0.063204	0.949604	0.99801
SYDE1	153.5073	0.079644562	0.277052	0.287472	0.773751	0.960624
tubgcp5	283.5728	0.277756391	0.154414	1.79878	0.072054	0.456627
si:dkey-234i14.14	0	NA	NA	NA	NA	NA
si:ch211-113a14.12	25.09437	1.340519707	0.626553	2.139514	0.032394	0.319256
dhx32a	60.05694	0.086724654	0.269954	0.321257	0.748016	0.955074
sorcs2	263.7572	-0.322003157	0.159273	-2.0217	0.043207	0.364174
sox10	520.8902	-0.195047603	0.158034	-1.23421	0.217124	0.69815
si:ch73-206p6.1	145.8859	-0.166694965	0.257012	-0.64859	0.516604	0.886933
polr1b	173.0245	-0.309202394	0.215269	-1.43635	0.150902	0.615085
si:dkey-193b15.8	24.6744	0.141596297	0.445769	0.317645	0.750754	0.955074
hmces	36.02706	-0.097058832	0.409994	-0.23673	0.812864	0.970328
mych	954.4726	-0.205039271	0.475501	-0.43121	0.666318	0.932664
pla2r1	59.4785	0.371595266	0.397149	0.935658	0.349449	0.801408
zgc:174353	0	NA	NA	NA	NA	NA

msh5	12.41924	-0.651900377	0.555165	-1.17425	0.240297	0.721295
si:dkey-76k16.6	31.38828	0.363967136	0.407728	0.892672	0.372033	0.819592
spsb3b	214.9053	-1.098083367	0.695045	-1.57987	0.114136	0.553175
si:ch73-289h5.4	49.90617	0.677546317	0.30606	2.213769	0.026845	0.290786
lrfn2b	90.51592	-0.08533644	0.309669	-0.27557	0.782876	0.962328
atp8a2	977.9424	0.035096416	0.11284	0.311027	0.75578	0.956532
ptpn9a	138.2329	0.160298498	0.190448	0.841693	0.39996	0.831277
igsf10	98.01193	0.023858934	0.376505	0.06337	0.949472	0.99801
plekho1b	184.497	-0.213742425	0.187245	-1.14151	0.253657	0.732873
si:ch211-103n10.5	70.30527	-0.463045963	0.48585	-0.95306	0.340558	0.795538
tjp1a	1681.105	0.186772517	0.115817	1.612654	0.10682	0.536763
ppp1r3da	83.44845	-0.145002457	0.268931	-0.53918	0.589762	0.908601
letm2	946.5847	0.078544826	0.127747	0.614845	0.538657	0.892777
recql4	72.19742	0.373320629	0.245442	1.521017	0.128256	0.57725
si:dkey-6f10.4	0.292935	0.892420518	5.241767	0.170252	0.864812	NA
tdrd7b	106.2896	0.09914277	0.220181	0.450278	0.65251	0.92891
babam1	304.2692	0.177957383	0.126913	1.402197	0.160857	0.627788
ahctf1	707.3386	0.046736637	0.132446	0.352873	0.724184	0.948273
bag6l	340.3418	-0.168559267	0.124602	-1.35278	0.176126	0.649415
pign	60.95294	0.311589661	0.239892	1.298872	0.193988	0.674122
eif3f	5183.844	-0.317661524	0.131561	-2.41457	0.015754	0.221358
snrnp200	1272.714	0.115480355	0.150935	0.765098	0.444213	0.854431
nudt19	159.1238	-0.74340897	0.235645	-3.15478	0.001606	0.061411
f2rl1.2	134.4066	0.629226039	0.300218	2.0959	0.036091	0.334091
si:dkeyp-75b4.8	3.660884	0.690018502	1.517805	0.454616	0.649385	0.927756
toe1	137.7041	-0.259552441	0.169271	-1.53335	0.125189	0.572897
si:dkey-16p21.7	359.8036	0.306271481	0.20874	1.46724	0.142311	0.601598
zdhhc1	197.4222	0.33203088	0.157836	2.103639	0.03541	0.330833
tmlhe	59.5404	-0.426372865	0.273238	-1.56044	0.118655	0.561771
aifm2	590.3204	0.22469416	0.113953	1.971816	0.048631	0.383778
gcnt4b	0.380616	0	5.048128	0	1	NA
rap2aa	48.0402	0.144701262	0.337538	0.428696	0.668144	0.933086
nitr5	2.046424	1.193012731	1.342074	0.888932	0.374039	NA
LO017815.1	131.0069	-0.142247932	0.611912	-0.23246	0.816177	0.971207
grin2cb	34.14389	-0.627599246	0.513285	-1.22271	0.221439	0.702841
angel1	127.3405	-0.21821222	0.306497	-0.71195	0.476493	0.869292
lrrn3a	130.9618	-0.036574497	0.293113	-0.12478	0.900698	0.99045
cdh16	5.403531	-0.044717885	0.794062	-0.05632	0.955091	0.998611
zgc:174862	8.471906	-2.981091138	1.035232	-2.87964	0.003981	0.105306
si:ch211-193k19.2	4.545127	0.203845697	1.023463	0.199173	0.842128	0.97643
tmprss13b	52.20899	0.349068868	0.335682	1.03988	0.298396	0.765416
ntsr1	2.102405	0.29529792	1.318532	0.22396	0.822789	NA
tspeara	13.42286	-0.665201655	0.580354	-1.1462	0.251712	0.732003
zzz3	554.612	0.012822737	0.111361	0.115146	0.908329	0.991812
ank3b	184.9966	0.165899286	0.290218	0.571637	0.567568	0.901809
b4gat1	159.4218	0.202472028	0.171631	1.179692	0.238123	0.719146
cln6a	182.3081	-0.011388908	0.171168	-0.06654	0.946951	0.997371
pdgfc	182.0482	-0.073189225	0.154124	-0.47487	0.634878	0.924272
CR847803.1	0	NA	NA	NA	NA	NA
arsj	115.3694	-0.229942591	0.208809	-1.10121	0.270806	0.746845
phf24	398.4623	0.405466132	0.172156	2.355229	0.018511	0.240697
CR394546.1	0.430162	1.055427995	2.693338	0.391866	0.695157	NA
frrs1b	180.1714	0.221417476	0.147176	1.504435	0.132469	0.5853
arsia	36.19066	0.156238212	0.518076	0.301574	0.762977	0.958842

dus2	206.9496	-0.109303184	0.186694	-0.58547	0.558234	0.899409
gpr137bb	101.2758	-0.121098766	0.339724	-0.35646	0.721494	0.947343
elfn2a	3.628316	-0.503339541	0.931109	-0.54058	0.588797	0.908018
hykk.1	1.48933	-0.950746523	1.704307	-0.55785	0.576947	NA
si:ch211-206k20.5	238.7779	-0.000504176	0.156615	-0.00322	0.997431	1
rin3	366.4252	0.024243336	0.152574	0.158896	0.873751	0.983831
cdca7a	452.5343	-0.063828938	0.214861	-0.29707	0.766413	0.960229
si:ch211-263p13.7	5.969507	1.070067565	0.841424	1.271734	0.203468	0.684359
tafa4a	0.646282	0.060657175	2.563679	0.02366	0.981124	NA
CU459089.1	22.29311	-0.281549932	0.356171	-0.79049	0.429242	0.846956
magixb	19.77764	0.343198714	0.428988	0.800019	0.4237	0.844048
mal2	55.1006	0.115658291	0.275295	0.420124	0.674395	0.934877
zgc:171965	4.677511	-0.372066462	0.745601	-0.49902	0.617769	0.916478
intu	295.7922	-0.086746769	0.13259	-0.65425	0.512952	0.885889
thbs3a	146.5469	0.105849388	0.287376	0.36833	0.712627	0.945821
lypd6b	76.6938	-0.193519389	0.216914	-0.89215	0.372314	0.819778
zgc:172079	1.396979	0.113467206	1.644545	0.068996	0.944993	NA
gpr78b	1.932737	2.421425525	1.568761	1.543528	0.122703	NA
si:ch211-42i9.8	102.7244	-0.183450176	0.277541	-0.66098	0.508623	0.883333
tnksa	550.4024	-0.319526571	0.122891	-2.60009	0.00932	0.165999
lratb.2	37.72064	0.590739087	0.689758	0.856444	0.391752	0.827902
rab16b	378.0421	-0.065823915	0.118063	-0.55753	0.577165	0.904798
grm8a	162.9513	0.333516668	0.322806	1.033179	0.30152	0.767562
arfgef3	50.94596	0.238490175	0.401044	0.594674	0.552062	0.8974
zgc:110239	738.7427	-0.177634166	0.1226	-1.44889	0.147368	0.607891
pimr140	0.561789	-1.768624254	3.397497	-0.52057	0.602669	NA
evi5a	31.37002	0.440828401	0.408233	1.079846	0.280211	0.752951
nlrp16	42.33707	-0.607113202	0.323881	-1.87449	0.060862	0.423588
pdgfd	62.07878	0.059861239	0.24868	0.240716	0.809775	0.969828
aak1b	67.46603	0.432260527	0.265356	1.628985	0.103316	0.530446
rp1	0.887266	1.93325255	2.264216	0.853829	0.3932	NA
cpo	9.927618	1.055421398	1.796688	0.587426	0.556918	0.899145
mb21d2	209.9509	0.315011814	0.165418	1.904334	0.056867	0.411856
slc13a5a	75.73783	0.370096852	0.282951	1.30799	0.190877	0.670774
dtnba	1481.264	-0.107336068	0.160406	-0.66915	0.503398	0.881759
si:dkey-30h22.11	72.14876	0.356363899	0.209266	1.702925	0.088582	0.499744
si:dkey-105i14.1	82.73984	1.007712479	0.404297	2.492503	0.012685	0.196197
rsad1	10.90336	-0.249397985	0.589344	-0.42318	0.672165	0.933953
CR749163.1	3.514354	2.555095078	1.326308	1.926472	0.054045	0.402389
iqsec2b	267.7861	-0.085994909	0.179543	-0.47897	0.631962	0.923139
nlgn1	15.44999	0.326599666	0.573615	0.569371	0.569104	0.902234
zgc:113886	1.161613	-4.891807191	4.661046	-1.04951	0.293944	NA
GRIK3	28.52163	0.224409296	0.405725	0.553107	0.58019	0.905618
rpl29	8914.337	-0.395089263	0.166857	-2.36783	0.017893	0.236508
knop1	208.4575	-0.401767587	0.187417	-2.14371	0.032057	0.318495
ppp2r3a	595.7796	0.00343406	0.219367	0.015654	0.98751	1
nocta	306.1105	0.074861368	0.297868	0.251324	0.801564	0.967776
supv3l1	258.6049	-0.111563983	0.164059	-0.68002	0.49649	0.878161
alyref	2638.913	0.046781608	0.101014	0.463119	0.643279	0.92593
spsb3a	475.6106	0.036348518	0.192577	0.188748	0.850291	0.978251
b3glctb	7.539789	-0.244905606	0.762469	-0.3212	0.748058	0.955074
zgc:175135	0.225112	-1.791433409	5.232815	-0.34235	0.732091	NA
si:ch73-38013.2	5.944429	-1.27732724	1.187774	-1.0754	0.282198	0.754654
AL935199.1	14.60853	0.182168971	0.522444	0.348686	0.727325	0.949095

usp54b	43.62567	-0.293120346	0.467912	-0.62644	0.531025	0.890545
nol9	401.7149	0.007166581	0.120369	0.059538	0.952523	0.998371
ascc1	139.2685	-0.011179083	0.384936	-0.02904	0.976832	1
nlgn4xb	31.61485	0.573947099	0.482513	1.189495	0.234245	0.714938
si:rp71-79p20.2	28.14518	-0.010611264	0.354397	-0.02994	0.976113	1
faah	59.48123	0.577015104	0.307282	1.877805	0.060408	0.422599
malt1	61.06111	-0.013860703	0.253585	-0.05466	0.95641	0.99899
cited1	242.7294	-0.391815429	0.14595	-2.6846	0.007262	0.144498
baalca	23.4074	-0.610718228	0.4861	-1.25636	0.208985	0.691152
csnk2b	3392.467	0.096631529	0.083456	1.15787	0.246917	0.727706
tmsb4x	31655.19	-0.059904395	0.153858	-0.38935	0.697019	0.94198
adamts9	114.9278	0.225938227	0.236131	0.956834	0.338651	0.795226
acer2	66.07128	-0.031461681	0.309559	-0.10163	0.919047	0.993892
SRMS	55.49335	0.279658764	0.252054	1.109518	0.267207	0.743418
atf5b	691.0428	-0.074461938	0.170383	-0.43703	0.662092	0.930742
arhgef10	222.8048	-0.038756762	0.156327	-0.24792	0.804195	0.968814
dcaf15	171.0795	-0.135476749	0.182875	-0.74082	0.458805	0.861537
brsk2b	462.1693	0.281113283	0.181218	1.551244	0.120843	0.566816
cdc42ep1b	37.20122	0.476834615	0.343165	1.389522	0.164674	0.634015
CU468012.1	6.013276	0.07884569	0.843987	0.093421	0.925569	0.994815
egr4	73.52982	-0.376501213	0.547533	-0.68763	0.491685	0.875929
hgsnat	691.0638	-0.077958414	0.124938	-0.62398	0.532643	0.890978
il13	3.893706	0	1.796583	0	1	1
otud4	74.37086	-0.298789616	0.299294	-0.99832	0.318126	0.779916
sox11a	2118.413	0.066672684	0.079452	0.839156	0.401382	0.832371
slc5a3b	37.6037	-0.106314717	0.407259	-0.26105	0.794054	0.965431
si:dkey-34f9.3	2.909612	-1.369464393	1.173937	-1.16656	0.243389	0.724437
cdc26	239.1264	-0.18277134	0.175348	-1.04234	0.297256	0.764844
cxxc4	143.9225	0.061317641	0.190817	0.321342	0.747951	0.955074
nrg2a	94.01303	0.081150523	0.192204	0.422209	0.672872	0.93414
si:dkey-6i22.5	76.80931	0.212759526	0.327692	0.649268	0.516165	0.88677
hmgxb3	85.02041	0.765112413	0.285755	2.67751	0.007417	0.145888
efcc1	130.6865	0.08287151	0.161646	0.512673	0.60818	0.915204
slc29a3	274.742	-0.418501646	0.178633	-2.3428	0.019139	0.243526
borcs5	74.25225	-0.433555801	0.251997	-1.72048	0.085345	0.490595
ttc22	60.08659	-0.251684502	0.251344	-1.00135	0.316655	0.778879
dhx30	135.5941	0.216542644	0.22737	0.95238	0.340904	0.795603
meis2a	1313.628	-0.157500364	0.13587	-1.1592	0.246374	0.727659
ajuba	537.9791	0.080905729	0.160177	0.505102	0.613487	0.915998
si:ch211-67f13.7	8.563767	0.175538862	0.877675	0.200004	0.841477	0.97643
olfm2a	485.1882	-0.029897666	0.202775	-0.14744	0.882783	0.986908
lgals3a	1498.901	-0.093544608	0.107896	-0.86699	0.385949	0.826939
samd11	506.6556	0.090237635	0.175113	0.515311	0.606336	0.915204
ttll2	7.581771	-0.041085943	0.827472	-0.04965	0.960399	0.999164
znrf2a	124.4563	-0.195045707	0.291514	-0.66908	0.503445	0.881759
asip1	48.21299	0.267714466	0.494447	0.541442	0.588203	0.907912
ndufaf4	127.0887	0.368406322	0.166021	2.219035	0.026484	0.288366
ankhd1	1370.077	0.092009338	0.156962	0.58619	0.557748	0.899184
sowahaa	34.36106	0.14265706	0.309873	0.460372	0.645249	0.926728
si:dkey-169i5.4	31.747	-6.405033687	1.049409	-6.10347	1.04E-09	7.76E-07
mettl22	97.83874	0.145027132	0.220238	0.658502	0.510216	0.883733
rasgrp3	28.18951	0.180773156	0.377597	0.478747	0.632119	0.923176
nostrin	8.542768	-0.068221831	0.728161	-0.09369	0.925355	0.994815
taf9	617.3575	-0.094077737	0.145236	-0.64776	0.517141	0.887148

CR626907.1	346.9158	-0.608131101	0.532255	-1.14256	0.253223	0.732759
vwa10.1	32.45213	0.523388474	0.555224	0.942663	0.345854	0.799025
slc2a5	39.49233	-0.533630894	0.515106	-1.03596	0.300219	0.766629
znf11h	0.578453	3.878207498	3.188963	1.216134	0.223934	NA
si:dkey-192l18.9	28.41583	0.386837489	0.458362	0.843955	0.398694	0.830925
si:ch211-255i20.3	96.12227	-0.412353506	0.751188	-0.54894	0.58305	0.90582
chl1a	220.6223	0.427005108	0.197956	2.157073	0.031	0.313376
ccn5	10.12856	0.139671854	0.579401	0.241062	0.809507	0.969828
fam83d	140.0366	0.271840036	0.314217	0.865134	0.386965	0.827592
znf296	324.4297	-0.240709036	0.129812	-1.85429	0.063698	0.430571
wu:fj20b03	0.652784	2.894477	2.679122	1.080383	0.279972	NA
si:dkey-28d5.11	0.046941	0	5.267649	0	1	NA
si:ch211-141o9.10	11.30396	-0.185262475	0.490511	-0.37769	0.705659	0.944274
herc1	1145.627	0.114633569	0.198048	0.578818	0.562712	0.900772
csmd3a	17.59921	0.311458721	0.555139	0.561046	0.574766	0.904516
rnf165a	330.2026	0.199830117	0.169098	1.181741	0.237308	0.718568
si:dkey-183p4.10	8.350086	-0.37793233	1.003767	-0.37651	0.706535	0.944683
rce1a	15.16313	0.31908515	0.413887	0.770948	0.440738	0.852579
si:zfos-1897c11.1	2.429914	0.142872262	1.247777	0.114501	0.90884	0.992033
treh	7.953953	-1.230806608	0.945389	-1.3019	0.192949	0.672855
bcorl1	177.9166	-0.110198776	0.210644	-0.52315	0.600869	0.913756
lmx1al	17.59183	-0.290318751	0.451051	-0.64365	0.519803	0.887148
dnase2b	9.412789	-0.855674651	0.778382	-1.0993	0.271638	0.747976
csmd3a	16.75247	-0.122116048	0.564338	-0.21639	0.828685	0.974157
si:dkey-48p11.3	96.07371	-0.154027956	0.211257	-0.7291	0.465938	0.865047
ccdc173	30.03834	-0.016560035	0.334895	-0.04945	0.960562	0.999243
tegt	2862.092	-0.279412963	0.125154	-2.23255	0.025579	0.283893
cd248b	445.6923	0.118197599	0.146676	0.805843	0.420333	0.842383
ints5	137.5705	-0.033916521	0.169967	-0.19955	0.841835	0.97643
smarcc2	608.5965	0.14160506	0.160973	0.879684	0.37903	0.823145
usp54a	338.2006	0.240618822	0.160102	1.502905	0.132864	0.586148
tor1	399.8217	-0.120903447	0.14856	-0.81384	0.415738	0.839167
rnf6	256.8488	0.124322283	0.149892	0.829413	0.406871	0.835226
CU207281.2	0.652988	0	2.729457	0	1	NA
si:ch211-186e20.7	166.6039	0.239850052	0.387078	0.619642	0.535493	0.891993
gltpb	6.282655	-0.011590805	0.878239	-0.0132	0.98947	1
si:ch211-140m22.7	1301.816	0.063669729	0.114755	0.554834	0.579008	0.905417
si:ch73-343l4.8	2.088087	-0.967394673	1.56676	-0.61745	0.536938	NA
tmem26a	34.50137	-0.187705786	0.395953	-0.47406	0.635457	0.924322
elf3	526.8199	-0.111011886	0.196542	-0.56483	0.572192	0.903461
GPR89B	49.79914	0.447293343	0.252441	1.771874	0.076415	0.468274
kiss2	3.137276	-1.246940629	1.420946	-0.87754	0.380192	0.823648
TMEM151A	81.00049	-0.147715008	0.33173	-0.44529	0.656112	0.929684
zfat	145.3801	-0.004754378	0.147205	-0.0323	0.974235	1
abcc10	146.4393	0.00403973	0.222188	0.018182	0.985494	1
b4galt2	27.72239	0.208768648	0.338629	0.616512	0.537557	0.892498
cdh24b	21.39503	0.302272575	0.509645	0.593104	0.553112	0.897809
nitr3a	2.834672	3.484172146	1.32578	2.628017	0.008588	0.158814
kbtbd7	33.2213	0.290132588	0.504048	0.575605	0.564882	0.901538
si:dkey-13n15.11	16.03556	-0.469001426	0.485194	-0.96663	0.333731	0.790676
drl	21.00939	0.395860002	0.394637	1.0031	0.315813	0.778379
wscd1b	133.0665	0.342662459	0.213146	1.607644	0.107913	0.539565
NAV1 (1 of many)	122.541	-0.119913808	0.235972	-0.50817	0.611334	0.915331
zgc:162060	0.086618	0	5.267649	0	1	NA

pacsin2	730.8932	0.244196506	0.176842	1.38087	0.167319	0.638363
dtx4b	123.0678	-0.352316941	0.236913	-1.48711	0.136985	0.5925
si:dkey-79d12.4	39.2207	-0.27846496	0.271685	-1.02495	0.305385	0.770528
fyco1b	77.31593	-0.001640642	0.210526	-0.00779	0.993782	1
zcchc24	741.3252	0.159526647	0.150723	1.058409	0.289869	0.760705
zgc:112001	92.33063	0.416581675	0.312884	1.331424	0.18305	0.659373
chpfb	56.31193	0.054390488	0.386017	0.140902	0.887948	0.988077
xpo1a	1219.982	-0.035196269	0.178324	-0.19737	0.843536	0.976791
il10rb	721.1913	-0.084529235	0.163445	-0.51717	0.605037	0.914985
phf19	85.42877	0.287202979	0.201973	1.421984	0.155031	0.619776
emilin3a	171.3259	-1.665846197	0.256256	-6.5007	7.99E-11	8.05E-08
acd	57.67168	-0.012002372	0.235297	-0.05101	0.959318	0.999164
RNF208	139.6543	0.602969922	0.34697	1.737816	0.082243	0.482668
snx8b	75.55815	0.106035995	0.612626	0.173084	0.862585	0.981202
dip2a	414.0603	-0.024567417	0.175059	-0.14034	0.888393	0.988188
nudcd2	215.5936	-0.135687348	0.179504	-0.7559	0.449708	0.856979
syt7b	73.62198	-0.529614988	0.431114	-1.22848	0.219267	0.700266
ftr05	15.17885	-1.306562104	0.52499	-2.48874	0.01282	0.197628
elk1	96.13664	-0.084784019	0.225041	-0.37675	0.70636	0.944623
abcg4b	33.87397	-1.094083321	0.36838	-2.96999	0.002978	0.090034
rrm2	872.4596	-0.146416575	0.274988	-0.53245	0.594416	0.910768
si:ch211-232i5.1	146.5949	-0.173079204	0.224826	-0.76984	0.441397	0.852874
nudt5	458.2885	-0.110630407	0.145376	-0.76099	0.44666	0.855582
ilrun	588.3099	-0.158738508	0.09787	-1.62192	0.10482	0.533188
nab2	36.75259	0.039201407	0.351668	0.111473	0.911241	0.992208
dph2	140.4792	-0.136609366	0.20798	-0.65684	0.511285	0.884659
lrfn4b	148.0794	0.708508815	0.292755	2.420142	0.015514	0.219725
armc5	151.2192	0.159444341	0.174414	0.91417	0.360627	0.811237
CNNM1	26.79829	-0.611898429	0.575089	-1.06401	0.287325	0.758355
si:ch211-186j3.6	170.0698	0.144650529	0.362308	0.399248	0.689711	0.939648
limd2	182.9169	-0.016476227	0.210089	-0.07842	0.93749	0.997305
zgc:172065	8.753633	-0.069022975	0.887783	-0.07775	0.938029	0.997331
lmf2a	56.72766	-0.889208759	0.283939	-3.13168	0.001738	0.064499
cipcb	489.9385	-0.220759312	0.401522	-0.54981	0.582452	0.90571
nlrc9	1.145507	-0.447479623	1.975687	-0.22649	0.820818	NA
psda	103.8779	0.235014594	0.191917	1.224564	0.22074	0.701955
depdc5	578.2458	-0.237400952	0.128709	-1.84448	0.065113	0.435355
ocrl	393.5481	0.108391561	0.124947	0.8675	0.385668	0.826939
dok1a	57.59788	-0.23333749	0.266777	-0.87465	0.381762	0.824957
usp12a	368.2709	-0.078080191	0.132602	-0.58883	0.555974	0.899002
ap5z1	133.771	0.00914183	0.178148	0.051316	0.959074	0.999164
atp5mea	774.1854	-0.182980192	0.184969	-0.98925	0.322541	0.78246
si:ch73-237c6.1	421.3818	-0.040764751	0.24893	-0.16376	0.86992	0.983365
sinhcafl	732.7616	0.08131488	0.148569	0.54732	0.584159	0.906402
sh3bp1	10.24701	1.101443565	0.571985	1.925651	0.054148	0.402505
slitrk3a	99.06526	0.514350363	0.324495	1.585082	0.112948	0.55033
rusc1	77.43913	0.514817243	0.292035	1.762859	0.077924	0.472646
neurl4	210.7263	0.129287361	0.176156	0.733939	0.462986	0.864191
CR391986.1	51.21188	0.308482764	0.279444	1.103917	0.269629	0.746036
tex2l	42.45313	-0.68116306	0.64081	-1.06297	0.287795	0.758441
kdm2ab	1007.634	0.165672528	0.122645	1.350825	0.176751	0.650052
crygm2f	60.21894	0.236679194	0.559331	0.423147	0.672188	0.933953
mrc2	106.1057	0.321569571	0.29904	1.075341	0.282222	0.754654
wdr47a	98.93997	0.049890881	0.229939	0.216974	0.828228	0.97403

si:ch211-202h22.8	58.22471	0.103438176	0.301548	0.343024	0.73158	0.950365
appl2	94.43755	-0.013163881	0.179419	-0.07337	0.941512	0.997371
si:dkey-18j18.3	35.22951	-0.174465977	0.334722	-0.52123	0.602209	0.914001
SPAG9	674.1926	-0.105744066	0.157706	-0.67051	0.50253	0.881359
si:ch211-218g4.2	129.5229	-0.128542739	0.201045	-0.63937	0.522579	0.887826
il10	2.000763	-2.364258798	1.692965	-1.39652	0.162558	NA
grin2ca	78.3692	0.039403863	0.41982	0.093859	0.925221	0.994815
si:ch211-264f5.8	87.78176	-0.301834304	0.374288	-0.80642	0.419999	0.842383
si:dkey-56i24.1	23.4577	0.517292413	0.422028	1.225729	0.220301	0.701707
inavaa	169.1187	0.030036182	0.173194	0.173425	0.862318	0.980994
SLC18A1	1.942952	-2.119766801	1.711739	-1.23837	0.215579	NA
zgc:175284	93.95939	-0.136871192	0.292768	-0.46751	0.640137	0.924877
pimr208	2.59391	1.361926206	1.21929	1.116983	0.264001	0.741984
hpse2	23.31335	0.085105309	0.348058	0.244515	0.806832	0.969726
znf576.2	505.2013	0.063751983	0.111457	0.571985	0.567332	0.901809
si:dkey-78p8.1	139.3669	-0.06125053	0.210283	-0.29128	0.77084	0.960484
si:dkeyp-100h4.1	12.30211	-0.56888923	0.777422	-0.73176	0.464313	0.864593
cacna2d2b	78.79283	0.283848047	0.307985	0.921631	0.356721	0.807388
CU984600.1	346.7548	0.113486494	0.206357	0.549952	0.582352	0.90571
cxcr3.1	0.863727	0	2.54136	0	1	NA
art4	3.075075	-0.828139183	1.926336	-0.4299	0.667266	0.933049
fndc3ba	384.1013	-0.020355865	0.168513	-0.1208	0.903852	0.991537
sc:d0202	0.899442	-3.353421406	2.089079	-1.60522	0.108446	NA
zgc:194443	0.322205	1.055369067	4.550563	0.231921	0.8166	NA
zgc:165453	1.609318	-2.536651411	1.665595	-1.52297	0.127766	NA
pacs2	55.07521	0.464739181	0.288299	1.612005	0.106961	0.537
tex2	318.7975	-0.000513285	0.162338	-0.00316	0.997477	1
slc12a5b	74.40267	0.424402611	0.363401	1.167865	0.242861	0.724396
fam160a2	170.0606	-0.064361619	0.142002	-0.45325	0.650372	0.927844
crygm3	5.411024	0.093643167	1.745384	0.053652	0.957213	0.999109
zbtb46	78.65368	0.270202906	0.213269	1.266955	0.205171	0.687166
si:ch211-67e16.3	16.6359	-0.505130524	0.510517	-0.98945	0.322443	0.78246
fhit	125.3121	0.081050232	0.254747	0.31816	0.750364	0.955074
vcam1a	1.289576	-0.86814291	2.186891	-0.39698	0.691385	NA
BX072576.1	3.976929	-1.089466945	1.20668	-0.90286	0.366598	0.816033
ccl34b.9	1.353646	0	4.963249	0	1	NA
zgc:171506	1.789493	-0.554802739	1.568351	-0.35375	0.723527	NA
tulp1b	23.79815	-0.250157289	0.564396	-0.44323	0.657599	0.930421
espn1a	22.04462	-0.282314139	0.51999	-0.54292	0.587183	0.907261
mybbp1a	781.7511	-0.070668056	0.191769	-0.36851	0.712496	0.945821
rbsn	54.81014	-0.827044735	0.270404	-3.05856	0.002224	0.074795
eps15	551.6816	0.132158766	0.10333	1.279002	0.200896	0.681045
cblc	94.49001	-0.132159049	0.186896	-0.70713	0.479488	0.86985
BX323458.1	10.3746	-0.144071178	0.526664	-0.27355	0.784427	0.962605
si:ch211-204d2.4	27.79543	0.270317857	0.387576	0.697457	0.485517	0.87303
aatka	181.6023	-0.066039259	0.178413	-0.37015	0.711271	0.945821
ora4	2.02421	0.922462741	1.641306	0.56203	0.574096	NA
cdh12a	11.88195	0.048523364	0.522698	0.092833	0.926037	0.994815
cspg4	141.3459	0.100186104	0.203796	0.491601	0.623002	0.918894
ctnnd1	1077.459	0.099268485	0.13949	0.711654	0.476679	0.869292
nlrc7	6.244818	-1.735505064	1.101357	-1.57579	0.115075	0.554651
nacc1b	6.413785	0.363720927	0.711181	0.511432	0.609049	0.915204
map7a	17.97635	-0.538139189	0.694734	-0.7746	0.438578	0.851718
si:ch211-197l9.2	96.78397	0.180073032	0.301674	0.596913	0.550566	0.8974

gal3st1b	36.94197	-0.450868407	0.393687	-1.14525	0.252107	0.732759
si:ch211-114l13.4	5.388713	2.968304008	1.452868	2.043065	0.041046	0.353808
vip	100.3552	0.089081294	0.430307	0.207018	0.835996	0.976007
unc13d	32.3549	-0.392730285	0.339328	-1.15738	0.247118	0.727871
zgc:194398	89.671	0.223332157	0.262502	0.850782	0.394891	0.828825
TESK1	162.7634	0.409673892	0.202989	2.018208	0.04357	0.365542
ftr14l	10.71681	-0.039909938	0.497926	-0.08015	0.936116	0.996958
ovol1b	21.91503	0.289854576	0.328716	0.881779	0.377896	0.82265
ora5	0.905017	-0.472519938	2.012534	-0.23479	0.814373	NA
CABZ01049847.1	61.56465	0.573178918	0.42041	1.36338	0.172763	0.645664
bnip1a	26.9293	-0.288698559	0.372078	-0.77591	0.437803	0.851283
nphp3	97.69406	0.007185061	0.224882	0.03195	0.974512	1
twist2	267.9109	-0.075213805	0.162545	-0.46273	0.64356	0.925997
si:dkey-181m9.8	98.48467	-0.139587514	0.200299	-0.69689	0.485869	0.873244
CU467961.1	88.48511	-0.162861697	0.263722	-0.61755	0.536871	0.892421
si:ch211-57m13.8	0.11354	0	5.267649	0	1	NA
FO704871.1	5.352557	0.74182262	0.876954	0.845908	0.397604	0.830253
foxp3b	0.788243	-1.450840058	2.030366	-0.71457	0.474874	NA
nkx3-1	31.03404	-0.177818893	0.374936	-0.47426	0.635311	0.924272
zgc:173575	73.25836	-0.342998564	0.212235	-1.61612	0.106068	0.534956
si:zfos-323e3.4	60.52542	0.036443687	0.241225	0.151077	0.879915	0.986339
phkb	897.0515	-0.080822281	0.155016	-0.52138	0.602103	0.914001
pik3ap1	37.51336	-0.014244226	0.384267	-0.03707	0.97043	1
CABZ01046425.1	0.397008	1.597148302	3.183833	0.501643	0.615918	NA
ptprh	0.702893	-0.868144273	2.615464	-0.33193	0.739944	NA
si:ch211-13f8.2	11.48189	0.525428419	0.752704	0.698055	0.485143	0.872911
zgc:162255	125.31	-0.215924641	0.183214	-1.17854	0.238581	0.719595
brinp1	84.6288	0.620635652	0.333264	1.862296	0.062561	0.427724
spry2	930.257	-0.118430091	0.124874	-0.9484	0.342927	0.797432
fam83b	69.83605	-0.237675762	0.227751	-1.04358	0.296681	0.764495
cecr2	342.9422	0.142322251	0.164626	0.864518	0.387303	0.827592
dcaf6	570.0091	0.314903988	0.11585	2.718213	0.006564	0.136957
pimr22	0.351992	0	4.374921	0	1	NA
MYO9B	485.7824	-0.044596239	0.163493	-0.27277	0.785028	0.962605
zgc:110063	255.0111	0.148850674	0.118493	1.256199	0.209044	0.691152
si:dkey-175m17.7	26.10854	-0.586171434	0.442338	-1.32516	0.185116	0.662704
tmod1	56.63119	-0.482247448	0.261451	-1.8445	0.06511	0.435355
col12a1a	4214.224	0.334268152	0.166	2.013663	0.044045	0.367392
si:ch211-263k4.2	137.3664	-0.187079227	0.226124	-0.82733	0.40805	0.835879
arhgap10	319.7373	-0.071337663	0.140162	-0.50897	0.610775	0.915331
CEP170B (1 of many)	34.00166	0.997177745	0.314173	3.173979	0.001504	0.058515
sec22a	263.3445	0.039926582	0.134929	0.295908	0.7673	0.960296
rec8a	0.564566	0	3.38654	0	1	NA
best1	4.991151	0.075635758	0.746392	0.101335	0.919284	0.993892
tmem234	56.4019	0.252476169	0.280651	0.899609	0.368328	0.817272
amot	336.9146	-0.024999639	0.136753	-0.18281	0.854948	0.979161
tcf20	880.1034	0.129941517	0.146705	0.885731	0.375763	0.822237
zmp:0000000845	8.218392	-0.630863926	0.749456	-0.84176	0.399921	0.831277
lyrm4	173.0686	-0.011018203	0.155308	-0.07094	0.943442	0.997371
zc3h4	790.6681	0.106493604	0.128695	0.827486	0.407962	0.835879
zmp:0000000846	603.5304	-0.013115506	0.156038	-0.08405	0.933014	0.996264
mcoln3a	50.03426	0.190535213	0.270189	0.705191	0.480691	0.870179
AL954361.1	1.551369	3.237580329	2.148026	1.507235	0.13175	NA
robo2	50.21223	0.189242648	0.298282	0.634442	0.525792	0.888982

unm_sa808	13.80829	-0.282488191	0.656581	-0.43024	0.66702	0.933039
trip11	647.5525	-0.059176843	0.127185	-0.46528	0.64173	0.925586
mcrip1	366.2872	0.09250686	0.119133	0.776502	0.437452	0.851283
dync1i2a	1262.333	-0.139338364	0.109353	-1.27421	0.20259	0.683051
si:dkey-92i15.4	65.08082	0.016924352	0.237204	0.071349	0.94312	0.997371
glis2a	159.6497	0.175640901	0.219011	0.801975	0.422568	0.843493
ifi46	87.75888	1.573835311	0.44944	3.501768	0.000462	0.026072
fam98a	252.9868	0.017184856	0.174314	0.098586	0.921467	0.994085
chpfa	66.97122	-0.419646772	0.218589	-1.9198	0.054883	0.405196
CABZ01059627.1	1.988457	1.974194773	1.363207	1.448199	0.147562	NA
si:ch211-163m17.4	0.092827	0	5.267649	0	1	NA
acsl2	115.2217	-0.276312309	0.234172	-1.17996	0.238017	0.718924
larp4aa	337.1828	0.277890955	0.174291	1.594411	0.110844	0.54583
cdh26.1	83.71543	-0.316348411	0.255164	-1.23979	0.215054	0.697086
hspb15	105.4503	-1.442910819	0.386413	-3.73412	0.000188	0.014357
lrrc3	8.036086	1.861495716	0.96158	1.935872	0.052883	0.399547
zeb2b	427.4525	0.155038284	0.148911	1.041145	0.297808	0.765407
plxdc1	26.38235	-0.80392164	0.467089	-1.72113	0.085227	0.490367
filip1a	18.8251	0.659881599	0.530746	1.24331	0.213754	0.696284
zgc:194285	97.50831	1.123371953	0.29624	3.792099	0.000149	0.012187
oat	251.2147	0.572761735	0.465647	1.230033	0.218685	0.699445
BX901889.2	0.193546	0	5.267649	0	1	NA
si:ch211-256e16.4	9.802429	1.906602247	0.818177	2.330305	0.01979	0.248394
vtg4	0.194876	0	5.267649	0	1	NA
tiam1a	696.3938	-0.010380504	0.144053	-0.07206	0.942554	0.997371
spc25	277.101	0.035205035	0.269133	0.130809	0.895926	0.98926
znf692	25.29658	-0.973976531	0.464322	-2.09763	0.035938	0.332937
anapc13	182.0693	-0.058459641	0.220871	-0.26468	0.791258	0.964766
ccdc88aa	60.75629	-0.102191844	0.352941	-0.28954	0.772165	0.960624
ppip5k2	432.8555	0.259023931	0.110819	2.337364	0.01942	0.245214
AL954696.1	228.236	0.067052298	0.213499	0.314064	0.753473	0.955861
ftr07	0	NA	NA	NA	NA	NA
tctn1	82.97312	0.182398803	0.230471	0.791416	0.428701	0.846502
gpr137ba	33.52252	0.926552271	0.469	1.975593	0.048201	0.382078
gzm3	1.23348	-1.274370805	2.010852	-0.63375	0.526246	NA
hmox2b	73.29909	0.190359863	0.20975	0.907554	0.364114	0.814549
best4	6.171644	-3.558182291	1.462983	-2.43214	0.01501	0.214412
ppp1r37	650.4984	-0.063131667	0.135885	-0.4646	0.642221	0.92567
cux1a	761.6176	0.038077192	0.148726	0.256022	0.797934	0.966447
fap	664.2666	-0.159648737	0.167579	-0.95268	0.340754	0.795603
nucks1a	4612.077	0.167332465	0.163774	1.021731	0.306908	0.772019
myo15ab	20.3353	0.384633648	0.586947	0.655313	0.512266	0.885459
klhl23	17.87567	0.98030455	0.637981	1.536572	0.124398	0.571885
znf990	20.09979	0.434836901	0.412245	1.054801	0.291516	0.761454
mocs1	56.12802	-1.011353534	0.336144	-3.00869	0.002624	0.083393
zgc:195081	27.27828	0.122598282	0.334891	0.366084	0.714303	0.945821
snpha	0.684091	2.999126888	2.926736	1.024734	0.305489	NA
si:ch211-284d12.3	0.601809	0	3.892613	0	1	NA
swi5	119.7511	-0.03535088	0.203308	-0.17388	0.861961	0.980696
adamts14	84.87102	-0.221302058	0.264352	-0.83715	0.402509	0.833401
timeless	454.692	0.032773111	0.127343	0.257361	0.7969	0.966447
dap3	524.1055	0.020896839	0.102757	0.203361	0.838853	0.976417
si:ch211-150o23.3	61.22269	0.230339086	0.265662	0.867038	0.385921	0.826939
si:dkey-76b14.2	10.3935	-0.431958977	0.651653	-0.66287	0.507416	0.883007

si:ch73-60h1.1	124.5832	0.459359149	0.289886	1.584618	0.113053	0.550449
wnt8a	0	NA	NA	NA	NA	NA
si:dkey-266m15.6	273.5326	0.286374924	0.201158	1.423629	0.154554	0.619425
socs4	2.899256	0.710195026	1.161848	0.611263	0.541025	0.893546
acaca	577.156	0.237642022	0.210709	1.127822	0.259395	0.736127
kazald2	118.4346	-0.35999261	0.206861	-1.74026	0.081813	0.481484
si:ch73-256j6.4	0.498768	0	3.545969	0	1	NA
ikbip	310.5264	0.134564814	0.238617	0.563937	0.572797	0.90359
si:ch211-80h18.1	1009.101	0.429288242	0.125167	3.429725	0.000604	0.031889
ddr1	484.83	-0.029195616	0.158523	-0.18417	0.853878	0.978905
noctb	145.3042	-0.23485612	0.196996	-1.19219	0.233188	0.714569
lingo4b	77.48415	0.43089771	0.251264	1.714919	0.08636	0.493048
adgrb1b	351.2712	-0.133435871	0.250999	-0.53162	0.59499	0.910869
poc1a	50.5217	-0.103171249	0.314702	-0.32784	0.743035	0.9547
lrrcc1	86.93855	0.107612385	0.188321	0.57143	0.567708	0.901809
si:ch211-158d24.2	69.24593	0.080481136	0.275226	0.292418	0.769967	0.960484
cers3a	57.86961	0.14110861	0.415104	0.339936	0.733905	0.95093
inpp5ja	9.021688	-0.187048516	0.767751	-0.24363	0.807516	0.969736
cars2	119.4351	0.456459816	0.187303	2.437014	0.014809	0.213551
si:ch211-264f5.2	14.50412	-0.172122807	0.727713	-0.23653	0.813025	0.970328
zgc:194839	133.709	-0.362039827	0.229801	-1.57545	0.115153	0.554815
zgc:171242	38.85834	-1.242793892	0.446792	-2.78159	0.005409	0.123724
grhl3	144.3876	-0.184081969	0.225264	-0.81718	0.413823	0.838564
si:ch211-57n23.4	6.25882	0.509286776	0.6729	0.756853	0.449138	0.856796
si:dkey-22i16.10	0.456571	-0.321977337	4.628244	-0.06957	0.944538	NA
slc9a5	76.69206	1.070567512	0.280599	3.815291	0.000136	0.011673
lrig2	532.0497	-0.137980094	0.159632	-0.86436	0.387388	0.827592
lonrf1l	898.6169	-0.289049357	0.188813	-1.53088	0.1258	0.573743
mpeg1.3	0.371558	0.815308977	4.32522	0.188501	0.850484	NA
wdr6	149.1252	-0.266718558	0.275033	-0.96977	0.332162	0.789602
stox2b	593.8606	0.013574989	0.108195	0.125468	0.900153	0.99045
si:dkey-21c19.3	410.2064	0.030547477	0.1263	0.241864	0.808885	0.969828
setd5	137.9011	0.388760349	0.175503	2.215125	0.026751	0.290183
fam222bb	260.7795	-0.103221436	0.139065	-0.74225	0.457934	0.860993
myo16	100.2872	0.352208332	0.274726	1.282033	0.199831	0.6804
mon1a	104.4281	-0.029088849	0.204404	-0.14231	0.886834	0.987671
nomo	930.2674	0.245492703	0.134023	1.831717	0.066994	0.441957
vwa8	183.8264	0.033717861	0.184298	0.182953	0.854835	0.979161
si:ch1073-184j22.2	118.6844	0.209926305	0.320398	0.655204	0.512336	0.885459
micu3a	151.3801	0.422111312	0.216588	1.948918	0.051305	0.394059
ldlrad3	360.1571	-0.309583534	0.169717	-1.82411	0.068135	0.445203
ptger1c	0.583777	0	2.762265	0	1	NA
myo1hb	29.41183	0.449336036	0.49641	0.905171	0.365375	0.815339
tbc1d10b	523.1826	-0.020161944	0.129341	-0.15588	0.876126	0.984856
ptger1a	37.83424	0.151919847	0.329507	0.461052	0.644761	0.926639
zgc:194224	51.79441	-0.336627552	0.35207	-0.95614	0.339003	0.795226
CT573423.1	0.184528	0	5.267649	0	1	NA
lrriq3	0.463007	-0.366115313	3.535242	-0.10356	0.917517	NA
vhll	32.61305	0.317659966	0.312705	1.015845	0.309703	0.774621
CABZ01085419.1	114.4784	0.108679782	0.189011	0.574993	0.565296	0.901538
inpp5kb	69.84301	0.221382236	0.249917	0.885822	0.375713	0.822237
pnp5a	2350.953	-0.706744246	0.185715	-3.80553	0.000142	0.011879
si:dkey-156m2.3	11.44119	1.212173598	0.803902	1.507862	0.13159	0.583305
scpp5	111.5092	-2.233800431	0.81106	-2.75417	0.005884	0.129841

arhgef9b	308.7816	-0.315955682	0.22028	-1.43433	0.151477	0.615372
fgf9	3.412919	-2.140981054	1.762908	-1.21446	0.224572	0.706495
BX322577.1	31.75702	-0.043165555	0.423104	-0.10202	0.91874	0.993884
zgc:171750	0	NA	NA	NA	NA	NA
fam117aa	266.097	-0.262570882	0.218922	-1.19938	0.23038	0.712099
vtcn1	22.49431	0.168470895	0.464989	0.362311	0.717119	0.94627
theg	11.12449	-0.49616851	0.497288	-0.99775	0.318401	0.779923
si:ch73-62l21.1	37.34074	0.746718213	0.368069	2.028746	0.042484	0.360968
samsn1b	70.24278	-0.672870661	0.519443	-1.29537	0.195192	0.67483
il21r.2	1.547366	1.055427497	2.410967	0.437761	0.66156	NA
kcna4	25.3207	-0.02951487	0.44373	-0.06652	0.946967	0.997371
zfyve1	161.647	-0.129445223	0.15478	-0.83632	0.402976	0.833582
ralbp1	148.0227	0.052571799	0.186142	0.282428	0.777615	0.961073
tpx2	1021.576	0.223194027	0.257257	0.867591	0.385619	0.826939
FNDC10	95.04715	0.092663481	0.23966	0.386646	0.699018	0.942499
supt20	136.3199	-0.000569819	0.21269	-0.00268	0.997862	1
tmem176	889.6933	0.005475022	0.155698	0.035164	0.971949	1
cdk5r2b	154.805	0.057324792	0.309259	0.185362	0.852945	0.978794
hspb9	7.647438	0.470087295	0.858446	0.547603	0.583965	0.906376
dock7	1002.22	0.087507959	0.156003	0.560936	0.574841	0.904516
myrf	132.5576	-0.102652494	0.224985	-0.45626	0.6482	0.927337
zmp:0000000524	183.961	0.354987171	0.243326	1.458895	0.144594	0.603539
rufy1	158.3808	0.388241897	0.2081	1.865648	0.062091	0.42715
arl8a	1429.94	-0.070559453	0.097652	-0.72256	0.469949	0.867349
frs3	20.36199	0.011390416	0.449373	0.025347	0.979778	1
gigyf1b	784.2824	0.035217833	0.121305	0.290325	0.771568	0.960624
phlpp1	463.6758	0.178590529	0.148732	1.200755	0.229846	0.711763
synpo2lb	234.3629	-0.27436965	0.192997	-1.42163	0.155135	0.619776
prdm13	166.0021	-0.191116602	0.268493	-0.71181	0.476581	0.869292
unm_hu7910	177.1734	-0.10466636	0.170701	-0.61316	0.539772	0.893049
sema7a	51.23218	0.23751987	0.384393	0.617909	0.536635	0.892421
itga8	325.3254	-0.087944721	0.18768	-0.46859	0.639363	0.924779
clip1a	301.6196	-0.05673382	0.159165	-0.35645	0.721506	0.947343
nos1apa	254.4468	-0.30879266	0.135915	-2.27195	0.02309	0.26812
znf1068	9.276194	0.27062016	0.693525	0.39021	0.696381	0.941759
ackr4a	25.17491	-0.37504542	0.428369	-0.87552	0.381291	0.824438
si:dkey-85k7.12	10.78111	0.892101183	0.701624	1.27148	0.203558	0.684359
cnnm2b	56.43664	0.004721304	0.33752	0.013988	0.988839	1
myo1f	81.79941	-0.10021878	0.24959	-0.40153	0.688027	0.938955
doc2a	21.0477	0.290236801	0.80865	0.358915	0.719658	0.94694
si:dkey-145c18.3	0.624469	0	3.045935	0	1	NA
FRMD1	319.2666	-0.027632237	0.141657	-0.19506	0.845342	0.976791
oxgr1a.2	0.049519	0	5.267649	0	1	NA
pnpla8	401.606	-0.123369303	0.13061	-0.94456	0.344884	0.79837
si:ch211-137a8.4	3141.056	0.0770815	0.078997	0.975749	0.329189	0.787409
b4galnt3a	12.15493	0.921410007	0.69947	1.317298	0.187739	0.666964
tpp2	666.6449	0.113913256	0.1926	0.591449	0.55422	0.898306
rubcn	45.28623	0.038317821	0.307745	0.124512	0.90091	0.99045
gra	39.35954	0.418792737	0.268417	1.560232	0.118705	0.561771
plppr4b	29.23533	0.283759543	0.476843	0.59508	0.55179	0.8974
si:ch211-212c13.8	261.9938	-0.21353822	0.455794	-0.4685	0.639429	0.924779
dna2	122.7428	0.115618597	0.234611	0.492811	0.622146	0.918461
cacna2d3	170.6493	-0.096121138	0.213479	-0.45026	0.652522	0.92891
rmdn2	40.0858	-0.441072496	0.301881	-1.46108	0.143994	0.603144

tmem169b	26.90563	-0.032968657	0.37095	-0.08888	0.92918	0.995381
igl3v3	0	NA	NA	NA	NA	NA
yeats2	124.36	0.026679262	0.19645	0.135807	0.891974	0.988566
abhd15a	123.1009	-0.074355352	0.21272	-0.34955	0.72668	0.949095
si:ch73-21k16.5	70.31519	-0.143653927	0.362493	-0.39629	0.691888	0.940274
nbr1b	690.6962	0.203690123	0.107515	1.89452	0.058156	0.41653
tmem70	121.2181	-0.140916154	0.184994	-0.76174	0.446218	0.855403
RASA2	33.55665	-0.205178506	0.287359	-0.71401	0.475218	0.869292
lrch1	264.4223	0.053691011	0.133817	0.401226	0.688254	0.93909
si:ch211-161c3.6	275.8488	-0.095106468	0.213323	-0.44583	0.655717	0.929606
mgaa	432.278	-0.195626283	0.206636	-0.94672	0.343781	0.798147
tmem258	631.0226	-0.077011906	0.17058	-0.45147	0.651649	0.928625
tmem184c	350.0611	-0.122885319	0.191249	-0.64254	0.520521	0.887165
lrrc18a	4.07687	0.072453146	0.879837	0.082348	0.93437	0.996793
dennd3a	250.1385	-0.207141975	0.168591	-1.22867	0.219196	0.700266
slc26a10	32.38436	-0.673369272	0.292519	-2.30197	0.021337	0.258949
msantd1	75.23113	0.024760321	0.282515	0.087642	0.930161	0.995707
rnf111	694.6342	-0.007493833	0.112744	-0.06647	0.947005	0.997371
gas2l1	296.3497	-0.178521083	0.188845	-0.94533	0.34449	0.798147
ap5b1	124.9447	0.113926042	0.190533	0.597933	0.549885	0.897373
paxx	113.0125	0.191401887	0.269771	0.709497	0.478016	0.869292
zgc:174320	0.314655	0	5.267649	0	1	NA
si:dkey-34m19.3	118.7098	-0.307228514	0.186071	-1.65114	0.09871	0.521402
grk5l	138.3514	0.024122322	0.179631	0.134288	0.893175	0.988566
rnf165b	93.25697	-0.107151552	0.205029	-0.52262	0.601242	0.913756
si:ch211-225k7.5	0	NA	NA	NA	NA	NA
ccdc18	101.2042	0.234445388	0.19917	1.177114	0.23915	0.719781
si:ch211-66e2.3	41.68432	-0.053759939	0.245392	-0.21908	0.82659	0.973383
bub1ba	161.155	0.117177327	0.218121	0.537211	0.591122	0.909388
npb	103.6905	0.147339027	0.282636	0.521304	0.602155	0.914001
ccdc149a	17.52777	-1.348566574	0.518015	-2.60333	0.009232	0.165181
si:dkey-73p2.3	25.44429	1.077128256	0.752989	1.430471	0.152582	0.61658
mrpl58	97.75726	-0.014895497	0.255635	-0.05827	0.953535	0.998436
si:dkey-28o19.1	5.064015	0.473440971	0.868992	0.544816	0.58588	0.906655
si:ch211-195b13.6	3.859257	0.073683368	1.310241	0.056237	0.955153	0.998611
tns1a	495.0858	0.195401577	0.136954	1.426766	0.153647	0.61823
BX571839.1	1.724628	-0.080903997	1.390903	-0.05817	0.953616	NA
si:dkey-238o13.4	157.1281	-0.245907366	0.164869	-1.49154	0.135821	0.591131
plekhm2	162.1999	-0.252531564	0.176887	-1.42764	0.153394	0.617897
arhgef19	106.3912	-0.017787145	0.322204	-0.0552	0.955975	0.998845
tusc3	506.1977	0.128341561	0.196269	0.653906	0.513173	0.886073
slc22a23	287.6023	0.011929686	0.182005	0.065546	0.947739	0.997371
g0s2	31.78186	0.58729028	0.409232	1.435105	0.151257	0.615279
lats2	367.4025	0.067650759	0.178452	0.379097	0.704616	0.943785
cxxc5a	880.3927	-0.056921042	0.146474	-0.38861	0.697566	0.942152
sdk1a	198.5506	-0.09568415	0.212478	-0.45032	0.652476	0.92891
tmem74b	8.301413	0.234209565	0.686277	0.341276	0.732896	0.950636
adm2b	6.201035	-0.415466315	0.677472	-0.61326	0.539704	0.893049
si:ch73-244f7.3	13.87541	-0.533937549	0.669282	-0.79778	0.425	0.844714
slc22a31	353.0095	-0.299324364	0.198253	-1.50981	0.131091	0.582816
atp10d	184.7818	-0.198421482	0.170115	-1.16639	0.243456	0.724437
iqgap1	957.1907	-0.055349062	0.10479	-0.52819	0.597369	0.911953
wdfy3	272.0057	0.267508449	0.189003	1.415368	0.15696	0.622766
map1l	270.8326	-0.687204374	0.169879	-4.04526	5.23E-05	0.005794

hemk1	213.6788	-0.116149338	0.182094	-0.63785	0.523569	0.888595
pcdh7a	488.0782	0.274434091	0.145421	1.887164	0.059138	0.419032
anks1ab	31.93256	-0.341373397	0.376418	-0.9069	0.364459	0.814648
rims1b	56.76846	0.701184187	0.309837	2.263076	0.023631	0.271997
brd4	605.1697	0.375367345	0.193913	1.935752	0.052898	0.399547
fbxo41	25.9212	0.429315153	0.364853	1.17668	0.239323	0.719781
zgc:163098	403.9389	0.09664006	0.118069	0.818507	0.413068	0.838274
si:ch73-24k9.2	32.01101	0.317637586	0.402396	0.789365	0.429899	0.847418
mmp17a	23.39638	0.149054367	0.477124	0.312402	0.754735	0.956283
chrdl2	18.36848	-0.539460904	0.556138	-0.97001	0.33204	0.789602
zgc:195245	23.97068	0.823967631	1.094532	0.752804	0.451568	0.857676
comtd1	395.2712	-0.615493226	0.406152	-1.51543	0.129665	0.579651
CR790388.1	3.144686	-1.622378638	1.089776	-1.48873	0.136559	0.59191
tmco4	95.71474	0.053363373	0.231914	0.230099	0.818015	0.971518
BX323994.1	0.132469	0	5.267649	0	1	NA
abhd16a	495.8752	-0.060670923	0.139211	-0.43582	0.662967	0.931141
ttll4	686.2914	0.204145988	0.128208	1.592298	0.111318	0.547032
CR936408.1	0	NA	NA	NA	NA	NA
ano9a	0	NA	NA	NA	NA	NA
enox2	248.8345	0.010160542	0.15327	0.066292	0.947145	0.997371
sdccag8	60.28084	-0.08169449	0.221037	-0.3696	0.711684	0.945821
epn3a	438.8216	0.020255498	0.109947	0.184229	0.853834	0.978905
rxrba	461.0957	0.095058882	0.156144	0.608789	0.542664	0.894595
tpcn3	184.3807	0.134948954	0.173927	0.775893	0.437812	0.851283
duox2	9.944904	-1.016737662	0.822423	-1.23627	0.216358	0.69815
BX901881.1	0.188555	-0.86811591	5.267649	-0.1648	0.8691	NA
esyt2b	179.543	0.408767617	0.174313	2.345027	0.019026	0.242745
rbm15b	387.1763	-0.046566759	0.140679	-0.33101	0.740634	0.953996
il7r	10.11894	-0.06629532	0.796312	-0.08325	0.93365	0.996576
uckl1b	277.7877	-0.175579649	0.210226	-0.83519	0.403608	0.833888
COA4	110.0486	0.384442491	0.210561	1.825804	0.06788	0.444287
igl1c3	0.684591	0	3.111234	0	1	NA
arhgef40	93.5269	0.223506363	0.310073	0.720819	0.471021	0.868025
faxcb	68.85463	0.350272744	0.254427	1.376711	0.168602	0.63999
fam118b	108.5301	0.036895098	0.1862	0.198148	0.842929	0.97668
mplkip	232.5643	-0.062662889	0.149922	-0.41797	0.675969	0.935448
alpk3a	270.1936	-0.084589089	0.23446	-0.36078	0.718262	0.946598
si:ch211-195e19.1	361.8513	-0.061506438	0.116423	-0.5283	0.59729	0.911953
wnk1a	1129.115	0.153398151	0.155616	0.985747	0.324257	0.783308
muc2.2	0.139193	-0.868103029	5.267649	-0.1648	0.869102	NA
tex11	11.15121	0.33396724	0.555998	0.600663	0.548064	0.896644
dclk3	6.246418	0.633807591	0.782485	0.809993	0.417944	0.841312
tp53bp1	787.2858	-0.018117313	0.132363	-0.13688	0.891129	0.988566
si:dkey-7f3.9	196.1398	0.577733047	0.270352	2.136964	0.032601	0.319477
abca1b	1430.193	0.083803002	0.215441	0.388984	0.697288	0.942027
si:dkey-7i4.5	15.49298	0.343045157	0.482685	0.710702	0.477269	0.869292
col17a1b	296.1671	-0.013878662	0.187705	-0.07394	0.941059	0.997371
cbfa2t3	90.17578	0.360748121	0.206208	1.749437	0.080215	0.478492
dpy19l3	425.3618	0.211060145	0.205953	1.024796	0.30546	0.770634
brca2	112.8775	0.177202739	0.196721	0.900783	0.367704	0.817078
tlnrd1	132.9315	0.115064894	0.187975	0.612129	0.540453	0.893173
gcfc2	145.6184	-0.116129269	0.181381	-0.64025	0.52201	0.88764
rps6kl1	57.70235	1.176326853	0.552169	2.130376	0.033141	0.322362
chsy1	288.7417	0.132586472	0.150273	0.882306	0.377611	0.822478

dhx32b	208.9872	0.164983979	0.190311	0.866916	0.385988	0.826939
ftr39p	30.67945	0.102454998	0.45788	0.22376	0.822944	0.971966
si:ch211-22d5.2	27.8123	-1.243899024	0.450631	-2.76035	0.005774	0.128995
zpax4	9.252823	-0.997873508	0.583418	-1.71039	0.087193	0.495407
zgc:174573	17.67353	0.074692875	0.382585	0.195232	0.845211	0.976791
znf1lk	1.361228	-2.113778409	1.676391	-1.26091	0.207341	NA
si:dkeyp-75b4.10	71.73812	1.530907248	0.964458	1.587325	0.112439	0.549251
opn8b	51.06081	0.13921794	0.242772	0.573452	0.566339	0.901739
rabl6a	68.86366	0.437055019	0.209454	2.086639	0.036921	0.337141
cercam	279.944	0.03137485	0.165705	0.189342	0.849825	0.97824
adgre8	0.093881	0	5.267649	0	1	NA
si:dkey-85a20.4	100.696	-0.065068533	0.239869	-0.27127	0.786186	0.962605
si:ch211-194c3.5	439.1023	0.083532803	0.145153	0.575479	0.564967	0.901538
retreg1	182.7055	-0.510898731	0.338932	-1.50738	0.131714	0.583645
samd1b	80.03801	-0.113605514	0.241596	-0.47023	0.638191	0.924779
CABZ01079080.1	21.31941	-0.108343642	0.496439	-0.21824	0.827241	0.973459
zgc:172215	55.16496	0.048460738	0.297631	0.162822	0.870659	0.983365
mms19	240.1838	-0.147371092	0.173642	-0.84871	0.396045	0.8295
adam12	21.20971	-0.706312566	0.402761	-1.75368	0.079486	0.477246
jam2b	241.9855	-0.413556236	0.250046	-1.65392	0.098144	0.521076
frmpd1a	10.59565	-0.160257176	0.703884	-0.22768	0.819898	0.971788
si:ch211-71m22.3	8.776932	-4.465747356	1.351597	-3.30405	0.000953	0.042958
hgfb	21.60593	0.428076396	0.456205	0.938342	0.348068	0.800392
si:ch211-5k11.8	3.037451	-1.231415955	1.063472	-1.15792	0.246897	0.727706
sult1st7	18.84028	-4.971451934	0.927033	-5.36275	8.20E-08	4.32E-05
etfbkmt	42.30037	0.225471804	0.29996	0.751674	0.452247	0.857994
fbxw8	211.8007	0.046431883	0.206857	0.224463	0.822397	0.971966
rin1a	38.21492	0.330378959	0.311788	1.059629	0.289314	0.760367
tanc2a	533.2269	0.127785696	0.147311	0.867453	0.385694	0.826939
PRRG3	10.21031	-0.144879855	0.600385	-0.24131	0.809314	0.969828
mfhas1	289.0208	-0.118227051	0.191757	-0.61655	0.537535	0.892498
mhc2dab	17.20861	1.055540128	0.531747	1.985043	0.04714	0.378022
clec11a	22.82732	-0.143521349	0.444063	-0.3232	0.746543	0.955074
cluap1	108.0166	-0.155556362	0.240811	-0.64597	0.5183	0.887148
actc1c	3542.861	-0.067333824	0.175988	-0.38261	0.702013	0.943292
si:ch211-229d2.5	479.3693	-0.334085395	0.196873	-1.69696	0.089705	0.502623
tmx2a	93.11652	0.502882769	0.220499	2.280661	0.022569	0.265653
ikbkb	78.10971	-0.204773144	0.285726	-0.71668	0.473573	0.869146
MTERF4	131.2383	-0.108481559	0.197765	-0.54854	0.583323	0.905948
sowahab	57.125	-0.255993348	0.242943	-1.05372	0.292012	0.761655
znf1li	0.641312	0.084152151	2.587642	0.032521	0.974057	NA
becn1	302.411	-0.080926652	0.142932	-0.56619	0.571265	0.902817
opn4xa	6.6941	0.60368507	0.755467	0.799089	0.424239	0.844355
si:dkeyp-118b1.2	156.0219	-0.042298358	0.166763	-0.25364	0.799771	0.967206
si:dkey-10c21.1	11.89792	0.234504435	0.768831	0.305014	0.760355	0.957886
si:ch73-278m9.1	53.22319	0.000305883	0.293856	0.001041	0.999169	1
bcl2l11	20.50861	-0.3240579	0.428723	-0.75587	0.449728	0.856979
CABZ01077217.1	97.3309	0.544406637	0.263134	2.068933	0.038552	0.344223
nckap5l	163.4097	0.350856396	0.260266	1.34807	0.177636	0.65136
si:ch211-276i12.9	612.576	-0.083267821	0.141699	-0.58764	0.556775	0.899063
si:ch211-67f13.8	7.525535	1.992066832	0.927414	2.14798	0.031715	0.31688
thpo	3.088479	0.543844877	1.174013	0.463236	0.643195	0.92593
srxn1	16.26359	-0.31270321	0.386665	-0.80872	0.418677	0.84177
bsnb	280.6262	0.274595088	0.290798	0.944283	0.345025	0.79837

best2	5.413673	-1.333337649	1.14437	-1.16513	0.243967	0.724522
galr1b	16.54809	-0.15662457	0.466219	-0.33595	0.736911	0.952272
ace	332.2551	-0.226198179	0.336686	-0.67184	0.501687	0.881077
si:ch211-230g15.5	250.402	-0.080187826	0.160265	-0.50034	0.616833	0.916365
si:ch211-79k12.1	355.6906	-0.21743164	0.179935	-1.20839	0.226898	0.707983
sod3b	79.76337	0.496659122	0.278585	1.782795	0.07462	0.46402
si:ch211-163m16.7	1.165625	-0.797482786	2.157465	-0.36964	0.711652	NA
tbx19	4.807256	0.655324674	1.118025	0.586145	0.557778	0.899184
nxph2b	46.372	-0.254297885	0.491475	-0.51742	0.604865	0.914985
zgc:172122	37.95132	0.261482902	0.348466	0.750382	0.453025	0.858265
usp13	749.458	-0.249245369	0.183908	-1.35527	0.175331	0.649279
megf6a	77.36584	0.287569279	0.275215	1.044888	0.296075	0.764246
tshz2	397.2579	0.037064291	0.147642	0.251041	0.801782	0.967776
parp2	268.3122	-0.082854328	0.135251	-0.6126	0.540142	0.893049
adam11	56.54768	0.797052244	0.275226	2.895988	0.00378	0.102427
dpep2	50.37603	-0.304096306	0.313841	-0.96895	0.33257	0.789756
bbs9	81.59816	0.194278988	0.255879	0.759261	0.447697	0.856366
mfn1b	557.2601	0.125760278	0.111973	1.123131	0.261382	0.738975
wdr20a	201.1743	-0.072627191	0.164725	-0.4409	0.659285	0.930581
si:ch211-162k9.6	4.831913	-0.208097118	0.773581	-0.269	0.787926	0.963339
zgc:174315	71.93266	-0.261420735	0.271294	-0.96361	0.335242	0.791962
plekhs1	21.36475	0.456792446	0.713615	0.640111	0.522101	0.887728
znf414	2.390027	-0.15103591	1.349413	-0.11193	0.910881	0.992208
slc7a14b	9.25791	-0.339330087	0.787171	-0.43108	0.666414	0.932664
si:ch211-160f23.5	130.7714	0.269682363	0.197064	1.368501	0.171155	0.643325
si:ch211-285c6.2	2.099956	-3.754649522	1.635278	-2.29603	0.021674	NA
si:dkey-253d23.11	27.61562	-0.112674768	0.372207	-0.30272	0.762103	0.958345
trim59	79.10298	0.089265493	0.272613	0.327444	0.743332	0.954856
wdr90	84.76252	0.011858954	0.240552	0.049299	0.960681	0.999243
BX901889.3	0	NA	NA	NA	NA	NA
pigw	65.55125	0.254188033	0.287622	0.883756	0.376828	0.822258
si:dkey-73p2.2	28.9595	0.818141631	0.742341	1.10211	0.270414	0.746683
mn1b	389.0308	-0.270134418	0.149316	-1.80915	0.070428	0.452779
ogdhl	50.85558	-0.185966683	0.567462	-0.32772	0.743126	0.954765
BX323590.1	25.98641	-0.379110203	0.461292	-0.82184	0.411165	0.837797
nlgn2b	89.2361	0.483958646	0.323451	1.496236	0.134592	0.589174
fam117ab	23.93082	0.306349162	0.345064	0.887805	0.374646	0.821243
plch1	165.1988	0.294573606	0.209196	1.408123	0.159095	0.624783
zgc:174935	144.4213	-0.529350669	0.199228	-2.657	0.007884	0.150098
gemin5	177.1429	0.196793871	0.1819	1.08188	0.279306	0.752477
si:dkeyp-2e4.2	11.21775	-0.71767053	0.490602	-1.46284	0.143512	0.602607
baiap3	29.39159	0.717624331	0.45113	1.590725	0.111672	0.547596
foxe1	39.17955	-0.035654002	0.379894	-0.09385	0.925226	0.994815
im:7154036	37.22057	0.755699277	0.29397	2.570665	0.01015	0.174988
CR589947.1	11.5896	0.244259152	0.675179	0.361769	0.717524	0.946379
dcxr	225.7735	-0.213095261	0.190243	-1.12012	0.262661	0.740207
prss59.1	47478.27	0.09358879	1.678631	0.055753	0.955539	0.998611
ube2ql1	91.4371	0.084077497	0.217739	0.38614	0.699393	0.942499
zgc:174877	150.3405	-0.026108484	0.157214	-0.16607	0.868102	0.982801
dhrsx	180.9622	0.280461981	0.188814	1.485385	0.137442	0.593264
si:dkeyp-75h12.2	136.5933	-0.207521949	0.188194	-1.1027	0.270156	0.746532
si:ch211-247j9.1	359.3728	-0.133256751	0.175722	-0.75834	0.448248	0.856741
bcr	522.8715	0.203265721	0.150529	1.350342	0.176906	0.650415
si:ch211-162k9.5	5.151253	-1.074336031	0.795222	-1.35099	0.176699	0.650047

rapgef3	82.34535	-0.119789819	0.273231	-0.43842	0.661082	0.930581
gdpd4b	44.29337	-0.043943355	0.294779	-0.14907	0.881497	0.986908
or115-15	4.155302	-0.265154771	1.093984	-0.24238	0.808489	0.969828
cacna1bb	26.66197	0.630965159	0.463894	1.360149	0.173783	0.646897
gcga	94.82389	0.535045555	0.295924	1.808048	0.070599	0.453373
and2	11037.98	0.006192201	0.130263	0.047536	0.962086	0.999451
hbae3	47136.63	0.017346574	0.275884	0.062876	0.949865	0.99801
rlim	545.6737	0.062829996	0.092718	0.677648	0.497995	0.878926
si:dkey-205h13.1	79.27504	-0.574898195	0.470525	-1.22182	0.221775	0.703656
tor3a	176.1685	0.340895109	0.195327	1.745257	0.08094	0.479801
st3gal1l	9.524633	0.256884617	0.709754	0.361935	0.717401	0.946272
kmt2ca	571.5806	-0.04414846	0.164023	-0.26916	0.787806	0.963294
pelp1	824.7269	0.086262939	0.105114	0.820661	0.41184	0.838075
lpxn	20.89725	-0.375044365	0.439936	-0.8525	0.393938	0.828374
dlc1	370.4977	-0.046323736	0.167454	-0.27664	0.78206	0.962311
rgmb	711.0416	-0.007710713	0.145853	-0.05287	0.957838	0.999109
ano9b	144.75	0.088255777	0.181615	0.485949	0.627003	0.920988
CABZ01046432.1	0.07132	0	5.267649	0	1	NA
cabin1	76.98935	0.124603275	0.245896	0.506731	0.612343	0.915709
hmcn2	661.149	0.12425602	0.198853	0.624864	0.532061	0.890978
pgbd4	37.48319	0.101969957	0.276148	0.369258	0.711935	0.945821
cop1	254.8443	-0.078365935	0.154135	-0.50842	0.611157	0.915331
AL928650.1	3.99444	-0.532954765	0.911447	-0.58473	0.558726	0.899409
cd40lg	1.450825	0.093647464	2.133879	0.043886	0.964995	NA
strc1	47.62543	0.586375795	0.44127	1.328835	0.183902	0.660725
slc47a4	37.98549	-1.110523697	0.544468	-2.03965	0.041385	0.355014
si:ch211-217k17.10	4.425841	0.875076162	0.957771	0.913659	0.360896	0.811253
zgc:194659	193.3872	-0.946079618	0.588677	-1.60713	0.108026	0.539897
GRIN2B (1 of many)	21.67843	0.210623443	0.508191	0.414457	0.67854	0.936026
znf654	421.4508	0.071759082	0.114007	0.629429	0.529068	0.890325
sap130b	193.0374	0.160854325	0.159926	1.005804	0.31451	0.777479
si:ch211-165d12.4	112.7819	0.090935046	0.249695	0.364185	0.71572	0.9461
flrt2	151.2906	0.212705117	0.185712	1.145349	0.252065	0.732759
abcg2b	21.15715	-1.76638372	1.590057	-1.11089	0.266614	0.743108
CR377211.1	2.226042	0.66568286	1.205236	0.552326	0.580725	0.905618
ppp1r9ba	715.6895	0.256345908	0.15456	1.658549	0.097207	0.519839
si:ch211-212g7.6	272.0712	0.268155186	0.135576	1.97789	0.047941	0.380801
lrrc3ca	12.23195	0.514948383	0.522609	0.985341	0.324457	0.783308
si:ch211-264f5.6	342.0604	0.135634194	0.169033	0.80241	0.422316	0.843493
fosl1b	2.229073	-0.338338781	1.409979	-0.23996	0.810361	0.969828
tjp1b	739.2835	-0.02664839	0.127045	-0.20976	0.833858	0.975487
zgc:174153	70.53486	0.686318876	0.470559	1.458518	0.144698	0.603718
arhgef38	80.66603	-0.20846993	0.235696	-0.88449	0.376434	0.822258
phldb1b	207.8	-0.126945325	0.153086	-0.82924	0.406969	0.835261
hdx	192.9942	-0.150897615	0.151872	-0.99358	0.320426	0.780821
si:ch211-235m3.5	34.53555	-0.184503325	0.322451	-0.57219	0.567193	0.901795
si:ch211-102c2.4	75.12991	0.518320739	0.276602	1.873888	0.060946	0.423677
agrn	1095.88	-0.00747553	0.165002	-0.04531	0.963864	0.999842
mrpl42	482.2969	-0.384953165	0.209542	-1.83712	0.066192	0.439276
tmprss15	7.814222	-1.79718306	1.453891	-1.23612	0.216414	0.69815
lrfn5b	223.5807	0.181978686	0.212625	0.855868	0.392071	0.827902
cerkl	33.66324	0.510420587	0.384955	1.325922	0.184865	0.662235
F2RL3	51.72518	-0.142896168	0.294254	-0.48562	0.627235	0.921095
si:dkey-197c15.6	131.6167	0.139464217	0.174615	0.798698	0.424466	0.844412

tapbp.1	33.60452	-0.828324496	0.382103	-2.16781	0.030173	0.309482
si:dkey-204l11.1	3.760356	0.449649797	1.126082	0.399305	0.689669	0.939646
ftr02	33.3912	-0.233445796	0.360491	-0.64758	0.517258	0.887148
sez6b	33.39086	-0.151561458	0.348655	-0.4347	0.663778	0.931603
dnajc30b	148.6656	-0.054444181	0.210239	-0.25896	0.795664	0.966097
zgc:174895	103.147	0.162204042	0.252815	0.641591	0.521139	0.887473
dpp4	169.3567	0.394267219	0.208785	1.888393	0.058973	0.418576
npbwr2a	8.681031	0.276761366	0.58227	0.475314	0.634563	0.924272
si:dkey-12f6.5	0.272039	0	5.267649	0	1	NA
tmem131l	603.2795	-0.171966981	0.12526	-1.37288	0.16979	0.641498
pkp3b	351.3857	-0.170538198	0.136036	-1.25363	0.209978	0.691821
coro2ba	237.4976	-0.278455848	0.182867	-1.52272	0.127828	0.577117
BX649448.1	3.060781	-4.219523993	1.567407	-2.69204	0.007102	0.14271
vipb	84.59735	-1.238356268	0.512528	-2.41617	0.015685	0.221082
si:dkey-238f9.1	100.9776	0.462226371	0.308184	1.499838	0.133656	0.587056
rbm46	3.963592	-0.260560366	1.050784	-0.24797	0.80416	0.968814
atp8b4	23.98281	-0.417194901	0.360294	-1.15793	0.246893	0.727706
nlgn4xa	234.6571	0.254922682	0.242838	1.049764	0.293827	0.763054
si:ch211-149a19.3	111.5959	0.711806178	0.222296	3.202068	0.001364	0.054641
si:dkey-57h18.1	42.26902	-0.208523566	0.277958	-0.7502	0.453136	0.858335
pimr126	0	NA	NA	NA	NA	NA
si:ch211-131k2.3	126.8788	0.231087106	0.252855	0.913912	0.360763	0.811253
man2b2	255.8014	0.26371644	0.203898	1.293374	0.195882	0.676219
si:ch73-15b2.5	194.9684	0.377853354	0.273776	1.380156	0.167539	0.638828
rhobtb1	15.68059	0.697858179	0.414848	1.682203	0.092529	0.508758
trpc7b	7.330894	-0.385422682	0.677864	-0.56858	0.569639	0.902322
cdk17	232.5952	-0.0920036	0.148465	-0.6197	0.535455	0.891993
si:dkey-88j15.4	0.716164	1.055423907	3.018678	0.349631	0.726616	NA
CABZ01071020.1	27.4046	0.018692135	0.446974	0.041819	0.966643	1
map3k21	124.2065	0.095949712	0.191221	0.501774	0.615827	0.916365
MSANTD2	59.72109	-0.004076676	0.258932	-0.01574	0.987438	1
nckipsd	597.8419	-0.401110895	0.153438	-2.61416	0.008945	0.16209
mvb12bb	16.86888	0.046478618	0.515111	0.09023	0.928104	0.995052
gpr4	6.018076	1.024779465	0.774196	1.323668	0.185613	0.663476
GID4	72.29585	-0.005690851	0.190473	-0.02988	0.976165	1
CABZ01072096.1	31.47453	-0.135983923	0.370236	-0.36729	0.713403	0.945821
kcnt1	20.10134	1.195637946	0.738356	1.619324	0.105378	0.534618
fam20a	140.0107	-0.769711796	0.279308	-2.75578	0.005855	0.129451
zgc:195282	8.016974	-0.453411261	0.621289	-0.72979	0.465518	0.864997
bicd1a	61.18219	0.115788717	0.235574	0.491516	0.623061	0.918894
tcima	813.0968	-0.052802626	0.246355	-0.21434	0.830285	0.974908
stk32a	0	NA	NA	NA	NA	NA
kif3cb	244.4991	0.300786048	0.149616	2.010381	0.044391	0.368519
si:ch211-149l1.2	69.91155	0.640070847	0.228492	2.801282	0.00509	0.119231
gal3st2	34.27672	0.419752526	0.303759	1.38186	0.167015	0.637452
mfn2	615.9684	0.12546397	0.13443	0.9333	0.350665	0.802215
fam114a2	508.0204	-0.036224839	0.113321	-0.31966	0.749222	0.955074
ptx3b	10.28195	0.372103141	0.564961	0.658636	0.51013	0.883733
cisd1	749.5153	-0.079902252	0.119808	-0.66692	0.504826	0.88195
nitr1f	0	NA	NA	NA	NA	NA
eys	1.296305	-0.719267892	1.800419	-0.3995	0.689525	NA
si:ch211-184m13.4	4.553965	1.584436654	0.980267	1.616332	0.106022	0.534956
znf1lg	0.61388	0.642657839	2.44125	0.263249	0.792358	NA
slc39a5	215.4651	-0.120472957	0.251386	-0.47923	0.631772	0.923003

si:dkey-17m8.1	274.7811	0.102925627	0.138718	0.741976	0.458102	0.860993
zgc:194242	34.80671	-0.489912063	0.368389	-1.32988	0.183559	0.660414
zgc:175135	0.043309	0	5.267649	0	1	NA
ftr13	24.02676	0.000760448	0.459091	0.001656	0.998678	1
stard13a	270.9311	-0.005751454	0.125335	-0.04589	0.963399	0.999842
dpys	221.0916	-0.239781277	0.289105	-0.82939	0.406883	0.835226
si:ch1073-464p5.5	1122.913	-0.137850246	0.169136	-0.81502	0.415058	0.838732
zbtb47b	9.170477	1.165711289	0.691435	1.685929	0.091809	0.507669
cdc42ep1a	79.51092	-0.162632376	0.303911	-0.53513	0.592559	0.909996
maml3	268.4896	0.131392095	0.151418	0.867745	0.385534	0.826939
cd83	23.10378	0.113251296	0.529633	0.21383	0.83068	0.974908
ccdc85cb	119.6365	0.027900603	0.199521	0.139838	0.888788	0.988188
ptprh	15.46874	0.070708384	0.750882	0.094167	0.924976	0.994815
myh7	872.1281	0.207715973	0.194704	1.066831	0.286048	0.757455
rspo2	157.5539	0.059824538	0.165263	0.361995	0.717356	0.946272
mfsd4b	113.9881	-0.336787726	0.193665	-1.73902	0.082031	0.482272
plcd3b	88.85328	0.339194762	0.226095	1.500229	0.133555	0.586903
aff3	209.6284	-0.16680324	0.243199	-0.68587	0.492794	0.876558
rbpms2b	494.5274	-0.250136382	0.129206	-1.93595	0.052874	0.399547
nyap2a	32.93044	0.340893392	0.368904	0.924071	0.355449	0.806546
zgc:194930	19.49306	-0.202263983	0.402797	-0.50215	0.615563	0.916365
pkn3	139.8992	0.199960412	0.176952	1.130026	0.258465	0.735636
gabrb2a	183.8911	1.076779327	0.393205	2.738469	0.006173	0.132917
castor1	101.2628	-0.287699923	0.216389	-1.32955	0.183667	0.66056
si:dkeyp-73d8.6	20.37546	0	1.805556	0	1	1
si:ch211-203d1.3	107.9005	-0.37036114	0.31648	-1.17025	0.241899	0.72348
calhm3	14.6017	0.818691248	1.120054	0.730939	0.464816	0.864823
si:ch211-256e16.6	20.65551	0.058960883	0.356221	0.165518	0.868536	0.982801
prmt5	318.401	-0.177577695	0.147344	-1.20519	0.22813	0.709324
SGPP2	30.95537	0.122735349	0.335611	0.365708	0.714583	0.945821
akap9	1133.584	-0.289559621	0.140289	-2.06403	0.039015	0.345405
sema4c	230.3435	-0.192154041	0.140981	-1.36298	0.17289	0.645664
sall3a	101.9957	0.046710674	0.247361	0.188836	0.850221	0.978251
ftr34	2.205951	-1.760183476	1.964205	-0.89613	0.370183	0.818484
cramp1	453.0771	-0.133218789	0.12877	-1.03455	0.300881	0.767015
sik2a	22.97104	0.001748243	0.537333	0.003254	0.997404	1
amigo1	36.48921	-0.002676759	0.429193	-0.00624	0.995024	1
frmd5	101.1838	-0.179367407	0.189855	-0.94476	0.344782	0.798302
ubtd1b	43.10457	0.108222744	0.351932	0.30751	0.758455	0.957865
amer1	430.9863	0.123661389	0.129607	0.954126	0.34002	0.795302
atr	133.8141	-0.233254423	0.27345	-0.85301	0.393656	0.828374
elf2b	513.0959	0.041855381	0.111846	0.374223	0.708238	0.945344
si:ch211-113e8.10	372.4826	0.253998498	0.127016	1.999729	0.045529	0.373513
si:dkey-28b4.7	378.7311	-0.144123673	0.148377	-0.97134	0.331381	0.789035
filip1b	136.7372	-0.034258192	0.231866	-0.14775	0.88254	0.986908
fam177a1	638.8743	-0.03996108	0.115662	-0.3455	0.729718	0.950056
alpk2	340.6873	-0.271336897	0.260089	-1.04325	0.296834	0.764495
PRRC2B	1908.898	0.017328263	0.163626	0.105901	0.915661	0.99311
sc:d217	12.65003	-7.264993327	3.142075	-2.31216	0.020769	0.255762
muc13b	156.7646	-0.780528166	0.413455	-1.88782	0.05905	0.418792
si:ch1073-174d20.2	608.4633	0.121811728	0.163198	0.746404	0.455424	0.860008
caap1	307.4621	0.129184069	0.163879	0.78829	0.430527	0.847542
cyp2x6	3.406604	-0.108738432	0.994587	-0.10933	0.912941	0.99273
st3gal1	1.100106	0.131056273	1.917614	0.068343	0.945512	NA

fam171a2b	53.9373	0.045816429	0.273821	0.167323	0.867116	0.982582
si:dkey-53k12.1	0.375525	0	4.315831	0	1	NA
wdr53	191.1332	0.242372378	0.129486	1.871807	0.061233	0.424867
CU855821.1	1.323972	1.0531366	2.041168	0.515948	0.605891	NA
zgc:172341	97.06807	0.920266721	0.335086	2.746356	0.006026	0.131072
gpr158b	89.80081	0.869383139	0.390751	2.224901	0.026088	0.286608
lrrc7	152.3029	0.358980751	0.26192	1.370576	0.170507	0.64208
plppr4a	197.2456	0.084599974	0.259792	0.325645	0.744693	0.95491
efl1	93.92215	-0.004501596	0.218715	-0.02058	0.983579	1
cep112	91.73636	0.395560268	0.225998	1.750284	0.080069	0.478179
CABZ01059627.2	0.932069	-2.352350848	2.287025	-1.02856	0.303685	NA
taf6l	250.852	-0.01751962	0.183242	-0.09561	0.923831	0.994713
naa30	392.972	-0.037416647	0.135996	-0.27513	0.783216	0.962406
rnf10	5571.05	-0.296161883	0.07962	-3.71969	0.000199	0.014952
tnrc6a	1774.909	0.05122283	0.182219	0.281106	0.778629	0.961073
zgc:194908	1.280857	-0.868143195	2.079344	-0.41751	0.676307	NA
asb13a.1	75.5188	-0.270037333	0.295212	-0.91472	0.360337	0.811057
zgc:162612	122.7426	0.089170817	0.170944	0.521636	0.601923	0.914001
b3galt1a	19.2811	-0.448406988	0.403721	-1.11069	0.266704	0.743178
wdr81	323.1457	-0.348640496	0.162416	-2.14659	0.031826	0.317164
si:dkey-18p12.4	33.09691	2.086200562	1.80197	1.157733	0.246973	0.727706
si:ch211-152p11.4	68.09431	-0.111173682	0.259463	-0.42848	0.668304	0.933086
gal3st4	489.561	0.448938209	0.129724	3.460707	0.000539	0.028963
ccl27b	2.605181	0.486175547	1.280308	0.379733	0.704143	0.943741
sin3aa	694.4269	-0.016052613	0.143953	-0.11151	0.911209	0.992208
rbm12b	399.2819	0.013023387	0.119864	0.108651	0.913479	0.992796
si:dkey-46g23.1	202.2416	0.175469313	0.203533	0.862118	0.388622	0.827613
dhx9	934.5614	0.184689083	0.188219	0.981245	0.326472	0.785445
selenop2	667.9544	-1.328229293	0.275672	-4.81815	1.45E-06	0.000386
fuz	44.00504	0.421453145	0.349332	1.206455	0.227642	0.708829
kif13ba	475.7916	0.051899996	0.201822	0.257157	0.797058	0.966447
znf219	423.5137	0.252500177	0.112126	2.251936	0.024326	0.276024
efna2b	42.3001	-0.151634688	0.266258	-0.5695	0.569016	0.902234
CSRNP3	16.99215	0.226405537	0.462684	0.489331	0.624607	0.919918
mcf2l2	56.75108	-0.181217627	0.32761	-0.55315	0.58016	0.905618
si:ch211-166a6.5	2601.582	-0.02114849	0.133473	-0.15845	0.874104	0.98385
megf8	613.7111	0.058540867	0.134068	0.43665	0.662365	0.930742
col6a4a	1215.735	0.106036318	0.221148	0.479482	0.631596	0.923003
kiz	118.2695	-0.116806296	0.20057	-0.58237	0.560316	0.899928
peak1	152.82	0.072816638	0.292159	0.249236	0.803178	0.968418
zgc:112998	26.86098	-0.387678066	0.361305	-1.07299	0.283274	0.755028
si:dkey-253d23.4	436.3379	0.547067034	0.205321	2.664447	0.007712	0.148279
si:ch211-112f3.4	40.69449	0.403941437	0.366342	1.102635	0.270186	0.746532
tap1	11.40741	0.082362797	0.619365	0.13298	0.89421	0.988777
si:ch73-296e2.3	0.260891	-1.733484505	4.769356	-0.36346	0.716259	NA
peal5	47.35209	0.128173622	0.273731	0.468247	0.639608	0.924779
nox5	8.602262	0.078379971	1.128317	0.069466	0.944618	0.997371
map6d1	44.885	1.60396873	0.504752	3.177734	0.001484	0.057996
radil	51.20528	0.522329654	0.294935	1.771	0.076561	0.468545
hnrnpul1l	2123.428	0.078447608	0.116191	0.675161	0.499574	0.879603
slitrk4	182.6982	0.508850552	0.243089	2.093266	0.036325	0.335171
myh7l	230.0532	-0.505279252	0.343245	-1.47207	0.141003	0.599658
isg20	490.4857	-0.219385325	0.225319	-0.97366	0.330223	0.788126
si:ch211-235o23.1	316.2193	0.143669762	0.15681	0.916206	0.359559	0.810644

si:dkeyp-73a2.2	0.147936	0	5.267649	0	1	NA
fam171a1	330.8038	0.041342326	0.175025	0.236209	0.813271	0.970328
LO018102.1	55.3605	0.31937619	0.281399	1.134958	0.256393	0.73401
si:ch211-176g6.2	10.62525	0.1118585	0.778531	0.143679	0.885754	0.987533
CABZ01033206.1	101.1251	-0.291788193	0.276928	-1.05366	0.292039	0.761655
ldah	65.28502	0.006946302	0.274628	0.025293	0.979821	1
aanat2	52.86795	-0.651034818	0.34189	-1.90422	0.056881	0.411856
tagln3a	15.16396	0.090334119	0.515946	0.175084	0.861013	0.980388
si:ch73-208g10.1	15.92775	0.074259427	0.556552	0.133428	0.893855	0.988675
micall1a	57.41641	0.292130944	0.272087	1.073669	0.282971	0.755028
lyrm7	168.4705	0.365675867	0.183136	1.996744	0.045853	0.374848
rhbdd3	96.98416	0.124850835	0.186886	0.66806	0.504096	0.881759
helq	49.99064	0.363018874	0.260134	1.39551	0.162862	0.630534
mrps36	1055.487	-0.050287696	0.12092	-0.41588	0.677501	0.936026
ninj2	21.47352	-1.905432884	0.748933	-2.5442	0.010953	0.182182
gpr174	6.131878	1.157901333	0.845845	1.368928	0.171022	0.643066
srbd1	21.68159	-0.276013601	0.433032	-0.6374	0.523865	0.888642
ccr2	3.736701	-1.458300121	1.002294	-1.45496	0.14568	0.604973
kdf1a	95.15604	-0.414204635	0.26867	-1.54169	0.12315	0.569695
man2c1	213.2679	0.043111585	0.182381	0.236382	0.813136	0.970328
arrdc1b	66.22198	0.003454092	0.210997	0.01637	0.986939	1
kcnma1a	214.3556	0.071953213	0.168071	0.428113	0.668569	0.933086
igl4v8	0.203413	0	5.165707	0	1	NA
zgc:194578	389.8337	0.505783111	0.172795	2.927072	0.003422	0.096214
gmps	938.3859	-0.271666684	0.119266	-2.27782	0.022737	0.266612
nrtn	6.971879	0.49507044	1.206828	0.410225	0.681641	0.937418
dchs1b	441.552	0.130676222	0.161607	0.808606	0.418742	0.84177
scyl1	594.3596	0.052470696	0.123666	0.424294	0.671352	0.933953
si:ch211-189k9.2	217.4524	0.34922039	0.228706	1.526942	0.126775	0.575696
tmem163a	37.76528	0.09930249	0.339694	0.29233	0.770035	0.960484
ppp1r16b	45.60618	0.336536941	0.266622	1.262226	0.206868	0.689246
kl	23.40115	-0.842810386	0.472802	-1.78259	0.074653	0.464106
si:ch211-218o21.4	23.9256	0.69109907	0.464173	1.488883	0.136518	0.591902
rapgef4	35.44963	0.078065294	0.419576	0.186058	0.8524	0.978648
SLC29A4 (1 of many)	66.41408	0.90890081	0.358621	2.534429	0.011263	0.183909
CU855878.1	77.51405	-0.290754603	0.241022	-1.20634	0.227686	0.708829
tmem151bb	9.384096	-0.654376941	0.69239	-0.9451	0.344608	0.798147
tas2r200.1	0.460706	0	4.72486	0	1	NA
tcaim	209.5811	0.102521154	0.204955	0.500212	0.616926	0.916365
trim107	43.3987	-0.236313259	0.321072	-0.73601	0.461723	0.863402
otulinb	148.028	0.042027012	0.175423	0.239575	0.81066	0.969828
phldb2a	144.2171	-0.251652108	0.255232	-0.98597	0.324146	0.783161
dnajc6	491.2497	0.268782814	0.180954	1.485366	0.137447	0.593264
si:dkey-183c2.4	13.41304	0.362292091	0.75584	0.479324	0.631708	0.923003
ehbp1l1b	217.773	0.032141214	0.269483	0.11927	0.905061	0.991631
si:dkey-7k24.5	7.805803	-2.100596119	1.538338	-1.3655	0.172097	0.644494
shroom4	367.6849	-0.066342578	0.141232	-0.46974	0.63854	0.924779
si:ch73-15n24.1	52.15585	-4.218778937	1.285753	-3.28117	0.001034	0.045108
si:ch73-343l4.2	6.646869	-0.814760046	0.86692	-0.93983	0.347303	0.799992
mark2a	769.8692	-0.134622959	0.113871	-1.18224	0.237112	0.718568
ptger4c	4.994973	-1.317236224	1.14666	-1.14876	0.250655	0.730618
tmem151a	78.41429	0.051435811	0.235552	0.218363	0.827147	0.973459
rapgef1l	77.11282	0.192694434	0.262995	0.732693	0.463745	0.864593
stoml3a	27.98545	0.357681078	0.641379	0.557675	0.577066	0.904798

pot1	56.68453	0.174418864	0.228945	0.761839	0.446156	0.855403
rassf7a	150.5473	0.132955771	0.210847	0.63058	0.528315	0.889948
klf4	21.7312	-0.198209593	0.515126	-0.38478	0.700401	0.942841
AL954695.2	1.083077	0.759446354	2.034883	0.373214	0.708989	NA
syt15	77.02164	-0.634538504	0.422886	-1.50049	0.133486	0.586903
si:dkeyp-68b7.5	36.95357	-0.324801826	0.296495	-1.09547	0.27331	0.749148
snx19b	257.2126	-0.204073791	0.164334	-1.24182	0.214302	0.696474
zgc:152830	1856.639	0.315000529	0.125195	2.516086	0.011867	0.18962
SLC46A3 (1 of many)	89.85137	0.107826152	0.278404	0.3873	0.698534	0.942433
zgc:162200	289.2598	0.323008676	0.140534	2.298431	0.021537	0.260177
jmjd1cb	1503.316	0.051951098	0.176103	0.295004	0.767991	0.960296
pjvk	8.7485	-0.708525678	0.646768	-1.09549	0.273304	0.749148
sqlea	66.86675	0.500846068	0.278974	1.795312	0.072604	0.457864
gfi1b	21.81314	0.146945052	0.349	0.421046	0.673721	0.934514
supt16h	1406.889	-0.083668129	0.148081	-0.56502	0.572062	0.903461
eloal	4.69333	-0.765965121	0.78614	-0.97434	0.329889	0.788084
doc2d	85.18505	-0.561749946	0.30952	-1.8149	0.069539	0.450071
dlx2a	253.51	0.060998717	0.12654	0.48205	0.629771	0.92285
prkd3	125.8818	-0.065460499	0.177868	-0.36803	0.712852	0.945821
nfatc2b	8.219173	0.986465388	0.704492	1.40025	0.161438	0.628449
pus10	120.9382	0.158069509	0.167868	0.941627	0.346383	0.799463
nhsb	243.7462	0.11190914	0.200041	0.559431	0.575868	0.904739
samd10a	111.8894	-0.063357132	0.239197	-0.26487	0.791107	0.964766
fam89a	78.33897	-0.356049742	0.30682	-1.16045	0.245866	0.727177
si:ch211-202h22.9	9.568321	1.055428896	1.751871	0.602458	0.546869	0.896107
agbl2	24.6954	0.080560693	0.446702	0.180345	0.856881	0.979558
nrip2	241.6301	0.309289596	0.187182	1.652351	0.098463	0.521103
BX927329.1	9.82772	-1.983513676	0.790087	-2.5105	0.012056	0.190742
igl3v5	0	NA	NA	NA	NA	NA
senp6b	0.402307	-0.86813702	4.302287	-0.20178	0.840085	NA
akap13	412.4819	0.021308793	0.162918	0.130794	0.895938	0.98926
si:zfos-223e1.2	14.8985	-1.021218004	0.447605	-2.28151	0.022518	0.265534
si:dkey-178e17.1	1.130718	1.891336941	2.288066	0.826609	0.408459	NA
tmem41b	78.50448	-0.262426408	0.185905	-1.41161	0.158063	0.623766
bahcc1b	667.176	0.299018183	0.151626	1.972076	0.048601	0.383778
adh5	2663.898	-0.068939988	0.12543	-0.54963	0.582574	0.90571
lrrtm4l1	16.16412	0.370413049	0.787789	0.470193	0.638217	0.924779
si:cabz01032454.3	34.86646	0.776730432	0.382522	2.030551	0.042301	0.359936
kif16bb	50.23793	-1.414027303	0.385433	-3.66867	0.000244	0.016928
UBA6	232.2095	-0.018624055	0.158062	-0.11783	0.906204	0.991631
il13ra1	133.6092	-0.097699931	0.232739	-0.41978	0.674643	0.934877
zmat3	7.748078	0.699305458	0.782131	0.894103	0.371267	0.818945
RF00001	0.517384	1.055415165	3.061407	0.344748	0.730284	NA
dre-mir-125b-2	0	NA	NA	NA	NA	NA
RF00001	0.269807	1.055353695	5.267649	0.200346	0.84121	NA
dre-let-7d-2	0	NA	NA	NA	NA	NA
RF00001	0.307786	1.055340904	5.267649	0.200344	0.841212	NA
dre-mir-192	1.90489	-0.868148283	2.313381	-0.37527	0.707458	NA
RF00001	0	NA	NA	NA	NA	NA
dre-mir-459	0	NA	NA	NA	NA	NA
RF00001	0	NA	NA	NA	NA	NA
dre-mir-133c	4.934564	-0.425387731	0.968944	-0.43902	0.660646	0.930581
RF00001	0	NA	NA	NA	NA	NA
RF00001	0.045161	0	5.267649	0	1	NA

dre-mir-135c-1	0	NA	NA	NA	NA	NA
RF00001	0	NA	NA	NA	NA	NA
RF00001	0	NA	NA	NA	NA	NA
NC_002333.1	3.438334	0.868796272	0.955853	0.908922	0.363391	0.813662
RF00026	0	NA	NA	NA	NA	NA
RF00001	19.4945	0.7178623	0.501254	1.432132	0.152106	0.615705
NC_002333.2	10.94595	-0.738448513	0.750893	-0.98343	0.325397	0.784089
dre-mir-29b-1	18.25811	2.564829509	1.569413	1.63426	0.102204	0.528963
RF00088	0.261706	0	5.267649	0	1	NA
RF00093	0.050624	1.055396947	5.267649	0.200354	0.841203	NA
dre-mir-15a-1	0.046941	0	5.267649	0	1	NA
RF00001	0	NA	NA	NA	NA	NA
RF00440	0.053506	-0.86807634	5.267649	-0.16479	0.869106	NA
dre-mir-124-5	32.98597	0.281709153	0.559566	0.503442	0.614653	0.915998
dre-mir-499	0	NA	NA	NA	NA	NA
RF00571	2.480231	-0.638450426	1.185527	-0.53854	0.590206	0.908901
mir338-2	0	NA	NA	NA	NA	NA
RF00001	0.138104	-0.86810168	5.267649	-0.1648	0.869102	NA
RF00001	0	NA	NA	NA	NA	NA
RF00612	0	NA	NA	NA	NA	NA
RF00001	0.08847	0	5.267649	0	1	NA
RF00001	0.847031	-2.341569717	2.847329	-0.82237	0.410864	NA
RF00001	0	NA	NA	NA	NA	NA
dre-let-7c-2	0	NA	NA	NA	NA	NA
RF00001	0	NA	NA	NA	NA	NA
RF00001	0	NA	NA	NA	NA	NA
RF00001	0.237859	-1.791188523	4.951468	-0.36175	0.71754	NA
RF00001	0.546985	-0.868142213	3.163063	-0.27446	0.783729	NA
RF00026	0	NA	NA	NA	NA	NA
RF00001	0.086616	0	5.267649	0	1	NA
RF00001	0.101446	-1.755425691	5.23528	-0.33531	0.737394	NA
RF00001	0	NA	NA	NA	NA	NA
RF00001	0	NA	NA	NA	NA	NA
RF00001	0.04222	0	5.267649	0	1	NA
RF00001	0	NA	NA	NA	NA	NA
NC_002333.3	12.02514	-0.779248293	0.675433	-1.1537	0.248623	0.728723
NC_002333.4	11048.85	-0.115898097	0.230935	-0.50186	0.615763	0.916365
RF00001	0	NA	NA	NA	NA	NA
dre-mir-130b	0.847426	1.185918438	2.00651	0.591035	0.554497	NA
dre-mir-456	0	NA	NA	NA	NA	NA
RF00413	0.973869	-2.38179113	1.867908	-1.27511	0.20227	NA
RF00001	0	NA	NA	NA	NA	NA
RF00581	0.423469	3.21668106	3.499536	0.919174	0.358005	NA
RF00001	1.103177	0.064430824	1.872775	0.034404	0.972555	NA
NC_002333.5	0.629146	1.904905651	2.629175	0.724526	0.468743	NA
RF00026	0	NA	NA	NA	NA	NA
RF00001	0	NA	NA	NA	NA	NA
RF00548	0.043308	0	5.267649	0	1	NA
RF00001	0	NA	NA	NA	NA	NA
RF00211	0.087179	0	5.267649	0	1	NA
RF00001	0	NA	NA	NA	NA	NA
RF00604	0	NA	NA	NA	NA	NA
RF00001	0.060442	0	5.267649	0	1	NA
RF00003	0	NA	NA	NA	NA	NA

RF00001	0	NA	NA	NA	NA	NA
RF00566	1.528061	1.08476814	1.28162	0.846404	0.397327	NA
mir140	1.026904	0.952204451	2.220623	0.428801	0.668068	NA
RF00342	0.179442	0	5.267649	0	1	NA
dre-mir-183	0.04222	0	5.267649	0	1	NA
dre-mir-21-2	1.193586	-0.255532298	1.605339	-0.15918	0.87353	NA
RF00001	0	NA	NA	NA	NA	NA
dre-mir-141	0.223858	1.055358154	5.267649	0.200347	0.841209	NA
RF00377	0.046941	0	5.267649	0	1	NA
RF00396	2.313002	-0.488063972	1.24226	-0.39288	0.694405	0.941093
RF00001	0	NA	NA	NA	NA	NA
dre-mir-125c	0.204566	0.117895018	5.207558	0.022639	0.981938	NA
RF00003	0	NA	NA	NA	NA	NA
dre-let-7j	1.189803	1.978481967	2.141952	0.923682	0.355652	NA
RF01299	0.18577	-0.86811591	5.267649	-0.1648	0.8691	NA
RF00089	0.835464	2.996048059	2.522951	1.187517	0.235024	NA
RF00056	0.232063	0	5.267649	0	1	NA
dre-mir-23a-1	0	NA	NA	NA	NA	NA
dre-mir-153a	0	NA	NA	NA	NA	NA
RF00619	0.563131	-1.823196973	4.032311	-0.45215	0.651163	NA
dre-mir-212	0.04222	0	5.267649	0	1	NA
dre-mir-731	0	NA	NA	NA	NA	NA
RF00001	0.147133	1.055383603	5.267649	0.200352	0.841205	NA
dre-mir-135c-2	0	NA	NA	NA	NA	NA
dre-mir-455-2	0.089161	0	5.267649	0	1	NA
dre-mir-26b	1.473703	1.011306237	1.98134	0.510415	0.609761	NA
mir223	1.038139	-2.172594633	2.478376	-0.87662	0.380693	NA
dre-mir-26a-1	0	NA	NA	NA	NA	NA
dre-mir-9-3	0.160586	0	5.267649	0	1	NA
dre-mir-24-3	0.353443	0	3.754398	0	1	NA
dre-mir-724	0	NA	NA	NA	NA	NA
dre-mir-9-2	0.195607	1.055362173	5.267649	0.200348	0.841209	NA
NC_002333.6	4.967964	-0.608605289	0.896156	-0.67913	0.497056	0.878572
RF00096	0.043308	0	5.267649	0	1	NA
RF00093	0.258045	1.055368603	4.829836	0.21851	0.827032	NA
dre-mir-10b-1	0	NA	NA	NA	NA	NA
RF00001	0	NA	NA	NA	NA	NA
RF00396	1.238305	-0.298098158	2.499846	-0.11925	0.90508	NA
dre-mir-124-1	0.61067	1.055386397	4.301	0.245382	0.806161	NA
RF00001	0.167824	0	5.267649	0	1	NA
RF00001	0	NA	NA	NA	NA	NA
RF00001	0	NA	NA	NA	NA	NA
si:dkey-71b5.7	183.0887	0.830187345	0.291156	2.851347	0.004353	0.108751
dre-mir-730	0.046941	0	5.267649	0	1	NA
RF00001	0	NA	NA	NA	NA	NA
RF00407	1.405081	0.117864211	1.28822	0.091494	0.9271	NA
RF00070	0.094679	0	5.267649	0	1	NA
dre-mir-375-2	9.727635	0.454471887	0.656677	0.692078	0.488888	0.873964
dre-mir-200c	0	NA	NA	NA	NA	NA
NC_002333.7	0.837286	-0.76171964	2.689716	-0.2832	0.777026	NA
RF00001	0	NA	NA	NA	NA	NA
RF00001	0.439406	0	4.174686	0	1	NA
RF00001	0	NA	NA	NA	NA	NA
dre-mir-17a-2	0.190607	-1.755450757	5.235279	-0.33531	0.73739	NA

RF00611	0.050624	1.055396947	5.267649	0.200354	0.841203	NA
RF00007	0.11354	0	5.267649	0	1	NA
dre-mir-181a-2	0.090321	0	5.267649	0	1	NA
RF00004	0	NA	NA	NA	NA	NA
RF00571	5.374964	1.309865877	0.929715	1.40889	0.158868	0.624456
RF01225	0.245644	-1.755465446	5.235278	-0.33531	0.737388	NA
RF00001	0	NA	NA	NA	NA	NA
mir16c	0	NA	NA	NA	NA	NA
RF00003	0	NA	NA	NA	NA	NA
RF00001	0	NA	NA	NA	NA	NA
RF00001	0	NA	NA	NA	NA	NA
RF00045	2.482061	-0.108987169	1.175381	-0.09272	0.926122	0.994815
dre-mir-27b	0.046941	0	5.267649	0	1	NA
RF00001	0.08553	0	5.267649	0	1	NA
dre-mir-152	0.043308	0	5.267649	0	1	NA
RF00396	0	NA	NA	NA	NA	NA
RF00001	0.102781	1.055383603	5.267649	0.200352	0.841205	NA
dre-mir-194a	0.353928	0	4.390747	0	1	NA
RF00056	1.039583	0	2.31356	0	1	NA
dre-mir-1788	0.170632	1.11785931	5.226359	0.213889	0.830634	NA
dre-let-7a-5	0.44549	0.093656397	2.937245	0.031886	0.974563	NA
dre-mir-722	0	NA	NA	NA	NA	NA
RF01291	1.947181	-0.868139122	1.994647	-0.43523	0.663392	NA
RF00003	0	NA	NA	NA	NA	NA
RF00001	0.136135	0	5.267649	0	1	NA
RF00001	0.655036	0	2.868673	0	1	NA
dre-mir-24-1	0	NA	NA	NA	NA	NA
RF00152	0.137987	0	5.267649	0	1	NA
dre-mir-18b	0	NA	NA	NA	NA	NA
RF00001	0.04222	0	5.267649	0	1	NA
RF00001	0.043309	0	5.267649	0	1	NA
RF00049	0	NA	NA	NA	NA	NA
RF00575	0	NA	NA	NA	NA	NA
RF00572	0.059954	1.055396947	5.267649	0.200354	0.841203	NA
RF00001	0	NA	NA	NA	NA	NA
dre-mir-30c	0	NA	NA	NA	NA	NA
dre-mir-31	0.253118	3.171067785	5.177695	0.612448	0.540242	NA
RF00001	0.240709	-1.769377521	5.234318	-0.33803	0.735338	NA
RF00030	5.698298	0.666664017	1.526286	0.436788	0.662265	0.930742
RF00001	0	NA	NA	NA	NA	NA
RF00066	0.114342	-0.868089684	5.267649	-0.1648	0.869104	NA
RF00020	0.197341	0	5.236633	0	1	NA
RF00273	0.086618	0	5.267649	0	1	NA
RF00001	0	NA	NA	NA	NA	NA
dre-mir-125a-2	0.904373	-1.746339453	2.174451	-0.80312	0.421907	NA
dre-mir-135a	2.095477	1.055436053	1.851589	0.570016	0.568667	NA
RF00001	0	NA	NA	NA	NA	NA
RF00001	0	NA	NA	NA	NA	NA
RF00001	0	NA	NA	NA	NA	NA
RF00001	0.04222	0	5.267649	0	1	NA
RF00190	0	NA	NA	NA	NA	NA
dre-mir-728	0	NA	NA	NA	NA	NA
dre-mir-182	0.131779	0	5.267649	0	1	NA
RF00001	1.300478	-0.385891604	1.479527	-0.26082	0.794231	NA

mir196d	0.127238	0	5.267649	0	1	NA
RF01290	0	NA	NA	NA	NA	NA
RF00001	0	NA	NA	NA	NA	NA
dre-mir-204-1	0	NA	NA	NA	NA	NA
RF00001	0.131779	0	5.267649	0	1	NA
dre-mir-7b	2.268099	3.056819205	1.673075	1.827066	0.06769	0.443563
RF00001	0.049519	0	5.267649	0	1	NA
RF01296	0.983514	0	2.90126	0	1	NA
dre-mir-181b-1	3.348307	-0.802493034	1.703912	-0.47097	0.637662	0.924779
RF00001	0	NA	NA	NA	NA	NA
RF00001	0	NA	NA	NA	NA	NA
dre-mir-153c	0.091739	0	5.267649	0	1	NA
RF00001	0	NA	NA	NA	NA	NA
dre-mir-15b	0	NA	NA	NA	NA	NA
dre-mir-132-1	0.344401	-1.71401976	3.879152	-0.44185	0.658595	NA
RF00020	0	NA	NA	NA	NA	NA
RF00001	0	NA	NA	NA	NA	NA
RF00001	0.04222	0	5.267649	0	1	NA
RF00001	0	NA	NA	NA	NA	NA
RF00002	6.989299	0.640721483	1.682463	0.380824	0.703334	0.943622
dre-mir-34a	0	NA	NA	NA	NA	NA
dre-mir-26a-3	0	NA	NA	NA	NA	NA
dre-mir-454b	0.73406	0.013260851	2.250295	0.005893	0.995298	NA
RF00001	0	NA	NA	NA	NA	NA
RF00001	0	NA	NA	NA	NA	NA
dre-mir-16b	16.96057	0.003972791	0.421745	0.00942	0.992484	1
RF00001	0	NA	NA	NA	NA	NA
rn7sk	133.1326	0.22708224	0.313927	0.723359	0.469459	0.867224
NC_002333.8	20.97122	0.212408353	0.436054	0.487115	0.626177	0.92063
dre-mir-29b-2	0.256263	0	5.267649	0	1	NA
RF00001	0	NA	NA	NA	NA	NA
BX936308.1	39.2182	-0.078544287	0.346776	-0.2265	0.820813	0.971788
mir363	8.933826	-0.361096777	0.608958	-0.59297	0.553198	0.897843
dre-mir-124-2	11.79612	-0.1339178	0.552719	-0.24229	0.808556	0.969828
dre-mir-9-7	0	NA	NA	NA	NA	NA
mir99-2	0.045161	0	5.267649	0	1	NA
RF00001	0	NA	NA	NA	NA	NA
dre-mir-138-1	0	NA	NA	NA	NA	NA
RF00001	0.049519	0	5.267649	0	1	NA
RF00001	0	NA	NA	NA	NA	NA
mir196b	0	NA	NA	NA	NA	NA
dre-mir-101b	0	NA	NA	NA	NA	NA
dre-mir-125b-1	0.096814	-0.868089684	5.267649	-0.1648	0.869104	NA
RF00001	0	NA	NA	NA	NA	NA
dre-mir-9-5	0.15117	-1.791424827	5.232816	-0.34234	0.732092	NA
dre-mir-15c	0	NA	NA	NA	NA	NA
NC_002333.9	7.121488	1.447500948	0.919218	1.574709	0.115324	0.555404
dre-mir-457b	1.308162	2.94724688	1.747114	1.686923	0.091618	NA
RF00096	0.084441	0	5.267649	0	1	NA
dre-mir-200a	0	NA	NA	NA	NA	NA
RF00001	0	NA	NA	NA	NA	NA
RF00001	0	NA	NA	NA	NA	NA
dre-mir-124-3	2.451422	-1.055440154	1.154294	-0.91436	0.360528	0.811237
RF00004	0	NA	NA	NA	NA	NA

dre-mir-429a	0	NA	NA	NA	NA	NA
RF00072	0	NA	NA	NA	NA	NA
dre-let-7c-1	0.094679	0	5.267649	0	1	NA
NC_002333.10	0.243448	0	5.267649	0	1	NA
RF00020	0	NA	NA	NA	NA	NA
RF00581	0.21807	0	5.267649	0	1	NA
mir100-2	0	NA	NA	NA	NA	NA
RF00001	0.152088	0	5.267649	0	1	NA
CR847953.2	0	NA	NA	NA	NA	NA
NC_002333.11	0.611346	0.057013627	2.344336	0.02432	0.980598	NA
dre-mir-126a	0	NA	NA	NA	NA	NA
dre-mir-23a-2	0	NA	NA	NA	NA	NA
RF00001	0	NA	NA	NA	NA	NA
dre-mir-301c	0	NA	NA	NA	NA	NA
RF00001	0	NA	NA	NA	NA	NA
dre-mir-375-1	1.102339	1.176705477	1.923281	0.611822	0.540656	NA
dre-mir-100-1	0	NA	NA	NA	NA	NA
RF00001	0	NA	NA	NA	NA	NA
dre-mir-204-2	0.092101	0	5.267649	0	1	NA
dre-mir-23b	0	NA	NA	NA	NA	NA
RF00001	0.073968	0	5.267649	0	1	NA
dre-mir-30d	0	NA	NA	NA	NA	NA
dre-mir-181b-2	0.861895	-0.71537127	1.721412	-0.41557	0.677723	NA
RF00001	0.043309	0	5.267649	0	1	NA
RF00133	0	NA	NA	NA	NA	NA
RF00003	0.174358	0	5.267649	0	1	NA
RF00608	0	NA	NA	NA	NA	NA
dre-mir-103	0	NA	NA	NA	NA	NA
RF00001	0	NA	NA	NA	NA	NA
dre-mir-144	0	NA	NA	NA	NA	NA
mir150	0	NA	NA	NA	NA	NA
dre-mir-734	0	NA	NA	NA	NA	NA
dre-mir-216a	0	NA	NA	NA	NA	NA
RF00001	0	NA	NA	NA	NA	NA
RF00577	0	NA	NA	NA	NA	NA
RF00377	0	NA	NA	NA	NA	NA
dre-mir-7a-3	0.206287	-0.868115716	5.164683	-0.16809	0.866515	NA
dre-mir-190a	0.128837	0	5.267649	0	1	NA
dre-mir-10c	0.232924	0	5.267649	0	1	NA
RF00001	0.4517	1.481414034	3.598071	0.411725	0.680541	NA
dre-mir-205	1.497681	-0.578867485	1.332777	-0.43433	0.664048	NA
RF00410	0.246408	0	5.267649	0	1	NA
RF00186	2.355967	-2.625737823	1.275254	-2.05899	0.039495	0.34755
mir206-2	0	NA	NA	NA	NA	NA
dre-mir-133a-2	0	NA	NA	NA	NA	NA
RF00092	1.657103	-2.361761456	1.954752	-1.20822	0.226964	NA
dre-mir-107b	0.142903	-0.868103008	5.267649	-0.1648	0.869102	NA
RF00001	0	NA	NA	NA	NA	NA
RF00001	0	NA	NA	NA	NA	NA
dre-mir-218a-1	0.043308	0	5.267649	0	1	NA
RF00001	0	NA	NA	NA	NA	NA
NC_002333.12	13.25648	0.552759527	0.573221	0.964304	0.334894	0.79181
dre-mir-184-1	0.288485	-0.749604582	4.644132	-0.16141	0.871771	NA
mir24-4	0.04222	0	5.267649	0	1	NA

dre-mir-203a	2.527007	-0.58183001	1.107021	-0.52558	0.599179	0.913173
dre-mir-155	0	NA	NA	NA	NA	NA
dre-let-7a-2	0	NA	NA	NA	NA	NA
RF00288	0.508535	-1.820603885	3.550568	-0.51276	0.608116	NA
CU929237.1	4.734592	-0.99713525	0.871537	-1.14411	0.252578	0.732759
RF00581	0.575965	3.472677838	3.312142	1.048469	0.294423	NA
dre-let-7b	1.420609	0.039495456	1.494452	0.026428	0.978916	NA
dre-let-7h	0	NA	NA	NA	NA	NA
RF00001	0.073968	0	5.267649	0	1	NA
dre-mir-15a-2	0	NA	NA	NA	NA	NA
RF00001	0	NA	NA	NA	NA	NA
mir29a-1	0.447142	2.439683462	4.196018	0.581428	0.560952	NA
RF00001	0	NA	NA	NA	NA	NA
RF00151	0.310787	-1.039034368	5.157212	-0.20147	0.840329	NA
RF00425	0	NA	NA	NA	NA	NA
RF00001	0.094679	0	5.267649	0	1	NA
dre-mir-200b	0	NA	NA	NA	NA	NA
dre-mir-199-3	8.122642	0.016633845	0.933463	0.01782	0.985783	1
RF00609	0	NA	NA	NA	NA	NA
RF00001	0.109473	1.055383603	5.267649	0.200352	0.841205	NA
CABZ01078244.1	0.087179	0	5.267649	0	1	NA
dre-mir-338-3	0	NA	NA	NA	NA	NA
RF00411	0.140685	-0.868089684	5.267649	-0.1648	0.869104	NA
dre-mir-128-1	0.04222	0	5.267649	0	1	NA
dre-mir-1306	0	NA	NA	NA	NA	NA
CR847548.1	0	NA	NA	NA	NA	NA
RF00133	1.366528	0.93112971	1.765146	0.527508	0.597841	NA
RF00190	0	NA	NA	NA	NA	NA
RF00421	8.085896	-0.363722458	0.713092	-0.51006	0.610007	0.915331
NC_002333.13	1761.535	-0.401823168	0.231834	-1.73324	0.083054	0.483628
RF00091	0.053506	-0.86807634	5.267649	-0.16479	0.869106	NA
RF00581	0	NA	NA	NA	NA	NA
RF00001	0	NA	NA	NA	NA	NA
RF00613	0.160274	-0.864880641	5.230461	-0.16535	0.868665	NA
RF00001	0	NA	NA	NA	NA	NA
RF00001	0	NA	NA	NA	NA	NA
RF00288	0.113703	1.055383603	5.267649	0.200352	0.841205	NA
dre-let-7e	0.049519	0	5.267649	0	1	NA
RF00001	0.04222	0	5.267649	0	1	NA
dre-mir-729	3.276927	0	1.900555	0	1	1
dre-mir-457a	0	NA	NA	NA	NA	NA
RF00001	0	NA	NA	NA	NA	NA
RF00001	0	NA	NA	NA	NA	NA
RF00001	0	NA	NA	NA	NA	NA
RF00001	0.564265	1.875125866	2.838247	0.660663	0.508828	NA
RF00026	0.468123	1.859598885	4.104984	0.45301	0.650542	NA
RF00016	0.350871	0	4.454105	0	1	NA
dre-mir-9-1	0	NA	NA	NA	NA	NA
RF00265	0	NA	NA	NA	NA	NA
RF00001	0.049519	0	5.267649	0	1	NA
RF00001	0	NA	NA	NA	NA	NA
RF00001	0	NA	NA	NA	NA	NA
NC_002333.14	3.350224	-3.255207646	1.492981	-2.18034	0.029232	0.304273
RF00089	3.17417	-0.094647069	0.878102	-0.10779	0.914166	0.992972

RF00001	0	NA	NA	NA	NA	NA
RF00001	0.249675	0	4.878677	0	1	NA
RF00001	0	NA	NA	NA	NA	NA
NC_002333.15	8.377183	0.426162882	0.559282	0.761982	0.446071	0.855403
RF00001	0.131777	0	5.267649	0	1	NA
RF00066	0.263478	0	5.267649	0	1	NA
dre-mir-101a	0	NA	NA	NA	NA	NA
dre-mir-92a-2	4.155377	-2.304023215	0.935874	-2.4619	0.013821	0.205798
RF00001	0	NA	NA	NA	NA	NA
RF00001	0	NA	NA	NA	NA	NA
RF00001	0	NA	NA	NA	NA	NA
dre-mir-142a	0.264461	0.093661915	4.127007	0.022695	0.981894	NA
CU442763.1	0.131761	0	5.267649	0	1	NA
dre-mir-301b	0.452197	-1.821041661	3.620252	-0.50302	0.614954	NA
dre-let-7a-6	2.470391	-0.562310133	1.504642	-0.37372	0.708615	0.94551
dre-mir-20b	0.100142	1.055383603	5.267649	0.200352	0.841205	NA
dre-mir-21-1	38.54237	0.763170579	0.474614	1.607981	0.107839	0.539429
dre-mir-181c	0.565764	-0.114554677	2.829034	-0.04049	0.9677	NA
dre-mir-99-1	0	NA	NA	NA	NA	NA
RF00211	0.249239	-1.331752152	5.204055	-0.25591	0.798023	NA
RF00001	0	NA	NA	NA	NA	NA
RF00001	0.04222	0	5.267649	0	1	NA
dre-mir-137-1	6.17732	-0.635391881	0.780747	-0.81383	0.415745	0.839167
RF00045	12.75752	0.301007152	0.483483	0.622581	0.53356	0.891181
RF00001	0.103263	1.055383603	5.267649	0.200352	0.841205	NA
dre-mir-1388	0	NA	NA	NA	NA	NA
RF00001	0	NA	NA	NA	NA	NA
RF00003	0.073968	0	5.267649	0	1	NA
dre-mir-219-1	1.945222	0.055045975	1.83193	0.030048	0.976029	NA
dre-mir-454a	1.557156	-0.481152119	1.681995	-0.28606	0.774832	NA
RF00003	0	NA	NA	NA	NA	NA
dre-let-7a-3	0.234085	0	5.267649	0	1	NA
RF01229	0.050624	1.055396947	5.267649	0.200354	0.841203	NA
RF00001	0	NA	NA	NA	NA	NA
RF00001	0	NA	NA	NA	NA	NA
dre-mir-10a	0.119909	2.079562922	5.22636	0.397899	0.690705	NA
RF00001	0	NA	NA	NA	NA	NA
RF00004	1.90881	1.055433186	2.011774	0.524628	0.599842	NA
RF00001	0	NA	NA	NA	NA	NA
dre-mir-34b	0	NA	NA	NA	NA	NA
dre-mir-462	0	NA	NA	NA	NA	NA
RF00026	0	NA	NA	NA	NA	NA
RF00001	0.063619	0	5.267649	0	1	NA
RF00066	0.621722	2.005156301	2.397906	0.836212	0.403036	NA
RF00001	0	NA	NA	NA	NA	NA
dre-mir-130c-1	0.53343	0.013582049	3.58106	0.003793	0.996974	NA
RF00001	0	NA	NA	NA	NA	NA
dre-mir-206-1	5.967495	-0.65829765	0.911822	-0.72196	0.47032	0.867552
CR354430.1	0.960561	-0.549069874	2.06779	-0.26553	0.790598	NA
RF00001	0.084441	0	5.267649	0	1	NA
RF00001	0.172145	0	5.267649	0	1	NA
RF00409	4.172465	0.498730669	0.856555	0.582252	0.560397	0.899928
dre-mir-19c	0.762442	-0.550081441	2.238843	-0.2457	0.805915	NA
dre-mir-740	0.342372	0.00403594	4.462997	0.000904	0.999278	NA

RF00001	0	NA	NA	NA	NA	NA
RF00089	0	NA	NA	NA	NA	NA
mir199-3a	0.452729	-2.386665428	3.570312	-0.66848	0.50383	NA
dre-mir-27a	0.453739	1.527103969	3.122241	0.489105	0.624767	NA
dre-mir-129-4	0.04222	0	5.267649	0	1	NA
dre-mir-27d	0.073968	0	5.267649	0	1	NA
dre-mir-142b	0	NA	NA	NA	NA	NA
RF00026	0	NA	NA	NA	NA	NA
RF00001	0.679768	2.5564091	2.570827	0.994392	0.320032	NA
RF00001	0.060442	0	5.267649	0	1	NA
RF00191	0.045161	0	5.267649	0	1	NA
dre-mir-726	0	NA	NA	NA	NA	NA
RF00001	0.098043	-1.71005882	5.238474	-0.32644	0.74409	NA
RF00093	0.819698	0.093656276	2.223767	0.042116	0.966406	NA
dre-mir-143	0.51142	-1.823253975	4.048125	-0.45039	0.652426	NA
RF00001	0	NA	NA	NA	NA	NA
RF00001	0.049519	0	5.267649	0	1	NA
RF00001	0	NA	NA	NA	NA	NA
RF00001	0.086618	0	5.267649	0	1	NA
RF00001	0	NA	NA	NA	NA	NA
RF00001	0	NA	NA	NA	NA	NA
dre-mir-135b	2.052024	2.572568874	1.714352	1.500607	0.133457	NA
dre-mir-460	0	NA	NA	NA	NA	NA
RF00001	0	NA	NA	NA	NA	NA
RF00068	0.151871	2.436401064	5.206324	0.46797	0.639806	NA
RF00319	0.567336	3.038865297	3.297004	0.921705	0.356682	NA
RF00001	0	NA	NA	NA	NA	NA
RF00001	0.126661	0	5.267649	0	1	NA
dre-mir-34c	0	NA	NA	NA	NA	NA
RF00003	0.04222	0	5.267649	0	1	NA
RF01299	1.212646	-0.864149892	1.849592	-0.46721	0.640349	NA
RF00003	0	NA	NA	NA	NA	NA
RF00270	0.185688	1.85412583	5.241768	0.353721	0.723548	NA
RF00001	0	NA	NA	NA	NA	NA
RF00001	0.096459	0	5.267649	0	1	NA
RF00001	0	NA	NA	NA	NA	NA
RF00001	0.56419	1.897761748	3.030372	0.626247	0.531153	NA
dre-mir-187	0.106524	1.92158077	5.236909	0.36693	0.713671	NA
NC_002333.16	14.89251	0.175176449	0.567753	0.308543	0.757669	0.957364
RF00001	0.08553	0	5.267649	0	1	NA
RF00004	0	NA	NA	NA	NA	NA
dre-let-7i	0.981138	-0.868148976	2.523328	-0.34405	0.730809	NA
RF00049	0	NA	NA	NA	NA	NA
RF00001	0	NA	NA	NA	NA	NA
NC_002333.17	6372.558	-0.828594712	0.364082	-2.27585	0.022855	0.266968
dre-let-7a-4	0	NA	NA	NA	NA	NA
dre-mir-9-6	0	NA	NA	NA	NA	NA
RF00001	0	NA	NA	NA	NA	NA
dre-mir-18c	0.04222	0	5.267649	0	1	NA
RF00001	0	NA	NA	NA	NA	NA
NC_002333.18	2790.918	-0.585928961	0.240999	-2.43125	0.015047	0.21481
RF00003	0	NA	NA	NA	NA	NA
dre-mir-458	0	NA	NA	NA	NA	NA
RF00151	0.084441	0	5.267649	0	1	NA

RF00001	0	NA	NA	NA	NA	NA
RF00001	0	NA	NA	NA	NA	NA
dre-mir-129-1	0.050723	-0.86807634	5.267649	-0.16479	0.869106	NA
dre-let-7d-1	0	NA	NA	NA	NA	NA
RF00273	0	NA	NA	NA	NA	NA
RF00020	0	NA	NA	NA	NA	NA
RF00096	0.367783	1.055397308	3.81253	0.276823	0.781916	NA
RF00001	0	NA	NA	NA	NA	NA
RF00001	0	NA	NA	NA	NA	NA
RF00015	0	NA	NA	NA	NA	NA
RF00001	0.045161	0	5.267649	0	1	NA
RF00150	0	NA	NA	NA	NA	NA
RF00001	0.04222	0	5.267649	0	1	NA
dre-mir-451	0	NA	NA	NA	NA	NA
RF00001	0	NA	NA	NA	NA	NA
RF00001	0	NA	NA	NA	NA	NA
CR847944.3	0.049519	0	5.267649	0	1	NA
RF00093	1.66954	-2.110626997	1.727887	-1.22151	0.221894	NA
dre-mir-10b-2	1.595265	0.868190238	1.809451	0.479809	0.631363	NA
dre-mir-27e	0.220576	0	5.267649	0	1	NA
RF00090	0.10878	0	5.267649	0	1	NA
dre-mir-130a	0.043309	0	5.267649	0	1	NA
dre-let-7f	0	NA	NA	NA	NA	NA
RF00068	1.623314	0.675909695	1.651882	0.409176	0.682411	NA
dre-mir-429b	7.000675	0.058890821	0.669257	0.087994	0.929881	0.995626
RF00001	0.136102	0	5.267649	0	1	NA
RF00001	0	NA	NA	NA	NA	NA
RF00001	0.129927	0	5.267649	0	1	NA
RF00001	0.049519	0	5.267649	0	1	NA
RF00001	0.184661	-1.710083148	5.238472	-0.32645	0.744086	NA
dre-mir-218a-2	0.1434	0	5.267649	0	1	NA
dre-mir-732	0	NA	NA	NA	NA	NA
mir214a	0.050723	-0.86807634	5.267649	-0.16479	0.869106	NA
NC_002333.19	23.7254	0.146962238	0.380999	0.385728	0.699698	0.942499
dre-mir-10d	4.706884	0.59801586	0.892593	0.669976	0.502873	0.881453
RF00001	5.439159	-0.466577903	1.040094	-0.44859	0.653726	0.929269
RF01299	0	NA	NA	NA	NA	NA
RF00619	0	NA	NA	NA	NA	NA
RF00001	0	NA	NA	NA	NA	NA
RF00003	0.049519	0	5.267649	0	1	NA
dre-mir-129-2	0.087179	0	5.267649	0	1	NA
RF00001	0.099037	0	5.267649	0	1	NA
RF00340	0.08553	0	5.267649	0	1	NA
dre-mir-145	8.367164	-0.140538619	0.927317	-0.15155	0.879539	0.986187
RF00045	6.495241	0.28558637	0.694385	0.411279	0.680868	0.937189
NC_002333.20	4.940971	0.73323993	0.909745	0.805984	0.420252	0.842383
dre-mir-190b	0	NA	NA	NA	NA	NA
dre-mir-125a-1	0.257725	0	5.267649	0	1	NA
mir96	0.364308	-2.410040694	3.783361	-0.63701	0.524118	NA
dre-mir-7a-2	0.225897	0.093667548	5.010069	0.018696	0.985084	NA
RF00137	0	NA	NA	NA	NA	NA
RF01277	0.727987	-0.820020644	2.358053	-0.34775	0.728026	NA
RF00001	0	NA	NA	NA	NA	NA
RF00270	0.570469	-1.379512748	3.05452	-0.45163	0.651536	NA

dre-mir-199-2	0.146408	1.854137197	5.241768	0.353724	0.723546	NA
dre-mir-27c	0.700234	-1.416817577	2.291149	-0.61839	0.53632	NA
arhgap19	184.5324	0.275868717	0.191291	1.442144	0.149262	0.611024
dre-mir-16a	0	NA	NA	NA	NA	NA
RF00001	0	NA	NA	NA	NA	NA
dre-mir-130c-2	0	NA	NA	NA	NA	NA
RF00001	1.140236	0.710965082	2.04239	0.348104	0.727762	NA
RF00001	0	NA	NA	NA	NA	NA
dre-mir-727	0	NA	NA	NA	NA	NA
RF00152	0	NA	NA	NA	NA	NA
RF00613	0.091242	-0.868089684	5.267649	-0.1648	0.869104	NA
RF00581	2.477889	0.757186694	1.280235	0.591443	0.554223	0.898306
RF00611	0	NA	NA	NA	NA	NA
dre-mir-218b	0.137587	0	5.267649	0	1	NA
CR354430.2	0.13069	0	5.267649	0	1	NA
dre-mir-217	1.501613	-1.516571386	1.583782	-0.95756	0.338283	NA
dre-let-7g-1	2.050609	1.055434009	1.86063	0.567245	0.570547	NA
RF00001	0.36136	2.962433098	3.720933	0.796153	0.425943	NA
dre-mir-489	0.133922	1.055383603	5.267649	0.200352	0.841205	NA
mir196a-1	0	NA	NA	NA	NA	NA
dre-mir-146a	1.125868	0	2.400962	0	1	NA
NC_002333.21	2.892187	-0.374900833	1.289402	-0.29076	0.771238	0.960484
RF00001	0.621447	-1.785485327	2.797255	-0.6383	0.523279	NA
RF00001	0.043309	0	5.267649	0	1	NA
RF01296	0.849746	0.711918008	1.995099	0.356833	0.721217	NA
RF00001	0	NA	NA	NA	NA	NA
RF00001	0	NA	NA	NA	NA	NA
dre-mir-210	3.170684	1.05628998	1.000668	1.055585	0.291158	0.761299
RF00001	0.163222	1.055370259	5.267649	0.200349	0.841207	NA
RF00001	0.310657	1.631374499	4.526054	0.360441	0.718518	NA
dre-mir-203b	2.89357	-1.11351179	1.450601	-0.76762	0.442712	0.853879
dre-mir-216b	0.050624	1.055396947	5.267649	0.200354	0.841203	NA
dre-mir-146b	0	NA	NA	NA	NA	NA
dre-mir-148	0	NA	NA	NA	NA	NA
RF00001	0	NA	NA	NA	NA	NA
RF00001	0	NA	NA	NA	NA	NA
RF00092	0.291087	-2.364631889	4.601776	-0.51385	0.607355	NA
RF00020	0	NA	NA	NA	NA	NA
RF00001	0.092828	0	5.267649	0	1	NA
RF00191	4.612408	-0.520533765	0.947353	-0.54946	0.582689	0.90571
RF00001	0	NA	NA	NA	NA	NA
dre-mir-153b	0.864487	-1.66792265	2.000942	-0.83357	0.404524	NA
RF00045	8.758605	-0.164492089	0.729302	-0.22555	0.821553	0.971788
RF00001	11.6286	0.863094702	0.636249	1.356536	0.174929	0.648869
RF00001	0	NA	NA	NA	NA	NA
RF00548	0.171618	-1.769364778	5.234319	-0.33803	0.735339	NA
dre-mir-301a	0	NA	NA	NA	NA	NA
RF00001	0.045161	0	5.267649	0	1	NA
dre-mir-107a	0	NA	NA	NA	NA	NA
RF01234	12.14049	0.563531191	0.657052	0.857666	0.391077	0.827902
dre-let-7g-2	0	NA	NA	NA	NA	NA
RF00001	0.093881	0	5.267649	0	1	NA
RF00001	0.30631	0	5.267649	0	1	NA
RF00015	0	NA	NA	NA	NA	NA

dre-mir-22b	0	NA	NA	NA	NA	NA
RF00377	0	NA	NA	NA	NA	NA
RF00284	0	NA	NA	NA	NA	NA
NC_002333.22	0.17618	0	5.267649	0	1	NA
NC_002333.23	2.286011	0.131013548	1.465501	0.089398	0.928765	0.995163
RF00001	0	NA	NA	NA	NA	NA
dre-mir-128-2	0.099037	0	5.267649	0	1	NA
dre-mir-9-4	0.133629	0	5.267649	0	1	NA
dre-mir-184-2	0	NA	NA	NA	NA	NA
dre-mir-30e-2	0	NA	NA	NA	NA	NA
RF00001	0	NA	NA	NA	NA	NA
NC_002333.24	6.565567	1.133105871	0.832477	1.361126	0.173474	0.64634
RF00026	0.091739	0	5.267649	0	1	NA
dre-mir-23a-3	0	NA	NA	NA	NA	NA
dre-mir-30b	0	NA	NA	NA	NA	NA
RF00001	0	NA	NA	NA	NA	NA
dre-mir-133b	0	NA	NA	NA	NA	NA
RF00001	0	NA	NA	NA	NA	NA
dre-mir-125b-3	1.974005	2.988673009	1.694746	1.763493	0.077817	NA
RF00001	0	NA	NA	NA	NA	NA
RF00001	0	NA	NA	NA	NA	NA
RF00001	0	NA	NA	NA	NA	NA
RF00396	0.043308	0	5.267649	0	1	NA
RF00001	0	NA	NA	NA	NA	NA
RF00072	0.219649	0	5.060338	0	1	NA
RF00001	0.046941	0	5.267649	0	1	NA
RF00001	0	NA	NA	NA	NA	NA
dre-mir-181b-3	21.40046	-0.175001042	0.381581	-0.45862	0.646506	0.926728
RF00430	3.561808	0.494687029	0.862306	0.573679	0.566185	0.901739
dre-mir-22a	6.78159	-0.217515367	1.136377	-0.19141	0.848203	0.977912
RF00001	0	NA	NA	NA	NA	NA
dre-mir-124-6	11.66856	-0.34815325	0.525717	-0.66225	0.507814	0.883056
dre-mir-736	0	NA	NA	NA	NA	NA
dre-mir-430a-1	0	NA	NA	NA	NA	NA
dre-mir-122	0.199578	0	5.267649	0	1	NA
RF00066	1.982494	-0.286234831	1.498781	-0.19098	0.848543	NA
RF00001	0	NA	NA	NA	NA	NA
RF00001	0	NA	NA	NA	NA	NA
RF00001	0	NA	NA	NA	NA	NA
RF00100	0	NA	NA	NA	NA	NA
RF00001	0.043308	0	5.267649	0	1	NA
RF00001	0.949829	0	2.635992	0	1	NA
RF00001	0	NA	NA	NA	NA	NA
RF00001	0	NA	NA	NA	NA	NA
RF01241	0.471686	1.055417662	3.102218	0.340214	0.733695	NA
dre-mir-2193	0	NA	NA	NA	NA	NA
RF00001	0	NA	NA	NA	NA	NA
RF00001	0	NA	NA	NA	NA	NA
RF00001	0	NA	NA	NA	NA	NA
RF00231	13.53867	0.09109853	0.518298	0.175765	0.860479	0.980264
RF00001	0.16893	1.11785931	5.226359	0.213889	0.830634	NA
RF00478	0.523189	1.898709793	3.090424	0.614385	0.538961	NA
dre-mir-2198	0.11056	0	5.267649	0	1	NA
RF00001	0.107013	-1.826650979	5.230461	-0.34923	0.726914	NA

RF00001	0.1369	0	5.267649	0	1	NA
RF00099	0	NA	NA	NA	NA	NA
RF00001	0	NA	NA	NA	NA	NA
RF00001	0.409549	-0.868133433	3.271284	-0.26538	0.790717	NA
RF00001	0.175088	0	5.267649	0	1	NA
RF00001	0	NA	NA	NA	NA	NA
RF00001	0.153953	-1.826663989	5.230461	-0.34924	0.726912	NA
RF00417	0.861967	2.524004608	2.821213	0.894652	0.370973	NA
RF00001	0.091739	0	5.267649	0	1	NA
RF00429	0.585198	-0.868140258	2.815585	-0.30833	0.757828	NA
RF00001	0	NA	NA	NA	NA	NA
RF00001	0	NA	NA	NA	NA	NA
RF00001	0	NA	NA	NA	NA	NA
RF00001	0	NA	NA	NA	NA	NA
dre-mir-2187	0	NA	NA	NA	NA	NA
dre-mir-2192	0	NA	NA	NA	NA	NA
RF01225	0	NA	NA	NA	NA	NA
dre-mir-2196	0	NA	NA	NA	NA	NA
RF00001	0	NA	NA	NA	NA	NA
RF00302	0.893987	1.202789721	1.909394	0.629933	0.528739	NA
RF00001	0.133559	0	5.267649	0	1	NA
RF00001	0.049021	-0.86807634	5.267649	-0.16479	0.869106	NA
RF00001	0	NA	NA	NA	NA	NA
RF00001	0	NA	NA	NA	NA	NA
RF00302	1.326152	-0.446879314	1.730322	-0.25826	0.796203	NA
RF00001	0.301084	1.055373291	4.602166	0.229321	0.818619	NA
RF00593	0.584178	-2.090997952	2.639606	-0.79216	0.428266	NA
RF00001	0	NA	NA	NA	NA	NA
RF00001	0	NA	NA	NA	NA	NA
RF00001	0	NA	NA	NA	NA	NA
RF00001	0.588853	1.055380887	3.900034	0.270608	0.786692	NA
RF00001	0	NA	NA	NA	NA	NA
RF00001	0	NA	NA	NA	NA	NA
RF00026	0	NA	NA	NA	NA	NA
RF01241	0.448444	-1.484220665	4.016379	-0.36954	0.711724	NA
RF00001	0.138182	0	5.267649	0	1	NA
RF00001	0	NA	NA	NA	NA	NA
RF00001	0	NA	NA	NA	NA	NA
RF00001	0	NA	NA	NA	NA	NA
RF00001	0	NA	NA	NA	NA	NA
RF00428	0.092827	0	5.267649	0	1	NA
RF00001	0	NA	NA	NA	NA	NA
RF00001	0	NA	NA	NA	NA	NA
RF00001	0.086616	0	5.267649	0	1	NA
RF00001	0	NA	NA	NA	NA	NA
RF00001	0.128836	0	5.267649	0	1	NA
RF00431	0	NA	NA	NA	NA	NA
RF00001	0.049021	-0.86807634	5.267649	-0.16479	0.869106	NA
RF00001	0.1775	0	5.267649	0	1	NA
RF00001	0.149233	-1.826663989	5.230461	-0.34924	0.726912	NA
RF00001	0	NA	NA	NA	NA	NA
RF00001	0.17872	0	5.267649	0	1	NA
RF00001	0	NA	NA	NA	NA	NA
RF00003	0	NA	NA	NA	NA	NA

RF00001	0	NA	NA	NA	NA	NA
RF00001	0	NA	NA	NA	NA	NA
RF00001	0	NA	NA	NA	NA	NA
RF00006	1.510834	-3.090046848	1.809771	-1.70742	0.087743	NA
RF00001	0	NA	NA	NA	NA	NA
RF00001	0	NA	NA	NA	NA	NA
RF00001	0.04222	0	5.267649	0	1	NA
RF00001	0	NA	NA	NA	NA	NA
RF00001	0	NA	NA	NA	NA	NA
RF00001	0.882818	0	2.427409	0	1	NA
RF00001	0.043309	0	5.267649	0	1	NA
RF00001	0	NA	NA	NA	NA	NA
RF00154	0	NA	NA	NA	NA	NA
RF00001	0.045161	0	5.267649	0	1	NA
RF00001	0	NA	NA	NA	NA	NA
RF00001	0	NA	NA	NA	NA	NA
RF00001	0.087179	0	5.267649	0	1	NA
RF00001	0	NA	NA	NA	NA	NA
RF00001	0	NA	NA	NA	NA	NA
RF00411	0.89751	-0.167037341	1.702046	-0.09814	0.921822	NA
RF00001	0	NA	NA	NA	NA	NA
RF00001	0	NA	NA	NA	NA	NA
dre-mir-2191	0	NA	NA	NA	NA	NA
RF00001	0	NA	NA	NA	NA	NA
RF00001	0	NA	NA	NA	NA	NA
RF00001	0	NA	NA	NA	NA	NA
RF00001	0	NA	NA	NA	NA	NA
RF00001	0.089161	0	5.267649	0	1	NA
RF00001	0	NA	NA	NA	NA	NA
RF00001	0	NA	NA	NA	NA	NA
RF00001	0	NA	NA	NA	NA	NA
RF00001	1.012028	1.055434849	2.193106	0.481251	0.630338	NA
RF00001	0.049519	0	5.267649	0	1	NA
RF00001	0	NA	NA	NA	NA	NA
RF00001	0	NA	NA	NA	NA	NA
RF00001	0.069091	0	5.267649	0	1	NA
RF00001	0	NA	NA	NA	NA	NA
RF00001	0	NA	NA	NA	NA	NA
RF00001	0.049519	0	5.267649	0	1	NA
RF00001	0.252278	1.055368498	4.854545	0.217398	0.827898	NA
RF00001	0.450593	-1.756791033	3.231153	-0.5437	0.586645	NA
RF00572	0	NA	NA	NA	NA	NA
RF00001	0	NA	NA	NA	NA	NA
RF00001	0.185949	0	5.267649	0	1	NA
RF00001	0.152169	-2.340093811	5.201855	-0.44986	0.652813	NA
RF00026	0	NA	NA	NA	NA	NA
RF00001	0	NA	NA	NA	NA	NA
RF00001	0.700445	1.035294295	2.17828	0.475281	0.634587	NA
RF00429	0.908281	2.994961112	1.869042	1.602405	0.109066	NA
RF00001	0	NA	NA	NA	NA	NA
RF00001	0.098043	-1.71005882	5.238474	-0.32644	0.74409	NA
RF00001	0.090321	0	5.267649	0	1	NA
RF00001	0	NA	NA	NA	NA	NA
RF00001	0.171507	1.055373006	5.267649	0.20035	0.841207	NA

RF00001	0	NA	NA	NA	NA	NA
RF00001	0	NA	NA	NA	NA	NA
RF00001	0.059954	1.055396947	5.267649	0.200354	0.841203	NA
RF00001	0	NA	NA	NA	NA	NA
RF00001	0	NA	NA	NA	NA	NA
RF00322	0.582661	1.055411275	3.058005	0.345131	0.729996	NA
RF00001	0	NA	NA	NA	NA	NA
RF00001	0	NA	NA	NA	NA	NA
RF01233	0.805282	-2.166369918	2.258046	-0.9594	0.337357	NA
RF00001	0.186616	1.055364149	5.267649	0.200348	0.841208	NA
RF01233	0.178945	-0.868109386	5.267649	-0.1648	0.869101	NA
RF00001	0.059954	1.055396947	5.267649	0.200354	0.841203	NA
RF00001	0	NA	NA	NA	NA	NA
RF00001	0.154861	-0.868103029	5.267649	-0.1648	0.869102	NA
RF00001	0	NA	NA	NA	NA	NA
RF00001	0	NA	NA	NA	NA	NA
RF00443	1.325309	-0.379632923	1.5346	-0.24738	0.804612	NA
RF00001	0	NA	NA	NA	NA	NA
RF00001	0	NA	NA	NA	NA	NA
RF00001	0	NA	NA	NA	NA	NA
RF00009	1.015123	2.576517861	2.024014	1.272974	0.203027	NA
RF00001	0.160584	1.055370259	5.267649	0.200349	0.841207	NA
RF00431	0.053262	1.055396947	5.267649	0.200354	0.841203	NA
RF00001	0	NA	NA	NA	NA	NA
RF00001	4.445792	-1.993372478	0.813768	-2.44956	0.014303	0.209352
RF00001	0	NA	NA	NA	NA	NA
RF00001	0	NA	NA	NA	NA	NA
RF00001	0	NA	NA	NA	NA	NA
RF00001	0	NA	NA	NA	NA	NA
RF00001	0	NA	NA	NA	NA	NA
RF00001	0	NA	NA	NA	NA	NA
RF00001	0.129927	0	5.267649	0	1	NA
RF00001	0	NA	NA	NA	NA	NA
RF00001	0.043308	0	5.267649	0	1	NA
RF00001	0	NA	NA	NA	NA	NA
RF00001	14.47691	-0.266541953	0.526494	-0.50626	0.612675	0.915754
RF00001	0	NA	NA	NA	NA	NA
RF00001	0.472562	3.371044602	3.426524	0.983809	0.325209	NA
RF00001	0	NA	NA	NA	NA	NA
RF00001	0	NA	NA	NA	NA	NA
RF00001	0.091739	0	5.267649	0	1	NA
RF00001	0.043309	0	5.267649	0	1	NA
RF00001	0	NA	NA	NA	NA	NA
RF00001	0.191864	0	5.267649	0	1	NA
RF00263	0.514706	-1.379915675	2.747894	-0.50217	0.615546	NA
RF00001	0	NA	NA	NA	NA	NA
RF00001	0	NA	NA	NA	NA	NA
RF00001	0	NA	NA	NA	NA	NA
RF00001	0	NA	NA	NA	NA	NA
RF00001	0.043308	0	5.267649	0	1	NA
RF00001	0	NA	NA	NA	NA	NA
RF00001	0	NA	NA	NA	NA	NA
RF00618	0	NA	NA	NA	NA	NA
RF00001	0	NA	NA	NA	NA	NA

RF00001	0	NA	NA	NA	NA	NA
RF00001	0	NA	NA	NA	NA	NA
RF00001	0	NA	NA	NA	NA	NA
RF00001	0	NA	NA	NA	NA	NA
RF00001	0	NA	NA	NA	NA	NA
RF00001	0.043308	0	5.267649	0	1	NA
RF00001	0.318349	3.49922331	5.121877	0.683192	0.494486	NA
RF00001	0.166965	1.921567426	5.236909	0.366928	0.713673	NA
RF00001	0.049519	0	5.267649	0	1	NA
RF00001	0	NA	NA	NA	NA	NA
RF00001	0	NA	NA	NA	NA	NA
RF00001	0.04222	0	5.267649	0	1	NA
RF00001	0.093881	0	5.267649	0	1	NA
RF00091	0.573891	1.117970416	2.750553	0.406453	0.68441	NA
RF00408	1.371992	-2.77219443	1.606924	-1.72516	0.084499	NA
RF00001	0	NA	NA	NA	NA	NA
RF00001	0	NA	NA	NA	NA	NA
RF00001	0	NA	NA	NA	NA	NA
RF00001	0.18065	-0.868109387	5.267649	-0.1648	0.869101	NA
RF00001	0	NA	NA	NA	NA	NA
RF00001	0.059954	1.055396947	5.267649	0.200354	0.841203	NA
RF00001	0.223698	0	5.267649	0	1	NA
RF00001	0	NA	NA	NA	NA	NA
RF00001	0	NA	NA	NA	NA	NA
RF00001	0	NA	NA	NA	NA	NA
RF00001	0	NA	NA	NA	NA	NA
RF00001	0	NA	NA	NA	NA	NA
RF00001	0	NA	NA	NA	NA	NA
RF00026	0.564747	2.921417033	3.897244	0.749611	0.453489	NA
RF00001	0	NA	NA	NA	NA	NA
RF00001	0	NA	NA	NA	NA	NA
RF00001	0.090249	0	5.267649	0	1	NA
RF00001	0.069091	0	5.267649	0	1	NA
RF00001	0.141258	0	5.267649	0	1	NA
RF00001	0	NA	NA	NA	NA	NA
RF00020	0	NA	NA	NA	NA	NA
RF00001	0	NA	NA	NA	NA	NA
RF00004	0	NA	NA	NA	NA	NA
RF00001	0.069091	0	5.267649	0	1	NA
RF00001	0	NA	NA	NA	NA	NA
RF00001	0	NA	NA	NA	NA	NA
RF00001	0	NA	NA	NA	NA	NA
RF00001	0	NA	NA	NA	NA	NA
RF00001	1.094327	-3.359917295	2.11689	-1.58719	0.112469	NA
RF00582	0	NA	NA	NA	NA	NA
RF00001	0.174358	0	5.267649	0	1	NA
RF00001	0	NA	NA	NA	NA	NA
RF00001	0.177268	0	5.267649	0	1	NA
RF00001	0.092827	0	5.267649	0	1	NA
RF01192	0.045161	0	5.267649	0	1	NA
RF00001	0.049519	0	5.267649	0	1	NA
RF00001	0	NA	NA	NA	NA	NA
RF00322	0	NA	NA	NA	NA	NA
RF00001	0	NA	NA	NA	NA	NA

RF00001	0.043309	0	5.267649	0	1	NA
RF00001	0	NA	NA	NA	NA	NA
RF00020	0.097564	1.055383603	5.267649	0.200352	0.841205	NA
RF00001	0	NA	NA	NA	NA	NA
RF00001	0	NA	NA	NA	NA	NA
RF00001	0	NA	NA	NA	NA	NA
RF00218	0	NA	NA	NA	NA	NA
RF00001	0	NA	NA	NA	NA	NA
RF00001	0.043308	0	5.267649	0	1	NA
RF00001	0	NA	NA	NA	NA	NA
RF00001	0.060442	0	5.267649	0	1	NA
RF00001	0	NA	NA	NA	NA	NA
RF00001	0.119909	2.079562922	5.22636	0.397899	0.690705	NA
RF00001	0.046941	0	5.267649	0	1	NA
RF00020	0.04222	0	5.267649	0	1	NA
RF00026	0	NA	NA	NA	NA	NA
RF00001	0.131274	1.055383603	5.267649	0.200352	0.841205	NA
RF00001	0	NA	NA	NA	NA	NA
RF00001	0	NA	NA	NA	NA	NA
RF00001	0	NA	NA	NA	NA	NA
RF00001	3.70598	1.553729181	1.031806	1.505834	0.13211	0.584623
RF00001	0	NA	NA	NA	NA	NA
RF00001	0	NA	NA	NA	NA	NA
RF00001	0	NA	NA	NA	NA	NA
RF00001	0.101247	1.854150541	5.241768	0.353726	0.723544	NA
RF00001	0	NA	NA	NA	NA	NA
RF00001	0	NA	NA	NA	NA	NA
RF00001	0	NA	NA	NA	NA	NA
RF00568	7.88184	0.369041138	0.628758	0.586936	0.557247	0.899165
RF00009	2.41544	0.091114364	1.38739	0.065673	0.947638	0.997371
RF00001	0	NA	NA	NA	NA	NA
RF00001	0	NA	NA	NA	NA	NA
RF00001	0	NA	NA	NA	NA	NA
RF00001	0.049519	0	5.267649	0	1	NA
dre-mir-2195	0	NA	NA	NA	NA	NA
RF00001	2.39956	0.487446714	1.211413	0.402379	0.687405	0.938835
RF00001	0.086618	0	5.267649	0	1	NA
RF01192	0.129927	0	5.267649	0	1	NA
RF00001	9.801681	1.221261454	0.591047	2.066269	0.038803	0.344867
RF00001	0.493502	0	3.184213	0	1	NA
RF00001	0.043308	0	5.267649	0	1	NA
RF00001	0	NA	NA	NA	NA	NA
RF00001	0	NA	NA	NA	NA	NA
RF00440	0	NA	NA	NA	NA	NA
RF00001	0	NA	NA	NA	NA	NA
RF00001	0.07132	0	5.267649	0	1	NA
RF00001	0	NA	NA	NA	NA	NA
RF00001	0	NA	NA	NA	NA	NA
RF00001	0.050723	-0.86807634	5.267649	-0.16479	0.869106	NA
RF00001	0	NA	NA	NA	NA	NA
RF00001	0.059954	1.055396947	5.267649	0.200354	0.841203	NA
RF00001	0.595543	0	2.985052	0	1	NA
RF00001	0	NA	NA	NA	NA	NA
RF00001	0	NA	NA	NA	NA	NA

RF00270	0.04222	0	5.267649	0	1	NA
RF00001	0	NA	NA	NA	NA	NA
RF00302	0	NA	NA	NA	NA	NA
RF00001	0.153868	0	5.267649	0	1	NA
RF00001	0	NA	NA	NA	NA	NA
RF00001	0	NA	NA	NA	NA	NA
RF00001	0	NA	NA	NA	NA	NA
dre-mir-2197	0	NA	NA	NA	NA	NA
RF00001	0	NA	NA	NA	NA	NA
RF00001	0	NA	NA	NA	NA	NA
RF00001	0	NA	NA	NA	NA	NA
RF00001	0	NA	NA	NA	NA	NA
RF00001	0	NA	NA	NA	NA	NA
RF00613	1.656476	0.967187287	1.834427	0.527242	0.598025	NA
RF00553	0.273815	0.930265321	4.043812	0.230047	0.818056	NA
RF00001	0	NA	NA	NA	NA	NA
RF00001	0	NA	NA	NA	NA	NA
RF00001	0.049021	-0.86807634	5.267649	-0.16479	0.869106	NA
RF00001	0	NA	NA	NA	NA	NA
dre-mir-2184	0.897198	0	2.429092	0	1	NA
RF00564	0.090321	0	5.267649	0	1	NA
RF00191	0.55407	-1.774169865	2.684164	-0.66098	0.508627	NA
RF00001	0.043309	0	5.267649	0	1	NA
RF00001	0.097664	-0.868089684	5.267649	-0.1648	0.869104	NA
RF00001	0.046941	0	5.267649	0	1	NA
RF00001	0	NA	NA	NA	NA	NA
RF00001	0.281084	-0.868129415	5.267649	-0.1648	0.869098	NA
RF00001	0	NA	NA	NA	NA	NA
dre-mir-735	0	NA	NA	NA	NA	NA
RF00001	0	NA	NA	NA	NA	NA
RF00001	0	NA	NA	NA	NA	NA
RF00001	0	NA	NA	NA	NA	NA
RF00001	0	NA	NA	NA	NA	NA
RF00001	0	NA	NA	NA	NA	NA
RF00001	0.073968	0	5.267649	0	1	NA
RF00001	0	NA	NA	NA	NA	NA
RF00001	0.043308	0	5.267649	0	1	NA
RF00001	0	NA	NA	NA	NA	NA
RF00001	0	NA	NA	NA	NA	NA
RF00001	0.043309	0	5.267649	0	1	NA
RF00001	2.184425	0	2.019014	0	1	NA
RF00599	0.207516	-0.748301491	5.238472	-0.14285	0.886411	NA
RF00001	0	NA	NA	NA	NA	NA
BX537109.1	10.14076	1.282318861	0.792894	1.617263	0.105821	0.534827
zgc:153681	4.417778	0.32699958	1.034359	0.316137	0.751898	0.955391
si:dkey-238d18.7	0	NA	NA	NA	NA	NA
si:ch211-168d23.3	23.04983	0.03354472	0.49545	0.067706	0.94602	0.997371
igfn1.4	0.76912	1.066637458	2.196328	0.485646	0.627218	NA
tspan5a	212.0574	0.053785221	0.184175	0.292033	0.770262	0.960484
nectin1b	214.1842	0.109465119	0.190828	0.573634	0.566216	0.901739
si:ch211-229i14.2	5.042147	-0.80249562	1.704167	-0.4709	0.637711	0.924779
si:ch211-106k21.5	19.06771	-0.366486698	0.512965	-0.71445	0.47495	0.869292
si:dkey-7e14.3	2.735614	-1.817008259	1.54034	-1.17962	0.238153	0.719146
plekhg6	62.93228	-0.291968774	0.271381	-1.07586	0.281988	0.754378

plxnd1	87.19394	-0.37173658	0.25623	-1.45079	0.146837	0.606781
hmcn2	4.686224	0.130352729	0.958165	0.136044	0.891786	0.988566
fgf20b	44.77046	0.297267689	0.310867	0.956254	0.338944	0.795226
ttc3	680.0331	-0.211875958	0.153434	-1.38089	0.167312	0.638363
kcnn4	27.33259	-0.284423776	0.423654	-0.67136	0.501992	0.881211
RF00001	0	NA	NA	NA	NA	NA
BX539307.2	1.120285	0.679245904	1.91445	0.3548	0.72274	NA
cd302	184.1953	-0.262021602	0.166423	-1.57443	0.115388	0.555596
slc37a1	130.0608	-0.170981922	0.184639	-0.92603	0.354429	0.805425
slco5a1a	635.1933	0.142785189	0.144892	0.985461	0.324398	0.783308
RF00006	0	NA	NA	NA	NA	NA
MTERF1	199.4241	0.006633479	0.161184	0.041155	0.967172	1
si:ch211-266i6.3	310.9518	0.22210543	0.329855	0.673343	0.500729	0.880413
CR792417.1	20.40003	0.134021448	0.507808	0.263921	0.791841	0.964835
umod	0.158299	0	5.267649	0	1	NA
trim33l	84.1916	0.164058405	0.214265	0.765682	0.443866	0.854255
RF00017	0	NA	NA	NA	NA	NA
si:ch211-42i6.2	6.659515	1.338355675	0.788403	1.697552	0.089592	0.502507
si:ch73-267c23.10	4.807998	-0.329169093	1.262619	-0.2607	0.794321	0.965431
wrap53	168.5342	0.054871235	0.153844	0.356667	0.721341	0.947343
ccbe1	61.02981	0.121181982	0.286173	0.423456	0.671962	0.933953
si:ch73-206d17.1	31.6203	-0.645192718	0.783949	-0.823	0.410506	0.8372
znf385d	49.65606	0.068336157	0.286938	0.238157	0.811759	0.97012
si:ch211-286b5.9	5.578516	-0.785110754	0.727799	-1.07875	0.280701	0.753147
rskrb	38.02675	0.260333594	0.428782	0.607147	0.543753	0.894925
ACVR1C	6.477254	1.580606063	1.526457	1.035474	0.300448	0.766629
relb	308.4817	-0.203768855	0.182808	-1.11466	0.264995	0.742063
si:dkey-3h2.4	2.543983	-2.097254656	1.688147	-1.24234	0.214111	0.696474
vkorc1	75.75831	0.172903556	0.239114	0.7231	0.469618	0.867224
FBXO48	414.5363	-0.031535112	0.206185	-0.15295	0.878441	0.985761
opn8a	0.614771	0	3.359454	0	1	NA
mgp	3.924449	0.119423499	1.13453	0.105263	0.916167	0.993244
gsg1l2a	12.25951	2.950461832	1.443853	2.043464	0.041007	0.353808
grin2da	129.0221	0.943136674	0.296088	3.185329	0.001446	0.056975
ntrk3b	150.0722	0.829686582	0.266383	3.114643	0.001842	0.065953
gpr156	32.20353	-0.346689101	0.383604	-0.90377	0.366118	0.815747
prr13	419.2121	-0.113145389	0.174878	-0.647	0.517635	0.887148
nkd3	0.173005	0	5.267649	0	1	NA
si:ch211-226h8.4	1.911901	0.971512125	1.66228	0.584445	0.558921	NA
nat16	596.3863	-0.121920887	0.197496	-0.61733	0.537015	0.89244
si:ch73-144d13.4	45.44442	-0.046865191	0.339117	-0.1382	0.890084	0.98835
si:dkey-120c6.5	31.62092	-0.821035747	0.440048	-1.86579	0.062071	0.42715
F2R (1 of many)	19.30441	0.01685276	0.444739	0.037894	0.969773	1
si:dkeyp-121d2.7	46.54079	0.050469614	0.279105	0.180826	0.856504	0.979558
zgc:171781	0.460877	0.563522553	2.949773	0.191039	0.848495	NA
kcnj1a.3	85.81741	-0.856266964	0.489197	-1.75035	0.080058	0.478179
hhla2b.2	289.298	-0.151511118	0.23735	-0.63834	0.523249	0.888312
si:rp71-1c10.10	4.422996	-1.290997027	0.753157	-1.71411	0.086508	0.493048
si:ch211-236p5.2	50.24733	-0.053894588	0.316519	-0.17027	0.864796	0.982074
CU469420.1	14.83871	0.630278297	0.691658	0.911258	0.36216	0.812082
fhdc4	144.533	-0.175884333	0.221	-0.79586	0.426115	0.844714
F2R (1 of many)	0.151871	2.436401064	5.206324	0.46797	0.639806	NA
bsnd	12.89677	-1.712830788	0.619481	-2.76494	0.005693	0.127699
CR925773.1	9.481931	-1.588527952	0.707392	-2.24561	0.024729	0.27868

taar12i	0.309689	0.093656381	3.97855	0.02354	0.981219	NA
ndst2a	538.659	0.190528251	0.123647	1.540904	0.12334	0.570077
acyp2	1.594923	1.662489947	1.521989	1.092314	0.274695	NA
si:dkey-4p15.5	535.8666	-0.237478181	0.239733	-0.9906	0.321883	0.782075
si:ch211-282j17.5	0	NA	NA	NA	NA	NA
cnsta	106.1754	0.129724633	0.230304	0.563275	0.573247	0.903952
scg3	1099.506	-0.020376916	0.174046	-0.11708	0.906799	0.991644
znf1137	7.518668	-3.101761011	0.704991	-4.39972	1.08E-05	0.001993
FAM107A	75.30783	-0.035555857	0.254827	-0.13953	0.889032	0.988224
si:dkey-27j5.6	0.221298	0	5.267649	0	1	NA
proser1	515.6611	0.115863931	0.143916	0.80508	0.420774	0.842383
si:ch211-63b16.4	32.27928	0.016938246	0.418185	0.040504	0.967691	1
erlec1	524.6508	0.237578863	0.10971	2.165513	0.030348	0.310589
cabcoco1	12.7431	-0.021816317	0.59935	-0.0364	0.970963	1
CU633991.1	9.590747	0.283506466	0.571387	0.496172	0.619773	0.91747
si:ch211-67e16.4	295.0638	0.078779686	0.145781	0.540397	0.588924	0.908056
si:dkey-274m17.3	42.95926	-0.064148171	0.321802	-0.19934	0.841996	0.97643
CABZ01071939.1	88.43536	-0.389486858	0.252862	-1.54032	0.123483	0.570142
srrm4	292.169	0.484102505	0.153125	3.161482	0.00157	0.060215
bri3	1346.065	-0.205260595	0.125788	-1.6318	0.102721	0.529448
si:ch211-157b11.12	248.432	-0.260117328	0.19955	-1.30352	0.192397	0.672283
si:dkey-102g19.3	27.82897	0.648975232	0.451367	1.437799	0.150491	0.6142
zgc:101566	734.4208	-0.08487354	0.118561	-0.71587	0.474074	0.869292
cux2b	22.91414	0.526698681	0.491349	1.071945	0.283745	0.755095
zp2.1	0.734663	-2.599065727	2.536691	-1.02459	0.305557	NA
si:dkey-83f18.9	0	NA	NA	NA	NA	NA
pou6f2	20.80119	0.461101465	0.42772	1.078044	0.281014	0.753425
si:ch211-167j9.5	7.162189	0.180356111	0.70933	0.254263	0.799293	0.967111
isg15	65.73273	-0.074463886	0.617758	-0.12054	0.904056	0.991581
or111-8	0.069091	0	5.267649	0	1	NA
RF00026	0.043308	0	5.267649	0	1	NA
si:dkey-246e3.4	0.808873	0.913316212	2.402959	0.38008	0.703886	NA
cald1b	42.03845	-0.693081104	0.44404	-1.56085	0.118558	0.561771
dbx1a	148.9767	0.25977028	0.186749	1.391017	0.16422	0.63332
mterf2	111.2597	-0.022246037	0.224503	-0.09909	0.921067	0.994085
BX950205.1	0	NA	NA	NA	NA	NA
srsf10b	596.6419	-0.126184391	0.154047	-0.81913	0.412714	0.838123
taar12c	0.692989	2.967613073	2.533744	1.171236	0.241504	NA
med11	234.5843	-0.186936648	0.170828	-1.0943	0.273824	0.749234
si:ch211-236p5.3	2.57537	-0.382737915	1.735838	-0.22049	0.825488	0.972729
si:ch211-282j17.13	13.24069	1.760419308	0.864371	2.036647	0.041685	0.356684
CABZ01046949.1	0	NA	NA	NA	NA	NA
si:ch211-212k18.8	392.7817	0.1793477	0.156918	1.142942	0.253063	0.732759
prpf3	341.0294	-0.126622338	0.123142	-1.02826	0.303827	0.769473
adgrf7	19.16472	-1.38327332	1.481996	-0.93339	0.350621	0.802215
znf1055	13.07103	-1.342531069	0.510925	-2.62765	0.008598	0.15886
tdg.2	258.2788	0.02031872	0.138102	0.147128	0.883031	0.986908
dthd1	5.773221	0.243768523	0.867361	0.281046	0.778675	0.961073
cx52.9	19.33316	-0.532488117	0.62935	-0.84609	0.397501	0.830253
tnfaip8l1	164.452	-0.043224657	0.220226	-0.19627	0.844396	0.976791
hdac10	130.0332	0.52517763	0.184358	2.848678	0.00439	0.109024
si:ch211-165g14.1	164.9512	-0.375787131	0.164956	-2.27811	0.02272	0.266612
si:dkey-83f18.10	0	NA	NA	NA	NA	NA
klhl13	10.03135	2.415330519	1.033324	2.337437	0.019416	0.245214

pkig	318.1764	0.053917074	0.163538	0.329691	0.741633	0.954276
mogat3a	220.0634	-0.215216811	0.26996	-0.79722	0.425324	0.844714
si:ch211-132p1.4	7.539253	0.545147733	0.791009	0.68918	0.49071	0.875539
fbxw10	4.752015	-0.08973048	0.779863	-0.11506	0.908398	0.991812
CU179643.1	2.909596	-0.361459205	1.132886	-0.31906	0.749681	0.955074
si:dkey-92f12.2	173.9045	0.478558136	0.166842	2.868324	0.004127	0.106542
mtnr1bb	6.279933	2.537203621	1.517171	1.672325	0.09446	0.513368
znf970	12.63117	-0.022863948	0.478082	-0.04782	0.961856	0.999451
BX546500.1	0.24537	0	4.236004	0	1	NA
si:ch211-116o3.3	0	NA	NA	NA	NA	NA
BX323559.1	0.190341	0	5.267649	0	1	NA
mast3b	122.891	0.426465226	0.20033	2.128818	0.033269	0.323073
ptprjb.2	0.069091	0	5.267649	0	1	NA
prodhb	316.2054	0.103426461	0.285758	0.361937	0.717399	0.946272
si:ch211-155o21.3	11.7978	-0.8917666	0.69761	-1.27832	0.201137	0.681045
RF00001	0	NA	NA	NA	NA	NA
zp2.5	2.917519	-0.385128395	1.284287	-0.29988	0.764271	0.959228
ch25hl3	199.0721	-0.46185018	0.218435	-2.11436	0.034484	0.327793
si:dkey-90l23.2	3.068054	-0.201527524	1.143532	-0.17623	0.860111	0.980191
CABZ01115881.1	138.2574	-0.091734548	0.181771	-0.50467	0.613789	0.915998
dbf4b	67.1121	0.229573442	0.268688	0.854425	0.392869	0.827902
irs4a	114.3498	0.275104627	0.227106	1.21135	0.225761	0.707068
hist1h2a11	1.630117	-0.443100614	1.74586	-0.2538	0.799649	NA
nlrc3l1	341.5704	-0.095079384	0.129166	-0.7361	0.46167	0.863402
si:dkey-201l21.2	0	NA	NA	NA	NA	NA
zgc:172051	362.0644	-0.859763004	0.273121	-3.14792	0.001644	0.06246
kcna7	86.13838	-0.005852388	0.30941	-0.01891	0.984909	1
omga	3.522244	0.820166141	1.422895	0.576407	0.56434	0.901433
si:cabz01069012.2	23.4331	0.571258412	0.452377	1.262794	0.206663	0.688976
CABZ01077217.2	1.74756	-1.210011483	1.522778	-0.79461	0.426842	NA
nrg2b	183.7586	0.908281669	0.232728	3.902753	9.51E-05	0.008792
si:ch211-113a14.11	11.25139	1.003856514	0.649649	1.54523	0.122291	0.569468
dre-mir-26a-2	0.958404	-2.796193989	1.973599	-1.4168	0.156542	NA
zgc:136963	20.02457	2.268309987	0.687885	3.297513	0.000975	0.043444
si:ch73-269m14.4	0	NA	NA	NA	NA	NA
si:dkey-266f7.10	3.845102	0.093645018	1.83745	0.050965	0.959354	0.999164
CR846087.1	10.15147	-0.535780029	0.610854	-0.8771	0.380432	0.823655
cabz01093075.1	1.84884	-2.32932003	1.817967	-1.28128	0.200096	NA
psma3	1929.057	0.134976819	0.131296	1.028038	0.303932	0.76949
gckr	2.739486	0	1.895404	0	1	1
vav3a	28.15373	0.337084889	0.34215	0.985198	0.324527	0.783308
im:7147486	333.093	0.010728581	0.182966	0.058637	0.953241	0.998436
stk19	53.70318	0.023770798	0.260021	0.091419	0.92716	0.994973
rnh1	13.96602	0.353734823	0.412995	0.856511	0.391715	0.827902
or111-3	13.14703	0.656822363	0.536118	1.225146	0.22052	0.701837
RF00017	0	NA	NA	NA	NA	NA
hs3st3b1b	676.5338	-0.098488882	0.132677	-0.74232	0.457894	0.860993
chrng	101.1221	0.405964799	0.253572	1.600984	0.10938	0.542487
ttc41	4.908908	0.205664557	0.754027	0.272755	0.785042	0.962605
cbln11	259.9654	0.794348927	0.535166	1.484303	0.137728	0.593771
mkrn2os.1	22.81529	-0.485290365	0.41575	-1.16726	0.243103	0.724396
crygm2d21	11315.26	0.189392379	0.361333	0.524149	0.600175	0.913756
si:ch211-125m10.6	11.22871	-1.059629818	0.705489	-1.50198	0.133102	0.586514
si:dkey-175g6.2	102.1897	0.130073196	0.417847	0.311294	0.755577	0.956532

edn3b	12.39891	0.331490285	0.584679	0.566962	0.57074	0.902481
ice2	105.8783	-0.170587799	0.196035	-0.87019	0.384196	0.826565
ascc3	316.05	0.559298091	0.195042	2.867582	0.004136	0.106542
si:ch211-197l9.5	5.114463	-1.332351783	1.132392	-1.17658	0.239363	0.719781
trim63b	1137.475	-0.065098107	0.215272	-0.3024	0.762347	0.958467
dnajc12	71.60368	-0.125996252	0.232457	-0.54202	0.587805	0.907608
taar14a	0.452216	0	4.748743	0	1	NA
smim15	622.6719	0.033455235	0.168009	0.199127	0.842163	0.97643
si:dkey-126g1.7	19.52023	-0.34967254	0.561084	-0.62321	0.533148	0.890978
prob1	69.46022	0.463111359	0.311159	1.488344	0.13666	0.591964
BMERB1	100.2247	0.819888097	0.287491	2.851875	0.004346	0.108751
CR847543.1	56.65119	-0.174129988	0.311436	-0.55912	0.57608	0.904749
si:dkeyp-97b10.3	102.8559	-0.28330772	0.198104	-1.4301	0.152689	0.61677
CU075735.1	0.214417	0	5.267649	0	1	NA
znf1023	1.492244	-1.257458779	1.557975	-0.80711	0.419602	NA
im:7151449	83.45545	-0.01100359	0.242831	-0.04531	0.963857	0.999842
si:ch211-238p8.24	0.561375	0.695554886	3.148552	0.220913	0.82516	NA
RF00274	0.197578	-0.86811193	5.267649	-0.1648	0.869101	NA
SAMD4B	570.4615	0.103504941	0.150812	0.686319	0.492512	0.876385
tmem68	168.6552	-0.009970519	0.182474	-0.05464	0.956425	0.99899
si:ch1073-513e17.1	68.61451	-0.353920146	0.340568	-1.0392	0.29871	0.765669
phyh	869.4617	0.049718234	0.156189	0.31832	0.750242	0.955074
znf1152	76.05989	-0.01380141	0.364963	-0.03782	0.969834	1
prodha	341.2448	-0.636095443	0.44464	-1.43058	0.152549	0.61658
LO018205.1	10.62224	0.580341804	0.706974	0.820882	0.411714	0.837966
gb:bc139872	186.7219	0.184514231	0.172389	1.070336	0.284468	0.75547
RGS9BP	75.58344	-0.067782389	0.366424	-0.18498	0.853242	0.978839
gsdmea	44.92947	-0.156879174	0.383855	-0.40869	0.682764	0.938006
mdc1	213.5292	0.172514583	0.149098	1.157052	0.247251	0.727871
top1	27.66742	0.524176835	0.336467	1.557884	0.119261	0.562753
pdgfba	70.84578	-0.235384505	0.270406	-0.87049	0.384035	0.826565
mfsd10	131.6184	0.166049829	0.197388	0.841237	0.400215	0.831472
bahd1	273.6723	-0.168001653	0.169585	-0.99067	0.321849	0.782075
gucy1b1	156.1142	-0.689483946	0.27238	-2.53133	0.011363	0.185148
itprid1	54.76138	0.003119777	0.286919	0.010873	0.991324	1
ddhd1a	173.1057	0.096416811	0.188676	0.511017	0.609339	0.915204
MYADM	337.7391	-0.230722658	0.186303	-1.23842	0.215559	0.697157
scn11aa	10.39063	0.375061548	0.532719	0.704052	0.4814	0.870594
znf521	22.50115	-0.052140218	0.41797	-0.12475	0.900724	0.99045
sult6b1	1286.823	-0.01910586	0.149069	-0.12817	0.898016	0.990171
zmp:0000001228	49.8407	-0.200673161	0.315125	-0.63681	0.524252	0.888801
itga2.3	20.47988	-2.013714005	0.877232	-2.29553	0.021703	0.261221
si:ch211-266k8.4	43.8656	-0.076552017	0.384058	-0.19932	0.842009	0.97643
dap1b	2004.482	-0.081332124	0.184764	-0.44019	0.659796	0.930581
irgq1	16.26189	-0.309966324	0.742666	-0.41737	0.676408	0.93557
atad3	261.6391	0.049318458	0.201929	0.244236	0.807048	0.969726
si:dkey-187k19.2	0.421212	-1.824031643	4.242233	-0.42997	0.667218	NA
si:dkey-264d12.5	93.86075	-0.312312015	0.26042	-1.19926	0.230426	0.712099
dpyda.3	37.03031	-1.069101713	0.324325	-3.29639	0.000979	0.043444
stk35	144.5637	0.118002713	0.229941	0.513188	0.60782	0.915204
qdprb2	0.470533	0.086913969	2.555827	0.034006	0.972872	NA
si:ch211-232i5.3	105.1095	-0.187229978	0.19656	-0.95253	0.340826	0.795603
mhc1zda	183.1461	-1.026910742	0.290507	-3.5349	0.000408	0.024112
lins1	40.16967	0.001845805	0.291836	0.006325	0.994954	1

AL831726.1	2.148814	1.99525532	1.846045	1.080827	0.279774	NA
ier2b	368.0899	-0.078997599	0.542261	-0.14568	0.884172	0.987047
RF00426	0	NA	NA	NA	NA	NA
erfl1	84.23121	0.089134757	0.238861	0.373166	0.709025	0.945546
fubp3	695.5868	0.106387256	0.158776	0.670045	0.502829	0.881453
si:dkey-7i4.1	9.038409	0.474781359	0.798337	0.594713	0.552035	0.8974
gig2o	28.6471	-0.264143184	0.430832	-0.6131	0.53981	0.893049
stambpb	201.1456	-0.318831809	0.188175	-1.69434	0.090201	0.50413
si:dkey-100n23.5	262.5077	-0.029144571	0.181302	-0.16075	0.872289	0.983447
crygm2d2	12176.29	0.04712265	0.418726	0.112538	0.910397	0.992208
CR381646.1	0.050723	-0.86807634	5.267649	-0.16479	0.869106	NA
pik3c2b	577.0273	-0.103004742	0.127527	-0.80771	0.419258	0.842286
CABZ01029822.1	8.973092	0.377844825	0.576227	0.655722	0.512003	0.885217
tnni3k	18.38094	-0.29177771	0.403486	-0.72314	0.469592	0.867224
pimr14	0	NA	NA	NA	NA	NA
TCF24	34.91191	0.301450405	0.298304	1.010549	0.312232	0.776164
si:ch211-269k10.2	206.227	0.30048641	0.184368	1.629819	0.10314	0.530217
si:dkey-24117.3	0.050723	-0.86807634	5.267649	-0.16479	0.869106	NA
si:dkeyp-118a3.2	48.77671	-0.763704137	0.361919	-2.11015	0.034845	0.32873
BX649442.1	1.076466	-2.606736662	2.374519	-1.0978	0.272294	NA
FP102309.1	8.498204	-0.499200329	0.643348	-0.77594	0.437784	0.851283
atxn1l	18.88816	-0.459996611	0.481025	-0.95628	0.338929	0.795226
mbd6	180.3071	-0.099329463	0.211883	-0.46879	0.639217	0.924779
klc4	482.0783	0.210333836	0.190639	1.103307	0.269894	0.746532
carmil3	167.5986	0.386212696	0.205336	1.880878	0.059989	0.421883
si:ch73-234b20.5	280.1133	0.083103238	0.163147	0.509376	0.610489	0.915331
zgc:64002	55.37584	-0.268761572	0.47031	-0.57146	0.56769	0.901809
BX004816.2	0	NA	NA	NA	NA	NA
cubn	99.95318	-0.896430167	0.420894	-2.12982	0.033186	0.322535
RF00017	0	NA	NA	NA	NA	NA
micall1b.2	26.38407	-0.826018252	0.615253	-1.34257	0.179412	0.654677
si:dkey-11k2.7	65.04409	-0.091063026	0.60423	-0.15071	0.880205	0.986408
FO704748.1	15.33621	-0.27469543	0.467475	-0.58762	0.556791	0.899063
si:ch73-334d15.2	26.49815	-0.493621695	0.388419	-1.27085	0.203783	0.684589
RF00281	0	NA	NA	NA	NA	NA
rbbp8l	94.54593	-0.407181654	0.220894	-1.84333	0.065281	0.435359
rhbdl1	16.91663	0.3940674	0.490344	0.803655	0.421596	0.842901
si:ch211-222k6.2	69.19357	-0.101743884	0.291763	-0.34872	0.727299	0.949095
si:dkey-165n16.1	144.5983	0.034172475	0.221758	0.154098	0.877533	0.985433
ggt1l2.2	0	NA	NA	NA	NA	NA
ftr36	2.113615	1.219981704	1.36015	0.896947	0.369747	NA
selenou1b	31.1751	0.609378847	0.51573	1.181584	0.237371	0.718568
RF00017	0.043308	0	5.267649	0	1	NA
si:ch211-71k14.1	1.403802	-2.121825063	1.965267	-1.07966	0.280293	NA
znf977	1.742562	0.409279472	1.875479	0.218227	0.827253	NA
chrna4a	1.794396	1.958135903	2.025006	0.966978	0.333555	NA
znf574	119.4322	0.273997874	0.157082	1.744294	0.081108	0.479923
RF00001	0	NA	NA	NA	NA	NA
hcar1-4	17.8407	-0.259374938	0.45261	-0.57307	0.5666	0.901739
col28a1b	17.64653	0.405021991	0.686002	0.590409	0.554916	0.898617
si:ch211-274p24.4	7.6001	-0.931564569	0.795919	-1.17043	0.241829	0.72348
si:ch211-157c3.4	616.7986	-0.157145531	0.131615	-1.19398	0.232487	0.713923
foxa	33.21874	-0.044358458	0.383812	-0.11557	0.907991	0.991745
si:ch73-106k19.5	0.727025	0	3.049743	0	1	NA

ntng2b	62.89393	0.088129684	0.343909	0.256258	0.797751	0.966447
si:ch1073-164k15.3	15.83438	-2.907437969	0.933277	-3.1153	0.001838	0.065916
si:ch73-222h13.1	14.1434	0.41884717	0.438788	0.954555	0.339803	0.795226
akna	104.2737	0.057190015	0.233046	0.245402	0.806145	0.969726
RF00001	0	NA	NA	NA	NA	NA
slc5a8	25.71903	-0.275283983	0.506127	-0.5439	0.586508	0.906711
ifnlr1	24.75262	0.833347326	0.48507	1.717995	0.085798	0.491332
RF00001	0	NA	NA	NA	NA	NA
serpina7	52.2364	0	1.740839	0	1	1
fam183a	11.06916	-0.200373173	0.521515	-0.38421	0.70082	0.942918
si:ch1073-296d18.1	16.80321	-0.019974104	0.460793	-0.04335	0.965425	1
CR788255.1	0.393939	0	4.992835	0	1	NA
sowahd	33.86725	0.194076588	0.39277	0.494123	0.621219	0.918461
tmem102	139.233	0.193196954	0.216104	0.894001	0.371321	0.818945
crygm2d4	6754.751	0.396514893	0.346521	1.144275	0.252509	0.732759
si:dkeyp-69c1.7	83.57943	0.058226869	0.44553	0.130691	0.89602	0.98926
figla	0.476256	-0.795521116	2.882002	-0.27603	0.782524	NA
si:ch211-162i8.4	4.102589	1.721364778	0.880807	1.954304	0.050665	0.391589
rskra	40.19054	-0.418334734	0.386046	-1.08364	0.278525	0.752148
scpp9	28.71763	-2.753532876	1.457274	-1.88951	0.058824	0.418455
ubxn2a	106.7494	0.359345162	0.295127	1.217595	0.223378	0.70531
cbx7b	29.84405	-0.608588143	0.618863	-0.9834	0.325412	0.784089
si:ch211-232b12.5	111.18	-0.379137881	0.240573	-1.57598	0.115031	0.554651
grip2a	75.92115	0.024526306	0.302117	0.081181	0.935298	0.996829
nfil3-6	128.129	-0.864759779	0.295132	-2.93008	0.003389	0.095869
si:ch73-105b23.6	21.28508	-0.288739212	0.577666	-0.49984	0.617189	0.916365
wdr93	19.61213	0.899585953	0.53896	1.669116	0.095094	0.51461
prrg2	210.7823	-0.052452277	0.197568	-0.26549	0.790632	0.964764
rorc	9.560816	-0.334037845	0.663747	-0.50326	0.614781	0.915998
fibinb	373.9109	-0.253980764	0.145407	-1.74669	0.080692	0.479638
ros1	30.65386	-1.115237101	0.617006	-1.8075	0.070685	0.453582
cthrc1a	428.8045	0.363014731	0.151957	2.388932	0.016897	0.230481
stk39	9.778271	0.432369762	0.621312	0.695897	0.486493	0.873244
akt3b	183.5678	0.127723255	0.150862	0.846623	0.397205	0.830253
cct2	1328.355	-0.26567992	0.196854	-1.34963	0.177135	0.650742
vwa3a	8.847763	0.751313039	0.646022	1.162984	0.244836	0.726138
si:ch73-380n15.2	60.35956	0.540650489	0.612818	0.882237	0.377649	0.822478
si:ch73-361p23.3	2.390112	1.030223242	1.407701	0.731848	0.464261	0.864593
kbtbd2	358.4409	0.01756495	0.110755	0.158594	0.873989	0.98385
oit3	34.21287	0.599133697	0.461197	1.299084	0.193915	0.674122
exoc7	752.9835	0.008820838	0.14877	0.059292	0.95272	0.998395
si:ch211-161h7.4	340.5002	0.160564934	0.143739	1.117055	0.263971	0.741984
ucn3l	10.81959	-1.490348426	0.8647	-1.72354	0.08479	0.488883
zgc:100918	858.8135	-0.211893167	0.113469	-1.8674	0.061845	0.426649
CR847844.1	4.496379	0	1.863517	0	1	1
kcnab2a	27.16955	0.60213328	0.409129	1.471745	0.14109	0.599658
znf1011	21.00381	-0.508845305	0.377669	-1.34733	0.177874	0.651389
si:ch73-50f9.4	51.47428	0.134163854	0.3179	0.422032	0.673002	0.93414
si:dkeyp-3f10.17	4.537228	0.181393802	0.929369	0.19518	0.845252	0.976791
ypel2b	13.07484	-0.423217477	0.563308	-0.75131	0.452468	0.857994
mtss1lb	57.45822	0.185533751	0.36395	0.509778	0.610207	0.915331
zgc:63568	74.39771	0.598202259	0.400927	1.492048	0.135687	0.591131
BX942819.2	0	NA	NA	NA	NA	NA
selenoj	1273.4	-0.167453107	0.154125	-1.08648	0.277268	0.751426

tmem182b	12.38187	0.443531129	0.479828	0.924354	0.355302	0.806546
zgc:66473	6.147475	0.720007091	0.822601	0.875281	0.381421	0.824626
si:ch211-202h22.10	22.66358	0.080055205	0.432991	0.184889	0.853316	0.978876
si:dkey-84h14.1	0.77145	0	2.549587	0	1	NA
si:dkey-152b24.8	2.492202	1.851878931	1.423118	1.301283	0.193162	0.673175
armc9	144.6637	-0.384790301	0.236609	-1.62627	0.103893	0.532096
crygm2d14	4020.537	0.262635756	0.379518	0.692025	0.488922	0.873964
cebpd	3903.02	-0.270686416	0.244129	-1.10878	0.267524	0.743943
CABZ01038521.1	30.08666	-0.424153153	0.440792	-0.96225	0.335922	0.792139
lgals9l5	1.179104	0.76469372	2.277092	0.33582	0.737006	NA
jac10	27.64294	-1.605262044	0.48233	-3.32814	0.000874	0.040034
tmem121b	12.21563	-0.168271224	0.485088	-0.34689	0.728675	0.949787
crygm2d1	13630.56	0.085659185	0.346326	0.247337	0.804647	0.969208
cbx8a	160.6264	-0.134507247	0.181204	-0.7423	0.457907	0.860993
maza	202.2317	0.474178864	0.183879	2.578756	0.009916	0.172469
si:dkey-77f5.3	148.323	-0.228897161	0.170042	-1.34612	0.178264	0.652303
scoca	367.485	-0.055517644	0.119703	-0.4638	0.642793	0.925707
unc5c	129.9762	-0.205407591	0.168402	-1.21975	0.222561	0.704536
rap1ab	367.5685	0.176881452	0.185437	0.953862	0.340154	0.795345
reep6	36.57163	-0.401645939	0.547402	-0.73373	0.463112	0.864191
dnah2	61.6792	0.001342852	0.301254	0.004458	0.996443	1
c3a.2	83.15561	0.111986677	0.483767	0.231489	0.816935	0.971207
si:dkeyp-46h3.6	0.565466	1.199557893	2.623539	0.457229	0.647507	NA
cep162	67.34907	-0.052085868	0.252767	-0.20606	0.836742	0.976007
dnah5	61.89849	-0.073212763	0.286097	-0.2559	0.798026	0.966458
zgc:66473	11.13795	0.148811514	0.522421	0.28485	0.775759	0.961073
tcf23	0.767917	0	3.104234	0	1	NA
si:dkey-29b11.3	4.144263	1.891341093	1.340965	1.410432	0.158412	0.623794
hbbe1.3	15524.74	-0.175055427	0.34528	-0.507	0.612158	0.915668
si:ch73-160p18.3	0.140973	-0.868103029	5.267649	-0.1648	0.869102	NA
prr11	82.17301	0.336673746	0.29541	1.139683	0.254418	0.733426
tshz3a	231.9105	-0.21897421	0.135717	-1.61346	0.106644	0.536345
slc25a34	498.3743	0.278006496	0.292224	0.951347	0.341428	0.796175
tpm1	4612.971	0.056103221	0.149439	0.375425	0.707345	0.944981
si:ch211-214p13.3	75.89426	0.71919259	0.341416	2.106497	0.035161	0.330358
si:ch73-304f21.1	11.96588	-1.015836271	0.657821	-1.54425	0.122529	0.569468
bean1	112.2586	0.419758531	0.288277	1.456095	0.145366	0.604476
CR790388.2	1.266153	0.625374822	1.850644	0.337923	0.735421	NA
zdhhc5b	171.9339	0.041095814	0.179016	0.229564	0.81843	0.971656
taar12g	0	NA	NA	NA	NA	NA
serbp1b	2400.357	0.06687725	0.098241	0.680745	0.496033	0.878093
si:dkey-187j14.6	7.726535	-1.698633618	0.950652	-1.78681	0.073968	0.461828
si:ch211-151p13.8	285.1334	0.241528161	0.270948	0.89142	0.372704	0.819953
RF00017	0	NA	NA	NA	NA	NA
spi2	13.47042	-0.714279216	0.465411	-1.53473	0.124851	0.572491
ponzr4	299.8609	0.303677608	0.232951	1.303614	0.192365	0.672283
ptp4a2a	774.5164	0.164907775	0.135759	1.214706	0.224478	0.706406
BX664625.2	0.190066	-1.73291671	5.236853	-0.33091	0.740714	NA
mmd2a	26.17438	0.213702867	0.35335	0.604791	0.545318	0.895275
peli3	494.1331	0.018018082	0.186522	0.0966	0.923044	0.994525
si:ch73-57f22.2	26.8885	-0.804119096	0.476033	-1.68921	0.091179	0.505533
NDUFB1	784.2063	0.037430147	0.142541	0.262592	0.792865	0.965312
ecscr	50.60484	-0.799926371	0.237297	-3.37099	0.000749	0.036381
si:dkey-21c1.4	34.96298	-0.599860132	0.311244	-1.9273	0.053942	0.402007

RF00001	0	NA	NA	NA	NA	NA
F2R (1 of many)	0	NA	NA	NA	NA	NA
dnajb12b	223.0577	0.201120997	0.187016	1.07542	0.282187	0.754654
ccr6a	9.177049	0.814515093	0.682479	1.193465	0.232687	0.714183
cbln20	171.8479	-0.670249151	0.353468	-1.89621	0.057932	0.415699
si:dkey-226l10.6	163.164	-0.084869969	0.150311	-0.56463	0.572326	0.903461
fam124b	41.63205	0.112736584	0.296717	0.379947	0.703985	0.943735
FO704810.1	97.88237	0.208701297	0.258576	0.807118	0.419599	0.842383
tradv30.0.6	0	NA	NA	NA	NA	NA
si:ch73-322b17.4	0.368432	1.055353197	5.126048	0.20588	0.836884	NA
CU693494.1	10.90582	-0.868156864	1.753966	-0.49497	0.620623	0.918135
btbd3b	96.88661	-0.135988135	0.310474	-0.438	0.661385	0.930607
si:dkey-261p22.1	7.896608	-0.904257657	0.907739	-0.99617	0.31917	0.779923
CU693446.1	7.08558	0.058738176	0.727169	0.080777	0.93562	0.996829
CABZ01045212.1	34.14902	-0.703173083	0.299076	-2.35115	0.018716	0.241927
snip1	276.3731	-0.218820755	0.172658	-1.26737	0.205024	0.686873
zgc:85936	194.3065	0.517992122	0.15454	3.351824	0.000803	0.037962
wu:fe05a04	142.4897	0.058148641	0.197598	0.294278	0.768546	0.960395
si:ch211-278p9.1	11.68238	1.190035341	0.807919	1.472965	0.140761	0.599658
znf407	109.0259	-0.049680084	0.205731	-0.24148	0.809183	0.969828
kif3a	686.6157	0.162092785	0.139058	1.165649	0.243756	0.724437
BX510934.1	15.66773	0.050101553	0.470431	0.106501	0.915185	0.993066
fbxo42	203.7726	-0.073593751	0.158317	-0.46485	0.642038	0.925586
cdk1	807.3997	0.108856669	0.296386	0.36728	0.71341	0.945821
si:ch73-308l14.2	215.6009	-0.092562969	0.167061	-0.55407	0.579533	0.905618
pacrg	29.14658	0.257395178	0.302096	0.852031	0.394197	0.828374
si:ch73-105b23.1	11.44021	-1.224795497	0.724147	-1.69136	0.090768	0.505206
nek11	4.869387	-0.537861127	1.075096	-0.50029	0.61687	0.916365
hdac11	20.44285	0.106932348	0.441876	0.241996	0.808783	0.969828
nox1	82.43815	0.000990368	0.336527	0.002943	0.997652	1
zmp:0000000912	2.691155	1.864525821	1.694632	1.100254	0.271221	0.747344
zgc:171717	0.343647	-3.291895274	5.169337	-0.63681	0.524247	NA
si:dkeyp-98a7.9	0.704776	1.684213415	2.322861	0.72506	0.468415	NA
si:ch211-242f23.8	10.85056	-0.036842149	0.620299	-0.05939	0.952638	0.998395
BX469925.1	1.241357	-0.648372699	1.524267	-0.42537	0.670569	NA
si:ch73-95l15.5	457.3528	-0.284785047	0.149851	-1.90045	0.057374	0.413587
si:dkey-51d8.3	1.732346	-2.387471022	1.967136	-1.21368	0.22487	NA
TENM3	155.6188	0.046691194	0.19416	0.240478	0.80996	0.969828
gpr153	58.00671	1.081244717	0.411327	2.628676	0.008572	0.158655
FP085398.1	45.29293	-0.275987929	0.306788	-0.8996	0.368331	0.817272
RF00001	0	NA	NA	NA	NA	NA
maptb	717.2687	-0.090807148	0.153701	-0.59081	0.554651	0.89855
BX649485.1	9.215098	1.406368977	0.671919	2.093064	0.036343	0.335171
si:ch211-173a9.6	49.07934	-1.57445341	0.435614	-3.61433	0.000301	0.019507
nitr1m	4.85185	-5.253979714	1.254374	-4.18853	2.81E-05	0.00362
si:dkey-11o18.5	13.03744	2.260102368	0.870341	2.5968	0.00941	0.166683
or128-5	12.02312	0.482405394	0.557112	0.865903	0.386543	0.827433
hmgn6	4588.109	0.046428497	0.142509	0.325794	0.74458	0.95491
si:dkey-33o22.1	0	NA	NA	NA	NA	NA
nsfl1c	1412.926	0.261210938	0.145757	1.792095	0.073118	0.460113
cdh26.2	115.1714	0.071856561	0.226328	0.317489	0.750873	0.955074
znf1064	11.77435	1.112044739	0.574616	1.935285	0.052955	0.399666
runx1	20.60859	-0.062948053	0.427935	-0.1471	0.883055	0.986908
sept7a	1675.86	-0.109115601	0.099798	-1.09337	0.274232	0.749612

ctslb	0.050723	-0.86807634	5.267649	-0.16479	0.869106	NA
fasn	654.0692	0.4588736	0.194207	2.362811	0.018137	0.237957
si:ch211-219a15.3	36.28332	0.052289339	0.41905	0.124781	0.900697	0.99045
si:ch211-248l17.3	32.19099	0.966905114	0.540603	1.788567	0.073685	0.461398
RF00001	0	NA	NA	NA	NA	NA
fibpb	31.29308	0.639836205	0.352631	1.814461	0.069607	0.450374
si:ch1073-127d16.1	20.70613	-0.457809257	0.536439	-0.85342	0.393425	0.828374
BX005012.1	4.422138	2.516772057	1.573222	1.599757	0.109653	0.543222
si:dkey-1c11.1	84.44289	0.256830131	0.263681	0.97402	0.330047	0.788084
klhl29	174.1205	0.072432	0.15086	0.480128	0.631136	0.923003
atox1	841.3672	0.067710116	0.183697	0.368597	0.712428	0.945821
lpl	2100.494	0.007417875	0.250588	0.029602	0.976385	1
si:dkey-256i11.3	0.094679	0	5.267649	0	1	NA
gfra3	74.54118	0.36279437	0.271475	1.336382	0.181424	0.657873
dicp3.3	12.83144	0.270627321	0.573731	0.471697	0.637143	0.924779
FQ311930.1	24.24947	1.429414939	0.904547	1.580255	0.114048	0.55294
si:ch211-226o13.1	15.10761	-1.209492867	0.705831	-1.71357	0.086607	0.493145
tmprss5	62.39643	-0.41462234	0.273335	-1.5169	0.129291	0.578874
RF00017	140.5502	0.050961086	0.375427	0.135742	0.892026	0.988566
si:dkey-118k5.3	51.48212	-0.278722151	0.294937	-0.94502	0.344647	0.798147
BX664618.1	27.26218	-1.945393391	0.539561	-3.60551	0.000312	0.020043
asb14b	142.3862	0.233814208	0.343527	0.680629	0.496106	0.878093
map4k3a	197.2477	0.150403604	0.177407	0.847791	0.396555	0.830053
si:dkey-156k2.8	0.419162	0.093654781	2.980464	0.031423	0.974932	NA
lrrc38b	37.43804	-0.364953221	0.371072	-0.98351	0.325356	0.784089
ccser2a	341.152	0.065602826	0.127529	0.514415	0.606962	0.915204
CR382337.1	3.364438	-1.822352686	1.626535	-1.12039	0.262548	0.740129
rfwd3	103.5217	-0.053612908	0.188232	-0.28482	0.775779	0.961073
CR388166.1	5.096889	1.696639171	1.196757	1.417697	0.156279	0.621949
RF00001	0	NA	NA	NA	NA	NA
si:ch211-173d10.4	4.716721	-0.600345183	1.17529	-0.51081	0.609487	0.915204
pimr190	3.209859	0.388111587	1.001617	0.387485	0.698397	0.942433
cnih4	53.64553	0.124162113	0.217586	0.570635	0.568247	0.902146
or122-1	0	NA	NA	NA	NA	NA
rab27b	50.14974	-0.425604338	0.319165	-1.33349	0.18237	0.658899
ponzr10	9.322296	0	1.820697	0	1	1
crygm2d10	15462.33	0.239949531	0.33704	0.711931	0.476507	0.869292
CABZ01074298.1	10.30006	-0.514983441	0.649396	-0.79302	0.427767	0.845893
RF00017	0	NA	NA	NA	NA	NA
pum3	505.3477	0.087623673	0.171155	0.511955	0.608682	0.915204
tiam2a	399.5172	0.045838318	0.157921	0.290261	0.771616	0.960624
rnf152	62.49069	0.759280255	0.310793	2.443045	0.014564	0.211168
si:dkeyp-110a12.4	128.7754	0.362957368	0.233001	1.55775	0.119293	0.562753
si:ch211-22k7.9	12.94008	0.253001562	0.665508	0.380163	0.703825	0.943735
si:dkey-54n8.2	215.3492	0.083882275	0.131793	0.636471	0.524469	0.888808
pdyn	24.08357	0.602265695	0.63486	0.94866	0.342794	0.79738
tnfrsf11a	164.249	-0.588817242	0.232756	-2.52977	0.011414	0.185332
FP236735.1	28.38533	-0.423958978	0.381345	-1.11175	0.266247	0.742912
mfrp	7.391655	-0.069151515	0.6507	-0.10627	0.915366	0.993068
si:dkey-30g5.1	0	NA	NA	NA	NA	NA
agap3	77.20738	0.174317524	0.293646	0.593631	0.552759	0.897444
si:dkey-211g8.9	0.116481	0	5.267649	0	1	NA
bcl3	25.56806	0.877023146	0.462796	1.895054	0.058085	0.416281
si:ch211-113p18.3	145.6563	-0.178796839	0.209013	-0.85543	0.39231	0.827902

si:dkey-33c14.6	20.06283	-0.332205091	0.491835	-0.67544	0.499396	0.879603
cntn1a	332.8434	0.026516839	0.146759	0.180683	0.856616	0.979558
plekhn1	99.74003	-0.520548888	0.267432	-1.94647	0.051598	0.394954
si:ch211-180a12.2	178.7537	-2.026016911	0.939862	-2.15565	0.031111	0.313664
cd44a	32.45455	-0.607364226	0.484066	-1.25471	0.209583	0.691743
SLC2A13 (1 of many)	11.59909	0.4876559	0.656617	0.74268	0.457676	0.860993
si:ch211-11k18.4	499.3842	0.314618078	0.17192	1.830027	0.067246	0.442764
adgrf3a	3.17262	1.403011875	1.480217	0.947842	0.34321	0.797757
eevs	245.7671	0.911810962	0.344793	2.644518	0.008181	0.153717
rnf216	385.3553	-0.318877429	0.138267	-2.30625	0.021097	0.25794
h2afy2	3281.776	0.172659336	0.100882	1.711494	0.08699	0.494706
CU896691.1	1.145049	1.094763633	1.520251	0.720121	0.471451	NA
ndufa4l2b	3.146516	0.511453581	1.371528	0.372908	0.709217	0.945546
RF00001	0	NA	NA	NA	NA	NA
il4	3.545264	-1.251817667	1.328053	-0.9426	0.345888	0.799025
dact3a	162.3479	-0.304890939	0.214345	-1.42243	0.154902	0.619752
psme4a	2121.493	0.135615314	0.205314	0.660526	0.508917	0.883442
tmem240a	34.99812	-0.358751035	0.387272	-0.92635	0.354262	0.805403
nitr10a	0	NA	NA	NA	NA	NA
plce1	209.3312	-0.113779891	0.217421	-0.52332	0.600754	0.913756
nudt9	276.2496	0.074561631	0.135498	0.550277	0.582129	0.90571
RF00001	0	NA	NA	NA	NA	NA
zgc:174972	22.49635	-0.157588621	0.432976	-0.36397	0.715884	0.946208
cdk4	324.2752	-0.412269313	0.177185	-2.32677	0.019977	0.250067
CR933559.1	20.63421	-0.023586269	0.44528	-0.05297	0.957756	0.999109
pde8a	192.0845	-0.029641637	0.150557	-0.19688	0.843921	0.976791
RF00017	0	NA	NA	NA	NA	NA
CABZ01044053.1	1293.888	-0.059079865	0.078337	-0.75418	0.450741	0.857162
rac1b	346.2419	-0.084301272	0.198669	-0.42433	0.671325	0.933953
she	104.5819	-0.082105381	0.196262	-0.41835	0.675694	0.935422
avd	4.290389	-0.646926938	0.833915	-0.77577	0.437885	0.851283
rpap3	484.7148	-0.118010574	0.111695	-1.05655	0.290718	0.76078
or122-2	0.43915	0.095639674	3.499665	0.027328	0.978198	NA
CU657980.2	0.344432	-1.71201628	4.422325	-0.38713	0.69866	NA
CT573433.1	86.05618	0.055548773	0.260453	0.213278	0.831111	0.974908
slc6a11b	206.5896	0.14715866	0.263843	0.557751	0.577014	0.904798
igsf5a	40.93489	-0.001168259	0.4846	-0.00241	0.998076	1
znf365	6.024792	0.092632828	0.768885	0.120477	0.904105	0.991581
bada	219.465	-0.471186775	0.213284	-2.2092	0.027161	0.293072
si:ch211-114c17.1	35.99296	0.250265129	0.342031	0.731703	0.46435	0.864593
pabpn1l	3.390765	-0.666796746	0.967212	-0.6894	0.490571	0.875539
BX664625.3	13.27298	-0.147083979	0.772861	-0.19031	0.849065	0.978067
znf1057	18.94554	1.396505565	0.402401	3.470437	0.00052	0.028195
wu:fc23c09	1.564909	0.028568832	1.641251	0.017407	0.986112	NA
CYP27A1 (1 of many)	36.22266	-3.740588578	0.550815	-6.79101	1.11E-11	1.36E-08
si:ch211-114l13.12	3.571721	1.303861805	1.177952	1.106888	0.268342	0.744748
mhc1zfa	27.29825	1.594529026	0.663557	2.403004	0.016261	0.225339
si:dkey-26c10.5	43.97096	0.527660166	0.402778	1.310051	0.190179	0.669977
si:ch73-125k17.2	0.720416	2.537678861	2.269874	1.117982	0.263574	NA
rpl35a	21681.33	-0.304850233	0.135948	-2.2424	0.024935	0.279833
si:dkey-106g10.7	150.8282	-0.050706599	0.188826	-0.26854	0.788286	0.963471
CU929391.1	17.78903	0.430897381	0.61047	0.705846	0.480284	0.870001
si:dkeyp-27c8.2	60.17752	-0.246385359	0.296217	-0.83177	0.405537	0.834645
si:ch73-139j3.4	12.26385	-3.866299453	1.131083	-3.41823	0.00063	0.032718

fgf18a	68.57485	0.496702047	0.244793	2.029074	0.042451	0.360949
AL935044.1	3.57878	0.769970304	0.890953	0.86421	0.387473	0.827592
pimr70	3.033703	1.935448117	1.142793	1.693613	0.090339	0.504496
pimr187	3.269315	0.871319356	1.402027	0.621471	0.53429	0.891573
CR759879.1	67.10656	0.848831714	0.326993	2.595868	0.009435	0.166881
usp43b	30.55353	-0.007151992	0.404413	-0.01768	0.98589	1
fam110b	350.8853	0.167047167	0.144267	1.1579	0.246905	0.727706
usp18	8.15873	-0.542485602	0.969261	-0.55969	0.575691	0.904739
tmed6	118.7917	-2.608311839	1.311749	-1.98842	0.046765	0.376754
si:ch211-214c20.1	5.964036	0.103119571	1.087754	0.0948	0.924473	0.994815
sik3	1198.289	-0.033278339	0.128738	-0.2585	0.796023	0.966305
si:dkey-66i24.8	22.29951	-0.055324761	0.443486	-0.12475	0.900722	0.99045
kdm6ba	1594.309	-0.063732326	0.105256	-0.6055	0.544848	0.895206
pfn1	22791.45	-0.038540655	0.178815	-0.21553	0.829351	0.974609
KCNIP4	26.48236	0.634969176	0.409252	1.551538	0.120773	0.566816
gstm.3	573.5404	0.010889661	0.180851	0.060213	0.951986	0.998032
fut8b	23.73589	0.524014873	0.5886	0.890274	0.373319	0.820558
pkd1l2b	0	NA	NA	NA	NA	NA
znf217	436.2584	0.1209511	0.129347	0.935092	0.349741	0.801408
adrb2a	102.0097	-0.124089357	0.195537	-0.63461	0.525684	0.888928
si:dkey-30c15.10	234.721	0.165798092	0.456096	0.363516	0.716219	0.94627
pcdh2aa15	1.563488	-0.536535771	1.839292	-0.29171	0.77051	NA
adgrg2a	87.95753	0.315427329	0.302058	1.044262	0.296364	0.764246
hsd17b7	192.2726	-0.011174709	0.198925	-0.05618	0.955202	0.998611
sema4gb	10.0457	0.257396209	0.720126	0.357432	0.720768	0.947343
atg4db	20.10764	0.367863417	0.441039	0.834084	0.404233	0.833888
smoc2	182.6368	-0.037654129	0.207131	-0.18179	0.855749	0.979532
CABZ01056629.1	28.60326	-0.530938478	0.38986	-1.36187	0.173239	0.646158
BX470188.1	6.356683	1.588525206	0.816381	1.945814	0.051677	0.395168
si:ch211-112g6.4	6.163128	-0.266025362	0.868564	-0.30628	0.75939	0.957886
ablim3	382.371	-0.230106379	0.145652	-1.57984	0.114144	0.553175
ciartb	111.8469	0.270691529	0.224236	1.207173	0.227366	0.708598
tp53inp2	524.9298	0.205223286	0.156415	1.312045	0.189505	0.669129
mpped1	148.7395	0.232015069	0.251126	0.923899	0.355539	0.806546
CR381544.1	145.2914	-0.05061234	0.158157	-0.32001	0.748958	0.955074
si:ch211-135n15.3	0.749217	-1.437528546	2.44396	-0.5882	0.556401	NA
v2rh13	0.049519	0	5.267649	0	1	NA
si:ch211-168f7.5	314.3403	-0.022138781	0.144386	-0.15333	0.878138	0.98573
CABZ01077220.1	0.277731	0	4.708733	0	1	NA
RF00001	0	NA	NA	NA	NA	NA
sh3d19	207.3239	-0.02568981	0.187792	-0.1368	0.891189	0.988566
shisa8b	28.53846	-0.271030876	0.780052	-0.34745	0.728251	0.949448
rce1b	3.16172	-0.449216112	1.524331	-0.2947	0.768225	0.960296
si:dkey-23a23.3	60.2092	-0.361186394	0.290101	-1.24503	0.213119	0.695467
zmp:0000000924	4.704821	0.910836727	0.931494	0.977823	0.328162	0.786542
fam78ab	57.32508	0.038869473	0.240059	0.161916	0.871372	0.983365
FO082877.1	172.884	0.299272147	0.158174	1.89204	0.058486	0.418172
tmem163b	53.12784	0.503935224	0.330166	1.526309	0.126933	0.575884
shisa2b	77.61633	0.110773571	0.237542	0.466333	0.640977	0.925364
si:ch211-217k17.12	2.739453	-0.86814215	1.920503	-0.45204	0.651241	0.928512
kansl1b	745.4414	0.10936785	0.123377	0.886456	0.375372	0.822237
si:dkey-16p6.1	5.465882	-1.117808865	1.154852	-0.96792	0.333082	0.790126
thoc1	645.5092	0.007299571	0.118228	0.061742	0.950769	0.99801
si:ch1073-303k11.2	222.4621	0.502282164	0.137779	3.645563	0.000267	0.017992

lgals17	16.8582	-0.47271319	2.060931	-0.22937	NA	NA
si:dkey-83f18.7	0	NA	NA	NA	NA	NA
RF00017	0.101446	-1.755425691	5.23528	-0.33531	0.737394	NA
smoc1	18.775	-0.30744943	0.531575	-0.57837	0.563011	0.900772
CABZ01038494.1	58.58991	-0.100690282	0.245191	-0.41066	0.681321	0.937331
gig2p	10.38798	-0.738695319	0.738017	-1.00092	0.316866	0.778879
zgc:174356	24.86024	-1.230434199	0.547537	-2.24722	0.024626	0.278337
si:ch211-181d7.1	0.129927	0	5.267649	0	1	NA
si:ch211-190p8.2	2.335292	1.548024765	1.331137	1.162934	0.244856	0.726138
susd5	18.41976	0.193377823	0.427545	0.452298	0.651054	0.928395
si:ch73-248e21.5	16.77068	0.050124273	0.549996	0.091136	0.927385	0.994973
dre-mir-194b	1.404407	0	2.182305	0	1	NA
foxj1b	44.44434	0.464443514	0.324203	1.432569	0.151981	0.615522
doc2b	134.3278	0.263998497	0.265392	0.99475	0.319858	0.780475
si:ch211-235i11.4	109.6707	0.015271061	0.213909	0.071391	0.943087	0.997371
si:dkey-254e13.6	12.34208	0.674097921	0.604244	1.115606	0.264591	0.742063
mrtfba	66.20478	0.00245182	0.307822	0.007965	0.993645	1
notch1	93.8849	-0.021937926	0.246621	-0.08895	0.929119	0.995361
si:ch211-103f14.3	152.6953	-0.118501937	0.206345	-0.57429	0.565771	0.901634
si:ch73-302o18.2	6.628152	-0.04747282	0.765995	-0.06198	0.950582	0.99801
ubap2a	1951.395	0.044299174	0.100864	0.439198	0.660518	0.930581
mir1-1	0.501603	0	3.471837	0	1	NA
si:ch211-132e22.4	0.137587	0	5.267649	0	1	NA
hbae1.3	353.4708	-0.13042334	0.491431	-0.26539	0.790705	0.964766
znf1028	28.24552	-0.617918622	0.481813	-1.28249	0.199672	0.6804
alx4a	465.205	-0.22917334	0.171059	-1.33973	0.180332	0.656129
tmem145	63.85771	-0.279018915	0.447282	-0.62381	0.532753	0.890978
prrt4	17.02952	-0.537546205	0.629599	-0.85379	0.39322	0.828219
miip	86.61726	-0.578124499	0.231478	-2.49754	0.012506	0.194482
sp1	215.1479	-0.325214662	0.185121	-1.75676	0.078958	0.475183
RF00001	0	NA	NA	NA	NA	NA
tmem117	21.46675	-0.257758843	0.5014	-0.51408	0.607198	0.915204
acadl	917.1366	-0.135752878	0.146441	-0.92701	0.35392	0.8049
CR925817.1	6.763076	0.303811209	0.755162	0.402313	0.687454	0.938835
zgc:77938	348.2769	0.353536871	0.192622	1.835393	0.066447	0.440066
si:cabz01069013.3	11.0462	0.234970492	0.803323	0.292498	0.769906	0.960484
RF00001	0.30721	1.0553616	4.651421	0.22689	0.820509	NA
rpl11b	15.02781	1.954194468	1.635033	1.195202	0.232008	0.713202
znf1000	2.17507	1.695897849	1.31062	1.293966	0.195677	NA
cox5aa	4721.282	0.015773882	0.138661	0.113759	0.909429	0.99213
nme4	158.7782	-1.186423202	0.245839	-4.82601	1.39E-06	0.000375
tmem107l	54.81553	-0.275958852	0.285984	-0.96494	0.334572	0.79151
rubcnl	16.35909	0.067373364	0.408088	0.165095	0.868869	0.982801
si:ch211-171h4.7	0	NA	NA	NA	NA	NA
notum2	272.3585	-0.144666684	0.164215	-0.88096	0.37834	0.822672
si:ch211-66k16.28	20.36695	0.378835611	0.49897	0.759235	0.447712	0.856366
si:ch211-189a15.5	42.58254	-0.42180623	0.316544	-1.33253	0.182685	0.659278
gm2a	329.3386	-0.349799865	0.158346	-2.20908	0.027169	0.293072
ssh2a	585.1197	0.062954304	0.173287	0.363294	0.716385	0.94627
si:dkey-190j3.3	0	NA	NA	NA	NA	NA
selenol	32.6693	-4.06964018	1.286287	-3.16387	0.001557	0.059822
si:dkey-24i24.3	8.115718	0.806483093	0.60652	1.329688	0.183621	0.660534
or111-7	3.150198	1.41080249	1.046922	1.347572	0.177796	0.651389
taar12j	1.80264	0.849678237	1.262327	0.673105	0.500881	NA

CLPB	29.27157	0.132328801	0.462293	0.286244	0.774691	0.96088
tmx3b	139.1989	-0.145436295	0.218005	-0.66712	0.504693	0.88195
pcdh1gb9	13.953	-0.365834588	0.551906	-0.66286	0.507423	0.883007
kcnj1a.6	92.13166	-0.748760528	0.378839	-1.97646	0.048103	0.38156
CR385078.1	3.074076	-0.755756836	0.904273	-0.83576	0.403289	0.833803
cntrl	146.6701	-0.139192115	0.230486	-0.60391	0.545906	0.895711
si:ch211-149k12.3	1.884553	0.759525821	1.417488	0.535825	0.592079	NA
BX571757.1	14.07113	0.53008318	0.532122	0.996168	0.319169	0.779923
pimr137	0.308392	0	5.267649	0	1	NA
fam217b	34.60922	0.038967089	0.338932	0.11497	0.908469	0.991812
znf982	30.94688	-0.25245594	0.35127	-0.71869	0.472329	0.868767
and1	7515.249	0.140213192	0.135624	1.033838	0.301212	0.767371
st6gal2b	55.66503	0.015615861	0.322286	0.048453	0.961355	0.999354
acot17	250.8399	-0.144012351	0.280796	-0.51287	0.608041	0.915204
ponzr2	63.85801	0.024520236	0.279144	0.087841	0.930003	0.995689
myct1b	21.05332	-0.238837754	0.447699	-0.53348	0.593702	0.910427
si:ch1073-390k14.1	114.5105	0.238432955	0.335602	0.710463	0.477417	0.869292
si:dkey-23f9.13	0	NA	NA	NA	NA	NA
si:dkey-1c7.2	0.411857	-1.826141375	4.964773	-0.36782	0.713008	NA
tmem104	130.8957	0.257158964	0.187053	1.374791	0.169196	0.640541
vps72b	38.37806	0.602307677	0.399313	1.508359	0.131463	0.583069
si:ch211-151o1.4	23.79068	-0.16715975	0.445351	-0.37534	0.707405	0.944981
f10	688.7147	-0.994573101	0.232288	-4.28163	1.86E-05	0.002746
si:ch73-352p18.4	77.69503	-2.07007604	0.519016	-3.98846	6.65E-05	0.006729
ponzr3	496.4882	0.322344728	0.56386	0.571675	0.567542	0.901809
pxna	842.0316	-0.028461088	0.123879	-0.22975	0.818286	0.971594
CR759791.1	0.71309	1.093467541	2.896623	0.377497	0.705804	NA
chst1	149.9743	0.162982039	0.197923	0.823462	0.410245	0.837066
crispld2	104.6095	-0.169701514	0.279735	-0.60665	0.544083	0.89515
si:dkey-175m17.6	16.6232	0.440977923	0.518042	0.851239	0.394636	0.828825
si:ch211-153b23.7	19.8055	-0.327646	0.551844	-0.59373	0.552693	0.8974
ltb4r2a	20.14765	-0.092796158	0.477979	-0.19414	0.846064	0.977039
si:ch211-152l15.2	7.888337	1.773587597	0.728499	2.434579	0.014909	0.213765
rexo4	173.6319	-0.282675742	0.202177	-1.39816	0.162065	0.629618
si:ch211-142d6.2	11.59386	0	4.483752	0	1	1
arhgef10la	185.097	0.425676159	0.235971	1.803936	0.071241	0.455342
BX000363.1	5.280139	0.06345389	0.916129	0.069263	0.94478	0.997371
adcy1b	18.56812	-0.000848799	0.714161	-0.00119	0.999052	1
BX927260.1	18.80773	-0.905059005	1.290647	-0.70124	0.483151	0.871988
cdca9	2.10684	1.086175915	1.240397	0.875668	0.381211	NA
znf1115	4.123529	0.182243197	0.781095	0.233318	0.815515	0.971158
grn2	19.09473	1.05543391	1.73315	0.608969	0.542545	0.894595
CU855711.1	0	NA	NA	NA	NA	NA
si:ch211-208k15.1	73.29902	-0.421246019	0.316309	-1.33175	0.182941	0.659278
si:ch211-212k18.6	15.21861	-0.630552696	0.718917	-0.87709	0.380439	0.823655
si:ch211-209f22.3	0	NA	NA	NA	NA	NA
lman2lb	376.3308	0.351605344	0.151347	2.323178	0.02017	0.251701
RF00001	0	NA	NA	NA	NA	NA
zmp:0000000936	32.82611	-0.525466808	0.299774	-1.75287	0.079624	0.477652
hhla2b.1	32.68736	0.412425082	0.436231	0.945427	0.344441	0.798147
si:dkey-192d15.3	0	NA	NA	NA	NA	NA
hist1h2a11	1.998915	-1.340508902	1.701379	-0.7879	0.430758	NA
si:ch211-201o1.1	27.46642	-0.54742013	0.642467	-0.85206	0.394181	0.828374
ddias	36.19501	-0.279258548	0.303974	-0.91869	0.358256	0.809596

dcst2	2.227611	-1.226953709	1.21484	-1.00997	0.312509	0.776164
pdzd8	173.937	-0.136282556	0.186733	-0.72983	0.465496	0.864997
tlr20.2	0.299226	-2.340130225	5.201853	-0.44986	0.652808	NA
ngfra	297.5016	0.151004714	0.189412	0.797229	0.425318	0.844714
tnfaip8l3	129.0848	-0.243857137	0.171549	-1.4215	0.155171	0.619776
lgals1l1	1806.151	0.41710707	0.363007	1.149034	0.250542	0.730471
si:ch211-226h8.14	2.627331	-0.479873442	1.313483	-0.36534	0.714854	0.945821
ecrg4b	275.1816	-0.498902328	0.504994	-0.98794	0.323183	0.783034
zmp:0000000937	1.214525	2.577902485	1.872399	1.376791	0.168577	NA
si:ch211-250n8.1	2.527405	0	1.910067	0	1	1
CR382327.1	10.9813	0.384373802	0.67505	0.569401	0.569084	0.902234
hmgcll1	4.115718	0.964336854	1.741405	0.553769	0.579737	0.905618
BX322530.1	246.497	-2.284340358	0.71594	-3.19068	0.001419	0.056314
zgc:174193	0.261032	3.213945857	5.176429	0.620881	0.534678	NA
MFAP4 (1 of many)	99.2053	0.837250364	0.654117	1.27997	0.200556	0.68081
otogl	44.36315	0.709772819	0.413044	1.718396	0.085724	0.491277
cfap299	13.44089	-0.229475354	0.521139	-0.44033	0.659695	0.930581
si:ch211-224l10.4	4.197952	-2.816023477	1.173098	-2.4005	0.016373	0.225867
lyplal1	70.15358	-0.059926453	0.227888	-0.26296	0.792578	0.965266
CABZ01059406.1	0.62888	-2.349276392	2.143367	-1.09607	0.273049	NA
RF00017	0	NA	NA	NA	NA	NA
mast1b	239.4322	0.012242036	0.166615	0.073475	0.941428	0.997371
RF00001	0	NA	NA	NA	NA	NA
si:ch73-180n10.1	9.474541	-0.868151849	1.769731	-0.49056	0.623741	0.919343
plcxd1	51.89982	-0.04150128	0.345957	-0.11996	0.904514	0.991581
xxylt1	35.55755	-0.050949582	0.368979	-0.13808	0.890175	0.98835
fgd5a	287.8077	-0.17826276	0.184719	-0.96505	0.334521	0.79151
nes	170.7836	0.168906757	0.179349	0.941774	0.346308	0.799463
klhl22	140.8055	0.028060568	0.274835	0.1021	0.918678	0.993884
prox3	172.1272	0.083513024	0.205914	0.405573	0.685057	0.938486
ppp1r3ab	175.8575	-0.163144775	0.29257	-0.55763	0.577099	0.904798
trabd2b	201.0542	-0.002655939	0.17427	-0.01524	0.98784	1
wdr59	185.4563	0.120205479	0.177646	0.676658	0.498623	0.87923
CR318646.1	0	NA	NA	NA	NA	NA
zgc:174288	90.05816	-0.496307873	0.325581	-1.52438	0.127415	0.576964
crygm2d3	12004.9	0.571201847	0.272051	2.099616	0.035763	0.33238
citb	19.25217	0.271415036	0.843463	0.321787	0.747614	0.955074
paqr8	21.16267	-0.220553689	0.4009	-0.55015	0.582219	0.90571
si:dkey-81j8.6	2.455456	-2.107916507	1.613627	-1.30632	0.191443	0.671456
si:ch211-237i5.4	21.28598	0.859156265	0.485188	1.770768	0.076599	0.468597
efs	394.8954	0.05761856	0.187958	0.30655	0.759186	0.957886
si:ch211-76m11.5	17.55747	-0.30345264	0.636461	-0.47678	0.633518	0.923924
BX571952.1	41.65508	0.104767681	0.399688	0.262124	0.793226	0.965312
dnal4b	83.47884	0.138484022	0.214609	0.645286	0.518742	0.887148
KCNB2	5.620465	2.782101676	1.417104	1.96323	0.049619	0.387751
deaf1	99.44834	0.043930772	0.18681	0.235163	0.814082	0.970483
or111-4	5.016128	0.77471729	0.712428	1.087432	0.276846	0.751311
znf1089	2.850537	-1.041243222	1.030432	-1.01049	0.31226	0.776164
taar14f	0.092828	0	5.267649	0	1	NA
znf1021	15.84122	-0.849518401	0.503617	-1.68683	0.091635	0.507334
stxbp6	578.5398	0.005852889	0.156501	0.037398	0.970167	1
si:ch211-135n15.1	0.073968	0	5.267649	0	1	NA
si:ch211-22k7.9	9.827576	0.212382371	0.696829	0.304784	0.760531	0.957886
si:busm1-228j01.4	1.189762	1.068617089	2.260872	0.472657	0.636458	NA

smarcd2	250.3187	-0.134389793	0.200918	-0.66888	0.503573	0.881759
c1qtnf1	87.51194	-0.083391766	0.247793	-0.33654	0.736465	0.951919
si:dkeyp-69b9.3	407.3988	0.424039179	0.277416	1.528532	0.126381	0.574727
si:ch211-149b19.2	37.41196	1.419197601	0.580367	2.445346	0.014471	0.210484
si:ch1073-340i21.3	606.8032	0.569707029	0.224291	2.540033	0.011084	0.18325
slc23a3	41.89481	0.339276331	0.336517	1.008198	0.313359	0.776777
si:dkey-238d18.3	1.063577	1.521176842	1.982305	0.767378	0.442857	NA
si:dkeyp-98a7.7	0.361489	-1.8266867	5.230459	-0.34924	0.726909	NA
caln1	13.46605	0.346048117	0.499482	0.692814	0.488426	0.873955
ralgapb	421.3108	0.097094746	0.159741	0.607828	0.543302	0.894925
si:dkey-21o19.2	62.2831	-0.525957714	0.270764	-1.94249	0.052077	0.396527
CABZ01059403.1	10.38454	-1.567287724	0.630475	-2.48588	0.012923	0.198143
CU570691.1	15.52447	-1.218521907	0.780838	-1.56053	0.118634	0.561771
cbln17	27.15389	-0.412322695	0.580979	-0.7097	0.477888	0.869292
si:ch211-241n15.3	16.43382	-0.108782835	0.441653	-0.24631	0.805444	0.96936
RF00001	0	NA	NA	NA	NA	NA
si:ch211-12h2.8	1.069853	-0.73203175	1.702785	-0.4299	0.667266	NA
si:dkey-185e18.7	91.70972	0.599700318	0.253938	2.361602	0.018196	0.238026
CR450793.1	9.41372	0.93084731	0.920343	1.011414	0.311818	0.776164
ypel5	474.0796	0.022528799	0.15463	0.145695	0.884162	0.987047
golt1a	32.8039	0.00787697	0.466581	0.016882	0.98653	1
si:dkey-259j3.5	29.34354	-0.959725023	0.408821	-2.34755	0.018898	0.242305
RF00001	0.049519	0	5.267649	0	1	NA
sycp2	1.875796	-4.439540444	1.947279	-2.27987	0.022615	NA
pdxka	251.8015	-0.147125153	0.141397	-1.04051	0.298103	0.765416
si:dkey-16p19.5	4.856176	0.972241648	1.014202	0.958627	0.337747	0.794799
wnk4b	270.9409	-0.54590919	0.188739	-2.89241	0.003823	0.10304
si:dkey-238d18.4	65.64635	-0.439736087	0.286088	-1.53707	0.124277	0.571556
si:ch211-14k19.8	341.0591	-0.424652437	0.200759	-2.11523	0.03441	0.327696
tmem220	40.48615	-0.292657732	0.339887	-0.86105	0.389213	0.827725
cep131	372.21	0.246647788	0.139459	1.768607	0.076959	0.469467
si:ch1073-228j22.2	2.967704	-1.386816004	1.618284	-0.85697	0.391463	0.827902
si:dkey-241l7.5	0.64352	1.552144561	2.566967	0.604661	0.545404	NA
mettl4	63.06017	0.306548231	0.224742	1.363999	0.172568	0.645319
taar1b	0.112399	0	5.267649	0	1	NA
si:ch211-207e14.4	96.96026	0.104679311	0.261473	0.400345	0.688903	0.93924
AL772298.1	0.043309	0	5.267649	0	1	NA
frmpd1b	385.0944	-0.227034618	0.173425	-1.30913	0.190492	0.670633
si:ch211-175m2.5	207.5246	-0.331504135	0.225781	-1.46826	0.142034	0.601083
si:dkey-7f16.3	0.916123	-1.758083437	2.527257	-0.69565	0.486649	NA
si:dkey-183k8.2	2.277426	-0.140567791	1.251441	-0.11232	0.910566	0.992208
si:ch211-154o6.2	230.2009	-0.032042226	0.118875	-0.26955	0.787509	0.963263
rrnad1	43.91606	-0.143049748	0.297259	-0.48123	0.630354	0.922869
zgc:173585	3.687249	-0.409301851	1.016762	-0.40255	0.687276	0.938835
foxb1a	139.7916	0.426696117	0.217429	1.962462	0.049709	0.388057
ptpn6	126.5856	-0.042481376	0.188873	-0.22492	0.822041	0.971966
cuzd1.1	17.5293	0	1.705853	0	1	1
RF00001	0	NA	NA	NA	NA	NA
ube2ka	358.5709	-0.195157374	0.188167	-1.03715	0.299665	0.766192
kcnj1a.5	118.5612	-0.765181889	0.303329	-2.52261	0.011649	0.187683
lactb11a	96.02316	0.689632614	0.478993	1.439754	0.149937	0.613246
nhsl2	339.2394	-0.396821775	0.166994	-2.37626	0.017489	0.233637
si:dkey-83f18.5	0	NA	NA	NA	NA	NA
ushbp1	160.7429	-0.294635242	0.187219	-1.57375	0.115546	0.555766

si:ch73-173p19.1	323.0042	0.001452516	0.113871	0.012756	0.989823	1
gphna	534.7602	-0.161859549	0.123419	-1.31146	0.189702	0.669289
rgs1	29.49143	-1.760864471	1.609788	-1.09385	0.274021	0.749284
si:ch211-160j14.3	115.0634	0.149586932	0.180347	0.829441	0.406855	0.835226
si:ch211-214b16.3	3.380853	-1.126344956	1.275481	-0.88307	0.377196	0.822476
mustn1b	136.0351	0.355639912	0.2236	1.590522	0.111717	0.547596
hbba1	0.669912	-4.477510958	3.886416	-1.15209	0.249283	NA
BX510940.1	14.68644	-0.21873593	0.526739	-0.41526	0.677949	0.936026
mmd2b	5.118424	0.150962269	0.76799	0.196568	0.844166	0.976791
si:cabz01054394.5	17.28615	0.923023171	0.521905	1.768567	0.076966	0.469467
si:dkey-160o24.3	31.31386	0.267027002	0.321144	0.831488	0.405698	0.834645
plrdgb	49.18393	-0.375395612	0.57634	-0.65134	0.514824	0.886401
mlxipl	21.89379	-0.889624582	0.462697	-1.92269	0.054519	0.403705
bcl2b	147.7875	0.724604088	0.310312	2.335081	0.019539	0.246447
si:ch211-198b3.4	4.682191	0.626029745	0.827225	0.756782	0.44918	0.856796
zmp:0000000951	13.77046	-0.088502947	0.597898	-0.14802	0.882324	0.986908
BX663516.1	0.951092	1.055410693	2.768628	0.381204	0.703052	NA
negaly6	113.3023	-0.187940398	0.25611	-0.73383	0.463054	0.864191
hbae1.3	3259.451	-0.506865513	0.365569	-1.38651	0.165591	0.635141
RF00001	0	NA	NA	NA	NA	NA
baxa	10.6185	-0.13294074	0.953719	-0.13939	0.88914	0.988253
il17rel	9.237574	1.358983527	0.890774	1.525621	0.127104	0.576323
si:ch1073-440b2.1	453.6748	0.060380683	0.124767	0.483947	0.628424	0.921864
im:7145024	48.51622	0.143762371	0.276305	0.520303	0.602853	0.914226
zgc:113119	54.79173	-0.016542619	0.339504	-0.04873	0.961138	0.99935
gpx2	54.93999	-0.624468202	0.342061	-1.8256	0.06791	0.44436
egr3	69.18206	0.010098271	0.384713	0.026249	0.979059	1
si:dkeyp-104f11.6	9.751854	-0.900802625	0.543701	-1.6568	0.09756	0.520966
acot14	213.4444	0.046254478	0.242256	0.190933	0.848578	0.977912
afap1l1a	306.7637	0.041986733	0.127128	0.330272	0.741195	0.954103
RF00325	0.182502	-0.868114638	5.267649	-0.1648	0.8691	NA
si:ch1073-391i24.1	33.79465	-0.549060508	0.397922	-1.37982	0.167642	0.638828
cyp46a1.3	89.05008	0.37730088	0.277266	1.360792	0.173579	0.646597
si:dkey-16p6.1	26.70726	0.638251597	0.55964	1.140468	0.254092	0.733163
adad1	11.3579	0.437018295	0.726085	0.601883	0.547252	0.896121
ppp1r32	1.76106	2.91703993	1.715689	1.700215	0.089091	NA
si:ch73-264i18.2	1.113223	1.024126874	2.273134	0.450535	0.652325	NA
wfdc2	227.1123	-0.002028655	0.195413	-0.01038	0.991717	1
tanc1b	183.865	0.287893857	0.169081	1.702695	0.088625	0.499864
zmp:0000001267	4.791603	0.834918956	1.332821	0.62643	0.531033	0.890545
CR384099.1	12.02084	-1.291902826	0.864177	-1.49495	0.134927	0.589926
dre-mir-92b	0	NA	NA	NA	NA	NA
si:dkey-103g5.4	21.93414	0.793117293	0.400482	1.980408	0.047658	0.380113
RF00017	0.838412	1.055413342	2.893185	0.364793	0.715266	NA
nkl.3	1.217249	0	4.636399	0	1	NA
smim13	846.9885	0.003796597	0.131759	0.028815	0.977012	1
si:dkey-73n8.3	94.33627	0.456331234	0.382987	1.191506	0.233455	0.714569
ankrd27	283.4825	-0.033196701	0.141492	-0.23462	0.814504	0.970536
htr3a	65.802	0.381732578	0.240865	1.584838	0.113003	0.550406
adam15	53.47353	0.012718328	0.253437	0.050183	0.959976	0.999164
zmp:0000001268	0.781187	-2.768897272	2.803812	-0.98755	0.323374	NA
ubap1la	15.48089	0.247037957	0.632444	0.390609	0.696087	0.941657
si:ch211-195o20.7	178.7264	-0.101564179	0.184545	-0.55035	0.582081	0.90571
ngfrb	76.16881	0.042594638	0.203852	0.208949	0.834488	0.975731

ro60	223.2147	0.200902592	0.149916	1.340098	0.180213	0.656017
si:ch211-91p5.3	55.6904	-0.317791634	0.252651	-1.25783	0.208453	0.69072
si:ch211-253b8.2	204.0002	-0.30226022	0.145411	-2.07865	0.037649	0.340171
prr14	423.9198	-0.028473379	0.116226	-0.24498	0.80647	0.969726
csgalnact1b	27.98382	-2.138529922	0.713224	-2.9984	0.002714	0.085055
tspearb	103.4075	-0.362742946	0.241797	-1.50019	0.133564	0.586903
mettl15	41.42367	-0.118046659	0.321221	-0.36749	0.713251	0.945821
vsig10l	21.52807	0.577362241	0.575015	1.004083	0.315339	0.777834
r3hcc1l	363.0622	0.095255272	0.124088	0.767644	0.442698	0.853879
dusp23b	216.752	0.333083816	0.212471	1.567665	0.116959	0.557831
FP885542.1	0.634658	2.998917251	2.890585	1.037478	0.299513	NA
kcnj11l	296.0722	0.017174918	0.261799	0.065604	0.947693	0.997371
dus1l	104.2789	0.134736073	0.244297	0.551526	0.581273	0.905618
pbx3a	17.28936	0.112060816	0.447783	0.250257	0.802389	0.968181
nexmifa	554.1276	0.076822112	0.157619	0.487391	0.625981	0.920625
tmem235b	7.224999	1.062861639	0.78262	1.358081	0.174438	0.647905
cenpx	117.4863	-0.542049108	0.23016	-2.3551	0.018518	0.240697
adgrl1a	48.50259	-0.594101916	0.396552	-1.49817	0.134089	0.587972
si:dkey-240n22.6	101.9542	0.052388706	0.452467	0.115785	0.907823	0.991745
si:dkeyp-115d7.2	1.854597	-0.332102146	1.323212	-0.25098	0.801828	NA
hnrnpa0a	2869.789	0.134241272	0.131111	1.023877	0.305893	0.771308
BX572619.1	155.8696	0.086687262	0.167531	0.51744	0.604849	0.914985
si:dkeyp-4f2.1	24.88381	0.93592946	0.405804	2.306357	0.021091	0.25794
pmaip1	202.7673	0.156862258	0.220646	0.710922	0.477133	0.869292
gc	980.167	-2.646598715	0.526669	-5.02516	5.03E-07	0.000166
si:ch73-106l15.4	57.235	2.520096699	1.513897	1.664642	0.095984	0.517378
mapta	604.2072	0.282799803	0.184665	1.531418	0.125666	0.573618
BX088538.1	16.64304	-1.238177967	0.519387	-2.38392	0.017129	0.231689
ccdc175	4.432572	0.126361981	0.84556	0.149442	0.881205	0.986892
arhgap20	48.48381	-0.378279181	0.387747	-0.97558	0.329271	0.787409
BX120005.1	1.045894	2.914554323	2.199561	1.325062	0.185151	NA
urahb	4.915328	-2.395772773	1.426624	-1.67933	0.093088	0.510737
si:ch211-198c19.1	0.750094	0	2.537751	0	1	NA
ednrba	105.3086	-0.525115922	0.214067	-2.45304	0.014165	0.208294
si:ch211-110e21.3	269.691	-0.233610908	0.203031	-1.15062	0.249891	0.729721
rhobtb2b	55.24767	0.969817894	0.429473	2.258158	0.023936	0.274217
si:ch73-109d9.4	8.768227	-0.606169923	0.618895	-0.97944	0.327363	0.786256
tspan4a	287.3781	-0.07690902	0.149606	-0.51408	0.607199	0.915204
si:dkeyp-121d4.3	298.3977	-0.1665146	0.171791	-0.96928	0.332404	0.789726
miga2	40.37848	0.889751641	0.328642	2.70736	0.006782	0.139038
wu:fb59d01	266.9781	-1.345821444	0.313937	-4.28692	1.81E-05	0.002746
grn1	22.55514	-0.274016647	0.838657	-0.32673	0.74387	0.95491
hopx	759.3901	-0.109992374	0.172636	-0.63714	0.524036	0.888694
fdxr	112.0747	-0.181076154	0.184991	-0.97884	0.327659	0.786438
si:dkey-238d18.10	0	NA	NA	NA	NA	NA
clk4a	2170.567	0.013510633	0.117161	0.115317	0.908194	0.991745
si:ch211-232p21.6	0.585017	-1.76756262	2.569553	-0.68789	0.491524	NA
zgc:158463	3217.96	-0.136272553	0.239051	-0.57006	0.568639	0.902234
il1fma	9.071889	-1.454798308	0.950845	-1.53001	0.126015	0.574193
RF00001	0.223379	-0.868105092	5.267649	-0.1648	0.869102	NA
ftr35	2.263876	0.973463552	2.116287	0.459987	0.645526	0.926728
pnpla2	123.989	-0.172895231	0.335067	-0.516	0.605853	0.915055
tmem176l.2	157.779	-0.454123383	0.423217	-1.07303	0.283258	0.755028
si:ch73-281i18.3	7.660418	-1.698239894	0.933447	-1.81932	0.068863	0.447809

si:dkey-24p1.7	14.04553	-0.556300158	0.450472	-1.23493	0.216857	0.69815
mbpb	1499.458	0.097959604	0.122434	0.800102	0.423652	0.844048
si:dkey-164f24.2	905.0618	-0.028997027	0.161188	-0.1799	0.857235	0.979685
rab12	291.4478	-0.225825771	0.169635	-1.33125	0.183108	0.659373
si:dkey-205h13.2	3140.231	-0.050469818	0.223236	-0.22608	0.821137	0.971788
si:ch211-212k18.15	7.421421	-0.867871533	0.901269	-0.96294	0.335575	0.792139
etv7	7.474819	2.251561067	1.241595	1.813442	0.069764	0.450821
macf1b	19.81354	0.575412904	0.367126	1.567343	0.117035	0.557961
si:ch211-105c13.3	992.7376	0.371699171	0.101175	3.673839	0.000239	0.016725
omgb	25.39206	-0.692708669	0.694384	-0.99759	0.318479	0.779923
men1	254.5622	0.175794982	0.135511	1.297279	0.194535	0.67483
rpl11a	60.5395	0.373607164	0.44939	0.831365	0.405768	0.834645
ciz1b	53.67929	-0.284141333	0.290252	-0.97895	0.327607	0.786438
dhx58	17.56879	-1.252618832	0.860659	-1.45542	0.145554	0.60482
fam161b	24.63295	-0.175570874	0.365983	-0.47972	0.631424	0.923003
CR936459.1	1.316058	-1.78269767	2.441897	-0.73005	0.465362	NA
hbae1.1	329.2344	-1.278785884	0.68511	-1.86654	0.061966	0.42715
si:ch211-132g1.3	155.9094	0.028648128	0.180914	0.158352	0.874179	0.98385
si:dkey-24117.4	0.199489	-2.731538732	5.185882	-0.52673	0.598384	NA
si:dkey-12j5.1	324.4075	0.134835269	0.155402	0.867655	0.385583	0.826939
tecrl2a	15.13015	0.133731057	0.485763	0.275301	0.783085	0.962328
cplx3b	88.52383	0.910978087	0.337322	2.700616	0.006921	0.140177
fam83e	174.3029	0.026094389	0.173121	0.150729	0.880189	0.986408
kcnq4	6.364812	0.713096763	0.721908	0.987794	0.323254	0.783113
pimr23	0.069091	0	5.267649	0	1	NA
si:ch73-367f21.5	22.333	0.035056574	0.43124	0.081292	0.935209	0.996829
bod1l1	451.3959	0.212920912	0.156044	1.364493	0.172412	0.645319
ugt1b5	219.0285	0.142356905	0.136931	1.039628	0.298513	0.765528
si:ch73-78o10.1	89.98553	-0.146089888	0.231758	-0.63036	0.528462	0.88999
si:ch211-198e20.10	3.73327	-2.321949971	1.174452	-1.97705	0.048036	0.381163
si:dkeyp-46h3.5	0	NA	NA	NA	NA	NA
zgc:92594	110.1774	0.383604567	0.256242	1.497038	0.134383	0.588706
fbln7	122.9287	0.116160151	0.192786	0.602536	0.546818	0.896107
si:dkeyp-98a7.4	0	NA	NA	NA	NA	NA
desi1b	212.0531	0.16958797	0.22312	0.760077	0.447209	0.856073
erbb4b	14.81548	-0.640247373	0.515484	-1.24203	0.214225	0.696474
rnf166	22.19505	0.437153501	0.391499	1.116616	0.264159	0.742063
BX571825.5	0	NA	NA	NA	NA	NA
baalcb	76.25021	0.222162877	0.314295	0.706861	0.479653	0.869938
si:ch211-93n23.7	4.425531	-0.513737513	1.463343	-0.35107	0.725535	0.948653
si:dkey-22114.11	2.692809	0.083926971	1.542258	0.054418	0.956602	0.999074
si:dkey-63j12.4	12.16036	-0.503792029	0.708876	-0.71069	0.477276	0.869292
BX957322.1	15.88155	0.486386871	0.496004	0.980611	0.326785	0.785579
sfrp2l	7.640598	-0.130630865	0.739498	-0.17665	0.859785	0.980066
MDFI	255.1226	0.373717104	0.192216	1.944255	0.051865	0.395819
patl2	2.030009	0.97791445	1.864029	0.524624	0.599845	NA
pstpip2	14.05521	-1.618837825	0.623055	-2.59823	0.009371	0.166502
ccdc169	5.291062	-0.971987487	0.760046	-1.27885	0.200949	0.681045
tfpt	289.5764	-0.157940366	0.123188	-1.28211	0.199806	0.6804
xrra1	12.52209	-0.003375185	0.560055	-0.00603	0.995192	1
nhlrc2	220.7298	0.18785916	0.144887	1.296591	0.194772	0.67483
si:dkey-265e15.2	27.59067	-1.963120993	0.502152	-3.90942	9.25E-05	0.008679
ncam3	25.5301	-0.669928473	0.507747	-1.31941	0.187031	0.665362
pcdh2ab11	0	NA	NA	NA	NA	NA

si:cabz01054396.2	82.76447	0.194143134	0.229145	0.847249	0.396856	0.830253
si:dkey-217f16.1	2.481754	-2.259377829	1.750576	-1.29065	0.196826	0.67714
CU929037.1	0.04222	0	5.267649	0	1	NA
coro7	81.19551	0.45530806	0.295715	1.539685	0.123637	0.570652
RF00009	9.433886	0.444593798	0.687822	0.646379	0.518034	0.887148
fgd5b	14.12435	-0.0321523	0.51704	-0.06219	0.950415	0.99801
ptges3b	1040.138	-0.041886927	0.136506	-0.30685	0.758957	0.957886
si:ch211-160d20.5	0.429725	-3.079608879	3.489987	-0.88241	0.377554	NA
dre-mir-2188	0.092101	0	5.267649	0	1	NA
BX322587.1	2.162361	-0.892741825	1.315595	-0.67858	0.497401	NA
rnaseh2c	221.4425	0.196801106	0.181495	1.084335	0.278216	0.751971
si:dkey-117n7.5	21.20936	-0.151701746	0.457103	-0.33188	0.739982	0.953684
ppm1lb	33.69843	-0.140587679	0.331711	-0.42383	0.671693	0.933953
mcama	605.4664	0.06313112	0.11341	0.556661	0.577759	0.905055
si:ch1073-406l10.2	287.8322	-1.090546733	0.193156	-5.64593	1.64E-08	9.76E-06
ctdspl3	329.8075	0.037116697	0.128907	0.287935	0.773397	0.960624
BX927136.1	0	NA	NA	NA	NA	NA
FO704622.1	7.160589	-0.837252569	0.9059	-0.92422	0.355371	0.806546
lsm3	511.9541	-0.214511727	0.226733	-0.9461	0.344099	0.798147
MFAP4 (1 of many)	34.0803	-1.298562175	0.339389	-3.82618	0.00013	0.011294
btf3l4	815.4357	0.045518772	0.160352	0.283868	0.776512	0.961073
tex26	4.546714	-0.650179427	0.897652	-0.72431	0.468875	0.867025
ism2a	57.60919	-0.04898409	0.30926	-0.15839	0.874148	0.98385
nfe2l2b	97.29084	-0.135085886	0.291107	-0.46404	0.642617	0.92567
atf7ip2	147.9923	0.038557834	0.190898	0.201982	0.839931	0.976417
trabd2a	125.5179	0.191504237	0.205823	0.93043	0.352149	0.802776
abcf3	236.8328	0.146081493	0.196621	0.742959	0.457506	0.860993
si:ch211-276a23.5	67.24236	0.20362177	0.2561	0.795086	0.426563	0.845247
gfral	48.22427	-0.076329934	0.277805	-0.27476	0.7835	0.96245
BX004785.1	2.628041	-0.890940401	1.46633	-0.6076	0.543454	0.894925
si:cabz01054394.7	13.52997	1.217759762	0.541803	2.247604	0.024601	0.278328
qpct	206.4337	-0.286907767	0.152744	-1.87835	0.060333	0.422591
CABZ01077218.1	43.44154	0.449816213	0.278038	1.617825	0.1057	0.534827
cyldb	56.26351	0.470785729	0.628194	0.749428	0.453599	0.858722
fam161a	69.75024	-0.682469541	0.273151	-2.4985	0.012472	0.194346
si:zfos-754c12.2	0.133559	0	5.267649	0	1	NA
aqp8b	109.558	-0.399110821	0.252304	-1.58187	0.11368	0.551905
si:dkey-26g8.5	115.8732	-0.355134597	0.46379	-0.76572	0.443842	0.854255
si:dkey-11o15.7	0.379948	0	5.267649	0	1	NA
si:ch211-286b5.4	0	NA	NA	NA	NA	NA
nrg3b	172.2592	0.550514749	0.215576	2.553692	0.010659	0.179908
rnf130	439.9168	-0.008687257	0.141075	-0.06158	0.950898	0.99801
hapln1a	2658.943	0.036080483	0.214414	0.168275	0.866367	0.982582
si:dkey-288a3.2	9.668244	0.215980031	0.699393	0.308811	0.757466	0.957364
ciz1a	85.92298	-0.859403215	0.214662	-4.00351	6.24E-05	0.006506
crebzf	522.0146	-0.068208702	0.130747	-0.52169	0.601889	0.914001
efna5a	416.9033	0.165294067	0.125357	1.318584	0.187308	0.66594
slc25a32a	564.4394	-0.139424018	0.15424	-0.90394	0.366025	0.815714
si:dkeyp-101e12.1	0.157736	-2.389175838	5.199609	-0.45949	0.645881	NA
sertad4	193.6158	0.019559677	0.15949	0.122639	0.902393	0.99094
si:ch73-41e3.7	3.51244	0.085087354	1.148834	0.074064	0.940959	0.997371
prr7	19.6389	-0.226474818	0.539887	-0.41949	0.674861	0.935024
akap1a	10.0131	-0.273856348	0.718839	-0.38097	0.703225	0.943622
pcdh18a	425.1381	-0.220807315	0.151338	-1.45904	0.144555	0.603539

si:dkey-239j18.3	536.9522	-0.296934179	0.41746	-0.71129	0.476906	0.869292
znf1042	20.97442	-0.658117503	0.436658	-1.50717	0.131767	0.583645
si:dkey-9k7.3	33.63758	-0.055498835	0.401016	-0.1384	0.889928	0.98835
lpar4	4.377772	-0.610004453	0.984657	-0.61951	0.535581	0.891993
si:rp71-19m20.1	285.0429	0.083041387	0.137135	0.605544	0.544817	0.895206
si:ch211-189a21.1	5.303062	-0.172019656	1.007516	-0.17074	0.864431	0.981867
si:dkey-207m2.4	6.655517	0.539474705	1.106884	0.487382	0.625988	0.920625
asb13b	60.22843	-0.240687857	0.282225	-0.85282	0.393758	0.828374
MRPS12	536.7631	0.095257076	0.136624	0.697222	0.485664	0.87303
slc35d2	30.83619	0.495141001	0.479792	1.031992	0.302076	0.76803
si:dkey-262k9.4	68.55666	-0.671281154	0.301939	-2.22324	0.0262	0.287022
BX296541.1	1.371429	-2.432688787	2.216822	-1.09738	0.272477	NA
scarb2c	354.5268	-0.069307345	0.216669	-0.31988	0.749062	0.955074
RF00017	0	NA	NA	NA	NA	NA
RF00017	0	NA	NA	NA	NA	NA
si:dkey-51d8.1	3.478767	-0.642365855	1.300089	-0.49409	0.62124	0.918461
si:dkey-22n8.3	74.27462	-3.102715286	0.606537	-5.11546	3.13E-07	0.000119
cita	317.946	0.242056858	0.166107	1.457233	0.145052	0.604004
cobl11a	71.80499	-0.287921946	0.42001	-0.68551	0.493021	0.876715
atxn7l1	30.25802	0.050261025	0.406798	0.123553	0.901669	0.990643
usp44	493.0437	-0.298333778	0.158154	-1.88635	0.059248	0.419296
si:dkey-16j16.4	29.70376	0.48880379	0.320627	1.524526	0.127377	0.576964
si:ch211-112c15.8	2.362733	-1.387228101	1.864414	-0.74406	0.456843	0.860783
zgc:172218	0.086618	0	5.267649	0	1	NA
jcada	559.527	0.068627701	0.132998	0.516006	0.60585	0.915055
socs1b	1.196638	0.087688588	2.069499	0.042372	0.966202	NA
pcdh2ab8	5.156311	0.808004134	0.834217	0.968578	0.332756	0.789823
zgc:173705	5.84032	-2.260819638	0.847214	-2.66853	0.007618	0.147466
si:dkeyp-75h12.5	720.1356	0.093845472	0.149529	0.627607	0.530261	0.890545
nedd9	327.0139	0.126433958	0.16396	0.771127	0.440632	0.852523
soul5l	3.682777	0.103289275	1.505957	0.068587	0.945318	0.997371
si:dkey-210j14.3	44.63615	-0.327575966	0.24866	-1.31736	0.187717	0.666964
slc16a12b	740.3266	0.039952646	0.177087	0.225611	0.821504	0.971788
si:dkey-19e4.5	1142.129	-0.054907722	0.13244	-0.41459	0.678445	0.936026
mmp20a	7.298505	0.077901636	0.698123	0.111587	0.911151	0.992208
dag1b	397.7272	0.007737357	0.135688	0.057023	0.954527	0.998611
rpz6	17.36869	-0.085012553	0.475322	-0.17885	0.858053	0.979774
RF00001	0.144555	1.854137197	5.241768	0.353724	0.723546	NA
cd44b	11.34855	0.723380913	0.670579	1.078741	0.280703	0.753147
tasora	368.0906	-0.063750234	0.146179	-0.43611	0.662757	0.93101
zmp:0000001289	0.872733	1.055430918	2.495012	0.423016	0.672283	NA
si:ch211-261n11.8	0	NA	NA	NA	NA	NA
CABZ01039863.1	47.98757	0.936033609	0.278379	3.362442	0.000773	0.036984
adgre5b.3	69.03879	-0.044642182	0.277898	-0.16064	0.872375	0.983447
si:ch211-265o23.1	96.63569	0.370410981	0.230437	1.607431	0.10796	0.539683
cacna1g	185.3528	-0.02382832	0.19001	-0.12541	0.900202	0.99045
clint1b	212.8201	0.050613302	0.162616	0.311244	0.755615	0.956532
sh3tc2	607.3929	-0.026856274	0.140576	-0.19105	0.84849	0.977912
si:ch211-161h7.8	124.4976	0.365166206	0.246411	1.48194	0.138356	0.594956
mlip	599.5147	0.03315871	0.255709	0.129674	0.896824	0.98939
smim4	237.9691	0.049008334	0.183084	0.267682	0.788944	0.963568
dsn1	190.9417	0.513012309	0.181656	2.824093	0.004741	0.114199
aldh2.1	736.0328	-0.620022606	0.172118	-3.60231	0.000315	0.020188
iqce	128.4571	-0.109114324	0.170115	-0.64141	0.521254	0.887473

rasal3	38.71416	-0.217899587	0.341826	-0.63746	0.523826	0.888642
si:ch211-64i20.3	5.88265	-1.101603232	1.035653	-1.06368	0.287474	0.758397
selenow2b	1005.888	0.222597089	0.196994	1.129971	0.258488	0.735636
znf1008	52.20952	-0.321601505	0.359428	-0.89476	0.370915	0.818486
npy2rl	9.865234	0.534822081	0.513998	1.040513	0.298101	0.765416
BX005085.1	113.496	-0.061847276	0.195696	-0.31604	0.751975	0.955417
si:dkey-210j14.5	33.14363	-0.14966627	0.344989	-0.43383	0.664413	0.931811
si:ch211-147k9.8	7.195287	0.917512929	0.773509	1.18617	0.235555	0.716683
si:ch73-367f21.6	16.37456	0.087693713	0.466678	0.187911	0.850947	0.978532
si:ch211-189e2.3	0.806491	1.560675295	2.047162	0.76236	0.445845	NA
si:dkey-88n24.10	0.124061	0	5.267649	0	1	NA
CR388164.1	0.483837	-0.868138064	3.213043	-0.27019	0.787013	NA
si:cabz01007807.1	45.5592	-0.699711285	0.363363	-1.92565	0.054148	0.402505
si:zfos-1505d6.3	0.195968	-1.791427895	5.221084	-0.34311	0.731513	NA
si:ch211-59o9.10	138.6424	0.15685486	0.179518	0.873755	0.382252	0.825191
SLC15A5	1.682442	-2.382745862	1.617882	-1.47276	0.140817	NA
spcs3	782.0329	0.019896423	0.154166	0.129058	0.897312	0.989719
rnf146	522.4915	-0.511665722	0.106723	-4.79433	1.63E-06	0.00042
si:ch73-109i22.2	10.08874	-0.506786557	0.564083	-0.89842	0.368959	0.817763
or116-2	3.569876	0.686633668	1.176504	0.583622	0.559475	0.899571
BX511080.1	0.566492	0	3.286668	0	1	NA
cln6b	1.630947	-0.868146615	2.061493	-0.42113	0.673664	NA
ccdc9b	29.56591	-0.225316949	0.392	-0.57479	0.565435	0.901538
CR388047.1	4.777092	-4.737255819	1.545853	-3.06449	0.00218	0.073859
BX682550.1	35.72815	0.063334961	0.304743	0.207831	0.835361	0.976007
si:dkey-16p6.1	10.66728	2.289078547	0.777772	2.943122	0.003249	0.093414
rtn4r	138.9345	0.533952132	0.249593	2.139289	0.032412	0.319299
si:dkey-6b12.5	141.1534	0.205374242	0.203088	1.011256	0.311894	0.776164
BX248410.1	1.897368	-1.785402158	1.715494	-1.04075	0.297991	NA
reck	1473.486	0.292336482	0.188329	1.552261	0.1206	0.56645
F2R (1 of many)	3.243716	-1.18764564	1.120173	-1.06023	0.289038	0.76024
si:dkey-165a24.9	39.32853	-0.759848749	0.30232	-2.51339	0.011958	0.190417
znf318	699.8571	-0.098172955	0.145316	-0.67558	0.499305	0.879603
CR394546.2	2.902282	1.965018558	1.803739	1.089414	0.275971	0.750674
rac3a	318.5526	-0.264714624	0.178562	-1.48248	0.138213	0.594797
fa2h	444.6873	0.011240189	0.143922	0.078099	0.937749	0.997305
CABZ01084273.1	58.53449	0.238594168	0.384918	0.619858	0.535351	0.891993
rxfp1	45.79068	0.58655909	0.426674	1.374725	0.169217	0.640541
CU462878.1	197.1959	-0.631170817	0.167965	-3.75775	0.000171	0.013466
si:dkey-1m11.5	3.588521	-0.575713845	1.220363	-0.47176	0.637101	0.924779
rab11bb	246.7923	-0.274726547	0.144437	-1.90205	0.057164	0.412874
CR626886.1	6.950281	0.400549564	0.861302	0.465051	0.641895	0.925586
dre-mir-30a	0	NA	NA	NA	NA	NA
si:dkeyp-67a8.4	5.437553	1.479155688	0.913823	1.618645	0.105524	0.534773
slc17a7b	48.54939	0.08144165	0.801721	0.101584	0.919087	0.993892
zmp:0000000984	4.689852	0.187530198	0.797159	0.235248	0.814016	0.970483
si:ch1073-174d20.1	53.41052	-0.330378126	0.402961	-0.81988	0.412287	0.838123
znf1136	42.87793	0.043539076	0.283183	0.153749	0.877808	0.985613
qdprb2	0.495157	0.642059814	2.49177	0.257672	0.79666	NA
si:dkey-248g21.1	82.1987	0.095498222	0.273074	0.349716	0.726552	0.949095
si:ch211-105f12.2	11.0239	-0.269323941	0.658356	-0.40909	0.682477	0.938006
tmem196b	14.04653	0.448984016	0.627971	0.714975	0.474624	0.869292
si:dkey-94l16.4	145.9006	-0.195170005	0.198798	-0.98175	0.326222	0.785142
tmem240b	64.92107	0.352548673	0.362777	0.971804	0.331148	0.788766

anapc10	123.2075	-0.149449436	0.208026	-0.71842	0.472501	0.868786
cyldl	56.66508	0.053583756	0.282386	0.189753	0.849502	0.978182
pttg1ipa	266.3213	-0.455867925	0.189183	-2.40967	0.015967	0.222864
BX005105.1	14.70746	0.497715019	1.248241	0.398733	0.69009	0.939785
zgc:173709	67.08461	0.214277969	0.277499	0.772174	0.440011	0.852079
si:ch73-362m14.2	14.24712	0.741143755	0.872622	0.849329	0.395698	0.829389
FO904898.1	0	NA	NA	NA	NA	NA
rab11fip4a	201.4509	0.213891775	0.243995	0.876625	0.380691	0.823893
BX323556.1	9.339511	1.613961377	0.926263	1.742445	0.081431	0.480419
BX548044.3	0	NA	NA	NA	NA	NA
si:ch211-117c9.1	4.876952	-0.548301539	0.933971	-0.58706	0.55716	0.899165
BX072576.2	42.95687	-0.379980374	0.293255	-1.29574	0.195067	0.67483
gapvd1	396.4793	-0.024792991	0.177163	-0.13994	0.888704	0.988188
si:dkeyp-73b11.8	586.2999	-0.669516111	0.258201	-2.593	0.009514	0.167766
bcam	426.3621	0.095861684	0.148013	0.647658	0.517206	0.887148
si:dkey-16p6.1	3.659494	0.050533573	1.047304	0.048251	0.961516	0.999434
si:ch73-286h23.4	2.258507	-4.890919333	3.409579	-1.43446	0.15144	0.615372
lamtor5	290.0013	-0.161602986	0.148615	-1.0874	0.276862	0.751311
mtx3	101.8771	0.081106324	0.228076	0.355612	0.722131	0.94744
RF00001	0	NA	NA	NA	NA	NA
rbm11	2.482439	-2.363894137	1.329062	-1.77862	0.075302	0.465238
rheb	215.9991	0.030679337	0.179497	0.170919	0.864288	0.981867
RF00001	0	NA	NA	NA	NA	NA
wdr45	663.5151	-0.105303509	0.145274	-0.72486	0.468538	0.866951
msl1b	527.6266	-0.030073205	0.102466	-0.29349	0.769145	0.960484
gsta.1	2683.491	0.257505537	0.187829	1.370961	0.170387	0.642035
tmem108	194.0016	0.174469643	0.161777	1.078456	0.28083	0.753193
clpb	113.6014	-0.279851576	0.265115	-1.05559	0.291157	0.761299
zp2.3	0.268168	-0.456832401	4.668313	-0.09786	0.922045	NA
zgc:113223	61.17477	0.047605007	0.246145	0.193402	0.846644	0.977214
phlpp2	17.15852	-0.211083336	0.48917	-0.43151	0.666095	0.932664
CU062628.1	1.578633	2.250330014	1.892751	1.18892	0.234471	NA
krtt1c19e	35108.77	-0.06901173	0.256261	-0.2693	0.787697	0.963263
BX248410.2	4.902284	-0.074986067	0.778134	-0.09637	0.92323	0.994525
serpina1	5028.803	-0.467020943	0.254978	-1.83161	0.067009	0.441957
raly1	38.06833	-0.560645711	0.429781	-1.30449	0.192066	0.672283
ldlrad2	69.9243	0.359074962	0.222293	1.615322	0.106241	0.53548
usp48	383.0643	0.019559095	0.131736	0.148472	0.88197	0.986908
CU929391.2	45.0902	0.033805846	0.334089	0.101188	0.919401	0.993892
ubxn1	468.007	0.142963421	0.124583	1.147538	0.251159	0.731476
CABZ01065328.1	0.213959	0	5.267649	0	1	NA
rnf212	3.321684	1.04349395	1.264238	0.825394	0.409148	0.836637
MAPK8IP1 (1 of many	71.43626	-0.124211096	0.418095	-0.29709	0.766399	0.960229
pprc1	228.5305	-0.236919569	0.276754	-0.85607	0.391961	0.827902
fam20cb	99.99606	-0.200323117	0.228116	-0.87816	0.379854	0.823607
si:dkey-27o4.1	29.97492	-0.662939493	0.429696	-1.54281	0.122877	0.569468
il19l	0.583753	0	4.028913	0	1	NA
RF00001	0	NA	NA	NA	NA	NA
zmp:0000000991	113.7885	0.010437878	0.278922	0.037422	0.970148	1
dre-mir-222a	0	NA	NA	NA	NA	NA
pcdh2ab5	1.877628	0.294364493	1.153679	0.255153	0.798605	NA
CR855311.1	551.8519	-0.051351621	0.309446	-0.16595	0.868199	0.982801
pimr168	0	NA	NA	NA	NA	NA
znf1065	35.18981	0.571890138	0.412296	1.387087	0.165415	0.634653

RF00001	0.043308	0	5.267649	0	1	NA
dre-mir-132-2	1.928194	0	2.117253	0	1	NA
zgc:86896	4040.623	-0.158769475	0.139399	-1.13896	0.254719	0.733542
si:dkey-46i9.6	257.6768	0.153938436	0.23431	0.656985	0.511191	0.884562
taar14e	0.09025	0	5.267649	0	1	NA
gzma	0.733979	-0.868137438	2.265393	-0.38322	0.701559	NA
CABZ01072157.1	6.513012	-0.419288275	0.953706	-0.43964	0.660197	0.930581
cd3eap	201.398	-0.390554188	0.177207	-2.20394	0.027529	0.29475
onecut2	20.35842	0.028035149	0.382845	0.073228	0.941624	0.997371
ndufv3	812.732	-0.27235367	0.183384	-1.48515	0.137503	0.593397
si:dkey-7l14.2	10.37318	-1.682815038	0.825272	-2.0391	0.04144	0.355219
si:cabz01074946.1	19.17166	-0.399624398	0.46351	-0.86217	0.388594	0.827613
CABZ01067973.1	2.211782	-1.403783986	1.728871	-0.81197	0.416811	0.840369
CABZ01020840.1	45.02151	-0.093775762	0.32234	-0.29092	0.771111	0.960484
ERBB4 (1 of many)	15.37068	0.608884903	0.543482	1.12034	0.262569	0.740129
scpp1	37.44586	-0.263357226	0.783042	-0.33633	0.736625	0.952008
wdsub1	228.1189	-0.132941567	0.18621	-0.71393	0.475268	0.869292
SH2B2	41.00783	-0.07604639	0.27065	-0.28098	0.778728	0.961073
si:dkey-246i14.3	34.67149	-0.069247658	0.334648	-0.20693	0.836067	0.976007
ctrb1	48399.69	-1.321654586	1.003787	-1.31667	0.18795	0.667259
si:dkeyp-7a3.1	6.005163	0.507742004	0.598598	0.848218	0.396316	0.829808
ponzr1	260.7887	-0.295700403	0.190713	-1.5505	0.121022	0.566931
mtbp	69.1784	0.135845782	0.249942	0.543509	0.586779	0.906862
sp100.3	1.095176	0.093653363	2.124539	0.044082	0.964839	NA
gnb1a	2758.183	0.160152761	0.104638	1.530545	0.125882	0.573811
RF00001	12.92331	0.367226675	0.457858	0.802054	0.422521	0.843493
si:ch211-197f20.1	2.010177	-3.359545809	1.708968	-1.96583	0.049318	NA
mrpl20	497.235	-0.061844334	0.191615	-0.32275	0.746882	0.955074
taar14h	2.374006	1.159020099	1.369716	0.846176	0.397455	0.830253
ppp1r3aa	87.79828	-0.317355292	0.325539	-0.97486	0.329629	0.788
sycn.1	66.7767	0	1.723233	0	1	1
BX530037.2	0.049021	-0.86807634	5.267649	-0.16479	0.869106	NA
ttll10	34.68854	0.163023179	0.404004	0.403519	0.686567	0.938732
si:ch211-269k10.5	19.45709	-0.286283235	0.481319	-0.59479	0.551985	0.8974
ccdc17	2.888572	1.043929597	1.45432	0.717813	0.472873	0.868965
trarg1a	221.0717	0.29628355	0.224271	1.321096	0.186469	0.664968
lipcb	94.95726	0.549600833	0.238653	2.302929	0.021283	0.258857
ANKRD66	1.253884	3.983667136	2.163165	1.841592	0.065535	NA
thsd7ab	15.79229	-0.993175424	0.636119	-1.56131	0.118452	0.561566
tmem121ab	13.14619	0.808264074	0.714539	1.131168	0.257984	0.735569
zmp:0000000997	0	NA	NA	NA	NA	NA
oma1	43.73097	0.306408048	0.243828	1.256656	0.208878	0.691152
trim8a	269.033	-0.081264805	0.139103	-0.5842	0.559082	0.899454
f2rl2	36.77378	-0.094374991	0.281285	-0.33551	0.737237	0.952312
zgc:158404	73.57823	-0.07363862	0.421268	-0.1748	0.861235	0.980484
ifit11	20.76404	0.867094331	0.448764	1.932184	0.053337	0.400175
tmem138	67.51402	-0.086106959	0.390609	-0.22044	0.825526	0.972729
CABZ01085658.1	155.5929	0.056869198	0.195175	0.291376	0.770764	0.960484
RF00001	0.086617	0	5.267649	0	1	NA
CR753886.1	198.0013	-0.104780867	0.158011	-0.66312	0.507251	0.883007
FP102786.1	0.597455	0	3.334683	0	1	NA
si:dkey-7j14.6	1118.569	0.196238346	0.133977	1.464717	0.142998	0.602002
si:ch1073-110a20.7	73.43982	-1.377298153	0.46518	-2.96079	0.003069	0.091315
mfap5	64.1984	-0.35467958	0.410771	-0.86345	0.387891	0.827592

PDZD4	149.7096	0.178321465	0.190644	0.935364	0.349601	0.801408
mrpl52	141.4425	0.071955037	0.188144	0.382446	0.70213	0.943292
naa40	2.357018	-0.344765109	1.421773	-0.24249	0.808401	0.969828
si:dkey-63j12.4	1.719141	0.084878711	1.391843	0.060983	0.951373	NA
rassf11	0.946721	3.67426604	2.584548	1.421628	0.155134	NA
wdr4	155.5883	0.004165111	0.188489	0.022097	0.98237	1
gpc1b	183.5662	0.211904739	0.13885	1.52614	0.126975	0.575962
taar10b	0.699798	0	3.246322	0	1	NA
pkp1a	12.9013	0.637870059	0.521953	1.222082	0.221676	0.703499
si:ch211-213a13.1	234.9285	0.382798041	0.28884	1.325293	0.185074	0.662675
FO904898.2	0.197941	0	5.212249	0	1	NA
pimr105	0	NA	NA	NA	NA	NA
si:dkeyp-117b11.1	7.482975	0.102952939	0.855412	0.120355	0.904202	0.991581
FO904898.3	0.649041	-2.762547314	2.922077	-0.94541	0.344452	NA
cd59	293.258	-0.285218592	0.364949	-0.78153	0.434491	0.850002
ctif	37.95822	0.296904504	0.331433	0.895821	0.370348	0.818484
malrd1	38.59505	-0.947073642	0.380507	-2.48898	0.012811	0.197626
si:dkeyp-106c3.1	303.0453	0.293390622	0.157723	1.860167	0.062862	0.429016
ADGRL3	256.5357	-0.034047371	0.1561	-0.21811	0.827342	0.973459
si:ch73-248e21.1	76.37684	0.003923238	0.240623	0.016305	0.986991	1
si:dkey-197j19.6	1.503472	-0.868144537	2.075451	-0.41829	0.675734	NA
tmtops3b	4.034186	0.529240719	1.179008	0.448887	0.653513	0.929269
si:dkey-32n7.8	0.205377	1.474697114	5.206324	0.283251	0.776984	NA
BX601644.1	0.464852	-0.868140828	3.264922	-0.2659	0.790317	NA
smim29	369.4527	-0.672389935	0.187301	-3.5899	0.000331	0.02066
kcnj1a.4	81.03433	0.211925172	0.381231	0.555897	0.578282	0.905417
myh6	62.95074	-0.207029308	0.280335	-0.73851	0.460206	0.862369
gp9	41.51535	0.084095107	0.249092	0.337607	0.73566	0.951543
si:dkey-54n8.4	29.84832	0.767827691	0.363355	2.113164	0.034587	0.328029
tnk2a	51.34368	-0.000390589	0.309685	-0.00126	0.998994	1
ly86	1.444178	1.055424914	2.182777	0.483524	0.628724	NA
gak	817.7635	0.232722392	0.134377	1.731859	0.083299	0.484434
tomm20a	62.89534	-0.311493546	0.279304	-1.11525	0.264743	0.742063
moto	2.001658	-0.664632771	1.530267	-0.43432	0.664053	NA
si:dkey-201i6.8	0.537694	2.894255816	2.701466	1.071365	0.284005	NA
si:ch1073-104i17.1	53.33427	0.076794275	0.222693	0.344844	0.730212	0.950056
si:ch73-40i7.5	217.763	0.168661032	0.136974	1.231333	0.218198	0.699358
si:ch211-166i24.1	0	NA	NA	NA	NA	NA
si:ch211-79k12.2	182.7949	-0.160591087	0.183099	-0.87707	0.380446	0.823655
tmem241	65.8156	-0.108867918	0.228782	-0.47586	0.634176	0.924077
slc35f2l	7.984362	-0.0024107	0.596135	-0.00404	0.996773	1
rcbtb2	146.2182	0.030033592	0.164404	0.182682	0.855048	0.979166
nell2a	564.603	0.570316657	0.168584	3.382992	0.000717	0.035767
BX005329.1	1.098428	-2.564036306	2.542265	-1.00856	0.313184	NA
si:dkey-15b23.3	4.608788	0.492372751	0.973131	0.505967	0.61288	0.915979
eif3ea	7808.727	-0.373396685	0.111916	-3.33641	0.000849	0.039393
si:dkey-52j6.3	3.38583	1.499407352	1.105019	1.356907	0.174811	0.648577
nlrb5	7.293201	0.977915805	0.913682	1.070302	0.284483	0.75547
si:dkey-28g23.6	45.30197	1.139853142	0.355774	3.203867	0.001356	0.054449
gaa	14.6037	0.507107349	0.41255	1.229201	0.218997	0.699984
cltcb	2369.266	0.174885729	0.119	1.469629	0.141662	0.600284
grem1a	0	NA	NA	NA	NA	NA
prdm2a	130.4365	0.313502072	0.276478	1.133912	0.256832	0.734393
leg1.1	2757.757	-0.911176616	0.228442	-3.98865	6.65E-05	0.006729

scn12aa	32.34819	-0.466073497	0.451261	-1.03282	0.301686	0.767693
celf5b	8.724427	0.378086369	0.706614	0.535068	0.592603	0.909996
tnfrsf9b	2.163604	-1.368439662	1.28098	-1.06828	0.285396	NA
cfbl	65.36959	0.167492787	0.283603	0.590589	0.554796	0.89855
si:dkey-16p6.1	4.381166	2.088576102	1.133479	1.842625	0.065384	0.435359
zmp:0000001003	34.4911	-0.135821988	0.300908	-0.45137	0.65172	0.928625
tor1l2	6.485147	0.00746251	0.914861	0.008157	0.993492	1
si:ch73-269m14.2	289.7816	-0.002126011	0.158742	-0.01339	0.989314	1
zc3h3	119.5187	-0.007655303	0.222712	-0.03437	0.97258	1
plk2a	245.5289	0.092264091	0.143142	0.644563	0.51921	0.887148
si:dkey-13p1.3	6.540229	-0.823728321	0.866312	-0.95084	0.341683	0.796379
bcas3	283.9245	-0.027772669	0.132265	-0.20998	0.833686	0.975482
si:ch211-136m16.8	57.36703	-0.123181281	0.274691	-0.44844	0.653838	0.929269
zgc:173556	10.00913	-1.599119912	0.554277	-2.88505	0.003913	0.103866
si:dkey-51d8.6	3.471219	1.188827722	1.044446	1.138237	0.255021	0.733542
FO834800.1	136.2354	-0.191640419	0.189742	-1.01001	0.312493	0.776164
ttll1	161.7411	0.33023014	0.293768	1.124117	0.260963	0.738533
mfap4	85.95303	-0.519084267	0.282722	-1.83602	0.066354	0.439894
diaph3	81.28473	0.164335557	0.291289	0.564166	0.572641	0.903562
klhdc8b	6.743118	0.087180081	0.753233	0.115741	0.907858	0.991745
cstf2	960.3187	0.080028516	0.098734	0.810546	0.417627	0.841149
RF00001	0.281702	-1.82550716	4.709956	-0.38758	0.698323	NA
si:ch211-207j7.2	94.0995	-0.14963492	0.258845	-0.57809	0.563205	0.90092
fam222a	139.3537	0.119055557	0.186192	0.639424	0.522547	0.887826
gpr155a	429.161	0.008167946	0.150799	0.054164	0.956804	0.999109
taar14g	0.166794	0	5.267649	0	1	NA
si:dkey-18a10.3	45.6203	-0.13618672	0.460362	-0.29583	0.767363	0.960296
kcnj10a	13.03892	2.45241675	0.74949	3.272114	0.001067	0.045972
si:dkey-85k15.11	0.299905	0.093660982	4.601732	0.020353	0.983761	NA
CACFD1	63.61194	0.18254588	0.210568	0.86692	0.385986	0.826939
slc2a10	103.3016	0.388925055	0.226812	1.714744	0.086392	0.493048
zgc:171517	0.454209	1.015477127	4.68076	0.216947	0.82825	NA
si:dkey-51d8.3	0.968278	1.925327285	1.884463	1.021685	0.30693	NA
tmem132e	590.4619	0.225385283	0.120459	1.871048	0.061338	0.424867
igfbp6b	107.2226	0.365750884	0.3271	1.118164	0.263497	0.741465
epor	79.22324	-0.557696816	0.318747	-1.74965	0.080178	0.478425
si:ch211-191d2.2	128.0993	-0.139301741	0.18278	-0.76213	0.445983	0.855403
zmp:0000001006	3.921265	1.397723092	0.994521	1.405424	0.159895	0.626123
CABZ01054965.1	36.75617	-1.963754487	0.473363	-4.14852	3.35E-05	0.004143
si:ch211-209l18.4	261.0626	0.834182668	0.175611	4.75017	2.03E-06	0.000506
serpina1l	5504.115	0.273552039	0.245039	1.116359	0.264269	0.742063
ciita	5.380522	0.581816903	0.831479	0.699737	0.484091	0.872532
lsm11	39.14457	0.065373532	0.274837	0.237863	0.811988	0.97012
CABZ01087514.1	39.96655	-0.07201876	0.323472	-0.22264	0.823813	0.972005
CR847545.1	0.70459	0	2.470394	0	1	NA
kcnj1a.2	86.40708	0.000530916	0.227022	0.002339	0.998134	1
FO904898.4	0.808513	-3.636528817	4.065836	-0.89441	0.371102	NA
si:ch1073-263o8.2	1.595689	1.280537151	1.420876	0.901231	0.367466	NA
si:dkey-210j14.4	217.5085	0.128539483	0.172318	0.745943	0.455702	0.860177
si:dkey-276j7.1	1828.178	0.199570781	0.123951	1.610078	0.107381	0.538065
ccl34a.4	28.51713	-0.247616889	0.509829	-0.48569	0.62719	0.921095
si:dkey-217f16.5	10.80581	-1.014525386	0.785622	-1.29137	0.196577	0.676952
si:rp71-36a1.5	7.560733	1.302328304	1.137196	1.14521	0.252122	0.732759
gabra3	7.568446	0.61236746	0.858936	0.712937	0.475885	0.869292

zmp:0000001301	23.07315	0.330731217	0.54241	0.609744	0.542031	0.894241
si:ch211-132p1.3	30.95852	0.23264599	0.716154	0.324855	0.745291	0.954956
cmklr1	39.54966	-0.512869197	0.395415	-1.29704	0.194618	0.67483
or117-1	1.314071	0.773303904	1.877431	0.411895	0.680417	NA
kansl1a	881.7659	-0.040520886	0.091063	-0.44498	0.656336	0.929684
sycn.3	58.46066	0	1.682981	0	1	1
apnl	4.496611	-0.954450988	1.521927	-0.62713	0.530572	0.890545
si:ch211-256e16.11	11.83805	0.246999237	0.739928	0.333815	0.738519	0.952968
creb3l3b	87.42149	-0.530338241	0.356247	-1.48868	0.136571	0.59191
RNF219	22.32818	-0.079420889	0.407765	-0.19477	0.845572	0.976909
si:ch211-152f22.4	0.672987	2.07105313	3.881527	0.533567	0.593641	NA
npc2	2714.158	-0.262562764	0.146536	-1.7918	0.073165	0.460285
si:ch211-117k10.3	580.2801	-0.423364739	0.236469	-1.79036	0.073396	0.46112
CR559930.1	0.259948	0	5.267649	0	1	NA
si:dkeyp-98a7.3	0	NA	NA	NA	NA	NA
si:ch211-120g10.1	146.1358	-1.03019414	0.340401	-3.02641	0.002475	0.079972
or128-4	0.600723	-0.868130891	3.292387	-0.26368	0.792028	NA
usp16	20.35249	0.063944359	0.418683	0.152727	0.878613	0.985761
CABZ01054394.1	72.32482	0.37038764	0.303449	1.220592	0.222241	0.703972
CABZ01033205.1	68.62319	-0.57962814	0.863212	-0.67148	0.501916	0.881148
si:ch211-170d8.8	13.88611	-1.984221889	0.948772	-2.09136	0.036496	0.335935
si:zfos-364h11.2	10.355	-5.55297206	1.543166	-3.59843	0.00032	0.020379
si:dkey-29j8.1	4.213211	1.079165675	1.514492	0.71256	0.476118	0.869292
si:ch73-29c22.1	42.30423	0.5556428	0.90383	0.614765	0.53871	0.892777
dlg5b.1	507.2365	-0.044113788	0.17449	-0.25282	0.800411	0.967364
igsf5b	24.58555	-0.427794086	0.50049	-0.85475	0.392689	0.827902
CU469420.2	13.78525	0.03075051	0.568109	0.054128	0.956833	0.999109
atp6ap1lb	213.1467	0.150442484	0.236708	0.635561	0.525063	0.888928
ripk3	25.73695	-0.567063037	0.399937	-1.41788	0.156225	0.621949
cbln18	122.1281	-0.322835402	0.66538	-0.48519	0.627542	0.921429
cc2d2a	188.3344	0.303370716	0.194716	1.55802	0.119228	0.562753
si:dkey-264d12.1	66.61425	-0.297310298	0.278992	-1.06566	0.286578	0.757624
si:dkey-62k3.5	76.28938	2.391122626	1.182668	2.021804	0.043197	0.364174
NABP2	137.0325	-0.258776396	0.174207	-1.48545	0.137424	0.593264
ifit12	4.773296	-0.672854883	0.912298	-0.73754	0.460795	0.862409
apof	477.7433	0.271404247	0.195311	1.389601	0.16465	0.634015
si:ch211-191o15.6	7.176976	0.954083504	0.862676	1.105959	0.268744	0.744925
slc44a1b	327.9916	0.153399232	0.158677	0.966742	0.333673	0.790676
si:dkeyp-115e12.6	316.213	0.014144209	0.155647	0.090874	0.927593	0.995015
larsa	147.1728	0.215294359	0.234506	0.918075	0.35858	0.80995
rnf4	211.0134	-0.268774357	0.136614	-1.96739	0.049138	0.386041
vegfba	52.70769	-0.7919143	0.37942	-2.08717	0.036873	0.337141
si:cabz01071911.3	139.962	-0.012956065	0.159155	-0.08141	0.93512	0.996829
mycbp	177.0772	0.563693685	0.177461	3.176442	0.001491	0.058157
il34	83.69335	-0.123097126	0.280933	-0.43817	0.661261	0.930581
cobl	473.5749	0.013544004	0.131007	0.103384	0.917658	0.993777
si:ch211-28p3.4	242.2875	0.024123721	0.221353	0.108983	0.913216	0.99273
si:dkey-84h14.2	13.29013	0.721153663	0.539878	1.335771	0.181624	0.657968
CR381544.2	6.303022	0.150428754	0.737997	0.203834	0.838483	0.976395
znf1124	2.707649	-2.444439409	1.334038	-1.83236	0.066898	0.44184
si:ch211-218h8.1	4.921282	-1.131596603	1.156748	-0.97826	0.327947	0.786438
aqp1a.2	1.336563	-0.102907321	2.062324	-0.0499	0.960203	NA
phox2bb	225.5486	-0.145075754	0.142104	-1.02091	0.307297	0.772541
sacs	111.1448	0.276867909	0.26092	1.061121	0.288635	0.759703

pcdh2ab6	4.919266	-1.107399391	0.73743	-1.5017	0.133174	0.586514
tmem173	18.84601	-1.737463688	0.629927	-2.7582	0.005812	0.129012
onecut3b	2.900319	0.544246313	1.159832	0.469246	0.638894	0.924779
slc38a3b	337.0964	0.087383429	0.220467	0.396357	0.691842	0.940274
si:dkey-199k11.6	0.55406	0	3.396163	0	1	NA
cib3	9.716041	-0.121455641	0.550007	-0.22083	0.825228	0.97261
alkal2a	13.46545	-0.054413424	0.529817	-0.1027	0.918199	0.993884
si:zfos-1192g2.3	539.3857	-0.07136358	0.155235	-0.45971	0.645721	0.926728
si:ch211-195j11.27	0.087381	0	5.267649	0	1	NA
lepa	4.732393	-0.873762407	1.045676	-0.8356	0.403382	0.833803
xirp2b	337.9383	-0.095962317	0.351131	-0.2733	0.784626	0.962605
zcchc10	186.5813	-0.112547345	0.148876	-0.75598	0.449661	0.856979
si:dkey-88n24.8	0	NA	NA	NA	NA	NA
fabp10b	56.84949	0.187410136	0.226752	0.8265	0.408521	0.836443
si:ch211-15b10.6	884.1501	-0.093037815	0.171081	-0.54382	0.586564	0.906711
pkhd1l1	322.12	-0.636235182	0.275335	-2.31076	0.020846	0.256424
fbxo40.2	132.844	0.279359226	0.315458	0.885567	0.375851	0.822237
rps19bp1	423.8089	-0.302172069	0.17628	-1.71416	0.0865	0.493048
rnf207a	30.2519	-2.124651803	1.403283	-1.51406	0.130011	0.580752
klf15	448.1611	-0.144053865	0.23218	-0.62044	0.534968	0.891993
lrrc75bb	19.54269	0.809646934	0.547039	1.480052	0.138859	0.596141
kcnq2b	28.79604	-0.074636161	0.386724	-0.193	0.846962	0.9773
cry3b	1418.362	0.363457381	0.364884	0.99609	0.319206	0.779923
si:ch211-253p18.5	9.614439	2.301274579	1.407319	1.635219	0.102003	0.528846
zgc:174259	35.82567	-3.541414813	1.188339	-2.98014	0.002881	0.088391
actr3	2507.577	-0.248962495	0.13631	-1.82645	0.067783	0.443779
pik3r6b	3.134799	-0.914117586	1.503323	-0.60806	0.543145	0.894925
or106-10	9.897901	1.351086485	0.742747	1.819039	0.068906	0.447963
CR749162.1	0.060442	0	5.267649	0	1	NA
crygm2d20	6540.215	0.587772937	0.23849	2.464562	0.013718	0.204799
mki67	1753.316	0.37703585	0.298386	1.263582	0.20638	0.688825
rpz	278.16	0.267464699	0.137126	1.950496	0.051117	0.393482
tor4ab	3.385516	0.680036388	1.6183	0.420217	0.674327	0.934877
or110-2	7.457082	0.930419464	0.693439	1.341746	0.179678	0.655282
si:dkey-30k22.5	0.045161	0	5.267649	0	1	NA
FP017295.1	8.169674	1.098016538	0.90304	1.215912	0.224019	0.705807
BX649250.1	235.006	0.186087448	0.158443	1.174474	0.240205	0.721177
urm1	239.7664	-0.132612826	0.164714	-0.80511	0.420756	0.842383
or110-1	0	NA	NA	NA	NA	NA
BX511123.1	0.166655	1.055370259	5.267649	0.200349	0.841207	NA
mlnr	1.50766	-0.41368651	1.775935	-0.23294	0.815808	NA
ucp3	1018.136	-0.834635657	0.266738	-3.12905	0.001754	0.064556
adh8a	38.73431	0.386810361	0.74228	0.521111	0.602289	0.914001
si:dkey-192p21.6	5.784902	-0.741139574	0.979013	-0.75703	0.449033	0.856796
rpz2	230.3173	-0.037856818	0.191959	-0.19721	0.843661	0.976791
or111-10	0.090321	0	5.267649	0	1	NA
ttc34	9.838869	0.806682093	0.555715	1.451611	0.14661	0.606437
si:dkey-19018.2	57.27411	0.002326283	0.3039	0.007655	0.993892	1
CR450686.1	6.528155	0.417672507	0.893444	0.467486	0.640152	0.924877
kcnj1a.1	45.62347	-0.605644565	0.508207	-1.19173	0.233368	0.714569
FP236356.1	2.276371	2.481356457	1.900734	1.305473	0.191732	0.672077
si:ch73-335l21.4	255.2362	-0.116788205	0.378652	-0.30843	0.757754	0.957397
CABZ01015525.1	12.47664	-4.525471959	1.325987	-3.41291	0.000643	0.032986
glt1d1	14.89844	-1.072440492	0.468186	-2.29063	0.021985	0.262571

znf975	4.935199	0.417133619	0.817912	0.509998	0.610053	0.915331
fars2	82.34906	-0.057121273	0.197035	-0.2899	0.771889	0.960624
CR381686.1	8.962699	-1.279736695	0.738505	-1.73288	0.083118	0.483758
slc22a7b.2	11.04323	-1.918606435	1.080762	-1.77523	0.075859	0.46659
smyd1b	799.1237	0.357707083	0.201726	1.773232	0.07619	0.467585
si:ch73-59p9.2	0.782338	0	3.98605	0	1	NA
mylk4a	264.7572	0.184607017	0.350388	0.526865	0.598288	0.912715
si:ch211-71m22.5	4.89608	-0.314687676	0.894965	-0.35162	0.725123	0.948653
si:ch211-225g23.1	134.6547	0.379179506	0.22602	1.677637	0.093418	0.511267
dre-mir-221	0.108976	0.093693664	5.267649	0.017787	0.985809	NA
adra2da	71.70364	0.323478883	0.22215	1.456131	0.145356	0.604476
si:ch211-66k16.27	53.93287	-1.936806232	0.575144	-3.36751	0.000758	0.036636
ebf3b	10.44365	-0.199624178	0.570041	-0.35019	0.726194	0.948987
dmxl2	909.4049	0.059194414	0.165845	0.356927	0.721147	0.947343
pmela	3075.722	-0.116619382	0.172545	-0.67588	0.499118	0.879596
card19	277.3592	-0.164668498	0.11331	-1.45326	0.146151	0.605672
kcnn1a	365.0967	0.135754207	0.17635	0.7698	0.441418	0.852874
hunk	577.1388	-0.074475558	0.19836	-0.37546	0.707322	0.944981
RF00001	0.4741	0	3.659205	0	1	NA
nlrc6	83.78058	1.018149674	0.408782	2.490691	0.01275	0.196806
CABZ01088229.1	13.18056	0.492631399	0.495644	0.993922	0.320261	0.780807
cep120	229.6272	0.153801076	0.175997	0.873885	0.382181	0.825191
si:ch73-134f24.1	0	NA	NA	NA	NA	NA
RF00017	0.259704	-2.707766391	5.186735	-0.52206	0.601631	NA
adcy5	77.87717	-0.221473881	0.220438	-1.0047	0.315042	0.777786
rtn3	2173.291	-0.040243628	0.117116	-0.34362	0.731131	0.950056
plekhd1	40.16447	0.888546749	0.379651	2.340429	0.019262	0.244673
CAMSAP3	255.9396	0.273838847	0.242282	1.130249	0.258372	0.735636
si:dkey-84k17.2	21.45656	0.29473895	0.440222	0.669524	0.503161	0.881598
prpf6	1088.591	0.158656647	0.095831	1.655585	0.097806	0.521071
AL954327.1	10.40775	-0.105774639	0.568814	-0.18596	0.852479	0.978672
galn	72.2728	0.094191718	0.300209	0.313754	0.753708	0.955912
si:ch211-214c20.1	5.935879	-0.270203135	0.834279	-0.32388	0.746032	0.955074
zgc:165409	145.8959	-0.107577989	0.188961	-0.56931	0.569143	0.902234
si:ch73-380l3.5	0.348715	0	5.267649	0	1	NA
RF00001	0	NA	NA	NA	NA	NA
si:ch211-161m3.6	54.03915	-0.614716706	0.358659	-1.71393	0.086542	0.493048
eif2b1	371.2576	-0.001114355	0.149621	-0.00745	0.994058	1
mtcl1	24.2085	0.086475503	0.362561	0.238513	0.811483	0.97012
zp2.6	0.092844	1.055383603	5.267649	0.200352	0.841205	NA
si:ch211-214p13.9	11.66416	1.751751187	0.879783	1.991117	0.046468	0.376263
TIMM22	173.1883	0.029587531	0.200849	0.147312	0.882886	0.986908
RF00001	0.043308	0	5.267649	0	1	NA
snx17	352.9024	0.014595716	0.130156	0.112141	0.910712	0.992208
nrarpa	1074.906	-0.093948968	0.106174	-0.88486	0.376232	0.822258
BX511100.2	0.633789	0	4.474334	0	1	NA
si:ch211-176g13.8	34.59571	-0.422844793	0.427874	-0.98825	0.323033	0.782754
abhd17aa	135.8067	0.062152843	0.171527	0.36235	0.717091	0.94627
pimr27	0	NA	NA	NA	NA	NA
gabra2a	19.6861	0.319484373	0.676633	0.472168	0.636807	0.924779
si:dkey-66a8.7	214.5658	-0.164037703	0.210387	-0.77969	0.435571	0.850467
RF00001	0	NA	NA	NA	NA	NA
ccdc28a	15.68828	-0.186615379	0.408714	-0.45659	0.647965	0.927245
znf1066	11.85778	0.874651688	0.488889	1.789058	0.073605	0.461398

foxc1a	590.5543	0.106773282	0.17891	0.5968	0.550641	0.8974
RF00003	0	NA	NA	NA	NA	NA
cnbd1	6.292507	-0.610206501	0.766447	-0.79615	0.425945	0.844714
si:dkey-83f18.11	0.049519	0	5.267649	0	1	NA
zmp:0000001020	16.6199	-0.296481389	0.54078	-0.54825	0.583522	0.906058
CABZ01073954.1	110.1362	0.610399099	0.268091	2.276839	0.022796	0.266874
atp6ap1la	40.21699	0.314897789	0.363689	0.865844	0.386576	0.827433
gpx7	272.6391	0.043670103	0.194657	0.224344	0.822489	0.971966
nfrkb	284.7051	-0.20272271	0.149578	-1.3553	0.175322	0.649279
si:dkey-22o12.2	386.1292	0.228726937	0.111559	2.050286	0.040337	0.351088
CT737190.1	36.22923	0.116830476	0.37937	0.307959	0.758113	0.95756
BX511084.1	2.148974	-0.649806246	1.351198	-0.48091	0.63058	NA
MAP3K11	56.683	0.091945464	0.252192	0.364585	0.715421	0.945988
carm1l	36.99754	-0.892933145	0.328772	-2.71597	0.006608	0.136957
ccser2b	13.75094	-0.00082153	0.534271	-0.00154	0.998773	1
zranb3	63.04541	-0.150742199	0.244042	-0.61769	0.536779	0.892421
ptprjb.1	8.407797	0.390251062	1.087613	0.358814	0.719734	0.94694
stard9	232.263	-0.034920868	0.204526	-0.17074	0.864428	0.981867
RF00003	0	NA	NA	NA	NA	NA
ostf1	476.3644	-0.052968081	0.105593	-0.50162	0.615933	0.916365
tnfsf13	0	NA	NA	NA	NA	NA
mmp19	0.344485	1.055412578	3.372428	0.312953	0.754316	NA
si:ch211-251f6.7	24.61831	-0.715425073	0.469447	-1.52397	0.127515	0.576964
slc6a19a.2	185.2602	3.400918188	1.111501	3.059754	0.002215	0.07471
si:dkey-156k2.4	19.52919	0.506601293	0.490338	1.033167	0.301526	0.767562
snw1	1058.376	-0.050623643	0.091584	-0.55276	0.580431	0.905618
si:ch1073-90m23.1	10.44747	-2.814056548	1.169408	-2.40639	0.016111	0.223897
suox	293.973	-0.193047184	0.145421	-1.32751	0.184341	0.661379
TMEM216	52.12923	0.0606034	0.300686	0.201551	0.840268	0.976417
si:ch211-66e2.5	196.9139	0.501387677	0.198775	2.522382	0.011656	0.187683
si:ch73-217n20.1	145.1066	-1.350645252	0.308956	-4.37164	1.23E-05	0.002154
kif28	9.661024	-1.112857427	0.728689	-1.52721	0.12671	0.57566
si:ch211-170p16.1	0	NA	NA	NA	NA	NA
peli1a	37.41071	-0.170746476	0.293872	-0.58102	0.561224	0.90039
LO018154.1	145.9679	0.016131419	0.146881	0.109826	0.912547	0.992523
si:ch73-91k6.2	99.66791	-0.339800733	0.194876	-1.74367	0.081216	0.479923
si:dkey-163f14.6	67.79733	-0.34179313	0.292482	-1.16859	0.242567	0.724345
RF00026	0	NA	NA	NA	NA	NA
arhgef28b	13.19528	-0.200658158	0.660404	-0.30384	0.761249	0.958159
spink4	385.4729	-0.673212527	0.368544	-1.82668	0.067748	0.443773
si:dkey-62k3.6	6.577203	0.889851013	0.839374	1.060136	0.289083	0.76024
prok2	1.420421	-3.083726519	1.866342	-1.65228	0.098477	NA
cltcl1	185.3741	0.483426416	0.245465	1.96943	0.048904	0.385015
mvb12ba	67.86885	-0.075005597	0.244935	-0.30623	0.759432	0.957886
ugt2a7	49.21791	-3.113521737	0.449387	-6.92837	4.26E-12	5.48E-09
si:dkeyp-67f1.2	72.09858	-0.336384098	0.252671	-1.33131	0.183085	0.659373
si:dkey-271j15.3	45.13071	0.142244627	0.451242	0.315229	0.752588	0.955756
cspp1b	148.2619	0.001163897	0.176864	0.006581	0.994749	1
si:ch211-197h24.6	476.5501	0.154417122	0.132936	1.161589	0.245403	0.726909
si:dkey-177p2.18	35.22916	0.464058116	0.345966	1.34134	0.17981	0.655282
pip4k2cb	9.164555	-0.57804725	0.988992	-0.58448	0.558897	0.899409
CABZ01072989.1	1.529427	0.367071795	1.362208	0.269468	0.787569	NA
si:ch73-352p4.5	73.35225	-0.432884149	0.222154	-1.94858	0.051346	0.394196
igflr1	73.59209	-2.939215504	0.533744	-5.50679	3.65E-08	2.07E-05

xrn1	617.7425	0.068458627	0.189074	0.362073	0.717297	0.946272
gcsha	445.5076	0.048968291	0.144264	0.339435	0.734282	0.951134
lsm8	328.3635	0.117636848	0.176504	0.666481	0.505103	0.882097
CT573256.1	13.1512	0.954893727	0.465499	2.051335	0.040234	0.350648
sptssb	123.3413	0.390735687	0.249184	1.568059	0.116867	0.557831
fgfbp1b	132.9738	0.192597099	0.224395	0.858294	0.39073	0.827902
si:dkey-234i14.3	9.721993	-0.080827514	0.578762	-0.13966	0.888932	0.988188
bicdl2l	238.8013	-0.584275358	0.657342	-0.88885	NA	NA
ints12	211.8117	-0.035643021	0.16409	-0.21722	0.828039	0.973905
FO704858.1	1.271065	0.077801642	1.909696	0.04074	0.967503	NA
cnrip1a	491.5908	0.451506985	0.151891	2.972581	0.002953	0.089911
havcr2	18.745	-0.509119272	0.539122	-0.94435	0.344991	0.79837
RF00017	0	NA	NA	NA	NA	NA
capn2a	1507.625	-0.167059053	0.109578	-1.52456	0.127368	0.576964
csnk2a4	18.91863	1.401002003	0.529485	2.645971	0.008146	0.153194
taar14d	0	NA	NA	NA	NA	NA
si:dkey-238d18.5	3.835891	0.676742773	1.544711	0.438103	0.661312	0.930581
BX649490.1	0.955202	1.917134636	2.118365	0.905007	0.365462	NA
si:ch211-202f3.4	20.65775	0.115593689	0.876466	0.131886	0.895074	0.989187
aebp1	540.2452	0.048553075	0.143524	0.338292	0.735143	0.951543
CABZ01061592.1	28.66745	0.590478751	0.487598	1.210995	0.225897	0.707208
zgc:114046	92.14547	0.419744195	0.241662	1.736909	0.082403	0.482825
gig2l	1.380556	0	5.267649	0	1	NA
taar14b	0	NA	NA	NA	NA	NA
traf3ip2l	148.43	-0.279938796	0.223833	-1.25066	0.211058	0.692821
si:dkey-16p6.1	3.697546	-2.116388277	1.137136	-1.86116	0.062722	0.428693
tbx22	28.96721	-0.183012395	0.308178	-0.59385	0.55261	0.8974
RF00001	0	NA	NA	NA	NA	NA
CT573476.1	34.66673	-0.234401804	0.418508	-0.56009	0.575418	0.904739
insm1a	685.805	0.035948093	0.165949	0.216622	0.828503	0.974137
adgrf6	127.8557	-0.400132116	0.310222	-1.28982	0.197112	0.677304
cwf19l2	212.9611	0.326386848	0.134765	2.421895	0.01544	0.218848
zbtb40	123.7715	0.24799015	0.21922	1.131239	0.257954	0.735569
dclre1a	111.4096	0.228983059	0.255592	0.895894	0.370309	0.818484
lysmd2	379.8949	0.322672522	0.146254	2.20624	0.027367	0.293836
CR524827.1	0.576274	0	4.506452	0	1	NA
mapkap1	88.31314	0.213014718	0.279454	0.762253	0.445909	0.855403
BX890608.2	0.325339	0.181757575	5.131276	0.035422	0.971744	NA
si:dkeyp-71f10.5	20.56161	0.416540356	0.57989	0.71831	0.472566	0.868786
si:ch211-203b20.4	0.043309	0	5.267649	0	1	NA
or111-5	4.209684	-0.426203708	1.118553	-0.38103	0.70318	0.943622
akap12a	207.3094	-0.671277895	0.233839	-2.87068	0.004096	0.106542
taar18c	0	NA	NA	NA	NA	NA
si:ch211-266k22.6	43.83858	0.021802401	0.342385	0.063678	0.949227	0.99801
zgc:174260	57.24853	-4.100279244	1.301563	-3.15027	0.001631	0.06206
serpinb14	480.8806	0.208739278	0.398641	0.523627	0.600538	0.913756
si:dkey-21a6.5	352.7275	-0.271098263	0.159348	-1.7013	0.088886	0.500363
CR391998.1	28.18443	0.474151691	0.442335	1.071928	0.283752	0.755095
smc6	33.73078	0.440164274	0.364739	1.206793	0.227512	0.70878
ddx27	490.4107	-0.076900601	0.169139	-0.45466	0.649355	0.927756
MDFIC2	3.598716	0.777122573	0.987988	0.786571	0.431533	0.848419
sv2	180.9557	-0.330892725	0.232913	-1.42067	0.155412	0.620161
si:ch211-198m17.1	161.5911	0.078505993	0.19536	0.401853	0.687792	0.938835
si:ch73-264p11.1	23.53212	0.579531461	0.378172	1.532456	0.12541	0.573278

casp7	400.7483	0.295494836	0.150374	1.965067	0.049406	0.387262
si:cabz01071909.2	19.61499	0.434953725	0.591728	0.735056	0.462305	0.863631
pimr21	0.208907	0	5.267649	0	1	NA
si:ch211-181d7.1	16.80034	-6.378100384	3.827323	-1.66647	0.095621	0.516418
tsen2	49.61549	-0.149386651	0.292276	-0.51111	0.609271	0.915204
si:ch73-233m11.2	9.720536	0.634913836	0.639173	0.993336	0.320546	0.780821
zgc:194007	22.71905	-0.073213468	0.526867	-0.13896	0.889482	0.988318
BX942825.1	0.059954	1.055396947	5.267649	0.200354	0.841203	NA
zgc:158701	4.603588	0.263580284	0.9946	0.265011	0.791001	0.964766
BX950194.1	0	NA	NA	NA	NA	NA
CR936300.1	4.868255	0.116094475	0.93085	0.124719	0.900746	0.99045
zgc:162958	86.97662	-0.06572062	0.234941	-0.27973	0.779683	0.961073
CU469539.1	0.236338	1.05539181	4.243249	0.248723	0.803575	NA
si:ch211-102c2.7	128.7623	-0.027050361	0.185407	-0.1459	0.884002	0.987036
CR354402.1	0.168647	0	5.267649	0	1	NA
or137-7	0.707363	0	2.992628	0	1	NA
si:dkey-29d8.3	72.53808	-0.316790166	0.267446	-1.1845	0.236215	0.717364
grnas	0.849449	0	2.372316	0	1	NA
BX005355.1	0.043308	0	5.267649	0	1	NA
si:dkeyp-69c1.9	28.33422	-0.215842559	0.344515	-0.62651	0.53098	0.890545
CR812481.1	9.407172	-1.946040545	0.688489	-2.82654	0.004705	0.113804
si:dkeyp-67e1.3	0.102662	0	5.267649	0	1	NA
AL953893.1	0.262872	-0.868116091	5.267649	-0.1648	0.8691	NA
znf1172	17.37557	0.35997697	0.431534	0.834179	0.40418	0.833888
BX323884.1	66.25614	1.362510173	0.31736	4.293261	1.76E-05	0.00272
si:ch73-54n14.2	0	NA	NA	NA	NA	NA
CR388209.1	0.454697	1.055394109	3.665504	0.287926	0.773403	NA
b3gnt2b	406.8324	-0.495214454	0.168257	-2.9432	0.003248	0.093414
si:dkeyp-51f12.3	5.5213	-1.288173078	0.819396	-1.5721	0.115927	0.556115
si:dkey-108k21.21	13.84288	0.815237852	0.501341	1.626114	0.103925	0.532096
rbp7a	0.08553	0	5.267649	0	1	NA
si:ch73-59c19.1	10.7985	-1.428352103	0.522056	-2.73601	0.006219	0.133542
CR974461.1	0	NA	NA	NA	NA	NA
si:ch211-15j1.5	6.343414	0.709644501	1.544451	0.45948	0.64589	0.926728
BX530075.1	0	NA	NA	NA	NA	NA
ugt5b4	269.8995	-0.086727206	0.272774	-0.31794	0.750527	0.955074
CU571079.1	22.98231	-0.041263828	0.362997	-0.11368	0.909495	0.99213
BX548073.1	7.68621	1.506649871	0.794316	1.896789	0.057856	0.415278
BX284679.1	0.210955	1.055364264	5.123777	0.205974	0.836811	NA
si:dkey-103e21.5	15.14662	-0.691089123	0.576437	-1.1989	0.230568	0.712206
or126-1	5.200218	-0.102480707	1.076269	-0.09522	0.924141	0.994813
si:ch211-12e13.1	10.23899	0.293095408	0.750664	0.390448	0.696205	0.941657
gpatch4	193.1742	0.072947311	0.246541	0.295882	0.76732	0.960296
si:ch73-25f10.6	461.7084	-0.231448673	0.115357	-2.00637	0.044817	0.370861
BX649388.1	12.00626	-0.589622164	0.575119	-1.02522	0.30526	0.770382
BX005429.1	0	NA	NA	NA	NA	NA
CU633804.1	0.316633	0	4.549245	0	1	NA
GBGT1 (1 of many)	3.596171	0.115071456	1.225063	0.093931	0.925164	0.994815
BX927308.1	132.1577	-0.134186731	0.257189	-0.52174	0.601849	0.914001
BX890576.1	5.483039	-0.493726473	0.743897	-0.6637	0.50688	0.883007
CR847971.1	1.038802	-2.309028232	2.268042	-1.01807	0.308644	NA
BX005313.1	0.495842	2.068161195	3.619801	0.571347	0.567765	NA
GBGT1 (1 of many)	1.817678	-0.868136768	1.988061	-0.43668	0.662347	NA
CR391940.1	24.64403	-0.535366523	0.412145	-1.29898	0.193953	0.674122

znf106b	33.51208	-0.498129857	0.295578	-1.68528	0.091935	0.507669
CU694486.1	0	NA	NA	NA	NA	NA
si:dkeyp-4c7.3	0.574205	0.939435634	2.876751	0.326561	0.744	NA
nuggc.2	8.317383	0.179817839	0.696703	0.258098	0.796331	0.966425
si:dkeyp-122a9.1	0.83963	-2.074204515	2.611096	-0.79438	0.426974	NA
tmem184bb	30.92652	1.082975588	0.480553	2.253604	0.024221	0.275306
si:dkey-172o10.8	0	NA	NA	NA	NA	NA
BX677668.1	1.21917	2.502601507	2.042051	1.225533	0.220374	NA
pth1b	42.25434	-1.468022629	0.469622	-3.12596	0.001772	0.06487
RUNDC1	70.40868	0.200092941	0.244872	0.817132	0.413853	0.838564
CR925863.1	0.282371	1.05536736	4.731485	0.223052	0.823495	NA
ttf1	54.73668	-0.152350773	0.287266	-0.53035	0.595871	0.911428
gbgt1l3	2.582965	-0.922396254	1.297794	-0.71074	0.477244	0.869292
ms4a17a.3	11.49049	-1.491911103	0.837895	-1.78055	0.074987	0.464308
si:dkey-58f10.7	47.47512	-0.549689107	0.315227	-1.74379	0.081196	0.479923
CR356233.1	19.7687	0.982174583	0.423707	2.318051	0.020447	0.253747
AL929229.1	0.927048	4.065046016	2.21317	1.836753	0.066246	NA
CR848717.1	0.097564	1.055383603	5.267649	0.200352	0.841205	NA
BX470189.2	4.607871	2.579449523	1.163161	2.217621	0.026581	0.288909
CR450716.1	0.119814	-0.868089684	5.267649	-0.1648	0.869104	NA
BX085193.1	0.36806	1.921543831	5.236909	0.366923	0.713676	NA
BX005012.2	0.259886	0	5.267649	0	1	NA
BX469885.1	4.941185	-5.713747554	1.597194	-3.57737	0.000347	0.021388
BX511112.1	0.997337	-3.101904659	2.710491	-1.14441	0.252455	NA
v2ra16	0.583805	0	2.760401	0	1	NA
mrap	0	NA	NA	NA	NA	NA
dicp1.1	10.07178	2.997279762	1.40235	2.137327	0.032571	0.319477
znf1048	7.222487	-0.515534303	0.711347	-0.72473	0.468618	0.866951
tcnbb	4076.988	0.557838074	0.417314	1.336736	0.181309	0.657731
si:ch211-284e13.11	0.060442	0	5.267649	0	1	NA
znf1020	9.366282	-1.203666301	0.629779	-1.91125	0.055972	0.409108
CR735121.1	0.245107	-3.029187758	5.176306	-0.5852	0.558411	NA
ccs	50.24241	0.84674771	0.481942	1.756948	0.078927	0.475183
CR396590.1	0	NA	NA	NA	NA	NA
CT027611.1	0.742148	4.392704439	2.813184	1.561471	0.118413	NA
CR381686.2	1.609494	-0.742697281	2.563699	-0.2897	0.772048	NA
BX530407.1	0	NA	NA	NA	NA	NA
BX294129.1	0.258471	-0.868123757	5.267649	-0.1648	0.869099	NA
si:ch211-139a5.1	35.83254	0.055788749	0.272178	0.204972	0.837594	0.97621
CR855277.1	1.662263	-2.387454763	1.703284	-1.40168	0.161012	NA
CR513782.1	0.23529	-0.868124374	4.979613	-0.17434	0.861602	NA
si:dkey-256e7.8	3.682504	1.8893193	1.030255	1.833836	0.066678	0.441033
ftr10	0.229766	-1.769373768	5.234318	-0.33803	0.735338	NA
nlrc8	1.885106	-5.229656611	3.999943	-1.30743	0.191066	NA
zgc:173545	0.129927	0	5.267649	0	1	NA
si:dkey-20i20.9	7.826377	0.972738453	0.613101	1.586588	0.112606	0.549758
si:dkey-4c23.3	1.431344	-0.519278244	1.824168	-0.28467	0.7759	NA
si:dkey-239h2.3	668.7399	-0.372374616	0.308578	-1.20674	0.227531	0.70878
BX649633.1	0	NA	NA	NA	NA	NA
si:ch211-156j16.1	1371.554	0.038529977	0.148968	0.258647	0.795908	0.966247
CR925731.1	12.34681	-1.163754425	0.649626	-1.79142	0.073225	0.460541
si:dkey-70p6.1	154.1074	0.306454467	0.236554	1.295496	0.195149	0.67483
CR391910.1	0.358818	0	4.446814	0	1	NA
AL935153.1	11.50132	-3.624718362	2.205965	-1.64314	0.100353	0.524753

si:dkey-22f5.9	25.27464	-0.321044135	0.796446	-0.4031	0.686878	0.93875
si:dkey-225k4.1	30.26746	-0.134277728	0.390699	-0.34369	0.731083	0.950056
gstk4	3.048865	-1.877321888	1.495143	-1.25561	0.209256	0.691286
BX649502.1	0	NA	NA	NA	NA	NA
BX927365.1	0.475406	-0.868146279	4.183999	-0.20749	0.835626	NA
si:dkey-90l23.1	6.810408	-0.081196705	0.724173	-0.11212	0.910726	0.992208
tbx5b	8.731983	0.445654533	0.590258	0.755016	0.450239	0.857151
si:dkey-112g5.13	0.046941	0	5.267649	0	1	NA
BX511123.2	2.047469	-0.448562164	1.387041	-0.3234	0.746396	NA
si:dkey-117n7.4	19.68851	-0.338052642	0.452289	-0.74743	0.454806	0.859845
BX005174.1	1.434578	0.516140283	1.530124	0.337319	0.735876	NA
CT583708.1	4.736109	-2.330295019	1.145329	-2.03461	0.04189	0.35789
atnl	530.014	0.123160955	0.13528	0.910412	0.362605	0.81253
si:ch211-15j1.3	2.39582	0	2.225662	0	1	1
si:ch211-117n7.6	52.68736	-0.289246017	0.376139	-0.76899	0.441901	0.853308
CT997819.1	0.623252	2.00003102	2.975284	0.672215	0.501447	NA
znf1165	35.19885	-0.126206633	0.368622	-0.34237	0.732069	0.950617
hcst	9.797237	-1.137612355	0.651013	-1.74745	0.08056	0.479386
pimr49	0.14264	0	5.267649	0	1	NA
si:dkey-147f3.4	87.38102	0.680151677	0.255043	2.666813	0.007657	0.147606
AL954134.1	1.190803	-0.868145468	2.217455	-0.39151	0.695424	NA
znf1044	5.968625	0.239722075	0.812733	0.294958	0.768026	0.960296
BX649639.1	16.31144	-1.765293163	1.606451	-1.09888	0.271821	0.748006
BX899181.1	4.963485	0.617808456	0.762316	0.810437	0.417689	0.841149
CR392002.1	4.583935	-0.510789889	1.14528	-0.446	0.6556	0.929606
BX294006.1	0.282251	-0.868127258	4.045521	-0.21459	0.830087	NA
CR318650.1	0	NA	NA	NA	NA	NA
si:dkey-4c15.8	10.9325	0.101562132	0.543077	0.187012	0.851651	0.978548
BX927234.1	0.137262	0	5.267649	0	1	NA
si:ch211-125e6.12	1.09666	-0.25873759	5.037776	-0.05136	0.959039	NA
cyp2x12	39.3956	1.428879266	1.306298	1.093839	0.274026	0.749284
si:dkey-112g5.11	0.867377	-2.310711983	2.183146	-1.05843	0.289858	NA
CR788254.1	0.04222	0	5.267649	0	1	NA
BX294661.1	0.175683	-0.868109138	5.267649	-0.1648	0.869101	NA
CR749763.1	0	NA	NA	NA	NA	NA
si:ch211-133n4.6	354.938	0.641630373	0.366523	1.750585	0.080017	0.478179
BX571955.1	0	NA	NA	NA	NA	NA
si:ch211-214p13.8	1.002815	1.055430458	2.253052	0.468445	0.639467	NA
CU633823.1	0.137802	1.055383603	5.267649	0.200352	0.841205	NA
BX511093.1	0	NA	NA	NA	NA	NA
CU639436.1	0	NA	NA	NA	NA	NA
tomm40	985.3261	0.074139898	0.147029	0.504254	0.614083	0.915998
eif4a1a	6329.946	0.709009596	0.189544	3.740597	0.000184	0.014175
BX571701.1	0.087179	0	5.267649	0	1	NA
si:ch211-235f12.2	0	NA	NA	NA	NA	NA
BX470185.1	34.34079	-0.490298704	0.30903	-1.58657	0.112609	0.549758
si:ch211-229n2.6	0.045161	0	5.267649	0	1	NA
si:dkey-20d21.12	134.9664	0.176952324	0.193795	0.913091	0.361195	0.81142
cox14	369.8875	0.203825624	0.183703	1.109541	0.267197	0.743418
CU468041.1	0	NA	NA	NA	NA	NA
vtg5	0.409253	0	4.275376	0	1	NA
CR925709.1	13.79093	0.080288412	0.486612	0.164995	0.868948	0.982801
AL954694.1	2.223755	-0.332919055	1.249698	-0.2664	0.789931	0.964214
si:dkey-84o3.7	0.40223	1.055396228	3.738238	0.282324	0.777695	NA

rad54b	67.08791	0.039868318	0.27929	0.142749	0.886488	0.987533
BX649502.2	0.073968	0	5.267649	0	1	NA
cenpw	51.40417	0.141442554	0.306767	0.461075	0.644745	0.926639
si:dkey-4i23.5	22.13172	-0.315766219	1.388393	-0.22743	0.820087	0.971788
si:dkey-247i3.6	6.707233	-0.14562755	0.834235	-0.17456	0.861422	0.9805
si:ch211-231i17.3	0	NA	NA	NA	NA	NA
si:dkeyp-100a1.6	29.77227	-0.273322432	0.32123	-0.85086	0.394847	0.828825
BX663503.1	102.4609	-0.320968346	0.337219	-0.95181	0.341193	0.796037
ank1a	770.1198	0.285263595	0.160394	1.778513	0.07532	0.465238
CR847899.1	0	NA	NA	NA	NA	NA
FP085398.2	23.72221	-0.980410329	0.390598	-2.51002	0.012072	0.190742
BX649522.1	11.25733	-0.297635648	0.549655	-0.5415	0.588166	0.907912
si:dkey-4c15.9	7.630831	-0.28050891	0.612385	-0.45806	0.646909	0.926728
tex264a	297.8325	0.18566583	0.163245	1.137346	0.255394	0.733542
apoc2	8761.025	-0.028247605	0.223409	-0.12644	0.899384	0.99045
cbx3b	692.9572	0.000220161	0.13786	0.001597	0.998726	1
CR788249.1	1.33189	-3.821025121	5.158606	-0.74071	0.45887	NA
ferd3l	0.354213	0.148986837	3.687855	0.040399	0.967775	NA
si:dkey-261e22.4	16.57857	0.825724513	0.568251	1.453098	0.146197	0.605672
mhc1zaa	1.901861	-1.730102803	2.314081	-0.74764	0.454676	NA
si:dkey-147f3.8	102.1487	-0.309331455	0.207457	-1.49106	0.135946	0.591131
si:ch211-272h9.5	2.422131	-0.346283708	1.502486	-0.23047	0.817724	0.971441
si:dkey-250k15.4	7.730411	-0.214088762	0.660221	-0.32427	0.745735	0.955074
apoc1	23012.59	-0.162494698	0.263137	-0.61753	0.536886	0.892421
si:dkey-96n2.1	0	NA	NA	NA	NA	NA
si:ch211-59d15.4	8.598003	0.159129907	0.764494	0.208151	0.835111	0.976007
BX321875.1	0.850522	-0.797673173	2.112677	-0.37757	0.705754	NA
BX908388.1	0	NA	NA	NA	NA	NA
BX511187.1	0.660532	2.446295201	3.237946	0.755508	0.449944	NA
si:dkey-40m6.11	0	NA	NA	NA	NA	NA
im:7140055	159.6227	0.045844819	0.159828	0.286838	0.774236	0.960695
BX957292.1	0.274719	1.055346338	5.267649	0.200345	0.841211	NA
CR318588.1	100.4579	0.637513154	0.63932	0.997175	0.31868	0.779923
LO018289.1	1.067582	0	2.627934	0	1	NA
si:ch211-250c4.4	287.5118	0.173504418	0.124493	1.393692	0.163411	0.631353
CHST12 (1 of many)	5.516315	-2.32191891	1.812973	-1.28072	0.20029	0.680711
si:dkey-112g5.16	1.599496	1.332031738	1.442016	0.923729	0.355628	NA
ms4a17a.5	30.60607	0.386753022	0.539673	0.716644	0.473594	0.869146
BX511089.1	0	NA	NA	NA	NA	NA
CR932978.1	0.095482	1.055383603	5.267649	0.200352	0.841205	NA
AL929092.1	1.027072	2.667596542	5.195716	0.513422	0.607656	NA
or103-3	0	NA	NA	NA	NA	NA
si:ch211-271e10.3	0.662005	0.637898046	2.585106	0.246759	0.805095	NA
si:dkeyp-13a3.10	121.0538	-0.679186189	0.234463	-2.89677	0.00377	0.102406
CT573163.1	0.046941	0	5.267649	0	1	NA
si:dkey-27p18.3	28.0903	0.222671277	0.404145	0.550968	0.581655	0.905618
FO904898.5	0.324815	-0.86813434	4.571789	-0.18989	0.849396	NA
BX571825.6	0.050723	-0.86807634	5.267649	-0.16479	0.869106	NA
vtg1	4.738558	0.975748205	0.880968	1.107587	0.26804	0.744423
or111-9	0	NA	NA	NA	NA	NA
si:ch211-243a20.3	245.0999	-0.889081656	0.204081	-4.3565	1.32E-05	0.002278
znf1009	30.69118	-0.003509721	0.389471	-0.00901	0.99281	1
si:dkey-26i13.7	0.227407	1.921556829	5.236909	0.366926	0.713674	NA
BX005065.1	48.25666	-1.818944372	0.451104	-4.03221	5.53E-05	0.006008

BX000447.1	60.22029	0.244562502	0.314034	0.778777	0.436111	0.850763
si:dkey-20i20.3	9.619877	0.209434179	0.587464	0.356506	0.721462	0.947343
BX548061.1	0.106927	0	5.267649	0	1	NA
irgf2	0.332701	-0.868129238	3.402075	-0.25518	0.798587	NA
si:dkey-103k4.1	4.556054	0.99940055	1.736869	0.575404	0.565018	0.901538
CR769769.1	7.553476	-1.334265936	0.803979	-1.65958	0.096999	0.519526
si:dkey-102f14.7	1.266142	0	2.757032	0	1	NA
BX510992.1	11.02655	-0.060768457	0.582132	-0.10439	0.91686	0.99353
si:dkey-211g8.5	0.207254	-2.389188515	5.199609	-0.45949	0.64588	NA
si:ch73-290k24.6	40.56832	-0.318990677	0.328805	-0.97015	0.331971	0.789602
si:ch211-220m6.4	0.356385	0	5.1926	0	1	NA
CR382296.1	1.374722	1.574316962	1.821508	0.864293	0.387427	NA
si:ch73-27e22.2	0.315207	1.854115399	5.241768	0.353719	0.723549	NA
CR788230.1	0.124582	1.055383603	5.267649	0.200352	0.841205	NA
si:dkeyp-41f9.4	76.27478	-0.230875313	0.336398	-0.68632	0.492513	0.876385
BX511161.1	44.31978	0.174628489	0.442453	0.394682	0.693077	0.94055
si:dkey-3k20.1	4.875211	-0.636938245	1.00251	-0.63534	0.525204	0.888928
si:dkey-234d14.2	0.328627	-2.707785434	5.186734	-0.52206	0.601629	NA
BX663611.1	1.082632	1.952511727	2.21814	0.880247	0.378725	NA
BX548015.1	0	NA	NA	NA	NA	NA
AL929266.1	2.274997	0.542003077	1.365312	0.396981	0.691381	0.940173
flnb	305.2468	-0.12553349	0.221906	-0.56571	0.571593	0.903152
BX000991.1	0.04222	0	5.267649	0	1	NA
cxl34b.11	234.3922	-0.098683161	0.336996	-0.29283	0.769651	0.960484
cenpv	64.44459	0.227967109	0.354744	0.642625	0.520468	0.887165
CU861651.1	0.408873	-1.717626595	3.261805	-0.52659	0.59848	NA
CR392026.1	0.452045	-1.335348704	3.633693	-0.36749	0.713253	NA
AL954142.1	5.78179	0.083515533	1.349186	0.061901	0.950642	0.99801
si:dkeyp-26a9.2	157.5156	0.797438343	0.186276	4.280958	1.86E-05	0.002746
si:dkey-222h21.6	0.586059	1.05542239	2.791347	0.378105	0.705353	NA
BX537166.1	6.006481	-1.487047579	1.318726	-1.12764	0.259472	0.736167
CT027772.1	0.61031	0	2.68068	0	1	NA
CT574549.1	0.137988	0	5.267649	0	1	NA
si:dkey-11o15.8	1.78831	0	4.994699	0	1	NA
si:dkey-59l11.7	0	NA	NA	NA	NA	NA
poldip2	890.1126	-0.009964334	0.124639	-0.07995	0.93628	0.997032
CR456642.1	4.240344	1.742646055	1.449089	1.202581	0.229139	0.711257
si:dkey-65l23.2	0	NA	NA	NA	NA	NA
BX927288.1	3.554547	0.131422569	1.234819	0.106431	0.915241	0.993068
si:ch211-199g17.2	44.81318	-0.884848613	0.274346	-3.22531	0.001258	0.051788
si:dkey-65b12.10	0.973367	1.634639115	2.377855	0.687443	0.491804	NA
BX004981.1	8.900126	-0.082008544	0.577781	-0.14194	0.88713	0.987739
pimr152	0	NA	NA	NA	NA	NA
BX323074.1	3.380418	1.231192496	1.178638	1.044589	0.296213	0.764246
CT583646.1	0	NA	NA	NA	NA	NA
si:ch211-152f2.1	0	NA	NA	NA	NA	NA
CR383669.1	0.188219	0	5.267649	0	1	NA
si:dkeyp-2e4.3	24.93863	-0.450845278	0.36573	-1.23273	0.217678	0.698365
pimr136	1.482074	-3.729708813	1.789429	-2.0843	0.037133	NA
CR383672.1	0.279436	-0.868125659	4.061755	-0.21373	0.830756	NA
BX649499.1	0.534552	-0.868144478	3.189021	-0.27223	0.785446	NA
CU207311.1	0	NA	NA	NA	NA	NA
CU467861.1	236.6671	-0.896711552	0.278148	-3.22386	0.001265	0.051937
pimr61	0	NA	NA	NA	NA	NA

AL590150.1	18.53543	-0.269308479	0.415274	-0.64851	0.516656	0.886933
si:dkey-57a22.13	0.165707	0	5.267649	0	1	NA
nfil3-4	0.384755	1.858590153	4.260742	0.436213	0.662682	NA
si:dkey-161j23.6	111.5875	-0.762697889	0.243302	-3.13478	0.00172	0.064499
CT027702.1	7.08261	1.122397511	0.62162	1.8056	0.070981	0.454693
si:dkey-222h21.12	0.147936	0	5.267649	0	1	NA
BX649478.1	17.32031	-4.337619285	1.553848	-2.79153	0.005246	0.121252
jac4	0.383595	1.055380736	4.28659	0.246205	0.805523	NA
si:ch211-284e13.6	545.6739	-0.419559725	0.29409	-1.42664	0.153684	0.61823
BX469930.1	49.24409	-3.118413541	1.362108	-2.2894	NA	NA
si:dkeyp-113d7.10	78.47373	0.142300612	0.245511	0.57961	0.562177	0.900715
si:ch211-51h9.6	138.0029	0.047397131	0.180622	0.262411	0.793005	0.965312
si:dkey-79f11.7	11.14733	-2.97527645	1.037562	-2.86756	0.004136	0.106542
hsp70.2	287.1792	-0.027431725	0.529174	-0.05184	0.958657	0.999109
si:ch211-218c6.8	244.5188	0.162707552	0.190346	0.854798	0.392663	0.827902
si:dkeyp-77c8.3	8.436658	0.36970494	0.659364	0.560699	0.575003	0.904516
BX511023.1	24.95758	-0.327244107	0.459775	-0.71175	0.476621	0.869292
BX005340.1	0	NA	NA	NA	NA	NA
dlgap2b	3.782453	0.66173593	1.244729	0.531631	0.594982	0.910869
BX571809.1	4.839328	-0.162889121	1.007947	-0.1616	0.871617	0.983365
si:dkeyp-51b9.3	9.722687	-5.387114731	1.944462	-2.77049	0.005597	0.126154
si:dkey-33c12.14	0.092844	1.055383603	5.267649	0.200352	0.841205	NA
zgc:153759	1.070236	-0.841577088	2.291485	-0.36726	0.713423	NA
snorc	194.7494	-0.261515535	0.336469	-0.77723	0.43702	0.851125
si:dkey-28d5.3	0.063619	0	5.267649	0	1	NA
zgc:193742	166.7907	0.143309846	0.199816	0.717208	0.473246	0.869064
CU207217.1	0.683999	-2.303256474	2.681699	-0.85888	0.390407	NA
si:dkey-6f10.4	0	NA	NA	NA	NA	NA
dcun1d4	487.0756	0.005948242	0.130108	0.045718	0.963535	0.999842
BX120005.2	0.093881	0	5.267649	0	1	NA
BX936320.1	22.94765	-0.135573721	0.462781	-0.29295	0.769557	0.960484
taar20d1	0	NA	NA	NA	NA	NA
BX901930.1	0.053506	-0.86807634	5.267649	-0.16479	0.869106	NA
CR396590.2	0.04222	0	5.267649	0	1	NA
BX569787.1	71.57311	-0.122783039	0.235662	-0.52101	0.602358	0.914001
si:dkey-79p17.3	6.415734	0.215646189	0.681758	0.316309	0.751768	0.955391
or115-8	0.094031	-0.868089684	5.267649	-0.1648	0.869104	NA
zmp:0000001316	88.06745	-0.467722001	0.276631	-1.69078	0.090879	0.505206
CU467861.2	3.690649	-0.755411245	1.044638	-0.72313	0.469599	0.867224
CR762493.1	0.100142	1.055383603	5.267649	0.200352	0.841205	NA
FP103004.1	30.41318	-0.46698261	0.415561	-1.12374	0.261123	0.738733
vtg7	5.987363	-4.372070252	1.642568	-2.66173	0.007774	0.148987
si:ch1073-394i4.1	0.189841	0	5.267649	0	1	NA
CR854838.1	0.65176	-0.926326792	2.420502	-0.3827	0.701942	NA
cxcl11.5	0.662214	-1.786032032	2.88988	-0.61803	0.536556	NA
BX677668.2	4.626338	1.742476822	1.037863	1.678908	0.09317	0.510759
si:dkey-24c2.7	3.152719	-0.853248953	1.583124	-0.53897	0.589911	0.908732
BX649599.1	0	NA	NA	NA	NA	NA
CU041345.1	0	NA	NA	NA	NA	NA
BX537137.1	1.668413	-0.652077922	1.65101	-0.39496	0.692875	NA
BX649502.3	0	NA	NA	NA	NA	NA
BX005364.1	0	NA	NA	NA	NA	NA
BX548032.1	1.454745	1.538019245	1.791891	0.858322	0.390715	NA
BX088710.1	0	NA	NA	NA	NA	NA

zgc:153352	38.32965	0.187020225	0.329023	0.568411	0.569756	0.902322
BX571825.7	0	NA	NA	NA	NA	NA
CR762431.1	36.00618	-0.070432539	0.373985	-0.18833	0.850618	0.978251
si:dkey-95p16.1	12.50827	0.630954362	0.662088	0.952977	0.340602	0.795538
BX469925.2	0.053262	1.055396947	5.267649	0.200354	0.841203	NA
si:ch211-246e12.3	10.44989	0.272372491	0.555845	0.490015	0.624123	0.919498
BX284635.1	27.57142	0.480667326	0.351356	1.368034	0.171301	0.64335
BX548073.2	0.145978	0	5.267649	0	1	NA
si:dkey-282h22.5	3.359083	1.055430727	1.82123	0.579515	0.562241	0.900715
si:dkey-9i23.5	0.531455	0	3.118622	0	1	NA
kif1	7.690609	-1.423731241	0.827378	-1.72077	0.085292	0.490552
rhbdd2	62.67863	-0.111870159	0.235848	-0.47433	0.635264	0.924272
si:dkeyp-20g2.1	40.13441	0.042445517	0.289928	0.1464	0.883606	0.987014
si:ch73-46j18.5	4408.003	0.30207574	0.132774	2.275105	0.0229	0.267028
fndc7rs1	25.11196	0.090226993	0.567338	0.159036	0.873641	0.983828
thbd	44.8438	0.54570057	0.330029	1.653491	0.098231	0.521076
si:dkey-7j22.1	9.663296	1.024527966	0.60064	1.705727	0.088059	0.498295
AL953896.1	2.844991	0.093650907	1.866575	0.050173	0.959985	0.999164
apopt1	198.9604	-0.205965851	0.182557	-1.12823	0.259223	0.736127
si:rp71-1f1.4	3.26069	0.761056277	1.078891	0.705406	0.480558	0.870179
BX284666.1	10.73645	0.02964103	0.586946	0.0505	0.959724	0.999164
sdhaf1	225.562	0.158263352	0.225348	0.702305	0.482489	0.871721
vegfbb	3.12944	-1.112731084	0.976732	-1.13924	0.254603	0.733441
si:ch211-207c7.2	0.32794	0	4.545699	0	1	NA
si:dkeyp-110g5.4	77.51169	-0.154482518	0.228191	-0.67699	0.498413	0.879132
iqcc	53.28519	-0.448806543	0.336285	-1.3346	0.182007	0.658775
si:ch211-215m21.22	0	NA	NA	NA	NA	NA
khnyn	212.2987	-0.327718059	0.206816	-1.58459	0.113059	0.550449
si:ch73-103b9.2	16.81421	-0.091166791	0.465546	-0.19583	0.844745	0.976791
BX323457.1	0.481162	1.897515574	3.211011	0.59094	0.554561	NA
si:dkey-149i17.8	0	NA	NA	NA	NA	NA
si:dkeyp-44a8.2	1387.197	0.37880853	0.12687	2.985795	0.002828	0.087379
BX927275.1	0.046941	0	5.267649	0	1	NA
si:dkeyp-46h3.2	2.191153	-0.772811841	1.277853	-0.60477	0.545329	NA
si:ch211-183d21.1	176.4824	0.343952437	0.262145	1.312069	0.189497	0.669129
si:dkey-222h21.1	1.382345	-0.258506096	2.167766	-0.11925	0.905077	NA
si:dkey-80c24.5	0.337093	-0.826435836	3.389613	-0.24381	0.807375	NA
si:ch73-343l4.3	0	NA	NA	NA	NA	NA
arid1b	500.139	0.04156501	0.131355	0.316432	0.751675	0.955365
znf1013	63.30847	-0.212094201	0.250383	-0.84708	0.396952	0.830253
si:ch73-376l24.2	106.6948	-0.013684095	0.227094	-0.06026	0.951951	0.998032
CR405715.1	3.373383	-0.94092127	1.014316	-0.92764	0.353594	0.804513
CR376835.1	0.313985	1.055366024	4.598443	0.229505	0.818476	NA
si:dkey-93l1.6	0.729411	0	2.764842	0	1	NA
pku300	0.277579	0	5.267649	0	1	NA
gpsm1a	388.8887	0.208546675	0.173803	1.199902	0.230178	0.712045
si:dkey-21e2.11	0	NA	NA	NA	NA	NA
si:dkey-250k15.10	8.971901	-0.586351065	1.359792	-0.43121	0.666318	0.932664
jac1	47.85598	-0.462788589	0.887144	-0.52166	0.601906	0.914001
nnt2	72.86709	0.088958858	0.329217	0.270214	0.786996	0.963065
si:dkey-47k20.1	0.467824	1.868581693	3.212815	0.581603	0.560834	NA
si:dkey-88l16.4	0	NA	NA	NA	NA	NA
BX663522.1	0	NA	NA	NA	NA	NA
BX005153.1	0.605498	2.557252711	2.732073	0.936012	0.349267	NA

si:dkey-21e2.8	136.0374	-0.896226823	1.075966	-0.83295	0.404872	0.834284
si:ch211-133l5.4	10.12288	-1.785633354	1.57671	-1.13251	0.257422	0.735125
si:dkey-242h9.5	0.497904	0	4.614194	0	1	NA
BX571825.8	0	NA	NA	NA	NA	NA
BX927394.1	0.469428	-0.868134558	3.151305	-0.27548	0.782944	NA
CR855328.1	0	NA	NA	NA	NA	NA
BX649502.4	0	NA	NA	NA	NA	NA
BX005068.1	0.404943	-2.363386471	3.201586	-0.73819	0.460397	NA
pdap1a	1608.38	-0.100253092	0.122519	-0.81827	0.413205	0.838317
si:dkey-85a20.4	0.603877	0	3.080998	0	1	NA
si:dkey-56e3.2	10.43962	-1.517988164	0.762429	-1.99099	0.046482	0.376263
fndc7rs2	8.937525	-1.777463323	0.987857	-1.79931	0.071969	0.4564
slc25a5	83808.85	-0.225155495	0.1198	-1.87943	0.060186	0.422294
si:ch211-214j8.12	41.91958	0.106112014	0.320984	0.330584	0.740959	0.954103
AL929315.1	0.677147	1.055424451	2.547493	0.414299	0.678655	NA
or123-1	0.957238	2.199115853	1.996418	1.101531	0.270666	NA
pimr201	0.862876	0.20849952	2.126209	0.098062	0.921883	NA
BX927385.1	0.824749	-2.339723529	2.192451	-1.06717	0.285894	NA
si:dkey-61l1.4	80.04913	-0.234182033	0.37016	-0.63265	0.526961	0.889861
BX571681.1	0.101446	-1.755425691	5.23528	-0.33531	0.737394	NA
CU394254.1	0	NA	NA	NA	NA	NA
BX323586.1	0.43494	1.055345477	4.942951	0.213505	0.830933	NA
si:ch211-198d23.1	12.2312	0.330154945	0.844235	0.39107	0.695745	0.941558
BX927388.1	0.873188	0	2.548011	0	1	NA
or125-6	0	NA	NA	NA	NA	NA
si:ch211-222e20.4	71.23326	0.511461953	0.35161	1.454627	0.145772	0.60501
si:ch211-95j8.3	0.467481	0	4.909862	0	1	NA
si:dkey-16p19.5	19.76124	-0.205386965	0.477816	-0.42985	0.667308	0.933049
si:ch1073-385f13.3	19.0341	-0.200406548	0.446855	-0.44848	0.653805	0.929269
BX957274.1	0.184352	-0.868115637	5.267649	-0.1648	0.8691	NA
CU861651.2	0.41891	0	4.25238	0	1	NA
pimr150	0	NA	NA	NA	NA	NA
BX322660.1	0.184282	-0.868114638	5.267649	-0.1648	0.8691	NA
CR925763.1	0.618271	-0.74994631	3.345044	-0.2242	0.822605	NA
si:dkey-174k12.3	0.050723	-0.86807634	5.267649	-0.16479	0.869106	NA
si:dkeyp-44d3.1	3.016836	-0.527019329	0.954291	-0.55226	0.580768	0.905618
BX005058.1	0.416155	-0.868122166	5.267649	-0.1648	0.869099	NA
si:dkey-256e7.5	15.49632	0.333705922	0.416227	0.80174	0.422703	0.843493
BX546499.1	10.93425	0.481392168	0.543805	0.88523	0.376033	0.822258
si:ch211-15j1.4	12.36797	1.625329227	0.812538	2.000312	0.045467	0.373435
znf1003	22.66142	-0.585811178	0.38257	-1.53125	0.125707	0.573691
CU855900.1	0	NA	NA	NA	NA	NA
BX936298.1	0.043308	0	5.267649	0	1	NA
si:dkey-262g12.12	52.91291	0.474686852	0.282763	1.678745	0.093202	0.510759
aup1	978.9266	0.121320137	0.138222	0.877723	0.380094	0.823607
lamp1b	566.3426	-0.264362373	0.112498	-2.34992	0.018777	0.241964
CR848741.1	0.632413	-0.584708349	2.752289	-0.21244	0.83176	NA
si:dkey-204f11.3	0.060442	0	5.267649	0	1	NA
BX322665.1	6.143505	-0.680022354	0.935013	-0.72729	0.46705	0.866065
sptbn4b	39.98135	-0.212273986	0.359835	-0.58992	0.555244	0.898723
zgc:174193	1.240812	2.22942841	1.706758	1.306236	0.191472	NA
zgc:174931	14.97049	-0.466700293	0.514634	-0.90686	0.364481	0.814648
pimr29	0.069091	0	5.267649	0	1	NA
CR954294.1	0.14264	0	5.267649	0	1	NA

CR812470.1	22.29133	2.233590851	0.82248	2.715678	0.006614	0.136957
CR753844.1	0.178356	0	5.267649	0	1	NA
EBF1 (1 of many)	0	NA	NA	NA	NA	NA
AL935295.1	6.41619	-0.673318249	0.679139	-0.99143	0.321476	0.781986
ptprua	347.2944	0.169704563	0.201162	0.843622	0.398881	0.831047
BX649641.1	0	NA	NA	NA	NA	NA
BX571701.2	0	NA	NA	NA	NA	NA
BX323080.1	1.197882	-2.78090866	1.993566	-1.39494	0.163033	NA
ago1	64.49851	0.093309818	0.329385	0.283285	0.776959	0.961073
CR381531.1	0.31275	0	4.562366	0	1	NA
si:ch211-223p8.8	0.247453	-0.864893651	5.23046	-0.16536	0.868663	NA
si:dkey-57a22.11	80.76989	0.29356527	0.341417	0.859843	0.389876	0.827843
qrfpr4	22.61159	-0.445620498	0.430776	-1.03446	0.300921	0.767035
si:dkey-1h24.6	15.49204	-0.562556253	0.648808	-0.86706	0.385908	0.826939
CR457445.1	14.62729	-0.60515368	0.511521	-1.18305	0.23679	0.718119
ppp3r1a	325.6867	0.365785944	0.127253	2.874475	0.004047	0.106027
cyp27c1	107.4525	0.060471092	0.170696	0.354262	0.723143	0.947898
dnaaf3	5.994115	1.728699809	0.925156	1.86855	0.061685	0.42588
si:dkey-93m18.3	5.223612	-0.183870284	0.929907	-0.19773	0.843257	0.976791
BX005156.1	0	NA	NA	NA	NA	NA
si:ch211-271e10.2	2.343008	-1.061056663	1.170031	-0.90686	0.36448	0.814648
tlr20.3	0.446466	-0.868138808	3.245513	-0.26749	0.789093	NA
pard3ba	110.7648	0.633280614	0.197574	3.205277	0.001349	0.054277
BX248497.1	236.0339	0.526349159	0.142112	3.703751	0.000212	0.015576
coa6	65.92199	0.463216875	0.304433	1.521574	0.128116	0.57717
AL954134.2	0	NA	NA	NA	NA	NA
si:dkey-58f10.12	19.69694	-0.345508287	0.48936	-0.70604	0.480162	0.870001
AL845369.1	0.595712	-0.609683177	3.071567	-0.19849	0.84266	NA
BX649292.1	0	NA	NA	NA	NA	NA
si:ch211-130h14.6	0.266973	-0.307582766	5.162256	-0.05958	0.952488	NA
CU682811.1	0.325523	-2.31014535	4.43305	-0.52112	0.602284	NA
pimr133	0.129045	1.055383603	5.267649	0.200352	0.841205	NA
tpt1	42835.32	-0.477477263	0.139405	-3.42512	0.000615	0.032141
CT025775.1	0	NA	NA	NA	NA	NA
cyp11a2	14.31736	-0.021387283	0.579051	-0.03694	0.970537	1
pimr200	0.139879	1.055371994	5.267649	0.20035	0.841207	NA
si:dkeyp-20e4.8	0.70614	-2.449589095	4.176418	-0.58653	0.55752	NA
BX649384.2	2.000895	-0.905338432	1.559031	-0.58071	0.561439	NA
or124-1	0.829704	-0.868137704	2.547655	-0.34076	0.733285	NA
BX537280.1	0.413789	-0.868129363	4.936118	-0.17587	0.860394	NA
CU463109.1	0.22096	-2.413182047	5.198538	-0.4642	0.642502	NA
BX571681.2	0.043308	0	5.267649	0	1	NA
si:dkey-31b16.7	0.151871	2.436401064	5.206324	0.46797	0.639806	NA
si:ch211-266a5.12	6.639372	-1.019518451	1.112717	-0.91624	0.35954	0.810644
CR847503.1	24.73719	0.450247136	0.313956	1.43411	0.151541	0.615372
VASH1	66.09781	-0.21025165	0.262055	-0.80232	0.422368	0.843493
BX569790.1	2.448602	-2.593118704	1.78123	-1.4558	0.145447	0.604596
gbgt1l2	11.11112	0.080864587	0.781361	0.103492	0.917573	0.993777
AL954655.2	25.82377	0.830206912	1.104865	0.751411	0.452406	0.857994
si:ch211-67f24.7	15.95291	0.664046984	0.52657	1.261079	0.20728	0.689456
unc5da	155.395	0.388617326	0.304704	1.275391	0.202171	0.682294
si:ch73-381f5.2	0	NA	NA	NA	NA	NA
si:dkey-188i13.8	17.28622	0.98225561	0.605834	1.621328	0.104947	0.533388
si:dkey-184p9.7	321.7907	-0.05989768	0.228766	-0.26183	0.793453	0.965366

si:dkey-266f7.4	0.891438	-2.336797338	2.670581	-0.87501	0.381566	NA
enam	266.8323	-0.809376553	0.379062	-2.13521	0.032744	0.320251
mhc1uka	1.257555	-2.312224218	2.116121	-1.09267	0.274538	NA
si:ch73-256j6.5	3.320245	1.524341776	1.830053	0.832949	0.404873	0.834284
si:ch211-263k4.2	12.71114	-0.003787444	0.452962	-0.00836	0.993329	1
BX510917.1	0.974758	-1.490082465	1.814575	-0.82117	0.411547	NA
si:ch211-57m13.7	0.092943	-0.868089684	5.267649	-0.1648	0.869104	NA
BX571825.9	0	NA	NA	NA	NA	NA
ftr37	3.61458	-0.425565333	0.930919	-0.45715	0.647566	0.927091
si:ch211-150o23.2	219.6142	-0.659331574	0.181889	-3.62491	0.000289	0.019027
si:ch211-133n4.12	0	NA	NA	NA	NA	NA
CR396590.3	0	NA	NA	NA	NA	NA
BX248399.1	0.651172	0	3.3344	0	1	NA
caiap	1.658795	-0.12548959	1.551757	-0.08087	0.935546	NA
si:dkey-61p9.9	67.9452	0.648639175	0.245385	2.64335	0.008209	0.153944
si:dkey-5i3.1	0	NA	NA	NA	NA	NA
si:dkey-57a22.14	0.060442	0	5.267649	0	1	NA
znf1151	26.01483	-0.005012047	0.336965	-0.01487	0.988133	1
si:dkeyp-68b7.7	32.34563	0.261916585	0.381308	0.68689	0.492152	0.87629
si:ch211-253p18.2	25.85895	-2.144176259	0.408268	-5.25189	1.51E-07	6.51E-05
BX530064.1	0	NA	NA	NA	NA	NA
CR769772.1	0.217647	0	5.267649	0	1	NA
tarsl2	456.9015	0.444697067	0.131519	3.381237	0.000722	0.035767
BX640547.1	1.744176	-4.552308855	1.818558	-2.50325	0.012306	NA
si:ch73-338o16.4	1.289923	1.055432578	2.12446	0.496801	0.61933	NA
si:rp71-23d18.4	0	NA	NA	NA	NA	NA
si:ch1073-170o4.1	30.77813	-0.155503894	0.409873	-0.3794	0.704394	0.943785
BX323550.1	0.087179	0	5.267649	0	1	NA
znf1109	15.6039	1.152301193	0.603061	1.910753	0.056036	0.409191
si:dkey-88p24.11	319.1102	0.139739956	0.208231	0.671082	0.502169	0.881277
si:dkey-21e2.15	379.5747	0	1.685968	0	1	1
BX323038.1	0.709895	1.055423089	2.650454	0.398205	0.690479	NA
rprm3	38.00832	0.316860426	0.362906	0.873121	0.382597	0.825435
BX470224.1	0.727633	1.927566651	2.929639	0.657954	0.510568	NA
ppib	6126.198	0.023177479	0.127143	0.182295	0.855352	0.97932
camta2	29.95635	0.245703477	0.335735	0.731838	0.464268	0.864593
CU075735.2	0.189278	0	5.267649	0	1	NA
CR855277.2	7.392385	0.960208641	1.00978	0.950909	0.341651	0.796379
BX957328.1	0.141258	0	5.267649	0	1	NA
si:ch1073-110a20.2	19.05184	0.947474075	0.789026	1.200815	0.229823	0.711763
si:dkey-151g10.6	40014.07	-0.390055555	0.152775	-2.55315	0.010675	0.179908
CU466285.1	1.049406	0	2.459065	0	1	NA
hoxc9a	193.7538	-0.05786057	0.154063	-0.37557	0.70724	0.944981
stap2a	240.6926	0.221185566	0.154254	1.433907	0.151599	0.615372
fndc7rs4	125.5286	0.28783359	0.313158	0.919132	0.358027	0.809315
si:dkey-21e2.3	0.372981	0	5.085146	0	1	NA
si:dkey-93n13.2	82.67628	0.072208161	0.246105	0.293403	0.769214	0.960484
CR388168.1	0.310373	0	5.267649	0	1	NA
si:dkeyp-67a8.4	12.31073	0.718766094	0.594021	1.210001	0.226279	0.707597
si:dkey-42p14.3	8.655876	0.279597022	0.627189	0.445794	0.655746	0.929606
BX248511.1	9.864471	-0.801107035	0.622214	-1.28751	0.197917	0.678663
CR589943.1	7.943268	0.452236082	0.752221	0.601201	0.547706	0.896211
BX548075.1	0	NA	NA	NA	NA	NA
CR318588.2	10.29191	-18.81516661	5.1472	-3.65542	NA	NA

si:ch211-191d15.2	1.444966	1.537619902	2.184333	0.703931	0.481476	NA
si:ch211-93g21.2	2.659942	-1.736063885	1.451125	-1.19636	0.231557	0.71317
si:dkey-66g10.2	3.115348	-1.320631987	1.170881	-1.1279	0.259364	0.736127
zgc:162611	44.87549	0.076066807	0.312103	0.243723	0.807445	0.969736
AL845320.1	1.792831	-0.338893895	1.837635	-0.18442	0.853685	NA
si:ch73-92e7.4	72.92796	0.054710996	0.240084	0.227883	0.819737	0.971785
CR788322.1	0	NA	NA	NA	NA	NA
BX510324.1	0.087381	0	5.267649	0	1	NA
SRBD1	81.38694	0.174831426	0.212114	0.824232	0.409808	0.836939
si:ch211-210c8.7	281.3762	0.080705596	0.157781	0.511502	0.608999	0.915204
si:ch1073-126c3.2	50.1876	-0.85525667	0.548462	-1.55937	0.118908	0.562318
si:ch211-260p9.3	9.416407	0.633601057	0.62261	1.017654	0.308843	0.77371
CU138515.1	1.060383	-0.830148546	1.968832	-0.42165	0.673284	NA
BX936337.1	2104.145	0.159005287	0.264038	0.602205	0.547037	0.896121
upk3b	8.428229	-0.561358121	0.725942	-0.77328	0.439355	0.852079
zgc:193541	3269.131	0.079639827	0.152783	0.521261	0.602185	0.914001
BX537288.1	0.37155	1.055396887	3.80277	0.277534	0.78137	NA
si:dkeyp-85e10.3	5.74314	-0.856556943	1.052655	-0.81371	0.415811	0.839227
CU074419.1	0.550004	-0.774813818	2.966261	-0.26121	0.793931	NA
BX649294.1	192.0581	-0.145295544	0.232626	-0.62459	0.532242	0.890978
si:dkey-161j23.7	117.5148	-0.418361468	0.219299	-1.90772	0.056427	0.410349
si:dkey-193b15.5	2.248252	1.584805273	1.775359	0.892667	0.372035	0.819592
zgc:171977	0.55364	-1.551678655	3.729423	-0.41606	0.677363	NA
si:dkeyp-46h3.3	0.784597	3.345065004	2.16959	1.541796	0.123123	NA
zgc:194246	171.0796	1.146487085	0.337148	3.400543	0.000673	0.034247
si:ch73-44m9.5	63.70136	0	5.267649	0	1	1
si:dkey-23c22.5	6.586279	-1.73373368	0.916741	-1.89119	0.058599	0.418278
CT027589.1	0.239892	-2.29353506	5.204055	-0.44072	0.659415	NA
si:ch211-114113.9	4.395053	0.080197678	1.685162	0.04759	0.962043	0.999451
si:dkey-188i13.11	283.2293	-0.859542769	0.207244	-4.14748	3.36E-05	0.004143
si:ch211-67f24.7	8.676767	0.54975757	0.694223	0.791903	0.428417	0.846133
si:ch211-157j23.3	25.77714	-0.3379536	0.478786	-0.70585	0.480279	0.870001
BX927258.1	5.0213	0.409216916	0.739926	0.553051	0.580228	0.905618
zgc:153317	14.0359	-0.03155192	0.564635	-0.05588	0.955437	0.998611
AL954320.1	0.185122	0	5.267649	0	1	NA
si:dkey-201l21.2	0.120883	0	5.267649	0	1	NA
BX323586.2	0.102662	0	5.267649	0	1	NA
BX547930.1	0	NA	NA	NA	NA	NA
BX323812.1	0.14351	1.055370259	5.267649	0.200349	0.841207	NA
mlnl	52.66577	0.206081076	0.437183	0.471384	0.637367	0.924779
crhr2	11.57472	2.590029224	0.776997	3.333382	0.000858	0.0396
zp3c	0.289653	-2.751193222	3.886168	-0.70795	0.478979	NA
si:ch211-106h4.12	356.5539	-0.342143682	0.344676	-0.99265	0.320879	0.781284
si:dkey-95h12.2	0	NA	NA	NA	NA	NA
zgc:171474	0.256199	-0.604297191	5.174495	-0.11678	0.907031	NA
BX649641.2	0.04222	0	5.267649	0	1	NA
si:dkey-66k12.3	0	NA	NA	NA	NA	NA
si:dkey-44g23.2	0	NA	NA	NA	NA	NA
si:ch211-132b12.4	0	NA	NA	NA	NA	NA
si:ch211-114c12.5	1.150383	-1.806596954	2.421333	-0.74612	0.455597	NA
si:dkey-32e6.3	53.69022	0.25310457	0.293977	0.860967	0.389256	0.827741
znf1047	7.092643	1.749296646	0.82799	2.112704	0.034626	0.328035
BX537338.1	0.049519	0	5.267649	0	1	NA
BX000981.1	0.196204	-1.732939949	5.215678	-0.33226	0.739696	NA

si:dkey-222h21.3	0.647492	2.997568758	2.6953	1.112147	0.266075	NA
BX001050.1	0.092844	1.055383603	5.267649	0.200352	0.841205	NA
si:dkey-117n7.3	24.5496	-0.449123922	0.438881	-1.02334	0.306148	0.771472
si:ch211-250g4.3	10.12115	-0.425660459	0.696979	-0.61072	0.541384	0.893819
CU681845.1	0.262826	0	5.267649	0	1	NA
cytl	67279.92	-0.267435206	0.207239	-1.29047	0.196888	0.67714
gna15.3	14.659	0.625693197	0.498761	1.254496	0.209662	0.691766
si:ch73-366i20.1	4.470484	-2.344906042	1.348585	-1.73879	0.082072	0.482272
BX294379.1	9.03481	-3.606415614	1.355198	-2.66117	0.007787	0.149109
BX548044.4	0	NA	NA	NA	NA	NA
mrpl34	590.0222	0.014424924	0.161344	0.089405	0.92876	0.995163
BX649388.2	0.060442	0	5.267649	0	1	NA
si:dkeyp-27c8.1	0.195752	0	5.267649	0	1	NA
ftr26	2.652298	3.939471306	1.569658	2.509765	0.012081	0.190742
BX004981.2	1.96751	2.522657207	1.618719	1.558428	0.119132	NA
BX321877.1	13.97375	0.172496683	0.451335	0.382192	0.702319	0.943402
CU207275.1	3.834773	0.325613273	0.927787	0.350957	0.725621	0.948653
si:dkey-93m18.4	8.328013	-4.685481658	1.169567	-4.00617	6.17E-05	0.00647
znf1128	7.545273	-0.90362039	0.737701	-1.22491	0.220608	0.701837
tmsb2	572.3943	0.064127836	0.291155	0.220253	0.825674	0.972839
si:dkeyp-82a1.3	0.48927	0	3.091692	0	1	NA
AL935198.1	1.39423	-2.63778469	1.784752	-1.47796	0.13942	NA
si:ch211-127i16.2	121.082	0.103242212	0.218851	0.471748	0.637107	0.924779
pias2	461.0122	-0.139984588	0.121183	-1.15515	0.24803	0.728489
AL929058.1	141.0592	-0.301215608	0.214903	-1.40163	0.161025	0.627788
si:dkey-222h21.11	0.814898	-0.868140994	2.575063	-0.33713	0.736016	NA
CU499330.1	71.90043	-0.080161291	0.364403	-0.21998	0.825887	0.972962
zmat4a	37.72189	0.760854254	0.459638	1.655333	0.097857	0.521071
si:ch211-161n3.3	1.988265	-2.355278992	1.723705	-1.36641	0.171812	NA
BX294113.1	0	NA	NA	NA	NA	NA
BX005329.2	6.70332	-2.693463316	1.122504	-2.39951	0.016417	0.226114
si:ch211-202f5.3	257.4313	0.028753683	0.145558	0.197541	0.843404	0.976791
tnni4b.1	9.624224	-0.233042259	0.522616	-0.44592	0.655658	0.929606
si:dkeyp-77c8.2	21.00211	-0.258940002	0.509631	-0.50809	0.611388	0.915331
BX323543.1	0.050723	-0.86807634	5.267649	-0.16479	0.869106	NA
acy3.1	50.76586	0.11984418	0.361928	0.331127	0.740548	0.953939
si:dkey-33c12.11	10.554	0.783566265	0.590217	1.32759	0.184313	0.661379
rarres3	16.46068	0.016469432	0.673651	0.024448	0.980495	1
mphosph6	226.413	-0.055126832	0.171808	-0.32086	0.748315	0.955074
adgrf3b	36.67859	-0.156860655	0.491279	-0.31929	0.749506	0.955074
BX511259.1	0.267102	2.987673653	4.637639	0.644223	0.519431	NA
BX890551.1	0	NA	NA	NA	NA	NA
BX294181.1	0	NA	NA	NA	NA	NA
CR383662.1	3.262819	0.491778712	1.326683	0.370683	0.710874	0.945821
si:ch211-174j14.2	0.467431	1.509718951	2.816171	0.536089	0.591897	NA
CR847870.1	0	NA	NA	NA	NA	NA
si:dkey-83k24.5	70.36159	-0.134023881	0.306865	-0.43675	0.662292	0.930742
CR356233.2	0	NA	NA	NA	NA	NA
FAM163A	11.37391	0.961692877	0.805037	1.194594	0.232246	0.713489
selenoh	912.9111	-0.294893482	0.182407	-1.61667	0.105949	0.534956
CT737131.1	0.155545	0.959877153	5.236908	0.183291	0.85457	NA
si:ch211-213a13.2	14.51191	0.30038214	0.460904	0.651723	0.51458	0.88631
si:dkeyp-77c8.4	6.428917	-0.913213986	0.769411	-1.1869	0.235267	0.716312
si:ch211-222n4.2	1.002407	1.967252439	2.087865	0.942232	0.346074	NA

BX950184.1	1.483906	-1.036174979	1.51537	-0.68378	0.494116	NA
BX640520.1	1.24086	1.383290222	1.75336	0.788937	0.430149	NA
CR513782.2	0.217342	0	5.267649	0	1	NA
AL935144.1	0	NA	NA	NA	NA	NA
si:ch211-234c11.2	3.553295	-0.379762968	0.940648	-0.40373	0.686415	0.938732
si:dkey-104n9.1	48.85289	0.306298495	0.469422	0.652502	0.514077	0.88631
ugt2a7	101.8766	-1.326172251	0.423088	-3.13451	0.001721	0.064499
si:ch211-161h7.5	143.6559	-0.355736477	0.713466	-0.4986	0.618059	0.916507
BX465186.1	0.07132	0	5.267649	0	1	NA
si:ch211-158d24.4	58.47764	-0.250297364	0.306175	-0.8175	0.413644	0.83842
c6	20.60567	0.42346635	0.637829	0.663919	0.506742	0.883007
vap	0.535148	-0.868131075	3.878691	-0.22382	0.822897	NA
BX548073.3	26.20894	-0.725686906	0.507314	-1.43045	0.152588	0.61658
STMP1	790.6939	0.091420783	0.140832	0.649147	0.516244	0.886815
lgals9l6	0.12723	1.055383603	5.267649	0.200352	0.841205	NA
BX682548.1	1.979288	0.877300708	1.759446	0.498623	0.618045	NA
CR847870.2	0.346683	0	4.441013	0	1	NA
FP016056.1	10.38299	-0.943462181	0.699999	-1.3478	0.177721	0.65136
SMIM18	63.0083	-0.104417864	0.347074	-0.30085	0.763527	0.959055
ubac2	122.9461	0.118673996	0.16381	0.724462	0.468782	0.866951
c3b.1	476.1381	-1.13312572	0.408719	-2.77239	0.005565	0.126154
thumpd2	58.85527	-0.074444391	0.297599	-0.25015	0.802472	0.968181
CR925713.1	7.218027	-0.068240005	0.684577	-0.09968	0.920597	0.994085
si:ch211-139g16.8	13.65795	-0.428467618	0.555658	-0.7711	0.440648	0.852523
BX897740.1	0.238366	-0.86811668	5.267649	-0.1648	0.8691	NA
plscr3a	64.8495	0.44949387	0.411639	1.091961	0.27485	0.750181
proca	59.78139	0.402652684	0.413759	0.973158	0.330475	0.788126
si:dkey-163f12.10	0.709881	-2.326545301	3.524216	-0.66016	0.509151	NA
si:ch73-92i20.1	9.995378	-0.025929576	0.661392	-0.0392	0.968727	1
LO018605.1	3.599766	0.427639056	1.035358	0.413035	0.679581	0.936344
CT573467.1	0	NA	NA	NA	NA	NA
BX323543.2	0.779381	-3.958256042	3.450905	-1.14702	0.251373	NA
BX649502.5	0	NA	NA	NA	NA	NA
BX649388.3	4.162536	-0.849901773	1.271357	-0.6685	0.503815	0.881759
BX511310.1	17.34165	0.313466678	1.009081	0.310646	0.75607	0.956532
tac3a	60.59896	-0.497307659	0.424288	-1.1721	0.241157	0.72266
iqsec3b	27.37768	1.150262407	0.402093	2.860691	0.004227	0.107395
adam17b	85.70744	0.000103945	0.275432	0.000377	0.999699	1
ccl34b.8	0.317989	0	4.541757	0	1	NA
BX005313.2	3.095045	1.055433326	1.843786	0.572427	0.567033	0.901747
tmprss12	0.07132	0	5.267649	0	1	NA
si:ch211-132g1.6	0	NA	NA	NA	NA	NA
BX000444.1	0.053262	1.055396947	5.267649	0.200354	0.841203	NA
BX004774.1	3.791202	-0.868154129	1.971012	-0.44046	0.659603	0.930581
CT573344.1	39.84557	-0.009323989	0.310029	-0.03007	0.976008	1
si:ch211-209l18.2	40.11194	0.878165237	0.407439	2.155327	0.031136	0.313664
espnlb	23.23858	-0.486954661	0.401748	-1.21209	0.225478	0.70685
BX511265.1	1.888135	-1.587708267	1.592269	-0.99714	0.318699	NA
si:dkeyp-122a9.2	0.253619	0	4.817845	0	1	NA
si:dkey-266m15.7	23.11812	0.079329372	0.383794	0.206698	0.836246	0.976007
CR847509.1	0.268678	0	4.115396	0	1	NA
gstk4	1.849953	2.524235838	1.850362	1.364185	0.172509	NA
si:dkey-117m1.4	206.3517	0.176383558	0.221592	0.795985	0.426041	0.844714
scpp8	28.86536	-0.511348441	0.490847	-1.04177	0.297519	0.765351

RANBP2	944.6157	0.291929637	0.15651	1.86524	0.062148	0.427289
si:ch73-112l6.1	285.5984	0.02251667	0.16026	0.1405	0.888265	0.988188
si:rp71-45k5.2	0.18478	0.09368032	5.267649	0.017784	0.985811	NA
CR788254.2	1.409313	0.012680196	1.720651	0.007369	0.99412	NA
si:ch211-57f7.7	4.510269	-4.098637322	1.429252	-2.86768	0.004135	0.106542
si:dkey-117n7.2	45.74106	-0.909544162	0.320596	-2.83705	0.004553	0.111673
BX936323.1	0	NA	NA	NA	NA	NA
BX294189.1	3.331134	-2.377189679	1.148981	-2.06895	0.03855	0.344223
BX004800.1	0	NA	NA	NA	NA	NA
znf1010	85.85119	-0.100400068	0.242798	-0.41351	0.679231	0.936209
CR932983.1	0.148188	1.854137197	5.241768	0.353724	0.723546	NA
fndc7rs3	20.27386	-2.605013771	1.155984	-2.2535	0.024227	0.275306
CU137717.1	1.267229	-4.076597362	5.154668	-0.79086	0.429028	NA
pimr83	0.049021	-0.86807634	5.267649	-0.16479	0.869106	NA
si:ch73-21g5.7	376.836	0.193593773	0.261312	0.740853	0.458783	0.861537
si:ch73-236c18.8	180.5334	-0.192507219	0.144334	-1.33376	0.182281	0.658775
si:dkeyp-106c3.3	18.51203	0.529975506	0.353212	1.500448	0.133499	0.586903
CR853291.1	0.084441	0	5.267649	0	1	NA
si:rp71-23d18.8	1.850942	1.055427329	2.048379	0.51525	0.606378	NA
si:dkey-16p6.2	3.154542	-0.021635108	0.954362	-0.02267	0.981914	1
BX510336.1	0	NA	NA	NA	NA	NA
CR749763.2	0.502735	-0.868138973	3.585408	-0.24213	0.808679	NA
mef2b	25.57795	0.99796918	0.61276	1.628647	0.103388	0.530446
CR759927.1	2.328531	0.410645105	1.254957	0.327218	0.743503	0.954856
si:dkey-93m18.6	23.45721	-8.274093882	1.238856	-6.67882	2.41E-11	2.66E-08
zgc:77118	1.771643	0.485824069	1.376257	0.353004	0.724086	NA
si:ch211-15j1.1	7.286159	0.660396974	0.853069	0.774143	0.438847	0.851954
si:dkey-79c1.1	1.063273	0.704695428	1.772078	0.397666	0.690876	NA
CR354547.1	1.846375	-1.900015045	1.705089	-1.11432	0.265142	NA
CT027756.1	0.160481	0	5.267649	0	1	NA
eif2ak1	325.8845	-0.097939689	0.166874	-0.58691	0.557265	0.899165
or125-7	2.896685	0.107412359	1.068473	0.100529	0.919925	0.993892
GUCY1A2	31.60165	0.148211723	0.385923	0.384044	0.700946	0.942918
si:dkey-30j10.5	2.370242	0	2.354481	0	1	1
si:dkey-75a21.2	139.2813	0.436176955	0.170648	2.55601	0.010588	0.179908
BX571724.1	4.00162	0.347754231	0.821447	0.423344	0.672045	0.933953
si:ch211-198m1.1	0.09854	-0.868089684	5.267649	-0.1648	0.869104	NA
chia.6	125.9785	-0.509111131	0.257065	-1.98047	0.04765	0.380113
CR788285.1	0.242938	-1.769300839	4.877572	-0.36274	0.716798	NA
c3a.4	42.54614	-0.747989665	0.789998	-0.94682	0.343728	0.798147
si:ch211-212c13.10	137.6646	0.27955604	0.187812	1.488489	0.136622	0.591964
si:dkey-112e17.1	59.23893	0.046334082	0.394385	0.117484	0.906476	0.991636
ftr32	0.046941	0	5.267649	0	1	NA
AL845362.1	4.611478	-0.887507531	1.007457	-0.88094	0.378351	0.822672
CR786562.1	45.65112	0.18953784	0.281643	0.672971	0.500965	0.880413
CT573148.1	8.731291	1.272596539	0.628285	2.02551	0.042815	0.362408
si:ch211-284e13.9	1.209921	-0.868139942	2.060165	-0.42139	0.673468	NA
si:ch211-146l10.7	0.100242	-0.868089684	5.267649	-0.1648	0.869104	NA
BX936308.2	10.74912	0.208694549	0.695136	0.300221	0.764008	0.959228
znf1127	1.90821	-0.063084084	1.128306	-0.05591	0.955413	NA
CR749762.2	1.627106	-2.106874651	1.648428	-1.27811	0.20121	NA
CR456628.1	3.744383	-0.327849856	1.10003	-0.29804	0.765675	0.959942
AL928845.1	1.111406	2.483779009	2.173039	1.142998	0.25304	NA
si:ch211-139n6.3	33.93472	-0.408367177	0.370279	-1.10286	0.270086	0.746532

CR847566.1	0.55589	0.112766428	2.754869	0.040933	0.967349	NA
si:ch211-215d8.2	85.78374	0.187964823	0.232703	0.807747	0.419236	0.842286
si:dkey-65123.2	0	NA	NA	NA	NA	NA
TST	399.5115	-0.113567426	0.169054	-0.67178	0.501723	0.881077
si:ch211-89o9.4	165.4061	-0.029683695	0.285451	-0.10399	0.917178	0.993735
BX649641.3	0.41982	-2.769475485	4.142824	-0.6685	0.503815	NA
GMEB2	183.8511	-0.109801125	0.152051	-0.72214	0.470211	0.86742
BX530024.1	0.235174	0	5.267649	0	1	NA
CU681836.1	673.7436	-0.068852693	0.162633	-0.42336	0.672031	0.933953
BX537358.1	7.826052	-0.696300778	0.682772	-1.01982	0.307816	0.772996
si:dkey-238m4.4	5.751332	-1.511225439	0.954246	-1.58369	0.113265	0.551017
si:ch211-226h8.11	0	NA	NA	NA	NA	NA
BX936438.1	0.181111	1.055370259	5.267649	0.200349	0.841207	NA
CR753837.1	0.801898	-1.779889031	2.202407	-0.80816	0.419001	NA
MFSD3	38.22719	0.322238999	0.322054	1.000574	0.317033	0.778879
znf5691	31.26564	-0.180749521	0.315499	-0.5729	0.566713	0.901739
si:dkey-229d11.3	0.490686	-3.896728841	3.82244	-1.01943	0.307997	NA
BX901930.2	0	NA	NA	NA	NA	NA
taar19o	0.259925	0	5.267649	0	1	NA
cngk	2.054206	1.166003834	1.53121	0.761492	0.446363	NA
si:ch73-62b13.1	8.069684	1.778699884	0.858354	2.072222	0.038245	0.342672
AL954694.2	0.495787	-1.825198689	4.599947	-0.39679	0.691525	NA
igic1s1	0.551887	-2.340335995	4.590905	-0.50978	0.610208	NA
or137-6	0.376131	0	4.305707	0	1	NA
si:dkey-204f11.51	0	NA	NA	NA	NA	NA
BX530037.3	0	NA	NA	NA	NA	NA
si:dkey-11o1.3	3.528846	-1.751634674	1.722512	-1.01691	0.309198	0.773791
CR786582.1	0.520155	0.038049622	3.412377	0.01115	0.991103	NA
si:ch211-173m16.2	0.768323	-3.325984033	2.870231	-1.15879	0.246543	NA
CABZ01039782.1	7.069365	-2.974167492	1.386392	-2.14526	0.031932	0.317405
BX510924.1	0.218858	-1.791258993	5.034195	-0.35582	0.721977	NA
CR513782.3	0	NA	NA	NA	NA	NA
si:dkey-9i23.14	9.03128	3.097727791	0.711687	4.352656	1.34E-05	0.002278
CU570889.1	1.397927	0.0456103	1.293195	0.035269	0.971865	NA
ifitm1	690.9839	0.1612887	0.322434	0.500222	0.616919	0.916365
si:dkey-221j11.3	217.0164	-0.455944684	0.301716	-1.51117	0.130744	0.582151
BX957257.1	0.163217	2.079549577	5.22636	0.397896	0.690707	NA
CT025585.1	0.924449	1.982341133	2.288818	0.866098	0.386436	NA
BX890559.1	5.359177	-1.364479136	0.853223	-1.59921	0.109775	0.543595
si:dkey-83f18.4	0	NA	NA	NA	NA	NA
ubb	3759.589	0.045887312	0.143202	0.320438	0.748637	0.955074
adgrf8	31.88899	0.159225665	0.558366	0.285164	0.775519	0.961073
si:ch211-209j10.6	93.71762	0.627422485	0.950058	0.660404	0.508994	0.883442
si:dkey-57a22.15	927.0661	-0.4449527	0.533375	-0.83422	0.404156	0.833888
BX323028.1	0.193511	1.05535865	5.267649	0.200347	0.841209	NA
si:dkeyp-87a6.2	0.17634	0	5.267649	0	1	NA
si:ch211-114l13.1	38.81899	0.245374312	0.696382	0.352356	0.724571	0.9484
BX640512.1	0	NA	NA	NA	NA	NA
si:rp71-1h3.1	7.288254	0.538955037	0.904217	0.596046	0.551144	0.8974
CR524821.1	0.298573	0	3.956496	0	1	NA
si:dkey-38n4.2	0.602108	-2.408247991	3.379625	-0.71258	0.476107	NA
si:ch73-249k16.4	4.994683	1.134766567	0.907368	1.250613	0.211076	0.692821
si:ch211-195h23.4	1.128815	0	2.274317	0	1	NA
BX088709.1	3.144066	-4.93501676	1.787306	-2.76115	0.00576	0.128818

si:ch211-13f8.1	51.63702	0.320229413	0.85136	0.376139	0.706814	0.944838
si:ch73-27e22.7	0.871682	-1.710118194	5.23847	-0.32645	0.744081	NA
si:ch211-239f4.6	0.445522	0	3.612915	0	1	NA
CR396590.4	0.084441	0	5.267649	0	1	NA
si:ch211-141e20.2	22.70892	1.220240196	0.508527	2.399558	0.016415	0.226114
si:dkey-58f10.7	1.086404	-1.401569064	1.948133	-0.71944	0.471868	NA
si:ch211-263m18.4	0.479522	-3.800058476	4.544742	-0.83614	0.403074	NA
si:ch211-57i17.2	32.14065	-1.061227428	0.439148	-2.41656	0.015668	0.221082
CU633785.1	0	NA	NA	NA	NA	NA
nyap2b	103.1749	-0.185686212	0.27366	-0.67853	0.497436	0.878744
BX284697.1	0.355296	0	3.80152	0	1	NA
inpp5jb	10.60237	0.113562023	0.58723	0.193386	0.846657	0.977214
CU469531.2	2.269741	1.322789568	1.444145	0.915967	0.359684	0.810668
mtmr3	0	NA	NA	NA	NA	NA
acer3	31.73562	-0.636605874	0.421906	-1.50888	0.13133	0.583069
si:ch1073-272j17.3	0.987524	3.028941223	2.43149	1.245714	0.212869	NA
BX842702.1	0	NA	NA	NA	NA	NA
lrrc66	2.012376	-2.435030121	1.831588	-1.32946	0.183695	NA
BX649476.1	0.049021	-0.86807634	5.267649	-0.16479	0.869106	NA
EPB41L2	1638.436	0.048384717	0.098573	0.490851	0.623532	0.919171
si:ch211-28e16.5	0	NA	NA	NA	NA	NA
si:ch211-235i11.5	41.86535	-0.014879296	0.261172	-0.05697	0.954568	0.998611
tgm2l	88.21334	0.226229974	0.369353	0.612504	0.540204	0.893049
uqcc3	347.8341	-0.116506504	0.16695	-0.69785	0.48527	0.872911
BX640539.1	1.179191	-3.565265696	2.021026	-1.76409	0.077717	NA
si:ch211-150d5.3	1.882818	0	2.169996	0	1	NA
CU469314.1	0.495227	-0.856002561	3.189581	-0.26837	0.788411	NA
si:dkeyp-82a1.1	5.503728	0.083357676	0.921594	0.090449	0.92793	0.995052
si:dkeyp-20g2.3	0	NA	NA	NA	NA	NA
CR392330.1	0.235041	1.055363809	4.965841	0.212525	0.831698	NA
CT583651.1	0	NA	NA	NA	NA	NA
AL953894.1	6.772707	1.107213725	0.896426	1.235142	0.216778	0.69815
BX511184.1	0	NA	NA	NA	NA	NA
crha	3.091693	0	1.869838	0	1	1
si:dkey-286j17.4	47.62372	0.512427009	0.977981	0.523964	0.600304	0.913756
si:ch73-376l24.4	0.555981	-0.850318996	2.715914	-0.31309	0.754214	NA
BX323822.1	0	NA	NA	NA	NA	NA
casp6	66.47322	0.113249012	0.279774	0.404788	0.685634	0.938486
zgc:111986	2860.624	0.297895047	0.134075	2.221854	0.026293	0.2875
BX530075.2	0	NA	NA	NA	NA	NA
pimr49	0.241766	0	5.267649	0	1	NA
BX936421.1	0.273594	-1.75547092	5.235277	-0.33532	0.737387	NA
gad1a	14.96861	-0.159625485	0.519692	-0.30715	0.758726	0.957882
sertad2a	120.6517	-0.290188961	0.208803	-1.38977	0.164597	0.634007
edil3a	547.0637	-0.056601642	0.151394	-0.37387	0.708501	0.94551
si:dkey-21e2.12	4.740767	0	1.832825	0	1	1
BX470229.1	7.288277	-0.01452329	0.817375	-0.01777	0.985824	1
si:dkey-216e24.9	3.927324	1.706474257	1.163103	1.467174	0.142329	0.601598
CR933734.1	0.188916	-0.868115997	5.267649	-0.1648	0.8691	NA
creb1b	289.7784	0.206564021	0.161966	1.27535	0.202185	0.682294
si:ch211-229b6.1	1.292849	-2.82534334	1.657329	-1.70476	0.08824	NA
swsap1	12.19135	-0.232205947	0.472027	-0.49193	0.622766	0.918894
znf1135	8.818609	0.278263482	0.617419	0.450688	0.652214	0.928768
BX539332.1	0.043309	0	5.267649	0	1	NA

si:dkey-69c1.1	0.046941	0	5.267649	0	1	NA
BX465862.1	19.14501	0.089794091	0.479176	0.187393	0.851353	0.978548
CT027825.1	0.354596	-0.868133336	3.835254	-0.22636	0.820924	NA
si:dkeyp-34f6.4	0.049519	0	5.267649	0	1	NA
iqcb1	5.100637	1.0569857	1.193105	0.885912	0.375665	0.822237
si:ch211-9n13.3	0	NA	NA	NA	NA	NA
CU467110.1	198.1027	0.211328195	0.246451	0.857484	0.391177	0.827902
tnfaip6	112.2556	-0.206807426	0.216236	-0.95639	0.338873	0.795226
BX927377.1	0	NA	NA	NA	NA	NA
BX323559.2	1.733279	-4.883251874	1.712212	-2.85201	0.004344	NA
si:dkey-10613.7	82.25967	-0.200215083	0.262394	-0.76303	0.445444	0.855403
BX649502.6	7.201581	1.752033034	0.732094	2.393181	0.016703	0.229135
mpc1	1572.765	-0.034326341	0.147534	-0.23267	0.81602	0.971207
CR388143.1	15.02626	0.16305285	0.462772	0.35234	0.724584	0.9484
si:dkey-78l4.10	0	NA	NA	NA	NA	NA
BX511129.1	52.34165	-0.601163827	0.268597	-2.23816	0.025211	0.281915
ahdc1	268.4554	0.497066653	0.156262	3.180984	0.001468	0.057739
oip5-as1	2462.449	0.107784421	0.116602	0.924381	0.355288	0.806546
si:ch211-14a11.2	0.245733	0.929637512	4.186256	0.222069	0.82426	NA
si:dkey-76d14.2	4.252862	0.482827323	0.787741	0.612926	0.539925	0.893049
gpr52	25.60587	0.706150371	0.458257	1.540949	0.123329	0.570077
si:dkey-56m15.6	1.387854	-0.184006284	1.397592	-0.13166	0.895254	NA
CR384062.1	0.176938	0	5.267649	0	1	NA
CU693484.1	45.56337	-0.398044094	0.298454	-1.33369	0.182306	0.658775
si:ch73-7i4.3	1.745872	-2.402732877	1.870986	-1.28421	0.19907	NA
znf653	86.53584	0.116473162	0.269736	0.431804	0.665884	0.932664
CT009596.1	0.11861	0	5.267649	0	1	NA
CR361551.1	36.0113	0.65110487	0.611408	1.064928	0.286909	0.757915
CU466287.1	0.288391	0.093661709	4.019962	0.023299	0.981412	NA
si:dkey-175d9.2	9.430091	0.661018896	0.972991	0.679368	0.496905	0.878572
CYP46A1	11.01215	-2.839125791	0.858607	-3.30667	0.000944	0.042765
si:ch211-197e7.3	2.698328	0.537370225	1.264873	0.424841	0.670953	0.933953
BX571825.10	0	NA	NA	NA	NA	NA
pamr1	207.2305	-0.38050724	0.25749	-1.47776	0.139473	0.597667
BX530034.1	4.890824	0.951867875	0.908965	1.047199	0.295008	0.763708
si:dkey-95p16.2	69.57396	-0.355827417	0.256844	-1.38538	0.165936	0.635475
BX571701.3	0.060442	0	5.267649	0	1	NA
BX664716.1	1.339614	-2.588920812	1.730598	-1.49597	0.134662	NA
BX004785.2	1.126128	2.773351204	2.374629	1.167909	0.242843	NA
CR450740.1	0.484046	1.055386708	4.049432	0.260626	0.794381	NA
si:dkey-78l4.8	0.405907	1.055395059	3.76203	0.280539	0.779064	NA
CR677617.1	4.59272	1.971049558	1.571558	1.254201	0.209769	0.691766
urp1	25.23045	-1.011432966	0.396157	-2.55311	0.010676	0.179908
si:ch211-217k17.9	122.3469	-0.389589268	0.340444	-1.14436	0.252476	0.732759
CU651633.1	0	NA	NA	NA	NA	NA
si:zfos-905g2.1	114.2519	-0.253364134	0.204118	-1.24126	0.214508	0.69658
zgc:194281	30.06395	0.183855113	0.3503	0.52485	0.599687	0.913468
BX649448.2	3.962604	-0.327486722	1.103523	-0.29676	0.766646	0.960296
rsf1b.1	0.107013	-1.826650979	5.230461	-0.34923	0.726914	NA
zgc:77614	0	NA	NA	NA	NA	NA
si:ch211-135f11.6	0.093881	0	5.267649	0	1	NA
si:dkey-238j22.1	0	NA	NA	NA	NA	NA
BX324218.1	2.934346	0.473372739	1.131059	0.418522	0.675566	0.935422
BX323812.2	0.129399	0	5.267649	0	1	NA

CT573204.1	11.34009	-0.140397816	0.607649	-0.23105	0.817275	0.971207
b3gnt3.3	39.59895	-0.363628513	0.42459	-0.85642	0.391764	0.827902
BX548044.5	0.196845	1.921554817	5.236909	0.366925	0.713675	NA
slc37a4b	10.57338	-0.001387469	0.603259	-0.0023	0.998165	1
si:dkey-230i18.2	1.391482	0.660173528	1.88985	0.349326	0.726845	NA
BX005309.1	267.1059	0.225205326	0.147871	1.522986	0.127762	0.577117
BX539321.1	0.44617	1.055388429	3.731094	0.282863	0.777282	NA
CR450686.2	3.926239	-0.610650194	1.127064	-0.54181	0.587952	0.907732
BX294165.1	5.844675	-1.09294631	1.01427	-1.07757	0.281226	0.753557
si:ch73-56d11.5	33.01994	0.124705463	0.529255	0.235625	0.813724	0.970397
si:dkey-111e8.4	0.894672	-3.248062192	2.14834	-1.51189	0.130561	NA
BX649641.4	0.312679	0	3.933089	0	1	NA
selenop	34720.04	0.209119044	0.208911	1.000997	0.316828	0.778879
BX005438.1	59.92561	-0.264504727	0.327595	-0.80741	0.419428	0.842347
BX842699.1	3.200657	1.383890253	0.975783	1.418235	0.156122	0.621751
BX324007.1	0.046941	0	5.267649	0	1	NA
BX571854.1	5.914254	-0.089002278	0.958805	-0.09283	0.926042	0.994815
si:dkey-20i20.7	17.14985	0.335184699	0.453154	0.739671	0.4595	0.862103
si:ch211-225b11.4	79.72008	0.020453858	0.183475	0.11148	0.911235	0.992208
BX294388.1	0.461427	0	3.664946	0	1	NA
bckdhbl	399.761	-0.394831146	0.20649	-1.9121	0.055863	0.408826
si:dkey-25o1.7	13.58004	-1.072856897	1.733317	-0.61896	0.535942	0.892102
lamc3	797.364	-0.102911165	0.175409	-0.58669	0.55741	0.899184
si:ch73-256j6.4	0.751934	0.120635228	2.112627	0.057102	0.954464	NA
AL929493.1	0.04222	0	5.267649	0	1	NA
BX000981.2	13.91943	-0.581653323	0.606071	-0.95971	0.3372	0.794158
CT025897.1	10.50507	0.340012069	0.561005	0.606076	0.544464	0.895206
si:dkey-80c24.4	0.049519	0	5.267649	0	1	NA
FP236157.1	3.682851	-0.974579185	0.941137	-1.03553	0.30042	0.766629
zgc:193505	1188.731	-0.148821965	0.152978	-0.97283	0.330636	0.788126
CU207343.1	0	NA	NA	NA	NA	NA
si:dkey-234l24.8	5.056048	0.674622117	0.858971	0.785384	0.432228	0.848419
si:ch211-282k23.2	7.747788	-0.918532654	1.072236	-0.85665	0.391638	0.827902
CR391910.2	4.24982	-1.186044275	1.068764	-1.10973	0.267114	0.743418
CR536604.1	10.63843	0.223527733	0.586453	0.381152	0.703091	0.943622
si:dkey-88n24.10	0.140822	0	5.267649	0	1	NA
oip5	67.75214	-0.224914537	0.245592	-0.9158	0.35977	0.810703
BX470224.2	0.613233	0.090405095	2.176001	0.041546	0.96686	NA
omd	53.46592	-0.608170109	0.366538	-1.65923	0.09707	0.519526
si:ch211-93g21.1	15.35403	1.51539685	0.587774	2.578198	0.009932	0.172469
or124-3	0.053262	1.055396947	5.267649	0.200354	0.841203	NA
BX255912.1	1.058809	1.922704314	1.968845	0.976565	0.328785	NA
si:dkey-159f12.2	74.40665	0.381109716	0.478441	0.796565	0.425704	0.844714
ccl34b.1	229.3901	0.038770071	0.330121	0.117442	0.90651	0.991636
BX664750.1	0	NA	NA	NA	NA	NA
si:dkey-92i17.2	80.81769	0.453628041	0.226635	2.001583	0.04533	0.372707
CT025909.1	1.122505	-1.396015449	1.913318	-0.72963	0.465616	NA
si:ch211-234c11.3	5.083466	1.13501404	0.909593	1.247826	0.212095	0.693739
BX005419.1	0.264416	0	5.267649	0	1	NA
si:dkey-45h7.1	10.21111	-0.416312763	0.577229	-0.72123	0.47077	0.86783
sult3st2	129.0097	0.341427625	0.328314	1.039941	0.298367	0.765416
si:dkey-27b3.4	5.036453	-1.502221953	1.355027	-1.10863	0.26759	0.74397
spc24	221.3681	0.367704359	0.242677	1.5152	0.129722	0.579795
CR392341.1	6.730816	-1.059094074	1.045693	-1.01282	0.311148	0.775936

BX537143.1	0	NA	NA	NA	NA	NA
s100a11	1080.101	0.463233554	0.242208	1.912545	0.055806	0.408826
si:ch211-106h4.4	12.65534	-0.630833547	0.609186	-1.03554	0.300419	0.766629
si:ch211-249h16.8	2.564495	-0.847676755	1.526085	-0.55546	0.578581	0.905417
BX000981.3	0.687458	1.427059234	2.146649	0.664785	0.506188	NA
si:dkey-26i13.5	0.337453	0	4.477553	0	1	NA
BX537282.1	1.468689	-0.272791445	1.675988	-0.16276	0.870704	NA
ugt5a2	5.681834	-0.799322258	1.809059	-0.44184	0.658602	0.930581
AL954868.1	0.507111	-0.868138091	3.113926	-0.27879	0.780404	NA
BX511034.3	7.95092	1.249636268	0.587376	2.12749	0.033379	0.323127
BX294113.2	0.132542	0	5.267649	0	1	NA
AL954359.1	9.069406	-0.010328056	0.686164	-0.01505	0.987991	1
CR513785.1	0.046941	0	5.267649	0	1	NA
si:ch211-287n14.3	0.837076	0.873762748	2.081226	0.419831	0.674609	NA
ftr25	5.190022	-0.406355226	0.812803	-0.49994	0.617115	0.916365
si:dkeyp-2e4.6	2.87623	-2.450848552	1.142227	-2.14568	0.031899	0.317397
tm7sf3	563.7836	-0.067209468	0.123255	-0.54529	0.585555	0.906655
entpd5b	530.3408	0.172417687	0.14126	1.220567	0.22225	0.703972
si:dkey-6f10.3	0.722748	1.055434089	2.370686	0.445202	0.656174	NA
CR318646.2	0.216219	0	5.100278	0	1	NA
CT573186.1	0.063619	0	5.267649	0	1	NA
si:dkey-285e18.5	2.410741	-1.166259661	1.218889	-0.95682	0.338657	0.795226
BX005283.1	1.735212	0.595427102	1.280036	0.465164	0.641814	NA
ankmy2b	1.615047	1.958180311	2.028292	0.965433	0.334328	NA
BX001030.1	327.5689	-0.128101405	0.225419	-0.56828	0.569844	0.902322
zgc:171435	5.44839	-1.819601118	0.899311	-2.02333	0.043039	0.363552
si:dkey-28d5.4	0.365801	1.055347551	5.147862	0.205007	0.837567	NA
si:ch211-56a11.2	41.43047	0.133388701	0.327478	0.407321	0.683772	0.938486
AL845312.1	0.04222	0	5.267649	0	1	NA
si:dkey-218h11.6	0.105115	1.055383603	5.267649	0.200352	0.841205	NA
tmem238a	312.6302	-0.169499394	0.173648	-0.97611	0.329009	0.787268
CU467633.2	0.378152	0	4.997252	0	1	NA
si:rp71-36a1.2	89.5447	0.562432953	0.327963	1.714929	0.086358	0.493048
si:dkey-193c22.1	80.43242	-0.008729685	0.260984	-0.03345	0.973316	1
CU074419.2	1.811694	-1.916789758	1.514013	-1.26603	0.205502	NA
FP016018.1	0	NA	NA	NA	NA	NA
BX005301.1	0.363303	1.055410559	3.351162	0.314939	0.752808	NA
BX649502.7	0	NA	NA	NA	NA	NA
BX537166.2	0.931521	0	2.21625	0	1	NA
BX255917.1	0.312203	2.667673267	5.195716	0.513437	0.607646	NA
si:dkey-217m5.8	0.226028	-0.793668545	5.235279	-0.1516	0.879502	NA
si:ch73-27e22.4	29.53957	0.075302581	0.456694	0.164886	0.869034	0.982843
si:dkey-126g1.9	528.9199	-0.361559896	0.26332	-1.37308	0.169727	0.641486
si:ch211-122l14.4	0.063619	0	5.267649	0	1	NA
si:dkeyp-46h3.8	0.144198	0	5.267649	0	1	NA
CR356222.1	1.856552	2.522447157	2.046336	1.232665	0.217701	NA
CR385053.1	0.147064	-2.293512936	5.204056	-0.44072	0.659418	NA
arhgap20b	12.39168	-0.007146273	0.652677	-0.01095	0.991264	1
si:dkeyp-7a3.1	2.524149	0.5299908	1.131671	0.468326	0.639551	0.924779
CR854927.1	0.536304	0	3.51923	0	1	NA
si:dkey-256i11.2	4.905761	-0.228726489	0.80345	-0.28468	0.775889	0.961073
artnb	13.08877	-0.612496585	0.478919	-1.27891	0.200927	0.681045
BX901930.3	0.063619	0	5.267649	0	1	NA
BX470224.3	0.159412	0	5.267649	0	1	NA

rnf212b	1.021881	-3.316248662	2.064325	-1.60646	0.108174	NA
si:dkey-54g2.2	0.270892	0	5.267649	0	1	NA
BX276096.1	0	NA	NA	NA	NA	NA
CR931802.1	2.583118	2.933095558	1.604426	1.828128	0.06753	0.443563
BX539307.3	19.50098	-1.37038912	0.441192	-3.10611	0.001896	0.067264
CR396583.1	7.807992	0.271262098	0.732804	0.37017	0.711256	0.945821
si:ch211-209a2.2	2.013499	1.220258893	1.405864	0.867978	0.385406	NA
pth1a	5.916046	-1.0725014	0.793906	-1.35092	0.176722	0.650047
si:dkey-83m22.7	10.09493	0.587751565	0.550336	1.067986	0.285527	0.756853
BX276103.1	0.314664	0.093655399	3.965699	0.023616	0.981159	NA
CU207257.1	0	NA	NA	NA	NA	NA
htr1aa	59.59395	0.315644464	0.383074	0.823977	0.409953	0.836939
si:dkey-122c11.1	0	NA	NA	NA	NA	NA
si:ch211-217k17.11	1.851117	0.093653786	2.145882	0.043643	0.965189	NA
FP102171.1	0.166911	-0.868103029	5.267649	-0.1648	0.869102	NA
cd180	7.284699	-2.143511537	1.462902	-1.46525	0.142854	0.602002
rimbp2	0	NA	NA	NA	NA	NA
BX004774.2	85.94788	0.079941818	0.451416	0.177091	0.859437	0.979975
FP101875.1	0.396016	1.055337996	5.084507	0.20756	0.835573	NA
si:ch211-197h24.9	256.5519	-0.239380656	0.14103	-1.69738	0.089626	0.50251
si:ch211-250k18.7	11.26465	0.555925874	0.722919	0.769002	0.441892	0.853308
si:ch211-271e10.6	0.060442	0	5.267649	0	1	NA
si:dkey-145c18.5	6.391587	0.350445691	0.66363	0.528074	0.597448	0.911953
prr18	701.9284	-0.008036428	0.199583	-0.04027	0.967881	1
si:ch1073-296i8.2	53.17973	0.405409138	0.331984	1.22117	0.222022	0.703829
rbp2b	673.4663	-0.534770074	0.299417	-1.78604	0.074093	0.462232
si:ch211-272h9.6	0	NA	NA	NA	NA	NA
BX547993.1	0	NA	NA	NA	NA	NA
BX663499.1	0	NA	NA	NA	NA	NA
cxcl11.7	0.688746	-0.868133489	3.633176	-0.23895	0.811147	NA
BX548078.1	25.7275	0.827753937	0.836024	0.990107	0.322122	0.782237
lrriq1	13.2637	-0.233321002	0.540287	-0.43185	0.665853	0.932664
si:dkey-70b23.2	38.49918	-0.027036632	0.302965	-0.08924	0.928891	0.995164
taar17a	0	NA	NA	NA	NA	NA
BX255915.1	0.221787	-2.32472863	5.202573	-0.44684	0.654989	NA
CR450686.3	9.881798	1.653880907	0.568023	2.911642	0.003595	0.09929
CT583625.1	0.120341	-0.868089684	5.267649	-0.1648	0.869104	NA
si:rp71-79p20.2	0	NA	NA	NA	NA	NA
itln2	0.207272	0	5.267649	0	1	NA
si:dkey-81p22.11	4.537354	-0.477874528	0.759501	-0.6292	0.529221	0.890406
slx4	2.802922	-1.010883949	0.987977	-1.02319	0.30622	0.771472
setbp1	115.9099	-0.216751652	0.219936	-0.98552	0.324367	0.783308
si:dkey-196n19.2	8.40293	0.680531355	0.754191	0.902332	0.36688	0.816111
sst6	31.91067	0.356203784	0.3615	0.98535	0.324452	0.783308
ccdc73	0.519153	2.559365536	3.367174	0.760093	0.447199	NA
pimr113	1.466776	0	2.480518	0	1	NA
si:dkeyp-2c8.3	0.894078	0	2.219031	0	1	NA
CR762470.1	0.137239	1.055371994	5.267649	0.20035	0.841207	NA
si:ch211-142c4.1	0.294451	0	5.267649	0	1	NA
ch25hl3	3.036265	-0.228851664	1.026268	-0.22299	0.82354	0.972005
CT027772.2	0.326589	1.055365306	4.56713	0.231078	0.817254	NA
si:ch211-110p13.9	94.44661	0.150092127	0.263747	0.569077	0.569304	0.902303
si:dkey-259j3.5	5.059998	-1.214472103	0.984111	-1.23408	0.217173	0.69815
si:ch211-102c2.8	20.89003	-0.337569332	0.436944	-0.77257	0.439778	0.852079

BX085193.2	2.092421	-1.258707717	1.456886	-0.86397	0.387604	NA
znf1121	12.80318	-0.099682614	0.510348	-0.19532	0.84514	0.976791
si:ch73-382f3.1	69.76036	-0.158635738	0.21165	-0.74952	0.453544	0.858722
CR628410.1	3.211733	0.698609699	1.568056	0.445526	0.65594	0.929606
si:ch211-165f21.4	1.065563	-3.087773908	1.984636	-1.55584	0.119746	NA
si:dkey-83f18.14	0.874113	-0.868140618	2.534873	-0.34248	0.731991	NA
BX571825.11	0	NA	NA	NA	NA	NA
zgc:152936	3.905903	-3.118566558	1.211227	-2.57472	0.010032	0.173583
zgc:136461	14214.18	-3.084018947	1.398666	-2.20497	0.027456	0.294109
si:ch211-1f22.12	0.87383	-1.808798589	2.525686	-0.71616	0.473892	NA
si:dkey-57c22.1	2.585603	-0.855435526	1.374888	-0.62219	0.53382	0.891181
BX649457.1	0.087179	0	5.267649	0	1	NA
CU639468.1	40.97708	1.47563407	0.431898	3.416628	0.000634	0.032718
si:dkey-19a16.1	0.131761	0	5.267649	0	1	NA
BX248512.1	0	NA	NA	NA	NA	NA
igl3v2	0	NA	NA	NA	NA	NA
or125-1	1.173314	0.085038781	1.971922	0.043125	0.965602	NA
si:dkey-79f11.10	5.141189	2.140949523	2.294721	0.932989	0.350826	0.802215
BX957345.1	0	NA	NA	NA	NA	NA
si:ch211-274k16.2	3.021412	-1.668378726	1.10836	-1.50527	0.132255	0.584912
si:dkey-248f6.3	1.160971	1.05542546	2.40247	0.439308	0.660438	NA
wee1	314.98	0.051733865	0.192179	0.269196	0.787779	0.963294
CR356223.1	1.311245	0.062134485	1.92891	0.032212	0.974303	NA
BX004840.1	0	NA	NA	NA	NA	NA
BX901878.1	0.073968	0	5.267649	0	1	NA
CU137716.1	1.502049	-0.275302013	1.582095	-0.17401	0.861857	NA
BX324213.1	5.066084	-1.653480169	0.919343	-1.79854	0.072091	0.456627
si:dkey-196j8.2	51.00823	-0.548141528	0.275354	-1.99068	0.046516	0.376263
zgc:174224	27.74127	-0.187870782	0.323859	-0.5801	0.561847	0.900715
si:dkey-28d5.10	0.920911	-0.868144833	2.373957	-0.3657	0.714593	NA
si:zfos-2330d3.7	128.1207	-0.194199772	0.403811	-0.48092	0.630575	0.923003
pimr69	0.517689	-0.85568782	3.156124	-0.27112	0.786299	NA
CT027791.1	0.230869	0	4.974856	0	1	NA
BX908800.1	0.337263	-0.868130865	3.870918	-0.22427	0.822547	NA
tmsb5	6.311031	0.265421652	0.902309	0.294158	0.768637	0.960395
CR677513.1	1.700024	1.964468739	1.711633	1.147716	0.251086	NA
CT027756.2	0.053506	-0.86807634	5.267649	-0.16479	0.869106	NA
CR376740.1	0.043308	0	5.267649	0	1	NA
BX530037.4	0	NA	NA	NA	NA	NA
BX510657.1	222.5258	-0.191632908	0.186054	-1.02999	0.303016	0.769158
trhr2	3.971099	-0.188650985	1.214376	-0.15535	0.876547	0.985007
CU694380.1	0.353276	0	5.193539	0	1	NA
CT030031.1	0.98422	0.093649672	2.42547	0.038611	0.969201	NA
BX323559.3	27.28015	0.952820883	0.865399	1.101019	0.270888	0.746845
CR456628.2	0	NA	NA	NA	NA	NA
BX284666.2	0	NA	NA	NA	NA	NA
BX324137.1	0.085528	0	5.267649	0	1	NA
BX276103.2	4.174324	0.074748748	0.979641	0.076302	0.939179	0.997371
CR354542.1	0	NA	NA	NA	NA	NA
BX890562.1	1.799832	1.43869419	1.24852	1.152319	0.24919	NA
CR855277.3	13.12099	0.857332257	0.752225	1.139729	0.254399	0.733426
BX005032.1	0	NA	NA	NA	NA	NA
CR392341.2	0	NA	NA	NA	NA	NA
rflnb	285.2153	0.442537188	0.199483	2.218424	0.026526	0.288683

ftr04	1.002445	0.100892857	2.255225	0.044737	0.964317	NA
si:dkeyp-1h4.6	639.0335	-0.283735043	0.396158	-0.71622	0.473858	0.869292
BX004770.1	3.893005	-3.082511716	1.54312	-1.99758	0.045762	0.374664
si:dkeyp-77c8.1	33.23651	-0.301146512	0.446938	-0.6738	0.500439	0.880222
BX294181.2	1.167347	3.514230267	2.299868	1.528014	0.126509	NA
vma21	1026.435	-0.256983875	0.158252	-1.62389	0.1044	0.53263
BX546499.2	2.942208	0.09069404	1.072144	0.084591	0.932586	0.996175
BX530407.2	0.049519	0	5.267649	0	1	NA
AL929321.1	0	NA	NA	NA	NA	NA
si:dkey-9i23.16	263.9042	-0.207228396	0.158201	-1.30991	0.190227	0.670045
si:dkey-251i10.2	4172.1	-0.44246216	0.188054	-2.35285	0.01863	0.241555
BX530064.2	1.202566	0.795233747	1.905277	0.417385	0.676397	NA
si:dkey-111e8.5	25.89735	-0.136256179	0.4661	-0.29233	0.770032	0.960484
CR396590.5	0	NA	NA	NA	NA	NA
si:ch211-250k18.6	14.07632	-0.200041517	0.645577	-0.30986	0.756664	0.956668
BX571825.12	0.147064	-2.293512936	5.204056	-0.44072	0.659418	NA
BX548247.1	0.376026	-2.295240395	4.248933	-0.54019	0.589065	NA
ftr79	8.273341	0.282638061	0.643911	0.43894	0.660705	0.930581
BX950224.1	2.551721	-0.314961122	1.322504	-0.23816	0.811761	0.97012
CR933734.2	6.662885	-4.53724342	1.350428	-3.35985	0.00078	0.037179
si:dkey-222h21.9	1.927194	-0.664206933	1.434409	-0.46305	0.643327	NA
CR925803.1	1.417523	-1.203873655	1.959752	-0.6143	0.539018	NA
si:ch211-220f16.1	9.154606	1.788840063	0.579151	3.088729	0.00201	0.069723
zgc:77486	302.3671	0.113323703	0.157876	0.717801	0.47288	0.868965
CR749763.3	4.777728	-0.315640554	1.266557	-0.24921	0.803197	0.968418
si:dkeyp-72a4.1	0.69325	-2.401374178	2.581918	-0.93007	0.352333	NA
si:ch211-165f21.7	0.732247	3.561678507	2.124165	1.676743	0.093593	NA
BX323543.3	0.184281	-0.868114638	5.267649	-0.1648	0.8691	NA
gzm3.3	3.264253	1.099819071	1.400442	0.785337	0.432256	0.848419
si:ch211-279g13.1	23.98519	-1.795992717	1.432751	-1.25353	0.210014	0.691821
BX465838.1	0	NA	NA	NA	NA	NA
znf1058	1.238214	1.539842397	2.08232	0.739484	0.459613	NA
si:dkey-9i23.15	167.1486	-0.541235851	0.19254	-2.81103	0.004938	0.117227
si:ch73-7i4.2	5.403991	-0.848430656	0.763568	-1.11114	0.266508	0.74299
si:ch73-376l24.6	39.23993	0.455068049	0.308652	1.47437	0.140382	0.599499
si:dkey-6a5.3	0.727197	-0.850823175	2.748502	-0.30956	0.756896	NA
ccl34b.4	23.95861	1.845846464	1.060621	1.740345	0.081798	0.481484
serp2	73.31187	0.092282426	0.283417	0.325606	0.744722	0.95491
FP102120.1	70.62197	0.323469897	0.251694	1.285173	0.198732	0.679447
si:dkey-98j1.5	1.944592	-0.245268189	1.298709	-0.18886	0.850206	NA
CU467633.3	0.616109	0.093655884	2.719452	0.034439	0.972527	NA
CR388373.1	0.291326	-0.793909104	4.652539	-0.17064	0.864507	NA
swt1	153.8676	0.151256583	0.182818	0.827362	0.408032	0.835879
si:dkey-192g7.3	55.66129	0.045064641	0.265483	0.169746	0.86521	0.982162
si:ch211-130h14.4	6.044144	0.42811677	0.638183	0.670837	0.502324	0.881277
znf1137	6.553832	-3.522225088	0.829333	-4.24706	2.17E-05	0.003005
si:dkey-111e8.1	170.6286	0.505971786	0.187102	2.704254	0.006846	0.139692
BX649641.5	0.647075	0.026201197	2.670959	0.00981	0.992173	NA
AL831745.2	3.071751	0.095368409	1.017777	0.093703	0.925345	0.994815
AL935183.1	65.7916	0.012407633	0.238822	0.051953	0.958566	0.999109
CR387989.1	0	NA	NA	NA	NA	NA
si:dkey-237j10.2	14.04908	-0.216192725	0.474903	-0.45524	0.64894	0.927649
BX890544.1	0	NA	NA	NA	NA	NA
si:dkeyp-3f10.16	0	NA	NA	NA	NA	NA

znf1045	2.615022	-0.021318171	1.777162	-0.012	0.990429	1
si:dkey-76p14.2	4.514491	0.778964486	1.152662	0.675796	0.49917	0.879596
si:ch211-226f6.1	0	NA	NA	NA	NA	NA
krt17	15183.76	0.658437003	0.180628	3.645264	0.000267	0.017992
si:ch73-376l24.3	176.9741	-0.057692969	0.145272	-0.39714	0.691267	0.940173
si:dkeyp-73d8.8	127.61	-0.339401679	0.525471	-0.6459	0.518344	0.887148
si:dkeyp-26a9.7	0.227845	2.001906196	4.969659	0.402826	0.687076	NA
CR759830.1	0.712634	0.093656858	2.26075	0.041427	0.966955	NA
BX004962.1	0.181295	0	5.267649	0	1	NA
taok3b	87.41397	0.775886475	0.282866	2.742945	0.006089	0.131486
BX248332.1	0	NA	NA	NA	NA	NA
si:dkey-88l16.3	5.448795	-2.358651222	1.254941	-1.87949	0.060177	0.422294
cyp2j20	23.72284	-1.319071966	0.561911	-2.34748	0.018901	0.242305
si:ch211-191j22.7	4.843167	2.310404809	1.140403	2.025955	0.042769	0.362408
CR749774.1	0	NA	NA	NA	NA	NA
CU570972.1	0	NA	NA	NA	NA	NA
CR788236.1	0.246404	2.505641223	5.20297	0.481579	0.630105	NA
si:dkey-121n8.7	1.970527	-3.514144778	5.129048	-0.68515	0.493252	NA
AL954149.1	0	NA	NA	NA	NA	NA
si:dkey-81h8.1	7.814399	0.845476687	0.832596	1.01547	0.309882	0.774621
BX936317.1	0.869907	1.10587883	1.888936	0.585451	0.558245	NA
si:dkey-21e2.16	317.7564	1.055410093	1.690044	0.624487	0.532308	0.890978
CR626882.1	0.049519	0	5.267649	0	1	NA
or103-1	0.3577	0	3.757432	0	1	NA
BX323880.1	0	NA	NA	NA	NA	NA
si:dkey-223d7.6	0	NA	NA	NA	NA	NA
CR759846.1	1.826081	-0.613955326	1.544278	-0.39757	0.690949	NA
CR548634.1	3.94191	1.080685662	0.933805	1.157292	0.247153	0.727871
si:ch211-229n2.7	88.38407	-0.021379408	0.258202	-0.0828	0.93401	0.996638
sult3st3	0.471628	0	3.581016	0	1	NA
BX248089.1	0.688461	-1.380310389	2.529591	-0.54567	0.585296	NA
CR450817.1	12.37876	-1.007979511	0.602288	-1.67358	0.094213	0.51266
CR936321.1	209.7937	-0.419971273	0.262127	-1.60217	0.109118	0.541965
si:ch211-278p9.3	3.021068	-0.249498404	1.274138	-0.19582	0.844753	0.976791
BX323559.4	4.410139	-0.393019663	1.167394	-0.33666	0.73637	0.951919
BX248118.1	5.867955	1.686445634	0.757289	2.226951	0.025951	0.285912
BX469925.3	4.929672	0.998656022	0.824828	1.210745	0.225993	0.707411
AL929237.1	178.4957	0.325251029	1.337922	0.243102	0.807927	0.969828
BX005064.1	5.420781	0.927075707	0.808857	1.146156	0.251731	0.732003
si:ch73-37h15.2	106.4935	-0.052504886	0.192084	-0.27334	0.784589	0.962605
BX571827.1	0	NA	NA	NA	NA	NA
znf1168	24.67667	-0.062992647	0.358232	-0.17584	0.860417	0.980264
AL954145.1	148.5456	-0.075345027	0.239882	-0.31409	0.753451	0.955861
slc22a21	56.18604	0.161339414	0.529265	0.304837	0.760491	0.957886
BX571945.1	0.098043	-1.71005882	5.238474	-0.32644	0.74409	NA
BX914211.1	15.65808	0.425255951	0.480599	0.884845	0.37624	0.822258
si:dkey-21o22.2	21.04951	0.52484238	0.649834	0.807655	0.419289	0.842286
CR846087.2	2.985375	0.970953168	1.245127	0.779803	0.435507	0.850467
CR318674.1	0.049519	0	5.267649	0	1	NA
CR589874.1	15.88548	0.654579997	0.76359	0.85724	0.391312	0.827902
or103-2	0.163218	2.079549577	5.22636	0.397896	0.690707	NA
si:ch211-202c21.7	2.12954	1.844792763	1.573272	1.172583	0.240963	NA
AL954695.3	1.334031	-0.460525374	1.496412	-0.30775	0.75827	NA
CR751602.1	88.53029	-0.358547863	0.214373	-1.67255	0.094417	0.513368

ftr19	17.39203	-1.418860095	0.512489	-2.76857	0.00563	0.126656
igf1	18.87854	-0.313051095	0.48431	-0.64639	0.518029	0.887148
wu:fc21g02	1469.758	0.179079097	0.11071	1.617557	0.105758	0.534827
BX936305.1	0.381577	-2.326223789	3.709917	-0.62703	0.53064	NA
CT027756.3	5.433319	0.484343768	0.804044	0.602384	0.546918	0.896121
BX511265.2	3.071348	4.110564747	1.489501	2.759693	0.005786	0.128995
BX936391.1	3.509395	-1.008376796	1.359055	-0.74197	0.458106	0.860993
CR391990.1	1.162904	0.102842445	1.908981	0.053873	0.957036	NA
pimr143	0	NA	NA	NA	NA	NA
pimr134	0.501428	-1.715383025	3.607883	-0.47545	0.634463	NA
BX465864.1	0.512606	-1.334105194	4.005105	-0.3331	0.739058	NA
BX323875.1	0.236521	0	5.267649	0	1	NA
CR812469.1	4.060654	-1.213847896	0.945398	-1.28395	0.199158	0.679605
hist2h3c	26.87711	0.572239297	0.457711	1.25022	0.211219	0.692983
si:ch73-256j6.2	1.513192	-0.868140445	2.004999	-0.43299	0.665023	NA
si:dkey-27j5.10	0.073968	0	5.267649	0	1	NA
BX072561.1	6.015058	0.030437787	0.683351	0.044542	0.964472	0.999948
si:ch73-160h15.3	71.13454	-0.118322426	0.254504	-0.46491	0.641994	0.925586
si:dkeyp-19e1.4	0.086617	0	5.267649	0	1	NA
BX649434.1	1.231226	1.985452117	1.753131	1.132518	0.257417	NA
AL807389.1	0	NA	NA	NA	NA	NA
lrp1ba	39.57187	-0.929223164	0.465056	-1.99809	0.045707	0.374363
si:ch73-263f13.1	0	NA	NA	NA	NA	NA
BX927282.1	1.673472	3.275530401	2.042493	1.603692	0.108782	NA
znf1027	2.246344	-0.210806487	1.030186	-0.20463	0.837862	0.976301
cdkn2d	47.62759	0.167444333	0.290557	0.576288	0.564421	0.901433
si:ch211-208f21.2	5.285297	-0.223257998	0.966349	-0.23103	0.81729	0.971207
shroom1	62.24975	-0.115512392	0.289198	-0.39942	0.689581	0.939582
si:ch211-125e6.14	0	NA	NA	NA	NA	NA
AL954149.2	18.91652	-0.530944564	0.424844	-1.24974	0.211395	0.692983
BX322661.1	0.66794	-2.55695519	2.677194	-0.95509	0.339533	NA
CU207281.3	0.601215	1.989945227	2.719641	0.731694	0.464355	NA
nup37	182.1389	-0.094014654	0.145378	-0.64669	0.517832	0.887148
si:dkeyp-82a1.2	0.453566	-0.868133531	2.829495	-0.30682	0.758984	NA
BX649484.1	4.476038	1.022576312	1.036961	0.986128	0.32407	0.783161
CR936371.1	1.412996	-1.523417338	1.41805	-1.0743	0.282686	NA
si:dkey-27d5.10	0.136698	0	5.267649	0	1	NA
znf111	1.956551	-2.464365436	1.799244	-1.36967	0.170791	NA
CR354556.1	203.4202	-0.221288258	0.237199	-0.93292	0.35086	0.802215
si:ch211-226h8.8	0	NA	NA	NA	NA	NA
CT583646.2	24.3272	0.305179048	0.383979	0.79478	0.426741	0.845352
elp1	286.5486	-0.121840034	0.156303	-0.77951	0.435677	0.850467
si:ch73-191k20.3	11.13534	-1.239366872	0.887775	-1.39604	0.162703	0.630455
fthl31	359.0234	-0.341161371	0.248096	-1.37512	0.169095	0.640442
CR545465.1	0	NA	NA	NA	NA	NA
si:dkey-179k24.1	0.531004	-0.50530645	3.473106	-0.14549	0.884323	NA
BX601644.2	0.135482	0	5.267649	0	1	NA
si:dkeyp-117b8.4	241.6832	0.005495094	0.150349	0.036549	0.970845	1
BX005417.1	27.97316	-1.907670452	0.693195	-2.752	0.005923	0.130335
si:ch1073-186o8.3	12.77684	-0.600771647	0.50844	-1.1816	0.237365	0.718568
si:dkey-17e16.15	91.22081	-0.992310736	0.367201	-2.70237	0.006885	0.139692
CR854965.1	0	NA	NA	NA	NA	NA
si:dkey-219e20.2	0.179483	0	5.267649	0	1	NA
si:dkey-28d5.8	0.836572	1.055426899	2.447032	0.431309	0.666244	NA

pimr125	1.261069	-0.868141506	2.126508	-0.40825	0.683092	NA
si:dkey-211g8.4	2.488859	1.055430666	1.88538	0.559797	0.575618	0.904739
BX908395.1	1.27941	-2.082390375	1.767025	-1.17847	0.238608	NA
or115-14	0.227575	-1.710083394	5.238472	-0.32645	0.744086	NA
si:dkey-112g5.15	0	NA	NA	NA	NA	NA
taar12h	0.262068	0.09365822	4.82001	0.019431	0.984497	NA
CR450777.1	2.132377	1.622015174	1.29514	1.252386	0.210429	NA
BX649502.8	0.049021	-0.86807634	5.267649	-0.16479	0.869106	NA
znf1171	51.96908	0.262206687	0.23397	1.120683	0.262423	0.74006
shda	39.70544	-0.287349558	0.370207	-0.77619	0.43764	0.851283
BX085193.3	0	NA	NA	NA	NA	NA
si:dkey-222h21.10	3.12423	4.267628509	1.495239	2.854145	0.004315	0.108751
CU693368.1	0.606644	2.48957171	3.012296	0.82647	0.408538	NA
wu:fc17b08	61.24979	-0.274940056	0.249598	-1.10153	0.270665	0.746683
si:ch211-119d14.2	17.18708	0.017182829	0.437815	0.039247	0.968694	1
BX649448.3	7.969627	0.102316017	0.82112	0.124605	0.900836	0.99045
B3GNT10	5.19787	0.669424299	1.119408	0.598016	0.549829	0.897373
BX324164.1	1.312911	-0.868136239	2.140188	-0.40564	0.68501	NA
commd9	162.2458	0.011232335	0.18357	0.061188	0.951209	0.99801
CR388077.1	4.06867	-1.633159075	0.944848	-1.72849	0.083901	0.486647
taar19g	0.04222	0	5.267649	0	1	NA
BX544876.1	0.270925	-0.868110153	5.267649	-0.1648	0.869101	NA
CU539058.1	170.9405	-0.021589216	0.20309	-0.1063	0.915342	0.993068
si:dkey-183n20.15	81.55812	-0.027776214	0.231879	-0.11979	0.904652	0.991581
si:ch73-337l15.2	23.53149	0.018173473	0.373398	0.048671	0.961182	0.99935
CR855393.2	0.39929	2.074056514	4.225058	0.490894	0.623501	NA
si:ch73-222f22.2	0	NA	NA	NA	NA	NA
map3k19	13.91653	0.785834792	0.532459	1.475861	0.139981	0.598517
peak3	3.391053	1.086822941	0.985684	1.102607	0.270198	0.746532
BX005156.2	0.768598	2.881324153	2.526912	1.140255	0.25418	NA
mrpl44	558.8896	-0.167523186	0.16164	-1.0364	0.300017	0.766629
si:dkeyp-20g2.3	0.10996	0	5.267649	0	1	NA
usp21	88.35688	-0.750728881	1.034007	-0.72604	0.467815	0.866174
si:ch73-196i15.3	29.81837	0.277441315	0.492467	0.56337	0.573183	0.903937
dnah11	37.80915	0.170040778	0.426856	0.398356	0.690368	0.93988
si:dkey-58f10.10	0.52116	-2.797660983	3.454099	-0.80995	0.417967	NA
si:ch211-117m20.4	7.712229	-2.337329274	1.553084	-1.50496	0.132334	0.585048
CT997819.2	2.043151	1.134193922	1.319679	0.859447	0.390094	NA
si:rp71-45g20.11	1.544824	-0.777158106	1.999434	-0.38869	0.697506	NA
CR318599.1	0.266722	0	4.809193	0	1	NA
CR855337.1	20.16081	0.087317223	0.360954	0.241907	0.808853	0.969828
si:dkey-222h21.2	36.46582	0.756943551	0.40942	1.848821	0.064484	0.433491
CT573178.1	1.055443	-0.868142922	2.464566	-0.35225	0.724651	NA
BX649350.1	0.352216	-0.86813429	3.875469	-0.22401	0.822751	NA
nupr1a	1954.787	-0.656096355	0.221724	-2.95906	0.003086	0.091315
BX537274.1	5.238671	-0.211669586	0.716939	-0.29524	0.76781	0.960296
BX296552.1	0.60259	1.596351935	2.939026	0.543157	0.587022	NA
si:dkey-237j11.3	0.82582	0.14793365	2.915885	0.050734	0.959538	NA
BX000981.4	1.011552	-1.377243869	2.097748	-0.65653	0.51148	NA
si:ch211-226o13.3	0	NA	NA	NA	NA	NA
si:dkey-247i3.6	3.214205	-2.403352811	1.013005	-2.3725	0.017668	0.234598
si:ch73-92e7.6	21.60239	-0.06861179	0.338787	-0.20252	0.839509	0.976417
si:ch211-255g12.6	3369.907	0.629395529	0.360734	1.744765	0.081026	0.479801
ndufa9b	82.17288	0.027834293	0.356324	0.078115	0.937737	0.997305

CR753837.2	0.130689	0	5.267649	0	1	NA
CR788285.2	0.183441	1.055370259	5.267649	0.200349	0.841207	NA
si:rp71-80o10.4	43.25078	0.62216784	0.402329	1.546416	0.122004	0.568897
CU326349.1	0.322307	-1.848372801	4.434248	-0.41684	0.676795	NA
BX322647.1	0.68081	0.935052981	2.164957	0.431904	0.665811	NA
CR788303.1	2.414676	-1.574182423	1.068674	-1.47302	0.140745	0.599658
BX294389.1	0.298613	-3.313063164	5.168828	-0.64097	0.521542	NA
efemp2a	442.095	0.378306086	0.174847	2.163644	0.030492	0.311199
or124-2	0.137189	0	5.267649	0	1	NA
SPATA1	11.28433	0.55054533	0.54515	1.009898	0.312544	0.776164
si:dkey-93n13.1	25.71999	-0.592698058	0.455821	-1.30029	0.193503	0.673698
si:dkey-9p24.5	0.367428	0	3.832074	0	1	NA
BX901918.1	7.012078	-2.597448238	1.006302	-2.58118	0.009846	0.171403
vgll4a	119.401	-0.387794888	0.203628	-1.90443	0.056854	0.411856
si:dkey-23f9.12	0	NA	NA	NA	NA	NA
si:dkeyp-82a1.6	127.8818	-0.230030145	0.175386	-1.31156	0.189667	0.669289
pimr88	0.374881	-0.511261258	4.301068	-0.11887	0.90538	NA
CR545466.1	0.043309	0	5.267649	0	1	NA
si:dkey-20i20.8	13.74277	-0.190266759	0.550322	-0.34574	0.72954	0.950056
si:dkey-222h21.8	0.915509	0	5.267649	0	1	NA
si:dkey-261l7.2	53.80312	0.12923027	0.335008	0.385753	0.69968	0.942499
si:ch211-114l13.13	2.255256	1.975471662	1.568249	1.259667	0.20779	0.689888
CR388079.1	0.050723	-0.86807634	5.267649	-0.16479	0.869106	NA
si:ch211-187g4.1	0.649262	2.607981229	3.223256	0.809114	0.41845	NA
si:ch73-343l4.1	0	NA	NA	NA	NA	NA
CR762497.1	2.932539	-0.779038384	1.178911	-0.66081	0.508733	0.883333
si:dkey-260g12.1	15.4217	0.60811335	0.747494	0.813536	0.415911	0.839284
AL935300.1	0.562267	0	2.967166	0	1	NA
CR759893.1	0.043309	0	5.267649	0	1	NA
si:ch211-125e6.13	0.865278	-3.798417576	3.039313	-1.24976	0.211386	NA
si:ch211-165f21.2	18.26548	0.586359576	0.693737	0.845219	0.397989	0.830737
samd10b	14.61433	0.481188045	0.50959	0.944265	0.345034	0.79837
BX927413.1	2.539107	-2.367826058	1.779784	-1.3304	0.183386	0.659894
zzef1	434.6465	-0.241752333	0.197301	-1.2253	0.220463	0.701764
BX649266.1	2.034013	-0.714336549	1.471729	-0.48537	0.627412	NA
si:dkey-85k15.6	1.759092	1.588716423	1.565604	1.014763	0.310219	NA
adgrg4b	10.71148	-1.598747794	0.915851	-1.74564	0.080873	0.479801
si:dkeyp-30e7.2	0.478053	-1.768362316	3.121428	-0.56652	0.571038	NA
celsr2	43.82208	0.139553182	0.274848	0.507747	0.611631	0.915331
si:dkey-201k7.1	0.084441	0	5.267649	0	1	NA
si:dkey-229d11.5	0.369443	2.308187444	4.212147	0.547984	0.583703	NA
CR848841.1	15.01514	0.413817999	0.477837	0.866024	0.386477	0.827374
si:dkey-78k11.4	1.018036	-0.433015152	2.329291	-0.1859	0.852523	NA
si:dkey-199f5.6	4.668891	-0.631750607	0.926171	-0.68211	0.495169	0.87806
BX545856.1	0.563724	0.09364933	2.891807	0.032384	0.974166	NA
si:dkey-33c12.10	0.874931	4.137611272	2.17739	1.900262	0.057399	NA
CU633933.1	0	NA	NA	NA	NA	NA
BX548075.2	0	NA	NA	NA	NA	NA
MFAP4 (1 of many)	45.29399	-0.488796834	0.419641	-1.1648	0.244101	0.724735
CU463033.1	0.22127	-0.868112681	5.267649	-0.1648	0.869101	NA
si:dkey-4c15.14	89.58104	0.136479135	0.213754	0.638488	0.523156	0.888312
aknad1	14.11612	0.337244824	0.652865	0.516562	0.605462	0.914985
si:ch211-264e16.2	4.881565	-0.042097962	0.775229	-0.0543	0.956693	0.999109
si:dkey-13n15.2	26.32519	-0.251203218	0.541636	-0.46379	0.642801	0.925707

si:dkey-27p18.7	1.435471	-3.582684943	2.305157	-1.5542	0.120136	NA
BX640465.1	0.389158	-2.411692805	4.336748	-0.55611	0.578138	NA
si:dkey-12l12.1	320.1886	0.275966755	0.215273	1.28194	0.199864	0.6804
AL954359.2	6.946607	-0.763998893	0.915185	-0.8348	0.403829	0.833888
si:ch211-222m18.4	0	NA	NA	NA	NA	NA
her4.2	345.6014	-0.004421172	0.169327	-0.02611	0.979169	1
si:dkey-253d23.8	13.86124	0.919952771	0.515655	1.784047	0.074416	0.463251
si:dkey-31f5.8	52.25837	-0.168044441	0.326882	-0.51408	0.607194	0.915204
caspbl	31.03822	0.726763224	0.576495	1.260659	0.207432	0.689456
CR848723.1	3.623731	-1.348086584	1.152154	-1.17006	0.241978	0.723529
BX571803.1	2.198351	0.769924111	1.215111	0.633625	0.526326	NA
plac8.2	1.12519	0	5.242183	0	1	NA
si:dkey-161j23.5	105.5134	0.443161399	0.202645	2.186886	0.028751	0.301537
si:dkeyp-73g8.5	0	NA	NA	NA	NA	NA
znf1036	2.443608	1.090511032	1.170941	0.931311	0.351693	0.802513
CR354556.2	0.515096	1.870006064	3.118395	0.599669	0.548727	NA
AL929520.1	0.41597	0	4.284894	0	1	NA
BX511231.1	0.148744	1.921567426	5.236909	0.366928	0.713673	NA
BX571945.2	0	NA	NA	NA	NA	NA
cfp	281.8983	0.239756257	0.19947	1.201969	0.229376	0.711579
cdh23	2.770751	2.053162586	1.197895	1.713975	0.086533	0.493048
CR293531.1	2.194904	1.252489067	1.244809	1.00617	0.314334	NA
AL928824.1	0.854822	-0.820333541	1.939579	-0.42294	0.672336	NA
BX005210.1	0.590936	-1.785294955	2.43511	-0.73315	0.463468	NA
si:dkey-65b12.12	4.881492	0.51278037	0.799467	0.641403	0.521261	0.887473
CR354556.3	366.1219	-0.166838491	0.243046	-0.68645	0.49243	0.876385
BX465186.2	5.187213	0.660097553	0.852551	0.774262	0.438776	0.85189
or128-8	0.87906	1.671076635	2.002669	0.834425	0.404042	NA
si:dkeyp-80c12.8	1.272184	-0.603913628	1.555796	-0.38817	0.69789	NA
si:ch73-199e17.1	124.9683	-0.471223157	0.239677	-1.96607	0.04929	0.386671
BX957308.1	0	NA	NA	NA	NA	NA
wu:fc63a07	100.0032	-0.140133045	0.198874	-0.70463	0.481038	0.870339
txnrd2.1	0	NA	NA	NA	NA	NA
CU137648.1	0.681989	-3.75430689	4.16927	-0.90047	0.36787	NA
BX927218.1	7.840433	-0.330796538	0.574327	-0.57597	0.564634	0.901445
si:dkey-163f12.6	9.232045	0.456904054	0.720261	0.634359	0.525847	0.889009
BX569801.1	67.3028	-0.186047054	0.253395	-0.73422	0.462816	0.864104
si:ch1073-75o15.4	0	NA	NA	NA	NA	NA
BX927121.1	0.147936	0	5.267649	0	1	NA
si:ch211-162i8.4	1.160318	-1.307475276	2.045212	-0.63929	0.522637	NA
si:dkey-27h10.2	24.36149	-0.426095925	0.380532	-1.11974	0.262826	0.740207
si:ch1073-412h12.3	0.653983	2.962133074	3.304826	0.896305	0.37009	NA
BX510923.1	249.4987	-0.132993399	0.207308	-0.64152	0.521182	0.887473
si:dkey-58f10.6	10.69035	0.64742597	0.681927	0.949406	0.342414	0.797179
taar19k	0.098043	-1.71005882	5.238474	-0.32644	0.74409	NA
si:ch211-202m22.1	2.545959	1.055432371	1.920763	0.549486	0.582672	0.90571
si:ch211-284e13.12	0.332856	1.055395646	3.893185	0.271088	0.786323	NA
CR933017.1	0.894727	0.098195554	1.737512	0.056515	0.954932	NA
BX548075.3	0.297677	-1.402438903	3.937686	-0.35616	0.721722	NA
CR376762.1	2.103243	0.996789747	1.194412	0.834545	0.403974	NA
BX927101.1	1.804727	-1.185869938	1.695526	-0.69941	0.484295	NA
si:ch211-238e22.4	0.846547	-0.436556702	2.366755	-0.18445	0.853658	NA
BX323457.2	3.322921	-0.185179181	1.289714	-0.14358	0.885831	0.987533
CR376748.1	0.060442	0	5.267649	0	1	NA

AL954767.1	24.57178	1.090498789	0.778016	1.401641	0.161023	0.627788
plekha1b	80.86665	0.00407654	0.228899	0.017809	0.985791	1
si:ch211-218m3.9	3.697069	0.966932918	1.71554	0.563632	0.573005	0.903718
CR925709.2	1.884106	1.465658848	1.694782	0.864807	0.387145	NA
CR354538.1	0.728332	0	2.792538	0	1	NA
ccl20b	15.07562	2.31177721	1.391023	1.661926	0.096528	0.518799
gabrz	19.21155	-1.011301911	0.786264	-1.28621	0.198369	0.679109
si:dkey-7l6.3	93.12003	-0.161833694	0.236387	-0.68461	0.493588	0.877027
or132-2	3.785865	2.640430367	1.182375	2.233158	0.025539	0.28373
es1	1.553009	3.52549646	1.911841	1.844032	0.065178	NA
si:ch211-125e6.8	48.16938	0.837889299	0.530487	1.579471	0.114228	0.553295
si:ch211-152p23.2	2.545104	1.44759716	1.016971	1.42344	0.154609	0.619425
si:ch73-194h10.3	0.431522	1.005256549	3.207015	0.313456	0.753935	NA
si:ch211-14c7.2	181.3039	0.460138525	0.184329	2.496285	0.01255	0.194767
BX511089.2	4.319049	-3.321148334	1.310122	-2.53499	0.011245	0.183909
si:ch211-1f22.11	0.247052	-0.868112681	5.267649	-0.1648	0.869101	NA
ksr2	135.0565	0.366261872	0.31246	1.172188	0.241122	0.722648
BX927181.1	0	NA	NA	NA	NA	NA
BX548157.1	2.865924	0.62664933	1.123907	0.557563	0.577143	0.904798
CR388168.2	5.876069	-4.002592155	1.517094	-2.63833	0.008332	0.155679
si:dkey-246e1.3	18.37647	-0.638495007	0.829206	-0.77001	0.441295	0.852874
CR786571.2	5.091029	-0.525443261	0.687898	-0.76384	0.444963	0.855159
BX957326.1	2.093655	0.104543438	1.359932	0.076874	0.938724	NA
si:ch211-137i24.12	28.6583	1.588856076	0.407981	3.894441	9.84E-05	0.009014
CT027623.1	1.164139	-3.512424734	1.935961	-1.81431	0.069631	NA
si:dkey-96l17.6	3.99023	-2.577549267	1.554322	-1.65831	0.097255	0.519934
si:dkey-121h17.7	4.057618	-1.900221089	0.960989	-1.97736	0.048001	0.381054
BX649349.1	0.363403	-0.868136285	3.409689	-0.25461	0.799025	NA
si:ch211-132b12.6	0.060442	0	5.267649	0	1	NA
CU462914.1	0	NA	NA	NA	NA	NA
taar12a	0	NA	NA	NA	NA	NA
BX649490.2	15.27002	-6.053683993	1.290926	-4.68941	2.74E-06	0.000648
BX855614.1	49.4536	0.106501382	0.271481	0.392298	0.694838	0.941197
si:ch73-7i4.1	1.448634	-1.826961951	1.806645	-1.01125	0.311899	NA
BX470218.1	0.08847	0	5.267649	0	1	NA
si:dkey-24h22.5	0.391435	-2.340746098	3.702992	-0.63212	0.527307	NA
nupr1b	2612.959	-0.18350728	0.182616	-1.00488	0.314953	0.777649
si:dkeyp-53e4.1	4.829663	-1.557214717	0.919084	-1.69431	0.090206	0.50413
zgc:174855	611.7337	0.480681349	0.353742	1.358846	0.174195	0.647434
BX005254.1	17.54344	-0.179026825	0.933179	-0.19185	0.847863	0.977782
si:ch211-139a5.9	371.3215	-0.041838079	0.180593	-0.23167	0.816794	0.971207
znf1144	50.0042	-0.510151174	0.246848	-2.06666	0.038766	0.344867
AL953841.1	0.645581	1.055418909	2.748377	0.384015	0.700967	NA
AL928920.1	0	NA	NA	NA	NA	NA
CU652528.1	0.260124	-0.868119338	5.267649	-0.1648	0.8691	NA
CT025748.1	5.293028	-0.260264316	0.911365	-0.28558	0.775203	0.960899
cdc42se2	42.71382	-0.209024288	0.360159	-0.58037	0.561667	0.900673
epsti1	7.112653	0.101231495	0.839825	0.120539	0.904056	0.991581
b4galnt2.1	4.048145	0.412571517	1.276393	0.323232	0.746519	0.955074
si:ch211-92l17.1	0.744985	0.188316628	2.382407	0.079045	0.936997	NA
CT573357.1	4.02319	-0.235342615	1.136452	-0.20709	0.835943	0.976007
si:dkey-35m8.1	40.10719	0.403783496	0.373934	1.079825	0.28022	0.752951
c2cd5	517.26	0.056254896	0.112859	0.498451	0.618166	0.916585
BX571803.2	1.71007	0.303125204	1.3074	0.231853	0.816652	NA

BX890572.1	0.416437	0	3.648901	0	1	NA
BX649502.9	0	NA	NA	NA	NA	NA
CR383662.2	0.609675	-2.126496636	2.557934	-0.83133	0.405785	NA
BX957257.2	0	NA	NA	NA	NA	NA
si:ch73-113g13.3	0.304414	2.438158698	4.582478	0.532061	0.594684	NA
si:dkey-7n6.2	77.11878	-0.073582076	0.371219	-0.19822	0.842875	0.976666
si:dkey-21h14.12	0.142347	0	5.267649	0	1	NA
si:ch73-256j6.3	0.262248	0	4.777449	0	1	NA
si:ch211-154e10.1	15.92591	-1.131207948	1.148417	-0.98501	0.324617	0.783313
si:zfos-364h11.2	1.041795	2.02903552	2.238202	0.906547	0.364646	NA
si:dkey-222h21.10	1.919555	-1.759478615	2.112218	-0.833	0.404844	NA
si:ch73-68b22.2	3.137184	0.559590654	1.073203	0.521421	0.602073	0.914001
CR392012.1	0	NA	NA	NA	NA	NA
si:dkey-262g12.3	26.6471	0.05302098	0.36779	0.144161	0.885373	0.987533
BX323458.2	91.19987	0.659823224	0.354927	1.859038	0.063022	0.429313
si:dkey-9p20.18	5.512354	0.088607946	0.863214	0.102649	0.918242	0.993884
zgc:113984	0.343149	-3.514029006	5.164352	-0.68044	0.496226	NA
CR318624.1	0.558916	3.269633526	2.397055	1.364021	0.172561	NA
BX323543.4	0	NA	NA	NA	NA	NA
AL935198.2	2.599179	0.077864894	1.028202	0.075729	0.939635	0.997371
si:dkey-84j12.1	0	NA	NA	NA	NA	NA
taar16b	0.198623	0	5.267649	0	1	NA
si:dkey-148f10.4	16.74519	0.273040058	0.464526	0.587782	0.556678	0.899063
BX323854.2	0	NA	NA	NA	NA	NA
CR626935.1	0.375516	-1.826371407	5.071071	-0.36015	0.718731	NA
si:ch211-120e1.7	14.79337	-0.097956873	0.499849	-0.19597	0.844631	0.976791
CR391921.1	4.09051	0.76963881	1.072795	0.717414	0.473118	0.868983
ms4a17c.1	2.861937	-2.754129819	1.669482	-1.64969	0.099006	0.522138
si:dkey-68l7.2	0.591969	1.690995343	3.007412	0.562276	0.573928	NA
dyrk2	697.4262	-0.187194775	0.118916	-1.57418	0.115447	0.555689
BX465844.1	0.783405	0.956387827	2.088027	0.458034	0.646928	NA
si:ch211-194g2.4	15.37915	0.191370351	0.504851	0.379063	0.704641	0.943785
si:dkey-149m13.5	26.86492	-0.382665234	0.390989	-0.97871	0.327723	0.786438
AL928977.1	1.016614	-1.141061065	1.76113	-0.64791	0.51704	NA
CU570781.1	0	NA	NA	NA	NA	NA
BX571681.3	1.153668	1.650952324	1.604902	1.028694	0.303624	NA
BX510324.2	0.529107	1.055418406	2.874749	0.367134	0.713519	NA
si:ch211-218d20.15	38.2579	-0.493436123	0.414122	-1.19152	0.233448	0.714569
si:dkey-21e2.8	0.060442	0	5.267649	0	1	NA
BX842684.1	0.04222	0	5.267649	0	1	NA
si:dkey-24f15.2	0.086616	0	5.267649	0	1	NA
CU571324.1	1.295562	-4.179231536	4.60231	-0.90807	0.36384	NA
BX470247.1	0	NA	NA	NA	NA	NA
si:ch211-51a19.5	2.294572	-3.087039601	1.661259	-1.85825	0.063133	0.429313
BX510337.1	0.265626	0.093657469	4.808949	0.019476	0.984462	NA
rhoab	1526.109	0.047310537	0.158472	0.298542	0.76529	0.959771
si:dkeyp-98a7.8	0.257148	2.594206969	5.198905	0.498991	0.617786	NA
trarg1b	53.05571	0.23144276	0.240113	0.963892	0.3351	0.79185
CR769772.2	0.046941	0	5.267649	0	1	NA
si:dkey-92j12.5	51.67128	-0.728479675	0.456353	-1.59631	0.11042	0.545275
si:dkey-9i23.6	20.03798	0.445392697	0.459591	0.969107	0.332492	0.789726
hbl2	14.31284	-0.904569273	0.608198	-1.48729	0.136937	0.5925
CR392012.2	1.435695	0.161289847	1.510245	0.106797	0.91495	NA
si:dkey-149i17.7	0.053506	-0.86807634	5.267649	-0.16479	0.869106	NA

si:dkey-267j14.7	0.20156	0	5.203891	0	1	NA
BX005414.1	0.65426	-3.033897945	2.952644	-1.02752	0.304176	NA
CR384089.1	0.302502	1.999112478	3.967811	0.503833	0.614379	NA
si:dkey-220k22.3	5.742628	-0.335000892	0.79763	-0.42	0.674489	0.934877
BX005329.3	0.101247	1.854150541	5.241768	0.353726	0.723544	NA
si:dkey-204f11.64	591.1608	-0.130987864	0.128193	-1.0218	0.306876	0.772019
si:dkey-201c13.2	208.4339	-0.653803854	0.451707	-1.44741	0.147783	0.608687
BX510928.1	0.144555	1.854137197	5.241768	0.353724	0.723546	NA
bcl2a	59.93183	0.09924543	0.333982	0.297158	0.766346	0.960229
cxcl11.6	0.376087	2.486304095	3.656608	0.679948	0.496537	NA
sftpba	64.132	-0.491650818	0.634107	-0.77534	0.438136	0.851454
arl8	757.2434	-0.552402402	0.102476	-5.39056	7.02E-08	3.78E-05
si:dkey-79i2.4	7.445087	1.038173685	0.72284	1.436243	0.150933	0.615085
BX294165.2	0	NA	NA	NA	NA	NA
BX548044.6	0.271634	1.499226878	4.669906	0.32104	0.74818	NA
or125-2	0.630791	0	3.080905	0	1	NA
si:dkey-83f18.6	1.164987	-2.730085853	2.209751	-1.23547	0.216655	NA
zgc:77614	0	NA	NA	NA	NA	NA
CR318588.3	393.6646	-1.039482063	0.307878	-3.37628	0.000735	0.036144
si:ch211-284e13.5	63.67963	-0.218405916	0.233935	-0.93362	0.350501	0.802215
apela	31.23877	-0.910668513	0.353835	-2.57371	0.010062	0.173845
acbd7	521.197	0.182693374	0.160533	1.13804	0.255104	0.733542
mical3b	283.3127	-0.353043176	0.190714	-1.85117	0.064146	0.432457
CR392363.1	5.069393	-0.310772356	0.936554	-0.33183	0.740021	0.953684
v2rc1	0.785616	0	3.536331	0	1	NA
znf1157	29.88595	-0.076081764	0.381076	-0.19965	0.841754	0.97643
si:ch211-12e13.12	11.25387	0.570365252	0.509765	1.118879	0.263192	0.740967
loxhd1a	4.994561	-0.252396469	0.960911	-0.26266	0.79281	0.965312
BX530079.1	0.175069	0	5.267649	0	1	NA
zgc:173425	0.494418	2.061957577	3.207825	0.64279	0.52036	NA
si:ch211-197g15.6	4.159943	-0.16571258	1.183145	-0.14006	0.888612	0.988188
BX927218.2	0	NA	NA	NA	NA	NA
si:dkey-84o3.2	1.198022	-3.102044291	2.018193	-1.53704	0.124283	NA
rpe65b	404.7124	-0.223223699	0.157717	-1.41534	0.156968	0.622766
si:dkey-71d15.2	61.66689	0.064319151	0.284935	0.225733	0.821409	0.971788
BX640536.1	0	NA	NA	NA	NA	NA
CR788311.1	0	NA	NA	NA	NA	NA
si:dkey-125i10.3	222.1419	-0.01268974	0.200358	-0.06334	0.9495	0.99801
npy2r	4.744881	1.001487201	0.908182	1.102739	0.270141	0.746532
fyba	1.571842	0.045787888	1.793501	0.02553	0.979632	NA
CT583625.2	2.9164	-0.916005339	1.513696	-0.60514	0.545083	0.895206
si:dkey-280e8.1	7.959131	-0.187301399	0.687794	-0.27232	0.785374	0.962605
BX248385.1	0	NA	NA	NA	NA	NA
BX571803.3	0.707437	0.208696817	2.378885	0.087729	0.930092	NA
CR626923.1	0.043309	0	5.267649	0	1	NA
si:dkey-192d15.1	2.215074	1.055433786	1.946779	0.542144	0.58772	0.907608
CU914617.1	0.157559	0	5.267649	0	1	NA
si:ch211-196h16.4	19.04886	1.332918269	0.542997	2.454744	0.014098	0.208027
si:dkey-193n17.9	28.51018	-0.376935049	0.759781	-0.49611	0.619817	0.91747
twf2a	687.2835	0.373861937	0.153135	2.441389	0.014631	0.212006
ovch2	2.618448	0.656303214	0.990702	0.662463	0.507675	0.883056
si:ch211-171b20.3	1.10608	0.827712901	1.879644	0.440356	0.659679	NA
si:ch211-133n4.11	0	NA	NA	NA	NA	NA
si:dkey-181m19.11	0	NA	NA	NA	NA	NA

BX294387.1	0.07132	0	5.267649	0	1	NA
plxnb2a	19.67425	0.493833904	0.454564	1.086389	0.277307	0.751426
l3mbtl1	51.38336	0.47634024	0.403	1.181987	0.237211	0.718568
CR392026.2	0.101446	-1.755425691	5.23528	-0.33531	0.737394	NA
si:ch211-208g1.1	0.586398	-1.716268233	3.459477	-0.49611	0.619819	NA
ms4a17a.11	629.3029	0.393870139	0.195199	2.017788	0.043613	0.3656
si:dkey-111i23.1	0.590186	2.065775445	3.4418	0.600202	0.548372	NA
CU179758.1	11.00559	0.134929369	0.481903	0.279993	0.779483	0.961073
or133-10	0	NA	NA	NA	NA	NA
si:dkey-58f10.4	2.342835	-1.734939988	1.630436	-1.0641	0.287285	0.758355
si:ch73-44m9.2	4.502027	-2.280175387	1.009751	-2.25816	0.023936	0.274217
AL953907.1	0.424991	1.055359511	4.901986	0.215292	0.829539	NA
si:dkey-23a23.1	0.61668	-3.150708296	4.431873	-0.71092	0.477134	NA
F13A1 (1 of many)	0.52642	0	3.118302	0	1	NA
coa5	242.3278	0.198666548	0.194879	1.019433	0.307997	0.772996
si:ch211-195b15.8	622.1695	0.35395782	0.173474	2.040408	0.04131	0.354893
BX663515.1	0.522138	0.010001289	3.563527	0.002807	0.997761	NA
CR749748.1	11.64352	-0.148225366	0.496888	-0.29831	0.765468	0.959831
si:dkey-21e2.13	151.0722	0	1.693338	0	1	1
BX927394.2	0	NA	NA	NA	NA	NA
BX323457.3	4.928245	2.188307179	1.05212	2.079902	0.037534	0.340115
zmp:0000001323	11.80927	0	1.876128	0	1	1
BX936461.1	4.378658	2.091864582	1.346849	1.553155	0.120386	0.565852
BX640520.2	0	NA	NA	NA	NA	NA
si:dkey-88l16.5	3.798583	0.089111691	0.87867	0.101417	0.91922	0.993892
BX119902.1	0.050624	1.055396947	5.267649	0.200354	0.841203	NA
si:ch211-147m20.3	0.116481	0	5.267649	0	1	NA
ms4a17a.9	445.6769	0.398985955	0.198989	2.005066	0.044956	0.371348
BX511089.3	0	NA	NA	NA	NA	NA
dio2	20.38176	-0.676555164	0.5261	-1.28598	0.19845	0.679184
si:dkey-11o15.10	0.347232	0	4.408206	0	1	NA
gpr186	38.14749	-1.130823176	0.553726	-2.04221	0.041131	0.353918
si:dkey-163f12.11	1.613231	0.939533936	1.193946	0.786915	0.431332	NA
v2ra20	0.165352	-0.868103029	5.267649	-0.1648	0.869102	NA
CR407561.1	0.25048	-0.066140403	5.204391	-0.01271	0.98986	NA
pimr152	0	NA	NA	NA	NA	NA
si:ch73-313l22.2	0	NA	NA	NA	NA	NA
CR396590.6	0	NA	NA	NA	NA	NA
BX649434.2	0.922433	0.067491398	2.173786	0.031048	0.975231	NA
si:dkey-258f14.2	23.87696	-0.318876709	0.333572	-0.95595	0.339099	0.795226
si:dkey-84o3.6	1.198871	0.60515862	1.713153	0.353243	0.723907	NA
si:dkey-277m11.2	14.33731	-0.307694843	0.651551	-0.47225	0.636748	0.924779
si:ch211-197h24.8	36.34481	-0.159455256	0.347183	-0.45928	0.646031	0.926728
il1r2	0	NA	NA	NA	NA	NA
BX470185.2	0.103025	-0.868089684	5.267649	-0.1648	0.869104	NA
pimr178	0.24655	2.436374376	5.206324	0.467964	0.63981	NA
si:dkey-266f7.5	4.638403	-1.589553493	0.892556	-1.7809	0.074929	0.464197
si:ch211-170d8.2	18.32095	0.240485867	0.41312	0.582121	0.560485	0.899928
znf1061	0.640021	-0.791259046	2.420142	-0.32695	0.743708	NA
cplx4c	108.6993	0.157065434	0.27344	0.574406	0.565693	0.901634
si:ch211-163b2.4	0.867138	-4.000317502	5.155772	-0.77589	0.437813	NA
cenph	49.43807	0.065917005	0.249889	0.263786	0.791945	0.964842
tyrobp	4.452141	-1.812694665	0.883795	-2.05104	0.040263	0.350654
rbm33b	9.50118	1.234984828	0.579325	2.131766	0.033026	0.321751

si:ch1073-143l10.2	43.5752	-0.037507753	0.376544	-0.09961	0.920654	0.994085
abcc6b.2	46.88779	-0.191464226	0.369339	-0.5184	0.604181	0.91484
si:ch211-231i17.4	0.73786	1.000603034	2.394239	0.417921	0.676005	NA
lingo4a	5.906893	-1.111560148	1.082171	-1.02716	0.304346	0.769752
si:dkey-22i16.7	8.920483	0	1.790196	0	1	1
CR749775.1	0.456584	1.055379606	4.142258	0.254784	0.79889	NA
or133-2	7.946613	1.293714013	0.608439	2.126285	0.03348	0.323151
si:ch73-196i15.5	2.61289	0.9725953	1.739167	0.55923	0.576004	0.904739
CR936403.1	0.508661	2.875448212	3.056118	0.940883	0.346765	NA
ungb	4.677415	0.208863178	0.979478	0.213239	0.83114	0.974908
CU469568.2	1.720664	0	2.199157	0	1	NA
CR735104.1	0.248325	0	5.267649	0	1	NA
BX248322.1	0.709603	-0.86814605	2.853738	-0.30421	0.760965	NA
si:dkey-93l1.4	0.099037	0	5.267649	0	1	NA
si:ch211-53m15.2	0.092101	0	5.267649	0	1	NA
apoa4b.2	871.6515	0.586668614	0.454366	1.29118	0.196641	0.676996
si:dkey-58f10.11	3.695602	2.544734822	1.707158	1.490627	0.13606	0.591131
CR450833.1	8.28144	-0.255751363	0.746721	-0.3425	0.731975	0.950617
FO834814.1	7.688252	0.369190339	0.79185	0.466238	0.641045	0.925364
CR855257.1	0.049519	0	5.267649	0	1	NA
BX005153.2	0.16003	0.959877153	5.236908	0.183291	0.85457	NA
AL929210.1	0.347478	-1.807578344	3.727823	-0.48489	0.627755	NA
si:ch211-132b12.2	18.90649	0.123421444	0.399656	0.308819	0.757459	0.957364
BX511265.3	0.318543	2.665446361	4.529697	0.588438	0.556238	NA
si:ch211-74m13.1	138.9855	0.108110113	0.197114	0.548465	0.583373	0.905948
BX284697.2	0	NA	NA	NA	NA	NA
CR392341.3	2.889017	-0.652138157	1.325063	-0.49216	0.622609	0.918894
si:ch73-379f5.5	26.77955	0	1.717684	0	1	1
AL929032.1	0.102175	1.055383603	5.267649	0.200352	0.841205	NA
BX530407.3	0	NA	NA	NA	NA	NA
CR377211.2	0.608305	-0.575770725	3.235925	-0.17793	0.858777	NA
nfil3-5	1551.13	-0.300641672	0.558438	-0.53836	0.590327	0.908901
BX255915.2	0.128839	0	5.267649	0	1	NA
wnt16	46.30901	-0.427770273	0.283972	-1.50638	0.131969	0.584315
BX530068.1	84.36775	-1.358163179	0.26972	-5.03546	4.77E-07	0.000161
si:ch211-214p16.2	0	NA	NA	NA	NA	NA
si:ch211-156j22.4	196.1452	0.2012793	0.291251	0.691085	0.489512	0.874615
si:ch73-18b11.2	58.28599	-0.191012352	0.256622	-0.74433	0.456674	0.860698
CR792454.1	0.544298	0	2.86717	0	1	NA
chst12b	3.08114	0	2.02341	0	1	1
ccl34a.3	0.753966	1.055427804	2.475174	0.426405	0.669812	NA
si:ch211-197g15.8	3.928695	0.821602309	1.245767	0.659515	0.509565	0.883733
CR377211.3	1.094575	-1.339614516	1.667981	-0.80314	0.421897	NA
si:dkey-91f15.1	39.29375	-0.025692092	0.679012	-0.03784	0.969817	1
otx1	239.4079	0.327551855	0.134433	2.436552	0.014828	0.213592
si:dkey-106c17.2	3.369742	-2.48067787	1.075724	-2.30606	0.021108	0.25794
si:dkey-84o3.4	3.778121	-3.350863987	1.571132	-2.13277	0.032944	0.321256
sult1st9	5.68152	-3.308110877	1.374813	-2.40623	0.016118	0.223897
taar18b	0.319903	2.517922993	3.840332	0.655652	0.512048	NA
FP015823.1	0.381753	0	3.307398	0	1	NA
pimr98	1.426463	2.275189368	1.74078	1.306995	0.191215	NA
si:dkey-194e6.2	0.161641	0	5.267649	0	1	NA
si:rp71-17i16.4	302.3866	0.454461537	0.372337	1.220564	0.222251	0.703972
si:dkey-21e2.10	63.16602	0	2.165945	0	1	1

CR388079.2	1.001648	-2.101082676	1.953474	-1.07556	0.282123	NA
sinhcafl	574.1495	0.01711956	0.11558	0.148118	0.882249	0.986908
si:ch211-67n3.9	26.73067	-0.30087142	0.543688	-0.55339	0.579997	0.905618
AL953903.1	0	NA	NA	NA	NA	NA
si:dkey-240n22.3	8.807943	0.772537784	0.79522	0.971477	0.331311	0.788949
BX571955.2	7.301899	0.113943958	1.058127	0.107685	0.914246	0.992972
CR932983.2	2.084766	0.689963046	1.621346	0.425549	0.670436	NA
BX927275.2	1.737319	1.382192672	1.209888	1.142413	0.253282	NA
lmo2	223.1546	-0.177469743	0.18865	-0.94074	0.34684	0.799748
thsd7ba	134.2975	0.425144469	0.362351	1.173295	0.240678	0.722178
si:dkeyp-68b7.10	31.50214	-0.360291501	0.377202	-0.95517	0.339492	0.795226
CR388166.2	29.55069	-0.453858271	0.386405	-1.17457	0.240169	0.721177
si:dkey-58f10.14	4.333636	2.501317738	1.183314	2.113825	0.03453	0.328029
si:ch73-120g24.4	71.8737	0.42233532	0.283091	1.491872	0.135733	0.591131
ftr33	1.780472	0.064775992	2.009046	0.032242	0.974279	NA
FP067425.1	0.209102	1.055362417	5.137509	0.205423	0.837242	NA
zgc:162928	68.19469	0.093518587	0.396576	0.235815	0.813576	0.970328
taar20t	0.197454	0	5.267649	0	1	NA
CR318668.1	0	NA	NA	NA	NA	NA
CR388189.1	0.063619	0	5.267649	0	1	NA
cbln7	0.09025	0	5.267649	0	1	NA
BX322661.2	81.7727	0.0419344	0.295563	0.14188	0.887175	0.987739
CU468826.1	0.320553	2.461492708	4.460611	0.551829	0.581066	NA
pold2	152.486	0.040509287	0.189584	0.213675	0.830801	0.974908
si:dkey-85k15.4	6.524022	-1.339788716	0.63135	-2.1221	0.033829	0.325104
si:dkey-250k15.7	28.05376	0.006881764	0.608599	0.011308	0.990978	1
si:dkey-7f3.14	0.40487	0	5.267649	0	1	NA
si:ch211-236l14.1	0	NA	NA	NA	NA	NA
pimr148	0	NA	NA	NA	NA	NA
CT573133.1	1.835178	-0.917045424	1.625822	-0.56405	0.57272	NA
si:dkey-103d23.3	0.75172	-1.753759352	2.662265	-0.65875	0.510058	NA
CT025776.1	0.043309	0	5.267649	0	1	NA
si:ch211-106h4.5	15.29389	0.953776659	0.829008	1.150503	0.249937	0.729721
or127-1	2.714458	0.457980299	1.168181	0.392046	0.695024	0.941197
CU394254.2	0.376674	-3.354576721	4.904049	-0.68404	0.493948	NA
si:dkey-238m4.3	0	NA	NA	NA	NA	NA
BX005445.1	2.081394	-1.713039563	1.201555	-1.42569	0.153959	NA
si:ch211-113e8.9	12.07546	0.188199147	0.578992	0.325046	0.745146	0.954956
BX088713.1	0.23603	-1.71064224	4.961755	-0.34477	0.730271	NA
TC2N	20.7283	0.400395989	0.456071	0.877925	0.379985	0.823607
si:dkey-10h3.3	0.937555	3.946499895	2.150111	1.835487	0.066434	NA
BX908726.1	3.99614	1.681108789	0.981375	1.713013	0.08671	0.493509
cd28	18.60328	0.146628446	0.539491	0.271791	0.785783	0.962605
ctslb	0.157736	-2.389175838	5.199609	-0.45949	0.645881	NA
si:dkey-73p2.3	3.303607	0.041501669	1.557411	0.026648	0.978741	1
BX548073.4	0.050723	-0.86807634	5.267649	-0.16479	0.869106	NA
CR545476.1	0	NA	NA	NA	NA	NA
CR450782.1	6.714486	2.749473526	0.849918	3.234988	0.001216	0.050523
CU657976.1	0.389481	0	5.007512	0	1	NA
ITLN1 (1 of many)	144.5786	-2.408522161	0.612751	-3.93067	8.47E-05	0.008144
ftr10	0	NA	NA	NA	NA	NA
cfdp1	217.1767	0.047594566	0.161969	0.29385	0.768873	0.960395
CU928069.1	0	NA	NA	NA	NA	NA
BX649508.1	0	NA	NA	NA	NA	NA

tspan37	118.1778	-0.298581078	0.266485	-1.12044	0.262525	0.740129
si:ch211-232m8.3	22.64444	1.092992966	0.571976	1.910908	0.056016	0.409174
pimr50	0.301596	0	5.267649	0	1	NA
pimr127	0.08847	0	5.267649	0	1	NA
BX649516.1	0.22126	-2.340106499	5.201854	-0.44986	0.652811	NA
si:ch211-229g14.3	0.106927	0	5.267649	0	1	NA
si:ch211-132g1.4	1.917183	-5.013962568	1.868738	-2.68307	0.007295	NA
CR749763.4	0.060442	0	5.267649	0	1	NA
CR847899.2	0	NA	NA	NA	NA	NA
si:dkey-28k24.2	3.100805	-4.658362078	1.487942	-3.13074	0.001744	0.064499
AL953855.1	0	NA	NA	NA	NA	NA
si:dkey-192k22.2	2.473022	-0.717608894	1.388737	-0.51673	0.605341	0.914985
BX640512.2	0	NA	NA	NA	NA	NA
btr04	0.806468	0	3.061762	0	1	NA
BX005024.1	0	NA	NA	NA	NA	NA
CU207311.2	0.559468	1.055381168	4.47416	0.235884	0.813523	NA
CU694808.1	0.99726	-0.29471275	1.692274	-0.17415	0.861746	NA
ftr97	37.67554	-0.726031116	0.384363	-1.88892	0.058903	0.418455
si:dkey-82i20.2	87.46289	0.265941191	0.402826	0.660188	0.509133	0.883443
si:ch73-343l4.6	0	NA	NA	NA	NA	NA
CT583652.1	0.223518	0	5.055639	0	1	NA
si:ch211-227e10.6	4.725202	0.072427302	0.995612	0.072747	0.942008	0.997371
si:ch211-165f21.2	3.012694	0.092965978	1.110595	0.083708	0.933288	0.996472
si:dkey-250k15.9	0.820115	1.055419243	2.697383	0.391275	0.695594	NA
si:ch73-266f23.1	63.55809	-0.348197433	0.287147	-1.21261	0.225278	0.70685
si:ch211-130m23.2	34.07607	-0.097182094	0.304815	-0.31882	0.749861	0.955074
si:dkey-88l16.2	0	NA	NA	NA	NA	NA
CR388373.2	20.16795	0.15658023	0.344955	0.453915	0.64989	0.927756
BX004962.2	0.049519	0	5.267649	0	1	NA
si:ch211-208h16.4	206.8947	0.220111492	0.258329	0.852058	0.394182	0.828374
AL954816.1	1.771506	-0.461883891	1.694014	-0.27266	0.785117	NA
BX571724.2	0.179442	0	5.267649	0	1	NA
krt96	105.4267	-0.316849916	0.224427	-1.41181	0.158004	0.623766
CT583625.3	0.615056	-0.868138675	2.728519	-0.31817	0.750354	NA
znf1062	0.786746	-1.045013107	2.877481	-0.36317	0.716478	NA
si:dkey-114c15.5	13.21692	0.549061793	0.518754	1.058425	0.289862	0.760705
AL953903.2	0	NA	NA	NA	NA	NA
si:ch211-255i3.4	88.91942	-0.10926504	0.303995	-0.35943	0.719273	0.94694
CR388363.1	0.413109	0	4.30197	0	1	NA
taar20w	0.043308	0	5.267649	0	1	NA
zgc:163080	187.351	-0.101683258	0.187603	-0.54201	0.58781	0.907608
CT025913.1	0.277549	-2.792433852	5.183761	-0.53869	0.590102	NA
BX649500.1	4.987845	-1.211987463	0.945341	-1.28206	0.19982	0.6804
AL590134.1	4.602353	0.592034541	0.79402	0.745616	0.455899	0.860324
CR847563.1	5.591664	2.260505884	0.857628	2.635766	0.008395	0.156734
CU469530.1	2.767935	0.068935123	1.248542	0.055212	0.955969	0.998845
CR450836.1	48.97557	0.695297135	0.25393	2.738148	0.006179	0.132924
lrrfip1b	100.0287	0.05694773	0.239297	0.23798	0.811897	0.97012
CU695074.1	0	NA	NA	NA	NA	NA
si:dkey-78l4.12	0	NA	NA	NA	NA	NA
BX640478.1	2.231912	0.105183263	1.400135	0.075124	0.940116	0.997371
si:ch211-235f1.3	1.270024	1.055456057	2.069454	0.510017	0.61004	NA
abr	231.6619	0.132602976	0.136051	0.974657	0.32973	0.788011
si:ch73-52p7.1	18.9882	0.447119935	0.602378	0.742258	0.457931	0.860993

si:ch211-274p24.2	19.91231	0.307434922	0.41134	0.747398	0.454823	0.859845
BX324164.2	5.513731	-1.504323064	1.236037	-1.21705	0.223584	0.705397
si:dkey-189h5.6	50.28503	-0.500985224	0.459245	-1.09089	0.275322	0.750424
si:ch211-127n13.2	0.241766	0	5.267649	0	1	NA
BX901918.2	0.40922	-1.826692069	5.230459	-0.34924	0.726908	NA
si:dkey-57k2.7	91.23668	-0.552519924	0.231353	-2.38821	0.016931	0.230621
BX957317.1	143.1614	-0.42888034	0.333336	-1.28663	0.198223	0.679109
si:dkey-23c22.7	4.406677	-2.496214054	1.165685	-2.14141	0.032241	0.318747
si:dkey-19a16.11	0	NA	NA	NA	NA	NA
CR854927.2	0.262548	2.079535926	5.22636	0.397894	0.690708	NA
znf1132	0.546974	-0.199744923	2.938455	-0.06798	0.945805	NA
AL732515.1	0.094032	-0.868089684	5.267649	-0.1648	0.869104	NA
si:ch211-197e7.1	51.14028	1.159516291	0.984363	1.177935	0.238822	0.719781
AL935117.1	2.014148	0.050279608	1.246952	0.040322	0.967836	NA
BX649502.10	0	NA	NA	NA	NA	NA
BX649610.1	0	NA	NA	NA	NA	NA
AL845306.1	0.267184	0	5.267649	0	1	NA
si:ch211-210b2.4	11.06951	-0.268250254	0.546277	-0.49105	0.62339	0.919118
CU469584.1	9.094539	-0.098093982	0.694595	-0.14122	0.887692	0.988077
zgc:66156	34.22124	0	1.703057	0	1	1
CT030044.1	3.453402	0.898431494	1.076864	0.834304	0.40411	0.833888
or128-7	0.046941	0	5.267649	0	1	NA
si:ch211-242f23.3	0.597135	-2.773597679	3.882806	-0.71433	0.475024	NA
camkmt	95.27695	0.298969301	0.21288	1.404402	0.160199	0.626611
CR769772.3	0.351146	-0.868132897	4.416119	-0.19658	0.844154	NA
CR759970.1	0	NA	NA	NA	NA	NA
CT990618.1	171.9289	0.010764119	0.156846	0.068628	0.945285	0.997371
CR759887.2	0.050624	1.055396947	5.267649	0.200354	0.841203	NA
mybl2a	138.3737	-0.188696274	0.209809	-0.89937	0.368454	0.817272
si:ch211-157j23.2	2.127395	-1.646534664	1.36137	-1.20947	0.226483	NA
CU468920.1	2.122384	-0.691937419	1.276627	-0.542	0.587816	NA
si:ch211-225k7.3	0.586432	1.490324766	2.793945	0.533412	0.593748	NA
si:dkey-40h20.1	6.273136	1.226927857	0.694395	1.766903	0.077244	0.46958
si:dkey-239j18.2	8.913191	0.133702597	0.692063	0.193194	0.846807	0.977263
tmem119a	22.75749	0.171961875	0.561696	0.306148	0.759492	0.957886
CU694757.1	0	NA	NA	NA	NA	NA
si:dkey-145c18.4	0	NA	NA	NA	NA	NA
CR396590.7	0.837533	4.20932268	2.216604	1.898996	0.057565	NA
AL928866.1	0.400469	-0.868137916	3.324672	-0.26112	0.794	NA
BX927385.2	1.753027	2.520620622	1.836172	1.372759	0.169827	NA
si:dkey-261h17.1	1068.511	-0.257536805	0.170663	-1.50903	0.13129	0.583069
htr7c	5.107027	-0.657665309	1.233368	-0.53323	0.593876	0.910482
cox8a	4418.685	0.125974389	0.110675	1.138242	0.255019	0.733542
si:ch211-278p9.2	2.88547	-1.80771757	1.64547	-1.0986	0.271941	0.748056
si:dkey-269p2.1	5.433065	-0.244724987	1.105305	-0.22141	0.824774	0.972272
BX936318.1	0.609232	-2.77879581	2.658682	-1.04518	0.295941	NA
AL954142.2	1.305681	-1.02114651	1.664558	-0.61346	0.53957	NA
CU682811.2	10.19479	-1.282910165	0.708138	-1.81167	0.070038	0.451397
zgc:152652	0.153006	-0.771121057	5.236853	-0.14725	0.882936	NA
BX950187.1	5.813574	-0.382713981	0.949785	-0.40295	0.686987	0.938807
si:dkeyp-2e4.3	27.64434	-1.553118193	0.420302	-3.69525	0.00022	0.015758
rusc2	1044.877	0.112375171	0.168406	0.667287	0.504589	0.88195
CU595864.1	0.18486	0	5.267649	0	1	NA
CR394546.3	19.26578	1.277460936	0.621775	2.054538	0.039924	0.3492

si:dkey-265e7.1	3.261543	-1.472602756	1.310714	-1.12351	0.26122	0.738737
si:dkey-200l5.4	61.99612	-0.646578874	0.817257	-0.79116	0.428852	0.846597
si:rp71-36a1.1	112.3227	-0.004350984	0.267517	-0.01626	0.987024	1
si:ch73-274k23.1	7.384781	-0.354066846	0.604347	-0.58587	0.557965	0.899301
adgre17	0.491655	2.922009193	3.546198	0.823984	0.409949	NA
ftr73	1.154307	-3.521284481	2.310384	-1.52411	0.127481	NA
mxra7	114.1251	0.050907548	0.214802	0.236997	0.812659	0.970328
BX247868.1	4.940595	-0.562288056	0.825248	-0.68136	0.495646	0.87806
BX957322.2	13.54155	0.138796602	0.538198	0.257891	0.796491	0.966447
CR926469.1	0	NA	NA	NA	NA	NA
si:ch211-207c6.2	37.81648	-0.058195795	0.332514	-0.17502	0.861066	0.980388
si:dkey-22p6.2	0	NA	NA	NA	NA	NA
si:ch211-215c18.3	22.52586	0.873452114	0.574783	1.519619	0.128607	0.578162
si:dkey-79f11.9	0.060442	0	5.267649	0	1	NA
sc:d0202	36.22022	-0.22784988	0.400154	-0.56941	0.569081	0.902234
si:dkey-19p15.4	0.273395	1.85410415	5.241768	0.353717	0.723551	NA
CR626907.2	18.37017	0.318312143	0.362652	0.877735	0.380087	0.823607
CR769777.1	3.305798	-2.638960732	1.264016	-2.08776	0.03682	0.336931
BX004840.2	0	NA	NA	NA	NA	NA
fkbpl	75.91831	0.183094946	0.202884	0.902462	0.366812	0.816111
galnt8b.2	13.33431	0.128013104	0.484549	0.26419	0.791633	0.964817
si:dkey-49n23.3	0.140685	-0.868089684	5.267649	-0.1648	0.869104	NA
si:dkey-88n24.3	0.087381	0	5.267649	0	1	NA
si:ch211-191i18.4	8.376127	-0.652784841	0.771412	-0.84622	0.39743	0.830253
si:dkey-14d8.1	26.38808	0.244189781	0.455708	0.535847	0.592064	0.90969
si:ch211-157j23.3	3.071204	0.69320396	1.164313	0.595376	0.551592	0.8974
CR318646.3	0.152169	-2.340093811	5.201855	-0.44986	0.652813	NA
si:dkeyp-44a8.4	274.257	0.107479022	0.180866	0.594246	0.552347	0.8974
BX248512.2	1.347173	1.703205165	1.439753	1.182985	0.236815	NA
ftr09	0.208377	0	5.267649	0	1	NA
si:dkeyp-72h1.1	94.39404	-0.637160449	0.4675	-1.36291	0.172911	0.645664
sh2d1aa	1.62049	-0.711332106	1.546198	-0.46005	0.645479	NA
si:dkey-4c15.12	5.70848	-0.702269018	0.765563	-0.91732	0.358973	0.810031
CU914776.1	403.5881	1.703963693	0.203961	8.354365	6.58E-17	1.91E-13
CR936240.1	0.130689	0	5.267649	0	1	NA
si:dkey-78l4.7	0.138182	0	5.267649	0	1	NA
si:ch211-213b8.4	3.356202	0	1.862921	0	1	1
si:ch211-77g15.32	189.0097	-0.868161223	1.676596	-0.51781	0.60459	0.914985
BX640520.3	0.099037	0	5.267649	0	1	NA
BX323065.1	1.500335	0.64593194	1.843755	0.350335	0.726087	NA
AL590146.1	0.453942	1.05542085	3.1273	0.337486	0.73575	NA
zgc:112966	11.73442	-0.277467747	0.53613	-0.51754	0.604781	0.914985
FP016012.1	0	NA	NA	NA	NA	NA
CR388207.1	18.203	0.989323333	0.500337	1.977316	0.048006	0.381054
gdf10a	313.2605	0.368189101	0.195076	1.887416	0.059104	0.419032
BX294163.1	0.046941	0	5.267649	0	1	NA
si:dkey-78l4.2	5.406209	0	1.850347	0	1	1
BX640481.1	5.760791	-0.811359055	0.960296	-0.84491	0.398164	0.830737
pimr179	1.976873	-2.389650645	1.724683	-1.38556	0.165881	NA
BX323590.2	126.7221	0.173198189	0.202019	0.857337	0.391259	0.827902
BX901878.2	2.339628	-0.868145949	1.907343	-0.45516	0.648994	0.927649
BX537288.2	43.15799	0.608504429	0.439878	1.383349	0.166558	0.636904
BX664750.2	0.050624	1.055396947	5.267649	0.200354	0.841203	NA
CR376741.1	5.251752	-0.353043635	0.735432	-0.48005	0.631192	0.923003

si:ch1073-75f15.2	0.331578	-2.413216571	5.198537	-0.46421	0.642497	NA
CU468730.1	6.862631	-2.212222297	1.020414	-2.16797	0.030161	0.309482
znf1170	22.87365	0.190344144	0.340762	0.558583	0.576446	0.90475
si:ch73-256j6.6	0.878478	-0.58229253	2.18129	-0.26695	0.789509	NA
si:ch73-52f15.5	212.1147	-0.213430933	0.177724	-1.20092	0.229784	0.711763
si:rp71-1i20.1	2.97298	0.364607572	0.99268	0.367296	0.713398	0.945821
BX897741.1	3.394089	1.055428615	1.869856	0.564444	0.572452	0.903461
si:dkey-9i23.8	0.315682	0	5.267649	0	1	NA
AL935184.2	0.318586	-1.332881143	4.510018	-0.29554	0.767583	NA
si:ch211-226h7.8	8.099764	0.982113385	1.687898	0.581856	0.560664	0.899982
si:ch211-285c6.4	1.227785	-0.868142441	2.137322	-0.40618	0.684609	NA
CR847543.2	14.85794	-0.19534196	0.444614	-0.43935	0.660407	0.930581
RNF224	16.90999	-0.031348478	0.444337	-0.07055	0.943755	0.997371
or125-4	16.95032	0.400139095	0.419524	0.953792	0.340189	0.795345
CR388363.2	0.045161	0	5.267649	0	1	NA
si:ch211-14a11.1	0.350216	-2.335655781	3.312055	-0.7052	0.480687	NA
BX324123.2	253.6959	-0.081216844	0.350014	-0.23204	0.816508	0.971207
si:ch211-133n4.7	6.43763	-0.327927765	0.869732	-0.37704	0.70614	0.944592
AL929520.2	0.696393	0.093654412	2.956628	0.031676	0.97473	NA
FP101887.1	175.3055	-0.653760228	0.226463	-2.88683	0.003891	0.103477
BX323074.2	102.5187	0.500352584	0.216339	2.312815	0.020733	0.255657
si:ch211-217k17.8	53.30669	-0.06509283	0.354447	-0.18365	0.854291	0.979073
si:dkeyp-117b11.3	24.66405	-0.189359125	0.393887	-0.48075	0.630698	0.923003
si:dkey-78l4.4	0	NA	NA	NA	NA	NA
BX649490.3	0.233191	1.055358154	5.267649	0.200347	0.841209	NA
si:dkeyp-53e4.2	1.866771	-2.559015258	1.802212	-1.41993	0.155628	NA
BX927216.1	0	NA	NA	NA	NA	NA
si:dkey-191c17.2	0.980665	-0.367988649	2.09135	-0.17596	0.860327	NA
CR383662.3	14.75435	-0.295640418	0.610961	-0.48389	0.628461	0.921864
ftr30	2.967181	2.031178988	1.302905	1.558962	0.119005	0.562496
AL929017.2	6.663959	0.794556364	0.735328	1.080547	0.279899	0.752787
AL929237.2	0.043309	0	5.267649	0	1	NA
si:ch211-196l7.4	823.09	-0.004103114	0.155431	-0.0264	0.97894	1
si:rp71-17i16.6	28.17164	0.264645216	0.304222	0.869908	0.384351	0.826565
si:dkey-19a16.13	12.88131	-0.348563612	0.585531	-0.5953	0.551646	0.8974
si:dkey-20i20.10	8.478319	0.978168454	0.663343	1.474604	0.140319	0.599409
si:ch211-147d7.5	22.43993	0.084415169	0.349017	0.241865	0.808885	0.969828
si:dkey-240h12.3	12.71953	-0.237884572	0.536285	-0.44358	0.657347	0.930235
si:ch211-191j22.3	553.2532	-0.046877728	0.132612	-0.35349	0.723717	0.947964
BX000705.1	0	NA	NA	NA	NA	NA
pimr171	6.334335	0	1.909239	0	1	1
cela1.2	629.7341	0	1.698113	0	1	1
si:ch73-90k17.1	0.398206	-0.868147553	4.363994	-0.19893	0.842314	NA
gstt2	114.8021	-1.734973723	0.32901	-5.27331	1.34E-07	6.21E-05
BX649377.1	3.601204	0.964006908	1.744808	0.5525	0.580606	0.905618
BX470183.1	0.352911	-0.868128313	4.428365	-0.19604	0.84458	NA
CU657977.1	2.26661	2.083354873	1.464727	1.42235	0.154925	0.619752
CR392001.1	0.049519	0	5.267649	0	1	NA
CR933734.3	17.91259	-5.281760865	1.556177	-3.39406	0.000689	0.034762
si:ch211-282j17.10	0.093881	0	5.267649	0	1	NA
CHST12 (1 of many)	0.871622	1.055426087	2.533782	0.416542	0.677014	NA
AL929457.1	4.337838	-0.535069304	0.941695	-0.5682	0.569901	0.902322
CU467646.2	3.042934	1.488988483	1.099727	1.353962	0.175749	0.649413
CU570881.1	0.234799	0	5.267649	0	1	NA

adgre6	0.13412	0	5.267649	0	1	NA
CU459065.1	0	NA	NA	NA	NA	NA
si:dkeyp-2e4.5	23.69156	-0.184125867	0.376572	-0.48895	0.624875	0.920195
si:dkey-286h21.1	0.200022	-0.868115203	5.213564	-0.16651	0.867755	NA
si:ch211-167j6.3	0.257995	-0.868110878	5.267649	-0.1648	0.869101	NA
CU986305.1	1.721615	3.298961352	1.756152	1.878517	0.06031	NA
BX649622.1	7.623314	-0.061200172	0.665194	-0.092	0.926695	0.994815
CR759885.1	20.29581	-1.893018809	0.703392	-2.69127	0.007118	0.142826
si:ch211-10d23.5	40.97442	-0.836592236	0.322464	-2.59437	0.009476	0.167353
rca2.2	24.64879	-0.85961218	0.636891	-1.3497	0.177112	0.650742
CR396590.8	0	NA	NA	NA	NA	NA
si:dkeyp-77c8.5	4.837453	0.246197428	0.80836	0.304564	0.760698	0.957886
entr1	181.9642	-0.307557806	0.202544	-1.51848	0.128894	0.578173
si:dkey-204f11.13	0	NA	NA	NA	NA	NA
BX323839.1	6.859296	0.433935527	0.868225	0.499796	0.617218	0.916365
BX950188.2	0	NA	NA	NA	NA	NA
znf1090	6.277927	-0.782967552	0.722302	-1.08399	0.27837	0.752078
si:dkey-71b5.3	24.9105	0.709144252	1.183951	0.598964	0.549197	0.897092
AL845369.2	0.504104	-0.868144804	3.638534	-0.2386	0.811418	NA
CR392363.2	2.310829	-0.096807324	1.27602	-0.07587	0.939525	0.997371
CR854897.1	3.040068	-2.483320191	1.85126	-1.34142	0.179784	0.655282
BX322661.3	0.40762	0.585858489	4.985846	0.117504	0.90646	NA
si:dkey-58f10.13	2.857376	-1.708074673	1.390582	-1.22832	0.219328	0.700266
si:ch211-198c19.3	39.52732	0.484012407	0.451291	1.072507	0.283492	0.755095
BX005462.1	0	NA	NA	NA	NA	NA
trim62.2	4.225575	-0.321766962	0.946596	-0.33992	0.733917	0.95093
senp3a	162.6386	0.051132832	0.164472	0.310891	0.755884	0.956532
si:dkeyp-82a1.8	0.873491	1.554166618	2.131862	0.729018	0.46599	NA
sid1	0.605483	1.98394108	2.360779	0.840375	0.400698	NA
si:dkey-33c12.12	0.964868	3.504428765	2.205755	1.588766	0.112113	NA
CR931802.2	0.274667	-1.402866893	4.6602	-0.30103	0.763391	NA
BX247877.1	1.768539	1.327297491	1.400675	0.947612	0.343327	NA
si:dkeyp-106c3.2	0.145303	1.055370259	5.267649	0.200349	0.841207	NA
lenep	54.8835	-0.162301226	0.6694	-0.24246	0.808425	0.969828
si:ch211-163g19.2	0.04222	0	5.267649	0	1	NA
CR318588.4	1637.467	0.356551284	0.231618	1.539392	0.123709	0.570837
BX664610.1	113.0193	-0.261053777	0.250906	-1.04044	0.298134	0.765416
si:dkey-246j7.1	27.23532	-0.036353045	0.46952	-0.07743	0.938285	0.997371
si:ch73-138i16.1	4.61572	0.211815519	1.018263	0.208017	0.835216	0.976007
CABZ01118770.1	3.999401	0.585032485	0.969143	0.60366	0.54607	0.895875
si:dkey-65b13.1	1.003543	3.239747371	2.000405	1.619545	0.10533	NA
AL929576.1	1.95703	2.13142378	1.526924	1.395893	0.162747	NA
BX537162.1	0	NA	NA	NA	NA	NA
si:dkey-21e2.14	3.401186	0	1.949698	0	1	1
si:ch211-122l14.7	0.053506	-0.86807634	5.267649	-0.16479	0.869106	NA
cybrd1	215.8255	0.286197143	0.20362	1.405544	0.15986	0.626123
si:dkey-222h21.7	0.318445	1.05534377	5.267649	0.200344	0.841211	NA
si:ch211-67e16.11	207.8725	0.259716187	0.210095	1.236182	0.216391	0.69815
BX663525.1	3.976423	0.670987828	1.194879	0.561553	0.574421	0.904516
CR925798.1	75.34705	-0.577355664	0.286725	-2.01362	0.044049	0.367392
CT583727.1	3.458694	-1.689790274	1.197075	-1.4116	0.158068	0.623766
si:dkey-11e23.9	0.51688	1.05542101	2.820606	0.374182	0.708269	NA
BX548073.5	0	NA	NA	NA	NA	NA
pimr100	0.301123	-0.868131582	4.003593	-0.21684	0.828334	NA

spp2	3.11301	0.792762266	1.548468	0.511965	0.608675	0.915204
si:dkey-11o15.5	30.9787	1.891074719	0.821458	2.302095	0.02133	0.258949
si:ch211-134c9.2	11.52841	0.102408412	0.522839	0.19587	0.844712	0.976791
si:dkey-184a18.5	53.51622	0.135692416	0.252494	0.537408	0.590986	0.9093
BX322575.1	16.88264	1.856051748	0.55615	3.337321	0.000846	0.039393
si:dkeyp-4f2.6	0.225296	0	5.046189	0	1	NA
AL627088.1	0.683332	-0.868147046	3.041094	-0.28547	0.775283	NA
BX465206.1	0.827436	-2.63772688	2.320307	-1.1368	0.255622	NA
grid2ipb	87.42883	-0.597547071	0.260792	-2.29127	0.021948	0.262532
CR846087.3	3.073219	0.317738998	0.85121	0.373279	0.708941	0.945546
si:dkeyp-51f12.2	15.72028	0.746548971	0.496937	1.5023	0.13302	0.58639
si:ch211-209f23.3	0.205064	0.959863809	5.236908	0.183288	0.854572	NA
si:dkey-57k2.6	36.18123	0.236656786	0.37359	0.633467	0.526429	0.889539
ftr60	2.692924	1.172212042	1.587052	0.73861	0.460144	0.862369
BX248510.2	0.589497	2.490157423	2.931198	0.849536	0.395583	NA
cacna1eb	16.87544	0.541024927	0.439615	1.230679	0.218443	0.699445
si:dkeyp-86h10.3	343.4857	-0.160236821	0.217897	-0.73538	0.462109	0.863402
znf1157	14.36305	-0.632115257	0.483476	-1.30744	0.191064	0.67094
BX927243.1	0	NA	NA	NA	NA	NA
taar19b	0.487683	0	3.602716	0	1	NA
dpm2	338.2766	-0.30810691	0.182919	-1.68439	0.092106	0.507702
c1qc	23.73676	0.899978749	0.547051	1.645146	0.09994	0.524166
CR927456.1	0.053506	-0.86807634	5.267649	-0.16479	0.869106	NA
si:ch211-133l5.7	172.0971	-2.762572261	1.392731	-1.98357	0.047304	0.378859
si:ch211-66k16.2	0.69093	2.50830567	3.403205	0.737042	0.461097	NA
drl1.3	3.159807	-0.307722738	1.180433	-0.26069	0.794334	0.965431
cycsa	0	NA	NA	NA	NA	NA
si:dkey-284p5.3	17.95908	-0.76703174	0.487398	-1.57373	0.11555	0.555766
BX248515.2	0	NA	NA	NA	NA	NA
si:ch1073-15f19.2	1.223948	0	2.407185	0	1	NA
CT025588.1	0.83433	0	2.457052	0	1	NA
si:dkey-253d23.3	59.61493	0.802644646	0.278929	2.87759	0.004007	0.105636
CU207238.1	0.099037	0	5.267649	0	1	NA
BX545856.2	0	NA	NA	NA	NA	NA
si:dkey-97a13.12	11.09946	0.667725327	1.436389	0.464864	0.642029	0.925586
hmx1	130.7861	0.201317051	0.208312	0.966423	0.333833	0.790692
CU681836.2	0	NA	NA	NA	NA	NA
tns3.1	16.92582	-0.019293816	0.394088	-0.04896	0.960953	0.999249
BX005024.2	0	NA	NA	NA	NA	NA
FP016213.1	1.117065	-0.543185089	1.810511	-0.30002	0.764164	NA
si:ch211-191a16.2	48.2393	-0.748276842	0.411734	-1.81738	0.069159	0.448617
znf1175	31.50632	-0.325535731	0.4075	-0.79886	0.424372	0.844412
BX855614.2	0	NA	NA	NA	NA	NA
si:ch211-221n20.2	0.223809	0	5.267649	0	1	NA
FP016142.1	50.85008	0.203368544	0.316611	0.64233	0.520659	0.887165
si:dkey-79f11.8	0.809553	0	2.773404	0	1	NA
tmem123	118.1203	0.047182388	0.248532	0.189844	0.849431	0.978148
ccdc141	141.1179	-0.0098228	0.228416	-0.043	0.965698	1
ftr38	7.994978	-0.481480874	0.975557	-0.49354	0.621628	0.918461
AL845324.2	0.165201	0	5.267649	0	1	NA
BX537103.1	0.580518	2.489819673	2.977168	0.836305	0.402984	NA
BX296526.2	1.541253	0.690516944	2.113258	0.326755	0.743853	NA
BX571694.1	3.814796	-1.106486728	0.909911	-1.21604	0.22397	0.705807
si:dkey-84o3.3	4.320153	3.14717491	1.177818	2.672039	0.007539	0.14728

zgc:162509	0.878559	0.392573684	2.187015	0.179502	0.857544	NA
AL953867.2	0	NA	NA	NA	NA	NA
BX927401.1	0.365242	0	3.830778	0	1	NA
CT573231.1	1.203191	0	2.522284	0	1	NA
ms4a17a.10	15.4935	0.580482252	0.582188	0.997069	0.318731	0.779923
trpm2	59.19645	0.06178726	0.315708	0.19571	0.844837	0.976791
CR774194.1	2.213848	-2.43699216	1.680343	-1.45029	0.146976	0.60703
si:dkey-228a15.1	83.89625	-0.646873255	0.28265	-2.2886	0.022102	0.263263
CR792453.1	0.466337	0	5.267649	0	1	NA
si:dkey-4c15.13	73.72741	0.013265357	0.211371	0.062759	0.949959	0.99801
CDPF1	172.7357	-0.326920973	0.211767	-1.54378	0.122642	0.569468
BX322665.2	16.20998	-2.985802628	0.714239	-4.1804	2.91E-05	0.003705
si:dkey-7i4.9	81.46738	0.039864447	0.29609	0.134636	0.892899	0.988566
BX284666.3	0.28788	0	5.267649	0	1	NA
BX465834.1	404.0374	0.14215804	0.169494	0.838718	0.401628	0.832651
si:dkey-172k15.6	17.18563	0.197621745	0.71576	0.276101	0.782471	0.962328
si:dkey-222h21.2	0.206413	0	5.267649	0	1	NA
CR931763.1	1.107058	0.071898557	1.8492	0.038881	0.968985	NA
BX005108.1	0.391234	1.002787289	2.687391	0.373145	0.70904	NA
CR925709.3	0	NA	NA	NA	NA	NA
si:ch211-196c10.15	19.55355	-2.32571326	0.816123	-2.84971	0.004376	0.108843
CU657925.1	0.04222	0	5.267649	0	1	NA
BX957357.1	0	NA	NA	NA	NA	NA
si:dkey-21e2.12	0	NA	NA	NA	NA	NA
si:ch211-234p6.9	0.472678	-0.868140854	3.231737	-0.26863	0.788215	NA
BX890591.1	2.345947	0.788715548	1.197506	0.658632	0.510132	0.883733
znf423	0.21916	0	5.267649	0	1	NA
CT573344.2	0	NA	NA	NA	NA	NA
pimr186	2.467996	0.044767884	1.164146	0.038456	0.969324	1
si:ch211-271e10.1	0.732804	-0.868143901	2.623149	-0.33095	0.740679	NA
AL953903.3	0.136698	0	5.267649	0	1	NA
si:dkey-73p2.1	3.616588	-0.466025308	1.305905	-0.35686	0.721196	0.947343
si:ch1073-75o15.3	0.229727	0	5.018867	0	1	NA
sox11b	934.1082	0.039775709	0.15146	0.262616	0.792847	0.965312
si:dkey-269i1.4	146.951	-0.606338547	0.310552	-1.95246	0.050884	0.392525
si:ch73-138e16.3	33.71539	-0.228159933	0.284743	-0.80128	0.422967	0.843821
si:zfos-2330d3.7	1.7578	0.039619257	1.863036	0.021266	0.983033	NA
cxcl11.8	0.152173	-1.826663989	5.230461	-0.34924	0.726912	NA
taar18j	0.043308	0	5.267649	0	1	NA
fdxacb1	44.98305	0.244205452	0.31398	0.777773	0.436703	0.851093
leg1.2	180.1029	-1.125199205	0.407102	-2.76393	0.005711	0.127973
CR377211.4	1.20416	0.030194598	1.693189	0.017833	0.985772	NA
si:dkey-234h16.7	142.5885	0.08850192	0.188392	0.469775	0.638516	0.924779
BX465864.2	0	NA	NA	NA	NA	NA
si:ch211-190k17.19	0	NA	NA	NA	NA	NA
si:dkey-28d5.1	0.043309	0	5.267649	0	1	NA
si:ch73-222f22.2	0	NA	NA	NA	NA	NA
dio3b	160.512	-0.407296945	0.253766	-1.60501	0.108492	0.541057
si:dkey-11o1.2	1.048048	-1.758618003	2.34069	-0.75132	0.452457	NA
zgc:194906	1.725014	-0.868148624	2.318993	-0.37436	0.708133	NA
emd	348.0622	0.024680761	0.133853	0.184387	0.85371	0.978905
si:ch211-122l14.6	0	NA	NA	NA	NA	NA
hccsa.2	1.454434	3.935783609	2.031907	1.93699	0.052747	NA
si:ch211-218h8.3	2.504629	-0.521354908	1.689699	-0.30855	0.757665	0.957364

AL928725.1	0	NA	NA	NA	NA	NA
CR769777.2	0.144882	0	5.267649	0	1	NA
CR854897.2	0.297961	1.05539711	3.996504	0.26408	0.791718	NA
BX908740.1	0.094679	0	5.267649	0	1	NA
CR352226.1	0.713355	-0.868137432	2.878562	-0.30159	0.762967	NA
si:dkey-249d8.1	22.65303	0.040747436	0.412214	0.09885	0.921257	0.994085
BX511100.3	0.318485	0	5.267649	0	1	NA
AL928685.1	0.060442	0	5.267649	0	1	NA
BX936406.1	0.045161	0	5.267649	0	1	NA
ghrhrl	1.612664	2.58067748	1.923478	1.341672	0.179702	NA
ccr8.1	6.002385	0.998751151	0.728481	1.371005	0.170374	0.642035
FP067424.1	24.06197	0.127680489	0.388203	0.328902	0.74223	0.954329
si:dkey-85n7.8	12.72692	-0.562171196	0.678863	-0.82811	0.40761	0.83577
FP103048.1	0	NA	NA	NA	NA	NA
si:dkey-87o1.2	460.0812	-0.14818578	0.196364	-0.75465	0.450461	0.857151
BX666064.1	4.23748	-1.35725836	1.885331	-0.7199	0.471584	0.86809
CU633916.1	11.85607	-0.519557135	0.631059	-0.82331	0.410332	0.837066
si:ch211-105n15.1	0.69507	0	3.129301	0	1	NA
BX649485.2	22.80818	-0.947387545	0.572295	-1.65542	0.09784	0.521071
si:ch1073-155h21.2	182.1414	0.878180826	0.387712	2.265035	0.023511	0.270879
BX649485.3	0.536453	0	3.503913	0	1	NA
CT027631.1	0.142363	1.055370259	5.267649	0.200349	0.841207	NA
si:dkey-16n15.6	8.069225	0.986888252	0.766635	1.287299	0.19799	0.678714
si:ch73-362i18.1	0.302374	0	5.267649	0	1	NA
hp	1098.427	-0.40638859	0.223694	-1.81672	0.06926	0.449134
BX470242.1	2.664815	0.364075132	1.320231	0.275766	0.782728	0.962328
dlg4b	6.616737	1.934001357	1.66655	1.160482	0.245853	0.727177
BX005108.2	211.0097	0.218090464	0.2288	0.953191	0.340494	0.795526
BX649481.1	0	NA	NA	NA	NA	NA
znhit6	185.4957	-0.329359642	0.168468	-1.95503	0.050579	0.391331
CU855786.1	0	NA	NA	NA	NA	NA
si:dkey-231j24.3	11.6038	1.118300062	0.750722	1.489632	0.136321	0.591569
BX649485.4	25.28048	-0.314247924	0.53871	-0.58333	0.559668	0.899585
si:dkey-122a22.2	73.13072	0.314580159	0.228662	1.375743	0.168901	0.640393
BX324132.2	13.19537	1.025541688	0.699504	1.466098	0.142622	0.601892
BX649295.1	0	NA	NA	NA	NA	NA
ftr92	3.1515	0.013596781	1.17292	0.011592	0.990751	1
smdt1a	180.4025	0.018852887	0.213833	0.088166	0.929745	0.995576
otulina	0	NA	NA	NA	NA	NA
serpinb1l1	59.09696	-0.383931445	0.326856	-1.17462	0.240147	0.721177
si:ch211-175f12.2	61.25399	0.507185335	0.680257	0.745579	0.455922	0.860324
CU682777.2	197.1033	-0.529970235	0.23538	-2.25155	0.024351	0.276029
alk	84.36782	-0.757376049	0.400337	-1.89185	0.058512	0.418172
AL954317.1	0.049519	0	5.267649	0	1	NA
CR354611.1	0.304068	-2.810267428	4.5283	-0.6206	0.534862	NA
si:dkey-185p13.1	2.527972	0.349655302	1.30581	0.267769	0.788877	0.963568
CR626882.2	0.141351	-1.710071829	5.238473	-0.32644	0.744088	NA
CU929426.1	1.121161	-0.994055669	2.061868	-0.48211	0.629725	NA
BX511173.1	0.871586	-0.682805381	1.976474	-0.34547	0.729744	NA
col24a1	158.5116	-0.1876268	0.222566	-0.84302	0.399219	0.831196
FP102167.1	53.30764	0.587477649	0.27779	2.114828	0.034445	0.327696
BX511100.4	0.180296	0.09368032	5.267649	0.017784	0.985811	NA
si:dkey-239n17.6	4.87593	0.076375586	0.896858	0.085159	0.932135	0.996053
CU672266.1	0.043308	0	5.267649	0	1	NA

CR352226.2	0.95918	1.055428652	2.379397	0.44357	0.657354	NA
CT574585.1	0.531359	0.130129317	2.81448	0.046236	0.963122	NA
si:ch211-57m13.3	0.046941	0	5.267649	0	1	NA
si:dkey-42i9.16	3.280359	-0.130277324	1.216212	-0.10712	0.914696	0.992972
si:ch211-163l21.11	3.588715	1.055429305	1.829394	0.576928	0.563988	0.901338
CR407550.1	0.043308	0	5.267649	0	1	NA
CU683879.1	1.538641	-0.481302774	1.659882	-0.28996	0.771845	NA
ptch1	0	NA	NA	NA	NA	NA
BX465849.1	0.043308	0	5.267649	0	1	NA
si:ch1073-441p17.1	2.551789	0.409963573	1.446019	0.283512	0.776785	0.961073
afp4	9181.283	-0.055301946	0.256502	-0.2156	0.8293	0.974609
ftr59	2.264869	1.698365573	1.346894	1.26095	0.207327	0.689456
oxr1a	703.6422	-0.010013976	0.122686	-0.08162	0.934947	0.996829
si:ch73-132k15.2	0.329184	-2.325990657	3.864576	-0.60187	0.547258	NA
AL844185.1	0	NA	NA	NA	NA	NA
AL845419.1	2.240509	0.701807338	1.349738	0.519958	0.603093	0.914226
FP067424.2	0	NA	NA	NA	NA	NA
CR352226.3	0.825242	-2.815325584	2.248323	-1.25219	0.210501	NA
CU993818.1	9.87245	-0.168830866	0.561661	-0.30059	0.763726	0.959055
wdr46	672.6345	-0.026384448	0.126077	-0.20927	0.834235	0.975731
FP325358.1	0	NA	NA	NA	NA	NA
BX001026.1	0.559687	-1.376215052	2.30037	-0.59826	0.549668	NA
si:ch211-57m13.9	0.52508	-2.828385393	4.625947	-0.61142	0.540923	NA
si:ch211-158m24.12	0	NA	NA	NA	NA	NA
BX248517.1	0.282968	2.077021334	4.683647	0.443462	0.657431	NA
znf236	220.407	0.07763559	0.14637	0.530407	0.59583	0.911427
si:ch73-30l9.1	5.858664	0.219567674	0.763986	0.287397	0.773808	0.960624
CR339054.1	0.087179	0	5.267649	0	1	NA
si:dkey-85n7.7	2.124895	-2.00182572	1.268697	-1.57786	0.114598	NA
BX511250.1	1.337988	2.51379282	2.334105	1.076984	0.281488	NA
BX571828.1	0.085528	0	5.267649	0	1	NA
pou3f3b	912.4331	0.167217657	0.087283	1.915802	0.05539	0.407556
atp5f1e	2515.829	-0.028792355	0.194392	-0.14812	0.882252	0.986908
BX664742.1	0.47721	1.923541921	4.000208	0.480861	0.630616	NA
BX088696.2	0.624505	0	2.852833	0	1	NA
col18a1b	169.9953	0.022536826	0.204697	0.110098	0.912331	0.992381
si:ch211-57m13.10	0.676598	0	3.150887	0	1	NA
prpf31	560.5006	0.07439489	0.127559	0.583221	0.559744	0.899644
BX927181.2	0.396107	0.093645742	3.82345	0.024492	0.98046	NA
CR848047.1	817.5831	0.216441229	0.10372	2.086775	0.036908	0.337141
AL928685.2	43.83412	0.05056124	0.266266	0.18989	0.849395	0.978148
sat1a.2	1272.669	-0.221898836	0.196732	-1.12793	0.259351	0.736127
BX539325.1	3.587483	0.47566083	1.497622	0.317611	0.75078	0.955074
si:ch211-149b19.4	13.64589	-1.760935525	1.607839	-1.09522	0.273421	0.749148
si:dkey-229b18.3	234.7312	0.228367244	0.163492	1.396808	0.162471	0.629825
si:ch1073-324l1.1	14.28906	0.418244351	0.475796	0.879042	0.379378	0.823515
si:dkey-209n16.2	9.589096	3.16142579	0.834527	3.788287	0.000152	0.012332
col6a3	31.92289	0.206386071	0.356369	0.579136	0.562497	0.900758
CR450738.1	0.086618	0	5.267649	0	1	NA
si:ch211-274k16.9	0.533582	-3.106564955	3.809798	-0.81541	0.414835	NA
si:ch73-236e11.2	15.18299	-0.144102043	0.550678	-0.26168	0.793567	0.965431
AL929171.1	1.179122	0.871751937	1.840126	0.473746	0.635681	NA
si:ch1073-325m22.2	3286.932	0.143199948	0.128133	1.117587	0.263743	0.741708
CR855300.1	92.10398	0.295939292	0.227942	1.298309	0.194181	0.674395

si:dkey-258f14.7	0.04222	0	5.267649	0	1	NA
si:ch211-57m13.1	0.195927	1.854123853	5.241768	0.353721	0.723548	NA
BX664742.2	0.433479	1.055407094	3.313963	0.318473	0.750126	NA
myha	280.1773	0.3743452	0.304448	1.229588	0.218852	0.699709
CU856621.1	0.175851	0	5.267649	0	1	NA
si:dkey-151m22.8	0.561692	-0.94203747	3.379706	-0.27873	0.780449	NA
tmem265	50.88432	0.450511371	0.312218	1.44294	0.149037	0.610726
si:ch73-226l13.2	20.44069	0	1.697092	0	1	1
si:dkey-19a16.12	1.575172	1.055432142	2.004185	0.526614	0.598462	NA
CT573247.1	2.118967	0.54962172	1.346339	0.408234	0.683102	NA
BX324207.1	4.987079	-0.996719191	0.777196	-1.28246	0.199683	0.6804
si:ch73-103b2.1	4.397208	0.550780422	0.9713	0.567055	0.570677	0.902481
CR936359.1	0	NA	NA	NA	NA	NA
si:ch73-341k19.1	37.16423	0.075444066	0.325356	0.231881	0.81663	0.971207
adkb	552.912	0.30571659	0.167191	1.828547	0.067467	0.443563
BX511100.5	0.803005	2.031833615	2.285813	0.888889	0.374063	NA
si:dkey-22i16.9	37.93432	-5.78150601	0.727237	-7.94996	1.87E-15	4.80E-12
BX465868.1	0.060442	0	5.267649	0	1	NA
BX890562.2	0.531174	1.869553699	3.147267	0.594025	0.552496	NA
CR352226.4	0	NA	NA	NA	NA	NA
si:ch211-57m13.6	0.310497	-0.933440633	4.421172	-0.21113	0.832786	NA
BX571828.2	0.045161	0	5.267649	0	1	NA
BX927257.1	0.049519	0	5.267649	0	1	NA
CU467655.1	11.47567	-1.762593636	1.617388	-1.08978	0.275811	0.750674
fhdc5	9.733997	-0.772015658	0.545957	-1.41406	0.157345	0.623263
si:ch73-361h17.1	178.9615	-1.269322791	0.668464	-1.89887	0.057582	0.413955
BX005108.3	4.623345	1.082092095	1.487203	0.727602	0.466857	0.865851
zgc:171699	0.45629	-1.715264651	3.628388	-0.47273	0.636402	NA
AL590145.1	0	NA	NA	NA	NA	NA
erlin2	0.059954	1.055396947	5.267649	0.200354	0.841203	NA
si:ch73-106g13.1	4.345898	-1.249236621	1.431546	-0.87265	0.382855	0.825721
CU856621.2	1.677683	0	2.233115	0	1	NA
BX511100.6	0.237432	-0.868128673	4.998236	-0.17369	0.862111	NA
si:ch73-211l13.2	275.7246	-0.273153739	0.167044	-1.63522	0.102003	0.528846
si:ch73-261i21.2	2.627418	0.473643924	1.387276	0.34142	0.732787	0.950617
CU469420.3	2.140698	-0.435049939	1.328783	-0.3274	0.743362	NA
si:ch211-161n3.5	2.281543	0	2.058893	0	1	1
CR391978.1	0	NA	NA	NA	NA	NA
AL807749.1	0	NA	NA	NA	NA	NA
si:ch211-64i20.5_2	0	NA	NA	NA	NA	NA
AL935166.1	57.04397	-0.253003689	0.288067	-0.87828	0.379791	0.823607
BX469910.1	0	NA	NA	NA	NA	NA
znf1073	9.030149	-4.285088331	0.950212	-4.50961	6.49E-06	0.001309
CR383662.4	2.451637	0.169292265	1.427365	0.118605	0.905588	0.991631
BX323801.1	0.516557	0	3.973174	0	1	NA
znf1112	20.07755	-0.420500887	0.391596	-1.07381	0.282907	0.755028
CR388052.1	16.54476	1.098592511	0.4936	2.225673	0.026036	0.286174
si:dkey-29j8.2	13.3026	0.861583541	0.658987	1.307437	0.191064	0.67094
si:dkey-240n22.8	11.12233	-0.061467593	0.517561	-0.11876	0.905462	0.991631
atp5md	1510.371	-0.079480448	0.182758	-0.43489	0.663639	0.931571
CT027801.1	0.890696	0	2.515107	0	1	NA
BX901962.1	0.15627	0	5.267649	0	1	NA
si:dkey-21h14.15	0	NA	NA	NA	NA	NA
si:dkeyp-4f2.3	22.02793	0.584839111	0.422072	1.385639	0.165857	0.635405

znf1115	10.31926	-0.981104249	0.824249	-1.1903	0.233928	0.71479
znf1146	0.792175	-1.742683259	2.462924	-0.70757	0.479214	NA
BX649337.1	1.453781	0.359899246	1.725682	0.208555	0.834796	NA
BX005012.3	2.834232	0	2.092065	0	1	1
si:ch211-178j18.4	0	NA	NA	NA	NA	NA
si:dkey-4e4.1	12.04784	0.96401803	0.554567	1.738325	0.082153	0.482431
si:dkey-262g12.7	16.04539	0.776891463	0.649709	1.195752	0.231793	0.713202
si:ch73-6k14.2	65.54366	1.400760496	0.743766	1.883336	0.059655	0.421147
si:ch73-289h5.5	0.147936	0	5.267649	0	1	NA
si:ch211-214j24.14	455.5257	0.117378214	0.164788	0.7123	0.476279	0.869292
BX784029.1	0	NA	NA	NA	NA	NA
si:ch211-162i8.2	2.909167	-1.142203373	1.089524	-1.04835	0.294477	0.763544
BX548032.2	0.207516	-0.748301491	5.238472	-0.14285	0.886411	NA
si:ch73-263o4.4	118.9113	0.312637357	0.212519	1.471106	0.141262	0.599899
BX649307.1	61.22213	0.510990469	0.728136	0.701779	0.482817	0.871821
si:dkey-21h14.3	0.136136	0	5.267649	0	1	NA
CR788248.1	0.049021	-0.86807634	5.267649	-0.16479	0.869106	NA
ccl39a.10	0.050624	1.055396947	5.267649	0.200354	0.841203	NA
si:dkey-191j3.1	2.332707	0.274004633	1.260267	0.217418	0.827883	0.973883
si:dkeyp-100h4.4	0.137262	0	5.267649	0	1	NA
si:dkey-156k2.7	0.377645	1.055386755	4.298731	0.245511	0.806061	NA
BX957333.1	0.221904	0	5.267649	0	1	NA
si:dkeyp-100h4.7	0	NA	NA	NA	NA	NA
taar20i	0.471389	0	3.677211	0	1	NA
BX927253.1	0.308813	-1.826689736	5.230459	-0.34924	0.726909	NA
col19a1	33.12268	1.231582714	0.453465	2.715936	0.006609	0.136957
BX649516.2	0.045161	0	5.267649	0	1	NA
BX649516.3	0.265949	-1.769045486	4.094714	-0.43203	0.665719	NA
CR381686.3	0.545889	-1.865340283	3.954916	-0.47165	0.637176	NA
LRRC56	9.294773	0.710605585	0.609873	1.16517	0.24395	0.724522
si:dkey-21h14.8	29.92633	0.946784994	0.380035	2.491307	0.012727	0.196596
si:dkey-256i11.6	0.421608	-2.340217508	4.955678	-0.47223	0.636763	NA
si:ch211-208g1.1	0.529233	1.055408041	3.458948	0.305124	0.760272	NA
taar20j	0.174358	0	5.267649	0	1	NA
taar20a	0.158701	0	5.267649	0	1	NA
BX927253.2	42.79113	-1.395090316	0.60279	-2.31439	0.020646	0.255657
BX548032.3	30.96384	0.980221086	0.370158	2.648115	0.008094	0.152599
buc	1.444447	-1.785496341	1.712012	-1.04292	0.296984	NA
si:dkeyp-98a7.4	0.18396	-0.868103029	5.267649	-0.1648	0.869102	NA
BX649399.1	0	NA	NA	NA	NA	NA
BX465862.2	4.95631	-0.538523261	1.03409	-0.52077	0.602527	0.914023
BX000981.5	0.1434	0	5.267649	0	1	NA
BX957333.2	0	NA	NA	NA	NA	NA
CU326364.1	3.317765	1.994230847	1.837613	1.085229	0.27782	0.751752
BX942819.3	1.107407	-0.203729587	2.100694	-0.09698	0.922741	NA
BX649516.4	6.637839	-0.147888196	0.676836	-0.2185	0.82704	0.973458
si:dkeyp-100h4.2	0	NA	NA	NA	NA	NA
zgc:123278	34.91952	-1.486616622	0.736436	-2.01866	0.043522	0.365455
CU570689.1	0	NA	NA	NA	NA	NA
BX784029.2	0	NA	NA	NA	NA	NA
ost4	348.7411	0.18112268	0.229467	0.789318	0.429926	0.847418
CR392001.2	0.04222	0	5.267649	0	1	NA
si:ch73-266f23.3	107.1263	-0.240605037	0.206165	-1.16705	0.24319	0.72442
CR381686.4	7.020356	-0.115624934	0.778504	-0.14852	0.881931	0.986908

BX469910.2	0.09233	-0.868089684	5.267649	-0.1648	0.869104	NA
BX649349.2	0.149233	-1.826663989	5.230461	-0.34924	0.726912	NA
BX119963.1	26.25086	0.873253131	0.598985	1.457888	0.144872	0.603718
CR388190.1	0	NA	NA	NA	NA	NA
zgc:173624	36.11857	0.132746599	0.41152	0.322577	0.747016	0.955074
CR388378.1	1.390914	-0.548650691	1.289449	-0.42549	0.670478	NA
CR388052.2	0.618333	1.559601936	2.933611	0.531632	0.594981	NA
BX465862.3	4.38131	-1.243228797	1.333193	-0.93252	0.351068	0.80227
CU469420.4	5.313049	-1.063078005	0.920666	-1.15468	0.24822	0.728489
si:dkey-21h14.9	1.191724	0	2.254207	0	1	NA
CU695141.1	0.073968	0	5.267649	0	1	NA
si:dkey-21h14.12	0.353276	0	5.193539	0	1	NA
BX469918.1	0.094182	-0.868089684	5.267649	-0.1648	0.869104	NA
BX927135.1	0.099037	0	5.267649	0	1	NA
BX664605.1	0.155545	0.959877153	5.236908	0.183291	0.85457	NA
taar20g	0	NA	NA	NA	NA	NA
taar20b	0	NA	NA	NA	NA	NA
FP015809.1	0	NA	NA	NA	NA	NA
znf1113	11.05019	-1.059776672	0.739251	-1.43358	0.151692	0.615406
CU469524.1	0	NA	NA	NA	NA	NA
si:dkey-21h14.12	0.394539	2.904065046	4.261813	0.681415	0.495609	NA
si:dkey-247i3.5	5.544449	-0.468196733	0.9297	-0.5036	0.614543	0.915998
si:dkey-21h14.10	0	NA	NA	NA	NA	NA
CT027801.2	1.808955	-0.074522636	1.347675	-0.0553	0.955902	NA
si:dkey-54j5.2	6.817213	-4.421046607	1.075766	-4.10967	3.96E-05	0.004708
CR381664.1	0	NA	NA	NA	NA	NA
si:ch211-112b1.2	2.823689	1.47402341	1.326406	1.111292	0.266443	0.742912
CR381664.2	0	NA	NA	NA	NA	NA
BX649518.1	0	NA	NA	NA	NA	NA
si:ch73-263o4.3	0.145246	-0.868103029	5.267649	-0.1648	0.869102	NA
si:dkeyp-100h4.8	0	NA	NA	NA	NA	NA
znf1050	11.96415	-0.109922305	0.607671	-0.18089	0.856453	0.979558
si:ch211-197k17.3	233.9266	-0.033512278	0.1656	-0.20237	0.839629	0.976417
CR293502.1	2.221215	0.796514805	1.058493	0.752499	0.451751	0.857676
si:dkey-51d8.9	11.78297	0.582954499	0.545253	1.069146	0.285004	0.756073
BX784029.3	0.209607	-0.868114638	5.267649	-0.1648	0.8691	NA
si:ch211-245n8.4	46.07251	-0.16743223	0.403974	-0.41446	0.678535	0.936026
nr6a1a	0	NA	NA	NA	NA	NA
znf1072	2.906838	-2.21435053	1.30092	-1.70214	0.088729	0.500206
si:dkey-260c8.8	18.96559	0.50990078	0.436964	1.166917	0.243244	0.72442
BX548046.1	0.842814	-3.359513753	2.020385	-1.66281	0.096351	NA
si:dkey-260c8.6	4.344136	-0.960294029	0.82875	-1.15873	0.246568	0.727706
tac4	0.229121	0.959863809	5.236908	0.183288	0.854572	NA
BX936397.1	1.250273	2.957736553	1.833334	1.61331	0.106677	NA
pimr206	0	NA	NA	NA	NA	NA
si:dkey-3d18.5	1.936365	-2.298253771	1.779779	-1.29131	0.196595	NA
FP017151.1	7.08944	-1.637364614	0.719478	-2.27577	0.02286	0.266968
ighv7-1	0	NA	NA	NA	NA	NA
si:ch211-76l23.4	303.5717	0.338101538	0.267673	1.263112	0.206549	0.688892
ighv1-3	0	NA	NA	NA	NA	NA
ighv4-3	0.050723	-0.86807634	5.267649	-0.16479	0.869106	NA
CU633478.1	0.266101	0	4.777809	0	1	NA
ighd	0.143059	0	5.267649	0	1	NA
ap1m1	994.6634	0.420843663	0.149897	2.807556	0.004992	0.117782

CT573429.2	3.707085	-0.349414236	0.961153	-0.36354	0.716204	0.94627
artna	3.330325	1.026529285	1.230543	0.834208	0.404164	0.833888
ighv8-1	0.174358	0	5.267649	0	1	NA
BX548060.1	1.750341	0.024056653	1.612301	0.014921	0.988095	NA
si:ch73-367p23.2	19655.77	0.150925273	0.206282	0.731644	0.464386	0.864593
si:dkey-15f23.1	0.40189	-1.067630608	4.961748	-0.21517	0.829633	NA
ighv4-1	0	NA	NA	NA	NA	NA
BX908803.1	0.04222	0	5.267649	0	1	NA
CU862078.1	0.21985	0	5.032833	0	1	NA
or124-4	0.123574	1.055383603	5.267649	0.200352	0.841205	NA
BX247899.1	0	NA	NA	NA	NA	NA
BX510989.1	4.737769	1.930467513	0.994297	1.941539	0.052193	0.396885
ighv5-2	0	NA	NA	NA	NA	NA
ighv4-7	0	NA	NA	NA	NA	NA
CR388364.1	6.387286	1.49770202	1.117976	1.339654	0.180358	0.656129
BX548046.2	61.7859	-0.434485902	0.300487	-1.44594	0.148194	0.609127
7-Mar	63.78067	0.14104483	0.236824	0.595568	0.551464	0.8974
si:dkey-3n22.9	8.287589	0.380543343	0.818616	0.464862	0.64203	0.925586
si:ch211-251g8.5	0.069091	0	5.267649	0	1	NA
ighv4-2	0	NA	NA	NA	NA	NA
ighv6-1	0.188426	1.854137197	5.241768	0.353724	0.723546	NA
CR786578.1	0	NA	NA	NA	NA	NA
nkd3	0.746712	0.831972576	3.222305	0.258192	0.796259	NA
ighz	0.411789	0	3.664566	0	1	NA
CU695199.1	0.205443	1.055361174	5.167193	0.204243	0.838164	NA
BX005368.1	0.791263	1.05541335	2.735644	0.385801	0.699644	NA
BX510989.2	0.060442	0	5.267649	0	1	NA
BX323555.3	0.284867	-1.733628439	4.675395	-0.3708	0.710788	NA
ighv4-8	0	NA	NA	NA	NA	NA
AL929243.1	39.54369	-0.209627815	0.307331	-0.68209	0.495182	0.87806
ighj1-2	0	NA	NA	NA	NA	NA
BX649612.1	0.760521	1.055406598	3.050842	0.345939	0.729388	NA
CR936968.1	0.485788	0	2.891063	0	1	NA
CR954285.1	0.249408	0.093666669	5.267649	0.017781	0.985813	NA
CR786578.2	0.648776	1.16265387	2.786033	0.417315	0.676448	NA
CR847785.1	4.553306	-1.42997944	0.839431	-1.70351	0.088472	0.499597
ighv1-4	0	NA	NA	NA	NA	NA
ighv6-2	0	NA	NA	NA	NA	NA
btr31	13.57839	0.23782696	0.588719	0.403974	0.686232	0.938732
si:ch73-269m14.3	0	NA	NA	NA	NA	NA
si:ch1073-342h5.2	87.55526	-0.077269268	0.220641	-0.3502	0.726186	0.948987
si:ch73-379f7.5	8.652125	0.615569214	0.647024	0.951386	0.341408	0.796175
ighv5-4	0	NA	NA	NA	NA	NA
AL627258.1	0	NA	NA	NA	NA	NA
wu:fj64h06	65.12165	0.117201771	0.306908	0.381879	0.702551	0.943591
si:dkey-211g8.8	0.617624	2.602160247	2.688897	0.967743	0.333173	NA
BX957317.2	16.66744	-0.593711964	0.393648	-1.50823	0.131495	0.583069
FP236454.1	0.817014	-3.111539303	2.004824	-1.55203	0.120656	NA
ighv10-1	0	NA	NA	NA	NA	NA
CU571254.1	2.182699	0.013847968	1.603721	0.008635	0.99311	NA
ighv5-8	0	NA	NA	NA	NA	NA
si:ch211-239j9.1	379.7267	0.077429301	0.214391	0.361159	0.717981	0.946533
CR352253.1	0.101247	1.854150541	5.241768	0.353726	0.723544	NA
ighj2-1	0	NA	NA	NA	NA	NA

si:ch73-379f7.4	2.797648	-0.51647736	1.05349	-0.49025	0.623954	0.919482
BX571681.4	0.699119	3.549621534	2.737656	1.296592	0.194772	NA
CU695077.1	0	NA	NA	NA	NA	NA
ighv4-9	0	NA	NA	NA	NA	NA
cd16412	79.13752	0.061364566	0.319921	0.191812	0.84789	0.977782
ighj2-5	0	NA	NA	NA	NA	NA
si:ch73-248e21.7	55.69189	0.073012234	0.268856	0.271567	0.785955	0.962605
ighv4-6	0	NA	NA	NA	NA	NA
BX323079.1	0.043309	0	5.267649	0	1	NA
ighv5-1	0	NA	NA	NA	NA	NA
CR792438.1	4.327245	-1.31999857	1.258411	-1.04894	0.294205	0.763323
ighv8-2	0	NA	NA	NA	NA	NA
ighv5-5	0	NA	NA	NA	NA	NA
ighv4-5	0	NA	NA	NA	NA	NA
si:ch211-85n16.3	141.0849	0.1877986	0.177852	1.055926	0.291002	0.761178
CR792426.1	0.202006	2.505652832	5.20297	0.481581	0.630103	NA
ighv1-2	0	NA	NA	NA	NA	NA
ighj1-1	0	NA	NA	NA	NA	NA
BX957260.1	0.090321	0	5.267649	0	1	NA
ighv1-4	2.4657	-2.038310864	1.405234	-1.45051	0.146915	0.606995
FP236809.1	0	NA	NA	NA	NA	NA
ighv3-2	0	NA	NA	NA	NA	NA
BX957344.1	0.192986	1.854123853	5.241768	0.353721	0.723548	NA
eppk1	177.3619	-0.358527413	0.311936	-1.14936	0.250406	0.730168
si:ch211-229c8.14	0.049021	-0.86807634	5.267649	-0.16479	0.869106	NA
BX927298.1	1.927143	0.700005387	1.662649	0.421018	0.673742	NA
si:ch211-195m9.3	53.01146	-0.754363051	0.327399	-2.30411	0.021216	0.258321
ighj2-3	0	NA	NA	NA	NA	NA
fnbp1b	3.603513	-1.117382472	1.006647	-1.11	0.266997	0.743418
neu3.5	11.51539	0.439375298	0.833344	0.527243	0.598025	0.912615
CT573133.2	0	NA	NA	NA	NA	NA
ighv13-2	0	NA	NA	NA	NA	NA
BX936397.2	11.15377	0.199373595	0.584922	0.340855	0.733213	0.95078
7-Mar	113.9769	0.14496556	0.233971	0.619587	0.53553	0.891993
ighv2-3	0.049519	0	5.267649	0	1	NA
BX890590.1	0.043308	0	5.267649	0	1	NA
defbl2	4.665613	1.946204408	1.526234	1.275168	0.20225	0.682413
neu3.4	5.333752	1.055436107	1.772987	0.595287	0.551652	0.8974
neu3.1	51.06126	-0.554510886	0.271212	-2.04457	0.040898	0.353713
BX572622.1	0	NA	NA	NA	NA	NA
si:ch211-162i14.1	0.240643	1.921554081	5.236909	0.366925	0.713675	NA
ighv9-3	0	NA	NA	NA	NA	NA
ighv1-1	0	NA	NA	NA	NA	NA
ighv5-3	0	NA	NA	NA	NA	NA
sox4a	0	NA	NA	NA	NA	NA
ighv4-3	0	NA	NA	NA	NA	NA
ighv11-1	0	NA	NA	NA	NA	NA
BX511272.1	1.250609	0.516753579	1.730646	0.29859	0.765253	NA
si:ch211-276a17.5	38.08558	-2.490385461	0.705941	-3.52776	0.000419	0.024584
AL935145.1	0.243482	2.667686611	5.195716	0.51344	0.607644	NA
CU570787.1	0.622842	0	3.284356	0	1	NA
CT027638.1	0.043309	0	5.267649	0	1	NA
ighv2-1	0	NA	NA	NA	NA	NA
ighv11-2	0	NA	NA	NA	NA	NA

CR792438.2	1.096736	-1.442745291	1.691743	-0.85282	0.393761	NA
si:dkey-261j15.2	167.4964	-0.233470668	0.161244	-1.44793	0.147637	0.608558
si:ch211-15j1.7	5.66686	0.933093231	0.846369	1.102466	0.270259	0.746532
CU074421.1	0	NA	NA	NA	NA	NA
raf1a	148.4489	-0.334061305	0.175564	-1.90279	0.057068	0.412539
BX470238.1	6.375115	-0.489087831	0.745095	-0.65641	0.51156	0.884872
ighv14-1	0	NA	NA	NA	NA	NA
CU659670.1	238.2291	0.022529432	0.150408	0.149788	0.880932	0.986846
CR936968.2	32.64474	-0.142552171	0.65219	-0.21857	0.826981	0.973458
BX324146.1	109.7947	-0.194892476	0.289304	-0.67366	0.500527	0.880311
pimr205	0.15032	-1.826663989	5.230461	-0.34924	0.726912	NA
ighv2-2	0	NA	NA	NA	NA	NA
si:dkey-217d24.6	220.4991	-0.44046639	0.176262	-2.49893	0.012457	0.194346
BX663606.1	2.044705	-0.205380333	1.418864	-0.14475	0.884908	NA
ighv9-1	0.335504	0	5.267649	0	1	NA
rock2b	42.42083	1.259936301	0.292565	4.306513	1.66E-05	0.002614
BX901907.1	0.267064	0	5.267649	0	1	NA
AL845295.1	0.046941	0	5.267649	0	1	NA
BX005275.1	0	NA	NA	NA	NA	NA
si:dkey-26h11.1	0	NA	NA	NA	NA	NA
si:ch211-214p16.3	99.1942	-0.573882006	0.492146	-1.16608	0.243582	0.724437
AL929009.1	0	NA	NA	NA	NA	NA
BX005461.1	1.215117	-2.732171448	1.989883	-1.37303	0.169743	NA
si:ch211-194e1.7	4.902434	-3.082714208	1.487569	-2.07232	0.038236	0.342672
CR391935.1	0.203741	-0.868114637	5.267649	-0.1648	0.8691	NA
si:ch211-196f5.9	31.63254	0.128159022	0.356771	0.359219	0.719431	0.94694
CR383672.2	1.092936	-2.748373088	2.189292	-1.25537	0.209344	NA
ap1m2	182.7332	0.205581382	0.239067	0.859931	0.389827	0.827843
BX005275.2	1.557197	-0.508172274	1.58308	-0.321	0.748209	NA
si:dkey-118j18.2	470.333	-0.176135597	0.108546	-1.62269	0.104656	0.53308
si:dkey-26h11.2	0	NA	NA	NA	NA	NA
CR450696.1	0	NA	NA	NA	NA	NA
CR382294.1	0.745369	-1.133810501	1.945312	-0.58284	0.559999	NA
BX571664.1	0	NA	NA	NA	NA	NA
si:dkeyp-82h4.1	0.743924	2.477575949	2.428188	1.020339	0.307568	NA
FP103106.1	1.106688	0	2.132309	0	1	NA
si:ch211-246n15.2	0	NA	NA	NA	NA	NA
BX571825.13	0	NA	NA	NA	NA	NA
AL845295.2	0	NA	NA	NA	NA	NA
mcat	0	NA	NA	NA	NA	NA
BX571825.14	0	NA	NA	NA	NA	NA
BX901907.2	0.996098	0.928067734	2.063746	0.4497	0.652926	NA
CU914550.1	1.546309	3.89505644	1.835498	2.12207	0.033832	NA
si:dkey-16p19.8	0.509713	-2.293556462	5.204054	-0.44072	0.659412	NA
BX571825.15	0	NA	NA	NA	NA	NA
si:ch211-181p13.2	1.51433	-0.537465344	1.93498	-0.27776	0.781194	NA
BX901907.3	0	NA	NA	NA	NA	NA
BX248324.1	82.69917	0.028607889	0.232329	0.123135	0.902	0.990847
si:ch211-73m21.1	0.439923	1.055404357	3.598262	0.293309	0.769286	NA
BX901907.4	0.088469	0	5.267649	0	1	NA
BX571825.16	0	NA	NA	NA	NA	NA
BX571705.1	7.448158	-0.248946009	0.671492	-0.37074	0.710834	0.945821
si:dkey-22h13.3	4.107246	0.354271132	1.102603	0.321304	0.74798	0.955074
BX324003.1	0.640068	1.400786846	2.24735	0.623306	0.533083	NA

BX088688.1	55.36799	-0.657237653	0.502849	-1.30703	0.191203	0.671109
BX927068.1	0	NA	NA	NA	NA	NA
BX890559.2	0	NA	NA	NA	NA	NA
si:ch73-103l1.2	33.19599	-1.37614066	0.427078	-3.22222	0.001272	0.052006
si:dkey-58j15.11	2.800878	0.717362153	1.050345	0.682978	0.494621	0.877987
si:ch211-1f22.8	5.95174	0	1.815775	0	1	1
BX322618.1	931.3803	-0.391371651	0.261851	-1.49463	0.13501	0.59
si:dkey-27j5.9	0.617628	0	2.821866	0	1	NA
BX842692.1	0.307223	1.942013074	4.494807	0.432057	0.6657	NA
BX547930.2	0.043308	0	5.267649	0	1	NA
si:ch211-286b5.2	3.804076	-0.987098217	1.293668	-0.76302	0.44545	0.855403
si:dkey-58j15.10	0.769607	1.05542199	2.522313	0.418434	0.67563	NA
CT033796.1	4.641218	-2.982991687	1.429747	-2.08638	0.036944	0.337141
irbpl	7.289103	0.087203109	1.138597	0.076588	0.938951	0.997371
AL929349.1	0	NA	NA	NA	NA	NA
CR626889.1	1.506505	0	2.352113	0	1	NA
si:ch211-176g6.1	0.126661	0	5.267649	0	1	NA
si:ch73-94k4.5	0.279694	3.310194291	5.173718	0.63981	0.522296	NA
AL928650.2	0.742333	0.722526553	2.529771	0.285609	0.775177	NA
BX548015.2	7.661026	-0.029956418	2.342259	-0.01279	0.989796	1
si:ch73-236c18.9	76.36588	-0.050121484	0.246002	-0.20374	0.838553	0.976395
AL929396.1	1.213068	-0.96188675	1.60847	-0.59801	0.549831	NA
si:dkey-202c14.3	3.067512	0.530783192	1.026721	0.516969	0.605178	0.914985
rltgr	294.4098	-0.179474982	0.22138	-0.81071	0.417533	0.841149
si:dkey-27j5.10	0.272039	0	5.267649	0	1	NA
CR854964.1	0	NA	NA	NA	NA	NA
CU469541.1	0.089161	0	5.267649	0	1	NA
BX088688.2	4.992502	0.616223611	0.86669	0.711008	0.477079	0.869292
BX901898.1	0.088469	0	5.267649	0	1	NA
BX901942.1	0.346254	-1.714183198	3.842555	-0.44611	0.655521	NA
BX649533.1	0.23509	0	4.959557	0	1	NA
si:ch211-1f22.7	0	NA	NA	NA	NA	NA
CR774189.1	2.449516	2.360650482	1.367537	1.726206	0.08431	0.487271
si:ch211-191a16.5	14.34961	-1.744257498	0.793005	-2.19955	0.027839	0.296561
nrp2a	831.9437	0.108628357	0.11348	0.95725	0.338441	0.795226
CR925879.1	1.46783	-0.878976719	1.561255	-0.56299	0.573439	NA
rac1l	1.724243	0	2.107664	0	1	NA
CR774192.1	0.150865	0.09368032	5.267649	0.017784	0.985811	NA
BX681416.1	0	NA	NA	NA	NA	NA
BX248126.1	0	NA	NA	NA	NA	NA
si:dkey-25o16.4	332.7387	0.270914589	0.251074	1.079021	0.280578	0.753147
CT573186.2	0.188905	-0.868102747	5.267649	-0.1648	0.869102	NA
si:dkey-19f4.2	1.940126	-4.847072152	1.863301	-2.60134	0.009286	NA
CR556696.1	7.26145	0.221104991	0.884322	0.250028	0.802566	0.968181
CU986306.1	0	NA	NA	NA	NA	NA
BX649231.1	1.728306	0.12240984	1.485019	0.08243	0.934305	NA
wu:fj29h11	198.6582	0.129389758	0.301793	0.428737	0.668115	0.933086
BX322603.1	11.28126	1.838084797	3.182417	0.577575	0.563551	0.901075
CU915827.1	6.226793	0.12926397	0.883265	0.146348	0.883647	0.987014
CR392024.1	1.066491	1.371955454	1.922708	0.713554	0.475503	NA
zgc:165656	30.4578	0.467909044	0.292094	1.601911	0.109175	0.542034
si:ch211-155o21.4	3.645493	-1.366796445	1.099134	-1.24352	0.213676	0.696139
BX470128.1	0.127749	0	5.267649	0	1	NA
BX248418.1	58.14703	-0.135241334	0.254742	-0.5309	0.595491	0.911376

si:dkey-27j5.7	0.294079	2.614137966	4.562882	0.572914	0.566703	NA
CR382370.1	0	NA	NA	NA	NA	NA
FP067451.1	0.449944	-0.868144008	3.71787	-0.23351	0.815369	NA
si:ch211-152f2.4	0.337756	-0.8681335	3.90303	-0.22243	0.823983	NA
si:dkey-182i3.9	16.39515	-0.65389319	0.511598	-1.27814	0.2012	0.681045
si:ch211-286b5.6	1.348465	-0.974337773	1.671079	-0.58306	0.559853	NA
adgre9	1.249704	0.074985839	2.047917	0.036616	0.970791	NA
cul4a	782.8302	-0.000885026	0.118106	-0.00749	0.994021	1
si:dkey-9c18.3	0.202494	2.850015501	5.18846	0.549299	0.5828	NA
BX323590.3	0.486913	-2.32655697	3.520269	-0.6609	0.508674	NA
AL928835.1	0.507831	2.538433637	2.715037	0.934954	0.349812	NA
BX004779.1	0.292408	-0.8072091	3.987657	-0.20243	0.839583	NA
si:ch211-1f22.9	0.134939	0	5.267649	0	1	NA
MFAP4 (1 of many)	10.00276	2.523477233	1.554024	1.623834	0.104411	0.53263
itgam	2.868382	0.923600205	1.61762	0.570963	0.568025	0.902004
adgre15	0.995486	-1.734653529	2.133652	-0.813	0.41622	NA
BX088594.1	0	NA	NA	NA	NA	NA
BX908780.2	0.045161	0	5.267649	0	1	NA
CR478288.1	0	NA	NA	NA	NA	NA
lratb.1	80.73985	-0.137624954	0.262474	-0.52434	0.600044	0.913756
BX296516.1	0	NA	NA	NA	NA	NA
si:ch1073-228b5.2	85.40489	-0.195328495	0.17991	-1.0857	0.277611	0.751607
BX322618.2	0.059954	1.055396947	5.267649	0.200354	0.841203	NA
AL928650.3	8.379573	-3.22012094	1.076449	-2.99143	0.002777	0.086456
si:ch1073-357b18.4	119.4853	-0.158123237	0.219097	-0.7217	0.470477	0.867743
bmb	30.91976	0.520960143	0.607824	0.857091	0.391395	0.827902
si:ch73-94k4.4	0	NA	NA	NA	NA	NA
CR626902.1	1.069528	2.775322139	1.988441	1.395728	0.162796	NA
BX649499.2	0	NA	NA	NA	NA	NA
BX465225.1	0.049021	-0.86807634	5.267649	-0.16479	0.869106	NA
CU929161.1	0	NA	NA	NA	NA	NA
si:ch73-212j7.3	11.85506	-0.160228048	0.637334	-0.2514	0.801502	0.967776
si:dkey-182i3.10	70.35052	0.198631865	0.297357	0.667991	0.50414	0.881759
BX469930.2	0	NA	NA	NA	NA	NA
si:ch211-255p10.3	2226.913	-0.195598407	0.198663	-0.98457	0.324834	0.783673
BX469908.1	2.207125	0.036993982	1.243676	0.029746	0.97627	1
BX000534.1	1.031451	-1.419523693	2.537108	-0.5595	0.575817	NA
si:ch211-1f22.10	0.238995	-0.868109223	5.267649	-0.1648	0.869101	NA
tube1	0.50067	0.093651319	2.924105	0.032027	0.97445	NA
si:dkey-27j5.6	0.08553	0	5.267649	0	1	NA
adgre16	1.165748	0.093647777	2.266904	0.041311	0.967048	NA
si:ch211-1f22.13	0.307608	2.506241582	4.563305	0.549216	0.582857	NA
si:dkey-187i8.2	0.918266	1.055408269	2.773997	0.380465	0.7036	NA
si:ch211-286b5.8	3.21795	-0.090368993	1.213712	-0.07446	0.940647	0.997371
CU914144.1	11.35745	-2.744421172	1.092729	-2.51153	0.012021	0.190742
BX908780.3	0	NA	NA	NA	NA	NA
buc2l	1.074914	1.055430091	2.137113	0.493858	0.621407	NA
BX908756.1	0	NA	NA	NA	NA	NA
MED9	208.4492	-0.096269713	0.181136	-0.53148	0.595089	0.910894
CR382370.2	457.8953	-0.002298055	0.125934	-0.01825	0.985441	1
si:ch73-94k4.1	0	NA	NA	NA	NA	NA
si:ch211-122h15.4	1.780395	-2.389645675	1.724025	-1.38609	0.165721	NA
si:dkey-11c5.11	56.45903	0.208395828	0.284774	0.731793	0.464295	0.864593
BX510992.2	2.417534	0.354906048	1.078463	0.329085	0.742091	0.954329

BX276101.1	34.89739	-0.010555358	0.346418	-0.03047	0.975692	1
BX649471.1	0	NA	NA	NA	NA	NA
AL954187.1	0.101247	1.854150541	5.241768	0.353726	0.723544	NA
BX908780.4	0.426838	1.055406637	3.570826	0.295564	0.767563	NA
adgre18	0.312476	0	3.464643	0	1	NA
si:ch211-131k2.2	10.95508	-0.668104415	0.712974	-0.93707	0.348724	0.801264
BX296520.1	0.063619	0	5.267649	0	1	NA
BX908780.5	0	NA	NA	NA	NA	NA
adgre19	0.384226	-0.868135737	3.317312	-0.2617	0.793554	NA
si:dkey-27j5.11	0	NA	NA	NA	NA	NA
gpr108	701.2731	-0.088305952	0.103647	-0.85199	0.394219	0.828374
si:dkey-119m7.8	96.61533	0.175941602	0.223111	0.788585	0.430355	0.847541
BX901920.1	547.7793	0.762565632	0.206789	3.687646	0.000226	0.016037
si:ch211-1f22.6	0.111164	-0.868089684	5.267649	-0.1648	0.869104	NA
BX547930.3	0.045161	0	5.267649	0	1	NA
si:dkey-4p15.4	0.098667	-0.868089684	5.267649	-0.1648	0.869104	NA
si:ch211-286b5.5	487.725	-0.312838549	0.139445	-2.24345	0.024868	0.279374
abcc3	406.6006	-0.010709232	0.1836	-0.05833	0.953486	0.998436
si:dkey-249p7.4	1.677911	1.427149816	1.545828	0.923227	0.355889	NA
cxl34c	0.191244	0	5.267649	0	1	NA
si:ch211-202n12.2	2.212458	0.06555025	1.367323	0.047941	0.961764	0.999451
si:ch211-1f22.2	1.025863	-2.771940152	2.294604	-1.20803	0.227037	NA
hemgn	608.1885	0.157008124	0.314162	0.499768	0.617239	0.916365
MZT1	363.6653	-0.014473642	0.207648	-0.0697	0.94443	0.997371
si:ch73-301j1.1	2.91182	0.244614309	1.209491	0.202246	0.839725	0.976417
si:ch211-1f22.16	0.601082	1.928305839	2.810498	0.686108	0.492645	NA
si:dkey-27j5.10	0	NA	NA	NA	NA	NA
BX296562.1	0.120883	0	5.267649	0	1	NA
AL845365.1	0	NA	NA	NA	NA	NA
CR450698.1	0.086617	0	5.267649	0	1	NA
BX004780.1	2.913439	-1.740957217	1.945541	-0.89484	0.37087	0.818484
AL929237.3	2.177696	0.820680966	1.38516	0.592481	0.553529	NA
BX927123.1	0.426853	-0.859650083	3.68709	-0.23315	0.815644	NA
si:dkey-1f1.3	17.12971	-0.057342142	0.544442	-0.10532	0.91612	0.993238
BX928755.1	0.043309	0	5.267649	0	1	NA
CR385078.2	0	NA	NA	NA	NA	NA
BX927300.1	6.295644	0.610603072	0.945503	0.645797	0.518411	0.887148
CU565528.1	0.050723	-0.86807634	5.267649	-0.16479	0.869106	NA
CR382372.1	32.71905	-0.191560196	0.412481	-0.46441	0.642354	0.92567
CT573003.1	16.17159	-0.485285416	1.365852	-0.3553	0.722366	0.947586
rgs7b	28.77119	0.114428216	0.40901	0.279769	0.779655	0.961073
CU660013.1	3.540718	1.498989407	0.946844	1.583142	0.113389	0.551243
si:dkey-66i24.9	76.55362	0.401927736	0.238471	1.685437	0.091904	0.507669
CR376838.1	0.073968	0	5.267649	0	1	NA
CU929359.1	0.086616	0	5.267649	0	1	NA
CR388157.1	0.653528	-0.868145332	3.437627	-0.25254	0.800622	NA
CU914156.1	0.240446	2.56148978	5.200379	0.492558	0.622325	NA
AL928716.1	0	NA	NA	NA	NA	NA
CR932360.1	0	NA	NA	NA	NA	NA
BX072570.1	0.186781	0	5.267649	0	1	NA
BX119915.1	0.101446	-1.755425691	5.23528	-0.33531	0.737394	NA
AL928920.2	0.219256	0	5.267649	0	1	NA
FP565451.1	2.603151	-0.235026582	1.223855	-0.19204	0.847712	0.97772
si:dkey-21e13.3	69.97724	-1.070116637	0.391715	-2.73188	0.006297	0.134482

CU550702.1	0.449774	0	2.925764	0	1	NA
si:dkey-1h6.8	12.09874	0.308106316	0.603494	0.510537	0.609675	0.915204
BX001014.1	0.907788	-0.739988641	1.923509	-0.38471	0.700454	NA
CR847530.1	0.276258	-0.749440561	4.708684	-0.15916	0.873542	NA
CR388421.1	0.272563	0	5.267649	0	1	NA
BX469896.1	10.26544	-0.432366557	0.59853	-0.72238	0.470061	0.867349
si:dkeyp-104f11.8	3.142294	-0.794405842	1.071214	-0.74159	0.458333	0.861001
BX255913.1	0.119909	2.079562922	5.22636	0.397899	0.690705	NA
BX470246.1	0.302632	0	4.608093	0	1	NA
si:ch211-194k22.8	588.3268	-0.12148491	0.110475	-1.09966	0.271481	0.74783
si:dkey-193p11.2	618.9376	-0.910367846	0.199393	-4.56571	4.98E-06	0.001059
CR388040.1	24.72558	-0.050265496	0.63992	-0.07855	0.937391	0.997305
si:dkey-66i24.7	88.55571	0.076459003	0.192379	0.39744	0.691043	0.940173
si:dkeyp-104f11.9	0.312002	-1.710093081	5.238471	-0.32645	0.744085	NA
hmx3b	5.089303	-0.668279631	1.155353	-0.57842	0.56298	0.900772
AL732488.1	201.6418	0.202128805	0.192316	1.051023	0.293248	0.762581
BX323889.1	13.8126	-0.383058009	0.517894	-0.73965	0.459515	0.862103
CR352342.1	0.063619	0	5.267649	0	1	NA
si:rp71-1c10.11	648.8068	-0.176316401	0.216183	-0.81559	0.414736	0.838698
si:dkey-16i5.8	15.77355	0.100499462	1.101917	0.091204	0.92733	0.994973
CU660013.2	1.081873	2.461394167	2.367617	1.039608	0.298522	NA
BX248238.1	0.24034	2.436374376	5.206324	0.467964	0.63981	NA
si:dkey-21c1.1	323.7805	-0.154496801	0.135502	-1.14018	0.254212	0.733304
si:ch211-250m6.2	0.156447	0	5.267649	0	1	NA
CT737227.1	3.779178	3.287147761	1.511709	2.174459	0.029671	0.306672
cpn1	0	NA	NA	NA	NA	NA
CR385078.3	0	NA	NA	NA	NA	NA
BX569783.1	0.990448	-1.79575856	2.058899	-0.87219	0.383103	NA
CR847530.2	0.045161	0	5.267649	0	1	NA
BX255897.1	0	NA	NA	NA	NA	NA
BX855623.1	1.370666	1.055419222	2.432682	0.43385	0.664397	NA
BX255963.1	0.677079	2.76544505	2.199751	1.257163	0.208695	NA
BX248390.1	2.319933	1.968032009	1.863283	1.056217	0.290869	0.761002
CR749163.2	2.524312	-0.634210905	1.416558	-0.44771	0.654361	0.929488
si:zfos-911d5.4	1.43247	-0.868142501	2.05371	-0.42272	0.6725	NA
si:dkey-219e21.2	61.02843	-0.920889704	0.717858	-1.28283	0.199552	0.680344
CR405682.1	0.198989	0.093667081	5.22139	0.017939	0.985687	NA
BX088644.1	0.093931	1.055383603	5.267649	0.200352	0.841205	NA
CU469493.1	0	NA	NA	NA	NA	NA
si:dkey-22l1.1	0	NA	NA	NA	NA	NA
AL928824.2	0	NA	NA	NA	NA	NA
CR385078.4	0	NA	NA	NA	NA	NA
CR381684.1	0	NA	NA	NA	NA	NA
BX296562.2	0	NA	NA	NA	NA	NA
asb2a.1	2.993733	2.796127502	1.490394	1.876099	0.060642	0.423018
NDUFAF8	87.35758	0.00500586	0.277117	0.018064	0.985588	1
BX001014.2	25.62047	0.323464942	0.33715	0.95941	0.337352	0.794355
BX842669.1	1.170433	0.39813646	1.4277	0.278866	0.780348	NA
CU459210.1	0.087179	0	5.267649	0	1	NA
AL928824.3	0.084441	0	5.267649	0	1	NA
CU570682.1	0.043309	0	5.267649	0	1	NA
si:ch211-202p1.5	5.747954	-0.704155148	0.917644	-0.76735	0.442873	0.853998
si:ch211-196c10.13	24.71395	0.899840721	0.740083	1.215864	0.224037	0.705807
si:ch73-205h11.1	2.229629	-1.652437983	1.316443	-1.25523	0.209395	0.691505

BX901952.1	0	NA	NA	NA	NA	NA
BX255913.2	0.284691	0	5.267649	0	1	NA
si:rp71-1c10.8	1.334577	2.307608539	1.94183	1.188368	0.234688	NA
CT573006.1	1.076119	1.92416512	1.934727	0.994541	0.31996	NA
si:dkey-61p9.11	19.39065	1.776858813	0.665198	2.671172	0.007559	0.14728
CU694525.1	0	NA	NA	NA	NA	NA
BX510919.1	0.084441	0	5.267649	0	1	NA
pimr124	0.175084	0	5.267649	0	1	NA
BX510920.1	0.060442	0	5.267649	0	1	NA
BX255897.2	0.341812	2.079522788	5.22636	0.397891	0.69071	NA
CR932077.1	6.134229	-0.677678525	0.786569	-0.86156	0.388928	0.827725
CU571074.1	347.8871	0.232886726	0.161895	1.438507	0.15029	0.613933
BX510319.1	4.395569	-1.479189169	1.101912	-1.34238	0.179471	0.654677
pimr128	0.773165	-3.312697549	2.512414	-1.31853	0.187326	NA
BX088686.1	2.929713	-2.137204119	1.070444	-1.99656	0.045873	0.374848
BX119992.1	0.326167	-2.340632107	3.894584	-0.601	0.547842	NA
CCDC189	3.323645	-0.159167303	0.814265	-0.19547	0.845022	0.976791
AL928920.3	0.837832	2.891982939	2.874946	1.005926	0.314451	NA
BX294177.1	0.170532	2.594231922	5.198905	0.498996	0.617782	NA
CR932360.2	0.176993	-0.868103029	5.267649	-0.1648	0.869102	NA
CR932362.1	0.630144	-3.349637719	2.383876	-1.40512	0.159985	NA
si:dkey-148d16.5	0.104229	-1.791411807	5.232816	-0.34234	0.732094	NA
CU929359.2	4.866016	-0.788334952	1.748862	-0.45077	0.652155	0.928768
CU660013.3	0.090249	0	5.267649	0	1	NA
si:ch211-28p3.3	42.42525	-0.545337426	0.295436	-1.84587	0.06491	0.434811
selp	0.084441	0	5.267649	0	1	NA
CR352243.1	0	NA	NA	NA	NA	NA
BX248238.2	0.152284	0.09368032	5.267649	0.017784	0.985811	NA
BX569801.2	0.333187	2.076152944	4.536068	0.457699	0.647169	NA
si:ch73-344o19.1	61.1829	0.415413464	0.30739	1.351423	0.17656	0.649967
BX004999.1	21.92483	0.152145021	0.513418	0.296337	0.766972	0.960296
BX842240.1	0.321822	0.007697392	4.497805	0.001711	0.998635	NA
BX119915.2	1.529219	0.996981786	2.069027	0.48186	0.629905	NA
CR848736.1	7.655783	-1.195105787	0.840437	-1.42201	0.155025	0.619776
CR751608.1	0.08847	0	5.267649	0	1	NA
si:ch73-127m5.2	239.1044	-0.058274362	0.138297	-0.42137	0.673485	0.934323
BX322665.3	0.04222	0	5.267649	0	1	NA
BX004803.1	0	NA	NA	NA	NA	NA
si:ch211-277e21.1	2.775979	0.318301651	1.228615	0.259074	0.795578	0.966077
si:dkeyp-13d11.1	0	NA	NA	NA	NA	NA
CU660013.4	148.1307	-0.022140653	0.19053	-0.11621	0.90749	0.991745
si:dkey-219e21.4	60.87886	-0.703516433	0.376025	-1.87093	0.061355	0.424867
BX119915.3	0.264541	0	4.783328	0	1	NA
BX957286.1	0	NA	NA	NA	NA	NA
si:dkey-287g12.6	97.60832	-0.223535404	0.256834	-0.87035	0.38411	0.826565
si:ch211-168b3.2	54.82245	0.049121589	0.388403	0.126471	0.899359	0.99045
BX279523.1	15.14951	-3.181452124	0.772749	-4.11706	3.84E-05	0.004607
AL833789.1	0.336111	0	5.19772	0	1	NA
AL928920.4	0.049519	0	5.267649	0	1	NA
BX901968.1	0	NA	NA	NA	NA	NA
si:dkeyp-106c3.2	1.704093	-0.070381366	1.388043	-0.05071	0.95956	NA
CR854832.1	0.136102	0	5.267649	0	1	NA
zgc:136683	0.575064	-2.413213343	5.198537	-0.46421	0.642497	NA
CR396584.1	2.158765	-1.760512733	1.905118	-0.9241	0.355436	NA

si:ch211-256e16.7	0.84372	3.467620215	2.414848	1.435958	0.151014	NA
CR847898.1	5.025185	-1.324226569	0.779937	-1.69786	0.089534	0.502318
BX571796.1	0.086618	0	5.267649	0	1	NA
CU914157.1	11.42637	-4.046979481	1.151144	-3.51561	0.000439	0.025327
BX323575.1	0.050723	-0.86807634	5.267649	-0.16479	0.869106	NA
BX279523.2	0	NA	NA	NA	NA	NA
si:ch73-144d13.7	13.93284	0.399653173	0.484093	0.825571	0.409048	0.836637
blvrb	751.8636	0.16830821	0.180661	0.931623	0.351531	0.802382
mhc1lja	9.513196	0.186359383	1.060586	0.175714	0.860519	0.980264
CR774195.1	0	NA	NA	NA	NA	NA
BX569793.1	17.89385	1.1661906	0.52806	2.208441	0.027214	0.293137
CR391963.1	28.69696	0.025041091	0.444622	0.05632	0.955087	0.998611
si:ch211-155k24.9	64.34833	-0.119576528	0.27238	-0.43901	0.660658	0.930581
CR450814.1	0.14609	1.055370722	5.267649	0.200349	0.841207	NA
AL807792.1	3.118644	0.926609179	1.266509	0.731624	0.464398	0.864593
CU013548.1	1.100862	-1.800316565	2.187737	-0.82291	0.410558	NA
CR848791.1	0	NA	NA	NA	NA	NA
CU929322.1	0.451585	-2.708159125	4.877299	-0.55526	0.578718	NA
CU929206.1	1.276791	0.645215853	1.947584	0.33129	0.740425	NA
CT573157.1	0.098043	-1.71005882	5.238474	-0.32644	0.74409	NA
zgc:193593	75.71712	0	1.678109	0	1	1
BX927351.1	0	NA	NA	NA	NA	NA
BX649487.1	0	NA	NA	NA	NA	NA
CU855854.1	0.132469	0	5.267649	0	1	NA
CR855311.2	0.793479	-1.801967316	2.241986	-0.80374	0.421549	NA
si:dkey-16p21.8	4395.783	0.005252158	0.11224	0.046794	0.962677	0.999607
BX957346.1	367.5073	-0.056444404	0.18613	-0.30325	0.761697	0.958242
znf1143	96.10214	-0.581711097	0.288164	-2.01868	0.043521	0.365455
BX572077.1	11.28908	-0.818314596	0.602355	-1.35853	0.174297	0.647604
si:ch73-1f23.1	0.86837	0	2.541178	0	1	NA
BX649324.1	6.035656	0.106861803	0.890151	0.120049	0.904444	0.991581
znf1012	8.622096	-0.577000639	0.636489	-0.90654	0.364652	0.814648
CU469362.1	74.95145	-0.403028832	0.231871	-1.73816	0.082183	0.482436
BX927361.1	0.579344	-2.808183135	3.391649	-0.82797	0.407688	NA
si:ch211-286b4.4	13.44647	-0.7501005	0.746319	-1.00507	0.314865	0.777649
CU207301.1	0	NA	NA	NA	NA	NA
CR936975.1	3.307789	-1.409604068	1.261618	-1.1173	0.263867	0.741875
si:ch73-380l3.4	3.952906	-0.490462867	1.543825	-0.31769	0.750718	0.955074
CR790362.1	1.170813	-0.317274516	1.934698	-0.16399	0.869738	NA
CR457444.1	0	NA	NA	NA	NA	NA
CR931780.1	1.14243	-0.868137038	2.210571	-0.39272	0.694526	NA
clmn	66.44014	0.299857694	0.266236	1.126287	0.260044	0.73712
CR385026.1	0.045161	0	5.267649	0	1	NA
CT583650.1	0.135482	0	5.267649	0	1	NA
si:dkey-33c3.1	1.454244	1.05542438	2.192591	0.48136	0.630261	NA
BX511245.1	0	NA	NA	NA	NA	NA
CU467963.1	0.459699	-0.794534619	3.680099	-0.2159	0.829065	NA
CR626887.1	1.952089	1.95410045	1.737969	1.124359	0.260861	NA
tmem176l.3b	350.7661	-0.467409466	0.220359	-2.12113	0.033911	0.325295
BX294110.1	0.315506	2.506272461	4.536194	0.552506	0.580602	NA
CR847984.1	20.42608	0.289956838	0.329603	0.879716	0.379013	0.823145
CR759918.1	127.4927	-0.169097855	0.190029	-0.88985	0.373546	0.820566
BX323807.1	0	NA	NA	NA	NA	NA
CU462980.1	19.9113	0.417972847	0.518445	0.806205	0.420125	0.842383

pimr28	0.229326	0	4.977387	0	1	NA
BX322538.1	0.201699	0	5.267649	0	1	NA
CU638693.1	0.557252	1.984271485	2.376588	0.834925	0.40376	NA
AL953866.1	1.29571	1.905215335	2.011231	0.947288	0.343492	NA
CR388164.2	7.992064	0.103830988	0.91027	0.114066	0.909185	0.992067
CR381618.1	361.1116	0.14215659	0.234937	0.605084	0.545123	0.895209
serpinb1l4	8.901476	-3.070896789	1.44416	-2.12642	0.033468	0.323151
CT033825.1	71.82344	0.92717851	0.397459	2.332768	0.01966	0.247302
FP102794.1	0	NA	NA	NA	NA	NA
CR774184.1	0	NA	NA	NA	NA	NA
CR450848.1	6.822573	1.535126291	1.701262	0.902345	0.366873	0.816111
CU628002.1	0	NA	NA	NA	NA	NA
BX004991.1	35.4771	-0.465895859	0.34823	-1.3379	0.18093	0.656975
si:dkey-90a13.10	2.071878	-3.438397535	1.677677	-2.0495	0.040413	NA
CR925728.1	6.088142	0.093649273	1.79237	0.052249	0.95833	0.999109
CU929451.1	24.72727	-1.648464843	0.442355	-3.72656	0.000194	0.014684
CT961047.1	0	NA	NA	NA	NA	NA
si:dkey-7i4.10	22.52524	-0.56485013	0.441549	-1.27925	0.200811	0.681045
si:ch211-180n24.3	1.445713	1.055428922	2.099828	0.502626	0.615227	NA
CU062633.1	4.876025	0.338626985	1.084702	0.312184	0.7549	0.956283
si:dkeyp-100a5.4	6.875966	-1.290454189	0.816921	-1.57966	0.114186	0.553258
si:ch73-23124.1	421.8506	-0.075138039	0.216	-0.34786	0.727944	0.949325
si:dkey-7i4.24	3.064852	2.371867328	1.362421	1.740922	0.081697	0.481171
si:ch73-185c24.2	55.5737	-1.1064791	0.428568	-2.58181	0.009828	0.171403
BX005128.1	0.620096	-0.54615362	2.075403	-0.26316	0.792431	NA
CR759878.1	0.67843	1.962582357	2.417656	0.811771	0.416923	NA
vps50	0.043309	0	5.267649	0	1	NA
BX005129.1	14.34209	-0.035520504	0.48844	-0.07272	0.942027	0.997371
CR391963.2	0.043308	0	5.267649	0	1	NA
si:ch211-107e6.6	0.214816	1.055359662	5.267649	0.200347	0.841209	NA
si:ch211-227m13.1	117.1038	-0.244171285	0.229115	-1.06571	0.286553	0.757624
AL954322.1	0.870782	-0.739033681	1.593101	-0.4639	0.642722	NA
CR790366.1	0	NA	NA	NA	NA	NA
si:zfos-375h5.1	14.88898	-1.176802218	0.611853	-1.92334	0.054437	0.403361
CR790377.1	0	NA	NA	NA	NA	NA
si:ch211-147k10.6	50.53897	-0.270752768	0.533089	-0.50789	0.611528	0.915331
CU638693.2	0.346147	0	5.267649	0	1	NA
BX927282.2	0.599358	0.093650399	2.643818	0.035422	0.971743	NA
BX005392.1	16.2128	-1.476452583	0.510799	-2.89047	0.003847	0.103374
si:ch211-210b2.3	1.913927	1.072336429	1.590953	0.674021	0.500298	NA
CR388163.1	0.383837	-0.19183096	4.145447	-0.04628	0.963091	NA
CU639412.1	0.314142	1.65007198	3.868722	0.426516	0.669732	NA
CR385086.1	0	NA	NA	NA	NA	NA
si:ch211-37e10.2	143.4725	-0.103983882	0.233259	-0.44579	0.655751	0.929606
si:ch211-135n15.2	20.95406	-0.254828387	0.372561	-0.68399	0.49398	0.877455
CR589875.1	0.086617	0	5.267649	0	1	NA
BX005461.2	2.575106	-0.196751213	1.503897	-0.13083	0.895912	0.98926
BX323035.1	0	NA	NA	NA	NA	NA
CU207354.1	0.562537	0.093655929	2.590056	0.03616	0.971155	NA
si:dkey-52p2.5	0.043309	0	5.267649	0	1	NA
si:dkey-77f5.8	75.24933	-0.105483224	0.244806	-0.43089	0.666552	0.932664
BX957258.1	8.966684	0.285737383	0.710319	0.402266	0.687488	0.938835
CR759863.1	0	NA	NA	NA	NA	NA
BX897682.1	3.480842	2.31339078	1.539841	1.502357	0.133005	0.58639

CR545469.1	0	NA	NA	NA	NA	NA
im:7143333	36.61608	-0.42942533	0.346863	-1.23802	0.215707	0.697271
si:dkey-197j19.5	10.65989	0.798873211	0.684328	1.167384	0.243055	0.724396
BX927244.1	12.87756	-1.874795127	0.711442	-2.6352	0.008409	0.156847
CR848812.1	17.45003	-0.064336567	0.397326	-0.16192	0.871366	0.983365
si:dkeyp-67f1.1	81.77747	-0.177069219	0.273914	-0.64644	0.517994	0.887148
CR848791.2	0.349803	1.055407845	3.421792	0.308437	0.75775	NA
BX005254.2	7.276727	-0.648450937	0.707491	-0.91655	0.359378	0.810472
BX005351.1	0	NA	NA	NA	NA	NA
si:dkey-29h14.10	7.905358	-0.820386028	0.775696	-1.05761	0.290232	0.76078
CLIP4	54.19051	0.05360545	0.281851	0.19019	0.84916	0.978098
CT025690.1	90.4449	-0.48037699	0.191665	-2.50634	0.012199	0.191703
CU179699.1	0	NA	NA	NA	NA	NA
AL844141.1	1.15026	0.527685378	2.471437	0.213514	0.830926	NA
si:dkey-238d18.13	0	NA	NA	NA	NA	NA
CR383662.5	3.038943	-2.987444766	1.423656	-2.09843	0.035867	0.332937
BX004812.1	8.386311	0.919592473	0.737381	1.247106	0.212359	0.694082
CR759863.2	0.045161	0	5.267649	0	1	NA
BX119902.2	2.612091	1.103993035	1.282507	0.860809	0.389343	0.827843
si:dkey-206p8.1	29.5636	-0.278649052	0.354807	-0.78535	0.432246	0.848419
CU633991.2	50.82456	-0.212311942	0.257675	-0.82395	0.409967	0.836939
CR926463.1	9.850717	1.583786002	0.74324	2.130922	0.033096	0.322195
BX248113.1	3.565485	0.71377708	1.23881	0.576179	0.564494	0.901433
si:dkeyp-93a5.3	3.99994	0	1.973842	0	1	1
mhc1lla	0.161568	0	5.267649	0	1	NA
CU972512.1	26.9407	-1.987999499	0.97823	-2.03224	0.042129	0.358873
NPC2 (1 of many)	39.60916	-4.350299212	0.889746	-4.88937	1.01E-06	0.0003
CR626962.1	0	NA	NA	NA	NA	NA
BX957326.2	0.389886	0	4.336758	0	1	NA
si:dkeyp-19h3.8	0	NA	NA	NA	NA	NA
si:dkey-14d8.22	27.22212	0.030514147	0.384155	0.079432	0.936689	0.997044
CU915818.1	0.686588	0	2.77366	0	1	NA
CR589944.1	96.18687	0.379613494	0.28434	1.33507	0.181853	0.658469
fam110c	91.54391	-0.017295241	0.222718	-0.07766	0.938102	0.997331
si:ch1073-340i21.2	168.6201	0.218420902	0.231685	0.942751	0.345808	0.799001
CR847968.1	132.4268	0.615987844	0.214711	2.868915	0.004119	0.106542
zgc:153405	3.15206	1.687844644	0.921649	1.831331	0.067051	0.441984
CR847923.1	0	NA	NA	NA	NA	NA
AL929222.1	2.239156	-2.060439895	1.418701	-1.45234	0.146406	0.605984
fbxl22	79.35577	0.19528785	0.30966	0.630652	0.528268	0.889948
BX005392.2	12.897	-0.506181309	0.604083	-0.83793	0.402068	0.832968
CR589876.1	41.47076	-0.284842318	0.366406	-0.77739	0.436926	0.851125
CT737162.1	0.507601	-3.110011307	2.644136	-1.17619	0.239518	NA
BX537308.1	11.43036	0.279644562	0.719558	0.388634	0.697547	0.942152
BX890558.1	50.93103	1.478660594	0.547602	2.700249	0.006929	0.140209
FP245462.1	21.71027	0.001786044	0.482264	0.003703	0.997045	1
CR855375.1	1.326711	1.068283349	2.065658	0.517164	0.605042	NA
CR854839.1	312.9654	-0.016066558	0.169053	-0.09504	0.924284	0.994815
CU694256.1	0.04222	0	5.267649	0	1	NA
opn1mw1	3921.788	0.081142983	1.027962	0.078936	0.937084	0.997163
FP102888.1	0.323665	-2.769503347	4.396809	-0.62989	0.528767	NA
hbaa1	7.909825	-2.471903173	0.90562	-2.72951	0.006343	0.134704
BX649300.1	0	NA	NA	NA	NA	NA
CU694202.1	0	NA	NA	NA	NA	NA

FP016018.2	2.183285	-2.390600888	1.738375	-1.37519	0.169072	NA
BX004972.1	0.351685	2.464049193	3.774873	0.65275	0.513917	NA
mical3a	203.8154	0.029309199	0.240211	0.122015	0.902887	0.991179
CR628341.1	9.606233	6.384595959	1.277729	4.996831	5.83E-07	0.000188
dhrs13b.1	7.272172	-1.305874555	0.931578	-1.40179	0.160979	0.627788
CU682780.1	0.099037	0	5.267649	0	1	NA
CU655829.1	0.149833	1.921567426	5.236909	0.366928	0.713673	NA
CR786571.3	1.15791	-2.100288017	2.021146	-1.03916	0.298732	NA
ugt1b4	1.91462	-0.662954503	1.312051	-0.50528	0.613361	NA
CR354383.1	3.158246	-0.214977309	1.03666	-0.20737	0.835717	0.976007
si:dkey-16m19.1	28.95143	-0.242722481	0.382813	-0.63405	0.526049	0.889156
CR352292.1	1.885428	0.849102338	1.477139	0.574829	0.565407	NA
AL845435.1	0.29106	2.962897837	5.184402	0.571502	0.567659	NA
si:ch73-38013.3	2.118998	-1.786697826	1.864574	-0.95823	0.337945	NA
si:ch211-256a21.4	25.80787	0.073664213	0.398755	0.184735	0.853437	0.978887
CU466278.1	13.19334	-0.448075389	0.626564	-0.71513	0.474528	0.869292
CR450813.1	0.553364	-0.754299688	3.486953	-0.21632	0.828738	NA
pimr42	0.045161	0	5.267649	0	1	NA
CR759845.1	4.842645	-1.169692648	0.967325	-1.2092	0.226585	0.707798
CT025745.1	11.6414	0.074472677	0.751413	0.09911	0.921051	0.994085
BX927349.1	0.613606	-0.868130521	3.279592	-0.26471	0.791235	NA
CR759845.2	1.724268	0.052384063	1.497087	0.034991	0.972087	NA
CU468915.1	56.89801	-1.077175498	0.306521	-3.5142	0.000441	0.025327
AL935145.2	1.592866	-0.402497502	1.489356	-0.27025	0.786968	NA
CR936518.1	3.208639	0.040883685	0.933	0.04382	0.965048	1
BX629346.1	21.08726	-0.158730288	0.535197	-0.29658	0.766785	0.960296
CU571318.1	290.9425	-0.093207339	0.187724	-0.49651	0.619532	0.917435
CR759845.3	0.881782	-0.850560102	2.730851	-0.31146	0.755448	NA
CR855375.2	2.38183	1.418053691	1.456978	0.973284	0.330412	0.788126
BX897714.1	0.045161	0	5.267649	0	1	NA
FP236594.1	0	NA	NA	NA	NA	NA
CU927934.1	5.64044	1.237174121	0.973173	1.271278	0.20363	0.684359
si:ch211-284f22.3	411.9352	0.182121331	0.146555	1.242682	0.213985	0.696474
fxyd6l	2675.265	-0.123650729	0.133834	-0.92391	0.355532	0.806546
CU929339.1	5.708231	-0.5614818	0.697844	-0.80459	0.421054	0.842517
si:dkey-159n16.4	0	NA	NA	NA	NA	NA
CR388369.1	4.372551	-0.152713927	0.820442	-0.18614	0.852338	0.978626
BX547934.1	1.019805	0	2.312653	0	1	NA
sparcl2	31.11192	0.540045064	0.414169	1.303925	0.192259	0.672283
CR759845.4	0.450486	1.481373992	3.603823	0.411056	0.681031	NA
si:ch73-223p23.2	0.632471	0	3.19276	0	1	NA
BX322555.1	0.28951	-2.754991261	5.185055	-0.53133	0.595188	NA
CR626887.2	0	NA	NA	NA	NA	NA
si:ch211-66i15.4	7.703866	0.476818204	0.631363	0.75522	0.450117	0.85711
AL954322.2	95.38576	-0.274114902	0.208288	-1.31604	0.188161	0.667333
CU928073.1	0.750624	0.14778532	2.902538	0.050916	0.959393	NA
BX322555.2	0.101247	1.854150541	5.241768	0.353726	0.723544	NA
CU855801.1	0.649612	0.524963053	2.521956	0.208157	0.835106	NA
CR318607.1	2.210701	-1.517381914	1.555309	-0.97561	0.329256	0.787409
CR848665.1	1.036232	0.0936551	2.167812	0.043203	0.96554	NA
si:ch73-181m17.1	5.809358	0.733223246	0.919842	0.797118	0.425382	0.844714
si:dkey-149i17.11	0	NA	NA	NA	NA	NA
im:7152348	144.3967	0.293494321	0.247446	1.186092	0.235586	0.716683
BX005462.2	0.191497	1.854123853	5.241768	0.353721	0.723548	NA

si:zfos-1714f5.4	1.022489	0	2.487893	0	1	NA
si:ch211-261a10.5	6.838142	0.119911497	0.73788	0.162508	0.870906	0.983365
si:dkey-172k15.4	50.83282	0.307247921	0.290741	1.056776	0.290614	0.76078
CR812472.1	0.698474	0	2.541282	0	1	NA
si:dkey-60d5.3	19.43197	0.05575441	0.465456	0.119785	0.904654	0.991581
BX120006.1	0.087179	0	5.267649	0	1	NA
si:dkey-7j22.2	2.617451	-3.606526557	1.411341	-2.55539	0.010607	0.179908
si:ch211-273k1.5	0.27882	0	4.700212	0	1	NA
CR293508.1	0.401666	0.104170968	2.890476	0.036039	0.971251	NA
CU927934.2	0	NA	NA	NA	NA	NA
AL954669.1	0.736687	2.660403691	2.412025	1.102975	0.270038	NA
CR394545.1	8.883301	-2.210380672	0.74574	-2.96401	0.003037	0.090902
znf1004	0.617996	0	2.832449	0	1	NA
si:ch211-108d22.1	1.179126	-3.584021836	2.292514	-1.56336	0.117968	NA
BX511075.1	0.294625	-3.029200167	5.176305	-0.58521	0.55841	NA
si:ch73-281n10.2	9908.317	0.041003671	0.154017	0.266228	0.790064	0.964274
si:dkey-52d15.2	0.502068	0	4.715286	0	1	NA
CR790366.2	2.963162	-0.168099142	1.12933	-0.14885	0.881673	0.986908
AL928927.1	0.181988	0	5.267649	0	1	NA
BX649442.2	5.658603	-0.327724408	0.916333	-0.35765	0.720607	0.947263
si:dkey-56f14.4	181.7403	0.260942441	0.255318	1.022029	0.306767	0.772
CR762407.2	0.841352	-0.868144777	2.503569	-0.34676	0.72877	NA
BX571737.1	0	NA	NA	NA	NA	NA
si:dkey-27l15.1	0.147561	-1.710071829	5.238473	-0.32644	0.744088	NA
si:dkey-33c14.3	764.7173	-0.94517608	0.546276	-1.73022	0.083591	0.485416
AL928657.1	27.13464	-0.65946093	0.367593	-1.794	0.072814	0.458474
AL935115.1	0	NA	NA	NA	NA	NA
FP102921.1	0.073968	0	5.267649	0	1	NA
AL772382.1	11.03122	0.371875805	0.543181	0.684626	0.49358	0.877027
FP102170.1	18.30858	0.06939278	0.437361	0.158663	0.873935	0.98385
FP015862.1	0.060442	0	5.267649	0	1	NA
si:dkey-7i4.23	0	NA	NA	NA	NA	NA
CR854927.3	0	NA	NA	NA	NA	NA
BX957346.2	0	NA	NA	NA	NA	NA
BX005242.1	0.189359	0	5.267649	0	1	NA
CU695222.1	0.087381	0	5.267649	0	1	NA
CR318658.1	0	NA	NA	NA	NA	NA
si:dkey-77f5.11	50.36291	0.162825283	0.3432	0.474433	0.635191	0.924272
CR848683.1	1.240727	1.282374251	1.54724	0.828814	0.40721	NA
CR545465.2	0	NA	NA	NA	NA	NA
CR753876.1	107.4864	-0.301321361	0.350254	-0.86029	0.389628	0.827843
si:ch211-191c10.3	1.576563	3.298973906	1.903948	1.732702	0.083149	NA
BX465205.1	0.163218	2.079549577	5.22636	0.397896	0.690707	NA
BX664705.1	0.049519	0	5.267649	0	1	NA
CR848742.1	0.08847	0	5.267649	0	1	NA
FP565432.1	4.329298	0.430290147	1.106492	0.388878	0.697366	0.942046
CU459174.1	2.365597	-2.065676496	1.44117	-1.43333	0.151763	0.615406
BX005254.3	10.14506	0.329334563	0.612897	0.537341	0.591032	0.909311
BX324227.1	0.092101	0	5.267649	0	1	NA
si:dkey-7i4.11	5.56084	0.388825801	0.763346	0.50937	0.610493	0.915331
CR954963.1	2.376942	1.825488252	1.427936	1.27841	0.201105	0.681045
GDPGP1	30.76115	0.521097801	0.318481	1.636197	0.101798	0.528581
CR944667.1	0	NA	NA	NA	NA	NA
si:ch211-207n23.2	331.1097	-0.483392344	0.254543	-1.89906	0.057557	0.4139

BX294100.1	40.86574	0.899044483	0.278906	3.223467	0.001266	0.051937
BX664738.1	0.403614	-1.807681634	4.147845	-0.43581	0.662973	NA
si:ch211-121j5.4	64.38356	-0.533634635	0.592914	-0.90002	0.368109	0.817272
CU861891.1	4.686767	0.726301357	0.844297	0.860244	0.389655	0.827843
CR385035.1	9.814607	0.089669264	0.533009	0.168232	0.866401	0.982582
CR848746.1	9.628171	-0.540073233	0.67329	-0.80214	0.422471	0.843493
BX936386.1	0.485256	0.746822993	3.068274	0.243402	0.807694	NA
BX005484.1	0.845057	0	2.485449	0	1	NA
CR749752.1	0.176435	1.055370259	5.267649	0.200349	0.841207	NA
si:ch73-330k17.3	8.849768	-0.340274148	0.88927	-0.38264	0.701984	0.943292
pcdh1a4	13.89489	-0.091681891	0.548833	-0.16705	0.867332	0.982582
CR381579.1	0	NA	NA	NA	NA	NA
CR388129.1	0.28898	0	5.267649	0	1	NA
BX546459.1	0.104229	-1.791411807	5.232816	-0.34234	0.732094	NA
CR383662.6	9.664719	-0.221840574	0.621649	-0.35686	0.721198	0.947343
BX927308.2	21.7965	-0.351060465	0.494256	-0.71028	0.47753	0.869292
CR538726.1	10.62752	0.538182341	0.526084	1.022996	0.30631	0.771472
si:dkey-7i4.21	0	NA	NA	NA	NA	NA
defbl3	5.355908	-0.492766045	0.973388	-0.50624	0.61269	0.915754
CR848757.1	69.16111	0.09746001	0.457327	0.213108	0.831243	0.974908
CU570976.1	1.530644	1.055423957	2.166519	0.487152	0.626151	NA
CU695073.1	0.633652	-2.745348195	4.351576	-0.63089	0.528115	NA
CR847799.1	0	NA	NA	NA	NA	NA
si:ch211-153l6.6	65.06083	-0.249019438	0.481293	-0.5174	0.604879	0.914985
CU929037.2	0.46016	0	4.800285	0	1	NA
FP101861.1	24.86818	-0.098828119	0.318349	-0.31044	0.756227	0.956532
BX640398.1	0.401312	1.646145155	3.212441	0.512428	0.608351	NA
CU861453.1	48.51357	0.347625983	0.302231	1.150199	0.250062	0.729807
si:dkey-28n18.9	134.2707	-0.020491784	0.188037	-0.10898	0.91322	0.99273
FP236850.1	0	NA	NA	NA	NA	NA
BX649398.1	0.980844	-1.814720939	2.90238	-0.62525	0.531805	NA
CR847531.1	531.9314	0.276962702	0.247339	1.119771	0.262811	0.740207
CR392042.1	1.90582	-3.242504587	1.928556	-1.68131	0.092702	NA
BX120006.2	0.120883	0	5.267649	0	1	NA
si:ch211-176l24.4	40.78229	0.172601609	0.313703	0.550206	0.582178	0.90571
BX000524.1	0.060442	0	5.267649	0	1	NA
ulk2	956.5674	-0.212764713	0.222673	-0.9555	0.339324	0.795226
BX324206.1	4.340446	-1.301147985	1.290316	-1.0084	0.313265	0.776663
CR759882.1	0.191882	1.05535617	5.267649	0.200347	0.841209	NA
alkbh3	578.7569	-0.087875011	0.263837	-0.33307	0.739085	0.95338
cox8b	302.7203	-0.638433092	0.211771	-3.01474	0.002572	0.082312
CR381647.1	77.32151	0.311736962	0.362456	0.860068	0.389752	0.827843
si:dkey-24p1.6	0	NA	NA	NA	NA	NA
AL808121.1	0.053262	1.055396947	5.267649	0.200354	0.841203	NA
CR388029.1	1.003593	-1.699911558	2.295016	-0.7407	0.458877	NA
si:dkey-172k15.3	62.4526	-0.307969343	0.241615	-1.27463	0.20244	0.682855
BX957355.1	0.502953	-0.868141633	3.186701	-0.27243	0.785294	NA
si:dkey-229e3.2	28.78448	0.240877322	0.320934	0.750552	0.452923	0.858256
CT027582.1	0.283522	-0.868134432	4.745832	-0.18293	0.854856	NA
BX005079.1	0.043309	0	5.267649	0	1	NA
AL954387.1	0.375372	1.858244586	4.3164	0.430508	0.666826	NA
CR854827.1	0	NA	NA	NA	NA	NA
CR925728.2	0.209501	0	5.267649	0	1	NA
prr33	1959.88	0.016849546	0.23302	0.072309	0.942356	0.997371

si:dkey-79d12.6	2.478835	0.079158331	1.250605	0.063296	0.949531	0.99801
CR847887.1	6.284057	1.179295967	0.758174	1.555442	0.119841	0.564602
CR854827.2	0	NA	NA	NA	NA	NA
si:dkey-234i14.12	0.132542	0	5.267649	0	1	NA
si:ch211-226h8.15	0.908488	-2.353169444	2.188824	-1.07508	0.282337	NA
ugt1b2	20.22687	1.275848563	0.931717	1.369352	0.170889	0.642777
AL929435.1	0.060442	0	5.267649	0	1	NA
CT737162.2	58.74943	-0.942387167	0.319908	-2.94581	0.003221	0.093414
si:ch73-147o17.1	329.3467	-0.180658217	0.164177	-1.10039	0.271163	0.747344
BX005012.4	0.505127	0	3.631139	0	1	NA
hbba1	4.073387	-2.87444738	1.443168	-1.99176	0.046397	0.376263
si:ch73-31d8.2	72.94542	0.05473115	0.342676	0.159717	0.873104	0.983537
BX323076.1	3.262901	-1.680687967	1.152384	-1.45844	0.144718	0.603718
BX120016.1	0.621035	3.076457663	3.34451	0.919853	0.35765	NA
CR457447.1	0.588718	2.07621036	4.547321	0.456579	0.647974	NA
zgc:174263	311.4798	-0.085202563	0.124343	-0.68522	0.493203	0.876787
soga3a	0	NA	NA	NA	NA	NA
BX322555.3	154.9856	-0.0083724	0.23956	-0.03495	0.97212	1
si:dkey-117a8.2	0	NA	NA	NA	NA	NA
BX537104.1	0.801334	1.055432194	2.309693	0.456958	0.647701	NA
si:dkey-42l23.9	2.992693	0.223378595	1.041949	0.214385	0.830247	0.974908
CR354429.1	0.154952	-2.364821159	5.200714	-0.45471	0.649317	NA
yeats2	52.30052	0.413264369	0.297258	1.390257	0.164451	0.633787
si:dkey-77f5.13	40.75907	-0.256444346	0.33736	-0.76015	0.447165	0.856073
si:ch211-270g19.5	97.55929	-0.589678225	0.24772	-2.38042	0.017293	0.232084
BX908753.1	7.230185	1.227805338	0.642692	1.91041	0.05608	0.409255
si:dkey-4c15.16	45.67448	-0.216673673	0.34767	-0.62322	0.533142	0.890978
AL808129.1	98.21401	-0.306562439	0.353501	-0.86722	0.385823	0.826939
CR925802.1	0	NA	NA	NA	NA	NA
si:ch211-40k21.5	198.5311	-0.167235592	0.155895	-1.07275	0.283384	0.755095
CR847898.2	17.75629	-1.005453217	0.456581	-2.20213	0.027656	0.29556
AL954322.3	13.09537	-2.456379588	0.775559	-3.16724	0.001539	0.05933
si:ch211-266k8.6	2.774895	-0.568386836	1.133243	-0.50156	0.615978	0.916365
CU929145.2	44.70794	0.267865127	0.374014	0.716191	0.473874	0.869292
si:ch211-222n22.1	7.97431	0.137898213	0.64846	0.212655	0.831596	0.974908
CR405687.1	0.431263	0	3.177544	0	1	NA
CU571382.2	0	NA	NA	NA	NA	NA
CR848759.1	0.507354	0	4.096455	0	1	NA
CU929145.3	0.183188	1.055363901	5.267649	0.200348	0.841208	NA
si:ch211-147g22.4	48.54524	-0.09027339	1.36541	-0.06611	0.947287	0.997371
CR381689.1	1.51614	0.096235	1.560615	0.061665	0.95083	NA
CR352232.1	0.046941	0	5.267649	0	1	NA
CU856222.1	248.699	0.029070486	0.223497	0.130071	0.89651	0.98939
zgc:174574	93.2451	0.018723178	0.201744	0.092806	0.926057	0.994815
BX119902.3	0.44142	3.26325136	3.505953	0.930774	0.35197	NA
si:ch211-261d7.3	426.1121	-0.034525533	0.115991	-0.29766	0.765964	0.959993
ANPEP	241.7533	-0.968663946	0.227779	-4.25265	2.11E-05	0.003003
FP102892.1	0	NA	NA	NA	NA	NA
BX323076.2	12.19091	0.736099785	0.618158	1.190795	0.233734	0.714749
BX629344.1	151.3946	-0.153710023	0.236524	-0.64987	0.515776	0.886634
CU855917.1	0.184203	0	5.267649	0	1	NA
slc26a6	14.121	0.40530482	0.619653	0.654084	0.513058	0.886007
CU855758.1	0.551168	-0.380661876	2.747893	-0.13853	0.889823	NA
BX571762.1	1.886753	1.575894524	1.4819	1.063428	0.287588	NA

FP016018.3	0	NA	NA	NA	NA	NA
CR792422.2	3.114232	-0.69290884	0.971377	-0.71333	0.475644	0.869292
flrt2	104.0709	0.382698362	0.242415	1.578688	0.114408	0.553523
BX530017.1	0	NA	NA	NA	NA	NA
CR925755.1	0	NA	NA	NA	NA	NA
tbpl2	0.694043	-0.871329861	2.116333	-0.41172	0.680547	NA
BX000358.1	0	NA	NA	NA	NA	NA
si:dkey-238d18.10	0.68315	0	2.898718	0	1	NA
BX901883.1	0	NA	NA	NA	NA	NA
zgc:193812	47.82499	-0.411902826	0.334945	-1.22976	0.218786	0.699596
CT573316.1	0	NA	NA	NA	NA	NA
CR812470.2	0.789218	1.558175431	2.57086	0.606091	0.544454	NA
si:zfos-1714f5.3	2.246859	-0.912527225	1.339422	-0.68128	0.495691	0.87806
BX927078.1	0	NA	NA	NA	NA	NA
CT027805.1	0.609359	0.110490628	2.33819	0.047255	0.96231	NA
si:dkey-276j7.3	6.598594	-1.31264857	1.135543	-1.15597	0.247695	0.728489
BX649242.1	0.675517	2.911803424	2.425079	1.200704	0.229866	NA
CR848791.3	0.238803	-2.293535501	5.204055	-0.44072	0.659415	NA
BX005183.1	0	NA	NA	NA	NA	NA
si:dkey-81l17.6	139.013	0.517625175	0.264606	1.956211	0.05044	0.39074
si:ch211-14i3.2	5.854531	0	1.766246	0	1	1
BX546473.1	0	NA	NA	NA	NA	NA
BX957270.1	0.147561	-1.710071829	5.238473	-0.32644	0.744088	NA
BX005060.1	0.063619	0	5.267649	0	1	NA
CR759826.1	0	NA	NA	NA	NA	NA
si:dkey-170g13.2	0.81717	1.055419063	2.836974	0.372023	0.709876	NA
CU571161.1	0	NA	NA	NA	NA	NA
CT573342.1	10.64436	-0.405709093	0.532451	-0.76197	0.44608	0.855403
CT573316.2	3.084529	-4.275416285	1.910603	-2.23773	0.025239	0.282092
CR759841.1	1.156841	0.194276116	2.060016	0.094308	0.924864	NA
CT583687.1	2.71408	-5.074762908	1.692647	-2.99812	0.002716	0.085055
BX000434.1	0.050723	-0.86807634	5.267649	-0.16479	0.869106	NA
slc9a1b	87.12049	-0.603262496	0.326912	-1.84534	0.064988	0.434945
si:ch211-33e4.2	11.28706	0.126604864	0.607693	0.208337	0.834966	0.975994
CR847994.1	33.33939	0.134149307	0.313238	0.428266	0.668457	0.933086
AL845550.1	0.232264	0	5.267649	0	1	NA
AL772300.1	7.662712	0.23323485	0.7779	0.299826	0.76431	0.959228
CR387932.1	0.327635	2.505626164	5.20297	0.481576	0.630107	NA
AL935186.2	0.321502	-0.794332909	3.91497	-0.2029	0.839216	NA
ten1	78.72057	0.36139968	0.359861	1.004275	0.315246	0.777834
CR854965.2	0.09854	-0.868089684	5.267649	-0.1648	0.869104	NA
BX649628.1	0.396775	0.635061758	4.207024	0.150953	0.880013	NA
si:ch211-147g22.7	6.938305	0.91568637	0.7693	1.190286	0.233934	0.71479
BX324164.3	50.26301	0.258785031	0.356071	0.726779	0.467361	0.86616
CT027551.1	0.101247	1.854150541	5.241768	0.353726	0.723544	NA
BX901883.2	41.12111	0.326420921	0.527893	0.618346	0.536347	0.892371
CU638693.3	0.046941	0	5.267649	0	1	NA
BX279523.3	1.876378	1.025199575	1.356859	0.755568	0.449908	NA
CR790363.1	0.401923	0	3.713601	0	1	NA
CU655820.1	2.17914	-3.078585214	1.682258	-1.83003	0.067245	NA
CU896541.1	16.68316	-0.214360846	0.508247	-0.42176	0.673197	0.93414
si:dkey-244a7.1	56.42802	-0.08405821	0.255447	-0.32906	0.742108	0.954329
FP017274.1	55.6009	0.294161958	0.354822	0.829041	0.407081	0.835278
mcf2b	19.82645	-0.59735669	0.409317	-1.4594	0.144456	0.603539

si:ch211-139d20.3	31.98432	-1.056891108	0.387815	-2.72525	0.006425	0.135464
ftr90	14.05303	0.848525085	0.605114	1.402256	0.160839	0.627788
si:dkey-108k21.7	24.67676	-0.154553799	0.482379	-0.3204	0.748666	0.955074
BX248418.2	0.13271	0	5.267649	0	1	NA
CR790366.3	0.342138	1.631053342	4.379444	0.372434	0.70957	NA
CR387933.1	0.510368	-1.774014312	2.717375	-0.65284	0.513859	NA
CR936416.1	0.501458	-0.868136423	2.848739	-0.30474	0.760561	NA
CR812472.2	0	NA	NA	NA	NA	NA
CR382363.1	5.080899	-1.427024364	1.313861	-1.08613	0.277421	0.751426
BX005183.2	0.141849	-0.868102566	5.267649	-0.1648	0.869102	NA
BX465853.1	0	NA	NA	NA	NA	NA
AL929182.1	0.151871	2.436401064	5.206324	0.46797	0.639806	NA
si:ch211-15d5.12	94.20731	-0.076207139	0.218417	-0.34891	0.72716	0.949095
CR854907.1	1.532813	-0.31320319	1.528059	-0.20497	0.837597	NA
FP101990.1	0	NA	NA	NA	NA	NA
BX571853.1	2.533746	-2.271614508	1.397478	-1.62551	0.104054	0.532096
BX530407.4	3.949071	-0.597926072	1.06539	-0.56123	0.574642	0.904516
si:dkey-33c14.7	7.259917	0.019721354	0.627709	0.031418	0.974936	1
BX465208.1	0	NA	NA	NA	NA	NA
BX897720.1	0	NA	NA	NA	NA	NA
si:dkey-77f5.6	51.66611	-0.328165704	0.284382	-1.15396	0.248517	0.728723
BX005459.1	0	NA	NA	NA	NA	NA
BX539313.1	1.412233	0	2.177035	0	1	NA
ugt5a4	2.980594	-0.475085678	1.324239	-0.35876	0.719774	0.94694
si:ch73-288o11.4	1.362008	2.083154999	1.600405	1.301643	0.193039	NA
FP340271.1	0.189354	1.055370259	5.267649	0.200349	0.841207	NA
BX470254.1	0.04222	0	5.267649	0	1	NA
BX511308.1	1.671171	0.612350715	1.390144	0.440495	0.659579	NA
si:dkey-273g18.1	2.045657	2.026363748	1.687818	1.200582	0.229913	NA
si:dkey-81e3.2	18.49781	-0.267594618	0.505173	-0.52971	0.596314	0.911626
BX547992.2	0	NA	NA	NA	NA	NA
BX548045.1	0.069091	0	5.267649	0	1	NA
CU571319.1	2.32828	1.100912734	1.329974	0.82777	0.407801	0.835802
AL928870.1	0.143468	1.854137197	5.241768	0.353724	0.723546	NA
si:ch211-106e7.2	70.61456	-0.235706705	0.27235	-0.86546	0.386788	0.827592
lrrc53	3.471578	2.900991204	1.357807	2.136527	0.032636	0.319477
si:ch73-306e8.2	97.0504	-1.502458621	0.555236	-2.70598	0.00681	0.139148
CR847793.1	0.927219	4.117133064	2.089141	1.97073	0.048755	NA
si:ch211-63b16.3	10.65025	-0.890796501	0.780927	-1.14069	0.253999	0.733071
CR786571.4	0.623697	0.093646477	3.456595	0.027092	0.978386	NA
si:ch73-250a16.5	41.3245	-0.201847457	0.336202	-0.60038	0.548256	0.896644
si:dkeyp-50b9.1	5.250774	-0.512797077	0.819644	-0.62563	0.531555	0.890835
CU929360.1	3.399792	-0.502357694	1.511414	-0.33238	0.739605	0.953573
CU638870.1	50.61734	-1.289635856	0.326671	-3.94781	7.89E-05	0.00771
BX569790.2	0	NA	NA	NA	NA	NA
BX470242.2	0	NA	NA	NA	NA	NA
BX005461.3	6.942551	-0.105638882	0.598014	-0.17665	0.859784	0.980066
CR392003.1	4.837282	-0.764147539	0.772656	-0.98899	0.322669	0.782525
CT027718.1	1.116242	-0.262392876	1.920271	-0.13664	0.891312	NA
BX649398.2	8.048636	1.025229926	0.730263	1.403919	0.160343	0.626923
CU928222.1	0.060442	0	5.267649	0	1	NA
AL928908.1	0	NA	NA	NA	NA	NA
BX510360.1	0	NA	NA	NA	NA	NA
CR788230.2	0.084441	0	5.267649	0	1	NA

CU855930.1	19.25999	-0.141338147	0.434883	-0.325	0.745179	0.954956
si:dkey-77f5.15	8.338203	-0.179167504	0.554983	-0.32283	0.746821	0.955074
si:dkey-195m11.8	495.2247	-0.018647294	0.223055	-0.0836	0.933375	0.996472
si:ch211-66i15.5	0.844374	-0.355947494	2.557797	-0.13916	0.889322	NA
BX890600.1	0.561775	3.502063332	2.782439	1.258631	0.208164	NA
CR925728.3	0.719739	-3.091452451	2.54531	-1.21457	0.224531	NA
CR381647.2	0.194106	0	5.267649	0	1	NA
chmp6a	4.552366	-0.552436043	1.200184	-0.46029	0.645306	0.926728
CU929070.1	175.9634	-0.232020464	0.183703	-1.26302	0.206583	0.688906
si:ch211-225b7.5	49.85116	0.119433703	0.323652	0.369018	0.712114	0.945821
BX539313.2	3.270075	-0.568184404	0.974534	-0.58303	0.559872	0.899668
CR931782.1	0.657451	0	3.748456	0	1	NA
coa3b	71.61037	-1.360611587	0.299003	-4.55049	5.35E-06	0.001127
BX119963.2	6.33216	0.651291456	0.84646	0.769429	0.441638	0.853157
si:dkey-208m12.3	0	NA	NA	NA	NA	NA
si:dkey-125e8.3	9.830665	0	1.968384	0	1	1
CU929339.2	0	NA	NA	NA	NA	NA
BX957326.3	3.685156	2.77564849	1.173434	2.365408	0.01801	0.236966
CR788249.2	0.084441	0	5.267649	0	1	NA
trmt61b	145.1375	-0.214511136	0.237165	-0.90448	0.365741	0.815695
CU207343.2	0.501884	-1.787388385	3.165841	-0.56459	0.572356	NA
CU693502.1	132.3348	-0.005193094	0.176585	-0.02941	0.976539	1
si:ch73-1f23.1	2.033164	0.537728818	1.481108	0.363058	0.716561	NA
BX927362.1	0.753691	-1.729949368	2.320458	-0.74552	0.455957	NA
FP015862.2	5.619181	-2.09824648	1.365871	-1.5362	0.12449	0.572138
BX004999.2	0	NA	NA	NA	NA	NA
zgc:113340	82.36076	0.046897608	0.219053	0.214092	0.830475	0.974908
CR396585.1	0.188628	1.854125201	5.241768	0.353721	0.723548	NA
si:dkey-4c2.11	4.544119	1.66018416	1.128509	1.47113	0.141256	0.599899
qkia	2477.865	-0.009040641	0.12845	-0.07038	0.943889	0.997371
si:dkey-263j23.1	9.054338	-1.886742862	0.817583	-2.30771	0.021015	0.257618
si:dkey-77f5.11	34.02694	0.617485466	0.324111	1.905168	0.056758	0.411608
si:ch211-213d14.2	125.9647	0.043273464	0.161523	0.267909	0.78877	0.963568
cfap99	17.517	0.465490399	0.492973	0.94425	0.345042	0.79837
si:ch211-175f11.5	2.24631	-0.828106776	1.599173	-0.51783	0.604574	0.914985
si:ch73-390p7.2	132.1372	0.097328588	0.182343	0.533766	0.593503	0.910427
AL928908.2	0	NA	NA	NA	NA	NA
ugt1b1	295.6109	-0.086753295	0.15926	-0.54473	0.585942	0.906655
AL772314.1	153.967	0.564781443	0.347721	1.624239	0.104325	0.532541
si:dkey-148h10.5	23.43865	-0.84901373	0.416552	-2.0382	0.04153	0.355733
CR382327.2	21.76507	0.164144654	0.446885	0.367308	0.713389	0.945821
CU467639.1	0.543156	-1.276037138	3.383132	-0.37718	0.706043	NA
si:dkey-6e2.2	298.3169	-0.086845304	0.138362	-0.62767	0.530222	0.890545
BX511253.1	0.356852	-0.85733973	3.347209	-0.25614	0.797846	NA
CR450813.2	0	NA	NA	NA	NA	NA
BX511311.1	0	NA	NA	NA	NA	NA
si:dkey-111k8.3	134.8224	-0.206485022	0.289974	-0.71208	0.476414	0.869292
CU694807.1	0.073968	0	5.267649	0	1	NA
si:ch73-288o11.5	211.748	-0.32476631	0.653646	-0.49685	0.619293	0.917397
CR626907.3	299.235	0.931311316	0.294201	3.165558	0.001548	0.059575
DNAL1 (1 of many)	0	NA	NA	NA	NA	NA
BX571780.1	0.353459	1.05539299	3.833035	0.275341	0.783054	NA
BX324164.4	74.12602	-0.28977736	0.291461	-0.99422	0.320114	0.780709
CR848836.1	0.059954	1.055396947	5.267649	0.200354	0.841203	NA

si:dkey-245f22.3	10.33616	0.429564687	0.690521	0.622088	0.533884	0.891181
BX890629.1	1.578445	0.904628344	1.564244	0.578317	0.56305	NA
BX649529.1	43.06652	0.042266253	0.287225	0.147154	0.883011	0.986908
CT573383.1	35.35946	-1.35098698	0.530923	-2.5446	0.01094	0.182182
CR854989.1	1.842822	-0.103130507	1.27243	-0.08105	0.935402	NA
CR751227.1	1.146042	-2.116301646	1.736904	-1.21843	0.223059	NA
BX323016.1	2.035913	0.281849316	1.161648	0.242629	0.808293	NA
BX537110.1	17.88643	1.82709071	0.625581	2.92063	0.003493	0.097752
AL935300.2	0.2617	0	5.267649	0	1	NA
si:dkey-111k8.5	44.53989	-0.272306686	0.279343	-0.97481	0.329654	0.788
si:ch73-364h19.1	31.27661	0.112036991	0.449323	0.249346	0.803093	0.968418
BX005129.2	4.175851	1.661230734	0.957291	1.735345	0.08268	0.483014
AL928724.1	0.738387	-0.8681478	3.377995	-0.257	0.797178	NA
CU466287.2	33.88641	0.25541129	0.343317	0.743951	0.456906	0.860832
ms4a17a.5	3.230178	-1.764580106	1.746797	-1.01018	0.312409	0.776164
si:dkey-7j14.5	585.7456	0.20849502	0.236903	0.880087	0.378812	0.823136
CR847983.1	0	NA	NA	NA	NA	NA
xkr5b	59.61047	0.059726808	0.272587	0.219111	0.826564	0.973383
pimr26	0	NA	NA	NA	NA	NA
si:ch73-93k15.5	0.274222	-1.451411383	5.198538	-0.2792	0.780094	NA
BX571880.1	294.3826	0.222414707	0.246166	0.903514	0.366253	0.81589
si:ch211-252f13.5	53.46609	-1.347500373	0.276188	-4.87893	1.07E-06	0.000308
CR848844.1	13.76228	0.089850903	0.551508	0.162919	0.870582	0.983365
wwp1	1397.859	0.057034772	0.088062	0.647666	0.517201	0.887148
CR627496.1	0.645149	1.932482802	2.329852	0.829444	0.406853	NA
si:ch211-39f2.3	4.41986	0.503007567	1.291597	0.389446	0.696946	0.94198
pimr62	0	NA	NA	NA	NA	NA
CU137680.1	0.511897	-3.729962545	3.281832	-1.13655	0.255727	NA
CU633855.1	0	NA	NA	NA	NA	NA
cbx6b	43.03651	-0.156496087	0.310757	-0.5036	0.614545	0.915998
si:ch211-40k21.9	103.9756	-0.212816791	0.226484	-0.93965	0.347395	0.800035
AL954861.1	0.638645	-1.522574232	3.284924	-0.4635	0.643003	NA
CR450727.1	37.15662	-0.249882577	0.38593	-0.64748	0.517321	0.887148
CT573103.1	0.107013	-1.826650979	5.230461	-0.34923	0.726914	NA
si:dkey-7i4.15	41.79307	-0.117339153	0.321419	-0.36507	0.715062	0.945823
AL772362.1	0.291846	0	5.267649	0	1	NA
BX908750.1	38.29452	0.791032169	0.4791	1.65108	0.098722	0.521402
cyp8b2	5.448531	-0.50322252	1.092828	-0.46048	0.645174	0.926728
BX511311.2	0.04222	0	5.267649	0	1	NA
cyp8b3	7.875548	0.498664988	0.771721	0.646173	0.518167	0.887148
BX511311.3	45.52545	0.31138591	0.485925	0.640811	0.521645	0.887551
CR847503.2	0	NA	NA	NA	NA	NA
BX005083.1	0.160481	0	5.267649	0	1	NA
si:ch211-151m7.6	0.997239	-0.922006588	1.772938	-0.52004	0.603033	NA
si:ch73-190f9.4	0.092101	0	5.267649	0	1	NA
BX072576.3	0	NA	NA	NA	NA	NA
si:ch211-256e16.10	0	NA	NA	NA	NA	NA
si:dkey-208c12.2	10.82059	0.391494881	0.635742	0.615808	0.538022	0.892498
CR391977.1	0.270512	1.055379111	4.73504	0.222887	0.823623	NA
FP102921.2	0.410996	-1.821717162	3.735268	-0.48771	0.625757	NA
si:dkey-114l24.2	5.113301	-2.799809934	1.460647	-1.91683	0.05526	0.406983
ptprt	8.626894	1.662132176	1.019816	1.629835	0.103136	0.530217
si:ch211-107e6.5	12.16699	0.321597156	0.803573	0.400209	0.689003	0.93924
CR847898.3	4.295284	0.402195375	0.897771	0.447993	0.654158	0.929434

CU914602.1	21.46517	0.224034534	0.374019	0.598993	0.549178	0.897092
zgc:92912	55.37635	1.061288929	0.404929	2.620927	0.008769	0.160701
BX294100.2	0.129925	0	5.267649	0	1	NA
CT573048.1	1.65315	-3.59256157	1.68245	-2.13532	0.032735	NA
BX663519.1	6.286967	0.340192643	0.753202	0.451662	0.651512	0.928562
CR376740.2	0.440994	0	4.202794	0	1	NA
arfgap3	321.516	-0.116905128	0.189711	-0.61623	0.537744	0.892498
si:dkey-238d18.6	0	NA	NA	NA	NA	NA
CR548634.2	2.457531	0.567184462	1.464422	0.38731	0.698527	0.942433
BX664750.3	0	NA	NA	NA	NA	NA
si:dkey-175d9.1	0.046941	0	5.267649	0	1	NA
proser3	76.50094	-0.013811319	0.189994	-0.07269	0.94205	0.997371
si:dkey-40c23.1	10.81472	-0.93935543	0.765015	-1.22789	0.219488	0.700391
BX548024.1	0.589917	3.694571166	3.277129	1.127381	0.259582	NA
FP236741.1	5.366805	-0.131077983	0.780801	-0.16788	0.866681	0.982582
BX682556.1	3.794673	-0.185544702	0.928346	-0.19987	0.841586	0.97643
tac3b	26.45354	-0.145227864	0.549595	-0.26425	0.791591	0.964817
BX088688.3	1.068007	0	2.609806	0	1	NA
CR925804.1	1.143609	-1.797625366	2.108387	-0.85261	0.393877	NA
si:ch211-66h3.4	0.981764	0.714365058	1.98083	0.360639	0.718369	NA
CT868708.1	0.636317	0.065054852	2.334079	0.027872	0.977764	NA
BX072559.1	0.639715	-0.909301596	2.368625	-0.38389	0.701057	NA
CU571382.3	0	NA	NA	NA	NA	NA
BX465205.2	0.36806	1.921543831	5.236909	0.366923	0.713676	NA
CU137681.1	23.98147	-1.755022259	0.677211	-2.59154	0.009555	0.168223
tmem82	127.1477	-0.371918256	0.224946	-1.65337	0.098257	0.521076
BX324166.1	0	NA	NA	NA	NA	NA
CU929312.1	6.41316	-0.975402871	0.989984	-0.98527	0.324491	0.783308
BX005183.3	104.648	0.757407994	0.482537	1.569638	0.116499	0.557474
CU896691.2	3.754679	1.188438948	1.018531	1.166817	0.243284	0.724437
si:dkey-24l11.2	0	NA	NA	NA	NA	NA
igfn1.3	2451.407	0.485608735	0.180146	2.695639	0.007025	0.141889
si:ch211-108d22.2	217.7538	1.817054706	0.283429	6.410972	1.45E-10	1.40E-07
BX908796.1	0.707918	-2.327536014	3.094285	-0.7522	0.451928	NA
si:ch211-178n15.1	148.6694	0.006114462	0.196762	0.031075	0.975209	1
FP102073.1	0.270999	2.436374376	5.206324	0.467964	0.63981	NA
BX571794.1	0.106895	1.055383603	5.267649	0.200352	0.841205	NA
si:ch211-210b2.1	152.4658	0.021680861	0.236353	0.091731	0.926912	0.994883
BX936342.1	0.347674	2.070609412	3.834877	0.539942	0.589237	NA
si:dkey-42p14.6	0	NA	NA	NA	NA	NA
CU137681.2	0	NA	NA	NA	NA	NA
BX323450.1	0	NA	NA	NA	NA	NA
CR762400.1	0.36899	0	4.411156	0	1	NA
si:dkey-7i4.14	8.541687	0.285199023	0.739217	0.385812	0.699636	0.942499
FP102794.2	0.049519	0	5.267649	0	1	NA
si:ch211-282b22.1	396.6014	-0.438846984	0.244394	-1.79566	0.072549	0.457864
BX005101.1	0	NA	NA	NA	NA	NA
CU306817.1	3.46316	1.466243311	1.014539	1.445231	0.148393	0.60973
wu:fb18f06	1154.55	-0.224874797	0.236122	-0.95237	0.34091	0.795603
AL928908.3	5.319608	0.829325025	1.204764	0.688371	0.491219	0.875595
si:ch211-246i5.5	174.4834	0.126355071	0.177612	0.71141	0.47683	0.869292
CR848661.1	0.094182	-0.868089684	5.267649	-0.1648	0.869104	NA
si:ch73-265h17.4	6.112625	-0.472409798	0.881198	-0.5361	0.59189	0.909603
BX120006.3	0.262372	0.093661053	4.135387	0.022649	0.981931	NA

CR589941.1	0.043308	0	5.267649	0	1	NA
CU469568.3	0.114252	0	5.267649	0	1	NA
CU138509.1	1.989001	-0.104087844	1.436518	-0.07246	0.942237	NA
CU571091.1	0.087179	0	5.267649	0	1	NA
BX936393.1	0.753303	0	2.866228	0	1	NA
BX511021.2	11.72609	-0.779504576	0.541485	-1.43957	0.149989	0.613319
si:dkey-35i13.1	459.6809	0.291589671	0.233395	1.249338	0.211542	0.693044
FP243382.1	0.172567	1.854137197	5.241768	0.353724	0.723546	NA
atg2b	27.90328	0.641999135	0.409738	1.566852	0.117149	0.558278
tgm8	0.717438	-0.778337603	2.755301	-0.28249	0.77757	NA
si:dkey-234i14.2	61.16441	-0.172347936	0.412088	-0.41823	0.675778	0.935422
si:dkey-23k10.3	31.42471	-0.415505198	0.411788	-1.00903	0.312962	0.776457
CR556709.1	0.21483	1.055364289	5.092479	0.20724	0.835823	NA
CR381579.2	0.084441	0	5.267649	0	1	NA
si:dkey-7i4.16	10.21538	-0.501035079	0.757587	-0.66136	0.508384	0.883268
BX001030.2	0	NA	NA	NA	NA	NA
BX936406.2	0.262472	0.960394109	4.787858	0.20059	0.84102	NA
trappc2	501.1275	-0.018744126	0.146612	-0.12785	0.898269	0.990199
BX511124.1	0	NA	NA	NA	NA	NA
tesmin	1.508377	0.450046128	1.779416	0.252918	0.800332	NA
BX324206.2	166.1374	0.128471468	0.264108	0.486436	0.626658	0.920665
si:ch211-235m3.10	48.01838	-0.118049391	0.392123	-0.30105	0.763375	0.958926
CU076099.1	14.26507	0.552035947	0.459887	1.200373	0.229994	0.711763
BX569785.1	5.403746	-0.488501899	0.703691	-0.6942	0.487557	0.873291
si:ch211-139l2.1	0.340457	-1.789608901	3.894807	-0.45949	0.645885	NA
BX005048.1	5.615151	-0.99601514	1.240786	-0.80273	0.422131	0.843389
CR381579.3	0	NA	NA	NA	NA	NA
FP016235.1	6.325724	0.217854352	0.822321	0.264926	0.791066	0.964766
CU633978.1	0.132469	0	5.267649	0	1	NA
CU207301.2	0.129927	0	5.267649	0	1	NA
CR450824.1	0.701204	-2.332503037	2.155762	-1.08199	0.279259	NA
ftr90	2.644316	0.526481653	1.451938	0.362606	0.716899	0.94627
si:ch211-241f5.3	16.14028	-0.430810345	0.598728	-0.71954	0.471807	0.868289
si:ch211-244k5.1	0.183043	0	5.267649	0	1	NA
CR383683.1	0	NA	NA	NA	NA	NA
sptbn5	118.551	0.672893542	0.26322	2.55639	0.010576	0.179908
si:ch211-235i11.3	54.09866	0.061458171	0.243763	0.252123	0.800946	0.96768
FP325112.1	0.329126	0	4.539679	0	1	NA
CT573316.3	0	NA	NA	NA	NA	NA
si:ch73-265h17.5	0.61786	-0.868139815	3.051938	-0.28446	0.776062	NA
CT867948.1	0.181296	0	5.267649	0	1	NA
BX629346.2	0	NA	NA	NA	NA	NA
pclaf	148.0866	-0.058256208	0.211256	-0.27576	0.782732	0.962328
si:ch211-116o3.5	230.7096	0.036382925	0.147406	0.246822	0.805046	0.969348
si:ch211-248a14.8	246.3701	-1.787754494	0.301107	-5.93728	2.90E-09	2.10E-06
CU137681.3	0.200285	1.854123109	5.241768	0.353721	0.723548	NA
si:ch73-71c20.5	219.1184	-0.05963728	0.165145	-0.36112	0.718008	0.946533
si:dkey-117a8.4	2.629725	0.096244608	1.27572	0.075443	0.939862	0.997371
si:dkey-23k10.2	13.95423	0.229772062	0.779483	0.294775	0.768166	0.960296
AL954831.1	6.892907	-1.810685157	0.979456	-1.84866	0.064506	0.433491
si:ch1073-66l23.1	0.334658	-2.38723419	3.795184	-0.62902	0.529338	NA
ccdc36	0.619886	0.093658216	2.3847	0.039275	0.968671	NA
birc6-as2	112.5655	0.28128234	0.242633	1.159294	0.246337	0.727641
BX323807.2	0.049519	0	5.267649	0	1	NA

CR786576.1	0.045161	0	5.267649	0	1	NA
BX957311.1	0.178355	0	5.267649	0	1	NA
CR848667.1	69.2982	-0.190859829	0.277331	-0.6882	0.491325	0.875595
BX927318.1	0	NA	NA	NA	NA	NA
FP017215.1	109.1826	0.259640781	0.368109	0.705338	0.4806	0.870179
BX571794.2	0	NA	NA	NA	NA	NA
stard10	284.8374	0.002819632	0.187409	0.015045	0.987996	1
CU469514.1	3.608318	-2.752692314	1.62825	-1.69058	0.090916	0.505285
BX005129.3	2.694821	-0.388153716	1.216918	-0.31896	0.749753	0.955074
CU207301.3	39.05469	0.587885987	0.37122	1.58366	0.113271	0.551017
BX927282.3	0.35503	0	3.828502	0	1	NA
BX936342.2	6.355686	-0.895146439	0.822729	-1.08802	0.276586	0.751172
si:dkey-96g2.1	74.12779	0.313518418	0.84894	0.369306	0.7119	0.945821
si:ch211-149e23.4	6.409187	-0.907910359	1.140038	-0.79639	0.425808	0.844714
CR388132.1	3.053245	-3.394508468	1.60337	-2.11711	0.034251	0.326714
CR352243.2	0.527625	1.03103158	2.967467	0.347445	0.728257	NA
CR936482.1	27.61782	0.005115056	0.422356	0.012111	0.990337	1
CR932000.1	2.396205	-1.088439622	1.146667	-0.94922	0.342508	0.797179
BX957346.3	0.293749	1.055364293	4.698171	0.224633	0.822265	NA
CR628365.1	5.412131	0.855647692	0.838686	1.020224	0.307622	0.772975
si:dkey-188h10.3	67.06628	0.164515445	0.401927	0.409317	0.682307	0.937945
AL929222.2	110.0357	0.393820595	0.279121	1.41093	0.158265	0.623766
si:dkey-238d18.9	0	NA	NA	NA	NA	NA
elob	88.29706	-0.268094733	0.218562	-1.22663	0.219962	0.701105
CT954240.1	6.849191	-0.087361111	0.922503	-0.0947	0.924553	0.994815
si:dkey-276j7.2	2.880358	2.550396112	1.432585	1.780276	0.075031	0.464411
CU861501.1	0.496398	-1.756061451	4.009128	-0.43802	0.661375	NA
BX323987.1	0	NA	NA	NA	NA	NA
si:rp71-77l1.1	288.1601	-0.474538251	0.174887	-2.7134	0.00666	0.137282
CU464118.1	19.01221	-0.093628383	0.389517	-0.24037	0.810043	0.969828
CR381621.1	0.240728	0.093657633	4.949328	0.018923	0.984902	NA
BX511195.1	0.04222	0	5.267649	0	1	NA
CR854827.3	2.86776	0.453861711	1.23606	0.367184	0.713482	0.945821
si:rp71-77l1.2	4.37842	-0.893212568	1.065876	-0.83801	0.402026	0.832956
slirp	484.5378	0.177804055	0.168848	1.053039	0.292323	0.761879
CR749763.5	0	NA	NA	NA	NA	NA
CR626882.3	0.628948	0	3.246811	0	1	NA
BX511010.1	0.043308	0	5.267649	0	1	NA
CU929206.2	1.476993	-2.679591587	1.807301	-1.48265	0.138168	NA
CR628327.1	1.302449	-1.258647422	1.738249	-0.72409	0.469011	NA
BX005234.1	17.97016	0.913263905	0.411252	2.22069	0.026372	0.287876
CU693494.2	64.61291	-0.055761901	0.471443	-0.11828	0.905846	0.991631
CT573392.1	29.9639	-0.677192825	0.360431	-1.87884	0.060266	0.422591
BX936364.1	0.496077	0	3.925157	0	1	NA
BX537342.1	80.32268	-0.041841893	0.205316	-0.20379	0.838515	0.976395
mhc1lia	18.63516	0.003161488	0.658995	0.004797	0.996172	1
BX000438.1	10.70481	1.573882052	1.489358	1.056752	0.290625	0.76078
BX649397.1	0.091739	0	5.267649	0	1	NA
si:ch211-167j9.4	104.3725	1.042941058	0.298955	3.488624	0.000486	0.027042
BX571786.1	0.233091	-1.710599731	4.981801	-0.34337	0.73132	NA
si:ch211-185a18.2	77.98065	0.437890591	0.240319	1.822121	0.068437	0.445916
CR391910.3	21.34394	-0.086740511	0.479143	-0.18103	0.856342	0.979558
CR387996.1	7.857447	2.048228977	0.602912	3.397226	0.000681	0.034589
BX465205.3	0	NA	NA	NA	NA	NA

si:ch1073-80i24.3	4.301849	0.141014139	0.854694	0.164988	0.868953	0.982801
BX005183.4	0	NA	NA	NA	NA	NA
CR385053.2	0.111164	-0.868089684	5.267649	-0.1648	0.869104	NA
BX649559.1	3.05307	-0.643078474	1.078847	-0.59608	0.551122	0.8974
CU683879.2	0.995285	-0.796517858	2.442836	-0.32606	0.744377	NA
si:dkey-42l23.4	4.737493	-0.396425163	0.948109	-0.41812	0.675858	0.935422
ccdc162	0.937948	-0.374057828	2.124836	-0.17604	0.860262	NA
CU467862.1	0.043308	0	5.267649	0	1	NA
BX572077.2	0.208087	0	5.267649	0	1	NA
pon3.2	182.6845	-0.284652079	0.181109	-1.57172	0.116016	0.556131
si:dkey-234i14.6	41.71532	0.698452318	0.378695	1.844369	0.065129	0.435355
FP325112.2	2.079999	-0.489247382	1.378223	-0.35498	0.722601	NA
si:ch211-33e4.3	1.887136	-3.704790401	1.718586	-2.15572	0.031106	NA
CR626944.1	52.6911	0.073574188	0.320046	0.229886	0.81818	0.971518
zgc:162952	109.4475	-1.655461723	0.330157	-5.01416	5.33E-07	0.000174
AL845357.1	0.087179	0	5.267649	0	1	NA
tomm6	493.7774	-0.249438453	0.133038	-1.87494	0.060801	0.423559
CU929037.3	1.811476	0	1.982418	0	1	NA
zgc:194207	27.00026	-0.930390453	0.582376	-1.59758	0.110137	0.544691
CR788235.1	10.94489	-0.445116869	0.570337	-0.78044	0.435129	0.850085
BX470154.1	0.438963	1.055395012	3.686554	0.286282	0.774662	NA
si:dkey-238d18.12	0.858824	0	2.78436	0	1	NA
si:ch211-275j6.5	153.2448	0.001358402	0.171116	0.007938	0.993666	1
lypc	427.0083	-0.444918423	0.252655	-1.76097	0.078243	0.473959
CR376747.1	0.656783	1.871738599	3.014365	0.62094	0.534639	NA
BX530017.2	1.765465	0.481578049	1.278811	0.376583	0.706484	NA
si:dkey-77f5.4	61.64139	-0.017068409	0.251945	-0.06775	0.945987	0.997371
si:ch211-256e16.8	0.337919	0.895605158	4.480211	0.199902	0.841557	NA
CR925777.1	0.899516	-0.97528653	1.948704	-0.50048	0.616737	NA
BX324126.1	0.448341	1.863533462	3.632489	0.513018	0.607939	NA
si:ch73-144d13.5	28.20869	0.250277007	0.344843	0.725771	0.467979	0.866337
BX927323.1	0.995963	0	2.374524	0	1	NA
CU928129.1	45.42437	-0.924515509	0.332258	-2.78252	0.005394	0.123615
si:dkey-117a8.1	15.07923	0.608946961	0.476654	1.277544	0.20141	0.681266
CT027582.2	0	NA	NA	NA	NA	NA
znf576.1	300.8628	0.038240251	0.137563	0.277984	0.781025	0.961659
BX005129.4	2.941793	-1.754958612	1.608178	-1.09127	0.275153	0.750424
CR933839.1	0	NA	NA	NA	NA	NA
CU464125.1	0	NA	NA	NA	NA	NA
smco4	280.1967	0.219676363	0.16361	1.342681	0.179375	0.654677
BX465227.1	155.4833	-0.258673943	0.189766	-1.36312	0.172845	0.645664
si:dkey-239b22.2	320.4826	-0.435666218	0.202035	-2.15639	0.031053	0.313376
lrp1aa	1279.465	0.117144184	0.177786	0.658904	0.509957	0.883733
CR848825.1	0.175305	0.09368032	5.267649	0.017784	0.985811	NA
CU463231.1	1.458042	0.852947867	1.517862	0.56194	0.574157	NA
CR382327.3	41.68365	0.168761116	0.3505	0.481487	0.630171	0.922869
BX571712.1	0.667534	-1.784102088	2.60179	-0.68572	0.492889	NA
CR388050.1	0.046941	0	5.267649	0	1	NA
pimr40	0	NA	NA	NA	NA	NA
si:dkey-42l23.1	2.204192	0	1.93627	0	1	1
si:ch211-235i11.6	31.25195	-0.755326021	0.331095	-2.2813	0.022531	0.265534
si:dkey-42l23.6	0.740102	-3.101945121	2.728507	-1.13687	0.255595	NA
CR933730.1	3.275633	-0.509962359	1.027155	-0.49648	0.619556	0.917435
znf1134	90.02683	-0.145955219	0.247938	-0.58868	0.556078	0.899002

BX088540.1	2.291329	0.430031462	1.496331	0.287391	0.773813	0.960624
si:ch211-105j21.9	151.9468	-0.059512911	0.199746	-0.29794	0.765747	0.959951
si:dkey-74h17.3	1.326393	0	2.324627	0	1	NA
si:ch211-269k10.4	14.16616	-0.147822061	0.540083	-0.2737	0.784313	0.962605
BX927395.1	0.049021	-0.86807634	5.267649	-0.16479	0.869106	NA
CT583687.2	0.471899	0.093652264	3.620339	0.025868	0.979362	NA
CR792439.1	0.741928	0	2.929882	0	1	NA
BX323586.3	102.9065	-0.16506674	0.230041	-0.71755	0.473033	0.868965
CR377210.1	2.654492	2.207114938	1.414226	1.560652	0.118606	0.561771
BX255963.2	0.913746	-0.868145771	2.503502	-0.34677	0.728762	NA
CT025854.1	0.04222	0	5.267649	0	1	NA
BX324134.1	0.170537	-1.755438701	5.23528	-0.33531	0.737392	NA
CR376776.1	0.224621	1.05535601	5.063182	0.208437	0.834888	NA
CU041346.1	0	NA	NA	NA	NA	NA
si:dkeyp-84f3.9	267.4089	-0.007961082	0.121273	-0.06565	0.94766	0.997371
CR847983.2	0.188905	-0.868102747	5.267649	-0.1648	0.869102	NA
CR381579.4	0.160585	0	5.267649	0	1	NA
BX294160.1	14.11696	-0.090884996	0.59518	-0.1527	0.878633	0.985761
si:dkey-1m11.6	8.191084	0.404291438	0.831042	0.486488	0.626621	0.920665
pimr13	0.050624	1.055396947	5.267649	0.200354	0.841203	NA
si:ch1073-145m9.1	28.83612	-0.492056052	0.341017	-1.44291	0.149046	0.610726
si:ch211-137j23.6	1.0096	-1.766146594	2.060667	-0.85708	0.391403	NA
BX530721.1	0.736391	-2.726906014	2.516842	-1.08346	0.278603	NA
BX548170.1	0	NA	NA	NA	NA	NA
CR786577.1	33.65951	0.633812563	0.57359	1.104991	0.269163	0.745285
si:dkey-40g16.5	5.516447	-0.79876772	0.702015	-1.13782	0.255195	0.733542
si:ch211-167b20.8	251.7388	0.040355041	0.143423	0.281371	0.778426	0.961073
CU694532.1	15.29691	0.487785625	0.713348	0.683798	0.494103	0.877558
BX323824.1	13.03452	0.26533901	0.477953	0.555158	0.578787	0.905417
CR936518.2	0.566522	-0.86813813	2.804695	-0.30953	0.756918	NA
CR759927.2	8.677043	0.864692263	0.520688	1.660673	0.096779	0.519309
CU928013.1	117.4827	-0.07342351	0.167102	-0.43939	0.660376	0.930581
CU104777.1	39.65723	-0.237537477	0.378812	-0.62706	0.530621	0.890545
BX293559.1	0	NA	NA	NA	NA	NA
si:ch73-265h17.2	2.5492	-2.106044832	1.592346	-1.32261	0.185967	0.663819
si:ch1073-100f3.2	45.32126	0.431700385	0.444858	0.970423	0.331836	0.789531
CR751227.2	7.268584	-0.760463277	0.697877	-1.08968	0.275854	0.750674
BX470264.1	84.57215	0.272048902	0.30995	0.877719	0.380096	0.823607
CR352228.1	1.368205	0.274717639	1.511079	0.181802	0.855738	NA
BX000451.1	1.480046	-1.079167303	1.567417	-0.6885	0.491138	NA
sgip1a	462.9327	-0.326074286	0.211538	-1.54144	0.123209	0.569695
BX649266.2	0	NA	NA	NA	NA	NA
si:ch73-90p23.1	3.657579	0.790372469	1.08725	0.726946	0.467259	0.86616
si:ch73-204p21.2	322.5416	0.104168299	0.147339	0.706996	0.479569	0.869929
BX927184.1	105.1499	0.358223236	0.246668	1.452248	0.146433	0.605984
CR387921.1	0.476589	0	3.192576	0	1	NA
CU928083.1	8.837575	-2.370245009	1.307327	-1.81305	0.069825	0.450824
BX322555.4	3.18987	0.089535624	1.0248	0.087369	0.930378	0.995707
si:dkey-195m11.11	17.32244	0.744246685	0.617735	1.204799	0.228281	0.709397
BX293567.1	1.878708	1.633813956	1.763168	0.926636	0.354116	NA
CU179756.1	0.511304	-0.868141097	3.522064	-0.24649	0.805306	NA
BX649356.1	0	NA	NA	NA	NA	NA
CT574587.1	0.885011	2.533983064	2.135877	1.18639	0.235468	NA
AL929309.1	0.400277	0.935346867	3.25707	0.287174	0.773979	NA

si:ch211-137j23.7	0.053506	-0.86807634	5.267649	-0.16479	0.869106	NA
AL954666.1	2.791319	-0.316010276	1.464094	-0.21584	0.829112	0.97456
tex36	1.798833	-0.127741437	1.29362	-0.09875	0.921339	NA
BX465857.1	0.254466	0	4.878514	0	1	NA
CR385026.2	0	NA	NA	NA	NA	NA
BX663528.1	0	NA	NA	NA	NA	NA
CU464134.1	7.58918	-0.868144469	1.824608	-0.4758	0.634219	0.924077
si:ch73-40a17.4	16.0769	-0.36778934	0.599329	-0.61367	0.539435	0.893049
BX005392.3	4.306244	-0.730868295	0.966745	-0.75601	0.449643	0.856979
BX323557.1	15.6822	-2.73460811	0.696723	-3.92496	8.67E-05	0.008237
BX510649.1	4.414415	-0.66932221	0.770466	-0.86872	0.384998	0.826939
CR762392.1	0.053262	1.055396947	5.267649	0.200354	0.841203	NA
si:dkey-22l14.10	20.60115	-0.898434635	0.453719	-1.98016	0.047686	0.380207
FP102895.1	0	NA	NA	NA	NA	NA
CT955996.1	0	NA	NA	NA	NA	NA
asb5a	438.0162	0.116254421	0.222184	0.523235	0.600811	0.913756
CU467879.1	0	NA	NA	NA	NA	NA
pimr175	0.415615	0.020830635	2.860336	0.007283	0.994189	NA
si:ch211-207d10.2	0.390582	1.055357499	5.030621	0.209787	0.833834	NA
CR926464.1	0.819228	-2.3828817	2.731943	-0.87223	0.383083	NA
BX664614.1	13.47248	0.077434416	0.583682	0.132665	0.894458	0.98891
CU137681.4	0	NA	NA	NA	NA	NA
CR354430.3	1.178976	-0.472149471	1.889238	-0.24992	0.802653	NA
mespbb	0.342502	0	4.433361	0	1	NA
BX901883.3	0.214368	0	5.267649	0	1	NA
BX663520.1	1.795559	0.116867865	1.385018	0.08438	0.932754	NA
CU469568.4	1.648337	-0.072849139	1.166047	-0.06248	0.950184	NA
si:ch211-284e13.14	17.64209	-0.034164137	0.46008	-0.07426	0.940806	0.997371
BX469902.1	62.56321	0.260063626	0.271996	0.956131	0.339006	0.795226
CT956066.1	1.358297	-0.89614026	1.562831	-0.57341	0.566368	NA
CR391915.1	0.103263	1.055383603	5.267649	0.200352	0.841205	NA
CU137680.2	0.049021	-0.86807634	5.267649	-0.16479	0.869106	NA
si:dkey-248g15.3	1565.667	0.007915041	0.285745	0.0277	0.977902	1
BX511132.1	1.580047	1.84971349	1.41303	1.30904	0.190521	NA
BX571762.2	73.56802	0.092028153	0.288231	0.319286	0.74951	0.955074
CR589875.2	8.934951	2.230998599	0.78527	2.841058	0.004496	0.110596
arhgef33	88.50955	-0.796934686	0.227214	-3.50743	0.000452	0.025763
BX936420.1	1.20785	0.80522972	1.592309	0.505699	0.613068	NA
BX950217.1	0.418883	1.05541144	3.261385	0.323608	0.746235	NA
BX942813.1	8.140392	-0.954202304	0.583899	-1.63419	0.102219	0.528963
FP089511.1	2.351383	0.578552649	1.146927	0.504437	0.613954	0.915998
BX004769.1	1.80385	0.043253409	1.667678	0.025936	0.979308	NA
BX957251.1	0.139769	0	5.267649	0	1	NA
CT033842.1	0.537975	2.559520614	3.439466	0.744162	0.456778	NA
si:ch1073-190k2.1	267.9589	0.515152819	0.300542	1.71408	0.086514	0.493048
CU855685.1	10.01547	1.732335414	0.755134	2.294078	0.021786	0.261665
CR812464.1	0.453472	-2.7551039	4.815364	-0.57215	0.567221	NA
BX000364.1	0	NA	NA	NA	NA	NA
CR387984.1	0	NA	NA	NA	NA	NA
ugt1b3	2.082418	0.093648129	1.946403	0.048113	0.961626	NA
BX005375.1	9.036816	-0.686570214	0.85916	-0.79912	0.424222	0.844355
BX957228.1	0	NA	NA	NA	NA	NA
BX323067.1	3.960351	-0.22454116	0.774681	-0.28985	0.771931	0.960624
BX571712.2	7.299415	3.057306429	1.053301	2.902594	0.003701	0.100884

EIF3J (1 of many)	0.090321	0	5.267649	0	1	NA
CR293498.1	0	NA	NA	NA	NA	NA
BX005450.1	4.129956	0.51879994	0.943703	0.549749	0.582492	0.90571
si:ch211-191c10.1	5.342257	0.739581875	0.825662	0.895744	0.370389	0.818484
FP102071.1	0.201801	0	5.267649	0	1	NA
BX571774.1	0.425495	2.559849746	3.606515	0.709785	0.477838	NA
CR848784.1	0.336436	0	3.892515	0	1	NA
si:ch211-272b8.7	1.557384	-1.284357598	1.641473	-0.78244	0.433955	NA
CR376785.1	0	NA	NA	NA	NA	NA
BX640549.1	0.637474	2.302492566	2.352704	0.978658	0.327749	NA
ftr91	4.878447	-0.215947173	0.872318	-0.24756	0.804478	0.969054
BX004996.1	0.415768	1.473771565	3.448138	0.427411	0.66908	NA
AL929523.1	0.517884	0	3.327515	0	1	NA
CU633486.1	1.07977	0.969193268	2.474881	0.391612	0.695345	NA
BX000438.2	880.0932	-0.214548053	0.238229	-0.9006	0.367804	0.817078
itgae.1	42.01833	-2.369308157	0.402826	-5.88171	4.06E-09	2.77E-06
si:ch211-130m23.5	196.0071	-0.11783444	0.161082	-0.73152	0.464463	0.864594
CU571179.1	0	NA	NA	NA	NA	NA
CT573139.2	0.573447	-0.868140729	2.831786	-0.30657	0.759171	NA
BX539313.3	2.508795	1.145753728	1.298911	0.882088	0.377729	0.822478
pet100	186.481	-0.106807549	0.251289	-0.42504	0.670808	0.93394
BX005129.5	2.741643	-1.480170792	1.428596	-1.0361	0.300155	0.766629
si:dkey-111k8.2	44.43968	-0.809557693	0.269052	-3.00893	0.002622	0.083393
CR936378.1	19.67725	-0.054350625	0.574343	-0.09463	0.924608	0.994815
AL928707.1	0.043309	0	5.267649	0	1	NA
si:dkey-262k9.2	787.0626	-0.014825682	0.13796	-0.10746	0.914421	0.992972
CR548622.1	1.996156	-1.76410009	1.591111	-1.10872	0.26755	NA
si:dkey-238d18.8	0	NA	NA	NA	NA	NA
si:dkey-77f5.14	57.5702	-0.127280968	0.294581	-0.43207	0.665687	0.932664
BX890602.1	1.081905	-2.373900405	2.011817	-1.17998	0.238009	NA
si:ch211-129o18.4	5.013801	-0.820847865	0.914805	-0.89729	0.369563	0.817994
BX571794.3	1.552758	0.152543299	2.165224	0.070452	0.943834	NA
si:ch73-144d13.8	22.38523	0.026352843	0.372129	0.070816	0.943544	0.997371
si:dkey-7i4.8	54.03538	0.019699767	0.291421	0.067599	0.946105	0.997371
CR848840.1	1.451619	-0.144572637	1.267601	-0.11405	0.909196	NA
si:ch211-69l10.4	22.24042	-0.660455906	0.627079	-1.05323	0.292237	0.761879
CU927934.3	14.0411	0.70748089	0.849524	0.832797	0.404959	0.834284
FP015862.3	1.075848	0.093653055	2.519167	0.037176	0.970345	NA
AL928707.2	0.043309	0	5.267649	0	1	NA
CR391995.1	0.129924	0	5.267649	0	1	NA
BX005332.1	0	NA	NA	NA	NA	NA
BX663503.2	60.81612	-0.378447117	0.549037	-0.68929	0.490639	0.875539
CR751602.2	77.92894	-0.208262032	0.270372	-0.77028	0.441135	0.852823
FP017161.1	20.79806	1.139713408	0.597034	1.90896	0.056267	0.410018
CU855758.2	0	NA	NA	NA	NA	NA
CT025912.1	0.045161	0	5.267649	0	1	NA
BX248121.1	212.4761	-0.059208936	0.261241	-0.22664	0.8207	0.971788
BX571712.3	1.042903	-4.348357639	2.150483	-2.02204	0.043173	NA
opn6b	61.20985	0.414845837	0.622643	0.666266	0.505241	0.882188
BX548024.2	3.393866	0.984889457	0.844171	1.166694	0.243334	0.724437
CT009746.1	0	NA	NA	NA	NA	NA
dscaml1	97.32316	0.433151194	0.270919	1.598821	0.10986	0.543786
fkbp3	554.1738	-0.027614351	0.15657	-0.17637	0.860003	0.980167
alp3	56.11315	2.058725396	0.8223	2.503619	0.012293	0.192713

si:ch1073-205c8.5	0.444056	0	4.17434	0	1	NA
dre-mir-365-1	0.046941	0	5.267649	0	1	NA
CU302323.1	1.200453	-0.803414405	1.855174	-0.43307	0.664966	NA
AL929457.2	0.930148	-1.811213798	2.659664	-0.68099	0.495876	NA
CU207354.2	0	NA	NA	NA	NA	NA
CABZ01085140.1	30.74918	-0.574273225	0.374118	-1.53501	0.124782	0.572486
znf1049	1.596592	-1.02849449	1.516925	-0.67801	0.497763	NA
trip4	127.7073	0.199836698	0.215035	0.929322	0.352722	0.803517
sh3bgrl3	1193.067	0.117037666	0.120299	0.972888	0.330609	0.788126
kcnq1.2	7.649441	1.615901265	0.740058	2.183478	0.029001	0.303224
si:dkey-238o14.3	3.567012	4.113214007	1.44603	2.844488	0.004448	0.109917
dre-mir-18a	0	NA	NA	NA	NA	NA
rab3gap1	571.3818	0.005525044	0.153167	0.036072	0.971225	1
gnl2	948.0836	-0.246600845	0.151935	-1.62307	0.104575	0.53308
GABRR1 (1 of many)	61.83057	-1.390072001	0.470187	-2.95642	0.003112	0.091566
CU459093.1	29.70346	-0.028357193	0.389195	-0.07286	0.941917	0.997371
RF00001	0.594252	-2.356782597	2.876273	-0.81939	0.412565	NA
si:dkey-30f3.2	9.795167	-0.832464637	0.757409	-1.09909	0.271727	0.747999
CU929178.1	0.046941	0	5.267649	0	1	NA
BX000463.1	0.230035	1.05536091	5.010242	0.210641	0.833168	NA
si:dkey-146c18.5	4.188386	0.099506242	0.801203	0.124196	0.90116	0.990512
CT027696.1	4.017905	-3.934633721	1.335736	-2.94567	0.003223	0.093414
slc12a1	30.74928	1.241089586	0.548313	2.26347	0.023607	0.271853
jac3	0.551851	0.041568842	2.80419	0.014824	0.988173	NA
RF00001	0.159785	2.50566729	5.20297	0.481584	0.630101	NA
zgc:64065	29.02961	-0.770144338	0.524149	-1.46932	0.141745	0.600409
BX248243.1	0.333426	-3.0388486	5.176027	-0.5871	0.557136	NA
tefb	548.2812	-0.087593823	0.222944	-0.3929	0.694397	0.941093
tmem106c	249.1275	-0.400096583	0.167445	-2.38942	0.016875	0.230481
si:ch211-215e19.8	0.099037	0	5.267649	0	1	NA
dusp2	131.4143	-0.348691532	0.387498	-0.89985	0.368198	0.817272
CABZ01079550.1	0.13396	0	5.267649	0	1	NA
btc	131.9617	-0.045352292	0.190888	-0.23759	0.812203	0.970129
WSCD1	34.23873	0.325921971	0.422256	0.771859	0.440198	0.852079
ctsa	674.7447	0.2873782	0.143908	1.996961	0.045829	0.374848
si:dkey-178j11.5	33.22947	0.710595697	0.470514	1.510254	0.130979	0.582602
trappc10	686.9788	0.069866151	0.11054	0.632042	0.52736	0.889948
CABZ01012959.1	0.633443	2.282723939	2.996058	0.761909	0.446114	NA
RF00001	0.317945	1.03462284	3.418329	0.302669	0.762142	NA
FP101882.1	160.5042	-0.601242628	0.246054	-2.44354	0.014544	0.211145
BX005085.2	35.79603	-0.567109711	0.351813	-1.61197	0.10697	0.537
si:dkey-44g17.6	20.21461	-0.47792231	0.508523	-0.93982	0.347308	0.799992
BX324212.1	0.458848	-0.868124148	4.016319	-0.21615	0.828871	NA
si:dkeyp-97a10.2	84.63651	-0.162954172	0.198919	-0.8192	0.412674	0.838123
bola3	135.0283	0.036280682	0.203822	0.178001	0.858722	0.979934
BX511136.1	2.631837	1.691070227	0.965861	1.750842	0.079973	0.478179
si:ch211-281k19.2	0.66612	1.05539008	3.817793	0.27644	0.78221	NA
stk40	260.8016	0.057031518	0.189999	0.300167	0.76405	0.959228
ankrd13d	168.6662	-0.294339309	0.279997	-1.05122	0.293155	0.762581
supt3h	292.2985	-0.496817163	0.189755	-2.61821	0.008839	0.16123
CR391944.1	15.37794	0.760716473	0.410975	1.851006	0.064169	0.432457
si:dkey-57l19.1	1.362938	-4.291084018	5.129881	-0.83649	0.40288	NA
si:dkey-285e18.6	0.212123	-1.378323151	5.201854	-0.26497	0.791034	NA
BX572628.1	0.802255	-0.999413956	2.38456	-0.41912	0.675129	NA

uts2d	13.4821	0.037938705	0.551276	0.06882	0.945133	0.997371
BX255896.1	3.99156	0.577978389	1.088893	0.530795	0.595561	0.911376
CR925797.1	0.569099	0.08400201	2.725441	0.030821	0.975412	NA
lhfpl2b	282.6608	0.261568856	0.175275	1.49233	0.135613	0.591012
CT583728.1	0.119909	2.079562922	5.22636	0.397899	0.690705	NA
CR792416.1	0.493054	-1.713183691	4.083937	-0.41949	0.674856	NA
cpxm1b	138.4982	-0.439261066	0.259549	-1.6924	0.09057	0.505206
cntn4	63.16228	0.999387349	0.295523	3.381753	0.00072	0.035767
zgc:77748	254.1883	-0.576462205	0.432426	-1.33309	0.182503	0.659083
traj56	0	NA	NA	NA	NA	NA
aipl2	21.85573	0.38411911	0.608202	0.631565	0.527671	0.889948
si:dkey-176f19.4	3.035763	0.324451963	1.240977	0.261449	0.793746	0.965431
zgc:162193	402.2642	0.381523433	0.210826	1.809659	0.070349	0.452395
tradv19.0	0	NA	NA	NA	NA	NA
BX537115.1	0.090249	0	5.267649	0	1	NA
dgkab	27.55519	0.627875113	0.597375	1.051056	0.293233	0.762581
si:rp71-1h20.5	85.30838	-0.218205356	0.222164	-0.98218	0.326011	0.784919
si:dkey-28i19.3	4.420811	-0.487963779	0.784478	-0.62202	0.533927	0.891181
or109-4	0	NA	NA	NA	NA	NA
si:dkeyp-86c4.1	1.268676	0.772885075	1.87281	0.412687	0.679836	NA
zgc:113184	221.0224	-0.047421657	0.190924	-0.24838	0.803841	0.968812
si:dkey-27n6.1	0.719738	1.027106654	2.642545	0.388681	0.697512	NA
BX663612.1	1.252987	-1.093559012	1.867919	-0.58544	0.55825	NA
si:dkey-16p6.1	10.71549	0.939200432	0.704928	1.332336	0.18275	0.659278
si:dkey-16l2.20	302.7317	-0.169063193	0.118954	-1.42125	0.155243	0.61985
znf998	1.307524	-0.891804986	1.527172	-0.58396	0.559248	NA
SLC39A11	73.16664	0.284084877	0.218151	1.302241	0.192834	0.672855
znf1077	35.28937	0.476279234	0.437867	1.087726	0.276716	0.75129
CU633999.1	0.76192	0.081628114	2.331521	0.035011	0.972071	NA
RF00001	0.617367	0	3.106827	0	1	NA
BX928743.1	0.111311	0	5.267649	0	1	NA
mtmr3	201.3458	-0.316833157	0.163277	-1.94047	0.052323	0.397352
AL954698.1	0	NA	NA	NA	NA	NA
APCDD1	6.741032	0.012373822	0.771166	0.016046	0.987198	1
CU984600.2	13.43627	-0.676313725	0.804955	-0.84019	0.400803	0.8318
ptrh1	65.99904	0.229448479	0.227685	1.007743	0.313578	0.777207
fn3krp	22.90567	-0.502130954	0.433423	-1.15853	0.24665	0.727706
si:ch211-199b20.3	67.34289	-0.394328857	0.274017	-1.43907	0.150131	0.613498
wrn	79.51578	0.026141972	0.20761	0.125919	0.899796	0.99045
BX465836.1	1.296837	0.516154057	1.53097	0.337142	0.73601	NA
BICD2	216.9332	0.169455701	0.224758	0.753947	0.450881	0.857288
si:ch211-215e19.3	0.129532	0	5.267649	0	1	NA
si:dkey-261j11.1	4.350008	-1.325419803	1.282583	-1.0334	0.301417	0.767562
si:ch211-209j12.6	3.010382	-0.532810295	1.212888	-0.43929	0.660451	0.930581
st8sia1	352.7505	0.111034887	0.152912	0.726137	0.467755	0.866174
AL773558.1	18.12249	-2.623922187	0.700737	-3.74452	0.000181	0.014053
tmem56b	536.5249	0.086386259	0.127972	0.675043	0.499649	0.879634
olfcg7	1.04696	0.76103096	2.307274	0.32984	0.741521	NA
smpd3	40.97911	0.975541613	0.298194	3.271501	0.00107	0.045987
BX649608.1	0	NA	NA	NA	NA	NA
thrap3b	1541.831	-0.028065717	0.089831	-0.31243	0.754715	0.956283
BX649416.1	43.8627	-0.115156538	0.271968	-0.42342	0.67199	0.933953
hhex	48.35289	-0.091783703	0.235791	-0.38926	0.697084	0.94198
tiam2b	61.6511	0.026451651	0.210607	0.125597	0.900051	0.99045

actr5	161.6991	-0.122149836	0.156271	-0.78166	0.434417	0.850002
zgc:174698	1.690647	0.517144603	1.37272	0.37673	0.706374	NA
fbn2b	985.0686	0.238093554	0.288382	0.825617	0.409021	0.836637
CR626866.1	0.337991	0.038445182	4.413528	0.008711	0.99305	NA
zgc:85932	439.5902	0.128473164	0.147811	0.86917	0.384754	0.826588
meis2a	2185.176	0.266331633	0.102477	2.598935	0.009351	0.166286
brcc3	351.4394	0.186059824	0.137431	1.353839	0.175788	0.649413
CR762475.1	0.155358	0	5.267649	0	1	NA
CR388206.1	0.111311	0	5.267649	0	1	NA
cidea	6.030433	-1.481043977	1.297239	-1.14169	0.253583	0.732873
CU928222.2	0	NA	NA	NA	NA	NA
det1	71.10409	0.223775654	0.204367	1.09497	0.27353	0.749148
asmt	51.06566	0.939466046	0.340132	2.762061	0.005744	0.128583
cpne9	8.416797	-1.294137619	1.136764	-1.13844	0.254937	0.733542
si:ch73-329n5.6	65.8976	-0.945943572	0.601488	-1.57267	0.115795	0.555893
pcdh15a	67.94277	0.77603302	0.288833	2.68679	0.007214	0.144046
RF00001	0	NA	NA	NA	NA	NA
si:dkeyp-85h7.1	0.090321	0	5.267649	0	1	NA
CU929294.1	46.53415	0.104364228	0.546633	0.190922	0.848587	0.977912
RF00001	0.046941	0	5.267649	0	1	NA
znf1053	15.37617	-0.687042543	0.428571	-1.6031	0.108912	0.541756
nipbla	766.7706	0.068733661	0.154002	0.446315	0.65537	0.929606
zbtb14	348.372	-0.038521493	0.145337	-0.26505	0.790971	0.964766
si:dkeyp-13d12.16	0.046941	0	5.267649	0	1	NA
CR550307.1	0.225485	-0.868115537	5.267649	-0.1648	0.8691	NA
si:zfos-451d2.2	3.857583	-0.054553506	1.010159	-0.054	0.956931	0.999109
rbm5	1809.685	-0.220888104	0.160427	-1.37688	0.168551	0.639966
si:dkey-237m9.1	4.571617	-1.662180388	0.870717	-1.90898	0.056265	0.410018
ctxn1	4.613483	1.198480066	0.855235	1.401346	0.161111	0.627852
CU695232.1	178.2064	0.152747642	0.219928	0.694534	0.487347	0.873291
tax1bp1a	242.3002	-0.07325162	0.177781	-0.41203	0.680315	0.936798
vstm2l	24.82765	0.017576612	0.488158	0.036006	0.971278	1
unc80	48.75547	0.197231684	0.457145	0.431443	0.666147	0.932664
trpc3	8.214444	-0.66172785	0.764319	-0.86577	0.386614	0.827438
si:dkey-27i16.2	94.1137	-0.097758697	0.205819	-0.47497	0.634806	0.924272
col5a3a	40.0848	-0.168363373	0.326705	-0.51534	0.606317	0.915204
efna3b	61.84626	0.235673627	0.246368	0.956591	0.338774	0.795226
gnsb	181.7717	-0.034645238	0.151536	-0.22863	0.819158	0.971785
CU928205.1	0.07132	0	5.267649	0	1	NA
taok1b	992.2552	-0.130525275	0.123236	-1.05915	0.289532	0.760499
si:ch211-76m11.8	1.471345	-0.906973625	1.462113	-0.62032	0.535049	NA
CR936540.1	48.92767	0.101799063	0.411762	0.247228	0.804732	0.969259
si:zfos-932h1.3	335.0069	0.415249197	0.154894	2.680869	0.007343	0.145171
scarb2a	1268.735	0.332189483	0.098333	3.3782	0.00073	0.036014
nadka	63.73652	-0.228145249	0.242215	-0.94191	0.346237	0.799463
cyp1a	753.5664	-0.332955973	0.23442	-1.42034	0.155509	0.620161
BX908386.1	1.537465	0.433311312	1.609084	0.269291	0.787706	NA
dync1li1	1091.949	0.018174294	0.111882	0.162442	0.870958	0.983365
BX005396.1	0	NA	NA	NA	NA	NA
dre-mir-193a-1	0	NA	NA	NA	NA	NA
grm1b	4.138489	0.098198969	1.635006	0.06006	0.952108	0.998066
si:cabz01054394.6	43.85192	0.546786436	0.284786	1.919991	0.054859	0.405196
CU280645.1	4.445874	-6.137495173	1.492122	-4.11327	3.90E-05	0.004659
RF00001	0.049519	0	5.267649	0	1	NA

spef2	34.01528	0.149452923	0.454234	0.329022	0.742139	0.954329
exosc3	412.0308	-0.339546594	0.237855	-1.42754	0.153426	0.617916
BX537137.2	0.450028	-3.594525293	3.952524	-0.90943	0.363126	NA
CR381673.1	0.361251	1.05539986	3.793065	0.278245	0.780825	NA
si:ch73-340n8.4	109.0717	-0.319353105	0.218473	-1.46175	0.14381	0.603089
RF00001	0	NA	NA	NA	NA	NA
RF00003	0	NA	NA	NA	NA	NA
CABZ01065679.1	22.81639	1.276270865	0.44721	2.85385	0.004319	0.108751
rab18b	1041.235	-0.000404579	0.15575	-0.0026	0.997927	1
mamdc2a	88.8186	0.100309304	0.31693	0.316503	0.751621	0.955356
si:ch1073-181h11.4	0.14264	0	5.267649	0	1	NA
znf326	44.45711	-0.532825914	0.361335	-1.4746	0.140319	0.599409
hdac4	100.2995	0.200839905	0.28515	0.704331	0.481227	0.87045
mxd3	523.554	0.012855725	0.237172	0.054204	0.956772	0.999109
si:ch211-66c13.1	2.457685	-1.385897389	1.49335	-0.92805	0.353384	0.804432
BX322574.1	8.898978	0.166605094	0.538825	0.309201	0.757169	0.95715
atp5pd	4775.004	0.009624214	0.123898	0.077678	0.938084	0.997331
si:ch211-229l10.4	6.559756	-0.087187106	0.684092	-0.12745	0.898585	0.990199
il17rd	317.9936	0.046731438	0.160673	0.290847	0.771168	0.960484
cyp19a1b	10.18445	0.708102357	0.727911	0.972787	0.330659	0.788126
nudcd3	1171.389	-0.076283903	0.136923	-0.55713	0.57744	0.904842
BX901957.1	111.4177	-0.399123995	0.200253	-1.9931	0.04625	0.375744
lrrc74b	24.26888	0.432803103	0.352685	1.227167	0.21976	0.700776
srp54	1322.877	-0.024063114	0.086837	-0.27711	0.781698	0.962077
aplp1	2057.679	0.183932959	0.174805	1.05222	0.292699	0.762191
si:dkey-165o8.1	7.517308	-5.291544711	1.418404	-3.73063	0.000191	0.01451
BX276102.1	17.38537	7.397015535	1.255997	5.889355	3.88E-09	2.72E-06
FLNB	491.9406	0.174944629	0.174089	1.004916	0.314937	0.777649
polr1a	477.9528	-0.306943557	0.178957	-1.71518	0.086313	0.493048
si:ch211-207l14.1	126.6539	-0.842792551	0.537152	-1.569	0.116648	0.557506
tnfrsf11b	63.42994	0.025768661	0.263081	0.097949	0.921972	0.994346
CU694197.1	339.5705	-0.071912083	0.160917	-0.44689	0.654954	0.929606
si:dkey-38l22.2	42.80739	0.32470111	0.36598	0.887209	0.374966	0.82179
si:dkey-14o6.1	4.302575	-0.706120472	0.975055	-0.72419	0.468952	0.867083
ctps1b	613.9802	-0.264540687	0.269853	-0.98031	0.326932	0.785707
tmem176l.3a	247.2868	-1.950468804	0.186666	-10.449	1.48E-25	1.14E-21
znf1015	42.06438	0.011974802	0.298871	0.040067	0.96804	1
si:ch211-76m11.8	28.24627	-3.443456285	1.082833	-3.18004	0.001473	0.057828
psmg3	406.1401	-0.01831359	0.137588	-0.1331	0.894111	0.988762
ly6pge	487.5253	0.074268179	0.231575	0.32071	0.748431	0.955074
tradv24.0.2	0	NA	NA	NA	NA	NA
CU929451.2	99.85054	-0.031481108	0.188014	-0.16744	0.867024	0.982582
FP016106.1	4.130951	0	1.803735	0	1	1
CABZ01079480.1	132.0259	-0.49212304	0.235309	-2.09139	0.036493	0.335935
fam136a	234.8651	0.06859317	0.175816	0.390142	0.696432	0.941772
LO017877.1	2.867132	-1.8532139	1.404154	-1.31981	0.186899	0.665301
CR925723.1	15.38669	0.739975514	0.452825	1.634132	0.102231	0.528963
map3k1	203.0803	0.167381092	0.187982	0.890412	0.373245	0.820501
si:dkey-22a18.2	15.36707	-0.229392943	0.679011	-0.33783	0.735489	0.951543
BX936393.2	0.411425	1.055369942	4.324761	0.24403	0.807208	NA
stbd1	131.7991	-0.234585197	0.228086	-1.0285	0.303717	0.769473
BX005376.1	19.77697	-0.13818757	0.537353	-0.25716	0.797053	0.966447
si:dkey-19b23.10	4.545131	1.551030055	1.143535	1.356346	0.174989	0.648927
ninl	154.9528	0.554079026	0.214088	2.588088	0.009651	0.169148

CT956093.1	15.23753	-6.223428752	1.283344	-4.84938	1.24E-06	0.000346
RF00067	0	NA	NA	NA	NA	NA
SNTA1	65.98786	0.329564083	0.295217	1.116343	0.264275	0.742063
trgv2	0.639831	0	3.758021	0	1	NA
CABZ01079241.1	204.4251	-0.007195321	0.168905	-0.0426	0.96602	1
si:dkey-56m15.8	2.471099	-2.665334191	1.28442	-2.07513	0.037975	0.342098
RF00003	0	NA	NA	NA	NA	NA
asic1c	6.099287	-0.688281505	0.773247	-0.89012	0.373402	0.820558
RF00026	0	NA	NA	NA	NA	NA
znf1078	5.113064	0.938520838	0.710167	1.32155	0.186318	0.664561
comp	401.4013	0.183030982	0.203922	0.897555	0.369423	0.817841
paf1	321.8545	-0.135363586	0.16229	-0.83408	0.404234	0.833888
utp6	427.1872	-0.10360595	0.16111	-0.64308	0.520175	0.887148
BX005003.1	237.8265	0.004926206	0.232791	0.021162	0.983117	1
BX537156.1	0.657398	-2.301184519	2.909033	-0.79105	0.428916	NA
si:ch211-9d9.8	0.640984	0	3.329819	0	1	NA
slc6a6b	149.6766	-0.186354735	0.297901	-0.62556	0.531604	0.890835
nr1h3	488.562	-0.106939483	0.131872	-0.81093	0.417404	0.841149
si:ch211-62a1.4	10.97117	0.425156442	0.585291	0.726401	0.467593	0.866174
nars2	54.36223	0.190887388	0.318855	0.598665	0.549396	0.897192
zfand6	532.824	-0.036493777	0.128543	-0.2839	0.776484	0.961073
si:dkey-3h2.4	5.367607	-0.243746906	1.060187	-0.22991	0.818162	0.971518
si:ch211-1o7.2	5.914977	0.630214386	0.820951	0.767664	0.442687	0.853879
tmem56a	202.1248	0.090951202	0.215779	0.421502	0.673389	0.934276
si:dkey-151g22.1	1.657483	0.775295669	1.572082	0.493165	0.621896	NA
pank4	419.9356	0.039183826	0.134333	0.291691	0.770523	0.960484
BX957236.1	0	NA	NA	NA	NA	NA
ccl39.5	0.305906	1.05535837	5.267649	0.200347	0.841209	NA
si:dkey-11f4.14	20.37525	-0.453823592	0.489057	-0.92796	0.35343	0.804432
CU570782.1	0.793111	1.916359627	2.142197	0.894577	0.371013	NA
gig2g	0.905191	1.104609955	2.041959	0.540956	0.588538	NA
si:ch73-366l1.5	2263.545	0.065062917	0.162641	0.40004	0.689127	0.93924
ube2a	1149.733	0.062854803	0.113109	0.555703	0.578414	0.905417
RF00001	0	NA	NA	NA	NA	NA
arr3b	1248.778	0.177557327	0.285821	0.621219	0.534455	0.891711
tec	23.74742	-0.60167829	0.411484	-1.46222	0.143682	0.602773
FO704758.1	44.59037	0.767269906	0.624592	1.228433	0.219284	0.700266
anxa1d	340.729	0.153955466	0.140425	1.096351	0.272925	0.748807
CR792424.1	1.055229	0.027250167	1.717987	0.015862	0.987345	NA
RF00017	0.043308	0	5.267649	0	1	NA
CU929365.1	0	NA	NA	NA	NA	NA
tollip	450.1235	-0.086799864	0.119116	-0.7287	0.466185	0.865297
traj32	0	NA	NA	NA	NA	NA
BX682557.1	14.67228	-0.32302011	1.19546	-0.27021	0.787002	0.963065
olfcd2	0.161192	0	5.267649	0	1	NA
si:ch211-145h19.3	0.227619	1.055368195	5.267649	0.200349	0.841208	NA
tradv25.0.1	0.060442	0	5.267649	0	1	NA
hrasa	259.0822	-0.161004041	0.138082	-1.16601	0.243612	0.724437
si:dkey-161l11.79	0	NA	NA	NA	NA	NA
CT583728.2	0.21483	1.055364289	5.092479	0.20724	0.835823	NA
lyar	280.5947	0.212799477	0.144379	1.473894	0.14051	0.599658
BX005286.1	4.687152	1.366657002	0.970491	1.408211	0.159068	0.624783
zgc:174650	2.072204	0.06498004	1.403585	0.046296	0.963075	NA
ntrk2b	22.37668	0.36794506	0.444216	0.828302	0.4075	0.83577

si:ch73-215f7.1	52.17347	-0.379184669	0.31591	-1.20029	0.230026	0.711766
CU855694.1	0.173415	1.11785931	5.226359	0.213889	0.830634	NA
si:ch211-59d8.3	10.8447	1.222564342	0.63403	1.928243	0.053825	0.401878
si:dkey-16p6.1	22.48734	0.642540326	0.794097	0.809146	0.418431	0.841565
RF00001	0.135047	0	5.267649	0	1	NA
BX321870.1	0	NA	NA	NA	NA	NA
BX649431.1	0.124591	1.055383603	5.267649	0.200352	0.841205	NA
CU856358.1	0.240682	-1.710085139	5.238472	-0.32645	0.744086	NA
gpr75	153.0752	0.136792518	0.225193	0.607447	0.543555	0.894925
rtn4rl1b	70.06067	-0.173653223	0.419191	-0.41426	0.678685	0.936026
ccne2	206.1992	-0.007525961	0.194663	-0.03866	0.96916	1
si:dkey-106c17.2	0.183431	-0.868109386	5.267649	-0.1648	0.869101	NA
RF00067	2.141784	0.40324367	1.276479	0.315903	0.752076	NA
BX004823.1	26.00891	1.563762051	0.474332	3.29677	0.000978	0.043444
si:dkey-25i10.1	0.788805	-3.343111305	2.101193	-1.59105	0.111597	NA
spata22	146.6386	0.18177182	0.192531	0.944118	0.345109	0.79837
zc3h7ba	154.8168	0.251524663	0.260124	0.96694	0.333574	0.790676
CR388378.2	0.452822	1.642388564	2.836408	0.579038	0.562563	NA
ncdn	358.1462	-0.12043831	0.169678	-0.7098	0.477826	0.869292
olfcg6	1.425117	1.660291598	1.792719	0.92613	0.354378	NA
CABZ01085700.1	21.75761	-0.238622629	0.390105	-0.61169	0.540744	0.893429
BX323564.1	6.734812	0.186911668	0.727881	0.256789	0.797342	0.966447
BX005082.1	0.107013	-1.826650979	5.230461	-0.34923	0.726914	NA
zgc:194202	1.543703	-1.653866696	1.710982	-0.96662	0.333735	NA
CR381534.1	0.645816	1.055420946	2.729357	0.386692	0.698984	NA
RF00001	0	NA	NA	NA	NA	NA
si:ch73-233f7.4	5.769171	0.334023241	0.719729	0.464096	0.642579	0.92567
si:ch1073-286c18.5	0	NA	NA	NA	NA	NA
BX927336.1	24.82277	0.489806364	0.376188	1.302024	0.192908	0.672855
si:ch211-8c17.2	12.84317	-0.54312078	0.538472	-1.00863	0.31315	0.776599
FP016142.2	0	NA	NA	NA	NA	NA
sgcd	384.4953	0.251387063	0.175223	1.434672	0.151381	0.615372
srsf11	2009.932	-0.020584272	0.118184	-0.17417	0.861731	0.980588
BX511079.1	0.049021	-0.86807634	5.267649	-0.16479	0.869106	NA
CU914631.1	0	NA	NA	NA	NA	NA
BX908763.1	0.761109	0.771195822	2.762959	0.279119	0.780153	NA
pdgfab	152.8926	0.385945832	0.273924	1.40895	0.15885	0.624456
CR457444.2	37.47206	0	2.746345	0	1	1
wbp1	645.4689	-0.190193943	0.11005	-1.72824	0.083945	0.486647
si:ch211-237a4.2	0.2875	1.05537143	4.681433	0.225438	0.821639	NA
zgc:194887	39.75641	0.077129496	0.530201	0.145472	0.884338	0.987047
CR318595.1	0.145978	0	5.267649	0	1	NA
gchfr	252.5285	-0.376705185	0.235178	-1.60179	0.109202	0.542034
cyb5a	1180.708	-0.118479423	0.140322	-0.84434	0.398481	0.830887
tubb2b	0	NA	NA	NA	NA	NA
zgc:92140	645.2326	-0.134113359	0.143606	-0.9339	0.350357	0.802215
FP102843.1	0	NA	NA	NA	NA	NA
hist1h2a11	1.101656	-0.262299901	1.714165	-0.15302	0.878383	NA
CU570791.1	17.75582	-0.276793249	0.391835	-0.7064	0.479937	0.870001
RF00001	0.238328	1.970286354	4.936483	0.399128	0.689799	NA
pithd1	44.32025	-0.370527417	0.299203	-1.23838	0.215575	0.697157
ora1	1.826672	0.07901004	1.243179	0.063555	0.949325	NA
BX914220.1	0.989592	1.047714158	2.658393	0.394116	0.693496	NA
si:dkey-14o6.4	3.055933	-1.596402767	1.21196	-1.31721	0.187769	0.666964

slx1b	79.56846	0.038821523	0.231632	0.1676	0.866898	0.982582
AL840638.1	110.325	-0.510229403	0.288196	-1.77042	0.076656	0.468636
si:dkey-4c15.8	8.734991	0.124187246	0.572402	0.216958	0.828241	0.97403
si:ch211-207e19.2	5.380448	-0.77799692	1.019792	-0.7629	0.445524	0.855403
RF00001	0	NA	NA	NA	NA	NA
chrm2a	10.26864	0.464987162	0.718689	0.646993	0.517636	0.887148
si:dkey-15h8.16	41.75922	0.393535244	0.326528	1.205212	0.228122	0.709324
BX901954.1	0	NA	NA	NA	NA	NA
ly6m4	1.609409	0	2.323126	0	1	NA
erap2	41.37875	-0.55183376	0.411408	-1.34133	0.179814	0.655282
si:ch211-188p14.2	20.8867	0.657888682	0.577904	1.138406	0.254951	0.733542
CR759843.1	12.73773	-0.839434642	0.549594	-1.52737	0.126669	0.575586
ccne1	85.14597	0.287612579	0.249477	1.15286	0.248968	0.728918
si:dkey-23f9.4	68.20854	0.190115157	0.206821	0.919226	0.357977	0.809315
traj54	0	NA	NA	NA	NA	NA
efna5b	15.42464	0.268920899	0.431025	0.62391	0.532687	0.890978
nitr12	0.237798	2.56148978	5.200379	0.492558	0.622325	NA
gspt1l	3645.67	-0.118872647	0.10659	-1.11524	0.264749	0.742063
BX914214.1	0.376424	-0.857089456	3.31597	-0.25847	0.796042	NA
tradv6.0.1	0	NA	NA	NA	NA	NA
RF00001	0.17618	0	5.267649	0	1	NA
CABZ01044277.1	110.9168	0.385023645	0.33644	1.144404	0.252456	0.732759
AL929586.1	0.319504	0	5.267649	0	1	NA
irf2bp2b	1027.152	-0.032625131	0.111693	-0.2921	0.770212	0.960484
pycr1b	930.4386	0.343556429	0.148302	2.316601	0.020525	0.254454
CU984600.3	23.51053	-0.327680203	0.424217	-0.77244	0.439856	0.852079
nbeal2	63.55114	-0.37466338	0.238202	-1.57288	0.115746	0.555893
si:ch211-126j24.1	758.8807	0.135434533	0.142896	0.947786	0.343239	0.797757
rgn	515.1577	0.136447184	0.178946	0.762506	0.445758	0.855403
mthfd2	730.7688	-0.04039031	0.18892	-0.2138	0.830706	0.974908
ankdd1a	53.6571	0.406739104	0.315265	1.290149	0.196999	0.677235
iqgap3	332.7068	0.327018086	0.318336	1.027274	0.304292	0.769725
si:dkey-21e2.7	2.313962	0.80465369	1.674695	0.480478	0.630888	0.923003
pcdh11	164.3662	0.527063138	0.24869	2.119355	0.034061	0.326054
BX957252.1	0.633926	-0.823668307	2.793356	-0.29487	0.768096	NA
FQ976914.1	5.572362	0.590250283	0.74902	0.78803	0.430679	0.847603
KIF1C	70.0369	-0.034177425	0.309271	-0.11051	0.912005	0.992371
CR788237.1	0	NA	NA	NA	NA	NA
CU469526.1	190.4753	-0.087979339	0.255927	-0.34377	0.731021	0.950056
AL935300.3	29.2005	-0.681191997	0.42123	-1.61715	0.105846	0.534827
si:ch73-359m17.9	71.8252	-0.061387223	0.309472	-0.19836	0.842763	0.9766
fgf19	17.415	0.822087136	0.8567	0.959597	0.337258	0.794213
si:dkeyp-53d3.3	17.55234	-0.119924263	0.466833	-0.25689	0.797265	0.966447
CT583690.1	0.772893	0.144595801	2.666703	0.054223	0.956758	NA
si:dkey-93h22.8	158.2416	-0.066363476	0.245918	-0.26986	0.787268	0.963145
grin3bb	30.23744	-1.372784513	0.612759	-2.24033	0.025069	0.280742
BX323031.1	2.254385	1.603517325	1.397575	1.147357	0.251234	0.731477
exd1	8.604016	0.921735134	0.668548	1.378711	0.167984	0.639267
map4k4	1596.581	0.046588028	0.13628	0.341856	0.73246	0.950617
prelid1a	1202.563	-0.139283058	0.115064	-1.21048	0.226095	0.707444
BX001023.1	31.81354	0.359417486	0.362804	0.990665	0.321849	0.782075
ppfia1	276.5742	0.139234094	0.16168	0.861172	0.389143	0.827725
traj55	0	NA	NA	NA	NA	NA
CU467043.1	0	NA	NA	NA	NA	NA

zgc:174180	0.659767	0.908935957	3.07539	0.295551	0.767573	NA
nup85	218.0053	0.16341124	0.174101	0.938601	0.347935	0.800324
si:zfos-2326c3.2	496.331	0.175260291	0.131903	1.328704	0.183946	0.660778
CU207221.1	314.556	-0.140922167	0.135407	-1.04073	0.298002	0.765416
RF00017	0	NA	NA	NA	NA	NA
zmiz2	224.0346	0.071351173	0.193045	0.36961	0.711673	0.945821
CU855895.1	0.315222	3.276401662	5.174649	0.633164	0.526627	NA
tmprss2	115.4796	-0.310851253	0.287439	-1.08145	0.279497	0.752477
pygo1	9.278817	-0.067482321	0.573787	-0.11761	0.906378	0.991631
CABZ01101739.1	0	NA	NA	NA	NA	NA
ptpn23b	260.1584	-0.222684699	0.171046	-1.3019	0.192951	0.672855
CT583672.1	169.4973	0.066970985	0.164143	0.408003	0.683271	0.938212
FQ311908.1	20.96361	-0.074435414	0.4019	-0.18521	0.853065	0.978831
vclb	294.9259	0.120910474	0.218655	0.552975	0.580281	0.905618
rela	117.651	-0.304856491	0.178982	-1.70328	0.088516	0.499597
si:ch211-11c3.9	4.059659	-3.399356713	1.759589	-1.9319	0.053371	0.400199
il1b	89.65792	0.515986529	0.693342	0.744202	0.456754	0.860698
zgc:165603	4.319256	1.071034492	1.138	0.941155	0.346626	0.79947
si:ch73-236c18.7	7.17688	-0.713560504	0.924327	-0.77198	0.440127	0.852079
dre-mir-365-2	0.875346	0	2.415912	0	1	NA
CR388231.1	0.497202	-1.756080566	3.982524	-0.44095	0.659252	NA
CABZ01078350.1	4.832091	1.055440421	1.782362	0.592158	0.553745	0.89821
CR925761.1	12.86899	0.119552191	0.546691	0.218683	0.826897	0.973458
parp11	2.259408	-0.14371749	1.245408	-0.1154	0.90813	0.991745
CU655844.1	0.44703	1.055392172	3.691833	0.285872	0.774976	NA
FP245456.1	22.7314	-0.145192772	0.47735	-0.30416	0.761003	0.958019
BX649331.1	2.496229	-4.333823835	1.596214	-2.71507	0.006626	0.136957
si:ch73-170d6.3	5.226412	0.843225523	0.829604	1.016419	0.30943	0.774075
rgrb	508.4072	-0.436668868	0.237003	-1.84246	0.065407	0.435359
rptor	286.1069	0.261097429	0.144095	1.811978	0.06999	0.451265
BX511018.1	0.160481	0	5.267649	0	1	NA
si:dkey-108k24.2	0	NA	NA	NA	NA	NA
en1b	251.7019	0.056987201	0.123473	0.461534	0.644415	0.926639
CU929403.1	1.626068	1.055430535	2.02425	0.521393	0.602093	NA
pxdn	172.1007	-0.174982955	0.239633	-0.73021	0.465261	0.864997
ccdc14	36.27178	0.614396271	0.28255	2.174466	0.02967	0.306672
si:dkey-201i2.4	4.079416	0.059903441	1.652962	0.03624	0.971091	1
scn4aa	7.416677	-0.127116476	1.129739	-0.11252	0.910412	0.992208
CABZ01046427.2	0.815481	0.093634378	5.267649	0.017775	0.985818	NA
CT737182.1	1.998594	0.4097673	1.087117	0.37693	0.706226	NA
lrtm1	11.49547	0.990828734	1.614819	0.613585	0.53949	0.893049
BX548018.1	0	NA	NA	NA	NA	NA
tfec	79.92424	-1.059201146	0.215698	-4.91058	9.08E-07	0.000281
dhrs13l1	350.3545	0.003338813	0.249787	0.013367	0.989335	1
MYH6 (1 of many)	83.46783	-0.126889973	0.353733	-0.35872	0.719807	0.94694
tbcd	172.8682	0.245294828	0.160513	1.52819	0.126465	0.575001
lzts3a	35.00067	0.415196563	0.355541	1.167789	0.242892	0.724396
csf3b	14.71839	-2.808118851	1.159509	-2.42182	0.015443	0.218848
elmo1	1367.305	0.057327691	0.122465	0.468114	0.639703	0.924779
si:ch211-156p11.1	3.362504	-1.543207835	1.344645	-1.14767	0.251105	0.731476
znf1125	0.336691	0.09365499	3.905184	0.023982	0.980867	NA
rgs2	76.6383	-0.534097643	0.370336	-1.4422	0.149246	0.611024
musk	97.19385	0.117312313	0.259244	0.452517	0.650897	0.928364
BX544876.2	0.069091	0	5.267649	0	1	NA

pcxa	983.5372	-0.150275578	0.098783	-1.52127	0.128191	0.57717
CABZ01007829.1	0.043309	0	5.267649	0	1	NA
BX005124.1	0.423726	0.644861176	3.593096	0.179472	0.857567	NA
slitrk6	335.7234	-0.156248146	0.119721	-1.3051	0.191858	0.672212
cbx8b	145.959	0.060697043	0.21083	0.287896	0.773426	0.960624
gig2d	2.386421	-0.22659944	1.483724	-0.15272	0.878616	0.985761
si:zfos-741a10.3	16.06073	-0.355957985	0.582485	-0.6111	0.541132	0.893658
RF00003	0	NA	NA	NA	NA	NA
BX640547.2	8.316043	0.836206272	0.764062	1.094422	0.27377	0.749234
CU693379.1	520.0185	-0.041369818	0.160699	-0.25744	0.796841	0.966447
BX547930.4	1.240149	1.512311477	2.031277	0.744513	0.456566	NA
nsmce1	52.83952	-0.127712166	0.267801	-0.47689	0.633439	0.923885
cratb	213.8902	-0.127650317	0.151998	-0.83982	0.401011	0.831893
polr2b	1199.264	-0.216580214	0.089685	-2.41489	0.01574	0.221337
glrx3	812.8271	0.127938615	0.091973	1.391045	0.164212	0.63332
rangrf	104.5671	0.018768381	0.192919	0.097286	0.922499	0.994477
cd7al	43.96413	-0.726429035	0.339688	-2.13852	0.032475	0.319445
pcdh2g28	1988.468	0.061994033	0.13453	0.460821	0.644927	0.926728
sowahca	9.524835	0.316624444	0.689862	0.458968	0.646257	0.926728
cdhr5b	25.82106	1.055417614	1.704585	0.619164	0.535808	0.892102
znrf2b	75.02337	-0.054428082	0.255909	-0.21268	0.831573	0.974908
CABZ01078120.1	0.59696	0	3.493893	0	1	NA
traj7	0	NA	NA	NA	NA	NA
CR382300.1	120.0703	0.01551529	0.202988	0.076435	0.939073	0.997371
si:ch211-194m7.5	4.094324	1.055438349	1.810274	0.583027	0.559875	0.899668
znf1156	112.5474	-2.570190603	0.291917	-8.80454	1.31E-18	5.07E-15
sbno2a	72.3204	0.110761919	0.2974	0.372434	0.70957	0.945793
map7d1a	382.9927	0.079092337	0.152988	0.516984	0.605167	0.914985
cyp2aa4	146.1191	-0.679239527	0.542875	-1.25119	0.210865	0.692523
vwa1l	29.93379	0.85008739	0.790081	1.075949	0.28195	0.754378
RF00001	0	NA	NA	NA	NA	NA
snap91a	654.0048	0.142979116	0.20245	0.706245	0.480036	0.870001
VSTM2B	115.1386	0.04128472	0.318219	0.129737	0.896775	0.98939
LO018021.1	56.10341	-0.362954642	0.241782	-1.50117	0.133312	0.586883
RF00001	0	NA	NA	NA	NA	NA
stx12	668.6421	-0.044820234	0.11909	-0.37636	0.706652	0.944735
CU571064.1	0.349601	-2.388311782	4.377897	-0.54554	0.585383	NA
kcnc3b	10.89632	1.40870271	0.728769	1.932989	0.053238	0.400175
CABZ01055522.1	180.5202	-0.225311903	0.170681	-1.32008	0.186808	0.665301
si:dkey-58i8.5	0.601206	-0.851862417	2.821387	-0.30193	0.762705	NA
ftr67	395.1338	-0.2800885	0.147304	-1.90143	0.057245	0.413213
sec14l1	535.2599	-0.324276707	0.136924	-2.3683	0.01787	0.236508
tcerg1a	680.2942	0.117327318	0.116553	1.006641	0.314107	0.777479
CT583651.2	91.464	0.375411963	0.252585	1.486281	0.137205	0.59288
cdh22	5.492841	0.074973854	0.947315	0.079143	0.936918	0.997079
farsa	1153.787	0.112276796	0.16568	0.677674	0.497978	0.878926
plcl2	73.38759	-0.108148213	0.29493	-0.36669	0.71385	0.945821
CT573793.1	0	NA	NA	NA	NA	NA
gcdhb	446.8825	0.098935494	0.238601	0.414648	0.678399	0.936026
fam168a	150.7551	0.820531605	0.2036	4.030125	5.57E-05	0.006008
sox4b	1531.212	0.259673396	0.192051	1.352104	0.176342	0.649785
BX664606.1	0	NA	NA	NA	NA	NA
tgm5l	25.97906	0.830092925	0.488105	1.700643	0.08901	0.500695
sdr42e1	28.4594	0.015976249	0.362389	0.044086	0.964836	1

CABZ01044108.1	0.212191	0	5.267649	0	1	NA
BX005073.1	0.101247	1.854150541	5.241768	0.353726	0.723544	NA
FQ311879.1	3.889067	0	2.023609	0	1	1
lrp12	512.4997	0.028570867	0.12562	0.227438	0.820083	0.971788
SLC25A29	35.06036	0.467211108	0.316354	1.476861	0.139713	0.598033
si:dkey-11d20.1	0.098043	-1.71005882	5.238474	-0.32644	0.74409	NA
smdt1b	2237.82	-0.139374655	0.194344	-0.71716	0.473278	0.869064
ehd1a	615.0665	-0.283829113	0.14127	-2.00912	0.044524	0.369362
RF00001	0	NA	NA	NA	NA	NA
oxsm	208.206	-0.471644408	0.175255	-2.69119	0.00712	0.142826
RYR2 (1 of many)	260.7357	0.886166727	0.274549	3.227718	0.001248	0.051445
lifra	90.97242	0.302570727	0.321911	0.93992	0.347258	0.799992
nop14	623.2284	-0.117695691	0.172922	-0.68063	0.496106	0.878093
fam57a	72.39421	0.135494639	0.274726	0.4932	0.621872	0.918461
plpp4	2.16944	-0.833881055	1.352497	-0.61655	0.537532	NA
RF00001	0	NA	NA	NA	NA	NA
BX548044.7	3.336192	1.266458295	1.259206	1.00576	0.314531	0.777479
PRDM15	58.0677	0.272566188	0.212841	1.280609	0.200331	0.680711
XPO5	109.6848	0.021800773	0.185941	0.117246	0.906665	0.991644
gga3b	54.53303	0.36242465	0.247564	1.463965	0.143204	0.602183
exoc6b	438.49	-0.238155145	0.112261	-2.12144	0.033885	0.325235
si:dkey-199m13.5	7.496402	0.421767025	0.65546	0.643467	0.519921	0.887148
BX927164.1	0.043308	0	5.267649	0	1	NA
si:dkey-4j21.2	4.755354	-2.487455948	1.197628	-2.07699	0.037803	0.340947
pclob	440.509	0.44044682	0.42522	1.035809	0.300291	0.766629
si:dkey-238i5.3	2.781211	-2.649420153	1.132257	-2.33995	0.019287	0.244673
stac3	1182.154	0.01221663	0.189904	0.064331	0.948707	0.99777
BX649355.1	0.877524	-3.114819891	5.154668	-0.60427	0.545663	NA
dre-mir-735	0	NA	NA	NA	NA	NA
CU861459.1	0	NA	NA	NA	NA	NA
si:zfos-128g4.2	94.43504	0.103331263	0.33661	0.306977	0.758861	0.957886
cyp2aa9	96.70155	-0.992643882	0.406872	-2.4397	0.0147	0.212735
si:ch211-223a21.1	14.25758	-0.133852342	0.463349	-0.28888	0.772673	0.960624
BX324119.1	0.2075	0	5.267649	0	1	NA
RF00001	0	NA	NA	NA	NA	NA
BX323544.1	0.389977	0	4.279676	0	1	NA
pds5b	1311.888	0.170694632	0.111429	1.531864	0.125556	0.573341
si:ch211-255f4.2	42.71726	-1.144498469	0.360075	-3.1785	0.00148	0.057996
zmp:0000001082	141.887	0.217354572	0.20965	1.036751	0.299852	0.766586
ubxn8	108.5595	0.381976945	0.256199	1.490941	0.135977	0.591131
tfe3a	57.90815	-0.125450912	0.315428	-0.39772	0.690839	0.940147
pex1	132.2387	0.112783658	0.182108	0.619323	0.535704	0.89207
herc7	35.68959	0.042078826	0.388852	0.108213	0.913827	0.992796
abraxas2	159.5245	0.234284253	0.162988	1.437435	0.150595	0.614417
CU062501.1	28.30914	-0.258615342	0.373966	-0.69155	0.489221	0.874296
SHC3	28.85498	0.688096552	0.501294	1.37264	0.169864	0.641525
zgc:153293	185.7048	-0.532790456	0.225935	-2.35815	0.018366	0.239337
CU633999.2	14.94661	0.68536265	0.506209	1.353913	0.175764	0.649413
faslg	1.330844	0	2.412297	0	1	NA
si:ch211-216p19.5	4.106464	-1.708316367	1.335062	-1.27958	0.200694	0.68103
krit1	290.9995	-0.065941674	0.128523	-0.51307	0.607902	0.915204
mcoln2	14.47066	-0.545789868	0.609938	-0.89483	0.370879	0.818484
smarcc1b	298.4899	0.151062854	0.130791	1.154991	0.248094	0.728489
zgc:173577	12.28044	-0.46474098	0.495071	-0.93874	0.347866	0.800274

suz12b	598.8079	-0.006820453	0.119787	-0.05694	0.954594	0.998611
prdm1b	110.634	0.05239047	0.270203	0.193893	0.84626	0.97711
si:ch211-196h24.1	2.164237	-1.754979978	1.339932	-1.30975	0.190279	NA
tex15	4.681861	0.146885724	0.906104	0.162107	0.871222	0.983365
gigyf1a	197.2279	-0.10535783	0.173395	-0.60762	0.543442	0.894925
rrp7a	309.6637	-0.130769726	0.217462	-0.60135	0.54761	0.896211
bxdc2	442.0058	-0.08133668	0.134185	-0.60615	0.544413	0.895206
kif5aa	1852.07	0.228260387	0.201054	1.135319	0.256242	0.73401
si:ch73-315f9.2	26.39305	0.256104548	0.438348	0.58425	0.559052	0.899454
trdj1	0.283508	0	5.267649	0	1	NA
BX323855.1	0.128839	0	5.267649	0	1	NA
CU856520.1	311.4528	-0.380420929	0.167227	-2.27487	0.022914	0.267056
rp9	283.4184	-0.013974061	0.194042	-0.07202	0.942589	0.997371
dre-mir-7148	0	NA	NA	NA	NA	NA
nme8	19.5152	-0.408683563	0.374095	-1.09246	0.274631	0.750181
CT583726.1	0	NA	NA	NA	NA	NA
paldla	637.5998	-0.093663752	0.11494	-0.81489	0.415134	0.838738
mslna	88.58661	-0.312063772	0.388652	-0.80294	0.42201	0.843292
si:dkeyp-92c9.4	14.84365	-0.797392684	1.608459	-0.49575	0.620071	0.917671
BX957297.1	2.739333	0.897596925	1.48007	0.606456	0.544212	0.895206
gata4	50.43692	-0.157272038	0.411043	-0.38262	0.702004	0.943292
stard13b	475.6836	-0.072772237	0.110659	-0.65763	0.510777	0.884139
ssh2b	312.2947	-0.00527166	0.179893	-0.0293	0.976622	1
znf1100	1.662715	-1.240269571	1.822286	-0.68061	0.496117	NA
RF00003	0	NA	NA	NA	NA	NA
pcdh1g13	24.60824	0.784091442	0.488679	1.604514	0.108601	0.541057
si:ch211-9d9.7	4.749035	4.053417928	1.392336	2.911236	0.0036	0.099301
CABZ01064972.1	5.153006	-1.510018803	1.319911	-1.14403	0.252611	0.732759
rabgap1	555.5152	-0.043309237	0.119549	-0.36227	0.71715	0.94627
RSBN1	161.9249	0.013223131	0.209785	0.063032	0.949741	0.99801
CU459186.1	3.1638	-1.468230123	1.442788	-1.01763	0.308852	0.77371
FP003590.1	1.61908	-3.099076741	2.001963	-1.54802	0.121618	NA
mrps16	374.7976	0.171720453	0.148608	1.155529	0.247874	0.728489
CR759816.1	3.173697	-1.214633331	1.127799	-1.07699	0.281483	0.753734
si:ch211-76m11.15	0.059954	1.055396947	5.267649	0.200354	0.841203	NA
si:ch211-153f2.3	47.21138	-0.036663319	0.239042	-0.15338	0.878102	0.98573
dre-mir-126b	0	NA	NA	NA	NA	NA
CABZ01112656.1	520.1412	0.00667166	0.128457	0.051937	0.958579	0.999109
pid1	117.4958	-0.093552911	0.171013	-0.54705	0.584343	0.906412
BX649431.2	0.119909	2.079562922	5.22636	0.397899	0.690705	NA
znf1040	24.83028	-0.449285287	0.512361	-0.87689	0.380545	0.823655
znf1071	0.821862	3.070586319	2.834423	1.08332	0.278666	NA
si:ch211-193i15.1	2.98845	0.585010795	1.227585	0.476554	0.63368	0.923974
CU459012.1	43.85931	-0.44853524	0.26172	-1.7138	0.086565	0.493048
zgc:162858	196.6588	0.479350179	0.191964	2.497088	0.012522	0.194587
znf1095	5.266899	-1.390533515	0.754185	-1.84376	0.065219	0.435355
zgc:114119	224.1831	0.152315924	0.166709	0.913666	0.360893	0.811253
CR352263.1	7.104985	-0.217791199	0.873237	-0.24941	0.803046	0.968418
cyp2k6	20.31042	0.822153149	1.108525	0.741664	0.458291	0.861001
RF01190	0	NA	NA	NA	NA	NA
si:ch73-311h14.2	275.4739	-0.207860332	0.131288	-1.58324	0.113367	0.551243
RF00001	0.046941	0	5.267649	0	1	NA
CR847947.1	0.940056	0	2.703509	0	1	NA
creb5a	28.00116	-0.225527716	0.399481	-0.56455	0.572378	0.903461

cyp2aa1	137.6034	-0.810377612	0.27377	-2.96006	0.003076	0.091315
palm2	24.72939	-0.353176976	0.454587	-0.77692	0.437207	0.851125
EGFLAM	18.35343	0.103179091	0.524377	0.196765	0.844011	0.976791
si:dkey-50i6.6	55.84189	-0.479394562	0.238863	-2.00699	0.044751	0.370491
fxyd1	1318.426	-0.047264058	0.146253	-0.32317	0.746569	0.955074
wacb	72.10122	-0.011184599	0.204679	-0.05464	0.956422	0.99899
CR749168.1	0.507339	-1.74783001	3.995116	-0.43749	0.661755	NA
RF00001	0.04222	0	5.267649	0	1	NA
setdb1b	725.1263	0.057437197	0.166874	0.344195	0.7307	0.950056
faua	18777.11	-0.369625837	0.13393	-2.75985	0.005783	0.128995
CABZ01034698.1	3.307168	-6.120539461	2.831698	-2.16144	0.030662	0.312139
si:ch211-188p14.5	0	NA	NA	NA	NA	NA
CU915778.1	9.318042	-0.576530132	0.599986	-0.96091	0.3366	0.793309
si:dkey-185m8.2	17.52672	0.174673218	0.425792	0.410232	0.681636	0.937418
tradv25.0.2	0	NA	NA	NA	NA	NA
trdj2	0.211204	0	5.267649	0	1	NA
scn4ba	59.5936	0.833625104	0.346432	2.406315	0.016114	0.223897
FO818704.1	1.387034	1.561531118	1.941268	0.804387	0.421173	NA
unc5a	9.162565	0.528150757	0.642592	0.821907	0.41113	0.837797
pcdh1g33	124.1067	0.168671169	0.277488	0.60785	0.543287	0.894925
CU138542.1	0.126661	0	5.267649	0	1	NA
kcng1	23.27833	-0.496269019	0.605033	-0.82023	0.412083	0.838123
pcbp2	3548.909	-0.039871815	0.091661	-0.43499	0.66357	0.931556
cfap157	8.435338	0.71600476	0.652004	1.09816	0.272135	0.748056
BX548000.1	1.61833	-0.53949911	1.514234	-0.35629	0.721627	NA
cacnb2a	152.6471	-0.007862841	0.179975	-0.04369	0.965153	1
si:ch211-227p7.5	9.010628	-0.986857204	0.782727	-1.26079	0.207383	0.689456
BX323797.1	0.045161	0	5.267649	0	1	NA
znf1070	5.106489	0.440647762	0.843793	0.522222	0.601516	0.913965
rragcb	80.32987	-0.209337246	0.205064	-1.02084	0.307331	0.772541
tradv2.0.3	0.248325	0	5.267649	0	1	NA
BX088596.1	20.25031	0.832057896	0.477546	1.742362	0.081445	0.480419
CR790365.1	0	NA	NA	NA	NA	NA
CU929262.1	0.141258	0	5.267649	0	1	NA
zgc:111868	6.393964	-0.498172725	0.721183	-0.69077	0.489709	0.874629
znf1154	9.774388	-0.50848816	0.59461	-0.85516	0.392461	0.827902
tradv6.0.2	0	NA	NA	NA	NA	NA
BX323051.1	0.816753	0.477157219	2.141354	0.22283	0.823668	NA
si:dkey-161l11.67	0	NA	NA	NA	NA	NA
si:ch211-147m6.3	0	NA	NA	NA	NA	NA
bsk146	132.5585	0.388232944	0.215199	1.804064	0.071221	0.455342
FP074867.1	7.932083	1.483125486	0.584928	2.535568	0.011227	0.183909
si:zfos-741a10.4	4.157381	0.111840674	0.999488	0.111898	0.910904	0.992208
si:ch211-106g8.54	0	NA	NA	NA	NA	NA
CABZ01032982.1	0.17763	0	5.267649	0	1	NA
CU467646.3	2.162107	2.403906208	3.279746	0.732955	0.463586	NA
FO905040.1	29.57299	-0.225072226	0.330683	-0.68063	0.496107	0.878093
aclya	1548.283	0.118977014	0.091771	1.296456	0.194818	0.67483
sbno1	1108.053	0.21936067	0.122299	1.793638	0.072871	0.458685
CR376766.1	0	NA	NA	NA	NA	NA
BX572078.1	1.178184	-2.32396134	2.039063	-1.13972	0.254403	NA
pcif1	141.9769	-0.137363804	0.158003	-0.86938	0.384642	0.826588
RF00001	0	NA	NA	NA	NA	NA
BX511142.1	3.079537	-0.81290109	0.923704	-0.88005	0.378835	0.823136

edar	27.93708	-0.084375143	0.41949	-0.20114	0.840591	0.97643
znf1087	7.738996	-0.165101169	0.723193	-0.22829	0.819417	0.971785
CU929418.1	2.000242	-4.224810506	1.831726	-2.30646	0.021085	NA
RASGRP2	13.81943	0.10698103	0.572749	0.186785	0.851829	0.978558
dock1	560.7138	0.042489658	0.141338	0.300624	0.763701	0.959055
CR974475.1	0.14264	0	5.267649	0	1	NA
gabrb4	16.38253	0.639739226	0.542392	1.179477	0.238208	0.719219
LO017849.1	0.389018	-0.868141166	3.818391	-0.22736	0.820145	NA
CABZ01015815.1	66.98391	0.339580951	0.34225	0.992202	0.321099	0.781417
gch2	7945.569	-0.418830791	0.27567	-1.51932	0.128682	0.578173
dre-mir-19a	0.053262	1.055396947	5.267649	0.200354	0.841203	NA
rpl24	25283.54	-0.396794771	0.138123	-2.87277	0.004069	0.106406
afmid	66.47968	-0.068871054	0.32567	-0.21147	0.832517	0.975149
FP016019.1	0.31506	0	3.954076	0	1	NA
clic6	62.40963	0.008126267	0.328202	0.02476	0.980246	1
CU929444.1	384.1497	-0.299289849	0.178484	-1.67684	0.093574	0.511703
zgc:175280	69.23013	-0.056763255	0.520585	-0.10904	0.913173	0.99273
si:dkey-71l4.3	9.885154	1.421434301	0.855812	1.660919	0.09673	0.519163
UNC13B	98.36332	-0.057628146	0.210657	-0.27356	0.78442	0.962605
FP017279.1	0.26542	0	5.267649	0	1	NA
nagk	318.7594	-0.047318018	0.163122	-0.29008	0.771757	0.960624
si:dkey-33i11.4	1047.898	0.024470248	0.183733	0.133183	0.894048	0.988762
si:dkey-218f9.10	120.3173	0.229602846	0.203819	1.126502	0.259953	0.73712
si:dkey-6d5.1	0.539865	1.004308421	2.983668	0.336602	0.736417	NA
naa38	303.1474	0.146173195	0.197755	0.739162	0.459808	0.862369
CU459186.2	3.173183	-0.807753801	0.975418	-0.82811	0.407608	0.83577
f3a	133.0298	0.068527537	0.23819	0.287701	0.773575	0.960624
RF01848	0	NA	NA	NA	NA	NA
CABZ01055168.1	0	NA	NA	NA	NA	NA
phlda1	62.20605	0.003173655	0.230435	0.013772	0.989012	1
si:ch73-72b7.1	19.79141	0.185360781	0.557912	0.33224	0.739708	0.953588
zgc:195356	20.67001	0.238923363	0.399698	0.59776	0.55	0.8974
BX663503.3	614.978	0.144190535	0.151044	0.954624	0.339768	0.795226
btr29	3.683384	0.591763125	1.071826	0.552108	0.580875	0.905618
fbxw5	515.5785	-0.042614224	0.100086	-0.42578	0.670271	0.933809
tradv25.0.3	0	NA	NA	NA	NA	NA
CR762408.1	0	NA	NA	NA	NA	NA
fnta	439.7731	-0.06594802	0.113515	-0.58096	0.561265	0.90039
igfbp3	423.6024	0.006654371	0.136924	0.048599	0.961239	0.99935
BX511066.1	0	NA	NA	NA	NA	NA
bzw1b	2086.297	-0.274359162	0.192572	-1.42471	0.154241	0.61894
hs3st3b1a	36.19348	-0.169650258	0.32864	-0.51622	0.605702	0.915055
si:dkey-190j3.2	3.927616	0.774000073	1.110093	0.697239	0.485653	0.87303
tpst2	53.37149	-0.601887026	0.286413	-2.10147	0.0356	0.331266
katnal1	195.4303	0.190132988	0.143957	1.320761	0.186581	0.664968
CR450748.1	0.120909	0	5.267649	0	1	NA
si:ch211-186j3.3	1.988825	-0.409086309	1.192699	-0.34299	0.731604	NA
CR749168.2	2.376464	0.975172244	1.302443	0.748726	0.454023	0.858944
dnaaf4	27.94431	-0.166894587	0.398318	-0.419	0.675217	0.935297
RF00017	0.049519	0	5.267649	0	1	NA
naccla	67.39524	0.320723521	0.227945	1.407023	0.15942	0.6257
RF00001	0	NA	NA	NA	NA	NA
cxcl8b.3	58.31105	-0.966064192	0.581047	-1.66263	0.096387	0.518287
si:dkey-161l11.78	0	NA	NA	NA	NA	NA

BX000701.1	26.65584	0.22318689	0.339127	0.658122	0.51046	0.884024
BX530097.1	45.5192	-0.023094814	0.280875	-0.08222	0.934468	0.99682
BX666065.1	0.310936	1.055365242	4.611357	0.228862	0.818976	NA
hmgb1a	7003.103	0.071946734	0.151332	0.475422	0.634486	0.924245
dok1b	119.8625	-0.024826978	0.185584	-0.13378	0.893578	0.988566
CR927050.1	0.996131	-3.335260647	2.746162	-1.21452	0.22455	NA
LO018568.1	5.47174	1.965659214	0.920824	2.134673	0.032788	0.32041
ankrd55	65.40953	0.128425525	0.311062	0.412861	0.679708	0.936428
fkbp10a	115.9286	0.326110384	0.218317	1.493745	0.135242	0.590585
map2k6	771.703	0.153548951	0.107835	1.423926	0.154468	0.619425
chia.2	2330.757	-0.369052309	0.706322	-0.5225	0.601323	0.913756
slc1a5	1512.031	0.081580758	0.160652	0.507811	0.611586	0.915331
purg	44.00145	0.293374332	0.357593	0.820415	0.41198	0.838123
CU856516.1	29.38583	0.442230695	0.370634	1.193173	0.232802	0.714439
CABZ01058261.1	640.0265	0.278312152	0.393555	0.707175	0.479457	0.86985
ier2a	518.7268	-0.203694581	0.505154	-0.40323	0.686777	0.93875
actc1b	400397.6	-0.064155373	0.192463	-0.33334	0.738879	0.953273
BX927287.1	4.04997	-1.293533272	1.087428	-1.18953	0.23423	0.714938
itchb	98.38478	-0.024432214	0.243085	-0.10051	0.91994	0.993892
zgc:123103	6570.548	-0.486994585	0.244881	-1.9887	0.046734	0.376639
BX908731.1	2.165094	0.649176652	1.322439	0.490894	0.623502	NA
atp1b2a	2219.347	0.1885731	0.171958	1.096623	0.272806	0.748747
CU466285.2	0	NA	NA	NA	NA	NA
CR855379.1	0.102175	1.055383603	5.267649	0.200352	0.841205	NA
si:dkey-11f4.7	2.658694	-1.362533238	1.759786	-0.77426	0.438777	0.85189
BX681417.1	0	NA	NA	NA	NA	NA
si:dkey-220o5.5	2.103405	0	1.998242	0	1	NA
glyr1	980.1502	0.140220886	0.089403	1.568418	0.116784	0.557831
CR318603.1	0.222721	1.055360606	5.267649	0.200348	0.841209	NA
atp8b2	2.752687	0.927538045	1.257136	0.737819	0.460625	0.862369
zgc:109982	15.58848	0.305087344	0.681394	0.44774	0.654341	0.929488
ghrhr2	1.121274	1.055422086	2.455593	0.429803	0.667339	NA
BX571942.1	41.18375	-0.131846776	0.257766	-0.5115	0.609002	0.915204
ppp2r2bb	139.5372	-0.004012558	0.195413	-0.02053	0.983618	1
prpsap1	106.4344	-0.102195657	0.222465	-0.45938	0.645962	0.926728
CABZ01076667.1	825.1258	-0.035116361	0.129251	-0.27169	0.78586	0.962605
galnt13	132.6939	0.830058308	0.242542	3.422328	0.000621	0.0324
RF00001	0.059954	1.055396947	5.267649	0.200354	0.841203	NA
CU929119.1	7.09261	-0.535612425	0.69708	-0.76837	0.44227	0.853736
si:zfos-2372e4.1	7.949645	0.453661436	0.986039	0.460085	0.645455	0.926728
hook2	165.2959	0.083266247	0.245442	0.339251	0.734421	0.951134
10-Sep	1453.722	0.00026294	0.094777	0.002774	0.997786	1
rasd4	267.7424	0.196775016	0.255707	0.769533	0.441577	0.853109
traj47	0	NA	NA	NA	NA	NA
acap3b	85.09037	-0.076915819	0.196187	-0.39205	0.695019	0.941197
ppp2cb	2255.285	-0.009507649	0.084979	-0.11188	0.910916	0.992208
si:dkey-68o6.5	20.00181	0.668244672	0.413135	1.617499	0.105771	0.534827
CR450749.1	0	NA	NA	NA	NA	NA
CT030005.1	0	NA	NA	NA	NA	NA
ipcef1	90.49986	-0.32489575	0.178773	-1.81737	0.069161	0.448617
CU861469.1	0.81363	-1.922790693	2.417907	-0.79523	0.42648	NA
BX465836.2	0.20403	0	5.267649	0	1	NA
pum1	2038.51	0.051695096	0.067568	0.765079	0.444225	0.854431
si:ch73-119p20.1	613.0368	0.589189577	0.238072	2.474834	0.01333	0.200944

vps52	354.5461	0.004694795	0.129807	0.036167	0.971149	1
tradv15.0	1.190781	-1.721342413	2.863482	-0.60114	0.547749	NA
PITPNC1	23.65825	-0.371177791	0.441543	-0.84064	0.400551	0.831725
si:dkey-16p6.1	32.65564	0.155273874	0.454265	0.341814	0.732491	0.950617
slc7a5	151.5797	0.065676577	0.268296	0.244792	0.806618	0.969726
vasna	432.3176	-0.114590987	0.170699	-0.6713	0.502028	0.881211
si:dkeyp-13d12.11	0	NA	NA	NA	NA	NA
si:dkeyp-115e12.7	133.2281	0.07803842	0.221751	0.351918	0.724899	0.948578
zgc:103508	231.9884	0.07893272	0.164599	0.479547	0.63155	0.923003
atxn3	186.5014	-0.173481059	0.183896	-0.94337	0.345493	0.79876
ugt5b3	99.47271	-0.690621185	0.426427	-1.61955	0.105329	0.534575
znf1039	0.295227	-2.780498223	3.862701	-0.71983	0.471628	NA
utp20	279.6388	0.054918253	0.180367	0.304481	0.760761	0.957886
si:dkey-112e7.2	869.9636	-0.327465156	0.149516	-2.19017	0.028512	0.300554
si:ch73-359m17.6	8.575632	1.099573169	0.997169	1.102694	0.27016	0.746532
epb41a	286.109	-0.071091841	0.17566	-0.40471	0.685688	0.938486
si:dkey-228b2.6	0	NA	NA	NA	NA	NA
st3gal1l3	1.848727	0	2.428945	0	1	NA
RF00001	0	NA	NA	NA	NA	NA
lsr	334.1026	0.00339908	0.169823	0.020015	0.984031	1
CR847534.1	3.967058	2.301750792	0.898885	2.560672	0.010447	0.178114
BX914214.2	4.782738	0.307290105	0.854466	0.359628	0.719125	0.94694
si:ch73-199g24.2	378.0716	-0.132749829	0.121772	-1.09015	0.275646	0.750653
BX511034.4	1.022687	0	2.927951	0	1	NA
xrcc5	429.0664	-0.108906567	0.2098	-0.5191	0.603693	0.914588
cdc37	1029.774	0.145757688	0.123102	1.184044	0.236396	0.717674
AL954741.1	42.63507	-0.642384235	0.293997	-2.185	0.028889	0.302602
RF00001	0.092101	0	5.267649	0	1	NA
si:ch211-51f19.1	14.95453	0.225601863	0.508138	0.443977	0.657059	0.929939
RF00001	0.20119	-2.743296963	5.185466	-0.52904	0.596781	NA
zgc:175088	1690.042	0.073796547	0.161148	0.457941	0.646995	0.926792
si:dkey-10p5.10	131.3785	0.467934481	0.17437	2.683565	0.007284	0.144623
si:zfos-169g10.3	122.9718	-0.391445446	0.196678	-1.99029	0.04656	0.376263
jpt1b	1616.663	0.128443507	0.161171	0.796941	0.425486	0.844714
naga	365.9603	-0.293143159	0.156193	-1.8768	0.060546	0.423018
nsdhl	98.81455	0.244111429	0.204097	1.196056	0.231675	0.713202
si:ch211-255f4.9	1.820537	-2.597122552	1.516497	-1.71258	0.08679	NA
CU651657.1	0.158409	0	5.267649	0	1	NA
CU138553.1	0	NA	NA	NA	NA	NA
btbd17a	102.6314	0.46517209	0.344118	1.351781	0.176445	0.649859
dlg2	79.82342	-0.365182836	0.275959	-1.32332	0.185727	0.663476
gig2i	0.625745	-0.868140758	3.04588	-0.28502	0.775628	NA
zgc:174944	7.328918	-2.130359296	1.080827	-1.97105	0.048719	0.384211
BX927130.1	6.873026	0.571413904	0.95404	0.598941	0.549212	0.897092
h2afy	261.5938	-0.42174601	0.183817	-2.29438	0.021768	0.261665
si:ch211-246b8.5	0	NA	NA	NA	NA	NA
CABZ01064859.1	132.6005	-0.02131285	0.234825	-0.09076	0.927683	0.995016
ly6m5	19.0997	0.05945281	0.363386	0.163608	0.87004	0.983365
mvda	99.69299	0.479795409	0.185873	2.58131	0.009843	0.171403
si:ch211-209j12.2	5.877356	0.224648887	1.106589	0.20301	0.839127	0.976417
pacsin3	799.2182	-0.204078256	0.125781	-1.62249	0.104697	0.53308
RF00003	0	NA	NA	NA	NA	NA
crfb15	6.102264	-1.647964186	1.070757	-1.53906	0.123788	0.570895
zgc:92162	12.41548	-1.185811376	0.67147	-1.76599	0.077397	0.470172

si:rp71-1d10.8	66.29538	0.353150401	0.229609	1.538053	0.124036	0.57117
znf1079	7.007638	0.239917121	0.768305	0.312268	0.754837	0.956283
si:dkey-220o21.4	0	NA	NA	NA	NA	NA
CU469503.1	0	NA	NA	NA	NA	NA
igfbp1a	1211.141	-0.314922437	0.207007	-1.52131	0.128181	0.57717
si:rp71-62i8.1	46.15783	-0.299847012	0.355935	-0.84242	0.399553	0.831196
slc25a13	4.280526	2.197864322	1.166651	1.883909	0.059577	0.420855
BX890548.1	401.1536	0.208736842	0.1819	1.147535	0.25116	0.731476
hccsb	505.2767	0.070147948	0.205951	0.340605	0.733401	0.950808
CR855320.1	0.704215	-2.341864618	2.655564	-0.88187	0.377847	NA
si:dkey-30e9.6	12.57178	0.031963617	0.485916	0.06578	0.947553	0.997371
CABZ01088346.1	189.4692	0.160470316	0.246379	0.651316	0.514843	0.886401
lhb	1.517088	-0.508938554	1.952979	-0.2606	0.794404	NA
BX511034.5	28.81132	0.178971405	0.416681	0.429516	0.667548	0.933086
evx1	0	NA	NA	NA	NA	NA
slc25a42	162.2796	-0.073724936	0.246464	-0.29913	0.76484	0.959571
AL954861.2	11.28163	-3.078427957	1.527266	-2.01565	0.043837	0.366547
cygb1	1417.012	0.174768988	0.100093	1.746064	0.0808	0.479801
CT583728.3	0	NA	NA	NA	NA	NA
zgc:158320	42.84601	-0.485097449	0.330239	-1.46893	0.141853	0.600644
sp9	345.6725	-0.044227696	0.153548	-0.28804	0.773318	0.960624
poldip3	497.7858	-0.102606463	0.120074	-0.85453	0.392814	0.827902
CABZ01050249.1	158.3689	0.106094281	0.19957	0.531613	0.594994	0.910869
si:cabz01030277.1	2.832755	0.679263862	1.71917	0.395111	0.692761	0.940444
ppp4r1l	74.87594	-0.122473642	0.195691	-0.62585	0.531412	0.890835
rpl13	28340.19	-0.301051913	0.121941	-2.46883	0.013556	0.20316
si:dkey-27n6.1	4.948979	-1.169183659	1.232481	-0.94864	0.342803	0.79738
si:ch211-209n20.1	6.22745	-0.030003832	1.04244	-0.02878	0.977038	1
dnajb1a	148.5522	0.374383484	0.209446	1.787493	0.073858	0.461761
si:dkey-238k10.1	12.99952	-1.739754727	1.142614	-1.52261	0.127857	0.577117
si:ch211-22312.4	523.1124	-0.159703517	0.124152	-1.28635	0.19832	0.679109
lrrtm4l2	8.016542	-1.019489523	0.810646	-1.25763	0.208527	0.690728
jpt1a	125.328	0.204421327	0.242571	0.842728	0.399381	0.831196
BX957256.1	0.354455	-0.868133593	4.450344	-0.19507	0.845337	NA
lama2	183.1938	0.099772111	0.528134	0.188915	0.85016	0.978251
si:dkey-161l11.76	0	NA	NA	NA	NA	NA
cables1	39.9327	-0.425707527	0.316892	-1.34338	0.179148	0.654401
rbpms	17.73426	-0.06315425	0.442404	-0.14275	0.886486	0.987533
cfap410	77.06976	-0.777666671	0.270271	-2.87736	0.00401	0.105636
ccl33.3	53.53153	0	1.701299	0	1	1
CR753902.1	0.184567	0	5.267649	0	1	NA
CU694952.1	1.482062	0.506851715	1.593709	0.318033	0.75046	NA
lrit1b	189.8269	0.270230177	0.327445	0.825269	0.409219	0.836637
FP085432.1	0.377278	-1.768541645	3.301313	-0.53571	0.59216	NA
BX640537.1	12.35942	0.10983414	0.548744	0.200156	0.841359	0.97643
zgc:162182	14.36316	-0.367492141	0.811486	-0.45286	0.650647	0.928065
si:dkeyp-79b7.3	1.248751	1.559390663	2.029025	0.768542	0.442165	NA
zgc:158343	412.8107	-0.137814698	0.190587	-0.72311	0.469615	0.867224
bcan	224.2788	0.274821759	0.259882	1.057485	0.29029	0.76078
BX914205.1	0.687345	-2.427541662	2.37881	-1.02049	0.307498	NA
CR759907.1	0.460658	-0.868135665	4.075402	-0.21302	0.831313	NA
ly6m3	6.828777	-4.335075171	1.511652	-2.86777	0.004134	0.106542
nme2b.2	43310.9	-0.058359186	0.157822	-0.36978	0.711547	0.945821
si:ch73-236c18.9	0.591957	0	3.728853	0	1	NA

CR391930.1	0.462384	-0.868136105	3.577307	-0.24268	0.808254	NA
lcat	182.089	-0.784511048	0.284446	-2.75803	0.005815	0.129012
CABZ01073265.1	728.2103	-0.50717437	0.23463	-2.1616	0.030649	0.312139
CU179702.1	0.060442	0	5.267649	0	1	NA
nkx1.2lb	99.5444	0.075810386	0.299139	0.253429	0.799937	0.967255
si:dkey-15h8.17	8.36085	-6.915059123	3.316352	-2.08514	0.037057	0.337668
atg16l1	554.2585	-0.026446311	0.124595	-0.21226	0.831905	0.974925
med24	391.8629	0.099434889	0.1339	0.742607	0.457719	0.860993
si:ch211-246b8.1	1.209821	1.893951403	2.226707	0.850562	0.395013	NA
BX927335.1	0.050624	1.055396947	5.267649	0.200354	0.841203	NA
tradv23.1.1	0	NA	NA	NA	NA	NA
AL844563.1	0	NA	NA	NA	NA	NA
si:ch1073-268j14.1	26.21706	-0.249950627	0.44	-0.56807	0.569988	0.902338
CABZ01073736.1	11.52075	-0.08916721	0.491623	-0.18137	0.856075	0.979558
serpl	1148.699	0.155495742	0.111248	1.397739	0.162192	0.629748
CR936249.1	2.700896	-1.37080718	1.263964	-1.08453	0.27813	0.751971
cbx4	235.1521	-0.021922448	0.161014	-0.13615	0.891701	0.988566
znf1122	1.31475	-3.746258017	1.786744	-2.0967	0.036021	NA
cc2d1b	325.6236	0.095863563	0.223935	0.428086	0.668589	0.933086
slit1b	296.3549	0.135937316	0.205678	0.660923	0.508662	0.883333
pcdh1g18	30.87666	-0.194961078	0.456959	-0.42665	0.669635	0.933428
sh3d21	671.6298	0.136875419	0.135232	1.012149	0.311467	0.77613
atrxl	655.7182	0.094932615	0.116373	0.815763	0.414636	0.838683
dre-mir-455-1	0.04222	0	5.267649	0	1	NA
elof1	2215.781	-0.116674385	0.154893	-0.75326	0.451295	0.857606
tradv14.0	3.184858	-1.844325153	1.425149	-1.29413	0.195621	0.675764
ogt.1	874.1721	0.04836642	0.168753	0.286611	0.77441	0.960818
BX293991.1	2.218893	0	1.948201	0	1	1
ddx46	1062.282	-0.086320793	0.117988	-0.73161	0.46441	0.864593
asb10	310.8858	0.116814599	0.22872	0.510733	0.609538	0.915204
prlh	11.74766	-0.424183113	0.548512	-0.77333	0.439325	0.852079
rnf213a	191.554	-0.557537836	0.293429	-1.90008	0.057423	0.413602
si:dkey-190j3.2	1.164489	0.531771887	1.624191	0.327407	0.74336	NA
RF00026	0	NA	NA	NA	NA	NA
pcdh2ab2	0	NA	NA	NA	NA	NA
BX000985.1	0	NA	NA	NA	NA	NA
muc5.3	82.66451	1.662873219	0.875974	1.898313	0.057655	0.41428
CT476811.1	7.455178	0.171100011	0.707283	0.241912	0.808849	0.969828
FQ312013.1	0.879967	-0.940571629	2.469878	-0.38082	0.703339	NA
CR855306.1	0.167824	0	5.267649	0	1	NA
zgc:174863	312.3798	0.298024748	0.159548	1.867929	0.061772	0.42635
hs2st1a	199.1116	-0.058823931	0.187017	-0.31454	0.753112	0.955777
SYDE2	203.6596	-0.031274231	0.176366	-0.17733	0.859252	0.979975
vdra	27.80752	-0.639164229	0.447663	-1.42778	0.153355	0.617847
m1ap	0.447731	0.907016445	3.199822	0.283458	0.776825	NA
si:ch73-233f7.7	6.477609	0.711719718	0.766936	0.928004	0.353406	0.804432
tmem176l.1	509.7611	-0.246564284	0.162477	-1.51753	0.129132	0.578579
CR847906.1	2.208859	-0.111700207	1.371046	-0.08147	0.935068	0.996829
si:ch73-309g22.1	1.424413	0	2.34477	0	1	NA
FP016247.1	0.26896	1.922155056	4.750613	0.404612	0.685763	NA
ndufa4l	5724.887	-0.081755956	0.120775	-0.67693	0.498453	0.879132
clec3bb	24.5721	0.621772572	0.515836	1.205368	0.228061	0.709324
FQ311879.2	0.045161	0	5.267649	0	1	NA
traj5	0	NA	NA	NA	NA	NA

CABZ01049362.1	0	NA	NA	NA	NA	NA
btr23	17.98047	0.159343339	0.524692	0.303689	0.761365	0.958159
cnn3a	1384.156	-0.117887014	0.134251	-0.87811	0.379884	0.823607
CABZ01034698.2	11.70987	-1.547579079	0.679729	-2.27676	0.022801	0.266874
zgc:112234	5.12813	-2.223635922	1.268665	-1.75274	0.079647	0.477652
ephx4	102.4945	0.819047349	0.333931	2.452747	0.014177	0.208295
mettl23	93.96741	-0.195747585	0.197277	-0.99225	0.321077	0.781417
adssl1	3456.237	-0.167723382	0.194784	-0.86107	0.389197	0.827725
si:ch211-119e14.1	48.28587	-0.302531995	0.295634	-1.02333	0.30615	0.771472
zgc:66475	511.6249	-0.056009562	0.153467	-0.36496	0.715141	0.945873
CU571163.1	0.580382	0.069106523	2.484379	0.027816	0.977809	NA
si:ch73-252p3.1	87.68091	0.236255365	0.225635	1.04707	0.295067	0.763708
gbe1b	161.5994	-0.009854372	0.21598	-0.04563	0.963608	0.999842
si:ch1073-13h15.3	88.83789	-0.957827509	0.710193	-1.34869	0.177438	0.651026
tradv4.0.2	0	NA	NA	NA	NA	NA
nkiras1	176.6545	0.258239273	0.145418	1.775836	0.07576	0.466355
dus3l	293.8862	-0.326270088	0.177993	-1.83305	0.066795	0.441428
BX957252.2	0.725038	2.457968723	2.498829	0.983648	0.325289	NA
pimr58	0	NA	NA	NA	NA	NA
ubl3a	900.8234	0.037038863	0.123489	0.299937	0.764225	0.959228
zgc:113279	76.85189	-0.617599893	0.363379	-1.6996	0.089206	0.501424
CABZ01066926.1	145.3536	-0.643609841	0.53243	-1.20882	0.226734	0.707913
si:ch73-379j16.2	1.280205	-0.271382716	2.266532	-0.11973	0.904693	NA
si:ch211-188p14.3	6.733944	0.603565733	0.714457	0.844789	0.398229	0.830737
dicp2.2	0.089161	0	5.267649	0	1	NA
FQ311924.1	6.186176	0.47003179	0.719524	0.653254	0.513592	0.88631
CR388156.1	0	NA	NA	NA	NA	NA
si:dkey-165b20.3	3.385159	1.779374345	1.053151	1.689571	0.09111	0.50549
snx25	419.9777	0.139901796	0.173665	0.805582	0.420484	0.842383
kynu	268.7765	0.463005863	0.246172	1.880824	0.059996	0.421883
nbeal1	230.8831	-0.196487465	0.177306	-1.10819	0.267782	0.744189
sec16b	136.8078	0.060007654	0.185567	0.323374	0.746412	0.955074
foxo1a	493.2408	0.072346073	0.147537	0.490359	0.62388	0.919431
CU929079.1	0.060442	0	5.267649	0	1	NA
CT027744.1	6.266514	-0.695029103	0.845658	-0.82188	0.411145	0.837797
NPC1L1	49.94508	-1.23718478	0.752024	-1.64514	0.099941	0.524166
mir196a-2	0.254477	0	5.267649	0	1	NA
lyrm5a	215.8593	-0.075568602	0.162758	-0.4643	0.642432	0.92567
BX088603.1	2.418701	-0.79473427	1.551608	-0.5122	0.608511	0.915204
lygl2	1.603726	-1.488502527	1.487562	-1.00063	0.317004	NA
inpp5ka	271.5666	-0.151234986	0.167561	-0.90257	0.366756	0.816111
lmbrd2a	498.2233	0.140899792	0.147213	0.957116	0.338509	0.795226
CU856423.1	14.77296	-0.081381679	0.469436	-0.17336	0.862368	0.981003
RF00001	0.809372	-1.811037223	2.648876	-0.6837	0.494165	NA
CU468012.2	0.192158	0	5.267649	0	1	NA
hmgn2	22602.41	0.007106402	0.183277	0.038774	0.96907	1
CR759927.3	37.22776	-0.414192358	0.38544	-1.0746	0.282556	0.754808
CU929203.1	0	NA	NA	NA	NA	NA
RF00001	0	NA	NA	NA	NA	NA
si:dkey-182g1.10	50.02857	-0.268163721	0.322526	-0.83145	0.405721	0.834645
BX005124.2	0.193702	1.921567426	5.236909	0.366928	0.713673	NA
aldh18a1	672.1429	0.002846836	0.103773	0.027433	0.978114	1
CR762480.1	0.129926	0	5.267649	0	1	NA
CR382383.2	4.528428	-1.471988747	1.048587	-1.40378	0.160383	0.626951

tradv23.3.8	0.147083	1.055370259	5.267649	0.200349	0.841207	NA
BX914205.2	0.124582	1.055383603	5.267649	0.200352	0.841205	NA
r3hcc1	108.4575	-0.250583751	0.196787	-1.27337	0.202885	0.683508
CU468012.3	4.727429	-0.499742069	1.527647	-0.32713	0.743568	0.954856
BX088603.2	0.406437	-1.791190913	4.953241	-0.36162	0.717636	NA
btd	75.27155	-1.058414668	0.281157	-3.7645	0.000167	0.013152
si:dkey-211i20.5	4.443607	4.416888453	1.446795	3.052878	0.002267	0.075801
CABZ01067558.1	12.6511	0.318676103	0.632367	0.503942	0.614302	0.915998
pigrl2.3	1.775759	0.993565878	1.859639	0.534279	0.593149	NA
si:dkey-93h22.7	95.21286	0.093187774	0.279975	0.332843	0.739253	0.953422
BX842700.1	2.908768	2.572271785	1.784243	1.44166	0.149398	0.611366
clca5.1	0.202892	-2.754967866	5.185055	-0.53133	0.595191	NA
si:ch73-160p18.4	0.539167	-2.18695978	3.468689	-0.63049	0.528377	NA
tpbga	116.6221	0.203392132	0.263354	0.772314	0.439928	0.852079
RF00001	0.984196	-1.803293754	2.288855	-0.78786	0.43078	NA
zgc:66483	4.299513	-0.131557035	0.830706	-0.15837	0.874167	0.98385
purab	248.2937	-0.275306191	0.162392	-1.69531	0.090016	0.504028
RF00001	0	NA	NA	NA	NA	NA
FP236734.1	60.81737	0.380576512	0.281684	1.351074	0.176672	0.650047
sdr39u1	293.534	0.287246419	0.115975	2.476798	0.013257	0.200494
si:dkey-1b17.6	28.55305	0.502688303	0.392649	1.280249	0.200457	0.68081
CABZ01083166.1	116.4446	0.319092918	0.195404	1.632992	0.102471	0.529139
si:ch211-69b22.5	113.2066	0.243730595	0.213368	1.142303	0.253328	0.732759
chmp1b	1276.79	-0.079536151	0.12244	-0.64959	0.515956	0.886651
BX908721.1	4.360953	0.080357494	1.328721	0.060477	0.951775	0.99801
CR855307.1	21.62959	0.043500918	0.351644	0.123707	0.901547	0.990643
BX510915.1	10.9109	1.010599159	0.779327	1.296759	0.194714	0.67483
cmtm6	382.691	-0.178400536	0.128269	-1.39083	0.164278	0.633351
sec14l7	288.3096	-0.987226495	0.3782	-2.61033	0.009046	0.163229
akap8l	2364.96	0.107080739	0.079664	1.344163	0.178896	0.653788
si:ch211-11c3.11	0.238114	0	4.934757	0	1	NA
szl	0.467871	0	3.596789	0	1	NA
tradv8.0	0	NA	NA	NA	NA	NA
trim35-19	0.676424	0.093650571	2.595594	0.036081	0.971218	NA
eed	466.9341	0.079429577	0.168449	0.471536	0.637258	0.924779
dcun1d1	267.403	0.080991005	0.119889	0.675549	0.499327	0.879603
si:ch211-76m11.9	0.203895	0	5.267649	0	1	NA
zgc:153184	166.7658	-0.104035678	0.191145	-0.54428	0.586251	0.906711
pcdh1g3	20.41795	0.119149263	0.418832	0.28448	0.776043	0.961073
LO018363.1	71.03429	0.696878849	0.269061	2.590044	0.009596	0.168452
CABZ01079663.1	98.7043	0.403351518	0.214626	1.879325	0.0602	0.422294
bin3	373.8404	-0.08596357	0.123712	-0.69487	0.487138	0.873291
xpot	848.8962	0.153057448	0.095926	1.595579	0.110583	0.545461
marveld3	52.16752	-0.315439073	0.267008	-1.18139	0.23745	0.718568
pimr207	0.178247	0	5.267649	0	1	NA
CR381657.1	0.842746	-2.384171899	2.93964	-0.81104	0.417342	NA
akt1	58.63593	0.27584225	0.226892	1.215742	0.224083	0.705812
CU915256.1	3.314874	-1.79809065	1.654475	-1.0868	0.277123	0.751426
cox5ab	3103.284	-0.030514798	0.144611	-0.21101	0.832877	0.975379
selenof	1829.982	-0.035951576	0.20517	-0.17523	0.8609	0.980388
snrpg	1192.581	-0.089848865	0.206898	-0.43427	0.664095	0.93177
CR927050.2	0.849807	-1.757179066	2.958581	-0.59393	0.552561	NA
znf1074	7.763242	1.152771251	0.738231	1.561533	0.118398	0.561566
capgb	1213.508	-0.205904118	0.105437	-1.95287	0.050835	0.392352

si:dkey-203a12.5	5.550164	0	2.62272	0	1	1
dicp3.1	17.40672	-0.13110901	0.513522	-0.25531	0.798481	0.966705
kdm4b	1571.113	-0.212709084	0.101631	-2.09296	0.036352	0.335171
BX927335.2	0.889749	-0.426487287	2.566005	-0.16621	0.867994	NA
bambib	258.184	-0.002386085	0.202542	-0.01178	0.990601	1
BX569789.1	31.97967	0.152513036	0.348138	0.438082	0.661327	0.930581
tent2	293.5132	0.037149123	0.187632	0.197989	0.843054	0.976775
BX004980.1	0	NA	NA	NA	NA	NA
trim35-24	4.111232	0.955285548	1.078873	0.885448	0.375915	0.822237
gnb5a	226.8293	-0.115503136	0.222179	-0.51986	0.603158	0.914226
CT009597.1	0.240305	2.667686611	5.195716	0.51344	0.607644	NA
FP016142.3	0	NA	NA	NA	NA	NA
spink2.3	19.78036	0	2.065996	0	1	1
vaspa	27.95678	0.421765224	0.391387	1.077618	0.281204	0.753557
CR550302.1	0.049519	0	5.267649	0	1	NA
ccnyl1	198.4033	0.023072065	0.134937	0.170984	0.864236	0.981867
AL935065.1	1.535533	0	1.99684	0	1	NA
neurl1b	306.1807	-0.005750842	0.216987	-0.0265	0.978856	1
si:dkey-61n16.5	137.1831	-1.514569015	0.549061	-2.75847	0.005807	0.129012
BX548059.1	6.975378	0.063822488	0.928095	0.068767	0.945175	0.997371
BX649620.1	4.986599	-0.01863852	1.037802	-0.01796	0.985671	1
ahrra	77.40677	0.010150906	0.561451	0.01808	0.985575	1
foxred1	68.39467	0.280118258	0.205571	1.362635	0.172997	0.645798
CR385021.1	0.148556	0	5.267649	0	1	NA
cacna1hb	11.80789	1.091872739	0.592426	1.843054	0.065321	0.435359
BX284696.1	0	NA	NA	NA	NA	NA
si:dkey-29j8.1	7.49552	0.258575136	0.979714	0.263929	0.791835	0.964835
zgc:163073	157.489	3.264155215	1.085208	3.007861	0.002631	0.083505
si:dkey-152b24.7	15.69849	-1.384207106	0.603312	-2.29435	0.021771	0.261665
si:rp71-1h20.9	24.43611	-0.316815141	0.452164	-0.70066	0.483512	0.872099
CU929391.3	2.119532	0.882686604	1.12669	0.783434	0.433372	NA
evlb	525.1694	0.245423274	0.190322	1.289519	0.197218	0.67737
zgc:153993	310.5365	0.003296283	0.136923	0.024074	0.980794	1
relt	147.646	0.250158189	0.158602	1.577266	0.114734	0.554525
FP236161.1	0.457097	1.055416406	3.174469	0.33247	0.739534	NA
ssbp2	162.6393	0.174975787	0.179031	0.977352	0.328395	0.786776
zgc:56622	70.67723	-0.266283221	0.315522	-0.84395	0.398699	0.830925
pcdh10a	855.9985	-0.309697535	0.102455	-3.02276	0.002505	0.08071
pkma	3283.407	0.073348072	0.182505	0.401897	0.68776	0.938835
gfra1a	154.4485	0.141347219	0.193136	0.731855	0.464257	0.864593
CIR1	206.5702	-0.160441208	0.146497	-1.09519	0.273435	0.749148
gig2f	0.049021	-0.86807634	5.267649	-0.16479	0.869106	NA
ccr9b	16.77722	-3.089411438	1.411075	-2.1894	0.028568	0.300821
mvb12a	187.5405	0.045149422	0.189794	0.237886	0.811969	0.97012
heatr1	441.1954	-0.099548305	0.198657	-0.50111	0.616297	0.916365
zgc:113019	164.3385	-0.268643793	0.167415	-1.60465	0.10857	0.541057
gap43	944.3993	0.271135675	0.205268	1.320885	0.18654	0.664968
baz1a	220.7631	-0.206306147	0.190546	-1.08271	0.278937	0.752417
rgs3a	261.3361	-0.261632627	0.150232	-1.74152	0.081592	0.480918
CABZ01111953.1	123.3594	-0.1341488	0.233992	-0.5733	0.566438	0.901739
si:ch73-46n24.1	0.086616	0	5.267649	0	1	NA
ube2d2l	399.3812	0.232800626	0.111806	2.082185	0.037326	0.339151
BX682557.2	11.626	-0.155378469	0.881049	-0.17636	0.860014	0.980167
si:dkey-250k10.4	1.828943	-0.848322182	1.805507	-0.46985	0.63846	NA

si:dkey-19b23.7	70.11326	0.322198147	0.259755	1.240392	0.21483	0.696864
zgc:63863	167.8919	0.142703115	0.209803	0.680176	0.496393	0.878161
pfklb	470.8433	-0.381050212	0.232338	-1.64007	0.10099	0.526541
wdr47b	70.19865	0.569114938	0.331304	1.7178	0.085833	0.491414
igf2bp2b	447.4418	-0.09309168	0.130024	-0.71596	0.474018	0.869292
BX510342.1	0.091242	-0.868089684	5.267649	-0.1648	0.869104	NA
vox	14.65975	0.097330423	0.439457	0.221479	0.82472	0.972272
CR855389.1	57.79273	0.255908523	0.310556	0.824033	0.409921	0.836939
BX323861.1	0.553226	-2.294792793	4.453114	-0.51532	0.606327	NA
myl12.1	7132.552	0.021298036	0.103357	0.206062	0.836742	0.976007
fhdc2	77.59054	-0.120964867	0.216822	-0.5579	0.576913	0.904798
si:ch211-272n13.3	238.6898	0.15386858	0.170599	0.90193	0.367094	0.81643
tradv18.0	0.251885	1.055363221	5.267649	0.200348	0.841208	NA
CU104782.1	1.07815	-3.402572195	5.166758	-0.65855	0.510184	NA
gne	273.5694	0.0712807	0.132485	0.538028	0.590557	0.908942
CU928046.1	46.31393	-1.511461852	0.685008	-2.20649	0.02735	0.293836
olfcg4	2.801767	-1.380080374	1.606849	-0.85887	0.39041	0.827902
cyb5b	357.7153	0.263371345	0.174139	1.512418	0.130428	0.581523
glula	5254.396	-0.191162163	0.224946	-0.84982	0.395428	0.829221
si:ch211-11n16.2	326.5532	0.035661674	0.13296	0.268214	0.788535	0.963567
CT737196.1	0	NA	NA	NA	NA	NA
CR383669.2	0.451866	0	3.270151	0	1	NA
pcdh2ac	0	NA	NA	NA	NA	NA
si:dkey-146m20.13	0.063619	0	5.267649	0	1	NA
actn4	4227.121	0.013825688	0.089249	0.154911	0.876892	0.985067
echdc3	35.14836	0.459739323	0.376982	1.219525	0.222645	0.704573
si:ch211-76m11.7	0.14264	0	5.267649	0	1	NA
pdk3b	11.10838	0.152084208	0.661146	0.230031	0.818068	0.971518
BX908776.1	0.682922	-1.800841234	2.441731	-0.73753	0.460802	NA
cspg5b	398.2825	0.387354945	0.173891	2.227578	0.025909	0.285638
si:dkey-285b23.3	1.246405	-0.929007139	1.538463	-0.60385	0.545941	NA
RF00001	0	NA	NA	NA	NA	NA
mon2	848.4295	0.05723343	0.101987	0.561185	0.574672	0.904516
si:dkey-11f4.16	48.10192	0.168816006	0.332651	0.507487	0.611813	0.915448
topaz1	22.12227	0.765044448	0.622398	1.229189	0.219001	0.699984
pnp5b	84.10034	-0.224560263	0.338293	-0.6638	0.506816	0.883007
pik3c2g	78.27225	0.447779405	0.223925	1.999685	0.045534	0.373513
CABZ01059119.1	78.52649	-0.042760788	0.216955	-0.1971	0.843753	0.976791
dre-mir-202	0	NA	NA	NA	NA	NA
BX572620.1	17.94547	0.55326121	0.403147	1.372356	0.169952	0.641753
si:dkey-203a12.7	6.950362	0	1.948858	0	1	1
si:dkey-31f5.11	186.4961	0.030769698	0.198011	0.155394	0.876511	0.985007
FBXO46	409.7404	-0.107646209	0.112759	-0.95466	0.339749	0.795226
tusc2a	146.4087	-0.077423791	0.19654	-0.39393	0.693629	0.940668
RF00001	0	NA	NA	NA	NA	NA
sb:cb1058	420.0543	-0.060826201	0.275446	-0.22083	0.825226	0.97261
tymp	41.67486	-2.763898774	1.397234	-1.97812	0.047915	0.380801
nots	11.23082	-0.892453095	0.642825	-1.38833	0.165037	0.634185
zgc:174944	12.53611	0.659342945	0.627668	1.050464	0.293505	0.76277
BX927336.2	7.46172	0.294161743	0.72371	0.406464	0.684402	0.938486
BX005396.2	7.007286	0.030358715	0.730234	0.041574	0.966838	1
BX255908.1	0.209715	0.093666232	5.267649	0.017781	0.985813	NA
fbxo7	291.786	-0.07308842	0.137893	-0.53004	0.596087	0.911517
si:dkey-199m13.7	16.23811	0.057053672	0.427874	0.133342	0.893923	0.988696

si:ch211-9d9.1	43.83999	-1.266315118	0.337291	-3.75437	0.000174	0.013603
lpar1	145.4362	0.085340356	0.195357	0.436842	0.662226	0.930742
prkcab	13.40796	-0.624221058	0.53347	-1.17011	0.241955	0.723529
CU138533.1	53.86919	-0.369728096	0.392662	-0.94159	0.346401	0.799463
nedd4a	492.3435	0.103278176	0.194137	0.531986	0.594736	0.910869
GNAZ	87.99588	0.20784392	0.292145	0.711441	0.476811	0.869292
zgc:174275	11.41271	-2.637286262	0.707811	-3.72597	0.000195	0.014684
si:ch211-154o6.3	170.6912	-0.034269477	0.170061	-0.20151	0.840298	0.976417
ebf3a	168.014	0.360000492	0.157134	2.291036	0.021961	0.262534
iqsec3a	290.4768	0.142029116	0.238036	0.596671	0.550727	0.8974
GPR180	36.14617	0.208623405	0.269066	0.775361	0.438126	0.851454
FP103005.1	1.494917	1.055430447	2.132385	0.494953	0.620633	NA
HTR7	3.49191	0.794990876	1.416994	0.56104	0.57477	0.904516
BX677667.1	0	NA	NA	NA	NA	NA
RF01684	1.058855	-1.437459537	2.439372	-0.58927	0.555677	NA
pkmb	8480.284	0.183449644	0.13463	1.362619	0.173003	0.645798
RF00001	0	NA	NA	NA	NA	NA
si:ch73-170d6.2	13.60607	0.418342296	1.155482	0.36205	0.717315	0.946272
hnrnpabb	4583.472	0.098053979	0.105504	0.92939	0.352687	0.803516
si:ch73-103b2.1	97.57609	0.409266542	0.373126	1.096859	0.272703	0.748552
traj4	0	NA	NA	NA	NA	NA
tnni4a	88.09421	-0.710764711	0.441966	-1.60819	0.107794	0.539319
myo7aa	156.2722	-0.319522597	0.232304	-1.37545	0.168992	0.640442
zgc:163083	30.56368	0.423494774	0.516554	0.819847	0.412303	0.838123
agap2	274.3633	0.167509806	0.179924	0.931001	0.351853	0.802563
CT956056.1	0	NA	NA	NA	NA	NA
BX908401.1	20.30989	-0.140335767	0.417961	-0.33576	0.73705	0.952291
si:dkeyp-72e1.6	78.4866	-0.279962586	0.281722	-0.99375	0.320343	0.780807
sp6	101.0834	0.012759309	0.205779	0.062005	0.950559	0.99801
CU302200.1	1.332757	0.454690976	1.554468	0.292506	0.7699	NA
trim105	175.6678	-0.124395691	0.14769	-0.84228	0.399634	0.831196
si:ch211-25d12.7	1.203021	-1.36949421	2.298151	-0.59591	0.551234	NA
snn	51.26104	0.295049852	0.321589	0.917475	0.358894	0.80995
CU207221.2	16.49782	-0.068855638	0.498741	-0.13806	0.890194	0.98835
si:ch211-57n23.1	51.02759	-0.123965343	0.318283	-0.38948	0.69692	0.94198
CU571336.1	2.150337	-0.635318851	1.418231	-0.44797	0.654178	NA
BX640537.2	32.41892	0.083351703	0.348957	0.23886	0.811214	0.97002
CR388184.1	4.905601	0.034026466	0.75351	0.045157	0.963982	0.999842
si:ch211-27e6.1	5.218912	1.576967967	0.83619	1.885896	0.059309	0.419405
si:dkeyp-51g9.4	1.987338	-1.375732089	1.788114	-0.76938	0.44167	NA
twnk	158.578	-0.169572263	0.152093	-1.11493	0.264882	0.742063
il17rc	308.5326	0.204655393	0.156925	1.304158	0.19218	0.672283
si:ch211-19312.10	32.40811	0.137515657	0.337386	0.407591	0.683574	0.938405
MTFP1	472.6698	0.15060654	0.176486	0.853363	0.393458	0.828374
RF00001	0.295196	0	5.267649	0	1	NA
si:ch211-208f21.3	0.085528	0	5.267649	0	1	NA
lrrc8c	195.5274	-0.008369283	0.180672	-0.04632	0.963053	0.999842
snrnp35	103.1651	0.018104032	0.201711	0.089752	0.928484	0.995052
BX323064.1	0	NA	NA	NA	NA	NA
p2ry2.1	7.460173	-1.222495128	0.761534	-1.60531	0.108427	0.54085
BX649345.1	3.693914	3.800122149	1.631826	2.328754	0.019872	0.248885
znf1005	68.60702	0.343550844	0.235075	1.461454	0.143891	0.603144
arl4aa	230.8894	0.003211999	0.180264	0.017818	0.985784	1
CR407584.1	2.754197	0.140152544	1.744773	0.080327	0.935977	0.996901

zgc:163040	2.772095	0.126185004	1.401376	0.090044	0.928253	0.995052
tbce	164.052	0.057670994	0.207019	0.278578	0.780569	0.961421
mir222b	0	NA	NA	NA	NA	NA
zgc:154054	460.637	-0.091044288	0.170585	-0.53372	0.593536	0.910427
BX072564.1	0.234379	0	5.267649	0	1	NA
si:dkey-26m3.1	0	NA	NA	NA	NA	NA
dpf1	90.69565	-0.067928447	0.292203	-0.23247	0.816173	0.971207
chrm5a	3.279893	-0.316014787	1.311216	-0.24101	0.809548	0.969828
col27a1b	102.7794	0.220819127	0.265625	0.831319	0.405793	0.834645
pcdh1g26	30.50325	0.030012427	0.425803	0.070484	0.943808	0.997371
BX005245.1	0.696502	-1.767952766	2.799244	-0.63158	0.52766	NA
ppa1a	68.8867	-0.771031475	0.599755	-1.28558	0.198591	0.679281
CU862015.1	0.279584	0	4.699872	0	1	NA
nlrc3l	91.29656	0.159090099	0.240285	0.662089	0.507914	0.883056
CABZ01112864.1	3.155969	1.128589831	1.343402	0.840098	0.400853	0.8318
pigrl3.5	0.39738	-0.868138352	5.041156	-0.17221	0.863272	NA
mapre2	914.4258	-0.110860678	0.08871	-1.2497	0.211408	0.692983
RF00001	0	NA	NA	NA	NA	NA
CU467646.4	33.47168	0.701002653	0.339019	2.067739	0.038665	0.344438
fam189a2	122.1879	0.202354239	0.186893	1.082728	0.278929	0.752417
si:ch211-209f23.7	0.049021	-0.86807634	5.267649	-0.16479	0.869106	NA
FP236551.1	0.690414	1.05542605	2.48092	0.425417	0.670533	NA
cldn17	8.301894	-0.414704261	0.827778	-0.50098	0.616382	0.916365
PRLHR	9.744966	-0.421134566	0.815956	-0.51612	0.605768	0.915055
plekhm1	431.8851	0.041860614	0.189063	0.221411	0.824772	0.972272
CT955967.1	0.250074	0.960281311	4.872023	0.197101	0.843748	NA
BX957318.1	6.272386	1.371370353	0.707079	1.939486	0.052442	0.397997
smyhc1	14424.62	-0.055781507	0.14787	-0.37723	0.705999	0.944512
elovl1a	161.5491	-0.458020123	0.263781	-1.73637	0.082499	0.482825
bnip3	291.1182	0.278929549	0.208281	1.339196	0.180507	0.656202
si:dkey-27n6.1	6.518296	-0.629933477	0.966567	-0.65172	0.51458	0.88631
BX005073.2	0.107013	-1.826650979	5.230461	-0.34923	0.726914	NA
FP085428.1	9.821788	-1.67941903	0.692522	-2.42508	0.015305	0.217692
polr2a	1625.02	0.202340016	0.186857	1.082858	0.278872	0.752417
CU929143.1	0.39745	-0.402180623	3.529312	-0.11395	0.909274	NA
zgc:152863	49.58383	0.634191024	0.340804	1.860867	0.062763	0.42872
CR383676.1	13221.28	0.128975416	0.146638	0.879547	0.379105	0.823173
nup153	762.4144	0.131163271	0.173956	0.754001	0.450849	0.857288
mia2	610.1205	0.115564713	0.125486	0.920941	0.357081	0.807809
ldb3b	7613.39	0.250540067	0.149556	1.675223	0.09389	0.511703
tgfbr3	59.83696	-0.329910512	0.370895	-0.8895	0.373736	0.820644
bpifc1	23.218	-0.603361979	0.449802	-1.34139	0.179792	0.655282
si:ch1073-228h2.2	29.19882	0.355787786	0.343681	1.035226	0.300564	0.766629
ccl39.7	13.41942	-0.19373478	0.58811	-0.32942	0.741839	0.954329
CABZ01023255.1	47.61066	0.080145526	0.382668	0.209439	0.834106	0.975678
ccnl12	136.5454	0.0515021	0.180307	0.285636	0.775157	0.960899
si:ch211-207i20.2	31.35647	0.026671699	0.311718	0.085564	0.931813	0.995854
FP236356.2	0.234145	-1.710092121	5.238472	-0.32645	0.744085	NA
RF00001	0	NA	NA	NA	NA	NA
creld1a	138.3204	0.305390749	0.273732	1.115657	0.264569	0.742063
RF00001	0	NA	NA	NA	NA	NA
apbb2b	250.949	-0.033204724	0.145528	-0.22817	0.819517	0.971785
rhot1a	611.7424	0.215452453	0.164866	1.306834	0.191269	0.671109
cox20	252.3089	0.18580249	0.167518	1.109148	0.267366	0.743683

tcf3b	1268.341	0.01869049	0.151874	0.123066	0.902055	0.990847
CR352226.5	22.09784	-8.039956043	1.286398	-6.24998	4.11E-10	3.40E-07
znf1096	9.07896	-1.934898598	0.678642	-2.85113	0.004356	0.108751
ctbs	153.1769	0.361253384	0.514499	0.702146	0.482588	0.871721
glulb	8066.164	-0.232148164	0.106115	-2.1877	0.028691	0.301484
si:ch211-76m11.5	11.59387	-0.303084705	0.729091	-0.4157	0.677628	0.936026
arhgap39	74.03695	0.543035126	0.214677	2.529542	0.011421	0.185332
RF00001	0	NA	NA	NA	NA	NA
strn	315.0059	0.032366122	0.144587	0.223853	0.822872	0.971966
CR855860.1	127.4298	-0.255085211	0.339584	-0.75117	0.45255	0.857994
dhx38	282.3761	-0.080168466	0.157759	-0.50817	0.611334	0.915331
tradv13.0	0	NA	NA	NA	NA	NA
arhgap11a	204.5024	0.32872838	0.187605	1.752233	0.079734	0.477867
pim1	1537.403	0.09022699	0.177877	0.507244	0.611984	0.915644
plaat1	109.9093	0.841352674	0.359047	2.343291	0.019114	0.243342
hist1h2a6	2.127575	1.073126083	1.494065	0.718259	0.472597	NA
si:ch211-231f6.6	161.0436	0.235978568	0.206159	1.144645	0.252356	0.732759
olfch1	0	NA	NA	NA	NA	NA
si:dkeyp-13d12.21	0.049519	0	5.267649	0	1	NA
pole2	103.7268	0.35871674	0.19309	1.857769	0.063202	0.429313
TADA1	103.7922	-0.198235659	0.208836	-0.94924	0.342498	0.797179
GABRB2 (1 of many)	5.946493	0.052182622	0.994661	0.052463	0.95816	0.999109
CU929317.1	0	NA	NA	NA	NA	NA
BX901923.1	8.436044	-0.248583379	0.602792	-0.41239	0.680056	0.93674
CU571069.1	67.75087	-1.531725316	0.585984	-2.61394	0.008951	0.16209
CABZ01111454.1	59.98038	0.165364895	0.292909	0.564561	0.572372	0.903461
prtga	1.106224	-0.825171955	1.90089	-0.4341	0.664218	NA
pimr184	0.379085	-0.752516851	3.817574	-0.19712	0.843734	NA
si:dkey-206d17.15	0	NA	NA	NA	NA	NA
ergic1	229.758	0.373614143	0.148572	2.514692	0.011914	0.190109
tradv25.0.4	0	NA	NA	NA	NA	NA
BX571954.1	0	NA	NA	NA	NA	NA
spink2.4	18.73348	0	2.291669	0	1	1
CABZ01044099.1	71.91578	-0.094538527	0.256837	-0.36809	0.712808	0.945821
tox2	195.5821	-0.12221344	0.157427	-0.77632	0.437562	0.851283
CABZ01088109.1	845.1919	0.15164293	0.114342	1.326217	0.184768	0.661987
ppfibp1b	498.9331	0.042599692	0.123554	0.344786	0.730256	0.950056
RF00001	0.045161	0	5.267649	0	1	NA
exosc7	194.1122	-0.001942748	0.162775	-0.01194	0.990477	1
si:dkey-16p6.1	3.895736	-2.49771525	1.173846	-2.1278	0.033353	0.323127
CU467837.1	6.557401	-0.577643993	0.873976	-0.66094	0.508652	0.883333
RF00001	0.043308	0	5.267649	0	1	NA
dre-mir-137-2	0	NA	NA	NA	NA	NA
mgrn1a	482.8983	-0.409075793	0.174095	-2.34973	0.018787	0.241964
BX908757.1	0	NA	NA	NA	NA	NA
abcg2a	1455.122	-0.241507841	0.205184	-1.17703	0.239184	0.719781
CU929568.1	0.188326	1.888174718	5.239287	0.360388	0.718557	NA
RF00026	0	NA	NA	NA	NA	NA
dnai1.1	1.084513	1.634010181	2.35124	0.694957	0.487082	NA
ap3b1a	274.5708	-0.176534544	0.142388	-1.23982	0.215043	0.697086
sugt1	467.759	0.078647053	0.138429	0.568142	0.569939	0.902322
CABZ01084603.1	220.2099	0.206007369	0.185131	1.112767	0.265809	0.742605
AL929114.1	0.570739	-0.805386326	2.395534	-0.3362	0.736718	NA
adipoqb	16.01333	-1.489205311	1.053935	-1.41299	0.157657	0.623766

evx1	22.59221	-0.021660293	0.320667	-0.06755	0.946146	0.997371
zmp:0000001114	408.9177	0.037678811	0.134534	0.280069	0.779424	0.961073
camk2b2	18.85684	-0.227823296	0.553483	-0.41162	0.68062	0.937071
si:dkey-11d20.1	0.09233	-0.868089684	5.267649	-0.1648	0.869104	NA
ppp2r2ca	487.037	0.151859051	0.19182	0.791675	0.42855	0.846289
anxa1b	1620.409	-0.784506318	0.199496	-3.93243	8.41E-05	0.008118
CU855936.1	0.193998	0	5.267649	0	1	NA
mafgb	34.75163	-0.197880535	0.317478	-0.62329	0.533095	0.890978
CR382281.1	1.816759	-2.34425431	1.885901	-1.24304	0.213852	NA
si:dkey-238o14.1	3.125885	-1.648987696	1.375135	-1.19915	0.230471	0.712099
RF00001	0	NA	NA	NA	NA	NA
RF00001	0.053506	-0.86807634	5.267649	-0.16479	0.869106	NA
zgc:101785	25.31727	-0.433807754	0.434034	-0.99948	0.317563	0.779441
impg2b	4.79989	-0.633618873	1.308697	-0.48416	0.628272	0.921864
CR385054.1	33.73708	2.805789881	1.407181	1.993908	0.046162	0.375668
cul3b	704.2086	-0.006413128	0.106391	-0.06028	0.951934	0.998032
si:ch211-237a4.2	14.44826	0.089254273	1.042815	0.08559	0.931793	0.995854
znf1118	9.794575	-2.560429773	0.780718	-3.27958	0.00104	0.045197
AL935044.2	3.526631	-3.101797549	1.558873	-1.98977	0.046616	0.376263
sf3a3	961.1614	0.00864282	0.110945	0.077902	0.937906	0.997305
dnah1	47.16427	0.582960618	0.322447	1.807925	0.070618	0.453373
mrps24	495.7844	0.111368371	0.198621	0.560708	0.574997	0.904516
CR925761.2	2.294286	-0.357832521	1.406222	-0.25446	0.799137	0.967045
caskb	474.3028	0.126137778	0.140715	0.896409	0.370034	0.818446
si:dkey-21c1.8	47.31211	0.453967746	0.258085	1.758984	0.07858	0.474455
znf1178	15.43142	0.275201048	0.475425	0.578853	0.562688	0.900772
CU861477.1	95.11263	0.341009015	0.245704	1.387886	0.165172	0.634423
brd3b	1200.089	0.084644198	0.119389	0.708979	0.478337	0.869292
cdh29	12.56625	-2.604793562	1.387043	-1.87795	0.060388	0.422591
CU929402.1	2.307649	0.232782928	1.369386	0.169991	0.865017	0.982136
cnmd	1989.753	0.398807941	0.16252	2.453895	0.014132	0.208027
dre-mir-7146	0.045161	0	5.267649	0	1	NA
dcp1a	174.2972	0.0036785	0.239507	0.015359	0.987746	1
il17ra2	0	NA	NA	NA	NA	NA
CR749168.3	0	NA	NA	NA	NA	NA
si:ch211-113d22.2	196.5468	-1.127038045	0.589596	-1.91154	0.055935	0.409094
CR626874.1	2.249899	0.077244189	1.196619	0.064552	0.948531	0.997738
znf1083	28.96248	-0.401033919	0.350603	-1.14384	0.25269	0.732759
lrp6	413.4289	-0.06351542	0.133566	-0.47554	0.634405	0.924185
CR936977.1	0	NA	NA	NA	NA	NA
CABZ01092282.1	407.0498	0.421732807	0.254929	1.654312	0.098064	0.521071
RF00026	0	NA	NA	NA	NA	NA
znf1150	0.086618	0	5.267649	0	1	NA
axin2	995.7234	0.075563952	0.114784	0.658312	0.510338	0.883879
FQ377903.1	32.62224	-0.285583018	0.400293	-0.71343	0.475577	0.869292
appl2	588.119	0.102066973	0.103	0.990943	0.321713	0.782075
C12orf75	55.96975	-0.509813065	0.27426	-1.85887	0.063046	0.429313
FP067440.1	0	NA	NA	NA	NA	NA
si:ch211-217i17.1	5.349162	-2.851567788	1.264477	-2.25514	0.024125	0.274967
tamm41	199.0151	0.118283707	0.18203	0.649805	0.515818	0.886634
tradv6.0.4	0.087179	0	5.267649	0	1	NA
angptl1b	27.01911	-0.901421106	0.713987	-1.26252	0.206763	0.689109
si:dkey-205i10.2	1.087204	-2.116623259	2.393964	-0.88415	0.376615	NA
CR382337.2	1.718928	2.256515612	1.764831	1.278602	0.201037	NA

CABZ01059120.1	72.90787	0.146968605	0.227782	0.645217	0.518786	0.887148
tox4b	54.13473	-0.178421081	0.331091	-0.53889	0.589964	0.908753
si:dkey-16p6.1	8.054691	-0.838183465	0.758012	-1.10577	0.268828	0.744925
CR749167.1	0	NA	NA	NA	NA	NA
si:dkeyp-9d4.3	593.4466	0.120114668	0.227394	0.528222	0.597345	0.911953
CSDC2 (1 of many)	18.85645	-0.78541628	0.513248	-1.53028	0.125946	0.573992
pam	1264.352	0.074135408	0.106528	0.695923	0.486477	0.873244
CU638702.1	0	NA	NA	NA	NA	NA
CT573484.1	3.666482	1.308858011	1.221422	1.071585	0.283906	0.755095
gb:ai877918	70.55001	-0.269765854	0.222303	-1.2135	0.224937	0.70685
lamp1a	507.0477	-0.192346529	0.157725	-1.2195	0.222654	0.704573
BX908782.1	79.57199	0.093628031	1.688906	0.055437	0.95579	0.998767
CU655842.1	132.1441	0.091267283	0.19498	0.468086	0.639723	0.924779
si:ch211-188p14.4	60.77983	0.739946147	0.358417	2.064486	0.038972	0.345405
trgv6	0.628731	0	2.847174	0	1	NA
znf1155	51.36827	0.173604301	0.324337	0.535259	0.59247	0.909996
AL953858.1	0	NA	NA	NA	NA	NA
znf1160	25.98561	-1.3878994	0.414	-3.35241	0.000801	0.037962
zgc:171929	1.352052	0.093648013	2.071644	0.045205	0.963944	NA
sncaip	142.1159	-0.086202156	0.191801	-0.44943	0.653118	0.929197
si:ch1073-185p12.2	3.750905	-2.371915045	1.265586	-1.87416	0.060908	0.423604
BX537249.1	0.181263	0	5.267649	0	1	NA
CABZ01056637.1	5.798532	-1.159483497	0.815158	-1.4224	0.154909	0.619752
RF00001	0.045161	0	5.267649	0	1	NA
klhdc4	266.7139	0.037376384	0.165669	0.225608	0.821506	0.971788
prpf39	1580.795	-0.038438939	0.104254	-0.3687	0.712348	0.945821
RF00001	0.257269	0	5.267649	0	1	NA
si:dkey-247i3.6	2.889322	-0.98438888	1.058351	-0.93012	0.352311	0.802897
si:ch211-117l17.7	0.56469	0	5.267649	0	1	NA
AL935294.1	0	NA	NA	NA	NA	NA
si:dkey-264f17.1	28.83652	0.53432903	0.346642	1.541445	0.123208	0.569695
BX511018.2	2.933378	-0.145691008	0.908139	-0.16043	0.872544	0.983447
utrn	1304.271	-0.200564816	0.160417	-1.25027	0.2112	0.692983
gpa33a	611.0346	-0.642273405	0.194863	-3.29603	0.000981	0.043444
kif16ba	152.4423	-0.006540459	0.164617	-0.03973	0.968307	1
SLC7A1 (1 of many)	342.6456	0.117419262	0.195074	0.601921	0.547227	0.896121
si:ch211-238e22.5	2.24889	1.296310513	1.264363	1.025268	0.305237	0.770382
ccdc135	75.77821	-0.694329403	0.336417	-2.0639	0.039028	0.345405
glis2b	82.49638	-0.268656843	0.247323	-1.08626	0.277365	0.751426
CU633762.1	0	NA	NA	NA	NA	NA
BX511246.1	13.34206	0.881269799	0.624956	1.410131	0.158501	0.623932
CU929203.2	0	NA	NA	NA	NA	NA
csppla	45.50476	0.39482983	0.2658	1.485437	0.137428	0.593264
tradv23.1.2	0	NA	NA	NA	NA	NA
CT737123.1	7.945405	0.999662672	0.868965	1.150406	0.249977	0.72973
dph5	201.4921	-0.102007756	0.169117	-0.60318	0.546391	0.896107
ebf3a	1576.623	0.243684937	0.084334	2.889536	0.003858	0.103374
FP016234.1	140.7035	0.155998463	0.227505	0.685693	0.492906	0.876623
CU929199.1	6.202156	-0.446293721	0.77924	-0.57273	0.566828	0.901747
c1r	5.044364	-1.267735805	0.913126	-1.38835	0.165031	0.634185
si:ch211-266k2.1	29.36217	-0.38729473	0.378562	-1.02307	0.306275	0.771472
si:ch211-152c8.2	3.154976	-0.569361356	0.913968	-0.62296	0.533314	0.891164
capzb	5235.803	-0.127690787	0.117353	-1.08809	0.276553	0.751172
CU571169.1	0	NA	NA	NA	NA	NA

si:ch73-29l19.1	80.23145	0.372367728	0.435296	0.855436	0.39231	0.827902
si:dkey-88e18.8	44.69906	0.394250992	0.332772	1.184748	0.236117	0.717364
zgc:174314	5.585452	-1.996384955	0.814304	-2.45164	0.01422	0.208802
si:dkeyp-104b3.2	0.11056	0	5.267649	0	1	NA
zgc:171759	7.883862	-0.281839994	0.781131	-0.36081	0.718241	0.946598
them6	221.6722	0.444528653	0.217565	2.043203	0.041032	0.353808
si:ch1073-266p11.2	45.05775	0.585852896	0.359548	1.629414	0.103225	0.530435
BX936382.1	12.20586	-0.441453265	0.496359	-0.88938	0.373797	0.820644
si:ch211-161c3.5	51.56061	0.103929861	0.275867	0.376739	0.706367	0.944623
thumpd1	243.6464	-0.032827323	0.171286	-0.19165	0.848015	0.977829
rhcgb	210.9882	0.264135918	0.723369	0.365147	0.715002	0.945821
GRM7	137.8943	0.394495291	0.285892	1.379877	0.167624	0.638828
or109-7	0	NA	NA	NA	NA	NA
FP085414.1	450.086	0.012141908	0.177299	0.068482	0.945402	0.997371
or109-3	0	NA	NA	NA	NA	NA
dre-mir-19b	0.053262	1.055396947	5.267649	0.200354	0.841203	NA
WDR7	260.3582	0.100271919	0.194617	0.515227	0.606394	0.915204
CU457819.1	1.401365	0.883707753	1.691983	0.522291	0.601468	NA
znfx1	17.48475	-0.484150798	0.582308	-0.83143	0.405728	0.834645
si:dkeyp-6a3.1	0.343654	1.055364631	4.512522	0.233875	0.815082	NA
BX571843.1	0.314559	1.055381606	4.527408	0.233109	0.815676	NA
dnai2a	3.735162	-2.094305698	1.386882	-1.51008	0.131023	0.582686
SLA (1 of many)	3.799106	0.215471826	0.912448	0.236147	0.813319	0.970328
impg2b	20.74508	-2.343860462	1.481653	-1.58192	0.113667	0.551905
fam166b	22.78038	0.002383425	0.563239	0.004232	0.996624	1
arhgef15	8.283199	0.487603523	0.98323	0.49592	0.619951	0.91761
zgc:113209	144.2867	-0.057378646	0.232871	-0.2464	0.805375	0.969348
ccl39.3	12.89463	0.157723342	0.973968	0.161939	0.871354	0.983365
pja2	302.6263	0.057740402	0.130023	0.444078	0.656986	0.929939
CABZ01065328.2	0.191661	-0.868103335	5.267649	-0.1648	0.869102	NA
si:dkey-237g15.2	19.74625	0.097582061	0.414069	0.235666	0.813692	0.970397
CU638714.1	0.060442	0	5.267649	0	1	NA
chdh	179.2578	-0.148120613	0.203806	-0.72677	0.467364	0.86616
adamts5	62.26085	-0.079645914	0.291201	-0.27351	0.784462	0.962605
SEMA4F	69.92684	0.171431745	0.261388	0.655852	0.511919	0.885162
si:dkey-165e24.1	23.48356	-3.176433599	1.407488	-2.25681	0.02402	0.27455
hoxa13a	79.40603	0.023373186	0.202188	0.115601	0.907969	0.991745
pip5k1cb	273.3253	-0.158179427	0.28341	-0.55813	0.576756	0.904769
slc15a1a	54.82058	-1.261496309	0.673892	-1.87196	0.061213	0.424867
ahsa1b	405.3883	0.050784856	0.133702	0.379836	0.704067	0.943735
RF00324	4.586725	-0.670930946	0.846287	-0.79279	0.427898	0.845893
BX248331.1	0.15979	0	5.267649	0	1	NA
fut10	26.71561	-0.14761577	0.379315	-0.38916	0.697154	0.94198
AL627305.1	28.77534	0.414545747	0.399504	1.037651	0.299433	0.766128
FQ377896.1	1.94204	2.145436449	1.445711	1.484001	0.137809	NA
CR354444.1	0.442906	-1.756781023	3.239487	-0.5423	0.58761	NA
znf1056	11.97203	-0.308849496	0.528421	-0.58448	0.5589	0.899409
SMIM10	173.1166	-0.074603282	0.169386	-0.44043	0.659623	0.930581
lat	7.238081	-1.38663841	1.546172	-0.89682	0.369815	0.818318
znf1104	2.783585	0.805114743	0.960312	0.838388	0.401813	0.832807
pik3r6a	115.3069	0.518161462	0.198022	2.61669	0.008879	0.161475
RF00001	0.957365	0	2.469437	0	1	NA
BX936384.1	3.004521	3.947206589	1.506756	2.619672	0.008801	0.160701
arf4b	25.86994	0.97838741	0.729479	1.341213	0.179851	0.655316

si:ch211-196h24.2	2.852882	-2.803147288	1.556933	-1.80043	0.071793	0.4564
zgc:158464	133.9362	-0.103852303	0.209093	-0.49668	0.619415	0.917435
tradv24.0.3	0.073968	0	5.267649	0	1	NA
si:dkey-71l4.1	4.780252	0.090827583	1.195452	0.075978	0.939437	0.997371
prkacaa	356.0869	-0.110138678	0.15512	-0.71002	0.477691	0.869292
aglb	693.4988	0.53785151	0.220895	2.434872	0.014897	0.213725
si:ch1073-398f15.1	205.0109	-0.455155497	0.317616	-1.43304	0.151847	0.615406
AL844192.1	5.124045	-0.220624833	0.932228	-0.23666	0.812917	0.970328
si:ch73-44m9.3	235.4337	0.228428883	0.284033	0.804234	0.421262	0.842742
CR382300.2	0	NA	NA	NA	NA	NA
AREL1	342.1146	0.034841956	0.12278	0.283777	0.776582	0.961073
si:ch211-125c23.3	3.974753	-2.824665128	1.569667	-1.79953	0.071935	0.4564
znf1174	23.66454	-0.332733722	0.352764	-0.94322	0.345569	0.798766
dcun1d5	494.3002	0.020480774	0.130675	0.156731	0.875457	0.984383
FO907110.1	2.949555	1.380056868	1.161753	1.187909	0.234869	0.715533
si:dkey-102m7.3	195.0485	-0.425818455	0.226457	-1.88035	0.06006	0.422205
BX276088.1	0.215663	1.055358649	5.267649	0.200347	0.841209	NA
CT955963.1	5.321294	-1.145507264	0.940593	-1.21786	0.223278	0.70531
eef2b	77110.32	-0.43493351	0.140978	-3.08511	0.002035	0.070205
alad	414.104	0.10988194	0.198049	0.554823	0.579016	0.905417
si:ch211-175l6.1	1.277582	0.943479355	2.6086	0.36168	0.717591	NA
txnrd3	2028.06	0.181865588	0.1175	1.547787	0.121674	0.568382
timm23b	46.61549	0.03648408	0.315909	0.115489	0.908057	0.991745
amdhd2	535.0047	-0.137237521	0.133374	-1.02897	0.303495	0.769363
CABZ01012885.1	0	NA	NA	NA	NA	NA
si:dkey-83h2.3	1.316061	-0.009386635	1.854861	-0.00506	0.995962	NA
si:dkey-34d22.2	1.756071	-1.806124751	2.132382	-0.847	0.396996	NA
rock2bl	5.129971	-0.51066737	0.734001	-0.69573	0.486597	0.873244
il10ra	12.08998	0.378062515	0.566414	0.667467	0.504474	0.881897
CT990567.1	0.04222	0	5.267649	0	1	NA
slc25a19	82.83091	0.277059582	0.242556	1.142249	0.253351	0.732759
si:dkey-61p9.9	1.587926	-0.812211419	1.880088	-0.43201	0.665736	NA
si:dkey-78o7.3	0	NA	NA	NA	NA	NA
abhd5b	1.244696	0	2.16554	0	1	NA
nitr11a	0.120883	0	5.267649	0	1	NA
rps18	19606.77	-0.287205482	0.112996	-2.54173	0.011031	0.183078
lnpep	312.5698	-0.091539875	0.127145	-0.71996	0.471547	0.86809
RF00001	0	NA	NA	NA	NA	NA
pip5k1aa	192.431	-0.030014907	0.177509	-0.16909	0.865726	0.982364
pde6c	1735.627	-0.26832034	0.399528	-0.67159	0.501843	0.881148
pax7a	100.1392	-0.087487699	0.22021	-0.39729	0.691152	0.940173
FO704610.1	250.4416	0.283489618	0.179516	1.579191	0.114292	0.553312
fgd1	101.9757	0.203758135	0.228069	0.893406	0.37164	0.819115
CT027704.1	0.147823	0	5.267649	0	1	NA
BX957252.3	5.026085	-3.967945857	1.447431	-2.74137	0.006118	0.131913
zgc:112265	2123.102	-0.184300071	0.38193	-0.48255	0.629416	0.92266
si:ch211-166e11.5	252.4874	-0.069780142	0.144917	-0.48152	0.630148	0.922869
camlg	504.7926	-0.509215489	0.180404	-2.82264	0.004763	0.114459
or106-5	0	NA	NA	NA	NA	NA
si:ch73-106n3.1	1.689873	-0.612623541	1.502758	-0.40767	0.683519	NA
si:ch211-171l17.14	4.387005	0.644794646	0.945961	0.681629	0.495474	0.87806
BX323861.2	0	NA	NA	NA	NA	NA
si:ch211-227e10.2	0.069091	0	5.267649	0	1	NA
ints1	659.8933	-0.200789903	0.165377	-1.21413	0.224697	0.706634

tpcn2	136.451	-0.220221753	0.223167	-0.9868	0.323739	0.783161
znf1129	12.35669	0.161834187	0.657757	0.246039	0.805652	0.96946
ptpro	458.3541	0.038071975	0.119393	0.318879	0.749818	0.955074
BX323994.2	0.043308	0	5.267649	0	1	NA
si:ch73-233f7.8	5.48839	-0.391693147	0.840848	-0.46583	0.641336	0.925554
fam167b	101.6583	-0.184196743	0.196693	-0.93647	0.349032	0.801306
BX547934.2	97.66107	0.141600697	0.255684	0.553811	0.579708	0.905618
irgf4	1.405054	0.054926614	1.564578	0.035106	0.971995	NA
CU634008.1	92.03813	0.086835129	0.237074	0.366279	0.714157	0.945821
CR376738.1	1.906454	1.055430738	1.952571	0.540534	0.588829	NA
znf1091	3.586723	0.060976399	1.082684	0.05632	0.955087	0.998611
sec22bb	657.5741	-0.049715749	0.128543	-0.38676	0.698931	0.942456
cnga2b	21.61365	0.822487623	0.429045	1.91702	0.055235	0.406983
zgc:153383	104.795	0.591979527	0.443074	1.336075	0.181525	0.657892
fastkd3	171.5771	-0.118288542	0.141515	-0.83588	0.403225	0.833799
cfh	1051.075	-0.365215168	0.231486	-1.5777	0.114635	0.554279
CR376766.2	245.4713	-0.19865003	0.27842	-0.71349	0.475542	0.869292
si:dkeyp-13d12.17	0	NA	NA	NA	NA	NA
si:ch211-286o17.1	978.2032	-0.109374811	0.19195	-0.56981	0.568808	0.902234
BX322631.1	10.74269	-0.868155033	1.722677	-0.50396	0.614292	0.915998
btr21	5.897501	-3.73249539	2.615547	-1.42704	0.153568	0.618187
olfcs2	3.136495	-0.521081153	1.311345	-0.39736	0.691099	0.940173
nop53	3902.155	-0.738212438	0.155339	-4.75228	2.01E-06	0.000506
BX649313.1	0.444703	0.093653772	3.657799	0.025604	0.979573	NA
pnkp	171.7538	-0.00210792	0.168356	-0.01252	0.99001	1
ptk2aa	334.2387	0.033933229	0.165208	0.205397	0.837262	0.976007
si:dkey-217g21.3	3.672131	-0.057702964	1.07728	-0.05356	0.957283	0.999109
CABZ01078737.1	69.19594	0.18953029	0.278618	0.680251	0.496345	0.878161
paqr7a	12.94399	0.523126653	0.50388	1.038198	0.299178	0.766047
BX908799.1	0.101247	1.854150541	5.241768	0.353726	0.723544	NA
si:dkey-193i10.1	7.14103	-1.840412293	0.864684	-2.12842	0.033302	0.323127
clrn3	25.79614	0.980980476	1.618367	0.606155	0.544412	0.895206
nrl	19.7859	-0.256466222	0.504561	-0.5083	0.611246	0.915331
fkrp	93.77544	0.091560524	0.235403	0.388952	0.697311	0.942027
znf1182	17.98887	-0.367702504	0.438948	-0.83769	0.402204	0.833156
BX004768.1	0.174583	1.055370259	5.267649	0.200349	0.841207	NA
LO017721.1	7.127109	1.997168872	0.758123	2.63436	0.00843	0.156879
fgf10b	20.32636	0.195920165	0.437613	0.447702	0.654368	0.929488
si:dkeyp-110c7.4	202.6831	0.171876465	0.497756	0.345303	0.729867	0.950056
si:dkey-43f9.4	33.05522	-0.507741515	0.36744	-1.38184	0.167022	0.637452
smg6	444.6248	0.041945033	0.135911	0.308621	0.75761	0.957364
pmt	211.3226	0.36915173	0.251845	1.465791	0.142705	0.601981
vent	2.335403	0.522061378	1.338524	0.390028	0.696516	0.941831
CR450808.1	14.34308	0.936371765	0.677633	1.381826	0.167025	0.637452
foxo6a	15.2225	-0.149381496	0.505492	-0.29552	0.767599	0.960296
nploc4	692.8818	0.069013677	0.138315	0.498958	0.617809	0.916478
RF00001	0	NA	NA	NA	NA	NA
BX511111.1	3.917622	-2.232808414	0.998136	-2.23698	0.025288	0.282476
CT025669.1	1.561709	-4.680128108	2.130883	-2.19633	0.028068	NA
pbx1a	594.4978	-0.041603834	0.12104	-0.34372	0.731056	0.950056
hic2	198.1511	-0.424948227	0.240392	-1.76773	0.077106	0.469501
CABZ01079251.1	38.59355	-1.450617455	0.379313	-3.82433	0.000131	0.011295
cpeb4b	721.5832	0.113260165	0.125317	0.903791	0.366106	0.815747
CR848025.1	4.491064	-1.574354137	0.846197	-1.8605	0.062814	0.428925

sos1	656.1271	-0.138536537	0.13418	-1.03246	0.301854	0.767977
ppp1r27b	406.8769	-0.267125494	0.763103	-0.35005	0.7263	0.948987
ddx52	278.9527	-0.224235127	0.233408	-0.9607	0.336703	0.793455
crb1	65.35678	0.009165658	0.243977	0.037568	0.970032	1
RF00001	0	NA	NA	NA	NA	NA
ubn2b	291.1195	0.009846834	0.139666	0.070503	0.943794	0.997371
si:dkey-82i20.2	0.635309	0.719246233	2.433651	0.295542	0.76758	NA
CR855379.2	1.000215	0	2.677596	0	1	NA
rps27l	868.5797	-0.593584864	0.198678	-2.98767	0.002811	0.087078
cfap20	289.5275	0.072233654	0.14199	0.508724	0.610945	0.915331
dusp1	1188.782	-0.125476634	0.459415	-0.27312	0.784759	0.962605
tradv5.0.1	0	NA	NA	NA	NA	NA
CR387919.1	60.45567	-0.056225173	0.293762	-0.1914	0.848215	0.977912
spop	603.3674	0.142332641	0.127182	1.119122	0.263088	0.740765
si:dkey-25o1.7	3.26759	-0.618441906	1.391211	-0.44453	0.656656	0.929824
abca3b	1378.666	0.018024598	0.210314	0.085703	0.931702	0.995827
map1lc3c	20.79181	0.520179217	0.477798	1.088702	0.276285	0.750674
sh3bp5a	667.373	0.002645639	0.1101	0.024029	0.980829	1
abhd14b	258.8562	0.503882435	0.19207	2.623433	0.008705	0.1602
CU639471.1	0.144395	1.055372236	5.267649	0.20035	0.841207	NA
si:ch1073-178p5.3	0.371572	1.556794327	3.248997	0.479161	0.631824	NA
irak4	150.9771	-0.723019122	0.258433	-2.79771	0.005147	0.12009
ecm1a	0.530794	0.093647308	3.578571	0.026169	0.979123	NA
nkrf	438.7183	0.029854219	0.147006	0.203081	0.839071	0.976417
CR388413.1	0	NA	NA	NA	NA	NA
si:dkeyp-88h4.2	0	NA	NA	NA	NA	NA
btr22	2.629998	0.948560498	1.291838	0.734272	0.462783	0.864104
si:ch211-106g8.62	0	NA	NA	NA	NA	NA
zgc:173486	13.89503	-1.560319447	0.474681	-3.28709	0.001012	0.044337
or108-2	0.325628	-1.332943469	4.479007	-0.2976	0.76601	NA
CR383680.1	1.087612	0.093651096	2.205331	0.042466	0.966127	NA
tefm	104.7163	-0.155012696	0.189811	-0.81667	0.414118	0.838631
CABZ01079427.1	158.5542	-0.430728249	0.225738	-1.90809	0.056379	0.410319
si:dkey-112a7.5	3.920655	-0.875944604	1.428784	-0.61307	0.53983	0.893049
lsm14ab	1454.689	-0.042870274	0.086381	-0.49629	0.619688	0.91747
trgv1	0.103751	0	5.267649	0	1	NA
aqp11	420.8871	0.112096385	0.173146	0.64741	0.517367	0.887148
si:dkeyp-5g9.1	4.044738	-0.519776635	1.464447	-0.35493	0.722642	0.947632
slbp	473.6442	0.071675237	0.168782	0.42466	0.671084	0.933953
tor2a	144.5656	0.193976587	0.182183	1.064737	0.286995	0.757955
zfpm2b	159.9449	0.2787061	0.238134	1.170375	0.24185	0.72348
BX323453.1	8.253948	-0.269375188	0.707752	-0.38061	0.703495	0.943668
sil1	201.9711	0.366382404	0.155313	2.358989	0.018325	0.239068
CR556708.1	0.11354	0	5.267649	0	1	NA
CR318647.1	0.306793	0	5.267649	0	1	NA
si:ch211-209j12.3	14.12987	0.160361045	0.4462	0.359393	0.719301	0.94694
BX927122.1	0.668139	0.908375134	3.109858	0.292095	0.770214	NA
FP085400.1	0.427313	-2.409491936	3.643416	-0.66133	0.508402	NA
ckap2l	208.8318	0.236961814	0.228184	1.03847	0.299051	0.765976
CT573787.1	0.680029	2.577506146	2.27791	1.131522	0.257835	NA
CU694481.1	70.9045	0.32483107	0.252676	1.285564	0.198595	0.679281
si:dkeyp-90h9.1	5.277554	-1.029609744	1.03275	-0.99696	0.318784	0.779923
RF00001	0	NA	NA	NA	NA	NA
si:ch211-195b11.3	5049.884	-0.568833831	0.163708	-3.47469	0.000511	0.027902

hid1b	69.04318	0.566147057	0.24752	2.287277	0.02218	0.263608
ccdc40	66.49272	0.637439452	0.334883	1.903472	0.056979	0.412306
coa3a	430.0402	0.146254865	0.136984	1.067681	0.285664	0.757084
BX324007.2	0.193221	0	5.267649	0	1	NA
zgc:113886	28.54189	0.025080689	0.353126	0.071025	0.943378	0.997371
rpl36	18227.78	-0.62476351	0.170909	-3.65553	0.000257	0.017645
BX649421.1	8.370499	1.520301025	0.760581	1.998868	0.045623	0.373922
sox32	0.6273	0.638751241	2.210284	0.288991	0.772589	NA
slc8a2b	22.74248	0.586026948	0.543092	1.079056	0.280563	0.753147
gnrhr1	2.614017	-0.410788309	1.112486	-0.36925	0.71194	0.945821
sez6l	177.4112	-0.095717846	0.278563	-0.34361	0.731137	0.950056
CR626884.1	0	NA	NA	NA	NA	NA
si:ch73-43g23.1	481.9242	0.12038085	0.242643	0.496123	0.619808	0.91747
ubxn4	0	NA	NA	NA	NA	NA
CABZ01109081.1	62.253	-0.426986669	0.271953	-1.57008	0.116397	0.557474
c1s	7.09859	-1.725372323	0.773338	-2.23107	0.025676	0.284447
RF00001	0	NA	NA	NA	NA	NA
CU019562.1	0.046941	0	5.267649	0	1	NA
olfcw1	0.274316	0	5.267649	0	1	NA
FP016142.4	0.324825	-2.324743267	5.202572	-0.44684	0.654987	NA
BX539336.1	174.0451	-0.144824646	0.200183	-0.72346	0.469396	0.867224
zbtb38	40.43295	-0.035952791	0.455344	-0.07896	0.937067	0.997163
CR812468.1	0.409476	1.055393469	3.746124	0.281729	0.778151	NA
si:ch211-163c2.1	2.274027	-1.712254915	4.357447	-0.39295	0.694357	0.941093
CABZ01089777.1	50.31824	-0.908482343	1.038895	-0.87447	0.381863	0.824957
CU019663.1	0.092827	0	5.267649	0	1	NA
si:ch1073-209h18.1	0.698851	0.919735906	2.576891	0.356917	0.721154	NA
taok2b	75.73628	0.176354702	0.207662	0.849241	0.395747	0.829389
CABZ01080550.1	2.84197	0	1.865312	0	1	1
trrap	762.2584	0.10460207	0.122279	0.855441	0.392307	0.827902
CABZ01066312.1	257.4588	-0.157334891	0.192739	-0.81631	0.414324	0.838631
si:ch73-197b13.1	138.0564	0.285110394	0.250773	1.136924	0.25557	0.733544
CR354537.1	0.448562	-0.868132127	3.505918	-0.24762	0.804429	NA
klhl12	101.356	0.235460031	0.226215	1.040867	0.297937	0.765407
BX649328.1	1.698087	-0.22552544	1.836578	-0.1228	0.902268	NA
zgc:114200	436.017	-0.044881549	0.119919	-0.37426	0.708208	0.945344
chia.1	424.0747	-0.400381105	0.653813	-0.61238	0.540287	0.893049
rbm12	367.9562	0.174946053	0.135191	1.294068	0.195642	0.675764
tradv26.0	0.319441	0	5.267649	0	1	NA
CR847939.1	3.483568	-0.335665683	1.055793	-0.31793	0.75054	0.955074
BX321870.2	0.122043	-0.868089684	5.267649	-0.1648	0.869104	NA
plppr1	145.5976	-0.006750705	0.210619	-0.03205	0.974431	1
BX005454.1	19.60984	0.302594153	0.426673	0.709195	0.478204	0.869292
si:dkey-211i20.4	0.07132	0	5.267649	0	1	NA
tlr1	2.356722	-1.941211762	1.401427	-1.38517	0.166001	0.635533
znf1147	0.662786	-2.300576942	2.98942	-0.76957	0.441553	NA
si:dkeyp-82b4.6	0	NA	NA	NA	NA	NA
si:dkey-207m2.4	8.852527	-0.256547945	0.832244	-0.30826	0.757884	0.957427
CR356223.2	2.968421	2.298642771	1.396057	1.646525	0.099656	0.524166
egfl7	178.9411	-0.397495957	0.196248	-2.02547	0.042819	0.362408
si:ch211-51h4.2	6.386391	-0.983315346	0.861579	-1.14129	0.253747	0.732873
si:ch73-252i11.1	62.99277	0.182699397	0.335076	0.545248	0.585583	0.906655
cxcl11.1	0.323644	0	5.267649	0	1	NA
or120-1	4.256763	0.002470814	0.994429	0.002485	0.998018	1

ep300a	447.0303	0.204904891	0.151349	1.353853	0.175783	0.649413
RF00001	0.053506	-0.86807634	5.267649	-0.16479	0.869106	NA
si:dkey-61p9.9	10.9652	-0.562490859	0.794068	-0.70837	0.478718	0.869292
pcdh1g31	47.67872	-0.194292853	0.304348	-0.63839	0.52322	0.888312
CR790382.1	0.527071	-2.71109143	3.472043	-0.78083	0.4349	NA
FQ311920.1	0.045161	0	5.267649	0	1	NA
znf974	1.528777	0.812403377	1.476216	0.550328	0.582094	NA
UBE2O	204.1244	0.277445222	0.170806	1.624331	0.104305	0.532541
tmem205	222.7618	0.277023249	0.12931	2.14232	0.032168	0.318495
ifnphi4	1.513692	-1.503273047	1.571675	-0.95648	0.338831	NA
lrch3	80.61691	-0.019898278	0.278908	-0.07134	0.943124	0.997371
si:ch211-241b2.4	20.70559	0.471233813	0.469352	1.00401	0.315374	0.777834
CU929154.1	0.07132	0	5.267649	0	1	NA
CR450749.2	0	NA	NA	NA	NA	NA
si:ch211-256e16.11	42.52695	0.701817092	0.35326	1.986685	0.046957	0.377647
prss35	1014.564	0.1113186	0.137594	0.809036	0.418494	0.841565
ddr2a	191.321	0.0140116	0.206085	0.067989	0.945794	0.997371
tnnt2b	78.71913	1.901385152	1.169333	1.626043	0.103941	0.532096
CR407585.1	0	NA	NA	NA	NA	NA
FQ323156.1	2315.252	-0.01878007	0.246939	-0.07605	0.939378	0.997371
eva1bb	139.8923	-0.215877744	0.146828	-1.47028	0.141487	0.600284
BX950868.1	0.263036	1.055362802	5.267649	0.200348	0.841208	NA
CABZ01066725.1	21.32436	-0.509138373	0.353287	-1.44115	0.149543	0.611852
si:ch211-285j22.3	4.829277	-1.769010677	1.704762	-1.03769	0.299415	0.766128
traj26	0	NA	NA	NA	NA	NA
si:dkey-48g21.7	6.680381	-3.156297143	1.079714	-2.92327	0.003464	0.097161
jakmip3	116.0128	0.358171661	0.3256	1.100036	0.271316	0.747491
agxt2	492.0857	0.704061421	0.206313	3.41259	0.000643	0.032986
CR855206.1	0.04222	0	5.267649	0	1	NA
si:dkey-19b23.12	508.9011	-0.017506056	0.122651	-0.14273	0.886503	0.987533
RF00001	0	NA	NA	NA	NA	NA
tlk1b	892.694	-0.082138412	0.10057	-0.81673	0.414084	0.838631
si:dkey-285b23.4	6.923281	-1.228542967	0.741123	-1.65768	0.097383	0.520377
socs5a	250.1759	-0.094652379	0.120552	-0.78516	0.432362	0.848419
me2	897.9963	-0.039144888	0.207084	-0.18903	0.85007	0.978251
uraha	684.5779	0.016794546	0.190255	0.088274	0.929659	0.995576
si:ch211-17516.2	1.832487	1.055429654	2.132816	0.494853	0.620704	NA
si:ch211-160f23.7	193.506	0.294973947	0.288399	1.0228	0.306403	0.771574
znf1097	4.010963	-1.15379594	0.855307	-1.34898	0.177342	0.650985
ephb4a	455.9252	-0.02610596	0.166855	-0.15646	0.875671	0.98444
mkrn2os.2	26.14881	1.398973785	0.428962	3.261303	0.001109	0.047149
tln1	1630.159	0.062619174	0.139148	0.45002	0.652696	0.929043
zgc:101540	101.3555	-0.502098535	0.232835	-2.15645	0.031048	0.313376
si:dkeyp-93h6.1	2.761862	-2.053102557	1.21388	-1.69135	0.090769	0.505206
epob	10.98722	0.503717782	0.526884	0.956031	0.339057	0.795226
osmr	3.656293	-0.261101098	1.358718	-0.19217	0.847611	0.977705
CABZ01052573.1	122.5026	-0.277255941	0.26763	-1.03597	0.300217	0.766629
CU179702.2	3.18158	0.544115781	0.949654	0.572962	0.566671	0.901739
cdc20	706.6399	0.349967135	0.268115	1.30529	0.191794	0.672149
reep5	1822.056	-0.050245022	0.114679	-0.43813	0.661289	0.930581
si:dkey-190j3.4	11.50266	-0.481841366	0.634673	-0.7592	0.447735	0.856366
RF00415	0	NA	NA	NA	NA	NA
si:ch211-103a14.5	20.20554	-1.062200162	0.691694	-1.53565	0.124624	0.572298
rab21	714.1711	0.010683754	0.149316	0.071552	0.942959	0.997371

CR450764.1	0	NA	NA	NA	NA	NA
CU074330.1	21.93679	-0.521899798	0.451858	-1.15501	0.248086	0.728489
RF00003	0	NA	NA	NA	NA	NA
RF00001	0	NA	NA	NA	NA	NA
BX293993.1	0.129045	1.055383603	5.267649	0.200352	0.841205	NA
zfyve9b	21.425	0.680727129	0.545273	1.248415	0.211879	0.693489
traj37	0	NA	NA	NA	NA	NA
ube2q1	807.2819	-0.096917312	0.1423	-0.68108	0.495824	0.87806
BX248131.1	0	NA	NA	NA	NA	NA
si:ch211-160d14.15	0.163104	0	5.267649	0	1	NA
si:ch211-154c21.1	27.84231	-0.099891681	0.3173	-0.31482	0.7529	0.955756
zmynd19	232.6235	-0.007804149	0.183189	-0.0426	0.966019	1
cmc4	44.3437	0.095365931	0.319195	0.29877	0.765115	0.959622
si:ch211-238e22.7	1.166663	2.044428732	1.805808	1.132141	0.257575	NA
slco1e1	5.508629	-2.106205904	1.472357	-1.4305	0.152574	0.61658
col28a2b	72.24625	0.879503699	0.436306	2.015798	0.043821	0.366547
f7l	78.24537	0.331274002	0.286291	1.157124	0.247222	0.727871
PLCB4	3.985818	-0.735090684	1.021795	-0.71941	0.471888	0.868369
CR855274.1	0.043309	0	5.267649	0	1	NA
trip10b	79.74601	-0.205556848	0.236699	-0.86843	0.385158	0.826939
plgrkt	372.8725	0.40079982	0.120392	3.329115	0.000871	0.039973
tril	147.1825	0.083763218	0.197624	0.423851	0.671675	0.933953
zgc:154142	45.78079	0.208260868	0.391667	0.53173	0.594913	0.910869
CR450785.1	7.57587	-0.30433601	0.956501	-0.31818	0.750351	0.955074
mmp13b	3.09836	-0.524964307	1.34397	-0.39061	0.696088	0.941657
timp4.3	595.2271	0.984162171	1.579176	0.623212	0.533145	0.890978
CABZ01076996.1	54.65171	0.044372886	0.289734	0.15315	0.87828	0.985761
CR381540.1	1.124852	0	2.197036	0	1	NA
si:ch211-106g8.20	0	NA	NA	NA	NA	NA
zgc:173517	24.31899	-0.528696106	0.407056	-1.29883	0.194003	0.674122
st6galnac4	10.86271	0.700622804	0.564179	1.241845	0.214294	0.696474
si:ch1073-209e23.1	46.81946	-0.203015988	0.351437	-0.57767	0.563484	0.901075
si:ch211-213c4.5	0.795927	-2.784636344	2.364173	-1.17785	0.238857	NA
znf1183	14.04124	-2.789142301	0.739291	-3.77272	0.000161	0.012946
g6fl	2.82808	-0.987397125	1.235443	-0.79923	0.42416	0.844355
BX927130.2	0	NA	NA	NA	NA	NA
nudt12	361.3709	-0.190124292	0.142182	-1.33719	0.18116	0.657295
srsf3a	1.236948	0	2.120449	0	1	NA
CT990625.1	0.635304	0	3.259268	0	1	NA
si:ch73-97h19.2	28.78762	-0.920795911	1.317316	-0.69899	0.484556	0.872911
BX548164.1	0.321756	0	3.910283	0	1	NA
RF00003	0	NA	NA	NA	NA	NA
scrt1a	223.6157	0.256183065	0.142344	1.799741	0.071902	0.4564
mapk15	7.141875	1.189372659	0.723235	1.644518	0.100069	0.524334
selenok	617.3507	0.293280967	0.121967	2.40459	0.016191	0.224498
calm3a	1625.674	0.206057347	0.170647	1.207507	0.227237	0.708529
hif1al	4748.236	0.013312086	0.123829	0.107504	0.914389	0.992972
CU929150.2	3.932342	0.772528784	1.021259	0.756447	0.449381	0.856979
pcdh1g32	25.29585	0.19376122	0.352312	0.549971	0.582339	0.90571
RF00001	0.354718	-0.868126271	5.166465	-0.16803	0.866559	NA
si:dkey-269i1.4	374.9215	0.453544555	0.375234	1.208698	0.226779	0.707959
FP016208.1	0.086618	0	5.267649	0	1	NA
RGS13 (1 of many)	4.404376	0.238700072	0.90783	0.262935	0.792601	0.965266
CR790380.1	59.48321	0.060268241	0.250941	0.240169	0.8102	0.969828

BX649384.3	10.04585	-0.131784252	0.570318	-0.23107	0.817259	0.971207
RF00003	0	NA	NA	NA	NA	NA
si:ch211-223a21.4	8.476647	0.24413193	0.583221	0.418593	0.675514	0.935422
cldn15lb	140.0605	-1.152930491	0.341893	-3.37219	0.000746	0.036381
esrrb	65.15118	0.081670938	0.233289	0.350085	0.726275	0.948987
RF00001	0	NA	NA	NA	NA	NA
rnf103	409.2209	-0.165059267	0.158922	-1.03862	0.298982	0.765883
gphnb	288.4901	0.180366738	0.209069	0.862714	0.388295	0.827592
ago4	685.3627	-0.351010234	0.143968	-2.43811	0.014764	0.213272
BX640584.1	0.819583	3.732169232	5.163742	0.722764	0.469825	NA
FP101914.1	180.8607	-0.48073488	0.242146	-1.98531	0.04711	0.378022
zgc:171695	0.053262	1.055396947	5.267649	0.200354	0.841203	NA
BX465868.2	0.053262	1.055396947	5.267649	0.200354	0.841203	NA
gprc5c	56.50052	-0.118953911	0.294467	-0.40396	0.686239	0.938732
BX004873.1	2.585468	-0.828138951	1.188638	-0.69671	0.485983	0.873244
si:dkey-269o24.1	5.143277	-4.050709433	1.590647	-2.54658	0.010878	0.181938
rictora	244.3213	-0.083870459	0.199429	-0.42055	0.674081	0.934789
adcy9	125.0399	-0.266517989	0.160016	-1.66557	0.095798	0.516953
RF00001	0	NA	NA	NA	NA	NA
otud1	61.1306	0.155751916	0.353801	0.440224	0.659775	0.930581
RF00001	0	NA	NA	NA	NA	NA
GAN	53.06665	-0.119056792	0.268956	-0.44266	0.65801	0.930581
sez6a	70.38961	-0.272420865	0.364018	-0.74837	0.454236	0.859224
dre-mir-19d	0	NA	NA	NA	NA	NA
znf1154	23.417	-0.135295814	0.423095	-0.31978	0.749138	0.955074
CU896630.1	33.34228	-0.177687549	0.282045	-0.63	0.528696	0.890094
si:ch211-285j22.5	0	NA	NA	NA	NA	NA
si:dkeyp-97a10.1	19.30679	-1.038196019	0.777098	-1.33599	0.181552	0.657892
si:dkey-11f4.20	5.013038	0.476015153	1.060229	0.448974	0.653451	0.929269
RF00001	0.053506	-0.86807634	5.267649	-0.16479	0.869106	NA
galnt18a	35.35477	-0.287645593	0.366379	-0.7851	0.432393	0.848419
si:zfos-1069f5.1	3.973254	0.877763615	1.114246	0.787764	0.430835	0.847629
CR589944.2	0.36894	-2.311622452	3.697499	-0.62519	0.531849	NA
cfap53	20.00677	0.454377751	0.420104	1.081584	0.279437	0.752477
znf973	16.69442	0.942370679	0.540325	1.74408	0.081145	0.479923
pdgfrb	340.1897	-0.243067032	0.147748	-1.64514	0.099941	0.524166
RF00001	0	NA	NA	NA	NA	NA
si:ch211-241b2.5	11.07899	-0.537736553	0.540729	-0.99447	0.319996	0.780618
si:ch211-183d5.2	6.086431	0.286209575	1.235426	0.231669	0.816795	0.971207
FP085399.1	0.890183	-1.437003214	2.412692	-0.5956	0.551441	NA
hist1h2a11	0.740533	-0.936916535	3.004787	-0.31181	0.755186	NA
lcor	34.89009	0.143743194	0.325348	0.441813	0.658625	0.930581
zgc:173581	16.51714	0.261079808	0.632776	0.412594	0.679904	0.936586
si:ch211-195p4.4	0.191369	0	5.267649	0	1	NA
BX294383.1	0.326478	1.055367829	4.549706	0.231964	0.816566	NA
zgc:162297	26.85727	0.796491412	0.478362	1.665041	0.095905	0.517372
arhgef12b	580.1301	0.137260991	0.126728	1.083113	0.278758	0.752299
si:ch211-255f4.7	144.6626	-0.5017707	0.174447	-2.87635	0.004023	0.105636
uvssa	125.7129	-0.02404532	0.167775	-0.14332	0.886039	0.987533
slc30a8	260.7734	-0.012823088	0.16044	-0.07992	0.936297	0.997032
prc1a	306.0747	0.320740518	0.266171	1.205019	0.228196	0.709324
slc5a2	25.19864	-0.91487567	0.931228	-0.98244	0.325883	0.784773
AL831768.1	0.375092	-1.712377852	4.306778	-0.3976	0.690925	NA
CU104709.1	0.102528	-1.76935176	5.23432	-0.33803	0.735341	NA

nfatc2a	16.33517	-0.165166871	0.503446	-0.32807	0.742857	0.954655
RF00001	0	NA	NA	NA	NA	NA
BX088583.1	0.060442	0	5.267649	0	1	NA
RF00003	0	NA	NA	NA	NA	NA
RF00026	0	NA	NA	NA	NA	NA
CR790368.1	0.583789	1.055421384	2.746155	0.384327	0.700736	NA
hint2	208.2796	-0.544133097	0.203182	-2.67806	0.007405	0.145771
CABZ01117603.1	0.643236	0.132180244	2.339284	0.056505	0.95494	NA
nr5a2	99.89592	0.154417497	0.210335	0.734151	0.462857	0.864104
clca1	9.274329	0	2.57801	0	1	1
zgc:194655	2.965966	-3.923190804	1.56348	-2.50927	0.012098	0.19082
RF00001	1.960507	-1.765100141	1.834326	-0.96226	0.335919	NA
glrx2	161.2294	-0.10378934	0.206063	-0.50368	0.614488	0.915998
RF00001	0	NA	NA	NA	NA	NA
si:ch211-212k18.9	206.3612	-0.375655371	0.176829	-2.1244	0.033636	0.323787
mafk	58.88589	-0.58511027	0.338871	-1.72664	0.084232	0.487221
si:ch73-47f2.1	1.114329	1.989635611	2.697605	0.737556	0.460784	NA
vap	0	NA	NA	NA	NA	NA
CU468012.4	0.043308	0	5.267649	0	1	NA
tradv22.0	0	NA	NA	NA	NA	NA
wu:fj16a03	1252.387	-0.311099078	0.201948	-1.54049	0.123441	0.570142
si:dkeyp-93a5.2	3.926246	0	3.290583	0	1	1
wars	169.2392	0.158974968	0.197082	0.806645	0.419871	0.842383
stk24a	24.61546	-3.938475304	1.316993	-2.99051	0.002785	0.086504
si:ch211-276i12.4	65.2629	1.020078238	0.660991	1.543255	0.122769	0.569468
si:ch211-188f17.1	71.89825	0.25996965	0.267204	0.972927	0.33059	0.788126
si:dkey-3p4.7	0.744376	3.72933281	2.79555	1.334025	0.182196	NA
si:ch211-155m12.5	8.546172	0.121039106	0.619384	0.195419	0.845065	0.976791
zgc:174653	2.959424	-1.324970328	0.974321	-1.35989	0.173865	0.646897
ttc39b	79.39048	-0.098107104	0.283629	-0.3459	0.729418	0.950056
CU468826.2	0	NA	NA	NA	NA	NA
si:ch211-57b15.2	1.023332	-2.755026148	5.185053	-0.53134	0.595183	NA
ep400	772.3778	-0.041117954	0.205393	-0.20019	0.841331	0.97643
BX569788.1	8.041658	0.584164052	0.581763	1.004128	0.315317	0.777834
si:ch211-1a19.3	97.74882	-0.013072832	0.301814	-0.04331	0.965451	1
tmprss13a	116.8118	-0.84951744	0.297247	-2.85795	0.004264	0.107992
pimr197	0.194921	-0.868117117	5.267649	-0.1648	0.8691	NA
fxyd6	116.5175	-0.045826618	0.320359	-0.14305	0.886252	0.987533
myh11b	29.31221	-0.95729243	0.513712	-1.86348	0.062395	0.427718
arhgap24	176.6291	0.115887264	0.154634	0.749427	0.4536	0.858722
CCDC39	158.0956	1.002824633	0.208926	4.799902	1.59E-06	0.000413
dre-mir-93	0	NA	NA	NA	NA	NA
zbtb7c	7.161797	-0.493656827	0.828999	-0.59549	0.551519	0.8974
INHBC	16.15123	0.083329287	0.655245	0.127173	0.898804	0.990265
CR388413.2	0.046941	0	5.267649	0	1	NA
RF00001	0.173236	0	5.267649	0	1	NA
nme3	612.3468	-0.454428304	0.192045	-2.36626	0.017969	0.2367
chrna3	45.68106	-0.238170989	0.373481	-0.63771	0.523665	0.888628
dre-mir-725	2.656732	-1.545180265	1.393578	-1.10879	0.267522	0.743943
si:dkey-16b10.1	3.896728	-0.789183871	0.892963	-0.88378	0.376814	0.822258
BX901898.2	1.135669	1.413422068	2.079488	0.679697	0.496696	NA
traj11	0	NA	NA	NA	NA	NA
RF00026	0	NA	NA	NA	NA	NA
soul4	401.9464	0.024047958	0.202165	0.118952	0.905313	0.991631

cited4b	995.9082	-0.041021004	0.117575	-0.34889	0.727171	0.949095
hpdl	34.44524	0.337034032	0.320123	1.052826	0.292421	0.761879
RF00001	0.050624	1.055396947	5.267649	0.200354	0.841203	NA
slco1f4	0.660251	0	3.819063	0	1	NA
CT583642.1	8.036971	-0.974865735	0.688252	-1.41644	0.156647	0.622452
ptdss2	382.9841	0.239742605	0.146896	1.632055	0.102668	0.529448
ext1b	62.31113	-0.117137734	0.242107	-0.48383	0.628509	0.921864
slc22a5	20.305	0.389192232	0.485446	0.801721	0.422714	0.843493
msx2b	271.9603	-0.007486263	0.14646	-0.05111	0.959234	0.999164
nufip1	119.6307	-0.712756785	0.241069	-2.95665	0.00311	0.091566
fnbp1b	65.08989	0.115698843	0.217201	0.532681	0.594255	0.910731
si:ch73-347e22.4	179.2742	0.126080759	0.226141	0.557531	0.577165	0.904798
si:ch73-158p21.3	22.47982	0.135205246	0.542376	0.249283	0.803142	0.968418
iqcb1	83.85207	-0.09134617	0.220493	-0.41428	0.678668	0.936026
tradv4.0.3	0	NA	NA	NA	NA	NA
si:dkey-206d17.12	49.38446	-0.310694889	0.260945	-1.19065	0.23379	0.714749
CU655870.1	0.320865	-2.387349099	3.845563	-0.62081	0.534727	NA
traf2b	99.11308	-0.195102988	0.231933	-0.8412	0.400234	0.831472
asf1ba	259.0274	0.140620017	0.184897	0.760531	0.446937	0.85583
zdhhc15a	21.33614	-0.332016332	0.410154	-0.80949	0.418232	0.84155
ccl20a.3	10.23133	-2.063505236	1.272156	-1.62205	0.104792	0.533164
ccl39.1	0	NA	NA	NA	NA	NA
ppl	2582.751	-0.078919449	0.14101	-0.55967	0.575702	0.904739
si:ch73-138e16.6	227.4116	-0.061601915	0.139971	-0.44011	0.659861	0.930581
si:dkeyp-107f9.1	0.326089	-1.755783668	4.463829	-0.39334	0.694071	NA
rtcb	298.5722	0.212223403	0.128658	1.649516	0.099042	0.522139
FQ790208.1	12.21192	0.582420419	0.597976	0.973987	0.330063	0.788084
CABZ01075274.1	32.99905	0.022531108	0.60541	0.037216	0.970313	1
tmc8	23.61056	-1.03290383	0.532799	-1.93864	0.052546	0.398274
ctsbb	86.5431	1.055414869	1.705991	0.618652	0.536146	0.892277
RF02041	0.151	0	5.267649	0	1	NA
syn2b	321.6414	0.225953108	0.231789	0.974823	0.329648	0.788
trim35-25	2.084029	-1.760221606	1.957452	-0.89924	0.368524	NA
BX890539.1	20.06875	-0.122249516	0.397741	-0.30736	0.758569	0.957865
srsf7b	158.4614	-0.694926568	0.263954	-2.63276	0.008469	0.157114
traj3	0	NA	NA	NA	NA	NA
dhx36	291.7612	0.203205622	0.140881	1.442387	0.149193	0.610959
gcna	71.87917	0.370343059	0.272515	1.358981	0.174153	0.647379
eif2a	697.7522	0.062205877	0.11292	0.550882	0.581714	0.905618
fdft1	138.7182	0.287136145	0.229419	1.251582	0.210722	0.692347
CABZ01058650.1	467.7879	-0.117236908	0.170528	-0.68749	0.491772	0.87595
BX511223.1	1.961867	2.551631508	1.635623	1.560036	0.118751	NA
CR812471.1	0	NA	NA	NA	NA	NA
shoc1	1.809037	1.360070632	1.635397	0.831646	0.405609	NA
CU651669.1	7.830417	0.099948053	1.058729	0.094404	0.924788	0.994815
si:dkeyp-80d11.14	12.4172	0.797859257	0.643963	1.238983	0.215352	0.697157
cnot6b	153.3403	-0.172578165	0.211668	-0.81533	0.414886	0.838732
si:zfos-1011f11.2	55.158	-0.140508071	0.338509	-0.41508	0.678084	0.936026
glud1b	11636.74	0.085511034	0.138979	0.61528	0.53837	0.892596
irx3a	772.393	-0.02326542	0.111948	-0.20782	0.835367	0.976007
sctr	9.149038	-1.377824301	1.567719	-0.87887	0.379471	0.823607
HECTD4	262.7332	0.149275299	0.23799	0.627235	0.530505	0.890545
pwwp2b	710.1446	0.155409957	0.111932	1.388437	0.165004	0.634185
ptprub	224.6468	0.033129677	0.18173	0.182302	0.855346	0.97932

eif3s6ip	3866.341	-0.270856577	0.119837	-2.26021	0.023808	0.273495
olfcg2	0.508637	0.756252135	3.02672	0.249859	0.802697	NA
ss18l2	263.7953	0.391894325	0.232544	1.68525	0.09194	0.507669
si:dkeyp-80d11.1	6.043551	-1.863396537	0.816498	-2.28218	0.022479	0.265534
si:ch73-359m17.5	22.00316	-0.601635091	0.491218	-1.22478	0.220657	0.701898
gart	829.2092	0.086922159	0.128441	0.676746	0.498567	0.879201
pappaa	27.41697	0.286061124	0.480237	0.595667	0.551398	0.8974
RF00001	0.043308	0	5.267649	0	1	NA
zgc:162971	44.77051	-0.491338779	0.256534	-1.9153	0.055455	0.407706
ATP11B	384.9546	0.04416909	0.166218	0.26573	0.790447	0.964589
cpne4a	206.225	-0.169025727	0.243335	-0.69462	0.487293	0.873291
cebpl	9.588024	0.211434112	0.655656	0.322477	0.747091	0.955074
RF00017	0	NA	NA	NA	NA	NA
tprn	56.80261	0.092637358	0.246964	0.375104	0.707583	0.945103
FP102192.1	4.810584	-0.87408566	0.915567	-0.95469	0.339733	0.795226
si:ch73-109d9.3	91.34878	-0.72352418	0.232808	-3.10782	0.001885	0.067081
cldn15b	113.8934	0.28740923	0.237404	1.210631	0.226037	0.707444
si:ch211-152c8.4	37.38183	-0.141034274	0.315354	-0.44723	0.654712	0.929527
FO704777.1	29.52716	-0.095745901	0.458153	-0.20898	0.834462	0.975731
med30	301.0601	-0.425792631	0.180642	-2.35711	0.018418	0.239743
vps33a	376.5939	0.130941678	0.135369	0.967292	0.333398	0.790506
CABZ01066813.1	88.70764	0.123878309	0.227527	0.544457	0.586127	0.906711
kif3b	194.741	0.15454471	0.15136	1.021044	0.307234	0.772541
si:dkey-161l11.62	0	NA	NA	NA	NA	NA
BX649345.2	0.050723	-0.86807634	5.267649	-0.16479	0.869106	NA
CR854945.1	30.00119	-0.767948291	0.644662	-1.19124	0.233559	0.71468
atad5b	74.33855	0.220708554	0.214943	1.026822	0.304504	0.769885
map1lc3b	2658.468	-0.176724898	0.116061	-1.52269	0.127837	0.577117
si:dkey-211f22.5	687.6313	0.358620532	0.148428	2.416127	0.015687	0.221082
si:dkeyp-104b3.18	0.48597	0	3.546047	0	1	NA
coq8b	194.7435	0.119792746	0.188097	0.636867	0.524211	0.888801
pbx1b	50.78455	-0.349810636	0.262781	-1.33119	0.183127	0.659373
BX510960.1	6.792182	0.354519955	0.781875	0.453423	0.650244	0.927835
CABZ01064859.2	47.65209	-0.092792727	0.274087	-0.33855	0.734947	0.951434
si:dkey-85k7.7	1207.222	-0.211960791	0.480719	-0.44092	0.659268	0.930581
BX927317.1	0	NA	NA	NA	NA	NA
znf999	19.39494	0.419408891	0.413264	1.01487	0.310168	0.775062
BX323596.1	0	NA	NA	NA	NA	NA
agpat2	396.5739	0.464835765	0.165964	2.800817	0.005097	0.119231
zgc:114173	81.92489	0.154651047	0.210293	0.735409	0.462091	0.863402
zdhhc13	372.688	0.326042684	0.118412	2.753465	0.005897	0.129875
lipeb	75.36574	-0.143486029	0.27447	-0.52278	0.60113	0.913756
CR293511.1	114.2755	-0.189991328	0.227869	-0.83377	0.404408	0.833939
RAB19	19.66467	0.309085776	0.435536	0.709668	0.47791	0.869292
si:dkeyp-110c7.8	0	NA	NA	NA	NA	NA
slco1f2	0.043309	0	5.267649	0	1	NA
si:ch73-338a16.3	12.12726	-1.51800795	0.88322	-1.71872	0.085665	0.491277
apoa4a	801.3943	-0.852853146	0.244662	-3.48584	0.000491	0.027259
orc4	232.6286	-0.151599817	0.153568	-0.98719	0.323552	0.783161
BX001023.2	16.65029	-0.271653402	0.464188	-0.58522	0.558398	0.899409
nansb	177.3367	0.136479122	0.238425	0.57242	0.567037	0.901747
si:dkey-157e10.8	0.204491	1.055360845	5.175029	0.203933	0.838406	NA
BX005396.3	1.474007	-3.763842201	2.322916	-1.62031	0.105166	NA
RF00001	0	NA	NA	NA	NA	NA

grap2b	73.64376	-0.469134879	0.303908	-1.54367	0.122667	0.569468
rab42b	82.614	-0.088058204	0.264873	-0.33245	0.739546	0.953573
FO704786.1	58.76689	0.02237408	0.345624	0.064735	0.948385	0.997733
TMEM200C	18.08746	-0.383402691	0.480736	-0.79753	0.425142	0.844714
RF00001	0.307339	1.055380564	4.558399	0.231524	0.816907	NA
BX649556.1	0.203992	-0.868116142	5.188233	-0.16732	0.867115	NA
mcu	95.46647	-1.094387511	0.23002	-4.7578	1.96E-06	0.000498
ugt1b7	40.29116	-0.071210176	0.274444	-0.25947	0.795272	0.965909
sh3rf2	98.34905	-0.441781176	0.228836	-1.93056	0.053538	0.400752
mcm7	1341.134	0.027185284	0.113856	0.238768	0.811285	0.97002
s100w	312.062	-0.078097475	0.151968	-0.51391	0.607316	0.915204
CR855860.2	0.229405	2.001884741	4.958565	0.403723	0.686417	NA
BX510922.1	23.10826	-0.023269989	0.385251	-0.0604	0.951835	0.99801
CABZ01095001.1	24.16892	-0.291382426	0.416923	-0.69889	0.484622	0.872911
CABZ01087566.1	9.103429	-0.351465659	0.830281	-0.42331	0.672069	0.933953
CABZ01048053.1	58.11223	0.539206611	0.290792	1.854266	0.063701	0.430571
CR589947.2	14.62779	-0.981309061	0.58744	-1.67048	0.094824	0.513949
cyp2k8	50.965	-0.132215141	0.285889	-0.46247	0.643745	0.925997
si:ch211-255f4.13	4.564428	-0.336690057	0.877958	-0.38349	0.701355	0.943145
BX855614.3	22.4004	-0.316082269	0.455169	-0.69443	0.487413	0.873291
si:dkey-238d18.15	0.07132	0	5.267649	0	1	NA
BX927181.3	0.546858	1.903689986	2.703366	0.704192	0.481313	NA
rbp4	25433.4	0.077154517	0.208599	0.36987	0.711479	0.945821
zgc:112964	450.5543	-0.202814872	0.232604	-0.87193	0.383245	0.825809
si:dkey-207b20.2	3.449151	-2.475188493	1.347422	-1.83698	0.066213	0.439276
CU302413.1	1.229736	1.715463575	1.928903	0.889347	0.373817	NA
znf513b	31.66153	-0.392986856	0.332893	-1.18052	0.237794	0.718607
mbd1a	331.7738	0.0184114	0.1163	0.15831	0.874213	0.98385
zgc:113363	44.71718	0.023487212	0.268378	0.087516	0.930262	0.995707
lamb1a	1464.857	0.095995457	0.156399	0.613787	0.539356	0.893049
glg1b	64.5066	0.591442925	0.251502	2.351646	0.018691	0.241927
BX537130.1	0.439966	0	4.235619	0	1	NA
CU681841.1	116.5411	-0.325004717	0.278163	-1.16839	0.242648	0.724345
CU278559.1	4.026296	-1.210316931	0.947103	-1.27791	0.20128	0.681123
pkd1l2b	7.800048	-0.915871485	0.854813	-1.07143	0.283976	0.755095
sgip1b	138.6521	0.413042316	0.269249	1.534052	0.125017	0.572594
meaf6	811.4464	0.021539296	0.147164	0.146362	0.883635	0.987014
fam78aa	22.9918	-0.23152991	0.373623	-0.61969	0.535463	0.891993
si:ch73-345f18.3	16.38552	-0.972422808	0.635961	-1.52906	0.12625	0.574654
BX511128.1	0.451024	0.961480393	4.145116	0.231955	0.816573	NA
polrmt	160.1254	-0.199687169	0.167067	-1.19525	0.231989	0.713202
tradv3.0.3	0	NA	NA	NA	NA	NA
oscp1a	0.652533	-0.868143844	2.853471	-0.30424	0.760944	NA
si:ch211-229l10.11	0.392639	-1.807339498	3.152685	-0.57327	0.566462	NA
cngb3.1	32.89167	-0.944745625	0.47936	-1.97085	0.048741	0.384258
CR790382.2	0	NA	NA	NA	NA	NA
pcdh1g2	21.18262	-0.114665411	0.409954	-0.2797	0.779705	0.961073
nsmfb	238.8974	0.093996162	0.229536	0.409506	0.682168	0.93781
ccdc166	1.521583	0.686748797	1.455464	0.471842	0.63704	NA
cep83	151.6708	0.115154543	0.215813	0.533585	0.593628	0.910427
hsd17b4	707.0796	0.005967284	0.171738	0.034746	0.972282	1
CU651662.1	27.95741	-0.606560312	0.459262	-1.32073	0.186592	0.664968
zcchc14	90.27232	-0.308565218	0.199823	-1.5442	0.122541	0.569468
CABZ01049825.1	0.607193	0	3.319777	0	1	NA

gli1	291.1697	0.008316756	0.197803	0.042046	0.966462	1
znf1063	8.582099	0.475938093	0.729446	0.652466	0.514101	0.88631
hist1h4l	0.87416	0.093653905	2.309887	0.040545	0.967659	NA
ldha	4080.961	0.083540528	0.195149	0.428085	0.668589	0.933086
si:dkey-238n5.3	0	NA	NA	NA	NA	NA
RF00001	0	NA	NA	NA	NA	NA
BX649411.1	24.69893	-0.496918841	0.322838	-1.53922	0.123751	0.570837
hgh1	116.1801	-0.002861319	0.239308	-0.01196	0.99046	1
GPATCH2	446.0588	-0.149071968	0.136616	-1.09117	0.275196	0.750424
BX927317.2	2.139327	0	2.080073	0	1	NA
trim35-12	64.3486	-0.214504965	0.246214	-0.87121	0.383638	0.826106
RF00001	0	NA	NA	NA	NA	NA
klhl8	238.194	0.040910612	0.15076	0.271363	0.786112	0.962605
si:ch211-227n13.3	49.5396	-0.370679501	0.492258	-0.75302	0.451439	0.857676
si:dkey-29j8.1	8.456145	1.601162853	0.931548	1.71882	0.085647	0.491277
dre-mir-365-3	0	NA	NA	NA	NA	NA
si:dkey-16p6.4	0.219506	-0.868116949	5.056954	-0.17167	0.863699	NA
BX936433.1	4.552432	0.819690538	0.893314	0.917584	0.358837	0.80995
znf207b	763.8602	0.054680242	0.09986	0.547572	0.583986	0.906376
ndr2	3.193677	0.225679072	1.184053	0.190599	0.84884	0.978002
vat1l	14.53313	0.666787039	0.487639	1.367377	0.171507	0.643534
corin	83.91671	0.490701175	0.220115	2.229299	0.025794	0.285137
zswim6	228.759	0.146642746	0.163282	0.898093	0.369136	0.817763
CT737129.1	2.479894	-1.78083465	1.676552	-1.0622	0.288145	0.759103
anapc15	222.3014	-0.035623324	0.165798	-0.21486	0.829877	0.974816
RF00001	1.744532	1.055436196	2.094324	0.503951	0.614296	NA
slc29a1a	1820.074	0.081742541	0.082629	0.989277	0.322527	0.78246
spink2.5	22.36381	0	2.232861	0	1	1
txndc11	217.896	0.260869943	0.143372	1.819534	0.06883	0.447723
si:dkey-201g16.1	3.439442	0.205416056	1.132927	0.181314	0.856121	0.979558
PKN2 (1 of many)	337.3588	0.043996493	0.123688	0.355704	0.722062	0.947403
psmd4a	479.8246	0.104356567	0.192879	0.541046	0.588476	0.90797
si:ch211-170p16.1	0.829128	3.226605003	2.668039	1.209354	0.226527	NA
tmem266	123.1318	0.144460147	0.277705	0.520193	0.602929	0.914226
CABZ01064248.1	0.163129	0	5.267649	0	1	NA
ssna1	68.52911	0.420877864	0.237936	1.768873	0.076915	0.469467
si:dkey-261o4.1	0.323805	-1.756127425	3.906568	-0.44953	0.653048	NA
CR388172.1	0.049519	0	5.267649	0	1	NA
mrpl15	606.6668	0.030992099	0.115497	0.268336	0.788441	0.963562
pcdh1gb2	19.67275	0.478780693	0.496998	0.963346	0.335374	0.79203
smug1	124.4117	-0.305523648	0.177421	-1.72203	0.085064	0.489675
si:ch73-100l22.3	310.0719	-0.023344293	0.144946	-0.16106	0.87205	0.983447
RF00001	0	NA	NA	NA	NA	NA
si:dkey-31n13.4	21.74411	-1.371595265	0.428449	-3.20131	0.001368	0.054652
ppp4r1	553.3292	0.071094318	0.122615	0.579817	0.562038	0.900715
grinaa	150.2371	0.063819603	0.238394	0.267706	0.788926	0.963568
pcdh2ab9	8.053063	-1.049114804	0.591049	-1.77501	0.075897	0.46659
si:ch73-299h12.6	81.70772	-0.36111813	0.228298	-1.58178	0.113699	0.551905
si:dkey-50i6.5	58.58584	0.074109447	0.333116	0.222473	0.823945	0.972032
CR376800.1	0.212312	2.43638772	5.206324	0.467967	0.639808	NA
tfr1a	229.9608	-0.289611372	0.297276	-0.97422	0.32995	0.788084
trnau1apa	214.2114	-0.412503391	0.141714	-2.91081	0.003605	0.099319
apoa1b	86214	-0.134128589	0.181322	-0.73973	0.459466	0.862103
znf1076	7.361499	0.757856659	0.743815	1.018878	0.308261	0.773155

CR382300.3	5.328918	0.851841327	0.743942	1.145038	0.252194	0.732759
tekt1	25.69656	0.220349598	0.41311	0.533393	0.593762	0.910427
uba2	1118.214	0.079411428	0.094803	0.837644	0.40223	0.833156
AL928808.1	0.784186	0.09365198	2.377325	0.039394	0.968576	NA
ctsc	569.4077	0.149967369	0.141506	1.059795	0.289238	0.760276
BX088728.1	0.35123	1.95911441	4.395797	0.445679	0.655829	NA
CR450808.2	2.199898	-1.531989632	1.37729	-1.11232	0.266	NA
BX005294.1	3.365056	-0.701979062	1.178385	-0.59571	0.551367	0.8974
CR589882.1	0.915642	1.964911738	2.748911	0.714796	0.474735	NA
si:ch211-262i1.6	377.2955	-0.164239523	0.216192	-0.75969	0.447438	0.856081
CR293511.2	1.941945	-2.308027539	2.325028	-0.99269	0.320862	NA
CU468723.1	3.585167	1.449416489	1.035573	1.399627	0.161625	0.628859
plppr5b	21.01396	0.422049781	0.631711	0.668106	0.504066	0.881759
arhgap28	112.4615	0.462940661	0.158888	2.913624	0.003573	0.099143
si:dkey-16p6.1	6.392203	0.501113093	0.846721	0.591828	0.553966	0.89821
si:dkey-34d22.3	0.853461	-0.245577676	2.543632	-0.09655	0.923087	NA
f13b	9.391188	2.779587883	1.542478	1.802028	0.071541	0.456244
CABZ01044156.1	4.364092	-0.988821171	0.855876	-1.15533	0.247955	0.728489
pard3aa	16.05919	-0.304494154	0.490234	-0.62112	0.53452	0.891711
si:ch211-215e19.6	0.131778	0	5.267649	0	1	NA
znf644a	269.8583	-0.130517407	0.141357	-0.92332	0.355841	0.806879
mibp	840.3395	-0.076066743	0.164295	-0.46299	0.643372	0.925953
ano2b	134.4672	0.117021732	0.252663	0.463154	0.643254	0.92593
wfikkn1	5.003753	0.807905693	0.787023	1.026534	0.30464	0.769932
si:cabz01021430.2	71.75412	0.374642074	0.219198	1.709152	0.087423	0.496224
gpr1	156.7435	0.299007955	0.227839	1.312368	0.189396	0.668949
si:ch73-193i22.1	78.27016	-0.465489635	0.37505	-1.24114	0.214554	0.69658
cngb3.2	122.6072	-0.060865594	0.501701	-0.12132	0.903439	0.991365
BX321912.1	0.073968	0	5.267649	0	1	NA
si:dkey-11n14.1	40.69081	0.933129616	0.526041	1.773873	0.076084	0.467233
ccdc142	173.1349	0.066034792	0.162369	0.406696	0.684231	0.938486
nadsyn1	78.81709	0.185958169	0.292307	0.636174	0.524663	0.888928
mfsd1	79.95235	-0.62637666	0.221201	-2.8317	0.00463	0.112688
cryabb	18.65186	-0.068588338	0.500468	-0.13705	0.890993	0.988566
CU019562.2	0	NA	NA	NA	NA	NA
CR388148.1	0.50552	1.984971856	2.274228	0.872811	0.382766	NA
BX088524.1	0.069091	0	5.267649	0	1	NA
f8	55.56686	-0.14618217	0.27739	-0.52699	0.5982	0.912715
CAMK2N1	240.0568	0.458191796	0.363748	1.259642	0.207799	0.689888
si:ch211-171l17.14	16.22848	0.453601242	0.828251	0.547662	0.583924	0.906376
zgc:174180	2.990542	-4.216563716	1.546445	-2.72662	0.006399	0.135026
pimr168	0	NA	NA	NA	NA	NA
si:dkey-31g6.6	2216.03	-0.126359302	0.279599	-0.45193	0.651319	0.928562
CR788226.1	22.88148	1.974711925	0.538248	3.66878	0.000244	0.016928
BX908401.2	0	NA	NA	NA	NA	NA
RF00003	0.235552	1.055386653	4.289141	0.24606	0.805636	NA
CR376730.1	1.037233	-2.290827494	1.861637	-1.23054	0.218493	NA
CABZ01065131.1	268.569	-0.132834325	0.154652	-0.85892	0.390383	0.827902
rplp2	11082.03	-0.13603565	0.142668	-0.95351	0.340329	0.795464
tgm1l4	59.25676	-0.30053715	0.263282	-1.1415	0.253661	0.732873
CABZ01012957.1	0.48614	2.999685455	3.02688	0.991016	0.321678	NA
wbp11	346.6501	0.031487559	0.139346	0.225966	0.821228	0.971788
FP102969.1	0	NA	NA	NA	NA	NA
dusp7	508.2686	-0.134524361	0.10473	-1.28449	0.198972	0.679593

drp2	6.239027	1.445301793	1.223899	1.180899	0.237643	0.718568
si:dkeyp-80d11.10	5.230041	-1.623456322	1.020433	-1.59095	0.111621	0.547577
hdac12	36.32436	-1.031242658	0.378551	-2.72419	0.006446	0.135777
cyp2y3	1001.292	0.061462733	0.13553	0.4535	0.650189	0.927835
BX901974.2	0	NA	NA	NA	NA	NA
si:dkey-1b17.5	11.3817	0.284791814	0.570243	0.499422	0.617482	0.916375
si:ch73-208h1.1	0.972087	0	2.925418	0	1	NA
gas8	128.525	0.093494343	0.196513	0.475766	0.634241	0.924077
rab11fip2	47.98192	0.321496566	0.282533	1.137908	0.255159	0.733542
AL831745.3	0.292935	0.892420518	5.241767	0.170252	0.864812	NA
fsbp	16.0603	0.298951286	0.535142	0.558639	0.576408	0.90475
lonp2	476.8199	-0.242945837	0.119525	-2.03259	0.042093	0.358832
lima1a	861.3141	0.046600146	0.118209	0.39422	0.693419	0.940668
relch	271.3306	-0.100410722	0.142094	-0.70665	0.479784	0.870001
CU856523.1	0	NA	NA	NA	NA	NA
klhl7	172.5664	0.093040095	0.155391	0.59875	0.54934	0.897174
si:dkey-46i9.1	2184.096	0.20993825	0.116688	1.799145	0.071996	0.4564
BX511066.2	0.277673	0	4.734464	0	1	NA
grik5	138.8919	-0.073437481	0.260287	-0.28214	0.777836	0.961073
si:ch211-215p11.3	4.942362	-0.249980825	0.888145	-0.28146	0.778354	0.961073
SUSD2	34.54999	-0.14737242	0.274657	-0.53657	0.591566	0.909437
si:ch211-284k5.2	19.56174	-0.596930285	0.548199	-1.08889	0.276201	0.750674
acp5b	25.66266	0.048476535	0.467984	0.103586	0.917498	0.993777
cdc42ep4a	178.7599	0.048725082	0.160497	0.30359	0.761441	0.958159
r3hdm1	1118.631	0.165361127	0.135767	1.217975	0.223234	0.70531
si:dkey-279j5.1	5.549066	0.247173381	0.912147	0.27098	0.786407	0.962701
emp3b	25.02763	-0.168847612	0.381868	-0.44216	0.658371	0.930581
si:dkey-176f19.1	1.401917	-0.899044852	1.635972	-0.54955	0.582629	NA
orc2	93.20148	-0.038769083	0.210197	-0.18444	0.853667	0.978905
si:dkey-56m15.2	8.880974	-0.217066393	0.991113	-0.21901	0.82664	0.973383
CR749168.4	0.053506	-0.86807634	5.267649	-0.16479	0.869106	NA
fgd4a	187.0918	0.178669509	0.14927	1.196957	0.231323	0.712847
pop4	147.4184	-0.207432692	0.241175	-0.86009	0.389739	0.827843
snapc3	184.1986	-0.153481715	0.16447	-0.93319	0.350722	0.802215
BX649328.2	0.329704	0	4.570482	0	1	NA
si:dkey-65j4.2	5.440814	1.291996458	0.750245	1.722099	0.085052	0.489675
plppr2a	39.40642	0.581524932	0.31781	1.829788	0.067282	0.442874
BX908782.2	57.35242	-1.805260398	1.41234	-1.27821	0.201177	0.681045
rbp5	882.394	-0.073420634	0.135745	-0.54087	0.588596	0.908014
hk2	226.8125	0.1759312	0.205317	0.856878	0.391512	0.827902
CT027586.1	0	NA	NA	NA	NA	NA
si:dkey-264f17.2	54.93657	0.66318079	0.477472	1.388942	0.16485	0.634185
si:ch1073-469d17.2	128.1208	0.330643074	0.689725	0.479384	0.631666	0.923003
RF00003	0	NA	NA	NA	NA	NA
si:ch73-281i18.6	16.5454	-0.126652617	0.466014	-0.27178	0.785792	0.962605
RF00001	0	NA	NA	NA	NA	NA
BX511128.2	1.972785	-0.325217574	1.301673	-0.24985	0.802707	NA
RF00001	0.101446	-1.755425691	5.23528	-0.33531	0.737394	NA
ugt5b2	171.4744	0.067739411	0.204514	0.331221	0.740477	0.953939
znf1047	1.96443	-0.512804012	1.402463	-0.36565	0.71463	NA
si:ch73-305o9.3	50.75485	-0.391250532	0.63153	-0.61953	0.535569	0.891993
ATG2A	489.8365	-0.010383643	0.207895	-0.04995	0.960165	0.999164
nr6a1a	6.706231	-0.153592648	0.740438	-0.20743	0.83567	0.976007
cetn2	178.9548	-0.052467301	0.187441	-0.27991	0.779544	0.961073

CU972516.1	0.230978	-1.755450266	5.235279	-0.33531	0.73739	NA
tmx4	52.21897	0.62407032	0.261238	2.388893	0.016899	0.230481
ftr64	13.56181	0.883723781	0.600057	1.472732	0.140823	0.599658
CT583728.4	13.32855	-1.029651988	0.527096	-1.95344	0.050767	0.392092
CR854829.1	0.142345	0	5.267649	0	1	NA
CR786582.2	0.21173	0	5.267649	0	1	NA
UBE2M	1382.154	0.079059823	0.100884	0.78367	0.433234	0.849392
FO907089.1	16.86356	1.171193512	0.754993	1.551264	0.120838	0.566816
arxb	19.56307	0.203965673	0.519363	0.392722	0.694524	0.941093
znf1080	3.807732	-0.317932128	1.255607	-0.25321	0.800106	0.967256
si:dkey-82o10.4	59.74047	0.585411527	0.353801	1.654637	0.097998	0.521071
CU855885.1	1.862564	0	1.991588	0	1	NA
si:ch211-194h1.2	2.05504	-1.737978842	2.027392	-0.85725	0.391308	NA
prr16	10.40382	0.98460769	0.640467	1.537328	0.124213	0.571374
si:ch211-1f22.3	0.043309	0	5.267649	0	1	NA
rab3db	59.97158	-1.002309147	0.265621	-3.77345	0.000161	0.012946
col10a1b	11.07295	1.675906596	1.039853	1.611676	0.107032	0.537
rpp25a	21.824	-0.234403761	0.602324	-0.38917	0.697154	0.94198
fgf3	110.7842	0.153854389	0.26527	0.579993	0.56192	0.900715
si:dkeyp-53d3.5	66.23701	-0.188478421	0.228917	-0.82335	0.410311	0.837066
washc5	355.2279	-0.056740475	0.121531	-0.46688	0.640586	0.925162
CR354395.1	0.838144	0.093656336	2.309555	0.040552	0.967653	NA
nkx2.2b	21.38509	-0.25286915	0.398221	-0.635	0.52543	0.888928
si:dkey-261j11.2	8.040904	0.132106698	1.035076	0.12763	0.898442	0.990199
CABZ01111555.1	0.140122	-0.868101293	5.267649	-0.1648	0.869102	NA
or106-8	8.11694	-0.548101281	0.749915	-0.73089	0.464849	0.864823
BX088583.2	7.149405	-1.695723756	0.908674	-1.86615	0.06202	0.42715
si:ch1073-475a24.1	8.552013	-2.027397503	1.060898	-1.91102	0.056002	0.409174
scarb1	404.5429	-0.119496039	0.15858	-0.75354	0.451127	0.857606
dre-mir-7149	0.47539	-0.373414692	2.564643	-0.1456	0.884236	NA
zhx2b	18.45224	1.050439348	0.529822	1.982625	0.047409	0.379101
psma1	2498.418	-0.044111468	0.15228	-0.28967	0.772067	0.960624
dbt	857.6142	0.042560725	0.110112	0.386521	0.699111	0.942499
znf1014	183.5283	0.02604072	0.168922	0.154158	0.877485	0.985427
CR354430.4	3.743711	1.068925498	1.045672	1.022238	0.306668	0.771918
si:dkey-226n24.1	0.254477	0	5.267649	0	1	NA
CU468917.1	27.34095	-0.559334839	0.480113	-1.16501	0.244017	0.724576
CR769772.4	2.151022	2.823536147	1.69808	1.662781	0.096356	NA
guca1e	18.944	1.09787684	0.971535	1.130044	0.258458	0.735636
CR352338.1	3.511506	-1.566774231	1.069733	-1.46464	0.143019	0.602002
ppargc1b	599.0062	0.028964214	0.16343	0.177227	0.85933	0.979975
si:ch211-106j21.4	4.519073	-0.722712	1.009836	-0.71567	0.474193	0.869292
CU928046.2	0	NA	NA	NA	NA	NA
tbxta	63.96254	-0.086545633	0.336283	-0.25736	0.796901	0.966447
si:ch211-17l8.4	79.78001	0.045293187	0.306837	0.147613	0.882648	0.986908
e2f4	366.3458	0.084473737	0.175186	0.482195	0.629667	0.92285
epc1a	418.7056	0.080521352	0.135026	0.596341	0.550947	0.8974
RF00001	0	NA	NA	NA	NA	NA
CU927920.1	0.099037	0	5.267649	0	1	NA
CU467822.1	906.1362	0.079114215	0.135584	0.583509	0.559551	0.899571
tecra	67.24059	0.877126752	0.328313	2.671618	0.007549	0.14728
si:ch73-389b16.1	120.0162	-0.508566665	0.251695	-2.02057	0.043325	0.364898
or109-13	0.514441	1.10472165	3.454367	0.319804	0.749117	NA
cacna1db	15.41884	0.301997032	0.890171	0.339257	0.734416	0.951134

CU695215.1	0.295871	0	5.267649	0	1	NA
zgc:163077	107.5749	-0.406684728	0.223095	-1.82292	0.068316	0.445546
tgm1l2	126.6288	0.123612258	0.20143	0.613673	0.539431	0.893049
RF00001	0	NA	NA	NA	NA	NA
si:dkeyp-57d7.4	97.91028	1.603335183	0.744782	2.152757	0.031338	0.314904
si:ch211-215p11.1	4.465851	0.556906765	1.08734	0.512173	0.60853	0.915204
BX640576.1	2.751624	-0.749486464	1.318697	-0.56835	0.569795	0.902322
preb	447.407	-0.166856315	0.110464	-1.51051	0.130913	0.582503
rims2a	253.1873	-0.093307012	0.267685	-0.34857	0.727412	0.949141
si:dkey-109a10.2	3.84079	1.335888305	1.247504	1.070849	0.284237	0.75523
zgc:112966	2.168786	0.669702448	1.601139	0.418266	0.675752	NA
si:dkey-238i5.2	100.7811	0.069220676	0.296773	0.233244	0.815572	0.971158
BX469901.1	0	NA	NA	NA	NA	NA
arfgap1	735.1088	-0.297166045	0.165088	-1.80005	0.071853	0.4564
BX530032.1	8.105513	2.238850777	0.689527	3.246936	0.001167	0.048965
ube2c	152.8322	-0.184773861	0.14877	-1.24201	0.214233	0.696474
micos10	876.1831	0.118921279	0.16	0.74326	0.457324	0.860993
RF00001	0.271185	0	5.267649	0	1	NA
mrps7	449.7767	0.211015441	0.135451	1.557876	0.119263	0.562753
znf1109	5.345253	-1.735171816	0.994192	-1.74531	0.080931	0.479801
lmnb2	1590.422	0.516867452	0.143157	3.610493	0.000306	0.019725
si:ch73-208h1.2	0.325862	0	5.263602	0	1	NA
arf6a	555.8966	0.169241884	0.135673	1.247423	0.212242	0.69394
ssr1	1826.101	0.142865976	0.145034	0.985048	0.3246	0.783313
ascl1b	296.2019	0.008131977	0.165823	0.04904	0.960887	0.999249
CABZ01079192.1	231.785	-1.763738115	0.66535	-2.65084	0.008029	0.151864
si:dkey-8l13.5	16.6846	-1.136825309	0.422059	-2.69352	0.00707	0.142573
etfa	3284.671	-0.164011356	0.204368	-0.80253	0.422247	0.843493
cyp2k20	1.778942	1.055436724	2.041605	0.516964	0.605181	NA
LO017682.1	6.925843	-0.8018923	0.958048	-0.83701	0.402589	0.833401
si:ch211-209j12.1	7.506397	-1.47263726	0.74079	-1.98793	0.04682	0.376932
dact1	343.3203	-0.04382519	0.1887	-0.23225	0.816345	0.971207
zgc:64201	45.89218	0.944615785	0.292395	3.23062	0.001235	0.051016
ccnd1	2308.727	0.055736028	0.122727	0.454147	0.649723	0.927756
trpm2	30.63017	-0.666940542	0.392341	-1.6999	0.08915	0.501359
si:ch211-155m12.1	8.74776	-3.847444533	1.056195	-3.64274	0.00027	0.018116
ARHGEF17	276.1709	0.203097837	0.13637	1.489318	0.136404	0.591738
myrf1	90.75146	-4.589433868	0.701817	-6.53936	6.18E-11	6.51E-08
si:ch73-359m17.8	0.197783	-0.868114638	5.267649	-0.1648	0.8691	NA
RF00003	0	NA	NA	NA	NA	NA
CU929240.1	39.48362	-0.220196048	0.310066	-0.71016	0.477605	0.869292
si:zfos-1056e6.1	19.43617	-0.223435592	0.422646	-0.52866	0.597042	0.911837
haus6	210.0738	0.241342418	0.242639	0.994656	0.319904	0.780475
zgc:110171	5.284222	-0.016632348	0.961115	-0.01731	0.986193	1
si:ch211-188c18.1	5.478347	1.337973112	1.213704	1.102388	0.270293	0.746536
sstr5	90.59281	0.29396672	0.233427	1.25935	0.207904	0.689937
BX323882.1	0.063619	0	5.267649	0	1	NA
BX005396.4	0.086616	0	5.267649	0	1	NA
RF00560	0.52487	2.489258021	3.05908	0.813728	0.415801	NA
sntg2	47.84972	0.563010084	0.385502	1.46046	0.144164	0.603155
ulk3	85.52267	-0.438347454	0.226953	-1.93145	0.053427	0.40036
CABZ01078663.1	0.414655	0	3.651284	0	1	NA
BX957361.1	0.295271	2.436361052	5.206324	0.467962	0.639812	NA
pcdh1g11	3.973941	-2.232683974	1.446198	-1.54383	0.122629	0.569468

si:ch73-233f7.6	2.728359	0.471427464	1.204413	0.391417	0.695489	0.9414
BX681417.2	2.996337	3.199630215	1.949311	1.641416	0.100711	0.525794
elmod2	538.3356	-0.150707101	0.145738	-1.0341	0.30109	0.767371
kntc1	305.4914	0.404206751	0.209652	1.927986	0.053857	0.401889
zgc:158417	21.13011	-0.285952616	0.615497	-0.46459	0.642226	0.92567
trgv7	0.376839	-2.744373636	3.660061	-0.74982	0.453365	NA
RF00001	0.086616	0	5.267649	0	1	NA
RF00001	0	NA	NA	NA	NA	NA
zgc:123107	5.657857	-1.090016928	1.141942	-0.95453	0.339816	0.795226
KLHL28	144.7642	-0.230938555	0.188444	-1.2255	0.220386	0.701707
chrnb4	30.37263	-0.160589596	0.400816	-0.40066	0.688673	0.939118
cfap52	32.31243	0.03904096	0.313125	0.124682	0.900776	0.99045
arl1	590.3702	0.176099658	0.105983	1.661583	0.096597	0.518929
tradv7.0	0	NA	NA	NA	NA	NA
CT990567.2	2.200291	-0.003732147	1.195743	-0.00312	0.99751	1
FP340292.1	0	NA	NA	NA	NA	NA
tradv16.0	0	NA	NA	NA	NA	NA
si:ch73-171o20.2	0.073968	0	5.267649	0	1	NA
CU929259.1	132.7915	0.439148299	0.250871	1.750495	0.080033	0.478179
si:dkey-207124.2	21.01235	0.235844318	0.580942	0.405969	0.684765	0.938486
si:dkey-16p6.1	3.282464	-3.101745771	1.55778	-1.99113	0.046466	0.376263
si:ch73-158p21.2	41.40269	0.532343542	0.355209	1.498677	0.133958	0.58783
znf1184	31.04263	0.232696265	0.339844	0.684716	0.493523	0.877027
si:dkey-26m3.3	19.36662	1.167933306	0.57583	2.028259	0.042534	0.361124
zgc:158316	193.9367	-0.06822782	0.249768	-0.27317	0.784726	0.962605
si:dkey-30e9.7	94.34445	-0.114059643	0.258389	-0.44143	0.658905	0.930581
si:ch1073-181h11.1	0.11861	0	5.267649	0	1	NA
bmp5	319.713	0.045553426	0.166431	0.273708	0.784309	0.962605
chrna7	9.935497	-0.160625595	0.82824	-0.19394	0.846226	0.97711
zgc:158260	6.648396	0.553276925	0.755893	0.731952	0.464198	0.864593
lrrc24	15.68297	-0.157533024	0.540146	-0.29165	0.770555	0.960484
csrp3	30.93527	0.429863902	0.348577	1.233196	0.217503	0.69815
si:ch211-156b7.4	286.0787	0.316802949	0.22163	1.42942	0.152883	0.617235
si:ch211-106g8.56	0	NA	NA	NA	NA	NA
arid1aa	1370.247	0.104424296	0.136954	0.76248	0.445774	0.855403
scn2b	49.7846	0.549329147	0.335852	1.635627	0.101918	0.528757
RF00026	0	NA	NA	NA	NA	NA
arsg	32.45822	-0.366535876	0.373937	-0.98021	0.326983	0.785751
bace1	118.5745	0.48492957	0.195017	2.486608	0.012897	0.198116
sox17	3.24521	-1.456110175	1.166964	-1.24778	0.212113	0.693739
CR394556.1	0.324891	0	4.589924	0	1	NA
pargl	85.96065	-0.345748745	0.31379	-1.10185	0.270528	0.746683
CU459186.3	6.366349	-0.007612575	0.700467	-0.01087	0.991329	1
LO018584.1	2.594785	0.716328387	1.662192	0.430954	0.666502	0.932664
larp1	734.0144	-0.128907089	0.162283	-0.79433	0.427002	0.845491
parietopsin	166.9652	-0.355828222	0.198059	-1.79658	0.072403	0.457602
si:ch211-93f2.1	524.0093	0.318126692	0.298185	1.066876	0.286028	0.757455
CU467822.2	3.566621	-0.891506903	1.01372	-0.87944	0.379162	0.8232
si:dkeyp-80d11.12	2.982979	-1.255389353	1.442945	-0.87002	0.38429	0.826565
BX649341.1	0.37335	-0.868139708	3.830603	-0.22663	0.820709	NA
fam168b	750.2631	0.037661112	0.111601	0.337463	0.735768	0.951543
chn1	596.1391	-0.014290705	0.148755	-0.09607	0.923466	0.994525
BX842687.1	0.643704	-1.957467507	3.058124	-0.64009	0.522116	NA
tradv24.0.5	0.25058	0	5.267649	0	1	NA

gb:eh456644	11.00462	-0.024701259	0.571019	-0.04326	0.965496	1
elavl1b	978.5747	-0.168505184	0.11518	-1.46298	0.143474	0.602607
AL953886.1	18.88242	0.83073181	0.502857	1.652025	0.098529	0.521133
si:ch211-24o8.4	1.344944	-0.285607833	1.657608	-0.1723	0.863201	NA
zgc:92161	167.233	1.57173278	0.324956	4.836759	1.32E-06	0.000364
si:ch211-196p9.1	174.0552	0.052612934	0.147565	0.356541	0.721436	0.947343
plekhg5b	93.77054	-0.130692313	0.27888	-0.46863	0.639332	0.924779
CABZ01085195.1	14.69457	-0.391690181	0.447236	-0.8758	0.381138	0.824296
prkar1ab	116.8438	-0.190000829	0.215017	-0.88365	0.376883	0.822258
im:7141269	61.65114	0.915233176	0.322587	2.837165	0.004552	0.111673
si:dkey-27n6.4	49.93646	0.374974982	1.128104	0.332394	0.739592	0.953573
traj41	0	NA	NA	NA	NA	NA
homer1b	552.9312	-0.074452097	0.120574	-0.61748	0.536918	0.892421
znf1016	2.063599	-0.015571057	1.218747	-0.01278	0.989806	NA
RF00001	0	NA	NA	NA	NA	NA
fah	455.7611	0.050252169	0.248598	0.202142	0.839806	0.976417
CU467646.5	1.599077	2.429223862	1.813669	1.339398	0.180441	NA
ptmab	17712.16	0.066272878	0.158986	0.416848	0.676789	0.935664
si:dkey-183i3.6	5.786478	1.049540627	0.898628	1.167936	0.242832	0.724396
trgv3	0	NA	NA	NA	NA	NA
cysltr3	5.609877	-0.295687755	0.909209	-0.32521	0.745019	0.954956
CT956002.1	0.251029	2.482881251	5.204054	0.477105	0.633287	NA
si:ch211-241j8.2	0.398533	1.055402733	3.682096	0.286631	0.774395	NA
zgc:172014	20.17734	-0.096767338	0.482377	-0.20061	0.841007	0.97643
syt12	223.6606	0.118344024	0.278807	0.424466	0.671226	0.933953
pdlim5a	100.6838	0.157359863	0.233815	0.673011	0.50094	0.880413
ca5a	556.7662	-0.234501074	0.160621	-1.45997	0.144299	0.603393
pimr181	0.126661	0	5.267649	0	1	NA
RF00001	0	NA	NA	NA	NA	NA
laptm5	115.4648	-0.018011352	0.296999	-0.06064	0.951642	0.99801
si:ch211-134a4.6	1.153509	-3.383354884	2.322724	-1.45663	0.145218	NA
zgc:162592	0.485081	0	4.777108	0	1	NA
BX784032.1	0.060442	0	5.267649	0	1	NA
cyp1c2	77.64428	0.208018695	0.2871	0.72455	0.468728	0.866951
si:ch73-299h12.3	37.65565	-0.225992012	0.300855	-0.75117	0.452553	0.857994
kyat3	400.3797	0.165182242	0.153854	1.073631	0.282988	0.755028
si:dkey-237i9.1	195.1956	-0.02936077	0.183962	-0.1596	0.873194	0.983537
atp6v0e1	552.9451	0.518192312	0.199359	2.599296	0.009342	0.166239
CABZ01033394.1	0	NA	NA	NA	NA	NA
RF00001	0.144983	-1.710071829	5.238473	-0.32644	0.744088	NA
or130-1	3.760758	1.11901485	1.285142	0.870732	0.3839	0.82644
si:ch1073-82l19.1	40.03218	-0.136465528	0.347266	-0.39297	0.694341	0.941093
LO018493.1	1.870446	0	2.104674	0	1	NA
tradv36.2.12	0.073968	0	5.267649	0	1	NA
or104-1	0.13984	0	5.267649	0	1	NA
si:ch211-120c15.3	30.28406	0.911992183	0.522606	1.745087	0.08097	0.479801
si:ch211-271b14.1	689.9394	-0.048534448	0.115067	-0.42179	0.673177	0.93414
zgc:114120	9.032161	0.160365311	0.74931	0.214017	0.830534	0.974908
nap1l1	3489.985	0.00437291	0.08476	0.051592	0.958854	0.999164
lpar3	65.03211	-0.334500735	0.308788	-1.08327	0.278688	0.752299
col5a3b	1500.924	0.316608315	0.177709	1.781611	0.074813	0.464151
si:dkey-5i16.5	12.44794	0.845692029	0.575376	1.469806	0.141614	0.600284
BX294178.1	9.719168	1.19933489	0.643764	1.863004	0.062462	0.427718
CU984583.1	0.191496	1.854123853	5.241768	0.353721	0.723548	NA

traj51	0	NA	NA	NA	NA	NA
si:ch211-243p7.3	32.88838	-0.031506984	0.343186	-0.09181	0.926851	0.994864
AL929114.2	2.121371	-0.776761058	1.680133	-0.46232	0.643851	NA
zwi	333.1377	0.200127919	0.254768	0.785529	0.432144	0.848419
pimr59	0.049519	0	5.267649	0	1	NA
si:dkey-16p6.1	26.92974	-1.161063979	0.51708	-2.24543	0.024741	0.27868
si:dkey-16b10.2	4.564279	-2.240338034	0.966709	-2.31749	0.020477	0.25399
si:dkeyp-13d12.22	0.308101	0	3.950428	0	1	NA
irx1a	364.576	0.212068244	0.128196	1.654247	0.098077	0.521071
AL627305.2	3.343076	-2.732690577	1.275219	-2.14292	0.03212	0.318495
RF00003	0.043309	0	5.267649	0	1	NA
CR788237.2	0.14162	0	5.267649	0	1	NA
znf1085	12.31446	0.574148821	0.504749	1.137493	0.255332	0.733542
znf1106	4.068457	-0.753171955	1.048822	-0.71811	0.472688	0.868807
mibp2	12763.21	-0.411733947	0.180744	-2.278	0.022727	0.266612
FP016142.5	0.869139	1.4039812	2.442389	0.574839	0.5654	NA
BX088718.1	0.382489	-0.794422626	3.810237	-0.2085	0.834841	NA
dydc2	19.80347	-0.328726002	0.376139	-0.87395	0.382146	0.825191
adipor2	2301.991	-0.052556285	0.110488	-0.47567	0.634308	0.924102
RF00001	0.069091	0	5.267649	0	1	NA
FQ311894.1	1209.233	0.135238939	0.182681	0.7403	0.459118	0.861818
mnta	371.9597	-0.103375751	0.126715	-0.81581	0.414609	0.838683
cyp2k19	109.7727	1.065292881	0.621931	1.71288	0.086735	0.493527
CR382337.3	3.571543	0.685400356	1.602655	0.427666	0.668895	0.933115
pcdh1g30	106.094	-0.014241527	0.249914	-0.05699	0.954556	0.998611
asic1b	84.2439	-0.273978095	0.289528	-0.94629	0.343999	0.798147
si:ch1073-416d2.4	93.14061	0.062749914	0.190848	0.328795	0.742311	0.954329
dennd1b	21.74402	0.300378125	0.369545	0.812832	0.416315	0.839732
magi3a	318.9772	-0.213864103	0.165458	-1.29256	0.196165	0.676562
CR854963.1	0	NA	NA	NA	NA	NA
BX842701.1	7.500969	-0.303771231	0.881584	-0.34457	0.730414	0.950056
dtwd2	34.66361	-1.138078834	0.481019	-2.36597	0.017983	0.236739
rbm34	268.8211	0.028023854	0.191533	0.146313	0.883674	0.987014
kdf1b	34.74254	0.192206233	0.33182	0.579248	0.562422	0.900715
traj58	0	NA	NA	NA	NA	NA
zgc:172128	56.93466	0.171219615	0.361634	0.473461	0.635884	0.924669
si:dkey-20i20.11	6.522026	1.605726547	0.641708	2.502269	0.01234	0.193188
pdxdc1	325.3713	-0.011702669	0.132568	-0.08828	0.929657	0.995576
OSCP1 (1 of many)	123.0471	0.064280999	0.224978	0.285722	0.775091	0.960899
dre-mir-25	0.515928	0.645074711	3.536687	0.182395	0.855273	NA
pigr	153.3697	0.000626738	0.209861	0.002986	0.997617	1
arid1ab	1408.095	0.144207229	0.12964	1.112365	0.265981	0.742773
ino80e	232.2982	0.228010624	0.172105	1.324831	0.185227	0.662813
CR388178.1	12.73343	2.424463259	0.895894	2.706193	0.006806	0.139148
stk10	194.1901	-0.216775831	0.16264	-1.33286	0.182578	0.659237
kncn	11.69909	0.508307885	0.632623	0.803493	0.42169	0.842943
xrn2	885.0065	0.148481457	0.11016	1.347874	0.177699	0.65136
zgc:193711	76.3806	0.123090239	0.225362	0.546188	0.584936	0.906655
CR376730.2	0.86503	-0.333485079	2.115585	-0.15763	0.874746	NA
zfyve9a	365.2299	0.113867859	0.120255	0.946887	0.343696	0.798147
RF00001	0	NA	NA	NA	NA	NA
zgc:113442	30.58204	-0.087357339	0.4459	-0.19591	0.844679	0.976791
CR848667.2	0	NA	NA	NA	NA	NA
rab3ip	707.1066	0.139501633	0.140588	0.99227	0.321066	0.781417

pcdh20	134.2546	0.188902565	0.26707	0.707315	0.479371	0.86985
si:dkeyp-86d6.2	10.33851	-0.536577757	0.576709	-0.93041	0.352157	0.802776
si:dkey-65l23.2	0.053262	1.055396947	5.267649	0.200354	0.841203	NA
metrnla	937.24	-0.06386262	0.160337	-0.3983	0.690407	0.93988
pin1	872.4995	0.021078888	0.132135	0.159526	0.873255	0.983537
si:dkeyp-44d3.5	20.90442	0.318814037	0.489318	0.651548	0.514693	0.886385
pmfbp1	20.72955	0.157628997	0.456539	0.345269	0.729892	0.950056
si:ch211-111e20.1	3.471941	0.090505298	1.118019	0.080951	0.935481	0.996829
foxj1a	105.4801	-0.050833177	0.247434	-0.20544	0.837227	0.976007
RF00001	0.053506	-0.86807634	5.267649	-0.16479	0.869106	NA
si:dkey-65b13.13	56.62824	1.302029592	0.760512	1.712043	0.086889	0.494282
si:ch1073-204b8.1	1.164394	-2.307761583	2.340074	-0.98619	0.324039	NA
zgc:101130	72.54772	-0.209244311	0.227649	-0.91915	0.358015	0.809315
FQ377605.1	9.690876	-0.294836184	0.531396	-0.55483	0.579008	0.905417
gorasp1b	7.334884	1.071230812	0.759262	1.410885	0.158278	0.623766
CU914622.1	19.28791	1.923856753	0.782872	2.457434	0.013993	0.207377
si:dkey-205h23.2	4.935647	0.777569263	0.833411	0.932996	0.350822	0.802215
si:ch73-358j7.2	0.113138	0	5.267649	0	1	NA
BX088707.1	1.592651	-1.730852481	1.731593	-0.99957	0.317518	NA
tmem106ba	925.1819	-0.021737899	0.115891	-0.18757	0.851212	0.978548
RF02209	0	NA	NA	NA	NA	NA
BX324216.1	0.36202	0	5.175955	0	1	NA
si:dkey-260j18.2	473.0243	-0.084223068	0.119537	-0.70458	0.481072	0.870339
BX511111.2	9.613391	0.261258915	0.617262	0.423255	0.672109	0.933953
si:dkey-92j16.2	4.81703	0.173363741	0.813941	0.212993	0.831332	0.974908
ptbp2a	260.7589	0.070741019	0.130067	0.54388	0.586524	0.906711
si:ch211-223g7.6	3.248184	0.44707554	1.227385	0.36425	0.715671	0.9461
numbl	60.23024	0.21085914	0.23915	0.881703	0.377937	0.82265
pigv	50.60062	-0.138945485	0.290104	-0.47895	0.631973	0.923139
hoxc6b	73.79913	0.059512381	0.251872	0.23628	0.813215	0.970328
znf1159	21.74109	0.388946207	0.429075	0.906476	0.364684	0.814648
CU571315.2	16.97897	1.891006238	2.295275	0.823869	0.410014	0.836939
etv1	144.4096	0.125135568	0.182662	0.685068	0.493301	0.876787
si:dkeyp-44d3.3	14.25493	0.419499224	0.4454	0.941848	0.34627	0.799463
abhd18	235.9022	0.246142126	0.150367	1.636939	0.101643	0.52828
tmem141	100.9407	-0.332746308	0.233372	-1.42582	0.15392	0.618475
zgc:171592	0.693153	0	3.032878	0	1	NA
BX664606.2	0	NA	NA	NA	NA	NA
sult4a1	479.7209	0.239155593	0.225057	1.062643	0.287944	0.758746
si:ch211-227p7.1	0	NA	NA	NA	NA	NA
CABZ01084347.1	3.561841	0.89603492	1.348272	0.66458	0.506319	0.883007
RF00026	0	NA	NA	NA	NA	NA
si:dkey-225f5.4	195.1112	0.321534513	0.214357	1.499998	0.133615	0.587004
si:ch211-209n20.59	2.315622	3.472261276	1.552422	2.236674	0.025308	0.282476
eps8l3a	213.2526	-0.356974115	0.266896	-1.3375	0.181058	0.657233
bmper	341.6663	0.066415538	0.163647	0.405848	0.684855	0.938486
spryd3	128.931	-0.41053204	0.209097	-1.96336	0.049605	0.387751
pkn2	179.7972	-0.167991917	0.155025	-1.08364	0.278523	0.752148
pde6gb	497.9402	0.265812088	0.19714	1.34834	0.177549	0.651332
socs9	84.65719	-0.005167351	0.228037	-0.02266	0.981921	1
irf6	166.538	-0.375163034	0.215542	-1.74056	0.081761	0.481425
CU633987.1	0	NA	NA	NA	NA	NA
thrap3a	179.3836	0.036993382	0.199117	0.185787	0.852611	0.978672
CU571315.3	16.99593	-2.404449276	1.489265	-1.61452	0.106415	0.535773

si:dkey-238k10.3	24.85701	-1.449421205	0.852979	-1.69925	0.089273	0.501424
CR354398.1	0	NA	NA	NA	NA	NA
gabrg1	8.443523	1.396291343	0.720425	1.938148	0.052605	0.398451
cxxc1a	341.4876	-0.061616023	0.135189	-0.45578	0.64855	0.927501
si:dkey-191j3.2	16.7239	-0.532794357	0.670378	-0.79477	0.426749	0.845352
si:dkey-1b17.2	9.042472	-0.974681422	0.71272	-1.36755	0.171453	0.643433
cog3	412.0397	0.145974243	0.156157	0.93479	0.349897	0.801572
si:dkeyp-13d12.24	0.159785	2.50566729	5.20297	0.481584	0.630101	NA
CU695215.2	4.567322	0.003052291	0.769519	0.003966	0.996835	1
apoea	7522.156	-0.704898523	0.248068	-2.84156	0.004489	0.11054
RF00001	0.046941	0	5.267649	0	1	NA
si:dkey-72l17.6	16.84861	-0.124136062	0.625862	-0.19834	0.842776	0.9766
ppp2r2d	357.0037	0.17229933	0.120234	1.433039	0.151847	0.615406
ppp1cbl	2694.669	-0.090501764	0.111957	-0.80836	0.418883	0.841915
FP009765.1	0.27541	-0.868110153	5.267649	-0.1648	0.869101	NA
CABZ01037298.1	141.1999	0.02404859	0.188763	0.127401	0.898623	0.990199
trpc4b	18.14742	0.215415489	0.460127	0.468166	0.639666	0.924779
btr25	13.97715	-0.845695058	0.6708	-1.26073	0.207408	0.689456
AL627305.3	14.53575	-0.834549891	0.573041	-1.45635	0.145295	0.604476
pnpla3	501.2171	-0.674537697	0.216325	-3.11817	0.00182	0.06578
C14orf28	65.45859	-0.287252813	0.271679	-1.05732	0.290364	0.76078
phf13	163.1516	-0.023699425	0.169715	-0.13964	0.888942	0.988188
chmp6b	1037.826	-0.168321399	0.096551	-1.74334	0.081274	0.480142
znf644b	128.2957	-0.05053417	0.20467	-0.24691	0.804982	0.969348
gpsm2l	101.6351	-0.198845663	0.183351	-1.08451	0.27814	0.751971
si:dkey-172k15.11	81.91429	0.261988263	0.224062	1.169267	0.242296	0.724106
si:dkey-183c6.7	13.77813	0.036996764	0.611697	0.060482	0.951772	0.99801
BX950220.1	0	NA	NA	NA	NA	NA
zgc:193790	36.74632	-0.106434586	0.286878	-0.37101	0.71063	0.945821
si:ch211-214k5.3	1.69946	0.814405349	1.590648	0.511996	0.608654	NA
slc52a2	87.0698	-0.58358733	0.199851	-2.92011	0.003499	0.097796
RF00001	0	NA	NA	NA	NA	NA
CR356236.1	33.51425	0.070170306	0.377744	0.185762	0.852632	0.978672
FP326652.1	0.173005	0	5.267649	0	1	NA
mab21l1	1787.057	0.060449536	0.109898	0.55005	0.582285	0.90571
slc25a43	473.1451	-0.087440511	0.239691	-0.36481	0.715256	0.945917
CU550719.1	0	NA	NA	NA	NA	NA
MCOLN3	170.9301	-0.237063944	0.268688	-0.8823	0.377614	0.822478
mtbl	281.3012	0.075086594	0.244257	0.307408	0.758533	0.957865
tradv28.0	0.138182	0	5.267649	0	1	NA
znf1164	38.71388	-0.276459082	0.331502	-0.83396	0.404305	0.833888
CR848705.1	0.227876	0	5.267649	0	1	NA
hadhb	2239.699	0.182647995	0.141842	1.287686	0.197855	0.678554
svild	693.8401	-0.042263826	0.108413	-0.38984	0.696654	0.941908
si:dkeyp-11e3.1	168.723	0.047342759	0.173818	0.27237	0.785338	0.962605
RGS13 (1 of many)	49.52663	-0.551699061	0.451184	-1.22278	0.221412	0.702841
zgc:92873	168.9922	0.121373262	0.21104	0.575121	0.56521	0.901538
b3galt2	785.025	0.027058496	0.147963	0.182874	0.854897	0.979161
si:dkeyp-82h4.3	11.63389	-2.378722089	0.542639	-4.38362	1.17E-05	0.00208
kcna2b	17.21864	0.509655247	0.503437	1.012352	0.31137	0.77613
ntn4	57.31062	-0.579672968	0.286925	-2.0203	0.043353	0.365001
grm5b	6.211379	0.118308392	0.965907	0.122484	0.902515	0.990964
shcbp1	172.8572	0.100871126	0.160202	0.629649	0.528924	0.890325
neo1b	0	NA	NA	NA	NA	NA

tbc1d10aa	273.8207	-0.136743267	0.17647	-0.77488	0.438409	0.851685
slc25a47b	52.65975	0.621109101	0.319155	1.946103	0.051642	0.395032
RNF122	68.01477	0.400833191	0.276548	1.449414	0.147222	0.607612
spink2.1	219.1767	-0.713761462	0.391273	-1.8242	0.068121	0.445203
CABZ01072548.1	73.66493	0.051603324	0.200208	0.257748	0.796601	0.966447
mif4gda	86.6519	-0.023044178	0.215083	-0.10714	0.914677	0.992972
mprip	905.5465	0.029496027	0.11208	0.26317	0.792419	0.965217
nr3c2	41.8076	0.032062078	0.344773	0.092995	0.925908	0.994815
CT573793.2	0.103025	-0.868089684	5.267649	-0.1648	0.869104	NA
pop1	50.72692	-0.391274822	0.285152	-1.37216	0.170013	0.641773
CABZ01052576.1	131.5089	0.375136686	0.157136	2.387345	0.016971	0.230743
RF00001	0	NA	NA	NA	NA	NA
wu:fd46c06	0.643795	1.867132574	3.318083	0.562714	0.573629	NA
si:ch73-233f7.5	4.588308	-0.403014933	0.709224	-0.56825	0.569867	0.902322
adra2b	39.01891	-0.226029107	0.324908	-0.69567	0.486635	0.873244
nfkbiz	171.9145	0.109416166	0.179663	0.609007	0.54252	0.894595
CR954226.1	0.170783	-0.868103029	5.267649	-0.1648	0.869102	NA
si:ch211-255f4.11	33.77039	0.265389279	0.336723	0.788152	0.430608	0.847542
mrc1a	467.2657	0.20806722	0.205461	1.012683	0.311212	0.775936
si:ch73-174h16.5	37.67008	0.950528761	0.605822	1.56899	0.11665	0.557506
CU855889.1	0	NA	NA	NA	NA	NA
dad1	1616.896	0.535867748	0.205449	2.608282	0.0091	0.16357
tgm1l1	732.6884	-0.004394932	0.156533	-0.02808	0.977601	1
tradv2.0.4	0	NA	NA	NA	NA	NA
zbtb49	123.3097	0.087275391	0.203957	0.427912	0.668715	0.933101
ptdss1b	7.672882	-0.433051901	0.788003	-0.54956	0.582624	0.90571
ndufc2	1276.416	0.207357419	0.149848	1.38378	0.166426	0.636527
slf1	79.82987	0.348278288	0.234896	1.482693	0.138156	0.594663
si:ch73-359m17.2	96.81096	0.042244212	0.381029	0.110869	0.91172	0.992336
RF00090	0	NA	NA	NA	NA	NA
phtf2	180.5923	-0.044142584	0.218755	-0.20179	0.840081	0.976417
BX000696.1	0.390199	3.222190182	4.215984	0.764279	0.444701	NA
iqsec2a	109.6528	0.402458812	0.321346	1.252417	0.210418	0.692133
RF00001	0	NA	NA	NA	NA	NA
NHLRC3	300.9456	0.413233504	0.180699	2.286856	0.022204	0.263608
eps8a	294.658	-0.30571634	0.172978	-1.76738	0.077165	0.469517
crygn1	37.49037	-0.526484918	0.574817	-0.91592	0.35971	0.810668
BX666065.2	0.156168	-0.868103029	5.267649	-0.1648	0.869102	NA
AL954698.2	0.545585	1.055395577	3.506877	0.30095	0.763452	NA
foxa1	149.7199	0.2051559	0.232734	0.881503	0.378045	0.82265
ptpn12	232.6705	0.171986999	0.169319	1.015756	0.309746	0.774621
ece2b	85.48904	0.203883976	0.243672	0.836715	0.402753	0.833401
btr24	5.354103	-0.908045022	0.953174	-0.95265	0.340766	0.795603
eef1akmt2	100.4712	0.071780076	0.288185	0.249076	0.803302	0.968443
ccdc77	131.6751	0.020290133	0.207864	0.097612	0.92224	0.994346
si:ch211-69b7.6	99.39431	-0.036309405	0.264043	-0.13751	0.890625	0.988446
fem1c	374.2034	-0.099320318	0.124753	-0.79613	0.425954	0.844714
si:ch211-249o11.5	0.745742	-0.714912542	2.213315	-0.32301	0.746691	NA
nrp1a	647.179	-0.102841643	0.103124	-0.99726	0.318639	0.779923
si:dkey-262g12.10	111.953	-0.270398701	0.186183	-1.45233	0.146411	0.605984
si:dkey-264f17.4	11.00031	1.143881191	0.601141	1.90285	0.05706	0.412539
si:ch211-117i20.2	0.579097	1.881690254	2.570663	0.731986	0.464177	NA
ly6m6	0.266177	-2.334611233	4.705852	-0.49611	0.619818	NA
si:dkeyp-61b2.1	15.13457	0.624784833	0.616632	1.013221	0.310955	0.775796

SDK2 (1 of many)	34.9881	0.269425161	0.343039	0.785407	0.432215	0.848419
BX005043.1	5.629356	1.33729517	0.741864	1.802614	0.071449	0.456052
RF00001	0.207272	0	5.267649	0	1	NA
ppox	146.1833	0.107249576	0.250154	0.428734	0.668117	0.933086
fnip1	548.5406	-0.186576591	0.187223	-0.99655	0.318984	0.779923
CABZ01065425.1	0.167133	-1.710071829	5.238473	-0.32644	0.744088	NA
CR381700.1	1.806795	-2.94212339	1.675906	-1.75554	0.079167	NA
hamp	344.8577	0.233123036	0.468537	0.497555	0.618797	0.917015
pla1a	62.29114	-1.202125485	0.332493	-3.61549	0.0003	0.019507
emilin2b	535.1648	0.011305925	0.122225	0.092501	0.9263	0.994815
shroom3	105.4716	0.708950508	0.233442	3.036946	0.00239	0.078211
mib1	810.3032	0.107790365	0.120387	0.895364	0.370593	0.818484
PCDH8	106.6732	0.333474651	0.284149	1.17359	0.240559	0.72199
CU694264.1	0.65229	0	3.256751	0	1	NA
tradv20.0	0	NA	NA	NA	NA	NA
CPNE8	350.9841	-0.296102783	0.18833	-1.57226	0.115891	0.556115
zc3h7a	453.6723	-0.056957947	0.099385	-0.5731	0.566574	0.901739
si:ch73-233f7.3	3.432833	2.90016178	0.998096	2.905693	0.003664	0.100272
SIKE1	334.011	-0.173071657	0.172805	-1.00154	0.316564	0.778879
man2a1	25.40086	-0.043790428	0.397409	-0.11019	0.912259	0.992371
phospho2	198.0279	-0.186351826	0.175063	-1.06448	0.287111	0.758016
jph3	222.2606	0.318407316	0.180081	1.768133	0.077039	0.469486
stc2b	40.42793	-0.277726527	0.536105	-0.51805	0.604427	0.914972
LO018612.1	0	NA	NA	NA	NA	NA
csf3a	15.61659	-0.244579625	0.64889	-0.37692	0.706233	0.944606
si:dkey-16p6.1	11.02379	1.080924594	0.789001	1.369991	0.17069	0.642339
si:ch211-227e10.6	5.300211	-0.101428868	0.9922	-0.10223	0.918577	0.993884
dlg1l	740.0154	0.09110988	0.145598	0.625764	0.53147	0.890835
RF00026	0	NA	NA	NA	NA	NA
si:dkey-31g6.4	1.766686	-0.150620205	1.416306	-0.10635	0.915307	NA
si:ch211-12p12.2	1.631354	-0.5475836	2.201618	-0.24872	0.803578	NA
RF00026	0	NA	NA	NA	NA	NA
HACD4	68.22014	-0.067739767	0.267365	-0.25336	0.79999	0.967256
BX511079.2	0	NA	NA	NA	NA	NA
si:ch211-205a14.7	7.543259	0.802684949	1.041628	0.770606	0.44094	0.852687
gpr19	97.72154	0.039329581	0.231823	0.169653	0.865283	0.982197
CR450749.3	0	NA	NA	NA	NA	NA
si:dkey-171c9.3	19.17364	0.595974497	0.385989	1.54402	0.122584	0.569468
mapk8ip1a	319.0356	0.238420834	0.221254	1.077591	0.281216	0.753557
si:ch211-8c17.3	1.229998	0.081959005	1.92316	0.042617	0.966007	NA
si:ch211-71n6.4	115.9545	0.122441934	0.217522	0.562895	0.573507	0.90414
RF00001	0	NA	NA	NA	NA	NA
CR559945.1	0.045161	0	5.267649	0	1	NA
CU695223.1	56.08292	0.020350699	0.222856	0.091318	0.92724	0.994973
zgc:136410	1312.634	-0.116425358	0.212749	-0.54724	0.584213	0.906402
si:ch211-180m24.3	12.39458	0.38845752	0.57636	0.673984	0.500322	0.880145
tk1	308.2877	0.199783367	0.216032	0.924786	0.355077	0.806266
traj42	0	NA	NA	NA	NA	NA
CU463038.2	4.682998	0.966651806	0.999291	0.967337	0.333375	0.790506
pepd	757.6999	0.059383247	0.164216	0.361617	0.717638	0.946421
traj44	0	NA	NA	NA	NA	NA
pdzph1	3.641835	-0.673833416	1.395366	-0.48291	0.629161	0.922403
USP34	294.9131	-0.278669775	0.211745	-1.31606	0.188154	0.667333
pycr1a	124.5981	-0.883557978	0.301818	-2.92746	0.003417	0.096212

si:dkey-157e10.6	2.3823	0.12385935	1.160101	0.106766	0.914975	0.993066
soul3	651.6265	-0.204858983	0.149091	-1.37406	0.169424	0.640975
si:dkey-1b17.1	24.78071	-0.018371484	0.345287	-0.05321	0.957567	0.999109
tradv40.0	0	NA	NA	NA	NA	NA
RSPH1	74.40119	-0.04019339	0.197715	-0.20329	0.838909	0.976417
si:dkey-179k24.5	4.811468	-1.288426398	1.037847	-1.24144	0.214443	0.69658
trim35-22	0.223191	2.43638772	5.206324	0.467967	0.639808	NA
smarcal1	60.76697	0.188775717	0.319632	0.590603	0.554786	0.89855
isg20l2	117.5199	-0.182475893	0.19553	-0.93324	0.350697	0.802215
znf750	51.60586	-0.215396104	0.338984	-0.63542	0.525157	0.888928
si:dkey-203a12.2	13.67056	0	1.763	0	1	1
coro6	118.3135	0.606004664	0.195601	3.098161	0.001947	0.068449
si:ch211-229l10.9	0.977142	1.507659843	2.13444	0.706349	0.479971	NA
aaas	305.3289	-0.043111347	0.150423	-0.2866	0.774418	0.960818
lama1	167.9993	0.299670373	0.255591	1.172461	0.241012	0.722599
CR388413.3	0	NA	NA	NA	NA	NA
zmiz1b	71.31728	-0.462362691	0.228159	-2.0265	0.042714	0.362408
CR391937.1	0.440438	0	4.176919	0	1	NA
slco1f1	0	NA	NA	NA	NA	NA
CABZ01056321.1	86.35744	0.683517141	0.599573	1.140006	0.254284	0.733353
eef1da	9755.306	-0.378637604	0.117609	-3.21946	0.001284	0.052391
CU013526.1	0.094679	0	5.267649	0	1	NA
fbxo11a	730.389	0.094972206	0.122858	0.773024	0.439508	0.852079
dnaja3b	160.3492	0.022708151	0.159867	0.142044	0.887045	0.987739
zgc:175177	4.618451	0.380224167	0.906239	0.419563	0.674805	0.93501
CU929307.1	0.07132	0	5.267649	0	1	NA
cxcl8b.1	26.23257	-1.695261545	0.750927	-2.25756	0.023973	0.274302
ca9	23.10266	-0.166294191	0.419533	-0.39638	0.691825	0.940274
CR855379.3	1.176787	0	4.744846	0	1	NA
trim35-21	1.536191	0.607716274	1.749648	0.347336	0.728339	NA
svbp	215.3806	-0.142452177	0.202225	-0.70443	0.481168	0.870445
taf2	415.08	-0.000973058	0.112647	-0.00864	0.993108	1
ttc39c	346.1766	0.374599806	0.149589	2.504198	0.012273	0.192528
si:ch211-156l18.8	12.01866	0.613586372	0.634463	0.967095	0.333496	0.790659
scn5lab	13.12441	1.464916943	0.545559	2.685166	0.007249	0.144427
si:dkey-237m9.2	0.767505	-2.985691181	2.534754	-1.1779	0.238836	NA
rpl26	4.293385	-5.034131219	1.614265	-3.11853	0.001818	0.06578
il6	3.172813	-1.761135299	1.8051	-0.97564	0.329241	0.787409
si:dkey-196h17.9	1.834766	-0.880183414	1.595614	-0.55163	0.581204	NA
mob1a	641.8113	0.016198359	0.119471	0.135584	0.89215	0.988566
si:ch73-299h12.1	51.3855	-0.005293677	0.348948	-0.01517	0.987896	1
ehmt2	257.3303	-0.020255367	0.139196	-0.14552	0.884303	0.987047
raf1a	0	NA	NA	NA	NA	NA
or106-11	5.240533	-0.446610344	0.904368	-0.49384	0.621421	0.918461
spint1a	1209.31	0.14730196	0.125286	1.175729	0.239703	0.72026
fcf1	402.1558	-0.030146781	0.154344	-0.19532	0.845141	0.976791
SH3RF3	18.15725	-0.173100367	0.477121	-0.3628	0.716753	0.94627
ano1	135.3831	-0.211642449	0.188692	-1.12163	0.262021	0.739695
SLC5A7	8.136633	1.678517834	0.925361	1.813907	0.069692	0.450548
ptar1	152.911	-0.067185219	0.169447	-0.3965	0.691738	0.940256
si:dkey-5n7.2	4.551682	1.162485339	0.877672	1.32451	0.185334	0.663092
ptn	644.0695	0.26532785	0.158308	1.676025	0.093733	0.511703
sugct	21.27751	0.582316711	0.4721	1.233461	0.217404	0.69815
CR925800.1	0	NA	NA	NA	NA	NA

si:dkey-243k1.3	4.202483	-0.241797067	1.20939	-0.19993	0.841533	0.97643
cep55l	149.6571	0.523185196	0.279125	1.874379	0.060878	0.423588
ddx47	525.198	-0.001593508	0.199644	-0.00798	0.993632	1
scp2b	565.7741	0.411198314	0.15834	2.596932	0.009406	0.166683
CR854899.1	0	NA	NA	NA	NA	NA
si:dkey-261o4.9	0.323087	-2.792020934	4.401559	-0.63433	0.525868	NA
si:ch211-11p18.6	0.358742	2.611181782	3.732765	0.69953	0.484221	NA
slc25a12	1447.844	0.139513849	0.12336	1.130951	0.258076	0.735569
si:ch73-138e16.4	35.91569	0.074321952	0.32922	0.225752	0.821395	0.971788
si:dkey-202l22.6	394.4339	-0.599064632	0.513743	-1.16608	0.243583	0.724437
eloa	981.8214	-0.059988653	0.129848	-0.46199	0.644088	0.926408
micall2a	99.31562	-0.093776695	0.246886	-0.37984	0.704066	0.943735
ndfip1l	496.5296	0.056398862	0.107839	0.522993	0.600979	0.913756
trim35-23	4.150324	-0.932405754	1.520295	-0.61331	0.539674	0.893049
znf1094	7.82596	-0.150633811	0.679294	-0.22175	0.824508	0.972227
CU570769.1	69.34573	-0.2515213	0.244433	-1.029	0.303481	0.769363
col4a3bpb	440.3257	-0.157292153	0.132008	-1.19154	0.233443	0.714569
si:dkeyp-13d12.20	0.207272	0	5.267649	0	1	NA
si:ch211-204c21.1	4.766362	0.950162875	0.880747	1.078814	0.280671	0.753147
slc6a16b	79.42684	-0.255120931	0.201341	-1.26711	0.205117	0.687085
si:zfos-80g12.1	420.8221	0.165819795	0.161113	1.029217	0.303378	0.769363
cnnm4a	7.643626	0.133345353	0.83171	0.160327	0.872624	0.983447
slc44a4	70.88345	-0.179723216	0.256377	-0.70101	0.483296	0.871988
CR774179.2	3.185925	0.870632958	1.351056	0.644409	0.51931	0.887148
slc39a14	19.93456	0.316090953	0.420799	0.751169	0.452551	0.857994
ved	50.07773	-0.327940127	0.323251	-1.01451	0.310342	0.775101
RF00003	0	NA	NA	NA	NA	NA
CU062645.1	1.526934	-0.868139367	2.157044	-0.40247	0.68734	NA
ap1g1	552.4486	0.13674519	0.128582	1.063486	0.287561	0.758397
CU658285.1	0	NA	NA	NA	NA	NA
cnn1b	35.5192	-0.224845322	0.581844	-0.38644	0.699174	0.942499
znf1001	2.046513	1.611581063	1.425475	1.130557	0.258241	NA
fut11	90.24493	0.200620998	0.228024	0.879826	0.378954	0.823145
cry2	411.2715	-0.312872037	0.202617	-1.54415	0.122552	0.569468
si:ch211-165e15.1	80.69774	0.19881173	0.307425	0.6467	0.517826	0.887148
cep97	228.5151	0.079660217	0.18337	0.434424	0.663981	0.93174
traj46	0	NA	NA	NA	NA	NA
CR381540.2	0	NA	NA	NA	NA	NA
CR847850.1	2.718982	-1.458798618	1.679622	-0.86853	0.385105	0.826939
eci2	849.8861	0.25678848	0.190876	1.345318	0.178522	0.652732
myhz1.1	1975.046	-0.497777001	0.307909	-1.61664	0.105957	0.534956
scin1a	3444.967	0.164424367	0.159684	1.029683	0.303159	0.769249
psmd7	737.482	0.064892786	0.1421	0.45667	0.647908	0.927241
enc2	137.0509	-0.114678125	0.244179	-0.46965	0.638607	0.924779
azi2	98.46153	-0.623448584	0.27109	-2.29978	0.021461	0.259793
RF00001	0	NA	NA	NA	NA	NA
si:ch211-114m9.1	14.23119	0.250558609	0.47091	0.532073	0.594675	0.910869
zgc:165519	2.557306	-1.231818007	1.239352	-0.99392	0.320261	0.780807
BX088544.1	24.45245	-0.138872832	0.442043	-0.31416	0.753399	0.955861
opn6a	104.219	-0.462932497	0.352464	-1.31342	0.189043	0.668516
CT027744.2	15.75812	0.588621569	0.589518	0.998479	0.318047	0.779872
si:dkey-261j11.3	1.168517	-0.552663745	1.743372	-0.31701	0.751237	NA
sytl4	110.6926	-0.079217236	0.215974	-0.36679	0.713776	0.945821
si:dkey-13m1.2	1.159549	0.093653455	2.021622	0.046326	0.963051	NA

plekhf1	300.1139	0.840925615	0.281623	2.986002	0.002827	0.087379
si:ch211-194b1.1	43.26518	0.737884628	0.32406	2.277	0.022786	0.266874
CABZ01080434.1	3.825458	2.209128665	1.007007	2.193756	0.028253	0.298982
ctnna1	1590.959	0.120597849	0.121792	0.990197	0.322078	0.782237
folr	64.37218	-1.357514966	0.623248	-2.17813	0.029396	0.304886
shank2a	92.46994	-0.230762325	0.527808	-0.43721	0.66196	0.930742
si:dkey-22o22.2	163.5502	0.349684793	0.223346	1.565662	0.117428	0.558915
BX511025.1	0.14264	0	5.267649	0	1	NA
gdpd4a	33.94387	-0.504983644	0.344677	-1.46509	0.142895	0.602002
BX322657.1	3.264055	2.073055171	1.27194	1.629837	0.103136	0.530217
CU571328.1	2.329196	0.049934204	2.024637	0.024663	0.980324	1
eif3ha	3076.71	-0.253978029	0.138732	-1.83071	0.067144	0.442345
slc1a2b	4969.019	0.186808056	0.148232	1.260245	0.207581	0.689655
FP016142.6	0.323174	-0.868131879	4.562233	-0.19029	0.849085	NA
znf1133	6.266956	-0.551879441	0.654413	-0.84332	0.39905	0.831196
cfhl4	3089.322	-0.415849012	0.215091	-1.93337	0.053191	0.400175
hip1rb	236.8097	0.180106479	0.157049	1.14682	0.251456	0.731717
olfcg5	0.116481	0	5.267649	0	1	NA
si:ch73-170d6.4	1.024549	-0.868142488	2.105992	-0.41223	0.680174	NA
CR354372.1	46.61417	0.429976568	0.357799	1.201726	0.22947	0.711596
BX088535.1	0	NA	NA	NA	NA	NA
cox18	178.1228	0.413324232	0.189716	2.178652	0.029357	0.304886
wnt5b	315.3288	-0.037884356	0.151039	-0.25082	0.80195	0.96782
tradv24.0.1	0.138182	0	5.267649	0	1	NA
si:dkey-71l4.5	6.689804	0.313398648	0.878462	0.356758	0.721273	0.947343
mpp7a	292.4088	-0.094310205	0.115981	-0.81315	0.416131	0.839647
trak2	154.1278	0.222635319	0.202851	1.097534	0.272408	0.748416
btbd17b	55.57407	0.416273768	0.447202	0.930841	0.351936	0.802591
zgc:171673	98.36608	-0.762143663	0.455035	-1.67491	0.093951	0.511719
dusp16	647.8831	0.001115273	0.169741	0.00657	0.994758	1
si:dkey-3p4.1	6.761101	-0.173653585	1.086326	-0.15985	0.872996	0.983537
stard4	138.4718	0.846121	0.566732	1.492983	0.135442	0.590943
ADAMTS7	47.96719	0.775102998	0.285109	2.718619	0.006556	0.136957
BX005417.2	4.192209	-0.25217527	0.994019	-0.25369	0.799733	0.967206
zgc:165573	479.7905	-0.09032826	0.135469	-0.66678	0.50491	0.88195
numa1	376.7973	0.202012199	0.298026	0.677835	0.497876	0.878926
si:ch73-359m17.11	0.730431	-3.103841716	2.324928	-1.33503	0.181867	NA
FO704745.1	0.425587	0	4.226899	0	1	NA
asb2b	1067.801	0.339746033	0.231129	1.46994	0.141578	0.600284
gpr142	6.177878	-0.054839233	0.810342	-0.06767	0.946045	0.997371
ticam1	114.7093	-0.348751672	0.206177	-1.69152	0.090738	0.505206
CR847898.4	4.491511	-0.141554939	0.823164	-0.17196	0.863466	0.981766
BX511227.1	1.616057	-0.780472476	1.226993	-0.63609	0.524721	NA
hoxa5a	70.06912	0.479616528	0.222513	2.15545	0.031127	0.313664
lrp2a	449.1108	0.101046521	0.186391	0.542121	0.587735	0.907608
cript	600.7284	-0.184622879	0.152692	-1.20912	0.226616	0.707798
kdm8	51.58185	-0.178249178	0.261921	-0.68054	0.49616	0.878095
FO704822.1	11.68183	0.926782982	0.51373	1.804029	0.071227	0.455342
BX323596.2	4.82067	0	1.917592	0	1	1
CR847504.1	0.332581	0.093659852	5.267649	0.01778	0.985814	NA
crtac1b	14.478	-0.313304815	0.5906	-0.53049	0.595775	0.911427
CR855206.2	0.049519	0	5.267649	0	1	NA
ccl33.2	0.103751	0	5.267649	0	1	NA
si:dkey-225f23.4	7.879146	0.553235867	0.639808	0.86469	0.387209	0.827592

si:dkey-161l11.74	0	NA	NA	NA	NA	NA
lck	6.710293	-1.412344228	0.858663	-1.64482	0.100008	0.524248
AL935126.1	3.099491	-2.168112574	1.817565	-1.19287	0.232922	0.714492
pimr182	2.022914	0.662347051	1.719991	0.385088	0.700173	NA
anapc11	336.6242	-0.16541532	0.226764	-0.72946	0.465721	0.864997
homer3a	6.035566	1.304382013	0.781467	1.669146	0.095088	0.51461
si:ch73-281i18.4	26.04221	0.342316221	0.371242	0.922083	0.356485	0.807342
si:cabz01007816.2	2.497021	-4.895563789	1.663409	-2.94309	0.00325	0.093414
mccc1	903.992	0.065241602	0.098452	0.662672	0.507541	0.883055
CR450832.1	5.395482	0.346642905	0.782745	0.442855	0.65787	0.930581
si:ch211-268b14.2	14.82379	-0.672363769	0.535808	-1.25486	0.20953	0.691743
CR762475.2	0	NA	NA	NA	NA	NA
AL928951.1	0.364217	2.888615941	3.673224	0.786398	0.431634	NA
cdk11b	806.8123	0.156930287	0.107522	1.459512	0.144424	0.603539
jazf1a	34.48723	0.146053304	0.315374	0.463112	0.643284	0.92593
traj6	0	NA	NA	NA	NA	NA
zgc:112433	25.67271	0.709695336	0.385127	1.842758	0.065364	0.435359
si:dkey-33i11.9	304.8721	0.203831069	0.263068	0.774822	0.438445	0.851685
nfat5b	49.88545	-0.50244013	0.360119	-1.39521	0.162954	0.630534
ftr87	15.2601	0.619115846	0.545464	1.135027	0.256364	0.73401
pde6ha	2870.125	0.480382831	0.31657	1.517462	0.12915	0.578579
si:dkey-16p19.5	6.020006	0.820687226	0.951225	0.862769	0.388265	0.827592
AL645691.1	1.380863	-0.642504848	1.624033	-0.39562	0.692383	NA
RF00001	0	NA	NA	NA	NA	NA
gfra1b	43.291	-0.077787758	0.356901	-0.21795	0.827465	0.973513
vasnb	368.6217	0.020108014	0.132818	0.151395	0.879664	0.986201
fam102aa	49.12734	-0.208281064	0.270369	-0.77036	0.441087	0.852823
si:ch73-106k19.2	3.236246	0.053754058	1.333757	0.040303	0.967852	1
polr3a	173.1709	-0.140996913	0.19932	-0.70739	0.479325	0.86985
CR678435.1	0	NA	NA	NA	NA	NA
metap2b	2643.051	-0.19637668	0.155267	-1.26477	0.205954	0.688314
si:ch211-260e23.9	1660.402	-0.143164091	0.12051	-1.18799	0.234837	0.715533
si:ch73-21d24.1	1.814622	1.941867647	1.49251	1.301075	0.193233	NA
si:ch211-106j21.2	5.163435	-1.906651913	1.335126	-1.42807	0.153272	0.617847
mif4gdb	108.8051	-0.289323534	0.320344	-0.90316	0.366439	0.81589
BX323855.2	0.298807	0	5.267649	0	1	NA
CU571310.1	21.83742	-0.154786115	0.482476	-0.32082	0.74835	0.955074
si:dkeyp-44b5.5	9.085811	0.776506808	0.560052	1.386491	0.165597	0.635141
il4r.1	116.9372	-0.067974148	0.223838	-0.30368	0.761375	0.958159
cpne7	23.66322	0.434105439	0.58415	0.743141	0.457397	0.860993
fam160a1b	42.08346	-0.107763696	0.283108	-0.38065	0.703466	0.943668
zgc:152938	87.90343	-0.623709502	0.229257	-2.72057	0.006517	0.136774
mios	288.3559	-0.018809013	0.128904	-0.14592	0.883988	0.987036
sp100.2	1.907733	0.051033865	1.568673	0.032533	0.974047	NA
polr3h	267.3397	-0.325041642	0.199601	-1.62845	0.103429	0.530538
btr30	32.27979	-0.319070923	0.483839	-0.65946	0.509602	0.883733
tradv17.0	0.145288	0	5.267649	0	1	NA
si:ch211-214b16.2	70.49546	1.07630064	0.338661	3.178105	0.001482	0.057996
zgc:194627	111.5325	0.360613269	0.307633	1.172217	0.24111	0.722648
si:dkey-250k10.1	11.95676	-0.34575272	0.649846	-0.53205	0.594689	0.910869
si:ch1073-392o20.2	599.8037	-0.032930042	0.124385	-0.26474	0.791207	0.964766
si:dkey-15h8.15	72.63166	0.265934758	0.205047	1.296945	0.19465	0.67483
si:ch211-220m17.4	3.643613	0	1.986331	0	1	1
BX323837.1	0.04222	0	5.267649	0	1	NA

CU929054.1	2.649004	-0.913067546	1.239507	-0.73664	0.461343	0.863016
znf1158	61.80642	0.363709367	0.257675	1.411506	0.158095	0.623766
pmpca	553.7351	0.155679401	0.152185	1.022965	0.306325	0.771472
zgc:109986	106.4695	-0.26102132	0.191517	-1.36292	0.172908	0.645664
r3hdm2	52.14279	-0.092467345	0.37779	-0.24476	0.806643	0.969726
stmnd1	2.370969	0.68722519	1.142779	0.601363	0.547598	0.896211
slc15a4	80.23881	-0.157321121	0.281556	-0.55876	0.576329	0.90475
or106-4	0.341576	0.093658844	3.85475	0.024297	0.980616	NA
fra10ac1	100.3585	-0.310360329	0.242164	-1.28161	0.199979	0.6804
si:dkey-3h2.3	4.322234	-0.715327725	1.084911	-0.65934	0.509676	0.883733
timp4.2	19.74657	-2.338310899	1.541331	-1.51707	0.129248	0.578874
utp18	53.29161	0.097919947	0.266837	0.366965	0.713645	0.945821
kif22	544.3176	0.242453692	0.224681	1.079101	0.280543	0.753147
tradv21.0	0	NA	NA	NA	NA	NA
frem2b	460.5403	0.324478588	0.19102	1.69866	0.089383	0.501698
hoxc11b	86.58236	-0.049166465	0.258673	-0.19007	0.849253	0.978102
ubc	19642.97	0.043737694	0.165128	0.264872	0.791108	0.964766
parn	711.3874	0.031851883	0.120085	0.265245	0.790821	0.964766
znf1037	2.574255	-0.774775928	1.005054	-0.77088	0.440778	0.852579
trmt9b	60.95259	0.082895706	0.292625	0.283283	0.77696	0.961073
RF00001	0.129924	0	5.267649	0	1	NA
si:rp71-7119.2	3.835495	-0.57420102	1.068019	-0.53763	0.590831	0.909183
si:dkey-29d5.2	0.455768	-0.868139604	3.603039	-0.24095	0.809597	NA
pdia3	2887.103	0.005027919	0.189946	0.02647	0.978882	1
CR925761.3	23.20072	-0.104101323	0.458598	-0.227	0.820424	0.971788
chmp1a	886.6221	0.083696515	0.142809	0.586071	0.557828	0.899184
BX927193.1	0.156531	-1.826663989	5.230461	-0.34924	0.726912	NA
usp47	763.2399	-0.031707069	0.094952	-0.33393	0.738435	0.952912
BX248331.2	157.5622	0.682037543	0.271645	2.510764	0.012047	0.190742
lman2la	206.3384	0.471257784	0.219573	2.146246	0.031853	0.317164
zgc:174624	55.80777	-0.822357258	0.356826	-2.30464	0.021187	0.258321
si:dkeyp-107f9.2	0.338736	0.960862071	4.474749	0.21473	0.829978	NA
BX511077.1	0.59135	0.370166833	3.3839	0.109391	0.912893	NA
or106-12	0.04222	0	5.267649	0	1	NA
slc16a5b	10.23877	1.94070059	0.876095	2.215172	0.026748	0.290183
or129-1	5.746603	1.395758949	1.005967	1.38748	0.165295	0.634423
dpyda.1	15.55781	-0.294494093	0.526892	-0.55893	0.576212	0.90475
si:dkey-26i24.1	14.20462	-0.642009839	0.434173	-1.4787	0.139222	0.597365
ckap5	1838.199	0.350661998	0.121067	2.896419	0.003774	0.102406
FO818743.1	6.325861	-0.762698045	0.657756	-1.15955	0.246234	0.727532
RF00001	0	NA	NA	NA	NA	NA
znf1181	38.19661	-1.551579978	0.357045	-4.34561	1.39E-05	0.002321
FP074874.1	54.51973	0.051773335	0.306097	0.169141	0.865686	0.982364
CR847844.2	1.938546	1.046754498	1.969842	0.53139	0.595149	NA
CR626941.1	0.890466	1.05542248	2.476864	0.426112	0.670026	NA
lrrc1	283.5728	-0.218645727	0.142602	-1.53326	0.125211	0.572897
CABZ01085139.1	113.8849	0.062364865	0.222752	0.279974	0.779497	0.961073
BX663516.2	11.0309	-2.76146145	1.495875	-1.84605	0.064885	0.434811
rims2b	283.2152	-0.066200362	0.211293	-0.31331	0.754045	0.955912
BX005336.1	1.74669	-0.482730308	1.578272	-0.30586	0.759711	NA
CU570769.2	5.100376	-1.103495756	0.711946	-1.54997	0.121148	0.567072
dnajc11b	170.2719	0.142877445	0.199118	0.717552	0.473034	0.868965
BX005124.3	0	NA	NA	NA	NA	NA
eva1ba	186.817	0.238509001	0.137523	1.734317	0.082862	0.483014

lrrn3b	325.6433	0.231031703	0.200063	1.154794	0.248175	0.728489
CR848025.2	1.638492	-2.974081748	1.637755	-1.81595	0.069378	NA
si:ch1073-376c22.1	62.10258	1.97532264	1.460491	1.352505	0.176214	0.649415
otud6b	230.9225	0.040902112	0.153065	0.26722	0.7893	0.963748
BX511128.3	47.75475	-0.496198487	0.585947	-0.84683	0.397089	0.830253
CR848032.1	7.243897	-2.783497213	1.526933	-1.82293	0.068314	0.445546
CU138533.2	14.22376	0.049310401	0.494692	0.099679	0.920599	0.994085
CR376766.3	0	NA	NA	NA	NA	NA
si:ch211-205a14.1	75.83355	1.028095715	0.770525	1.33428	0.182112	0.658775
si:dkey-13n23.3	0.043309	0	5.267649	0	1	NA
CU469532.1	6.763591	2.782246412	1.377272	2.020114	0.043372	0.365027
AL645691.2	10.62134	0.741212797	0.652222	1.136442	0.255772	0.733642
tmem42a	70.10695	-0.225212411	0.213719	-1.05378	0.291985	0.761655
pcdh2ab3	0	NA	NA	NA	NA	NA
zgc:123284	19.07279	-0.558607318	0.757083	-0.73784	0.460611	0.862369
itih3b	301.6442	-0.288909074	0.407606	-0.7088	0.478451	0.869292
zgc:153039	95.12402	0.078853561	0.230752	0.341724	0.732559	0.950617
CT737184.1	0.65984	0	3.018058	0	1	NA
si:ch73-182e20.4	1.003775	0	5.267649	0	1	NA
CR762480.2	2.180971	0.695794844	1.123675	0.619214	0.535776	NA
SAMD9	0.842521	0.063327414	2.458599	0.025758	0.979451	NA
mapk10	98.62779	0.389965112	0.209887	1.857976	0.063172	0.429313
si:ch73-299h12.2	14.44743	-0.93142403	0.467486	-1.99241	0.046326	0.376018
dre-mir-7147	0.271837	-1.710096158	5.238471	-0.32645	0.744084	NA
wdr48a	88.2062	-0.146754283	0.234238	-0.62652	0.530976	0.890545
sars2	127.6426	0.225454532	0.183954	1.225604	0.220348	0.701707
sumo2b	1626.374	-0.146868206	0.124666	-1.17809	0.23876	0.719781
bmpr1bb	143.5961	0.027086433	0.236514	0.114524	0.908823	0.992033
mgst3a	1122.082	-0.134189821	0.223893	-0.59935	0.548941	0.897091
tmem39a	408.3176	-0.072188585	0.137365	-0.52552	0.599219	0.913175
cdh1	1131.06	-0.000793063	0.134614	-0.00589	0.995299	1
BX663521.1	0	NA	NA	NA	NA	NA
[truncated: 382,622 more chars]
