# Supplementary material for: Mycn regulates intestinal development through ribosomal biogenesis in a zebrafish model of Feingold syndrome 1
Source: PLoS Biol. 2022 Nov 1;20(11):e3001856. doi: 10.1371/journal.pbio.3001856 (PMC9624419; doi:10.1371/journal.pbio.3001856)
Supplement: S4 Table — (PDF) [file pbio.3001856.s011.pdf]

external_gene_name	baseMean	log2FoldChange	lfcSE	stat	pvalue	padj
slc35a5	110.7843772	-0.089832458	0.1935898	-0.464035	0.642623	0.959994
ccdc80	238.4015724	-0.099075092	0.1637249	-0.605131	0.545092	0.945463
nrf1	1098.943144	0.024786055	0.0906902	0.2733047	0.784619	0.97627
ube2h	2872.066754	0.036277141	0.0694333	0.5224745	0.60134	0.956045
slc9a3r1a	578.595402	-0.284462678	0.1065059	-2.670864	0.007566	0.138393
dap	1360.164668	-0.293162476	0.0823357	-3.560574	0.00037	0.013938
itsn1	774.8533231	-0.095268376	0.1074052	-0.887	0.375079	0.894316
myh10	3307.760608	0.068453167	0.0781422	0.8760074	0.381026	0.895192
tmem267	81.46602461	0.131059734	0.2149771	0.609645	0.542097	0.944974
thraa	88.48352469	0.140161917	0.2225947	0.6296731	0.528908	0.942109
itsn2a	366.4213905	0.397075794	0.1190188	3.3362436	0.000849	0.027171
hoxb2a	140.2560912	-0.049079599	0.1803371	-0.272155	0.785503	0.97627
ptpn4b	802.4027832	0.106561404	0.095044	1.1211801	0.262211	0.830166
sema6e	739.9901406	0.100717075	0.092404	1.0899644	0.275729	0.839541
krt97	2059.823256	-0.676745372	0.0924589	-7.319415	2.49E-13	9.66E-11
usp49	401.5704819	-0.029108567	0.1175779	-0.247568	0.804468	0.979705
slc40a1	1183.376349	0.128762988	0.0818194	1.5737467	0.115546	0.637206
dcaf8	401.6524669	0.057448599	0.118443	0.4850316	0.627654	0.957354
cfap300	7.784043597	-0.218924592	0.7252682	-0.301853	0.762764	NA
prkag3b	96.12103045	0.526670851	0.2249433	2.3413495	0.019214	0.259523
triob	864.0831659	-0.019338327	0.1038091	-0.186287	0.852219	0.985174
pde6a	353.3519912	0.278874884	0.1547859	1.801681	0.071596	0.523122
matr3l1.1	1376.713683	-0.084307277	0.0841554	-1.001805	0.316438	0.867129
si:ch73-314g15.3	93.7200388	-0.132475597	0.2153881	-0.615055	0.538518	0.943336
slc39a13	328.5883075	0.003580145	0.1194459	0.0299729	0.976089	0.996762
nitr2b	1.006818213	1.048314579	1.9690503	0.532396	0.594452	NA
cntn2	296.1828382	-0.188920677	0.1313701	-1.43808	0.150411	0.698669
tnni1a	4.397434008	0.02757987	0.9176952	0.0300534	0.976024	NA
pms1	104.97284	-0.108703159	0.1977083	-0.549816	0.582446	0.952117
upf3b	465.065988	-0.043735922	0.1140997	-0.383313	0.701487	0.968134
stx1b	8003.781798	0.064561879	0.074053	0.8718337	0.383299	0.895192
ankar	12.86079713	0.219441444	0.5263733	0.4168932	0.676757	0.965257
ofd1	174.4339804	0.013153708	0.1532147	0.0858515	0.931584	0.992702
asdurf	300.490504	0.115808834	0.1326246	0.8732077	0.38255	0.895192
gtf2e1	376.6806551	0.056242916	0.1150516	0.4888495	0.624948	0.957354
slc1a4	1746.627161	-0.09240065	0.0770089	-1.19987	0.23019	0.797857
ttn.1	13346.33684	0.304783266	0.0762461	3.9973647	6.41E-05	0.003387
znf281a	746.6925073	-0.068077337	0.0922662	-0.737836	0.460614	0.921978
ell	598.5937215	0.100333733	0.0966998	1.0375795	0.299466	0.855609
grasp	107.0856978	0.021736325	0.1909394	0.1138389	0.909366	0.990702
dnah6	78.07753066	0.057905481	0.2589872	0.2235844	0.823081	0.982189
ptpn4a	913.5432219	0.031042228	0.0889276	0.3490731	0.727034	0.970402
opn1mw4	0.474778155	0.852397662	2.898154	0.2941175	0.768668	NA
osgepl1	22.65022457	-0.608842854	0.4360455	-1.396283	0.162629	0.7175
psmb9a	5.887001654	0.632335053	0.7932828	0.7971117	0.425386	NA
creg2	481.9932817	-0.024179885	0.1126938	-0.214563	0.830108	0.982908
sypl2b	175.092547	0.218191829	0.1614916	1.351103	0.176662	0.737285
daxx	319.5469013	0.135330847	0.122562	1.1041824	0.269514	0.834705
slc6a22.1	266.5483062	0.022588176	0.1311058	0.1722896	0.86321	0.985841
lcmt2	43.40201887	-0.063908538	0.2872047	-0.222519	0.82391	0.982196
znf511	41.61059353	-0.001113897	0.3007513	-0.003704	0.997045	0.99906
spi1b	29.56747846	0.372973101	0.3584987	1.040375	0.298166	0.85401
scfd1	936.7749391	0.089621814	0.0921628	0.9724296	0.330837	0.87417

nr4a1	401.3114029	0.372618462	0.1230352	3.0285509	0.002457	0.062216
rassf6	128.4309205	-0.345900203	0.1789822	-1.932596	0.053286	0.452416
telo2	232.5201317	0.041032495	0.1438114	0.2853215	0.775398	0.975687
snx9a	270.3385627	0.092605209	0.1398326	0.6622577	0.507806	0.935734
dstyk	852.8509651	0.006084669	0.0985729	0.0617276	0.95078	0.99381
mapk14a	749.986437	0.073814286	0.0969076	0.7616973	0.446241	0.917154
scrib	2573.037459	0.043702037	0.0767198	0.5696317	0.568928	0.948983
zgc:56699	72.93390087	-0.230119246	0.2300728	-1.000202	0.317213	0.867335
skap2	42.43366628	0.057118343	0.3152409	0.1811895	0.856219	0.985384
unk	114.8647547	-0.060953196	0.1875968	-0.324916	0.745245	0.974094
dzip1	370.3432914	-0.236951074	0.1286091	-1.842412	0.065415	0.500198
ncor2	2700.208108	-0.052613342	0.0716074	-0.734747	0.462494	0.922698
myh9b	3039.369443	-0.101014677	0.1083356	-0.932423	0.351118	0.880473
znf384l	555.6286181	0.005529337	0.1024366	0.0539781	0.956953	0.994543
bysl	562.3259108	-0.050627637	0.1054751	-0.479996	0.63123	0.957356
slc17a6a	44.3201432	0.572425611	0.2986549	1.9166794	0.055279	0.46072
dicer1	1075.463057	0.07079635	0.0994577	0.7118236	0.476574	0.926716
rimbp2	157.1394167	0.191772474	0.1770207	1.0833336	0.27866	0.842672
matr3l1.1	694.6645765	0.00265084	0.1109899	0.0238836	0.980945	0.996944
hsd17b8	124.4266191	-0.123737808	0.1810732	-0.683358	0.494381	0.931926
arl14ep	391.9395496	-0.124131401	0.1150469	-1.078963	0.280604	0.84355
mycbp2	3230.525754	0.051198056	0.0747081	0.6853081	0.49315	0.931926
map4k2	210.3395507	0.2078427	0.1466596	1.4171777	0.156431	0.706639
puf60b	2228.094294	-0.01130198	0.0725392	-0.155805	0.876187	0.987881
srrm1	3340.460728	-0.078067371	0.0942018	-0.828725	0.40726	0.901305
pcgf1	66.98506237	-0.182920997	0.2507305	-0.729552	0.465664	0.923865
gas2a	29.25283633	0.484811658	0.3797375	1.2767022	0.201707	0.767736
psmb8a	1.138501641	-0.373253155	1.7712222	-0.210732	0.833096	NA
g2e3	318.9474736	-0.043687197	0.1345315	-0.324736	0.745381	0.974094
slc6a22.2	97.74071568	0.528032817	0.2203896	2.3959065	0.016579	0.237453
mctp2a	102.4931772	-0.000720927	0.2074903	-0.003475	0.997228	0.999097
actn3b	6620.348673	0.26517875	0.0906117	2.9265402	0.003428	0.078354
slc2a1a	111.8786856	0.858882861	0.1969089	4.3618276	1.29E-05	0.000886
inppl1b	100.2505867	-0.014328779	0.2035146	-0.070407	0.94387	0.993364
mier1b	281.3105896	0.043000165	0.1430582	0.3005782	0.763736	0.975374
adam8a	131.5394087	-0.077055193	0.1830362	-0.420983	0.673767	0.965257
tdh2	1318.217197	0.007618699	0.0813355	0.09367	0.925371	0.992676
mhc1zea	166.8526748	0.032496824	0.1771458	0.1834468	0.854447	0.985174
skap1	13.92563701	1.165861264	0.5225563	2.2310732	0.025676	0.310555
sp3a	138.4706356	0.070632835	0.1748377	0.4039908	0.686219	0.966376
tomm34	313.2013299	0.196752836	0.1285525	1.5305257	0.125887	0.654803
kifc1	897.5802833	-0.110729979	0.0930311	-1.190247	0.233949	0.802633
csmd2	64.30211894	0.292496897	0.2597656	1.1260032	0.260164	0.828288
prf1.3	0.481032184	-2.398473301	2.80721	-0.854398	0.392885	NA
echs1	1062.157946	-0.044472418	0.0837103	-0.531265	0.595235	0.954434
nfyal	230.7591545	-0.251967145	0.156409	-1.61095	0.107191	0.617056
kirrel1a	840.4616487	0.089798797	0.0907743	0.9892536	0.322539	0.870226
psmb13a	1.178070337	0.530310831	1.9330889	0.2743334	0.783828	NA
gpm6bb	702.7035254	0.077508857	0.1022011	0.7583957	0.448214	0.917683
uckl1a	75.72106679	0.39071551	0.2345785	1.6656068	0.095792	0.589171
flot1a	3112.543381	0.012694734	0.0694421	0.1828104	0.854947	0.985174
si:ch211-247n2.1	45.5287406	0.168263293	0.3035107	0.5543899	0.579312	0.951257
oc90	12.87319814	-0.006099792	0.541003	-0.011275	0.991004	0.997679
strn3	1639.417939	0.064096901	0.0743046	0.8626237	0.388344	0.896271

gulp1a	322.5623346	0.057499911	0.1270261	0.4526622	0.650792	0.960708
fkbp8	593.8735953	0.033605942	0.1001333	0.3356121	0.737163	0.972535
noc2l	701.9320025	-0.031924336	0.0990223	-0.322395	0.747153	0.97419
tnxba	1208.657835	0.112284508	0.0946382	1.1864606	0.23544	0.804771
slc29a2	429.1365493	-0.193175891	0.1143131	-1.689885	0.09105	0.578171
ptpra	309.578729	-0.133863824	0.1317783	-1.015826	0.309712	0.86172
otol1a	19.94280981	0.332589742	0.427698	0.7776276	0.436789	0.914408
stim2b	53.43132692	0.223681436	0.271798	0.8229694	0.410525	0.903084
nup155	660.2582362	-0.087951272	0.1158794	-0.75899	0.447859	0.917154
tspan11	81.80578632	0.034733467	0.2212723	0.1569716	0.875267	0.987881
prkacba	75.4426244	0.058756927	0.260303	0.2257252	0.821415	0.981845
irx2a	456.9776852	-0.084886437	0.1054259	-0.805177	0.420718	0.90651
atp5po	6711.565159	0.043561318	0.1009107	0.4316818	0.665973	0.964736
kcnh6a	157.0960653	-0.035822677	0.1637748	-0.218731	0.826859	0.982473
zgc:112148	117.1952421	-0.600638039	0.1923566	-3.122524	0.001793	0.049451
tnfrsf21	603.0250382	0.110467637	0.1120818	0.9855983	0.32433	0.870928
zmp:0000000521	7.880354907	0.446028997	0.6840558	0.652036	0.514378	NA
c3b.2	535.5350531	0.426381207	0.1269911	3.3575677	0.000786	0.025573
cfap43	23.10187312	0.081256691	0.4054669	0.2004028	0.841166	0.985174
zgc:112982	1009.947658	0.000181499	0.1046737	0.0017339	0.998617	0.999375
nalcn	871.2356201	0.029413267	0.0959383	0.3065854	0.759159	0.975374
aff4	194.8252949	0.170056088	0.1545107	1.1006104	0.271066	0.835943
dbx1b	125.3027712	-0.266110243	0.1957281	-1.359591	0.173959	0.733946
atp1a1a.4	6658.899427	-0.490698912	0.0719285	-6.822042	8.98E-12	2.57E-09
phgdh	1272.428888	-0.154074796	0.0801818	-1.921568	0.05466	0.457793
nav2b	702.5894408	-0.253753208	0.0964969	-2.629652	0.008547	0.151445
stx3a	436.9570086	0.0311626	0.1138416	0.2737365	0.784287	0.97627
cacnb3a	9.499928775	0.154693372	0.6081313	0.2543749	0.799206	NA
kbtbd12	414.6719128	0.149594339	0.1164038	1.285133	0.198746	0.763848
ppm1ba	235.6937074	0.131959229	0.1352246	0.975852	0.329138	0.873436
tuba1a	8391.637863	-0.046282812	0.0849219	-0.545005	0.58575	0.952847
stt3b	2093.974105	0.081464951	0.0784557	1.0383564	0.299104	0.855189
syt14b	73.77316123	0.417221299	0.2298062	1.8155357	0.069442	0.514708
man2b1	634.1833849	-0.096394051	0.1055204	-0.913511	0.360974	0.885686
manea	169.2225577	0.150434685	0.1668129	0.9018167	0.367154	0.887458
si:dkey-247m21.3	17.01402108	0.209429984	0.4682502	0.4472608	0.654687	0.961305
vill	221.2488874	-0.148748579	0.1429153	-1.040816	0.297961	0.85401
rorab	2521.960065	0.029034909	0.0800176	0.3628567	0.716712	0.96909
drd4-rs	151.4235636	-0.103245251	0.1673606	-0.616903	0.537299	0.943336
palmda	249.661805	0.239988861	0.1368216	1.754027	0.079426	0.547489
pink1	304.1548956	0.030298401	0.1307498	0.2317281	0.816749	0.981594
klhl15	494.3340476	-0.02742287	0.1035045	-0.264944	0.791053	0.977461
prkag2b	73.70025362	0.072237787	0.2366857	0.3052055	0.76021	0.975374
LO018422.1	68.61364514	0.311377706	0.2607254	1.1942747	0.232371	0.800644
b3gat3	387.2573296	0.139940178	0.1128497	1.2400579	0.214954	0.782493
dnajc1	673.6495071	0.048365918	0.0980931	0.4930614	0.621969	0.957354
ak1	5244.840595	0.101522408	0.0959135	1.0584784	0.289837	0.846246
pfkfb3	1285.663133	-0.109564169	0.1102522	-0.99376	0.32034	0.868202
dock5	20.02823285	-0.2723532	0.4401023	-0.618841	0.536021	0.943336
txlng	351.3955109	-0.164980654	0.1243535	-1.326707	0.184605	0.747376
cthrc1b	65.34068245	0.264514301	0.2487136	1.0635296	0.287542	0.845979
hsd11b2	604.6605456	-0.03497882	0.1049863	-0.333175	0.739002	0.972722
fkbp16	265.7850726	0.060745051	0.1354927	0.4483272	0.653917	0.961214
myhb	3476.077612	0.017573703	0.0793651	0.2214285	0.824759	0.982196

stxbp1a	9751.213735	0.059911238	0.0726769	0.8243505	0.40974	0.902351
adamts18	393.700987	0.010424844	0.1180589	0.0883021	0.929637	0.992702
lmcd1	58.6058179	0.28975283	0.2630477	1.101522	0.27067	0.835351
rxrbb	505.8873758	0.058813212	0.1216342	0.4835253	0.628723	0.957354
grb10a	119.6711323	0.083731998	0.194154	0.4312658	0.666275	0.964736
ppil2	392.9230751	-0.069148022	0.1164876	-0.593609	0.552774	0.947265
rictorb	402.5313488	-0.034990059	0.113506	-0.308266	0.75788	0.975374
pygb	739.0591287	-0.04235201	0.0919165	-0.460766	0.644966	0.96029
qkib	31.5323724	0.089733113	0.34574	0.2595393	0.795219	0.978466
tox4a	413.536087	0.144023341	0.1098621	1.3109462	0.189876	0.754269
pfkfb2b	168.3001269	0.118425923	0.1646392	0.7193059	0.471952	0.924838
brf2	22.59060022	0.687222807	0.4071399	1.687928	0.091425	0.57849
adss	227.4476459	0.497757166	0.1421352	3.5019974	0.000462	0.016755
mnat1	117.4331271	0.109721498	0.2016929	0.5440027	0.58644	0.953319
csnk2a1	2699.372842	-0.04060286	0.0705964	-0.575141	0.565196	0.948739
lamb2	1779.841567	0.232368213	0.0874378	2.6575245	0.007872	0.142411
si:dkeyp-52c3.7	1.675486141	2.010499125	1.6659854	1.2067928	0.227512	NA
stam	1421.810152	0.11794035	0.0793892	1.4855962	0.137386	0.674358
cwf19l1	354.0023131	-0.116694557	0.1193731	-0.977561	0.328291	0.872883
celf2	175.9815985	-0.169360301	0.1598208	-1.059689	0.289286	0.845979
psme1	80.87599158	-0.258307887	0.2211363	-1.168094	0.242769	0.811583
cacnb1	214.4521868	0.246846956	0.1492957	1.6534097	0.098248	0.597502
tra2b	790.0884397	-0.017341314	0.09383	-0.184816	0.853373	0.985174
aplnra	203.934979	-0.15324565	0.1466719	-1.044819	0.296107	0.853273
itfg2	134.2786044	-0.286720732	0.1749659	-1.638724	0.101271	0.604628
igbp1	531.9667527	-0.143400604	0.1120476	-1.279819	0.200609	0.766801
chmp2bb	1082.678112	-0.037782849	0.0968949	-0.389936	0.696584	0.96736
aspn	490.8816744	0.395264233	0.1133169	3.4881307	0.000486	0.01747
rho	14675.76992	0.336027253	0.2025155	1.6592667	0.097062	0.593844
rhd	23.845769	0.447160754	0.3893198	1.1485692	0.250734	0.820469
bach1b	626.2581394	0.056490147	0.1059024	0.5334171	0.593745	0.954434
pygl	1557.845007	0.379684415	0.0788617	4.8145634	1.48E-06	0.000139
hspb11	150.1213509	-0.351011432	0.1766199	-1.987383	0.04688	0.425112
stk25a	324.3293996	-0.157247744	0.1298146	-1.211326	0.22577	0.793245
invs	58.47573931	-0.057765724	0.2674147	-0.216016	0.828976	0.982721
rtca	487.4794605	-0.072171739	0.1086332	-0.664361	0.506459	0.935724
tbx3a	633.3176694	-0.071106603	0.1014614	-0.700824	0.483413	0.929461
scocb	1339.808851	0.046090598	0.0817283	0.5639492	0.572789	0.949717
si:dkey-103j14.5	37.81463232	-1.066487374	0.3282915	-3.248599	0.00116	0.034642
zgc:103692	232.8413972	0.007645912	0.1466374	0.0521416	0.958416	0.994893
sypb	4911.215393	0.102810473	0.08131	1.2644259	0.206077	0.773826
plpp2a	18.72329438	0.076417033	0.4542098	0.1682417	0.866393	0.986673
mmp14a	1333.127059	0.13684144	0.0841642	1.6258865	0.103974	0.610807
psmb6	1122.008042	-0.163428044	0.106559	-1.533686	0.125107	0.654648
kcna2a	8.309041893	0.239449177	0.6544951	0.3658533	0.714475	NA
tbxas1	246.2379656	-0.134852069	0.1482079	-0.909885	0.362883	0.885944
epb41l3a	1375.359136	0.078582266	0.0925788	0.8488152	0.395984	0.899505
ca15c	22.04917881	1.301128248	0.4605642	2.8250744	0.004727	0.099016
mrpl41	369.5774427	0.168863883	0.1247829	1.3532609	0.175972	0.736505
dcaf13	566.2713902	-0.036776475	0.0980409	-0.375114	0.707576	0.968664
zfand5b	1729.01347	-0.09155009	0.093864	-0.975348	0.329388	0.873436
mcoln1a	418.5047083	-0.064049927	0.110742	-0.578371	0.563014	0.948728
si:ch211-197g15.6	33.48565203	0.52517006	0.3670214	1.4308975	0.15246	0.70162
si:dkey-21p1.3	20.99644497	0.486381365	0.4313679	1.1275325	0.259517	0.827693

ankrd22	82.18190749	0.410916848	0.2562719	1.6034412	0.108837	0.621829
arsh	59.37291573	0.394324264	0.2649485	1.4883052	0.13667	0.673303
p2rx2	72.10378835	-0.343210636	0.2294253	-1.495958	0.134664	0.670689
gins2	118.3259788	0.110825399	0.2002915	0.5533204	0.580044	0.951257
me3	1927.457073	0.096426386	0.0790379	1.2200015	0.222464	0.788748
fabp11b	161.5706302	-0.779254154	0.1698882	-4.586864	4.50E-06	0.000369
lhx8a	115.1059038	0.041975945	0.1914187	0.2192887	0.826425	0.982196
frmd6	373.1063361	-0.022707706	0.1167502	-0.194498	0.845786	0.985174
fam102bb	140.1249383	-0.207042871	0.1731005	-1.196085	0.231663	0.799752
dlc	859.9822345	-0.054326405	0.0912774	-0.595179	0.551724	0.947156
sgf29	239.8234937	-0.212340086	0.1485159	-1.429747	0.15279	0.701776
tubb4b	19425.59572	-0.019210827	0.1075057	-0.178696	0.858177	0.985773
cyp11a1	5.562617533	-0.233442977	0.8435458	-0.27674	0.78198	NA
tagapa	38.43241389	0.066445869	0.3255899	0.2040784	0.838292	0.984797
shpk	20.49459969	-0.333108854	0.4230367	-0.787423	0.431034	0.91234
ubald1a	758.2223526	-0.068008729	0.0932818	-0.729068	0.46596	0.923865
cers5	602.0443148	-0.375873039	0.1088547	-3.45298	0.000554	0.019228
UBB	2964.169296	0.144897558	0.0770756	1.8799404	0.060116	0.478687
mief2	313.7822661	0.029187709	0.1257489	0.2321111	0.816452	0.981594
atm	257.6160605	-0.128380925	0.1357313	-0.945846	0.344227	0.878434
tlcd1	137.2218255	-0.053429018	0.1748785	-0.305521	0.75997	0.975374
ugt5d1	94.7968435	-0.418032956	0.2044797	-2.044374	0.040917	0.394957
cry-dash	865.022848	0.16191616	0.0968732	1.6714243	0.094638	0.585169
gale	454.1667052	-0.185245529	0.1067882	-1.7347	0.082794	0.554784
ercc3	440.8344852	-0.058638635	0.1121712	-0.52276	0.601141	0.956045
nusap1	664.3622683	-0.06179984	0.100275	-0.616304	0.537694	0.943336
si:ch211-225b11.1	474.6080237	0.033431302	0.1403776	0.2381528	0.811763	0.981081
zmynd10	27.97061365	0.106170334	0.3851015	0.2756944	0.782783	0.97627
pde4cb	45.3205063	-0.196580309	0.2916169	-0.674105	0.500245	0.934224
prdm1a	328.657408	-0.266817807	0.1231235	-2.167075	0.030229	0.336193
arhgef37	124.4772892	0.104977587	0.2142141	0.4900592	0.624092	0.957354
rdh14b	300.1577982	0.011113456	0.1273867	0.0872419	0.930479	0.992702
ercc6l	270.3157055	-0.094632922	0.132556	-0.713909	0.475283	0.925754
wnt7ab	3.000751058	1.03490228	1.1903426	0.8694155	0.38462	NA
noc3l	444.3874664	0.056910013	0.1128844	0.5041443	0.61416	0.95688
itgb6	315.2464051	-0.110945833	0.1273723	-0.871036	0.383735	0.895192
zgc:92818	492.6289311	0.038144265	0.1138746	0.3349672	0.73765	0.972535
itga10	401.1101831	-0.075175727	0.1199624	-0.626661	0.530882	0.942209
ankrd33ab	10.83254859	0.50741217	0.5955521	0.852003	0.394212	NA
zgc:153911	38.25335488	-0.155663195	0.3926668	-0.396426	0.691791	0.967305
dmbx1b	45.48537184	-0.116447934	0.2831828	-0.411211	0.680918	0.965476
hsdl2	1934.177222	-0.026571638	0.0791912	-0.335538	0.737219	0.972535
adora2b	12.80861116	0.196006934	0.5253285	0.3731131	0.709064	0.968664
tm9sf4	1130.78324	0.042604726	0.0834269	0.5106834	0.609573	0.956718
smu1a	941.5954464	-0.071307352	0.085839	-0.83071	0.406138	0.901305
rundc3ab	1171.922262	-0.073866278	0.0814094	-0.907344	0.364225	0.886494
eif3eb	252.023594	-0.000276353	0.1447635	-0.001909	0.998477	0.999364
mapkapk2a	878.3955815	0.054553918	0.0996104	0.5476727	0.583917	0.952214
syngr1a	575.6177334	0.065085143	0.1021078	0.6374162	0.523854	0.939336
tmod2	43.80321449	0.250330994	0.2933289	0.853414	0.39343	0.898076
cabp5a	101.3826759	0.483775033	0.2040469	2.3709015	0.017745	0.246928
tbx15	304.2748962	0.126338077	0.1338716	0.9437255	0.34531	0.878434
dpysl3	5245.135785	0.03983175	0.0671757	0.5929486	0.553216	0.947265
mylpfb	15592.24733	0.29530827	0.0846403	3.4889808	0.000485	0.017464

ruvbl1	1043.362705	-0.068041975	0.0875747	-0.776959	0.437183	0.914408
slc45a2	353.6672933	-0.377432194	0.145228	-2.598895	0.009352	0.162082
pabpc1l	0.341366344	1.891903201	3.2128156	0.5888614	0.555954	NA
pcsk1	26.28032607	0.228676326	0.3853649	0.5934021	0.552912	0.947265
irx7	155.7978676	-0.238464846	0.171785	-1.388159	0.165089	0.721223
unm_sa1614	126.319167	-0.033394282	0.1751148	-0.190699	0.848761	0.985174
rnf145a	328.1550519	0.193539499	0.1278439	1.5138733	0.130058	0.662656
rpa3	230.7344448	-0.074376092	0.1541814	-0.482394	0.629526	0.957354
anxa11b	2180.879495	-0.018344884	0.0718095	-0.255466	0.798363	0.978884
b4galt1	161.9859719	0.050479762	0.1677444	0.3009327	0.763466	0.975374
zgc:85722	798.9130635	0.119085435	0.0887871	1.3412471	0.17984	0.741062
sh3gl1b	404.3696953	0.105812648	0.1204536	0.8784516	0.379699	0.895192
rgs5a	731.4905637	0.064648478	0.0952336	0.6788408	0.497239	0.93322
stxbp5a	573.8192484	-0.012146297	0.1066924	-0.113844	0.909361	0.990702
mapre1b	3578.592917	0.096270789	0.0807221	1.1926194	0.233018	0.80134
optn	432.0882869	-0.035390907	0.1158894	-0.305385	0.760073	0.975374
kcnj1b	48.26354606	0.0714043	0.2778685	0.2569716	0.797201	0.978577
ATG14	505.380216	-0.089485911	0.1049451	-0.852692	0.39383	0.898496
tbce1a	439.0499009	-0.251839583	0.1119249	-2.250076	0.024444	0.3019
fam113	0	NA	NA	NA	NA	NA
rabif	266.6735537	0.029535892	0.1372892	0.2151363	0.829661	0.982805
gnb3b	12793.44419	0.272394714	0.0821818	3.3145366	0.000918	0.029089
rasl12	30.12937291	0.411317221	0.3468732	1.1857857	0.235707	0.804824
her11	7.079546724	0.164783122	0.7547281	0.2183344	0.827169	NA
ncl	11731.72513	-0.112961986	0.0682945	-1.654042	0.098119	0.59749
utp15	371.1609399	-0.007385852	0.1222376	-0.060422	0.951819	0.993864
ankrd2	0.325573202	0.005883621	3.3172783	0.0017736	0.998585	NA
kiss1ra	4.707980623	0.850762253	0.9189779	0.92577	0.354565	NA
sdc2	1212.677141	0.090929631	0.0862459	1.0543061	0.291743	0.847498
spon2b	282.4570924	0.049015299	0.142721	0.3434343	0.731272	0.971557
tdh	3940.034196	0.192075544	0.0768412	2.4996438	0.012432	0.197293
sema6d	385.2735759	-0.310217589	0.114393	-2.711858	0.006691	0.126122
phf20a	250.398233	0.003489467	0.1370565	0.0254601	0.979688	0.99687
pdcd2l	58.90419741	-0.127654406	0.2602922	-0.490427	0.623832	0.957354
dedd1	1192.352643	-0.028228501	0.0842531	-0.335044	0.737592	0.972535
gdf5	59.96744433	-0.047846277	0.2542517	-0.188185	0.850732	0.985174
miox	202.9140373	0.135286239	0.1627616	0.8311928	0.405865	0.901305
pvalb2	72004.53101	0.180503075	0.0992211	1.8192011	0.068881	0.513259
si:ch211-48m9.1	226.1946382	0.158327356	0.1541027	1.0274146	0.304225	0.858657
pdx1	92.72568519	-0.712465084	0.2081225	-3.423296	0.000619	0.020853
tle3a	1543.328091	-0.017323537	0.0918215	-0.188665	0.850355	0.985174
ap2m1a	6193.741383	-0.028193764	0.080226	-0.351429	0.725266	0.970402
atp1a1a.1	9372.332129	0.042079895	0.0702513	0.5989906	0.549179	0.946312
arcn1a	1340.166032	0.016049609	0.0853012	0.1881523	0.850757	0.985174
meis3	599.3057547	-0.034147564	0.0969125	-0.352355	0.724572	0.970402
BX908804.1	0.323844819	-1.825095364	3.274639	-0.557342	0.577293	NA
cog8	155.2118954	-0.132681604	0.1680626	-0.789477	0.429833	0.911615
rasgrf2b	12.75425079	1.325274695	0.55017	2.4088459	0.016003	0.233194
cep57l1	170.9396726	0.866815006	0.1691414	5.1247935	2.98E-07	3.57E-05
trmt2a	178.7941446	-0.016107197	0.1611439	-0.099955	0.92038	0.992049
col4a4	134.6111826	0.034550377	0.1756024	0.1967535	0.84402	0.985174
si:dkey-28b4.8	2024.461674	0.119736729	0.0870935	1.3748063	0.169191	0.726198
fndc1	91.71523874	0.299874087	0.2113402	1.4189167	0.155923	0.705963
zgc:113210	197.5781653	-0.115389532	0.1508601	-0.764878	0.444344	0.916726

reps1	764.6306361	-0.086729188	0.0975409	-0.889157	0.373919	0.893677
vps50	395.9307799	0.002435222	0.1244074	0.0195746	0.984383	0.996944
aven	198.0586592	0.010720326	0.1525752	0.0702626	0.943985	0.993364
si:ch211-160o17.2	393.8124136	0.295234264	0.1252089	2.3579329	0.018377	0.25227
tjp3	505.0544869	-0.299719003	0.1319462	-2.271524	0.023115	0.291648
adipor1a	1460.879893	0.103237539	0.084287	1.2248333	0.220638	0.787641
sec31b	117.1117	-0.195765321	0.1984869	-0.986288	0.323992	0.870928
gls2b	59.71353971	0.150586332	0.2853218	0.5277772	0.597654	0.95534
gbx2	442.893434	-0.108159894	0.1077696	-1.003622	0.315561	0.866773
meis1a	596.850293	-0.145829214	0.0961656	-1.516438	0.129409	0.661221
bgnb	268.6157037	0.223360824	0.1513908	1.4753924	0.140107	0.679809
abcd3b	213.3866056	0.11298964	0.1426437	0.7921109	0.428296	0.911082
ppp6c	1759.358966	-0.079199111	0.0798824	-0.991447	0.321467	0.869201
smo	522.7446843	0.020762788	0.1028836	0.2018085	0.840066	0.985029
si:dkey-32n7.4	18.91687221	0.583680407	0.4510785	1.2939665	0.195677	0.761077
gpr34b	0.158915748	0.967652056	4.0804729	0.2371421	0.812547	NA
pdap1b	376.7657982	0.042854661	0.1235649	0.3468191	0.728727	0.970631
a1cf	337.7298275	-0.570885846	0.1300183	-4.390812	1.13E-05	0.000809
ss18	1416.182799	-0.102564865	0.0771194	-1.329949	0.183535	0.747284
phc1	295.8510509	-0.114880728	0.1293011	-0.888474	0.374286	0.893914
gda	77.07080119	0.011302934	0.2262775	0.0499516	0.960161	0.995388
tnnt2d	730.3799624	0.311124734	0.1051187	2.959748	0.003079	0.073439
cep135	221.1679905	-0.298974828	0.1425346	-2.09756	0.035944	0.369801
itpkca	715.5833411	-0.080116309	0.0945771	-0.8471	0.396939	0.899672
prkcea	996.8822163	0.058996837	0.0891735	0.6615964	0.50823	0.935735
ube2al	950.2916722	0.135423386	0.0867717	1.5606868	0.118598	0.642318
lrrc4.2	264.4487876	0.166540464	0.1312568	1.2688136	0.204508	0.772352
hdac8	260.7731905	-0.07106329	0.1313016	-0.541222	0.588355	0.953319
limk1b	125.3806642	-0.08325514	0.1846087	-0.450982	0.652003	0.960708
scnm1	82.69278278	-0.332246041	0.2214482	-1.500333	0.133528	0.667982
eif4a1b	9020.002017	-0.126946642	0.0793407	-1.60002	0.109594	0.623766
hspa9	5375.623417	-0.005043985	0.0632412	-0.079758	0.93643	0.992702
CABZ01111496.1	6.43614444	0.085850899	0.7365922	0.1165515	0.907216	NA
sorbs2a	131.0434294	-0.10265721	0.1732129	-0.592665	0.553405	0.947265
tacr3l	33.23832108	-0.33364283	0.3386957	-0.985081	0.324584	0.871063
fgfr1op	439.1485072	0.055402509	0.1101311	0.5030597	0.614922	0.95688
cd276	449.647135	0.122872433	0.108611	1.1313072	0.257926	0.825413
gorasp1a	826.6773227	0.030577858	0.0931459	0.3282791	0.742701	0.973303
btbd7	39.3244835	0.32593308	0.3398383	0.9590828	0.337517	0.876395
gpx9	282.4055221	0.118391159	0.1343107	0.8814723	0.378062	0.895192
mybphb	7352.719449	0.35616089	0.2047694	1.7393266	0.081977	0.552725
spire2	117.0321659	0.359928339	0.1844926	1.9509097	0.051068	0.443485
stambpl1	233.4761521	0.185369941	0.1441361	1.2860755	0.198417	0.763597
ubxn6	418.908505	0.105237669	0.1246762	0.8440877	0.39862	0.900359
or103-4	0	NA	NA	NA	NA	NA
oclnb	421.2812558	-0.061789879	0.1206565	-0.512114	0.608571	0.956718
kdm5bb	4824.123038	-0.159775278	0.0660846	-2.417739	0.015617	0.229092
rcan1a	1036.769444	0.138664719	0.0893238	1.5523827	0.120571	0.645578
ada	1019.546673	-0.335195827	0.089559	-3.742736	0.000182	0.007911
zgc:123105	1775.14999	-0.005839294	0.075772	-0.077064	0.938573	0.992849
apip	522.1147793	-0.111610557	0.1211583	-0.921196	0.356948	0.883467
dachc	387.6248971	-0.138933013	0.1160383	-1.197304	0.231188	0.799444
pxmp2	86.69983432	0.165778188	0.2224158	0.7453525	0.456059	0.921215
si:dkey-27m7.4	34.68774013	0.223936359	0.3379831	0.6625668	0.507608	0.935734

skp1	4767.322085	-0.039000104	0.0821499	-0.474743	0.63497	0.957881
nudt21	797.0040948	-0.042092737	0.0913397	-0.460837	0.644915	0.96029
nfkbil1	158.4170041	-0.015858702	0.1621029	-0.097831	0.922066	0.992269
nr2f6b	694.9655613	-0.039055911	0.0957642	-0.407834	0.683396	0.965476
mlst8	267.5256762	0.087937648	0.1439939	0.6107041	0.541395	0.944954
magi1b	2487.567975	-0.038502688	0.0760994	-0.505953	0.61289	0.95688
stx5al	403.4819898	-0.101113515	0.1173894	-0.861352	0.389044	0.896303
sult1st4	105.2791378	-0.526601197	0.2017992	-2.609531	0.009067	0.159222
psmd1	2847.542995	-0.106813702	0.1034734	-1.032282	0.30194	0.856944
rassf7b	180.8551406	0.076160218	0.153067	0.4975612	0.618793	0.957354
wdr21	40.44436524	-0.857534039	0.3154826	-2.718166	0.006564	0.124228
foxm1	196.7198251	-0.045266204	0.1500787	-0.301616	0.762944	0.975374
rhcga	851.0714139	0.781913195	0.1354148	5.7742098	7.73E-09	1.34E-06
ngly1	436.6868466	0.140333265	0.1177314	1.1919787	0.23327	0.801695
chchd6a	346.4200813	-0.291095131	0.1200727	-2.424324	0.015337	0.22717
mterf3	143.7723433	0.148727249	0.1795052	0.8285402	0.407365	0.901305
pomk	212.1916368	-0.083518115	0.1469589	-0.568309	0.569825	0.948983
ano2a	182.2701751	0.001604712	0.1545018	0.0103864	0.991713	0.997679
naa15a	1451.044306	-0.002182001	0.0851299	-0.025631	0.979551	0.996866
anxa2a	992.3531469	-0.023623445	0.0960064	-0.246061	0.805635	0.979893
urb2	269.2606644	0.19190363	0.1335915	1.4364956	0.150861	0.698912
bin2a	735.456157	-0.581592418	0.09661	-6.020002	1.74E-09	3.38E-07
gdf9	2.29457288	-3.568136068	1.6189121	-2.204033	0.027522	NA
nup88	621.8757339	-0.028392968	0.0976582	-0.290738	0.771252	0.975687
gbp3	61.25560459	0.103274559	0.2652826	0.3893001	0.697054	0.96736
pik3cd	121.7699962	0.448490682	0.1882332	2.3826331	0.017189	0.242176
zbtb22b	44.39068615	-0.233969689	0.3055886	-0.765636	0.443893	0.916631
zgc:101559	173.6270324	0.200813609	0.1552529	1.2934609	0.195852	0.76113
loxa	90.89855377	0.185962103	0.2039892	0.911627	0.361965	0.885944
clcn3	1442.617062	0.094634957	0.0775328	1.2205802	0.222245	0.788551
dhps	354.5750985	-0.063292467	0.1220299	-0.518664	0.603995	0.956467
pik3ip1	1094.162703	-0.13656601	0.0884626	-1.543771	0.122644	0.650831
wdr12	355.2019886	-0.209630361	0.1234558	-1.698019	0.089504	0.572876
dab1b	31.76303633	-0.193063433	0.3599816	-0.536315	0.591741	0.954434
sox9a	758.9273123	-0.188252618	0.1060336	-1.775406	0.075831	0.536487
stc1	0.679983498	-1.527277543	2.5551009	-0.597737	0.550016	NA
stx16	631.09439	0.059944278	0.0980132	0.6115939	0.540806	0.944633
emc2	1782.805289	-0.041189175	0.0748606	-0.550212	0.582174	0.952028
pank2	502.9679591	-0.05352427	0.1117046	-0.479159	0.631826	0.957356
eps8l1b	156.130968	-0.38115098	0.1723299	-2.211752	0.026984	0.316663
fam91a1	876.7532029	0.039935315	0.0937399	0.4260225	0.670091	0.965223
cacng5a	98.82808754	-0.172034488	0.2104336	-0.817524	0.413629	0.905864
slf2	183.1291635	-0.180958272	0.1591622	-1.136942	0.255562	0.824118
trpc2b	17.80182988	0.0510655	0.44696	0.1142507	0.909039	0.990702
commd2	301.057066	-0.186924691	0.1295746	-1.442603	0.149132	0.696454
gng12a	360.3828042	-0.123683765	0.1207568	-1.024238	0.305723	0.860027
si:dkey-275b16.2	0	NA	NA	NA	NA	NA
si:ch211-266g18.6	178.677939	-0.219080504	0.1578217	-1.388152	0.165091	0.721223
cwc25	676.4257433	-0.111857414	0.1008229	-1.109444	0.267239	0.833191
asb8	626.2416343	-0.089969467	0.0998761	-0.90081	0.367689	0.887476
col4a3	111.5988247	-0.064611177	0.1931192	-0.334566	0.737952	0.972535
rbpja	398.811914	0.180219581	0.1133769	1.5895623	0.111934	0.627242
fgf8a	32.27970706	-0.463642706	0.3345761	-1.385762	0.16582	0.721626
tenm1	875.8314272	0.100077652	0.088748	1.127661	0.259463	0.827693

foxa2	257.0897724	-0.379002613	0.1526953	-2.482084	0.013062	0.204336
capslb	23.05668006	0.083596019	0.4532036	0.1844558	0.853656	0.985174
chrna5	236.3863704	-0.22640691	0.1432149	-1.580889	0.113903	0.632675
igf2bp2a	75.31671283	-0.141627121	0.2354093	-0.601621	0.547427	0.945526
hnrnpdl	3571.923157	0.024354925	0.076462	0.3185231	0.750088	0.97419
dhrs7	334.1224798	0.020340098	0.137116	0.1483422	0.882073	0.988344
ippk	178.4154112	-0.086187449	0.1598712	-0.539106	0.589814	0.953762
pde10a	14.32886898	0.695605744	0.5048907	1.3777354	0.168285	0.725919
fech	29.78021582	0.144582712	0.3515173	0.4113104	0.680845	0.965476
neurod4	1386.048097	-0.134456385	0.0871354	-1.543074	0.122813	0.650905
sult1st5	29.31961538	-0.288461707	0.3542772	-0.814226	0.415516	0.906237
ppp1caa	3792.610793	-0.040152332	0.083044	-0.483507	0.628736	0.957354
nup93	1467.345071	-0.128676831	0.0858685	-1.498533	0.133995	0.669349
madd	2202.768517	0.006955167	0.0767589	0.0906106	0.927802	0.992702
anks1b	1275.537614	0.01203728	0.0882143	0.136455	0.891462	0.989291
impa1	568.9086527	-0.039319689	0.0988358	-0.397829	0.690757	0.967157
aprt	168.0033455	-0.23402367	0.1687415	-1.386877	0.165479	0.721223
tbc1d14	199.8640059	0.169894931	0.1484172	1.1447117	0.252329	0.821887
itln3	149.5151109	-0.10839333	0.165568	-0.654676	0.512677	0.936253
psma5	1526.33432	-0.055207995	0.0871705	-0.633333	0.526516	0.940506
mak16	457.1193043	-0.003251359	0.1110851	-0.029269	0.97665	0.996762
tcea1	1581.128999	-0.100619061	0.0783895	-1.283578	0.19929	0.764601
col8a1b	162.3748196	0.560641815	0.1796248	3.1211825	0.001801	0.049493
adarb1b	37.59156002	0.001069187	0.3423966	0.0031227	0.997508	0.999174
gnrhr2	3.338151239	0.885070687	1.0490776	0.8436656	0.398856	NA
dohh	387.2005281	0.034243774	0.1274126	0.2687629	0.788112	0.976703
hsp90b1	5716.14638	0.016859847	0.0742985	0.2269205	0.820486	0.981594
dctn5	490.0082511	-0.103674119	0.1068717	-0.970081	0.332006	0.874653
klhl32	397.9488295	-0.060280983	0.1175472	-0.512824	0.608075	0.956718
pla2g7	451.0106916	-0.003177545	0.1097129	-0.028962	0.976895	0.996762
surf1	285.0394478	-0.068797301	0.1399849	-0.491462	0.623099	0.957354
rpl3	29544.10675	-0.34635411	0.0770028	-4.497941	6.86E-06	0.000521
nr2f6a	617.0552819	-0.255114342	0.0978725	-2.606599	0.009145	0.160082
slc26a3.2	132.7485266	-0.443071468	0.1777774	-2.492282	0.012693	0.200185
arih1	474.9110182	0.057766006	0.1107599	0.5215427	0.601989	0.956045
clockb	335.1318207	-0.056944514	0.1241878	-0.458535	0.646568	0.960362
zgc:172270	45.78354241	0.154750292	0.3019596	0.5124867	0.60831	0.956718
mogat3b	173.3600591	-0.177625592	0.1586466	-1.119631	0.262871	0.830166
tfg	1368.563629	-0.011265972	0.098104	-0.114837	0.908574	0.990702
zfyve19	322.3689247	-0.072864703	0.1313503	-0.554736	0.579075	0.951052
tmem59l	3630.327357	0.082688126	0.0701636	1.1785053	0.238595	0.807659
cryl1	244.8510629	-0.049601405	0.1397819	-0.354848	0.722703	0.970375
runx1t1	1821.412994	-0.066054861	0.0749982	-0.880752	0.378452	0.895192
eif5	4234.329953	0.039719066	0.0657937	0.6036907	0.546049	0.945526
obsl1a	136.7568419	0.319323654	0.1844502	1.7312193	0.083413	0.556666
hars	1628.649391	-0.053563178	0.0822487	-0.651234	0.514895	0.937646
vdac3	7515.519019	0.039460929	0.0794351	0.4967693	0.619352	0.957354
slc5a8l	94.62494592	-0.419330304	0.2068337	-2.027379	0.042624	0.405993
atp7a	474.1274441	-0.07933576	0.1085904	-0.730597	0.465026	0.923671
cldng	1.794681846	-3.19748812	1.7151324	-1.864281	0.062282	NA
prnprs3	2605.75927	0.055056811	0.071389	0.7712228	0.440575	0.916336
ryr2b	48.13979216	-0.24647379	0.2791031	-0.883092	0.377186	0.895192
mitfa	13.47214527	-0.094935005	0.5136947	-0.184808	0.853379	0.985174
polb	459.226659	0.156937775	0.1087667	1.4428842	0.149053	0.696454

pfas	464.8998547	0.168013482	0.1081328	1.5537697	0.120239	0.645215
lats1	390.7801279	0.128276658	0.1149201	1.1162248	0.264326	0.830245
tspan2a	1076.4509	-0.088715561	0.084329	-1.052018	0.292791	0.848607
cnpy1	263.436096	0.103074423	0.1334248	0.7725281	0.439802	0.916082
tada2b	150.6916191	0.110696859	0.1736647	0.6374171	0.523853	0.939336
pip4k2aa	839.7617389	-0.068124171	0.0968984	-0.703047	0.482026	0.928561
ctnnd2b	1189.994921	0.00217148	0.0876407	0.0247771	0.980233	0.996879
dennd5b	1677.629846	-0.005508683	0.0798218	-0.069012	0.94498	0.993409
idh2	6788.287005	0.067784183	0.0708459	0.9567833	0.338677	0.877373
asb2a.1	155.5952107	0.212732177	0.1763272	1.2064624	0.227639	0.794934
LHX3	85.44805583	-0.098772564	0.2114633	-0.467091	0.640435	0.959994
aqp3a	2251.28309	0.329018893	0.0793515	4.1463461	3.38E-05	0.001942
plxnb2a	1187.533757	-0.039479021	0.0827783	-0.476925	0.633416	0.957581
prkag1	485.1712627	0.154868716	0.1097474	1.4111379	0.158204	0.709224
nr1d2a	2252.956948	-0.041128616	0.086152	-0.477396	0.63308	0.957356
ankrd16	55.77492741	-0.282709021	0.2574926	-1.097931	0.272235	0.837242
snapc1b	141.4325632	-0.243586249	0.1725785	-1.411452	0.158111	0.709224
galnt2	1177.637809	0.110278516	0.0830371	1.3280626	0.184157	0.747376
stom	1590.533564	-0.035823962	0.081089	-0.441786	0.658644	0.962545
usb1	129.4548951	-0.151277242	0.1789836	-0.845202	0.397998	0.899672
chm	557.1783353	0.16389509	0.1104958	1.4832695	0.138003	0.67563
mrpl21	344.1547218	0.127296298	0.1218355	1.044821	0.296106	0.853273
acsl1b	957.4133227	-0.222145009	0.0915101	-2.427547	0.015201	0.225685
cbx3a	1875.120693	-0.100842285	0.0810089	-1.24483	0.213194	0.781631
pard6b	30.64551475	-0.650296309	0.350586	-1.854884	0.063613	0.4929
tm9sf2	3045.544542	0.069420151	0.0694891	0.9990073	0.317791	0.867607
cdk20	39.5859712	-0.0640295	0.3052734	-0.209745	0.833867	0.983583
decr1	403.5756329	-0.091762392	0.1141039	-0.8042	0.421281	0.90651
ccdc102a	849.8799882	-0.07907248	0.0906843	-0.871953	0.383234	0.895192
pbrm1	582.0860791	0.113407828	0.1007862	1.1252314	0.260491	0.828546
zdhhc23b	46.97095576	-0.154187937	0.3084502	-0.499879	0.61716	0.95723
gpr61l	12.71835886	0.276367923	0.5307465	0.5207155	0.602565	0.956045
ctsl.1	147.0070148	-0.815992796	0.1943901	-4.197707	2.70E-05	0.001621
hapln2	16.66069699	-0.117543822	0.4672361	-0.251573	0.801371	0.979377
sycp1	22.5981051	-0.032048179	0.4058832	-0.078959	0.937065	0.99279
ftr01	27.14163818	-1.998273133	0.4105467	-4.867347	1.13E-06	0.000111
med13b	926.1199856	0.044779564	0.1136536	0.3940004	0.693581	0.96736
setb	5222.878983	-0.046670552	0.0681663	-0.684657	0.493561	0.931926
cx28.6	14.4409229	-0.260033306	0.5233145	-0.496897	0.619262	0.957354
cldn12	707.999907	0.098196774	0.0944039	1.0401772	0.298258	0.85401
cndp2	799.0309579	-0.202831568	0.0949611	-2.135943	0.032684	0.352204
nxnl2	203.0169374	-0.068570273	0.158682	-0.432124	0.665651	0.964736
rpa1	1747.194181	-0.088609366	0.0782427	-1.132494	0.257427	0.825314
mtfr1l	1086.124155	0.016549767	0.0879408	0.1881922	0.850726	0.985174
rrs1	351.1416707	0.180659699	0.1231446	1.4670534	0.142362	0.684315
nmur1b	1.829494234	-2.205461113	1.6346402	-1.349203	0.177272	NA
pfn2	7566.991758	-0.094008398	0.0718325	-1.308716	0.19063	0.755212
parp3	231.4518728	-0.052848977	0.1364381	-0.387348	0.698499	0.967998
traf4a	818.4015525	-0.084873258	0.0889745	-0.953905	0.340132	0.87805
tlx1	26.35919222	-0.64469705	0.3992295	-1.614853	0.106343	0.615567
larp6b	1.020882972	1.110886363	2.0729323	0.5359009	0.592027	NA
trim35-13	51.88051056	0.468236494	0.2720825	1.7209357	0.085262	0.560519
isl2a	550.3607766	-0.133586852	0.1034123	-1.291789	0.19643	0.761835
chordc1a	69.78830645	0.035647994	0.2356063	0.1513032	0.879737	0.987881

spop1b	546.1527812	-0.028063123	0.109093	-0.25724	0.796993	0.978569
zgc:153896	30.59114883	-0.507921591	0.3621106	-1.40267	0.160715	0.713833
LTN1	509.4672755	0.051094454	0.1150963	0.4439279	0.657095	0.962166
crhr1	30.00626531	-0.071744735	0.3462623	-0.207198	0.835856	0.984089
fhl2b	157.5681575	0.026651692	0.1706476	0.1561797	0.875891	0.987881
syt9a	633.66861	0.019969596	0.1029839	0.1939099	0.846246	0.985174
phyhip1a	95.11603897	0.048308908	0.213071	0.2267269	0.820636	0.981594
adcyap1a	160.8728251	-0.505805271	0.1647792	-3.069593	0.002144	0.056364
spag1a	540.7372903	0.184248153	0.1071709	1.7191998	0.085578	0.561521
CHST13	104.6971566	0.073574789	0.2019689	0.3642877	0.715643	0.96909
isl1	1841.709547	-0.118762936	0.0742639	-1.599202	0.109776	0.623766
arhgdig	2008.153742	0.09894005	0.077402	1.2782626	0.201157	0.766855
cog2	505.4568383	0.084581524	0.108424	0.7800997	0.435332	0.914408
phf6	355.9273595	0.003302574	0.1175898	0.0280856	0.977594	0.996762
marcksa	620.2639177	0.056825962	0.1058152	0.5370305	0.591247	0.954193
CR354540.1	21.3074331	-0.22428427	0.4094664	-0.547748	0.583865	0.952214
uhrf1bp1l	602.7528912	-0.221619269	0.1046252	-2.11822	0.034156	0.359261
galk2	50.92832141	-0.33327025	0.2757433	-1.208625	0.226807	0.794528
bhlhe40	2792.744124	0.120181443	0.0755845	1.5900279	0.111829	0.627242
bach2b	314.2817904	-0.10367726	0.1223612	-0.847305	0.396825	0.899672
acsl4a	1149.387134	-0.107606157	0.0843876	-1.275142	0.202259	0.768575
cx39.9	87.14113296	0.254811723	0.2217054	1.149326	0.250422	0.820164
acsbg2	2055.553312	-0.048611743	0.0830481	-0.585345	0.558316	0.948478
tie1	281.2059593	-0.135859277	0.1480535	-0.917636	0.358809	0.884602
esr1	10.43939753	0.413505945	0.6039094	0.6847152	0.493524	NA
tti1	147.8584733	-0.330151413	0.1850741	-1.783887	0.074442	0.531413
mgat4b	616.1782856	0.061463753	0.0997316	0.6162916	0.537702	0.943336
mvk	103.1685091	0.055715047	0.1947415	0.2860974	0.774804	0.975687
rttn	96.00281198	-0.19706443	0.2038397	-0.966762	0.333663	0.874933
usp19	1098.63681	0.017821205	0.0915659	0.194627	0.845685	0.985174
DHRS11 (1 of many)	256.4823707	-0.448973251	0.1393004	-3.223058	0.001268	0.037265
grm2a	392.1936946	0.112879937	0.1189681	0.9488254	0.342709	0.878434
rab23	44.76519049	-0.012821108	0.29727	-0.043129	0.965598	0.996315
orai1b	38.24750342	0.294852927	0.3240534	0.90989	0.362881	0.885944
tmem63c	181.9534804	-0.199530977	0.1654572	-1.205937	0.227842	0.794963
reep3b	784.5190528	0.008954117	0.0900703	0.0994125	0.920811	0.992138
ik	1633.529106	0.045634815	0.0796761	0.5727544	0.566811	0.948739
zgc:110366	108.3648369	0.077844049	0.1972171	0.3947125	0.693055	0.96736
stmn1a	956.7172347	-0.120713259	0.0974768	-1.238379	0.215575	0.783025
rabac1	697.7590053	0.20884437	0.0929615	2.2465675	0.024668	0.302262
copa	4745.276994	-0.038887762	0.0642239	-0.605503	0.544845	0.945463
cnot1	4361.957319	-0.006092825	0.0897641	-0.067876	0.945884	0.993759
ulk4	70.56633732	-0.151085803	0.2322247	-0.650602	0.515304	0.9377
fam169ab	190.1176413	0.239383986	0.1478529	1.6190684	0.105433	0.614144
nf1b	96.42018368	0.321958241	0.203709	1.5804812	0.113997	0.632839
zgc:122979	148.8778715	0.57513953	0.1788487	3.2157881	0.001301	0.038109
cbx1a	1613.370742	0.07393737	0.0791134	0.9345748	0.350007	0.880195
tnfsf10l	469.8196622	0.032960769	0.1070163	0.3079975	0.758084	0.975374
spam1	84.50558642	0.585431331	0.2491242	2.3499581	0.018776	0.255518
efhc2	35.72594089	0.083968201	0.3309649	0.2537072	0.799722	0.979377
ccdc106a	765.4153261	-0.052257091	0.0914701	-0.571303	0.567794	0.948739
rnd1b	608.8632496	0.118030343	0.1068019	1.1051335	0.269102	0.834439
slc6a3	169.6360531	-0.126117447	0.1758491	-0.717191	0.473256	0.924993
pde3a	60.04956578	0.026880726	0.2765078	0.0972151	0.922556	0.99233

dlb	441.0154731	0.125134343	0.1113072	1.1242249	0.260918	0.829083
asic4b	9.592033464	0.204727655	0.6157048	0.3325094	0.739505	NA
slit2	1380.921704	0.13209624	0.0824103	1.6029103	0.108954	0.622318
dhfr	275.0427456	-0.103155631	0.1282989	-0.804026	0.421382	0.90651
wnt11	33.43779923	-0.063868767	0.3625442	-0.176168	0.860162	0.985773
cul5b	73.89015281	0.009482979	0.2304202	0.0411552	0.967172	0.996315
tmed9	2197.101513	0.095592856	0.075257	1.2702188	0.204007	0.771764
cyp46a1.2	23.47147787	0.405038928	0.3938915	1.0283007	0.303808	0.858093
atl3	747.5294466	-0.018934976	0.0937107	-0.202058	0.839871	0.984979
zgc:112496	52.49323285	-0.072140976	0.2787976	-0.258757	0.795822	0.978498
seh1l	763.8950197	-0.032151728	0.095185	-0.337781	0.735528	0.972535
zgc:77375	136.3421606	-0.192236911	0.1885012	-1.019818	0.307815	0.861088
hecw1b	102.3699666	-0.0283135	0.2201828	-0.128591	0.897681	0.990121
ambp	1372.917329	0.185547312	0.1073945	1.7277172	0.084039	0.557646
irf2bpl	1973.932052	-0.021085944	0.0808967	-0.260653	0.79436	0.977937
rhogb	153.9574911	-0.219149442	0.1799312	-1.217963	0.223238	0.789115
slc45a1	248.4794994	0.087951303	0.137417	0.640032	0.522152	0.939056
vangl1	224.9067545	-0.015516331	0.1378657	-0.112547	0.91039	0.990702
klhl18	263.9735111	0.26666949	0.1350682	1.974332	0.048344	0.433147
lypd6	206.5506039	-0.083702276	0.1516061	-0.552103	0.580877	0.951731
fam8a1b	247.4372032	0.03607308	0.1475579	0.2444672	0.806869	0.980254
ldlrap1a	180.0085513	-0.026902535	0.1663385	-0.161734	0.871516	0.987754
cbwd	201.9635937	0.02769322	0.1523208	0.1818085	0.855733	0.985174
ush1ga	114.7344135	0.106915771	0.1926583	0.5549501	0.578929	0.951052
casp9	342.946452	-0.287780188	0.1288467	-2.233509	0.025515	0.309193
LIN28A	1.16600141	0.393120273	1.8307582	0.2147309	0.829977	NA
exoc4	403.6741746	0.126527394	0.1162757	1.0881674	0.276521	0.839541
nelfe	435.5317665	-0.038585252	0.1128053	-0.342052	0.732312	0.971719
gnb3a	1729.862248	-0.183326607	0.087073	-2.105436	0.035253	0.365357
lhfpl6	401.3950452	0.110466367	0.1158736	0.9533351	0.34042	0.87805
fmnl3	363.9342444	0.162957962	0.119934	1.35873	0.174232	0.734375
ppp1r13ba	731.345311	0.099189303	0.1058859	0.9367565	0.348884	0.880195
yrk	272.7338918	0.030223245	0.1339949	0.2255552	0.821547	0.981928
si:dkey-153k10.9	1430.942877	-0.00275848	0.0774793	-0.035603	0.971599	0.996315
hdr	74.10418119	0.082326597	0.246942	0.3333843	0.738844	0.972722
b3gnt5b	73.83526372	-0.132640403	0.2250968	-0.589259	0.555687	0.947621
elovl6	823.7492143	0.141631838	0.0984267	1.4389571	0.150163	0.698387
snx10a	58.69958559	-0.299382851	0.2965578	-1.009526	0.312722	0.864656
spra	246.9667221	0.123311631	0.1366134	0.9026317	0.366721	0.88734
tcf7l2	3178.157148	-0.114927831	0.0722846	-1.589936	0.111849	0.627242
dpy30	443.9702525	-0.368636341	0.1334655	-2.762035	0.005744	0.113814
btk	34.28199078	-0.045449459	0.3329833	-0.136492	0.891433	0.989291
fam92a1	101.9157726	-0.160448619	0.2039978	-0.786521	0.431562	0.91277
grm5a	162.1851935	0.036726469	0.1642704	0.2235733	0.823089	0.982189
aplnr2	21.62864778	0.278824061	0.4158009	0.670571	0.502494	0.935304
tnfrsfa	372.0440857	0.042099032	0.1291942	0.3258586	0.744531	0.974094
tardbpl	4833.347464	-0.047972679	0.0735161	-0.652546	0.514049	0.936851
p2rx5	92.41068755	0.205622558	0.2039553	1.0081745	0.313371	0.865132
unc119.2	36.4831017	0.625474288	0.3563531	1.7552093	0.079224	0.547489
desi2	412.2349839	-0.09486415	0.113049	-0.839142	0.40139	0.90084
prkcsh	2006.412619	-0.053847023	0.0716101	-0.751948	0.452082	0.918781
atat1	392.6015515	-0.001334576	0.1193026	-0.011186	0.991075	0.997679
tbx21	21.14713949	-0.528484338	0.4311616	-1.225722	0.220303	0.787184
pcyt1ab	240.172381	-0.239753046	0.1391138	-1.723432	0.084811	0.560519

tspan33a	222.0704168	0.119891365	0.1402578	0.8547926	0.392666	0.897688
xkr8.1	14.60142818	1.028940314	0.5006347	2.0552717	0.039853	0.391511
rcor3	67.80641108	0.134037482	0.2477406	0.5410397	0.58848	0.953319
ppat	323.6023244	-0.195155004	0.1222396	-1.596495	0.110378	0.624676
zgc:63882	171.2785796	-0.029025245	0.1576706	-0.184088	0.853945	0.985174
pin4	317.4822018	-0.038676777	0.1295382	-0.298574	0.765265	0.975374
zgc:173742	20.63038643	-0.293340752	0.441426	-0.66453	0.506351	0.935724
kmt2a	399.7030421	-0.088710184	0.1311918	-0.676187	0.498922	0.934006
ptgs2a	986.0434347	0.154930568	0.1020122	1.5187451	0.128827	0.659982
prkcg	12.56655507	0.228225114	0.532612	0.4285017	0.668286	0.964736
gtf3c2	139.4433272	-0.215796168	0.1751087	-1.232356	0.217816	0.785256
plk4	283.6462378	-0.17217965	0.1273392	-1.352134	0.176332	0.73663
zgc:194665	296.7559133	-0.084460544	0.1265812	-0.667244	0.504616	0.935617
nmnat2	1010.79407	-0.016004334	0.0959567	-0.166787	0.867538	0.987231
sell1	934.2795986	0.315152988	0.0883104	3.5686958	0.000359	0.013616
ntrk1	20.62521113	-0.344113968	0.4659368	-0.738542	0.460185	0.921978
kat5a	512.3474403	-0.048117635	0.1087633	-0.442407	0.658195	0.962545
sox4a	6693.96462	-0.13870806	0.0645682	-2.148241	0.031695	0.345305
gpr22a	284.9157523	-0.011319369	0.1384606	-0.081752	0.934844	0.992702
znf800a	307.5659062	-0.112820468	0.1267347	-0.89021	0.373353	0.893571
lrrc4ba	72.46390524	-0.001623152	0.2271039	-0.007147	0.994297	0.998694
or102-2	3.783978736	-0.660031124	0.9780924	-0.674815	0.499794	NA
mtmr2	384.8043384	0.116027084	0.1189373	0.9755312	0.329297	0.873436
stx2a	109.7133686	0.038638943	0.2031234	0.190224	0.849134	0.985174
gpm6ab	6160.542793	0.030488174	0.0700915	0.4349771	0.663579	0.964736
panx3	64.1883146	-0.015984375	0.2546928	-0.062759	0.949958	0.99381
egln1b	315.9930924	-0.20465794	0.1287419	-1.589676	0.111908	0.627242
epn3b	13.65522621	0.411445572	0.55859	0.7365788	0.461379	0.922161
osbp	145.0705976	0.200543043	0.1771263	1.1322037	0.257549	0.825314
epha7	665.4911177	-0.367481054	0.103152	-3.56252	0.000367	0.013861
ccdc9	690.3227797	0.034689987	0.1071575	0.323729	0.746143	0.97419
cdhr1a	397.8935721	0.28028602	0.113695	2.4652441	0.013692	0.210066
spice1	94.96101173	-0.024683213	0.2116552	-0.11662	0.907161	0.990702
kctd16a	7.489171937	-0.074394173	0.705033	-0.105519	0.915964	NA
zgc:101810	529.763858	0.248550294	0.1139326	2.181556	0.029142	0.330614
ptk2ab	807.5120991	0.046984978	0.0926383	0.5071873	0.612023	0.95688
dnajb6a	1638.269354	-0.044865822	0.0870312	-0.515514	0.606194	0.956718
acaa1	387.3282821	-0.29052128	0.1334417	-2.177141	0.02947	0.332709
nabp1a	281.8795773	0.183409851	0.1347676	1.3609348	0.173534	0.732906
six4a	113.7061777	-0.26064808	0.1927422	-1.352315	0.176275	0.736614
eefsec	144.5649246	-0.049774915	0.1688728	-0.294748	0.768186	0.975687
rxrgb	255.5437577	-0.443592057	0.1408126	-3.150229	0.001631	0.046066
irf2bp2a	968.4819032	0.048472963	0.1005195	0.4822244	0.629647	0.957354
syf2	1038.216347	-0.199891075	0.088382	-2.261672	0.023718	0.297019
tbr1b	411.8349321	-0.059268226	0.1156651	-0.512412	0.608362	0.956718
mad2l1	384.8769012	-0.252307381	0.1198896	-2.104498	0.035335	0.36572
tcf12	1604.60548	-0.011187142	0.08196	-0.136495	0.89143	0.989291
mpp5b	54.8410105	-0.492315127	0.2725204	-1.806526	0.070836	0.520712
tcea3	117.7665071	-0.3740184	0.1934253	-1.933658	0.053155	0.452412
trappc11	1104.531413	-0.016355251	0.0842612	-0.194102	0.846096	0.985174
hnrnpub	4853.976963	-0.007577748	0.0667213	-0.113573	0.909576	0.990702
lmbr1l	161.2743817	-0.003319942	0.1655639	-0.020052	0.984002	0.996944
zgc:100868	2152.442561	-0.083849071	0.1181628	-0.709606	0.477948	0.926857
mylkb	58.25997256	-0.060352215	0.2618299	-0.230502	0.817702	0.981594

hspa4a	1383.390682	0.175931993	0.0826831	2.1277855	0.033355	0.355558
ybx1	14079.64573	-0.139634637	0.0796178	-1.753813	0.079463	0.547489
hhla2a.2	5.238384069	-0.538652683	0.8605235	-0.625959	0.531342	NA
ankrd10b	327.4483591	0.119390976	0.1215048	0.9826033	0.325803	0.871896
wdr36	483.7722165	-0.149939391	0.1083657	-1.383643	0.166468	0.722981
fgfr3	2164.442176	0.083698503	0.0738554	1.1332754	0.257099	0.825303
cops2	950.738817	-0.044602191	0.0922844	-0.483312	0.628874	0.957354
lrp1ba	27.8301871	-0.208853644	0.3587357	-0.582194	0.560436	0.948728
ccdc146	18.22131731	0.329278311	0.4461867	0.7379833	0.460525	0.921978
mgrn1b	842.1966607	0.05723573	0.0940343	0.6086689	0.542744	0.944974
grwd1	336.2072209	-0.046916896	0.1232042	-0.380806	0.703347	0.968134
asz1	3.944434732	-0.971028866	0.9739744	-0.996976	0.318776	NA
flot2a	4681.439058	-0.004878748	0.0695829	-0.070114	0.944103	0.993364
dnajc5ab	2686.330595	-0.052900616	0.0816297	-0.648056	0.516949	0.938543
rassf1	506.3614364	0.112885704	0.1117211	1.0104244	0.312292	0.864401
foxp1a	737.0438542	-0.384521433	0.0959486	-4.007578	6.13E-05	0.003262
hinfp	127.2894776	-0.022039035	0.1853391	-0.118912	0.905345	0.990702
esrrga	472.8549042	0.020947553	0.1059227	0.1977626	0.843231	0.985174
zgc:171734	0.325452849	0.005883619	3.3177086	0.0017734	0.998585	NA
mindy1	516.4946996	0.018773493	0.1085132	0.1730066	0.862646	0.985841
penka	221.7654243	-0.371145938	0.1427922	-2.599203	0.009344	0.162078
psen1	875.3297129	-0.076757161	0.092525	-0.829583	0.406774	0.901305
mst1rb	9.901199558	0.312305982	0.6293513	0.4962347	0.619729	NA
spata6l	24.28844944	-0.237072722	0.4029084	-0.588404	0.556261	0.947902
tmc6b	322.4577218	-0.299037839	0.1230038	-2.431127	0.015052	0.224971
rock2b	478.7578154	-0.08022105	0.1179426	-0.68017	0.496397	0.932436
acsm3	104.6011671	-0.176819467	0.2182008	-0.810352	0.417738	0.906237
aga	188.1511244	-0.286874088	0.1536509	-1.867051	0.061895	0.485483
pex19	998.801918	0.108163269	0.0958606	1.1283391	0.259177	0.82755
zp2l2	0.995966258	-1.056292575	2.0854203	-0.506513	0.612497	NA
gys2	274.8513235	0.441990819	0.1390194	3.1793459	0.001476	0.04224
stip1	1767.985804	0.023669949	0.0832523	0.284316	0.776168	0.975826
immp2l	83.46454079	-0.353935079	0.2174055	-1.627995	0.103526	0.609625
lmo7a	1258.420511	-0.01754846	0.1082094	-0.162171	0.871171	0.987754
slc8a3	32.41313122	0.239144371	0.3539508	0.6756429	0.499267	0.934006
anos1b	336.5333478	0.161758256	0.1236455	1.3082422	0.190791	0.755212
skp2	159.2599437	0.030027135	0.1636018	0.1835379	0.854376	0.985174
mtdhb	542.898567	0.217142122	0.1084259	2.0026783	0.045212	0.417766
bccip	966.7793603	-0.191122514	0.093113	-2.052586	0.040113	0.392085
rsad2	4.251712716	0.186102543	0.979076	0.1900798	0.849247	NA
mxb	3.758706303	0.835972124	1.0212801	0.8185532	0.413041	NA
grna	44.78493679	0.595861004	0.2996118	1.9887768	0.046726	0.424613
zmat2	725.5632423	-0.116065571	0.09331	-1.24387	0.213547	0.781737
cyp4t8	130.6645327	-0.409843965	0.1773976	-2.310312	0.020871	0.27277
elovl5	104.345925	0.218605848	0.1982114	1.1028926	0.270074	0.834705
ppp3ca	2085.620425	-0.039498563	0.0889221	-0.444193	0.656903	0.96205
brf1b	154.5703366	-0.050873153	0.1642091	-0.309807	0.756708	0.975374
slc5a12	114.4282454	0.426431785	0.1938676	2.1996029	0.027835	0.323391
si:dkeyp-2e4.8	32.19979392	-0.477069197	0.3428687	-1.391405	0.164103	0.71945
tspy	775.3114978	0.056452614	0.0901465	0.6262322	0.531163	0.942209
fkbp9	712.502517	0.303004475	0.3238949	0.9355024	0.349529	0.880195
tshz1	1210.318286	-0.104522753	0.0855351	-1.221987	0.221713	0.78829
crppa	247.8190827	0.044552233	0.1399171	0.3184189	0.750167	0.97419
rab20	92.11586539	-0.304545538	0.2109048	-1.443996	0.14874	0.69636

wnt2ba	22.33694951	0.033331173	0.4208727	0.0791954	0.936877	0.992702
dimt1l	214.0462914	0.27134789	0.149468	1.8154252	0.069459	0.514708
ncapd2	992.1591889	-0.070206371	0.0920838	-0.762419	0.44581	0.917154
eif4e3	781.2313181	-0.018397797	0.1084881	-0.169583	0.865338	0.986385
ggctb	561.1160281	-0.003488171	0.1101964	-0.031654	0.974748	0.996605
cep89	62.66545779	0.27381791	0.2570007	1.0654365	0.286678	0.84596
glcea	333.4270293	0.292285454	0.1217628	2.4004485	0.016375	0.236243
zgc:86764	311.8649685	0.004936016	0.1280075	0.0385604	0.969241	0.996315
limk2	920.3976392	-0.138001577	0.0882583	-1.563611	0.117909	0.641446
oclna	873.104332	-0.189825459	0.1076969	-1.762591	0.07797	0.544026
cdh17	2256.76186	-0.185891069	0.0781068	-2.379959	0.017315	0.243166
calub	1049.990414	0.16376667	0.0861038	1.9019683	0.057175	0.469122
atp2a2b	2721.1384	0.108103608	0.0752075	1.437405	0.150603	0.698912
large1	36.15009427	0.306055626	0.3240757	0.9443955	0.344968	0.878434
rnaseh2b	214.8381003	0.214569996	0.1405408	1.5267457	0.126824	0.656588
psmd11b	1092.30968	0.118430724	0.0890606	1.3297765	0.183592	0.747284
hhatlb	520.5081488	0.351619475	0.1027187	3.4231315	0.000619	0.020853
camkvb	462.9177225	0.039357018	0.1186515	0.3317026	0.740114	0.972943
tbx20	32.22591182	-0.513625401	0.3416762	-1.503252	0.132774	0.667381
aspa	9.49816035	0.074451669	0.623755	0.1193604	0.90499	NA
cyth1a	379.6077936	-0.090182516	0.1240083	-0.72723	0.467085	0.924328
tpm3	8487.799683	0.073087949	0.0674426	1.0837065	0.278495	0.842429
efr3a	74.29368087	-0.099955154	0.2327393	-0.429473	0.667579	0.964736
dynll2b	622.1463632	0.021547101	0.1048045	0.2055932	0.837109	0.984645
zgc:101040	86.75963091	-0.202820931	0.2169504	-0.934872	0.349854	0.880195
dglucy	299.8105473	-0.08088678	0.140251	-0.576729	0.564123	0.948739
cpn1	6.002436204	-0.10804383	0.8428652	-0.128186	0.898001	NA
sp4	812.7831496	0.096068492	0.092722	1.0360922	0.300159	0.856224
ahcy	8165.752217	-0.14880889	0.0925862	-1.607248	0.108	0.619371
coro2a	394.7089557	0.021460123	0.1225017	0.1751823	0.860936	0.985773
zgc:158328	20.20780674	0.717414017	0.435363	1.6478527	0.099383	0.599511
wrnip1	71.99578898	-0.0741574	0.2294956	-0.323132	0.746595	0.97419
pdcd6	564.900524	0.023209705	0.1076659	0.2155716	0.829322	0.982805
desmb	182.0417806	-0.075644229	0.1566943	-0.48275	0.629273	0.957354
ssr2	2179.625252	-0.136577126	0.0744636	-1.834147	0.066632	0.504944
ccdc85ca	53.79203053	0.17768126	0.2943737	0.6035907	0.546116	0.945526
srcap	1394.216647	0.057446249	0.0863843	0.6650078	0.506046	0.935724
asb3	99.69510182	-0.049133352	0.2227975	-0.220529	0.825459	0.982196
rpe	483.6413613	-0.011063592	0.1090844	-0.101422	0.919215	0.991623
kdelr2a	1481.871906	0.223338147	0.0835041	2.6745756	0.007482	0.136997
zc3h12a	27.94025694	-0.115201707	0.3572938	-0.322428	0.747128	0.97419
rad52	67.80904276	-0.04001343	0.2355971	-0.169838	0.865137	0.986367
fbxl15	86.65666166	0.267623037	0.2101685	1.2733735	0.202886	0.769849
CALHM1 (1 of many)	5.353635404	0.374080631	0.8723163	0.428836	0.668043	NA
celf1	2026.681829	0.045017615	0.0752323	0.5983813	0.549586	0.946312
stam2	722.0328944	0.009725892	0.0920603	0.1056469	0.915862	0.991314
nipsnap1	83.384457	-0.49762644	0.222318	-2.238354	0.025198	0.30686
ftr66	1.964116229	0.510643648	1.3857694	0.3684911	0.712507	NA
lipib	56.79085244	-0.011248342	0.2691719	-0.041789	0.966667	0.996315
slc22a4	14.29416894	0.321073427	0.5175513	0.6203702	0.535014	0.943336
ccdc85al	283.4019452	0.01283163	0.126579	0.1013725	0.919255	0.991623
dip2ba	1157.969733	-0.120127297	0.0897662	-1.338224	0.180824	0.741776
larp6a	370.7944637	-0.002540396	0.1289559	-0.0197	0.984283	0.996944
tyro3	18.18024045	0.74298171	0.4489989	1.6547517	0.097975	0.59749

sucla2	3355.220151	0.144115869	0.0818512	1.7607063	0.078288	0.545287
grin3ba	56.19937916	-0.041635393	0.2721998	-0.152959	0.878431	0.987881
mcamb	718.5700529	-0.016010099	0.0917426	-0.174511	0.861464	0.985841
gprc6a	23.85216812	0.3006779	0.4037998	0.7446212	0.456501	0.921215
camk4	361.2147403	-0.042125802	0.1168305	-0.360572	0.718419	0.96909
tubgcp4	428.5587745	0.088328435	0.1099865	0.8030843	0.421926	0.90651
prdm9	235.1923701	-0.1728005	0.1417764	-1.218825	0.222911	0.789115
slc5a5	15.9482868	0.104704929	0.5954641	0.1758375	0.860422	0.985773
dnmt3aa	27.99884209	0.455369056	0.372119	1.2237187	0.221058	0.787641
trim3b	1098.73895	0.115617244	0.0835421	1.3839397	0.166377	0.722981
grapa	112.150711	-0.041177574	0.204991	-0.200875	0.840796	0.985092
map3k5	677.3043966	0.009701631	0.1015008	0.0955818	0.923853	0.992628
pgam1a	2215.314985	-0.098884752	0.0724862	-1.364187	0.172509	0.731348
wdfy1	75.65759057	0.111869712	0.2345226	0.4770105	0.633355	0.957562
mtfmt	109.1259079	0.148189481	0.1899158	0.7802904	0.43522	0.914408
gdi2	6163.273672	-0.012121774	0.0671885	-0.180414	0.856827	0.985597
foxp2	462.496036	-0.084648172	0.1112966	-0.760564	0.446918	0.917154
tacc3	818.8750005	-0.16559694	0.0908671	-1.822409	0.068393	0.512556
katnb1	645.1637069	-0.076003067	0.0977089	-0.777852	0.436656	0.914408
csnk1g2a	908.579515	0.012590388	0.0865613	0.1454506	0.884355	0.98879
kif4	775.3704415	-0.039810244	0.0962823	-0.413474	0.679259	0.965257
slc30a1a	561.5758703	0.309768428	0.1122372	2.7599442	0.005781	0.11443
dnase1l1	161.3614182	-0.091688374	0.1710651	-0.535985	0.591969	0.954434
irf2bp1	628.4588498	-0.004559762	0.1078538	-0.042277	0.966278	0.996315
nav3	219.9646607	0.186386954	0.1446762	1.288304	0.19764	0.762928
tenm3	2309.561242	0.030312166	0.0820724	0.3693345	0.711878	0.969006
nfkbiaa	539.0759905	-0.477320808	0.136487	-3.497189	0.00047	0.017028
rapgef2	1973.292948	0.027428056	0.080027	0.3427352	0.731798	0.971572
ucmab	686.0649199	-0.17750974	0.1065326	-1.666248	0.095664	0.588752
dgcr6	198.6092736	-0.126513786	0.1459353	-0.866917	0.385988	0.89551
gdf6b	8.150218759	-0.157537014	0.66955	-0.235288	0.813985	NA
naca	9559.047831	-0.227225946	0.0696682	-3.261542	0.001108	0.033401
slc5a9	73.56678439	-0.084777857	0.2245651	-0.37752	0.705787	0.968412
galr1a	11.60527518	0.05223557	0.5676247	0.0920248	0.926678	0.992702
acy3.2	225.3101824	-0.077522494	0.1452914	-0.533566	0.593642	0.954434
igfn1.1	815.2468871	0.374856399	0.112966	3.3183119	0.000906	0.028837
ubr7	651.966982	-0.070231228	0.0975281	-0.720112	0.471456	0.924838
xpo7	1319.582158	-0.148738011	0.0791092	-1.880161	0.060086	0.478687
wif1	556.8867893	0.074217704	0.1075605	0.6900089	0.490189	0.931926
btbd3a	10.70312144	-0.940361848	0.6505447	-1.445499	0.148318	NA
rint1	192.5867232	-0.215641217	0.1483889	-1.453217	0.146164	0.691942
znf710b	1136.099824	-0.180210239	0.0806076	-2.235649	0.025375	0.308303
hnrnph1l	2654.190254	-0.077289939	0.0769079	-1.004967	0.314913	0.866054
pou4f1	402.1610523	-0.027446242	0.1136839	-0.241426	0.809225	0.980642
ywhah	5885.549624	0.002230465	0.0676159	0.0329873	0.973685	0.996315
gfm2	268.9074417	-0.006579065	0.1351867	-0.048667	0.961185	0.995729
entpd8	49.11500768	-0.756112306	0.2805671	-2.694943	0.00704	0.13096
vsx2	450.0205464	0.017488686	0.115577	0.1513164	0.879726	0.987881
mphosph8	1101.231742	0.049630053	0.0841868	0.5895231	0.55551	0.947607
ugp2a	46.72632779	0.026551182	0.3094003	0.085815	0.931614	0.992702
zbtb20	3.701774465	-1.046137009	1.0561667	-0.990504	0.321928	NA
mtf2	425.5202331	0.070165586	0.1127508	0.622307	0.53374	0.943276
rxrga	148.2046609	-0.398792302	0.1728129	-2.307654	0.021018	0.273449
adgb	76.40437836	0.04388623	0.2278144	0.1926403	0.847241	0.985174

paxip1	500.5220816	-0.059094621	0.1041885	-0.567189	0.570586	0.949061
aph1b	829.1306965	0.045459286	0.0911421	0.4987738	0.617939	0.957354
bfb	44.1627867	-0.201825932	0.3282005	-0.614947	0.53859	0.943336
nek2	84.51825427	0.123247793	0.2266562	0.5437654	0.586603	0.953319
maco1a	297.1342627	0.146507655	0.1474212	0.9938031	0.320319	0.868202
RHOU (1 of many)	58.38383253	0.058364644	0.2565498	0.2274983	0.820036	0.981594
zmp:0000000527	2.411113467	1.926067046	1.3632399	1.4128599	0.157697	NA
smyd2b	399.1286354	0.235591498	0.1211095	1.9452762	0.051742	0.446202
adat3	37.96732317	-0.178659602	0.338587	-0.527662	0.597734	0.95534
gcat	415.7752574	0.249859457	0.1284683	1.9449113	0.051786	0.446386
robo3	1235.140622	-0.161598369	0.0811739	-1.990768	0.046506	0.423787
olfml2a	133.2727902	0.244137638	0.1834801	1.3305949	0.183322	0.747003
hrasb	932.4127038	-0.002383812	0.0892442	-0.026711	0.97869	0.996762
man1a1	1018.957233	-0.04973942	0.0834541	-0.596009	0.551169	0.946571
ttbk2a	27.71335758	-0.328671023	0.3612847	-0.909729	0.362966	0.885944
f3b	181.9851639	0.464034109	0.1855019	2.5015062	0.012367	0.196732
sec61a1l	601.318943	0.043405625	0.0964373	0.4500919	0.652644	0.960708
trip10a	369.0537696	0.047622192	0.1201147	0.3964726	0.691756	0.967305
slc25a23a	546.524046	0.27812107	0.1124943	2.4723133	0.013424	0.207707
ddx19	509.3402931	0.166759647	0.1030002	1.6190226	0.105442	0.614144
eps8l3b	356.5959108	-0.828285672	0.129479	-6.397067	1.58E-10	3.64E-08
ethe1	317.1777244	-0.496427064	0.1384542	-3.585496	0.000336	0.012973
olfml2ba	64.00834751	-0.084356313	0.2466965	-0.341944	0.732393	0.971719
rpf1	279.729202	0.153348461	0.1438272	1.0661995	0.286333	0.845942
gpm6ba	1350.934499	0.047303977	0.0831451	0.5689326	0.569402	0.948983
cand2	278.9349347	0.274579319	0.1296078	2.1185399	0.034129	0.359261
ptprfb	3378.557961	0.003863117	0.0801315	0.0482097	0.961549	0.995845
col14a1a	348.7621297	-0.170883386	0.1299886	-1.314602	0.188644	0.753288
tsr2	279.2065142	-0.178526388	0.1322541	-1.349874	0.177056	0.738098
ddx3xb	5754.333006	0.015035967	0.0740447	0.2030661	0.839083	0.984979
scn8aa	679.2829568	0.04925187	0.0946713	0.5202408	0.602896	0.956045
guk1b	714.328614	0.157898311	0.0960019	1.6447422	0.100023	0.601211
npy8br	8.332631478	0.393599298	0.6651216	0.5917704	0.554004	NA
ncanb	47.52420612	-0.160779788	0.3022605	-0.531925	0.594778	0.954434
ppm1f	8.40315104	0.351639882	0.6783088	0.5184068	0.604175	NA
enpp1	733.105576	0.208697242	0.0928509	2.2476588	0.024598	0.302262
rpl28	13064.15975	-0.266920187	0.0773231	-3.452012	0.000556	0.019235
smim14	160.1768279	-0.166637471	0.1647137	-1.011679	0.311691	0.864257
ampd3a	130.2497045	0.440717646	0.2019473	2.1823404	0.029084	0.330497
klhl10a	1.148909967	1.312490708	1.8001617	0.729096	0.465943	NA
ncf2	16.9058725	0.299071902	0.4694756	0.6370339	0.524103	0.939336
slc39a10	754.0573728	0.122545637	0.0968047	1.2659058	0.205547	0.773646
eif2s1a	720.3526456	0.004188407	0.0930273	0.0450234	0.964089	0.995927
faima	114.3298795	-0.092914711	0.1849258	-0.502443	0.615356	0.95688
gatad2b	47.53705761	0.444350926	0.3063579	1.4504307	0.146938	0.693731
got2b	2649.335867	0.13081872	0.0842299	1.5531154	0.120396	0.645215
tnni2a.2	521.5621118	0.170818875	0.1167615	1.4629732	0.143475	0.686804
cd9a	634.9791158	-0.143728837	0.1030287	-1.395037	0.163005	0.717875
SP5	4.881132812	0.31244379	0.8528734	0.3663425	0.71411	NA
slc25a6	176.0068059	0.066111843	0.1646395	0.4015552	0.688011	0.966493
ralgapa2	1094.32934	-0.002892745	0.0872014	-0.033173	0.973537	0.996315
or131-1	0.837291216	-1.914743452	2.2412189	-0.854331	0.392921	NA
gon4l	775.8202115	0.033248106	0.0953549	0.3486773	0.727332	0.970402
mmadhc	613.4335139	0.002215426	0.1103539	0.0200756	0.983983	0.996944

rfx1a	391.6229974	-0.095338853	0.1179548	-0.808266	0.418938	0.906406
cyb5r3	543.915363	0.016724815	0.1046938	0.1597499	0.873078	0.987846
slc7a9	48.74677187	-1.259355635	0.2875064	-4.380271	1.19E-05	0.000834
dera	128.7075918	0.173500835	0.1767097	0.9818409	0.326178	0.872073
clk4b	690.8444304	-0.157181462	0.0975922	-1.610595	0.107268	0.617322
tgm1l3	49.44315385	-1.943051723	0.3611582	-5.380057	7.45E-08	1.07E-05
tcf3a	34.21981993	0.18302083	0.3377397	0.5418991	0.587888	0.953319
srpk3	464.3403197	0.031430981	0.1069705	0.2938284	0.768889	0.975687
serpina10a	1408.580364	0.021372873	0.0804312	0.2657285	0.790448	0.977359
ak2	2207.907586	-0.459606288	0.0918588	-5.003402	5.63E-07	6.22E-05
tbc1d17	632.389326	-0.124695146	0.1037017	-1.202441	0.229193	0.796702
clul1	1434.150778	0.05676396	0.0773426	0.7339285	0.462992	0.922698
v2rl1	21.62084185	-0.484014729	0.4086931	-1.184299	0.236295	0.805463
htra4	67.64436833	0.456503408	0.2368614	1.9273015	0.053942	0.455056
sart1	1636.048863	0.092979297	0.0751326	1.2375357	0.215888	0.783484
ankmy2a	173.0960201	0.060041808	0.173907	0.3452525	0.729905	0.971143
ric8b	661.8368826	0.028937776	0.0992675	0.2915132	0.770659	0.975687
sfswap	1433.489975	-0.090895474	0.0774351	-1.173828	0.240464	0.808525
rgl1	858.1247783	-0.280591884	0.0928244	-3.022824	0.002504	0.063006
prc1b	188.7138473	-0.185536446	0.1575891	-1.177343	0.239058	0.807768
mycla	164.6715089	0.010206957	0.161998	0.0630067	0.949761	0.99381
dct	1763.945184	-0.033886084	0.0889	-0.381171	0.703076	0.968134
bmi1a	303.2873667	-0.069255327	0.1424877	-0.486044	0.626936	0.957354
klhl42	38.73505427	-0.133730273	0.3190296	-0.419178	0.675086	0.965257
tktb	4467.339672	-0.26045772	0.0775579	-3.358237	0.000784	0.025553
tp53i11b	696.9445069	-0.02870215	0.1040481	-0.275855	0.78266	0.97627
lta4h	886.7912989	-0.578693779	0.0958069	-6.040209	1.54E-09	3.11E-07
abat	3849.417712	0.00098583	0.0898729	0.0109692	0.991248	0.997679
aebp2	521.8972309	0.020433134	0.1121637	0.1821724	0.855447	0.985174
cyp20a1	409.8652078	-0.023668177	0.1133031	-0.208893	0.834532	0.983992
usta	141.8747341	-0.306539543	0.1716516	-1.785824	0.074128	0.530917
malt2	78.51204459	-0.065025885	0.2206775	-0.294665	0.76825	0.975687
crybg1b	296.9383188	-0.043389037	0.1542744	-0.281246	0.778522	0.975942
akap1b	1320.454535	0.018670198	0.0804273	0.2321375	0.816431	0.981594
znf385b	106.5272113	-0.053028378	0.1978589	-0.268011	0.788691	0.976703
uck2a	601.6551919	0.008089871	0.1026698	0.078795	0.937196	0.99279
gpr37l1a	34.68729099	0.391812734	0.3559527	1.1007439	0.271008	0.835943
rad21a	6157.09067	-0.071326038	0.0662471	-1.076667	0.281629	0.84357
cdk15	48.26180266	0.428754795	0.2810784	1.5253924	0.127161	0.656588
igf2r	160.1948353	0.250736524	0.1864647	1.3446865	0.178726	0.739486
pimr185	2.296881697	0.424419626	1.2199724	0.3478928	0.727921	NA
chrd	76.67547542	-0.172694421	0.2511201	-0.687696	0.491644	0.931926
myof	477.1262626	-0.154498911	0.1125829	-1.372312	0.169966	0.727155
tbx2b	1189.949232	-0.165194301	0.0845986	-1.952684	0.050857	0.44241
kdm5c	3790.65965	-0.10089593	0.0699638	-1.442116	0.14927	0.696454
csnk1db	3205.271969	-0.005907903	0.0693877	-0.085143	0.932147	0.992702
cep170aa	1257.726684	-0.054263015	0.0799902	-0.678371	0.497536	0.933353
star	86.87845939	0.385399752	0.2555283	1.5082469	0.131491	0.66499
pde11al	24.52078131	0.319521452	0.381131	0.8383506	0.401834	0.90084
lrrk2	293.7260503	-0.138692787	0.1470293	-0.9433	0.345527	0.878434
lrrc23	22.87557575	-0.150784519	0.4013431	-0.3757	0.70714	0.968664
hif1aa	271.2966664	0.229033425	0.1325827	1.7274757	0.084082	0.557747
gatad2ab	683.1314967	-0.051929729	0.1004273	-0.517088	0.605095	0.95651
sav1	464.8637007	0.124661056	0.1164571	1.0704461	0.284419	0.844955

eif4g1a	4698.112234	0.067023458	0.0697805	0.9604899	0.336809	0.876088
erbb3a	614.2922031	-0.373043933	0.1029738	-3.622706	0.000292	0.011553
pou4f3	81.52697056	0.476529738	0.2243448	2.1240955	0.033662	0.357045
gpx1b	217.6439158	-0.46576608	0.1585128	-2.93835	0.0033	0.076671
akr1b1.1	426.4402145	-0.036037557	0.1103236	-0.326653	0.74393	0.974094
gnl3	709.6530273	0.043371429	0.0959015	0.4522495	0.651089	0.960708
ugt1ab	1731.453604	-0.530735974	0.07914	-6.706295	2.00E-11	5.41E-09
ddx39aa	2633.551247	-0.181421609	0.0750693	-2.416723	0.015661	0.229392
brms1la	657.1624003	-0.072843964	0.1098017	-0.663414	0.507066	0.935734
slc27a1a	632.7085926	0.042654141	0.1024193	0.4164659	0.677069	0.965257
ptp4a1	5524.140023	-0.028311034	0.0738819	-0.383193	0.701577	0.968134
ell2	591.0514475	0.182701647	0.0971527	1.8805625	0.060031	0.478654
mier3a	461.1730703	-0.057951499	0.1102524	-0.525626	0.599148	0.955891
vldlr	1735.077725	0.043326088	0.0848121	0.5108479	0.609458	0.956718
tuba8l4	9323.859507	-0.046346989	0.1074301	-0.431415	0.666166	0.964736
stat1a	664.8621553	-0.081053063	0.0991026	-0.81787	0.413431	0.905838
mpp5a	168.5982995	-0.129965703	0.1737294	-0.748093	0.454404	0.920544
rragd	52.97890481	0.260645647	0.3004322	0.8675688	0.38563	0.89551
waslb	592.0010963	-0.160360612	0.1075103	-1.491584	0.135808	0.672208
prdm5	61.16875224	0.136220135	0.2556838	0.5327679	0.594194	0.954434
ndufs5	2008.575559	-0.075940898	0.1962739	-0.386913	0.698821	0.967998
maats1	44.95707412	0.258003963	0.2973033	0.867814	0.385496	0.89551
arhgef7a	1122.598505	0.070712918	0.0811663	0.87121	0.383639	0.895192
psmd9	243.9405205	-0.266476121	0.139599	-1.908868	0.056279	0.465438
RAPH1 (1 of many)	987.2091769	-0.004809403	0.1087473	-0.044225	0.964725	0.996004
shisa4	396.5106316	0.001583425	0.1371699	0.0115435	0.99079	0.997679
ptpn18	17.06121104	0.442387044	0.4728532	0.9355696	0.349495	0.880195
itgav	2148.592597	0.206526474	0.0776739	2.6588906	0.00784	0.142065
rpl23a	16859.79678	-0.348215895	0.0811132	-4.292962	1.76E-05	0.001125
snx14	931.6448837	-0.010792253	0.0870612	-0.123962	0.901346	0.990121
tas1r3	2.952202733	-1.831067659	1.2689793	-1.442945	0.149036	NA
med17	260.4170752	-0.21655892	0.1353601	-1.599873	0.109627	0.623766
nkx1.2la	15.81143197	-0.445878682	0.5170435	-0.862362	0.388488	0.896271
itga5	368.3945405	0.019143361	0.1181965	0.1619622	0.871336	0.987754
slc18a3a	864.4162527	0.193403157	0.0907489	2.1311906	0.033073	0.354192
crlf1b	30.14985461	-0.00047302	0.3682755	-0.001284	0.998975	0.999632
atp6v1h	2668.358836	0.014063281	0.0702665	0.2001419	0.84137	0.985174
ugt5c2	4.33346125	0.014887252	0.8984707	0.0165695	0.98678	NA
stxbp5l	333.6195953	0.024940196	0.1249947	0.1995301	0.841848	0.985174
triobpb	351.7599854	-0.164950542	0.1188187	-1.388254	0.16506	0.721223
smad2	874.0817844	0.02262727	0.094768	0.2387648	0.811288	0.981081
exosc9	221.2938546	0.043944217	0.1442945	0.3045453	0.760712	0.975374
rbm25b	1701.372787	-0.040136714	0.0807983	-0.496752	0.619364	0.957354
nrcama	246.1294356	0.011850605	0.1359345	0.0871788	0.930529	0.992702
laptm4a	2770.299717	0.016176961	0.0712099	0.2271728	0.820289	0.981594
ywhae1	4816.074097	0.011105368	0.0751088	0.147857	0.882456	0.988344
hmgb3b	1081.867582	-0.128784517	0.086201	-1.494003	0.135175	0.670689
mapk12b	15.60658749	-0.051047287	0.5238122	-0.097453	0.922366	0.99233
rpl38	3045.811881	-0.30148704	0.1141575	-2.640974	0.008267	0.147176
atrnl1b	1467.692696	0.053776172	0.0857577	0.6270712	0.530613	0.942209
esyt3	200.0014897	0.161653527	0.1456704	1.1097211	0.267119	0.833191
fabp2	2097.296366	-0.919807631	0.1004552	-9.156397	5.37E-20	4.82E-17
polr1e	228.6445989	0.016776503	0.1597585	0.1050116	0.916367	0.991314
slc19a3a	11.00283241	0.014780194	0.6073078	0.0243372	0.980584	NA

pdgfrl	367.3030301	0.227073226	0.1247102	1.8208071	0.068636	0.513124
pcdh8	227.5483266	0.445931028	0.1478384	3.016342	0.002558	0.064039
grap2a	38.60215959	0.089216844	0.3122717	0.2857026	0.775106	0.975687
saxo2	11.25041867	-0.930583492	0.651083	-1.429286	0.152922	0.702086
ddi2	1199.477964	0.093032946	0.0819409	1.1353669	0.256222	0.824633
anp32a	8789.5026	-0.142111252	0.0677799	-2.096657	0.036024	0.370238
agpat9l	279.6323191	-0.264810674	0.136367	-1.941898	0.05215	0.44874
asb7	243.5815639	-0.025025793	0.136402	-0.183471	0.854429	0.985174
rtn1a	9223.066355	0.048220854	0.0633111	0.7616493	0.446269	0.917154
cyp2x10.2	1.012669898	-0.989442317	1.8898446	-0.523558	0.600586	NA
pip5k1bb	234.4395109	-0.170972089	0.1373295	-1.244977	0.21314	0.781631
her6	1022.993407	-0.139281177	0.090549	-1.538186	0.124003	0.652658
otop2	34.32680789	-0.782254643	0.3322687	-2.354284	0.018558	0.253962
supt6h	2516.027899	0.008185257	0.0735977	0.1112162	0.911445	0.991066
fn1b	4847.688647	0.034214695	0.0811427	0.4216608	0.673273	0.965257
brd3a	987.9348863	0.054911295	0.0866969	0.6333708	0.526492	0.940506
c1qtnf6b	75.95694077	0.237667841	0.2372941	1.0015751	0.316549	0.867129
rgp1	276.987119	0.061818214	0.1394568	0.4432784	0.657564	0.96252
ak4	645.0252541	-0.036742372	0.0961257	-0.382233	0.702289	0.968134
rras	603.645735	-0.167179551	0.1019301	-1.640139	0.100976	0.604004
irf4a	4.272475133	-0.195838938	0.9343848	-0.209591	0.833987	NA
nlk1	1582.710443	0.051577151	0.0758196	0.6802617	0.496339	0.932415
dus4l	94.20559064	0.019559993	0.2091078	0.0935402	0.925474	0.992676
kcnmb2	23.49231658	0.032636729	0.4414577	0.0739295	0.941067	0.992857
nadka	18.35575191	-0.21905312	0.4855097	-0.451182	0.651859	0.960708
cldnd	4.552851705	-0.30246027	0.8915766	-0.339242	0.734427	NA
phf8	1022.58731	0.010779435	0.084761	0.1271745	0.898802	0.990121
DMWD	27.04716774	-0.28161997	0.3753379	-0.75031	0.453068	0.919811
zgc:111983	2747.823718	0.320640935	0.1909021	1.6796096	0.093033	0.580695
sgk2b	320.2919371	0.338535508	0.1320811	2.5630875	0.010375	0.17519
lsm14aa	703.7356155	-0.035178983	0.0972031	-0.361912	0.717418	0.96909
kaznb	219.7611419	-0.006620184	0.1494824	-0.044287	0.964675	0.996004
chrna2a	46.46349634	0.151123041	0.3036332	0.4977158	0.618684	0.957354
csrp1a	332.7047207	0.059708159	0.118983	0.5018209	0.615794	0.95688
nectin3b	148.9951157	-0.22669946	0.16464	-1.37694	0.168531	0.725919
gpr27	342.8201108	0.014531074	0.1234807	0.1176789	0.906322	0.990702
map2k2a	1115.253269	0.032969637	0.0914389	0.3605647	0.718425	0.96909
hnf1ba	411.9210575	-0.481554774	0.1276667	-3.771969	0.000162	0.00723
napga	554.834718	-0.070386046	0.1005896	-0.699735	0.484093	0.929461
pspc1	753.4191739	-0.091221212	0.0897982	-1.015846	0.309703	0.86172
ppp2r2ab	1019.630046	0.121631867	0.094525	1.2867692	0.198175	0.76333
slitrk2	265.3169652	-0.012171886	0.1468151	-0.082906	0.933926	0.992702
lnpk	375.7316698	-0.03564496	0.1184512	-0.300925	0.763472	0.975374
eomesa	396.9979976	-0.188824669	0.1163911	-1.622329	0.104733	0.612652
drg2	458.0409451	-0.040045533	0.110942	-0.360959	0.71813	0.96909
bud13	241.4643905	-0.070007706	0.1384186	-0.505768	0.61302	0.95688
fam49a	503.4503518	-0.183562959	0.1262184	-1.454328	0.145855	0.691641
ripk1l	39.30113542	0.180856561	0.3116564	0.5803075	0.561707	0.948728
pth2r	19.32556115	-0.002884734	0.4506789	-0.006401	0.994893	0.998694
arhgef6	437.2338173	-0.036561509	0.1088406	-0.335918	0.736933	0.972535
rpl12	17308.03098	-0.414720974	0.0786846	-5.270678	1.36E-07	1.82E-05
wipf2b	651.1523377	-0.111252231	0.0987882	-1.126169	0.260094	0.828288
speccl1a	785.0583491	-0.00880547	0.0896923	-0.098174	0.921794	0.992138
tmem178b	561.9277918	0.027414919	0.1115233	0.2458222	0.80582	0.979908

mllt3	330.3099598	0.140524133	0.1311709	1.0713056	0.284032	0.844697
klhl41b	1095.106387	0.124793124	0.1126857	1.1074441	0.268102	0.83343
fam234b	523.4265908	0.012475769	0.1059957	0.1177007	0.906305	0.990702
slc24a3	60.24628482	0.312542593	0.2605944	1.1993451	0.230394	0.798424
snd1	3590.783254	-0.149212565	0.07865	-1.897172	0.057805	0.471676
elovl4a	142.644092	0.006728665	0.1676055	0.0401459	0.967977	0.996315
cass4	22.70800178	0.355901001	0.4153443	0.8568819	0.39151	0.897688
rb1	489.1686445	0.065341294	0.1053391	0.6202947	0.535064	0.943336
copz2	740.0224563	0.058063258	0.095677	0.6068674	0.543939	0.945438
arntl1a	608.6353475	-0.133692302	0.0960324	-1.392159	0.163874	0.71945
nt5dc1	140.7228392	0.020187699	0.1823781	0.1106915	0.911861	0.991066
sult1st6	123.9905369	-0.146640079	0.1809584	-0.810352	0.417738	0.906237
wdr26b	1400.205911	-0.089056229	0.0798958	-1.114654	0.264999	0.830836
gtf2f2b	0.349228403	0.005884014	3.2372587	0.0018176	0.99855	NA
urod	165.3844148	0.183734175	0.1687611	1.0887233	0.276276	0.839541
galnt9	781.8181277	0.059623849	0.1006146	0.5925966	0.553451	0.947265
mycn	2130.824851	0.972317845	0.0784082	12.400723	2.59E-35	1.28E-31
eef1a2	38.39117075	0.163036187	0.3307921	0.4928661	0.622107	0.957354
gnmt	756.0963196	0.095812043	0.0988164	0.9695962	0.332248	0.874653
parp9	203.0340818	-0.248839256	0.1490026	-1.670033	0.094913	0.585587
asic2	113.5039116	0.032438126	0.1980058	0.1638241	0.86987	0.987395
nkain1	690.0975091	-0.089583971	0.0944732	-0.948248	0.343003	0.878434
kifap3b	1115.615611	-0.009575813	0.0900913	-0.10629	0.915352	0.991171
si:ch1073-349o24.2	69.46429725	0.422675622	0.2492755	1.6956166	0.089959	0.57419
glra4a	251.0425727	0.227141041	0.141479	1.605475	0.108389	0.620165
trh	148.4996028	0.322493589	0.1737838	1.8557177	0.063494	0.49217
stx17	71.17957387	-0.289000258	0.2306691	-1.252878	0.21025	0.778043
enpp4	1.292891743	2.681696034	1.8542769	1.446222	0.148115	NA
phf21aa	587.6640791	-0.04257693	0.1153096	-0.36924	0.711949	0.969006
mpped2a	925.7492766	-0.064650665	0.0894454	-0.722794	0.469806	0.924741
kcns3a	125.7880363	0.364804271	0.1988532	1.8345408	0.066574	0.504929
myo3b	177.0397844	0.206292904	0.1694318	1.2175575	0.223392	0.789519
oprk1	12.44576503	0.542877233	0.5627675	0.9646563	0.334717	0.875699
lhx6	242.5528803	-0.069955846	0.134798	-0.518968	0.603783	0.956207
far1	203.2601759	0.049293088	0.1542931	0.3194769	0.749365	0.97419
impdh2	751.4952161	0.072014213	0.0943767	0.7630508	0.445433	0.917117
si:ch1073-459j12.1	806.8669291	0.219350895	0.1027137	2.1355563	0.032716	0.352352
wnt8b	54.85333849	-0.174322399	0.2710744	-0.64308	0.520172	0.939056
arl8ba	579.3598032	-0.121548217	0.1145009	-1.061548	0.288441	0.845979
lrp5	1240.342293	-0.061409945	0.0854965	-0.718274	0.472588	0.924838
cacna1ab	79.57386197	0.2004276	0.2284438	0.8773605	0.380291	0.895192
fbxo38	416.4018174	-0.015925662	0.1152523	-0.138181	0.890097	0.989291
tbx16l	4.477879896	-0.543281226	0.8943932	-0.60743	0.543566	NA
gdpd3b	55.71316196	-0.760155637	0.2833865	-2.682399	0.00731	0.134958
csel1	1606.009489	0.070378067	0.0770832	0.9130143	0.361235	0.885686
zak	25.67069769	-0.21996413	0.4090918	-0.537689	0.590792	0.954193
ccdc113	14.56857614	0.191006562	0.5014414	0.380915	0.703266	0.968134
celf3b	657.7707934	0.045338154	0.0969639	0.4675779	0.640087	0.959994
creld1b	321.7409957	0.012231682	0.1397382	0.0875328	0.930248	0.992702
hoxa11b	85.25744094	-0.029050441	0.2131165	-0.136313	0.891574	0.989291
ms4a17a.6	4.621741319	-0.211505468	0.8690414	-0.243378	0.807713	NA
uox	916.8109836	0.402994464	0.0929044	4.3377349	1.44E-05	0.000972
ttc9c	77.29777694	-0.262284942	0.2325177	-1.128021	0.259311	0.827577
hnrpkl	1563.721033	0.082265351	0.0809122	1.0167232	0.309285	0.861381

tmigd1	21.68657553	-1.010167047	0.4398489	-2.296623	0.02164	0.278385
cnot4a	388.7919095	-0.034858715	0.1263226	-0.27595	0.782587	0.97627
mrps18b	293.146816	0.099138729	0.1272308	0.7792038	0.43586	0.914408
si:dkey-19a16.16	0	NA	NA	NA	NA	NA
ankrd50l	280.2217057	0.221121349	0.1545878	1.4303935	0.152604	0.701776
rhcgl1	227.3036038	0.945229799	0.1882643	5.0207608	5.15E-07	5.78E-05
pias4a	1145.313722	0.069926916	0.0810083	0.8632066	0.388024	0.896144
aqp10a	20.80455487	0.816563496	0.443574	1.8408731	0.06564	0.500566
xab2	500.2886875	-0.175064264	0.117922	-1.484577	0.137656	0.675209
her13	95.91855796	0.023950059	0.2120953	0.1129212	0.910093	0.990702
itm2ba	3893.418513	0.032571415	0.0721681	0.4513271	0.651754	0.960708
cx43.4	953.3706508	0.038397304	0.0919298	0.4176808	0.67618	0.965257
lipia	233.7116207	-0.150406151	0.1420585	-1.058762	0.289708	0.846246
asb6	125.7708345	-0.061668123	0.1773393	-0.347741	0.728035	0.970402
acat2	493.714183	-0.811775629	0.1288669	-6.299332	2.99E-10	6.57E-08
slc6a16a	75.55652013	0.294045053	0.2337001	1.2582153	0.208314	0.775404
mrto4	187.0989118	0.123357923	0.1597666	0.7721135	0.440047	0.916082
cnot3a	1032.299694	0.010009217	0.0848088	0.118021	0.906051	0.990702
gtf2h4	118.953651	0.241536965	0.1925467	1.2544329	0.209685	0.777044
psmc3	3358.260306	0.015198262	0.0774732	0.1961746	0.844474	0.985174
l1cama	418.3806976	0.032463709	0.1277947	0.2540302	0.799472	0.979366
kcnk2b	15.44270366	0.031028487	0.486636	0.0637612	0.94916	0.99381
itgb1bp1	396.9378821	-0.020503952	0.1194151	-0.171703	0.863671	0.985952
aig1	185.3387701	-0.194530271	0.1674945	-1.161413	0.245474	0.815388
plxna3	578.7140999	-0.062526421	0.1060165	-0.58978	0.555338	0.947452
recql	239.2967951	-0.037680919	0.145465	-0.259038	0.795606	0.978498
sash1a	736.9283012	-0.324648949	0.0906487	-3.581399	0.000342	0.013122
slc30a4	357.824557	-0.18510561	0.1395215	-1.326717	0.184602	0.747376
nadk2	275.155108	-0.128777197	0.1335154	-0.964512	0.334789	0.875699
zbtb16a	763.9870208	-0.23080381	0.091098	-2.533578	0.01129	0.184823
ppp1r8a	59.20336127	-0.193479904	0.2498887	-0.774264	0.438775	0.915997
grm2b	857.6923152	0.1158302	0.0918971	1.2604337	0.207513	0.775175
rae1	762.5264825	0.018861581	0.0928738	0.2030883	0.839066	0.984979
mta1	510.3977462	-0.105528359	0.1072672	-0.983789	0.325219	0.871629
CR846090.1	0.166657454	-0.955901296	4.0804729	-0.234262	0.814781	NA
cnpy2	270.6971015	0.188110217	0.1351512	1.39185	0.163968	0.71945
abce1	2658.013137	-0.018402448	0.0771692	-0.238469	0.811517	0.981081
wdr55	140.0990577	0.228151182	0.1691164	1.3490776	0.177312	0.738201
actn1	1438.413122	0.111293306	0.0814404	1.3665618	0.171763	0.730416
ncam1b	573.5755885	0.037191425	0.1023769	0.3632795	0.716396	0.96909
pbk	307.7011987	-0.246691043	0.1266233	-1.948228	0.051388	0.444245
ryk	644.8089374	0.140777633	0.0988256	1.4245053	0.1543	0.70398
mycb	589.1991125	0.115278171	0.1058675	1.0888906	0.276202	0.839541
acp2	456.4773856	-0.071911866	0.1219005	-0.589923	0.555243	0.947449
rundc3aa	37.82043399	0.362617613	0.3266962	1.1099535	0.267019	0.833191
ric8a	582.9493911	0.105265633	0.101268	1.0394762	0.298583	0.854441
use1	416.9958231	-0.023680582	0.1194976	-0.198168	0.842914	0.985174
rab5ab	461.0957272	-0.102666814	0.107567	-0.954445	0.339858	0.87805
nipsnap2	570.2036172	0.072713014	0.1092634	0.6654837	0.505741	0.935724
mynn	484.4084503	-0.03648875	0.1093592	-0.33366	0.738636	0.972722
si:ch211-251b21.1	8100.669951	0.210512137	0.0639694	3.2908231	0.000999	0.030961
ela3l	132.7559563	-0.721788767	0.2137083	-3.377448	0.000732	0.023992
myf5	39.568704	-0.176763308	0.3111251	-0.568142	0.569938	0.948983
ect2	326.2303885	-0.09764471	0.1249383	-0.781543	0.434483	0.914278

yipf5	622.391728	0.132045004	0.1082414	1.2199125	0.222498	0.788748
mrpl57	450.6635387	-0.033281824	0.1115504	-0.298357	0.765431	0.975374
metrnlb	36.93258875	-0.086238575	0.3139839	-0.274659	0.783578	0.97627
aco2	9423.54146	0.088104541	0.0635651	1.3860514	0.165731	0.721576
sh3gl3b	190.3223862	-0.621032914	0.1522351	-4.079434	4.51E-05	0.002487
tmpoa	437.8220793	-0.135285765	0.12313	-1.098723	0.271889	0.836816
rpl7	21923.34914	-0.362203619	0.0906812	-3.994252	6.49E-05	0.0034
chmp4bb	1197.679138	-0.12172215	0.0840681	-1.447899	0.147645	0.694905
tbx16	6.739927876	0.503079728	0.7582505	0.6634743	0.507027	NA
tcap	427.548985	-0.367166687	0.157901	-2.325297	0.020056	0.266383
degs1	823.2495079	0.078309614	0.0994604	0.787345	0.43108	0.91234
dmrt1	3.640076028	-1.146635083	1.0659907	-1.075652	0.282083	NA
fgf20a	67.56214406	-0.034904258	0.2372009	-0.147151	0.883013	0.988659
cxcr3.1	2.665287728	-1.125376781	1.1680178	-0.963493	0.3353	NA
UBL4A	256.3053814	-0.047895112	0.1365628	-0.350719	0.7258	0.970402
sft2d1	504.62287	0.004844884	0.107449	0.0450901	0.964036	0.995927
AMDHD1	68.6935162	-0.11671258	0.2436676	-0.478983	0.631951	0.957356
tcf7l1b	593.1851087	-0.099819692	0.1263383	-0.790099	0.42947	0.911489
dysf	282.830811	0.040572501	0.147569	0.2749393	0.783363	0.97627
slc26a3.1	0.839019598	-0.671793185	2.356059	-0.285134	0.775541	NA
odc1	3797.657058	0.226022798	0.0933474	2.4213084	0.015465	0.228575
ubtd1a	252.0472854	0.341654907	0.1353947	2.5233985	0.011623	0.189002
kcnk6	275.777184	-0.168657025	0.1568575	-1.075224	0.282274	0.843666
cct7	5680.448834	-0.058053623	0.1001923	-0.579422	0.562304	0.948728
irf2a	238.7295902	0.009635834	0.13669	0.0704941	0.9438	0.993364
rnd3b	172.7219373	0.131291456	0.1581714	0.8300582	0.406506	0.901305
lrrk1	16.84437373	0.064144893	0.4604787	0.1393005	0.889213	0.989291
strap	1052.445045	-0.058012851	0.0894585	-0.648489	0.516669	0.938543
phox2a	444.8420847	-0.248563394	0.1145944	-2.16907	0.030077	0.335259
barx1	296.2612607	-0.168173267	0.1265989	-1.328395	0.184048	0.747376
ppifa	106.8455371	-0.16456251	0.201664	-0.816023	0.414487	0.906237
slc2a1b	255.3773369	-0.168261245	0.1568398	-1.072822	0.283351	0.84466
arid2	1048.556434	-0.008383775	0.0984835	-0.085129	0.932159	0.992702
chmp4c	90.96099354	0.097200608	0.2116655	0.4592179	0.646078	0.96029
ftcd	599.8366698	0.043236963	0.1117534	0.3868963	0.698833	0.967998
apol1	114.3368435	-0.248202104	0.1976611	-1.255695	0.209227	0.776073
ndor1	225.4285823	0.041132059	0.1441769	0.2852888	0.775423	0.975687
prdm12b	367.6373378	0.066078003	0.1177684	0.5610843	0.57474	0.950037
avpr2aa	36.80254798	-0.153661374	0.3226378	-0.476266	0.633885	0.95776
ube2ib	2044.699411	-0.091468814	0.0855804	-1.068805	0.285157	0.845129
fbxl18	110.0837487	-0.041809196	0.2093375	-0.199721	0.841698	0.985174
ift81	73.32075891	-0.202938653	0.2341354	-0.866758	0.386075	0.89551
slc25a39	523.2792199	0.048500941	0.1064017	0.4558285	0.648513	0.960708
srgap1a	49.10452111	0.212442167	0.285784	0.7433662	0.45726	0.921679
tdrd1	26.2448842	-0.066970736	0.381707	-0.175451	0.860726	0.985773
ifit10	5.462608657	-0.826996813	0.8225468	-1.00541	0.314699	NA
fbxo8	238.7632575	0.306610305	0.1438763	2.1310685	0.033084	0.354192
rpe65a	3202.571386	-0.151647976	0.1097589	-1.381647	0.16708	0.723651
epc2	541.8843625	-0.140217968	0.1098348	-1.276626	0.201734	0.767736
adrb1	19.21627181	-0.1997051	0.4270132	-0.467679	0.640014	0.959994
st3gal8	161.4323228	0.179271721	0.1824413	0.9826267	0.325791	0.871896
ufl1	616.1083569	-0.151721328	0.0958999	-1.58208	0.113631	0.631874
kmt2e	1641.760292	-0.156173485	0.0995061	-1.569487	0.116534	0.639148
ndufs2	3937.723237	0.076646641	0.0749362	1.022825	0.306391	0.860027

zcchc9	160.4517549	0.1699508	0.1581134	1.0748662	0.282435	0.843666
itln1	3.459158892	0.170002151	1.0220201	0.1663393	0.86789	NA
opn4.1	500.9303093	0.179359494	0.1305557	1.3738161	0.169499	0.726744
sema3bl	157.4549576	0.057588828	0.1696999	0.3393569	0.734341	0.972021
cdh23	9.555134028	-1.278876097	0.6802839	-1.879915	0.06012	NA
appbp2	818.2911587	0.090944703	0.0919102	0.9894949	0.322421	0.870226
crybb1l1	30709.61321	-0.125949877	0.0788796	-1.596735	0.110325	0.624637
si:ch1073-155h21.1	7.164081389	-0.039148791	0.7430108	-0.052689	0.957979	NA
bloc1s4	160.9983117	-0.097562688	0.1636449	-0.596185	0.551051	0.946549
zmynd8	328.2005596	0.127504001	0.1227248	1.0389423	0.298832	0.85478
stxbp2	589.8654677	-0.132594908	0.1009942	-1.312897	0.189218	0.753954
wwox	153.8753649	-0.209076548	0.1671366	-1.250932	0.210959	0.778412
gins1	127.9310959	-0.020591645	0.1810058	-0.113762	0.909426	0.990702
mkrn2	417.1198203	-0.102583462	0.1112741	-0.921899	0.356581	0.883422
cnot4b	457.6253509	0.075060645	0.1130571	0.663918	0.506743	0.935734
nsfa	4446.62419	0.072720804	0.0720162	1.0097839	0.312599	0.864642
crybb1l3	122.6192105	-0.017071636	0.1923354	-0.08876	0.929273	0.992702
ccnh	109.4951022	-0.080892824	0.1943294	-0.416266	0.677215	0.965257
amph	2660.231448	0.05899996	0.0743853	0.7931674	0.42768	0.91075
ghrb	321.7007615	0.091503502	0.1240726	0.7374994	0.460819	0.921978
ttyh3b	3719.469383	0.085365313	0.069974	1.2199579	0.222481	0.788748
ppdpfa	1893.008582	0.140991128	0.093217	1.5125038	0.130406	0.662797
cul1b	632.2025832	-0.228584505	0.0959228	-2.383004	0.017172	0.242176
nfkbiab	1361.571687	-0.412441822	0.1002138	-4.115621	3.86E-05	0.002188
fabp7a	11931.62973	0.053412179	0.065513	0.8152915	0.414906	0.906237
nsa2	3951.24262	-0.397104342	0.091976	-4.317477	1.58E-05	0.001037
adamts8a	60.24414993	0.222404166	0.2537315	0.8765336	0.38074	0.895192
rab3il1	141.5870594	-0.104519961	0.1717004	-0.608735	0.5427	0.944974
lgsn	1894.001707	0.186667591	0.0759956	2.4562949	0.014038	0.214185
sub1b	571.7184647	-0.08300847	0.1099224	-0.755155	0.450156	0.918761
nudt8	135.0305915	0.020450911	0.1703274	0.1200683	0.904429	0.99032
efnb1	176.3694374	0.241573349	0.1572968	1.5357804	0.124592	0.653845
rgl2	30.7308749	-0.381328496	0.3430096	-1.111714	0.266261	0.832419
stn1	89.68809231	-0.13556973	0.2171093	-0.624431	0.532345	0.942651
zyg11	683.2049652	0.120393043	0.0933253	1.2900367	0.197038	0.761944
atp1a1a.2	3077.397451	-0.021490017	0.094138	-0.228282	0.819427	0.981594
il15l	20.4824893	-0.161944965	0.4276664	-0.378671	0.704932	0.968134
tsr1	1048.244596	-0.099284799	0.0910162	-1.090847	0.27534	0.839541
uqcrfs1	4121.122388	0.006841077	0.0717631	0.0953286	0.924054	0.992676
lmnl3	91.8246231	-0.002252514	0.2065637	-0.010905	0.991299	0.997679
cpne2	596.9169579	-0.040693708	0.1038382	-0.391895	0.695136	0.96736
wdr61	363.5702998	0.085980123	0.1270708	0.6766316	0.49864	0.934006
spg21	251.4055573	0.101661086	0.1414765	0.7185723	0.472404	0.924838
klhdc10	542.4945921	0.004587929	0.1030787	0.044509	0.964499	0.995927
sult5a1	22.72197104	-0.360910393	0.4046026	-0.892012	0.372386	0.892843
vash2	592.5749792	-0.319516756	0.1033488	-3.091636	0.001991	0.053335
blk	3.808222402	-0.886825723	1.0837986	-0.818257	0.413211	NA
tmx2b	1298.694265	0.038566917	0.0786029	0.4906549	0.623671	0.957354
zgc:112965	1.727511566	2.104037042	1.7084701	1.2315328	0.218124	NA
atp2b1b	1230.644855	0.086780355	0.1005086	0.8634121	0.387911	0.896133
ppp2r1ba	4952.986846	-0.001013219	0.0677105	-0.014964	0.988061	0.997369
cst3	2579.653545	-0.02314166	0.0734242	-0.315177	0.752627	0.97481
gas6	396.895015	0.10429034	0.1216465	0.8573227	0.391267	0.897688
cyth3a	347.4285183	0.105876223	0.1168698	0.9059328	0.364971	0.886714

zdhhc16a	377.9411398	0.048588045	0.1184984	0.4100313	0.681783	0.965476
sp3b	884.5663964	-0.064067106	0.094504	-0.67793	0.497816	0.933482
rnpepl1	294.0498863	0.036672303	0.1346882	0.2722755	0.78541	0.97627
palm1b	1863.574786	0.127597804	0.0763934	1.6702728	0.094865	0.585477
atf3	819.8448444	0.286502667	0.1161702	2.4662322	0.013654	0.20979
camta1b	1665.202168	0.028354854	0.0788621	0.3595497	0.719184	0.969183
map2k1	947.1321485	0.152751472	0.0863831	1.7683023	0.07701	0.540058
ctsla	17394.22345	0.284580663	0.0782446	3.637065	0.000276	0.011106
MCHR2 (1 of many)	12.74084292	-0.421911399	0.5607783	-0.752368	0.45183	0.918761
rtn4rl1a	9.5932182	-0.718600694	0.6379388	-1.126441	0.259979	NA
tra2a	1628.934481	-0.081855454	0.0810262	-1.010234	0.312383	0.864401
pimr195	0	NA	NA	NA	NA	NA
dazap2	2686.62247	-0.075763514	0.0709351	-1.068069	0.28549	0.845164
ehd3	21.06494903	0.405812782	0.4403468	0.9215754	0.35675	0.883422
fancl	16.84381509	0.50919413	0.4666962	1.0910613	0.275246	0.839541
slc35b2	305.8638273	-0.18998375	0.1279991	-1.484259	0.13774	0.675209
meox1	212.1134795	-0.136765419	0.1468311	-0.931447	0.351622	0.880769
pdcd2	32.76028341	-0.536678227	0.3550452	-1.511577	0.130641	0.662797
crnkl1	445.8118757	-0.023895211	0.1150569	-0.207682	0.835478	0.984089
lrp2b	60.00960839	-2.926418003	0.3302418	-8.861439	7.90E-19	6.01E-16
si:ch211-196i2.1	624.6035421	0.319727356	0.1026179	3.1157089	0.001835	0.050201
xpnpep3	272.0718139	-0.059272544	0.1344349	-0.440901	0.659284	0.962839
ttc27	491.8081952	-0.039201004	0.1082908	-0.361997	0.717354	0.96909
ptpn1	709.0535964	0.039708486	0.0956558	0.4151186	0.678055	0.965257
hmx4	371.9510555	0.097793271	0.1250175	0.7822367	0.434075	0.914001
gabpb2a	390.6634382	-0.044050953	0.1189187	-0.370429	0.711063	0.968985
lhx1b	127.4261214	0.064754586	0.1854608	0.3491551	0.726973	0.970402
itga11b	27.10516861	-0.1930393	0.3638085	-0.530607	0.595691	0.954434
iars	1147.932881	-0.077680686	0.1023261	-0.759149	0.447764	0.917154
hibadhb	1467.010252	0.074418583	0.0851649	0.8738173	0.382218	0.895192
hnrnpaba	14118.50802	-0.088873508	0.0707215	-1.25667	0.208873	0.776073
cks1b	238.8054091	0.178494901	0.1423218	1.2541638	0.209782	0.77726
fth1b	586.3795822	-0.136098623	0.0991282	-1.372955	0.169766	0.726965
si:ch211-220f16.2	329.076335	-0.016966903	0.1291598	-0.131364	0.895488	0.989814
onecut1	80.17984846	-0.04741022	0.2343907	-0.20227	0.839706	0.984979
masp2	150.191159	0.047171644	0.1778965	0.2651634	0.790884	0.977402
nedd8	694.418251	0.007596964	0.1069245	0.0710498	0.943358	0.993364
wt1b	26.63191858	-0.30688994	0.3669666	-0.836289	0.402993	0.90084
pou2f1b	218.1371432	0.04612418	0.1456103	0.3167645	0.751422	0.97419
zgc:194189	13.5644973	-0.592630444	0.5410046	-1.095426	0.27333	0.838214
nudt17	278.3581481	-0.250667485	0.1325431	-1.891215	0.058596	0.473924
mxg	2.299317655	0.341892505	1.3878956	0.2463388	0.80542	NA
med31	275.6864082	-0.017061013	0.1301862	-0.131051	0.895735	0.989814
kif18a	193.2071315	-0.042639673	0.1500936	-0.284087	0.776344	0.975826
fam129bb	384.4979378	-0.174448777	0.1247543	-1.398339	0.162011	0.716531
mep1a.2	14.01425296	-1.497971949	0.5432484	-2.757435	0.005826	0.114852
myl9b	1188.12115	0.167874541	0.0888098	1.8902709	0.058722	0.474103
sart3	1291.415545	0.0081961	0.0805349	0.1017707	0.918939	0.991623
skib	813.7803005	0.079322427	0.0942842	0.8413121	0.400173	0.90084
si:dkey-42i9.4	1656.865434	-0.486920204	0.2505658	-1.943283	0.051982	0.447688
scara3	110.5919207	0.087988084	0.1876926	0.4687882	0.639221	0.959994
tma16	245.6753127	0.058607191	0.137977	0.4247606	0.671011	0.965257
il11r4	4.398787045	-0.130072405	0.9865019	-0.131852	0.895101	NA
olfcd1	1.013460044	-2.21473548	2.0108742	-1.101379	0.270732	NA

srsf9	966.2116597	-0.084130215	0.0845655	-0.994852	0.319808	0.86809
ifit9	1.205810811	1.348074313	1.7970906	0.7501427	0.453169	NA
slc7a10a	728.7442625	0.237975927	0.1065044	2.2344233	0.025455	0.308994
mettl2a	209.8947153	0.074646175	0.1444029	0.5169298	0.605205	0.95651
src	78.27424237	0.342073643	0.2285006	1.497036	0.134384	0.670529
si:ch211-195d17.2	356.9071867	-0.06343606	0.1188194	-0.533887	0.59342	0.954434
pcdh15b	205.4742297	-0.075808296	0.151377	-0.500792	0.616518	0.95688
sox1b	574.0121329	-0.036509975	0.1372359	-0.266038	0.79021	0.977338
foxl1	181.6341128	-0.180786349	0.1628197	-1.110347	0.26685	0.833073
kcnab1a	25.70913512	0.241457652	0.3753324	0.6433168	0.520019	0.938995
rbl1	330.7364474	-0.127249872	0.1341824	-0.948335	0.342959	0.878434
stxbp3	601.7996178	-0.021643185	0.1044569	-0.207197	0.835856	0.984089
serinc5	811.7679594	0.096628946	0.0889083	1.0868385	0.277108	0.840549
sms	1045.262861	0.072035688	0.0965792	0.7458714	0.455745	0.921215
btr33	31.61585753	-0.230672649	0.3431408	-0.672239	0.501432	0.934613
caspa	108.7940339	0.157827684	0.1955849	0.8069524	0.419694	0.90651
dbn1a	246.5612398	0.024761428	0.1424292	0.1738508	0.861983	0.985841
hoxb1a	11.48682009	-0.807552524	0.569048	-1.419129	0.155861	0.705963
si:ch73-111e15.1	1.03982315	1.062941397	2.0584146	0.5163884	0.605583	NA
mia3	904.0121165	-0.034875541	0.0867065	-0.402225	0.687518	0.966376
syk	160.9591456	-0.025244565	0.1703506	-0.148192	0.882191	0.988344
sf1	2470.284315	0.022414279	0.0915775	0.2447576	0.806644	0.980254
tmeff1a	22.7489891	0.497579568	0.413295	1.2039331	0.228615	0.796028
pank1a	203.8627498	0.333424158	0.1442572	2.311318	0.020815	0.272701
ugp2b	1002.911432	-0.11796847	0.0955097	-1.235147	0.216776	0.784252
pax1a	57.95012039	0.54256333	0.2672762	2.0299722	0.042359	0.404449
myt1la	973.6582071	-0.037814981	0.0988927	-0.382384	0.702177	0.968134
kcnk13a	13.25701375	0.205789206	0.5549675	0.3708131	0.710777	0.968985
znf410	560.9450447	0.058207024	0.1135075	0.5128036	0.608089	0.956718
crygm4	1.121798001	-0.423136943	1.8510039	-0.228599	0.819181	NA
vps35	2206.954535	0.023632325	0.0730227	0.3236298	0.746218	0.97419
cog5	380.7173681	0.066593833	0.1251993	0.5319027	0.594793	0.954434
sars	2499.724712	0.011813639	0.072492	0.1629648	0.870546	0.987505
manba	86.47562632	-0.327123677	0.2195385	-1.490051	0.136211	0.673089
mocs3	88.16558797	-0.307970414	0.2141036	-1.438418	0.150316	0.698669
cct8	4693.948116	-0.104480366	0.0797515	-1.310074	0.190171	0.754837
vezf1a	67.67940146	-0.38725203	0.2491822	-1.554092	0.120162	0.645215
ptchd4	25.83415239	0.348257705	0.384047	0.9068102	0.364507	0.886564
cnot6a	1313.63391	0.157191618	0.0779762	2.0158914	0.043811	0.411165
mfsd4ab	203.6410168	-0.151167803	0.1449865	-1.042634	0.297118	0.853945
klhl24b	851.5008069	-0.218345549	0.0896948	-2.434317	0.01492	0.223742
rcor2	834.8852599	-0.081931545	0.0975001	-0.840323	0.400728	0.90084
map2k7	7.504147194	-0.191471541	0.7026898	-0.272484	0.78525	NA
fam114a1	286.8454805	-0.091758089	0.1253257	-0.732157	0.464073	0.923197
eif2s3	5531.191275	-0.204607878	0.0746355	-2.741428	0.006117	0.118579
hand2	260.4960271	-0.092295777	0.1360336	-0.678478	0.497469	0.933353
rdh5	478.9576242	0.044818755	0.1310681	0.3419501	0.732388	0.971719
ip6k2a	1761.904568	0.030478596	0.0780381	0.3905605	0.696122	0.96736
ambra1a	187.48083	-0.198248243	0.1795144	-1.104358	0.269438	0.834705
asic1a	69.8616576	0.358829434	0.2532123	1.4171089	0.156451	0.706639
znfl2a	38.59627537	0.443961951	0.3067498	1.4473097	0.14781	0.695242
mcl1b	1665.770665	-0.117508106	0.0926953	-1.267681	0.204912	0.772822
csnk1da	4700.83227	0.009338412	0.0663688	0.1407049	0.888103	0.989291
spred2b	91.19695829	-0.007506265	0.2108302	-0.035603	0.971599	0.996315

epn2	1631.312883	0.115868898	0.07725	1.4999217	0.133635	0.66814
brd7	943.3119715	0.03470682	0.090176	0.3848788	0.700327	0.967998
arpc1a	3366.073002	0.089226799	0.0761898	1.1711119	0.241554	0.810471
hdac6	388.6659342	-0.029082288	0.1145395	-0.253906	0.799568	0.979366
mmp14b	1445.153431	0.20980846	0.081692	2.568285	0.01022	0.17288
snupn	290.2094963	-0.106012605	0.1404306	-0.754911	0.450302	0.918761
ftr24	90.89654543	0.06065115	0.2151272	0.2819316	0.777996	0.975942
cacna1c	41.84825732	0.062008651	0.310219	0.1998867	0.841569	0.985174
phospho1	71.22650445	0.309251081	0.2550829	1.2123552	0.225376	0.792847
tspan7b	3092.205804	0.092496603	0.0727014	1.2722814	0.203273	0.77034
atp1la	1223.718352	0.091757402	0.0824865	1.1123927	0.265969	0.831945
exoc3l2a	76.50601108	-0.27807148	0.2206027	-1.260508	0.207486	0.775175
unc45b	433.9667588	0.474513062	0.1152437	4.1174762	3.83E-05	0.00218
bcl2l1	590.6598433	-0.011967877	0.098636	-0.121334	0.903427	0.990154
atxn10	84.52446997	0.019657494	0.2156632	0.091149	0.927374	0.992702
fkbp4	1499.262605	0.071704135	0.0791873	0.9055	0.365201	0.886714
pcolce2a	63.26460516	0.13391843	0.2498622	0.5359692	0.59198	0.954434
cpeb1b	22.65875599	-0.061274106	0.4410712	-0.138921	0.889512	0.989291
faah2a	241.2740463	-0.426817383	0.1439995	-2.964019	0.003036	0.07278
anapc5	236.9843185	0.036828154	0.1481216	0.2486345	0.803644	0.979643
apex2	143.6108273	-0.172777223	0.1684845	-1.025478	0.305138	0.859759
asb14a	0.489712128	-0.887840729	2.693685	-0.329601	0.741702	NA
trmt61a	167.3006911	-0.1289684	0.1600334	-0.805884	0.42031	0.90651
lrp3	68.66008604	-0.049369136	0.2381993	-0.20726	0.835807	0.984089
dmd	839.9032162	0.033022339	0.0921708	0.3582734	0.720139	0.969318
ltv1	609.610548	-0.015191424	0.0978681	-0.155223	0.876645	0.987881
si:ch211-107o10.3	265.147837	-0.289089395	0.1637036	-1.765932	0.077407	0.542016
myl6	773.6457336	-0.050675465	0.0967056	-0.524018	0.600266	0.956045
pno1	906.8107042	-0.052026407	0.0903407	-0.575891	0.564689	0.948739
glcci1a	147.5234445	-0.16115554	0.1739546	-0.926423	0.354226	0.882064
sox21b	216.6845539	0.124491019	0.1456734	0.8545899	0.392778	0.897688
chia.4	6.450964848	-1.171706352	0.7787125	-1.504671	0.132409	NA
yod1	841.7147279	-0.032238222	0.1009643	-0.319303	0.749497	0.97419
arhgap12a	479.5012146	0.062295363	0.1068048	0.5832637	0.559716	0.948478
pole3	555.8697589	-0.026778464	0.1063209	-0.251865	0.801146	0.979377
zgc:136872	139.439571	-0.926698896	0.1843548	-5.026715	4.99E-07	5.67E-05
brap	262.6119866	-0.076151281	0.1345815	-0.565837	0.571504	0.949418
slc25a24	307.9940673	-0.308837058	0.1289734	-2.394579	0.016639	0.2379
fam20b	591.6126865	0.071665024	0.1030381	0.6955199	0.48673	0.930882
lyst	165.06455	0.142155613	0.1603491	0.886538	0.375328	0.894316
mtmr8	137.6441116	-0.068725577	0.172757	-0.397816	0.690766	0.967157
nbas	153.1224081	0.06019407	0.1755486	0.3428912	0.73168	0.971557
cops3	757.3661321	-0.065870364	0.0971112	-0.678298	0.497583	0.933353
mttp	820.6455141	-0.213774446	0.0910044	-2.349057	0.018821	0.255627
kifap3a	872.9982128	-0.024779646	0.0933682	-0.265397	0.790704	0.977402
coro1b	1471.054072	0.038615025	0.0770477	0.5011835	0.616242	0.95688
fign	94.78153234	0.10174377	0.2117416	0.480509	0.630865	0.957354
snx3	980.8576642	0.013885895	0.0946077	0.1467734	0.883311	0.988706
epas1a	919.1432567	0.107238074	0.0853855	1.2559284	0.209142	0.776073
apeh	473.5246065	-0.154574003	0.1161217	-1.331138	0.183144	0.746697
paip2b	3391.37626	-0.168244038	0.0732583	-2.296585	0.021642	0.278385
lmo3	1977.311984	-0.073134788	0.082638	-0.885002	0.376156	0.894698
prkcba	329.1166143	0.192009753	0.127138	1.5102467	0.13098	0.663795
ranbp3a	15.13618897	-0.205640261	0.4953213	-0.415165	0.678021	0.965257

zgc:66479	846.0089214	0.274266296	0.0921826	2.9752501	0.002927	0.070768
atp6ap2	2858.522056	-0.042646233	0.07547	-0.565075	0.572023	0.949494
esf1	237.513184	-0.024054049	0.1465233	-0.164165	0.869601	0.987282
ube2na	2270.537603	0.01918521	0.0821104	0.2336515	0.815256	0.981185
entpd6	686.9869955	0.191423093	0.0945308	2.0249822	0.042869	0.406764
tmed5	410.7502574	0.176960732	0.1133398	1.5613294	0.118446	0.641861
tle2b	131.7619969	-0.243673829	0.1959053	-1.243835	0.21356	0.781737
cacng1a	366.1320406	0.189804905	0.1217397	1.5591045	0.118972	0.642772
prpf4ba	267.3762986	0.052624058	0.1379043	0.3815985	0.702759	0.968134
dldh	3691.945152	-0.073210883	0.0660826	-1.107869	0.267918	0.833349
camk1gb	636.8101939	0.301307434	0.1027873	2.9313694	0.003375	0.077672
actr3b	16.52442822	0.24797586	0.4891986	0.5069023	0.612223	0.95688
her5	1.961408324	-0.984996006	1.4132582	-0.696968	0.485823	NA
marcksb	8671.159679	-0.039863013	0.075058	-0.531096	0.595352	0.954434
orc3	188.9085373	-0.108094528	0.1538069	-0.702794	0.482184	0.928585
bokb	12.47883558	0.444042547	0.5441157	0.8160812	0.414454	0.906237
eml2	1566.452526	-0.035832232	0.0852757	-0.420193	0.674345	0.965257
cmtm3	81.32753476	0.454863707	0.2241555	2.0292333	0.042435	0.404971
glud1a	1204.742317	0.021108216	0.0870184	0.2425719	0.808337	0.980642
hsf1	207.3478304	0.053857649	0.1422539	0.3786022	0.704983	0.968134
chga	712.7959955	0.294889159	0.0963428	3.0608313	0.002207	0.057504
neu1	203.0726305	-0.068613807	0.1461407	-0.469505	0.638709	0.959628
si:dkey-46g23.5	4.643520045	-1.051399954	0.8957239	-1.173799	0.240475	NA
hmbsa	290.1959735	-0.033813915	0.1292055	-0.261707	0.793548	0.977937
ptprq	13.21240591	-0.157879299	0.5756758	-0.27425	0.783892	0.97627
elp4	112.0601681	-0.206572656	0.1890651	-1.092601	0.274569	0.839136
cyp7b1	32.05197796	0.101214831	0.3778456	0.2678735	0.788797	0.976747
mylipa	387.9020586	0.048082722	0.1282194	0.3750036	0.707658	0.968664
tfap2e	314.532752	-0.038883477	0.1220449	-0.3186	0.75003	0.97419
cabp4	32.07310461	0.162213934	0.343229	0.4726114	0.63649	0.958565
rap1b	1405.757053	0.153652207	0.0881196	1.7436774	0.081215	0.551495
usp28	471.3301235	0.155003574	0.1209075	1.2820009	0.199842	0.765237
hprt1	800.2283009	0.139131579	0.0923342	1.5068253	0.131855	0.665566
msx1b	164.811155	0.030170998	0.1612525	0.1871041	0.851579	0.985174
ctns	101.5601874	0.016765321	0.19578	0.0856335	0.931758	0.992702
smarca2	561.3304923	-0.100984455	0.1073407	-0.940784	0.346815	0.8794
si:ch211-241e1.5	81.57446556	-0.469487155	0.2244764	-2.091477	0.036485	0.373234
slc8a2a	62.62013047	0.004016403	0.2510839	0.0159963	0.987237	0.997136
mrpl53	192.4083492	-0.096159175	0.1599138	-0.601319	0.547628	0.945581
renbp	78.1754744	-0.083632622	0.2373196	-0.352405	0.724534	0.970402
sec11a	568.5100647	0.031449322	0.1148041	0.2739389	0.784132	0.97627
ccdc127b	73.85337563	-0.017605015	0.2358619	-0.074641	0.9405	0.992857
rtf1	1721.465383	-0.071286598	0.0739278	-0.964273	0.334909	0.875876
pla2g3	6.105745906	-0.406344983	0.7861147	-0.516903	0.605224	NA
pofut1	281.6574281	-0.164420053	0.1264803	-1.299966	0.193613	0.757219
tbl1xr1b	776.1716398	-0.045028595	0.0980988	-0.459013	0.646225	0.96032
fgb	2786.832644	0.143942198	0.1118518	1.2869012	0.198129	0.76333
mdn1	903.0106913	-0.173114498	0.0963334	-1.797036	0.07233	0.525828
golga1	464.9384101	-0.027075608	0.106952	-0.253157	0.800147	0.979377
casq2	2737.28515	0.138720881	0.0845846	1.6400245	0.101	0.604004
sh3glb2a	16.04426972	-0.408654704	0.486037	-0.840789	0.400466	0.90084
e2f7	187.1187686	-0.06122354	0.1621735	-0.377519	0.705788	0.968412
pdia6	2115.384718	-0.302173203	0.0819808	-3.685904	0.000228	0.009487
col11a1b	5500.520081	0.270584703	0.0764163	3.5409301	0.000399	0.014765

rhbg	3505.45612	0.362261781	0.0779085	4.649837	3.32E-06	0.000287
cnr1	20.90309981	0.649145164	0.4234417	1.5330215	0.125271	0.654648
chrna1	730.9919112	0.174287365	0.1128254	1.544753	0.122406	0.650311
ankrd28b	868.6523856	-0.028784054	0.0920117	-0.31283	0.754409	0.975114
ank2a	668.9667841	0.055021344	0.0965059	0.5701347	0.568586	0.948983
tnikb	586.8951205	-0.008293232	0.1001943	-0.082772	0.934033	0.992702
si:ch73-141c7.1	427.2453559	0.427308018	0.1394934	3.0632846	0.002189	0.057186
nphp1	73.71451121	-0.075712907	0.2255312	-0.335709	0.73709	0.972535
cbr4	78.9940297	0.045048025	0.222783	0.2022058	0.839756	0.984979
BX296551.1	16.33773155	0.37939973	0.4792697	0.7916205	0.428582	0.911378
cd74a	2.290009204	0.394129153	1.2682449	0.3107674	0.755977	NA
gata2b	12.80350592	0.246602111	0.5475144	0.450403	0.65242	0.960708
serinc1	5473.151366	-0.037717661	0.0697847	-0.540486	0.588862	0.953345
pigc	118.8400392	0.172853916	0.1848031	0.9353408	0.349613	0.880195
sele	101.4644872	-0.114942459	0.2647617	-0.434135	0.66419	0.964736
slc10a4	25.67003767	0.915966991	0.3879338	2.3611426	0.018219	0.25105
myo1eb	536.0250783	-0.059795874	0.1000165	-0.59786	0.549933	0.94648
hlx1	193.6500896	-0.01165229	0.1515179	-0.076904	0.9387	0.992857
tp53bp2a	1136.919757	-0.198512833	0.08578	-2.31421	0.020656	0.271399
ppp1r13bb	1386.138637	0.142333463	0.0777284	1.8311631	0.067076	0.506251
pla2g1b	4.323238798	0.196117318	0.9489974	0.2066574	0.836277	NA
kmo	225.7636993	-0.38880151	0.1428748	-2.721275	0.006503	0.12329
ftr55	1.110561499	2.435091206	2.0673778	1.1778646	0.238851	NA
mob1ba	610.7356243	-0.059526208	0.0966518	-0.615883	0.537972	0.943336
ctr9	1242.948368	-0.129337416	0.0809801	-1.59715	0.110232	0.62463
zfand1	146.535928	0.123536733	0.1653776	0.746998	0.455065	0.921022
col16a1	2218.951137	0.27581169	0.0861577	3.2012442	0.001368	0.039791
anxa3a	246.9597666	-0.339571427	0.1382194	-2.456757	0.01402	0.214075
prkcda	404.1321698	0.094088285	0.1108359	0.8488973	0.395938	0.899504
slc9a6a	285.8646968	0.100338152	0.1284178	0.7813417	0.434602	0.914333
ppiaa	24346.59455	-0.365148858	0.0971177	-3.759858	0.00017	0.007454
mfsd13a	651.5559997	0.041811335	0.0982564	0.425533	0.670448	0.965257
zgc:112437	96.17719164	0.09114826	0.2032201	0.4485198	0.653778	0.961168
toml12	62.88767567	-0.362532708	0.254115	-1.426648	0.153681	0.702578
mars2	73.9422379	-0.106470543	0.2309649	-0.460982	0.644812	0.96029
fev	71.66397349	-0.340422352	0.2326516	-1.463228	0.143405	0.686804
etfb	2143.604555	-0.096281937	0.084116	-1.144632	0.252361	0.821887
napepld	88.63441995	0.254047381	0.2231011	1.1387095	0.254824	0.823484
rtraf	553.3007691	0.091971859	0.1170504	0.7857457	0.432016	0.912876
FAR2	17.68039789	0.261301504	0.4678127	0.55856	0.576462	0.950611
uev1d	148.6257227	0.01043443	0.1709363	0.0610428	0.951325	0.99381
zgc:136439	0	NA	NA	NA	NA	NA
ppm1da	591.3171798	-0.004475533	0.1029555	-0.043471	0.965326	0.996257
smyd1a	1494.754658	0.270451102	0.1148437	2.3549486	0.018525	0.253683
dnm1b	460.5782228	0.08427756	0.1258395	0.6697227	0.503035	0.935304
rpl15	11149.07732	-0.348027384	0.0809617	-4.298667	1.72E-05	0.0011
dusp8a	597.2357774	-0.137195205	0.1135793	-1.207924	0.227076	0.794587
pvalb6	1101.14414	0.129809035	0.097797	1.327331	0.184399	0.747376
napsa	93.58269068	-0.343868126	0.230151	-1.494098	0.13515	0.670689
clgn	2097.193287	0.049681378	0.0724613	0.6856261	0.492949	0.931926
aif1l	1043.447215	0.038530692	0.0862695	0.4466317	0.655141	0.961614
nrap	593.5915583	0.200123884	0.1119878	1.7870154	0.073935	0.530091
txndc5	2833.298781	0.100250027	0.0749216	1.3380656	0.180875	0.741776
caprin1a	1016.068658	0.071684897	0.0849768	0.8435825	0.398903	0.900446

atg3	674.3274009	-0.07250397	0.0998929	-0.725817	0.467951	0.924328
fgf6a	72.49259023	0.108955828	0.269693	0.4039994	0.686213	0.966376
diras1b	4.644179547	0.200413293	0.865203	0.2316373	0.81682	NA
mpp4l	141.8382683	0.16368518	0.1691328	0.9677911	0.333149	0.874926
robo4	576.7009438	-0.086862201	0.1026859	-0.845902	0.397607	0.899672
npl	213.7254761	-0.29857867	0.1545746	-1.931615	0.053407	0.45286
vcanb	1493.447139	0.393668234	0.0814254	4.8347119	1.33E-06	0.000128
si:ch211-210b2.2	47.06843358	0.767231633	0.2867488	2.6756225	0.007459	0.136696
mef2cb	1454.207445	0.052037724	0.081314	0.6399602	0.522198	0.939056
tmem30c	125.3406242	-0.345109318	0.1808602	-1.908155	0.056371	0.46581
ubxn7	412.4004899	-0.176755543	0.1105651	-1.598656	0.109897	0.624178
myog	52.50035832	0.086142354	0.2876055	0.2995157	0.764547	0.975374
zgc:92137	51.5298633	-0.568019821	0.3555833	-1.597431	0.11017	0.62463
atp5mc3b	5999.57972	0.031654243	0.2077875	0.1523395	0.878919	0.987881
slc35e4	14.89114182	-0.390989811	0.52477	-0.745069	0.45623	0.921215
rgs9bp	53.29853907	0.359694694	0.2898208	1.2410934	0.214571	0.782493
hnf1a	61.25990364	0.01532509	0.2617301	0.058553	0.953308	0.994184
nid2b	1.657494491	-1.086853109	1.7352975	-0.626321	0.531104	NA
med19b	197.0723363	0.018908387	0.1458527	0.1296403	0.896851	0.989971
ppardb	589.9901695	0.087914613	0.1009947	0.8704878	0.384034	0.895192
prkar2ab	167.9906099	0.221537346	0.1701804	1.3017792	0.192992	0.756795
pdcl	2064.969206	-0.082382116	0.0823115	-1.000857	0.316896	0.867308
slc38a8a	11.35148787	0.259915734	0.5698708	0.4560959	0.648321	0.960708
arf1	2078.812487	-0.054325424	0.0755935	-0.718652	0.472355	0.924838
gipc2	475.8856668	-0.215024371	0.1180207	-1.82192	0.068467	0.512635
MAP3K13	26.84599291	-0.090381886	0.3933526	-0.229773	0.818268	0.981594
xrcc1	650.0533623	-0.036470854	0.0988139	-0.369086	0.712063	0.969006
syne1a	2490.570917	0.081111903	0.0859589	0.9436122	0.345368	0.878434
prelid3b	1487.083861	0.114988383	0.087107	1.3200827	0.186807	0.750951
tnfa	5.578901287	-1.277412656	0.8446407	-1.512374	0.130439	NA
rnf150b	184.6591714	-0.267287811	0.1545062	-1.729948	0.08364	0.557158
tial1	1884.578375	0.044026089	0.0787309	0.559197	0.576027	0.950611
wls	1587.079185	0.172557913	0.081817	2.1090729	0.034938	0.363807
cldnb	1721.363598	-0.045322851	0.0875627	-0.517605	0.604734	0.95651
ubr4	3068.563003	-0.035671105	0.0798724	-0.446601	0.655163	0.961614
foxi3b	260.0368026	-0.030689262	0.1585307	-0.193586	0.8465	0.985174
gng3	3484.185111	0.027326811	0.0862022	0.3170083	0.751237	0.97419
wrap73	163.8515852	0.107667755	0.1643986	0.6549191	0.51252	0.936253
spegb	812.4013055	0.200172132	0.0902162	2.2188056	0.0265	0.313216
ca10b	113.8274296	-0.113477059	0.1966008	-0.577195	0.563808	0.948739
papola	2874.966015	-0.044419997	0.0764549	-0.580996	0.561243	0.948728
nr1d2b	3548.237638	0.122583659	0.0712315	1.7209188	0.085266	0.560519
ccnc	822.4609704	-0.167906455	0.0967423	-1.735605	0.082634	0.554782
rgs12a	306.8080884	-0.327521714	0.1320631	-2.480039	0.013137	0.204864
chia.3	9.141476782	-1.043981109	0.6365	-1.64019	0.100966	NA
CABZ01027646.1	46.26437534	0.021695837	0.2981841	0.0727599	0.941997	0.993099
nrbp2b	125.1776298	-0.054137849	0.1882055	-0.287653	0.773612	0.975687
unc119.1	389.2739866	0.044380674	0.1139798	0.3893733	0.697	0.96736
rcvrn3	9553.467501	0.380395111	0.0909038	4.1845896	2.86E-05	0.001686
psmb1	2423.920275	-0.169966574	0.0828619	-2.051203	0.040247	0.392394
lhfpl2a	264.3983895	-0.424552759	0.1477226	-2.873986	0.004053	0.087787
fgfr1op2	309.3869305	-0.231090487	0.1344219	-1.719143	0.085588	0.561521
si:ch211-197g15.6	5.341097347	0.558257031	0.8101373	0.6890894	0.490767	NA
dlg1	1081.451045	0.040277913	0.0859433	0.4686569	0.639315	0.959994

med27	303.9993898	-0.135475897	0.1243729	-1.089272	0.276034	0.839541
ppa2	163.4578807	-0.02801418	0.159552	-0.17558	0.860624	0.985773
daam1b	994.0774526	0.050222059	0.0866517	0.5795857	0.562194	0.948728
llgl1	567.0803069	0.105510043	0.1125425	0.9375132	0.348495	0.880195
ascl1b	25.99093951	0.313199579	0.3784391	0.827609	0.407892	0.901493
rpn2	3555.149345	-0.00147969	0.078419	-0.018869	0.984946	0.996944
twf2b	51.37621503	0.409196595	0.2859764	1.4308755	0.152466	0.70162
kif21b	43.09945469	0.105521854	0.3023061	0.3490563	0.727047	0.970402
gigyf2	1200.470432	0.025179226	0.0873089	0.2883925	0.773046	0.975687
cacnb4a	3.960697999	-0.000779022	0.9284538	-0.000839	0.999331	NA
ppp1r7	1140.517617	0.043701858	0.0856598	0.5101794	0.609926	0.95688
efhc1	75.57661684	-0.36260558	0.2280903	-1.589746	0.111892	0.627242
fbxw2	633.0342316	0.14583556	0.0958384	1.5216822	0.128089	0.658954
dffb	93.68819843	-0.354398902	0.2139761	-1.656254	0.09767	0.596644
sf3b6	500.6537764	0.071723831	0.1091815	0.6569227	0.511231	0.936253
zc3h11a	1775.396164	-0.088385441	0.0818552	-1.079779	0.280241	0.843423
srek1	254.562324	-0.090545241	0.1327802	-0.681918	0.495291	0.931928
ppme1	2037.073844	-0.063263131	0.0861234	-0.734564	0.462605	0.922698
mcl1a	1301.307057	-0.16001723	0.1042435	-1.535033	0.124776	0.654423
myh11a	677.5291937	-0.162748991	0.1087241	-1.496898	0.13442	0.670539
slc49a4	550.7094503	0.0301054	0.1038788	0.2898126	0.77196	0.975687
klc1a	869.3136396	0.218860635	0.0960173	2.2793869	0.022644	0.287397
smfn	162.6996218	0.114515314	0.1754146	0.6528267	0.513868	0.936778
her4.4	216.3615164	0.011008186	0.1512382	0.0727871	0.941976	0.993099
pou3f1	1658.318192	0.027088709	0.0875994	0.3092341	0.757143	0.975374
mmp23bb	207.9120692	-0.086013674	0.1801028	-0.477581	0.632949	0.957356
hdlbpa	14506.98947	-0.028713941	0.0685637	-0.418792	0.675368	0.965257
agk	493.563858	-0.074210672	0.1075621	-0.689933	0.490236	0.931926
fam117bb	122.4019979	-0.145125106	0.185944	-0.780478	0.43511	0.914408
dusp23a	58.33456577	-0.533940636	0.2905885	-1.837446	0.066144	0.502802
wdr41	504.296352	0.017261408	0.1064539	0.1621491	0.871188	0.987754
dcps	363.2504285	0.020519574	0.1248842	0.1643088	0.869488	0.987282
ddx55	199.186006	0.01317013	0.1493625	0.0881756	0.929737	0.992702
dcaf11	803.5321519	-0.13207306	0.0916941	-1.440366	0.149764	0.697935
mapk8b	63.39332024	-0.082673935	0.2514874	-0.32874	0.742352	0.973154
snrpc	680.7903734	-0.03707434	0.1106852	-0.334953	0.737661	0.972535
cyp2k6	0	NA	NA	NA	NA	NA
ier5	1488.279556	-0.233112078	0.0803084	-2.902713	0.003699	0.082473
sult3st1	5.81544542	0.090369413	0.7961875	0.1135027	0.909632	NA
rnf114	125.1756774	0.394693448	0.1929656	2.045408	0.040815	0.394957
znf385a	57.69627879	0.105535932	0.2686914	0.3927775	0.694484	0.96736
slc38a5a	640.9989236	0.120386718	0.0979074	1.2295972	0.218848	0.785734
dmbx1a	848.2528677	-0.032810405	0.0996874	-0.329133	0.742055	0.973154
cadm2a	36.30652208	0.5967617	0.3234194	1.8451637	0.065014	0.497901
micu2	638.0132713	-0.210441528	0.1115297	-1.886866	0.059178	0.475427
erc1a	734.974053	-0.00451496	0.0953943	-0.047329	0.962251	0.995907
cdc6	363.7215695	-0.100259493	0.1298069	-0.772374	0.439893	0.916082
fndc4b	504.6452311	0.030202423	0.1161398	0.2600523	0.794823	0.978206
edf1	1545.44327	-0.222478265	0.0821054	-2.709667	0.006735	0.126476
med14	947.7436029	-0.090462503	0.1039661	-0.870115	0.384238	0.895192
cbl	211.8844867	0.0273448	0.1425151	0.1918731	0.847842	0.985174
rundc3b	6.996471156	-0.400611888	0.7068341	-0.566769	0.570871	NA
icn	3734.592075	-0.282817195	0.2380123	-1.188246	0.234736	0.803509
fam199x	993.1237015	0.014794337	0.0937227	0.1578522	0.874573	0.987881

gna11a	165.5918394	-0.053153279	0.1602234	-0.331745	0.740082	0.972943
vim	1053.701939	-0.104078305	0.0953127	-1.091967	0.274848	0.839487
trim13	754.1186084	0.240399057	0.0996512	2.4124055	0.015848	0.231784
qki2	315.5488652	-0.144054949	0.1215641	-1.185013	0.236012	0.805056
grpel1	658.7266663	0.108429996	0.1019777	1.0632717	0.287659	0.845979
CU856539.1	378.2810359	0.302746643	0.1176419	2.5734603	0.010069	0.171342
spon1a	26.78556806	0.448716679	0.377038	1.1901101	0.234003	0.802633
ssh1b	481.8323132	0.006523993	0.1070677	0.0609333	0.951412	0.99381
cmtm7	224.3945923	-0.022369211	0.1438053	-0.155552	0.876386	0.987881
engase	125.978898	-0.315639573	0.183396	-1.721082	0.085236	0.560519
dnm1a	108.0454467	0.202983605	0.1904846	1.0656169	0.286597	0.845942
neu3.2	82.69898935	0.139107127	0.2258024	0.6160568	0.537857	0.943336
ufd1l	1054.352676	-0.016245489	0.0851309	-0.19083	0.848659	0.985174
ndrg3b	1946.281509	-0.122053548	0.074043	-1.648415	0.099267	0.599058
itpkb	25.61693933	0.043424113	0.4001423	0.1085217	0.913582	0.991119
adam9	297.2262468	0.084684278	0.1368417	0.6188484	0.536016	0.943336
CR792422.1	17.61669547	-0.015370592	0.467876	-0.032852	0.973793	0.996315
nup133	566.947561	0.049785481	0.0985461	0.5051999	0.613418	0.95688
rbfox3a	1250.566376	0.053776331	0.0821313	0.6547603	0.512622	0.936253
p4ha2	473.7697174	-0.306394212	0.1169345	-2.620221	0.008787	0.155004
slc1a6	63.12450108	0.052322548	0.2425025	0.2157609	0.829174	0.982743
f9a	10.46341279	0.049464476	0.588174	0.0840984	0.932978	NA
pank1b	642.2201454	-0.00958332	0.0993206	-0.096489	0.923132	0.992588
bri3bp	252.9712262	0.046479314	0.133201	0.3489411	0.727134	0.970402
ndufb8	2299.39148	-0.007368597	0.0888806	-0.082904	0.933928	0.992702
smpdl3b	150.072267	0.233840638	0.1697747	1.3773584	0.168401	0.725919
dacha	831.1331732	-0.12465737	0.0894835	-1.393077	0.163597	0.71945
ldb1a	3091.959067	-0.013611386	0.0702482	-0.193761	0.846363	0.985174
plppr3a	981.2256267	-0.152816433	0.0989453	-1.544454	0.122478	0.65052
tor4aa	131.3666461	-0.035653525	0.1843817	-0.193368	0.846671	0.985174
cpa2	42.80333399	-0.068993687	0.3228666	-0.213691	0.830788	0.982994
atp5fa1	16976.72923	-0.076089948	0.1041016	-0.73092	0.464828	0.923584
vstm4a	108.1457517	-0.097195095	0.1988978	-0.488668	0.625076	0.957354
abi1a	1003.527975	-0.034459594	0.0829071	-0.415641	0.677673	0.965257
rps15a	6655.31657	-0.250185638	0.083747	-2.987399	0.002814	0.068857
myd88	153.0896157	0.183311896	0.1720726	1.0653175	0.286732	0.845979
asap2a	353.7894649	0.041648459	0.1291761	0.3224161	0.747137	0.97419
si:ch73-265h17.1	7.551498882	-0.168037114	0.7158308	-0.234744	0.814407	NA
myo3a	185.9129441	0.114372113	0.1560231	0.7330462	0.46353	0.92286
pax3a	26.83469037	-0.340676505	0.3771283	-0.903344	0.366343	0.887239
eif4bb	2286.369006	-0.127206495	0.0734732	-1.731332	0.083393	0.556666
smad3b	171.9429656	0.042584509	0.1596148	0.2667955	0.789627	0.977238
ppm1na	141.5134406	0.792629744	0.1982331	3.9984738	6.38E-05	0.00338
rbm22	608.8958608	-0.180486228	0.1011595	-1.784174	0.074395	0.531273
rpl22l1	2713.282516	-0.331750202	0.1001182	-3.313585	0.000921	0.029142
prmt1	3298.370241	0.045557187	0.1054684	0.4319509	0.665777	0.964736
wdr54	310.9527742	-0.077136231	0.1295138	-0.595583	0.551454	0.946978
nqo1	778.144595	-0.007538883	0.0944326	-0.079833	0.93637	0.992702
jak3	24.67801901	0.026303519	0.3792649	0.069354	0.944708	0.993409
bin2b	124.6404765	0.226155269	0.2093866	1.0800846	0.280104	0.843423
igf2bp3	1560.788669	0.041592884	0.0787999	0.5278294	0.597618	0.95534
dpydb	709.3601545	-0.281184333	0.1104293	-2.546284	0.010888	0.180962
fezf1	206.7184128	-0.061363268	0.163882	-0.374436	0.70808	0.968664
ptgs2b	320.6284335	0.167324285	0.3747261	0.4465242	0.655219	0.961614

scamp2	696.5951717	-0.014924981	0.1064527	-0.140203	0.8885	0.989291
clasp1a	1075.563047	0.070870218	0.0858521	0.8254923	0.409092	0.901769
xpo4	524.294957	-0.141254423	0.1052892	-1.341585	0.179731	0.741062
fmodb	59.26623736	0.346872688	0.262274	1.3225585	0.185982	0.75038
kcnh6b	24.58801001	0.3790134	0.4093576	0.9258735	0.354512	0.882105
traf3ip1	59.82232373	-0.102752328	0.2522039	-0.407418	0.683701	0.965679
b4galt6	932.7500463	0.099815108	0.0913411	1.0927731	0.274493	0.839136
cp	649.3569842	-0.19457168	0.3796381	-0.512519	0.608288	0.956718
qars	1008.342107	0.183121252	0.0915978	1.999189	0.045588	0.418697
gpr183a	10.18334145	-0.015150499	0.601732	-0.025178	0.979913	NA
srpx	764.787834	0.039066658	0.0998429	0.3912811	0.695589	0.96736
radx	19.14479226	0.400947553	0.4384267	0.9145145	0.360447	0.885686
zgc:56231	4.517547615	0.994466447	0.9500911	1.0467064	0.295235	NA
acer1	78.15355015	-0.397118273	0.2320487	-1.711358	0.087015	0.565255
c1ql3a	25.05747316	-0.20211581	0.3759571	-0.537603	0.590851	0.954193
slc5a3a	0.166657454	-0.955901296	4.0804729	-0.234262	0.814781	NA
rnf2	1753.138008	-0.131426775	0.07529	-1.745607	0.080879	0.550801
lpar5a	4.203063818	0.613822546	1.0389519	0.5908094	0.554648	NA
sept4a	324.0083652	0.017917559	0.1338643	0.1338486	0.893522	0.989291
lrrfip2	434.2181959	-0.07298256	0.1185701	-0.615522	0.53821	0.943336
ppp4r4	170.3239736	-0.268041416	0.1565792	-1.711858	0.086923	0.565027
igsf9b	287.8142436	-0.157300726	0.1327761	-1.184706	0.236134	0.805191
epn1	1404.245842	-0.01802851	0.0943675	-0.191046	0.84849	0.985174
sirt4	99.1950784	0.133321304	0.2167179	0.6151836	0.538434	0.943336
ndrg1b	2029.337837	-0.336698313	0.0869961	-3.870268	0.000109	0.005269
npsn	157.9864483	-0.20461485	0.208991	-0.97906	0.32755	0.872701
scara5	134.8630398	0.12150268	0.176532	0.6882757	0.491279	0.931926
eaf2	374.9667193	-0.020970533	0.1183537	-0.177185	0.859363	0.985773
clu	984.3327628	-0.108132255	0.1038174	-1.041562	0.297615	0.85401
tent5c	160.1582312	0.266564775	0.1581372	1.6856547	0.091862	0.57849
rnf11a	497.3921826	-0.191615885	0.1067197	-1.795506	0.072573	0.52617
trabd	218.3391986	0.079452079	0.1516116	0.52405	0.600244	0.956045
guca1a	26.4621021	0.685192819	0.3798381	1.8039077	0.071246	0.522712
atp1a2a	1867.115221	0.420994906	0.0913867	4.6067422	4.09E-06	0.000344
p2rx3a	31.2436411	0.7164833	0.3631578	1.9729255	0.048504	0.433449
hsp90aa1.1	799.4950884	0.606233598	0.0936732	6.4717935	9.68E-11	2.34E-08
bzw1a	3182.956764	0.055727211	0.0735143	0.7580457	0.448424	0.917768
prg4a	28.88745106	0.57141622	0.3775975	1.5132944	0.130205	0.662797
sae1	934.2178181	-0.042688135	0.1052336	-0.405651	0.684999	0.966146
cxcr5	6.844348586	0.070134917	0.7132977	0.0983249	0.921674	NA
dvl1a	37.88041769	-0.006001416	0.3543434	-0.016937	0.986487	0.996965
rpl21	8582.898836	-0.175029613	0.0762883	-2.294318	0.021772	0.279561
per3	1926.590315	0.007473788	0.0743211	0.1005607	0.919899	0.991857
si:ch211-282j22.3	133.6976816	-0.102547649	0.1783683	-0.574921	0.565345	0.948739
scamp4	685.6713835	0.123811611	0.0957017	1.2937248	0.19576	0.76113
mmgt1	738.4154793	0.048477946	0.1073744	0.4514851	0.65164	0.960708
pdha1b	144.9318668	0.268986947	0.1742517	1.543669	0.122669	0.650831
mmp25b	49.73655354	-0.111128103	0.2849355	-0.390011	0.696528	0.96736
spop1a	329.5308318	-0.0970161	0.1357005	-0.714928	0.474653	0.925287
aqp4	5.542848337	-0.013514283	0.8207689	-0.016465	0.986863	NA
ezh2	533.5146235	0.203528215	0.1177813	1.7280184	0.083985	0.557646
slc25a25a	621.2973247	0.353577466	0.099729	3.545382	0.000392	0.014572
pard3ab	327.0806108	-0.137041731	0.1296131	-1.057314	0.290368	0.846262
tex9	63.93333284	-0.100681171	0.2450661	-0.410833	0.681195	0.965476

foxn4	303.6712303	-0.126545189	0.1462127	-0.865487	0.386771	0.89551
slc7a6os	850.1235557	0.080298143	0.0966589	0.8307374	0.406122	0.901305
mtmr10	278.5994464	0.117323862	0.1293625	0.9069386	0.364439	0.886564
zgc:112294	49.33630628	0.178523622	0.3059152	0.5835723	0.559508	0.948478
kctd9b	294.4699694	-0.079486773	0.136931	-0.580488	0.561586	0.948728
sarm1	424.3331149	0.118565335	0.1273991	0.9306605	0.352029	0.880844
themis	5.115703613	0.647519033	0.8773865	0.7380089	0.460509	NA
clic2	138.8505469	0.227578869	0.1700111	1.338612	0.180697	0.741776
hoxb6a	176.7483146	0.206462405	0.1583991	1.3034318	0.192427	0.756795
slc20a1b	4918.65391	0.131357023	0.0780967	1.6819783	0.092573	0.579468
arhgap42b	14.58836681	-0.259445759	0.4934243	-0.525807	0.599023	0.955806
ppm1k	575.6605924	-0.292659982	0.1002817	-2.918378	0.003519	0.079772
insig1	975.6822398	0.046577114	0.0974407	0.4780045	0.632647	0.957356
fbxo36a	5.693566548	0.578595846	0.877211	0.6595857	0.50952	NA
trim16	213.3683187	-0.183048876	0.1535384	-1.192203	0.233182	0.801623
gngt2a	819.8097519	-0.160849743	0.1118463	-1.438132	0.150397	0.698669
tjap1	653.9543708	-0.184083013	0.1268713	-1.450943	0.146796	0.693524
il17d	3.539432946	0.175466513	1.0211105	0.1718389	0.863564	NA
crygs4	0.854974378	0.638273715	2.0970274	0.3043707	0.760845	NA
msi1	1370.427507	-0.039567897	0.0794948	-0.497742	0.618666	0.957354
npffr1l3	13.00732743	0.545107397	0.5438938	1.0022313	0.316232	0.866931
chchd10	838.1701423	0.10640694	0.0999492	1.0646099	0.287053	0.845979
6-Sep	2313.813396	0.02154524	0.0740475	0.2909652	0.771078	0.975687
ttyh2l	519.4826385	-0.098175343	0.1068618	-0.918713	0.358245	0.884398
scin	267.7354395	-0.22227091	0.1367832	-1.624987	0.104165	0.611388
CABZ01073795.1	0.157187365	-0.955901296	4.0804729	-0.234262	0.814781	NA
xrcc4	115.2270104	0.046220218	0.1860418	0.2484399	0.803794	0.979643
zgc:101716	53.45447581	-0.08666435	0.2713167	-0.319421	0.749407	0.97419
dnaja2b	2020.588612	0.022102768	0.0782429	0.2824891	0.777568	0.975942
acsl4b	419.7757772	-0.032689785	0.1275237	-0.256343	0.797686	0.978668
nod2	69.52476963	0.062850909	0.2343098	0.2682385	0.788516	0.976703
capn12	282.738652	0.544883177	0.1554383	3.5054634	0.000456	0.01663
flj13639	93.51511	0.422817	0.2240228	1.8873839	0.059109	0.475427
epha4l	218.2520275	0.0239252	0.141295	0.1693279	0.865539	0.986465
sox19a	858.1079717	0.063146675	0.1024369	0.6164446	0.537601	0.943336
pnpla6	1125.668006	-0.385740618	0.2101565	-1.835493	0.066433	0.504618
tlk2	828.533638	0.008922763	0.0965025	0.0924614	0.926331	0.992702
zgc:55621	0	NA	NA	NA	NA	NA
ppp1r12a	1087.382531	-0.035956605	0.0835084	-0.430575	0.666778	0.964736
thbs1b	5896.502469	0.304671844	0.06944	4.3875563	1.15E-05	0.000811
homer3b	520.6962678	0.087324008	0.1023722	0.8530048	0.393657	0.898242
dla	577.2459883	-0.138509155	0.1103286	-1.255424	0.209325	0.776293
cdc25b	442.9009739	0.064935911	0.1163037	0.5583308	0.576619	0.950611
tfeb	221.4330691	0.077625478	0.1474336	0.5265115	0.598533	0.955488
slc7a14a	726.1467258	0.193695424	0.1077413	1.7977827	0.072211	0.525546
memo1	400.948965	0.170530112	0.1125812	1.51473	0.129841	0.662436
churc1	182.1495324	-0.475930883	0.1576408	-3.019085	0.002535	0.063623
fshb	0.158795395	0.967652056	4.0804729	0.2371421	0.812547	NA
kras	112.1575549	0.19604057	0.2004092	0.9782015	0.327975	0.872701
amt	340.7639137	-0.243658697	0.1307164	-1.864026	0.062318	0.487498
DDX17	1977.797223	-0.269226811	0.0747366	-3.602344	0.000315	0.0123
cdkn1ca	315.140921	0.268178708	0.143083	1.8742881	0.060891	0.482482
mettl13	153.1497328	-0.018946235	0.1681527	-0.112673	0.91029	0.990702
nprl3	197.1373389	-0.243662064	0.1643981	-1.482146	0.138301	0.676087

HTRA3	99.83442323	-0.139249724	0.2185028	-0.63729	0.523936	0.939336
cacna1fa	42.03723224	-0.323573997	0.310649	-1.041607	0.297594	0.85401
syt14a	16.6681055	0.674073112	0.4732559	1.4243311	0.154351	0.70398
abcb4	394.9317915	-0.18946567	0.1307185	-1.449417	0.147221	0.69409
BX957278.1	83.5419369	-0.090625496	0.2235165	-0.405453	0.685144	0.966146
pomgnt2	112.6375351	-0.020154198	0.1947909	-0.103466	0.917593	0.991623
rapgef6	393.6007352	-0.07891907	0.1426186	-0.553357	0.580019	0.951257
cbsb	1232.486901	0.042225925	0.0813172	0.5192742	0.60357	0.956045
kif11	1203.56477	-0.121642751	0.0853209	-1.425708	0.153953	0.702738
ndel1a	831.4834973	0.028696716	0.0880468	0.3259258	0.74448	0.974094
sh3glb1b	316.8407702	0.012518336	0.1310614	0.095515	0.923906	0.992628
mpp2b	915.7736871	0.105746916	0.0916544	1.153757	0.2486	0.817939
glmnb	229.8171708	0.236033525	0.1418977	1.6634062	0.096231	0.590771
fkbp7	245.8583744	0.303028771	0.1536769	1.9718569	0.048626	0.433734
psma8	2923.742789	-0.025704083	0.0800468	-0.321113	0.748125	0.97419
rab34b	48.89895294	-0.232124544	0.2781981	-0.834386	0.404064	0.901191
trmt1	183.9930992	-0.014623892	0.1648877	-0.08869	0.929328	0.992702
zdhhc3a	87.74061747	-0.052967051	0.2219606	-0.238633	0.81139	0.981081
gtf2a1	865.4085867	0.022859476	0.089192	0.2562951	0.797723	0.978668
hnrnpa1a	7814.655518	-0.092723638	0.0930638	-0.996345	0.319082	0.867746
pdlim3a	24.42373501	-0.100306497	0.416487	-0.240839	0.80968	0.980648
zgc:92907	70.76856614	0.167324346	0.2363893	0.7078339	0.479048	0.926978
fgfr1a	1079.838677	0.042072486	0.0989037	0.4253885	0.670554	0.965257
pgbd5	44.10976039	0.057342381	0.2924644	0.1960662	0.844558	0.985174
slc17a9b	164.6075692	0.188037965	0.1594721	1.1791277	0.238347	0.807449
fbxo9	963.0147624	-0.062116141	0.0953988	-0.651121	0.514969	0.937646
camk2b1	2940.38999	0.020177339	0.0747447	0.2699499	0.787199	0.976596
glra3	407.0468497	0.113062771	0.1346451	0.8397093	0.401071	0.90084
ddx11	147.1357836	-0.106147642	0.1744926	-0.608322	0.542974	0.944974
zgc:91968	169.3630232	0.103957229	0.1637422	0.6348835	0.525504	0.94013
wdr3	823.2690442	-0.299631871	0.0987187	-3.03521	0.002404	0.061172
si:dkey-42l23.2	3.046851276	-0.627152543	1.1344861	-0.552808	0.580395	NA
si:ch211-288d18.1	507.5914828	-0.144827416	0.1018365	-1.422156	0.154981	0.704624
drd2b	51.74981565	0.127813154	0.281672	0.4537659	0.649997	0.960708
ccna2	935.648923	-0.061893748	0.0943276	-0.656157	0.511723	0.936253
chrna10a	50.29172855	0.12706709	0.2802959	0.4533319	0.65031	0.960708
pptc7a	1028.409718	0.042172755	0.0875607	0.4816402	0.630062	0.957354
snrpb	2453.683551	-0.3358609	0.0805105	-4.171639	3.02E-05	0.001759
dpysl5a	2340.065017	0.115584899	0.0844455	1.3687507	0.171077	0.729069
uqcrb	20.11827902	0.04209115	0.5416611	0.0777075	0.938061	0.99279
rpp14	185.7458823	-0.089379253	0.1514379	-0.590204	0.555054	0.947449
arhgef18a	399.3982081	-0.102491098	0.1137203	-0.901256	0.367452	0.887458
sema3fa	909.2972153	0.056546739	0.0935939	0.6041709	0.54573	0.945526
cahz	1202.5711	0.191343966	0.0952755	2.0083219	0.044609	0.41583
ndrg2	4537.364934	0.138801389	0.0725094	1.9142525	0.055588	0.461579
si:ch211-12m10.1	1363.29004	0.056147581	0.0874431	0.6421041	0.520806	0.939056
atp6v1d	2691.148842	-0.175126559	0.0781741	-2.240213	0.025077	0.306095
acvr2aa	1908.594624	0.013985184	0.0759787	0.1840671	0.853961	0.985174
fgfr1b	238.3716772	-0.105418245	0.161723	-0.651844	0.514502	0.937328
dnajc11a	1081.267409	-0.148572414	0.0819957	-1.811954	0.069993	0.516704
rplp2l	5015.218578	-0.413031484	0.103348	-3.996511	6.43E-05	0.003387
akt2	2147.205338	-0.097877036	0.0729978	-1.340822	0.179978	0.741126
ccdc28b	142.0756576	-0.068044316	0.1775219	-0.383301	0.701497	0.968134
pcyt1aa	205.8258342	-0.009207071	0.1463765	-0.0629	0.949846	0.99381

otx2b	1349.657911	-0.079908457	0.0831014	-0.961578	0.336262	0.876088
ppp1r14aa	155.5577306	0.124730641	0.1695206	0.7357845	0.461862	0.922466
ift46	354.8870761	-0.05544026	0.1201982	-0.461241	0.644626	0.96029
esrp1	528.7649448	-0.125141547	0.1102984	-1.134573	0.256554	0.824839
krr1	239.2003353	-0.034573633	0.1536118	-0.225071	0.821924	0.982156
rnpc3	374.6645228	-0.11105954	0.1159093	-0.958159	0.337982	0.876609
enpp2	221.5564676	-0.094984981	0.1500167	-0.633163	0.526627	0.940528
trpm1a	266.907525	-0.058040822	0.1414534	-0.410318	0.681573	0.965476
traip	85.48391307	-0.040256193	0.2207614	-0.182352	0.855307	0.985174
uqcc2	444.3861986	0.132354325	0.1125288	1.1761817	0.239522	0.808091
tlx2	234.9844502	-0.031684834	0.1385992	-0.228608	0.819174	0.981594
si:dkey-110k5.10	12.71807647	0.531985701	0.5739902	0.9268202	0.35402	0.881994
atf7a	169.182158	-0.094918688	0.1726483	-0.549781	0.58247	0.952117
blzf1	549.0545795	-0.050276719	0.1032343	-0.487015	0.626247	0.957354
nae1	941.5484888	-0.025464791	0.090339	-0.28188	0.778035	0.975942
stk3	715.1555495	0.167949673	0.0969929	1.7315662	0.083351	0.556629
adra1ba	8.810354836	0.283827104	0.6311747	0.4496807	0.652941	NA
ankrd45	15.23023151	-0.310776331	0.4796275	-0.647954	0.517015	0.938543
arhgap33	134.5029618	0.075171124	0.1948486	0.3857924	0.69965	0.967998
ncaldb	449.4743255	0.401095622	0.1064267	3.7687517	0.000164	0.007299
nfatc2ip	283.9869608	-0.050076408	0.127772	-0.39192	0.695117	0.96736
fyna	1501.880814	-0.015235668	0.0874083	-0.174305	0.861626	0.985841
zgc:64106	273.6650695	-0.483699925	0.1476577	-3.275819	0.001054	0.032299
mknk2a	266.708569	0.214879257	0.1405621	1.5287142	0.126335	0.655241
dnase1l4.2	72.70127768	-0.12664849	0.225789	-0.560915	0.574855	0.950037
tnnc1a	89.20644978	0.166281506	0.2322606	0.7159264	0.474037	0.924993
fen1	606.2501622	-0.069514117	0.0978526	-0.710397	0.477458	0.926857
rps9	16967.24699	-0.324505509	0.093607	-3.466681	0.000527	0.018573
col2a1b	16304.30597	0.171967727	0.0809632	2.1240236	0.033668	0.357045
aadat	29.45802224	-0.044334043	0.3619959	-0.122471	0.902526	0.990121
sigmar1	357.7744649	-0.136378386	0.1195328	-1.140928	0.2539	0.823394
ryr1a	1074.78626	-0.021736468	0.0914855	-0.237595	0.812196	0.981081
ptger2a	47.93634975	-0.029555666	0.3002418	-0.09844	0.921583	0.992138
elmsan1a	134.8690032	-0.485799416	0.1726715	-2.813431	0.004902	0.102006
fes	99.86025548	0.22090477	0.1994603	1.1075123	0.268072	0.83343
gsna	722.7120653	0.082312668	0.0952269	0.8643845	0.387377	0.895778
otub1b	704.308565	0.068206482	0.1029361	0.6626097	0.507581	0.935734
CABZ01044048.1	16.63682557	-0.182580965	0.5049894	-0.361554	0.717685	0.96909
afg1la	199.2729197	0.041299013	0.1522941	0.2711793	0.786253	0.976471
calcrla	39.86439068	0.2140811	0.317929	0.6733613	0.500717	0.93428
gnaq	207.609042	-0.010838037	0.1639685	-0.066098	0.9473	0.99381
sirt2	580.4922982	0.089599546	0.1171707	0.7646924	0.444455	0.916726
scube3	80.37908478	0.201788501	0.2196777	0.9185663	0.358322	0.884398
dcun1d3	313.2963852	-0.072786996	0.1245094	-0.58459	0.558823	0.948478
ppm1bb	2660.960994	0.07628493	0.0707975	1.0775091	0.281253	0.84357
si:ch211-212o1.2	266.1148745	-0.032627238	0.1296545	-0.251648	0.801313	0.979377
lpcat1	146.2663663	-0.087536617	0.1684673	-0.519606	0.603338	0.956045
rcc2	2881.145839	-0.03266637	0.0697178	-0.468551	0.639391	0.959994
orai1a	102.629136	0.247174537	0.1982385	1.2468544	0.212451	0.780421
myl4	693.0811978	0.042486628	0.0948911	0.447741	0.65434	0.961305
upb1	257.3794809	0.220768964	0.1426051	1.548114	0.121595	0.648265
sema6dl	689.6337733	-0.047249728	0.096372	-0.490285	0.623932	0.957354
ugt2a5	164.9680724	-0.052779701	0.1692876	-0.311775	0.755211	0.975318
arrb2b	1856.146063	-0.085666914	0.0796913	-1.074984	0.282382	0.843666

c1ql4a	34.76670179	0.447056644	0.3491182	1.2805308	0.200359	0.766727
atp5pb	6621.81382	-0.0349562	0.074589	-0.468651	0.639319	0.959994
spag7	1094.882717	0.019775688	0.0814231	0.2428757	0.808102	0.980642
sfpq	4790.734619	-0.087057372	0.0826585	-1.053217	0.292241	0.848075
pou6f1	542.1054169	0.059634047	0.1044094	0.5711558	0.567894	0.948761
calcr1b	9.15920123	-0.792480448	0.6358967	-1.246241	0.212676	NA
abcb11a	217.8220947	-1.240285514	0.151023	-8.212562	2.17E-16	1.22E-13
hoxb10a	94.77201111	0.051174525	0.2116483	0.2417904	0.808943	0.980642
si:dkey-23k10.5	32.89183108	-1.132254797	0.3508469	-3.227204	0.00125	0.036865
sox5	1742.942191	-0.034490468	0.0775529	-0.444735	0.656512	0.961833
cry1b	536.2106685	-0.098657103	0.1020545	-0.96671	0.333689	0.874933
smarcb1b	677.2585909	-0.091958408	0.0938622	-0.979717	0.327226	0.872658
atxn2l	774.6083859	0.085948714	0.1140148	0.7538384	0.450946	0.918761
epha4b	45.10392866	0.104811298	0.310571	0.3374794	0.735756	0.972535
si:dkeyp-117h8.2	849.9508959	-0.039759661	0.0972549	-0.408819	0.682672	0.965476
dennd6b	393.1470995	-0.042268156	0.1177959	-0.358825	0.719726	0.969318
FOXK2	991.6986242	-0.222138398	0.0843395	-2.633859	0.008442	0.14985
actr1	2503.33824	-0.028315482	0.072701	-0.389479	0.696922	0.96736
rbm39a	3245.131746	-0.037582437	0.0832903	-0.451222	0.651829	0.960708
mybpc3	1355.624217	0.230792789	0.0829609	2.7819465	0.005403	0.109079
slc26a2	352.345657	0.187794661	0.1187775	1.581062	0.113864	0.632634
scxb	10.21285159	0.169898004	0.6008817	0.2827479	0.77737	NA
syt5b	2909.909158	0.026233671	0.0804528	0.3260755	0.744367	0.974094
snrpd1	1022.699167	-0.197942723	0.0891345	-2.220721	0.02637	0.312638
ftr52p	12.80386263	-0.048799743	0.5474407	-0.089142	0.928969	0.992702
twf1b	577.37266	0.013069678	0.1124767	0.116199	0.907495	0.990702
chuk	912.0952706	0.020139467	0.0870016	0.2314838	0.816939	0.981594
aldoaa	12372.66427	0.007378003	0.0693552	0.10638	0.915281	0.991171
pde6b	323.1719514	0.26538699	0.1349061	1.9671982	0.04916	0.435115
sema3b	400.2556672	0.088863935	0.1170541	0.75917	0.447751	0.917154
si:ch211-246m6.5	94.51182696	-0.096906769	0.2107282	-0.459866	0.645612	0.96029
prtfdc1	171.0187325	-0.105167197	0.1646221	-0.63884	0.522927	0.939336
ttc36	81.22469886	0.468107281	0.2245512	2.0846352	0.037102	0.377011
esrrgb	15.77672947	0.155142157	0.5018927	0.3091142	0.757235	0.975374
ctsll	20.80676903	-0.0346876	0.4409745	-0.078661	0.937302	0.99279
clocka	84.78478905	0.106759828	0.2113375	0.5051627	0.613445	0.95688
zgc:56556	101.1714541	-0.046229784	0.1956128	-0.236333	0.813174	0.981081
marveld1	145.8016505	0.244404146	0.1720414	1.4206126	0.155429	0.705158
b9d1	215.20989	-0.320127052	0.1582027	-2.023525	0.043019	0.407353
u2af2b	2394.653111	-0.002688636	0.0698756	-0.038477	0.969307	0.996315
pigo	540.7425638	0.191818284	0.1201364	1.596671	0.110339	0.624637
ints13	406.2320498	-0.007007188	0.1115328	-0.062826	0.949905	0.99381
cpm	227.7603343	0.114426019	0.1519691	0.7529559	0.451476	0.918761
dhrs12	561.0220808	0.048291678	0.1038342	0.4650847	0.641871	0.959994
cttn	1756.077811	-0.091466357	0.0802632	-1.13958	0.254461	0.823475
stag2a	831.4192886	-0.065076649	0.0907782	-0.716876	0.473451	0.924993
tbx6	44.37359782	-0.063952711	0.3347117	-0.191068	0.848472	0.985174
tent5bb	228.8655845	0.253500855	0.1379313	1.8378774	0.06608	0.502802
wars2	90.48775771	-0.095829046	0.2215183	-0.432601	0.665305	0.964736
mical1	12.88937808	-0.304962108	0.5434181	-0.561192	0.574666	0.950037
batf	4.83452673	-0.740067727	0.9020005	-0.820474	0.411946	NA
pcnx1	977.6582478	0.007296513	0.1020095	0.0715277	0.942978	0.993364
plod2	710.1014777	0.228241273	0.0933229	2.4457165	0.014456	0.218716
pbxip1b	621.7017504	0.098057312	0.1017567	0.9636447	0.335224	0.876088

cetn3	255.2606149	0.141406991	0.1464065	0.9658517	0.334118	0.875263
zgc:100846	180.4354044	0.044506066	0.1591666	0.2796193	0.77977	0.976146
atp5l	4313.354677	-0.109595882	0.0922137	-1.188499	0.234637	0.803509
chn2	23.1741752	-0.143621973	0.4111095	-0.349352	0.726825	0.970402
map2k5	196.9057948	0.068104943	0.1489247	0.4573113	0.647447	0.960581
aak1a	34.96114877	-0.032751086	0.3364721	-0.097337	0.922459	0.99233
inaa	368.6092439	0.457385469	0.1226656	3.7287196	0.000192	0.008264
ptk7a	718.984489	0.047805534	0.0945363	0.5056843	0.613078	0.95688
galnt8a.2	1.820454227	-0.279786658	1.4495387	-0.193018	0.846945	NA
sp8a	274.2984598	0.2079615	0.1328624	1.5652392	0.117527	0.641232
ednraa	447.4201707	0.132963688	0.1096951	1.2121208	0.225466	0.793022
foxn1	43.82572263	0.628578507	0.3115542	2.0175577	0.043637	0.410116
MFN1	37.73731187	0.05987885	0.3110992	0.1924751	0.84737	0.985174
mrpl19	558.9440132	-0.163816814	0.102802	-1.593518	0.111044	0.626294
pdca	210.8538203	0.445336416	0.1545494	2.8815151	0.003958	0.086474
cry4	778.0602527	-0.278281099	0.0955989	-2.910924	0.003604	0.081067
tsg101b	414.44298	-0.006100353	0.1123201	-0.054312	0.956686	0.994543
itpr2	146.5707551	-0.332415878	0.1766884	-1.881368	0.059922	0.478654
txnl1	1323.587856	-0.04642378	0.079118	-0.586766	0.557361	0.948478
spns1	1374.291715	0.270729876	0.0907238	2.9841096	0.002844	0.069229
tpgs2	141.0914407	-0.333210357	0.1691747	-1.969623	0.048882	0.434811
plp1b	352.5103422	0.442601108	0.1239939	3.5695386	0.000358	0.013599
prkcbp1l	1568.128529	-0.178211679	0.0765837	-2.327019	0.019964	0.266381
gyg1a	799.9663341	-0.040626639	0.1096134	-0.370636	0.710909	0.968985
bmpr2a	559.3747453	-0.052199006	0.1127753	-0.462858	0.643466	0.959994
slc35e1	668.2235813	0.15346199	0.0950245	1.6149726	0.106317	0.615567
insra	1326.017589	-0.050227048	0.082544	-0.608488	0.542864	0.944974
dlx4a	177.7240171	-0.255007706	0.1616777	-1.57726	0.114736	0.635336
ctnnbl1	722.7809149	-0.118708469	0.1029447	-1.153129	0.248857	0.81797
csrp2	442.2239506	0.105505508	0.1113019	0.9479222	0.343169	0.878434
slc35d1a	211.8416087	0.150282868	0.1443403	1.0411704	0.297796	0.85401
fmn1	13.72813377	-1.06581564	0.5297994	-2.011734	0.044248	0.413497
zgc:136908	281.6620074	-0.0434536	0.145526	-0.298597	0.765248	0.975374
crx	2431.078052	-0.064210978	0.0845685	-0.759277	0.447687	0.917154
bfsp2	3470.373126	0.172197951	0.0763195	2.2562775	0.024053	0.299515
ybey	0	NA	NA	NA	NA	NA
slc38a7	313.6063972	0.142749094	0.1330818	1.0726414	0.283432	0.84466
mob2b	617.048184	0.055181542	0.107475	0.5134362	0.607646	0.956718
cpa6	56.7972674	0.231205139	0.2589059	0.8930085	0.371853	0.892248
hpgd	241.357374	-0.04109903	0.1372354	-0.299478	0.764575	0.975374
glra1	840.7117494	0.131706275	0.095924	1.3730277	0.169744	0.726965
kdsr	350.9229203	-0.073860676	0.1211622	-0.609602	0.542126	0.944974
hs6st2	571.3684781	0.017305044	0.1008527	0.1715874	0.863762	0.985952
dnaaf1	13.51074814	-0.338685797	0.5232083	-0.647325	0.517422	0.938592
zgc:100832	135.765	-0.070123093	0.1710997	-0.409838	0.681925	0.965476
ttc26	196.7439519	0.073990883	0.1533016	0.4826491	0.629345	0.957354
rnf19a	983.9160576	-0.10767121	0.0863381	-1.247088	0.212365	0.780251
polr3gla	67.90260717	-0.168093844	0.2453615	-0.685087	0.493289	0.931926
mettl21a	34.30761669	0.213492552	0.3614991	0.5905757	0.554805	0.947449
mtnr1al	8.125492417	0.414438443	0.6661674	0.6221236	0.533861	NA
thbs3b	168.9181718	0.361752248	0.1699216	2.1289357	0.03326	0.355427
dcn	2318.750002	0.298541791	0.0704902	4.2352213	2.28E-05	0.001407
angptl1a	334.4328206	0.076342649	0.1251273	0.6101201	0.541782	0.944974
dmac2l	131.9600102	-0.112257066	0.1785375	-0.628759	0.529507	0.942209

kif15	379.434948	-0.035689313	0.1156027	-0.308724	0.757532	0.975374
apoa1a	13026.05938	0.197436549	0.205437	0.9610565	0.336524	0.876088
meis1b	4117.423222	-0.072594338	0.0674141	-1.076843	0.28155	0.84357
c19h1orf109	79.00545265	-0.336306926	0.2273939	-1.478962	0.13915	0.677963
cnga1b	120.9777766	0.171242295	0.2066923	0.8284891	0.407394	0.901305
zgc:109965	496.8479028	-0.142675753	0.1273448	-1.120389	0.262548	0.830166
fbxl2	169.9356726	0.045568181	0.1587854	0.2869796	0.774128	0.975687
cyp46a1.1	152.2368453	-1.004990678	0.1813791	-5.540831	3.01E-08	4.54E-06
sgce	949.4848574	0.075644366	0.0851824	0.8880279	0.374526	0.894213
ccnl1b	984.5587328	0.150904902	0.0866907	1.7407276	0.081731	0.552725
papolg	1856.372901	-0.097551342	0.0751199	-1.298609	0.194078	0.75784
emc7	1257.356412	0.075037874	0.0820832	0.914168	0.360629	0.885686
rab4b	24.86632554	0.275321525	0.3792573	0.7259493	0.46787	0.924328
zgc:162872	48.17511708	0.140777798	0.2877213	0.4892853	0.62464	0.957354
cant1a	443.085922	-0.299940659	0.1119461	-2.679332	0.007377	0.135568
scp2a	1229.829373	-0.044466437	0.0866683	-0.513065	0.607906	0.956718
fer	24.25021592	-0.019213127	0.4226256	-0.045461	0.96374	0.995927
gpt2	664.3431806	0.286880994	0.1030881	2.7828721	0.005388	0.109051
CDK18	62.23362705	-0.03774659	0.2472177	-0.152686	0.878646	0.987881
umps	276.2681277	-0.183090356	0.1338244	-1.368139	0.171269	0.729413
dpf2	243.6747341	0.032407921	0.1452477	0.2231217	0.823441	0.982196
nup35	288.8143982	0.199737949	0.1262218	1.5824367	0.11355	0.631853
psme3	644.80364	0.023977115	0.0996212	0.2406829	0.809801	0.980648
ube2e3	1890.215481	-0.116599257	0.0864485	-1.348772	0.17741	0.738316
def6a	9.200782712	-0.511313697	0.6548971	-0.780754	0.434947	NA
rgma	2646.940576	0.060116399	0.0721462	0.8332577	0.404699	0.901305
eftud2	1530.70197	-0.078969658	0.082313	-0.959382	0.337366	0.876394
clcn1b	51.19235866	0.24050811	0.2687337	0.8949681	0.370804	0.890813
tmem206	71.88842988	0.09794981	0.2542278	0.3852837	0.700027	0.967998
eif4e1c	1098.897599	0.280101201	0.0813003	3.4452684	0.00057	0.019517
katnal2	9.90845297	1.371124574	0.6697222	2.0473035	0.040628	NA
zgc:65851	224.2212777	0.119252415	0.1421181	0.839108	0.401409	0.90084
bcs1l	238.965117	-0.076761177	0.135469	-0.566633	0.570964	0.949079
cnga3b	40.16379524	0.49412699	0.318516	1.551341	0.12082	0.645876
syt13	502.6284211	0.144262766	0.109214	1.3209187	0.186528	0.750917
myoz2a	246.5245954	0.2750619	0.1419532	1.9376937	0.052661	0.450394
usp25	1200.01476	-0.025761802	0.0857386	-0.300469	0.763819	0.975374
zgc:110269	196.1940184	-0.294927204	0.1681541	-1.75391	0.079446	0.547489
ptpn11b	246.6466008	0.241586412	0.1351311	1.7877924	0.07381	0.529749
capn9	535.3067825	-0.204561649	0.1035821	-1.974875	0.048282	0.432986
nacad	550.892203	0.09005377	0.104966	0.8579328	0.39093	0.897643
vipr2	61.57977826	-0.022662752	0.2543256	-0.089109	0.928995	0.992702
galnt8a.1	8.997498262	-0.77029437	0.6539289	-1.177948	0.238817	NA
fbp2	1263.592432	-1.091897573	0.0958812	-11.38802	4.80E-30	1.05E-26
trim46a	144.3890892	0.01584052	0.1799258	0.0880392	0.929846	0.992702
ttc21b	285.7611573	-0.002318001	0.1306173	-0.017747	0.985841	0.996944
rdh10b	184.1049161	0.111645156	0.1626418	0.6864479	0.492431	0.931926
naa35	861.4732652	0.05901889	0.0923083	0.6393668	0.522584	0.939331
hsc70	1175.217716	0.618786293	0.1009689	6.1284858	8.87E-10	1.87E-07
pdha1a	4151.689974	0.084503144	0.0676245	1.2495938	0.211448	0.778917
cox4i1l	118.3076279	-0.632857816	0.216606	-2.9217	0.003481	0.079216
adar	663.4864453	-0.041947016	0.0979586	-0.428212	0.668497	0.96478
kcnk5b	155.8909948	0.508354199	0.1677228	3.0309191	0.002438	0.061888
mmp13a	100.2876542	-0.028577925	0.2477171	-0.115365	0.908156	0.990702

eya4	580.0384622	0.106173287	0.1011708	1.0494458	0.293973	0.849999
adssl	1310.509212	-0.094017948	0.0800317	-1.174758	0.240091	0.808111
ercc6l2	120.0497877	-0.082747145	0.1848857	-0.447558	0.654472	0.961305
col1a1a	32753.84354	0.303986352	0.1065754	2.8523128	0.00434	0.093084
mgat1a	410.7377422	-0.049315192	0.1146475	-0.430146	0.667089	0.964736
rnf207b	88.32446759	0.367320823	0.2093683	1.7544246	0.079358	0.547489
col11a2	16012.18306	0.115733682	0.0969749	1.1934401	0.232697	0.80121
nef1b	951.8861649	0.140249888	0.0915893	1.5312916	0.125697	0.654803
fam76b	1035.404585	-0.025353157	0.0957527	-0.264778	0.791181	0.977461
spic	0.679983498	-1.527277543	2.5551009	-0.597737	0.550016	NA
zgc:113057	1.118581941	-2.434763583	2.0644676	-1.179366	0.238252	NA
si:ch211-169p10.1	8.631167378	-0.79246538	0.6798098	-1.165716	0.243729	NA
vmp1	785.086903	0.071798923	0.0993917	0.7223832	0.470059	0.924787
rnls	331.4241397	-0.19265427	0.1273675	-1.512586	0.130385	0.662797
zgc:77849	2322.312733	-0.154228296	0.0783475	-1.968515	0.049009	0.435115
rassf4	73.81074726	-0.210466147	0.2298378	-0.915716	0.359816	0.885611
spint1b	589.2834385	0.041199251	0.1017831	0.4047751	0.685643	0.966307
aacs	62.18546058	-0.172548197	0.2556256	-0.675004	0.499674	0.934065
aurka	251.968794	-0.163343022	0.145655	-1.121438	0.262102	0.830166
mphosph10	323.8565449	-0.171503032	0.1277139	-1.342869	0.179314	0.740258
GPR62 (1 of many)	18.80100641	0.666648698	0.4418961	1.5086097	0.131399	0.664861
per1b	2258.65797	0.035129372	0.072055	0.4875355	0.625879	0.957354
rlbp1a	1084.662761	0.082508942	0.09234	0.893534	0.371571	0.892003
u2af2a	1365.711287	0.030850539	0.0843433	0.3657734	0.714534	0.96909
rnpep	315.0836423	0.234198757	0.1249776	1.8739263	0.060941	0.482482
sdcbp2	2169.000402	-0.114897515	0.0812597	-1.413955	0.157375	0.708089
hcfc1b	1812.87192	-0.035882412	0.0848854	-0.422716	0.672503	0.965257
slc6a6a	20.06383268	-0.268755364	0.4820912	-0.557478	0.577201	0.950788
epx	8.933588736	0.762742446	0.6371652	1.1970875	0.231272	NA
dnase1	2.496544088	0.208764915	1.2037135	0.1734341	0.86231	NA
rap1aa	1124.808803	0.068215787	0.0930066	0.733451	0.463283	0.922698
ak5	358.5591925	0.082756834	0.1191566	0.6945214	0.487355	0.931584
pdss2	245.8934356	0.313999652	0.1470965	2.134651	0.03279	0.352808
polr3f	156.9699162	-0.182733312	0.1624183	-1.125079	0.260556	0.828546
slkb	586.9526078	0.071768981	0.112294	0.6391169	0.522747	0.939336
waca	1466.352029	0.015597612	0.0773805	0.2015703	0.840253	0.985029
AL590149.1	322.5575348	0.120644413	0.1238069	0.9744563	0.32983	0.873938
fmnl2a	51.57364239	0.255664755	0.2861417	0.8934899	0.371595	0.892003
ptdss1a	227.0613532	-0.181104565	0.1530407	-1.183375	0.23666	0.805854
pdcd10b	424.2838667	-0.113408366	0.1156905	-0.980274	0.326951	0.872658
col5a1	8947.404373	0.251704645	0.0644027	3.9082944	9.29E-05	0.004595
si:dkey-97a13.6	31.73373582	-0.470564078	0.3605431	-1.305154	0.191841	0.75597
eif3ja	433.3832395	-0.053092288	0.1134016	-0.468179	0.639656	0.959994
hpxa	7.149207444	-0.654652183	0.7686859	-0.851651	0.394408	NA
saga	1119.289906	0.169041549	0.0943643	1.7913724	0.073234	0.527933
prkag2a	554.8996679	0.055819801	0.0999003	0.5587551	0.576329	0.950611
cdc34b	1016.652441	-0.09132527	0.0958085	-0.953206	0.340486	0.87805
zgc:92749	32.12167228	-0.010007364	0.334331	-0.029933	0.976121	0.996762
cideb	26.98113868	0.222975542	0.3876846	0.5751468	0.565192	0.948739
si:ch211-266d19.4	133.4237008	-0.014400098	0.1724608	-0.083498	0.933456	0.992702
smad4b	125.3449309	-0.239697424	0.1839946	-1.302742	0.192663	0.756795
atf6	1133.125849	0.229817007	0.0826147	2.7817927	0.005406	0.109079
tfap2b	1420.607492	0.007882581	0.0938851	0.0839599	0.933088	0.992702
inhbaa	140.9100247	-0.153772638	0.1692759	-0.908414	0.363659	0.886174

gtf2e2	571.7969484	-0.010212368	0.1060855	-0.096265	0.92331	0.992588
rpusd4	123.9434042	-0.13931398	0.1876815	-0.742289	0.457912	0.921978
pfn2l	5728.958752	0.133736667	0.0747746	1.7885295	0.073691	0.529749
atp2b1a	1652.806508	0.201976186	0.0957175	2.1101283	0.034847	0.363434
eif1b	9868.765122	-0.042251633	0.0768162	-0.550036	0.582295	0.95207
c3a.1	4899.546986	0.107476056	0.2230253	0.4819007	0.629877	0.957354
bbx	17.07227908	-0.215210373	0.4783476	-0.449904	0.65278	0.960708
wee2	1.18109702	-0.359546947	1.8242229	-0.197096	0.843752	NA
rbm14a	992.9310246	-0.073980998	0.0940229	-0.78684	0.431376	0.912692
calhm5.2	33.90246786	-0.067567587	0.3302257	-0.20461	0.837877	0.984701
hcls1	276.2232072	0.180713787	0.130749	1.3821432	0.166928	0.723549
maco1b	883.6153103	0.014706703	0.0887004	0.165802	0.868313	0.987245
poc1bl	121.3155778	-0.250939082	0.194506	-1.290135	0.197004	0.761944
rer1	1029.758589	0.118727223	0.0915534	1.2968085	0.194697	0.758758
arl13b	328.9305889	0.022335039	0.1238208	0.1803819	0.856853	0.985597
smarca1	193.9908164	-0.084219225	0.1485407	-0.566977	0.57073	0.949061
nucks1b	2050.656148	-0.02989988	0.0872394	-0.342734	0.731799	0.971572
foxa3	359.4731113	-0.215529865	0.1240814	-1.737003	0.082387	0.553931
plek2	223.9974112	0.682576353	0.3232469	2.1116252	0.034719	0.362665
tmem161a	520.8136113	0.230405533	0.1051561	2.1910813	0.028446	0.327409
hnmt	164.8789736	-0.500973667	0.177389	-2.824153	0.004741	0.099196
pfkpb	537.6040771	-0.014333388	0.1220664	-0.117423	0.906525	0.990702
dipk2aa	73.3250648	-0.067241086	0.2439016	-0.275689	0.782787	0.97627
csnk2a2a	891.7135612	-0.004103755	0.0898725	-0.045662	0.96358	0.995927
nop56	2181.740745	-0.159366611	0.0891257	-1.78811	0.073758	0.529749
dlgap4b	33.07303079	-0.303326659	0.3469875	-0.874172	0.382025	0.895192
itga3b	371.7177901	0.379798353	0.1207104	3.1463605	0.001653	0.046434
mettl3	208.9319712	0.193313932	0.1417437	1.3638276	0.172622	0.731348
asah2	78.09455046	-0.532897899	0.2587186	-2.059758	0.039422	0.389957
foxn3	35.97636336	0.107524194	0.3292309	0.326592	0.743976	0.974094
alg9	732.9816685	0.013827721	0.0941361	0.1468907	0.883218	0.988706
arih2	1537.787262	-0.071870045	0.095332	-0.753892	0.450914	0.918761
tmprss4b	29.66825743	0.382377866	0.3666183	1.0429864	0.296955	0.853798
picalma	1351.336321	0.061346346	0.0776	0.7905458	0.429209	0.911406
npepl1	1241.160623	-0.062885356	0.0849914	-0.739903	0.459359	0.921978
snap23.1	735.5376672	-0.118532953	0.1022304	-1.159468	0.246265	0.816376
slc4a1a	281.8051091	0.282785092	0.1653688	1.7100272	0.087261	0.566108
znf143a	461.8929035	-0.120840485	0.1082634	-1.116172	0.264349	0.830245
ammecr1	145.3099122	-0.084213931	0.1783053	-0.472302	0.636711	0.958679
anos1a	1133.181481	0.115175787	0.089472	1.2872833	0.197996	0.763261
ddx49	158.8394797	-0.110956933	0.1673332	-0.66309	0.507273	0.935734
slc34a2a	38.59776013	1.315978732	0.3241309	4.0600231	4.91E-05	0.002688
arhgef4	2276.078689	0.048860682	0.0724432	0.6744685	0.500013	0.934224
ech1	338.6304853	-0.138924722	0.1429702	-0.971704	0.331198	0.874339
tcea2	345.5821143	0.113303044	0.1167169	0.970751	0.331672	0.874653
eif2d	287.4622844	0.017928976	0.1292019	0.1387671	0.889634	0.989291
mat2b	445.9304601	-0.17852354	0.1185372	-1.506055	0.132053	0.665787
itgb5	563.3988215	0.145063832	0.1037398	1.3983426	0.16201	0.716531
myhz2	22414.59667	0.270403573	0.0999399	2.7056623	0.006817	0.127648
tnfsf13b	18.99502116	0.164196013	0.4404167	0.3728197	0.709283	0.968736
syap1	948.5523837	-0.070189833	0.0890657	-0.788068	0.430657	0.912304
scml2	733.9796332	-0.044621526	0.0979696	-0.455463	0.648776	0.960708
mob1bb	318.8844296	0.199878553	0.1305963	1.530507	0.125891	0.654803
rhoub	841.971884	0.13319096	0.0881998	1.5101055	0.131016	0.663795

cfl1l	3074.943398	0.049434405	0.1023246	0.4831134	0.629015	0.957354
osbpl7	1227.387489	0.280192699	0.0815479	3.4359265	0.000591	0.020129
nf1a	795.3084945	-0.043811333	0.0938083	-0.467031	0.640478	0.959994
adal	38.37535784	0.256045937	0.3255922	0.7864008	0.431633	0.91277
gpia	5575.278349	-0.115611289	0.0934833	-1.236705	0.216197	0.783589
ppfia2	604.4840973	0.051659703	0.1386823	0.372504	0.709518	0.968856
tdg.1	996.9575696	0.170556075	0.0897335	1.9006951	0.057342	0.469987
opcml	60.49768396	0.405104695	0.2528429	1.602199	0.109112	0.622856
pibf1	0	NA	NA	NA	NA	NA
ubiad1	507.4078517	0.065879171	0.1187278	0.5548756	0.57898	0.951052
or101-1	9.957374935	0.321386327	0.6538438	0.4915338	0.623049	NA
pole4	74.79512173	0.07543458	0.2357557	0.3199692	0.748992	0.97419
dtnbb	12.24225332	0.227637057	0.5717674	0.3981288	0.690535	0.967111
si:ch211-59h6.1	35.22095894	-0.461703836	0.3330392	-1.386335	0.165645	0.721576
nsun3	62.1674877	-0.190981488	0.2587133	-0.738197	0.460394	0.921978
erap1a	116.658759	-0.120292822	0.183541	-0.6554	0.51221	0.936253
ints6	214.5101713	0.141319149	0.1473893	0.9588154	0.337652	0.876395
mta2	3436.252308	0.044551518	0.0680459	0.6547278	0.512643	0.936253
ndufv2	2148.038402	0.092849226	0.0963073	0.9640936	0.334999	0.875906
abcc12	447.7857781	-0.563458905	0.1219036	-4.622168	3.80E-06	0.000321
zgc:158412	32.97265455	-0.692667704	0.3482279	-1.989122	0.046688	0.424613
itpk1a	284.5948513	-0.722045368	0.1295833	-5.572055	2.52E-08	3.89E-06
hoxb5a	696.2209216	-0.140732317	0.0956875	-1.470749	0.141359	0.681968
ascc2	265.7825411	0.019513642	0.1431398	0.1363258	0.891564	0.989291
oxct1a	1352.449363	0.046257885	0.0784937	0.5893198	0.555647	0.947621
mmp15b	74.6331344	0.180010307	0.2395817	0.7513525	0.452441	0.919104
mrpl11	435.6942059	-0.189983409	0.1166186	-1.6291	0.103292	0.609625
bmi1b	476.0790449	0.005762885	0.1063088	0.0542089	0.956769	0.994543
ywhaba	13869.92532	0.043326516	0.0666369	0.6501884	0.515571	0.9377
tubgcp2	437.9197467	0.001466039	0.1137881	0.0128839	0.98972	0.997612
uap1l1	285.7203716	-0.148677462	0.1340397	-1.109205	0.267342	0.833191
ndrg3a	4079.241045	0.059327938	0.0663179	0.8945993	0.371001	0.891178
gclc	1019.504254	-0.166671717	0.1119126	-1.489303	0.136408	0.673303
dmtn	363.5643183	0.008230003	0.1169255	0.0703867	0.943886	0.993364
hiat1a	726.7912302	0.020478509	0.0952192	0.2150671	0.829715	0.982805
maf1	625.3100695	0.035534047	0.096305	0.368974	0.712147	0.969006
dlx1a	294.5385224	-0.12556819	0.1242466	-1.010637	0.31219	0.864401
pdk1	80.34750703	0.23222797	0.2240855	1.0363363	0.300045	0.856146
prph2la	85.35005908	0.003394031	0.2251869	0.0150721	0.987975	0.997369
atp1b1a	6492.026993	-0.078637674	0.0672826	-1.168767	0.242497	0.811298
dhx16	428.1832035	-0.122879736	0.1145866	-1.072374	0.283552	0.84466
dennd3b	9.631224402	0.108760252	0.5994864	0.1814224	0.856036	NA
zgc:55558	1511.846243	0.054621112	0.0834476	0.6545558	0.512754	0.936253
jag1b	538.4582901	0.064276754	0.1091141	0.5890785	0.555809	0.947625
nkd3	90.36026649	0.116236341	0.2092535	0.555481	0.578566	0.951052
si:ch211-1f22.1	1.804341312	-0.79774002	1.3966279	-0.57119	0.567871	NA
zeb1b	897.2230013	-0.077319209	0.0898708	-0.860337	0.389603	0.896421
htr2cl2	4.276171362	0.171508951	1.0482094	0.1636209	0.87003	NA
pde4ca	227.6805797	0.290990495	0.1415703	2.0554481	0.039836	0.391511
slc35a3a	187.3418017	0.152430174	0.1606891	0.9486028	0.342823	0.878434
phc2b	715.3891116	-0.121864179	0.0959657	-1.269872	0.20413	0.771764
glulc	32.7935041	0.086007373	0.3381414	0.2543533	0.799223	0.979293
dhcr24	105.3999302	-0.67274117	0.2086386	-3.224433	0.001262	0.037142
zgc:172271	36.54176542	0.613776625	0.3449897	1.7791157	0.075221	0.534207

wbp11a	68.91346767	-0.097461475	0.2380457	-0.409423	0.682229	0.965476
tars	1954.214893	-0.011469221	0.0732469	-0.156583	0.875573	0.987881
hsf4	204.1371149	0.259207408	0.1686159	1.537266	0.124228	0.652801
tmc5	102.324285	-0.73086126	0.2076672	-3.519388	0.000433	0.015869
colec11	29.14392821	-0.400035236	0.3591731	-1.113767	0.265379	0.831302
eif4eb	280.8360973	0.137650165	0.1359996	1.0121364	0.311473	0.863945
znf76	105.1175731	-0.138449417	0.1977229	-0.700219	0.48379	0.929461
tmem179aa	335.1205465	0.08854324	0.1267925	0.6983316	0.48497	0.93001
crispld1b	258.5335278	0.347708439	0.133317	2.6081333	0.009104	0.159622
slc2a3a	1931.479482	0.240586785	0.0863306	2.7868067	0.005323	0.108022
gpx8	99.6656093	-0.158668881	0.2107093	-0.753023	0.451436	0.918761
rpl19	9345.740492	-0.296460283	0.079819	-3.714157	0.000204	0.008689
map3k15	198.1155432	0.165592307	0.146549	1.1299452	0.258499	0.826711
cadps2	267.9109644	-0.101088957	0.1372499	-0.736532	0.461407	0.922161
pygmb	5048.714022	0.066316182	0.0899324	0.7374003	0.460879	0.921978
ttc5	328.2529949	-0.057442654	0.1252954	-0.458458	0.646624	0.960362
ndufa10	2847.409448	-0.026830727	0.0749808	-0.357835	0.720467	0.969318
anxa6	895.1162085	0.121280982	0.0942186	1.2872303	0.198014	0.763261
kif26ba	996.6133274	-0.141111061	0.1036823	-1.360995	0.173515	0.732906
tasorb	1369.633638	0.002600972	0.0972266	0.0267516	0.978658	0.996762
cirbpb	49381.62539	-0.1501913	0.0615943	-2.438394	0.014753	0.222347
sh3gl3a	397.4159034	0.20631315	0.1141239	1.8078001	0.070638	0.520155
isoc2	123.8782436	-0.034384182	0.1788776	-0.192222	0.847568	0.985174
ppp6r3	1786.17416	0.136153217	0.0851204	1.5995368	0.109701	0.623766
cwc15	176.3255539	0.155581965	0.1612982	0.9645611	0.334765	0.875699
gabra4	14.62992455	0.270563333	0.4899932	0.5521777	0.580827	0.951731
CABZ01088367.1	12.89817512	-0.533629819	0.5396879	-0.988775	0.322773	0.870524
guca1b	56.33699157	0.213512321	0.264516	0.8071812	0.419562	0.90651
bnip2	215.3502017	0.395291022	0.143979	2.745477	0.006042	0.117821
bmp2a	78.72231401	0.049377298	0.2196523	0.2247975	0.822137	0.982186
lin7a	1239.055723	0.138950691	0.0865833	1.6048205	0.108533	0.62063
lmna	414.6177304	-0.046378775	0.1088851	-0.425942	0.67015	0.965223
slc8a1a	26.81765875	0.043071946	0.3705286	0.1162446	0.907459	0.990702
bhmt	11348.38868	0.078848491	0.1010457	0.7803252	0.4352	0.914408
hira	639.7701927	-0.055413518	0.1130671	-0.490094	0.624067	0.957354
sycp3	5.408925958	0.209545457	0.8233119	0.2545153	0.799098	NA
hey2	89.15148485	0.225394137	0.2189167	1.0295888	0.303203	0.85729
atp6v1ba	4850.685525	0.047455189	0.064303	0.7379931	0.460519	0.921978
tdrd9	28.13210182	0.041344387	0.360073	0.1148222	0.908586	0.990702
mtrex	799.3389858	-0.152121456	0.0914086	-1.664191	0.096074	0.590174
wfikkn2a	112.6113916	0.203246564	0.1906403	1.0661262	0.286367	0.845942
supt7l	69.48762395	-0.032548317	0.2616851	-0.12438	0.901015	0.990121
nedd1	238.7043475	-0.096032041	0.1362988	-0.70457	0.481078	0.927927
cct4	4716.529072	-0.16266412	0.0874933	-1.859161	0.063004	0.489719
arhgef39	85.73472553	-0.527601649	0.2190892	-2.408159	0.016033	0.233377
gata1a	8.932222654	0.682851863	0.6764834	1.009414	0.312776	NA
rrp15	260.4534115	-0.065965901	0.143157	-0.460794	0.644946	0.96029
map3k9	54.53025363	-0.033026977	0.2686735	-0.122926	0.902166	0.990121
zbtb34	33.48243425	-0.078098868	0.3351215	-0.233046	0.815725	0.981571
abca2	531.3925041	0.103042348	0.1157704	0.8900574	0.373435	0.893659
ube2kb	1467.152113	-0.078168032	0.0897629	-0.870828	0.383848	0.895192
pck1	2061.085698	0.207637554	0.107205	1.9368273	0.052766	0.450909
cyp2ae1	26.00920377	-0.538559159	0.3717964	-1.448533	0.147468	0.694625
svep1	388.6113041	0.085565018	0.113052	0.7568641	0.449131	0.91823

mcm9	77.05870016	-0.233011493	0.2398544	-0.97147	0.331314	0.874362
hoxb4a	347.5380275	0.106440789	0.1226822	0.8676138	0.385606	0.89551
ikzf1	35.83253005	0.360734101	0.3370266	1.0703431	0.284465	0.844955
lpgat1	910.8111373	-0.041750256	0.1003894	-0.415883	0.677496	0.965257
pgm1	2206.952016	0.094836278	0.0764305	1.2408171	0.214673	0.782493
rfx2	500.7222843	-0.181836041	0.1179505	-1.54163	0.123164	0.651012
gadd45bb	231.6566297	0.19647756	0.1457173	1.3483475	0.177547	0.738343
csnk2a2b	1049.742601	0.00572961	0.0852288	0.0672262	0.946402	0.99381
brd1a	545.6367555	0.072817065	0.1145906	0.6354541	0.525132	0.940066
tnfb	72.56852186	-0.543415435	0.2627369	-2.068287	0.038613	0.385234
sema3gb	198.4575588	0.154392083	0.1495652	1.0322728	0.301944	0.856944
anxa13l	298.1734225	0.244035532	0.1254259	1.9456554	0.051696	0.446003
pbx3b	3043.305483	-0.021991951	0.0788408	-0.278941	0.78029	0.97627
vdac2	11972.2057	-0.05571085	0.0787705	-0.707255	0.479408	0.927091
cd164	1787.983687	-0.297080983	0.07517	-3.952119	7.75E-05	0.003948
cntn3a.2	5.342165981	0.345964861	0.8537707	0.4052199	0.685316	NA
casr	0	NA	NA	NA	NA	NA
adgrl4	57.7495268	-0.376527696	0.2638887	-1.426843	0.153625	0.702578
tpd52l2b	1850.633446	-0.001347048	0.0759714	-0.017731	0.985853	0.996944
brox	364.7705529	-0.087546265	0.1167952	-0.749571	0.453513	0.920041
tbck	378.1643642	-0.011868864	0.1154882	-0.102771	0.918145	0.991623
napba	529.0918784	0.079100368	0.1088912	0.7264167	0.467583	0.924328
hyou1	1364.283625	0.122516294	0.0894654	1.369426	0.170866	0.728484
si:dkey-42i9.6	1140.385267	0.007452796	0.0896718	0.0831119	0.933763	0.992702
vsig8b	23.39282667	-0.126574981	0.4079719	-0.310254	0.756368	0.975374
cilp2	91.54896507	0.378615178	0.2138898	1.7701417	0.076704	0.539626
stimate	300.6142721	0.003812161	0.1294614	0.0294463	0.976509	0.996762
cers2a	68.47806352	-0.066648771	0.2351362	-0.283448	0.776834	0.975826
ccm2	131.0177356	0.296514154	0.1735206	1.7088125	0.087486	0.566637
usp9	3526.765426	0.068794912	0.0724963	0.9489435	0.342649	0.878434
zgc:77486	331.5624045	-0.292676516	0.12622	-2.318782	0.020407	0.268884
gtse1	220.8214288	-0.266599616	0.1417512	-1.880757	0.060005	0.478654
g6pca.2	2135.883196	0.333175127	0.0917589	3.6309849	0.000282	0.011325
ap4b1	184.7223402	-0.145789327	0.1581848	-0.921639	0.356717	0.883422
srsf6a	2141.572929	-0.174580832	0.085356	-2.045325	0.040823	0.394957
slc4a4a	2646.234451	0.07146273	0.0727165	0.9827575	0.325727	0.871896
vta1	551.3851743	-0.170408231	0.1096722	-1.553796	0.120233	0.645215
polr3glb	273.2994219	-0.210028574	0.1394039	-1.506619	0.131908	0.665566
lancl1	502.0032316	0.011811888	0.1030512	0.1146215	0.908745	0.990702
slc25a55b	312.5833516	-0.089866655	0.1535003	-0.585449	0.558246	0.948478
eno1b	1455.054761	0.236309659	0.088618	2.6666097	0.007662	0.139512
tnni2a.3	100.1660354	-0.202088283	0.2290329	-0.882355	0.377585	0.895192
actn3a	11681.81288	0.418813025	0.0815434	5.1360736	2.81E-07	3.42E-05
rrbp1a	1085.444916	0.061100094	0.090196	0.6774143	0.498143	0.933765
ctss2.2	71.63367102	0.258916098	0.2322546	1.1147941	0.264939	0.830836
si:dkey-246g23.4	28.35242357	0.286316417	0.3766903	0.7600844	0.447204	0.917154
thop1	1472.440467	0.043749705	0.0840755	0.5203622	0.602811	0.956045
ppp1r13l	529.3310287	0.152612535	0.1054965	1.4466118	0.148006	0.695665
zgc:77158	121.9902662	0.181762582	0.1834172	0.9909789	0.321696	0.869375
gucy1a1	293.5901894	0.018347674	0.1318112	0.1391966	0.889295	0.989291
sim1b	19.45181463	-0.601265977	0.446627	-1.346237	0.178226	0.739299
klf11b	697.7583669	-0.209546371	0.1210093	-1.731656	0.083335	0.556629
hps4	84.94217546	-0.216126037	0.2268072	-0.952906	0.340637	0.878134
snrpd3	0.650476611	1.436325931	2.5890223	0.5547754	0.579048	NA

pcid2	350.2646868	-0.132002377	0.1285539	-1.026826	0.304503	0.858905
capns1b	1603.826713	-0.024294123	0.0828782	-0.29313	0.769422	0.975687
fam219ab	441.5849689	0.045310915	0.1149661	0.3941242	0.693489	0.96736
anapc16	414.4384623	-0.288838819	0.1252465	-2.306164	0.021101	0.273793
snx13	37.17977286	0.343786329	0.3263424	1.0534529	0.292133	0.848075
osbpl2b	499.252904	0.000353043	0.1211181	0.0029149	0.997674	0.999174
arhgef3	39.70420386	-0.180233534	0.3227317	-0.558462	0.576529	0.950611
spo11	1.814481193	-1.399828196	1.5816487	-0.885044	0.376133	NA
sulf2b	3254.494198	0.112397376	0.0729492	1.5407619	0.123375	0.651258
aldh3b1	235.0003673	-0.087148324	0.1428898	-0.609899	0.541929	0.944974
abl2	510.2382947	0.096813566	0.1044911	0.9265241	0.354174	0.882044
tbc1d19	120.4618624	0.19361505	0.1811872	1.0685914	0.285254	0.845164
sept5a	626.6167142	-0.017191294	0.1039124	-0.16544	0.868597	0.987245
ttc14	380.0501568	-0.253293445	0.1224548	-2.068465	0.038596	0.385234
egfra	461.5137909	-0.218938697	0.1124965	-1.946183	0.051633	0.445651
lmo4a	543.7291842	0.003618717	0.1003845	0.0360486	0.971244	0.996315
slc12a3	358.8535363	0.076829214	0.1364859	0.5629097	0.573496	0.949869
amy2a	131.5831375	-0.502146531	0.2267923	-2.214125	0.02682	0.31568
cdc14aa	45.23828103	0.226833318	0.3054836	0.7425383	0.457761	0.921978
ptpmt1	147.0109706	-0.133941211	0.177177	-0.755974	0.449665	0.918526
armc1	433.7586583	0.129318895	0.1221484	1.0587028	0.289735	0.846246
fam133b	458.0921673	-0.16605296	0.1216789	-1.364681	0.172353	0.731348
slc5a1	274.0743377	-0.297929415	0.1304657	-2.283585	0.022396	0.285346
spata20	205.0029184	-0.212536506	0.1613862	-1.316944	0.187858	0.752419
pi4k2b	293.7615587	0.007461524	0.1257199	0.0593504	0.952673	0.993993
sorl1	1044.859733	0.0148489	0.0947131	0.1567777	0.87542	0.987881
si:ch211-253p14.2	0.158795395	0.967652056	4.0804729	0.2371421	0.812547	NA
frya	804.6335983	0.189954229	0.0896713	2.1183381	0.034146	0.359261
slc16a9a	353.1311111	0.039970146	0.1242846	0.3216017	0.747754	0.97419
plekhb2	307.3875802	-0.08429602	0.1289515	-0.653703	0.513303	0.936525
eif3m	3232.376279	-0.237581861	0.0868191	-2.736515	0.006209	0.119726
XRCC2	19.55905002	0.100427416	0.4272427	0.2350594	0.814163	0.981081
psmb3	1939.219319	0.014005042	0.073737	0.1899324	0.849362	0.985174
ivns1abpb	2048.499172	-0.238111703	0.0867645	-2.744346	0.006063	0.117889
pdf	89.75439326	-0.284288431	0.2096395	-1.356082	0.175073	0.735545
slc31a1	724.3943215	-0.343838577	0.1095919	-3.137444	0.001704	0.047485
mipb	3669.664422	0.002326532	0.0696511	0.0334026	0.973353	0.996315
cul2	798.2877232	0.027051109	0.091531	0.2955404	0.767581	0.975687
psma6b	717.5127133	-0.112530478	0.0994426	-1.131613	0.257797	0.825314
psap	7019.239659	-0.075018553	0.0760737	-0.98613	0.324069	0.870928
pex3	217.6097532	0.179333636	0.1419242	1.2635876	0.206378	0.774011
anxa13	1820.172903	0.053859009	0.0764873	0.704156	0.481336	0.928307
ndfip1	2441.835474	0.118100506	0.0725102	1.6287425	0.103368	0.609625
ube2q2	1749.70148	0.084141807	0.0768477	1.0949161	0.273553	0.838253
ern1	348.7171241	0.043542081	0.1350537	0.3224056	0.747145	0.97419
stard8	278.5416839	-0.012643613	0.1281441	-0.098667	0.921403	0.992138
eif2b4	987.3590957	-0.024014792	0.0967459	-0.248225	0.80396	0.979643
lrrc45	217.3233923	-0.076242042	0.1476659	-0.516314	0.605635	0.95651
cwc22	830.3965414	-0.10369967	0.0892524	-1.16187	0.245288	0.815319
lbr	1054.623933	0.060635089	0.0877543	0.6909642	0.489588	0.931926
pdia7	1308.289454	0.064297121	0.0899881	0.7145069	0.474914	0.925399
rrm1	2409.280304	-0.079705654	0.085076	-0.936876	0.348822	0.880195
lhx1a	1323.238481	-0.015304754	0.0841988	-0.181769	0.855764	0.985174
ms4a17a.4	9.839069868	-0.018319919	0.6182797	-0.02963	0.976362	NA

abcc2	1588.070564	-0.425964737	0.1071824	-3.974206	7.06E-05	0.003655
si:dkeyp-93d12.1	31.54644964	-0.121923379	0.3576814	-0.340871	0.7332	0.971947
smarcad1a	523.2318381	0.127572601	0.1068889	1.1935062	0.232671	0.80121
cldn7b	2713.636239	0.000487213	0.0834091	0.0058412	0.995339	0.998723
ngfb	66.43153768	-0.109514698	0.2367585	-0.462559	0.643681	0.960014
olfm1b	1643.656451	0.050317254	0.0785173	0.6408426	0.521625	0.939056
gabrr2b	35.13998545	0.772026448	0.3434062	2.2481437	0.024567	0.302262
rab36	33.37120026	0.045032886	0.3746033	0.1202149	0.904313	0.990306
cldn5b	352.9866188	-0.17145067	0.1307447	-1.311339	0.189743	0.754269
pgam1b	3742.274027	0.03234184	0.0826382	0.3913667	0.695526	0.96736
fam184a	601.3441698	-0.11205199	0.1040902	-1.076489	0.281708	0.84357
wdr82	1175.218324	0.033715513	0.0920641	0.3662178	0.714203	0.96909
atp6v1e1a	317.1713836	0.101097916	0.125815	0.803544	0.42166	0.90651
osr1	213.4648787	-0.012634082	0.1538899	-0.082098	0.934569	0.992702
pkd2	67.84923127	0.278457311	0.2584559	1.077388	0.281307	0.84357
pyroxd2	382.8889571	-0.034992746	0.1176647	-0.297394	0.766166	0.975374
dkk1a	18.6680356	-0.517953007	0.464176	-1.115855	0.264484	0.830245
cfl2	1171.097308	0.163861788	0.1047989	1.5635838	0.117915	0.641446
wasla	638.9874502	-0.111627178	0.1017973	-1.096563	0.272832	0.838096
picalmb	1907.279014	0.033190409	0.0781564	0.4246664	0.67108	0.965257
acot8	79.60733267	-0.275894101	0.2269909	-1.215441	0.224198	0.791484
srp72	1225.698269	-0.175805336	0.0925966	-1.898616	0.057615	0.470971
med22	212.9779163	0.062263186	0.1617849	0.3848517	0.700347	0.967998
mtdha	1994.352912	-0.115705132	0.0768989	-1.504639	0.132417	0.666729
ercc4	205.2587293	-0.022307121	0.144606	-0.154261	0.877404	0.987881
ssr3	1333.326866	-0.168156475	0.0926402	-1.815156	0.0695	0.514708
CABZ01041610.1	14.07898536	0.508421761	0.5051804	1.0064162	0.314215	0.865176
bloc1s1	168.2329091	-0.083409495	0.1631451	-0.51126	0.609169	0.956718
pfkma	1294.448749	0.377168294	0.0896431	4.2074444	2.58E-05	0.001562
ildr2	223.4069033	0.054131371	0.1494766	0.3621394	0.717248	0.96909
foxp1b	349.3261315	-0.16702212	0.1191033	-1.40233	0.160817	0.713963
sst2	47.05530451	0.205307137	0.389469	0.5271463	0.598092	0.955459
myl1	13852.24059	0.185341273	0.0865128	2.1423574	0.032165	0.349081
otpa	1240.712391	-0.073556082	0.0975006	-0.754417	0.450599	0.918761
zgc:55413	14.51667131	-0.064687119	0.521637	-0.124008	0.901309	0.990121
odf3l2a	16.74360555	0.310262719	0.463914	0.6687937	0.503627	0.935304
cdh13	1339.473522	0.065768971	0.080864	0.8133287	0.41603	0.906237
mecp2	10.3452359	0.204232757	0.6462947	0.3160056	0.751998	NA
zgc:86598	1381.74952	-0.095573409	0.0852774	-1.120735	0.2624	0.830166
dlst	3178.99002	-0.027442464	0.0814481	-0.336932	0.736168	0.972535
si:dkey-121j17.5	69.77119113	-0.970275269	0.2488336	-3.899293	9.65E-05	0.004757
sept8b	454.9212744	-0.131209106	0.1175427	-1.116268	0.264308	0.830245
esyt1b	109.3704969	0.089953074	0.1977709	0.4548348	0.649228	0.960708
rbmx	1846.666371	-0.029186904	0.0795509	-0.366896	0.713697	0.96909
jag2a	9.67055918	-0.492760797	0.6074876	-0.811145	0.417282	NA
pdlim3b	88.05227901	0.203305846	0.2171569	0.9362164	0.349162	0.880195
eya1	1143.730813	-0.094665773	0.0836515	-1.131668	0.257774	0.825314
camk2d2	1802.138022	0.090769335	0.0897318	1.0115626	0.311747	0.864257
rfc2	244.6581803	-0.164208701	0.1330435	-1.234249	0.21711	0.784302
dlgap1a	17.49695172	-0.370623868	0.5483454	-0.675895	0.499107	0.934006
eno2	2090.030377	0.160528012	0.0746158	2.1513932	0.031445	0.343534
med15	314.2311669	-0.072217589	0.1265422	-0.5707	0.568203	0.948983
brinp2	158.2360791	-0.058750392	0.1644481	-0.357258	0.720899	0.969534
vac14	421.6105728	0.007771692	0.1125237	0.0690671	0.944936	0.993409

spaw	1.33588621	-0.674429397	1.6626744	-0.405629	0.685015	NA
pgghg	161.1357231	0.199188271	0.1783743	1.1166869	0.264128	0.830245
atp5pf	2451.309396	0.055382749	0.0861922	0.6425493	0.520517	0.939056
gucy2c	200.6866075	-0.749010914	0.1607992	-4.658052	3.19E-06	0.000278
en1a	30.65969262	0.176331844	0.3553137	0.4962709	0.619703	0.957354
sh2d5	91.31406201	-0.114876223	0.2067718	-0.55557	0.578505	0.951052
npm1a	2745.727596	-0.187122826	0.0779153	-2.401617	0.016323	0.235941
ormdl1	383.7964994	0.20997639	0.1136435	1.8476757	0.064649	0.497355
rab11al	175.8350894	-0.044250891	0.1584744	-0.279231	0.780068	0.976146
stk17b	177.1104562	0.065754848	0.1581193	0.4158559	0.677515	0.965257
krt95	1.363667969	0.069617777	1.7253717	0.0403494	0.967815	NA
amh	5.614567051	1.258156318	0.8265472	1.5221832	0.127963	NA
optc	23.01453252	-0.061554004	0.4019278	-0.153147	0.878282	0.987881
tmbim4	1363.314075	0.058141733	0.0839732	0.6923842	0.488696	0.931926
luc7l3	908.4876219	-0.026843414	0.0896688	-0.299362	0.764664	0.975374
ddx4	57.97376513	0.460051619	0.2738315	1.6800537	0.092947	0.580357
slc12a4	458.8044173	0.115804846	0.1086158	1.0661876	0.286339	0.845942
galnt6	509.0109482	-0.225077724	0.1025391	-2.195042	0.028161	0.325071
selenoo2	16.44993448	-0.05058359	0.5205351	-0.097176	0.922587	0.99233
smc1b	3.986040298	0.222753268	0.9418183	0.2365141	0.813034	NA
elavl3	1695.848264	-0.157820293	0.0927479	-1.701606	0.088829	0.570481
mxe	1.818018269	-0.180966542	1.4787402	-0.122379	0.902599	NA
ppp2r5d	1057.845487	0.061391253	0.0905441	0.6780261	0.497755	0.93348
rabgef1	849.0619079	0.041678677	0.0869232	0.4794886	0.631591	0.957356
dgkza	503.8573789	0.051433355	0.1090676	0.4715731	0.637232	0.958872
rab3c	450.5417837	-0.143952198	0.1116847	-1.288916	0.197427	0.762635
usf1l	405.4482922	-0.041500051	0.1138166	-0.364622	0.715393	0.96909
arhgef25b	177.0273955	0.388190347	0.1552481	2.500451	0.012404	0.19716
yipf1	276.4980873	-0.050969242	0.1345649	-0.378771	0.704858	0.968134
sstr3	7.505388373	1.462424117	0.7377828	1.9821878	0.047458	NA
ptf1a	45.83490506	-0.309757062	0.2916094	-1.062233	0.28813	0.845979
nr6a1b	38.5461855	-0.086927284	0.3504251	-0.248062	0.804086	0.979643
ca2	557.2783455	0.104178749	0.1191659	0.8742331	0.381991	0.895192
tac1	349.3677634	0.178564839	0.1224929	1.4577561	0.144908	0.688986
trpv6	451.6731181	0.2441315	0.1455572	1.6772203	0.093499	0.581585
ythdf2	801.1618721	0.20010664	0.0929995	2.151696	0.031421	0.343463
nutf2l	458.6092235	-0.25332452	0.1087594	-2.32922	0.019847	0.265495
tmem126a	347.4132894	0.045391355	0.1252021	0.3625446	0.716945	0.96909
usp5	2338.117799	0.037518841	0.071787	0.5226409	0.601224	0.956045
cdh6	1413.502938	0.042793078	0.0773051	0.5535612	0.579879	0.951257
pdk3a	54.95591531	-0.014145364	0.2878897	-0.049135	0.960812	0.9956
atg4a	227.0471247	0.056757559	0.1376169	0.4124315	0.680023	0.965311
aida	185.5633317	0.085912881	0.1664602	0.5161166	0.605773	0.956524
pfdn1	647.1890268	-0.078515171	0.1099724	-0.713953	0.475256	0.925754
golim4b	84.63364729	-0.004890176	0.2106751	-0.023212	0.981481	0.996944
rfx3	216.6851551	-0.161708411	0.1496676	-1.08045	0.279942	0.843423
ppp1r3cb	563.7741446	0.25175885	0.105213	2.3928495	0.016718	0.238173
serpinb1l3	439.575448	-0.491802227	0.1335282	-3.683133	0.00023	0.009571
eif4e1b	2.134591507	1.167970524	1.3957492	0.8368054	0.402702	NA
hnrnpr	2297.647407	-0.129923027	0.0826985	-1.571044	0.116172	0.63841
ctnnb1	9011.738855	-0.035085834	0.0712049	-0.492744	0.622193	0.957354
chpt1	1160.753252	-0.178760488	0.0892453	-2.003024	0.045175	0.417766
rhpn2	555.7203934	-0.090946945	0.1089364	-0.834863	0.403795	0.900979
exoc3	814.6542701	-0.046155204	0.0997179	-0.462858	0.643466	0.959994

slc38a5b	1980.840048	-0.179812013	0.0823737	-2.18288	0.029045	0.330497
adcy2b	365.7786013	-0.123154177	0.1137796	-1.082392	0.279078	0.842806
ilf2	3833.017447	-0.10340504	0.0772273	-1.338969	0.180581	0.741768
dennd1a	115.1406231	-0.090988065	0.1915917	-0.474906	0.634854	0.9578
slc5a6b	57.29295923	-0.060745615	0.2589276	-0.234605	0.814516	0.981081
si:ch211-212d10.1	8.468518367	-0.141189745	0.6618745	-0.213318	0.831079	NA
si:dkeyp-94g1.1	2.507124096	-0.208616168	1.3526392	-0.154229	0.877429	NA
dlx3b	456.3227877	-0.112270436	0.10924	-1.027741	0.304071	0.858423
mbtps1	1317.607291	-0.023190492	0.0919859	-0.252109	0.800957	0.979377
aoc2	1784.545285	0.317784404	0.0896805	3.5435187	0.000395	0.014648
svilb	30.04269453	-0.119165289	0.3542096	-0.336426	0.73655	0.972535
ankrd13b	177.0150677	0.100532882	0.1529324	0.657368	0.510944	0.936253
itpr1a	137.7395108	0.134997835	0.171351	0.7878438	0.430788	0.91234
zgc:171731	1.467810344	-1.756356131	1.7046236	-1.030348	0.302846	NA
colgalt2	68.51431951	0.220584816	0.2360894	0.9343273	0.350135	0.880195
fzd9b	391.4584372	0.065156288	0.1134401	0.5743672	0.565719	0.948739
acsl3b	1735.302306	0.074005124	0.0789778	0.9370367	0.34874	0.880195
efna3a	254.3961717	0.032788244	0.1411192	0.2323443	0.816271	0.981594
bckdhb	809.5825303	0.015082604	0.1002149	0.1505026	0.880368	0.987881
znf710a	413.2553914	0.02259398	0.1207229	0.1871558	0.851539	0.985174
tmem26b	10.44913941	0.688520106	0.6273325	1.0975362	0.272407	NA
esco2	479.5633877	-0.100188124	0.1083972	-0.924268	0.355347	0.882347
rps4x	19768.2758	-0.249185519	0.0731434	-3.406807	0.000657	0.021954
znf622	626.5775583	-0.123170925	0.0979296	-1.25775	0.208482	0.775404
pgm2l1	175.4504725	0.006257277	0.1586873	0.0394315	0.968546	0.996315
dync1h1	803.4549607	-0.027657778	0.2378015	-0.116306	0.90741	0.990702
her1	0.488104098	-2.415107183	2.7936704	-0.864493	0.387317	NA
acox1	737.8784075	-0.095168037	0.0921522	-1.032726	0.301732	0.856944
cacybp	298.6555091	-0.128284024	0.1331931	-0.963143	0.335476	0.876088
rbfox1	3686.855157	0.022969072	0.0677251	0.3391514	0.734496	0.972021
mfsd6l	45.37997783	-0.091829891	0.2879178	-0.318945	0.749768	0.97419
arf2a	7524.964677	0.013044814	0.0648051	0.201293	0.840469	0.985042
usp4	1221.581311	0.21527432	0.0845587	2.5458578	0.010901	0.180962
zgc:113220	92.90854475	-0.081069254	0.2077261	-0.39027	0.696337	0.96736
g3bp2	1038.981148	-0.004298044	0.0846489	-0.050775	0.959505	0.995187
lrrc4bb	545.3324471	-0.034767185	0.1218003	-0.285444	0.775304	0.975687
ehd1b	1175.865858	0.099834727	0.0885352	1.1276277	0.259477	0.827693
uqcrc2a	2633.716654	0.035899197	0.0956461	0.3753336	0.707412	0.968664
wnt11r	943.5614323	0.197617705	0.0995572	1.984967	0.047148	0.42638
cryba1l2	429.5259701	0.176528978	0.112381	1.5708084	0.116227	0.63841
cacna2d1a	271.1186943	0.151604766	0.1527515	0.9924926	0.320957	0.868715
fhl5	115.746951	0.317486892	0.1967605	1.6135701	0.106621	0.615567
hacd2	1728.314309	-0.009239391	0.0819012	-0.112811	0.91018	0.990702
ranbp1	2137.546092	-0.117872806	0.0886454	-1.329711	0.183613	0.747284
si:dkeyp-69b9.6	1082.650877	-0.032085387	0.0829357	-0.38687	0.698852	0.967998
l3mbtl1a	302.6035051	0.083666436	0.1315109	0.6361939	0.52465	0.939713
prph2b	4297.737787	0.321677655	0.0893275	3.6011056	0.000317	0.01233
pih1d2	21.12442869	0.131155089	0.4111995	0.3189573	0.749759	0.97419
reep1	7.190041156	-0.177602826	0.7194662	-0.246854	0.805022	NA
drd2l	4.390364507	0.103195798	0.9955929	0.1036526	0.917445	NA
hprt1l	251.2672002	0.033583039	0.1484151	0.2262778	0.820985	0.981628
rpl8	23450.33591	-0.233909553	0.0838451	-2.789783	0.005274	0.107431
syngr3a	1174.59714	0.102553519	0.0807324	1.2702897	0.203981	0.771764
abi2b	1111.06648	0.02011583	0.0833426	0.2413632	0.809274	0.980642

robo2	1442.802837	-0.00881909	0.091775	-0.096095	0.923445	0.992588
ncbp2	362.0891636	-0.065276902	0.1171427	-0.557243	0.577362	0.950805
htra1b	267.7892906	0.104240722	0.1408016	0.7403378	0.459095	0.921978
panx1b	189.2569459	-0.004233333	0.148686	-0.028472	0.977286	0.996762
lamp2	794.5773514	0.084249307	0.0896409	0.9398533	0.347293	0.880123
ndufab1b	715.9189988	0.062620781	0.1006637	0.622079	0.53389	0.943276
slc10a2	78.14585895	-1.108631545	0.2427938	-4.566144	4.97E-06	0.000396
uts1	77.55834493	0.504138301	0.2463889	2.0461077	0.040746	0.394957
p2ry11	7.870089529	-1.050133034	0.7537328	-1.393243	0.163546	NA
KCNN2	27.26285685	0.26241387	0.4188312	0.6265385	0.530962	0.942209
tprkb	47.30287266	0.056603398	0.2870857	0.1971655	0.843698	0.985174
kif23	700.8005094	-0.119482708	0.0913368	-1.308155	0.190821	0.755212
znf598	723.2589426	0.048460013	0.0967201	0.5010333	0.616348	0.95688
angpt2a	30.03628931	-0.265901339	0.359528	-0.739584	0.459552	0.921978
svopb	5.352746236	-0.524393567	0.8487112	-0.61787	0.536661	NA
cnih3	182.7598705	-0.169853452	0.1499829	-1.132485	0.25743	0.825314
snx33	49.25083578	0.114882993	0.2720709	0.4222538	0.67284	0.965257
diablob	247.8255539	-0.154745706	0.1517316	-1.019865	0.307793	0.861088
ppp3cca	601.1255944	0.118738416	0.1055704	1.1247321	0.260703	0.828666
triap1	166.6121216	0.173886495	0.1694698	1.0260619	0.304862	0.859575
ccdc82	339.0786496	-0.056467707	0.1202528	-0.469575	0.638659	0.959628
g6pcb	4.466523042	0.092251977	0.9019498	0.1022806	0.918534	NA
ankhb	701.6232932	0.140891479	0.0946237	1.4889668	0.136496	0.673303
ntng1a	300.8268267	0.109836307	0.1365015	0.804653	0.42102	0.90651
irge1	7.610606417	-0.496826131	0.6958887	-0.713945	0.475261	NA
lims2	190.746158	0.209086227	0.1539667	1.3579966	0.174465	0.734611
acvr1l	1168.250075	0.038264603	0.080636	0.4745348	0.635119	0.957927
tbp	323.0693896	0.055723783	0.1282043	0.4346481	0.663818	0.964736
jagn1b	290.5686982	-0.064686302	0.140858	-0.45923	0.646069	0.96029
cdh4	1075.457708	0.048797053	0.0868395	0.5619226	0.574169	0.950037
arhgap4b	785.9955365	0.003635968	0.0915281	0.0397252	0.968312	0.996315
dnm1l	1832.560009	0.000154206	0.0795597	0.0019382	0.998454	0.999364
ccdc114	63.23047282	0.413849764	0.2465743	1.678398	0.093269	0.580702
tspan18b	1044.243987	-0.023156424	0.0844059	-0.274346	0.783819	0.97627
kif26ab	760.0960243	-0.126593568	0.0941451	-1.344665	0.178734	0.739486
l1camb	202.8169203	-0.01982723	0.1621041	-0.122312	0.902652	0.990121
slc23a1	62.24585707	0.454266321	0.267536	1.6979636	0.089515	0.572876
calm2b	12309.32592	-0.089446867	0.0761835	-1.174097	0.240356	0.808437
grip1	721.4175293	0.041320049	0.0956407	0.432034	0.665717	0.964736
ube2d4	33.21177656	0.403971208	0.3364566	1.2006637	0.229882	0.79764
daam1a	1103.204187	-0.063658577	0.0845718	-0.752716	0.45162	0.918761
esrrd	7.698704653	-0.081081308	0.7607691	-0.106578	0.915124	NA
c4	9.423376676	0.242203668	0.6270979	0.3862294	0.699327	NA
txk	0.356300316	0.005884122	3.214964	0.0018302	0.99854	NA
pola2	323.1410566	-0.029564529	0.126392	-0.233911	0.815054	0.981081
dmrt2a	235.8853799	-0.053141414	0.145585	-0.36502	0.715097	0.96909
gja8b	267.5983134	0.098781572	0.1427228	0.692122	0.488861	0.931926
dnajb11	1062.586922	0.14890895	0.0877504	1.6969613	0.089704	0.573763
slc18a2	6.401808966	-0.693027725	0.7817929	-0.88646	0.37537	NA
ddx39ab	3547.836647	-0.154497208	0.1038734	-1.487361	0.136919	0.673303
dnase1l4.1	260.3604716	-0.2483394	0.1632239	-1.521465	0.128143	0.658954
gorasp2	1057.301958	-0.004205114	0.0944479	-0.044523	0.964487	0.995927
rpl27	8697.134713	-0.262065029	0.0935206	-2.802217	0.005075	0.104214
camkk1b	284.6766295	0.440104034	0.1265972	3.4764116	0.000508	0.018073

sh2d4bb	11.06970948	-0.39389348	0.5760486	-0.683785	0.494111	0.931926
wasa	7.165984607	0.340310202	0.7158037	0.4754239	0.634485	NA
wdr91	141.5782563	-0.027237756	0.1800326	-0.151293	0.879744	0.987881
fbln2	857.9120105	0.21453435	0.0931417	2.3033124	0.021261	0.275144
slc50a1	40.79836063	-0.132807851	0.3252645	-0.408307	0.683048	0.965476
zgc:92664	235.1202367	-0.05934151	0.1375714	-0.431351	0.666214	0.964736
mknk2b	3875.341382	-0.067131949	0.0891638	-0.752906	0.451507	0.918761
atp6v0a1b	1740.560362	0.188820459	0.0865386	2.1819225	0.029115	0.330497
ror1	266.5787905	0.031827557	0.1360343	0.2339671	0.815011	0.981081
mpp3a	519.0850683	0.067036208	0.104498	0.6415073	0.521193	0.939056
cblb	425.1653852	-0.097773788	0.1100568	-0.888394	0.374329	0.893914
pcmt	833.0193444	0.007980198	0.0936474	0.0852154	0.93209	0.992702
hnrnpua	1565.14761	0.041645324	0.0849283	0.4903584	0.62388	0.957354
rbb4l	1431.819881	-0.098475985	0.0867743	-1.134851	0.256438	0.824773
nsrp1	244.9990677	-0.253139616	0.1538007	-1.645894	0.099786	0.600955
cbll1	179.3515067	-0.116190602	0.1724205	-0.673879	0.500388	0.934237
cd2ap	813.1400165	-0.144839762	0.0938678	-1.543019	0.122826	0.650905
ero1a	95.59649091	-0.746602357	0.2073506	-3.600676	0.000317	0.01233
pycr3	96.86569097	-0.943399266	0.222737	-4.235485	2.28E-05	0.001407
prpf19	1343.554718	-0.058323397	0.0874295	-0.66709	0.504714	0.935617
cdkl5	429.526024	0.071870015	0.1147847	0.6261291	0.53123	0.942209
st3gal3b	81.28849003	0.085834982	0.2184699	0.3928915	0.6944	0.96736
fzr1a	1003.582298	-0.165832324	0.0903714	-1.83501	0.066504	0.50468
adma	211.4399653	0.206685612	0.1600842	1.2911056	0.196667	0.761944
mab21l2	2128.27066	-0.077134047	0.0767602	-1.00487	0.314959	0.866054
alpi.1	300.6667174	0.03280967	0.1286118	0.2551062	0.798641	0.978884
plxnc1	90.14341769	0.040456163	0.2230348	0.1813894	0.856062	0.985261
sacm1la	399.6854517	-0.094698994	0.124441	-0.760995	0.44666	0.917154
ube2g1a	676.935668	0.194018788	0.1014693	1.9120936	0.055864	0.462781
fam110a	89.98427741	-0.167604613	0.2306497	-0.726663	0.467432	0.924328
pitpnbl	1629.476039	0.129820234	0.0802614	1.6174669	0.105778	0.615371
klf12a	310.9373983	0.084877586	0.130599	0.6499097	0.515751	0.9377
zc4h2	3004.365154	-0.091816584	0.0805233	-1.140248	0.254183	0.823394
psmc5	2525.612478	-0.017667682	0.0786274	-0.224701	0.822212	0.982186
u2af1	1840.026931	-0.188881963	0.0793873	-2.379245	0.017348	0.243465
comta	316.0034432	-0.123952297	0.1211369	-1.023241	0.306194	0.860027
pgd	1743.770194	-0.249488669	0.099332	-2.511664	0.012016	0.193023
mfge8a	1127.796279	0.048707636	0.0878037	0.5547334	0.579077	0.951052
lsg1	514.3306899	-0.22663046	0.1087919	-2.083156	0.037237	0.377602
fosl1a	972.5261638	0.628038127	0.2611075	2.4052856	0.01616	0.234787
fcho1	203.1540142	-0.190243301	0.1514985	-1.255743	0.209209	0.776073
st3gal3a	401.0993348	0.041554424	0.1185572	0.3505011	0.725963	0.970402
ndufs3	1574.83145	-0.083840423	0.0871039	-0.962533	0.335782	0.876088
dhx15	2350.609596	-0.066518557	0.0735696	-0.904158	0.365911	0.887239
thoc7	260.2829042	0.086128696	0.1344262	0.6407137	0.521709	0.939056
foxf1	313.9417182	-0.309341803	0.1252787	-2.46923	0.01354	0.208689
arl3l2	289.2592248	-0.035936722	0.1457821	-0.24651	0.805288	0.979883
ppil1	322.2868679	-0.231673092	0.1378326	-1.68083	0.092796	0.579945
slc24a4a	25.44867412	0.480148777	0.4241801	1.1319456	0.257657	0.825314
hdac1	6763.3975	-0.014829761	0.0683465	-0.216979	0.828225	0.982721
lim2.4	2946.019266	0.025350334	0.094378	0.2686044	0.788234	0.976703
fut8a	1657.081682	-0.106776701	0.0818391	-1.304716	0.19199	0.756256
racgap1	407.1302487	-0.003982055	0.1164046	-0.034209	0.972711	0.996315
chd1l	252.058825	-0.208488706	0.137818	-1.512783	0.130335	0.662797

gpc4	1852.004706	0.146432639	0.072997	2.00601	0.044855	0.416612
ppp2r5ea	1179.310913	0.164908434	0.0902915	1.8264012	0.06779	0.509881
iqch	17.58659681	0.792965302	0.4695795	1.6886711	0.091282	0.578443
zgc:113518	222.4031888	-0.12323791	0.143663	-0.857827	0.390988	0.897643
klf3	488.8063893	-0.208274495	0.1119697	-1.860097	0.062872	0.48946
adam10b	723.6572751	-0.101910079	0.1122301	-0.908046	0.363854	0.886174
pibf1	596.2442353	-0.401506628	0.0992946	-4.043591	5.26E-05	0.002867
mc4r	26.15623107	-0.136199444	0.4051855	-0.336141	0.736765	0.972535
prps1a	1567.438014	0.091481812	0.0940288	0.9729128	0.330597	0.87417
CU929145.1	0.990234925	0.997648457	1.9937656	0.500384	0.616805	NA
slc4a8	689.3257623	0.274520492	0.0943843	2.9085399	0.003631	0.081502
sox6	1269.365065	0.00340864	0.0838605	0.0406466	0.967578	0.996315
gad2	3068.053847	0.143497155	0.0812791	1.7654859	0.077482	0.542062
znf207a	843.4561869	-0.074888197	0.1013284	-0.739064	0.459868	0.921978
psen2	309.5856093	0.036935795	0.1359891	0.2716085	0.785923	0.97627
s100a1	23.18433283	-0.451507518	0.4205054	-1.073726	0.282946	0.844523
alpl	348.3208649	0.066332879	0.1201229	0.5522083	0.580806	0.951731
fth1a	10011.66896	-0.323075159	0.0724726	-4.457891	8.28E-06	0.000608
phactr4a	507.8605708	-0.067993484	0.1016594	-0.668836	0.5036	0.935304
zic2a	1118.956174	-0.126901787	0.0851176	-1.490899	0.135988	0.672424
zgc:100829	574.06076	-0.100931253	0.1176274	-0.858059	0.39086	0.897643
fstl1a	216.3320811	0.190149535	0.141197	1.3466969	0.178078	0.738995
zgc:123305	365.7491114	-0.183585514	0.1214482	-1.511636	0.130627	0.662797
dnmt3ab	2055.635292	-0.037102094	0.0772163	-0.480496	0.630875	0.957354
zic1	1253.934881	-0.104087997	0.0886472	-1.174183	0.240322	0.808437
cyp3c1	747.7641266	0.062945304	0.1042669	0.6036938	0.546047	0.945526
itprip	71.63107818	-0.052129848	0.2297906	-0.226858	0.820534	0.981594
ankrd33bb	16.99766721	0.366822171	0.4672872	0.7850036	0.432451	0.91326
c1ql2	2.805266554	0.528268623	1.1526052	0.4583257	0.646718	NA
si:ch211-220f21.2	7.681608807	-1.037978168	0.6949188	-1.493668	0.135262	NA
tubg1	741.0041904	0.01221112	0.0901424	0.1354647	0.892244	0.989291
rasl11b	248.8204377	-0.091593799	0.1366691	-0.670187	0.502739	0.935304
ada2b	17.52150676	-0.492463842	0.4837855	-1.017938	0.308707	0.861088
rgs6	78.72330603	0.140376786	0.2385864	0.5883687	0.556285	0.947902
psmd10	164.4219393	0.141740926	0.1959125	0.7234909	0.469378	0.924505
gemin2	240.5342365	-0.001888118	0.1490016	-0.012672	0.98989	0.997612
rap2c	1003.615581	0.036105229	0.0862999	0.4183693	0.675677	0.965257
tnip1	2116.846391	0.048079742	0.0781417	0.6152888	0.538364	0.943336
ca15a	434.5396239	0.332441716	0.1192998	2.7866086	0.005326	0.108022
zgc:77112	62.87622129	-0.027218912	0.276454	-0.098457	0.921569	0.992138
pla2g12b	1590.007764	-0.050742532	0.1033289	-0.491078	0.623371	0.957354
msx3	197.345827	0.061480192	0.15477	0.3972358	0.691194	0.967295
nmd3	767.302959	0.098564236	0.1001168	0.9844924	0.324873	0.871294
si:dkeyp-75b4.9	0	NA	NA	NA	NA	NA
gsk3ab	2193.124606	-0.089346914	0.0807228	-1.106836	0.268365	0.833634
bmp6	55.7961141	0.003565538	0.2777269	0.0128383	0.989757	0.997612
dvl3a	379.1622991	-0.040613378	0.1141294	-0.355854	0.72195	0.970024
hsd17b12a	1357.315232	-0.044828425	0.0922933	-0.485717	0.627168	0.957354
kctd5a	248.6544869	0.098923958	0.1364989	0.7247236	0.468622	0.924328
ano5a	25.20854993	-0.209121331	0.3854512	-0.542536	0.587449	0.953319
cax2	82.61172761	0.260421075	0.2361238	1.1029006	0.27007	0.834705
aicda	0.173729368	-0.955901296	4.0804729	-0.234262	0.814781	NA
aarsd1	230.5265813	-0.126868	0.1575026	-0.805498	0.420532	0.90651
hps3	154.3797417	0.192685001	0.1755038	1.0978964	0.27225	0.837242

zap70	5.381017058	1.394093654	0.8763573	1.5907823	0.111659	NA
tmem50a	1457.448554	-0.040713087	0.0895522	-0.45463	0.649376	0.960708
tspan7	1471.145838	0.054462043	0.0790967	0.6885503	0.491106	0.931926
iah1	45.19507258	0.045458646	0.3093648	0.1469419	0.883178	0.988706
ankrd46b	547.7265806	-0.043264729	0.1115247	-0.387939	0.698061	0.967998
atp1b3a	2560.875453	0.079436193	0.0719785	1.1036094	0.269763	0.834705
creb3l1	268.6390898	0.114484278	0.1573568	0.7275456	0.466892	0.924328
smcr8b	164.802158	0.064731694	0.1966981	0.3290916	0.742086	0.973154
cgnl1	415.7093724	-0.101850335	0.1178801	-0.864016	0.387579	0.895884
rab22a	228.9502225	-0.12125024	0.139143	-0.871408	0.383532	0.895192
sesn3	701.6426868	-0.009338286	0.0961056	-0.097167	0.922594	0.99233
vps26b	504.6716539	0.090570369	0.1035728	0.8744613	0.381867	0.895192
lemd3	781.3614789	0.039929902	0.0996748	0.4006017	0.688713	0.966672
mov10a	403.6074573	-0.071839478	0.1166958	-0.615613	0.53815	0.943336
eif4e2rs1	563.5992654	0.018391243	0.0999787	0.1839516	0.854051	0.985174
pnn	2092.508339	-0.119942349	0.085672	-1.400018	0.161508	0.715364
chata	577.5413786	0.197893659	0.1120759	1.7657109	0.077444	0.542062
slc25a40	195.0023906	0.021986935	0.14644	0.150143	0.880652	0.987917
mtmr9	233.0069573	0.129733334	0.1432343	0.9057423	0.365072	0.886714
rpl5b	16184.72136	-0.295937731	0.0957106	-3.092006	0.001988	0.053335
socs6b	430.0520303	-0.060958834	0.1179455	-0.516839	0.605269	0.95651
apoa2	33076.33788	0.076864166	0.2304555	0.3335315	0.738733	0.972722
slc22a16	276.6771854	-0.113478172	0.1321337	-0.858813	0.390443	0.897304
npas1	137.6892651	-0.150286835	0.1814813	-0.828112	0.407607	0.901327
pax8	163.374987	-0.146639224	0.1734428	-0.845462	0.397853	0.899672
b2ml	1025.472999	-0.066481404	0.095044	-0.699481	0.484252	0.929579
zc3h15	760.8669935	0.069770143	0.0908675	0.7678226	0.442593	0.916336
mafa	249.3919957	-0.033708545	0.1331967	-0.253073	0.800211	0.979377
ptprea	221.0097624	0.011872918	0.1412139	0.0840776	0.932995	0.992702
ptbp2b	1649.596019	0.014161898	0.0827312	0.1711796	0.864083	0.98601
stat6	86.51496731	0.173386929	0.2162383	0.8018326	0.42265	0.906865
lztr1	623.9282934	0.052743697	0.1055872	0.4995273	0.617408	0.957354
mxtx2	1.204202781	0.475444534	1.7483234	0.2719431	0.785666	NA
dcbld1	298.3856629	0.057463093	0.1252368	0.4588355	0.646352	0.960362
slc25a36b	692.4061988	-0.273160849	0.0988639	-2.762999	0.005727	0.113592
rnft1	418.456778	-0.050146017	0.1195182	-0.419568	0.674801	0.965257
dlat	2366.748973	0.067869894	0.0784813	0.8647904	0.387154	0.895778
pwp1	571.0146129	-0.127715028	0.0990018	-1.290028	0.197041	0.761944
stk17al	148.8027397	-0.145443621	0.1733879	-0.838834	0.401563	0.90084
rwdd1	612.1621206	-0.050971867	0.1091122	-0.467151	0.640392	0.959994
snap91b	3011.134792	0.132139357	0.0853259	1.5486431	0.121468	0.647929
kptn	268.8435218	0.033338236	0.1279152	0.2606276	0.79438	0.977937
slc44a1a	337.4478029	-0.220871451	0.1229683	-1.796165	0.072468	0.526156
matn4	10578.72814	0.180317053	0.0679644	2.653109	0.007975	0.143761
cldnc	839.6148431	-0.531612562	0.0939454	-5.658739	1.52E-08	2.53E-06
zgc:153311	9.757986629	-0.458348339	0.6200427	-0.739221	0.459773	NA
yaf2	479.803617	0.084053597	0.1058148	0.7943463	0.426994	0.910533
rabgef1l	64.54308506	-0.103641163	0.2572617	-0.402863	0.687049	0.966376
phip	51.92011331	0.119217641	0.2879185	0.4140673	0.678825	0.965257
rapgef1b	168.0879552	-0.095100568	0.1603235	-0.593179	0.553061	0.947265
COX5B	7214.241106	-0.104397998	0.0854855	-1.221236	0.221997	0.788551
abcc9	263.7133329	0.154809588	0.1289101	1.2009117	0.229785	0.79764
zdhhc16b	303.3592118	0.158081655	0.1238305	1.2765966	0.201745	0.767736
hcfc1a	1793.803449	0.114981421	0.0802817	1.4322249	0.152079	0.700984

mfsd5	185.2319499	0.088192389	0.15531	0.5678475	0.570139	0.948983
wdhd1	309.527145	-0.138068244	0.1268437	-1.088491	0.276378	0.839541
nup107	1215.046711	-0.070935068	0.0931903	-0.761185	0.446546	0.917154
gpcpd1	914.0437506	-0.002065111	0.1143661	-0.018057	0.985593	0.996944
dbnlb	1221.883603	-0.02024131	0.0959666	-0.21092	0.832949	0.983557
zic5	489.0411613	-0.154340842	0.1051121	-1.468345	0.142011	0.683406
hacd3	415.4290085	0.068280919	0.1151534	0.5929562	0.55321	0.947265
gins4	200.2316334	-0.195769016	0.1583894	-1.235998	0.216459	0.783724
gpat3	608.7167306	0.394712673	0.0965922	4.0863814	4.38E-05	0.00242
mhc1laa	87.47004469	-0.199123445	0.2094542	-0.950678	0.341768	0.878134
fibpa	516.7779896	-0.011447198	0.1034341	-0.110671	0.911877	0.991066
rab5b	263.2366024	0.147018638	0.1401434	1.0490588	0.294151	0.849999
tlr3	8.649500151	0.487375384	0.6847989	0.7117059	0.476647	NA
cryz	217.5632599	0.002194337	0.140132	0.0156591	0.987506	0.997189
nob1	299.419325	0.017178471	0.1282942	0.133899	0.893482	0.989291
cldn15la	707.4736093	-1.315153436	0.0975693	-13.47917	2.07E-41	4.10E-37
spsb4a	351.7399552	-0.088365596	0.1218155	-0.725405	0.468203	0.924328
mbnl3	707.3514753	0.084426194	0.0978956	0.8624104	0.388462	0.896271
smurf1	708.3942649	0.045136455	0.0929188	0.4857626	0.627135	0.957354
rtn2a	152.8362874	0.535606232	0.1719358	3.1151516	0.001839	0.050214
bicral	244.0477876	-0.087734177	0.1375245	-0.637953	0.523504	0.939336
qtrt2	133.2447053	-0.136611925	0.1765919	-0.773602	0.439166	0.916082
htr6	13.70858486	-0.254274593	0.5655715	-0.449589	0.653007	0.960792
bbs12	33.20573074	-0.772629027	0.3482058	-2.218886	0.026494	0.313216
exd2	303.8042985	0.04306656	0.1267566	0.3397581	0.734039	0.971993
ptrhd1	107.2411825	0.24313998	0.1993975	1.2193732	0.222703	0.789115
ap3m2	1002.07488	0.075210335	0.0911632	0.8250079	0.409367	0.90193
keap1a	254.1944506	0.094200745	0.1590947	0.592105	0.55378	0.947449
nme6	63.33945334	0.104800575	0.2624215	0.3993598	0.689628	0.966799
mkks	18.62477444	-0.247882111	0.4527615	-0.547489	0.584043	0.952214
slc6a2	179.7199682	0.255130731	0.1553007	1.6428171	0.100421	0.602464
xiap	407.6032657	0.130737889	0.1211091	1.0795053	0.280363	0.843423
dag1	1949.690584	0.181270642	0.0872941	2.0765512	0.037843	0.381012
zfp36l1a	1928.059334	0.141623745	0.0773411	1.8311585	0.067077	0.506251
fam53b	437.4594244	-0.039265653	0.1150741	-0.341221	0.732938	0.971844
gmpr	887.2293238	0.268337668	0.0885801	3.029321	0.002451	0.062137
ccdc170	13.11137404	0.210463953	0.5451957	0.3860338	0.699472	0.967998
usp33	1306.66815	0.092469684	0.0844094	1.0954902	0.273302	0.838214
cct3	5639.393356	-0.168334479	0.076583	-2.198067	0.027944	0.323709
eif4enif1	443.1453071	0.068988603	0.141905	0.4861605	0.626853	0.957354
trim33	1034.464599	-0.041464576	0.0938127	-0.441993	0.658494	0.962545
SBNO2	509.4648389	-0.133769056	0.1252855	-1.067714	0.28565	0.845448
trib3	721.4129284	0.046012801	0.1078898	0.4264795	0.669758	0.965223
zgc:112408	3.483480383	-0.159046647	1.0297922	-0.154445	0.877259	NA
nup214	302.1262794	-0.11746949	0.1588002	-0.739732	0.459463	0.921978
zmp:0000001048	65.95367516	0.35356449	0.3100488	1.140351	0.25414	0.823394
mhc1lba	6.092782264	-0.052752198	0.8137563	-0.064826	0.948313	NA
bcap29	161.0619538	0.129606411	0.160356	0.8082417	0.418951	0.906406
sdr16c5a	379.5711153	-0.036415454	0.1128761	-0.322614	0.746987	0.97419
arl6ip5b	445.8772096	0.115847143	0.114526	1.0115358	0.31176	0.864257
psmd13	1346.282797	-0.1055685	0.0960279	-1.099352	0.271615	0.83659
ralab	549.9245068	-0.079937353	0.100123	-0.798392	0.424643	0.9085
chmp4ba	620.5718244	0.106052795	0.1057885	1.0024985	0.316103	0.866931
nudt3a	205.66064	-0.177502779	0.1481538	-1.198098	0.230879	0.799177

fxr2	1560.927453	0.162700577	0.0762433	2.1339646	0.032846	0.353176
zdhhc5a	859.2877677	0.020406336	0.1043832	0.1954944	0.845006	0.985174
eif1ad	222.005532	-0.126728917	0.1523498	-0.831828	0.405506	0.901305
clca1	1.814708353	0.186512888	1.5197095	0.1227293	0.902321	NA
zgc:65894	3305.527524	0.215446661	0.066698	3.23018	0.001237	0.036676
upf1	1773.290866	-0.076920679	0.0890297	-0.863989	0.387594	0.895884
tfdp1b	254.3493486	-0.2688112	0.1386475	-1.938811	0.052524	0.449813
mrpl46	272.2822307	0.055533922	0.132725	0.4184134	0.675645	0.965257
dock9b	513.9347242	-0.045861858	0.1028347	-0.445976	0.655614	0.96173
med7	293.0602969	0.041265021	0.1241486	0.3323842	0.739599	0.972943
c9	2113.576894	0.282460164	0.2441969	1.1566903	0.247399	0.81686
tmco6	93.37999824	-0.129019237	0.2282407	-0.565277	0.571885	0.949494
lctlb	551.7433394	-0.020072191	0.1014547	-0.197844	0.843167	0.985174
zgc:92518	149.80898	0.103889473	0.1796952	0.5781429	0.563168	0.948728
ncoa5	1309.84308	-0.03907237	0.0849588	-0.459898	0.645589	0.96029
dip2bb	39.74420244	0.037852225	0.3067988	0.123378	0.901808	0.990121
cap1	246.2894182	0.054890233	0.1394523	0.3936129	0.693867	0.96736
fmo5	238.4750574	-0.245101501	0.137864	-1.77785	0.075429	0.534788
kpna1	1275.524568	0.198493578	0.0892716	2.223479	0.026184	0.311899
her8a	140.6429085	-0.281982904	0.172161	-1.637903	0.101442	0.605353
gna15.1	156.1805126	-0.203985294	0.173505	-1.175674	0.239725	0.808111
zbtb8os	242.168713	0.050125191	0.1354211	0.3701431	0.711276	0.969006
asns	396.276767	0.046170435	0.1117668	0.4130962	0.679536	0.965257
calcoco1b	23.97010765	0.231059742	0.4059909	0.5691255	0.569271	0.948983
arf2b	3.135393141	0.464836258	1.0787995	0.4308829	0.666554	NA
fam219aa	20.7557085	-0.008462454	0.4214044	-0.020082	0.983978	0.996944
riok1	312.1109434	-0.040614303	0.127651	-0.318167	0.750358	0.97419
ptch1	730.9740952	-0.107414801	0.1111107	-0.966737	0.333675	0.874933
agt	1112.874422	0.321505405	0.1275884	2.5198648	0.01174	0.190597
dhtkd1	969.2559156	0.213894185	0.0854062	2.5044352	0.012265	0.195612
crygm1	0	NA	NA	NA	NA	NA
fignl1	85.01601984	-0.334160209	0.2161175	-1.546197	0.122057	0.649408
farp2	490.3293564	0.058154321	0.1034879	0.5619429	0.574155	0.950037
slc7a1	22.57960972	-0.153600873	0.4549674	-0.337609	0.735658	0.972535
eif3c	6866.192317	-0.194459814	0.0717523	-2.710156	0.006725	0.12641
ythdf1	1034.783048	0.021539568	0.0848075	0.2539819	0.79951	0.979366
vtg3	1.852202149	0.869537402	1.455503	0.5974137	0.550231	NA
esr2a	6.744681524	1.058429093	0.7737921	1.3678468	0.17136	NA
irf9	126.8999088	-0.488545538	0.1922438	-2.541282	0.011045	0.18215
fut9a	574.3048332	-0.008688076	0.0997605	-0.087089	0.9306	0.992702
trappc6b	267.3607213	-0.043426903	0.1403365	-0.309448	0.75698	0.975374
cdc42bpab	308.743468	-0.263066404	0.1248664	-2.106783	0.035136	0.364718
anxa5b	981.2961939	-0.422584162	0.090962	-4.645719	3.39E-06	0.000291
galcb	410.9100046	-0.000450498	0.1172863	-0.003841	0.996935	0.999057
eif4a2	735.4758243	-0.153938599	0.0969872	-1.587205	0.112466	0.627868
ugt5a1	24.23391455	-0.026781561	0.3930245	-0.068142	0.945672	0.993609
slc17a7a	311.6182175	0.191433861	0.124943	1.532169	0.125481	0.654803
ptpn2a	168.2579662	0.035651606	0.1659265	0.2148639	0.829873	0.982821
baiap2l2a	79.02261466	-1.132435865	0.2594067	-4.365484	1.27E-05	0.000877
dkc1	1012.103042	0.009184957	0.0942068	0.0974978	0.922331	0.99233
rab11fip3	194.4881364	0.205836727	0.1530669	1.3447505	0.178706	0.739486
ddc	220.5306495	0.055017638	0.165234	0.3329681	0.739158	0.972722
cdk8	501.0530282	0.11291112	0.104782	1.0775812	0.281221	0.84357
apbb1ip	171.0998465	0.032854197	0.1574112	0.2087157	0.83467	0.984067

setd3	718.5205474	0.106902415	0.0996284	1.073011	0.283266	0.84466
gtf2h2	217.2424461	0.069160793	0.143143	0.4831587	0.628983	0.957354
rbm8a	1809.965471	-0.156456307	0.0769772	-2.032501	0.042103	0.402974
ctdnep1a	208.424733	0.095241461	0.1539282	0.6187395	0.536088	0.943336
gata3	1570.004568	-0.143267305	0.0852095	-1.681354	0.092694	0.579675
helz2	10.57283484	0.234548431	0.6343906	0.3697225	0.711589	NA
wdcp	178.4345607	0.411364755	0.1582374	2.5996682	0.009331	0.162001
klhl4	96.73169896	0.094014595	0.2126133	0.4421859	0.658355	0.962545
suco	2024.943234	-0.004632202	0.0905846	-0.051137	0.959217	0.995097
npas2	33.74023418	0.006942382	0.4000952	0.0173518	0.986156	0.996965
zgc:55888	1.356046616	0.792643957	1.7222439	0.4602391	0.645345	NA
sash3	29.59129399	-0.376302106	0.3690909	-1.019538	0.307948	0.861088
mrm3a	70.28347819	-0.265192049	0.2340864	-1.132881	0.257264	0.825314
zgc:154046	424.5642956	0.254924395	0.1165732	2.1868187	0.028756	0.328909
eif5b	1377.184988	-0.1768124	0.0919068	-1.923824	0.054377	0.457162
iqsec1b	1366.074687	0.06841719	0.0817265	0.8371486	0.402509	0.90084
prlra	264.7088906	0.314087002	0.1446963	2.1706637	0.029957	0.335036
mroh1	628.1395122	-0.104520447	0.1009416	-1.035454	0.300457	0.856251
rgs7a	503.8020585	-0.026046866	0.1141537	-0.228174	0.819511	0.981594
haus4	105.3290348	-0.179393303	0.1953143	-0.918485	0.358365	0.884398
ckmt1	5250.889968	-0.026099181	0.0703995	-0.370729	0.710839	0.968985
pimr110	0	NA	NA	NA	NA	NA
echdc2	164.9260653	-0.35319836	0.1585354	-2.227883	0.025888	0.310878
scrn3	294.9294589	-0.130329586	0.1367113	-0.95332	0.340428	0.87805
mcat	31.22397708	-0.286154766	0.366095	-0.781641	0.434426	0.914254
si:ch211-195b13.1	919.6734741	0.326730695	0.086552	3.7749648	0.00016	0.007176
tprb	1480.293731	-0.0793484	0.0871169	-0.910827	0.362387	0.885944
akr7a3	262.0105172	0.018697182	0.1344187	0.1390966	0.889374	0.989291
znf106a	1296.42606	0.158327329	0.0804906	1.9670293	0.04918	0.435115
mgme1	211.5823708	0.032348201	0.1473904	0.219473	0.826282	0.982196
rnf20	954.1609687	0.035000293	0.093319	0.3750607	0.707615	0.968664
gabbr1b	62.03804675	-0.075276719	0.252246	-0.298426	0.765378	0.975374
si:ch73-166o21.1	5.06953012	-0.476320871	0.8800689	-0.541231	0.588348	NA
gnao1a	3962.283857	0.03615641	0.0699864	0.5166206	0.605421	0.95651
acadvl	3697.99478	-0.067532259	0.0716624	-0.942367	0.346005	0.878857
cd9b	1177.955809	-0.263479613	0.0915867	-2.876832	0.004017	0.08719
p2rx1	54.32499257	0.309223755	0.2894633	1.0682657	0.285401	0.845164
trpa1a	232.1843835	-0.114809278	0.1353979	-0.84794	0.396471	0.899672
pbld	118.4489958	-0.194018665	0.1808009	-1.073107	0.283223	0.84466
atic	1338.988755	-0.158893285	0.0936809	-1.696113	0.089865	0.573834
rchy1	287.4078431	-0.141526865	0.1263734	-1.11991	0.262752	0.830166
mmp11b	162.7420977	0.340334188	0.1787019	1.9044799	0.056848	0.467988
sdha	5406.330803	-0.017063418	0.0762424	-0.223805	0.822909	0.982189
zgc:162339	121.8875633	0.083029421	0.1786551	0.4647469	0.642113	0.959994
gadd45gb.1	408.0952466	-0.145429661	0.1106473	-1.314353	0.188727	0.753461
rfxank	15.30589444	-0.686349486	0.4910359	-1.397758	0.162186	0.716781
psat1	1064.51547	-0.102611875	0.0925193	-1.109086	0.267393	0.833191
lrrc4cb	303.0040486	-0.012331523	0.1377334	-0.089532	0.928659	0.992702
rgs7bpb	248.4539917	0.162334438	0.1394397	1.1641908	0.244347	0.813858
angel2	353.2390721	0.105243202	0.1230892	0.8550156	0.392543	0.897688
slc35f6	718.1879504	0.064732484	0.0973835	0.6647169	0.506232	0.935724
slco1c1	122.6968391	0.043062707	0.191624	0.224725	0.822193	0.982186
abcc6a	62.17500112	0.009424945	0.2560462	0.0368095	0.970637	0.996315
ipo9	738.374614	0.006590264	0.1014373	0.0649688	0.948199	0.99381

tbca	805.546915	-0.071424269	0.0954679	-0.74815	0.45437	0.920544
znf292a	431.6730818	-0.061846965	0.1228864	-0.503285	0.614764	0.95688
scamp3	704.4767871	0.054889351	0.0940053	0.5838965	0.55929	0.948478
sgtb	154.4275057	0.161699983	0.174269	0.9278757	0.353472	0.881844
tfa	16546.30535	0.265466161	0.0913066	2.9074147	0.003644	0.081518
cishb	54.85639429	0.049657875	0.2732275	0.1817455	0.855782	0.985174
plekho2	192.012359	0.166332066	0.1550726	1.0726076	0.283447	0.84466
aqr	831.197809	-0.08306317	0.1001393	-0.829476	0.406835	0.901305
huwe1	4461.302595	-0.027082418	0.100397	-0.269753	0.78735	0.976596
srsf6b	3944.746169	-0.113439463	0.0718035	-1.57986	0.114139	0.633095
zeb1a	19.35235049	-0.264419163	0.4395405	-0.601581	0.547453	0.945526
zgc:152891	26.83771295	0.277547853	0.3764886	0.7372013	0.461	0.922038
crybb1l2	36544.6359	-0.038588694	0.0653393	-0.59059	0.554795	0.947449
mtmr6	561.9892423	-0.098303416	0.1018045	-0.96561	0.334239	0.875298
prkacab	993.2124654	0.101145625	0.0853046	1.1856999	0.235741	0.804824
ints3	584.2263865	0.029523573	0.1011032	0.2920141	0.770276	0.975687
casc1	22.43180172	0.505595925	0.4196605	1.2047737	0.228291	0.795541
abcg2d	44.49277136	-0.329900635	0.2932526	-1.124971	0.260601	0.828546
vtg6	0.339758314	0.005883863	3.2682461	0.0018003	0.998564	NA
rimk1a	7160.859677	-0.156728417	0.0796429	-1.967888	0.049081	0.435115
tcirg1a	248.5350507	-0.239910653	0.1340313	-1.78996	0.07346	0.528705
glipr2l	123.0465232	-0.015120401	0.1781774	-0.084862	0.932371	0.992702
neurod2	166.9563425	-0.097035419	0.1777618	-0.545873	0.585153	0.952487
sf3b5	831.0586893	-0.038381829	0.101248	-0.379087	0.704623	0.968134
pon2	208.0742489	0.033434723	0.1480718	0.2258008	0.821356	0.981845
smad7	223.1173672	0.021956566	0.1459205	0.1504693	0.880394	0.987881
farsb	930.7120347	-0.011885405	0.0928107	-0.128061	0.898101	0.990121
fam102ab	53.47208784	0.029873295	0.2641844	0.1130775	0.909969	0.990702
rnf128a	1073.620902	-0.078152194	0.0865095	-0.903394	0.366317	0.887239
rhobtb4	27.52138641	-0.116499944	0.3650685	-0.319118	0.749637	0.97419
smarce1	3870.11107	-0.042828248	0.0731191	-0.585732	0.558055	0.948478
gys1	1118.860209	0.074141469	0.0923523	0.8028114	0.422084	0.90651
dnajb9b	12.79896174	-0.260775367	0.5282247	-0.493683	0.62153	0.957354
eif3g	2078.42831	-0.179958866	0.0744318	-2.417767	0.015616	0.229092
fbxo15	7.379408691	0.993558481	0.7089492	1.4014523	0.161079	NA
wdr5	1420.21003	0.038773861	0.0840306	0.4614252	0.644494	0.96029
bckdk	1270.554227	0.129938018	0.0787638	1.6497181	0.099001	0.598346
zp3e	8.491664171	-0.046092154	0.6422365	-0.071768	0.942786	NA
ace2	482.3314359	-0.345023405	0.1192246	-2.893895	0.003805	0.08416
si:dkey-94f20.4	50.433148	0.360246376	0.2774995	1.2981876	0.194223	0.758049
ttyh2	55.23131648	0.008111241	0.2680454	0.0302607	0.975859	0.996762
hmcn1	497.9547028	-0.243107427	0.1107325	-2.195447	0.028132	0.325071
itgb2	78.38602551	0.334405755	0.2393989	1.3968561	0.162457	0.717058
neurog3	2.194007624	-0.64329868	1.3164545	-0.48866	0.625082	NA
slc16a1a	207.629015	-0.232095687	0.1465542	-1.583685	0.113266	0.631061
MCUR1	135.4313012	-0.276952774	0.1838159	-1.506685	0.131891	0.665566
tmem214	1248.485644	0.006452275	0.0793382	0.0813262	0.935183	0.992702
snx27b	225.5881132	0.089444603	0.1498094	0.5970559	0.55047	0.946549
pmpcb	307.1411682	-0.082226621	0.1306741	-0.629249	0.529186	0.942209
bcar3	466.941049	-0.127927188	0.1110735	-1.151734	0.24943	0.819036
ssrp1b	314.6762689	0.028087376	0.1214881	0.2311944	0.817164	0.981594
lin28a	55.02026638	-0.265264389	0.2645979	-1.002519	0.316093	0.866931
myo10	777.0117534	0.013205562	0.0911673	0.1448498	0.88483	0.988838
dennd5a	273.1749977	-0.004666676	0.1339154	-0.034848	0.972201	0.996315

nr4a2a	635.299841	-0.046839068	0.1042645	-0.449233	0.653263	0.960919
scamp2l	194.9994754	0.192082897	0.1494051	1.2856517	0.198565	0.763597
ywhae2	2459.38518	0.130504266	0.0743413	1.755474	0.079178	0.547489
ak7b	34.72605863	-0.000282075	0.321329	-0.000878	0.9993	0.999704
sqor	514.7224431	-0.274201751	0.1288192	-2.128578	0.033289	0.355433
svilc	612.5323686	0.268015101	0.1101036	2.4342083	0.014924	0.223742
ikbkg	587.4582685	-0.074527174	0.0994798	-0.749169	0.453755	0.920074
zgc:152670	0.863695607	1.965711988	2.22386	0.8839189	0.37674	NA
slc27a4	31.0586259	-0.694844173	0.3497446	-1.986719	0.046954	0.425314
adsl	888.0203608	0.079645256	0.0996245	0.7994546	0.424027	0.908025
zmynd12	9.676535717	-0.269526576	0.6243926	-0.431662	0.665987	NA
large2	1053.131373	-0.028899579	0.0942112	-0.306753	0.759031	0.975374
chordc1b	296.5629254	-0.021202256	0.1277063	-0.166024	0.868138	0.987245
mespaa	0.166657454	-0.955901296	4.0804729	-0.234262	0.814781	NA
bud31	729.0984678	-0.089688672	0.0940873	-0.953249	0.340464	0.87805
asb12a	264.053498	-0.241503433	0.1345589	-1.794779	0.072689	0.52617
emc8	507.5770407	0.104248955	0.1197754	0.8703706	0.384098	0.895192
vaspb	2288.298622	-0.002152531	0.0718152	-0.029973	0.976088	0.996762
nr2e1	282.45249	-0.114520614	0.1379738	-0.830017	0.406529	0.901305
kcna1b	3.586196321	0.547534267	1.0285946	0.532313	0.594509	NA
fam8a1a	29.78580582	0.121248224	0.3516603	0.344788	0.730254	0.971335
kctd10	795.2072749	0.032032162	0.0935924	0.3422518	0.732161	0.971665
vps37a	656.4077311	-0.017615655	0.0998362	-0.176446	0.859944	0.985773
mafba	638.1805409	0.045910288	0.1043655	0.439899	0.66001	0.963432
ilvbl	581.7615923	-0.156724304	0.1036477	-1.512086	0.130512	0.662797
pdzk1ip1	172.5422038	-0.135406016	0.1549126	-0.87408	0.382075	0.895192
myof1	235.5862865	0.176984247	0.1369845	1.2920021	0.196356	0.761835
zgc:63587	948.1088418	0.019328663	0.1003995	0.1925176	0.847337	0.985174
pla2g4ab	7.827990996	-0.268957477	0.7473282	-0.359892	0.718928	NA
brd9	563.3131839	-0.083330258	0.1009943	-0.825098	0.409316	0.901918
cables2b	30.24686556	0.221479277	0.3535186	0.6264997	0.530987	0.942209
wnt10a	10.20982516	-0.07777377	0.6008434	-0.129441	0.897009	NA
kif3ca	185.3227548	0.302164457	0.1630659	1.853021	0.063879	0.493997
sinup	0.473170125	-0.833888596	2.9014002	-0.287409	0.773799	NA
slc3a1	102.6103532	-0.494426371	0.2161215	-2.287724	0.022154	0.283074
nr2f1b	410.5194322	0.084321311	0.1184992	0.711577	0.476727	0.926808
si:dkey-29p10.4	76.2592327	0.065337258	0.2256914	0.2894983	0.7722	0.975687
dlx2b	80.23546142	-0.085755169	0.2193393	-0.39097	0.695819	0.96736
npc1	2122.251091	0.028189399	0.078588	0.3586986	0.719821	0.969318
zpax2	2.985198621	0.675276084	1.0940658	0.6172171	0.537092	NA
cnn1a	5.360834459	-0.934114195	0.834904	-1.118828	0.263213	NA
foxf2a	343.0821971	-0.225509077	0.1298618	-1.736532	0.08247	0.554303
elp2	307.3123832	0.100183167	0.1219846	0.8212771	0.411488	0.903483
plxnb1b	998.8274171	0.131469507	0.090998	1.4447521	0.148528	0.696298
pabpc1a	12349.2733	-0.184623238	0.0815846	-2.262966	0.023638	0.296445
otud7b	58.87215296	-0.184403192	0.2781636	-0.662931	0.507375	0.935734
megf10	238.0099275	0.209402164	0.1393875	1.5023027	0.133019	0.667628
fbxw11b	1446.884954	0.078964901	0.0792298	0.9966572	0.318931	0.867746
npy8ar	1.364726559	1.599180424	1.7155395	0.9321735	0.351247	NA
eif5a	14139.6226	-0.186184312	0.0693384	-2.685155	0.00725	0.134075
traf5	17.22404726	0.061305131	0.4542957	0.1349454	0.892655	0.989291
garem	318.969594	0.072220612	0.1389921	0.5196024	0.603341	0.956045
chd6	902.658411	0.173407159	0.0935128	1.8543686	0.063686	0.493277
prx	1123.273618	0.230171984	0.1011249	2.2761168	0.022839	0.289129

smtlb	5.605107253	0.027539366	0.8149955	0.0337908	0.973044	NA
kcnk1b	9.601136086	-0.692596581	0.6214646	-1.114459	0.265082	NA
tmed1b	145.0795276	-0.18830047	0.1794318	-1.049427	0.293982	0.849999
gdpd1	892.7810169	0.213285507	0.0887665	2.4027704	0.016271	0.235374
scml4	41.10096493	0.321471027	0.315132	1.0201154	0.307674	0.861088
eapp	185.2192522	-0.005435162	0.1489288	-0.036495	0.970888	0.996315
opn1sw2	11787.23073	0.23263115	0.083881	2.7733474	0.005548	0.111157
gnai2b	963.8880407	-0.046321265	0.0854113	-0.542332	0.58759	0.953319
fabp11a	1287.978435	-0.11392552	0.102022	-1.116676	0.264133	0.830245
gpsm2	549.3367951	0.111057494	0.1098661	1.010844	0.312091	0.864401
lmbrd2b	835.176304	0.063615842	0.0977333	0.6509124	0.515103	0.937646
cela1.6	492.5934285	-0.325200617	0.2629868	-1.236566	0.216248	0.783589
larp7	251.6850753	0.005165303	0.1567493	0.0329526	0.973712	0.996315
bag5	81.12416558	-0.114564366	0.2356235	-0.486218	0.626813	0.957354
f11r.1	1333.152093	-0.161999578	0.0778698	-2.080391	0.03749	0.378999
kdr	83.30212381	0.182969051	0.2305591	0.7935884	0.427435	0.91075
cdkl1	140.5414503	0.184530545	0.1773559	1.0404537	0.298129	0.85401
zgc:77929	222.5899011	-0.091313572	0.1552948	-0.588002	0.556531	0.94804
kidins220b	7771.943724	0.046863909	0.0707015	0.6628414	0.507432	0.935734
uts2b	8.809704998	1.092840121	0.6929814	1.5770122	0.114793	NA
epha2a	410.0580767	0.197696566	0.1211563	1.6317477	0.102733	0.609031
rsph9	32.35709848	-0.129691727	0.3675155	-0.352888	0.724173	0.970402
mettl8	38.24575892	0.035149739	0.3276517	0.1072777	0.914569	0.991119
slc23a2	18.06350494	0.431873242	0.4866214	0.8874933	0.374813	0.894316
prdm4	280.4319453	-0.119031043	0.1343397	-0.886045	0.375593	0.894316
rhbdf1b	118.2797656	-0.12553443	0.213891	-0.586908	0.557265	0.948478
sema3d	620.8739864	0.235737722	0.0964478	2.4441995	0.014517	0.21947
b9d2	67.81310808	-0.112833145	0.2426029	-0.465094	0.641864	0.959994
sobpb	337.9225772	0.084911663	0.1355749	0.6263082	0.531113	0.942209
gstt1b	386.6721075	0.023669588	0.1213222	0.1950969	0.845317	0.985174
unc13ba	67.86639963	0.323105407	0.2457028	1.3150256	0.188501	0.753276
smarcc1a	2640.289945	0.005378219	0.1010849	0.053205	0.957569	0.99467
slc44a5a	8.270187561	-0.860188336	0.6949826	-1.237712	0.215823	NA
klf1	8.386535638	0.876694464	0.6694712	1.3095328	0.190354	NA
apmap	1224.423395	-0.023426737	0.0933484	-0.25096	0.801845	0.979492
patl1	1474.915777	0.01049894	0.0769232	0.136486	0.891437	0.989291
crocc2	49.82091718	0.201241755	0.289828	0.6943489	0.487463	0.931584
phf20l1	488.0001218	0.061066973	0.1057621	0.5773996	0.56367	0.948739
sphkap	644.0500814	0.016568236	0.1000472	0.1656042	0.868468	0.987245
mindy2	96.53180946	0.115829832	0.1989435	0.5822248	0.560415	0.948728
adcy3b	22.30014471	0.694546553	0.4416648	1.572565	0.11582	0.637943
eif4ba	657.7953028	0.045291569	0.0956292	0.4736163	0.635774	0.958142
mylz3	42033.62551	0.147872009	0.0911378	1.6225106	0.104694	0.612652
hal	336.3987075	-0.154472206	0.1666449	-0.926954	0.35395	0.881976
eif3i	4292.46027	-0.225637225	0.0763452	-2.955485	0.003122	0.073687
camk1db	558.2664411	0.12044281	0.112362	1.0719181	0.283757	0.84466
nup50	1025.88008	-0.045778497	0.091924	-0.498004	0.618481	0.957354
ncam2	889.9971416	0.052471942	0.0906151	0.5790642	0.562546	0.948728
zgc:110699	56.3316431	0.037636715	0.2630049	0.1431027	0.886209	0.988936
ifi45	23.38600063	0.225496156	0.3972231	0.5676813	0.570251	0.948989
cel.1	345.7492619	-0.3312137	0.131536	-2.518045	0.011801	0.191113
tgfbr1a	17.62279235	0.105091515	0.4540154	0.2314713	0.816949	0.981594
rock2a	696.5395271	-0.0992995	0.093427	-1.062857	0.287847	0.845979
si:ch211-1o7.3	10.06796382	0.532308536	0.5957581	0.8934978	0.371591	NA

ctdp1	275.8454025	-0.220521934	0.1399358	-1.57588	0.115054	0.635519
ccnt1	70.15445595	0.602373718	0.2404926	2.5047496	0.012254	0.195612
kif25	144.8462086	-0.320476531	0.1715631	-1.86798	0.061765	0.485049
prkag3a	60.32127826	0.412241329	0.2567372	1.6056937	0.108341	0.620165
fgf1a	25.96978037	0.109111386	0.3824699	0.285281	0.775429	0.975687
banp	234.0742881	-0.117067263	0.1405616	-0.832854	0.404927	0.901305
itk	1.135343319	3.640271539	2.1311155	1.7081531	0.087608	NA
reep3a	259.4689785	-0.11880601	0.1357327	-0.875294	0.381414	0.895192
mccc2	1948.602028	0.007274498	0.0796432	0.0913386	0.927224	0.992702
si:ch211-195b15.7	23.88455635	0.393089656	0.3987985	0.9856849	0.324288	0.870928
nit2	271.5708533	0.09990383	0.1302167	0.7672121	0.442955	0.916336
fat1a	3022.14534	0.020883548	0.0700138	0.2982777	0.765491	0.975374
ccdc6b	302.2358054	-0.113226454	0.129117	-0.876929	0.380525	0.895192
ccng2	1355.721805	-0.249779009	0.0816402	-3.059511	0.002217	0.05751
rpp40	100.6156208	0.157862641	0.1955115	0.8074339	0.419417	0.906454
sys1	281.4552803	-0.032845226	0.1260648	-0.260542	0.794445	0.977937
pnpo	356.8979465	0.087722404	0.1299247	0.6751788	0.499562	0.934006
tlx3b	95.11733921	0.095819395	0.2128772	0.4501158	0.652627	0.960708
alg6	304.5938654	-0.06014239	0.1258629	-0.477841	0.632764	0.957356
krt4	86679.40241	-0.053501526	0.0822386	-0.650565	0.515327	0.9377
pdcb	2564.48967	0.173977875	0.0873763	1.9911337	0.046466	0.423616
rnf167	166.0294432	0.100948827	0.1757374	0.57443	0.565677	0.948739
cdh7b	9.665998011	-0.178520614	0.6617611	-0.269766	0.78734	NA
rgs13	25.63765606	0.415071554	0.4093507	1.0139755	0.310594	0.86284
zgc:112163	34.96735355	0.103394728	0.3378924	0.3059989	0.759605	0.975374
gmppb	1057.654232	-0.045427051	0.0900822	-0.504285	0.614061	0.95688
sephs2	1357.444876	0.079991373	0.0841071	0.9510653	0.341571	0.878134
braf	383.300722	-0.02437598	0.1236731	-0.1971	0.843749	0.985174
snrkb	320.0574662	0.23146305	0.1301034	1.7790694	0.075228	0.534207
nova2	4323.961055	-0.173207893	0.0684729	-2.529584	0.01142	0.186316
utp4	434.419762	-0.183702303	0.1104787	-1.662784	0.096356	0.59086
mmp2	3701.501008	0.370922988	0.0756256	4.9047255	9.36E-07	9.54E-05
dnajc5gb	332.5692357	-0.058332368	0.1269762	-0.459396	0.64595	0.96029
utp25	389.168468	-0.051743324	0.1348517	-0.383706	0.701197	0.968134
paqr3a	209.8645157	0.116714883	0.1484347	0.7863044	0.431689	0.91277
lrrc40	190.0504694	-0.043285374	0.1490361	-0.290435	0.771483	0.975687
ppp1r14ab	157.297746	0.102796373	0.1774928	0.5791581	0.562482	0.948728
si:ch211-106h11.1	94.11801521	-0.022975457	0.2269814	-0.101222	0.919374	0.991639
si:ch73-151m17.5	4.263712467	-0.542464614	1.0023179	-0.54121	0.588363	NA
phb2b	720.2583224	0.132444116	0.1090619	1.2143945	0.224597	0.791954
ak5l	75.87142378	-0.09928795	0.2380418	-0.417103	0.676603	0.965257
sec63	1049.367946	-0.046546339	0.085755	-0.542783	0.58728	0.953319
g3bp1	1898.713832	0.182514125	0.0744076	2.4528975	0.014171	0.214979
grm6a	5.195400888	-0.343213929	0.8690009	-0.394952	0.692878	NA
smc2	1030.946853	-0.104775827	0.0851539	-1.230428	0.218537	0.785734
arhgap29b	472.8347174	-0.142635773	0.1084332	-1.315425	0.188367	0.75324
pik3cg	95.63063457	-0.214256647	0.2140054	-1.001174	0.316743	0.867129
srrt	1569.029437	-0.062943047	0.0808748	-0.778278	0.436405	0.914408
mdh1aa	7018.474554	-0.07241352	0.0858168	-0.843815	0.398773	0.900446
slc16a12a	78.87378343	0.234369916	0.2321643	1.0095002	0.312735	0.864656
atp5mc3a	2027.69609	-0.02681481	0.0981149	-0.2733	0.784623	0.97627
rorcb	310.1822484	-0.129891649	0.1222474	-1.062531	0.287995	0.845979
hsd17b10	763.9465514	-0.013048128	0.0993701	-0.131308	0.895531	0.989814
zgc:158689	6.265280117	-0.008930827	0.7435444	-0.012011	0.990417	NA

chrnb2	496.2334649	0.034902048	0.1098875	0.3176161	0.750776	0.97419
sdr16c5b	692.3461077	-0.297087019	0.0984855	-3.016554	0.002557	0.064039
bcor	1844.592744	-0.120353745	0.1006911	-1.195277	0.231979	0.800126
tgm1	117.6271293	0.255906877	0.1898047	1.3482644	0.177573	0.738343
gsk3ba	1862.289477	0.083083875	0.0832912	0.9975107	0.318517	0.867746
stard3	413.1960477	-0.064938032	0.1136575	-0.571348	0.567763	0.948739
si:ch211-210c8.6	185.5005436	-0.072797903	0.1593468	-0.456852	0.647778	0.960708
pigs	366.3096335	0.03175699	0.1183015	0.2684412	0.78836	0.976703
gata5	266.8410551	-0.368754473	0.1382161	-2.667956	0.007631	0.13921
micall2b	119.3085583	-0.040409087	0.2159075	-0.187159	0.851536	0.985174
brf1a	106.5205217	-0.249423617	0.1936076	-1.288295	0.197643	0.762928
cand1	1634.566441	0.031110382	0.0746557	0.416718	0.676885	0.965257
syvn1	637.9351172	0.015019608	0.0971011	0.15468	0.877074	0.987881
srsf1b	1983.443664	-0.087550535	0.0841294	-1.040665	0.298031	0.85401
copz1	1256.160268	-0.011990025	0.0837645	-0.14314	0.88618	0.988936
prepl	80.51178019	-0.173209483	0.2229309	-0.776965	0.43718	0.914408
rgs5b	97.95817364	0.037115737	0.2288756	0.1621656	0.871175	0.987754
dnajc3b	508.3536378	0.186359784	0.1100783	1.6929742	0.09046	0.576277
kcnip3b	120.6212338	0.053739912	0.1911449	0.2811475	0.778597	0.975942
rdh1	160.4822391	-0.295736205	0.1647801	-1.794732	0.072696	0.52617
bgna	55.49905287	-0.442233473	0.2589017	-1.708114	0.087615	0.566637
zbtb11	621.711515	-0.118830037	0.101129	-1.175035	0.239981	0.808111
tcp1	4694.085483	-0.11863178	0.0704542	-1.683815	0.092218	0.57849
tln2a	586.426195	0.119600999	0.1093351	1.0938935	0.274002	0.838846
casp3a	1727.696096	-0.132222245	0.076498	-1.728441	0.083909	0.557501
pdss1	477.0278531	0.045158638	0.1066742	0.4233321	0.672053	0.965257
her7	2.31319428	-0.77869527	1.3558072	-0.574341	0.565737	NA
cx36.7	1.030311776	0.994458412	1.953155	0.5091549	0.610644	NA
xrcc3	50.95375406	0.140146349	0.2736525	0.5121326	0.608558	0.956718
ncoa2	1559.694595	-0.036522567	0.0799214	-0.456981	0.647685	0.960645
ints6l	85.76052591	0.343642348	0.2148178	1.5996924	0.109667	0.623766
ptk6a	40.13320289	-0.283680855	0.3364643	-0.843123	0.39916	0.900683
tp73	23.88767788	0.403961442	0.3885227	1.039737	0.298462	0.85439
sfxn2	587.3321764	-0.117058456	0.1144243	-1.023021	0.306298	0.860027
cyp39a1	25.55104539	0.008727464	0.3889865	0.0224364	0.9821	0.996944
rsph14	21.90550172	0.248554067	0.4234544	0.5869678	0.557225	0.948478
zgc:172106	1.055856997	2.296593324	2.1028377	1.0921401	0.274772	NA
zgc:77739	77.3872871	-0.103598823	0.2213731	-0.467983	0.639797	0.959994
vps13d	476.9884305	-0.021993269	0.1070812	-0.205389	0.837268	0.984645
dse	407.7380403	0.17636229	0.1256329	1.403791	0.160381	0.71328
tssk6	0	NA	NA	NA	NA	NA
ubp1	199.1073298	0.053986439	0.1574008	0.3429871	0.731608	0.971557
eci1	499.1971041	-0.221201711	0.1154117	-1.916631	0.055285	0.46072
nkx2.5	26.78151143	-0.357344456	0.3802224	-0.93983	0.347305	0.880123
vrk1	255.9267199	-0.033125348	0.1646351	-0.201205	0.840539	0.985042
mdh1b	13.24931224	0.287465529	0.526351	0.5461479	0.584964	0.952436
crtap	693.2782698	0.202629881	0.105108	1.9278257	0.053877	0.454699
msh2	448.1197281	0.022288677	0.1128712	0.1974701	0.84346	0.985174
tbx2a	545.9164849	-0.003007716	0.1143937	-0.026293	0.979024	0.996802
scn8ab	77.91411211	0.216686698	0.2327668	0.9309174	0.351896	0.88081
msantd4	16.91896112	-0.465538604	0.4879365	-0.954097	0.340035	0.87805
brms11b	288.1780562	0.14294133	0.1253267	1.1405496	0.254057	0.823394
zgc:171704	22.19275186	-0.273613538	0.418204	-0.654259	0.512945	0.936253
sf3b2	2009.798005	-0.152209538	0.0907041	-1.678089	0.09333	0.580895

pik3r2	757.7487389	0.165888603	0.0926818	1.7898729	0.073474	0.528705
neil1	197.4277988	0.148141853	0.1473007	1.0057104	0.314555	0.865694
ntm	26.22430841	-0.194740518	0.3759668	-0.517973	0.604477	0.95651
ptchd1	164.3763544	0.224378341	0.1677188	1.3378244	0.180954	0.741776
rdh12	275.3580704	0.040074547	0.1344212	0.2981267	0.765606	0.975374
mrps22	546.8075105	0.001745397	0.1037578	0.0168218	0.986579	0.996965
rbp1.1	2.175737239	-1.104521527	1.3680625	-0.807362	0.419458	NA
scap	79.86873368	-0.189207953	0.2180633	-0.867674	0.385573	0.89551
casq1b	1053.905703	0.116809772	0.1099853	1.0620487	0.288214	0.845979
eif2b3	500.7330957	-0.053660473	0.1076576	-0.498436	0.618177	0.957354
rab3da	120.2598463	-0.111814196	0.188742	-0.592418	0.553571	0.947322
mafga	3.813022008	0.137853889	0.9858993	0.1398255	0.888798	NA
pak4	510.0267864	-0.052098324	0.1019639	-0.510949	0.609387	0.956718
kdm7aa	390.8731515	-0.09291382	0.1262189	-0.736132	0.46165	0.922466
CR392347.1	6.036594409	0.522008205	0.8273196	0.6309631	0.528065	NA
cenpj	79.3243449	0.077730507	0.2313174	0.3360339	0.736845	0.972535
psmd3	1764.753942	-0.00775175	0.0810002	-0.0957	0.923759	0.992628
mid1ip1l	513.9706524	0.243143743	0.1106084	2.1982394	0.027932	0.323709
gpx1a	675.5667147	-0.095161439	0.102201	-0.93112	0.351791	0.88081
slc38a4	2411.517636	0.118835242	0.0817442	1.4537444	0.146017	0.69189
nt5c1ba	188.8148819	-0.009343593	0.1525418	-0.061253	0.951158	0.99381
ano10b	201.739842	0.016472789	0.1447872	0.1137724	0.909418	0.990702
ctnnal1	119.7076594	0.095395071	0.182763	0.5219605	0.601698	0.956045
depdc1a	171.0314582	-0.094681522	0.1560168	-0.606868	0.543939	0.945438
gnai2a	1798.290664	-0.07431629	0.0854765	-0.869436	0.384609	0.89551
pgm2	759.5540807	-0.000626272	0.0912662	-0.006862	0.994525	0.998694
acvrl1	353.3141165	0.053974177	0.1331703	0.405302	0.685256	0.966146
dnajc28	84.18483869	0.294941474	0.2218957	1.3291899	0.183785	0.747376
asna1	1424.943487	0.037275196	0.0998199	0.3734245	0.708833	0.968664
ubr5	3328.619809	-0.038391292	0.1039893	-0.369185	0.71199	0.969006
nck2a	512.5394604	-0.012875179	0.1022337	-0.125939	0.89978	0.990121
htr2cl1	191.1106719	-0.027677968	0.1540065	-0.179719	0.857373	0.985703
mfap1	789.5645207	-0.145865821	0.0953607	-1.529622	0.12611	0.654806
ncoa1	135.7060293	-0.121626992	0.1749713	-0.695126	0.486977	0.931104
atp1a3a	9921.620458	0.111487864	0.0801018	1.3918265	0.163975	0.71945
bmp7a	8.735090993	0.49773576	0.6500576	0.7656794	0.443867	NA
pdia2	350.7239558	-0.459977281	0.1341327	-3.429271	0.000605	0.020492
trim101	387.0791178	0.332709869	0.1155697	2.8788685	0.003991	0.08682
mthfd1a	717.4448842	-0.054814339	0.1312147	-0.417745	0.676133	0.965257
olfm1a	343.1358071	0.115813807	0.1220036	0.9492659	0.342485	0.878434
wdr33	1726.123654	-0.03430625	0.0790185	-0.434155	0.664176	0.964736
cfap161	6.222159629	-0.077966947	0.7877289	-0.098977	0.921157	NA
cyba	158.0799489	-0.078026991	0.1677117	-0.465245	0.641756	0.959994
pdpk1b	1430.343581	-0.101873813	0.0818883	-1.244058	0.213478	0.781725
rev1	419.2216697	0.072171289	0.1101788	0.655038	0.512443	0.936253
si:ch73-186j5.2	1.022491002	2.265845936	2.0916203	1.083297	0.278677	NA
etv4	425.9219455	-0.063527303	0.1156226	-0.549437	0.582706	0.952214
rell1	306.2259381	-0.066482432	0.1268023	-0.5243	0.60007	0.956045
arglu1b	1155.744297	0.086899657	0.0899768	0.9658008	0.334144	0.875263
lbx1a	135.4297995	-0.087746994	0.1980782	-0.442992	0.657772	0.962538
taf5	398.4771389	0.061067086	0.1163751	0.5247435	0.599762	0.956045
il1r3	0.365141898	1.973577737	3.3927314	0.5817076	0.560764	NA
rhocb	930.0488324	-0.098382525	0.0889044	-1.10661	0.268463	0.833634
gc2	22.98964705	-0.019134956	0.3919912	-0.048815	0.961067	0.995659

rpl35	11313.21445	-0.393498002	0.0952065	-4.133101	3.58E-05	0.002051
ggt1b	79.32341832	0.410201341	0.2343603	1.750302	0.080066	0.549631
hpda	314.0039573	0.280994522	0.1367674	2.0545434	0.039923	0.391511
sult1st3	30.67350902	-0.107099239	0.3629345	-0.295093	0.767923	0.975687
prkcha	21.15744391	-0.176056087	0.4117318	-0.427599	0.668943	0.964961
frzb	460.6408371	0.14006521	0.1208228	1.1592616	0.24635	0.816376
kank2	39.44484711	0.22127918	0.3286236	0.6733514	0.500724	0.93428
hpca	2531.620684	0.067306737	0.0776933	0.8663134	0.386318	0.89551
rps6kb1a	623.9584543	-0.090476891	0.1034147	-0.874894	0.381632	0.895192
krt18a.1	2527.196719	-0.173273212	0.0744754	-2.326585	0.019987	0.266383
mknk1	442.6530644	0.079900035	0.1128796	0.7078345	0.479048	0.926978
pth1rb	31.77964671	0.145100809	0.3523594	0.4117977	0.680488	0.965431
sulf2a	1347.074786	0.148010369	0.0793859	1.8644408	0.06226	0.487382
ddx61	2036.923011	0.165103503	0.07597	2.1732712	0.02976	0.334058
aldh7a1	2535.082509	-0.029585379	0.0702084	-0.421393	0.673468	0.965257
m6pr	363.4079378	-0.091397605	0.1154579	-0.79161	0.428588	0.911378
heg1	80.04064395	0.398572299	0.2220674	1.7948259	0.072681	0.52617
msrb2	650.3148256	-0.11817026	0.1066235	-1.108295	0.267734	0.833349
mbnl2	905.6040134	-0.000754405	0.0914206	-0.008252	0.993416	0.998618
zgc:56095	529.0433631	-0.159349988	0.1015523	-1.569143	0.116615	0.639148
pak2b	1751.626296	-0.001019835	0.075094	-0.013581	0.989164	0.997589
agxtb	1243.798334	0.602522113	0.084384	7.1402438	9.32E-13	3.37E-10
cyp2v1	275.9192591	-0.250876702	0.1368213	-1.833608	0.066712	0.505041
pdia4	1519.555043	0.119336997	0.0843764	1.4143404	0.157262	0.707876
znf503	1383.643129	-0.149293593	0.0886302	-1.684456	0.092094	0.57849
smn1	215.549239	-0.11258469	0.1608974	-0.699729	0.484096	0.929461
ftr06	0	NA	NA	NA	NA	NA
zdhhc8b	312.7202538	0.026288569	0.1278461	0.2056267	0.837083	0.984645
stk38b	143.4374059	0.23054561	0.1775719	1.2983225	0.194177	0.758049
or115-1	0.797014944	-1.848753454	2.2697209	-0.814529	0.415342	NA
midn	2403.551128	0.186024085	0.0744776	2.4977201	0.012499	0.198049
lipf	785.6084724	0.01088319	0.1031407	0.1055179	0.915965	0.991314
mapkapk2b	56.97271711	0.022177335	0.2691864	0.0823865	0.934339	0.992702
slc6a9	2252.629303	0.135236451	0.075649	1.7876827	0.073827	0.529749
hapln4	62.74633342	0.286842374	0.2514259	1.1408625	0.253927	0.823394
ifih1	8.411256217	0.273714931	0.6490946	0.4216873	0.673253	NA
kdm7ab	570.2024427	-0.077156454	0.1067065	-0.723072	0.469636	0.924505
cope	1797.894327	-0.040346089	0.0768575	-0.524947	0.59962	0.956045
flnca	550.3723711	-0.067622957	0.1164719	-0.580595	0.561514	0.948728
tnfrsf1a	410.1006739	-0.183629961	0.1268193	-1.447966	0.147627	0.694905
sf3b4	404.6792548	-0.133441208	0.1132056	-1.178751	0.238497	0.807659
zgc:152658	0.547712847	2.559541715	2.9076406	0.8802813	0.378707	NA
parp16	243.524602	-0.024737813	0.137798	-0.179522	0.857528	0.985703
retsat	526.1373265	-0.245638734	0.1034395	-2.37471	0.017563	0.245605
rab5aa	1779.433221	-0.062921766	0.0847538	-0.742406	0.457841	0.921978
lbx1b	38.54533832	-0.020108311	0.3150774	-0.06382	0.949113	0.99381
agtr1a	10.87661311	-0.264743073	0.567877	-0.466198	0.641074	NA
cpsf6	1756.796147	-0.069950459	0.0791431	-0.883848	0.376779	0.895192
cers4b	64.97440459	0.146427119	0.2646498	0.5532864	0.580067	0.951257
slc6a19a.1	83.59310631	0.511464849	0.2355864	2.1710286	0.029929	0.335036
rad54l	207.5509272	-0.055748632	0.1512586	-0.368565	0.712452	0.969087
arl5a	286.2789708	-0.027106281	0.1412713	-0.191874	0.847841	0.985174
sec61g	1316.193391	-0.00434281	0.2202511	-0.019718	0.984269	0.996944
igf2a	175.2078083	0.080763843	0.1562826	0.5167807	0.605309	0.95651

gtpbp3	38.64795433	0.087143605	0.3065355	0.2842855	0.776192	0.975826
acot7	309.9601876	0.09796197	0.1290042	0.7593706	0.447631	0.917154
aar2	238.232792	0.052071327	0.1374739	0.3787724	0.704857	0.968134
si:ch211-262i1.4	0	NA	NA	NA	NA	NA
saal1	92.4320217	-0.026285987	0.2312778	-0.113655	0.909511	0.990702
itga2b	4.420944525	0.341149016	0.9591105	0.3556931	0.72207	NA
elk3	424.2991166	-0.017195026	0.1217623	-0.141218	0.887698	0.989291
phf2	816.1609406	0.083265886	0.0952946	0.8737737	0.382242	0.895192
cdh2	4751.954031	-0.060497576	0.0703827	-0.859552	0.390036	0.897023
carm1	1494.504833	0.131211321	0.0843427	1.5556935	0.119781	0.644854
dgkh	97.82112414	-0.281916886	0.2232692	-1.262677	0.206705	0.774273
npnta	358.9209487	0.086596038	0.1239888	0.6984185	0.484916	0.93001
slco3a1	69.04703764	0.06702855	0.2313006	0.2897897	0.771977	0.975687
zw10	439.3848646	-0.095561372	0.1204964	-0.793064	0.42774	0.91075
psme4b	1142.718198	0.128108528	0.0847694	1.5112587	0.130723	0.662797
scamp5a	113.915423	0.017417385	0.2084944	0.0835389	0.933423	0.992702
prl2	11.73069903	0.707106069	0.596522	1.1853814	0.235867	0.804837
npr1b	220.3078344	-0.327359943	0.1419241	-2.306584	0.021078	0.273668
klf5l	489.2576164	-0.512060656	0.1130719	-4.528627	5.94E-06	0.00046
pknox1.1	537.9925323	-0.043091175	0.1054826	-0.408514	0.682896	0.965476
hivep2b	10.2173327	-0.488242347	0.6091723	-0.801485	0.422851	NA
kdm4aa	231.2428159	-0.149181581	0.1577053	-0.945952	0.344173	0.878434
efna1b	688.4409229	-0.008062752	0.0967797	-0.08331	0.933605	0.992702
st8sia2	12.78768382	0.89601388	0.5749708	1.5583642	0.119147	0.64321
crygmxl1	75.86646473	-0.311029454	0.2381322	-1.306121	0.191511	0.755886
abhd3	251.1889989	0.409465285	0.1457567	2.8092379	0.004966	0.102608
esrp2	687.1597291	-0.141146679	0.0953752	-1.47991	0.138897	0.677395
bdnf	196.7760671	0.525397007	0.1704463	3.0824779	0.002053	0.054414
flncb	991.7403882	0.164255908	0.0838896	1.9580003	0.05023	0.438746
tmem248	109.1880484	0.218374262	0.1900018	1.1493271	0.250421	0.820164
trpc4apa	1109.034272	0.095432598	0.0805284	1.1850806	0.235986	0.805056
dgat2	65.76583625	0.502185868	0.2448845	2.0507053	0.040296	0.392394
si:dkey-199f5.8	756.0600512	0.111516667	0.1023615	1.0894396	0.27596	0.839541
scaf4b	612.6002745	-0.017525009	0.1124262	-0.15588	0.876128	0.987881
dclk1a	621.0847429	0.20714217	0.1052175	1.968705	0.048987	0.435115
henmt1	26.36030419	1.571851971	0.3981242	3.9481446	7.88E-05	0.003993
apobec2a	250.9432722	0.207603425	0.1328861	1.5622663	0.118225	0.641526
jak2b	643.1065145	-0.070908979	0.0986744	-0.718616	0.472378	0.924838
snrpa	1078.961955	-0.041372069	0.0895049	-0.462232	0.643915	0.96009
rnaseh2a	359.0417947	-0.086041676	0.1229584	-0.699763	0.484076	0.929461
mmp24	25.10850986	-0.01489086	0.3950812	-0.037691	0.969934	0.996315
zfand5a	2224.627639	0.038470575	0.0883993	0.4351909	0.663424	0.964736
pes	715.9204424	-0.035600746	0.096729	-0.368046	0.712839	0.96909
aimp2	760.5090337	-0.101322979	0.0963443	-1.051676	0.292948	0.848631
cstf3	1706.649347	-0.14885043	0.0773059	-1.925473	0.05417	0.455813
kat6a	1909.354366	-0.040228893	0.0878639	-0.457855	0.647057	0.960362
hnrnpk	2407.102318	-0.122123588	0.0724204	-1.686315	0.091735	0.57849
rad17	187.620141	0.09854177	0.1562772	0.6305574	0.52833	0.941868
lratd1	440.9736211	-0.111711759	0.1087961	-1.026799	0.304515	0.858905
fat2	328.3427023	0.141185152	0.1279579	1.1033722	0.269866	0.834705
zcchc17	307.5962253	-0.113359791	0.1222835	-0.927025	0.353914	0.881976
hoga1	211.832413	0.122178461	0.145808	0.8379408	0.402064	0.90084
gclm	376.4590096	-0.082744965	0.117969	-0.701413	0.483045	0.929338
tmem39b	314.3665529	-0.079253539	0.1276966	-0.62064	0.534837	0.943336

nrarpb	64.20144261	-0.09486924	0.246608	-0.384696	0.700462	0.967998
gtpbp4	2885.801207	-0.336366304	0.0788703	-4.264803	2.00E-05	0.001256
cyb5r1	373.0088246	-0.31989426	0.1175202	-2.722036	0.006488	0.123125
gabbr1a	33.10109216	0.175521939	0.3293257	0.5329737	0.594052	0.954434
acvr1ba	1872.598101	-0.064114024	0.089659	-0.715088	0.474555	0.925287
b3gnt5a	505.6511438	0.06851245	0.1053674	0.6502246	0.515547	0.9377
clptm1	1860.826127	0.128194273	0.0775006	1.6541069	0.098106	0.59749
sptlc2a	239.4118173	-0.053120885	0.1512423	-0.35123	0.725416	0.970402
zgc:91890	169.6386002	0.030988066	0.1637731	0.1892134	0.849926	0.985174
eya2	666.4012831	-0.126672271	0.0948489	-1.335516	0.181708	0.743756
castor2	1088.439911	-0.125367649	0.0890671	-1.407564	0.15926	0.710139
hspa4b	2951.240023	-0.041018759	0.0816948	-0.502097	0.615599	0.95688
cplx2l	2382.458765	0.114542435	0.0719345	1.5923165	0.111314	0.627048
smc3	3386.314178	-0.069431421	0.0720307	-0.963915	0.335089	0.875906
ak7a	85.32395897	-0.040360681	0.2153335	-0.187433	0.851321	0.985174
smarcd1	1949.878345	-0.030890028	0.0755793	-0.40871	0.682753	0.965476
erp44	1259.169513	-0.078312089	0.0786202	-0.996081	0.319211	0.867746
barhl1b	360.8793247	0.263535481	0.1320733	1.9953733	0.046002	0.421189
podn	4.90162669	0.193126438	0.9311158	0.207414	0.835687	NA
tmem59	1147.964473	0.017419051	0.0880821	0.1977594	0.843233	0.985174
sgsm3	841.9225445	0.004074293	0.0910922	0.0447272	0.964325	0.995927
snx12	2139.069733	0.014736286	0.0775136	0.1901123	0.849221	0.985174
arpc5b	807.5534515	-0.026530729	0.093583	-0.2835	0.776794	0.975826
fat1b	284.3042424	0.106440048	0.1356531	0.784649	0.432659	0.91326
DHDH	229.0668941	-0.103989523	0.1450502	-0.716921	0.473423	0.924993
si:ch73-160i9.2	1.805520255	0.249416744	1.3925451	0.1791086	0.857852	NA
myl7	728.4431724	0.354402734	0.0992773	3.5698252	0.000357	0.013599
cd82a	1931.650052	0.071690124	0.0782229	0.9164847	0.359413	0.884844
kdm4ab	757.3273288	-0.234881302	0.0991332	-2.36935	0.017819	0.247181
mob3a	470.2552941	-0.092911614	0.1136553	-0.817486	0.413651	0.905864
ing1	246.3058337	0.069732008	0.1391128	0.5012622	0.616187	0.95688
parvb	551.3540165	0.022703309	0.0986954	0.2300341	0.818065	0.981594
he1.2	7.26236758	-0.670590954	0.7764834	-0.863626	0.387794	NA
klhl40b	74.81404064	0.159320998	0.2757185	0.5778394	0.563373	0.948739
tpm4b	693.5199076	-0.089076198	0.1048	-0.849964	0.395345	0.899293
plk2b	489.4747732	-0.071347202	0.1079415	-0.66098	0.508625	0.935807
ankef1a	17.80394716	0.04190542	0.4561301	0.0918716	0.9268	0.992702
tram1	598.539518	-0.118131163	0.1033011	-1.143562	0.252805	0.822335
fam185a	76.93548985	0.228007528	0.2438533	0.9350194	0.349778	0.880195
rab4a	233.2313212	0.092100839	0.1507147	0.611094	0.541137	0.944921
haus5	149.1100875	0.061191338	0.1651974	0.3704135	0.711074	0.968985
sh3bp5la	322.0070132	0.040381599	0.1203812	0.3354478	0.737287	0.972535
lrit1a	313.8149077	0.216831151	0.1280016	1.693972	0.090271	0.575253
rpsa	27996.68312	-0.268888516	0.075223	-3.574554	0.000351	0.013418
slain1a	205.3771729	-0.156978092	0.1488461	-1.054634	0.291593	0.847361
12-Sep	984.2668567	-0.07917366	0.0879618	-0.900092	0.368071	0.887482
vps9d1	259.4359926	0.227697366	0.1351011	1.6853855	0.091914	0.57849
fam120c	1184.988438	0.069779101	0.0872142	0.8000886	0.423659	0.907829
abo	7.632769443	-1.204982094	0.7241508	-1.663993	0.096114	NA
camsap1a	673.3237381	0.152108204	0.1021837	1.4885759	0.136599	0.673303
CTBP1	1000.196774	0.066879812	0.0940674	0.7109773	0.477098	0.926857
mrpl24	455.7398224	-0.147495853	0.1057389	-1.394907	0.163044	0.717888
arglu1a	2445.482259	-0.053519162	0.0899026	-0.595302	0.551642	0.947138
mogat2	154.0895744	-0.660167674	0.1769617	-3.730569	0.000191	0.00823

rpl7a	33032.31291	-0.365929454	0.080024	-4.572745	4.81E-06	0.000387
sptan1	8673.788949	-0.015366109	0.0815253	-0.188483	0.850498	0.985174
nln	162.3425204	0.127778673	0.1644113	0.7771889	0.437047	0.914408
sema3aa	1901.706922	0.103090478	0.073224	1.407878	0.159167	0.710139
gsr	558.1156366	-0.234715055	0.1167037	-2.011205	0.044304	0.413823
cul1a	1270.037605	0.120691126	0.0784313	1.5388138	0.12385	0.652372
fancf	33.78491667	0.609178712	0.3349632	1.8186436	0.068966	0.513259
rhag	2725.960324	0.096873249	0.0748874	1.2935854	0.195809	0.76113
dhrs9	173.5816326	0.009094221	0.1812095	0.0501862	0.959974	0.995388
zbtb39	223.4707955	-0.136432367	0.1417262	-0.962647	0.335724	0.876088
rasd1	290.4980086	-0.02506889	0.1355348	-0.184963	0.853258	0.985174
GNB4	211.340057	0.063585751	0.1526293	0.4166025	0.676969	0.965257
larsb	981.8425421	-0.008139962	0.0880713	-0.092425	0.926361	0.992702
tfdp1a	909.5974453	-0.029865557	0.0870619	-0.343038	0.73157	0.971557
cbln8	3.648571093	-0.520663666	0.9957019	-0.522911	0.601036	NA
znf687b	980.3539831	-0.171301061	0.0873201	-1.96176	0.04979	0.437386
ints7	229.6532663	-0.167722924	0.1444803	-1.160871	0.245694	0.815435
mid1ip1b	895.8589671	0.28704953	0.096964	2.9603734	0.003073	0.07338
phactr3b	494.7307268	-0.001998825	0.1113778	-0.017946	0.985682	0.996944
dusp5	190.8749616	0.163945781	0.1625749	1.0084321	0.313247	0.865104
fbxo30b	66.84251284	0.114252915	0.2581108	0.4426507	0.658018	0.962545
rab39ba	240.4166948	0.111889843	0.1344116	0.8324418	0.40516	0.901305
fer1l6	45.9090626	0.184738075	0.3069892	0.601774	0.547325	0.945526
prpsap2	784.2735642	0.042178596	0.0994879	0.4239571	0.671597	0.965257
plxna4	1730.611131	0.080993605	0.0750107	1.0797605	0.280249	0.843423
ndufb4	0.988894344	-1.041944082	1.9792901	-0.526423	0.598594	NA
hes6	388.8056522	0.053929214	0.1152962	0.4677448	0.639967	0.959994
adprhl2	363.2672793	0.052998651	0.1181728	0.4484842	0.653804	0.961168
uxt	74.31517632	-0.303337889	0.2253022	-1.34636	0.178186	0.73929
gpc1a	65.14056464	0.143045777	0.2494537	0.5734361	0.566349	0.948739
chrnd	354.3099548	0.278297186	0.1249827	2.2266851	0.025968	0.311101
mark3a	1254.542634	0.025207265	0.092168	0.2734926	0.784475	0.97627
sparc	20430.65821	0.273563895	0.086139	3.175841	0.001494	0.042508
coa7	183.8389717	-0.123153901	0.1514174	-0.813341	0.416023	0.906237
fabp1a	0	NA	NA	NA	NA	NA
sec23b	1527.92959	0.280774091	0.078898	3.5586964	0.000373	0.014011
ptbp1a	1330.170217	-0.105715463	0.0814396	-1.298084	0.194259	0.758049
mbip	171.7500145	0.08957	0.1555089	0.5759798	0.564629	0.948739
krt99	48.20882846	-0.256536624	0.2843676	-0.90213	0.366988	0.887458
tgfb3	969.6699513	0.106421982	0.0952718	1.1170357	0.263979	0.830245
flt1	173.9086123	-0.056815929	0.1621296	-0.350435	0.726012	0.970402
cdc42bpb	1966.517578	0.009992898	0.0870516	0.1147928	0.908609	0.990702
stat5a	17.5841477	-0.161092518	0.4635248	-0.347538	0.728187	0.970402
rergla	90.24090527	-0.09231519	0.2156692	-0.428041	0.668621	0.96478
psma6a	1013.435076	0.020960376	0.0851565	0.2461395	0.805574	0.979893
atp5f1d	3670.593658	0.106237335	0.0805598	1.3187392	0.187256	0.751302
zgc:63470	229.8528167	-0.217259749	0.1394165	-1.55835	0.11915	0.64321
gadd45ga	692.3118008	-0.262352126	0.097934	-2.678868	0.007387	0.13563
kcnj2a	94.93105025	0.326612704	0.2206678	1.4801101	0.138844	0.677395
etnk1	1200.581453	-0.028122069	0.0862413	-0.326086	0.744359	0.974094
trioa	401.6834489	-0.122332824	0.1154344	-1.059761	0.289253	0.845979
rnf13	751.742352	0.160862842	0.0928005	1.7334268	0.08302	0.555556
CABZ01086041.1	8.590758203	-0.007820484	0.6479569	-0.012069	0.99037	NA
ssr4	1794.223144	-0.046055948	0.0869402	-0.529743	0.59629	0.954499

pcsk2	181.9512481	-0.030158108	0.1547942	-0.194827	0.845528	0.985174
tfe3b	7.235474989	-0.139899784	0.7014302	-0.199449	0.841911	NA
elf2a	427.9342595	0.159066121	0.112835	1.4097228	0.158622	0.709303
zdhhc7	57.03976238	-0.238311932	0.27464	-0.867725	0.385545	0.89551
tmx1	294.8620832	-0.094176925	0.1289541	-0.730314	0.465199	0.923671
shbg	160.4189173	0.348681765	0.1705122	2.0449079	0.040864	0.394957
esd	650.485943	-0.02533141	0.1022107	-0.247835	0.804262	0.979643
cry5	748.1382754	0.395491807	0.1139523	3.4706787	0.000519	0.018364
pcnx3	59.17729481	0.168976097	0.2523925	0.6694972	0.503178	0.935304
zgc:103759	28.46230871	0.046157581	0.3605085	0.1280347	0.898122	0.990121
mcm5	1044.40971	0.056210634	0.086752	0.647946	0.51702	0.938543
sp7	162.7904563	-0.349906347	0.1784715	-1.960573	0.049929	0.437835
mpx	241.0155322	-0.165992885	0.3877693	-0.428071	0.668599	0.96478
tbce1b	99.61653332	0.153217336	0.2037063	0.7521482	0.451962	0.918761
agfg1b	381.5473036	0.035410986	0.1225606	0.2889264	0.772638	0.975687
parp1	1450.081419	-0.021720666	0.0814767	-0.266587	0.789787	0.977238
hdgfl2	800.9404482	0.077638117	0.0955969	0.8121406	0.416711	0.906237
fads2	273.1655924	-0.240721682	0.1313415	-1.832792	0.066834	0.505573
gpt2l	1497.959694	0.014762223	0.0785172	0.1880125	0.850867	0.985174
cds1	159.8233776	-0.213925113	0.1739614	-1.229728	0.218799	0.785734
clcn7	467.0769914	0.080262021	0.1162819	0.6902367	0.490045	0.931926
asap2b	558.2566477	0.088131168	0.1116927	0.7890502	0.430083	0.911615
neurod1	2844.921881	-0.184378506	0.0762015	-2.419619	0.015537	0.228768
taf7	841.5024769	0.042150938	0.0923891	0.4562326	0.648223	0.960708
ldb2a	846.6856691	-0.044267716	0.0898659	-0.492598	0.622297	0.957354
uso1	1161.59265	-0.110578965	0.0811502	-1.362645	0.172994	0.732033
gstk2	0.63196552	0.01215153	2.613686	0.0046492	0.99629	NA
chico	27.27462155	0.192415897	0.4145867	0.464115	0.642565	0.959994
igl4v9	0	NA	NA	NA	NA	NA
senp8	46.06123513	-0.199513164	0.2967735	-0.672274	0.501409	0.934613
nrd1a	1339.440092	-0.000297281	0.0829269	-0.003585	0.99714	0.99906
col12a1b	1293.076045	0.361727198	0.0795838	4.5452348	5.49E-06	0.000429
ip6k2b	1886.203496	-0.047468379	0.079272	-0.598804	0.549304	0.946312
jade3	465.8897208	0.155367452	0.1071429	1.4500951	0.147032	0.693893
yif1a	555.6767021	0.110026317	0.1033645	1.0644501	0.287125	0.845979
ldhba	12071.77078	-0.091250115	0.0954135	-0.956365	0.338888	0.87745
twist3	125.1780105	-0.227469416	0.1810712	-1.256243	0.209028	0.776073
zgc:162239	0.84841062	-0.578696897	2.0997862	-0.275598	0.782857	NA
pou2f2a	162.8260641	-0.206813263	0.1722889	-1.200387	0.229989	0.797721
rxfp2b	13.29907339	0.094582762	0.5333929	0.1773229	0.859255	0.985773
fgl2b	17.83727711	0.695148905	0.4697193	1.4799239	0.138894	0.677395
colq	182.2612422	0.432950744	0.1526435	2.8363528	0.004563	0.096402
clcn5a	925.4056633	0.220771674	0.09021	2.4473075	0.014393	0.218087
aldocb	12180.50308	0.012848355	0.0674034	0.1906188	0.848824	0.985174
zar1	6.310033961	0.605374281	0.7572477	0.7994403	0.424035	NA
spout1	111.4996196	-0.024103555	0.1899038	-0.126925	0.899	0.990121
rhoua	82.69535541	0.213260392	0.221925	0.9609572	0.336574	0.876088
oatx	78.90598311	0.116756246	0.2231176	0.5232948	0.600769	0.956045
metap1d	83.90752053	-0.213328917	0.2140921	-0.996435	0.319039	0.867746
pbx2	580.3344378	0.101826832	0.1023906	0.9944939	0.319982	0.868153
celsr2	646.8020336	-0.018487613	0.1195726	-0.154614	0.877126	0.987881
bmpr1aa	1064.521766	-0.031681791	0.0836199	-0.378879	0.704778	0.968134
mtch2	1785.86402	0.028434548	0.0737531	0.385537	0.69984	0.967998
ccndx	9.795902477	0.640519034	0.6039782	1.0605002	0.288917	NA

tlr4ba	5.49699546	-0.432993302	0.8031657	-0.539108	0.589812	NA
dctn1a	122.3095449	-0.110035743	0.1787936	-0.615435	0.538268	0.943336
rfng	209.1269051	-0.200091848	0.1540556	-1.298829	0.194003	0.75784
hsd3b2	7.405768389	0.821271326	0.7248611	1.1330051	0.257212	NA
rom1a	963.7503401	0.126439426	0.1039628	1.2161986	0.223909	0.790781
kcnn3	400.5460393	0.096404468	0.1151872	0.8369376	0.402628	0.90084
acp5a	19.43422855	-0.423053247	0.4967568	-0.851631	0.394419	0.898841
zgc:158803	929.78237	-0.286735053	0.0994425	-2.883424	0.003934	0.086142
si:ch1073-280e3.1	67.65840622	0.304919475	0.2417424	1.2613406	0.207186	0.775175
zhx3	607.7493289	-0.2718821	0.1024376	-2.654125	0.007951	0.143459
rps6	19456.83466	-0.366777638	0.0801061	-4.578646	4.68E-06	0.000379
impg2a	99.43559107	0.105316075	0.2236163	0.4709678	0.637664	0.958872
prmt3	260.5853139	0.125938614	0.1450342	0.8683371	0.38521	0.89551
qrich1	379.0891349	-0.00318827	0.1156969	-0.027557	0.978015	0.996762
evpla	417.5932634	-0.021022119	0.1181896	-0.177868	0.858827	0.985773
nfe2l3	774.1251476	0.067106315	0.0907434	0.7395175	0.459593	0.921978
fn1a	2145.454338	0.071587141	0.07456	0.9601285	0.336991	0.876088
edrf1	253.5682127	-0.035692115	0.1326183	-0.269134	0.787826	0.976596
nkx2.1	151.9990092	0.062869628	0.1721841	0.3651302	0.715014	0.96909
ugdh	966.2826869	-0.241193768	0.1049885	-2.297335	0.0216	0.278249
cnot3b	606.628728	-0.065098353	0.101462	-0.641603	0.521131	0.939056
pdlim1	541.8459301	0.023431596	0.1066457	0.2197144	0.826094	0.982196
atp1a1b	8438.455379	0.140424539	0.0656917	2.1376303	0.032547	0.351491
fgl2a	50.85288554	0.040367375	0.2770379	0.1457107	0.88415	0.988784
hsph1	57.81554411	0.100406929	0.2545293	0.3944808	0.693226	0.96736
rbks	74.31317012	-0.349729708	0.2363267	-1.479857	0.138911	0.677395
bsg	11576.90562	0.031010234	0.0617781	0.5019613	0.615695	0.95688
gpat4	966.4345127	0.082331111	0.0858995	0.9584589	0.337831	0.876447
rcvrn2	5406.057306	0.229614668	0.0789344	2.9089292	0.003627	0.081493
dscc1	90.09868804	-0.022026913	0.2140098	-0.102925	0.918023	0.991623
epb41l3b	1109.091348	0.012541179	0.100412	0.1248972	0.900605	0.990121
lft1	1.005556699	-1.067025392	1.9900474	-0.536181	0.591834	NA
cmpk	1744.337805	-0.123371478	0.0741044	-1.664833	0.095946	0.589937
tal1	561.9403129	-0.024682879	0.1015167	-0.243141	0.807896	0.980642
slc5a11	47.34379437	-0.19069827	0.2838369	-0.671859	0.501674	0.934712
sgta	1415.617543	0.004045595	0.0821177	0.0492658	0.960707	0.995547
ptprdb	118.701654	0.321729007	0.1832037	1.7561275	0.079067	0.547489
serpinh1b	861.0166338	0.500923336	0.1026768	4.8786431	1.07E-06	0.000106
carf	293.8989871	-0.007466709	0.1419882	-0.052587	0.958061	0.99467
sec62	1048.616893	0.113508719	0.0848941	1.3370626	0.181202	0.742302
znf513a	395.8965648	0.13314702	0.1200392	1.109196	0.267346	0.833191
dop1a	839.8420478	0.081316689	0.0910097	0.8934951	0.371592	0.892003
sfxn1	30.91945387	0.178521661	0.3541914	0.5040259	0.614243	0.95688
smarcd3b	948.2250159	0.050021632	0.0861351	0.5807346	0.561419	0.948728
stk38a	91.02897293	0.014607792	0.2215775	0.0659263	0.947436	0.99381
idi1	1135.899253	0.011151824	0.0914651	0.1219243	0.902959	0.990121
grhprb	1227.549095	-0.059349999	0.2183757	-0.271779	0.785792	0.97627
cabp1a	55.21165707	0.410145833	0.2679953	1.5304217	0.125912	0.654803
bmp4	249.8972225	-0.006870671	0.1454382	-0.047241	0.962321	0.995907
slc30a7	512.2459294	-0.099882175	0.1041573	-0.958955	0.337582	0.876395
sh3bp4a	524.8381734	-0.053808619	0.1011892	-0.531762	0.594891	0.954434
rad23aa	420.979026	-0.070769622	0.1173953	-0.602832	0.546621	0.945526
col1a2	21699.74887	0.301877098	0.1039983	2.9027108	0.003699	0.082473
vcp	9072.735269	0.019451993	0.0669602	0.2905009	0.771433	0.975687

bmp2k	486.3308087	-0.012380752	0.1129999	-0.109564	0.912755	0.991119
mrps33	290.5399926	0.044727335	0.1314814	0.34018	0.733721	0.971993
fam151b	72.12798523	0.197332102	0.2557284	0.7716473	0.440323	0.916329
cps1	189.9074597	-0.279795869	0.1559684	-1.793927	0.072825	0.526522
cldn11a	446.3078022	0.103779356	0.1228444	0.8448033	0.398221	0.899933
vps35l	636.213187	-0.107478766	0.0966902	-1.111578	0.26632	0.83247
cnot8	625.881083	-0.059460486	0.1029386	-0.577631	0.563513	0.948739
ahr1a	8.395699377	-0.819000684	0.7109602	-1.151964	0.249336	NA
nkd1	728.5661722	0.040348955	0.092402	0.4366674	0.662353	0.964665
aox6	139.7546181	0.081325352	0.1751525	0.4643118	0.642424	0.959994
bmpr2b	67.44546133	-0.108208876	0.2537371	-0.426461	0.669772	0.965223
thbs4b	2239.139155	0.337750274	0.0797938	4.2327894	2.31E-05	0.001417
nol10	510.1709418	0.0492909	0.1045917	0.4712696	0.637448	0.958872
neil3	41.65353552	-0.120804062	0.3154298	-0.382982	0.701733	0.968134
ek1	300.8730633	0.014031572	0.1335803	0.1050422	0.916342	0.991314
tg	781.8880618	0.218916818	0.1226294	1.7851906	0.07423	0.530917
slc33a1	266.0263689	0.078895688	0.1351529	0.5837513	0.559388	0.948478
nuak1a	244.6564718	0.023293663	0.132837	0.1753552	0.860801	0.985773
psmc2	2033.55498	-0.012228369	0.0749271	-0.163204	0.870358	0.987421
kctd16b	55.74847599	0.047750385	0.2834583	0.1684564	0.866224	0.986673
usp2a	1194.136615	0.214505096	0.0823495	2.6048134	0.009192	0.160717
slc20a1a	504.6791031	0.281986049	0.1210063	2.3303415	0.019788	0.265102
endou2	531.9326915	-0.379577292	0.1133948	-3.347395	0.000816	0.026229
coq8aa	550.2667611	-0.315063667	0.1024157	-3.076322	0.002096	0.055328
fnbp1l	729.4891917	-0.090410223	0.0939327	-0.9625	0.335798	0.876088
jdp2b	146.6829836	0.192149484	0.2232071	0.8608575	0.389317	0.896407
sipa1l1	688.4969423	0.024509534	0.1020559	0.2401579	0.810208	0.980841
ptges	89.93529103	-0.065316921	0.2074816	-0.314808	0.752907	0.97481
pah	538.4110039	0.073892372	0.1139178	0.6486465	0.516567	0.938543
acox1	11.52221415	-0.193720364	0.5564355	-0.348145	0.727731	0.970402
spag6	51.45062658	0.063176172	0.2835666	0.2227913	0.823698	0.982196
efnb2a	1649.17957	-0.015396233	0.0818905	-0.18801	0.850869	0.985174
zbtb22a	118.3020484	-0.119014631	0.2023207	-0.588247	0.556366	0.947918
nova1	614.0753952	0.058244662	0.1012166	0.5754457	0.56499	0.948739
GCA	910.7603365	-0.059549085	0.1048625	-0.567878	0.570118	0.948983
ppef1	10.13662262	0.237141342	0.6121672	0.38738	0.698475	NA
rpl5a	17181.36955	-0.168691006	0.0891718	-1.891754	0.058524	0.473924
cdc73	666.6613898	-0.165828206	0.1029811	-1.610278	0.107337	0.617344
nf2a	72.50640967	0.187806525	0.2442452	0.7689261	0.441937	0.916336
slc1a1	576.3272121	-0.276115826	0.1085819	-2.542926	0.010993	0.181902
cpsf4	352.0012493	-0.053972146	0.1229348	-0.439031	0.660639	0.963881
amfra	1084.466395	0.056654247	0.0827996	0.6842334	0.493828	0.931926
dld	316.858357	-0.199868745	0.1207911	-1.654664	0.097993	0.59749
si:ch211-225b10.3	51.05495424	0.202402177	0.279352	0.7245418	0.468733	0.924328
usf2	1196.767698	0.078766793	0.0843646	0.9336473	0.350486	0.880195
mapre3a	116.9987514	0.033303864	0.1886331	0.1765536	0.859859	0.985773
eif6	771.2297354	-0.046041555	0.0984726	-0.467557	0.640101	0.959994
sept9a	977.8343745	-0.061982664	0.0872228	-0.710625	0.477317	0.926857
mc1r	18.90125124	0.811858552	0.4767982	1.7027298	0.088619	0.569498
lpin1	809.5786767	0.014774849	0.1062605	0.1390437	0.889416	0.989291
icmt	427.3113119	0.08396929	0.1148513	0.7311134	0.46471	0.923535
fermt2	25.24797809	0.222556746	0.3941661	0.5646268	0.572328	0.949494
rhoj	27.60034037	-0.523805333	0.3660063	-1.431138	0.152391	0.701601
btbd10b	886.1939549	-0.048665506	0.0900324	-0.540533	0.588829	0.953345

ewsr1a	7839.529174	0.006127306	0.0680918	0.0899859	0.928298	0.992702
rab2a	4498.161512	0.035880739	0.0671972	0.5339622	0.593368	0.954434
angptl6	38.55077196	-0.024808701	0.3203413	-0.077445	0.93827	0.99279
si:dkeyp-120h9.1	359.5784824	0.132039606	0.1254851	1.0522333	0.292693	0.848607
efcab7	223.2863314	-0.249098495	0.1416921	-1.758026	0.078743	0.546914
si:ch211-278j3.3	381.3990272	0.286166312	0.1156563	2.4742823	0.01335	0.207377
pif1	89.95875323	0.063888353	0.2079745	0.3071933	0.758696	0.975374
TAFA3	301.6120377	0.082786827	0.1288777	0.6423672	0.520635	0.939056
btg2	1390.670568	0.241689887	0.1149632	2.1023243	0.035525	0.367016
os9	685.345171	0.00073767	0.0972403	0.007586	0.993947	0.998694
cnih1	1380.548002	0.12493978	0.0791297	1.5789236	0.114354	0.633752
tyk2	132.7308419	-0.497800028	0.1864676	-2.669633	0.007593	0.138645
plekha6	1253.137211	-0.112012269	0.0816461	-1.371925	0.170087	0.727199
nkx2.9	7.400077252	1.13043017	0.7267868	1.5553807	0.119855	NA
ptpn11a	822.2346163	-0.097014597	0.0912279	-1.063431	0.287587	0.845979
clasp2	4010.773182	0.003512796	0.0691482	0.050801	0.959484	0.995187
lmx1a	10.73004729	-0.656043543	0.5956094	-1.101466	0.270694	NA
snrnp25	96.77149344	-0.284639269	0.2561942	-1.111029	0.266556	0.832634
fbp1b	890.478417	-0.011861912	0.0893149	-0.13281	0.894344	0.989689
g6pc3	99.60531399	0.170979878	0.2040457	0.8379491	0.402059	0.90084
eif4g2a	3905.853127	0.190194692	0.0690188	2.7556951	0.005857	0.115121
nipsnap3a	180.9393473	-0.300135167	0.1572253	-1.908949	0.056269	0.465438
mical2b	361.1450876	-0.065645632	0.1165027	-0.563469	0.573116	0.949762
snx15	195.5338424	-0.105657962	0.1464995	-0.721217	0.470776	0.924838
napab	721.0057521	-0.056219496	0.105227	-0.534269	0.593156	0.954434
emx3	147.2698949	-0.167819412	0.1708377	-0.982333	0.325936	0.871896
nceh1a	1.140109671	0.438235627	1.7543529	0.249799	0.802743	NA
snx5	560.5115339	0.098721149	0.1060337	0.9310352	0.351835	0.88081
xkr6a	3.8268738	0.914323011	1.0801868	0.846449	0.397302	NA
tmtc4	440.9475403	0.060312361	0.1182291	0.5101312	0.60996	0.95688
adi1	242.0789127	-0.112632194	0.1553924	-0.724825	0.46856	0.924328
yipf3	1132.336322	0.047739829	0.0885759	0.5389708	0.589907	0.953762
cacng8a	489.7257403	0.180622805	0.1120481	1.6120108	0.10696	0.616444
psmd8	1958.177186	0.274786711	0.0963026	2.8533688	0.004326	0.092876
si:ch211-171h4.3	26.61337541	0.822513687	0.3715821	2.2135452	0.02686	0.315961
ewsr1b	4178.720793	-0.159716471	0.0711195	-2.245747	0.02472	0.302486
safb	2320.614576	-0.020917992	0.0769589	-0.271807	0.78577	0.97627
map3k7	840.4790606	0.109322255	0.0908784	1.2029511	0.228995	0.796691
hddc3	178.797863	-0.278577303	0.1747619	-1.594039	0.110927	0.626294
znrf1	529.9439509	-0.049508725	0.1035432	-0.478146	0.632547	0.957356
nono	2607.457145	0.027209458	0.0754911	0.3604325	0.718524	0.96909
lta	0.982922306	1.012515237	1.9057885	0.5312842	0.595222	NA
arhgap17a	70.02410173	-0.17290078	0.2330517	-0.741899	0.458149	0.921978
lgi1a	869.6440032	-0.043668248	0.087789	-0.497423	0.618891	0.957354
znf330	453.5463092	-0.104523394	0.1100664	-0.94964	0.342295	0.878434
rab7a	1667.205203	-0.153158715	0.0862238	-1.776293	0.075685	0.535835
h3f3a	7421.987477	-0.059717777	0.0819807	-0.728437	0.466346	0.923908
srd5a1	11.43522723	-0.509519608	0.6034013	-0.844412	0.398439	0.900324
exoc6	639.9436859	0.013459944	0.0957528	0.1405698	0.88821	0.989291
nup62l	790.8673947	-0.167535922	0.0909085	-1.842907	0.065343	0.500198
hacl1	193.2929886	-0.203063897	0.1501535	-1.352375	0.176255	0.736614
ism1	510.3461752	0.233470553	0.104824	2.2272619	0.02593	0.311101
rem1	180.5439625	0.25197943	0.1576944	1.5978967	0.110066	0.624518
si:dkey-77f5.10	0.8136773	-1.876897407	2.1384966	-0.877671	0.380122	NA

ddx3xa	2912.447633	0.172989527	0.069708	2.4816316	0.013078	0.204434
atp2a1	77184.51976	0.263918211	0.1912895	1.3796797	0.167685	0.724848
otofb	397.0347806	0.116610299	0.1205376	0.9674184	0.333335	0.874926
txlnba	677.9394561	0.360180983	0.0948411	3.7977299	0.000146	0.006653
gnl3l	424.4884319	0.071033066	0.1201929	0.590992	0.554526	0.947449
miga1	324.7371805	-0.025443254	0.1223079	-0.208026	0.835208	0.984089
rnf144aa	14.19882396	-0.023408976	0.5236702	-0.044702	0.964345	0.995927
grk7a	2686.525099	0.505605881	0.0942345	5.3653999	8.08E-08	1.15E-05
nfe2	20.48399843	0.152661154	0.4399043	0.3470326	0.728567	0.970604
emc3	1556.190753	0.10081117	0.0788626	1.2783136	0.201139	0.766855
snap25a	6045.341569	0.163267067	0.0732069	2.2302132	0.025733	0.310555
tnnt2a	235.8497578	-0.104248104	0.1604124	-0.649876	0.515773	0.9377
si:ch211-160d20.3	83.63130091	-0.022603944	0.219673	-0.102898	0.918044	0.991623
gatd3a	252.8493248	0.027095689	0.1398662	0.1937258	0.846391	0.985174
ap1b1	1738.454047	-0.010785094	0.0753758	-0.143084	0.886224	0.988936
baxa	509.7116622	-0.176745054	0.1093444	-1.616408	0.106006	0.615567
jak1	1410.457412	-0.097000013	0.080346	-1.207279	0.227325	0.794617
gtdc1	107.089662	-0.112496588	0.1996941	-0.563345	0.5732	0.949762
slc7a3a	3737.664549	-0.027021761	0.078017	-0.346358	0.729074	0.970822
trpc4apb	341.9176517	-0.177942795	0.1298075	-1.370821	0.170431	0.728041
INSM2	130.5233677	0.131025675	0.2002806	0.6542106	0.512976	0.936253
prkg1a	37.96355875	0.369570067	0.3142765	1.1759391	0.239619	0.808091
dpp3	1235.73446	-0.104077766	0.0876503	-1.18742	0.235062	0.803894
clpp	635.2289569	-0.087295327	0.1039071	-0.840129	0.400836	0.90084
sesn1	2139.345297	-0.053413952	0.0813589	-0.656522	0.511488	0.936253
slc9a8	276.8948894	0.065632001	0.1292465	0.5078048	0.61159	0.95688
odf2b	91.21119911	0.200925471	0.2155184	0.9322891	0.351187	0.880484
mdkb	7299.53471	0.032110553	0.0707243	0.4540246	0.649811	0.960708
rrm2	645.7740664	-1.320746883	0.1047104	-12.61333	1.78E-36	1.18E-32
slc25a22a	14.42242285	-0.263469612	0.5086723	-0.517956	0.604489	0.95651
smpd4	319.8885075	-0.023033298	0.129704	-0.177584	0.85905	0.985773
efcab6	31.56137925	-0.141302048	0.3498578	-0.403884	0.686298	0.966376
zgc:153921	2.585714461	1.122985608	1.2658028	0.8871726	0.374986	NA
fga	3019.979018	0.173543319	0.1012613	1.7138176	0.086562	0.563981
bdkrb1	22.5676768	-0.607036712	0.4410624	-1.376306	0.168727	0.725919
gfi1aa	28.40959204	-0.120368836	0.3636308	-0.331019	0.74063	0.973154
caprin2	21.36112093	0.570022656	0.4727063	1.2058707	0.227867	0.794963
tmem178	324.4877492	0.182264546	0.1269603	1.4356028	0.151115	0.699147
elf1	713.8391147	-0.138140224	0.0929769	-1.485748	0.137346	0.674358
arrdc2	2247.193105	-0.135036691	0.0730888	-1.847571	0.064664	0.497355
slmapb	601.7204091	0.094476121	0.1017979	0.9280751	0.353369	0.881808
tnr	161.3917942	0.335611515	0.1720768	1.9503593	0.051133	0.443542
cep85l	377.4753002	0.120283879	0.1207394	0.9962274	0.31914	0.867746
lama4	2035.145579	0.140595243	0.0763314	1.8419058	0.065489	0.500378
sla2	7.695958813	-0.481497479	0.7000658	-0.687789	0.491586	NA
neurod6b	922.558794	-0.276451469	0.0923525	-2.993439	0.002759	0.067771
rac3b	2079.957068	0.081723084	0.0843226	0.9691715	0.33246	0.874653
necap1	1111.828847	0.080006976	0.0825732	0.9689222	0.332584	0.874653
efemp2b	148.521814	-0.037729034	0.1715725	-0.219901	0.825948	0.982196
si:ch73-177h5.2	1.966872208	-1.561998219	1.4395963	-1.085025	0.277911	NA
faimb	172.1157396	0.226197008	0.1618935	1.3971965	0.162354	0.716781
srp68	1478.929106	-0.042014833	0.083932	-0.500582	0.616666	0.95688
ift22	63.12462956	-0.112249284	0.2566648	-0.437338	0.661866	0.964504
si:dkey-230p4.1	90.44209892	-0.172980829	0.2079569	-0.831811	0.405516	0.901305

mcrs1	570.4592097	-0.007932684	0.1001499	-0.079208	0.936867	0.992702
rbm7	303.3147886	0.033273561	0.129279	0.2573779	0.796887	0.978569
tns1b	58.86728467	0.017437647	0.26236	0.0664646	0.947008	0.99381
atp6v0a1a	1742.145752	0.183337651	0.0896967	2.0439732	0.040956	0.395057
eef1a1l1	99614.27981	-0.256754882	0.0742918	-3.456034	0.000548	0.019137
galr2a	2.29303713	0.062133857	1.3334868	0.046595	0.962836	NA
ccdc149b	311.3542591	0.067686737	0.1312274	0.5157972	0.605996	0.956686
trim25l	57.88481865	-0.240280955	0.2569269	-0.935212	0.349679	0.880195
apoa4b.2	1312.626177	0.271035487	0.1251724	2.1652979	0.030365	0.336756
ptprz1b	1475.071221	0.035758345	0.0773106	0.4625284	0.643702	0.960014
nfat5a	24.71151977	-0.38378858	0.4088227	-0.938765	0.347851	0.880195
pdk2a	1664.328376	-0.198911424	0.0881795	-2.255757	0.024086	0.299543
gli2b	173.8668159	-0.319231924	0.1651668	-1.932785	0.053263	0.452412
armc1l	230.6211794	-0.158640982	0.1374581	-1.154104	0.248457	0.817939
tmod4	1895.35347	0.336366094	0.085178	3.9489806	7.85E-05	0.00399
LO018197.1	179.8352582	-0.043847934	0.1827226	-0.23997	0.810354	0.980867
slc25a55a	2700.410282	0.092628186	0.0793714	1.1670222	0.243201	0.812077
bag1	357.8206555	0.005462549	0.1249921	0.0437031	0.965141	0.996154
cyb5r4	189.1696624	0.10726185	0.1493612	0.7181373	0.472673	0.924838
gabrp	349.6602457	0.072370555	0.1192178	0.6070451	0.543821	0.945438
ddx56	378.9656367	0.100640137	0.1154822	0.8714772	0.383494	0.895192
myo1ca	212.0874375	0.245581964	0.1460758	1.6811958	0.092725	0.579684
creb3l3l	1999.066624	0.099020708	0.0811301	1.2205173	0.222269	0.788551
fam49ba	290.6391116	0.039680633	0.1352084	0.2934775	0.769157	0.975687
ezra	1002.960464	-0.506250914	0.0938238	-5.395762	6.82E-08	9.85E-06
auts2b	539.3627579	-0.073290299	0.1072307	-0.683483	0.494302	0.931926
gmcl1	594.7120198	0.038546905	0.10404	0.3705009	0.711009	0.968985
si:ch211-214j8.1	169.1895155	-0.823951573	0.1914917	-4.302805	1.69E-05	0.001088
dnajb6b	584.821678	0.106152477	0.1019016	1.0417154	0.297544	0.85401
pck2	2234.3437	-0.022769717	0.0777424	-0.292887	0.769609	0.975687
pth1ra	2.65892339	-1.628192357	1.2695507	-1.282495	0.199669	NA
hgs	680.4160355	-0.022863022	0.1056222	-0.21646	0.828629	0.982721
thap12b	185.4100213	0.04795156	0.15293	0.3135524	0.753861	0.975056
ubald1b	651.4293723	-0.420273306	0.1097836	-3.828199	0.000129	0.006006
ripor3	149.9912644	0.122501349	0.1780682	0.6879461	0.491487	0.931926
slc35a3b	50.62716857	-0.251458006	0.2887593	-0.870822	0.383851	0.895192
prickle2a	487.7884991	-0.001141766	0.1078285	-0.010589	0.991552	0.997679
slc16a10	748.4121467	-0.205750037	0.0940429	-2.187832	0.028682	0.328405
ctu1	105.646918	-0.477945501	0.1975538	-2.419318	0.01555	0.228768
c5	546.4725794	0.163382349	0.1247146	1.3100499	0.190179	0.754837
trim35-28	271.2714682	-0.002161444	0.1309074	-0.016511	0.986827	0.997062
ift57	141.0738695	-0.191708487	0.1721648	-1.113517	0.265486	0.831302
foxd3	257.853352	-0.024050308	0.1387668	-0.173315	0.862404	0.985841
herpud2	472.9717583	-0.093936355	0.1232981	-0.761864	0.446141	0.917154
rhbdd1	118.5280001	-0.089591708	0.1854617	-0.483074	0.629043	0.957354
clptm1l	659.9101406	-0.077016709	0.1040788	-0.739985	0.459309	0.921978
isl1l	6.66172181	-0.188326266	0.8221694	-0.22906	0.818822	NA
alas1	1680.928754	-0.269886379	0.0886227	-3.04534	0.002324	0.059686
sec31a	844.0144279	0.200044149	0.0937542	2.1337077	0.032867	0.35321
znf367	130.275643	0.133081776	0.1968427	0.6760819	0.498989	0.934006
nelfcd	417.1266291	-0.20885132	0.125605	-1.662763	0.09636	0.59086
sf3a2	974.9146787	-0.070945003	0.0892619	-0.794796	0.426732	0.910217
poc1b	144.2969689	-0.313237154	0.1695497	-1.847465	0.06468	0.497355
c1d	169.2292447	0.09967091	0.1708288	0.583455	0.559587	0.948478

ptmaa	13180.14093	-0.055702319	0.0658914	-0.845365	0.397907	0.899672
spata6	37.00745626	-0.144340888	0.3364157	-0.429055	0.667883	0.964736
ndc1	561.9746341	-0.07980113	0.1118624	-0.713387	0.475607	0.92611
cfl1	13824.47689	-0.144540267	0.0653636	-2.211325	0.027013	0.316822
dhrs4	366.3919878	-0.427046576	0.1306345	-3.269017	0.001079	0.032882
adgrv1	737.3860101	0.047916266	0.0947958	0.505468	0.61323	0.95688
pabpc1b	1881.893848	0.071589784	0.0912293	0.7847233	0.432616	0.91326
plekhf2	225.7073733	0.146523639	0.1438467	1.0186094	0.308388	0.861088
rtn1b	2856.096497	-0.144712006	0.0838028	-1.726816	0.084201	0.55797
nipal3	554.207482	-0.006064953	0.0996357	-0.060871	0.951462	0.99381
cbr1l	551.6301039	-0.406622783	0.1054918	-3.854544	0.000116	0.005551
valopa	9.501739881	0.303172457	0.6204921	0.4886	0.625125	NA
ptpreb	686.4280922	-0.043654043	0.0976196	-0.447185	0.654741	0.961305
zgc:92040	206.6806189	0.261506539	0.1562965	1.6731438	0.094299	0.583986
thrb	543.1558348	0.025465708	0.1041042	0.2446176	0.806753	0.980254
cyp2ad2	72.07212537	0.85678206	0.271396	3.1569447	0.001594	0.045166
cept1b	282.8719739	0.293703455	0.1434687	2.0471601	0.040642	0.394726
rbfox1l	875.2816777	0.256438346	0.0969141	2.6460387	0.008144	0.14587
si:dkeyp-4h4.1	10.75533943	-0.28813493	0.6394149	-0.450623	0.652261	NA
coro1cb	2630.616502	0.099552409	0.0820674	1.2130571	0.225108	0.792185
st8sia6	567.6617409	0.190485868	0.1031696	1.8463372	0.064843	0.497901
nebl	18.93916446	0.156398315	0.4492322	0.3481458	0.727731	0.970402
noto	9.966889318	1.113162095	0.6628075	1.6794652	0.093061	NA
serpind1	120.3527987	0.237063433	0.1883217	1.2588218	0.208095	0.775404
kctd9a	12.26460366	-0.331464995	0.558295	-0.593709	0.552706	0.947265
hmgcl	671.2654326	-0.09888686	0.0935744	-1.056773	0.290615	0.846337
prkci	1367.400951	-0.035658224	0.0808468	-0.441059	0.65917	0.962839
nkx2.7	17.95832185	-0.716449643	0.4611488	-1.553619	0.120275	0.645215
pgls	214.3827963	-0.074448496	0.1535756	-0.484768	0.627841	0.957354
apaf1	659.9318646	-0.152416059	0.0981237	-1.553305	0.12035	0.645215
zgc:165604	146.7967539	0.134958772	0.1757921	0.7677181	0.442655	0.916336
mvp	2392.827685	-0.48264575	0.0943734	-5.114213	3.15E-07	3.75E-05
slc25a48	96.89948548	0.022496838	0.2018295	0.1114646	0.911248	0.991066
cct6a	5386.317318	-0.103065486	0.0781578	-1.318685	0.187275	0.751302
arhgap22	38.70317285	-0.200744018	0.311007	-0.645465	0.518626	0.938803
eif3d	5209.160857	-0.113561683	0.0825285	-1.37603	0.168812	0.725919
mybpc2b	1553.093635	0.068670572	0.094155	0.7293351	0.465797	0.923865
rab7b	238.201374	-0.077097983	0.1375576	-0.560478	0.575154	0.950291
mier3b	115.1927183	-0.155479272	0.210726	-0.737827	0.46062	0.921978
slc30a2	7.203950818	-0.641270173	0.7593474	-0.844502	0.398389	NA
rhoca	590.1924791	-0.019203805	0.1078486	-0.178063	0.858674	0.985773
nsun4	44.94208633	-0.003040336	0.2848879	-0.010672	0.991485	0.997679
pax4	8.152861173	-1.610958585	0.7000581	-2.301178	0.021382	NA
cpa5	1133.033094	-0.1530627	0.1076832	-1.421417	0.155196	0.704997
wsb1	3235.250278	-0.194252553	0.0874388	-2.221582	0.026312	0.312554
prph2lb	122.0242811	0.319646012	0.1842316	1.7350228	0.082737	0.554784
pdhb	4061.647684	0.048366725	0.0653292	0.7403544	0.459085	0.921978
nefma	1540.314413	0.032847315	0.088813	0.369848	0.711496	0.969006
gria1a	759.4152487	0.039720804	0.0999539	0.3973912	0.691079	0.967256
fbp1a	395.5222577	-0.206229907	0.1282065	-1.608576	0.107709	0.6186
mc3r	4.841135029	0.293851338	0.8663717	0.3391747	0.734478	NA
actr6	387.4111413	0.059579904	0.1139902	0.5226757	0.6012	0.956045
tob1b	2419.192164	0.140387589	0.0811527	1.7299188	0.083645	0.557158
cab39l1	611.0910891	0.215827304	0.0959031	2.2504732	0.024419	0.3019

phf21ab	186.0490677	-0.076583829	0.1499101	-0.510865	0.609446	0.956718
ppm1db	281.8231175	-0.050148956	0.1257789	-0.398707	0.690109	0.966857
naalad2	273.8553001	0.169351762	0.12847	1.3182205	0.18743	0.75162
jag2b	678.5283277	0.024442212	0.0983475	0.2485291	0.803725	0.979643
chrnb5b	32.1325613	0.286624999	0.3415312	0.8392353	0.401337	0.90084
mul1a	232.245461	-0.136212755	0.1386774	-0.982228	0.325988	0.871917
yipf2	671.3427743	-0.165676112	0.0996496	-1.662587	0.096395	0.59086
mcm3ap	716.4618689	0.063923258	0.0994945	0.6424801	0.520562	0.939056
zgc:110319	378.9969989	-0.034073333	0.1162425	-0.293123	0.769428	0.975687
chd3	560.2351185	0.01192552	0.1100716	0.1083433	0.913723	0.991119
poglut3	243.9746587	0.266028421	0.1475688	1.8027417	0.071429	0.522931
odf3l2b	4.342931339	0.011093838	0.9081496	0.0122159	0.990253	NA
asmtl	319.272351	0.229780097	0.1285205	1.7878862	0.073794	0.529749
ncoa4	1976.582337	-0.023775485	0.0760594	-0.312591	0.754591	0.975114
cdh11	2538.523837	0.110155494	0.071887	1.5323414	0.125438	0.654803
zfp36l1b	1701.781954	-0.092945321	0.0847579	-1.096598	0.272817	0.838096
prf1.2	0	NA	NA	NA	NA	NA
strbp	722.1948346	-0.026390828	0.0922134	-0.286193	0.77473	0.975687
daw1	56.9149797	0.474423399	0.2816248	1.6845939	0.092067	0.57849
ireb2	906.0867023	-0.146864508	0.0907295	-1.618707	0.10551	0.614178
olfml2bb	120.322858	0.612651092	0.1834919	3.338846	0.000841	0.026962
cdc14b	810.2118762	-0.060665114	0.0888805	-0.682547	0.494893	0.931926
cyth1b	329.2039073	-0.080960345	0.1297745	-0.623854	0.532723	0.942805
hnf4a	213.148799	-0.574580512	0.1509143	-3.80733	0.00014	0.00646
gpr132a	0.157187365	-0.955901296	4.0804729	-0.234262	0.814781	NA
tab2	391.1422363	0.002988925	0.115495	0.0258793	0.979354	0.996847
rgs14a	8.068632858	0.417687586	0.6696232	0.6237651	0.532782	NA
ephb2b	945.2120986	-0.04863448	0.086765	-0.560531	0.575117	0.950291
vrk2	46.06732032	-0.262432351	0.2944946	-0.891128	0.372861	0.892868
rad23b	2292.586492	-0.180492963	0.0747851	-2.413488	0.015801	0.231267
prkn	49.09465593	0.210890162	0.287338	0.7339446	0.462983	0.922698
ubl3b	1523.558996	-0.018543581	0.0789646	-0.234834	0.814337	0.981081
wdr45b	657.2498731	-0.059687204	0.1094507	-0.545334	0.585524	0.95262
OTOP1	28.64298939	0.152596386	0.3542029	0.4308164	0.666602	0.964736
gdap2	344.0815631	-0.04089149	0.1305975	-0.313111	0.754197	0.975056
cgas	3.517623225	-0.67208193	1.1053986	-0.608	0.543188	NA
slc16a7	50.11343731	-0.1069979	0.2690153	-0.397739	0.690823	0.967157
orc5	127.9111206	-0.278445435	0.1971312	-1.412488	0.157806	0.709035
dynlrb1	142.7554402	0.230004263	0.1682263	1.367231	0.171553	0.729995
cntn5	718.1970989	0.0844071	0.1038837	0.8125153	0.416496	0.906237
tmem60	139.5660496	0.050190875	0.1758373	0.2854392	0.775308	0.975687
magi2a	180.1983432	-0.012609655	0.151123	-0.08344	0.933502	0.992702
lhfpl3	1137.923885	0.137551871	0.082721	1.662841	0.096344	0.59086
negr1	354.8189588	-0.000333469	0.1175947	-0.002836	0.997737	0.999174
si:dkey-151g10.3	156.4112263	-0.056335212	0.1695293	-0.332304	0.73966	0.972943
sh3bgr	547.7031702	0.356276791	0.110866	3.2135804	0.001311	0.038289
gnai1	1678.272644	-0.003297107	0.0774918	-0.042548	0.966062	0.996315
fzd3a	740.8902402	0.092781551	0.0975092	0.9515157	0.341343	0.878134
sec61a1	5037.797532	-0.020954661	0.0816678	-0.256584	0.7975	0.978577
phf11	10.13646222	0.253878448	0.595237	0.4265166	0.669731	NA
asrgl1	138.544076	-0.432407592	0.17875	-2.419064	0.015561	0.228768
mxa	4.327757656	1.177407368	0.9204148	1.2792138	0.200822	NA
morn3	43.83924783	0.13884457	0.3110976	0.4463055	0.655377	0.961632
pdcd4a	1849.758768	-0.200381438	0.0849185	-2.359692	0.01829	0.251621

col7a1	1119.850582	0.153560827	0.0964965	1.5913615	0.111528	0.627242
cacna1ba	141.0644101	0.07122905	0.1766082	0.4033168	0.686715	0.966376
klhl24a	106.0417267	0.150655141	0.1969493	0.7649439	0.444305	0.916726
ccdc25	356.136229	0.110987895	0.1230832	0.9017307	0.3672	0.887458
abcb5	1899.973233	0.251909163	0.0871151	2.8916805	0.003832	0.084661
myoc	517.8189023	0.480876868	0.1060738	4.5334198	5.80E-06	0.000452
hmmr	424.9667767	-0.126563839	0.1087544	-1.163758	0.244522	0.814189
tle5	70.67007951	-0.156942219	0.2933935	-0.534921	0.592705	0.954434
zfp36l2	1185.111008	0.001814903	0.0987739	0.0183743	0.98534	0.996944
nmt2	745.1873821	0.032544963	0.0935695	0.347816	0.727978	0.970402
calm1a	9608.299742	0.028391583	0.0652975	0.4348032	0.663705	0.964736
cherp	801.1436894	-0.142205913	0.1052151	-1.351573	0.176512	0.736982
katna1	106.756016	-0.25744667	0.2010867	-1.280277	0.200448	0.766801
ahr2	1388.681022	-0.107299321	0.0801085	-1.339426	0.180432	0.741452
rps23	7160.378549	-0.465952403	0.079233	-5.880788	4.08E-09	7.41E-07
hid1a	72.11304554	0.01594746	0.2396517	0.0665443	0.946944	0.99381
si:dkey-256h2.1	27.73697839	-0.022498284	0.3875957	-0.058046	0.953712	0.994213
ntpcr	73.95172765	0.22125551	0.2345169	0.9434521	0.34545	0.878434
arnt	186.9309046	-0.052105456	0.1630046	-0.319656	0.749229	0.97419
erap1b	72.40212616	-0.431785407	0.2479051	-1.741737	0.081555	0.552315
rplp1	17731.66107	-0.462839068	0.0929501	-4.979438	6.38E-07	6.85E-05
upk1a	8.359966201	-0.007345468	0.6829795	-0.010755	0.991419	NA
rcan2	138.5337471	0.13182334	0.1698452	0.7761382	0.437667	0.914942
fbxl3b	199.6016889	0.085902665	0.1628174	0.5276014	0.597776	0.95534
cacul1	5.54116887	-0.786313	0.8194644	-0.959545	0.337284	NA
gja3	90.51578259	0.192341371	0.2303003	0.8351765	0.403618	0.900979
zbtb7b	188.8694017	-0.222713237	0.1682674	-1.323568	0.185647	0.749638
disc1	10.76532075	0.319144904	0.5693946	0.5604987	0.575139	NA
asap3	388.9925205	-0.171362204	0.1182991	-1.44855	0.147463	0.694625
b4galt7	191.9193453	0.461681715	0.159842	2.8883629	0.003873	0.085098
derl1	1053.433308	0.074570344	0.0862154	0.8649309	0.387077	0.895735
VSTM2A	2.25145806	-0.856143254	1.3297808	-0.643823	0.51969	NA
ak9	38.4180825	-0.612606997	0.3243343	-1.888813	0.058917	0.475038
vax1	251.5173752	-0.05972513	0.1311775	-0.4553	0.648893	0.960708
hsp70.3	1287.925253	-0.099335748	0.3314351	-0.299714	0.764395	0.975374
smad9	178.3044644	0.202292848	0.1546573	1.3080074	0.190871	0.755212
ddx23	1459.173636	-0.018109048	0.0854241	-0.21199	0.832115	0.983517
tnc	3511.660359	0.309402444	0.0807307	3.8325261	0.000127	0.005957
zgc:101100	91.91728671	0.047952953	0.2108553	0.2274211	0.820096	0.981594
stl3	2237.700193	-0.141443887	0.0740602	-1.909849	0.056153	0.464781
sfr1	19.64908433	-0.210379249	0.4365348	-0.48193	0.629856	0.957354
ginm1	1146.777161	-0.127881768	0.089601	-1.427236	0.153512	0.702578
mical3a	166.8368802	-0.133373591	0.1715762	-0.777343	0.436956	0.914408
sh3bp2	4.300095255	0.009458212	0.9137502	0.010351	0.991741	NA
ndufa2	1007.498975	-0.045228973	0.1107124	-0.408527	0.682887	0.965476
ercc2	248.6889711	-0.158773503	0.137883	-1.151509	0.249523	0.819204
plecb	2238.67227	0.148063831	0.0742782	1.9933696	0.046221	0.422549
erlin1	2016.516598	-0.060878033	0.0714617	-0.851897	0.394271	0.898711
ppp2r2aa	64.50879905	0.080044403	0.2449898	0.3267255	0.743876	0.974094
cfap206	10.9955599	0.018555574	0.5726951	0.0324004	0.974153	NA
traf3	255.4251907	-0.339864363	0.1524473	-2.229389	0.025788	0.310555
zgc:66426	9.82132409	0.349255984	0.6053316	0.5769664	0.563962	NA
focad	221.9832898	0.038065373	0.149857	0.2540114	0.799487	0.979366
map1ab	502.2143132	0.19565485	0.1034561	1.8911874	0.058599	0.473924

tlr4bb	0	NA	NA	NA	NA	NA
si:dkey-34e4.1	69.44569629	0.292049056	0.2410526	1.2115572	0.225682	0.793245
opn4a	2.385997333	0.877555654	1.3090177	0.6703925	0.502608	NA
obscnb	181.2612448	0.476946117	0.1550601	3.0758788	0.002099	0.055336
rbm4.3	3454.64453	-0.115046199	0.0814274	-1.412869	0.157694	0.709014
ocstamp	13.58409161	-0.43046686	0.5396388	-0.797694	0.425048	0.908819
ttc23	34.00556268	-0.167527747	0.3317469	-0.504987	0.613568	0.95688
mgst1.2	536.9500984	0.110018247	0.1075919	1.0225515	0.30652	0.860027
IGLON5	426.1911903	0.120141665	0.111273	1.0797017	0.280275	0.843423
ddx59	158.9403834	-0.249979802	0.1653315	-1.511991	0.130536	0.662797
gsto1	61.21547192	-0.356463447	0.2738621	-1.301616	0.193048	0.756795
usp6nl	510.4943244	-0.04552015	0.1070279	-0.425311	0.67061	0.965257
gtf2ird1	815.5682819	-0.049174737	0.0975232	-0.504237	0.614095	0.95688
uck2b	798.6850868	-0.437066	0.1036429	-4.217038	2.48E-05	0.001506
uck1	101.2087859	0.14540395	0.2004876	0.7252516	0.468298	0.924328
ppan	296.1245172	-0.15145243	0.1537573	-0.98501	0.324619	0.871063
prkcbb	903.2441208	0.179738521	0.0902502	1.991558	0.04642	0.423579
si:ch211-57i17.1	119.8344841	-0.181698573	0.1953213	-0.930255	0.352239	0.880896
pdzk1	476.393172	-0.520211187	0.1092416	-4.762026	1.92E-06	0.000175
trim35-34	1.189776963	0.42714694	1.8029205	0.2369195	0.812719	NA
brd2a	1711.511375	0.079014738	0.0881542	0.896324	0.37008	0.889876
hnf1bb	71.09432103	-0.400218043	0.2656941	-1.506312	0.131987	0.665721
higd1a	1276.814421	0.280684012	0.095797	2.9299876	0.00339	0.077847
dspa	1718.889417	-0.293281725	0.0734318	-3.993933	6.50E-05	0.0034
atp6v1g1	4081.224299	-0.070573534	0.0756119	-0.933366	0.350631	0.880195
or115-5	0	NA	NA	NA	NA	NA
si:ch211-156l18.7	0.173729368	-0.955901296	4.0804729	-0.234262	0.814781	NA
ufc1	359.5046509	0.011091019	0.1287511	0.0861431	0.931353	0.992702
nek1	528.0131325	0.327828346	0.1013813	3.2336171	0.001222	0.036292
kng1	548.2977845	0.07712124	0.1149671	0.6708112	0.502341	0.935304
mtmr1b	104.8913631	0.130599935	0.1932902	0.6756675	0.499252	0.934006
ntmt1	467.6726503	0.078914264	0.1045804	0.7545801	0.450501	0.918761
rrp12	1203.698932	-0.056361546	0.0848259	-0.664438	0.50641	0.935724
ing5a	91.65006946	-0.348776502	0.2122626	-1.643137	0.100355	0.602451
faf1	794.2824364	0.024293486	0.0900097	0.2698987	0.787238	0.976596
slc26a5	958.5222572	-0.093113123	0.1023258	-0.909967	0.36284	0.885944
ppp1r8b	662.4950487	0.001850707	0.1098574	0.0168465	0.986559	0.996965
cd81b	344.58305	-0.114942301	0.1187828	-0.967668	0.33321	0.874926
cox6a1	1456.266078	0.031316461	0.1007035	0.3109769	0.755818	0.975374
eno1a	14907.62942	0.062916694	0.0839443	0.749505	0.453553	0.920041
clcn5b	207.0631956	0.13838184	0.1660851	0.8331984	0.404733	0.901305
rad21l1	6.152422007	1.660469865	0.8468956	1.9606547	0.049919	NA
pkd2l1	94.61086254	0.119064496	0.2088087	0.5702086	0.568536	0.948983
cox4i2	690.545095	-0.129928113	0.1140499	-1.139222	0.254611	0.823484
zgc:109913	7.14142241	0.505583639	0.7568262	0.6680314	0.504114	NA
pex5la	15.01752291	-0.152080766	0.5105293	-0.297888	0.765788	0.975374
mchr1b	254.6331886	0.085536076	0.1420084	0.6023311	0.546954	0.945526
ntn1b	885.9760656	0.194632441	0.093426	2.0832784	0.037226	0.377602
chrnb1l	24.846314	-0.434434997	0.4117283	-1.05515	0.291357	0.847
gxylt1b	88.43721161	0.106108371	0.2146692	0.4942878	0.621103	0.957354
clcnk	103.4575028	-0.037486927	0.2031416	-0.184536	0.853593	0.985174
zgc:56106	278.587553	-0.108942829	0.1309988	-0.831632	0.405617	0.901305
nkx6.1	332.7901461	0.019065493	0.1305015	0.146094	0.883847	0.988706
nmur3	32.23873045	0.073504111	0.3626014	0.2027133	0.839359	0.984979

dnpep	541.4855851	-0.117318266	0.1023	-1.146807	0.251462	0.820927
ddx43	80.71007586	-0.19166995	0.2364573	-0.81059	0.417601	0.906237
si:ch211-241e1.3	428.9487452	0.063171981	0.1328034	0.4756803	0.634302	0.95776
fbxo44	265.8206654	-0.31025345	0.1438182	-2.157261	0.030985	0.34077
cyp2p9	191.234088	2.356418971	0.1829016	12.883536	5.57E-38	5.51E-34
otud3	212.0427517	-0.134477812	0.1439057	-0.934485	0.350054	0.880195
cyp2ad3	835.6633001	0.554248152	0.0934209	5.9328073	2.98E-09	5.50E-07
psmf1	231.8024076	-0.144748054	0.1395185	-1.037483	0.299511	0.855609
snx4	802.2842942	0.049978911	0.0980959	0.5094904	0.610408	0.95688
armc2	56.82712117	-0.572884943	0.2760429	-2.075347	0.037954	0.381357
grapb	28.81841488	0.305776665	0.3639109	0.8402515	0.400767	0.90084
he1.3	0	NA	NA	NA	NA	NA
fkbp1aa	9131.614689	-0.0289228	0.0773639	-0.373854	0.708513	0.968664
itgb1b.2	838.1883935	0.417696963	0.0964087	4.3325648	1.47E-05	0.000988
chic2	435.3440877	-0.121256499	0.1147243	-1.056938	0.29054	0.846262
lad1	168.7688204	-0.920611512	0.175914	-5.233303	1.67E-07	2.15E-05
stat3	407.9787597	-0.121113263	0.114368	-1.058979	0.289609	0.846246
casp8ap2	184.2797789	0.119552607	0.1528097	0.7823629	0.434001	0.914001
epha2b	238.102449	0.116814744	0.1629781	0.7167513	0.473528	0.924993
aasdh	26.65507778	0.327943826	0.3899818	0.8409209	0.400392	0.90084
hpcal1	122.0490046	-0.469277405	0.1868925	-2.510948	0.012041	0.193258
apobb.1	28812.56979	0.218656318	0.0846338	2.5835588	0.009779	0.167691
gid8a	456.8077064	-0.020484234	0.1050922	-0.194917	0.845458	0.985174
si:ch211-106a19.1	288.2898302	0.108432899	0.1273426	0.8515052	0.394489	0.898861
cops7a	734.6503665	0.134583126	0.0962151	1.3987737	0.161881	0.716275
golt1ba	68.49949667	0.390586587	0.2396035	1.6301375	0.103072	0.609625
pitpnc1b	127.6219335	-0.010242909	0.1754929	-0.058367	0.953457	0.994213
fzr1b	682.2512237	0.024333187	0.0939173	0.2590916	0.795565	0.978498
dnd1	1.795620083	-1.434666831	1.4695598	-0.976256	0.328938	NA
pvalb3	195.5346197	0.118186265	0.1799182	0.656889	0.511252	0.936253
bnip4	107.5372372	-0.165677173	0.1923239	-0.861449	0.388991	0.896295
ptk2ba	17.3792285	0.417283012	0.4507624	0.9257272	0.354588	0.882105
lias	349.2190548	-0.097480611	0.1165056	-0.836703	0.402759	0.90084
eogt	57.82178337	0.039746427	0.262043	0.151679	0.87944	0.987881
wnt7ba	20.73463297	0.460455543	0.4269648	1.078439	0.280838	0.84357
zgc:175214	887.1239089	-0.201927778	0.1205997	-1.674364	0.094059	0.583414
mcf2a	131.4268241	0.035462091	0.1790428	0.1980649	0.842994	0.985174
skilb	198.0205689	0.201987738	0.147918	1.3655381	0.172084	0.731144
znf1163	29.73704697	-0.402835515	0.3673348	-1.096644	0.272797	0.838096
tbcc	150.1194303	-0.205233928	0.1631948	-1.257601	0.208536	0.775404
taar11	1.348572358	0.736885736	1.7227585	0.427736	0.668843	NA
ino80e	170.2425605	0.13535559	0.1689415	0.8011979	0.423017	0.90714
ecd	203.6769736	0.078044081	0.1507589	0.5176749	0.604685	0.95651
pth2	25.95819211	-0.181534171	0.401029	-0.452671	0.650786	0.960708
zgc:66448	563.0897917	-0.025288454	0.1095292	-0.230883	0.817406	0.981594
fxr1	1229.241848	0.047018644	0.0912616	0.5152073	0.606408	0.956718
tm9sf1	523.7772625	0.089454434	0.1048406	0.8532426	0.393525	0.898076
epha6	448.7771508	0.126144587	0.1286848	0.9802602	0.326958	0.872658
wdr13	645.6131635	0.032427896	0.0987049	0.3285337	0.742508	0.973232
tmpob	581.139929	0.129268805	0.1084193	1.1923039	0.233142	0.801623
ccdc28a	13.37910249	-0.247072977	0.5183256	-0.476675	0.633593	0.957703
clic4	1720.253942	-0.10279003	0.083941	-1.224552	0.220744	0.787641
setx	256.7644879	-0.003066325	0.1412199	-0.021713	0.982677	0.996944
dtl	101.7096902	0.008481648	0.2062652	0.0411201	0.9672	0.996315

ccdc12	162.5802546	-0.063431391	0.1717411	-0.369343	0.711872	0.969006
pkp2	145.818216	0.040173134	0.1710106	0.234916	0.814274	0.981081
cdv3	1899.613465	0.050084709	0.0732442	0.6838041	0.494099	0.931926
hoxa2b	144.3328985	-0.049096475	0.1669192	-0.294133	0.768656	0.975687
si:dkey-23a23.2	207.3018685	-0.142586862	0.1461876	-0.975369	0.329377	0.873436
zbtb1	212.6994324	0.041541627	0.1487561	0.27926	0.780045	0.976146
fam129ab	17.66004569	-0.028191676	0.4718787	-0.059743	0.95236	0.993904
foxo3a	2655.58222	0.041919831	0.0733461	0.5715342	0.567638	0.948739
ccn1	838.2437846	0.145946327	0.1021496	1.4287512	0.153076	0.702423
krt1-19d	1179.675248	-0.434603579	0.1004304	-4.327409	1.51E-05	0.001002
scaper	658.1962669	-0.136871749	0.0946001	-1.446846	0.14794	0.695523
mapk7	349.4698429	-0.246351117	0.1229105	-2.004313	0.045037	0.417316
plg	1826.71451	-0.229332618	0.1219156	-1.881076	0.059962	0.478654
gopc	170.1937785	0.017097996	0.1603463	0.1066317	0.915081	0.991123
ucp1	457.2516193	0.320843055	0.1141461	2.8108096	0.004942	0.102322
wdr73	80.37731062	-0.185168911	0.22649	-0.817559	0.413609	0.905864
cbln10	51.73802035	0.326637327	0.3089642	1.0572012	0.29042	0.846262
snrnp40	385.8259482	-0.071052567	0.1151524	-0.617031	0.537214	0.943336
v2rx4	0.63357355	1.435201493	2.6108194	0.5497131	0.582516	NA
fez1	3446.537617	0.027553174	0.0749623	0.3675605	0.713201	0.96909
tdo2b	26.04080314	-1.369920118	0.4032907	-3.396856	0.000682	0.02273
pcp4l1	395.3516554	-0.021294081	0.1142498	-0.186382	0.852145	0.985174
si:rp71-1g18.13	29.8858943	-0.277935255	0.3564492	-0.779733	0.435548	0.914408
lcp1	171.964323	0.138467515	0.1833659	0.7551433	0.450163	0.918761
kpna4	2931.625083	-0.014115396	0.0699019	-0.201932	0.83997	0.985029
mhc1lda	1.186560904	-1.24155368	1.8444777	-0.673119	0.500871	NA
mob2a	251.1716279	-0.082530138	0.1524553	-0.54134	0.588273	0.953319
crema	328.9936355	0.148150669	0.134418	1.102164	0.27039	0.835161
selenot2	1697.409956	0.051862846	0.0815667	0.6358333	0.524885	0.939846
vsnl1a	3112.957308	0.258190063	0.0807671	3.1967219	0.00139	0.040243
kcnh1b	13.88243659	-0.030425623	0.5177712	-0.058763	0.953141	0.994157
syne3	465.419999	0.077886117	0.108428	0.7183211	0.472559	0.924838
atad1a	109.2261396	-0.10790971	0.2005503	-0.538068	0.59053	0.954193
tfap2d	254.8963036	0.018380403	0.1364192	0.1347347	0.892822	0.989291
psmd4b	1064.493925	-0.044009026	0.0856769	-0.513663	0.607488	0.956718
hsd17b3	16.42902313	-0.335129282	0.4932159	-0.679478	0.496835	0.932829
fabp3	3196.991508	-0.029080534	0.0808407	-0.359726	0.719052	0.969183
rps27.1	7760.874451	-0.357326798	0.07582	-4.712833	2.44E-06	0.000216
snu13b	812.9173166	-0.098893607	0.0973881	-1.015459	0.309887	0.861992
gpbar1	2.834371097	-0.188591653	1.1411324	-0.165267	0.868734	NA
zgc:66427	619.0885268	0.177073438	0.0982493	1.8022863	0.0715	0.522931
spryd4	288.3048453	-0.112610961	0.1308666	-0.860502	0.389513	0.896421
sim1a	147.5354155	-0.186271537	0.1732087	-1.075417	0.282188	0.843666
fmn2a	217.6731566	0.042111137	0.1453822	0.2896581	0.772078	0.975687
ywhaqb	9926.79484	-0.019250763	0.0747948	-0.257381	0.796885	0.978569
anp32b	4424.044153	-0.132228464	0.0845363	-1.564162	0.11778	0.641446
nr5a1b	5.592287053	0.126250285	0.8228191	0.1534363	0.878054	NA
mxd	4.244197523	-0.905085036	1.0393549	-0.870814	0.383856	NA
slc37a2	1117.598922	-0.06934003	0.083655	-0.828881	0.407172	0.901305
atg5	372.700968	-0.02400373	0.1226943	-0.195639	0.844893	0.985174
tjp2b	936.5308658	-0.101951126	0.1067387	-0.955147	0.339503	0.87805
atp2b3b	1616.964634	0.178019164	0.0929333	1.9155582	0.055421	0.461238
galnt14	313.3617165	-0.129512918	0.1322109	-0.979593	0.327287	0.872658
ctnnb2	4339.211051	0.02143583	0.07297	0.2937622	0.76894	0.975687

lrp13	3.626592945	0.525147765	0.9956437	0.5274455	0.597884	NA
rflna	16.41653246	0.206635356	0.5142003	0.4018577	0.687789	0.966442
ift74	557.0364816	0.119822946	0.1224671	0.9784091	0.327872	0.872701
gmppab	359.4594892	0.029330456	0.1161671	0.2524849	0.800666	0.979377
ankrd49	250.3697145	0.132625245	0.1390948	0.9534883	0.340343	0.87805
BX323555.1	0	NA	NA	NA	NA	NA
ggt1a	32.95606256	-0.118675757	0.3786155	-0.313447	0.753941	0.975056
pinx1	255.9643715	-0.199228925	0.1456033	-1.368299	0.171218	0.729413
nnt	11210.59765	0.137659622	0.0680862	2.0218437	0.043192	0.407874
ahr1b	229.8529303	-0.088072201	0.1406238	-0.626297	0.53112	0.942209
cdh7a	417.3299115	-0.088601166	0.1154491	-0.767448	0.442815	0.916336
kcnn1b	43.1911689	-0.062886963	0.2947985	-0.213322	0.831076	0.982994
lpp	449.5906065	-0.101052036	0.1080513	-0.935223	0.349674	0.880195
coq9	572.6189589	-0.034962502	0.1100227	-0.317775	0.750655	0.97419
cdc7	108.2298392	-0.356790834	0.1971331	-1.809898	0.070312	0.518591
kcnk5a	53.89082015	-0.308125317	0.2764229	-1.114688	0.264984	0.830836
nrbf2b	414.3760537	-0.267834233	0.1150977	-2.327016	0.019964	0.266381
sh3gl2a	1720.655152	0.26993418	0.080254	3.363499	0.00077	0.025154
AL845324.1	471.4409648	-0.048006594	0.1072481	-0.447622	0.654426	0.961305
ggps1	764.6632961	-0.102864443	0.1112341	-0.924757	0.355092	0.882321
kyat1	432.8991633	-0.008056333	0.1101882	-0.073114	0.941715	0.993099
idh3g	1101.989727	0.049353183	0.0845491	0.583722	0.559407	0.948478
he1.1	32.17684941	-0.53601709	0.4314974	-1.242226	0.214153	0.782493
gabpb1	97.79221369	0.068892896	0.2050376	0.3360013	0.73687	0.972535
timm8a	317.4853186	0.007357511	0.1546867	0.047564	0.962064	0.995907
ercc5	198.9599385	-0.185988009	0.1478127	-1.258268	0.208295	0.775404
cacna1fb	443.7964905	0.054888887	0.1138922	0.4819372	0.629851	0.957354
spon1b	2092.803085	0.027604466	0.0723414	0.3815862	0.702768	0.968134
kctd6b	290.1501875	0.051387146	0.1254556	0.4096041	0.682096	0.965476
mao	691.8825294	0.098611379	0.1080754	0.9124311	0.361542	0.88578
aqp1a.1	1107.090989	-0.114975804	0.0822673	-1.397589	0.162237	0.716781
hcrtr2	2.767134206	1.689980048	1.2117395	1.3946727	0.163115	NA
cdc42se1	222.587667	-0.071272512	0.156094	-0.4566	0.647959	0.960708
zgc:73226	132.282919	0.321442471	0.1843427	1.7437219	0.081208	0.551495
gnsa	550.5382104	0.046750819	0.1084802	0.4309617	0.666496	0.964736
mfsd4aa	18.76920425	0.263613922	0.4475664	0.588994	0.555865	0.94764
gabrb3	960.0132653	0.150081773	0.0893664	1.6793987	0.093074	0.580702
ryr1b	1331.49921	0.233798374	0.0924061	2.5301197	0.011402	0.186316
dyrk1ab	1142.100861	0.101555453	0.087152	1.1652686	0.24391	0.813336
uvrag	79.6025541	-0.166491676	0.2223821	-0.748674	0.454054	0.920295
faxdc2	528.0991275	0.277609236	0.105046	2.6427399	0.008224	0.147048
pfkfb2a	68.54123855	-0.177072004	0.2368005	-0.747769	0.4546	0.920747
lsm4	858.0977338	-0.209319698	0.0976672	-2.143193	0.032098	0.348544
ccdc174	319.4716574	-0.058255839	0.1372531	-0.424441	0.671244	0.965257
dnase1l1l	515.8338482	0.169952506	0.105579	1.6097192	0.107459	0.617344
si:dkey-172h23.2	361.3678566	0.154564539	0.1209174	1.2782659	0.201156	0.766855
stk24b	966.2904801	-0.021916326	0.0852159	-0.257186	0.797035	0.978569
cacna2d4b	387.543018	0.27896106	0.141442	1.9722647	0.048579	0.433684
casd1	474.2297983	0.062083133	0.1070579	0.5799025	0.56198	0.948728
atf2	9.507969078	0.684675278	0.6493331	1.0544285	0.291687	NA
mark4a	52.148034	0.212839937	0.2803259	0.7592588	0.447698	0.917154
phactr1	286.131146	0.307492112	0.1368754	2.246511	0.024671	0.302262
llgl2	1203.610578	-0.080015636	0.0888943	-0.900121	0.368056	0.887482
dnajc24	50.81544093	0.179566457	0.2786156	0.6444953	0.519254	0.938945

skila	58.11696668	-0.511843865	0.2624027	-1.950604	0.051104	0.443542
si:ch73-362m14.4	161.1992678	-0.063679601	0.1622526	-0.392472	0.69471	0.96736
znhit3	34.59363546	-0.120259005	0.3326621	-0.361505	0.717722	0.96909
fam167ab	84.86113318	-0.17554095	0.215199	-0.815715	0.414663	0.906237
rnf181	90.88313344	-0.098567622	0.211942	-0.465069	0.641882	0.959994
tpm4a	1612.138576	-0.048978243	0.0975126	-0.502276	0.615473	0.95688
atp6v1c1a	2087.954877	0.023193973	0.0732323	0.3167179	0.751458	0.97419
dctn4	532.0641624	-0.31304721	0.1311982	-2.386063	0.01703	0.241051
pef1	319.0958601	-0.010611979	0.1261467	-0.084124	0.932958	0.992702
heatr5a	484.2829011	0.024379989	0.1094052	0.2228412	0.823659	0.982196
rprmb	71.14620938	-0.18189367	0.2321328	-0.783576	0.433289	0.913654
mdga2a	350.8608065	0.009423434	0.1206164	0.0781273	0.937727	0.99279
sdf2	344.5655681	0.092926665	0.1176508	0.7898517	0.429614	0.911615
angptl2a	87.39732555	0.407476504	0.2182257	1.8672249	0.06187	0.485483
coch	303.4432061	0.504243425	0.137703	3.6618195	0.00025	0.010194
tmem38a	2652.177823	0.156120314	0.0966401	1.6154825	0.106206	0.615567
dnajc4	268.3737298	0.076837186	0.1447718	0.5307471	0.595594	0.954434
lmbr1	105.8168218	-0.079972872	0.1997672	-0.40033	0.688913	0.966672
adra1d	75.59939005	0.316481058	0.2361325	1.3402691	0.180158	0.74128
naa40	747.9751278	0.034734249	0.0943988	0.3679522	0.712909	0.96909
gmppaa	261.2030304	0.098875529	0.1393462	0.7095674	0.477972	0.926857
vamp8	342.116542	0.079933054	0.1304289	0.6128477	0.539977	0.944206
sde2	499.7854549	-0.059773148	0.1026334	-0.582395	0.560301	0.948728
p2rx8	10.91936421	-0.312897344	0.6111265	-0.512001	0.60865	NA
cav3	696.0948624	0.090158791	0.1034985	0.8711117	0.383693	0.895192
hao1	319.3464633	0.069785828	0.1350386	0.5167842	0.605307	0.95651
kcnh1a	8.941904365	0.424299762	0.6446308	0.6582058	0.510406	NA
narf	470.8292156	0.016220913	0.1182272	0.1372012	0.890872	0.989291
pnocb	68.10848578	-0.360524915	0.2433003	-1.481811	0.138391	0.676189
znf395b	915.8739386	-0.244345662	0.0970828	-2.516879	0.01184	0.191129
mcm3	873.8776239	0.049027506	0.0876179	0.5595601	0.57578	0.950373
opn7a	0.166657454	-0.955901296	4.0804729	-0.234262	0.814781	NA
wasf2	795.9428571	-0.163152962	0.0987791	-1.651695	0.098597	0.59799
ifng1	0.166657454	-0.955901296	4.0804729	-0.234262	0.814781	NA
cdk5rap2	294.6911642	0.024572875	0.1285034	0.1912236	0.84835	0.985174
ubl7a	199.88778	0.246650217	0.1485326	1.66058	0.096798	0.592778
pcbp4	1315.86852	-0.031814986	0.0820165	-0.387909	0.698083	0.967998
pigm	75.7569571	0.208985611	0.2399341	0.8710124	0.383747	0.895192
adh8b	1373.706641	-0.763554281	0.1272994	-5.998096	2.00E-09	3.77E-07
slc11a2	545.2689166	-0.12674301	0.1178237	-1.0757	0.282061	0.843666
c1qtnf4	1123.109899	-0.006122505	0.0951006	-0.064379	0.948668	0.99381
zbed4	506.3876227	-0.088685039	0.1052154	-0.84289	0.39929	0.900683
nsmce4a	130.6024673	0.026338215	0.2056535	0.1280709	0.898093	0.990121
herpud1	765.6058362	-0.102628583	0.0977805	-1.049581	0.293911	0.849999
trap1	1250.178208	-0.000371762	0.0809856	-0.00459	0.996337	0.998902
arhgap1	2481.005451	0.041925562	0.0890011	0.4710678	0.637592	0.958872
col4a3bpa	309.8497299	-0.077476449	0.1273506	-0.608371	0.542941	0.944974
crlf1a	158.4278436	-0.124631998	0.1703158	-0.73177	0.464309	0.923325
msto1	284.2837739	-0.188789175	0.1488697	-1.268151	0.204744	0.772783
get4	963.8977625	-0.044142218	0.0924438	-0.477503	0.633004	0.957356
twistnb	147.229682	-0.073293329	0.1710484	-0.428495	0.668291	0.964736
ets1	75.55604517	0.088445169	0.2346164	0.3769778	0.70619	0.968459
pvalb4	4690.81289	-0.197670864	0.2017454	-0.979804	0.327183	0.872658
gnpda2	990.2141089	0.009269812	0.0987475	0.0938739	0.925209	0.992676

mrps18a	415.8653584	0.020767468	0.1150843	0.1804544	0.856796	0.985597
nsl1	61.28432474	-0.089547158	0.2482468	-0.360718	0.71831	0.96909
mpc2	1855.543076	0.090913369	0.0849653	1.0700061	0.284617	0.844955
pigk	114.6783195	-0.153006015	0.1848261	-0.827838	0.407762	0.901408
top2a	1996.270206	-0.045117923	0.0723438	-0.62366	0.532851	0.942805
col9a2	19015.82359	0.088845677	0.091365	0.972426	0.330839	0.87417
c6ast3	13.41041854	1.153050786	0.542942	2.1237089	0.033694	0.357045
prf1.6	0.158795395	0.967652056	4.0804729	0.2371421	0.812547	NA
emilin1a	334.9265807	0.454011685	0.1239874	3.6617577	0.00025	0.010194
tspan36	1205.856825	-0.146397694	0.1251088	-1.170163	0.241935	0.810713
pla2g4aa	45.66178187	0.047446617	0.2889637	0.1641958	0.869577	0.987282
cryba4	22644.52237	-0.015284536	0.0782095	-0.195431	0.845056	0.985174
slc4a1b	92.66394332	-0.140657599	0.2174217	-0.646934	0.517674	0.938592
nolc1	882.9591958	0.00062735	0.1087314	0.0057697	0.995396	0.99873
sccpdhb	288.9663266	-0.419981238	0.1269638	-3.307883	0.00094	0.029552
kif26bb	84.55624227	0.570966044	0.2202176	2.5927353	0.009522	0.163896
gpc5b	192.3888098	0.215725847	0.1651508	1.3062359	0.191472	0.755886
homezb	728.0797013	0.087768728	0.1069479	0.8206684	0.411835	0.903944
zgc:162964	157.6226596	-0.276727156	0.1814129	-1.5254	0.127159	0.656588
foxo6b	371.8518992	0.061487649	0.1158681	0.5306693	0.595648	0.954434
nlrc5	4.954201555	0.037603004	0.8876391	0.0423629	0.966209	NA
pip5k1ab	585.6231582	0.02106724	0.09793	0.2151254	0.82967	0.982805
snrpa1	717.7192022	-0.072918592	0.1031613	-0.706841	0.479666	0.927091
pgm3	250.1253436	-0.145136594	0.1363421	-1.064503	0.287101	0.845979
oser1	591.6162435	-0.035299052	0.1079807	-0.326902	0.743742	0.974094
pane1	100.9899952	-0.010520695	0.2370113	-0.044389	0.964594	0.995973
polr3g	2.855122227	0.188712634	1.2366611	0.1525985	0.878715	NA
lysmd3	145.0723743	-0.056606498	0.171328	-0.330398	0.741099	0.973154
myo1b	1380.66401	-0.016991127	0.0800446	-0.212271	0.831896	0.98349
slc25a23b	35.22828482	0.211792462	0.3323211	0.6373128	0.523921	0.939336
selenbp1	358.5775135	-0.329530675	0.1511265	-2.180495	0.029221	0.331124
hdhd2	134.843495	-0.050145172	0.1727773	-0.29023	0.77164	0.975687
gng13a	58.10648628	-0.407095242	0.2661571	-1.52953	0.126133	0.654806
dpp6b	766.2858352	0.145849595	0.0950289	1.5347924	0.124835	0.654423
hsp90aa1.2	2365.25625	0.208573767	0.1003081	2.0793311	0.037587	0.379013
inhbab	44.76673016	0.106430298	0.3116203	0.3415384	0.732698	0.971719
slc24a5	44.6872525	0.020540864	0.2946319	0.069717	0.944419	0.993364
si:ch211-129c21.1	466.8593684	-0.107238052	0.1146112	-0.935668	0.349444	0.880195
ctnna2	1364.627193	0.033803933	0.0944772	0.3577997	0.720493	0.969318
mxc	18.49227967	-0.450743277	0.4532116	-0.994554	0.319953	0.868153
ogfrl2	19.49294087	-0.167187976	0.4421403	-0.378133	0.705332	0.968412
rilpl2	154.8952973	0.18655878	0.1610506	1.1583861	0.246707	0.816669
rnf150a	11.77537225	-0.570218383	0.5849051	-0.97489	0.329615	0.873583
tmem134	387.001309	-0.009415012	0.117204	-0.08033	0.935975	0.992702
tnn	5176.13656	0.286205299	0.0782436	3.657875	0.000254	0.010326
crhbp	335.7303371	0.060104327	0.1213478	0.4953064	0.620384	0.957354
max	3144.7418	0.008810496	0.0687934	0.1280719	0.898092	0.990121
cops8	895.4437955	0.046501017	0.0874434	0.5317843	0.594875	0.954434
col5a2b	231.9331093	0.079201004	0.1666905	0.475138	0.634689	0.9578
dscama	1308.724476	-0.02755749	0.1038999	-0.265231	0.790831	0.977402
dock4b	489.2355227	-0.096671261	0.1057946	-0.913764	0.360841	0.885686
ptgr1	1480.873442	-0.314778579	0.0986058	-3.192292	0.001411	0.040686
tbx5a	19.05688665	0.595804173	0.4423575	1.3468839	0.178018	0.738995
fam50a	599.4576662	-0.150945856	0.1089065	-1.386013	0.165743	0.721576

stil	110.4245367	-0.115528451	0.2134991	-0.541119	0.588425	0.953319
tmem136a	330.9417504	0.144141067	0.125558	1.148004	0.250967	0.820469
spast	336.3370063	-0.002939404	0.134846	-0.021798	0.982609	0.996944
rnf144b	277.7310413	-0.092045812	0.1283147	-0.717344	0.473162	0.924993
tmed4	472.7373346	-0.067108821	0.1112962	-0.602975	0.546525	0.945526
patj	274.2248339	0.045200831	0.1300406	0.3475901	0.728148	0.970402
MARK4	1068.177003	-0.023758657	0.090488	-0.262561	0.792889	0.977937
fancg	89.88475589	-0.105743459	0.2079549	-0.508492	0.611108	0.95688
synj1	1645.771625	-0.020351051	0.0884176	-0.23017	0.81796	0.981594
tpi1a	342.3869153	0.147549903	0.1244671	1.1854533	0.235838	0.804837
gna14a	18.52309135	-0.022564803	0.4704256	-0.047967	0.961743	0.995907
prdm8	116.4598079	-0.062811315	0.1855472	-0.338519	0.734972	0.972129
pnoca	100.6044508	-0.009022412	0.2126093	-0.042437	0.966151	0.996315
nr3c1	1141.394892	-0.025040902	0.0884669	-0.283054	0.777136	0.975826
stx5a	977.9986539	0.139449932	0.0857012	1.6271651	0.103702	0.610299
syngr3b	473.7948715	0.174488959	0.1187556	1.4693115	0.141748	0.682643
trip13	119.1880055	-0.120820623	0.1996747	-0.605087	0.545121	0.945463
AL935186.1	50.7234559	-1.777677465	0.3334923	-5.330491	9.79E-08	1.36E-05
brd8	1187.431262	-0.08697378	0.0827455	-1.0511	0.293213	0.849024
rpl18a	14702.59545	-0.282897549	0.0856665	-3.302311	0.000959	0.029926
slc25a10	757.8872884	-0.00947423	0.0923043	-0.102641	0.918248	0.991623
marveld2a	274.8961065	-0.108504559	0.1290916	-0.840524	0.400615	0.90084
grnb	483.8269779	-0.047836673	0.113122	-0.422877	0.672385	0.965257
loxl4	280.26812	0.060973223	0.1267387	0.4810941	0.63045	0.957354
ezrb	2483.712969	0.032017746	0.073496	0.4356396	0.663098	0.964736
edem1	747.6167458	-0.005923798	0.1032052	-0.057398	0.954228	0.994409
ppp3cb	2326.761941	0.027166629	0.0767936	0.3537618	0.723517	0.970402
magixa	26.73458957	-0.236745635	0.3708445	-0.638396	0.523216	0.939336
lbx2	212.4992732	0.169192633	0.1456342	1.1617647	0.245331	0.815324
cd63	2209.666616	-0.083493521	0.0864884	-0.965373	0.334358	0.875361
si:dkey-181c13.1	2.314256126	1.926318878	1.5343758	1.2554414	0.209319	NA
asic4a	288.6372449	0.021748409	0.1266977	0.1716559	0.863708	0.985952
mpp7b	3.289981667	0.248328898	1.0717886	0.2316958	0.816774	NA
zgc:103482	12.18831098	0.190032011	0.5578548	0.3406478	0.733369	0.971993
six6a	73.389827	0.040011635	0.2315315	0.172813	0.862798	0.985841
cpne8	16.59244255	0.27855979	0.4816363	0.5783613	0.56302	0.948728
syt2a	35.44702746	-0.155319171	0.3225219	-0.481577	0.630106	0.957354
ints11	301.090042	-0.015122493	0.1232581	-0.12269	0.902353	0.990121
myo5ab	208.5823691	0.606330984	0.1445476	4.1946812	2.73E-05	0.001635
sppl3	747.098501	0.030929264	0.1123665	0.2752534	0.783122	0.97627
znf668	143.9433661	-0.143858004	0.1668808	-0.862041	0.388665	0.896271
lipt1	64.69927505	0.014752373	0.2434268	0.0606029	0.951675	0.993864
s100a10b	1227.030089	0.187696854	0.0910444	2.061598	0.039246	0.388998
pdcd6ip	2739.44802	-0.073650191	0.0898751	-0.819473	0.412517	0.904974
gbgt1l1	32.18839943	-0.370609146	0.3417656	-1.084396	0.278189	0.841891
panx1a	363.956663	-0.000631616	0.1181477	-0.005346	0.995735	0.998888
tspan9a	9.430526784	1.043318484	0.6887063	1.5148961	0.129799	NA
gfap	6050.459381	0.114820997	0.0669092	1.7160728	0.086149	0.563157
dixdc1a	800.8449243	-0.02154595	0.0894887	-0.240767	0.809735	0.980648
dpf3	132.5201606	-0.452435849	0.1866005	-2.424623	0.015324	0.227153
cuzd1.2	1.998351438	0.004139829	1.402095	0.0029526	0.997644	NA
fynb	92.81306093	-0.025960875	0.2088198	-0.124322	0.90106	0.990121
col21a1	46.24820241	0.464095517	0.297198	1.56157	0.118389	0.641743
ccsapa	194.8759266	-0.224800249	0.1504001	-1.494682	0.134998	0.670689

myl12.2	692.3959039	-0.109307393	0.1012516	-1.079562	0.280337	0.843423
rbm28	375.6441805	0.085059797	0.1209814	0.7030815	0.482005	0.928561
nek10	10.66052892	-0.257559968	0.5900396	-0.436513	0.662465	NA
hagh	444.9731765	-0.051821568	0.1063947	-0.487069	0.62621	0.957354
tusc2b	351.6775585	0.100468903	0.1193664	0.8416846	0.399964	0.90084
znf277	120.7695133	-0.197164995	0.1884186	-1.04642	0.295367	0.851935
igfbp5b	1081.605544	0.293363395	0.0888523	3.3016966	0.000961	0.029926
prdx2	5534.372551	-0.233769194	0.0980422	-2.384373	0.017108	0.241859
admp	0.804086858	-1.860428069	2.2645275	-0.821552	0.411332	NA
idh1	1860.05478	-0.088895633	0.0837097	-1.061952	0.288258	0.845979
arl2bp	123.3203834	0.281097291	0.1841324	1.5266038	0.12686	0.656588
lsm14b	3.378709501	0.883999793	1.086412	0.8136874	0.415824	NA
pfdn2	1076.352699	0.080077398	0.1036698	0.7724274	0.439861	0.916082
srsf3a	40.53762756	0.109911885	0.3150876	0.3488297	0.727217	0.970402
ftr83	326.69645	-0.232469026	0.1539452	-1.510077	0.131024	0.663795
ube2g2	496.3878195	-0.018120214	0.1086605	-0.16676	0.867559	0.987231
fzd5	180.4567351	0.040498624	0.1546841	0.261815	0.793464	0.977937
tdrd3	516.6852012	-0.040488231	0.1067553	-0.379262	0.704493	0.968134
socs3a	555.3619764	-0.062371924	0.33564	-0.18583	0.852578	0.985174
msrb1a	217.1208099	-0.003026516	0.1417843	-0.021346	0.98297	0.996944
spon2a	242.3286087	0.177744056	0.1484617	1.1972384	0.231214	0.799444
aatf	267.8318059	-0.01017479	0.1326117	-0.076726	0.938841	0.992857
bnip3la	192.7524784	0.159590889	0.1540835	1.0357426	0.300322	0.856251
gipr	28.44222948	-0.035442461	0.3694217	-0.09594	0.923568	0.992588
nhlh2	681.7446824	0.087469336	0.1068565	0.818568	0.413033	0.905281
mtx1a	279.7225944	-0.011657338	0.1372506	-0.084935	0.932313	0.992702
gucy2f	141.6383305	0.100121569	0.182874	0.5474893	0.584043	0.952214
si:ch73-335d12.2	0.491560863	0.853615855	2.865157	0.2979299	0.765757	NA
wdr24	236.42926	-0.239929018	0.1386754	-1.730149	0.083604	0.557158
dip2cb	916.3848471	0.000891899	0.1039799	0.0085776	0.993156	0.998554
zgc:101679	346.6904412	-0.038692514	0.1217641	-0.317766	0.750662	0.97419
sgk1	2633.362078	0.175189375	0.0772235	2.2686023	0.023293	0.292994
clint1a	1137.809031	-0.043072412	0.0861011	-0.500254	0.616896	0.956957
si:ch73-34314.6	0	NA	NA	NA	NA	NA
ndufaf1	612.5774304	0.077446762	0.1056964	0.7327286	0.463724	0.922875
wdr83os	559.8474051	0.096542541	0.1034515	0.9332156	0.350709	0.880195
ormdl3	17.16966353	0.261988754	0.4606699	0.5687125	0.569551	0.948983
pex11g	46.56093474	-0.611138024	0.3024163	-2.02085	0.043295	0.40826
slc25a3b	8476.688078	-0.002446743	0.0689675	-0.035477	0.9717	0.996315
nf2b	471.2866265	-0.057438019	0.1117177	-0.514135	0.607157	0.956718
mkln1	582.2024971	0.099273397	0.1012443	0.9805327	0.326823	0.872658
zgc:66447	240.215545	0.015629321	0.1344693	0.1162296	0.907471	0.990702
rpl10	16456.67716	-0.359275302	0.0730548	-4.917889	8.75E-07	9.01E-05
plekhj1	237.3949894	-0.068073895	0.1474002	-0.46183	0.644203	0.960129
agmo	150.5755358	-0.286174915	0.1685434	-1.69793	0.089521	0.572876
or102-5	0.157187365	-0.955901296	4.0804729	-0.234262	0.814781	NA
trim35-14	0.166657454	-0.955901296	4.0804729	-0.234262	0.814781	NA
nvl	494.2702305	-0.087994931	0.1037402	-0.848224	0.396313	0.899672
ifnphi1	1.679609444	0.005089711	1.4178403	0.0035898	0.997136	NA
casp6l1	23.29958211	0.245680829	0.408898	0.6008365	0.547949	0.945768
prr15la	67.99733791	-0.0725624	0.2349442	-0.30885	0.757436	0.975374
gli2a	629.0731196	-0.059421952	0.0986595	-0.602294	0.546979	0.945526
or111-2	2.237462172	0.271244177	1.4380036	0.1886255	0.850386	NA
adgrb2	99.96007379	0.07060038	0.2070554	0.3409733	0.733124	0.971947

fstl3	326.7676128	0.151868494	0.1363821	1.1135515	0.265472	0.831302
grm6b	340.9545411	0.059243422	0.1279135	0.4631522	0.643255	0.959994
antxr1a	20.51853486	-0.504117879	0.4214763	-1.196077	0.231667	0.799752
comtb	268.203609	0.071526477	0.1392692	0.5135844	0.607543	0.956718
tmem9b	758.3187703	-0.051267433	0.11098	-0.461952	0.644116	0.960129
mbd1b	593.0985643	-0.178821471	0.1010818	-1.769077	0.076881	0.540051
dmgdh	767.4435767	-0.004950569	0.1312603	-0.037716	0.969914	0.996315
cxxc1b	321.2538659	0.064039443	0.1325207	0.4832412	0.628925	0.957354
grin1b	3842.544718	-0.012494557	0.0708677	-0.176308	0.860052	0.985773
ube2f	515.2757862	-0.067567375	0.1092154	-0.618662	0.536139	0.943336
tspan35	235.9570425	0.069169823	0.144837	0.47757	0.632956	0.957356
zgc:66440	188.4968513	0.134284346	0.1618578	0.829644	0.40674	0.901305
si:ch211-125e6.11	0	NA	NA	NA	NA	NA
chp2	158.7998526	0.01614856	0.1663698	0.0970642	0.922675	0.99233
chd4b	3632.742716	-0.072746556	0.0705043	-1.031803	0.302164	0.857048
abhd2a	26.50537784	-0.155188707	0.3732523	-0.415774	0.677575	0.965257
taf5l	102.5127374	-0.042319106	0.2005732	-0.210991	0.832894	0.983557
chek2	68.2985003	-0.217621177	0.2669686	-0.815157	0.414983	0.906237
dmtf1	368.1243203	-0.080272806	0.1284093	-0.625132	0.531884	0.942359
gna12a	494.8682093	-0.097492153	0.1067518	-0.91326	0.361106	0.885686
sox12	1221.626274	-0.102414208	0.0869424	-1.177954	0.238815	0.807768
rps21	8655.006871	-0.176848229	0.1014163	-1.743785	0.081197	0.551495
ddx51	192.8176405	-0.029505123	0.1539174	-0.191695	0.847981	0.985174
camk2n1a	1808.472619	0.085930862	0.0816048	1.053012	0.292336	0.848099
zgc:56525	1206.15571	-0.042964836	0.0841217	-0.510746	0.609529	0.956718
lmf2b	653.1639242	-0.079923211	0.1112312	-0.718532	0.472429	0.924838
ankdd1b	17.41631029	-0.227227422	0.4718395	-0.481578	0.630106	0.957354
kctd2	57.90724293	-0.104598859	0.2538891	-0.411986	0.680349	0.965431
usp14	1199.312195	-0.013625432	0.0857529	-0.158892	0.873754	0.987881
shox	426.3822051	-0.077322569	0.1232232	-0.6275	0.530332	0.942209
pnx	1.57818626	1.909813316	1.774008	1.0765528	0.28168	NA
plekhg5a	593.6295314	0.072368425	0.0986253	0.733771	0.463088	0.922698
lgals9l1	74.99283039	-0.082340432	0.2255269	-0.365103	0.715035	0.96909
tecrb	2231.263664	0.01552602	0.0733593	0.2116436	0.832385	0.983557
bscl2l	158.6623596	-0.172464621	0.1695671	-1.017088	0.309112	0.861381
si:dkey-190g11.3	10.53229137	0.003928453	0.5838309	0.0067288	0.994631	NA
tiam1b	165.9234291	0.119146959	0.1622033	0.7345531	0.462612	0.922698
crygs3	2.775474421	-0.182679766	1.1959654	-0.152747	0.878598	NA
mlh1	119.9211034	-0.352466506	0.1828023	-1.92813	0.053839	0.454574
irak1	81.18694861	-0.206173245	0.2209211	-0.933244	0.350694	0.880195
rhoq	255.8586141	0.120564121	0.1460714	0.8253781	0.409157	0.901769
mcts1	592.8375852	0.053879118	0.1033632	0.5212603	0.602185	0.956045
magi3b	23.46970761	0.059793108	0.438462	0.1363701	0.891529	0.989291
blmh	1347.531327	-0.112330595	0.1174576	-0.956351	0.338895	0.87745
l3mbtl3	14.90890863	0.023916039	0.5038642	0.0474652	0.962142	0.995907
xpnpep2	93.04934369	0.438406249	0.211226	2.0755314	0.037937	0.381357
mospd2	277.2134678	-0.06188859	0.1291023	-0.479376	0.631671	0.957356
ankrd44	57.71383784	0.092644869	0.2790584	0.331991	0.739896	0.972943
ube2b	483.7585122	0.051819635	0.1077711	0.4808307	0.630637	0.957354
tsc1a	741.8204003	0.184484208	0.0912192	2.0224274	0.043132	0.407581
mxf	4.880823235	-0.212633457	0.900311	-0.236178	0.813295	NA
ccdc3a	30.36096465	-0.084844684	0.4046464	-0.209676	0.83392	0.983583
hltf	246.3896547	-0.148906492	0.1397457	-1.065553	0.286626	0.845942
trappc5	569.5214046	-0.011109809	0.1040114	-0.106813	0.914937	0.991119

cd82b	994.1395632	0.006197119	0.0909291	0.0681533	0.945664	0.993609
pdcd5	647.3078348	-0.053112218	0.1001532	-0.53031	0.595897	0.954434
lhcgr	1.614149009	-0.60141512	1.5131948	-0.397447	0.691038	NA
si:dkey-43k4.5	50.18280395	-0.017786763	0.2923924	-0.060832	0.951493	0.99381
zgc:162324	19.09294205	-0.314658627	0.475523	-0.661711	0.508157	0.935735
adprm	64.74898501	0.134247443	0.2775572	0.483675	0.628617	0.957354
si:dkey-13a21.4	16.11744768	-0.295226924	0.4810881	-0.613665	0.539437	0.943908
slc48a1b	303.24774	-0.17932929	0.1332992	-1.345314	0.178524	0.739486
sfxn5b	192.8102578	0.007993182	0.1483232	0.0538903	0.957023	0.994543
slc46a1	90.40657725	-0.036429173	0.2210206	-0.164823	0.869084	0.987245
col11a1a	23716.96052	0.169749053	0.0921882	1.841331	0.065573	0.500566
emilin1b	556.1977353	0.063592623	0.1051691	0.60467	0.545398	0.945526
hps1	140.8593356	-0.010330122	0.1724428	-0.059905	0.952232	0.993904
ube3d	110.9103138	-0.237092497	0.19723	-1.202112	0.22932	0.796938
prpf8	4716.225242	-0.011229082	0.0727272	-0.1544	0.877294	0.987881
polr2eb	724.4695826	-0.056922736	0.1076182	-0.528932	0.596853	0.954913
yju2	466.8815198	0.03243107	0.119642	0.2710675	0.786339	0.976476
enoph1	630.5592062	-0.079649193	0.0964534	-0.825779	0.40893	0.90171
fanci	170.1241877	-0.157673108	0.1583707	-0.995595	0.319447	0.867905
nsd2	784.5843298	-0.159261934	0.1061751	-1.499993	0.133616	0.66814
prnpa	216.8021463	0.746544889	0.1556076	4.7976127	1.61E-06	0.000148
zgc:56585	94.71242766	0.354591696	0.2082773	1.7024984	0.088662	0.569591
CR847944.1	0	NA	NA	NA	NA	NA
insyn1	371.7950695	0.01130452	0.1399026	0.0808028	0.935599	0.992702
slc1a7b	72.62663202	-0.234174263	0.2334199	-1.003232	0.315749	0.866931
lyrm1	54.36103435	-0.091289864	0.2598941	-0.351258	0.725395	0.970402
erbb2	45.16739295	0.227055473	0.2948977	0.7699466	0.441332	0.916336
zgc:112994	28.89246203	-0.193690733	0.3569762	-0.542587	0.587414	0.953319
adgre5a	0	NA	NA	NA	NA	NA
dhrs13a.1	312.0917494	0.166315266	0.1264917	1.3148312	0.188567	0.753276
mmp11a	76.64810325	0.377608897	0.237691	1.5886548	0.112138	0.627454
arhgap29a	334.6348113	-0.004796796	0.1276579	-0.037575	0.970026	0.996315
xkr7	105.7656976	0.110766946	0.2005383	0.5523481	0.58071	0.951731
susd4	227.4256647	0.015116394	0.1376753	0.1097974	0.91257	0.991119
styxl1	1.965054466	1.588801095	1.4409003	1.1026447	0.270181	NA
csad	188.8020165	0.262853458	0.1541945	1.7046876	0.088253	0.568811
wasb	20.17321665	-0.381140948	0.4621142	-0.824777	0.409498	0.902019
pbld2	140.5991339	0.44215612	0.1694265	2.6097223	0.009062	0.159222
dbi	2186.340459	-0.207800183	0.0703431	-2.954094	0.003136	0.073769
aco1	51.696548	0.117351744	0.2715192	0.4322043	0.665593	0.964736
si:dkey-27j5.5	33.53595485	0.295941317	0.3316885	0.8922267	0.372271	0.892711
pip4p1b	57.96291531	0.228736927	0.2628612	0.8701813	0.384201	0.895192
rfx4	226.3241991	0.159639462	0.1610856	0.9910224	0.321675	0.869375
rad51d	45.26001922	-0.025256563	0.2898565	-0.087135	0.930564	0.992702
hephl1b	10.4597713	0.017930438	0.6429472	0.0278879	0.977752	NA
anxa5a	107.3757749	0.172809549	0.2040391	0.8469431	0.397027	0.899672
ccr12b.2	0.639947932	-1.440068751	2.4365394	-0.59103	0.5545	NA
apoob	338.5387495	0.097185171	0.1378277	0.705121	0.480735	0.927601
med29	223.0059941	0.01069106	0.1399325	0.0764016	0.9391	0.992857
zgc:66474	312.220772	-0.003022046	0.1301606	-0.023218	0.981477	0.996944
mis12	119.4446144	0.06293661	0.2135474	0.2947196	0.768208	0.975687
six1b	362.0665167	0.173122211	0.1156883	1.4964536	0.134535	0.670689
tial1	2715.040082	0.089827614	0.0757388	1.1860184	0.235615	0.804824
arhgap12b	980.9432783	0.076588856	0.0870361	0.8799664	0.378877	0.895192

rab15	401.8611451	0.226272566	0.1147027	1.9726871	0.048531	0.433449
khsrp	1846.375086	-0.193548947	0.0965387	-2.004886	0.044975	0.417005
ppm1e	91.5398169	0.287416489	0.2143323	1.3409855	0.179925	0.741062
xkr9	18.42927739	0.02340009	0.4397484	0.0532124	0.957563	0.99467
si:ch211-285c6.3	1.121918354	-0.422914697	1.8509454	-0.228486	0.819269	NA
hoxb6b	217.0891432	-0.154831008	0.156975	-0.986342	0.323965	0.870928
ralgps2	683.2367387	0.106655845	0.0963795	1.1066233	0.268457	0.833634
alcama	1351.844647	-0.021984781	0.0797362	-0.275719	0.782764	0.97627
ppp4r2a	644.6855571	0.016732696	0.0978253	0.1710467	0.864187	0.98601
cyp2u1	27.30774682	0.345472307	0.3790413	0.9114371	0.362065	0.885944
pimr196	0.323844819	-1.825095364	3.274639	-0.557342	0.577293	NA
cdk2	313.9804355	-0.047491624	0.1240646	-0.382798	0.70187	0.968134
iscub	94.34978156	0.037379412	0.2082733	0.1794729	0.857566	0.985703
pafah1b1b	3194.815625	0.10076359	0.0708103	1.4230067	0.154734	0.704573
en2a	315.9155309	-0.135330248	0.1376698	-0.983006	0.325604	0.871896
socs3b	795.4313337	-0.196293529	0.2844399	-0.690105	0.490128	0.931926
zgc:113425	9.345930974	0.133771826	0.6345412	0.2108166	0.83303	NA
gmds	1228.432478	-0.792051509	0.0802122	-9.874453	5.37E-23	8.17E-20
mark3b	1125.980453	0.015260144	0.0852242	0.1790588	0.857892	0.985703
ehmt1b	1527.117127	0.030798619	0.079695	0.386456	0.699159	0.967998
asb1	14.83915064	0.30445121	0.4924116	0.618286	0.536387	0.943336
gosr1	303.6102487	-0.084346119	0.1266287	-0.66609	0.505354	0.935724
tspo	611.7856483	-0.225559441	0.1150673	-1.960239	0.049968	0.437835
tlr19	0.666939546	1.487980453	2.2924711	0.6490727	0.516291	NA
uri1	874.5753348	-0.176592714	0.0861026	-2.050956	0.040271	0.392394
slc25a14	876.9600272	0.208946566	0.0873136	2.3930579	0.016709	0.238173
zgc:101583	128.7293715	0.245567278	0.1978961	1.2408902	0.214646	0.782493
gtf2h1	255.8496903	0.118462848	0.1363349	0.8689104	0.384896	0.89551
ftr72	1.97020862	-0.487641474	1.3234626	-0.368459	0.712531	NA
rab5c	2485.939782	0.045723452	0.0818851	0.5583854	0.576581	0.950611
syncripl	785.3361034	0.214395287	0.0899937	2.3823361	0.017203	0.242176
anxa1a	1228.815289	0.028121304	0.0979469	0.2871078	0.77403	0.975687
il11ra	36.36828821	-0.351966059	0.3259064	-1.07996	0.28016	0.843423
grik4	231.0323454	0.150385674	0.1439948	1.0443824	0.296309	0.853286
ldlrb	546.4512971	-0.016226057	0.1092464	-0.148527	0.881927	0.988344
fam126a	455.7877454	-0.136940065	0.1147558	-1.193317	0.232745	0.801236
ahsg1	12.73445141	-0.089931799	0.5954764	-0.151025	0.879956	0.987881
bcl2l10	877.4877327	0.019988451	0.0892904	0.2238589	0.822867	0.982189
nol11	649.8690263	-0.069470977	0.0992606	-0.699885	0.483999	0.929461
tmem41ab	110.2969978	0.607098872	0.1952616	3.1091563	0.001876	0.050962
robo1	1616.175719	-0.067064338	0.0914395	-0.733429	0.463297	0.922698
aqp7	68.45185649	-0.280533089	0.2501076	-1.12165	0.262011	0.830166
grm1a	575.4409548	-0.198622955	0.1031127	-1.926271	0.054071	0.455557
suv39h1a	4.618752265	-0.100389309	0.9766782	-0.102786	0.918132	NA
stau2	5162.421152	0.035344005	0.0699458	0.5053057	0.613344	0.95688
taf8	189.2645777	-0.184877291	0.1497537	-1.234543	0.217001	0.784302
extl3	237.3575638	0.177680928	0.1381103	1.286515	0.198263	0.76333
gucy2d	1516.850588	0.261806335	0.09121	2.8703696	0.0041	0.0887
cotl1	5002.678398	-0.12538572	0.0758712	-1.652614	0.098409	0.597834
cdr2l	700.3000627	-0.08402196	0.0943299	-0.890725	0.373077	0.893126
slc25a32b	175.9756148	0.056942856	0.1579281	0.3605618	0.718427	0.96909
med21	192.339163	0.035078517	0.1674692	0.2094625	0.834087	0.983673
arhgap23b	76.04940676	0.286818932	0.2352388	1.2192669	0.222743	0.789115
acin1b	2768.470367	0.05737202	0.076567	0.7493045	0.453674	0.920041

rhoaa	3596.490486	0.00909255	0.0731602	0.1242827	0.901091	0.990121
cacna2d4a	50.34106134	-0.060855054	0.2973115	-0.204685	0.837819	0.984701
aktip	1041.159877	0.060960234	0.0973363	0.6262849	0.531128	0.942209
si:ch211-226m16.3	0.182570949	0.967652056	4.0804729	0.2371421	0.812547	NA
fam107b	1174.271931	-0.078873548	0.0844369	-0.934112	0.350246	0.880195
uchl1	595.3750733	-0.001193664	0.1035655	-0.011526	0.990804	0.997679
fam57ba	525.5250304	0.161144351	0.1059637	1.5207505	0.128322	0.658954
palm1a	439.8956433	0.102048364	0.115475	0.8837268	0.376844	0.895192
gpatch11	262.6816436	-0.23678971	0.138541	-1.709167	0.08742	0.566637
tor1l3	3.85538121	-1.847265497	1.0767748	-1.715554	0.086244	NA
cryzl1	207.690116	0.0880503	0.1654115	0.5323105	0.594511	0.954434
cbln13	7.875624602	-0.259406474	0.7119429	-0.364364	0.715586	NA
slc48a1a	48.51307711	-0.320827322	0.2757475	-1.163482	0.244634	0.814376
tmed2	2211.921875	-0.022730173	0.0710978	-0.319703	0.749194	0.97419
nos2a	1.161995204	-0.406585265	1.814365	-0.224092	0.822685	NA
rom1b	610.4172864	0.181067502	0.1094917	1.6537094	0.098187	0.59749
fbxl4	203.1101633	0.050368265	0.1471685	0.3422489	0.732164	0.971665
ap2s1	2448.860453	0.060834674	0.0772718	0.787282	0.431117	0.91234
si:ch211-119o8.6	2.262897238	-0.437223843	1.3142383	-0.332682	0.739374	NA
krt1-c5	265.0583585	0.100485392	0.1402258	0.7165968	0.473623	0.924993
lims1	1093.451553	-0.025689544	0.0950483	-0.270279	0.786946	0.976596
rexo1	242.234755	-0.186008708	0.1354085	-1.373686	0.169539	0.726744
tbc1d22b	5.486143505	-0.818290548	0.8318949	-0.983646	0.325289	NA
klhl6	9.863743544	0.172433752	0.6204363	0.2779234	0.781071	NA
zgc:85777	720.2821075	-0.152543276	0.0950479	-1.604909	0.108514	0.62063
gbf1	1056.93761	0.109544792	0.0853327	1.2837374	0.199234	0.764601
ppp2r5a	24.73225736	-0.228693608	0.4014344	-0.569691	0.568887	0.948983
pcdh17	2167.129254	0.099114965	0.0772813	1.2825227	0.199659	0.76498
scyl3	452.9025899	0.076866033	0.1067776	0.7198705	0.471605	0.924838
arpc1b	633.7298151	0.193162472	0.1019753	1.8942091	0.058197	0.472993
slc38a3a	1479.164939	0.222422285	0.0797653	2.7884608	0.005296	0.107627
acbd4	72.70198577	-0.07334827	0.2533044	-0.289566	0.772149	0.975687
cnppd1	1183.83294	-0.039135675	0.0865969	-0.451929	0.65132	0.960708
znf804a	181.2031439	-0.04505744	0.1579513	-0.285262	0.775444	0.975687
hmbox1a	35.95509905	0.075065812	0.3169055	0.2368713	0.812757	0.981081
tgfb2	72.22653857	-0.189461194	0.2314771	-0.818488	0.413079	0.905281
ptgdsb.1	3829.331794	0.614128505	0.0899635	6.8264219	8.71E-12	2.53E-09
psmc4	2807.232217	-0.014171684	0.0828819	-0.170986	0.864234	0.98601
zfr	4343.796781	0.040685657	0.0678701	0.5994633	0.548864	0.946312
ephb4b	394.4292671	-0.022335818	0.1222376	-0.182725	0.855014	0.985174
tmem218	35.94876151	-0.401883661	0.3439884	-1.168306	0.242683	0.811434
tmem169a	93.44051437	-0.347931799	0.2064861	-1.685013	0.091986	0.57849
nup160	961.167711	-0.264665326	0.0999393	-2.64826	0.008091	0.1451
ube2l3b	1864.893065	0.048355501	0.0811312	0.5960164	0.551164	0.946571
aptx	140.0603899	0.020043936	0.1774675	0.1129443	0.910075	0.990702
gabrr3a	44.90630201	0.190442398	0.3129389	0.6085609	0.542816	0.944974
tpd52l2a	872.9312455	0.071368871	0.0863303	0.8266954	0.40841	0.90171
fbxo28	155.6474657	0.046264543	0.1697805	0.2724962	0.78524	0.97627
igl3v2	0.158795395	0.967652056	4.0804729	0.2371421	0.812547	NA
kpna7	4.676865706	1.089650127	0.8837111	1.2330389	0.217561	NA
phactr2	196.0575074	0.158450197	0.1519403	1.0428449	0.29702	0.853798
rnf25	548.4109467	-0.251384934	0.1048029	-2.398646	0.016456	0.236409
namptb	255.5291102	-0.178840403	0.138475	-1.291499	0.196531	0.761835
kat8	552.5045441	-0.024371464	0.1019703	-0.239006	0.811101	0.981081

hcn3	27.27279517	0.651610811	0.3769648	1.7285721	0.083886	0.557501
si:dkey-16l2.17	84.14026322	-0.04800472	0.2170366	-0.221183	0.82495	0.982196
smad1	949.9863917	-0.169305263	0.0863663	-1.960316	0.049959	0.437835
gabarapl2	817.5153195	-0.108102759	0.0915791	-1.18043	0.237829	0.806522
adgrg1	289.2443032	-0.219584917	0.1341751	-1.636555	0.101723	0.605868
ift88	290.4997398	0.059780593	0.1287116	0.4644537	0.642323	0.959994
rs1a	3632.410563	0.127470446	0.0869726	1.4656385	0.142747	0.684998
btg1	5487.796106	-0.171176218	0.0644203	-2.657178	0.00788	0.142427
numb	483.4515477	0.111445196	0.1061708	1.049678	0.293866	0.849999
kcnf1b	63.01286987	-0.105230561	0.2713149	-0.387854	0.698124	0.967998
nrp1b	101.603955	0.252381287	0.1987567	1.2698	0.204156	0.771764
lsp1	802.362024	-0.063503702	0.1074165	-0.591191	0.554392	0.947449
tcp11l2	1167.961813	-0.070181834	0.0863815	-0.812464	0.416526	0.906237
lin7c	1610.056615	0.11059677	0.0846808	1.3060433	0.191538	0.755886
mpzl2b	401.7106392	-0.101784528	0.1160232	-0.877277	0.380336	0.895192
zmym2	1316.917346	-0.093239446	0.0810381	-1.150563	0.249912	0.819849
slc25a4	7169.700744	0.361607054	0.0835647	4.3272683	1.51E-05	0.001002
cd2bp2	419.6053094	-0.106877212	0.1292478	-0.826917	0.408284	0.90171
si:ch211-106h11.3	92.02360107	0.065767986	0.2158695	0.3046654	0.760621	0.975374
ccdc171	92.7824909	0.197542017	0.2161971	0.9137128	0.360868	0.885686
ccnd3	2.941894425	0.989209447	1.1194072	0.8836904	0.376863	NA
si:ch211-150g13.3	174.303894	0.039239118	0.1531949	0.2561386	0.797844	0.978695
vangl2	1340.450064	-0.008595628	0.0860187	-0.099927	0.920402	0.992049
zgpat	333.5221752	0.108747808	0.1197463	0.9081517	0.363798	0.886174
trappc4	505.494247	0.012139434	0.1108663	0.1094962	0.912809	0.991119
gad1b	3796.09525	0.217193101	0.0737326	2.9456879	0.003222	0.07565
igf1ra	70.99555177	0.21801433	0.2351483	0.9271355	0.353856	0.881976
slc25a3a	1109.689426	0.30937864	0.1037462	2.9820732	0.002863	0.06955
rpp30	84.03765071	0.084244402	0.2156159	0.3907151	0.696008	0.96736
mettl26	207.6264136	-0.071250283	0.1477561	-0.482216	0.629653	0.957354
rmnd1	200.3597808	0.07150721	0.1488475	0.4804058	0.630939	0.957354
hsd17b1	0.166657454	-0.955901296	4.0804729	-0.234262	0.814781	NA
gphb5	6.41666387	-0.837675077	0.7629314	-1.097969	0.272218	NA
uros	383.9988803	-0.083648365	0.1275486	-0.655816	0.511943	0.936253
elovl4b	1446.538219	0.170691783	0.1145706	1.4898391	0.136267	0.673089
mast1a	49.56260527	-0.612879722	0.2878991	-2.128801	0.033271	0.355427
oxsr1b	417.7313092	0.042156348	0.1136574	0.3709072	0.710707	0.968985
usp20	481.7070384	0.088838553	0.1080646	0.8220878	0.411027	0.903291
trpc7a	10.44642062	0.346090168	0.6088096	0.5684703	0.569716	NA
hmox1a	200.0015083	0.17105517	0.4732525	0.3614459	0.717766	0.96909
noxred1	4.344220591	1.188606194	0.9282832	1.2804348	0.200392	NA
TPGS1	116.6589367	-0.457407853	0.1897109	-2.411078	0.015905	0.232457
mapk1	145.0693731	0.225632565	0.1693848	1.3320707	0.182837	0.745944
pak6a	9.103067784	0.122908312	0.6440096	0.1908486	0.848644	NA
as3mt	139.6591318	-0.19381563	0.1733239	-1.118228	0.26347	0.830245
angptl7	501.1005856	0.352315081	0.117316	3.0031295	0.002672	0.066215
ttpa	161.7965821	-0.191471481	0.1601147	-1.195839	0.231759	0.799932
top3b	321.9034961	-0.147560155	0.127465	-1.157652	0.247006	0.816669
htr7b	3.769007976	0.661154778	0.9918423	0.6665926	0.505032	NA
fzd7b	803.3543019	-0.060819114	0.0925918	-0.656852	0.511276	0.936253
2-Sep	1603.065428	0.135496004	0.0855595	1.5836464	0.113274	0.631061
selenot1b	480.1118328	-0.196810989	0.1097524	-1.793227	0.072937	0.526902
fam221a	11.16497974	0.395857481	0.6028865	0.6566037	0.511436	0.936253
pdlim5b	418.3687208	-0.149752565	0.1250176	-1.197852	0.230975	0.799177

lrfn1	41.58739526	0.022315013	0.2995983	0.0744831	0.940626	0.992857
hpn	51.87295781	-0.107459753	0.2942094	-0.365249	0.714925	0.96909
cavin2a	977.1146086	-0.026386641	0.0880665	-0.299622	0.764466	0.975374
gatad1	283.7915243	0.172599734	0.1291284	1.3366525	0.181336	0.742543
grip2b	12.29678042	0.055350815	0.5620326	0.0984833	0.921549	0.992138
bco2l	32.4157934	-0.134193118	0.3480881	-0.385515	0.699856	0.967998
adra1ab	55.4520092	-0.320848229	0.271758	-1.180639	0.237746	0.806474
zgc:165539	2.158525443	-0.615437506	1.3478612	-0.456603	0.647956	NA
cpsf3	1102.45833	-0.067924333	0.0826418	-0.821912	0.411127	0.903291
crhb	76.92551945	-0.085227766	0.2217032	-0.384423	0.700665	0.967998
irf10	7.487835011	-0.254442795	0.726632	-0.350167	0.726213	NA
si:ch211-13315.8	27.74674781	-0.235271401	0.3727895	-0.631111	0.527968	0.941647
tmem230a	68.49507497	0.086631859	0.2402174	0.3606395	0.718369	0.96909
pold1	767.1104869	-0.051024942	0.0981332	-0.519956	0.603094	0.956045
zgc:103510	5.929972633	0.828624947	0.8334086	0.9942602	0.320096	NA
btbd16	2.984019679	0.040462297	1.1147069	0.0362986	0.971044	NA
dgkb	15.68216541	0.257970987	0.4964672	0.5196133	0.603333	0.956045
srsf5b	4883.144568	-0.072338068	0.0642299	-1.126237	0.260065	0.828288
si:ch211-13c6.2	915.1383675	0.031026913	0.0911128	0.3405329	0.733455	0.971993
adcyap1b	1562.021112	0.096177489	0.0823158	1.1683968	0.242647	0.811434
gadd45ba	741.4038669	-0.068665002	0.1263244	-0.543561	0.586744	0.953319
hirip3	329.64455	-0.153985801	0.1216632	-1.265673	0.20563	0.77367
dpp7	432.7442853	0.118148288	0.1341309	0.880843	0.378403	0.895192
tnfaip3	19.57656429	-0.41324449	0.4362287	-0.947312	0.34348	0.878434
adcy6b	155.5190887	0.030366347	0.162926	0.1863813	0.852146	0.985174
ucmaa	39.7989858	0.414108088	0.3154801	1.3126282	0.189308	0.753954
sbds	302.6838588	-0.042106145	0.1219423	-0.345296	0.729872	0.971143
frk	160.4308962	0.077035344	0.1634176	0.4714019	0.637354	0.958872
nus1	782.160283	0.006035858	0.1007706	0.059897	0.952238	0.993904
tmtops2b	17.32814056	0.08845322	0.4707741	0.1878889	0.850964	0.985174
top1mt	408.0975146	0.085907997	0.1116793	0.7692384	0.441752	0.916336
naa50	1432.720245	-0.026491352	0.0921659	-0.287431	0.773782	0.975687
pudp	80.83902266	-0.274558661	0.2212335	-1.241035	0.214593	0.782493
grin1a	3625.850916	0.162797504	0.0699938	2.3258853	0.020025	0.266383
ccdc191	30.42855682	-0.28401931	0.38211	-0.743292	0.457305	0.921679
paplna	349.9069806	0.28239483	0.1238226	2.2806397	0.02257	0.286638
zfyve21	381.8791422	-0.059594902	0.1241686	-0.479951	0.631262	0.957356
sem1	1864.595975	-0.083150979	0.0901321	-0.922546	0.356244	0.883307
adamtsl3	12.87308652	0.682787293	0.5792836	1.1786754	0.238527	0.807659
si:ch211-216p19.6	10.83317595	0.026148754	0.6340309	0.0412421	0.967103	NA
zc2hc1a	619.3730579	0.055559873	0.1051717	0.5282779	0.597307	0.955244
fntb	575.6134482	-0.059789488	0.1092199	-0.547423	0.584088	0.952214
naprt	381.6070187	-0.16244779	0.1160119	-1.400268	0.161433	0.715364
glis1b	31.09510505	-0.321412483	0.3408459	-0.942985	0.345689	0.87848
rad18	179.4515918	-0.048952241	0.1529576	-0.320038	0.74894	0.97419
kcnf1a	45.65244925	-0.831038701	0.2940422	-2.826257	0.00471	0.09895
fgf12a	444.5172773	0.097870214	0.1083883	0.9029593	0.366548	0.88734
camkva	173.0093495	-0.024133068	0.1590257	-0.151756	0.87938	0.987881
ap1s3b	165.6154008	-0.062458113	0.1636598	-0.381634	0.702733	0.968134
yy1b	721.3062423	-0.155343436	0.0947357	-1.639756	0.101056	0.604075
gstz1	236.7765852	0.157964328	0.1665908	0.9482178	0.343019	0.878434
slc35d1b	186.5297522	-0.544226417	0.1567875	-3.471109	0.000518	0.018364
hao2	335.1694066	-0.045739205	0.1279274	-0.35754	0.720687	0.969381
pfkpa	3186.8235	0.04576353	0.069544	0.6580515	0.510505	0.936253

tpmt.2	4.743827517	0.948095335	0.9263831	1.0234376	0.306101	NA
tp53inp1	392.5497221	0.242403669	0.1111603	2.1806682	0.029208	0.331124
trim63a	551.9514488	0.026741378	0.1366699	0.195664	0.844873	0.985174
rdh8a	665.8396106	0.054244496	0.0954	0.5686006	0.569627	0.948983
bmp1b	109.4176691	0.401130348	0.2008003	1.9976577	0.045754	0.419441
traf6	265.8001418	0.071073227	0.1359667	0.5227251	0.601166	0.956045
diras1a	1871.879524	0.055945247	0.085266	0.6561258	0.511743	0.936253
bnip3lb	1251.035829	-0.151906257	0.0931379	-1.630983	0.102894	0.609169
bmp1a	961.259483	0.092033125	0.0971154	0.9476675	0.343299	0.878434
mapkapk5	247.5686339	-0.041065895	0.1328917	-0.309018	0.757308	0.975374
zbtb2b	606.7386234	0.0209601	0.1006678	0.2082106	0.835065	0.984089
aldh2.2	1547.991252	-0.189100412	0.0825735	-2.290085	0.022016	0.281963
galk1	67.97290343	-0.03822238	0.2492594	-0.153344	0.878127	0.987881
cldn23a	466.4501947	0.318534221	0.1118026	2.8490761	0.004385	0.093935
fut9d	1416.627993	0.391193541	0.0964684	4.0551453	5.01E-05	0.002737
sh2d3ca	113.1052446	-0.041963021	0.1902071	-0.220617	0.82539	0.982196
glrx	239.2599189	-0.065469984	0.1367013	-0.478927	0.63199	0.957356
dscamb	1256.411788	-0.010970328	0.100088	-0.109607	0.912721	0.991119
sumo3a	4451.142864	-0.041107366	0.0894243	-0.459689	0.645739	0.96029
hccsa.1	226.7639373	-0.057496906	0.1486533	-0.386785	0.698915	0.967998
pax2a	773.8402629	-0.015870289	0.0930253	-0.170602	0.864537	0.98601
tead1a	39.21060242	-0.170226714	0.3112962	-0.546832	0.584494	0.952406
prg4b	52.45249064	0.447795943	0.3063439	1.4617428	0.143812	0.687224
cst14a.1	11.55475337	0.265415043	0.59623	0.4451555	0.656207	0.961833
slc4a2a	352.4272401	-0.137464226	0.1201831	-1.14379	0.252711	0.822335
cradd	27.08011148	0.507804648	0.3861179	1.3151544	0.188458	0.753276
ube2v2	2557.076265	-0.03478833	0.0825446	-0.421449	0.673427	0.965257
commd8	249.913681	-0.023184365	0.1392116	-0.16654	0.867732	0.987245
ttn.2	12557.01062	0.288328816	0.0808565	3.5659329	0.000363	0.013708
itgb8	39.38620665	-0.009983859	0.3095133	-0.032257	0.974267	0.99644
zbtb18	1304.703765	-0.005937545	0.0956807	-0.062056	0.950518	0.99381
cyb561a3b	6.168052516	1.071921123	0.7713767	1.3896207	0.164644	NA
aldh3a2a	289.8198855	-0.288394703	0.1280472	-2.252252	0.024306	0.301184
sult1st1	895.9050311	-0.343625762	0.098069	-3.50392	0.000458	0.016695
si:ch211-196h16.12	21.554232	-0.823469752	0.4788045	-1.719846	0.085461	0.561427
mkrn4	200.5665588	-0.323256635	0.149514	-2.162049	0.030614	0.338573
prph	626.2100126	0.129635954	0.0999343	1.2972112	0.194559	0.758506
synj2bp	253.3643242	0.211961622	0.1437677	1.4743342	0.140392	0.680585
pex14	393.8867322	-0.06738555	0.116852	-0.576674	0.56416	0.948739
gmfb	2234.584364	0.060813255	0.0746199	0.8149742	0.415087	0.906237
nitr9	0	NA	NA	NA	NA	NA
hmga1a	26455.74253	-0.10887013	0.0680192	-1.60058	0.10947	0.623766
dhdhl	745.6625255	-0.291356537	0.100846	-2.889124	0.003863	0.085067
tesca	87.12216908	-0.167196637	0.2146796	-0.778819	0.436086	0.914408
pax3b	9.050933292	-0.196853081	0.6302434	-0.312345	0.754779	NA
stxbp6l	93.59950667	0.35915034	0.2346123	1.5308246	0.125813	0.654803
sult2st3	123.4896905	0.287634054	0.1794611	1.6027653	0.108986	0.622321
trub2	247.3932427	0.086111879	0.150513	0.5721227	0.567239	0.948739
htatip2	207.0972698	-0.194700543	0.1511482	-1.288144	0.197696	0.762928
rab44	3.351597534	0.290618934	1.0156842	0.2861312	0.774778	NA
daam2	13.27041622	1.214244849	0.5465376	2.2217043	0.026303	0.312554
fkbp5	1075.970459	0.422497188	0.0924873	4.5681649	4.92E-06	0.000394
rhogc	538.3353686	-0.139543076	0.1057047	-1.320121	0.186795	0.750951
adnp2b	859.2059684	0.065852111	0.090461	0.7279617	0.466637	0.924218

cplane2	61.37467353	-0.152036651	0.2574654	-0.590513	0.554847	0.947449
edem2	323.9564345	-0.2151117	0.1404992	-1.531053	0.125756	0.654803
rps6ka2	32.57713707	-0.018374234	0.3464729	-0.053032	0.957706	0.99467
lin9	267.0048482	0.057736426	0.1442811	0.4001662	0.689034	0.966721
si:ch211-173n18.3	72.17183491	0.287127997	0.2447709	1.1730481	0.240777	0.809163
cabp5b	162.6174957	0.298422178	0.169515	1.7604466	0.078332	0.545401
itgb4	1152.838054	-0.02883621	0.0880318	-0.327566	0.74324	0.973687
hbp1	693.2390416	-0.217303659	0.098544	-2.205144	0.027444	0.320231
c1ql3b	49.94443213	0.293648102	0.2775015	1.0581858	0.289971	0.846246
inip	190.6914657	0.038685354	0.1480425	0.2613125	0.793852	0.977937
macf1a	4891.59491	0.060465107	0.0800753	0.755103	0.450187	0.918761
morc2	1243.084977	-0.061758782	0.0831796	-0.742475	0.457799	0.921978
si:ch211-121a2.4	19.3829915	0.27621819	0.4284031	0.6447623	0.519081	0.938945
si:ch211-114c12.2	1930.708697	-0.058691268	0.0868372	-0.675877	0.499119	0.934006
ndufs1	4.858587777	-0.655516282	0.9120754	-0.718708	0.472321	NA
plppr3b	713.7545073	0.189450408	0.0978736	1.9356639	0.052909	0.451736
march5l	446.4355434	0.010517383	0.112153	0.0937771	0.925286	0.992676
jkamp	347.8086615	0.145561181	0.120817	1.2048074	0.228278	0.795541
si:ch211-234h8.7	80.94429689	0.118410806	0.2263583	0.5231122	0.600896	0.956045
gpr135	44.11078557	0.535459262	0.2924069	1.8312128	0.067069	0.506251
mospd1	592.3474171	-0.182779493	0.1135427	-1.609786	0.107445	0.617344
krt18b	1048.92341	-0.157316341	0.1242716	-1.265907	0.205546	0.773646
jph2	1071.453264	0.283048883	0.1035609	2.7331635	0.006273	0.120609
gdap1l1	41.2137742	-0.379467849	0.2968196	-1.278446	0.201092	0.766855
ms4a17c.2	0	NA	NA	NA	NA	NA
cntfr	1141.626222	-0.044294522	0.0868485	-0.51002	0.610037	0.95688
tek	94.31589936	0.05652221	0.2234971	0.2528991	0.800346	0.979377
ahsa1a	116.5344442	0.61381863	0.1875541	3.2727552	0.001065	0.032585
nuak1b	1435.227581	-0.044920046	0.0821781	-0.546618	0.584641	0.952411
exosc2	252.3705962	0.086218682	0.1468167	0.5872538	0.557033	0.948478
crkl	1383.916067	0.101787849	0.0777785	1.3086895	0.19064	0.755212
mindy3	723.3282302	0.003547859	0.1003841	0.0353428	0.971806	0.996315
mapk14b	524.6900152	0.245255363	0.1034722	2.370253	0.017776	0.247053
adra1aa	1.863520974	1.446597312	1.5038905	0.9619034	0.336098	NA
stat4	122.7895263	-0.596632122	0.2128224	-2.803427	0.005056	0.10404
msnb	47.20466362	-0.285055186	0.3060306	-0.93146	0.351616	0.880769
tm9sf3	1462.92412	0.101648711	0.0800703	1.2694938	0.204265	0.772006
gzmk	2.054383069	0.550735228	1.428156	0.3856268	0.699773	NA
si:dkey-24p1.6	37.41444665	0.139735887	0.3362672	0.4155501	0.677739	0.965257
chst12a	11.01325852	0.357061978	0.5960496	0.5990475	0.549141	NA
nlk2	146.1696388	0.020776218	0.1659454	0.1251991	0.900366	0.990121
CYTH2	31.96546021	0.297893693	0.3417181	0.8717527	0.383343	0.895192
ankrd9	376.1004002	0.198658009	0.1139053	1.7440626	0.081148	0.551495
zgc:162945	160.4026944	-0.027243742	0.1807235	-0.150748	0.880174	0.987881
tmed3	489.8030619	0.157335856	0.1126471	1.396715	0.162499	0.717086
slc34a1a	103.966052	0.414816424	0.272524	1.5221283	0.127977	0.658954
calcr	69.27700428	0.091585262	0.2513722	0.3643412	0.715603	0.96909
lsm12a	529.2068654	-0.025269467	0.1159883	-0.217862	0.827536	0.982721
btr16	4.581753827	-1.870778342	0.9780539	-1.912756	0.055779	NA
sgsm1a	9.282884172	0.209780783	0.7016689	0.298974	0.76496	NA
rdh20	23.90809798	0.108379954	0.4003535	0.2707106	0.786614	0.976596
vipr1a	69.6250049	0.098040419	0.2375666	0.4126861	0.679837	0.965311
ndufb10	1998.893351	-0.131602233	0.0797634	-1.649907	0.098962	0.598346
mrgbp	378.8506607	-0.006608535	0.1201075	-0.055022	0.956121	0.994543

plpp2b	222.6835823	0.04083968	0.1419755	0.2876531	0.773612	0.975687
tekt4	44.35204615	0.098413255	0.2882545	0.3414109	0.732794	0.971719
odf2a	98.64574362	0.098820649	0.2000193	0.4940557	0.621267	0.957354
si:dkey-10h3.2	2.945908661	-0.971398854	1.1481508	-0.846055	0.397522	NA
taf1b	107.6408985	0.041538867	0.1965991	0.2112872	0.832663	0.983557
mtss1la	551.0709636	0.058666447	0.1054313	0.5564422	0.577909	0.951024
ZBTB26	112.2362349	-0.211049206	0.2105661	-1.002294	0.316201	0.866931
maff	848.3308608	-0.095053056	0.0933903	-1.017805	0.308771	0.861088
cpsf2	898.6540863	-0.100904167	0.0959798	-1.051306	0.293118	0.848999
tekt2	14.06139806	0.436297576	0.5247933	0.8313703	0.405764	0.901305
pus3	74.30386691	-0.101297552	0.2242365	-0.451744	0.651453	0.960708
pcca	948.01599	-0.058357111	0.0853414	-0.683808	0.494097	0.931926
eif1axa	370.0782607	-0.320159694	0.132473	-2.416793	0.015658	0.229392
xpnpep1	770.9608363	-0.3422985	0.0904671	-3.783681	0.000155	0.006976
cdkn1ba	536.6987471	-0.101858232	0.1041449	-0.978044	0.328053	0.872701
epb41b	96.42518664	0.336490169	0.2347398	1.4334604	0.151726	0.700137
rab32a	116.8464453	-0.025570762	0.1938853	-0.131886	0.895074	0.989814
zdhhc23a	21.11253113	0.735028312	0.4315775	1.7031199	0.088546	0.569447
nup12	105.0203684	0.189508902	0.2045351	0.9265348	0.354168	0.882044
peli2	264.978851	-0.104622876	0.1317726	-0.793965	0.427216	0.91075
gbe1a	6.972967549	0.034583252	0.7466146	0.0463201	0.963055	NA
tm6sf2	233.172279	-0.296009122	0.1757153	-1.684595	0.092067	0.57849
rbbp4	2142.059943	0.031942728	0.0911639	0.3503881	0.726047	0.970402
clpxa	1961.674585	0.042311558	0.0755784	0.5598369	0.575591	0.950373
uqcrq	1729.740058	-0.027374421	0.2254156	-0.12144	0.903343	0.990121
tnni2a.4	17601.41689	0.214307582	0.1765554	1.2138266	0.224814	0.792185
creld2	156.5417807	0.136828114	0.1729406	0.7911854	0.428836	0.911406
klf6a	2019.332183	-0.010147928	0.088375	-0.114828	0.908581	0.990702
pfkfb4b	1173.158887	0.044366505	0.0873315	0.5080243	0.611436	0.95688
guf1	164.6331177	0.120483598	0.1712839	0.7034146	0.481797	0.928392
chchd1	410.97083	-0.005616713	0.1392131	-0.040346	0.967817	0.996315
ftr51	116.3888569	-0.26640428	0.1984047	-1.342732	0.179359	0.740258
ube2d1a	350.4460062	0.068054433	0.1230359	0.5531268	0.580177	0.951357
gem	204.7587782	0.07404447	0.1520275	0.4870467	0.626225	0.957354
si:dkey-40m6.8	1345.100593	0.091690727	0.0785103	1.1678821	0.242854	0.811584
adamts15a	95.02791179	-0.015192606	0.2028554	-0.074894	0.940299	0.992857
tubgcp3	816.3657128	0.154186732	0.0894174	1.724349	0.084645	0.560351
lrp1ab	2053.636437	-0.065655258	0.0933083	-0.703638	0.481658	0.928386
hsp90ab1	58549.49358	-0.264230689	0.0645678	-4.092296	4.27E-05	0.002379
med23	575.6864905	-0.119834493	0.1128736	-1.06167	0.288386	0.845979
ppfibp2b	437.3728808	-0.092650023	0.1085473	-0.853545	0.393357	0.898076
si:ch211-200p22.4	1828.339682	0.058997421	0.0784346	0.7521865	0.451939	0.918761
lnx2a	687.694241	-0.092063839	0.1017161	-0.905106	0.365409	0.886903
foxd1	256.103583	-0.128800592	0.136472	-0.943788	0.345278	0.878434
tyrp1a	312.1228091	-0.255197781	0.1363947	-1.871024	0.061342	0.482931
ube2z	642.7232186	0.067075135	0.1023966	0.6550521	0.512434	0.936253
avpr2ab	2.414620719	-0.211494448	1.2703758	-0.166482	0.867778	NA
pnp4b	129.4829271	0.679561901	0.1952834	3.479875	0.000502	0.017905
FO704779.1	20.84329809	-0.185516437	0.4540568	-0.408575	0.682851	0.965476
stxbp5b	107.0305526	0.07492194	0.2014318	0.3719468	0.709932	0.968928
syt9b	474.5226543	-0.007983981	0.106369	-0.075059	0.940168	0.992857
fbxo3	427.2139853	-0.180058663	0.1228148	-1.466099	0.142621	0.684998
fubp1	6034.807156	-0.043266786	0.0706208	-0.612663	0.540099	0.944206
poli	105.7337672	0.002811687	0.2053599	0.0136915	0.989076	0.997589

ssb	1406.935588	-0.110816814	0.0805367	-1.375979	0.168828	0.725919
zgc:136493	74.05310751	-0.025342159	0.232271	-0.109106	0.913118	0.991119
hoxb3a	985.8588457	-0.036343151	0.0884346	-0.410961	0.681101	0.965476
stx11b.1	245.194197	0.217200837	0.13774	1.5768905	0.114821	0.635451
ipmkb	449.6048266	-0.061786584	0.1085687	-0.569101	0.569287	0.948983
zgc:55943	650.69849	0.043692997	0.0974292	0.4484592	0.653822	0.961168
nexmifb	554.6762384	0.145606529	0.1140403	1.2767989	0.201673	0.767736
baiap2l1a	192.0884809	0.046930552	0.1597564	0.2937631	0.768939	0.975687
rmc1	172.9902127	-0.047890055	0.164245	-0.291577	0.77061	0.975687
prr15lb	68.15575239	-0.207480279	0.237753	-0.872672	0.382842	0.895192
atxn7l3	443.1084222	-0.074599529	0.1254327	-0.594738	0.552019	0.947215
serpine2	555.3199213	-0.09681164	0.1301109	-0.74407	0.456834	0.921215
mboat1	155.8412531	0.114345462	0.1638728	0.6977694	0.485321	0.930188
ankrd6b	185.0362316	-0.208288013	0.1788026	-1.164905	0.244057	0.813415
rasa4	285.5815926	-0.039021929	0.1293077	-0.301776	0.762823	0.975374
aldh3a2b	27.97881943	-0.655085454	0.3755566	-1.744305	0.081106	0.551495
si:dkey-90m5.4	104.3598206	0.932203814	0.2120159	4.3968577	1.10E-05	0.000793
sort1a	550.7339259	0.005116791	0.103371	0.0494993	0.960521	0.995421
h2afx	586.337296	-0.121323027	0.1023406	-1.185483	0.235826	0.804837
mtx2	867.8139759	0.040885705	0.0918479	0.4451456	0.656215	0.961833
si:ch211-147k10.5	6.651179934	-0.732036924	0.7367814	-0.993561	0.320437	NA
tph1a	51.3432195	-0.297800251	0.2762338	-1.078073	0.281001	0.84357
atp2a2a	779.8662111	0.064332001	0.0963199	0.6678995	0.504198	0.935448
zgc:92242	557.1929398	-0.10549128	0.1021935	-1.032269	0.301946	0.856944
zgc:56676	1594.816041	-0.045662857	0.0910032	-0.501772	0.615828	0.95688
cacna1sa	336.4281153	0.128625222	0.1293169	0.9946514	0.319906	0.868153
cd209	0.349228403	0.005884014	3.2372587	0.0018176	0.99855	NA
asap1a	1657.913006	0.02535559	0.0771414	0.3286898	0.74239	0.973154
afdna	1273.061171	-0.109627449	0.0827476	-1.324842	0.185224	0.749153
gstm.2	134.2768548	-0.054644727	0.1750682	-0.312134	0.754939	0.97522
camk1b	527.3176951	0.096592144	0.1184662	0.8153563	0.414868	0.906237
ldlra	518.2046399	-0.20090195	0.1187395	-1.691956	0.090654	0.576398
zgc:86609	245.7117411	-0.008999748	0.1347237	-0.066802	0.94674	0.99381
specc1	150.0686342	-0.05772196	0.1702865	-0.33897	0.734633	0.972021
ush2a	55.88638938	0.147047419	0.2571525	0.5718296	0.567437	0.948739
f9b	93.71835718	-0.258680129	0.2095639	-1.234373	0.217064	0.784302
tfcp2l1	89.65651001	0.597901634	0.2181478	2.7408094	0.006129	0.118579
rpl34	11380.80053	-0.117052356	0.0767952	-1.524214	0.127455	0.657527
gsk3aa	328.5667859	-0.029837007	0.126494	-0.235877	0.813528	0.981081
timm17a	1972.451053	0.163928497	0.0822673	1.9926333	0.046302	0.422877
kcns3b	0.317831496	1.808837541	3.5779923	0.5055454	0.613176	NA
impdh1b	1427.433755	-0.246687457	0.0793603	-3.108451	0.001881	0.051014
rpl18	17104.94237	-0.287131776	0.068843	-4.170819	3.04E-05	0.00176
id2b	287.0048435	0.058539299	0.1264789	0.4628383	0.64348	0.959994
grem1a	11.82522161	2.046450219	0.662139	3.0906657	0.001997	0.053438
kansl3	1200.447682	0.023307042	0.0879073	0.265132	0.790908	0.977402
snapc1a	1.920153526	0.036725403	1.5303945	0.0239973	0.980855	NA
tbc1d7	392.0428258	-0.018455773	0.1246999	-0.148001	0.882342	0.988344
msra	236.0516817	0.10926954	0.1543494	0.7079361	0.478985	0.926978
trim55a	138.6180107	0.15447438	0.1787038	0.8644159	0.387359	0.895778
wdr32	418.7220589	-0.04654301	0.1182104	-0.39373	0.69378	0.96736
macrod1	51.84653906	-0.260954466	0.2677989	-0.974442	0.329837	0.873938
gpkow	531.9638226	0.050299147	0.102893	0.4888493	0.624948	0.957354
zgc:77056	358.2976036	-0.034423371	0.1204099	-0.285885	0.774966	0.975687

sgms1	289.3423774	0.069703794	0.1281161	0.5440674	0.586395	0.953319
si:dkey-47k20.3	0.809821446	0.539374789	2.2312571	0.2417358	0.808985	NA
tomm70a	1823.62622	-0.083582805	0.1051306	-0.795038	0.426592	0.910189
commd5	118.5040839	0.072875764	0.1838948	0.3962905	0.691891	0.96733
rab1ab	1745.625669	-0.042184394	0.0782576	-0.539045	0.589856	0.953762
criml	49.82547129	0.066065257	0.2800706	0.2358879	0.81352	0.981081
xpr1b	978.3476617	-0.000264963	0.096057	-0.002758	0.997799	0.999174
hsp70.1	410.9562	0.0062844	0.3782485	0.0166145	0.986744	0.997062
tkta	2689.931142	0.107215554	0.0725496	1.4778237	0.139455	0.678238
rufy3	1074.664648	0.074488451	0.0939726	0.7926614	0.427975	0.910858
pgp	460.2945583	0.086401387	0.1124422	0.7684073	0.442245	0.916336
lrrc30a	42.12513299	-0.091916967	0.298953	-0.307463	0.758491	0.975374
p2rx3b	142.579741	-0.119947793	0.1775562	-0.675548	0.499327	0.934006
si:dkeyp-9d4.2	2.990821905	-1.027487153	1.2464268	-0.824346	0.409743	NA
hmgb2a	11.53039839	-0.25247504	0.5653764	-0.446561	0.655192	0.961614
pqbp1	368.7563955	-0.196106296	0.1268587	-1.545863	0.122138	0.649408
clybl	424.7162549	-0.093336486	0.1103581	-0.84576	0.397687	0.899672
mep1a.1	25.49099782	0.792267305	0.3950753	2.0053579	0.044925	0.417005
wbp11b	102.9169165	0.064437945	0.2021547	0.3187556	0.749912	0.97419
mpp6b	1101.698385	0.070977006	0.0998848	0.7105884	0.477339	0.926857
mef2ca	1242.24574	0.192123186	0.0907348	2.1174143	0.034225	0.359596
nr1i2	151.0170655	0.006572292	0.1721561	0.0381764	0.969547	0.996315
wapl b	1130.000232	-0.107002734	0.0884733	-1.209435	0.226496	0.793959
dedd	793.7560305	0.01158159	0.0892402	0.12978	0.896741	0.989971
prxl2b	605.4320719	-0.323930706	0.1049152	-3.087547	0.002018	0.053856
oxgr1b	0.515216064	0.926382026	2.6525479	0.3492423	0.726907	NA
srsf5a	6985.361626	-0.105499383	0.0695562	-1.51675	0.12933	0.661083
cel.2	179.4055394	-0.09605189	0.1704635	-0.563475	0.573112	0.949762
myf6	29.93966577	0.318192466	0.379518	0.838412	0.401799	0.90084
slc26a1	234.5977368	-0.143645437	0.1351069	-1.063198	0.287692	0.845979
si:dkey-23a13.11	0.316103113	0.005883447	3.3519121	0.0017553	0.9986	NA
tmprss9	161.3111445	0.104267854	0.176979	0.5891538	0.555758	0.947621
zgc:161973	32.76611113	0.141692245	0.3463237	0.4091324	0.682443	0.965476
cnn3b	579.5347465	0.074761233	0.1019078	0.7336165	0.463183	0.922698
armh1	16.32196314	0.037634048	0.4747735	0.0792674	0.93682	0.992702
rassf2a	665.5412076	0.102886782	0.0928652	1.1079156	0.267898	0.833349
slc6a14	62.70191219	-0.061981695	0.2452553	-0.252723	0.800482	0.979377
khk	159.4204609	-0.559789335	0.1619869	-3.45577	0.000549	0.019137
kcnh2a	22.92485402	-0.296755138	0.4367948	-0.679393	0.496889	0.932829
rab41	2194.808586	0.101083632	0.0765838	1.3199089	0.186865	0.750967
si:dkeyp-86f7.4	4.292228698	-0.737206766	1.0665499	-0.691207	0.489435	NA
slc2a15a	73.61668058	-0.361960435	0.2333025	-1.551464	0.120791	0.645876
cnga1a	102.8729371	0.141681491	0.2290625	0.6185276	0.536228	0.943336
phyhd1	399.3895572	0.04975669	0.119476	0.4164577	0.677075	0.965257
trim54	11.51608967	-0.594389103	0.5628666	-1.056003	0.290967	0.846568
casc3	1069.422723	0.090560268	0.0892272	1.0149406	0.310134	0.862409
ap3m1	312.5754163	0.025411898	0.1256263	0.2022816	0.839697	0.984979
SPDEF	23.78945361	-0.588950061	0.408036	-1.443378	0.148914	0.696454
atp13a1	799.8877481	0.112511122	0.1040747	1.0810609	0.27967	0.843169
grtp1a	319.6808119	0.056314234	0.1216778	0.4628142	0.643498	0.959994
parpbp	168.6279777	-0.38191157	0.1578613	-2.419285	0.015551	0.228768
ampd2b	626.2510031	-0.050365175	0.0964287	-0.522305	0.601458	0.956045
glb1l	238.4683501	0.035650708	0.1589295	0.2243178	0.82251	0.982189
si:dkey-42l23.9	0	NA	NA	NA	NA	NA

tnni2b.2	3177.030259	0.363357394	0.0909293	3.9960442	6.44E-05	0.003387
BX511121.1	4.854766203	-0.351511331	0.9707965	-0.362085	0.717288	NA
slc6a7	248.5806117	0.099000905	0.1366028	0.7247356	0.468614	0.924328
lrrfip1a	434.375699	0.011368463	0.1208657	0.0940587	0.925063	0.992676
agfg1a	366.801705	0.085072541	0.1165949	0.729642	0.465609	0.923865
nup188	1068.237648	-0.151030807	0.0841024	-1.795797	0.072527	0.52617
pimr135	0.332645115	0.005883744	3.2924249	0.0017871	0.998574	NA
grcc10	403.932866	0.007747847	0.1216354	0.0636973	0.949211	0.99381
eef1db	2802.076706	-0.311083456	0.0826538	-3.763694	0.000167	0.007357
tbx4	61.40248708	0.239412545	0.2707167	0.8843657	0.376499	0.89493
plekha1a	591.1211831	-0.063897632	0.102699	-0.622184	0.533821	0.943276
glipr1b	189.2293399	0.240805254	0.1675774	1.436979	0.150724	0.698912
nfkbib	1371.603791	-0.183681308	0.0917742	-2.001449	0.045344	0.418148
tram2	264.5081424	0.259392408	0.1323751	1.9595255	0.050051	0.43812
malb	74.31122367	-0.341003916	0.2384788	-1.429913	0.152742	0.701776
ccdc78	16.13399982	-0.739446691	0.5003658	-1.477812	0.139458	0.678238
sdcbp	126.2983944	-0.031896145	0.1809546	-0.176266	0.860085	0.985773
sh3bp4	100.6057082	0.685295752	0.2002901	3.4215157	0.000623	0.020942
stmn4	1760.664316	0.083536461	0.084408	0.9896743	0.322333	0.870226
si:ch211-207i1.2	568.0663336	-0.053023351	0.1023253	-0.518184	0.60433	0.95651
sec22ba	159.990318	0.072953824	0.1772305	0.4116324	0.680609	0.965476
myod1	227.2560617	0.074350131	0.1398336	0.5317042	0.594931	0.954434
vps4a	182.6674229	-0.099715121	0.1539117	-0.647872	0.517067	0.938543
susd6	1653.480601	0.151144511	0.0805767	1.8757851	0.060685	0.48134
sox7	216.7655183	-0.437140088	0.1468145	-2.9775	0.002906	0.070337
si:ch211-87m7.2	30.62632347	-0.079399174	0.3743782	-0.212083	0.832042	0.98349
fkbp6	3.287703845	0.249847362	1.0749957	0.2324171	0.816214	NA
sdhdb	991.596357	-0.050683974	0.0892905	-0.56763	0.570286	0.948989
gnptab	628.5905444	-0.008968598	0.1010108	-0.088788	0.92925	0.992702
pak7	399.9577898	-0.05019959	0.1135106	-0.442246	0.658311	0.962545
ssbp3b	2026.247662	0.001517672	0.0767459	0.0197753	0.984223	0.996944
naglu	157.6985188	0.179046207	0.1793528	0.9982904	0.318139	0.867746
mybpc2a	237.2314492	0.211099244	0.1408867	1.4983621	0.134039	0.669349
ppp1r14bb	631.6681475	0.129108135	0.1033296	1.2494784	0.21149	0.778917
cdk21	1.556139089	2.947626016	1.7933025	1.6436859	0.100241	NA
hyi	240.147613	-0.053782841	0.1398599	-0.384548	0.700572	0.967998
traf2a	99.21473833	0.059713457	0.202105	0.2954576	0.767644	0.975687
zgc:92429	144.0933009	0.291710522	0.1921098	1.5184576	0.128899	0.659982
uchl3	619.2465807	0.124967592	0.1036804	1.2053159	0.228081	0.795505
sap30l	861.3727281	-0.089027694	0.1089778	-0.816935	0.413966	0.905905
matn1	21340.00448	0.016246201	0.0790784	0.2054442	0.837225	0.984645
ppp2ca	567.5588907	0.051447457	0.105849	0.4860457	0.626935	0.957354
tmem30aa	815.4358779	0.205956757	0.0891142	2.3111562	0.020824	0.272701
pgrmc2	874.8460075	0.134029821	0.0911596	1.4702769	0.141487	0.682048
dennd6aa	21.57062474	0.271824085	0.4088458	0.6648573	0.506142	0.935724
vbp1	917.4887953	-0.054933676	0.0875283	-0.62761	0.530259	0.942209
dennd2db	44.8277097	-0.386694142	0.30432	-1.270682	0.203842	0.771579
klhl36	66.14630542	-0.02150367	0.2474087	-0.086916	0.930739	0.992702
cbln9	0.157187365	-0.955901296	4.0804729	-0.234262	0.814781	NA
si:ch211-220i18.4	40.09460489	-0.31252328	0.3055285	-1.022894	0.306358	0.860027
mfsd2b	171.0570149	0.037662256	0.1573611	0.2393365	0.810845	0.981081
scdb	1391.447361	0.178304766	0.0944427	1.8879671	0.05903	0.475271
gtf3aa	193.0665056	0.187034994	0.1509946	1.238687	0.215461	0.783025
grtp1b	14.49503185	-0.226056273	0.4957826	-0.455958	0.64842	0.960708

tnnt3a	4066.842918	0.067193883	0.0943676	0.7120442	0.476437	0.926541
idh3a	1409.507326	0.040360775	0.0816212	0.4944889	0.620961	0.957354
jag1a	187.0451281	-0.050518972	0.1584745	-0.318783	0.749891	0.97419
sox13	570.1462254	0.08401693	0.1062229	0.790949	0.428974	0.911406
hspa12b	67.11498117	0.123065487	0.2392814	0.5143128	0.607033	0.956718
tmc2b	13.84726086	0.706344808	0.5379534	1.3130223	0.189175	0.753954
efna1a	570.0762586	0.064955633	0.1210967	0.5363948	0.591686	0.954434
zc3h10	358.1086823	-0.039039229	0.1278138	-0.305438	0.760032	0.975374
guk1a	843.9670389	-0.062151643	0.0911058	-0.682192	0.495118	0.931928
mespba	0	NA	NA	NA	NA	NA
cryba2a	12297.35249	0.02453412	0.0744484	0.3295453	0.741744	0.973154
mnx2b	25.2049369	0.621705858	0.3832948	1.6220045	0.104802	0.612652
zgc:66313	30.85141093	-0.434316984	0.3490173	-1.2444	0.213353	0.781631
angpt1	64.00714735	0.1072619	0.279006	0.3844429	0.70065	0.967998
metrn	448.8691374	0.033482771	0.1076265	0.3111014	0.755724	0.975374
naa15b	1009.279912	0.088442518	0.0871057	1.0153466	0.309941	0.861992
grin2bb	698.368122	0.063243857	0.0987321	0.6405602	0.521808	0.939056
prf1.1	1.22848649	-0.350511206	1.8878393	-0.185668	0.852705	NA
twist1a	214.4798739	-0.060496001	0.1650545	-0.366521	0.713976	0.96909
rps26l	8923.849353	-0.19256921	0.089953	-2.140776	0.032292	0.349696
crygn2	24739.47617	0.19920185	0.0795966	2.5026442	0.012327	0.196417
pkd1a	397.2615646	-0.117999555	0.1315844	-0.89676	0.369847	0.889487
rsrp1	2324.753731	0.139280533	0.0978944	1.4227629	0.154805	0.704573
ppa1b	1896.643777	-0.061510236	0.0788823	-0.779773	0.435525	0.914408
sppl2	855.2368028	0.002729517	0.0936708	0.0291395	0.976753	0.996762
crabp2b	901.3408969	0.052878417	0.0904545	0.5845858	0.558826	0.948478
tppp3	109.3068261	0.068090232	0.1951336	0.3489416	0.727133	0.970402
tmem263	532.4027805	0.245713302	0.1039141	2.3645815	0.01805	0.249253
slc16a5a	33.36033795	0.297197255	0.3393632	0.8757497	0.381166	0.895192
zgc:66484	258.167811	-0.497100117	0.1347935	-3.687864	0.000226	0.009434
hmgb1b	6747.990559	-0.054338331	0.0654389	-0.830367	0.406331	0.901305
sptb	497.7712533	0.182611208	0.1174428	1.5548953	0.119971	0.645215
hjv	107.1085394	0.226311992	0.2024335	1.1179572	0.263585	0.830245
si:dkey-167k11.5	0	NA	NA	NA	NA	NA
tsnaxip1	28.36361228	0.020182023	0.362875	0.055617	0.955647	0.994543
acsl1a	540.6057281	-0.030324036	0.1054724	-0.287507	0.773724	0.975687
slc22a2	150.858741	0.216910474	0.1780831	1.2180298	0.223213	0.789115
arhgef12a	745.0796723	-0.109706637	0.097703	-1.122859	0.261498	0.829593
psmc1a	2352.916686	-0.129930944	0.07969	-1.630454	0.103005	0.609625
rnd1a	1515.84689	0.005609621	0.0839398	0.0668291	0.946718	0.99381
helz	896.4885377	0.038384754	0.1047378	0.3664844	0.714004	0.96909
zgc:77880	247.7101083	0.06071563	0.1374036	0.441878	0.658577	0.962545
nudt1	56.11045358	0.174543873	0.2760426	0.6323077	0.527186	0.941186
foxk2	1096.753951	-0.039013362	0.0898237	-0.434333	0.664047	0.964736
slc10a1	22.55424046	-0.47596262	0.400802	-1.187525	0.23502	0.803894
lrrc42	85.05117258	-0.171911964	0.2294022	-0.749391	0.453621	0.920041
nampta	328.3469692	0.038385978	0.1225498	0.3132277	0.754108	0.975056
rps19	14091.2773	-0.231763397	0.0857858	-2.701652	0.0069	0.128953
phkg1a	334.5805352	0.410442571	0.1320911	3.1072689	0.001888	0.051148
rhogd	129.307861	0.108405328	0.189533	0.5719602	0.567349	0.948739
syt1a	6407.391851	0.051618121	0.0652996	0.7904816	0.429247	0.911406
nfe2l1a	296.8386173	0.022901104	0.1268664	0.1805135	0.856749	0.985597
th	123.782626	0.238085421	0.1881637	1.2653103	0.20576	0.773784
lrit2	52.96141837	0.148873502	0.2689003	0.5536382	0.579826	0.951257

mfsd2aa	161.6421855	0.435074815	0.1617948	2.6890533	0.007165	0.132793
ccdc3b	290.4195945	0.246530359	0.1294664	1.9042032	0.056884	0.468089
plekhm3	236.4105957	0.047630122	0.1375115	0.346372	0.729063	0.970822
pimr189	2.49942042	-1.435466538	1.2329926	-1.164213	0.244338	NA
tex30	18.97709376	0.627028627	0.4354173	1.4400638	0.149849	0.697935
letmd1	144.6593973	0.08977036	0.1652824	0.5431332	0.587038	0.953319
gnai3	60.99220448	-0.20370051	0.2492923	-0.817115	0.413863	0.905864
r3hdml	21.52317375	0.680903551	0.4320519	1.5759764	0.115031	0.635519
3-Sep	1482.402374	-0.078902303	0.0882768	-0.893806	0.371426	0.891981
CU929230.1	610.6535247	-0.08276046	0.1046464	-0.790858	0.429027	0.911406
phka2	1043.211004	0.09096834	0.0972896	0.9350266	0.349775	0.880195
atp6v1e1b	3803.728803	0.002183224	0.0730193	0.0298993	0.976147	0.996762
ctps1a	1056.856895	0.0307454	0.098432	0.3123518	0.754773	0.975207
otx2a	113.2727706	0.072592683	0.1915204	0.3790338	0.704663	0.968134
ing4	1844.296847	-0.079967143	0.076291	-1.048186	0.294553	0.850664
xirp1	169.2015726	-0.218502484	0.1639242	-1.332949	0.182549	0.745308
cldn11b	203.8540156	0.215174343	0.1531873	1.4046486	0.160126	0.712495
b3gat2	317.5392036	0.025501837	0.1280237	0.1991962	0.842109	0.985174
sptlc3	252.8244887	-0.41856371	0.1351067	-3.098024	0.001948	0.052555
gabrr3b	78.7074804	0.087748914	0.2236707	0.392313	0.694827	0.96736
prrg4	321.8307565	-0.155847103	0.1267734	-1.229336	0.218946	0.785734
dnmt1	1805.032233	-0.072967312	0.0902259	-0.808718	0.418678	0.906406
guca1c	111.0996392	0.265441058	0.1965058	1.3508053	0.176758	0.737285
melk	175.0279322	-0.137154152	0.160378	-0.855193	0.392444	0.897688
syt16	5.142382242	-0.297337992	0.8383955	-0.354651	0.722851	NA
hadh	853.1101057	-0.130809269	0.087011	-1.503364	0.132745	0.667381
sybl1	487.1306495	0.049650239	0.1084732	0.4577189	0.647154	0.960435
BX323854.1	0.981152638	-0.049279921	1.9490317	-0.025284	0.979828	NA
acads	558.4142122	0.066548431	0.1041638	0.6388825	0.522899	0.939336
exoc3l2b	184.9461722	-0.234185286	0.1574365	-1.487491	0.136885	0.673303
mpi	107.7355016	-0.133116782	0.2059286	-0.646422	0.518006	0.938592
ddx18	813.2848742	0.003858051	0.1078537	0.0357712	0.971465	0.996315
zgc:110006	25.03278261	-0.32985649	0.3799106	-0.868248	0.385259	0.89551
fam120b	126.8470121	0.17706552	0.1767242	1.0019316	0.316377	0.867129
znhit2	61.82023236	-0.029975484	0.2539876	-0.118019	0.906052	0.990702
esyt2a	453.4124595	-0.035961623	0.1078333	-0.333493	0.738762	0.972722
cmtr1	628.8759009	-0.154154608	0.1116706	-1.380441	0.167451	0.724546
otofa	377.5568798	-0.06217603	0.1202021	-0.517263	0.604973	0.95651
tescb	75.76247404	-0.051001321	0.2307051	-0.221067	0.82504	0.982196
klf11a	1463.380624	-0.221583008	0.092057	-2.407019	0.016083	0.233847
opn7c	13.68905764	-0.447038587	0.5330434	-0.838653	0.401664	0.90084
siah1	958.7629308	0.024880848	0.08704	0.2858552	0.774989	0.975687
cetp	122.9206754	0.22984925	0.2146093	1.0710123	0.284164	0.844697
baxb	57.95112112	-0.031830297	0.2677867	-0.118864	0.905383	0.990702
phf23a	312.7545494	0.073608215	0.1251457	0.5881799	0.556412	0.947918
foxq1a	202.9858389	0.27876234	0.1842692	1.5127996	0.130331	0.662797
cited2	702.5860745	-0.296208263	0.1033451	-2.866205	0.004154	0.089484
cluha	1636.846483	0.034547242	0.0820675	0.4209615	0.673783	0.965257
tmem120a	157.7838462	0.21732483	0.1668111	1.3028202	0.192636	0.756795
cpa1	56.91505938	-0.228748049	0.2808539	-0.814473	0.415374	0.906237
fgf10a	116.4340325	-0.055981535	0.1903657	-0.294074	0.768702	0.975687
ksr1b	145.2738321	0.224591433	0.165193	1.3595698	0.173966	0.733946
fermt3b	8.226191573	-0.442148224	0.6803323	-0.6499	0.515757	NA
si:ch211-259g3.4	664.5677582	0.134555447	0.1071031	1.2563169	0.209001	0.776073

srprb	671.2049757	-0.073275594	0.0961554	-0.762054	0.446028	0.917154
ftr56	28.32919935	-0.261170097	0.4011495	-0.651054	0.515011	0.937646
yipf4	877.2990064	0.018122331	0.0862488	0.210117	0.833576	0.983583
ak8	28.4324582	0.018768754	0.3727428	0.0503531	0.959841	0.995379
pde12	199.2559608	0.044951545	0.1505149	0.2986518	0.765206	0.975374
si:ch211-51a6.2	158.3511773	0.206333457	0.1663619	1.2402687	0.214876	0.782493
dnaja1	189.2060469	0.041167615	0.157662	0.2611131	0.794005	0.977937
ccdc80l1	112.0742667	0.389423043	0.1917152	2.0312581	0.042229	0.403776
csrp1b	74.46558996	-0.691675954	0.2394742	-2.888311	0.003873	0.085098
tmem127	269.7159201	0.145356168	0.1278097	1.1372857	0.255419	0.823924
mybl1	200.7679949	-0.033738992	0.152698	-0.220952	0.825129	0.982196
dtna	79.86185227	0.034885228	0.2210823	0.157793	0.87462	0.987881
pip4k2ca	1092.788584	-0.118528624	0.0861513	-1.37582	0.168877	0.725919
lipg	302.91557	0.260130345	0.1296555	2.0063188	0.044822	0.416612
nr1h5	59.85187282	0.196268449	0.2524656	0.7774068	0.436919	0.914408
gspt1	598.7474652	0.178182561	0.1010547	1.7632284	0.077862	0.543659
igsf21a	597.6940442	0.010390195	0.098436	0.1055528	0.915937	0.991314
cadm1a	338.7454026	0.246826253	0.1186721	2.0799016	0.037535	0.379013
veph1	10.45330074	0.037535833	0.619974	0.0605442	0.951722	NA
timm50	482.791335	0.014482109	0.1150752	0.125849	0.899851	0.990121
ivns1abpa	4337.382656	-0.106550078	0.0732552	-1.454505	0.145806	0.691641
lrba	1224.218913	-0.018222397	0.0839	-0.217192	0.828059	0.982721
dnal4a	19.9493037	0.061617857	0.4289452	0.1436497	0.885777	0.988936
baiap2l1b	133.4819358	-0.339054043	0.1764041	-1.922031	0.054602	0.457793
notum1a	105.8116517	0.26332026	0.2039947	1.2908194	0.196766	0.761944
moxd1	100.2603654	-0.285210203	0.2041236	-1.397243	0.162341	0.716781
irx3b	46.71013288	-0.146054186	0.2840319	-0.514218	0.6071	0.956718
gba3	21.89186869	-0.502651643	0.4184664	-1.201176	0.229683	0.79764
nr1d4a	74.64823908	-0.283083173	0.228829	-1.237095	0.216052	0.783484
tuba8l2	1683.41635	0.073522079	0.1014765	0.7245231	0.468745	0.924328
hmg20a	385.771878	-0.080629613	0.113781	-0.708638	0.478549	0.926978
ppp2r5cb	1249.008392	-0.023895559	0.0844113	-0.283085	0.777112	0.975826
alg11	216.2156816	0.030758288	0.1429527	0.215164	0.829639	0.982805
commd1	225.5641322	-0.141101322	0.1548763	-0.911058	0.362265	0.885944
arcn1b	1586.108603	0.029109271	0.0835593	0.3483666	0.727565	0.970402
ssu72	762.6100351	-0.024101155	0.1038468	-0.232084	0.816473	0.981594
lhx2b	320.1968097	-0.188467969	0.1359489	-1.386315	0.165651	0.721576
podxl	685.6912126	0.174969201	0.09745	1.7954767	0.072578	0.52617
kidins220a	402.1642273	0.04112303	0.112942	0.3641076	0.715778	0.96909
snai3	4.118754577	-0.360553045	0.9440361	-0.381927	0.702515	NA
hbegfb	93.75717813	-0.141586079	0.2106977	-0.671987	0.501592	0.934648
enkur	59.22780067	-0.034616756	0.2567835	-0.134809	0.892763	0.989291
sap30bp	509.2090585	0.002180515	0.1181062	0.0184623	0.98527	0.996944
rnf123	56.83103782	-0.351345739	0.257974	-1.361943	0.173216	0.732435
si:ch73-193i2.2	7.540720479	-0.994698797	0.7830061	-1.270359	0.203957	NA
vamp1	1770.213196	0.136413131	0.0783104	1.7419535	0.081517	0.552315
dnajc9	337.5839895	-0.011833754	0.1211504	-0.097678	0.922188	0.992292
dhrs11b	276.1750518	-0.616189613	0.134718	-4.573923	4.79E-06	0.000386
smap1	1964.40787	-0.000378404	0.0763085	-0.004959	0.996043	0.998888
zic4	337.4612004	-0.074905374	0.1238933	-0.604596	0.545448	0.945526
six6b	418.8237318	-0.00068434	0.1314216	-0.005207	0.995845	0.998888
ppdpfb	7886.070615	-0.209013143	0.08555	-2.44317	0.014559	0.219929
tmed1a	93.18864812	-0.206223772	0.2129946	-0.968211	0.332939	0.874926
hsd20b2	163.9751387	0.100496771	0.1690958	0.5943184	0.552299	0.947265

hoxa10b	141.8320966	0.021872765	0.1868333	0.117071	0.906804	0.990702
rab6bb	1274.481473	0.073891139	0.0856624	0.862585	0.388366	0.896271
RTKN	64.07361094	-0.062549665	0.2486234	-0.251584	0.801363	0.979377
rbm17	459.1120979	-0.016062785	0.1058639	-0.151731	0.879399	0.987881
mc5ra	57.90111392	-0.119196778	0.2625271	-0.454036	0.649803	0.960708
cmpk2	2.351582791	-1.306968304	1.303324	-1.002796	0.315959	NA
rtn4ip1	261.7471094	-0.129399566	0.1368253	-0.945729	0.344287	0.878434
efna2a	393.0110818	-0.041179528	0.1233658	-0.3338	0.73853	0.972722
reep2	774.1783006	0.051880701	0.0931524	0.556944	0.577566	0.95086
klhl38a	32.25911429	-0.273522781	0.3352598	-0.815853	0.414584	0.906237
cers4a	243.4251617	0.210644201	0.149283	1.4110394	0.158233	0.709224
dctn2	1912.4497	-0.034494448	0.0763294	-0.451916	0.65133	0.960708
wt1a	57.72195159	0.070374988	0.2578209	0.2729607	0.784883	0.97627
igfbp2b	29.65478995	0.654795776	0.3582614	1.827704	0.067594	0.508988
csrnp1a	350.0910832	-0.028428092	0.1264617	-0.224796	0.822138	0.982186
calm2a	4260.914011	0.069579079	0.0779746	0.8923301	0.372216	0.892686
rcor1	785.2440885	0.024059623	0.0915866	0.262698	0.792783	0.977937
TXN	3949.199139	-0.173397703	0.086115	-2.013559	0.044056	0.412803
kcnj11l	249.332635	0.234860249	0.1405905	1.6705272	0.094815	0.585466
syt6b	193.6901204	-0.072448255	0.1503293	-0.48193	0.629855	0.957354
col9a1b	3888.790585	0.206851781	0.2614462	0.7911829	0.428837	0.911406
ognb	4.813156262	0.172530225	0.9608674	0.1795567	0.857501	NA
prrc2a	4057.229724	0.086699536	0.0662653	1.3083701	0.190748	0.755212
seta	6062.557573	-0.055931655	0.0689999	-0.810605	0.417593	0.906237
aplar	313.6549195	0.277785444	0.1327756	2.0921419	0.036426	0.372818
flvcr2b	212.3124888	-0.037201335	0.1491502	-0.249422	0.803034	0.979643
psmb2	1741.123169	-0.114842669	0.0784683	-1.463555	0.143316	0.686804
ap5m1	91.40839121	0.048126251	0.2069372	0.2325645	0.8161	0.981594
CU571315.1	39.07633662	-1.006992777	0.3155231	-3.191502	0.001415	0.040718
tmem200a	64.61379509	-0.065398757	0.2581936	-0.253293	0.800041	0.979377
ephb3a	1088.850142	-0.057651805	0.0829368	-0.695129	0.486974	0.931104
srpk1b	608.1867476	-0.005349926	0.0992308	-0.053914	0.957004	0.994543
tada2a	339.5445635	-0.260801883	0.120925	-2.156724	0.031027	0.340851
flvcr1	432.6029142	0.192025716	0.1109568	1.7306357	0.083517	0.557158
si:dkey-239b22.1	984.5550421	0.331671985	0.3247143	1.0214272	0.307052	0.860982
calb1	339.1290763	0.073979699	0.1305438	0.566704	0.570915	0.949078
rd3	124.3242874	0.068082532	0.2059796	0.3305304	0.740999	0.973154
g6pca.1	136.6798332	0.349912034	0.1740785	2.0100819	0.044423	0.41454
unm_sa1261	97.66874889	-0.00013675	0.2060445	-0.000664	0.99947	0.999704
chst10	253.9804265	0.02141117	0.1469513	0.1457025	0.884156	0.988784
pan3	645.7524677	0.007104212	0.0973866	0.0729486	0.941847	0.993099
stat2	220.3723472	-0.339902379	0.141463	-2.402764	0.016272	0.235374
cort	340.2548309	0.300008687	0.1191734	2.5174137	0.011822	0.191129
fahd1	170.6375097	-0.295124349	0.1612283	-1.830475	0.067179	0.506828
si:ch211-207d6.2	255.7312557	0.00433089	0.131782	0.032864	0.973783	0.996315
sox21a	80.04690451	0.234323222	0.2394115	0.9787466	0.327705	0.872701
col5a2a	6780.851469	0.308623814	0.082946	3.7207806	0.000199	0.008482
ercc1	187.4282571	-0.334014801	0.1684754	-1.982573	0.047415	0.427732
atp6v0b	2375.434182	0.100748268	0.072208	1.3952515	0.16294	0.717875
fosab	512.8442281	0.361525487	0.3701454	0.976712	0.328712	0.872945
kif9	11.91561838	-0.31291303	0.5439226	-0.57529	0.565095	0.948739
mylk2	33.69982449	0.092842975	0.3405538	0.2726235	0.785143	0.97627
prkg1b	300.9947125	0.389324339	0.1427709	2.7269169	0.006393	0.122178
mllt1b	189.7557334	0.228332371	0.1528735	1.4936036	0.135279	0.670689

grm3	66.08198633	-0.459403109	0.26822	-1.712785	0.086752	0.564768
clstn1	8050.42157	0.102131855	0.076459	1.3357733	0.181623	0.743565
si:ch73-27e22.6	0	NA	NA	NA	NA	NA
pthlha	81.54673663	-0.275523388	0.2460968	-1.119573	0.262896	0.830166
si:busm1-266f07.2	15.05728342	-0.141321013	0.5264892	-0.268421	0.788375	0.976703
C3AR1	5.201428251	0.292323701	0.8980163	0.3255216	0.744786	NA
npr1a	84.2272015	0.168980406	0.2172777	0.7777161	0.436736	0.914408
mef2aa	118.2785069	-0.124705908	0.1935627	-0.644266	0.519403	0.938945
tmc4	56.08508643	-0.403528872	0.2684834	-1.502994	0.132841	0.667545
dab2	271.6586157	-0.385650963	0.1415287	-2.724897	0.006432	0.122415
smad6b	380.7180452	0.144066448	0.1162936	1.2388161	0.215414	0.783013
roraa	2110.959657	0.010153236	0.0870347	0.1166574	0.907132	0.990702
kat7b	719.0028301	-0.056683446	0.0998991	-0.567407	0.570438	0.949061
pus7	470.1433128	0.008218601	0.1112545	0.0738721	0.941112	0.992857
ube2s	993.6404521	0.042546549	0.0853609	0.4984313	0.61818	0.957354
fthl27	5800.424858	-0.077266556	0.0950195	-0.813165	0.416124	0.906237
pparaa	62.20077939	0.254863277	0.2449279	1.0405644	0.298078	0.85401
crybg1a	735.108635	-0.023120488	0.0931501	-0.248207	0.803974	0.979643
adcy8	271.8964853	-0.080846985	0.1303888	-0.620046	0.535228	0.943336
abcf1	2252.693071	-0.019620376	0.079163	-0.247848	0.804252	0.979643
ache	1810.447774	0.195677627	0.0745546	2.6246214	0.008675	0.153289
rbm24b	21.8317574	0.291132831	0.4563271	0.6379915	0.523479	0.939336
dhrs13b.1	112.2163408	-0.022547736	0.1854321	-0.121596	0.903219	0.990121
trim2a	44.3875197	0.084774247	0.2953923	0.2869887	0.774121	0.975687
eif4ebp2	4047.428287	0.064384602	0.0742858	0.8667144	0.386099	0.89551
vps37c	879.9908829	-0.026062014	0.0873786	-0.298265	0.765501	0.975374
mboat2a	28.76519169	0.073014096	0.3613657	0.2020505	0.839877	0.984979
pparg	71.51472461	-0.9685406	0.2384487	-4.061841	4.87E-05	0.002674
trpa1b	84.59565935	-0.302534318	0.2225189	-1.35959	0.17396	0.733946
pard6ga	1.806499777	1.423624041	1.4738733	0.9659067	0.334091	NA
psmb12	2.004323476	-1.017052804	1.4085527	-0.722055	0.470261	NA
ift140	285.1035748	0.02530335	0.1263945	0.2001935	0.841329	0.985174
mapk8a	286.4992455	0.092352667	0.1282307	0.7202069	0.471398	0.924838
tcp11l1	492.3030255	0.044543247	0.1049165	0.4245592	0.671158	0.965257
tbx1	423.6314166	0.016301268	0.1183609	0.1377251	0.890458	0.989291
lef1	938.8033225	-0.056559445	0.0874784	-0.646553	0.517921	0.938592
ptbp1b	1150.800428	-0.017512635	0.080837	-0.216641	0.828488	0.982721
tns3.2	627.6879353	-0.033442804	0.100656	-0.332248	0.739702	0.972943
hif1an	815.3116477	-0.383806885	0.1124208	-3.414019	0.00064	0.021454
stard14	430.7839814	0.002284026	0.1194709	0.0191178	0.984747	0.996944
erc1b	0.484248244	0.852636534	2.8793247	0.2961238	0.767136	NA
mb	1568.096508	-0.362612805	0.1049883	-3.45384	0.000553	0.019206
rbck1	68.00911025	-0.250333222	0.2431179	-1.029678	0.303161	0.85729
tmem63a	149.5148918	-0.050478906	0.1671843	-0.301936	0.762701	0.975374
cpped1	178.5937446	0.051671034	0.167452	0.3085722	0.757647	0.975374
poglut2	204.0895413	0.133483389	0.1598502	0.8350528	0.403688	0.900979
nos2b	25.49451827	0.671346146	0.3853011	1.7423934	0.08144	0.552315
pcbd1	194.9875161	-0.101528444	0.1637006	-0.620208	0.535121	0.943336
six4b	223.7306698	-0.121752111	0.1470323	-0.828064	0.407634	0.901327
ptpa	692.1754661	-0.079848507	0.0936755	-0.852395	0.393995	0.898496
ccdc65	28.7021781	0.05118254	0.3538417	0.1446481	0.884989	0.988911
slc15a2	177.1357943	-0.044106348	0.1544034	-0.285657	0.775141	0.975687
pafah1b1a	1630.293876	-0.024655684	0.0787355	-0.313146	0.75417	0.975056
bet1l	122.1237445	-0.025215	0.186593	-0.135134	0.892506	0.989291

mxd1	18.1735812	-0.22633839	0.4504955	-0.502421	0.615371	0.95688
ftr16	14.11145778	-0.401916502	0.5204765	-0.772209	0.439991	0.916082
enah	1426.228782	-0.003663619	0.0774307	-0.047315	0.962262	0.995907
arl6	455.7060538	0.140028291	0.1084812	1.2908068	0.196771	0.761944
cdk16	322.2337806	0.083066707	0.1331955	0.6236448	0.532861	0.942805
BX323082.1	8.855239477	0.012933078	0.6472384	0.0199819	0.984058	NA
acsl3a	70.34720798	-0.264320687	0.2324077	-1.137315	0.255407	0.823924
dpysl2b	1189.158127	0.006621458	0.0870811	0.0760378	0.939389	0.992857
sgsh	144.5177628	-0.113972273	0.1968166	-0.579078	0.562536	0.948728
c8g	152.4181414	0.24179757	0.1999049	1.2095627	0.226447	0.793959
prdx3	1186.658599	-0.062431243	0.0859779	-0.726131	0.467758	0.924328
mapk6	1331.388913	0.155975704	0.09563	1.6310332	0.102883	0.609169
slain2	400.9629305	0.262944048	0.1169686	2.2479882	0.024577	0.302262
cnot7	73.79565565	-0.03171709	0.2430461	-0.130498	0.896172	0.989971
ddx1	964.3180647	0.152990581	0.0878394	1.7417079	0.08156	0.552315
scg5	307.9607764	0.198993117	0.1292245	1.5399025	0.123584	0.651905
gtf2f1	752.1757141	-0.15350307	0.0988515	-1.552865	0.120455	0.645323
drd3	55.65697351	-0.285589926	0.2573155	-1.109882	0.26705	0.833191
ppm1aa	277.1276702	-0.041067356	0.1305935	-0.314467	0.753166	0.97481
zgc:171776	0.48983248	-0.887840757	2.6934859	-0.329625	0.741683	NA
grk6	21.84988901	1.002220313	0.4142569	2.4193208	0.01555	0.228768
srgap2	979.8876349	-0.060469596	0.0864361	-0.699587	0.484185	0.929542
puf60a	1485.020068	-0.107571389	0.0859163	-1.252048	0.210552	0.778338
lrrc8aa	54.93892741	0.164510215	0.2605709	0.6313452	0.527815	0.941629
klf12b	28.62278318	0.320499423	0.3538115	0.905848	0.365016	0.886714
gpc3	11.45009976	-0.093831349	0.5774403	-0.162495	0.870916	0.987697
cthl	56.74617254	-0.627804577	0.2895443	-2.168251	0.03014	0.335575
acss3	292.9959768	-0.380053032	0.1286597	-2.95394	0.003137	0.073769
phactr4b	392.5759763	-0.099554617	0.1121894	-0.88738	0.374874	0.894316
wnt2bb	30.33312978	0.205489889	0.343288	0.5985934	0.549444	0.946312
anapc4	224.3315206	-0.050077819	0.1407015	-0.355915	0.721904	0.970024
dnm3a	64.82849102	0.110363495	0.2629659	0.4196875	0.674714	0.965257
tnnt2c	171.7731422	-0.157222261	0.1713553	-0.917522	0.358869	0.884602
tmtopsb	38.50275633	0.290857844	0.3263109	0.891352	0.37274	0.892868
txndc16	113.6810335	-0.071899226	0.2055734	-0.34975	0.726527	0.970402
rngtt	564.8444785	-0.027179066	0.1142825	-0.237823	0.812018	0.981081
zgc:110224	53.88457458	-0.025197827	0.2876312	-0.087605	0.930191	0.992702
mybl2b	451.9236989	-0.09266588	0.11072	-0.836939	0.402627	0.90084
satlb	87.42258741	-0.216711844	0.2306617	-0.939522	0.347463	0.880195
rars2	91.51040458	-0.03154488	0.2082172	-0.1515	0.879581	0.987881
zgc:113294	57.22415619	-0.186388736	0.2894776	-0.64388	0.519653	0.938945
pomp	1735.210522	-0.039545529	0.0762852	-0.51839	0.604186	0.95651
tbc1d20	108.961629	-0.069740228	0.1911632	-0.36482	0.715246	0.96909
tox	2540.540778	-0.098820166	0.0803679	-1.229598	0.218848	0.785734
mfsd6a	164.9224972	-0.371129537	0.1712541	-2.167128	0.030225	0.336193
mest	181.4683648	-0.211809599	0.1536596	-1.378434	0.168069	0.725792
epb41l5	772.4207871	-0.050798623	0.0908522	-0.559135	0.57607	0.950611
mecr	169.1589357	0.043553501	0.1729774	0.2517872	0.801206	0.979377
usp36	1242.112842	-0.115647892	0.0841529	-1.374259	0.169361	0.726744
serhl	202.836043	0.126083803	0.1502556	0.8391287	0.401397	0.90084
pde4a	539.9016223	0.13963963	0.1015412	1.3752023	0.169069	0.726144
tiprl	316.6600241	-0.109953846	0.123501	-0.890308	0.373301	0.893554
btbd6b	1066.212271	-0.027040363	0.0838568	-0.322459	0.747105	0.97419
ccdc180	20.18856039	0.796385778	0.4644059	1.7148485	0.086373	0.563305

rnf145b	776.7867358	0.203424559	0.0898955	2.2629006	0.023642	0.296445
ompb	54.19806449	0.156370373	0.309284	0.5055882	0.613146	0.95688
tspan12	128.9965825	0.276986119	0.181216	1.528486	0.126392	0.655289
ckap4	848.7321133	0.185011747	0.1011979	1.8282166	0.067517	0.508678
coq8ab	72.44905026	0.050877999	0.2365796	0.2150566	0.829723	0.982805
ppp2r1bb	5065.453824	0.077944299	0.0774797	1.0059969	0.314417	0.865529
mpv17	167.2312872	-0.353660838	0.1573195	-2.248043	0.024573	0.302262
itga9	54.33673317	0.248713114	0.2856407	0.8707201	0.383907	0.895192
mark2b	1276.305929	-0.064724559	0.0780676	-0.829083	0.407057	0.901305
slc1a8b	48.83774121	0.949503549	0.3068529	3.0943282	0.001973	0.052997
ampd3b	222.7528767	-0.031056541	0.1468967	-0.211418	0.832561	0.983557
si:dkey-40c11.2	242.8130853	0.031094926	0.1370014	0.2269679	0.820449	0.981594
pon1	213.9782886	0.077540431	0.1724527	0.4496329	0.652975	0.960792
rrp36	131.5047565	-0.180095654	0.1787719	-1.007405	0.31374	0.865132
arpp19a	1928.523215	0.030739399	0.0761263	0.403795	0.686364	0.966376
praf2	381.6751834	0.018979574	0.114266	0.1660999	0.868078	0.987245
5-Mar	92.4781952	0.066865209	0.209713	0.3188414	0.749847	0.97419
egln3	843.6339407	-0.398383005	0.2415072	-1.64957	0.099031	0.598346
cacng2a	564.4107908	-0.080038249	0.1048067	-0.763675	0.445061	0.916958
il20ra	93.98563507	-0.490798592	0.2261227	-2.170497	0.029969	0.335036
ywhaz	4601.52413	0.036742104	0.0791709	0.464086	0.642586	0.959994
clcn6	80.69014286	0.09649607	0.2210758	0.4364841	0.662486	0.964665
pax2b	21.50680085	-0.461084892	0.4366128	-1.05605	0.290945	0.846568
thnsl2	33.940834	-0.280454747	0.3272531	-0.856996	0.391447	0.897688
macrod2	676.1016732	-0.044708576	0.0985686	-0.453578	0.650132	0.960708
st7l	214.9076849	-0.303845276	0.1613591	-1.883037	0.059695	0.478288
sept8a	1098.303993	-0.121162395	0.0859293	-1.410024	0.158533	0.709224
msi2b	404.5520038	-0.026064145	0.1120358	-0.232641	0.81604	0.981594
fgfrl1a	1037.959209	0.047228448	0.0855077	0.55233	0.580722	0.951731
mgst1.1	209.8078279	-0.02582048	0.1416504	-0.182283	0.855361	0.985174
tob1a	1976.268691	0.12606233	0.0733151	1.7194592	0.085531	0.561521
rcan3	966.9170101	0.089594286	0.0925769	0.967782	0.333153	0.874926
SCYL2	553.909734	-0.029492456	0.1008951	-0.292308	0.770051	0.975687
neb	10842.20208	0.306343573	0.0805744	3.8019948	0.000144	0.006578
ltb4r	23.70461936	-0.124757463	0.3956187	-0.315348	0.752498	0.974708
si:dkey-261m9.12	5.805654315	0.060763996	0.9100331	0.0667712	0.946764	NA
cd36	362.421438	-0.357832843	0.1201388	-2.978496	0.002897	0.070195
fuk	168.173498	-0.054053097	0.1554849	-0.347642	0.728109	0.970402
ppp1r10	1758.87049	0.246664356	0.0869073	2.8382468	0.004536	0.09614
sinhcaf	452.0922119	-0.064418841	0.116375	-0.553545	0.57989	0.951257
qrsl1	151.0297194	-0.036390689	0.1877931	-0.193781	0.846348	0.985174
foxg1b	1172.386041	-0.052130139	0.1005858	-0.518265	0.604273	0.95651
arl4cb	475.7023867	-0.084390931	0.1070114	-0.788616	0.430336	0.911957
gria1b	74.04009262	-0.214446015	0.2271893	-0.943909	0.345216	0.878434
rps27a	17264.42165	-0.24926128	0.0747519	-3.334513	0.000854	0.027297
gria3a	1417.133545	0.114795614	0.0830395	1.3824225	0.166842	0.723339
si:dkey-11p23.7	3.192094564	0.509355849	1.0778534	0.4725651	0.636524	NA
tnfsf10l3	41.38072069	0.258657362	0.3186654	0.8116895	0.41697	0.906237
pde4d	12.3050101	0.708067413	0.5594569	1.2656335	0.205644	0.77367
net1	1606.92309	-0.071218848	0.092859	-0.766957	0.443107	0.916336
slc38a9	96.022799	-0.062763024	0.2114073	-0.296882	0.766557	0.97561
pigf	292.1047969	0.033477812	0.1336845	0.250424	0.802259	0.979492
tmem8a	58.208002	-0.241934418	0.2720189	-0.889403	0.373787	0.893677
kcnd2	485.9695519	0.042889712	0.1081938	0.3964155	0.691799	0.967305

grk5	26.01279579	0.336229125	0.3929041	0.8557537	0.392134	0.897688
ktn1	2677.754445	-0.083835736	0.073307	-1.143625	0.252779	0.822335
tdrd7a	10.33711334	-0.230200057	0.589614	-0.390425	0.696222	NA
tm7sf2	190.5973077	-0.059049854	0.1595924	-0.370004	0.711379	0.969006
rxfp2a	53.68240873	0.130873502	0.2689084	0.4866842	0.626482	0.957354
htra1a	373.8143639	0.273082913	0.122489	2.2294492	0.025784	0.310555
pvalb5	698.4260806	-0.009506181	0.1113314	-0.085386	0.931954	0.992702
si:dkey-206f10.1	371.8253157	0.176014987	0.1266497	1.3897784	0.164596	0.72071
ndrg1a	2955.974389	-0.169094135	0.0844605	-2.00205	0.045279	0.418148
ccdc120	151.4175016	0.076558146	0.1688651	0.4533688	0.650283	0.960708
lratd2	47.3133346	-0.195256252	0.2877743	-0.678505	0.497452	0.933353
ugt5g1	107.1264943	0.193986486	0.2035122	0.9531935	0.340492	0.87805
pacsin1a	606.6515097	0.047171172	0.1135042	0.4155896	0.67771	0.965257
erh	1784.332916	-0.105096049	0.0832068	-1.26307	0.206564	0.774129
pde4ba	2856.902008	0.027977736	0.0793565	0.3525578	0.72442	0.970402
trit1	290.4699904	-0.086304456	0.1344263	-0.64202	0.52086	0.939056
si:ch211-226m7.4	30.0124533	0.151785783	0.3854596	0.3937787	0.693744	0.96736
xgb	4.806469737	-0.099160752	0.8520856	-0.116374	0.907356	NA
chchd3a	781.8562821	0.056498768	0.0987923	0.5718942	0.567394	0.948739
cryba1l1	25698.837	-0.027382854	0.0842839	-0.324888	0.745266	0.974094
cnksr1	89.8076796	-0.061169975	0.2099869	-0.291304	0.770819	0.975687
dtx2	412.6366759	-0.06002611	0.1117945	-0.536933	0.591314	0.954193
nrbp2a	287.5196942	-0.096422428	0.1413109	-0.682342	0.495022	0.931926
kcnc1b	163.7190058	0.196076727	0.1629878	1.2030147	0.228971	0.796691
her12	188.3197714	-0.415976551	0.1734059	-2.39886	0.016446	0.236409
cox4i1	4100.221049	-0.031918541	0.0894306	-0.356909	0.72116	0.969602
cmlc1	278.2987896	-0.008561685	0.1383776	-0.061872	0.950665	0.99381
mrpl27	310.5679773	-0.116656316	0.1295481	-0.900486	0.367861	0.887482
plxnb3	34.64003062	-0.105702535	0.3269127	-0.323336	0.746441	0.97419
ccl34b.3	1.369090567	-0.712833094	1.6541989	-0.430923	0.666524	NA
rnf185	363.1745881	-0.107782837	0.1252381	-0.860624	0.389445	0.896421
h3f3c	21326.88863	0.07868136	0.0683153	1.1517387	0.249428	0.819036
pcnt	846.4534782	-0.077299653	0.0875053	-0.883371	0.377036	0.895192
ppcdc	30.5331885	-0.191123706	0.3550822	-0.538252	0.590403	0.954193
pkd1b	220.3340512	0.04565226	0.1386523	0.3292572	0.741961	0.973154
ptprja	30.1268431	0.504044483	0.3475344	1.450344	0.146963	0.693731
ccni2	48.2522336	-0.312242178	0.2918601	-1.069835	0.284694	0.844955
zgc:171927	7.309455989	0.107317492	0.7431221	0.1444144	0.885173	NA
trim35-10	1.497897664	1.006742206	1.6084322	0.6259152	0.531371	NA
CR735102.1	191.6831343	0.453242904	0.1564263	2.8974859	0.003762	0.083577
kif7	233.3620579	-0.003058071	0.1475234	-0.020729	0.983462	0.996944
tmc2a	31.93590553	0.116044836	0.358899	0.3233357	0.746441	0.97419
prkrip1	168.7504776	0.050123228	0.1599366	0.3133944	0.753981	0.975056
ndufaf7	178.6671329	-0.22299565	0.1518278	-1.46874	0.141903	0.683173
lyrm2	212.5596059	0.038099073	0.1459399	0.2610601	0.794046	0.977937
desi1a	948.48995	0.120881097	0.0871547	1.3869709	0.165451	0.721223
psme2	27.67697367	-0.016188587	0.3823865	-0.042336	0.966231	0.996315
nr1d1	1662.544858	0.085704271	0.0914359	0.9373158	0.348596	0.880195
sst1.2	141.3430779	-0.417367859	0.187115	-2.230542	0.025711	0.310555
sult2st1	323.5918564	0.222624271	0.1256842	1.7712986	0.076511	0.538876
trappc6bl	279.2476611	-0.034680649	0.1304291	-0.265897	0.790319	0.977338
nr2f5	675.8371948	0.061667028	0.1006763	0.612528	0.540188	0.944279
snrpe	656.2489996	0.033671876	0.1129239	0.2981821	0.765564	0.975374
tmem243b	175.0081647	-0.142035269	0.1640815	-0.865638	0.386688	0.89551

arl4ab	334.785747	-0.105029397	0.1215116	-0.864357	0.387392	0.895778
prkar2aa	2092.44984	-0.019352602	0.0798345	-0.242409	0.808463	0.980642
zgc:101840	675.7468001	-0.054862987	0.0983674	-0.557735	0.577025	0.950762
lect2l	205.9658817	-0.669853798	0.3833451	-1.747391	0.080569	0.550664
mcm6l	3.540424925	-0.022458673	1.0842534	-0.020713	0.983474	NA
stmn2a	4968.272881	0.066508372	0.0781343	0.8512056	0.394655	0.898861
osbpl3a	171.7297952	-0.007181059	0.1598078	-0.044936	0.964159	0.995927
dis3l2	292.4126985	-0.044194273	0.1380925	-0.320034	0.748943	0.97419
primpol	209.3047416	0.039886829	0.1434512	0.2780516	0.780973	0.97627
gsto2	560.3420737	-0.220957689	0.1199235	-1.842488	0.065404	0.500198
gpr37b	156.7047866	-0.023012681	0.1684585	-0.136607	0.891341	0.989291
igf2b	687.3324407	0.130669958	0.1061017	1.2315536	0.218116	0.785379
sar1ab	367.8421663	0.262034627	0.1281527	2.0447056	0.040884	0.394957
unc5b	93.37777911	0.024748511	0.2313623	0.1069686	0.914814	0.991119
cntln	93.38814542	-0.375933325	0.2066784	-1.818929	0.068922	0.513259
zgc:101699	7.579023286	-0.142453756	0.7710738	-0.184747	0.853427	NA
asl	141.6806486	-0.0990081	0.170871	-0.579432	0.562298	0.948728
mgst3b	1985.175364	-0.197784492	0.0949721	-2.082554	0.037292	0.377702
rrm2b	720.6465691	0.158681027	0.0954888	1.6617755	0.096558	0.591675
ppil3	44.43830173	0.079579001	0.3167954	0.2512	0.80166	0.979492
chchd4a	182.6145449	0.140167937	0.1504753	0.9315015	0.351594	0.880769
gyg2	20.71329797	0.46120942	0.4337659	1.0632681	0.28766	0.845979
grifin	357.7076979	-0.059729231	0.1235166	-0.483573	0.628689	0.957354
cabp1b	27.70749319	-0.032995752	0.367775	-0.089717	0.928512	0.992702
acot18	223.2685626	-0.211171073	0.1466289	-1.440174	0.149818	0.697935
gpatch1	519.6999825	-0.07743627	0.1083173	-0.714902	0.474669	0.925287
mboat7	441.1613024	0.041992861	0.1119928	0.3749605	0.70769	0.968664
rps6ka1	576.1444474	-0.052843998	0.0994135	-0.531558	0.595032	0.954434
metap1	1174.176486	-0.189719151	0.0872271	-2.175002	0.02963	0.333089
mdm2	671.7239835	0.221880057	0.1042065	2.1292335	0.033235	0.355427
map4k6	243.52125	-0.054970856	0.1469628	-0.374046	0.70837	0.968664
tap2t	50.72050073	-0.11990889	0.2902733	-0.41309	0.679541	0.965257
nxph1	1100.533745	-0.015706932	0.0853412	-0.184049	0.853975	0.985174
nkap	539.424474	-0.112118834	0.1102728	-1.01674	0.309277	0.861381
atpsckmt	81.42491999	0.236114604	0.2196849	1.0747876	0.28247	0.843666
pimr111	0	NA	NA	NA	NA	NA
tagln2	1010.02956	-0.042947337	0.0976538	-0.439792	0.660088	0.963432
ube2j1	529.0083952	-0.029776352	0.1082118	-0.275167	0.783188	0.97627
rorb	17.8390004	0.108855372	0.4471151	0.2434616	0.807648	0.980642
zgc:64189	6.146881346	-0.097578686	0.7816034	-0.124844	0.900647	NA
efr3ba	174.4937479	0.006283455	0.1545486	0.0406568	0.967569	0.996315
ccdc115	175.3108949	0.101727073	0.1522426	0.6681905	0.504012	0.93532
p4ha1a	382.8041076	0.252248721	0.1228019	2.0541114	0.039965	0.391511
paics	2251.348741	-0.095654092	0.1120457	-0.853706	0.393268	0.898076
adamts15b	134.9088725	0.25965973	0.1850915	1.4028719	0.160655	0.713726
fbxw4	264.3588046	-0.020808619	0.1422612	-0.146271	0.883708	0.988706
cyp17a1	197.6517499	0.312531775	0.1612315	1.9384044	0.052574	0.449847
fkbp1ab	142.7518691	-0.064287813	0.1729598	-0.371692	0.710122	0.968928
mtrf1	42.68201385	0.081739003	0.2903387	0.2815298	0.778304	0.975942
CABZ01088134.1	14.9676207	-0.882862438	0.5191858	-1.700475	0.089042	0.571473
ido1	13.89243067	-1.142653014	0.553977	-2.062636	0.039147	0.388646
polr2c	432.5415914	0.033754111	0.1211807	0.2785436	0.780595	0.97627
api5	2079.744236	0.074197211	0.0753812	0.9842928	0.324972	0.87132
pdia8	528.5974512	0.099550312	0.1089157	0.9140128	0.36071	0.885686

lrpap1	657.2162685	-0.054050212	0.1061535	-0.50917	0.610633	0.95688
map1lc3a	497.0096428	-0.094603465	0.1044853	-0.905424	0.365241	0.886714
morn5	6.617055783	1.034623976	0.801545	1.2907871	0.196778	NA
rasgef1ba	1038.420538	-0.010972912	0.0846397	-0.129643	0.896849	0.989971
selenon	691.4174344	0.071916214	0.0977462	0.7357443	0.461886	0.922466
NPFFR2	17.30381756	0.587008398	0.479579	1.2240077	0.220949	0.787641
smarcb1a	875.4934014	-0.107240545	0.0944323	-1.135634	0.25611	0.824633
stmn1b	7487.064134	0.063404789	0.0620014	1.0226354	0.30648	0.860027
scd	24.42843932	0.471609098	0.3933638	1.1989133	0.230562	0.798726
pi4k2a	383.0496135	0.094908779	0.1187723	0.7990815	0.424243	0.908243
tpma	73449.63554	0.307305791	0.1761713	1.7443582	0.081097	0.551495
oxgr1a.1	1.67359612	1.199668891	1.5082627	0.7953978	0.426382	NA
adora2aa	43.06665539	0.10806634	0.2994892	0.3608355	0.718222	0.96909
jade1	699.1174773	-0.19068306	0.0921284	-2.069754	0.038475	0.384637
tshba	22.13121135	0.363663044	0.4199022	0.866066	0.386454	0.89551
il12ba	0.643043639	1.435364982	2.5984886	0.5523846	0.580685	NA
ncf1	168.717135	-0.667512963	0.173777	-3.841204	0.000122	0.005778
zgc:153867	4763.131446	-0.129444913	0.0721907	-1.793098	0.072957	0.526902
nt5c1bb	66.13761225	0.108729855	0.2645255	0.4110374	0.681045	0.965476
rbm48	106.615	-0.433237476	0.1985484	-2.182024	0.029108	0.330497
ncaph2	241.605188	-0.137410798	0.1417198	-0.969595	0.332249	0.874653
pmelb	726.9066287	-0.422258772	0.1420745	-2.972094	0.002958	0.071412
trappc13	502.5105287	-0.068123843	0.1089656	-0.625187	0.531848	0.942359
hspbap1	26.02955684	0.375384282	0.408311	0.9193588	0.357908	0.884398
ndufb7	1648.592417	-0.070009577	0.10418	-0.672006	0.50158	0.934648
cyp27a7	135.8785706	0.323023254	0.1724887	1.8727209	0.061107	0.482482
snx27a	61.25018866	-0.105040686	0.2496598	-0.420735	0.673948	0.965257
ampd1	5101.154273	0.49505485	0.0954671	5.1856093	2.15E-07	2.69E-05
fat3a	138.2992401	0.086973672	0.1714219	0.5073661	0.611898	0.95688
igsf9ba	1070.690447	0.021191168	0.0911556	0.2324726	0.816171	0.981594
mad1l1	267.5528645	0.030987177	0.1323563	0.2341194	0.814892	0.981081
abrab	190.6616526	0.203961825	0.1622433	1.2571353	0.208705	0.775884
cnot9	779.0301558	0.035511623	0.0962125	0.3690958	0.712056	0.969006
sepsecs	94.53861106	-0.081031576	0.2010325	-0.403077	0.686892	0.966376
derl3	178.0095251	-0.113441937	0.1539718	-0.736771	0.461262	0.922161
spty2d1	454.528543	-0.072095969	0.1102248	-0.654081	0.513059	0.936253
ap2m1b	3951.416995	0.050872595	0.0767229	0.6630692	0.507286	0.935734
msi2a	660.7812412	0.009660409	0.0945159	0.1022094	0.91859	0.991623
si:ch211-243j20.2	506.689671	-0.520702395	0.1099772	-4.734637	2.19E-06	0.000195
ndfip2	2100.668971	0.132326542	0.0721856	1.8331434	0.066781	0.50537
fbxo16	74.2310539	-0.463651596	0.2396012	-1.935098	0.052978	0.451939
lamb2l	760.8985635	-0.010354914	0.1054311	-0.098215	0.921762	0.992138
entpd2a.2	59.84609598	-0.081253294	0.264741	-0.306916	0.758907	0.975374
CABZ01088025.1	2.661404136	-1.558289373	1.2647212	-1.232121	0.217904	NA
oxtr	3.987679569	1.29972836	1.0108459	1.2857829	0.198519	NA
trmt44	139.1122461	-0.037883583	0.1792722	-0.211319	0.832638	0.983557
nitr1i	0.166657454	-0.955901296	4.0804729	-0.234262	0.814781	NA
nup58	483.562898	-0.038074633	0.1076838	-0.353578	0.723655	0.970402
trmt2b	33.83757766	0.148937001	0.3368443	0.4421538	0.658378	0.962545
prrx1a	540.9573515	-0.037144538	0.1047894	-0.354469	0.722988	0.970375
mrps23	225.0287152	0.208372226	0.1424732	1.4625363	0.143594	0.686804
nxn	768.8347899	0.035719634	0.0935715	0.3817364	0.702657	0.968134
rsrc1	421.3169528	-0.158499933	0.126974	-1.248287	0.211926	0.77986
slc19a1	67.80321394	0.239652843	0.251302	0.9536447	0.340264	0.87805

cyp26a1	686.592787	-0.085065024	0.0964241	-0.882197	0.37767	0.895192
tango6	159.7925396	-0.243879717	0.1689412	-1.443578	0.148858	0.69636
prom1b	2163.776811	0.1054497	0.0736401	1.4319611	0.152155	0.701049
rgmd	986.6514484	0.015094902	0.0894202	0.1688086	0.865947	0.986517
dnal1	52.0476008	-0.146521936	0.2918805	-0.501993	0.615673	0.95688
irx5a	664.8551579	-0.150162717	0.0973684	-1.542212	0.123022	0.65095
zgc:92275	81.94103658	0.155660357	0.2549113	0.6106452	0.541434	0.944954
csnk1g2b	840.0832711	0.134998629	0.0948873	1.4227262	0.154816	0.704573
elac2	343.2304979	0.028172175	0.124035	0.2271308	0.820322	0.981594
unm_sa911	86.1553219	-0.338030318	0.253463	-1.333648	0.182319	0.744719
lrp11	127.8793621	0.183502441	0.1772205	1.0354469	0.30046	0.856251
plcd1b	489.1431006	-0.083681154	0.1085205	-0.771109	0.440642	0.916336
zgc:65895	937.8142662	0.020959072	0.1083709	0.1934013	0.846645	0.985174
dclk2a	495.8585457	-0.105859429	0.1100532	-0.961893	0.336103	0.876088
si:dkey-59l11.10	1.01990345	-0.910958137	1.9547665	-0.466019	0.641202	NA
mtus1b	870.0011319	0.046614694	0.0924867	0.5040151	0.614251	0.95688
actl6b	48.82944647	0.128222871	0.2969738	0.4317649	0.665912	0.964736
rarga	1331.533085	-0.039974784	0.0877353	-0.455629	0.648657	0.960708
shisa2a	8.023954674	-0.595637322	0.6745508	-0.883013	0.377229	NA
ccnk	278.0575396	-0.052375473	0.1283554	-0.408051	0.683237	0.965476
slc4a1ap	379.173874	0.042485271	0.1182305	0.3593427	0.719339	0.969183
lingo1a	34.99106152	-0.194347629	0.3507241	-0.554132	0.579488	0.951257
serpini1	1364.452325	-0.026831384	0.0901005	-0.297794	0.765861	0.975374
prkcq	45.91756958	0.088072347	0.2970155	0.2965244	0.76683	0.975687
mrrf	321.2651156	-0.075980203	0.126957	-0.598472	0.549525	0.946312
cpsf1	1211.579232	-0.138065657	0.0855833	-1.613231	0.106694	0.615633
esr2b	27.96088722	0.068341295	0.3831028	0.1783889	0.858418	0.985773
calm3a	4526.044145	0.111164584	0.0689025	1.6133606	0.106666	0.615633
oxsr1a	760.222527	-0.062399185	0.1015486	-0.614476	0.538901	0.943697
top2b	2548.279967	-0.066073685	0.0723067	-0.913797	0.360823	0.885686
cx44.2	75.34657435	0.224530871	0.2333495	0.9622087	0.335945	0.876088
si:dkey-240h12.4	17.77452839	-0.237209361	0.4554927	-0.520775	0.602523	0.956045
capn2l	766.1957085	0.038410905	0.100227	0.3832392	0.701542	0.968134
rab42a	176.1895773	-0.010558399	0.1545143	-0.068333	0.945521	0.993589
hs1bp3	34.11741862	-0.253104471	0.3338964	-0.758033	0.448431	0.917768
si:dkey-243i1.1	265.2575492	0.031578407	0.1381756	0.2285382	0.819228	0.981594
kcnip3a	456.077507	0.172648513	0.1240977	1.391231	0.164155	0.71945
capza1a	2657.116979	0.007249036	0.0709054	0.1022353	0.91857	0.991623
cdk10	262.5179016	0.023544673	0.1487268	0.1583082	0.874214	0.987881
pld2	234.5432849	-0.010559428	0.1422152	-0.07425	0.940812	0.992857
slit3	2162.496216	0.069630803	0.0845123	0.8239131	0.409989	0.902615
ogdha	4726.755475	0.087615568	0.0673069	1.3017332	0.193008	0.756795
rpl37	9000.164147	-0.336401106	0.2373579	-1.417274	0.156403	0.706639
hif1ab	2911.044048	0.091175704	0.0955025	0.9546939	0.339733	0.87805
sema3c	98.57357761	0.255193045	0.2017062	1.2651721	0.20581	0.773784
chrne	516.9794309	0.401899385	0.1155429	3.4783576	0.000504	0.017975
cox10	735.9377734	0.056685627	0.0916959	0.6181915	0.536449	0.943336
ppp5c	1716.689477	-0.016116302	0.0947367	-0.170117	0.864918	0.986212
palmdb	60.82376969	0.267701865	0.253082	1.0577671	0.290162	0.846262
ing3	940.2587184	-0.156155844	0.092744	-1.683729	0.092234	0.57849
si:dkey-88n24.7	0	NA	NA	NA	NA	NA
pcdh19	1654.367135	0.06003861	0.0796917	0.7533863	0.451218	0.918761
hexb	1481.518006	0.003746848	0.0771463	0.0485681	0.961263	0.995758
chst11	246.3037991	-0.06303019	0.1497195	-0.420989	0.673763	0.965257

mars	714.1631668	-0.049381921	0.135026	-0.365722	0.714573	0.96909
zgc:173443	44.10488423	-0.03879662	0.2864126	-0.135457	0.89225	0.989291
pik3r3b	1933.848266	0.160936529	0.0719669	2.2362576	0.025335	0.308291
irx6a	185.7858791	-0.030105506	0.1543809	-0.195008	0.845387	0.985174
sncga	976.5675184	0.171907264	0.1130068	1.5212117	0.128207	0.658954
atp1b2b	1009.153848	0.290684009	0.1050751	2.7664395	0.005667	0.113081
jpt2	1419.144832	-0.036857812	0.0832324	-0.44283	0.657889	0.962545
zgc:172121	60.95444113	-0.201034425	0.2487876	-0.808056	0.419058	0.906406
igf1rb	541.8308915	0.02245838	0.1094907	0.2051168	0.837481	0.984665
mpped2	415.0487493	-0.213743783	0.1128366	-1.894277	0.058188	0.472993
unc119a	129.3490056	0.054882777	0.1828068	0.3002228	0.764007	0.975374
si:ch211-163121.7	11.72713087	0.284079139	0.5553602	0.5115223	0.608985	0.956718
aldoab	4945.810232	0.187620675	0.0939921	1.9961323	0.04592	0.420766
ttyh3a	895.8384808	0.05108224	0.0912492	0.5598103	0.575609	0.950373
grin2aa	722.1423457	0.119331478	0.0970845	1.2291502	0.219015	0.785734
fbxl14a	316.9687712	0.045243864	0.1267148	0.3570527	0.721052	0.969602
per2	3881.140663	0.122582103	0.0809141	1.514965	0.129781	0.662436
lmo1	1164.458191	-0.064407227	0.0913788	-0.704838	0.480911	0.92776
trdmt1	93.12869068	-0.139716953	0.2037709	-0.685657	0.492929	0.931926
rab6ba	816.6949067	0.212265639	0.0971702	2.1844719	0.028928	0.329881
chodl	174.1277123	0.307135087	0.1687209	1.8203734	0.068702	0.513248
atp6v1aa	5254.451318	0.045954415	0.0696503	0.6597877	0.50939	0.936123
tgfbr2b	531.220258	0.017967496	0.1167141	0.1539445	0.877653	0.987881
pard3bb	141.8837881	0.118339882	0.171266	0.6909714	0.489584	0.931926
srpx2	8.445758875	0.033948888	0.6630463	0.0512014	0.959165	NA
fitm2	227.6144723	-0.077691562	0.1449422	-0.536017	0.591947	0.954434
gpr143	112.643377	-0.246799389	0.1875304	-1.31605	0.188157	0.753063
si:dkey-23o4.6	49.44078022	0.08881641	0.2774268	0.3201436	0.748859	0.97419
scn4ab	471.1580384	0.251382446	0.1089267	2.3078138	0.021009	0.273449
tmem165	970.6258858	-0.048176174	0.0873838	-0.551317	0.581416	0.951731
zgc:153169	65.60973448	-0.067649478	0.2477327	-0.273074	0.784796	0.97627
insb	1.930681209	-1.008161712	1.4919778	-0.675722	0.499217	NA
mlf2	2208.903346	0.041877235	0.0777747	0.5384427	0.590271	0.954143
nuf2	200.7063143	-0.301589363	0.1487828	-2.027045	0.042658	0.406074
emc9	307.776919	0.033711638	0.134157	0.251285	0.801594	0.979492
fhl3a	508.1402801	-0.105253094	0.1056116	-0.996605	0.318956	0.867746
fabp7b	155.9171718	-0.162829046	0.1671549	-0.974121	0.329997	0.874009
CABZ01090041.1	26.41477269	-0.509194043	0.3749525	-1.358023	0.174456	0.734611
celf3a	1897.485379	-0.069836439	0.0893679	-0.781449	0.434538	0.914297
ube2e2	1595.919287	-0.079740915	0.0971094	-0.821145	0.411564	0.903549
scel	609.1012887	-0.083479099	0.1013587	-0.823601	0.410166	0.902747
tmem198b	627.8786253	0.032262657	0.1054505	0.3059506	0.759642	0.975374
mysm1	274.5641213	0.071767269	0.1445059	0.4966391	0.619444	0.957354
vegfab	446.8945438	-0.209911128	0.1280576	-1.639194	0.101173	0.604409
pvalb7	739.8923171	0.025666914	0.1027871	0.2497094	0.802812	0.979643
ftr85	26.15819207	-0.012198744	0.3778086	-0.032288	0.974242	0.99644
esyt1a	29.65901947	-0.14022973	0.3732242	-0.375725	0.707121	0.968664
def6c	38.95821218	-0.627812728	0.3086376	-2.034142	0.041937	0.401972
tfpia	689.8366787	0.025555487	0.0969811	0.2635101	0.792157	0.977937
med6	382.6951567	0.038099032	0.1147853	0.3319156	0.739953	0.972943
tspan3a	1872.814909	0.011064042	0.0812595	0.1361569	0.891697	0.989291
zdhhc2	107.8688389	0.0685371	0.1980182	0.3461151	0.729256	0.970822
phf3	688.4335548	-0.162039668	0.1005754	-1.611126	0.107152	0.617015
rab13	183.7327283	0.25960894	0.1614359	1.6081238	0.107808	0.618989

ncapd3	467.9378072	-0.194478854	0.1109698	-1.752539	0.079681	0.548406
txn2	459.6984871	-0.080707513	0.110402	-0.731033	0.464759	0.92354
dachb	271.4704294	-0.080468734	0.1328968	-0.605498	0.544848	0.945463
mylka	355.9495064	0.121795602	0.116396	1.0463899	0.295381	0.851935
kcnip1b	615.4636977	-0.086443048	0.0986807	-0.875988	0.381037	0.895192
asah1b	478.6908623	-0.034360016	0.1111322	-0.309182	0.757183	0.975374
copg2	2821.570717	0.02191079	0.0717401	0.3054191	0.760047	0.975374
uspl1	540.2630263	0.045929433	0.1115213	0.4118446	0.680453	0.965431
si:dkey-222n6.2	116.9126224	0.085709022	0.191599	0.4473354	0.654633	0.961305
nt5c2l1	445.4121607	0.257709948	0.1493519	1.7255222	0.084433	0.559138
ccnb3	337.6603391	0.028936655	0.1378333	0.2099395	0.833715	0.983583
f7	195.11155	0.093244488	0.154719	0.6026699	0.546728	0.945526
mid2	104.9490901	0.061787063	0.2043964	0.3022904	0.762431	0.975374
acbd5a	476.8028639	0.052293392	0.1048269	0.4988547	0.617882	0.957354
bet1	321.961883	0.029604341	0.1244757	0.2378324	0.812011	0.981081
rarab	1078.490089	-0.013288336	0.0868021	-0.153088	0.878329	0.987881
tgfb1b	19.0206672	-0.471489711	0.4356808	-1.082191	0.279168	0.842806
ldb2b	887.2372549	0.069986368	0.0984987	0.7105307	0.477375	0.926857
rps10	12912.06614	-0.337844157	0.079742	-4.236714	2.27E-05	0.001407
paqr7b	27.09076034	0.005760784	0.3797466	0.0151701	0.987896	0.997369
si:ch211-152f2.2	0.647019846	-1.455099716	2.4289208	-0.599073	0.549125	NA
hat1	1213.450624	-0.053101151	0.0952949	-0.55723	0.577371	0.950805
cx52.6	20.79686644	-0.187264134	0.4201671	-0.44569	0.655821	0.961833
chchd3b	228.4595404	-0.009611727	0.1373736	-0.069968	0.944219	0.993364
slc1a7a	39.10837918	-0.131020207	0.3179691	-0.412053	0.6803	0.965431
si:dkey-224e22.2	94.09447812	0.021807799	0.2325905	0.0937605	0.925299	0.992676
tm9sf5	755.8049298	-0.011569607	0.0952742	-0.121435	0.903347	0.990121
myclb	523.5556854	0.066993421	0.1070087	0.6260556	0.531278	0.942209
hddc2	206.4783498	-0.128953096	0.1425212	-0.904799	0.365572	0.887073
gpr157	55.52945458	-0.240181254	0.262002	-0.916715	0.359292	0.884762
zcchc8	486.9951941	-0.08444114	0.1067306	-0.791162	0.42885	0.911406
asb4	44.82021051	-0.107901329	0.314004	-0.34363	0.731124	0.971557
retsatl	1.151545593	-2.472760569	2.0114878	-1.229319	0.218952	NA
bag2	162.9327992	0.038406955	0.1628332	0.2358669	0.813536	0.981081
trim35-27	12.36503124	0.712002534	0.5584662	1.274925	0.202336	0.768682
thy1	553.4316541	0.124671876	0.131308	0.9494616	0.342386	0.878434
khdc4	1064.753574	0.015261824	0.0979595	0.1557973	0.876193	0.987881
lpcat4	188.4306229	-0.181441805	0.1538615	-1.179254	0.238297	0.807449
pfdn5	972.0776642	-0.133650733	0.0899454	-1.485909	0.137303	0.674358
or115-10	0	NA	NA	NA	NA	NA
pdcd10a	763.840579	-0.150650679	0.0931536	-1.617229	0.105829	0.615489
fgf13a	1247.717198	0.002289129	0.0825704	0.0277234	0.977883	0.996762
ubtf	2905.894162	0.00451546	0.0694833	0.0649863	0.948185	0.99381
rtel1	181.0095679	-0.112126651	0.1576814	-0.711096	0.477025	0.926857
hsf2bp	1.329752533	0.767635872	1.650511	0.4650898	0.641867	NA
si:ch211-254c8.3	0.182570949	0.967652056	4.0804729	0.2371421	0.812547	NA
tdgf1	21.39216049	-0.15855901	0.4334458	-0.36581	0.714506	0.96909
dicp2.1	0.330916733	-1.849579229	3.2489867	-0.569279	0.569167	NA
gldc	793.1835263	-0.166589217	0.115284	-1.445033	0.148449	0.696298
camsap1b	612.3669688	-0.036232645	0.0986324	-0.36735	0.713358	0.96909
rxrab	5.887710319	0.43350323	0.7858465	0.5516386	0.581196	NA
zgc:101858	294.2120831	-0.216955681	0.1384687	-1.566822	0.117156	0.640585
surf4l	209.2833216	0.224169388	0.157051	1.4273668	0.153474	0.702578
rgs3b	325.3457182	0.074369475	0.1275356	0.5831272	0.559808	0.948478

pho	84.42913956	0.07296273	0.2189762	0.3331994	0.738984	0.972722
selenow1	2414.740272	0.046743354	0.0760286	0.6148124	0.538679	0.943391
ehd2a	2.075640094	0.572762562	1.4177906	0.4039825	0.686226	NA
polr2j	299.9162823	-0.004434688	0.1402879	-0.031611	0.974782	0.996605
lrwd1	130.7286215	0.045002416	0.1936049	0.2324446	0.816193	0.981594
orai2	53.52873779	0.137272452	0.2675742	0.5130257	0.607933	0.956718
pex12	60.78908709	-0.110023167	0.2521903	-0.43627	0.662641	0.964665
drll.2	0.94955631	1.001959126	2.2550603	0.4443159	0.656814	NA
drll.1	3.521124531	1.023895559	1.0368561	0.9875001	0.323398	NA
ap2b1	3142.700913	-0.022397297	0.0687346	-0.325852	0.744536	0.974094
pkdcca	89.60037056	0.650013533	0.2173477	2.9906616	0.002784	0.06821
tmem136b	109.3068756	0.140942	0.1909637	0.7380567	0.46048	0.921978
mrpl48	264.886069	-0.060434668	0.1457441	-0.414663	0.678389	0.965257
layna	9.060547097	0.922873293	0.6393997	1.4433434	0.148924	NA
btg4	0	NA	NA	NA	NA	NA
schip1	1334.632506	0.006364272	0.0781834	0.0814018	0.935122	0.992702
gna14	115.7317235	0.147312825	0.1927715	0.7641837	0.444758	0.916777
slc25a46	340.5541215	0.086859497	0.1202658	0.7222292	0.470154	0.924787
tmem150ab	18.88608119	0.280740945	0.4470293	0.6280147	0.529994	0.942209
fzd9a	39.9199466	0.147762268	0.3114334	0.4744586	0.635173	0.957927
abl1	379.5024013	0.194894136	0.1314621	1.4825124	0.138204	0.675945
rab14l	880.771039	0.033951062	0.0985894	0.3443684	0.730569	0.97149
fam81b	1.275648953	-2.653845067	1.9988247	-1.327703	0.184276	NA
gcnt4a	187.5478867	0.229423802	0.1615028	1.4205564	0.155446	0.705158
si:dkey-47k20.8	1.161805827	-3.660681204	1.934818	-1.892003	0.058491	NA
zgc:110249	27.72638471	0.062497302	0.380246	0.1643602	0.869448	0.987282
npr3	509.5597203	0.017684761	0.1174989	0.15051	0.880362	0.987881
eef2l2	5184.497046	0.262728398	0.0843258	3.1156344	0.001835	0.050201
akr1a1a	68.30067983	0.513220244	0.2715094	1.8902487	0.058725	0.474103
riok2	423.9246242	-0.037921928	0.1115645	-0.33991	0.733924	0.971993
tmtc2b	14.50390572	-0.728258054	0.5266498	-1.382813	0.166722	0.723339
emc6	885.4880454	0.039481888	0.0913299	0.4322999	0.665523	0.964736
ncor1	2200.641252	-0.018857857	0.0916687	-0.205717	0.837012	0.984645
dmrt3a	51.49428565	-0.107513976	0.2774044	-0.387571	0.698333	0.967998
grb10b	46.70757006	-0.306447919	0.2792017	-1.097586	0.272385	0.837519
entpd3	738.8830699	0.35544157	0.0963418	3.6893817	0.000225	0.009418
myh7bb	638.3923207	-0.103861994	0.0958042	-1.084107	0.278317	0.84215
hnrnpl	4616.640357	-0.079985769	0.0889833	-0.898885	0.368714	0.888057
srsf7a	2198.633909	-0.110555118	0.0724361	-1.526243	0.126949	0.656588
nccrp1	504.4208466	-0.102879855	0.117832	-0.873106	0.382605	0.895192
ckma	31894.38711	0.193639969	0.1059803	1.8271315	0.06768	0.509442
capns1a	1904.302011	-0.078899374	0.0804438	-0.980801	0.326691	0.872642
taf1	1150.892615	0.003715859	0.0912955	0.0407014	0.967534	0.996315
cfap298	176.8359599	0.149693364	0.1516653	0.9869982	0.323644	0.870928
mis18a	69.03777269	-0.134576386	0.2481695	-0.542276	0.587628	0.953319
tppp2	350.1015415	0.239564187	0.1289346	1.8580289	0.063165	0.490775
ftr99	5.729467772	0.942180337	0.8054694	1.1697283	0.24211	NA
ins	401.4358231	0.202388649	0.158149	1.2797341	0.200639	0.766801
pafah1b2	138.0307906	0.289547654	0.1802629	1.6062521	0.108219	0.620087
gnb2	1613.50764	0.061873181	0.0829681	0.7457463	0.455821	0.921215
mink1	992.8643295	-0.072109644	0.0843689	-0.854694	0.39272	0.897688
fgf5	10.68569519	-0.254745924	0.6312963	-0.403528	0.68656	NA
tmem174	20.91067354	0.042291909	0.4268905	0.0990697	0.921083	0.992138
fcho2	654.0976699	-0.000173409	0.095514	-0.001816	0.998551	0.999364

tnpo1	1013.593289	-0.117445406	0.0949057	-1.237495	0.215903	0.783484
ptcd2	59.9765326	0.232580291	0.2546631	0.9132864	0.361092	0.885686
enc1	1208.53634	0.040168606	0.0799961	0.5021318	0.615575	0.95688
ankra2	700.0606541	0.142868964	0.1103058	1.2952077	0.195249	0.760159
btf3	7308.645749	-0.287111959	0.2503109	-1.147021	0.251373	0.820814
ttc33	230.9942663	-0.081103357	0.1370562	-0.591753	0.554016	0.947449
ptger4b	16.18188731	-0.360052	0.4743876	-0.758983	0.447863	0.917154
ophn1	196.3824398	-0.031112619	0.1550859	-0.200615	0.840999	0.985174
ccn1l1	83.10189089	-0.085887548	0.2200069	-0.390386	0.696251	0.96736
surf6	30.01990967	0.106758183	0.4052692	0.2634253	0.792223	0.977937
arpc5lb	299.2597777	-0.129514278	0.1248307	-1.037519	0.299494	0.855609
zmat5	199.9762914	0.075817788	0.1553146	0.4881561	0.625439	0.957354
ml7	6.956593726	-0.414851316	0.7334017	-0.565654	0.571629	NA
myhc4	565.72056	0.677693323	0.3733908	1.8149706	0.069528	0.514708
camkk1a	41.05807289	0.019783533	0.3050321	0.0648572	0.948288	0.99381
atp2a1l	7767.008865	0.579192188	0.1038007	5.5798507	2.41E-08	3.78E-06
spns3	58.17496945	-0.529097784	0.2661142	-1.988236	0.046786	0.424961
slc25a25b	49.96348116	0.587934279	0.2939328	2.0002336	0.045475	0.418148
sh3glb2b	1056.42921	0.091744814	0.0846546	1.0837545	0.278474	0.842429
si:dkey-220k22.1	375.7766055	0.094355226	0.1194168	0.7901335	0.42945	0.911489
nelfb	475.8520594	-0.131980791	0.1075251	-1.227442	0.219657	0.786189
entpd2a.1	66.50191572	-0.371891059	0.2577928	-1.442597	0.149134	0.696454
ddx31	144.3903456	-0.100621613	0.179269	-0.561288	0.574601	0.950037
barhl1a	252.4589627	-0.25585176	0.1332996	-1.919374	0.054937	0.459334
adam28	201.2984158	0.002864015	0.1565513	0.0182944	0.985404	0.996944
tm2d2	290.5702883	-0.003654435	0.1280002	-0.02855	0.977223	0.996762
histh1l	30342.84999	-0.29890524	0.0799064	-3.74069	0.000184	0.007935
sfrp1a	290.5050992	0.189874501	0.1416819	1.3401467	0.180198	0.74128
zgc:110329	183.2686814	-0.091859865	0.1565993	-0.586592	0.557478	0.948478
tacr1a	20.08649692	-0.592532922	0.4367514	-1.356682	0.174882	0.735545
rasa1a	786.9982332	-0.09990792	0.0933985	-1.069695	0.284757	0.844955
atp6v0a2a	522.9853799	0.085064603	0.1153618	0.7373725	0.460896	0.921978
brwd3	681.2373693	-0.00764621	0.0985404	-0.077595	0.93815	0.99279
etnppl	337.9487106	0.445019502	0.1181305	3.767187	0.000165	0.007304
jmjd7	96.25690486	-0.111131107	0.1985707	-0.559655	0.575715	0.950373
uprt	513.505003	0.000446111	0.1091887	0.0040857	0.99674	0.998936
cfap73	6.019629955	-0.50373326	0.7955628	-0.633179	0.526617	NA
iqcd	11.65437111	0.274028117	0.5687198	0.4818333	0.629924	0.957354
agtr2	160.4136065	-0.03541318	0.1582048	-0.223844	0.822879	0.982189
cx27.5	33.44633159	-0.240630627	0.3396224	-0.708524	0.47862	0.926978
gbgt1l3	78.14393356	-0.332223323	0.2206632	-1.505567	0.132178	0.666055
rps6ka3a	954.7560419	0.046162242	0.0876402	0.5267247	0.598385	0.955488
gabarapa	1609.31227	0.068003729	0.0779178	0.8727627	0.382792	0.895192
gps2	581.6641722	-0.033629484	0.1019321	-0.329921	0.74146	0.973154
tp53	891.0741354	0.035749782	0.0957153	0.3735014	0.708775	0.968664
capga	13.12428292	0.263628697	0.5605212	0.4703278	0.638121	0.959194
mpdu1a	147.6785088	0.193107396	0.1640579	1.1770683	0.239168	0.807768
znf703	1164.069927	-0.090100815	0.0913416	-0.986416	0.323929	0.870928
dgcr8	1042.314734	-0.013662506	0.085884	-0.159081	0.873605	0.987858
atp6v0a2b	304.3272264	-0.059478163	0.1298548	-0.458036	0.646927	0.960362
cyp1d1	272.9379826	0.092691989	0.1311518	0.7067537	0.47972	0.927091
zgc:101016	145.2614948	0.109004392	0.1804799	0.6039697	0.545864	0.945526
abhd17b	415.0981712	0.105329006	0.1103913	0.9541427	0.340011	0.87805
AK6	201.2119411	0.061980486	0.1453757	0.4263469	0.669855	0.965223

cds2	1584.683214	0.059556453	0.0771375	0.7720822	0.440066	0.916082
hs3st1l2	180.6223366	-0.17617664	0.1611282	-1.093394	0.274221	0.839127
si:dkey-174n20.1	92.1164919	-0.060030787	0.2043042	-0.29383	0.768887	0.975687
ficd	157.2980634	0.523339765	0.1668043	3.1374472	0.001704	0.047485
ISCU (1 of many)	494.6359486	-0.039779721	0.1151783	-0.345375	0.729812	0.971143
coro1ca	1538.451765	-0.106754332	0.0856566	-1.246306	0.212652	0.780725
dao.3	4.418772514	0.092866402	0.892773	0.1040202	0.917153	NA
dao.1	23.47197977	0.011592158	0.3988213	0.029066	0.976812	0.996762
dao.2	126.2377491	0.132743472	0.1921655	0.6907768	0.489706	0.931926
tchp	222.6835673	-0.133635875	0.1393304	-0.959129	0.337494	0.876395
aldh3b2	49.35218318	-0.078102804	0.2748373	-0.284178	0.776274	0.975826
ankle2	72.90738658	0.148192838	0.240774	0.6154853	0.538234	0.943336
pgam5	747.2699591	-0.024168073	0.09766	-0.247472	0.804543	0.979705
mtnr1c	2.794455884	-0.163811091	1.100302	-0.148878	0.88165	NA
zgc:113208	363.0724184	-0.018422938	0.1164566	-0.158196	0.874303	0.987881
coq5	316.7668347	-0.157378472	0.1256345	-1.252669	0.210326	0.778043
xbp1	4673.393986	0.041385416	0.0931362	0.4443537	0.656787	0.96205
snrnp27	522.1167941	0.049524311	0.1034958	0.4785153	0.632283	0.957356
parvab	94.41834059	0.197867304	0.2084134	0.9493981	0.342418	0.878434
ypel1	182.5741364	-0.278999363	0.1751931	-1.592525	0.111267	0.627048
sdf2l1	598.7822956	-0.046597997	0.1238398	-0.376277	0.706711	0.968636
ccl19a.2	0	NA	NA	NA	NA	NA
tctn2	127.4566388	-0.297126838	0.1798446	-1.652131	0.098508	0.597984
rfc5	511.2185712	-0.027858155	0.1130492	-0.246425	0.805353	0.979883
pimr123	0.157187365	-0.955901296	4.0804729	-0.234262	0.814781	NA
irx4a	233.4933618	-0.125052512	0.1363329	-0.917258	0.359007	0.884725
ext1c	352.6913115	-0.13406258	0.1243696	-1.077937	0.281062	0.84357
evla	738.1987192	0.055300903	0.0953339	0.580076	0.561863	0.948728
sat1a.1	566.0646926	-0.054879379	0.1159874	-0.47315	0.636106	0.958498
rad21b	571.1725698	0.049491421	0.125325	0.3949045	0.692913	0.96736
BX649384.1	0.182570949	0.967652056	4.0804729	0.2371421	0.812547	NA
si:ch211-193k19.1	139.9117889	-0.106993104	0.1839836	-0.581536	0.560879	0.948728
zgc:103438	19.46419554	-0.44092352	0.4415951	-0.998479	0.318047	0.867746
si:dkey-267n13.1	9.647646204	0.138166811	0.6435076	0.2147089	0.829994	NA
ptp4a2b	2095.29743	0.025851158	0.0864649	0.2989788	0.764956	0.975374
bmp8a	40.74326913	-0.225946876	0.3121858	-0.723758	0.469214	0.924496
zgc:91910	982.9402637	0.135680342	0.1152	1.1777806	0.238884	0.807768
si:rp71-45k5.4	1285.427961	-0.009382041	0.0926866	-0.101223	0.919373	0.991639
zgc:113424	0.688986717	0.114008068	2.399209	0.047519	0.9621	NA
FP102018.1	580.156157	0.141040553	0.1027703	1.3723858	0.169943	0.727155
mon1ba	9.598511244	-0.140859765	0.6565765	-0.214537	0.830129	NA
rps3a	21389.87662	-0.302487006	0.0803193	-3.766056	0.000166	0.007304
stm	3275.960146	0.224167223	0.0768199	2.9180879	0.003522	0.079772
scxa	101.7415934	0.045399696	0.2064168	0.2199419	0.825916	0.982196
spinb	33.95972126	-0.426651288	0.3325348	-1.283027	0.199482	0.764896
srp19	362.6968329	-0.132010092	0.1204342	-1.096118	0.273027	0.838141
zgc:101664	128.6542535	0.279221227	0.1812084	1.5408845	0.123345	0.651258
dock6	316.5738292	-0.248993163	0.1386313	-1.796082	0.072482	0.526156
marcksl1b	14645.34273	0.048275744	0.0630104	0.7661554	0.443584	0.916631
cbln6	0	NA	NA	NA	NA	NA
arl5c	208.6213273	0.066670101	0.1464228	0.4553261	0.648875	0.960708
arntl1b	215.4061308	0.096410545	0.1441246	0.6689388	0.503535	0.935304
gsx1	189.7629905	-0.128126141	0.1523285	-0.841117	0.400282	0.90084
slc25a11	2201.504836	0.169847929	0.0762722	2.2268651	0.025956	0.311101

ipo7	3550.383786	-0.159612695	0.0703092	-2.270154	0.023198	0.292366
stx18	246.6115895	-0.247455608	0.1378979	-1.794485	0.072736	0.526264
CU633984.1	12.39482494	0.59999009	0.5719065	1.0491053	0.29413	0.849999
si:ch1073-157b13.1	360.5487376	-0.045013319	0.1250244	-0.360036	0.71882	0.969092
ctdnep1b	235.4304465	0.095518756	0.1388894	0.6877323	0.491621	0.931926
cdh31	0.489712128	-0.887840729	2.693685	-0.329601	0.741702	NA
si:dkey-17m8.2	0	NA	NA	NA	NA	NA
gngt1	1169.484885	0.226132091	0.1223087	1.8488636	0.064478	0.496874
clcn4	570.4525757	0.00284314	0.1139751	0.0249453	0.980099	0.996879
col1a1b	24425.24927	0.302513317	0.1804292	1.6766313	0.093615	0.582118
rgcc	917.5282218	0.179142291	0.0956398	1.8730933	0.061056	0.482482
sirt3	157.763201	0.225513289	0.1766667	1.2764902	0.201782	0.767736
drd4b	15.66494091	0.735684888	0.5138907	1.431598	0.152259	0.701157
si:ch211-173p18.3	1341.097117	-0.035127396	0.0805422	-0.436137	0.662738	0.964665
zmym4	515.3381818	-0.201259498	0.1057946	-1.902361	0.057124	0.468896
pyyb	74.90209154	-0.79841942	0.2558182	-3.121042	0.001802	0.049493
eef2k	1529.932693	-0.209412401	0.0839698	-2.493901	0.012635	0.199712
mosmob	122.5712642	0.023121087	0.1885978	0.1225947	0.902428	0.990121
cart3	49.38022207	-0.563454055	0.294047	-1.916204	0.055339	0.460942
cdkn2aip	131.3078957	-0.05381335	0.1727286	-0.311549	0.755384	0.975318
cnn2	1452.255347	0.088715018	0.0818091	1.0844156	0.278181	0.841891
angptl4	2820.828928	0.012725239	0.0834041	0.1525733	0.878735	0.987881
rps28	6078.463899	-0.344473406	0.1866891	-1.845172	0.065013	0.497901
si:rp71-39b20.4	121.0344612	0.445760999	0.1917044	2.3252523	0.020058	0.266383
insl3	1.695240948	0.547460725	1.6714392	0.3275385	0.743261	NA
spire1a	333.9815304	-0.276362683	0.1332737	-2.073648	0.038112	0.382552
azin1b	7047.988435	0.019053283	0.0834502	0.2283193	0.819398	0.981594
laptm4b	1088.548372	0.162111275	0.0846208	1.9157377	0.055398	0.461238
rpl30	11598.17475	-0.428287654	0.0855712	-5.005042	5.58E-07	6.20E-05
hsd17b12b	662.4809373	0.010894398	0.097746	0.1114562	0.911255	0.991066
fjx1	57.31713551	-0.058294609	0.2689774	-0.216727	0.828421	0.982721
tmem54b	9.450117642	0.678979344	0.6705313	1.012599	0.311252	NA
fuca1.2	217.4355621	-0.136542503	0.1492772	-0.914691	0.360354	0.885686
atp6v1c1b	996.3707516	0.066562853	0.0912484	0.7294689	0.465715	0.923865
rida	428.7370409	-0.187538377	0.1193199	-1.571728	0.116014	0.63841
nt5c1aa	135.1483844	0.349790176	0.1850957	1.8897796	0.058787	0.474281
zgc:91944	314.0067643	-0.060101021	0.1320176	-0.45525	0.648929	0.960708
zbtb8b	324.1588776	-0.027396057	0.1215645	-0.225362	0.821697	0.982004
fuca1.1	951.636247	-0.155345597	0.0960809	-1.616821	0.105917	0.615567
acana	2042.10585	0.153276964	0.0844741	1.8144855	0.069603	0.514708
fndc5b	139.8965916	0.096581935	0.172063	0.5613173	0.574581	0.950037
lingo1b	448.9626701	0.071666554	0.1069542	0.6700679	0.502814	0.935304
crabp1b	280.6867503	0.088878299	0.1404971	0.6325988	0.526996	0.941016
slc25a44b	270.7539674	0.033689668	0.1324388	0.2543792	0.799203	0.979293
fam49al	2634.107822	0.047284893	0.0799071	0.5917483	0.554019	0.947449
mfsd2ab	944.0922444	0.266282362	0.0918047	2.9005295	0.003725	0.082862
znf281b	710.1068963	-0.0923196	0.0921676	-1.001649	0.316513	0.867129
yars	1008.518916	0.107886041	0.0844625	1.2773247	0.201488	0.767524
tmem167a	393.5483969	-0.187165795	0.1409724	-1.327677	0.184285	0.747376
bzw2	910.4937262	0.152632476	0.0891604	1.7118867	0.086918	0.565027
dync1li2	1081.514591	0.142009641	0.0994473	1.4279889	0.153295	0.702578
hrh3	52.52880471	0.52209264	0.2715551	1.9226031	0.05453	0.457793
sync	40.2997506	0.077190691	0.3083207	0.2503584	0.80231	0.979492
tmem106bb	172.6338912	0.005135748	0.1561725	0.0328851	0.973766	0.996315

cdr2a	22.14088844	0.755307001	0.4229757	1.7856982	0.074148	0.530917
tdp2b	107.2536573	0.209548615	0.1926748	1.0875764	0.276782	0.840075
gmnn	151.5693199	-0.227806516	0.1757695	-1.296053	0.194957	0.759324
tnni2b.1	1143.610844	-0.041892536	0.1039636	-0.402954	0.686982	0.966376
kcng2	45.72293694	0.094446826	0.2862607	0.3299329	0.741451	0.973154
ccdc96	23.05268582	0.233807717	0.4065303	0.5751298	0.565204	0.948739
pgr	5.806923612	-0.095873505	0.7779919	-0.123232	0.901923	NA
ptprn2	1913.857317	0.059335952	0.0721913	0.8219262	0.411119	0.903291
ube3c	555.0847083	-0.045755533	0.0994338	-0.460161	0.645401	0.96029
mnx1	80.37551493	0.241349763	0.2304596	1.0472543	0.294982	0.851519
fam210aa	387.1023077	0.194480273	0.1268823	1.5327607	0.125335	0.654648
ptpn2b	432.8962414	-0.06628821	0.1126554	-0.588416	0.556253	0.947902
psmg2	298.6894335	-0.254127542	0.1302433	-1.951176	0.051036	0.443404
cited4a	1579.245688	0.047321638	0.0957957	0.4939849	0.621317	0.957354
sumo3b	1328.50363	0.011398604	0.1011489	0.1126913	0.910275	0.990702
pitpnb	189.1291285	0.13692202	0.1558992	0.8782727	0.379796	0.895192
caly	926.3828671	0.136235361	0.0957094	1.4234266	0.154613	0.704307
arrdc3b	7017.827578	-0.091748988	0.0808296	-1.135091	0.256337	0.824633
sept5b	98.77849349	0.143874404	0.2032367	0.7079154	0.478998	0.926978
mdka	8024.308945	0.149363211	0.0657218	2.2726603	0.023047	0.291198
harbi1	196.9755498	-0.1519982	0.1587101	-0.95771	0.338209	0.876778
atg13	1331.916971	-0.048834147	0.0876058	-0.557431	0.577233	0.950788
f2	1741.683771	0.384834235	0.0855275	4.4995384	6.81E-06	0.00052
znf408	31.89224422	0.364162772	0.3378367	1.0779254	0.281067	0.84357
rps20	15728.25926	-0.381267769	0.0860454	-4.431007	9.38E-06	0.000687
penkb	188.5053293	-0.131039412	0.1554469	-0.842985	0.399237	0.900683
irx4b	105.9333299	-0.009477228	0.1905648	-0.049732	0.960336	0.995421
thap11	219.4402195	-0.079581854	0.143995	-0.552671	0.580489	0.951621
vrk3	192.7365606	0.020127514	0.1484999	0.1355389	0.892186	0.989291
tradd	138.7403448	-0.28998844	0.1755963	-1.651449	0.098647	0.59799
gnao1b	3445.723713	0.063844064	0.086616	0.7370933	0.461066	0.922038
tmppe	221.3096848	-0.02207279	0.1428557	-0.154511	0.877207	0.987881
exosc6	174.9953947	0.303581157	0.1673063	1.8145231	0.069597	0.514708
ogfod1	130.868922	-0.139396199	0.1782114	-0.782196	0.434099	0.914001
ppp1r11	568.7626054	0.09279122	0.0996086	0.9315585	0.351565	0.880769
cnep1r1	242.5854829	-0.020616026	0.1360838	-0.151495	0.879585	0.987881
mgat1b	295.5113376	0.107418546	0.124753	0.8610499	0.389211	0.896362
si:dkey-78a14.4	18.49194307	0.085881997	0.4591797	0.1870335	0.851634	0.985174
ddx39b	537.6849243	0.067916202	0.1067774	0.6360538	0.524741	0.939791
znrd1	35.59758815	-0.057155649	0.3474257	-0.164512	0.869328	0.987274
gtf2h4	0.878629579	0.686795053	2.2010162	0.3120354	0.755014	NA
cebpg	1637.115459	-0.060321226	0.0837291	-0.720433	0.471258	0.924838
cebpa	706.5715793	-0.069495512	0.0928338	-0.748602	0.454097	0.920295
heatr3	335.2954066	-0.077469759	0.123697	-0.626287	0.531127	0.942209
cd81a	12915.05454	0.083714581	0.0653986	1.2800663	0.200522	0.766801
tph1b	65.03190566	-0.40228566	0.2431763	-1.654296	0.098067	0.59749
itln2	3.996604593	-0.338518989	1.1163666	-0.303233	0.761712	NA
itga11a	232.2842768	0.208503591	0.1416064	1.4724168	0.140908	0.681252
pard6gb	321.7548881	-0.037611485	0.1297196	-0.289945	0.771859	0.975687
sdhaf3	181.7243823	0.141538839	0.151248	0.9358061	0.349373	0.880195
pias1a	322.7401748	-0.128075704	0.1267219	-1.010683	0.312168	0.864401
smad3a	418.4933004	0.083391026	0.1210822	0.6887144	0.491003	0.931926
eya3	564.7770387	0.049742858	0.102173	0.4868496	0.626365	0.957354
ctdsp2	2739.278451	-0.076305793	0.0755015	-1.010653	0.312182	0.864401

myo1g	3.387788532	0.01564599	1.0558498	0.0148184	0.988177	NA
si:dkeyp-92c9.2	3.857622245	-0.599625394	1.0155758	-0.590429	0.554903	NA
rgs18	13.62286564	0.21607484	0.5188069	0.4164842	0.677056	0.965257
txnipa	10098.1058	-0.084069121	0.0816341	-1.029829	0.30309	0.85729
mtx1b	130.8109273	-0.135046672	0.1878893	-0.718757	0.472291	0.924838
eif3hb	246.0667624	-0.127721441	0.1405676	-0.908612	0.363555	0.886101
mchr2	3.620070472	0.266801763	0.9818007	0.2717474	0.785816	NA
nitr13	0.166657454	-0.955901296	4.0804729	-0.234262	0.814781	NA
bbox1	540.4590575	-0.099454193	0.1150887	-0.864153	0.387504	0.895884
tipin	168.8571146	0.030073636	0.1554908	0.1934111	0.846637	0.985174
lctla	891.876563	0.259888152	0.1027347	2.5297024	0.011416	0.186316
crybgx	3230.925751	-0.013696973	0.0786802	-0.174084	0.861799	0.985841
tssc4	546.2178205	0.057660283	0.1244175	0.463442	0.643048	0.959994
ano5b	600.2971093	0.162344646	0.1074003	1.5115849	0.130639	0.662797
gas2b	30.20299339	-0.027133905	0.3433246	-0.079033	0.937007	0.992786
gla	66.40277066	0.171475408	0.2405873	0.7127366	0.476009	0.926338
fnbp1a	14.79347469	0.538325068	0.5519758	0.9752693	0.329427	0.873436
nudcd1	287.2091381	0.003152154	0.127407	0.0247408	0.980262	0.996879
trhrb	5.141155378	0.705224634	0.94431	0.7468147	0.455175	NA
hnrnpa01	7828.145469	-0.171417358	0.065485	-2.61766	0.008853	0.156033
hnrnpa0b	144.8131953	0.239016813	0.1848352	1.2931346	0.195964	0.761148
cars	864.9525141	0.024356215	0.0916195	0.265841	0.790362	0.977338
nfatc1	202.8547274	-0.139402543	0.1519781	-0.917254	0.35901	0.884725
rnasel3	265.8087248	0.703707405	0.1427305	4.9303242	8.21E-07	8.54E-05
pcdh1b	889.4402005	-0.0091982	0.1023558	-0.089865	0.928395	0.992702
myo1ea	585.1266275	-0.142461219	0.097667	-1.458643	0.144663	0.688975
ccnb2	100.7299182	-0.189482765	0.2102847	-0.901077	0.367547	0.887458
mbpa	915.414934	0.224129248	0.088351	2.5368063	0.011187	0.183548
spata4	15.69817846	-0.270081473	0.4722542	-0.571898	0.567391	0.948739
txnl4a	415.5387521	0.113757428	0.1194067	0.9526889	0.340748	0.878134
pitx2	649.40057	-0.051492106	0.0980657	-0.525077	0.599529	0.956045
tbc1d5	781.5900865	0.014679351	0.0931356	0.1576126	0.874762	0.987881
dazl	6.721575763	0.896369832	0.7484045	1.1977077	0.231031	NA
lcp2b	3.249393254	-0.740375907	1.1675185	-0.634145	0.525986	NA
npy	78.78881066	0.29929389	0.2225624	1.3447638	0.178702	0.739486
npvf	90.66554345	0.052963373	0.2124461	0.2493027	0.803127	0.979643
trpm7	897.3705932	0.095376699	0.0923681	1.0325719	0.301804	0.856944
zbtb3	163.309103	0.047977192	0.1630908	0.2941747	0.768624	0.975687
slc27a2a	664.0244862	0.067233525	0.1180671	0.5694516	0.56905	0.948983
gatm	3189.814182	0.092280991	0.0944751	0.9767762	0.32868	0.872945
sb:cb649	51.4923668	-0.180433156	0.2720542	-0.663225	0.507186	0.935734
tmem144b	1.999048969	0.975922653	1.3685926	0.7130849	0.475793	NA
rras2	641.0498406	0.148869723	0.0959307	1.5518461	0.120699	0.645578
hoxa13b	19.82217617	-0.045519769	0.4333557	-0.10504	0.916344	0.991314
rasal2	909.2765766	-0.066386831	0.0899063	-0.7384	0.460271	0.921978
gcgrb	27.90390589	0.19537566	0.3800181	0.514122	0.607167	0.956718
lamc1	3609.719755	0.049088438	0.0715361	0.6862049	0.492584	0.931926
CC2D1A	150.1908449	0.127677946	0.1698954	0.751509	0.452346	0.919102
rnaset2	545.8726177	-0.003635598	0.1061747	-0.034242	0.972684	0.996315
nucb2b	281.2460372	-0.230715817	0.1456507	-1.584035	0.113186	0.631061
cdx4	12.02654714	0.364450955	0.5533108	0.6586731	0.510106	0.936253
gnb1l	24.31356122	0.63930459	0.4056861	1.5758604	0.115058	0.635519
golga7ba	616.7209573	0.153445296	0.0992044	1.5467586	0.121921	0.649101
rps13	9187.232908	-0.343211151	0.0861208	-3.985231	6.74E-05	0.003508

nucb2a	1854.448803	0.107179923	0.0837768	1.2793506	0.200774	0.766855
dnaaf3l	12.0448701	0.220719095	0.5752578	0.3836873	0.70121	0.968134
phf23b	292.0934712	0.044454141	0.133479	0.3330422	0.739102	0.972722
nod1	3.149578253	0.483808077	1.1210099	0.4315823	0.666045	NA
rpl39	9418.477417	-0.312311335	0.1111039	-2.810985	0.004939	0.102322
ndufa1	1097.738302	-0.009045462	0.1196831	-0.075578	0.939754	0.992857
c1galt1c1	435.384513	0.067111425	0.1079581	0.6216434	0.534176	0.943276
cers3b	51.33161818	-0.238119715	0.2742275	-0.868329	0.385214	0.89551
vps11	509.7416884	-0.042858957	0.1095397	-0.391264	0.695602	0.96736
calb2b	4149.288071	0.058830713	0.0793685	0.7412353	0.458551	0.921978
cmtr2	178.1089941	-0.279417933	0.1605621	-1.740248	0.081815	0.552725
riox2	103.1769683	0.003929872	0.192092	0.0204583	0.983678	0.996944
acta1a	3813.02881	0.126324246	0.096861	1.3041803	0.192172	0.756673
cyfip2	2945.314734	0.03420618	0.0746164	0.4584272	0.646646	0.960362
cldn7a	975.142405	0.112555678	0.1002246	1.123034	0.261423	0.829593
ponzr6	57.9007738	-0.54338247	0.2734264	-1.987308	0.046888	0.425112
slc24a6a	539.736304	0.177054151	0.1057239	1.6746845	0.093996	0.58339
rbm4.1	540.829899	0.006053285	0.1142272	0.0529934	0.957737	0.99467
pop7	566.061554	0.102537114	0.1002343	1.0229748	0.30632	0.860027
si:ch211-81a5.5	22.81840159	0.345856196	0.4257208	0.8124014	0.416561	0.906237
si:ch73-335l21.1	831.4429766	0.113186648	0.1003592	1.1278158	0.259398	0.827693
glb1	196.5772029	0.254054132	0.1554775	1.6340249	0.102254	0.607744
si:dkey-33c9.6	22.23597324	-0.15870962	0.4146104	-0.382792	0.701874	0.968134
ntn5	282.3771741	-0.010972148	0.1384695	-0.079239	0.936843	0.992702
pcdh20	195.8037749	0.247896123	0.1535828	1.6140874	0.106508	0.615567
zgc:103586	178.7526449	0.211458261	0.1623576	1.3024231	0.192772	0.756795
slc3a2a	2312.443312	-0.039348432	0.0858536	-0.45832	0.646722	0.960362
si:dkey-264d12.4	127.6023788	-0.195068187	0.1888296	-1.033038	0.301586	0.856944
sat2a	0	NA	NA	NA	NA	NA
ndufv1	4983.424477	0.079831768	0.0812524	0.9825153	0.325846	0.871896
atp1lc	233.9800828	0.257121965	0.1403824	1.8315823	0.067014	0.506251
serpinh2	261.4395394	0.265014392	0.1321701	2.0051006	0.044952	0.417005
PRKAR2B	255.5214947	0.067773725	0.1614554	0.4197674	0.674655	0.965257
anxa4	1679.68595	-0.445492591	0.0881889	-5.051571	4.38E-07	5.04E-05
cacng6a	195.0753388	0.379931571	0.1491039	2.5480988	0.010831	0.180681
prlhr2b	3.29685416	0.27107151	1.0351481	0.2618674	0.793424	NA
tmtops3a	7.788023212	0.074835965	0.6869267	0.1089432	0.913248	NA
rab11fip1b	27.52116728	-0.456904244	0.3757246	-1.216061	0.223962	0.790824
dand5	3.422964639	-0.680587392	1.0643958	-0.639412	0.522555	NA
snapc2	134.1536674	-0.193505488	0.1722615	-1.123324	0.2613	0.829527
si:dkeyp-80c12.4	2.200210324	-0.168395489	1.3110895	-0.128439	0.897801	NA
tcn2	1344.620755	-0.320366391	0.1017811	-3.147601	0.001646	0.046303
hexim1	1015.365432	0.078362544	0.0881441	0.8890273	0.373988	0.893685
c1ql3b	27.03553014	-0.170098885	0.3680454	-0.462168	0.643961	0.96009
zgc:112356	2037.614885	-0.052304025	0.0848272	-0.616595	0.537502	0.943336
si:ch73-174h16.4	178.5576768	0.077616701	0.1581535	0.4907682	0.62359	0.957354
gstcd	89.75318361	-0.018022144	0.2093612	-0.086082	0.931402	0.992702
zgc:162171	22.04469244	-0.534526599	0.4085099	-1.308479	0.190711	0.755212
fam122b	545.8232609	-0.017683423	0.1031352	-0.171459	0.863863	0.985952
rab39bb	348.4342034	0.027741038	0.1355284	0.204688	0.837816	0.984701
syt4	1289.987458	-0.013206675	0.0801449	-0.164785	0.869113	0.987245
ccnt2b	1186.97025	0.074772947	0.0850586	0.8790757	0.37936	0.895192
rhbdf1a	568.55187	0.010301456	0.124648	0.0826444	0.934134	0.992702
pknox1.2	145.6508024	0.078496325	0.1730986	0.4534774	0.650205	0.960708

tmprss3b	8.658108658	0.223657562	0.6466522	0.34587	0.72944	NA
pdxkb	53.40906296	0.371385494	0.2770878	1.340317	0.180142	0.74128
s1pr2	343.2244035	0.093315293	0.1346588	0.692976	0.488325	0.93189
agpat3	2180.156069	0.055541361	0.0823191	0.6747079	0.499861	0.934224
col18a1a	5909.841402	0.14193449	0.0808212	1.7561543	0.079062	0.547489
gpr185b	10.45034184	0.391100294	0.6069167	0.6444053	0.519313	NA
aanat1	38.7272989	0.343465004	0.3090529	1.1113468	0.266419	0.832634
bach2a	59.83655816	-0.26166064	0.2673923	-0.978564	0.327795	0.872701
sema3e	238.1190913	0.094001578	0.1359182	0.6916038	0.489186	0.931926
si:ch73-71d17.2	90.70254905	-0.23221627	0.2149291	-1.080432	0.27995	0.843423
atp6v0cb	8108.633239	0.132610506	0.075532	1.7556857	0.079142	0.547489
st8sia5	278.0350583	0.092688553	0.1363282	0.6798926	0.496572	0.932588
cbr1	417.121542	0.02872445	0.1152718	0.2491888	0.803215	0.979643
mhc1zba	540.1312915	0.271926958	0.1344292	2.0228267	0.043091	0.4075
setd4	117.4156673	-0.14575333	0.2020833	-0.721254	0.470753	0.924838
kdm2ba	1966.504252	-0.009910267	0.0801466	-0.123652	0.901591	0.990121
tab1	287.6934541	-0.219677408	0.1366387	-1.607724	0.107896	0.619311
gpr182	61.45878333	0.1414214	0.2638067	0.5360796	0.591904	0.954434
mgat3b	82.97390653	0.00152686	0.2485665	0.0061427	0.995099	0.998694
polr2f	581.9772464	-0.267971672	0.0980508	-2.732988	0.006276	0.120609
sema6ba	5.289353913	0.546020313	0.8487747	0.6433042	0.520027	NA
cd74b	5.656557411	0.510670674	0.8385901	0.6089634	0.542549	NA
rps14	13612.0504	-0.481959569	0.0709401	-6.793894	1.09E-11	3.08E-09
plp2	726.3495281	-0.08027229	0.1053228	-0.762155	0.445968	0.917154
ebp1	51.48719525	-0.264480832	0.2841946	-0.930633	0.352044	0.880844
arl11	35.75138902	0.050933315	0.3206104	0.1588636	0.873776	0.987881
rcbtb1	240.7586567	0.122495481	0.14059	0.8712959	0.383593	0.895192
cdadc1	187.0389791	-0.034848141	0.1659514	-0.20999	0.833675	0.983583
cab39l	1590.53996	-0.079542019	0.0753114	-1.056176	0.290888	0.846568
aplnrb	252.3860781	-0.156705201	0.1392232	-1.125568	0.260348	0.828473
tnni4b.2	317.990272	0.396904405	0.135486	2.9294856	0.003395	0.077847
hnrnpa1b	6402.587256	-0.146730436	0.0867174	-1.692052	0.090636	0.576398
trmt10a	96.79572276	0.216475041	0.2154194	1.0049006	0.314945	0.866054
ndufa7	608.7579077	-0.165811624	0.1277678	-1.297758	0.194371	0.758233
ccnt2a	852.0671409	0.062584418	0.0926091	0.6757912	0.499173	0.934006
bcl9	2024.109686	-0.038219372	0.0870518	-0.439042	0.660631	0.963881
calcoco1a	768.5734247	-0.112078918	0.0987367	-1.135129	0.256321	0.824633
znf865	1680.523818	0.022431782	0.0766171	0.2927776	0.769692	0.975687
si:ch211-114n24.6	1034.618262	0.169663894	0.0929146	1.8260193	0.067847	0.509884
tmem18	325.4850369	-0.18619891	0.119671	-1.555924	0.119726	0.644854
tomm40l	733.0576282	0.057547281	0.0974474	0.5905468	0.554824	0.947449
slc9a3.2	23.02664648	0.462245041	0.4134925	1.1179042	0.263608	0.830245
si:dkey-211g8.1	0	NA	NA	NA	NA	NA
nitr7a	0.158795395	0.967652056	4.0804729	0.2371421	0.812547	NA
nitr8	0.157187365	-0.955901296	4.0804729	-0.234262	0.814781	NA
hmgn3	3840.743442	0.036287779	0.0697533	0.5202303	0.602903	0.956045
prmt9	119.628876	0.106416008	0.1903677	0.5590023	0.57616	0.950611
hax1	504.9092059	-0.088842692	0.1099669	-0.807904	0.419146	0.906406
urgcp	70.9284736	-0.094204636	0.2533459	-0.371842	0.71001	0.968928
pygo2	1044.58088	0.042118589	0.0846532	0.4975431	0.618806	0.957354
s100s	337.1385292	0.065036789	0.1214605	0.5354561	0.592335	0.954434
rgs12b	773.6045662	0.037692108	0.0942823	0.3997792	0.689319	0.966763
dtd2	61.9927229	-0.287709222	0.2649742	-1.085801	0.277567	0.84125
aldh8a1	350.9111127	-0.778669962	0.118483	-6.571997	4.96E-11	1.23E-08

hbs1l	958.1776344	-0.015537506	0.0851906	-0.182385	0.855281	0.985174
si:dkey-170l10.1	49.16169538	0.348720813	0.2958747	1.17861	0.238554	0.807659
commd7	201.9305027	0.031219307	0.1539116	0.2028392	0.839261	0.984979
si:ch211-226m16.2	420.4024412	-0.119490774	0.1096444	-1.089803	0.2758	0.839541
tap2a	1.338122747	-0.741451151	1.6474665	-0.450055	0.652671	NA
dnmt3bb.1	559.5626352	0.014642337	0.1153349	0.1269549	0.898976	0.990121
ttll5	22.34127966	0.410581378	0.4133544	0.9932913	0.320568	0.868418
si:ch211-106j24.1	397.0408832	0.427989974	0.1174219	3.6448898	0.000268	0.010817
ifrd2	1025.955517	0.039008332	0.0850818	0.4584805	0.646607	0.960362
pou2f2a	750.8696317	-0.061457478	0.0934368	-0.657743	0.510703	0.936253
hyal3	11.63145114	0.413427179	0.6073194	0.6807409	0.496035	0.932377
mgll	950.5792815	-0.001222372	0.0945623	-0.012927	0.989686	0.997612
ankrd52a	22.3435766	0.651506254	0.4283401	1.5210024	0.128259	0.658954
krt91	14164.52311	0.094215946	0.0868633	1.0846461	0.278078	0.841891
rnf41	836.4450004	0.087558717	0.0905775	0.9666718	0.333708	0.874933
cytl1	10174.75681	-0.071781714	0.0982418	-0.730663	0.464985	0.923671
upp2	1.185891111	-0.36621692	1.8036008	-0.203048	0.839098	NA
krt92	4455.225075	-0.603390794	0.0911633	-6.61879	3.62E-11	9.18E-09
krt15	1600.872649	0.106604819	0.0978296	1.0896985	0.275846	0.839541
tsen54	108.4012569	-0.116502257	0.1892707	-0.615532	0.538203	0.943336
anks4b	320.4089237	-0.718915664	0.1244771	-5.775486	7.67E-09	1.34E-06
slc43a2a	886.5902781	-0.121013934	0.1038813	-1.164925	0.244049	0.813415
DXO	47.61192559	-0.066334593	0.2996257	-0.221392	0.824788	0.982196
plagx	879.4726811	-0.09548818	0.0917383	-1.040876	0.297933	0.85401
myo1d	422.4861358	-0.119479583	0.11187	-1.068021	0.285511	0.845164
slc34a2b	343.0268306	-1.199766908	0.1395214	-8.599158	8.03E-18	5.12E-15
slc2a12	207.3135849	0.056620568	0.1507621	0.3755623	0.707242	0.968664
tbpl1	140.333327	0.276632075	0.1823142	1.5173367	0.129182	0.660844
tcf21	42.41056817	-0.298013232	0.3226086	-0.923761	0.355611	0.882511
arih1l	322.5348289	0.088964131	0.1326193	0.6708232	0.502333	0.935304
rps12	20921.91153	-0.397054736	0.0743834	-5.337945	9.40E-08	1.32E-05
zgc:153284	202.2571103	0.237050594	0.1603155	1.4786509	0.139234	0.677963
sh3bgrl2	599.4988132	0.079583698	0.100325	0.7932588	0.427627	0.91075
zgc:153935	64.63993599	0.01642324	0.2486173	0.0660583	0.947331	0.99381
f13a1b	291.5663683	-0.03949957	0.1452454	-0.271951	0.78566	0.97627
aimp1b	249.8684202	-0.072099917	0.1419781	-0.507824	0.611577	0.95688
sgms2b	38.68850504	0.25633079	0.3241623	0.7907483	0.429091	0.911406
irak1bp1	48.11983867	-0.113475553	0.2761475	-0.410924	0.681129	0.965476
scamp1	341.8694178	0.183338581	0.1298262	1.4121843	0.157896	0.709114
edn1	36.17320493	-0.088897086	0.3293462	-0.26992	0.787222	0.976596
st6galnac6	7.5261108	-0.866445861	0.7293429	-1.187981	0.234841	NA
dolpp1	79.75004317	0.122375052	0.2210052	0.5537203	0.57977	0.951257
tatdn1	57.3053916	0.137036577	0.2526483	0.5424006	0.587543	0.953319
rnf139	279.4204442	0.046639048	0.1478338	0.3154829	0.752395	0.974684
tbx18	71.34108328	-0.18388263	0.2409113	-0.763279	0.445297	0.917117
ctssl	13.53794159	-0.178151224	0.5466146	-0.325917	0.744487	0.974094
gpd1c	528.7645677	-0.357728497	0.124304	-2.877853	0.004004	0.087004
golph3l	410.9203475	-0.159130799	0.1133171	-1.404297	0.16023	0.7128
ensaa	121.6761608	0.176853442	0.1920193	0.9210189	0.357041	0.883467
zgc:198371	56.24419443	-0.001264663	0.2603373	-0.004858	0.996124	0.998888
asxl1	1943.651949	-0.032883939	0.0894491	-0.367627	0.713151	0.96909
rnf24	150.4983925	-0.110632234	0.1942142	-0.56964	0.568922	0.948983
hsd3b7	373.9481461	0.041007	0.1172981	0.3495964	0.726642	0.970402
smox	2573.891437	0.021293317	0.0875452	0.2432265	0.80783	0.980642

si:ch1073-416d2.3	31.95383797	0.064553473	0.3498394	0.1845232	0.853603	0.985174
ripply3	5.184685586	0.262485464	0.9347461	0.2808094	0.778857	NA
plxnb2b	62.09834054	0.465750877	0.2528618	1.8419184	0.065487	0.500378
has2	84.56559987	0.045061627	0.2180838	0.2066253	0.836302	0.984231
dctd	113.0039253	-0.094730537	0.1970624	-0.480713	0.63072	0.957354
erbb3b	300.1968894	-0.327610575	0.1348969	-2.428601	0.015157	0.225353
tbc1d16	200.4576486	-0.204968076	0.1462405	-1.401582	0.16104	0.714313
lsm6	541.5833806	-0.028303078	0.1379284	-0.205201	0.837415	0.984665
arf3b	14.42499429	0.077620785	0.5064782	0.1532559	0.878196	0.987881
DISP3	23.8416	0.229215199	0.4121935	0.5560864	0.578152	0.951024
fkbp11	178.1182005	-0.141941133	0.1565601	-0.906624	0.364606	0.886586
zgc:152968	4.082403184	-0.097672661	0.9419125	-0.103696	0.917411	NA
bscl2	452.7979221	0.134996635	0.1072911	1.2582274	0.20831	0.775404
banf1	1692.590365	-0.104441805	0.0765737	-1.363938	0.172587	0.731348
slc3a2b	4110.533671	-0.054717172	0.0679666	-0.80506	0.420785	0.90651
ube4b	1580.697424	0.003750056	0.0880678	0.0425814	0.966035	0.996315
gab1	446.2771837	-0.060317627	0.11009	-0.547894	0.583765	0.952214
kif1b	3343.263185	0.049260303	0.0736071	0.6692333	0.503347	0.935304
casz1	263.09008	-0.017660959	0.1419019	-0.124459	0.900952	0.990121
ptger2b	13.33230899	0.322566467	0.5708098	0.5651032	0.572004	0.949494
psmc6	2300.839383	-0.078651894	0.0866084	-0.908133	0.363808	0.886174
cgrrf1	58.94662156	0.106005428	0.2576432	0.4114427	0.680748	0.965476
ddhd1b	235.0610125	0.212797445	0.1397547	1.5226492	0.127846	0.658686
rad51b	51.94688841	0.051506955	0.2662349	0.1934643	0.846595	0.985174
zte38	1.308375155	0.736523946	1.6727564	0.4403056	0.659716	NA
gcdha	1083.841369	0.366380852	0.092507	3.9605735	7.48E-05	0.00383
slc44a2	1165.689533	-0.020279425	0.0840597	-0.24125	0.809361	0.980642
kmt2d	572.3274037	-0.008055046	0.1031163	-0.078116	0.937736	0.99279
aldh9a1b	304.0903965	-0.23709169	0.1236643	-1.91722	0.05521	0.460643
dhh	12.36756021	0.192910191	0.5443542	0.3543836	0.723051	0.970383
galca	379.2791099	-0.007178893	0.1336645	-0.053708	0.957168	0.994589
lgals8a	95.9707878	0.095756812	0.2054216	0.4661477	0.64111	0.959994
dnajc22	30.54041982	-0.322877964	0.3563663	-0.906028	0.364921	0.886714
ikzf4	85.81754952	0.428240699	0.2206921	1.9404442	0.052326	0.449084
zdhhc14	27.02208851	-0.087442963	0.3796661	-0.230315	0.817847	0.981594
tmem242	198.5638295	0.115950848	0.1557054	0.744681	0.456465	0.921215
rps26	4006.332266	-0.428556859	0.0772857	-5.545096	2.94E-08	4.47E-06
si:dkey-60a16.1	177.255314	0.228691033	0.1602967	1.4266736	0.153674	0.702578
cfap36	450.4281289	0.019900647	0.1136452	0.175112	0.860992	0.985773
asah1a	237.4501339	0.1212984	0.1412496	0.8587521	0.390477	0.897304
slc7a2	532.6489596	0.275183661	0.1074928	2.5600206	0.010467	0.176292
irs2a	668.5711058	-0.131149183	0.107184	-1.223589	0.221107	0.787641
ankrd10a	201.2969626	0.028269114	0.1514805	0.1866188	0.85196	0.985174
mtmr7a	337.5712563	0.128123668	0.1185209	1.0810217	0.279687	0.843169
pfdn6	414.1565853	-0.218400242	0.1231068	-1.774071	0.076051	0.537342
pwp2h	536.9800883	-0.053634012	0.1086859	-0.493477	0.621675	0.957354
cxcl12a	1035.192362	0.089469035	0.101638	0.8802717	0.378712	0.895192
mat2ab	485.0001759	0.186812219	0.1142553	1.6350415	0.10204	0.607206
TENM2	1211.889396	0.005768701	0.0832106	0.0693265	0.94473	0.993409
pfkfb1	103.1172033	-0.056383972	0.2026949	-0.278172	0.780881	0.97627
zgc:153146	29.41163762	0.105281622	0.3514356	0.2995758	0.764501	0.975374
cpb2	81.77907821	0.541846707	0.2551425	2.1237019	0.033695	0.357045
slc8a4b	251.4675417	0.022533591	0.1362658	0.165365	0.868657	0.987245
atp6ap1b	743.9855608	0.004391491	0.0975781	0.0450049	0.964103	0.995927

hivep3b	300.6958322	-0.108891623	0.144715	-0.752456	0.451777	0.918761
si:dkey-78k11.9	5.303158258	-1.289041984	0.9165824	-1.406357	0.159618	NA
rcc1	381.6700111	-0.260362731	0.138931	-1.874044	0.060924	0.482482
oprd1b	34.98046101	-0.444563684	0.3323091	-1.337802	0.180961	0.741776
ddrgk1	74.85792332	-0.028446216	0.2305256	-0.123397	0.901793	0.990121
nek12	24.33159102	0.063534936	0.4030848	0.1576218	0.874755	0.987881
ggh	91.79157835	0.11247568	0.2133568	0.5271717	0.598074	0.955459
zc3h13	1619.181103	-0.105146703	0.0938366	-1.12053	0.262488	0.830166
zic2b	179.4672299	-0.151364261	0.1558264	-0.971365	0.331367	0.874362
tpbgb	72.7593205	0.320133596	0.2361523	1.3556232	0.175219	0.735698
rpa2	587.0234631	-0.173323899	0.1028654	-1.684959	0.091997	0.57849
ttr	128.2116998	0.46343652	0.1924552	2.4080228	0.016039	0.233377
zgc:113364	32.81121314	-0.358147049	0.3375428	-1.061042	0.288671	0.845979
tshr	2.571911483	-0.994166215	1.2942635	-0.768133	0.442408	NA
arid5b	835.3006856	0.040232617	0.0961898	0.4182627	0.675755	0.965257
zgc:101851	90.14192941	-0.094782127	0.2043752	-0.463765	0.642816	0.959994
denr	973.7033055	-0.148743473	0.1173551	-1.267465	0.204989	0.772822
smad5	563.8529936	-0.030655615	0.1002275	-0.30586	0.759711	0.975374
gpr137	102.8676678	-0.028876703	0.1994207	-0.144803	0.884866	0.988838
trmo	28.01480986	-0.137871693	0.3659403	-0.37676	0.706352	0.968459
pimr212	0.158915748	0.967652056	4.0804729	0.2371421	0.812547	NA
si:ch211-145b13.5	47.27462142	0.361476023	0.281535	1.283947	0.19916	0.764592
ndufb6	1190.90889	-0.062333065	0.0910874	-0.684322	0.493772	0.931926
toporsa	123.0807582	-0.194629617	0.2007676	-0.969427	0.332332	0.874653
mtap	299.2131223	-0.247322982	0.130487	-1.895384	0.058042	0.472311
cdkn2a/b	5.490155204	0.830785744	0.8173799	1.016401	0.309438	NA
myoz2b	190.5628661	0.291827823	0.1674979	1.7422779	0.08146	0.552315
zgc:158263	174.0405867	-0.364957843	0.1571066	-2.322995	0.020179	0.266908
map9	79.01954713	-0.115989238	0.2251523	-0.515159	0.606442	0.956718
lrata	34.11939304	-0.093150744	0.3417152	-0.272598	0.785163	0.97627
fgg	1672.885274	0.356122786	0.1013344	3.5143342	0.000441	0.016114
plrg1	726.4017151	0.109407324	0.0917082	1.1929933	0.232872	0.801255
ptges3a	1358.70121	0.090606487	0.0792851	1.1427929	0.253125	0.822746
mipa	2817.073674	0.104704333	0.0727298	1.439634	0.149971	0.697935
si:dkey-30k22.7	2.182878176	0.28030228	1.3988912	0.2003746	0.841188	NA
dpf2l	1022.991201	-0.145177449	0.091191	-1.592015	0.111381	0.627123
chrm1a	4.193767824	-0.004935696	0.9627293	-0.005127	0.995909	NA
gnpda1	308.5772118	-0.693736198	0.1337083	-5.188429	2.12E-07	2.67E-05
gatb	151.5772206	-0.131547111	0.1777391	-0.740114	0.459231	0.921978
ddost	3560.277482	-0.015232336	0.0713456	-0.213501	0.830936	0.982994
fam131c	36.40292238	-0.074407912	0.3141607	-0.236847	0.812776	0.981081
klhl17	484.0842347	0.02475041	0.105323	0.2349954	0.814212	0.981081
wdr92	194.0434237	-0.224802248	0.1464649	-1.534854	0.12482	0.654423
cnrip1b	374.331837	0.050452574	0.1155703	0.4365532	0.662435	0.964665
rpl9	151.6358866	-0.167443943	0.2104773	-0.795544	0.426297	0.909969
frs2b	270.3963358	-0.003123821	0.1350981	-0.023123	0.981552	0.996944
polr3e	924.4891783	-0.040516891	0.0893977	-0.453221	0.65039	0.960708
si:ch1073-272o11.3	10.28199829	0.750182978	0.625484	1.199364	0.230386	NA
kdelr2b	813.0174744	0.021356465	0.0966489	0.2209695	0.825116	0.982196
scube2	276.5957389	-0.002428051	0.1385847	-0.01752	0.986022	0.996944
st5	682.829482	0.138320413	0.0978422	1.4137095	0.157447	0.708089
ephb2a	68.14632115	-0.196120575	0.2383404	-0.822859	0.410588	0.903084
qdprb1	107.4411691	-0.330913613	0.2136497	-1.548861	0.121415	0.647929
pax5	15.7108714	-0.590622598	0.4783287	-1.234763	0.216919	0.784302

gsg1l	111.561335	0.026421174	0.1947057	0.135698	0.89206	0.989291
med19a	413.1716641	0.062610679	0.1252956	0.4997039	0.617284	0.957346
slc43a1a	524.7164242	-0.045860261	0.1000661	-0.4583	0.646737	0.960362
ssrp1a	1023.993777	-0.018458229	0.09364	-0.197119	0.843734	0.985174
zgc:113229	17.53707234	-0.406453277	0.4844409	-0.839015	0.401461	0.90084
lim2.3	686.6238934	-0.052989829	0.0970053	-0.546257	0.584889	0.952436
si:dkey-4p15.3	104.3312343	0.338618754	0.1935951	1.7491077	0.080272	0.549641
zgc:112083	144.3774309	-0.109050235	0.1674176	-0.651367	0.51481	0.937646
wdr19	216.1882127	0.118547622	0.1544603	0.7674959	0.442787	0.916336
tbc1d10c	8.720810113	0.274862277	0.6490432	0.4234884	0.671939	NA
tmem144a	139.4314223	0.01564743	0.1752136	0.0893049	0.92884	0.992702
med28	276.4269379	0.013204135	0.1269957	0.1039731	0.917191	0.991572
npy1r	11.01718254	-1.003933939	0.5934918	-1.691572	0.090728	NA
dlgap1b	17.9688245	0.306714303	0.4821049	0.6361983	0.524647	0.939713
egr1	943.69669	0.152726125	0.1231034	1.2406333	0.214741	0.782493
smim19	354.8889903	0.066996866	0.1206111	0.5554783	0.578568	0.951052
s100a10a	588.3388544	-1.2978135	0.1122038	-11.56657	6.09E-31	1.72E-27
si:ch211-39a7.1	18.47946087	-0.388529896	0.4484823	-0.866322	0.386314	0.89551
tll1	13.70237413	0.148979009	0.5099998	0.2921158	0.770198	0.975687
haus7	137.4936256	-0.243027027	0.173334	-1.402074	0.160893	0.714142
fmr1	883.0227957	-0.099249292	0.0857308	-1.157686	0.246992	0.816669
ugt8	388.5366765	0.020789556	0.1133493	0.1834115	0.854475	0.985174
cd79a	1.172093801	-1.384729205	1.9581194	-0.707173	0.479459	NA
foxb2	35.88553264	0.076715863	0.3268753	0.2346946	0.814446	0.981081
sorbs3	357.8835814	-0.094888096	0.121802	-0.779036	0.435959	0.914408
tmem192	242.0437006	-0.078325157	0.1381951	-0.566772	0.570869	0.949078
1-Mar	30.59993876	0.159790356	0.3968473	0.4026495	0.687206	0.966376
canx	7325.960712	0.011212822	0.0738111	0.1519125	0.879256	0.987881
bmp15	3.483668765	-0.430260855	1.0336215	-0.416265	0.677216	NA
rtn4rl2b	39.72227109	0.231552054	0.3033508	0.7633146	0.445276	0.917117
gria4a	526.4427714	0.007131049	0.1032816	0.0690447	0.944954	0.993409
gria3b	733.209196	0.055417834	0.0948584	0.5842162	0.559075	0.948478
aasdhppt	43.53842391	0.085112369	0.3017379	0.2820739	0.777887	0.975942
thoc2	1751.542432	-0.089835282	0.0769226	-1.167867	0.242861	0.811584
prps1b	425.2067843	-0.070691073	0.121009	-0.58418	0.559099	0.948478
kctd12b	40.96082156	0.382481317	0.3072668	1.2447856	0.213211	0.781631
hdac3	766.6741278	0.089289372	0.0953172	0.9367606	0.348882	0.880195
slc35c2	1255.405798	0.315295255	0.0842962	3.7403244	0.000184	0.007935
CR749762.1	2.159892768	-0.17488655	1.3304961	-0.131445	0.895424	NA
si:rp71-17i16.5	2.645869394	0.380416939	1.1724021	0.3244765	0.745577	NA
si:ch211-191a24.4	142.5881215	-0.304938321	0.1670086	-1.825884	0.067868	0.509884
mcm3l	11.92036114	-0.070804887	0.545576	-0.12978	0.89674	0.989971
mep1b	0.347458735	-1.908042286	3.4571486	-0.551912	0.581009	NA
ccnq	197.4737576	-0.058287837	0.1487142	-0.391945	0.695099	0.96736
tnnc1b	1833.122803	0.061934887	0.1029657	0.60151	0.5475	0.945526
mtnr1aa	12.59765402	0.341961199	0.5661984	0.60396	0.54587	0.945526
cyp21a2	43.7544475	0.290340273	0.2873351	1.0104588	0.312276	0.864401
pm20d1.1	51.35066045	-1.691804715	0.3027349	-5.588404	2.29E-08	3.63E-06
il1rapl2	91.36050141	0.135857994	0.2130533	0.6376713	0.523688	0.939336
arhgef3l	51.43247274	-0.615722288	0.276834	-2.224157	0.026138	0.311732
atoh8	280.1120658	0.029957258	0.1345969	0.2225702	0.82387	0.982196
st3gal5	492.7133729	0.061043921	0.1059592	0.5761076	0.564542	0.948739
asb12b	29.17958042	0.157460352	0.3651506	0.4312202	0.666308	0.964736
uba1	6502.496639	-0.005512626	0.0735906	-0.074909	0.940287	0.992857

mtm1	270.7164743	-0.076470079	0.1386447	-0.551554	0.581254	0.951731
polr1d	120.0600098	-0.086784132	0.1845067	-0.470358	0.6381	0.959194
rps6ka1	1422.111537	0.081037965	0.0895826	0.9046177	0.365668	0.887084
BX530018.1	2.000307227	0.047416512	1.4426464	0.0328677	0.97378	NA
SYNPR	512.6310477	-0.076859808	0.1116386	-0.68847	0.491157	0.931926
bhlhe23	463.5541229	0.156985384	0.125775	1.2481447	0.211978	0.77986
gid8b	595.2373775	-0.02506883	0.0994448	-0.252088	0.800973	0.979377
prickle2b	559.9190729	0.072478172	0.1106346	0.6551131	0.512395	0.936253
prf1.5	59.15083395	-0.041228149	0.2524611	-0.163305	0.870278	0.987421
zgc:162144	36.6085796	0.322822099	0.3146666	1.0259179	0.30493	0.859575
nol4lb	60.19912163	-0.350234981	0.2529928	-1.384367	0.166246	0.722887
lgals8b	78.47554668	0.062739687	0.2399912	0.2614249	0.793765	0.977937
ddit4	1049.164954	-0.08952256	0.2771342	-0.32303	0.746673	0.97419
BX569798.1	5.21314942	0.487430593	0.8251875	0.5906907	0.554728	NA
trpt1	155.7137735	-0.031331328	0.1647949	-0.190123	0.849213	0.985174
leng1	186.9708746	-0.176738581	0.148668	-1.188814	0.234513	0.803509
nkx3-2	114.1873562	0.003915102	0.187264	0.0209069	0.98332	0.996944
aurkb	534.2423975	-0.31135474	0.1081389	-2.879211	0.003987	0.08682
zgc:173570	87.74435468	-0.188989483	0.2132999	-0.886027	0.375603	0.894316
rgs11	260.4241903	-0.006302156	0.1499176	-0.042037	0.966469	0.996315
rnf175	605.1525774	-0.028800866	0.114434	-0.251681	0.801288	0.979377
zgc:56409	104.0491678	0.123378887	0.1916424	0.6437975	0.519707	0.938945
pmm2	232.6579482	0.073269216	0.1352076	0.5419016	0.587886	0.953319
pls3	1995.001374	0.213345179	0.0763554	2.7941058	0.005204	0.106533
npm1b	498.2873405	-0.063033854	0.1175603	-0.536183	0.591832	0.954434
fgf24	109.899114	0.337595365	0.1953241	1.7283853	0.083919	0.557501
zgc:103678	330.393226	-0.088353578	0.1312443	-0.6732	0.50082	0.93428
pcdh2ab2	0.63357355	1.435201493	2.6108194	0.5497131	0.582516	NA
pcdh2aa1	0.365141898	1.973577737	3.3927314	0.5817076	0.560764	NA
gss	96.34967753	-0.202403682	0.2137555	-0.946893	0.343693	0.878434
exosc10	585.0850639	-0.064814488	0.1031182	-0.628546	0.529647	0.942209
pcnp	824.6865645	-0.031252985	0.096379	-0.324272	0.745732	0.97419
fbxl31	44.86286745	-0.901862113	0.3000019	-3.006188	0.002645	0.065718
zgc:112980	241.4926328	-0.045660727	0.1416067	-0.322448	0.747114	0.97419
actb1	60669.78951	-0.011865034	0.0954311	-0.124331	0.901053	0.990121
fscn1b	431.1208333	-0.036758445	0.1362932	-0.269701	0.78739	0.976596
slc43a1b	270.1877029	0.329649562	0.151998	2.1687754	0.0301	0.33532
tlr2	0.967129164	-0.023635743	1.9568585	-0.012078	0.990363	NA
uncx4.1	336.0884924	-0.161730248	0.1274972	-1.2685	0.204619	0.77258
rybpa	777.0411286	0.063675455	0.0962459	0.6615917	0.508233	0.935735
stk16	241.2315029	0.078001727	0.135878	0.5740569	0.565929	0.948739
acss2	1245.966517	-0.111497922	0.0858486	-1.298774	0.194021	0.75784
sox8b	13.12027219	0.723129645	0.5256171	1.3757726	0.168892	0.725919
proza	1904.970217	-0.041464632	0.0822203	-0.504311	0.614043	0.95688
pvalb1	57152.72873	0.192679488	0.2302824	0.8367096	0.402756	0.90084
pvalb8	925.6022322	0.137579603	0.1048688	1.3119213	0.189547	0.754269
nptx2a	29.97111046	-0.189413785	0.3702066	-0.511643	0.608901	0.956718
phlda3	195.2144674	0.235666693	0.1651341	1.4271233	0.153544	0.702578
lgals3bpa	23.16866246	-0.479879855	0.4056482	-1.182995	0.236811	0.805854
adtrp1	141.9254769	0.195846676	0.1706106	1.1479163	0.251003	0.820469
zgc:113278	10.44280438	0.531101631	0.6103516	0.8701568	0.384215	NA
b4galt5	7.147545795	1.217365225	0.7426242	1.639275	0.101156	NA
mitfb	3.631200915	0.79205473	1.056526	0.7496784	0.453448	NA
polr3k	132.7328904	0.02409269	0.1797107	0.1340637	0.893352	0.989291

igfals	10.86040238	1.71788314	0.6804402	2.5246642	0.011581	NA
ogfr	570.0935491	-0.083617738	0.1031071	-0.81098	0.417377	0.906237
unkl	759.6975892	-0.146356813	0.0941166	-1.555059	0.119932	0.645215
col9a3	14735.84866	0.084118615	0.0837017	1.0049807	0.314906	0.866054
hml3	1372.53998	0.042747491	0.0844296	0.5063091	0.61264	0.95688
zgc:101663	375.6352587	-0.287815219	0.116521	-2.470072	0.013509	0.208523
taf11	550.9295229	-0.043997674	0.1025624	-0.428985	0.667934	0.964736
il11a	35.21711941	0.173646125	0.3740448	0.4642388	0.642477	0.959994
cox6b2	5384.484382	0.056727911	0.0828215	0.6849421	0.49338	0.931926
slc2a3b	151.8191471	-0.085642722	0.1793251	-0.477584	0.632947	0.957356
cln3	237.9988438	-0.080441504	0.140778	-0.571407	0.567724	0.948739
atp5mf	2775.176229	-0.014568289	0.1002644	-0.145299	0.884475	0.98879
actb2	49018.02051	-0.100674544	0.0900903	-1.117485	0.263787	0.830245
wipi2	1091.404553	-0.034003612	0.0961903	-0.353503	0.723711	0.970402
foxk1	442.654741	-0.22091631	0.109079	-2.025286	0.042838	0.406662
cyp3c3	43.22675154	-0.648943782	0.3535783	-1.835361	0.066452	0.504618
cyp3c3	14.8410172	-0.240675915	0.5552824	-0.43343	0.664703	0.964736
lfng	972.0871182	0.115138467	0.0890974	1.2922765	0.196261	0.761702
prcp	70.94140793	-0.147000733	0.2424016	-0.606435	0.544226	0.945438
rab30	101.1183286	-0.06114073	0.2046945	-0.298693	0.765175	0.975374
wnt9b	266.6717015	0.001329947	0.1547535	0.008594	0.993143	0.998554
mlx	259.494457	-0.093668808	0.1357476	-0.690022	0.49018	0.931926
psmc3ip	67.53621764	0.019861778	0.2404802	0.0825922	0.934176	0.992702
ezh1	188.1288311	-0.222596792	0.1617576	-1.376113	0.168787	0.725919
ramp2	225.9993495	0.10100107	0.1555733	0.6492185	0.516197	0.937997
cacna1aa	338.6639847	0.087353436	0.1201331	0.7271386	0.467141	0.924328
alkbh7	136.8611411	-0.105814022	0.1822823	-0.580495	0.561581	0.948728
timm29	224.8315086	-0.00262695	0.1389585	-0.018905	0.984917	0.996944
nmrk1	24.58119703	0.003109741	0.3861481	0.0080532	0.993575	0.998672
znfl1	1.314079744	0.007937271	1.657586	0.0047885	0.996179	NA
cdk5r1a	108.8352334	0.087167659	0.2340744	0.3723929	0.7096	0.968902
itga3a	153.3925097	-0.056664934	0.172224	-0.329019	0.742141	0.973154
rbbp6	990.4227211	-0.139199265	0.0963674	-1.444465	0.148608	0.69636
gng13b	1582.506305	0.119934968	0.0895027	1.340015	0.18024	0.74128
gna13b	276.9450345	0.012038871	0.1395158	0.0862904	0.931236	0.992702
rgs9a	256.4031539	0.242302707	0.1457269	1.6627176	0.096369	0.59086
ddx42	1164.164164	0.036789597	0.081075	0.4537724	0.649993	0.960708
strada	394.0895524	0.1830131	0.1106622	1.653799	0.098168	0.59749
rnf113a	229.8253162	0.012216975	0.1494377	0.0817529	0.934843	0.992702
lin7b	1244.534758	0.045166412	0.0836529	0.5399265	0.589248	0.953345
aldh16a1	344.390992	0.014258881	0.1262571	0.1129352	0.910082	0.990702
syt5a	745.1166722	0.367353396	0.0985267	3.7284637	0.000193	0.008264
cpt1cb	54.94621219	0.18381164	0.2687443	0.6839648	0.493997	0.931926
prl	59.44823356	0.258306711	0.2589201	0.9976309	0.318458	0.867746
tnnt1	194.0538364	0.01804351	0.1639817	0.1100337	0.912383	0.991119
pih1d1	110.4672147	0.195666683	0.1962518	0.9970186	0.318755	0.867746
nosip	481.5277658	-0.07368967	0.1156939	-0.636937	0.524166	0.939336
lrrc17	705.5813537	0.373839991	0.0975827	3.8310057	0.000128	0.005966
rcn3	916.2153311	0.226827063	0.0863857	2.6257492	0.008646	0.152919
psmb7	2344.233987	-0.043457577	0.0893662	-0.486286	0.626764	0.957354
nek6	125.8638795	-0.341457339	0.1820058	-1.87608	0.060644	0.481212
lhx2a	6.967183645	0.70548449	0.7190412	0.9811461	0.326521	NA
srrm2	1352.971808	-0.103322645	0.0907697	-1.138294	0.254998	0.82364
thoc6	169.3897523	-0.098255398	0.1596925	-0.615279	0.538371	0.943336

si:dkey-16l2.16	275.4115483	0.049251754	0.1318139	0.373646	0.708668	0.968664
fus	3873.1748	-0.105235181	0.0715125	-1.471564	0.141139	0.681968
elob	4776.961431	-0.031501171	0.078833	-0.399594	0.689456	0.966763
gdf3	113.6879832	-0.118774555	0.1920638	-0.618412	0.536304	0.943336
tubb5	8176.117827	0.100136742	0.0863691	1.1594042	0.246291	0.816376
flot1b	4345.22664	-0.081596089	0.0691145	-1.180593	0.237764	0.806474
crls1	270.0010409	0.052596862	0.139197	0.3778591	0.705535	0.968412
med25	512.8219881	0.018491727	0.1067005	0.173305	0.862412	0.985841
osr2	90.56611174	-0.51750114	0.2064999	-2.50606	0.012209	0.195183
CT573429.1	3.588601285	-1.435280134	1.0697566	-1.341689	0.179697	NA
rac2	429.8850575	0.140317931	0.1086356	1.2916385	0.196482	0.761835
cyth4a	38.10263101	0.14097928	0.3176596	0.4438061	0.657183	0.962175
fam83fa	46.3863489	-0.143569478	0.3095781	-0.463759	0.642821	0.959994
prph2a	2873.440793	0.354945628	0.078821	4.5031838	6.69E-06	0.000513
flr	221.0001524	0.150992656	0.1400106	1.078437	0.280839	0.84357
cbx7a	398.8343601	-0.129031464	0.1405877	-0.9178	0.358723	0.884602
nol12	290.828918	0.124249047	0.1311043	0.9477111	0.343277	0.878434
ndufa6	1140.767436	0.005235978	0.104824	0.0499502	0.960162	0.995388
rbx1	832.1569383	-0.254162216	0.099421	-2.556425	0.010575	0.17767
tpk1	133.109915	-0.125964161	0.1734883	-0.726067	0.467798	0.924328
si:ch211-149k23.9	6.674286691	0.005197358	0.8043335	0.0064617	0.994844	NA
fgfbp2b	1820.579171	-0.004792086	0.109316	-0.043837	0.965034	0.996154
grb2b	1329.722608	0.030098287	0.0859545	0.3501654	0.726215	0.970402
chmp3	275.0023394	0.023650017	0.1278328	0.1850074	0.853223	0.985174
kpna2	1537.246359	-0.265076085	0.0790393	-3.353725	0.000797	0.025763
smurf2	552.3088188	-0.067176671	0.1073032	-0.626045	0.531285	0.942209
ddx5	12482.75868	0.124170318	0.069429	1.7884494	0.073704	0.529749
mgat3a	9.145768386	-0.436446394	0.6682754	-0.653094	0.513696	NA
ptx4	2.758622689	-1.277645537	1.1971822	-1.067211	0.285877	NA
ergic3	391.0307876	0.070446305	0.1177383	0.5983293	0.54962	0.946312
cycl	4719.877047	-0.062902209	0.0742484	-0.847186	0.396891	0.899672
romo1	1351.491661	-0.112299889	0.0793705	-1.414882	0.157103	0.707805
TMEM114	17.58258528	0.057836565	0.4901546	0.1179966	0.90607	0.990702
clec16a	625.1923096	-0.014466594	0.100771	-0.143559	0.885849	0.988936
socs1a	50.22372162	-0.313124379	0.306996	-1.019962	0.307746	0.861088
pigq	409.003276	-0.038532391	0.1100401	-0.350167	0.726213	0.970402
gskip	689.15626	0.068410981	0.0947427	0.7220715	0.470251	0.924787
slc37a4a	382.7690619	-0.217846251	0.1260882	-1.72773	0.084037	0.557646
sgcg	307.9703744	0.108053453	0.124679	0.8666535	0.386132	0.89551
si:dkeyp-59c12.1	6.668544319	0.171714089	0.7338569	0.2339885	0.814994	NA
rprd1b	367.0789996	-0.082387602	0.122028	-0.675153	0.499578	0.934006
xpa	69.10634249	-0.198875079	0.2491756	-0.798132	0.424794	0.908585
gnrhr4	1.678080482	-0.611659387	1.5253744	-0.40099	0.688428	NA
si:ch211-13k12.2	21.1484171	0.040475152	0.4290541	0.0943358	0.924842	0.992676
myl9a	516.9077958	-0.310457448	0.1106315	-2.80623	0.005012	0.103462
zgc:113411	71.61347264	0.119140251	0.2354283	0.5060575	0.612816	0.95688
pdgfbb	118.8484284	0.130203137	0.1951795	0.6670943	0.504712	0.935617
atf4b	5918.43087	0.078899065	0.0642433	1.228129	0.219399	0.78594
hbbe3	6.662672083	-1.584440211	0.8533851	-1.856653	0.063361	NA
zgc:92360	486.1400459	-0.076268744	0.1189585	-0.641137	0.521434	0.939056
lgals2b	3125.298759	-0.826568629	0.0713842	-11.57915	5.26E-31	1.72E-27
isca2	354.4618648	0.124816949	0.1215564	1.0268236	0.304504	0.858905
dusp11	416.6115696	0.157097124	0.1192638	1.3172241	0.187764	0.752195
tcf7l1a	665.2873247	-0.254400248	0.119771	-2.124056	0.033665	0.357045

hyal6	62.76589237	0.06924579	0.2571856	0.2692444	0.787742	0.976596
cyb561	28.69023328	-0.069204004	0.3635403	-0.190361	0.849026	0.985174
gh1	181.1357752	0.43910655	0.1627577	2.6979158	0.006978	0.130041
zgc:103564	35.73366935	-0.298183699	0.326207	-0.914094	0.360668	0.885686
smtla	5.428185067	-0.458439517	0.819975	-0.55909	0.576101	NA
cdab	72.38185471	0.13406935	0.2314173	0.5793401	0.56236	0.948728
her2	112.1762793	-0.009769514	0.19363	-0.050455	0.95976	0.995348
aldh4a1	497.8036024	0.255696409	0.1097442	2.3299318	0.01981	0.265212
slc35b1	672.8853687	0.057818184	0.0989514	0.5843087	0.559013	0.948478
tyw3	50.63178664	-0.50780888	0.2716147	-1.869593	0.06154	0.483664
nras	558.2152313	0.023829976	0.1012449	0.2353697	0.813922	0.981081
CABZ01074994.1	1.313718686	0.007433509	1.6576955	0.0044842	0.996422	NA
selenow2a	2377.465801	0.00166549	0.0853516	0.0195133	0.984432	0.996944
pkdccb	121.9024064	-0.05649289	0.1823479	-0.309808	0.756707	0.975374
tnpo2	1729.785876	-0.049613151	0.079833	-0.621461	0.534296	0.943336
zgc:113333	182.7748291	0.141561871	0.1538032	0.9204091	0.357359	0.883904
e4f1	225.780805	0.014132892	0.1399048	0.1010179	0.919536	0.991651
ggact.2	365.9252858	0.00692501	0.1231116	0.0562499	0.955143	0.994543
proca	80.16467282	0.059794769	0.2472306	0.2418583	0.80889	0.980642
PIGG	178.0487654	0.145548743	0.1837013	0.7923122	0.428179	0.911082
zgc:113293	1.93598671	0.44942462	1.4946638	0.3006861	0.763654	NA
ptgir	17.13293338	-0.012221413	0.4588805	-0.026633	0.978752	0.996762
natd1	978.93694	-0.096639563	0.0933914	-1.03478	0.300772	0.856365
wnt6a	21.75236971	-0.504642072	0.4386564	-1.150427	0.249968	0.819849
cd151	147.6653717	-0.007763347	0.1949	-0.039832	0.968227	0.996315
thoc5	598.6000719	-0.225453519	0.1136077	-1.984491	0.047201	0.426579
zgc:103559	24.78738139	1.312803408	0.396963	3.3071179	0.000943	0.029586
tmem86b	357.0583007	-0.033029453	0.1438841	-0.229556	0.818437	0.981594
aspdh	50.50109365	-0.189225467	0.2770409	-0.683024	0.494592	0.931926
rnf34a	197.3419264	0.354536278	0.1474947	2.4037222	0.016229	0.235102
ccar2	405.3297231	0.049985774	0.1164237	0.4293434	0.667673	0.964736
vgll3	17.49011196	-0.462021484	0.4624362	-0.999103	0.317745	0.867607
tsc22d1	3080.845264	-0.016053607	0.0702091	-0.228654	0.819138	0.981594
ykt6	318.6831546	0.166629904	0.1297556	1.2841824	0.199078	0.764531
dnajc15	358.6167823	-0.090448888	0.1262972	-0.716159	0.473893	0.924993
rcl1	304.3392347	-0.185329913	0.1314128	-1.410288	0.158455	0.709224
zgc:172053	3.405432823	0.678101267	1.0612736	0.6389505	0.522855	NA
pigh	225.4410798	-0.008499285	0.1554124	-0.054689	0.956387	0.994543
slc27a1b	0	NA	NA	NA	NA	NA
enosf1	410.5790679	0.387374955	0.1182459	3.2760118	0.001053	0.032299
thgl1	91.62621031	0.096446726	0.2135104	0.4517192	0.651471	0.960708
drd4a	112.1603154	0.281682641	0.1914879	1.4710203	0.141286	0.681968
sagb	3195.989831	0.130034315	0.0891208	1.4590795	0.144543	0.688888
th2	18.59416164	0.518218976	0.4468445	1.1597299	0.246159	0.816291
ascl1a	487.8964712	-0.236220636	0.1174075	-2.011973	0.044223	0.413497
si:dkeyp-52c3.7	0.356300316	0.005884122	3.214964	0.0018302	0.99854	NA
rpap2	121.8460357	0.002153652	0.1897378	0.0113507	0.990944	0.997679
pmm1	2.163469886	0.697330298	1.2645909	0.5514276	0.581341	NA
zgc:153921	6.939014926	1.298379274	0.7797102	1.6652076	0.095871	NA
b3galt6	314.4669435	0.066341053	0.1339913	0.4951145	0.620519	0.957354
entpd4	391.9004239	0.179575947	0.1244286	1.4432043	0.148963	0.696454
c4b	285.8653442	0.086050641	0.1706438	0.5042707	0.614071	0.95688
sulf1	1005.100736	0.132228437	0.0849649	1.5562712	0.119644	0.644641
csrnp1b	472.6481796	0.053827594	0.107217	0.5020432	0.615637	0.95688

actr10	1541.049152	0.007783067	0.0849233	0.0916482	0.926978	0.992702
fabp10a	801.4921675	0.657831681	0.11284	5.8297721	5.55E-09	9.98E-07
plcg1	1805.823142	-0.028059908	0.0905417	-0.309911	0.756628	0.975374
spire1b	12.82598133	-0.171098648	0.546764	-0.31293	0.754334	0.975114
nrp2b	1769.822747	-0.021686472	0.0756225	-0.286773	0.774286	0.975687
mrps35	481.5113931	-0.093395852	0.1216571	-0.767697	0.442667	0.916336
stmn3	630.4166405	0.355675413	0.0979435	3.6314355	0.000282	0.011325
igsf8	205.8772418	0.300338583	0.1559771	1.9255298	0.054163	0.455813
ube2d3	1307.582824	0.107627166	0.0938903	1.1463078	0.251668	0.821198
acy1	557.5307774	-0.236484182	0.1114387	-2.122101	0.033829	0.357857
si:dkeyp-68b7.7	112.4967856	0.101210145	0.1882177	0.5377291	0.590764	0.954193
b3gnt7	239.3008419	0.041599834	0.1521176	0.2734715	0.784491	0.97627
cep41	80.63251845	0.053020839	0.2246383	0.2360276	0.813411	0.981081
polr2h	229.0555972	0.109242443	0.1461689	0.7473715	0.454839	0.921022
chrnb3b	37.34651791	0.635141883	0.3211055	1.9779851	0.04793	0.43124
snap29	596.8231383	0.038204103	0.1046153	0.3651865	0.714972	0.96909
pik3r1	2488.640031	0.124181608	0.0727173	1.7077304	0.087686	0.566637
gga1	857.754267	0.125220859	0.0943573	1.3270931	0.184478	0.747376
ccr12a	7.688520048	0.939604773	0.717563	1.3094386	0.190386	NA
wdr48b	241.8271645	0.069763259	0.1376261	0.5069041	0.612222	0.95688
BX890608.1	0.816385205	1.891093756	2.136903	0.8849694	0.376173	NA
zgc:112185	64.98503074	0.0972131	0.2440115	0.3983956	0.690339	0.966973
anks3	135.3674743	0.029445945	0.2010313	0.1464744	0.883547	0.988706
h1f0	21675.90848	-0.264058476	0.0714997	-3.69314	0.000222	0.009319
klf6b	6.958032334	0.134945368	0.7216237	0.1870024	0.851659	NA
pitrm1	762.5304971	0.034467434	0.0976251	0.3530591	0.724044	0.970402
gnptg	203.4223768	-0.130457217	0.1569773	-0.831058	0.405941	0.901305
tsr3	199.2191399	-0.223944154	0.1488906	-1.504085	0.132559	0.666979
fzd8b	51.64455991	-0.331106338	0.2741626	-1.207701	0.227162	0.794617
BX548047.1	0	NA	NA	NA	NA	NA
scg2b	2530.118474	0.206334222	0.0733584	2.8126852	0.004913	0.102047
ube2d1b	1029.814702	0.187502307	0.0846152	2.2159422	0.026695	0.315011
cox6c	2856.35095	-0.001037125	0.1162058	-0.008925	0.992879	0.998437
abraa	199.3891278	0.338966736	0.1654258	2.0490558	0.040457	0.393309
eif4e2	214.4407881	0.097575915	0.1475439	0.6613348	0.508398	0.93575
CU929150.1	2.45544643	-0.624210837	1.2425962	-0.502344	0.615426	NA
rspo4	5.495958816	1.906568984	0.8710639	2.188782	0.028613	NA
sdhc	1320.805389	0.037873451	0.1005716	0.3765821	0.706484	0.968459
mpz	168.1813251	0.263099067	0.1630467	1.6136422	0.106605	0.615567
pih1d3	20.37383823	0.163528149	0.4271192	0.382863	0.701821	0.968134
pip4p1a	406.4267042	-0.164050569	0.1203679	-1.362909	0.172911	0.731956
cpt2	585.8877767	-0.035177437	0.1089593	-0.322849	0.746809	0.97419
magoh	918.5663519	-0.033070225	0.0921636	-0.358821	0.719729	0.969318
elovl6l	231.5314291	-0.097350691	0.1784222	-0.54562	0.585327	0.952585
alas2	350.3572118	0.207521942	0.1513816	1.3708527	0.170421	0.728041
ftr11	1.323817282	-0.795924658	1.8294525	-0.435062	0.663518	NA
ajap1	65.93006257	0.107479084	0.2530876	0.4246715	0.671076	0.965257
cavin4a	741.8192604	0.194976949	0.0940866	2.0723129	0.038236	0.383215
igfbp1b	124.8423103	-0.410543046	0.2052203	-2.000499	0.045446	0.418148
fggy	112.630787	-0.104595388	0.2038767	-0.513032	0.607929	0.956718
gbp1	41.6763806	-0.137428669	0.3181349	-0.431982	0.665754	0.964736
gbp2	25.88640544	-0.281614388	0.3865889	-0.72846	0.466332	0.923908
tcf7	62.39347756	-0.019949157	0.2564267	-0.077797	0.93799	0.99279
MFAP4 (1 of many)	1.144594032	-2.465288488	1.9220008	-1.282668	0.199608	NA

zgc:171687	2.768282155	-0.177533434	1.1445404	-0.155113	0.876732	NA
si:zfos-2330d3.1	26.96658885	1.223043555	0.3963537	3.085738	0.00203	0.054039
si:ch211-134m17.9	126.1238441	-0.150253652	0.1997107	-0.752357	0.451837	0.918761
nfkb2	755.4617042	-0.411667837	0.0996425	-4.13145	3.60E-05	0.00206
gng7	499.7835446	0.223988627	0.1148714	1.9499069	0.051187	0.443542
zgc:101744	633.9910187	-0.132844123	0.1106526	-1.200551	0.229925	0.79764
elavl1a	3909.429606	0.068315071	0.0689145	0.9913019	0.321538	0.869201
gpr160	32.92091285	-0.439975406	0.3380262	-1.301601	0.193053	0.756795
hkdc1	282.9116405	-0.181913078	0.1315806	-1.382522	0.166811	0.723339
si:dkeyp-66d1.7	55.04278051	-0.373935123	0.2608796	-1.433363	0.151754	0.700137
tep1	70.99421344	-0.517849529	0.2421774	-2.138306	0.032492	0.35109
casq1a	2578.595829	0.42485311	0.0932145	4.5578006	5.17E-06	0.000411
zp3f.1	1.142078759	1.327282915	1.913709	0.6935657	0.487955	NA
ch25hl2	9.626468093	0.092499056	0.6085993	0.1519868	0.879197	NA
s100z	78.31942824	-0.245846385	0.2303806	-1.067131	0.285912	0.845942
slc25a36a	1204.930436	-0.089299898	0.0924344	-0.966089	0.333999	0.875256
phf20b	996.1345514	0.046729681	0.0910778	0.5130742	0.607899	0.956718
rbp1.2	237.8567969	-0.085772992	0.165876	-0.517091	0.605093	0.95651
copb2	3533.540085	0.019645897	0.0756018	0.2598602	0.794972	0.978328
plk3	811.8719152	0.194136575	0.1176918	1.6495334	0.099038	0.598346
mrpl12	970.9627321	0.030754665	0.0987048	0.3115823	0.755358	0.975318
zgc:103625	101.7892208	-0.02312074	0.1942358	-0.119034	0.905248	0.990702
ubtfl	1237.173849	0.109628357	0.0833932	1.3145957	0.188646	0.753288
faim2a	95.59730832	0.372765327	0.2136917	1.744407	0.081088	0.551495
abcf2a	1343.023517	0.508327327	0.0803476	6.3265994	2.51E-10	5.63E-08
smarcd3a	618.087504	0.046794345	0.0987613	0.4738125	0.635634	0.958142
tmub1	510.0659491	-0.045424916	0.1156429	-0.392803	0.694465	0.96736
dnai1.2	45.50623537	0.054225737	0.2860712	0.1895533	0.849659	0.985174
tmem53	177.7074723	-0.124327227	0.1532882	-0.811069	0.417326	0.906237
klf17	67.94563889	-0.181153813	0.2394892	-0.756418	0.449399	0.918383
ccdc24	17.83788934	0.103746938	0.4854238	0.2137244	0.830762	0.982994
zgc:113531	166.6094518	0.129512433	0.1614355	0.8022551	0.422405	0.906561
klhl20	348.0355969	0.032006213	0.1201407	0.2664061	0.789926	0.977338
cenpl	86.46136079	0.05968183	0.2145629	0.2781554	0.780893	0.97627
pigp	171.5867862	-0.307643908	0.2151052	-1.430202	0.152659	0.701776
e2f5	38.91722545	-0.105014265	0.3046236	-0.344735	0.730294	0.971335
myrip	707.5533976	-0.044714972	0.0964936	-0.463398	0.643079	0.959994
mrc1b	91.98538657	0.298639714	0.2124217	1.4058814	0.159759	0.711986
jph1b	1258.568138	0.312755462	0.1098872	2.8461491	0.004425	0.094496
etfdh	1206.2447	-0.101738052	0.0924886	-1.100006	0.271329	0.836239
ppid	1102.617861	-0.125336269	0.0888158	-1.411193	0.158188	0.709224
ldhd	238.3538588	-0.002837545	0.1422216	-0.019952	0.984082	0.996944
gins3	84.41708055	-0.132647367	0.2148248	-0.617468	0.536926	0.943336
chmp5a	149.1840866	0.049373004	0.1763705	0.2799391	0.779524	0.976146
setd7	451.9121984	-0.110511486	0.1072022	-1.030869	0.302602	0.85729
rgs20	542.215007	0.160269436	0.1188313	1.348714	0.177429	0.738316
kcnb2	411.0670791	0.470878845	0.1185331	3.9725504	7.11E-05	0.003671
babam2	457.3632228	-0.096139448	0.110322	-0.871444	0.383512	0.895192
acox3	258.1169125	-0.085035202	0.1321142	-0.643649	0.519803	0.938945
trappc8	688.9320616	-0.036494024	0.1048186	-0.348164	0.727717	0.970402
shhb	170.4901208	0.040572614	0.170527	0.2379249	0.811939	0.981081
en2b	123.1136009	-0.084221428	0.1867588	-0.450964	0.652016	0.960708
htr5aa	4.083257855	-0.622733897	0.996426	-0.624968	0.531992	NA
slu7	907.965675	-0.002431509	0.0888455	-0.027368	0.978166	0.996762

zgc:101569	229.749414	-0.051460249	0.1374783	-0.374316	0.70817	0.968664
il12a	3.97817145	2.805255335	1.1908943	2.3555871	0.018493	NA
ift80	354.7680336	-0.00985225	0.1236362	-0.079687	0.936486	0.992702
acaa2	1406.144974	-0.095811501	0.0811132	-1.181207	0.23752	0.806474
smc4	1043.023767	-0.183025481	0.097274	-1.881546	0.059898	0.478654
AL954146.1	38.1315079	0.203818781	0.3171406	0.6426765	0.520434	0.939056
tmx3a	550.9427372	0.084163698	0.1251905	0.672285	0.501402	0.934613
zgc:113691	4.17226768	-0.095969777	0.9076483	-0.105735	0.915793	NA
acadm	2829.951096	0.007927946	0.0841166	0.0942494	0.924911	0.992676
tmem256	277.0942501	-0.189483479	0.1335261	-1.419074	0.155877	0.705963
hectd3	959.3435746	-0.073206466	0.086915	-0.842277	0.399633	0.90084
msl2a	444.6122786	0.049026085	0.1077132	0.455154	0.648998	0.960708
pccb	1318.018759	0.027163967	0.0885466	0.306776	0.759014	0.975374
rad50	176.4713113	-0.102055495	0.1553523	-0.656929	0.511226	0.936253
drd1b	25.79525641	0.560870685	0.3887688	1.4426846	0.149109	0.696454
itprid2	368.4725884	-0.107382598	0.1288677	-0.833278	0.404688	0.901305
ppp1r1c	175.4266813	-0.044806922	0.171924	-0.26062	0.794385	0.977937
ctnnbip1	1611.245016	-0.019195232	0.0798281	-0.240457	0.809976	0.980764
pimr68	3.992991858	0.219330585	0.9460919	0.231828	0.816672	NA
fdx1b	7.445147861	0.591907423	0.6929886	0.8541374	0.393029	NA
flvcr2a	26.81462618	-0.092785725	0.381956	-0.242923	0.808065	0.980642
traf4b	90.93204274	-0.423134979	0.211497	-2.000666	0.045428	0.418148
cul3a	1489.354853	0.070286836	0.0773013	0.9092584	0.363214	0.885944
ccr6b	3.521787566	-0.449322884	1.0713528	-0.419398	0.674926	NA
zgc:113142	40.00623454	-0.26880704	0.3623506	-0.741842	0.458183	0.921978
DPYSL2 (1 of many)	124.2259581	0.293138459	0.2014223	1.4553426	0.145574	0.691067
pdcd7	299.0835589	0.01739536	0.1326149	0.131172	0.895639	0.989814
slc25a24l	5.639837318	-0.484894395	0.8154215	-0.594655	0.552074	NA
dnajb4	330.2224357	-0.060639558	0.1240036	-0.489014	0.624831	0.957354
si:dkey-24c2.9	6.531145168	1.038287266	0.7692416	1.3497544	0.177095	NA
txndc12	1016.66055	0.050875484	0.0875848	0.5808711	0.561327	0.948728
zgc:153615	670.5860886	0.067648808	0.0977581	0.6920024	0.488936	0.931926
cdyl	123.6891719	-0.14288045	0.1953558	-0.731386	0.464543	0.923483
si:dkey-31m5.5	0.157187365	-0.955901296	4.0804729	-0.234262	0.814781	NA
wnt3	88.90504761	0.164181753	0.2352449	0.6979184	0.485228	0.930188
nsfb	498.0972126	-0.141909886	0.106598	-1.331263	0.183103	0.746684
egln1a	57.09328391	-0.21669187	0.2602457	-0.832644	0.405046	0.901305
trappc1	471.1243126	0.096856836	0.1171189	0.8269957	0.40824	0.90171
eno3	7314.796874	-0.323810617	0.1065957	-3.037746	0.002384	0.06083
zgc:85858	437.5384664	0.064338728	0.1326708	0.4849503	0.627712	0.957354
fbxo5	132.1609055	-0.147689572	0.1763119	-0.837661	0.402221	0.90084
stk25b	1137.756967	0.044505455	0.0818257	0.5439055	0.586507	0.953319
marcksl1a	4535.114224	-0.00196965	0.0718884	-0.027399	0.978142	0.996762
sfrp5	244.3150973	0.032405106	0.1368021	0.2368757	0.812753	0.981081
hhatla	3146.359218	0.358820589	0.0832889	4.3081455	1.65E-05	0.001071
klhl40a	76.88881877	0.670930835	0.2356809	2.8467761	0.004416	0.094496
morn4	345.5354434	0.132635865	0.1183225	1.120969	0.262301	0.830166
CR847936.1	68.25172752	-0.193170923	0.248477	-0.77742	0.436911	0.914408
st3gal7	239.0391092	-0.306665069	0.1617625	-1.895773	0.05799	0.47228
trpc2a	0.682853041	2.891318346	2.3286279	1.2416403	0.214369	NA
klhl31	1175.888211	0.173375757	0.1090836	1.5893846	0.111974	0.627242
srd5a2b	11.36106503	0.450931352	0.601529	0.749642	0.45347	0.920041
slx4ip	64.51772717	-0.008696602	0.249568	-0.034847	0.972202	0.996315
im:7138239	8.413927182	1.108697015	0.6872028	1.6133477	0.106669	NA

tyr	151.8371014	0.138694827	0.1863893	0.7441137	0.456808	0.921215
zgc:123010	412.6234051	0.067524583	0.1268233	0.5324304	0.594428	0.954434
got1	5001.711255	0.253002516	0.0690846	3.6622129	0.00025	0.010194
nkx2.3	97.43447949	-0.30487723	0.2262222	-1.34769	0.177758	0.738343
si:dkey-24p1.1	0.476626891	2.395187592	2.815932	0.8505843	0.395	NA
ca8	291.3465749	-0.137381385	0.1356135	-1.013036	0.311043	0.863236
aep1	2178.926438	0.102223231	0.0823014	1.2420598	0.214214	0.782493
jac7	2.188742165	-2.456045621	1.6217617	-1.514431	0.129917	NA
trim35-9	12.41354373	-0.148238815	0.5669706	-0.261458	0.79374	0.977937
nr5a5	17.60883465	0.053418286	0.4573794	0.1167921	0.907025	0.990702
tefa	3667.277673	0.173823934	0.0705804	2.4627776	0.013787	0.211002
trim9	640.619839	-0.028045962	0.102807	-0.272802	0.785005	0.97627
map4k5	660.3451403	0.079326772	0.0946081	0.8384773	0.401763	0.90084
cdkn3	82.56459442	0.102581001	0.2230946	0.4598094	0.645653	0.96029
atp1a1a.3	1159.966641	0.241685722	0.1079776	2.238295	0.025202	0.30686
lamb4	542.010756	0.316013167	0.1051858	3.0043329	0.002662	0.066037
cox16	134.7757806	-0.044360102	0.17537	-0.252952	0.800306	0.979377
arpc5a	1032.075547	0.004728905	0.0840255	0.0562794	0.955119	0.994543
plaub	44.45475103	0.227769718	0.2946592	0.7729936	0.439526	0.916082
lgmn	3133.992901	0.175346819	0.0755374	2.3213247	0.020269	0.267561
fbxo36b	3.941248424	0.19767726	1.0260733	0.1926541	0.84723	NA
rhebl1	918.1836159	-0.092196236	0.0854249	-1.079266	0.280469	0.843423
mhc1uma	0	NA	NA	NA	NA	NA
ctslb	2.263167942	0.797165978	1.5012195	0.5310123	0.59541	NA
olfm3b	21.29374737	0.588294256	0.4482637	1.3123843	0.189391	0.75413
trpm3	64.38216711	-0.164919317	0.2569092	-0.641936	0.520915	0.939056
nfyba	1091.363648	-0.088677156	0.0880057	-1.00763	0.313632	0.865132
greb1l	155.7701883	-0.058527253	0.1634981	-0.357969	0.720366	0.969318
zgc:110130	417.7840827	0.103140993	0.1182198	0.8724507	0.382963	0.895192
nasp	1311.335839	-0.062061879	0.0901214	-0.688648	0.491045	0.931926
zgc:77439	467.126985	0.136021054	0.1142281	1.1907843	0.233738	0.80256
prpf38a	259.3761634	-0.159246765	0.131236	-1.213438	0.224962	0.792185
arrb2a	510.3562307	-0.061970988	0.1073853	-0.57709	0.563879	0.948739
orc1	202.9013081	0.129066457	0.1621522	0.7959589	0.426056	0.909755
gpr101	5.513902177	-0.322885782	0.8626367	-0.374301	0.70818	NA
st6galnac5a	5.790887504	-0.268900339	0.8486815	-0.316845	0.751361	NA
mpl	1.331480916	1.593955787	1.7098336	0.9322286	0.351218	NA
vps13a	1374.603519	0.003327655	0.0905012	0.0367692	0.970669	0.996315
cep78	129.652536	-0.077876036	0.1887667	-0.412552	0.679935	0.965311
zgc:171579	2.962721368	-0.003197833	1.0730994	-0.00298	0.997622	NA
cacng5b	6.288348096	0.172105691	0.7522594	0.228785	0.819036	NA
zgc:152791	2.26281817	-0.399153687	1.3056015	-0.305724	0.759815	NA
klhl21	796.8242721	-0.164448323	0.096728	-1.700111	0.08911	0.571727
spega	215.3352901	0.18184211	0.1475532	1.2323833	0.217806	0.785256
zbtb48	97.82522089	-0.228612179	0.2099821	-1.088722	0.276276	0.839541
igfbp5a	95.80472208	0.213980657	0.2152961	0.9938902	0.320276	0.868202
arhgap4a	32.1015779	-0.114500662	0.3420508	-0.334747	0.737816	0.972535
rbm25a	538.6029875	-0.089718731	0.1314577	-0.682491	0.494928	0.931926
emc4	254.490444	0.182314068	0.1640686	1.1112063	0.26648	0.832634
arg2	776.0138165	0.248629146	0.1244004	1.9986206	0.045649	0.41876
vti1b	159.3420282	-0.148585612	0.1631012	-0.911002	0.362294	0.885944
pls1	291.6485619	-0.834674021	0.1518274	-5.497518	3.85E-08	5.77E-06
fbln5	251.5147589	0.126173239	0.1430748	0.8818692	0.377848	0.895192
golga5	813.5614301	-0.078843932	0.0905375	-0.870842	0.38384	0.895192

rdh14a	204.0530804	-0.046210547	0.1450433	-0.318598	0.750031	0.97419
terfa	361.6064406	-0.119251082	0.1345062	-0.886584	0.375303	0.894316
six1a	150.4695031	0.197925257	0.1872315	1.0571154	0.290459	0.846262
hook2	32.64334771	-0.037264435	0.3310004	-0.112581	0.910363	0.990702
parla	166.5113769	0.283154018	0.159986	1.7698676	0.076749	0.539626
vps37b	349.7814791	-0.016229251	0.1174164	-0.13822	0.890067	0.989291
pmepa1	25.24776777	-0.272155556	0.3836311	-0.70942	0.478064	0.926857
ccndbp1	332.7886997	0.104656266	0.1344854	0.7781982	0.436452	0.914408
st6gal2a	2009.296209	-0.069678708	0.0777488	-0.896204	0.370144	0.889876
gadd45gip1	499.3718238	-0.098990202	0.1098856	-0.900848	0.367669	0.887476
tmem209	491.5192044	-0.03603602	0.103661	-0.347633	0.728116	0.970402
ahcyl2	1601.217277	-0.013987142	0.081075	-0.172521	0.863028	0.985841
drg1	704.0955066	-0.051014124	0.1006379	-0.506908	0.612219	0.95688
ndufa5	780.5475217	-0.085500845	0.1128556	-0.757613	0.448683	0.917876
rps24	9878.391516	-0.228937968	0.0895721	-2.555908	0.010591	0.177783
qrfpra	4.154436425	-1.343937924	0.9602299	-1.3996	0.161633	NA
ssbp1	179.8358686	-0.153015752	0.1575798	-0.971037	0.33153	0.874556
ccl19b	43.8091922	1.128933917	0.314646	3.5879494	0.000333	0.012922
pald1b	212.3811784	0.211031287	0.1423749	1.4822226	0.138281	0.676087
ska1	89.18035292	-0.305789681	0.2133809	-1.43307	0.151838	0.700196
zgc:194209	256.224284	-0.131056864	0.1375585	-0.952735	0.340724	0.878134
dnajb12a	495.0192751	0.042820936	0.1180837	0.362632	0.71688	0.96909
cuedc2	278.9445788	-0.05150377	0.1354539	-0.380231	0.703774	0.968134
epha3	50.85794337	0.303897915	0.2705576	1.1232281	0.261341	0.829527
apool	397.9069112	-0.048122364	0.1141321	-0.421638	0.67329	0.965257
rcn2	114.3632651	0.186355725	0.1864933	0.9992622	0.317668	0.867607
ch25hl1.1	198.5197532	-0.198895984	0.155392	-1.279963	0.200558	0.766801
dlg4b	400.1978886	-0.027056389	0.1170628	-0.231127	0.817216	0.981594
sdhaf4	169.8811668	-0.120754034	0.1960093	-0.616063	0.537853	0.943336
wnk1b	1163.031278	0.031014297	0.0887649	0.3493983	0.72679	0.970402
si:ch211-240l19.5	69.0051001	-0.585435455	0.2537228	-2.307382	0.021034	0.273449
nt5c2a	1069.341677	-0.038765746	0.0936992	-0.413725	0.679075	0.965257
polr1c	174.3220033	-0.120815054	0.157249	-0.768304	0.442307	0.916336
prom2	132.0410562	0.181794714	0.20184	0.9006872	0.367755	0.887476
cep68	87.67612134	0.034339528	0.2129578	0.1612504	0.871896	0.987846
dmrta2	111.8060095	-0.359985065	0.1887908	-1.906794	0.056547	0.46635
fuom	16.67580165	-0.585969115	0.4747331	-1.234313	0.217086	0.784302
AL831745.1	104.0867984	-1.171878673	0.2291767	-5.113429	3.16E-07	3.75E-05
snrpb2	463.8462719	-0.024272078	0.1123166	-0.216104	0.828907	0.982721
adka	1037.653206	-0.042183513	0.0841922	-0.501038	0.616344	0.95688
msl2b	688.3278866	0.003897875	0.0923663	0.0422002	0.966339	0.996315
oprm1	7.139216251	-1.322277762	0.7807892	-1.693514	0.090358	NA
rgs17	169.4262139	-0.064824645	0.1563602	-0.414585	0.678445	0.965257
il13ra2	88.13169898	0.287854623	0.2395182	1.2018067	0.229438	0.797069
il22ra2	0.157187365	-0.955901296	4.0804729	-0.234262	0.814781	NA
zgc:110353	27.72255117	-0.046427694	0.3564856	-0.130237	0.896379	0.989971
hk1	3979.737158	0.014014345	0.0737502	0.1900245	0.84929	0.985174
dhrs7ca	56.85529315	0.180406003	0.2754353	0.6549849	0.512477	0.936253
tspan15	239.6033275	-0.15141471	0.1383232	-1.094644	0.273672	0.838381
acbd6	566.2072639	0.060805096	0.1025373	0.5930048	0.553178	0.947265
lhx4	458.4047187	-0.041839321	0.1082313	-0.386573	0.699072	0.967998
qsox1	780.0423907	0.245765097	0.1071847	2.2929123	0.021853	0.280334
hnrnph3	237.6987815	0.026664334	0.1353796	0.1969597	0.843859	0.985174
terb2	5.595572136	1.023793726	0.8477353	1.2076809	0.22717	NA

calhm2.1	62.039972	0.477236027	0.2548461	1.8726444	0.061118	0.482482
si:dkey-51a16.9	545.9690455	-0.160327219	0.1044951	-1.534304	0.124955	0.654531
bag3	268.8117295	0.180931473	0.1437271	1.2588542	0.208083	0.775404
git1	700.6994325	-0.015070238	0.0914017	-0.164879	0.869039	0.987245
pitpnaa	844.3607168	-0.257871512	0.1015046	-2.540491	0.01107	0.18241
naa20	455.7250031	0.128790319	0.111457	1.1555162	0.247879	0.81711
ccdc85a	99.63930484	0.153227446	0.2082534	0.7357742	0.461868	0.922466
soul2	427.5254217	0.280221445	0.1107264	2.5307561	0.011382	0.186155
si:cabz01090165.1	134.2039583	0.218072823	0.1748058	1.2475148	0.212209	0.780111
ugt2a6	79.59271727	-0.372988239	0.2418728	-1.542085	0.123053	0.65095
eef1a1a	1273.145404	0.199795197	0.0809603	2.4678158	0.013594	0.209269
ap1m3	149.1543246	0.088830693	0.1848436	0.4805721	0.630821	0.957354
zgc:153119	17.07870559	0.40521262	0.5267558	0.7692608	0.441739	0.916336
c8a	438.6327748	0.358140009	0.1224407	2.9250084	0.003444	0.078559
c8b	427.7633835	0.320877008	0.1354557	2.3688711	0.017842	0.247192
ssx2ipa	309.8024116	-0.113127089	0.1238929	-0.913104	0.361188	0.885686
tubb2	1144.241099	0.177246923	0.0867038	2.0442814	0.040926	0.394957
rrh	72.10922489	-0.302341631	0.2497166	-1.210739	0.225995	0.793556
dapp1	20.45138457	-0.393881813	0.4604312	-0.855463	0.392295	0.897688
mrps6	299.0868906	-0.11915129	0.1424612	-0.836377	0.402943	0.90084
loxl3b	1058.152525	0.113999411	0.0858293	1.3282116	0.184108	0.747376
emx1	44.25687749	-0.046161548	0.3024204	-0.15264	0.878682	0.987881
fstl1b	5613.48127	0.189285954	0.0631735	2.9962887	0.002733	0.067381
ptk2bb	439.3301769	0.005767177	0.1088269	0.0529941	0.957737	0.99467
pa2g4a	3084.995669	-0.071482618	0.0695367	-1.027984	0.303957	0.858391
cfd	2397.151838	0.51702901	0.0912626	5.6652907	1.47E-08	2.46E-06
ier3ip1	226.477409	-0.07211235	0.1489752	-0.484056	0.628346	0.957354
atoh1c	10.17697371	-0.537365672	0.6079394	-0.883913	0.376743	NA
mat1a	2474.639913	0.414173405	0.071811	5.7675466	8.04E-09	1.38E-06
poll	163.2485656	-0.176835873	0.1597867	-1.106699	0.268424	0.833634
fgf8b	42.01999925	0.439880029	0.3030456	1.4515309	0.146632	0.692998
cenpk	91.00660762	0.027879201	0.2112063	0.1319999	0.894984	0.989814
nrgna	740.3353466	-0.148769142	0.1000237	-1.487339	0.136925	0.673303
slc6a1b	4418.106918	0.151937763	0.0703984	2.1582567	0.030908	0.340296
mtif3	163.3982668	-0.101994194	0.159096	-0.641086	0.521467	0.939056
itm2cb	694.4181226	0.17697435	0.1003553	1.7634771	0.07782	0.543558
si:dkey-235d18.5	268.9034851	-0.416999855	0.135035	-3.088088	0.002014	0.05383
fank1	10.06611409	0.05661832	0.6028124	0.0939236	0.92517	NA
slc36a4	460.0406358	0.028742846	0.1116236	0.2574979	0.796794	0.978569
tsta3	191.1141193	0.176501276	0.1550468	1.1383744	0.254964	0.82364
mrpl38	612.930002	-0.106874967	0.1112603	-0.960585	0.336761	0.876088
TSTA3 (1 of many)	1414.745277	-0.401049507	0.0815879	-4.915549	8.85E-07	9.07E-05
dsc2l	573.5878515	-0.060059733	0.10955	-0.54824	0.583527	0.952214
samm50l	713.7822171	0.156265961	0.0921781	1.6952618	0.090026	0.574248
si:ch211-121a2.2	3.78754581	0.628178154	0.9752731	0.6441048	0.519507	NA
sirt5	274.4524884	-0.012124929	0.1273697	-0.095195	0.92416	0.992676
zgc:194221	71.00207054	0.388344547	0.2639965	1.4710214	0.141285	0.681968
shtn1	78.27667587	0.000921614	0.2263311	0.004072	0.996751	0.998936
fam204a	297.8154039	0.079850904	0.1239774	0.6440764	0.519526	0.938945
emx2	621.7394843	-0.055582816	0.1058523	-0.525098	0.599515	0.956045
mrps26	534.3715053	-0.09372087	0.1141155	-0.821281	0.411486	0.903483
OSBPL8	15.39218813	0.01409696	0.5096971	0.0276575	0.977935	0.996762
zgc:123321	64.40568623	0.331560481	0.2452456	1.3519531	0.17639	0.73663
asap1b	1064.483827	0.047797003	0.091143	0.5244177	0.599988	0.956045

zgc:112160	436.7737797	-0.585578	0.1507288	-3.884978	0.000102	0.004972
BX914200.1	3.709035756	-0.215227669	1.0854222	-0.198289	0.842819	NA
ldlrap1b	59.19572964	-0.015791834	0.27251	-0.05795	0.953789	0.994213
si:ch73-308m11.1	151.5143546	-1.100958949	0.3755967	-2.931227	0.003376	0.077672
xpc	545.1197117	-0.114059987	0.1114861	-1.023087	0.306267	0.860027
prop1	4.711259298	0.027028854	0.86858	0.0311184	0.975175	NA
mcfd2	536.9011073	-0.093642132	0.1055054	-0.887558	0.374779	0.894316
dync2li1	36.4830353	-0.187321071	0.323842	-0.578434	0.562971	0.948728
tbxtb	9.85639073	0.159320349	0.601898	0.2646966	0.791243	NA
mea1	910.805137	0.061285088	0.0972272	0.6303286	0.52848	0.941896
zgc:172182	3.375294021	0.326934744	1.0341058	0.3161521	0.751887	NA
bbs5	157.2357518	0.247830221	0.163182	1.5187352	0.128829	0.659982
zp3b	30.13493436	-0.18359867	0.344571	-0.532833	0.594149	0.954434
gng5	734.3042816	-0.136492774	0.1039545	-1.313005	0.189181	0.753954
gsta.2	136.9167103	-0.838180778	0.197858	-4.236275	2.27E-05	0.001407
znf800b	121.3971073	-0.058425914	0.2003342	-0.291642	0.77056	0.975687
dusp22b	86.81591337	-0.186946771	0.2159459	-0.865711	0.386649	0.89551
dhdds	339.3585021	-0.083987143	0.1272765	-0.65988	0.509331	0.936123
ftr93	23.74960362	0.312478108	0.3959551	0.7891757	0.430009	0.911615
lifrb	135.2233291	-0.048263964	0.1791071	-0.26947	0.787568	0.976596
rpz4	92.93597289	-0.195691572	0.205468	-0.952419	0.340885	0.878134
lamtor2	346.0660933	-0.10779699	0.1212365	-0.889146	0.373924	0.893677
ambra1b	680.9756501	-0.102458264	0.0978786	-1.046789	0.295197	0.851652
arpp19b	708.1149501	-0.090258409	0.1020209	-0.884705	0.376316	0.894814
cemip	545.7491702	0.209688541	0.1025714	2.0443183	0.040922	0.394957
ap3s2	667.1531173	-0.146268477	0.1010269	-1.447817	0.147668	0.694905
c1qbp	1266.415725	-0.129428029	0.1079889	-1.19853	0.230711	0.798822
CR855393.1	0	NA	NA	NA	NA	NA
BX323555.2	0	NA	NA	NA	NA	NA
gnpnat1	160.808247	0.097577178	0.1657851	0.5885763	0.556146	0.947902
zbtb7a	5.38134588	0.012665187	0.830315	0.0152535	0.98783	NA
si:ch73-168d20.1	0.831706979	-0.653219184	2.2172216	-0.294612	0.768291	NA
sh3gl2b	16.88525527	0.075215825	0.4759353	0.1580379	0.874427	0.987881
rbm42	384.5809359	0.035835493	0.1203689	0.2977138	0.765922	0.975374
tmem147	1052.271494	-0.103397078	0.0959995	-1.077058	0.281454	0.84357
gapdhs	25549.01398	0.021203188	0.0589388	0.3597493	0.719035	0.969183
bola1	240.1848563	-0.067229357	0.1371225	-0.490287	0.623931	0.957354
ckmt2b	2696.049843	0.375683769	0.0935557	4.0156172	5.93E-05	0.003192
slc25a33	746.0197278	0.3486924	0.0991979	3.515119	0.00044	0.016096
hlcs	91.84570991	0.145711224	0.2133631	0.6829262	0.494653	0.931926
CABZ01064671.1	14.01734076	0.023189006	0.5209118	0.0445162	0.964493	0.995927
ccdc172	3.468470599	1.322507468	1.1253223	1.1752255	0.239904	NA
tent5ba	681.6883797	0.059684881	0.0949838	0.6283689	0.529762	0.942209
fibina	314.7912137	-0.133379858	0.1293899	-1.030837	0.302617	0.85729
si:ch211-87j1.4	16.00797814	0.250727671	0.5139337	0.48786	0.625649	0.957354
utp11	124.929255	0.026888398	0.1780691	0.1509998	0.879976	0.987881
gdnfa	36.48000055	0.656944141	0.3390947	1.9373471	0.052703	0.450561
st7	72.84696444	-0.215729837	0.2488048	-0.867065	0.385907	0.89551
fgfbp1b	22.41102415	-0.327109739	0.4531606	-0.721841	0.470393	0.924838
fgfbp2a	51.27227626	0.109593613	0.2857225	0.3835666	0.7013	0.968134
prom1a	777.718268	0.086439915	0.0916763	0.9428822	0.345741	0.87848
akirin1	926.2654376	0.113503745	0.1017172	1.1158753	0.264476	0.830245
cnr2	7.90778906	0.721894595	0.6878325	1.0495209	0.293938	NA
rai14	348.2608244	-0.104630357	0.1179778	-0.886865	0.375152	0.894316

tecrl2b	38.18701365	0.393846942	0.3246001	1.2133296	0.225004	0.792185
ppt1	865.308647	-0.058796853	0.1019884	-0.576505	0.564274	0.948739
hivep2a	598.2000815	0.171006937	0.1001982	1.7066874	0.08788	0.567519
snrnp48	134.2756503	-0.148650611	0.1898375	-0.783041	0.433603	0.913667
xkr8.3	66.38445347	0.099268236	0.2413833	0.4112474	0.680891	0.965476
ptp4a3a	1104.271028	0.174463529	0.0834831	2.0898057	0.036635	0.374263
slc13a5b	32.26465987	0.398458138	0.3368283	1.182971	0.236821	0.805854
fryl	1478.245364	-0.013778082	0.0869799	-0.158405	0.874137	0.987881
pnpla7b	581.4430551	0.016787658	0.1049927	0.1598936	0.872965	0.987846
si:ch211-244a23.1	39.466882	-0.132474355	0.3569456	-0.371133	0.710538	0.968985
scfd2	173.0552491	-0.226866189	0.1727201	-1.313491	0.189018	0.753954
neurod6a	343.773089	-0.101060611	0.1399015	-0.72237	0.470067	0.924787
palld	1458.90361	0.055961414	0.093224	0.6002895	0.548313	0.945871
ebag9	656.5934265	-0.181712842	0.1040067	-1.747127	0.080615	0.550664
gpd1l	424.7545897	0.139633881	0.1162931	1.2007064	0.229865	0.79764
osbpl10b	397.2767993	-0.008708477	0.1163106	-0.074873	0.940316	0.992857
tardbp	4793.297571	-0.069485192	0.0717085	-0.968995	0.332548	0.874653
trmt11	113.180112	0.042537271	0.1866558	0.2278915	0.819731	0.981594
tcf19l	37.39098639	-0.078591903	0.3134968	-0.250694	0.80205	0.979492
pttg1ipb	281.231755	-0.325169829	0.1630596	-1.994178	0.046133	0.421936
mcm4	1629.512041	0.034672848	0.0844318	0.4106611	0.681321	0.965476
cldn1	299.3461776	-0.008073267	0.1369022	-0.058971	0.952975	0.994151
snai2	1183.457431	-0.186749003	0.0836452	-2.232632	0.025573	0.309666
GALNTL6	650.8560921	-0.104545578	0.1000727	-1.044696	0.296163	0.853273
gbp	549.9575823	-0.166531933	0.1032808	-1.612419	0.106871	0.616112
acp6	175.7783676	0.374649736	0.1796036	2.085981	0.03698	0.376157
gja5a	21.71152185	0.28937891	0.4076755	0.7098267	0.477812	0.926857
coil	146.4686796	0.024067844	0.1800423	0.1336788	0.893657	0.989291
scpep1	421.4693294	-0.180881115	0.1157668	-1.562462	0.118179	0.641526
pycard	333.1057409	0.017143178	0.1392605	0.1231015	0.902027	0.990121
fli1b	58.9812827	-0.005380466	0.264646	-0.020331	0.983779	0.996944
zp3d.1	6.499959062	0.122487162	0.7923073	0.1545955	0.87714	NA
ccsapb	536.7833136	-0.084995798	0.1033592	-0.822334	0.410887	0.903176
epb41l4a	369.0631659	-0.052119224	0.1150925	-0.452846	0.650659	0.960708
si:dkey-95h12.1	274.2279031	0.487168652	0.1277275	3.8141264	0.000137	0.006329
kif20bb	152.2396799	-0.190285966	0.1704055	-1.116666	0.264137	0.830245
pi4kb	1069.928061	0.077375615	0.092797	0.833816	0.404385	0.901305
znf366	9.457027917	0.667737315	0.6477067	1.0309255	0.302576	NA
zgc:113232	1626.979049	0.108838681	0.3984242	0.2731728	0.78472	0.97627
psma2	970.1784466	-0.166838309	0.0964662	-1.729499	0.08372	0.557399
zfpm2a	703.6108454	-0.050776963	0.0976961	-0.519744	0.603242	0.956045
si:ch211-215k15.4	1.841160817	-2.158421599	1.5579239	-1.385447	0.165916	NA
zfyve26	323.9594989	0.15550542	0.1234795	1.2593623	0.2079	0.775404
ackr4b	70.43999051	-0.207501263	0.2374882	-0.873733	0.382264	0.895192
fosaa	52.14793814	0.670356727	0.3107174	2.157448	0.030971	0.34077
jdp2a	71.38413058	0.151471515	0.2285288	0.6628114	0.507451	0.935734
si:ch73-138e16.5	25.38929209	0.285061053	0.4156199	0.6858696	0.492795	0.931926
nek4	202.1910269	-0.118442384	0.1462129	-0.810068	0.417901	0.906293
grm4	579.062094	0.015668883	0.106259	0.1474594	0.882769	0.988508
glt8d1	261.6821475	0.186723718	0.1307384	1.4282241	0.153227	0.702508
cdc42l	2129.874691	-0.111536488	0.0780486	-1.429064	0.152986	0.702214
wnt4b	376.2004817	0.101408023	0.1182083	0.8578754	0.390961	0.897643
zgc:92287	494.029835	0.241807736	0.1117635	2.1635659	0.030498	0.337781
zgc:165461	1476.19776	0.173588823	0.0805686	2.1545478	0.031197	0.341959

prim1	269.6847482	-0.143811556	0.1291613	-1.113426	0.265526	0.831302
rgs16	272.0398178	0.065419177	0.1351639	0.4839989	0.628387	0.957354
havcr1	36.24462076	0.91034366	0.3221865	2.8255177	0.00472	0.098984
zgc:101562	65.85944133	0.075897107	0.2392719	0.3172002	0.751092	0.97419
slc35a1	380.5559558	-0.080235713	0.1187465	-0.675689	0.499238	0.934006
syncrip	5318.303433	-0.023209489	0.0768301	-0.302088	0.762585	0.975374
fam210ab	205.0776386	-0.113389798	0.1484767	-0.763687	0.445054	0.916958
qdpra	405.5433753	0.136241995	0.1336228	1.0196014	0.307918	0.861088
nenf	279.0775258	-0.097892018	0.1464028	-0.668648	0.50372	0.935314
fstl5	3107.944952	0.074727183	0.0749368	0.9972021	0.318666	0.867746
BX465228.1	24.64652824	0.166183985	0.3863029	0.4301909	0.667057	0.964736
scrt1b	207.0006218	0.155189844	0.1459096	1.0636026	0.287509	0.845979
krcp	478.7109242	-0.071817196	0.111931	-0.64162	0.52112	0.939056
si:dkey-37m8.11	4.604889587	-0.850997253	0.8792092	-0.967912	0.333088	NA
mef2d	1813.671017	-0.065654089	0.0765627	-0.857521	0.391157	0.897688
kpnb3	3535.208763	-0.03601801	0.0769617	-0.467999	0.639785	0.959994
rap2ab	7.142916875	-0.320655302	0.7814927	-0.410311	0.681578	NA
oxgr1a.3	0.173729368	-0.955901296	4.0804729	-0.234262	0.814781	NA
crata	753.7398512	-0.087531461	0.104249	-0.839638	0.401111	0.90084
tlr18	34.87955918	0.396437772	0.3243421	1.2222828	0.221601	0.78829
si:ch211-132g1.1	0.365141898	1.973577737	3.3927314	0.5817076	0.560764	NA
ctsk	574.9112848	0.112894943	0.1133321	0.996143	0.319181	0.867746
atp1a1a.5	129.4826068	0.211282792	0.1959912	1.0780219	0.281024	0.84357
onecut1	390.0772726	0.145516056	0.1154595	1.2603213	0.207553	0.775175
hormad1	4.671020809	0.006990199	0.8696997	0.0080375	0.993587	NA
ephx2	389.0835397	-0.466391032	0.124363	-3.750239	0.000177	0.007729
slc38a11	22.76206072	-1.375256278	0.4171397	-3.296872	0.000978	0.030397
si:ch73-340m8.2	24.62501821	0.285262929	0.3887666	0.733764	0.463093	0.922698
runx2a	2.149092142	-3.475601723	1.6113237	-2.156985	0.031007	NA
mpdu1b	690.8004989	0.020971589	0.095381	0.2198718	0.825971	0.982196
sox19b	402.4060501	-0.066995814	0.1255779	-0.5335	0.593687	0.954434
scamp5b	1230.578859	0.243999272	0.0894724	2.7270902	0.00639	0.122178
fbxo32	83.26503506	-0.264512537	0.2314689	-1.142756	0.25314	0.822746
klhl38b	43.00869348	-0.040431921	0.330672	-0.122272	0.902684	0.990121
zgc:110182	135.3962905	-0.091290639	0.1774044	-0.514591	0.606839	0.956718
zgc:92590	2.913453164	-1.356989124	1.1796197	-1.150362	0.249995	NA
si:dkey-79d12.5	382.6288442	0.285638803	0.1480996	1.9286937	0.053769	0.454191
ubl7b	456.5182569	-0.136937191	0.1108695	-1.235121	0.216786	0.784252
ywhab1	12836.51532	-0.031461513	0.0663994	-0.473822	0.635627	0.958142
pon3.2	224.8577869	-0.046865282	0.152603	-0.307106	0.758763	0.975374
cadm4	1785.061142	0.102615894	0.0774939	1.3241806	0.185443	0.74928
apoeb	10150.08991	0.339682849	0.0767443	4.4261619	9.59E-06	0.000697
apoa4b.1	7769.358496	0.369221206	0.0857854	4.3040091	1.68E-05	0.001087
gtpbp10	258.4465651	-0.018935792	0.1424921	-0.13289	0.89428	0.989689
osgin2	7.138672972	0.591938345	0.7365673	0.8036446	0.421602	NA
nbn	186.7437787	-0.078495294	0.1707748	-0.459642	0.645773	0.96029
otomp	744.6232737	0.244562609	0.0982282	2.4897402	0.012784	0.200782
pxdc1b	142.7450451	-0.172048449	0.1745662	-0.985577	0.324341	0.870928
prpf4bb	738.7460715	-0.105713557	0.0982248	-1.076241	0.281819	0.843666
psph	950.7927311	-0.033142503	0.105211	-0.31501	0.752754	0.97481
CABZ01041494.1	0.31598276	0.005883445	3.3523626	0.001755	0.9986	NA
rx2	142.9571285	0.093204912	0.1757937	0.5301947	0.595977	0.954434
dhx29	350.2564719	-0.024705015	0.1282992	-0.192558	0.847305	0.985174
crfb2	61.53147455	-0.019037113	0.2536581	-0.07505	0.940175	0.992857

mat2aa	2906.287405	0.242098719	0.089702	2.6989234	0.006956	0.12977
hvcn1	282.7659151	-0.073437928	0.1274234	-0.57633	0.564392	0.948739
rpp38	121.7596072	-0.295893995	0.182422	-1.62203	0.104797	0.612652
crot	670.0243882	-0.162646721	0.1132511	-1.436161	0.150957	0.698912
spred3	591.4101115	0.131994304	0.1004751	1.3137016	0.188947	0.753954
ehd2b	555.1689631	0.093804334	0.1067846	0.878444	0.379703	0.895192
tmem182a	603.2387123	0.349948325	0.1044379	3.3507779	0.000806	0.025953
arhgef1a	316.1519415	-0.066531661	0.1344383	-0.494886	0.62068	0.957354
mmd	204.5937628	0.148411952	0.1468787	1.010439	0.312285	0.864401
bcl7bb	182.2931424	0.068431289	0.1860514	0.3678085	0.713016	0.96909
slc25a20	743.1984872	-0.122699722	0.1089382	-1.126324	0.260028	0.828288
usp22	604.7127707	-0.016227523	0.1142637	-0.142018	0.887066	0.989231
acot19	11.32637891	-2.221993094	0.648217	-3.427854	0.000608	0.020564
nptxra	230.5205417	0.203331722	0.1576783	1.2895355	0.197212	0.762204
klf2b	697.8465589	-0.106570693	0.0948301	-1.123807	0.261095	0.829247
cyp2k22	0	NA	NA	NA	NA	NA
rsl24d1	3193.4939	-0.262106824	0.0850972	-3.080089	0.002069	0.054779
snrpd2	1117.967879	-0.109123692	0.1032311	-1.057082	0.290474	0.846262
prtgb	3.942894484	-0.509877687	0.9551967	-0.533793	0.593484	NA
polr2i	253.227599	-0.083538328	0.1369772	-0.60987	0.541948	0.944974
si:ch211-219a4.3	45.08690056	-0.146199711	0.2854301	-0.512208	0.608505	0.956718
fam214a	805.2678198	-0.052764216	0.107408	-0.491251	0.623249	0.957354
wdfy2	611.9502608	-0.042701689	0.1036647	-0.411921	0.680397	0.965431
unc50	297.9688498	0.059334311	0.1424122	0.4166377	0.676943	0.965257
slc25a51b	303.7348779	-0.230138223	0.1464772	-1.571154	0.116147	0.63841
irf2	146.1122649	-0.343745877	0.168149	-2.044294	0.040925	0.394957
vil1	2200.501873	-0.752315887	0.0769409	-9.77784	1.40E-22	1.75E-19
enpp6	173.6159168	0.107158921	0.1582619	0.6770987	0.498343	0.933963
snx10b	6.681189336	0.481663238	0.7989515	0.6028692	0.546596	NA
lrp2bp	8.59619519	-0.037383103	0.6725287	-0.055586	0.955672	NA
cga	63.36302388	0.488153526	0.2590165	1.8846428	0.059478	0.476934
osbpl3b	187.604262	0.071699375	0.1565217	0.4580794	0.646895	0.960362
gsdmeb	82.45943204	-0.285371179	0.2197412	-1.29867	0.194057	0.75784
zgc:113176	101.8295198	-0.330019322	0.1975653	-1.670431	0.094834	0.585466
dusp19a	7.885724441	-0.357933085	0.6823692	-0.524545	0.5999	NA
mthfd1b	1026.309654	-0.27742988	0.0933619	-2.971553	0.002963	0.071451
BX004816.1	0	NA	NA	NA	NA	NA
sb:cb81	591.1013524	-0.105132841	0.1120125	-0.938581	0.347946	0.880195
yif1b	305.3178584	0.053627271	0.1299494	0.4126782	0.679842	0.965311
ca15b	3.023708727	0.386810357	1.1341993	0.3410427	0.733071	NA
zgc:92313	383.3405662	-0.051704641	0.1310074	-0.394669	0.693087	0.96736
smpd2a	106.163724	0.47280812	0.2122303	2.227807	0.025893	0.310878
s1pr5a	160.3376708	-0.022006417	0.1624585	-0.135459	0.892249	0.989291
siae	159.5388854	0.215041562	0.1782671	1.2062886	0.227706	0.794934
lgals3bpb	79.23032867	0.871829838	0.2521079	3.458162	0.000544	0.019035
epcam	6966.429534	0.039719949	0.0920348	0.4315754	0.66605	0.964736
csgalnact1a	260.2781407	0.256169286	0.1406654	1.821125	0.068588	0.513124
ftr90	0.999705014	0.975110312	1.9871203	0.4907153	0.623628	NA
spsb4b	188.9986057	-0.476689574	0.1555503	-3.064537	0.00218	0.057157
b3gat1a	78.14420295	0.378577594	0.2243346	1.6875575	0.091496	0.57849
tmem97	167.461055	-0.074072679	0.1741583	-0.425318	0.670605	0.965257
bckdha	1176.796073	0.088547618	0.0799733	1.1072154	0.268201	0.833475
ift20	198.3116101	0.08655472	0.1482533	0.5838299	0.559335	0.948478
exosc5	209.4387512	-0.235843954	0.1489043	-1.583862	0.113225	0.631061

rad1	111.4614685	-0.123827945	0.1981103	-0.625045	0.531941	0.942359
hnrnpul1	2001.715558	-0.1530226	0.0721787	-2.120051	0.034002	0.359162
ckmb	48874.75142	0.209636205	0.1066419	1.9657951	0.049322	0.435504
phykpl	92.56659149	-0.073102623	0.2202728	-0.331873	0.739985	0.972943
pdzd3a	51.91923269	-0.470134357	0.2780831	-1.690626	0.090908	0.577827
ube4a	445.0908709	0.103724128	0.1126468	0.9207907	0.35716	0.883583
c18h3orf33	32.19906941	-0.014765823	0.3381156	-0.043671	0.965167	0.996154
eef1akmt1	170.8167376	0.136180276	0.157028	0.8672357	0.385813	0.89551
v2rh32	2.980751047	0.298946829	1.1109496	0.2690913	0.787859	NA
CU570684.1	0	NA	NA	NA	NA	NA
tfap2c	958.0358273	-0.026389772	0.0910767	-0.289753	0.772005	0.975687
rtf2	747.3631258	-0.063613572	0.0907749	-0.700784	0.483438	0.929461
gcnt7	125.2378476	-0.094781295	0.1901237	-0.498524	0.618114	0.957354
si:ch73-352p4.8	208.7226034	0.127606415	0.1465068	0.8709933	0.383758	0.895192
sestd1	1129.307327	-0.050514829	0.0836813	-0.603658	0.546071	0.945526
ilkap	137.0915984	0.157019354	0.1784048	0.8801295	0.378789	0.895192
psmg1	362.9283533	-0.114785154	0.1178188	-0.974252	0.329932	0.874009
lrrc32	32.59023459	-0.042140388	0.3448165	-0.122211	0.902732	0.990121
fosl2	556.9992562	0.161141528	0.1022163	1.5764751	0.114916	0.635519
grik1b	191.6694124	0.169674114	0.1499446	1.1315787	0.257812	0.825314
zgc:110333	363.434473	-0.141257356	0.1199018	-1.178109	0.238753	0.807768
v2rx1	3.662393904	-0.578186097	1.0920696	-0.529441	0.5965	NA
PRMT8	9.05958791	-0.366392585	0.6352599	-0.57676	0.564101	NA
rnf11b	174.2772974	0.136420659	0.1623493	0.8402908	0.400745	0.90084
si:ch73-44m9.3	0.339758314	0.005883863	3.2682461	0.0018003	0.998564	NA
ccr10	0.173729368	-0.955901296	4.0804729	-0.234262	0.814781	NA
pxylp1	345.0090606	0.154045903	0.1221638	1.2609785	0.207317	0.775175
prickle1a	290.5744149	-0.038393862	0.1257633	-0.305287	0.760148	0.975374
gps1	862.4538192	0.045532064	0.0885055	0.5144549	0.606934	0.956718
wipi1	485.6225404	-0.134771561	0.1090854	-1.235468	0.216656	0.78407
nifk	397.9673637	0.059722666	0.1340331	0.4455816	0.6559	0.961833
lrrc51	4.528134411	-0.800971598	0.9491602	-0.843874	0.39874	NA
ctdspl	734.5196111	-0.077264554	0.0961192	-0.803841	0.421489	0.90651
ccdc93	277.2728485	-0.167281463	0.1313938	-1.273131	0.202972	0.769914
MEP1B	20.12876503	-1.034087526	0.4340806	-2.382248	0.017207	0.242176
plcd4b	68.99671931	-0.054547839	0.2488949	-0.21916	0.826525	0.982253
fkbp14	195.0418667	0.190928659	0.1626145	1.174118	0.240348	0.808437
plekha8	271.1356691	0.144964329	0.1414219	1.0250485	0.30534	0.85982
nab1b	13.41437195	-0.309180978	0.5322916	-0.580849	0.561342	0.948728
si:ch211-113j13.2	1.528634823	-1.028202071	1.6599902	-0.619402	0.535651	NA
BX936415.1	11.7883464	0.597436271	0.5824876	1.0256635	0.30505	0.859636
glsb	506.1982576	0.16784627	0.10838	1.5486828	0.121458	0.647929
chchd4b	117.3853322	-0.233678399	0.201616	-1.159027	0.246445	0.816419
adprh	123.5685869	-0.088743475	0.1790322	-0.495684	0.620117	0.957354
rbbp9	281.007172	-0.279174983	0.133944	-2.084266	0.037136	0.377158
macc1	242.4732966	0.077620869	0.1347213	0.5761589	0.564508	0.948739
zgc:114130	228.8485589	-0.157336398	0.1391778	-1.13047	0.258278	0.826273
tfb1m	66.0821544	-0.050142106	0.237766	-0.210889	0.832974	0.983557
wdr75	626.5193519	-0.084996948	0.1036083	-0.820369	0.412006	0.904218
elavl2	5.091563745	-0.114664671	0.8490789	-0.135046	0.892576	NA
crygm6	0	NA	NA	NA	NA	NA
crygm7	2.238892264	-1.864255946	1.5779697	-1.181427	0.237433	NA
zgc:153846	0.982760668	0.918233209	2.0855726	0.4402787	0.659735	NA
kcnab1b	230.2656091	0.240943962	0.1431271	1.683426	0.092293	0.57849

tm4sf4	659.4679375	-0.652393577	0.108169	-6.031243	1.63E-09	3.19E-07
id1	362.4292529	-0.410377569	0.1264051	-3.246527	0.001168	0.034842
drd6b	4.767032681	-0.909906495	0.8890603	-1.023447	0.306096	NA
inhbb	269.5750616	-0.010300131	0.1287915	-0.079975	0.936257	0.992702
ralba	182.3931182	0.143068206	0.1691427	0.8458432	0.39764	0.899672
med16	314.9372284	0.03549129	0.128955	0.2752224	0.783145	0.97627
sult3st4	57.08994082	-0.852949505	0.3310426	-2.576555	0.009979	0.170402
sst1.1	398.7720153	0.031796897	0.1195348	0.2660054	0.790235	0.977338
lactb	234.1380919	-0.101373877	0.1381362	-0.733869	0.463029	0.922698
ncf4	26.7703315	-0.04506753	0.3699338	-0.121826	0.903037	0.990121
tsku	299.7730154	-0.102864904	0.1311068	-0.784588	0.432695	0.91326
si:dkey-27n14.1	21.52544847	0.509642686	0.4182781	1.2184302	0.223061	0.789115
fundc1	967.0029532	0.041894554	0.0960531	0.4361603	0.66272	0.964665
dcun1d2a	717.4251087	0.246252038	0.0918614	2.6806924	0.007347	0.13527
adra2a	57.79083828	-0.048457723	0.2552768	-0.189824	0.849447	0.985174
plekhh2	72.15860604	-0.03130234	0.2267471	-0.13805	0.890201	0.989291
shoc2	420.2686886	0.120460503	0.1136515	1.0599113	0.289185	0.845979
tsg101a	846.5565614	0.142854256	0.1110864	1.2859743	0.198452	0.763597
tcf25	377.3984378	-0.032805768	0.1146421	-0.286158	0.774757	0.975687
si:dkey-286j15.1	966.5752336	-0.227855541	0.0951857	-2.393799	0.016675	0.2379
upp1	7.597698275	-0.011071086	0.6939936	-0.015953	0.987272	NA
add3a	1152.880238	-0.006384417	0.0850334	-0.075081	0.94015	0.992857
hnrnph1	324.1781701	0.073024682	0.1328991	0.5494744	0.58268	0.952214
mxi1	2042.363852	-0.0678701	0.0780788	-0.869251	0.38471	0.89551
fdps	746.6131943	-0.032379021	0.0974262	-0.332344	0.739629	0.972943
gpa33b	28.67319868	-0.945176073	0.39463	-2.395094	0.016616	0.237747
si:dkey-11d18.4	0.515216064	0.926382026	2.6525479	0.3492423	0.726907	NA
si:ch73-113g13.1	0.158795395	0.967652056	4.0804729	0.2371421	0.812547	NA
gcgb	180.9175222	0.492063809	0.1678791	2.931061	0.003378	0.077672
ildr1b	208.0533993	-0.39738132	0.1548887	-2.565592	0.0103	0.174078
meox2a	17.91527377	0.271849063	0.4494236	0.6048838	0.545256	0.945526
kdelr3	270.0077629	-0.049743277	0.1308702	-0.380096	0.703874	0.968134
cbfb	934.1631652	-0.14906006	0.1065329	-1.399193	0.161755	0.716022
si:dkey-49n23.1	580.8287907	0.147743502	0.1115713	1.3242072	0.185434	0.74928
zmp:0000000606	1.642507947	1.187327128	1.5663803	0.7580069	0.448447	NA
wnt10b	34.40468365	-0.333671554	0.3329056	-1.002301	0.316198	0.866931
nr2f2	3055.120537	0.08840521	0.0770346	1.147604	0.251132	0.820469
deptor	359.6419118	-0.046681235	0.1250842	-0.373199	0.709001	0.968664
ssuh2.1	4.457643678	-0.094544898	0.8778982	-0.107695	0.914238	NA
pnp6	285.2633007	0.217992521	0.1478732	1.4741856	0.140432	0.680611
ntd5	544.8268121	0.006614221	0.10738	0.0615964	0.950884	0.99381
olig2	261.4422516	0.005409466	0.1427901	0.037884	0.96978	0.996315
olig1	76.82562879	0.214865718	0.2349237	0.9146192	0.360392	0.885686
dharma	0.476747244	2.393381959	3.0525537	0.7840589	0.433006	NA
rab13	96.30756793	0.149521813	0.2059154	0.7261322	0.467758	0.924328
zgc:101731	53.2063026	0.042633152	0.2664921	0.159979	0.872898	0.987846
zgc:113149	378.8255138	0.074722126	0.1154999	0.6469453	0.517667	0.938592
zgc:92606	1522.918092	-0.361815913	0.0921368	-3.926943	8.60E-05	0.004322
fgf14	150.4477176	0.093755723	0.1640534	0.5714951	0.567664	0.948739
hspa13	163.3975762	0.257600038	0.166758	1.5447542	0.122406	0.650311
itgbl1	388.6626875	0.275974253	0.1155946	2.3874317	0.016967	0.240499
tpi1b	7595.493201	-0.084152288	0.070413	-1.195124	0.232039	0.800126
usp37	478.5453629	0.160199542	0.1071254	1.4954395	0.1348	0.670689
cttnbp2	1216.775276	0.118806822	0.0858254	1.3842853	0.166271	0.722887

or102-4	0.514116189	-2.475533565	2.7461255	-0.901464	0.367342	NA
or102-1	0.173729368	-0.955901296	4.0804729	-0.234262	0.814781	NA
st3gal4	173.3988072	0.235327147	0.1621067	1.4516801	0.146591	0.692967
AL845428.1	0.792850356	1.849779641	2.4264239	0.7623481	0.445852	NA
gemin7	135.7279901	-0.208753441	0.1832606	-1.139107	0.254658	0.823484
or111-11	10.8414807	-0.009929081	0.5769028	-0.017211	0.986268	NA
pdcd4b	1008.704197	-0.089911371	0.0922077	-0.975096	0.329513	0.873467
or111-6	3.383973964	0.608146579	1.0352029	0.5874661	0.556891	NA
or111-1	6.216622346	0.765143446	0.7970714	0.9599434	0.337084	NA
or107-1	5.392844037	1.370583886	0.8564365	1.6003334	0.109525	NA
or119-2	18.53612172	0.613988385	0.4531916	1.3548096	0.175478	0.736242
zgc:152857	5.798168009	0.469225196	0.861735	0.5445122	0.586089	NA
piga	124.9971723	0.199448791	0.1958592	1.0183276	0.308522	0.861088
cxcr3.2	28.7237848	1.125470237	0.3822028	2.9446942	0.003233	0.07565
mid1ip1a	955.5778374	0.060966362	0.0900663	0.6769054	0.498466	0.934006
lgals9l3	216.9402976	-0.288234765	0.1404844	-2.051721	0.040197	0.392132
calb2a	3500.471698	-0.034337879	0.0728346	-0.47145	0.637319	0.958872
hspb1	331.2810145	-0.05275264	0.1461815	-0.360871	0.718196	0.96909
got2a	1291.368562	0.253486302	0.0857252	2.9569638	0.003107	0.073687
jagn1a	248.2017283	-0.054250657	0.1371526	-0.39555	0.692437	0.96736
mier1a	339.3132963	-0.029808999	0.1203411	-0.247704	0.804363	0.979643
si:dkey-12e7.4	25.92280921	0.020398515	0.3762047	0.0542219	0.956758	0.994543
chka	809.5295328	-0.026828456	0.0970832	-0.276345	0.782283	0.97627
thap3	43.35362915	-0.160913359	0.3137587	-0.512857	0.608051	0.956718
kmt5b	1196.939827	0.103348237	0.0971494	1.063807	0.287416	0.845979
anpepa	91.32373791	-1.051588283	0.2125791	-4.946808	7.54E-07	7.89E-05
arpin	129.2765139	-0.315990895	0.1904582	-1.659109	0.097094	0.593855
LO018380.1	11.18543119	-0.036288092	0.5697619	-0.06369	0.949217	0.99381
pgpep1l	10.52685606	0.706790513	0.5883733	1.2012621	0.22965	NA
barx2	85.9749261	0.054118203	0.2313481	0.2339254	0.815043	0.981081
cftr	96.1128936	-0.083001443	0.2131189	-0.389461	0.696935	0.96736
ctsh	236.1396542	0.030012185	0.1457406	0.2059288	0.836847	0.984636
dnajc3a	414.0351849	-0.024424187	0.1129522	-0.216235	0.828805	0.982721
wdr18	327.1463103	-0.08082518	0.1186649	-0.681121	0.495795	0.932377
cnfn	171.5058641	0.123453428	0.1706411	0.7234682	0.469392	0.924505
wnt2	47.41501638	-0.227623728	0.2799237	-0.813164	0.416124	0.906237
ceacam1	1516.308445	0.17364832	0.0857806	2.0243309	0.042936	0.407008
rapsn	239.2728677	0.216528714	0.1453466	1.4897405	0.136292	0.673089
dhrs13a.3	345.5871768	0.19642337	0.1188188	1.6531332	0.098304	0.597502
ddb2	600.532925	0.012473961	0.109379	0.1140434	0.909203	0.990702
cryba1a	97.64339286	0.158950775	0.2023144	0.785662	0.432066	0.912882
paqr5a	75.40081048	-0.162779938	0.2333172	-0.697677	0.485379	0.930209
slc17a6b	1801.425853	0.2310114	0.0868904	2.6586527	0.007845	0.142065
morf4l1	956.3766641	-0.075672701	0.0891218	-0.849093	0.39583	0.899464
si:ch211-63p21.4	2.851356728	-0.140069982	1.1048656	-0.126776	0.899118	NA
rab32b	5.148286498	-1.266885892	0.8578842	-1.476756	0.139741	NA
aagab	321.5819203	0.079589116	0.1233794	0.6450762	0.518878	0.938919
crygs2	0.804086858	-1.860428069	2.2645275	-0.821552	0.411332	NA
adra1bb	12.8009953	0.915852817	0.5665037	1.6166757	0.105948	0.615567
crygm5	495.4747322	0.035417563	0.1050449	0.337166	0.735992	0.972535
rpl4	27581.11548	-0.247866871	0.0729997	-3.395449	0.000685	0.02277
alg12	168.283354	-0.060562781	0.1639359	-0.36943	0.711807	0.969006
drap1	1558.578937	-0.081169175	0.0954061	-0.850775	0.394894	0.899087
slc6a11	133.2640838	-0.117954499	0.1734435	-0.680075	0.496457	0.932461

cetn4	186.6366618	-0.098934006	0.1527392	-0.647732	0.517158	0.938592
xpo6	851.3691299	-0.022884875	0.1161995	-0.196945	0.843871	0.985174
ftr53	10.73237355	0.860974012	0.5826149	1.4777756	0.139468	NA
zmp:0000000608	0.814536469	0.637862482	2.230678	0.28595	0.774916	NA
fam173a	152.6916144	-0.051601751	0.1692688	-0.304851	0.76048	0.975374
cdc40	773.8290355	-0.365652123	0.1027935	-3.557151	0.000375	0.014067
rps29	6730.201574	-0.309091221	0.1804707	-1.712695	0.086769	0.564768
med18	65.93971116	0.234648593	0.2475364	0.9479357	0.343162	0.878434
wdr20b	122.0782021	-0.131396244	0.2007527	-0.654518	0.512778	0.936253
setdb1a	590.4257254	-0.107140908	0.1005504	-1.065545	0.28663	0.845942
si:dkey-24117.6	0.840359184	-3.190376554	2.3269423	-1.37106	0.170356	NA
gemin4	6.674862874	-0.289532775	0.7248148	-0.399458	0.689556	NA
smtnl1	72.52738764	0.304344246	0.2434116	1.2503276	0.21118	0.77879
si:ch211-231m23.4	1.197759375	-0.38361194	1.8179428	-0.211014	0.832876	NA
noxo1a	421.8016773	-0.862426262	0.1500582	-5.74728	9.07E-09	1.55E-06
lim2.5	491.530909	0.044677466	0.110759	0.4033755	0.686672	0.966376
zgc:110796	68.28013773	-0.036338038	0.2412171	-0.150645	0.880256	0.987881
drc3	66.76165782	0.106124869	0.2519549	0.4212058	0.673605	0.965257
crybb3	4.106728178	-0.314439161	0.9580361	-0.328212	0.742751	NA
trak1a	448.6774456	-0.013935856	0.1271887	-0.109568	0.912752	0.991119
mrps25	841.1417233	-0.056985195	0.0955134	-0.59662	0.550761	0.946549
ndufb9	2091.311358	-0.024234127	0.0811082	-0.298788	0.765102	0.975374
rangap1a	847.8873376	-0.206893222	0.1002149	-2.064496	0.038971	0.387434
csdc2a	864.7166134	-0.027796272	0.0910713	-0.305214	0.760203	0.975374
tmem33	915.7351375	-0.013060156	0.1022367	-0.127744	0.898351	0.990121
si:dkey-125e8.4	6.507432229	0.140654576	0.7632028	0.1842951	0.853782	NA
zgc:92380	1062.671474	0.112156482	0.0861097	1.3024835	0.192751	0.756795
mrpl51	678.5334483	0.03030715	0.1004121	0.3018277	0.762783	0.975374
cyp19a1a	1.504272046	-0.28893243	1.4975093	-0.192942	0.847004	NA
ankrd39	56.35459437	-0.090852489	0.2729182	-0.332893	0.739215	0.972732
lgi3	61.78701651	0.252205809	0.2479951	1.016979	0.309163	0.861381
zgc:161969	477.434875	-0.003122619	0.1033825	-0.030205	0.975904	0.996762
ttk	377.7211697	-0.03859304	0.121377	-0.31796	0.750515	0.97419
chmp7	449.1436598	0.016567517	0.1090237	0.1519625	0.879217	0.987881
dctn3	988.8015041	-0.021958648	0.0890731	-0.246524	0.805277	0.979883
zgc:136564	190.2135825	-0.579290986	0.1804328	-3.210564	0.001325	0.038636
si:ch211-57i17.5	3.351088384	-0.556567768	1.1081597	-0.502245	0.615495	NA
rmi1	85.15189643	-0.039576188	0.2238056	-0.176833	0.85964	0.985773
sh2d4a	71.60692567	-0.538044829	0.2368593	-2.27158	0.023112	0.291648
si:ch211-245j22.3	14.11102531	0.447889203	0.5052068	0.8865463	0.375323	0.894316
arntl2	173.4811457	-0.192972266	0.1612076	-1.197042	0.23129	0.799569
si:dkey-283b15.2	203.671369	-0.042007864	0.1459302	-0.287863	0.773452	0.975687
si:dkey-81n2.1	0.815164977	-0.617697568	2.1259573	-0.29055	0.771395	NA
tmed10	2910.445926	-0.081886445	0.0734797	-1.11441	0.265103	0.831033
dnajb1b	296.8533627	-0.09333498	0.1283996	-0.72691	0.467281	0.924328
eif2b2	456.6366186	0.256487223	0.1171522	2.18935	0.028571	0.328246
adgre7	0	NA	NA	NA	NA	NA
ndufa3	970.6639793	0.009802477	0.1230956	0.079633	0.936529	0.992702
zc3h14	618.0410763	-0.27510988	0.1049947	-2.620226	0.008787	0.155004
rmdn3	331.9269746	-0.004091926	0.1286242	-0.031813	0.974621	0.996579
rad51	397.8158496	-0.505818234	0.1235206	-4.095012	4.22E-05	0.002358
adgrg11	5.290010159	0.904667154	0.8594737	1.0525827	0.292532	NA
bmf2	168.8276241	-0.022037627	0.1587769	-0.138796	0.889611	0.989291
atp6ap1a	3291.262052	0.052225985	0.0770437	0.677875	0.497851	0.933482

taz	308.6021936	-0.057741863	0.1347538	-0.428499	0.668288	0.964736
yrdc	204.9508422	0.322682351	0.1459855	2.210372	0.027079	0.317032
ints14	446.8701194	-0.07585216	0.1119116	-0.677786	0.497907	0.933499
bmp2b	213.6781436	0.067394333	0.1483894	0.4541721	0.649705	0.960708
slc24a1	88.20656621	0.286749479	0.2333966	1.2285934	0.219224	0.785734
si:dkey-7c18.24	198.5728601	-0.418394929	0.1958097	-2.136742	0.032619	0.352078
uba52	9959.880505	-0.237445608	0.102704	-2.311942	0.020781	0.272632
spred1	773.6233072	0.107064579	0.0914855	1.1702898	0.241884	0.810713
rab11a	4482.396939	0.024722035	0.0711567	0.347431	0.728268	0.970402
taar12b	0.656489935	-1.4754824	2.3018704	-0.640993	0.521527	NA
pimr183	0.48983248	-0.887840757	2.6934859	-0.329625	0.741683	NA
smg5	1242.743615	0.012499992	0.0840629	0.1486981	0.881792	0.988344
paqr6	205.0944052	0.459954046	0.1561751	2.9451186	0.003228	0.07565
afp4	216.6667932	-0.471282164	0.5641705	-0.835354	0.403518	0.900979
si:ch211-196f5.2	71.33159104	-0.070596191	0.2495811	-0.282859	0.777285	0.975826
ipo4	554.5583943	0.060841182	0.1049082	0.5799471	0.56195	0.948728
tgfb1a	578.7097088	0.17350206	0.1047452	1.6564197	0.097637	0.596644
med4	219.4081284	-0.125892776	0.1557875	-0.808106	0.41903	0.906406
itm2bb	1839.910689	0.037792795	0.1068283	0.3537715	0.72351	0.970402
pex10	57.34277409	0.039716497	0.255624	0.1553708	0.876529	0.987881
rasal1b	7.700903255	-0.112788871	0.6879283	-0.163954	0.869767	NA
ppih	306.1863265	-0.25362322	0.1313526	-1.930858	0.053501	0.453265
ccdc130	128.0735059	-0.281126343	0.1864923	-1.507442	0.131697	0.665187
mrps28	215.3496227	-0.00941193	0.149391	-0.063002	0.949765	0.99381
sult1st2	15.79534637	0.141144021	0.4901397	0.2879669	0.773372	0.975687
gypc	123.0827119	0.221414438	0.1929021	1.1478076	0.251048	0.820469
pimr138	4.978701137	0.964804048	0.9076507	1.0629684	0.287796	NA
unc93a	7.698531823	-0.245720592	0.7036695	-0.349199	0.72694	NA
si:dkey-102f14.5	103.1929575	-0.027475168	0.1934401	-0.142035	0.887053	0.989231
tnfaip1	194.8756979	0.317774352	0.1517312	2.0943241	0.036231	0.37198
ces2	4278.817847	0.036322816	0.2143195	0.1694798	0.865419	0.986385
mrpl39	454.4844839	-0.112165863	0.1062611	-1.055568	0.291166	0.846582
zfpm1	241.2958295	-0.448767766	0.1526436	-2.939971	0.003282	0.076541
trmt10c	119.6784221	-0.081241449	0.1861205	-0.436499	0.662475	0.964665
nudt18	100.3666823	-0.194110609	0.2100698	-0.924029	0.355471	0.882386
znf143b	427.4811738	0.196204731	0.1107016	1.772375	0.076332	0.538373
dhx40	134.2254437	-0.073159051	0.176757	-0.413896	0.67895	0.965257
adprhl1	38.54405737	-0.124034579	0.3242705	-0.382503	0.702088	0.968134
dcun1d2b	771.0918705	-0.145254277	0.109328	-1.32861	0.183977	0.747376
ces3	1957.294547	-0.204369533	0.2893122	-0.706398	0.479941	0.927154
zgc:91860	1472.144785	0.008763193	0.0816271	0.1073564	0.914506	0.991119
wdr83	112.1093858	-0.017457992	0.189031	-0.092355	0.926416	0.992702
zgc:92335	304.3669323	-0.117790355	0.1263082	-0.932563	0.351046	0.880473
eif4ebp3l	1055.089363	-0.303620767	0.1177297	-2.578966	0.00991	0.169473
adarb1a	616.1369962	0.080133948	0.1051758	0.761905	0.446117	0.917154
b3gnt9	32.10438204	0.725225528	0.3545431	2.0455216	0.040803	0.394957
nme5	40.35101122	-0.207493419	0.3109127	-0.667369	0.504537	0.935617
rack1	31085.24002	-0.279433479	0.0824026	-3.391076	0.000696	0.023059
bbs2	663.6627794	0.179613107	0.0999856	1.7963904	0.072432	0.526156
mt2	892.1054631	0.010379975	0.1135453	0.091417	0.927161	0.992702
fam192a	506.8190437	-0.126095658	0.1025337	-1.229798	0.218773	0.785734
si:dkey-194e6.1	89.07259207	0.616772146	0.2228601	2.7675304	0.005648	0.112817
sb:cb37	420.0405269	-0.018832265	0.1566614	-0.12021	0.904317	0.990306
oprd1a	8.121643199	-0.236601982	0.7103749	-0.333066	0.739084	NA

mkrn1	788.751785	0.007401541	0.0922241	0.080256	0.936034	0.992702
si:dkey-105h12.2	89.63157254	0.469908628	0.234389	2.0048241	0.044982	0.417005
usf1	630.9204691	-0.052162682	0.0948017	-0.55023	0.582162	0.952028
bhlhe41	630.0615452	0.033052185	0.095348	0.3466481	0.728856	0.970662
piwil1	29.41727656	-0.398997024	0.3523367	-1.132431	0.257453	0.825314
rfx6	1.7000073	-1.196582402	1.5272193	-0.783504	0.433331	NA
rrbp1b	1039.055196	0.115591855	0.0904007	1.2786607	0.201017	0.766855
si:busm1-228j01.6	0.341486697	1.892317624	3.2124068	0.5890654	0.555817	NA
vgll2a	14.16945041	-1.447907103	0.5272709	-2.74604	0.006032	0.117745
kat14	698.1050051	0.031075867	0.0984106	0.3157776	0.752171	0.974577
bco2b	7.372171731	0.396049169	0.7148633	0.5540209	0.579565	NA
arpc5la	545.730935	0.085050953	0.1003072	0.8479044	0.396491	0.899672
TUBB4B	681.2301082	0.251891031	0.0965164	2.6098275	0.009059	0.159222
glipr2	37.268426	0.394447461	0.3474296	1.1353305	0.256237	0.824633
man1a2	1225.669867	0.061134181	0.08821	0.6930531	0.488276	0.93189
sntb1	290.0649874	-0.177563483	0.138985	-1.277573	0.2014	0.767338
tbc1d32	131.5794866	-0.401068827	0.1827465	-2.194673	0.028187	0.325187
tmem237a	490.7744026	0.193600398	0.1049216	1.8451906	0.06501	0.497901
hsdl1	102.3251471	-0.042259912	0.2001232	-0.211169	0.832755	0.983557
sspn	138.3163811	-0.077099898	0.1814618	-0.424882	0.670923	0.965257
ccdc92	464.9727712	-0.107201587	0.1138922	-0.941255	0.346574	0.8794
ndst3	564.9027115	0.05634892	0.0988001	0.5703329	0.568452	0.948983
trdn	237.4745449	0.04025787	0.1411909	0.2851308	0.775544	0.975687
cx32.3	185.3782518	-0.162693984	0.1533016	-1.061268	0.288568	0.845979
mgat4c	13.62587549	1.12610265	0.5742209	1.9610967	0.049868	0.437835
cx28.1	2.072144456	-0.224981179	1.4581081	-0.154297	0.877376	NA
cx28.9	148.8350393	-0.231744536	0.1954386	-1.185766	0.235715	0.804824
cx43	3239.540419	0.055523635	0.0709657	0.782401	0.433979	0.914001
rnf141	226.9205146	0.023659954	0.1473915	0.1605245	0.872468	0.987846
rps25	9894.080863	-0.427459306	0.1106427	-3.863419	0.000112	0.005406
si:dkeyp-28d2.4	1.133855642	2.462671251	1.9506495	1.2624878	0.206773	NA
tsn	407.6253813	0.060204945	0.1277903	0.4711229	0.637553	0.958872
ccl38a.5	3.106153703	0.767946707	1.0707303	0.7172177	0.47324	NA
tsc22d2	667.1788111	0.035061731	0.0949278	0.3693518	0.711866	0.969006
rh50	7.996757599	0.997870109	0.702841	1.4197665	0.155676	NA
rbm39b	1667.91056	-0.117998306	0.0773541	-1.525432	0.127152	0.656588
ren	24.3119866	-0.063706531	0.4431003	-0.143775	0.885679	0.988936
capn3a	5632.082869	-0.018700118	0.0646925	-0.289062	0.772534	0.975687
tnfrsf19	452.1568471	0.138011532	0.112034	1.2318714	0.217997	0.785379
ift172	394.8595396	-0.109172779	0.1211632	-0.901039	0.367568	0.887458
si:dkey-221h15.4	5.100487897	0.114664748	0.8967764	0.1278632	0.898257	NA
ube2v1	1695.629585	-0.055477572	0.0974485	-0.569302	0.569152	0.948983
rab11ba	1351.229466	0.098450504	0.0815278	1.2075704	0.227213	0.794617
sf3a1	2190.560987	-0.063319994	0.0949173	-0.667107	0.504704	0.935617
cad	1637.986629	-0.250134039	0.0839251	-2.980444	0.002878	0.069835
dnajc5ga	835.6421733	-0.007700704	0.0979795	-0.078595	0.937355	0.99279
ankzf1	134.1924894	-0.02877085	0.1757027	-0.163747	0.86993	0.987395
usp39	585.188752	0.040524167	0.0981326	0.412953	0.679641	0.965311
znf512	35.71345248	0.333563875	0.3580563	0.9315961	0.351545	0.880769
si:ch211-245h14.1	12.92946071	-0.829078142	0.5498494	-1.507828	0.131599	0.665022
stum	39.06489654	-0.140175076	0.3141507	-0.446203	0.65545	0.961632
ccl38a.4	1.609902098	-1.227764369	1.6314598	-0.752556	0.451717	NA
ccl38.1	3.990407809	-0.442504236	1.0041402	-0.44068	0.659445	NA
hsbp1b	954.5022718	-0.065551838	0.0904561	-0.724681	0.468648	0.924328

ccl38.6	8.72585095	-1.457173164	0.6787396	-2.146881	0.031803	NA
cryba2b	15422.38286	0.024918282	0.0737109	0.3380543	0.735322	0.972528
dlg4a	30.92532827	0.16899022	0.3508639	0.4816404	0.630061	0.957354
adat2	72.45007714	-0.2675202	0.2315157	-1.155517	0.247879	0.81711
styk1b	267.6804861	-0.022899039	0.1316981	-0.173875	0.861964	0.985841
selenoo1	362.8145739	0.028107988	0.1278239	0.2198962	0.825952	0.982196
prox2	110.723228	0.18525195	0.2020241	0.9169795	0.359153	0.884751
cxcr4b	112.4714743	-0.113713237	0.1971114	-0.576898	0.564008	0.948739
si:ch211-173a9.6	59.5841638	-1.564148833	0.2726642	-5.73654	9.66E-09	1.63E-06
brk1	2720.390761	-0.020590904	0.0880083	-0.233966	0.815012	0.981081
il17a/f3	2.652710646	-0.759080918	1.159807	-0.654489	0.512797	NA
calhm6	5.277394124	1.131486415	0.9102389	1.2430653	0.213844	NA
adamts6	25.0231691	-0.428452623	0.4004601	-1.069901	0.284664	0.844955
rrp9	282.8360553	-0.223218733	0.135955	-1.641857	0.10062	0.603272
zgc:113337	84.93538682	0.145724548	0.2255295	0.6461442	0.518186	0.938592
si:ch211-63o20.7	75.12842248	-0.26514796	0.2433356	-1.089639	0.275872	0.839541
nfya	1317.352373	-0.043755816	0.087087	-0.502438	0.615359	0.95688
elp3	287.2052153	-0.317988622	0.1333674	-2.384306	0.017111	0.241859
pklr	1106.930156	0.077670395	0.1128809	0.6880741	0.491406	0.931926
cyp11c1	106.8771472	-0.102137832	0.1931382	-0.528833	0.596921	0.954913
fhl2a	789.7639272	0.22202768	0.0957546	2.3187167	0.02041	0.268884
mapk12a	546.5846529	0.002747436	0.1073096	0.0256029	0.979574	0.996866
si:dkeyp-118h3.6	188.6499613	-0.060048432	0.1523035	-0.394268	0.693383	0.96736
prrx1b	384.3778994	-0.094582809	0.1205781	-0.784411	0.432799	0.91326
kpna5	1391.022629	0.002152775	0.0806334	0.0266983	0.9787	0.996762
gorab	99.90923848	0.019556864	0.2199567	0.0889123	0.929152	0.992702
pou3f3a	609.1396541	-0.093850117	0.097646	-0.961126	0.336489	0.876088
mettl11b	32.21688505	1.336160702	0.3520937	3.7949008	0.000148	0.006714
tal2	408.7168102	-0.110116492	0.1163217	-0.946655	0.343815	0.878434
hmg20b	621.0868133	-0.10091584	0.1088722	-0.92692	0.353968	0.881976
zgc:171971	122.989126	0.091635582	0.1824057	0.5023722	0.615406	0.95688
fam129aa	60.98636358	0.165611522	0.2635744	0.6283294	0.529788	0.942209
mau2	718.8405732	-0.03069847	0.0957956	-0.320458	0.748621	0.97419
trmt1l	230.0565041	-0.189746979	0.1595099	-1.189562	0.234219	0.802815
si:ch211-145c1.1	1.939172771	-0.030264765	1.5009051	-0.020164	0.983912	NA
eef2a.2	15.00672274	0.791314263	0.5050683	1.5667472	0.117174	0.640585
pebp1	583.9576237	0.021802512	0.1096809	0.1987814	0.842434	0.985174
fbxl17	29.89398294	-0.596658748	0.3611632	-1.652047	0.098525	0.597984
dmap1	654.2712294	0.041190474	0.1045959	0.3938059	0.693724	0.96736
si:ch73-55i23.1	15.64796612	-0.961088158	0.508338	-1.890648	0.058671	0.474103
btbd2a	937.7545758	-0.007175699	0.0869204	-0.082555	0.934205	0.992702
eef2a.1	476.314322	-0.157520278	0.1104848	-1.42572	0.153949	0.702738
mnx2a	26.88728062	-0.198464746	0.3704146	-0.535791	0.592103	0.954434
si:dkey-44k1.5	210.0649619	0.116109215	0.144496	0.8035465	0.421659	0.90651
dio1	74.94991247	-0.027015601	0.2244687	-0.120354	0.904203	0.990291
bin1a	27.00194764	0.327828831	0.3698902	0.8862868	0.375463	0.894316
tmem198a	90.03040532	-0.268979749	0.2189509	-1.228494	0.219262	0.785734
trim35-40	118.1807352	0.100646752	0.1920685	0.5240148	0.600268	0.956045
si:dkey-97o5.1	167.9163218	-0.106115969	0.1599169	-0.663569	0.506966	0.935734
acot11b	91.45665666	0.277782761	0.2289786	1.2131387	0.225077	0.792185
nudt3b	18.84385957	-0.049787531	0.4322468	-0.115183	0.9083	0.990702
si:dkey-4e7.3	150.5065367	0.034472225	0.1885657	0.1828128	0.854945	0.985174
elmo3	44.39919819	0.458214487	0.29224	1.5679391	0.116895	0.639958
pacsin1b	668.16215	0.000282788	0.1005104	0.0028135	0.997755	0.999174

zp3a.1	0.158795395	0.967652056	4.0804729	0.2371421	0.812547	NA
zp3a.2	3.286247409	0.919792463	1.0722727	0.8577971	0.391004	NA
si:dkey-51e6.1	60.13081291	0.319045933	0.2566546	1.2430943	0.213833	0.782251
selp	3.410047828	-2.583702156	1.2306593	-2.099446	0.035778	NA
fpgt	49.88426882	-0.2735948	0.2843289	-0.962247	0.335925	0.876088
myo6b	172.5431237	-0.136500191	0.1542711	-0.884808	0.37626	0.894789
lhx8b	7.562731881	-0.585371665	0.7048506	-0.83049	0.406262	NA
rbbp5	516.2162653	-0.05164121	0.1093083	-0.472436	0.636615	0.958607
skia	1292.883033	-0.017297003	0.0850682	-0.203331	0.838876	0.984979
ppil6	10.71092272	0.205255168	0.6006714	0.3417096	0.732569	NA
RFESD (1 of many)	36.30202602	-0.099516823	0.3336906	-0.298231	0.765527	0.975374
c7a	191.3923303	0.420701443	0.1763145	2.3860856	0.017029	0.241051
foxl2a	46.77732732	-0.16000976	0.3297606	-0.48523	0.627513	0.957354
drc1	24.62131842	0.162359049	0.3890472	0.4173248	0.676441	0.965257
paplnb	17.92226122	0.696685277	0.5006868	1.3914593	0.164086	0.71945
smek1	2191.474541	-0.01710094	0.0775715	-0.220454	0.825518	0.982196
ttc38	69.7399271	-0.273860551	0.2387632	-1.146997	0.251383	0.820814
tspan33b	34.4996656	0.029728171	0.3692454	0.0805106	0.935831	0.992702
sema3ab	40.40917116	-0.163588028	0.298779	-0.547522	0.58402	0.952214
si:ch211-233h19.2	57.02453224	0.348173705	0.2779401	1.2526931	0.210317	0.778043
tmem243a	28.41585107	-0.185791722	0.3698077	-0.502401	0.615386	0.95688
pias4b	8.709460295	-0.59326102	0.6516516	-0.910396	0.362614	NA
mthfd1l	930.0199711	-0.753703924	0.2523548	-2.986683	0.00282	0.068933
stx11b.2	33.89478214	-1.167566048	0.3657172	-3.192538	0.00141	0.040686
specc1lb	66.33894723	-0.071583277	0.2504673	-0.285799	0.775032	0.975687
fgf16	35.89095161	0.033472548	0.3341272	0.1001791	0.920202	0.992044
atrx	4238.69063	0.028188556	0.0659924	0.4271487	0.669271	0.965082
myl13	2455.744353	0.219926884	0.0855332	2.5712445	0.010133	0.171998
FRMD4A	69.31555878	-0.100631382	0.2501497	-0.402285	0.687475	0.966376
eif4h	2154.112795	-0.033113168	0.080161	-0.413083	0.679546	0.965257
tgfbr1b	49.12622019	-0.087340598	0.2781846	-0.313966	0.753547	0.974981
cdan1	138.6043587	0.108314511	0.1826203	0.5931131	0.553105	0.947265
arsa	131.6913352	-0.058338734	0.1935512	-0.301412	0.7631	0.975374
tmem231	119.8056078	0.208180492	0.2087965	0.9970498	0.31874	0.867746
efnb3b	166.8143457	0.011724823	0.1568826	0.0747363	0.940425	0.992857
itga6a	363.6770508	0.087795125	0.1189032	0.738375	0.460287	0.921978
atp8b3	535.8466728	0.114027604	0.0999449	1.1409045	0.25391	0.823394
dlx6a	135.3203708	-0.367574697	0.1871223	-1.964356	0.049489	0.436093
ca4b	99.25138044	-0.990793187	0.2295878	-4.315531	1.59E-05	0.001043
dlx5a	369.3717396	-0.094508474	0.1208743	-0.781874	0.434289	0.914161
arhgef18b	72.36880449	-0.008648466	0.2378943	-0.036354	0.971	0.996315
cdca4	1166.072679	0.055801662	0.0830818	0.6716476	0.501808	0.934874
si:dkey-177p2.6	710.9411901	0.353564676	0.0988519	3.5767111	0.000348	0.013334
si:dkey-91m11.5	302.5662595	-0.082169673	0.1421461	-0.578065	0.56322	0.948728
plin2	176.4428952	-0.164448459	0.1821432	-0.902853	0.366604	0.88734
impdh1a	475.6700646	0.136194941	0.1056171	1.2895164	0.197219	0.762204
chrac1	224.8963071	-0.02796227	0.1506544	-0.185605	0.852754	0.985174
lpar2a	84.09857601	-0.321278089	0.2187573	-1.468651	0.141928	0.683173
tmem251	327.98462	-0.01288668	0.1198922	-0.107486	0.914404	0.991119
trmt6	156.2664528	0.100795196	0.165393	0.6094284	0.542241	0.944974
si:dkeyp-55f12.3	92.59866665	-0.207330298	0.2277604	-0.9103	0.362664	0.885944
syt1b	15.93141451	0.710186109	0.5035018	1.4104937	0.158394	0.709224
cldnk	220.1199847	0.281162993	0.1534753	1.831975	0.066955	0.506105
kif26aa	255.917481	0.085913674	0.1515418	0.5669305	0.570761	0.949061

ptafr	9.642815051	-0.658116845	0.6551517	-1.004526	0.315125	NA
zgc:103681	56.87956347	-0.334001165	0.2669686	-1.251088	0.210902	0.778412
zbtb25	60.96278449	-0.041552296	0.2538049	-0.163717	0.869954	0.987395
zgc:77752	77.61105626	0.127940534	0.2356772	0.5428635	0.587224	0.953319
syndig1l	46.53624779	-0.125134582	0.2885843	-0.433615	0.664568	0.964736
trim35-30	34.68691986	0.04350216	0.3238492	0.1343284	0.893143	0.989291
derl2	303.4283416	0.067356401	0.1278831	0.5267029	0.5984	0.955488
RALGDS	65.05274773	0.033342561	0.2462765	0.1353867	0.892306	0.989291
rab40b	313.929656	-0.127988905	0.1267617	-1.009681	0.312648	0.864656
galnt17	228.317309	-0.018717659	0.138266	-0.135374	0.892316	0.989291
gstt1a	851.9663178	-0.31110109	0.1010323	-3.079223	0.002075	0.054865
p2rx7	60.16029056	-0.268003631	0.2569054	-1.0432	0.296856	0.853798
p2rx4b	4.588660569	0.635759845	0.9109362	0.6979192	0.485228	NA
mad2l2	169.5598357	-0.115235637	0.1624787	-0.709235	0.478178	0.926857
rfc4	339.183708	-0.17810306	0.1233158	-1.444284	0.148659	0.69636
tlk1a	774.0558904	0.047100611	0.0977491	0.4818519	0.629911	0.957354
ndufa12	1677.780042	-0.080613404	0.1209247	-0.666641	0.505001	0.935724
s1pr3a	45.66598809	-0.108576421	0.2863103	-0.379226	0.70452	0.968134
nr2c2	610.4545028	0.001549918	0.0986826	0.0157061	0.987469	0.997189
tle2a	1205.460833	0.004130238	0.0807478	0.0511498	0.959206	0.995097
foxd5	9.551722696	-0.508145158	0.6237886	-0.814611	0.415295	NA
thap12a	333.6059347	-0.177420276	0.1224938	-1.448402	0.147505	0.694631
si:dkey-250d21.1	7.03717977	0.004865287	0.7191793	0.0067651	0.994602	NA
parp12a	38.86465507	-0.057553797	0.3508394	-0.164046	0.869695	0.987304
ubr3	754.7108244	-0.04709929	0.1080574	-0.435873	0.662929	0.964736
glod4	147.7518099	-0.195152464	0.1723772	-1.132124	0.257582	0.825314
acot20	102.4509302	0.323251604	0.2014479	1.6046411	0.108573	0.620676
acot21	2.725876	0.779510417	1.1753579	0.6632111	0.507195	NA
gpr176	108.1889504	0.252128421	0.1909628	1.320301	0.186735	0.750951
hipk2	38.04047429	0.384772805	0.3424136	1.1237077	0.261137	0.829247
UTP14C	386.8298969	-0.07630184	0.1123081	-0.679397	0.496886	0.932829
si:dkey-251i10.1	57.33152404	0.13619407	0.2527149	0.5389237	0.58994	0.953762
katnbl1	234.9393739	-0.109524609	0.1364436	-0.802709	0.422143	0.90651
ebf2	288.5038434	0.105567124	0.14041	0.7518489	0.452142	0.918781
sebox	74.98094789	-0.087892761	0.2298163	-0.382448	0.702129	0.968134
unga	125.2858041	-0.04701314	0.1901414	-0.247254	0.804712	0.979705
gnat2	11658.35824	0.375790951	0.0819176	4.5874276	4.49E-06	0.000369
nup205	1228.858552	-0.147609278	0.0953233	-1.548513	0.121499	0.647929
gstm.1	696.0980906	-0.056473894	0.106373	-0.530904	0.595485	0.954434
rsrc2	1135.012464	-0.051819137	0.0845169	-0.613121	0.539796	0.944178
actc1a	2089.277405	0.27355934	0.0979287	2.7934545	0.005215	0.106637
si:dkey-48j7.3	0.333314908	-1.858695402	3.5126701	-0.52914	0.596708	NA
ywhaqa	4031.230722	0.140225465	0.075702	1.8523356	0.063978	0.49437
degs2	3.466110453	0.165937656	1.0111213	0.1641125	0.869643	NA
sema3ga	82.32143772	-0.08782831	0.2268577	-0.387152	0.698644	0.967998
tpd52l1	353.0490414	-0.009999258	0.1247447	-0.080158	0.936112	0.992702
mboat2b	517.257621	-0.049079067	0.1163057	-0.421983	0.673037	0.965257
cacna1sb	853.6974829	0.361003908	0.0934876	3.8615144	0.000113	0.005422
pex7	95.87092917	0.102684451	0.2132258	0.4815762	0.630107	0.957354
dym	204.467713	0.008729222	0.1436061	0.0607859	0.95153	0.99381
rnf144ab	390.9996628	-0.224393282	0.1118371	-2.00643	0.04481	0.416612
tnni1c	236.2662577	0.150090903	0.1413607	1.0617581	0.288346	0.845979
lpar2b	185.5188229	-0.064298141	0.1555522	-0.413354	0.679347	0.965257
allc	44.90138098	0.08688512	0.310484	0.2798377	0.779602	0.976146

mis18bp1	80.15519107	-0.226433194	0.2275805	-0.994959	0.319756	0.86809
rps7	18748.08436	-0.293314336	0.0772007	-3.799372	0.000145	0.006625
rnaseh1	250.7518493	0.222707829	0.1439825	1.5467699	0.121919	0.649101
batf3	5.550107214	0.451791535	0.8613234	0.5245318	0.599909	NA
rbm19	329.7477552	0.029659502	0.1208005	0.2455246	0.80605	0.980105
ica1	131.4088307	-0.136558221	0.1754248	-0.778443	0.436308	0.914408
crp3	122.9770529	0.34190788	0.2291252	1.4922318	0.135638	0.671761
sema3h	76.01591248	0.232065936	0.2224354	1.0432958	0.296811	0.853798
gstr	1082.937257	-0.316481972	0.0853703	-3.707166	0.00021	0.008913
cryaba	0.173729368	-0.955901296	4.0804729	-0.234262	0.814781	NA
acbd3	696.0296428	-0.079396657	0.0999748	-0.794167	0.427098	0.910658
nhsl1b	611.5980715	-0.118946691	0.1112474	-1.069209	0.284976	0.845094
hebp2	746.4021564	0.30093729	0.107462	2.8004069	0.005104	0.104597
tert	148.1102911	-0.372297625	0.1993243	-1.867798	0.06179	0.485049
cyp51	491.4717884	-0.429068272	0.1347982	-3.183042	0.001457	0.041826
wtap	1255.379334	0.071078874	0.0878407	0.8091792	0.418412	0.906406
si:ch1073-440p11.2	0.547712847	2.559541715	2.9076406	0.8802813	0.378707	NA
sod2	736.3717221	-0.116633512	0.1044201	-1.116965	0.26401	0.830245
zgc:77784	665.0832932	-0.065191442	0.113351	-0.575129	0.565204	0.948739
rreb1b	493.3165133	-0.220351511	0.1049102	-2.100382	0.035695	0.368007
nrn1b	36.28557163	0.459427342	0.3314592	1.3860752	0.165724	0.721576
acad8	489.0916916	0.096573056	0.1152848	0.8376908	0.402204	0.90084
thyn1	108.9528959	-0.265446491	0.1938767	-1.369151	0.170952	0.728693
fam32a	906.4552168	0.011574889	0.1123852	0.102993	0.917969	0.991623
tsta3l2	0.340386822	-1.882368278	3.2159502	-0.585323	0.558331	NA
si:dkeyp-70f9.7	0.508023798	0.926122713	2.6636728	0.3476864	0.728076	NA
klf2a	390.999517	-0.102739017	0.1344226	-0.764299	0.444689	0.916777
eps15l1a	708.3264031	0.023171795	0.1053839	0.2198799	0.825965	0.982196
odr4	126.0357674	0.30823952	0.1828877	1.685403	0.091911	0.57849
cadm1b	12.11149168	0.047980522	0.5477556	0.0875948	0.930199	0.992702
si:dkey-15j16.3	5.639631857	2.546785026	0.9569556	2.6613408	0.007783	NA
serpinc1	1032.706202	0.222750446	0.1018771	2.1864615	0.028782	0.328978
bora	85.24009196	0.096983988	0.2177486	0.4453943	0.656035	0.961833
zbtb37	30.23128089	-0.039824032	0.3528724	-0.112857	0.910144	0.990702
s1pr1	1615.15792	0.07742767	0.0819583	0.94472	0.344802	0.878434
si:dkey-15j16.6	9.379760131	1.756168322	0.7596786	2.3117254	0.020793	NA
parp6a	339.0106795	0.117801433	0.1351776	0.8714567	0.383505	0.895192
cx30.3	267.5547124	-0.003938027	0.1452913	-0.027104	0.978376	0.996762
tuba8l	7186.913546	-0.053781132	0.0807005	-0.666429	0.505137	0.935724
adipor1b	449.7591291	0.00796179	0.1142331	0.0696977	0.944434	0.993364
blnk	25.93375236	0.125265671	0.38538	0.3250445	0.745147	0.974094
gfra2a	31.59991778	0.021006294	0.3495427	0.0600965	0.952079	0.993904
supt5h	2612.282036	-0.123197354	0.0733822	-1.678845	0.093182	0.580702
cebpb	1972.945549	0.018071414	0.1116972	0.1617893	0.871472	0.987754
grb7	48.66643885	-0.221770276	0.2851824	-0.777644	0.436779	0.914408
exo5	79.86261142	-0.386605046	0.2173681	-1.778573	0.07531	0.534207
plaa	528.18057	0.048079878	0.1145612	0.4196873	0.674714	0.965257
tmem189	831.5452388	-0.005956872	0.098745	-0.060326	0.951896	0.993864
slc52a3	61.28907051	-0.347954018	0.2580352	-1.348475	0.177506	0.738343
stx6	902.6935761	-0.073307275	0.0871239	-0.841414	0.400116	0.90084
coasy	312.4313925	-0.036384066	0.1267425	-0.287071	0.774058	0.975687
riok3	1321.574335	0.039926858	0.0796276	0.5014201	0.616076	0.95688
si:ch211-282j17.11	0	NA	NA	NA	NA	NA
ctsl2	125.76109	0.124383859	0.1784362	0.6970776	0.485754	0.930598

cnstb	45.87246326	0.013123166	0.2981032	0.0440222	0.964887	0.996119
ndufa11	711.5266624	-0.128803356	0.1320282	-0.975574	0.329275	0.873436
apoba	4651.342289	0.291200275	0.0998154	2.9173884	0.00353	0.07986
gdf7	20.80817834	0.758824762	0.4435083	1.7109595	0.087089	0.565361
git2b	243.7957308	0.141944851	0.1344486	1.0557553	0.29108	0.846568
tpp1	377.6620129	-0.032413756	0.1162882	-0.278736	0.780447	0.97627
yy1a	1500.19982	-0.111817444	0.0851714	-1.312852	0.189233	0.753954
atpaf1	530.8862261	-0.021099103	0.1004402	-0.210066	0.833616	0.983583
slc16a4	414.6758069	0.266838776	0.1101724	2.4220118	0.015435	0.22845
or128-10	2.997695393	0.316882015	1.0955809	0.2892365	0.7724	NA
fgf1b	36.42528378	0.092004396	0.3204876	0.2870763	0.774054	0.975687
alkal2b	21.8918963	0.306784244	0.4116541	0.7452476	0.456122	0.921215
mmp9	1149.050459	-0.558679783	0.2516935	-2.219683	0.02644	0.313072
agps	142.7918022	-0.113875682	0.1708673	-0.666457	0.505119	0.935724
spcs2	994.3571698	-0.131318027	0.1029069	-1.276086	0.201925	0.76786
nfe2l2a	1703.386238	-0.086729246	0.0791291	-1.096047	0.273058	0.838141
rnf169	267.0922442	0.220701561	0.1280759	1.7232086	0.084851	0.560519
egr2b	72.21357561	-0.637060185	0.2336283	-2.726811	0.006395	0.122178
adoa	275.5699255	-0.233560094	0.1286279	-1.815781	0.069404	0.514708
si:dkey-30j22.1	34.19931267	-0.300755103	0.3862441	-0.778666	0.436177	0.914408
rffl	172.1077334	-0.054863796	0.1572777	-0.348834	0.727214	0.970402
tfdp2	675.5465896	-0.055228352	0.0954585	-0.578559	0.562887	0.948728
atp1b3b	4655.184998	0.115467543	0.0709889	1.6265567	0.103831	0.610348
dph6	105.977665	-0.270558191	0.1987157	-1.361534	0.173345	0.732435
eml1	1223.235896	-0.018576175	0.0846534	-0.219438	0.826309	0.982196
oxt	79.64734354	0.437197051	0.2361625	1.8512552	0.064133	0.495295
disp2	383.9700342	0.062912888	0.1137732	0.5529677	0.580286	0.951457
pcmtl	220.9354174	-0.13466438	0.1603078	-0.840036	0.400888	0.90084
nphs2	38.13745698	-0.191462973	0.3203078	-0.597747	0.550009	0.94648
srp9	285.2268202	-0.407517628	0.1407772	-2.89477	0.003794	0.08411
ivd	1314.720183	0.046873457	0.0781325	0.5999226	0.548558	0.946195
ephx1	362.7516183	-0.603676201	0.1259706	-4.792197	1.65E-06	0.000152
itpka	181.8307433	-0.065136634	0.1518758	-0.428881	0.66801	0.964736
si:ch211-246m6.4	62.60168149	0.380646557	0.2570467	1.480846	0.138648	0.676943
slc5a6a	12.46304923	-0.183316817	0.528169	-0.34708	0.728531	0.970604
ltk	73.34444519	-0.165113905	0.2409322	-0.685313	0.493147	0.931926
rpl7l1	753.6056451	-0.078487529	0.1172644	-0.669321	0.503291	0.935304
cx35.4	285.3438938	0.170078538	0.1348666	1.2610868	0.207278	0.775175
zdhhc8a	92.56677213	-0.028203387	0.2306533	-0.122276	0.90268	0.990121
slc25a27	38.08034997	-0.145638393	0.3344615	-0.435441	0.663242	0.964736
phlda2	375.2961673	0.088775613	0.1441073	0.6160385	0.537869	0.943336
abracl	885.841536	0.148869621	0.0966778	1.5398529	0.123596	0.651905
heca	191.8515671	-0.045387121	0.1509715	-0.300634	0.763694	0.975374
si:ch211-222f23.6	12.04956038	0.359678576	0.6011775	0.5982902	0.549646	0.946312
nthl1	119.1006387	-0.217812849	0.1901356	-1.145566	0.251975	0.821478
TNPO2	846.0851825	0.07350873	0.0942984	0.7795334	0.435666	0.914408
paip1	822.7792812	-0.096652222	0.0949145	-1.018308	0.308531	0.861088
tyms	414.1542999	0.006802407	0.1116993	0.0608993	0.951439	0.99381
si:ch211-163l21.8	136.8665634	0.026663205	0.1794293	0.1486	0.881869	0.988344
gtpbp1l	184.408263	0.234152641	0.1658269	1.4120302	0.157941	0.709157
afg1lb	77.15648821	-0.370289548	0.2306411	-1.605479	0.108388	0.620165
foxo3b	1451.307646	0.112178549	0.076708	1.4624099	0.143629	0.686804
rpl10a	22013.80005	-0.287890917	0.0988063	-2.91369	0.003572	0.080595
hsh2d	31.39007013	-0.170193362	0.370024	-0.459952	0.64555	0.96029

sult3st3	113.8816406	-0.700586056	0.1953994	-3.585405	0.000337	0.012973
gpr173	51.00063953	0.010544791	0.2745427	0.0384086	0.969362	0.996315
mfng	57.66653745	0.492713604	0.2643834	1.8636327	0.062373	0.487498
mapre1a	148.7528485	0.172632411	0.166204	1.0386778	0.298955	0.855008
tmem120b	56.42128478	0.323968593	0.2822369	1.1478604	0.251026	0.820469
bloc1s6	594.5872155	0.015503163	0.1120812	0.1383209	0.889987	0.989291
ccn2a	3400.720863	-0.190634859	0.07793	-2.446231	0.014436	0.218572
fam228a	24.1401468	0.638293359	0.3905103	1.634511	0.102152	0.607531
si:dkey-256e7.5	19.95316065	-0.036033223	0.4211162	-0.085566	0.931811	0.992702
nab1a	202.4613104	0.31259077	0.1599843	1.9538841	0.050715	0.44139
hook1	532.6189489	-0.369106643	0.1022956	-3.608237	0.000308	0.012095
mstna	0.158795395	0.967652056	4.0804729	0.2371421	0.812547	NA
dnajc5aa	216.7513451	-0.004789806	0.1477996	-0.032407	0.974147	0.99644
cyp2n13	86.36875076	-0.980217502	0.2441655	-4.014561	5.96E-05	0.003192
slc17a9a	32.14673482	0.142216402	0.349308	0.4071375	0.683907	0.965679
cyp2ad6	122.2746553	0.714346866	0.1999766	3.5721516	0.000354	0.013516
zgc:100920	193.7779154	-0.610042323	0.1799758	-3.38958	0.0007	0.023147
dgcr2	377.2830449	0.073861868	0.1251198	0.5903289	0.55497	0.947449
znf1035	921.4588544	-0.072168979	0.095683	-0.754251	0.450699	0.918761
gng8	88.48497023	-0.101995651	0.2091887	-0.487577	0.625849	0.957354
zgc:136605	1.167462343	2.488720452	2.0131823	1.2362121	0.21638	NA
auh	661.1544352	-0.070507764	0.1023449	-0.688923	0.490872	0.931926
nfil3	782.1985373	-0.193926873	0.126176	-1.536955	0.124304	0.653027
cyp2p6	176.3626312	0.234134321	0.1579151	1.4826598	0.138165	0.675945
cyp2p7	104.5876589	-0.141006041	0.1958941	-0.719808	0.471644	0.924838
cyp2p8	175.6598213	-0.245306136	0.156324	-1.569216	0.116598	0.639148
has1	3.257128324	1.236982754	1.1095362	1.1148647	0.264908	NA
fpr1	65.6639515	-0.226546308	0.2701043	-0.838736	0.401617	0.90084
slc24a2	1335.266293	0.27756057	0.1196813	2.3191634	0.020386	0.268884
si:dkeyp-84f3.5	474.7035447	0.158577555	0.1068755	1.4837592	0.137873	0.675313
cyp2p10	94.78116033	-0.477382325	0.2072822	-2.303055	0.021276	0.275152
crygs1	0.340386822	-1.882368278	3.2159502	-0.585323	0.558331	NA
prss1	1584.786914	-0.563605697	0.2286242	-2.465206	0.013693	0.210066
sptlc1	512.6410204	-0.087110115	0.1133487	-0.768514	0.442182	0.916336
vmo1a	7.326777918	0.939289759	0.7994518	1.1749173	0.240028	NA
pcyt2	933.3688885	0.002786646	0.0843528	0.0330356	0.973646	0.996315
si:dkeyp-117h8.4	72.30160218	0.198091106	0.24732	0.8009507	0.42316	0.907185
gnav1	126.0636652	-0.078490316	0.181601	-0.432213	0.665587	0.964736
fam43a	986.7568203	-0.041916223	0.0860147	-0.487315	0.626035	0.957354
camk2d1	561.3314134	0.153860983	0.1012485	1.5196366	0.128602	0.659646
chst14	58.31483053	-0.321559585	0.2551436	-1.260308	0.207558	0.775175
napgb	433.3348022	-0.027473845	0.1268874	-0.216522	0.828581	0.982721
exoc1	1250.05021	-0.207001424	0.0843328	-2.454578	0.014105	0.214639
slc26a11	161.3941231	-0.148565625	0.1638278	-0.90684	0.364491	0.886564
ttbk2b	650.3336175	0.170424787	0.1058662	1.6098123	0.107439	0.617344
ints10	270.6379941	-0.004224172	0.1356094	-0.03115	0.97515	0.996762
capn3b	273.1505696	0.223647494	0.1374594	1.6270074	0.103736	0.610314
fbxl5	1136.833689	-0.127603045	0.0899942	-1.417903	0.156219	0.70658
hiat1b	709.5755931	-0.065226436	0.097533	-0.668763	0.503647	0.935304
nog2	253.2702265	-0.075552416	0.14636	-0.516209	0.605708	0.95651
grp	21.42787689	0.185564037	0.4139452	0.4482816	0.65395	0.961214
nisch	718.5414237	-0.01667577	0.0950642	-0.175416	0.860753	0.985773
MMP23B	63.07009917	0.271627443	0.2506782	1.0835703	0.278555	0.842483
ctsz	655.7374188	0.060417282	0.1008034	0.5993578	0.548934	0.946312

abhd17ab	99.47847421	-0.108369201	0.202242	-0.535839	0.59207	0.954434
alox5b.1	3.33596603	0.018100104	1.0488862	0.0172565	0.986232	NA
alox5b.2	1.161136035	-2.48301767	1.9168088	-1.295391	0.195185	NA
mpeg1.2	279.0641239	-0.317312255	0.1605563	-1.97633	0.048117	0.432341
gnas	571.4049074	-0.002585508	0.1014932	-0.025475	0.979676	0.99687
kctd6a	190.5102853	-0.223223955	0.1486646	-1.501528	0.133219	0.667628
lxn	314.906444	-0.361346635	0.1418205	-2.547916	0.010837	0.180681
qtrt1	91.4121251	-0.222509885	0.2066763	-1.07661	0.281654	0.84357
blf	31.62677405	0.15301396	0.4174703	0.3665266	0.713972	0.96909
cldne	2033.327074	0.027356251	0.0754098	0.3627679	0.716778	0.96909
notch2	1314.526236	4.55E-05	0.091397	0.0004976	0.999603	0.999704
BX664625.1	2.487393773	0.182899978	1.1958309	0.152948	0.878439	NA
cldnf	308.1843085	-0.07620493	0.1216985	-0.626178	0.531198	0.942209
cux1b	394.9196602	0.212591146	0.1237832	1.7174474	0.085897	0.562802
pomca	74.56639792	0.207919445	0.2454268	0.8471748	0.396898	0.899672
cdca8	424.2928294	-0.367037381	0.1127268	-3.25599	0.00113	0.033958
si:ch211-132f19.7	295.7089923	0.319390628	0.1366866	2.3366639	0.019457	0.261725
or108-3	0.809821446	0.539374789	2.2312571	0.2417358	0.808985	NA
slc1a3b	768.8284848	0.0813639	0.100614	0.8086741	0.418703	0.906406
ucp2	2977.497094	0.107260046	0.0929615	1.1538112	0.248578	0.817939
dnajb13	14.49276504	0.592815484	0.5393611	1.0991068	0.271721	0.836643
cela1.5	8.73201562	-0.824044292	0.648548	-1.270599	0.203871	NA
cela1.4	234.4829651	-0.040331071	0.1482309	-0.272083	0.785558	0.97627
cela1.3	5.548164129	-0.693459255	0.8325453	-0.832939	0.404879	NA
cela1.1	5.713761576	-0.474640653	0.8313196	-0.570948	0.568035	NA
lsm1	133.5813534	-0.091253121	0.1864375	-0.489457	0.624518	0.957354
bag4	205.9929593	-0.097178086	0.161291	-0.602502	0.54684	0.945526
gpd1b	803.6087747	0.154715822	0.1079152	1.4336799	0.151664	0.700137
hhla2a.1	511.6203683	-0.083156145	0.1160254	-0.716707	0.473555	0.924993
mllt1a	487.6009574	0.075075178	0.1090622	0.6883701	0.49122	0.931926
rnasel2	0.513487682	-0.887846295	2.6557956	-0.334305	0.738149	NA
si:rp71-1i20.2	4.438662215	-0.105377682	0.9002366	-0.117056	0.906816	NA
nsun5	113.0207917	-0.094861965	0.1862004	-0.509462	0.610429	0.95688
nfic	10.73460268	-0.315673057	0.5937973	-0.531618	0.594991	NA
ripk4	266.6299138	0.133176985	0.1422518	0.9362059	0.349167	0.880195
adam17a	769.0037967	0.067077143	0.0900061	0.7452508	0.45612	0.921215
kcnh5a	8.972699763	0.259953327	0.6519258	0.3987468	0.69008	NA
nfixa	9.018502278	-0.41923497	0.6657586	-0.62971	0.528884	NA
CT573139.1	6.038970216	-0.49434415	0.8035236	-0.61522	0.538409	NA
ccna1	5.941363019	-1.013224254	0.7878984	-1.285983	0.198449	NA
atg16l2	2.988813769	0.039232244	1.1513535	0.0340749	0.972817	NA
arrb1	27.23858445	0.651055058	0.3767002	1.728311	0.083932	0.557501
si:dkey-222f2.1	595.2406822	0.177183446	0.0998298	1.7748553	0.075922	0.536938
prkchb	101.2634646	0.397479497	0.2210037	1.7985197	0.072095	0.525083
gtf3c3	292.831324	-0.07830026	0.1284012	-0.609809	0.541988	0.944974
irf1b	324.5677757	-0.653723328	0.3323071	-1.967227	0.049157	0.435115
ppm1ab	411.2185593	0.290392784	0.1127768	2.5749331	0.010026	0.171049
ckbb	30104.32821	0.110268725	0.084102	1.3111312	0.189813	0.754269
xylb	90.72672518	-0.287935169	0.2244058	-1.2831	0.199457	0.764896
morc3b	7.878828358	0.352611845	0.6838461	0.5156304	0.606113	NA
mgmt	83.76390722	-0.580458954	0.222699	-2.606473	0.009148	0.160082
aqp12	129.4062951	-0.017806907	0.2120201	-0.083987	0.933067	0.992702
stap2b	194.9953311	-0.433216269	0.1496376	-2.895102	0.00379	0.08411
prrg1	437.752823	0.036373225	0.1143356	0.3181268	0.750389	0.97419

zbtb21	34.50443014	0.050628456	0.3368264	0.1503103	0.88052	0.987881
atg101	351.1663171	-0.046428451	0.1276175	-0.363809	0.716	0.96909
impact	182.3146517	-0.271563638	0.1611113	-1.685566	0.091879	0.57849
nmu	32.17955113	-0.183641528	0.3582864	-0.512555	0.608263	0.956718
gtf3c6	81.45129484	0.199261099	0.2280729	0.8736727	0.382297	0.895192
nop2	512.8090862	0.041068964	0.1075954	0.3816981	0.702685	0.968134
srd5a3	57.54198809	-0.169714164	0.2641298	-0.642541	0.520522	0.939056
obscna	184.0738443	-0.000814915	0.1545303	-0.005273	0.995792	0.998888
ank2b	3850.05399	-0.018356992	0.0710389	-0.258408	0.796092	0.978498
kita	51.24798641	-0.184512265	0.285468	-0.64635	0.518053	0.938592
gsx2	77.0994489	-0.140775473	0.22586	-0.623287	0.533096	0.942969
lnx1	609.179704	0.026415585	0.0981259	0.269201	0.787775	0.976596
fip1l1a	15.07870021	1.411664576	0.5545404	2.545648	0.010908	0.180962
prkcz	44.21562136	0.079233737	0.2866552	0.2764078	0.782235	0.97627
ccdc6a	771.9126927	-0.125238823	0.0955373	-1.31089	0.189895	0.754269
abraxas1	57.34633644	0.099053254	0.2797578	0.3540678	0.723288	0.970402
npy4r	2.783216127	-0.524146356	1.1458538	-0.457429	0.647363	NA
gpx3	139.4958366	0.138908232	0.1833327	0.7576837	0.44864	0.917876
tbl2	352.397452	0.069013966	0.1225436	0.563179	0.573313	0.949869
rmnd5b	1077.113823	-0.062637828	0.0913113	-0.685981	0.492725	0.931926
dph3	20.42882256	-0.007540337	0.430251	-0.017525	0.986017	0.996944
nck2b	261.1898461	0.108079428	0.1308603	0.8259143	0.408853	0.90171
mdh2	9158.103826	0.047134401	0.0658969	0.7152755	0.474439	0.925203
atraid	0	NA	NA	NA	NA	NA
si:dkey-71h2.2	151.821691	0.108651155	0.1756497	0.6185671	0.536202	0.943336
crip3	84.42697223	-0.112915462	0.2333016	-0.483989	0.628393	0.957354
fndc4a	75.05071177	0.155687999	0.2260247	0.6888095	0.490943	0.931926
nitr2a	0.476626891	2.395187592	2.815932	0.8505843	0.395	NA
zgc:171480	316.7664652	-0.112124628	0.1288457	-0.870224	0.384178	0.895192
paox1	100.1806712	0.178395855	0.1952393	0.913729	0.360859	0.885686
rpp21	86.05538086	-0.04530877	0.2110957	-0.214636	0.830051	0.982899
slc8a1b	572.9329862	0.004907593	0.123769	0.0396512	0.968371	0.996315
xkr6b	39.02438506	0.064142992	0.3037837	0.2111469	0.832773	0.983557
tnfaip2a	183.4062928	0.463615377	0.1528269	3.0335983	0.002417	0.06142
eipr1	545.2798158	0.009519171	0.1108688	0.0858598	0.931578	0.992702
b3gntl1	99.29986108	-0.070790452	0.1969796	-0.35938	0.719311	0.969183
si:dkey-5n18.1	20.79051077	0.166144507	0.4352655	0.3817084	0.702678	0.968134
zgc:153665	292.9671651	-0.095100251	0.1459505	-0.651593	0.514664	0.937537
kcnj13	185.3234642	0.238030265	0.1660548	1.4334442	0.151731	0.700137
si:ch211-152f2.3	1.015858219	-2.218611177	2.2570181	-0.982983	0.325616	NA
efhd1	526.2317732	0.205103131	0.1265656	1.620528	0.105119	0.613317
gpr55a	3.166508057	0.146153233	1.0623197	0.1375793	0.890573	NA
itm2ca	2342.126637	0.019305371	0.078659	0.245431	0.806123	0.980105
cab39	115.7166535	0.144353153	0.1980599	0.7288359	0.466102	0.923865
rps5	13316.52554	-0.314251883	0.0814306	-3.859136	0.000114	0.005461
gapdh	20754.38044	-0.126946979	0.2148628	-0.590828	0.554636	0.947449
wu:fj39g12	107.016013	-0.084622779	0.191552	-0.441775	0.658652	0.962545
atp6v1b2	3563.115483	0.025489093	0.0728296	0.3499827	0.726352	0.970402
kcnk3a	3.157587408	-0.144960835	1.0694863	-0.135542	0.892183	NA
ndufb11	843.0827716	0.031123038	0.101031	0.3080543	0.758041	0.975374
sytl3	1.695522939	0.603725578	1.4373587	0.4200243	0.674468	NA
rsph3	27.89635523	0.187814992	0.3588585	0.5233678	0.600718	0.956045
atp2b3a	2982.979486	0.104637264	0.1023411	1.0224367	0.306574	0.860027
tagapb	62.86262106	0.057437884	0.2425634	0.2367953	0.812816	0.981081

opn8c	4.255420478	-0.92993266	0.9468578	-0.982125	0.326038	NA
rbbp8	74.53100619	-0.270183296	0.233706	-1.156082	0.247648	0.81711
b3gnt3.1	83.34728857	0.29529689	0.2179368	1.3549657	0.175428	0.736233
otx5	1075.813496	-0.126094681	0.0909088	-1.387046	0.165428	0.721223
ube2d2	2234.100686	0.042815166	0.0789288	0.5424527	0.587507	0.953319
si:ch211-22i13.2	423.356233	-0.106803914	0.112464	-0.949672	0.342279	0.878434
irf1a	14.74935522	-0.519159388	0.5382702	-0.964496	0.334797	0.875699
cltca	6121.749414	-0.056576772	0.0917041	-0.61695	0.537268	0.943336
scrn2	682.1787862	0.111841835	0.1095955	1.020497	0.307493	0.861088
ccdc124	972.6825765	0.069958013	0.0865554	0.8082452	0.418949	0.906406
rpl11	9720.162231	-0.329353865	0.2193893	-1.50123	0.133296	0.667628
vamp4	717.7672065	0.006765958	0.0976245	0.069306	0.944746	0.993409
prdx6	1283.728129	-0.126300741	0.0840954	-1.501874	0.13313	0.667628
si:dkey-239i20.4	35.59662573	-0.062535258	0.3211524	-0.194721	0.845611	0.985174
si:dkey-239i20.2	59.18509683	-0.720853918	0.2706456	-2.66346	0.007734	0.140565
plpp6	24.92564367	0.368560971	0.3941354	0.9351126	0.34973	0.880195
jun	2231.327765	-0.363483233	0.078068	-4.655985	3.22E-06	0.00028
si:dkey-86e18.1	64.93687891	-0.196774968	0.2530066	-0.777746	0.436719	0.914408
zpr1	275.4403898	0.092292908	0.1274175	0.7243348	0.46886	0.924328
atg4da	320.4730915	-0.019200061	0.14064	-0.136519	0.891411	0.989291
ENKD1	71.77499642	-0.004834616	0.2289555	-0.021116	0.983153	0.996944
ches1	14.96132146	0.183044685	0.5034802	0.3635589	0.716187	0.96909
mrpl40	366.5520815	0.066537399	0.1177876	0.564893	0.572147	0.949494
tmem30ab	846.2729685	0.187373809	0.0965562	1.9405672	0.052311	0.449084
kcnk13b	0.325573202	0.005883621	3.3172783	0.0017736	0.998585	NA
gle1	439.2888625	-0.029508494	0.1094838	-0.269524	0.787527	0.976596
psmc1b	1410.391186	-0.106890524	0.0999891	-1.069021	0.28506	0.845094
zgc:65997	136.373858	-0.186441391	0.1800767	-1.035345	0.300508	0.856251
nepro	78.85477285	-0.290185559	0.229529	-1.264265	0.206135	0.773855
dram2a	8.403413498	-1.267445112	0.6847565	-1.850943	0.064178	NA
KIF2A	936.4675674	-0.067145419	0.0942543	-0.712386	0.476226	0.926403
gadd45aa	202.7543951	-0.185806466	0.1570851	-1.18284	0.236873	0.805854
ifit15	1.87232127	2.165498693	1.697339	1.2758198	0.202019	NA
fas	17.44839295	0.392017587	0.510222	0.7683275	0.442293	0.916336
srd5a2a	194.7792206	-0.274709024	0.1523535	-1.803103	0.071372	0.522931
ca4a	27.15739445	-0.048903919	0.3821105	-0.127984	0.898162	0.990121
rapgef1a	81.00308297	-0.506615706	0.2281344	-2.22069	0.026372	0.312638
eif4ebp1	1380.772716	-0.174266606	0.084306	-2.067073	0.038727	0.38611
pqlc2	451.4434186	0.016409919	0.1300767	0.1261557	0.899609	0.990121
tbc1d9	17.63856922	-0.364449783	0.4684523	-0.777987	0.436577	0.914408
bcas2	658.6410695	-0.157670342	0.1232216	-1.279567	0.200697	0.766855
cmc2	208.8253183	0.122010969	0.1528027	0.7984872	0.424588	0.9085
cenpn	104.6609111	-0.066732102	0.1975517	-0.337796	0.735517	0.972535
ehbp1	791.6566837	0.074753383	0.0921563	0.8111587	0.417275	0.906237
vkorc1l1	162.2778223	-0.259880917	0.1708917	-1.520735	0.128326	0.658954
slc6a8	55.87132222	0.606158762	0.293895	2.0625008	0.03916	0.388646
pnck	4.104508093	1.090391271	0.9725146	1.1212081	0.262199	NA
cxadr	366.8254797	-0.079153874	0.1222196	-0.647636	0.51722	0.938592
cadpsa	101.2305804	0.168913827	0.2245664	0.7521776	0.451944	0.918761
cnih2	544.3056112	0.100236978	0.102326	0.9795848	0.327291	0.872658
faub	154.9923813	-0.118927705	0.1723374	-0.690087	0.49014	0.931926
si:dkey-63b1.1	3.306994042	-0.018682317	1.0894819	-0.017148	0.986319	NA
glrx5	583.7985756	-0.06285959	0.1050864	-0.598171	0.549726	0.946312
abt1	124.2132439	0.295912708	0.1860961	1.590107	0.111811	0.627242

zgc:100997	89.91247832	-0.277986817	0.219706	-1.265267	0.205776	0.773784
si:dkey-174m14.3	628.212093	0.132707283	0.1016518	1.3055088	0.19172	0.755945
ylpm1	788.6741521	0.063270322	0.0977614	0.6471914	0.517508	0.938592
bpnt1	286.9302447	-0.735504565	0.1320381	-5.570395	2.54E-08	3.90E-06
nabp1b	0.809272006	1.852129802	2.2597637	0.8196122	0.412437	NA
si:ch211-59d15.9	64.12454911	0.044890212	0.2425205	0.1850987	0.853152	0.985174
nefmb	1231.887551	0.041650506	0.1069511	0.3894351	0.696954	0.96736
gpd1a	303.4172327	0.257671612	0.1361534	1.89251	0.058423	0.473924
mob3c	73.32118484	-0.415791082	0.2277089	-1.825976	0.067854	0.509884
parvaa	171.8290141	0.070520179	0.1734048	0.4066795	0.684243	0.965824
asf1bb	595.5643199	0.016390569	0.1004468	0.1631765	0.870379	0.987421
cldn5a	81.82172988	0.04524783	0.2182486	0.2073224	0.835758	0.984089
c3a.6	472.4801467	0.200169898	0.3596891	0.5565081	0.577864	0.951024
cdc45	175.7252824	-0.102008501	0.1655027	-0.616356	0.53766	0.943336
cpa4	315.5431427	-0.344027649	0.1491172	-2.307096	0.021049	0.273477
cwc27	242.5446388	-0.084772849	0.1419262	-0.597302	0.550305	0.946549
srek1ip1	96.31233945	-0.219807115	0.2011635	-1.092679	0.274535	0.839136
plac8.1	411.2037912	-0.796520252	0.1150603	-6.922631	4.43E-12	1.39E-09
cops4	1107.656028	-0.049824569	0.0862071	-0.577964	0.563289	0.948739
trmu	99.38789889	-0.135680027	0.203009	-0.668345	0.503913	0.93532
si:ch211-282j17.8	0.515216064	0.926382026	2.6525479	0.3492423	0.726907	NA
efcab11	18.08144405	0.228368259	0.4598438	0.4966214	0.619456	0.957354
nrxn3a	1552.711194	0.07895596	0.0932149	0.8470316	0.396978	0.899672
ptbp3	43.11466748	0.415016352	0.3430977	1.2096156	0.226426	0.793959
si:ch211-282j17.3	0	NA	NA	NA	NA	NA
si:ch211-282j17.2	0	NA	NA	NA	NA	NA
si:ch211-282j17.1	0.31598276	0.005883445	3.3523626	0.001755	0.9986	NA
si:dkey-192d15.2	76.14133813	0.536368945	0.247816	2.1643843	0.030435	0.337343
psmb10	114.7363785	-0.253990749	0.1894014	-1.341018	0.179915	0.741062
si:rp71-7l19.2	2.027271102	-0.399650865	1.4669433	-0.272438	0.785285	NA
arhgdia	2670.299785	-0.120241864	0.0874054	-1.37568	0.168921	0.725919
ms4a17a.7	140.1259597	0.259577473	0.2046478	1.2684108	0.204651	0.77258
cdc5l	1655.097198	-0.102297342	0.0798567	-1.281011	0.20019	0.766271
ms4a17a.1	23.08740213	0.890265023	0.4335773	2.0533016	0.040043	0.391989
zmp:0000000619	32.97878645	0.196197507	0.3420213	0.5736411	0.566211	0.948739
ms4a17a.8	76.54286134	0.286993217	0.2438623	1.1768661	0.239249	0.807768
postna	1094.137872	0.16979944	0.0890538	1.9067069	0.056559	0.46635
st6galnac1.1	99.58296937	-0.261162527	0.2111816	-1.236672	0.216209	0.783589
st6galnac1.2	2.801407445	-0.167989194	1.1069243	-0.151762	0.879375	NA
si:ch211-119c20.2	31.17978186	0.05443708	0.3464706	0.1571189	0.875151	0.987881
nme2a	620.8151454	0.221642653	0.1259665	1.7595369	0.078486	0.545813
klf7b	939.7344293	-0.03107598	0.0928783	-0.334588	0.737936	0.972535
rab3ab	72.13979541	0.238297066	0.2397709	0.9938531	0.320294	0.868202
si:ch73-111k22.2	1098.247036	0.127801182	0.083375	1.5328476	0.125313	0.654648
akap7	165.9234814	-0.338517439	0.1663927	-2.034448	0.041906	0.401871
tmem244	68.52112272	-0.307467612	0.2372992	-1.295696	0.19508	0.759653
sod1	887.9992825	-0.403477522	0.1048903	-3.846664	0.00012	0.005664
ftr54	4.731700852	-0.285175349	0.8805195	-0.323872	0.746035	NA
ppil4	319.6710865	0.002780087	0.1289225	0.021564	0.982796	0.996944
amd1	5037.102523	0.198487853	0.0724277	2.7404966	0.006135	0.118579
ufm1	732.803213	0.055615677	0.0949095	0.5859863	0.557885	0.948478
cdk19	20.08136757	0.637456425	0.4435578	1.437144	0.150677	0.698912
nptnb	1190.391549	0.056588613	0.0854213	0.6624651	0.507673	0.935734
timm9	388.6597048	-0.223047496	0.1199341	-1.859751	0.062921	0.489621

arid4a	1458.084911	-0.107783664	0.0798511	-1.349807	0.177078	0.738098
mmachc	38.91533217	-0.229372592	0.3108591	-0.737867	0.460595	0.921978
nup43	221.8556127	0.009177907	0.1470762	0.0624024	0.950242	0.99381
si:ch211-214k5.3	0.657159727	-2.841717825	2.5166161	-1.129182	0.258821	NA
timm10b	149.994827	0.154535232	0.1699599	0.9092455	0.363221	0.885944
psma6l	52.03732577	-0.484823946	0.2837867	-1.70841	0.08756	0.566637
ugt5g2	0.484368597	0.855487352	2.7040894	0.316368	0.751723	NA
gabrr1	243.4994449	0.260470801	0.1484493	1.7546114	0.079326	0.547489
pnrc1	37.82816399	0.120302847	0.3571106	0.3368784	0.736209	0.972535
rnf14	373.6701966	0.132100443	0.1154328	1.1443925	0.252461	0.82189
fgf11b	433.4846931	0.048371205	0.1172814	0.412437	0.680019	0.965311
mei4	2.259900554	1.31216772	1.3339459	0.9836739	0.325276	NA
FO904943.1	3.554644412	-1.317807944	1.0656499	-1.236624	0.216227	NA
sox9b	936.2172577	0.049279245	0.0890638	0.5533025	0.580056	0.951257
gpn1	237.1537512	-0.011482808	0.1353459	-0.08484	0.932388	0.992702
stmn4l	1309.469755	-0.279330018	0.1028636	-2.715538	0.006617	0.124967
il17a/f1	0.173729368	-0.955901296	4.0804729	-0.234262	0.814781	NA
il17a/f2	0	NA	NA	NA	NA	NA
lrrc57	217.6384042	0.019803767	0.1609063	0.1230764	0.902047	0.990121
rpf2	549.366375	-0.035437805	0.1212061	-0.292376	0.769999	0.975687
fgf18b	44.31325591	0.558338129	0.2905183	1.9218692	0.054622	0.457793
si:ch211-241j12.3	292.3556335	0.074769694	0.1274477	0.5866697	0.557426	0.948478
lrpprc	2426.03382	-0.070767117	0.0742974	-0.952485	0.340851	0.878134
ppp2r3c	284.6860284	-0.054003146	0.1369312	-0.394382	0.693299	0.96736
znf292b	1232.257787	-0.181885591	0.0858272	-2.119207	0.034073	0.359261
etf1b	3713.863704	0.041417635	0.0722745	0.5730603	0.566604	0.948739
tube1	0	NA	NA	NA	NA	NA
setd6	106.3649258	0.076468066	0.1944974	0.3931573	0.694203	0.96736
cyp2x8	19.53431681	0.697734492	0.4783168	1.4587288	0.14464	0.688975
lgals3b	1243.298122	0.014593157	0.0828771	0.1760819	0.86023	0.985773
cyp2x7	164.6985182	0.638020656	0.1629622	3.9151456	9.03E-05	0.004478
loxl2a	561.8508314	0.252123435	0.0991077	2.5439349	0.010961	0.181681
xkrx	306.6576868	-0.035870709	0.1265501	-0.283451	0.776831	0.975826
gfra2b	118.6172736	0.230104279	0.2001929	1.1494128	0.250386	0.820164
myo6a	1831.259155	-0.306459668	0.076395	-4.011516	6.03E-05	0.003224
nkl.4	0.158795395	0.967652056	4.0804729	0.2371421	0.812547	NA
tmem86a	15.43388681	-0.331389976	0.5416907	-0.61177	0.54069	0.944633
zbtb2a	23.82753227	-0.41350377	0.4012605	-1.030512	0.30277	0.85729
stx11a	28.98394276	0.144334936	0.3840316	0.3758413	0.707035	0.968664
rassf2b	9.767585071	-0.012740553	0.6431717	-0.019809	0.984196	NA
slc18b1	82.73535315	-0.887332126	0.2170999	-4.087207	4.37E-05	0.002418
prnpb	51.81445488	0.094113857	0.2675156	0.351807	0.724983	0.970402
vsnl1b	1501.720318	0.119740319	0.0780387	1.5343702	0.124939	0.654531
ahi1	334.2109683	-0.025094347	0.1214386	-0.206642	0.836289	0.984231
lft2	4.927306551	0.763554967	0.8970892	0.8511472	0.394688	NA
adora4a	0.339758314	0.005883863	3.2682461	0.0018003	0.998564	NA
ctbp2a	2203.584456	-0.005233502	0.0801788	-0.065273	0.947957	0.99381
TATDN3	122.2680852	-0.001405656	0.1801253	-0.007804	0.993774	0.998672
fuca2	919.5679037	-0.111682586	0.0977002	-1.143115	0.252991	0.822599
loxl2b	1098.543241	0.303002259	0.0840093	3.6067694	0.00031	0.012137
peli1b	191.3178087	0.143049686	0.1513002	0.9454696	0.344419	0.878434
zmpste24	834.4931636	0.128750514	0.0933233	1.3796186	0.167704	0.724848
pitpnab	892.7993734	0.205037773	0.0961633	2.132183	0.032992	0.353977
atp5if1b	1502.409704	-0.007373287	0.0830366	-0.088796	0.929244	0.992702

rpl13a	16898.13444	-0.223101047	0.0818745	-2.724914	0.006432	0.122415
gfpt2	206.4983357	-0.34512959	0.1435089	-2.404934	0.016175	0.23484
egr2a	43.23510956	0.531150218	0.3139238	1.6919717	0.090651	0.576398
znf593	340.6471442	-0.073925188	0.1244524	-0.594004	0.55251	0.947265
cyb561d2	15.79552787	0.775644194	0.4805797	1.6139761	0.106533	0.615567
txn	448.8568756	-0.056982087	0.1265977	-0.450104	0.652636	0.960708
si:ch211-214p16.1	1.512134105	-0.953111348	1.578721	-0.603724	0.546027	NA
ogna	668.1959944	0.262608642	0.0988912	2.6555296	0.007918	0.142994
iars2	826.5826442	0.050644461	0.0875335	0.578572	0.562878	0.948728
cenpp	146.4496823	0.005761348	0.1692184	0.0340468	0.97284	0.996315
rab3gap2	651.8810203	0.035185415	0.099492	0.3536508	0.723601	0.970402
acss1	300.7076262	-0.390205534	0.1590889	-2.452751	0.014177	0.214979
nol8	170.499866	0.107101987	0.1624902	0.6591288	0.509813	0.936253
wapla	128.1791311	-0.010863338	0.1773696	-0.061247	0.951163	0.99381
ppp1cb	4291.527436	-0.079732311	0.0711317	-1.120911	0.262326	0.830166
mafaa	390.8567851	0.153844884	0.1113774	1.3812931	0.167189	0.723887
grid1b	122.5609471	0.125464617	0.193042	0.6499343	0.515735	0.9377
padi2	1374.18897	0.338982618	0.0809825	4.1858751	2.84E-05	0.001682
prelid1b	53.81635351	0.073814514	0.2926545	0.2522241	0.800868	0.979377
oxtrl	4.177494113	0.603235684	0.9250875	0.652085	0.514346	NA
rbms2a	906.7547151	-0.008691001	0.0939797	-0.092477	0.926319	0.992702
stau1	2566.035569	0.006278115	0.0774055	0.0811069	0.935357	0.992702
prkab1a	650.5744325	-0.002404345	0.1019526	-0.023583	0.981185	0.996944
gxylt2	161.6457413	0.108403852	0.1623991	0.6675151	0.504443	0.935552
tcta	388.3090433	-0.030204388	0.1190448	-0.253723	0.79971	0.979377
gnat1	2102.357003	0.302277079	0.1100651	2.7463492	0.006026	0.117745
sumf1	252.6269488	-0.251790684	0.1379283	-1.825519	0.067923	0.510097
endou	35.60607137	-0.340212599	0.3199559	-1.063311	0.287641	0.845979
fto	241.0239053	-0.091765628	0.1476817	-0.621374	0.534353	0.943336
si:dkey-27b3.2	733.100068	0.000988305	0.0936675	0.0105512	0.991582	0.997679
BX005421.1	0.666939546	1.487980453	2.2924711	0.6490727	0.516291	NA
dram2b	370.0614138	0.084611335	0.1277403	0.6623698	0.507734	0.935734
snx18b	42.37585499	-0.200484072	0.2944882	-0.680788	0.496006	0.932377
rasgef1bb	405.9340145	-0.063933293	0.1222399	-0.523015	0.600964	0.956045
paqr3b	117.9362915	0.391143917	0.4899472	0.798339	0.424674	0.9085
anxa3b	735.363216	-0.076726419	0.0993042	-0.77264	0.439735	0.916082
rbm18	340.8397816	-0.172927407	0.1249786	-1.383656	0.166464	0.722981
si:ch211-243g18.2	819.3836356	0.073885041	0.1003977	0.7359234	0.461777	0.922466
pimr71	1.352428212	-0.694422538	1.6416302	-0.423008	0.67229	NA
cdc37l1	93.59241976	0.123912168	0.2064123	0.6003139	0.548297	0.945871
sumo1	1291.380023	-0.032883257	0.0858228	-0.383153	0.701606	0.968134
spp1	65.97555244	-0.092805542	0.2481716	-0.373957	0.708436	0.968664
synpr	60.47576771	0.071321041	0.2521465	0.2828556	0.777288	0.975826
opn1mw3	0.641435609	-0.01573847	2.4541227	-0.006413	0.994883	NA
opn1mw2	436.3961539	0.79352688	0.1362204	5.825314	5.70E-09	1.02E-06
erbin	1287.488765	-0.178446017	0.0927172	-1.924626	0.054276	0.456511
smc5	350.3029915	-0.288600894	0.1216889	-2.371628	0.01771	0.246724
rph3al	0	NA	NA	NA	NA	NA
pip5k1ba	105.1579932	-0.092901494	0.1980982	-0.468967	0.639093	0.959927
phax	384.462303	0.042509417	0.1376397	0.3088457	0.757439	0.975374
lmnb1	2489.568166	0.06351035	0.076185	0.8336338	0.404487	0.901305
atf1	840.5207866	-0.093063701	0.0975656	-0.953857	0.340156	0.87805
prrc1	567.1546914	-0.002960646	0.102463	-0.028895	0.976949	0.996762
itgb7	24.42359329	-0.193116616	0.3901006	-0.495043	0.62057	0.957354

fstl4	132.7538454	-0.095138853	0.1794126	-0.53028	0.595918	0.954434
zgc:193690	59.05304919	-0.440036732	0.253771	-1.733991	0.08292	0.555438
BX950188.1	13.55923507	-0.218680205	0.5382357	-0.406291	0.684529	0.966043
tmem185	448.1140375	0.003846185	0.1118636	0.0343828	0.972572	0.996315
ankrd46a	1198.703789	-0.019199293	0.0849209	-0.226084	0.821136	0.981748
zranb2	797.5053386	-0.105062739	0.0950386	-1.105474	0.268954	0.834243
si:ch211-218m3.16	4.283202237	-0.685089387	0.9268254	-0.739178	0.459799	NA
or115-6	0.50625413	-0.940106488	2.8357787	-0.331516	0.740255	NA
or115-7	1.499238245	2.909600561	1.7684723	1.6452622	0.099916	NA
rp2	448.874928	0.020090906	0.1179329	0.1703588	0.864728	0.986052
chst7	227.591244	0.100606594	0.1459139	0.6894928	0.490513	0.931926
or115-13	0	NA	NA	NA	NA	NA
cyfip1	1797.914358	-0.012933076	0.0804785	-0.160702	0.872328	0.987846
CT030712.1	3.346133651	0.580591656	1.0341509	0.5614187	0.574512	NA
tp63	637.1118026	0.117429463	0.1039478	1.1296969	0.258604	0.826732
tbc1d23	951.0410007	0.060091612	0.0882537	0.6808966	0.495937	0.932377
unc119b	1207.235431	0.077175083	0.0867952	0.8891634	0.373915	0.893677
angptl3	209.0255385	0.67520781	0.1597923	4.225534	2.38E-05	0.001457
atg4c	233.4750057	-0.05075559	0.1498281	-0.338759	0.734791	0.972021
zgc:158291	894.8094467	-0.007484749	0.0944075	-0.079281	0.936809	0.992702
rbmx2	132.1789483	-0.426507453	0.207855	-2.051947	0.040175	0.392132
siah2l	576.6560767	0.179109052	0.1214041	1.4753128	0.140128	0.679809
cldn2	227.5022604	0.097101635	0.1375731	0.7058184	0.480301	0.927397
wdr78	19.42751227	0.304828307	0.4400386	0.6927308	0.488479	0.931926
nop16	217.9285229	0.061209669	0.1716152	0.3566682	0.72134	0.969666
pars2	70.18918183	-0.034308721	0.2449593	-0.140059	0.888613	0.989291
ttc4	201.8944769	0.122887975	0.146855	0.836798	0.402706	0.90084
csmd1a	5.453372486	0.439849199	0.8063143	0.5455059	0.585406	NA
tlr5a	24.74425639	-0.039238148	0.4084574	-0.096064	0.92347	0.992588
disp1	199.4031139	0.124054865	0.1528655	0.8115297	0.417062	0.906237
dnajc19	129.9779252	0.002392075	0.187593	0.0127514	0.989826	0.997612
acvr2ba	84.01489564	-0.340897161	0.2180455	-1.563422	0.117953	0.641477
ppig	577.2176375	-0.059606843	0.1057963	-0.563411	0.573155	0.949762
sned1	165.9317083	0.245590524	0.1753661	1.400445	0.16138	0.715364
rps6ka4	49.50664259	-0.245538415	0.2936374	-0.836196	0.403045	0.900848
cdca5	235.6956813	-0.226997693	0.1439763	-1.576632	0.11488	0.635519
si:ch73-194h10.2	237.4486255	-1.325186437	0.1446539	-9.161081	5.14E-20	4.82E-17
gfi1ab	60.14132514	-0.010550703	0.2589896	-0.040738	0.967505	0.996315
cdc23	275.3188166	0.022011481	0.1330987	0.1653772	0.868647	0.987245
sall4	274.7476077	-0.124333792	0.1279456	-0.97177	0.331165	0.874339
pak1ip1	390.1886655	-0.146200641	0.1211324	-1.206949	0.227452	0.794775
tlr9	0.31759079	1.807926197	3.579045	0.5051421	0.613459	NA
kif20a	409.5657329	-0.073689508	0.111241	-0.662431	0.507695	0.935734
ublcp1	308.9988866	0.13110319	0.1212059	1.0816566	0.279405	0.842882
viml	41.62579076	-0.44651068	0.3217993	-1.387544	0.165276	0.721223
etv5b	929.9029192	0.036026662	0.096627	0.3728427	0.709266	0.968736
ca4c	31.72786612	0.028520122	0.3525932	0.0808868	0.935532	0.992702
tpst1l	451.1774087	0.09084164	0.1093478	0.8307586	0.40611	0.901305
st6gal1	24.77081981	0.2634935	0.3839212	0.6863219	0.49251	0.931926
eef1b2	5690.359838	-0.376999751	0.1913201	-1.970518	0.048779	0.434204
def6b	78.35146374	-0.01551548	0.2364934	-0.065606	0.947691	0.99381
pparda	60.24343134	0.208770578	0.2603723	0.8018156	0.42266	0.906865
camk1ga	242.4102678	0.326677049	0.139291	2.3452848	0.019013	0.257244
slc15a1b	140.8627304	-0.703809052	0.2123269	-3.314742	0.000917	0.029089

nr4a2b	516.9359625	0.025556152	0.1012895	0.252308	0.800803	0.979377
CR631122.1	5.614026907	-0.49250602	0.8243752	-0.597429	0.550221	NA
fkbp2	905.7546394	-0.009593162	0.0874118	-0.109747	0.91261	0.991119
ppp1r14ba	1669.546022	-0.00153182	0.0892274	-0.017168	0.986303	0.996965
zgc:110782	36.93055239	-0.079734215	0.3357287	-0.237496	0.812272	0.981081
zgc:77262	1456.796668	-0.019899884	0.0839011	-0.237183	0.812515	0.981081
zgc:112416	7.142103242	0.492349808	0.7213601	0.6825298	0.494904	NA
hif1al2	54.10475247	0.271250238	0.3070968	0.8832729	0.377089	0.895192
atg9a	647.1352492	0.029789251	0.0999029	0.2981819	0.765564	0.975374
pacs1a	322.2555501	0.103189211	0.1340746	0.76964	0.441513	0.916336
ccr7	2.200169039	-0.208059758	1.2753711	-0.163137	0.870411	NA
cycsb	4614.360418	0.171363553	0.0732301	2.3400703	0.01928	0.259881
ola1	2868.83093	-0.133112824	0.0729541	-1.824609	0.06806	0.51036
fabp6	184.7446125	-1.915979857	0.2178374	-8.795461	1.42E-18	1.04E-15
ttc1	171.6259169	-0.127097819	0.1578839	-0.805008	0.420815	0.90651
cldn19	243.7535321	0.248585554	0.1417139	1.7541372	0.079407	0.547489
cdc42	3799.053598	0.004165281	0.0716591	0.0581263	0.953648	0.994213
rbms3	40.30085763	0.146444686	0.3098523	0.4726273	0.636479	0.958565
vars	1383.152346	-0.125047674	0.0799729	-1.563626	0.117905	0.641446
tex261	228.2846402	0.257217823	0.1454545	1.7683726	0.076999	0.540058
emp2	1269.247086	0.100416192	0.0801865	1.2522824	0.210467	0.778338
pgap2	75.60400563	0.008378379	0.2380965	0.035189	0.971929	0.996315
ncbp3	566.2245227	0.033063587	0.0991474	0.3334792	0.738773	0.972722
plppr2b	33.65599086	-0.034139701	0.3301636	-0.103402	0.917644	0.991623
rtn4a	4262.259116	0.163846753	0.0690909	2.3714668	0.017718	0.246724
stx12l	451.7908393	-0.189017329	0.1065579	-1.773847	0.076088	0.537348
c1qb	1.819038829	4.314240719	3.5266802	1.223315	0.221211	NA
c1qa	3.702316027	0.603058782	1.0916209	0.5524434	0.580645	NA
zak	311.0666103	0.104330847	0.1304886	0.7995397	0.423978	0.908025
birc2	2047.639039	-0.05282397	0.0747581	-0.706598	0.479816	0.927094
polq	59.25973589	-0.014512409	0.251835	-0.057627	0.954046	0.994271
pcf11	1217.167609	-0.008453139	0.0984182	-0.08589	0.931554	0.992702
ccdc90b	62.81463973	-0.29334631	0.2562362	-1.144828	0.25228	0.821887
alg8	143.2674585	-0.166286396	0.1707626	-0.973787	0.330162	0.874097
dtd1	277.6328238	-0.124880606	0.1399469	-0.892343	0.372209	0.892686
guca1d	14.681825	1.046610776	0.5046994	2.073731	0.038104	0.382552
myo7ab	164.3906371	0.124873585	0.1676031	0.7450551	0.456238	0.921215
sc5d	120.1846979	-0.32110956	0.1855753	-1.730347	0.083568	0.557158
st14b	263.6877556	-0.387003033	0.140464	-2.755176	0.005866	0.115189
hp1bp3	8225.158551	-0.062340889	0.0613422	-1.016281	0.309496	0.861604
parapinopsinb	93.26220074	0.148330701	0.2129464	0.6965635	0.486076	0.930833
rbp4l	402.1410148	0.212864467	0.1387639	1.5340044	0.125029	0.654648
nr0b2a	79.07640256	-0.194335067	0.2501363	-0.776917	0.437208	0.914408
dusp4	422.1039891	0.092300251	0.1157088	0.7976941	0.425048	0.908819
ppp1r3b	114.4429539	0.072262315	0.2003745	0.3606363	0.718371	0.96909
eri1	185.5346195	0.093936833	0.1602478	0.5861974	0.557743	0.948478
fybb	37.64750933	0.010868801	0.3121491	0.0348193	0.972224	0.996315
ccdc80l2	200.5346063	0.299072401	0.1461915	2.045758	0.04078	0.394957
or137-2	0.658957134	2.851427774	2.5143902	1.1340434	0.256776	NA
vav2	445.4633203	0.05609205	0.10675	0.5254525	0.599269	0.955949
or115-12	7.589833803	0.071009569	0.7144599	0.0993892	0.920829	NA
ddt	647.8838147	-0.071996375	0.0994755	-0.72376	0.469213	0.924496
p2rx4a	50.94999243	-1.36356122	0.2820089	-4.83517	1.33E-06	0.000128
idh3b	1503.960443	0.050402727	0.0780214	0.6460118	0.518272	0.938661

gnrh2	43.39485038	-0.439421393	0.307918	-1.427073	0.153559	0.702578
si:dkeyp-87d8.8	26.85536519	-0.004535823	0.400111	-0.011336	0.990955	0.997679
gnaia	853.0491716	-0.071152134	0.0931404	-0.763923	0.444913	0.916906
mitd1	103.2536363	0.178923701	0.201893	0.8862303	0.375493	0.894316
tspan10	32.73935658	-0.298569398	0.3627929	-0.822975	0.410522	0.903084
kctd13	570.8044269	-0.093599966	0.0994968	-0.940734	0.346841	0.8794
pou5f3	9.137955491	-1.131795659	0.6621651	-1.709235	0.087407	NA
fut7	5.801381656	0.754985306	0.7856989	0.9609093	0.336598	NA
clic3	130.5773244	-0.076463795	0.1833245	-0.417095	0.676609	0.965257
bace2	204.6865563	-0.139388002	0.1573499	-0.885847	0.3757	0.894316
entpd2b	4.373150299	0.252795547	0.9042089	0.2795765	0.779802	NA
cd8a	0	NA	NA	NA	NA	NA
wdr38	6.652040099	0.15139751	0.7361272	0.2056676	0.837051	NA
aadacl4	3.596934559	-0.907275404	1.2165545	-0.745775	0.455804	NA
dhrs3b	300.346437	-0.004176423	0.1239481	-0.033695	0.97312	0.996315
dck	139.382871	-0.042721862	0.1751409	-0.243928	0.807286	0.980545
slc4a4b	184.1737583	0.248158584	0.1654055	1.5003043	0.133536	0.667982
fpgs	417.8972105	-0.137715152	0.1121994	-1.227414	0.219667	0.786189
cdk9	752.6755339	-0.054385738	0.0903224	-0.602129	0.547088	0.945526
ttc17	502.2395166	0.076910141	0.1159954	0.6630449	0.507302	0.935734
vps33b	254.1322536	-0.081062056	0.1320995	-0.613644	0.539451	0.943908
serinc4	234.3471438	-0.002721065	0.1385524	-0.019639	0.984331	0.996944
cstf1	475.7364582	-0.122566362	0.1091723	-1.122687	0.26157	0.829691
wnt7aa	621.5613906	0.300129128	0.0956598	3.1374643	0.001704	0.047485
zgc:171599	133.8492331	-0.30732337	0.1818372	-1.690102	0.091009	0.578093
wbp2nl	953.0382851	-0.123535405	0.1157819	-1.066966	0.285987	0.845942
opn1lw2	19854.6338	0.312969501	0.0933204	3.3537095	0.000797	0.025763
opn1lw1	4.7098439	1.349805194	0.9216147	1.464609	0.143028	NA
zgc:92107	585.6421407	-0.121855421	0.1031981	-1.180791	0.237686	0.806474
crygm2e	15.4708169	-0.564511894	0.5373267	-1.050593	0.293445	0.849325
zgc:113307	205.3823135	0.123116739	0.1454867	0.8462405	0.397419	0.899672
fmoda	1623.102551	0.211985068	0.0776014	2.7317178	0.006301	0.120958
tmem183a	466.7725018	0.101623569	0.1167033	0.8707858	0.383871	0.895192
prlhr2a	15.07566495	0.347987956	0.5039948	0.6904594	0.489905	0.931926
atp2b4	3722.434171	0.080987899	0.0706571	1.1462099	0.251708	0.821198
suclg2	1136.589822	-0.180689094	0.0838249	-2.155555	0.031118	0.341651
ube2w	216.7836246	-0.093728644	0.1642796	-0.570543	0.568309	0.948983
gdf11	6.297148392	0.318472818	0.7416264	0.4294248	0.667614	NA
cdk5rap1	100.2736126	-0.227934276	0.2007531	-1.135396	0.256209	0.824633
hpdb	184.3653601	0.116020278	0.1712045	0.6776708	0.49798	0.933548
cbx2	419.7966981	-0.443455379	0.134993	-3.285025	0.00102	0.031507
mrpl45	345.8265176	-0.105656126	0.1208923	-0.873969	0.382135	0.895192
npepps	1895.042143	0.055755383	0.0773464	0.7208534	0.471	0.924838
rnf8	340.8875285	-0.134066686	0.1192201	-1.124531	0.260788	0.828804
slit1a	214.9474629	0.15594992	0.1504293	1.0366989	0.299876	0.855995
zgc:114123	1.839504714	1.42777393	1.4235548	1.0029638	0.315878	NA
vcla	438.8051155	0.314194793	0.1130904	2.7782627	0.005465	0.109711
bcap31	802.7692385	0.049945856	0.0910314	0.5486663	0.583234	0.952214
krt98	14.8038423	0.170507815	0.5083909	0.3353872	0.737333	0.972535
krt94	1017.549343	0.070282364	0.0913948	0.7689975	0.441895	0.916336
krt93	18.02101918	-2.318428905	0.5193457	-4.464134	8.04E-06	0.000593
kpna6	63.0156505	-0.191115353	0.2469554	-0.773886	0.438998	0.916082
dhrs3a	208.2625566	0.027198387	0.1593166	0.1707191	0.864445	0.98601
plvapb	816.5983942	-0.095922824	0.093003	-1.031395	0.302355	0.85729

ptprga	105.7072739	0.11805139	0.2168039	0.5445077	0.586092	0.953231
tapbp.2	22.03214295	-0.380984411	0.4127039	-0.923142	0.355933	0.882844
tuba2	5871.463193	0.190499315	0.0747014	2.5501453	0.010768	0.179985
cyp27b1	21.92669827	1.249540542	0.4297363	2.9076915	0.003641	0.081518
npffl	120.3662332	-0.097087458	0.1830926	-0.530264	0.595929	0.954434
pdrg1	161.8217993	-0.05408791	0.1624183	-0.333016	0.739122	0.972722
aamp	763.79472	-0.316711391	0.0996623	-3.177846	0.001484	0.042398
lhfpl5a	19.27285176	0.375850074	0.4642829	0.8095281	0.418211	0.906406
ift52	273.3816042	-0.039504101	0.129836	-0.304262	0.760929	0.975374
rab9a	58.3839336	-0.226871759	0.2511832	-0.903212	0.366413	0.887239
cdc42ep4b	744.757034	0.124345122	0.0952267	1.3057798	0.191627	0.755886
tekt3	33.83957464	0.268522924	0.33719	0.7963549	0.425826	0.909514
kcnj16	5.946820114	0.004311976	0.7579672	0.0056889	0.995461	NA
slc16a3	1825.983794	-0.074222804	0.0870512	-0.852633	0.393863	0.898496
ablim1b	876.5736916	-0.020843964	0.0920517	-0.226438	0.820861	0.981628
vti1a	202.727355	0.01997019	0.1489201	0.1341	0.893323	0.989291
entpd1	586.8494648	0.213847488	0.0992171	2.1553482	0.031135	0.341651
kcnk1a	24.01155045	0.761962541	0.4031269	1.8901307	0.05874	0.474103
itgb3b	20.02138698	0.412876165	0.4326967	0.9541929	0.339986	0.87805
chad	293.7810402	-0.101721541	0.1554844	-0.654224	0.512968	0.936253
arl4d	788.8406625	-0.017829073	0.1035191	-0.17223	0.863257	0.985841
tmem106a	220.8350352	-0.22828163	0.1536594	-1.485634	0.137376	0.674358
cdk5r1b	611.8386054	0.015088735	0.1036389	0.1455895	0.884245	0.98879
si:ch211-270n8.1	97.5082542	0.640482204	0.2700505	2.3717124	0.017706	0.246724
mrpl10	244.3585424	0.114486123	0.1352613	0.846407	0.397326	0.899672
cdk5rap3	382.5017464	-0.087297091	0.113986	-0.765858	0.443761	0.916631
si:dkey-222b8.1	13.00049722	0.856797136	0.5366157	1.5966681	0.11034	0.624637
elac1	153.2515407	0.105702694	0.183189	0.5770143	0.56393	0.948739
foxn2b	573.084893	-0.074057983	0.0989276	-0.748608	0.454094	0.920295
oplah	515.4542185	-0.277803415	0.1143763	-2.428854	0.015147	0.225353
FAM53C	609.3562907	0.057796858	0.1028795	0.561792	0.574258	0.950037
fkbp10b	471.071668	0.193834577	0.1084799	1.786824	0.073966	0.53012
klhl11	211.6633667	0.043796394	0.140989	0.310637	0.756077	0.975374
id4	664.6834562	0.065129463	0.0977036	0.6666028	0.505026	0.935724
vdac1	6369.400422	-0.05307031	0.0712489	-0.744858	0.456358	0.921215
CABZ01084564.1	3.143591673	0.768833306	1.1366807	0.6763846	0.498796	NA
ca7	45.09105839	-0.148512872	0.2929235	-0.507002	0.612153	0.95688
lcmt1	238.2821525	-0.020390007	0.1596275	-0.127735	0.898359	0.990121
aqp8a.1	252.908889	-0.301689632	0.1365042	-2.210113	0.027097	0.317055
hbae5	40.49415602	-0.627018374	0.3120413	-2.009409	0.044494	0.41501
hbbe2	2311.798758	-0.015040429	0.0837239	-0.179643	0.857433	0.985703
zgc:163057	22.74136561	0.185589614	0.4171516	0.4448973	0.656394	0.961833
shisa9a	377.9221637	-0.08478946	0.1280628	-0.662093	0.507912	0.935734
tomm22	989.784681	-0.064423925	0.0900219	-0.715647	0.474209	0.925066
tmem184ba	1013.077282	0.179395534	0.0844232	2.1249548	0.03359	0.357045
csnk1e	991.8854379	-0.108764363	0.0859762	-1.265052	0.205853	0.773784
slc16a8	30.01521258	-0.122324593	0.409773	-0.298518	0.765308	0.975374
phf5a	196.4195709	-0.103825086	0.1808994	-0.573938	0.56601	0.948739
rgs9b	271.7333835	-0.041046624	0.1322028	-0.310482	0.756194	0.975374
si:ch211-147h1.4	1.479747632	0.372261087	1.6452828	0.2262596	0.820999	NA
zdhhc4	266.592628	-0.064142758	0.1350431	-0.47498	0.634801	0.9578
msrb1b	0	NA	NA	NA	NA	NA
nptx2b	17.0064386	0.252845772	0.4767639	0.5303375	0.595878	0.954434
bhlha15	5.334386492	0.561092373	0.8236099	0.6812598	0.495707	NA

dlgap5	611.6423492	-0.101012011	0.1146652	-0.88093	0.378356	0.895192
minpp1b	55.9626411	-0.028828304	0.2629353	-0.10964	0.912695	0.991119
pofut2	183.8266612	0.041327411	0.1499948	0.2755256	0.782912	0.97627
acta2	1541.445857	-0.379939845	0.0865994	-4.387329	1.15E-05	0.000811
ch25h	30.03746104	-0.233377273	0.3715044	-0.628195	0.529876	0.942209
zgc:55262	521.5152659	-0.040728223	0.1020395	-0.399142	0.689789	0.966819
dusp19b	125.1549516	0.012964294	0.1874748	0.0691522	0.944868	0.993409
dkk1b	29.31839532	0.067770419	0.3888614	0.1742791	0.861646	0.985841
glipr1a	10.26642731	-0.26945528	0.6048592	-0.445484	0.65597	NA
stub1	688.7114634	0.094254899	0.0980649	0.9611478	0.336478	0.876088
cox6b1	1816.407621	-0.459082211	0.0883452	-5.196458	2.03E-07	2.59E-05
h3f3b.1	12077.25864	-0.158574856	0.0690534	-2.29641	0.021652	0.278385
nif3l1	223.9632166	-0.282499804	0.1559292	-1.811719	0.07003	0.516704
zmp:0000000624	92.59186824	0.04725195	0.2154305	0.2193373	0.826387	0.982196
decr2	206.9301523	-0.353467391	0.1547706	-2.283815	0.022382	0.285346
gsnb	1279.300369	-0.053076298	0.0996823	-0.532455	0.594411	0.954434
gsgl12b	69.13695309	0.246523257	0.2331624	1.0573027	0.290373	0.846262
tectb	165.0053482	0.308744644	0.1566906	1.9704099	0.048791	0.434204
pts	142.3485376	-0.414209444	0.1967542	-2.105213	0.035273	0.365366
tmem204	122.4260227	-0.011327293	0.1814994	-0.06241	0.950237	0.99381
klhl14	204.1325127	-0.068174855	0.156288	-0.436213	0.662682	0.964665
rdh12l	31.23509875	-0.254142165	0.3668269	-0.692812	0.488427	0.931926
gpr137c	121.4582053	-0.244697794	0.1827873	-1.338702	0.180668	0.741776
sh3glb1a	287.9884989	0.135104494	0.1543552	0.8752829	0.38142	0.895192
cops9	488.0631003	-0.067183786	0.1386672	-0.484497	0.628034	0.957354
phb2a	1627.290817	-0.018770065	0.0749911	-0.250297	0.802358	0.979492
wipf2a	775.0173249	0.099400894	0.1071996	0.9272507	0.353796	0.881976
vmo1b	139.5119112	-0.142923275	0.2100337	-0.680478	0.496202	0.932415
st8sia3	7.487307365	1.821628108	0.7443825	2.4471667	0.014398	NA
smpx	346.1896436	-0.010049882	0.1220976	-0.08231	0.9344	0.992702
mtfr1	229.1198588	0.212213484	0.138421	1.5331021	0.125251	0.654648
pde7a	1164.600733	-0.016299453	0.0837456	-0.194631	0.845682	0.985174
slc51a	234.7430065	0.412515208	0.1408249	2.9292781	0.003398	0.077847
pola1	655.4741767	-0.208797209	0.1077144	-1.938433	0.05257	0.449847
map7d2b	1996.250419	-0.045510725	0.0744335	-0.611428	0.540916	0.944633
pkp4	720.9088407	0.01643117	0.0949482	0.173054	0.862609	0.985841
si:ch211-140b10.6	149.9708899	-0.163198874	0.1640259	-0.994958	0.319757	0.86809
usp46	4.428642533	0.037005916	0.9852832	0.0375587	0.97004	NA
ccdc58	133.2567284	-0.056097133	0.1756067	-0.319448	0.749387	0.97419
cstl4a.2	441.9481881	-0.123327303	0.1332025	-0.925863	0.354517	0.882105
eloca	182.768136	-0.18976527	0.1603729	-1.183275	0.2367	0.805854
elocb	1007.171079	0.086539196	0.0991637	0.8726905	0.382832	0.895192
flj110111	299.3753871	0.196658275	0.1320875	1.4888487	0.136527	0.673303
trappc3	638.0248479	0.046500012	0.0970191	0.4792872	0.631734	0.957356
tuba1b	1836.321408	-0.225858329	0.0812452	-2.77996	0.005437	0.10944
prdm14	15.55591818	0.379877881	0.5128919	0.7406588	0.4589	0.921978
ngdn	147.6457975	-0.003455863	0.1705693	-0.020261	0.983835	0.996944
pabpn1	3716.698484	-0.01760715	0.0746471	-0.235872	0.813532	0.981081
pi15a	4.510926119	-0.518191062	0.8876956	-0.583749	0.559389	NA
gpr22b	184.813521	0.215640358	0.1624711	1.327254	0.184425	0.747376
cd226	40.78116619	-0.104300046	0.3265137	-0.319435	0.749396	0.97419
mtrr	165.1559682	-0.181480218	0.1565942	-1.158921	0.246489	0.816426
cct5	4822.529551	-0.098108962	0.0737338	-1.330584	0.183326	0.747003
mllt10	1658.215992	-0.14610997	0.0909167	-1.607075	0.108038	0.619409

commd3	236.0916114	0.019072155	0.1438713	0.132564	0.894538	0.989716
tagln	325.3850753	-0.470278508	0.1248622	-3.766379	0.000166	0.007304
acadl1	249.3401067	-0.190820162	0.1347561	-1.416041	0.156764	0.707081
gcm2	183.2211098	-0.021892848	0.1530317	-0.143061	0.886242	0.988936
elovl2	237.7677036	-0.176004716	0.1452709	-1.211562	0.22568	0.793245
gnal	97.35437395	0.159744224	0.198053	0.8065731	0.419912	0.90651
mppe1	201.5600113	-0.031327063	0.1510409	-0.207408	0.835691	0.984089
fam49bb	329.5907306	-0.030385671	0.1391397	-0.218382	0.827131	0.982678
epdr1	360.1369548	0.060027018	0.120704	0.4973074	0.618972	0.957354
stard3nl	372.664346	-0.152188586	0.1149359	-1.324117	0.185464	0.74928
otulina	206.9232223	0.282715104	0.159137	1.7765515	0.075642	0.535726
si:ch211-146l10.8	0	NA	NA	NA	NA	NA
zgc:173856	0	NA	NA	NA	NA	NA
si:dkey-96n2.3	1.471026404	-0.306609088	1.5202188	-0.201687	0.840161	NA
mcee	218.8896652	0.013676228	0.1516292	0.0901952	0.928132	0.992702
slc35b3	254.6711766	0.249499794	0.1486143	1.6788409	0.093183	0.580702
cpb1	888.678764	-0.459717425	0.1301309	-3.532731	0.000411	0.015173
agtr1b	30.05099266	-0.028846725	0.356252	-0.080973	0.935464	0.992702
fzd8a	546.1901123	-0.067674617	0.1182951	-0.572083	0.567266	0.948739
slc35g2b	798.7087439	-0.072365026	0.0924017	-0.783157	0.433535	0.913667
f13a1a.1	1.929313885	-1.585101638	1.6063823	-0.986752	0.323764	NA
ralaa	554.0864614	-0.033327488	0.1058446	-0.314872	0.752859	0.97481
lancl2	968.1795383	-0.130006698	0.0861301	-1.509423	0.131191	0.663979
gsap	79.65255111	-0.155875077	0.218121	-0.714627	0.47484	0.925399
stk38l	409.5503912	-0.019424157	0.1145745	-0.169533	0.865377	0.986385
lrtm2a	167.8432129	-0.165030067	0.1642422	-1.004797	0.314995	0.866054
dcp1b	193.1555878	0.109190002	0.1631869	0.6691101	0.503425	0.935304
rassf8b	169.6962851	0.179589903	0.1574198	1.1408346	0.253939	0.823394
pawr	381.4704003	-0.12708327	0.1163575	-1.092179	0.274754	0.839332
rps16	10064.98331	-0.36464431	0.0972606	-3.749149	0.000177	0.007745
ndufb2	1768.41285	0.028573112	0.0807318	0.3539265	0.723394	0.970402
slc35e3	231.4956477	0.04469834	0.1531139	0.2919287	0.770341	0.975687
gramd4b	544.188569	0.046460446	0.1088227	0.426937	0.669425	0.965093
tbc1d22a	242.6742561	-0.084781	0.1360699	-0.623069	0.533239	0.942971
taf3	788.7819071	-0.052704626	0.1233484	-0.427283	0.669173	0.965037
atp5f1c	6440.004032	0.014359318	0.0851912	0.168554	0.866148	0.986647
kin	139.689452	-0.016965695	0.1770995	-0.095798	0.923681	0.992628
itih2	1587.107493	0.185803682	0.1042672	1.7819953	0.07475	0.532583
itih5	51.27967902	-0.032621061	0.2739909	-0.119059	0.905229	0.990702
tmem110l	320.4432055	0.105538943	0.1340405	0.7873659	0.431068	0.91234
sfmbt2	20.04070328	-0.435236755	0.4491145	-0.9691	0.332495	0.874653
acot15	324.7417193	-0.17038193	0.1560815	-1.091622	0.274999	0.839541
lamb1b	503.2109566	0.047424008	0.105677	0.4487637	0.653602	0.961112
tmcc3	723.5725034	-0.095738136	0.0932424	-1.026767	0.30453	0.858905
nr2c1	573.4518543	-0.122011384	0.0976199	-1.249862	0.21135	0.778917
fgd6	219.3357437	0.037167259	0.1420203	0.2617039	0.79355	0.977937
si:ch211-244b2.4	23.32238252	-0.139757731	0.400242	-0.349183	0.726952	0.970402
ppp6r2a	829.0999174	0.129003093	0.0883979	1.4593461	0.14447	0.688868
gcc1	314.5674432	-0.12958285	0.1213093	-1.068202	0.285429	0.845164
lamtor4	509.6468606	-0.199125642	0.1088581	-1.829222	0.067366	0.508048
atp6v1f	913.8642745	0.139364742	0.0886906	1.5713593	0.116099	0.63841
hgfa	61.10322083	0.37647734	0.2477834	1.519381	0.128667	0.659646
lepb	48.07301449	0.430116261	0.3635581	1.1830745	0.23678	0.805854
bik	67.3009804	0.007439474	0.257015	0.0289457	0.976908	0.996762

si:dkey-202b22.6	11.25515564	0.287995353	0.5876458	0.4900832	0.624075	0.957354
hsd17b2	27.74157067	-0.179491398	0.3615499	-0.49645	0.619577	0.957354
snx20	23.58375401	0.544796907	0.3919681	1.3899012	0.164559	0.720706
sco2	0	NA	NA	NA	NA	NA
OTUD7A	147.5684913	0.159887462	0.1754894	0.9110944	0.362246	0.885944
socs2	59.89574863	0.130237211	0.2523969	0.5160015	0.605853	0.956575
mybpc1	1013.058389	0.064253855	0.0871075	0.7376385	0.460734	0.921978
dram1	39.78870389	-0.094129297	0.3158374	-0.298031	0.76568	0.975374
pus1	140.8819728	-0.097335486	0.1753987	-0.554939	0.578937	0.951052
washc3	299.336	-0.07154663	0.1260757	-0.56749	0.570382	0.949061
noc4l	291.1146182	-0.228948742	0.1270456	-1.802099	0.07153	0.522931
phpt1	254.8584353	-0.127745914	0.1343672	-0.950722	0.341745	0.878134
bcat1	458.3970656	0.039775194	0.1204499	0.3302218	0.741232	0.973154
lrmp	408.4318439	0.247806166	0.1143751	2.1666097	0.030265	0.33626
lum	1731.248933	0.244080309	0.0738459	3.3052659	0.000949	0.029735
zgc:172145	48.9474431	0.36828726	0.2906941	1.2669238	0.205183	0.773257
kcnj8	24.44269688	0.469456662	0.4272844	1.0986984	0.2719	0.836816
tnni2a.1	744.0255433	-0.090486044	0.0935234	-0.967523	0.333282	0.874926
shisal1a	141.1252125	-0.252770339	0.1699721	-1.487129	0.136981	0.673303
si:dkey-14k9.3	108.6886916	-0.063465023	0.1887319	-0.336271	0.736667	0.972535
rassf8a	66.82998543	0.057675816	0.2515761	0.229258	0.818668	0.981594
akr1b1.2	110.4719064	0.472790164	0.1910137	2.475164	0.013318	0.207028
cax1	45.42660027	-0.104723727	0.2907272	-0.360213	0.718688	0.969092
ticrr	219.6253413	-0.20559046	0.1428786	-1.438917	0.150174	0.698387
alkbh3	118.6316127	-0.160210972	0.1915562	-0.836366	0.402949	0.90084
tafa5b	175.0129205	-0.071519432	0.1561615	-0.457984	0.646964	0.960362
nfybb	265.776299	-0.30240259	0.1429303	-2.115735	0.034367	0.360397
lrrc10	12.36412099	0.488775439	0.54829	0.8914542	0.372686	0.892868
clta	5152.19943	0.023007571	0.0683493	0.3366173	0.736405	0.972535
nansa	597.4792723	-0.036289763	0.0967984	-0.3749	0.707735	0.968664
nek8	66.57920087	0.136186529	0.2425485	0.5614817	0.574469	0.950037
rab34a	137.2651079	0.114380414	0.1756308	0.6512548	0.514882	0.937646
asb15a	150.523323	0.195296746	0.1822293	1.0717085	0.283851	0.844676
lmod2a	1.49754665	-1.062363946	1.7496145	-0.607199	0.543719	NA
rbl2	288.240974	0.019737804	0.1334485	0.1479057	0.882417	0.988344
slc13a1	165.2316395	-0.745850185	0.1936836	-3.850869	0.000118	0.005608
elavl4	6413.52884	0.018500874	0.0678247	0.272775	0.785026	0.97627
usp3	195.499836	-0.144100994	0.1536611	-0.937785	0.348355	0.880195
cal2	21.27044499	0.36220087	0.4358681	0.8309874	0.405981	0.901305
msrb3	456.9480844	0.314533543	0.1105575	2.8449777	0.004441	0.094639
hic1l	305.7521698	-0.05960833	0.1301732	-0.457915	0.647013	0.960362
irf7	110.6291201	-0.281003466	0.1944847	-1.444862	0.148497	0.696298
ANKRD50	225.8579655	0.2730202	0.155817	1.7521851	0.079742	0.548458
si:dkey-39a18.1	33.80233532	-0.308880855	0.3280103	-0.94168	0.346356	0.879249
ifng1r	0	NA	NA	NA	NA	NA
il26	1.805681893	0.297287591	1.5041014	0.1976513	0.843318	NA
il22	4.484643323	-1.189880749	1.0142747	-1.173135	0.240742	NA
mdm1	46.38827173	0.087593745	0.3022586	0.2897974	0.771971	0.975687
calua	1675.719534	0.198347752	0.0778791	2.5468681	0.010869	0.180962
opn1sw1	45416.23535	0.371200098	0.0814901	4.5551552	5.23E-06	0.000414
tnpo3	1020.493604	-0.021476283	0.0837615	-0.256398	0.797643	0.978668
irf5	91.83072314	-0.298026972	0.2103655	-1.416711	0.156568	0.706842
ccdc87	8.625234117	-0.122881884	0.6472275	-0.189859	0.84942	NA
cntn1b	832.5561626	0.139982607	0.0913007	1.5332038	0.125226	0.654648

pdzrn4	479.6014303	-0.058014296	0.1109552	-0.522863	0.60107	0.956045
zcrb1	118.4554394	-0.181131175	0.2007104	-0.90245	0.366818	0.88734
pphln1	781.0537012	0.021666116	0.089743	0.2414241	0.809226	0.980642
prickle1b	750.098246	0.050620556	0.0974577	0.5194104	0.603475	0.956045
myca	885.0480232	-0.030300338	0.0936613	-0.32351	0.746309	0.97419
mrpl23	750.9877826	-0.162697277	0.0926779	-1.755513	0.079172	0.547489
dclre1c	245.3709117	-0.127725661	0.1342141	-0.951656	0.341272	0.878134
meig1	64.03792225	-0.070388008	0.2389942	-0.294518	0.768362	0.975687
tspan9b	251.7404111	0.056281719	0.1340765	0.4197732	0.674651	0.965257
adm2a	75.39296939	-0.222640272	0.235698	-0.9446	0.344863	0.878434
ciapin1	656.4889205	0.072724789	0.0998356	0.7284454	0.466341	0.923908
guca1g	60.41337696	0.40661553	0.249088	1.632417	0.102592	0.608473
cped1	382.2512987	0.102801307	0.1151178	0.8930096	0.371852	0.892248
stab2	118.9885967	-0.007621209	0.2113421	-0.036061	0.971234	0.996315
ppfibp1a	473.7228479	-0.144229458	0.108527	-1.328973	0.183857	0.747376
syt10	195.4496807	0.121863268	0.1513047	0.8054161	0.42058	0.90651
si:ch211-161n3.4	2.512227917	0.978540643	1.3795503	0.7093186	0.478127	NA
tmtc2a	267.4739311	-0.002544952	0.1311748	-0.019401	0.984521	0.996944
mettl25	53.94899065	0.040358037	0.2647136	0.1524593	0.878825	0.987881
ccdc59	33.74727933	0.161356227	0.333889	0.4832631	0.628909	0.957354
cracr2ab	21.44296897	-0.580595847	0.4281657	-1.356008	0.175097	0.735545
prmt8b	209.3016501	0.263221772	0.1468128	1.7929079	0.072988	0.526929
asb13a.2	9.003724326	0.461328018	0.6606333	0.6983118	0.484982	NA
cry1a	2679.484868	-0.02658284	0.0710034	-0.374388	0.708116	0.968664
PYURF	141.8787624	-0.091843081	0.1892161	-0.485387	0.627402	0.957354
cnbpa	3015.81896	-0.011982548	0.0687248	-0.174356	0.861586	0.985841
zgc:103499	160.3613892	0.135876858	0.1592175	0.8534041	0.393435	0.898076
avpr1ab	88.36400764	0.043164457	0.2362647	0.1826953	0.855037	0.985174
srgap1b	812.7144722	-0.040692457	0.0946788	-0.429795	0.667345	0.964736
si:ch211-125a15.1	160.5661003	-0.208542371	0.1664864	-1.252609	0.210348	0.778043
si:dkey-106n21.1	24.52295866	0.68734934	0.4218173	1.6294953	0.103208	0.609625
si:ch211-68a17.7	228.3425657	0.123441399	0.1620091	0.7619414	0.446095	0.917154
acanb	148.4021397	0.240340701	0.1669445	1.4396447	0.149968	0.697935
fbxl14b	716.364812	0.127777507	0.0943649	1.3540794	0.175711	0.736411
hapln3	132.8353114	0.288338279	0.1739791	1.6573155	0.097456	0.595884
mfge8b	350.5091292	0.114011273	0.1300499	0.876673	0.380664	0.895192
abhd2b	221.7163871	0.101680073	0.1381935	0.7357806	0.461864	0.922466
rlbp1b	244.4756894	-0.258215404	0.1752979	-1.473009	0.140749	0.681252
lrtm2b	93.53485048	0.211645714	0.220975	0.9577813	0.338173	0.876778
samm50	1055.676529	0.105945072	0.0855663	1.2381641	0.215655	0.78305
mcm10	106.5838931	0.003583471	0.1978522	0.0181119	0.98555	0.996944
tnnt2e	832.0517687	0.112510174	0.1050062	1.0714626	0.283961	0.844697
zgc:101783	239.4651528	-0.237138537	0.137634	-1.722965	0.084895	0.560519
lyrm5b	36.62646242	-0.744843651	0.323363	-2.303429	0.021255	0.275144
cart2	72.71391365	-0.004541799	0.2272415	-0.019987	0.984054	0.996944
si:dkey-14d8.7	16.93009187	-0.310652359	0.4925394	-0.630716	0.528226	0.941768
si:dkey-14d8.6	34.89832624	-0.198296656	0.3415978	-0.580497	0.561579	0.948728
mapk11	296.8593937	0.014945885	0.1412398	0.1058192	0.915726	0.991314
si:dkey-14d8.20	53.03661746	-0.058217816	0.281199	-0.207034	0.835983	0.984089
zgc:113263	674.0569012	-0.287916747	0.0973618	-2.957184	0.003105	0.073687
apex1	1888.807955	-0.096772825	0.079652	-1.214946	0.224387	0.79161
osgep	184.0957243	-0.142652969	0.1651644	-0.863703	0.387751	0.896133
si:ch211-214j24.10	998.5001817	-0.125086551	0.0999152	-1.251927	0.210596	0.778338
ccdc167	233.2413229	-0.127510871	0.145964	-0.873578	0.382348	0.895192

galnt8b.1	41.37787251	-0.291204287	0.3186659	-0.913823	0.36081	0.885686
rad51ap1	111.874285	-0.106327181	0.1861229	-0.571274	0.567814	0.948739
fgf23	7.185158702	1.376123048	0.7314625	1.8813309	0.059927	NA
fgf6b	5.507163081	-0.092962368	0.8216518	-0.113141	0.909919	NA
cebpz	560.4022728	-0.220629751	0.1031002	-2.139954	0.032359	0.350031
tigarb	107.1082597	0.078212901	0.210191	0.3721039	0.709815	0.968928
asb15b	7.314660455	-0.794068057	0.7183358	-1.105427	0.268974	NA
lmod2b	43.1390607	0.269521534	0.3089869	0.872275	0.383058	0.895192
plekha5	1103.564675	-0.095751601	0.1002094	-0.955515	0.339317	0.87805
frs2a	33.11758962	0.056793536	0.3325041	0.1708055	0.864377	0.98601
yeats4	396.68644	-0.21760231	0.1136343	-1.914935	0.055501	0.461579
ube2nb	1053.63828	0.005093422	0.0962932	0.0528949	0.957816	0.99467
nudt4b	417.6632875	0.23689035	0.1144319	2.0701427	0.038439	0.384468
gas2l3	148.4912919	0.154405619	0.180395	0.8559305	0.392036	0.897688
scaf11	1131.021079	-0.166634747	0.085914	-1.939553	0.052434	0.449428
slc38a2	2565.007154	0.212990733	0.0875638	2.4324058	0.014999	0.224348
mmp30	423.6527592	0.303485154	0.1366274	2.221261	0.026333	0.312554
acat1	1832.302761	0.116842418	0.077312	1.511311	0.130709	0.662797
kctd15a	732.6780352	0.237815921	0.1004542	2.3674058	0.017913	0.247704
si:ch211-152c2.3	480.0831694	0.174205979	0.1090184	1.5979497	0.110054	0.624518
agbl5	303.1834059	-0.157977816	0.1360718	-1.160988	0.245647	0.815431
nr2e3	271.1354031	-0.031866654	0.158664	-0.200844	0.840821	0.985092
dynlt1	71.26167812	-0.36073552	0.2659491	-1.356408	0.174969	0.735545
tmem181	674.8932037	-0.031549307	0.1121575	-0.281295	0.778484	0.975942
tulp4a	642.7594296	-0.017292336	0.1077036	-0.160555	0.872444	0.987846
mrps10	245.9540878	-0.100825555	0.1648886	-0.611477	0.540884	0.944633
si:ch211-51e12.7	3005.258935	-0.10065532	0.0736823	-1.366071	0.171917	0.730757
crabp1a	494.4082899	0.046141384	0.1131013	0.4079651	0.683299	0.965476
slc25a44a	27.46381231	0.31206238	0.3905458	0.7990417	0.424266	0.908243
psma4	1547.083152	-0.068410554	0.0799514	-0.855651	0.392191	0.897688
oaz2a	2359.65603	0.104610279	0.0780967	1.3394962	0.180409	0.741452
rbpms2a	2268.946473	-0.058225368	0.0740709	-0.786076	0.431823	0.912787
cpeb1a	19.5091976	0.345235739	0.4423823	0.7804013	0.435155	0.914408
immp1l	42.68429697	-0.352750419	0.2996907	-1.177048	0.239176	0.807768
pax6b	857.1228166	0.074326722	0.111005	0.6695797	0.503126	0.935304
slc6a1a	489.4824377	0.120069657	0.1129982	1.0625801	0.287972	0.845979
syn2a	2627.155619	0.10761449	0.0719803	1.4950557	0.1349	0.670689
sec24d	1106.085307	0.006823904	0.0980958	0.0695637	0.944541	0.993409
hrc	2843.318856	0.141538753	0.0964323	1.4677532	0.142171	0.683847
kat5b	496.8470288	-0.123487998	0.1081812	-1.141492	0.253665	0.823394
prlrb	10.15374304	0.445497854	0.5916944	0.7529188	0.451499	NA
kcnk10b	7.686117375	0.380410401	0.6774592	0.5615252	0.57444	NA
gpr65	0.31437473	-1.788572922	3.5930657	-0.497785	0.618636	NA
egfl6	1834.395296	0.271405494	0.0868732	3.1241552	0.001783	0.049384
csf3r	8.444168541	0.614344503	0.6930188	0.886476	0.375361	NA
spg11	448.0234893	-0.012638359	0.1201196	-0.105215	0.916205	0.991314
sidt2	902.0016154	-0.199341437	0.1068916	-1.864893	0.062196	0.487078
chchd6b	606.9497261	0.008664937	0.1039689	0.0833416	0.93358	0.992702
zgc:153704	754.7704502	0.040851256	0.1029296	0.3968855	0.691452	0.967305
si:ch73-55i23.1	1.343078476	-0.679794512	1.7232066	-0.394494	0.693216	NA
zbtb43	4.864267533	0.283942516	0.8819076	0.321964	0.74748	NA
arf4a	397.2472891	0.093289444	0.1142949	0.8162169	0.414376	0.906237
saa	5.833548158	-2.036597977	0.9082933	-2.242225	0.024947	NA
necap2	729.3729016	-0.084568897	0.1177056	-0.718478	0.472462	0.924838

med20	231.8829274	-0.105093373	0.1371707	-0.76615	0.443587	0.916631
tmcc2	306.2952872	-0.036977156	0.1410572	-0.262143	0.793211	0.977937
kdm2bb	742.59613	-0.071864885	0.0929025	-0.773552	0.439196	0.916082
slc47a1	102.5678677	0.087679388	0.2002944	0.4377525	0.661566	0.964237
rasl11a	148.7463832	-0.004516365	0.1675056	-0.026962	0.97849	0.996762
kcna6a	74.70238047	0.18401947	0.2320329	0.7930749	0.427734	0.91075
snai1b	481.2745975	-0.26600231	0.1079814	-2.463409	0.013762	0.210896
CU914487.1	19.07597195	-0.223408306	0.4723593	-0.472963	0.63624	0.958561
tmed7	1339.409066	0.049852663	0.087554	0.5693936	0.569089	0.948983
pym1	202.0512668	-0.167069015	0.1580575	-1.057014	0.290505	0.846262
zgc:110339	1143.53748	0.02978306	0.0953901	0.3122237	0.754871	0.97522
slc27a6	44.43512221	0.026359665	0.2941793	0.0896041	0.928602	0.992702
CT027815.1	200.2438007	0.086716511	0.1604768	0.540368	0.588943	0.953345
zgc:77838	129.8196746	-0.02262726	0.1944209	-0.116383	0.907349	0.990702
snx16	264.6138913	0.159254684	0.1411541	1.1282329	0.259222	0.82755
stk11	777.0207255	-0.022350086	0.0918349	-0.243372	0.807717	0.980642
cacng6b	42.04325517	-0.102975124	0.31284	-0.329162	0.742033	0.973154
bloc1s2	180.6664459	-0.118139929	0.154305	-0.765626	0.443899	0.916631
brd2b	101.8190433	-0.154634633	0.2076095	-0.744834	0.456372	0.921215
dhrs11a	60.56986198	-0.056371363	0.2570679	-0.219286	0.826427	0.982196
si:ch211-283g2.1	74.47125055	0.004687306	0.2325239	0.0201584	0.983917	0.996944
si:ch211-283g2.3	0	NA	NA	NA	NA	NA
ebp	138.1490983	-0.308821055	0.1706973	-1.809173	0.070424	0.519033
rab10	2262.950345	0.173098227	0.0735468	2.35358	0.018594	0.254199
caskin1	1362.028845	-0.028097975	0.0863676	-0.32533	0.744931	0.974094
rab11fip1a	141.863227	0.152393855	0.1709337	0.891538	0.372641	0.892868
tk2	198.8750915	0.008817943	0.1546873	0.057005	0.954541	0.994533
dynlt3	1084.445881	-0.012111664	0.0922584	-0.13128	0.895554	0.989814
b3galnt2	115.878128	-0.131676571	0.2026172	-0.649879	0.515771	0.9377
zgc:112038	7.117663688	-0.900968083	0.7193983	-1.252391	0.210427	NA
zgc:162025	33.95329789	-0.189548745	0.3518454	-0.538727	0.590075	0.953903
si:ch211-170d8.5	32.33584251	0.67896273	0.3575538	1.8989105	0.057576	0.470971
prkab1b	661.6578531	-0.137571334	0.1017029	-1.352678	0.176159	0.736595
tnfaip8l2b	130.6820911	-0.198919138	0.1932111	-1.029543	0.303224	0.85729
b4galnt4a	133.9106387	0.118287828	0.1769368	0.6685314	0.503794	0.935314
RPS17	10080.69746	-0.296328048	0.081902	-3.618081	0.000297	0.011738
CR339041.1	2.625644215	1.597784907	1.3735324	1.163267	0.244721	NA
CR339041.2	4.751885588	-0.166843382	0.9489733	-0.175815	0.86044	NA
mhc1lfa	8.061286832	-0.448565471	0.703349	-0.637757	0.523632	NA
mhc1lga	3.475778967	-0.997344656	1.0431524	-0.956087	0.339028	NA
si:ch211-113a14.24	4.296999548	-0.425853306	0.9034068	-0.471386	0.637365	NA
nfatc3b	162.3244442	-0.015992952	0.1619695	-0.09874	0.921344	0.992138
slc7a10b	209.4609677	0.073840571	0.1518893	0.4861473	0.626863	0.957354
faap24	14.22963842	-0.755561158	0.5548453	-1.361751	0.173276	0.732435
si:ch211-113a14.18	10.7217266	1.065594984	0.607412	1.7543198	0.079376	NA
hist1h2a4	2.698914385	1.161843446	2.6284126	0.4420324	0.658466	NA
zgc:173552	4.87880366	0.939791974	0.9472679	0.9921079	0.321145	NA
ada2a	352.5460279	-0.148933389	0.1176116	-1.266315	0.2054	0.773635
ccnd2a	190.3407823	0.294258597	0.1592862	1.8473573	0.064695	0.497355
tigara	51.26516584	0.245028225	0.2794641	0.8767789	0.380607	0.895192
rpgrip1l	60.71356253	0.07116864	0.2503222	0.2843081	0.776174	0.975826
pdhx	540.1339509	0.05899583	0.1081755	0.5453713	0.585498	0.95262
igl4v10	0.513487682	-0.887846295	2.6557956	-0.334305	0.738149	NA
trim44	185.8236739	-0.054122265	0.1508718	-0.35873	0.719797	0.969318

CABZ01080568.1	293.5476642	0.495428928	0.1514279	3.2717153	0.001069	0.03262
sntb2	289.2883853	0.020060907	0.1341574	0.1495327	0.881133	0.988177
fhod1	215.056885	0.012987144	0.1468364	0.0884463	0.929522	0.992702
rplp0	20794.14485	-0.248624075	0.1762422	-1.410696	0.158334	0.709224
dhx33	56.88943441	0.010627177	0.2598896	0.0408911	0.967383	0.996315
zgc:113426	31.12109925	-0.356318035	0.366021	-0.973491	0.330309	0.874098
si:dkeyp-110c12.3	1.9678137	1.585325189	1.440232	1.1007429	0.271009	NA
brd1b	611.9718115	0.022601318	0.1025601	0.2203715	0.825582	0.982196
fnbp4	2703.558027	-0.058192771	0.0746686	-0.779348	0.435775	0.914408
BX664721.1	0.981193923	0.036684775	1.9489489	0.0188229	0.984982	NA
CU469568.1	53.23714502	0.325342438	0.2750772	1.1827314	0.236916	0.805854
btr12	62.01060059	-0.201886278	0.2531881	-0.797377	0.425232	0.908835
ptprz1a	1044.520621	0.035000486	0.0841091	0.4161319	0.677313	0.965257
aass	430.3904739	0.257488941	0.1308463	1.9678739	0.049083	0.435115
swap70a	60.6197305	0.241972229	0.246443	0.9818588	0.326169	0.872073
hyal4	256.8612582	0.136312607	0.1381885	0.9864248	0.323925	0.870928
tmeff2a	907.7295092	-0.096642025	0.0857725	-1.126726	0.259858	0.828288
si:dkeyp-19e1.3	112.7248514	-0.1435601	0.1986485	-0.722684	0.469874	0.924783
ctu2	118.7215517	-0.324727036	0.1878117	-1.729003	0.083809	0.557501
kcnc1a	592.5544186	0.12732285	0.1057538	1.2039554	0.228607	0.796028
galns	206.2851268	-0.218950109	0.1553735	-1.409185	0.15878	0.709382
cdt1	171.6957855	-0.184958846	0.1668718	-1.108389	0.267694	0.833349
tes	279.90892	-0.052270878	0.13769	-0.379627	0.704222	0.968134
ccdc33	1.535978683	1.070276215	1.659346	0.6449988	0.518928	NA
pkp3a	1316.95423	0.037540224	0.0828841	0.4529245	0.650603	0.960708
zgc:158222	288.8321113	-0.024701622	0.135426	-0.182399	0.855269	0.985174
stra6	852.5153534	0.169975678	0.0896771	1.8954183	0.058037	0.472311
islr2	6366.784494	0.024383597	0.0690524	0.3531174	0.724	0.970402
ush1c	244.2697559	-0.882847956	0.1398702	-6.311911	2.76E-10	6.12E-08
abcc8	519.1466593	0.031701983	0.1053737	0.3008528	0.763527	0.975374
kcnj11	147.7526174	0.37145879	0.1793685	2.0709251	0.038366	0.384124
ankrd11	272.5977553	-0.1620995	0.1272278	-1.274089	0.202632	0.769545
ist1	1791.424053	-0.053088176	0.076804	-0.691216	0.48943	0.931926
dhodh	293.8963194	-0.173636483	0.1269931	-1.36729	0.171534	0.729995
hp	6.153177061	-0.931696183	0.8440224	-1.103876	0.269647	NA
rsph10b	28.56684289	0.31842911	0.3687333	0.8635756	0.387821	0.896133
kcng4b	40.1137704	0.501345202	0.3212346	1.5606823	0.118599	0.642318
fbn2a	82.18263249	0.092547557	0.2294995	0.4032581	0.686758	0.966376
exoc3l1	144.1588895	-0.042336732	0.1912632	-0.221353	0.824817	0.982196
prmt7	292.7297437	0.108622357	0.1269422	0.8556833	0.392173	0.897688
hpxb	171.9793032	-0.314974142	0.1814865	-1.735524	0.082648	0.554782
slc14a2	33.22189234	0.430643562	0.3412455	1.2619758	0.206958	0.774778
pde5ab	16.11778994	-0.857306196	0.4929889	-1.738997	0.082035	0.552886
cdk7	311.4856294	0.178470666	0.1230147	1.4508076	0.146833	0.693524
nsmaf	295.6690186	-0.304824149	0.1298757	-2.347046	0.018923	0.256465
slc30a5	627.8259746	0.081802957	0.0972811	0.8408925	0.400408	0.90084
ccnb1	715.8243711	-0.087931818	0.0986065	-0.891745	0.37253	0.892868
cyb5r2	261.7589621	-0.703223534	0.1368185	-5.139828	2.75E-07	3.40E-05
plag1	151.0381496	-0.039913642	0.1683014	-0.237156	0.812536	0.981081
edc3	384.3178999	-0.078435397	0.1171862	-0.669323	0.503289	0.935304
kxd1	84.72733571	-0.277100215	0.2235744	-1.239409	0.215194	0.78279
ergic2	322.6487597	-0.002049256	0.1292774	-0.015852	0.987353	0.997136
pcxb	1097.469089	-0.744388579	0.1049703	-7.091421	1.33E-12	4.45E-10
ugt2a4	698.3340111	-0.622040952	0.1133822	-5.486232	4.11E-08	6.10E-06

ythdc1	1288.663874	-0.093951262	0.089163	-1.053702	0.292019	0.848075
brms1	231.4872051	0.058040806	0.1379878	0.4206227	0.674031	0.965257
isca1	803.5223645	0.046590477	0.1025966	0.4541133	0.649747	0.960708
selenom	275.1070861	0.056611562	0.1419678	0.3987634	0.690068	0.966857
zpd	0	NA	NA	NA	NA	NA
mmp15a	134.1823438	-0.062041986	0.1768378	-0.350841	0.725707	0.970402
btr01	55.48350643	-0.173313156	0.2700231	-0.641846	0.520973	0.939056
smu1b	254.3300637	-0.018297357	0.1471849	-0.124315	0.901065	0.990121
cd99	738.1914635	0.176524265	0.0918515	1.9218451	0.054625	0.457793
STX3	174.9758798	0.030930206	0.1530678	0.2020687	0.839863	0.984979
ndufs8a	3926.147745	0.04766578	0.0723188	0.6591066	0.509827	0.936253
tmem187	47.5896638	0.558153682	0.2892777	1.9294736	0.053672	0.45394
si:dkey-9i23.4	0.667059899	1.488443644	2.4079024	0.6181495	0.536477	NA
cav2	247.5875372	-0.206829639	0.1366686	-1.513366	0.130187	0.662797
ciao2b	285.39082	0.142185257	0.1306537	1.0882607	0.27648	0.839541
rrad	196.863565	0.081159912	0.1631385	0.4974907	0.618843	0.957354
rtn4rl2a	88.87500728	0.592877969	0.2663095	2.226274	0.025996	0.311101
cabp2a	153.5653601	0.067315498	0.1718903	0.3916189	0.69534	0.96736
si:ch73-109d9.1	106.8439213	-0.078722213	0.1992805	-0.395032	0.692819	0.96736
cmtm4	17.41853229	-0.296723184	0.4577586	-0.648209	0.51685	0.938543
pomgnt1	464.3197051	0.041639286	0.1066129	0.3905651	0.696119	0.96736
tspan1	37.47111543	0.051003674	0.3339023	0.1527503	0.878595	0.987881
akr1a1b	349.7960352	-0.154374058	0.1173074	-1.315979	0.188181	0.753063
nxnl1	96.32675832	0.44361529	0.2050524	2.1634239	0.030509	0.337781
trim35-1	35.32724513	0.279611675	0.3287267	0.8505901	0.394997	0.899122
caspb	804.7234059	-0.113896831	0.1266902	-0.899019	0.368643	0.888057
ggt5a	265.3234908	0.226444874	0.132962	1.7030798	0.088553	0.569447
parietopsin	1.157631196	2.488108944	1.9178096	1.2973702	0.194504	NA
pcolceb	690.6141732	0.019752372	0.1164023	0.1696905	0.865254	0.986367
PAQR9	95.17921442	0.205973859	0.2242558	0.9184773	0.358369	0.884398
col4a6	4483.418735	0.175940487	0.0709292	2.4805077	0.01312	0.204756
col4a5	11458.28736	0.206352011	0.0867033	2.3799778	0.017314	0.243166
si:dkeyp-93a5.2	0	NA	NA	NA	NA	NA
dnajb9a	50.11965994	-0.212543027	0.2763701	-0.769052	0.441862	0.916336
blcap	1021.145979	-0.034098898	0.0853726	-0.399413	0.689589	0.966799
si:ch211-122f10.4	218.618697	-0.251606554	0.1420426	-1.771346	0.076503	0.538876
gabarapb	2583.885267	-0.086664308	0.0755275	-1.147453	0.251194	0.820469
cxcr1	10.90994557	0.455650919	0.6052672	0.7528095	0.451564	NA
CCKAR	24.22563079	0.060119126	0.4207618	0.1428816	0.886384	0.988936
rbpjb	48.31463032	-0.113538731	0.2780434	-0.408349	0.683018	0.965476
notch1b	284.9137619	0.075211313	0.1303434	0.5770242	0.563923	0.948739
trim35-31	2.348514823	-0.844313062	1.2558901	-0.672283	0.501404	NA
agxta	256.1913341	0.275753637	0.1452272	1.8987746	0.057594	0.470971
dtymk	134.6482253	-0.235659392	0.1748093	-1.348094	0.177628	0.738343
atg4b	351.6336887	-0.090469652	0.1189274	-0.760713	0.446828	0.917154
zgc:114041	32.17353636	0.223038573	0.35163	0.6342991	0.525886	0.940223
mboat4	0.824006558	0.61083159	2.1185808	0.2883211	0.773101	NA
hexa	440.3892221	0.039127428	0.1136624	0.3442424	0.730664	0.97149
crtc3	44.4722316	0.190724641	0.3008596	0.6339325	0.526125	0.94048
ehf	13.43098832	0.47587525	0.5558251	0.8561601	0.391909	0.897688
adamtsl5	95.1032321	0.136014769	0.2032535	0.6691878	0.503376	0.935304
rag2	18.1705254	0.309313248	0.475803	0.6500868	0.515636	0.9377
rag1	46.24534224	-0.31675626	0.2808086	-1.128015	0.259314	0.827577
accs	245.5586023	0.207361036	0.1386879	1.4951629	0.134872	0.670689

boka	167.6461025	0.022391451	0.163389	0.1370438	0.890996	0.989291
gli3	220.3263509	-0.097180229	0.1516845	-0.640673	0.521735	0.939056
slc1a2a	730.302228	0.346907538	0.1063239	3.2627424	0.001103	0.033311
notch3	2206.979951	0.020039134	0.0766051	0.2615899	0.793638	0.977937
acvr1bb	21.35143808	0.003590866	0.4114184	0.008728	0.993036	0.99854
ptgs1	206.387023	0.178961406	0.1492876	1.1987695	0.230618	0.79878
pbx4	3278.544978	-0.008280432	0.0702865	-0.11781	0.906218	0.990702
lix1	365.9944469	-0.26162159	0.1274281	-2.053092	0.040064	0.391993
lrrc8ab	455.4333061	-0.147273661	0.1092729	-1.347761	0.177735	0.738343
il12rb2	17.5774267	0.056445733	0.4678099	0.1206596	0.903961	0.990291
il23r	2.874060145	0.175591628	1.1472468	0.1530548	0.878355	NA
znf507	44.52523646	-0.126233025	0.2989816	-0.42221	0.672872	0.965257
si:ch211-236k19.2	0.173729368	-0.955901296	4.0804729	-0.234262	0.814781	NA
uap1	452.8225926	-0.232222329	0.1109132	-2.093731	0.036284	0.372062
zgc:171711	6.434388318	-0.49593674	0.7744227	-0.640395	0.521916	NA
eif2s1b	2438.596621	-0.187120248	0.0808837	-2.313449	0.020698	0.271768
rbfox2	3055.652662	0.070498237	0.0752125	0.9373204	0.348594	0.880195
znf541	3.817778069	1.173373341	1.0877206	1.0787451	0.280701	NA
fdx2	160.6791747	0.026950515	0.1646754	0.1636584	0.87	0.987395
s1pr5b	5.12827744	-0.276188339	0.8231363	-0.335532	0.737224	NA
c3a.3	799.3797375	0.575700258	0.3057092	1.8831631	0.059678	0.478288
btr02	113.0997796	-0.033164522	0.1894312	-0.175074	0.861021	0.985773
zgc:114118	8.916297873	0.889534008	0.6408344	1.3880871	0.165111	NA
rcvrna	1183.193031	0.242944245	0.0990106	2.4537184	0.014139	0.214731
aff2	317.111153	-0.027825227	0.1282874	-0.216898	0.828288	0.982721
zgc:158564	1322.240237	0.230362605	0.0833359	2.7642665	0.005705	0.113266
alkbh4	126.9070979	-0.12023762	0.1794847	-0.669904	0.502919	0.935304
trim35-3	1.914913393	-0.225421391	1.5437063	-0.146026	0.883901	NA
rab1aa	416.6675015	-0.012541399	0.1263598	-0.099251	0.920939	0.992138
slc22a18	395.2044544	-0.165740877	0.1106116	-1.498404	0.134028	0.669349
cabp2b	108.2810248	-0.012815448	0.2043141	-0.062724	0.949986	0.99381
osgn1	430.1837838	-0.095401117	0.1167482	-0.817152	0.413841	0.905864
zmp:0000000634	78.10805576	0.384087131	0.236846	1.6216742	0.104873	0.612814
rab33ba	281.3700719	0.002500194	0.1314167	0.0190249	0.984821	0.996944
pimr198	0	NA	NA	NA	NA	NA
zgc:171489	35.84139838	0.445280828	0.3241175	1.3738254	0.169496	0.726744
uqcrc1	5101.207651	0.021699033	0.0742535	0.292229	0.770112	0.975687
pimr196	0.31437473	-1.788572922	3.5930657	-0.497785	0.618636	NA
tlr5b	100.243556	0.506745305	0.2087501	2.4275214	0.015202	0.225685
slc4a2b	92.61404641	-0.220112349	0.2019172	-1.090112	0.275664	0.839541
abcf2b	85.04118815	0.274047557	0.2329113	1.1766179	0.239348	0.807932
ftr98	1.452835086	-1.011714448	1.6795834	-0.60236	0.546934	NA
ociad1	660.4563608	-0.02411107	0.0933564	-0.258269	0.796199	0.978498
ociad2	304.6476382	-1.367291519	0.1328876	-10.28908	7.89E-25	1.30E-21
sgcb	349.6729265	-1.228622711	0.1357819	-9.048501	1.45E-19	1.19E-16
spata18	52.71319218	0.397938602	0.2883484	1.3800619	0.167568	0.724734
tbl3	615.1528012	-0.182110758	0.117591	-1.54868	0.121459	0.647929
smarca5	2698.149653	-0.008791616	0.0759935	-0.115689	0.907899	0.990702
si:dkey-156n14.3	662.1558572	0.057896201	0.0991517	0.5839151	0.559277	0.948478
cacng1b	2.049235703	-0.962029482	1.3853896	-0.694411	0.487425	NA
il15	56.02194096	0.034528864	0.262555	0.131511	0.895371	0.989814
cltb	147.948757	0.409950503	0.1764259	2.3236418	0.020145	0.266806
higd2a	533.2218138	0.047135534	0.1122543	0.4198995	0.674559	0.965257
faf2	924.5151926	-0.01296486	0.0871639	-0.148741	0.881758	0.988344

zgc:66455	4.31299211	-0.212183118	0.8982527	-0.236218	0.813264	NA
b3gnt2a	3.927366378	-1.187204437	1.0923885	-1.086797	0.277127	NA
psmd12	1712.749356	0.108420913	0.0786964	1.3777109	0.168293	0.725919
ccdc22	507.9141096	-0.138627547	0.1096107	-1.264726	0.205969	0.773798
cldnd1b	269.2047406	-0.10534118	0.1360276	-0.77441	0.438688	0.915997
pou2f3	160.8768414	-0.235784197	0.1611821	-1.462844	0.14351	0.686804
pcgf5b	32.74853622	0.026981984	0.3336331	0.0808732	0.935543	0.992702
tmed8	493.0561314	0.163911349	0.1033257	1.5863555	0.112659	0.628587
pkz	17.98130938	0.401065875	0.4604204	0.8710861	0.383707	0.895192
gpr6	7.246993234	-0.028323449	0.7563899	-0.037446	0.97013	NA
dnmt3ba	930.2458612	0.029866094	0.0849118	0.3517307	0.72504	0.970402
pak6b	69.51946785	0.251378526	0.2605724	0.9647167	0.334687	0.875699
mgat2	324.8262701	-0.190831552	0.1292158	-1.476844	0.139718	0.678481
si:dkey-71l1.1	53.04895954	0.287041291	0.2786166	1.0302377	0.302898	0.85729
crfb12	88.46305656	0.471205598	0.233346	2.0193428	0.043452	0.409149
ankrd12	923.0774149	-0.205610722	0.0919741	-2.235528	0.025383	0.308303
ppp1r12c	526.0349797	-0.057336952	0.1020837	-0.561666	0.574344	0.950037
si:ch211-256e16.3	245.2963259	-0.092793992	0.1491518	-0.622145	0.533847	0.943276
bicdl2	152.0049884	-0.251272058	0.167221	-1.502634	0.132933	0.667628
si:dkey-43k4.3	27.17695176	-0.397812467	0.3732594	-1.06578	0.286523	0.845942
syngr2a	518.6574469	-0.201403153	0.1135875	-1.773111	0.07621	0.538016
inha	0.365141898	1.973577737	3.3927314	0.5817076	0.560764	NA
spred2a	447.010446	0.065110557	0.116515	0.5588169	0.576287	0.950611
mia	813.9876737	-0.057597624	0.0913056	-0.630823	0.528157	0.941768
actr2a	1595.270911	-0.038014567	0.0886603	-0.428766	0.668093	0.964736
hspb2	67.95578403	0.25470919	0.2725859	0.934418	0.350088	0.880195
rhpn1	5.940875815	0.824204419	0.7893904	1.0441024	0.296438	NA
porcnl	156.4010779	-0.154440779	0.1627662	-0.948851	0.342697	0.878434
pisd	813.0985177	0.101625761	0.0966293	1.051708	0.292934	0.848631
dusp10	4.281636488	0.017568423	0.910727	0.0192905	0.984609	NA
gsk3bb	36.62339484	-0.114592384	0.3309498	-0.346253	0.729153	0.970822
igfbp2a	1149.244205	0.275683823	0.0999282	2.7588179	0.005801	0.114711
taf1a	106.8846062	0.101311673	0.1910145	0.5303873	0.595843	0.954434
pdcd11	984.1898613	-0.087393231	0.0885473	-0.986967	0.323659	0.870928
arhgef11	917.3549507	0.018029896	0.0964244	0.1869848	0.851673	0.985174
pcdh18b	1433.832484	-0.029113787	0.0820152	-0.35498	0.722604	0.970375
arhgap45a	13.99275157	0.433112939	0.5468006	0.7920857	0.428311	0.911082
zgc:110789	148.5591981	-0.503205111	0.1747074	-2.880273	0.003973	0.086636
lin28b	4.977601262	0.543509786	0.9033323	0.6016721	0.547392	NA
tsga10	65.31635023	0.080445226	0.2575827	0.3123084	0.754806	0.975207
calcoco2	55.80291821	0.275824607	0.2698183	1.0222605	0.306658	0.86012
stx4	358.2739134	0.145324012	0.123573	1.1760179	0.239588	0.808091
sgms2a	63.83326225	-0.176276718	0.243608	-0.723608	0.469306	0.924505
snx11	248.3498702	-0.027882828	0.1419985	-0.19636	0.844328	0.985174
mul1b	84.10743973	-0.100492019	0.2204581	-0.455833	0.64851	0.960708
CR762407.1	20.32133164	-0.019196108	0.4186222	-0.045855	0.963425	0.995927
ubxn10	7.132604418	-0.439663095	0.7382231	-0.595569	0.551463	NA
tia1	1586.285065	0.14514812	0.0825281	1.7587727	0.078616	0.546417
cptp	244.701865	0.185979765	0.1424284	1.3057776	0.191628	0.755886
lig3	880.4199519	0.033839625	0.0883523	0.383008	0.701714	0.968134
fgfrl1b	469.8352812	0.011653901	0.1184392	0.0983957	0.921618	0.992138
porcn	182.1701363	0.017684126	0.1636918	0.1080331	0.913969	0.991119
cenpi	199.7236031	-0.11820422	0.1621506	-0.728978	0.466015	0.923865
tmem35	2432.355097	0.058918746	0.0817794	0.7204592	0.471242	0.924838

arl13a	226.9474743	-0.129212163	0.1590272	-0.812516	0.416495	0.906237
c6ast4	137.0948735	-0.077989156	0.5324691	-0.146467	0.883553	0.988706
nkiras2	455.948905	-0.059635238	0.1061069	-0.56203	0.574096	0.950037
cpeb2	14.09848427	-0.300681467	0.5371295	-0.559793	0.57562	0.950373
dpp9	1598.211967	0.085763156	0.0788036	1.0883148	0.276456	0.839541
CU468164.1	0.846012444	-0.57358332	2.1013569	-0.272959	0.784885	NA
olig4	41.11840304	-0.353523029	0.3105063	-1.138537	0.254896	0.823581
tnmd	693.4285549	0.371624291	0.0968544	3.8369389	0.000125	0.005865
raver1	388.3559595	0.014801962	0.1122973	0.1318105	0.895134	0.989814
ahrrb	2.297362113	-1.312358568	1.3717981	-0.95667	0.338734	NA
bsdc1	1115.494072	-0.062907878	0.0848821	-0.74112	0.45862	0.921978
spryd7a	384.2217794	0.054507193	0.1133538	0.4808592	0.630617	0.957354
fkbp1b	516.6669933	0.422429277	0.1031487	4.0953424	4.22E-05	0.002358
AL954655.1	17.61165038	0.355286615	0.5060257	0.7021118	0.482609	0.929042
si:ch211-106n13.3	117.7703736	-0.094664974	0.2029016	-0.466556	0.640817	0.959994
fam210b	217.8710976	-0.271803776	0.1838578	-1.478337	0.139318	0.678204
kpna3	1557.167594	0.158556295	0.0817013	1.9406817	0.052297	0.449084
shisa9b	5.943211754	-0.840791973	0.790625	-1.063452	0.287577	NA
ca10a	570.1545212	0.186585592	0.099611	1.873142	0.061049	0.482482
ftr69	2.946489095	1.015072657	1.1238134	0.9032395	0.366399	NA
hs3st4	20.17770528	-0.503897431	0.4568212	-1.103052	0.270005	0.834705
ube2ia	1337.918202	-0.002511636	0.0804796	-0.031208	0.975103	0.996762
fermt1	468.9490202	-0.235440615	0.1082097	-2.175781	0.029572	0.332811
thrab	93.14550401	-0.151361593	0.2108964	-0.717706	0.472939	0.924993
si:ch211-193e13.5	2.156906127	0.220425787	1.2793919	0.1722895	0.86321	NA
pptc7b	43.2609523	-0.191745952	0.2912123	-0.65844	0.510255	0.936253
sept7b	1955.016263	-0.063799537	0.0772598	-0.825779	0.408929	0.90171
csnk1a1	3552.661769	0.017929324	0.0673447	0.266232	0.790061	0.977338
si:ch211-138g9.2	0.650115553	1.435486287	2.5894636	0.5543566	0.579335	NA
si:dkey-182g1.2	332.6250848	-0.159222052	0.130833	-1.216987	0.223609	0.790003
paqr5b	60.32926786	-0.25427674	0.2775019	-0.916306	0.359506	0.884959
arrdc3a	4854.295018	0.136563092	0.0733345	1.862194	0.062576	0.487923
si:dkey-172m14.1	283.5660808	-0.186077051	0.1374336	-1.353942	0.175755	0.736411
nr2f1a	2353.250308	0.072171876	0.0751196	0.9607602	0.336673	0.876088
bdh2	0	NA	NA	NA	NA	NA
fam172a	259.5308422	0.055279574	0.143477	0.3852852	0.700026	0.967998
SLC9B2	299.8273725	-0.065619785	0.1349651	-0.486198	0.626827	0.957354
smpd2b	119.1893478	-0.021206957	0.2037743	-0.104071	0.917113	0.991572
capn1a	815.6440964	0.121023388	0.0952092	1.2711316	0.203682	0.771276
cisd2	172.7793042	-0.005824395	0.1553136	-0.037501	0.970086	0.996315
pkp1b	186.3176671	-0.16559702	0.1656173	-0.999878	0.31737	0.867404
tnni1b	178.9102019	-0.11289862	0.1627361	-0.693753	0.487837	0.931809
suclg1	3216.730504	-0.069112008	0.0767277	-0.900744	0.367724	0.887476
lrrtm1	660.9419666	0.219393799	0.093932	2.3356651	0.019509	0.262069
prim2	382.5812731	-0.044054661	0.1204799	-0.36566	0.714619	0.96909
sltm	2526.868827	-0.045728979	0.0743621	-0.61495	0.538588	0.943336
ankrd31	1.84649756	1.471731511	1.5572461	0.945086	0.344615	NA
hmgcra	734.0114823	-0.100932286	0.1110364	-0.909002	0.363349	0.885944
egf	67.97554988	-0.101083792	0.2678208	-0.377431	0.705854	0.968412
card11	110.1141445	0.016784106	0.1919557	0.0874374	0.930324	0.992702
grinab	1169.659579	-0.088234273	0.0908439	-0.971273	0.331412	0.874362
gpatch3	255.8233799	-0.280938534	0.1437655	-1.954145	0.050684	0.44139
capn1b	415.1590269	0.075797143	0.1102283	0.687638	0.491681	0.931926
pimr130	1.302602537	2.681479841	1.8879348	1.4203244	0.155513	NA

chrnb3a	338.2139982	-0.01547032	0.1227973	-0.125983	0.899746	0.990121
gria2b	1079.68118	-0.00287036	0.0867595	-0.033084	0.973608	0.996315
si:ch211-239f4.1	36.89029941	0.270671253	0.313961	0.8621175	0.388623	0.896271
glrbb	1493.439894	0.067895194	0.0793595	0.8555394	0.392253	0.897688
opn3	94.92202421	0.135156212	0.2018822	0.6694807	0.503189	0.935304
zgc:153932	205.8183645	0.722998693	0.3639615	1.9864703	0.046981	0.425369
glrba	260.2122829	0.234374414	0.1316679	1.7800418	0.075069	0.533746
cdc42ep3	325.6070922	0.282602249	0.1223647	2.3095075	0.020915	0.273172
zdhhc12b	97.04727354	0.132286064	0.2074559	0.6376587	0.523696	0.939336
azin1a	545.5665671	0.019334326	0.1055519	0.1831737	0.854662	0.985174
shmt1	847.1614528	-0.237912765	0.093617	-2.54134	0.011043	0.18215
smcr8a	201.8974511	0.300599219	0.1527722	1.9676304	0.049111	0.435115
runx3	198.4012373	-0.187718784	0.1470147	-1.276871	0.201648	0.767736
top3a	149.608818	-0.159436832	0.1671118	-0.954073	0.340047	0.87805
zfand3	1891.625628	-0.0375857	0.0945351	-0.397585	0.690936	0.967157
ndufs4	866.851656	0.124296525	0.1059177	1.1735198	0.240587	0.808802
ppifb	546.474859	-0.008444921	0.1095633	-0.077078	0.938562	0.992849
fsta	530.8339252	-0.08296961	0.1036521	-0.800462	0.423443	0.90766
golph3	1201.388239	-0.011454543	0.0811343	-0.14118	0.887728	0.989291
ctso	66.62969089	0.115164738	0.2734969	0.4210825	0.673695	0.965257
khdrbs1a	14196.39998	-0.050307989	0.0667878	-0.753251	0.451299	0.918761
chp1	107.3702005	-0.1121362	0.2039515	-0.549818	0.582444	0.952117
htt	830.0501618	0.030667784	0.1043061	0.2940173	0.768745	0.975687
CR933791.1	2.141233338	-0.239702095	1.3293395	-0.180317	0.856904	NA
CR933791.2	0.166657454	-0.955901296	4.0804729	-0.234262	0.814781	NA
dnajb5	25.66562571	0.70295893	0.3759255	1.8699421	0.061492	0.483475
rx3	254.9361785	0.043519108	0.1418214	0.3068585	0.758951	0.975374
znf532	118.1497443	0.021647486	0.2038586	0.1061887	0.915433	0.991171
htra3a	5.91287694	-0.314279182	0.7736277	-0.406241	0.684566	NA
atxn2	449.7967099	0.223543714	0.1229004	1.8189021	0.068926	0.513259
kcnk3b	8.637162451	0.559197357	0.7288272	0.7672565	0.442929	NA
CU550714.1	0.182570949	0.967652056	4.0804729	0.2371421	0.812547	NA
zgc:153642	0	NA	NA	NA	NA	NA
rtkn2b	60.70170955	0.109264887	0.2617868	0.4173812	0.6764	0.965257
zgc:165423	670.5146567	-0.143304226	0.1142162	-1.254675	0.209597	0.776996
bcl7a	961.6874614	0.085978187	0.0885936	0.9704788	0.331808	0.874653
wnt8a	0	NA	NA	NA	NA	NA
si:dkey-57n24.6	122.2900998	-0.071288272	0.1953955	-0.364841	0.71523	0.96909
si:ch211-202f3.3	387.2450358	0.223050343	0.1143936	1.9498494	0.051194	0.443542
adrb3b	0.158915748	0.967652056	4.0804729	0.2371421	0.812547	NA
zgc:113162	211.1962356	-0.182757312	0.1485915	-1.229931	0.218723	0.785734
arf6b	1141.688284	0.243585519	0.0865389	2.8147502	0.004882	0.101715
aspg	496.5341152	-0.092394662	0.1034952	-0.892744	0.371994	0.89248
nppal	106.1171504	-0.037312135	0.192781	-0.193547	0.846531	0.985174
arhgap23a	1268.427591	-0.083592345	0.0807586	-1.035088	0.300628	0.856282
rab25b	28.56615168	-0.76239504	0.3767933	-2.023377	0.043034	0.407353
plcd3a	331.7022437	0.032597487	0.1181504	0.2758981	0.782626	0.97627
nppb	8.466021169	-0.833179915	0.7138148	-1.167221	0.243121	NA
nppa	152.3737868	-0.096873915	0.1801691	-0.537683	0.590796	0.954193
nmt1a	2264.335531	0.024330866	0.0813034	0.2992601	0.764742	0.975374
ubqln4	2087.694977	0.036175039	0.0863698	0.4188388	0.675334	0.965257
mbnl1	43.99512322	0.227873512	0.2957678	0.7704472	0.441035	0.916336
gabrr2a	117.154629	0.427858149	0.1859447	2.3009972	0.021392	0.276471
xcr1b.1	0	NA	NA	NA	NA	NA

slc25a28	1051.004747	0.081427167	0.0872555	0.9332036	0.350715	0.880195
sema4e	270.237294	0.270504464	0.1322065	2.0460751	0.040749	0.394957
slc35f1	248.1037958	0.056719177	0.1347782	0.4208334	0.673877	0.965257
cd22	10.81495379	-0.660752148	0.5875273	-1.124632	0.260745	NA
lpcat2	177.127342	-0.107889671	0.1659928	-0.649966	0.515714	0.9377
rnft2	324.8162649	0.182815785	0.1435027	1.273954	0.20268	0.769545
grsf1	386.7210037	0.084418887	0.1135841	0.7432278	0.457344	0.921679
kif19	466.5335493	-0.308050749	0.1143338	-2.69431	0.007053	0.131086
si:dkey-33i11.1	5.07439649	0.860185401	0.8698884	0.9888457	0.322739	NA
si:ch211-203b20.7	3.36019841	0.597187268	1.032289	0.5785079	0.562921	NA
eif2s2	4057.777448	0.009906662	0.0697263	0.1420793	0.887017	0.989231
rps11	12341.0561	-0.372917877	0.1045452	-3.56705	0.000361	0.013676
CR628323.2	3.655522653	-0.523832987	0.9791613	-0.534981	0.592663	NA
tubb1	17.93004819	0.426819089	0.4582655	0.9313795	0.351657	0.880769
cyp8b1	338.3623168	-0.403192444	0.1277556	-3.155966	0.0016	0.045253
gosr2	433.1874492	-0.222704093	0.1177206	-1.891802	0.058517	0.473924
gipc3	49.95477581	-0.171065902	0.2738772	-0.624608	0.532228	0.942546
thada	178.7278815	-0.450224764	0.1581724	-2.846418	0.004421	0.094496
mthfr	493.708897	-0.058477795	0.1112059	-0.525851	0.598991	0.955806
si:ch211-204c21.1	708.1539752	-0.139919244	0.1002312	-1.395965	0.162725	0.717762
hsf2	1255.02297	-0.250785331	0.086644	-2.894433	0.003798	0.08411
prpf38b	983.1027374	-0.051396068	0.0899405	-0.571445	0.567698	0.948739
fam102ba	88.48552611	0.541761991	0.2131512	2.5416797	0.011032	0.18215
cep44	64.55229778	-0.009148901	0.2492357	-0.036708	0.970718	0.996315
pkib	298.3733616	-0.037789772	0.130353	-0.289903	0.77189	0.975687
ly75	315.3184752	0.139390504	0.1281204	1.0879652	0.27661	0.839683
si:ch211-272h9.3	0.166657454	-0.955901296	4.0804729	-0.234262	0.814781	NA
smpdl3a	26.50437426	-0.735903943	0.394527	-1.865281	0.062142	0.486844
clvs2	18.08049711	0.257394001	0.4593982	0.5602851	0.575285	0.95035
si:dkey-148a17.6	1.753484786	2.139621533	1.6668905	1.2836005	0.199282	NA
helb	96.54582456	-0.19552869	0.2070924	-0.944161	0.345087	0.878434
carhsp1	437.5633869	-0.083462643	0.1183654	-0.705127	0.480731	0.927601
pcp4a	1106.459822	0.058678338	0.0983939	0.5963615	0.550934	0.946549
irak3	35.1974825	-0.09695623	0.3352242	-0.289228	0.772407	0.975687
b3gnt7l	143.3780287	0.22393653	0.1934054	1.1578607	0.246921	0.816669
tas1r2.1	0	NA	NA	NA	NA	NA
b2m	29.80143922	0.035157754	0.3778003	0.0930591	0.925857	0.992702
lrrc47	519.6224439	-0.176945966	0.103125	-1.715839	0.086191	0.563157
pcmtd1	616.3360547	-0.323189696	0.0976503	-3.309663	0.000934	0.029459
alg3	364.1715812	0.066551957	0.1197736	0.5556481	0.578451	0.951052
prss56	8.224589799	0.44339293	0.7057208	0.6282838	0.529818	NA
zgc:110626	280.1114132	0.096740219	0.1302887	0.7425063	0.457781	0.921978
zgc:113377	21.64846739	-0.223406587	0.4491102	-0.497443	0.618877	0.957354
chic1	162.9630502	-0.264675214	0.1613879	-1.639994	0.101006	0.604004
cttnbp2nla	75.1886995	-0.070366911	0.2306389	-0.305096	0.760293	0.975374
vstm4b	40.18065587	-0.061131235	0.3079032	-0.19854	0.842622	0.985174
galnt16	332.8149725	0.169505517	0.1260517	1.34473	0.178712	0.739486
pdzd11	487.3446501	0.177280716	0.1094566	1.6196438	0.105309	0.613727
mtor	1209.628509	-0.045551129	0.0816466	-0.557906	0.576908	0.950728
dis3l	136.2381188	-0.242886749	0.1840886	-1.319401	0.187035	0.751002
zgc:172323	44.37115338	0.204628153	0.3007674	0.6803534	0.496281	0.932415
snx22	47.58599251	-0.155857255	0.2868355	-0.543368	0.586876	0.953319
ppfia4	163.6775495	0.08759401	0.1650826	0.5306073	0.595691	0.954434
smad6a	400.0366222	-0.124904393	0.119389	-1.046197	0.29547	0.852067

me1	90.38011226	0.175170758	0.241608	0.7250206	0.468439	0.924328
cox7a2a	1691.300415	-0.043316613	0.1177013	-0.368021	0.712857	0.96909
asb5b	262.2655139	0.149014523	0.1414849	1.0532183	0.292241	0.848075
itgb1b.1	380.6985186	0.218535727	0.1139123	1.9184558	0.055053	0.459919
kif6	13.24242881	0.153695332	0.5518669	0.2785007	0.780628	0.97627
sts	62.03931868	0.014409213	0.2577622	0.0559012	0.955421	0.994543
depdc7b	2.12476036	1.178327444	1.3385161	0.8803237	0.378684	NA
inab	599.4206033	0.205277813	0.1044853	1.9646581	0.049454	0.436093
mylpfa	75609.85044	0.133837241	0.1087918	1.2302143	0.218617	0.785734
zgc:113090	69.83427903	0.165327667	0.2355606	0.7018476	0.482774	0.929133
atp1b4	3212.953801	0.219099739	0.0731193	2.99647	0.002731	0.067381
zgc:113372	78.68021722	-0.169135553	0.2188435	-0.772861	0.439605	0.916082
slc2a15b	123.9146874	-0.037031421	0.1776334	-0.208471	0.834861	0.984089
RAP1GAP	96.29337912	-0.161859576	0.2305702	-0.701997	0.482681	0.929089
apln	1848.329368	0.080972786	0.0777861	1.0409666	0.297891	0.85401
pnrc2	6797.395364	-0.059083508	0.0715083	-0.826247	0.408664	0.90171
ftr14	46.50315792	-0.112054795	0.3032004	-0.369573	0.7117	0.969006
nkx2.2a	289.5342571	0.103883765	0.1247485	0.8327458	0.404988	0.901305
insm1b	605.6276203	-0.102602755	0.1078101	-0.951699	0.341249	0.878134
tespa1	18.57301846	0.100012858	0.4359862	0.2293945	0.818562	0.981594
tmprss3a	48.14505518	0.03402756	0.2853189	0.1192615	0.905068	0.990702
lrrc6	33.38481884	0.142781118	0.3280431	0.4352511	0.66338	0.964736
zgc:112285	1148.681011	0.030674139	0.0877694	0.3494855	0.726725	0.970402
gna11b	81.9367352	-0.122136317	0.2346264	-0.520556	0.602676	0.956045
si:ch211-138g9.3	0.158915748	0.967652056	4.0804729	0.2371421	0.812547	NA
adgrd1	15.75846478	-0.330906703	0.4814507	-0.687312	0.491886	0.931926
si:dkey-56i24.1	15.24652252	0.116603563	0.4875445	0.239165	0.810978	0.981081
basp1	8204.150736	0.060231352	0.0691412	0.8711351	0.38368	0.895192
cilp	1307.646228	0.352864167	0.080731	4.3708625	1.24E-05	0.000862
pax10	297.8023952	0.060267116	0.1437949	0.4191186	0.675129	0.965257
rpl31	10042.59225	-0.472108287	0.1073421	-4.398165	1.09E-05	0.000791
ftr12	7.387239509	0.418454123	0.7541435	0.5548733	0.578981	NA
eif3jb	855.9496905	0.183845184	0.0950835	1.9335125	0.053173	0.452412
nptxrb	493.7063354	0.100587294	0.1192354	0.8436025	0.398892	0.900446
fam189a1	280.6790505	-0.175136853	0.1331054	-1.315776	0.188249	0.753073
plpp1a	612.5759154	0.076946109	0.1113581	0.6909787	0.489579	0.931926
rprma	62.14899762	0.016543617	0.2502801	0.0661004	0.947298	0.99381
wdr27	5.828665551	-0.043629462	0.8162126	-0.053454	0.957371	NA
CU207281.1	0	NA	NA	NA	NA	NA
jade2	6.836486527	0.209480812	0.7143129	0.293262	0.769322	NA
cdkn2aipnl	514.9221017	-0.124127726	0.1245232	-0.996824	0.31885	0.867746
PPP2CA	1537.012389	-0.066889782	0.0762917	-0.876763	0.380615	0.895192
sord	323.5746221	-0.118886595	0.132253	-0.898933	0.368688	0.888057
myl2b	83.61928398	-0.187598564	0.2304264	-0.814137	0.415567	0.906237
serinc2l	12.42329956	0.818825678	0.5699089	1.4367658	0.150785	0.698912
si:ch211-257p13.3	360.1515742	0.147536507	0.1295339	1.1389798	0.254712	0.823484
sub1a	1204.80062	-0.054758505	0.1172432	-0.467051	0.640464	0.959994
ppp4r2b	944.7017192	-0.046505032	0.088233	-0.52707	0.598145	0.955466
si:ch211-251f6.6	349.1110808	-0.105219031	0.1375381	-0.765018	0.444261	0.916726
pyya	285.3254635	0.041239736	0.1383104	0.298168	0.765575	0.975374
ftr06	1.108833117	1.320027098	2.0128984	0.6557843	0.511963	NA
pop5	119.8134717	-0.148794834	0.1875619	-0.793311	0.427597	0.91075
mpp2a	143.7182937	-0.081438924	0.1670254	-0.487584	0.625844	0.957354
cntn3b	18.86245448	0.020449376	0.4481308	0.0456326	0.963603	0.995927

ccdc103	28.76218851	0.046065749	0.3707085	0.1242641	0.901106	0.990121
GK3P	567.2415161	0.111294109	0.1017986	1.0932776	0.274272	0.839136
rpl23	12085.0902	-0.377917343	0.1068048	-3.538393	0.000403	0.014879
rybpb	740.1068355	-0.027059046	0.0910334	-0.297243	0.766281	0.975437
si:dkey-283b1.7	11.86521276	0.188378187	0.5829563	0.3231429	0.746587	0.97419
c3a.5	0.158795395	0.967652056	4.0804729	0.2371421	0.812547	NA
poglut1	218.9909491	0.185562958	0.1499934	1.237141	0.216035	0.783484
timmdc1	400.7344742	0.058745157	0.1175837	0.4996029	0.617355	0.957354
gtpbp1	1185.125638	0.039175819	0.0803414	0.4876168	0.625821	0.957354
adam10a	2449.109876	-0.037025149	0.0714857	-0.517938	0.604502	0.95651
tdrkh	290.6472494	0.18683805	0.1273319	1.4673313	0.142286	0.684232
ngb	524.40735	0.032698277	0.1063613	0.3074265	0.758519	0.975374
lipca	12.43931118	0.995377319	0.6015614	1.6546563	0.097994	0.59749
gdf6a	151.5091309	-0.402522087	0.1750236	-2.299816	0.021459	0.276973
aqp9b	1354.20449	0.247751346	0.0916693	2.7026646	0.006879	0.128683
entpd5a	153.2313153	-0.010659435	0.1710294	-0.062325	0.950304	0.99381
zgc:113054	513.9396292	0.133638526	0.1047384	1.2759272	0.201981	0.76789
syne2b	818.6140917	-0.100652315	0.100006	-1.006462	0.314193	0.865176
aldh6a1	3644.860663	-0.087324032	0.0767625	-1.137588	0.255293	0.823868
osbp2	386.362997	0.365880672	0.1149768	3.1822123	0.001462	0.041885
si:ch73-22a13.3	5.333327902	0.204593977	0.8345108	0.2451664	0.806328	NA
aldh1a2	1161.150346	-0.244918031	0.0977321	-2.506015	0.01221	0.195183
gemin8	259.4131809	-0.1100746	0.1439382	-0.764735	0.444429	0.916726
ednrbb	1.141958407	1.326526475	1.8207251	0.7285704	0.466264	NA
isl2b	492.7437696	-0.147377223	0.112518	-1.30981	0.19026	0.754967
cbsa	176.0258864	0.158569355	0.1667904	0.9507102	0.341752	0.878134
cryaa	1002.698708	-0.079005897	0.0975245	-0.810113	0.417875	0.906293
fbxo22	659.03709	0.017965792	0.1041307	0.1725311	0.86302	0.985841
kazald3	206.3569583	0.33785217	0.1702127	1.9848828	0.047158	0.42638
crybb2	12.99196775	-0.153758546	0.5367507	-0.286462	0.774524	0.975687
si:dkey-1f12.3	785.85654	-0.072368026	0.1015054	-0.712947	0.475878	0.926338
adhfe1	146.7872108	-0.061000499	0.1659369	-0.367613	0.713162	0.96909
LO017829.1	25.79618856	0.046175916	0.3797066	0.1216095	0.903208	0.990121
enpp7.2	0.158915748	0.967652056	4.0804729	0.2371421	0.812547	NA
enpp7.1	4.495563059	-0.090510502	0.8823123	-0.102583	0.918294	NA
snx24	38.19081545	-0.071572342	0.319539	-0.223986	0.822768	0.982189
nog3	221.0061387	-0.026861801	0.1437052	-0.186923	0.851721	0.985174
cyp4f3	7.878436152	-0.536736202	0.7328783	-0.732367	0.463944	NA
lmo7b	244.0781516	0.131251789	0.1510359	0.8690104	0.384841	0.89551
kctd12.2	244.9604427	0.243866002	0.1343861	1.8146672	0.069575	0.514708
hspa4l	1112.593151	0.303948652	0.0972583	3.1251708	0.001777	0.049283
jakmip2	1207.16898	0.10142385	0.0910704	1.1136865	0.265414	0.831302
wdr76	407.2722697	0.161031851	0.1232409	1.3066431	0.191334	0.755886
rtkn2a	127.4408341	0.095187062	0.1817583	0.5237012	0.600486	0.956045
tspan3b	524.528742	-0.055511268	0.1065172	-0.521148	0.602264	0.956045
ms4a17a.12	16.18649451	-0.509032787	0.5152324	-0.987967	0.323169	0.870607
pstpip1b	2.610036199	-0.735004429	1.1858366	-0.619819	0.535377	NA
sox3	865.0648845	-0.179103838	0.0946844	-1.891588	0.058546	0.473924
AL590151.1	9.782438733	-0.129684519	0.6238728	-0.20787	0.83533	NA
adob	556.9324543	-0.273932728	0.1032647	-2.652724	0.007985	0.143794
fancd2	373.9188617	0.000968093	0.1153291	0.0083942	0.993302	0.998554
gpat2	18.90481449	0.428521565	0.4871842	0.8795885	0.379082	0.895192
htr1fb	1.50674929	-0.34759043	1.6384219	-0.21215	0.83199	NA
cers6	253.3364045	0.048724479	0.1377005	0.3538438	0.723456	0.970402

creb1a	901.0874695	-0.003727911	0.0949303	-0.03927	0.968675	0.996315
gatd1	325.2647165	-0.194621735	0.1205226	-1.614816	0.106351	0.615567
camk2a	142.1877128	0.174639797	0.1767124	0.9882714	0.32302	0.870607
sra1	223.8599085	-0.045516671	0.1497645	-0.303922	0.761188	0.975374
ebi3	14.61964724	1.333673757	0.5144019	2.5926687	0.009523	0.163896
csf1rb	3.67853806	0.519303748	0.9984485	0.5201107	0.602986	NA
anxa2b	739.5221279	-0.899031339	0.0939364	-9.570642	1.06E-21	1.17E-18
si:dkey-182o15.5	0.173729368	-0.955901296	4.0804729	-0.234262	0.814781	NA
cracr2b	87.56251095	-0.317501422	0.2149786	-1.476898	0.139703	0.678481
got1l1	10.79061851	0.038493526	0.5865342	0.0656288	0.947673	NA
adrb3a	60.32104327	0.068038132	0.2702502	0.2517598	0.801227	0.979377
or135-1	1.163603234	0.397890555	1.8318468	0.2172073	0.828047	NA
foxb1b	49.43936102	-0.317883166	0.2847706	-1.116278	0.264303	0.830245
ndufaf6	119.2452236	-0.069032132	0.1882888	-0.366629	0.713896	0.96909
lypla2	1835.61399	0.13030136	0.0898888	1.4495833	0.147175	0.69407
gabrg2	15.04041077	0.78622352	0.510214	1.540968	0.123325	0.651258
myb	439.7897994	-0.147186204	0.1111742	-1.323924	0.185528	0.749313
stag2b	2260.005139	-0.051434893	0.0836253	-0.615064	0.538513	0.943336
aldob	7379.785354	-0.253752525	0.2032349	-1.248567	0.211823	0.779854
gtf2a2	248.9712119	0.015779452	0.1334178	0.118271	0.905853	0.990702
maea	1069.161892	-0.059217998	0.0854173	-0.693279	0.488135	0.93189
zgc:162948	89.23648529	-0.057757118	0.2088457	-0.276554	0.782123	0.97627
il2rgb	12.9560187	-0.063705574	0.5236669	-0.121653	0.903174	0.990121
ghrhrb	17.56622833	-0.043369239	0.4601429	-0.094252	0.924909	0.992676
si:ch73-89b15.3	66.19557929	-0.828253365	0.2429799	-3.408731	0.000653	0.021837
GPR68	3.527337275	0.427371899	1.0327804	0.4138071	0.679015	NA
ccdc88c	667.282847	0.074473485	0.0948077	0.7855215	0.432148	0.912959
zgc:153990	369.784995	-0.1499748	0.122045	-1.228848	0.219129	0.785734
adcyap1r1a	117.4496443	-0.046124666	0.1838384	-0.250898	0.801893	0.979492
ttll3	23.41114739	-0.377061855	0.3936373	-0.957891	0.338117	0.876778
AL954695.1	4.891743218	0.007533135	0.9055065	0.0083193	0.993362	NA
dcdc2b	46.77091627	0.112866943	0.2810646	0.4015694	0.688001	0.966493
CABZ01084566.1	199.364252	0.111376216	0.1514985	0.7351639	0.46224	0.922698
mff	599.3069404	0.010689921	0.096988	0.110219	0.912236	0.991119
si:dkey-187j14.4	91.59105281	0.093130905	0.20688	0.4501688	0.652589	0.960708
apbb3	36.79034395	0.209750711	0.3193423	0.656821	0.511296	0.936253
vgll2b	53.70942439	0.077951608	0.275439	0.2830086	0.77717	0.975826
alpi.2	47.01786869	-1.21123605	0.3107935	-3.897238	9.73E-05	0.004774
si:ch211-218m3.13	2.705409119	0.403021954	1.2517504	0.3219667	0.747478	NA
si:rp71-1g18.1	230.7006941	-0.284679258	0.1541127	-1.847215	0.064716	0.497355
si:ch211-276c2.4	467.3103872	-0.062145304	0.1171149	-0.530636	0.595671	0.954434
cbln5	9.303091386	0.105770925	0.6165626	0.1715494	0.863792	NA
klhl43	398.5203178	0.370589242	0.1425199	2.600263	0.009315	0.162001
osbpl2a	20.40875535	-0.040754866	0.4617477	-0.088262	0.929668	0.992702
trub1	90.75400073	0.156232156	0.2086804	0.748667	0.454058	0.920295
hnrnpc	1530.615463	-0.090628026	0.0850517	-1.065564	0.286621	0.845942
or115-11	5.987295806	-0.022475656	0.8032585	-0.027981	0.977678	NA
or115-2	0	NA	NA	NA	NA	NA
pcmtd2a	224.1710017	-0.311839167	0.1530977	-2.036863	0.041664	0.400593
ubxn11	14.54741991	-0.429849046	0.5299522	-0.811109	0.417303	0.906237
pax9	246.0174616	-0.030163855	0.153342	-0.19671	0.844055	0.985174
vtnb	958.6632852	0.21032799	0.0986261	2.1325797	0.032959	0.35382
mtnr1ba	1.800015086	0.733377371	1.542912	0.4753203	0.634559	NA
plekhh1	37.01455161	0.53912523	0.3201448	1.6840042	0.092181	0.57849

si:ch211-284o19.8	52.9329911	-1.048287623	0.2902207	-3.612035	0.000304	0.011943
ei24	242.0186379	0.176946343	0.1352449	1.3083401	0.190758	0.755212
cbln14	21.42614485	0.115619137	0.4045994	0.285762	0.77506	0.975687
slc13a2	633.5173211	-0.080463262	0.1460539	-0.550915	0.581692	0.951868
uts2a	6.43477612	-0.660941272	0.7538791	-0.87672	0.380638	NA
ccdc187	256.8883158	-0.100507731	0.1510609	-0.665346	0.505829	0.935724
crip1	829.403796	-0.15890177	0.088343	-1.798691	0.072068	0.525083
ism2b	4.594972336	1.568955315	0.9620126	1.6309092	0.102909	NA
crygmx	5602.853677	0.089887461	0.0711697	1.2630019	0.206589	0.774129
elmsan1b	617.229459	0.14470349	0.1111656	1.3016928	0.193021	0.756795
etv2	66.59561551	-0.338472503	0.2819184	-1.200605	0.229905	0.79764
cryba1b	19194.8472	-0.01901612	0.0707011	-0.268965	0.787957	0.976685
klhl26	51.29964744	0.034225796	0.2804069	0.1220576	0.902853	0.990121
cpdb	1.495310112	-0.994696088	1.5684186	-0.634203	0.525948	NA
inpp5l	462.6493478	0.125520636	0.1084387	1.1575264	0.247057	0.816669
med13a	1778.076594	-0.008877039	0.0974968	-0.09105	0.927453	0.992702
tmem62	47.81917898	0.079381361	0.2822591	0.2812358	0.77853	0.975942
slc30a1b	40.20484231	0.522712156	0.3104726	1.6836017	0.092259	0.57849
angpt2b	46.68731384	0.667872646	0.2867607	2.3290247	0.019858	0.265495
si:dkey-42l23.3	0	NA	NA	NA	NA	NA
fbl	2310.392096	-0.193394418	0.0863771	-2.238954	0.025159	0.30686
alg10	132.8503118	-0.048794808	0.1810059	-0.269576	0.787487	0.976596
srfa	34.30652804	0.395973571	0.3419441	1.1580068	0.246861	0.816669
si:dkey-117a8.3	1.657414181	-1.213759884	1.5969087	-0.760068	0.447214	NA
opn4b	29.65797698	0.236749262	0.3585702	0.6602592	0.509088	0.936123
si:dkey-42l23.5	0	NA	NA	NA	NA	NA
hdhd3	94.03891108	-0.151092484	0.2138613	-0.706498	0.479879	0.927125
si:ch211-137j23.8	0	NA	NA	NA	NA	NA
tgfa	107.281484	0.329187781	0.1989255	1.6548296	0.097959	0.59749
btr32	8.399494854	0.138607217	0.7145032	0.193991	0.846183	NA
lin37	214.1914607	-0.138232125	0.1707546	-0.809537	0.418207	0.906406
myadml2	17.12740463	0.549888821	0.5374192	1.0232028	0.306212	0.860027
sptbn2	1249.13016	-0.021266356	0.0896867	-0.237118	0.812565	0.981081
cdc42ep2	39.14290756	-0.202321086	0.3069195	-0.659199	0.509768	0.936253
slc2a11a	17.93309923	1.192638065	0.4585698	2.6007777	0.009301	0.162001
npm2b	4.944556129	0.518728999	0.8953113	0.5793839	0.56233	NA
cyp17a2	15.81318029	0.433287396	0.5326106	0.8135162	0.415922	0.906237
fetub	7379.94389	0.296884674	0.2094799	1.4172467	0.156411	0.706639
chmp2a	608.3525398	-0.112488006	0.1045558	-1.075865	0.281988	0.843666
htr1b	53.13162804	0.332625297	0.263301	1.2632893	0.206485	0.774129
hmgb2b	6133.828744	-0.276993055	0.0661156	-4.189527	2.80E-05	0.001665
sfi1	156.9529493	-0.032603122	0.1747762	-0.186542	0.85202	0.985174
gpank1	153.7570228	-0.182293764	0.174863	-1.042495	0.297182	0.853945
rargb	6.155671599	0.541202192	0.7707005	0.7022212	0.482541	NA
ppp1r2	317.7805036	-0.022409121	0.1277752	-0.175379	0.860782	0.985773
scaf1	2468.551931	0.004437131	0.0953563	0.0465321	0.962886	0.995927
ttll6	63.81730287	-0.041324743	0.2488624	-0.166055	0.868114	0.987245
hoxb8b	92.42771119	0.01485889	0.2175652	0.0682963	0.94555	0.993589
mustn1a	13.5201232	-0.157891939	0.5194387	-0.303966	0.761153	0.975374
hoxb5b	527.570692	-0.072427413	0.1071929	-0.675673	0.499248	0.934006
mxd4	361.5960626	-0.284140317	0.1204865	-2.358276	0.01836	0.25227
rab24	187.9036465	0.003729582	0.1494317	0.0249584	0.980088	0.996879
hoxb1b	147.0524077	-0.036614939	0.1703724	-0.214911	0.829837	0.982821
rnf34b	8.221721251	0.550238281	0.6747004	0.8155298	0.414769	NA

plin1	8.286729563	-0.863858166	0.7030091	-1.228801	0.219146	NA
vsig8a	122.2990482	0.024400843	0.1884634	0.1294726	0.896984	0.990006
zgc:153219	9.167597045	0.543278231	0.6733417	0.8068389	0.419759	NA
ostn	38.26702138	0.133591344	0.3086265	0.4328576	0.665118	0.964736
h1fx	3360.591014	-0.364794547	0.0730752	-4.992042	5.97E-07	6.51E-05
pof1b	570.9103864	0.086100268	0.099482	0.8654857	0.386772	0.89551
arpc4	943.3419624	0.031877267	0.099097	0.3216776	0.747697	0.97419
tatdn3	23.35068222	-0.248448735	0.4019454	-0.618116	0.536499	0.943336
irs1	364.8108253	0.033451888	0.1267941	0.2638284	0.791912	0.977937
rbm41	39.81125805	-0.066445497	0.3294316	-0.201697	0.840153	0.985029
ripply1	37.07217314	0.249333403	0.3262164	0.764319	0.444677	0.916777
tmem30b	359.7545665	0.067975174	0.1142956	0.5947315	0.552023	0.947215
htr1d	142.1654043	-0.029386236	0.1770057	-0.166019	0.868142	0.987245
slc26a6l	69.80685852	0.491039012	0.2364028	2.0771283	0.03779	0.380669
im:7136021	74.66671167	0.036375161	0.2300504	0.1581182	0.874364	0.987881
ccdc105	11.90825301	0.225894928	0.5658667	0.3992016	0.689745	0.966819
adgrg6	468.0691096	0.133277904	0.1076857	1.2376569	0.215843	0.783484
cx23	289.2728546	0.097466255	0.1429457	0.6818409	0.49534	0.931928
nmbr	10.53962832	-0.125784773	0.5987156	-0.210091	0.833597	NA
ftr50	0.809151653	1.852283317	2.2398915	0.8269523	0.408264	NA
bms1	763.6233897	0.023487944	0.0905829	0.2592977	0.795406	0.978498
pcna	4292.065529	-0.071681705	0.0937953	-0.764235	0.444727	0.916777
zgc:113625	9.013449487	1.04595687	0.7389907	1.4153857	0.156955	NA
nfatc4	101.879934	0.086107037	0.2080311	0.4139143	0.678937	0.965257
mrps17	178.0965266	-0.208766521	0.1553363	-1.343965	0.17896	0.73986
ltb4r2b	45.63341694	0.545173123	0.2925252	1.863679	0.062367	0.487498
pgrmc1	782.4437079	-0.244306348	0.1031932	-2.367467	0.01791	0.247704
adgra1a	483.5634997	0.045743716	0.1120854	0.4081147	0.683189	0.965476
valopb	16.79395408	0.1100941	0.4647875	0.2368697	0.812758	0.981081
btr06	19.83091811	-1.32752586	0.4542182	-2.922661	0.003471	0.079063
ncs1b	109.3119006	-0.174747967	0.1918065	-0.911064	0.362262	0.885944
pgk1	9420.156242	-0.043233136	0.0798808	-0.541221	0.588356	0.953319
slc38a8b	30.01719479	0.608555915	0.3625704	1.678449	0.093259	0.580702
mthfd2l	42.0174381	0.162492292	0.2931065	0.5543797	0.579319	0.951257
rnf40	1521.998416	-0.015646432	0.0774267	-0.202081	0.839854	0.984979
hbl4	19.42971131	-0.280849511	0.4797758	-0.585377	0.558295	0.948478
npffr1l1	2.713391684	0.16139052	1.2526068	0.1288437	0.897481	NA
CU682777.1	9.3105948	0.038250223	0.6430733	0.0594803	0.95257	NA
phkg2	245.4936309	0.07820637	0.1422786	0.5496707	0.582545	0.952161
st8sia7.1	49.16665296	-0.894297797	0.2946055	-3.035577	0.002401	0.061172
hectd1	4199.920981	0.045054322	0.0823485	0.5471174	0.584298	0.952332
si:ch73-269m23.5	57.98615334	-0.180304253	0.2550577	-0.706916	0.479619	0.927091
ap4s1	95.29376324	-0.201423268	0.2196138	-0.917171	0.359053	0.884725
fbxl8	52.56441519	-0.256994056	0.2698635	-0.952311	0.340939	0.878134
ipo11	331.37547	0.127811704	0.1312899	0.9735078	0.330301	0.874098
taar16a	0	NA	NA	NA	NA	NA
ghrl	3.921838121	-0.479920225	0.9699521	-0.494788	0.62075	NA
crbn	273.0277529	-0.223819696	0.1430522	-1.564602	0.117676	0.641446
pqlc3	78.50395075	0.048307119	0.220831	0.2187515	0.826844	0.982473
taar18d	0	NA	NA	NA	NA	NA
sobpa	13.44097452	-0.109429524	0.5134031	-0.213145	0.831214	0.983098
taar18a	0.332645115	0.005883744	3.2924249	0.0017871	0.998574	NA
tctex1d2	68.77155612	0.207556006	0.2370098	0.8757275	0.381178	0.895192
nat10	544.8691952	-0.021620873	0.1041567	-0.20758	0.835557	0.984089

taar20c	0.349228403	0.005884014	3.2372587	0.0018176	0.99855	NA
cog4	388.8134729	0.156727477	0.1178387	1.3300175	0.183513	0.747284
cdkn1bb	1415.224729	-0.019971494	0.089791	-0.222422	0.823985	0.982196
caprin1b	2874.549715	-0.237917027	0.0740199	-3.214231	0.001308	0.038259
zgc:63972	66.13575038	-0.265061107	0.2393219	-1.107551	0.268056	0.83343
acin1a	3886.547152	-0.160974156	0.0991749	-1.623134	0.104561	0.612567
si:ch211-14a17.11	246.0105284	-0.132291859	0.1418761	-0.932446	0.351106	0.880473
dhrs1	639.3280346	-0.702789558	0.1118502	-6.283311	3.31E-10	7.12E-08
kti12	121.3057199	0.035787824	0.182001	0.1966353	0.844113	0.985174
ehd4	117.3831822	0.253560044	0.1977725	1.2820795	0.199815	0.765237
homeza	504.5035456	-0.141038223	0.1099455	-1.282801	0.199562	0.764903
zgc:77151	442.4620021	0.153005438	0.1114591	1.3727499	0.16983	0.726965
slc38a6	139.6739251	-0.060906566	0.172745	-0.352581	0.724403	0.970402
zp3f.2	2.103902422	-0.656949399	1.354709	-0.484938	0.627721	NA
stk33	12.99557385	0.22577704	0.5348326	0.4221452	0.672919	0.965257
oxct1b	624.5411262	0.346693492	0.1048079	3.3078956	0.00094	0.029552
ap2a1	4844.774968	0.061373036	0.0655782	0.9358755	0.349337	0.880195
ngs	1682.537943	0.115198268	0.2777926	0.4146916	0.678368	0.965257
pparab	2.795153415	0.504690138	1.1403369	0.4425799	0.65807	NA
rerglb	63.94856886	0.215284618	0.2472943	0.8705602	0.383994	0.895192
ccn4a	6.142744744	0.235571285	0.7773595	0.3030403	0.761859	NA
trim66	147.2415686	-0.054119035	0.1699476	-0.318445	0.750147	0.97419
dctpp1	0	NA	NA	NA	NA	NA
ap1g2	761.942651	-0.077679265	0.0941672	-0.824908	0.409424	0.901955
sla1a	5.217945923	0.541303091	0.8470004	0.6390824	0.522769	NA
slc7a8b	26.08712731	0.214489167	0.3935641	0.5449917	0.585759	0.952847
gabpb2b	52.71016253	-0.21917495	0.2779651	-0.788498	0.430405	0.912006
lrrc9	13.52582996	0.060678551	0.531042	0.1142632	0.909029	0.990702
dcaf7	3069.577728	0.025647478	0.0728185	0.3522111	0.72468	0.970402
ccdc47	1804.236872	0.158159481	0.0767648	2.0603137	0.039369	0.389822
si:zfos-464b6.2	66.24804376	0.146580135	0.2414445	0.6070965	0.543787	0.945438
b3gnt3.2	5.180212511	0.30019736	0.8741576	0.3434133	0.731288	NA
alg13	3.564428728	0.756334518	1.040583	0.7268373	0.467326	NA
pcbp3	87.33823343	0.031692263	0.2219459	0.1427927	0.886454	0.988936
rhof	37.36464535	-0.193229528	0.3267406	-0.591385	0.554262	0.947449
si:ch211-198n5.11	220.824765	-0.107417587	0.1439098	-0.746423	0.455412	0.921215
spata17	6.207080973	0.292385364	0.7770112	0.3762949	0.706698	NA
ssuh2.2	10.58036393	-0.004486668	0.6107945	-0.007346	0.994139	NA
rpe65c	60.01173692	-0.079956822	0.2531919	-0.315795	0.752158	0.974577
slc7a6	360.3869653	0.278360458	0.1211249	2.2981279	0.021555	0.278029
yipf6	515.7049084	-0.219007734	0.1052954	-2.079936	0.037531	0.379013
fzd2	1172.721976	0.179369575	0.0830346	2.1601792	0.030759	0.339614
cbx1b	1349.867022	0.086479697	0.0798898	1.0824879	0.279036	0.842806
ccdc43	247.511572	-0.029086447	0.1365414	-0.213023	0.831309	0.983152
slc29a1b	91.5443483	-0.184513516	0.2210078	-0.834873	0.403789	0.900979
kif1c	5.441514265	-0.102225447	0.8264866	-0.123687	0.901563	NA
loxl1	678.5695245	0.227863713	0.0984055	2.3155586	0.020582	0.270609
epha4a	1340.828675	-0.003392418	0.0799923	-0.042409	0.966172	0.996315
clip3	327.9573876	0.049161714	0.1398933	0.351423	0.725271	0.970402
slmapa	672.2767438	0.187798548	0.1181929	1.5889154	0.11208	0.627454
tinf2	121.2295764	-0.078424859	0.1818774	-0.431196	0.666326	0.964736
stambpa	361.671132	-0.187419964	0.122531	-1.529572	0.126123	0.654806
adrb2b	40.51453565	0.275949974	0.3329055	0.8289139	0.407153	0.901305
rars	969.7988317	-0.048139976	0.0878665	-0.547876	0.583777	0.952214

araf	779.4185067	0.091832804	0.0936322	0.9807822	0.3267	0.872642
ACOT12	39.46050931	-0.23835838	0.3331167	-0.71554	0.474275	0.925066
nhsl1a	960.9791123	0.028658797	0.0871141	0.32898	0.742171	0.973154
lrp10	789.0578139	-0.089728686	0.104969	-0.854811	0.392656	0.897688
imp4	226.0332772	-0.245129952	0.1385342	-1.769455	0.076818	0.539801
il12bb	10.24873411	-0.175475095	0.5888979	-0.297972	0.765725	NA
samsn1a	679.1061845	-0.098740336	0.1009637	-0.977979	0.328085	0.872701
si:ch73-285p12.4	0	NA	NA	NA	NA	NA
her15.2	160.5483387	-0.364665373	0.1623218	-2.246559	0.024668	0.302262
her15.1	530.9125006	-0.240900774	0.1096797	-2.196402	0.028063	0.324895
smg1	2981.381532	-0.000667112	0.0835206	-0.007987	0.993627	0.998672
wdr95	15.68106243	-0.089895282	0.4992368	-0.180065	0.857101	0.985597
znhit1	176.0314532	-0.190230164	0.1629614	-1.167332	0.243076	0.812061
waif2	15.69597533	0.176247483	0.4971787	0.3544952	0.722968	0.970375
arl6ip1	2894.210132	0.024148085	0.0801439	0.3013089	0.763179	0.975374
mfsd6b	165.8864193	0.009703116	0.1793802	0.0540924	0.956862	0.994543
FO681288.1	76.77646053	-0.157376005	0.2299456	-0.684405	0.493719	0.931926
cox6a2	62.31127329	-0.19825491	0.2584658	-0.767045	0.443055	0.916336
cnot6l	371.2537808	0.200436348	0.1178502	1.7007721	0.088986	0.5713
mrpl1	215.0919298	0.086158542	0.1415702	0.6085923	0.542795	0.944974
slco2b1	303.1217503	0.116503027	0.1276625	0.9125864	0.36146	0.885719
coro1a	84.83847928	0.062201527	0.2462325	0.2526129	0.800567	0.979377
cldni	2609.404541	0.15770302	0.0775593	2.0333213	0.04202	0.402375
fras1	1280.085136	0.186993212	0.0850246	2.1992836	0.027858	0.323464
fli1a	567.0279364	-0.094064098	0.1161545	-0.809819	0.418044	0.906305
tent5ab	451.8506635	0.544555277	0.1263173	4.3110096	1.63E-05	0.001061
si:ch211-51c14.1	266.6165994	-0.116134233	0.1414046	-0.821291	0.411481	0.903483
rac1l	2.159222975	0.25875996	1.3309414	0.1944187	0.845848	NA
si:ch211-1f22.5	0.173729368	-0.955901296	4.0804729	-0.234262	0.814781	NA
slc34a1b	8.213551875	0.344756064	0.6634678	0.5196274	0.603323	NA
pgpep1	122.870269	-0.068878702	0.1895743	-0.363334	0.716356	0.96909
adra2db	9.291545155	0.099136476	0.6166034	0.1607784	0.872268	NA
chrna9	34.49018729	-0.112887473	0.326134	-0.346138	0.729239	0.970822
prdm8b	964.1104278	-0.006131867	0.0921368	-0.066552	0.946939	0.99381
si:dkey-166k12.1	12.73504446	0.173782907	0.539289	0.3222445	0.747268	0.97419
psmb4	2496.531917	-0.086917542	0.0953012	-0.91203	0.361753	0.885944
prlh2r	1.313649662	-2.692304005	1.8571878	-1.449667	0.147151	NA
dhx8	1137.067194	0.0182758	0.0893315	0.2045841	0.837897	0.984701
or118-2	0.158795395	0.967652056	4.0804729	0.2371421	0.812547	NA
or118-3	0	NA	NA	NA	NA	NA
or118-1	0	NA	NA	NA	NA	NA
si:ch211-242b18.1	2537.583192	0.045821326	0.081164	0.5645524	0.572378	0.949494
or116-1	3.708839591	0.869803025	1.0954538	0.7940116	0.427189	NA
prkg2	14.77179693	-0.089559025	0.488896	-0.183186	0.854652	0.985174
si:ch211-244e12.7	24.92795943	0.372988934	0.4009565	0.9302479	0.352243	0.880896
uggt1	453.9891993	0.133977164	0.1108592	1.2085341	0.226842	0.794528
cuedc1b	35.11341597	-0.197472617	0.324151	-0.609199	0.542392	0.944974
lmo4b	1253.979472	-0.053178292	0.0836823	-0.635479	0.525116	0.940066
col10a1a	4271.665557	0.004941488	0.0878566	0.0562449	0.955147	0.994543
hs6st1a	86.10101097	-0.295546355	0.218317	-1.353749	0.175817	0.736411
alox5ap	8.736636662	1.235165363	0.7199249	1.7156863	0.086219	NA
ghra	25.78832183	0.071068208	0.3991225	0.1780611	0.858675	0.985773
si:dkey-46a10.3	436.367303	-0.069991626	0.1075031	-0.651066	0.515004	0.937646
pogza	207.4807902	-0.066087981	0.1549578	-0.42649	0.669751	0.965223

faah2b	61.11367097	-1.191526618	0.2771238	-4.299618	1.71E-05	0.001098
lysmd1	66.00316027	-0.048985296	0.2429792	-0.201603	0.840227	0.985029
emc10	900.388917	-0.10575604	0.0945467	-1.118559	0.263328	0.830245
plcxd3	467.4603754	0.046026271	0.1098203	0.4191051	0.675139	0.965257
zgc:153441	195.1303708	-0.204716422	0.1578129	-1.29721	0.194559	0.758506
rfc1	496.611336	-0.007702866	0.1065923	-0.072265	0.942391	0.993276
anp32e	11714.14971	0.052963372	0.0683933	0.7743938	0.438698	0.915997
btr09	3.819234752	-0.377846935	0.9737702	-0.388025	0.697998	NA
sec13	1260.207503	0.014081995	0.0962576	0.1462949	0.883689	0.988706
ptp4a3b	1257.440809	0.135674325	0.0799655	1.69666	0.089761	0.573763
rpl32	12077.77838	-0.411683198	0.0729312	-5.644813	1.65E-08	2.70E-06
nubpl	115.6562968	-0.040179208	0.1898189	-0.211671	0.832364	0.983557
id3	674.5753494	-0.004551443	0.0980045	-0.046441	0.962959	0.995927
ssuh2.4	30.16572974	-0.093854817	0.3836043	-0.244666	0.806715	0.980254
pik3c3	598.3565176	-0.006460861	0.0993309	-0.065044	0.948139	0.99381
nucb1	965.3029038	0.215574657	0.0887594	2.4287526	0.015151	0.225353
ugt5f1	63.29746171	-0.076501954	0.2490974	-0.307117	0.758755	0.975374
zgc:136870	18.09131017	-0.457300017	0.4762954	-0.960119	0.336996	0.876088
hsd17b14	156.8255723	-0.267728712	0.172518	-1.551889	0.120689	0.645578
CU657980.1	5.241171884	0.681075426	0.8615153	0.7905552	0.429204	NA
xcr1a.1	0	NA	NA	NA	NA	NA
pdk4	294.993929	-0.024397471	0.134356	-0.181588	0.855906	0.985174
bcat2	1131.202143	0.183036332	0.0880233	2.079408	0.03758	0.379013
CSKMT	60.36433709	-0.289136714	0.2500624	-1.156258	0.247576	0.817092
tp53bp2b	711.9996299	0.0097057	0.0993061	0.0977352	0.922143	0.992292
aplp2	5178.496192	0.158687047	0.0703692	2.2550628	0.024129	0.299742
hibch	450.9967788	0.166258847	0.110007	1.511348	0.1307	0.662797
gpsm1b	158.0300518	0.101564445	0.176676	0.5748628	0.565384	0.948739
six2b	56.89334053	0.192455629	0.2714168	0.7090779	0.478276	0.926927
six3b	901.7827672	0.016057401	0.0976187	0.164491	0.869345	0.987274
FP015808.1	1.641419359	-1.960714519	1.6347667	-1.199385	0.230378	NA
rgra	542.3220687	0.172582768	0.2834503	0.6088643	0.542614	0.944974
ms4a17a.16	17.60092595	-2.20261706	0.5164464	-4.264948	2.00E-05	0.001256
mta3	499.4134737	-0.01774389	0.10405	-0.170532	0.864592	0.98601
ier5l	1438.917874	0.02044	0.0902069	0.2265903	0.820742	0.981594
cox7a2l	1012.820337	0.036360466	0.0996038	0.365051	0.715073	0.96909
tmsb	342.5247912	-0.099337382	0.1733393	-0.573081	0.56659	0.948739
dgat1b	260.0255046	-0.090731351	0.1408364	-0.644232	0.519425	0.938945
eif4ebp3	348.8651494	-0.350242554	0.1357947	-2.579207	0.009903	0.169473
zgc:110540	258.1828985	-0.023435382	0.1449659	-0.161661	0.871573	0.987762
tpra1	94.68294	-0.149078976	0.2070087	-0.720158	0.471428	0.924838
ppp2r5b	772.463883	0.039566614	0.0899544	0.4398517	0.660045	0.963432
zgc:101765	32.41725604	0.014546751	0.3762203	0.0386655	0.969157	0.996315
badb	110.3680242	-0.097026332	0.1878185	-0.516596	0.605438	0.95651
ldlrad4b	5.290058233	-0.38956333	0.8333003	-0.467495	0.640146	NA
lgals2a	410.7118723	0.160744976	0.1211358	1.3269812	0.184515	0.747376
mc5rb	4.50601267	0.340803361	0.8782391	0.388053	0.697977	NA
mc2r	0.341486697	1.892317624	3.2124068	0.5890654	0.555817	NA
eef2kmt	48.30574209	-0.208368581	0.3043992	-0.684524	0.493644	0.931926
znf1015	61.5262955	-0.226125206	0.2485897	-0.909632	0.363017	0.885944
alg1	193.7832736	-0.048286764	0.1677111	-0.287916	0.773411	0.975687
cd40	20.90681656	-0.996837493	0.4472058	-2.229035	0.025812	0.31065
itsn2b	1040.961738	-0.032524843	0.1009521	-0.322181	0.747316	0.97419
cxcr2	0.499972362	-2.44370697	3.0013188	-0.814211	0.415524	NA

kifc3	1845.232529	-0.032216579	0.0761126	-0.423275	0.672094	0.965257
ebna1bp2	444.7033304	0.15436129	0.1126523	1.3702454	0.17061	0.728116
zbtb41	216.8331008	-0.159795022	0.1427261	-1.119592	0.262888	0.830166
col4a1	376.226011	0.236028748	0.1228306	1.9215786	0.054659	0.457793
si:dkey-33m11.7	1.487688759	-0.324299566	1.5016004	-0.215969	0.829012	NA
si:dkey-33m11.8	75.39647327	0.007979211	0.2271275	0.035131	0.971975	0.996315
pds5a	1552.48747	-0.065817229	0.0799459	-0.823272	0.410353	0.902997
ptch2	1167.694579	-0.003570421	0.0893606	-0.039955	0.968129	0.996315
pomt2	310.0191996	-0.013213942	0.1267555	-0.104248	0.916973	0.991572
itih3a	298.8567122	0.442741475	0.1636064	2.7061377	0.006807	0.127586
depdc7a	118.9707521	-0.132798813	0.1810705	-0.733409	0.463309	0.922698
casp3b	224.280163	0.243536472	0.1443945	1.6866052	0.091679	0.57849
ponzr5	241.8504464	-0.717275595	0.1488333	-4.819321	1.44E-06	0.000136
map2	1288.365804	0.018152926	0.0865505	0.2097379	0.833872	0.983583
itih1	370.6104838	-0.178366691	0.11984	-1.488374	0.136652	0.673303
bcl9l	826.6122958	0.01743174	0.1041002	0.1674516	0.867015	0.986978
prdx5	1165.54759	0.146147349	0.1066742	1.3700351	0.170676	0.728116
zdhhc20a	22.8437361	0.517195703	0.4291161	1.2052583	0.228104	0.795505
borcs7	104.1884266	-0.049227803	0.1968538	-0.250073	0.802531	0.979583
svila	1134.590951	-0.004641383	0.0827575	-0.056084	0.955275	0.994543
nxf1	1192.48519	0.014592991	0.0888409	0.1642598	0.869527	0.987282
chac2	93.56843633	0.555532143	0.202679	2.7409456	0.006126	0.118579
rbm4.2	1059.111328	-0.032491795	0.097816	-0.332173	0.739759	0.972943
ggnbp2	777.111034	-0.01198424	0.0929686	-0.128906	0.897432	0.990121
pora	1527.006703	-0.013918823	0.0757222	-0.183814	0.854159	0.985174
cdh27	237.3558716	0.070722265	0.1578837	0.4479391	0.654197	0.961305
fam184b	506.9405662	-0.107025288	0.1200871	-0.89123	0.372806	0.892868
cxcl12b	418.8665571	0.072193685	0.1152222	0.6265605	0.530947	0.942209
znf148	413.2490057	0.084742749	0.1147244	0.7386638	0.460111	0.921978
gde1	357.6280897	0.03007802	0.115851	0.2596269	0.795152	0.978466
pom121	301.7513219	0.120452077	0.1424118	0.8458015	0.397664	0.899672
dtnbp1a	484.9816954	-0.156682765	0.1072937	-1.460317	0.144203	0.688428
mylipb	195.1070732	0.352471802	0.1578141	2.2334622	0.025518	0.309193
ctsba	2961.128394	-0.077699466	0.0954225	-0.814268	0.415492	0.906237
kcnk2a	39.4243415	0.132698294	0.3187515	0.4163064	0.677186	0.965257
ccnl1a	1533.97165	-0.092549763	0.0793481	-1.166377	0.243462	0.81239
armc4	71.74998426	-0.107178108	0.2295355	-0.466935	0.640547	0.959994
pim3	1020.296889	0.021392173	0.0943809	0.2266578	0.82069	0.981594
lrfn4a	294.4300733	-0.010204215	0.1279263	-0.079766	0.936423	0.992702
cenpf	461.8376939	0.044564041	0.11088	0.4019124	0.687748	0.966442
si:dkey-101k6.5	49.98039304	0.137769201	0.2866721	0.4805811	0.630814	0.957354
zgc:110410	51.90162308	-0.895882245	0.2756547	-3.250016	0.001154	0.034522
smyd2a	721.4382265	0.069147838	0.0959039	0.721012	0.470902	0.924838
slc8a4a	27.17683888	-0.055361059	0.4495347	-0.123152	0.901987	0.990121
prox1a	515.9919828	-0.17985473	0.1037981	-1.732736	0.083143	0.55599
cyp27a1.4	14.89562056	-0.12150684	0.488167	-0.248904	0.803435	0.979643
chad1a	13.16766745	-0.099098382	0.5188233	-0.191006	0.848521	0.985174
zhx2a	550.7430919	-0.043840889	0.1068643	-0.410248	0.681624	0.965476
epoa	36.50545148	-0.482299542	0.3318382	-1.453418	0.146108	0.691942
si:ch211-256m1.8	505.0729259	-0.611610159	0.1225307	-4.991484	5.99E-07	6.51E-05
c1qtnf6a	7.104730888	0.315382746	0.7376501	0.4275506	0.668978	NA
pxdc1a	84.05292637	0.013686853	0.2353859	0.0581464	0.953632	0.994213
tm4sf5	95.43144667	-0.949000222	0.2087818	-4.545417	5.48E-06	0.000429
ccr9a	36.02289088	0.135532974	0.324211	0.4180394	0.675918	0.965257

slc17a5	672.6723712	0.193077981	0.1093223	1.766136	0.077373	0.541968
zgc:136930	10729.9172	0.338943816	0.0924102	3.6678172	0.000245	0.010078
dtnbp1b	153.9923378	0.086005979	0.2122355	0.4052384	0.685302	0.966146
tuba1c	15988.5246	0.065540015	0.0961218	0.6818437	0.495338	0.931928
ablim2	125.5381265	-0.122075809	0.1843435	-0.662219	0.507831	0.935734
dock8	478.8376034	0.006398958	0.1066555	0.0599965	0.952158	0.993904
slc7a7	263.1781351	0.030428557	0.1400986	0.2171938	0.828057	0.982721
ncs1a	193.5643345	0.086309679	0.147432	0.5854204	0.558265	0.948478
fancc	74.40933123	-0.220279723	0.2287134	-0.963126	0.335484	0.876088
kif17	17.27616264	0.096585314	0.4711438	0.2050018	0.837571	0.984665
xdh	230.048328	0.123467121	0.1396247	0.8842786	0.376546	0.89493
zmp:0000000650	5.59289123	0.182943211	0.8517598	0.2147826	0.829937	NA
cntd2	6.24596968	1.086381684	0.7945179	1.3673471	0.171517	NA
snap23.2	47.61858729	0.073782998	0.3134323	0.2354033	0.813896	0.981081
slc12a10.3	51.10949224	0.409048681	0.2756193	1.4841078	0.13778	0.675209
pitpnm3	127.3548648	-0.149273181	0.1802289	-0.828242	0.407533	0.901305
si:ch1073-358c10.1	101.6020603	-0.094408389	0.2107019	-0.448066	0.654105	0.961305
nkd2a	47.95577329	-0.043870599	0.2794836	-0.15697	0.875268	0.987881
rel	208.4913199	-0.407974835	0.1693077	-2.409665	0.015967	0.233052
cfb	900.7574788	0.130201626	0.2636865	0.4937743	0.621466	0.957354
b3gat1b	160.087403	0.125421199	0.1754122	0.7150082	0.474604	0.925287
id2a	2829.160982	-0.182569295	0.071036	-2.570095	0.010167	0.172274
afap1	237.7328675	0.128468231	0.1481873	0.8669315	0.38598	0.89551
prelid3a	204.865817	0.345796612	0.1617051	2.1384399	0.032481	0.35109
mpeg1.1	63.79177501	0.689237365	0.2634371	2.6163261	0.008888	0.156504
rab18a	680.2162872	-0.020972566	0.0974446	-0.215226	0.829591	0.982805
atg9b	693.3661088	-0.131265599	0.112074	-1.17124	0.241502	0.810471
atoh1a	45.30127122	-0.55879122	0.2921142	-1.91292	0.055758	0.462291
cyb561d1	11.94988483	-0.13904129	0.558383	-0.249007	0.803355	0.979643
atxn7l2a	126.5896104	-0.118017237	0.1835721	-0.642893	0.520293	0.939056
grid2	270.7951191	-0.07436117	0.1440553	-0.516199	0.605715	0.95651
ret	759.7424978	0.089391854	0.1005687	0.8888639	0.374076	0.893787
sypl2a	1572.556907	0.014222707	0.0762011	0.186647	0.851937	0.985174
slc12a10.1	25.30549254	-0.773462035	0.4183398	-1.848885	0.064474	0.496874
mcmbp	841.0662697	0.090913974	0.0908522	1.0006794	0.316982	0.867335
c1ql4b	86.00854597	0.086651338	0.2082233	0.4161461	0.677303	0.965257
aldh9a1a.2	32.75374734	-0.518830121	0.350851	-1.478776	0.1392	0.677963
si:dkey-28d5.11	0	NA	NA	NA	NA	NA
zmp:0000000652	1.33749424	0.038256826	1.6335383	0.0234196	0.981316	NA
si:dkeyp-50d11.2	70.35577532	0.446050526	0.2421945	1.8417036	0.065519	0.500411
si:ch73-1a9.4	766.7111972	-0.088194111	0.0949863	-0.928493	0.353152	0.881552
slc16a13	170.217373	-0.057376679	0.1671947	-0.343173	0.731468	0.971557
mipep	262.2660767	0.094004841	0.1350084	0.6962888	0.486248	0.930833
si:dkey-83f18.2	0.182570949	0.967652056	4.0804729	0.2371421	0.812547	NA
pknox2	511.8272466	0.004433601	0.1176503	0.0376846	0.969939	0.996315
sun1	1128.966729	0.142385423	0.0904734	1.5737813	0.115538	0.637206
leo1	705.7964351	-0.080358755	0.0954795	-0.841634	0.399993	0.90084
sp100.1	7.150104519	1.226483222	0.7671403	1.598773	0.109871	NA
llph	490.0195959	-0.064349019	0.1132109	-0.568399	0.569764	0.948983
si:dkey-25e12.3	50.2885796	0.584193752	0.319781	1.8268555	0.067721	0.50956
zgc:163121	56.90054206	-0.254403017	0.2550671	-0.997397	0.318572	0.867746
sema3fb	582.1143994	0.13530437	0.1106779	1.2225056	0.221516	0.788243
irf4b	3.143405551	1.097834121	1.1545954	0.9508388	0.341686	NA
gnb5b	1282.537867	0.249679756	0.1017126	2.4547576	0.014098	0.214639

bambia	499.9974114	-0.062539764	0.103577	-0.6038	0.545977	0.945526
zgc:66160	403.3079421	0.176649614	0.1137857	1.5524767	0.120548	0.645578
vtna	554.053063	0.257462524	0.1182878	2.1765777	0.029512	0.332709
si:dkey-67c22.2	997.7494805	-0.051440437	0.0897818	-0.57295	0.566679	0.948739
foxq1b	192.6737347	-0.073457904	0.1768101	-0.415462	0.677804	0.965257
foxc1b	526.8161975	0.015163131	0.1123591	0.1349524	0.89265	0.989291
si:dkey-56f14.7	677.2661816	0.153362875	0.0965932	1.5877197	0.11235	0.627572
mylk4b	63.02907261	0.168626959	0.2503961	0.673441	0.500667	0.93428
zp2l1	0.499182217	-0.916838237	2.6774436	-0.34243	0.732027	NA
serpinb1	543.9037332	-0.730525599	0.1023401	-7.138213	9.46E-13	3.37E-10
rwdd2b	95.79137175	0.440253655	0.2081351	2.1152304	0.03441	0.360397
kat6b	898.9217791	-0.046839961	0.0930655	-0.503301	0.614753	0.95688
BX248515.1	11.75086739	0.280190202	0.5501094	0.5093354	0.610517	0.95688
vps26bl	45.3156318	-0.113466528	0.2940111	-0.385926	0.699551	0.967998
ftr97	42.65403847	-0.11611401	0.3057667	-0.379747	0.704133	0.968134
METTL21C	3.492242897	0.151951359	0.9860217	0.1541055	0.877527	NA
adamtsl7	65.42872511	0.288495889	0.2679973	1.076488	0.281709	0.84357
zcchc7	178.8908076	-0.22535031	0.1601044	-1.407521	0.159273	0.710139
slc22a15	235.1034757	0.050747274	0.1508423	0.3364259	0.73655	0.972535
gpm6aa	22410.5064	0.026302411	0.064151	0.4100078	0.6818	0.965476
dlgap3	149.0761688	-0.03879033	0.176832	-0.219363	0.826368	0.982196
lrit3a	262.8087703	0.274671723	0.1421079	1.9328386	0.053256	0.452412
si:dkeyp-3f10.14	6.815621801	0.660837381	0.7289493	0.9065616	0.364639	NA
cdc16	291.4910309	-0.158049171	0.1243916	-1.270578	0.203879	0.771579
nubp1	230.756188	-0.239418375	0.1414448	-1.692663	0.09052	0.576398
dexi	0	NA	NA	NA	NA	NA
rps27.2	4369.08858	-0.424231667	0.1088802	-3.896314	9.77E-05	0.00478
CR352329.1	5.052041705	-1.002279979	1.0087353	-0.993601	0.320417	NA
pelo	504.3350151	0.02266145	0.106221	0.2133424	0.83106	0.982994
mmp20b	2.57129993	0.015701013	1.2908427	0.0121634	0.990295	NA
atf7b	1032.360591	0.018125195	0.0854378	0.2121449	0.831994	0.98349
galnt12	44.06611959	-0.221086526	0.3050163	-0.724835	0.468553	0.924328
hic1	202.6795574	0.018721013	0.1443281	0.1297115	0.896795	0.989971
si:ch1073-184j22.1	74.32866701	-0.021416817	0.2424788	-0.088324	0.929619	0.992702
cicb	1530.864308	0.003436938	0.0808984	0.0424846	0.966112	0.996315
si:ch211-212k18.7	905.6002991	-0.15804313	0.0977642	-1.616574	0.10597	0.615567
si:ch211-37e10.1	1.999598409	0.473439221	1.4046542	0.3370504	0.736079	NA
ypel3	728.085916	-0.284287335	0.1035709	-2.744857	0.006054	0.117821
icn2	636.2367831	-0.22646998	0.2425284	-0.933787	0.350414	0.880195
pygma	1604.074166	0.511171244	0.1063619	4.8059629	1.54E-06	0.000143
ftr61	6.296258228	-1.09238699	0.7712966	-1.4163	0.156688	NA
slc22a6l	204.2977564	-0.715584163	0.1714574	-4.17354	3.00E-05	0.001755
rnf7	1016.625	-0.092820687	0.0877756	-1.057477	0.290294	0.846262
lgals1b	8.120085531	0.685308937	0.7583604	0.9036719	0.366169	NA
cmn	1331.580751	0.256507177	0.2350645	1.0912203	0.275176	0.839541
sertad2b	432.2961016	-0.00742446	0.1086253	-0.068349	0.945508	0.993589
grk7b	75.42044248	0.458317431	0.231909	1.9762815	0.048123	0.432341
otub1a	750.3528673	-0.075000834	0.0926083	-0.809872	0.418014	0.906305
avl9	514.6407592	0.102949014	0.1052742	0.9779133	0.328117	0.872701
epdl2	466.0498	-1.240293061	0.1502897	-8.252679	1.55E-16	9.01E-14
pfkfb4a	263.2233401	-0.054914822	0.1346569	-0.407813	0.683411	0.965476
appb	6941.097904	0.10565125	0.074201	1.4238512	0.15449	0.704213
npat	430.5628574	-0.064340634	0.1091017	-0.589731	0.555371	0.947452
FO834888.1	8.098377195	-0.293205148	0.7185673	-0.408041	0.683243	NA

dvl1b	103.2808422	-0.008866023	0.1969676	-0.045013	0.964097	0.995927
wnt1	17.91032257	-0.111392484	0.4471535	-0.249115	0.803272	0.979643
chrna6	334.1198741	0.24686363	0.1226147	2.0133289	0.04408	0.412803
p2ry8	3.125210979	-0.480644452	1.0717111	-0.448483	0.653805	NA
c1galt1b	311.8158046	0.163811433	0.1252929	1.3074278	0.191067	0.755785
drosha	447.1993761	0.023881075	0.1116862	0.213823	0.830685	0.982994
cacnb2b	131.8959414	0.218629069	0.184879	1.1825523	0.236987	0.805854
mastl	224.1948972	-0.135263949	0.1381513	-0.9791	0.327531	0.872701
ghdc	64.9313776	0.12206766	0.2453467	0.4975314	0.618814	0.957354
gpr61	81.52699287	-0.187683561	0.2216295	-0.846835	0.397087	0.899672
FOXN2 (1 of many)	18.56916136	0.185466769	0.4728084	0.3922663	0.694861	0.96736
pcolce2b	359.5251215	0.037798717	0.1260736	0.2998147	0.764319	0.975374
c1galt1a	105.0473935	0.169638411	0.2010471	0.8437747	0.398795	0.900446
stat5b	132.3864508	-0.111542176	0.184085	-0.605928	0.544563	0.945438
s100t	375.4895264	0.279970791	0.1177529	2.377613	0.017425	0.244372
pipox	139.5160758	-0.663437229	0.191564	-3.463266	0.000534	0.018777
capn2b	417.1783972	0.009842448	0.1213893	0.0810817	0.935377	0.992702
clul1	53.06102668	1.441756129	0.3042703	4.7384065	2.15E-06	0.000192
cgna	189.9264942	-0.047581029	0.1698714	-0.2801	0.779401	0.976146
exoc2	978.9410978	-0.249351298	0.0931304	-2.677444	0.007419	0.136082
zgc:123238	208.3444446	0.020540346	0.1517512	0.1353554	0.892331	0.989291
acta1b	12239.05989	0.492039678	0.2205692	2.2307727	0.025696	0.310555
acad9	337.9703699	-0.102989274	0.1367497	-0.753122	0.451376	0.918761
zgc:153345	24.506176	0.064464512	0.3950284	0.1631896	0.870369	0.987421
dipk2ab	257.6725332	0.206949923	0.1381198	1.4983367	0.134046	0.669349
foxh1	5.863347542	0.622815112	0.8200778	0.7594586	0.447578	NA
smtnl	213.8171766	-0.096528381	0.1457443	-0.662313	0.50777	0.935734
crk	1447.124585	-0.024792731	0.080522	-0.3079	0.758158	0.975374
ankrd33aa	112.9860758	0.229137434	0.2267362	1.0105905	0.312212	0.864401
ruvbl2	723.8763683	0.052541837	0.1010919	0.5197432	0.603243	0.956045
si:ch211-217a12.1	132.3834361	0.368767062	0.1797312	2.0517699	0.040192	0.392132
prss60.2	203.1004685	-0.335922519	0.1493409	-2.249368	0.024489	0.3019
ftr82	443.5247677	-0.297370417	0.1075605	-2.76468	0.005698	0.113266
cpda	664.5129648	0.158503294	0.094019	1.6858645	0.091822	0.57849
aclyb	65.48751337	0.57400944	0.2832134	2.0267734	0.042686	0.406074
nubp2	68.23780325	-0.504105961	0.2391815	-2.107629	0.035063	0.364435
birc5b	3.263608517	0.264125569	1.0571213	0.2498536	0.802701	NA
pdzd3b	42.6854123	-0.266755135	0.3096151	-0.86157	0.388924	0.896295
gpr161	80.79777517	0.371964748	0.2303043	1.6151013	0.106289	0.615567
map3k10	9.857809783	-0.366430541	0.6371881	-0.575074	0.565241	NA
armt1	211.6130709	-0.177269342	0.1474277	-1.202415	0.229203	0.796702
akap12b	2434.976083	0.004245971	0.0702423	0.0604475	0.951799	0.993864
mtol	259.5395052	-0.189661707	0.1334633	-1.421078	0.155294	0.705118
pimr191	9.020079067	-0.664005148	0.6619874	-1.003048	0.315838	NA
CU655961.1	42.82928233	-0.124995044	0.2934545	-0.425943	0.670149	0.965223
pcnx2	756.0780764	0.133887781	0.0969977	1.3803185	0.167489	0.72455
f5	356.1368591	0.115072772	0.1411619	0.815183	0.414968	0.906237
timm8b	405.87089	-0.043770965	0.1162275	-0.376597	0.706473	0.968459
sdhda	30.58204029	0.510012029	0.3576064	1.4261824	0.153816	0.702599
fmnl1a	638.1495047	0.149626188	0.1005331	1.4883274	0.136665	0.673303
capn8	472.9954929	0.04530216	0.1063773	0.4258631	0.670208	0.965236
ino80c	274.585549	-0.007481949	0.1353287	-0.055287	0.95591	0.994543
bco2a	140.3514424	-0.434202789	0.1725174	-2.516863	0.01184	0.191129
hsp70l	2059.851604	0.081258653	0.2681716	0.30301	0.761882	0.975374

trim35-29	10.67185418	-0.400459885	0.6557485	-0.610691	0.541404	NA
si:dkeyp-69e1.8	70.85314636	0.059738297	0.2305956	0.2590609	0.795588	0.978498
ube3a	1435.131401	0.059510397	0.0872168	0.6823273	0.495032	0.931926
ribc1	18.6639712	-0.055740786	0.4583086	-0.121623	0.903198	0.990121
9-Mar	314.7877637	0.117096908	0.1361183	0.8602581	0.389647	0.896421
ankrd54	421.4171714	-0.034930432	0.1076271	-0.32455	0.745521	0.974094
si:ch211-238g23.1	1.623378393	-0.611067996	1.5115622	-0.404263	0.68602	NA
foxp3a	5.346760651	0.361013354	0.8247349	0.4377326	0.66158	NA
fosb	75.73878446	0.868875414	0.2894778	3.0015265	0.002686	0.066466
npas4a	75.29106254	1.344490079	0.5908917	2.275358	0.022884	0.289518
suv39h1b	304.6307306	0.122915608	0.1322696	0.9292808	0.352744	0.881552
smc1a	23.3686461	0.298198463	0.4313677	0.691286	0.489386	0.931926
tmem9	283.2834499	0.25688912	0.1337252	1.9210226	0.054729	0.458175
efhd2	1121.365455	0.161320235	0.0862228	1.8709705	0.061349	0.482931
srm	525.5919018	0.119391678	0.1136293	1.0507124	0.293391	0.849291
tmem115	767.7154575	-0.036298684	0.0979978	-0.370403	0.711082	0.968985
dffa	165.0720697	-0.131703925	0.1624812	-0.81058	0.417607	0.906237
ptger3	3.528313542	-0.108814872	1.0291318	-0.105735	0.915793	NA
ptpn3	422.8462281	-0.291759294	0.1128842	-2.584588	0.00975	0.167496
prss23	371.1017169	0.152974354	0.1174639	1.3023092	0.192811	0.756795
dzank1	41.86655041	0.294673226	0.3391937	0.8687462	0.384986	0.89551
zgc:158423	453.3336154	-0.023824565	0.120405	-0.19787	0.843147	0.985174
foxo4	1535.685842	-0.078923513	0.0766545	-1.0296	0.303198	0.85729
cnpy4	219.9703432	-0.06036285	0.1396593	-0.432215	0.665585	0.964736
hey1	35.07906468	0.393471999	0.3335454	1.1796653	0.238133	0.807277
vtg2	0.854974378	0.638273715	2.0970274	0.3043707	0.760845	NA
si:dkey-225f5.5	40.67400494	0.20312138	0.3057873	0.664257	0.506526	0.935729
celsr3	219.5326034	-0.037624059	0.1439171	-0.261429	0.793762	0.977937
si:dkey-182g1.6	4.925230562	0.802106568	0.8898173	0.9014283	0.367361	NA
lectin	0.173729368	-0.955901296	4.0804729	-0.234262	0.814781	NA
slc26a6	90.16517121	0.049597195	0.2105651	0.2355433	0.813787	0.981081
arhgef1b	1039.296182	0.046151715	0.0852256	0.5415239	0.588146	0.953319
mettl16	132.0149595	-0.013823127	0.181767	-0.076049	0.93938	0.992857
march4l	78.17806472	-0.032748019	0.2240618	-0.146156	0.883798	0.988706
CABZ01046427.1	0	NA	NA	NA	NA	NA
cdh10a	169.0071454	0.109797328	0.1754721	0.6257254	0.531495	0.942358
mtnr1ab	75.47531103	0.209852554	0.2522112	0.8320509	0.40538	0.901305
nr4a3	373.6888589	0.229433792	0.1262734	1.8169603	0.069223	0.514556
kcnc3a	130.2224673	0.064736239	0.202467	0.3197373	0.749167	0.97419
tor1l1	0.499182217	-0.916838237	2.6774436	-0.34243	0.732027	NA
dop1b	1062.576975	0.016221699	0.0900506	0.1801398	0.857043	0.985597
rsl1d1	528.0309662	-0.271680497	0.11069	-2.454426	0.014111	0.214639
cpe	9999.014269	0.127691525	0.0734414	1.7386854	0.08209	0.553066
pag1	823.0690215	0.101101845	0.0980926	1.0306777	0.302692	0.85729
msmo1	320.9619958	-0.217744553	0.1241131	-1.754405	0.079361	0.547489
dmc1	1.950601905	2.338222269	1.59317	1.467654	0.142198	NA
tex10	528.6928444	-0.004024128	0.1091007	-0.036885	0.970577	0.996315
zgc:153215	32.72775041	0.106964637	0.3440011	0.3109428	0.755844	0.975374
nmnat1-rbp7a	0	NA	NA	NA	NA	NA
zgc:154093	396.3535517	0.304432107	0.123422	2.4665959	0.01364	0.20974
lzic	162.1884628	0.097611454	0.1621839	0.6018566	0.54727	0.945526
zswim5	2236.789151	-0.0439054	0.0740414	-0.592984	0.553192	0.947265
steap4	353.7308101	-0.677011752	0.1407113	-4.811355	1.50E-06	0.00014
luc7l	1844.711144	-0.042914236	0.0822136	-0.521985	0.601681	0.956045

nipa2	470.2075046	-0.074511797	0.1097755	-0.678765	0.497286	0.93322
nipa1	69.36525198	-0.647795304	0.2403738	-2.694949	0.00704	0.13096
si:dkey-47k20.9	0	NA	NA	NA	NA	NA
foxi3a	154.5464246	0.017251421	0.1846855	0.0934097	0.925578	0.992676
zc3h7bb	859.3655559	0.029783308	0.0993433	0.2998019	0.764328	0.975374
si:ch1073-416j23.1	11.10284253	0.321662393	0.5718706	0.562474	0.573793	0.949988
tspan14	496.8087737	0.058663301	0.1047574	0.5599917	0.575485	0.950373
asph	2628.612567	0.125150948	0.0682427	1.8339085	0.066668	0.504944
v2rc2	2.852923473	0.160116477	1.1096438	0.1442954	0.885267	NA
lcp2a	12.63289941	0.336827062	0.5550085	0.6068863	0.543926	0.945438
klc3	68.63373917	0.025893903	0.2443363	0.1059765	0.915601	0.9913
cflara	153.8589899	-0.088203408	0.1720092	-0.512783	0.608103	0.956718
rfc3	205.8896864	-0.088418185	0.1547591	-0.571328	0.567777	0.948739
zgc:136971	119.7160274	-0.077896152	0.1935633	-0.402432	0.687366	0.966376
zar1l	1.767051434	0.777420258	1.5123228	0.5140571	0.607212	NA
tpmt.1	154.6709278	-0.327143242	0.1633379	-2.002862	0.045192	0.417766
pecr	205.7126839	-0.058002264	0.1526879	-0.379875	0.704039	0.968134
gzm3.2	0.789393591	-0.56601807	2.4000363	-0.235837	0.813559	NA
tmem161b	509.9856197	0.188411375	0.1045139	1.802739	0.071429	0.522931
hmbsb	203.0894444	0.091891509	0.1465534	0.6270174	0.530648	0.942209
v2rh1	0.341366344	1.891903201	3.2128156	0.5888614	0.555954	NA
rps8a	8866.399604	-0.235851521	0.0800854	-2.944999	0.00323	0.07565
si:ch211-59d17.3	230.490866	-0.046364774	0.1541446	-0.300787	0.763577	0.975374
v2rh7	0.325573202	0.005883621	3.3172783	0.0017736	0.998585	NA
fryb	2032.280776	-1.03E-06	0.0862956	-1.20E-05	0.99999	0.99999
sprn	320.1894139	0.128434262	0.135904	0.9450369	0.34464	0.878434
dnajc18	834.2544336	0.167592312	0.0905944	1.8499189	0.064325	0.496087
si:dkey-47k20.2	4.27066418	-0.454803258	0.9342506	-0.486811	0.626392	NA
cyb5d1	17.457542	-0.243533341	0.4987693	-0.488268	0.62536	0.957354
atp5mc1	4290.901061	0.03580436	0.0741252	0.4830257	0.629077	0.957354
v2rh9	0	NA	NA	NA	NA	NA
eve1	7.834511232	-0.686723042	0.6778856	-1.013037	0.311043	NA
si:dkey-47k20.7	0	NA	NA	NA	NA	NA
hoxb13a	12.88093216	-0.69267732	0.5817707	-1.190636	0.233796	0.802571
itga6l	40.52039986	0.09304739	0.3071696	0.3029186	0.761952	0.975374
ttbk1a	573.481055	-0.155128101	0.1046315	-1.482614	0.138177	0.675945
sostdc1b	13.90731468	-0.11965891	0.5113782	-0.233993	0.81499	0.981081
hoxb9a	294.5524834	0.097949411	0.1257558	0.7788857	0.436047	0.914408
eva1c	7.802018947	-0.288219985	0.6880965	-0.418866	0.675314	NA
tprg1	307.9535322	-0.505889616	0.1259015	-4.018139	5.87E-05	0.003169
hoxb8a	488.6582387	0.072327063	0.1172218	0.6170106	0.537228	0.943336
slc22a7a	254.7461049	-0.1836535	0.140033	-1.311501	0.189689	0.754269
cyp26c1	235.8018843	-0.061256492	0.1413172	-0.433468	0.664675	0.964736
hoxb7a	109.4568952	0.098105252	0.2004241	0.4894882	0.624496	0.957354
pip4p2	104.2666316	-0.408631622	0.1938797	-2.107655	0.035061	0.364435
stxbp1b	1849.940168	0.118250681	0.0829096	1.4262608	0.153793	0.702599
itih6	155.2625268	-0.211003244	0.1707149	-1.235998	0.216459	0.783724
si:ch73-139e5.4	101.2024557	-0.975035849	0.2222534	-4.387047	1.15E-05	0.000811
noxa1	19.61276633	-0.41644755	0.4574685	-0.91033	0.362648	0.885944
kctd17	533.85963	0.170176479	0.0995753	1.7090231	0.087447	0.566637
grem2a	10.13649138	0.18356281	0.6135379	0.2991874	0.764797	NA
rangap1b	452.6714864	0.105323215	0.1178835	0.8934517	0.371615	0.892003
adgre14	2.293146196	1.316308218	1.3179731	0.9987368	0.317922	NA
mov10b.2	4.254053154	-1.176451322	0.9419986	-1.248889	0.211706	NA

rca2.1	922.4343826	-0.05000418	0.0942872	-0.530339	0.595877	0.954434
dcst1	4.229962077	0.685820229	0.9566703	0.7168825	0.473447	NA
rftn2	522.3897114	0.079062333	0.1073208	0.7366917	0.46131	0.922161
l3mbtl2	454.4134199	0.098105484	0.1305642	0.7513963	0.452414	0.919104
si:dkey-191g9.5	103.4867232	0.219317798	0.2304891	0.9515322	0.341334	0.878134
igsf21b	482.7079199	0.039119018	0.1114926	0.3508664	0.725689	0.970402
mob4	1871.736632	-0.084950397	0.0907226	-0.936376	0.34908	0.880195
ecrg4a	403.8296567	0.164928604	0.120935	1.363779	0.172637	0.731348
nrbp1	1019.079848	0.303725121	0.0929611	3.2672294	0.001086	0.032938
capza1b	3996.334553	0.110371938	0.0803924	1.3729154	0.169779	0.726965
cttnbp2nlb	146.2476527	0.127332144	0.1702098	0.7480893	0.454406	0.920544
si:dkey-12h9.6	584.6052076	0.058275052	0.1011809	0.575949	0.56465	0.948739
gtf2h5	313.0344714	0.4300464	0.1307141	3.2899766	0.001002	0.031006
kcnd3	153.4079351	-0.081513387	0.1834886	-0.444242	0.656867	0.96205
uxs1	619.9626124	-0.011443425	0.0982788	-0.116438	0.907305	0.990702
syt11b	258.848093	0.045318784	0.1386468	0.3268649	0.74377	0.974094
ndufa4	4187.118385	-0.033332659	0.069561	-0.479186	0.631806	0.957356
lmod1a	39.17115398	0.566789151	0.3428788	1.6530306	0.098325	0.597502
pikfyve	866.82247	-0.018895816	0.0954195	-0.198029	0.843023	0.985174
eef1g	16625.92837	-0.280845724	0.083098	-3.379691	0.000726	0.023836
serac1	248.524456	0.104470645	0.1395474	0.748639	0.454075	0.920295
gdi1	4211.154489	0.10056835	0.0832182	1.2084905	0.226859	0.794528
myct1a	8.354064181	1.082291194	0.6871472	1.57505	0.115245	NA
polr2gl	569.3223016	-0.059331715	0.1035358	-0.573055	0.566608	0.948739
fastkd1	268.6815847	-0.015442674	0.1343923	-0.114907	0.908518	0.990702
neurog1	155.0063421	-0.197558316	0.1688905	-1.169742	0.242105	0.810733
kitb	23.73387075	-0.101851944	0.3866967	-0.26339	0.79225	0.977937
c1qtnf5	34.38846033	0.665613564	0.3349874	1.986981	0.046925	0.425245
sf3b1	6744.403036	-0.039866764	0.0618134	-0.644953	0.518957	0.938945
coq10b	1001.660701	0.457585014	0.0935999	4.8887329	1.01E-06	0.000102
zgc:123244	25.6132229	-0.105474258	0.3759825	-0.28053	0.779071	0.976146
si:dkey-159a18.1	42.76423097	0.561697617	0.3082806	1.8220334	0.06845	0.512635
rbms2b	409.4128675	-0.057633773	0.1421342	-0.405488	0.685119	0.966146
tyrp1b	4510.923555	-0.097333622	0.0792764	-1.227775	0.219531	0.786189
fam3c	295.3306618	0.161330187	0.1280134	1.26026	0.207576	0.775175
si:ch211-229c8.4	0.499182217	-0.916838237	2.6774436	-0.34243	0.732027	NA
npdc1b	1124.920845	0.218842909	0.0870657	2.5135369	0.011953	0.192313
hspd1	4572.514081	-0.057298607	0.0646344	-0.886504	0.375346	0.894316
nr2c2ap	38.25434898	-0.368643511	0.3132166	-1.17696	0.239211	0.807768
slc25a51a	8.799780374	-0.401242308	0.653565	-0.613929	0.539262	NA
si:ch211-215a10.4	304.4665335	0.238144129	0.1356823	1.7551593	0.079232	0.547489
jac2	0.339758314	0.005883863	3.2682461	0.0018003	0.998564	NA
hspe1	1413.976588	-0.176030864	0.089678	-1.962921	0.049655	0.436976
scrt2	353.7114863	-0.04503875	0.1209874	-0.37226	0.709699	0.968928
dpt	9.393896533	0.338808835	0.6297985	0.5379639	0.590602	NA
v2ra17	0.856462055	1.928072848	2.2076658	0.8733536	0.38247	NA
ncam1a	806.938123	0.130421381	0.096306	1.3542393	0.17566	0.736411
dvl2	539.2095275	-0.109769491	0.1026774	-1.069072	0.285037	0.845094
eif5a2	7567.077113	-0.092807579	0.0728881	-1.273289	0.202916	0.769849
si:ch211-168k14.2	25.60527089	0.033195938	0.3926609	0.084541	0.932626	0.992702
nme7	121.1893489	-0.197268728	0.1889407	-1.044077	0.29645	0.853523
slc2a2	261.1848001	0.449685525	0.1357466	3.3126837	0.000924	0.029189
abcb9	141.2096878	-0.244073293	0.1689353	-1.444774	0.148521	0.696298
si:ch211-201h21.5	143.2212253	-0.43325384	0.1841112	-2.353219	0.018612	0.254199

zgc:113436	168.5514936	0.013568524	0.1643375	0.082565	0.934197	0.992702
camk2g2	3486.537678	0.075672188	0.0704093	1.0747473	0.282488	0.843666
myoz1a	803.7048698	0.236091701	0.1005784	2.3473392	0.018908	0.256439
si:ch211-199o1.2	39.37016892	0.345809202	0.3133966	1.1034235	0.269843	0.834705
ogfod2	33.75640049	0.184529202	0.3296408	0.5597888	0.575624	0.950373
si:ch73-130a3.4	42.07489348	0.409259137	0.3021428	1.3545223	0.17557	0.736411
gnrh3	40.80475969	-0.320274632	0.3222267	-0.993942	0.320251	0.868202
tnika	1058.061201	-0.076921377	0.0836726	-0.919314	0.357931	0.884398
creb3l3a	31.15106309	-0.522349511	0.408503	-1.278692	0.201006	0.766855
pld1a	279.6039178	-0.104155926	0.1347987	-0.772678	0.439713	0.916082
ghsra	2.945908661	-0.971398854	1.1481508	-0.846055	0.397522	NA
zgc:110591	275.7037668	-0.279884193	0.1275988	-2.193471	0.028273	0.325989
smndc1	1298.431387	-0.004368271	0.0795252	-0.054929	0.956195	0.994543
tmem45b	351.9134746	-0.212325527	0.1189172	-1.785491	0.074182	0.530917
zgc:171740	46.08209492	0.064024116	0.3085802	0.2074797	0.835635	0.984089
zgc:162952	125.7059571	-0.171678574	0.1765125	-0.972614	0.330745	0.87417
bricd5	104.5141906	0.147386735	0.2134421	0.6905231	0.489865	0.931926
si:dkey-183i3.5	12842.72584	0.421356635	0.0824284	5.111791	3.19E-07	3.76E-05
add3b	123.9102569	0.144770806	0.1793917	0.8070095	0.419661	0.90651
sort1b	771.759863	0.099642729	0.1002118	0.9943217	0.320066	0.868153
cdc27	975.7244287	0.028592502	0.0871979	0.3279037	0.742984	0.973486
tmem131	1364.715144	-0.028031381	0.0806764	-0.347455	0.72825	0.970402
slc35g2a	388.4747779	0.238438806	0.1171013	2.0361764	0.041733	0.400593
dixdc1b	263.4211987	-0.171450643	0.1395167	-1.22889	0.219113	0.785734
atxn7l2b	241.3276535	-0.141460188	0.144793	-0.976982	0.328578	0.872945
atpaf2	232.496421	0.07957826	0.1434335	0.5548095	0.579025	0.951052
or132-5	6.689329977	-0.268341122	0.7258	-0.369718	0.711593	NA
ccdc157	18.98372592	-0.184255313	0.4395669	-0.419175	0.675088	0.965257
cyb561a3a	67.15077893	-0.169052903	0.2574251	-0.656707	0.511369	0.936253
drd7	10.93648011	0.511413543	0.5924927	0.8631558	0.388052	NA
vsx1	1849.583134	0.06818684	0.0802042	0.850166	0.395233	0.899244
si:ch211-183d21.3	62.09344987	-0.041144038	0.2559141	-0.160773	0.872272	0.987846
znf706	1254.804855	-0.173677254	0.0861704	-2.015509	0.043851	0.411346
a2ml	1802.766892	0.107646363	0.112418	0.9575547	0.338287	0.876778
kansl2	3.324203577	-0.308248442	1.0461689	-0.294645	0.768265	NA
ldb3a	1394.752968	0.281718944	0.0906016	3.1094243	0.001875	0.050962
zgc:123295	52.53071184	0.075430924	0.277746	0.2715824	0.785943	0.97627
rhot2	149.166271	0.165972453	0.1793597	0.9253611	0.354778	0.882105
mhc2dbb	9.930974889	0.400720408	0.5975986	0.6705512	0.502507	NA
ahcyl1	1807.37677	0.170726482	0.0769717	2.2180409	0.026552	0.313644
mlh3	62.34444043	0.042316675	0.2465155	0.1716593	0.863705	0.985952
szrd1	688.0010356	0.079589394	0.1031611	0.7715056	0.440407	0.916336
stk31	5.287512961	-0.196423064	0.8987257	-0.218557	0.826995	NA
acap1	89.68552703	-0.201805145	0.230433	-0.875765	0.381158	0.895192
rab3aa	787.1409055	0.108354553	0.0898265	1.2062644	0.227716	0.794934
mpv17l2	134.64804	-0.114596838	0.1743096	-0.657433	0.510903	0.936253
ufsp2	400.2927234	-0.207754135	0.1102259	-1.884804	0.059456	0.476934
noxo1b	21.04401606	-0.468314138	0.4743565	-0.987262	0.323514	0.870837
ankrd37	486.3318336	-0.319229031	0.1299252	-2.457021	0.014009	0.214075
ifi30	420.3703417	0.103731198	0.1297449	0.7995014	0.424	0.908025
si:ch73-86n18.1	257.9773748	-0.287153956	0.3069305	-0.935567	0.349496	0.880195
cfap97	137.1332237	-0.178011854	0.1712207	-1.039663	0.298496	0.85439
TMC1	11.43014001	-0.245242925	0.5982055	-0.409964	0.681832	0.965476
rnf17	31.96732058	0.105272054	0.37126	0.2835535	0.776753	0.975826

TOM1L2	477.0091991	-0.075834914	0.1064473	-0.712418	0.476206	0.926403
onecut3a	5.579558776	-0.865121708	0.8377554	-1.032666	0.30176	NA
dusp22a	162.8609277	-0.242261948	0.1740042	-1.392276	0.163839	0.71945
helt	103.4019996	-0.255632714	0.1965917	-1.300323	0.19349	0.757219
irf8	29.58740534	0.096973641	0.3649867	0.2656909	0.790477	0.977359
fdx1	743.8424986	-0.085138838	0.0931573	-0.913926	0.360756	0.885686
usp1	633.0425901	0.089672451	0.0986429	0.9090614	0.363318	0.885944
auts2a	1974.595577	-0.050205904	0.0876535	-0.572777	0.566796	0.948739
her9	783.8770749	-0.296543593	0.1003069	-2.956362	0.003113	0.073687
4-Mar	8.298626656	-0.524882547	0.7216724	-0.727314	0.467034	NA
zgc:152753	0.341366344	1.891903201	3.2128156	0.5888614	0.555954	NA
MANEAL	418.2899274	0.128409676	0.112634	1.1400613	0.254261	0.823394
afap1l1b	102.7179215	0.097507936	0.194258	0.5019508	0.615702	0.95688
lhfpl5b	51.51961394	-0.270842997	0.2750572	-0.984679	0.324782	0.871284
crp2	7.881532855	0.193501185	0.6808742	0.2841952	0.776261	NA
fitm1l	207.6464274	0.50431412	0.1446499	3.4864472	0.000489	0.017534
tmub2	187.1067222	-0.016456458	0.1525995	-0.107841	0.914122	0.991119
asb16	37.63347924	0.092197058	0.3251673	0.2835373	0.776765	0.975826
chaf1b	350.8512265	-0.080157127	0.1327778	-0.603694	0.546047	0.945526
trnau1apb	1577.844581	-0.165745197	0.0882339	-1.878475	0.060316	0.479378
ccdc125	75.87095024	-0.252733695	0.2465148	-1.025227	0.305256	0.85982
si:ch211-168b3.1	291.3892163	0.110883425	0.136801	0.8105455	0.417627	0.906237
rab1bb	740.5912783	-0.056863906	0.1007152	-0.564601	0.572345	0.949494
cpvl	656.0260453	-0.10216421	0.0997049	-1.024666	0.305521	0.859981
vat1	3666.353502	0.03190087	0.0768025	0.4153626	0.677877	0.965257
ssbp4	2891.013321	-0.140069564	0.0704229	-1.988978	0.046704	0.424613
zgc:110158	1682.285419	-0.057114457	0.0807703	-0.707122	0.479491	0.927091
ikzf5	430.1802901	-0.155450598	0.1106425	-1.404981	0.160027	0.712495
crp2	79.68417496	1.427670042	0.2689124	5.3090516	1.10E-07	1.51E-05
ca6	1186.684956	0.16291155	0.0912437	1.7854547	0.074188	0.530917
si:ch211-208g24.8	0.476747244	2.393381959	3.0525537	0.7840589	0.433006	NA
si:ch73-334d15.4	8.933434133	-0.423004344	0.6323016	-0.668991	0.503501	NA
frg1	317.5365308	-0.189786255	0.1267659	-1.497139	0.134357	0.670529
si:ch73-113g13.2	0.513487682	-0.887846295	2.6557956	-0.334305	0.738149	NA
pdik1l	192.625308	-0.19342276	0.1570365	-1.231706	0.218059	0.785379
gstk1	376.0384994	0.003114496	0.1169897	0.026622	0.978761	0.996762
arr3a	15587.04961	0.383634103	0.0730848	5.2491616	1.53E-07	2.00E-05
spsb1	511.8464057	-0.130586313	0.1132395	-1.153187	0.248834	0.81797
thoc3	337.362161	-0.075640048	0.1299641	-0.582007	0.560562	0.948728
si:dkey-280e21.3	496.137089	0.081208419	0.1094627	0.7418819	0.458159	0.921978
bcar1	753.8530587	-0.05123838	0.0949516	-0.539626	0.589455	0.953498
cpamd8	676.1616141	0.135094847	0.095214	1.4188546	0.155941	0.705963
nutf2	1044.401055	0.099853999	0.0901106	1.1081269	0.267807	0.833349
serinc2	477.7138105	0.047762309	0.1058072	0.4514089	0.651695	0.960708
pla2g4f.1	101.6291812	-0.107751268	0.2046581	-0.526494	0.598545	0.955488
nr0b1	70.92565592	0.046247549	0.2440069	0.1895338	0.849674	0.985174
vps26a	780.2505918	0.035909389	0.0916422	0.3918433	0.695174	0.96736
tango2	384.9327658	0.066066896	0.1174656	0.5624362	0.573819	0.949988
slc22a13a	1.861362508	-0.749752561	1.4509934	-0.516717	0.605354	NA
rap1gap	36.95426841	0.279980937	0.3416822	0.8194191	0.412547	0.904974
copb1	3634.257854	0.026949623	0.0686688	0.392458	0.69472	0.96736
ccz1	606.4549397	0.056076447	0.1021716	0.5488455	0.583111	0.952214
asb11	0.158795395	0.967652056	4.0804729	0.2371421	0.812547	NA
necab1	181.5270834	-0.065561679	0.1667311	-0.393218	0.694158	0.96736

nrsn1l	135.5279174	-0.34253437	0.1962337	-1.745543	0.08089	0.550801
arf2b	106.5063583	0.382929837	0.2009491	1.9056061	0.056701	0.467172
ndufs6	887.1029798	0.007635702	0.1081692	0.0705904	0.943724	0.993364
cyp2r1	41.74588363	0.589769107	0.3231287	1.8251835	0.067973	0.510097
calca	205.2302723	0.323563867	0.1641264	1.9714313	0.048675	0.433734
gucy1b2	2.445347834	-0.19730206	1.1940522	-0.165237	0.868757	NA
irx1b	264.0109528	-0.277209726	0.1358706	-2.040248	0.041326	0.398192
pimr47	0.182570949	0.967652056	4.0804729	0.2371421	0.812547	NA
papss2b	1176.043529	0.119088091	0.0839898	1.4178871	0.156224	0.70658
plekha3	210.8193609	-0.17308894	0.151897	-1.139515	0.254488	0.823475
mcf2b	12.1927588	-0.012511484	0.5646502	-0.022158	0.982322	0.996944
wbp2	605.9586807	0.184497868	0.1036163	1.780587	0.07498	0.533634
atad1b	251.4868018	-0.047609279	0.1352976	-0.351886	0.724924	0.970402
cybb	77.56610246	0.042883862	0.2237778	0.1916359	0.848027	0.985174
pimr48	0.173729368	-0.955901296	4.0804729	-0.234262	0.814781	NA
rpgra	32.24737276	-0.385310222	0.3400353	-1.133148	0.257152	0.825314
xk	102.6360424	0.062889286	0.1985196	0.3167913	0.751402	0.97419
arhgap42a	451.8706516	-0.141883589	0.1073204	-1.322056	0.18615	0.750443
grpr	16.24583418	0.673837426	0.4794081	1.4055613	0.159854	0.712089
ctcf	2855.843725	0.052413897	0.071899	0.7289929	0.466006	0.923865
ptenb	1206.417755	0.157417346	0.0830292	1.895928	0.05797	0.47228
vegfd	30.11668242	0.22294948	0.3492969	0.6382807	0.523291	0.939336
trpc6a	69.71362252	-0.185500771	0.2448485	-0.757614	0.448682	0.917876
cxcl14	607.3396979	0.084919635	0.1058087	0.8025773	0.422219	0.90651
tmem170a	171.0408518	-0.14885498	0.1662141	-0.895562	0.370487	0.890489
angptl5	157.0027538	0.025374592	0.1683403	0.1507339	0.880186	0.987881
fgf13b	783.8117497	0.020743167	0.0942962	0.2199788	0.825888	0.982196
pir	311.4424541	-0.062548234	0.1351195	-0.46291	0.643429	0.959994
carmil2	700.1283268	-0.079439912	0.1024365	-0.775504	0.438042	0.915284
rpia	815.0596752	-0.099383561	0.1063802	-0.93423	0.350185	0.880195
hdac9b	485.5409344	0.137774466	0.1054068	1.3070734	0.191188	0.755886
slc22a7b.1	52.51806908	0.534817206	0.2781537	1.9227402	0.054513	0.457793
klhl3	424.0388488	0.08037235	0.1094095	0.7346015	0.462582	0.922698
ext2	1193.817494	0.056566096	0.0841687	0.672056	0.501548	0.934648
htatsf1	525.3284156	0.042406798	0.1102639	0.3845936	0.700539	0.967998
zgc:113276	70.98194811	0.339124132	0.2324944	1.4586336	0.144666	0.688975
gfra4a	135.5822932	0.147194862	0.1908449	0.77128	0.440541	0.916336
SLC22A7 (1 of many)	28.33447636	0.398014483	0.3624661	1.0980738	0.272172	0.837242
fhl1b	138.609074	-0.01571961	0.1774462	-0.088588	0.929409	0.992702
gna15.3	9.431709949	0.427921693	0.6366695	0.6721253	0.501504	NA
tspan18a	347.5603106	0.010816004	0.115618	0.0935495	0.925467	0.992676
ttbk1b	217.0910136	0.243867899	0.1420798	1.7164154	0.086086	0.563109
arfip2b	0.998646424	-0.967051882	1.9878844	-0.486473	0.626632	NA
nsun2	480.210967	-0.196490071	0.1048921	-1.87326	0.061033	0.482482
sp8b	10.67293407	0.267840565	0.5910701	0.4531452	0.650444	NA
abcb8	424.2774774	0.134733498	0.1227644	1.0974962	0.272425	0.837519
BX530018.2	78.62307148	0.012335229	0.2219627	0.0555734	0.955682	0.994543
trim47	360.3240906	-0.024191269	0.1193445	-0.202701	0.839369	0.984979
morc3a	207.7377351	-0.011275531	0.1597577	-0.070579	0.943733	0.993364
stc2a	339.1608957	-0.064697994	0.130199	-0.496916	0.619248	0.957354
cdk5	131.2878898	-0.006746356	0.1921099	-0.035117	0.971986	0.996315
mtmr1a	895.7064867	0.030090692	0.087194	0.3451006	0.730019	0.971177
cpeb4a	704.1437214	0.167786175	0.1030591	1.6280583	0.103513	0.609625
phc2a	839.3334747	-0.186686428	0.0897212	-2.080738	0.037458	0.378871

RAMP1	306.625716	0.1820505	0.1475669	1.2336815	0.217322	0.784755
pskh1	141.6682671	0.167166431	0.1714409	0.9750671	0.329527	0.873467
vars2	251.3047728	0.047620762	0.1434678	0.3319265	0.739945	0.972943
slc6a19b	54.09782653	0.394229341	0.2833723	1.3912063	0.164163	0.71945
utp3	157.3610336	-0.122740912	0.172906	-0.709871	0.477784	0.926857
cd99l2	5217.57253	0.079417166	0.0773884	1.026215	0.30479	0.859515
fhad1	27.09351209	0.139056747	0.3694349	0.3764039	0.706617	0.968573
hmgb3a	14116.931	-0.010943241	0.0623651	-0.175471	0.86071	0.985773
mettl7a	100.7454559	0.310826073	0.2019905	1.5388155	0.123849	0.652372
her4.2	168.8217659	-0.203169524	0.1592096	-1.276113	0.201915	0.76786
her4.1	101.2926009	-0.052273766	0.2047317	-0.255328	0.79847	0.978884
tas1r1	4.518619751	0.785731542	0.909626	0.8637963	0.3877	NA
tmeff1b	2899.583833	-0.038878473	0.0863598	-0.450192	0.652572	0.960708
senp3b	428.4428237	-0.081220531	0.1078999	-0.752739	0.451607	0.918761
crmp1	980.0491877	0.138844872	0.1019477	1.3619226	0.173222	0.732435
cavin4b	1119.789281	0.311535679	0.1021966	3.0483952	0.002301	0.059236
ela2	269.8774877	-0.448941498	0.1795863	-2.499865	0.012424	0.197293
necab2	2007.873402	0.082061237	0.0728951	1.1257449	0.260273	0.828368
si:ch211-254p10.2	75.23282335	0.074988942	0.2385285	0.3143814	0.753231	0.974828
dctn1b	873.2246322	-0.082122467	0.0891055	-0.921632	0.356721	0.883422
slc39a8	60.96389318	-1.17021733	0.2821241	-4.147881	3.36E-05	0.001935
zgc:172079	4.847388063	-0.917460447	0.8805877	-1.041873	0.297471	NA
hydin	65.23250169	-0.686945075	0.2544347	-2.699887	0.006936	0.129517
ela2l	326.293963	-0.589941135	0.1576169	-3.742881	0.000182	0.007911
itgb3a	121.3473493	0.36024837	0.19411	1.8558977	0.063468	0.492164
rprml	79.41991842	0.380067672	0.2364674	1.6072729	0.107995	0.619371
zgc:163079	0.63357355	1.435201493	2.6108194	0.5497131	0.582516	NA
gpr37l1b	57.29183853	-0.066528499	0.2529445	-0.263016	0.792538	0.977937
sshla	8.742577554	0.162778524	0.6633914	0.2453733	0.806167	NA
zanl	396.0132232	-0.649184496	0.1344703	-4.827717	1.38E-06	0.000131
raraa	543.1851169	0.050593421	0.1100511	0.4597268	0.645712	0.96029
aire	2.732878889	-0.315671489	1.2060257	-0.261745	0.793518	NA
pde6ga	23.0691388	0.198890382	0.4370847	0.4550386	0.649082	0.960708
jazf1b	102.9644275	0.153856849	0.2186429	0.7036901	0.481626	0.928386
arl3a	22.19529572	-0.163325357	0.4072075	-0.401086	0.688357	0.966672
serpine1	354.2471033	0.451788192	0.149475	3.0224999	0.002507	0.063006
ppp2r2ca	283.031027	0.281891553	0.1273882	2.212854	0.026908	0.316334
zgc:123297	0.513487682	-0.887846295	2.6557956	-0.334305	0.738149	NA
sufu	577.8065001	-0.044383136	0.1036504	-0.4282	0.668505	0.96478
ap1s1	887.939782	0.031589324	0.0902468	0.3500327	0.726314	0.970402
si:dkey-40g16.6	6.041875551	-1.394248442	0.8663289	-1.609375	0.107534	NA
plch2b	45.93531848	0.34619219	0.2948226	1.1742388	0.240299	0.808437
pde5aa	36.84268341	0.815690548	0.3400849	2.3984907	0.016463	0.236409
hoxa9b	154.6575696	-0.119544743	0.1645305	-0.726581	0.467483	0.924328
zmp:0000000662	291.6287614	0.028243752	0.133303	0.2118764	0.832203	0.983557
si:ch211-107m4.1	25.99022642	0.2082337	0.3743675	0.5562281	0.578055	0.951024
gng2	1688.28929	-0.061852324	0.0866189	-0.714075	0.475181	0.925737
exo1	76.15186191	0.056569979	0.2296717	0.246308	0.805444	0.979883
svopa	22.44588403	0.765074956	0.4270765	1.7914236	0.073225	0.527933
laynb	23.43152816	0.112716043	0.3899232	0.2890724	0.772526	0.975687
si:ch211-125o16.4	737.0027598	-0.045778598	0.1036136	-0.441821	0.658619	0.962545
pimr93	1.685032042	-0.618937429	1.5371164	-0.402661	0.687197	NA
otud5b	926.2515907	0.052565724	0.0894405	0.5877174	0.556722	0.94812
usp30	65.86760081	-0.076579428	0.2574566	-0.297446	0.766126	0.975374

si:dkey-27c15.3	84.62582498	0.222859311	0.2126846	1.0478392	0.294713	0.851
gatc	203.7472572	-0.092960298	0.1446091	-0.642838	0.520329	0.939056
tax1bp1b	2441.566397	-0.014533583	0.073077	-0.19888	0.842356	0.985174
spx	35.54836225	0.372600106	0.3221298	1.1566769	0.247404	0.81686
cep63	150.7171987	-0.129354648	0.1685167	-0.767608	0.44272	0.916336
ap4m1	148.562918	0.21462295	0.1691544	1.2687989	0.204513	0.772352
and3	946.2520266	0.395411084	0.099934	3.9567212	7.60E-05	0.003883
lygl1	89.32596527	0.165017585	0.2105648	0.7836904	0.433222	0.913654
vamp2	1215.68652	0.217744106	0.0921427	2.3631177	0.018122	0.250046
per1a	464.3684737	-0.017628461	0.1154739	-0.152662	0.878665	0.987881
tbccd1	152.1987906	0.104389142	0.1614041	0.6467565	0.51779	0.938592
dnah5l	117.5871438	0.197792464	0.2036403	0.9712835	0.331407	0.874362
mpp6a	465.9562221	0.199905714	0.1086967	1.8391144	0.065898	0.501955
ttc12	25.91236059	0.093051008	0.3715189	0.250461	0.802231	0.979492
mrpl13	618.2812184	0.030496897	0.1078323	0.2828179	0.777316	0.975826
si:rp71-45g20.10	4.100628876	-0.960013326	1.0337181	-0.928699	0.353045	NA
LO018309.1	12.53461784	-0.149705686	0.5743862	-0.260636	0.794373	0.977937
CU234171.1	12.58420373	-0.158231086	0.5320979	-0.297372	0.766182	0.975374
or133-1	2.826952666	0.205963614	1.1178571	0.1842486	0.853818	NA
pcolcea	483.3139323	0.335001292	0.1264435	2.6494147	0.008063	0.144946
emg1	182.3519068	0.112384044	0.1563319	0.7188809	0.472214	0.924838
si:ch211-237l4.6	85.63703137	0.449385266	0.2181432	2.0600471	0.039394	0.389879
si:rp71-45g20.4	9.130570961	0.685234516	0.6304808	1.0868443	0.277106	NA
tmem88a	34.38755191	-0.196582719	0.3456674	-0.568705	0.569556	0.948983
ankk1	29.70464176	-0.156119803	0.3490996	-0.447207	0.654726	0.961305
ltbp1	861.9900613	0.108831603	0.0897069	1.2131907	0.225057	0.792185
dbr1	540.5727824	0.031230517	0.1043817	0.2991952	0.764791	0.975374
sap130a	483.7702058	-0.071070679	0.1142081	-0.622291	0.53375	0.943276
drd2a	24.37426564	-0.095204409	0.4351265	-0.218797	0.826808	0.982473
kdm6bb	391.5546833	-0.072336513	0.133945	-0.540046	0.589165	0.953345
tfip11	801.0782323	-0.106873017	0.0960625	-1.112537	0.265907	0.831945
hepacama	510.5517364	0.212972609	0.1170263	1.8198695	0.068779	0.513259
kera	988.2157865	0.422502757	0.0862086	4.9009373	9.54E-07	9.67E-05
or125-8	1.314938914	1.626939831	1.7740828	0.9170597	0.359111	NA
htr7a	7.267901752	-0.948659861	0.7198822	-1.317799	0.187571	NA
epyc	4860.883653	0.049147912	0.078567	0.6255541	0.531607	0.942358
si:dkeyp-38g8.5	23.23757045	-0.07390351	0.4166781	-0.177364	0.859223	0.985773
ccdc30	86.0647421	-0.119522122	0.2225686	-0.537012	0.591259	0.954193
ilk	1002.883298	0.041883462	0.0876587	0.4778016	0.632791	0.957356
nek7	481.056121	0.033667655	0.1051006	0.3203374	0.748713	0.97419
ifit16	37.12782251	0.116161582	0.3197853	0.3632487	0.716419	0.96909
letm1	142.0358082	0.293851239	0.1753397	1.6758971	0.093758	0.582645
lhx9	977.8676289	-0.129761208	0.0915569	-1.417274	0.156403	0.706639
rad9a	60.27642884	0.023090785	0.257487	0.0896775	0.928544	0.992702
tpte	263.9494811	-0.170260451	0.1376993	-1.236466	0.216285	0.783589
dact2	173.788248	-0.052474283	0.166696	-0.31479	0.752921	0.97481
snai1a	332.3748407	-0.170032086	0.1215309	-1.399085	0.161788	0.716022
kirrel1b	490.1432194	0.067229249	0.1043192	0.6444571	0.519279	0.938945
camkvl	463.8405378	0.11478375	0.1153004	0.9955187	0.319484	0.867905
si:ch211-287a12.9	51.80666135	0.01560632	0.2687118	0.0580783	0.953686	0.994213
fam241a	245.6803327	0.017027782	0.1482615	0.1148497	0.908564	0.990702
ctbp1	4645.848239	-0.05629722	0.0772311	-0.728945	0.466035	0.923865
si:dkey-154b15.1	24.2599469	-0.429429644	0.4257206	-1.008712	0.313113	0.865039
dpm1	322.0636288	-0.205967036	0.1269142	-1.622884	0.104614	0.612567

cadm3	442.6154104	0.071134309	0.1164722	0.6107406	0.541371	0.944954
cdc14ab	489.3424362	0.016122406	0.1089652	0.1479592	0.882375	0.988344
slc6a4b	14.91817564	-0.010341181	0.4964378	-0.020831	0.983381	0.996944
vps36	278.4869369	-0.057529592	0.1292687	-0.445039	0.656292	0.961833
chrna2b	165.556833	0.053554651	0.1588705	0.3370964	0.736044	0.972535
ran	9276.779342	-0.158066028	0.0797089	-1.98304	0.047363	0.427455
rtn2b	368.9794088	0.642766763	0.1353292	4.7496542	2.04E-06	0.000185
htr2aa	28.33698247	0.106320237	0.3605981	0.2948441	0.768113	0.975687
ppm1nb	578.3765462	0.157596276	0.1035665	1.5216918	0.128086	0.658954
stoml3b	455.2166979	-1.107596299	0.1269156	-8.727032	2.61E-18	1.78E-15
kcnk12l	2.021294566	-1.600702766	1.5254152	-1.049355	0.294015	NA
si:ch211-191j22.8	11.95018327	0.330417685	0.5535305	0.5969277	0.550556	0.946549
tm2d1	167.6827292	-0.030199178	0.1645222	-0.183557	0.854361	0.985174
kcnk18	27.5355069	0.070696146	0.3664624	0.1929151	0.847025	0.985174
zgc:91976	526.2716819	-0.037765789	0.1051594	-0.359129	0.719499	0.969299
lrit3b	8.707771954	-0.724943862	0.6581652	-1.101462	0.270696	NA
ndufs8b	11.52322382	0.247487647	0.55668	0.4445779	0.656625	0.961928
minpp1a	247.5716634	-0.087720543	0.1456547	-0.60225	0.547008	0.945526
tvp23b	291.3542797	-0.23602545	0.1299111	-1.816823	0.069244	0.514556
si:dkey-157g16.6	149.4930394	-0.24321713	0.1734251	-1.402433	0.160786	0.713963
enpep	298.1841821	-0.905392678	0.1298235	-6.974026	3.08E-12	1.02E-09
rpgrb	221.2800227	-0.182007681	0.1487928	-1.223229	0.221243	0.787842
lamtor3	224.4526359	-0.221911598	0.1672694	-1.326672	0.184617	0.747376
ftsj1	161.8664678	0.048674478	0.168496	0.2888761	0.772676	0.975687
zmp:0000001103	2.73055215	0.876411384	1.2575846	0.6969006	0.485865	NA
kdm5ba	2568.283657	-0.251631894	0.0781522	-3.219765	0.001283	0.03764
ndr1	1.705233733	0.592496448	1.5482586	0.3826857	0.701953	NA
htr1ab	41.96837498	0.098156308	0.3155674	0.311047	0.755765	0.975374
papss1	424.5610449	0.250590947	0.1149888	2.1792647	0.029312	0.331778
zwilch	76.25113592	0.285446111	0.2288795	1.2471456	0.212344	0.780251
rnf180	72.89026228	-0.192894032	0.2291949	-0.841616	0.400003	0.90084
nle1	221.0290632	0.090098869	0.1432423	0.6289963	0.529351	0.942209
si:ch73-383g2.1	7.100483977	0.183982003	0.7525841	0.244467	0.806869	NA
serpine3	94.46800489	-0.564473611	0.2256027	-2.502069	0.012347	0.196577
slc25a1a	391.9072866	-0.11635301	0.1201922	-0.968058	0.333015	0.874926
gpr25	0.649418021	-1.460710349	2.4263694	-0.602015	0.547164	NA
c6	330.3582866	0.159613511	0.1399368	1.1406116	0.254032	0.823394
hpf1	397.5804049	-0.118449869	0.124054	-0.954825	0.339666	0.87805
ghsrb	6.206610695	-0.197663374	0.7732343	-0.255632	0.798235	NA
c7b	246.3980369	-0.535637167	0.1905365	-2.811206	0.004936	0.102322
pld1b	252.9865578	-0.214237036	0.1415387	-1.513629	0.13012	0.662797
hadhaa	832.3438786	0.374045794	0.0878283	4.2588297	2.05E-05	0.001286
zgc:174164	97.34593262	-0.05271299	0.2051364	-0.256966	0.797205	0.978577
zgc:110045	377.6162427	-0.114503545	0.1161444	-0.985872	0.324196	0.870928
zgc:194252	6.37585911	0.851291968	0.7816695	1.0890689	0.276124	NA
nradd	88.1004335	0.294504996	0.2064431	1.4265676	0.153705	0.702578
mocos	65.04652071	0.208662307	0.24998	0.8347161	0.403878	0.900979
prep	248.4442373	-0.366679354	0.1367245	-2.681886	0.007321	0.135037
ankrd29	109.5100942	0.002601929	0.1913618	0.0135969	0.989152	0.997589
lrmda	16.10789511	-0.554596813	0.4787041	-1.158538	0.246645	0.816669
eif4g2b	5592.432273	0.01425746	0.0659066	0.2163283	0.828732	0.982721
abca4a	210.5091331	0.17688176	0.1584753	1.1161471	0.264359	0.830245
ifit8	0.48448895	0.85344211	2.878836	0.2964539	0.766883	NA
il17c	2.937845939	-0.686529056	1.1522742	-0.595804	0.551306	NA

zgc:55781	176.20794	0.16368116	0.1560804	1.0486976	0.294317	0.850231
nmt1b	44.92271122	0.230543619	0.3242009	0.7111134	0.477014	0.926857
plppr5a	59.8723466	-0.033483054	0.2594036	-0.129077	0.897297	0.990121
has3	12.94258345	0.148344374	0.5212269	0.2846061	0.775946	0.975826
tbc1d15	431.1229134	-0.210299784	0.1216447	-1.728803	0.083844	0.557501
si:dkey-222b8.4	77.75401802	-0.002447436	0.229201	-0.010678	0.99148	0.997679
rorca	238.9190623	0.193454257	0.1346256	1.4369798	0.150724	0.698912
chtopa	2176.380571	-0.080728334	0.0723126	-1.11638	0.264259	0.830245
si:dkey-30k6.5	100.7545891	0.104838997	0.2030457	0.5163321	0.605622	0.95651
tph2	112.5187252	-0.125105193	0.2190038	-0.571247	0.567833	0.948739
tnfsf10	377.1663575	-0.20790353	0.1338504	-1.553253	0.120363	0.645215
im:7138535	96.65316117	-0.223618359	0.208451	-1.072762	0.283378	0.84466
sclt1	258.7068525	-0.129362183	0.1357621	-0.95286	0.340661	0.878134
zmynd11	1958.984459	-0.08810445	0.0738484	-1.193044	0.232852	0.801255
pimr141	0	NA	NA	NA	NA	NA
emc1	1917.869888	0.15877183	0.0871747	1.8213073	0.06856	0.513124
si:dkey-91i10.3	199.3987395	-0.667673545	0.1529977	-4.363946	1.28E-05	0.00088
zgc:173729	13.05624299	-0.866305138	0.5262792	-1.646094	0.099744	0.600955
pimr52	0	NA	NA	NA	NA	NA
slc30a9	933.5774141	0.053282069	0.0880307	0.6052671	0.545002	0.945463
alox5a	13.53087551	1.005639394	0.5284148	1.9031251	0.057024	0.46866
iqca1	21.87011327	-0.065714301	0.4681246	-0.140378	0.888361	0.989291
pimr54	0	NA	NA	NA	NA	NA
swap70b	316.0309609	-0.094412421	0.1303539	-0.724278	0.468895	0.924328
slc25a16	362.794798	-0.068569523	0.1160235	-0.590997	0.554523	0.947449
cbln1	780.3209396	0.134240949	0.0900345	1.490994	0.135963	0.672424
pbdc1	616.4502545	0.009070612	0.0957633	0.0947191	0.924538	0.992676
galnt7	1306.517008	-0.113465751	0.0831983	-1.363799	0.172631	0.731348
rwdd3	60.15098747	0.041438914	0.2676177	0.1548437	0.876945	0.987881
nexn	887.8825802	0.238853022	0.0980803	2.4352806	0.01488	0.223418
tut1	258.8136243	-0.237336033	0.1317511	-1.801397	0.07164	0.523122
tmem47	1164.136032	0.141015807	0.0886761	1.5902352	0.111782	0.627242
e2f8	355.6811269	-0.025471382	0.1167048	-0.218255	0.827231	0.982682
cth1	0.473290478	-0.833892061	2.901156	-0.287434	0.77378	NA
kcnk7	3.708846379	-1.049014492	1.0561306	-0.993262	0.320582	NA
cmasa	781.3370441	-0.053812285	0.0964796	-0.557758	0.57701	0.950762
slc25a29	9.083767543	0.55724257	0.6633713	0.8400161	0.400899	NA
ehbp1l1a	170.2795226	0.129342664	0.1774788	0.728778	0.466138	0.923865
elovl8b	79.77145825	-0.065334559	0.2529535	-0.258287	0.796186	0.978498
rps8b	109.7154651	0.025860019	0.1892637	0.1366348	0.891319	0.989291
sfxn3	118.5954207	-0.048878508	0.1915838	-0.255129	0.798624	0.978884
selenou1a	1148.62533	0.122131282	0.0905213	1.3491991	0.177273	0.738201
lrrc31	47.27186634	-0.611324967	0.2976026	-2.054165	0.03996	0.391511
rab33a	215.6327541	-0.087290371	0.1506828	-0.579299	0.562387	0.948728
si:ch211-130m23.3	44.2206408	-0.615456601	0.3134096	-1.963745	0.04956	0.436328
CR847953.1	344.2904241	0.068895672	0.1204104	0.572174	0.567204	0.948739
CABZ01067232.1	173.60225	0.084566574	0.1712145	0.4939217	0.621361	0.957354
rxfp3	28.35198815	0.021904817	0.3969926	0.0551769	0.955998	0.994543
phb	3414.040487	-0.035935201	0.0836999	-0.429334	0.66768	0.964736
nudt13	30.05471445	-0.376239529	0.3924681	-0.95865	0.337735	0.876395
slc44a5b	197.2410941	-0.112512647	0.148757	-0.756352	0.449438	0.918383
oard1	46.47875679	-0.283289995	0.3041444	-0.931432	0.35163	0.880769
sv2ba	2071.184389	0.347862885	0.1009227	3.4468259	0.000567	0.019439
arg1	19.53210259	0.430671086	0.4469502	0.9635773	0.335258	0.876088

st6galnac5b	62.82062466	0.344782833	0.250163	1.3782328	0.168131	0.725902
amacr	104.9097561	0.074433908	0.1963235	0.379139	0.704585	0.968134
apodb	701.4482144	-0.294260462	0.0982739	-2.99429	0.002751	0.06774
phf1	4.944206532	-0.81969037	0.9344343	-0.877205	0.380375	NA
si:dkey-228d14.5	51.42474976	0.102398381	0.2741171	0.373557	0.708734	0.968664
ston2	573.0091692	0.071974122	0.0992327	0.7253062	0.468264	0.924328
ppp3ccb	573.0901665	0.187988603	0.1023026	1.8375737	0.066125	0.502802
crygm2d13	15961.4855	0.050531939	0.0671707	0.7522916	0.451876	0.918761
gfpt1	459.2094383	0.04626012	0.1069928	0.4323668	0.665475	0.964736
kcnc2	71.99969745	-0.026111637	0.2317637	-0.112665	0.910296	0.990702
lrrc28	51.84261169	-0.29056942	0.2702022	-1.075378	0.282206	0.843666
hrh2b	2.795754184	-0.88380829	1.145882	-0.771291	0.440535	NA
RNF157	474.8797118	0.049619697	0.1072835	0.4625101	0.643716	0.960014
srsf2a	3503.501402	0.026551661	0.0707192	0.3754522	0.707324	0.968664
n4bp1	380.4209411	0.090495298	0.1287234	0.7030211	0.482043	0.928561
stk35l	550.0504286	0.073896278	0.1032224	0.7158938	0.474057	0.924993
habp2	126.4149605	0.427278725	0.1821968	2.3451498	0.019019	0.257244
si:dkey-202g17.3	137.5419662	-0.203450162	0.2083474	-0.976495	0.328819	0.873113
mdm4	1258.860361	-0.042109372	0.0834708	-0.50448	0.613924	0.95688
pcdh1g9	272.86081	0.00789079	0.1392788	0.0566546	0.95482	0.994543
mef2ab	89.51857151	-0.027961596	0.2112512	-0.132362	0.894698	0.989716
itpa	194.2175903	0.218691225	0.1524922	1.4341146	0.15154	0.700127
pgap3	9.798531314	0.966462855	0.6105219	1.5830109	0.113419	NA
crygm2a	0.158795395	0.967652056	4.0804729	0.2371421	0.812547	NA
rpl17	12542.42306	-0.278909807	0.0813165	-3.429927	0.000604	0.020478
polr3c	92.79196606	-0.113253756	0.2056167	-0.5508	0.581771	0.95187
nefla	267.5442499	0.014249334	0.1295529	0.1099886	0.912418	0.991119
pgam2	1610.191728	0.230065264	0.0892161	2.5787422	0.009916	0.169473
pnp4a	307.4819134	-0.707915678	0.1255989	-5.636321	1.74E-08	2.82E-06
mbtps2	342.7894436	0.15310392	0.1240587	1.2341248	0.217156	0.784302
prrc2c	1841.890424	0.050706813	0.1050861	0.4825263	0.629432	0.957354
sall3b	348.2371456	0.046011225	0.1176091	0.3912217	0.695633	0.96736
si:ch1073-396h14.1	291.2978406	0.225062153	0.1279164	1.7594467	0.078502	0.545813
s100b	272.0243241	-0.003531184	0.1470268	-0.024017	0.980839	0.996944
arl4ca	296.0027646	0.141497888	0.1331261	1.0628863	0.287834	0.845979
cdkn2c	67.46063185	0.319473577	0.2511674	1.2719547	0.203389	0.770611
nitr3c	4.777460962	0.720588887	0.9107214	0.7912287	0.428811	NA
cops5	624.3545505	0.19774167	0.1005905	1.9658083	0.049321	0.435504
dusp14	167.8216746	0.009305503	0.1609773	0.0578063	0.953903	0.994255
josd2	411.6241106	-0.159035129	0.1213216	-1.310856	0.189907	0.754269
galm	221.3381961	-0.532277574	0.1725726	-3.084369	0.00204	0.054215
ppp1r42	8.885086934	0.856694258	0.6440546	1.3301579	0.183466	NA
cxcr4a	98.22553326	0.002330211	0.203466	0.0114526	0.990862	0.997679
wdr34	38.29932702	-0.020249919	0.3305635	-0.061259	0.951153	0.99381
adam8b	66.29085817	-0.029404554	0.2578822	-0.114023	0.909219	0.990702
dnttip2	455.8697026	-0.057402807	0.1110856	-0.516744	0.605335	0.95651
satb1a	150.978095	0.0810679	0.1936182	0.4186998	0.675436	0.965257
dbpb	612.7782526	-0.010777665	0.1092679	-0.098635	0.921428	0.992138
dhx57	354.5223006	-0.114307016	0.1217146	-0.93914	0.347659	0.880195
aldoca	226.2135346	-0.632996665	0.1410474	-4.487828	7.20E-06	0.000541
sprn2	146.7073512	0.231067552	0.167324	1.3809592	0.167292	0.724173
epas1b	419.2899966	0.255178722	0.1181293	2.160164	0.03076	0.339614
plpp5	490.4031477	0.132422327	0.1161625	1.1399746	0.254297	0.823394
golga7	1355.045849	0.032720757	0.0782344	0.41824	0.675772	0.965257

neu4	11.05849294	0.383888707	0.5763584	0.666059	0.505373	NA
sfrp1b	72.41419816	0.466966181	0.2457343	1.900289	0.057395	0.470146
sh2b1	785.986261	-0.002793313	0.1148639	-0.024318	0.980599	0.996944
foxj2	128.2428436	-0.08781852	0.1784438	-0.492135	0.622624	0.957354
si:ch211-266o15.1	853.9432475	-0.148813882	0.089592	-1.661018	0.09671	0.592423
mcm6	1160.219254	0.065793992	0.0901872	0.7295267	0.46568	0.923865
sbk3	56.7318761	0.250057129	0.2657591	0.9409165	0.346748	0.8794
ical1	360.7344206	-0.119655381	0.1228275	-0.974174	0.32997	0.874009
srsf1a	4453.567111	-0.051906875	0.0645713	-0.803869	0.421472	0.90651
ctsd	3494.370969	-0.040386127	0.0888687	-0.454447	0.649507	0.960708
styx	199.4709035	-0.205222783	0.1575976	-1.302195	0.19285	0.756795
si:ch211-137i24.10	394.1960322	-0.626033561	0.1187551	-5.271637	1.35E-07	1.82E-05
zgc:66443	158.8333968	-0.023881811	0.1705102	-0.140061	0.888612	0.989291
zgc:175264	553.6928496	-0.088920309	0.1018786	-0.872807	0.382768	0.895192
cmah	307.9743781	-0.396753218	0.131065	-3.027148	0.002469	0.062293
atl2	497.7110578	0.170169863	0.104204	1.6330456	0.102459	0.608235
slc17a8	28.37701771	-0.307058338	0.3711614	-0.827291	0.408072	0.90171
ephb6	162.7942741	-0.04173061	0.1755827	-0.237669	0.812138	0.981081
ano10a	618.4084308	0.004076097	0.0977178	0.041713	0.966728	0.996315
rxraa	172.037626	-0.090289283	0.1569845	-0.575148	0.565191	0.948739
hells	560.9246823	0.171566755	0.1076653	1.5935199	0.111044	0.626294
nr1h4	96.79105008	-0.395592528	0.2163158	-1.828774	0.067434	0.508361
si:dkey-10f21.4	118.247474	0.020399277	0.1850933	0.1102108	0.912242	0.991119
zbtb47a	67.55045599	0.264442013	0.2631637	1.0048575	0.314965	0.866054
nudt4a	335.9317703	0.183814007	0.1317904	1.3947447	0.163093	0.717943
pimr55	5.795488932	0.451157181	0.8084433	0.5580567	0.576806	NA
7-Mar	259.3178548	-0.251232437	0.1477995	-1.699819	0.089165	0.571894
baz2ba	2373.48534	-0.227630578	0.0907545	-2.5082	0.012135	0.194451
itgae.2	12.54988257	0.78730634	0.5816745	1.353517	0.175891	0.736505
lyz	4.628387648	0.042308788	0.9006947	0.0469735	0.962534	NA
ankrd6a	130.4533526	-0.098440318	0.1916059	-0.513764	0.607417	0.956718
cx52.7	27.12642126	0.071374707	0.3613023	0.1975484	0.843398	0.985174
mrpl28	693.0783398	-0.04962378	0.095899	-0.517459	0.604836	0.95651
si:ch73-61d6.3	168.784246	-0.453352828	0.1623487	-2.792464	0.005231	0.106854
dnmt3bb.2	181.6568468	-0.025201854	0.1605476	-0.156974	0.875265	0.987881
gulp1b	55.13704948	0.182059488	0.2761865	0.6591903	0.509774	0.936253
zgc:171470	2.585752491	-0.352319945	1.1984029	-0.293991	0.768765	NA
atp6v0ca	4997.289063	-0.032263341	0.0668665	-0.482504	0.629448	0.957354
sap18	0	NA	NA	NA	NA	NA
mrps31	500.711951	0.072509661	0.1034291	0.7010569	0.483267	0.92946
arfrp1	298.6820901	0.296810123	0.1285945	2.3081092	0.020993	0.273449
hoxd10a	93.53442598	0.172331012	0.2183588	0.7892102	0.429989	0.911615
dnmt3bb.3	125.1942027	-0.099272149	0.1872502	-0.530158	0.596003	0.954434
lasp1	2485.896711	0.005706086	0.0700827	0.0814193	0.935109	0.992702
cdc42l2	37.75234999	-0.204500066	0.3337319	-0.612768	0.54003	0.944206
p3h3	278.0683969	0.278532724	0.1378164	2.0210424	0.043275	0.40826
si:dkey-33c12.3	1176.370371	0.243389277	0.0897917	2.7105989	0.006716	0.126361
arpc3	1616.696032	0.033118599	0.0925262	0.3579374	0.72039	0.969318
zgc:114174	47.11702908	-0.110412686	0.2798097	-0.394599	0.693139	0.96736
BX901922.1	0.853874503	-1.902668424	2.1134959	-0.900247	0.367989	NA
nts	26.56943658	0.010187934	0.3708491	0.0274719	0.978083	0.996762
si:dkey-33c12.4	403.6770216	-0.140314987	0.1212066	-1.157652	0.247006	0.816669
zgc:114081	65.76570843	-0.270279598	0.2524106	-1.070793	0.284262	0.844697
si:ch211-266g18.10	178.4059677	0.339471009	0.1573843	2.1569554	0.031009	0.340842

si:ch73-181d5.4	351.1152195	-0.115828848	0.1236688	-0.936606	0.348961	0.880195
mrps34	719.1020797	0.059453868	0.0980661	0.6062634	0.54434	0.945438
zgc:86709	984.000127	0.182220932	0.1184114	1.5388798	0.123834	0.652372
eif1axb	2548.670241	-0.006284121	0.077604	-0.080977	0.93546	0.992702
syt11a	2445.249252	0.154233229	0.0811435	1.9007464	0.057335	0.469987
tsc1b	661.0815725	-0.26411209	0.0957661	-2.757888	0.005818	0.114852
ccdc136b	178.2193217	0.062633517	0.1598271	0.3918829	0.695145	0.96736
rps6ka3b	82.03168871	-0.189251859	0.2264239	-0.83583	0.403251	0.900901
mzt2b	390.5833292	0.039060317	0.1114621	0.3504359	0.726012	0.970402
naf1	192.5899647	-0.056392529	0.1477732	-0.381616	0.702747	0.968134
lhx5	713.1619133	-0.127819082	0.1049307	-1.218128	0.223175	0.789115
ythdf3	264.4293247	0.251282319	0.1366937	1.8382879	0.06602	0.502687
dido1	1493.064287	-0.040384264	0.1002834	-0.402701	0.687168	0.966376
glt8d2	22.33028603	0.720408347	0.420798	1.7120052	0.086896	0.565027
slc43a3b	274.3577121	0.121410887	0.1288921	0.9419576	0.346214	0.87905
dph1	185.7674532	-0.14691605	0.1532436	-0.958709	0.337705	0.876395
parp12b	206.8750874	-0.03770802	0.1418361	-0.265856	0.79035	0.977338
plcd4a	71.13619778	0.14467644	0.2467028	0.5864402	0.55758	0.948478
svopl	193.3215312	-0.319522612	0.1576891	-2.026282	0.042736	0.406083
eps8l1a	147.8622388	-0.024836682	0.1663227	-0.149328	0.881295	0.988245
ints8	484.7925391	0.010017927	0.1134574	0.0882968	0.929641	0.992702
uba3	742.1777012	0.068705558	0.0930494	0.738377	0.460285	0.921978
fstb	296.3314835	0.344801843	0.1419078	2.4297598	0.015109	0.225333
arl15b	292.1049833	-0.021561471	0.1288332	-0.16736	0.867087	0.986978
tmf1	757.9202867	-0.086940108	0.0932448	-0.932385	0.351137	0.880473
cep350	730.8372819	0.022695272	0.1016967	0.2231662	0.823406	0.982196
wfdc1	534.6610717	0.23287097	0.1221102	1.9070553	0.056513	0.46635
six2a	281.3709443	-0.107226089	0.1311819	-0.817385	0.413708	0.905864
hgd	430.9018604	-0.015952631	0.1239274	-0.128726	0.897575	0.990121
six3a	915.7324056	0.049681212	0.0928946	0.5348128	0.592779	0.954434
arxa	264.2030229	0.021202044	0.1399618	0.1514845	0.879594	0.987881
ano3	35.6723296	-0.092571675	0.3291724	-0.281225	0.778537	0.975942
fbxw9	41.83424917	-0.354748454	0.3063332	-1.158048	0.246845	0.816669
ormdl2	237.6102068	-0.045066284	0.149771	-0.300901	0.76349	0.975374
si:dkey-11o15.7	18.8559827	0.837241261	0.4422515	1.8931336	0.05834	0.473924
hspa14	486.3888486	0.030984416	0.1045254	0.2964294	0.766902	0.975687
si:dkey-6n6.2	158.7050154	-0.155934495	0.1679209	-0.928619	0.353087	0.881552
eml3	141.8337368	0.065426613	0.1772637	0.369092	0.712059	0.969006
bhlhe22	942.7793893	-0.006953122	0.0871953	-0.079742	0.936442	0.992702
ndufa8	1510.868202	-0.082837967	0.0757054	-1.094214	0.273861	0.838546
kitlgb	7.762814812	-0.385711156	0.7718078	-0.49975	0.617251	NA
rab1ba	4544.206522	0.037008873	0.0707702	0.5229446	0.601013	0.956045
tlr21	4.756920385	-0.170260487	0.9029324	-0.188564	0.850435	NA
pafah1b3	805.8361719	0.026912425	0.1005609	0.2676232	0.788989	0.976924
ugt5e1	0	NA	NA	NA	NA	NA
smyd3	58.57172326	-0.04545798	0.2587827	-0.175661	0.86056	0.985773
serping1	213.3139956	0.107420116	0.1409102	0.7623303	0.445863	0.917154
igf3	0.482640214	-0.866525639	2.7061322	-0.320208	0.748811	NA
magt1	543.2403604	-0.071487046	0.1058647	-0.675268	0.499505	0.934006
cx31.7	2.204457235	0.257115366	1.2965921	0.1983009	0.84281	NA
tnfsf10l4	19.45649937	0.005960957	0.4306905	0.0138405	0.988957	0.997589
snf8	230.5396161	0.035255921	0.1641454	0.2147847	0.829935	0.982821
txndc17	239.2304828	-0.174331464	0.1369529	-1.27293	0.203043	0.770037
birc7	196.3411407	0.33177337	0.1515467	2.1892491	0.028579	0.328246

aifm1	1532.396842	-0.044265952	0.0801997	-0.551947	0.580985	0.951731
rprd2a	601.0547182	-0.052897543	0.1157405	-0.457036	0.647645	0.960645
cfap57	15.65381499	0.165485078	0.5360497	0.3087122	0.75754	0.975374
ciarta	1683.060826	-0.165514363	0.0933765	-1.772549	0.076303	0.538373
b3gnt3.4	67.00660724	-0.276988799	0.2389743	-1.159073	0.246426	0.816419
sardh	699.7266024	-0.025808146	0.1127037	-0.228991	0.818876	0.981594
glra4b	117.4825637	0.051473602	0.2088946	0.2464095	0.805365	0.979883
rpl36a	11368.00085	-0.374565051	0.1911052	-1.959994	0.049996	0.437835
eps8l2	195.3871213	-0.392427323	0.145779	-2.691933	0.007104	0.1319
impad1	276.4684109	0.112635848	0.1429351	0.788021	0.430684	0.912304
fgfr2	3005.322996	0.047641396	0.0697497	0.6830341	0.494585	0.931926
snap25b	4517.933563	0.028283973	0.0677763	0.4173136	0.676449	0.965257
GPR83	3.663765726	0.820395966	1.0153666	0.8079801	0.419102	NA
hcfc2	128.2664403	-0.105331422	0.18015	-0.584687	0.558758	0.948478
msna	2090.556874	0.07962586	0.0722414	1.1022185	0.270367	0.835161
foxd2	300.246289	-0.155828935	0.124138	-1.255288	0.209374	0.77633
ttc25	34.89450437	0.282156095	0.3501969	0.805707	0.420412	0.90651
atp7b	43.77321824	-0.144672132	0.3252602	-0.444789	0.656472	0.961833
dnajc5b	21.96996956	0.64547703	0.423145	1.5254275	0.127153	0.656588
dnajc7	2228.736748	0.046674364	0.0759878	0.6142351	0.53906	0.943733
znf385c	215.305553	0.23156418	0.1480111	1.564506	0.117699	0.641446
trim55b	113.7900884	-0.255000407	0.190241	-1.340407	0.180113	0.74128
ipo8	424.90834	-0.039749997	0.1091115	-0.364306	0.715629	0.96909
tnfaip2b	91.39199724	-0.125654912	0.2356226	-0.533289	0.593834	0.954434
pcyt1ba	81.20445097	-0.026955124	0.2237878	-0.120449	0.904127	0.990291
htr2ab	10.12096752	0.723570399	0.5934553	1.2192501	0.222749	NA
zdhhc20b	188.4649128	0.050606303	0.1533847	0.3299305	0.741452	0.973154
ackr3b	1023.316082	0.054137359	0.0892359	0.6066769	0.544065	0.945438
exoc3l4	33.8384218	-0.75179188	0.3327014	-2.259659	0.023842	0.298015
plcxd2	32.74053473	0.230065098	0.3443157	0.6681807	0.504018	0.93532
pex16	185.9252136	0.034562174	0.1586044	0.2179143	0.827496	0.982721
smc1a1	2023.487658	-0.134662728	0.0816947	-1.648366	0.099278	0.599058
si:ch211-153b23.5	1459.351621	-0.240756571	0.1203164	-2.001029	0.045389	0.418148
abi2a	259.9813119	-0.005118327	0.1407873	-0.036355	0.970999	0.996315
slc25a26	195.8978056	-0.144475266	0.1530411	-0.944029	0.345155	0.878434
ndnl2	168.8183765	0.109714517	0.1716337	0.6392364	0.522669	0.939336
fam151a	33.13943083	0.130652321	0.3650624	0.3578904	0.720425	0.969318
tubd1	63.88752354	0.101010372	0.2441369	0.4137449	0.679061	0.965257
tada3l	659.1662707	0.029281249	0.0929532	0.3150107	0.752754	0.97481
ugcg	240.6706634	0.003022457	0.1386263	0.0218029	0.982605	0.996944
mpp4a	63.09747857	-0.143850522	0.2592079	-0.554962	0.578921	0.951052
arpc4l	834.3334027	-0.041913353	0.1033155	-0.405683	0.684975	0.966146
ak3	490.4962438	-0.429715402	0.1050899	-4.089027	4.33E-05	0.002406
acot11a	66.63045945	-0.271015109	0.2527865	-1.072111	0.28367	0.84466
rps6kb1b	936.9272939	0.071597428	0.0953591	0.7508194	0.452761	0.919551
nt5c2b	597.3814925	0.074279991	0.1153225	0.6441064	0.519506	0.938945
fbrsl1	1595.052931	-0.051855213	0.0855584	-0.60608	0.544462	0.945438
ssbp3a	182.844177	0.021910396	0.1628541	0.13454	0.892976	0.989291
phactr3a	427.6382498	-0.054672818	0.1342503	-0.407245	0.683828	0.965679
il17ra1a	105.8911268	-0.274721794	0.2013802	-1.364195	0.172506	0.731348
cysltr1	25.55203182	0.257236865	0.4021364	0.6396756	0.522384	0.939056
si:dkeyp-77h1.4	48.9004028	0.259513642	0.3158164	0.8217232	0.411234	0.903427
adamts15b	8.861389452	0.389546944	0.656136	0.5936984	0.552714	NA
si:ch211-213d14.1	10.31950354	0.216735753	0.6050382	0.3582183	0.72018	NA

draxin	370.505414	0.032173795	0.1165294	0.2761003	0.782471	0.97627
slc39a1	111.3771211	-0.242657482	0.1984911	-1.222511	0.221515	0.788243
celsr1b	587.6590464	-0.043326154	0.1006675	-0.430389	0.666913	0.964736
cip2a	145.3721503	-0.125969628	0.1686507	-0.746926	0.455108	0.921022
glmna	290.2936296	0.089683885	0.1349979	0.6643355	0.506476	0.935724
COLGALT1	139.7859109	0.061178952	0.1817348	0.3366387	0.736389	0.972535
si:dkey-208b23.5	7.322085643	-0.372656425	0.6975823	-0.534211	0.593195	NA
CT990561.1	21.25793764	-0.344133617	0.4286762	-0.802782	0.422101	0.90651
cpt1b	483.8275326	0.227863632	0.1033339	2.2051206	0.027446	0.320231
gpalpp1	311.382507	-0.01684243	0.125058	-0.134677	0.892867	0.989291
sephs1	992.6185666	0.041674571	0.0900333	0.4628795	0.643451	0.959994
timm13	1042.64779	0.06949571	0.0854842	0.8129658	0.416238	0.906237
zgc:173737	4.429116314	0.791898059	0.9612827	0.8237931	0.410057	NA
sh3bgrl	597.5529844	0.216434664	0.115053	1.8811732	0.059948	0.478654
prpf18	467.7542343	0.076281557	0.1079529	0.7066187	0.479803	0.927094
c1qtnf9	252.1494611	0.34098641	0.1430758	2.3832563	0.01716	0.242176
cep76	121.0282162	-0.11714414	0.1860115	-0.629768	0.528846	0.942109
enc3	522.5236565	0.03304279	0.1084026	0.3048155	0.760507	0.975374
tmbim1a	187.1132361	0.002420954	0.1553769	0.0155812	0.987569	0.997201
casp8	108.3603868	-0.149983718	0.2016856	-0.743651	0.457088	0.921539
neu3.3	22.6630209	-0.59321089	0.414655	-1.430613	0.152541	0.701776
lsm7	962.0711031	0.164891121	0.1209567	1.3632238	0.172812	0.731888
btr21	4.095447136	-0.583210865	0.9481378	-0.615112	0.538481	NA
krt18a.2	50.75931056	-1.476162014	0.2877966	-5.129186	2.91E-07	3.53E-05
slc25a22b	13.72076049	0.746376318	0.5149853	1.4493159	0.147249	0.69409
grhl2a	168.8316414	-0.05613478	0.1699233	-0.330354	0.741133	0.973154
si:dkey-5i3.5	147.6427482	0.045355262	0.1824219	0.2486283	0.803648	0.979643
sass6	70.82839942	-0.071984052	0.2391662	-0.300979	0.76343	0.975374
scin1b	700.3138939	-0.104188919	0.1000239	-1.04164	0.297579	0.85401
polr2l	445.6253424	-0.224123152	0.1380786	-1.623157	0.104556	0.612567
tapbpl	619.2261184	0.090272388	0.0991549	0.9104175	0.362602	0.885944
acot9.1	284.7407602	-0.037493616	0.1260516	-0.297446	0.766126	0.975374
selenot1a	1430.833598	-0.044792504	0.0857725	-0.522224	0.601514	0.956045
ankrd33ba	79.11406295	-0.043281087	0.2182165	-0.19834	0.842779	0.985174
krt8	8407.817685	-0.092056242	0.0842448	-1.092723	0.274516	0.839136
hspb8	325.9944404	0.381744589	0.1256367	3.0384807	0.002378	0.060825
si:dkey-222f8.3	377.7474559	-0.186225412	0.1281881	-1.452751	0.146293	0.692388
abhd14a	47.69132986	0.035528629	0.2987873	0.1189094	0.905347	0.990702
mex3b	1262.555511	-0.046233944	0.0852128	-0.542571	0.587425	0.953319
ropn1l	15.38754998	0.463465801	0.5163703	0.8975454	0.369428	0.889127
krt5	29471.66177	0.299087062	0.0776564	3.8514154	0.000117	0.005608
mcph1	26.02018599	-0.119959892	0.3754586	-0.319502	0.749346	0.97419
otpb	835.2088894	-0.02428316	0.0933585	-0.260106	0.794782	0.978206
zgc:171775	565.0737055	0.112822643	0.1093102	1.0321327	0.30201	0.856944
pimr57	0	NA	NA	NA	NA	NA
ccl19a.1	10.1269726	-1.071934415	0.636949	-1.68292	0.092391	NA
acot9.2	66.8469053	0.07411347	0.2431318	0.3048284	0.760497	0.975374
adcy2a	151.0630502	0.104594725	0.1784137	0.5862483	0.557709	0.948478
tagln3b	588.5460279	0.099444187	0.1038469	0.9576037	0.338263	0.876778
hce2l1	7.667675308	0.648106188	0.704076	0.920506	0.357308	NA
CR956623.1	39.32041214	0.151447413	0.3546738	0.4270048	0.669376	0.965092
rnaset2l	6.274189727	0.000853396	0.801417	0.0010649	0.99915	NA
bglap	0.173729368	-0.955901296	4.0804729	-0.234262	0.814781	NA
si:dkey-11m19.5	1.457700461	1.008854538	1.6187964	0.6232127	0.533145	NA

gcn1	2309.059319	-0.189498029	0.0965724	-1.962238	0.049735	0.437286
lgi1b	137.8726884	0.208149736	0.1741731	1.1950737	0.232058	0.800126
rab35b	213.260896	0.013514412	0.1529624	0.0883512	0.929598	0.992702
snx6	155.1939264	-0.08274233	0.1645852	-0.502732	0.615152	0.95688
zgc:136472	107.1575975	-0.892036045	0.1997843	-4.464996	8.01E-06	0.000593
zrsr2	173.2337307	0.181871166	0.1605284	1.1329531	0.257234	0.825314
rpl6	32077.5143	-0.33510426	0.0908532	-3.688413	0.000226	0.009434
abhd17c	872.0899952	-0.012532662	0.0908125	-0.138006	0.890236	0.989291
dynll1	2795.377147	0.056943154	0.1040065	0.5474962	0.584038	0.952214
plekhg2	277.5187544	-0.185331175	0.1313993	-1.410443	0.158409	0.709224
cacng3a	3.435813421	1.031635984	1.1080511	0.9310365	0.351835	NA
ice1	196.4853731	-0.00227052	0.1478373	-0.015358	0.987746	0.997329
zgc:158846	3365.404171	-1.002533176	0.0877118	-11.42986	2.97E-30	7.33E-27
ndufab1a	1006.850317	0.04198762	0.0882145	0.475972	0.634094	0.95776
rasd3	15.29544145	-0.083591209	0.4966203	-0.16832	0.866331	0.986673
prpf40a	1197.690429	-0.004004845	0.0839956	-0.047679	0.961972	0.995907
mapk13	88.20145482	0.080622529	0.2157845	0.3736252	0.708683	0.968664
plk1	869.1772039	-0.078475861	0.086602	-0.906166	0.364848	0.886714
st6galnac3	35.66647562	0.267561398	0.3290704	0.8130826	0.416171	0.906237
ern2	46.33925964	-0.193020593	0.2895373	-0.666652	0.504995	0.935724
stc1l	130.4762997	0.676077921	0.1940754	3.4835836	0.000495	0.017691
pimr72	0.356300316	0.005884122	3.214964	0.0018302	0.99854	NA
zgc:110425	25.01136882	0.589104826	0.4462255	1.3201953	0.18677	0.750951
chtf18	171.5089969	-0.196167916	0.1612381	-1.216635	0.223743	0.790335
caps2	5.32051111	1.142112206	0.8395294	1.3604195	0.173697	NA
adora2ab	12.49453087	0.069743535	0.5487312	0.1270996	0.898862	0.990121
si:dkey-30c15.17	28.79488821	0.088296261	0.3654892	0.2415838	0.809103	0.980642
dnaja3a	834.2042613	-2.65E-05	0.1003	-0.000265	0.999789	0.999839
slc9a3.1	170.7558735	0.105832882	0.1550481	0.6825811	0.494872	0.931926
olah	83.81073175	-0.405225663	0.2166623	-1.87031	0.061441	0.483266
flt3	3.025275472	0.676030926	1.0972996	0.616086	0.537838	NA
apls2	1055.026056	0.110340746	0.0979943	1.1259912	0.260169	0.828288
cfap70	29.99791577	-0.070764809	0.3671155	-0.192759	0.847148	0.985174
shq1	91.8026722	-0.17868206	0.2277513	-0.784549	0.432718	0.91326
cnot10	384.15987	-0.055158769	0.1164352	-0.473729	0.635693	0.958142
pimr209	0.340386822	-1.882368278	3.2159502	-0.585323	0.558331	NA
pole	474.5332464	-0.131233147	0.1057422	-1.241067	0.214581	0.782493
si:dkey-211g8.7	0.173729368	-0.955901296	4.0804729	-0.234262	0.814781	NA
si:dkey-102c8.2	1.209546312	-0.321199246	1.8955078	-0.169453	0.86544	NA
alcamb	1664.413905	-0.074535263	0.0811733	-0.918224	0.358501	0.884398
lama5	2895.844269	0.024186353	0.0715643	0.3379666	0.735388	0.972535
si:dkey-30c15.12	6.138991796	-1.0219059	0.8051476	-1.269216	0.204364	NA
si:ch73-160i9.3	2.299589602	1.892552949	1.3542231	1.3975194	0.162257	NA
bves	254.2866463	0.061079094	0.1408015	0.4337957	0.664437	0.964736
popdc3	97.7676641	0.27982878	0.1994537	1.4029762	0.160624	0.713726
sprb	9.834634998	-0.772781541	0.6484454	-1.191745	0.233361	NA
muc5.2	638.4118947	0.738814044	0.1142807	6.4649079	1.01E-10	2.41E-08
il11b	5.275865162	0.911663632	0.878431	1.0378318	0.299348	NA
lzts3b	33.28520311	0.246221517	0.3273738	0.7521113	0.451984	0.918761
adrm1	1381.297579	-0.064728348	0.0923422	-0.700962	0.483327	0.92946
fbxo30a	39.34964939	0.386472371	0.3071941	1.2580723	0.208366	0.775404
znf991	80.33880048	-0.039075649	0.2243048	-0.174208	0.861702	0.985841
ubox5	116.1329784	-0.2892752	0.1981811	-1.459651	0.144386	0.688868
si:ch73-368j24.17	2.38097032	-0.410107889	1.3118331	-0.312622	0.754568	NA

socs6a	8.643563483	0.670497985	0.6605725	1.0150256	0.310094	NA
si:dkey-253d23.5	35.67207878	0.072444959	0.3279291	0.2209166	0.825157	0.982196
avp	30.42629721	0.668134271	0.3803583	1.7565919	0.078987	0.547489
ccl27a	1.641648778	-0.058871685	1.5562357	-0.03783	0.969824	NA
pdp2	41.55891671	0.058510108	0.2959341	0.1977133	0.843269	0.985174
abcg2c	298.9949794	-0.15881203	0.1381389	-1.149654	0.250286	0.820164
ccdc106b	16.79379445	0.315338328	0.4996036	0.6311771	0.527925	0.941647
si:ch211-274f20.2	32.4716028	-1.137254188	0.3641768	-3.122808	0.001791	0.049451
chst2b	188.449124	0.097898372	0.1530255	0.6397521	0.522334	0.939056
terb1	0.965400781	-1.006309521	1.9906007	-0.505531	0.613186	NA
sri	857.5971645	-0.081675903	0.0868152	-0.940802	0.346806	0.8794
nt5c3a	982.0064659	0.150840946	0.0869409	1.7349821	0.082744	0.554784
sox18	192.1804721	-0.262890353	0.1614552	-1.628256	0.103471	0.609625
gdap1	272.93326	0.08302648	0.1307242	0.6351269	0.525346	0.94013
jph1a	53.39723676	-0.275587867	0.2638043	-1.044668	0.296176	0.853273
vsig10	250.6503129	-0.057712833	0.1354039	-0.426227	0.669942	0.965223
sik1	2438.691469	0.313129726	0.0781367	4.0074583	6.14E-05	0.003262
wsb2	53.230138	0.142276921	0.278423	0.5110099	0.609344	0.956718
zgc:162472	288.2026794	-0.157785338	0.1320439	-1.194946	0.232108	0.800157
CABZ01030107.1	52.6998617	-0.143044916	0.2783394	-0.513923	0.607306	0.956718
si:ch211-71m22.1	386.393105	-0.702902393	0.1144327	-6.142497	8.12E-10	1.73E-07
dnajb2	89.85402909	0.023304257	0.2059664	0.1131459	0.909915	0.990702
ptprna	1421.935304	0.117138903	0.0794374	1.474606	0.140318	0.680564
hck	56.27501301	-0.124421037	0.2602485	-0.478085	0.632589	0.957356
trim46b	305.9495885	0.065003369	0.1278439	0.508459	0.611131	0.95688
dpm3	210.7634462	0.028566164	0.1418703	0.2013541	0.840422	0.985042
desma	2802.393891	0.16542516	0.1086094	1.5231197	0.127729	0.658251
si:dkey-178e17.3	46.63635404	0.475904273	0.2859113	1.6645175	0.096009	0.590141
ilf3a	1690.47161	-0.039376727	0.0772703	-0.509597	0.610334	0.95688
pimr94	0.173729368	-0.955901296	4.0804729	-0.234262	0.814781	NA
dennd2da	264.6152349	-0.061743444	0.1359106	-0.454295	0.649617	0.960708
nuggc.3	14.28507033	0.653735584	0.5341068	1.2239792	0.22096	0.787641
nkl.2	0.31759079	1.807926197	3.579045	0.5051421	0.613459	NA
aqp10b	21.17716253	0.252518528	0.4180595	0.6040254	0.545827	0.945526
zgc:114104	55.91138091	-0.422065927	0.2752406	-1.533444	0.125167	0.654648
cd8b	0.341366344	1.891903201	3.2128156	0.5888614	0.555954	NA
pimr204	0.944852573	-2.150786085	2.3013054	-0.934594	0.349998	NA
si:dkey-204a24.11	0.332524763	0.005883742	3.292841	0.0017868	0.998574	NA
cgnb	220.0642042	0.004278987	0.1411838	0.0303079	0.975821	0.996762
fzd3b	98.78007725	-0.053122944	0.2052685	-0.258797	0.795792	0.978498
cast	2898.487774	0.078412891	0.090811	0.8634731	0.387877	0.896133
ddr2b	349.175931	-0.103828349	0.1222547	-0.849279	0.395726	0.899378
tbl1xr1a	795.6435571	-0.269719517	0.0896162	-3.009719	0.002615	0.065122
tmeff2b	486.0281623	-0.223132183	0.1046918	-2.131325	0.033062	0.354192
si:dkey-73p2.2	1.281503894	-0.740244924	1.7458823	-0.423995	0.67157	NA
nos1apb	12.15635685	0.593719656	0.5851871	1.0145809	0.310306	0.862656
vax2	6.249455981	-1.299364645	0.7809723	-1.663778	0.096157	NA
terf1	96.68005841	-0.11488319	0.2145377	-0.535492	0.59231	0.954434
cept1a	18.99940512	0.316979167	0.453668	0.6987029	0.484738	0.93001
si:dkey-119f1.1	178.3945537	0.098324247	0.1609642	0.6108456	0.541302	0.944954
or126-7	0	NA	NA	NA	NA	NA
rfesd	19.94339909	1.057270825	0.4670479	2.263731	0.023591	0.296275
nudt2	208.7767599	0.197586274	0.15167	1.3027377	0.192664	0.756795
akirin2	689.977522	-0.071407084	0.0943756	-0.756627	0.449273	0.918313

rdh10a	1216.710747	0.151295717	0.0878739	1.7217367	0.085117	0.560519
slc2a6	51.96615744	0.20798936	0.2786476	0.7464244	0.455411	0.921215
scgn	81.07346672	-0.022655677	0.215425	-0.105167	0.916243	0.991314
ihha	66.83595187	-0.318288499	0.2607207	-1.220803	0.222161	0.788551
prdx1	1438.627749	-0.235952869	0.2674704	-0.882164	0.377688	0.895192
gabra6b	19.27390127	0.043294503	0.4682571	0.0924588	0.926333	0.992702
si:ch211-117c9.2	29.05451211	-0.233110433	0.4211444	-0.553517	0.57991	0.951257
ube2r2	238.2586509	-0.010976687	0.1356854	-0.080898	0.935523	0.992702
ubap1	725.1326838	-0.007039273	0.093371	-0.07539	0.939904	0.992857
si:ch73-213k20.5	0	NA	NA	NA	NA	NA
or128-3	0.166657454	-0.955901296	4.0804729	-0.234262	0.814781	NA
or128-2	0.158915748	0.967652056	4.0804729	0.2371421	0.812547	NA
nav1b	45.28033946	0.369780148	0.3095356	1.1946288	0.232232	0.800306
CABZ01053221.1	0	NA	NA	NA	NA	NA
2-Oct	34.0778057	0.143160453	0.3418102	0.4188302	0.67534	0.965257
slc9a2	89.82536275	0.006525879	0.2098165	0.0311028	0.975188	0.996762
si:ch73-242m19.1	29.80821771	0.110783339	0.3556195	0.3115221	0.755404	0.975318
zgc:171887	2.358063979	-0.889056567	1.3159448	-0.675603	0.499293	NA
si:dkey-19a16.2	39.38711148	-0.138820501	0.3036054	-0.45724	0.647499	0.960585
mybpha	462.0998362	0.114938834	0.1127899	1.0190529	0.308178	0.861088
rab25a	125.8747469	-0.128373557	0.1873184	-0.685323	0.49314	0.931926
rev3l	731.5866921	-0.148797203	0.0906585	-1.641293	0.100737	0.603443
grk1a	684.1455549	0.239575738	0.1138643	2.1040468	0.035374	0.365843
naxe	347.4587195	-0.006248865	0.1216591	-0.051364	0.959036	0.995095
ihhb	19.45778097	-0.194071736	0.4592121	-0.422619	0.672573	0.965257
rbm38	324.0286795	-0.175826049	0.1229512	-1.430048	0.152703	0.701776
nog1	113.4930413	0.060507369	0.1859977	0.3253125	0.744945	0.974094
bin1b	122.4898146	0.277262655	0.1800321	1.5400735	0.123542	0.651905
sema5a	927.5475213	-0.001885292	0.0943878	-0.019974	0.984064	0.996944
wnt3a	1.832752574	1.384701176	1.5765857	0.8782911	0.379786	NA
mrpl4	650.251783	-0.062726184	0.0988406	-0.63462	0.525676	0.940145
ccdc51	178.9760327	0.051492365	0.1650272	0.3120235	0.755023	0.975231
zgc:171424	0.325452849	0.005883619	3.3177086	0.0017734	0.998585	NA
zdhhc3b	260.6697381	0.000869479	0.1316924	0.0066023	0.994732	0.998694
si:ch73-212j7.3	95.86233689	0.009661804	0.2145772	0.0450272	0.964086	0.995927
susd1	28.46490209	-0.614492404	0.3772373	-1.628928	0.103328	0.609625
mcoln1b	10.69437963	0.212333558	0.6098275	0.3481863	0.7277	NA
sash1b	305.5553706	-0.047978334	0.1236765	-0.387934	0.698065	0.967998
zgc:112052	333.3546917	-0.115988512	0.1286938	-0.901275	0.367442	0.887458
ncln	902.1909196	-0.029678989	0.103533	-0.286662	0.774371	0.975687
endog	188.2598016	0.106001341	0.1559977	0.679506	0.496817	0.932829
apc	3639.316952	0.025278029	0.0692024	0.3652766	0.714905	0.96909
CABZ01040556.1	46.0932975	-0.254228565	0.2891892	-0.879108	0.379343	0.895192
zgc:171857	88.20129721	-0.121132076	0.2092377	-0.578921	0.562643	0.948728
ptpdc1b	235.7322084	0.413991484	0.1407473	2.941381	0.003268	0.076374
zgc:113227	42.19362918	0.031328884	0.3012831	0.1039849	0.917181	0.991572
mmp16b	28.05066497	-0.0947819	0.368946	-0.256899	0.797257	0.978577
cldnj	54.15190297	-0.086784564	0.2925367	-0.296662	0.766724	0.975687
ttc29	4.11563754	0.57185956	0.9477924	0.6033595	0.54627	NA
cep170ab	110.7495935	0.086579836	0.1901881	0.4552326	0.648942	0.960708
nhej1	155.7903795	-0.146048616	0.1767072	-0.826501	0.40852	0.90171
si:ch211-237c6.4	126.5327351	0.08650856	0.1861374	0.4647565	0.642106	0.959994
si:ch211-18l15.2	11.95059464	0.111076124	0.5851246	0.1898333	0.84944	0.985174
nnr	0	NA	NA	NA	NA	NA

pou1f1	6.686965056	0.742541738	0.7755315	0.9574617	0.338334	NA
si:busm1-52i16.2	2.125027809	0.652244066	1.4183524	0.4598604	0.645616	NA
cldn10a	7.457685948	-1.042779094	0.7916971	-1.317144	0.18779	NA
b4galnt3b	162.2291716	0.162157549	0.1593055	1.0179029	0.308724	0.861088
si:ch211-248e11.2	45.58652876	-0.237902093	0.2959675	-0.803811	0.421506	0.90651
cdcp1a	121.5646155	-0.583625212	0.1876106	-3.110832	0.001866	0.050813
si:dkey-8e10.3	18.83402022	0.502337635	0.449003	1.1187846	0.263232	0.830245
acot22	0.341366344	1.891903201	3.2128156	0.5888614	0.555954	NA
CU856539.2	500.5790745	-0.338578287	0.1145663	-2.955305	0.003124	0.073687
spina	28.09869571	0.039024252	0.3826287	0.1019899	0.918765	0.991623
abcc4	671.0914418	0.271126536	0.0960649	2.8223265	0.004768	0.099657
trim35-7	14.80477513	-0.018709727	0.4998856	-0.037428	0.970144	0.996315
otol1b	37.97359295	0.491170566	0.3301033	1.48793	0.136769	0.673303
ngfa	53.46552398	0.142872241	0.2870817	0.4976709	0.618716	0.957354
txnl4b	145.4059116	0.047969867	0.1739158	0.2758224	0.782684	0.97627
themis2	7.383380494	-0.373659935	0.7448963	-0.501627	0.61593	NA
zgc:112332	113.8731111	-0.104235629	0.2002609	-0.520499	0.602716	0.956045
haus1	88.29788855	-0.101626675	0.2194411	-0.463116	0.643281	0.959994
cntnap2a	724.6484247	0.012928305	0.1017977	0.127	0.89894	0.990121
zgc:113423	115.7358674	0.068149231	0.1859606	0.3664714	0.714013	0.96909
rad23ab	212.4737371	-0.012412518	0.1541901	-0.080501	0.935839	0.992702
KCNJ6	112.5084796	0.137490536	0.2080647	0.6608066	0.508736	0.935875
KCNJ15	67.69064148	0.389069982	0.2447159	1.5898846	0.111861	0.627242
slc35a2	143.8271964	0.168231298	0.1739032	0.9673849	0.333352	0.874926
cers2b	597.1719106	0.097832121	0.1068867	0.9152878	0.360041	0.885686
rock1	755.6602511	-0.0455117	0.0922331	-0.493442	0.6217	0.957354
stx1a	12.60078377	-0.460181923	0.5285335	-0.870677	0.383931	0.895192
jmjd4	73.13151163	-0.044360886	0.2344338	-0.189226	0.849916	0.985174
jam2a	170.2466576	0.03740903	0.1620571	0.2308386	0.81744	0.981594
pim2	774.890215	-0.297404428	0.1084923	-2.74125	0.006121	0.118579
strip2	392.3443185	0.03607004	0.1201677	0.3001642	0.764052	0.975374
otud5a	938.9460196	-0.206080418	0.0934792	-2.204559	0.027485	0.320429
lim2.2	16.92672947	-0.346924709	0.4657074	-0.744941	0.456307	0.921215
thap1	118.6577503	-0.092469035	0.1845922	-0.500937	0.616416	0.95688
zgc:123217	10.21052003	0.426209715	0.6149815	0.6930448	0.488281	NA
srrd	40.29188914	-0.096209322	0.3232686	-0.297614	0.765998	0.975374
mmp28	128.6775143	0.330381373	0.2063446	1.6011147	0.109352	0.623408
porb	1778.534017	-0.150251275	0.0740151	-2.030008	0.042356	0.404449
FO904903.1	0	NA	NA	NA	NA	NA
ntf3	14.01865158	-0.290880101	0.514813	-0.565021	0.57206	0.949494
epm2a	13.03910118	0.581790785	0.5679278	1.0244097	0.305642	0.860027
psmd5	420.6461132	-0.209473581	0.1270333	-1.648966	0.099155	0.598682
mpzl1l	245.1450234	-0.055694262	0.1428465	-0.389889	0.696619	0.96736
zgc:174904	192.4223991	0.305641015	0.1546331	1.9765559	0.048092	0.432341
slc13a4	439.1740166	0.368908478	0.1120746	3.2916341	0.000996	0.03092
pdk2b	867.7801708	-0.086509607	0.2400354	-0.360404	0.718545	0.96909
ft1	7.542301735	0.768059168	0.7427994	1.0340062	0.301133	NA
atpv0e2	837.2126018	0.022433365	0.0906899	0.2473634	0.804627	0.979705
vipr1b	13.31280396	-0.885232089	0.5290632	-1.673207	0.094287	0.583986
emsy	635.0971512	-0.140994332	0.1038513	-1.357656	0.174573	0.734625
lgals1a	94.1137937	-0.003862204	0.2146651	-0.017992	0.985645	0.996944
rassf3	165.6260926	0.146488844	0.1627481	0.9000953	0.36807	0.887482
jupb	22.82749608	0.296146485	0.4197794	0.7054812	0.480511	0.927601
gars	3304.692103	-0.123711992	0.0738507	-1.675164	0.093902	0.582989

gsc	214.8028621	-0.339884068	0.1457859	-2.331393	0.019733	0.264538
nip7	169.9122976	-0.020304901	0.1563176	-0.129895	0.896649	0.989971
vps25	1042.278352	0.058497043	0.0849571	0.688548	0.491108	0.931926
LO018513.1	16.39087005	0.61784788	0.4754238	1.2995729	0.193747	0.757596
ppp2r5ca	380.4125029	-0.103358208	0.1160079	-0.890959	0.372951	0.892934
sstr2a	40.39813245	0.683413184	0.3299828	2.071057	0.038353	0.384124
ank1a	184.1139738	0.141293724	0.1642512	0.8602297	0.389662	0.896421
nktr	789.3496234	-0.204003931	0.1108397	-1.840532	0.06569	0.500755
si:ch73-126o18.1	0	NA	NA	NA	NA	NA
si:dkeyp-113d7.1	726.3495285	-0.09735696	0.0936941	-1.039094	0.298761	0.85478
heatr5b	349.6426933	0.045808988	0.143928	0.3182772	0.750275	0.97419
EFEMP1	10.82878085	0.04006098	0.5740182	0.0697904	0.94436	NA
plcd1a	458.00131	0.013086931	0.1070061	0.1223008	0.902661	0.990121
prkacbb	1102.974171	0.067003723	0.087966	0.7616999	0.446239	0.917154
uqcrh	2658.089189	-0.19063215	0.0903994	-2.108776	0.034964	0.363882
gata1b	0.523937293	2.50362281	2.7291625	0.9173594	0.358954	NA
spdya	4.720609034	0.023352169	0.868723	0.026881	0.978555	NA
wfikkn2b	5.911568596	0.520605778	0.8346852	0.6237151	0.532815	NA
tmem107	175.4491673	-0.285376124	0.160559	-1.777391	0.075504	0.535131
rbp3	3580.869174	0.409659448	0.0839838	4.8778408	1.07E-06	0.000106
slc22a13b	14.4136096	0.349024815	0.5323415	0.6556408	0.512055	0.936253
gdf2	14.78250204	-0.122942261	0.5307727	-0.231629	0.816826	0.981594
cep19	69.85702506	-0.18656046	0.2432273	-0.767021	0.443069	0.916336
tax1bp3	484.6117178	0.05280111	0.1104106	0.4782249	0.63249	0.957356
CU467633.1	0.997976631	0.006464214	1.862781	0.0034702	0.997231	NA
tspan2b	251.6384855	-0.33396289	0.1397448	-2.389806	0.016857	0.23981
abhd13	15.6423672	0.266247541	0.4829343	0.5513121	0.58142	0.951731
ciao1	504.125935	-0.061586604	0.1134097	-0.543045	0.587099	0.953319
zbtb8a	80.6724307	0.084390853	0.2167059	0.3894257	0.696961	0.96736
fabp1b.1	1602.480668	-0.880392862	0.100392	-8.769555	1.79E-18	1.27E-15
hephl1a	116.587872	0.136340213	0.1939935	0.7028081	0.482175	0.928585
runx2b	14.58718462	-0.659343061	0.5057121	-1.303791	0.192305	0.756743
mrps27	476.7643283	-0.061564594	0.117377	-0.524503	0.599929	0.956045
zgc:113274	81.45944034	-0.171785142	0.2181667	-0.787403	0.431046	0.91234
ptger4a	5.206943464	-0.453211532	0.8347752	-0.542914	0.587189	NA
arfip2a	812.3654118	0.065073686	0.093504	0.6959456	0.486463	0.930833
hnrnpd	1558.660727	-0.006421472	0.0913515	-0.070294	0.94396	0.993364
tmem54a	314.8363573	0.127517588	0.1345696	0.9475958	0.343335	0.878434
zpcx	0.673073222	-1.460830855	2.4012698	-0.608358	0.54295	NA
il1r1	0.508023798	0.926122713	2.6636728	0.3476864	0.728076	NA
evx2	255.4488935	0.200081771	0.1302375	1.536284	0.124469	0.653544
hoxd13a	7.970273145	0.115923529	0.6606196	0.175477	0.860705	NA
pabpc4	2935.723578	0.085913549	0.0974227	0.8818639	0.37785	0.895192
hoxd12a	23.49585628	0.407316735	0.4273012	0.953231	0.340473	0.87805
hoxd11a	50.50160829	0.022826425	0.28724	0.0794681	0.93666	0.992702
hoxd9a	125.5558149	0.023521425	0.1847049	0.1273459	0.898667	0.990121
hoxd4a	135.4316014	-0.124436194	0.1794421	-0.693461	0.48802	0.93189
tfap2a	3227.789109	0.02030305	0.074584	0.2722173	0.785455	0.97627
hoxd3a	477.4242154	0.128585127	0.114401	1.1239857	0.261019	0.829139
mak	480.8023327	0.004881018	0.1074789	0.0454137	0.963778	0.995927
marco	46.20891908	-0.35707337	0.2901592	-1.230612	0.218468	0.785734
spcs1	330.5733154	-0.064594772	0.1426187	-0.452919	0.650607	0.960708
hnrnpl2	3075.865118	-0.09009925	0.0736209	-1.223827	0.221017	0.787641
chchd2	1065.85913	0.162715832	0.0994115	1.6367907	0.101674	0.605756

tmem37	67.23125991	0.239059129	0.2418685	0.9883849	0.322964	0.870607
golt1bb	334.5998911	-0.210081708	0.1220428	-1.721378	0.085182	0.560519
dpysl5b	3105.306619	0.086215267	0.0825859	1.0439461	0.29651	0.853573
ora3	10.06646486	0.71787245	0.5991498	1.1981518	0.230858	NA
topbp1	304.2272602	-0.189974526	0.1301889	-1.459222	0.144504	0.688868
rpn1	2562.246872	-0.115723411	0.093442	-1.238451	0.215549	0.783025
gata2a	442.0864419	-0.155063606	0.1201569	-1.29051	0.196874	0.761944
si:ch211-285f17.1	428.2870008	-0.027953884	0.1255606	-0.222633	0.823821	0.982196
tgif1	1056.895685	-0.102540552	0.092694	-1.106227	0.268628	0.833906
dlgap2a	25.7629538	0.065469202	0.3711161	0.1764117	0.859971	0.985773
avil	78.27459901	-0.233396767	0.2236965	-1.043364	0.29678	0.853798
calml4b	2.164486196	-0.25998089	1.3544931	-0.19194	0.847789	NA
rxfp3.3b	29.81050573	0.022103755	0.3549514	0.0622726	0.950346	0.99381
homer2	196.6478162	-0.032337567	0.1488064	-0.217313	0.827964	0.982721
hnrnpa3	410.7220989	-0.125668611	0.1121863	-1.120178	0.262638	0.830166
glmp	384.6432359	-0.251846059	0.1173194	-2.14667	0.03182	0.34636
sarnp	779.2304522	-0.150017168	0.0988914	-1.516989	0.129269	0.661022
srsf3b	3746.790216	-0.140317835	0.0728532	-1.926034	0.0541	0.455611
slc39a4	10.75345366	0.323211739	0.6284353	0.5143119	0.607034	NA
cavin1b	443.0012615	0.039649068	0.111272	0.3563257	0.721597	0.969747
tgfbr2a	43.90153863	-0.310094703	0.3023254	-1.025699	0.305034	0.859636
nipal4	182.8539499	0.145076225	0.155714	0.931684	0.3515	0.880769
mfap2	1612.076215	0.079423015	0.0950742	0.8353792	0.403504	0.900979
gria4b	783.5288753	0.137992425	0.0943656	1.462317	0.143654	0.686804
si:ch1073-15f12.3	4.999139283	-0.201662175	0.8523507	-0.236595	0.812971	NA
nr1d4b	78.84362576	-0.532912313	0.2225849	-2.394198	0.016657	0.2379
dnajc8	1317.347816	-0.096313196	0.0925567	-1.040586	0.298068	0.85401
fgf7	11.40202804	0.218445126	0.5725412	0.3815361	0.702806	0.968134
bdh1	185.7407343	0.043298644	0.1661462	0.2606056	0.794397	0.977937
gyg1b	598.7577087	0.275093843	0.1064776	2.5835842	0.009778	0.167691
tas1r2.2	0.158915748	0.967652056	4.0804729	0.2371421	0.812547	NA
myef2	2363.068049	-0.170077384	0.0717071	-2.371834	0.0177	0.246724
si:ch211-160o17.6	106.2216545	0.034849157	0.2228758	0.1563614	0.875748	0.987881
tmem167b	657.926961	-0.008228338	0.0949643	-0.086647	0.930952	0.992702
rab43	245.7991371	-0.005431381	0.1331553	-0.04079	0.967463	0.996315
raf1b	446.580643	-0.309542659	0.1082961	-2.858298	0.004259	0.091545
abcd2	9.832025715	-0.148087016	0.6778401	-0.218469	0.827064	NA
ccr12b.1	0.488104098	-2.415107183	2.7936704	-0.864493	0.387317	NA
impa2	86.31538544	0.218131096	0.222265	0.9814011	0.326395	0.87218
zgc:111976	6.672265278	-0.276178939	0.7546886	-0.365951	0.714402	NA
prune2	894.5172413	0.205956636	0.0937952	2.195812	0.028105	0.325071
rnf215	20.25257608	-0.154811078	0.4317564	-0.358561	0.719923	0.969318
fsd1l	174.0309765	0.024479156	0.1620952	0.1510171	0.879962	0.987881
fktn	413.2839966	0.042560461	0.1119094	0.3803119	0.703714	0.968134
galnt18b	248.8726147	0.112942244	0.1331948	0.8479479	0.396467	0.899672
smtnb	1019.723848	0.14963287	0.0923188	1.6208279	0.105055	0.613317
dqx1	74.0016092	-0.015154069	0.2274064	-0.066639	0.946869	0.99381
si:ch211-283h6.4	112.1148941	-0.082465638	0.1923716	-0.428679	0.668157	0.964736
sbf2	845.2767154	-0.199645801	0.0926613	-2.154575	0.031195	0.341959
mepce	577.0793902	-0.087850239	0.0987213	-0.889881	0.37353	0.893669
wasf3a	156.4805317	0.040417435	0.1775619	0.2276244	0.819938	0.981594
arhgap31	38.96176881	0.079005066	0.3327193	0.2374526	0.812306	0.981081
kank4	89.6308031	0.109467659	0.2217596	0.4936322	0.621566	0.957354
mlxip	176.8395504	-0.00099629	0.1641978	-0.006068	0.995159	0.998694

camsap3	473.7216612	-0.163664707	0.1175083	-1.392793	0.163683	0.71945
tead1b	473.2581529	0.004714322	0.1092976	0.0431329	0.965596	0.996315
emb	66.71432451	0.117637889	0.2438712	0.4823771	0.629538	0.957354
cplx4b	0.538871265	0.999144257	2.7784799	0.359601	0.719146	NA
arl16	225.4336298	-0.115234726	0.1382319	-0.833634	0.404488	0.901305
ACSF3	135.1822995	-0.181033316	0.1744007	-1.038031	0.299256	0.855386
si:dkey-163m14.2	37.11180979	0.245324541	0.3339556	0.7346022	0.462582	0.922698
coq2	251.1682388	0.019467432	0.1415816	0.1374998	0.890636	0.989291
rims4	23.01633544	0.138579941	0.4440243	0.3120999	0.754965	0.97522
KIAA0895L	88.28496968	0.186340721	0.2116225	0.8805338	0.37857	0.895192
si:ch211-146m13.3	600.6169054	0.076834537	0.0996876	0.7707533	0.440853	0.916336
akap10	249.5676669	0.113764911	0.1477235	0.7701207	0.441228	0.916336
sympk	1089.791424	-0.105766875	0.0924403	-1.144164	0.252556	0.822063
recql5	205.6787739	-0.196411651	0.1549172	-1.26785	0.204852	0.772822
xylt2	21.93113506	-0.018610105	0.4168417	-0.044646	0.96439	0.995927
tecta	137.8402241	0.201052777	0.184934	1.0871594	0.276966	0.840247
si:ch73-281f12.4	179.4771017	-0.106169028	0.1588985	-0.668156	0.504034	0.93532
fscn2a	97.3206327	0.357992611	0.2286226	1.5658674	0.11738	0.640853
uhmk1	23.89196974	-0.629463059	0.4019958	-1.565845	0.117385	0.640853
mbd5	219.1253844	0.256546432	0.1778767	1.4422713	0.149226	0.696454
sowahcb	145.6701022	-0.044525864	0.1703832	-0.261328	0.79384	0.977937
rcc1l	388.0882066	-0.018150331	0.1192414	-0.152215	0.879017	0.987881
CU928117.1	473.1902874	-0.057957861	0.1102225	-0.525826	0.599009	0.955806
clip2	233.2241591	0.0515787	0.142065	0.3630642	0.716557	0.96909
rabep1	643.3449486	0.065364795	0.0939547	0.6957058	0.486613	0.930859
map1aa	1981.227719	0.000595466	0.0736442	0.0080857	0.993549	0.998672
mtss1	531.3836351	-0.167466341	0.1064345	-1.573421	0.115621	0.637206
ccdc84	117.5217944	0.011715358	0.1970032	0.0594679	0.952579	0.99398
gpr146	930.9363814	-0.00208685	0.0878746	-0.023748	0.981054	0.996944
nprl2	176.0890086	0.222601888	0.1577042	1.4115153	0.158093	0.709224
hs3st2	42.39036313	-0.11097178	0.304392	-0.364569	0.715433	0.96909
asphd2	437.2430492	0.131377334	0.1141421	1.150998	0.249733	0.819758
mlec	166.7728747	0.113323376	0.162914	0.6956024	0.486678	0.930882
thumpd3	236.9297147	0.065317729	0.1433635	0.4556093	0.648671	0.960708
si:ch211-127d4.3	67.97470844	-0.2347711	0.264831	-0.886494	0.375351	0.894316
mtmr14	506.2711805	0.054761582	0.104151	0.5257901	0.599034	0.955806
nt5dc2	2191.730204	-0.352269454	0.0726035	-4.851965	1.22E-06	0.000119
cidec	21.89384868	0.120586676	0.408363	0.2952928	0.76777	0.975687
kdm2aa	1318.760474	-0.093460737	0.0861846	-1.084425	0.278176	0.841891
eif3ba	6819.676537	-0.217459639	0.0788618	-2.757476	0.005825	0.114852
mks1	135.900224	-0.145218435	0.1747257	-0.831122	0.405905	0.901305
mrm1	11.49553468	-0.078288477	0.5597151	-0.139872	0.888761	0.989291
arhgap36	160.4632694	-0.201852178	0.1614077	-1.250573	0.21109	0.778604
fscn1a	4002.611765	-0.005748324	0.0673771	-0.085316	0.93201	0.992702
slc43a3a	150.5251263	0.090948237	0.1831321	0.4966264	0.619453	0.957354
mblac2	26.19722414	0.725901704	0.42967	1.6894399	0.091135	0.578217
slc29a4	452.4393146	0.121359102	0.1088575	1.1148441	0.264917	0.830836
adam19a	345.3337755	-0.016589515	0.1255574	-0.132127	0.894884	0.989814
dusp28	11.76992817	-0.025996973	0.5834373	-0.044558	0.964459	0.995927
dnajc16	27.94098495	-0.645449158	0.3781729	-1.706757	0.087867	0.567519
agmat	31.95729032	-0.472702234	0.3595155	-1.314831	0.188567	0.753276
flii	1075.048071	0.065068828	0.0917242	0.7093968	0.478078	0.926857
vwa5b1	5.132469766	-0.277975632	0.9028984	-0.30787	0.758181	NA
si:ch211-216l23.1	1424.975883	-0.101805881	0.0813658	-1.251213	0.210857	0.778412

nol6	556.1693229	0.019200403	0.1044043	0.1839044	0.854088	0.985174
slc19a2	8.332402059	0.069106299	0.6799053	0.1016411	0.919042	NA
arsk	42.65293188	0.001831338	0.3009089	0.006086	0.995144	0.998694
fam169aa	8.36753648	0.161612613	0.6775342	0.2385306	0.81147	NA
ubac1	602.4107737	0.210110346	0.0992659	2.1166411	0.03429	0.360094
hypk	1051.701337	-0.17991026	0.0979079	-1.837546	0.066129	0.502802
sec14l8	240.3249826	-0.238786035	0.1367654	-1.745953	0.080819	0.550801
ptprsa	211.3915684	0.127955457	0.1448156	0.8835749	0.376926	0.895192
tmem98	42.37506896	0.277978454	0.2931547	0.9482314	0.343012	0.878434
plod1a	1196.236904	0.002877241	0.0983099	0.029267	0.976652	0.996762
kcnj19a	47.22708588	0.2323326	0.3001956	0.7739374	0.438968	0.916082
abtb2a	171.0533338	0.22785844	0.1596623	1.4271275	0.153543	0.702578
wdtc1	462.3789174	0.034305668	0.1119305	0.3064909	0.759231	0.975374
gabrd	348.1300186	0.06389634	0.1344619	0.4752003	0.634644	0.9578
gpatch8	3584.43768	-0.07920267	0.0776686	-1.019751	0.307846	0.861088
cpt1aa	708.9424202	0.099695962	0.0979594	1.0177271	0.308808	0.861088
SRCIN1	209.3151595	0.190565371	0.1628218	1.1703923	0.241843	0.810713
dub	91.20892997	-0.12092423	0.2165966	-0.558292	0.576645	0.950611
slc32a1	2841.83826	0.167773879	0.0777108	2.1589528	0.030854	0.33998
atcayb	220.4672769	0.188493092	0.1428626	1.3194009	0.187035	0.751002
parp8	208.8533943	0.063411311	0.146254	0.4335698	0.664601	0.964736
trpm5	112.8870264	-0.120820417	0.1995609	-0.605431	0.544893	0.945463
kdm6al	760.5972654	0.037463927	0.1153429	0.3248047	0.745329	0.974094
kcnq1.1	41.8731629	0.184258044	0.3033485	0.6074138	0.543576	0.945438
pdzd2	203.8992291	-0.051231384	0.1444574	-0.354647	0.722854	0.970375
retreg3	704.9690795	0.138335883	0.0981167	1.4099114	0.158566	0.709224
slc25a38a	58.78003433	0.201438074	0.3085385	0.6528783	0.513835	0.936778
sacm1lb	281.2572398	-0.03913525	0.1278068	-0.306206	0.759448	0.975374
caska	839.0409774	0.040522613	0.0914873	0.4429318	0.657815	0.962538
smpd5	328.3470059	-0.010482854	0.1221003	-0.085854	0.931582	0.992702
sin3ab	1179.584428	0.000525316	0.0884563	0.0059387	0.995262	0.998696
oaz2b	194.566382	0.039286177	0.1483553	0.2648114	0.791155	0.977461
zgc:136864	0	NA	NA	NA	NA	NA
mtmr12	157.5529245	0.117196847	0.1666132	0.7034067	0.481802	0.928392
kif5ab	135.557689	0.170646929	0.1858892	0.9180033	0.358617	0.884532
soat2	109.7021201	-0.14050904	0.2012913	-0.698038	0.485153	0.930136
crtac1a	614.0781302	0.373084554	0.2387803	1.5624592	0.11818	0.641526
adgrb3	1161.537691	0.097244451	0.090155	1.0786358	0.28075	0.84357
zfyve27	333.4130616	0.053743815	0.1225081	0.4386959	0.660882	0.964022
ddit3	562.1778537	-0.135135693	0.1171873	-1.15316	0.248845	0.81797
exosc1	207.0370483	-0.278185016	0.1727772	-1.61008	0.10738	0.617344
fam135a	851.5750992	0.096737406	0.093595	1.0335742	0.301335	0.856944
epg5	89.48044821	0.140273649	0.223292	0.6282074	0.529868	0.942209
slc35f3b	4.723116429	0.670406075	0.9209164	0.7279772	0.466628	NA
manba1	376.9270018	-0.125823644	0.115462	-1.089741	0.275827	0.839541
CABZ01041604.1	59.85671275	0.016282624	0.2502707	0.06506	0.948126	0.99381
wdr66	25.48146043	0.548657938	0.3893138	1.4092949	0.158748	0.709382
alkbh2	64.10313118	-0.264587616	0.2619531	-1.010057	0.312468	0.864401
blvra	97.11340785	-0.018014196	0.2076471	-0.086754	0.930867	0.992702
pappab	8.906064382	0.88220253	0.6491611	1.3589887	0.17415	NA
cactin	669.5799955	0.061825672	0.0990745	0.6240322	0.532606	0.942805
clk2b	276.2358925	-0.0361975	0.1305951	-0.277173	0.781647	0.97627
shrprbck1r	426.4390871	-0.001998612	0.1143399	-0.01748	0.986054	0.996944
trpv1	30.40141989	0.216652423	0.3556287	0.6092097	0.542385	0.944974

frmd3	24.90748856	0.454235903	0.3905012	1.1632124	0.244743	0.814435
fam222ba	485.2972728	0.044922494	0.1079762	0.4160409	0.67738	0.965257
erall	225.5381562	0.068465478	0.1428018	0.4794441	0.631623	0.957356
ago3a	15.45552709	0.509358211	0.5239349	0.9721784	0.330962	0.874224
AL929217.1	9.835483876	0.100766885	0.6193342	0.162702	0.870753	NA
ntrk2a	245.8788807	0.031957091	0.1349577	0.2367934	0.812817	0.981081
CR847944.2	5.022867759	2.306622102	0.9962416	2.3153241	0.020595	NA
tbc1d9	41.62067692	-0.151789087	0.30273	-0.501401	0.616089	0.95688
vps16	967.2677138	-0.065757507	0.085039	-0.773263	0.439367	0.916082
hs3st3l	103.9795822	0.175534798	0.1969612	0.8912151	0.372814	0.892868
sdc4	3828.084042	0.06562574	0.0772666	0.8493413	0.395691	0.899378
si:ch211-217k17.7	364.1012761	-0.063783872	0.1196049	-0.533288	0.593834	0.954434
bbs7	298.2943046	0.154364586	0.1449506	1.0649465	0.2869	0.845979
sdr42e2	22.84408573	-0.332649457	0.435606	-0.763648	0.445077	0.916958
hyal1	49.60010947	0.281991268	0.2748737	1.0258938	0.304942	0.859575
ccnj	21.39539015	-0.00095909	0.4130659	-0.002322	0.998147	0.999281
zgc:162613	191.7652075	-0.118264436	0.1491233	-0.793065	0.42774	0.91075
slc25a47a	146.9352921	0.361657504	0.1744516	2.0731109	0.038162	0.382665
usp24	750.9415458	-0.073122594	0.1150857	-0.635375	0.525184	0.940073
hyal2a	36.10725342	-0.042597496	0.3465818	-0.122907	0.90218	0.990121
plpp3	2301.618231	0.085442202	0.0785072	1.0883361	0.276447	0.839541
herc4	98.54170987	0.082385208	0.2024775	0.4068858	0.684092	0.965679
dab1a	27.44523553	0.315114457	0.3650329	0.8632495	0.388	0.896144
c1qtnf2	25.89588713	0.330386504	0.4060269	0.813706	0.415813	0.906237
sv2a	5863.149843	0.120911067	0.0690134	1.7519942	0.079775	0.548493
si:dkey-121b10.7	4.41628836	-0.925631282	1.0254689	-0.902642	0.366716	NA
plxdc2	690.6432158	0.14168516	0.0959611	1.4764856	0.139814	0.678781
pld6	2.322814721	0.439458333	1.2953654	0.3392543	0.734418	NA
ptk6b	86.02807216	-0.774107528	0.2377993	-3.255297	0.001133	0.033989
plch2a	1072.29825	-0.00745621	0.0924856	-0.08062	0.935744	0.992702
chpf2	415.2101904	0.256817824	0.1183449	2.1700788	0.030001	0.335162
polk	84.66293468	0.079290259	0.2134783	0.3714208	0.710324	0.968985
camsap2b	96.02195359	-0.149194398	0.2049976	-0.727786	0.466745	0.924327
raly	480.578102	-0.050725546	0.1053078	-0.481688	0.630027	0.957354
cplx4a	1455.60923	0.177877129	0.091524	1.9435027	0.051955	0.447654
poc5	52.23454228	-0.235727388	0.2726464	-0.86459	0.387264	0.895778
zufsp	128.290923	-0.17314877	0.1870734	-0.925566	0.354672	0.882105
dnah12	76.63414587	0.347169957	0.2417989	1.4357799	0.151065	0.699078
trpm4a	882.9322845	-0.691519199	0.1049996	-6.585922	4.52E-11	1.13E-08
sv2ca	4.460818453	0.304542591	0.8847307	0.3442207	0.73068	NA
mettl9	231.8161993	0.052659543	0.1442667	0.3650152	0.7151	0.96909
ogg1	26.31245785	-0.617850535	0.3843851	-1.607374	0.107972	0.619371
fzd7a	1540.152913	0.022386468	0.0821648	0.272458	0.78527	0.97627
mtrf1l	74.99389826	-0.698989166	0.2289466	-3.053066	0.002265	0.058495
nedd4l	724.4385241	-0.098817999	0.0923906	-1.069568	0.284814	0.844998
n4bp3	25.3924127	0.02940968	0.3872575	0.0759435	0.939464	0.992857
hhipl2	190.7260218	0.472872652	0.1568263	3.0152628	0.002568	0.064186
iqgap2	355.540111	0.030595051	0.1367988	0.2236501	0.82303	0.982189
f2r	20.38231001	-0.363099455	0.428828	-0.846725	0.397148	0.899672
nsd1a	753.6864415	-0.016206418	0.0983534	-0.164777	0.869119	0.987245
si:ch1073-44g3.1	539.8119158	-0.034196712	0.1053637	-0.324559	0.745515	0.974094
vcpkmt	138.2952732	-0.241499006	0.1787869	-1.350764	0.176771	0.737285
ncapg2	262.6435809	-0.017632517	0.1332404	-0.132336	0.894718	0.989716
nsmfa	21.61012615	0.572492042	0.4532137	1.2631834	0.206523	0.774129

nom1	166.0523559	0.030704032	0.1665203	0.1843861	0.853711	0.985174
fam160b2	245.9303947	0.024305809	0.1412375	0.1720917	0.863365	0.985841
setd1a	1058.963422	-0.071687813	0.0942504	-0.76061	0.44689	0.917154
tmem151ba	79.42879984	0.620120421	0.2181853	2.8421732	0.004481	0.09535
rnf32	23.74171601	-0.188833431	0.4060062	-0.4651	0.64186	0.959994
aimp1a	1067.919945	-0.02768554	0.087598	-0.316052	0.751963	0.974526
lig1	580.5580322	-0.105711469	0.1087905	-0.971697	0.331201	0.874339
zgc:195075	4.205472037	-0.109311544	0.9150919	-0.119454	0.904916	NA
slc47a2.1	52.14524709	0.656146496	0.2984795	2.1982966	0.027928	0.323709
epc1b	772.1933672	-0.069710931	0.1112858	-0.626413	0.531044	0.942209
cylda	708.7450272	-0.029546318	0.0979929	-0.301515	0.763022	0.975374
lrrc74a	6.580054931	-0.135430198	0.825733	-0.164012	0.869722	NA
ubap2b	2237.723126	-0.090217025	0.0953181	-0.946484	0.343902	0.878434
fam98b	251.9628249	0.045559786	0.1607984	0.2833348	0.77692	0.975826
isoc1	111.3115625	-0.110543836	0.1874004	-0.58988	0.555271	0.947449
adcy7	290.6390549	0.320761071	0.1408994	2.2765258	0.022815	0.289004
tent4b	622.8000712	0.197509593	0.0980539	2.0142971	0.043978	0.412341
abi3a	199.3171249	0.16628135	0.1635789	1.0165206	0.309382	0.861497
crb2b	0	NA	NA	NA	NA	NA
kcnq3	17.43236626	0.383575168	0.4594714	0.8348183	0.40382	0.900979
btaf1	1107.762187	0.046990509	0.0869	0.5407423	0.588685	0.953319
zgc:165481	99.95686739	-0.072587731	0.2044853	-0.354978	0.722606	0.970375
dapk1	633.3071466	-0.061384062	0.0969013	-0.63347	0.526427	0.940506
ptgis	184.3879369	-0.179867082	0.1601866	-1.12286	0.261497	0.829593
kcnb1	7.559324306	0.278773465	0.7212232	0.3865287	0.699105	NA
tbc1d10ab	24.94812177	0.596054114	0.387889	1.5366616	0.124376	0.653231
trappc12	667.92957	0.073468553	0.1039524	0.706752	0.479721	0.927091
kank1a	785.3434882	-0.040623128	0.0912377	-0.445245	0.656143	0.961833
cpeb3	18.09133561	0.308182094	0.4904133	0.628413	0.529733	0.942209
crb2a	42.94717198	0.173301009	0.3080062	0.5626544	0.57367	0.949904
aggf1	214.0423311	-0.155593216	0.1556086	-0.999901	0.317358	0.867404
sybu	345.1150375	-0.019550902	0.117708	-0.166097	0.868081	0.987245
znf395a	3162.380598	-0.448411306	0.0994305	-4.509797	6.49E-06	0.000501
lrrn1	1600.646694	-0.004261163	0.079254	-0.053766	0.957122	0.994589
camk1a	35.00010889	-0.152846248	0.3217958	-0.474979	0.634802	0.9578
glyctk	255.0587388	0.135814249	0.1332635	1.0191408	0.308136	0.861088
ca16b	780.475018	0.026641237	0.0910631	0.2925579	0.76986	0.975687
timm17b	30.42850648	-0.16374933	0.3463629	-0.472768	0.636379	0.958565
acyp1	144.2639568	0.035748813	0.1863706	0.1918157	0.847887	0.985174
adamts3	35.67618167	0.252569573	0.3218502	0.7847426	0.432604	0.91326
dusp3b	1.030002046	-2.236497097	2.086282	-1.072001	0.283719	NA
sh3pxd2aa	51.92785489	-0.114503127	0.2769708	-0.413412	0.679305	0.965257
ablim1a	410.6011142	0.243404028	0.1112067	2.1887543	0.028615	0.328246
psmd11a	1178.167165	-0.055063099	0.0833102	-0.66094	0.50865	0.935807
fam155b	144.1319996	-0.020311877	0.1776589	-0.114331	0.908976	0.990702
h6pd	480.4426076	-0.209950097	0.1196029	-1.755393	0.079192	0.547489
dnah7	24.04279629	-0.040091272	0.3936234	-0.101852	0.918874	0.991623
fam160b1	86.37504772	-0.149524626	0.2176291	-0.687062	0.492044	0.931926
mns1	91.03068378	-0.273775567	0.2078238	-1.317345	0.187723	0.752195
trmt10b	44.16117103	0.371662738	0.2978523	1.2478088	0.212101	0.77986
vcpip1	60.15364691	-0.410459301	0.2523384	-1.626622	0.103817	0.610348
pkn1a	624.11712	0.057532552	0.0981076	0.5864228	0.557591	0.948478
si:ch211-214c7.5	0.182570949	0.967652056	4.0804729	0.2371421	0.812547	NA
rpusd1	46.91337574	0.226493045	0.2870373	0.7890719	0.43007	0.911615

lhpp	267.0504374	0.063240241	0.1361707	0.4644187	0.642348	0.959994
atp2c1	400.8267561	-0.060155934	0.12203	-0.49296	0.622041	0.957354
mrpl18	357.2592256	-0.063404769	0.1314096	-0.482497	0.629453	0.957354
CU019662.1	12.32198548	-0.626626614	1.2213276	-0.51307	0.607902	0.956718
traf7	252.2344188	0.039104961	0.1393627	0.2805984	0.779018	0.976146
d2hgdh	94.49640316	-0.044399102	0.2112707	-0.210153	0.833549	0.983583
ankef1b	2.125400153	-0.630319348	1.3838149	-0.455494	0.648754	NA
cep85	205.1308135	0.038414428	0.1527793	0.2514374	0.801476	0.979444
scn1ba	196.039807	0.209043005	0.148263	1.4099468	0.158555	0.709224
mst1ra	149.631421	-0.168054617	0.1714764	-0.980045	0.327064	0.872658
dok7	109.4486221	0.322816516	0.2013203	1.6034968	0.108825	0.621829
uacab	955.8101644	0.081066605	0.0864996	0.937191	0.34866	0.880195
enox1	371.7774321	-0.143037797	0.1149093	-1.244789	0.213209	0.781631
slc16a6b	1835.369151	0.07312357	0.0827999	0.8831359	0.377163	0.895192
fgd4b	52.89905593	0.019934317	0.2818919	0.0707162	0.943624	0.993364
wdr44	1040.323066	0.017450238	0.086209	0.2024179	0.83959	0.984979
mogs	717.5544284	0.043431707	0.0967242	0.4490261	0.653413	0.960996
si:ch211-216b21.2	71.01970859	0.081228076	0.2501223	0.3247534	0.745368	0.974094
alkbh8	71.88280559	0.421487701	0.2394759	1.7600426	0.078401	0.545524
zranb1a	1153.218347	-0.072912808	0.0946375	-0.770443	0.441037	0.916336
zgc:162160	17.267358	-0.174126016	0.4646549	-0.374743	0.707852	0.968664
pecam1	227.0696192	-0.256765884	0.1467176	-1.750069	0.080106	0.549631
si:dkey-37g12.1	4.473732008	0.528557643	0.989856	0.5339743	0.593359	NA
pde9a	48.45097033	0.192000347	0.2853156	0.6729402	0.500985	0.934398
rnf121	667.8616646	0.054857279	0.0991265	0.5534069	0.579985	0.951257
plpbp	1126.070365	0.083168488	0.0879783	0.9453295	0.344491	0.878434
arhgap35b	705.4675157	0.164622749	0.0956461	1.721166	0.085221	0.560519
nin	199.7003864	0.056546689	0.1469464	0.3848117	0.700377	0.967998
slc4a10b	49.25383627	0.501785164	0.2853817	1.758295	0.078697	0.546789
tfcp2	1025.196531	-0.090295905	0.0848965	-1.0636	0.28751	0.845979
urb1	349.9568275	-0.090511402	0.1188578	-0.76151	0.446352	0.917154
srgap3	41.26840727	0.379488749	0.3391227	1.1190309	0.263127	0.830245
wdr62	311.3204267	-0.009586493	0.1209156	-0.079283	0.936808	0.992702
rabgap1l2	211.0726038	-0.196623245	0.1793601	-1.096248	0.27297	0.838141
cish	263.7741706	-0.110712621	0.1677398	-0.660026	0.509237	0.936123
scn4bb	5.556164236	0.17937605	0.8608549	0.2083697	0.83494	NA
dcaf5	345.4220607	0.016176197	0.1302289	0.1242136	0.901146	0.990121
zgc:153654	10.52343624	0.165583289	0.5994783	0.2762123	0.782385	NA
exoc5	259.0441889	-0.110255857	0.1385468	-0.795802	0.426147	0.909807
fam20cl	1.157520887	-0.478318508	1.9214732	-0.248933	0.803412	NA
map1sb	340.1822704	0.024367544	0.1299415	0.187527	0.851247	0.985174
si:ch211-284e13.4	110.372498	-0.224394833	0.2171632	-1.0333	0.301463	0.856944
trmt12	37.86287348	-0.18329856	0.3445012	-0.532069	0.594678	0.954434
si:dkey-157l19.2	28.19646409	-0.131139091	0.3863229	-0.339455	0.734267	0.972021
apoda.1	318.898485	-0.542201359	0.1251384	-4.332814	1.47E-05	0.000988
map3k3	169.8313476	-0.144233097	0.1624726	-0.887738	0.374682	0.894316
wasf1	437.6137359	0.133735686	0.1132455	1.1809365	0.237628	0.806474
apoda.2	1129.022273	-0.286208267	0.2797532	-1.023074	0.306273	0.860027
samd7	285.8881395	0.035756218	0.1410332	0.2535305	0.799858	0.979377
cep104	17.46141632	0.165672344	0.4581159	0.3616385	0.717622	0.96909
nadkb	427.9241729	0.050302329	0.126254	0.3984216	0.690319	0.966973
slc12a9	209.3696868	-0.211732473	0.1463124	-1.447126	0.147862	0.695319
syn1	2076.736883	0.103841474	0.0768079	1.3519641	0.176387	0.73663
plxna2	63.25355395	0.284720861	0.2615361	1.0886483	0.276309	0.839541

ttll11	20.13254254	-0.177901628	0.4493913	-0.395872	0.692199	0.96736
coq6	508.5707235	0.00326714	0.1069141	0.0305585	0.975622	0.996762
mmaa	252.1293342	-0.145036442	0.131679	-1.101439	0.270706	0.835351
stk26	465.6992736	-0.001522767	0.1064804	-0.014301	0.98859	0.997519
ash2l	1015.926718	-0.074955908	0.0859843	-0.871739	0.383351	0.895192
fibcd1	66.75064529	0.015131155	0.2483104	0.0609364	0.95141	0.99381
mtfr2	149.5936128	-0.165406409	0.1730572	-0.955791	0.339178	0.877867
si:dkey-57k2.7	186.6035043	-0.368029189	0.157523	-2.336352	0.019473	0.261766
hhip	996.2197082	0.072159577	0.0853771	0.8451863	0.398007	0.899672
rft1	159.6025132	0.227297788	0.1630061	1.3944126	0.163193	0.718029
gpr185a	5.028729735	0.2124081	0.8404738	0.2527242	0.800481	NA
thbs2a	239.5538492	0.263090397	0.139044	1.8921381	0.058473	0.473924
ddx28	149.4665322	0.069732302	0.1645535	0.4237667	0.671736	0.965257
si:ch211-157b11.8	9.008529456	0.556234059	0.6782506	0.8201011	0.412159	NA
arhgef28a	419.1639412	0.072559828	0.114549	0.6334394	0.526447	0.940506
zfyve28	47.59253093	0.06476143	0.2829557	0.2288748	0.818966	0.981594
map1b	941.7891098	0.088349125	0.0955369	0.924764	0.355089	0.882321
bnipl	97.5623128	0.151882014	0.2014949	0.753776	0.450984	0.918761
tbl1x	149.3930467	0.045409186	0.1717261	0.264428	0.79145	0.977645
clcn2c	331.2770688	0.37902788	0.127689	2.9683674	0.002994	0.071933
tmem259	649.1455653	0.082579076	0.0951701	0.8677001	0.385559	0.89551
elp6	110.8537708	-0.073120982	0.1898875	-0.385075	0.700182	0.967998
sdk2b	493.3411453	0.019847565	0.1084535	0.1830052	0.854794	0.985174
pmp22b	2763.951692	0.143315018	0.0759538	1.8868697	0.059178	0.475427
pik3r4	627.6414613	0.154100151	0.1059516	1.4544392	0.145825	0.691641
gcnt3	22.26363208	-0.145928928	0.4123663	-0.353882	0.723427	0.970402
vps8	794.2985291	0.063507514	0.1011613	0.6277845	0.530145	0.942209
ubr1	60.73267656	0.086781752	0.2601206	0.3336212	0.738665	0.972722
atl1	910.46405	-0.068991341	0.0935257	-0.737672	0.460714	0.921978
mid1	16.20928101	-0.205962351	0.4984853	-0.413176	0.679477	0.965257
selenoi	160.3997647	0.272220336	0.174453	1.5604219	0.11866	0.642318
mrpl32	415.1061031	0.113341525	0.1191118	0.951556	0.341322	0.878134
eprs	3611.97999	-0.058544963	0.0708994	-0.825747	0.408948	0.90171
cacna1ha	282.6377243	0.224368545	0.1287116	1.743188	0.081301	0.551885
tnfrsf9a	65.00605483	-0.163608857	0.2487659	-0.657682	0.510743	0.936253
l2hgdh	247.673964	-0.274511212	0.1517139	-1.8094	0.070389	0.518967
dpcd	72.4403901	0.1706338	0.2437087	0.7001548	0.483831	0.929461
pfkla	95.84258042	0.147524054	0.2125866	0.693948	0.487715	0.931809
sos2	662.2188963	0.171588197	0.1222699	1.4033565	0.160511	0.713404
cul4b	1264.512773	-0.034658744	0.0842862	-0.411203	0.680924	0.965476
kbtbd3	69.44642163	-0.086640033	0.2335545	-0.370963	0.710665	0.968985
zdhhc9	895.5924341	-0.019428787	0.0897033	-0.21659	0.828528	0.982721
pcsk5b	420.0874123	0.062661984	0.1130976	0.5540525	0.579543	0.951257
rbm27	234.6620004	0.09464029	0.1501478	0.6303143	0.528489	0.941896
rfk	50.55140154	-0.041063046	0.299762	-0.136985	0.891042	0.989291
bmp3	243.5226706	0.048647697	0.1388114	0.3504589	0.725994	0.970402
nkx6.3	37.8527074	-0.32006311	0.3483908	-0.91869	0.358258	0.884398
adam22	63.29371313	0.102879497	0.252141	0.4080236	0.683256	0.965476
sgpp1	41.75708721	0.472113031	0.313189	1.5074379	0.131698	0.665187
atad2b	194.6692309	-0.103564634	0.1614303	-0.641544	0.521169	0.939056
shank1	111.0305642	-0.082616903	0.2471753	-0.334244	0.738195	0.972535
lgr4	1157.445811	0.005317774	0.079999	0.066473	0.947001	0.99381
ecel1	155.2380975	0.027828008	0.1690037	0.1646592	0.869212	0.987274
rps6ka5	13.19812033	0.501313363	0.5269807	0.9512935	0.341455	0.878134

mut	1016.250046	-0.14223854	0.0900093	-1.580265	0.114046	0.632852
dis3	255.165587	0.106295504	0.1325018	0.8022195	0.422426	0.906561
slc25a21	109.0178872	-0.108248781	0.193841	-0.558441	0.576543	0.950611
cluhb	71.0468533	-0.23438085	0.2593789	-0.903623	0.366195	0.887239
tmem229b	564.5908957	0.113966938	0.1291098	0.8827136	0.377391	0.895192
si:dkey-226m8.10	98.77888151	0.106899038	0.2027348	0.527285	0.597996	0.955459
taspl	258.3414798	0.025300099	0.1336658	0.1892788	0.849874	0.985174
kank1b	9.38591521	0.438077966	0.6529681	0.6709025	0.502283	NA
ctdspl2b	1192.042013	0.003505787	0.1014502	0.0345567	0.972433	0.996315
hadhab	2873.162119	-0.061401139	0.0720279	-0.852463	0.393957	0.898496
prkaa1	845.2642854	0.047337815	0.0956494	0.4949096	0.620664	0.957354
zgc:158659	29.37835864	-0.23077918	0.4016047	-0.574643	0.565533	0.948739
capn15	240.177281	-0.002836238	0.1414943	-0.020045	0.984008	0.996944
rgs7bpa	379.7284102	0.122276033	0.1175094	1.0405641	0.298078	0.85401
cd109	154.5559812	0.16495118	0.1683101	0.9800432	0.327065	0.872658
pcdh7b	397.2184868	0.050224419	0.1230384	0.4082012	0.683126	0.965476
ipo13	151.2256812	0.159349985	0.1646961	0.9675396	0.333274	0.874926
lig4	91.48462691	-0.285719184	0.2102765	-1.358779	0.174217	0.734375
si:ch73-14h1.2	86.42356577	-0.808623231	0.2189911	-3.692493	0.000222	0.009323
dgkaa	668.3204467	-0.125538021	0.0995775	-1.260707	0.207415	0.775175
hip1ra	40.29821891	-0.245572539	0.3095062	-0.793433	0.427525	0.91075
GARNL3	212.9010976	0.062786952	0.1611437	0.3896333	0.696808	0.96736
usp16	6.419480842	0.051006877	0.7625664	0.0668884	0.946671	NA
clstn2	829.5514206	0.054011676	0.098402	0.548888	0.583082	0.952214
CLSTN2 (1 of many)	92.46993614	0.158307384	0.2122264	0.7459363	0.455706	0.921215
apba2b	1725.292317	0.000582351	0.0785837	0.0074106	0.994087	0.998694
prkaa2	11.30910996	0.374261414	0.5862642	0.6383835	0.523224	0.939336
sirt7	64.99641017	0.32131877	0.2492161	1.2893178	0.197288	0.762244
senp5	198.4799025	-0.06375139	0.1507348	-0.422937	0.672341	0.965257
BX005421.2	0.491560863	0.853615855	2.865157	0.2979299	0.765757	NA
si:ch211-10a23.2	552.0721074	0.119103323	0.1125636	1.0580984	0.290011	0.846246
klf8	522.9409525	0.040075238	0.1103196	0.3632649	0.716407	0.96909
prf1.9	2.917889451	0.024714463	1.1427822	0.0216266	0.982746	NA
tm4sf18	152.0691226	-0.219037235	0.1635132	-1.339569	0.180385	0.741452
lct	7.662426681	-1.069017592	0.717689	-1.489528	0.136348	NA
ndst2b	67.07870682	0.143989705	0.2464581	0.584236	0.559062	0.948478
kdm1a	3596.685888	-0.089166413	0.0694655	-1.283607	0.199279	0.764601
si:ch73-127m5.1	88.45197092	-0.149512527	0.2248503	-0.664943	0.506087	0.935724
agr1	1073.525843	-0.135133232	0.0924796	-1.461222	0.143955	0.687741
lrsam1	107.8657558	0.058338192	0.2040766	0.2858642	0.774982	0.975687
chd2	1804.166187	-0.079904867	0.0792554	-1.008195	0.313361	0.865132
trim69	36.01335913	-0.325812726	0.3313225	-0.98337	0.325425	0.871896
znf346	1401.92672	0.197303147	0.0886508	2.2256216	0.02604	0.311101
kmt2bb	1205.555281	0.115625868	0.0871194	1.3272112	0.184439	0.747376
npffr2b	0.482760567	-0.866527181	2.7059286	-0.320233	0.748792	NA
nsd1b	136.2346903	0.055619478	0.1758812	0.3162331	0.751826	0.974458
pex11a	36.94386704	0.13518477	0.3330377	0.4059143	0.684806	0.966146
sv2bb	1709.518963	0.152977693	0.0924688	1.6543701	0.098052	0.59749
prkx	187.0769299	0.032306565	0.1778832	0.1816167	0.855884	0.985174
stim1a	1096.093252	0.132563079	0.0850797	1.5581038	0.119209	0.643307
dnajc16l	786.1082897	0.048441037	0.0988225	0.4901823	0.624005	0.957354
trim8b	1307.489472	0.038725343	0.0874309	0.442925	0.65782	0.962538
appl1	126.7165546	-0.085286808	0.1808646	-0.471551	0.637248	0.958872
syt17	5.135462918	1.088375455	0.8492936	1.2815067	0.200016	NA

pgm5	20.95018184	-0.02126162	0.4336156	-0.049033	0.960893	0.9956
tsfm	309.220295	0.109390252	0.1322928	0.8268802	0.408305	0.90171
taok3a	439.9182704	-0.01382068	0.12037	-0.114818	0.908589	0.990702
pear1	177.5303274	-0.115174352	0.1574271	-0.731604	0.46441	0.923403
abhd6a	35.47605155	-0.140353877	0.3367137	-0.416834	0.6768	0.965257
nphs1	69.38157548	0.036664922	0.2454087	0.1494035	0.881235	0.988235
pxnb	10.08049862	0.054072065	0.5867551	0.0921544	0.926575	NA
smg7	2591.874072	-0.047974853	0.0788592	-0.608361	0.542948	0.944974
ankib1a	86.38882878	-0.047187636	0.2403274	-0.196347	0.844338	0.985174
map7d3	910.2619157	-0.003623481	0.0889691	-0.040727	0.967513	0.996315
acap2	240.7414579	0.086269817	0.1461605	0.5902402	0.55503	0.947449
pcnx4	502.6789056	-0.045877834	0.11086	-0.413836	0.678994	0.965257
lrrc14b	31.94299385	0.252488273	0.3379432	0.7471323	0.454984	0.921022
slc20a2	998.5933746	0.179306272	0.0885154	2.0257081	0.042795	0.406447
pfkmb	512.9855607	0.248175582	0.1117939	2.2199388	0.026423	0.313054
map1sa	364.1788509	-0.109609809	0.1173415	-0.93411	0.350247	0.880195
mecom	207.7701824	-0.234731518	0.1490139	-1.575233	0.115203	0.636141
plekha7a	252.5633574	0.109829876	0.131677	0.8340857	0.404233	0.901305
car15	16.94193286	0.639401499	0.4580846	1.3958154	0.16277	0.7178
fam83hb	288.1189549	0.094011383	0.1430411	0.6572333	0.511031	0.936253
tecpr2	70.15742935	0.031679508	0.2333768	0.1357441	0.892024	0.989291
trpc5b	75.66436624	0.180928227	0.2266165	0.7983896	0.424644	0.9085
trim35-33	3.861240648	-0.083449989	0.9943487	-0.083924	0.933117	NA
pik3c2a	753.8273644	0.030474182	0.0943076	0.3231362	0.746592	0.97419
antxr1d	25.61265879	0.466654355	0.3765554	1.2392715	0.215245	0.782831
cabp7b	96.33863792	0.257506337	0.1992452	1.292409	0.196216	0.761674
setd1ba	453.058072	0.017831242	0.1142088	0.1561284	0.875932	0.987881
dlgap4a	544.5981722	-0.106231446	0.1077095	-0.986278	0.323997	0.870928
nckap1	4145.487005	-0.05029327	0.0655095	-0.767725	0.442651	0.916336
kctd3	88.82166151	-0.095169108	0.2072462	-0.459208	0.646085	0.96029
pstpip1a	3.095745377	0.160940701	1.0756697	0.1496191	0.881065	NA
dars2	179.6111177	0.043299654	0.1541523	0.2808888	0.778796	0.976037
atxn1b	792.5484245	-0.055892548	0.0891467	-0.626973	0.530677	0.942209
scai	9.532074543	-0.048425085	0.6184058	-0.078306	0.937584	NA
mbtd1	274.1239514	0.066487727	0.1295834	0.5130883	0.60789	0.956718
mctp1b	121.1455712	0.05959104	0.1874886	0.3178382	0.750608	0.97419
pde1a	75.53237341	0.183212924	0.2596775	0.7055401	0.480474	0.927601
edil3b	15.71083337	-0.347517078	0.4851202	-0.716353	0.473774	0.924993
slc28a1	46.17073641	-1.010652495	0.2885023	-3.503101	0.00046	0.016716
nmur1a	3.040768928	-0.989439688	1.1508932	-0.859715	0.389946	NA
znf592	260.0995791	-0.061762845	0.1411155	-0.437676	0.661621	0.964247
mettl7a	0	NA	NA	NA	NA	NA
col8a2	599.7310855	0.04711513	0.0982194	0.4796928	0.631446	0.957356
znf362a	622.1005008	0.075446263	0.1078067	0.6998291	0.484034	0.929461
trim62.1	52.33843982	-0.21260262	0.2723869	-0.780517	0.435087	0.914408
si:ch211-126i22.5	290.3378328	-0.101870047	0.1256944	-0.810458	0.417677	0.906237
fbxl16	170.0297463	0.294349965	0.1693139	1.7384867	0.082125	0.553114
anln	687.1200133	-0.05157007	0.0925778	-0.557046	0.577496	0.950825
pla2g6	667.9406432	0.000726114	0.1135902	0.0063924	0.9949	0.998694
ess2	619.5379899	-0.017459448	0.1041042	-0.167711	0.86681	0.986958
jarid2a	505.6190588	-0.129856128	0.1164365	-1.115253	0.264742	0.830616
rnf19b	47.7255623	0.310413566	0.2867409	1.0825577	0.279005	0.842806
si:ch211-136a13.1	300.6463834	0.067290126	0.1484328	0.4533373	0.650306	0.960708
rnf220a	1075.79936	-0.038690232	0.0821489	-0.470977	0.637657	0.958872

baiap2l2b	23.11386527	-0.378675482	0.4004165	-0.945704	0.3443	0.878434
adnp2a	292.6294634	0.074903847	0.1304402	0.574239	0.565806	0.948739
ANKFN1	3.651158645	0.273853582	0.9678786	0.2829421	0.777221	NA
rnf217	13.33964371	0.13927299	0.5610818	0.2482223	0.803962	0.979643
pip5kl1	46.0376109	0.032960539	0.3016971	0.1092504	0.913004	0.991119
dync1i1	842.1421706	0.01138987	0.0921893	0.1235488	0.901673	0.990121
polg	193.6902746	0.106517668	0.1789789	0.5951408	0.551749	0.947156
si:ch1073-214b20.2	3.308105203	-0.248998081	1.0527917	-0.236512	0.813035	NA
tmcc1b	701.8226402	-0.025913971	0.0988083	-0.262265	0.793117	0.977937
btr04	144.5961514	-0.141147432	0.1724515	-0.818476	0.413085	0.905281
zgc:153018	600.1656514	0.120846044	0.0988969	1.2219395	0.22173	0.78829
atp2a3	1128.190404	0.004037092	0.0852465	0.0473579	0.962228	0.995907
atp8b1	288.9765459	-0.083672256	0.1281487	-0.652931	0.513801	0.936778
smyd4	98.29659186	-0.116910929	0.2181173	-0.536	0.591958	0.954434
zgc:154077	222.3417471	-0.063719767	0.1507537	-0.422675	0.672533	0.965257
mief1	373.7226196	-0.015815032	0.1157501	-0.136631	0.891323	0.989291
rpusd2	93.17547586	0.239602247	0.2174728	1.1017575	0.270567	0.835315
fbxw7	869.4336053	0.102876762	0.0908708	1.132121	0.257584	0.825314
klhdc8a	8.250642086	0.45055773	0.6736084	0.6688719	0.503577	NA
cox11	271.8813778	0.19712686	0.144508	1.3641239	0.172529	0.731348
cyb5d2	126.695034	0.134605365	0.1771726	0.7597416	0.447409	0.917154
hlfb	53.07076415	0.61039039	0.2693316	2.2663158	0.023432	0.294562
ankfy1	945.2158298	0.067160676	0.0973163	0.690128	0.490114	0.931926
kcnj5	13.27595718	0.496258105	0.5459455	0.9089884	0.363356	0.885944
spns2	481.2846525	0.053639144	0.1068386	0.5020579	0.615627	0.95688
ccdc186	494.3978361	-0.042920152	0.1071654	-0.400504	0.688785	0.966672
fam160a1a	92.07949636	0.222292902	0.2096445	1.0603325	0.288993	0.845979
si:dkey-72l14.4	582.8078971	-0.104918542	0.0997437	-1.051881	0.292854	0.848607
rabggta	502.2663019	0.046286234	0.1238207	0.3738167	0.708541	0.968664
vezf1b	50.5585324	-0.082176735	0.2690291	-0.305457	0.760018	0.975374
dner	50.07345794	0.37199397	0.2713244	1.3710301	0.170366	0.728041
atp10a	47.28080779	-0.04656703	0.2776817	-0.167699	0.86682	0.986958
pum2	47.65780229	0.1805316	0.2856005	0.6321124	0.527313	0.941244
gabbr2	17.92388019	-0.066309932	0.4687196	-0.14147	0.887498	0.989291
si:dkey-72l14.3	88.34228975	0.201539979	0.2132601	0.945043	0.344637	0.878434
abcg4a	13.6799439	0.331208639	0.5689905	0.5820987	0.5605	0.948728
ranbp9	985.9071658	-0.128018841	0.0897308	-1.426699	0.153667	0.702578
rnf182	102.7118807	-0.316788864	0.2065435	-1.533763	0.125088	0.654648
brinp3a.2	223.7045661	0.124517045	0.1527299	0.8152763	0.414914	0.906237
nipblb	1583.996431	0.071536153	0.0774641	0.9234747	0.35576	0.882675
cyhr1	467.3185118	-0.077887942	0.1180987	-0.659516	0.509565	0.936253
stpg2	16.41096822	0.136290621	0.4765766	0.2859784	0.774895	0.975687
dpagt1	240.4742257	0.056886098	0.1375638	0.4135251	0.679222	0.965257
nemp2	263.5185823	-0.084998651	0.1290588	-0.658604	0.51015	0.936253
c2cd2l	467.1027797	0.183111916	0.1166862	1.5692678	0.116586	0.639148
chst3a	16.74147823	0.497450648	0.481817	1.0324474	0.301863	0.856944
arpp21	441.5016015	0.12929935	0.1257139	1.0285205	0.303705	0.857923
igl4v8	0.339758314	0.005883863	3.2682461	0.0018003	0.998564	NA
gtpbp6	67.68451114	-0.131054348	0.2460799	-0.532568	0.594332	0.954434
phldb1a	343.607974	0.198990335	0.1253022	1.5880827	0.112268	0.627572
stk36	67.91824546	0.025235268	0.2660821	0.0948402	0.924442	0.992676
dapk2a	84.90946635	0.068479187	0.2270032	0.3016662	0.762907	0.975374
nfasca	1059.99276	0.03604958	0.0896101	0.4022938	0.687468	0.966376
nars	1357.163716	-0.332379608	0.1178116	-2.821281	0.004783	0.099772

snx19a	175.5400284	-0.101968988	0.1619044	-0.62981	0.528819	0.942109
slc19a3b	10.63334079	0.347185188	0.5980782	0.5805013	0.561577	NA
ep300b	1608.542738	0.034977685	0.0869091	0.4024629	0.687343	0.966376
zbtb44	55.8908786	-0.087768412	0.2732592	-0.321191	0.748066	0.97419
parga	605.19854	-0.069463084	0.0998056	-0.695984	0.486439	0.930833
slc43a2b	3215.302059	0.071528866	0.1066775	0.6705148	0.50253	0.935304
adgrl3.1	1830.259498	0.025053084	0.0837368	0.2991884	0.764796	0.975374
srpra	1200.429024	0.050804018	0.102857	0.4939285	0.621357	0.957354
kif21a	552.7219513	0.181479344	0.1026848	1.7673434	0.077171	0.540934
senp7b	340.3183525	-0.259191209	0.1313439	-1.973379	0.048452	0.433449
colec12	936.8606817	0.237861214	0.0934054	2.5465461	0.010879	0.180962
tapt1b	548.4992097	-0.006366471	0.1011472	-0.062943	0.949812	0.99381
gfod2	270.6434258	0.122856534	0.1482642	0.8286323	0.407313	0.901305
zfp64	144.5362244	-0.087087019	0.180401	-0.482741	0.629279	0.957354
nrn1lb	113.3522448	0.176420471	0.1948487	0.9054227	0.365241	0.886714
bicra	744.181121	-0.152429687	0.1213836	-1.255768	0.2092	0.776073
ube2j2	47.42145557	0.014930019	0.2979083	0.0501162	0.96003	0.995388
slc6a4a	137.1644361	0.237551646	0.1766203	1.344985	0.17863	0.739486
dhx34	190.3527383	-0.018957184	0.1809847	-0.104745	0.916578	0.991381
larp1b	1303.873198	-0.091598207	0.085392	-1.072679	0.283415	0.84466
st14a	1352.755985	-0.085589391	0.084695	-1.01056	0.312227	0.864401
znf740b	303.4477596	-0.046184446	0.1284986	-0.359416	0.719284	0.969183
mov10b.1	61.86936808	0.068064372	0.2453171	0.2774546	0.781431	0.97627
vps45	470.5867138	0.018269571	0.1058184	0.1726502	0.862926	0.985841
nat15	433.6243991	0.097399225	0.1162001	0.8382026	0.401917	0.90084
cbx5	4510.925968	0.035409079	0.0724147	0.4889761	0.624859	0.957354
plekho1a	334.1100786	-0.00083854	0.119015	-0.007046	0.994378	0.998694
hectd2	20.73808999	0.377144851	0.4374531	0.8621377	0.388612	0.896271
cnnm2a	22.81161745	-0.246689324	0.4255451	-0.579702	0.562116	0.948728
emilin2a	168.8428048	0.097119971	0.16281	0.5965232	0.550826	0.946549
armc7	52.71463557	0.57927536	0.2767177	2.0933801	0.036315	0.372071
acsf2	834.0015178	-0.11807869	0.097978	-1.205155	0.228144	0.795505
trpc1	240.9583303	0.258090675	0.1427491	1.8080018	0.070606	0.520155
ddx20	222.9761037	-0.139516019	0.1448222	-0.963361	0.335367	0.876088
stag1a	815.2494695	0.00569875	0.1035273	0.0550459	0.956102	0.994543
ttc7b	988.7114632	-0.032291104	0.0912663	-0.353812	0.72348	0.970402
rab40c	1036.518146	0.102019193	0.0859574	1.1868573	0.235284	0.804375
rabep2	308.1514636	0.199778427	0.1248316	1.6003836	0.109514	0.623766
lpin2	1221.685873	-0.025192017	0.0869491	-0.289733	0.772021	0.975687
fam149b1	481.9869196	0.001016202	0.1035986	0.009809	0.992174	0.997927
hexdc	102.4789495	-0.17196071	0.2023759	-0.849709	0.395487	0.899378
si:ch211-157j23.2	5.862770517	0.347551963	0.8383044	0.4145892	0.678443	NA
uba7	12.5802364	-0.253582072	0.5546985	-0.457153	0.647561	0.960606
ogfrl1	98.0132309	-0.03428343	0.2141202	-0.160113	0.872792	0.987846
timp2a	1629.369992	0.023451661	0.0782493	0.2997042	0.764403	0.975374
pfdn4	438.7208855	-0.126531918	0.1192425	-1.061131	0.28863	0.845979
tinagl1	32.53136808	0.025828307	0.3648757	0.0707866	0.943568	0.993364
abcc5	3289.928552	-0.03818428	0.0688041	-0.554971	0.578914	0.951052
alg5	157.6676987	0.106153142	0.1749641	0.6067139	0.544041	0.945438
cbln4	47.61484205	-0.296277008	0.3000274	-0.9875	0.323398	0.870761
tuft1a	306.5894209	0.195308455	0.1249781	1.5627418	0.118113	0.641526
ubash3ba	23.06021598	0.161663446	0.4010498	0.4031007	0.686874	0.966376
xylt1	41.09168887	-0.131939115	0.3048836	-0.432752	0.665195	0.964736
myom1a	3360.661271	0.463858375	0.0884937	5.2417093	1.59E-07	2.07E-05

dusp3a	175.0963202	-0.02432683	0.1704783	-0.142698	0.886529	0.988936
si:dkey-44g23.5	555.4641997	-0.06539707	0.1087886	-0.601139	0.547747	0.945705
zgc:153240	533.4210498	0.134539877	0.1108079	1.2141721	0.224682	0.791954
sost	311.8205457	0.003195137	0.1228967	0.0259986	0.979258	0.996802
si:dkey-202e22.2	169.0460868	0.392926132	0.1611305	2.4385587	0.014746	0.222347
lmln	6.431231239	0.132769129	0.846728	0.1568026	0.8754	NA
ago2	5.81815432	1.327966744	0.8082458	1.6430234	0.100378	NA
BX294434.1	859.5572745	0.165915501	0.0879664	1.8861229	0.059278	0.475912
lss	120.7418129	-0.105437503	0.1872508	-0.563082	0.573379	0.949869
glg1a	2693.928102	0.008054786	0.0770565	0.1045309	0.916748	0.99151
kcnc4	32.39279008	0.016341154	0.3729513	0.0438158	0.965051	0.996154
pi15b	16.37804486	-0.087352985	0.4625808	-0.188838	0.85022	0.985174
arhgap5	1239.373925	0.076786633	0.0889372	0.8633805	0.387928	0.896133
gmpr2	752.5171915	-0.00934882	0.0906101	-0.103176	0.917823	0.991623
oca2	109.4139577	-0.184286136	0.209602	-0.87922	0.379282	0.895192
ppp1r9a	26.19559015	0.007676391	0.3814516	0.0201242	0.983944	0.996944
znf438	119.773781	-0.224117735	0.181712	-1.233368	0.217439	0.784786
ngrn	182.169443	-0.180333175	0.1502787	-1.199992	0.230143	0.797833
cdon	1400.862585	-0.016415766	0.0871019	-0.188466	0.850511	0.985174
galnt1	966.0452154	0.026488879	0.0950316	0.2787375	0.780446	0.97627
ddx6	758.6783418	-0.022462812	0.0983701	-0.22835	0.819374	0.981594
wwp2	2803.97447	-0.028042638	0.0717869	-0.390637	0.696065	0.96736
adat1	44.34074185	0.122933198	0.312531	0.3933472	0.694063	0.96736
tfpi2	57.77264703	-0.062072859	0.2613586	-0.237501	0.812268	0.981081
bcl11aa	852.0619299	-0.104174287	0.0891308	-1.16878	0.242492	0.811298
foxred2	513.2688567	0.117385451	0.1080566	1.0863332	0.277332	0.840857
aoc1	44.33346703	-0.492717703	0.3118089	-1.580191	0.114063	0.632852
chst6	31.93239614	0.104060623	0.3404609	0.3056463	0.759874	0.975374
dipk1ab	58.83489497	0.020773571	0.2605885	0.0797179	0.936462	0.992702
GFOD1	7.8909762	0.32229604	0.6824991	0.4722293	0.636763	NA
hepacam2	70.30412758	-0.115777282	0.2365993	-0.489339	0.624602	0.957354
klf13	23.82745972	-0.087856249	0.4065574	-0.216098	0.828911	0.982721
tsen34	150.3608724	-0.193883063	0.1688707	-1.148116	0.250921	0.820469
cdh18a	433.568378	0.032533883	0.1088877	0.298784	0.765105	0.975374
cog7	581.6110415	0.040377096	0.1000512	0.4035641	0.686533	0.966376
znf142	193.0157805	-0.141592037	0.1473696	-0.960795	0.336655	0.876088
sgpl1	1071.322933	0.149735301	0.0986797	1.5173878	0.129169	0.660844
efcab1	15.84344028	0.541946984	0.4817101	1.125048	0.260569	0.828546
smg8	317.6924259	-0.145080562	0.1208941	-1.200063	0.230115	0.797833
cmya5	200.1798506	0.472362417	0.1590911	2.969131	0.002986	0.071842
serpinf2b	1079.369739	0.131418522	0.0994913	1.3209053	0.186533	0.750917
haus3	145.3480982	0.036921511	0.1679631	0.2198191	0.826012	0.982196
grhl1	258.1895598	-0.1109322	0.1380687	-0.803457	0.421711	0.90651
trip12	3198.869403	-0.032213122	0.0785378	-0.410161	0.681688	0.965476
epb41l4b	169.765838	0.14355045	0.1658862	0.865355	0.386844	0.89551
csgalnact2	344.4388556	-0.001924362	0.1210701	-0.015895	0.987318	0.997136
eva1a	144.7663928	0.13861436	0.1740256	0.7965173	0.425731	0.909437
bivm	146.4821291	0.007129341	0.1674517	0.0425755	0.96604	0.996315
nelfa	277.4452862	-0.130606781	0.1337072	-0.976812	0.328662	0.872945
sec23ip	824.6419222	0.112139442	0.0956961	1.1718283	0.241266	0.810257
slx4	309.9730405	0.1891869	0.1458529	1.2971079	0.194594	0.758506
c2cd4a	111.5560291	-0.139648084	0.2007994	-0.695461	0.486767	0.930882
nub1	57.7195117	0.00520294	0.2699394	0.0192745	0.984622	0.996944
zmat4b	28.35255614	-0.452871389	0.3859755	-1.173317	0.240669	0.808939

FQ311928.1	295.6776052	0.024719503	0.130863	0.188896	0.850174	0.985174
znf646	696.7340622	0.132139948	0.0963517	1.3714339	0.17024	0.727696
zgc:194330	18.0112097	-0.373794782	0.4552768	-0.821027	0.411631	0.903595
col6a2	2857.152804	0.12729857	0.0679918	1.8722625	0.06117	0.482482
inpp5f	134.5507418	-0.013781351	0.1869611	-0.073712	0.941239	0.992857
ugt5c3	138.8030122	-0.057532814	0.17518	-0.328421	0.742593	0.973232
ugt5c1	5.255648189	-0.543503156	0.8480037	-0.640921	0.521574	NA
adcy6a	220.5903978	-0.173704822	0.1550273	-1.120479	0.26251	0.830166
smap2	6.42620057	-0.431266452	0.7854749	-0.549052	0.58297	NA
tesk2	417.0263901	-0.040993543	0.1114306	-0.367884	0.71296	0.96909
n4bp2	264.7784446	-0.154425127	0.1399063	-1.103775	0.269691	0.834705
nrxn2a	1675.497012	0.024256495	0.0931159	0.2604978	0.79448	0.977937
phf14	289.7575494	-0.005853022	0.127279	-0.045986	0.963322	0.995927
si:dkey-253i9.4	990.1084606	-0.073663415	0.0860016	-0.856535	0.391702	0.897688
dsela	31.21828451	0.012479853	0.3745477	0.0333198	0.97342	0.996315
vwa7	55.4849852	0.094185326	0.2578932	0.3652106	0.714954	0.96909
cpne5a	19.5567632	-0.318278951	0.4645606	-0.685118	0.493269	0.931926
ncaph	387.8754202	0.014382552	0.1121783	0.1282115	0.897982	0.990121
sema5ba	59.43267111	-0.143990549	0.2530273	-0.569071	0.569308	0.948983
gba2	563.1991107	-0.007228769	0.1018605	-0.070967	0.943424	0.993364
tbkbp1	346.2765836	0.129633497	0.1176464	1.101891	0.270509	0.835266
igf2bp1	5079.454423	-0.039265528	0.0671002	-0.585177	0.558428	0.948478
thsd7aa	331.695356	-0.078867121	0.1371996	-0.574835	0.565403	0.948739
si:ch211-132b12.1	9.358345328	0.038782243	0.6234137	0.0622095	0.950396	NA
wu:fj05g07	222.662397	0.456785461	0.1496207	3.0529572	0.002266	0.058495
DNAH10	169.6792227	-0.282735565	0.1986215	-1.423489	0.154594	0.704307
u2surp	1648.107472	-0.139378759	0.0899361	-1.549753	0.121201	0.647458
si:ch211-250e5.2	90.38601119	-0.0385365	0.2099231	-0.183574	0.854347	0.985174
thsd7bb	20.94423525	0.744115337	0.4278268	1.7392911	0.081984	0.552725
dusp13a	1.054757122	0.07669138	2.0202577	0.0379612	0.969719	NA
kdm4c	466.7328993	0.026326056	0.1127337	0.2335243	0.815354	0.981244
mrps9	669.9481118	-0.051271313	0.0972206	-0.527371	0.597936	0.955459
tgfbrap1	114.6622239	-0.065073301	0.1968399	-0.33059	0.740954	0.973154
tbx3b	14.84025067	0.45820757	0.5042216	0.9087424	0.363486	0.886042
tmtops2a	81.36041403	-0.279788876	0.2274968	-1.229858	0.21875	0.785734
lrp1bb	386.7675741	0.0723467	0.1184212	0.6109268	0.541248	0.944954
b4galnt1a	42.19580471	-0.025326732	0.3263441	-0.077607	0.93814	0.99279
slka	656.659859	0.089885098	0.1033502	0.869714	0.384457	0.89551
nxt2	135.4760733	0.103324425	0.1787295	0.5781049	0.563193	0.948728
unc5db	18.64292562	0.02384467	0.4468499	0.0533617	0.957444	0.99467
ccdc85b	1181.871902	0.097445241	0.0853105	1.1422427	0.253353	0.823169
ano6	20.16540238	-0.101229213	0.4371508	-0.231566	0.816875	0.981594
zgc:153409	22.28015583	0.456694357	0.4402551	1.0373404	0.299577	0.855675
si:ch211-145o7.3	49.47792219	0.286816393	0.2815358	1.0187563	0.308319	0.861088
foxo1b	9.919771919	-0.102081883	0.5968332	-0.171039	0.864193	NA
rap1gap2a	65.70575222	-0.750131721	0.2581211	-2.906123	0.003659	0.081763
tmem246	216.7061204	-0.161076385	0.1446397	-1.113639	0.265434	0.831302
stim1b	65.71070573	0.0597669	0.2411693	0.2478213	0.804273	0.979643
tmem121aa	147.6118532	0.192554995	0.1755622	1.096791	0.272733	0.838096
slc7a3b	195.6357032	-0.210356322	0.1569719	-1.340089	0.180216	0.74128
gldn	17.97510466	0.146252367	0.4763563	0.307023	0.758826	0.975374
myo1cb	1343.844095	0.030570271	0.0790117	0.3869084	0.698824	0.967998
kctd7	278.8373544	0.141988351	0.1266417	1.1211819	0.26221	0.830166
cyp4v7	40.27555377	-0.625208579	0.3356728	-1.862553	0.062525	0.487868

ctdspl2a	911.0261621	0.026453918	0.0897452	0.2947669	0.768172	0.975687
cachd1	1115.730651	-0.016962328	0.0856861	-0.197959	0.843077	0.985174
abcb10	171.0272366	0.130307535	0.1680371	0.775469	0.438063	0.915284
cemip2	416.4467096	0.099631827	0.1192833	0.8352541	0.403575	0.900979
ssx2ipb	87.64976114	-0.260607377	0.2134745	-1.220789	0.222166	0.788551
sorbs2b	1025.252091	0.047024933	0.0861169	0.5460591	0.585025	0.952436
ufsp1	26.10844376	0.015967932	0.4101267	0.0389341	0.968943	0.996315
si:dkey-85k7.11	0.83328727	-3.180757216	2.5122726	-1.266088	0.205482	NA
them4	257.1141345	0.296146524	0.1316446	2.2495908	0.024475	0.3019
kcng3	5.790581277	-0.736493882	0.8030103	-0.917166	0.359056	NA
ndufaf5	402.7643397	-0.089000979	0.1176384	-0.756564	0.449311	0.918313
fbxo11a	881.2812951	0.07600382	0.09254	0.8213077	0.411471	0.903483
aifm4	448.0379764	-0.494217986	0.1176988	-4.199007	2.68E-05	0.001616
myo5aa	95.41227858	0.193220954	0.2056286	0.9396598	0.347392	0.880195
kcnj9	11.14951105	0.324568618	0.5884629	0.5515532	0.581255	0.951731
dcakd	71.04357895	-0.070411405	0.2502354	-0.281381	0.778418	0.975942
sipa1l2	1610.426473	-0.145122661	0.0814044	-1.782737	0.074629	0.532003
ankrd34bb	27.34415554	0.219395947	0.4130927	0.5311059	0.595345	0.954434
ensab	31.43260028	-0.016366208	0.3668526	-0.044612	0.964416	0.995927
nrxn1a	3292.056173	0.082051059	0.0707283	1.1600881	0.246013	0.816218
marveld2b	155.7441393	0.127058532	0.1671702	0.7600551	0.447222	0.917154
wwc3	364.4531897	0.157679891	0.1140336	1.3827492	0.166742	0.723339
ptpdc1a	254.1893662	0.021214365	0.1377456	0.1540112	0.877601	0.987881
CABZ01055347.1	34.7531907	0.228920298	0.3308803	0.6918524	0.48903	0.931926
zgc:162707	249.8495627	0.076081354	0.1318681	0.5769504	0.563973	0.948739
hcn2b	215.304804	0.055218569	0.1586316	0.3480931	0.72777	0.970402
si:ch73-334d15.1	151.0349302	0.065737893	0.1622668	0.4051222	0.685388	0.966198
bicc1a	231.7735425	-0.034902526	0.1484775	-0.235069	0.814155	0.981081
slc25a17	103.0628722	-0.265345199	0.1990079	-1.33334	0.18242	0.744977
hcn4	5.363388479	1.157604006	0.9048561	1.2793239	0.200783	NA
atxn1a	42.49217711	0.519701209	0.3146854	1.6514945	0.098638	0.59799
arvcfb	1204.784379	0.144398193	0.0811849	1.7786332	0.0753	0.534207
nxpe3	111.2417227	0.16261295	0.1976991	0.8225275	0.410777	0.90316
qpctla	241.1578144	0.115932511	0.1434425	0.8082158	0.418966	0.906406
yjefn3	384.7130101	0.107757281	0.1194169	0.9023624	0.366864	0.88734
cal4	82.97557858	0.26254572	0.2182571	1.2029197	0.229007	0.796691
sipa1l3	46.42284812	-0.053039351	0.2859232	-0.185502	0.852835	0.985174
map3k8	45.96982242	-0.254813121	0.2972039	-0.857368	0.391242	0.897688
tpd52	203.852497	0.111878893	0.157603	0.7098778	0.47778	0.926857
si:dkey-45k15.1	17.39236039	0.122893477	0.4969942	0.2472735	0.804697	0.979705
cytip	6.726836383	-0.425082548	0.8943929	-0.475275	0.634591	NA
znf704	14.01745785	-0.248114415	0.5119193	-0.484675	0.627907	0.957354
washc4	475.5887029	-0.02241367	0.1231704	-0.181973	0.855604	0.985174
tmem64	42.37365499	-0.208774929	0.3062166	-0.681789	0.495373	0.931928
mast3a	382.4437005	-0.082761065	0.1216449	-0.68035	0.496283	0.932415
acot13	245.9541022	0.012623812	0.1399381	0.09021	0.92812	0.992702
raph1b	648.8254605	-0.098759764	0.1142119	-0.864706	0.3872	0.895778
hnrnpm	6143.291402	-0.147806693	0.0738984	-2.000134	0.045486	0.418148
ank3a	1570.575663	-0.002136154	0.0782933	-0.027284	0.978233	0.996762
ece1	380.3949386	-0.140903917	0.1211289	-1.163256	0.244726	0.814435
2-Mar	28.92677082	0.157446329	0.3803713	0.4139279	0.678927	0.965257
itpr3	259.826807	0.014478465	0.1333202	0.1085992	0.91352	0.991119
clasrp	590.4091003	0.010874086	0.1069837	0.1016424	0.919041	0.991623
arhgef9a	568.5815268	0.142109641	0.1039101	1.3676206	0.171431	0.729863

dipk2b	12.06017591	0.255931541	0.5624015	0.4550691	0.64906	0.960708
htr3b	47.0064641	0.179970167	0.2925842	0.6151055	0.538485	0.943336
ripor2	188.6216386	0.019344936	0.1496824	0.1292399	0.897168	0.990121
rabgap1l	1169.921753	-0.127947124	0.0845672	-1.512964	0.130289	0.662797
sh3pxd2ab	135.9995256	-0.04377146	0.1908306	-0.229373	0.818579	0.981594
kdm6a	1111.823491	-0.039141972	0.084647	-0.462414	0.643784	0.960045
opa3	174.4879787	0.150540942	0.159216	0.9455137	0.344397	0.878434
trim108	18.52558904	-0.099032052	0.4837632	-0.204712	0.837797	0.984701
spata5l1	111.5788345	-0.00604858	0.190182	-0.031804	0.974628	0.996579
ahnak	2803.768736	0.024004882	0.0775082	0.3097078	0.756783	0.975374
bmp10	2.905940877	-0.026843586	1.1457431	-0.023429	0.981308	NA
abcd4	180.1768016	-0.193173209	0.1645644	-1.173846	0.240457	0.808525
neurl1ab	60.02910588	0.03792042	0.2488126	0.1524055	0.878867	0.987881
mbd3a	1271.564308	-0.069142219	0.081752	-0.845756	0.397689	0.899672
fmn2b	941.48439	-0.008529813	0.1015731	-0.083977	0.933075	0.992702
arhgap25	6.973177013	-0.406650297	0.723892	-0.561755	0.574283	NA
klhl2	332.7199161	0.023012266	0.1272146	0.1808933	0.856451	0.985537
gnl1	326.2974726	-0.209055909	0.1241136	-1.684392	0.092106	0.57849
nyx	160.4469932	0.183614697	0.169176	1.0853473	0.277768	0.841388
jam3b	1063.211873	0.087327782	0.0909615	0.9600521	0.337029	0.876088
rasgrp4	369.2188392	0.100483063	0.1216304	0.8261342	0.408728	0.90171
zgc:154006	23.57220027	0.050359528	0.3918262	0.1285252	0.897733	0.990121
arhgap27l	289.1513041	-0.180566511	0.1366656	-1.321229	0.186425	0.750788
si:dkey-208k22.6	21.96997439	-0.522795284	0.4107658	-1.272733	0.203113	0.770099
cnot2	1230.235602	-0.132819161	0.0836296	-1.588184	0.112245	0.627572
si:ch211-194e15.5	162.1665901	0.007335238	0.1792007	0.0409331	0.967349	0.996315
si:dkey-13n15.2	102.5889755	-0.006900371	0.203323	-0.033938	0.972927	0.996315
mbd3b	2037.278889	0.033201542	0.086288	0.384776	0.700403	0.967998
kif1aa	1942.699777	0.1462547	0.0763858	1.9146841	0.055533	0.461579
meox2b	41.6351005	-0.248438905	0.2988572	-0.831296	0.405806	0.901305
wscd2	26.84194055	-0.156894384	0.3810123	-0.411783	0.680499	0.965431
UNC13A	136.6329676	-0.110209361	0.1727234	-0.638069	0.523429	0.939336
prss12	294.1422204	0.027428848	0.1318718	0.2079964	0.835232	0.984089
sntg1	4.034113107	-0.702332574	1.0563031	-0.664897	0.506116	NA
zpld1a	36.55682292	-0.112638378	0.3297571	-0.34158	0.732667	0.971719
nfixb	42.31548052	-0.375033819	0.3024291	-1.240072	0.214949	0.782493
tiparp	939.1924197	-0.196645097	0.089785	-2.190178	0.028511	0.327971
si:dkey-38p12.3	426.6764114	-0.227791995	0.1084464	-2.100504	0.035685	0.368007
pld7	292.011754	0.111356265	0.1267066	0.878851	0.379482	0.895192
rxfp3.2b	33.68650516	0.059430175	0.3351133	0.1773435	0.859239	0.985773
col15a1b	1254.393582	0.092371031	0.0859662	1.0745036	0.282597	0.843865
si:dkey-19b23.13	126.6910889	0.023094985	0.1817951	0.1270386	0.89891	0.990121
olfml3a	70.16851845	0.368038096	0.2444698	1.5054544	0.132207	0.666055
lman2	1240.981548	-0.094524553	0.0833481	-1.134093	0.256755	0.824902
zgc:153968	120.139912	0.345553374	0.2185012	1.581471	0.11377	0.632292
myo18ab	1704.920684	0.05919584	0.0833722	0.7100186	0.477693	0.926857
hdlbpb	903.4402283	-0.036722022	0.0864718	-0.42467	0.671077	0.965257
ddx10	374.9462952	0.015664742	0.1150194	0.1361921	0.891669	0.989291
v2rx3	3.095824445	0.133928205	1.0962878	0.1221652	0.902768	NA
v2rh10	0	NA	NA	NA	NA	NA
satb2	120.066495	-0.111272527	0.1809062	-0.615084	0.538499	0.943336
ppcs	148.5466482	-0.044489308	0.1701354	-0.261494	0.793712	0.977937
atp13a2	198.6544989	-0.172006448	0.153021	-1.124071	0.260983	0.829139
usp32	932.6284577	0.053669291	0.0930136	0.5770047	0.563936	0.948739

slco2a1	269.2497308	-0.006426132	0.1289406	-0.049838	0.960252	0.995388
iqub	22.89438202	-0.03364106	0.3973862	-0.084656	0.932535	0.992702
ankrd13a	120.5854668	-0.144489772	0.2017637	-0.716134	0.473909	0.924993
v2ra18	3.216739331	0.198727244	1.1224662	0.1770452	0.859473	NA
fhod3b	72.67005334	0.225470889	0.2404758	0.9376031	0.348448	0.880195
mfsd12a	148.8522987	-0.208357588	0.1661897	-1.253733	0.209939	0.777416
si:ch73-233f7.1	1342.092085	0.017680068	0.0935072	0.189077	0.850032	0.985174
git2a	336.1631311	0.082661833	0.1210527	0.6828583	0.494696	0.931926
tmem51b	117.9897583	-0.353413622	0.191975	-1.840936	0.065631	0.500566
cplx2	1019.02476	0.03593116	0.0921636	0.3898627	0.696638	0.96736
gltpa	207.6879078	-0.256364681	0.1468133	-1.746195	0.080777	0.550801
amotl2a	1324.390391	-0.080941794	0.0851337	-0.950761	0.341726	0.878134
fbxo10	22.77621822	-0.249332215	0.4260802	-0.585177	0.558429	0.948478
htr4	7.975389269	0.102815582	0.7102632	0.144757	0.884903	NA
trpv4	186.6324394	0.038480093	0.1533074	0.2509996	0.801814	0.979492
commd4	203.7190668	0.151547659	0.1553754	0.9753646	0.329379	0.873436
amotl2b	497.9392886	-0.04843052	0.1260952	-0.384079	0.70092	0.968134
golgb1	994.253522	-0.021156757	0.0838827	-0.252218	0.800872	0.979377
ecpas	1108.610841	-0.013883772	0.0871742	-0.159265	0.87346	0.987858
sytl2a	82.80399843	0.066295046	0.2517917	0.2632933	0.792325	0.977937
ube3b	34.36308483	-0.000203351	0.3394439	-0.000599	0.999522	0.999704
vrtn	7.318082693	0.025836884	0.7147288	0.0361492	0.971163	NA
myo1ha	7.353111201	0.401253564	0.7041633	0.5698303	0.568793	NA
lingo3a	140.7645829	0.273288588	0.167494	1.6316325	0.102757	0.609031
grhl2b	185.0719768	-0.056617233	0.1652711	-0.342572	0.731921	0.971665
sema6bb	50.88258389	0.192838654	0.280957	0.6863636	0.492484	0.931926
ppfibp2a	338.4934674	0.078922517	0.1333792	0.5917152	0.554041	0.947449
lap3	457.3763534	0.034386388	0.1062315	0.323693	0.74617	0.97419
rbm47	1007.099136	-0.253100345	0.0852105	-2.970296	0.002975	0.071657
tbc1d2b	94.03217945	0.067568008	0.2019855	0.3345191	0.737988	0.972535
mpnd	551.0957042	-0.103459274	0.1111927	-0.93045	0.352138	0.880879
kcnh4b	55.6387792	0.226704929	0.2660822	0.852011	0.394208	0.898673
dot1l	39.20646267	0.426076486	0.3280295	1.298897	0.193979	0.75784
wdr74	148.4975991	-0.094361387	0.1805649	-0.52259	0.60126	0.956045
acacb	728.3858416	0.093533524	0.0969432	0.9648281	0.334631	0.875699
abtb2b	195.3547438	-0.150872809	0.1461783	-1.032115	0.302018	0.856944
adnpa	0	NA	NA	NA	NA	NA
myo5b	952.7131657	-0.373905018	0.0897206	-4.167439	3.08E-05	0.001781
hs2st1b	235.4509052	-0.095804946	0.1385101	-0.691682	0.489137	0.931926
gpr18	0.794338034	3.131709106	2.3335617	1.3420297	0.179586	NA
vps51	437.4981485	0.130899601	0.113489	1.153412	0.248741	0.81797
fsd1	212.7401348	-0.020812689	0.1517188	-0.137179	0.890889	0.989291
rufy2	1069.807631	-0.056399004	0.0931131	-0.605704	0.544711	0.945438
gse1	1670.162083	-0.113158019	0.0857359	-1.319844	0.186887	0.750967
ccdc181	71.55064882	0.062866519	0.2307718	0.2724185	0.7853	0.97627
fndc3bb	139.7341904	-0.080999477	0.1702847	-0.475671	0.634309	0.95776
kif1ab	333.4612032	0.081147218	0.1481066	0.5478976	0.583762	0.952214
cpox	470.9157035	-0.529384618	0.1144475	-4.625567	3.74E-06	0.000317
gpr17	19.78800758	0.287319599	0.4378823	0.6561571	0.511723	0.936253
atp8b5a	49.21144991	-0.227983327	0.2987319	-0.76317	0.445362	0.917117
mthfsd	14.00914446	0.2485652	0.5225394	0.475687	0.634297	0.95776
il1rapl1a	649.5701833	-0.113481915	0.1068261	-1.062305	0.288097	0.845979
arhgap45b	33.94148495	-0.034392631	0.336443	-0.102224	0.918579	0.991623
casp2	250.436266	0.079269394	0.1342334	0.5905341	0.554833	0.947449

kif1bp	568.8275035	0.081421752	0.0993369	0.8196524	0.412414	0.904913
cpt1ab	1981.249326	0.025864502	0.0753355	0.343324	0.731355	0.971557
rnf38	288.7450314	-0.114295569	0.1279698	-0.893145	0.371779	0.892248
elmod1	2355.990694	0.037696715	0.0779029	0.4838936	0.628461	0.957354
zgc:174945	1.306217685	0.741218782	1.6736071	0.442887	0.657847	NA
slc12a7b	2243.191748	0.368350179	0.0823372	4.4736803	7.69E-06	0.000576
si:ch211-236l14.4	250.4810326	0.420104136	0.136124	3.0861863	0.002027	0.05403
gmeb1	437.9857799	-0.091815018	0.1162911	-0.789527	0.429804	0.911615
tab3	412.5605319	0.061742773	0.129511	0.4767376	0.633549	0.957703
acsbg1	8.862418044	0.282399899	0.6339079	0.4454904	0.655965	NA
shc2	48.97172935	0.178911704	0.2866991	0.6240399	0.532601	0.942805
tbc1d1	673.852073	0.028310132	0.1020102	0.2775225	0.781379	0.97627
hipk3b	73.57446916	0.806347296	0.257247	3.1345261	0.001721	0.047873
dph7	207.0583101	-0.141952735	0.1503371	-0.94423	0.345052	0.878434
clcn1a	88.91245966	0.341186147	0.2147561	1.5887144	0.112125	0.627454
tkfc	280.7366712	-0.346781804	0.1353806	-2.561532	0.010421	0.175676
kiaa1549lb	16.54936359	0.043293429	0.470777	0.0919616	0.926729	0.992702
nceh1b.1	67.07886177	-0.151848338	0.2411471	-0.629692	0.528896	0.942109
uggt2	857.4766323	0.206238802	0.094312	2.1867723	0.028759	0.328909
pm20d1.2	182.0030884	-0.020578383	0.1528508	-0.134631	0.892904	0.989291
iffo2a	18.62497727	-0.028652368	0.4374985	-0.065491	0.947783	0.99381
ankrd24	156.782574	0.025195533	0.1635537	0.1540505	0.87757	0.987881
klf5b	38.14362801	0.124079128	0.3099813	0.4002794	0.688951	0.966672
iffo1b	45.72846087	-0.062675324	0.2839934	-0.220693	0.825332	0.982196
shdb	383.2836232	-0.128433583	0.1131458	-1.135116	0.256327	0.824633
rxfp3.3a2	7.312814974	-0.381719226	0.6987637	-0.546278	0.584875	NA
tent4a	72.8601412	-0.144297891	0.2342831	-0.615912	0.537952	0.943336
ctdspl1b	618.6695123	0.204438696	0.1039293	1.9670935	0.049172	0.435115
klhl5	8.286961067	-0.062327203	0.7090238	-0.087906	0.929952	NA
agap1	1730.869444	-0.051295845	0.0809504	-0.63367	0.526296	0.940506
mxra8b	383.311624	0.153956457	0.1190081	1.2936639	0.195782	0.76113
cyp4v8	204.0290848	-0.120088867	0.1695032	-0.708476	0.47865	0.926978
kcnab2b	45.19565281	0.068007164	0.3076648	0.2210431	0.825059	0.982196
sytl5	17.41342862	-0.442790439	0.4746704	-0.932838	0.350904	0.880443
stag1b	1179.895681	0.017665034	0.0820789	0.2152201	0.829596	0.982805
ranbp10	414.0156135	0.084101413	0.113011	0.7441879	0.456763	0.921215
eif2ak3	597.076397	-0.125358956	0.1008639	-1.242853	0.213922	0.782251
stoml1	133.9118793	0.151573662	0.1861453	0.8142759	0.415487	0.906237
larp4b	488.7152556	0.073723304	0.1077856	0.6839808	0.493987	0.931926
otc	198.2048353	0.118453038	0.1545528	0.7664246	0.443424	0.916631
SENP7	310.5437283	0.008679077	0.1257543	0.0690161	0.944977	0.993409
chaf1a	1292.325804	-0.158006677	0.0882675	-1.790089	0.07344	0.528705
dip2ca	519.289476	0.013427175	0.1030933	0.130243	0.896374	0.989971
abi3bpa	27.34681848	-0.095786754	0.3724664	-0.257169	0.797048	0.978569
atrn	698.6933273	0.047169566	0.0979339	0.481647	0.630057	0.957354
tub	485.794439	0.178141576	0.1103663	1.6140945	0.106507	0.615567
tmem168b	18.9985393	-0.046445169	0.4499003	-0.103234	0.917777	0.991623
olfml3b	486.8506613	0.218844759	0.1047521	2.089168	0.036693	0.374386
camsap2a	343.9124624	0.013223213	0.1270111	0.1041107	0.917081	0.991572
cacnb4b	2040.258485	0.129428652	0.0798456	1.6209862	0.105021	0.613313
dcbld2	149.017877	-0.134704058	0.1653476	-0.814672	0.41526	0.906237
ripor1	657.5791158	-0.063367429	0.0979642	-0.646843	0.517734	0.938592
bmt2	58.29537729	0.334149515	0.265847	1.2569242	0.208781	0.776022
slc22a7b.3	17.11714906	0.445745447	0.4676776	0.9531041	0.340537	0.878053

kif14	243.6740537	0.038838195	0.1375974	0.2822597	0.777744	0.975942
PDE3B	593.0605086	-0.150486215	0.1139889	-1.320183	0.186774	0.750951
TBC1D8B	546.4949864	0.122008281	0.1025623	1.1896021	0.234203	0.802815
fbxo31	102.5222685	0.156667041	0.1969515	0.79546	0.426346	0.909969
pcm1	1251.312407	-0.095017664	0.0864234	-1.099444	0.271575	0.83659
si:dkey-217l24.1	44.53518961	0.144385076	0.2958412	0.4880492	0.625515	0.957354
sigirr	76.89633026	-0.109979462	0.2195341	-0.500967	0.616394	0.95688
skiv2l	317.4148695	-0.099009377	0.126769	-0.781022	0.434789	0.914408
prrt1	140.3943712	0.360129446	0.1940328	1.8560235	0.06345	0.492164
mrps5	556.3193973	0.0592897	0.1082189	0.5478683	0.583782	0.952214
narfl	299.7107083	-0.02979597	0.1261558	-0.236184	0.81329	0.981081
kcnj3b	372.6282753	0.117464056	0.1330813	0.8826486	0.377426	0.895192
plekha7b	306.0512718	-0.09484358	0.1380628	-0.68696	0.492108	0.931926
arap1a	110.3452309	0.493746393	0.197153	2.5043824	0.012267	0.195612
mxra8a	230.1252832	0.231890178	0.1449999	1.5992434	0.109767	0.623766
ino80db	513.0319265	-0.081943758	0.1143923	-0.71634	0.473781	0.924993
adipoqa	5.859313351	0.594349931	0.8332962	0.7132517	0.47569	NA
znf362b	72.84106767	-0.05313384	0.2460168	-0.215976	0.829006	0.982721
lrrc34	6.524363123	-0.029159367	0.7760261	-0.037575	0.970026	NA
nfs1	467.8429935	0.053217158	0.1144122	0.4651353	0.641835	0.959994
si:ch211-15p9.2	0	NA	NA	NA	NA	NA
setd2	1435.633247	-0.023076774	0.082407	-0.280034	0.779451	0.976146
ptpn21	42.11340349	0.409355297	0.299182	1.3682486	0.171234	0.729413
fbxl7	66.56915255	0.276329345	0.2414422	1.144495	0.252418	0.821887
armc8	310.5591435	-0.05909439	0.1312715	-0.450169	0.652588	0.960708
ednrab	5.971464881	-0.159169712	0.7651623	-0.208021	0.835213	NA
arhgap17b	286.7820583	-0.088355079	0.1376627	-0.641823	0.520988	0.939056
edc4	867.6612584	0.220822284	0.088605	2.4922113	0.012695	0.200185
kdm3b	2736.688066	-0.169744491	0.0785093	-2.162094	0.030611	0.338573
jarid2b	64.61277594	0.195679945	0.2437916	0.8026526	0.422176	0.90651
fan1	210.5835628	-0.129874417	0.146023	-0.889411	0.373782	0.893677
slc25a15b	129.8800644	-0.103211948	0.1770179	-0.583059	0.559853	0.948478
afg3l2	1126.885465	0.065755931	0.0885582	0.7425164	0.457775	0.921978
ppp2r2cb	184.0885564	-0.182794024	0.1633389	-1.119109	0.263094	0.830245
si:dkey-202e17.1	15.73668277	0.352241965	0.4803319	0.7333304	0.463357	0.922701
hace1	160.6488214	-0.178299538	0.1682915	-1.059468	0.289387	0.846123
edaradd	19.0070758	0.086454967	0.4612997	0.187416	0.851334	0.985174
qpctlb	26.11992961	-0.227140317	0.3769774	-0.60253	0.546821	0.945526
soat1	35.22690594	-0.699212684	0.3380078	-2.068629	0.038581	0.385234
znf652	297.3725183	0.177953055	0.1294261	1.3749396	0.16915	0.726198
pdpk1a	213.5715126	-0.144707371	0.1458945	-0.991863	0.321264	0.868938
rims3	4.833314255	0.511670522	0.8506974	0.6014719	0.547526	NA
ccdc61	237.3567138	-0.000961225	0.1353058	-0.007104	0.994332	0.998694
si:ch211-1f22.14	0.500161739	2.450146464	2.7711133	0.8841741	0.376602	NA
wdr26a	319.184748	0.124315882	0.1242078	1.0008704	0.316889	0.867308
rasl10a	3.961605242	-1.273296815	0.9680552	-1.315314	0.188404	NA
sik2b	1250.967895	0.154452893	0.0854429	1.8076745	0.070657	0.520155
si:dkey-103g5.3	307.6102398	0.236022221	0.1364401	1.7298596	0.083655	0.557158
adam23a	1652.070416	0.045434758	0.0746115	0.608951	0.542557	0.944974
shank2b	309.7450175	0.155209528	0.124724	1.2444242	0.213343	0.781631
hmgn7	7224.084219	-0.071400626	0.0701052	-1.018478	0.308451	0.861088
rasgrp2	8.23504096	-1.23875974	0.7332036	-1.689517	0.09112	NA
b4galt3	60.64760396	-0.226253049	0.2602937	-0.869222	0.384726	0.89551
si:ch211-233a24.2	1895.918086	-0.000885921	0.082822	-0.010697	0.991465	0.997679

nudt14	59.97283017	0.389084962	0.2608468	1.4916226	0.135798	0.672208
zeb2a	2053.173618	-0.031126092	0.0722716	-0.430682	0.6667	0.964736
wfs1a	111.0461256	0.191262522	0.209482	0.913026	0.361229	0.885686
cacna1ea	70.76276545	0.020094935	0.2398359	0.0837862	0.933226	0.992702
mtus2a	102.0681994	0.157629144	0.1975831	0.7977867	0.424994	0.908819
arhgap15	5.913214255	0.60422254	0.7934203	0.7615416	0.446334	NA
synm	171.0864467	-0.001922833	0.1564973	-0.012287	0.990197	0.997679
sema4ab	358.1868825	-0.00251228	0.1182723	-0.021241	0.983053	0.996944
axdnd1	42.23809224	0.268089143	0.2988661	0.897021	0.369708	0.889476
tafa1a	194.488616	-0.058162748	0.1542715	-0.377016	0.706162	0.968459
scn3b	227.6897325	-0.069222053	0.1580857	-0.437877	0.661476	0.964234
PAXBP1	970.1001297	0.011334666	0.0911173	0.1243964	0.901001	0.990121
tpcn1	271.2378501	0.200588792	0.1332389	1.5054819	0.1322	0.666055
phex	578.5486534	0.105865741	0.1118932	0.9461321	0.344081	0.878434
bcl2l13	698.3219639	0.287702299	0.0998887	2.8802299	0.003974	0.086636
nrd1b	327.4266375	0.058578372	0.125041	0.4684735	0.639446	0.959994
leng9	114.8737625	-0.10030814	0.1902601	-0.527216	0.598044	0.955459
nlgn3b	1100.391209	0.001001824	0.0919287	0.0108978	0.991305	0.997679
CRHR2	17.08768559	-0.092017913	0.4687149	-0.19632	0.84436	0.985174
slc35a4	484.9612734	-0.13491902	0.1047392	-1.288143	0.197696	0.762928
si:ch211-238n5.4	478.6157333	0.005741973	0.1062016	0.0540667	0.956882	0.994543
snapin	480.6476312	0.081419967	0.107712	0.7559043	0.449707	0.918526
flcn	541.2485537	-0.250058234	0.1041522	-2.400892	0.016355	0.236236
cdc42ep5	240.1268904	0.014197645	0.1400626	0.1013664	0.91926	0.991623
anks1aa	49.41627217	-0.005151258	0.2734054	-0.018841	0.984968	0.996944
ndst1a	336.4602929	0.024949938	0.1262359	0.1976453	0.843323	0.985174
znf319b	205.2622365	0.053199675	0.1476265	0.3603668	0.718573	0.96909
frem1b	364.0028586	0.253912584	0.1194711	2.1253062	0.033561	0.357045
ctnnd2a	686.0199261	-0.025942901	0.1052059	-0.246592	0.805224	0.979883
ttl	64.96248616	0.145076315	0.2432329	0.5964502	0.550875	0.946549
nfia	123.7384249	-0.197416228	0.1904393	-1.036636	0.299906	0.855995
pex2	45.69531047	0.373222616	0.2863881	1.3032057	0.192505	0.756795
zgc:153901	54.30986512	-0.213485361	0.2754656	-0.774998	0.438341	0.915672
zgc:153521	220.0885802	0.021991825	0.1417074	0.1551918	0.87667	0.987881
clcn2a	21.50449436	0.255593083	0.4162189	0.6140833	0.53916	0.943733
trim109	45.19789166	0.161406187	0.2895328	0.5574712	0.577206	0.950788
gpd2	79.42191742	0.037019635	0.2345921	0.1578043	0.874611	0.987881
msrb1b	6.706896195	0.657116941	0.7578947	0.8670293	0.385926	NA
pias1b	145.2365352	-0.06982186	0.1656992	-0.421377	0.67348	0.965257
neurl2	47.99653582	0.604909374	0.2908692	2.0796609	0.037557	0.379013
hecw2a	413.8791472	0.02158008	0.1161261	0.1858331	0.852576	0.985174
skor1b	300.1817534	-0.109262965	0.1391675	-0.785118	0.432384	0.91326
xpr1a	770.1004322	0.170014276	0.0990463	1.7165125	0.086068	0.563109
rnf115	526.6870139	-0.110279458	0.113318	-0.973186	0.330461	0.874098
las1l	523.1155248	-0.102715927	0.1114201	-0.92188	0.356591	0.883422
sgk3	366.1950314	-0.007966274	0.1227007	-0.064924	0.948234	0.99381
shisa7a	99.47272188	0.115870472	0.1994576	0.580928	0.561289	0.948728
zc3h12b	13.34284771	0.235152923	0.5223165	0.4502116	0.652558	0.960708
pgap1	40.38339937	-0.08281801	0.3157567	-0.262284	0.793102	0.977937
stra6l	66.14240547	0.294155052	0.2490962	1.1808896	0.237647	0.806474
hipk3a	844.5772387	-0.031990647	0.0912242	-0.350682	0.725827	0.970402
tafa4b	80.83670686	-0.028243136	0.2275201	-0.124135	0.901209	0.990121
sin3b	833.7475751	0.061604828	0.1083249	0.5687044	0.569557	0.948983
snapc5	366.6339974	0.207954536	0.1165179	1.7847438	0.074303	0.530996

kiaa1549la	1147.094776	0.146491973	0.0980672	1.4937917	0.13523	0.670689
ackr3a	28.21807685	0.028982404	0.3930716	0.0737331	0.941223	0.992857
vcam1b	539.9476044	0.018616239	0.1049426	0.1773945	0.859199	0.985773
mlphb	87.47696291	-0.593353563	0.2176846	-2.725749	0.006416	0.122335
lyve1b	23.32611585	-0.38954319	0.4160367	-0.936319	0.349109	0.880195
dock11	252.3000721	-0.003866284	0.1410589	-0.027409	0.978134	0.996762
si:dkey-6n6.1	522.6879747	0.021557581	0.1290345	0.1670683	0.867316	0.987125
8-Mar	746.8207964	0.133567466	0.0915863	1.4583776	0.144736	0.688986
hhipl1	233.229752	-0.159608993	0.1370727	-1.164411	0.244257	0.813808
si:ch73-196l6.5	6.685284747	-0.422152443	0.736138	-0.573469	0.566327	NA
zc3h18	1226.55275	-0.051635962	0.0946449	-0.545576	0.585358	0.952585
cplx3a	21.37995931	-0.034899467	0.4275191	-0.081633	0.934939	0.992702
bcl11ba	1695.977431	-0.058024106	0.0916179	-0.633327	0.52652	0.940506
golga3	495.7079526	-0.194163344	0.1075179	-1.805869	0.070939	0.521075
tecpr1a	142.3484949	-0.007515279	0.1990488	-0.037756	0.969882	0.996315
si:ch211-107n13.1	51.58930651	0.048086868	0.2723336	0.1765734	0.859844	0.985773
ulk1a	208.09428	-0.017883642	0.1457677	-0.122686	0.902356	0.990121
abcc13	62.07509274	0.221709889	0.2463546	0.8999625	0.36814	0.88754
atp9b	46.3411669	0.348238277	0.2913115	1.1954154	0.231925	0.800084
ASTE1	34.25707887	0.227846167	0.3524981	0.6463756	0.518036	0.938592
mapk8ip3	2349.69571	0.06197481	0.0802464	0.7723065	0.439933	0.916082
washc2c	839.8022762	-0.105510793	0.0993464	-1.062049	0.288213	0.845979
si:dkey-117i10.1	28.9487939	-0.617907527	0.3692786	-1.673283	0.094272	0.583986
TMEM132C	101.2801972	-0.201137304	0.2127957	-0.945213	0.34455	0.878434
acmsd	186.0694802	-0.463384437	0.1552967	-2.983865	0.002846	0.069229
rc3h1a	20.39234352	0.588168357	0.4357852	1.3496748	0.17712	0.738119
lpar6a	520.4950361	-0.014588851	0.1125012	-0.129677	0.896822	0.989971
bach1a	102.8590447	-0.017485744	0.1944557	-0.089921	0.92835	0.992702
rspry1	639.9096324	0.126626232	0.0969157	1.3065604	0.191362	0.755886
adck1	152.7884583	0.04589124	0.1655674	0.2771756	0.781645	0.97627
egln2	40.301933	0.243195576	0.3156787	0.7703897	0.441069	0.916336
kcnh4a	39.55171349	0.1027917	0.3117706	0.329703	0.741624	0.973154
mrpl2	889.2966882	0.004994688	0.0947224	0.0527298	0.957947	0.99467
znrf3	227.8532779	0.161956612	0.1374308	1.1784591	0.238614	0.807659
si:ch211-210g13.5	38.20192741	0.230334777	0.3117685	0.7388007	0.460028	0.921978
arhgap35a	1177.349564	-0.080468706	0.0834249	-0.964565	0.334763	0.875699
kremen1	14.72278174	-0.549865671	0.5128161	-1.072247	0.283609	0.84466
brat1	211.8881369	-0.102144548	0.1563822	-0.653172	0.513645	0.936778
pleca	1676.681507	0.246831405	0.0776838	3.1773877	0.001486	0.042404
myl10	4234.090062	0.153233613	0.1086295	1.4106072	0.15836	0.709224
stox1	261.2936363	-0.018914651	0.1441592	-0.131207	0.895612	0.989814
coq7	273.6653857	-0.035220797	0.135142	-0.260621	0.794385	0.977937
ltc4s	5.270330169	-0.206427804	0.8462949	-0.243919	0.807293	NA
piwil2	22.70341272	0.049653204	0.4117852	0.1205804	0.904023	0.990291
si:dkey-26i13.8	123.663493	0.034236144	0.1910309	0.1792178	0.857767	0.985703
dhx37	387.1325765	-0.120577698	0.1136937	-1.060549	0.288895	0.845979
kcnj12b	211.5808972	0.381129078	0.1529994	2.4910491	0.012737	0.200229
satb1b	79.97695706	-0.065869808	0.2323627	-0.283478	0.77681	0.975826
dnah9l	23.05657846	-0.020726127	0.4059822	-0.051052	0.959284	0.995115
npffr1l2	10.30465418	-0.384379168	0.6020053	-0.638498	0.52315	NA
duox	22.57138423	-0.47741158	0.4108374	-1.162045	0.245217	0.815227
cadm2b	197.4858304	0.028244512	0.1480373	0.1907933	0.848688	0.985174
kat2b	24.41240544	-0.189755676	0.3909993	-0.485309	0.627457	0.957354
fem1b	290.5892078	-0.007908242	0.1297852	-0.060933	0.951412	0.99381

kcnh8	20.45551806	-0.330888816	0.4575133	-0.723233	0.469537	0.924505
tet3	3392.541335	-0.117940745	0.0807393	-1.46076	0.144081	0.68818
rif1	865.3471173	-0.109990633	0.0929408	-1.183448	0.236632	0.805854
zc3hc1	405.391716	-0.02752817	0.1192653	-0.230815	0.817459	0.981594
ydjc	103.6156123	0.084609758	0.2206472	0.3834618	0.701377	0.968134
abca4b	755.3394392	0.273458118	0.0994503	2.7496972	0.005965	0.116901
lmod3	101.3145158	0.648851511	0.2211044	2.9345931	0.00334	0.077242
oxnad1	276.2832021	-0.109284645	0.1319984	-0.827924	0.407713	0.9014
mpp3b	212.324504	0.169371147	0.144037	1.1758866	0.23964	0.808091
kcnj19b	112.6077538	0.035271217	0.1994381	0.1768529	0.859624	0.985773
btbd10a	1130.174981	-0.061345084	0.0818062	-0.749883	0.453325	0.920041
megf11	246.5598196	0.062856027	0.1614376	0.3893519	0.697016	0.96736
kcnh7	19.75536019	0.119766482	0.4364977	0.2743806	0.783792	0.97627
gpnmb	1285.037931	-0.642137713	0.0868455	-7.394026	1.42E-13	5.63E-11
nrxn3b	407.1390744	0.142748082	0.1292685	1.1042757	0.269474	0.834705
ccdc126	106.654302	0.003393585	0.1936597	0.0175234	0.986019	0.996944
dgkg	8.739866393	-0.608634811	0.6408705	-0.9497	0.342265	NA
nkain4	124.1082104	0.182631134	0.1903814	0.9592908	0.337412	0.876394
ankmy1	24.11961343	-0.130368064	0.3833603	-0.340067	0.733806	0.971993
plat	38.47914385	0.603072005	0.3352452	1.798898	0.072035	0.525083
gal3st1a	129.1875167	0.473325182	0.1772701	2.6700796	0.007583	0.138588
tmem264	40.23436849	-0.133132472	0.3017863	-0.441148	0.659106	0.962839
pcdh1a	493.7812404	0.106971244	0.1157008	0.9245509	0.3552	0.882342
dennd4a	202.7392673	0.100098712	0.1476204	0.6780818	0.49772	0.93348
cep290	527.1048758	0.012686614	0.1076257	0.1178772	0.906165	0.990702
scn1lab	152.1355428	0.006235975	0.1721023	0.0362341	0.971096	0.996315
socs5b	145.0452673	0.244758806	0.180861	1.353298	0.17596	0.736505
pkn1b	49.25554472	-0.124526235	0.283395	-0.439409	0.660365	0.963766
ino80b	301.2991406	0.003353056	0.1350491	0.0248284	0.980192	0.996879
si:ch73-74h11.1	491.4896031	0.209894874	0.103497	2.0280293	0.042557	0.405751
mfap3l	1027.722544	0.065074564	0.0899843	0.7231766	0.469571	0.924505
naif1	9.716820657	0.609795567	0.6634065	0.9191884	0.357997	NA
pllp	179.2826684	-0.522546655	0.1564738	-3.339515	0.000839	0.02694
zgc:158403	220.5268085	-0.157701619	0.1549054	-1.018051	0.308654	0.861088
CR352265.1	46.31371161	0.261262718	0.2899145	0.9011715	0.367497	0.887458
nsd3	92.44661561	-0.018699828	0.2116046	-0.088372	0.929581	0.992702
snx21	20.62507138	0.097928817	0.4225745	0.2317433	0.816737	0.981594
aifm3	9.330820821	0.10901423	0.6622963	0.1646004	0.869259	NA
glsl	24.49009777	-0.121505654	0.3833837	-0.31693	0.751297	0.97419
adpgk2	29.52862003	-0.177526686	0.3527959	-0.503199	0.614824	0.95688
irg1l	641.2084377	-0.49342672	0.2597848	-1.899367	0.057516	0.470942
CU639469.1	2598.151115	0.047073695	0.073377	0.6415323	0.521177	0.939056
trim36	600.7567787	-0.025827344	0.1006362	-0.256641	0.797456	0.978577
abcb7	698.1207532	-0.088529083	0.0938704	-0.943099	0.34563	0.878469
nell2b	3115.540821	0.082315108	0.079179	1.0396075	0.298522	0.85439
baiap2a	416.1209708	0.080333338	0.1140211	0.7045477	0.481092	0.927927
erfl3	1149.388098	0.033925708	0.0808968	0.4193701	0.674946	0.965257
ttll9	23.26735583	-0.164899096	0.4103699	-0.40183	0.687809	0.966442
CR786571.1	20.94828419	0.087958538	0.4186758	0.2100874	0.833599	0.983583
p2ry10	0.714370301	1.588951002	2.5174818	0.6311668	0.527931	NA
glsa	31.94511089	0.75012436	0.3445834	2.176902	0.029488	0.332709
crym	82.96823416	-0.690525879	0.2197876	-3.141787	0.001679	0.047099
irgf3	3.775917256	-0.382487127	1.0567128	-0.361959	0.717382	NA
slc6a15	101.5699783	-0.241096165	0.2010589	-1.199132	0.230477	0.798571

pi4kab	109.4905568	-0.182381408	0.2170405	-0.840311	0.400734	0.90084
alx1	72.46192291	-0.537689468	0.2551318	-2.107497	0.035074	0.364435
si:ch73-171o20.1	0.157187365	-0.955901296	4.0804729	-0.234262	0.814781	NA
tent5d	14.46285919	-0.043598514	0.5468598	-0.079725	0.936456	0.992702
si:ch73-22o12.1	1138.702057	-0.077024426	0.0863627	-0.891872	0.372462	0.892843
ecsit	400.4325688	0.289397841	0.1131977	2.5565697	0.010571	0.17767
spata13	932.8236422	0.04684783	0.0866398	0.5407192	0.588701	0.953319
tmtc3	1009.266739	0.090918826	0.0900022	1.0101849	0.312407	0.864401
kcnk10a	20.60258812	-0.572272145	0.4349967	-1.315578	0.188316	0.753187
sdk1b	77.01597758	0.010739479	0.2281145	0.0470793	0.96245	0.995907
plekhg7	218.6047008	-0.187322815	0.1541945	-1.214848	0.224424	0.79161
gk5	419.9896049	-0.018524963	0.1209135	-0.153208	0.878234	0.987881
mras	678.1736578	0.061192152	0.0990684	0.6176756	0.536789	0.943336
eea1	84.67955249	-0.002929195	0.2162818	-0.013543	0.989194	0.997589
mrs2	147.6247778	-0.031035304	0.1733446	-0.179038	0.857908	0.985703
slc2a11l	15.61043974	0.710405241	0.5325793	1.3338958	0.182238	0.74454
hcar1-3	10.94439365	0.182146435	0.5631852	0.3234219	0.746376	NA
cntn3a.1	389.4325015	0.054700474	0.1227106	0.4457682	0.655765	0.961833
map3k2	7.811705999	0.404265151	0.7090621	0.5701406	0.568582	NA
steap2	139.9372553	0.238079096	0.1902306	1.2515287	0.210742	0.778412
foxe3	34.76518999	-0.398478468	0.3301285	-1.207041	0.227417	0.794775
si:dkey-103i16.6	25.4425752	-0.216993373	0.3783968	-0.573455	0.566337	0.948739
tnksb	1327.61333	-0.081607589	0.0834537	-0.977878	0.328135	0.872701
si:ch211-81a5.1	42.65410902	0.490771088	0.3076225	1.5953681	0.11063	0.625514
si:dkey-220f10.4	137.0183912	-0.113378604	0.1807674	-0.627207	0.530524	0.942209
idua	56.76403922	-0.272846149	0.2548061	-1.070799	0.28426	0.844697
kcnv2b	31.90918857	0.365803962	0.3386962	1.0800356	0.280126	0.843423
furina	958.7010459	0.134480017	0.0899768	1.4946082	0.135017	0.670689
polr3b	514.2907744	-0.030138068	0.1089659	-0.276583	0.782101	0.97627
mrpl37	742.2062191	-0.104661692	0.0940724	-1.112566	0.265895	0.831945
tceanc2	109.7534931	0.016168184	0.1989969	0.0812484	0.935244	0.992702
si:ch211-243o19.4	6.893171651	-0.524638879	0.7589439	-0.691275	0.489393	NA
FAM83G	456.8103847	0.266605164	0.1095505	2.4336275	0.014948	0.223931
cmip	1967.657404	0.054926042	0.0732998	0.7493343	0.453656	0.920041
gpr12	13.52673682	0.859803948	0.532583	1.6144038	0.10644	0.615567
ndnf	1499.704697	0.017303249	0.0769207	0.2249493	0.822019	0.982186
kcna1a	15.64582505	0.383113402	0.4761226	0.8046528	0.42102	0.90651
taco1	139.1154954	-0.371780948	0.1740256	-2.136358	0.03265	0.352204
amn	56.65904527	-1.328053079	0.267792	-4.959271	7.08E-07	7.48E-05
wasf3b	819.567122	-0.084033055	0.1002956	-0.837854	0.402113	0.90084
nt5dc3	6.856417514	0.091347151	0.7206579	0.1267552	0.899134	NA
meak7	0	NA	NA	NA	NA	NA
clk2a	1551.441813	-0.066598808	0.0783275	-0.850261	0.39518	0.899244
dipk1aa	207.2945899	0.043484763	0.1549792	0.2805846	0.779029	0.976146
dagla	620.4046258	0.07890552	0.1016302	0.7763982	0.437514	0.914814
zgc:158785	164.2668563	0.076452183	0.165481	0.4619998	0.644081	0.960129
armc10	192.2716913	0.196744375	0.1563879	1.258054	0.208372	0.775404
kmt2ba	926.7493228	-0.041541012	0.085503	-0.485843	0.627079	0.957354
si:dkey-246g23.2	5.969952619	-0.003133983	0.8040674	-0.003898	0.99689	NA
fam45a	224.1976916	-0.005818907	0.1437829	-0.04047	0.967718	0.996315
kcng4a	88.68544136	0.201143219	0.2217704	0.9069886	0.364413	0.886564
sbf1	1720.156383	0.048385644	0.0832897	0.5809318	0.561286	0.948728
fam129ba	482.6409767	-0.053944913	0.1059549	-0.509131	0.61066	0.95688
sdhaf2	547.018925	0.063595192	0.1051561	0.6047696	0.545332	0.945526

hip1	895.3444034	-0.083138362	0.0854842	-0.972558	0.330773	0.87417
ttll7	603.9614004	0.072932695	0.1005146	0.7255929	0.468088	0.924328
itga2.2	21.98192721	-0.162288765	0.4114986	-0.394385	0.693297	0.96736
iffo1a	9.553064119	0.06381561	0.6185144	0.1031756	0.917824	NA
samd13	297.3213181	0.039976087	0.1236694	0.3232497	0.746506	0.97419
ccdc151	29.2510855	0.234856806	0.3615246	0.6496289	0.515932	0.93786
fbxo18	391.4126972	-0.081263947	0.112243	-0.724	0.469066	0.92448
pnpla7a	923.8415673	-0.065712794	0.0959813	-0.684642	0.49357	0.931926
tyw1	314.3987991	-0.266881175	0.1260144	-2.117862	0.034187	0.359388
abi1b	29.30184626	-0.09222736	0.3791375	-0.243256	0.807807	0.980642
si:dkey-14o18.2	8.81485339	0.586731841	0.7206492	0.8141712	0.415547	NA
hyal2b	390.5591096	0.207889152	0.1197785	1.735613	0.082632	0.554782
pglyrp2	19.9291249	0.626183664	0.4343464	1.4416688	0.149396	0.696734
atp8a1	44.79107178	0.124138709	0.3316313	0.3743275	0.708161	0.968664
alkbh5	182.5678037	-0.124385973	0.1569032	-0.792756	0.42792	0.910858
anapc7	263.5741472	-0.04915567	0.1466403	-0.335212	0.737465	0.972535
cacng7a	50.1968861	0.145713234	0.2791172	0.5220504	0.601635	0.956045
apc2	302.8539667	-0.160513368	0.1225548	-1.309728	0.190288	0.754967
si:dkeyp-27e10.3	567.4677516	0.110067434	0.1081878	1.0173742	0.308975	0.861246
catip	41.24346198	0.245086465	0.3006254	0.8152554	0.414926	0.906237
antxr2b	48.58621393	0.170744692	0.2807489	0.6081759	0.543071	0.944974
dbpa	641.7853818	0.159841991	0.1061201	1.5062372	0.132006	0.665721
fam78ba	41.13627376	-0.070031384	0.3177019	-0.220431	0.825535	0.982196
panx2	76.88563369	0.19790513	0.2328175	0.850044	0.395301	0.899293
rpain	92.79743544	-0.038292116	0.2109191	-0.181549	0.855937	0.985174
mlc1	124.4825959	0.235004716	0.1865007	1.260074	0.207643	0.775175
mesd	406.628729	0.044531421	0.1169215	0.380866	0.703303	0.968134
rad54l2	1248.00341	0.074424661	0.0939553	0.7921281	0.428286	0.911082
dok6	15.70907789	-0.064747279	0.5442641	-0.118963	0.905305	0.990702
psd2	630.0262915	0.199453153	0.100277	1.9890222	0.046699	0.424613
lipea	30.27666605	-0.042937321	0.3436773	-0.124935	0.900575	0.990121
btbd11b	33.92407341	0.005927652	0.3341429	0.0177399	0.985846	0.996944
thtpa	119.2815869	-0.041880465	0.1839658	-0.227653	0.819916	0.981594
rc3h2	40.87018625	0.078176646	0.3055647	0.2558432	0.798072	0.978793
bap1	521.1256738	0.088766034	0.1128644	0.7864838	0.431584	0.91277
shank3b	217.6573713	0.014496641	0.158969	0.0911916	0.92734	0.992702
tulp4b	766.3906325	0.089127832	0.0950178	0.9380119	0.348238	0.880195
abcg8	28.79990832	0.038504648	0.3508091	0.1097595	0.9126	0.991119
ubn2a	627.2688007	-0.179682734	0.1141907	-1.573532	0.115596	0.637206
syne1b	1372.11636	0.020324762	0.101041	0.2011537	0.840578	0.985042
gripap1	80.86804281	0.102775976	0.2212309	0.4645644	0.642243	0.959994
soga1	196.9072803	-0.156934102	0.1707273	-0.919209	0.357986	0.884398
atrip	183.6028121	-0.211841015	0.1538729	-1.376727	0.168597	0.725919
abcg5	55.20093025	-0.584414317	0.2670512	-2.188398	0.028641	0.328313
ago3b	24.91379987	-0.154732178	0.3801839	-0.406993	0.684013	0.965679
si:dkey-211g8.6	1.621341276	-0.610147117	1.5935892	-0.382876	0.701812	NA
ccm2l	83.04167144	-0.349063302	0.2401315	-1.453634	0.146048	0.69189
ctsf	3498.844252	0.113943985	0.0718163	1.5866023	0.112603	0.628452
scube1	69.76598671	-0.097468383	0.235927	-0.413129	0.679512	0.965257
psmd14	1455.547511	-0.093076841	0.0815299	-1.141628	0.253609	0.823394
man2a2	810.8459065	0.083951529	0.0919234	0.9132766	0.361097	0.885686
CABZ01059392.1	1.11484644	-0.41260322	1.8539026	-0.222559	0.823879	NA
si:ch73-160i9.1	0	NA	NA	NA	NA	NA
gusb	289.4400416	-0.189068596	0.1399598	-1.350878	0.176735	0.737285

si:ch211-38m6.7	9.793403288	-0.745472884	0.6174453	-1.207351	0.227297	NA
slc4a10a	3013.438204	0.048344312	0.0721055	0.6704662	0.502561	0.935304
exd3	200.1735986	0.020414251	0.1627076	0.1254659	0.900155	0.990121
shisa7b	1474.27488	0.054264789	0.0770812	0.7039951	0.481436	0.928386
tfam	412.1181373	-0.189656735	0.1306329	-1.45183	0.146549	0.692967
tmtc1	468.6420158	0.065197094	0.1126432	0.5787932	0.562729	0.948728
nrxn2b	1025.030047	0.017933525	0.0932163	0.1923861	0.84744	0.985174
bcl11ab	256.4043568	-0.193740182	0.134889	-1.436293	0.150919	0.698912
dcaf12	681.0708989	0.01714805	0.1040233	0.1648482	0.869063	0.987245
mapk8ip2	35.87429688	-0.142022977	0.3349679	-0.42399	0.671573	0.965257
CABZ01081780.1	13.83827897	0.20440074	0.5426985	0.3766377	0.706443	0.968459
si:dkey-32e23.4	54.60187014	-0.136777287	0.267456	-0.511401	0.60907	0.956718
ppwd1	301.6126941	0.045317308	0.1327041	0.3414913	0.732734	0.971719
cmtm8b	8.794289994	-0.589428108	0.6530308	-0.902604	0.366736	NA
chkb	396.8751305	0.132931671	0.1118842	1.188118	0.234787	0.803509
ubn1	56.44936607	0.121865487	0.2653827	0.4592065	0.646086	0.96029
manf	543.5820348	0.173936783	0.106497	1.6332556	0.102415	0.608235
dock3	47.8929571	-0.196970194	0.3044827	-0.646901	0.517696	0.938592
rc3h1b	626.0292918	0.121640835	0.0980767	1.2402627	0.214878	0.782493
si:ch211-220f12.4	27.57881602	-0.076984712	0.3666434	-0.209972	0.83369	0.983583
agpat5	435.2997107	0.077257794	0.1224403	0.6309835	0.528051	0.941711
zranb1b	949.9905774	-0.028719068	0.0956828	-0.300149	0.764064	0.975374
extl2	65.94139578	-0.136376387	0.2455037	-0.555496	0.578555	0.951052
lin54	469.1433876	-0.082466288	0.1112863	-0.741028	0.458676	0.921978
pxk	459.35518	-0.054554002	0.1088917	-0.500993	0.616376	0.95688
golga2	537.9500984	-0.015217052	0.1056461	-0.144038	0.88547	0.988936
erbb4a	12.43686772	0.052712527	0.568389	0.0927402	0.92611	0.992702
exoc8	218.7777359	0.315675248	0.1582081	1.9953172	0.046008	0.421189
prmt2	38.49695954	-0.691672952	0.315106	-2.195049	0.02816	0.325071
mtif2	260.436969	0.075860393	0.1332643	0.5692479	0.569188	0.948983
lrrc3b	2.492228153	-1.4304165	1.2737087	-1.123033	0.261424	NA
ppm1la	936.1074404	-0.031873296	0.0875073	-0.364236	0.715682	0.96909
ubap2l	1982.998729	0.028641201	0.0961715	0.2978137	0.765845	0.975374
arl14	128.2980968	0.004228863	0.1906906	0.0221766	0.982307	0.996944
nxph2a	147.0123777	-0.003851888	0.1678256	-0.022952	0.981689	0.996944
xpo1b	4971.767542	-0.186221855	0.0784746	-2.373022	0.017643	0.246556
bmp7b	95.59182239	0.043934623	0.2059031	0.2133753	0.831034	0.982994
baz1a	325.6494497	-0.254103063	0.1236139	-2.055619	0.039819	0.391511
ttc13	360.2237881	0.092977805	0.1232077	0.7546427	0.450463	0.918761
lix1l	197.5523418	0.311862076	0.159056	1.9607065	0.049913	0.437835
mrpl9	405.8798874	0.035681826	0.1185283	0.3010404	0.763384	0.975374
spata2	372.7256123	-0.059187347	0.1151881	-0.513832	0.607369	0.956718
prcc	1071.300821	0.048504466	0.0932744	0.520019	0.60305	0.956045
hecw2b	207.9569416	-0.08934254	0.1458129	-0.61272	0.540061	0.944206
r3hdm4	598.3536965	0.048613222	0.1043716	0.4657706	0.64138	0.959994
btbd11a	376.0445013	0.150161792	0.1193523	1.2581389	0.208342	0.775404
si:ch211-194e18.2	13.05849369	0.404107283	0.5278013	0.7656429	0.443889	0.916631
rfx5	249.123206	0.043252807	0.1347275	0.3210392	0.748181	0.97419
pcdh9	82.3833646	0.450228885	0.2306538	1.9519679	0.050942	0.442781
SLC35G1	57.72228409	0.432721839	0.2568425	1.6847749	0.092032	0.57849
retreg2	146.4824788	-0.302610725	0.1741922	-1.737224	0.082348	0.553931
msh3	142.6905456	0.303326912	0.1743205	1.7400535	0.08185	0.552725
abi3b	90.37081615	0.122437385	0.2075648	0.5898755	0.555274	0.947449
ube2t	104.3547418	-0.218971932	0.1919688	-1.140664	0.25401	0.823394

slc2a11b	215.7294576	0.067240834	0.1500852	0.4480176	0.65414	0.961305
ssuh2rs1	1997.825265	0.008048129	0.0753094	0.1068676	0.914894	0.991119
neto2b	27.15787223	0.375369691	0.3838581	0.9778866	0.32813	0.872701
myh9a	2601.523282	0.20967538	0.0701013	2.9910334	0.00278	0.06821
abcb6a	114.2697385	0.192347588	0.185367	1.0376584	0.299429	0.855609
pcloa	1669.199982	0.136051746	0.0761138	1.7874787	0.07386	0.529749
CU655845.1	53.66762287	0.124494314	0.2839168	0.4384886	0.661032	0.964099
sgsm2	41.76714415	-0.102770271	0.3115554	-0.329862	0.741504	0.973154
tjp2a	855.7205975	-0.086298538	0.0897894	-0.961122	0.336491	0.876088
oxr1b	917.9676965	0.163116803	0.0875241	1.8636785	0.062367	0.487498
soga3a	0	NA	NA	NA	NA	NA
plbd1	28.89392385	0.035507544	0.3556264	0.0998451	0.920467	0.99206
apba2a	321.9472461	0.042366159	0.1221148	0.3469371	0.728639	0.970631
FP085394.1	27.94856195	0.138210984	0.3866458	0.3574615	0.720746	0.969395
rabl2	254.8665993	0.075384619	0.1348407	0.5590645	0.576118	0.950611
mgat4a	886.6367556	-0.050130028	0.0933641	-0.53693	0.591316	0.954193
shank3a	33.09961325	0.379319475	0.3626457	1.0459782	0.295571	0.852234
nup210	1167.219254	-0.04032178	0.0857623	-0.470157	0.638243	0.959231
czib	407.2204015	-0.048932593	0.1150074	-0.425473	0.670492	0.965257
fam162a	338.3949072	-0.047007974	0.1221656	-0.384789	0.700394	0.967998
hrc	10.62539425	0.004436727	0.5970158	0.0074315	0.994071	NA
inpp4aa	1083.873957	-0.055199909	0.0836622	-0.659795	0.509385	0.936123
abtb1	280.7318269	0.127541344	0.1301031	0.9803099	0.326933	0.872658
GPR45	15.71244239	-0.045139889	0.4982411	-0.090598	0.927812	0.992702
micu1	1134.864775	-0.206732245	0.0977343	-2.115248	0.034409	0.360397
si:ch211-1e14.1	27.13908904	0.216521003	0.385016	0.5623689	0.573865	0.949988
sgk2a	81.77800914	0.073808063	0.2284951	0.3230181	0.746682	0.97419
rasa3	534.9205449	0.052966172	0.1198391	0.4419774	0.658506	0.962545
zpld1b	22.66901054	-0.244043796	0.4079679	-0.598194	0.549711	0.946312
pter	125.2582162	-0.185328446	0.1870708	-0.990686	0.321839	0.869463
cenpe	1033.407345	-0.05738376	0.0866739	-0.662065	0.507929	0.935734
sec22c	22.86304285	0.250933143	0.4025602	0.6233432	0.533059	0.942969
pvrl2l	66.56257121	0.059800798	0.2630487	0.2273373	0.820161	0.981594
LO018340.1	389.7835903	0.052254344	0.1212738	0.430879	0.666556	0.964736
lrrc73	240.3426684	-0.044225895	0.1433155	-0.308591	0.757632	0.975374
cers1	172.9045487	-0.04253382	0.1666749	-0.25519	0.798576	0.978884
rxylt1	81.18455043	-0.206122687	0.2204325	-0.935083	0.349746	0.880195
ptprk	1214.563157	-0.068030005	0.0909099	-0.748323	0.454265	0.920541
erf	619.5210662	0.020969634	0.1057562	0.1982829	0.842824	0.985174
DHX35	234.1337237	-0.148962174	0.1410897	-1.055798	0.291061	0.846568
atp2b2	1404.889082	0.095397255	0.0931871	1.0237173	0.305969	0.860027
trpm4b.2	80.67083034	-0.251158876	0.2320721	-1.082245	0.279144	0.842806
rph3aa	31.23564681	0.080011891	0.3917089	0.2042637	0.838147	0.984797
wnt9a	2.514423416	-0.557624855	1.2516174	-0.445523	0.655942	NA
srebf2	1585.316863	-0.097144703	0.0901778	-1.077257	0.281365	0.84357
si:dkey-32n7.7	1.804420379	-0.844592863	1.4389097	-0.586967	0.557226	NA
snap47	183.415839	0.149707274	0.1788501	0.8370543	0.402562	0.90084
iba57	167.1904895	-0.195400692	0.1668758	-1.170935	0.241625	0.810499
ccdc13	6.470450933	0.379577446	0.7818535	0.4854841	0.627333	NA
wash1	84.64742616	-0.147338957	0.211666	-0.696092	0.486371	0.930833
isy1	456.9787139	0.026543302	0.1086329	0.2443394	0.806968	0.980279
arfgef1	1021.820347	-0.112468201	0.0888054	-1.266456	0.20535	0.773635
abcg1	101.4411171	0.128609192	0.2066679	0.622299	0.533745	0.943276
si:ch211-214j24.15	21.01696122	-1.271556361	0.4745739	-2.679364	0.007376	0.135568

lrrc58b	376.0722632	0.261092952	0.12512	2.0867409	0.036912	0.376038
si:ch211-15d5.11	308.9317735	0.059395799	0.1233896	0.4813679	0.630255	0.957354
zgc:153913	406.7661447	0.341238776	0.1476984	2.310375	0.020867	0.27277
PLEKHB1	244.7664527	0.269978069	0.1486714	1.8159385	0.06938	0.514708
bbs4	196.0825932	0.275358142	0.1872519	1.4705227	0.14142	0.68201
adpgk	400.3264793	0.294704688	0.1200936	2.4539586	0.014129	0.214731
elmo2	1396.522027	0.063410276	0.0793091	0.7995335	0.423981	0.908025
chd4a	3604.463414	0.009867249	0.0828437	0.1191068	0.905191	0.990702
kalrnb	1089.560276	0.041673672	0.0925416	0.4503236	0.652477	0.960708
slc25a15a	100.8905036	-0.171362835	0.1999312	-0.857109	0.391385	0.897688
pip4k2ab	14.30298387	0.191076288	0.5261103	0.3631867	0.716465	0.96909
rlf	481.0726299	-0.221394506	0.1061667	-2.085348	0.037038	0.376547
si:ch211-166g5.4	18.34764839	-0.178813309	0.4913294	-0.363938	0.715905	0.96909
pigx	119.3775342	-0.009546521	0.2059248	-0.046359	0.963024	0.995927
creb3l2	473.0468069	0.165162303	0.1070878	1.5423068	0.122999	0.65095
syt7a	43.64128729	0.364398733	0.295508	1.2331264	0.217529	0.784786
dyrk1aa	1128.235421	-0.005085291	0.0921374	-0.055192	0.955985	0.994543
perp	3567.657838	0.052803529	0.0715747	0.7377405	0.460672	0.921978
vps41	591.7173361	-0.08741657	0.1014723	-0.861482	0.388973	0.896295
mtg2	171.1039561	-0.088846209	0.1622626	-0.547546	0.584004	0.952214
klhl35	8.469643568	0.391010538	0.7373176	0.5303149	0.595894	NA
DGKI	358.2745278	0.108503825	0.1269358	0.854793	0.392666	0.897688
ppp1r9ala	299.978821	0.005518719	0.1317127	0.0418997	0.966579	0.996315
map2k4a	40.67993243	0.225658752	0.3080671	0.7324987	0.463864	0.922875
uba5	420.1758116	0.02850171	0.1127282	0.2528357	0.800395	0.979377
hipk1a	70.14752558	-0.170985895	0.2353335	-0.726569	0.46749	0.924328
bud23	235.9735584	-0.03981546	0.1374809	-0.289607	0.772117	0.975687
coro2bb	60.32656473	-0.009440199	0.2601242	-0.036291	0.97105	0.996315
antxr1c	552.702651	0.114097019	0.1018996	1.1197009	0.262841	0.830166
si:ch211-133n4.10	2.237463168	2.473652019	1.6243684	1.5228393	0.127799	NA
skor2	236.0430323	-0.115459757	0.1487397	-0.776254	0.437599	0.914896
aadac	72.44904257	0.040204889	0.275338	0.1460202	0.883905	0.988706
gfm1	806.3076546	0.085297178	0.1064889	0.8009959	0.423134	0.907185
ddx21	1538.179379	-0.265783215	0.0924492	-2.874911	0.004041	0.087626
bop1	589.1105288	-0.239070088	0.1052552	-2.271338	0.023127	0.291648
VIT	1193.050536	0.124145105	0.0986257	1.2587495	0.208121	0.775404
hpse	195.2311269	-0.183826589	0.1554793	-1.182322	0.237078	0.80591
nrxn1b	311.1925045	0.04108027	0.1424527	0.2883783	0.773057	0.975687
galnt11	780.8602808	0.078648388	0.0928068	0.8474424	0.396749	0.899672
lnpa	106.3246664	0.051387008	0.1992317	0.2579259	0.796464	0.97852
tead3b	46.01744752	-0.192304117	0.2804983	-0.68558	0.492978	0.931926
nuak2	267.076245	-0.263661607	0.1396113	-1.888541	0.058953	0.475038
clp1	182.8664059	0.159851276	0.1594475	1.0025325	0.316086	0.866931
mat2al	242.3258718	-0.528564522	0.1397447	-3.782357	0.000155	0.006997
gtf2a1l	16.04815392	0.871280869	0.4895117	1.7798981	0.075093	0.533746
zmp:0000000711	454.6377607	0.070851631	0.1105763	0.6407486	0.521686	0.939056
ccny	75.7474762	-0.113097967	0.2342055	-0.482901	0.629166	0.957354
fhod3a	587.7802303	0.181325258	0.1039445	1.7444429	0.081082	0.551495
ppm1h	362.244988	-0.044023033	0.1191711	-0.36941	0.711822	0.969006
atp23	169.2541319	0.217309655	0.1747806	1.243328	0.213747	0.78213
nrm	119.6012583	-0.032355659	0.2028888	-0.159475	0.873295	0.987846
rreb1a	536.3198499	-0.27067384	0.1024937	-2.640882	0.008269	0.147176
pde8b	14.30701698	0.205699504	0.5110876	0.4024741	0.687335	0.966376
trim3a	348.2615528	-0.090596226	0.1200357	-0.754744	0.450403	0.918761

syngap1a	339.4715064	0.152030983	0.1737151	0.8751741	0.381479	0.895192
vps72a	559.4251573	0.056690586	0.1038078	0.5461113	0.584989	0.952436
usp8	1370.72376	-0.155352476	0.0941602	-1.649875	0.098969	0.598346
cdk12	990.7603804	0.026732053	0.0996261	0.2683239	0.78845	0.976703
osbpl6	11.62654491	-0.482616522	0.5634408	-0.856552	0.391692	0.897688
rbm45	17.1866806	-0.404607028	0.4675796	-0.865322	0.386862	0.89551
pde11a	18.13430866	0.634709893	0.4493525	1.4124988	0.157803	0.709035
cox19	96.96363217	0.128428599	0.2076979	0.6183433	0.536349	0.943336
mt-nd1	33221.42279	0.093155491	0.0723143	1.2882026	0.197675	0.762928
mt-nd2	22548.7548	0.024613477	0.1019747	0.2413684	0.80927	0.980642
mt-co1	256080.8098	0.002460716	0.066161	0.0371929	0.970331	0.996315
mt-co2	68688.27516	-0.029315883	0.0646942	-0.453146	0.650444	0.960708
mt-atp8	3700.989045	0.005765716	0.3480236	0.016567	0.986782	0.997062
mt-atp6	43698.99309	-0.091730027	0.0910063	-1.007953	0.313477	0.865132
mt-co3	76946.86712	-0.026262516	0.0713983	-0.367831	0.712999	0.96909
mt-nd3	4292.730446	0.181300037	0.0940786	1.9271125	0.053966	0.45506
mt-nd4l	8138.164985	0.168322103	0.0861474	1.9538855	0.050715	0.44139
mt-nd4	22302.67422	0.01919201	0.0797367	0.2406922	0.809794	0.980648
mt-nd5	19288.20675	0.037052307	0.0837392	0.4424728	0.658147	0.962545
mt-nd6	4058.217009	-0.090863539	0.2078337	-0.437193	0.661971	0.964543
mt-cyb	62928.38624	0.049119227	0.0922794	0.5322879	0.594527	0.954434
kctd8	496.1055271	-0.140037946	0.114301	-1.225168	0.220512	0.787502
slc24a4b	8.858614761	0.196456089	0.649829	0.3023197	0.762408	NA
ccdc15	16.35744632	0.267146513	0.4783729	0.5584483	0.576538	0.950611
zgc:158482	175.4547186	-0.058411141	0.1619938	-0.360576	0.718416	0.96909
fut9b	84.2749206	-0.034396057	0.2482946	-0.138529	0.889822	0.989291
pcsk5a	101.2429675	-0.162537102	0.2015729	-0.806344	0.420044	0.90651
tcf15	34.75399781	0.56922885	0.331879	1.7151698	0.086314	0.563258
adam19b	335.1696613	0.171943727	0.1253284	1.3719453	0.17008	0.727199
si:ch211-193c2.2	5.651352308	-1.053804975	0.833442	-1.264401	0.206086	NA
adamts12	267.4133802	0.481081583	0.1354113	3.5527443	0.000381	0.014224
gtf2h3	156.6938977	-0.152480828	0.1631713	-0.934483	0.350055	0.880195
kiss1rb	3.750735178	0.343820837	1.0779658	0.3189534	0.749762	NA
zgc:153044	26.06386693	0.049962308	0.3993007	0.1251245	0.900425	0.990121
sftpbb	66.338965	0.064185804	0.2366455	0.2712319	0.786213	0.976471
SLC6A13	25.0586808	0.577001353	0.3897445	1.4804605	0.13875	0.677277
puraa	117.3926947	0.289392217	0.1857887	1.5576419	0.119318	0.643589
BX901974.1	1.038053482	0.018230632	1.9186971	0.0095016	0.992419	NA
sco1	294.1675737	0.028611805	0.1338755	0.2137195	0.830766	0.982994
fancb	29.08138829	-0.277986523	0.3571175	-0.778417	0.436323	0.914408
arfgap2	1090.704837	0.07873126	0.0955996	0.8235519	0.410194	0.902747
napaa	481.3662828	-0.014544574	0.1111873	-0.130811	0.895924	0.989866
rsph4a	53.53959762	0.047288518	0.2747777	0.1720974	0.863361	0.985841
srebf1	79.36207206	-0.111293031	0.2457974	-0.452784	0.650705	0.960708
zswim7	22.40375149	-0.340616928	0.4220173	-0.807116	0.4196	0.90651
dnajc25	123.3154313	0.293886707	0.1829917	1.6060114	0.108271	0.620153
ywhag1	777.2952417	0.095933457	0.1041662	0.9209656	0.357068	0.883467
zgc:154075	260.2919548	-0.440765639	0.1344015	-3.27947	0.00104	0.031984
prpf4	458.130057	-0.153536348	0.1141939	-1.344523	0.178779	0.739486
tmem232	7.005556698	0.293822161	0.7464943	0.3936027	0.693874	NA
kcmf1	423.3822939	-0.048582259	0.1256725	-0.386578	0.699068	0.967998
BX470189.1	160.8804296	0.085629499	0.1591671	0.537985	0.590587	0.954193
ftr80	0.316103113	0.005883447	3.3519121	0.0017553	0.9986	NA
si:dkey-172m14.2	36.08510355	0.361397836	0.3541347	1.0205095	0.307487	0.861088

fam167aa	6.65593274	-0.745853789	0.7517668	-0.992135	0.321132	NA
cep57	39.18962761	0.09495955	0.3269118	0.2904746	0.771453	0.975687
zglp1	2.284665674	0.821641665	1.3203789	0.6222772	0.53376	NA
pomt1	287.0931751	-0.049065854	0.1312778	-0.373756	0.708586	0.968664
card9	18.1839342	-0.414888261	0.4562239	-0.909396	0.363141	0.885944
snapc4	137.9484854	-0.094581548	0.1981437	-0.477338	0.633121	0.957356
plcl1	56.67714015	0.220530718	0.2735042	0.8063157	0.420061	0.90651
myoz3a	106.2449689	-0.002713663	0.2300919	-0.011794	0.99059	0.997679
shisal1b	86.24510159	0.006480293	0.2208381	0.0293441	0.97659	0.996762
parvg	22.88373724	0.219508166	0.4015315	0.5466774	0.5846	0.952411
snx18a	534.7341864	0.018012679	0.103413	0.174182	0.861722	0.985841
hspb3	25.55259461	-0.453475536	0.3930866	-1.153628	0.248653	0.81797
tomm7	498.7987604	0.012124121	0.1091142	0.1111141	0.911526	0.991066
si:dkey-3h3.3	25.85815716	0.01761434	0.4057381	0.0434131	0.965372	0.996257
wwtr1	39.69624638	0.013628585	0.3190848	0.0427115	0.965932	0.996315
tank	78.12494054	-0.199861844	0.2280624	-0.876347	0.380841	0.895192
arid3c	79.2787653	0.193607599	0.2238228	0.865004	0.387037	0.895735
trpc6b	11.78362296	-0.44183588	0.5560761	-0.79456	0.426869	0.910366
itpkcb	574.2783937	0.044297211	0.1116789	0.3966479	0.691627	0.967305
si:rp71-68n21.9	64.76952282	-0.265165583	0.2436717	-1.088208	0.276503	0.839541
pigl	106.1135237	0.077689697	0.1902712	0.4083103	0.683046	0.965476
zgc:158640	39.69617606	-0.344199562	0.3021749	-1.139074	0.254672	0.823484
urp2	34.05515525	-0.055562612	0.3291172	-0.168823	0.865936	0.986517
CABZ01088428.1	16.39454598	-0.290672461	0.4917476	-0.591101	0.554453	0.947449
cabz01076234.2	15.8095329	-0.077184751	0.4922883	-0.156788	0.875412	0.987881
ifngr2	490.5488858	0.249406124	0.1044452	2.3879133	0.016944	0.240499
clpxb	590.610273	0.018974764	0.1017695	0.1864485	0.852093	0.985174
spi1a	37.8108859	0.388872598	0.3167796	1.2275808	0.219604	0.786189
CABZ01064472.1	123.04374	-0.064320013	0.1827993	-0.351861	0.724942	0.970402
ggcx	559.936623	0.168430191	0.100642	1.6735584	0.094217	0.583986
zgc:158432	11.57078072	-0.671887338	0.5691217	-1.180569	0.237774	0.806474
si:dkeyp-27c8.1	0.31759079	1.807926197	3.579045	0.5051421	0.613459	NA
zgc:171844	47.84203799	-0.004881931	0.3024068	-0.016144	0.98712	0.997136
zmp:0000001127	1.106743675	0.427540854	3.8920321	0.1098503	0.912528	NA
CABZ01060891.1	7.049077015	0.440847734	0.7281322	0.6054501	0.54488	NA
CABZ01083448.1	306.7641962	0.210562917	0.1283743	1.6402268	0.100958	0.604004
cntnap3	491.9955425	0.015017194	0.119922	0.1252247	0.900346	0.990121
si:ch211-229c8.13	1.550520356	-1.831174474	1.7752598	-1.031497	0.302308	NA
ppargc1a	124.2658678	0.101729168	0.1901687	0.5349416	0.59269	0.954434
tbata	19.98229064	0.555122251	0.4474928	1.2405166	0.214784	0.782493
tle2c	190.2655716	-0.067865556	0.1724834	-0.393461	0.693979	0.96736
riox1	355.1815432	-0.182879095	0.1247918	-1.465474	0.142792	0.684998
il21	0	NA	NA	NA	NA	NA
nmrk2	877.1008228	0.772314776	0.0944471	8.1772243	2.90E-16	1.60E-13
jund	549.2016151	-0.014943088	0.1011856	-0.14768	0.882595	0.988415
lcn15	123.095363	0.363476652	0.2058888	1.7654025	0.077496	0.542062
scospondin	47.42979725	0.392047371	0.2910933	1.3468099	0.178041	0.738995
CU469531.1	0.870416506	-1.929882401	2.2178997	-0.87014	0.384224	NA
gltpd2	27.51938283	-0.403182397	0.3697027	-1.090558	0.275467	0.839541
MAN1C1	19.50674084	-0.275706823	0.4876921	-0.56533	0.57185	0.949494
ints9	202.44705	0.035466218	0.1441594	0.2460208	0.805666	0.979893
lyn	45.92590196	0.373319855	0.2852234	1.3088681	0.190579	0.755212
elp5	201.3409926	-0.042348603	0.1515811	-0.279379	0.779954	0.976146
rab8a	79.63212194	0.028873297	0.2296817	0.1257101	0.899961	0.990121

sstr1b	62.63498067	0.107962245	0.2578339	0.4187279	0.675415	0.965257
EVA1A	1.803389528	-0.31515553	1.5317887	-0.205743	0.836991	NA
zcchc2	265.2784314	-0.158958364	0.1332814	-1.192652	0.233006	0.80134
sh3gl1a	235.6188295	0.088187639	0.1389691	0.6345847	0.525699	0.940145
slc6a5	767.5643371	0.267277509	0.1199484	2.2282707	0.025862	0.310878
fem1a	108.6012166	-0.179314912	0.1894871	-0.946317	0.343987	0.878434
atp5if1a	1243.44126	-0.166005895	0.090352	-1.837323	0.066162	0.502802
ar	46.95040886	-0.042914956	0.290313	-0.147823	0.882482	0.988344
nitr7b	0.158915748	0.967652056	4.0804729	0.2371421	0.812547	NA
gtf3c5	148.7225048	0.032594107	0.1816785	0.1794054	0.857619	0.985703
zgc:103697	273.9131944	-0.162554138	0.1327207	-1.224784	0.220657	0.787641
gas1b	261.6738305	-0.019539878	0.1396547	-0.139916	0.888727	0.989291
ftr23	40.72806375	-0.181153388	0.3050236	-0.5939	0.552579	0.947265
myhz1.1	175097.9459	0.409291906	0.0892684	4.5849609	4.54E-06	0.000369
si:dkey-31e10.1	10.75052383	0.596216055	0.6055969	0.9845097	0.324865	NA
myhz1.2	64889.44047	0.438056053	0.08948	4.8955771	9.80E-07	9.89E-05
csk	915.0916117	0.074791571	0.0905681	0.8258045	0.408915	0.90171
myhz1.3	16991.919	0.682894619	0.08871	7.6980583	1.38E-14	6.66E-12
CABZ01084793.1	222.8789157	0.006659484	0.145217	0.0458588	0.963423	0.995927
gck	154.0038036	1.233136352	0.185156	6.6599846	2.74E-11	7.13E-09
si:ch211-230g14.3	8.67979177	-0.377936211	0.6601057	-0.572539	0.566957	NA
ece2a	57.08917572	0.275503817	0.2742121	1.0047106	0.315036	0.866054
si:cabz01007802.1	211.9304549	0.099972532	0.1511946	0.6612178	0.508473	0.93575
tmem119b	238.2935879	0.092345152	0.1382001	0.6681988	0.504007	0.93532
agbl4	33.48012953	-0.05746331	0.3304684	-0.173884	0.861956	0.985841
sirt1	626.7783671	0.059111355	0.0989593	0.5973302	0.550287	0.946549
BX664721.2	0	NA	NA	NA	NA	NA
wbp4	141.3784382	0.058201213	0.1728109	0.3367913	0.736274	0.972535
CT573494.1	0.158915748	0.967652056	4.0804729	0.2371421	0.812547	NA
psmb11b	0.499972362	-2.44370697	3.0013188	-0.814211	0.415524	NA
tcnba	826.2375199	0.097128089	0.0903377	1.0751667	0.2823	0.843666
atf5a	1142.173322	-0.190150861	0.0910158	-2.089208	0.036689	0.374386
si:ch211-198o12.4	1.344727791	0.097265354	1.731498	0.0561741	0.955203	NA
tafa5l	178.3118176	0.106619467	0.1548815	0.6883939	0.491205	0.931926
cfap45	66.53801539	-0.02222937	0.2485426	-0.089439	0.928733	0.992702
CABZ01040999.1	1.305159095	-0.702694412	1.6729937	-0.420022	0.674469	NA
CABZ01079818.1	4.519419941	-0.099591983	0.9320512	-0.106852	0.914906	NA
f11r.2	96.90156562	0.494564076	0.2082019	2.3754062	0.01753	0.24549
CFAP77	30.5005775	-0.327919576	0.348466	-0.941037	0.346686	0.8794
gkap1	575.0854926	-0.114684856	0.1083403	-1.058562	0.289799	0.846246
opn7d	7.672242239	0.283556518	0.718644	0.3945716	0.693159	NA
nfu1	408.0012952	0.007991297	0.1136676	0.0703041	0.943952	0.993364
nppc	77.91549846	0.05355134	0.2287964	0.2340567	0.814941	0.981081
si:ch211-194e1.7	4.811775238	0.485549871	0.8636247	0.5622232	0.573964	NA
ppp1r15b	106.4482676	-0.396348368	0.1919196	-2.065179	0.038906	0.386986
spaca9	0	NA	NA	NA	NA	NA
ch25hl1.2	7.776421401	-0.54968565	0.7161515	-0.767555	0.442752	NA
si:dkey-24l11.2	130.8960488	-0.256660918	0.1766035	-1.453318	0.146136	0.691942
tnfsf11	9.141540013	0.479070665	0.6201419	0.7725178	0.439808	NA
slc39a6	574.5441706	0.0852894	0.1064887	0.8009244	0.423175	0.907185
nmba	11.01926073	0.386319141	0.6948666	0.5559616	0.578237	NA
pimr180	0.50625413	-0.940106488	2.8357787	-0.331516	0.740255	NA
arrdc1a	198.0822568	0.083366426	0.1576457	0.5288213	0.596929	0.954913
tprg1l	23.05070627	-0.146297306	0.4608463	-0.317454	0.750899	0.97419

malt3	65.27916933	0.117482258	0.2608808	0.4503293	0.652473	0.960708
chchd7	173.1774636	0.194124103	0.1597655	1.2150561	0.224345	0.79161
ehmt1a	367.0581167	-0.051453031	0.1200027	-0.428766	0.668094	0.964736
si:dkey-16p6.4	0.678375468	-2.878459328	2.4926399	-1.154783	0.248179	NA
whrnb	92.6380882	0.296084404	0.2081447	1.422493	0.154883	0.704624
hes2.2	58.40233711	0.059063874	0.2630476	0.2245369	0.82234	0.982189
ing5b	55.2149824	-0.063379336	0.2642357	-0.239859	0.810439	0.980868
uqcc1	892.9515758	-0.005370928	0.1184069	-0.04536	0.96382	0.995927
pak2a	1124.131758	0.104560248	0.0944734	1.1067693	0.268394	0.833634
trib2	641.1534036	0.269715455	0.110677	2.4369606	0.014811	0.222891
dpep1	124.66743	-1.036786726	0.1939778	-5.344872	9.05E-08	1.28E-05
crb3b	79.59146722	0.005387538	0.2498667	0.0215616	0.982798	0.996944
spg7	950.0992347	0.014303463	0.0899998	0.1589277	0.873726	0.987881
pimr188	0.97357257	1.035850083	1.9870616	0.5212974	0.60216	NA
cdh15	66.56064879	0.281056968	0.2532591	1.1097606	0.267102	0.833191
taf4a	191.2670699	0.004140901	0.1611906	0.0256895	0.979505	0.996866
tmem44	12.21256804	-0.123922742	0.6310578	-0.196373	0.844318	0.985174
klf9	239.2624013	-0.08757923	0.1910348	-0.458447	0.646632	0.960362
pld3	86.68552983	0.07008255	0.2259219	0.3102069	0.756404	0.975374
def8	496.7384843	0.124177811	0.115479	1.0753279	0.282228	0.843666
fzd10	391.5105376	0.189965943	0.1217793	1.5599195	0.118779	0.642608
ccni	5663.507645	-0.09408099	0.0717424	-1.311372	0.189732	0.754269
stx2b	120.5536557	0.360326548	0.1930899	1.8661077	0.062026	0.486324
b3gnt2l	106.8084019	0.064215624	0.2191797	0.2929816	0.769536	0.975687
mbl2	88.59542267	-1.690467372	0.2514234	-6.723587	1.77E-11	4.87E-09
MBL2 (1 of many)	18.82378599	0.262570938	0.4422727	0.5936856	0.552722	0.947265
tmem150aa	46.46710588	-0.236348009	0.285089	-0.829032	0.407086	0.901305
CRACR2A	44.89180861	-0.191727406	0.2876312	-0.666574	0.505044	0.935724
cbln12	52.01155963	0.020001417	0.2979523	0.0671296	0.946479	0.99381
zgc:64051	21.19556495	0.013311006	0.4287142	0.0310487	0.975231	0.996762
trim110	38.0185529	-0.074745456	0.3310206	-0.225803	0.821355	0.981845
cngb1a	202.8265356	-0.03535211	0.1463232	-0.241603	0.809088	0.980642
plcb3	559.7523114	-0.179973632	0.1015928	-1.77152	0.076474	0.538876
luzp2	650.7433976	0.013144798	0.1005165	0.1307726	0.895955	0.989866
si:ch211-151h10.2	3.474601019	-0.401616604	0.9912138	-0.405177	0.685348	NA
nanos3	4.363195296	0.197165335	0.9752159	0.2021761	0.839779	NA
rwdd	316.0115614	0.039088636	0.120744	0.3237316	0.746141	0.97419
kcne4	104.1908115	-0.251945357	0.2150868	-1.171366	0.241452	0.810468
lrch4	354.8950367	0.049655269	0.1265439	0.3923956	0.694766	0.96736
pros1	251.9993385	-0.036490777	0.151412	-0.241003	0.809553	0.980642
vamp5	356.7835709	-0.053749392	0.126609	-0.424531	0.671179	0.965257
csf1b	38.25800781	0.678652677	0.3192766	2.1255946	0.033537	0.357045
grhpra	52.8463285	-0.173892194	0.2681365	-0.648521	0.516648	0.938543
tomm5	595.7809142	0.003880236	0.1423958	0.0272497	0.978261	0.996762
si:ch211-203b8.6	80.67793039	0.124786834	0.2242346	0.5565013	0.577868	0.951024
ptx3a	551.415947	0.413214407	0.1198387	3.4480871	0.000565	0.019382
si:ch211-240b21.2	3.306524912	0.317669912	1.0901531	0.2913993	0.770746	NA
GRB14	20.45225604	-0.445159486	0.4307195	-1.033525	0.301358	0.856944
cyp2x10.2	1.803430813	-0.264503508	1.4521142	-0.182151	0.855465	NA
slc7a4	15.6717024	0.327516183	0.4971165	0.6588318	0.510004	0.936253
lamc2	112.747283	0.196787481	0.2304	0.8541123	0.393043	0.897878
eif3k	1020.753546	-0.129307775	0.0930704	-1.389355	0.164725	0.720954
cyp2x12	23.9837081	-0.495553174	0.4005058	-1.237318	0.215969	0.783484
TMEM208	302.4146952	-0.070580185	0.1245129	-0.56685	0.570816	0.949072

SLCO3A1 (1 of many)	368.9578056	0.174507485	0.1180164	1.4786716	0.139228	0.677963
zgc:171509	2.79202997	-1.229213255	1.2269429	-1.00185	0.316416	NA
mrps14	285.4070272	-0.037590106	0.1385993	-0.271214	0.786226	0.976471
astn1	1520.632051	0.002697931	0.08054	0.033498	0.973277	0.996315
si:dkey-21n10.3	0	NA	NA	NA	NA	NA
mmab	132.1916463	-0.157501965	0.175941	-0.895198	0.370681	0.890626
si:ch211-132b12.3	0	NA	NA	NA	NA	NA
lmx1bb	283.3827831	-0.047657932	0.125591	-0.379469	0.704339	0.968134
si:dkey-154p10.3	95.99792365	-0.134832103	0.2107609	-0.63974	0.522342	0.939056
nfkbie	180.2388652	-0.394085261	0.1772041	-2.223906	0.026155	0.311744
angptl2b	370.0422338	0.131083056	0.1286254	1.0191074	0.308152	0.861088
ralgps1	18.36193044	-0.071617245	0.4366543	-0.164014	0.86972	0.987304
zgc:158427	0.474778155	0.852397662	2.898154	0.2941175	0.768668	NA
si:ch211-132b12.7	581.3149793	0.500559878	0.112031	4.4680469	7.89E-06	0.000589
foxg1c	22.89862652	-0.007506095	0.4050212	-0.018533	0.985214	0.996944
pglyrp5	36.5286113	-0.203807914	0.3276679	-0.621995	0.533945	0.943276
slc6a18	19.07635738	0.281677396	0.4527284	0.6221774	0.533825	0.943276
tns2b	413.3194402	0.2479224	0.1209006	2.0506298	0.040303	0.392394
cep126	30.45209591	-0.131483724	0.3485583	-0.377222	0.706009	0.968412
znf131	426.9979248	0.008901015	0.118397	0.0751794	0.940072	0.992857
yap1	1755.83752	0.099228219	0.0795084	1.248022	0.212023	0.77986
tbcb	1081.532652	-0.078053688	0.0834033	-0.935858	0.349346	0.880195
six5	77.67367774	-0.039350455	0.2286321	-0.172113	0.863349	0.985841
six9	17.49558058	-0.484658794	0.5015042	-0.96641	0.333839	0.87516
vgll4l	220.4378826	-0.155712671	0.1545534	-1.007501	0.313694	0.865132
ptgesl	665.8086555	0.152426129	0.1035134	1.4725257	0.140879	0.681252
foxl2b	41.23486665	-0.02437944	0.3121045	-0.078113	0.937738	0.99279
ttc9b	25.47680327	0.182988733	0.3891237	0.4702586	0.63817	0.959196
si:ch211-153j24.3	17.53020071	0.484095681	0.4562941	1.060929	0.288722	0.845979
si:ch211-195h23.3	22.5874849	0.008194397	0.4270379	0.0191889	0.98469	0.996944
si:dkey-56m19.5	3210.36761	0.084234363	0.0725548	1.1609754	0.245652	0.815431
h3f3b.1	3023.064544	-0.114411221	0.0719828	-1.589425	0.111964	0.627242
si:ch1073-429i10.3	6022.000909	-0.147090954	0.0867077	-1.6964	0.08981	0.573799
gucd1	332.4474417	-0.092968185	0.1294118	-0.71839	0.472517	0.924838
CABZ01112732.1	9.359685756	0.213015616	0.6286886	0.3388253	0.734741	NA
tmem91	18.53991097	0.365322359	0.453596	0.8053914	0.420594	0.90651
tnnt3b	40912.08163	0.262920995	0.0861464	3.0520267	0.002273	0.058601
otog	149.4792558	0.236344658	0.1697281	1.3924896	0.163774	0.71945
aopep	132.2352867	0.073345298	0.1748947	0.4193683	0.674947	0.965257
zgc:136254	4.091282548	0.103892687	0.9769415	0.1063448	0.915309	NA
dnah3	17.20156976	0.152249858	0.4669832	0.3260285	0.744403	0.974094
si:dkey-94e7.2	136.0124801	0.140883101	0.195747	0.7197203	0.471697	0.924838
gpx4a	1810.390997	-0.286887315	0.2702954	-1.061384	0.288515	0.845979
map7d2a	439.2176646	0.090587069	0.1125736	0.804692	0.420997	0.90651
neto1	102.1917249	0.279232188	0.2006646	1.3915369	0.164063	0.71945
CABZ01100185.1	178.9114519	0.021689506	0.1594156	0.1360563	0.891777	0.989291
cyp2y3	162.7731669	-1.847497451	0.2135669	-8.650675	5.12E-18	3.37E-15
gbgt1l4	297.7763757	0.10988115	0.1299621	0.8454864	0.397839	0.899672
crybb1	19586.08882	-0.163828002	0.0989148	-1.656253	0.097671	0.596644
chs1	265.1338413	-1.072471308	0.1375599	-7.796395	6.37E-15	3.23E-12
hapln1b	649.8506218	0.118027318	0.1073931	1.0990216	0.271759	0.836643
v2rh14	3.85389	0.92204345	0.9952918	0.9264051	0.354235	NA
BX530037.1	0.817563152	-0.628689195	2.3745149	-0.264765	0.79119	NA
si:dkey-47k20.4	1.641260976	0.568773532	1.4684935	0.3873177	0.698521	NA

elovl8a	42.38144169	0.232916815	0.317025	0.7346953	0.462525	0.922698
htr5ab	50.29242766	-0.159849977	0.300081	-0.532689	0.594249	0.954434
rbm33a	1370.557968	-0.172625163	0.0819884	-2.105482	0.035249	0.365357
olfcq19	0.157187365	-0.955901296	4.0804729	-0.234262	0.814781	NA
shha	488.9013685	-0.060252978	0.1093971	-0.550773	0.581789	0.95187
slc16a1b	367.3097505	-0.00073159	0.1187104	-0.006163	0.995083	0.998694
tmem51a	109.0074621	0.033199201	0.2159882	0.1537084	0.87784	0.987881
olfcd3	1.837037515	-0.248740973	1.4062673	-0.17688	0.859602	NA
glis1a	14.44678672	-0.345028233	0.505675	-0.682312	0.495042	0.931926
rnf44	52.2401838	0.202007581	0.2732249	0.7393454	0.459697	0.921978
CU302253.1	177.9024685	-1.295990743	0.1824743	-7.10232	1.23E-12	4.18E-10
rabggtb	564.9558405	0.112958098	0.1104053	1.0231222	0.30625	0.860027
vimr1	818.6664903	0.097618888	0.1358682	0.7184824	0.47246	0.924838
naa80	32.76390463	-0.46726841	0.3653457	-1.278976	0.200906	0.766855
CU326366.2	1.003989955	-2.202606988	1.9969604	-1.10298	0.270036	NA
cnot11	364.291307	-0.141173044	0.1314537	-1.073938	0.282851	0.844367
atmin	54.69840719	-0.203230259	0.2582087	-0.787078	0.431236	0.912495
dalrd3	941.3251439	0.001915208	0.0888625	0.0215525	0.982805	0.996944
si:ch211-181d7.3	0	NA	NA	NA	NA	NA
sptbn4a	389.566182	0.18000349	0.1224716	1.4697564	0.141628	0.682394
rab8b	487.0480394	0.039121823	0.1046116	0.3739722	0.708425	0.968664
cd151l	552.6494641	0.042218916	0.1011809	0.4172615	0.676487	0.965257
si:ch211-281l24.3	447.0618513	-0.160676846	0.1318086	-1.219017	0.222838	0.789115
lpar5b	10.43476761	-0.998008309	0.5902064	-1.690948	0.090847	NA
rsf1b.1	767.3485622	-0.102403071	0.0956375	-1.070742	0.284285	0.844697
taf10	622.8323713	-0.048249513	0.1028152	-0.469284	0.638867	0.959659
fam214b	732.0208116	0.18531146	0.0929094	1.9945401	0.046093	0.42177
rhoga	3.605215567	0.513695185	0.9867095	0.5206144	0.602635	NA
dnlz	123.8906665	0.137901928	0.1808143	0.7626716	0.445659	0.917154
irgq2	39.85943527	-0.349037417	0.3254505	-1.072475	0.283507	0.84466
or104-2	0	NA	NA	NA	NA	NA
or105-1	0	NA	NA	NA	NA	NA
or106-1	0.481032184	-2.398473301	2.80721	-0.854398	0.392885	NA
zgc:152951	30.6529373	0.052663745	0.3597289	0.1463984	0.883607	0.988706
iqcg	5.118800562	1.096225225	0.8675081	1.2636484	0.206356	NA
ctrl	420.5818188	-0.193208803	0.2776476	-0.695878	0.486505	0.930833
crfb1	70.29661129	-0.223115835	0.2382631	-0.936426	0.349054	0.880195
chmp2ba	15.58914046	-0.254199856	0.5158157	-0.492811	0.622146	0.957354
kctd4	331.010858	-0.036885042	0.126286	-0.292076	0.770229	0.975687
psenen	501.0245717	-0.090092327	0.1209007	-0.745176	0.456165	0.921215
gpr85	775.6474638	-0.014124032	0.0986553	-0.143166	0.886159	0.988936
lysmd4	128.6185691	0.233333853	0.1808539	1.2901789	0.196989	0.761944
or108-1	0.333314908	-1.858695402	3.5126701	-0.52914	0.596708	NA
tmem168a	276.3685845	-0.051733226	0.1342318	-0.385402	0.69994	0.967998
ifrd1	2220.177551	0.2316788	0.0770276	3.0077388	0.002632	0.065465
fam174b	215.7396991	-0.094795038	0.1453719	-0.652086	0.514345	0.937216
nid1a	1974.897275	0.326952805	0.0838696	3.898345	9.69E-05	0.004764
crfb4	47.98709896	0.110636055	0.3157032	0.3504432	0.726006	0.970402
si:dkey-14o1.20	1.005048545	-0.006083169	1.9358873	-0.003142	0.997493	NA
ksr1a	53.97604986	-0.009866802	0.263909	-0.037387	0.970176	0.996315
cuedc1a	611.3141235	-0.000862045	0.1016188	-0.008483	0.993232	0.998554
gmnc	8.400895965	-0.756050537	0.6581355	-1.148776	0.250648	NA
masp1	92.72956078	0.210973074	0.2128266	0.9912909	0.321544	0.869201
eif2ak2	6.813700786	-0.47427364	0.7470528	-0.63486	0.52552	NA

rxfp2l	19.26506409	0.406179958	0.4279711	0.9490827	0.342579	0.878434
spry4	810.2541264	0.138830366	0.1122743	1.2365282	0.216262	0.783589
cox5b2	114.0116383	0.055938724	0.2035059	0.2748752	0.783412	0.97627
CR384085.1	1.451497761	0.290766955	1.6521281	0.1759954	0.860298	NA
map4l	1762.825564	0.118419777	0.0754689	1.5691195	0.11662	0.639148
si:ch211-250k18.5	0.959146752	0.977549673	2.0106641	0.4861825	0.626838	NA
exosc8	183.9374648	0.00561904	0.1567061	0.0358572	0.971396	0.996315
si:ch211-244o22.2	473.8621398	0.019502249	0.1060641	0.1838723	0.854114	0.985174
tnks1bp1	159.016009	-0.442057484	0.1674756	-2.639533	0.008302	0.14763
mespab	0.50625413	-0.940106488	2.8357787	-0.331516	0.740255	NA
plcg2	53.53417855	-0.131946618	0.269001	-0.490506	0.623776	0.957354
sparta	40.35794341	-0.252473877	0.3042764	-0.829752	0.406679	0.901305
vsir	80.56013392	-0.066727623	0.21876	-0.305027	0.760346	0.975374
slc6a17	61.13332595	0.092936257	0.250068	0.371644	0.710158	0.968928
BX324132.1	1.802691997	-1.439918492	1.4686844	-0.980414	0.326882	NA
BX548044.1	1.13982768	0.359745903	1.9566936	0.183854	0.854128	NA
tlr7	1.297015045	-0.037963565	1.8363812	-0.020673	0.983506	NA
spata2l	80.61748787	-0.102584528	0.2251514	-0.455625	0.64866	0.960708
pimr149	0	NA	NA	NA	NA	NA
BX548044.2	0.816144499	1.890298486	2.2559176	0.8379289	0.402071	NA
h2afva	2078.988396	-0.110428843	0.0815584	-1.353985	0.175741	0.736411
purba	2013.478522	0.130340274	0.0728105	1.7901292	0.073433	0.528705
bloc1s3	17.56027049	0.38391878	0.4531591	0.8472052	0.396881	0.899672
zgc:172139	18.07100473	-0.240121421	0.4506662	-0.532814	0.594162	0.954434
dclre1b	452.0377689	0.090028323	0.1115945	0.8067453	0.419813	0.90651
zgc:66024	10.66119886	-0.311732468	0.5877	-0.530428	0.595815	NA
zgc:66024	0.964851341	-0.019994509	2.0736065	-0.009642	0.992307	NA
fam83ha	96.99383435	-0.016200469	0.2257074	-0.071776	0.94278	0.993364
rnf183	496.8693224	-0.238005065	0.1099004	-2.165643	0.030339	0.336652
si:ch211-163l21.10	19.27136604	-0.05887469	0.4443016	-0.132511	0.89458	0.989716
f2rl1.1	39.87240303	-0.292214751	0.3175901	-0.9201	0.35752	0.884033
gbp4	24.77668333	-0.704554365	0.3879223	-1.816226	0.069336	0.514708
il2rga	10.06600831	-0.939811214	0.6320024	-1.487037	0.137005	NA
si:dkey-98f17.5	45.72665655	0.326027313	0.2953077	1.1040258	0.269582	0.834705
si:dkey-61p9.7	7.22984606	-0.60965712	0.7296858	-0.835506	0.403433	NA
nap1l4b	1204.906509	-0.015563016	0.0839805	-0.185317	0.85298	0.985174
si:ch211-250m6.4	0.847902465	0.655335913	2.102784	0.3116516	0.755305	NA
fance	80.91714745	0.14450182	0.2342258	0.6169338	0.537278	0.943336
si:ch211-250m6.7	9.219677085	-0.52430988	0.6810677	-0.769835	0.441398	NA
zgc:153031	199.5578477	-0.211196116	0.1454816	-1.451703	0.146584	0.692967
tspan13a	185.8204349	-0.417526201	0.1688783	-2.47235	0.013423	0.207707
mrpl14	322.1738056	-0.105835732	0.1442731	-0.733579	0.463205	0.922698
klhl41a	50.69350088	0.197494908	0.2801936	0.7048516	0.480903	0.92776
sostdc1a	183.3924216	0.26343937	0.1770883	1.4876159	0.136852	0.673303
mettl5	305.2335233	-0.098275657	0.1261683	-0.778925	0.436024	0.914408
nrip1b	741.2551642	-0.096718569	0.0963484	-1.003842	0.315455	0.866773
nos1	53.80622349	-0.227428359	0.2698568	-0.842774	0.399355	0.900683
zgc:153012	230.8272016	-0.224444378	0.1657783	-1.353883	0.175774	0.736411
si:ch73-42k18.1	2.087198782	-0.685701579	1.3572948	-0.505197	0.61342	NA
ppp1r35	30.69836166	-0.601218627	0.3546284	-1.695348	0.090009	0.574248
map2k2b	183.6563977	0.117792399	0.1616602	0.7286421	0.466221	0.923908
rad51c	30.25203736	0.249916584	0.3929921	0.6359329	0.52482	0.939846
umodl1	19.52656778	0.103574165	0.4328382	0.2392907	0.81088	0.981081
si:ch211-137a8.2	86.97822277	-0.234933877	0.2157877	-1.088727	0.276274	0.839541

cyp1b1	48.47360781	0.242698582	0.2774252	0.8748254	0.381669	0.895192
si:ch211-218m3.11	0	NA	NA	NA	NA	NA
xaf1	7.438488335	0.199835099	0.7110263	0.2810516	0.778671	NA
atp5meb	2789.672405	0.031245621	0.1095799	0.28514	0.775537	0.975687
zgc:113983	21.86409952	0.360801714	0.4734839	0.7620147	0.446051	0.917154
BX897692.1	0.81902083	-3.15978573	2.3136362	-1.365723	0.172026	NA
si:ch211-264e16.1	77.48166753	-0.003861917	0.2354339	-0.016403	0.986913	0.997073
si:ch211-219a15.4	14.97056978	0.592428117	0.5060022	1.1708015	0.241679	0.810541
frmd7	6.351420399	-0.141520162	0.7438248	-0.19026	0.849105	NA
nrip1a	545.6781919	-0.009488063	0.1120397	-0.084685	0.932512	0.992702
si:ch211-261n11.7	8.249328175	0.448658168	0.7080373	0.6336646	0.5263	NA
zgc:153372	93.43610875	0.203606937	0.2162279	0.9416313	0.346381	0.879249
zgc:153759	3.010900983	-0.316816742	1.0769331	-0.294184	0.768617	NA
pdlim4	238.7107413	0.248029491	0.1390005	1.7843786	0.074362	0.531227
lim2.1	785.1027588	0.026512848	0.0980325	0.2704495	0.786814	0.976596
bsx	56.53327945	-0.151969947	0.256071	-0.593468	0.552868	0.947265
glo1	959.664558	0.005590664	0.0969683	0.0576545	0.954024	0.994271
glceb	759.4349476	0.215282059	0.0929655	2.3157205	0.020574	0.270609
jhy	18.63252281	0.129075988	0.4461081	0.2893379	0.772323	0.975687
btbd9	463.3393145	-0.136243238	0.1147919	-1.186871	0.235278	0.804375
gabra1	91.12417471	0.013781001	0.2255155	0.0611089	0.951272	0.99381
zgc:158862	21.3441267	0.016299031	0.4206661	0.0387458	0.969093	0.996315
hspa8	47677.67138	-0.144269867	0.0809696	-1.781778	0.074785	0.532583
zgc:153631	172.3590876	-0.224848105	0.1599615	-1.405639	0.159831	0.712089
pimr129	0.663441495	-1.490281006	2.4117839	-0.617916	0.53663	NA
h2afx1	10285.78486	-0.127540053	0.0813471	-1.56785	0.116916	0.639958
hist2h2l	2103.830846	-0.081324046	0.1005689	-0.80864	0.418722	0.906406
pcyox1l	552.430994	0.047939091	0.1058422	0.4529297	0.650599	0.960708
tle3b	1382.182094	0.018607517	0.087417	0.2128592	0.831437	0.983245
si:ch211-147a11.3	172.3118812	0.120133893	0.1556171	0.7719837	0.440124	0.916107
eps8b	5.595396212	0.676061857	0.829405	0.8151167	0.415006	NA
ifnphi2	0	NA	NA	NA	NA	NA
prdx4	1075.465624	-0.133357972	0.0871087	-1.530938	0.125785	0.654803
nphp4	159.2305895	-0.056424029	0.1638607	-0.344341	0.73059	0.97149
elnb	467.6620824	-0.07951771	0.1442169	-0.551376	0.581376	0.951731
cyp7a1	224.5440523	-0.71587595	0.1738793	-4.117086	3.84E-05	0.00218
adcy1a	8.666410843	0.104194586	0.6425795	0.1621505	0.871187	NA
admb	210.699942	0.178590662	0.1500591	1.1901352	0.233993	0.802633
rxfp3.3a1	13.12506079	0.107312634	0.5234068	0.2050272	0.837551	0.984665
acss2l	30.72478126	-0.324761658	0.3442589	-0.943365	0.345494	0.878434
skor1a	256.545871	0.01172529	0.1398618	0.0838348	0.933188	0.992702
plac8l1	23.27101669	-0.074156415	0.3977024	-0.186462	0.852082	0.985174
pimr51	0	NA	NA	NA	NA	NA
akip1	27.34428626	-0.009069716	0.36069	-0.025145	0.979939	0.996879
si:ch211-242e8.1	140.722817	0.128733683	0.1716576	0.7499444	0.453288	0.920041
agpat4	153.9914386	-0.216514452	0.1631638	-1.326976	0.184517	0.747376
chtf8	99.17112207	-0.377133569	0.2000387	-1.885303	0.059389	0.476605
wu:fk65c09	3.666637559	-1.469815592	1.1273042	-1.303832	0.192291	NA
serpinf1	855.4707317	0.225478879	0.1074442	2.0985663	0.035855	0.369078
ranbp3b	1174.451401	0.045197392	0.084908	0.5323105	0.594511	0.954434
lgals9l4	0.984220606	-1.041956579	2.232304	-0.466763	0.64067	NA
btg3	554.1480819	-0.224649785	0.1057636	-2.124075	0.033664	0.357045
nufip2	1062.870709	0.198928663	0.083719	2.376148	0.017494	0.24517
cry3a	6954.986209	0.035628495	0.0663248	0.5371823	0.591142	0.954193

mrm3b	109.2683847	0.033726765	0.2048998	0.1646013	0.869258	0.987274
ints2	355.9033889	-0.008872238	0.1159028	-0.076549	0.938982	0.992857
agrp	64.77679783	0.079932895	0.2698876	0.2961711	0.767099	0.975687
atp6v0d1	3255.621356	0.039488155	0.0793339	0.4977461	0.618663	0.957354
col2a1a	79168.19202	0.13179626	0.0875824	1.5048256	0.132369	0.6667
gls2a	45.04648794	1.032988161	0.2990583	3.4541368	0.000552	0.019206
tmco1	681.8876866	0.115770075	0.1008891	1.1474982	0.251176	0.820469
aldh9a1a.1	5130.11041	-0.314867769	0.0859011	-3.66547	0.000247	0.010149
napbb	118.4865269	-0.372158888	0.185781	-2.003213	0.045154	0.417766
zgc:112962	233.3328569	0.023370972	0.1405024	0.1663386	0.86789	0.987245
si:dkeyp-82a1.4	0.832927207	1.89113355	2.1260675	0.8894983	0.373735	NA
fgfr4	1800.888164	0.099442666	0.0761483	1.3059079	0.191584	0.755886
fam89b	103.8615115	0.480648475	0.2096359	2.292778	0.021861	0.280334
znrd2	284.658379	-0.004889899	0.1297357	-0.037691	0.969934	0.996315
ikzf2	46.83820408	-0.046481539	0.3008122	-0.15452	0.8772	0.987881
dbn1	227.1687431	-0.163997947	0.1460091	-1.123204	0.261351	0.829527
unc93b1	45.70383669	-0.259868727	0.3106756	-0.836463	0.402894	0.90084
timm10	470.2958481	0.138339813	0.1069079	1.2940097	0.195662	0.761077
kcnh5b	43.87737067	0.231524921	0.2882101	0.8033198	0.42179	0.90651
ppp2r5eb	1167.827281	0.041294052	0.0850101	0.4857548	0.627141	0.957354
si:ch211-216l23.2	518.8720192	-0.064179684	0.1070322	-0.59963	0.548753	0.946312
fhdc3	231.3007591	0.058857691	0.1354907	0.4344041	0.663995	0.964736
mstnb	55.18038215	0.025189097	0.2836265	0.0888108	0.929232	0.992702
vwc2l	3.59056033	-0.256432997	1.0172173	-0.252093	0.800969	NA
ppp1r15a	2763.71524	-0.281893299	0.0862637	-3.267809	0.001084	0.032921
grik1a	254.024749	0.139314226	0.1581737	0.8807675	0.378444	0.895192
aars	3929.327937	-0.09763932	0.0696616	-1.401623	0.161028	0.714313
map3k7cl	22.89328525	0.033679199	0.4040087	0.0833625	0.933563	0.992702
pex11b	133.2205172	0.294053673	0.1754958	1.6755593	0.093825	0.582691
rit1	192.1754531	0.119567241	0.1479859	0.8079636	0.419112	0.906406
tmem135	409.4087618	0.207205142	0.1157585	1.7899782	0.073457	0.528705
tafa5a	254.6531691	0.065830411	0.1309331	0.5027789	0.61512	0.95688
si:dkey-73n10.1	187.2103034	-0.248059048	0.163351	-1.518565	0.128872	0.659982
cerk	102.2578469	0.176054984	0.2051598	0.8581358	0.390817	0.897643
gramd4a	24.4849413	-0.138277855	0.4069854	-0.339761	0.734036	0.971993
vps4b	1360.402298	-0.042381617	0.0809257	-0.52371	0.60048	0.956045
ubtd2	468.2697662	0.006721664	0.1066128	0.0630474	0.949729	0.99381
celsr1a	710.1514235	-0.146907763	0.1067109	-1.376689	0.168608	0.725919
cyp27a1.2	1.155163996	-0.396716192	1.7478367	-0.226976	0.820443	NA
si:dkey-242h9.3	40.16740971	0.065030796	0.3436021	0.1892619	0.849888	0.985174
zgc:172339	13.7916916	0.240416246	0.506196	0.474947	0.634825	0.9578
zgc:163030	414.1657162	0.124572543	0.1358504	0.9169831	0.359152	0.884751
mycbpap	21.44865211	0.340655202	0.4087037	0.8335017	0.404562	0.901305
ebf1b	102.302726	-0.188965115	0.1966867	-0.960742	0.336682	0.876088
zgc:172120	41.28519345	0.184958952	0.3134162	0.5901385	0.555098	0.947449
taar15	0	NA	NA	NA	NA	NA
stx7l	78.39743961	-0.307167741	0.2252382	-1.363746	0.172647	0.731348
taar14i	0	NA	NA	NA	NA	NA
taar14l	0.831198824	0.593716313	2.0278563	0.2927803	0.76969	NA
taar10	0.647689638	-2.824814845	2.5064236	-1.12703	0.25973	NA
c6ast1	2.504646853	-0.171240561	1.2078337	-0.141775	0.887258	NA
zgc:153738	21.13059119	0.171614402	0.4119925	0.4165474	0.677009	0.965257
ttc32	151.7267132	0.221863892	0.175386	1.2650034	0.20587	0.773784
pdia5	860.9206344	-0.082709645	0.0899618	-0.919387	0.357893	0.884398

si:ch211-244b2.3	13.88772606	-0.768264243	0.5276698	-1.455956	0.145405	0.690663
matn3a	1606.120267	0.072360268	0.1238496	0.5842591	0.559046	0.948478
rxfp3.3a3	7.911757483	0.869204811	0.6849794	1.2689503	0.204459	NA
aftpha	611.9477397	0.056997052	0.1033334	0.5515839	0.581233	0.951731
uacaa	191.6638543	-0.190059422	0.1577635	-1.204711	0.228315	0.795541
zpax1	7.933963037	0.263161145	0.6829414	0.3853349	0.699989	NA
mixl1	8.481785945	1.272756241	0.6659725	1.9111243	0.055989	NA
chrm4a	219.0516272	-0.233117064	0.1563869	-1.490643	0.136055	0.672589
rec8b	1.54286122	-0.921930898	1.6254026	-0.567202	0.570577	NA
metap2a	89.73492973	0.359571929	0.2186233	1.6447101	0.10003	0.601211
erich3	18.17941964	0.223294969	0.4702072	0.4748863	0.634868	0.9578
si:ch211-196h16.5	110.4923331	-0.268428321	0.2157047	-1.244425	0.213343	0.781631
matn3b	145.2965306	0.293043863	0.2231129	1.3134334	0.189037	0.753954
esrra	350.4636877	0.276981695	0.1232253	2.2477664	0.024591	0.302262
wdr35	316.5167852	-0.053808102	0.1214524	-0.443039	0.657738	0.962538
si:dkey-182i3.11	0.31598276	0.005883445	3.3523626	0.001755	0.9986	NA
kbtbd4	202.1206599	-0.009195063	0.1445755	-0.0636	0.949288	0.99381
ighmbp2	127.1284709	-0.178497126	0.1774457	-1.005926	0.314451	0.865529
pimr132	0.182570949	0.967652056	4.0804729	0.2371421	0.812547	NA
trmt5	162.6147237	0.051432932	0.1649873	0.3117388	0.755239	0.975318
elovl7a	630.9284284	-0.029531686	0.0977591	-0.302086	0.762586	0.975374
tsen15	141.2507068	-0.146319627	0.1819836	-0.804026	0.421382	0.90651
pimr109	0.681873518	0.003772382	2.2906421	0.0016469	0.998686	NA
bbc3	306.4119471	-0.499310522	0.1306446	-3.8219	0.000132	0.006147
ercc8	211.8470594	-0.150525565	0.1426029	-1.055557	0.29117	0.846582
ndufaf2	185.8773911	0.136961398	0.1547303	0.885162	0.376069	0.894658
gabpa	562.5130799	-0.084608796	0.1097163	-0.77116	0.440612	0.916336
bida	154.3912881	-0.093582362	0.1632281	-0.573322	0.566426	0.948739
acot16	74.06145895	0.111419365	0.2365703	0.4709779	0.637657	0.958872
ahsg2	12.54833337	1.20496748	0.5515193	2.1848146	0.028902	0.329785
insl5b	2.310327993	-0.396093506	1.2810772	-0.309188	0.757179	NA
bcl6ab	336.5428493	0.086580832	0.1208857	0.7162204	0.473855	0.924993
moxd1l	110.4899034	0.208243373	0.1973912	1.0549778	0.291435	0.847104
upf3a	798.3763493	0.129497584	0.0983416	1.3168133	0.187901	0.752442
osbpl9	848.471458	0.079060622	0.0934709	0.8458318	0.397647	0.899672
tmem177	235.3484631	0.154280169	0.1435259	1.0749289	0.282407	0.843666
snx9b	742.8582809	0.057320315	0.0993265	0.5770896	0.563879	0.948739
si:ch211-203k16.3	45.71635686	0.441968804	0.2945333	1.5005734	0.133466	0.667982
pomcb	13.84119086	0.491157895	0.5422829	0.9057227	0.365083	0.886714
il15ra	1.822370991	-0.282870896	1.4492794	-0.19518	0.845252	NA
oxa1l	650.7562399	-0.011778736	0.1017171	-0.115799	0.907812	0.990702
tmem199	84.09614275	-0.275359352	0.2272865	-1.211507	0.225701	0.793245
efr3bb	33.14079413	0.705417158	0.3414635	2.0658643	0.038841	0.386536
fam169b	7.128558941	-0.080601784	0.7910343	-0.101894	0.918841	NA
si:ch73-122g19.1	2.825381424	-1.708118189	1.2313843	-1.387153	0.165395	NA
vps53	599.4839381	0.107360918	0.0973313	1.1030465	0.270007	0.834705
mus81	142.0142574	-0.173053366	0.1744753	-0.99185	0.321271	0.868938
nkx3.3	86.70509342	0.042393448	0.2225424	0.190496	0.84892	0.985174
si:dkey-12e7.1	146.3406243	-0.046000096	0.1706162	-0.269611	0.787459	0.976596
drgx	112.8391193	-0.041814604	0.2106242	-0.198527	0.842633	0.985174
trip6	269.759244	0.051627205	0.1305432	0.3954797	0.692489	0.96736
tmem72	15.86246548	-0.12050743	0.4785467	-0.25182	0.801181	0.979377
FP102052.1	57.93287848	0.0530801	0.2622593	0.2023955	0.839608	0.984979
zgc:114045	350.7033982	0.038504236	0.1212669	0.3175165	0.750852	0.97419

bcl6b	222.4055643	0.115897821	0.1442842	0.8032609	0.421824	0.90651
tbc1d24	997.908775	0.086023469	0.0983097	0.8750256	0.38156	0.895192
zgc:153115	780.2996739	0.162305492	0.1020322	1.5907277	0.111671	0.627242
adgrl2a	1172.214482	-0.017342271	0.08733	-0.198583	0.842589	0.985174
ppp3r1b	2629.51893	0.073188221	0.0797628	0.9175739	0.358842	0.884602
spa17	418.6840294	0.157136232	0.1083775	1.4498974	0.147087	0.693916
CR847571.1	0.657349104	1.487939685	2.418178	0.6153144	0.538347	NA
zgc:153142	0	NA	NA	NA	NA	NA
nrn1la	184.856708	0.004939965	0.1617658	0.0305378	0.975638	0.996762
crcp	81.00652941	-0.041399377	0.2227098	-0.185889	0.852532	0.985174
sh2d4ba	8.26543575	-0.366286805	0.6682937	-0.548093	0.583628	NA
zgc:162608	546.42305	0.177336734	0.140484	1.2623267	0.206831	0.774452
tnfsf12	90.19222126	0.381980002	0.2122485	1.7996832	0.071911	0.524516
si:dkey-242g16.2	93.66306763	0.154959523	0.2181269	0.7104098	0.47745	0.926857
pard6a	488.9589273	-0.032366927	0.1039563	-0.311351	0.755534	0.975318
zgc:158868	14.87222931	-0.210125897	0.5035291	-0.417306	0.676454	0.965257
si:ch211-125e6.5	6.452957147	0.519574703	0.7458149	0.6966536	0.48602	NA
mxtx1	0.489712128	-0.887840729	2.693685	-0.329601	0.741702	NA
cnga4	28.27720203	0.104178269	0.3634099	0.2866688	0.774366	0.975687
mtg1	84.72340198	0.102036965	0.2241325	0.4552528	0.648927	0.960708
tmem88b	45.86055389	0.169770532	0.2872198	0.5910822	0.554465	0.947449
itga2.1	28.87201722	-0.275888013	0.358883	-0.768741	0.442047	0.916336
zswim8	1527.310326	0.050775172	0.0931933	0.5448369	0.585866	0.952941
si:dkeyp-114g9.1	3.808785141	-0.91111685	0.9985922	-0.912401	0.361557	NA
lrrc4.1	1138.037536	0.059814443	0.0912427	0.655553	0.512112	0.936253
pln	82.38014358	-0.031452081	0.2541863	-0.123736	0.901524	0.990121
zgc:194990	94.53557636	0.285249424	0.2165455	1.3172722	0.187747	0.752195
gkup	157.1075584	-0.158841104	0.1626179	-0.976775	0.32868	0.872945
cx34.5	2.618169958	1.567522295	1.2369838	1.2672132	0.205079	NA
col17a1a	6464.610833	0.4282877	0.0858836	4.9868402	6.14E-07	6.63E-05
trim23	279.7743872	0.201989907	0.1439114	1.4035717	0.160446	0.71328
grpel2	1143.286712	0.051369057	0.0818458	0.6276322	0.530245	0.942209
nhp2	542.5344236	-0.08174867	0.1043757	-0.783215	0.433501	0.913667
tmie	142.5549645	-0.218416219	0.1805264	-1.209885	0.226323	0.793959
hsbp1a	892.9273089	-0.007064778	0.0920592	-0.076742	0.938829	0.992857
nkain2	112.439202	-0.000800373	0.1930569	-0.004146	0.996692	0.998936
slbp2	1.84135345	2.16383757	1.5572676	1.3895091	0.164678	NA
tp53i11a	118.199546	0.032729921	0.182553	0.17929	0.85771	0.985703
slc26a4	9.629717685	-0.532062163	0.6529968	-0.814801	0.415186	NA
plscr3b	1544.665588	0.027879528	0.081044	0.3440049	0.730843	0.971497
tnk1	404.4411241	-0.254035356	0.1229041	-2.06694	0.03874	0.38611
cfap58	19.78621305	-0.254340075	0.4637123	-0.548487	0.583358	0.952214
neurl1aa	33.44930075	0.228216879	0.327732	0.6963522	0.486208	0.930833
ptgdsa	163.2636147	-0.007885136	0.158021	-0.049899	0.960203	0.995388
dachd	830.7316398	-0.086321691	0.0875602	-0.985855	0.324204	0.870928
lpar6b	41.53990122	-0.577087768	0.2989262	-1.930536	0.05354	0.453409
pimr128	2.654869111	0.707032785	1.1823403	0.5979943	0.549844	NA
dbh	81.77973379	0.089049263	0.234164	0.3802859	0.703733	0.968134
gja5b	8.456931846	0.293573226	0.6702955	0.4379758	0.661404	NA
gja8a	99.35190472	0.057831914	0.2009075	0.2878534	0.773459	0.975687
zgc:113314	60.19066804	-0.1935238	0.2588723	-0.747565	0.454723	0.920902
si:dkey-6n21.13	35.93716348	0.299729536	0.3226289	0.9290225	0.352877	0.881552
rnaseka	313.7804699	-0.1866809	0.1294236	-1.442402	0.149189	0.696454
alox12	556.062179	-0.025128813	0.1014354	-0.247732	0.804342	0.979643

cox7a1	36.29366154	-0.006510065	0.3641466	-0.017878	0.985737	0.996944
igsf9bb	21.58374776	0.175955382	0.4091399	0.4300617	0.667151	0.964736
khdrbs2	652.3791112	-0.027765045	0.1256876	-0.220905	0.825166	0.982196
mhc1zca	85.28104817	0.230202473	0.2270675	1.0138061	0.310675	0.862927
chsy3	668.3806273	0.195607509	0.1004409	1.9474892	0.051476	0.444687
frem1a	528.4275269	0.292731189	0.1040581	2.8131521	0.004906	0.102006
spint2	2090.620943	0.029675725	0.0789895	0.3756922	0.707146	0.968664
slc13a3	149.0279041	-0.132162048	0.1972393	-0.670059	0.50282	0.935304
ghrh	1.236658278	1.362380035	1.954156	0.6971706	0.485696	NA
traf1	17.43183024	-0.361949207	0.4674727	-0.774268	0.438772	0.915997
dab2ipa	45.61738018	-0.30210352	0.302778	-0.997772	0.31839	0.867746
sprydd7b	303.8712972	-0.058839932	0.1590805	-0.369875	0.711476	0.969006
tmem223	138.2986412	-0.057372796	0.1716428	-0.334257	0.738186	0.972535
tmem179b	49.81431502	-0.561152825	0.2805513	-2.000179	0.045481	0.418148
arl2	262.4218415	0.045900741	0.1309769	0.350449	0.726002	0.970402
sumf2	107.8222889	0.139816812	0.1908415	0.7326331	0.463782	0.922875
phkg1b	162.605733	0.444465814	0.1632218	2.7230782	0.006468	0.122855
spag1b	63.47650079	-0.281954566	0.2506441	-1.12492	0.260623	0.828546
hs6st3b	3.152604936	0.154343508	1.044796	0.147726	0.882559	NA
abhd11	189.4129179	0.065868999	0.1569426	0.4197013	0.674704	0.965257
cldnh	1372.201987	-0.206193744	0.0852101	-2.419827	0.015528	0.228768
si:dkey-88e18.2	3.41581341	0.392263494	1.0398581	0.3772279	0.706004	NA
zgc:136892	110.1963134	0.067138965	0.188137	0.3568621	0.721195	0.969602
mettl27	34.583953	0.165997478	0.3378581	0.4913231	0.623198	0.957354
bbs10	39.56923721	-0.026177016	0.3290484	-0.079554	0.936592	0.992702
aqp3b	8.056297746	0.049720713	0.7512999	0.0661796	0.947235	NA
vps29	798.8225799	-0.021216945	0.0933315	-0.227329	0.820168	0.981594
gpn3	108.7496975	0.161035656	0.1970946	0.8170474	0.413901	0.905864
ube2g1b	682.0522556	0.074591545	0.0982022	0.7595708	0.447511	0.917154
zgc:153981	131.9388419	0.102080466	0.1732504	0.5892077	0.555722	0.947621
si:dkey-238c7.16	248.0861253	0.195509702	0.1559405	1.2537459	0.209934	0.777416
gtf3c4	153.5394497	-0.091623568	0.1721595	-0.532202	0.594586	0.954434
si:dkey-30c15.2	30.38128125	0.733637612	0.3563275	2.0588857	0.039505	0.390393
si:dkey-8e10.2	0.840668913	0.571309826	2.0205965	0.2827432	0.777374	NA
galt	149.3152384	-0.060022332	0.1649153	-0.363959	0.715889	0.96909
atg12	98.0781863	-0.017483079	0.2258604	-0.077407	0.9383	0.99279
zgc:162780	106.7142731	-0.106513276	0.1932378	-0.551203	0.581494	0.951731
zgc:162396	275.1663485	-0.345225821	0.1285802	-2.684907	0.007255	0.134075
atoh7	57.14506953	-0.07827345	0.2557948	-0.306001	0.759604	0.975374
CR388165.1	1.181176088	-0.433914662	1.8067623	-0.240161	0.810205	NA
muc13a	126.707283	-0.374628205	0.1866876	-2.006712	0.04478	0.416612
kcnh3	37.42737556	-0.112050515	0.3112285	-0.360027	0.718827	0.969092
zgc:153151	22.51245584	-0.424786474	0.4431383	-0.958587	0.337767	0.876395
mucms1	40.64799282	-0.092157861	0.3450738	-0.267067	0.789417	0.977148
zgc:114181	2041.110676	0.396849098	0.0799176	4.96573	6.84E-07	7.28E-05
tmem222a	311.5820176	0.12690696	0.1275656	0.9948366	0.319816	0.86809
tmem150c	63.62021724	0.177623531	0.2407812	0.7376968	0.460699	0.921978
kif13a	244.1522633	-0.019045393	0.1375213	-0.138491	0.889853	0.989291
si:ch211-214c7.4	700.4902806	-0.106596621	0.095824	-1.112421	0.265957	0.831945
chrm5b	3.331169679	0.021389598	1.0675661	0.0200359	0.984015	NA
zgc:109889	1520.053446	0.004083129	0.077669	0.0525709	0.958074	0.99467
snx30	295.9280119	-0.039738554	0.1387249	-0.286456	0.774529	0.975687
taok1a	1264.151874	-0.03413498	0.078825	-0.433047	0.66498	0.964736
zgc:162331	143.5784003	0.148509357	0.1858907	0.7989069	0.424344	0.908312

palm2	13.63487924	-0.158796405	0.5277135	-0.300914	0.76348	0.975374
ckmt2a	4959.898664	0.259296626	0.084106	3.0829729	0.002049	0.054397
atf7ip	3298.888232	-0.116504161	0.0706178	-1.649785	0.098987	0.598346
mrps18c	346.8662963	-0.018445738	0.1449454	-0.12726	0.898735	0.990121
si:ch211-255g12.8	7.476152468	0.053872031	0.6905741	0.0780105	0.93782	NA
tat	170.372951	-0.883270766	0.1775125	-4.975823	6.50E-07	6.94E-05
emp1	127.4424198	0.243396744	0.1869111	1.3022063	0.192846	0.756795
vegfc	45.00740984	0.224307955	0.2967302	0.7559322	0.44969	0.918526
tmem160	88.00362617	-0.21017599	0.2104851	-0.998531	0.318022	0.867746
ppp6r2b	65.68669519	-0.105437578	0.2449644	-0.43042	0.66689	0.964736
chrnb1	346.0418865	0.357096169	0.1272974	2.8052118	0.005028	0.103574
si:dkey-121j17.6	54.65263565	0.103849866	0.2767131	0.375298	0.707439	0.968664
fgf11a	10.36232198	-0.226672378	0.6459556	-0.35091	0.725656	NA
adra2c	77.90461462	0.174392393	0.2401141	0.7262896	0.467661	0.924328
maip1	299.0156061	0.025156394	0.1344139	0.1871561	0.851538	0.985174
tyw5	161.9507568	-0.126276081	0.178199	-0.708624	0.478558	0.926978
chid1	307.8370433	-0.088566119	0.1290587	-0.686247	0.492558	0.931926
tmem230b	258.0486091	-0.185525023	0.1419671	-1.306817	0.191275	0.755886
her8.2	144.6052186	-0.066175678	0.1707034	-0.387665	0.698264	0.967998
nicn1	26.18544624	0.735398824	0.3930057	1.8712168	0.061315	0.482931
pcgf6	71.99215721	-0.324333218	0.2379868	-1.36282	0.172939	0.731956
col7a1l	397.5116496	0.251750374	0.1153305	2.1828607	0.029046	0.330497
si:ch211-261n11.5	412.7672713	-0.141750804	0.1155906	-1.226317	0.220079	0.786751
lrguk	8.872531182	0.290409394	0.6591425	0.4405867	0.659512	NA
lgi2b	195.542246	0.251125293	0.1492819	1.682222	0.092526	0.579378
efhb	5.905468051	0.059617435	0.8725437	0.068326	0.945526	NA
prmt6	132.9794096	0.125747377	0.1729978	0.7268729	0.467304	0.924328
si:ch211-113e8.5	0.182570949	0.967652056	4.0804729	0.2371421	0.812547	NA
cdc34a	755.5782891	0.06720127	0.0930316	0.722349	0.47008	0.924787
vwa10.2	12.80299242	-0.267940801	0.5312024	-0.504404	0.613977	0.95688
foxi2	40.0170108	-0.191476005	0.3057069	-0.626339	0.531093	0.942209
zbbx	7.479452094	0.860282611	0.7006484	1.2278378	0.219508	NA
glis3	43.23725775	-0.11469612	0.3169127	-0.361917	0.717414	0.96909
rpz3	65.50008709	-0.013232998	0.2416712	-0.054756	0.956333	0.994543
mpg	32.40412012	-0.04719529	0.343338	-0.13746	0.890667	0.989291
mavs	170.3459606	-0.040845415	0.1695212	-0.240946	0.809597	0.980642
hbba2	1.163885225	0.470408	1.7459696	0.2694251	0.787603	NA
hbaa2	0.488104098	-2.415107183	2.7936704	-0.864493	0.387317	NA
pou4f2	1024.072773	-0.134371853	0.0901222	-1.490996	0.135963	0.672424
fndc5a	10.80584993	0.1130536	0.610691	0.1851241	0.853132	NA
cul5a	246.3190419	0.017102316	0.1396367	0.1224772	0.902521	0.990121
mos	5.727264766	-0.427955906	0.8297999	-0.515734	0.60604	NA
slc35f2	119.726388	-0.109762307	0.1853951	-0.592045	0.55382	0.947449
capn5b	8.486334832	0.730450538	0.6963802	1.0489248	0.294213	NA
ckba	686.1866735	-0.116325573	0.1074847	-1.082253	0.27914	0.842806
mtmr11	501.9958015	-0.099461243	0.1045924	-0.950941	0.341634	0.878134
si:dkey-189g17.2	15.33347315	0.342958816	0.5022628	0.6828274	0.494716	0.931926
etv5a	927.8157328	0.078602307	0.0866343	0.9072885	0.364254	0.886494
syngap1b	574.625482	0.011711781	0.1026433	0.1141018	0.909157	0.990702
caln2	6.195252999	-0.301951635	0.7873074	-0.383524	0.701331	NA
nectin1a	91.72964205	-0.217171591	0.2110878	-1.028821	0.303564	0.857769
gig2j	0.522329263	0.999930388	2.8066578	0.3562709	0.721638	NA
nitr1b	0.332645115	0.005883744	3.2924249	0.0017871	0.998574	NA
flot2b	1348.917877	-0.093007696	0.0876718	-1.060862	0.288753	0.845979

fbxo40.1	73.97535295	-0.086342302	0.2310832	-0.373642	0.708671	0.968664
pus7l	88.7852316	-0.331622806	0.2142629	-1.547738	0.121685	0.648399
rec114	1.187378788	0.431861727	1.803976	0.2393944	0.8108	NA
tmc6a	3.268175202	0.515645311	1.2167851	0.4237768	0.671729	NA
dynll2a	1441.572987	0.033859836	0.0906305	0.373603	0.7087	0.968664
crygm2d5	3649.580376	-0.06328533	0.086728	-0.729699	0.465574	0.923865
zgc:154058	49.30434585	0.581359011	0.2882967	2.0165304	0.043745	0.410733
znf1124	275.8858987	-0.237984875	0.1265997	-1.879821	0.060132	0.478687
si:dkey-228l14.1	0	NA	NA	NA	NA	NA
crygm2d12	8890.706629	0.107220692	0.0730877	1.4670134	0.142372	0.684315
si:ch211-198k9.6	15.07488042	-0.046050154	0.4997144	-0.092153	0.926577	0.992702
sstr2b	11.7343678	-0.656632713	0.5776562	-1.136719	0.255656	0.824285
zdhhc18a	354.6573424	-0.074851607	0.1169923	-0.639799	0.522303	0.939056
ostm1	257.8805057	-0.115438203	0.1460214	-0.790557	0.429203	0.911406
dnali1	36.42725187	0.012877055	0.3223046	0.0399531	0.968131	0.996315
crygm2d17	14100.41529	0.126513018	0.0675978	1.8715566	0.061268	0.482931
procal	112.1100872	0.203848988	0.1882306	1.0829747	0.27882	0.842767
crygm2d15	8135.636159	0.002798789	0.1056559	0.0264897	0.978867	0.996762
crygm2d11	8064.059927	0.065504518	0.0850966	0.7697668	0.441438	0.916336
si:ch211-117n7.7	148.7272169	-1.092369336	0.1735573	-6.293998	3.09E-10	6.72E-08
rragca	730.0901981	-0.00110735	0.094454	-0.011724	0.990646	0.997679
sfxn4	171.1481195	-0.145350993	0.1563913	-0.929406	0.352679	0.881552
rhbdl2	202.9075272	-0.124506745	0.1432842	-0.86895	0.384875	0.89551
pnpla4	13.44740148	-0.448538357	0.5197187	-0.863041	0.388115	0.89625
CU571382.1	0.347458735	-1.908042286	3.4571486	-0.551912	0.581009	NA
kctd12.1	184.9403524	0.263321151	0.1563933	1.6837111	0.092238	0.57849
acod1	85.45155409	-0.058433532	0.2283307	-0.255916	0.798015	0.978793
zgc:162944	718.7294395	-0.119828288	0.103119	-1.162039	0.24522	0.815227
nitr1b	0.166657454	-0.955901296	4.0804729	-0.234262	0.814781	NA
mrpl30	645.944491	-0.126208792	0.1146884	-1.10045	0.271136	0.835995
lipt2	23.60469994	-0.067573718	0.4218701	-0.160177	0.872742	0.987846
txndc9	1319.618233	-0.013401153	0.0838171	-0.159886	0.872971	0.987846
pnisr	2219.491246	-0.00593901	0.0826565	-0.071852	0.94272	0.993364
coq3	261.7284302	-0.132951115	0.1645861	-0.807791	0.419211	0.906406
grxcr1b	9.149280724	-0.046954564	0.6385293	-0.073535	0.94138	NA
sox1a	559.8833902	0.001392574	0.1192662	0.0116762	0.990684	0.997679
zgc:113030	78.32959638	-0.054063068	0.2369092	-0.228202	0.819489	0.981594
snu13a	346.8431575	-0.109062931	0.1335982	-0.81635	0.4143	0.906237
cldna	115.0541829	-0.007598869	0.1952891	-0.038911	0.968961	0.996315
sphk2	448.7857384	-0.077949084	0.1054235	-0.73939	0.45967	0.921978
CR392036.1	1.293012095	2.68170335	1.8621377	1.4401209	0.149833	NA
pimr152	0	NA	NA	NA	NA	NA
CABZ01071903.1	134.4995945	0.148689258	0.1914485	0.776654	0.437363	0.914595
si:dkey-33c12.12	289.7531129	0.153165961	0.1298205	1.1798284	0.238068	0.807196
gtf2f2a	603.4169838	-0.020563551	0.1022902	-0.201031	0.840674	0.985092
hmga2	195.0527953	0.153034159	0.1725923	0.8866801	0.375251	0.894316
ska3	178.5512798	-0.226032506	0.1663639	-1.358663	0.174253	0.734375
cox17	827.8933865	0.033883158	0.1235105	0.2743343	0.783828	0.97627
pla1a	133.2822879	0.202209508	0.1936226	1.0443486	0.296324	0.853286
hsd3b1	9.579500747	1.158698935	0.6642222	1.7444447	0.081082	NA
prkceb	24.95212199	-0.279941121	0.3852243	-0.726696	0.467412	0.924328
CABZ01053976.1	150.332927	-0.009935964	0.1861562	-0.053374	0.957434	0.99467
parp4	331.4056202	-0.23754578	0.1283803	-1.850329	0.064266	0.496018
dnm2a	508.1996862	-0.050924851	0.1175377	-0.433264	0.664823	0.964736

ppap2d	668.54982	0.13322011	0.0946389	1.4076682	0.159229	0.710139
tdp2a	0.515095711	0.926015374	2.6527421	0.3490785	0.72703	NA
p2ry13	3.931139784	1.288570441	0.9863179	1.3064454	0.191401	NA
p2ry12	6.757961806	2.039088693	0.7999539	2.5490077	0.010803	NA
itga6b	923.4132982	0.067186843	0.10744	0.6253427	0.531746	0.942359
eef1a1l2	326.2290302	0.287221287	0.1253549	2.2912648	0.021948	0.281271
kcnq5b	117.8755251	-0.011106835	0.1837217	-0.060455	0.951794	0.993864
kcnq5a	8.669940135	0.693819915	0.6478101	1.0710236	0.284159	NA
pdlim2	484.4180056	-0.030308521	0.1107835	-0.273583	0.784405	0.97627
zgc:162936	59.90703802	-0.338955189	0.2569105	-1.319351	0.187052	0.751002
sh2b3	1.946594704	-0.468402419	1.4036711	-0.333698	0.738607	NA
il21r.1	16.08949083	-0.438557374	0.4728721	-0.927433	0.353702	0.881976
alox5b.3	10.21739478	0.433081887	0.6081032	0.7121849	0.47635	NA
pcsk7	365.3531206	-0.116909615	0.1221839	-0.956834	0.338651	0.877373
lingo2b	332.0505502	0.132714402	0.124443	1.0664677	0.286212	0.845942
mygl	643.6640381	-0.164766297	0.1017787	-1.618867	0.105476	0.614157
cd28l	97.8331574	0.167299159	0.1995047	0.8385724	0.401709	0.90084
lman1	678.9204105	0.046959429	0.0998196	0.4704429	0.638039	0.959194
cspg5a	4494.175852	0.013160467	0.0912159	0.1442782	0.885281	0.988936
nkd3	1.646484154	-0.008692931	1.423537	-0.006107	0.995128	NA
arid6	155.257074	-0.872520223	0.1697921	-5.138755	2.77E-07	3.40E-05
bnc2	575.1688679	-0.084831187	0.0984679	-0.861511	0.388956	0.896295
dpy19l1l	39.09617653	0.326879856	0.3315021	0.9860567	0.324105	0.870928
dnajb14	143.6355132	0.18750798	0.1670575	1.1224158	0.261686	0.82979
si:ch211-145b13.6	17.89314989	-0.27125193	0.4837121	-0.560771	0.574953	0.950119
txnipb	614.5796625	0.065484058	0.1010639	0.6479469	0.517019	0.938543
rcn1	753.3299452	0.210505055	0.0914955	2.3007159	0.021408	0.276496
hce2l2	14.4636594	0.522687553	0.5074895	1.0299474	0.303035	0.85729
sesn2	378.5623538	-0.046094403	0.1147489	-0.401698	0.687906	0.966493
dmrt2b	21.34687366	-0.035902067	0.4260563	-0.084266	0.932845	0.992702
pimr173	0.513487682	-0.887846295	2.6557956	-0.334305	0.738149	NA
taf15	5968.46764	-0.062583138	0.0716894	-0.872976	0.382676	0.895192
cyp3c4	54.12826079	-0.758801958	0.2680378	-2.830951	0.004641	0.097899
dchs1a	335.8547835	-0.066356667	0.1228749	-0.540034	0.589173	0.953345
tmem41aa	311.7805941	-0.014801617	0.1255232	-0.117919	0.906132	0.990702
ehhadh	260.5340787	-0.506304924	0.1377068	-3.676689	0.000236	0.009795
rgs8	534.7623893	0.21569257	0.1138257	1.894937	0.058101	0.472597
rbp2a	1038.371233	-0.06018515	0.0968919	-0.621158	0.534496	0.943336
AL935184.1	0	NA	NA	NA	NA	NA
zgc:162344	83.89469742	0.066317738	0.215038	0.3084001	0.757778	0.975374
zgc:153920	11.28376416	0.271645324	0.5688332	0.4775483	0.632972	0.957356
dars	2322.469395	-0.124971726	0.0855616	-1.460606	0.144124	0.688216
celf4	315.4277403	0.093702829	0.1405744	0.6665712	0.505046	0.935724
eny2	357.0125548	-0.131895461	0.1294211	-1.019118	0.308147	0.861088
rgs4	706.368004	-0.05158639	0.0950565	-0.542692	0.587342	0.953319
zgc:173548	29.13350404	0.006210629	0.3667712	0.0169333	0.98649	0.996965
sfrp2	337.8690089	0.206551168	0.124426	1.6600323	0.096908	0.593269
tdrd6	17.25138518	0.996597061	0.5072542	1.9646898	0.04945	0.436093
foxg1d	157.8520293	0.191909913	0.1751316	1.095804	0.273165	0.838141
arfip1	250.4911098	-0.011672356	0.1350964	-0.0864	0.931148	0.992702
adora1a	3.207571219	-1.493640465	1.2715492	-1.174662	0.24013	NA
si:dkey-69o16.5	154.4798454	-0.307959804	0.1705403	-1.805789	0.070951	0.521075
si:ch211-262i1.5	105.0411144	0.121591137	0.191544	0.6347947	0.525562	0.94013
gfer	249.4549748	-0.180131756	0.1337362	-1.346918	0.178007	0.738995

si:ch211-163c2.2	1.21367287	0.460988657	1.8129472	0.2542758	0.799282	NA
pitx3	276.6321136	-0.079425595	0.1362141	-0.583094	0.55983	0.948478
si:dkey-13m1.5	3.723288649	0.51596056	1.0861176	0.4750504	0.634751	NA
lrp8	1269.887702	-0.039719512	0.080134	-0.495664	0.620132	0.957354
actr2b	71.6702779	0.16708263	0.2395668	0.6974366	0.48553	0.930406
abcb11b	150.2766634	0.420661557	0.1780286	2.3628873	0.018133	0.250046
nbeaa	2206.613955	0.028289874	0.0956093	0.2958903	0.767314	0.975687
rspo3	133.2770133	-0.15707883	0.1883028	-0.834182	0.404178	0.901305
atp5f1b	20637.15837	-0.00546216	0.087874	-0.062159	0.950436	0.99381
phf10	312.0578623	-0.100187153	0.1236751	-0.810083	0.417892	0.906293
mettl6	77.27168901	0.374465257	0.2236301	1.6744854	0.094035	0.583414
KCNV1	5.015641243	-0.172486191	0.9003949	-0.191567	0.848081	NA
sytl1	95.58186427	0.043992294	0.2032493	0.216445	0.828641	0.982721
selenos	1068.339217	-0.02055644	0.0903636	-0.227486	0.820046	0.981594
lcorl	629.5461064	0.151448352	0.1029957	1.4704335	0.141444	0.68201
si:ch211-168d1.3	57.76525226	0.067752907	0.2624603	0.2581453	0.796295	0.978498
six7	1062.633823	0.173458046	0.0903189	1.9205065	0.054794	0.458525
dek	561.4262778	-0.03946209	0.1040671	-0.379199	0.70454	0.968134
ncapg	818.7650003	-0.116690907	0.0911327	-1.280451	0.200387	0.766727
opn5	6.758610648	0.206192222	0.7218808	0.2856319	0.77516	NA
nit1	71.46136023	-0.125985787	0.2419109	-0.520794	0.60251	0.956045
ctc1	383.038159	-0.282553477	0.1136704	-2.485726	0.012929	0.202578
rhoh	0.964730988	-0.018658301	1.9582344	-0.009528	0.992398	NA
stk11ip	352.4243814	-0.166136689	0.1203982	-1.379893	0.16762	0.7248
ptpn23a	937.3486433	0.020553026	0.1152312	0.1783634	0.858438	0.985773
si:dkey-22l11.6	41.54040974	-0.048638931	0.302841	-0.160609	0.872402	0.987846
nfxl1	273.3530369	-0.11270715	0.1402212	-0.803781	0.421523	0.90651
rpp25l	71.12340888	0.043354387	0.2376281	0.1824464	0.855232	0.985174
aste1a	3.334976464	0.32506539	1.0447486	0.3111422	0.755693	NA
si:dkey-84o3.3	1.695213209	-1.193025259	1.5279745	-0.780789	0.434927	NA
RETSAT	25.18384643	-0.551533809	0.38784	-1.422065	0.155007	0.704624
si:ch211-191i18.2	60.96162638	0.326399072	0.2666187	1.2242169	0.22087	0.787641
cart4	93.61265833	0.135430922	0.2147104	0.6307609	0.528197	0.941768
magi1a	779.675528	-0.009908095	0.1281335	-0.077326	0.938364	0.99279
cygb2	236.3795781	-0.017500682	0.1429424	-0.122432	0.902557	0.990121
jtb	704.7360361	0.099763729	0.0961861	1.0371952	0.299645	0.855745
nfyc	1763.929858	-0.029761589	0.0838535	-0.354924	0.722647	0.970375
tuba8l3	1252.269397	0.122118408	0.0839555	1.4545608	0.145791	0.691641
tgm2a	317.8746423	0.270702577	0.1299206	2.0835997	0.037197	0.37758
vamp3	1015.361349	-0.123459381	0.0873186	-1.413896	0.157393	0.708089
stox2a	1550.991776	0.04205615	0.0777297	0.5410563	0.588469	0.953319
ubr2	1215.735616	-0.012249919	0.0876568	-0.139749	0.888859	0.989291
tnfrsf1b	99.35535424	-0.19479214	0.1984792	-0.981423	0.326384	0.87218
zgc:153499	0.31598276	0.005883445	3.3523626	0.001755	0.9986	NA
sod3a	93.62103698	-0.081378951	0.2500388	-0.325465	0.744829	0.974094
si:ch211-225p5.8	82.38187839	0.571241565	0.2238257	2.5521718	0.010705	0.179263
gria2a	1427.936494	0.030754567	0.0779567	0.3945084	0.693206	0.96736
PLEKHH3	637.4085054	0.04381474	0.1008263	0.4345568	0.663884	0.964736
zp3d.2	2.118166602	-0.667368935	1.3153398	-0.507374	0.611893	NA
dyrk1b	1600.720344	0.022746772	0.0873075	0.2605362	0.79445	0.977937
si:dkey-13e3.1	0	NA	NA	NA	NA	NA
si:zfos-2070c2.1	0	NA	NA	NA	NA	NA
etnk2	3.987448907	0.48046754	0.9516837	0.5048605	0.613657	NA
slc41a1	37.7097983	0.20288765	0.3197898	0.6344407	0.525793	0.940223

si:ch211-120e1.1	0	NA	NA	NA	NA	NA
pou3f2a	291.5755177	0.11406118	0.1255832	0.9082519	0.363745	0.886174
fam43b	139.3862718	-0.167803685	0.1772934	-0.946475	0.343907	0.878434
nhsa	47.7054273	0.008255583	0.2777929	0.0297185	0.976292	0.996762
cdk6	49.60508536	0.103886231	0.2818134	0.3686348	0.7124	0.969087
zgc:158258	80.28941987	-0.36156563	0.2533488	-1.427145	0.153538	0.702578
aldh1l2	592.4576518	-0.014859965	0.1134019	-0.131038	0.895745	0.989814
polh	202.1218974	-0.008729759	0.1488013	-0.058667	0.953217	0.994157
knl1	318.641882	-0.262315783	0.1255812	-2.088815	0.036724	0.374517
suz12a	427.9836269	0.074786479	0.1158216	0.6457039	0.518471	0.938765
crlf3	418.4348785	-0.163223516	0.1137445	-1.435001	0.151287	0.699449
spock3	2865.847063	0.125496888	0.0739087	1.6979996	0.089508	0.572876
tut4	55.26571057	-0.227051956	0.2683655	-0.846055	0.397522	0.899672
casp8l2	28.26786858	0.01166195	0.4147828	0.0281158	0.97757	0.996762
mettl14	191.713369	-0.090791151	0.1647221	-0.551178	0.581512	0.951731
hist1h2a3	24.10159304	0.024549577	0.4377783	0.0560776	0.95528	0.994543
sft2d3	222.5417825	0.155745051	0.1482998	1.0502041	0.293624	0.849594
si:ch211-113a14.22	11.40266164	1.17091719	0.5939957	1.9712554	0.048695	0.433734
CR354435.1	13.80273161	0.584392624	0.5797533	1.0080023	0.313453	0.865132
BX942819.1	5.584943345	-0.361075493	0.853063	-0.423269	0.672099	NA
cald1a	645.2495464	0.102576112	0.1026546	0.9992358	0.31768	0.867607
irge4	2.788240727	0.441142802	1.2657093	0.3485341	0.727439	NA
arl8bb	106.7338605	0.07714212	0.1947416	0.3961255	0.692012	0.96733
si:dkey-245p14.4	53.48961772	-0.120211661	0.2827793	-0.425108	0.670758	0.965257
clrn2	39.08122992	0.443198264	0.339387	1.3058786	0.191594	0.755886
muc5.1	1376.354423	0.1535183	0.0912934	1.6815919	0.092648	0.57957
hoxc1a	147.0714875	0.039699028	0.1775383	0.2236083	0.823062	0.982189
hoxc4a	333.973953	-0.249169463	0.1192357	-2.089723	0.036643	0.374263
hoxc3a	461.724629	0.013679195	0.1069734	0.1278747	0.898248	0.990121
hoxc5a	205.6983993	-0.017402541	0.1536628	-0.113251	0.909831	0.990702
or134-1	3.81309782	-0.912658793	0.9725951	-0.938375	0.348052	NA
hoxc6a	236.1535358	0.086053923	0.1367864	0.6291118	0.529276	0.942209
or137-3	4.727879373	-0.835689022	0.9156709	-0.912652	0.361426	NA
hoxc8a	316.4008191	-0.023036415	0.1381029	-0.166806	0.867523	0.987231
hoxc10a	37.24240559	-0.219532049	0.3124622	-0.702587	0.482313	0.928726
hoxc11a	34.64961375	-0.018541798	0.3599252	-0.051516	0.958915	0.995095
hoxc12a	21.55103707	-0.476350951	0.4509472	-1.056334	0.290816	0.846568
hoxc13a	4.495253329	-0.750129482	0.8905347	-0.842336	0.3996	NA
cx39.4	18.83546562	0.340677739	0.4411298	0.7722846	0.439946	0.916082
smim12	297.0584407	0.043501983	0.1301679	0.3341991	0.738229	0.972535
inka1a	261.2758016	-0.202076303	0.1433001	-1.410161	0.158492	0.709224
cx30.9	6.595008859	0.59786581	0.7576264	0.7891301	0.430036	NA
casp6l2	1.34044285	2.725103408	1.8428697	1.4787282	0.139213	NA
kat7a	429.3648785	0.083229971	0.1077597	0.7723663	0.439898	0.916082
ccl35.2	0.812928439	-0.504524747	2.2480137	-0.224431	0.822422	NA
krtcap2	471.1788209	-0.12636082	0.1067479	-1.183731	0.23652	0.805854
nudt7	50.85484687	-0.097119481	0.2883862	-0.336769	0.736291	0.972535
foxf2b	91.82624333	-0.552704262	0.2092149	-2.641802	0.008247	0.147053
flad1	445.4671608	0.044273864	0.1120549	0.3951088	0.692763	0.96736
tspan4b	199.8521615	0.096570412	0.1555591	0.6207958	0.534734	0.943336
tlr19	1.00508983	0.079172592	1.9360604	0.0408937	0.967381	NA
COQ10A	229.6979733	0.470601255	0.1474673	3.191224	0.001417	0.040718
uroc1	441.006718	0.322170813	0.1262503	2.5518417	0.010716	0.179263
serpinb1l2	12.2621942	-0.106984006	0.5561764	-0.192356	0.847463	0.985174

alg2	210.6178724	-0.171763105	0.1610784	-1.066332	0.286273	0.845942
mala	0	NA	NA	NA	NA	NA
wdyhv1	0	NA	NA	NA	NA	NA
inka1b	80.62751604	-0.094667976	0.2249214	-0.420894	0.673833	0.965257
abitram	80.74071725	0.085446039	0.2333816	0.3661215	0.714274	0.96909
ccnd2b	29.32245847	0.102362283	0.3917795	0.2612752	0.79388	0.977937
mtmr7b	675.4185625	0.101508244	0.104962	0.9670949	0.333497	0.874933
si:dkey-61f9.1	2.388945101	0.575163589	1.4392443	0.3996289	0.68943	NA
zgc:162816	143.3539324	-0.284789222	0.1747744	-1.629467	0.103214	0.609625
nkd3l	0	NA	NA	NA	NA	NA
zgc:153157	70.01982457	-0.078249835	0.2429862	-0.322034	0.747427	0.97419
dll4	273.3879399	-0.010842181	0.1345129	-0.080603	0.935757	0.992702
chac1	0	NA	NA	NA	NA	NA
s100v1	680.2652998	-0.208217273	0.0926821	-2.246575	0.024667	0.302262
si:dkey-222b8.1	73.32528252	-0.192201455	0.2429493	-0.791118	0.428875	0.911406
chtopb	19.96431334	-0.198682318	0.4283548	-0.463827	0.642772	0.959994
fip1l1b	1236.062442	-0.127134882	0.0816882	-1.556343	0.119627	0.644641
ino80	531.7975924	-0.024626239	0.1021756	-0.241019	0.809541	0.980642
vps18	514.0042049	-0.08941423	0.1074732	-0.831967	0.405427	0.901305
rhov	114.1667494	0.117325041	0.1937065	0.6056846	0.544724	0.945438
vapb	901.4757978	0.106400996	0.0938387	1.1338717	0.256848	0.825036
rpl22	7889.813363	-0.249856648	0.080555	-3.101692	0.001924	0.051979
atp6v1c2	14.36582932	-0.092464077	0.5000378	-0.184914	0.853296	0.985174
zdhhc17	161.1296638	0.000439973	0.160283	0.002745	0.99781	0.999174
si:ch211-113g11.6	1106.647659	0.15791887	0.0884629	1.785143	0.074238	0.530917
slc39a9	601.2204091	0.045477971	0.1179517	0.3855643	0.699819	0.967998
grk4	958.3681094	-0.024010089	0.0929232	-0.258386	0.796109	0.978498
tspan5b	92.02500453	0.192213722	0.205733	0.9342874	0.350156	0.880195
saraf	365.4144834	0.019890434	0.124165	0.1601936	0.872729	0.987846
gch1	268.5437119	0.184108775	0.1299066	1.4172395	0.156413	0.706639
pla2g12a	206.2037496	0.286197514	0.1546049	1.8511544	0.064147	0.495295
tmem245	254.3798965	0.120218096	0.1365549	0.8803645	0.378662	0.895192
zgc:153292	110.7370659	-0.230924302	0.1897128	-1.217231	0.223516	0.789816
zgc:154061	60.62591695	-0.073180187	0.2499655	-0.292761	0.769705	0.975687
e2f3	328.7122434	-0.089830655	0.1206881	-0.744321	0.456682	0.921215
si:dkey-13n23.3	16.81531401	-0.443334359	0.4682803	-0.946729	0.343777	0.878434
lactbl1b	204.5880163	0.525260289	0.1517422	3.4615317	0.000537	0.018831
sh3rf1	555.0932166	0.184728037	0.1044378	1.7687847	0.07693	0.540058
tarbp2	385.0117349	0.07506892	0.1132101	0.6630938	0.50727	0.935734
arf5	3131.038915	0.005733984	0.0778103	0.0736918	0.941256	0.992857
parp6b	591.0271349	0.088754052	0.1034211	0.8581813	0.390792	0.897643
khdrbs1b	5002.729423	-0.028273991	0.0667078	-0.423849	0.671676	0.965257
dnajc2	324.5543814	0.198545463	0.1249111	1.5894943	0.111949	0.627242
amn1	102.1345309	-0.201575616	0.2063693	-0.976771	0.328682	0.872945
tspan13b	326.7929835	0.059082138	0.1306497	0.452218	0.651112	0.960708
agr2	1807.195404	-0.177393363	0.0763856	-2.32234	0.020215	0.267195
zgc:195001	682.4879503	0.355709515	0.1030268	3.4525932	0.000555	0.019228
cenps	78.81305331	0.087384885	0.218667	0.3996254	0.689432	0.966763
rbp7b	29.61326064	0.597080828	0.3618342	1.6501503	0.098912	0.598346
zgc:110783	171.0445245	0.086524744	0.1544012	0.560389	0.575214	0.950312
si:dkeyp-75b4.7	0.471562095	-2.373056629	3.0645586	-0.774355	0.438721	NA
hpcal4	446.5237578	0.124702697	0.1092939	1.1409852	0.253876	0.823394
pdgfra	1402.131609	-0.081189531	0.0806753	-1.006375	0.314235	0.865176
FRMD7	14.17334013	0.337831497	0.5073221	0.6659112	0.505468	0.935724

bicc1b	12.71572677	-0.256251277	0.5354434	-0.478578	0.632239	0.957356
phyhiplb	159.0175116	0.414562148	0.1749063	2.3701954	0.017779	0.247053
si:dkey-31e10.5	15.95104437	-0.386174261	0.4938733	-0.78193	0.434256	0.914161
trpc5a	8.717070362	-0.385296173	0.6445686	-0.597758	0.550001	NA
trpc4a	116.2531158	-0.128848496	0.1938835	-0.664567	0.506328	0.935724
si:dkey-183j2.10	322.5014457	-0.095941135	0.1206777	-0.79502	0.426602	0.910189
cnpy3	361.0681587	0.117405524	0.1170707	1.0028596	0.315929	0.866931
brpf3a	293.3278911	0.026187158	0.1313447	0.1993773	0.841968	0.985174
cacna1i	20.52030644	-0.235892282	0.4426708	-0.532884	0.594114	0.954434
zc2hc1c	12.33150952	0.747184385	0.5496905	1.3592819	0.174057	0.734137
ripply2	1.448040995	-1.007244037	1.6721265	-0.602373	0.546926	NA
creb5b	19.56306	-0.583282206	0.4662114	-1.251111	0.210894	0.778412
stmn2b	2985.540651	-0.068636026	0.0959376	-0.715424	0.474347	0.925115
hey1	923.2707968	0.020846923	0.1020884	0.2042046	0.838194	0.984797
arf3a	511.8215844	-0.064053962	0.1105744	-0.579284	0.562398	0.948728
si:dkeyp-97e7.9	4.354688277	-0.627318172	0.9927726	-0.631885	0.527462	NA
mafbb	189.0824076	-0.120273439	0.1653697	-0.7273	0.467042	0.924328
grin2ab	660.7973926	0.039127958	0.1136522	0.3442782	0.730637	0.97149
top1l	2204.54593	0.21465806	0.0784908	2.7348184	0.006241	0.120174
msgn1	3.134574261	-1.460934405	1.1545367	-1.265386	0.205733	NA
rnmt	697.145578	-0.034337947	0.0928483	-0.369829	0.71151	0.969006
ldlrad4a	488.0935499	0.195456162	0.1039331	1.880596	0.060027	0.478654
si:ch211-93g23.2	49.88543228	-0.051274192	0.2709677	-0.189226	0.849915	0.985174
nap1l4a	1246.249158	0.110744935	0.0862556	1.2839159	0.199171	0.764592
adap2	102.8184267	0.145625267	0.2017526	0.721801	0.470417	0.924838
cadpsb	2809.90207	0.026530192	0.0719127	0.3689221	0.712186	0.969006
atad5a	467.626274	0.008828406	0.1049855	0.0840917	0.932984	0.992702
ppp4ca	403.3394371	0.091731614	0.1105879	0.8294906	0.406827	0.901305
inavab	174.4795876	0.040777585	0.1735497	0.234962	0.814238	0.981081
mapk3	1750.916748	0.074806411	0.0729794	1.0250348	0.305347	0.85982
fam131bb	14.51353857	0.327521453	0.4945706	0.6622339	0.507821	0.935734
edn2	19.5488479	-0.346201275	0.4268449	-0.811071	0.417325	0.906237
ggact.3	161.8286198	-0.147186559	0.1581536	-0.930656	0.352032	0.880844
ggact.1	152.5784108	0.323907523	0.1802307	1.7971831	0.072307	0.525828
itpk1b	214.0517758	0.122096127	0.1679062	0.7271686	0.467123	0.924328
clic5b	646.3236372	-0.130427138	0.1126268	-1.158047	0.246845	0.816669
mrpl35	254.5033682	0.035852132	0.1555479	0.2304893	0.817712	0.981594
nt5c1ab	44.58712185	0.028838024	0.2894357	0.0996354	0.920634	0.992118
prelp	299.4049994	0.052964609	0.1277495	0.4145975	0.678437	0.965257
eno4	26.92109025	-0.189910598	0.3912472	-0.485398	0.627394	0.957354
dyrk3	1249.204657	-0.072551641	0.0783753	-0.925695	0.354604	0.882105
RASSF5	288.6829479	0.12516517	0.1311948	0.9540409	0.340063	0.87805
hspa12a	10.94770332	0.448411663	0.5914232	0.7581909	0.448337	NA
zgc:162509	21.94329901	-0.124497506	0.4015993	-0.310004	0.756558	0.975374
b4galnt2.2	4.192397244	-1.179862503	1.0612843	-1.111731	0.266254	NA
ikbke	113.3272305	-0.168236749	0.2131201	-0.789399	0.429879	0.911615
mrpl36	588.3963599	-0.089466022	0.1083768	-0.825509	0.409083	0.901769
zgc:152986	0	NA	NA	NA	NA	NA
vhl	149.7229442	-0.12868896	0.1704753	-0.754884	0.450319	0.918761
tatdn2	3.807318414	1.508664062	1.011838	1.4910135	0.135958	NA
grin2db	50.40286621	0.386254318	0.2921253	1.3222212	0.186094	0.750443
FAM72B	357.5274166	-0.066116254	0.1201652	-0.550211	0.582174	0.952028
med10	463.0856048	-0.099703259	0.1070701	-0.931196	0.351752	0.88081
cacng7b	79.53893671	-0.044152133	0.2270881	-0.194427	0.845841	0.985174

enpp5	231.2697819	-0.102667243	0.165138	-0.621706	0.534135	0.943276
cacng8b	696.8014853	0.214878599	0.0951832	2.2575265	0.023975	0.299022
pimr60	0.182570949	0.967652056	4.0804729	0.2371421	0.812547	NA
ttpal	305.9022105	0.053182292	0.1280141	0.415441	0.677819	0.965257
prkcdb	1040.836433	0.049870501	0.086874	0.5740559	0.56593	0.948739
pex5	660.1406593	-0.029211232	0.1019354	-0.286566	0.774445	0.975687
si:ch211-69g19.2	868.0625715	-0.118926721	0.0945618	-1.257662	0.208514	0.775404
pa2g4b	2604.582156	-0.156506971	0.0808513	-1.935738	0.0529	0.451736
zbtb12.2	147.1563329	0.043077147	0.1748312	0.2463928	0.805378	0.979883
si:ch211-182e10.4	0.182570949	0.967652056	4.0804729	0.2371421	0.812547	NA
rhol	114.95056	0.560543268	0.2026753	2.7657201	0.00568	0.113103
cd4-1	1.16278535	-1.292219773	1.7937845	-0.720387	0.471286	NA
cxcr3.3	222.2303476	-0.321203895	0.1586541	-2.024555	0.042913	0.406985
crip2	1027.38164	0.187785874	0.0846318	2.2188564	0.026496	0.313216
slc2a9l1	3.636414049	1.432833121	1.0618905	1.3493229	0.177233	NA
ptgr2	169.0781557	-0.110300605	0.1665457	-0.662284	0.507789	0.935734
psmd6	2214.310549	0.005836631	0.074716	0.0781176	0.937735	0.99279
fam3a	339.2633263	0.003988895	0.1175943	0.0339208	0.97294	0.996315
ifnphi3	2.572655968	1.532766764	1.4518113	1.0557617	0.291077	NA
fezf2	335.8555254	-0.008764664	0.1227259	-0.071417	0.943066	0.993364
c1qtnf12	170.9807275	0.090955756	0.1766262	0.5149617	0.60658	0.956718
sdf4	1094.035494	0.141221153	0.0917897	1.5385288	0.123919	0.652391
dkk3b	148.7109446	0.124075108	0.170047	0.7296517	0.465603	0.923865
CR759887.1	0.802320445	1.849899411	2.4175996	0.7651802	0.444164	NA
cdipt	790.294492	0.046814466	0.0925262	0.5059589	0.612886	0.95688
ncalda	1369.452843	0.050114712	0.0774033	0.6474492	0.517341	0.938592
si:dkey-80c24.1	0.499972362	-2.44370697	3.0013188	-0.814211	0.415524	NA
nxph3	9.918472624	-0.290774584	0.6079131	-0.478316	0.632425	NA
kbtbd8	207.3921086	0.223743845	0.143141	1.5631014	0.118029	0.641526
s100v2	589.6103978	0.089752661	0.1049708	0.8550253	0.392537	0.897688
thap7	273.2614443	-0.182349702	0.1365799	-1.335114	0.181839	0.743853
zgc:64022	50.14148748	0.185177614	0.2797375	0.6619692	0.507991	0.935734
wu:fi42e03	2.492658236	-0.163857423	1.1709947	-0.13993	0.888715	NA
prss60.3	46.24387426	0.697643556	0.2969921	2.3490304	0.018822	0.255627
prss60.1	1.642519234	-0.523298403	1.5434776	-0.339039	0.734581	NA
zgc:173544	0	NA	NA	NA	NA	NA
slc25a18	247.4593461	0.34921088	0.1444116	2.4181629	0.015599	0.229092
vdrb	9.444757557	0.273660504	0.619715	0.4415909	0.658785	NA
ndufa9a	2314.48642	0.0440559	0.083743	0.5260844	0.59883	0.955788
chrna4b	77.35287844	-0.3556041	0.2245995	-1.583281	0.113357	0.631061
cnga3a	194.7583082	0.172466104	0.1726388	0.9989996	0.317795	0.867607
gabra5	252.5318936	0.042255021	0.1366489	0.3092233	0.757152	0.975374
dyrk4	313.3004975	0.219896658	0.1258535	1.7472429	0.080595	0.550664
rnd2	211.5746585	-0.172466188	0.1498605	-1.150845	0.249796	0.819829
pimr89	0	NA	NA	NA	NA	NA
tmem45a	232.890239	-0.12494387	0.1392447	-0.897297	0.36956	0.889338
sft2d2a	48.12067938	-0.056212881	0.3065913	-0.183348	0.854525	0.985174
ccr11.1	0.991963308	2.204864061	2.1328402	1.033769	0.301244	NA
si:ch211-155i14.1	0.166657454	-0.955901296	4.0804729	-0.234262	0.814781	NA
foxg1a	518.9475521	-0.127305308	0.1043064	-1.220494	0.222278	0.788551
her4.3	120.2858453	-0.159292982	0.1969907	-0.808632	0.418727	0.906406
irgf1	9.283543499	1.308723082	0.6630074	1.9739193	0.048391	NA
cyp2x9	185.1770687	-0.567107728	0.1568068	-3.616601	0.000298	0.011781
rln3a	64.55362177	0.225546491	0.2550302	0.8843912	0.376485	0.89493

gjd2b	210.9684887	0.355672838	0.1476227	2.4093374	0.015982	0.233052
znf770	451.8953262	0.015618187	0.1209707	0.1291072	0.897273	0.990121
jupa	3241.413237	-0.112756679	0.0692104	-1.629186	0.103274	0.609625
lrrc15	51.44468502	0.155994309	0.2681032	0.5818442	0.560672	0.948728
greb1	129.6617614	-0.040654881	0.1818459	-0.223568	0.823094	0.982189
eaf1	280.4564303	0.028890813	0.1466761	0.1969701	0.843851	0.985174
pmvk	56.3482554	0.074140234	0.2669669	0.2777132	0.781233	0.97627
zgc:109744	98.87610394	0.112387735	0.2048849	0.5485409	0.583321	0.952214
opa1	1664.739764	0.004666655	0.0751792	0.0620737	0.950504	0.99381
znf516	666.7125206	-0.007708808	0.1046727	-0.073647	0.941291	0.992857
ccka	110.8227357	0.235736973	0.1897683	1.2422356	0.21415	0.782493
si:ch211-156l18.6	0.173729368	-0.955901296	4.0804729	-0.234262	0.814781	NA
pax7b	171.9320481	0.042911246	0.1774371	0.2418392	0.808905	0.980642
cnp	2034.827657	-0.058858368	0.0732156	-0.803905	0.421452	0.90651
ndufb5	1632.322829	0.006920899	0.0826259	0.0837619	0.933246	0.992702
bpgm	487.883743	0.089104512	0.1059908	0.8406814	0.400526	0.90084
actl6a	1237.829332	-0.095155851	0.0890954	-1.068022	0.285511	0.845164
zgc:136858	260.4442498	-0.313890289	0.144029	-2.179354	0.029305	0.331778
rftn1a	28.17673314	0.039982031	0.3812323	0.1048758	0.916474	0.991349
snrka	1156.017619	0.078733705	0.0875799	0.8989929	0.368656	0.888057
lin52	134.2022293	-0.183649417	0.1893051	-0.970124	0.331985	0.874653
taf13	696.2416592	0.003031863	0.1164337	0.0260394	0.979226	0.996802
tnnc2	27705.78058	0.1701261	0.0833575	2.0409215	0.041259	0.397781
evi5b	262.8809133	0.082092787	0.1391271	0.5900559	0.555153	0.947449
arid3a	53.12042005	-0.272860187	0.2700694	-1.010334	0.312335	0.864401
gamt	2574.895028	0.099052982	0.0986932	1.0036456	0.315549	0.866773
si:dkey-56d12.4	36.28032189	0.218990498	0.3266846	0.6703423	0.50264	0.935304
dazap1	207.0970593	-0.026013245	0.1541075	-0.168799	0.865954	0.986517
trim35-20	7.23675999	-0.531545098	0.7609191	-0.698557	0.484829	NA
rps15	7478.101461	-0.388741647	0.0885643	-4.389372	1.14E-05	0.000811
si:ch211-282j17.10	1.021631832	0.046422221	1.8514003	0.0250741	0.979996	NA
si:dkey-88n24.6	0.347458735	-1.908042286	3.4571486	-0.551912	0.581009	NA
zgc:195077	0.846012444	-0.57358332	2.1013569	-0.272959	0.784885	NA
si:dkey-32e6.6	93.43372383	0.200803644	0.2273534	0.8832226	0.377116	0.895192
si:busm1-105l16.2	16.72195846	0.369401451	0.4660056	0.7926974	0.427954	0.910858
bcl6aa	376.8211137	0.09203215	0.1312827	0.7010225	0.483289	0.92946
tmem11	542.4790561	-0.179882936	0.1026357	-1.752635	0.079665	0.548406
smx5	0	NA	NA	NA	NA	NA
cfap126	55.52116595	-0.072472206	0.275859	-0.262715	0.792771	0.977937
ccl25b	280.953815	0.003752895	0.140604	0.0266912	0.978706	0.996762
asmt2	148.7823242	-0.043260467	0.1840396	-0.235061	0.814162	0.981081
AL953867.1	0	NA	NA	NA	NA	NA
BX510941.1	0.635422286	2.809933352	2.5211574	1.114541	0.265047	NA
si:ch211-262h13.5	9.659578842	-0.021009179	0.6436475	-0.032641	0.973961	NA
abhd6b	26.10122056	0.615520446	0.3760265	1.6369073	0.10165	0.605756
si:ch211-262h13.3	224.5520671	0.111286751	0.1421157	0.7830715	0.433585	0.913667
met	148.8929928	-0.09548901	0.168672	-0.566122	0.571311	0.949418
sox2	1366.524049	-0.044104597	0.081561	-0.540756	0.588676	0.953319
dusp6	2046.665164	0.057196895	0.0749674	0.7629571	0.445489	0.917137
dnajc27	343.1918488	0.111001506	0.1270285	0.8738314	0.38221	0.895192
kitlga	259.9387889	0.122560233	0.1341304	0.9137397	0.360854	0.885686
si:ch211-284e20.8	476.8598989	0.369109763	0.1419403	2.6004578	0.00931	0.162001
cpne5b	29.30860191	0.022842824	0.3478776	0.0656634	0.947646	0.99381
cnbpb	3174.275457	-0.053114287	0.0736687	-0.720989	0.470916	0.924838

zgc:158296	208.7414739	0.237920167	0.1473971	1.6141439	0.106496	0.615567
sox14	383.8433148	0.000558498	0.1178504	0.004739	0.996219	0.998888
cart1	16.01062598	0.06509929	0.4844886	0.134367	0.893112	0.989291
si:ch211-232m10.6	152.4244678	0.105846673	0.1829906	0.578427	0.562976	0.948728
hcrt	17.83242861	0.420199798	0.460824	0.9118445	0.361851	0.885944
znf740a	555.5160391	-0.095164954	0.1007423	-0.944638	0.344844	0.878434
igfbp6a	1.030311776	0.994458412	1.953155	0.5091549	0.610644	NA
styk1a	94.73284331	-0.007368841	0.2178712	-0.033822	0.973019	0.996315
hmga1b	2464.490992	0.287345219	0.0749892	3.8318208	0.000127	0.00596
fam131ba	127.3944726	-0.266058568	0.1806438	-1.472835	0.140795	0.681252
pla2g4f.2	1.001591779	-2.198778422	2.0996481	-1.047213	0.295001	NA
hmx2	93.00558086	-0.070333663	0.2129629	-0.330263	0.741202	0.973154
hmx3a	225.8184082	-0.11593436	0.1435267	-0.807755	0.419232	0.906406
im:7160594	183.8203425	-0.077999285	0.169942	-0.458976	0.646251	0.96032
pex6	104.960498	-0.140777605	0.2127146	-0.661814	0.50809	0.935734
si:ch211-288g17.3	7708.80331	0.016440799	0.0681057	0.2414011	0.809244	0.980642
si:ch211-288g17.4	96.4339471	0.295069912	0.2176727	1.3555667	0.175237	0.735698
lepr	363.8033873	0.159468754	0.1256465	1.2691863	0.204375	0.772273
si:rp71-15k1.1	0.976668277	3.423229811	2.0853785	1.6415389	0.100686	NA
insl5a	15.76417996	-2.416850968	0.5597812	-4.317492	1.58E-05	0.001037
furinb	555.6771925	-0.001462435	0.1141145	-0.012816	0.989775	0.997612
si:ch211-81a5.8	571.6183173	0.038752429	0.1046924	0.3701552	0.711267	0.969006
si:dkey-88e12.3	2.097607108	0.193170451	1.3372151	0.1444573	0.885139	NA
ash1l	2036.552098	-0.048916071	0.0799367	-0.611935	0.540581	0.944631
mrps21	188.1142261	-0.135582616	0.1576013	-0.860289	0.38963	0.896421
sergef	116.4422004	-0.088459344	0.1892734	-0.467363	0.64024	0.959994
mlpha	93.65134103	-0.023889437	0.2097009	-0.113921	0.9093	0.990702
inpp4ab	69.37282882	-0.116524127	0.2575173	-0.45249	0.650916	0.960708
nuggc.1	3.350229215	-1.202399941	1.0828358	-1.110418	0.266819	NA
abhd12	423.2433975	0.058152855	0.1211603	0.4799664	0.631251	0.957356
ppp1r3ca	107.8551121	0.047994939	0.2024029	0.2371257	0.812559	0.981081
pcgf5a	23.76197494	-0.200334603	0.3930893	-0.509641	0.610303	0.95688
kif20ba	258.9299916	-0.102841268	0.1391109	-0.739275	0.45974	0.921978
tuft1b	175.1013798	0.15199103	0.1610603	0.9436903	0.345328	0.878434
cdk5r2a	1439.157461	-0.034542489	0.0862808	-0.400349	0.688899	0.966672
ifit14	10.41178707	1.669869279	0.6312694	2.6452562	0.008163	NA
arl6ip6	41.77837415	-0.281201913	0.3040095	-0.924977	0.354978	0.882321
s100u	773.8513459	-0.165598234	0.096958	-1.707937	0.087648	0.566637
pbxip1a	914.8374987	0.208451663	0.097408	2.1399851	0.032356	0.350031
nt5e	169.5468162	-0.135334067	0.175621	-0.770603	0.440942	0.916336
ptena	2036.971067	0.084323863	0.0830053	1.0158859	0.309684	0.86172
si:dkey-42l16.1	0.317831496	1.808837541	3.5779923	0.5055454	0.613176	NA
papss2a	764.9497763	-0.566809637	0.1033559	-5.484058	4.16E-08	6.13E-06
zgc:171679	310.6654632	-0.023612525	0.1298798	-0.181803	0.855737	0.985174
dytn	17.63206653	0.123045264	0.4546265	0.2706513	0.786659	0.976596
mllt11	1732.261719	0.138350336	0.0791236	1.748535	0.080371	0.549728
si:dkey-147f3.4	182.0922872	0.062702901	0.159707	0.3926122	0.694606	0.96736
snx1a	2361.183471	-0.019305591	0.0712263	-0.271046	0.786356	0.976476
pnkd	191.0842425	0.201041461	0.1691779	1.1883436	0.234698	0.803509
si:dkey-286j15.3	160.863731	0.210923333	0.174386	1.2095197	0.226463	0.793959
pex13	345.9781034	0.159941845	0.1229649	1.3007117	0.193357	0.757219
smim8	109.5430625	0.204989134	0.1900081	1.0788444	0.280657	0.84355
cx28.8	35.7503193	0.083738225	0.3158215	0.2651441	0.790898	0.977402
zgc:153722	13.26278312	0.003785271	0.5355804	0.0070676	0.994361	0.998694

bbip1	222.4495059	-0.077739847	0.14225	-0.546502	0.584721	0.952411
ccn6	542.3803073	0.070166402	0.115835	0.6057446	0.544684	0.945438
si:dkey-119m7.4	55.74812748	0.257424345	0.2718993	0.9467636	0.343759	0.878434
cbx6a	677.9325595	0.012469291	0.1002966	0.1243242	0.901059	0.990121
si:dkey-150i13.2	5.474159139	0.608692949	0.8319342	0.7316599	0.464376	NA
ZNF512B	29.63542661	-0.175140836	0.3533509	-0.495657	0.620136	0.957354
gtpbp2b	124.3868589	0.05551762	0.1854474	0.2993712	0.764657	0.975374
naa10	894.6124456	-0.138184048	0.0895656	-1.542825	0.122873	0.650905
hps5	267.6538757	-0.236482637	0.1380172	-1.713429	0.086634	0.56426
g6pd	755.2051067	-0.186880899	0.0944824	-1.977944	0.047935	0.43124
pthlhb	2.814722101	0.121046402	1.2109372	0.0999609	0.920375	NA
rccd1	58.15846771	0.112592007	0.2688488	0.4187931	0.675367	0.965257
ldhbb	433.1074876	0.033944843	0.1171515	0.2897516	0.772006	0.975687
p4ha1b	948.4526215	-0.44687794	0.1235937	-3.615702	0.0003	0.011799
si:dkeyp-34c12.1	13.48928336	-0.409148848	0.5585255	-0.732552	0.463832	0.922875
wipf1b	224.4465839	0.2546809	0.1507533	1.6893883	0.091145	0.578217
adgrg7.2	0.182570949	0.967652056	4.0804729	0.2371421	0.812547	NA
gpr155b	86.87631	-0.085118102	0.2229555	-0.381772	0.702631	0.968134
slc35b4	478.4954587	-0.193115963	0.1074965	-1.796486	0.072417	0.526156
adgrg7.1	0.31598276	0.005883445	3.3523626	0.001755	0.9986	NA
actn2b	708.8140311	0.104199605	0.0955897	1.0900718	0.275682	0.839541
chrm3a	88.80308448	-0.087448746	0.2093728	-0.41767	0.676188	0.965257
abi3bpb	1627.474438	0.252945292	0.0788778	3.2067995	0.001342	0.039088
si:ch73-367f21.4	102.731632	0.362580387	0.211344	1.7155932	0.086237	0.563157
si:dkey-222p3.1	55.04985085	-0.042529216	0.2969172	-0.143236	0.886104	0.988936
wnt7bb	116.9262735	0.107917823	0.1908343	0.5655054	0.57173	0.949494
xirp2a	935.1312632	0.266526489	0.1219747	2.1850968	0.028882	0.329785
eepd1	123.4107023	0.309743832	0.193675	1.5992966	0.109755	0.623766
zgc:64065	12.06245249	0.233451488	0.5755093	0.4056433	0.685005	0.966146
si:dkey-208m12.2	20.6899524	0.798161464	0.45035	1.7723135	0.076343	0.538373
cica	940.9218375	0.072487046	0.1122992	0.6454814	0.518615	0.938803
pick1	80.34332919	0.032299189	0.2230697	0.1447942	0.884873	0.988838
sft2d2b	136.6039191	-0.27324852	0.1803457	-1.515138	0.129738	0.662411
alkbh1	46.33455626	0.066012424	0.3069155	0.2150834	0.829702	0.982805
zmp:0000001138	0.841338706	-0.563977448	2.2263736	-0.253317	0.800024	NA
slc12a10.2	406.9700132	0.434005413	0.1209812	3.5873781	0.000334	0.012925
pimr73	1.539555802	2.928625969	1.7835936	1.6419806	0.100594	NA
cx40.8	8.524052039	-0.200252281	0.6967781	-0.287397	0.773808	NA
cavin2b	1373.069622	0.028185235	0.082809	0.3403644	0.733582	0.971993
usp40	485.3208007	-0.025240088	0.1042433	-0.242127	0.808682	0.980642
fopnl	65.30215959	-0.025315266	0.251831	-0.100525	0.919928	0.991857
sptssa	375.2610505	-0.170955658	0.1128331	-1.51512	0.129742	0.662411
tmem170b	11.72955437	0.501606541	0.5932823	0.845477	0.397845	0.899672
wnt4a	101.1074989	0.413656004	0.198777	2.0810059	0.037433	0.378816
oprl1	225.1558453	-0.01576756	0.1376275	-0.114567	0.908788	0.990702
zgc:112255	334.1829002	0.239137376	0.1191049	2.0077872	0.044666	0.41583
p3h1	372.1147754	0.046934094	0.1171569	0.4006088	0.688708	0.966672
rgl3a	242.5923213	0.163160896	0.1405945	1.1605073	0.245842	0.815789
palm3	93.33383537	0.102657257	0.2025196	0.5069003	0.612225	0.95688
si:ch211-133n4.9	0.316103113	0.005883447	3.3519121	0.0017553	0.9986	NA
hs6st3a	9.039078749	-0.933712259	0.7026031	-1.328933	0.18387	NA
zgc:158445	11.40510379	-0.12340896	0.5605361	-0.220162	0.825745	0.982196
si:ch211-133n4.4	1521.540622	0.166208184	0.0798149	2.0824194	0.037304	0.377702
lrfn5a	27.89066561	-0.001065461	0.4087373	-0.002607	0.99792	0.999234

zgc:158254	247.6978919	0.063916126	0.1545468	0.4135714	0.679188	0.965257
si:ch211-117c9.5	1079.283378	-0.090940468	0.0857349	-1.060717	0.288818	0.845979
ftr58	8.096461696	-0.780433464	0.6755984	-1.155174	0.248019	NA
trim35-39	63.68212042	0.079522531	0.2549155	0.3119564	0.755074	0.975234
tmem79a	34.99028879	-0.241715944	0.3782832	-0.638981	0.522835	0.939336
ppp1r18	315.9940283	0.084308467	0.1315747	0.6407651	0.521675	0.939056
fbxo34	1038.227052	-0.100249198	0.0887642	-1.129388	0.258734	0.826867
zmp:0000001139	14.35024793	0.462340313	0.5443647	0.8493209	0.395703	0.899378
si:zfos-452g4.1	63.91080603	0.324852041	0.2528707	1.2846569	0.198912	0.764191
ncoa6	1367.308161	-0.047568787	0.1125359	-0.422699	0.672515	0.965257
samhd1	168.7883587	0.164676499	0.1565186	1.0521211	0.292744	0.848607
tonsl	181.3216616	-0.120870727	0.1530595	-0.789698	0.429704	0.911615
chrm3b	0.173729368	-0.955901296	4.0804729	-0.234262	0.814781	NA
tbc1d2	60.94159629	0.244994005	0.256197	0.9562718	0.338935	0.87745
gip	2.821488783	0.546899828	1.1680579	0.4682129	0.639632	NA
si:dkey-7l4.5	2.137965949	0.236687056	1.2602923	0.1878033	0.851031	NA
cbarpb	88.43360321	0.163329478	0.2278634	0.7167869	0.473506	0.924993
ryr3	2498.12695	0.421608607	0.084307	5.0008722	5.71E-07	6.27E-05
si:ch211-212d10.2	264.438522	0.111231649	0.1346785	0.825905	0.408858	0.90171
mif	461.8818267	-0.208770704	0.1103683	-1.891581	0.058547	0.473924
borcs8	101.3655815	-0.000692777	0.2065319	-0.003354	0.997324	0.999143
abch1	75.73865269	0.195983097	0.2229569	0.879018	0.379392	0.895192
mgst2	113.1508839	-0.191579056	0.1963262	-0.97582	0.329154	0.873436
aftphb	248.6939122	0.105194355	0.1427576	0.7368739	0.461199	0.922161
si:ch211-235e9.8	3565.700067	0.211893339	0.0662128	3.2001862	0.001373	0.039879
si:ch211-197g15.7	28.04749763	0.135435566	0.3849247	0.3518495	0.724951	0.970402
map4k3b	108.2645623	0.113156912	0.1980395	0.5713856	0.567738	0.948739
morn2	23.71521706	0.074504502	0.3901458	0.1909658	0.848552	0.985174
si:ch211-197g15.10	0.810062152	0.536965757	2.3785369	0.2257546	0.821392	NA
lrrtm2	503.9418728	0.072211793	0.1033341	0.6988188	0.484665	0.93001
celf5a	40.00234035	0.152529028	0.308339	0.4946797	0.620826	0.957354
hsd11b1la	348.4572104	-0.004840731	0.1257079	-0.038508	0.969283	0.996315
micos13	563.6199437	0.169789839	0.1111939	1.5269708	0.126768	0.656588
si:dkey-7l4.11	0.491320158	0.8528132	2.8656354	0.2976	0.766008	NA
slc7a11	127.04621	0.206063155	0.1923639	1.0712155	0.284073	0.844697
si:dkey-21e2.3	0.802320445	1.849899411	2.4175996	0.7651802	0.444164	NA
si:dkey-7l4.6	0.166657454	-0.955901296	4.0804729	-0.234262	0.814781	NA
foxq2	46.18729884	-0.376637676	0.2871675	-1.311561	0.189668	0.754269
camk2g1	4032.42305	0.079598805	0.0650934	1.2228407	0.22139	0.788076
timm23a	1170.523185	0.030848903	0.0817404	0.3774008	0.705876	0.968412
BX247870.1	1.006818213	1.048314579	1.9690503	0.532396	0.594452	NA
si:dkey-172o19.2	1.134337053	2.462221963	2.0582167	1.196289	0.231584	NA
oaz1a	6340.315165	-0.005103405	0.0773082	-0.066014	0.947367	0.99381
tepsin	76.52593411	0.00953679	0.2339937	0.0407566	0.96749	0.996315
AL935279.1	0.157187365	-0.955901296	4.0804729	-0.234262	0.814781	NA
arl3b	910.9564896	0.06543805	0.0864199	0.7572107	0.448924	0.917995
cks2	85.0056761	-0.146642989	0.2215339	-0.661944	0.508007	0.935734
mier2	273.6105927	-0.074671233	0.1341919	-0.556451	0.577902	0.951024
cybc1	135.757554	-0.422826945	0.1818045	-2.325723	0.020033	0.266383
gna15.4	5.120854511	-0.875377763	0.8460345	-1.034683	0.300817	NA
gbx1	144.0109491	-0.001077454	0.1810322	-0.005952	0.995251	0.998696
ap3d1	985.2546178	0.047920733	0.0921141	0.5202321	0.602902	0.956045
nat9	367.3307899	0.115248084	0.1254904	0.918382	0.358419	0.884398
lrrc59	227.3812323	-0.147183295	0.1390402	-1.058566	0.289797	0.846246

tdo2a	264.7346819	-0.07461675	0.1315251	-0.56732	0.570497	0.949061
ptprc	48.70530961	-0.172699109	0.2823369	-0.611677	0.540751	0.944633
si:dkey-121a9.3	20.91303351	0.404132444	0.4236925	0.9538342	0.340168	0.87805
myoz1b	2681.00673	0.430965902	0.0891903	4.8319837	1.35E-06	0.000129
zgc:153659	0	NA	NA	NA	NA	NA
fam234a	17.3897021	0.013717096	0.4769118	0.0287623	0.977054	0.996762
tdrd5	7.178758871	-0.36776228	0.7584155	-0.484909	0.627741	NA
crp1	17.52883239	0.208304484	0.4537756	0.4590473	0.6462	0.96032
crp7	0.665049525	-0.08670226	2.5686305	-0.033754	0.973073	NA
crp6	0.339758314	0.005883863	3.2682461	0.0018003	0.998564	NA
si:dkey-121a11.3	325.4741415	0.147698598	0.1209513	1.2211413	0.222033	0.788551
si:ch211-234p6.5	116.0829239	0.155097603	0.1860822	0.8334896	0.404569	0.901305
CU896602.1	0.158795395	0.967652056	4.0804729	0.2371421	0.812547	NA
lama3	97.22594757	-0.839511089	0.2118474	-3.962811	7.41E-05	0.003804
zgc:154125	6.306608437	0.804624636	0.7890234	1.0197728	0.307836	NA
lrrc39	191.6483674	0.269951059	0.1707776	1.5807168	0.113943	0.632717
zbtb33	288.9597913	-0.047081273	0.1285813	-0.366159	0.714246	0.96909
mrpl54	326.5361802	-0.008364498	0.1254438	-0.066679	0.946837	0.99381
znf618	63.77113706	-0.163904018	0.2461125	-0.665972	0.505429	0.935724
aox5	226.2527499	0.065647037	0.143105	0.4587334	0.646426	0.960362
lnx2b	404.0650739	-0.200707452	0.117771	-1.704218	0.08834	0.569189
zgc:152948	45.23043171	-0.463670375	0.2927806	-1.583678	0.113267	0.631061
nrros	27.64926934	0.645665633	0.3651431	1.7682535	0.077019	0.540058
fbxo45	416.122627	0.105649185	0.1128973	0.9357988	0.349377	0.880195
olfm3a	254.7516546	0.036381392	0.1488415	0.2444304	0.806897	0.980254
fshr	0	NA	NA	NA	NA	NA
sult3st5	5.753004758	-1.082598878	0.94477	-1.145886	0.251842	NA
zic6	102.3999643	-0.092802653	0.2055945	-0.451387	0.651711	0.960708
zic3	964.1844996	0.116943969	0.0956325	1.2228474	0.221387	0.788076
fhl1a	198.9973507	0.02010771	0.1771116	0.1135313	0.909609	0.990702
cxcl32b.1	0	NA	NA	NA	NA	NA
fam207a	117.8692366	-0.170600603	0.1879237	-0.907818	0.363974	0.886249
hs6st1b	222.5361234	0.13864715	0.1595469	0.8690055	0.384844	0.89551
mgat5	744.9843427	0.127166378	0.0939738	1.3532104	0.175988	0.736505
krt222	401.8746602	-0.039830905	0.1326157	-0.300348	0.763911	0.975374
insrb	122.2467003	-0.105916693	0.1873916	-0.565216	0.571927	0.949494
si:dkey-42i9.8	1.639291888	-0.001819744	1.4395878	-0.001264	0.998991	NA
si:dkey-42i9.7	0.824205979	0.498048327	2.3641483	0.2106671	0.833147	NA
josd1	565.7326596	0.093372511	0.1005238	0.9288596	0.352962	0.881552
ecm2	243.6859471	0.361136858	0.1449765	2.4910026	0.012738	0.200229
xrcc6	510.3557813	-0.132426526	0.1025978	-1.290735	0.196796	0.761944
zgc:171500	0	NA	NA	NA	NA	NA
CU929149.1	0.158795395	0.967652056	4.0804729	0.2371421	0.812547	NA
zgc:153675	237.7933194	0.1128549	0.1403054	0.8043515	0.421194	0.90651
fblim1	127.9923767	-0.430237508	0.203849	-2.11057	0.034809	0.363229
dlx4b	73.18588072	-0.273199328	0.2293081	-1.191407	0.233494	0.802097
mtus1a	178.1276446	0.012287875	0.1579561	0.077793	0.937993	0.99279
hnf4g	92.69922191	-0.877005932	0.2406791	-3.643881	0.000269	0.010838
ppp1cab	1650.658364	0.026139441	0.0788681	0.3314322	0.740318	0.973082
TSTD1	158.9991068	0.126925199	0.2105905	0.602711	0.546701	0.945526
si:dkeyp-87e7.4	757.0966882	-0.159100567	0.0913397	-1.741855	0.081534	0.552315
lsm5	192.3886095	0.092174145	0.1703872	0.5409686	0.588529	0.953319
si:ch211-222k6.3	61.33806958	0.091993902	0.2580016	0.3565633	0.721419	0.969705
urad	63.1327361	0.297657923	0.2422366	1.2287899	0.219151	0.785734

si:ch211-222k6.1	26.66027806	0.635469108	0.3774938	1.6833895	0.0923	0.57849
gtf3ab	1.8162786	0.814953581	1.541792	0.5285756	0.5971	NA
stard15	103.8272194	0.207833819	0.209409	0.9924779	0.320964	0.868715
tgfbi	6049.877645	0.315157908	0.0751384	4.1943678	2.74E-05	0.001635
BX539307.1	0.966579724	0.959501961	2.1265385	0.4512037	0.651843	NA
si:dkey-253d23.2	69.74527469	-0.167540385	0.2406441	-0.696217	0.486293	0.930833
si:ch211-236g6.1	25.87192072	-0.629260395	0.4005937	-1.57082	0.116225	0.63841
aqp8a.2	284.811339	-1.079813314	0.1581756	-6.826674	8.69E-12	2.53E-09
TIMM21	106.811881	-0.002663773	0.2102858	-0.012667	0.989893	0.997612
netol1	230.6044443	0.024509169	0.1411832	0.1735984	0.862181	0.985841
pvalb9	841.1408609	0.343468408	0.0974787	3.5235231	0.000426	0.015652
si:ch211-156p11.1	3.471201992	0.686111577	1.1258632	0.6094094	0.542253	NA
si:ch211-213a13.5	4.915289106	0.187913586	0.8647192	0.2173117	0.827965	NA
ptgdsb.2	0	NA	NA	NA	NA	NA
BX511034.1	29.00154004	-0.068082214	0.3577487	-0.190307	0.849068	0.985174
BX511034.2	0.341486697	1.892317624	3.2124068	0.5890654	0.555817	NA
drd1a	10.03592966	-0.445821245	0.6216359	-0.717174	0.473267	NA
zgc:171490	0	NA	NA	NA	NA	NA
si:dkey-19a16.7	0	NA	NA	NA	NA	NA
zgc:113298	12.24264247	0.096464489	0.5467862	0.1764209	0.859963	0.985773
rrp8	98.55586753	-0.131471227	0.2091842	-0.628495	0.52968	0.942209
si:ch73-256j6.2	0.166657454	-0.955901296	4.0804729	-0.234262	0.814781	NA
si:dkey-19a16.5	1.909102993	0.89942414	1.4922532	0.6027289	0.546689	NA
si:dkey-19a16.4	56.06308831	-0.103046766	0.2719238	-0.378955	0.704722	0.968134
ywhag2	4969.955265	0.099649153	0.0689427	1.44539	0.148348	0.696298
si:rp71-36a1.3	0.500282092	2.45023829	2.5970012	0.9434876	0.345432	NA
si:dkey-222f2.7	0.831868617	-0.546811265	2.1107396	-0.259061	0.795588	NA
smyd5	161.0852701	-0.038783889	0.1675709	-0.231448	0.816967	0.981594
tma7	1330.496679	-0.001005775	0.2214531	-0.004542	0.996376	0.998902
nudt22	163.1212096	0.042645992	0.1588053	0.2685427	0.788282	0.976703
ctdspla	542.1217226	-0.096926957	0.1058718	-0.915513	0.359923	0.885686
atcaya	745.6876714	-0.168123523	0.0951906	-1.766177	0.077366	0.541968
mydgf	525.528232	-0.033255692	0.1190743	-0.279285	0.780026	0.976146
loxl5a	6.320950689	0.162674338	0.7828517	0.2077971	0.835387	NA
zfr2	761.7979447	0.148754832	0.1062614	1.3998959	0.161545	0.715364
rx1	950.0279109	0.12050282	0.1045656	1.1524139	0.249151	0.818463
slco5a1a	123.8452665	0.086484845	0.1949293	0.4436728	0.657279	0.962245
uqcrc2b	3936.386187	0.020045678	0.0803838	0.2493746	0.803071	0.979643
prex2	424.249461	0.037322889	0.1134268	0.3290481	0.742119	0.973154
ndc80	422.7847148	-0.246471177	0.1129247	-2.182615	0.029064	0.330497
pimr193	0.158915748	0.967652056	4.0804729	0.2371421	0.812547	NA
zgc:66433	640.5045562	-0.046966308	0.0963477	-0.487467	0.625928	0.957354
ids	115.4880881	-0.026898908	0.1885579	-0.142656	0.886562	0.988936
zgc:162941	0.157187365	-0.955901296	4.0804729	-0.234262	0.814781	NA
ppp1r9bb	179.7177795	-0.1107312	0.1776147	-0.623435	0.532999	0.942965
si:dkey-20i20.8	77.46088972	-0.353138502	0.2210742	-1.597376	0.110182	0.62463
si:dkey-20i20.2	77.74543098	-0.390457805	0.2391335	-1.632803	0.10251	0.608356
ankha	70.87179659	0.366728915	0.2464109	1.4882818	0.136677	0.673303
znf1167	40.94165241	-0.128392315	0.3123908	-0.410999	0.681073	0.965476
si:dkey-37o8.1	0	NA	NA	NA	NA	NA
si:ch211-207i20.3	63.10325655	-0.401486315	0.2524421	-1.59041	0.111742	0.627242
prlh2	43.5431448	-0.153379991	0.2930322	-0.523424	0.600679	0.956045
zgc:112977	26.51258027	0.154734534	0.3700799	0.4181112	0.675866	0.965257
pimr46	0.365141898	1.973577737	3.3927314	0.5817076	0.560764	NA

si:ch211-253p2.2	101.1351159	0.017556316	0.2263611	0.0775589	0.938179	0.99279
eef1e1	634.4569426	0.045442078	0.0987955	0.4599611	0.645544	0.96029
znf1153	58.986307	-0.139880336	0.2554076	-0.547675	0.583915	0.952214
znf1180	43.42754938	0.02127115	0.2961261	0.0718314	0.942736	0.993364
adarb2	20.87567631	-0.465562186	0.4298068	-1.083189	0.278724	0.842736
znf1162	29.54811591	0.167047064	0.3696332	0.4519266	0.651322	0.960708
si:dkey-32n7.9	0	NA	NA	NA	NA	NA
nrn1a	402.6657346	-0.041270983	0.1177169	-0.350595	0.725892	0.970402
itgb1a	2210.05132	0.203245493	0.072983	2.7848315	0.005356	0.108505
si:ch73-134f24.1	0	NA	NA	NA	NA	NA
znf711	289.0792096	-0.21970158	0.1261757	-1.741235	0.081642	0.552623
glod5	261.9859472	-0.029169389	0.1534702	-0.190066	0.849258	0.985174
zdhhc15b	430.5505842	0.063718287	0.1097124	0.5807756	0.561392	0.948728
rell2	32.14520387	-0.127717113	0.3400793	-0.375551	0.707251	0.968664
dhrs7cb	476.8206501	0.276275411	0.1060856	2.6042696	0.009207	0.16083
trmt112	332.6788395	-0.003412215	0.1211369	-0.028168	0.977528	0.996762
tgfb5	59.94996813	0.18939819	0.2812965	0.6733045	0.500754	0.93428
pimr56	0.157187365	-0.955901296	4.0804729	-0.234262	0.814781	NA
arl9	380.7176585	-0.047083403	0.148636	-0.31677	0.751418	0.97419
pcdh2ab1	4.538831639	0.620690438	0.9228968	0.6725459	0.501236	NA
CR847851.1	0.365141898	1.973577737	3.3927314	0.5817076	0.560764	NA
sec24b	987.5710639	0.003193061	0.115789	0.0275766	0.978	0.996762
rasa1b	76.86023587	-0.028631828	0.2210271	-0.12954	0.89693	0.990003
adgra3	233.5636844	-0.180019565	0.1462983	-1.230496	0.218511	0.785734
tlr8b	1.476211552	1.771389495	1.6751477	1.0574527	0.290305	NA
quo	544.2116617	-0.183639747	0.1023608	-1.794043	0.072806	0.526522
heatr6	205.3550198	-0.062245049	0.1449185	-0.429518	0.667547	0.964736
nav2a	780.268282	-0.054803359	0.0912969	-0.600277	0.548322	0.945871
mamdc2b	71.78377583	0.451300646	0.2352419	1.9184532	0.055054	0.459919
frmd8	563.6978436	-0.078029764	0.0997343	-0.782377	0.433993	0.914001
col9a1a	13802.84549	0.152635156	0.0871474	1.7514587	0.079867	0.548745
si:dkeyp-72g9.4	1001.038388	0.016200988	0.0941274	0.1721176	0.863345	0.985841
rbfa	75.87260976	-0.369214712	0.2254573	-1.637626	0.1015	0.605447
cnksr2b	622.4065287	0.0318254	0.1193649	0.2666228	0.78976	0.977238
zgc:171551	9.704676296	-0.598562596	0.6341418	-0.943894	0.345224	NA
fam149a	32.62336689	0.067550686	0.3498911	0.193062	0.84691	0.985174
usp7	1799.954761	-0.026736377	0.0729954	-0.366275	0.71416	0.96909
mmrn2b	75.08693174	-0.108372738	0.2478234	-0.437298	0.661895	0.964504
vav3b	270.1343088	0.018801194	0.1402015	0.1341013	0.893322	0.989291
cpxm1a	489.6793825	0.085433041	0.1066085	0.8013716	0.422917	0.90714
si:ch73-233k15.2	27.74127272	0.013101067	0.3948269	0.0331818	0.97353	0.996315
si:dkey-250123.4	173.630153	-0.105503384	0.1839061	-0.573681	0.566184	0.948739
smarcad1b	582.4719308	0.030886524	0.0999167	0.3091228	0.757228	0.975374
zgc:194679	68.84709724	-0.201668102	0.2406162	-0.838132	0.401957	0.90084
nlrx1	305.7636191	-0.050390349	0.1291392	-0.390202	0.696387	0.96736
zbtb32	0.500161739	2.450146464	2.7711133	0.8841741	0.376602	NA
gal3st3	166.3385702	0.208072861	0.1612986	1.2899855	0.197056	0.761944
dok4	349.1872311	0.039810194	0.1202048	0.3311863	0.740504	0.973154
myh14	963.6247513	0.027659117	0.0896163	0.3086393	0.757596	0.975374
ftr95	1.839552787	-3.211433563	1.6821863	-1.909083	0.056251	NA
suds3	542.6533432	-0.246588048	0.1067175	-2.310662	0.020852	0.27277
dennd2c	208.6708166	-0.149116364	0.150525	-0.990642	0.321861	0.869463
RAP1GDS1	14.6750534	0.124844766	0.5257435	0.2374632	0.812297	0.981081
prss59.2	2274.742095	-0.430465284	0.2483857	-1.733052	0.083086	0.555802

slc25a37	10.26393878	0.003699869	0.5800278	0.0063788	0.994911	NA
ip6k1	892.9247289	0.141288461	0.0955155	1.4792199	0.139082	0.677963
si:dkey-1j5.4	126.756371	-0.077396872	0.2085561	-0.371108	0.710557	0.968985
crygm2d18	13572.9664	-0.059560882	0.0892165	-0.667599	0.50439	0.935541
CACNA2D1	11.58028631	0.259847435	0.5562914	0.4671067	0.640424	0.959994
tacc1	142.1773468	-0.207993162	0.1875581	-1.108953	0.26745	0.833239
slc12a7a	244.0001332	-0.11777427	0.137719	-0.855178	0.392452	0.897688
pdzrn3a	26.52483987	-0.284785448	0.3671217	-0.775725	0.437911	0.915258
myo19	41.44454885	0.29294231	0.3025387	0.9682805	0.332904	0.874926
si:ch211-113j14.1	110.7853336	-0.440560926	0.1867159	-2.359526	0.018298	0.251621
gna13a	173.6541337	-0.141007091	0.1589716	-0.886996	0.375081	0.894316
tnni1d	276.3873712	-0.003114115	0.1395012	-0.022323	0.98219	0.996944
magi2b	23.85971424	0.602866833	0.3942846	1.5290143	0.126261	0.655241
tbc1d12b	181.9874023	0.17236555	0.1500557	1.1486775	0.250689	0.820469
tti2	115.6518012	0.044761917	0.1939474	0.2307942	0.817475	0.981594
si:dkey-17e16.9	5.428157328	-1.005811941	0.8272264	-1.215885	0.224029	NA
si:dkey-288i20.2	10.41395862	-0.18597124	0.671296	-0.277033	0.781755	NA
cmbl	222.8160331	-0.300259411	0.1983151	-1.514052	0.130013	0.662595
si:ch73-303b9.1	0.522329263	0.999930388	2.8066578	0.3562709	0.721638	NA
zmp:0000000735	16.61552552	-0.689204537	0.4939664	-1.395246	0.162942	0.717875
man1b1a	386.7050835	-0.049711326	0.1129655	-0.440058	0.659895	0.963364
zgc:194210	34.47149164	-0.112311106	0.3560499	-0.315436	0.75243	0.974684
si:dkeyp-104h9.5	320.4954892	-0.019755113	0.1319906	-0.149671	0.881025	0.988111
cntnap5l	346.9006342	0.173173645	0.1261563	1.3726916	0.169848	0.726965
WDR31	8.408443344	0.487124583	0.7477279	0.651473	0.514741	NA
thbs2b	155.9358468	0.016196517	0.1732478	0.0934876	0.925516	0.992676
or131-2	8.259543027	-0.576119796	0.6557711	-0.878538	0.379652	NA
scly	33.72742187	0.393055421	0.3318779	1.1843376	0.236279	0.805463
pax1b	85.78001156	0.412818622	0.2216542	1.8624448	0.06254	0.487868
zgc:174917	112.0530388	-0.888823556	0.5331721	-1.667048	0.095505	0.588012
znf1177	15.59836894	-0.770317643	0.5252742	-1.466506	0.142511	0.684646
LO017700.1	65.16824759	0.169045664	0.2648413	0.6382905	0.523285	0.939336
RASGRF1	225.9318083	0.080194009	0.1467645	0.5464127	0.584782	0.952433
senp2	620.8371457	0.131255962	0.0956715	1.3719445	0.170081	0.727199
herc2	2354.458109	0.101170287	0.1042046	0.9708807	0.331608	0.874645
myo9ab	1004.784138	0.039017073	0.0910576	0.4284878	0.668296	0.964736
si:dkey-85k7.10	0.173729368	-0.955901296	4.0804729	-0.234262	0.814781	NA
zgc:110843	1037.493927	-0.249481238	0.1009441	-2.471479	0.013456	0.20803
rbm15	454.467611	0.076951254	0.1114153	0.6906707	0.489772	0.931926
arhgef7b	840.5836516	-0.062072871	0.0886461	-0.700233	0.483782	0.929461
hdac7b	6.982877764	0.018749655	0.7100381	0.0264065	0.978933	NA
klf7a	30.24010068	-0.442480467	0.3449685	-1.282669	0.199608	0.764932
pex26	177.3803412	-0.034717192	0.1667022	-0.208259	0.835027	0.984089
kif13bb	247.0565433	0.013608403	0.143517	0.0948209	0.924457	0.992676
gas2l2	0.323844819	-1.825095364	3.274639	-0.557342	0.577293	NA
tdp1	91.38339578	-0.306367796	0.2037598	-1.503574	0.132691	0.667303
rex1bd	165.2881496	-0.136962574	0.1572278	-0.871109	0.383695	0.895192
pdzrn3b	564.3611265	-0.095213197	0.1012049	-0.940796	0.346809	0.8794
gdpd2	666.8527573	0.450831616	0.0981478	4.5933962	4.36E-06	0.000361
tpst1	788.0883728	0.107096249	0.1056641	1.0135533	0.310796	0.862927
crygm2d6	5079.468778	-0.055929102	0.0829809	-0.674	0.500312	0.934224
si:ch1073-165f9.2	42.65003126	-0.299722501	0.3017638	-0.993236	0.320595	0.868418
slc41a2b	404.1268805	0.09460015	0.1147856	0.8241464	0.409856	0.902506
clstn3	2862.242729	0.119770274	0.0695686	1.7216143	0.085139	0.560519

gdf10b	124.9022955	-0.085177625	0.1934944	-0.440207	0.659787	0.963278
secisbp2	102.396763	-0.401432578	0.1950804	-2.05778	0.039611	0.390659
dnase2	157.0310048	0.105191336	0.1812913	0.5802336	0.561757	0.948728
cx47.1	49.78407496	0.206272379	0.3054771	0.6752467	0.499519	0.934006
nemp1	93.38423571	-0.139306533	0.2284353	-0.609829	0.541975	0.944974
inka2	62.66609752	0.461376671	0.2823122	1.6342782	0.1022	0.60761
CABZ01046997.1	2.026250295	-1.556805599	1.4182557	-1.09769	0.27234	NA
ptpn9b	285.7348222	-0.047910649	0.1312761	-0.364961	0.715141	0.96909
zgc:92481	209.0053797	-0.735705953	0.1709947	-4.302509	1.69E-05	0.001088
ppip5k1b	378.9800798	0.006415734	0.1181356	0.0543082	0.95669	0.994543
cinp	112.3009931	0.097738485	0.1862444	0.5247861	0.599732	0.956045
tmem255a	783.2181538	0.095750657	0.0895381	1.0693847	0.284896	0.845094
si:ch211-202h22.7	30.11551903	0.188970704	0.3539186	0.5339383	0.593384	0.954434
pimr213	13.31306075	-2.783449086	0.658762	-4.225273	2.39E-05	0.001457
fam163ba	35.76692326	0.031454211	0.3317539	0.0948119	0.924464	0.992676
znf1085	14.81113372	-0.23814744	0.505071	-0.471513	0.637275	0.958872
b3glcta	274.9074951	-0.036107599	0.1270637	-0.284169	0.776281	0.975826
rnf41l	163.820911	0.206221378	0.1619534	1.2733379	0.202898	0.769849
cntnap5a	62.92270313	0.096270863	0.2589555	0.3717661	0.710067	0.968928
adck2	162.8838724	-0.088880016	0.1669021	-0.532528	0.594361	0.954434
synrg	751.0962284	-0.016198434	0.1065342	-0.152049	0.879148	0.987881
otulina	34.95045214	-0.355021398	0.3486312	-1.018329	0.308521	0.861088
gareml	18.56615272	0.316092574	0.44615	0.7084895	0.478641	0.926978
wnt6b	6.89810565	0.806563978	0.7412397	1.0881284	0.276538	NA
BX511021.1	9.746878233	0.650917801	0.6205613	1.0489178	0.294216	NA
si:ch73-386h18.1	1342.192631	-0.086656279	0.0833096	-1.040172	0.29826	0.85401
si:dkeyp-67e1.6	0.474898508	0.855240867	2.7202602	0.3143967	0.75322	NA
prok1	9.403542801	0.465050016	0.6185126	0.7518845	0.452121	NA
cd4-2.2	2.962102904	-0.669981969	1.1811811	-0.567214	0.570569	NA
slc4a7	149.9860512	0.023138014	0.1736455	0.1332485	0.893997	0.989527
tox3	708.106701	-0.022908291	0.0964007	-0.237636	0.812163	0.981081
pmch	29.04066486	0.084333096	0.3496628	0.2411841	0.809412	0.980642
si:ch211-160j14.2	48.87498722	-0.010499289	0.6177817	-0.016995	0.98644	0.996965
ccdc32	585.4433538	0.039553561	0.0986544	0.4009305	0.688471	0.966672
si:ch211-258f14.2	88.70931735	0.060528868	0.2204354	0.2745878	0.783633	0.97627
si:dkey-202b22.5	7.945078938	0.357981033	0.6628787	0.54004	0.589169	NA
mctp2b	51.83370844	0.160168148	0.265641	0.6029497	0.546542	0.945526
colgalt1	70.59286228	0.207633477	0.2398182	0.8657953	0.386602	0.89551
crabp2a	401.4695079	0.164296167	0.1125761	1.4594226	0.144449	0.688868
pctp	26.0446345	0.141372713	0.3766614	0.375331	0.707414	0.968664
mntb	472.0346673	0.012556838	0.104188	0.120521	0.90407	0.990291
si:ch211-276k2.1	0	NA	NA	NA	NA	NA
prickle3	60.85512745	0.119501709	0.2783898	0.4292604	0.667734	0.964736
srpk1a	609.7547304	0.175614695	0.1063811	1.6508071	0.098778	0.598346
si:ch211-23l10.3	35.82240232	0.380319468	0.3344517	1.1371431	0.255478	0.823982
taptla	118.373849	-0.176928637	0.2025503	-0.873505	0.382388	0.895192
crygmxl2	11211.98118	0.03062628	0.0967873	0.3164287	0.751677	0.974352
slc6a11a	76.43635354	0.505730185	0.2459463	2.0562624	0.039757	0.391511
stac	42.50976475	-0.174014566	0.2933854	-0.593126	0.553097	0.947265
znf1059	58.82475879	0.046882412	0.2717372	0.1725285	0.863022	0.985841
si:ch1073-159d7.7	16.12363355	0.371612167	0.4949844	0.7507554	0.4528	0.919551
zgc:194275	1.295689006	-0.690664441	1.7006876	-0.406109	0.684663	NA
ptgfr	0.48448895	0.85344211	2.878836	0.2964539	0.766883	NA
usp31	174.6830603	0.151262601	0.1595485	0.9480665	0.343096	0.878434

rbms1a	1506.087893	0.069071981	0.0819006	0.8433636	0.399025	0.900619
CT573248.1	20.85112967	-0.368303878	0.422664	-0.871387	0.383543	0.895192
myt1a	2106.223926	-0.144543985	0.0796592	-1.814529	0.069596	0.514708
fastkd2	319.4950185	-0.101765462	0.1294694	-0.786019	0.431856	0.912787
adamtsl2	47.82890894	-0.120514967	0.2784437	-0.432816	0.665148	0.964736
si:dkey-91i10.2	173.78509	0.351225096	0.1706678	2.0579456	0.039595	0.390659
ky	17.27698835	0.496764285	0.4897104	1.0144041	0.31039	0.862656
abca5	220.6333166	-0.025500662	0.1447677	-0.176149	0.860177	0.985773
efnb2b	177.8232922	0.015613248	0.1720879	0.0907283	0.927708	0.992702
si:ch211-281g13.5	1.297376104	-0.034812563	1.7279065	-0.020147	0.983926	NA
kctd1	220.1082054	-0.113868101	0.1456655	-0.78171	0.434385	0.914254
calm3b	16087.7906	0.023514157	0.068807	0.341741	0.732546	0.971719
dlg5a	1019.198937	0.024631598	0.0870731	0.2828842	0.777266	0.975826
map3k14a	317.7136242	-0.122912416	0.1269984	-0.967826	0.333131	0.874926
si:dkey-234i14.15	0	NA	NA	NA	NA	NA
rem2	27.23382058	0.173147503	0.3734617	0.4636285	0.642914	0.959994
tas2r200.2	0	NA	NA	NA	NA	NA
zgc:171452	5.307043864	-0.362911308	0.837682	-0.433233	0.664846	NA
irx5b	211.856686	-0.235202818	0.1695383	-1.387314	0.165346	0.721223
mrnip	18.04579038	0.13051198	0.4636725	0.2814745	0.778346	0.975942
BX511100.1	0.158915748	0.967652056	4.0804729	0.2371421	0.812547	NA
map6b	51.75954516	0.29593038	0.2680954	1.1038248	0.269669	0.834705
rnf26	84.36079104	-0.119290157	0.2145075	-0.556112	0.578134	0.951024
antxr1b	27.22113118	-0.034644758	0.3620874	-0.095681	0.923774	0.992628
si:dkey-3d4.3	36.17694704	-0.225240116	0.3211066	-0.70145	0.483022	0.929338
dnai2b	13.12114382	0.662862849	0.5370897	1.2341752	0.217138	0.784302
gpc5c	7.826691453	-0.053352037	0.7165594	-0.074456	0.940648	NA
strip1	996.874455	0.171607015	0.0867347	1.9785275	0.047869	0.43124
si:ch1073-188e1.1	0	NA	NA	NA	NA	NA
tecpr1b	168.4412125	0.003357687	0.1691514	0.0198502	0.984163	0.996944
nim1k	86.08919265	0.188975661	0.2152869	0.8777853	0.38006	0.895192
tgm2b	701.868318	-0.038567716	0.0953345	-0.404551	0.685807	0.966307
si:ch211-86h15.1	79.86095357	0.060308059	0.2218354	0.2718595	0.78573	0.97627
zgc:163014	2.120643845	-0.712338836	1.4157264	-0.503161	0.614851	NA
bcdin3d	52.0920163	-0.094354214	0.2877678	-0.327883	0.743	0.973486
si:dkey-1h24.2	31.21179606	0.08728549	0.3678258	0.2373012	0.812423	0.981081
ftr12	56.24011758	0.197970018	0.2572921	0.7694368	0.441634	0.916336
frmpd2	57.36193736	0.538021019	0.2792212	1.9268634	0.053997	0.455128
usp2b	88.16588716	-0.013330856	0.2268233	-0.058772	0.953134	0.994157
dnmbp	499.0304958	0.006991227	0.1058249	0.0660641	0.947327	0.99381
znf1161	45.24680252	-0.00683509	0.2959698	-0.023094	0.981575	0.996944
ttc39a	39.3619597	-0.473928586	0.3053683	-1.55199	0.120665	0.645578
edem3	868.5833786	0.110932605	0.0881946	1.2578159	0.208458	0.775404
kif5ba	1862.363338	-0.048827121	0.0756114	-0.645764	0.518432	0.938765
scpp7	0.524057646	2.503896172	2.7289583	0.9175282	0.358866	NA
slc47a2.2	2.460512468	0.622636951	1.2614463	0.4935897	0.621596	NA
c2cd3	214.5733733	0.224313714	0.1521349	1.4744397	0.140363	0.680585
myo1013	245.0981136	0.041592066	0.1447329	0.2873712	0.773828	0.975687
zgc:113452	113.3808606	-0.212032419	0.1934708	-1.09594	0.273105	0.838141
rbpjl	2.658336168	-1.12159655	1.1682715	-0.960048	0.337031	NA
itpr1b	559.5003746	-0.118822482	0.1103402	-1.076874	0.281537	0.84357
si:ch211-226h7.5	23.942247	0.037284005	0.4114816	0.0906092	0.927803	0.992702
slitrk5b	16.59983799	0.072583303	0.4652539	0.156008	0.876027	0.987881
sgca	446.7950203	0.293827953	0.1237151	2.3750367	0.017547	0.245562

paqr4b	68.40384453	0.402216111	0.2470606	1.6280057	0.103524	0.609625
tnrc6b	1357.256548	-0.031123105	0.0882554	-0.352648	0.724352	0.970402
zgc:162879	100.6459976	-0.097826436	0.2013768	-0.485788	0.627118	0.957354
birc6	2293.982622	-0.022391299	0.108517	-0.206339	0.836526	0.984386
tasor2	530.4196635	-0.084832659	0.1041048	-0.814877	0.415143	0.906237
gpam	503.4052107	0.326616243	0.1149444	2.8415151	0.00449	0.095365
fbxo4	38.59608396	-0.036913654	0.324037	-0.113918	0.909303	0.990702
calhm2.2	0.481032184	-2.398473301	2.80721	-0.854398	0.392885	NA
prokr1a	5.839500704	0.112838587	0.8057615	0.1400397	0.888629	NA
si:dkey-172j4.3	675.0523939	-0.02153993	0.1096707	-0.196406	0.844293	0.985174
arhgap32b	631.9046536	0.03783775	0.1046058	0.3617174	0.717563	0.96909
pcsk9	25.85602093	0.686550629	0.376743	1.8223315	0.068405	0.512556
zgc:172253	78.20789536	0.578428347	0.2455098	2.3560294	0.018471	0.253122
flna	3704.474921	0.039258778	0.0773293	0.507683	0.611676	0.95688
commd6	99.32283299	0.076886339	0.1968365	0.3906102	0.696085	0.96736
si:dkey-1c7.3	0	NA	NA	NA	NA	NA
zgc:110286	9.211674688	0.136227772	0.6952787	0.1959326	0.844663	NA
SLC5A10	17.08429544	-0.051525471	0.4826532	-0.106755	0.914984	0.991119
eif3bb	32.89166211	-0.203352129	0.3431045	-0.592683	0.553393	0.947265
wu:fc38h03	270.5005694	0.337975595	0.1342177	2.5181143	0.011799	0.191113
si:dkey-188i13.6	8.890570063	-0.337270929	0.6468607	-0.521397	0.602091	NA
map3k22	22.36783799	-0.481771279	0.4225651	-1.140111	0.25424	0.823394
zgc:172302	713.3380263	0.259472277	0.0999163	2.5968956	0.009407	0.162543
rap1gap2b	19.89415087	0.576323212	0.432432	1.3327486	0.182614	0.745308
MDFIC	149.935772	0.034873044	0.1655941	0.2105935	0.833204	0.983583
si:ch211-269e2.1	505.0157657	0.111492038	0.1059379	1.0524286	0.292603	0.848607
zgc:158398	297.9152191	0.004301493	0.1352379	0.0318069	0.974626	0.996579
prr12a	2638.550769	-0.066032248	0.0831932	-0.793722	0.427357	0.91075
CU138547.1	10.29563014	-0.331335125	0.634729	-0.52201	0.601663	NA
pde4bb	114.2474757	0.121445548	0.1974553	0.6150532	0.53852	0.943336
CR376783.1	18.46012157	0.408843311	0.4474558	0.9137065	0.360871	0.885686
brpf3b	200.7524898	0.200121777	0.1596712	1.2533368	0.210083	0.777646
serbp1a	13179.02916	-0.145355934	0.0620119	-2.344	0.019078	0.257862
arhgef10lb	168.0658357	0.022943103	0.1648122	0.1392075	0.889286	0.989291
spen	1385.420596	-0.023552271	0.0878563	-0.268077	0.78864	0.976703
rab14	2224.691954	0.038426898	0.0706478	0.5439219	0.586495	0.953319
tmem237b	218.8901145	-0.169021392	0.1514624	-1.11593	0.264452	0.830245
olig3	93.60829631	-0.221396596	0.2058491	-1.075528	0.282138	0.843666
abcb6b	33.3252744	-0.20522559	0.4033675	-0.508781	0.610906	0.95688
micu3b	636.7832967	0.30025385	0.1056522	2.8419069	0.004484	0.09535
gramd1bb	18.07271313	0.093064231	0.4472805	0.2080668	0.835177	0.984089
nck1a	474.8175422	-0.097915076	0.1123808	-0.87128	0.383601	0.895192
dzip1l	20.84723802	0.096841469	0.4128147	0.2345882	0.814528	0.981081
si:ch1073-314i13.4	85.46554247	-0.013375759	0.2294353	-0.058299	0.953511	0.994213
si:ch211-214e3.5	267.7642614	-0.034606629	0.1387089	-0.249491	0.802981	0.979643
wrb	328.7478422	0.090665058	0.1307206	0.6935788	0.487946	0.931877
POLR2E (1 of many)	0	NA	NA	NA	NA	NA
limch1a	121.9948625	0.273757938	0.1871131	1.4630612	0.143451	0.686804
si:ch73-12o23.1	360.226601	0.180362026	0.1223775	1.4738169	0.140531	0.680926
inpp5d	39.4256496	-0.205208717	0.3279947	-0.625646	0.531547	0.942358
si:ch73-335m24.2	155.8689029	0.232887125	0.17202	1.3538377	0.175788	0.736411
hint3	139.6258227	-0.006867042	0.1715339	-0.040033	0.968067	0.996315
sptlc2b	931.4953977	0.060829086	0.0858901	0.7082203	0.478808	0.926978
tuba4l	15.7029506	-1.183077739	0.4889447	-2.419656	0.015535	0.228768

adnpb	690.0999115	0.156893317	0.0971269	1.6153435	0.106236	0.615567
znf1015	207.1682669	-0.121806475	0.1586708	-0.767668	0.442685	0.916336
cth	895.3358509	0.06987201	0.1086133	0.6433097	0.520023	0.938995
gpr183b	0.998956154	2.235011873	2.0006611	1.1171367	0.263936	NA
phf12a	645.4630645	0.105230079	0.1016688	1.0350282	0.300656	0.856282
si:ch73-257c13.2	111.8246762	0.558959386	0.1994308	2.802774	0.005067	0.104143
ctslb	0	NA	NA	NA	NA	NA
rapgef5b	161.0227403	-0.127316211	0.1729865	-0.735989	0.461737	0.922466
lrrc75ba	218.2305675	0.137960356	0.1586621	0.8695229	0.384561	0.89551
cnnm4b	15.42055888	-0.508380603	0.5228989	-0.972235	0.330934	0.874224
rbfox3b	4.450622098	-1.792024237	1.0404761	-1.722312	0.085013	NA
mast2	916.5014056	0.016346058	0.085141	0.1919881	0.847752	0.985174
ttc37	238.6119064	-0.010594393	0.1452133	-0.072957	0.94184	0.993099
itga1	149.3960843	-0.024615976	0.1661557	-0.14815	0.882224	0.988344
fam20ca	9.077576304	-0.275205092	0.6307708	-0.4363	0.662619	NA
sall1a	937.1400794	0.179088882	0.0854065	2.0968989	0.036003	0.37021
CABZ01083937.1	23.85602382	0.209080079	0.3950509	0.5292485	0.596633	0.954903
tead3a	46.46452857	-0.11635491	0.2912398	-0.399516	0.689513	0.966775
si:ch211-194m7.3	88.69810965	-0.071305177	0.2601791	-0.274062	0.784037	0.97627
efcab2	8.894705115	1.214401325	0.6651506	1.825754	0.067887	NA
taar12m	0	NA	NA	NA	NA	NA
mcm8	80.34938839	-0.411397274	0.2160987	-1.903747	0.056943	0.468383
apba1b	1105.152918	0.039751838	0.0856656	0.4640352	0.642623	0.959994
arvcfa	349.360137	-0.014534269	0.1407051	-0.103296	0.917728	0.991623
eif4g3b	751.013119	0.225806279	0.1206032	1.8723081	0.061164	0.482482
si:ch1073-335m2.2	1542.910181	0.030256178	0.0874414	0.3460167	0.72933	0.970822
si:dkey-1c7.1	0.673073222	-1.460830855	2.4012698	-0.608358	0.54295	NA
boc	1816.996222	-0.070241357	0.0800965	-0.876959	0.380509	0.895192
cbfa2t2	315.7184261	-0.136951713	0.1258328	-1.088363	0.276435	0.839541
serf2	1797.03288	-0.014613733	0.1072742	-0.136228	0.891641	0.989291
hykk.2	58.93191425	-0.29152934	0.2550417	-1.143066	0.253011	0.822599
si:ch211-214b16.4	97.76561315	0.324610501	0.222673	1.4577898	0.144898	0.688986
l3mbtl1b	201.0622855	0.123409569	0.1535034	0.8039536	0.421424	0.90651
fxn	126.8041296	0.23050321	0.1841255	1.2518807	0.210613	0.778338
znf11c	0.801959387	1.851619607	2.2655353	0.8172989	0.413758	NA
ftr57	0.643163992	1.4381025	2.4329265	0.5910999	0.554454	NA
zgc:165582	7.769673512	0.430642437	0.706444	0.6095918	0.542132	NA
TTC9	483.3282549	0.078743544	0.1039783	0.7573075	0.448866	0.917972
zgc:171901	76.45326971	-0.409075541	0.2349485	-1.741129	0.081661	0.552623
usp12b	546.0695237	-0.066601496	0.1011805	-0.658245	0.510381	0.936253
tirap	5.00665157	-0.970450258	0.9097794	-1.066687	0.286113	NA
elfn1b	102.2995577	0.25781391	0.2274065	1.1337141	0.256914	0.82511
mdga1	19.05135225	0.506723784	0.4777286	1.0606939	0.288829	0.845979
junba	841.9729656	-0.059394976	0.1087231	-0.546296	0.584862	0.952436
knstrn	203.0021478	-0.274332783	0.1477114	-1.857221	0.06328	0.491281
farp1	872.0697759	-0.010842561	0.0890564	-0.121749	0.903098	0.990121
slc12a8	28.11759687	-0.20674597	0.3999132	-0.516977	0.605172	0.95651
alkal1	11.84571586	0.176135259	0.574077	0.3068147	0.758984	0.975374
gpr26	4.456344382	-0.516436266	0.9387367	-0.55014	0.582224	NA
tmem176l.4	1328.863155	0.077300548	0.0951108	0.8127418	0.416366	0.906237
sh3yl1	84.77457114	-0.012029882	0.2125682	-0.056593	0.954869	0.994543
fscn2b	375.9318988	0.340144991	0.1256555	2.7069642	0.00679	0.12739
gnpat	51.53708205	-0.382844629	0.2801785	-1.366431	0.171804	0.730433
pex5lb	26.48503304	-0.298803335	0.4175697	-0.715577	0.474253	0.925066

brip1	138.3277636	-0.185237252	0.1766334	-1.04871	0.294311	0.850231
sema4ba	19.32102454	0.111423001	0.4757288	0.2342154	0.814818	0.981081
zgc:171482	60.6902822	-0.069952146	0.3061713	-0.228474	0.819278	0.981594
hcn4l	32.96957115	-0.338527766	0.3404849	-0.994252	0.3201	0.868153
ibtk	588.0608761	0.220572637	0.1162038	1.8981528	0.057676	0.471275
si:busm1-57f23.1	235.4226591	-0.030345955	0.1536341	-0.197521	0.84342	0.985174
BX571825.1	0	NA	NA	NA	NA	NA
hhat	8.760178146	0.373389335	0.657266	0.5680947	0.569971	NA
ddb1	1678.694312	-0.043414743	0.0913838	-0.475081	0.634729	0.9578
ttc19	261.2701041	0.033084775	0.1397933	0.2366692	0.812913	0.981081
gp1bb	20.64104194	0.217768121	0.4455589	0.4887528	0.625017	0.957354
alx4b	34.10694787	-0.915162978	0.3589811	-2.549335	0.010793	0.180252
gas7a	134.8968528	0.075528897	0.1947208	0.3878831	0.698103	0.967998
dapk3	470.9093191	0.00112807	0.1146552	0.0098388	0.99215	0.997927
mnd1	120.7661549	-0.167704667	0.1900921	-0.882229	0.377653	0.895192
zfx	627.6709256	-0.160211356	0.1014413	-1.579351	0.114256	0.633565
setd9	42.01686809	-0.483486977	0.3026642	-1.597437	0.110168	0.62463
si:dkey-96f10.1	1.630611944	-0.56588234	1.5089763	-0.375011	0.707652	NA
vps13c	643.0878591	0.055405157	0.0986698	0.561521	0.574442	0.950037
gdpd3a	258.1293763	-0.136016476	0.1394198	-0.975589	0.329268	0.873436
prdm11	140.535218	0.027052452	0.1686065	0.1604473	0.872529	0.987846
amigo3	183.6585443	0.231992805	0.1622585	1.4297731	0.152782	0.701776
vps39	572.8175251	-0.118936761	0.1071761	-1.109732	0.267115	0.833191
odam	235.4953567	-0.393883295	0.13938	-2.825968	0.004714	0.09895
cnksr2a	28.63857923	-0.693949301	0.3554128	-1.952516	0.050877	0.44241
ulk1b	49.18044646	-0.005955156	0.285309	-0.020873	0.983347	0.996944
ifngr1l	32.79978681	0.195457163	0.344127	0.5679798	0.570049	0.948983
casc4	799.2794599	0.018969835	0.0886477	0.2139912	0.830554	0.982994
chd9	2240.395316	-0.09932697	0.0745252	-1.332797	0.182598	0.745308
tnip2	79.33635715	-0.788420427	0.2443202	-3.226996	0.001251	0.036865
zmiz1a	1798.55812	-0.124374408	0.0746587	-1.665908	0.095732	0.588986
mturn	60.10354541	-0.148066578	0.2561151	-0.578125	0.56318	0.948728
tmem94	651.0578283	-0.00839601	0.0976208	-0.086006	0.931461	0.992702
rmdn1	547.7380051	-0.504449817	0.1155517	-4.365578	1.27E-05	0.000877
si:dkey-28e7.3	503.1856052	0.261099436	0.1159305	2.2522068	0.024309	0.301184
tacr2	10.69120811	0.404994255	0.5827195	0.6950072	0.487051	NA
si:busm1-104n07.3	1.868864504	0.85083392	1.5020443	0.5664506	0.571088	NA
BX248501.1	19.06666222	0.339051412	0.4333043	0.7824788	0.433933	0.914001
map6a	189.0412238	-0.061135565	0.1508611	-0.405244	0.685298	0.966146
cntnap1	23.91724664	-0.222229866	0.3959754	-0.561221	0.574647	0.950037
zbtb16b	212.3498628	-0.124583419	0.1531212	-0.813626	0.415859	0.906237
chst15	54.68417585	0.051979803	0.2620719	0.1983417	0.842778	0.985174
unm_sa821	68.00395199	-1.132873191	0.2463238	-4.599121	4.24E-06	0.000353
KCNK12	1.459669549	1.801334326	1.6829353	1.0703527	0.284461	NA
spag9a	62.50140396	0.056367288	0.2589939	0.2176394	0.82771	0.982721
slc25a38b	697.6377563	0.133729327	0.0993909	1.3454888	0.178467	0.739486
lingo2a	444.7986527	0.080393885	0.1084553	0.7412627	0.458534	0.921978
si:dkey-179j5.5	0.158915748	0.967652056	4.0804729	0.2371421	0.812547	NA
gpr63	212.3215577	0.027985833	0.1658305	0.1687617	0.865984	0.986517
znf1007	198.7505907	-0.005460065	0.146654	-0.037231	0.970301	0.996315
si:ch211-213a13.2	55.2325677	-0.183890581	0.283623	-0.648363	0.51675	0.938543
si:ch211-240l19.8	0.856582408	1.927068658	2.1124105	0.9122605	0.361632	NA
zbtb17	382.5551956	-0.093886145	0.1154047	-0.813538	0.41591	0.906237
ndufs7	1481.22693	-0.10344499	0.0922981	-1.12077	0.262386	0.830166

tspan31	967.6878118	-0.026416247	0.0919616	-0.287253	0.773919	0.975687
ganc	82.14749139	0.069079625	0.217842	0.3171088	0.751161	0.97419
cntnap2b	254.4108396	0.001031503	0.1525435	0.006762	0.994605	0.998694
CHST8	129.0263696	0.088940525	0.179904	0.4943776	0.62104	0.957354
slc47a3	54.81810348	0.108982957	0.2655827	0.4103542	0.681546	0.965476
gpr37a	43.78529961	-0.046579744	0.3021111	-0.154181	0.877467	0.987881
fam57bb	30.70188916	0.182639116	0.3633914	0.5025962	0.615248	0.95688
pusl1	17.83726058	0.191143296	0.4624003	0.4133719	0.679334	0.965257
gpaa1	324.0901568	-0.084191434	0.1196856	-0.703438	0.481783	0.928392
zgc:56304	1478.300144	-0.103683415	0.086231	-1.202391	0.229212	0.796702
ppp1r9alb	313.7502537	-0.125703253	0.1261553	-0.996417	0.319048	0.867746
add2	869.7036637	0.007990155	0.0908718	0.0879278	0.929934	0.992702
gfra4b	110.5158057	0.099767046	0.2043253	0.4882756	0.625355	0.957354
grid1a	224.9112885	-0.072955459	0.1442427	-0.505783	0.613009	0.95688
METTL18	52.9990831	0.262137724	0.2693666	0.9731633	0.330472	0.874098
rin2	254.2493698	-0.051386755	0.1335127	-0.384883	0.700324	0.967998
wdpcp	74.21444084	0.097736611	0.2263227	0.4318463	0.665853	0.964736
eda	14.56005783	0.133772534	0.4983267	0.2684434	0.788358	0.976703
lzts2a	66.05458614	-0.334060449	0.2417942	-1.38159	0.167098	0.723651
frmd4ba	618.5548489	-0.003786958	0.1017884	-0.037204	0.970322	0.996315
gpr78a	27.26007453	0.323056787	0.3860581	0.8368087	0.4027	0.90084
cbln2a	62.31671806	0.117435353	0.2489762	0.471673	0.63716	0.958872
mrvi1	7.685662038	-0.28302022	0.8549809	-0.331025	0.740625	NA
phc3	7.933331869	0.829215796	0.717348	1.1559464	0.247703	NA
unm_sa1506	0.484248244	0.852636534	2.8793247	0.2961238	0.767136	NA
si:ch211-113e8.6	1.000564183	3.450262048	2.0260994	1.7029086	0.088585	NA
wdr37	966.5909914	0.084506767	0.0876673	0.9639484	0.335072	0.875906
si:ch211-240l19.6	31.55645934	-0.326675337	0.4166167	-0.784115	0.432973	0.913433
wfs1b	146.4069756	-0.088921126	0.1848078	-0.481155	0.630407	0.957354
tbc1d31	159.8661547	0.154329768	0.1707489	0.9038406	0.36608	0.887239
si:ch211-198a12.6	3179.414499	0.054013498	0.0694741	0.7774623	0.436886	0.914408
pidd1	60.19465037	-0.156761744	0.2702524	-0.580057	0.561876	0.948728
si:dkey-10o6.2	10.54078169	0.153315203	0.6187218	0.2477935	0.804294	NA
gpr35.1	15.10903135	0.133258603	0.5154751	0.2585161	0.796009	0.978498
keap1b	532.6257438	-0.132923228	0.110005	-1.208338	0.226917	0.794587
abca1a	1397.664966	-0.214790118	0.1047991	-2.049542	0.040409	0.39304
cep170b	96.44110098	0.027497944	0.2236658	0.1229421	0.902153	0.990121
loxhd1b	23.80626192	0.006369104	0.3901563	0.0163245	0.986976	0.997073
si:dkeyp-51b7.3	26.34614458	-0.170743226	0.3798277	-0.449528	0.653051	0.960792
spock1	12.19908285	0.387132191	0.5767184	0.6712673	0.50205	0.935237
CABZ01032476.1	133.5010306	0.136075129	0.1775023	0.7666105	0.443313	0.916631
adamts16	19.43245562	0.451894425	0.4639706	0.9739721	0.33007	0.874085
smtna	7.70172114	0.130699748	0.6797978	0.1922627	0.847536	NA
si:ch211-233m11.1	0	NA	NA	NA	NA	NA
ctss2.1	39.15847308	-0.060953602	0.3448779	-0.17674	0.859713	0.985773
rilp	51.04930473	-0.155768454	0.3070698	-0.507274	0.611963	0.95688
gper1	11.62945998	0.445471105	0.5893659	0.7558481	0.44974	0.918526
cntf	26.23443958	0.317987129	0.3845795	0.8268437	0.408326	0.90171
SAMD8	177.2727676	-0.006243666	0.1778608	-0.035104	0.971997	0.996315
cdk14	13.74660995	0.445707504	0.5392993	0.8264567	0.408545	0.90171
zgc:153760	26.25146399	0.465540172	0.3845246	1.2106902	0.226014	0.793556
akt1s1	2269.62402	-0.277067028	0.0867816	-3.192693	0.00141	0.040686
cacng4b	11.39871521	-0.445138163	0.5729786	-0.776884	0.437227	0.914408
matk	0.182570949	0.967652056	4.0804729	0.2371421	0.812547	NA

nptx1l	704.6515628	0.065636457	0.0973523	0.6742155	0.500174	0.934224
pan2	537.397036	-0.185476877	0.1103872	-1.680239	0.092911	0.580357
rprd2b	523.7961272	-0.039383646	0.1043347	-0.377474	0.705821	0.968412
frem3	416.1966331	0.198931039	0.110835	1.794839	0.072679	0.52617
rims1a	876.2974894	-0.039350903	0.1031806	-0.381379	0.702922	0.968134
mlsl	100.5079108	0.204805155	0.2005404	1.0212663	0.307128	0.861074
oga	3295.084109	-0.186540639	0.0703922	-2.650019	0.008049	0.144818
fbrs	1345.023458	0.020502483	0.0909166	0.2255087	0.821584	0.981928
ppm1j	81.69035664	0.108922643	0.2351281	0.463248	0.643187	0.959994
mms22l	185.2497895	-0.262867403	0.1514655	-1.735494	0.082653	0.554782
mertka	160.6433609	-0.149234308	0.1932052	-0.772414	0.439869	0.916082
zgc:194621	31.1594231	0.024202227	0.3700512	0.0654024	0.947854	0.99381
npdc1a	489.0882318	0.167817882	0.1071677	1.5659371	0.117363	0.640853
sdsl	86.7756258	-0.638495671	0.2190277	-2.915136	0.003555	0.080346
arfgef2	937.2164172	-0.119172111	0.0971551	-1.226617	0.219967	0.786668
ccdc97	180.3326284	-0.041473091	0.1623619	-0.255436	0.798386	0.978884
armc3	27.27050488	0.641148296	0.4019391	1.5951378	0.110681	0.625514
si:ch211-186e20.2	0.491560863	0.853615855	2.865157	0.2979299	0.765757	NA
stk17a	760.0867976	-0.077618251	0.1099385	-0.706015	0.480179	0.927347
rab38c	22.70911885	-0.819647181	0.4096953	-2.000626	0.045433	0.418148
si:ch211-51h9.7	44.93554406	-0.14410124	0.286473	-0.503019	0.614951	0.95688
rassf9	9.216839801	-0.542488485	0.6448317	-0.841287	0.400187	NA
myo10l1	73.84255968	0.166365278	0.2349928	0.707959	0.478971	0.926978
nrde2	169.0951188	-0.094941448	0.1560646	-0.608347	0.542957	0.944974
dnajc10	499.2941162	0.107191526	0.1090778	0.9827074	0.325751	0.871896
mfsd9	11.35406693	-0.568277418	0.5781583	-0.98291	0.325652	0.871896
pkmyt1	98.79776996	0.200729718	0.2020836	0.9933003	0.320564	0.868418
BX682234.1	1.495310112	-0.994696088	1.5684186	-0.634203	0.525948	NA
msl1a	157.8876126	-0.278043427	0.1625436	-1.710577	0.087159	0.565634
slitrk3b	168.7917888	0.122445579	0.1606977	0.7619624	0.446082	0.917154
arhgap18	88.44935518	-0.119267545	0.2113391	-0.564342	0.572521	0.949494
elmod3	5.147327927	0.295256162	0.8233738	0.3585931	0.7199	NA
zmp:0000000760	2035.538015	0.076666033	0.3167626	0.2420299	0.808757	0.980642
kcnd1	128.9143596	0.115957112	0.1794466	0.646193	0.518154	0.938592
brwd1	578.2654522	-0.109511258	0.1095459	-0.999683	0.317464	0.867541
abca12	2011.159381	0.087598268	0.0794587	1.102438	0.270271	0.835161
qser1	346.2543064	0.007599375	0.1222778	0.0621485	0.950445	0.99381
hlfa	1050.32691	0.287739718	0.0916371	3.1399911	0.00169	0.047321
si:dkey-188i13.9	1.690969553	0.610696504	1.5782705	0.3869403	0.6988	NA
tfb2m	608.3456274	-0.046615156	0.0971462	-0.479845	0.631337	0.957356
arv1	169.4199623	0.214155862	0.1636279	1.3087983	0.190603	0.755212
csde1	7286.634177	0.025793418	0.0621381	0.415098	0.67807	0.965257
ccar1	859.2113621	-0.073949991	0.0978857	-0.755473	0.449965	0.918761
ttc7a	221.1125766	0.024675332	0.1458517	0.1691809	0.865654	0.986517
bicdl1	18.95867202	0.221730429	0.4443291	0.4990229	0.617763	0.957354
BX571825.2	0	NA	NA	NA	NA	NA
mrap2a	62.54390755	0.220771981	0.2461887	0.8967592	0.369847	0.889487
zgc:171426	35.53211216	-0.023244066	0.3195918	-0.07273	0.942021	0.993099
mhc1zja	5.189349535	0.739063793	0.8903989	0.8300367	0.406518	NA
zgc:113295	63.5253059	-0.192351684	0.2504562	-0.768005	0.442484	0.916336
ifngr1	48.16619956	-0.030860906	0.2882437	-0.107065	0.914737	0.991119
ccl44	115.2123917	0.009458896	0.2049412	0.0461542	0.963187	0.995927
si:dkey-110g7.8	32.98542951	-0.249899095	0.3350111	-0.745943	0.455702	0.921215
tmem154	130.4486203	0.24120244	0.1912566	1.2611456	0.207256	0.775175

ank1b	326.3843441	0.095185097	0.1324607	0.7185914	0.472393	0.924838
alms1	361.284293	-0.273332364	0.1175605	-2.325036	0.02007	0.266383
ptprt	40.98917356	0.075486911	0.3076108	0.2453975	0.806149	0.980105
adgrg3	8.569034308	0.114828274	0.6416111	0.1789686	0.857962	NA
rab5if	1236.997932	-0.150714221	0.0964131	-1.563213	0.118003	0.641526
aldh3a1	94.23571342	-0.348408267	0.2078619	-1.676153	0.093708	0.582517
necab3	230.6233885	-0.043850333	0.1409692	-0.311063	0.755753	0.975374
dbf4	149.2631554	-0.122455896	0.1781414	-0.687408	0.491825	0.931926
cflarb	25.64031856	-0.232950115	0.3829468	-0.608309	0.542982	0.944974
atxn7	208.3999549	-0.079340861	0.1512171	-0.524682	0.599804	0.956045
afap1l2	405.0129691	-0.169556937	0.1224527	-1.384672	0.166153	0.722724
tbrg4	715.7696915	0.161531546	0.105987	1.5240694	0.127491	0.657542
megf6b	184.542742	0.165515348	0.1553019	1.0657648	0.28653	0.845942
zgc:194392	80.33367406	-0.093270477	0.2194575	-0.425005	0.670833	0.965257
fhdc1	84.57204751	0.096870956	0.2243169	0.4318486	0.665851	0.964736
btr05	28.76284662	-1.283773188	0.3797455	-3.380615	0.000723	0.023796
mxra5a	215.6671457	0.078344673	0.1413851	0.5541224	0.579495	0.951257
si:zfos-367g9.1	0	NA	NA	NA	NA	NA
si:cabz01036022.1	6.164764177	-0.367410062	0.758617	-0.484316	0.628162	NA
si:dkeyp-23e4.3	73.80419222	-0.476621449	0.2409493	-1.978098	0.047918	0.43124
si:dkey-253d23.9	27.86265032	0.503865953	0.3947565	1.276397	0.201815	0.767736
si:dkey-49c17.4	1.371719405	1.657057572	1.8000877	0.9205427	0.357289	NA
si:ch73-184c24.1	10.50709574	0.525299752	0.6172235	0.8510689	0.394731	NA
rhobtb2a	8.928345348	0.267579738	0.6721408	0.3981007	0.690556	NA
rasip1	124.4736776	-0.284854502	0.1908972	-1.492188	0.13565	0.671761
setmar	21.3649804	-0.021685314	0.4196004	-0.051681	0.958783	0.995027
impg1b	148.9081997	0.192881651	0.1706562	1.1302354	0.258377	0.826456
snx8a	55.50674904	-0.127959484	0.2687844	-0.476067	0.634026	0.95776
phldb2b	539.5327396	0.025725869	0.1042296	0.2468193	0.805048	0.979883
CABZ01061495.1	283.3165798	0.032758253	0.1311988	0.2496841	0.802832	0.979643
si:dkeyp-68b7.12	6.929092962	-1.146206302	0.738057	-1.553005	0.120422	NA
smim7	157.6411482	0.108403203	0.1809311	0.5991408	0.549079	0.946312
rac1a	1729.817971	-0.008125729	0.0812829	-0.099968	0.920369	0.992049
il12rb2l	125.7818651	0.053166992	0.186476	0.2851144	0.775556	0.975687
s1pr4	3.113266902	0.79006505	1.0743458	0.7353918	0.462101	NA
myo15b	215.8300288	-0.523726978	0.1656024	-3.162557	0.001564	0.044368
sh2d1ab	0.633934609	1.436045836	2.6103598	0.5501333	0.582228	NA
stard7	908.3726526	0.197419597	0.0885437	2.2296299	0.025772	0.310555
slc5a7a	184.9988478	0.395039818	0.1821215	2.1691008	0.030075	0.335259
si:dkey-112m2.1	242.4467636	0.11126093	0.1384653	0.8035296	0.421669	0.90651
frmpd3	498.3367079	0.011309075	0.1073515	0.1053462	0.916101	0.991314
ptpn5	595.6299208	0.059679317	0.1074168	0.5555866	0.578494	0.951052
stim2a	118.6673821	0.359448141	0.1863269	1.9291266	0.053715	0.45411
AL929017.1	97.96098949	-0.093151646	0.1978787	-0.470751	0.637818	0.959031
si:ch1073-291c23.2	360.5755667	-0.047468845	0.131231	-0.36172	0.717562	0.96909
pimr170	0	NA	NA	NA	NA	NA
abcd1	16.11169119	0.37956887	0.508756	0.7460724	0.455624	0.921215
CT025742.1	80.1178445	0.108240447	0.2388652	0.4531446	0.650445	0.960708
pde6d	391.0924912	0.038552523	0.1159907	0.332376	0.739605	0.972943
myorg	799.1299825	0.070959085	0.08901	0.7972035	0.425333	0.908894
tysnd1	37.93070529	0.153573724	0.3121863	0.4919297	0.622769	0.957354
hes2.1	19.4346636	0.07265291	0.4867592	0.1492584	0.88135	0.988251
taok2a	878.2452313	-0.046136626	0.0931148	-0.495481	0.620261	0.957354
nol4la	14.55290459	0.140538915	0.5000466	0.2810516	0.778671	0.975942

itcha	51.95112853	0.200131024	0.2651839	0.7546878	0.450436	0.918761
si:ch211-253b8.5	221.0907512	0.417995614	0.1518766	2.752205	0.00592	0.116124
camk1da	36.19376981	0.101923311	0.3253636	0.3132598	0.754083	0.975056
znf831	13.45215291	0.402171619	0.5517974	0.7288392	0.4661	0.923865
col6a1	3933.88821	0.168924728	0.0683168	2.4726691	0.013411	0.207707
dcaf17	49.90568654	-0.063704506	0.2763741	-0.230501	0.817702	0.981594
crtc2	192.8891412	-0.052752911	0.1481536	-0.356069	0.721789	0.969939
CU929346.1	4.144296544	-1.071983804	0.9367278	-1.144392	0.252461	NA
etaa1	68.68555743	-0.184554579	0.2414105	-0.764485	0.444579	0.916777
BFSP1	698.374815	0.117959385	0.1126592	1.0470466	0.295078	0.851519
fam117ba	369.4346796	-0.261724019	0.1146407	-2.282994	0.022431	0.285605
bub1bb	220.6614303	-0.102552322	0.1384006	-0.740982	0.458705	0.921978
si:dkey-234i14.13	0	NA	NA	NA	NA	NA
si:ch1073-70f20.1	23.87107906	0.193339456	0.394788	0.4897298	0.624325	0.957354
polg2	74.96716688	-0.0333837	0.2252953	-0.148178	0.882203	0.988344
calhm5.1	37.23165947	-0.332386694	0.3257515	-1.020369	0.307553	0.861088
c1qtnf7	1.330381041	-0.013681077	1.5829989	-0.008643	0.993104	NA
zgc:194114	1.301781397	-2.680907112	1.8600666	-1.441296	0.149501	NA
nup98	2194.168353	-0.13993332	0.0781064	-1.791573	0.073201	0.527933
or102-3	1.443756055	0.969583669	1.6798317	0.577191	0.56381	NA
zgc:162698	609.7655273	0.048600984	0.0995551	0.4881817	0.625421	0.957354
polr2m	120.7719939	0.12414147	0.1992717	0.622976	0.5333	0.942992
larp4ab	847.8610182	0.132672995	0.0893238	1.4853037	0.137463	0.674492
jac9	10.3352661	-0.339465938	0.7974212	-0.425705	0.670323	NA
LO017656.1	5.66167453	1.236346162	0.863434	1.4318943	0.152174	NA
aurkaip1	675.5632634	0.071577836	0.0935584	0.7650604	0.444236	0.916726
sparcl1	739.6076719	0.235522258	0.0925209	2.5456124	0.010909	0.180962
eif2b5	598.8939511	-0.059697281	0.1037994	-0.575122	0.565209	0.948739
tbrg1	87.43507596	0.220019636	0.2155757	1.0206144	0.307437	0.861088
chst2a	647.9671806	0.054880142	0.0967688	0.5671263	0.570628	0.949061
pask	100.9047181	-0.280470936	0.20074	-1.397185	0.162358	0.716781
glra2	186.1655495	-0.007328472	0.1602731	-0.045725	0.96353	0.995927
usp45	251.4489455	-0.007055111	0.1326974	-0.053167	0.957599	0.99467
sqstm1	210.5816474	-0.04404805	0.170385	-0.258521	0.796005	0.978498
soul5	777.0815129	0.320005962	0.2639115	1.2125504	0.225302	0.792725
apobb.2	461.7759294	0.106102626	0.1452218	0.7306247	0.465008	0.923671
myzap	237.981289	0.017404984	0.1376821	0.1264143	0.899404	0.990121
fam120a	947.2057442	0.045795429	0.0846309	0.5411195	0.588425	0.953319
si:ch211-93e11.8	2.657466954	-0.697277337	1.2049192	-0.578692	0.562797	NA
si:ch73-375g18.1	531.49183	-0.041928952	0.1034669	-0.40524	0.685301	0.966146
cdc123	331.6783689	-0.198776582	0.1201172	-1.654855	0.097954	0.59749
fgfr1bl	0	NA	NA	NA	NA	NA
stx10	92.12782196	-0.125457601	0.2060963	-0.608733	0.542701	0.944974
GTPBP8	55.81370838	0.423222659	0.2582204	1.638998	0.101214	0.60447
raver2	389.8288374	0.037705581	0.1172907	0.3214712	0.747853	0.97419
si:ch211-225k7.6	0	NA	NA	NA	NA	NA
fmnl2b	1233.551447	-0.042139207	0.0793281	-0.531202	0.595279	0.954434
si:dkeyp-123h10.2	525.8098434	-0.054852647	0.1110879	-0.493777	0.621464	0.957354
cxcl18b	274.4654202	-0.570493152	0.1750148	-3.259685	0.001115	0.033569
rnf151	20.02053007	0.314063428	0.4577127	0.6861584	0.492613	0.931926
lonrf1	1443.229827	0.172624109	0.0824532	2.0936015	0.036295	0.372062
sccpdha.2	15.27933932	-0.054179138	0.4880571	-0.11101	0.911609	0.991066
rasgrf2a	96.71604195	0.265255245	0.201071	1.3192121	0.187098	0.751002
ggt1l2.1	1.284290867	1.543812104	1.8520158	0.8335848	0.404515	NA

si:dkeyp-33b5.4	101.1625884	0.068989483	0.1955041	0.3528801	0.724178	0.970402
gabra6a	224.0824822	0.405029649	0.1621066	2.498539	0.012471	0.19775
aipl1	86.16973012	0.384378442	0.2132836	1.8021937	0.071515	0.522931
rpap1	268.6240575	0.063755316	0.1377762	0.4627455	0.643547	0.959994
prkdc	271.5968338	-0.174741476	0.1286432	-1.358342	0.174355	0.734611
CU467646.1	0	NA	NA	NA	NA	NA
si:dkey-52l18.4	71.15723876	-0.133036639	0.2291282	-0.580621	0.561496	0.948728
wdr17	326.4159232	0.144923548	0.1323604	1.0949161	0.273553	0.838253
neo1b	605.5047605	0.065001316	0.1026472	0.6332496	0.526571	0.940512
nkx2.4a	110.1678484	-0.052018746	0.1927776	-0.269838	0.787285	0.976596
tmco3	112.2828764	-0.213351414	0.1920413	-1.110967	0.266583	0.832634
dab2ipb	139.0766499	0.050360491	0.1826768	0.2756808	0.782793	0.97627
als2a	15.84460044	0.292925174	0.4959733	0.5906067	0.554784	0.947449
tacr3a	2.771377862	0.490503279	1.1713513	0.4187499	0.675399	NA
nanog	3.309441286	-0.915968951	1.0767869	-0.85065	0.394964	NA
sall2	299.7304683	-0.053928983	0.1364549	-0.395215	0.692684	0.96736
hbegfa	930.2637858	0.291010325	0.1101311	2.6423987	0.008232	0.147048
trafd1	358.4921648	-0.069499889	0.1186664	-0.585674	0.558094	0.948478
TMEM8B	415.0991122	-0.069808997	0.117671	-0.593256	0.55301	0.947265
or103-5	3.956921213	0.45238471	0.9787171	0.4622222	0.643922	NA
mrps11	447.146273	0.042944783	0.1127623	0.3808435	0.703319	0.968134
fh	1814.745593	-0.013037583	0.076152	-0.171205	0.864063	0.98601
adgrb1a	1395.193598	0.049532124	0.0859445	0.5763271	0.564394	0.948739
adgrf11	0	NA	NA	NA	NA	NA
hdac5	54.06328023	0.144723611	0.3025969	0.4782719	0.632457	0.957356
SAT2	325.9379505	0.064515529	0.1258956	0.5124526	0.608334	0.956718
gprc5bb	40.54181325	0.285574443	0.3323808	0.8591785	0.390242	0.897284
nanp	88.24594819	0.117735733	0.2121269	0.5550249	0.578878	0.951052
lrrc38a	67.95709402	0.210279028	0.2460445	0.8546382	0.392751	0.897688
nagpa	69.08574101	0.096847138	0.2393778	0.4045786	0.685787	0.966307
si:dkey-188i13.10	15.89332082	-0.559361707	0.5058521	-1.105781	0.268821	0.834131
tbc1d4	222.999192	0.03431267	0.1401966	0.2447468	0.806652	0.980254
igdcc3	903.1278187	-0.005205049	0.0917671	-0.05672	0.954768	0.994543
meltf	217.4049318	-0.30081439	0.1452954	-2.070365	0.038418	0.384454
defbl1	561.5335343	-0.823239628	0.1269737	-6.483543	8.96E-11	2.19E-08
cxcl20	55.3483305	-0.234130222	0.3027661	-0.773304	0.439343	0.916082
mylk5	559.5013822	-0.084569738	0.1006005	-0.840649	0.400545	0.90084
zc3h6	36.31418209	-0.058403141	0.3152169	-0.185279	0.85301	0.985174
bbs1	168.6980491	0.188004013	0.1649141	1.1400121	0.254281	0.823394
zdhhc22	80.73826111	0.195029239	0.2234912	0.8726482	0.382855	0.895192
fbxo25	586.7049825	-0.286931925	0.0992402	-2.891287	0.003837	0.084672
si:ch1073-322p19.1	270.0975556	-0.120834616	0.1321	-0.914721	0.360338	0.885686
ribc2	20.23607727	0.066954212	0.4471781	0.1497261	0.880981	0.988111
lpcat3	572.7401946	-0.376669163	0.1018065	-3.699854	0.000216	0.009128
si:dkey-59p5.2	0	NA	NA	NA	NA	NA
crfb16	69.08985876	0.157169792	0.2353141	0.6679148	0.504188	0.935448
rnf220b	159.5523557	-0.058660617	0.1643664	-0.356889	0.721175	0.969602
adamts10	95.05137285	0.095469183	0.2115003	0.4513902	0.651708	0.960708
cntnap5b	683.998521	0.03172752	0.0963941	0.3291439	0.742047	0.973154
oacyl	68.57863414	0.421546729	0.2463723	1.7110149	0.087078	0.565361
ymel1la	801.2779692	-0.011933874	0.107641	-0.110867	0.911722	0.991066
BX908780.1	0	NA	NA	NA	NA	NA
si:dkey-56e3.3	128.838038	-0.208706734	0.1929321	-1.081763	0.279358	0.842882
inpp4b	465.9331223	-0.016254691	0.110794	-0.146711	0.88336	0.988706

zgc:195212	3.675044508	1.148340064	1.0460035	1.0978357	0.272276	NA
si:dkey-100n19.2	10.79588647	-0.016608095	0.606182	-0.027398	0.978142	NA
p4htm	215.062823	0.039643659	0.1419591	0.2792611	0.780044	0.976146
chd7	3564.163161	-0.081958275	0.0881407	-0.929858	0.352445	0.881179
11-Mar	6.181786595	-1.032893152	0.7910481	-1.305727	0.191645	NA
vezt	117.1098939	0.305124591	0.1826528	1.6705172	0.094817	0.585466
tdrd12	2.170703437	0.732118802	1.3354977	0.5481992	0.583555	NA
amer2	1323.441893	0.008082272	0.0779024	0.1037487	0.917369	0.991585
si:ch211-223a10.1	82.24442355	-0.023900002	0.216667	-0.110308	0.912165	0.991119
smad4a	335.4310075	-0.058962563	0.1477188	-0.399154	0.68978	0.966819
slc46a2	25.34023672	0.493526211	0.3981508	1.239546	0.215143	0.78275
tet1	12.60715235	0.235744343	0.5380183	0.4381716	0.661262	0.964234
pimr74	1.959800294	-1.556886293	1.4551202	-1.069937	0.284648	NA
wdr11	398.4883382	0.027703813	0.118167	0.2344463	0.814639	0.981081
zgc:172133	2.457095745	-0.206129555	1.2111562	-0.170192	0.864859	NA
fam171a2a	851.7331123	0.055459949	0.0899514	0.6165544	0.537529	0.943336
phrf1	735.2210355	0.070795706	0.1092083	0.6482633	0.516815	0.938543
pik3cb	414.259623	-0.032480969	0.1130679	-0.287269	0.773906	0.975687
timp2b	630.1667853	0.181304269	0.127552	1.4214145	0.155196	0.704997
ankrd1a	33.27727411	0.301785701	0.3352189	0.9002647	0.367979	0.887482
si:dkeyp-74a11.1	111.5026861	0.067134364	0.2033167	0.330196	0.741252	0.973154
plaua	10.4945759	0.382555117	0.6315195	0.6057693	0.544668	NA
si:dkey-20i10.7	123.0095166	-0.390423098	0.196058	-1.991365	0.046441	0.423579
rapgef5a	20.80698732	0.049342013	0.4412751	0.1118169	0.910969	0.991059
thsd1	2.147944193	-0.244997877	1.3020838	-0.188158	0.850753	NA
si:dkeyp-14d3.1	153.0454783	0.397671047	0.1761638	2.2573935	0.023983	0.299022
tbc1d30	477.7414854	-0.09247293	0.1072248	-0.862421	0.388456	0.896271
irs2b	862.8221482	-0.122318841	0.0924817	-1.322627	0.185959	0.75038
kansl1l	22.57839349	0.255330868	0.411417	0.6206133	0.534854	0.943336
MRAS	10.94345782	0.041349268	0.5904385	0.0700315	0.944169	NA
nitr4a	0.365141898	1.973577737	3.3927314	0.5817076	0.560764	NA
myo15aa	102.8367157	-0.588906788	0.2123902	-2.772759	0.005558	0.111245
tulp1a	484.6400328	0.034605493	0.1178005	0.2937636	0.768939	0.975687
fam83c	6.428202071	0.206798224	0.7530385	0.2746184	0.783609	NA
trnt1	287.6779642	-0.132463392	0.138731	-0.954822	0.339668	0.87805
prxl2c	43.7481202	0.092182327	0.291545	0.3161856	0.751862	0.974458
gsdf	10.79824336	-0.02574103	0.5878244	-0.04379	0.965072	NA
kcnq2a	62.4167605	0.154337183	0.2688859	0.5739875	0.565976	0.948739
zgc:174906	294.4762177	-0.148504136	0.1338976	-1.109088	0.267392	0.833191
CABZ01071972.1	2.343401954	0.880621204	1.2998471	0.6774806	0.498101	NA
srfbp1	241.0785855	-0.137808301	0.1367793	-1.007523	0.313683	0.865132
mrpl16	943.8140266	-0.082075071	0.1016204	-0.807664	0.419284	0.906406
dock10	529.3613508	-0.066707585	0.1140719	-0.584785	0.558692	0.948478
phox2ba	3.143924992	-0.137077418	1.0698043	-0.128133	0.898044	NA
golga4	386.3992344	-0.034548754	0.1142372	-0.30243	0.762324	0.975374
si:ch211-119d14.3	19.79378273	-0.539421762	0.4288578	-1.25781	0.20846	0.775404
tbc1d25	519.4699576	-0.023292226	0.1018836	-0.228616	0.819167	0.981594
arhgap32a	402.8179601	0.250157604	0.113077	2.2122774	0.026948	0.316613
lars2	187.8429419	-0.18020987	0.1533567	-1.175103	0.239954	0.808111
ipp	140.4753156	0.084579346	0.1888296	0.4479137	0.654215	0.961305
med1	359.1994642	0.049968055	0.1180819	0.4231645	0.672175	0.965257
csf1a	35.08451366	-0.224346348	0.3323018	-0.675128	0.499594	0.934006
si:ch211-76l23.7	14.59863671	0.523718735	0.5001808	1.0470589	0.295072	0.851519
tmem129	188.4885703	-0.117483053	0.1616926	-0.726583	0.467482	0.924328

chfr	159.4832534	-0.167170407	0.165825	-1.008113	0.3134	0.865132
si:ch73-138n13.1	797.7739577	-0.082251218	0.0996494	-0.825406	0.409141	0.901769
brinp3b	169.916589	0.030808451	0.155125	0.1986041	0.842572	0.985174
espl1	457.5884141	-0.101555381	0.1098276	-0.92468	0.355132	0.882321
ftr29	1.316595017	-3.843590041	1.8868985	-2.036988	0.041651	NA
whrna	138.0231168	0.01281119	0.1862598	0.0687813	0.945164	0.993474
mrm2	23.17201408	-0.168921185	0.3933186	-0.429477	0.667576	0.964736
nck1b	750.6469302	0.083633889	0.0923851	0.9052747	0.36532	0.886795
zgc:171501	15.69449878	-0.074625323	0.4948224	-0.150812	0.880124	0.987881
sgsm1b	70.99412057	0.1259701	0.2407447	0.5232519	0.600799	0.956045
kri1	314.2124279	0.075486492	0.1296772	0.5821108	0.560492	0.948728
slc9a6b	337.4065443	-0.00956315	0.12211	-0.078316	0.937577	0.99279
gramd1ba	71.37304307	0.069290546	0.2428663	0.2853033	0.775412	0.975687
LO017852.1	439.1067995	-0.123185683	0.1191753	-1.033651	0.301299	0.856944
si:ch211-281g13.4	3.670459881	0.264020565	1.0757514	0.245429	0.806124	NA
spock2	2436.561939	0.030770885	0.0782715	0.3931301	0.694223	0.96736
dguok	122.1595406	0.076594482	0.1796546	0.4263431	0.669858	0.965223
cipca	222.259629	-0.125817283	0.1411941	-0.891094	0.372879	0.892868
fbxo33	150.6337155	-0.105427425	0.1684734	-0.625781	0.531459	0.942358
gpha2	2.130894036	0.243344004	1.3102391	0.1857249	0.852661	NA
adck5	158.8334808	-0.106913726	0.1635786	-0.653593	0.513374	0.93654
BX571825.3	0	NA	NA	NA	NA	NA
si:ch211-207k7.4	41.89755361	0.50229774	0.3099776	1.6204325	0.105139	0.613317
zgc:175107	68.10976163	0.076192853	0.2347629	0.3245524	0.74552	0.974094
pttg1	69.70408622	-0.053297785	0.233528	-0.228229	0.819469	0.981594
MRPL49	229.7440779	0.100680138	0.1389947	0.7243452	0.468854	0.924328
BX547992.1	0.488104098	-2.415107183	2.7936704	-0.864493	0.387317	NA
myom2a	5741.071916	0.344186297	0.1894405	1.8168568	0.069239	0.514556
akap11	784.3160493	0.124141111	0.0927608	1.3382932	0.180801	0.741776
sytl2b	104.0926052	-0.603606617	0.204201	-2.955943	0.003117	0.073687
shc1	972.6357989	0.008404124	0.0850005	0.0988714	0.92124	0.992138
vwa2	76.23282338	0.369856871	0.2259586	1.636835	0.101665	0.605756
cgref1	206.5540078	0.149556137	0.1482883	1.0085497	0.313191	0.865069
psmb5	2225.497697	-0.066615094	0.0765349	-0.870388	0.384088	0.895192
naa25	957.5825877	0.030905013	0.0959379	0.3221358	0.74735	0.97419
mkxa	363.6810395	0.171796029	0.1166496	1.4727526	0.140818	0.681252
hdc	67.53340549	0.123459692	0.2465488	0.5007516	0.616546	0.95688
soga3b	96.67142652	-0.030132307	0.218045	-0.138193	0.890088	0.989291
pik3ca	59.69784218	-0.056896238	0.2663845	-0.213587	0.830869	0.982994
ptpn20	146.4056453	0.255675577	0.1711384	1.4939698	0.135184	0.670689
kmt5c	530.7589473	0.007731043	0.103418	0.0747553	0.940409	0.992857
mss51	61.35787616	0.334282757	0.2567745	1.3018535	0.192966	0.756795
shisa3	423.5581132	0.067237477	0.1087345	0.618364	0.536335	0.943336
vwa1	395.0246078	0.069350569	0.115262	0.6016776	0.547389	0.945526
znf989	27.28398161	0.50813468	0.3867667	1.3138015	0.188913	0.753954
nlrp15	0.158795395	0.967652056	4.0804729	0.2371421	0.812547	NA
si:dkey-63d15.12	29.9630355	0.169448386	0.3749597	0.451911	0.651333	0.960708
zgc:173587	19.22837483	0.336316464	0.4559131	0.7376766	0.460711	0.921978
si:ch211-267e7.3	213.7489707	-0.329512434	0.1417421	-2.324733	0.020086	0.266389
si:ch211-26b3.4	1059.224529	0.12066997	0.0844841	1.4283158	0.153201	0.702508
ccdc160	65.79901607	-0.171375551	0.2447535	-0.700197	0.483804	0.929461
smim1	17.72385669	0.82108868	0.4661158	1.761555	0.078145	0.54467
cdnf	128.9742616	0.557205099	0.1903166	2.9277803	0.003414	0.078133
si:dkey-40c23.2	9.177940723	-0.350571941	0.6376523	-0.549785	0.582467	NA

si:ch211-132g1.7	13.62633734	0.196328215	0.5631225	0.3486421	0.727358	0.970402
si:dkey-108k21.10	14.21177183	0.826098883	0.5711492	1.4463802	0.148071	0.695805
phf12b	778.0898502	0.154845671	0.0899351	1.7217494	0.085115	0.560519
ccdc136b	346.0476189	0.126794067	0.1256802	1.0088625	0.313041	0.865017
cd37	15.10668674	0.213735773	0.5344711	0.3999014	0.689229	0.966763
si:ch211-120k19.1	37.72501015	0.576261132	0.3354235	1.7180105	0.085795	0.562501
cpz	179.5012924	0.56214331	0.1581668	3.5541172	0.000379	0.014176
cln8	54.5443517	0.110709604	0.265595	0.4168361	0.676798	0.965257
pwwp2a	403.4468353	0.036408924	0.1248675	0.2915804	0.770607	0.975687
zgc:174154	0.157187365	-0.955901296	4.0804729	-0.234262	0.814781	NA
lyrm9	40.8316031	-0.145802244	0.300397	-0.485365	0.627417	0.957354
pigu	137.7002128	0.038516506	0.174434	0.2208084	0.825242	0.982196
slc4a11	75.1236483	0.225422804	0.2307442	0.9769381	0.3286	0.872945
zbtb24	48.21702988	-0.63756405	0.2977587	-2.141211	0.032257	0.349508
cpne1	1642.7156	0.013663853	0.0786584	0.1737113	0.862092	0.985841
zgc:163061	35.54384532	0.35882223	0.3296458	1.0885086	0.276371	0.839541
snphb	14.91176571	0.401676934	0.4930944	0.8146046	0.415299	0.906237
BX649498.1	39.95447541	-0.104711334	0.3183218	-0.328948	0.742195	0.973154
si:ch73-236c18.3	0.81823394	3.165585784	2.3439761	1.3505196	0.176849	NA
zfhx4	2544.052541	-0.001595649	0.0784493	-0.02034	0.983772	0.996944
chd8	2016.799794	0.066736485	0.086984	0.7672267	0.442947	0.916336
si:dkey-89b17.4	2476.446093	-0.013380093	0.0719027	-0.186086	0.852377	0.985174
cdh5	488.7655126	-0.127691551	0.1063349	-1.200843	0.229812	0.79764
aifm5	52.7965188	0.364930252	0.2748684	1.3276544	0.184292	0.747376
adamts17	15.9290023	0.369123916	0.4780579	0.7721323	0.440036	0.916082
luzp1	709.9364624	-0.0412075	0.0940919	-0.43795	0.661423	0.964234
apcdd1l	233.6680527	0.141978024	0.1488855	0.9536057	0.340283	0.87805
ppm1g	1336.148655	-0.046608512	0.0931129	-0.500559	0.616682	0.95688
kmt2cb	898.7299747	-0.098844328	0.0919569	-1.074899	0.28242	0.843666
si:dkey-76k16.5	11.54929853	-0.240637679	0.5560292	-0.432779	0.665175	0.964736
si:ch211-119o8.4	1.150397644	2.462741491	1.944031	1.2668222	0.205219	NA
fam13a	862.6577683	-0.147262	0.0919434	-1.601659	0.109231	0.623132
lzts1	129.0746932	-0.215563256	0.1814563	-1.187962	0.234848	0.80358
fbxl20	39.15469136	-0.011630327	0.3092098	-0.037613	0.969996	0.996315
gpbp1l1	1362.574987	-0.041989723	0.0953614	-0.440322	0.659704	0.963227
ide	1459.256544	0.087804004	0.0803432	1.0928622	0.274454	0.839136
bicd2	22.89805904	1.15960704	0.4168169	2.7820539	0.005402	0.109079
lrch2	26.8351111	-0.005030911	0.3757448	-0.013389	0.989317	0.997589
itfg1	281.1486329	-0.026026233	0.1331762	-0.195427	0.845059	0.985174
tnfaip8l2a	40.93592025	0.102696107	0.3238092	0.31715	0.75113	0.97419
trim71	962.8687482	-0.030782051	0.0883193	-0.348531	0.727441	0.970402
flrt1b	427.1924936	-0.10412418	0.1111903	-0.93645	0.349041	0.880195
ambn	0.492230656	-0.896181115	2.8626633	-0.313059	0.754236	NA
cdc25d	24.84148799	0.031478945	0.3864459	0.0814576	0.935078	0.992702
si:dkeyp-41f9.3	47.72009065	0.653660498	0.2916815	2.2410081	0.025026	0.305655
RNF5	171.6790175	0.202809748	0.1612236	1.2579408	0.208413	0.775404
mical2a	46.14510225	-0.054778332	0.2960395	-0.185037	0.8532	0.985174
ercc6	532.983949	0.050505086	0.1078882	0.4681241	0.639696	0.959994
apoc4	1.852751589	0.34253551	1.4966504	0.2288681	0.818971	NA
polr2k	198.9385968	-0.071690454	0.1966765	-0.36451	0.715478	0.96909
slc36a1	200.7870173	0.226416592	0.1502161	1.5072729	0.131741	0.66523
cenpo	46.4087531	-0.103825639	0.2814693	-0.36887	0.712224	0.969006
birc5a	207.3375132	-0.056762829	0.1487335	-0.381641	0.702728	0.968134
zgc:174938	181.1337719	0.035877137	0.1845415	0.1944123	0.845853	0.985174

bend5	13.20077855	-0.520064729	0.5692933	-0.913527	0.360966	0.885686
lrig1	804.4836642	-0.013653482	0.0932773	-0.146375	0.883625	0.988706
zgc:172090	0	NA	NA	NA	NA	NA
si:dkey-197i20.6	85.26105709	-0.372285972	0.2337912	-1.592387	0.111298	0.627048
rad9b	0.31598276	0.005883445	3.3523626	0.001755	0.9986	NA
si:ch211-225k7.4	1.794442136	-0.254656755	1.5328234	-0.166136	0.86805	NA
si:ch211-89o9.6	229.8801895	-0.025289012	0.1456016	-0.173686	0.862112	0.985841
steap3	6.499320263	1.376334362	0.7800598	1.7643959	0.077665	NA
ifi35	32.7658148	-0.038820377	0.3352172	-0.115807	0.907806	0.990702
sema5bb	93.88366834	-0.201694691	0.2007207	-1.004852	0.314968	0.866054
vps26c	425.9641645	-0.109069522	0.1125569	-0.969017	0.332537	0.874653
si:ch1073-429i10.1	35.68964803	-0.272807655	0.3450871	-0.790547	0.429208	0.911406
tsc22d3	1683.855045	0.034015782	0.0755471	0.4502592	0.652524	0.960708
rereb	627.3466557	0.109027365	0.0991946	1.0991264	0.271713	0.836643
tlr4al	0	NA	NA	NA	NA	NA
pms2	95.77102152	0.274280012	0.2022794	1.3559462	0.175116	0.735545
arhgap21b	1655.387994	-0.060146965	0.074191	-0.810705	0.417535	0.906237
fndc7b	131.4846911	0.067432274	0.1797716	0.3750996	0.707586	0.968664
mrpl17	337.8773217	-0.074798852	0.1184571	-0.631442	0.527751	0.941629
orc6	104.8956917	0.327071517	0.2077243	1.5745461	0.115361	0.636838
tlr7	148.176082	-0.003986998	0.1849733	-0.021554	0.982803	0.996944
anapc1	417.2065998	-0.156421406	0.1151936	-1.357901	0.174495	0.734611
adora1b	16.1474913	-0.088213461	0.4718472	-0.186953	0.851697	0.985174
si:ch73-380l3.2	76.09933728	0.029505905	0.227393	0.1297573	0.896758	0.989971
nkpd1	28.44954623	-0.161601101	0.3761103	-0.429664	0.66744	0.964736
zswim2	5.438315747	0.569765357	0.845231	0.6740943	0.500251	NA
si:ch211-106h4.9	290.4845181	-0.026441304	0.1364262	-0.193814	0.846322	0.985174
nid2a	1681.238188	0.119456578	0.0925	1.2914224	0.196557	0.761835
ndufb3	1310.615638	-0.013539053	0.0957951	-0.141333	0.887606	0.989291
shox2	405.3088452	-0.008958635	0.1169583	-0.076597	0.938944	0.992857
si:dkey-27p23.3	16.90984995	0.204844796	0.4971745	0.4120179	0.680326	0.965431
TMEM236	5.153577458	-0.885753621	0.8594811	-1.030568	0.302744	NA
rpz5	378.7779755	0.027430449	0.1173615	0.2337262	0.815198	0.981175
il2rb	9.594604317	0.302337712	0.612289	0.4937827	0.62146	NA
zdhhc6	468.8246747	-0.018973217	0.1071067	-0.177143	0.859396	0.985773
si:dkey-242e21.3	5.72082136	-0.608338741	0.8404443	-0.72383	0.46917	NA
cep152	126.7075161	-0.065125055	0.1777077	-0.366473	0.714012	0.96909
dhrs7b	133.5457332	-0.149158015	0.178039	-0.837783	0.402153	0.90084
map1lc3cl	183.6600125	0.057864841	0.1658713	0.3488538	0.727199	0.970402
dnph1	83.50587649	-0.157144888	0.236172	-0.665383	0.505805	0.935724
zgc:174698	3.276395311	-2.481686758	1.2368187	-2.006508	0.044802	NA
taf4b	2.291345534	-2.593707058	1.4999462	-1.7292	0.083773	NA
zyx	397.0909119	0.080539931	0.1183936	0.6802727	0.496332	0.932415
cxxc5b	608.295941	0.083280287	0.0994929	0.8370475	0.402566	0.90084
zgc:165518	15.1697578	0.064705017	0.4872418	0.1327986	0.894353	0.989689
mrpl47	216.1665932	-0.048429464	0.1522772	-0.318035	0.750458	0.97419
ovgp1	5.827671487	-0.577192554	0.7986529	-0.722708	0.469859	NA
zfyve16	135.1411661	-0.145746961	0.1823943	-0.799076	0.424246	0.908243
NCKAP1L	57.88904003	-0.52067501	0.2650489	-1.964449	0.049478	0.436093
myo18aa	636.6409554	0.283568729	0.0999016	2.8384812	0.004533	0.09614
mri1	44.53883794	-0.090842644	0.3013469	-0.301455	0.763067	0.975374
gig2e	15.44190121	0.284710449	0.4874642	0.5840644	0.559177	0.948478
ywhabb	5622.765066	-0.069688137	0.0875447	-0.796029	0.426015	0.909755
samd4a	264.9029571	-0.190312844	0.1329068	-1.431928	0.152165	0.701049

si:ch1073-287p18.1	44.96180756	-0.049599013	0.315577	-0.157169	0.875111	0.987881
sccpdha.1	142.2022141	0.073035643	0.1830814	0.3989244	0.689949	0.966857
sdhb	2634.274005	0.03433447	0.0873088	0.3932532	0.694132	0.96736
foxj3	718.7702966	-0.04529386	0.0978835	-0.462732	0.643556	0.959994
si:ch211-57m13.5	0.498553709	0.926267934	2.6786929	0.345791	0.7295	NA
hmgxb4a	234.2666714	0.044568907	0.1421982	0.3134281	0.753955	0.975056
si:ch73-190m4.1	0.521188103	-2.493476766	2.9577741	-0.843025	0.399215	NA
si:dkey-79f11.5	0.833717353	0.587279662	2.212766	0.2654052	0.790697	NA
prex1	441.8146824	0.155719814	0.1118343	1.392415	0.163797	0.71945
nol7	324.8528552	0.224702141	0.1212467	1.8532643	0.063844	0.493997
usp38	368.3827557	-0.071033552	0.1198799	-0.592539	0.55349	0.947265
calml4a	76.55992321	-0.830419427	0.2266993	-3.663087	0.000249	0.010194
grin3a	105.0393291	0.215999564	0.2018654	1.0700177	0.284611	0.844955
slc41a2a	195.8110063	0.022088547	0.1622383	0.1361488	0.891704	0.989291
si:dkey-219c3.2	173.3480985	0.04920317	0.1643436	0.299392	0.764641	0.975374
kirrel3a	301.8050971	0.073196419	0.1356721	0.5395098	0.589535	0.953498
traj39	0	NA	NA	NA	NA	NA
kcnt2	25.96470026	0.210184644	0.3939771	0.5334946	0.593691	0.954434
asphd1	239.2275841	0.14683988	0.142644	1.029415	0.303285	0.85729
slc12a5a	33.73734396	-0.015574656	0.3798299	-0.041004	0.967292	0.996315
mettl24	57.15021352	0.164237321	0.2577944	0.6370865	0.524069	0.939336
dok2	26.75722258	0.383931501	0.3876652	0.9903688	0.321994	0.869586
arhgef2	197.6469813	-0.372868421	0.1534612	-2.429725	0.01511	0.225333
CU694442.1	3.439523094	0.941819321	1.1885374	0.7924188	0.428117	NA
plxnb1a	1027.473912	-0.039461774	0.0884266	-0.446266	0.655405	0.961632
virma	1197.073034	-0.007508077	0.0985277	-0.076203	0.939258	0.992857
dlec1	65.49401874	0.1270419	0.2529208	0.5022991	0.615457	0.95688
msh4	2.862823644	1.24171776	1.2018815	1.0331449	0.301536	NA
f7i	112.9716408	0.071577072	0.2042534	0.3504328	0.726014	0.970402
taar14j	0	NA	NA	NA	NA	NA
kiss1	2.033631938	0.014997283	1.3735429	0.0109187	0.991288	NA
syt3	276.8402844	0.098600335	0.1370409	0.7194955	0.471836	0.924838
slc7a8a	2086.816582	0.014764375	0.0777734	0.1898383	0.849436	0.985174
lyve1a	179.036331	-0.037545796	0.1733621	-0.216574	0.82854	0.982721
si:dkey-182i3.8	69.97636887	0.280075394	0.2382853	1.1753786	0.239843	0.808111
si:dkey-1d7.3	138.7316515	0.265101279	0.1716824	1.5441379	0.122555	0.650752
pigt	40.61585637	-0.003148498	0.3003997	-0.010481	0.991638	0.997679
kcnip2	6.430837696	-0.386923491	0.7446303	-0.519618	0.60333	NA
prr12b	2281.318886	-0.088380774	0.0932622	-0.947659	0.343303	0.878434
gnal2	330.8069573	0.076917992	0.1250673	0.6150128	0.538546	0.943336
oafa	156.9178411	0.179169157	0.1773782	1.0100969	0.312449	0.864401
sh3kbp1	656.229181	-0.029652709	0.1013057	-0.292705	0.769747	0.975687
cx34.4	58.56637739	-0.03153253	0.2593965	-0.121561	0.903247	0.990121
adgre10	13.91481519	0.324268076	0.5394884	0.6010659	0.547796	0.945706
pgs1	122.2323059	0.224756344	0.1886014	1.1917001	0.233379	0.801883
fam155a	30.0046712	0.56807409	0.3459032	1.6422923	0.100529	0.602933
mcf2la	1006.70979	0.085943738	0.0861676	0.9974025	0.318569	0.867746
igsf9a	375.2969696	-0.253727695	0.1234216	-2.055781	0.039804	0.391511
cdcp2	32.62097651	-0.241585431	0.3491676	-0.69189	0.489007	0.931926
mrtfaa	29.9601389	-0.113355091	0.3841867	-0.295052	0.767954	0.975687
triobpa	78.72596952	-0.224332336	0.2223086	-1.009103	0.312925	0.864884
si:ch211-39k3.2	127.802539	-0.044014415	0.2170285	-0.202805	0.839288	0.984979
bub3	473.1527511	-0.061629075	0.1189211	-0.518235	0.604294	0.95651
shprh	134.0270334	0.116860264	0.1747889	0.6685794	0.503764	0.935314

vwa5b2	22.57505048	0.006146747	0.4170022	0.0147403	0.988239	0.997369
herc3	236.2130399	-0.057480403	0.1364807	-0.421161	0.673637	0.965257
sall1b	44.24820542	0.117939311	0.3097241	0.3807883	0.70336	0.968134
bag6	1319.345416	0.036880048	0.0920273	0.4007511	0.688603	0.966672
spata7	231.3158571	0.141002828	0.1460439	0.9654827	0.334303	0.875332
si:ch1073-186i23.1	270.9885772	-0.026102211	0.1383914	-0.188612	0.850397	0.985174
chad1b	266.470045	0.08447535	0.1295287	0.6521748	0.514288	0.937198
rfx1b	357.5262774	-0.081498174	0.1442679	-0.564909	0.572136	0.949494
si:ch211-266g18.9	15.65168002	-0.19553504	0.5046023	-0.387503	0.698384	0.967998
slc35f3a	7.818979746	0.429168984	0.6762209	0.6346579	0.525652	NA
kcnj14	2.626818908	-0.743954913	1.1538115	-0.64478	0.51907	NA
si:ch73-70k4.1	9.664426499	0.102833278	0.6520894	0.1576981	0.874695	NA
zgc:66472	190.1650938	0.012800785	0.1569966	0.0815354	0.935016	0.992702
cacna1db	47.0474139	0.17530673	0.286304	0.6123098	0.540333	0.944448
ccser1	16.87853567	-0.626236161	0.4694653	-1.333935	0.182225	0.74454
txnrd2.2	25.26558674	-0.125900069	0.4027152	-0.312628	0.754563	0.975114
zgc:171558	652.4893505	0.096772608	0.1006842	0.9611501	0.336477	0.876088
igfn1.2	17.59741785	0.518506928	0.5095136	1.0176507	0.308844	0.861088
hivep3a	41.64295494	0.07257499	0.3186819	0.2277349	0.819852	0.981594
si:dkey-219c10.4	29.48185789	-0.215372935	0.3584374	-0.600866	0.547929	0.945768
adamts2	131.6950089	0.394708207	0.1858942	2.1232953	0.033729	0.357045
acsl5	359.6460788	-0.470002347	0.1422311	-3.304497	0.000951	0.02977
si:ch73-211l2.3	0.491320158	0.8528132	2.8656354	0.2976	0.766008	NA
cox15	375.8521044	-0.104574212	0.1189474	-0.879164	0.379313	0.895192
sugp1	341.1733698	-0.107476955	0.1231348	-0.87284	0.38275	0.895192
FRMD5	81.7129888	0.07386959	0.223875	0.3299591	0.741431	0.973154
si:ch211-250c4.5	6.891903349	0.166012865	0.7528372	0.2205163	0.825469	NA
slco4a1	37.63166136	0.054585448	0.3145958	0.1735098	0.862251	0.985841
prkd1	161.0776146	0.02533835	0.1675668	0.1512135	0.879807	0.987881
mbd2	133.1982523	0.032151272	0.1789694	0.1796468	0.85743	0.985703
serpinh1a	551.4502682	0.397874136	0.1075821	3.6983315	0.000217	0.00915
faxca	57.44034138	0.113679103	0.2834363	0.4010746	0.688365	0.966672
si:dkey-266f7.9	22.31348741	-0.530614046	0.4068251	-1.30428	0.192138	0.756673
cdh24a	45.53288835	-0.098790844	0.3107531	-0.317908	0.750555	0.97419
vav3b	44.64169394	0.022598122	0.2880668	0.0784475	0.937472	0.99279
mhc1uba	3.640429303	-0.281285031	1.0527066	-0.267202	0.789314	NA
plekhg4	1101.737602	0.049502346	0.0875599	0.565354	0.571833	0.949494
BX324188.1	3.48107542	2.090908957	1.1343628	1.8432454	0.065293	NA
csrnp2	6.191390234	-0.731952439	0.8312375	-0.880558	0.378557	NA
si:dkey-258f14.3	43.73470492	0.038100468	0.3022023	0.126076	0.899672	0.990121
slc45a3	26.10955027	-0.180917418	0.3817081	-0.473968	0.635523	0.958142
prr5a	82.60766331	0.198324452	0.2180891	0.9093736	0.363153	0.885944
tmem125b	98.82128074	0.259003596	0.2033012	1.2739896	0.202667	0.769545
hsbp1l1	33.05114136	-0.056432471	0.3592771	-0.157072	0.875188	0.987881
arpc2	2907.468963	-0.095609652	0.0775921	-1.232209	0.217871	0.785305
acap3a	152.9339325	0.050282144	0.1669552	0.3011715	0.763284	0.975374
clic5a	171.7875018	0.268343841	0.1557732	1.7226571	0.084951	0.560519
znf1lb	1.466888559	0.924909503	1.8132884	0.510073	0.61	NA
pip5k1ca	158.5874538	-0.070400489	0.1654833	-0.425423	0.670528	0.965257
osbpl5	178.7192687	-0.204650734	0.1555959	-1.315271	0.188419	0.753276
ppp2r3b	188.2613152	0.015281757	0.1699322	0.0899286	0.928344	0.992702
piezo2a.2	249.4523833	-0.058719146	0.1331016	-0.44116	0.659097	0.962839
umad1	49.52134429	0.486276584	0.2774933	1.7523906	0.079707	0.548406
twist1b	146.8822576	-0.096203159	0.1707409	-0.563445	0.573132	0.949762

si:ch211-149b19.3	353.6775022	0.026045886	0.1268322	0.205357	0.837293	0.984645
mab21l3	38.59315963	0.15997541	0.3110165	0.5143631	0.606998	0.956718
ERC1	30.7744344	0.350188353	0.3907361	0.8962273	0.370131	0.889876
si:ch73-139e5.2	15.47843907	-0.132188083	0.4901697	-0.269678	0.787408	0.976596
tmem69	139.8378956	0.06218506	0.1688974	0.3681824	0.712737	0.96909
ftr70	0.182570949	0.967652056	4.0804729	0.2371421	0.812547	NA
pappa2	225.2097476	0.392654634	0.1532305	2.5625094	0.010392	0.175332
dgkzb	138.5323803	0.066068202	0.1778978	0.3713829	0.710352	0.968985
kif5c	238.2058284	-0.072852339	0.1408487	-0.517238	0.60499	0.95651
cacnb3b	15.24041243	-0.132819721	0.4883735	-0.271963	0.78565	0.97627
arl15a	42.76802702	0.578817677	0.2958452	1.9564881	0.050408	0.439877
wwc1	18.58732102	-0.340413495	0.4634061	-0.73459	0.462589	0.922698
si:dkeyp-73d8.9	3.294968638	1.266007846	1.1343776	1.1160374	0.264406	NA
si:dkey-283j8.1	8.978507998	-0.402161836	0.6549141	-0.614068	0.53917	NA
sez6l2	719.0053046	0.128548713	0.0982813	1.3079669	0.190885	0.755212
rpgrip1	217.7212859	0.183361216	0.1608999	1.139598	0.254454	0.823475
IYD	27.24940578	0.161850591	0.4287902	0.3774587	0.705833	0.968412
gba	253.6775806	0.004214326	0.1418806	0.0297033	0.976304	0.996762
diaph2	796.909096	-0.023794196	0.1010648	-0.235435	0.813871	0.981081
6-Mar	1439.982313	0.071012052	0.0818449	0.8676413	0.385591	0.89551
crtc1a	11.52780179	0.085357308	0.5681286	0.1502429	0.880573	0.987884
dlgap2b	10.63040071	-0.947662551	0.6229074	-1.521354	0.128171	NA
dda1	1027.991848	-0.032001487	0.0931601	-0.343511	0.731214	0.971557
myh7ba	107.3632494	0.025593699	0.1909674	0.1340213	0.893386	0.989291
crtc1b	23.79636468	0.192858883	0.4211786	0.4579028	0.647022	0.960362
ush1gb	0.986657807	-0.948525167	1.9936381	-0.475776	0.634234	NA
CABZ01041962.1	190.0530588	-0.035843309	0.1529683	-0.234319	0.814738	0.981081
jakmip1	266.2067409	0.019362229	0.1327668	0.1458364	0.884051	0.988784
slc31a2	72.9540511	0.039179661	0.2297299	0.1705466	0.86458	0.98601
ftr76	2.769580455	-0.901262682	1.1748246	-0.767147	0.442994	NA
klhl30	22.76437506	0.020406257	0.4327248	0.0471576	0.962388	0.995907
grid2ipa	61.67648922	-0.129761245	0.2533476	-0.512187	0.60852	0.956718
sema4bb	282.2177257	-0.27077861	0.1419919	-1.907	0.056521	0.46635
ftr43	1.514601305	1.04180901	1.6547679	0.6295801	0.528969	NA
nwd1	19.70309429	-0.694953558	0.4462235	-1.557411	0.119373	0.643644
CABZ01072254.1	22.44831887	-0.464598419	0.4041787	-1.149488	0.250355	0.820164
stard5	4.633609583	-0.215817366	0.8622512	-0.250295	0.802359	NA
cdip1	203.7416543	-0.001910622	0.1469437	-0.013002	0.989626	0.997612
emid1	980.7098497	0.00549812	0.0869336	0.063245	0.949571	0.99381
foxp4	1937.175897	-0.049685339	0.0779509	-0.637393	0.523869	0.939336
smpd1	390.626191	0.143573501	0.1283926	1.1182385	0.263465	0.830245
si:ch211-160b11.4	1.201333238	-2.500792036	1.9278236	-1.29721	0.194559	NA
actc1a	727.5039112	-1.258059749	0.3203836	-3.92673	8.61E-05	0.004322
gabrb1	124.0450785	0.119093129	0.1934189	0.6157264	0.538075	0.943336
prkar1aa	3256.648724	-0.016124573	0.0700121	-0.230311	0.81785	0.981594
siglec15l	0	NA	NA	NA	NA	NA
zgc:162972	18.23425212	0.060207628	0.4631909	0.1299845	0.896579	0.989971
mmrn2a	324.55831	0.129675333	0.127954	1.013453	0.310844	0.862927
si:dkey-98f17.3	16.30305914	0.926091225	0.5152471	1.7973729	0.072276	0.525826
si:dkey-71p21.13	6.17219036	0.751676405	0.7948801	0.9456476	0.344328	NA
clcf1	57.92932683	0.017467336	0.2731835	0.0639399	0.949018	0.99381
ppip5k1a	499.5915066	0.162247413	0.106725	1.5202375	0.128451	0.659397
icam3	186.6424823	-0.226009219	0.1683166	-1.342763	0.179349	0.740258
zgc:172075	72.59067254	-0.073631171	0.2450095	-0.300524	0.763778	0.975374

si:ch211-1a19.2	0.325573202	0.005883621	3.3172783	0.0017736	0.998585	NA
lca5	53.1053913	-0.275292939	0.2813122	-0.978603	0.327776	0.872701
mtpap	180.2508546	0.216834178	0.1506144	1.4396645	0.149962	0.697935
zgc:165583	0.663561848	-1.490280258	2.4116568	-0.617949	0.536609	NA
si:dkey-285e18.2	1.622011068	-1.238012138	1.6067485	-0.770508	0.440999	NA
col19a1	228.095344	0.242920751	0.1488034	1.6324947	0.102575	0.608473
pold3	582.9533361	0.263659745	0.1184381	2.2261401	0.026005	0.311101
pcsk1nl	1395.154444	0.125454841	0.0944952	1.3276319	0.1843	0.747376
znf827	1346.92332	-0.084839424	0.0805242	-1.053589	0.292071	0.848075
trim2b	199.5767887	-0.079920805	0.1582888	-0.504905	0.613626	0.95688
ptcd1	228.035813	0.048444496	0.1407137	0.344277	0.730638	0.97149
ngef	273.1494361	-0.155091463	0.1340749	-1.156752	0.247374	0.81686
stat1b	2.66092372	0.016159258	1.1267473	0.0143415	0.988558	NA
frrs1a	6.17803076	0.884146649	0.7825504	1.1298271	0.258549	NA
lrrc3cb	1.830783485	0.799248267	1.3781958	0.5799236	0.561966	NA
ccdc88b	35.07183702	0.063402339	0.3287863	0.1928375	0.847086	0.985174
fbxl13	1.647824735	1.237995142	1.622278	0.7631214	0.445391	NA
ankrd1b	204.1836824	0.832386783	0.1584811	5.2522791	1.50E-07	1.98E-05
si:ch211-226h7.6	13.83727812	0.702281364	0.6422041	1.0935485	0.274153	0.839127
zdhhc11	14.80260729	0.202041667	0.5555454	0.3636817	0.716096	0.96909
DOCK4 (1 of many)	29.71322878	-0.053730296	0.3620725	-0.148397	0.88203	0.988344
zgc:165514	0.67370173	-2.870759253	2.497836	-1.149299	0.250433	NA
b3galt4	15.07939832	-0.777192953	0.5423107	-1.433114	0.151825	0.700196
fthl28	232.3590375	0.536988914	0.156357	3.4343769	0.000594	0.020179
SERP1	1154.206134	-0.27194466	0.0983715	-2.764465	0.005702	0.113266
tha1	128.8210008	-0.280789986	0.1781383	-1.576247	0.114969	0.635519
ror2	281.8810475	-0.177600273	0.1290532	-1.376179	0.168766	0.725919
kif2c	384.2675376	-0.158147025	0.1134171	-1.394384	0.163202	0.718029
mrtfab	819.7551637	-0.015660275	0.0978816	-0.159992	0.872887	0.987846
atp10b	119.9417424	0.097045132	0.1867497	0.5196535	0.603305	0.956045
lrrc18b	4.855800557	-0.49035951	0.8905935	-0.550599	0.581909	NA
gramd1c	151.5255506	-0.37937663	0.178517	-2.125157	0.033574	0.357045
si:ch211-74f19.2	716.7327317	0.059265763	0.1134467	0.5224105	0.601385	0.956045
txlnbb	488.8676447	0.295695224	0.1077754	2.7436255	0.006076	0.118032
prune	21.41965691	-0.113464542	0.4082273	-0.277945	0.781055	0.97627
dlk2	51.62852895	0.079421779	0.2740283	0.2898306	0.771946	0.975687
ppp1r3db	50.78267118	-0.211879249	0.2861281	-0.740505	0.458994	0.921978
rasef	21.49390685	-0.08793856	0.4071665	-0.215977	0.829006	0.982721
irf3	42.22555695	-0.421837362	0.3002664	-1.404877	0.160058	0.712495
si:dkey-247i3.1	0.158915748	0.967652056	4.0804729	0.2371421	0.812547	NA
znf1084	15.81648437	0.167575873	0.4832817	0.3467457	0.728782	0.970631
si:ch211-285c6.1	3.504929046	0.692918324	1.0151088	0.682605	0.494857	NA
tbr1a	20.21244619	-0.368237997	0.4975184	-0.740149	0.459209	0.921978
pou3f2b	1082.694818	0.027725026	0.0982615	0.2821556	0.777824	0.975942
zgc:195170	9.090780723	-0.499435796	0.6679326	-0.747734	0.454621	NA
ano8a	77.05937542	0.247997042	0.2320304	1.0688127	0.285154	0.845129
zgc:172131	10.53887292	0.573229508	0.6762718	0.8476318	0.396643	NA
adamts13	194.0140737	0.005540458	0.1645699	0.0336663	0.973143	0.996315
znf1041	67.41354377	0.034064765	0.2535771	0.1343369	0.893136	0.989291
CR385050.1	0.688945432	-0.015421672	2.3988056	-0.006429	0.994871	NA
ppp1r1b	522.2646297	0.101137569	0.1050667	0.9626038	0.335746	0.876088
snrpd3l	636.6838929	-0.040586998	0.0955953	-0.424571	0.671149	0.965257
si:dkey-125e8.1	0	NA	NA	NA	NA	NA
calr	2443.787679	0.017681333	0.0791521	0.2233844	0.823236	0.982196

tns2a	27.04242498	0.21027438	0.3911045	0.5376424	0.590824	0.954193
klb	17.64822851	0.010935336	0.4578793	0.0238826	0.980946	0.996944
triqk	376.7064572	-0.289243545	0.1252549	-2.309239	0.02093	0.273186
nfatc3a	234.5591211	0.151772183	0.1375274	1.1035775	0.269776	0.834705
FQ323119.1	436.078684	0.211611101	0.109002	1.9413505	0.052216	0.448919
dtx4a	858.4068218	0.028684173	0.1035352	0.2770476	0.781744	0.97627
usp11	148.4680258	0.144925528	0.1654488	0.8759541	0.381055	0.895192
loxl3a	39.86996404	0.252821778	0.3018048	0.8376997	0.402199	0.90084
si:zfos-2070c2.3	0.157187365	-0.955901296	4.0804729	-0.234262	0.814781	NA
pdp1	206.4394235	0.122788504	0.1528057	0.8035596	0.421651	0.90651
mxra5b	5.366298343	-1.134609969	0.8430778	-1.345795	0.178369	NA
si:ch73-290k24.5	16.68464326	0.385371534	0.4974044	0.7747651	0.438478	0.915831
myot	477.274326	0.347541979	0.1185273	2.9321694	0.003366	0.077672
si:ch211-240l19.7	0.173729368	-0.955901296	4.0804729	-0.234262	0.814781	NA
plod3	1546.590512	-0.006492835	0.0834225	-0.077831	0.937963	0.99279
atp6v1ab	1925.52527	-0.030768747	0.0775528	-0.396746	0.691555	0.967305
ano9a	156.1414917	-0.174218839	0.1640692	-1.061862	0.288298	0.845979
col28a2a	1744.98608	0.253984953	0.0999063	2.5422314	0.011015	0.182111
lmtk2	946.8304451	0.096971286	0.0868785	1.1161718	0.264349	0.830245
osbpl11	47.85090608	-0.387279624	0.2791007	-1.387598	0.165259	0.721223
dusp12	48.49511874	-0.030869711	0.2927253	-0.105456	0.916014	0.991314
si:ch211-159i8.4	497.4688427	0.236432802	0.1088994	2.1711114	0.029923	0.335036
mrpl43	189.6297454	0.149202285	0.1491199	1.0005528	0.317043	0.867335
cln5	33.16119289	-0.369258763	0.3352401	-1.101475	0.27069	0.835351
si:dkeyp-13d11.1	0	NA	NA	NA	NA	NA
taar19p	0.173729368	-0.955901296	4.0804729	-0.234262	0.814781	NA
mylk3	58.66808518	0.157390479	0.296128	0.5314947	0.595076	0.954434
brinp3a.1	515.2828958	0.053479456	0.1103754	0.4845232	0.628015	0.957354
CABZ01102039.1	207.7525486	0.025005618	0.1659684	0.150665	0.88024	0.987881
zmp:0000001161	0.158795395	0.967652056	4.0804729	0.2371421	0.812547	NA
rpl37	112.7814816	0.158621046	0.1897313	0.8360301	0.403138	0.900877
CABZ01039859.1	230.9583758	-0.196765295	0.1370012	-1.436231	0.150937	0.698912
tmem260	164.2151587	0.200102788	0.1602549	1.2486531	0.211792	0.779854
si:dkey-178k16.1	1917.650787	-0.045314937	0.0812033	-0.558043	0.576815	0.950654
CABZ01080074.1	396.1983615	-0.143727006	0.1191504	-1.206266	0.227715	0.794934
vopp1	63.59300771	0.127772749	0.2612972	0.488994	0.624846	0.957354
tmem175	60.8626487	0.02686576	0.2537681	0.1058674	0.915688	0.991314
nsmce2	87.51956529	-0.224280561	0.208324	-1.076595	0.281661	0.84357
hnrnpa01	87.77014738	-0.207706476	0.2132762	-0.973885	0.330114	0.874085
slc25a1b	238.067342	-0.275389103	0.1417319	-1.943028	0.052013	0.447758
epdl1	417.5750681	0.134639839	0.2827241	0.4762234	0.633915	0.95776
zgc:194686	4.278550445	3.587132677	1.2849569	2.7916366	0.005244	NA
si:dkey-217f16.6	1.006818213	1.048314579	1.9690503	0.532396	0.594452	NA
npm2a	1.385673855	-0.665060118	1.7096737	-0.388998	0.697277	NA
tmem65	106.9201728	0.037567802	0.206769	0.1816897	0.855826	0.985174
si:dkey-201l21.4	0	NA	NA	NA	NA	NA
cacng3b	741.2313113	-0.032372843	0.0984598	-0.328792	0.742313	0.973154
nat16l	27.78718849	-0.381853353	0.3755393	-1.016813	0.309242	0.861381
mctp1a	157.6031234	-0.085290371	0.1671476	-0.51027	0.609863	0.95688
ggcta	8.088991689	0.170037687	0.65758	0.258581	0.795959	NA
cavin1a	21.39440143	0.209700539	0.4073603	0.5147791	0.606707	0.956718
espn	42.17670403	0.160172623	0.3022467	0.5299399	0.596154	0.954444
shroom2a	730.1704185	0.015339043	0.0907068	0.1691058	0.865713	0.986517
si:dkeyp-117b11.2	92.21273498	0.164772372	0.212392	0.7757936	0.437871	0.915258

dkk2	13.01416868	0.311450737	0.5470581	0.5693193	0.569139	0.948983
lrrc29	104.8690523	0.186162884	0.1968594	0.9456642	0.34432	0.878434
ARHGAP22 (1 of many	313.8797666	-0.091738792	0.1389573	-0.660194	0.509129	0.936123
abhd10a	419.8744653	0.248498238	0.1207572	2.0578339	0.039606	0.390659
zgc:163143	53.1311244	0.015235416	0.2969985	0.051298	0.959088	0.995095
ppp4cb	1573.296232	0.027938756	0.0918412	0.3042073	0.76097	0.975374
btbd6a	361.6321449	-0.061566259	0.1182338	-0.520716	0.602564	0.956045
zgc:174310	122.5367346	-0.104584029	0.2204582	-0.474394	0.635219	0.957927
si:dkey-183c6.8	445.3241414	-0.089000193	0.1109274	-0.802328	0.422363	0.906561
serpinf2a	220.6306514	0.343967976	0.1569229	2.1919557	0.028383	0.326872
kbtbd13	6.005810399	0.170928057	0.7801454	0.2190977	0.826574	NA
prdm2b	905.9015283	0.029364847	0.0911761	0.3220673	0.747402	0.97419
ndnfl	476.7180904	-0.062344735	0.1111433	-0.56094	0.574838	0.950037
lamtor1	96.68270176	0.092579684	0.1998243	0.4633054	0.643145	0.959994
maml1	340.8753129	-0.13259134	0.1176848	-1.126665	0.259884	0.828288
ovol1a	216.047518	-0.175131365	0.1489034	-1.176141	0.239539	0.808091
trim37	516.3027946	0.005729623	0.1020399	0.0561508	0.955222	0.994543
org	2.644220079	-0.00327282	1.1345402	-0.002885	0.997698	NA
zgc:173770	0.158795395	0.967652056	4.0804729	0.2371421	0.812547	NA
mcf2lb	324.6990962	0.278020149	0.1254839	2.2155837	0.02672	0.315011
dhrs13b.2	8.049577873	-0.456432018	0.7380554	-0.618425	0.536295	NA
stab1	159.4240157	0.05259617	0.1636105	0.3214718	0.747853	0.97419
acp1	614.7587546	-0.24756251	0.0969872	-2.552529	0.010694	0.179263
haao	189.0381002	-0.283984346	0.1525485	-1.861601	0.062659	0.488383
ankrd40	475.7322758	0.146538199	0.1182738	1.2389741	0.215355	0.782987
vwc2	95.26393806	0.098826488	0.2038146	0.4848842	0.627759	0.957354
frmd4bb	140.0977286	0.396824921	0.1857689	2.1361217	0.032669	0.352204
usp53b	81.99749967	0.13803643	0.218981	0.630358	0.52846	0.941896
chgb	350.8960795	0.111187015	0.1269319	0.8759582	0.381053	0.895192
asxl2	41.88775638	0.019448251	0.3055572	0.0636485	0.94925	0.99381
grm8b	286.9237509	0.167867997	0.138246	1.2142706	0.224644	0.791954
polr2d	403.0577083	-0.175325424	0.2892635	-0.60611	0.544442	0.945438
fgf22	11.28359474	0.531753289	0.5751846	0.9244916	0.35523	0.882342
si:ch211-265g22.4	8.76703468	0.669636529	0.6898725	0.970667	0.331714	NA
mettl1	280.107826	-0.133515606	0.1389373	-0.960977	0.336564	0.876088
mafb	65.14881431	-0.013727256	0.2429661	-0.056499	0.954945	0.994543
gar1	1393.20393	-0.202448815	0.0919379	-2.202016	0.027664	0.321596
mn1a	648.8071602	0.117648799	0.1096968	1.0724906	0.2835	0.84466
jac6	2.849078905	-0.137979823	1.116776	-0.123552	0.90167	NA
si:ch211-222l21.1	17027.67482	-0.085424753	0.0868098	-0.984045	0.325093	0.871528
nfe2l1b	328.4236905	0.30529483	0.125311	2.4362978	0.014838	0.223115
si:ch211-14a17.10	7.119556964	-0.195865242	0.7119133	-0.275125	0.78322	NA
sc:d156	2.490128421	0.962595326	1.2991769	0.7409271	0.458738	NA
clec3ba	8.456497513	-0.988080905	0.6685547	-1.477936	0.139425	NA
pogzb	758.637692	0.061346838	0.0937308	0.6545005	0.512789	0.936253
aldh5a1	404.956556	0.017670316	0.1150153	0.1536345	0.877898	0.987881
trhra	2.855362933	0.187853003	1.1560515	0.1624954	0.870916	NA
si:ch211-221f10.2	306.5597436	0.415745837	0.1254006	3.3153404	0.000915	0.029089
si:dkey-266m15.5	29.2542565	-0.630144047	0.3575612	-1.762339	0.078012	0.54413
zgc:193801	75.30383885	-0.192122397	0.2315537	-0.82971	0.406703	0.901305
cdkn1a	145.3598547	0.328074596	0.1875526	1.7492408	0.080249	0.549641
bicc2	77.98854847	-0.21370434	0.234738	-0.910395	0.362614	0.885944
rb1cc1	984.0998246	-0.079694125	0.0857718	-0.929141	0.352816	0.881552
apbb1	28.81042125	0.443268355	0.3738379	1.1857235	0.235732	0.804824

rarres3l	78.31730408	-0.107175156	0.2303268	-0.465318	0.641704	0.959994
hspg2	393.4256657	0.273086444	0.1171236	2.3316093	0.019721	0.264538
kank3	279.6309961	-0.034492398	0.1296229	-0.266098	0.790164	0.977338
sec61b	1387.064848	-0.046549993	0.0974917	-0.477476	0.633023	0.957356
ndp	62.61847861	0.169597593	0.2432905	0.6970991	0.485741	0.930598
sp5a	237.829955	0.044721763	0.1411901	0.3167485	0.751434	0.97419
crygm2d7	13294.06547	0.268588313	0.0913518	2.9401532	0.003281	0.076541
si:dkey-88j15.3	2.781088656	0.484897148	1.1328522	0.4280321	0.668628	NA
si:ch73-160i9.3	19.74607523	0.032819064	0.4648265	0.070605	0.943712	0.993364
patz1	379.3853425	-0.025975132	0.1144838	-0.226889	0.82051	0.981594
si:ch211-113e8.11	663.0889923	0.150566781	0.1006878	1.495383	0.134815	0.670689
csf2rb	22.73232491	0.172350718	0.402652	0.4280389	0.668623	0.96478
atad2	745.8360425	-0.082598054	0.0946542	-0.872629	0.382865	0.895192
man1b1b	29.66459916	0.19381202	0.3578379	0.5416196	0.588081	0.953319
pcdh12	73.65165588	0.065022378	0.2296319	0.2831591	0.777055	0.975826
sema4ga	22.09226	-0.275246428	0.4067219	-0.676743	0.498569	0.934006
prss16	330.9618637	-0.227658198	0.1336457	-1.703445	0.088485	0.569447
si:ch211-199g17.9	68.49785792	0.211172214	0.2447254	0.8628945	0.388195	0.896271
fbxo21	154.9676283	-0.105287707	0.1795947	-0.586252	0.557706	0.948478
ssuh2.3	1.151545593	-2.472760569	2.0114878	-1.229319	0.218952	NA
tm2d3	825.3747434	-0.099207358	0.0989813	-1.002284	0.316206	0.866931
abhd8b	63.07284261	0.123632374	0.246743	0.5010573	0.616331	0.95688
atp13a3	34.46563314	-0.389583566	0.3586531	-1.086241	0.277373	0.840857
col14a1b	1268.195629	0.253386201	0.0856212	2.9593855	0.003083	0.073439
ptprb	357.1038647	0.231982194	0.1164023	1.992935	0.046269	0.422789
smg9	375.0935618	-0.03398884	0.1203053	-0.282522	0.777544	0.975942
zdhhc12a	79.05558669	-0.162099725	0.2234605	-0.725407	0.468203	0.924328
capn10	172.0510039	0.235415576	0.1633654	1.4410371	0.149574	0.697402
FO082781.1	24.21295292	-0.433863329	0.3980602	-1.089944	0.275738	0.839541
esamb	79.90310865	0.108618501	0.2197019	0.4943905	0.62103	0.957354
kcnv2a	57.04565522	0.445641679	0.2624105	1.6982616	0.089458	0.572876
pde9al	13.04825563	0.310554062	0.541087	0.5739448	0.566005	0.948739
zgc:193807	0.31437473	-1.788572922	3.5930657	-0.497785	0.618636	NA
plagl2	49.26487357	0.126208322	0.2861866	0.4410002	0.659213	0.962839
cdca7b	392.0797129	-0.285883467	0.1264391	-2.261036	0.023757	0.297136
ccng1	7301.338612	0.300708563	0.0796239	3.7766129	0.000159	0.007145
wu:fi04e12	583.6452122	0.154301357	0.1043312	1.4789576	0.139152	0.677963
rai1	314.8878019	-0.276049301	0.1409168	-1.958953	0.050118	0.438277
ccn4b	22.77574336	0.583312747	0.4232883	1.3780507	0.168188	0.725919
si:rp71-84d9.1	4.852440554	0.3067053	0.8967051	0.3420358	0.732324	NA
gramd1a	1019.138894	0.128156843	0.0882313	1.4525107	0.14636	0.692538
avpr2l	6.766806768	0.297453198	0.7589832	0.3919101	0.695125	NA
NAMPT (1 of many)	0.817752529	3.164885207	2.3443606	1.3499993	0.177016	NA
SIPA1	21.50650414	-0.024848589	0.4542184	-0.054706	0.956372	0.994543
ipmka	37.3096377	-1.016270033	0.3480581	-2.919829	0.003502	0.07951
b4galnt4b	227.6409336	0.057382712	0.1373745	0.41771	0.676159	0.965257
CABZ01068356.1	3.002290064	0.341515882	1.0727024	0.3183696	0.750205	NA
zfand4	273.5244619	-0.127436127	0.1363806	-0.934415	0.35009	0.880195
si:dkey-42l23.7	40.93935225	0.160496975	0.3401517	0.4718394	0.637041	0.958872
si:dkey-265c15.6	18.84612494	0.089894956	0.4404358	0.2041046	0.838272	0.984797
palb2	45.81511501	-0.249912229	0.2856882	-0.874773	0.381698	0.895192
prkra	276.1115474	-0.103198674	0.1342514	-0.768697	0.442073	0.916336
zgc:171686	6.729047935	0.312920287	0.7763224	0.4030803	0.686889	NA
pi4kaa	123.4890461	-0.121578937	0.2101189	-0.57862	0.562846	0.948728

si:ch211-247i17.1	3.575519304	0.242534074	1.0590916	0.229002	0.818867	NA
myo9aa	835.8751519	0.074943063	0.1154331	0.6492335	0.516187	0.937997
syt6a	129.1980588	-0.111254337	0.1809211	-0.614933	0.538599	0.943336
bard1	26.66512418	-0.299539684	0.3691756	-0.811374	0.417151	0.906237
si:dkey-193c22.2	236.9066742	0.256354359	0.1369152	1.8723581	0.061157	0.482482
cyth1a	1142.241065	-0.097254162	0.0829475	-1.172479	0.241005	0.809517
zgc:193811	5.961980251	-0.342652635	0.7888801	-0.434353	0.664032	NA
TMCC1	52.23871437	-0.070083823	0.277518	-0.252538	0.800625	0.979377
ntn2	19.09040002	-0.2202352	0.4377658	-0.503089	0.614902	0.95688
tmem67	213.7180104	-0.143996242	0.1513267	-0.951559	0.341321	0.878134
slc9a7	801.9999213	0.159157771	0.0926842	1.7172052	0.085942	0.562905
ap5s1	160.842077	-0.138455161	0.1707317	-0.810952	0.417393	0.906237
ephb1	13.25034068	0.193048692	0.52896	0.364959	0.715142	0.96909
sp2	499.8932678	-0.186541317	0.1063038	-1.754795	0.079294	0.547489
ddhd2	412.425485	0.23194151	0.11485	2.0195163	0.043434	0.409149
zgc:163061	27.85992986	0.528520582	0.3990903	1.3243134	0.185399	0.74928
pgfb	14.63298347	0.681536407	0.4994044	1.3646984	0.172348	0.731348
reps2	6.514023574	0.690303514	0.7397968	0.9330989	0.350769	NA
lrrc58a	62.06364834	0.264819207	0.2486346	1.0650941	0.286833	0.845979
gucy2g	9.107552145	-0.197996857	0.6434326	-0.30772	0.758296	NA
si:ch73-343l4.5	0	NA	NA	NA	NA	NA
fam13b	2907.201429	0.09885731	0.0712607	1.3872621	0.165362	0.721223
acadsb	611.9178865	-0.058659999	0.0995299	-0.589371	0.555613	0.947621
trim45	137.2075643	-0.007987313	0.1887427	-0.042319	0.966245	0.996315
angpt4	25.97598897	-0.294500506	0.3770412	-0.781083	0.434754	0.914408
BX571825.4	0	NA	NA	NA	NA	NA
cx32.2	13.57326421	0.085592394	0.5185327	0.1650665	0.868892	0.987245
crygm2d16	8178.153198	0.159361687	0.0875201	1.8208585	0.068628	0.513124
cdk13	518.8104546	0.200604977	0.1301941	1.5408148	0.123362	0.651258
dlg3	1586.580356	0.045719159	0.0768683	0.5947726	0.551995	0.947215
si:dkey-178o16.4	20.40570434	0.074800436	0.4382463	0.1706813	0.864474	0.98601
si:ch211-261p9.4	52.22189675	0.437323301	0.2882324	1.5172594	0.129201	0.660844
rnd3a	1531.942185	4.40E-05	0.0867769	0.0005067	0.999596	0.999704
tnfrsf18	3.697840539	0.578904965	1.002865	0.5772511	0.56377	NA
arap3	158.5998365	0.171584503	0.1711522	1.002526	0.31609	0.866931
tradv30.0.1	0	NA	NA	NA	NA	NA
ttyh1	751.9511718	0.122651999	0.0940569	1.3040193	0.192227	0.756683
leng8	2802.939902	-0.032207065	0.0899287	-0.35814	0.720239	0.969318
zgc:174268	81.88232501	0.02465073	0.215394	0.1144449	0.908885	0.990702
ganab	2075.419135	-0.077078843	0.0755303	-1.020502	0.30749	0.861088
gzm3.4	7.671063049	0.509086854	0.678102	0.7507526	0.452802	NA
eif3s10	8378.339143	-0.138325865	0.0673879	-2.052682	0.040103	0.392085
nlrc8	0	NA	NA	NA	NA	NA
xkr8.2	37.11705747	0.167622817	0.3164594	0.529682	0.596332	0.954499
CCKBR	7.264714427	-0.264018714	0.7087813	-0.372497	0.709523	NA
dpp6a	171.582875	-0.182945322	0.1612956	-1.134224	0.256701	0.824902
zgc:194101	1.818526424	-0.761925714	1.442107	-0.528342	0.597262	NA
ankib1b	453.6991623	-0.064402538	0.1100626	-0.585144	0.558451	0.948478
si:dkey-65b12.6	2661.612436	0.578248501	0.0841549	6.8712362	6.36E-12	1.94E-09
iqck	41.73061837	0.06669929	0.3118595	0.2138761	0.830644	0.982994
atp1b1b	2739.705932	0.163065765	0.0800798	2.0362899	0.041721	0.400593
LRRC75A	235.8327384	0.031578008	0.135393	0.2332322	0.815581	0.981457
gpx4b	2028.244562	-0.103750231	0.0904134	-1.14751	0.251171	0.820469
apom	1291.830453	0.124507419	0.1144073	1.0882823	0.276471	0.839541

ftr86	137.9353371	-0.172344381	0.2104278	-0.819019	0.412776	0.904974
si:dkey-174i8.1	69.12139068	0.044910549	0.2428247	0.1849505	0.853268	0.985174
plin6	129.664778	0.479192855	0.1809524	2.6481704	0.008093	0.1451
nitr6a	0	NA	NA	NA	NA	NA
tnrc6c1	1319.049236	0.037949166	0.0869901	0.4362471	0.662657	0.964665
zgc:112492	94.21476319	0.200850584	0.2211693	0.9081307	0.363809	0.886174
si:ch211-157b11.14	527.9461261	-0.082169192	0.1067914	-0.769437	0.441634	0.916336
areg	69.04050799	0.053634934	0.2604951	0.2058961	0.836872	0.984636
frem2a	241.9411899	0.280367716	0.1397529	2.0061673	0.044838	0.416612
her3	10.25485759	-0.099837689	0.6377266	-0.156552	0.875598	NA
TCIM (1 of many)	41.13365482	-0.120371673	0.3167734	-0.379993	0.703951	0.968134
tmem79b	324.0641803	-0.179499099	0.1239978	-1.447599	0.147729	0.695027
gask1a	216.5080865	0.229250277	0.1534325	1.4941445	0.135138	0.670689
si:ch73-193c12.2	52.03736993	-0.425614899	0.2713969	-1.568238	0.116826	0.639958
mrtfbb	204.6701465	-0.158289136	0.1514251	-1.04533	0.295871	0.852974
piezo1	92.11765207	0.300014848	0.2076425	1.4448627	0.148496	0.696298
mpp4l	269.9957951	-0.077217215	0.1446054	-0.533986	0.593351	0.954434
unm_hu7912	184.0650387	0.131352636	0.1565824	0.8388722	0.401541	0.90084
zgc:171967	556.4313219	0.008062887	0.1156306	0.0697297	0.944409	0.993364
ralgapa1	1041.744301	-0.069167513	0.0872902	-0.792386	0.428136	0.911082
rfxap	16.40027393	0.251298215	0.5410427	0.4644702	0.642311	0.959994
flrt3	1371.764674	0.030275636	0.0840777	0.360091	0.718779	0.969092
si:dkey-188g12.1	5.050734779	-0.679573034	0.9666478	-0.70302	0.482043	NA
slc2a13b	85.48520834	0.10819084	0.2386817	0.4532851	0.650343	0.960708
prozb	33.70590766	0.348758206	0.3498275	0.9969435	0.318792	0.867746
CR376751.1	0	NA	NA	NA	NA	NA
bend3	213.0334262	0.166729793	0.1465309	1.1378472	0.255184	0.823839
loxl5b	52.46713785	0.127260175	0.2903752	0.4382612	0.661197	0.964234
eme1	124.2477631	0.171889452	0.1848543	0.9298645	0.352441	0.881179
lacc1	68.97093139	-0.490313878	0.3187934	-1.53803	0.124041	0.652685
igdcc4	140.0478586	0.162293051	0.1747998	0.928451	0.353174	0.881552
ZNF335	387.2482265	-0.031924401	0.1139622	-0.280132	0.779377	0.976146
kazna	213.8286936	0.124529762	0.1428489	0.8717585	0.38334	0.895192
als2b	562.3072992	-0.055871854	0.1032488	-0.541138	0.588413	0.953319
tet2	940.2069539	0.121033474	0.0912342	1.3266245	0.184633	0.747376
aldh1a3	163.2771882	-0.01893503	0.1650071	-0.114753	0.908641	0.990702
wdr60	147.7846585	-0.10922835	0.193321	-0.56501	0.572067	0.949494
znf451	519.0753937	-0.143027718	0.1119224	-1.277919	0.201278	0.767169
lhfpl4b	105.7617807	0.115552033	0.1988613	0.5810684	0.561194	0.948728
dsg2.1	889.6948486	0.043244602	0.1116081	0.3874682	0.69841	0.967998
zgc:173837	0	NA	NA	NA	NA	NA
kel	23.60734682	0.117416867	0.4075457	0.2881072	0.773265	0.975687
ferl14	96.07485854	-0.081524397	0.2153282	-0.378605	0.704981	0.968134
jac8	5.220192752	-0.735103957	0.8523896	-0.862404	0.388465	NA
si:ch211-38m6.6	25.24940381	-0.430836771	0.4294758	-1.003169	0.315779	0.866931
paqr4a	15.20871444	0.567672661	0.4950869	1.1466122	0.251542	0.820927
gdpd5b	627.9112938	0.106660101	0.0986436	1.081267	0.279578	0.843127
cables2a	156.934288	0.192419826	0.1696053	1.1345154	0.256578	0.824839
cep70	109.6530987	-0.138720948	0.2051189	-0.676295	0.498853	0.934006
pagr1	362.187429	-0.141803679	0.1194121	-1.187515	0.235024	0.803894
ftr22	0.976358547	-0.047642605	2.2026384	-0.02163	0.982743	NA
si:ch211-165b10.3	12.58091102	-0.272139842	0.5578757	-0.487814	0.625681	0.957354
plvapa	149.6418194	0.245307034	0.1710972	1.4337287	0.15165	0.700137
pdzd7a	10.22904924	0.356266094	0.6536935	0.5450048	0.58575	NA

pmchl	59.48594858	0.077615315	0.2520428	0.307945	0.758124	0.975374
ppp1r16a	34.53464806	-0.261024459	0.3356395	-0.777693	0.43675	0.914408
zgc:198329	23.89473814	-0.436522907	0.3894873	-1.120763	0.262389	0.830166
rnasel4	0	NA	NA	NA	NA	NA
znf839	795.588698	-0.04411415	0.0884137	-0.498951	0.617814	0.957354
dolk	8.823588164	0.38852313	0.6344743	0.6123544	0.540303	NA
adgra2	119.9100428	-0.188057877	0.1902562	-0.988445	0.322935	0.870607
sharpin	317.7982958	-0.044945557	0.1225075	-0.36688	0.713708	0.96909
si:ch73-182a11.2	77.60884471	0.02417092	0.2253007	0.1072829	0.914565	0.991119
stxbp4	45.25057116	0.120672233	0.3250995	0.3711855	0.710499	0.968985
si:ch73-92i20.1	8.681777558	-0.047022477	0.6576338	-0.071503	0.942998	NA
igsf3	328.3802489	-0.007974747	0.1312304	-0.060769	0.951543	0.99381
aldh1l1	1801.349716	-0.251425843	0.0855311	-2.939583	0.003287	0.076547
abcc8b	210.7817314	0.099567958	0.1591558	0.6256004	0.531577	0.942358
wdfy4	199.1998409	0.021979472	0.1504923	0.1460504	0.883882	0.988706
uhrf1bp1	52.16816217	0.117567198	0.2747769	0.4278641	0.66875	0.964829
eif4ea	297.2431492	0.009703734	0.133554	0.0726577	0.942078	0.993099
znf280d	706.3485633	-0.003510966	0.0986813	-0.035579	0.971618	0.996315
socs7	484.9112341	0.106265426	0.1085561	0.978899	0.32763	0.872701
calhm1	17.01522097	0.907777189	0.4729289	1.9194794	0.054924	0.459334
fam131a	18.78192818	-1.081583884	0.4800859	-2.252896	0.024266	0.301022
pcdhb	5.786454719	-0.909434091	0.8355551	-1.088419	0.27641	NA
si:dkey-94e7.1	8.73905276	0.05745185	0.68344	0.0840628	0.933007	NA
si:ch211-81n22.1	30.9633375	0.211815088	0.3613427	0.5861889	0.557749	0.948478
bub1	454.745411	-0.08496765	0.1147169	-0.740672	0.458892	0.921978
nmnat3	81.86952038	0.078726857	0.2277834	0.3456216	0.729627	0.971036
pimr177	0.484248244	0.852636534	2.8793247	0.2961238	0.767136	NA
BX901889.1	0.330916733	-1.849579229	3.2489867	-0.569279	0.569167	NA
txlna	658.8103126	0.025547796	0.1004771	0.254265	0.799291	0.979316
pbrm1l	1345.773251	-0.059580801	0.0800322	-0.74446	0.456598	0.921215
esama	190.8502872	-0.034108374	0.1647373	-0.207047	0.835973	0.984089
si:ch211-85n16.4	42.34885084	-0.187777656	0.3048992	-0.615868	0.537982	0.943336
arl6ip5a	75.05695519	0.305407299	0.241584	1.2641869	0.206163	0.773855
scg5	17.4742876	-2.813714222	0.5935203	-4.740721	2.13E-06	0.000191
ptprnb	53.85127655	0.008813293	0.2618091	0.033663	0.973146	0.996315
tnrc18	1520.757851	-0.009310305	0.0818441	-0.113757	0.909431	0.990702
ppfia3	1480.137816	-0.006893567	0.0903818	-0.076272	0.939203	0.992857
gask1b	98.41901809	-0.160182788	0.2157333	-0.742504	0.457782	0.921978
esco1	314.6410654	-0.03215666	0.123163	-0.26109	0.794023	0.977937
cela1.5	0	NA	NA	NA	NA	NA
rbm6	815.6161947	0.087016221	0.0999379	0.8707025	0.383917	0.895192
si:ch211-11p18.6	21.68056646	-0.536959745	0.4160156	-1.29072	0.196801	0.761944
srgn	278.4652151	-0.117959756	0.139079	-0.848149	0.396355	0.899672
si:ch73-280o22.2	120.9924498	0.006706092	0.1913961	0.0350378	0.97205	0.996315
tanc1a	680.9684338	-0.018605265	0.1015748	-0.183168	0.854666	0.985174
fastk	604.0751815	0.068144129	0.1127063	0.6046169	0.545434	0.945526
zgc:173726	30.46349039	-0.241364232	0.3664752	-0.65861	0.510146	0.936253
gprc5ba	391.9650917	0.261117034	0.1146524	2.277466	0.022758	0.288662
slc38a10	895.3316775	0.026183927	0.088998	0.2942081	0.768599	0.975687
agtrap	61.59644392	-0.152991484	0.2674782	-0.571977	0.567337	0.948739
avpr1aa	32.10135303	-0.542527103	0.3416539	-1.587943	0.112299	0.627572
col28a1a	404.869116	0.298096802	0.1128117	2.6424278	0.008231	0.147048
zgc:110821	119.4929956	-0.168060767	0.180103	-0.933137	0.350749	0.880195
ankrd34ba	17.11509971	-0.314073061	0.4878498	-0.643791	0.519711	0.938945

si:ch211-196g2.4	65.11043502	-0.158801589	0.2431746	-0.653035	0.513734	0.936778
blm	192.2126637	-0.224208245	0.1492612	-1.50212	0.133066	0.667628
si:ch211-127b11.1	4.461742498	-0.516207622	1.0371528	-0.497716	0.618684	NA
elk4	28.97600347	0.434814138	0.38178	1.1389129	0.25474	0.823484
scaf4a	1284.748295	-0.089866781	0.0843291	-1.065667	0.286574	0.845942
si:ch211-160o17.4	2636.225551	-0.055161186	0.0755298	-0.730323	0.465193	0.923671
fndc7a	17.84484165	0.349191018	0.4559936	0.7657805	0.443807	0.916631
si:ch211-226h8.6	0	NA	NA	NA	NA	NA
trim65	20.45350615	0.515147648	0.4265736	1.2076406	0.227186	0.794617
zbtb10	154.5532658	0.208429667	0.1764053	1.1815387	0.237389	0.806412
sema4aa	9.037858099	-0.317246731	0.6834794	-0.464164	0.64253	NA
oafb	15.84741983	0.670010996	0.5344167	1.253724	0.209942	0.777416
si:ch211-180f4.1	156.909084	0.162634441	0.1679365	0.9684285	0.33283	0.874926
arhgef16	274.9772603	-0.235446825	0.1358281	-1.733417	0.083022	0.555556
si:ch73-44m9.1	0	NA	NA	NA	NA	NA
ccnjl	403.8520911	-0.018962429	0.1108462	-0.17107	0.864169	0.98601
naxd	829.5072101	-0.178135954	0.0871323	-2.044432	0.040911	0.394957
arid5a	7.258233238	-0.381066514	0.6994306	-0.544824	0.585875	NA
cyp26b1	1042.135042	0.089486905	0.0914837	0.9781737	0.327988	0.872701
si:ch211-255p10.4	22.53776379	0.112668356	0.4198051	0.2683825	0.788405	0.976703
snrnp70	3700.385873	-0.148780255	0.0769157	-1.934329	0.053073	0.452412
bcl10	235.0189521	0.005039634	0.1377683	0.0365805	0.970819	0.996315
gpr158a	21.18997594	0.551177923	0.4156721	1.3259922	0.184842	0.747917
si:dkey-28d5.14	0	NA	NA	NA	NA	NA
zgc:195173	69.11710588	-0.068990522	0.2519859	-0.273787	0.784248	0.97627
col6a3	3202.739048	0.334701743	0.0665762	5.0273488	4.97E-07	5.67E-05
znf526	231.9075799	-0.037172688	0.1541185	-0.241195	0.809404	0.980642
adcy3a	284.9442744	0.0639859	0.1386612	0.461455	0.644472	0.96029
cbln2b	657.0302596	0.017854605	0.0944405	0.1890566	0.850048	0.985174
si:ch211-157p22.10	65.76319115	0.025942791	0.2520722	0.1029181	0.918028	0.991623
synpo2b	108.8087595	0.419476353	0.1969908	2.1294209	0.033219	0.355427
ccdc153	9.048767196	-0.251216449	0.667813	-0.376178	0.706785	NA
nwd2	651.4882806	-0.156102689	0.0962439	-1.621948	0.104814	0.612652
kcnj3a	131.7165175	-0.005308723	0.1813947	-0.029266	0.976652	0.996762
nmbb	47.27605499	-0.603799166	0.305731	-1.974936	0.048275	0.432986
si:ch211-153b23.4	607.873747	-0.25353884	0.1398768	-1.812586	0.069896	0.516293
qsox2	85.12925617	0.066377353	0.2530657	0.2622929	0.793096	0.977937
zgc:174888	165.200012	0.276289974	0.1590477	1.7371522	0.08236	0.553931
map2k4b	527.6550698	-0.139156123	0.1015671	-1.370091	0.170659	0.728116
zgc:152977	91.51687127	-0.089832153	0.2076514	-0.43261	0.665298	0.964736
ccdc83	5.215158951	0.005396256	0.8399135	0.0064248	0.994874	NA
slc37a4b	716.0705862	0.301085651	0.0932504	3.2287858	0.001243	0.036745
impg1a	236.7538797	0.382898248	0.1507497	2.5399599	0.011087	0.182535
atrnl1a	40.55140859	-0.09213206	0.2990738	-0.308058	0.758038	0.975374
nags	57.41143407	-0.127957673	0.2594239	-0.493238	0.621845	0.957354
chst12b	11.13427746	-0.095612446	0.5908064	-0.161834	0.871437	0.987754
myo7bb	205.6995987	-0.76135892	0.1546147	-4.924234	8.47E-07	8.77E-05
mocs2	90.68132079	0.201989402	0.2058973	0.9810203	0.326583	0.872564
zgc:153116	27.39757111	-0.065255856	0.3658145	-0.178385	0.858421	0.985773
lzts2b	27.89214345	-0.272984168	0.3775324	-0.723075	0.469634	0.924505
or112-1	6.010392788	-0.001706254	0.8564731	-0.001992	0.99841	NA
eif4g3a	1303.883765	-0.036287628	0.0823375	-0.440718	0.659417	0.96288
zgc:162184	122.2126286	-0.026762543	0.1803514	-0.148391	0.882034	0.988344
si:ch211-106h4.6	19.30282119	-0.025874046	0.4591886	-0.056347	0.955065	0.994543

si:ch211-208f21.3	2.943499199	-0.020793767	1.1314159	-0.018379	0.985337	NA
ypel2a	73.70246311	-0.528168781	0.2402464	-2.198446	0.027917	0.323709
smarca4a	6113.864871	-0.072588894	0.0815902	-0.889676	0.37364	0.893677
fbxl6	148.3833609	-0.004545048	0.1639186	-0.027727	0.97788	0.996762
ntrk3a	594.0871444	0.027706897	0.099047	0.2797349	0.779681	0.976146
ano8b	29.03066879	0.32557591	0.3624536	0.8982554	0.369049	0.888432
vwf	53.82701546	-0.131768727	0.3071677	-0.42898	0.667938	0.964736
hspb6	210.8051314	0.638834153	0.1518227	4.2077657	2.58E-05	0.001562
rfx7a	526.5859673	-0.264390365	0.1107384	-2.387523	0.016962	0.240499
si:ch211-165i18.2	1.935316918	0.957568656	1.4448608	0.6627411	0.507496	NA
cd247l	3.634575357	0.259952073	0.9816467	0.2648123	0.791154	NA
setd1bb	7.76493075	0.057973587	0.7437928	0.0779432	0.937873	NA
si:dkey-225n22.4	21.31073369	0.757046583	0.4311926	1.7557041	0.079139	0.547489
zgc:171459	66.53279517	-0.336791245	0.2451591	-1.373766	0.169514	0.726744
gmip	142.7691725	0.169559373	0.1682927	1.0075267	0.313682	0.865132
si:rp71-36a1.5	36.72431745	0.07600177	0.3135005	0.2424295	0.808447	0.980642
alkbh6	132.6128842	0.074303944	0.1813685	0.409685	0.682037	0.965476
nat8l	2466.356712	0.074109727	0.0820942	0.9027399	0.366664	0.88734
timd4	57.81490402	0.542617792	0.2822355	1.9225708	0.054534	0.457793
si:dkey-28d5.13	1.202474398	-0.315413906	1.8153552	-0.173748	0.862064	NA
lmod1b	11.54892827	0.320509874	0.5660942	0.5661777	0.571273	0.949418
si:ch73-27e22.3	10.25725567	0.278218848	0.5871723	0.4738283	0.635622	NA
wdr43	1276.443802	-0.118208511	0.0853037	-1.385737	0.165827	0.721626
wdr89	92.93180321	-0.005722117	0.2055987	-0.027831	0.977797	0.996762
zgc:174698	9.025378027	0.016135654	0.6398346	0.0252185	0.979881	NA
crispld1a	80.89759104	0.131753423	0.2223295	0.5926043	0.553446	0.947265
gdpd5a	221.3781487	-0.378882765	0.145838	-2.597971	0.009378	0.16228
scarf1	46.59088241	0.068139017	0.2888019	0.2359369	0.813482	0.981081
si:ch211-188c16.1	14.08105835	0.178451176	0.5289447	0.3373721	0.735836	0.972535
mmp25a	2.604263582	-0.007810395	1.1679243	-0.006687	0.994664	NA
rps2	25923.4777	-0.380198867	0.0968846	-3.924246	8.70E-05	0.004355
synpo2la	884.8897519	0.319496123	0.1108646	2.881859	0.003953	0.086474
akap6	1030.849124	0.022656661	0.0943874	0.2400392	0.8103	0.980867
nbr1a	1390.920596	-0.129030963	0.0823	-1.567812	0.116925	0.639958
gas1a	730.4873293	0.063387866	0.0953093	0.6650752	0.506002	0.935724
yars2	72.79894723	0.199728107	0.2284926	0.874112	0.382057	0.895192
usp42	506.0609462	0.039325892	0.1053731	0.373206	0.708995	0.968664
mlf1	321.7672253	0.307515205	0.1243316	2.4733468	0.013385	0.207707
erg	20.94931696	-0.316835747	0.4514814	-0.701769	0.482823	0.929133
cutc	207.0268037	-0.182579797	0.1488344	-1.226731	0.219924	0.786668
tmem201	231.2047946	-0.143754235	0.1435713	-1.001274	0.316694	0.867129
gpr84	185.689193	-0.204089528	0.1937471	-1.053381	0.292166	0.848075
si:ch211-209f23.6	2.168693064	0.248812578	1.2709797	0.1957644	0.844795	NA
slc46a3	62.45142772	-0.046178154	0.2564497	-0.180067	0.8571	0.985597
mblac1	42.20836855	0.459225894	0.3058208	1.5016176	0.133196	0.667628
MB21D2	5.278392891	-0.354017659	0.8116764	-0.436156	0.662723	NA
snx29	330.2214805	-0.074524575	0.1374648	-0.542136	0.587725	0.953319
CR376737.1	169.5915147	-0.05306709	0.1666038	-0.318523	0.750088	0.97419
thap4	161.8991918	0.15390853	0.1699833	0.9054331	0.365236	0.886714
slc45a4	448.5640862	0.130742946	0.1093646	1.1954778	0.2319	0.800084
ano1l	186.5977015	0.112146124	0.156014	0.7188211	0.472251	0.924838
nlgn2a	849.9607279	-0.010830514	0.0995587	-0.108785	0.913373	0.991119
SH3TC1	60.89493552	-0.350185883	0.2753844	-1.271626	0.203506	0.770906
si:ch211-171h4.5	1.511852115	-1.010943093	1.5814422	-0.639254	0.522658	NA

ptrh2	158.0349657	0.176229812	0.1720809	1.0241101	0.305783	0.860027
ppp1r14c	3020.469664	-0.136206777	0.083162	-1.637848	0.101453	0.605353
sorcs3b	34.36381863	-0.173214484	0.3263561	-0.530753	0.59559	0.954434
b4galnt1b	122.200715	-0.161843904	0.1826821	-0.885932	0.375654	0.894316
rerea	1171.036898	-0.176077237	0.1022234	-1.722475	0.084983	0.560519
sypl1	42.51425944	-0.040495197	0.3028453	-0.133716	0.893627	0.989291
lrrc61	14.6947234	-0.300371214	0.5148943	-0.583365	0.559648	0.948478
fthl30	52.68572719	0.231840281	0.2773171	0.8360116	0.403148	0.900877
bptf	1729.884856	-0.034993024	0.0895529	-0.390753	0.69598	0.96736
mapk9	137.1092194	0.131636638	0.1784792	0.7375463	0.46079	0.921978
ntng2a	251.1482446	-0.04587932	0.1319171	-0.347789	0.727999	0.970402
slc30a6	178.5336964	-0.054771274	0.1635108	-0.33497	0.737647	0.972535
kcnk15	62.0281984	-0.302135723	0.252492	-1.196615	0.231457	0.799752
tfr1b	896.1980878	-0.446211083	0.102365	-4.359019	1.31E-05	0.000894
si:dkey-85n7.6	257.4918246	0.179910958	0.1315556	1.3675661	0.171448	0.729863
hcn5	46.28331077	0.359811489	0.2878233	1.2501123	0.211259	0.778917
anxa11a	1545.100244	-0.101423199	0.0921443	-1.1007	0.271027	0.835943
dnah11	2.238716927	-0.674951187	1.4062242	-0.479974	0.631246	NA
rgs19	34.69589474	0.223565303	0.3411009	0.6554228	0.512196	0.936253
tcte1	2.370754626	1.891439854	1.3582926	1.3925128	0.163767	NA
obsl1b	319.2458771	0.25431857	0.1351637	1.8815599	0.059896	0.478654
tradv27.0	0	NA	NA	NA	NA	NA
si:dkey-81n2.2	0	NA	NA	NA	NA	NA
tlcd2	415.7516912	0.108014396	0.1265943	0.8532325	0.39353	0.898076
ubash3bb	128.3703712	-0.001227367	0.1843024	-0.00666	0.994687	0.998694
rbm10	454.0977379	-0.068100573	0.1164752	-0.584679	0.558764	0.948478
col8a1a	428.5520451	0.15280555	0.1586478	0.963175	0.33546	0.876088
ncoa3	1227.672165	-0.0439675	0.0829013	-0.530359	0.595863	0.954434
psip1b	452.3611276	-0.312612094	0.1471644	-2.124237	0.03365	0.357045
si:dkey-184p18.2	176.5717606	-0.104197909	0.1565859	-0.665436	0.505772	0.935724
cby1	116.0100692	0.093644664	0.1905014	0.4915695	0.623024	0.957354
myo9b	417.3549024	-0.032249476	0.1115907	-0.288998	0.772583	0.975687
ftr08	0	NA	NA	NA	NA	NA
zgc:194312	171.9633972	-0.041037559	0.1611077	-0.254721	0.798938	0.979066
zmp:0000001175	3.928706958	-0.629856636	1.0310761	-0.610873	0.541284	NA
nectin4a	146.6245391	0.13536126	0.170929	0.7919153	0.42841	0.911195
gpr20	1.979569643	-2.234237259	1.6098219	-1.387879	0.165174	NA
camta1a	1235.121968	0.037157736	0.0797233	0.466084	0.641155	0.959994
usf3	356.174008	-0.025862519	0.1206672	-0.214329	0.83029	0.982947
mon1bb	274.1741688	-0.04876048	0.1303686	-0.37402	0.708389	0.968664
SYDE1	98.90587541	0.182582714	0.2172957	0.8402501	0.400768	0.90084
tubgcp5	507.5778512	0.112900286	0.10315	1.0945258	0.273724	0.838381
si:dkey-234i14.14	0	NA	NA	NA	NA	NA
si:ch211-113a14.12	6.774214414	-0.073242367	0.7261114	-0.100869	0.919654	NA
dhx32a	69.83605805	0.048445808	0.2375896	0.2039054	0.838427	0.984797
sorcs2	568.8658566	0.010757052	0.1048025	0.1026412	0.918248	0.991623
sox10	496.8778815	0.012097646	0.1088686	0.1111216	0.91152	0.991066
si:ch73-206p6.1	220.6428565	0.18129371	0.1707065	1.0620199	0.288227	0.845979
polr1b	222.9592719	-0.013300023	0.148118	-0.089793	0.928451	0.992702
si:dkey-193b15.8	58.41143366	0.174749548	0.2639248	0.6621187	0.507895	0.935734
hmces	45.75193499	-0.106697124	0.3015821	-0.353791	0.723495	0.970402
mych	615.2345554	0.240943227	0.1160929	2.0754351	0.037946	0.381357
pla2r1	11.98641446	0.264424916	0.5533443	0.4778669	0.632745	0.957356
zgc:174353	0	NA	NA	NA	NA	NA

msh5	12.91590611	-0.625105694	0.5590027	-1.118252	0.263459	0.830245
si:dkey-76k16.6	22.76417549	0.010103993	0.4050252	0.0249466	0.980098	0.996879
spsb3b	5.733666734	-0.32878225	0.8367924	-0.392908	0.694388	NA
si:ch73-289h5.4	29.45801493	-0.084761675	0.3736713	-0.226835	0.820552	0.981594
lrfn2b	247.8290828	0.076216209	0.133916	0.5691344	0.569265	0.948983
atp8a2	1981.557083	-0.121041965	0.0877933	-1.378715	0.167983	0.725719
ptpn9a	95.49896848	0.134849628	0.2107676	0.6398023	0.522301	0.939056
igsf10	75.97585417	0.355421646	0.223701	1.5888247	0.1121	0.627454
plekho1b	229.4633014	0.068973383	0.1401287	0.4922144	0.622568	0.957354
si:ch211-103n10.5	7.532362692	0.398584999	0.7355425	0.5418925	0.587893	NA
tjp1a	2520.069646	-0.078236548	0.0904183	-0.865273	0.386889	0.89551
ppp1r3da	99.64859828	-0.270515579	0.2193096	-1.233487	0.217394	0.784786
letm2	1104.261403	0.11171437	0.0856739	1.3039482	0.192251	0.756683
recql4	99.15811271	-0.17455075	0.2016975	-0.865409	0.386815	0.89551
si:dkey-6f10.4	0	NA	NA	NA	NA	NA
tdrd7b	171.2920836	0.067610554	0.1679668	0.4025233	0.687299	0.966376
babam1	436.1932802	0.022123084	0.109074	0.2028264	0.839271	0.984979
ahctf1	997.8502181	-0.043852583	0.0927632	-0.472737	0.636401	0.958565
bag6l	524.9852425	0.129881639	0.1111182	1.1688604	0.24246	0.811298
pign	106.1211174	-0.239268602	0.1985827	-1.204881	0.228249	0.795541
eif3f	3245.147098	-0.327654324	0.0869466	-3.768455	0.000164	0.007299
snrnp200	2473.155979	0.071407583	0.0756735	0.9436271	0.34536	0.878434
nudt19	149.7757497	0.168566765	0.2140282	0.7875913	0.430936	0.91234
f2rl1.2	300.1645977	0.191995991	0.152867	1.2559679	0.209128	0.776073
si:dkeyp-75b4.8	5.613748172	0.175034727	0.8514487	0.2055728	0.837125	NA
toe1	150.9884984	-0.060370609	0.1667796	-0.361978	0.717368	0.96909
si:dkey-16p21.7	863.8466233	0.02726467	0.1113452	0.2448661	0.80656	0.980254
zdhhc1	365.8348734	0.143043518	0.1173618	1.2188249	0.222911	0.789115
tmlhe	31.19454716	0.354842232	0.3647589	0.9728131	0.330646	0.87417
aifm2	648.1615185	-0.198660753	0.1119836	-1.774016	0.07606	0.537342
gcnt4b	0	NA	NA	NA	NA	NA
rap2aa	34.69192751	-0.117435671	0.3862575	-0.304035	0.761101	0.975374
nitr5	2.334532634	-0.798295812	1.2644475	-0.63134	0.527818	NA
LO017815.1	80.6397886	0.552967589	0.2534069	2.1821327	0.0291	0.330497
grin2cb	97.50379585	0.09614322	0.1992502	0.4825252	0.629433	0.957354
angel1	52.78762489	-0.313903689	0.2656015	-1.18186	0.237261	0.806257
lrrn3a	367.3485602	0.051974082	0.1285832	0.4042058	0.686061	0.966376
cdh16	7.323803735	0.662229569	0.7008473	0.9448985	0.344711	NA
zgc:174862	4.982876016	-0.981587193	0.9128854	-1.075258	0.282259	NA
si:ch211-193k19.2	3.427132235	0.663052847	1.0961842	0.6048735	0.545263	NA
tmprss13b	65.86575078	0.361304825	0.2445395	1.4774904	0.139544	0.678238
ntsr1	2.76346132	0.191801704	1.2935319	0.1482775	0.882124	NA
tspeara	22.0126753	0.090064113	0.421457	0.2136971	0.830783	0.982994
zzz3	967.3338109	-0.005058857	0.0936711	-0.054007	0.95693	0.994543
ank3b	177.0384574	-0.030468932	0.1837299	-0.165835	0.868286	0.987245
b4gat1	281.2643764	0.010882502	0.1335477	0.0814878	0.935054	0.992702
cln6a	307.6951286	-0.122380393	0.1330703	-0.919667	0.357747	0.884371
pdgfc	165.5845517	-0.020116959	0.1596217	-0.126029	0.899709	0.990121
CR847803.1	0	NA	NA	NA	NA	NA
arsj	128.2946181	0.198785737	0.1807492	1.0997874	0.271425	0.836396
phf24	992.018685	0.220014649	0.0936195	2.3500948	0.018769	0.255518
CR394546.1	0.838940531	-0.561526863	2.0217856	-0.277738	0.781213	NA
frrs1b	193.1088331	0.167059859	0.1495148	1.1173463	0.263846	0.830245
arsia	88.83202235	-0.153745355	0.2162976	-0.710805	0.477205	0.926857

dus2	243.7902054	0.009188904	0.1403915	0.065452	0.947814	0.99381
gpr137bb	229.1945225	0.252344495	0.1707859	1.477549	0.139529	0.678238
elfn2a	3.508784899	0.143582365	0.9858328	0.1456458	0.884201	NA
hykk.1	1.003361447	-0.877559612	2.0717967	-0.423574	0.671876	NA
si:ch211-206k20.5	235.2462617	0.035445269	0.1424428	0.2488386	0.803486	0.979643
rin3	288.57894	0.109917368	0.1356134	0.8105199	0.417641	0.906237
cdca7a	580.6786414	0.105406727	0.1065956	0.9888468	0.322738	0.870524
si:ch211-263p13.7	8.467060689	0.079800654	0.6725306	0.1186573	0.905547	NA
tafa4a	1.175083697	1.334695785	1.855189	0.7194392	0.47187	NA
CU459089.1	21.93346123	0.200296939	0.4080795	0.4908282	0.623548	0.957354
magixb	15.08263568	-0.290251928	0.5158029	-0.562719	0.573626	0.949904
mal2	41.26693952	-0.067082183	0.309164	-0.216979	0.828224	0.982721
zgc:171965	3.123795581	1.476674125	1.1145321	1.3249274	0.185195	NA
intu	317.3739099	0.120735205	0.124052	0.9732629	0.330423	0.874098
thbs3a	266.92531	0.314178335	0.1384488	2.2692749	0.023252	0.292665
lypd6b	75.93854521	-0.021213633	0.2260311	-0.093853	0.925226	0.992676
zgc:172079	0.665331516	0.047715978	2.3078578	0.0206754	0.983505	NA
gpr78b	2.028168054	0.489124736	1.316488	0.3715376	0.710237	NA
si:ch211-42i9.8	101.0546148	-0.193765539	0.1998524	-0.969543	0.332274	0.874653
tnksa	467.9396963	-0.074988982	0.1113938	-0.673188	0.500828	0.93428
lratb.2	92.14699593	-0.295015805	0.252628	-1.167788	0.242892	0.811584
rab16b	480.8392261	0.177799433	0.107752	1.6500804	0.098926	0.598346
grm8a	488.2280097	-0.048557853	0.1167838	-0.415793	0.677562	0.965257
arfgef3	38.05996535	-0.257735651	0.3280032	-0.785772	0.432001	0.912876
zgc:110239	687.7599373	-0.273905198	0.0982267	-2.788501	0.005295	0.107627
pimr140	0.157187365	-0.955901296	4.0804729	-0.234262	0.814781	NA
evi5a	27.19561747	-0.496050372	0.3831438	-1.294685	0.195429	0.760412
nlrp16	31.54046327	-0.465137679	0.3471663	-1.339812	0.180306	0.741397
pdgfd	61.39003575	-0.18852723	0.2694183	-0.699756	0.484079	0.929461
aak1b	42.62501544	0.481596889	0.296336	1.6251718	0.104126	0.611338
rp1	1.286727072	-1.584087934	1.825231	-0.867884	0.385458	NA
cpo	16.31798873	-1.027981641	0.5159368	-1.992457	0.046321	0.422877
mb21d2	448.0132799	-0.020618956	0.12148	-0.169731	0.865222	0.986367
slc13a5a	131.5363014	0.351292136	0.173195	2.0283042	0.042529	0.405679
dtnba	1335.509036	0.349860987	0.0923297	3.7892572	0.000151	0.006841
si:dkey-30h22.11	97.77383427	0.021332495	0.2095704	0.1017915	0.918922	0.991623
si:dkey-105i14.1	74.91955627	0.07971385	0.2772008	0.2875672	0.773678	0.975687
rsad1	11.62776489	-0.003432698	0.5569489	-0.006163	0.995082	0.998694
CR749163.1	4.351721592	0.939375682	0.9731163	0.9653272	0.334381	NA
iqsec2b	360.4911793	0.033819692	0.1314218	0.2573369	0.796919	0.978569
nlgn1	35.16222437	0.075114316	0.3273425	0.229467	0.818506	0.981594
zgc:113886	0.323844819	-1.825095364	3.274639	-0.557342	0.577293	NA
GRIK3	48.35181948	0.081857244	0.2799204	0.2924304	0.769958	0.975687
rpl29	2927.604872	-0.179442188	0.1092152	-1.643015	0.10038	0.602451
knop1	135.0820238	-0.151723888	0.1829258	-0.829429	0.406862	0.901305
ppp2r3a	817.9530756	0.351712586	0.0932418	3.7720494	0.000162	0.00723
nocta	431.1577837	0.535198635	0.1278509	4.1861163	2.84E-05	0.001682
supv3l1	294.0326402	0.138557646	0.1254952	1.1040876	0.269555	0.834705
alyref	3187.231453	-0.059854403	0.0862173	-0.694228	0.48754	0.93164
spsb3a	436.851675	-0.187824654	0.1243905	-1.50996	0.131054	0.663795
b3glctb	11.30992785	0.542546182	0.5769355	0.9403932	0.347016	0.879707
zgc:175135	0	NA	NA	NA	NA	NA
si:ch73-38o13.2	1.697251321	1.219455701	1.5047527	0.8104028	0.417709	NA
AL935199.1	30.7453503	0.178296382	0.4006957	0.444967	0.656344	0.961833

usp54b	64.67869137	-0.1237016	0.27975	-0.442186	0.658354	0.962545
nol9	497.0823806	0.003488386	0.1077609	0.0323715	0.974176	0.99644
ascc1	99.97303402	-0.09047215	0.209132	-0.432608	0.6653	0.964736
nlgn4xb	21.20439609	0.355555084	0.4204475	0.8456587	0.397743	0.899672
si:rp71-79p20.2	4.436696382	0.547112337	0.8923852	0.6130899	0.539817	NA
faah	80.97039795	-0.007899461	0.2309372	-0.034206	0.972713	0.996315
malt1	76.77561131	-0.130168967	0.2334293	-0.557638	0.577092	0.950788
cited1	223.1841848	-0.185349464	0.1406497	-1.317809	0.187568	0.75202
baalca	33.88202847	0.090706362	0.3324115	0.2728737	0.78495	0.97627
csnk2b	5822.542431	-0.001715916	0.0645551	-0.026581	0.978794	0.996762
tmsb4x	18100.49065	-0.078022701	0.1074792	-0.725933	0.46788	0.924328
adamts9	195.2017482	0.271800858	0.1481691	1.8343959	0.066595	0.504929
acer2	6.593806553	-1.286090635	0.8483297	-1.516027	0.129513	NA
SRMS	102.1660826	0.034073085	0.1946519	0.1750462	0.861043	0.985773
atf5b	768.7781941	-0.055256984	0.1039003	-0.531827	0.594846	0.954434
arhgef10	203.5296002	0.237193621	0.1676047	1.4151966	0.157011	0.707677
dcaf15	255.7336852	-0.40546088	0.1368841	-2.962075	0.003056	0.073064
brsk2b	1197.215523	-0.036546371	0.0866414	-0.421812	0.673162	0.965257
cdc42ep1b	68.85273019	-0.193420769	0.2367193	-0.817089	0.413878	0.905864
CU468012.1	5.007091696	-0.989118423	0.8769775	-1.127872	0.259374	NA
egr4	101.3614443	0.657374816	0.2576009	2.5519121	0.010713	0.179263
hgsnat	840.6464041	-0.075324808	0.0911807	-0.826105	0.408745	0.90171
il13	2.010646529	-0.478327043	1.3048551	-0.366575	0.713936	NA
otud4	108.6170114	-0.053341717	0.2420032	-0.220417	0.825546	0.982196
sox11a	3076.701384	-0.057435967	0.0720156	-0.797548	0.425133	0.908819
slc5a3b	57.77524383	0.272080297	0.2734754	0.9948987	0.319786	0.86809
si:dkey-34f9.3	6.153259384	-0.304038593	0.817008	-0.372137	0.709791	NA
cdc26	227.9090482	-0.284651632	0.1408939	-2.020327	0.04335	0.408576
cxxc4	274.6185362	0.035582064	0.1298336	0.2740591	0.784039	0.97627
nrg2a	100.6346007	0.121630991	0.2052408	0.5926257	0.553432	0.947265
si:dkey-6i22.5	83.97536399	-0.001588807	0.228362	-0.006957	0.994449	0.998694
hmgxb3	60.72041335	0.041426914	0.2596906	0.1595241	0.873256	0.987846
efcc1	112.1470748	-0.030506595	0.1910492	-0.159679	0.873134	0.987846
slc29a3	301.7204171	-0.221730985	0.1376859	-1.610412	0.107308	0.617344
borcs5	102.8048963	-0.194085852	0.2089803	-0.928728	0.35303	0.881552
ttc22	56.14737435	0.314987349	0.2692078	1.1700527	0.24198	0.810713
dhx30	219.2985931	0.184755843	0.1814605	1.0181603	0.308602	0.861088
meis2a	3137.526301	-0.089464306	0.0869531	-1.02888	0.303536	0.857769
ajuba	487.6668873	0.084960045	0.1144021	0.7426439	0.457697	0.921978
si:ch211-67f13.7	11.19279538	-0.624561589	0.6031121	-1.035565	0.300405	0.856251
olfm2a	1327.751758	-0.004071221	0.0861576	-0.047253	0.962311	0.995907
lgals3a	1202.226982	0.054566893	0.0862884	0.6323781	0.52714	0.941186
samd11	916.2045343	-0.097148063	0.0998046	-0.973383	0.330363	0.874098
ttll2	14.76337052	-1.764033822	0.5600225	-3.149934	0.001633	0.046066
znrf2a	88.70360498	-0.02863915	0.2401833	-0.119239	0.905086	0.990702
asip1	34.96510625	-0.049503795	0.3346576	-0.147924	0.882403	0.988344
ndufaf4	152.8216798	-0.002689581	0.1649165	-0.016309	0.986988	0.997073
ankhd1	1948.136238	-0.035548462	0.1049328	-0.338774	0.73478	0.972021
sowahaa	25.80833419	-0.549917635	0.3856738	-1.425862	0.153908	0.702738
si:dkey-169i5.4	60.20966984	-0.101463269	0.2502664	-0.405421	0.685168	0.966146
mettl22	101.4938682	0.291612108	0.204267	1.4276026	0.153406	0.702578
rasgrp3	13.68764224	-0.023718341	0.5181029	-0.045779	0.963486	0.995927
nostrin	8.692397609	-0.186155844	0.6768405	-0.275036	0.783288	NA
taf9	666.6263588	-0.111740088	0.0950982	-1.174997	0.239996	0.808111

CR626907.1	160.135731	-0.021076969	0.1808747	-0.116528	0.907234	0.990702
vwa10.1	33.21999571	-0.292243943	0.3437432	-0.850181	0.395224	0.899244
slc2a5	36.6657793	0.472600225	0.3292831	1.4352397	0.151219	0.699388
znf11h	0.356300316	0.005884122	3.214964	0.0018302	0.99854	NA
si:dkey-192l18.9	27.96681947	-0.013858488	0.3557005	-0.038961	0.968921	0.996315
si:ch211-255i20.3	31.2048984	0.014405241	0.342563	0.0420514	0.966458	0.996315
chl1a	386.5694767	0.044535207	0.1318883	0.3376736	0.735609	0.972535
ccn5	11.21942793	0.240348985	0.5787027	0.4153238	0.677905	0.965257
fam83d	101.449603	-0.06909967	0.2124646	-0.325229	0.745008	0.974094
znf296	435.0186161	0.174427334	0.1114218	1.5654691	0.117473	0.641157
wu:fj20b03	0.158915748	0.967652056	4.0804729	0.2371421	0.812547	NA
si:dkey-28d5.11	0.173729368	-0.955901296	4.0804729	-0.234262	0.814781	NA
si:ch211-141o9.10	7.537355208	-0.119373855	0.694377	-0.171915	0.863504	NA
herc1	1936.5903	0.056012717	0.105475	0.5310523	0.595383	0.954434
csmd3a	29.57451361	0.138319114	0.3531835	0.3916353	0.695328	0.96736
rnf165a	468.7546536	-0.202819489	0.1160891	-1.747102	0.08062	0.550664
si:dkey-183p4.10	10.27066177	0.079988321	0.6280715	0.1273554	0.898659	NA
rce1a	4.409274686	-0.538020283	0.9151813	-0.587884	0.55661	NA
si:zfos-1897c11.1	5.739527168	1.506912587	0.854667	1.7631576	0.077874	NA
treh	3.832200632	0.129192191	0.9969006	0.1295938	0.896888	NA
bcorl1	365.0355479	0.041781194	0.1203946	0.3470355	0.728565	0.970604
lmx1al	2.680813421	-0.313857236	1.2231765	-0.256592	0.797494	NA
dnase2b	12.27820272	-0.227096868	0.5471191	-0.415078	0.678085	0.965257
csmd3a	30.18542878	0.033469944	0.3962165	0.0844739	0.93268	0.992702
si:dkey-48p11.3	75.17279807	-0.170953546	0.2364288	-0.723066	0.469639	0.924505
ccdc173	30.32788364	-0.19714183	0.3432236	-0.574383	0.565709	0.948739
tegt	2913.456321	-0.137177255	0.079189	-1.732276	0.083224	0.556259
cd248b	314.3626674	0.147919339	0.1212235	1.2202204	0.222381	0.788748
ints5	201.0396206	-0.11904012	0.1511725	-0.787446	0.431021	0.91234
smarcc2	1416.02748	0.079822168	0.07843	1.0177503	0.308797	0.861088
usp54a	579.6972813	0.009060066	0.1046708	0.0865577	0.931023	0.992702
tor1	435.3869587	-0.08826338	0.1182729	-0.746269	0.455505	0.921215
rnf6	381.9605161	0.039843376	0.1179124	0.3379065	0.735434	0.972535
CU207281.2	1.709828404	0.638900379	1.5032996	0.4249987	0.670838	NA
si:ch211-186e20.7	103.1150464	0.225233605	0.2096891	1.0741314	0.282764	0.844235
gltpb	5.017881036	0.581047288	0.8482582	0.6849887	0.493351	NA
si:ch211-140m22.7	1034.347439	0.153860418	0.1064856	1.4448935	0.148488	0.696298
si:ch73-343l4.8	0.948309339	0.013153536	2.2227673	0.0059176	0.995278	NA
tmem26a	13.76220032	0.52571565	0.5632912	0.9332929	0.350669	0.880195
elf3	995.4027535	-0.646673564	0.1160345	-5.573116	2.50E-08	3.89E-06
GPR89B	38.78174868	-0.06397141	0.3141409	-0.203639	0.838635	0.984979
kiss2	2.641863189	0.029941083	1.1787311	0.0254011	0.979735	NA
TMEM151A	211.3115524	-0.039497472	0.1450401	-0.272321	0.785375	0.97627
zfat	143.7865933	0.367368686	0.1763796	2.0828295	0.037267	0.377702
abcc10	222.1466293	-0.011734332	0.1421932	-0.082524	0.93423	0.992702
b4galt2	22.37362418	-0.006912651	0.410026	-0.016859	0.986549	0.996965
cdh24b	37.36387699	-0.031094015	0.3529882	-0.088088	0.929807	0.992702
nitr3a	5.826696216	0.410620039	0.8124535	0.5054074	0.613273	NA
kbtbd7	37.00593163	-0.044951073	0.3344603	-0.134399	0.893087	0.989291
si:dkey-13n15.11	18.02694198	-0.216493556	0.4676098	-0.462979	0.643379	0.959994
drl	3.178925761	-0.157254919	1.1006051	-0.14288	0.886385	NA
wscd1b	318.6012538	-0.035268539	0.1239481	-0.284543	0.775994	0.975826
NAV1 (1 of many)	50.43229307	-0.261375143	0.2746767	-0.951574	0.341313	0.878134
zgc:162060	0.317831496	1.808837541	3.5779923	0.5055454	0.613176	NA

pacsin2	667.0398887	-0.072378115	0.1000904	-0.723127	0.469602	0.924505
dtx4b	12.41027378	-0.119926803	0.5649994	-0.21226	0.831904	0.98349
si:dkey-79d12.4	49.09063533	-0.214005346	0.2742087	-0.780447	0.435128	0.914408
fyco1b	39.94624546	-0.109873142	0.3043864	-0.360966	0.718125	0.96909
zcchc24	575.9665077	0.131299886	0.0995875	1.3184379	0.187357	0.751481
zgc:112001	229.6728834	-0.0221647	0.1454216	-0.152417	0.878858	0.987881
chpfb	117.4552645	0.002278454	0.1861818	0.0122378	0.990236	0.997679
xpo1a	2873.100748	-0.08239945	0.0717589	-1.148282	0.250852	0.820469
il10rb	438.4402806	-0.059253494	0.1188657	-0.498491	0.618138	0.957354
phf19	139.6512578	0.260898032	0.1734222	1.5044097	0.132476	0.666729
emilin3a	407.587575	0.079522013	0.1235913	0.6434273	0.519947	0.938995
acd	79.22606323	-0.19269972	0.2238156	-0.860975	0.389252	0.896362
RNF208	350.3135434	0.169011427	0.1213621	1.3926208	0.163734	0.71945
snx8b	239.9306962	0.293907061	0.1722939	1.7058469	0.088037	0.568063
dip2a	394.7191304	-0.035117197	0.1165576	-0.301286	0.763196	0.975374
nudcd2	236.5208258	0.077641223	0.1490723	0.5208294	0.602486	0.956045
syt7b	234.4506823	0.09870207	0.144333	0.6838497	0.49407	0.931926
ftr05	9.116757351	-0.014301885	0.6703193	-0.021336	0.982978	NA
elk1	72.58981999	-0.143817151	0.2364721	-0.608178	0.543069	0.944974
abcg4b	43.09243945	0.215006055	0.3000179	0.7166441	0.473594	0.924993
rrm2	560.2534587	1.400793692	0.1203798	11.636451	2.69E-31	1.06E-27
si:ch211-232i5.1	70.60438876	-0.248657276	0.249321	-0.997338	0.318601	0.867746
nudt5	444.7308677	-0.039710286	0.112562	-0.352786	0.724249	0.970402
ilrun	537.110857	0.014020198	0.1018917	0.137599	0.890557	0.989291
nab2	48.03189154	-0.279318615	0.296508	-0.942027	0.346179	0.87905
dph2	111.9963303	-0.232128093	0.1874802	-1.238147	0.215662	0.78305
lrfn4b	360.8495952	0.024585158	0.1163721	0.2112634	0.832682	0.983557
armc5	255.4353981	0.073806937	0.1322403	0.5581274	0.576757	0.950653
CNNM1	68.90896076	0.197674901	0.237273	0.8331116	0.404782	0.901305
si:ch211-186j3.6	491.3614756	0.170196649	0.1148684	1.4816669	0.138429	0.676209
limd2	241.4375777	0.235775723	0.1397609	1.6869939	0.091605	0.57849
zgc:172065	6.503892741	-0.221218798	0.7429939	-0.29774	0.765902	NA
lmf2a	56.83201158	-0.321229523	0.2602715	-1.23421	0.217125	0.784302
cipcb	1093.383148	0.046179639	0.0844936	0.5465462	0.584691	0.952411
nlrc9	0.846174082	-0.46494763	2.1994676	-0.211391	0.832582	NA
psda	120.2951922	0.240400888	0.1848455	1.3005504	0.193412	0.757219
depdc5	598.8405449	0.050603888	0.1220674	0.414557	0.678466	0.965257
ocrl	661.5088898	0.058759687	0.0963609	0.6097874	0.542003	0.944974
dok1a	75.77773244	0.446581204	0.2446659	1.8252694	0.06796	0.510097
usp12a	614.8673579	-0.23428755	0.0992654	-2.360215	0.018264	0.251504
ap5z1	134.2497443	0.038567515	0.1869228	0.2063285	0.836534	0.984386
atp5mea	702.9308531	0.094617684	0.1232903	0.7674379	0.442821	0.916336
si:ch73-237c6.1	248.7545752	0.426956625	0.1389075	3.0736769	0.002114	0.055672
sinhcafl	942.147905	-0.265648914	0.0974326	-2.726489	0.006401	0.122179
sh3bp1	3.060489361	-0.173789435	1.1622837	-0.149524	0.88114	NA
slitrk3a	277.428986	0.14268141	0.1282403	1.1126098	0.265876	0.831945
rusc1	131.3452239	0.379611937	0.1789925	2.1208265	0.033936	0.358663
neurl4	307.4254818	-0.009778704	0.1310914	-0.074595	0.940537	0.992857
CR391986.1	56.01616897	0.019957013	0.2690179	0.0741847	0.940863	0.992857
tex2l	113.2261139	0.218978924	0.2113888	1.0359058	0.300246	0.856251
kdm2ab	1604.805363	-0.042630903	0.0820808	-0.519378	0.603497	0.956045
crygm2f	39.0959287	-0.355396931	0.3355618	-1.05911	0.28955	0.846246
mrc2	53.37500229	-0.018938497	0.2808251	-0.067439	0.946232	0.99381
wdr47a	150.0656463	-0.156598371	0.1798749	-0.870596	0.383975	0.895192

si:ch211-202h22.8	73.65625258	-0.278752816	0.2315759	-1.203721	0.228697	0.796173
appl2	93.28114838	-0.009463847	0.2058769	-0.045968	0.963335	0.995927
si:dkey-18j18.3	30.75297053	0.506015255	0.3721182	1.3598238	0.173886	0.733946
SPAG9	1030.576859	-0.022418797	0.1019074	-0.219992	0.825878	0.982196
si:ch211-218g4.2	104.2289454	-0.267284628	0.2009145	-1.33034	0.183406	0.747151
il10	4.477210104	-0.330422478	0.9353167	-0.353273	0.723884	NA
grin2ca	230.3140073	-0.287136992	0.14353	-2.000536	0.045442	0.418148
si:ch211-264f5.8	261.4907768	-0.014481386	0.1332079	-0.108713	0.91343	0.991119
si:dkey-56i24.1	11.79735592	-0.565981091	0.6040858	-0.936922	0.348799	0.880195
inavaa	297.5315895	0.171018134	0.1398091	1.2232261	0.221244	0.787842
SLC18A1	2.756485174	0.88743765	1.2490485	0.710491	0.4774	NA
zgc:175284	65.94136827	-0.417818179	0.2391768	-1.746901	0.080654	0.550687
pimr208	2.149913281	0.186176459	1.3397624	0.1389623	0.88948	NA
hpse2	4.971410095	-0.960578608	0.883573	-1.087153	0.276969	NA
znf576.2	748.1816578	-0.050228818	0.0915283	-0.548779	0.583157	0.952214
si:dkey-78p8.1	86.13534285	-0.080893081	0.2176808	-0.371613	0.710181	0.968928
si:dkeyp-100h4.1	14.41050614	-0.331818584	0.5613017	-0.591159	0.554414	0.947449
cacna2d2b	185.5292242	0.026754419	0.1708449	0.1566006	0.87556	0.987881
CU984600.1	511.6161456	0.058262497	0.1042401	0.5589258	0.576212	0.950611
cxcr3.1	0.647689638	-2.824814845	2.5064236	-1.12703	0.25973	NA
art4	7.81656062	-0.82223139	0.7206247	-1.140998	0.253871	NA
fndc3ba	478.2671784	0.106469853	0.1075898	0.989591	0.322374	0.870226
sc:d0202	1.473262941	-0.365764327	1.5700333	-0.232966	0.815788	NA
zgc:194443	0.31437473	-1.788572922	3.5930657	-0.497785	0.618636	NA
zgc:165453	1.663695949	-0.5971764	1.4430799	-0.413821	0.679005	NA
pacs2	34.28589669	0.124945625	0.3268178	0.3823097	0.702232	0.968134
tex2	243.2796123	-0.001887195	0.1396039	-0.013518	0.989214	0.997589
slc12a5b	114.6188187	0.210898041	0.2112917	0.9981371	0.318213	0.867746
fam160a2	149.0157257	0.065683608	0.171741	0.3824574	0.702122	0.968134
crygm3	13.94186517	-0.493154413	0.518692	-0.950765	0.341724	0.878134
zbtb46	112.6692306	-0.108356902	0.1873284	-0.578433	0.562972	0.948728
si:ch211-67e16.3	4.39916239	0.241870285	0.9175928	0.2635922	0.792094	NA
fhit	99.91635955	-0.119108142	0.2086809	-0.570767	0.568158	0.948983
vcam1a	2.499850502	-0.167781508	1.1959875	-0.140287	0.888433	NA
BX072576.1	5.733879701	-0.844443711	0.8342363	-1.012236	0.311425	NA
ccl34b.9	0.173729368	-0.955901296	4.0804729	-0.234262	0.814781	NA
zgc:171506	0.983671166	-0.053965044	1.9476354	-0.027708	0.977895	NA
tulp1b	38.51999261	-0.150548131	0.3153423	-0.477412	0.633069	0.957356
espn1a	19.1281299	0.402637329	0.4377037	0.9198855	0.357633	0.884199
mybbp1a	903.9496164	-0.18297414	0.0929903	-1.967669	0.049106	0.435115
rbsn	22.3064494	0.139206024	0.4106267	0.3390087	0.734603	0.972021
eps15	596.9315389	-0.071769129	0.0995649	-0.720828	0.471016	0.924838
cblc	88.26904522	-0.046401292	0.2211304	-0.209837	0.833795	0.983583
BX323458.1	11.34854618	0.393092076	0.597778	0.6575888	0.510802	0.936253
si:ch211-204d2.4	20.11743529	0.216755883	0.4505296	0.4811135	0.630436	0.957354
aatka	188.9852462	0.19685045	0.1492268	1.3191357	0.187124	0.751002
ora4	1.331721622	1.594539771	1.8084122	0.8817347	0.37792	NA
cdh12a	17.40270362	-0.344487807	0.4691777	-0.734237	0.462804	0.922698
cspg4	108.8385431	0.071999734	0.19753	0.3645003	0.715484	0.96909
ctnnd1	1654.453175	-0.035101105	0.0774107	-0.45344	0.650232	0.960708
nlrc7	2.990509915	-2.352076253	1.2669899	-1.856429	0.063392	NA
nacc1b	7.278991651	-0.082609925	0.7847817	-0.105265	0.916166	NA
map7a	27.31336056	0.121120724	0.3721212	0.3254873	0.744812	0.974094
si:ch211-197l9.2	204.239924	0.000286985	0.1585389	0.0018102	0.998556	0.999364

gal3st1b	40.72322079	0.544141653	0.3044189	1.7874765	0.073861	0.529749
si:ch211-114l13.4	7.293945833	0.512988314	0.7300351	0.70269	0.482249	NA
vip	135.2456485	-0.118086108	0.1904054	-0.620182	0.535138	0.943336
unc13d	21.25263469	0.594038778	0.4291595	1.3841913	0.1663	0.722887
zgc:194398	82.11434494	0.27750893	0.2253529	1.2314417	0.218158	0.785379
TESK1	363.9183706	-0.026490833	0.1149415	-0.230472	0.817725	0.981594
ftr14l	12.88370772	0.9213478	0.5402868	1.705294	0.08814	0.568452
ovol1b	13.70839312	0.314012899	0.5130376	0.6120661	0.540494	0.944563
ora5	1.356046616	0.792643957	1.7222439	0.4602391	0.645345	NA
CABZ01049847.1	113.1733694	0.351413803	0.1931106	1.8197542	0.068796	0.513259
bnip1a	41.66895465	0.137314147	0.3151879	0.435658	0.663085	0.964736
nphp3	56.74570148	0.455833232	0.2605552	1.749469	0.08021	0.549641
twist2	185.835974	0.059072408	0.1551266	0.3808012	0.703351	0.968134
si:dkey-181m9.8	74.58146016	0.062913013	0.2426934	0.2592284	0.795459	0.978498
CU467961.1	85.27263094	0.198228928	0.2254748	0.8791622	0.379313	0.895192
si:ch211-57m13.8	0.157187365	-0.955901296	4.0804729	-0.234262	0.814781	NA
FO704871.1	3.321777663	-1.21677839	1.0491541	-1.159771	0.246142	NA
foxp3b	1.365706081	3.891476754	1.8557422	2.0969922	0.035994	NA
nkx3-1	13.82231315	0.063768111	0.5249816	0.1214673	0.903321	0.990121
zgc:173575	76.21742004	-0.05437742	0.2249758	-0.241703	0.80901	0.980642
si:zfos-323e3.4	91.49300226	-0.275387141	0.2221631	-1.239572	0.215134	0.78275
phkb	1362.879798	0.18218693	0.0877467	2.0762825	0.037868	0.381068
pik3ap1	44.57473755	0.205368574	0.2967925	0.6919601	0.488962	0.931926
CABZ01046425.1	0.157187365	-0.955901296	4.0804729	-0.234262	0.814781	NA
ptprh	0.964730988	-0.018658301	1.9582344	-0.009528	0.992398	NA
si:ch211-13f8.2	26.40673413	-0.271335563	0.4064322	-0.667604	0.504387	0.935541
zgc:162255	178.7924581	-0.158956545	0.1599814	-0.993594	0.320421	0.868301
brinp1	180.6753903	0.149389638	0.1645848	0.9076758	0.36405	0.886323
spry2	977.31521	0.038604359	0.0875066	0.4411596	0.659097	0.962839
fam83b	56.62669116	-0.237111937	0.2700033	-0.878182	0.379845	0.895192
cecr2	463.9486366	-0.249860338	0.1127826	-2.215416	0.026732	0.315011
dcaf6	605.3435199	0.181559962	0.0990039	1.8338659	0.066674	0.504944
pimr22	0.325573202	0.005883621	3.3172783	0.0017736	0.998585	NA
MYO9B	556.7384827	0.022051597	0.1090461	0.2022227	0.839743	0.984979
zgc:110063	448.9336608	0.206114955	0.105721	1.9496123	0.051222	0.443542
si:dkey-175m17.7	45.22644699	-0.278038642	0.3250642	-0.855335	0.392366	0.897688
tmod1	62.46067241	0.441894734	0.2539811	1.7398726	0.081881	0.552725
col12a1a	7732.843063	0.271366689	0.0786647	3.4496648	0.000561	0.019327
si:ch211-263k4.2	204.1492496	-0.271397605	0.1512114	-1.794822	0.072682	0.52617
arhgap10	421.4910178	0.052560752	0.1168189	0.4499335	0.652758	0.960708
CEP170B (1 of many)	36.34097078	-0.010655812	0.3149901	-0.033829	0.973013	0.996315
sec22a	332.9454865	-0.111870077	0.1186645	-0.942743	0.345813	0.878481
rec8a	0.706508242	2.93232624	2.4625218	1.1907818	0.233739	NA
best1	6.624116657	0.889067779	0.7553241	1.1770679	0.239168	NA
tmem234	55.1216727	0.06253692	0.2726093	0.2294013	0.818557	0.981594
amot	297.5539499	-0.050954141	0.1297762	-0.392631	0.694592	0.96736
tcf20	1688.377227	0.009969437	0.0929269	0.1072825	0.914565	0.991119
zmp:0000000845	2.445499181	3.680838207	1.6029974	2.2962222	0.021663	NA
lyrm4	131.5153638	0.040719201	0.1830474	0.2224517	0.823962	0.982196
zc3h4	1288.459448	-0.148765428	0.1046055	-1.422156	0.154981	0.704624
zmp:0000000846	755.3649534	0.218682228	0.0989904	2.2091251	0.027166	0.31767
mcoln3a	82.18020994	-0.149958498	0.2348061	-0.638648	0.523052	0.939336
AL954361.1	1.338473762	1.655884431	1.8743577	0.883441	0.376998	NA
robo2	106.6082949	-0.054980823	0.205165	-0.267984	0.788712	0.976703

unm_sa808	29.84348468	0.065581754	0.3644439	0.1799502	0.857192	0.985643
trip11	826.2260731	-0.013057151	0.0879858	-0.148401	0.882027	0.988344
mcrip1	440.758153	-0.107026766	0.1222007	-0.875828	0.381124	0.895192
dync1i2a	1485.880082	-0.096119929	0.0820502	-1.171477	0.241407	0.810468
si:dkey-92i15.4	25.86328619	-0.811193995	0.3947998	-2.054697	0.039908	0.391511
glis2a	164.2270269	0.015142495	0.1747644	0.0866452	0.930954	0.992702
ifi46	10.08023371	-1.365466547	0.6335813	-2.155156	0.03115	NA
fam98a	427.0082865	0.143973115	0.1260726	1.1419855	0.25346	0.823246
chpfa	65.60325387	0.041592918	0.2426358	0.1714212	0.863893	0.985952
CABZ01059627.1	2.516392505	-0.179090833	1.1950274	-0.149863	0.880872	NA
si:ch211-163m17.4	0.341366344	1.891903201	3.2128156	0.5888614	0.555954	NA
acsl2	132.3592352	-0.405161977	0.2009026	-2.016709	0.043726	0.410733
larp4aa	458.9271185	0.139649556	0.1079734	1.2933702	0.195883	0.76113
cdh26.1	103.5896615	-0.136693465	0.2068808	-0.660735	0.508782	0.935875
hspb15	172.3320871	0.157043958	0.1682995	0.9331219	0.350757	0.880195
lrrc3	23.06721094	-0.142905567	0.3985949	-0.358523	0.719952	0.969318
zeb2b	1043.894067	-0.009559277	0.0964312	-0.09913	0.921035	0.992138
plxdc1	38.22037911	-0.360577568	0.3303597	-1.09147	0.275066	0.839541
filip1a	31.27194161	0.489566428	0.3720402	1.3158967	0.188209	0.753063
zgc:194285	100.2109554	0.289473454	0.2037485	1.4207389	0.155393	0.705158
oat	243.6329428	0.439454601	0.1522909	2.8856256	0.003906	0.085637
BX901889.2	0.476747244	2.393381959	3.0525537	0.7840589	0.433006	NA
si:ch211-256e16.4	4.193097878	0.140116853	0.9380964	0.149363	0.881267	NA
vtg4	0.173729368	-0.955901296	4.0804729	-0.234262	0.814781	NA
tiam1a	1344.737427	0.02208987	0.0813321	0.271601	0.785929	0.97627
spc25	211.3264653	-0.035109951	0.1579931	-0.222225	0.824139	0.982196
znf692	46.67628176	0.160752659	0.2795111	0.5751208	0.56521	0.948739
anapc13	154.9161454	-0.17959101	0.1744158	-1.029672	0.303164	0.85729
ccdc88aa	101.9935749	-0.112422122	0.1975247	-0.569155	0.569251	0.948983
ppip5k2	812.2827953	0.001271974	0.094099	0.0135174	0.989215	0.997589
AL954696.1	180.8519555	-0.04371395	0.1655725	-0.264017	0.791767	0.977853
ftr07	0	NA	NA	NA	NA	NA
tctn1	139.4005002	-0.077574572	0.1724046	-0.449957	0.652742	0.960708
gpr137ba	82.30208205	0.428149148	0.2309478	1.8538781	0.063757	0.493627
gzm3	1.686094135	0.672481678	1.5803796	0.4255191	0.670458	NA
hmox2b	27.43171773	0.866602841	0.3858487	2.2459656	0.024706	0.302486
best4	4.4836638	-1.755957374	1.0247125	-1.71361	0.0866	NA
ppp1r37	1026.096151	0.007251293	0.0903799	0.0802312	0.936053	0.992702
cux1a	1322.934623	-0.032835921	0.0910801	-0.360517	0.71846	0.96909
fap	680.9367602	0.244554696	0.0970222	2.5206062	0.011715	0.190352
nucks1a	6539.21815	0.013961983	0.0711685	0.1961819	0.844468	0.985174
myo15ab	23.42441521	-0.042215117	0.4018192	-0.10506	0.916328	0.991314
klhl23	29.8391902	0.122727489	0.3801397	0.3228484	0.74681	0.97419
znf990	25.21461121	0.036786008	0.3788522	0.0970986	0.922648	0.99233
mocs1	19.42772906	-0.005052463	0.4570781	-0.011054	0.99118	0.997679
zgc:195081	21.55659476	0.05910156	0.4308983	0.137159	0.890905	0.989291
snpha	0.666819193	1.487924459	2.4081466	0.6178712	0.53666	NA
si:ch211-284d12.3	1.957363094	-1.608186298	1.6308555	-0.9861	0.324084	NA
swi5	104.8813698	-0.297918621	0.1943706	-1.532735	0.125341	0.654648
adamts14	38.56174719	0.133133769	0.3330368	0.399757	0.689336	0.966763
timeless	674.2274361	-0.048865301	0.0950905	-0.513882	0.607334	0.956718
dap3	645.0023346	-0.04213356	0.0985948	-0.427341	0.669131	0.965037
si:ch211-150o23.3	83.24766766	0.167130883	0.2448668	0.682538	0.494899	0.931926
si:dkey-76b14.2	9.164935971	-0.532949311	0.6398173	-0.832971	0.404861	NA

si:ch73-60h1.1	237.7802242	0.057318786	0.1410858	0.406269	0.684545	0.966043
wnt8a	0	NA	NA	NA	NA	NA
si:dkey-266m15.6	246.7048966	0.264511712	0.1443801	1.8320508	0.066944	0.506105
socs4	3.711597735	0.862629428	1.0885705	0.7924424	0.428103	NA
acaca	1302.25401	0.147400451	0.079277	1.8593093	0.062983	0.489719
kazald2	6.898132546	0.688778634	0.7584005	0.9081991	0.363773	NA
si:ch73-256j6.4	1.336676355	-1.486211065	1.8623398	-0.798034	0.424851	NA
ikbip	393.163521	0.150575304	0.1244905	1.2095329	0.226458	0.793959
si:ch211-80h18.1	2144.863968	0.354984763	0.0754412	4.7054472	2.53E-06	0.000223
ddr1	870.245887	0.192687825	0.0898436	2.1447023	0.031977	0.347421
noctb	132.2411722	-0.110663592	0.1859909	-0.594995	0.551847	0.947215
lingo4b	127.619088	-0.039997067	0.1799992	-0.222207	0.824153	0.982196
adgrb1b	877.8437687	0.16900765	0.1071681	1.5770328	0.114788	0.635447
poc1a	27.88300732	-0.078033813	0.3722569	-0.209624	0.833962	0.983583
lrrcc1	141.9660363	-0.362209268	0.1746974	-2.073352	0.03814	0.382634
si:ch211-158d24.2	78.53442195	-0.058410828	0.237156	-0.246297	0.805452	0.979883
cers3a	135.3614954	-0.533515237	0.1859499	-2.869135	0.004116	0.08895
inpp5ja	13.29024861	0.103442851	0.5548327	0.1864397	0.8521	0.985174
cars2	115.3332544	-0.1237961	0.1930714	-0.641193	0.521397	0.939056
si:ch211-264f5.2	21.83071871	-0.178619834	0.456122	-0.391605	0.69535	0.96736
zgc:194839	35.90595888	0.101293337	0.3378041	0.2998582	0.764285	0.975374
zgc:171242	45.63196594	-0.294881506	0.3190857	-0.924145	0.355411	0.882347
grhl3	111.1368748	0.020664613	0.2103251	0.0982508	0.921733	0.992138
si:ch211-57n23.4	1.203102906	-1.262120851	1.8388013	-0.686382	0.492472	NA
si:dkey-22i16.10	0	NA	NA	NA	NA	NA
slc9a5	184.0029427	-0.149142662	0.1579155	-0.944446	0.344942	0.878434
lrig2	757.8779642	-0.012530953	0.0894238	-0.14013	0.888557	0.989291
lonrf1l	1115.698198	0.194631356	0.1103005	1.7645561	0.077638	0.542865
mpeg1.3	0.332524763	0.005883742	3.292841	0.0017868	0.998574	NA
wdr6	272.256758	-0.025098115	0.1339053	-0.187432	0.851322	0.985174
stox2b	1103.800897	0.035400606	0.0848606	0.417162	0.67656	0.965257
si:dkey-21c19.3	541.3866031	-0.0649024	0.1064204	-0.609868	0.541949	0.944974
setd5	192.3051305	0.011925253	0.1490095	0.0800301	0.936213	0.992702
fam222bb	396.7508586	0.161275228	0.1208697	1.33429	0.182109	0.744502
myo16	205.0137101	0.135030335	0.1543697	0.8747205	0.381726	0.895192
mon1a	167.3632992	-0.017861518	0.1681574	-0.106219	0.915409	0.991171
nomo	2654.166042	0.037923061	0.0730338	0.5192536	0.603584	0.956045
vwa8	282.9280896	0.084996593	0.1328189	0.6399433	0.522209	0.939056
si:ch1073-184j22.2	92.50277913	0.401450065	0.2221217	1.807343	0.070709	0.520162
micu3a	309.4684153	0.112080778	0.122806	0.9126655	0.361419	0.885719
ldlrad3	220.2512742	0.161931679	0.1553785	1.0421755	0.29733	0.853945
ptger1c	1.637563505	-0.572814497	1.5077409	-0.379916	0.704008	NA
myo1hb	21.43395459	0.02766099	0.4183616	0.0661174	0.947284	0.99381
tbc1d10b	1155.177746	0.096383651	0.0842766	1.143659	0.252765	0.822335
ptger1a	29.50675629	0.298384954	0.3471411	0.8595495	0.390037	0.897023
zgc:194224	50.2160356	-0.057699433	0.2829538	-0.203918	0.838417	0.984797
CT573423.1	0.173729368	-0.955901296	4.0804729	-0.234262	0.814781	NA
lrriq3	0.158915748	0.967652056	4.0804729	0.2371421	0.812547	NA
vhll	19.54925111	0.455758821	0.4363198	1.0445523	0.29623	0.853273
CABZ01085419.1	157.8000351	-0.103277289	0.1679466	-0.614941	0.538594	0.943336
inpp5kb	171.3982559	0.094484549	0.169097	0.5587594	0.576326	0.950611
pnp5a	1808.460831	0.418633079	0.0921083	4.545008	5.49E-06	0.000429
si:dkey-156m2.3	17.51553568	0.464599941	0.4982927	0.9323837	0.351138	0.880473
scpp5	377.4399013	-0.147737086	0.1335896	-1.105903	0.268769	0.834131

arhgef9b	771.8626796	0.126322403	0.0903526	1.3981055	0.162081	0.716681
fgf9	0.474898508	0.855240867	2.7202602	0.3143967	0.75322	NA
BX322577.1	7.09719002	-0.05948655	0.7084583	-0.083966	0.933083	NA
zgc:171750	0	NA	NA	NA	NA	NA
fam117aa	285.851994	-0.072523405	0.1276312	-0.568226	0.569881	0.948983
vtcn1	18.23242642	0.756968379	0.4444486	1.703163	0.088538	0.569447
theg	6.439989007	0.216927896	0.7308752	0.2968057	0.766615	NA
si:ch73-62l21.1	13.82226058	0.254248778	0.5103331	0.4982016	0.618342	0.957354
samsn1b	216.1394613	0.155822922	0.150939	1.032357	0.301905	0.856944
il21r.2	0.31759079	1.807926197	3.579045	0.5051421	0.613459	NA
kcna4	26.91569521	-0.024441577	0.3878879	-0.063012	0.949757	0.99381
zfyve1	145.960564	-0.144128161	0.1753435	-0.821976	0.41109	0.903291
ralbp1	81.25015988	0.02058163	0.2396138	0.085895	0.93155	0.992702
tpx2	1349.406185	-0.097726122	0.0784767	-1.245288	0.213026	0.781631
FNDC10	154.2865672	0.01239918	0.1754714	0.0706621	0.943667	0.993364
supt20	167.8756921	-0.156157998	0.1735978	-0.899539	0.368366	0.887976
tmem176	257.4347813	0.166455087	0.1501463	1.1086191	0.267595	0.833349
cdk5r2b	431.8380983	0.428048668	0.1093316	3.9151399	9.04E-05	0.004478
hspb9	0.474778155	0.852397662	2.898154	0.2941175	0.768668	NA
dock7	1705.181376	-0.041410145	0.0782683	-0.529079	0.596751	0.954913
myrf	44.74478563	0.110399211	0.305732	0.3610979	0.718026	0.96909
zmp:0000000524	283.4959879	-0.198662308	0.1345765	-1.476204	0.139889	0.678981
rufy1	190.8941049	-0.075448529	0.1722758	-0.437952	0.661421	0.964234
arl8a	2272.901533	0.016464802	0.0796263	0.2067761	0.836185	0.984209
frs3	19.81017626	0.091687948	0.4465053	0.2053457	0.837302	0.984645
gigyf1b	1040.450056	-0.056978479	0.0858861	-0.663419	0.507062	0.935734
phlpp1	578.5958372	-0.177520444	0.1109699	-1.599717	0.109661	0.623766
synpo2lb	248.3268165	0.157267553	0.1442465	1.0902696	0.275594	0.839541
prdm13	413.7901931	0.004907927	0.1158163	0.0423768	0.966198	0.996315
unm_hu7910	216.8914747	0.061471634	0.1464927	0.4196225	0.674761	0.965257
sema7a	45.14069986	0.310886559	0.2962607	1.0493682	0.294009	0.849999
itga8	284.5103669	0.241598761	0.1423922	1.696714	0.089751	0.573763
clip1a	360.5565635	0.015263949	0.1190684	0.1281948	0.897995	0.990121
nos1apa	579.1143254	0.003690603	0.0985656	0.0374431	0.970132	0.996315
znf1068	4.196630456	-0.351037918	0.9205702	-0.381327	0.702961	NA
ackr4a	9.226063391	0.615291819	0.6294717	0.9774733	0.328335	NA
si:dkey-85k7.12	22.67478702	0.694448475	0.4251872	1.6332771	0.102411	0.608235
cnnm2b	75.08407378	0.044151166	0.2623508	0.1682905	0.866355	0.986673
myo1f	34.35167554	-0.061062767	0.3541156	-0.172437	0.863094	0.985841
doc2a	58.71066753	0.013788985	0.2689693	0.051266	0.959114	0.995095
si:dkey-145c18.3	1.487060251	0.360976966	1.5663761	0.2304536	0.817739	NA
FRMD1	198.5554436	0.078284268	0.1602997	0.488362	0.625293	0.957354
oxgr1a.2	0.182570949	0.967652056	4.0804729	0.2371421	0.812547	NA
pnpla8	623.6208996	0.136024376	0.1003127	1.3560041	0.175098	0.735545
si:ch211-137a8.4	5628.373593	-0.052206156	0.0640832	-0.814662	0.415266	0.906237
b4galnt3a	14.13793136	0.256616758	0.6030499	0.4255316	0.670449	0.965257
tpp2	849.5573387	0.006912157	0.0908708	0.0760658	0.939367	0.992857
rubcn	24.94586883	0.542084396	0.4186958	1.2946974	0.195425	0.760412
gra	37.74503	0.077227489	0.3203365	0.2410824	0.809491	0.980642
plppr4b	66.15220305	0.062393438	0.2467719	0.2528385	0.800393	0.979377
si:ch211-212c13.8	198.7842686	0.133018842	0.2033533	0.6541267	0.51303	0.936253
dna2	138.7596537	0.132171341	0.1771373	0.7461518	0.455576	0.921215
cacna2d3	517.7484219	0.092684579	0.1079118	0.8588921	0.3904	0.897304
rmdn2	43.83496922	0.049304495	0.2945406	0.1673946	0.86706	0.986978

tmem169b	20.41122977	-0.445932727	0.4477378	-0.995968	0.319266	0.867746
igl3v3	0	NA	NA	NA	NA	NA
yeats2	123.6834325	-0.05010726	0.180621	-0.277417	0.78146	0.97627
abhd15a	121.4186305	-0.038960392	0.186935	-0.208417	0.834904	0.984089
si:ch73-21k16.5	101.2003241	-0.5876567	0.2011621	-2.921309	0.003486	0.079224
nbr1b	1233.396926	-0.048917512	0.0854378	-0.572551	0.566949	0.948739
tmem70	116.0605899	0.036082952	0.1950405	0.1850024	0.853227	0.985174
RASA2	16.71402522	0.019435932	0.4698374	0.0413674	0.967003	0.996315
lrch1	323.8475664	-0.098763565	0.1259047	-0.784431	0.432787	0.91326
si:ch211-161c3.6	115.4342111	-0.094263118	0.2029259	-0.46452	0.642275	0.959994
mgaa	603.6608983	-0.102284452	0.1036574	-0.986755	0.323763	0.870928
tmem258	275.1041336	-0.171594521	0.1343081	-1.277619	0.201384	0.767338
tmem184c	396.3350781	0.016445164	0.1179775	0.1393924	0.88914	0.989291
lrrc18a	3.440647801	-0.084995799	1.1229163	-0.075692	0.939664	NA
dennd3a	223.9771253	0.003100023	0.155148	0.0199811	0.984058	0.996944
slc26a10	13.06609509	-0.110821591	0.5159442	-0.214794	0.829928	0.982821
msantd1	81.61241272	-0.112363005	0.2203256	-0.509986	0.610061	0.95688
rnf111	1071.5537	-0.061543077	0.0864187	-0.71215	0.476372	0.926505
gas2l1	418.8533749	-0.028440984	0.1226874	-0.231817	0.81668	0.981594
ap5b1	153.5241397	-0.06455418	0.1623785	-0.397554	0.690959	0.967157
paxx	49.81328184	0.199601916	0.2756372	0.7241473	0.468975	0.924394
zgc:174320	0.173729368	-0.955901296	4.0804729	-0.234262	0.814781	NA
si:dkey-34m19.3	143.77145	-0.08486598	0.1836184	-0.462187	0.643947	0.96009
grk5l	232.285424	0.184447893	0.1371999	1.3443736	0.178828	0.739486
rnf165b	128.5733467	-0.052092903	0.1858561	-0.280286	0.779258	0.976146
si:ch211-225k7.5	0	NA	NA	NA	NA	NA
ccdc18	106.4237695	0.122974696	0.2084166	0.5900428	0.555162	0.947449
si:ch211-66e2.3	22.815349	0.460443339	0.3989659	1.1540919	0.248463	0.817939
bub1ba	149.2144781	-0.150732559	0.1683745	-0.895222	0.370668	0.890626
npb	193.7884688	0.186137396	0.1506041	1.2359386	0.216481	0.783724
ccdc149a	14.73000416	-0.331790468	0.5193855	-0.638814	0.522944	0.939336
si:dkey-73p2.3	28.43645303	-0.138547155	0.4849639	-0.285685	0.775119	0.975687
mrpl58	49.1335896	-0.294158355	0.2792887	-1.053241	0.29223	0.848075
si:dkey-28o19.1	9.688435222	0.04922996	0.6758376	0.0728429	0.941931	NA
si:ch211-195b13.6	3.686878275	-0.806547985	0.9935615	-0.811775	0.416921	NA
tns1a	1019.213201	0.059122911	0.0833643	0.7092115	0.478193	0.926857
BX571839.1	0.157187365	-0.955901296	4.0804729	-0.234262	0.814781	NA
si:dkey-238o13.4	139.5018073	-0.019673051	0.1741513	-0.112965	0.910058	0.990702
plekhm2	185.2632891	-0.027820018	0.1602394	-0.173615	0.862168	0.985841
arhgef19	73.05784038	0.158090848	0.2494791	0.6336837	0.526287	0.940506
tusc3	1037.223124	0.078064803	0.0863186	0.9043793	0.365794	0.887084
slc22a23	407.0261569	0.089840554	0.1137704	0.7896657	0.429723	0.911615
g0s2	29.64869933	-0.430375924	0.3521696	-1.22207	0.221681	0.78829
lats2	125.0490743	0.212262468	0.1915236	1.1082836	0.267739	0.833349
cxxc5a	848.1519398	-0.044706944	0.0967997	-0.46185	0.644189	0.960129
sdk1a	404.2224254	0.030473803	0.1268278	0.2402769	0.810116	0.980789
tmem74b	1.923637035	0.46347727	1.4525078	0.3190876	0.74966	NA
adm2b	7.38397438	0.560865271	0.7499772	0.7478431	0.454555	NA
si:ch73-244f7.3	8.103132261	-0.304576328	0.6761921	-0.450429	0.652401	NA
slc22a31	290.2015774	-0.079586816	0.1341496	-0.593269	0.553001	0.947265
atp10d	288.8660815	-0.333187513	0.1406204	-2.369411	0.017816	0.247181
iqgap1	1164.730001	0.01868367	0.0813477	0.2296766	0.818343	0.981594
wdfy3	187.3418846	0.102655996	0.1565611	0.6556929	0.512022	0.936253
map1l	560.5169135	0.085018721	0.1142483	0.7441574	0.456781	0.921215

hemk1	161.5851578	-0.314089685	0.1602409	-1.96011	0.049983	0.437835
pcdh7a	1376.914601	0.089001234	0.0861897	1.0326202	0.301782	0.856944
anks1ab	51.72783836	0.037434846	0.2826786	0.132429	0.894645	0.989716
rims1b	63.18872878	0.318035725	0.2595276	1.2254409	0.220409	0.787278
brd4	1073.636232	0.044335643	0.1111444	0.3989013	0.689966	0.966857
fbxo41	26.39484143	0.069930873	0.3764426	0.1857677	0.852627	0.985174
zgc:163098	650.8540122	-0.181489176	0.0950167	-1.910077	0.056123	0.464732
si:ch73-24k9.2	14.55916441	-0.7301354	0.5190229	-1.40675	0.159502	0.710997
mmp17a	32.22755676	-0.25612243	0.3460741	-0.74008	0.459252	0.921978
chrdl2	14.86229685	0.138541911	0.4890881	0.2832657	0.776973	0.975826
zgc:195245	49.57066313	0.048813222	0.283473	0.1721971	0.863283	0.985841
comtd1	162.9633692	0.207624947	0.177297	1.1710576	0.241576	0.810471
CR790388.1	3.247854248	-0.114233917	1.0822121	-0.105556	0.915935	NA
tmco4	59.39259131	-0.268652161	0.2653271	-1.012532	0.311284	0.863542
BX323994.1	0.489712128	-0.887840729	2.693685	-0.329601	0.741702	NA
abhd16a	923.2369361	0.028604336	0.0852933	0.3353643	0.73735	0.972535
ttll4	1527.400384	0.104872602	0.0761496	1.377191	0.168453	0.725919
CR936408.1	0	NA	NA	NA	NA	NA
ano9a	0	NA	NA	NA	NA	NA
enox2	385.3455745	-0.013995126	0.1148578	-0.121847	0.90302	0.990121
sdccag8	75.35405695	-0.067328531	0.228312	-0.294897	0.768073	0.975687
epn3a	448.0625286	-0.031020073	0.1091887	-0.284096	0.776337	0.975826
rxrba	377.7497894	0.056165732	0.130843	0.4292606	0.667734	0.964736
tpcn3	108.8003841	-0.056489349	0.1904902	-0.296547	0.766812	0.975687
duox2	4.670875973	0.228338941	1.0304061	0.2216009	0.824625	NA
BX901881.1	0.498674062	0.926637565	2.6784892	0.3459553	0.729376	NA
esyt2b	204.5003299	0.211516069	0.1463297	1.4454764	0.148324	0.696298
rbm15b	508.7384443	0.017543169	0.1028103	0.1706362	0.86451	0.98601
il7r	7.529411668	-0.027144877	0.7035839	-0.038581	0.969225	NA
uckl1b	472.8510136	-0.04316957	0.1125819	-0.38345	0.701386	0.968134
COA4	77.16615071	-0.207581516	0.2340209	-0.887021	0.375067	0.894316
igl1c3	0.864444467	0.614560748	2.0893881	0.2941343	0.768655	NA
arhgef40	47.46623495	0.561731305	0.291128	1.9294993	0.053669	0.45394
faxcb	104.0443359	0.009591682	0.1932515	0.0496332	0.960415	0.995421
fam118b	90.75998319	-0.099365369	0.2067042	-0.480713	0.630721	0.957354
mplkip	252.2400619	0.018804123	0.1401479	0.1341735	0.893265	0.989291
alpk3a	116.5529499	0.486602339	0.2023811	2.4043861	0.0162	0.234928
si:ch211-195e19.1	469.9652814	-0.101142233	0.1080369	-0.936182	0.349179	0.880195
wnk1a	2074.792806	-0.069824884	0.0824436	-0.846941	0.397028	0.899672
muc2.2	0.325573202	0.005883621	3.3172783	0.0017736	0.998585	NA
tex11	16.85723571	0.509510729	0.4704686	1.0829857	0.278815	0.842767
dclk3	4.550041397	0.222136616	0.9937966	0.2235232	0.823128	NA
tp53bp1	1520.347729	-0.067700162	0.0813919	-0.831781	0.405533	0.901305
si:dkey-7f3.9	572.7011695	0.081440792	0.1016389	0.8012762	0.422972	0.90714
abca1b	1443.115868	0.469240394	0.0904312	5.188924	2.12E-07	2.67E-05
si:dkey-7i4.5	16.01975355	0.658434083	0.5072655	1.2980068	0.194285	0.758049
col17a1b	544.1593091	-0.02876409	0.1054394	-0.272802	0.785005	0.97627
cbfa2t3	76.89485744	-0.191826947	0.2305176	-0.832158	0.40532	0.901305
dpy19l3	920.1533108	0.110216963	0.0947936	1.1627047	0.244949	0.814877
brca2	150.7298529	-0.131323601	0.1721644	-0.76278	0.445594	0.917154
tlnrd1	83.70322442	0.017129119	0.2305542	0.0742954	0.940775	0.992857
gcfc2	179.2611465	0.076551642	0.170322	0.4494526	0.653105	0.960792
rps6kl1	91.36544541	-0.08245855	0.2175946	-0.378955	0.704721	0.968134
chsy1	572.8741141	0.030625961	0.1017842	0.300891	0.763498	0.975374

dhx32b	402.8057628	-0.089523316	0.1223421	-0.731746	0.464324	0.923325
ftr39p	13.24534282	-0.084169578	0.5416398	-0.155398	0.876508	0.987881
si:ch211-22d5.2	63.6967672	0.583604966	0.2586995	2.2559188	0.024076	0.299543
zpax4	13.08568823	0.566285191	0.5523002	1.0253214	0.305212	0.85982
zgc:174573	16.46694152	-0.570654937	0.4882592	-1.168754	0.242503	0.811298
znf1lk	1.312859516	-1.608377215	1.7182901	-0.936034	0.349256	NA
si:dkeyp-75b4.10	0.837332501	-1.876981437	2.1230279	-0.884106	0.376639	NA
opn8b	34.02452353	0.540901981	0.3307048	1.6356038	0.101923	0.60687
rabl6a	84.4449194	0.333825539	0.2187372	1.5261486	0.126973	0.656588
cercam	274.0918596	0.095766706	0.1319433	0.7258169	0.467951	0.924328
adgre8	0.347458735	-1.908042286	3.4571486	-0.551912	0.581009	NA
si:dkey-85a20.4	129.8716175	0.030923504	0.1803493	0.1714645	0.863859	0.985952
si:ch211-194c3.5	619.7777859	-0.069397712	0.0964993	-0.719152	0.472047	0.924838
retreg1	11.11186573	-0.039459008	0.5817824	-0.067824	0.945925	0.993759
samd1b	129.4076607	0.019793961	0.1904996	0.1039055	0.917244	0.991572
CABZ01079080.1	4.152147317	-0.605711483	0.9528364	-0.635693	0.524977	NA
zgc:172215	23.84902863	0.070245666	0.401158	0.1751072	0.860995	0.985773
mms19	241.2889203	0.090440017	0.139477	0.6484223	0.516712	0.938543
adam12	13.93306573	0.565419495	0.5230774	1.0809481	0.27972	0.843169
jam2b	126.8188523	0.153438691	0.2116636	0.7249176	0.468503	0.924328
frmpd1a	21.08659587	0.531063372	0.424314	1.251581	0.210723	0.778412
si:ch211-71m22.3	4.558195236	-0.51527898	0.9159303	-0.562574	0.573725	NA
hgfb	15.53033527	0.074519917	0.4921993	0.1514019	0.879659	0.987881
si:ch211-5k11.8	0.157187365	-0.955901296	4.0804729	-0.234262	0.814781	NA
sult1st7	0.341366344	1.891903201	3.2128156	0.5888614	0.555954	NA
etfbkmt	40.96168212	0.444656461	0.3022941	1.4709399	0.141307	0.681968
fbxw8	176.5981528	-0.106883979	0.1766304	-0.605128	0.545094	0.945463
rin1a	37.55129008	0.309565132	0.3405396	0.909043	0.363327	0.885944
tanc2a	1102.6012	-0.0585553	0.1011474	-0.57891	0.56265	0.948728
PRRG3	11.38454414	0.006600361	0.5708231	0.0115629	0.990774	0.997679
mfhas1	494.1139688	-0.040740057	0.1077871	-0.377968	0.705455	0.968412
mhc2dab	0.974750517	-1.023458015	2.0966828	-0.488132	0.625456	NA
clec11a	26.43369542	0.697545319	0.3988045	1.7490908	0.080275	0.549641
cluap1	138.9163979	-0.15454194	0.1947948	-0.793357	0.42757	0.91075
actc1c	1036.193193	0.09475663	0.0841311	1.1262978	0.26004	0.828288
si:ch211-229d2.5	502.281076	0.062619573	0.1049032	0.596927	0.550556	0.946549
tmx2a	60.22652258	0.633992451	0.2654305	2.388544	0.016915	0.240289
ikbkb	58.01829906	0.096535561	0.2847044	0.3390729	0.734555	0.972021
MTERF4	102.9163216	-0.106395707	0.2004156	-0.530875	0.595505	0.954434
sowahab	69.11880476	0.01796374	0.2385929	0.0752903	0.939984	0.992857
znf1li	0.341366344	1.891903201	3.2128156	0.5888614	0.555954	NA
becn1	338.8180387	-0.015261215	0.1253949	-0.121705	0.903132	0.990121
opn4xa	10.93833804	-0.002671833	0.5637858	-0.004739	0.996219	NA
si:dkeyp-118b1.2	85.43858879	0.080635473	0.2118334	0.3806551	0.703459	0.968134
si:dkey-10c21.1	6.858347577	0.21564233	0.7604692	0.2835648	0.776744	NA
si:ch73-278m9.1	80.11861197	-0.010567812	0.2261355	-0.046732	0.962727	0.995927
bcl2l11	5.485346572	0.619488097	0.8355098	0.7414493	0.458421	NA
CABZ01077217.1	119.8103003	-0.039045817	0.1851713	-0.210863	0.832994	0.983557
nckap5l	116.4888551	0.032798668	0.1842391	0.1780223	0.858705	0.985773
si:ch211-276i12.9	932.7988628	-0.039572646	0.0989574	-0.399896	0.689233	0.966763
si:ch211-67f13.8	7.433398707	0.560412357	0.7123264	0.7867354	0.431437	NA
thpo	1.432557583	0.317312701	1.6575224	0.191438	0.848182	NA
srxn1	18.4275548	-0.611111424	0.4437706	-1.377089	0.168485	0.725919
bsnb	661.5718887	-0.028718841	0.0961566	-0.298667	0.765194	0.975374

best2	4.790127156	-0.110031518	0.8495571	-0.129516	0.896949	NA
galr1b	26.33574545	-0.489473716	0.3751379	-1.304783	0.191967	0.756256
ace	751.8880255	-0.339710338	0.1013354	-3.352337	0.000801	0.025849
si:ch211-230g15.5	521.8427647	-0.218013861	0.1103323	-1.975974	0.048158	0.432457
si:ch211-79k12.1	344.1858052	0.085114718	0.1243162	0.6846634	0.493556	0.931926
sod3b	31.49651038	-0.089052939	0.3366553	-0.264523	0.791377	0.977616
si:ch211-163m16.7	0	NA	NA	NA	NA	NA
tbx19	9.840960812	0.449932468	0.7029656	0.6400491	0.522141	NA
nxph2b	129.2682272	0.149245827	0.212533	0.7022241	0.482539	0.928998
zgc:172122	57.64569409	0.045716189	0.259819	0.175954	0.86033	0.985773
usp13	419.7831611	0.162981195	0.1116229	1.4601053	0.144261	0.68854
megf6a	75.94807293	0.233934075	0.2364116	0.9895203	0.322409	0.870226
tshz2	918.5744613	-0.135278405	0.0919485	-1.471241	0.141226	0.681968
parp2	315.8655408	-0.045039308	0.1266312	-0.355673	0.722085	0.97014
adam11	87.61956265	0.020742117	0.2160619	0.0960008	0.92352	0.992588
dpep2	13.30772109	-0.355470795	0.5220807	-0.680873	0.495952	0.932377
bbs9	189.5060895	0.076292429	0.1600739	0.4766074	0.633642	0.957703
mfn1b	779.4550389	0.078213097	0.0950572	0.8228004	0.410622	0.903084
wdr20a	178.4306491	0.0566715	0.1572575	0.3603739	0.718568	0.96909
si:ch211-162k9.6	0.983001374	0.918633527	2.0854433	0.440498	0.659576	NA
zgc:174315	58.39341498	-0.017193409	0.2753711	-0.062437	0.950215	0.99381
plekhs1	41.78132788	-0.012483307	0.3451181	-0.036171	0.971146	0.996315
znf414	3.579873268	0.264645662	1.0101716	0.2619809	0.793336	NA
slc7a14b	4.200088464	0.160877098	1.0087392	0.1594833	0.873288	NA
si:ch211-160f23.5	165.6308284	0.003531667	0.1626071	0.021719	0.982672	0.996944
si:ch211-285c6.2	0.31598276	0.005883445	3.3523626	0.001755	0.9986	NA
si:dkey-253d23.11	33.68878383	0.163087679	0.3281366	0.4970115	0.619181	0.957354
trim59	70.12837957	-0.334608131	0.2397435	-1.395692	0.162807	0.717803
wdr90	131.742195	-0.004164053	0.1932707	-0.021545	0.982811	0.996944
BX901889.3	0	NA	NA	NA	NA	NA
pigw	97.96926125	-0.088573378	0.2061388	-0.429678	0.66743	0.964736
si:dkey-73p2.2	7.972719547	-0.691643629	0.7608207	-0.909076	0.36331	NA
mn1b	480.8087885	-0.00681123	0.1139741	-0.059761	0.952346	0.993904
ogdhl	112.7847175	0.031211379	0.1907896	0.1635906	0.870053	0.987399
BX323590.1	8.275786206	0.60353402	0.7194153	0.838923	0.401513	NA
nlgn2b	247.4009169	-0.018299747	0.134242	-0.136319	0.891569	0.989291
fam117ab	19.28695666	-0.183870463	0.4491438	-0.40938	0.682261	0.965476
plch1	259.4488917	-0.153279078	0.1436879	-1.06675	0.286085	0.845942
zgc:174935	82.89018489	-0.151526905	0.2253206	-0.672495	0.501269	0.934613
gemin5	305.3527067	-0.134415712	0.1350861	-0.995037	0.319718	0.86809
si:dkeyp-2e4.2	8.916533085	-0.019470973	0.6582864	-0.029578	0.976403	NA
baiap3	50.45088174	0.137583093	0.299416	0.4595048	0.645872	0.96029
foxe1	57.75554182	-0.007652846	0.2518096	-0.030391	0.975755	0.996762
im:7154036	19.99637549	0.254077089	0.4257174	0.596821	0.550627	0.946549
CR589947.1	12.06558348	-0.584057261	0.555247	-1.051887	0.292851	0.848607
dcxr	150.7866223	-0.170917018	0.1740982	-0.981728	0.326234	0.872104
prss59.1	514.7231201	-0.663638309	0.139816	-4.746513	2.07E-06	0.000186
ube2ql1	79.85983679	0.357553532	0.2341653	1.5269281	0.126779	0.656588
zgc:174877	178.4608238	0.143663579	0.1599152	0.8983733	0.368987	0.888432
dhrsx	457.4370597	0.094521965	0.1139226	0.8297031	0.406707	0.901305
si:dkeyp-75h12.2	175.2655681	0.020603774	0.163044	0.1263694	0.89944	0.990121
si:ch211-247j9.1	749.7272879	0.127201401	0.093586	1.3591927	0.174086	0.734137
bcr	1174.148756	0.03015692	0.084921	0.3551172	0.722502	0.970375
si:ch211-162k9.5	7.578549013	-1.824319501	0.7531535	-2.422241	0.015425	NA

rapgef3	46.07281686	0.100903783	0.2879446	0.3504278	0.726018	0.970402
gdpd4b	67.78228689	0.482010393	0.2406655	2.0028227	0.045196	0.417766
or115-15	2.93081305	-0.652912877	1.1963471	-0.545755	0.585234	NA
cacna1bb	53.88943539	0.430908572	0.276698	1.5573244	0.119393	0.643644
gcga	162.0072056	-0.129692749	0.2106451	-0.615693	0.538097	0.943336
and2	4164.100218	0.61062905	0.0808513	7.5524967	4.27E-14	1.84E-11
hbae3	12686.89491	0.154512396	0.2841662	0.5437396	0.586621	0.953319
rlim	989.9821207	0.051549995	0.0842728	0.611704	0.540734	0.944633
si:dkey-205h13.1	90.45670435	-0.158086435	0.2156899	-0.732934	0.463599	0.922875
tor3a	268.2847175	-0.11629153	0.1328126	-0.875606	0.381244	0.895192
st3gal1l	15.41622798	-0.351929807	0.4897269	-0.718625	0.472372	0.924838
kmt2ca	712.7362434	0.038231389	0.0948821	0.4029356	0.686996	0.966376
pelp1	1448.191877	-0.222879274	0.0878153	-2.538047	0.011147	0.183384
lpxn	12.13124659	-0.185549198	0.5692691	-0.325943	0.744468	0.974094
dlc1	274.6819103	-0.184386207	0.1324799	-1.391805	0.163981	0.71945
rgmb	1281.246668	-0.020824739	0.0841813	-0.24738	0.804614	0.979705
ano9b	205.1929191	-0.326576944	0.1477066	-2.210985	0.027037	0.31691
CABZ01046432.1	0	NA	NA	NA	NA	NA
cabin1	62.02689682	-0.155959855	0.2494458	-0.625226	0.531823	0.942359
hmcn2	972.272356	0.162719696	0.093668	1.737196	0.082353	0.553931
pgbd4	23.63058646	0.083862292	0.4081053	0.2054918	0.837188	0.984645
cop1	399.9221722	0.158249204	0.1140024	1.3881219	0.1651	0.721223
AL928650.1	3.446489196	1.311621177	1.0935374	1.1994296	0.230361	NA
cd40lg	1.529345901	-1.744664827	1.7410432	-1.00208	0.316305	NA
strc1	30.51487105	0.494471904	0.3956045	1.2499147	0.211331	0.778917
slc47a4	57.3034441	0.153470373	0.2787535	0.5505594	0.581936	0.951952
si:ch211-217k17.10	1.959600626	0.424286044	1.4640552	0.289802	0.771968	NA
zgc:194659	41.88303969	-1.021024024	0.858898	-1.18876	0.234534	0.803509
GRIN2B (1 of many)	21.45306297	0.44167984	0.4450717	0.9923792	0.321013	0.868715
znf654	522.3897686	-0.24002119	0.1068261	-2.246841	0.02465	0.302262
sap130b	388.0814376	0.072583509	0.1238724	0.5859539	0.557907	0.948478
si:ch211-165d12.4	59.96111203	-0.391272549	0.2842002	-1.37675	0.16859	0.725919
flrt2	292.7964756	0.18241567	0.1272527	1.4334913	0.151717	0.700137
abcg2b	62.73124551	-0.860228255	0.2628692	-3.272457	0.001066	0.032585
CR377211.1	1.006415869	0.95573537	2.0728081	0.4610824	0.644739	NA
ppp1r9ba	1207.792191	-0.063523183	0.0886078	-0.716903	0.473434	0.924993
si:ch211-212g7.6	481.2181908	0.195377905	0.1089432	1.7933932	0.07291	0.526902
lrrc3ca	19.86238215	-0.002042001	0.4357557	-0.004686	0.996261	0.998888
si:ch211-264f5.6	229.267214	0.155861487	0.1541288	1.0112418	0.311901	0.864401
fosl1b	4.462268347	0.554654817	0.9465966	0.5859463	0.557912	NA
tjp1b	1365.14224	-0.049106963	0.0977412	-0.502418	0.615373	0.95688
zgc:174153	0.515095711	0.926015374	2.6527421	0.3490785	0.72703	NA
arhgef38	74.84856182	-0.257667983	0.2371239	-1.086639	0.277196	0.840688
phldb1b	101.9449185	0.07549895	0.1971719	0.3829093	0.701787	0.968134
hdx	381.5196819	-0.116838278	0.1178498	-0.991417	0.321482	0.869201
si:ch211-235m3.5	33.04490782	-0.406473387	0.3489213	-1.164943	0.244042	0.813415
si:ch211-102c2.4	25.9005165	0.029510585	0.3805997	0.0775371	0.938196	0.99279
agrn	2850.524161	-0.003612751	0.0788926	-0.045793	0.963475	0.995927
mrpl42	222.0482765	-0.127883955	0.156604	-0.816607	0.414153	0.906114
tmprss15	5.310538659	-0.729024935	0.8154237	-0.894044	0.371298	NA
lrfn5b	418.6584803	0.014428745	0.1177459	0.1225414	0.90247	0.990121
cerkl	64.29221886	-0.05771875	0.2463773	-0.23427	0.814776	0.981081
F2RL3	34.06545559	0.034889637	0.3422124	0.1019532	0.918794	0.991623
si:dkey-197c15.6	170.698188	-0.210902818	0.1653046	-1.275843	0.202011	0.76789

tapbp.1	18.53362229	0.077021734	0.4759122	0.1618402	0.871432	0.987754
si:dkey-204l11.1	3.241575888	0.897200768	1.1249781	0.7975273	0.425145	NA
ftr02	35.06095982	-0.821918735	0.3634533	-2.261415	0.023734	0.29703
sez6b	60.46664452	-0.198897517	0.2485548	-0.800216	0.423586	0.907829
dnajc30b	52.35789889	0.13621199	0.2693289	0.505746	0.613035	0.95688
zgc:174895	58.05822363	0.580217713	0.2597654	2.2336222	0.025508	0.309193
dpp4	385.5287915	-0.916588254	0.1320252	-6.942524	3.85E-12	1.25E-09
npbwr2a	15.52844175	-0.006749838	0.4800143	-0.014062	0.988781	0.997589
si:dkey-12f6.5	0.157187365	-0.955901296	4.0804729	-0.234262	0.814781	NA
tmem131l	944.4057499	-0.192652666	0.0880213	-2.188706	0.028618	0.328246
pkp3b	359.5911701	-0.03921748	0.1220203	-0.321401	0.747906	0.97419
coro2ba	299.5966791	0.067976943	0.1330887	0.5107642	0.609516	0.956718
BX649448.1	5.633579786	-0.194298342	0.8591359	-0.226156	0.82108	NA
vipb	61.75765175	-0.661335621	0.2540393	-2.603281	0.009234	0.161152
si:dkey-238f9.1	210.6354894	0.110413057	0.1492904	0.7395858	0.459551	0.921978
rbm46	2.493245458	-0.612411926	1.1997045	-0.510469	0.609723	NA
atp8b4	21.29302423	-0.158618802	0.4101168	-0.386765	0.69893	0.967998
nlgn4xa	663.0336449	0.179193785	0.1020243	1.7563842	0.079023	0.547489
si:ch211-149a19.3	108.4565101	0.18692456	0.1911048	0.978126	0.328012	0.872701
si:dkey-57h18.1	57.27284746	0.101712754	0.260536	0.3903981	0.696242	0.96736
pimr126	0	NA	NA	NA	NA	NA
si:ch211-131k2.3	45.72056513	0.54764901	0.3236106	1.6923085	0.090587	0.576398
man2b2	292.0878263	-0.062198206	0.1524859	-0.407895	0.683351	0.965476
si:ch73-15b2.5	75.21180319	-0.120089766	0.230491	-0.521017	0.602355	0.956045
rhobtb1	3.858683095	-0.106030642	0.9572831	-0.110762	0.911805	NA
trpc7b	11.42569328	-0.03773704	0.5522072	-0.068339	0.945516	0.993589
cdk17	210.86551	-0.170242082	0.1555527	-1.094433	0.273765	0.838381
si:dkey-88j15.4	0	NA	NA	NA	NA	NA
CABZ01071020.1	20.34105896	-0.430966649	0.460774	-0.93531	0.349628	0.880195
map3k21	73.78018237	-0.155759027	0.2302019	-0.676619	0.498648	0.934006
MSANTD2	84.32495677	0.093545543	0.22113	0.4230341	0.67227	0.965257
nckipsd	304.9369685	0.06845894	0.1345128	0.50894	0.610794	0.95688
mvb12bb	16.29714242	-0.968397734	0.4775561	-2.02782	0.042579	0.405759
gpr4	1.60255145	1.190672731	1.6167718	0.7364507	0.461456	NA
GID4	49.94094379	0.737575512	0.2770957	2.6618077	0.007772	0.141128
CABZ01072096.1	14.9931221	0.1663423	0.5046069	0.3296473	0.741666	0.973154
kcnt1	15.20359732	0.317309614	0.5166615	0.6141538	0.539114	0.943733
fam20a	36.51900264	0.131375895	0.3198517	0.41074	0.681263	0.965476
zgc:195282	4.112858351	-0.851055301	0.9724183	-0.875195	0.381468	NA
bicd1a	107.2782631	0.061869333	0.1971254	0.3138578	0.753629	0.975024
tcima	1505.107528	-0.088928251	0.0801492	-1.109534	0.2672	0.833191
stk32a	0	NA	NA	NA	NA	NA
kif3cb	573.6106234	0.004207531	0.1030151	0.0408438	0.96742	0.996315
si:ch211-149l1.2	49.72441389	-0.113578733	0.2767943	-0.410336	0.681559	0.965476
gal3st2	36.51266939	0.176677957	0.3302317	0.535012	0.592642	0.954434
mfn2	1248.866541	0.060822123	0.0877424	0.6931893	0.488191	0.93189
fam114a2	439.3631234	-0.127534788	0.1104516	-1.154667	0.248227	0.817664
ptx3b	8.402266544	-0.177328041	0.6458811	-0.274552	0.78366	NA
cisd1	797.0951576	-0.000715678	0.0903139	-0.007924	0.993677	0.998672
nitr1f	0	NA	NA	NA	NA	NA
eys	2.713536368	-0.335873978	1.2215424	-0.274959	0.783348	NA
si:ch211-184m13.4	0.65919784	2.852228985	2.3514193	1.2129819	0.225137	NA
znf11g	0.513487682	-0.887846295	2.6557956	-0.334305	0.738149	NA
slc39a5	90.28551505	-0.1658435	0.2310988	-0.71763	0.472985	0.924993

si:dkey-17m8.1	208.4421886	-0.020460316	0.1531684	-0.133581	0.893734	0.989291
zgc:194242	5.164783683	0.873084348	0.9001936	0.9698851	0.332104	NA
zgc:175135	0.158915748	0.967652056	4.0804729	0.2371421	0.812547	NA
ftr13	12.60352719	-0.244544628	0.6184785	-0.395397	0.69255	0.96736
stard13a	406.3451085	0.060124208	0.111082	0.5412596	0.588329	0.953319
dpys	220.602476	0.614055275	0.1413774	4.3433763	1.40E-05	0.00095
si:ch1073-464p5.5	439.5644904	0.429260542	0.1115248	3.8490127	0.000119	0.005637
zbtb47b	1.451106703	-1.806925218	1.770077	-1.020817	0.307341	NA
cdc42ep1a	45.61357436	-0.000588225	0.2920883	-0.002014	0.998393	0.999364
maml3	507.3030137	-0.083087666	0.1060433	-0.783526	0.433319	0.913654
cd83	32.52410824	-0.508681373	0.3469902	-1.465982	0.142653	0.684998
ccdc85cb	171.0911908	-0.064478714	0.1632835	-0.394888	0.692925	0.96736
ptprh	24.68395717	-1.150749823	0.401284	-2.867669	0.004135	0.089168
myh7	1511.665598	-0.090517665	0.0833324	-1.086224	0.27738	0.840857
rspo2	199.1054275	0.06641918	0.151346	0.4388566	0.660765	0.963994
mfsd4b	164.4510649	-0.143700316	0.1585691	-0.906232	0.364813	0.886714
plcd3b	164.5604742	0.258637403	0.1689765	1.5306113	0.125865	0.654803
aff3	207.6201444	0.038244294	0.1522786	0.2511469	0.801701	0.979492
rbpms2b	945.8266625	0.08573153	0.0896588	0.9561977	0.338972	0.87745
nyap2a	61.64606288	-0.036180113	0.2548227	-0.141982	0.887095	0.989231
zgc:194930	9.963817193	0.008834805	0.6177047	0.0143026	0.988589	NA
pkn3	225.7055337	-0.04153534	0.1463569	-0.283795	0.776568	0.975826
gabrb2a	501.5082296	0.182364414	0.1163222	1.5677525	0.116939	0.639958
castor1	182.994792	0.063011573	0.1631712	0.3861685	0.699372	0.967998
si:dkeyp-73d8.6	2.476775735	2.681623393	1.4401944	1.8619872	0.062605	NA
si:ch211-203d1.3	84.68508524	0.016167068	0.2177823	0.074235	0.940823	0.992857
calhm3	15.7036261	0.759475726	0.4922719	1.5427973	0.12288	0.650905
si:ch211-256e16.6	11.49203647	-0.278390873	0.5642327	-0.493397	0.621732	0.957354
prmt5	593.9208158	-0.058454353	0.1127503	-0.518441	0.604151	0.95651
SGPP2	34.92487413	-0.037446903	0.3280255	-0.114159	0.909112	0.990702
akap9	1888.131525	-0.030852678	0.0861868	-0.357974	0.720363	0.969318
sema4c	414.6301214	-0.024718921	0.1126264	-0.219477	0.826278	0.982196
sall3a	259.0743272	0.117041475	0.1418229	0.8252647	0.409221	0.90181
ftr34	0.849390142	1.928131185	2.2127708	0.8713651	0.383555	NA
cramp1	805.6370365	-0.12297133	0.0937149	-1.312186	0.189457	0.754244
sik2a	39.87067499	-0.049117413	0.3039561	-0.161594	0.871626	0.987766
amigo1	52.0847983	-0.137604183	0.268078	-0.513299	0.607742	0.956718
frmd5	142.7321399	0.069057188	0.1716674	0.4022733	0.687483	0.966376
ubtd1b	22.43252843	0.516162279	0.4101155	1.2585778	0.208183	0.775404
amer1	668.7401465	-0.02836933	0.0970288	-0.292381	0.769996	0.975687
atr	211.8116877	0.038227573	0.1529187	0.2499862	0.802598	0.979592
elf2b	664.3714122	-0.121129113	0.1047941	-1.155877	0.247731	0.81711
si:ch211-113e8.10	546.1447057	-0.085936136	0.1080367	-0.795434	0.426361	0.909969
si:dkey-28b4.7	342.6939854	-0.155075739	0.1269956	-1.221111	0.222044	0.788551
filip1b	128.9878875	-0.055616885	0.1807441	-0.307711	0.758302	0.975374
fam177a1	828.0734125	-0.113899383	0.0926638	-1.229168	0.219009	0.785734
alpk2	174.959816	0.016669786	0.1658233	0.1005274	0.919926	0.991857
PRRC2B	2515.436824	0.006470221	0.1171601	0.0552255	0.955959	0.994543
sc:d217	0	NA	NA	NA	NA	NA
muc13b	289.2759405	-1.257670062	0.1357197	-9.266672	1.92E-20	1.90E-17
si:ch1073-174d20.2	1305.550428	0.162373912	0.0991761	1.6372283	0.101583	0.605756
caap1	273.2767066	0.031220091	0.1329639	0.2348013	0.814363	0.981081
cyp2x6	2.465707907	-0.926370273	1.3158955	-0.703985	0.481442	NA
st3gal1	1.630691012	-0.616903343	1.4938275	-0.412968	0.67963	NA

fam171a2b	49.68364661	0.406971571	0.2853449	1.4262445	0.153798	0.702599
si:dkey-53k12.1	0	NA	NA	NA	NA	NA
wdr53	193.8511528	0.144631962	0.1491802	0.9695119	0.33229	0.874653
CU855821.1	0.965400781	-1.006309521	1.9906007	-0.505531	0.613186	NA
zgc:172341	111.7155208	0.219135195	0.194601	1.1260742	0.260134	0.828288
gpr158b	233.797119	0.240857171	0.1479178	1.6283174	0.103458	0.609625
lrrc7	371.5624248	0.145750459	0.1178184	1.2370777	0.216058	0.783484
plppr4a	519.9323309	0.190205468	0.1049775	1.8118685	0.070007	0.516704
efl1	148.5810595	-0.064953245	0.1726939	-0.376118	0.706829	0.968664
cep112	121.5426277	0.011443182	0.1857536	0.0616041	0.950878	0.99381
CABZ01059627.2	1.797940186	0.8407671	1.545573	0.5439841	0.586452	NA
taf6l	364.6395904	0.011466485	0.121309	0.094523	0.924694	0.992676
naa30	653.8843674	0.173072261	0.0981247	1.7637989	0.077766	0.543372
rnf10	6544.872545	-0.035191188	0.0659413	-0.533674	0.593567	0.954434
tnrc6a	2524.51558	-0.028845053	0.0773138	-0.373091	0.709081	0.968664
zgc:194908	1.511182322	-0.351757116	1.5857123	-0.221829	0.824447	NA
asb13a.1	71.92419583	0.142237856	0.233717	0.6085901	0.542796	0.944974
zgc:162612	77.97010033	-0.113314549	0.2255662	-0.502356	0.615417	0.95688
b3galt1a	13.30169974	0.279153072	0.5450748	0.5121371	0.608555	0.956718
wdr81	307.1291096	0.051851261	0.1325254	0.3912553	0.695609	0.96736
si:dkey-18p12.4	9.488163816	-1.224801753	0.7413175	-1.652196	0.098495	NA
si:ch211-152p11.4	38.78652932	-0.094607242	0.3055345	-0.309645	0.756831	0.975374
gal3st4	1032.065737	0.058545318	0.0848979	0.6895972	0.490448	0.931926
ccl27b	5.692456476	0.716721644	0.8226503	0.8712349	0.383626	NA
sin3aa	980.7287643	-0.051793644	0.0865639	-0.598329	0.549621	0.946312
rbm12b	586.6589141	0.059747999	0.0998043	0.5986513	0.549405	0.946312
si:dkey-46g23.1	93.40129121	0.156241055	0.2182661	0.7158282	0.474097	0.924993
dhx9	1704.732454	0.03544826	0.0799433	0.4434175	0.657464	0.962444
selenop2	193.9958134	0.552661256	0.1988185	2.7797275	0.00544	0.10944
fuz	40.99608795	-0.356024094	0.3321314	-1.071938	0.283748	0.84466
kif13ba	696.4487739	-0.0610426	0.0924355	-0.66038	0.50901	0.936123
znf219	898.2237717	-0.048380395	0.088095	-0.549184	0.582879	0.952214
efna2b	40.49568666	0.220515167	0.3005075	0.7338093	0.463065	0.922698
CSRNP3	11.21184312	0.285899965	0.6250277	0.4574197	0.647369	0.960581
mcf2l2	30.40551985	0.191201738	0.3817225	0.500892	0.616447	0.95688
si:ch211-166a6.5	1712.011492	-0.075046245	0.0864324	-0.868265	0.385249	0.89551
megf8	1007.085989	0.114579689	0.0890886	1.2861315	0.198397	0.763597
col6a4a	1691.029991	0.187402886	0.0755916	2.4791509	0.01317	0.205051
kiz	182.7476578	-0.018676293	0.1526052	-0.122383	0.902596	0.990121
peak1	37.40868752	0.00668309	0.3331963	0.0200575	0.983997	0.996944
zgc:112998	23.92828121	-0.18747588	0.3902479	-0.480402	0.630942	0.957354
si:dkey-253d23.4	365.0740827	0.113189461	0.1176527	0.9620642	0.336017	0.876088
si:ch211-112f3.4	86.273426	-0.136712335	0.2256543	-0.605849	0.544615	0.945438
tap1	9.972711251	0.234237533	0.6339689	0.369478	0.711771	NA
si:ch73-296e2.3	0	NA	NA	NA	NA	NA
peal5	34.53012808	-0.058333809	0.3291734	-0.177213	0.859341	0.985773
nox5	13.22278319	-0.617889041	0.5663929	-1.090919	0.275308	0.839541
map6d1	99.96214129	0.218189376	0.201564	1.0824819	0.279038	0.842806
radil	108.0454367	0.231380896	0.1899145	1.2183424	0.223094	0.789115
hnrnpul1l	3944.006131	-0.026989832	0.0749054	-0.360319	0.718609	0.96909
slitrk4	445.3267031	0.181729595	0.1063907	1.7081348	0.087611	0.566637
myh7l	557.3446994	0.005736241	0.105545	0.0543488	0.956657	0.994543
isg20	271.7288235	0.052157266	0.143008	0.3647158	0.715324	0.96909
si:ch211-235o23.1	308.996519	-0.048028469	0.1366108	-0.351571	0.72516	0.970402

si:dkeyp-73a2.2	0	NA	NA	NA	NA	NA
fam171a1	442.3082585	0.035610803	0.1099009	0.3240266	0.745918	0.97419
LO018102.1	77.5521385	-0.073722584	0.2358234	-0.312618	0.754571	0.975114
si:ch211-176g6.2	14.76203357	0.089668959	0.4977835	0.1801364	0.857045	0.985597
CABZ01033206.1	62.44689818	-0.068424465	0.2827054	-0.242035	0.808753	0.980642
ldah	100.8700878	-0.045861238	0.2076551	-0.220853	0.825207	0.982196
aanat2	52.33983914	0.00408835	0.2710919	0.015081	0.987968	0.997369
tagln3a	11.96503142	-0.084380308	0.5471291	-0.154224	0.877433	0.987881
si:ch73-208g10.1	8.199427119	-0.342481134	0.7367463	-0.464856	0.642034	NA
micall1a	35.30497783	-0.066596127	0.3249511	-0.204942	0.837617	0.984665
lyrm7	159.2217184	-0.101468788	0.1740302	-0.583053	0.559858	0.948478
rhbdd3	92.80304619	0.075672725	0.2187288	0.3459659	0.729368	0.970822
helq	55.30701308	-0.448106197	0.2594842	-1.726911	0.084184	0.55797
mrps36	881.5897937	0.024907263	0.0933471	0.2668242	0.789605	0.977238
ninj2	15.82974228	0.106626111	0.5024487	0.2122129	0.831941	0.98349
gpr174	4.841161772	0.081035297	0.8820666	0.0918698	0.926801	NA
srbd1	29.6036639	-0.163442691	0.378992	-0.431256	0.666282	0.964736
ccr2	1.515470518	0.323632373	1.5591799	0.2075658	0.835568	NA
kdf1a	128.8893525	-0.047828234	0.1853249	-0.258078	0.796347	0.978498
man2c1	148.5671972	-0.219118095	0.1757886	-1.246486	0.212586	0.780627
arrdc1b	83.59906088	0.382934726	0.2148038	1.7827185	0.074632	0.532003
kcnma1a	484.9171384	0.01696998	0.1060878	0.1599617	0.872911	0.987846
igl4v8	0.497574187	-2.437446738	2.6012773	-0.937019	0.348749	NA
zgc:194578	982.2700158	0.062294842	0.0897857	0.6938166	0.487797	0.931809
gmps	1080.961327	-0.07808083	0.0850375	-0.918193	0.358518	0.884398
nrtn	17.83125746	0.147602612	0.4543743	0.3248481	0.745296	0.974094
dchs1b	1032.445111	0.030503732	0.0892316	0.3418488	0.732465	0.971719
scyl1	868.4055478	0.060074433	0.089105	0.6741983	0.500185	0.934224
si:ch211-189k9.2	484.7513189	0.165567499	0.103832	1.5945718	0.110808	0.625858
tmem163a	20.71840184	-0.124242049	0.4281829	-0.290161	0.771693	0.975687
ppp1r16b	30.29533782	-0.498748589	0.353851	-1.409488	0.158691	0.709303
kl	21.78648011	0.465015697	0.4115739	1.1298474	0.258541	0.826711
si:ch211-218o21.4	21.5449805	-0.04104144	0.4032907	-0.101766	0.918942	0.991623
rapgef4	54.28361055	0.411727571	0.2714668	1.5166777	0.129348	0.661083
SLC29A4 (1 of many)	138.1175584	0.062550476	0.1764684	0.354457	0.722996	0.970375
CU855878.1	125.5927283	0.131973868	0.1789234	0.7375996	0.460758	0.921978
tmem151bb	3.585485091	0.718358313	1.0865596	0.6611311	0.508528	NA
tas2r200.1	0	NA	NA	NA	NA	NA
tcaim	183.7282009	0.081980677	0.1654233	0.4955811	0.62019	0.957354
trim107	67.15651315	-0.017169043	0.2533283	-0.067774	0.945966	0.993759
otulinb	161.2645481	-0.141600162	0.1657424	-0.854339	0.392917	0.897836
phldb2a	26.11507803	-0.5842371	0.3734699	-1.564348	0.117736	0.641446
dnajc6	1299.210941	0.18165304	0.0857397	2.118656	0.03412	0.359261
si:dkey-183c2.4	22.31726519	1.350980199	0.422247	3.1995024	0.001377	0.039915
ehbp1l1b	225.9340427	0.059106622	0.1469741	0.4021568	0.687569	0.966379
si:dkey-7k24.5	20.55863567	0.328045156	0.4151579	0.7901696	0.429429	0.911489
shroom4	355.5906231	-0.120545484	0.1300294	-0.927063	0.353894	0.881976
si:ch73-15n24.1	11.95535837	0.83248352	0.5709175	1.4581504	0.144799	0.688986
si:ch73-343l4.2	1.829337094	0.319794686	1.4914512	0.2144185	0.830221	NA
mark2a	900.7714519	-0.07801916	0.1003813	-0.777228	0.437024	0.914408
ptger4c	11.22391446	0.327217641	0.5946502	0.5502691	0.582135	0.952028
tmem151a	168.6943742	0.080747975	0.1574053	0.5129941	0.607955	0.956718
rapgefl1	68.27472049	-0.230260152	0.2352464	-0.978804	0.327677	0.872701
stoml3a	13.19528941	0.534171551	0.5773497	0.9252132	0.354855	0.882185

pot1	75.36904769	-0.402152873	0.2294872	-1.752398	0.079705	0.548406
rassf7a	160.3177024	0.00625309	0.1637135	0.0381953	0.969532	0.996315
klf4	15.92329771	0.429375702	0.4704503	0.912691	0.361405	0.885719
AL954695.2	2.180330666	-1.193210315	1.3920002	-0.857191	0.391339	NA
syt15	23.14234004	-0.25524417	0.4105754	-0.621674	0.534156	0.943276
si:dkeyp-68b7.5	37.52486281	-0.121106989	0.3137435	-0.386006	0.699492	0.967998
snx19b	398.8553488	0.077970542	0.1155415	0.6748274	0.499785	0.934186
zgc:152830	1388.501799	-0.270120201	0.085093	-3.174413	0.001501	0.042656
SLC46A3 (1 of many)	72.4550638	0.516221166	0.2365416	2.1823698	0.029082	0.330497
zgc:162200	456.9045495	0.074269142	0.1106876	0.67098	0.502233	0.935304
jmjd1cb	2729.358346	-0.080076112	0.0839905	-0.953395	0.34039	0.87805
pjvk	3.888165651	-0.24817662	1.0128631	-0.245025	0.806437	NA
sqlea	134.9209098	-0.312793611	0.1857456	-1.683989	0.092184	0.57849
gfi1b	7.178842437	0.193498069	0.7118709	0.2718162	0.785763	NA
supt16h	2265.69363	-0.127353741	0.0729192	-1.746504	0.080723	0.550801
eloal	3.14001781	1.491919797	1.1146651	1.3384467	0.180751	NA
doc2d	217.4639448	-0.006982392	0.145864	-0.047869	0.96182	0.995907
dlx2a	391.2074037	-0.160357896	0.1162926	-1.378917	0.16792	0.725624
prkd3	91.23636132	-0.14403068	0.2095059	-0.687478	0.491782	0.931926
nfatc2b	9.487795352	-0.910052452	0.6610793	-1.376616	0.168631	NA
pus10	177.9060626	-0.036763239	0.1549453	-0.237266	0.81245	0.981081
nhsb	597.8923796	-0.180260579	0.1048451	-1.719303	0.085559	0.561521
samd10a	101.1044027	0.037681683	0.2098176	0.1795926	0.857472	0.985703
fam89a	39.51415813	-0.07153951	0.3666215	-0.195132	0.84529	0.985174
si:ch211-202h22.9	2.857879201	0.18673648	1.1359627	0.1643861	0.869427	NA
agbl2	39.14864948	0.236315119	0.3329854	0.7096861	0.477899	0.926857
nrip2	548.3168587	0.10264447	0.1125838	0.911716	0.361918	0.885944
BX927329.1	5.316339016	-0.546352055	0.8634332	-0.632767	0.526886	NA
igl3v5	0	NA	NA	NA	NA	NA
senp6b	0.488104098	-2.415107183	2.7936704	-0.864493	0.387317	NA
akap13	378.4210344	-0.055127986	0.1132188	-0.486915	0.626318	0.957354
si:zfos-223e1.2	16.64776335	0.179847437	0.4599396	0.391024	0.695779	0.96736
si:dkey-178e17.1	2.5015376	0.17069564	1.2767871	0.1336916	0.893646	NA
tmem41b	102.0474248	0.313029451	0.1982266	1.5791493	0.114302	0.633643
bahcc1b	1185.630104	-0.094234172	0.0871257	-1.081588	0.279436	0.842882
adh5	1715.206362	-0.279223687	0.0892138	-3.129825	0.001749	0.048577
lrrtm4l1	42.09664269	0.177566829	0.3097937	0.5731777	0.566524	0.948739
si:cabz01032454.3	46.97510189	-0.030759702	0.3029923	-0.10152	0.919138	0.991623
kif16bb	64.93121083	0.208690567	0.2672294	0.7809416	0.434837	0.914408
UBA6	307.6676903	0.026490488	0.1241806	0.2133222	0.831076	0.982994
il13ra1	182.4848118	-0.181901957	0.164163	-1.108057	0.267837	0.833349
zmat3	15.30073141	-0.222765484	0.494329	-0.450642	0.652247	0.960708
RF00001	1.450079108	1.819886428	1.7323838	1.0505099	0.293484	NA
dre-mir-125b-2	0	NA	NA	NA	NA	NA
RF00001	0.794578739	3.129658898	2.5486248	1.2279794	0.219455	NA
dre-let-7d-2	0	NA	NA	NA	NA	NA
RF00001	0.476386185	2.392464142	3.0533824	0.7835455	0.433307	NA
dre-mir-192	0.679516628	0.149584948	2.5505337	0.0586485	0.953232	NA
RF00001	0	NA	NA	NA	NA	NA
dre-mir-459	0	NA	NA	NA	NA	NA
RF00001	0	NA	NA	NA	NA	NA
dre-mir-133c	9.612367637	0.505598406	0.6792769	0.7443186	0.456684	NA
RF00001	0	NA	NA	NA	NA	NA
RF00001	0.166657454	-0.955901296	4.0804729	-0.234262	0.814781	NA

dre-mir-135c-1	0	NA	NA	NA	NA	NA
RF00001	0	NA	NA	NA	NA	NA
RF00001	0	NA	NA	NA	NA	NA
NC_002333.1	5.669769664	1.73759832	0.8785041	1.9779057	0.047939	NA
RF00026	0	NA	NA	NA	NA	NA
RF00001	38.72609277	0.146157328	0.3212878	0.4549109	0.649173	0.960708
NC_002333.2	27.44623464	-0.644579154	0.4502464	-1.431614	0.152254	0.701157
dre-mir-29b-1	6.201260407	-0.718930114	0.7987389	-0.900082	0.368077	NA
RF00088	0.961236193	2.170866656	2.290243	0.9478761	0.343193	NA
RF00093	0	NA	NA	NA	NA	NA
dre-mir-15a-1	0.173729368	-0.955901296	4.0804729	-0.234262	0.814781	NA
RF00001	0	NA	NA	NA	NA	NA
RF00440	0	NA	NA	NA	NA	NA
dre-mir-124-5	96.92911533	0.475943379	0.2018949	2.3573822	0.018404	0.252376
dre-mir-499	0	NA	NA	NA	NA	NA
RF00571	5.119707652	0.066909561	0.9493068	0.0704825	0.94381	NA
mir338-2	0	NA	NA	NA	NA	NA
RF00001	0.323844819	-1.825095364	3.274639	-0.557342	0.577293	NA
RF00001	0	NA	NA	NA	NA	NA
RF00612	0	NA	NA	NA	NA	NA
RF00001	0.325573202	0.005883621	3.3172783	0.0017736	0.998585	NA
RF00001	2.030514901	2.311190427	1.6633927	1.3894436	0.164698	NA
RF00001	0	NA	NA	NA	NA	NA
dre-let-7c-2	0	NA	NA	NA	NA	NA
RF00001	0	NA	NA	NA	NA	NA
RF00001	0	NA	NA	NA	NA	NA
RF00001	0.492110303	-0.89618148	2.8628934	-0.313033	0.754255	NA
RF00001	0.863695607	1.965711988	2.22386	0.8839189	0.37674	NA
RF00026	0	NA	NA	NA	NA	NA
RF00001	0.31759079	1.807926197	3.579045	0.5051421	0.613459	NA
RF00001	0	NA	NA	NA	NA	NA
RF00001	0	NA	NA	NA	NA	NA
RF00001	0	NA	NA	NA	NA	NA
RF00001	0.157187365	-0.955901296	4.0804729	-0.234262	0.814781	NA
RF00001	0	NA	NA	NA	NA	NA
NC_002333.3	16.9145956	0.148778326	0.5031951	0.2956673	0.767484	0.975687
NC_002333.4	15799.68608	0.633726969	0.2986533	2.1219489	0.033842	0.357857
RF00001	0	NA	NA	NA	NA	NA
dre-mir-130b	1.013460044	-2.21473548	2.0108742	-1.101379	0.270732	NA
dre-mir-456	0	NA	NA	NA	NA	NA
RF00413	1.133237178	0.375573287	1.8721312	0.2006127	0.841001	NA
RF00001	0	NA	NA	NA	NA	NA
RF00581	0.325452849	0.005883619	3.3177086	0.0017734	0.998585	NA
RF00001	2.528020063	-0.187679332	1.1943971	-0.157133	0.87514	NA
NC_002333.5	0.173729368	-0.955901296	4.0804729	-0.234262	0.814781	NA
RF00026	0	NA	NA	NA	NA	NA
RF00001	0	NA	NA	NA	NA	NA
RF00548	0.158795395	0.967652056	4.0804729	0.2371421	0.812547	NA
RF00001	0	NA	NA	NA	NA	NA
RF00211	0	NA	NA	NA	NA	NA
RF00001	0	NA	NA	NA	NA	NA
RF00604	0	NA	NA	NA	NA	NA
RF00001	0	NA	NA	NA	NA	NA
RF00003	0	NA	NA	NA	NA	NA

RF00001	0	NA	NA	NA	NA	NA
RF00566	3.304397441	1.229539124	1.0627073	1.1569875	0.247277	NA
mir140	3.219275555	2.458081228	1.265492	1.9423917	0.05209	NA
RF00342	0.658957134	2.851427774	2.5143902	1.1340434	0.256776	NA
dre-mir-183	0.157187365	-0.955901296	4.0804729	-0.234262	0.814781	NA
dre-mir-21-2	1.521673218	1.029857275	1.5956979	0.6453961	0.518671	NA
RF00001	0	NA	NA	NA	NA	NA
dre-mir-141	0.635422286	2.809933352	2.5211574	1.114541	0.265047	NA
RF00377	0.173729368	-0.955901296	4.0804729	-0.234262	0.814781	NA
RF00396	4.878267767	0.546166147	0.8752242	0.62403	0.532608	NA
RF00001	0	NA	NA	NA	NA	NA
dre-mir-125c	0	NA	NA	NA	NA	NA
RF00003	0	NA	NA	NA	NA	NA
dre-let-7j	2.187832662	0.192877281	1.3358529	0.1443851	0.885196	NA
RF01299	0.498553709	0.926267934	2.6786929	0.345791	0.7295	NA
RF00089	1.32527496	-2.703143513	1.9371225	-1.395443	0.162882	NA
RF00056	0.347458735	-1.908042286	3.4571486	-0.551912	0.581009	NA
dre-mir-23a-1	0	NA	NA	NA	NA	NA
dre-mir-153a	0	NA	NA	NA	NA	NA
RF00619	1.689949989	-0.450849563	1.7054564	-0.264357	0.791505	NA
dre-mir-212	0.157187365	-0.955901296	4.0804729	-0.234262	0.814781	NA
dre-mir-731	0	NA	NA	NA	NA	NA
RF00001	0	NA	NA	NA	NA	NA
dre-mir-135c-2	0	NA	NA	NA	NA	NA
dre-mir-455-2	0.330916733	-1.849579229	3.2489867	-0.569279	0.569167	NA
dre-mir-26b	1.493073575	-0.932799967	1.6069462	-0.58048	0.561591	NA
mir223	0	NA	NA	NA	NA	NA
dre-mir-26a-1	0	NA	NA	NA	NA	NA
dre-mir-9-3	0.317831496	1.808837541	3.5779923	0.5055454	0.613176	NA
dre-mir-24-3	1.300753802	1.583438483	1.7347538	0.9127742	0.361361	NA
dre-mir-724	0	NA	NA	NA	NA	NA
dre-mir-9-2	0.523937293	2.50362281	2.7291625	0.9173594	0.358954	NA
NC_002333.6	7.130473445	-0.079191027	0.7101226	-0.111517	0.911206	NA
RF00096	0.158795395	0.967652056	4.0804729	0.2371421	0.812547	NA
RF00093	0.5046461	-2.45370851	2.7629627	-0.888072	0.374502	NA
dre-mir-10b-1	0	NA	NA	NA	NA	NA
RF00001	0	NA	NA	NA	NA	NA
RF00396	2.579150702	0.696969378	1.3432688	0.5188607	0.603858	NA
dre-mir-124-1	0	NA	NA	NA	NA	NA
RF00001	0.173729368	-0.955901296	4.0804729	-0.234262	0.814781	NA
RF00001	0	NA	NA	NA	NA	NA
RF00001	0	NA	NA	NA	NA	NA
si:dkey-71b5.7	433.3745737	0.062406983	0.1269557	0.491565	0.623027	0.957354
dre-mir-730	0.173729368	-0.955901296	4.0804729	-0.234262	0.814781	NA
RF00001	0	NA	NA	NA	NA	NA
RF00407	0.996127896	-0.965496869	1.8966759	-0.509047	0.610719	NA
RF00070	0.349228403	0.005884014	3.2372587	0.0018176	0.99855	NA
dre-mir-375-2	4.90868856	0.810005845	0.9074219	0.8926453	0.372047	NA
dre-mir-200c	0	NA	NA	NA	NA	NA
NC_002333.7	0.811320409	-1.830272858	2.4089013	-0.759796	0.447377	NA
RF00001	0	NA	NA	NA	NA	NA
RF00001	1.618155215	-0.032684466	1.5883641	-0.020577	0.983583	NA
RF00001	0	NA	NA	NA	NA	NA
dre-mir-17a-2	0.330916733	-1.849579229	3.2489867	-0.569279	0.569167	NA

RF00611	0	NA	NA	NA	NA	NA
RF00007	0.157187365	-0.955901296	4.0804729	-0.234262	0.814781	NA
dre-mir-181a-2	0.333314908	-1.858695402	3.5126701	-0.52914	0.596708	NA
RF00004	0	NA	NA	NA	NA	NA
RF00571	13.74387229	-0.228262375	0.5434343	-0.420037	0.674459	0.965257
RF01225	0.531799352	0.999478841	2.790358	0.3581902	0.720201	NA
RF00001	0	NA	NA	NA	NA	NA
mir16c	0	NA	NA	NA	NA	NA
RF00003	0	NA	NA	NA	NA	NA
RF00001	0	NA	NA	NA	NA	NA
RF00001	0	NA	NA	NA	NA	NA
RF00045	1.430708847	-0.318075374	1.6858966	-0.188668	0.850353	NA
dre-mir-27b	0.173729368	-0.955901296	4.0804729	-0.234262	0.814781	NA
RF00001	0.316103113	0.005883447	3.3519121	0.0017553	0.9986	NA
dre-mir-152	0.158795395	0.967652056	4.0804729	0.2371421	0.812547	NA
RF00396	0	NA	NA	NA	NA	NA
RF00001	0.182570949	0.967652056	4.0804729	0.2371421	0.812547	NA
dre-mir-194a	0.166657454	-0.955901296	4.0804729	-0.234262	0.814781	NA
RF00056	1.154923291	-0.397128357	1.7479381	-0.227198	0.82027	NA
dre-mir-1788	0	NA	NA	NA	NA	NA
dre-let-7a-5	0.673073222	-1.460830855	2.4012698	-0.608358	0.54295	NA
dre-mir-722	0	NA	NA	NA	NA	NA
RF01291	6.758949206	1.094214529	0.7731084	1.4153442	0.156968	NA
RF00003	0	NA	NA	NA	NA	NA
RF00001	0.500161739	2.450146464	2.7711133	0.8841741	0.376602	NA
RF00001	1.410428931	0.838481333	1.7650076	0.4750582	0.634746	NA
dre-mir-24-1	0	NA	NA	NA	NA	NA
RF00152	0.508023798	0.926122713	2.6636728	0.3476864	0.728076	NA
dre-mir-18b	0	NA	NA	NA	NA	NA
RF00001	0.157187365	-0.955901296	4.0804729	-0.234262	0.814781	NA
RF00001	0.158915748	0.967652056	4.0804729	0.2371421	0.812547	NA
RF00049	0	NA	NA	NA	NA	NA
RF00575	0	NA	NA	NA	NA	NA
RF00572	0	NA	NA	NA	NA	NA
RF00001	0	NA	NA	NA	NA	NA
dre-mir-30c	0	NA	NA	NA	NA	NA
dre-mir-31	0	NA	NA	NA	NA	NA
RF00001	0	NA	NA	NA	NA	NA
RF00030	19.01839035	0.931446928	0.5459697	1.7060413	0.088	0.568063
RF00001	0	NA	NA	NA	NA	NA
RF00066	0	NA	NA	NA	NA	NA
RF00020	0.506415768	-0.866825728	2.6674086	-0.324969	0.745204	NA
RF00273	0.317831496	1.808837541	3.5779923	0.5055454	0.613176	NA
RF00001	0	NA	NA	NA	NA	NA
dre-mir-125a-2	2.451512504	1.983296058	1.3229665	1.499128	0.13384	NA
dre-mir-135a	5.81489598	0.251814402	0.7716281	0.3263417	0.744166	NA
RF00001	0	NA	NA	NA	NA	NA
RF00001	0	NA	NA	NA	NA	NA
RF00001	0	NA	NA	NA	NA	NA
RF00001	0.157187365	-0.955901296	4.0804729	-0.234262	0.814781	NA
RF00190	0	NA	NA	NA	NA	NA
dre-mir-728	0	NA	NA	NA	NA	NA
dre-mir-182	0.48448895	0.85344211	2.878836	0.2964539	0.766883	NA
RF00001	0.673031937	-1.510323851	2.4021096	-0.628749	0.529513	NA

mir196d	0	NA	NA	NA	NA	NA
RF01290	0	NA	NA	NA	NA	NA
RF00001	0	NA	NA	NA	NA	NA
dre-mir-204-1	0	NA	NA	NA	NA	NA
RF00001	0.48448895	0.85344211	2.878836	0.2964539	0.766883	NA
dre-mir-7b	5.727422749	-0.525682013	0.8270204	-0.635634	0.525015	NA
RF00001	0.182570949	0.967652056	4.0804729	0.2371421	0.812547	NA
RF01296	3.626781327	0.262713658	1.0782921	0.2436387	0.807511	NA
dre-mir-181b-1	11.33621417	-0.158374062	0.5614172	-0.282097	0.777869	0.975942
RF00001	0	NA	NA	NA	NA	NA
RF00001	0	NA	NA	NA	NA	NA
dre-mir-153c	0.339758314	0.005883863	3.2682461	0.0018003	0.998564	NA
RF00001	0	NA	NA	NA	NA	NA
dre-mir-15b	0	NA	NA	NA	NA	NA
dre-mir-132-1	0.655741074	0.077611818	2.4374018	0.031842	0.974598	NA
RF00020	0	NA	NA	NA	NA	NA
RF00001	0	NA	NA	NA	NA	NA
RF00001	0.157187365	-0.955901296	4.0804729	-0.234262	0.814781	NA
RF00001	0	NA	NA	NA	NA	NA
RF00002	24.80453346	1.214334441	1.0671853	1.1378853	0.255168	0.823839
dre-mir-34a	0	NA	NA	NA	NA	NA
dre-mir-26a-3	0	NA	NA	NA	NA	NA
dre-mir-454b	0.484368597	0.855487352	2.7040894	0.316368	0.751723	NA
RF00001	0	NA	NA	NA	NA	NA
RF00001	0	NA	NA	NA	NA	NA
dre-mir-16b	5.298700393	-0.18298347	0.8107874	-0.225686	0.821446	NA
RF00001	0	NA	NA	NA	NA	NA
rn7sk	313.6195769	0.587252098	0.4676292	1.2558071	0.209186	0.776073
NC_002333.8	24.98486023	0.057236006	0.4226647	0.135417	0.892282	0.989291
dre-mir-29b-2	0.95259428	-3.385964433	2.2372857	-1.513425	0.130172	NA
RF00001	0	NA	NA	NA	NA	NA
BX936308.1	69.85611105	0.150376761	0.243083	0.6186232	0.536165	0.943336
mir363	1.158779144	-2.454178177	2.015231	-1.217815	0.223294	NA
dre-mir-124-2	19.56098018	-0.406067699	0.4496112	-0.903153	0.366445	0.887239
dre-mir-9-7	0	NA	NA	NA	NA	NA
mir99-2	0.166657454	-0.955901296	4.0804729	-0.234262	0.814781	NA
RF00001	0	NA	NA	NA	NA	NA
dre-mir-138-1	0	NA	NA	NA	NA	NA
RF00001	0.182570949	0.967652056	4.0804729	0.2371421	0.812547	NA
RF00001	0	NA	NA	NA	NA	NA
mir196b	0	NA	NA	NA	NA	NA
dre-mir-101b	0	NA	NA	NA	NA	NA
dre-mir-125b-1	0.158795395	0.967652056	4.0804729	0.2371421	0.812547	NA
RF00001	0	NA	NA	NA	NA	NA
dre-mir-9-5	0.173729368	-0.955901296	4.0804729	-0.234262	0.814781	NA
dre-mir-15c	0	NA	NA	NA	NA	NA
NC_002333.9	13.90282923	0.092495702	0.5264614	0.1756932	0.860535	0.985773
dre-mir-457b	3.477775794	0.112172945	1.0077155	0.1113141	0.911367	NA
RF00096	0.31437473	-1.788572922	3.5930657	-0.497785	0.618636	NA
dre-mir-200a	0	NA	NA	NA	NA	NA
RF00001	0	NA	NA	NA	NA	NA
RF00001	0	NA	NA	NA	NA	NA
dre-mir-124-3	5.754700009	-0.065998096	0.794745	-0.083043	0.933817	NA
RF00004	0	NA	NA	NA	NA	NA

dre-mir-429a	0	NA	NA	NA	NA	NA
RF00072	0	NA	NA	NA	NA	NA
dre-let-7c-1	0.349228403	0.005884014	3.2372587	0.0018176	0.99855	NA
NC_002333.10	0	NA	NA	NA	NA	NA
RF00020	0	NA	NA	NA	NA	NA
RF00581	0.806485033	-1.864932549	2.2428596	-0.831498	0.405693	NA
mir100-2	0	NA	NA	NA	NA	NA
RF00001	0.325452849	0.005883619	3.3177086	0.0017734	0.998585	NA
CR847953.2	0	NA	NA	NA	NA	NA
NC_002333.11	0.648507523	-0.035374916	2.4454854	-0.014465	0.988459	NA
dre-mir-126a	0	NA	NA	NA	NA	NA
dre-mir-23a-2	0	NA	NA	NA	NA	NA
RF00001	0	NA	NA	NA	NA	NA
dre-mir-301c	0	NA	NA	NA	NA	NA
RF00001	0	NA	NA	NA	NA	NA
dre-mir-375-1	1.457659176	0.9469376	1.6749778	0.5653434	0.57184	NA
dre-mir-100-1	0	NA	NA	NA	NA	NA
RF00001	0	NA	NA	NA	NA	NA
dre-mir-204-2	0.340386822	-1.882368278	3.2159502	-0.585323	0.558331	NA
dre-mir-23b	0	NA	NA	NA	NA	NA
RF00001	0	NA	NA	NA	NA	NA
dre-mir-30d	0	NA	NA	NA	NA	NA
dre-mir-181b-2	0.830098949	-1.902766313	2.1285797	-0.893914	0.371368	NA
RF00001	0.158915748	0.967652056	4.0804729	0.2371421	0.812547	NA
RF00133	0	NA	NA	NA	NA	NA
RF00003	0	NA	NA	NA	NA	NA
RF00608	0	NA	NA	NA	NA	NA
dre-mir-103	0	NA	NA	NA	NA	NA
RF00001	0	NA	NA	NA	NA	NA
dre-mir-144	0	NA	NA	NA	NA	NA
mir150	0	NA	NA	NA	NA	NA
dre-mir-734	0	NA	NA	NA	NA	NA
dre-mir-216a	0	NA	NA	NA	NA	NA
RF00001	0	NA	NA	NA	NA	NA
RF00577	0	NA	NA	NA	NA	NA
RF00377	0	NA	NA	NA	NA	NA
dre-mir-7a-3	0.330916733	-1.849579229	3.2489867	-0.569279	0.569167	NA
dre-mir-190a	0.474898508	0.855240867	2.7202602	0.3143967	0.75322	NA
dre-mir-10c	0.861574924	-3.219550392	2.3111143	-1.393073	0.163598	NA
RF00001	0.476506538	2.394892832	2.8161716	0.8504073	0.395099	NA
dre-mir-205	1.96109959	-0.479788712	1.4100909	-0.340254	0.733665	NA
RF00410	0.643404698	1.436206627	2.5980397	0.552804	0.580398	NA
RF00186	3.221721803	-0.098073118	1.1145211	-0.087996	0.92988	NA
mir206-2	0	NA	NA	NA	NA	NA
dre-mir-133a-2	0	NA	NA	NA	NA	NA
RF00092	1.975822855	0.45282007	1.4368084	0.3151569	0.752643	NA
dre-mir-107b	0.347458735	-1.908042286	3.4571486	-0.551912	0.581009	NA
RF00001	0	NA	NA	NA	NA	NA
RF00001	0	NA	NA	NA	NA	NA
dre-mir-218a-1	0.158795395	0.967652056	4.0804729	0.2371421	0.812547	NA
RF00001	0	NA	NA	NA	NA	NA
NC_002333.12	23.27625586	-0.693625326	0.4012497	-1.728663	0.083869	0.557501
dre-mir-184-1	0.158915748	0.967652056	4.0804729	0.2371421	0.812547	NA
mir24-4	0.157187365	-0.955901296	4.0804729	-0.234262	0.814781	NA

dre-mir-203a	1.656194948	-0.02003601	1.5061657	-0.013303	0.989386	NA
dre-mir-155	0	NA	NA	NA	NA	NA
dre-let-7a-2	0	NA	NA	NA	NA	NA
RF00288	0.5046461	-2.45370851	2.7629627	-0.888072	0.374502	NA
CU929237.1	9.102050632	-0.037556884	0.6332932	-0.059304	0.95271	NA
RF00581	0.703759051	-1.526884723	2.5277341	-0.604053	0.545809	NA
dre-let-7b	0.481032184	-2.398473301	2.80721	-0.854398	0.392885	NA
dre-let-7h	0	NA	NA	NA	NA	NA
RF00001	0	NA	NA	NA	NA	NA
dre-mir-15a-2	0	NA	NA	NA	NA	NA
RF00001	0	NA	NA	NA	NA	NA
mir29a-1	0.825545564	-1.896597388	2.3991135	-0.790541	0.429212	NA
RF00001	0	NA	NA	NA	NA	NA
RF00151	0	NA	NA	NA	NA	NA
RF00425	0	NA	NA	NA	NA	NA
RF00001	0.349228403	0.005884014	3.2372587	0.0018176	0.99855	NA
dre-mir-200b	0	NA	NA	NA	NA	NA
dre-mir-199-3	13.1249596	-0.634174927	0.5920218	-1.071202	0.284079	0.844697
RF00609	0	NA	NA	NA	NA	NA
RF00001	0.182570949	0.967652056	4.0804729	0.2371421	0.812547	NA
CABZ01078244.1	0	NA	NA	NA	NA	NA
dre-mir-338-3	0	NA	NA	NA	NA	NA
RF00411	0	NA	NA	NA	NA	NA
dre-mir-128-1	0.157187365	-0.955901296	4.0804729	-0.234262	0.814781	NA
dre-mir-1306	0	NA	NA	NA	NA	NA
CR847548.1	0	NA	NA	NA	NA	NA
RF00133	2.166936942	-1.634451512	1.5496276	-1.054738	0.291545	NA
RF00190	0	NA	NA	NA	NA	NA
RF00421	11.90742958	0.679291354	0.6348961	1.0699253	0.284653	0.844955
NC_002333.13	455.4348111	-0.032685591	0.3493231	-0.093568	0.925452	0.992676
RF00091	0	NA	NA	NA	NA	NA
RF00581	0	NA	NA	NA	NA	NA
RF00001	0	NA	NA	NA	NA	NA
RF00613	0	NA	NA	NA	NA	NA
RF00001	0	NA	NA	NA	NA	NA
RF00001	0	NA	NA	NA	NA	NA
RF00288	0	NA	NA	NA	NA	NA
dre-let-7e	0.182570949	0.967652056	4.0804729	0.2371421	0.812547	NA
RF00001	0.157187365	-0.955901296	4.0804729	-0.234262	0.814781	NA
dre-mir-729	6.862821894	0.226582971	0.7236118	0.3131278	0.754184	NA
dre-mir-457a	0	NA	NA	NA	NA	NA
RF00001	0	NA	NA	NA	NA	NA
RF00001	0	NA	NA	NA	NA	NA
RF00001	0	NA	NA	NA	NA	NA
RF00001	1.229586365	1.377206388	1.8794026	0.7327894	0.463687	NA
RF00026	0.158795395	0.967652056	4.0804729	0.2371421	0.812547	NA
RF00016	1.30115289	-1.524277999	1.8450057	-0.826164	0.408711	NA
dre-mir-9-1	0	NA	NA	NA	NA	NA
RF00265	0	NA	NA	NA	NA	NA
RF00001	0.182570949	0.967652056	4.0804729	0.2371421	0.812547	NA
RF00001	0	NA	NA	NA	NA	NA
RF00001	0	NA	NA	NA	NA	NA
NC_002333.14	4.560168822	0.611080918	0.9786353	0.6244215	0.532351	NA
RF00089	1.321151658	-0.00170508	1.7199258	-0.000991	0.999209	NA

RF00001	0	NA	NA	NA	NA	NA
RF00001	0.657349104	1.487939685	2.418178	0.6153144	0.538347	NA
RF00001	0	NA	NA	NA	NA	NA
NC_002333.15	7.655128604	-0.907176017	0.6938673	-1.30742	0.19107	NA
RF00001	0.484248244	0.852636534	2.8793247	0.2961238	0.767136	NA
RF00066	0.182570949	0.967652056	4.0804729	0.2371421	0.812547	NA
dre-mir-101a	0	NA	NA	NA	NA	NA
dre-mir-92a-2	3.687589353	-1.067482214	1.0129471	-1.053838	0.291957	NA
RF00001	0	NA	NA	NA	NA	NA
RF00001	0	NA	NA	NA	NA	NA
RF00001	0	NA	NA	NA	NA	NA
dre-mir-142a	0.323844819	-1.825095364	3.274639	-0.557342	0.577293	NA
CU442763.1	0	NA	NA	NA	NA	NA
dre-mir-301b	0.492110303	-0.89618148	2.8628934	-0.313033	0.754255	NA
dre-let-7a-6	1.651679593	-3.043323949	1.7468161	-1.742212	0.081471	NA
dre-mir-20b	0.182570949	0.967652056	4.0804729	0.2371421	0.812547	NA
dre-mir-21-1	68.67329239	-0.286662407	0.2430981	-1.179205	0.238317	0.807449
dre-mir-181c	0.325452849	0.005883619	3.3177086	0.0017734	0.998585	NA
dre-mir-99-1	0	NA	NA	NA	NA	NA
RF00211	0.157187365	-0.955901296	4.0804729	-0.234262	0.814781	NA
RF00001	0	NA	NA	NA	NA	NA
RF00001	0.157187365	-0.955901296	4.0804729	-0.234262	0.814781	NA
dre-mir-137-1	11.38054922	-0.315937067	0.5724383	-0.551915	0.581007	0.951731
RF00045	10.06796961	-0.52807497	0.5910693	-0.893423	0.371631	NA
RF00001	0.158915748	0.967652056	4.0804729	0.2371421	0.812547	NA
dre-mir-1388	0	NA	NA	NA	NA	NA
RF00001	0	NA	NA	NA	NA	NA
RF00003	0	NA	NA	NA	NA	NA
dre-mir-219-1	4.38383736	-0.180872101	0.9374998	-0.19293	0.847014	NA
dre-mir-454a	4.010914731	-0.236911565	0.969921	-0.244259	0.807031	NA
RF00003	0	NA	NA	NA	NA	NA
dre-let-7a-3	0.863695607	1.965711988	2.22386	0.8839189	0.37674	NA
RF01229	0	NA	NA	NA	NA	NA
RF00001	0	NA	NA	NA	NA	NA
RF00001	0	NA	NA	NA	NA	NA
dre-mir-10a	0	NA	NA	NA	NA	NA
RF00001	0	NA	NA	NA	NA	NA
RF00004	6.185309129	-0.863997944	0.8002892	-1.079607	0.280317	NA
RF00001	0	NA	NA	NA	NA	NA
dre-mir-34b	0	NA	NA	NA	NA	NA
dre-mir-462	0	NA	NA	NA	NA	NA
RF00026	0	NA	NA	NA	NA	NA
RF00001	0	NA	NA	NA	NA	NA
RF00066	0.696687138	-1.510024977	2.3787501	-0.634798	0.52556	NA
RF00001	0	NA	NA	NA	NA	NA
dre-mir-130c-1	0.983121726	0.913084443	2.2265742	0.4100849	0.681744	NA
RF00001	0	NA	NA	NA	NA	NA
dre-mir-206-1	4.548137677	0.534682454	0.9412951	0.5680285	0.570016	NA
CR354430.1	1.640082033	-0.57523401	1.5155163	-0.379563	0.70427	NA
RF00001	0.31437473	-1.788572922	3.5930657	-0.497785	0.618636	NA
RF00001	0.633693903	1.437927987	2.4435336	0.5884625	0.556222	NA
RF00409	2.352641381	-0.426468192	1.2232374	-0.348639	0.72736	NA
dre-mir-19c	1.202474398	-0.315413906	1.8153552	-0.173748	0.862064	NA
dre-mir-740	0.474778155	0.852397662	2.898154	0.2941175	0.768668	NA

RF00001	0	NA	NA	NA	NA	NA
RF00089	0	NA	NA	NA	NA	NA
mir199-3a	0.31759079	1.807926197	3.579045	0.5051421	0.613459	NA
dre-mir-27a	0.645291463	-2.8201041	2.5306349	-1.114386	0.265114	NA
dre-mir-129-4	0.157187365	-0.955901296	4.0804729	-0.234262	0.814781	NA
dre-mir-27d	0	NA	NA	NA	NA	NA
dre-mir-142b	0	NA	NA	NA	NA	NA
RF00026	0	NA	NA	NA	NA	NA
RF00001	0.341486697	1.892317624	3.2124068	0.5890654	0.555817	NA
RF00001	0	NA	NA	NA	NA	NA
RF00191	0.166657454	-0.955901296	4.0804729	-0.234262	0.814781	NA
dre-mir-726	0	NA	NA	NA	NA	NA
RF00001	0	NA	NA	NA	NA	NA
RF00093	2.306231433	0.416484698	1.2264274	0.3395918	0.734164	NA
dre-mir-143	0.166657454	-0.955901296	4.0804729	-0.234262	0.814781	NA
RF00001	0	NA	NA	NA	NA	NA
RF00001	0.182570949	0.967652056	4.0804729	0.2371421	0.812547	NA
RF00001	0	NA	NA	NA	NA	NA
RF00001	0.317831496	1.808837541	3.5779923	0.5055454	0.613176	NA
RF00001	0	NA	NA	NA	NA	NA
RF00001	0	NA	NA	NA	NA	NA
dre-mir-135b	5.938060856	0.466203413	0.8008723	0.5821195	0.560486	NA
dre-mir-460	0	NA	NA	NA	NA	NA
RF00001	0	NA	NA	NA	NA	NA
RF00068	0	NA	NA	NA	NA	NA
RF00319	1.230565887	3.733090424	1.9706951	1.8943013	0.058185	NA
RF00001	0	NA	NA	NA	NA	NA
RF00001	0.471562095	-2.373056629	3.0645586	-0.774355	0.438721	NA
dre-mir-34c	0	NA	NA	NA	NA	NA
RF00003	0.157187365	-0.955901296	4.0804729	-0.234262	0.814781	NA
RF01299	1.523921042	-0.893741451	1.7449739	-0.51218	0.608525	NA
RF00003	0	NA	NA	NA	NA	NA
RF00270	0.31437473	-1.788572922	3.5930657	-0.497785	0.618636	NA
RF00001	0	NA	NA	NA	NA	NA
RF00001	0.356300316	0.005884122	3.214964	0.0018302	0.99854	NA
RF00001	0	NA	NA	NA	NA	NA
RF00001	0.356300316	0.005884122	3.214964	0.0018302	0.99854	NA
dre-mir-187	0	NA	NA	NA	NA	NA
NC_002333.16	31.50318652	0.279428087	0.4012557	0.696384	0.486188	0.930833
RF00001	0.316103113	0.005883447	3.3519121	0.0017553	0.9986	NA
RF00004	0	NA	NA	NA	NA	NA
dre-let-7i	1.495661127	1.077139194	1.7501629	0.6154508	0.538257	NA
RF00049	0	NA	NA	NA	NA	NA
RF00001	0	NA	NA	NA	NA	NA
NC_002333.17	6316.732984	0.668539354	0.3798483	1.7600166	0.078405	0.545524
dre-let-7a-4	0	NA	NA	NA	NA	NA
dre-mir-9-6	0	NA	NA	NA	NA	NA
RF00001	0	NA	NA	NA	NA	NA
dre-mir-18c	0.157187365	-0.955901296	4.0804729	-0.234262	0.814781	NA
RF00001	0	NA	NA	NA	NA	NA
NC_002333.18	242.4001844	-0.213985676	0.4401731	-0.48614	0.626868	0.957354
RF00003	0	NA	NA	NA	NA	NA
dre-mir-458	0	NA	NA	NA	NA	NA
RF00151	0.31437473	-1.788572922	3.5930657	-0.497785	0.618636	NA

RF00001	0	NA	NA	NA	NA	NA
RF00001	0	NA	NA	NA	NA	NA
dre-mir-129-1	0	NA	NA	NA	NA	NA
dre-let-7d-1	0	NA	NA	NA	NA	NA
RF00273	0	NA	NA	NA	NA	NA
RF00020	0	NA	NA	NA	NA	NA
RF00096	0.330916733	-1.849579229	3.2489867	-0.569279	0.569167	NA
RF00001	0	NA	NA	NA	NA	NA
RF00001	0	NA	NA	NA	NA	NA
RF00015	0	NA	NA	NA	NA	NA
RF00001	0.166657454	-0.955901296	4.0804729	-0.234262	0.814781	NA
RF00150	0	NA	NA	NA	NA	NA
RF00001	0.157187365	-0.955901296	4.0804729	-0.234262	0.814781	NA
dre-mir-451	0	NA	NA	NA	NA	NA
RF00001	0	NA	NA	NA	NA	NA
RF00001	0	NA	NA	NA	NA	NA
CR847944.3	0.182570949	0.967652056	4.0804729	0.2371421	0.812547	NA
RF00093	4.742081439	-0.487210118	0.9139108	-0.533105	0.593961	NA
dre-mir-10b-2	4.330756601	0.233064551	0.9691394	0.2404861	0.809953	NA
dre-mir-27e	0.814536469	0.637862482	2.230678	0.28595	0.774916	NA
RF00090	0.166657454	-0.955901296	4.0804729	-0.234262	0.814781	NA
dre-mir-130a	0.158915748	0.967652056	4.0804729	0.2371421	0.812547	NA
dre-let-7f	0	NA	NA	NA	NA	NA
RF00068	2.456666658	0.171472539	1.2263118	0.1398279	0.888796	NA
dre-mir-429b	8.948580077	-1.359752513	0.6734286	-2.019149	0.043472	NA
RF00001	0.5046461	-2.45370851	2.7629627	-0.888072	0.374502	NA
RF00001	0	NA	NA	NA	NA	NA
RF00001	0.476747244	2.393381959	3.0525537	0.7840589	0.433006	NA
RF00001	0.182570949	0.967652056	4.0804729	0.2371421	0.812547	NA
RF00001	0.317831496	1.808837541	3.5779923	0.5055454	0.613176	NA
dre-mir-218a-2	0.530029684	-0.939524117	2.7937867	-0.336291	0.736652	NA
dre-mir-732	0	NA	NA	NA	NA	NA
mir214a	0	NA	NA	NA	NA	NA
NC_002333.19	16.82842674	-0.175092226	0.4725024	-0.370564	0.710963	0.968985
dre-mir-10d	3.452956192	-0.119011933	1.0565086	-0.112646	0.910311	NA
RF00001	11.18204147	-0.356265669	0.6308528	-0.564737	0.572253	0.949494
RF01299	0	NA	NA	NA	NA	NA
RF00619	0	NA	NA	NA	NA	NA
RF00001	0	NA	NA	NA	NA	NA
RF00003	0.182570949	0.967652056	4.0804729	0.2371421	0.812547	NA
dre-mir-129-2	0	NA	NA	NA	NA	NA
RF00001	0.365141898	1.973577737	3.3927314	0.5817076	0.560764	NA
RF00340	0.316103113	0.005883447	3.3519121	0.0017553	0.9986	NA
dre-mir-145	21.81166475	-0.372861058	0.4382415	-0.850812	0.394874	0.899087
RF00045	8.898768595	-0.233683939	0.6447654	-0.362432	0.717029	NA
NC_002333.20	10.05050441	0.188680494	0.615502	0.3065474	0.759188	NA
dre-mir-190b	0	NA	NA	NA	NA	NA
dre-mir-125a-1	0.158795395	0.967652056	4.0804729	0.2371421	0.812547	NA
mir96	0.489712128	-0.887840729	2.693685	-0.329601	0.741702	NA
dre-mir-7a-2	0.166657454	-0.955901296	4.0804729	-0.234262	0.814781	NA
RF00137	0	NA	NA	NA	NA	NA
RF01277	1.172805874	1.340994147	1.8747961	0.7152747	0.474439	NA
RF00001	0	NA	NA	NA	NA	NA
RF00270	0.657159727	-2.841717825	2.5166161	-1.129182	0.258821	NA

dre-mir-199-2	0.166657454	-0.955901296	4.0804729	-0.234262	0.814781	NA
dre-mir-27c	0.706628594	2.93253102	2.4623982	1.1909248	0.233683	NA
arhgap19	203.4071735	0.046590429	0.1637634	0.2844985	0.776028	0.975826
dre-mir-16a	0	NA	NA	NA	NA	NA
RF00001	0	NA	NA	NA	NA	NA
dre-mir-130c-2	0	NA	NA	NA	NA	NA
RF00001	0	NA	NA	NA	NA	NA
RF00001	0	NA	NA	NA	NA	NA
dre-mir-727	0	NA	NA	NA	NA	NA
RF00152	0	NA	NA	NA	NA	NA
RF00613	0.157187365	-0.955901296	4.0804729	-0.234262	0.814781	NA
RF00581	3.784382322	-0.564770663	1.066158	-0.529725	0.596303	NA
RF00611	0	NA	NA	NA	NA	NA
dre-mir-218b	0	NA	NA	NA	NA	NA
CR354430.2	0.482760567	-0.866527181	2.7059286	-0.320233	0.748792	NA
dre-mir-217	2.330006987	0.432382524	1.2175105	0.3551366	0.722487	NA
dre-let-7g-1	4.946297216	-0.566432912	0.8543305	-0.663014	0.507322	NA
RF00001	0.52295777	-0.916487494	2.6406233	-0.347072	0.728537	NA
dre-mir-489	0	NA	NA	NA	NA	NA
mir196a-1	0	NA	NA	NA	NA	NA
dre-mir-146a	4.160597841	-1.077646608	0.949349	-1.135143	0.256316	NA
NC_002333.21	6.133047496	-0.204562088	0.7769143	-0.263301	0.792319	NA
RF00001	1.659102521	-0.481707176	1.6148571	-0.298297	0.765476	NA
RF00001	0.158915748	0.967652056	4.0804729	0.2371421	0.812547	NA
RF01296	1.887684329	0.826035529	1.4328941	0.5764805	0.56429	NA
RF00001	0	NA	NA	NA	NA	NA
RF00001	0	NA	NA	NA	NA	NA
dre-mir-210	4.930414715	0.58250929	0.8552164	0.681125	0.495792	NA
RF00001	0.182570949	0.967652056	4.0804729	0.2371421	0.812547	NA
RF00001	0.317831496	1.808837541	3.5779923	0.5055454	0.613176	NA
dre-mir-203b	6.246316196	0.887741531	0.8187012	1.0843291	0.278219	NA
dre-mir-216b	0	NA	NA	NA	NA	NA
dre-mir-146b	0	NA	NA	NA	NA	NA
dre-mir-148	0	NA	NA	NA	NA	NA
RF00001	0	NA	NA	NA	NA	NA
RF00001	0	NA	NA	NA	NA	NA
RF00092	0.500161739	2.450146464	2.7711133	0.8841741	0.376602	NA
RF00020	0	NA	NA	NA	NA	NA
RF00001	0.341486697	1.892317624	3.2124068	0.5890654	0.555817	NA
RF00191	2.651412346	-0.007849672	1.1463432	-0.006848	0.994536	NA
RF00001	0	NA	NA	NA	NA	NA
dre-mir-153b	1.504151694	-0.287778376	1.5448187	-0.186286	0.85222	NA
RF00045	6.108331292	-0.579581822	0.8860703	-0.654104	0.513045	NA
RF00001	27.08154465	-0.08178602	0.3614276	-0.226286	0.820979	0.981628
RF00001	0	NA	NA	NA	NA	NA
RF00548	0	NA	NA	NA	NA	NA
dre-mir-301a	0	NA	NA	NA	NA	NA
RF00001	0.166657454	-0.955901296	4.0804729	-0.234262	0.814781	NA
dre-mir-107a	0	NA	NA	NA	NA	NA
RF01234	11.14858835	0.604505743	0.5929432	1.0195003	0.307966	0.861088
dre-let-7g-2	0	NA	NA	NA	NA	NA
RF00001	0.347458735	-1.908042286	3.4571486	-0.551912	0.581009	NA
RF00001	0.365141898	1.973577737	3.3927314	0.5817076	0.560764	NA
RF00015	0	NA	NA	NA	NA	NA

dre-mir-22b	0	NA	NA	NA	NA	NA
RF00377	0	NA	NA	NA	NA	NA
RF00284	0	NA	NA	NA	NA	NA
NC_002333.22	0.654133044	-1.416854166	2.5841447	-0.548287	0.583495	NA
NC_002333.23	2.36486066	0.8593146	1.297465	0.6623027	0.507777	NA
RF00001	0	NA	NA	NA	NA	NA
dre-mir-128-2	0.365141898	1.973577737	3.3927314	0.5817076	0.560764	NA
dre-mir-9-4	0.492110303	-0.89618148	2.8628934	-0.313033	0.754255	NA
dre-mir-184-2	0	NA	NA	NA	NA	NA
dre-mir-30e-2	0	NA	NA	NA	NA	NA
RF00001	0	NA	NA	NA	NA	NA
NC_002333.24	9.887021234	-0.380338745	0.6112977	-0.622182	0.533822	NA
RF00026	0.339758314	0.005883863	3.2682461	0.0018003	0.998564	NA
dre-mir-23a-3	0	NA	NA	NA	NA	NA
dre-mir-30b	0	NA	NA	NA	NA	NA
RF00001	0	NA	NA	NA	NA	NA
dre-mir-133b	0	NA	NA	NA	NA	NA
RF00001	0	NA	NA	NA	NA	NA
dre-mir-125b-3	5.928270994	0.484135521	0.7748643	0.6248004	0.532102	NA
RF00001	0	NA	NA	NA	NA	NA
RF00001	0	NA	NA	NA	NA	NA
RF00001	0	NA	NA	NA	NA	NA
RF00396	0.158795395	0.967652056	4.0804729	0.2371421	0.812547	NA
RF00001	0	NA	NA	NA	NA	NA
RF00072	0.48983248	-0.887840757	2.6934859	-0.329625	0.741683	NA
RF00001	0.173729368	-0.955901296	4.0804729	-0.234262	0.814781	NA
RF00001	0	NA	NA	NA	NA	NA
dre-mir-181b-3	16.55108373	-0.879784139	0.4736596	-1.857419	0.063252	0.491256
RF00430	2.620924942	-1.609299439	1.2818151	-1.255485	0.209303	NA
dre-mir-22a	22.36856264	0.038741257	0.4119365	0.0940467	0.925072	0.992676
RF00001	0	NA	NA	NA	NA	NA
dre-mir-124-6	13.89424023	0.115575081	0.5506753	0.2098788	0.833762	0.983583
dre-mir-736	0	NA	NA	NA	NA	NA
dre-mir-430a-1	0	NA	NA	NA	NA	NA
dre-mir-122	0.158795395	0.967652056	4.0804729	0.2371421	0.812547	NA
RF00066	5.126070901	0.286874758	0.8238968	0.3481926	0.727696	NA
RF00001	0	NA	NA	NA	NA	NA
RF00001	0	NA	NA	NA	NA	NA
RF00001	0	NA	NA	NA	NA	NA
RF00100	0	NA	NA	NA	NA	NA
RF00001	0.158795395	0.967652056	4.0804729	0.2371421	0.812547	NA
RF00001	0.873165696	1.965547116	2.2171785	0.8865083	0.375344	NA
RF00001	0	NA	NA	NA	NA	NA
RF00001	0	NA	NA	NA	NA	NA
RF01241	1.325436598	-2.680967306	1.8525508	-1.447176	0.147848	NA
dre-mir-2193	0	NA	NA	NA	NA	NA
RF00001	0	NA	NA	NA	NA	NA
RF00001	0	NA	NA	NA	NA	NA
RF00001	0	NA	NA	NA	NA	NA
RF00231	7.046573029	0.605770739	0.7600407	0.7970241	0.425437	NA
RF00001	0	NA	NA	NA	NA	NA
RF00478	1.545407487	0.993496468	1.6449182	0.6039792	0.545857	NA
dre-mir-2198	0.173729368	-0.955901296	4.0804729	-0.234262	0.814781	NA
RF00001	0	NA	NA	NA	NA	NA

RF00001	0.506415768	-0.866825728	2.6674086	-0.324969	0.745204	NA	
RF00099	0	NA	NA	NA	NA	NA	
RF00001	0	NA	NA	NA	NA	NA	
RF00001	0.340386822	-1.882368278	3.2159502	-0.585323	0.558331	NA	
RF00001	0.643404698	1.436206627	2.5980397	0.552804	0.580398	NA	
RF00001	0	NA	NA	NA	NA	NA	
RF00001	0.173729368	-0.955901296	4.0804729	-0.234262	0.814781	NA	
RF00417	1.100311556	-3.586134366	3.9466188	-0.90866	0.36353	NA	
RF00001	0.339758314	0.005883863	3.2682461	0.0018003	0.998564	NA	
RF00429	1.188797441	-1.322727039	1.8673491	-0.708345	0.478731	NA	
RF00001	0	NA	NA	NA	NA	NA	
RF00001	0	NA	NA	NA	NA	NA	
RF00001	0	NA	NA	NA	NA	NA	
RF00001	0	NA	NA	NA	NA	NA	
dre-mir-2187	0	NA	NA	NA	NA	NA	
dre-mir-2192	0	NA	NA	NA	NA	NA	
RF01225	0	NA	NA	NA	NA	NA	
dre-mir-2196	0	NA	NA	NA	NA	NA	
RF00001	0	NA	NA	NA	NA	NA	
RF00302	0.633814256	1.438196278	2.4434004	0.5886044	0.556127	NA	
RF00001	0.491560863	0.853615855	2.865157	0.2979299	0.765757	NA	
RF00001	0	NA	NA	NA	NA	NA	
RF00001	0	NA	NA	NA	NA	NA	
RF00001	0	NA	NA	NA	NA	NA	
RF00302	2.113802594	0.707668695	1.3492795	0.5244789	0.599945	NA	
RF00001	0.680773643	-2.882718692	2.4687055	-1.167705	0.242926	NA	
RF00593	0.825614588	1.890321184	2.2486323	0.8406538	0.400542	NA	
RF00001	0	NA	NA	NA	NA	NA	
RF00001	0	NA	NA	NA	NA	NA	
RF00001	0	NA	NA	NA	NA	NA	
RF00001	1.990324486	-1.631932347	1.6062005	-1.01602	0.30962	NA	
RF00001	0	NA	NA	NA	NA	NA	
RF00001	0	NA	NA	NA	NA	NA	
RF00026	0	NA	NA	NA	NA	NA	
RF01241	0.158795395	0.967652056	4.0804729	0.2371421	0.812547	NA	
RF00001	0	NA	NA	NA	NA	NA	
RF00001	0	NA	NA	NA	NA	NA	
RF00001	0	NA	NA	NA	NA	NA	
RF00001	0	NA	NA	NA	NA	NA	
RF00001	0	NA	NA	NA	NA	NA	
RF00428	0.341366344	1.891903201	3.2128156	0.5888614	0.555954	NA	
RF00001	0	NA	NA	NA	NA	NA	
RF00001	0	NA	NA	NA	NA	NA	
RF00001	0.31759079	1.807926197	3.579045	0.5051421	0.613459	NA	
RF00001	0	NA	NA	NA	NA	NA	
RF00001	0.474778155	0.852397662	2.898154	0.2941175	0.768668	NA	
RF00431	0	NA	NA	NA	NA	NA	
RF00001	0	NA	NA	NA	NA	NA	
RF00001	0.333314908	-1.858695402	3.5126701	-0.52914	0.596708	NA	
RF00001	0.157187365	-0.955901296	4.0804729	-0.234262	0.814781	NA	
RF00001	0	NA	NA	NA	NA	NA	
RF00001	0.658218317	-0.061628513	2.4342948	-0.025317	0.979802	NA	
RF00001	0	NA	NA	NA	NA	NA	
RF00003	0	NA	NA	NA	NA	NA	

RF00001	0	NA	NA	NA	NA	NA
RF00001	0	NA	NA	NA	NA	NA
RF00001	0	NA	NA	NA	NA	NA
RF00006	0.838940531	-0.561526863	2.0217856	-0.277738	0.781213	NA
RF00001	0	NA	NA	NA	NA	NA
RF00001	0	NA	NA	NA	NA	NA
RF00001	0.157187365	-0.955901296	4.0804729	-0.234262	0.814781	NA
RF00001	0	NA	NA	NA	NA	NA
RF00001	0	NA	NA	NA	NA	NA
RF00001	3.034676537	-0.301987747	1.0944881	-0.275917	0.782612	NA
RF00001	0.158915748	0.967652056	4.0804729	0.2371421	0.812547	NA
RF00001	0	NA	NA	NA	NA	NA
RF00154	0	NA	NA	NA	NA	NA
RF00001	0.166657454	-0.955901296	4.0804729	-0.234262	0.814781	NA
RF00001	0	NA	NA	NA	NA	NA
RF00001	0	NA	NA	NA	NA	NA
RF00001	0	NA	NA	NA	NA	NA
RF00001	0	NA	NA	NA	NA	NA
RF00001	0	NA	NA	NA	NA	NA
RF00411	0.665839671	-1.495777257	2.4093426	-0.620824	0.534716	NA
RF00001	0	NA	NA	NA	NA	NA
RF00001	0	NA	NA	NA	NA	NA
dre-mir-2191	0	NA	NA	NA	NA	NA
RF00001	0	NA	NA	NA	NA	NA
RF00001	0	NA	NA	NA	NA	NA
RF00001	0	NA	NA	NA	NA	NA
RF00001	0	NA	NA	NA	NA	NA
RF00001	0.330916733	-1.849579229	3.2489867	-0.569279	0.569167	NA
RF00001	0	NA	NA	NA	NA	NA
RF00001	0	NA	NA	NA	NA	NA
RF00001	0	NA	NA	NA	NA	NA
RF00001	3.284958157	-0.577324491	1.0337626	-0.558469	0.576524	NA
RF00001	0.182570949	0.967652056	4.0804729	0.2371421	0.812547	NA
RF00001	0	NA	NA	NA	NA	NA
RF00001	0	NA	NA	NA	NA	NA
RF00001	0	NA	NA	NA	NA	NA
RF00001	0	NA	NA	NA	NA	NA
RF00001	0	NA	NA	NA	NA	NA
RF00001	0.182570949	0.967652056	4.0804729	0.2371421	0.812547	NA
RF00001	0.476506538	2.394892832	2.8161716	0.8504073	0.395099	NA
RF00001	1.014518634	-0.023592803	1.9308724	-0.012219	0.990251	NA
RF00572	0	NA	NA	NA	NA	NA
RF00001	0	NA	NA	NA	NA	NA
RF00001	0.158915748	0.967652056	4.0804729	0.2371421	0.812547	NA
RF00001	0	NA	NA	NA	NA	NA
RF00026	0	NA	NA	NA	NA	NA
RF00001	0	NA	NA	NA	NA	NA
RF00001	1.471026404	-0.306609088	1.5202188	-0.201687	0.840161	NA
RF00429	0.52295777	-0.916487494	2.6406233	-0.347072	0.728537	NA
RF00001	0	NA	NA	NA	NA	NA
RF00001	0	NA	NA	NA	NA	NA
RF00001	0.333314908	-1.858695402	3.5126701	-0.52914	0.596708	NA
RF00001	0	NA	NA	NA	NA	NA
RF00001	0	NA	NA	NA	NA	NA

RF00001	0	NA	NA	NA	NA	NA
RF00001	0	NA	NA	NA	NA	NA
RF00001	0	NA	NA	NA	NA	NA
RF00001	0	NA	NA	NA	NA	NA
RF00001	0	NA	NA	NA	NA	NA
RF00322	1.645037762	-0.524806043	1.568569	-0.334576	0.737945	NA
RF00001	0	NA	NA	NA	NA	NA
RF00001	0	NA	NA	NA	NA	NA
RF01233	1.21292401	1.413512392	1.8890573	0.7482634	0.454301	NA
RF00001	0.471562095	-2.373056629	3.0645586	-0.774355	0.438721	NA
RF01233	0.476386185	2.392464142	3.0533824	0.7835455	0.433307	NA
RF00001	0	NA	NA	NA	NA	NA
RF00001	0	NA	NA	NA	NA	NA
RF00001	0.157187365	-0.955901296	4.0804729	-0.234262	0.814781	NA
RF00001	0	NA	NA	NA	NA	NA
RF00001	0	NA	NA	NA	NA	NA
RF00443	0.365141898	1.973577737	3.3927314	0.5817076	0.560764	NA
RF00001	0	NA	NA	NA	NA	NA
RF00001	0	NA	NA	NA	NA	NA
RF00001	0	NA	NA	NA	NA	NA
RF00009	1.333689715	-0.736234712	1.8666381	-0.394417	0.693273	NA
RF00001	0.182570949	0.967652056	4.0804729	0.2371421	0.812547	NA
RF00431	0	NA	NA	NA	NA	NA
RF00001	0	NA	NA	NA	NA	NA
RF00001	2.454535932	-0.239433478	1.2394918	-0.193171	0.846825	NA
RF00001	0	NA	NA	NA	NA	NA
RF00001	0	NA	NA	NA	NA	NA
RF00001	0	NA	NA	NA	NA	NA
RF00001	0	NA	NA	NA	NA	NA
RF00001	0	NA	NA	NA	NA	NA
RF00001	0	NA	NA	NA	NA	NA
RF00001	0.476747244	2.393381959	3.0525537	0.7840589	0.433006	NA
RF00001	0	NA	NA	NA	NA	NA
RF00001	0.158795395	0.967652056	4.0804729	0.2371421	0.812547	NA
RF00001	0	NA	NA	NA	NA	NA
RF00001	25.21674435	0.089381908	0.3920707	0.227974	0.819666	0.981594
RF00001	0	NA	NA	NA	NA	NA
RF00001	0.664231641	-2.853946573	2.346554	-1.216229	0.223898	NA
RF00001	0	NA	NA	NA	NA	NA
RF00001	0	NA	NA	NA	NA	NA
RF00001	0.339758314	0.005883863	3.2682461	0.0018003	0.998564	NA
RF00001	0.158915748	0.967652056	4.0804729	0.2371421	0.812547	NA
RF00001	0	NA	NA	NA	NA	NA
RF00001	0.706508242	2.93232624	2.4625218	1.1907818	0.233739	NA
RF00263	1.148909967	1.312490708	1.8001617	0.729096	0.465943	NA
RF00001	0	NA	NA	NA	NA	NA
RF00001	0	NA	NA	NA	NA	NA
RF00001	0	NA	NA	NA	NA	NA
RF00001	0	NA	NA	NA	NA	NA
RF00001	0.158795395	0.967652056	4.0804729	0.2371421	0.812547	NA
RF00001	0	NA	NA	NA	NA	NA
RF00001	0	NA	NA	NA	NA	NA
RF00618	0	NA	NA	NA	NA	NA
RF00001	0	NA	NA	NA	NA	NA

RF00001	0	NA	NA	NA	NA	NA
RF00001	0	NA	NA	NA	NA	NA
RF00001	0	NA	NA	NA	NA	NA
RF00001	0	NA	NA	NA	NA	NA
RF00001	0	NA	NA	NA	NA	NA
RF00001	0.158795395	0.967652056	4.0804729	0.2371421	0.812547	NA
RF00001	0	NA	NA	NA	NA	NA
RF00001	0	NA	NA	NA	NA	NA
RF00001	0.182570949	0.967652056	4.0804729	0.2371421	0.812547	NA
RF00001	0	NA	NA	NA	NA	NA
RF00001	0	NA	NA	NA	NA	NA
RF00001	0.157187365	-0.955901296	4.0804729	-0.234262	0.814781	NA
RF00001	0.347458735	-1.908042286	3.4571486	-0.551912	0.581009	NA
RF00091	0.157187365	-0.955901296	4.0804729	-0.234262	0.814781	NA
RF00408	1.501522856	-2.90329683	1.7761284	-1.634621	0.102129	NA
RF00001	0	NA	NA	NA	NA	NA
RF00001	0	NA	NA	NA	NA	NA
RF00001	0	NA	NA	NA	NA	NA
RF00001	0.476747244	2.393381959	3.0525537	0.7840589	0.433006	NA
RF00001	0	NA	NA	NA	NA	NA
RF00001	0	NA	NA	NA	NA	NA
RF00001	0.356300316	0.005884122	3.214964	0.0018302	0.99854	NA
RF00001	0	NA	NA	NA	NA	NA
RF00001	0	NA	NA	NA	NA	NA
RF00001	0	NA	NA	NA	NA	NA
RF00001	0	NA	NA	NA	NA	NA
RF00001	0	NA	NA	NA	NA	NA
RF00001	0	NA	NA	NA	NA	NA
RF00026	1.298595336	-1.598743955	1.8947659	-0.843769	0.398799	NA
RF00001	0	NA	NA	NA	NA	NA
RF00001	0	NA	NA	NA	NA	NA
RF00001	0.332524763	0.005883742	3.292841	0.0017868	0.998574	NA
RF00001	0	NA	NA	NA	NA	NA
RF00001	0.522329263	0.999930388	2.8066578	0.3562709	0.721638	NA
RF00001	0	NA	NA	NA	NA	NA
RF00020	0	NA	NA	NA	NA	NA
RF00001	0	NA	NA	NA	NA	NA
RF00004	0	NA	NA	NA	NA	NA
RF00001	0	NA	NA	NA	NA	NA
RF00001	0	NA	NA	NA	NA	NA
RF00001	0	NA	NA	NA	NA	NA
RF00001	0	NA	NA	NA	NA	NA
RF00001	0	NA	NA	NA	NA	NA
RF00001	2.149603552	-1.218704386	1.4777127	-0.824724	0.409529	NA
RF00582	0	NA	NA	NA	NA	NA
RF00001	0	NA	NA	NA	NA	NA
RF00001	0	NA	NA	NA	NA	NA
RF00001	0.655741074	0.077611818	2.4374018	0.031842	0.974598	NA
RF00001	0.341366344	1.891903201	3.2128156	0.5888614	0.555954	NA
RF01192	0.166657454	-0.955901296	4.0804729	-0.234262	0.814781	NA
RF00001	0.182570949	0.967652056	4.0804729	0.2371421	0.812547	NA
RF00001	0	NA	NA	NA	NA	NA
RF00322	0	NA	NA	NA	NA	NA
RF00001	0	NA	NA	NA	NA	NA

RF00001	0.158915748	0.967652056	4.0804729	0.2371421	0.812547	NA
RF00001	0	NA	NA	NA	NA	NA
RF00020	0.173729368	-0.955901296	4.0804729	-0.234262	0.814781	NA
RF00001	0	NA	NA	NA	NA	NA
RF00001	0	NA	NA	NA	NA	NA
RF00001	0	NA	NA	NA	NA	NA
RF00218	0	NA	NA	NA	NA	NA
RF00001	0	NA	NA	NA	NA	NA
RF00001	0.158795395	0.967652056	4.0804729	0.2371421	0.812547	NA
RF00001	0	NA	NA	NA	NA	NA
RF00001	0	NA	NA	NA	NA	NA
RF00001	0	NA	NA	NA	NA	NA
RF00001	0	NA	NA	NA	NA	NA
RF00001	0.173729368	-0.955901296	4.0804729	-0.234262	0.814781	NA
RF00020	0.157187365	-0.955901296	4.0804729	-0.234262	0.814781	NA
RF00026	0	NA	NA	NA	NA	NA
RF00001	0	NA	NA	NA	NA	NA
RF00001	0	NA	NA	NA	NA	NA
RF00001	0	NA	NA	NA	NA	NA
RF00001	0	NA	NA	NA	NA	NA
RF00001	8.315364946	0.361869007	0.6682622	0.5415075	0.588158	NA
RF00001	0	NA	NA	NA	NA	NA
RF00001	0	NA	NA	NA	NA	NA
RF00001	0	NA	NA	NA	NA	NA
RF00001	0	NA	NA	NA	NA	NA
RF00001	0	NA	NA	NA	NA	NA
RF00001	0	NA	NA	NA	NA	NA
RF00001	0	NA	NA	NA	NA	NA
RF00568	4.483934258	-0.145670252	0.9869913	-0.14759	0.882666	NA
RF00009	4.031570988	0.745354741	1.0159156	0.7336778	0.463145	NA
RF00001	0	NA	NA	NA	NA	NA
RF00001	0	NA	NA	NA	NA	NA
RF00001	0	NA	NA	NA	NA	NA
RF00001	0.182570949	0.967652056	4.0804729	0.2371421	0.812547	NA
dre-mir-2195	0	NA	NA	NA	NA	NA
RF00001	2.485036883	0.218266609	1.2044806	0.1812122	0.856201	NA
RF00001	0.317831496	1.808837541	3.5779923	0.5055454	0.613176	NA
RF01192	0.476747244	2.393381959	3.0525537	0.7840589	0.433006	NA
RF00001	3.618300804	-0.011945638	0.9817127	-0.012168	0.990291	NA
RF00001	1.121918354	-0.422914697	1.8509454	-0.228486	0.819269	NA
RF00001	0.158795395	0.967652056	4.0804729	0.2371421	0.812547	NA
RF00001	0	NA	NA	NA	NA	NA
RF00001	0	NA	NA	NA	NA	NA
RF00440	0	NA	NA	NA	NA	NA
RF00001	0	NA	NA	NA	NA	NA
RF00001	0	NA	NA	NA	NA	NA
RF00001	0	NA	NA	NA	NA	NA
RF00001	0	NA	NA	NA	NA	NA
RF00001	0	NA	NA	NA	NA	NA
RF00001	0	NA	NA	NA	NA	NA
RF00001	0	NA	NA	NA	NA	NA
RF00001	1.968321855	0.965031282	1.4110895	0.6838909	0.494044	NA
RF00001	0	NA	NA	NA	NA	NA
RF00001	0	NA	NA	NA	NA	NA

RF00270	0.157187365	-0.955901296	4.0804729	-0.234262	0.814781	NA	
RF00001	0	NA	NA	NA	NA	NA	
RF00302	0	NA	NA	NA	NA	NA	
RF00001	0.332524763	0.005883742	3.292841	0.0017868	0.998574	NA	
RF00001	0	NA	NA	NA	NA	NA	
RF00001	0	NA	NA	NA	NA	NA	
RF00001	0	NA	NA	NA	NA	NA	
dre-mir-2197	0	NA	NA	NA	NA	NA	
RF00001	0	NA	NA	NA	NA	NA	
RF00001	0	NA	NA	NA	NA	NA	
RF00001	0	NA	NA	NA	NA	NA	
RF00001	0	NA	NA	NA	NA	NA	
RF00001	0	NA	NA	NA	NA	NA	
RF00613	3.436399648	-0.432229431	1.0343392	-0.41788	0.676035	NA	
RF00553	0.173729368	-0.955901296	4.0804729	-0.234262	0.814781	NA	
RF00001	0	NA	NA	NA	NA	NA	
RF00001	0	NA	NA	NA	NA	NA	
RF00001	0	NA	NA	NA	NA	NA	
RF00001	0	NA	NA	NA	NA	NA	
dre-mir-2184	2.348628387	2.615352647	1.4633998	1.7871758	0.073909	NA	
RF00564	0.333314908	-1.858695402	3.5126701	-0.52914	0.596708	NA	
RF00191	0.673891106	1.487913167	2.4008011	0.619757	0.535418	NA	
RF00001	0.158915748	0.967652056	4.0804729	0.2371421	0.812547	NA	
RF00001	0.173729368	-0.955901296	4.0804729	-0.234262	0.814781	NA	
RF00001	0.173729368	-0.955901296	4.0804729	-0.234262	0.814781	NA	
RF00001	0	NA	NA	NA	NA	NA	
RF00001	0.347458735	-1.908042286	3.4571486	-0.551912	0.581009	NA	
RF00001	0	NA	NA	NA	NA	NA	
dre-mir-735	0	NA	NA	NA	NA	NA	
RF00001	0	NA	NA	NA	NA	NA	
RF00001	0	NA	NA	NA	NA	NA	
RF00001	0	NA	NA	NA	NA	NA	
RF00001	0	NA	NA	NA	NA	NA	
RF00001	0	NA	NA	NA	NA	NA	
RF00001	0	NA	NA	NA	NA	NA	
RF00001	0	NA	NA	NA	NA	NA	
RF00001	0.158795395	0.967652056	4.0804729	0.2371421	0.812547	NA	
RF00001	0	NA	NA	NA	NA	NA	
RF00001	0	NA	NA	NA	NA	NA	
RF00001	0.158915748	0.967652056	4.0804729	0.2371421	0.812547	NA	
RF00001	8.065268707	-0.15980402	0.6734373	-0.237296	0.812427	NA	
RF00599	0.182570949	0.967652056	4.0804729	0.2371421	0.812547	NA	
RF00001	0	NA	NA	NA	NA	NA	
BX537109.1	7.011048568	0.050582143	0.7616843	0.0664083	0.947053	NA	
zgc:153681	6.14302574	-0.374967155	0.7806631	-0.480319	0.631001	NA	
si:dkey-238d18.7	0	NA	NA	NA	NA	NA	
si:ch211-168d23.3	28.62639026	-0.044205031	0.3907866	-0.113118	0.909937	0.990702	
igfn1.4	0.650905698	-0.045771139	2.5873401	-0.01769	0.985886	NA	
tspan5a	374.8752033	0.142087361	0.1211419	1.1729007	0.240836	0.809224	
nectin1b	371.5106436	0.163135916	0.1359418	1.200042	0.230123	0.797833	
si:ch211-229i14.2	0.49144051	0.855669345	2.6923358	0.3178167	0.750624	NA	
si:ch211-106k21.5	10.91077256	0.103042583	0.713832	0.1443513	0.885223	NA	
si:dkey-7e14.3	5.336619774	-0.179425931	0.8063783	-0.222508	0.823918	NA	
plekhg6	54.04979102	-0.054238519	0.2933107	-0.184918	0.853293	0.985174	

plxnd1	23.2310923	-0.261966256	0.4145438	-0.631939	0.527427	0.941361
hmcn2	3.395883513	0.630730556	1.1821412	0.5335493	0.593653	NA
fgf20b	46.78241523	-0.22473626	0.2870265	-0.782981	0.433638	0.913667
ttc3	1061.457317	-0.079630983	0.0907774	-0.877211	0.380372	0.895192
kcnn4	13.81646363	0.488648834	0.518082	0.9431881	0.345585	0.878467
RF00001	0	NA	NA	NA	NA	NA
BX539307.2	1.810746688	2.157278989	1.6475449	1.3093901	0.190402	NA
cd302	109.814547	0.049131947	0.1881697	0.2611044	0.794012	0.977937
slc37a1	222.0093661	0.310044989	0.1443199	2.148317	0.031689	0.345305
slco5a1a	1487.784454	0.127862654	0.1004685	1.2726639	0.203137	0.770099
RF00006	0	NA	NA	NA	NA	NA
MTERF1	116.1097694	-0.094809773	0.1894139	-0.500543	0.616693	0.95688
si:ch211-266i6.3	405.6127849	-0.012603847	0.114874	-0.109719	0.912632	0.991119
CR792417.1	35.42424776	0.013602681	0.3623619	0.0375389	0.970055	0.996315
umod	0.349228403	0.005884014	3.2372587	0.0018176	0.99855	NA
trim33l	66.14565283	0.009216202	0.2621788	0.0351524	0.971958	0.996315
RF00017	0	NA	NA	NA	NA	NA
si:ch211-42i6.2	3.500761202	0.393235041	1.0328891	0.3807137	0.703416	NA
si:ch73-267c23.10	9.662793144	-0.247837596	0.6313733	-0.392537	0.694661	NA
wrap53	193.2599042	-0.244258063	0.1538675	-1.587457	0.112409	0.627726
ccbe1	28.21762053	-0.180307106	0.3611591	-0.499246	0.617606	0.957354
si:ch73-206d17.1	35.60313336	0.454509256	0.3226568	1.4086463	0.15894	0.709613
znf385d	59.92512545	-0.178383647	0.2582801	-0.69066	0.48978	0.931926
si:ch211-286b5.9	1.528006315	-0.313383006	1.55636	-0.201356	0.84042	NA
rskrb	69.85715509	-0.067799699	0.2400862	-0.282397	0.777639	0.975942
ACVR1C	13.63599758	0.654941663	0.5390348	1.2150267	0.224356	0.79161
relb	342.5528615	-0.329876538	0.1227784	-2.686764	0.007215	0.133582
si:dkey-3h2.4	5.024967491	0.811117222	0.8799433	0.9217835	0.356642	NA
vkorc1	53.96107865	0.359700966	0.2883872	1.2472847	0.212293	0.780251
FBXO48	227.8215732	-0.039524902	0.1568328	-0.252019	0.801026	0.979377
opn8a	2.030325524	0.485585208	1.440622	0.3370663	0.736067	NA
mgp	5.388620869	0.102184602	0.8347452	0.1224141	0.902571	NA
gsg1l2a	36.15674008	0.118825941	0.3248038	0.3658392	0.714485	0.96909
grin2da	365.6391494	-0.047172953	0.1316244	-0.358391	0.720051	0.969318
ntrk3b	450.0741051	0.16326542	0.111999	1.4577402	0.144912	0.688986
gpr156	26.64423348	-0.024382718	0.3879614	-0.062848	0.949887	0.99381
prr13	386.6986218	-0.207978783	0.1390487	-1.495726	0.134725	0.670689
nkd3	0.365141898	1.973577737	3.3927314	0.5817076	0.560764	NA
si:ch211-226h8.4	0	NA	NA	NA	NA	NA
nat16	1275.636005	0.337624353	0.0922044	3.6616943	0.000251	0.010194
si:ch73-144d13.4	54.67389438	0.043935144	0.2979698	0.1474483	0.882778	0.988508
si:dkey-120c6.5	18.29212598	-0.245739561	0.4598698	-0.534368	0.593087	0.954434
F2R (1 of many)	24.60156568	0.437207599	0.392498	1.1139104	0.265318	0.831302
si:dkeyp-121d2.7	49.67465779	-0.038440285	0.2821135	-0.136258	0.891617	0.989291
zgc:171781	0.31759079	1.807926197	3.579045	0.5051421	0.613459	NA
kcnj1a.3	181.947858	-0.211746922	0.1745292	-1.213246	0.225036	0.792185
hhla2b.2	233.9746194	-0.183143807	0.1385687	-1.321682	0.186274	0.750697
si:rp71-1c10.10	4.59259124	-1.589265973	0.9352558	-1.699285	0.089266	NA
si:ch211-236p5.2	10.44274423	-0.036194943	0.5879862	-0.061557	0.950915	NA
CU469420.1	10.51531802	0.095429761	0.6292782	0.1516496	0.879463	NA
fhdc4	103.0180729	0.082015418	0.2105351	0.389557	0.696864	0.96736
F2R (1 of many)	0	NA	NA	NA	NA	NA
bsnd	18.43435682	0.024246908	0.4667037	0.0519535	0.958566	0.994935
CR925773.1	9.128624045	0.336149498	0.7087456	0.474288	0.635295	NA

taar12i	0.330916733	-1.849579229	3.2489867	-0.569279	0.569167	NA
ndst2a	848.3155508	-0.013672339	0.0998236	-0.136965	0.891058	0.989291
acyp2	2.124300278	-1.709794783	1.4128316	-1.21019	0.226206	NA
si:dkey-4p15.5	358.4482244	-0.138659044	0.1284801	-1.079226	0.280487	0.843423
si:ch211-282j17.5	0	NA	NA	NA	NA	NA
cnsta	112.8602538	0.040552719	0.2167913	0.1870588	0.851615	0.985174
scg3	2144.314245	0.025753648	0.0775637	0.3320323	0.739865	0.972943
znf1137	4.638486244	-0.182339519	0.8674735	-0.210196	0.833515	NA
FAM107A	30.87666812	-0.039593155	0.3492365	-0.113371	0.909737	0.990702
si:dkey-27j5.6	0.173729368	-0.955901296	4.0804729	-0.234262	0.814781	NA
proser1	1021.365601	0.013519315	0.0894333	0.1511665	0.879844	0.987881
si:ch211-63b16.4	23.54024174	0.744093305	0.4592984	1.6200652	0.105218	0.613382
erlec1	757.6238069	-0.01862036	0.0943204	-0.197416	0.843502	0.985174
cabcoco1	6.229597274	-0.027451606	0.7897174	-0.034761	0.97227	NA
CU633991.1	2.131481258	-0.271770984	1.3260227	-0.204952	0.83761	NA
si:ch211-67e16.4	369.1278643	-0.003196374	0.1155012	-0.027674	0.977922	0.996762
si:dkey-274m17.3	21.84411503	-0.159062944	0.4178591	-0.380662	0.703454	0.968134
CABZ01071939.1	100.5682914	0.018444195	0.2138639	0.0862427	0.931274	0.992702
srrm4	639.9714249	-0.047857418	0.0991779	-0.482541	0.629422	0.957354
bri3	1011.790334	-0.238155166	0.0963135	-2.472709	0.013409	0.207707
si:ch211-157b11.12	179.5489002	-0.335312331	0.1532605	-2.187859	0.02868	0.328405
si:dkey-102g19.3	20.15395685	-0.357196088	0.4606099	-0.775485	0.438053	0.915284
zgc:101566	629.7837537	-0.053552299	0.0991308	-0.540219	0.589046	0.953345
cux2b	29.43800286	-0.175686586	0.351141	-0.500331	0.616842	0.956957
zp2.1	1.180506295	0.366333622	1.928949	0.1899136	0.849377	NA
si:dkey-83f18.9	0	NA	NA	NA	NA	NA
pou6f2	26.98696801	0.630514523	0.3767943	1.6733656	0.094255	0.583986
si:ch211-167j9.5	5.252919949	0.72971976	0.8265429	0.8828577	0.377313	NA
isg15	6.424823202	-0.105571628	0.8002381	-0.131925	0.895043	NA
or111-8	0	NA	NA	NA	NA	NA
RF00026	0.158795395	0.967652056	4.0804729	0.2371421	0.812547	NA
si:dkey-246e3.4	0.157187365	-0.955901296	4.0804729	-0.234262	0.814781	NA
cald1b	35.59582354	0.032500678	0.3285298	0.0989277	0.921196	0.992138
dbx1a	216.7292979	0.018179297	0.1457734	0.1247093	0.900754	0.990121
mterf2	100.4724571	0.007203075	0.1974061	0.0364886	0.970893	0.996315
BX950205.1	0	NA	NA	NA	NA	NA
srsf10b	245.1957295	-0.232607032	0.149886	-1.551893	0.120688	0.645578
taar12c	0.316103113	0.005883447	3.3519121	0.0017553	0.9986	NA
med11	214.8477557	-0.287732313	0.1694296	-1.698242	0.089462	0.572876
si:ch211-236p5.3	0.841338706	-0.563977448	2.2263736	-0.253317	0.800024	NA
si:ch211-282j17.13	3.802837586	-0.646157724	1.0163545	-0.63576	0.524933	NA
CABZ01046949.1	0	NA	NA	NA	NA	NA
si:ch211-212k18.8	110.2582918	0.266309463	0.1879563	1.4168689	0.156521	0.706794
prpf3	488.4149005	0.101368473	0.1130278	0.8968457	0.369801	0.889487
adgrf7	12.9963927	1.658034394	0.5735885	2.8906339	0.003845	0.084754
znf1055	24.62849896	-0.001448385	0.3970814	-0.003648	0.99709	0.99906
tdg.2	495.6310506	0.106089786	0.1028235	1.0317663	0.302182	0.857048
dthd1	5.058281315	0.671938937	0.8843914	0.7597755	0.447389	NA
cx52.9	39.88216671	0.563151512	0.3606062	1.5616801	0.118363	0.641743
tnfaip8l1	225.2947626	-0.181616071	0.1522122	-1.193177	0.2328	0.801255
hdac10	164.9673974	-0.140808257	0.1632064	-0.862762	0.388268	0.896271
si:ch211-165g14.1	179.5695199	-0.180642498	0.1725363	-1.046982	0.295108	0.851519
si:dkey-83f18.10	0	NA	NA	NA	NA	NA
klhl13	28.1301933	0.612489765	0.3595529	1.703476	0.088479	0.569447

pkig	341.9290508	-0.021341195	0.1339583	-0.159312	0.873423	0.987858
mogat3a	160.8508424	-0.070587045	0.1655453	-0.426391	0.669823	0.965223
si:ch211-132p1.4	3.411566499	0.122552101	1.0306589	0.1189066	0.905349	NA
fbxw10	4.937366276	0.579792674	0.8448495	0.6862674	0.492544	NA
CU179643.1	2.865263104	1.272037305	1.2029355	1.0574443	0.290309	NA
si:dkey-92f12.2	336.54096	0.040045819	0.1179086	0.3396345	0.734132	0.971993
mtnr1bb	15.97618785	0.117017388	0.4745948	0.2465627	0.805247	0.979883
znf970	17.7569909	-0.212072521	0.4601837	-0.460843	0.644911	0.96029
BX546500.1	0.681753165	0.003445546	2.2907653	0.0015041	0.9988	NA
si:ch211-116o3.3	0	NA	NA	NA	NA	NA
BX323559.1	0.703759051	-1.526884723	2.5277341	-0.604053	0.545809	NA
mast3b	216.5559852	-0.040894822	0.1565825	-0.261171	0.793961	0.977937
ptprjb.2	0	NA	NA	NA	NA	NA
prodhb	49.773509	0.090103549	0.2814928	0.3200918	0.748899	0.97419
si:ch211-155o21.3	10.7646645	0.153431455	0.5953802	0.2577033	0.796636	NA
RF00001	0	NA	NA	NA	NA	NA
zp2.5	3.470233508	0.356531717	1.1190266	0.3186088	0.750023	NA
ch25hl3	257.4379907	-0.222695627	0.1428929	-1.55848	0.11912	0.64321
si:dkey-90l23.2	3.324643703	-0.338370208	1.1511016	-0.293953	0.768794	NA
CABZ01115881.1	108.4956628	0.099004738	0.2013972	0.4915894	0.62301	0.957354
dbf4b	84.14290216	0.001935567	0.2157478	0.0089714	0.992842	0.998437
irs4a	116.0193103	-0.218162137	0.2002314	-1.08955	0.275911	0.839541
hist1h2a11	4.550913611	0.997591157	0.9360277	1.065771	0.286527	NA
nlrc3l1	251.5593899	-0.1317414	0.1360678	-0.968204	0.332943	0.874926
si:dkey-201l21.2	0	NA	NA	NA	NA	NA
zgc:172051	165.4204824	0.501943976	0.1680933	2.9861028	0.002826	0.068979
kcna7	106.9021916	0.312961056	0.19617	1.5953563	0.110633	0.625514
omga	7.839758863	0.469734922	0.7455881	0.6300194	0.528682	NA
si:cabz01069012.2	16.63477426	-0.518562391	0.4668226	-1.110834	0.26664	0.832673
CABZ01077217.2	3.552211862	-0.002907174	1.0321791	-0.002817	0.997753	NA
nrg2b	510.9924481	0.022941066	0.1018359	0.2252748	0.821766	0.982026
si:ch211-113a14.11	23.01997756	0.166842761	0.4137114	0.4032829	0.68674	0.966376
dre-mir-26a-2	1.973157231	1.00524485	1.3446592	0.7475834	0.454711	NA
zgc:136963	40.89773392	0.027786701	0.2997543	0.0926983	0.926143	0.992702
si:ch73-269m14.4	0	NA	NA	NA	NA	NA
si:dkey-266f7.10	3.639086462	-0.835415989	1.0474994	-0.797534	0.425141	NA
CR846087.1	4.915670119	1.463763021	0.8990625	1.6280993	0.103504	NA
cabz01093075.1	0.979585893	-0.937756642	1.9970899	-0.469562	0.638668	NA
psma3	2191.045903	-0.012193695	0.0777565	-0.156819	0.875387	0.987881
gckr	0.965280428	-1.0079287	2.0820225	-0.48411	0.628308	NA
vav3a	3.861349715	0.633340446	0.9726405	0.6511557	0.514946	NA
im:7147486	592.7155526	-0.129616214	0.0996792	-1.300333	0.193487	0.757219
stk19	41.16518747	-0.106220434	0.298714	-0.355592	0.722146	0.970155
rnh1	14.69461759	-0.224530955	0.4935138	-0.454964	0.649135	0.960708
or111-3	4.020036043	0.035933245	0.9556071	0.0376025	0.970005	NA
RF00017	0	NA	NA	NA	NA	NA
hs3st3b1b	839.7837148	0.219286009	0.0972581	2.2546811	0.024153	0.299817
chrng	150.9791239	0.004681771	0.1842668	0.0254076	0.97973	0.99687
ttc41	3.951389548	0.02458885	0.9624272	0.0255488	0.979617	NA
cbln11	141.2541944	-0.084820879	0.2130062	-0.398209	0.690476	0.967097
mkrn2os.1	16.72213619	-0.662085897	0.4616464	-1.434184	0.15152	0.700127
crygm2d21	15307.06832	0.102705645	0.1014293	1.0125837	0.311259	0.863542
si:ch211-125m10.6	16.41021307	0.363936567	0.5264682	0.6912793	0.48939	0.931926
si:dkey-175g6.2	221.6346996	0.730059542	0.199014	3.6683836	0.000244	0.010076

edn3b	4.167473589	-0.093471996	0.9409874	-0.099334	0.920873	NA
ice2	168.1248391	-0.072735554	0.1611406	-0.451379	0.651716	0.960708
ascc3	504.420624	-0.017182508	0.1183756	-0.145152	0.884591	0.988801
si:ch211-197l9.5	3.623139435	1.107099738	1.013463	1.0923929	0.27466	NA
trim63b	244.5233622	0.244279521	0.1514772	1.6126485	0.106821	0.616004
dnajc12	62.63227439	-0.176521827	0.2503385	-0.705133	0.480728	0.927601
taar14a	0.173729368	-0.955901296	4.0804729	-0.234262	0.814781	NA
smim15	528.5552841	-0.228446032	0.1043568	-2.189086	0.028591	0.328246
si:dkey-126g1.7	17.4133631	-0.597703876	0.473915	-1.261205	0.207235	0.775175
prob1	53.41732332	0.179826871	0.2821639	0.6373135	0.523921	0.939336
BMERB1	200.7831153	-0.106533449	0.1484405	-0.717684	0.472952	0.924993
CR847543.1	14.12689411	0.216529493	0.5223836	0.4145028	0.678506	0.965257
si:dkeyp-97b10.3	118.6747077	-0.254578165	0.2101543	-1.211387	0.225747	0.793245
CU075735.1	0	NA	NA	NA	NA	NA
znf1023	2.670703538	1.14931245	1.1680276	0.9839771	0.325127	NA
im:7151449	66.43993356	0.177975218	0.2623914	0.6782815	0.497593	0.933353
si:ch211-238p8.24	0.173729368	-0.955901296	4.0804729	-0.234262	0.814781	NA
RF00274	0.547712847	2.559541715	2.9076406	0.8802813	0.378707	NA
SAMD4B	1297.650035	0.150976903	0.0855942	1.7638686	0.077754	0.543372
tmem68	308.5781182	0.359214403	0.1223263	2.9365264	0.003319	0.077033
si:ch1073-513e17.1	30.61335582	0.219707611	0.3758638	0.5845404	0.558857	0.948478
phyh	972.4374601	0.183769138	0.0976653	1.8816219	0.059887	0.478654
znf1152	44.68250214	-0.36674571	0.3228389	-1.136002	0.255956	0.824633
prodha	162.1290924	0.123327656	0.1599935	0.7708291	0.440808	0.916336
LO018205.1	8.597751049	0.000306059	0.6579053	0.0004652	0.999629	NA
gb:bc139872	255.3714345	-0.026895953	0.1327905	-0.202544	0.839491	0.984979
RGS9BP	181.6326694	0.556603358	0.1565583	3.5552457	0.000378	0.014142
gsdmea	9.173602215	-0.172635799	0.6452529	-0.267547	0.789048	NA
mdc1	248.6369352	-0.146423363	0.1358249	-1.078031	0.28102	0.84357
top1	21.58863797	0.057260795	0.4088881	0.1400402	0.888628	0.989291
pdgfba	76.36177963	0.238615152	0.2342844	1.018485	0.308448	0.861088
mfsd10	125.7137544	-0.226934311	0.1831678	-1.238942	0.215367	0.782987
bahd1	470.4178285	0.018576563	0.1106902	0.1678248	0.866721	0.986958
gucy1b1	329.8033107	0.040698573	0.1208689	0.3367168	0.73633	0.972535
itprid1	47.85962864	0.417180953	0.2779891	1.5007097	0.133431	0.667982
ddhd1a	342.876144	0.153786236	0.120266	1.2787171	0.200997	0.766855
MYADM	436.1189494	-0.055094169	0.1077198	-0.511458	0.60903	0.956718
scn11aa	4.934895821	-0.7959014	0.8661127	-0.918935	0.35813	NA
znf521	33.21138125	0.087247304	0.3378629	0.2582329	0.796227	0.978498
sult6b1	764.4129292	-0.13911481	0.1009902	-1.377508	0.168355	0.725919
zmp:0000001228	57.19903887	-0.096568106	0.2637083	-0.366193	0.714221	0.96909
itga2.3	54.45767413	-0.09830191	0.2981121	-0.329748	0.74159	0.973154
si:ch211-266k8.4	63.09293829	-0.055675253	0.2440063	-0.228171	0.819513	0.981594
dap1b	987.9101795	-0.395319464	0.1121481	-3.524975	0.000424	0.015595
irgq1	17.71091877	-0.027895298	0.4828439	-0.057773	0.95393	0.994255
atad3	219.9868285	-0.192178773	0.1626798	-1.181331	0.237471	0.806474
si:dkey-187k19.2	0.158795395	0.967652056	4.0804729	0.2371421	0.812547	NA
si:dkey-264d12.5	59.60160619	-0.072323523	0.2551302	-0.283477	0.776811	0.975826
dpyda.3	35.38787743	-0.404771914	0.3355438	-1.206316	0.227696	0.794934
stk35	109.5239095	0.059641455	0.2031202	0.2936264	0.769043	0.975687
qdprb2	0.989994219	0.997631756	1.9011773	0.5247442	0.599761	NA
si:ch211-232i5.3	107.7636401	-0.165310287	0.1928132	-0.85736	0.391246	0.897688
mhc1zda	245.8644377	-0.047584948	0.1643397	-0.289552	0.772159	0.975687
lins1	35.2893399	-0.304191299	0.3287149	-0.925396	0.35476	0.882105

AL831726.1	5.346020839	-0.739253794	0.8287147	-0.892049	0.372367	NA
ier2b	266.1356521	0.261341897	0.1686406	1.549697	0.121214	0.647458
RF00426	0	NA	NA	NA	NA	NA
erfl1	199.1986835	0.154977937	0.1527962	1.0142786	0.31045	0.862681
fubp3	939.3618494	-0.042372655	0.0942469	-0.449592	0.653005	0.960792
si:dkey-7i4.1	7.859430206	-0.410752579	0.7063001	-0.581555	0.560866	NA
gig2o	10.37207632	-0.21986347	0.624668	-0.351969	0.724862	NA
stambpb	219.257318	-0.196654096	0.1463555	-1.343674	0.179054	0.739994
si:dkey-100n23.5	147.4999158	-0.303005032	0.176534	-1.716412	0.086087	0.563109
crygm2d2	20405.00213	-0.016737986	0.2368398	-0.070672	0.943659	0.993364
CR381646.1	0	NA	NA	NA	NA	NA
pik3c2b	932.5283436	-0.055117086	0.089575	-0.615318	0.538345	0.943336
CABZ01029822.1	10.5200516	0.605961514	0.6050876	1.0014442	0.316612	NA
tnni3k	15.03310169	0.060697495	0.4972969	0.1220549	0.902856	0.990121
pimr14	0	NA	NA	NA	NA	NA
TCF24	45.24868285	-0.233127344	0.2926912	-0.796496	0.425744	0.909437
si:ch211-269k10.2	320.1858149	-0.280426157	0.147396	-1.902536	0.057101	0.468896
si:dkey-24l17.3	0	NA	NA	NA	NA	NA
si:dkeyp-118a3.2	81.870063	0.163337488	0.217984	0.7493094	0.453671	0.920041
BX649442.1	0.166657454	-0.955901296	4.0804729	-0.234262	0.814781	NA
FP102309.1	12.26126941	-0.161946994	0.547286	-0.295909	0.767299	0.975687
atxn1l	6.406261953	0.21271619	0.7697099	0.2763589	0.782272	NA
mbd6	81.96997443	0.093102227	0.2178009	0.4274648	0.669041	0.965031
klc4	690.4967717	-0.183325865	0.0987726	-1.85604	0.063448	0.492164
carmil3	432.0579377	0.128624531	0.1073726	1.197927	0.230945	0.799177
si:ch73-234b20.5	193.2030119	-0.105678102	0.1651574	-0.639863	0.522262	0.939056
zgc:64002	15.33902264	-0.133470711	0.4991653	-0.267388	0.789171	0.976965
BX004816.2	0	NA	NA	NA	NA	NA
cubn	257.4042794	-0.455549216	0.146829	-3.102583	0.001918	0.051894
RF00017	0	NA	NA	NA	NA	NA
micall1b.2	34.15353618	0.074551157	0.3670745	0.2030954	0.83906	0.984979
si:dkey-11k2.7	190.2802483	-0.241922895	0.5067472	-0.477404	0.633075	0.957356
FO704748.1	6.099371524	-0.097228816	0.7669566	-0.126772	0.899121	NA
si:ch73-334d15.2	11.94177277	0.827976844	0.5536757	1.4954183	0.134805	0.670689
RF00281	0	NA	NA	NA	NA	NA
rbbp8l	82.20226768	-0.29542362	0.2222126	-1.329464	0.183695	0.747284
rhbdl1	14.91308626	-0.072794733	0.5095012	-0.142875	0.886389	0.988936
si:ch211-222k6.2	58.89606989	-0.551741454	0.2598253	-2.12351	0.033711	0.357045
si:dkey-165n16.1	66.46140963	-0.19559163	0.2435624	-0.803045	0.421949	0.90651
ggt1l2.2	0	NA	NA	NA	NA	NA
ftr36	1.156651673	0.411122976	1.6895393	0.2433344	0.807746	NA
selenou1b	58.28675234	-0.115538366	0.2695827	-0.428582	0.668227	0.964736
RF00017	0.158795395	0.967652056	4.0804729	0.2371421	0.812547	NA
si:ch211-71k14.1	2.59577202	-0.727067354	1.3390184	-0.542985	0.58714	NA
znf977	1.160507527	-1.286691038	1.8769381	-0.685527	0.493012	NA
chrna4a	2.63078507	-0.313649245	1.2539163	-0.250136	0.802482	NA
znf574	137.0473291	-0.004971249	0.178803	-0.027803	0.977819	0.996762
RF00001	0	NA	NA	NA	NA	NA
hcar1-4	24.30441328	0.023806818	0.3890129	0.061198	0.951202	0.99381
col28a1b	47.10697579	-0.144888544	0.3044504	-0.475902	0.634144	0.95776
si:ch211-274p24.4	13.00785816	0.045970342	0.5255774	0.0874664	0.930301	0.992702
si:ch211-157c3.4	329.9588043	0.087856546	0.1222569	0.7186225	0.472374	0.924838
foxa	44.20565022	-0.196905004	0.31008	-0.635014	0.52542	0.94013
si:ch73-106k19.5	1.452168549	2.869798567	1.9414573	1.4781673	0.139363	NA

ntng2b	167.0254802	0.272411302	0.1762776	1.5453536	0.122261	0.649888
si:ch1073-164k15.3	28.45255837	0.686663727	0.3864334	1.7769262	0.07558	0.535481
si:ch73-222h13.1	15.90383068	-0.108970155	0.4888917	-0.222892	0.823619	0.982196
akna	146.0963162	-0.214485521	0.1771617	-1.210677	0.226019	0.793556
RF00001	0	NA	NA	NA	NA	NA
slc5a8	31.14300153	0.129651533	0.3420161	0.3790802	0.704628	0.968134
ifnlr1	47.69538431	-0.161954514	0.2861548	-0.565968	0.571415	0.949418
RF00001	0	NA	NA	NA	NA	NA
serpina7	5.169526445	1.533261189	0.8673027	1.7678502	0.077086	NA
fam183a	6.955945973	1.159178602	0.7895726	1.468109	0.142075	NA
si:ch1073-296d18.1	17.30727092	0.45369713	0.4573409	0.9920327	0.321182	0.868935
CR788255.1	0	NA	NA	NA	NA	NA
sowahd	31.60002673	-0.636711044	0.3627744	-1.755116	0.07924	0.547489
tmem102	198.5501834	0.179053858	0.1513699	1.1828891	0.236853	0.805854
crygm2d4	16608.27778	0.033849215	0.0709261	0.4772462	0.633187	0.957382
si:dkeyp-69c1.7	162.9091876	0.237377043	0.1935017	1.226744	0.219919	0.786668
figla	0.655861427	0.077956683	2.4372582	0.0319854	0.974484	NA
si:ch211-162i8.4	5.515847522	0.836438464	0.828704	1.0093332	0.312815	NA
rskra	73.58000502	-0.191552751	0.2402907	-0.797171	0.425352	0.908894
scpp9	85.76521317	-1.361428098	0.2668952	-5.100984	3.38E-07	3.93E-05
ubxn2a	101.331343	-0.626533285	0.2007418	-3.12109	0.001802	0.049493
cbx7b	9.758626298	0.676438773	0.6527119	1.0363512	0.300038	NA
si:ch211-232b12.5	156.6681538	-0.11775321	0.1663587	-0.707827	0.479053	0.926978
grip2a	201.8171055	0.028997715	0.1533656	0.1890757	0.850033	0.985174
nfil3-6	174.2805906	0.027775341	0.2006888	0.1384	0.889924	0.989291
si:ch73-105b23.6	32.655183	0.2034583	0.4111321	0.4948733	0.62069	0.957354
wdr93	28.44065782	0.048849817	0.3681361	0.132695	0.894435	0.989689
prrg2	162.3760545	-0.277270125	0.1798349	-1.541804	0.123121	0.651012
rorc	9.215979461	-0.782567654	0.6461646	-1.211096	0.225858	NA
fibinb	311.7354528	0.53188407	0.1323328	4.0192928	5.84E-05	0.003162
ros1	53.16643949	-0.936625101	0.2889721	-3.24123	0.00119	0.035443
cthrc1a	494.7598719	0.293131374	0.11096	2.641774	0.008247	0.147053
stk39	20.67878632	0.16185645	0.4254221	0.3804609	0.703603	0.968134
akt3b	289.9156563	0.002914768	0.1368307	0.021302	0.983005	0.996944
cct2	704.2628196	-0.114442189	0.0951045	-1.203331	0.228848	0.796558
vwa3a	10.61477404	0.090006542	0.6239829	0.1442452	0.885307	NA
si:ch73-380n15.2	175.6769536	0.129889201	0.1649242	0.7875693	0.430949	0.91234
si:ch73-361p23.3	1.782023436	-2.171780397	1.673072	-1.298079	0.19426	NA
kbtbd2	472.3696007	0.069330695	0.1088291	0.6370601	0.524086	0.939336
oit3	16.52809919	0.20752297	0.5091844	0.4075596	0.683597	0.96567
exoc7	852.1883353	-0.101930752	0.0900677	-1.131713	0.257755	0.825314
si:ch211-161h7.4	348.9722677	-0.121903597	0.1179085	-1.033883	0.301191	0.856944
ucn3l	21.34523001	0.110461401	0.421897	0.2618208	0.79346	0.977937
zgc:100918	733.9487463	0.021654766	0.1098232	0.1971784	0.843688	0.985174
CR847844.1	1.818127336	1.405806012	1.5330681	0.9169886	0.359149	NA
kcnab2a	21.5725603	0.688107137	0.4146107	1.6596462	0.096986	0.593561
znf1011	18.64680082	-0.808176515	0.4472189	-1.807116	0.070744	0.520229
si:ch73-50f9.4	57.23230979	0.207944596	0.2654857	0.7832609	0.433474	0.913667
si:dkeyp-3f10.17	6.740896112	1.259864322	0.8131148	1.5494298	0.121278	NA
ypel2b	5.247651235	-0.575062482	0.8656907	-0.664282	0.50651	NA
mtss1lb	16.00712055	-0.072570075	0.4939936	-0.146905	0.883207	0.988706
zgc:63568	101.0453617	-0.176543226	0.2171874	-0.812861	0.416298	0.906237
BX942819.2	0	NA	NA	NA	NA	NA
selenoj	720.1266005	-0.064186595	0.0993714	-0.645926	0.518327	0.938676

tmem182b	7.290337721	-0.664438463	0.6955182	-0.955314	0.339419	NA
zgc:66473	5.752579326	-0.770613114	0.7973876	-0.966422	0.333833	NA
si:ch211-202h22.10	38.9548051	-0.250838426	0.3044533	-0.823898	0.409998	0.902615
si:dkey-84h14.1	2.139814685	0.681223001	1.2737661	0.5348101	0.592781	NA
si:dkey-152b24.8	2.652443197	-0.362034262	1.2027068	-0.301016	0.763402	NA
armc9	230.921235	-0.430539931	0.1799852	-2.392085	0.016753	0.238497
crygm2d14	9249.411733	0.03142978	0.0664129	0.4732481	0.636036	0.958465
cebpd	2311.029503	0.034571419	0.1051101	0.3289067	0.742226	0.973154
CABZ01038521.1	9.459662701	-0.115823035	0.6455241	-0.179425	0.857604	NA
lgals9l5	1.253241566	1.40838817	1.9820666	0.7105655	0.477354	NA
jac10	32.52142401	0.278038718	0.3393476	0.8193331	0.412596	0.904974
tmem121b	11.33214461	-0.127066138	0.5681421	-0.223652	0.823028	0.982189
crygm2d1	14798.58762	0.144072628	0.0706326	2.0397463	0.041376	0.398326
cbx8a	187.6403178	0.11177371	0.1556521	0.7180994	0.472696	0.924838
maza	451.3311903	0.219887773	0.1087292	2.0223442	0.043141	0.407581
si:dkey-77f5.3	123.8016254	-0.138002687	0.1880945	-0.733688	0.463139	0.922698
scoca	433.7330491	-0.03186008	0.1082249	-0.294388	0.768462	0.975687
unc5c	157.2966411	0.02612581	0.170065	0.1536225	0.877907	0.987881
rap1ab	398.9634523	0.188387224	0.1212987	1.5530854	0.120403	0.645215
reep6	101.1233681	0.228958855	0.1991945	1.1494239	0.250381	0.820164
dnah2	108.9308686	0.060303815	0.1973374	0.3055874	0.759919	0.975374
c3a.2	221.3329422	0.573278714	0.3262447	1.7572047	0.078883	0.547489
si:dkeyp-46h3.6	0.856702761	1.928463937	2.2075106	0.8735921	0.38234	NA
cep162	97.23973546	-0.033227339	0.2064851	-0.160919	0.872157	0.987846
dnah5	76.99153258	0.160130927	0.2376528	0.6738019	0.500437	0.934237
zgc:66473	11.69269184	-0.043307099	0.5664547	-0.076453	0.939059	0.992857
tcf23	0.847740827	0.553245907	2.099006	0.2635752	0.792107	NA
si:dkey-29b11.3	8.826545824	-0.057083044	0.6360851	-0.089741	0.928493	NA
hbbe1.3	1787.507679	0.122158816	0.1979561	0.6171004	0.537169	0.943336
si:ch73-160p18.3	0.332645115	0.005883744	3.2924249	0.0017871	0.998574	NA
prr11	76.97187904	0.1179394	0.2248966	0.5244161	0.599989	0.956045
tshz3a	313.3604435	-0.003042582	0.123176	-0.024701	0.980293	0.996879
slc25a34	76.77274565	-0.194615702	0.229228	-0.849005	0.395879	0.899472
tpm1	3928.512074	0.00230294	0.0786657	0.029275	0.976645	0.996762
si:ch211-214p13.3	96.1510072	-0.088861446	0.1996792	-0.445021	0.656305	0.961833
si:ch73-304f21.1	8.777672085	0.305327524	0.6825948	0.4473042	0.654655	NA
bean1	293.5467052	0.020078688	0.1313218	0.1528969	0.87848	0.987881
CR790388.2	1.87546505	-0.809447507	1.4633859	-0.553133	0.580172	NA
zdhhc5b	308.6169862	0.142203556	0.1251271	1.1364729	0.255759	0.824482
taar12g	0	NA	NA	NA	NA	NA
serbp1b	2925.731199	-0.018126716	0.0705826	-0.256816	0.797321	0.978577
si:dkey-187j14.6	16.86032501	0.112918196	0.4557112	0.2477846	0.804301	0.979643
si:ch211-151p13.8	606.1627725	0.17745348	0.1233776	1.4382961	0.15035	0.698669
RF00017	0	NA	NA	NA	NA	NA
spi2	4.308164765	-2.028612087	1.0850909	-1.869532	0.061549	NA
ponzr4	440.4826405	0.105686116	0.1382	0.764733	0.444431	0.916726
ptp4a2a	1295.876221	0.182339075	0.0838978	2.173348	0.029754	0.334058
BX664625.2	0.333314908	-1.858695402	3.5126701	-0.52914	0.596708	NA
mmd2a	8.511855724	1.028828327	0.6762629	1.5213438	0.128174	NA
peli3	433.9088998	-0.184783055	0.1090446	-1.694563	0.090158	0.574908
si:ch73-57f22.2	20.4459659	-0.53899987	0.4587831	-1.174847	0.240056	0.808111
NDUFB1	548.5915668	0.032921785	0.1142683	0.2881095	0.773263	0.975687
ecscr	40.21995559	0.267016341	0.3014936	0.8856452	0.375809	0.894376
si:dkey-21c1.4	27.7788815	0.175451012	0.369779	0.4744753	0.635161	0.957927

RF00001	0	NA	NA	NA	NA	NA
F2R (1 of many)	0	NA	NA	NA	NA	NA
dnajb12b	382.5130021	0.036847302	0.1157103	0.3184445	0.750148	0.97419
ccr6a	12.87452626	0.353612587	0.544488	0.6494405	0.516054	0.937995
cbln20	87.20577479	0.112499272	0.2186999	0.5144003	0.606972	0.956718
si:dkey-226l10.6	206.8004473	0.089521476	0.1441149	0.6211811	0.53448	0.943336
fam124b	40.03792744	0.410781936	0.3044895	1.3490843	0.17731	0.738201
FO704810.1	159.0932025	-0.017643316	0.1646245	-0.107173	0.914652	0.991119
tradv30.0.6	0	NA	NA	NA	NA	NA
si:ch73-322b17.4	0.173729368	-0.955901296	4.0804729	-0.234262	0.814781	NA
CU693494.1	10.21842666	-0.359643624	0.6999044	-0.513847	0.607359	NA
btbd3b	207.5541325	0.068354822	0.188402	0.3628136	0.716744	0.96909
si:dkey-261p22.1	5.8927864	0.446720255	0.7905603	0.5650679	0.572028	NA
CU693446.1	0.831868617	-0.546811265	2.1107396	-0.259061	0.795588	NA
CABZ01045212.1	10.84899724	0.355900917	0.6115016	0.5820114	0.560559	NA
snip1	317.8890299	0.024715798	0.1298332	0.1903658	0.849023	0.985174
zgc:85936	196.7357283	-0.245566137	0.1626663	-1.509631	0.131138	0.66388
wu:fe05a04	212.9840116	-0.185016831	0.157375	-1.175643	0.239738	0.808111
si:ch211-278p9.1	11.52525428	-0.178986107	0.593095	-0.301783	0.762817	0.975374
znf407	152.3902519	-0.143645324	0.1666746	-0.861831	0.388781	0.896295
kif3a	1628.176953	0.090706369	0.0829844	1.0930534	0.27437	0.839136
BX510934.1	16.89402748	0.224807782	0.4595495	0.4891917	0.624706	0.957354
fbxo42	113.5388022	-0.434584314	0.1941255	-2.238677	0.025177	0.30686
cdk1	736.5138539	0.010344085	0.1012599	0.1021538	0.918635	0.991623
si:ch73-308l14.2	163.381858	-0.083423076	0.172227	-0.484379	0.628117	0.957354
pacrg	34.22309423	0.281280376	0.3400839	0.8270912	0.408185	0.90171
si:ch73-105b23.1	2.133130573	0.201771914	1.3031327	0.154836	0.876951	NA
nek11	4.249810494	0.450524148	1.0664116	0.4224674	0.672684	NA
hdac11	15.67857716	0.331922659	0.5007924	0.6627949	0.507462	0.935734
nox1	149.2083036	-0.78573669	0.1759356	-4.466048	7.97E-06	0.000592
zmp:0000000912	0.317711143	1.810477155	3.2978238	0.5489915	0.583011	NA
zgc:171717	0.182570949	0.967652056	4.0804729	0.2371421	0.812547	NA
si:dkeyp-98a7.9	0	NA	NA	NA	NA	NA
si:ch211-242f23.8	27.56695913	0.23012938	0.3724192	0.617931	0.536621	0.943336
BX469925.1	1.709357036	-1.205057426	1.5258566	-0.789758	0.429669	NA
si:ch73-95l15.5	493.3594883	-0.231618941	0.1079122	-2.146364	0.031844	0.34636
si:dkey-51d8.3	3.62227701	-0.532200361	0.9933508	-0.535763	0.592123	NA
TENM3	370.6835932	0.027264651	0.1156734	0.2357036	0.813663	0.981081
gpr153	150.8999843	0.142053409	0.1845506	0.7697263	0.441462	0.916336
FP085398.1	13.682995	0.161930735	0.5228372	0.3097154	0.756777	0.975374
RF00001	0	NA	NA	NA	NA	NA
maptb	1877.518662	0.010074824	0.0865126	0.116455	0.907292	0.990702
BX649485.1	6.823171871	0.087990584	0.722317	0.1218171	0.903044	NA
si:ch211-173a9.6	59.32253671	-1.092939028	0.2559814	-4.269604	1.96E-05	0.001237
nitr1m	0	NA	NA	NA	NA	NA
si:dkey-11o18.5	22.30128912	-0.728176378	0.4216398	-1.727011	0.084166	0.55797
or128-5	6.134276772	-1.035823825	0.7957675	-1.301666	0.19303	NA
hmgn6	5429.82089	-0.025752357	0.0790473	-0.325784	0.744588	0.974094
si:dkey-33o22.1	0	NA	NA	NA	NA	NA
nsfl1c	1722.111664	0.079778582	0.0849379	0.9392576	0.347599	0.880195
cdh26.2	210.7182864	-0.25933714	0.149077	-1.739619	0.081926	0.552725
znf1064	18.79806935	-0.045847948	0.4874704	-0.094053	0.925067	0.992676
runx1	24.82580406	0.114096777	0.3790424	0.3010133	0.763404	0.975374
sept7a	2382.418282	-0.039989933	0.0749236	-0.533743	0.593519	0.954434

ctslb	0	NA	NA	NA	NA	NA
fasn	1441.248678	0.173573931	0.0922252	1.8820666	0.059827	0.478654
si:ch211-219a15.3	109.4545796	0.266914925	0.1881498	1.41863	0.156007	0.706028
si:ch211-248l17.3	96.76271292	-0.175844861	0.2118338	-0.830108	0.406478	0.901305
RF00001	0	NA	NA	NA	NA	NA
fibpb	20.21795946	-0.23209183	0.4271711	-0.543323	0.586908	0.953319
si:ch1073-127d16.1	8.266698506	-0.087842991	0.6852336	-0.128194	0.897995	NA
BX005012.1	9.408882924	-0.078892919	0.6255362	-0.12612	0.899637	NA
si:dkey-1c11.1	177.624529	0.176224919	0.1558304	1.1308767	0.258107	0.825859
klhl29	270.889091	-0.111563678	0.1303657	-0.855775	0.392122	0.897688
atox1	403.7352767	0.063835085	0.1249804	0.5107607	0.609519	0.956718
lpl	1132.803746	0.132066685	0.0881513	1.4981819	0.134086	0.669381
si:dkey-256i11.3	0.349228403	0.005884014	3.2372587	0.0018176	0.99855	NA
gfra3	150.5453582	0.060373213	0.1644665	0.3670852	0.713555	0.96909
dicp3.3	3.337412422	0.27340267	1.0376681	0.263478	0.792182	NA
FQ311930.1	40.43431157	0.222871623	0.3144428	0.7087826	0.478459	0.926978
si:ch211-226o13.1	17.63213105	0.055573929	0.4596404	0.1209074	0.903764	0.990291
tmprss5	65.88073343	0.120521874	0.2454455	0.4910332	0.623403	0.957354
RF00017	372.4928545	0.521894221	0.4730903	1.1031598	0.269958	0.834705
si:dkey-118k5.3	20.27786222	-0.001429678	0.436092	-0.003278	0.997384	0.999153
BX664618.1	36.38222561	0.350120511	0.3245301	1.0788538	0.280653	0.84355
asb14b	64.0503404	0.272190548	0.2447736	1.1120094	0.266134	0.832153
map4k3a	323.8420623	0.066966915	0.1204621	0.5559168	0.578268	0.951052
si:dkey-156k2.8	0.648507523	-0.035374916	2.4454854	-0.014465	0.988459	NA
lrrc38b	46.23758436	0.157953266	0.3114096	0.5072203	0.612	0.95688
ccser2a	574.0362397	-0.018668805	0.1048089	-0.178122	0.858627	0.985773
CR382337.1	0.166657454	-0.955901296	4.0804729	-0.234262	0.814781	NA
rfwd3	96.93849795	-0.143782526	0.2045373	-0.702965	0.482078	0.928561
CR388166.1	4.421089209	0.038378663	0.9456459	0.0405846	0.967627	NA
RF00001	0	NA	NA	NA	NA	NA
si:ch211-173d10.4	1.22848649	-0.350511206	1.8878393	-0.185668	0.852705	NA
pimr190	2.783339735	0.865732034	1.1942046	0.7249445	0.468486	NA
cnih4	43.68803467	-0.149407543	0.2878414	-0.519062	0.603718	0.95618
or122-1	0	NA	NA	NA	NA	NA
rab27b	16.57004402	-0.488989794	0.5043293	-0.969584	0.332254	0.874653
ponzr10	3.940588922	-1.288292691	1.0074578	-1.278756	0.200983	NA
crygm2d10	15049.59001	-0.02846361	0.0713641	-0.398851	0.690003	0.966857
CABZ01074298.1	6.711070674	-0.258991858	0.7819192	-0.331226	0.740474	NA
RF00017	0	NA	NA	NA	NA	NA
pum3	571.2278186	0.008272111	0.1033019	0.080077	0.936176	0.992702
tiam2a	601.5098061	-0.086686097	0.1024032	-0.846518	0.397264	0.899672
rnf152	83.61470226	-0.126153805	0.2228802	-0.566016	0.571383	0.949418
si:dkeyp-110a12.4	244.5770407	-0.079398864	0.1406703	-0.564432	0.57246	0.949494
si:ch211-22k7.9	13.4100806	-0.74703608	0.5357715	-1.394318	0.163221	0.718029
si:dkey-54n8.2	308.3467512	0.085389256	0.1269164	0.6727995	0.501075	0.934477
pdyn	69.14122678	-0.085024918	0.2393742	-0.355197	0.722442	0.970375
tnfrsf11a	206.3054297	-0.099706806	0.1481331	-0.673089	0.50089	0.934309
FP236735.1	14.87095897	-0.978573797	0.5319915	-1.839454	0.065848	0.501768
mfrp	12.2901277	0.248547484	0.5521687	0.4501297	0.652617	0.960708
si:dkey-30g5.1	0	NA	NA	NA	NA	NA
agap3	32.98574138	0.080545988	0.3626177	0.2221237	0.824218	0.982196
si:dkey-211g8.9	0.166657454	-0.955901296	4.0804729	-0.234262	0.814781	NA
bcl3	59.7367174	-0.693166273	0.2669927	-2.596199	0.009426	0.162646
si:ch211-113p18.3	194.1484628	0.236698279	0.1555713	1.5214782	0.12814	0.658954

si:dkey-33c14.6	32.92362255	0.231935805	0.3523903	0.6581787	0.510423	0.936253
cntn1a	759.0471969	-0.129017373	0.0988181	-1.305605	0.191687	0.755945
plekhn1	153.0318917	-0.034034311	0.1909282	-0.178257	0.858521	0.985773
si:ch211-180a12.2	410.434814	0.011804011	0.1205149	0.0979465	0.921975	0.992224
cd44a	51.29592072	-0.205504394	0.2706261	-0.759366	0.447633	0.917154
SLC2A13 (1 of many)	10.76916122	-0.281723734	0.636112	-0.442884	0.65785	NA
si:ch211-11k18.4	637.4466493	0.163603615	0.1133245	1.4436737	0.148831	0.69636
adgrf3a	2.178953298	-0.191895525	1.2772276	-0.150244	0.880572	NA
eevs	665.8896705	0.763546197	0.1265511	6.0335006	1.60E-09	3.17E-07
rnf216	342.1665194	-0.160755851	0.1186449	-1.354933	0.175439	0.736233
h2afy2	5107.51857	-0.020711829	0.0653286	-0.317041	0.751213	0.97419
CU896691.1	1.975273416	0.957536214	1.3943287	0.6867364	0.492249	NA
ndufa4l2b	5.250292201	0.143719652	0.8619905	0.16673	0.867583	NA
RF00001	0	NA	NA	NA	NA	NA
il4	3.364537935	0.032386783	1.257576	0.0257533	0.979454	NA
dact3a	133.6904461	0.056533437	0.1899179	0.2976731	0.765953	0.975374
psme4a	336.6842511	0.12518099	0.1215304	1.0300386	0.302992	0.85729
tmem240a	75.53685647	0.09851911	0.2640094	0.3731651	0.709026	0.968664
nitr10a	0	NA	NA	NA	NA	NA
plce1	293.8239964	-0.025851699	0.1243853	-0.207836	0.835357	0.984089
nudt9	385.2515744	-0.053316281	0.1159507	-0.459818	0.645647	0.96029
RF00001	0	NA	NA	NA	NA	NA
zgc:174972	46.56421336	-0.362831248	0.2947023	-1.231179	0.218256	0.785404
cdk4	312.3506211	-0.327941271	0.1274063	-2.57398	0.010054	0.171232
CR933559.1	23.92677322	0.000657247	0.4002977	0.0016419	0.99869	0.999398
pde8a	276.2452899	0.08857997	0.1285589	0.6890224	0.490809	0.931926
RF00017	0	NA	NA	NA	NA	NA
CABZ01044053.1	2023.866455	-0.105403869	0.0762762	-1.381871	0.167011	0.723594
rac1b	240.3935131	0.06219518	0.1503855	0.4135717	0.679188	0.965257
she	67.04419946	-0.054686414	0.2415654	-0.226383	0.820903	0.981628
avd	1.386302362	-1.54102959	1.8481342	-0.83383	0.404377	NA
rpap3	333.9372563	-0.09482772	0.1206498	-0.785975	0.431882	0.912787
or122-2	0	NA	NA	NA	NA	NA
CU657980.2	0.158795395	0.967652056	4.0804729	0.2371421	0.812547	NA
CT573433.1	172.7304087	0.152108872	0.1666644	0.9126657	0.361418	0.885719
slc6a11b	414.4943067	0.150058667	0.1343095	1.1172605	0.263883	0.830245
igsf5a	35.80556382	-0.377082166	0.3267025	-1.154206	0.248416	0.817939
znf365	1.80091554	2.1734762	1.5664644	1.3875044	0.165288	NA
bada	239.6431377	-0.211973456	0.1398942	-1.515242	0.129711	0.662411
si:ch211-114c17.1	39.73266339	0.030365261	0.3375026	0.0899705	0.928311	0.992702
pabpn1l	1.812633453	0.287259488	1.3842345	0.2075223	0.835602	NA
BX664625.3	8.024057105	0.64559331	0.6934841	0.9309418	0.351884	NA
znf1057	17.14565997	-0.1027025	0.4615599	-0.222512	0.823916	0.982196
wu:fc23c09	2.975917756	1.763331744	1.309229	1.3468475	0.178029	NA
CYP27A1 (1 of many)	11.91416523	-0.738651817	0.557687	-1.324492	0.18534	0.74928
si:ch211-114l13.12	2.508059078	-1.026597373	1.2227302	-0.839594	0.401136	NA
mhc1zfa	32.77901528	0.328154813	0.3730755	0.8795935	0.37908	0.895192
si:dkey-26c10.5	96.03525939	-0.119046125	0.2095327	-0.568151	0.569933	0.948983
si:ch73-125k17.2	1.148092083	-0.385892966	1.819804	-0.212052	0.832067	NA
rpl35a	14335.13132	-0.270196581	0.075386	-3.584173	0.000338	0.013009
si:dkey-106g10.7	189.5208551	-0.021427166	0.1563747	-0.137024	0.891011	0.989291
CU929391.1	24.75459525	0.251861141	0.4081559	0.6170709	0.537188	0.943336
si:dkeyp-27c8.2	112.7029556	0.11801254	0.194585	0.6064832	0.544194	0.945438
si:ch73-139j3.4	16.30859929	-0.52844234	0.4825349	-1.095138	0.273456	0.838214

fgf18a	91.97880793	0.104948654	0.2209417	0.4750062	0.634783	0.9578
AL935044.1	0.356300316	0.005884122	3.214964	0.0018302	0.99854	NA
pimr70	3.541940341	1.056789025	1.1276868	0.9371299	0.348692	NA
pimr187	2.018457259	0.498928664	1.4424654	0.345886	0.729428	NA
CR759879.1	62.05043959	-0.088168576	0.2624315	-0.335968	0.736895	0.972535
usp43b	52.74397427	-0.066063372	0.2747702	-0.240431	0.809996	0.980764
fam110b	691.9362604	0.041728494	0.1026844	0.4063761	0.684466	0.966043
usp18	5.137081238	-0.045785776	0.881357	-0.051949	0.958569	NA
tmed6	27.06011307	-0.695389991	0.3685649	-1.88675	0.059194	0.475427
si:ch211-214c20.1	3.372114748	-1.563192064	1.125122	-1.389353	0.164725	NA
sik3	971.5927354	-0.029024048	0.1082533	-0.268112	0.788613	0.976703
si:dkey-66i24.8	19.60986891	-0.112008102	0.4365017	-0.256604	0.797484	0.978577
kdm6ba	2840.183601	-0.007113863	0.0714031	-0.09963	0.920638	0.992118
pfn1	6112.713759	0.109481567	0.1872448	0.5846975	0.558751	0.948478
KCNIP4	24.31385666	-0.382655868	0.3957297	-0.966963	0.333563	0.874933
gstm.3	492.3162878	-0.056163317	0.1111754	-0.505178	0.613434	0.95688
fut8b	58.85097309	0.179855447	0.2623807	0.6854752	0.493044	0.931926
pkd1l2b	0	NA	NA	NA	NA	NA
znf217	502.4821982	-0.185370417	0.1084154	-1.709816	0.0873	0.566175
adrb2a	71.54771905	-0.064175706	0.2335869	-0.27474	0.783516	0.97627
si:dkey-30c15.10	274.013673	-0.034777961	0.1348022	-0.257993	0.796413	0.978518
pcdh2aa15	1.143205378	2.461556447	2.0540176	1.1984106	0.230757	NA
adgrg2a	219.2532913	-0.178504075	0.1438515	-1.240892	0.214646	0.782493
hsd17b7	213.2602661	0.07357461	0.166111	0.4429242	0.657821	0.962538
sema4gb	16.38259583	-0.10877863	0.4705736	-0.231162	0.817189	0.981594
atg4db	8.686453556	-0.048120983	0.6717779	-0.071632	0.942895	NA
smoc2	237.8015801	0.181763161	0.1396554	1.301512	0.193083	0.756795
CABZ01056629.1	10.44188282	-0.622154788	0.6257706	-0.994222	0.320115	NA
BX470188.1	6.784777867	-0.944449686	0.7602699	-1.242256	0.214142	NA
si:ch211-112g6.4	5.310812772	0.587587712	0.9414443	0.6241344	0.532539	NA
ablim3	647.9477619	0.096297594	0.0966342	0.996517	0.318999	0.867746
ciartb	288.1368731	0.168323807	0.1424519	1.1816185	0.237357	0.806412
tp53inp2	481.6864732	0.129774933	0.1053067	1.2323516	0.217818	0.785256
mpped1	416.0864688	-0.192799872	0.1207963	-1.596074	0.110472	0.62503
CR381544.1	80.519926	-0.320747463	0.2186903	-1.466675	0.142465	0.684592
si:ch211-135n15.3	1.319972715	-1.5487922	1.7260877	-0.897285	0.369567	NA
v2rh13	0.182570949	0.967652056	4.0804729	0.2371421	0.812547	NA
si:ch211-168f7.5	500.8876727	-0.207987332	0.1072137	-1.939932	0.052388	0.449228
CABZ01077220.1	0.31598276	0.005883445	3.3523626	0.001755	0.9986	NA
RF00001	0	NA	NA	NA	NA	NA
sh3d19	330.1080221	-0.021735188	0.1336938	-0.162574	0.870854	0.987683
shisa8b	65.70824551	0.038419405	0.2527097	0.1520298	0.879163	0.987881
rce1b	8.724683509	0.700549036	0.6480696	1.0809781	0.279707	NA
si:dkey-23a23.3	67.42933008	0.177393541	0.2448538	0.7244877	0.468766	0.924328
zmp:0000000924	2.87064792	0.922616524	1.1908526	0.7747529	0.438486	NA
fam78ab	8.601731506	0.681106018	0.6694076	1.0174758	0.308927	NA
FO082877.1	321.5490174	0.015360652	0.1236634	0.1242134	0.901146	0.990121
tmem163b	89.04464877	0.025054237	0.2165303	0.1157078	0.907884	0.990702
shisa2b	39.77079109	0.255428692	0.3072523	0.831332	0.405786	0.901305
si:ch211-217k17.12	0.643404698	1.436206627	2.5980397	0.552804	0.580398	NA
kansl1b	1288.127286	-0.128643553	0.0850459	-1.512636	0.130372	0.662797
si:dkey-16p6.1	6.588857487	0.002581521	0.7442359	0.0034687	0.997232	NA
thoc1	973.9314646	-0.04685456	0.0881577	-0.531486	0.595082	0.954434
si:ch1073-303k11.2	402.793228	0.183701468	0.1149198	1.5985193	0.109927	0.624178

lgals17	0	NA	NA	NA	NA	NA
si:dkey-83f18.7	0	NA	NA	NA	NA	NA
RF00017	0	NA	NA	NA	NA	NA
smoc1	7.915448443	0.117724636	0.6840552	0.1720982	0.86336	NA
CABZ01038494.1	58.06083038	0.217237827	0.2597657	0.8362839	0.402995	0.90084
gig2p	6.78446789	-0.835155927	0.7911111	-1.055675	0.291117	NA
zgc:174356	11.59103598	-0.387136125	0.5743462	-0.674047	0.500282	0.934224
si:ch211-181d7.1	0.476747244	2.393381959	3.0525537	0.7840589	0.433006	NA
si:ch211-190p8.2	0.838940531	-0.561526863	2.0217856	-0.277738	0.781213	NA
susd5	24.49002404	0.190836622	0.3870781	0.4930183	0.622	0.957354
si:ch73-248e21.5	12.92800852	-0.052027582	0.5560998	-0.093558	0.92546	0.992676
dre-mir-194b	0.340386822	-1.882368278	3.2159502	-0.585323	0.558331	NA
foxj1b	60.93802685	0.179265743	0.2486863	0.7208508	0.471001	0.924838
doc2b	349.351231	0.197253827	0.1341868	1.4699947	0.141563	0.68225
si:ch211-235i11.4	193.6211492	-0.158733351	0.156164	-1.016453	0.309414	0.861497
si:dkey-254e13.6	9.470419629	0.359544146	0.6337294	0.5673465	0.570479	NA
mrtfba	28.39445087	0.020896212	0.3867493	0.0540304	0.956911	0.994543
notch1	89.87792377	-0.234872567	0.2265193	-1.036876	0.299793	0.855995
si:ch211-103f14.3	173.9066131	0.136776864	0.156825	0.8721624	0.38312	0.895192
si:ch73-302o18.2	5.50945219	-0.597050631	0.8172775	-0.730536	0.465063	NA
ubap2a	2998.346662	-0.025063794	0.0980927	-0.255511	0.798328	0.978884
mir1-1	1.852202149	0.869537402	1.455503	0.5974137	0.550231	NA
si:ch211-132e22.4	0	NA	NA	NA	NA	NA
hbae1.3	45.51589313	0.450693187	0.3155609	1.4282287	0.153226	0.702508
znf1028	29.22421218	0.008466448	0.3630538	0.0233201	0.981395	0.996944
alx4a	294.4078855	-0.260468049	0.1289996	-2.019138	0.043473	0.409154
tmem145	130.8525615	-0.004561832	0.1840864	-0.024781	0.98023	0.996879
prrt4	36.0682306	0.227353948	0.3243583	0.7009346	0.483344	0.92946
miip	70.05514283	-0.235699099	0.2491541	-0.945997	0.34415	0.878434
sp1	138.3719492	-0.127838254	0.1816768	-0.703657	0.481646	0.928386
RF00001	0	NA	NA	NA	NA	NA
tmem117	32.77236077	-0.065241492	0.3415288	-0.191028	0.848504	0.985174
acadl	967.1502844	-0.085546321	0.0925556	-0.92427	0.355346	0.882347
CR925817.1	9.04655815	0.462401643	0.6348978	0.7283088	0.466425	NA
zgc:77938	752.1752866	-0.691079071	0.0913957	-7.561392	3.99E-14	1.75E-11
si:cabz01069013.3	12.6317581	0.150832601	0.5569612	0.2708135	0.786535	0.976575
RF00001	0.948068633	0.012260313	2.0842059	0.0058825	0.995306	NA
rpl11b	22.74167111	0.645727789	0.4140731	1.5594537	0.118889	0.642772
znf1000	2.90133375	0.897359733	1.141004	0.786465	0.431595	NA
cox5aa	2758.048206	-0.084925514	0.0835115	-1.016932	0.309186	0.861381
nme4	33.7123717	-0.454949167	0.3331028	-1.365792	0.172004	0.730972
tmem107l	63.61567256	-0.047911535	0.2495085	-0.192024	0.847724	0.985174
rubcnl	15.77149531	0.42698411	0.5027557	0.8492874	0.395721	0.899378
si:ch211-171h4.7	0	NA	NA	NA	NA	NA
notum2	141.4584381	-0.267791424	0.179295	-1.49358	0.135285	0.670689
si:ch211-66k16.28	19.38065256	0.554573575	0.4538847	1.2218379	0.221769	0.78829
si:ch211-189a15.5	41.9254904	0.048118481	0.303741	0.1584195	0.874126	0.987881
gm2a	248.3092577	0.061362829	0.1507848	0.4069563	0.68404	0.965679
ssh2a	564.2585178	0.204513041	0.120994	1.6902736	0.090976	0.578069
si:dkey-190j3.3	0	NA	NA	NA	NA	NA
selenol	6.987361973	-0.446046544	0.8493687	-0.525151	0.599478	NA
si:dkey-24i24.3	13.27145411	0.295168525	0.5514043	0.5353032	0.59244	0.954434
or111-7	1.554290354	1.886980028	1.746668	1.0803312	0.279995	NA
taar12j	0.820749213	-1.887809118	2.2327507	-0.845508	0.397827	NA

CLPB	16.16167003	0.135429874	0.4944832	0.2738816	0.784176	0.97627
tmx3b	192.8224954	0.418180774	0.1526119	2.7401578	0.006141	0.118585
pcdh1gb9	29.74770724	0.072102842	0.3666723	0.1966411	0.844108	0.985174
kcnj1a.6	203.478317	-0.123763814	0.1783825	-0.693811	0.487801	0.931809
CR385078.1	2.519419188	-0.595573162	1.2058478	-0.493904	0.621374	NA
cntrl	151.6370058	-0.16012445	0.1756177	-0.911779	0.361885	0.885944
si:ch211-149k12.3	1.330260688	-0.014903156	1.6527429	-0.009017	0.992805	NA
BX571757.1	16.16556577	-0.011524947	0.4782868	-0.024096	0.980776	0.996944
pimr137	1.139799941	-2.459945758	2.024345	-1.215181	0.224297	NA
fam217b	19.80228731	0.175433404	0.4411786	0.3976472	0.69089	0.967157
znf982	26.64305493	-0.214457647	0.3775061	-0.568091	0.569973	0.948983
and1	1933.509061	0.677599747	0.0952072	7.117103	1.10E-12	3.82E-10
st6gal2b	54.56360143	0.232474466	0.2649144	0.8775455	0.38019	0.895192
acot17	49.85022772	0.155011097	0.2991676	0.5181414	0.60436	0.95651
ponzr2	37.98500978	0.164477448	0.3164624	0.5197378	0.603246	0.956045
myct1b	10.54609671	-0.514816618	0.6288708	-0.818637	0.412994	NA
si:ch1073-390k14.1	223.3194891	0.256803051	0.1492053	1.7211388	0.085226	0.560519
si:dkey-23f9.13	0	NA	NA	NA	NA	NA
si:dkey-1c7.2	0.158795395	0.967652056	4.0804729	0.2371421	0.812547	NA
tmem104	213.8421793	0.226092951	0.1462396	1.5460442	0.122094	0.649408
vps72b	79.88555936	-0.101077067	0.2286543	-0.442052	0.658452	0.962545
si:ch211-151o1.4	12.85977958	0.359036617	0.5322299	0.6745893	0.499937	0.934224
f10	402.7238098	0.361314151	0.1242429	2.908127	0.003636	0.081517
si:ch73-352p18.4	197.4588787	0.205419487	0.1528009	1.3443605	0.178832	0.739486
ponzr3	485.3261479	0.110089787	0.1547609	0.7113538	0.476865	0.926826
pxna	788.3807524	-0.040724712	0.0980148	-0.415495	0.677779	0.965257
CR759791.1	0.507044276	-2.459587493	2.7586536	-0.89159	0.372613	NA
chst1	368.69559	0.169436825	0.1234228	1.3728168	0.169809	0.726965
crispld2	63.79642415	-0.122406651	0.2476563	-0.49426	0.621122	0.957354
si:dkey-175m17.6	16.21561411	-0.328427704	0.4871587	-0.67417	0.500203	0.934224
si:ch211-153b23.7	15.4442536	0.013504366	0.5065302	0.0266605	0.97873	0.996762
ltb4r2a	33.42944478	-0.120624195	0.3447596	-0.349879	0.726429	0.970402
si:ch211-152l15.2	6.880892859	0.372141992	0.745793	0.4989883	0.617788	NA
rexo4	141.2754933	0.075698954	0.1937297	0.3907451	0.695986	0.96736
si:ch211-142d6.2	0	NA	NA	NA	NA	NA
arhgef10la	263.364477	0.00143034	0.1379205	0.0103708	0.991725	0.997679
BX000363.1	2.676434871	0.357483988	1.2400889	0.2882729	0.773138	NA
adcy1b	44.53658204	-0.060126415	0.3102154	-0.193822	0.846316	0.985174
BX927260.1	56.79185199	0.055425704	0.277209	0.1999419	0.841526	0.985174
cdca9	3.019501859	0.018473864	1.1013934	0.0167732	0.986618	NA
znf1115	4.086007046	0.106172476	0.9419315	0.1127178	0.910254	NA
grn2	0.654761552	-2.837720206	2.3559398	-1.204496	0.228398	NA
CU855711.1	0	NA	NA	NA	NA	NA
si:ch211-208k15.1	146.6255172	0.149165146	0.1681237	0.8872344	0.374953	0.894316
si:ch211-212k18.6	9.672279932	-0.049135852	0.628513	-0.078178	0.937687	NA
si:ch211-209f22.3	0	NA	NA	NA	NA	NA
lman2lb	605.1100872	0.110439319	0.1161433	0.9508881	0.341661	0.878134
RF00001	0	NA	NA	NA	NA	NA
zmp:0000000936	24.42219217	0.108856845	0.3812581	0.2855201	0.775246	0.975687
hhla2b.1	19.35084431	0.564567956	0.4446176	1.2697831	0.204162	0.771764
si:dkey-192d15.3	0	NA	NA	NA	NA	NA
hist1h2a11	5.271727317	1.309230631	0.8970595	1.4594691	0.144436	NA
si:ch211-201o1.1	4.556620613	0.828187869	0.9074315	0.9126726	0.361415	NA
ddias	28.14020217	-0.003921497	0.3614851	-0.010848	0.991344	0.997679

dcst2	4.132458278	-0.345974922	0.9248943	-0.37407	0.708352	NA
pdzd8	381.5463512	0.165764923	0.118399	1.4000538	0.161497	0.715364
tlr20.2	0.31759079	1.807926197	3.579045	0.5051421	0.613459	NA
ngfra	613.8331918	0.085245848	0.0986277	0.8643197	0.387412	0.895778
tnfaip8l3	203.4701354	0.0380002	0.1533321	0.2478293	0.804266	0.979643
lgals1l1	699.6634884	-0.029755675	0.2606806	-0.114146	0.909122	0.990702
si:ch211-226h8.14	0.491320158	0.8528132	2.8656354	0.2976	0.766008	NA
ecrg4b	168.201547	0.522039969	0.1907784	2.7363688	0.006212	0.119726
zmp:0000000937	3.343546099	-0.292492952	1.0269297	-0.284823	0.77578	NA
si:ch211-250n8.1	6.796843014	1.268606433	0.7648388	1.6586585	0.097185	NA
CR382327.1	7.151665102	-0.39154003	0.738056	-0.530502	0.595764	NA
hmgcll1	8.20791631	0.82767387	0.7136531	1.1597706	0.246142	NA
BX322530.1	252.6383924	0.05107629	0.1670672	0.305723	0.759816	0.975374
zgc:174193	0	NA	NA	NA	NA	NA
MFAP4 (1 of many)	48.86160784	0.670485044	0.2883064	2.3255993	0.02004	0.266383
otogl	116.1089347	0.187618188	0.1834772	1.0225694	0.306511	0.860027
cfap299	12.83138865	-0.682708382	0.5375964	-1.269927	0.20411	0.771764
si:ch211-224l10.4	6.396002561	0.948442407	0.776038	1.2221598	0.221647	NA
lyplal1	48.09636823	0.167215814	0.2780674	0.60135	0.547607	0.945581
CABZ01059406.1	0.506374483	-0.940103506	2.8355584	-0.331541	0.740236	NA
RF00017	0	NA	NA	NA	NA	NA
mast1b	589.5237415	0.038900293	0.1119301	0.3475408	0.728185	0.970402
RF00001	0	NA	NA	NA	NA	NA
si:ch73-180n10.1	2.796142981	0.137882125	1.1306565	0.1219487	0.90294	NA
plcxd1	139.0163619	0.086004825	0.1740181	0.4942293	0.621144	0.957354
xxylt1	20.41441983	-0.089654067	0.432002	-0.207532	0.835595	0.984089
fgd5a	308.9290861	-0.185857551	0.1238011	-1.501259	0.133289	0.667628
nes	279.8557405	-0.125731248	0.1293003	-0.972397	0.330853	0.87417
klhl22	146.4347464	-0.16381328	0.1783668	-0.918407	0.358406	0.884398
prox3	385.0067501	0.102819455	0.1156421	0.8891181	0.37394	0.893677
ppp1r3ab	103.7373279	0.203428089	0.2099043	0.9691471	0.332472	0.874653
trabd2b	259.8978067	0.104749106	0.1411677	0.7420191	0.458076	0.921978
wdr59	278.0126188	-0.086095376	0.1345879	-0.639696	0.52237	0.939056
CR318646.1	0	NA	NA	NA	NA	NA
zgc:174288	38.30648974	-0.574178755	0.3193289	-1.79808	0.072164	0.525397
crygm2d3	14135.48586	0.087352963	0.0733771	1.1904662	0.233863	0.802571
citb	20.71113344	0.23303156	0.4487426	0.519299	0.603552	0.956045
paqr8	18.39934308	-0.203928802	0.4544736	-0.448714	0.653638	0.961112
si:dkey-81j8.6	2.263515702	0.416243913	1.2766289	0.3260493	0.744387	NA
si:ch211-237i5.4	34.93221169	0.474379005	0.3335759	1.4221021	0.154997	0.704624
efs	611.6961666	0.082280846	0.1021631	0.8053872	0.420596	0.90651
si:ch211-76m11.5	19.93551994	-0.127886828	0.4318894	-0.29611	0.767146	0.975687
BX571952.1	94.67370008	-0.042450512	0.2028224	-0.209299	0.834215	0.983765
dnal4b	94.16545713	0.087188271	0.2191576	0.3978337	0.690753	0.967157
KCNB2	3.019271197	-1.008811366	1.1344165	-0.889278	0.373854	NA
deaf1	115.6697038	0.206652456	0.1895864	1.0900171	0.275706	0.839541
or111-4	1.543840742	0.401158302	1.5787306	0.2541018	0.799417	NA
znf1089	3.344215891	-0.580218537	1.0458694	-0.554772	0.579051	NA
taar14f	0.341486697	1.892317624	3.2124068	0.5890654	0.555817	NA
znf1021	15.75870653	0.416948964	0.503386	0.8282887	0.407507	0.901305
stxbp6	341.4998248	-0.292789448	0.1550324	-1.888569	0.05895	0.475038
si:ch211-135n15.1	0	NA	NA	NA	NA	NA
si:ch211-22k7.9	18.29198198	0.000402104	0.4458954	0.0009018	0.99928	0.999704
si:busm1-228j01.4	0	NA	NA	NA	NA	NA

smarcd2	205.7664248	-0.276648467	0.1486778	-1.860724	0.062783	0.489019
c1qtnf1	70.94945039	0.829052847	0.2375372	3.4902027	0.000483	0.017416
si:dkeyp-69b9.3	517.6727022	0.125059233	0.1358999	0.9202304	0.357452	0.883975
si:ch211-149b19.2	98.0646644	-0.178024635	0.2008869	-0.886193	0.375513	0.894316
si:ch1073-340i21.3	868.3772748	0.148534494	0.1180517	1.2582155	0.208314	0.775404
slc23a3	42.55513808	0.335412513	0.3060964	1.095774	0.273178	0.838141
si:dkey-238d18.3	0.865544342	3.23079022	2.2781012	1.4181944	0.156134	NA
si:dkeyp-98a7.7	0	NA	NA	NA	NA	NA
caln1	7.482420044	-0.568454076	0.695949	-0.816804	0.41404	NA
ralgapb	469.058032	-0.031741639	0.1255082	-0.252905	0.800342	0.979377
si:dkey-21o19.2	65.7171063	-0.210625582	0.2455854	-0.857647	0.391087	0.897688
CABZ01059403.1	13.49901253	-0.561238742	0.5500456	-1.020349	0.307563	0.861088
CU570691.1	12.84917367	0.813211241	0.5505224	1.4771629	0.139632	0.678399
cbln17	9.211177492	-0.013226484	0.6244472	-0.021181	0.983101	NA
si:ch211-241n15.3	9.178649562	-0.916630184	0.6573507	-1.394431	0.163187	NA
RF00001	0	NA	NA	NA	NA	NA
si:ch211-12h2.8	0.792730004	1.851900952	2.2729754	0.8147475	0.415217	NA
si:dkey-185e18.7	117.9456041	-0.170960985	0.1831051	-0.933677	0.350471	0.880195
CR450793.1	3.784239396	0.890460279	1.1045641	0.8061644	0.420148	NA
ypel5	522.9701646	-0.324596957	0.1254457	-2.58755	0.009666	0.166207
golt1a	48.1161418	-0.00558162	0.2804958	-0.019899	0.984124	0.996944
si:dkey-259j3.5	46.56053135	-0.299701853	0.286921	-1.044545	0.296233	0.853273
RF00001	0.182570949	0.967652056	4.0804729	0.2371421	0.812547	NA
sycp2	2.974872618	1.396688758	1.2888129	1.0837017	0.278497	NA
pdxka	347.810135	0.0476493	0.1281899	0.3717087	0.71011	0.968928
si:dkey-16p19.5	2.806163753	-0.105716368	1.2448892	-0.08492	0.932325	NA
wnk4b	348.5567803	0.226869767	0.1224554	1.852672	0.063929	0.494191
si:dkey-238d18.4	53.31069299	-0.340721126	0.2651569	-1.284979	0.198799	0.763906
si:ch211-14k19.8	77.22112015	-0.002743206	0.2327035	-0.011788	0.990594	0.997679
tmem220	21.45443492	0.128117874	0.4094318	0.3129163	0.754344	0.975114
cep131	377.8108464	-0.083855641	0.118043	-0.710382	0.477467	0.926857
si:ch1073-228j22.2	4.453908177	0.325742154	0.8904742	0.3658075	0.714509	NA
si:dkey-241l7.5	0.182570949	0.967652056	4.0804729	0.2371421	0.812547	NA
mettl4	59.98805923	0.279630973	0.2628532	1.0638293	0.287406	0.845979
taar1b	0.158795395	0.967652056	4.0804729	0.2371421	0.812547	NA
si:ch211-207e14.4	28.27580694	0.358360858	0.369375	0.9701816	0.331956	0.874653
AL772298.1	0.158915748	0.967652056	4.0804729	0.2371421	0.812547	NA
frmpd1b	705.2867279	-0.065737688	0.1058516	-0.621036	0.534576	0.943336
si:ch211-175m2.5	66.95946935	0.073673265	0.2496003	0.2951649	0.767868	0.975687
si:dkey-7f16.3	1.163885225	0.470408	1.7459696	0.2694251	0.787603	NA
si:dkey-183k8.2	4.888132446	-1.130721286	0.9012969	-1.254549	0.209642	NA
si:ch211-154o6.2	326.7267675	-0.002670033	0.1255637	-0.021264	0.983035	0.996944
rrnad1	48.91137047	-0.174673138	0.2880545	-0.606389	0.544256	0.945438
zgc:173585	10.32374128	-0.121097856	0.6360996	-0.190376	0.849015	NA
foxb1a	377.0456871	-0.014046291	0.1260441	-0.11144	0.911268	0.991066
ptpn6	73.0938197	-0.125086548	0.2367964	-0.528245	0.597329	0.955244
cuzd1.1	3.977449713	-1.910382811	1.051626	-1.816599	0.069279	NA
RF00001	0	NA	NA	NA	NA	NA
ube2ka	400.1403446	0.007409661	0.1186718	0.0624383	0.950214	0.99381
kcnj1a.5	206.7337742	-0.244252271	0.17168	-1.422718	0.154818	0.704573
lactb11a	278.5176114	0.113143797	0.1451622	0.77943	0.435726	0.914408
nhsl2	663.3163864	-0.024765182	0.1055082	-0.234723	0.814424	0.981081
si:dkey-83f18.5	0	NA	NA	NA	NA	NA
ushbp1	97.50665494	-0.414095494	0.2014873	-2.055194	0.03986	0.391511

si:ch73-173p19.1	430.1315677	0.09976813	0.1126517	0.8856334	0.375815	0.894376
gphna	1219.20156	0.099216934	0.0848942	1.1687123	0.24252	0.811298
rgs1	33.14896245	-0.844460868	0.3708304	-2.277216	0.022773	0.288667
si:ch211-160j14.3	103.3467961	0.008292296	0.2015169	0.0411494	0.967177	0.996315
si:ch211-214b16.3	4.066517275	0.268731488	0.9656602	0.2782878	0.780791	NA
mustn1b	76.90513149	0.106771074	0.2360368	0.4523493	0.651017	0.960708
hbba1	0	NA	NA	NA	NA	NA
BX510940.1	16.29128395	-0.398714136	0.4709785	-0.846565	0.397237	0.899672
mmd2b	1.900261412	0.337116562	1.4509205	0.2323467	0.816269	NA
si:cabz01054394.5	20.90415257	-0.013858113	0.4259729	-0.032533	0.974047	0.99644
si:dkey-160o24.3	21.81876781	-0.052013277	0.412339	-0.126142	0.899619	0.990121
plrdgb	135.5196661	0.148018494	0.1769589	0.8364569	0.402898	0.90084
mlxipl	13.44817442	-0.360062134	0.5500532	-0.654595	0.512728	0.936253
bcl2b	261.961248	0.238521835	0.1443963	1.651855	0.098564	0.59799
si:ch211-198b3.4	2.287301299	-0.881736487	1.3686099	-0.644257	0.519409	NA
zmp:0000000951	5.622235483	-0.876953324	0.8181059	-1.071931	0.283751	NA
BX663516.1	1.237328071	0.498012251	1.9151249	0.2600417	0.794832	NA
negaly6	46.02297943	-0.233344764	0.2819026	-0.82775	0.407812	0.901418
hbae1.3	189.7507091	-0.082843838	0.155877	-0.531469	0.595094	0.954434
RF00001	0	NA	NA	NA	NA	NA
baxa	4.160168753	-0.826239556	0.9383418	-0.880532	0.378571	NA
il17rel	13.3527056	0.156983086	0.5251124	0.2989514	0.764977	0.975374
si:ch1073-440b2.1	653.4639561	0.136222901	0.1020321	1.3350988	0.181844	0.743853
im:7145024	52.1756619	0.361294433	0.2841462	1.271509	0.203548	0.770916
zgc:113119	52.63720292	0.145927972	0.2753306	0.5300099	0.596105	0.954444
gpx2	17.52205067	-0.37427523	0.4635623	-0.807389	0.419442	0.906454
egr3	186.8994016	0.065087516	0.1574211	0.4134613	0.679269	0.965257
si:dkeyp-104f11.6	12.20823083	0.373336775	0.5567201	0.6706005	0.502475	0.935304
acot14	138.3709559	-0.160808863	0.1778125	-0.904373	0.365797	0.887084
afap1l1a	262.1970027	-0.264309971	0.1344158	-1.966361	0.049257	0.435457
RF00325	0.48448895	0.85344211	2.878836	0.2964539	0.766883	NA
si:ch1073-391i24.1	83.67799375	-0.362363144	0.222173	-1.630996	0.102891	0.609169
cyp46a1.3	122.2418736	-0.397714847	0.1980503	-2.00815	0.044627	0.41583
si:dkey-16p6.1	17.4409339	-0.171554725	0.4693013	-0.365553	0.714698	0.96909
adad1	7.919867036	-0.128396484	0.6739455	-0.190515	0.848906	NA
ppp1r32	1.622828953	-0.034616346	1.4878691	-0.023266	0.981438	NA
si:ch73-264i18.2	0.339758314	0.005883863	3.2682461	0.0018003	0.998564	NA
wfdc2	102.5787128	-0.051814953	0.2051192	-0.252609	0.80057	0.979377
tanc1b	231.8917518	-0.139277327	0.1464563	-0.950982	0.341613	0.878134
zmp:0000001267	11.98837351	0.594999225	0.5524592	1.0770012	0.28148	0.84357
CR384099.1	34.28952074	0.333732807	0.3390373	0.9843543	0.324941	0.87132
dre-mir-92b	0	NA	NA	NA	NA	NA
si:dkey-103g5.4	31.56525475	-0.039034969	0.3583728	-0.108923	0.913264	0.991119
RF00017	0.824635066	-0.63939233	2.222556	-0.287683	0.773589	NA
nkl.3	0.182570949	0.967652056	4.0804729	0.2371421	0.812547	NA
smim13	670.4250439	0.065786384	0.1076639	0.6110347	0.541177	0.944921
si:dkey-73n8.3	45.88269301	-0.06890868	0.2882693	-0.239043	0.811072	0.981081
ankrd27	308.4979162	-0.097322896	0.1247921	-0.77988	0.435461	0.914408
htr3a	161.7725301	-0.026313248	0.1613109	-0.163121	0.870423	0.987421
adam15	55.647161	-0.133551587	0.2645168	-0.504889	0.613637	0.95688
zmp:0000001268	0.63035749	-1.416391854	2.614809	-0.541681	0.588038	NA
ubap1la	32.70056266	-0.165121567	0.3382309	-0.488192	0.625414	0.957354
si:ch211-195o20.7	129.617216	0.092587741	0.1810787	0.511312	0.609133	0.956718
ngfrb	74.71132073	0.159043967	0.2327541	0.6833133	0.494409	0.931926

ro60	217.0218155	0.040789606	0.1558011	0.2618057	0.793471	0.977937
si:ch211-91p5.3	34.1004307	0.061201715	0.3281245	0.1865198	0.852037	0.985174
si:ch211-253b8.2	223.8917163	0.023644978	0.1421002	0.1663965	0.867845	0.987245
prr14	610.4082704	-0.04846077	0.1003925	-0.482713	0.6293	0.957354
csgalnact1b	37.75340033	-0.176540008	0.3201853	-0.551368	0.581381	0.951731
tspearb	29.53159351	0.629755516	0.3698123	1.7029059	0.088586	0.569471
mettl15	49.54642333	-0.155141596	0.2807292	-0.552638	0.580511	0.951621
vsig10l	20.3882885	0.658969369	0.4318994	1.5257475	0.127073	0.656588
r3hcc1l	365.1004142	-0.081537814	0.119418	-0.682793	0.494737	0.931926
dusp23b	149.2094844	0.355426372	0.2025692	1.7545922	0.079329	0.547489
FP885542.1	0.530029684	-0.939524117	2.7937867	-0.336291	0.736652	NA
kcnj11l	562.1582294	-0.054432469	0.0999815	-0.544425	0.586149	0.953245
dus1l	153.828995	0.159780373	0.172965	0.923773	0.355604	0.882511
pbx3a	28.8461992	-0.543349538	0.3602057	-1.508442	0.131441	0.664907
nexmifa	1409.393952	-0.013271098	0.0800322	-0.165822	0.868297	0.987245
tmem235b	7.627697859	-0.62793242	0.6805816	-0.922641	0.356194	NA
cenpx	62.2534092	-0.213573469	0.2581932	-0.827185	0.408132	0.90171
adgrl1a	128.1206255	-0.169299645	0.1945325	-0.87029	0.384142	0.895192
si:dkey-240n22.6	55.03963348	-0.657762099	0.2796055	-2.352465	0.018649	0.254502
si:dkeyp-115d7.2	0.356300316	0.005884122	3.214964	0.0018302	0.99854	NA
hnrnpa0a	5522.464884	0.056029073	0.0745217	0.7518487	0.452142	0.918781
BX572619.1	224.008693	-0.153356756	0.1481653	-1.035038	0.300651	0.856282
si:dkeyp-4f2.1	20.38473978	-0.778260646	0.4593522	-1.694257	0.090216	0.575093
pmaip1	75.65697318	0.287590843	0.246506	1.1666686	0.243344	0.812133
gc	171.2688793	0.044416418	0.2157541	0.205866	0.836896	0.984636
si:ch73-106l15.4	5.782524048	0.79242063	0.8234282	0.9623433	0.335877	NA
mapta	1372.126833	0.199060626	0.0970004	2.0521631	0.040154	0.392132
BX088538.1	31.29349574	0.299606968	0.348832	0.858886	0.390403	0.897304
ccdc175	4.714365049	-0.209821384	0.8986678	-0.23348	0.815388	NA
arhgap20	106.0156773	0.083653244	0.2038407	0.4103855	0.681523	0.965476
BX120005.1	0.808333769	-0.605422531	2.2351432	-0.270865	0.786495	NA
urahb	8.422815995	-0.041554932	0.648672	-0.064062	0.948921	NA
si:ch211-198c19.1	1.187499141	0.431586902	1.7362295	0.2485771	0.803688	NA
ednrba	22.19215383	-0.29091956	0.4208411	-0.691281	0.489389	0.931926
si:ch211-110e21.3	212.3522702	-0.125228605	0.1550343	-0.807748	0.419236	0.906406
rhobtb2b	77.85740353	0.245370218	0.23523	1.0431079	0.296898	0.853798
si:ch73-109d9.4	5.141993444	-0.865679132	0.8710899	-0.993788	0.320326	NA
tspan4a	301.8610999	-0.09901631	0.1241401	-0.797618	0.425092	0.908819
si:dkeyp-121d4.3	411.3370455	-0.033391487	0.1161212	-0.287557	0.773686	0.975687
miga2	38.18468617	0.105633834	0.3370063	0.3134477	0.753941	0.975056
wu:fb59d01	620.2298995	-0.887001462	0.1058693	-8.378266	5.37E-17	3.22E-14
grn1	22.43899049	-0.923670151	0.4494857	-2.054949	0.039884	0.391511
hopx	328.4090558	-0.017953578	0.1227813	-0.146224	0.883745	0.988706
fdxr	154.7723093	-0.52024589	0.1755078	-2.964233	0.003034	0.07278
si:dkey-238d18.10	0	NA	NA	NA	NA	NA
clk4a	2970.950248	-0.064287814	0.0808897	-0.794759	0.426754	0.910217
si:ch211-232p21.6	0.809941799	0.539629624	2.2311614	0.2418604	0.808888	NA
zgc:158463	8249.989484	0.555356803	0.4644805	1.1956516	0.231833	0.800045
il1fma	8.891916057	0.405172208	0.670659	0.6041405	0.54575	NA
RF00001	0	NA	NA	NA	NA	NA
ftr35	0.678375468	-2.878459328	2.4926399	-1.154783	0.248179	NA
pnpla2	53.27349453	-0.265426138	0.2747194	-0.966172	0.333958	0.875256
tmem176l.2	146.6922079	-1.733316662	0.1923258	-9.012397	2.02E-19	1.59E-16
si:ch73-281i18.3	3.551422559	1.343649893	1.0931499	1.2291543	0.219014	NA

si:dkey-24p1.7	4.763366204	0.111453985	0.8712877	0.1279187	0.898213	NA
mbpb	1824.581196	0.028675426	0.078598	0.3648366	0.715233	0.96909
si:dkey-164f24.2	1019.225838	0.389605341	0.0896373	4.3464664	1.38E-05	0.00094
rab12	76.06245863	0.279362286	0.2350578	1.1884831	0.234643	0.803509
si:dkey-205h13.2	1306.131503	0.048248664	0.0924862	0.5216849	0.60189	0.956045
si:ch211-212k18.15	1.303389427	-1.599233742	1.7364034	-0.921004	0.357049	NA
etv7	8.989397735	-0.194061873	0.6395516	-0.303434	0.761559	NA
macf1b	21.62106576	-0.536389535	0.4116162	-1.30313	0.19253	0.756795
si:ch211-105c13.3	746.2350355	0.091860552	0.0934956	0.982512	0.325848	0.871896
omgb	60.09344097	-0.086876817	0.2628754	-0.330487	0.741032	0.973154
men1	358.1066166	-0.220534013	0.1173002	-1.880082	0.060097	0.478687
rpl11a	121.888036	0.053222428	0.1917989	0.2774908	0.781403	0.97627
ciz1b	53.84501653	0.030725033	0.2760878	0.1112872	0.911389	0.991066
dhx58	13.0200462	0.037968414	0.5251302	0.0723029	0.942361	0.993276
fam161b	32.38509019	0.258373752	0.3473623	0.7438162	0.456988	0.921431
CR936459.1	2.685370062	1.173916984	1.3156641	0.8922619	0.372253	NA
hbae1.1	22.92221796	0.059522155	0.4616686	0.1289283	0.897414	0.990121
si:ch211-132g1.3	118.9981329	0.029799871	0.186861	0.1594762	0.873294	0.987846
si:dkey-24l17.4	0	NA	NA	NA	NA	NA
si:dkey-12j5.1	305.245073	-0.119116263	0.1261262	-0.944421	0.344954	0.878434
tecrl2a	32.5280209	0.168330799	0.3400628	0.4949992	0.620601	0.957354
cplx3b	233.688001	0.116363221	0.1357699	0.8570619	0.391411	0.897688
fam83e	170.0208416	0.098025395	0.1583826	0.6189152	0.535972	0.943336
kcnq4	2.472428806	-2.673971963	1.4411044	-1.855502	0.063525	NA
pimr23	0	NA	NA	NA	NA	NA
si:ch73-367f21.5	23.86669308	-0.07616164	0.3922172	-0.194182	0.846033	0.985174
bod1l1	861.2465025	-0.008607611	0.0933185	-0.092239	0.926508	0.992702
ugt1b5	173.9166522	0.293915655	0.1548847	1.897642	0.057743	0.471435
si:ch73-78o10.1	57.29629106	0.352470162	0.2539325	1.3880465	0.165123	0.721223
si:ch211-198e20.10	3.959907854	0.235082836	0.9284195	0.2532076	0.800108	NA
si:dkeyp-46h3.5	0	NA	NA	NA	NA	NA
zgc:92594	263.493311	0.072710988	0.1441806	0.5043049	0.614047	0.95688
fbln7	66.24885121	-0.059545581	0.2584274	-0.230415	0.817769	0.981594
si:dkeyp-98a7.4	0	NA	NA	NA	NA	NA
desi1b	129.2907139	0.299684064	0.1972276	1.5194834	0.128641	0.659646
erbb4b	16.24418363	0.592817338	0.4711406	1.2582598	0.208298	0.775404
rnf166	7.589112188	-0.467657244	0.7459051	-0.626966	0.530681	NA
BX571825.5	0	NA	NA	NA	NA	NA
baalcb	150.8650467	0.004403636	0.1628724	0.0270373	0.97843	0.996762
si:ch211-93n23.7	9.722078864	-0.328588805	0.6196579	-0.530275	0.595922	NA
si:dkey-22l14.11	7.972369242	0.874448729	0.7553576	1.1576619	0.247002	NA
si:dkey-63j12.4	11.71454712	-0.323807199	0.6094871	-0.531278	0.595226	0.954434
BX957322.1	18.85116078	0.89642354	0.4647861	1.9286796	0.053771	0.454191
sfrp2l	6.663599877	-0.24733034	0.7600054	-0.325432	0.744854	NA
MDFI	289.3008641	0.091101801	0.1274637	0.7147275	0.474777	0.925399
patl2	4.124764645	-2.002483718	1.0250323	-1.953581	0.050751	NA
pstpip2	11.05674785	0.122595239	0.5711108	0.214661	0.830032	NA
ccdc169	4.508447633	-0.554944054	0.9120944	-0.608428	0.542903	NA
tfpt	252.4712189	-0.107764202	0.1332386	-0.808806	0.418627	0.906406
xrra1	9.055560374	0.157837632	0.6476874	0.2436941	0.807468	NA
nhlrc2	339.1024126	0.020455851	0.124081	0.1648588	0.869055	0.987245
si:dkey-265e15.2	36.91870503	0.149795458	0.3371449	0.4443058	0.656821	0.96205
ncam3	50.92025167	-0.885610385	0.2812284	-3.149078	0.001638	0.046135
pcdh2ab11	0	NA	NA	NA	NA	NA

si:cabz01054396.2	96.22351758	0.061138315	0.2084025	0.2933665	0.769242	0.975687
si:dkey-217f16.1	1.152686753	-0.310571849	1.95009	-0.15926	0.873464	NA
CU929037.1	0.157187365	-0.955901296	4.0804729	-0.234262	0.814781	NA
coro7	102.1172779	-0.044322645	0.1981228	-0.223713	0.822981	0.982189
RF00009	18.23659998	0.216787849	0.534688	0.4054474	0.685149	0.966146
fgd5b	8.396280807	0.342389437	0.6918674	0.4948772	0.620687	NA
ptges3b	1312.418019	-0.237406191	0.0836879	-2.836804	0.004557	0.096369
si:ch211-160d20.5	0.325573202	0.005883621	3.3172783	0.0017736	0.998585	NA
dre-mir-2188	0.340386822	-1.882368278	3.2159502	-0.585323	0.558331	NA
BX322587.1	0.967129164	-0.023635743	1.9568585	-0.012078	0.990363	NA
rnaseh2c	325.5971272	-0.036708314	0.1270658	-0.288892	0.772664	0.975687
si:dkey-117n7.5	43.09661266	0.162287719	0.3000992	0.5407802	0.588659	0.953319
ppm1lb	49.4815807	-0.060368252	0.2802593	-0.215401	0.829454	0.982805
mcama	1070.244725	-0.034848936	0.0856468	-0.406891	0.684088	0.965679
si:ch1073-406l10.2	116.6610491	0.373889326	0.1962494	1.9051748	0.056757	0.467438
ctdspl3	528.4059239	0.080980403	0.1065203	0.7602346	0.447114	0.917154
BX927136.1	0	NA	NA	NA	NA	NA
FO704622.1	4.947286782	-0.782317766	0.8661206	-0.903243	0.366397	NA
lsm3	400.5895949	-0.110076822	0.1373477	-0.801447	0.422873	0.90714
MFAP4 (1 of many)	11.61678579	0.40972465	0.5522977	0.7418547	0.458175	0.921978
btf3l4	591.8152126	-0.008521147	0.1000759	-0.085147	0.932145	0.992702
tex26	5.432302424	0.944881106	0.8597096	1.0990701	0.271738	NA
ism2a	12.45951439	-0.019054766	0.5374127	-0.035456	0.971716	0.996315
nfe2l2b	253.4978353	-0.350786611	0.141274	-2.483022	0.013027	0.20396
atf7ip2	171.3143612	0.106969022	0.1754789	0.6095834	0.542138	0.944974
trabd2a	128.8493079	0.143334857	0.1883942	0.7608242	0.446762	0.917154
abcf3	278.207005	-0.099244544	0.1379183	-0.719589	0.471778	0.924838
si:ch211-276a23.5	45.14623263	0.327581928	0.296819	1.1036419	0.269748	0.834705
gfral	30.73966393	0.228808413	0.3537169	0.6468687	0.517717	0.938592
BX004785.1	0.158795395	0.967652056	4.0804729	0.2371421	0.812547	NA
si:cabz01054394.7	23.14297739	0.038602161	0.3988961	0.0967725	0.922907	0.992466
qpct	211.4706107	0.344009274	0.1433165	2.4003472	0.01638	0.236243
CABZ01077218.1	68.66787379	-0.152871537	0.2410407	-0.634214	0.525941	0.940236
cyldb	87.48282077	0.170257567	0.2117266	0.8041387	0.421317	0.90651
fam161a	153.2866582	0.120266013	0.162271	0.741143	0.458607	0.921978
si:zfos-754c12.2	0.491560863	0.853615855	2.865157	0.2979299	0.765757	NA
aqp8b	77.69215992	-0.803243315	0.262564	-3.059229	0.002219	0.05751
si:dkey-26g8.5	0.50625413	-0.940106488	2.8357787	-0.331516	0.740255	NA
si:dkey-11o15.7	0	NA	NA	NA	NA	NA
si:ch211-286b5.4	0	NA	NA	NA	NA	NA
nrg3b	466.5095692	0.090276836	0.1097592	0.8224988	0.410793	0.90316
rnf130	784.1900175	0.037320617	0.0885436	0.4214945	0.673394	0.965257
hapln1a	4023.626808	0.319644638	0.0688352	4.6436251	3.42E-06	0.000293
si:dkey-288a3.2	7.733560586	0.043659719	0.6955814	0.0627672	0.949952	NA
ciz1a	58.9034089	-0.04687231	0.2616564	-0.179137	0.85783	0.985703
crebzf	763.182833	-0.016116417	0.09953	-0.161925	0.871365	0.987754
efna5a	585.3543512	0.025725472	0.0970271	0.2651369	0.790904	0.977402
slc25a32a	667.838492	-0.134637537	0.0951405	-1.415144	0.157026	0.707677
si:dkeyp-101e12.1	0	NA	NA	NA	NA	NA
sertad4	299.6448613	0.000289103	0.1331945	0.0021705	0.998268	0.999329
si:ch73-41e3.7	2.296987508	-0.063120416	1.4247498	-0.044303	0.964663	NA
prr7	25.70921465	1.009105708	0.4050336	2.4914126	0.012724	0.200229
akap1a	10.29694556	0.738494086	0.658565	1.1213686	0.262131	NA
pcdh18a	949.1802561	0.021321021	0.0994328	0.2144264	0.830215	0.982916

si:dkey-239j18.3	14.33879302	-0.52829318	0.5044754	-1.047213	0.295001	0.851519
znf1042	19.77111158	-0.799746246	0.4681517	-1.708306	0.08758	0.566637
si:dkey-9k7.3	39.42236384	-0.12746977	0.3109599	-0.409924	0.681862	0.965476
lpar4	12.54547387	0.681598538	0.5449772	1.2506918	0.211047	0.77859
si:rp71-19m20.1	314.4962997	0.101671729	0.1319538	0.7705103	0.440997	0.916336
si:ch211-189a21.1	11.71446781	-0.010394284	0.5794827	-0.017937	0.985689	0.996944
si:dkey-207m2.4	6.166843574	0.269501592	0.7717837	0.3491932	0.726944	NA
asb13b	113.9535165	0.430065198	0.1905032	2.2575219	0.023975	0.299022
MRPS12	499.3244988	-0.074962703	0.1119185	-0.669797	0.502987	0.935304
slc35d2	32.0615949	-0.193610573	0.3604506	-0.537135	0.591174	0.954193
si:dkey-262k9.4	104.6694533	0.215165193	0.1993166	1.0795148	0.280358	0.843423
BX296541.1	0.173729368	-0.955901296	4.0804729	-0.234262	0.814781	NA
scarb2c	242.0025792	0.068272907	0.15703	0.4347762	0.663725	0.964736
RF00017	0	NA	NA	NA	NA	NA
RF00017	0	NA	NA	NA	NA	NA
si:dkey-51d8.1	3.766249585	1.810788472	1.0815698	1.6742224	0.094087	NA
si:dkey-22n8.3	21.8925439	-0.187910382	0.4544338	-0.413504	0.679237	0.965257
cita	467.9679847	-0.048643233	0.1046972	-0.464609	0.642212	0.959994
cobl11a	133.6285029	-0.036072477	0.1964601	-0.183612	0.854318	0.985174
atxn7l1	9.749008242	-1.183389181	0.6235102	-1.897947	0.057703	NA
usp44	517.3280233	-0.140514417	0.1035493	-1.35698	0.174787	0.735372
si:dkey-16j16.4	21.14101635	-0.203166512	0.4126779	-0.492313	0.622498	0.957354
si:ch211-112c15.8	0.964610636	-0.018889399	1.958304	-0.009646	0.992304	NA
zgc:172218	0.317831496	1.808837541	3.5779923	0.5055454	0.613176	NA
jcada	373.5216372	-0.108201723	0.1142732	-0.946869	0.343706	0.878434
socs1b	1.32527496	-2.703143513	1.9371225	-1.395443	0.162882	NA
pcdh2ab8	2.757753476	-0.863420747	1.1839024	-0.729301	0.465818	NA
zgc:173705	1.596696509	-0.007252485	1.5931558	-0.004552	0.996368	NA
si:dkeyp-75h12.5	1302.071578	0.008305368	0.094277	0.0880954	0.929801	0.992702
nedd9	613.9930218	-0.047091341	0.0984833	-0.478166	0.632532	0.957356
soul5l	6.769351299	-0.502222987	0.7962082	-0.630768	0.528192	NA
si:dkey-210j14.3	50.71615526	0.117278457	0.2735149	0.4287828	0.668081	0.964736
slc16a12b	531.0964759	0.070586196	0.1175814	0.6003177	0.548295	0.945871
si:dkey-19e4.5	1195.609949	0.070941836	0.0830489	0.8542174	0.392985	0.897848
mmp20a	7.146764698	0.33333732	0.7143281	0.4666445	0.640754	NA
daglb	340.8067114	-0.000300461	0.1234527	-0.002434	0.998058	0.999271
rpz6	42.15225513	0.147770683	0.3073595	0.4807747	0.630677	0.957354
RF00001	0.158795395	0.967652056	4.0804729	0.2371421	0.812547	NA
cd44b	15.2094171	-0.284149392	0.5259922	-0.540216	0.589048	0.953345
tasora	490.3210553	-0.152155388	0.1104408	-1.37771	0.168293	0.725919
zmp:0000001289	0.173729368	-0.955901296	4.0804729	-0.234262	0.814781	NA
si:ch211-261n11.8	0	NA	NA	NA	NA	NA
CABZ01039863.1	34.8573673	0.026857854	0.3229284	0.0831697	0.933717	0.992702
adgre5b.3	73.80721971	-0.372440119	0.2260679	-1.64747	0.099461	0.599802
si:ch211-265o23.1	101.759643	-0.066197885	0.198808	-0.332974	0.739154	0.972722
cacna1g	381.7021298	0.117602752	0.1285535	0.9148157	0.360288	0.885686
clint1b	405.6722925	0.194131726	0.1136149	1.7086827	0.08751	0.566637
sh3tc2	432.8707911	0.035595759	0.1151308	0.3091766	0.757187	0.975374
si:ch211-161h7.8	149.8732817	-0.591460822	0.1809807	-3.268088	0.001083	0.032921
mlip	219.6957026	0.044256286	0.1537293	0.2878845	0.773435	0.975687
smim4	197.2590403	0.120425242	0.1573494	0.7653364	0.444071	0.916699
dsn1	188.3819768	-0.157261283	0.1537919	-1.022559	0.306517	0.860027
aldh2.1	467.9241873	-0.10459267	0.1044407	-1.001455	0.316607	0.867129
iqce	154.0022677	0.024480274	0.1707084	0.1434041	0.885971	0.988936

rasal3	25.47690476	-0.045920452	0.3800081	-0.120841	0.903817	0.990291
si:ch211-64i20.3	8.714296688	-0.614654789	0.689318	-0.891685	0.372562	NA
selenow2b	551.8271102	0.166726651	0.1052994	1.5833587	0.11334	0.631061
znf1008	93.86563545	0.036632184	0.2147432	0.170586	0.864549	0.98601
npy2rl	8.203772057	-0.00356251	0.6668252	-0.005342	0.995737	NA
BX005085.1	82.84162298	-0.152095991	0.2202485	-0.690566	0.489839	0.931926
si:dkey-210j14.5	35.72805722	-0.361018453	0.3320063	-1.087385	0.276867	0.840075
si:ch211-147k9.8	5.335592178	0.728977828	0.8261993	0.8823268	0.3776	NA
si:ch73-367f21.6	26.29137344	-0.124194548	0.3897729	-0.318633	0.750005	0.97419
si:ch211-189e2.3	1.676273031	-1.177417679	1.5072204	-0.781185	0.434694	NA
si:dkey-88n24.10	0	NA	NA	NA	NA	NA
CR388164.1	1.118581941	-2.434763583	2.0644676	-1.179366	0.238252	NA
si:cabz01007807.1	26.00775375	-0.009196079	0.3970664	-0.02316	0.981523	0.996944
si:zfos-1505d6.3	0.339758314	0.005883863	3.2682461	0.0018003	0.998564	NA
si:ch211-59o9.10	202.4405573	0.01305891	0.1454963	0.0897542	0.928483	0.992702
SLC15A5	4.340815154	0.031549274	0.9116765	0.0346058	0.972394	NA
spcs3	865.0835612	-0.104236235	0.0994355	-1.04828	0.29451	0.850662
rnf146	730.8358881	-0.127996492	0.1047308	-1.222148	0.221652	0.78829
si:ch73-109i22.2	9.497193284	-0.407737101	0.623872	-0.653559	0.513396	NA
or116-2	5.301419585	-0.152288413	0.82111	-0.185467	0.852863	NA
BX511080.1	0.158795395	0.967652056	4.0804729	0.2371421	0.812547	NA
cln6b	2.833045057	-0.493546763	1.1007781	-0.448362	0.653892	NA
ccdc9b	12.22470164	-0.185396498	0.559212	-0.331532	0.740243	0.973048
CR388047.1	0.524057646	2.503896172	2.7289583	0.9175282	0.358866	NA
BX682550.1	20.36837435	0.209924588	0.4265467	0.4921492	0.622614	0.957354
si:dkey-16p6.1	7.689671405	0.843762925	0.7602213	1.1098912	0.267046	NA
rtn4r	380.2824891	0.087582237	0.1132571	0.7733044	0.439342	0.916082
si:dkey-6b12.5	180.5419718	0.065089697	0.1499999	0.4339316	0.664338	0.964736
BX248410.1	1.314749537	-0.712951519	1.7335279	-0.411272	0.680873	NA
reck	732.1709292	0.138287795	0.1351351	1.0233302	0.306152	0.860027
F2R (1 of many)	3.002888572	-0.969017615	1.1816225	-0.820074	0.412174	NA
si:dkey-165a24.9	23.40300309	-0.185793316	0.3943382	-0.471152	0.637532	0.958872
znf318	1110.293804	-0.057984661	0.0846576	-0.684931	0.493387	0.931926
CR394546.2	4.190940409	0.141720625	0.9486018	0.1493995	0.881238	NA
rac3a	736.7839963	0.005636408	0.1006879	0.055979	0.955359	0.994543
fa2h	877.4175884	-0.670527715	0.0897249	-7.473153	7.83E-14	3.29E-11
CABZ01084273.1	101.4162679	0.140796892	0.2062372	0.682694	0.4948	0.931926
rxfp1	123.6025155	-0.054444671	0.217466	-0.250359	0.802309	0.979492
CU462878.1	207.913713	-0.405498553	0.152162	-2.664913	0.007701	0.140088
si:dkey-1m11.5	3.764371025	-0.399910944	1.0174454	-0.393054	0.69428	NA
rab11bb	434.1396347	-0.095862147	0.1126054	-0.851311	0.394597	0.898861
CR626886.1	14.83579228	-0.744096557	0.5108607	-1.456555	0.145239	0.690044
dre-mir-30a	0	NA	NA	NA	NA	NA
si:dkeyp-67a8.4	11.21411005	0.237658341	0.5821781	0.4082227	0.68311	0.965476
slc17a7b	146.4969546	0.274453318	0.1849639	1.4838211	0.137856	0.675313
zmp:0000000984	5.79603713	0.243035194	0.8088975	0.3004524	0.763832	NA
si:ch1073-174d20.1	128.9117994	0.225033461	0.1810914	1.2426511	0.213996	0.782319
znf1136	61.6516816	0.0755591	0.2523114	0.2994677	0.764583	0.975374
qdprb2	0.847782112	0.655103352	2.1028616	0.3115295	0.755398	NA
si:dkey-248g21.1	35.62886452	0.024939625	0.3651899	0.0682922	0.945553	0.993589
si:ch211-105f12.2	18.8330198	0.467132137	0.4818024	0.9695514	0.33227	0.874653
tmem196b	22.83722919	0.081980558	0.4013394	0.2042674	0.838145	0.984797
si:dkey-94l16.4	62.0852937	-0.30023612	0.2459973	-1.220485	0.222281	0.788551
tmem240b	137.0078187	0.117504809	0.1792009	0.6557154	0.512007	0.936253

anapc10	137.8931787	0.145496807	0.2029108	0.7170482	0.473344	0.924993
cyldl	21.96219239	-0.304779256	0.4185188	-0.728233	0.466471	0.924063
pttg1ipa	282.3196991	0.270229914	0.1379565	1.9588046	0.050136	0.438277
BX005105.1	37.73996665	0.284523845	0.3349575	0.8494326	0.395641	0.899378
zgc:173709	53.62888586	0.381696446	0.2628301	1.4522554	0.146431	0.692708
si:ch73-362m14.2	18.75703515	-0.267865277	0.4628669	-0.578709	0.562786	0.948728
FO904898.1	0	NA	NA	NA	NA	NA
rab11fip4a	292.9931377	0.112925915	0.1353155	0.834538	0.403978	0.901101
BX323556.1	8.190583146	-1.051780064	0.7097076	-1.481991	0.138343	NA
BX548044.3	0	NA	NA	NA	NA	NA
si:ch211-117c9.1	8.379248971	-1.157131915	0.7563983	-1.529792	0.126068	NA
BX072576.2	29.18505514	0.554370773	0.3646956	1.5200916	0.128488	0.659414
gapvd1	581.8244145	0.098721588	0.1079276	0.9147021	0.360348	0.885686
si:dkeyp-73b11.8	564.509002	-0.998937652	0.1095879	-9.115405	7.84E-20	6.74E-17
bcam	296.1604632	0.054551564	0.1309449	0.4165993	0.676972	0.965257
si:dkey-16p6.1	4.141698948	-1.04756836	1.0037955	-1.043607	0.296667	NA
si:ch73-286h23.4	0.173729368	-0.955901296	4.0804729	-0.234262	0.814781	NA
lamtor5	264.6789188	-0.027807227	0.1385366	-0.200721	0.840917	0.985146
mtx3	149.0379293	-0.054095282	0.1815785	-0.297917	0.765767	0.975374
RF00001	0	NA	NA	NA	NA	NA
rbm11	1.493073575	-0.932799967	1.6069462	-0.58048	0.561591	NA
rheb	494.0251095	0.150205245	0.1040887	1.4430505	0.149006	0.696454
RF00001	0	NA	NA	NA	NA	NA
wdr45	488.9045494	0.053050003	0.1095982	0.4840406	0.628357	0.957354
msl1b	580.325358	0.0749387	0.0992594	0.7549781	0.450262	0.918761
gsta.1	3425.54299	-0.923723574	0.0974445	-9.479481	2.56E-21	2.66E-18
tmem108	259.8284697	0.079638527	0.1474493	0.5401078	0.589123	0.953345
clpb	202.2946934	-0.165612281	0.1521874	-1.088213	0.276501	0.839541
zp2.3	0	NA	NA	NA	NA	NA
zgc:113223	56.73902822	0.267803461	0.260163	1.0293679	0.303307	0.85729
phlpp2	15.48959576	0.18608643	0.5294202	0.351491	0.72522	0.970402
CU062628.1	2.897729046	-0.633951666	1.2323868	-0.51441	0.606966	NA
krtt1c19e	23151.50264	0.294713079	0.1926098	1.5301045	0.125991	0.654803
BX248410.2	0.959146752	0.977549673	2.0106641	0.4861825	0.626838	NA
serpina1	3802.183211	0.388860737	0.0991498	3.9219534	8.78E-05	0.004386
ralyl	70.81287135	0.219024759	0.2354007	0.9304337	0.352147	0.880879
ldlrad2	80.51964538	0.245793898	0.2294124	1.0714064	0.283987	0.844697
usp48	499.9135768	-0.039749928	0.1120632	-0.35471	0.722807	0.970375
CU929391.2	37.84422512	0.01684868	0.319848	0.0526771	0.957989	0.99467
ubxn1	577.5751723	-0.114643877	0.0992552	-1.155042	0.248073	0.817415
CABZ01065328.1	0	NA	NA	NA	NA	NA
rnf212	6.230536631	-1.651919814	0.8523702	-1.938031	0.052619	NA
MAPK8IP1 (1 of many	172.5514226	-0.274936272	0.1666963	-1.649325	0.099081	0.598421
pprc1	342.4918533	0.203198469	0.1451602	1.3998221	0.161567	0.715364
fam20cb	134.2249311	0.18708727	0.1769354	1.0573762	0.29034	0.846262
si:dkey-27o4.1	19.9118804	-0.633762906	0.4334372	-1.462179	0.143692	0.686818
il19l	0.839019598	-0.671793185	2.356059	-0.285134	0.775541	NA
RF00001	0	NA	NA	NA	NA	NA
zmp:0000000991	48.98739761	-0.192987105	0.2896294	-0.666324	0.505204	0.935724
dre-mir-222a	0	NA	NA	NA	NA	NA
pcdh2ab5	0.680145136	-1.475466752	2.2805885	-0.646968	0.517653	NA
CR855311.1	841.6851127	-0.775651369	0.3278081	-2.366175	0.017973	0.248356
pimr168	0	NA	NA	NA	NA	NA
znf1065	36.03462776	-0.531131644	0.3434086	-1.546646	0.121949	0.649101

RF00001	0.158795395	0.967652056	4.0804729	0.2371421	0.812547	NA
dre-mir-132-2	1.537947772	1.852333309	1.7347791	1.0677632	0.285627	NA
zgc:86896	1935.566117	0.241885333	0.0852596	2.8370456	0.004553	0.096369
si:dkey-46i9.6	72.11241622	-0.125012373	0.2445194	-0.511258	0.609171	0.956718
taar14e	0.332645115	0.005883744	3.2924249	0.0017871	0.998574	NA
gzma	2.010526176	-0.478445426	1.3048707	-0.366661	0.713872	NA
CABZ01072157.1	4.925628655	-0.75789046	0.8840231	-0.85732	0.391268	NA
cd3eap	133.0071939	0.159857707	0.1748811	0.9140937	0.360668	0.885686
onecut2	4.356057861	-0.40541178	0.9989783	-0.405826	0.68487	NA
ndufv3	892.7465571	0.023259499	0.1051125	0.2212819	0.824873	0.982196
si:dkey-71l4.2	8.961215892	-0.312923658	0.6262437	-0.499684	0.617298	NA
si:cabz01074946.1	8.14677219	-0.715202479	0.6966889	-1.026574	0.304621	NA
CABZ01067973.1	3.216216635	0.169789409	1.0681629	0.1589546	0.873705	NA
CABZ01020840.1	67.87114906	-0.342551544	0.2797812	-1.224355	0.220818	0.787641
ERBB4 (1 of many)	13.49436272	0.79830962	0.5696321	1.4014477	0.16108	0.714331
scpp1	29.63304898	0.258384462	0.3565971	0.7245838	0.468707	0.924328
wdsub1	241.0115975	-0.095005182	0.1471918	-0.645452	0.518635	0.938803
SH2B2	58.88212688	0.159568248	0.2533185	0.6299116	0.528752	0.942109
si:dkey-246i14.3	29.73793621	-0.235684615	0.3629866	-0.649293	0.516149	0.937997
ctrb1	1260.796959	-0.59130615	0.2721802	-2.17248	0.029819	0.334457
si:dkeyp-7a3.1	7.347576033	0.134256773	0.7015318	0.1913766	0.848231	NA
ponzr1	187.7443843	-0.474105391	0.1566182	-3.02714	0.002469	0.062293
mtbp	90.34790959	-0.061135899	0.2114063	-0.289187	0.772438	0.975687
sp100.3	1.164634085	-0.410115428	1.7445115	-0.235089	0.81414	NA
gnb1a	4613.219485	0.003244803	0.0908356	0.0357217	0.971504	0.996315
RF00001	8.120845422	0.288087608	0.6726453	0.4282905	0.66844	NA
si:ch211-197f20.1	3.366813498	0.027877911	1.0853313	0.0256861	0.979508	NA
mrpl20	374.4954771	-0.07965391	0.140755	-0.565905	0.571459	0.949418
taar14h	2.510577606	-1.028637339	1.2403737	-0.829296	0.406937	NA
ppp1r3aa	63.67363801	-0.068442907	0.270891	-0.252658	0.800532	0.979377
sycn.1	0.656369582	-1.475482481	2.3019817	-0.640962	0.521547	NA
BX530037.2	0	NA	NA	NA	NA	NA
ttll10	35.96262196	-0.302348364	0.3397286	-0.88997	0.373482	0.893663
si:ch211-269k10.5	14.54806016	0.609200917	0.516635	1.1791709	0.23833	0.807449
ccdc17	2.776388175	0.871243325	1.3254226	0.6573325	0.510967	NA
trarg1a	544.9797629	0.136359032	0.113048	1.2062048	0.227739	0.794934
lipcb	50.50485246	0.244337327	0.2797585	0.8733867	0.382452	0.895192
ANKRD66	0.330916733	-1.849579229	3.2489867	-0.569279	0.569167	NA
thsd7ab	11.82200025	-0.284094025	0.6183869	-0.459411	0.645939	0.96029
tmem121ab	36.91167288	0.052338957	0.3479951	0.1504014	0.880448	0.987881
zmp:0000000997	0	NA	NA	NA	NA	NA
oma1	51.85287987	0.354621209	0.2735282	1.2964705	0.194813	0.758937
trim8a	426.2825825	-0.00150921	0.1108927	-0.01361	0.989141	0.997589
f2rl2	57.75052144	-0.182269793	0.2531355	-0.720048	0.471495	0.924838
zgc:158404	58.26531435	-0.816471097	0.2668202	-3.060005	0.002213	0.05751
ifit11	15.6361547	0.163872206	0.4973024	0.3295223	0.741761	0.973154
tmem138	105.3057208	0.192704571	0.2052348	0.9389468	0.347758	0.880195
CABZ01085658.1	137.7445348	0.043511589	0.1813453	0.2399377	0.810379	0.980867
RF00001	0.317711143	1.810477155	3.2978238	0.5489915	0.583011	NA
CR753886.1	256.9784142	0.20935437	0.1332489	1.5711526	0.116147	0.63841
FP102786.1	0.515095711	0.926015374	2.6527421	0.3490785	0.72703	NA
si:dkey-7j14.6	740.3621602	-0.096396548	0.1154842	-0.834716	0.403878	0.900979
si:ch1073-110a20.7	40.81441247	-1.238629466	0.3250596	-3.810469	0.000139	0.006408
mfap5	18.75741189	-0.148775869	0.4657449	-0.319436	0.749396	0.97419

PDZD4	373.0975303	-0.056895021	0.1182844	-0.481002	0.630515	0.957354
mrpl52	128.93532	0.099819226	0.1978581	0.5044989	0.613911	0.95688
naa40	6.576068528	0.56051929	0.7622228	0.7353746	0.462111	NA
si:dkey-63j12.4	2.211219419	-1.128190456	1.3859967	-0.813992	0.415649	NA
rassf11	0.698456806	0.09172817	2.5265115	0.0363063	0.971038	NA
wdr4	145.0280393	0.142406518	0.1684938	0.8451734	0.398014	0.899672
gpc1b	236.0740303	0.107862696	0.1388029	0.7770925	0.437104	0.914408
taar10b	0.158915748	0.967652056	4.0804729	0.2371421	0.812547	NA
pkp1a	7.790651207	0.007668296	0.7451974	0.0102903	0.99179	NA
si:ch211-213a13.1	195.2852812	-0.138779279	0.1483255	-0.93564	0.349458	0.880195
FO904898.2	0.497574187	-2.437446738	2.6012773	-0.937019	0.348749	NA
pimr105	0	NA	NA	NA	NA	NA
si:dkeyp-117b11.1	10.44149288	0.557473338	0.6516386	0.8554947	0.392277	NA
FO904898.3	0.325452849	0.005883619	3.3177086	0.0017734	0.998585	NA
cd59	334.8123996	0.258911841	0.1350449	1.9172272	0.055209	0.460643
ctif	27.53052092	-0.015084608	0.3800133	-0.039695	0.968336	0.996315
malrd1	27.86099908	-0.59901209	0.3671472	-1.631531	0.102778	0.609031
si:dkeyp-106c3.1	593.4234214	0.175553954	0.1068453	1.6430673	0.100369	0.602451
ADGRL3	697.6138866	-0.007267651	0.09399	-0.077324	0.938366	0.99279
si:ch73-248e21.1	47.12034225	-0.04405956	0.279644	-0.157556	0.874807	0.987881
si:dkey-197j19.6	1.474483169	0.984722888	1.5416227	0.6387574	0.522981	NA
tmtops3b	8.425677384	-0.246728479	0.7366892	-0.334915	0.737689	NA
si:dkey-32n7.8	0	NA	NA	NA	NA	NA
BX601644.1	0.789152885	-0.566731584	2.4003003	-0.236109	0.813348	NA
smim29	245.7471433	-0.278570988	0.141992	-1.961879	0.049777	0.437386
kcnj1a.4	145.6258174	-0.038667093	0.1905234	-0.202952	0.839173	0.984979
myh6	43.0260921	0.082207734	0.3074472	0.2673881	0.78917	0.976965
gp9	7.878839491	-0.033185718	0.6855203	-0.04841	0.96139	NA
si:dkey-54n8.4	41.3976388	0.108506755	0.3164574	0.3428795	0.731689	0.971557
tnk2a	86.61379162	0.23207548	0.2190073	1.0596702	0.289295	0.845979
ly86	1.785871259	0.751392726	1.5456876	0.486122	0.626881	NA
gak	1741.865261	-0.021847149	0.084378	-0.25892	0.795697	0.978498
tomm20a	51.79636604	-0.313538403	0.2760939	-1.135622	0.256115	0.824633
moto	2.547233184	3.736360685	1.5705053	2.3790819	0.017356	NA
si:dkey-201i6.8	0.672283077	0.02892376	2.3005571	0.0125725	0.989969	NA
si:ch1073-104i17.1	43.08457655	-0.377130019	0.2986648	-1.26272	0.20669	0.774273
si:ch73-40i7.5	152.8335219	0.005316798	0.1737816	0.0305947	0.975593	0.996762
si:ch211-166i24.1	0	NA	NA	NA	NA	NA
si:ch211-79k12.2	277.8417293	-0.197480698	0.1284464	-1.537456	0.124182	0.652729
tmem241	61.92384888	0.114469792	0.2606761	0.4391265	0.66057	0.963881
slc35f2l	6.975726536	0.55042783	0.7460026	0.7378363	0.460614	NA
rcbtb2	120.8099127	0.026861934	0.1847578	0.14539	0.884403	0.98879
nell2a	1240.470393	-0.032382724	0.0961745	-0.336708	0.736337	0.972535
BX005329.1	0.491560863	0.853615855	2.865157	0.2979299	0.765757	NA
si:dkey-15b23.3	13.044478	0.177519162	0.5215544	0.3403656	0.733581	0.971993
eif3ea	5683.693905	-0.213949242	0.0776287	-2.756059	0.00585	0.115107
si:dkey-52j6.3	4.09163341	0.780225179	1.011024	0.7717177	0.440282	NA
nlrb5	8.295796261	0.49292929	0.702132	0.7020464	0.48265	NA
si:dkey-28g23.6	85.89825076	-0.228940059	0.2153934	-1.062893	0.287831	0.845979
gaa	22.49985585	0.005754388	0.3973991	0.0144801	0.988447	0.997476
cltcb	6079.825653	0.080343177	0.0774003	1.0380222	0.29926	0.855386
grem1a	0	NA	NA	NA	NA	NA
prdm2a	267.9389552	-0.155740498	0.132322	-1.176981	0.239203	0.807768
leg1.1	668.7898595	0.012979794	0.1278739	0.1015046	0.91915	0.991623

scn12aa	80.36226554	0.396838142	0.2186783	1.8147122	0.069568	0.514708
celf5b	7.046670346	-1.383826878	0.7699456	-1.797305	0.072287	NA
tnfrsf9b	0.173729368	-0.955901296	4.0804729	-0.234262	0.814781	NA
cfbl	33.8313909	0.258428621	0.3689722	0.7004013	0.483677	0.929461
si:dkey-16p6.1	2.960133816	-1.007892427	1.1053167	-0.911858	0.361843	NA
zmp:0000001003	23.55952187	0.308526574	0.4066021	0.7587923	0.447977	0.917292
torll2	6.813223626	0.661512266	0.7229447	0.9150247	0.360179	NA
si:ch73-269m14.2	373.9454993	0.013415773	0.1342641	0.0999208	0.920407	0.992049
zc3h3	132.8890651	-0.019347438	0.1810711	-0.10685	0.914908	0.991119
plk2a	372.8579361	0.00389439	0.1225817	0.0317698	0.974656	0.996579
si:dkey-13p1.3	4.956630725	1.481784928	0.9412856	1.5742139	0.115438	NA
bcas3	372.6237815	0.047055087	0.1162157	0.4048943	0.685555	0.966307
si:ch211-136m16.8	39.29232355	-0.34699594	0.3095952	-1.120805	0.262371	0.830166
zgc:173556	3.286363264	-0.343212532	1.103742	-0.310954	0.755836	NA
si:dkey-51d8.6	6.677891642	0.125512964	0.8084761	0.1552464	0.876627	NA
FO834800.1	203.5919514	0.109326354	0.1525253	0.7167752	0.473513	0.924993
ttll1	253.3222936	-0.179930857	0.1354265	-1.328624	0.183972	0.747376
mfap4	39.29845197	0.359931161	0.3154045	1.1411731	0.253798	0.823394
diaph3	120.7588635	0.131490434	0.1977055	0.6650822	0.505998	0.935724
klhdc8b	4.393120486	-0.773178429	0.9498508	-0.814	0.415645	NA
cstf2	1094.02021	-0.065582096	0.0840664	-0.780123	0.435319	0.914408
RF00001	0.646899493	-1.455098895	2.4290529	-0.59904	0.549146	NA
si:ch211-207j7.2	194.998447	0.166957954	0.1629938	1.0243209	0.305684	0.860027
fam222a	265.5254285	-0.037732645	0.132444	-0.284895	0.775724	0.975826
gpr155a	343.1336805	0.111684916	0.1173064	0.9520787	0.341057	0.878134
taar14g	0.341366344	1.891903201	3.2128156	0.5888614	0.555954	NA
si:dkey-18a10.3	131.4674421	0.320112504	0.1795424	1.7829354	0.074597	0.532003
kcnj10a	9.078433213	-0.060642019	0.6380292	-0.095046	0.924278	NA
si:dkey-85k15.11	0	NA	NA	NA	NA	NA
CACFD1	30.77770931	-0.124817313	0.3445661	-0.362245	0.717169	0.96909
slc2a10	113.8591279	0.150182259	0.199669	0.7521563	0.451957	0.918761
zgc:171517	0	NA	NA	NA	NA	NA
si:dkey-51d8.3	0.672283077	0.02892376	2.3005571	0.0125725	0.989969	NA
tmem132e	1413.162658	0.034960867	0.0856671	0.4081013	0.683199	0.965476
igfbp6b	108.5263179	0.264385188	0.1965918	1.3448436	0.178676	0.739486
epor	8.430516416	-0.163559966	0.6424007	-0.254607	0.799026	NA
si:ch211-191d2.2	157.4914991	-0.169136318	0.1638871	-1.032029	0.302058	0.856944
zmp:0000001006	3.050682799	-0.786358835	1.2760481	-0.616245	0.537733	NA
CABZ01054965.1	18.80952872	-0.911656334	0.4506779	-2.022856	0.043088	0.4075
si:ch211-209l18.4	341.3976298	0.171001335	0.1217384	1.4046626	0.160122	0.712495
serpina1l	3920.578871	0.125124424	0.0967708	1.2929979	0.196012	0.761182
ciita	9.820371566	-0.00776873	0.627467	-0.012381	0.990122	NA
lsm11	37.38126339	-0.020885448	0.3214446	-0.064974	0.948195	0.99381
CABZ01087514.1	37.58424285	0.285876589	0.3490583	0.8189937	0.41279	0.904974
CR847545.1	1.637134418	0.001080649	1.5015749	0.0007197	0.999426	NA
kcnj1a.2	151.4392789	0.076016209	0.1647987	0.461267	0.644607	0.96029
FO904898.4	0	NA	NA	NA	NA	NA
si:ch1073-263o8.2	0.678375468	-2.878459328	2.4926399	-1.154783	0.248179	NA
si:dkey-210j14.4	210.0814901	0.078979391	0.1510909	0.5227277	0.601164	0.956045
si:dkey-276j7.1	4033.6366	-0.007247973	0.0670852	-0.108041	0.913963	0.991119
ccl34a.4	44.21606954	-0.664096486	0.3111104	-2.134601	0.032794	0.352808
si:dkey-217f16.5	17.62374772	0.073234143	0.5363335	0.1365459	0.89139	0.989291
si:rp71-36a1.5	4.971251712	-0.175069817	0.839516	-0.208537	0.83481	NA
gabra3	7.506839387	0.04025133	0.7834947	0.0513741	0.959027	NA

zmp:0000001301	26.12765148	-0.055945942	0.3914369	-0.142925	0.88635	0.988936
si:ch211-132p1.3	50.7231885	-1.182282072	0.3259084	-3.627651	0.000286	0.011419
cmklr1	24.01438766	0.758477792	0.4012848	1.8901236	0.058741	0.474103
or117-1	1.834906788	-0.823777427	1.4572737	-0.565287	0.571879	NA
kansl1a	1276.019876	0.024859813	0.0897415	0.2770157	0.781768	0.97627
sycn.3	6.542991311	-0.4838659	0.7629407	-0.634212	0.525943	NA
apnl	0.530029684	-0.939524117	2.7937867	-0.336291	0.736652	NA
si:ch211-256e16.11	11.42831205	-0.581298497	0.6535605	-0.889433	0.37377	0.893677
creb3l3b	40.13627899	-0.494112999	0.3236902	-1.5265	0.126885	0.656588
RNF219	27.8403191	0.002647967	0.3750656	0.00706	0.994367	0.998694
si:ch211-152f22.4	0.953494487	3.392387971	2.405043	1.4105311	0.158383	NA
npc2	1081.616915	-0.099070657	0.1066785	-0.928684	0.353053	0.881552
si:ch211-117k10.3	417.0976982	-0.013257682	0.1129887	-0.117336	0.906593	0.990702
CR559930.1	0	NA	NA	NA	NA	NA
si:dkeyp-98a7.3	0	NA	NA	NA	NA	NA
si:ch211-120g10.1	66.91275313	-0.121386594	0.2898692	-0.418763	0.675389	0.965257
or128-4	0.173729368	-0.955901296	4.0804729	-0.234262	0.814781	NA
usp16	36.55908253	-0.375663868	0.3225263	-1.164754	0.244119	0.813482
CABZ01054394.1	85.51725549	-0.136070596	0.21966	-0.61946	0.535613	0.943336
CABZ01033205.1	68.07210723	0.23255467	0.2596759	0.8955573	0.370489	0.890489
si:ch211-170d8.8	2.81830725	-1.704798534	1.2904729	-1.321065	0.18648	NA
si:zfos-364h11.2	10.54629435	0.903013542	0.6248863	1.4450845	0.148434	NA
si:dkey-29j8.1	2.446447709	0.598496899	1.2224782	0.4895767	0.624433	NA
si:ch73-29c22.1	9.204184893	-0.408718686	0.6551707	-0.623835	0.532736	NA
dlg5b.1	210.419987	0.028646728	0.1458351	0.1964323	0.844272	0.985174
igsf5b	28.04763183	0.061707623	0.3681754	0.1676039	0.866895	0.986978
CU469420.2	15.08204287	-0.484065889	0.4876823	-0.992584	0.320913	0.868715
atp6ap1lb	520.2230312	0.218784608	0.1058948	2.0660568	0.038823	0.386536
ripk3	10.78831886	-0.16489061	0.6018961	-0.273952	0.784122	NA
cbln18	45.99469886	0.177065164	0.3018235	0.5866513	0.557438	0.948478
cc2d2a	282.6519061	-0.234147093	0.1400086	-1.672376	0.09445	0.584556
si:dkey-264d12.1	38.0986542	-0.0210815	0.3378852	-0.062392	0.95025	0.99381
si:dkey-62k3.5	157.0345245	0.100706991	0.1927092	0.5225854	0.601263	0.956045
NABP2	135.674221	-0.087539382	0.186511	-0.469352	0.638818	0.959658
ifit12	3.304115451	1.201411346	1.1260349	1.0669397	0.285999	NA
apof	612.6043659	-0.017137293	0.1088357	-0.15746	0.874882	0.987881
si:ch211-191o15.6	4.945319953	-0.988005682	1.0108875	-0.977365	0.328389	NA
slc44a1b	651.564394	0.01369413	0.0940938	0.1455369	0.884287	0.98879
si:dkeyp-115e12.6	464.8985576	-0.143160738	0.1199368	-1.193635	0.232621	0.80121
larsa	202.4160589	0.046230798	0.1447902	0.3192952	0.749503	0.97419
rnf4	251.4033781	0.076437733	0.1374492	0.5561163	0.578131	0.951024
vegfba	102.1992837	-0.035565054	0.1954822	-0.181935	0.855634	0.985174
si:cabz01071911.3	180.0012592	0.125447606	0.1506863	0.8325082	0.405122	0.901305
mycbp	179.9728813	-0.196100291	0.1590177	-1.233198	0.217502	0.784786
il34	86.60916685	-0.397702984	0.2523623	-1.575921	0.115044	0.635519
cobl	581.4216456	0.213506521	0.1007223	2.1197533	0.034027	0.359235
si:ch211-28p3.4	52.36253566	-0.081738461	0.2719459	-0.300569	0.763743	0.975374
si:dkey-84h14.2	6.132109012	0.07280439	0.7658792	0.0950599	0.924267	NA
CR381544.2	3.071969823	0.147948543	1.0953713	0.135067	0.892559	NA
znf1124	2.946777874	-1.372920967	1.2058418	-1.138558	0.254887	NA
si:ch211-218h8.1	10.27642651	0.569244877	0.5838662	0.9749578	0.329581	NA
aqp1a.2	0	NA	NA	NA	NA	NA
phox2bb	470.3820018	-0.029031272	0.1071598	-0.270916	0.786456	0.976539
sacs	224.0139545	0.174284076	0.14863	1.1726033	0.240955	0.809487

pcdh2ab6	2.196553891	0.666011611	1.3082987	0.5090669	0.610705	NA
tmem173	17.28812791	0.219658549	0.4845757	0.4533008	0.650332	0.960708
onecut3b	1.341930527	3.871810095	1.8532521	2.0891978	0.03669	NA
slc38a3b	413.0985572	0.238531589	0.1224262	1.9483708	0.051371	0.444245
si:dkey-199k11.6	2.044002481	1.058544983	1.4171311	0.7469633	0.455086	NA
cib3	9.24173618	0.727270435	0.6329353	1.149044	0.250538	NA
alkal2a	7.851595886	0.070240496	0.6740544	0.104206	0.917006	NA
si:zfos-1192g2.3	548.3427126	-0.101266157	0.1008921	-1.003708	0.31552	0.866773
si:ch211-195j11.27	0.323844819	-1.825095364	3.274639	-0.557342	0.577293	NA
lepa	12.33431566	0.186855408	0.5554521	0.3364024	0.736567	0.972535
xirp2b	71.86460769	0.296412729	0.2332627	1.270725	0.203826	0.771579
zcchc10	193.4160183	0.007156851	0.1483469	0.048244	0.961522	0.995845
si:dkey-88n24.8	0	NA	NA	NA	NA	NA
fabp10b	40.24259956	-0.323895152	0.3032166	-1.068197	0.285431	0.845164
si:ch211-15b10.6	716.3585162	-0.33062451	0.1329958	-2.485977	0.01292	0.202578
pkhd1l1	535.0808011	-0.211607455	0.1093328	-1.935443	0.052936	0.451772
fbxo40.2	117.2261844	0.407126949	0.1924605	2.1153791	0.034398	0.360397
rps19bp1	350.7375031	0.011598086	0.1309117	0.0885947	0.929404	0.992702
rnf207a	2.668975156	0.759922281	1.1584689	0.6559712	0.511843	NA
klf15	181.8437785	0.332132187	0.1525825	2.1767386	0.0295	0.332709
lrrc75bb	29.8923134	-0.675470994	0.3740763	-1.805704	0.070965	0.521075
kcnq2b	21.65515436	1.079402318	0.4289942	2.5161235	0.011865	0.191219
cry3b	595.1704959	0.257505219	0.1032702	2.4935089	0.012649	0.199773
si:ch211-253p18.5	24.24918683	0.313138684	0.3887987	0.8054006	0.420589	0.90651
zgc:174259	11.86962931	0.650803385	0.5748994	1.13203	0.257622	0.825314
actr3	1643.651446	-0.079194017	0.1029919	-0.768935	0.441932	0.916336
pik3r6b	0.878629579	0.686795053	2.2010162	0.3120354	0.755014	NA
or106-10	2.760392357	-0.864660802	1.1663532	-0.741337	0.458489	NA
CR749162.1	0	NA	NA	NA	NA	NA
crygm2d20	16229.59572	0.070551411	0.0818994	0.8614402	0.388996	0.896295
mki67	2041.127152	-0.07674644	0.080095	-0.958193	0.337966	0.876609
rpz	325.7628732	-0.004459623	0.1233536	-0.036153	0.97116	0.996315
tor4ab	3.37293713	0.356789418	1.0856906	0.328629	0.742436	NA
or110-2	2.157964717	1.142490925	1.3332228	0.8569392	0.391478	NA
si:dkey-30k22.5	0.166657454	-0.955901296	4.0804729	-0.234262	0.814781	NA
FP017295.1	22.84558536	-0.22988161	0.4061272	-0.566033	0.571371	0.949418
BX649250.1	349.0552725	-0.002169367	0.1194022	-0.018169	0.985504	0.996944
urm1	240.0522982	-0.199135755	0.1387839	-1.434862	0.151327	0.69947
or110-1	0	NA	NA	NA	NA	NA
BX511123.1	0.173729368	-0.955901296	4.0804729	-0.234262	0.814781	NA
mlnr	3.003399983	0.000619935	1.1508981	0.0005387	0.99957	NA
ucp3	578.8973748	0.153590379	0.1363543	1.1264062	0.259994	0.828288
adh8a	7.415057132	-0.486723496	0.7386283	-0.658956	0.509924	NA
si:dkey-192p21.6	6.038035482	-0.777280783	0.8106011	-0.958894	0.337612	NA
rpz2	316.5472861	-0.126229947	0.1267602	-0.995817	0.319339	0.867749
or111-10	0.333314908	-1.858695402	3.5126701	-0.52914	0.596708	NA
ttc34	7.855412714	-0.180149069	0.7096631	-0.253852	0.79961	NA
si:dkey-19018.2	37.32255475	0.07243478	0.3185148	0.2274142	0.820102	0.981594
CR450686.1	13.34145593	-0.581925111	0.5578297	-1.043195	0.296858	0.853798
kcnj1a.1	69.92960539	0.653153136	0.234104	2.7900126	0.005271	0.107431
FP236356.1	0.31598276	0.005883445	3.3523626	0.001755	0.9986	NA
si:ch73-335l21.4	742.4037616	0.086097185	0.2850876	0.3020025	0.76265	0.975374
CABZ01015525.1	0.824205979	0.498048327	2.3641483	0.2106671	0.833147	NA
gltld1	17.46293737	-0.247104476	0.4527907	-0.545737	0.585247	0.952562

znf975	5.47254007	0.275323192	0.8319435	0.3309398	0.74069	NA
fars2	93.17290839	-0.079527349	0.2020839	-0.393536	0.693923	0.96736
CR381686.1	4.464166152	0.112051415	0.9004303	0.1244421	0.900965	NA
slc22a7b.2	22.55208557	1.268038892	0.4168016	3.0423083	0.002348	0.060184
smyd1b	446.4037589	0.358189449	0.1265659	2.8300638	0.004654	0.098004
si:ch73-59p9.2	0	NA	NA	NA	NA	NA
mylk4a	183.8591138	0.51828007	0.1527034	3.3940314	0.000689	0.02285
si:ch211-71m22.5	5.611028805	0.847378674	0.8416145	1.006849	0.314007	NA
si:ch211-225g23.1	182.4556652	-0.137683604	0.1585485	-0.868401	0.385175	0.89551
dre-mir-221	0	NA	NA	NA	NA	NA
adra2da	70.62504234	-0.134466709	0.2388502	-0.562975	0.573452	0.949869
si:ch211-66k16.27	15.59911458	-0.091235136	0.4863613	-0.187587	0.8512	0.985174
ebf3b	9.410493366	0.055351331	0.6155677	0.0899192	0.928351	NA
dmxl2	2257.17233	0.043215764	0.0986892	0.4378975	0.661461	0.964234
pmela	3019.0402	-0.130213575	0.0850298	-1.531387	0.125674	0.654803
card19	227.3556299	0.149424483	0.1396873	1.0697073	0.284751	0.844955
kcnn1a	907.8086821	0.030393046	0.0950666	0.3197027	0.749194	0.97419
hunk	1078.089981	-0.23422909	0.086248	-2.715764	0.006612	0.124967
RF00001	0.983163012	1.013789546	1.9987523	0.5072112	0.612007	NA
nlrc6	61.30534844	-0.084568234	0.2464793	-0.343105	0.73152	0.971557
CABZ01088229.1	10.14821255	0.28165004	0.6150482	0.4579316	0.647002	NA
cep120	305.5176193	-0.190761878	0.1414006	-1.349088	0.177309	0.738201
si:ch73-134f24.1	0	NA	NA	NA	NA	NA
RF00017	0	NA	NA	NA	NA	NA
adcy5	93.37514755	-0.140647241	0.2183607	-0.644105	0.519507	0.938945
rtn3	4298.486285	0.119930146	0.0785906	1.5260122	0.127007	0.656588
plekhd1	103.7445353	0.240942906	0.210313	1.1456394	0.251944	0.821478
CAMSAP3	711.4394472	0.060602129	0.1305871	0.4640743	0.642595	0.959994
si:dkey-84k17.2	32.93924635	0.451049172	0.3440575	1.3109703	0.189868	0.754269
prpf6	1646.132718	-0.072213307	0.0837576	-0.86217	0.388594	0.896271
AL954327.1	6.632868264	-0.892798355	0.7371236	-1.211192	0.225822	NA
galn	110.9422319	0.372533692	0.2015826	1.848045	0.064596	0.497355
si:ch211-214c20.1	1.197130868	0.490080416	1.8201022	0.2692598	0.78773	NA
zgc:165409	194.2828713	-0.06536954	0.1541463	-0.424075	0.671511	0.965257
si:ch73-380l3.5	0	NA	NA	NA	NA	NA
RF00001	0	NA	NA	NA	NA	NA
si:ch211-161m3.6	70.57404887	-0.206577691	0.2653418	-0.778534	0.436254	0.914408
eif2b1	414.0152268	-0.025135622	0.1128355	-0.222763	0.82372	0.982196
mtcl1	12.76132193	-0.282941254	0.570758	-0.495729	0.620086	0.957354
zp2.6	0.157187365	-0.955901296	4.0804729	-0.234262	0.814781	NA
si:ch211-214p13.9	13.09106112	-0.447356809	0.5487053	-0.815295	0.414903	0.906237
TIMM22	148.0706512	-0.07747515	0.1681512	-0.460747	0.64498	0.96029
RF00001	0.158795395	0.967652056	4.0804729	0.2371421	0.812547	NA
snx17	443.5757401	0.000330491	0.1128949	0.0029274	0.997664	0.999174
nrarpa	1421.730716	0.074386409	0.0863718	0.8612345	0.389109	0.896347
BX511100.2	0.158795395	0.967652056	4.0804729	0.2371421	0.812547	NA
si:ch211-176g13.8	68.06326624	-0.177916044	0.2406919	-0.739186	0.459794	0.921978
abhd17aa	158.0359421	-0.435842235	0.1656706	-2.630775	0.008519	0.151081
pimr27	0	NA	NA	NA	NA	NA
gabra2a	48.90472488	0.423782488	0.2746611	1.5429289	0.122848	0.650905
si:dkey-66a8.7	93.70889763	0.276029134	0.2274354	1.2136597	0.224878	0.792185
RF00001	0	NA	NA	NA	NA	NA
ccdc28a	15.90428426	-0.23233742	0.4707799	-0.493516	0.621648	0.957354
znf1066	7.215462522	-0.06506326	0.7056017	-0.09221	0.926531	NA

foxc1a	491.5540383	-0.258016883	0.1128697	-2.285971	0.022256	0.283929
RF00003	0	NA	NA	NA	NA	NA
cnbd1	4.537800788	0.837033831	0.93892	0.8914858	0.372669	NA
si:dkey-83f18.11	0.182570949	0.967652056	4.0804729	0.2371421	0.812547	NA
zmp:0000001020	16.86095469	0.052759534	0.5191343	0.1016298	0.91905	0.991623
CABZ01073954.1	283.0405704	0.128185205	0.1335133	0.9600932	0.337008	0.876088
atp6ap1la	57.9447587	0.077181107	0.2555649	0.302002	0.762651	0.975374
gpx7	200.1337986	0.122419194	0.1571595	0.7789489	0.43601	0.914408
nfrkb	424.1928568	-0.214528428	0.1373809	-1.561559	0.118392	0.641743
si:dkey-22o12.2	443.6164843	0.050207714	0.1107715	0.4532548	0.650365	0.960708
CT737190.1	49.17970431	0.199914173	0.2987133	0.669251	0.503335	0.935304
BX511084.1	5.401907787	-0.26144541	0.8425321	-0.310309	0.756326	NA
MAP3K11	36.42778354	0.239937025	0.3471625	0.6911375	0.489479	0.931926
carm1l	31.20900338	0.341770616	0.3394226	1.0069176	0.313974	0.865176
ccser2b	11.55262815	0.510375313	0.5663103	0.9012291	0.367467	0.887458
zranb3	89.55219628	-0.133447124	0.2070515	-0.644512	0.519244	0.938945
ptprjb.1	14.29186037	-0.394570223	0.5183282	-0.761236	0.446516	0.917154
stard9	221.8088612	0.122294098	0.1381912	0.8849629	0.376177	0.894698
RF00003	0	NA	NA	NA	NA	NA
ostf1	464.9831986	-0.163747334	0.1053715	-1.554	0.120184	0.645215
tnfsf13	0	NA	NA	NA	NA	NA
mmp19	0.853874503	-1.902668424	2.1134959	-0.900247	0.367989	NA
si:ch211-251f6.7	13.82665638	-1.162710119	0.5455684	-2.13119	0.033073	0.354192
slc6a19a.2	257.4020115	-1.16332813	0.1388497	-8.378328	5.37E-17	3.22E-14
si:dkey-156k2.4	12.25127787	-0.146862111	0.5407146	-0.271607	0.785924	0.97627
snw1	1503.461845	-0.126939589	0.0848354	-1.496305	0.134574	0.670689
si:ch1073-90m23.1	17.48981834	-0.749506744	0.4989712	-1.502104	0.13307	0.667628
suox	342.2601194	0.149562697	0.1198561	1.2478518	0.212085	0.77986
TMEM216	38.13051125	-0.178447464	0.3395284	-0.525574	0.599184	0.955891
si:ch211-66e2.5	382.3358842	0.115723655	0.1177999	0.982375	0.325915	0.871896
si:ch73-217n20.1	111.4538896	-0.111836093	0.1943256	-0.575509	0.564947	0.948739
kif28	10.484989	-0.63163167	0.6096557	-1.036047	0.30018	NA
si:ch211-170p16.1	0	NA	NA	NA	NA	NA
peli1a	26.15784812	0.054901491	0.3785993	0.1450121	0.884701	0.988838
LO018154.1	118.8918097	0.160374572	0.1852799	0.8655798	0.386721	0.89551
si:ch73-91k6.2	33.31472924	-0.094567304	0.3388401	-0.279091	0.780175	0.976218
si:dkey-163f14.6	101.5615192	-0.049557774	0.2033643	-0.24369	0.807471	0.980642
RF00026	0	NA	NA	NA	NA	NA
arhgef28b	20.19423821	0.039710664	0.4372024	0.090829	0.927628	0.992702
spink4	47.31103089	-0.352825774	0.3047882	-1.15761	0.247023	0.816669
si:dkey-62k3.6	9.080924003	-0.370035234	0.6235433	-0.593439	0.552887	NA
prok2	2.387458266	1.912712991	1.3551631	1.4114264	0.158119	NA
cltcl1	136.4722667	0.018849395	0.1751592	0.1076129	0.914303	0.991119
mvb12ba	61.04994946	-0.318250215	0.259131	-1.228144	0.219393	0.78594
ugt2a7	27.89517669	-0.578135176	0.394595	-1.465136	0.142884	0.685108
si:dkeyp-67f1.2	37.30192508	-0.067301825	0.3245851	-0.207347	0.835739	0.984089
si:dkey-271j15.3	93.44750959	-0.044290822	0.2308127	-0.191891	0.847828	0.985174
cspp1b	230.2301116	-0.09846277	0.1427793	-0.689615	0.490436	0.931926
si:ch211-197h24.6	708.4808219	0.036316065	0.0952543	0.3812537	0.703015	0.968134
si:dkey-177p2.18	84.90379172	-0.018655656	0.2111652	-0.088346	0.929601	0.992702
pip4k2cb	24.34281965	-0.726250399	0.4347397	-1.670541	0.094812	0.585466
CABZ01072989.1	0.182570949	0.967652056	4.0804729	0.2371421	0.812547	NA
si:ch73-352p4.5	52.94826208	-0.164290424	0.2630356	-0.624594	0.532238	0.942546
igflr1	43.8593267	0.449421338	0.3176942	1.4146351	0.157176	0.707876

xrn1	994.6338237	0.029727287	0.0927514	0.3205049	0.748586	0.97419
gcsha	385.4624558	-0.124689729	0.1125565	-1.107797	0.267949	0.833349
lsm8	296.2840505	-0.127203367	0.1275046	-0.997638	0.318455	0.867746
CT573256.1	17.17318044	0.726886643	0.4659043	1.5601629	0.118721	0.642473
sptssb	241.7572461	-0.003588387	0.1416796	-0.025327	0.979794	0.996879
fgfbp1b	131.9652498	0.120465344	0.1843266	0.653543	0.513406	0.93654
si:dkey-234i14.3	19.75299339	-0.319316026	0.459742	-0.694555	0.487334	0.931584
bicdl2l	211.5228868	0.299241146	0.1594266	1.8769838	0.06052	0.480421
ints12	259.6652087	-0.109184406	0.1451546	-0.752194	0.451935	0.918761
FO704858.1	0.31598276	0.005883445	3.3523626	0.001755	0.9986	NA
cnrip1a	1122.414265	0.02636238	0.0936781	0.2814144	0.778393	0.975942
havcr2	17.81551989	0.188631735	0.4633199	0.4071307	0.683912	0.965679
RF00017	0	NA	NA	NA	NA	NA
capn2a	1342.409903	0.239866822	0.0865852	2.7702981	0.005601	0.111976
csnk2a4	27.46530981	0.21651954	0.4060131	0.5332821	0.593838	0.954434
taar14d	0	NA	NA	NA	NA	NA
si:dkey-238d18.5	1.356245041	-2.710785197	1.9732092	-1.373795	0.169505	NA
BX649490.1	2.09163507	1.173698227	1.3801324	0.8504244	0.395089	NA
si:ch211-202f3.4	28.67101336	0.177490009	0.4016541	0.4418976	0.658563	0.962545
aebp1	587.3284342	0.089988336	0.1015813	0.8858749	0.375685	0.894316
CABZ01061592.1	54.04877965	0.068927279	0.2837179	0.242943	0.80805	0.980642
zgc:114046	122.5277154	0.006633463	0.2042736	0.0324734	0.974095	0.99644
gig2l	0	NA	NA	NA	NA	NA
taar14b	0	NA	NA	NA	NA	NA
traf3ip2l	168.3024493	-0.003463922	0.1704334	-0.020324	0.983785	0.996944
si:dkey-16p6.1	4.973646385	-0.229014269	0.9155524	-0.250138	0.802481	NA
tbx22	19.50226386	-0.115616933	0.4287249	-0.269676	0.787409	0.976596
RF00001	0	NA	NA	NA	NA	NA
CT573476.1	67.31075947	0.017721782	0.2876062	0.0616182	0.950867	0.99381
insm1a	947.2635062	0.048017388	0.0909812	0.5277724	0.597657	0.95534
adgrf6	313.5065564	-0.202575899	0.1337963	-1.514062	0.13001	0.662595
cwf19l2	338.434507	-0.140146867	0.1215498	-1.152999	0.248911	0.818009
zbtb40	183.0639726	-0.116717759	0.1594397	-0.73205	0.464138	0.923204
dclre1a	172.4438886	0.069647438	0.1653448	0.4212254	0.67359	0.965257
lysmd2	541.0603821	0.149337456	0.1025167	1.4567136	0.145195	0.690034
CR524827.1	0	NA	NA	NA	NA	NA
mapkap1	108.3719762	-0.08040514	0.2058634	-0.390575	0.696111	0.96736
BX890608.2	0	NA	NA	NA	NA	NA
si:dkeyp-71f10.5	8.116196517	-0.379778582	0.7236806	-0.524788	0.599731	NA
si:ch211-203b20.4	0.158915748	0.967652056	4.0804729	0.2371421	0.812547	NA
or111-5	1.683107494	1.239144993	1.5746599	0.7869287	0.431324	NA
akap12a	184.5028437	-0.315941844	0.1536636	-2.056062	0.039777	0.391511
taar18c	0	NA	NA	NA	NA	NA
si:ch211-266k22.6	112.6166141	-0.03747618	0.1861335	-0.20134	0.840432	0.985042
zgc:174260	6.703079214	0.929083211	0.7568748	1.2275256	0.219625	NA
serpinb14	393.1670808	-0.093936351	0.1339598	-0.701228	0.483161	0.92946
si:dkey-21a6.5	204.9993492	-0.244108881	0.1566809	-1.558	0.119233	0.643307
CR391998.1	30.56110883	0.153607242	0.3605798	0.4260006	0.670107	0.965223
smc6	39.84010735	-0.145903124	0.3064659	-0.476083	0.634015	0.95776
ddx27	582.1231822	-0.188949358	0.0998404	-1.892513	0.058423	0.473924
MDFIC2	5.32122798	-1.122310059	0.8621679	-1.30173	0.193009	NA
sv2	117.9438403	-0.099609867	0.2048079	-0.486358	0.626714	0.957354
si:ch211-198m17.1	271.4220885	-0.292823616	0.1473166	-1.987716	0.046843	0.425112
si:ch73-264p11.1	31.17440098	-0.363593837	0.3440116	-1.056923	0.290547	0.846262

casp7	801.345305	0.162984196	0.0898131	1.8147048	0.069569	0.514708
si:cabz01071909.2	21.47071112	-0.727142815	0.4322562	-1.682203	0.092529	0.579378
pimr21	0	NA	NA	NA	NA	NA
si:ch211-181d7.1	0.182570949	0.967652056	4.0804729	0.2371421	0.812547	NA
tsen2	55.59764775	-0.268287125	0.2612815	-1.026812	0.304509	0.858905
si:ch73-233m11.2	5.365164936	0.019996082	0.9144928	0.0218658	0.982555	NA
zgc:194007	21.65149672	-0.089324875	0.4256832	-0.209839	0.833793	0.983583
BX942825.1	0	NA	NA	NA	NA	NA
zgc:158701	5.793260201	-0.767486554	0.8345837	-0.919604	0.35778	NA
BX950194.1	0	NA	NA	NA	NA	NA
CR936300.1	8.304189222	-0.869478396	0.714792	-1.216408	0.22383	NA
zgc:162958	69.10153249	-0.265794933	0.2480063	-1.071726	0.283843	0.844676
CU469539.1	0.674011459	1.487968231	2.2861421	0.6508643	0.515134	NA
si:ch211-102c2.7	123.3041528	0.070235198	0.1797889	0.3906536	0.696053	0.96736
CR354402.1	0.349228403	0.005884014	3.2372587	0.0018176	0.99855	NA
or137-7	0.635542639	2.809903053	2.5424041	1.1052149	0.269066	NA
si:dkey-29d8.3	66.63593968	-0.142931502	0.2367378	-0.603754	0.546007	0.945526
grnas	2.144848486	-1.167991318	1.3031737	-0.896267	0.37011	NA
BX005355.1	0.158795395	0.967652056	4.0804729	0.2371421	0.812547	NA
si:dkeyp-69c1.9	13.11116008	0.132876565	0.5418946	0.2452074	0.806296	0.980105
CR812481.1	7.437292437	-0.246509813	0.7167491	-0.343928	0.730901	NA
si:dkeyp-67e1.3	0.157187365	-0.955901296	4.0804729	-0.234262	0.814781	NA
AL953893.1	0	NA	NA	NA	NA	NA
znf1172	19.57081009	-0.420505877	0.4481262	-0.938365	0.348057	0.880195
BX323884.1	110.4683465	0.06238025	0.1908747	0.3268126	0.74381	0.974094
si:ch73-54n14.2	0	NA	NA	NA	NA	NA
CR388209.1	1.480214502	-0.378082517	1.6730776	-0.22598	0.821217	NA
b3gnt2b	554.5051844	0.269332062	0.1061642	2.5369386	0.011183	0.183548
si:dkeyp-51f12.3	3.345223152	0.88043495	1.0554391	0.8341883	0.404175	NA
si:dkey-108k21.21	31.09614939	0.434741963	0.3758094	1.1568151	0.247348	0.81686
rbp7a	0.316103113	0.005883447	3.3519121	0.0017553	0.9986	NA
si:ch73-59c19.1	2.803454605	-1.301381158	1.2031374	-1.081656	0.279405	NA
CR974461.1	0	NA	NA	NA	NA	NA
si:ch211-15j1.5	10.16097876	0.849142569	0.6187223	1.372413	0.169935	NA
BX530075.1	0	NA	NA	NA	NA	NA
ugt5b4	223.8938441	-0.431998696	0.1443175	-2.993392	0.002759	0.067771
CU571079.1	24.84901322	0.095949101	0.3872376	0.2477784	0.804306	0.979643
BX548073.1	11.99570064	-0.588886482	0.593287	-0.992583	0.320913	0.868715
BX284679.1	0.323844819	-1.825095364	3.274639	-0.557342	0.577293	NA
si:dkey-103e21.5	20.6064471	-1.062390881	0.4267615	-2.489425	0.012795	0.2008
or126-1	0.666939546	1.487980453	2.2924711	0.6490727	0.516291	NA
si:ch211-12e13.1	6.85570318	-0.343639781	0.8394049	-0.409385	0.682257	NA
gpatch4	183.6324213	0.107764161	0.1857092	0.5802845	0.561723	0.948728
si:ch73-25f10.6	469.6033658	0.151971796	0.1045131	1.4540935	0.14592	0.691682
BX649388.1	6.07543883	0.553803991	0.7666723	0.7223477	0.470081	NA
BX005429.1	0	NA	NA	NA	NA	NA
CU633804.1	1.165331617	1.275668599	1.8732058	0.6810083	0.495866	NA
GBGT1 (1 of many)	4.765213944	-0.495763531	0.8853109	-0.559988	0.575488	NA
BX927308.1	205.0513546	-0.259070457	0.1542051	-1.680038	0.09295	0.580357
BX890576.1	5.82537333	1.11732866	0.8259984	1.3527008	0.176151	NA
CR847971.1	2.497214877	1.479473532	1.2462217	1.1871672	0.235162	NA
BX005313.1	1.123285678	0.391074727	1.9652571	0.1989942	0.842267	NA
GBGT1 (1 of many)	0.641555962	-0.014184532	2.3335058	-0.006079	0.99515	NA
CR391940.1	31.20147959	-0.17014306	0.3439062	-0.494737	0.620786	0.957354

znf106b	48.85445061	0.026669879	0.2777781	0.0960114	0.923511	0.992588
CU694486.1	0	NA	NA	NA	NA	NA
si:dkeyp-4c7.3	1.021349841	-0.039072774	2.0390376	-0.019162	0.984712	NA
nuggc.2	11.30349802	-0.771774397	0.576399	-1.338959	0.180584	0.741768
si:dkeyp-122a9.1	0.792730004	1.851900952	2.2729754	0.8147475	0.415217	NA
tmem184bb	54.14176638	0.025840967	0.2771725	0.0932306	0.92572	0.992689
si:dkey-172o10.8	0	NA	NA	NA	NA	NA
BX677668.1	2.54255619	1.062823932	1.3018698	0.8163826	0.414281	NA
pth1b	0.988894344	-1.041944082	1.9792901	-0.526423	0.598594	NA
RUNDC1	123.2919476	0.233728773	0.1907156	1.2255355	0.220374	0.787278
CR925863.1	0.365141898	1.973577737	3.3927314	0.5817076	0.560764	NA
ttf1	57.42751233	-0.292178545	0.2657529	-1.099437	0.271577	0.83659
gbgt1l3	1.767669898	2.174064496	1.6432594	1.3230197	0.185829	NA
ms4a17a.3	4.248778647	-0.225709705	0.916524	-0.246267	0.805475	NA
si:dkey-58f10.7	12.78700535	0.279495156	0.5331052	0.5242776	0.600085	0.956045
CR356233.1	18.81128672	0.129158412	0.4719163	0.2736892	0.784323	0.97627
AL929229.1	1.465400882	0.273436181	1.5956724	0.1713611	0.86394	NA
CR848717.1	0.173729368	-0.955901296	4.0804729	-0.234262	0.814781	NA
BX470189.2	9.781421581	-0.275183938	0.6657049	-0.413372	0.679334	NA
CR450716.1	0	NA	NA	NA	NA	NA
BX085193.1	0	NA	NA	NA	NA	NA
BX005012.2	0.31598276	0.005883445	3.3523626	0.001755	0.9986	NA
BX469885.1	0	NA	NA	NA	NA	NA
BX511112.1	0	NA	NA	NA	NA	NA
v2ra16	1.664634186	0.538718286	1.5014365	0.3588019	0.719743	NA
mrap	0	NA	NA	NA	NA	NA
dicp1.1	2.25349602	0.732514473	1.3950446	0.5250832	0.599525	NA
znf1048	17.02762352	-0.360698387	0.4749376	-0.759465	0.447575	0.917154
tcnbb	4157.680964	0.244145079	0.0818155	2.9840923	0.002844	0.069229
si:ch211-284e13.11	0	NA	NA	NA	NA	NA
znf1020	3.961563957	-1.295144316	0.9810087	-1.320217	0.186763	NA
CR735121.1	0	NA	NA	NA	NA	NA
ccs	72.87260073	-0.296696006	0.2649872	-1.119662	0.262858	0.830166
CR396590.1	0	NA	NA	NA	NA	NA
CT027611.1	0	NA	NA	NA	NA	NA
CR381686.2	1.777700466	2.052228824	3.8880257	0.5278331	0.597615	NA
BX530407.1	0	NA	NA	NA	NA	NA
BX294129.1	0.499972362	-2.44370697	3.0013188	-0.814211	0.415524	NA
si:ch211-139a5.1	49.77547965	0.044630744	0.269379	0.1656801	0.868409	0.987245
CR855277.1	1.96965918	-0.012388435	1.3732927	-0.009021	0.992802	NA
CR513782.1	0.681245011	1.537021632	2.3941387	0.6419936	0.520877	NA
si:dkey-256e7.8	5.778543344	0.397395482	0.7966557	0.4988297	0.617899	NA
ftr10	0	NA	NA	NA	NA	NA
nlrc8	0.69766666	1.536459923	2.3782158	0.6460557	0.518243	NA
zgc:173545	0.476747244	2.393381959	3.0525537	0.7840589	0.433006	NA
si:dkey-20i20.9	4.470260803	-0.327876238	0.91496	-0.35835	0.720081	NA
si:dkey-4c23.3	0.999584661	0.976925822	1.8949579	0.5155396	0.606176	NA
si:dkey-239h2.3	95.26572665	-0.612119721	0.2615291	-2.340542	0.019256	0.25973
BX649633.1	0	NA	NA	NA	NA	NA
si:ch211-156j16.1	1643.180354	0.184838209	0.0860347	2.1484144	0.031681	0.345305
CR925731.1	7.235732395	0.127283879	0.7312516	0.174063	0.861816	NA
si:dkey-70p6.1	331.7664527	0.250840323	0.1266108	1.9811928	0.04757	0.42893
CR391910.1	0.855644171	-0.484665734	2.1924736	-0.221059	0.825047	NA
AL935153.1	0.158795395	0.967652056	4.0804729	0.2371421	0.812547	NA

si:dkey-22f5.9	36.07436657	1.825071922	0.3463647	5.2692196	1.37E-07	1.82E-05
si:dkey-225k4.1	18.16733597	-0.301619289	0.4697662	-0.642063	0.520833	0.939056
gstk4	2.790212228	0.891576422	1.1702567	0.7618639	0.446141	NA
BX649502.1	0	NA	NA	NA	NA	NA
BX927365.1	1.571742854	1.077963887	1.7704183	0.6088752	0.542607	NA
si:dkey-90l23.1	2.342741209	-1.88024215	1.3614357	-1.381073	0.167256	NA
tbx5b	8.666242664	0.470974361	0.6762531	0.6964469	0.486149	NA
si:dkey-112g5.13	0.173729368	-0.955901296	4.0804729	-0.234262	0.814781	NA
BX511123.2	3.580501776	-0.017168637	1.0101965	-0.016995	0.98644	NA
si:dkey-117n7.4	52.27518759	-0.075541994	0.2731183	-0.276591	0.782094	0.97627
BX005174.1	3.14648155	-1.43718534	1.1345648	-1.266728	0.205252	NA
CT583708.1	6.864456668	0.213288383	0.7878801	0.2707117	0.786613	NA
atn1	1137.384638	0.017983471	0.1009706	0.1781059	0.85864	0.985773
si:ch211-15j1.3	1.988211556	0.486847799	1.3953852	0.3488985	0.727166	NA
si:ch211-117n7.6	63.56328936	-0.209337132	0.2446577	-0.855633	0.392201	0.897688
CT997819.1	0.325573202	0.005883621	3.3172783	0.0017736	0.998585	NA
znf1165	32.36270272	-0.445381255	0.3363747	-1.324063	0.185482	0.74928
hcst	5.781983657	-0.235397148	0.8039727	-0.292792	0.769681	NA
pimr49	0	NA	NA	NA	NA	NA
si:dkey-147f3.4	130.1629217	-0.105690032	0.1771448	-0.596631	0.550754	0.946549
AL954134.1	2.918879017	-0.321560732	1.216958	-0.264233	0.7916	NA
znf1044	6.284230339	-0.492937018	0.8285748	-0.594922	0.551896	NA
BX649639.1	54.16362482	0.233465789	0.2727536	0.8559587	0.392021	0.897688
BX899181.1	1.804179674	-0.844890155	1.4389594	-0.587154	0.557101	NA
CR392002.1	4.201706538	-0.331340786	1.009439	-0.328243	0.742728	NA
BX294006.1	0.325573202	0.005883621	3.3172783	0.0017736	0.998585	NA
CR318650.1	0	NA	NA	NA	NA	NA
si:dkey-4c15.8	19.56189861	-0.238243711	0.4321697	-0.551274	0.581446	0.951731
BX927234.1	0.507044276	-2.459587493	2.7586536	-0.89159	0.372613	NA
si:ch211-125e6.12	0	NA	NA	NA	NA	NA
cyp2x12	43.54254915	-0.982473852	0.3825139	-2.568466	0.010215	0.17288
si:dkey-112g5.11	1.694663769	-0.575875761	1.4805886	-0.388951	0.697313	NA
CR788254.1	0.157187365	-0.955901296	4.0804729	-0.234262	0.814781	NA
BX294661.1	0.471562095	-2.373056629	3.0645586	-0.774355	0.438721	NA
CR749763.1	0	NA	NA	NA	NA	NA
si:ch211-133n4.6	672.7842518	0.356501107	0.098308	3.6263681	0.000287	0.011436
BX571955.1	0	NA	NA	NA	NA	NA
si:ch211-214p13.8	0.839689391	-1.918726865	2.219847	-0.864351	0.387395	NA
CU633823.1	0	NA	NA	NA	NA	NA
BX511093.1	0	NA	NA	NA	NA	NA
CU639436.1	0	NA	NA	NA	NA	NA
tomm40	1271.083694	0.137559958	0.0824397	1.6686134	0.095194	0.58671
eif4a1a	10230.71991	0.025420616	0.0729285	0.3485691	0.727413	0.970402
BX571701.1	0	NA	NA	NA	NA	NA
si:ch211-235f12.2	0	NA	NA	NA	NA	NA
BX470185.1	10.66727092	0.393374527	0.5917331	0.6647837	0.506189	NA
si:ch211-229n2.6	0.166657454	-0.955901296	4.0804729	-0.234262	0.814781	NA
si:dkey-20d21.12	59.23616068	0.044319049	0.2571275	0.1723621	0.863153	0.985841
cox14	218.6824293	-0.336835617	0.1565399	-2.151755	0.031417	0.343463
CU468041.1	0	NA	NA	NA	NA	NA
vtg5	0.802200093	1.852032281	2.2653592	0.8175446	0.413617	NA
CR925709.1	11.7906021	-0.232284804	0.5695527	-0.407837	0.683393	0.965476
AL954694.1	0.473170125	-0.833888596	2.9014002	-0.287409	0.773799	NA
si:dkey-84o3.7	0.50625413	-0.940106488	2.8357787	-0.331516	0.740255	NA

rad54b	65.72560458	-0.001083209	0.2507815	-0.004319	0.996554	0.998936
BX649502.2	0	NA	NA	NA	NA	NA
cenpw	29.74307001	-0.454288042	0.3645648	-1.246111	0.212724	0.780843
si:dkey-4i23.5	2.713144068	-1.62154707	1.284059	-1.262829	0.206651	NA
si:dkey-247i3.6	6.050454181	-0.280957676	0.8051431	-0.348954	0.727124	NA
si:ch211-231i17.3	0	NA	NA	NA	NA	NA
si:dkeyp-100a1.6	23.12813507	-0.689346292	0.4000139	-1.723306	0.084833	0.560519
BX663503.1	286.8472498	0.149768176	0.1266569	1.1824716	0.237019	0.805854
ank1a	465.4816537	0.231125694	0.1234137	1.8727725	0.0611	0.482482
CR847899.1	0	NA	NA	NA	NA	NA
FP085398.2	14.30878702	-0.81318332	0.5116254	-1.589412	0.111968	0.627242
BX649522.1	11.26716633	0.168735413	0.564539	0.2988906	0.765024	0.975374
si:dkey-4c15.9	11.17137093	0.325021576	0.5762652	0.5640139	0.572745	0.949717
tex264a	459.9073647	-0.017282837	0.1145573	-0.150866	0.880081	0.987881
apoc2	4565.399535	0.140740111	0.0899833	1.564069	0.117801	0.641446
cbx3b	719.1698848	0.145725454	0.0960066	1.5178692	0.129047	0.66057
CR788249.1	0	NA	NA	NA	NA	NA
ferd3l	0.158915748	0.967652056	4.0804729	0.2371421	0.812547	NA
si:dkey-261e22.4	20.2335922	-0.17466507	0.4221853	-0.413717	0.679082	0.965257
mhc1zaa	0.878629579	0.686795053	2.2010162	0.3120354	0.755014	NA
si:dkey-147f3.8	113.2274364	-0.077021628	0.1996926	-0.385701	0.699718	0.967998
si:ch211-272h9.5	5.49312606	0.407673175	0.8593108	0.4744188	0.635201	NA
si:dkey-250k15.4	6.340908172	0.441277195	0.7831007	0.5634999	0.573095	NA
apoc1	3986.78026	0.147669074	0.1867478	0.7907407	0.429095	0.911406
si:dkey-96n2.1	0	NA	NA	NA	NA	NA
si:ch211-59d15.4	18.09966686	0.423255202	0.4559182	0.9283577	0.353222	0.881562
BX321875.1	1.964074944	0.468159373	1.3311091	0.3517062	0.725059	NA
BX908388.1	0	NA	NA	NA	NA	NA
BX511187.1	0.158915748	0.967652056	4.0804729	0.2371421	0.812547	NA
si:dkey-40m6.11	0	NA	NA	NA	NA	NA
im:7140055	294.1831482	-0.091445726	0.1364344	-0.670254	0.502696	0.935304
BX957292.1	0.347458735	-1.908042286	3.4571486	-0.551912	0.581009	NA
CR318588.1	5.076764667	0.289286122	0.8536015	0.3389007	0.734685	NA
LO018289.1	1.212762372	1.332730815	1.9345871	0.6888968	0.490888	NA
si:ch211-250c4.4	189.942699	-0.39606725	0.1525261	-2.596717	0.009412	0.162543
CHST12 (1 of many)	2.0351609	0.524903868	1.3769596	0.381205	0.703051	NA
si:dkey-112g5.16	3.083504768	1.084665128	1.1574183	0.9371419	0.348686	NA
ms4a17a.5	43.32374042	-0.006255542	0.3236236	-0.01933	0.984578	0.996944
BX511089.1	0	NA	NA	NA	NA	NA
CR932978.1	0.157187365	-0.955901296	4.0804729	-0.234262	0.814781	NA
AL929092.1	3.119658826	2.387586659	2.5487976	0.9367502	0.348887	NA
or103-3	0	NA	NA	NA	NA	NA
si:ch211-271e10.3	0	NA	NA	NA	NA	NA
si:dkeyp-13a3.10	135.4282379	0.122753253	0.1798375	0.6825786	0.494873	0.931926
CT573163.1	0.173729368	-0.955901296	4.0804729	-0.234262	0.814781	NA
si:dkey-27p18.3	13.7525254	0.193262128	0.54184	0.3566775	0.721333	0.969666
FO904898.5	0.679983498	-1.527277543	2.5551009	-0.597737	0.550016	NA
BX571825.6	0	NA	NA	NA	NA	NA
vtg1	10.35863756	0.017505541	0.6197088	0.028248	0.977464	NA
or111-9	0	NA	NA	NA	NA	NA
si:ch211-243a20.3	137.8340102	-0.938753678	0.1774626	-5.289869	1.22E-07	1.67E-05
znf1009	28.93260706	-0.213425345	0.3576614	-0.596724	0.550691	0.946549
si:dkey-26i13.7	0	NA	NA	NA	NA	NA
BX005065.1	30.21079974	0.272183991	0.3470703	0.7842329	0.432903	0.913384

BX000447.1	90.0716294	0.131902914	0.2141289	0.6159977	0.537896	0.943336
si:dkey-20i20.3	15.97839814	-0.271255186	0.5026748	-0.539624	0.589457	0.953498
BX548061.1	0.158795395	0.967652056	4.0804729	0.2371421	0.812547	NA
irgf2	0.500282092	2.45023829	2.5970012	0.9434876	0.345432	NA
si:dkey-103k4.1	2.50397706	0.203701306	1.1829685	0.172195	0.863284	NA
CR769769.1	13.50496098	-0.280904245	0.5127258	-0.547864	0.583785	0.952214
si:dkey-102f14.7	2.437017663	0.5294138	1.3960977	0.3792097	0.704532	NA
BX510992.1	5.760823394	0.754771507	0.7842935	0.9623585	0.33587	NA
si:dkey-211g8.5	0.182570949	0.967652056	4.0804729	0.2371421	0.812547	NA
si:ch73-290k24.6	64.98190299	0.089874823	0.243919	0.3684617	0.712529	0.969087
si:ch211-220m6.4	1.310464596	1.562423165	1.9190939	0.8141463	0.415561	NA
CR382296.1	1.808588223	-0.269473476	1.4809264	-0.181963	0.855612	NA
si:ch73-27e22.2	0	NA	NA	NA	NA	NA
CR788230.1	0	NA	NA	NA	NA	NA
si:dkeyp-41f9.4	174.4337531	0.104777749	0.1695826	0.6178567	0.53667	0.943336
BX511161.1	35.0609368	0.331014674	0.3479555	0.9513131	0.341445	0.878134
si:dkey-3k20.1	9.444560426	-0.893119823	0.6438483	-1.387159	0.165393	NA
si:dkey-234d14.2	0.490502273	-2.421143305	2.7891168	-0.868068	0.385357	NA
BX663611.1	1.498766878	0.289100544	1.5459299	0.1870075	0.851655	NA
BX548015.1	0	NA	NA	NA	NA	NA
AL929266.1	4.304820321	-0.672832946	0.8984053	-0.748919	0.453906	NA
flnb	204.4285173	0.038574264	0.1531107	0.251937	0.80109	0.979377
BX000991.1	0.157187365	-0.955901296	4.0804729	-0.234262	0.814781	NA
cxl34b.11	119.2512982	0.136939874	0.2286376	0.5989386	0.549214	0.946312
cenpv	37.73778432	0.137838576	0.3281622	0.4200318	0.674462	0.965257
CU861651.1	0.667059899	1.488443644	2.4079024	0.6181495	0.536477	NA
CR392026.1	0.632206226	0.012903346	2.6133421	0.0049375	0.99606	NA
AL954142.1	1.627904039	-3.043274384	1.7521162	-1.736914	0.082402	NA
si:dkeyp-26a9.2	210.8960458	-0.100897589	0.148119	-0.681193	0.49575	0.932377
si:dkey-222h21.6	0.339758314	0.005883863	3.2682461	0.0018003	0.998564	NA
BX537166.1	15.49273567	0.068004485	0.4882329	0.139287	0.889223	0.989291
CT027772.1	2.01834695	-1.026639109	1.3661031	-0.751509	0.452346	NA
CT574549.1	0.508144151	0.926490632	2.6634746	0.3478504	0.727953	NA
si:dkey-11o15.8	0.635662991	2.808056561	2.7492814	1.0213784	0.307075	NA
si:dkey-59l11.7	0	NA	NA	NA	NA	NA
poldip2	1021.037329	0.082950043	0.0847015	0.9793219	0.327421	0.872701
CR456642.1	9.457176256	0.582467001	0.6185867	0.9416094	0.346393	NA
si:dkey-65l23.2	0	NA	NA	NA	NA	NA
BX927288.1	6.21184633	-0.783068134	0.774099	-1.011586	0.311736	NA
si:ch211-199g17.2	24.56175335	-0.563838152	0.3984438	-1.415101	0.157039	0.707677
si:dkey-65b12.10	1.024339737	3.476540236	2.0349298	1.7084325	0.087556	NA
BX004981.1	9.787968138	0.04286824	0.6000009	0.071447	0.943042	NA
pimr152	0	NA	NA	NA	NA	NA
BX323074.1	4.015626252	-0.282975642	1.0006248	-0.282799	0.777331	NA
CT583646.1	0	NA	NA	NA	NA	NA
si:ch211-152f2.1	0	NA	NA	NA	NA	NA
CR383669.1	0.166657454	-0.955901296	4.0804729	-0.234262	0.814781	NA
si:dkeyp-2e4.3	14.60274926	-0.521164502	0.5495887	-0.948281	0.342986	0.878434
pimr136	1.308375155	0.736523946	1.6727564	0.4403056	0.659716	NA
CR383672.1	0.341486697	1.892317624	3.2124068	0.5890654	0.555817	NA
BX649499.1	0.824875771	-0.638730095	2.222328	-0.287415	0.773795	NA
CU207311.1	0	NA	NA	NA	NA	NA
CU467861.1	278.085342	-0.174837732	0.1324429	-1.320099	0.186802	0.750951
pimr61	0	NA	NA	NA	NA	NA

AL590150.1	26.7487424	0.067257062	0.3674665	0.1830291	0.854775	0.985174
si:dkey-57a22.13	0.339758314	0.005883863	3.2682461	0.0018003	0.998564	NA
nfil3-4	0	NA	NA	NA	NA	NA
si:dkey-161j23.6	165.9443259	0.013572039	0.1702891	0.0797	0.936476	0.992702
CT027702.1	4.813864927	-0.066856026	0.8718717	-0.076681	0.938877	NA
si:dkey-222h21.12	0	NA	NA	NA	NA	NA
BX649478.1	6.59443168	0.309254869	0.7436972	0.4158344	0.677531	NA
jac4	1.190756486	2.514004893	1.9965149	1.2591967	0.207959	NA
si:ch211-284e13.6	490.258423	-0.114810486	0.1073721	-1.069277	0.284945	0.845094
BX469930.1	13.56159074	0.652999605	0.548808	1.1898508	0.234105	0.802753
si:dkeyp-113d7.10	62.37845559	-0.180779653	0.2507324	-0.721006	0.470906	0.924838
si:ch211-51h9.6	128.497171	-0.058716593	0.1777616	-0.330311	0.741165	0.973154
si:dkey-79f11.7	7.156211699	0.554007039	0.778353	0.7117684	0.476608	NA
hsp70.2	891.3155606	-0.013260716	0.2743226	-0.04834	0.961445	0.995842
si:ch211-218c6.8	262.2098004	-0.234134497	0.1406864	-1.66423	0.096066	0.590174
si:dkeyp-77c8.3	3.22558007	-0.57441694	1.1088593	-0.518025	0.604441	NA
BX511023.1	60.32494551	-0.044263891	0.2572394	-0.172073	0.86338	0.985841
BX005340.1	0	NA	NA	NA	NA	NA
dlgap2b	5.900158175	0.807723985	0.8558754	0.9437401	0.345302	NA
BX571809.1	2.705725637	-1.113233272	1.2097115	-0.920247	0.357444	NA
si:dkeyp-51b9.3	3.111658872	0.479487952	1.0763361	0.4454816	0.655972	NA
si:dkey-33c12.14	0.157187365	-0.955901296	4.0804729	-0.234262	0.814781	NA
zgc:153759	0.833476647	0.586781922	2.2129474	0.2651585	0.790887	NA
snorc	465.6483169	-0.07135001	0.1143656	-0.623876	0.532709	0.942805
si:dkey-28d5.3	0	NA	NA	NA	NA	NA
zgc:193742	128.3427314	0.018570821	0.1837201	0.1010821	0.919485	0.991651
CU207217.1	0.31598276	0.005883445	3.3523626	0.001755	0.9986	NA
si:dkey-6f10.4	0	NA	NA	NA	NA	NA
dcun1d4	690.8936899	0.071832023	0.0935254	0.7680484	0.442458	0.916336
BX120005.2	0.347458735	-1.908042286	3.4571486	-0.551912	0.581009	NA
BX936320.1	46.68914511	0.009804405	0.2952329	0.0332091	0.973508	0.996315
taar20d1	0	NA	NA	NA	NA	NA
BX901930.1	0	NA	NA	NA	NA	NA
CR396590.2	0.157187365	-0.955901296	4.0804729	-0.234262	0.814781	NA
BX569787.1	93.40143542	-0.478093348	0.2145603	-2.228247	0.025864	0.310878
si:dkey-79p17.3	6.581133323	1.02099344	0.7662987	1.3323701	0.182739	NA
or115-8	0.158795395	0.967652056	4.0804729	0.2371421	0.812547	NA
zmp:0000001316	31.17031317	0.073610556	0.3443494	0.213767	0.830729	0.982994
CU467861.2	7.726889003	-0.40403967	0.7080562	-0.570632	0.568249	NA
CR762493.1	0.182570949	0.967652056	4.0804729	0.2371421	0.812547	NA
FP103004.1	41.63491494	-0.031774846	0.3096737	-0.102608	0.918274	0.991623
vtg7	13.24977754	0.451331493	0.6000975	0.7520969	0.451993	0.918761
si:ch1073-394i4.1	0.157187365	-0.955901296	4.0804729	-0.234262	0.814781	NA
CR854838.1	0	NA	NA	NA	NA	NA
cxcl11.5	0.182570949	0.967652056	4.0804729	0.2371421	0.812547	NA
BX677668.2	7.681255502	0.036253148	0.6997066	0.0518119	0.958679	NA
si:dkey-24c2.7	3.542655794	-1.312436319	1.0922668	-1.201571	0.22953	NA
BX649599.1	0	NA	NA	NA	NA	NA
CU041345.1	0	NA	NA	NA	NA	NA
BX537137.1	0.365141898	1.973577737	3.3927314	0.5817076	0.560764	NA
BX649502.3	0	NA	NA	NA	NA	NA
BX005364.1	0	NA	NA	NA	NA	NA
BX548032.1	1.629942151	0.008362405	1.4412693	0.0058021	0.995371	NA
BX088710.1	0	NA	NA	NA	NA	NA

zgc:153352	33.66221243	0.16877361	0.3293565	0.5124344	0.608347	0.956718
BX571825.7	0	NA	NA	NA	NA	NA
CR762431.1	37.1147996	0.324046938	0.3200213	1.0125794	0.311261	0.863542
si:dkey-95p16.1	3.484341813	0.400398492	1.1218348	0.356914	0.721156	NA
BX469925.2	0	NA	NA	NA	NA	NA
si:ch211-246e12.3	8.207705983	-0.716652386	0.694328	-1.032152	0.302001	NA
BX284635.1	49.13055055	-0.215436332	0.2748788	-0.78375	0.433187	0.913654
BX548073.2	0.538871265	0.999144257	2.7784799	0.359601	0.719146	NA
si:dkey-282h22.5	4.19646203	1.348791886	0.994855	1.3557673	0.175173	NA
si:dkey-9i23.5	1.469259991	1.782268073	1.7611588	1.011986	0.311545	NA
kif1	4.843402808	0.93712474	0.8701069	1.0770226	0.28147	NA
rhbdd2	79.6679643	-0.303300777	0.2316042	-1.309565	0.190343	0.755033
si:dkeyp-20g2.1	60.9726118	-0.039891556	0.2513485	-0.15871	0.873897	0.987881
si:ch73-46j18.5	6553.379876	0.182747353	0.0664945	2.7483083	0.00599	0.117165
fndc7rs1	46.81278379	0.020468419	0.2817231	0.0726544	0.942081	0.993099
thbd	13.60918652	0.074694608	0.5401429	0.1382868	0.890014	0.989291
si:dkey-7j22.1	4.744476359	-1.378494707	0.9190505	-1.499912	0.133637	NA
AL953896.1	9.228632078	0.290242729	0.6355146	0.456705	0.647883	NA
apopt1	195.3052379	0.052010986	0.1534784	0.3388815	0.734699	0.972021
si:rp71-1f1.4	5.485650045	-0.346963642	0.9319233	-0.372309	0.709663	NA
BX284666.1	3.022729205	-0.266774883	1.1062384	-0.241155	0.809435	NA
sdhaf1	251.0346572	-0.143645389	0.1357155	-1.05843	0.289859	0.846246
vegfbb	2.986693087	-1.392035027	1.1691005	-1.190689	0.233776	NA
si:ch211-207c7.2	0.974991223	-1.022830706	2.0964916	-0.487877	0.625637	NA
si:dkeyp-110g5.4	67.96296112	-0.131232643	0.2462854	-0.532848	0.594139	0.954434
iqcc	79.22905172	-0.236514795	0.2320585	-1.019203	0.308107	0.861088
si:ch211-215m21.22	0	NA	NA	NA	NA	NA
khnyn	222.0865069	-0.289134562	0.1588586	-1.820075	0.068748	0.513259
si:ch73-103b9.2	21.99442214	-0.546369846	0.4094187	-1.334501	0.18204	0.744499
BX323457.1	0.688986717	0.114008068	2.399209	0.047519	0.9621	NA
si:dkey-149i17.8	0	NA	NA	NA	NA	NA
si:dkeyp-44a8.2	1830.490863	0.101653147	0.0986236	1.0307181	0.302673	0.85729
BX927275.1	0.173729368	-0.955901296	4.0804729	-0.234262	0.814781	NA
si:dkeyp-46h3.2	2.613225516	0.373494033	1.1968048	0.312076	0.754983	NA
si:ch211-183d21.1	101.451675	-0.044058148	0.2018839	-0.218235	0.827246	0.982682
si:dkey-222h21.1	0.349228403	0.005884014	3.2372587	0.0018176	0.99855	NA
si:dkey-80c24.5	0.639827579	-1.440067194	2.4366738	-0.590997	0.554522	NA
si:ch73-34l14.3	0	NA	NA	NA	NA	NA
arid1b	988.6815985	-0.184251361	0.0945103	-1.949537	0.051231	0.443542
znf1013	63.88934937	-0.024155682	0.2490241	-0.097001	0.922725	0.99233
si:ch73-376l24.2	80.37009268	-0.306763886	0.2594272	-1.182466	0.237021	0.805854
CR405715.1	3.106661858	0.428580749	1.0760283	0.3982988	0.69041	NA
CR376835.1	0.515885857	-0.89611015	2.8190897	-0.317872	0.750582	NA
si:dkey-93l1.6	1.818175409	-3.213124482	1.6789957	-1.913718	0.055656	NA
pku300	0	NA	NA	NA	NA	NA
gpsm1a	867.9734369	0.158829851	0.0921723	1.7231835	0.084855	0.560519
si:dkey-21e2.11	0	NA	NA	NA	NA	NA
si:dkey-250k15.10	11.42314364	-1.813637095	0.6598756	-2.748453	0.005988	0.117165
jac1	23.36751868	1.314140096	0.4477686	2.934864	0.003337	0.077242
nnt2	30.2959168	0.16649187	0.3442113	0.4836909	0.628605	0.957354
si:dkey-47k20.1	0.484368597	0.855487352	2.7040894	0.316368	0.751723	NA
si:dkey-88l16.4	0	NA	NA	NA	NA	NA
BX663522.1	0	NA	NA	NA	NA	NA
BX005153.1	1.364296476	-0.705781629	1.7443612	-0.404607	0.685766	NA

si:dkey-21e2.8	7.540660626	-0.762536321	0.7038242	-1.083419	0.278623	NA
si:ch211-133l5.4	4.805872224	1.646660299	0.9088466	1.8118132	0.070015	NA
si:dkey-242h9.5	0.157187365	-0.955901296	4.0804729	-0.234262	0.814781	NA
BX571825.8	0	NA	NA	NA	NA	NA
BX927394.1	0.845032922	-3.195649681	2.1776262	-1.467492	0.142242	NA
CR855328.1	0	NA	NA	NA	NA	NA
BX649502.4	0	NA	NA	NA	NA	NA
BX005068.1	0.670675047	-1.455259498	2.4036813	-0.605429	0.544894	NA
pdap1a	2029.503684	0.047335752	0.0724446	0.6534062	0.513494	0.936615
si:dkey-85a20.4	1.222514451	1.394641811	1.9626635	0.7105863	0.477341	NA
si:dkey-56e3.2	15.22204923	0.991711112	0.5329855	1.8606717	0.062791	0.489019
fndc7rs2	0.645291463	-2.8201041	2.5306349	-1.114386	0.265114	NA
slc25a5	55970.34653	-0.098478837	0.0826205	-1.191943	0.233284	0.801695
si:ch211-214j8.12	56.10372066	-0.535257155	0.2796867	-1.913774	0.055649	0.461579
AL929315.1	1.472246632	1.049390408	1.6667905	0.6295875	0.528965	NA
or123-1	0.964610636	-0.018889399	1.958304	-0.009646	0.992304	NA
pimr201	0.996127896	-0.965496869	1.8966759	-0.509047	0.610719	NA
BX927385.1	1.51295199	0.327014375	1.4960191	0.2185897	0.82697	NA
si:dkey-61l1.4	121.4677756	-0.228805812	0.2002776	-1.142443	0.25327	0.823033
BX571681.1	0	NA	NA	NA	NA	NA
CU394254.1	0	NA	NA	NA	NA	NA
BX323586.1	0.365141898	1.973577737	3.3927314	0.5817076	0.560764	NA
si:ch211-198d23.1	29.63774835	-0.699997103	0.3554857	-1.969129	0.048938	0.435115
BX927388.1	2.701980093	0.767053272	1.2236327	0.6268656	0.530747	NA
or125-6	0	NA	NA	NA	NA	NA
si:ch211-222e20.4	123.6921638	-0.162520358	0.1801065	-0.902357	0.366867	0.88734
si:ch211-95j8.3	1.198429168	-1.20658346	3.9052074	-0.308968	0.757346	NA
si:dkey-16p19.5	12.96434084	0.302814927	0.5352738	0.5657196	0.571584	0.949472
si:ch1073-385f13.3	16.59007957	-0.623896245	0.5151266	-1.211151	0.225837	0.79334
BX957274.1	0.492110303	-0.89618148	2.8628934	-0.313033	0.754255	NA
CU861651.2	0.635301933	2.809457917	2.5427008	1.1049109	0.269198	NA
pimr150	0	NA	NA	NA	NA	NA
BX322660.1	0.491560863	0.853615855	2.865157	0.2979299	0.765757	NA
CR925763.1	0.31759079	1.807926197	3.579045	0.5051421	0.613459	NA
si:dkey-174k12.3	0	NA	NA	NA	NA	NA
si:dkeyp-44d3.1	2.573214281	-0.113217404	1.2672649	-0.08934	0.928812	NA
BX005058.1	0	NA	NA	NA	NA	NA
si:dkey-256e7.5	17.11468186	0.275917587	0.4619907	0.5972362	0.55035	0.946549
BX546499.1	18.26031338	-0.313476133	0.4401264	-0.712241	0.476315	0.926486
si:ch211-15j1.4	8.216942997	-1.17792308	0.6972271	-1.68944	0.091135	NA
znf1003	25.57276854	-0.322955072	0.3793873	-0.851254	0.394628	0.898861
CU855900.1	0	NA	NA	NA	NA	NA
BX936298.1	0.158795395	0.967652056	4.0804729	0.2371421	0.812547	NA
si:dkey-262g12.12	82.21677328	-0.072573283	0.2209804	-0.328415	0.742598	0.973232
aup1	718.1831478	-0.06144513	0.1067899	-0.575383	0.565032	0.948739
lamp1b	820.8708558	-0.022834527	0.0918472	-0.248614	0.803659	0.979643
CR848741.1	0.158795395	0.967652056	4.0804729	0.2371421	0.812547	NA
si:dkey-204f11.3	0	NA	NA	NA	NA	NA
BX322665.1	3.70400521	2.200596521	1.1076107	1.986796	0.046945	NA
sptbn4b	53.72259179	-0.011314344	0.3140681	-0.036025	0.971262	0.996315
zgc:174193	1.342958123	-0.680000894	1.7232513	-0.394603	0.693136	NA
zgc:174931	14.58995362	0.136116724	0.5433557	0.2505113	0.802192	0.979492
pimr29	0	NA	NA	NA	NA	NA
CR954294.1	0	NA	NA	NA	NA	NA

CR812470.1	51.23397327	0.197308716	0.2725945	0.7238178	0.469178	0.924496
CR753844.1	0.657469457	1.487996951	2.3011149	0.6466417	0.517864	NA
EBF1 (1 of many)	0	NA	NA	NA	NA	NA
AL935295.1	7.486245519	0.881755192	0.7231124	1.2193888	0.222697	NA
ptprua	933.8181478	0.038283238	0.0889484	0.4303983	0.666906	0.964736
BX649641.1	0	NA	NA	NA	NA	NA
BX571701.2	0	NA	NA	NA	NA	NA
BX323080.1	0.990622727	-0.0667832	1.9438623	-0.034356	0.972593	NA
ago1	9.581502372	-0.020811096	0.6509551	-0.03197	0.974496	NA
CR381531.1	1.155713436	-1.281524989	1.8786642	-0.682147	0.495146	NA
si:ch211-223p8.8	0	NA	NA	NA	NA	NA
si:dkey-57a22.11	63.33126106	0.014795326	0.2551394	0.0579892	0.953757	0.994213
qrfpr4	10.19625259	0.050092628	0.6154828	0.0813875	0.935134	NA
si:dkey-1h24.6	1.521604194	-0.961730371	1.6012032	-0.60063	0.548087	NA
CR457445.1	27.94756295	-0.007862119	0.3852237	-0.020409	0.983717	0.996944
ppp3r1a	322.3642206	-0.028911686	0.1241311	-0.232912	0.815829	0.981594
cyp27c1	151.7262519	0.246939966	0.1653676	1.4932793	0.135364	0.670849
dnaaf3	3.952448138	0.498436688	0.9518073	0.5236739	0.600505	NA
si:dkey-93m18.3	4.812366959	1.154517939	0.8772038	1.3161342	0.188129	NA
BX005156.1	0	NA	NA	NA	NA	NA
si:ch211-271e10.2	1.143845172	-1.265201982	1.909223	-0.662679	0.507536	NA
tlr20.3	0.496945679	-0.834564081	2.8549631	-0.29232	0.770042	NA
pard3ba	92.07509697	-0.3053299	0.2125067	-1.436801	0.150774	0.698912
BX248497.1	259.8770446	0.093288861	0.1334009	0.6993121	0.484357	0.929601
coa6	40.60879918	-0.067623375	0.302861	-0.223282	0.823316	0.982196
AL954134.2	0	NA	NA	NA	NA	NA
si:dkey-58f10.12	5.290520399	1.545334811	0.9068263	1.7041134	0.08836	NA
AL845369.1	0.976719606	-0.046864593	2.2023826	-0.021279	0.983023	NA
BX649292.1	0	NA	NA	NA	NA	NA
si:ch211-130h14.6	0	NA	NA	NA	NA	NA
CU682811.1	0.182570949	0.967652056	4.0804729	0.2371421	0.812547	NA
pimr133	0	NA	NA	NA	NA	NA
tpt1	31420.76184	-0.336505295	0.0903635	-3.723908	0.000196	0.008396
CT025775.1	0	NA	NA	NA	NA	NA
cyp11a2	11.31026223	0.368475751	0.5959709	0.6182781	0.536392	0.943336
pimr200	0.317711143	1.810477155	3.2978238	0.5489915	0.583011	NA
si:dkeyp-20e4.8	0.173729368	-0.955901296	4.0804729	-0.234262	0.814781	NA
BX649384.2	1.157751549	2.488197843	1.9005833	1.3091759	0.190475	NA
or124-1	0.48448895	0.85344211	2.878836	0.2964539	0.766883	NA
BX537280.1	1.340331546	-3.866967578	3.7746737	-1.024451	0.305622	NA
CU463109.1	0	NA	NA	NA	NA	NA
BX571681.2	0.158795395	0.967652056	4.0804729	0.2371421	0.812547	NA
si:dkey-31b16.7	0	NA	NA	NA	NA	NA
si:ch211-266a5.12	8.086799692	0.673400475	0.7287727	0.9240199	0.355476	NA
CR847503.1	24.82160368	0.547006633	0.3892359	1.4053346	0.159922	0.712228
VASH1	142.4839082	0.293099716	0.1774065	1.6521363	0.098507	0.597984
BX569790.1	0.166657454	-0.955901296	4.0804729	-0.234262	0.814781	NA
gbgt1l2	17.00940447	-0.588669639	0.4863406	-1.210406	0.226123	0.793638
AL954655.2	28.15484169	0.053592852	0.3747717	0.1430013	0.886289	0.988936
si:ch211-67f24.7	20.10988102	-0.570246825	0.4432152	-1.286614	0.198229	0.76333
unc5da	444.9808559	0.152083016	0.1248219	1.2184001	0.223072	0.789115
si:ch73-381f5.2	0	NA	NA	NA	NA	NA
si:dkey-188i13.8	8.795978087	-0.050744706	0.6326358	-0.080212	0.936069	NA
si:dkey-184p9.7	92.63677558	-0.30614464	0.2338553	-1.30912	0.190494	0.755212

si:dkey-266f7.4	0.158795395	0.967652056	4.0804729	0.2371421	0.812547	NA
enam	487.6024595	-0.209913226	0.1333698	-1.573919	0.115506	0.637206
mhc1uka	1.290183837	-0.02344436	1.647742	-0.014228	0.988648	NA
si:ch73-256j6.5	5.054126771	0.63840888	0.9026668	0.7072476	0.479413	NA
si:ch211-263k4.2	13.00952431	-0.631876692	0.5328277	-1.185893	0.235665	0.804824
BX510917.1	0.831706979	-0.653219184	2.2172216	-0.294612	0.768291	NA
si:ch211-57m13.7	0.157187365	-0.955901296	4.0804729	-0.234262	0.814781	NA
BX571825.9	0	NA	NA	NA	NA	NA
ftr37	3.497706781	-0.118132249	1.0030523	-0.117773	0.906248	NA
si:ch211-150o23.2	160.6684542	0.10655027	0.1645836	0.6473929	0.517378	0.938592
si:ch211-133n4.12	0	NA	NA	NA	NA	NA
CR396590.3	0	NA	NA	NA	NA	NA
BX248399.1	1.130329852	-2.449181944	2.0196915	-1.212652	0.225263	NA
caiap	1.448230372	1.025641948	1.6993965	0.603533	0.546154	NA
si:dkey-61p9.9	67.81784204	-0.022616937	0.2384927	-0.094833	0.924448	0.992676
si:dkey-5i3.1	0	NA	NA	NA	NA	NA
si:dkey-57a22.14	0	NA	NA	NA	NA	NA
znf1151	27.35222313	-0.326942957	0.3663048	-0.892543	0.372102	0.892629
si:dkeyp-68b7.7	26.37844876	0.186642743	0.3746307	0.4982046	0.61834	0.957354
si:ch211-253p18.2	10.19874	0.866856572	0.6028628	1.4379002	0.150462	NA
BX530064.1	0	NA	NA	NA	NA	NA
CR769772.1	0.547712847	2.559541715	2.9076406	0.8802813	0.378707	NA
tarsl2	421.1444546	-0.017149963	0.111267	-0.154133	0.877505	0.987881
BX640547.1	2.321567749	0.03878383	1.3533206	0.0286583	0.977137	NA
si:ch73-338o16.4	2.679911971	-3.801724469	1.5404296	-2.467964	0.013588	NA
si:rp71-23d18.4	0	NA	NA	NA	NA	NA
si:ch1073-170o4.1	27.49496102	-0.030447393	0.3783812	-0.080468	0.935865	0.992702
BX323550.1	0	NA	NA	NA	NA	NA
znf1109	20.00762191	0.712809207	0.431142	1.653305	0.098269	0.597502
si:dkey-88p24.11	176.7134892	-0.229602389	0.1723833	-1.331929	0.182883	0.745944
si:dkey-21e2.15	13.30480162	-1.69698518	0.578183	-2.935031	0.003335	0.077242
BX323038.1	2.187365793	0.715941476	1.3414459	0.5337088	0.593543	NA
rprm3	67.52829859	0.01524555	0.2376682	0.0641464	0.948854	0.99381
BX470224.1	0.158915748	0.967652056	4.0804729	0.2371421	0.812547	NA
ppib	7157.286096	-0.122138201	0.0769261	-1.587734	0.112346	0.627572
camta2	23.57986604	-0.301031323	0.4008648	-0.750955	0.45268	0.919496
CU075735.2	0.474778155	0.852397662	2.898154	0.2941175	0.768668	NA
CR855277.2	9.71417778	-0.239654688	0.6133349	-0.39074	0.695989	NA
BX957328.1	0.522329263	0.999930388	2.8066578	0.3562709	0.721638	NA
si:ch1073-110a20.2	7.706780267	-0.045341864	0.7684577	-0.059004	0.952949	NA
si:dkey-151g10.6	15900.79219	-0.446044819	0.1066779	-4.18123	2.90E-05	0.001706
CU466285.1	0.474778155	0.852397662	2.898154	0.2941175	0.768668	NA
hoxc9a	116.3707196	0.075206944	0.189899	0.3960365	0.692078	0.967354
stap2a	492.5699874	-0.176603199	0.1132587	-1.559291	0.118928	0.642772
fndc7rs4	322.6830626	0.031353747	0.1249198	0.2509909	0.801821	0.979492
si:dkey-21e2.3	0	NA	NA	NA	NA	NA
si:dkey-93n13.2	147.9847486	0.174407013	0.1662107	1.0493127	0.294034	0.849999
CR388168.1	0.325573202	0.005883621	3.3172783	0.0017736	0.998585	NA
si:dkeyp-67a8.4	13.96605338	-0.083295161	0.5609039	-0.148502	0.881947	0.988344
si:dkey-42p14.3	5.098046023	0.036350714	0.8963052	0.0405562	0.96765	NA
BX248511.1	5.445308593	0.583949486	0.863358	0.67637	0.498806	NA
CR589943.1	4.629053938	-0.21440723	0.8970912	-0.239003	0.811103	NA
BX548075.1	0	NA	NA	NA	NA	NA
CR318588.2	0	NA	NA	NA	NA	NA

si:ch211-191d15.2	0.531799352	0.999478841	2.790358	0.3581902	0.720201	NA
si:ch211-93g21.2	1.123526383	0.393221643	1.8303296	0.2148365	0.829895	NA
si:dkey-66g10.2	2.280738783	-1.260458015	1.3534773	-0.931274	0.351712	NA
zgc:162611	74.76478849	0.427755807	0.255867	1.6717894	0.094566	0.584906
AL845320.1	0.665960023	-1.495776328	2.4092162	-0.620856	0.534694	NA
si:ch73-92e7.4	81.16936117	-0.184705999	0.2369857	-0.779397	0.435746	0.914408
CR788322.1	0	NA	NA	NA	NA	NA
BX510324.1	0.323844819	-1.825095364	3.274639	-0.557342	0.577293	NA
SRBD1	111.2428174	-0.093291129	0.1941553	-0.480498	0.630874	0.957354
si:ch211-210c8.7	169.2919922	0.012114537	0.1609921	0.0752493	0.940016	0.992857
si:ch1073-126c3.2	22.71136445	-0.254149959	0.4287737	-0.592737	0.553357	0.947265
si:ch211-260p9.3	4.780340549	0.719249351	0.8839647	0.813663	0.415838	NA
CU138515.1	1.507100304	1.842319674	1.6667457	1.1053394	0.269013	NA
BX936337.1	843.3685236	-0.041164178	0.1138024	-0.361716	0.717564	0.96909
upk3b	13.09125375	0.067983263	0.548906	0.1238523	0.901432	0.990121
zgc:193541	2677.864843	-0.027667132	0.0906039	-0.305364	0.760089	0.975374
BX537288.1	0.325573202	0.005883621	3.3172783	0.0017736	0.998585	NA
si:dkeyp-85e10.3	4.279974891	-0.481451002	0.9929285	-0.48488	0.627762	NA
CU074419.1	0.524057646	2.503896172	2.7289583	0.9175282	0.358866	NA
BX649294.1	86.32859808	-0.175822881	0.2120779	-0.829048	0.407077	0.901305
si:dkey-161j23.7	197.5860988	-0.179086679	0.1453994	-1.231688	0.218066	0.785379
si:dkey-193b15.5	3.035387768	-0.462580036	1.1821187	-0.391314	0.695565	NA
zgc:171977	0	NA	NA	NA	NA	NA
si:dkeyp-46h3.3	1.354156595	0.015823789	1.57664	0.0100364	0.991992	NA
zgc:194246	112.9633937	0.309304712	0.2073931	1.4913933	0.135858	0.672288
si:ch73-44m9.5	0	NA	NA	NA	NA	NA
si:dkey-23c22.5	5.110236474	0.078018599	0.8269527	0.0943447	0.924835	NA
CT027589.1	0.341486697	1.892317624	3.2124068	0.5890654	0.555817	NA
si:ch211-114l13.9	0.80742327	0.548673017	2.1289713	0.2577174	0.796625	NA
si:dkey-188i13.11	122.5072946	0.011947729	0.1889221	0.0632416	0.949574	0.99381
si:ch211-67f24.7	12.03082517	-0.236314766	0.546135	-0.432704	0.66523	0.964736
si:ch211-157j23.3	8.315656981	0.012790738	0.6460268	0.0197991	0.984204	NA
BX927258.1	5.905203015	0.287816496	0.8096378	0.355488	0.722224	NA
zgc:153317	12.18710204	-0.194100305	0.5398535	-0.359543	0.719189	0.969183
AL954320.1	0.173729368	-0.955901296	4.0804729	-0.234262	0.814781	NA
si:dkey-201l21.2	0	NA	NA	NA	NA	NA
BX323586.2	0.157187365	-0.955901296	4.0804729	-0.234262	0.814781	NA
BX547930.1	0	NA	NA	NA	NA	NA
BX323812.1	0.332524763	0.005883742	3.292841	0.0017868	0.998574	NA
mlnl	22.60579551	-0.429893377	0.4055858	-1.059932	0.289176	0.845979
crhr2	22.70348614	0.397126249	0.4310293	0.9213439	0.356871	0.883422
zp3c	0.323844819	-1.825095364	3.274639	-0.557342	0.577293	NA
si:ch211-106h4.12	115.4280388	0.374962945	0.2023413	1.8531212	0.063865	0.493997
si:dkey-95h12.2	0	NA	NA	NA	NA	NA
zgc:171474	0	NA	NA	NA	NA	NA
BX649641.2	0.157187365	-0.955901296	4.0804729	-0.234262	0.814781	NA
si:dkey-66k12.3	0	NA	NA	NA	NA	NA
si:dkey-44g23.2	0	NA	NA	NA	NA	NA
si:ch211-132b12.4	0	NA	NA	NA	NA	NA
si:ch211-114c12.5	1.941330241	-0.030104089	1.3853588	-0.02173	0.982663	NA
si:dkey-32e6.3	69.22531593	0.480113303	0.2450533	1.9592197	0.050087	0.43824
znf1047	11.07752006	-0.249766087	0.5899579	-0.423363	0.672031	0.965257
BX537338.1	0.182570949	0.967652056	4.0804729	0.2371421	0.812547	NA
BX000981.1	0.356300316	0.005884122	3.214964	0.0018302	0.99854	NA

si:dkey-222h21.3	1.045287033	0.090981892	1.9340789	0.0470415	0.96248	NA
BX001050.1	0.157187365	-0.955901296	4.0804729	-0.234262	0.814781	NA
si:dkey-117n7.3	54.5593462	-0.020736775	0.2746711	-0.075497	0.939819	0.992857
si:ch211-250g4.3	29.36937284	-0.020277567	0.4098019	-0.049481	0.960536	0.995421
CU681845.1	0.325452849	0.005883619	3.3177086	0.0017734	0.998585	NA
cytl	16282.17202	0.002910072	0.0947366	0.0307175	0.975495	0.996762
gna15.3	11.412439	-0.992700949	0.5763771	-1.722312	0.085013	0.560519
si:ch73-366i20.1	11.35488606	-0.377784147	0.5674414	-0.665768	0.50556	0.935724
BX294379.1	20.81309386	0.164902089	0.4502879	0.3662148	0.714205	0.96909
BX548044.4	0	NA	NA	NA	NA	NA
mrpl34	374.0155741	0.149580925	0.118375	1.2636191	0.206367	0.774011
BX649388.2	0	NA	NA	NA	NA	NA
si:dkeyp-27c8.1	0.471562095	-2.373056629	3.0645586	-0.774355	0.438721	NA
ftr26	1.332271062	0.763437842	1.6495887	0.462805	0.643504	NA
BX004981.2	2.003533331	-0.514167452	1.3416074	-0.383247	0.701536	NA
BX321877.1	15.6420563	0.077896932	0.4878576	0.1596715	0.87314	0.987846
CU207275.1	6.087781842	-0.790499252	0.8061772	-0.980553	0.326813	NA
si:dkey-93m18.4	4.051944361	0.512815854	0.9762804	0.5252752	0.599392	NA
znf1128	5.036133973	-1.174940285	0.9343531	-1.257491	0.208576	NA
tmsb2	854.4537994	0.153013138	0.0958069	1.5970986	0.110244	0.62463
si:dkeyp-82a1.3	0.317711143	1.810477155	3.2978238	0.5489915	0.583011	NA
AL935198.1	2.416967566	0.996613846	1.2873182	0.7741783	0.438825	NA
si:ch211-127i16.2	186.3201115	-0.210192681	0.1534323	-1.369938	0.170706	0.728116
pias2	616.5810679	0.018008088	0.1038754	0.1733624	0.862367	0.985841
AL929058.1	277.8638968	0.215582438	0.1336606	1.6129088	0.106764	0.615857
si:dkey-222h21.11	2.599548558	-0.038915716	1.2294682	-0.031652	0.974749	NA
CU499330.1	25.46119006	0.663391682	0.3975941	1.6685149	0.095214	0.58671
zmat4a	107.3532185	0.059549612	0.1930989	0.3083892	0.757786	0.975374
si:ch211-161n3.3	4.813421298	-0.101856546	0.8721019	-0.116794	0.907023	NA
BX294113.1	0	NA	NA	NA	NA	NA
BX005329.2	15.20172947	0.106656675	0.4940829	0.215868	0.829091	0.982737
si:ch211-202f5.3	252.3487918	0.326089931	0.146246	2.2297359	0.025765	0.310555
tnni4b.1	2.022075663	1.607502852	1.3874082	1.1586373	0.246604	NA
si:dkeyp-77c8.2	40.65956271	0.638413881	0.3459611	1.8453346	0.064989	0.497901
BX323543.1	0	NA	NA	NA	NA	NA
acy3.1	29.87808098	-0.33242003	0.3594296	-0.924854	0.355042	0.882321
si:dkey-33c12.11	16.87688409	-0.689103416	0.4615103	-1.493148	0.135398	0.670851
rarres3	16.54388201	-0.250032156	0.473737	-0.527787	0.597647	0.95534
mphosph6	224.0202898	0.094216424	0.141695	0.6649239	0.506099	0.935724
adgrf3b	48.27143387	0.014587014	0.2864228	0.0509283	0.959383	0.995165
BX511259.1	0.158795395	0.967652056	4.0804729	0.2371421	0.812547	NA
BX890551.1	0	NA	NA	NA	NA	NA
BX294181.1	0	NA	NA	NA	NA	NA
CR383662.1	2.65947283	-2.149878984	1.3195394	-1.629265	0.103257	NA
si:ch211-174j14.2	0.515885857	-0.89611015	2.8190897	-0.317872	0.750582	NA
CR847870.1	0	NA	NA	NA	NA	NA
si:dkey-83k24.5	73.77855362	-0.292065292	0.227415	-1.284283	0.199043	0.764531
CR356233.2	0	NA	NA	NA	NA	NA
FAM163A	29.42559266	0.247031908	0.3638875	0.6788689	0.497221	0.93322
selenoh	441.095077	-0.374635717	0.1280417	-2.925889	0.003435	0.078428
CT737131.1	0	NA	NA	NA	NA	NA
si:ch211-213a13.2	14.18269312	0.611766112	0.5296894	1.1549524	0.24811	0.817415
si:dkeyp-77c8.4	1.571932231	4.088279848	1.8298064	2.234269	0.025465	NA
si:ch211-222n4.2	1.49813837	1.006839534	1.522663	0.661236	0.508461	NA

BX950184.1	1.180145236	0.365759426	1.9291048	0.1896006	0.849622	NA
BX640520.1	1.019741812	-0.999014948	1.9781285	-0.50503	0.613538	NA
CR513782.2	0.356300316	0.005884122	3.214964	0.0018302	0.99854	NA
AL935144.1	0	NA	NA	NA	NA	NA
si:ch211-234c11.2	2.049586718	0.555428723	1.4044988	0.395464	0.6925	NA
si:dkey-104n9.1	51.86763713	0.608982881	0.2887424	2.1090872	0.034937	0.363807
ugt2a7	289.175261	-0.680154553	0.1478483	-4.600355	4.22E-06	0.000353
si:ch211-161h7.5	213.5519004	-0.486742998	0.1977601	-2.461281	0.013844	0.211721
BX465186.1	0	NA	NA	NA	NA	NA
si:ch211-158d24.4	55.05012715	-0.248294606	0.2754642	-0.901368	0.367393	0.887458
c6	23.24101268	-0.042103978	0.4074226	-0.103342	0.917691	0.991623
vap	0	NA	NA	NA	NA	NA
BX548073.3	28.09139891	0.339691114	0.4488766	0.7567584	0.449195	0.918265
STMP1	635.978163	0.17041572	0.1071767	1.5900449	0.111825	0.627242
lgals9l6	0	NA	NA	NA	NA	NA
BX682548.1	2.412184514	1.413354695	1.4114983	1.0013152	0.316674	NA
CR847870.2	0.31437473	-1.788572922	3.5930657	-0.497785	0.618636	NA
FP016056.1	21.72678466	0.483667457	0.4175705	1.1582894	0.246746	0.816669
SMIM18	104.4576767	-0.110919038	0.2143732	-0.517411	0.604869	0.95651
ubac2	171.0859687	0.068427411	0.1601548	0.4272579	0.669191	0.965037
c3b.1	662.8421821	0.194062677	0.112416	1.7262905	0.084295	0.55841
thumpd2	72.80185456	-0.118505546	0.2316601	-0.511549	0.608967	0.956718
CR925713.1	5.147699276	-1.016134835	0.8918844	-1.139312	0.254573	NA
si:ch211-139g16.8	6.759432066	-1.136043102	0.8004871	-1.41919	0.155844	NA
BX897740.1	0.157187365	-0.955901296	4.0804729	-0.234262	0.814781	NA
plscr3a	50.80208751	-1.234725838	0.2787494	-4.42952	9.44E-06	0.000689
proca	51.91247127	0.470748922	0.2945035	1.5984494	0.109943	0.624178
si:dkey-163f12.10	0	NA	NA	NA	NA	NA
si:ch73-92i20.1	3.18388149	-0.133375247	1.0396492	-0.128289	0.897921	NA
LO018605.1	5.550594294	1.253169387	0.8449072	1.4832035	0.13802	NA
CT573467.1	0	NA	NA	NA	NA	NA
BX323543.2	0	NA	NA	NA	NA	NA
BX649502.5	0	NA	NA	NA	NA	NA
BX649388.3	4.282615015	-0.418654325	0.977786	-0.428166	0.668531	NA
BX511310.1	5.686319545	0.361854388	0.7865784	0.460036	0.64549	NA
tac3a	63.06137585	0.070151587	0.254333	0.2758257	0.782682	0.97627
iqsec3b	38.80796794	0.057211398	0.3069434	0.1863907	0.852138	0.985174
adam17b	24.75507449	0.096241771	0.3903233	0.2465694	0.805241	0.979883
ccl34b.8	0.157187365	-0.955901296	4.0804729	-0.234262	0.814781	NA
BX005313.2	9.639007546	0.053166671	0.6296435	0.0844393	0.932707	NA
tmprss12	0	NA	NA	NA	NA	NA
si:ch211-132g1.6	0	NA	NA	NA	NA	NA
BX000444.1	0	NA	NA	NA	NA	NA
BX004774.1	9.603954053	0.300428158	0.6095837	0.4928416	0.622125	NA
CT573344.1	42.07016473	-0.686339429	0.3158228	-2.173179	0.029767	0.334058
si:ch211-209l18.2	48.83943898	-0.353463235	0.310384	-1.138793	0.254789	0.823484
espnlb	24.64456024	0.125833746	0.3876462	0.3246098	0.745476	0.974094
BX511265.1	0.794338034	3.131709106	2.3335617	1.3420297	0.179586	NA
si:dkeyp-122a9.2	0.173729368	-0.955901296	4.0804729	-0.234262	0.814781	NA
si:dkey-266m15.7	33.13588017	-0.089593565	0.3420096	-0.261962	0.793351	0.977937
CR847509.1	0.990784365	0.018336633	1.8663564	0.0098248	0.992161	NA
gstk4	0.50625413	-0.940106488	2.8357787	-0.331516	0.740255	NA
si:dkey-117m1.4	317.3463145	-0.29953099	0.1351847	-2.215717	0.026711	0.315011
scpp8	9.762289614	-0.611816087	0.6371343	-0.960262	0.336923	NA

RANBP2	1927.706414	-0.036788713	0.1019525	-0.360842	0.718218	0.96909
si:ch73-112l6.1	270.4859017	-0.02479888	0.1363061	-0.181935	0.855634	0.985174
si:rp71-45k5.2	0	NA	NA	NA	NA	NA
CR788254.2	1.371530028	-0.640785266	1.8369316	-0.348835	0.727214	NA
si:ch211-57f7.7	8.772506769	-0.63564935	0.6757714	-0.940628	0.346896	NA
si:dkey-117n7.2	92.16471466	-0.03426452	0.2050178	-0.167129	0.867268	0.987125
BX936323.1	0	NA	NA	NA	NA	NA
BX294189.1	2.50056158	-0.510623241	1.2885816	-0.396268	0.691908	NA
BX004800.1	0	NA	NA	NA	NA	NA
znf1010	129.9772434	-0.022288759	0.1861736	-0.11972	0.904705	0.990567
CR932983.1	0.173729368	-0.955901296	4.0804729	-0.234262	0.814781	NA
fndc7rs3	47.91491836	-0.154155882	0.2917193	-0.528439	0.597195	0.955244
CU137717.1	0	NA	NA	NA	NA	NA
pimr83	0	NA	NA	NA	NA	NA
si:ch73-21g5.7	63.80695224	-0.550062155	0.2672801	-2.057999	0.03959	0.390659
si:ch73-236c18.8	244.1696153	0.003250973	0.1412016	0.0230236	0.981631	0.996944
si:dkeyp-106c3.3	9.651219914	-0.09724788	0.6021238	-0.161508	0.871693	NA
CR853291.1	0.31437473	-1.788572922	3.5930657	-0.497785	0.618636	NA
si:rp71-23d18.8	4.252733524	0.673545725	0.9692227	0.6949339	0.487097	NA
si:dkey-16p6.2	2.188973823	1.191399035	1.3193453	0.9030229	0.366514	NA
BX510336.1	0	NA	NA	NA	NA	NA
CR749763.2	0.325573202	0.005883621	3.3172783	0.0017736	0.998585	NA
mef2b	46.1724475	-0.259216846	0.3033102	-0.854626	0.392758	0.897688
CR759927.1	3.172882439	-0.451648694	1.0701155	-0.422056	0.672984	NA
si:dkey-93m18.6	9.82008741	-0.416535716	0.6121008	-0.680502	0.496187	NA
zgc:77118	1.297537742	0.031968932	1.7469507	0.0182998	0.9854	NA
si:ch211-15j1.1	15.58813849	0.694675512	0.5206498	1.3342472	0.182123	0.744502
si:dkey-79c1.1	2.489280538	-0.980779674	1.2273236	-0.799121	0.42422	NA
CR354547.1	1.834906788	-0.823777427	1.4572737	-0.565287	0.571879	NA
CT027756.1	0.330916733	-1.849579229	3.2489867	-0.569279	0.569167	NA
eif2ak1	293.8186308	-0.128508738	0.1283464	-1.001265	0.316699	0.867129
or125-7	0.498553709	0.926267934	2.6786929	0.345791	0.7295	NA
GUCY1A2	58.21067233	-0.110725701	0.2783603	-0.397778	0.690794	0.967157
si:dkey-30j10.5	1.269155061	2.614569192	3.9472603	0.6623757	0.50773	NA
si:dkey-75a21.2	158.5477665	-0.050584486	0.1601701	-0.315817	0.752141	0.974577
BX571724.1	1.99638235	-0.467313187	1.3274943	-0.352027	0.724818	NA
si:ch211-198m1.1	0.182570949	0.967652056	4.0804729	0.2371421	0.812547	NA
chia.6	106.2353444	0.573060249	0.1938697	2.9559039	0.003118	0.073687
CR788285.1	0	NA	NA	NA	NA	NA
c3a.4	2.010766882	-0.477928131	1.3557981	-0.352507	0.724458	NA
si:ch211-212c13.10	211.3193317	-0.036914658	0.1505456	-0.245206	0.806297	0.980105
si:dkey-112e17.1	46.4663154	-0.379268736	0.2811413	-1.349033	0.177326	0.738201
ftr32	0.173729368	-0.955901296	4.0804729	-0.234262	0.814781	NA
AL845362.1	1.628213769	-0.563718013	1.4711157	-0.383191	0.701578	NA
CR786562.1	42.97687091	-0.412529057	0.3106738	-1.327853	0.184227	0.747376
CT573148.1	8.143504802	-0.584903656	0.7033365	-0.831613	0.405627	NA
si:ch211-284e13.9	1.330071311	-2.707674392	1.853274	-1.461022	0.144009	NA
si:ch211-146l10.7	0.182570949	0.967652056	4.0804729	0.2371421	0.812547	NA
BX936308.2	24.34763501	-0.052184678	0.3982893	-0.131022	0.895758	0.989814
znf1127	2.949636131	0.642693895	1.1063005	0.5809397	0.561281	NA
CR749762.2	2.135258044	-1.16186778	1.3038928	-0.891076	0.372888	NA
CR456628.1	4.586099761	-0.21712478	0.8893764	-0.244131	0.807129	NA
AL928845.1	2.167672256	-0.703178131	1.2973829	-0.541997	0.58782	NA
si:ch211-139n6.3	16.76157789	-0.163192465	0.4755265	-0.343183	0.731461	0.971557

CR847566.1	0.515216064	0.926382026	2.6525479	0.3492423	0.726907	NA
si:ch211-215d8.2	95.09800552	0.031003929	0.2090032	0.1483419	0.882073	0.988344
si:dkey-65l23.2	0	NA	NA	NA	NA	NA
TST	242.6034224	0.094012657	0.1511809	0.6218552	0.534037	0.943276
si:ch211-89o9.4	108.8772995	0.315745644	0.2067937	1.5268626	0.126795	0.656588
BX649641.3	0.347458735	-1.908042286	3.4571486	-0.551912	0.581009	NA
GMEB2	281.8012016	-0.219878886	0.1361436	-1.615051	0.1063	0.615567
BX530024.1	0.865544342	3.23079022	2.2781012	1.4181944	0.156134	NA
CU681836.1	176.5189841	-0.012877235	0.169916	-0.075786	0.939589	0.992857
BX537358.1	5.152010713	-1.071074671	0.849852	-1.260307	0.207559	NA
si:dkey-238m4.4	2.560008691	0.642131771	1.2235951	0.5247911	0.599728	NA
si:ch211-226h8.11	0	NA	NA	NA	NA	NA
BX936438.1	0.158915748	0.967652056	4.0804729	0.2371421	0.812547	NA
CR753837.1	0.974080725	-0.034512448	1.8748673	-0.018408	0.985313	NA
MFSD3	45.36780713	0.352063415	0.2975752	1.1831074	0.236767	0.805854
znf569l	25.43664278	0.140724612	0.3846845	0.3658183	0.714501	0.96909
si:dkey-229d11.3	0.158795395	0.967652056	4.0804729	0.2371421	0.812547	NA
BX901930.2	0	NA	NA	NA	NA	NA
taar19o	0.514116189	-2.475533565	2.7461255	-0.901464	0.367342	NA
cngk	4.960508824	-0.035481323	0.8789307	-0.040369	0.967799	NA
si:ch73-62b13.1	19.94839405	0.284710594	0.4285248	0.664397	0.506436	0.935724
AL954694.2	0	NA	NA	NA	NA	NA
igic1s1	0	NA	NA	NA	NA	NA
or137-6	0.182570949	0.967652056	4.0804729	0.2371421	0.812547	NA
si:dkey-204f11.51	0	NA	NA	NA	NA	NA
BX530037.3	0	NA	NA	NA	NA	NA
si:dkey-11o1.3	5.540068995	-1.184662756	0.8333886	-1.421501	0.155171	NA
CR786582.1	0.166657454	-0.955901296	4.0804729	-0.234262	0.814781	NA
si:ch211-173m16.2	0.633934609	1.436045836	2.6103598	0.5501333	0.582228	NA
CABZ01039782.1	7.593610742	-0.121237459	0.7369272	-0.164518	0.869324	NA
BX510924.1	0.158915748	0.967652056	4.0804729	0.2371421	0.812547	NA
CR513782.3	0	NA	NA	NA	NA	NA
si:dkey-9i23.14	10.07790428	0.443717976	0.6067942	0.7312495	0.464627	NA
CU570889.1	1.503172171	-1.79012084	1.6481094	-1.086166	0.277405	NA
ifitm1	149.2456165	-0.395244542	0.1963427	-2.013034	0.044111	0.412803
si:dkey-221j11.3	189.8075812	0.315007928	0.1867535	1.6867575	0.09165	0.57849
BX957257.1	0.158795395	0.967652056	4.0804729	0.2371421	0.812547	NA
CT025585.1	1.161805827	-3.660681204	1.934818	-1.892003	0.058491	NA
BX890559.1	8.293128398	0.120069171	0.6546737	0.1834031	0.854482	NA
si:dkey-83f18.4	0	NA	NA	NA	NA	NA
ubb	4189.488083	0.311342081	0.0727218	4.2812731	1.86E-05	0.001178
adgrf8	33.07556247	0.292941518	0.3325376	0.8809274	0.378357	0.895192
si:ch211-209j10.6	200.5710084	0.037770557	0.1535998	0.2459025	0.805758	0.979893
si:dkey-57a22.15	2003.783147	0.126178605	0.0766937	1.6452267	0.099923	0.601211
BX323028.1	0.491320158	0.8528132	2.8656354	0.2976	0.766008	NA
si:dkeyp-87a6.2	0.330916733	-1.849579229	3.2489867	-0.569279	0.569167	NA
si:ch211-114l13.1	115.0038892	0.009480144	0.201998	0.0469319	0.962568	0.995927
BX640512.1	0	NA	NA	NA	NA	NA
si:rp71-1h3.1	11.53234389	0.320160951	0.5622932	0.5693844	0.569095	0.948983
CR524821.1	0.825855294	1.891116598	2.1306575	0.8875742	0.37477	NA
si:dkey-38n4.2	1.63085265	-0.564911028	1.581708	-0.357153	0.720978	NA
si:ch73-249k16.4	2.113760313	-1.192408571	1.4535733	-0.820329	0.412029	NA
si:ch211-195h23.4	0.663561848	-1.490280258	2.4116568	-0.617949	0.536609	NA
BX088709.1	3.316974524	0.926811205	1.1009506	0.8418281	0.399884	NA

si:ch211-13f8.1	163.4093536	0.17513395	0.1726498	1.0143886	0.310397	0.862656
si:ch73-27e22.7	0	NA	NA	NA	NA	NA
si:ch211-239f4.6	0.854503011	-3.209619274	2.2865784	-1.403678	0.160415	NA
CR396590.4	0.31437473	-1.788572922	3.5930657	-0.497785	0.618636	NA
si:ch211-141e20.2	22.31625972	0.209195811	0.4221037	0.4956029	0.620175	0.957354
si:dkey-58f10.7	0.847782112	0.655103352	2.1028616	0.3115295	0.755398	NA
si:ch211-263m18.4	0	NA	NA	NA	NA	NA
si:ch211-57i17.2	6.838813419	-0.214714963	0.7428252	-0.289052	0.772542	NA
CU633785.1	0	NA	NA	NA	NA	NA
nyap2b	202.9476852	-0.201040771	0.151575	-1.326346	0.184725	0.747597
BX284697.1	1.308615861	0.736812935	1.6726917	0.4404953	0.659578	NA
inpp5jb	8.59336609	-0.795461588	0.6492796	-1.225145	0.220521	NA
CU469531.2	2.25628215	0.383766697	1.3225249	0.2901773	0.771681	NA
mtmr3	0	NA	NA	NA	NA	NA
acer3	6.484508934	-0.2422217	0.7530783	-0.321642	0.747724	NA
si:ch1073-272j17.3	1.043517365	-0.941794384	2.0508711	-0.459217	0.646079	NA
BX842702.1	0	NA	NA	NA	NA	NA
lrrc66	1.816168291	-0.859048062	1.5260785	-0.562912	0.573495	NA
BX649476.1	0	NA	NA	NA	NA	NA
EPB41L2	2851.420303	-0.058620189	0.0826512	-0.709248	0.478171	0.926857
si:ch211-28e16.5	0	NA	NA	NA	NA	NA
si:ch211-235i11.5	51.36795799	0.153846509	0.2703034	0.5691624	0.569246	0.948983
tgm2l	181.5551693	0.157396556	0.2068046	0.7610882	0.446604	0.917154
uqcc3	220.297507	-0.084386691	0.1444187	-0.58432	0.559005	0.948478
BX640539.1	2.519686637	-1.038754654	1.3660163	-0.760426	0.447	NA
si:ch211-150d5.3	0.476506538	2.394892832	2.8161716	0.8504073	0.395099	NA
CU469314.1	0.687217049	-1.490137326	2.387795	-0.624064	0.532585	NA
si:dkeyp-82a1.1	13.97927102	-0.176800304	0.5146265	-0.343551	0.731184	0.971557
si:dkeyp-20g2.3	0	NA	NA	NA	NA	NA
CR392330.1	0.643284345	1.438369819	2.4327963	0.5912414	0.554359	NA
CT583651.1	0	NA	NA	NA	NA	NA
AL953894.1	4.177020485	-0.117630255	1.0022356	-0.117368	0.906569	NA
BX511184.1	0	NA	NA	NA	NA	NA
crha	0.31598276	0.005883445	3.3523626	0.001755	0.9986	NA
si:dkey-286j17.4	30.31651404	-0.773239577	0.3552033	-2.176893	0.029489	0.332709
si:ch73-376l24.4	0.182570949	0.967652056	4.0804729	0.2371421	0.812547	NA
BX323822.1	0	NA	NA	NA	NA	NA
casp6	82.51861591	-0.283608274	0.2397684	-1.182843	0.236872	0.805854
zgc:111986	4032.833716	0.006182956	0.0669611	0.0923365	0.926431	0.992702
BX530075.2	0	NA	NA	NA	NA	NA
pimr49	0	NA	NA	NA	NA	NA
BX936421.1	0.633934609	1.436045836	2.6103598	0.5501333	0.582228	NA
gad1a	14.40433478	-0.462457291	0.5118867	-0.903437	0.366294	0.887239
sertad2a	82.56382437	-0.257906917	0.226149	-1.140429	0.254108	0.823394
edil3a	1257.910963	0.011724979	0.0873573	0.1342186	0.89323	0.989291
si:dkey-21e2.12	0	NA	NA	NA	NA	NA
BX470229.1	9.713339313	0.940415974	0.6357864	1.4791381	0.139103	NA
si:dkey-216e24.9	1.196340722	1.367701255	1.8023662	0.7588365	0.44795	NA
CR933734.1	0.499182217	-0.916838237	2.6774436	-0.34243	0.732027	NA
creb1b	405.7751641	0.044832674	0.1216005	0.3686883	0.71236	0.969087
si:ch211-229b6.1	0.330916733	-1.849579229	3.2489867	-0.569279	0.569167	NA
swsap1	9.193237171	-0.848542628	0.6326809	-1.341186	0.17986	NA
znf1135	8.521576809	0.224420297	0.6583759	0.3408696	0.733202	NA
BX539332.1	0.158915748	0.967652056	4.0804729	0.2371421	0.812547	NA

si:dkey-69c1.1	0.173729368	-0.955901296	4.0804729	-0.234262	0.814781	NA
BX465862.1	17.96630081	-0.153072364	0.4848039	-0.315741	0.752199	0.974577
CT027825.1	0.802200093	1.852032281	2.2653592	0.8175446	0.413617	NA
si:dkeyp-34f6.4	0.182570949	0.967652056	4.0804729	0.2371421	0.812547	NA
iqcb1	3.808425078	0.118521823	0.9798701	0.1209567	0.903725	NA
si:ch211-9n13.3	0	NA	NA	NA	NA	NA
CU467110.1	106.8944251	0.028192249	0.2017954	0.1397071	0.888891	0.989291
tnfaip6	116.1774509	0.249245558	0.1975432	1.2617269	0.207047	0.774967
BX927377.1	0	NA	NA	NA	NA	NA
BX323559.2	1.354276948	0.016538586	1.6286883	0.0101545	0.991898	NA
si:dkey-10613.7	63.1163442	0.400443211	0.2439161	1.641725	0.100647	0.603272
BX649502.6	4.015357808	-1.019395617	0.9774886	-1.042872	0.297008	NA
mpc1	722.5050042	-0.047227988	0.1093095	-0.432057	0.6657	0.964736
CR388143.1	17.03174897	-0.061732031	0.4896834	-0.126065	0.89968	0.990121
si:dkey-78l4.10	0	NA	NA	NA	NA	NA
BX511129.1	29.8167816	-0.103180155	0.3568834	-0.289114	0.772494	0.975687
ahdc1	719.4803288	0.013282897	0.095726	0.1387596	0.88964	0.989291
oip5-as1	2518.73116	0.088218686	0.0731513	1.2059759	0.227827	0.794963
si:ch211-14a11.2	0.341366344	1.891903201	3.2128156	0.5888614	0.555954	NA
si:dkey-76d14.2	6.097595068	0.870617931	0.7772759	1.1200887	0.262676	NA
gpr52	48.93091197	-0.313297427	0.2781166	-1.126497	0.259955	0.828288
si:dkey-56m15.6	0.812928439	-0.504524747	2.2480137	-0.224431	0.822422	NA
CR384062.1	0.651026051	-0.042574319	2.4423909	-0.017431	0.986092	NA
CU693484.1	72.24406954	-0.257793904	0.2522268	-1.022072	0.306747	0.860249
si:ch73-7i4.3	1.123767089	0.391854525	1.9650308	0.1994139	0.841939	NA
znf653	113.9510553	-0.215490951	0.1861939	-1.157347	0.247131	0.816774
CT009596.1	0.182570949	0.967652056	4.0804729	0.2371421	0.812547	NA
CR361551.1	56.691866	0.24772342	0.2680481	0.9241751	0.355395	0.882347
CU466287.1	0.157187365	-0.955901296	4.0804729	-0.234262	0.814781	NA
si:dkey-175d9.2	5.256225367	-0.184726319	0.8114459	-0.227651	0.819918	NA
CYP46A1	5.935344027	-1.50296269	0.9014579	-1.667258	0.095463	NA
si:ch211-197e7.3	4.482879323	-0.423003952	1.0056753	-0.420617	0.674035	NA
BX571825.10	0	NA	NA	NA	NA	NA
pamr1	171.7978587	0.042816591	0.175654	0.2437553	0.80742	0.980642
BX530034.1	5.663652914	-0.507253448	0.7976694	-0.635919	0.524829	NA
si:dkey-95p16.2	51.31887906	-0.159951106	0.2700539	-0.592293	0.553654	0.947383
BX571701.3	0	NA	NA	NA	NA	NA
BX664716.1	1.005718337	-0.979164561	1.8926618	-0.517348	0.604913	NA
BX004785.2	0	NA	NA	NA	NA	NA
CR450740.1	0	NA	NA	NA	NA	NA
si:dkey-78l4.8	0.513487682	-0.887846295	2.6557956	-0.334305	0.738149	NA
CR677617.1	8.950994705	-0.662609867	0.6920083	-0.957517	0.338306	NA
urp1	45.65844419	0.239257794	0.294907	0.8112992	0.417194	0.906237
si:ch211-217k17.9	131.6153689	0.093152851	0.2156274	0.4320085	0.665735	0.964736
CU651633.1	0	NA	NA	NA	NA	NA
si:zfos-905g2.1	69.16511733	-0.514452054	0.2449172	-2.100514	0.035684	0.368007
zgc:194281	14.85636386	0.002527847	0.5164566	0.0048946	0.996095	0.998888
BX649448.2	1.12805203	-2.447047822	1.9526379	-1.253201	0.210133	NA
rsf1b.1	0	NA	NA	NA	NA	NA
zgc:77614	0	NA	NA	NA	NA	NA
si:ch211-135f11.6	0.347458735	-1.908042286	3.4571486	-0.551912	0.581009	NA
si:dkey-238j22.1	0	NA	NA	NA	NA	NA
BX324218.1	0.521188103	-2.493476766	2.9577741	-0.843025	0.399215	NA
BX323812.2	0.157187365	-0.955901296	4.0804729	-0.234262	0.814781	NA

CT573204.1	18.19595259	0.600179288	0.4664114	1.2868024	0.198163	0.76333
b3gnt3.3	35.46349774	0.076272757	0.3672426	0.2076904	0.835471	0.984089
BX548044.5	0.333314908	-1.858695402	3.5126701	-0.52914	0.596708	NA
slc37a4b	22.90181375	-0.045186905	0.3991109	-0.113219	0.909857	0.990702
si:dkey-230i18.2	0.508023798	0.926122713	2.6636728	0.3476864	0.728076	NA
BX005309.1	333.4896994	0.077949001	0.1300821	0.5992295	0.54902	0.946312
BX539321.1	0.975568402	0.934212443	2.1194354	0.4407836	0.65937	NA
CR450686.2	7.423900879	0.18081293	0.6948505	0.2602185	0.794695	NA
BX294165.1	2.669534639	-0.737177766	1.1629507	-0.633886	0.526155	NA
si:ch73-56d11.5	31.0916954	-0.137239491	0.4207116	-0.326208	0.744267	0.974094
si:dkey-111e8.4	0.317711143	1.810477155	3.2978238	0.5489915	0.583011	NA
BX649641.4	1.155163996	-0.396716192	1.7478367	-0.226976	0.820443	NA
selenop	10566.34289	0.24752893	0.074483	3.3232951	0.00089	0.028373
BX005438.1	58.13563618	-0.036027474	0.2740045	-0.131485	0.895392	0.989814
BX842699.1	2.350764906	-2.532271048	1.508116	-1.679096	0.093133	NA
BX324007.1	0.173729368	-0.955901296	4.0804729	-0.234262	0.814781	NA
BX571854.1	10.19734144	0.456825776	0.6424198	0.7111016	0.477021	NA
si:dkey-20i20.7	23.77331584	-0.219572453	0.3973366	-0.552611	0.58053	0.951621
si:ch211-225b11.4	103.0998777	0.102977643	0.1931096	0.5332602	0.593853	0.954434
BX294388.1	1.465882293	0.270744345	1.6885553	0.1603408	0.872613	NA
bckdhbl	384.7382135	-0.0164538	0.1201466	-0.136948	0.891072	0.989291
si:dkey-25o1.7	15.3244803	-0.137442473	0.5486468	-0.250512	0.802192	0.979492
lamc3	877.3715713	0.194568362	0.1104185	1.7620995	0.078052	0.544221
si:ch73-256j6.4	1.477280433	-1.766093184	1.6754071	-1.054128	0.291824	NA
AL929493.1	0.157187365	-0.955901296	4.0804729	-0.234262	0.814781	NA
BX000981.2	15.34865362	-0.412145514	0.507431	-0.81222	0.416665	0.906237
CT025897.1	8.168485889	-0.520929859	0.6757454	-0.770897	0.440768	NA
si:dkey-80c24.4	0.182570949	0.967652056	4.0804729	0.2371421	0.812547	NA
FP236157.1	5.698355393	-1.548565783	0.8944531	-1.731299	0.083398	NA
zgc:193505	611.9527651	0.016095016	0.1203876	0.1336933	0.893645	0.989291
CU207343.1	0	NA	NA	NA	NA	NA
si:dkey-234l24.8	5.292096345	0.332179392	0.8473792	0.392008	0.695052	NA
si:ch211-282k23.2	11.96126721	-1.512098561	0.6302746	-2.399111	0.016435	0.236409
CR391910.2	2.377437742	0.465403164	1.2468402	0.3732661	0.70895	NA
CR536604.1	13.26614175	0.585386656	0.557019	1.0509275	0.293292	0.849129
si:dkey-88n24.10	0.521188103	-2.493476766	2.9577741	-0.843025	0.399215	NA
oip5	24.21546692	-0.151126218	0.382773	-0.394819	0.692976	0.96736
BX470224.2	0.182570949	0.967652056	4.0804729	0.2371421	0.812547	NA
omd	11.99348846	0.584642781	0.5490818	1.0647645	0.286982	0.845979
si:ch211-93g21.1	25.3840617	0.161897685	0.399949	0.4047958	0.685628	0.966307
or124-3	0	NA	NA	NA	NA	NA
BX255912.1	2.465697616	2.006541235	1.3352416	1.5027552	0.132902	NA
si:dkey-159f12.2	210.6887258	0.081454638	0.2023063	0.4026302	0.68722	0.966376
ccl34b.1	74.91678432	0.51973784	0.2309984	2.2499626	0.024451	0.3019
BX664750.1	0	NA	NA	NA	NA	NA
si:dkey-92i17.2	76.59129039	0.270620495	0.2226668	1.2153609	0.224228	0.791484
CT025909.1	1.868272784	-0.804119683	1.3949472	-0.576452	0.56431	NA
si:ch211-234c11.3	8.816735011	-1.199493689	0.659938	-1.817585	0.069128	NA
BX005419.1	0.166657454	-0.955901296	4.0804729	-0.234262	0.814781	NA
si:dkey-45h7.1	7.88877645	-0.176507842	0.6785829	-0.260112	0.794777	NA
sult3st2	60.66860892	-0.183818515	0.2579122	-0.712717	0.476021	0.926338
si:dkey-27b3.4	0.476386185	2.392464142	3.0533824	0.7835455	0.433307	NA
spc24	219.7622135	0.062494679	0.1431389	0.4366017	0.6624	0.964665
CR392341.1	6.74679396	-0.758872203	0.7663221	-0.990278	0.322038	NA

BX537143.1	0	NA	NA	NA	NA	NA
s100a11	406.1136682	0.309201602	0.3104715	0.9959097	0.319294	0.867746
si:ch211-106h4.4	21.97301043	0.864067923	0.4128967	2.0926978	0.036376	0.372502
si:ch211-249h16.8	2.475765219	-1.457410054	1.2361177	-1.179022	0.238389	NA
BX000981.3	0.651146404	-0.045019457	2.587009	-0.017402	0.986116	NA
si:dkey-26i13.5	0.968187754	2.173022767	2.1450023	1.0130632	0.31103	NA
BX537282.1	2.767855328	0.154517109	1.2181253	0.1268483	0.89906	NA
ugt5a2	13.74506441	-0.875603772	0.5472914	-1.599886	0.109624	0.623766
AL954868.1	1.672417178	0.012632872	1.4948056	0.0084512	0.993257	NA
BX511034.3	12.95381316	0.457507508	0.5217521	0.8768677	0.380559	0.895192
BX294113.2	0.490502273	-2.421143305	2.7891168	-0.868068	0.385357	NA
AL954359.1	13.67951133	0.077396782	0.5328649	0.1452465	0.884516	0.98879
CR513785.1	0.173729368	-0.955901296	4.0804729	-0.234262	0.814781	NA
si:ch211-287n14.3	0.82062886	-1.887809105	2.232843	-0.845473	0.397847	NA
ftr25	6.377356831	0.516890928	0.7591353	0.6808944	0.495938	NA
si:dkeyp-2e4.6	1.378911671	1.64127814	1.7384613	0.9440982	0.345119	NA
tm7sf3	638.1279114	-0.075008298	0.1051665	-0.713234	0.475701	0.926202
entpd5b	448.9108494	0.149763763	0.1254722	1.1936012	0.232634	0.80121
si:dkey-6f10.3	1.643848528	3.056427142	1.7262286	1.7705808	0.07663	NA
CR318646.2	0.798743327	-0.585351522	2.137483	-0.273851	0.784199	NA
CT573186.1	0	NA	NA	NA	NA	NA
si:dkey-285e18.5	3.846893898	-0.1218161	0.9698396	-0.125604	0.900045	NA
BX005283.1	2.487032714	0.183030945	1.1713673	0.1562541	0.875833	NA
ankmy2b	1.995471851	0.006123268	1.3624957	0.0044942	0.996414	NA
BX001030.1	324.8551014	0.06445068	0.142216	0.4531885	0.650413	0.960708
zgc:171435	8.819701317	-0.152336302	0.6489341	-0.234748	0.814404	NA
si:dkey-28d5.4	0	NA	NA	NA	NA	NA
si:ch211-56a11.2	60.01397181	-0.082836082	0.2728664	-0.303577	0.76145	0.975374
AL845312.1	0.157187365	-0.955901296	4.0804729	-0.234262	0.814781	NA
si:dkey-218h11.6	0.166657454	-0.955901296	4.0804729	-0.234262	0.814781	NA
tmem238a	203.9215442	0.245993977	0.1498621	1.6414689	0.1007	0.603407
CU467633.2	0.158915748	0.967652056	4.0804729	0.2371421	0.812547	NA
si:rp71-36a1.2	68.92438412	0.028709691	0.2521951	0.1138392	0.909365	0.990702
si:dkey-193c22.1	132.846661	0.094296381	0.172899	0.5453843	0.585489	0.95262
CU074419.2	2.81000832	0.188400883	1.1884005	0.1585332	0.874037	NA
FP016018.1	0	NA	NA	NA	NA	NA
BX005301.1	1.155163996	-0.396716192	1.7478367	-0.226976	0.820443	NA
BX649502.7	0	NA	NA	NA	NA	NA
BX537166.2	1.644635418	-0.579573079	1.506492	-0.384717	0.700447	NA
BX255917.1	0.166657454	-0.955901296	4.0804729	-0.234262	0.814781	NA
si:dkey-217m5.8	0	NA	NA	NA	NA	NA
si:ch73-27e22.4	35.15976405	0.116674783	0.3443504	0.3388257	0.734741	0.972021
si:dkey-126g1.9	38.93444507	0.080142164	0.3399554	0.2357432	0.813632	0.981081
si:ch211-122l14.4	0	NA	NA	NA	NA	NA
si:dkeyp-46h3.8	0.531799352	0.999478841	2.790358	0.3581902	0.720201	NA
CR356222.1	3.109964864	-1.319780657	1.2578927	-1.0492	0.294086	NA
CR385053.1	0	NA	NA	NA	NA	NA
arhgap20b	1.829442905	-0.28947141	1.4482335	-0.199879	0.841575	NA
si:dkeyp-7a3.1	2.694331001	0.060924456	1.2351292	0.0493264	0.960659	NA
CR854927.1	0.475018861	0.853207185	2.8976509	0.2944479	0.768416	NA
si:dkey-256i11.2	3.725553428	0.137062397	1.0344703	0.1324953	0.894593	NA
artnb	15.73691898	0.092604639	0.4704945	0.196824	0.843965	0.985174
BX901930.3	0	NA	NA	NA	NA	NA
BX470224.3	0.333314908	-1.858695402	3.5126701	-0.52914	0.596708	NA

rnf212b	2.028047701	0.488979081	1.3304427	0.3675311	0.713223	NA
si:dkey-54g2.2	0	NA	NA	NA	NA	NA
BX276096.1	0	NA	NA	NA	NA	NA
CR931802.1	7.041303072	-0.411000493	0.7226412	-0.568748	0.569527	NA
BX539307.3	24.43775694	-0.419410752	0.3892046	-1.07761	0.281208	0.84357
CR396583.1	3.820482719	0.896018778	0.9771635	0.9169589	0.359164	NA
si:ch211-209a2.2	0.823965273	0.503156481	2.2182458	0.2268263	0.820559	NA
pth1a	3.403505862	1.619516132	1.1109681	1.4577522	0.144909	NA
si:dkey-83m22.7	12.67005747	0.639048376	0.5709928	1.1191881	0.26306	0.830245
BX276103.1	0.317831496	1.808837541	3.5779923	0.5055454	0.613176	NA
CU207257.1	0	NA	NA	NA	NA	NA
htr1aa	107.2736459	-0.091212018	0.1925677	-0.473662	0.635741	0.958142
si:dkey-122c11.1	0	NA	NA	NA	NA	NA
si:ch211-217k17.11	0.365141898	1.973577737	3.3927314	0.5817076	0.560764	NA
FP102171.1	0.157187365	-0.955901296	4.0804729	-0.234262	0.814781	NA
cd180	1.820254807	-0.234595052	1.3683104	-0.171449	0.863871	NA
rimbp2	0	NA	NA	NA	NA	NA
BX004774.2	80.93219826	-0.027707433	0.2878346	-0.096262	0.923313	0.992588
FP101875.1	0.499972362	-2.44370697	3.0013188	-0.814211	0.415524	NA
si:ch211-197h24.9	164.7809734	-0.043055905	0.1578769	-0.272718	0.78507	0.97627
si:ch211-250k18.7	10.61715644	-0.042017686	0.6205664	-0.067709	0.946018	NA
si:ch211-271e10.6	0	NA	NA	NA	NA	NA
si:dkey-145c18.5	7.62871842	0.115109593	0.7101538	0.1620911	0.871234	NA
prr18	1309.962079	-0.349589119	0.0908513	-3.847926	0.000119	0.005649
si:ch1073-296i8.2	58.18917461	-0.178876088	0.289757	-0.617331	0.537016	0.943336
rbp2b	318.4410367	0.151463868	0.1428567	1.0602504	0.289031	0.845979
si:ch211-272h9.6	0	NA	NA	NA	NA	NA
BX547993.1	0	NA	NA	NA	NA	NA
BX663499.1	0	NA	NA	NA	NA	NA
cxcl11.7	0	NA	NA	NA	NA	NA
BX548078.1	19.73542691	0.724404475	0.4343092	1.6679465	0.095326	0.587222
lrriq1	20.59833855	-0.459559541	0.4202881	-1.093439	0.274201	0.839127
si:dkey-70b23.2	10.7124241	0.068578431	0.6166066	0.1112191	0.911443	NA
taar17a	0	NA	NA	NA	NA	NA
BX255915.1	0	NA	NA	NA	NA	NA
CR450686.3	10.20833157	-0.227525585	0.6272161	-0.362755	0.716788	NA
CT583625.1	0	NA	NA	NA	NA	NA
si:rp71-79p20.2	0	NA	NA	NA	NA	NA
itln2	0	NA	NA	NA	NA	NA
si:dkey-81p22.11	4.139183675	-0.081588804	0.9303637	-0.087696	0.930119	NA
slx4	4.3542824	2.108798521	1.0296822	2.0480091	0.040559	NA
setbp1	26.46299908	-0.23867628	0.3794644	-0.628982	0.529361	0.942209
si:dkey-196n19.2	8.090340301	-0.39670784	0.7030165	-0.564294	0.572554	NA
sst6	40.6612823	-0.199362007	0.30029	-0.663898	0.506755	0.935734
ccdc73	1.294259067	3.830954643	1.9403765	1.9743357	0.048344	NA
pimr113	4.470857299	-2.435244432	1.1997522	-2.029789	0.042378	NA
si:dkeyp-2c8.3	2.465546269	-0.967067841	1.2236668	-0.790303	0.429351	NA
CR762470.1	0.31759079	1.807926197	3.579045	0.5051421	0.613459	NA
si:ch211-142c4.1	0	NA	NA	NA	NA	NA
ch25hl3	2.773508588	0.873428394	1.1383686	0.7672633	0.442925	NA
CT027772.2	1.006776927	0.956324643	2.0726162	0.4614094	0.644505	NA
si:ch211-110p13.9	80.2707827	0.088237026	0.2249763	0.3922058	0.694906	0.96736
si:dkey-259j3.5	5.863090137	0.282114982	0.845027	0.3338532	0.73849	NA
si:ch211-102c2.8	14.02891898	0.788646862	0.5151514	1.530903	0.125793	0.654803

BX085193.2	2.586773051	2.084283134	1.3387196	1.5569228	0.119489	NA
znf1121	8.50154754	0.16143141	0.7046073	0.2291084	0.818785	NA
si:ch73-382f3.1	39.53989557	0.010807344	0.3092628	0.0349455	0.972123	0.996315
CR628410.1	7.554990174	-0.455173192	0.7020012	-0.648394	0.51673	NA
si:ch211-165f21.4	1.903597824	1.441005883	1.4863203	0.9695124	0.33229	NA
si:dkey-83f18.14	2.781689425	-0.909541844	1.2142429	-0.749061	0.453821	NA
BX571825.11	0	NA	NA	NA	NA	NA
zgc:152936	3.141756236	0.741279492	1.1364592	0.6522711	0.514226	NA
zgc:136461	79.02289469	0.549692592	0.2985941	1.8409362	0.065631	0.500566
si:ch211-1f22.12	1.105255998	-0.398330741	1.974669	-0.20172	0.840135	NA
si:dkey-57c22.1	6.124396134	0.69467171	0.8044546	0.8635313	0.387845	NA
BX649457.1	0	NA	NA	NA	NA	NA
CU639468.1	111.5762064	0.033380846	0.190373	0.1753445	0.860809	0.985773
si:dkey-19a16.1	0	NA	NA	NA	NA	NA
BX248512.1	0	NA	NA	NA	NA	NA
igl3v2	0	NA	NA	NA	NA	NA
or125-1	1.188986818	1.302846014	1.9437106	0.6702881	0.502674	NA
si:dkey-79f11.10	1.691124433	-2.06650781	1.8098741	-1.141796	0.253539	NA
BX957345.1	0	NA	NA	NA	NA	NA
si:ch211-274k16.2	1.943248001	2.296079959	1.5562465	1.4753961	0.140106	NA
si:dkey-248f6.3	0.689615224	-1.495598879	2.3854835	-0.626958	0.530687	NA
wee1	369.0638058	0.168713382	0.1145174	1.473256	0.140682	0.681252
CR356223.1	3.289620609	0.247625166	1.0822624	0.2288033	0.819022	NA
BX004840.1	0	NA	NA	NA	NA	NA
BX901878.1	0	NA	NA	NA	NA	NA
CU137716.1	2.587480874	0.003150506	1.1817579	0.0026659	0.997873	NA
BX324213.1	3.336862982	0.560077037	1.0747013	0.5211467	0.602265	NA
si:dkey-196j8.2	48.78284881	0.026091614	0.2764724	0.0943733	0.924813	0.992676
zgc:174224	24.48931004	-0.070417118	0.3998569	-0.176106	0.860211	0.985773
si:dkey-28d5.10	1.504351114	-0.343407952	1.5790382	-0.217479	0.827835	NA
si:zfos-2330d3.7	40.21673361	0.874000667	0.3087527	2.8307468	0.004644	0.097899
pimr69	1.062808558	2.296330608	2.0991908	1.0939123	0.273993	NA
CT027791.1	0.356300316	0.005884122	3.214964	0.0018302	0.99854	NA
BX908800.1	0.500402445	2.450713647	2.7706722	0.8845195	0.376416	NA
tmsb5	7.332816155	-0.083176772	0.7251366	-0.114705	0.908679	NA
CR677513.1	4.996043576	-0.582151711	0.8352224	-0.697002	0.485802	NA
CT027756.2	0	NA	NA	NA	NA	NA
CR376740.1	0.158795395	0.967652056	4.0804729	0.2371421	0.812547	NA
BX530037.4	0	NA	NA	NA	NA	NA
BX510657.1	270.6212845	0.07787373	0.1458118	0.53407	0.593293	0.954434
trhr2	5.9751881	-0.670117226	0.8484906	-0.789776	0.429659	NA
CU694380.1	0.339758314	0.005883863	3.2682461	0.0018003	0.998564	NA
CT030031.1	2.711423438	1.18318494	1.2608956	0.9383687	0.348055	NA
BX323559.3	59.12540776	-0.014004645	0.2652369	-0.052801	0.957891	0.99467
CR456628.2	0	NA	NA	NA	NA	NA
BX284666.2	0	NA	NA	NA	NA	NA
BX324137.1	0.31598276	0.005883445	3.3523626	0.001755	0.9986	NA
BX276103.2	7.314095353	0.702024885	0.7332387	0.9574303	0.33835	NA
CR354542.1	0	NA	NA	NA	NA	NA
BX890562.1	2.171909124	1.168434516	1.3246536	0.882068	0.37774	NA
CR855277.3	8.396133958	0.03712685	0.6805798	0.0545518	0.956496	NA
BX005032.1	0	NA	NA	NA	NA	NA
CR392341.2	0	NA	NA	NA	NA	NA
rflnb	271.0836339	0.245693916	0.1579474	1.5555426	0.119817	0.644872

ftr04	0.841528083	3.19809933	2.2959749	1.3929156	0.163645	NA
si:dkeyp-1h4.6	362.1039033	0.563615396	0.1302462	4.3273077	1.51E-05	0.001002
BX004770.1	10.95335093	-0.129619786	0.6216343	-0.208515	0.834827	NA
si:dkeyp-77c8.1	81.14725019	0.104855718	0.22384	0.4684405	0.63947	0.959994
BX294181.2	2.651518157	-0.438665464	1.4016649	-0.31296	0.754311	NA
vma21	970.0517944	-0.01705076	0.0985515	-0.173014	0.862641	0.985841
BX546499.2	5.446858043	-0.961867643	0.8636752	-1.113691	0.265412	NA
BX530407.2	0.182570949	0.967652056	4.0804729	0.2371421	0.812547	NA
AL929321.1	0	NA	NA	NA	NA	NA
si:dkey-9i23.16	82.27293707	0.18721352	0.21485	0.8713684	0.383553	0.895192
si:dkey-251i10.2	950.2388709	-0.102438553	0.1266191	-0.809029	0.418499	0.906406
BX530064.2	1.24549986	2.565897048	2.0172075	1.2720045	0.203372	NA
si:dkey-111e8.5	14.22144731	-0.224455418	0.530484	-0.423114	0.672212	0.965257
CR396590.5	0	NA	NA	NA	NA	NA
si:ch211-250k18.6	5.624585738	0.387384764	0.9130805	0.4242613	0.671375	NA
BX571825.12	0	NA	NA	NA	NA	NA
BX548247.1	0.841648436	3.197630988	2.1594651	1.4807514	0.138673	NA
ftr79	7.637882559	-2.719039168	0.8459943	-3.214016	0.001309	NA
BX950224.1	3.041535484	0.633570339	1.2158258	0.5211029	0.602295	NA
CR933734.2	0.158795395	0.967652056	4.0804729	0.2371421	0.812547	NA
si:dkey-222h21.9	0.689615224	-1.495598879	2.3854835	-0.626958	0.530687	NA
CR925803.1	0.792368945	1.848914193	2.4268398	0.7618608	0.446143	NA
si:ch211-220f16.1	8.261474333	-0.439387943	0.6699774	-0.655825	0.511937	NA
zgc:77486	479.351842	0.082282063	0.1218411	0.6753227	0.499471	0.934006
CR749763.3	5.136812547	0.10122001	0.8854532	0.1143144	0.908989	NA
si:dkeyp-72a4.1	1.473854661	1.840539378	1.7103251	1.0761343	0.281867	NA
si:ch211-165f21.7	0.824126911	0.609775617	2.0333139	0.2998925	0.764259	NA
BX323543.3	0.49144051	0.855669345	2.6923358	0.3178167	0.750624	NA
gzm3.3	4.481927387	-0.313450441	0.87407	-0.35861	0.719887	NA
si:ch211-279g13.1	4.376916794	1.455614913	0.9434212	1.5429109	0.122852	NA
BX465838.1	0	NA	NA	NA	NA	NA
znf1058	3.795084593	0.321302758	1.0658254	0.3014591	0.763064	NA
si:dkey-9i23.15	129.0119143	-0.017326352	0.1791482	-0.096715	0.922953	0.992466
si:ch73-7i4.2	9.054141721	0.374426573	0.6445202	0.5809384	0.561282	NA
si:ch73-376l24.6	37.38776478	0.467062329	0.3297497	1.4164145	0.156654	0.70691
si:dkey-6a5.3	1.545287134	0.991900992	1.7324568	0.5725401	0.566956	NA
ccl34b.4	1.830353403	-0.819163785	1.4009786	-0.584708	0.558744	NA
serp2	154.0837559	-0.222570712	0.1651548	-1.347649	0.177771	0.738343
FP102120.1	152.2062243	0.066569875	0.2001781	0.3325532	0.739472	0.972943
si:dkey-98j1.5	3.787009917	0.151516999	1.0201563	0.1485233	0.88193	NA
CU467633.3	0.166657454	-0.955901296	4.0804729	-0.234262	0.814781	NA
CR388373.1	0.476626891	2.395187592	2.815932	0.8505843	0.395	NA
swt1	162.2651824	-0.111145164	0.1653043	-0.672367	0.50135	0.934613
si:dkey-192g7.3	56.50598373	-0.415135583	0.2790439	-1.487707	0.136828	0.673303
si:ch211-130h14.4	5.108387738	-0.10329453	0.8240519	-0.12535	0.900247	NA
znf1137	5.744570171	-0.416908572	0.7933089	-0.525531	0.599214	NA
si:dkey-111e8.1	242.5855634	-0.203260901	0.1646398	-1.23458	0.216987	0.784302
BX649641.5	1.638501742	0.571100201	1.5154132	0.3768611	0.706277	NA
AL831745.2	6.578833708	-1.051582269	0.751882	-1.3986	0.161933	NA
AL935183.1	109.507968	0.094698212	0.1920378	0.4931227	0.621926	0.957354
CR387989.1	0	NA	NA	NA	NA	NA
si:dkey-237j10.2	11.83595712	-0.508601891	0.5669891	-0.897022	0.369707	0.889476
BX890544.1	0	NA	NA	NA	NA	NA
si:dkeyp-3f10.16	0	NA	NA	NA	NA	NA

znf1045	0.650905698	-0.045771139	2.5873401	-0.01769	0.985886	NA
si:dkey-76p14.2	2.822437064	0.149676251	1.157332	0.1293287	0.897098	NA
si:ch211-226f6.1	0	NA	NA	NA	NA	NA
krt17	3217.293613	0.256923122	0.2448824	1.0491693	0.2941	0.849999
si:ch73-376l24.3	134.5497063	-0.006237499	0.1838329	-0.03393	0.972933	0.996315
si:dkeyp-73d8.8	33.27508249	-0.128363431	0.3619	-0.354693	0.72282	0.970375
si:dkeyp-26a9.7	0.158915748	0.967652056	4.0804729	0.2371421	0.812547	NA
CR759830.1	1.986870975	-0.500293445	1.3080111	-0.382484	0.702102	NA
BX004962.1	0.666819193	1.487924459	2.4081466	0.6178712	0.53666	NA
taok3b	225.6659091	0.017586429	0.1383845	0.1270838	0.898874	0.990121
BX248332.1	0	NA	NA	NA	NA	NA
si:dkey-88l16.3	8.791530513	-0.175162431	0.6351654	-0.275775	0.782721	NA
cyp2j20	33.22606213	1.502121069	0.3786861	3.9666653	7.29E-05	0.003753
si:ch211-191j22.7	9.095768864	-0.138832499	0.6337648	-0.21906	0.826603	NA
CR749774.1	0	NA	NA	NA	NA	NA
CU570972.1	0	NA	NA	NA	NA	NA
CR788236.1	0.317831496	1.808837541	3.5779923	0.5055454	0.613176	NA
si:dkey-121n8.7	0.173729368	-0.955901296	4.0804729	-0.234262	0.814781	NA
AL954149.1	0	NA	NA	NA	NA	NA
si:dkey-81h8.1	16.76992328	0.040942379	0.4927629	0.0830874	0.933782	0.992702
BX936317.1	1.544940617	1.906194886	1.7511677	1.0885279	0.276362	NA
si:dkey-21e2.16	14.26109332	-0.443068345	0.5614517	-0.789148	0.430026	0.911615
CR626882.1	0.182570949	0.967652056	4.0804729	0.2371421	0.812547	NA
or103-1	0.31598276	0.005883445	3.3523626	0.001755	0.9986	NA
BX323880.1	0	NA	NA	NA	NA	NA
si:dkey-223d7.6	0	NA	NA	NA	NA	NA
CR759846.1	1.297805191	-0.760789523	1.7411822	-0.436938	0.662156	NA
CR548634.1	3.365315777	0.647393608	1.0750968	0.6021724	0.547059	NA
si:ch211-229n2.7	61.3979124	-0.464260165	0.2669096	-1.739391	0.081966	0.552725
sult3st3	1.504738916	-1.065827818	1.6676907	-0.639104	0.522755	NA
BX248089.1	0.807663976	0.546685639	2.2534268	0.2426019	0.808314	NA
CR450817.1	2.801500059	-1.234292008	1.2265752	-1.006291	0.314275	NA
CR936321.1	137.472669	-0.059243817	0.1737472	-0.340977	0.733121	0.971947
si:ch211-278p9.3	0.989014697	-1.039032001	1.9050484	-0.54541	0.585472	NA
BX323559.4	3.575582659	-0.105896711	1.1161563	-0.094876	0.924413	NA
BX248118.1	9.884337535	0.693929356	0.6313088	1.0991916	0.271684	NA
BX469925.3	0.814536469	0.637862482	2.230678	0.28595	0.774916	NA
AL929237.1	32.32852526	2.818854159	0.4371197	6.448701	1.13E-10	2.66E-08
BX005064.1	5.049330514	-0.131840545	0.887319	-0.148583	0.881883	NA
si:ch73-37h15.2	79.16017899	0.013497739	0.2246821	0.0600748	0.952096	0.993904
BX571827.1	0	NA	NA	NA	NA	NA
znf1168	26.91493186	-0.473380378	0.3812514	-1.241649	0.214366	0.782493
AL954145.1	138.6778368	0.19077314	0.1707287	1.1174052	0.263821	0.830245
slc22a21	51.38128072	0.256064813	0.276554	0.9259127	0.354491	0.882105
BX571945.1	0	NA	NA	NA	NA	NA
BX914211.1	14.84519071	0.10779956	0.4971224	0.2168471	0.828327	0.982721
si:dkey-21o22.2	52.7057806	0.676444561	0.3045106	2.2214152	0.026323	0.312554
CR846087.2	0.838899245	-0.667458734	2.2321093	-0.299026	0.76492	NA
CR318674.1	0.182570949	0.967652056	4.0804729	0.2371421	0.812547	NA
CR589874.1	42.88266688	-0.210193114	0.3142473	-0.668878	0.503573	0.935304
or103-2	0.158915748	0.967652056	4.0804729	0.2371421	0.812547	NA
si:ch211-202c21.7	2.024203134	0.067917171	1.3988728	0.0485514	0.961277	NA
AL954695.3	1.475032609	0.318592214	1.58558	0.200931	0.840753	NA
CR751602.1	102.7273737	-0.20598512	0.1997628	-1.031149	0.302471	0.85729

ftr19	20.06202031	-0.151753133	0.4244316	-0.357544	0.720684	0.969381
igf1	44.6998604	0.233570023	0.2859948	0.8166932	0.414104	0.906107
wu:fc21g02	2385.69194	0.108053295	0.0775921	1.3925817	0.163746	0.71945
BX936305.1	0.325573202	0.005883621	3.3172783	0.0017736	0.998585	NA
CT027756.3	3.306682052	-0.914332289	1.0575665	-0.864562	0.387279	NA
BX511265.2	2.667487479	0.396527576	1.1541597	0.3435639	0.731174	NA
BX936391.1	2.417366654	-0.564011318	1.2848541	-0.438969	0.660684	NA
CR391990.1	0.83198897	-0.546504737	2.1106392	-0.258929	0.79569	NA
pimr143	0	NA	NA	NA	NA	NA
pimr134	0.679983498	-1.527277543	2.5551009	-0.597737	0.550016	NA
BX465864.1	0	NA	NA	NA	NA	NA
BX323875.1	0.347458735	-1.908042286	3.4571486	-0.551912	0.581009	NA
CR812469.1	8.411345576	-0.515803188	0.6479093	-0.796104	0.425972	NA
hist2h3c	61.37478682	0.088218162	0.3016516	0.2924505	0.769942	0.975687
si:ch73-256j6.2	1.475152962	0.31880875	1.5686434	0.2032385	0.838949	NA
si:dkey-27j5.10	0	NA	NA	NA	NA	NA
BX072561.1	0.476747244	2.393381959	3.0525537	0.7840589	0.433006	NA
si:ch73-160h15.3	74.45658239	0.057252018	0.2302158	0.2486885	0.803602	0.979643
si:dkeyp-19e1.4	0.317711143	1.810477155	3.2978238	0.5489915	0.583011	NA
BX649434.1	1.838215462	-1.418452594	1.4563693	-0.973965	0.330074	NA
AL807389.1	0	NA	NA	NA	NA	NA
lrp1ba	69.28112803	0.147186346	0.2328134	0.6322074	0.527251	0.941218
si:ch73-263f13.1	0	NA	NA	NA	NA	NA
BX927282.1	3.307654786	2.018094058	1.1678469	1.7280468	0.08398	NA
znf1027	2.760931506	1.232510066	1.1984884	1.0283872	0.303768	NA
cdkn2d	76.73766368	-0.153824319	0.2334839	-0.658822	0.51001	0.936253
si:ch211-208f21.2	7.819056823	-0.570621593	0.7244429	-0.78767	0.43089	NA
shroom1	4.965787829	0.014544115	0.8372702	0.0173709	0.986141	NA
si:ch211-125e6.14	0	NA	NA	NA	NA	NA
AL954149.2	21.39027753	0.015697325	0.4343009	0.0361439	0.971168	0.996315
BX322661.1	0.515095711	0.926015374	2.6527421	0.3490785	0.72703	NA
CU207281.3	0.173729368	-0.955901296	4.0804729	-0.234262	0.814781	NA
nup37	225.7713388	-0.296813157	0.1422624	-2.086378	0.036944	0.376072
si:dkeyp-82a1.2	1.478888463	-0.978751762	1.5408752	-0.635192	0.525303	NA
BX649484.1	2.329697258	-0.832337136	1.2722313	-0.654234	0.512961	NA
CR936371.1	1.434165613	0.985213129	1.6745955	0.588329	0.556311	NA
si:dkey-27d5.10	0.182570949	0.967652056	4.0804729	0.2371421	0.812547	NA
znf111	0.31598276	0.005883445	3.3523626	0.001755	0.9986	NA
CR354556.1	216.8840383	0.083038154	0.1448611	0.5732261	0.566492	0.948739
si:ch211-226h8.8	0	NA	NA	NA	NA	NA
CT583646.2	8.375174913	-1.702663013	0.7137476	-2.385525	0.017055	NA
elp1	403.5627799	-0.00778609	0.1101448	-0.07069	0.943645	0.993364
si:ch73-191k20.3	5.005916008	-0.567605642	0.8828104	-0.642953	0.520255	NA
fthl31	261.8021494	0.350647167	0.1349747	2.597873	0.00938	0.16228
CR545465.1	0	NA	NA	NA	NA	NA
si:dkey-179k24.1	0.158915748	0.967652056	4.0804729	0.2371421	0.812547	NA
BX601644.2	0.499972362	-2.44370697	3.0013188	-0.814211	0.415524	NA
si:dkeyp-117b8.4	376.9043866	-0.168395257	0.1228538	-1.370697	0.17047	0.728048
BX005417.1	5.720045756	-0.246342175	0.7963634	-0.309334	0.757068	NA
si:ch1073-186o8.3	6.574081897	-0.419805136	0.7416722	-0.566025	0.571377	NA
si:dkey-17e16.15	203.4541663	-0.002521168	0.1750152	-0.014405	0.988507	0.997486
CR854965.1	0	NA	NA	NA	NA	NA
si:dkey-219e20.2	0.664231641	-2.853946573	2.346554	-1.216229	0.223898	NA
si:dkey-28d5.8	1.368970215	-0.71302941	1.6542401	-0.431031	0.666446	NA

pimr125	2.992899042	0.320450137	1.0841734	0.2955709	0.767558	NA
si:dkey-211g8.4	5.68035755	0.183455062	0.8134969	0.2255142	0.821579	NA
BX908395.1	0.330916733	-1.849579229	3.2489867	-0.569279	0.569167	NA
or115-14	0	NA	NA	NA	NA	NA
si:dkey-112g5.15	0	NA	NA	NA	NA	NA
taar12h	0.317831496	1.808837541	3.5779923	0.5055454	0.613176	NA
CR450777.1	2.099764578	0.189205728	1.382718	0.1368361	0.89116	NA
BX649502.8	0	NA	NA	NA	NA	NA
znf1171	68.76304552	-0.228871237	0.2384958	-0.959645	0.337234	0.876339
shda	14.85437364	-0.457843851	0.5148106	-0.889344	0.373818	0.893677
BX085193.3	0	NA	NA	NA	NA	NA
si:dkey-222h21.10	5.81628789	0.090755708	0.8152489	0.1113227	0.91136	NA
CU693368.1	0.332645115	0.005883744	3.2924249	0.0017871	0.998574	NA
wu:fc17b08	26.21057871	0.006545657	0.3663198	0.0178687	0.985744	0.996944
si:ch211-119d14.2	20.64616401	-0.286493367	0.4254479	-0.673392	0.500698	0.93428
BX649448.3	7.020280117	0.602509369	0.7254221	0.8305638	0.40622	NA
B3GNT10	8.399589634	0.266032454	0.6587168	0.4038647	0.686312	NA
BX324164.1	0.158795395	0.967652056	4.0804729	0.2371421	0.812547	NA
commd9	177.2296136	0.061067739	0.1540344	0.3964551	0.691769	0.967305
CR388077.1	5.817603885	0.763373238	0.7815995	0.9766808	0.328727	NA
taar19g	0.157187365	-0.955901296	4.0804729	-0.234262	0.814781	NA
BX544876.1	0	NA	NA	NA	NA	NA
CU539058.1	465.7496233	-0.033050143	0.1050997	-0.314465	0.753168	0.97481
si:dkey-183n20.15	55.68417596	-0.57367459	0.2655286	-2.1605	0.030734	0.339614
si:ch73-337l15.2	27.07614338	-0.254372125	0.3663264	-0.694387	0.48744	0.931584
CR855393.2	0	NA	NA	NA	NA	NA
si:ch73-222f22.2	0	NA	NA	NA	NA	NA
map3k19	13.3957134	-0.901581714	0.559551	-1.611259	0.107123	0.617015
peak3	1.01990345	-0.910958137	1.9547665	-0.466019	0.641202	NA
BX005156.2	0.647019846	-1.455099716	2.4289208	-0.599073	0.549125	NA
mrpl44	450.708999	-0.021711851	0.1175771	-0.184661	0.853495	0.985174
si:dkeyp-20g2.3	0.182570949	0.967652056	4.0804729	0.2371421	0.812547	NA
usp21	293.1476288	0.216717764	0.1317394	1.6450494	0.09996	0.601211
si:ch73-196i15.3	38.41303488	0.363050966	0.3157377	1.1498501	0.250206	0.820164
dnah11	51.69451896	-0.105842859	0.2847016	-0.371768	0.710066	0.968928
si:dkey-58f10.10	0.507044276	-2.459587493	2.7586536	-0.89159	0.372613	NA
si:ch211-117m20.4	23.64019089	0.971243019	0.4077946	2.3816968	0.017233	0.242366
CT997819.2	1.979517071	-0.540778015	1.4087084	-0.383882	0.701066	NA
si:rp71-45g20.11	2.89285564	0.622753531	1.1893566	0.5236054	0.600553	NA
CR318599.1	0.50625413	-0.940106488	2.8357787	-0.331516	0.740255	NA
CR855337.1	18.11822528	-0.304960686	0.4526355	-0.673745	0.500474	0.934237
si:dkey-222h21.2	29.41104686	-0.381814455	0.3615775	-1.055969	0.290983	0.846568
CT573178.1	0.482640214	-0.866525639	2.7061322	-0.320208	0.748811	NA
BX649350.1	0.474898508	0.855240867	2.7202602	0.3143967	0.75322	NA
nupr1a	819.4718255	-0.446933804	0.1321704	-3.381497	0.000721	0.023759
BX537274.1	4.320806096	1.181028392	0.9228066	1.2798222	0.200608	NA
BX296552.1	0.953253781	3.394542042	2.2020103	1.541565	0.123179	NA
si:dkey-237j11.3	1.43726132	2.849099914	1.9089963	1.4924596	0.135579	NA
BX000981.4	1.325436598	-2.680967306	1.8525508	-1.447176	0.147848	NA
si:ch211-226o13.3	0	NA	NA	NA	NA	NA
si:dkey-247i3.6	1.021160465	-3.467929953	2.1547914	-1.609404	0.107528	NA
si:ch73-92e7.6	27.55313874	0.24854438	0.3627587	0.6851508	0.493249	0.931926
si:ch211-255g12.6	10434.11898	-0.235656118	0.0683197	-3.449316	0.000562	0.019327
ndufa9b	155.3071107	-0.041370107	0.1662712	-0.248811	0.803507	0.979643

CR753837.2	0.482640214	-0.866525639	2.7061322	-0.320208	0.748811	NA
CR788285.2	0.182570949	0.967652056	4.0804729	0.2371421	0.812547	NA
si:rp71-80o10.4	59.41913365	-0.306898977	0.2555339	-1.201011	0.229747	0.79764
CU326349.1	0	NA	NA	NA	NA	NA
BX322647.1	1.495430465	-0.994616347	1.5232261	-0.652967	0.513778	NA
CR788303.1	1.646992309	-0.633760738	1.5074309	-0.420424	0.674175	NA
BX294389.1	0	NA	NA	NA	NA	NA
efemp2a	566.113454	0.167627818	0.1077444	1.5557918	0.119758	0.644854
or124-2	0.50625413	-0.940106488	2.8357787	-0.331516	0.740255	NA
SPATA1	5.974560588	0.152565272	0.7633659	0.1998586	0.841591	NA
si:dkey-93n13.1	40.41082683	-0.201482569	0.322555	-0.624646	0.532204	0.942546
si:dkey-9p24.5	0.506374483	-0.940103506	2.8355584	-0.331541	0.740236	NA
BX901918.1	0.531799352	0.999478841	2.790358	0.3581902	0.720201	NA
vgll4a	59.42139078	0.093449863	0.2577214	0.3626003	0.716903	0.96909
si:dkey-23f9.12	0	NA	NA	NA	NA	NA
si:dkeyp-82a1.6	171.033673	-0.205619898	0.1770124	-1.161613	0.245393	0.815388
pimr88	0.157187365	-0.955901296	4.0804729	-0.234262	0.814781	NA
CR545466.1	0.158915748	0.967652056	4.0804729	0.2371421	0.812547	NA
si:dkey-20i20.8	13.57145236	-0.567793038	0.6265037	-0.906288	0.364783	0.886714
si:dkey-222h21.8	0.333314908	-1.858695402	3.5126701	-0.52914	0.596708	NA
si:dkey-261l7.2	41.88753712	0.440157053	0.3048541	1.4438287	0.148787	0.69636
si:ch211-114l13.13	3.42269719	-0.374603775	1.0723984	-0.349314	0.726854	NA
CR388079.1	0	NA	NA	NA	NA	NA
si:ch211-187g4.1	0	NA	NA	NA	NA	NA
si:ch73-343l4.1	0	NA	NA	NA	NA	NA
CR762497.1	0.182570949	0.967652056	4.0804729	0.2371421	0.812547	NA
si:dkey-260g12.1	34.78839996	0.002065165	0.3386639	0.006098	0.995135	0.998694
AL935300.1	1.610482533	3.038710433	1.7590623	1.7274603	0.084085	NA
CR759893.1	0.158915748	0.967652056	4.0804729	0.2371421	0.812547	NA
si:ch211-125e6.13	0	NA	NA	NA	NA	NA
si:ch211-165f21.2	23.54792419	-0.048173894	0.3961491	-0.121605	0.903211	0.990121
samd10b	17.06115847	0.599996811	0.4836384	1.2405896	0.214757	0.782493
BX927413.1	0	NA	NA	NA	NA	NA
zzef1	704.9815721	-0.10523	0.0952154	-1.105178	0.269083	0.834439
BX649266.1	3.674411502	0.261807824	0.992143	0.2638811	0.791872	NA
si:dkey-85k15.6	1.185891111	-0.36621692	1.8036008	-0.203048	0.839098	NA
adgrg4b	17.06803964	-1.177017386	0.4967943	-2.369225	0.017825	0.247181
si:dkeyp-30e7.2	0	NA	NA	NA	NA	NA
celsr2	95.95138881	-0.002584117	0.2102878	-0.012288	0.990195	0.997679
si:dkey-201k7.1	0.31437473	-1.788572922	3.5930657	-0.497785	0.618636	NA
si:dkey-229d11.5	0.173729368	-0.955901296	4.0804729	-0.234262	0.814781	NA
CR848841.1	15.87505115	0.550040802	0.5034615	1.092518	0.274605	0.839136
si:dkey-78k11.4	0.521188103	-2.493476766	2.9577741	-0.843025	0.399215	NA
si:dkey-199f5.6	8.856293416	-0.275415281	0.6392929	-0.430812	0.666605	NA
BX545856.1	1.130880288	0.459759413	1.9539337	0.2352994	0.813976	NA
si:dkey-33c12.10	1.139989318	0.439423795	1.8430523	0.2384218	0.811554	NA
CU633933.1	0	NA	NA	NA	NA	NA
BX548075.2	0	NA	NA	NA	NA	NA
MFAP4 (1 of many)	12.54923421	0.088953975	0.5367577	0.1657246	0.868374	0.987245
CU463033.1	0.158915748	0.967652056	4.0804729	0.2371421	0.812547	NA
si:dkey-4c15.14	61.20319572	-0.177274915	0.2507461	-0.70699	0.479573	0.927091
aknad1	12.39173869	0.540109053	0.5927671	0.9111658	0.362208	0.885944
si:ch211-264e16.2	1.195240847	-0.382051374	1.7326308	-0.220504	0.825479	NA
si:dkey-13n15.2	73.9370508	0.197389476	0.2605837	0.7574897	0.448757	0.917876

si:dkey-27p18.7	0.499972362	-2.44370697	3.0013188	-0.814211	0.415524	NA
BX640465.1	0.846174082	-0.46494763	2.1994676	-0.211391	0.832582	NA
si:dkey-12l12.1	141.0792169	0.140409395	0.172823	0.8124462	0.416536	0.906237
AL954359.2	12.50663418	-0.264443852	0.5384532	-0.491118	0.623343	0.957354
si:ch211-222m18.4	0	NA	NA	NA	NA	NA
her4.2	385.1524804	-0.062654829	0.1340812	-0.46729	0.640292	0.959994
si:dkey-253d23.8	15.70757711	-0.482076018	0.4872897	-0.989301	0.322516	0.870226
si:dkey-31f5.8	47.87800684	-0.221605265	0.282437	-0.784618	0.432677	0.91326
caspbl	39.57164314	0.174137016	0.3099145	0.5618872	0.574193	0.950037
CR848723.1	1.000134101	0.001091541	2.0514706	0.0005321	0.999575	NA
BX571803.1	0.989873867	0.997084054	1.9939383	0.5000576	0.617034	NA
plac8.2	1.468480137	-2.855501588	1.8846635	-1.515125	0.129741	NA
si:dkey-161j23.5	160.302558	-0.203577819	0.158689	-1.282873	0.199537	0.764903
si:dkeyp-73g8.5	0	NA	NA	NA	NA	NA
znf1036	3.913115897	-1.938996374	1.0792982	-1.796534	0.07241	NA
CR354556.2	1.304877104	-0.769183969	1.7390479	-0.442302	0.658271	NA
AL929520.1	0.482760567	-0.866527181	2.7059286	-0.320233	0.748792	NA
BX511231.1	0.157187365	-0.955901296	4.0804729	-0.234262	0.814781	NA
BX571945.2	0	NA	NA	NA	NA	NA
cfp	213.6011641	0.012014483	0.1648372	0.072887	0.941896	0.993099
cdh23	3.839234762	0.169688405	0.9774517	0.1736029	0.862178	NA
CR293531.1	3.715203682	2.675174995	1.1953553	2.2379748	0.025223	NA
AL928824.1	0.356300316	0.005884122	3.214964	0.0018302	0.99854	NA
BX005210.1	0.316103113	0.005883447	3.3519121	0.0017553	0.9986	NA
si:dkey-65b12.12	2.554262817	1.013112256	1.3368711	0.7578235	0.448557	NA
CR354556.3	445.8640655	0.092649634	0.1088209	0.8513958	0.39455	0.898861
BX465186.2	4.618116127	0.855756014	0.8957444	0.9553574	0.339397	NA
or128-8	0.878629579	0.686795053	2.2010162	0.3120354	0.755014	NA
si:dkeyp-80c12.8	0.182570949	0.967652056	4.0804729	0.2371421	0.812547	NA
si:ch73-199e17.1	208.4076476	-0.093456603	0.1624726	-0.575215	0.565146	0.948739
BX957308.1	0	NA	NA	NA	NA	NA
wu:fc63a07	103.4541507	-0.04738534	0.208407	-0.227369	0.820137	0.981594
txnrd2.1	0	NA	NA	NA	NA	NA
CU137648.1	0	NA	NA	NA	NA	NA
BX927218.1	10.82159863	-0.234448283	0.5985491	-0.391694	0.695284	NA
si:dkey-163f12.6	8.309348622	0.064431356	0.7317677	0.0880489	0.929838	NA
BX569801.1	90.46169086	0.224569755	0.2336356	0.9611965	0.336453	0.876088
si:ch1073-75o15.4	0	NA	NA	NA	NA	NA
BX927121.1	0	NA	NA	NA	NA	NA
si:ch211-162i8.4	1.651951293	1.990872718	1.775745	1.1211479	0.262225	NA
si:dkey-27h10.2	13.4248463	-0.276990994	0.5456055	-0.507676	0.61168	0.95688
si:ch1073-412h12.3	0.31598276	0.005883445	3.3523626	0.001755	0.9986	NA
BX510923.1	618.1548399	0.344956896	0.0989007	3.4879122	0.000487	0.01747
si:dkey-58f10.6	8.19155362	0.062804622	0.6564777	0.0956691	0.923783	NA
taar19k	0	NA	NA	NA	NA	NA
si:ch211-202m22.1	7.487400256	-0.699813266	0.7305391	-0.957941	0.338093	NA
si:ch211-284e13.12	0.323844819	-1.825095364	3.274639	-0.557342	0.577293	NA
CR933017.1	1.304997457	-0.766489526	1.6584892	-0.462161	0.643966	NA
BX548075.3	0.340386822	-1.882368278	3.2159502	-0.585323	0.558331	NA
CR376762.1	2.401680166	0.010086734	1.2872093	0.0078361	0.993748	NA
BX927101.1	3.096759274	-0.623820686	1.1527972	-0.541137	0.588413	NA
si:ch211-238e22.4	0.473290478	-0.833892061	2.901156	-0.287434	0.77378	NA
BX323457.2	7.406729374	-0.191749105	0.7061224	-0.271552	0.785966	NA
CR376748.1	0	NA	NA	NA	NA	NA

AL954767.1	75.37090744	-0.017555435	0.2383313	-0.07366	0.941281	0.992857
plekha1b	62.21244526	-0.038028414	0.2521609	-0.15081	0.880125	0.987881
si:ch211-218m3.9	3.659847637	-1.437394816	1.0619798	-1.353505	0.175894	NA
CR925709.2	1.157159828	-0.479018393	1.9216574	-0.249274	0.803149	NA
CR354538.1	1.339181337	0.685980852	1.724488	0.3977881	0.690786	NA
ccl20b	10.95896656	0.094261623	0.5642342	0.1670612	0.867322	NA
gabrz	43.71436187	0.270675191	0.3037505	0.8911102	0.37287	0.892868
si:dkey-7l6.3	83.05389434	0.18213583	0.2302017	0.7912009	0.428827	0.911406
or132-2	3.900959696	0.940038804	0.9890752	0.950422	0.341898	NA
es1	3.536019632	0.774433352	1.1057814	0.7003494	0.483709	NA
si:ch211-125e6.8	37.57915847	0.27270315	0.3999396	0.6818608	0.495327	0.931928
si:ch211-152p23.2	1.995592204	0.006106639	1.3025092	0.0046884	0.996259	NA
si:ch73-194h10.3	0.678375468	-2.878459328	2.4926399	-1.154783	0.248179	NA
si:ch211-14c7.2	450.5128679	0.201356835	0.1149592	1.7515498	0.079851	0.548745
BX511089.2	7.294609958	-0.190336833	0.7475311	-0.254621	0.799016	NA
si:ch211-1f22.11	0	NA	NA	NA	NA	NA
ksr2	399.4827915	0.053215634	0.1215187	0.4379215	0.661443	0.964234
BX927181.1	0	NA	NA	NA	NA	NA
BX548157.1	2.45469757	-0.203670203	1.1789681	-0.172753	0.862846	NA
CR388168.2	1.133855642	2.462671251	1.9506495	1.2624878	0.206773	NA
si:dkey-246e1.3	46.62122259	0.07822211	0.2970543	0.263326	0.792299	0.977937
CR786571.2	3.634722454	-0.020626954	1.0024845	-0.020576	0.983584	NA
BX957326.1	5.403197038	0.635814207	0.8411809	0.7558591	0.449734	NA
si:ch211-137i24.12	63.12658252	-0.239478023	0.2495611	-0.959597	0.337258	0.876339
CT027623.1	0.687217049	-1.490137326	2.387795	-0.624064	0.532585	NA
si:dkey-96l17.6	3.89121766	0.12229975	0.991096	0.1233985	0.901792	NA
si:dkey-121h17.7	2.302705644	-1.857274169	1.3604565	-1.365185	0.172195	NA
BX649349.1	0.674681252	0.02288157	2.4148996	0.0094752	0.99244	NA
si:ch211-132b12.6	0	NA	NA	NA	NA	NA
CU462914.1	0	NA	NA	NA	NA	NA
taar12a	0	NA	NA	NA	NA	NA
BX649490.2	21.891941	-0.701800868	0.4403962	-1.593567	0.111033	0.626294
BX855614.1	34.39368411	0.105514559	0.3441034	0.3066362	0.75912	0.975374
si:ch73-7i4.1	1.046266556	2.29659065	2.1083099	1.0893041	0.27602	NA
BX470218.1	0.325573202	0.005883621	3.3172783	0.0017736	0.998585	NA
si:dkey-24h22.5	0.325452849	0.005883619	3.3177086	0.0017734	0.998585	NA
nupr1b	1738.112378	-0.500636847	0.0779827	-6.419843	1.36E-10	3.17E-08
si:dkeyp-53e4.1	5.097698417	0.264245652	0.8740485	0.3023238	0.762405	NA
zgc:174855	1.670767863	-0.603556225	1.4420043	-0.418554	0.675542	NA
BX005254.1	28.53014791	0.544709949	0.3698654	1.4727248	0.140825	0.681252
si:ch211-139a5.9	132.7346842	0.073084141	0.1848567	0.3953556	0.69258	0.96736
znf1144	66.20129386	-0.066113133	0.2406004	-0.274784	0.783482	0.97627
AL953841.1	1.648013116	0.612181948	1.5693341	0.3900903	0.69647	NA
AL928920.1	0	NA	NA	NA	NA	NA
CU652528.1	0.785936826	-3.109243185	2.5574139	-1.215776	0.22407	NA
CT025748.1	13.94010979	-0.452177542	0.5402173	-0.837029	0.402576	0.90084
cdc42se2	59.30806218	-0.229349451	0.281229	-0.815525	0.414772	0.906237
epsti1	1.019274942	0.15513021	2.1740357	0.0713559	0.943115	NA
b4galnt2.1	3.790343074	-0.365487563	0.9880281	-0.369916	0.711445	NA
si:ch211-92l17.1	1.284479248	0.705670405	1.8843222	0.3744956	0.708036	NA
CT573357.1	8.764920908	0.71346199	0.6401037	1.1146037	0.26502	NA
si:dkey-35m8.1	91.11683201	-0.017033531	0.2346158	-0.072602	0.942123	0.993099
c2cd5	968.895519	-0.029023332	0.0874783	-0.331777	0.740057	0.972943
BX571803.2	1.503643539	0.396624324	1.5798506	0.2510518	0.801774	NA

BX890572.1	1.536847897	0.353065782	1.5547275	0.2270917	0.820352	NA
BX649502.9	0	NA	NA	NA	NA	NA
CR383662.2	0.31437473	-1.788572922	3.5930657	-0.497785	0.618636	NA
BX957257.2	0	NA	NA	NA	NA	NA
si:ch73-113g13.3	0.340386822	-1.882368278	3.2159502	-0.585323	0.558331	NA
si:dkey-7n6.2	175.2824475	0.223249329	0.1676045	1.3320011	0.18286	0.745944
si:dkey-21h14.12	0.524057646	2.503896172	2.7289583	0.9175282	0.358866	NA
si:ch73-256j6.3	0.158795395	0.967652056	4.0804729	0.2371421	0.812547	NA
si:ch211-154e10.1	42.8988043	0.042774156	0.2996485	0.1427478	0.886489	0.988936
si:zfos-364h11.2	0.173729368	-0.955901296	4.0804729	-0.234262	0.814781	NA
si:dkey-222h21.10	1.368970215	-0.71302941	1.6542401	-0.431031	0.666446	NA
si:ch73-68b22.2	7.47636218	-0.855778129	0.6932919	-1.234369	0.217065	NA
CR392012.1	0	NA	NA	NA	NA	NA
si:dkey-262g12.3	28.58314528	0.315760196	0.360232	0.8765468	0.380733	0.895192
BX323458.2	156.805991	0.270723336	0.1948992	1.3890427	0.16482	0.72105
si:dkey-9p20.18	7.786912051	0.1733963	0.671553	0.258202	0.796251	NA
zgc:113984	0	NA	NA	NA	NA	NA
CR318624.1	0.824126911	0.609775617	2.0333139	0.2998925	0.764259	NA
BX323543.4	0	NA	NA	NA	NA	NA
AL935198.2	2.955100014	0.316496319	1.0785064	0.293458	0.769172	NA
si:dkey-84j12.1	0	NA	NA	NA	NA	NA
taar16b	0	NA	NA	NA	NA	NA
si:dkey-148f10.4	5.458595664	0.264755218	0.7973409	0.3320477	0.739853	NA
BX323854.2	0	NA	NA	NA	NA	NA
CR626935.1	0	NA	NA	NA	NA	NA
si:ch211-120e1.7	17.05539397	0.095161434	0.4555463	0.2088952	0.83453	0.983992
CR391921.1	9.482824034	0.167369111	0.6438222	0.2599617	0.794893	NA
ms4a17c.1	0	NA	NA	NA	NA	NA
si:dkey-68l7.2	1.015618509	2.235791211	2.0934924	1.067972	0.285533	NA
dyrk2	656.900724	-0.013933593	0.0967818	-0.143969	0.885525	0.988936
BX465844.1	1.322518982	0.714630552	1.6676242	0.4285321	0.668264	NA
si:ch211-194g2.4	0.508144151	0.926490632	2.6634746	0.3478504	0.727953	NA
si:dkey-149m13.5	27.84475763	0.285740975	0.3727788	0.7665163	0.443369	0.916631
AL928977.1	1.14957976	0.422186685	1.750283	0.2412105	0.809392	NA
CU570781.1	0	NA	NA	NA	NA	NA
BX571681.3	1.469418374	-0.969026147	1.5650397	-0.61917	0.535804	NA
BX510324.2	0.998526071	-0.967342786	1.9879686	-0.486599	0.626543	NA
si:ch211-218d20.15	62.29926393	0.166156026	0.2630483	0.631656	0.527612	0.941521
si:dkey-21e2.8	0	NA	NA	NA	NA	NA
BX842684.1	0.157187365	-0.955901296	4.0804729	-0.234262	0.814781	NA
si:dkey-24f15.2	0.31759079	1.807926197	3.579045	0.5051421	0.613459	NA
CU571324.1	0.173729368	-0.955901296	4.0804729	-0.234262	0.814781	NA
BX470247.1	0	NA	NA	NA	NA	NA
si:ch211-51a19.5	1.966682831	-4.424062371	1.6782987	-2.63604	0.008388	NA
BX510337.1	0.333314908	-1.858695402	3.5126701	-0.52914	0.596708	NA
rhoab	1404.475609	0.114359043	0.0823658	1.3884286	0.165007	0.721223
si:dkeyp-98a7.8	0.31759079	1.807926197	3.579045	0.5051421	0.613459	NA
trarg1b	74.90588752	0.137037308	0.2304177	0.5947342	0.552021	0.947215
CR769772.2	0.173729368	-0.955901296	4.0804729	-0.234262	0.814781	NA
si:dkey-92j12.5	107.2248771	0.174493716	0.2004937	0.87032	0.384126	0.895192
si:dkey-9i23.6	18.59590967	-0.118591644	0.4856119	-0.244211	0.807068	0.98034
hbl2	8.952575992	-0.436165404	0.6464213	-0.674739	0.499842	NA
CR392012.2	2.193097125	-0.203060173	1.2550789	-0.161791	0.871471	NA
si:dkey-149i17.7	0	NA	NA	NA	NA	NA

si:dkey-267j14.7	0.489712128	-0.887840729	2.693685	-0.329601	0.741702	NA
BX005414.1	1.022491002	2.265845936	2.0916203	1.083297	0.278677	NA
CR384089.1	0.69766666	1.536459923	2.3782158	0.6460557	0.518243	NA
si:dkey-220k22.3	9.525031611	0.275611879	0.6285379	0.4384968	0.661026	NA
BX005329.3	0	NA	NA	NA	NA	NA
si:dkey-204f11.64	391.8134709	0.014490233	0.114193	0.1268925	0.899025	0.990121
si:dkey-201c13.2	84.38576458	0.192219296	0.2613819	0.7353963	0.462098	0.922698
BX510928.1	0.158795395	0.967652056	4.0804729	0.2371421	0.812547	NA
bcl2a	14.595387	0.15977112	0.5320508	0.300293	0.763954	0.975374
cxcl11.6	0	NA	NA	NA	NA	NA
sftpba	186.5949811	-0.018567746	0.1676581	-0.110748	0.911816	0.991066
arl8	985.8155355	0.025864818	0.0894287	0.2892229	0.772411	0.975687
si:dkey-79i2.4	7.939038123	-0.206170715	0.7245953	-0.284532	0.776003	NA
BX294165.2	0	NA	NA	NA	NA	NA
BX548044.6	0	NA	NA	NA	NA	NA
or125-2	1.153153623	-1.360395608	1.9656115	-0.692098	0.488876	NA
si:dkey-83f18.6	0.332645115	0.005883744	3.2924249	0.0017871	0.998574	NA
zgc:77614	0	NA	NA	NA	NA	NA
CR318588.3	99.64615986	-0.505957454	0.2050337	-2.467679	0.013599	0.209269
si:ch211-284e13.5	57.1645427	-0.191680009	0.2542729	-0.753836	0.450948	0.918761
apela	10.65639331	0.295217893	0.5963069	0.4950771	0.620546	NA
acbd7	980.9756027	-0.072434009	0.0899546	-0.805229	0.420688	0.90651
mical3b	461.3419945	0.008825444	0.1095679	0.0805477	0.935802	0.992702
CR392363.1	1.47876811	-0.978269979	1.5879855	-0.616045	0.537865	NA
v2rc1	0.332645115	0.005883744	3.2924249	0.0017871	0.998574	NA
znf1157	27.75363321	-0.6362186	0.3866053	-1.645654	0.099835	0.600955
si:ch211-12e13.12	17.94693912	0.37862465	0.445838	0.8492426	0.395746	0.899378
loxhd1a	9.912270919	-0.006867754	0.6018966	-0.01141	0.990896	NA
BX530079.1	0.158795395	0.967652056	4.0804729	0.2371421	0.812547	NA
zgc:173425	0.855602886	-0.591621869	2.0951335	-0.282379	0.777653	NA
si:ch211-197g15.6	10.28374477	-0.511522218	0.6345223	-0.806153	0.420155	NA
BX927218.2	0	NA	NA	NA	NA	NA
si:dkey-84o3.2	1.80395351	-0.214910589	1.5039205	-0.1429	0.886369	NA
rpe65b	231.5786057	-0.014960689	0.1375415	-0.108772	0.913383	0.991119
si:dkey-71d15.2	32.52051417	0.191021753	0.3704716	0.5156179	0.606121	0.956692
BX640536.1	0	NA	NA	NA	NA	NA
CR788311.1	0	NA	NA	NA	NA	NA
si:dkey-125i10.3	116.9990328	-0.128033609	0.187556	-0.682642	0.494833	0.931926
npy2r	11.75118757	-0.002942629	0.5876341	-0.005008	0.996005	0.998888
fyba	2.29625319	0.887679114	1.2963275	0.6847646	0.493493	NA
CT583625.2	3.62718367	0.286432288	1.0098143	0.2836485	0.77668	NA
si:dkey-280e8.1	3.814908526	0.334624159	1.0782561	0.3103383	0.756304	NA
BX248385.1	0	NA	NA	NA	NA	NA
BX571803.3	0.341366344	1.891903201	3.2128156	0.5888614	0.555954	NA
CR626923.1	0.158915748	0.967652056	4.0804729	0.2371421	0.812547	NA
si:dkey-192d15.1	1.337304863	-2.692321341	1.849776	-1.455485	0.145535	NA
CU914617.1	0.325452849	0.005883619	3.3177086	0.0017734	0.998585	NA
si:ch211-196h16.4	12.41472028	-0.355319804	0.5538958	-0.641492	0.521203	0.939056
si:dkey-193n17.9	81.64640456	0.233111985	0.2174225	1.0721613	0.283648	0.84466
twf2a	256.2385842	0.629650784	0.143792	4.3788992	1.19E-05	0.000836
ovch2	3.105164137	1.127517924	1.1162607	1.0100848	0.312455	NA
si:ch211-171b20.3	0.632206226	0.012903346	2.6133421	0.0049375	0.99606	NA
si:ch211-133n4.11	0	NA	NA	NA	NA	NA
si:dkey-181m19.11	0	NA	NA	NA	NA	NA

BX294387.1	0	NA	NA	NA	NA	NA
plxnb2a	14.75227535	-1.05067011	0.5566958	-1.887333	0.059116	0.475427
l3mbtl1	127.8171759	-0.088108955	0.1761481	-0.500198	0.616936	0.956957
CR392026.2	0	NA	NA	NA	NA	NA
si:ch211-208g1.1	1.286568689	1.536509974	1.8937955	0.8113389	0.417171	NA
ms4a17a.11	443.2675444	0.005218722	0.1524152	0.0342402	0.972686	0.996315
si:dkey-111i23.1	0.339758314	0.005883863	3.2682461	0.0018003	0.998564	NA
CU179758.1	5.568352274	0.160213459	0.7996021	0.2003665	0.841194	NA
or133-10	0	NA	NA	NA	NA	NA
si:dkey-58f10.4	1.292341307	-0.031881014	1.858428	-0.017155	0.986313	NA
si:ch73-44m9.2	0.530029684	-0.939524117	2.7937867	-0.336291	0.736652	NA
AL953907.1	0.173729368	-0.955901296	4.0804729	-0.234262	0.814781	NA
si:dkey-23a23.1	0.173729368	-0.955901296	4.0804729	-0.234262	0.814781	NA
F13A1 (1 of many)	0.839569038	-1.918727829	2.2199361	-0.864317	0.387414	NA
coa5	204.5157446	-0.091759153	0.1525131	-0.601648	0.547409	0.945526
si:ch211-195b15.8	1425.35067	-0.48005518	0.0857008	-5.601527	2.12E-08	3.42E-06
BX663515.1	0.877488419	-1.943394526	2.3575695	-0.824321	0.409757	NA
CR749748.1	9.413677014	-0.079551279	0.6284597	-0.126581	0.899272	NA
si:dkey-21e2.13	8.098944432	0.199349832	0.6841956	0.2913638	0.770773	NA
BX927394.2	0	NA	NA	NA	NA	NA
BX323457.3	2.90121581	0.98935766	1.2050613	0.821002	0.411645	NA
zmp:0000001323	0.833476647	0.586781922	2.2129474	0.2651585	0.790887	NA
BX936461.1	7.824058241	0.47553187	0.7082762	0.6713933	0.50197	NA
BX640520.2	0	NA	NA	NA	NA	NA
si:dkey-88l16.5	2.011316322	-0.979272553	1.3394436	-0.731104	0.464716	NA
BX119902.1	0	NA	NA	NA	NA	NA
si:ch211-147m20.3	0.166657454	-0.955901296	4.0804729	-0.234262	0.814781	NA
ms4a17a.9	208.4599184	0.134284779	0.1879989	0.7142849	0.475051	0.925575
BX511089.3	0	NA	NA	NA	NA	NA
dio2	33.51426409	-0.10319662	0.3817499	-0.270325	0.78691	0.976596
si:dkey-11o15.10	1.276978248	1.558674568	1.8171775	0.8577448	0.391033	NA
gpr186	82.09253695	0.667905931	0.2195573	3.0420577	0.00235	0.060184
si:dkey-163f12.11	1.646645792	0.041181395	1.4832032	0.0277652	0.977849	NA
v2ra20	0.158915748	0.967652056	4.0804729	0.2371421	0.812547	NA
CR407561.1	0.157187365	-0.955901296	4.0804729	-0.234262	0.814781	NA
pimr152	0	NA	NA	NA	NA	NA
si:ch73-313l22.2	0	NA	NA	NA	NA	NA
CR396590.6	0	NA	NA	NA	NA	NA
BX649434.2	0.825545564	-1.896597388	2.3991135	-0.790541	0.429212	NA
si:dkey-258f14.2	43.58234408	0.098157474	0.2894056	0.3391693	0.734482	0.972021
si:dkey-84o3.6	2.485586322	-0.158818725	1.1839578	-0.134142	0.89329	NA
si:dkey-277m11.2	10.6894407	0.309066399	0.5862258	0.5272139	0.598045	NA
si:ch211-197h24.8	25.91578869	-0.081837147	0.3989765	-0.205118	0.83748	0.984665
il1lr2	0	NA	NA	NA	NA	NA
BX470185.2	0.182570949	0.967652056	4.0804729	0.2371421	0.812547	NA
pimr178	0.349228403	0.005884014	3.2372587	0.0018176	0.99855	NA
si:dkey-266f7.5	0.981272991	-0.049048874	1.9489635	-0.025167	0.979922	NA
si:ch211-170d8.2	10.45439627	-0.135725447	0.5987744	-0.226672	0.820679	NA
znf1061	0.339758314	0.005883863	3.2682461	0.0018003	0.998564	NA
cplx4c	221.9552681	0.275392401	0.1396806	1.9715863	0.048657	0.433734
si:ch211-163b2.4	1.420878542	2.793083696	1.9569603	1.4272562	0.153506	NA
cenph	40.87892667	0.163201952	0.3118775	0.5232887	0.600773	0.956045
tyrobp	2.319598661	-0.365565428	1.2950908	-0.28227	0.777736	NA
rbm33b	11.43450812	0.138804466	0.56008	0.2478297	0.804266	0.979643

si:ch1073-143l10.2	31.1057979	0.112063246	0.3639246	0.3079298	0.758136	0.975374
abcc6b.2	81.04119272	0.085102565	0.2216742	0.3839084	0.701046	0.968134
si:ch211-231i17.4	2.119495897	1.697032437	1.4259561	1.1901015	0.234007	NA
lingo4a	11.44118094	0.467595484	0.5573541	0.8389558	0.401494	0.90084
si:dkey-22i16.7	0.182570949	0.967652056	4.0804729	0.2371421	0.812547	NA
CR749775.1	0.841889141	3.198609757	2.2957028	1.3933031	0.163528	NA
or133-2	3.160415666	0.785550284	1.0661922	0.7367811	0.461255	NA
si:ch73-196i15.5	5.182370976	1.084727419	0.8586148	1.2633458	0.206465	NA
CR936403.1	1.121918354	-0.422914697	1.8509454	-0.228486	0.819269	NA
ungb	8.509317487	-0.098461305	0.7160021	-0.137515	0.890623	NA
CU469568.2	3.748764847	0.015947995	1.013496	0.0157356	0.987445	NA
CR735104.1	0	NA	NA	NA	NA	NA
BX248322.1	1.412036961	1.647750358	1.816016	0.9073435	0.364225	NA
si:dkey-93l1.4	0.365141898	1.973577737	3.3927314	0.5817076	0.560764	NA
si:ch211-53m15.2	0.340386822	-1.882368278	3.2159502	-0.585323	0.558331	NA
apoa4b.2	257.18632	0.357638694	0.3724962	0.9601138	0.336998	0.876088
si:dkey-58f10.11	0.475018861	0.853207185	2.8976509	0.2944479	0.768416	NA
CR450833.1	4.435194163	0.085846042	0.9003295	0.0953496	0.924037	NA
FO834814.1	8.26068394	0.107307028	0.6668286	0.1609215	0.872155	NA
CR855257.1	0.182570949	0.967652056	4.0804729	0.2371421	0.812547	NA
BX005153.2	0	NA	NA	NA	NA	NA
AL929210.1	0.330916733	-1.849579229	3.2489867	-0.569279	0.569167	NA
si:ch211-132b12.2	30.68224245	-0.144569747	0.3479294	-0.415515	0.677765	0.965257
BX511265.3	0.513487682	-0.887846295	2.6557956	-0.334305	0.738149	NA
si:ch211-74m13.1	136.0116716	-0.090859622	0.1773735	-0.51225	0.608476	0.956718
BX284697.2	0	NA	NA	NA	NA	NA
CR392341.3	4.581234386	-0.861992957	0.897424	-0.960519	0.336794	NA
si:ch73-379f5.5	0.521188103	-2.493476766	2.9577741	-0.843025	0.399215	NA
AL929032.1	0.157187365	-0.955901296	4.0804729	-0.234262	0.814781	NA
BX530407.3	0	NA	NA	NA	NA	NA
CR377211.2	0	NA	NA	NA	NA	NA
nfil3-5	1408.788615	-0.248963921	0.0849238	-2.931614	0.003372	0.077672
BX255915.2	0.475018861	0.853207185	2.8976509	0.2944479	0.768416	NA
wnt16	47.1737219	0.179620179	0.2828798	0.6349699	0.525448	0.94013
BX530068.1	50.94131755	-0.249109833	0.273479	-0.910892	0.362352	0.885944
si:ch211-214p16.2	0	NA	NA	NA	NA	NA
si:ch211-156j22.4	255.4580894	-0.235087693	0.1345848	-1.746762	0.080679	0.550687
si:ch73-18b11.2	36.3234807	-0.38365642	0.3449032	-1.11236	0.265984	0.831945
CR792454.1	1.166362468	0.393666232	1.8306178	0.2150456	0.829732	NA
chst12b	1.308694928	0.663383953	1.8738364	0.3540245	0.723321	NA
ccl34a.3	2.107758276	-1.698437439	1.4463464	-1.174295	0.240277	NA
si:ch211-197g15.8	0.990664012	0.018879338	1.9436762	0.0097132	0.99225	NA
CR377211.3	1.445522467	-1.005141956	1.6998642	-0.591307	0.554315	NA
si:dkey-91f15.1	22.33689252	1.859519565	0.472146	3.9384416	8.20E-05	0.004148
otx1	463.3384974	-0.087455768	0.1062836	-0.822853	0.410592	0.903084
si:dkey-106c17.2	4.891863324	0.742810705	0.9212946	0.8062684	0.420088	NA
si:dkey-84o3.4	10.35685653	0.173058151	0.6478592	0.2671231	0.789374	NA
sult1st9	3.551219883	0.201054133	1.1727366	0.1714401	0.863878	NA
taar18b	0.323844819	-1.825095364	3.274639	-0.557342	0.577293	NA
FP015823.1	0.670675047	-1.455259498	2.4036813	-0.605429	0.544894	NA
pimr98	2.833003772	-0.522160428	1.1125489	-0.469337	0.638829	NA
si:dkey-194e6.2	0.333314908	-1.858695402	3.5126701	-0.52914	0.596708	NA
si:rp71-17i16.4	474.6903065	0.120658438	0.1225513	0.9845544	0.324843	0.871294
si:dkey-21e2.10	0.499972362	-2.44370697	3.0013188	-0.814211	0.415524	NA

CR388079.2	2.322505987	0.862211419	1.2581021	0.685327	0.493138	NA
sinhcafl	828.0203103	-0.063665909	0.0886615	-0.718079	0.472709	0.924838
si:ch211-67n3.9	69.68359941	0.180482845	0.2382237	0.7576192	0.448679	0.917876
AL953903.1	0	NA	NA	NA	NA	NA
si:dkey-240n22.3	7.647215216	-0.379765726	0.680808	-0.557816	0.57697	NA
BX571955.2	21.21942534	0.023389599	0.4274462	0.0547194	0.956362	0.994543
CR932983.2	5.693599927	-0.880503935	0.8247154	-1.067646	0.28568	NA
BX927275.2	0.849510495	1.927128725	2.1168069	0.9103942	0.362615	NA
lmo2	104.8802745	0.075034158	0.1973418	0.3802244	0.703779	0.968134
thsd7ba	394.8375068	0.140891352	0.1121249	1.2565567	0.208914	0.776073
si:dkeyp-68b7.10	16.00872483	0.190772682	0.4887086	0.3903608	0.69627	0.96736
CR388166.2	21.24534282	-0.206060919	0.4568427	-0.451054	0.65195	0.960708
si:dkey-58f10.14	5.291707548	-0.196203305	0.8151219	-0.240704	0.809784	NA
si:ch73-120g24.4	109.8518596	-0.131919804	0.198942	-0.663107	0.507262	0.935734
ftr33	0.856462055	1.928072848	2.2076658	0.8733536	0.38247	NA
FP067425.1	0.31598276	0.005883445	3.3523626	0.001755	0.9986	NA
zgc:162928	188.1963072	0.055229625	0.1511643	0.3653615	0.714842	0.96909
taar20t	0.182570949	0.967652056	4.0804729	0.2371421	0.812547	NA
CR318668.1	0	NA	NA	NA	NA	NA
CR388189.1	0	NA	NA	NA	NA	NA
cbln7	0.332645115	0.005883744	3.2924249	0.0017871	0.998574	NA
BX322661.2	104.084452	0.186725384	0.2166306	0.8619529	0.388713	0.896271
CU468826.1	0.166657454	-0.955901296	4.0804729	-0.234262	0.814781	NA
pold2	198.3386255	0.05404397	0.1539566	0.3510338	0.725563	0.970402
si:dkey-85k15.4	2.661044073	0.016239213	1.1267419	0.0144125	0.988501	NA
si:dkey-250k15.7	25.48529861	-0.968061834	0.4051689	-2.38928	0.016881	0.239981
si:dkey-7f3.14	0.157187365	-0.955901296	4.0804729	-0.234262	0.814781	NA
si:ch211-236l14.1	0	NA	NA	NA	NA	NA
pimr148	0	NA	NA	NA	NA	NA
CT573133.1	2.814375584	0.520974681	1.1201875	0.4650781	0.641876	NA
si:dkey-103d23.3	0.697787013	1.536711783	2.3781027	0.6461924	0.518155	NA
CT025776.1	0.158915748	0.967652056	4.0804729	0.2371421	0.812547	NA
si:ch211-106h4.5	37.89631545	0.191882927	0.33579	0.5714372	0.567703	0.948739
or127-1	2.126808516	1.685638895	1.4758742	1.1421291	0.2534	NA
CU394254.2	0	NA	NA	NA	NA	NA
si:dkey-238m4.3	0	NA	NA	NA	NA	NA
BX005445.1	1.997801003	-1.583517413	1.4589375	-1.085391	0.277749	NA
si:ch211-113e8.9	10.02834817	0.006122309	0.6285095	0.009741	0.992228	NA
BX088713.1	0.508023798	0.926122713	2.6636728	0.3476864	0.728076	NA
TC2N	19.14440386	0.084561748	0.4695788	0.18008	0.85709	0.985597
si:dkey-10h3.3	1.622629532	0.016637092	1.5873147	0.0104813	0.991637	NA
BX908726.1	5.630329199	0.184411819	0.784098	0.2351897	0.814061	NA
cd28	8.835265882	0.052780949	0.7053753	0.0748268	0.940353	NA
ctslb	0	NA	NA	NA	NA	NA
si:dkey-73p2.3	1.155833789	-1.281252378	1.8785909	-0.682028	0.495221	NA
BX548073.4	0	NA	NA	NA	NA	NA
CR545476.1	0	NA	NA	NA	NA	NA
CR450782.1	12.35977381	-0.072515572	0.5409119	-0.134062	0.893354	0.989291
CU657976.1	0	NA	NA	NA	NA	NA
ITLN1 (1 of many)	41.04573298	0.036976196	0.4134022	0.0894436	0.928729	0.992702
ftr10	0	NA	NA	NA	NA	NA
cfdp1	275.2628277	0.124091411	0.1279413	0.9699089	0.332092	0.874653
CU928069.1	0	NA	NA	NA	NA	NA
BX649508.1	0	NA	NA	NA	NA	NA

tspan37	50.21975384	0.384495916	0.29385	1.3084769	0.190712	0.755212
si:ch211-232m8.3	19.23740169	0.155379661	0.4286505	0.3624857	0.716989	0.96909
pimr50	0	NA	NA	NA	NA	NA
pimr127	0.325573202	0.005883621	3.3172783	0.0017736	0.998585	NA
BX649516.1	0	NA	NA	NA	NA	NA
si:ch211-229g14.3	0.158795395	0.967652056	4.0804729	0.2371421	0.812547	NA
si:ch211-132g1.4	0	NA	NA	NA	NA	NA
CR749763.4	0	NA	NA	NA	NA	NA
CR847899.2	0	NA	NA	NA	NA	NA
si:dkey-28k24.2	2.827897692	-1.70872598	1.2328078	-1.386044	0.165733	NA
AL953855.1	0	NA	NA	NA	NA	NA
si:dkey-192k22.2	1.327123696	-1.628668251	1.8860251	-0.863545	0.387838	NA
BX640512.2	0	NA	NA	NA	NA	NA
btr04	0.521188103	-2.493476766	2.9577741	-0.843025	0.399215	NA
BX005024.1	0	NA	NA	NA	NA	NA
CU207311.2	0	NA	NA	NA	NA	NA
CU694808.1	0.862716084	-0.503809387	2.0872007	-0.24138	0.80926	NA
ftr97	60.95652275	-0.387369543	0.2547288	-1.520714	0.128332	0.658954
si:dkey-82i20.2	48.1156166	-0.245802886	0.2824015	-0.870402	0.384081	0.895192
si:ch73-343l4.6	0	NA	NA	NA	NA	NA
CT583652.1	0.824247264	0.61130654	2.1184171	0.2885676	0.772912	NA
si:ch211-227e10.6	4.552380337	-0.996942248	0.9323587	-1.069269	0.284948	NA
si:ch211-165f21.2	2.170541799	0.69101812	1.2637478	0.5468007	0.584516	NA
si:dkey-250k15.9	1.170647409	-2.468126443	1.9121268	-1.290776	0.196782	NA
si:ch73-266f23.1	70.47725572	0.146419636	0.2346779	0.6239175	0.532682	0.942805
si:ch211-130m23.2	47.75725625	-0.291765105	0.2908877	-1.003016	0.315853	0.866931
si:dkey-88l16.2	0	NA	NA	NA	NA	NA
CR388373.2	17.0686406	0.198537461	0.4563363	0.4350683	0.663513	0.964736
BX004962.2	0.182570949	0.967652056	4.0804729	0.2371421	0.812547	NA
si:ch211-208h16.4	235.1796922	0.260505941	0.1394689	1.8678428	0.061784	0.485049
AL954816.1	4.76135809	-0.100881375	0.9117364	-0.110648	0.911896	NA
BX571724.2	0.658957134	2.851427774	2.5143902	1.1340434	0.256776	NA
krt96	29.75376304	-0.123242205	0.3796702	-0.324603	0.745481	0.974094
CT583625.3	0.837961008	-3.186152199	2.1620582	-1.473666	0.140571	NA
znf1062	1.737730516	1.270550401	1.6042426	0.7919939	0.428364	NA
si:dkey-114c15.5	13.11135833	0.122040758	0.5191638	0.2350718	0.814153	0.981081
AL953903.2	0	NA	NA	NA	NA	NA
si:ch211-255i3.4	188.8291226	0.213579956	0.1656354	1.2894584	0.197239	0.762204
CR388363.1	0.666629816	-2.858789115	2.7069088	-1.056108	0.290919	NA
taar20w	0.158795395	0.967652056	4.0804729	0.2371421	0.812547	NA
zgc:163080	99.66123913	-0.514847425	0.2024412	-2.543195	0.010984	0.181902
CT025913.1	0	NA	NA	NA	NA	NA
BX649500.1	1.147851377	-0.386326166	1.8199142	-0.212277	0.831891	NA
AL590134.1	2.159971835	-0.214951541	1.2581686	-0.170845	0.864346	NA
CR847563.1	6.861081055	0.492861431	0.82952	0.5941526	0.55241	NA
CU469530.1	1.97126721	0.460325443	1.3758336	0.3345793	0.737942	NA
CR450836.1	68.82750839	-0.484605448	0.2400939	-2.0184	0.04355	0.409681
lrrfip1b	63.81391361	0.196777794	0.2597852	0.7574634	0.448772	0.917876
CU695074.1	0	NA	NA	NA	NA	NA
si:dkey-78l4.12	0	NA	NA	NA	NA	NA
BX640478.1	1.983575848	-1.525970255	1.4494796	-1.052771	0.292446	NA
si:ch211-235f1.3	2.362221779	0.861878636	1.3106976	0.6575724	0.510813	NA
abr	456.8422799	0.082926887	0.1058465	0.7834638	0.433355	0.913654
si:ch73-52p7.1	20.48712525	-0.154072491	0.4238918	-0.363471	0.716253	0.96909

si:ch211-274p24.2	18.9116447	0.223222008	0.4937229	0.4521201	0.651182	0.960708
BX324164.2	2.691932825	0.059712836	1.1762226	0.0507666	0.959511	NA
si:dkey-189h5.6	23.27566305	0.661721702	0.4388433	1.5078769	0.131586	0.665022
si:ch211-127n13.2	0	NA	NA	NA	NA	NA
BX901918.2	0	NA	NA	NA	NA	NA
si:dkey-57k2.7	59.83739863	-0.086286381	0.2615695	-0.329879	0.741491	0.973154
BX957317.1	229.9906256	0.241900037	0.1462839	1.6536343	0.098202	0.59749
si:dkey-23c22.7	4.436076923	-0.768132146	0.9371765	-0.819624	0.412431	NA
si:dkey-19a16.11	0	NA	NA	NA	NA	NA
CR854927.2	0	NA	NA	NA	NA	NA
znf1132	0.158915748	0.967652056	4.0804729	0.2371421	0.812547	NA
AL732515.1	0.158915748	0.967652056	4.0804729	0.2371421	0.812547	NA
si:ch211-197e7.1	41.6793661	-0.1848151	0.3049673	-0.606016	0.544504	0.945438
AL935117.1	2.421211221	-0.217403659	1.2865635	-0.16898	0.865812	NA
BX649502.10	0	NA	NA	NA	NA	NA
BX649610.1	0	NA	NA	NA	NA	NA
AL845306.1	0.341366344	1.891903201	3.2128156	0.5888614	0.555954	NA
si:ch211-210b2.4	10.88654002	-0.095202462	0.5779782	-0.164716	0.869167	NA
CU469584.1	13.12022907	0.144680705	0.5495979	0.2632483	0.792359	0.977937
zgc:66156	47.52540175	0.042793467	0.3300061	0.1296748	0.896824	0.989971
CT030044.1	1.755852962	0.253541306	1.5720048	0.1612853	0.871869	NA
or128-7	0.173729368	-0.955901296	4.0804729	-0.234262	0.814781	NA
si:ch211-242f23.3	0.476386185	2.392464142	3.0533824	0.7835455	0.433307	NA
camkmt	195.5166364	-0.115508557	0.150988	-0.765018	0.444261	0.916726
CR769772.3	0.182570949	0.967652056	4.0804729	0.2371421	0.812547	NA
CR759970.1	0	NA	NA	NA	NA	NA
CT990618.1	251.7075875	0.018273915	0.1347441	0.1356194	0.892122	0.989291
CR759887.2	0	NA	NA	NA	NA	NA
mybl2a	137.6631105	-0.059274748	0.1749574	-0.338795	0.734764	0.972021
si:ch211-157j23.2	2.714446866	-0.699506096	1.1675466	-0.599125	0.54909	NA
CU468920.1	2.803108088	-0.856910311	1.1640536	-0.736143	0.461643	NA
si:ch211-225k7.3	1.202474398	-0.315413906	1.8153552	-0.173748	0.862064	NA
si:dkey-40h20.1	7.026930821	-0.247241673	0.7333579	-0.337136	0.736014	NA
si:dkey-239j18.2	0.473170125	-0.833888596	2.9014002	-0.287409	0.773799	NA
tmem119a	22.8447928	-0.204273247	0.3960605	-0.515763	0.60602	0.956686
CU694757.1	0	NA	NA	NA	NA	NA
si:dkey-145c18.4	0	NA	NA	NA	NA	NA
CR396590.7	0.339758314	0.005883863	3.2682461	0.0018003	0.998564	NA
AL928866.1	0.829590794	-0.541313395	2.1122702	-0.256271	0.797742	NA
BX927385.2	2.532185647	0.2170996	1.2338553	0.1759522	0.860331	NA
si:dkey-261h17.1	746.4206501	0.100543657	0.0938704	1.0710903	0.284129	0.844697
htr7c	7.008289087	0.565756776	0.7343642	0.7704036	0.441061	NA
cox8a	2528.503705	0.066724951	0.0743376	0.8975939	0.369402	0.889127
si:ch211-278p9.2	1.69115893	3.089930315	1.7196362	1.7968512	0.072359	NA
si:dkey-269p2.1	4.120672337	0.570347797	0.9314512	0.6123217	0.540325	NA
BX936318.1	0.665960023	-1.495776328	2.4092162	-0.620856	0.534694	NA
AL954142.2	1.488788634	1.021546813	1.5869898	0.6437009	0.519769	NA
CU682811.2	3.80065588	-1.399519032	1.2132727	-1.153507	0.248702	NA
zgc:152652	0	NA	NA	NA	NA	NA
BX950187.1	5.444956489	0.030208912	0.9043527	0.0334039	0.973352	NA
si:dkeyp-2e4.3	30.09717751	-0.569779257	0.3703287	-1.538577	0.123908	0.652391
rusc2	1761.66391	0.087701172	0.0758528	1.1562017	0.247599	0.817092
CU595864.1	0.157187365	-0.955901296	4.0804729	-0.234262	0.814781	NA
CR394546.3	16.69512548	-0.181678331	0.4838042	-0.37552	0.707273	0.968664

si:dkey-265e7.1	3.694692261	0.802785408	0.9962928	0.8057726	0.420374	NA
si:dkey-200l5.4	106.3897916	0.365886895	0.1981888	1.8461529	0.06487	0.497901
si:rp71-36a1.1	115.2785507	0.257667526	0.1949774	1.3215248	0.186326	0.750697
si:ch73-274k23.1	4.252754854	-0.673542119	0.9439826	-0.713511	0.47553	NA
adgre17	0.531799352	0.999478841	2.790358	0.3581902	0.720201	NA
ftr73	0.489712128	-0.887840729	2.693685	-0.329601	0.741702	NA
mxra7	93.27517391	-0.120301774	0.2259566	-0.532411	0.594441	0.954434
BX247868.1	3.777498543	0.133032358	0.9561981	0.1391264	0.88935	NA
BX957322.2	27.91705581	3.26535236	0.4900749	6.6629664	2.68E-11	7.08E-09
CR926469.1	0	NA	NA	NA	NA	NA
si:ch211-207c6.2	16.13643392	-0.96391263	0.4947951	-1.948105	0.051402	0.444245
si:dkey-22p6.2	0	NA	NA	NA	NA	NA
si:ch211-215c18.3	42.94338438	0.181934918	0.3066243	0.593348	0.552948	0.947265
si:dkey-79f11.9	0	NA	NA	NA	NA	NA
sc:d0202	18.24591813	0.452561373	0.4925132	0.9188817	0.358157	0.884398
si:dkey-19p15.4	0.633934609	1.436045836	2.6103598	0.5501333	0.582228	NA
CR626907.2	16.59680681	-0.110893953	0.4575072	-0.242387	0.80848	0.980642
CR769777.1	1.157562172	-0.399802663	1.8346433	-0.217918	0.827493	NA
BX004840.2	0	NA	NA	NA	NA	NA
fkbpl	103.2618685	-0.370495809	0.1935828	-1.913888	0.055634	0.461579
galnt8b.2	18.05913278	0.332654847	0.4544479	0.7319978	0.46417	0.923204
si:dkey-49n23.3	0	NA	NA	NA	NA	NA
si:dkey-88n24.3	0.323844819	-1.825095364	3.274639	-0.557342	0.577293	NA
si:ch211-191i18.4	13.94827879	-0.089032152	0.5817093	-0.153053	0.878357	0.987881
si:dkey-14d8.1	34.95739816	-0.152568809	0.3426489	-0.445263	0.65613	0.961833
si:ch211-157j23.3	1.453504879	-1.807683458	1.6864857	-1.071864	0.283781	NA
CR318646.3	0	NA	NA	NA	NA	NA
si:dkeyp-44a8.4	250.8754566	0.086520732	0.1489685	0.580799	0.561376	0.948728
BX248512.2	0.996127896	-0.965496869	1.8966759	-0.509047	0.610719	NA
ftr09	0	NA	NA	NA	NA	NA
si:dkeyp-72h1.1	208.798265	-0.073792212	0.1497743	-0.492689	0.622232	0.957354
sh2d1aa	0.667059899	1.488443644	2.4079024	0.6181495	0.536477	NA
si:dkey-4c15.12	7.571916846	0.38717884	0.708456	0.5465108	0.584715	NA
CU914776.1	471.1973531	0.674791413	0.1238149	5.4500038	5.04E-08	7.38E-06
CR936240.1	0.482640214	-0.866525639	2.7061322	-0.320208	0.748811	NA
si:dkey-78l4.7	0	NA	NA	NA	NA	NA
si:ch211-213b8.4	6.241433527	-1.092539359	0.8135975	-1.34285	0.179321	NA
si:ch211-77g15.32	3.156377224	-1.943486649	1.183012	-1.642829	0.100418	NA
BX640520.3	0.365141898	1.973577737	3.3927314	0.5817076	0.560764	NA
BX323065.1	2.266611409	1.30416549	1.3562144	0.9616219	0.33624	NA
AL590146.1	0.166657454	-0.955901296	4.0804729	-0.234262	0.814781	NA
zgc:112966	9.495729938	-0.661801998	0.6143738	-1.077198	0.281392	NA
FP016012.1	0	NA	NA	NA	NA	NA
CR388207.1	19.1963919	-0.125482623	0.4787565	-0.262101	0.793243	0.977937
gdf10a	386.8047061	0.19053498	0.1195378	1.5939304	0.110952	0.626294
BX294163.1	0.173729368	-0.955901296	4.0804729	-0.234262	0.814781	NA
si:dkey-78l4.2	1.159600284	3.665293876	1.9627315	1.8674454	0.061839	NA
BX640481.1	2.487702507	-0.19355015	1.1912939	-0.162471	0.870935	NA
pimr179	1.958154234	-0.002462971	1.4893867	-0.001654	0.998681	NA
BX323590.2	121.4449354	-0.25020631	0.1909352	-1.310425	0.190052	0.754636
BX901878.2	6.656796408	0.175881869	0.7502026	0.2344458	0.814639	NA
BX537288.2	88.41544472	-0.109687831	0.220018	-0.49854	0.618103	0.957354
BX664750.2	0	NA	NA	NA	NA	NA
CR376741.1	4.251108794	-0.009283605	0.9793082	-0.00948	0.992436	NA

si:ch1073-75f15.2	0.632206226	0.012903346	2.6133421	0.0049375	0.99606	NA
CU468730.1	15.07579913	-0.808969173	0.5037443	-1.605912	0.108293	0.620153
znf1170	31.50575107	-0.306086184	0.3471712	-0.881658	0.377962	0.895192
si:ch73-256j6.6	0.349228403	0.005884014	3.2372587	0.0018176	0.99855	NA
si:ch73-52f15.5	136.5309501	0.101232143	0.1748947	0.5788178	0.562712	0.948728
si:rp71-1i20.1	3.357968661	-1.557257885	1.1169199	-1.394243	0.163244	NA
BX897741.1	8.658369851	-0.851456593	0.6815344	-1.249323	0.211547	NA
si:dkey-9i23.8	0.333314908	-1.858695402	3.5126701	-0.52914	0.596708	NA
AL935184.2	0.173729368	-0.955901296	4.0804729	-0.234262	0.814781	NA
si:ch211-226h7.8	25.89648637	0.038047629	0.4285398	0.0887844	0.929253	0.992702
si:ch211-285c6.4	1.15118779	1.305539583	1.8815949	0.6938473	0.487778	NA
CR847543.2	4.206613198	0.379276018	0.9205703	0.4120011	0.680339	NA
RNF224	14.06084775	-0.847287795	0.5247774	-1.614566	0.106405	0.615567
or125-4	5.085124837	0.487988137	0.862341	0.5658877	0.57147	NA
CR388363.2	0.166657454	-0.955901296	4.0804729	-0.234262	0.814781	NA
si:ch211-14a11.1	0.498674062	0.926637565	2.6784892	0.3459553	0.729376	NA
BX324123.2	790.6185619	0.066988423	0.0978317	0.6847313	0.493514	0.931926
si:ch211-133n4.7	0.839730676	-1.88066928	2.2379651	-0.840348	0.400713	NA
AL929520.2	0	NA	NA	NA	NA	NA
FP101887.1	198.5590271	0.067958267	0.1546941	0.4393073	0.660439	0.963802
BX323074.2	177.384665	0.190859834	0.1651971	1.1553464	0.247949	0.817156
si:ch211-217k17.8	30.78322245	0.122295687	0.3821463	0.3200232	0.748951	0.97419
si:dkeyp-117b11.3	25.19158123	0.005034329	0.4143552	0.0121498	0.990306	0.997679
si:dkey-78l4.4	0	NA	NA	NA	NA	NA
BX649490.3	0.635662991	2.808056561	2.7492814	1.0213784	0.307075	NA
si:dkeyp-53e4.2	0	NA	NA	NA	NA	NA
BX927216.1	0	NA	NA	NA	NA	NA
si:dkey-191c17.2	0.476506538	2.394892832	2.8161716	0.8504073	0.395099	NA
CR383662.3	7.816621613	0.405997032	0.719689	0.5641284	0.572667	NA
ftr30	3.669033475	-1.470131244	1.1224432	-1.30976	0.190277	NA
AL929017.2	9.330124285	0.319880153	0.6127778	0.5220166	0.601659	NA
AL929237.2	0.158915748	0.967652056	4.0804729	0.2371421	0.812547	NA
si:ch211-196l7.4	917.13758	-0.166638984	0.1083631	-1.537784	0.124101	0.652708
si:rp71-17i16.6	24.0679803	-0.010246482	0.3847396	-0.026632	0.978753	0.996762
si:dkey-19a16.13	5.256035991	-0.745087035	0.830586	-0.897062	0.369686	NA
si:dkey-20i20.10	8.433798346	-0.153262578	0.6905957	-0.221928	0.82437	NA
si:ch211-147d7.5	5.92958058	-0.304769043	0.784565	-0.388456	0.697679	NA
si:dkey-240h12.3	12.19775107	-0.041620219	0.5455451	-0.076291	0.939188	0.992857
si:ch211-191j22.3	479.9821942	-0.109441579	0.1103028	-0.992192	0.321104	0.868843
BX000705.1	0	NA	NA	NA	NA	NA
pimr171	4.045339317	0.235675287	1.0124807	0.2327702	0.81594	NA
cela1.2	3.374624228	0.613820888	1.0289564	0.596547	0.55081	NA
si:ch73-90k17.1	0.809701093	0.53615962	2.3788628	0.2253848	0.82168	NA
gstt2	148.6400347	-0.192820085	0.1673262	-1.15236	0.249173	0.818463
BX649377.1	5.637758916	-0.498875061	0.8317626	-0.599781	0.548652	NA
BX470183.1	0	NA	NA	NA	NA	NA
CU657977.1	3.540061454	-0.115458457	1.0132676	-0.113947	0.90928	NA
CR392001.1	0.182570949	0.967652056	4.0804729	0.2371421	0.812547	NA
CR933734.3	38.14597485	0.19970966	0.3098187	0.6446017	0.519185	0.938945
si:ch211-282j17.10	0.347458735	-1.908042286	3.4571486	-0.551912	0.581009	NA
CHST12 (1 of many)	0.846174082	-0.46494763	2.1994676	-0.211391	0.832582	NA
AL929457.1	7.155902812	0.649844262	0.7645825	0.8499335	0.395362	NA
CU467646.2	1.812993516	-2.135589317	1.6619004	-1.285028	0.198782	NA
CU570881.1	0	NA	NA	NA	NA	NA

adgre6	0.173729368	-0.955901296	4.0804729	-0.234262	0.814781	NA
CU459065.1	0	NA	NA	NA	NA	NA
si:dkeyp-2e4.5	17.33102227	-0.323893453	0.465384	-0.69597	0.486447	0.930833
si:dkey-286h21.1	0.323844819	-1.825095364	3.274639	-0.557342	0.577293	NA
si:ch211-167j6.3	0	NA	NA	NA	NA	NA
CU986305.1	2.639117254	0.354391793	1.1475719	0.3088188	0.757459	NA
BX649622.1	9.015832125	0.455476674	0.6406482	0.7109622	0.477108	NA
CR759885.1	12.23342737	0.23389837	0.5785762	0.4042654	0.686018	0.966376
si:ch211-10d23.5	39.64580085	-0.126827767	0.3155442	-0.401933	0.687733	0.966442
rca2.2	19.21948995	0.091972092	0.4366593	0.2106267	0.833179	0.983583
CR396590.8	0	NA	NA	NA	NA	NA
si:dkeyp-77c8.5	0.508144151	0.926490632	2.6634746	0.3478504	0.727953	NA
entr1	177.4996601	-0.033678739	0.1532597	-0.21975	0.826066	0.982196
si:dkey-204f11.13	0	NA	NA	NA	NA	NA
BX323839.1	8.87952219	-0.529958659	0.6640499	-0.79807	0.42483	NA
BX950188.2	0	NA	NA	NA	NA	NA
znf1090	4.837561166	0.71231575	0.8904311	0.7999673	0.42373	NA
si:dkey-71b5.3	37.50884205	0.266489974	0.3276816	0.8132588	0.41607	0.906237
AL845369.2	0.658218317	-0.061628513	2.4342948	-0.025317	0.979802	NA
CR392363.2	2.430842948	-0.188310594	1.292486	-0.145696	0.884161	NA
CR854897.1	0.972352342	-1.017118925	1.9872165	-0.511831	0.608769	NA
BX322661.3	0.521188103	-2.493476766	2.9577741	-0.843025	0.399215	NA
si:dkey-58f10.13	1.601761304	1.957946414	1.7038977	1.1490986	0.250515	NA
si:ch211-198c19.3	57.41540046	0.674202808	0.2717645	2.4808343	0.013108	0.20473
BX005462.1	0	NA	NA	NA	NA	NA
trim62.2	6.578575923	1.048996957	0.7524614	1.3940874	0.163291	NA
senp3a	300.638704	0.013178416	0.1268533	0.103887	0.917259	0.991572
si:dkeyp-82a1.8	0.832927207	1.89113355	2.1260675	0.8894983	0.373735	NA
sid1	1.006656575	0.95954179	1.9638723	0.4885968	0.625127	NA
si:dkey-33c12.12	0.347458735	-1.908042286	3.4571486	-0.551912	0.581009	NA
CR931802.2	0	NA	NA	NA	NA	NA
BX247877.1	3.991225693	0.027723206	0.9734589	0.0284791	0.97728	NA
si:dkeyp-106c3.2	0.349228403	0.005884014	3.2372587	0.0018176	0.99855	NA
lenep	134.280331	0.174263752	0.1768073	0.9856142	0.324322	0.870928
si:ch211-163g19.2	0.157187365	-0.955901296	4.0804729	-0.234262	0.814781	NA
CR318588.4	709.7202294	-0.067711841	0.1123039	-0.602934	0.546552	0.945526
BX664610.1	168.5154237	0.290092875	0.1638924	1.7700203	0.076724	0.539626
si:dkey-246j7.1	38.15299024	-0.338523864	0.3277102	-1.032998	0.301605	0.856944
si:ch73-138i16.1	5.019098008	0.199529435	0.8621608	0.2314295	0.816981	NA
CABZ01118770.1	4.042285048	-0.121966028	0.9948143	-0.122602	0.902422	NA
si:dkey-65b13.1	2.494224981	0.177872727	1.1834387	0.1503016	0.880527	NA
AL929576.1	3.221639233	-0.153325792	1.0931401	-0.140262	0.888453	NA
BX537162.1	0	NA	NA	NA	NA	NA
si:dkey-21e2.14	0	NA	NA	NA	NA	NA
si:ch211-122l14.7	0	NA	NA	NA	NA	NA
cybrd1	67.59246635	0.002475127	0.2406482	0.0102853	0.991794	0.997697
si:dkey-222h21.7	0.666629816	-2.858789115	2.7069088	-1.056108	0.290919	NA
si:ch211-67e16.11	213.7536622	0.388624776	0.1605046	2.4212687	0.015466	0.228575
BX663525.1	2.029388282	1.599396952	1.4420994	1.1090754	0.267398	NA
CR925798.1	83.58206661	-0.031435415	0.2221662	-0.141495	0.887479	0.989291
CT583727.1	2.234878028	0.830908077	1.4103303	0.5891585	0.555755	NA
si:dkey-11e23.9	1.487730044	-0.268459873	1.5651803	-0.17152	0.863815	NA
BX548073.5	0	NA	NA	NA	NA	NA
pimr100	0.689615224	-1.495598879	2.3854835	-0.626958	0.530687	NA

spp2	8.682147418	-0.422400955	0.7291765	-0.579285	0.562397	NA
si:dkey-11o15.5	20.19502132	0.512174429	0.43378	1.1807239	0.237712	0.806474
si:ch211-134c9.2	8.396869272	0.239969364	0.6568117	0.3653549	0.714847	NA
si:dkey-184a18.5	38.74684278	-0.213442339	0.3102728	-0.687918	0.491504	0.931926
BX322575.1	22.33288638	0.386829103	0.4369466	0.8853006	0.375995	0.894587
si:dkeyp-4f2.6	0.831078472	0.593707769	2.1126471	0.2810255	0.778691	NA
AL627088.1	1.315955223	-0.066570411	1.8293747	-0.03639	0.970972	NA
BX465206.1	0.974991223	-1.022830706	2.0964916	-0.487877	0.625637	NA
grid2ipb	200.0562185	0.006017564	0.1488618	0.0404238	0.967755	0.996315
CR846087.3	1.503962317	-2.886341382	1.8017439	-1.601971	0.109162	NA
si:dkeyp-51f12.2	12.65917895	0.252095628	0.5391022	0.4676212	0.640055	0.959994
si:ch211-209f23.3	0.182570949	0.967652056	4.0804729	0.2371421	0.812547	NA
si:dkey-57k2.6	17.78078341	-0.127459115	0.4535008	-0.281056	0.778667	0.975942
ftr60	1.471775264	-1.028353818	1.5759115	-0.652545	0.514049	NA
BX248510.2	0.854503011	-3.209619274	2.2865784	-1.403678	0.160415	NA
cacna1eb	10.16029452	0.435791086	0.6128539	0.7110848	0.477032	NA
si:dkeyp-86h10.3	373.3236017	0.443681178	0.1230287	3.6063235	0.000311	0.012137
znf1157	25.47602376	-0.209004683	0.3875804	-0.539255	0.589711	0.953704
BX927243.1	0	NA	NA	NA	NA	NA
taar19b	0.500402445	2.450713647	2.7706722	0.8845195	0.376416	NA
dpm2	210.4100696	-0.004311521	0.1428573	-0.030181	0.975923	0.996762
c1qc	4.936765507	1.452901288	0.9347017	1.5544011	0.120089	NA
CR927456.1	0	NA	NA	NA	NA	NA
si:ch211-133l5.7	71.293955	-0.589527872	0.2345063	-2.513911	0.01194	0.192266
si:ch211-66k16.2	0.632206226	0.012903346	2.6133421	0.0049375	0.99606	NA
drl1.3	1.451776496	-2.862465318	1.8134246	-1.578486	0.114454	NA
cycsa	0	NA	NA	NA	NA	NA
si:dkey-284p5.3	20.37293707	1.029800701	0.4566706	2.2550185	0.024132	0.299742
BX248515.2	0	NA	NA	NA	NA	NA
si:ch1073-15f19.2	0	NA	NA	NA	NA	NA
CT025588.1	2.803643982	-0.199242602	1.1306948	-0.176213	0.860127	NA
si:dkey-253d23.3	43.16616235	0.137486283	0.300142	0.4580708	0.646902	0.960362
CU207238.1	0.365141898	1.973577737	3.3927314	0.5817076	0.560764	NA
BX545856.2	0	NA	NA	NA	NA	NA
si:dkey-97a13.12	21.97599608	0.636483762	0.4168389	1.5269297	0.126778	0.656588
hmx1	198.1123265	0.108519574	0.1554068	0.6982938	0.484993	0.93001
CU681836.2	0	NA	NA	NA	NA	NA
tns3.1	14.60703529	-0.729378633	0.4953524	-1.472444	0.140901	0.681252
BX005024.2	0	NA	NA	NA	NA	NA
FP016213.1	1.322491243	-1.548905306	1.8087333	-0.856348	0.391805	NA
si:ch211-191a16.2	28.8717066	0.12856261	0.4052818	0.3172178	0.751078	0.97419
znf1175	38.35878597	-0.201635918	0.3231188	-0.62403	0.532608	0.942805
BX855614.2	0	NA	NA	NA	NA	NA
si:ch211-221n20.2	0.824205979	0.498048327	2.3641483	0.2106671	0.833147	NA
FP016142.1	85.30419962	-0.078196242	0.2284763	-0.342251	0.732162	0.971665
si:dkey-79f11.8	0.182570949	0.967652056	4.0804729	0.2371421	0.812547	NA
tmem123	43.86143341	0.65772658	0.3245319	2.0266929	0.042694	0.406074
ccdc141	123.0528091	-0.013011531	0.1979934	-0.065717	0.947603	0.99381
ftr38	4.199296081	-0.604442874	1.0003253	-0.604246	0.54568	NA
AL845324.2	0.347458735	-1.908042286	3.4571486	-0.551912	0.581009	NA
BX537103.1	1.561362267	1.873467306	1.7432854	1.0746762	0.28252	NA
BX296526.2	0.665049525	-0.08670226	2.5686305	-0.033754	0.973073	NA
BX571694.1	5.478365012	0.102082916	0.7940749	0.1285558	0.897709	NA
si:dkey-84o3.3	4.792646926	-0.057625585	0.8948861	-0.064394	0.948656	NA

zgc:162509	0.651146404	-0.045019457	2.587009	-0.017402	0.986116	NA
AL953867.2	0	NA	NA	NA	NA	NA
BX927401.1	0.800351357	0.565853532	2.1350944	0.2650251	0.79099	NA
CT573231.1	0.173729368	-0.955901296	4.0804729	-0.234262	0.814781	NA
ms4a17a.10	20.22150248	1.096246149	0.5214353	2.1023629	0.035521	0.367016
trpm2	61.98138624	-0.199953706	0.2556429	-0.78216	0.43412	0.914001
CR774194.1	2.735064098	0.738656946	1.2984618	0.5688708	0.569444	NA
si:dkey-228a15.1	30.67751299	0.415927034	0.3820925	1.0885506	0.276352	0.839541
CR792453.1	0	NA	NA	NA	NA	NA
si:dkey-4c15.13	85.00935929	-0.107945711	0.2192599	-0.492319	0.622494	0.957354
CDPF1	103.8380486	0.171046165	0.1962453	0.8715935	0.38343	0.895192
BX322665.2	10.20715938	-0.00926338	0.6182577	-0.014983	0.988046	NA
si:dkey-7i4.9	122.6352524	-0.125795871	0.1860436	-0.676164	0.498937	0.934006
BX284666.3	0.838778893	-0.672501496	2.3563046	-0.285405	0.775334	NA
BX465834.1	447.0387691	-0.244335469	0.1239154	-1.971793	0.048633	0.433734
si:dkey-172k15.6	25.92849183	-0.376355867	0.3842254	-0.979518	0.327324	0.872658
si:dkey-222h21.2	0.316103113	0.005883447	3.3519121	0.0017553	0.9986	NA
CR931763.1	0.317831496	1.808837541	3.5779923	0.5055454	0.613176	NA
BX005108.1	0.839060883	-0.561237798	2.0216937	-0.277608	0.781313	NA
CR925709.3	0	NA	NA	NA	NA	NA
si:ch211-196c10.15	6.796686174	-0.974709666	0.7855512	-1.240797	0.214681	NA
CU657925.1	0.157187365	-0.955901296	4.0804729	-0.234262	0.814781	NA
BX957357.1	0	NA	NA	NA	NA	NA
si:dkey-21e2.12	0	NA	NA	NA	NA	NA
si:ch211-234p6.9	0.814536469	0.637862482	2.230678	0.28595	0.774916	NA
BX890591.1	5.611007012	-1.68165883	0.8504692	-1.977331	0.048004	NA
znf423	0.808333769	-0.605422531	2.2351432	-0.270865	0.786495	NA
CT573344.2	0	NA	NA	NA	NA	NA
pimr186	2.691609549	-0.00363273	1.1561864	-0.003142	0.997493	NA
si:ch211-271e10.1	1.757887666	0.078280266	1.5913271	0.0491918	0.960766	NA
AL953903.3	0.182570949	0.967652056	4.0804729	0.2371421	0.812547	NA
si:dkey-73p2.1	1.964195297	0.468413351	1.323575	0.3539001	0.723414	NA
si:ch1073-75o15.3	0.847782112	0.655103352	2.1028616	0.3115295	0.755398	NA
sox11b	1189.021064	0.165768057	0.0874234	1.896151	0.05794	0.472262
si:dkey-269i1.4	0	NA	NA	NA	NA	NA
si:ch73-138e16.3	43.94553955	-0.020708433	0.2898803	-0.071438	0.943049	0.993364
si:zfos-2330d3.7	0.513487682	-0.887846295	2.6557956	-0.334305	0.738149	NA
cxcl11.8	0.166657454	-0.955901296	4.0804729	-0.234262	0.814781	NA
taar18j	0.158795395	0.967652056	4.0804729	0.2371421	0.812547	NA
fdxacb1	87.41163413	-0.134745634	0.2174038	-0.619794	0.535393	0.943336
leg1.2	98.29571808	-0.183392884	0.2489574	-0.736644	0.461339	0.922161
CR377211.4	2.308238551	-0.81758949	1.2920505	-0.632784	0.526874	NA
si:dkey-234h16.7	211.8671052	-0.148931294	0.1489476	-0.999891	0.317363	0.867404
BX465864.2	0	NA	NA	NA	NA	NA
si:ch211-190k17.19	0	NA	NA	NA	NA	NA
si:dkey-28d5.1	0.158915748	0.967652056	4.0804729	0.2371421	0.812547	NA
si:ch73-222f22.2	0	NA	NA	NA	NA	NA
dio3b	100.4829287	-0.055536795	0.2055041	-0.270247	0.786971	0.976596
si:dkey-11o1.2	1.539005366	0.350453901	1.6299364	0.2150108	0.829759	NA
zgc:194906	0.88633	-0.542564367	2.3109422	-0.234781	0.814379	NA
emd	297.4448437	0.092734096	0.1279582	0.7247221	0.468623	0.924328
si:ch211-122l14.6	0	NA	NA	NA	NA	NA
hccsa.2	0.981822431	-1.031556312	1.9826505	-0.520292	0.60286	NA
si:ch211-218h8.3	1.518295521	-0.302185166	1.6161124	-0.186983	0.851674	NA

AL928725.1	0	NA	NA	NA	NA	NA
CR769777.2	0.31437473	-1.788572922	3.5930657	-0.497785	0.618636	NA
CR854897.2	0.158795395	0.967652056	4.0804729	0.2371421	0.812547	NA
BX908740.1	0.349228403	0.005884014	3.2372587	0.0018176	0.99855	NA
CR352226.1	1.000443831	3.450160141	2.0458237	1.6864406	0.091711	NA
si:dkey-249d8.1	9.05961115	-0.510958221	0.6725015	-0.759787	0.447382	NA
BX511100.3	0.959666193	-3.394558403	2.232981	-1.520191	0.128463	NA
AL928685.1	0	NA	NA	NA	NA	NA
BX936406.1	0.166657454	-0.955901296	4.0804729	-0.234262	0.814781	NA
ghrhrl	1.86120087	-0.798548741	1.3883063	-0.575196	0.565158	NA
ccr8.1	7.920588898	-0.064926293	0.7133765	-0.091013	0.927483	NA
FP067424.1	12.94855815	-0.251850723	0.5460069	-0.461259	0.644613	0.96029
si:dkey-85n7.8	13.81135993	-0.811274587	0.5155316	-1.573666	0.115565	0.637206
FP103048.1	0	NA	NA	NA	NA	NA
si:dkey-87o1.2	247.1456375	-0.032362865	0.1359435	-0.238061	0.811834	0.981081
BX666064.1	1.011020583	-2.242667267	2.1244332	-1.055654	0.291126	NA
CU633916.1	11.27046255	0.32333583	0.5814138	0.55612	0.578129	0.951024
si:ch211-105n15.1	0	NA	NA	NA	NA	NA
BX649485.2	12.29096417	-0.711304026	0.6197278	-1.147769	0.251064	0.820469
si:ch1073-155h21.2	504.2940092	0.144446223	0.1245971	1.1593066	0.246331	0.816376
BX649485.3	1.450319813	1.821033431	1.7709124	1.0283024	0.303808	NA
CT027631.1	0.339758314	0.005883863	3.2682461	0.0018003	0.998564	NA
si:dkey-16n15.6	11.62255225	-0.811934446	0.5951057	-1.364353	0.172456	0.731348
si:ch73-362i18.1	0.158915748	0.967652056	4.0804729	0.2371421	0.812547	NA
hp	155.5211948	0.163560806	0.2110284	0.7750655	0.438301	0.915672
BX470242.1	1.820883314	-0.81111314	1.3805197	-0.587542	0.55684	NA
dlg4b	18.62995147	0.461432017	0.4894114	0.9428306	0.345768	0.87848
BX005108.2	635.2610158	0.021656936	0.0955861	0.2265699	0.820758	0.981594
BX649481.1	0	NA	NA	NA	NA	NA
znhit6	154.3272081	-0.358774637	0.1619837	-2.214881	0.026768	0.315256
CU855786.1	0	NA	NA	NA	NA	NA
si:dkey-231j24.3	12.82993281	0.040523548	0.5616393	0.0721523	0.942481	0.993317
BX649485.4	11.48430646	-0.719747972	0.6320311	-1.138786	0.254793	0.823484
si:dkey-122a22.2	104.9374692	0.034807856	0.2200056	0.1582135	0.874289	0.987881
BX324132.2	8.489836538	1.045563258	0.7292706	1.433711	0.151655	NA
BX649295.1	0	NA	NA	NA	NA	NA
ftr92	2.783795566	0.140307505	1.3029173	0.1076872	0.914244	NA
smdt1a	100.2617342	-0.008626223	0.2102303	-0.041032	0.96727	0.996315
otulina	0	NA	NA	NA	NA	NA
serpinb1l1	17.51810857	-0.076903708	0.4713145	-0.163169	0.870386	0.987421
si:ch211-175f12.2	190.135001	0.427353279	0.1619956	2.6380554	0.008338	0.148141
CU682777.2	314.9090506	-0.841787583	0.1270629	-6.624966	3.47E-11	8.92E-09
alk	232.404478	0.271600522	0.1480143	1.8349616	0.066511	0.50468
AL954317.1	0.182570949	0.967652056	4.0804729	0.2371421	0.812547	NA
CR354611.1	0.341366344	1.891903201	3.2128156	0.5888614	0.555954	NA
si:dkey-185p13.1	0.515216064	0.926382026	2.6525479	0.3492423	0.726907	NA
CR626882.2	0.158795395	0.967652056	4.0804729	0.2371421	0.812547	NA
CU929426.1	2.704028249	1.11939469	1.2386107	0.9037502	0.366128	NA
BX511173.1	0.173729368	-0.955901296	4.0804729	-0.234262	0.814781	NA
col24a1	203.6995499	0.333258718	0.1680024	1.9836549	0.047294	0.427031
FP102167.1	76.67553171	0.154342675	0.2331967	0.6618563	0.508063	0.935734
BX511100.4	0	NA	NA	NA	NA	NA
si:dkey-239n17.6	9.952828185	-0.391204007	0.5931453	-0.659542	0.509548	NA
CU672266.1	0.158795395	0.967652056	4.0804729	0.2371421	0.812547	NA

CR352226.2	0.48448895	0.85344211	2.878836	0.2964539	0.766883	NA
CT574585.1	0.522329263	0.999930388	2.8066578	0.3562709	0.721638	NA
si:ch211-57m13.3	0.173729368	-0.955901296	4.0804729	-0.234262	0.814781	NA
si:dkey-42i9.16	1.81297997	-0.32088687	1.4512486	-0.221111	0.825006	NA
si:ch211-163l21.11	9.045616411	0.222274027	0.6922602	0.3210845	0.748146	NA
CR407550.1	0.158795395	0.967652056	4.0804729	0.2371421	0.812547	NA
CU683879.1	1.118303205	1.297464276	1.92255	0.6748663	0.499761	NA
ptch1	0	NA	NA	NA	NA	NA
BX465849.1	0.158795395	0.967652056	4.0804729	0.2371421	0.812547	NA
si:ch1073-441p17.1	1.551499878	-0.340953829	1.6673473	-0.204489	0.837972	NA
afp4	2919.842658	0.207158751	0.1941192	1.0671731	0.285894	0.845942
ftr59	2.040197956	0.521653209	1.4801101	0.3524422	0.724507	NA
oxr1a	1471.680302	0.132975319	0.0822807	1.6161179	0.106069	0.615567
si:ch73-132k15.2	0.657977612	-0.062339112	2.4345873	-0.025606	0.979572	NA
AL844185.1	0	NA	NA	NA	NA	NA
AL845419.1	1.54332809	-1.824983617	1.7446718	-1.046033	0.295546	NA
FP067424.2	0	NA	NA	NA	NA	NA
CR352226.3	1.545569125	1.051525788	1.5511744	0.6778901	0.497841	NA
CU993818.1	7.466702714	-1.29655579	0.7090507	-1.82858	0.067463	NA
wdr46	577.0590895	-0.106666255	0.0977464	-1.091255	0.275161	0.839541
FP325358.1	0	NA	NA	NA	NA	NA
BX001026.1	0.31598276	0.005883445	3.3523626	0.001755	0.9986	NA
si:ch211-57m13.9	0.182570949	0.967652056	4.0804729	0.2371421	0.812547	NA
si:ch211-158m24.12	0	NA	NA	NA	NA	NA
BX248517.1	0.339758314	0.005883863	3.2682461	0.0018003	0.998564	NA
znf236	320.3841742	0.021014669	0.1295655	0.1621934	0.871154	0.987754
si:ch73-30l9.1	2.66359034	1.122956291	1.1792584	0.9522564	0.340967	NA
CR339054.1	0	NA	NA	NA	NA	NA
si:dkey-85n7.7	2.068526896	0.533299241	1.346359	0.3961048	0.692028	NA
BX511250.1	0.638219549	-2.807461932	2.539214	-1.105642	0.268881	NA
BX571828.1	0.31598276	0.005883445	3.3523626	0.001755	0.9986	NA
pou3f3b	1360.891357	-0.018315256	0.0827378	-0.221365	0.824808	0.982196
atp5f1e	1724.689364	0.105469559	0.1217555	0.8662405	0.386358	0.89551
BX664742.1	0	NA	NA	NA	NA	NA
BX088696.2	1.145453202	-0.38209434	1.8398115	-0.207681	0.835478	NA
col18a1b	299.1790176	0.340130715	0.1245509	2.7308574	0.006317	0.121156
si:ch211-57m13.10	1.477191074	4.017654067	1.8024171	2.2290369	0.025811	NA
prpf31	806.0850206	0.081560178	0.0883878	0.9227538	0.356136	0.883148
BX927181.2	0.810062152	0.536965757	2.3785369	0.2257546	0.821392	NA
CR848047.1	1270.908332	-0.130828066	0.0848236	-1.542355	0.122987	0.65095
AL928685.2	36.55183388	0.113400162	0.3170366	0.357688	0.720577	0.969364
sat1a.2	1655.139548	0.073251958	0.1115241	0.6568264	0.511293	0.936253
BX539325.1	1.338594115	1.65163228	1.7855437	0.9250024	0.354965	NA
si:ch211-149b19.4	48.50869174	0.080751221	0.2945566	0.274145	0.783973	0.97627
si:dkey-229b18.3	397.339112	0.08209392	0.1116103	0.7355409	0.46201	0.922621
si:ch1073-324l1.1	21.68001147	-0.162407812	0.4096275	-0.396477	0.691753	0.967305
si:dkey-209n16.2	14.49249091	0.169237652	0.5434806	0.311396	0.7555	0.975318
col6a3	10.29260504	0.538076874	0.6138624	0.8765431	0.380735	NA
CR450738.1	0.317831496	1.808837541	3.5779923	0.5055454	0.613176	NA
si:ch211-274k16.9	0	NA	NA	NA	NA	NA
si:ch73-236e11.2	7.912713642	-0.159561344	0.6986136	-0.228397	0.819338	NA
AL929171.1	0.356300316	0.005884122	3.214964	0.0018302	0.99854	NA
si:ch1073-325m22.2	2845.670048	-0.007972682	0.0808598	-0.098599	0.921457	0.992138
CR855300.1	108.7155776	-0.032096705	0.1996571	-0.160759	0.872283	0.987846

si:dkey-258f14.7	0.157187365	-0.955901296	4.0804729	-0.234262	0.814781	NA
si:ch211-57m13.1	0.349228403	0.005884014	3.2372587	0.0018176	0.99855	NA
BX664742.2	0.871557666	0.705131062	2.2070113	0.3194959	0.74935	NA
myha	389.448176	0.648962692	0.1584653	4.0952992	4.22E-05	0.002358
CU856621.1	0.649418021	-1.460710349	2.4263694	-0.602015	0.547164	NA
si:dkey-151m22.8	0.157187365	-0.955901296	4.0804729	-0.234262	0.814781	NA
tmem265	102.6768835	-0.194650864	0.1953519	-0.996411	0.31905	0.867746
si:ch73-226l13.2	14.43575834	-0.320411913	0.5223757	-0.613374	0.539629	0.94408
si:dkey-19a16.12	1.328773011	-0.730366326	1.5986317	-0.45687	0.647765	NA
CT573247.1	2.023223612	-0.941421312	1.4034182	-0.670806	0.502344	NA
BX324207.1	5.687724652	0.499790397	0.8256271	0.6053464	0.544949	NA
si:ch73-103b2.1	1.767480521	0.240005077	1.5017509	0.1598168	0.873025	NA
CR936359.1	0	NA	NA	NA	NA	NA
si:ch73-341k19.1	44.32376033	0.265683925	0.3035669	0.8752072	0.381461	0.895192
adkb	641.7708089	0.228635979	0.1059065	2.1588483	0.030862	0.33998
BX511100.5	1.779775612	-0.289480223	1.45627	-0.198782	0.842433	NA
si:dkey-22i16.9	3.005554197	-1.370733674	1.1560305	-1.185725	0.235731	NA
BX465868.1	0	NA	NA	NA	NA	NA
BX890562.2	1.139989318	0.439423795	1.8430523	0.2384218	0.811554	NA
CR352226.4	0	NA	NA	NA	NA	NA
si:ch211-57m13.6	0	NA	NA	NA	NA	NA
BX571828.2	0.166657454	-0.955901296	4.0804729	-0.234262	0.814781	NA
BX927257.1	0.182570949	0.967652056	4.0804729	0.2371421	0.812547	NA
CU467655.1	29.51288283	0.728269566	0.3724109	1.9555536	0.050518	0.440643
fhdc5	2.923459989	0.534733584	1.1796534	0.4532972	0.650335	NA
si:ch73-361h17.1	40.26668354	0.039051226	0.3179759	0.1228119	0.902256	0.990121
BX005108.3	13.13454251	-0.43441411	0.6293352	-0.690275	0.490021	0.931926
zgc:171699	0.341486697	1.892317624	3.2124068	0.5890654	0.555817	NA
AL590145.1	0	NA	NA	NA	NA	NA
erlin2	0	NA	NA	NA	NA	NA
si:ch73-106g13.1	10.07504237	-0.180639319	0.6145849	-0.293921	0.768818	NA
CU856621.2	5.922507671	-0.978355302	0.8800029	-1.111764	0.26624	NA
BX511100.6	0.688945432	-0.015421672	2.3988056	-0.006429	0.994871	NA
si:ch73-211l13.2	377.8694057	-0.018954367	0.1231727	-0.153884	0.877701	0.987881
si:ch73-261i21.2	1.710577264	0.023065194	1.4878009	0.0155029	0.987631	NA
CU469420.3	2.143268195	-0.241278784	1.3326509	-0.181052	0.856327	NA
si:ch211-161n3.5	4.81748333	0.340684153	0.9311622	0.3658698	0.714462	NA
CR391978.1	0	NA	NA	NA	NA	NA
AL807749.1	0	NA	NA	NA	NA	NA
si:ch211-64i20.5_2	0	NA	NA	NA	NA	NA
AL935166.1	102.3516636	-0.272780745	0.2023896	-1.3478	0.177723	0.738343
BX469910.1	0	NA	NA	NA	NA	NA
znf1073	8.694907088	0.597923794	0.6570651	0.9099917	0.362827	NA
CR383662.4	1.681028097	-1.234623942	1.5615249	-0.790653	0.429147	NA
BX323801.1	0.889079191	3.262746891	2.2913259	1.4239558	0.154459	NA
znf1112	14.30250916	-0.013723	0.5179249	-0.026496	0.978862	0.996762
CR388052.1	27.04932765	0.287397407	0.3935878	0.7301989	0.465269	0.923717
si:dkey-29j8.2	5.957158246	-0.177931236	0.8821441	-0.201703	0.840149	NA
si:dkey-240n22.8	7.923520214	-0.858212597	0.682005	-1.258367	0.208259	NA
atp5md	1069.322616	-0.183078853	0.2498251	-0.732828	0.463663	0.922875
CT027801.1	2.762120739	-0.512131646	1.1539238	-0.443818	0.657174	NA
BX901962.1	0	NA	NA	NA	NA	NA
si:dkey-21h14.15	0	NA	NA	NA	NA	NA
si:dkeyp-4f2.3	19.11052903	-0.299129819	0.4546901	-0.657876	0.510618	0.936253

znf1115	25.03062923	-0.037135541	0.395675	-0.093854	0.925225	0.992676
znf1146	1.662947089	0.022927706	1.4800892	0.0154908	0.987641	NA
BX649337.1	1.467249618	0.931888212	1.6630798	0.5603388	0.575248	NA
BX005012.3	2.584185499	0.694490974	1.3012088	0.5337275	0.59353	NA
si:ch211-178j18.4	0	NA	NA	NA	NA	NA
si:dkey-4e4.1	13.86895493	0.141352505	0.5058626	0.2794287	0.779916	0.976146
si:dkey-262g12.7	17.27581078	-0.278642961	0.4517966	-0.616744	0.537403	0.943336
si:ch73-6k14.2	97.36174994	0.341099821	0.2169004	1.5726101	0.115809	0.637943
si:ch73-289h5.5	0	NA	NA	NA	NA	NA
si:ch211-214j24.14	234.4860067	-0.068135479	0.1395856	-0.488127	0.62546	0.957354
BX784029.1	0	NA	NA	NA	NA	NA
si:ch211-162i8.2	2.276132826	-0.041240912	1.3267094	-0.031085	0.975202	NA
BX548032.2	0.182570949	0.967652056	4.0804729	0.2371421	0.812547	NA
si:ch73-263o4.4	75.216812	0.201013788	0.2288944	0.8781945	0.379838	0.895192
BX649307.1	40.45117788	-0.026401159	0.3212412	-0.082185	0.9345	0.992702
si:dkey-21h14.3	0.500282092	2.45023829	2.5970012	0.9434876	0.345432	NA
CR788248.1	0	NA	NA	NA	NA	NA
ccl39a.10	0	NA	NA	NA	NA	NA
si:dkey-191j3.1	5.937816895	-0.177563784	0.774843	-0.229161	0.818744	NA
si:dkeyp-100h4.4	0.507044276	-2.459587493	2.7586536	-0.89159	0.372613	NA
si:dkey-156k2.7	0	NA	NA	NA	NA	NA
BX957333.1	0	NA	NA	NA	NA	NA
si:dkeyp-100h4.7	0	NA	NA	NA	NA	NA
taar20i	0.698456806	0.09172817	2.5265115	0.0363063	0.971038	NA
BX927253.1	0	NA	NA	NA	NA	NA
col19a1	17.8601071	-0.27334975	0.4578418	-0.59704	0.550481	0.946549
BX649516.2	0.166657454	-0.955901296	4.0804729	-0.234262	0.814781	NA
BX649516.3	0.340386822	-1.882368278	3.2159502	-0.585323	0.558331	NA
CR381686.3	0	NA	NA	NA	NA	NA
LRRC56	16.49457978	0.248072504	0.4868443	0.5095521	0.610365	0.95688
si:dkey-21h14.8	31.85878696	0.434049532	0.3434771	1.2636927	0.20634	0.774011
si:dkey-256i11.6	0.547712847	2.559541715	2.9076406	0.8802813	0.378707	NA
si:ch211-208g1.1	0.166657454	-0.955901296	4.0804729	-0.234262	0.814781	NA
taar20j	0	NA	NA	NA	NA	NA
taar20a	0.323844819	-1.825095364	3.274639	-0.557342	0.577293	NA
BX927253.2	8.244327063	-0.466961488	0.6693078	-0.697678	0.485378	NA
BX548032.3	69.28712785	-0.054805517	0.2477234	-0.221237	0.824908	0.982196
buc	2.152391767	0.263881274	1.2940365	0.203921	0.838415	NA
si:dkeyp-98a7.4	0	NA	NA	NA	NA	NA
BX649399.1	0	NA	NA	NA	NA	NA
BX465862.2	2.23130091	-0.00402286	1.3767331	-0.002922	0.997669	NA
BX000981.5	0.530029684	-0.939524117	2.7937867	-0.336291	0.736652	NA
BX957333.2	0	NA	NA	NA	NA	NA
CU326364.1	1.782724223	1.405699597	1.4922252	0.9420157	0.346185	NA
BX942819.3	0.522329263	0.999930388	2.8066578	0.3562709	0.721638	NA
BX649516.4	5.633634618	-0.671646721	0.7939643	-0.845941	0.397586	NA
si:dkeyp-100h4.2	0	NA	NA	NA	NA	NA
zgc:123278	2.615221348	0.33986507	1.1563676	0.2939075	0.768829	NA
CU570689.1	0	NA	NA	NA	NA	NA
BX784029.2	0	NA	NA	NA	NA	NA
ost4	447.7131814	-0.410506945	0.1495495	-2.744957	0.006052	0.117821
CR392001.2	0.157187365	-0.955901296	4.0804729	-0.234262	0.814781	NA
si:ch73-266f23.3	131.2765409	-0.250987949	0.1836191	-1.366894	0.171658	0.730287
CR381686.4	1.694543416	-0.576433298	1.4371675	-0.40109	0.688354	NA

BX469910.2	0.158915748	0.967652056	4.0804729	0.2371421	0.812547	NA
BX649349.2	0.157187365	-0.955901296	4.0804729	-0.234262	0.814781	NA
BX119963.1	12.9939105	-0.251093296	0.5284202	-0.475177	0.634661	0.9578
CR388190.1	0	NA	NA	NA	NA	NA
zgc:173624	29.71046586	-0.304802959	0.3649373	-0.83522	0.403594	0.900979
CR388378.1	0.665211163	0.04738924	2.3079847	0.0205327	0.983618	NA
CR388052.2	0.514116189	-2.475533565	2.7461255	-0.901464	0.367342	NA
BX465862.3	3.805476467	-0.646204398	0.9647922	-0.669786	0.502994	NA
CU469420.4	5.975817603	-0.190220759	0.8076251	-0.235531	0.813797	NA
si:dkey-21h14.9	3.492510346	-0.139895615	1.0216297	-0.136934	0.891083	NA
CU695141.1	0	NA	NA	NA	NA	NA
si:dkey-21h14.12	0.339758314	0.005883863	3.2682461	0.0018003	0.998564	NA
BX469918.1	0.166657454	-0.955901296	4.0804729	-0.234262	0.814781	NA
BX927135.1	0.365141898	1.973577737	3.3927314	0.5817076	0.560764	NA
BX664605.1	0	NA	NA	NA	NA	NA
taar20g	0	NA	NA	NA	NA	NA
taar20b	0	NA	NA	NA	NA	NA
FP015809.1	0	NA	NA	NA	NA	NA
znf1113	2.356889534	0.084018267	1.2934489	0.0649568	0.948208	NA
CU469524.1	0	NA	NA	NA	NA	NA
si:dkey-21h14.12	0.679983498	-1.527277543	2.5551009	-0.597737	0.550016	NA
si:dkey-247i3.5	6.236430567	0.583597076	0.7714155	0.7565276	0.449333	NA
si:dkey-21h14.10	0	NA	NA	NA	NA	NA
CT027801.2	2.860267333	1.309207479	1.19596	1.0946917	0.273652	NA
si:dkey-54j5.2	3.622946803	-0.803326313	0.9844146	-0.816045	0.414475	NA
CR381664.1	0	NA	NA	NA	NA	NA
si:ch211-112b1.2	2.935021932	0.96305317	1.1805639	0.8157569	0.414639	NA
CR381664.2	0	NA	NA	NA	NA	NA
BX649518.1	0	NA	NA	NA	NA	NA
si:ch73-263o4.3	0.339758314	0.005883863	3.2682461	0.0018003	0.998564	NA
si:dkeyp-100h4.8	0	NA	NA	NA	NA	NA
znf1050	16.49645733	-0.197289767	0.4991575	-0.395246	0.692662	0.96736
si:ch211-197k17.3	297.6856411	0.019366601	0.1356611	0.1427572	0.886482	0.988936
CR293502.1	1.014439566	0.061150899	1.9310049	0.0316679	0.974737	NA
si:dkey-51d8.9	9.082260333	-1.034600036	0.6379788	-1.621684	0.104871	NA
BX784029.3	0.317831496	1.808837541	3.5779923	0.5055454	0.613176	NA
si:ch211-245n8.4	25.38632829	-0.19635723	0.3965397	-0.495177	0.620475	0.957354
nr6a1a	0	NA	NA	NA	NA	NA
znf1072	0.961274223	-2.176123588	2.1499397	-1.012179	0.311453	NA
si:dkey-260c8.8	21.64500636	-0.332157233	0.4165469	-0.797407	0.425215	0.908835
BX548046.1	0.173729368	-0.955901296	4.0804729	-0.234262	0.814781	NA
si:dkey-260c8.6	4.781996652	-0.501132016	0.8541198	-0.586723	0.55739	NA
tac4	0	NA	NA	NA	NA	NA
BX936397.1	3.299263622	-0.558861362	1.0363901	-0.539238	0.589722	NA
pimr206	0	NA	NA	NA	NA	NA
si:dkey-3d18.5	2.801139995	0.194813883	1.1550565	0.1686618	0.866063	NA
FP017151.1	8.804028527	-0.26553725	0.6311389	-0.420727	0.673954	NA
ighv7-1	0	NA	NA	NA	NA	NA
si:ch211-76l23.4	400.2766187	0.408637317	0.132027	3.0951033	0.001967	0.052931
ighv1-3	0	NA	NA	NA	NA	NA
ighv4-3	0	NA	NA	NA	NA	NA
CU633478.1	0.982063136	-1.030975913	1.9824747	-0.520045	0.603032	NA
ighd	0	NA	NA	NA	NA	NA
ap1m1	1491.423058	0.072555476	0.0975001	0.7441579	0.456781	0.921215

CT573429.2	2.594552787	-0.001580917	1.181351	-0.001338	0.998932	NA
artna	0	NA	NA	NA	NA	NA
ighv8-1	0	NA	NA	NA	NA	NA
BX548060.1	3.288253285	-0.03222391	1.045262	-0.030829	0.975406	NA
si:ch73-367p23.2	21310.93091	0.318054219	0.0812076	3.916559	8.98E-05	0.004474
si:dkey-15f23.1	0.317831496	1.808837541	3.5779923	0.5055454	0.613176	NA
ighv4-1	0	NA	NA	NA	NA	NA
BX908803.1	0.157187365	-0.955901296	4.0804729	-0.234262	0.814781	NA
CU862078.1	0.813556947	-1.876896976	2.1385771	-0.877638	0.38014	NA
or124-4	0	NA	NA	NA	NA	NA
BX247899.1	0	NA	NA	NA	NA	NA
BX510989.1	9.879866371	1.210214521	0.6287828	1.924694	0.054268	NA
ighv5-2	0	NA	NA	NA	NA	NA
ighv4-7	0	NA	NA	NA	NA	NA
CR388364.1	6.92428858	-0.148505188	0.723408	-0.205286	0.837349	NA
BX548046.2	63.39029898	-0.181643618	0.2496877	-0.727483	0.46693	0.924328
7-Mar	132.1409885	-0.154576943	0.1762366	-0.877099	0.380433	0.895192
si:dkey-3n22.9	11.94484752	0.160194825	0.5465126	0.2931219	0.769429	0.975687
si:ch211-251g8.5	0	NA	NA	NA	NA	NA
ighv4-2	0	NA	NA	NA	NA	NA
ighv6-1	0	NA	NA	NA	NA	NA
CR786578.1	0	NA	NA	NA	NA	NA
nkd3	0.476747244	2.393381959	3.0525537	0.7840589	0.433006	NA
ighz	0.987447952	-2.180143481	2.1065994	-1.034911	0.30071	NA
CU695199.1	0.339758314	0.005883863	3.2682461	0.0018003	0.998564	NA
BX005368.1	1.612179921	-1.230089424	1.6172772	-0.760593	0.4469	NA
BX510989.2	0	NA	NA	NA	NA	NA
BX323555.3	0.173729368	-0.955901296	4.0804729	-0.234262	0.814781	NA
ighv4-8	0	NA	NA	NA	NA	NA
AL929243.1	69.02452565	0.486716055	0.2431545	2.0016743	0.04532	0.418148
ighj1-2	0	NA	NA	NA	NA	NA
BX649612.1	0.967826695	2.172518087	2.1451904	1.012739	0.311185	NA
CR936968.1	0.807543623	0.548915946	2.1288858	0.2578419	0.796529	NA
CR954285.1	0	NA	NA	NA	NA	NA
CR786578.2	0.524057646	2.503896172	2.7289583	0.9175282	0.358866	NA
CR847785.1	3.480497245	0.159394757	1.0755009	0.1482051	0.882181	NA
ighv1-4	0	NA	NA	NA	NA	NA
ighv6-2	0	NA	NA	NA	NA	NA
btr31	19.26308425	-0.278594705	0.4481047	-0.621718	0.534127	0.943276
si:ch73-269m14.3	0	NA	NA	NA	NA	NA
si:ch1073-342h5.2	61.31363475	-0.042119762	0.268142	-0.15708	0.875182	0.987881
si:ch73-379f7.5	12.88992735	-0.087106564	0.5357048	-0.162602	0.870832	0.987683
ighv5-4	0	NA	NA	NA	NA	NA
AL627258.1	0	NA	NA	NA	NA	NA
wu:fj64h06	109.1654421	0.324798512	0.2001339	1.6229063	0.104609	0.612567
si:dkey-211g8.8	0.687845557	-2.894878771	2.482332	-1.166193	0.243536	NA
BX957317.2	14.38009576	-0.09430196	0.4933809	-0.191134	0.84842	0.985174
FP236454.1	0.497574187	-2.437446738	2.6012773	-0.937019	0.348749	NA
ighv10-1	0	NA	NA	NA	NA	NA
CU571254.1	4.015558471	-0.974719357	0.9927193	-0.981868	0.326165	NA
ighv5-8	0	NA	NA	NA	NA	NA
si:ch211-239j9.1	717.5989005	-0.002196445	0.0934311	-0.023509	0.981244	0.996944
CR352253.1	0	NA	NA	NA	NA	NA
ighj2-1	0	NA	NA	NA	NA	NA

si:ch73-379f7.4	1.679891435	0.058551884	1.5481587	0.0378203	0.969831	NA
BX571681.4	1.35741394	1.61342852	1.8633764	0.8658629	0.386565	NA
CU695077.1	0	NA	NA	NA	NA	NA
ighv4-9	0	NA	NA	NA	NA	NA
cd16412	112.6063265	0.076496592	0.2003255	0.3818615	0.702564	0.968134
ighj2-5	0	NA	NA	NA	NA	NA
si:ch73-248e21.7	13.46062677	0.108657648	0.5121791	0.2121478	0.831992	0.98349
ighv4-6	0	NA	NA	NA	NA	NA
BX323079.1	0.158915748	0.967652056	4.0804729	0.2371421	0.812547	NA
ighv5-1	0	NA	NA	NA	NA	NA
CR792438.1	4.773025669	0.723510502	0.9318318	0.7764389	0.43749	NA
ighv8-2	0	NA	NA	NA	NA	NA
ighv5-5	0	NA	NA	NA	NA	NA
ighv4-5	0	NA	NA	NA	NA	NA
si:ch211-85n16.3	128.0150553	0.087420724	0.1827459	0.478373	0.632385	0.957356
CR792426.1	0.157187365	-0.955901296	4.0804729	-0.234262	0.814781	NA
ighv1-2	0	NA	NA	NA	NA	NA
ighj1-1	0	NA	NA	NA	NA	NA
BX957260.1	0.333314908	-1.858695402	3.5126701	-0.52914	0.596708	NA
ighv1-4	0.954322662	-2.166551746	2.1536321	-1.005999	0.314416	NA
FP236809.1	0	NA	NA	NA	NA	NA
ighv3-2	0	NA	NA	NA	NA	NA
BX957344.1	0.339758314	0.005883863	3.2682461	0.0018003	0.998564	NA
eppk1	75.46879131	-0.438009757	0.2588404	-1.6922	0.090608	0.576398
si:ch211-229c8.14	0	NA	NA	NA	NA	NA
BX927298.1	0.862554446	-0.606014613	2.2108469	-0.27411	0.784	NA
si:ch211-195m9.3	54.62467203	0.112530017	0.2620891	0.4293579	0.667663	0.964736
ighj2-3	0	NA	NA	NA	NA	NA
fnbp1b	1.463792852	-0.354987176	1.5721664	-0.225795	0.821361	NA
neu3.5	5.165131442	0.694657888	0.8423384	0.824678	0.409554	NA
CT573133.2	0	NA	NA	NA	NA	NA
ighv13-2	0	NA	NA	NA	NA	NA
BX936397.2	13.54055121	-0.066043333	0.5277094	-0.125151	0.900404	0.990121
7-Mar	300.1539833	0.095683028	0.124914	0.7659912	0.443682	0.916631
ighv2-3	0.182570949	0.967652056	4.0804729	0.2371421	0.812547	NA
BX890590.1	0.158795395	0.967652056	4.0804729	0.2371421	0.812547	NA
defbl2	0	NA	NA	NA	NA	NA
neu3.4	0.831947685	-0.652557682	2.2169958	-0.294343	0.768496	NA
neu3.1	103.0882018	-0.194598306	0.1937184	-1.004542	0.315117	0.866156
BX572622.1	0	NA	NA	NA	NA	NA
si:ch211-162i14.1	0.173729368	-0.955901296	4.0804729	-0.234262	0.814781	NA
ighv9-3	0	NA	NA	NA	NA	NA
ighv1-1	0	NA	NA	NA	NA	NA
ighv5-3	0	NA	NA	NA	NA	NA
sox4a	0	NA	NA	NA	NA	NA
ighv4-3	0	NA	NA	NA	NA	NA
ighv11-1	0	NA	NA	NA	NA	NA
BX511272.1	1.354036242	0.016210196	1.6287563	0.0099525	0.992059	NA
si:ch211-276a17.5	89.71849477	0.48727667	0.2245047	2.170452	0.029973	0.335036
AL935145.1	0	NA	NA	NA	NA	NA
CU570787.1	2.040435407	-1.51651584	1.4625685	-1.036885	0.299789	NA
CT027638.1	0.158915748	0.967652056	4.0804729	0.2371421	0.812547	NA
ighv2-1	0	NA	NA	NA	NA	NA
ighv11-2	0	NA	NA	NA	NA	NA

CR792438.2	1.981998812	1.613635914	1.4278015	1.1301542	0.258411	NA
si:dkey-261j15.2	180.5685963	-0.132782706	0.1518485	-0.874442	0.381878	0.895192
si:ch211-15j1.7	4.506090743	-0.535077398	0.8932246	-0.59904	0.549146	NA
CU074421.1	0	NA	NA	NA	NA	NA
raf1a	72.26961864	-0.063448253	0.2308529	-0.274843	0.783437	0.97627
BX470238.1	10.01279136	0.622815704	0.608414	1.023671	0.305991	NA
ighv14-1	0	NA	NA	NA	NA	NA
CU659670.1	325.190044	0.060368076	0.1226454	0.4922163	0.622566	0.957354
CR936968.2	107.4686576	0.057116075	0.1889971	0.302206	0.762495	0.975374
BX324146.1	240.7046482	0.044000054	0.138892	0.3167934	0.7514	0.97419
pimr205	0.158795395	0.967652056	4.0804729	0.2371421	0.812547	NA
ighv2-2	0	NA	NA	NA	NA	NA
si:dkey-217d24.6	285.5499913	-0.311592294	0.127909	-2.436047	0.014849	0.223115
BX663606.1	3.557744617	0.458893979	1.038345	0.4419475	0.658527	NA
ighv9-1	0	NA	NA	NA	NA	NA
rock2b	46.45566167	1.643239735	0.3155886	5.2069046	1.92E-07	2.47E-05
BX901907.1	0.166657454	-0.955901296	4.0804729	-0.234262	0.814781	NA
AL845295.1	0.173729368	-0.955901296	4.0804729	-0.234262	0.814781	NA
BX005275.1	0	NA	NA	NA	NA	NA
si:dkey-26h11.1	0	NA	NA	NA	NA	NA
si:ch211-214p16.3	34.89081531	0.586935319	0.3388393	1.7321937	0.083239	0.556259
AL929009.1	0	NA	NA	NA	NA	NA
BX005461.1	0.31759079	1.807926197	3.579045	0.5051421	0.613459	NA
si:ch211-194e1.7	12.94675129	0.226454515	0.5255747	0.4308702	0.666563	0.964736
CR391935.1	0.31759079	1.807926197	3.579045	0.5051421	0.613459	NA
si:ch211-196f5.9	58.75562015	-0.230170217	0.2559934	-0.899126	0.368586	0.888057
CR383672.2	1.705852197	2.016319027	1.6838861	1.1974201	0.231143	NA
ap1m2	280.6348131	-0.323003866	0.1307933	-2.469575	0.013527	0.208651
BX005275.2	1.306338038	0.741623717	1.7640738	0.420404	0.67419	NA
si:dkey-118j18.2	543.9467649	-0.027757279	0.1078315	-0.257414	0.79686	0.978569
si:dkey-26h11.2	0	NA	NA	NA	NA	NA
CR450696.1	0	NA	NA	NA	NA	NA
CR382294.1	0.847782112	0.655103352	2.1028616	0.3115295	0.755398	NA
BX571664.1	0	NA	NA	NA	NA	NA
si:dkeyp-82h4.1	1.665706323	0.021681786	1.5779834	0.0137402	0.989037	NA
FP103106.1	1.842930485	-1.373238437	1.4574523	-0.942218	0.346081	NA
si:ch211-246n15.2	0	NA	NA	NA	NA	NA
BX571825.13	0	NA	NA	NA	NA	NA
AL845295.2	0	NA	NA	NA	NA	NA
mcat	0	NA	NA	NA	NA	NA
BX571825.14	0	NA	NA	NA	NA	NA
BX901907.2	1.288455455	-0.74775726	1.6799295	-0.445112	0.656239	NA
CU914550.1	1.80395351	-0.214910589	1.5039205	-0.1429	0.886369	NA
si:dkey-16p19.8	0	NA	NA	NA	NA	NA
BX571825.15	0	NA	NA	NA	NA	NA
si:ch211-181p13.2	2.945557646	-2.332905933	1.2703563	-1.836419	0.066296	NA
BX901907.3	0	NA	NA	NA	NA	NA
BX248324.1	132.3321085	0.272028641	0.1902772	1.4296437	0.152819	0.701776
si:ch211-73m21.1	0.157187365	-0.955901296	4.0804729	-0.234262	0.814781	NA
BX901907.4	0.325452849	0.005883619	3.3177086	0.0017734	0.998585	NA
BX571825.16	0	NA	NA	NA	NA	NA
BX571705.1	3.044446312	1.847553833	1.2127755	1.5234096	0.127656	NA
si:dkey-22h13.3	7.566820809	-0.384262059	0.6828715	-0.562715	0.573629	NA
BX324003.1	0.158795395	0.967652056	4.0804729	0.2371421	0.812547	NA

BX088688.1	121.6778762	-0.302845275	0.1876246	-1.614103	0.106505	0.615567
BX927068.1	0	NA	NA	NA	NA	NA
BX890559.2	0	NA	NA	NA	NA	NA
si:ch73-103l1.2	53.58392094	-0.552476935	0.2697197	-2.048337	0.040527	0.393799
si:dkey-58j15.11	2.487984498	-0.158914286	1.2417172	-0.127979	0.898165	NA
si:ch211-1f22.8	1.967972083	-0.443319144	1.3568954	-0.326716	0.743883	NA
BX322618.1	675.9060446	-0.032043927	0.1032712	-0.310289	0.756341	0.975374
si:dkey-27j5.9	1.030981568	0.030822112	1.9223158	0.0160338	0.987207	NA
BX842692.1	0.158795395	0.967652056	4.0804729	0.2371421	0.812547	NA
BX547930.2	0.158795395	0.967652056	4.0804729	0.2371421	0.812547	NA
si:ch211-286b5.2	4.8141847	-0.084259839	0.8647521	-0.097438	0.922378	NA
si:dkey-58j15.10	1.005048545	-0.006083169	1.9358873	-0.003142	0.997493	NA
CT033796.1	6.461493527	-0.848805139	0.7868554	-1.078731	0.280708	NA
irbpl	19.93614148	0.350177198	0.4238688	0.8261452	0.408722	0.90171
AL929349.1	0	NA	NA	NA	NA	NA
CR626889.1	1.291094336	-0.753462392	1.8405577	-0.409366	0.682271	NA
si:ch211-176g6.1	0.471562095	-2.373056629	3.0645586	-0.774355	0.438721	NA
si:ch73-94k4.5	0	NA	NA	NA	NA	NA
AL928650.2	0	NA	NA	NA	NA	NA
BX548015.2	3.67127677	-0.271420755	1.020737	-0.265907	0.790311	NA
si:ch73-236c18.9	70.01947614	-0.266047503	0.245104	-1.085448	0.277723	0.841383
AL929396.1	2.337748693	0.022497347	1.2351387	0.0182144	0.985468	NA
si:dkey-202c14.3	5.279695442	0.735023563	0.9096143	0.8080607	0.419056	NA
rltgr	495.7619458	-0.545498494	0.1117644	-4.88079	1.06E-06	0.000106
si:dkey-27j5.10	0.157187365	-0.955901296	4.0804729	-0.234262	0.814781	NA
CR854964.1	0	NA	NA	NA	NA	NA
CU469541.1	0.330916733	-1.849579229	3.2489867	-0.569279	0.569167	NA
BX088688.2	5.876191826	0.930866703	0.8158614	1.1409619	0.253886	NA
BX901898.1	0.325452849	0.005883619	3.3177086	0.0017734	0.998585	NA
BX901942.1	0.663603133	-1.440371613	2.4108786	-0.597447	0.550209	NA
BX649533.1	0.349228403	0.005884014	3.2372587	0.0018176	0.99855	NA
si:ch211-1f22.7	0	NA	NA	NA	NA	NA
CR774189.1	2.347886315	-0.386006999	1.2928486	-0.298571	0.765267	NA
si:ch211-191a16.5	7.930780261	-0.509134972	0.6905842	-0.737253	0.460969	NA
nrp2a	1515.56101	0.008083673	0.0785914	0.102857	0.918076	0.991623
CR925879.1	1.132127259	1.350278378	2.0029569	0.6741425	0.500221	NA
rac1l	2.366988131	-0.365285818	1.2345287	-0.295891	0.767313	NA
CR774192.1	0.182570949	0.967652056	4.0804729	0.2371421	0.812547	NA
BX681416.1	0	NA	NA	NA	NA	NA
BX248126.1	0	NA	NA	NA	NA	NA
si:dkey-25o16.4	342.9758172	-0.009296681	0.1231498	-0.075491	0.939824	0.992857
CT573186.2	0	NA	NA	NA	NA	NA
si:dkey-19f4.2	0	NA	NA	NA	NA	NA
CR556696.1	8.183972462	0.193679434	0.7163822	0.2703577	0.786885	NA
CU986306.1	0	NA	NA	NA	NA	NA
BX649231.1	0.865423989	3.229020164	2.1645875	1.4917485	0.135765	NA
wu:fj29h11	325.2324267	-0.173540955	0.1312082	-1.322638	0.185956	0.75038
BX322603.1	0.182570949	0.967652056	4.0804729	0.2371421	0.812547	NA
CU915827.1	4.962598513	-0.571460603	0.8798152	-0.649523	0.516	NA
CR392024.1	1.03927371	2.26599918	2.0825649	1.088081	0.276559	NA
zgc:165656	26.34219199	-0.744829139	0.3789141	-1.965694	0.049334	0.435504
si:ch211-155o21.4	6.536895418	-0.216701744	0.7657834	-0.28298	0.777192	NA
BX470128.1	0.473170125	-0.833888596	2.9014002	-0.287409	0.773799	NA
BX248418.1	70.52376965	0.042191886	0.2313362	0.1823834	0.855282	0.985174

si:dkey-27j5.7	0.173729368	-0.955901296	4.0804729	-0.234262	0.814781	NA
CR382370.1	0	NA	NA	NA	NA	NA
FP067451.1	1.206600957	0.476016095	1.9191448	0.2480355	0.804107	NA
si:ch211-152f2.4	0.347458735	-1.908042286	3.4571486	-0.551912	0.581009	NA
si:dkey-182i3.9	14.54116657	-0.084884344	0.4982036	-0.170381	0.864711	0.986052
si:ch211-286b5.6	1.134337053	2.462221963	2.0582167	1.196289	0.231584	NA
adgre9	1.778678993	0.758118305	1.6257845	0.4663092	0.640994	NA
cul4a	778.9768656	0.029883338	0.1006608	0.2968715	0.766565	0.97561
si:dkey-9c18.3	0	NA	NA	NA	NA	NA
BX323590.3	0.349228403	0.005884014	3.2372587	0.0018176	0.99855	NA
AL928835.1	1.006226492	-2.236625194	2.0993903	-1.065369	0.286709	NA
BX004779.1	0.476626891	2.395187592	2.815932	0.8505843	0.395	NA
si:ch211-1f22.9	0	NA	NA	NA	NA	NA
MFAP4 (1 of many)	0.158915748	0.967652056	4.0804729	0.2371421	0.812547	NA
itgam	1.809646813	0.774154744	1.4887092	0.5200174	0.603051	NA
adgre15	1.022611355	2.264378702	2.0087475	1.127259	0.259633	NA
BX088594.1	0	NA	NA	NA	NA	NA
BX908780.2	0.166657454	-0.955901296	4.0804729	-0.234262	0.814781	NA
CR478288.1	0	NA	NA	NA	NA	NA
lratb.1	86.22328933	-0.174831101	0.2361743	-0.740263	0.459141	0.921978
BX296516.1	0	NA	NA	NA	NA	NA
si:ch1073-228b5.2	96.20124849	0.129754826	0.2069446	0.6270027	0.530657	0.942209
BX322618.2	0	NA	NA	NA	NA	NA
AL928650.3	2.90638125	-1.353498497	1.1917493	-1.135724	0.256072	NA
si:ch1073-357b18.4	66.84742742	0.180299188	0.2473815	0.7288304	0.466105	0.923865
bmb	38.9284883	0.371843776	0.323216	1.1504497	0.249959	0.819849
si:ch73-94k4.4	0	NA	NA	NA	NA	NA
CR626902.1	0.682732688	2.892275314	2.4663977	1.1726719	0.240927	NA
BX649499.2	0	NA	NA	NA	NA	NA
BX465225.1	0	NA	NA	NA	NA	NA
CU929161.1	0	NA	NA	NA	NA	NA
si:ch73-212j7.3	14.63914403	-0.329892911	0.5113705	-0.645115	0.518853	0.938919
si:dkey-182i3.10	69.14745677	-0.142777006	0.2353294	-0.606711	0.544043	0.945438
BX469930.2	0	NA	NA	NA	NA	NA
si:ch211-255p10.3	1217.17886	0.06575682	0.1092161	0.6020797	0.547121	0.945526
BX469908.1	2.451900306	1.412415922	1.3018513	1.0849288	0.277953	NA
BX000534.1	0.476386185	2.392464142	3.0533824	0.7835455	0.433307	NA
si:ch211-1f22.10	0	NA	NA	NA	NA	NA
tube1	0.805815241	-0.598842605	2.1324592	-0.280823	0.778847	NA
si:dkey-27j5.6	0.316103113	0.005883447	3.3519121	0.0017553	0.9986	NA
adgre16	1.306605487	-0.046335721	1.6599707	-0.027914	0.977731	NA
si:ch211-1f22.13	0.323844819	-1.825095364	3.274639	-0.557342	0.577293	NA
si:dkey-187i8.2	1.630680968	1.266303401	1.7120746	0.739631	0.459524	NA
si:ch211-286b5.8	3.234958387	1.503826065	1.1225045	1.339706	0.180341	NA
CU914144.1	33.55848671	-0.031572878	0.339889	-0.092892	0.92599	0.992702
BX908780.3	0	NA	NA	NA	NA	NA
buc2l	2.005852438	-0.474736504	1.3528245	-0.350922	0.725647	NA
BX908756.1	0	NA	NA	NA	NA	NA
MED9	194.3074914	0.059135574	0.1525785	0.3875747	0.698331	0.967998
CR382370.2	748.2862484	0.249900273	0.1017898	2.4550619	0.014086	0.214639
si:ch73-94k4.1	0	NA	NA	NA	NA	NA
si:ch211-122h15.4	4.709802615	1.330412252	0.9266945	1.4356535	0.151101	NA
si:dkey-11c5.11	25.14130899	0.253241516	0.4177627	0.6061851	0.544392	0.945438
BX510992.2	1.51134396	-0.296330036	1.4961018	-0.198068	0.842992	NA

BX276101.1	16.03562176	0.190134515	0.4984382	0.3814605	0.702862	0.968134
BX649471.1	0	NA	NA	NA	NA	NA
AL954187.1	0	NA	NA	NA	NA	NA
BX908780.4	1.358520603	-2.711649253	1.8448862	-1.469819	0.141611	NA
adgre18	0.831198824	0.593716313	2.0278563	0.2927803	0.76969	NA
si:ch211-131k2.2	12.86258813	-0.142393672	0.532858	-0.267226	0.789295	0.977058
BX296520.1	0	NA	NA	NA	NA	NA
BX908780.5	0	NA	NA	NA	NA	NA
adgre19	0.973452217	1.035664467	1.98712	0.5211887	0.602235	NA
si:dkey-27j5.11	0	NA	NA	NA	NA	NA
gpr108	1172.76354	-0.03527904	0.0866319	-0.407229	0.68384	0.965679
si:dkey-119m7.8	132.9917948	0.661955694	0.1926376	3.4362749	0.00059	0.020129
BX901920.1	675.8220591	-0.088667449	0.1110174	-0.798681	0.424476	0.908494
si:ch211-1f22.6	0	NA	NA	NA	NA	NA
BX547930.3	0.166657454	-0.955901296	4.0804729	-0.234262	0.814781	NA
si:dkey-4p15.4	0.166657454	-0.955901296	4.0804729	-0.234262	0.814781	NA
si:ch211-286b5.5	472.932033	-0.030482206	0.1216901	-0.25049	0.802208	0.979492
abcc3	556.8210467	-0.05914098	0.11817	-0.500474	0.616742	0.956881
si:dkey-249p7.4	2.941743078	-1.370898796	1.1534601	-1.18851	0.234633	NA
cxl34c	0.158795395	0.967652056	4.0804729	0.2371421	0.812547	NA
si:ch211-202n12.2	4.114456338	0.11405482	0.9583265	0.1190146	0.905264	NA
si:ch211-1f22.2	0.490502273	-2.421143305	2.7891168	-0.868068	0.385357	NA
hemgn	220.9319447	-0.071851238	0.1543799	-0.465418	0.641632	0.959994
MZT1	260.1893382	0.106439604	0.1526999	0.6970508	0.485771	0.930598
si:ch73-301j1.1	4.320870622	0.868470345	1.0159595	0.8548277	0.392647	NA
si:ch211-1f22.16	0.974991223	-1.022830706	2.0964916	-0.487877	0.625637	NA
si:dkey-27j5.10	0	NA	NA	NA	NA	NA
BX296562.1	0	NA	NA	NA	NA	NA
AL845365.1	0	NA	NA	NA	NA	NA
CR450698.1	0.317711143	1.810477155	3.2978238	0.5489915	0.583011	NA
BX004780.1	0.659318192	2.852076546	2.5139723	1.1344901	0.256589	NA
AL929237.3	3.356044113	-0.534094346	1.0608196	-0.503473	0.614632	NA
BX927123.1	0.31598276	0.005883445	3.3523626	0.001755	0.9986	NA
si:dkey-1f1.3	6.576427501	-0.019938494	0.7451374	-0.026758	0.978653	NA
BX928755.1	0.158915748	0.967652056	4.0804729	0.2371421	0.812547	NA
CR385078.2	0	NA	NA	NA	NA	NA
BX927300.1	2.292325057	-1.309420028	1.3233486	-0.989475	0.322431	NA
CU565528.1	0	NA	NA	NA	NA	NA
CR382372.1	19.25422569	0.158087134	0.4273713	0.3699058	0.711453	0.969006
CT573003.1	47.12493821	0.522929186	0.2995058	1.7459734	0.080816	0.550801
rgs7b	55.05686637	-0.190536007	0.2673791	-0.712606	0.476089	0.926338
CU660013.1	4.918234308	-0.069690157	0.8737117	-0.079763	0.936425	NA
si:dkey-66i24.9	60.02844452	-0.379274762	0.2493964	-1.520771	0.128317	0.658954
CR376838.1	0	NA	NA	NA	NA	NA
CU929359.1	0.31759079	1.807926197	3.579045	0.5051421	0.613459	NA
CR388157.1	0.904013163	2.00662374	2.339454	0.8577316	0.391041	NA
CU914156.1	0	NA	NA	NA	NA	NA
AL928716.1	0	NA	NA	NA	NA	NA
CR932360.1	0	NA	NA	NA	NA	NA
BX072570.1	0.689615224	-1.495598879	2.3854835	-0.626958	0.530687	NA
BX119915.1	0	NA	NA	NA	NA	NA
AL928920.2	0	NA	NA	NA	NA	NA
FP565451.1	2.963278591	-1.417134629	1.2972411	-1.092422	0.274648	NA
si:dkey-21e13.3	102.8256196	-0.003017853	0.2081491	-0.014499	0.988432	0.997476

CU550702.1	0.80047171	0.566096181	2.1350079	0.2651495	0.790894	NA
si:dkey-1h6.8	13.33374311	0.530288725	0.5428993	0.9767717	0.328682	0.872945
BX001014.1	0.967008811	-0.023866834	1.9569279	-0.012196	0.990269	NA
CR847530.1	0.173729368	-0.955901296	4.0804729	-0.234262	0.814781	NA
CR388421.1	0.476386185	2.392464142	3.0533824	0.7835455	0.433307	NA
BX469896.1	9.713576858	0.120012174	0.6444203	0.1862328	0.852262	NA
si:dkeyp-104f11.8	7.792416224	-0.430122351	0.7026513	-0.612142	0.540444	NA
BX255913.1	0	NA	NA	NA	NA	NA
BX470246.1	0.792489298	1.851487174	2.2731551	0.814501	0.415358	NA
si:ch211-194k22.8	503.505139	-0.123079353	0.1090831	-1.128309	0.25919	0.82755
si:dkey-193p11.2	379.8228735	0.272789217	0.1394204	1.9565945	0.050395	0.439877
CR388040.1	60.65574596	0.095155708	0.2701756	0.3521995	0.724689	0.970402
si:dkey-66i24.7	58.48005867	0.129776018	0.2556341	0.5076631	0.61169	0.95688
si:dkeyp-104f11.9	0	NA	NA	NA	NA	NA
hmx3b	8.83207182	-0.342652619	0.7298524	-0.469482	0.638725	NA
AL732488.1	278.5449377	0.136021841	0.1341392	1.0140347	0.310566	0.86284
BX323889.1	20.78874019	0.089850848	0.4335422	0.2072482	0.835816	0.984089
CR352342.1	0	NA	NA	NA	NA	NA
si:rp71-1c10.11	118.1328774	0.362153438	0.1996181	1.8142316	0.069642	0.514805
si:dkey-16i5.8	15.89440675	-0.053745638	0.5318722	-0.10105	0.919511	0.991651
CU660013.2	2.338378196	1.308032533	1.3486442	0.969887	0.332103	NA
BX248238.1	0.325573202	0.005883621	3.3172783	0.0017736	0.998585	NA
si:dkey-21c1.1	282.1025956	0.227027163	0.1321579	1.7178478	0.085824	0.56251
si:ch211-250m6.2	0.341486697	1.892317624	3.2124068	0.5890654	0.555817	NA
CT737227.1	10.24927676	0.396434626	0.6068067	0.6533128	0.513555	NA
cpn1	0	NA	NA	NA	NA	NA
CR385078.3	0	NA	NA	NA	NA	NA
BX569783.1	1.513580497	-0.354123196	1.5602609	-0.226964	0.820452	NA
CR847530.2	0.166657454	-0.955901296	4.0804729	-0.234262	0.814781	NA
BX255897.1	0	NA	NA	NA	NA	NA
BX855623.1	3.554421656	0.254472898	1.1018926	0.2309416	0.81736	NA
BX255963.1	0.682853041	2.891318346	2.3286279	1.2416403	0.214369	NA
BX248390.1	2.338497554	-0.417012027	1.2361301	-0.337353	0.735851	NA
CR749163.2	2.769848899	0.119127006	1.2637627	0.0942637	0.9249	NA
si:zfos-911d5.4	3.927184907	0.250728274	0.9773726	0.2565329	0.797539	NA
si:dkey-219e21.2	31.19214741	-1.100355254	0.4373075	-2.516205	0.011863	0.191219
CR405682.1	0.31598276	0.005883445	3.3523626	0.001755	0.9986	NA
BX088644.1	0.158795395	0.967652056	4.0804729	0.2371421	0.812547	NA
CU469493.1	0	NA	NA	NA	NA	NA
si:dkey-22l1.1	0	NA	NA	NA	NA	NA
AL928824.2	0	NA	NA	NA	NA	NA
CR385078.4	0	NA	NA	NA	NA	NA
CR381684.1	0	NA	NA	NA	NA	NA
BX296562.2	0	NA	NA	NA	NA	NA
asb2a.1	5.305456784	1.359826017	0.8466096	1.6062019	0.10823	NA
NDUFAF8	66.20921099	-0.173855176	0.2462763	-0.705936	0.480228	0.927347
BX001014.2	24.01244137	-0.167922898	0.4043409	-0.4153	0.677922	0.965257
BX842669.1	1.35144869	-2.705106269	1.8223791	-1.484382	0.137708	NA
CU459210.1	0	NA	NA	NA	NA	NA
AL928824.3	0.31437473	-1.788572922	3.5930657	-0.497785	0.618636	NA
CU570682.1	0.158915748	0.967652056	4.0804729	0.2371421	0.812547	NA
si:ch211-202p1.5	1.665973772	-0.602291113	1.6154293	-0.372837	0.70927	NA
si:ch211-196c10.13	62.4988744	-0.616298279	0.2746954	-2.24357	0.02486	0.303822
si:ch73-205h11.1	4.973160271	2.30910207	1.0065379	2.2941034	0.021785	NA

BX901952.1	0	NA	NA	NA	NA	NA
BX255913.2	1.047874586	3.504465839	2.1494511	1.6304003	0.103017	NA
si:rp71-1c10.8	1.735091635	1.274647451	1.6049595	0.7941929	0.427083	NA
CT573006.1	2.333742488	-0.378236254	1.2239999	-0.309017	0.757309	NA
si:dkey-61p9.11	35.43856263	0.048262428	0.4298109	0.1122876	0.910595	0.990817
CU694525.1	0	NA	NA	NA	NA	NA
BX510919.1	0.31437473	-1.788572922	3.5930657	-0.497785	0.618636	NA
pimr124	0.643043639	1.435364982	2.5984886	0.5523846	0.580685	NA
BX510920.1	0	NA	NA	NA	NA	NA
BX255897.2	0	NA	NA	NA	NA	NA
CR932077.1	5.356351093	0.357352259	0.837478	0.4267005	0.669598	NA
CU571074.1	975.794548	-0.208746009	0.092494	-2.25686	0.024017	0.299249
BX510319.1	0.968977899	0.950295762	2.2382818	0.4245648	0.671154	NA
pimr128	0.839689391	-1.918726865	2.219847	-0.864351	0.387395	NA
BX088686.1	2.488331015	-0.610996786	1.2334899	-0.49534	0.62036	NA
BX119992.1	0.641555962	-0.014184532	2.3335058	-0.006079	0.99515	NA
CCDC189	2.289809784	0.430675828	1.2669057	0.3399431	0.733899	NA
AL928920.3	0.341486697	1.892317624	3.2124068	0.5890654	0.555817	NA
BX294177.1	0	NA	NA	NA	NA	NA
CR932360.2	0.182570949	0.967652056	4.0804729	0.2371421	0.812547	NA
CR932362.1	1.190997191	2.512992113	1.9069703	1.317793	0.187573	NA
si:dkey-148d16.5	0	NA	NA	NA	NA	NA
CU929359.2	15.48964421	0.442841692	0.515844	0.8584798	0.390628	0.897545
CU660013.3	0.332524763	0.005883742	3.292841	0.0017868	0.998574	NA
si:ch211-28p3.3	12.40542312	-0.570978556	0.5554945	-1.027874	0.304009	0.858414
selp	0.31437473	-1.788572922	3.5930657	-0.497785	0.618636	NA
CR352243.1	0	NA	NA	NA	NA	NA
BX248238.2	0.158795395	0.967652056	4.0804729	0.2371421	0.812547	NA
BX569801.2	0.789273238	-0.566394988	2.2706407	-0.249443	0.803018	NA
si:ch73-344o19.1	78.45845545	0.169273544	0.2206535	0.7671465	0.442994	0.916336
BX004999.1	30.5251381	0.099111373	0.4075526	0.2431867	0.807861	0.980642
BX842240.1	0.166657454	-0.955901296	4.0804729	-0.234262	0.814781	NA
BX119915.2	3.135949369	-2.43731969	1.2890138	-1.890841	0.058646	NA
CR848736.1	18.32122907	0.43175853	0.449655	0.9601996	0.336955	0.876088
CR751608.1	0.325573202	0.005883621	3.3172783	0.0017736	0.998585	NA
si:ch73-127m5.2	346.5930858	0.084405547	0.1188564	0.7101474	0.477613	0.926857
BX322665.3	0.157187365	-0.955901296	4.0804729	-0.234262	0.814781	NA
BX004803.1	0	NA	NA	NA	NA	NA
si:ch211-277e21.1	2.411311893	0.040878467	1.3279644	0.0307828	0.975443	NA
si:dkeyp-13d11.1	0	NA	NA	NA	NA	NA
CU660013.4	286.9257613	-0.028609632	0.1315863	-0.217421	0.82788	0.982721
si:dkey-219e21.4	19.61337959	0.43447446	0.4431049	0.9805228	0.326828	0.872658
BX119915.3	0.481032184	-2.398473301	2.80721	-0.854398	0.392885	NA
BX957286.1	0	NA	NA	NA	NA	NA
si:dkey-287g12.6	86.25255447	0.02310727	0.2140652	0.107945	0.914039	0.991119
si:ch211-168b3.2	62.07388616	-1.038687186	0.2639108	-3.93575	8.29E-05	0.004184
BX279523.1	35.00713949	-5.095868642	0.6570559	-7.755609	8.79E-15	4.35E-12
AL833789.1	0.971534457	-3.409585359	2.1825892	-1.562175	0.118247	NA
AL928920.4	0.182570949	0.967652056	4.0804729	0.2371421	0.812547	NA
BX901968.1	0	NA	NA	NA	NA	NA
si:dkeyp-106c3.2	1.315257692	-1.612412619	1.7573009	-0.917551	0.358854	NA
CR854832.1	0.5046461	-2.45370851	2.7629627	-0.888072	0.374502	NA
zgc:136683	0	NA	NA	NA	NA	NA
CR396584.1	1.65689248	1.165419669	1.5944899	0.7309044	0.464838	NA

si:ch211-256e16.7	1.971696297	-0.0155606	1.3945315	-0.011158	0.991097	NA
CR847898.1	3.877819438	-1.193215828	0.9964981	-1.197409	0.231147	NA
BX571796.1	0.317831496	1.808837541	3.5779923	0.5055454	0.613176	NA
CU914157.1	21.22097859	0.715003001	0.4754746	1.5037669	0.132641	0.667222
BX323575.1	0	NA	NA	NA	NA	NA
BX279523.2	0	NA	NA	NA	NA	NA
si:ch73-144d13.7	27.81183625	-0.260762046	0.3733915	-0.698361	0.484951	0.93001
blvrb	258.0161173	0.095204844	0.1590493	0.5985871	0.549448	0.946312
mhc1lja	6.213028528	0.140244165	0.8069313	0.1737994	0.862023	NA
CR774195.1	0	NA	NA	NA	NA	NA
BX569793.1	22.31549445	0.016728118	0.4624009	0.0361767	0.971141	0.996315
CR391963.1	62.45891502	0.394332134	0.2454985	1.6062508	0.108219	0.620087
si:ch211-155k24.9	47.7992164	-0.212311785	0.2898213	-0.732561	0.463826	0.922875
CR450814.1	0.341486697	1.892317624	3.2124068	0.5890654	0.555817	NA
AL807792.1	2.168184909	0.735148348	1.3438971	0.5470273	0.58436	NA
CU013548.1	3.347628116	-1.254966004	1.0912412	-1.150035	0.250129	NA
CR848791.1	0	NA	NA	NA	NA	NA
CU929322.1	0.946580956	-0.97691498	2.2566655	-0.432902	0.665086	NA
CU929206.1	2.853033782	1.282631816	1.1830709	1.0841546	0.278296	NA
CT573157.1	0	NA	NA	NA	NA	NA
zgc:193593	265.8292488	0.610754611	0.1490277	4.0982637	4.16E-05	0.002345
BX927351.1	0	NA	NA	NA	NA	NA
BX649487.1	0	NA	NA	NA	NA	NA
CU855854.1	0.489712128	-0.887840729	2.693685	-0.329601	0.741702	NA
CR855311.2	1.852710304	0.290918308	1.3642227	0.2132484	0.831133	NA
si:dkey-16p21.8	2026.288327	0.170832945	0.0903196	1.8914276	0.058567	0.473924
BX957346.1	291.2800169	-0.036478791	0.1318601	-0.276648	0.782051	0.97627
znf1143	67.06132607	-0.370932795	0.2534864	-1.463324	0.143379	0.686804
BX572077.1	9.108503929	-0.29234107	0.6558387	-0.445751	0.655777	NA
si:ch73-1f23.1	2.625372516	-1.101237991	1.2518451	-0.879692	0.379026	NA
BX649324.1	5.398601198	0.625845094	0.9398732	0.6658825	0.505486	NA
znf1012	11.59104769	0.041385845	0.6245322	0.066267	0.947165	0.99381
CU469362.1	72.34435951	0.109364837	0.2257818	0.4843828	0.628114	0.957354
BX927361.1	0.339758314	0.005883863	3.2682461	0.0018003	0.998564	NA
si:ch211-286b4.4	15.61189716	0.622379934	0.5061638	1.2296019	0.218846	0.785734
CU207301.1	0	NA	NA	NA	NA	NA
CR936975.1	0.325452849	0.005883619	3.3177086	0.0017734	0.998585	NA
si:ch73-380l3.4	2.917528393	0.025233912	1.1832348	0.0213262	0.982985	NA
CR790362.1	2.285885901	1.868135679	1.5016923	1.2440203	0.213492	NA
CR457444.1	0	NA	NA	NA	NA	NA
CR931780.1	4.025650279	0.503726018	0.931972	0.5404948	0.588856	NA
clmn	43.18628597	0.27439179	0.3042469	0.9018721	0.367125	0.887458
CR385026.1	0.166657454	-0.955901296	4.0804729	-0.234262	0.814781	NA
CT583650.1	0.499972362	-2.44370697	3.0013188	-0.814211	0.415524	NA
si:dkey-33c3.1	3.434871928	-0.657588907	1.1476359	-0.572994	0.566649	NA
BX511245.1	0	NA	NA	NA	NA	NA
CU467963.1	1.133906971	-0.442920623	1.9523852	-0.226861	0.820532	NA
CR626887.1	1.353218358	-1.510769436	1.7691256	-0.853964	0.393125	NA
tmem176l.3b	420.5744342	-0.160665006	0.1138748	-1.410892	0.158277	0.709224
BX294110.1	0.340386822	-1.882368278	3.2159502	-0.585323	0.558331	NA
CR847984.1	8.536301195	-0.869560073	0.6686732	-1.300426	0.193455	NA
CR759918.1	273.534345	0.030313116	0.1362661	0.2224553	0.823959	0.982196
BX323807.1	0	NA	NA	NA	NA	NA
CU462980.1	38.53546543	0.452212789	0.3235846	1.39751	0.16226	0.716781

pimr28	0.339758314	0.005883863	3.2682461	0.0018003	0.998564	NA
BX322538.1	0.522329263	0.999930388	2.8066578	0.3562709	0.721638	NA
CU638693.1	1.180306874	0.442800236	1.7391297	0.2546102	0.799024	NA
AL953866.1	3.90803161	0.935925957	0.9828249	0.9522815	0.340954	NA
CR388164.2	1.17819069	0.530495249	1.9330381	0.274436	0.78375	NA
CR381618.1	583.5085303	-0.006213597	0.0998782	-0.062212	0.950394	0.99381
serpinb1l4	22.78894717	-0.805683097	0.4365892	-1.845403	0.064979	0.497901
CT033825.1	218.8336572	-0.001548332	0.1442818	-0.010731	0.991438	0.997679
FP102794.1	0	NA	NA	NA	NA	NA
CR774184.1	0	NA	NA	NA	NA	NA
CR450848.1	0.332645115	0.005883744	3.2924249	0.0017871	0.998574	NA
CU628002.1	0	NA	NA	NA	NA	NA
BX004991.1	77.37770669	-0.013157952	0.2383169	-0.055212	0.95597	0.994543
si:dkey-90a13.10	0	NA	NA	NA	NA	NA
CR925728.1	0.846640952	-1.930134747	2.2349699	-0.863607	0.387804	NA
CU929451.1	19.57212435	-0.647447021	0.4381618	-1.477644	0.139503	0.678238
CT961047.1	0	NA	NA	NA	NA	NA
si:dkey-7i4.10	24.93251286	-0.101039007	0.3979621	-0.253891	0.79958	0.979366
si:ch211-180n24.3	1.538455926	1.005163047	1.5742941	0.6384849	0.523158	NA
CU062633.1	8.884322669	-0.967241571	0.6840403	-1.414013	0.157358	NA
si:dkeyp-100a5.4	5.109637965	0.843507798	0.904034	0.9330487	0.350795	NA
si:ch73-23124.1	429.4701543	-0.078654182	0.1475933	-0.532912	0.594095	0.954434
si:dkey-7i4.24	5.364680269	-0.712258055	0.845431	-0.842479	0.39952	NA
si:ch73-185c24.2	136.9758822	-0.445274077	0.1929594	-2.307606	0.021021	0.273449
BX005128.1	0.499302569	-0.916836436	2.6772503	-0.342455	0.732009	NA
CR759878.1	0.31437473	-1.788572922	3.5930657	-0.497785	0.618636	NA
vps50	0.158915748	0.967652056	4.0804729	0.2371421	0.812547	NA
BX005129.1	9.952064783	-0.400757148	0.6281293	-0.638017	0.523463	NA
CR391963.2	0.158795395	0.967652056	4.0804729	0.2371421	0.812547	NA
si:ch211-107e6.6	0.158915748	0.967652056	4.0804729	0.2371421	0.812547	NA
si:ch211-227m13.1	74.43657743	0.218756068	0.2254383	0.9703591	0.331868	0.874653
AL954322.1	0.482640214	-0.866525639	2.7061322	-0.320208	0.748811	NA
CR790366.1	0	NA	NA	NA	NA	NA
si:zfos-375h5.1	9.02063517	0.125863139	0.6793934	0.1852581	0.853027	NA
CR790377.1	0	NA	NA	NA	NA	NA
si:ch211-147k10.6	142.3603543	0.233733809	0.1822649	1.2823853	0.199708	0.765017
CU638693.2	1.275610923	0.724314706	1.8883685	0.3835664	0.7013	NA
BX927282.2	0.871516381	0.597595468	2.1830013	0.2737495	0.784277	NA
BX005392.1	7.925238305	0.104776962	0.6873041	0.1524463	0.878835	NA
si:ch211-210b2.3	2.839137449	-1.254938875	1.1459382	-1.095119	0.273464	NA
CR388163.1	0	NA	NA	NA	NA	NA
CU639412.1	0.332524763	0.005883742	3.292841	0.0017868	0.998574	NA
CR385086.1	0	NA	NA	NA	NA	NA
si:ch211-37e10.2	130.5439908	0.322707447	0.1812688	1.7802703	0.075032	0.533697
si:ch211-135n15.2	10.95378566	0.54260119	0.5802745	0.9350767	0.349749	NA
CR589875.1	0.317711143	1.810477155	3.2978238	0.5489915	0.583011	NA
BX005461.2	3.275567136	-0.597985638	1.0518396	-0.568514	0.569686	NA
BX323035.1	0	NA	NA	NA	NA	NA
CU207354.1	0.476506538	2.394892832	2.8161716	0.8504073	0.395099	NA
si:dkey-52p2.5	0.158915748	0.967652056	4.0804729	0.2371421	0.812547	NA
si:dkey-77f5.8	91.06747961	-0.125744567	0.2089615	-0.601759	0.547334	0.945526
BX957258.1	18.11223504	-0.291384211	0.4465229	-0.652563	0.514038	0.936851
CR759863.1	0	NA	NA	NA	NA	NA
BX897682.1	8.362368874	0.485968416	0.680715	0.7139087	0.475284	NA

CR545469.1	0	NA	NA	NA	NA	NA
im:7143333	41.28564518	-0.039623617	0.3016024	-0.131377	0.895477	0.989814
si:dkey-197j19.5	12.22270144	0.406163182	0.5536842	0.7335646	0.463214	0.922698
BX927244.1	5.38142845	0.047529847	0.8909903	0.053345	0.957457	NA
CR848812.1	30.33842007	0.12406366	0.3527277	0.3517265	0.725043	0.970402
si:dkeyp-67f1.1	31.03320065	-0.224326189	0.3519231	-0.63743	0.523845	0.939336
CR848791.2	0.84841062	-0.578696897	2.0997862	-0.275598	0.782857	NA
BX005254.2	4.298259818	-0.009689555	0.9580036	-0.010114	0.99193	NA
BX005351.1	0	NA	NA	NA	NA	NA
si:dkey-29h14.10	6.859561294	-0.627705354	0.7288889	-0.861181	0.389138	NA
CLIP4	71.71416264	0.128066831	0.2298642	0.5571414	0.577431	0.950805
CT025690.1	124.1798341	-0.282750986	0.1799675	-1.571123	0.116154	0.63841
CU179699.1	0	NA	NA	NA	NA	NA
AL844141.1	0.31598276	0.005883445	3.3523626	0.001755	0.9986	NA
si:dkey-238d18.13	0	NA	NA	NA	NA	NA
CR383662.5	1.816708682	3.220085527	1.6905176	1.9047927	0.056807	NA
BX004812.1	5.072486762	-1.262388368	0.8849996	-1.426428	0.153745	NA
CR759863.2	0.166657454	-0.955901296	4.0804729	-0.234262	0.814781	NA
BX119902.2	3.949812512	1.600184179	1.0385976	1.5407162	0.123386	NA
si:dkey-206p8.1	32.23763416	0.024216823	0.3514299	0.0689094	0.945062	0.993443
CU633991.2	71.05137248	-0.041391502	0.2386955	-0.173407	0.862331	0.985841
CR926463.1	18.05372587	0.68939171	0.4646619	1.4836415	0.137904	0.675313
BX248113.1	2.820347622	-0.179421044	1.1062596	-0.162187	0.871159	NA
si:dkeyp-93a5.3	0.498553709	0.926267934	2.6786929	0.345791	0.7295	NA
mhc1lla	0.332524763	0.005883742	3.292841	0.0017868	0.998574	NA
CU972512.1	9.048977656	-0.166899369	0.6472469	-0.25786	0.796515	NA
NPC2 (1 of many)	13.81595182	0.524685047	0.5373823	0.976372	0.32888	0.873157
CR626962.1	0	NA	NA	NA	NA	NA
BX957326.2	0.499182217	-0.916838237	2.6774436	-0.34243	0.732027	NA
si:dkeyp-19h3.8	0	NA	NA	NA	NA	NA
si:dkey-14d8.22	10.79376455	-0.412657404	0.5840507	-0.706544	0.47985	NA
CU915818.1	1.759897197	-1.409859507	1.6105821	-0.875373	0.381371	NA
CR589944.1	131.419419	-0.135601814	0.1938905	-0.699373	0.484319	0.929601
fam110c	106.4443819	0.446372383	0.1982811	2.2512103	0.024372	0.301776
si:ch1073-340i21.2	195.7454131	-0.537222477	0.1551726	-3.462096	0.000536	0.018825
CR847968.1	366.2841304	-0.112165426	0.1194349	-0.939134	0.347662	0.880195
zgc:153405	3.695523692	0.556923836	1.0174217	0.5473874	0.584113	NA
CR847923.1	0	NA	NA	NA	NA	NA
AL929222.1	1.989269151	-0.502873238	1.3290087	-0.378382	0.705147	NA
fbxl22	27.95202263	0.206645084	0.3922165	0.5268648	0.598287	0.955488
BX005392.2	11.94122149	0.27142109	0.5635784	0.481603	0.630088	0.957354
CR589876.1	74.4681097	0.169326421	0.2548471	0.6644236	0.506419	0.935724
CT737162.1	0.158795395	0.967652056	4.0804729	0.2371421	0.812547	NA
BX537308.1	33.13202074	-0.502520362	0.3393119	-1.480998	0.138607	0.676911
BX890558.1	77.29958319	-0.111794732	0.2311735	-0.483597	0.628672	0.957354
FP245462.1	20.16984427	-0.205169637	0.4815573	-0.426054	0.670068	0.965223
CR855375.1	3.47645301	1.002615667	1.1738322	0.8541388	0.393028	NA
CR854839.1	173.8087766	0.279624518	0.155022	1.8037729	0.071267	0.522712
CU694256.1	0.157187365	-0.955901296	4.0804729	-0.234262	0.814781	NA
opn1mw1	12004.99692	0.228011699	0.0950298	2.3993694	0.016423	0.236409
FP102888.1	0.182570949	0.967652056	4.0804729	0.2371421	0.812547	NA
hbaa1	1.008426242	2.234978276	1.9960971	1.1196742	0.262853	NA
BX649300.1	0	NA	NA	NA	NA	NA
CU694202.1	0	NA	NA	NA	NA	NA

FP016018.2	1.149459407	0.422305922	1.838616	0.2296869	0.818335	NA
BX004972.1	0.491320158	0.8528132	2.8656354	0.2976	0.766008	NA
mical3a	112.3961079	-0.054633725	0.1910382	-0.285983	0.774891	0.975687
CR628341.1	21.22929277	0.026387092	0.4252194	0.0620552	0.950519	0.99381
dhrs13b.1	5.599049389	-0.333747628	0.8209972	-0.406515	0.684364	NA
CU682780.1	0.365141898	1.973577737	3.3927314	0.5817076	0.560764	NA
CU655829.1	0.158915748	0.967652056	4.0804729	0.2371421	0.812547	NA
CR786571.3	1.470088166	-1.757445669	1.7465606	-1.006232	0.314304	NA
ugt1b4	2.451199519	-1.028780444	1.2504642	-0.822719	0.410668	NA
CR354383.1	6.177573934	0.561716721	0.7987615	0.7032346	0.48191	NA
si:dkey-16m19.1	31.01688278	0.075173481	0.3727906	0.2016507	0.84019	0.985029
CR352292.1	2.492145583	-1.503971322	1.2858653	-1.169618	0.242155	NA
AL845435.1	0	NA	NA	NA	NA	NA
si:ch73-38013.3	2.486722985	-1.014424677	1.2215208	-0.83046	0.406279	NA
si:ch211-256a21.4	37.91167527	-0.058134063	0.3377193	-0.172137	0.86333	0.985841
CU466278.1	9.830464494	-0.542033727	0.6133944	-0.883663	0.376878	NA
CR450813.1	0.471562095	-2.373056629	3.0645586	-0.774355	0.438721	NA
pimr42	0.166657454	-0.955901296	4.0804729	-0.234262	0.814781	NA
CR759845.1	8.73613539	0.078130415	0.6780909	0.1152212	0.90827	NA
CT025745.1	30.12364495	-1.172749134	0.3575478	-3.279979	0.001038	0.031976
BX927349.1	0	NA	NA	NA	NA	NA
CR759845.2	2.541535383	0.209225824	1.2009193	0.1742214	0.861691	NA
CU468915.1	17.18289965	-0.561178586	0.5011451	-1.119793	0.262802	0.830166
AL935145.2	1.16384394	0.399527086	1.7443472	0.229041	0.818837	NA
CR936518.1	3.335753063	0.886769592	1.055752	0.8399412	0.400941	NA
BX629346.1	40.15312957	0.502269882	0.3215232	1.5621576	0.118251	0.641526
CU571318.1	604.3951911	-0.16606743	0.0992817	-1.672689	0.094389	0.584358
CR759845.3	2.120406395	1.138056897	1.5038092	0.7567828	0.44918	NA
CR855375.2	6.691416163	-0.715232576	0.7519926	-0.951116	0.341545	NA
BX897714.1	0.166657454	-0.955901296	4.0804729	-0.234262	0.814781	NA
FP236594.1	0	NA	NA	NA	NA	NA
CU927934.1	10.09027277	-0.071894812	0.6399725	-0.11234	0.910553	NA
si:ch211-284f22.3	462.0086692	-0.076360376	0.1170195	-0.652544	0.51405	0.936851
fxyd6l	1781.564869	-0.065590128	0.0849468	-0.772132	0.440036	0.916082
CU929339.1	6.469156165	-0.996297602	0.7536656	-1.321936	0.18619	NA
si:dkey-159n16.4	0	NA	NA	NA	NA	NA
CR388369.1	5.213148577	-0.187784275	0.8724411	-0.21524	0.82958	NA
BX547934.1	1.172255439	-1.303351983	1.8073824	-0.721127	0.470831	NA
sparcl2	21.29667261	0.254727265	0.4295928	0.5929505	0.553214	0.947265
CR759845.4	0.158795395	0.967652056	4.0804729	0.2371421	0.812547	NA
si:ch73-223p23.2	0	NA	NA	NA	NA	NA
BX322555.1	0.317831496	1.808837541	3.5779923	0.5055454	0.613176	NA
CR626887.2	0	NA	NA	NA	NA	NA
si:ch211-66i15.4	6.68485566	-0.27932794	0.7399128	-0.377515	0.705791	NA
AL954322.2	73.00413637	0.055034934	0.2376092	0.2316195	0.816834	0.981594
CU928073.1	0.158915748	0.967652056	4.0804729	0.2371421	0.812547	NA
BX322555.2	0	NA	NA	NA	NA	NA
CU855801.1	0.347458735	-1.908042286	3.4571486	-0.551912	0.581009	NA
CR318607.1	5.885483825	-0.061807117	0.7993965	-0.077317	0.938371	NA
CR848665.1	3.195939131	0.788486769	1.0833462	0.7278253	0.466721	NA
si:ch73-181m17.1	7.093730994	-0.321487254	0.7328147	-0.438702	0.660878	NA
si:dkey-149i17.11	0	NA	NA	NA	NA	NA
im:7152348	172.2968667	0.016688619	0.182377	0.0915061	0.92709	0.992702
BX005462.2	0.332645115	0.005883744	3.2924249	0.0017871	0.998574	NA

si:zfos-1714f5.4	2.143027489	-0.243471598	1.4351233	-0.169652	0.865284	NA
si:ch211-261a10.5	5.755639088	0.900577898	0.8189164	1.099719	0.271455	NA
si:dkey-172k15.4	52.65720811	0.113894525	0.2658059	0.4284876	0.668296	0.964736
CR812472.1	2.321045053	-0.000352531	1.2231431	-0.000288	0.99977	NA
si:dkey-60d5.3	31.51786993	0.100614573	0.3446409	0.2919403	0.770332	0.975687
BX120006.1	0	NA	NA	NA	NA	NA
si:dkey-7j22.2	1.649390484	-0.63991731	1.5880575	-0.402956	0.686981	NA
si:ch211-273k1.5	0.317711143	1.810477155	3.2978238	0.5489915	0.583011	NA
CR293508.1	0.696687138	-1.510024977	2.3787501	-0.634798	0.52556	NA
CU927934.2	0	NA	NA	NA	NA	NA
AL954669.1	0.97432143	-0.03661469	1.971898	-0.018568	0.985186	NA
CR394545.1	6.646346796	-0.114671568	0.758717	-0.151139	0.879866	NA
znf1004	1.816829035	3.220170394	1.6788325	1.918101	0.055098	NA
si:ch211-108d22.1	0.849630847	1.928522947	2.2126134	0.8716041	0.383424	NA
BX511075.1	0.182570949	0.967652056	4.0804729	0.2371421	0.812547	NA
si:ch73-281n10.2	9979.413805	-0.042619303	0.0644668	-0.661104	0.508545	0.935788
si:dkey-52d15.2	0.794578739	3.129658898	2.5486248	1.2279794	0.219455	NA
CR790366.2	3.421005594	-2.09644369	1.1566651	-1.81249	0.069911	NA
AL928927.1	0.672283077	0.02892376	2.3005571	0.0125725	0.989969	NA
BX649442.2	8.109416289	-0.190348631	0.6709362	-0.283706	0.776636	NA
si:dkey-56f14.4	387.9103044	0.297582735	0.1556001	1.9124847	0.055814	0.46256
CR762407.2	1.114726087	-0.412825569	1.8539615	-0.222672	0.823791	NA
BX571737.1	0	NA	NA	NA	NA	NA
si:dkey-27l15.1	0.182570949	0.967652056	4.0804729	0.2371421	0.812547	NA
si:dkey-33c14.3	500.7721437	1.144168025	0.1564197	7.3147296	2.58E-13	9.81E-11
AL928657.1	37.63991506	-0.00264273	0.3452788	-0.007654	0.993893	0.998691
AL935115.1	0	NA	NA	NA	NA	NA
FP102921.1	0	NA	NA	NA	NA	NA
AL772382.1	6.418700987	-0.838255954	0.7512643	-1.115794	0.26451	NA
FP102170.1	10.70677989	-0.761179789	0.5970684	-1.274862	0.202358	NA
FP015862.1	0	NA	NA	NA	NA	NA
si:dkey-7i4.23	0	NA	NA	NA	NA	NA
CR854927.3	0	NA	NA	NA	NA	NA
BX957346.2	0	NA	NA	NA	NA	NA
BX005242.1	0.698456806	0.09172817	2.5265115	0.0363063	0.971038	NA
CU695222.1	0.323844819	-1.825095364	3.274639	-0.557342	0.577293	NA
CR318658.1	0	NA	NA	NA	NA	NA
si:dkey-77f5.11	63.61866519	0.082034029	0.3148825	0.2605227	0.794461	0.977937
CR848683.1	0.347458735	-1.908042286	3.4571486	-0.551912	0.581009	NA
CR545465.2	0	NA	NA	NA	NA	NA
CR753876.1	138.908427	0.297528352	0.1761991	1.6885913	0.091298	0.578443
si:ch211-191c10.3	0.805694888	-0.600710451	2.2372785	-0.268501	0.788314	NA
BX465205.1	0.158915748	0.967652056	4.0804729	0.2371421	0.812547	NA
BX664705.1	0.182570949	0.967652056	4.0804729	0.2371421	0.812547	NA
CR848742.1	0.325573202	0.005883621	3.3172783	0.0017736	0.998585	NA
FP565432.1	1.000134101	0.001091541	2.0514706	0.0005321	0.999575	NA
CU459174.1	0.698456806	0.09172817	2.5265115	0.0363063	0.971038	NA
BX005254.3	4.377860824	-0.679793102	0.9411061	-0.722334	0.470089	NA
BX324227.1	0.340386822	-1.882368278	3.2159502	-0.585323	0.558331	NA
si:dkey-7i4.11	5.698455258	0.176792532	0.8303935	0.2129021	0.831403	NA
CR954963.1	1.851060989	-0.261701952	1.428439	-0.183208	0.854635	NA
GDPGP1	35.2514642	0.328844149	0.3356308	0.9797795	0.327195	0.872658
CR944667.1	0	NA	NA	NA	NA	NA
si:ch211-207n23.2	229.3278104	0.36190545	0.1670423	2.1665501	0.030269	0.33626

BX294100.1	61.2320691	0.281940941	0.2526217	1.11606	0.264396	0.830245
BX664738.1	0	NA	NA	NA	NA	NA
si:ch211-121j5.4	174.2113002	0.04959393	0.1649464	0.300667	0.763668	0.975374
CU861891.1	7.215895018	0.380178559	0.7091973	0.5360688	0.591911	NA
CR385035.1	8.332459644	0.716824527	0.6790222	1.0556717	0.291118	NA
CR848746.1	4.088525574	0.104978333	0.9438483	0.1112237	0.911439	NA
BX936386.1	0.491320158	0.8528132	2.8656354	0.2976	0.766008	NA
BX005484.1	2.641945512	1.579544879	1.2360052	1.2779436	0.201269	NA
CR749752.1	0.166657454	-0.955901296	4.0804729	-0.234262	0.814781	NA
si:ch73-330k17.3	4.823170965	-0.155586685	0.9537804	-0.163126	0.870419	NA
pcdh1a4	23.30715512	-0.000398115	0.3983212	-0.000999	0.999203	0.999704
CR381579.1	0	NA	NA	NA	NA	NA
CR388129.1	0	NA	NA	NA	NA	NA
BX546459.1	0	NA	NA	NA	NA	NA
CR383662.6	6.328471778	-0.95993161	0.7831348	-1.225755	0.220291	NA
BX927308.2	29.15252855	0.151982884	0.3827584	0.3970727	0.691314	0.967305
CR538726.1	12.85981435	-0.228152727	0.5461806	-0.417724	0.676149	0.965257
si:dkey-7i4.21	0	NA	NA	NA	NA	NA
defbl3	10.7512387	-0.523162426	0.6218625	-0.841283	0.400189	NA
CR848757.1	206.7360903	0.148221228	0.1422189	1.0422051	0.297317	0.853945
CU570976.1	2.287652314	0.433314905	1.274729	0.3399271	0.733911	NA
CU695073.1	0	NA	NA	NA	NA	NA
CR847799.1	0	NA	NA	NA	NA	NA
si:ch211-153l6.6	170.5089672	0.00995478	0.1689983	0.0589046	0.953028	0.994154
CU929037.2	0	NA	NA	NA	NA	NA
FP101861.1	22.13867708	0.871756714	0.4113843	2.1190814	0.034084	0.359261
BX640398.1	0.663603133	-1.440371613	2.4108786	-0.597447	0.550209	NA
CU861453.1	90.28097114	-0.055027115	0.2120834	-0.25946	0.795281	0.978466
si:dkey-28n18.9	145.8998216	-1.005062101	0.1778371	-5.651587	1.59E-08	2.62E-06
FP236850.1	0	NA	NA	NA	NA	NA
BX649398.1	0.992082665	-2.218637874	2.2726308	-0.976242	0.328945	NA
CR847531.1	1678.300486	0.150663881	0.0925042	1.6287246	0.103371	0.609625
CR392042.1	0.157187365	-0.955901296	4.0804729	-0.234262	0.814781	NA
BX120006.2	0	NA	NA	NA	NA	NA
si:ch211-176l24.4	48.11927932	0.263051157	0.297465	0.8843095	0.376529	0.89493
BX000524.1	0	NA	NA	NA	NA	NA
ulk2	1098.966807	0.100671299	0.0865518	1.1631335	0.244775	0.814435
BX324206.1	0	NA	NA	NA	NA	NA
CR759882.1	0.522329263	0.999930388	2.8066578	0.3562709	0.721638	NA
alkbh3	367.2624903	-0.048002802	0.1511807	-0.317519	0.750849	0.97419
cox8b	324.2567614	-1.011557379	0.1397652	-7.237546	4.57E-13	1.70E-10
CR381647.1	127.3531743	0.101742699	0.1813625	0.5609909	0.574804	0.950037
si:dkey-24p1.6	0	NA	NA	NA	NA	NA
AL808121.1	0	NA	NA	NA	NA	NA
CR388029.1	0.809151653	1.852283317	2.2398915	0.8269523	0.408264	NA
si:dkey-172k15.3	71.38516062	-0.062840495	0.2373583	-0.264749	0.791202	0.977461
BX957355.1	0.63196552	0.01215153	2.613686	0.0046492	0.99629	NA
si:dkey-229e3.2	30.0947167	-0.240998071	0.3495176	-0.689516	0.490498	0.931926
CT027582.1	0.847620474	0.550669627	2.2003584	0.2502636	0.802383	NA
BX005079.1	0.158915748	0.967652056	4.0804729	0.2371421	0.812547	NA
AL954387.1	1.008546595	2.235815189	2.0974266	1.0659802	0.286433	NA
CR854827.1	0	NA	NA	NA	NA	NA
CR925728.2	0	NA	NA	NA	NA	NA
prr33	979.2280034	0.25524596	0.1127607	2.2636072	0.023598	0.296275

si:dkey-79d12.6	4.671879979	0.428092421	0.8717764	0.4910576	0.623386	NA
CR847887.1	10.22684392	-0.519986371	0.6133072	-0.84784	0.396527	NA
CR854827.2	0	NA	NA	NA	NA	NA
si:dkey-234i14.12	0.490502273	-2.421143305	2.7891168	-0.868068	0.385357	NA
si:ch211-226h8.15	1.135704377	3.642164013	1.9627362	1.8556564	0.063503	NA
ugt1b2	56.533812	-0.406880855	0.2731481	-1.489598	0.13633	0.673106
AL929435.1	0	NA	NA	NA	NA	NA
CT737162.2	29.55013545	-0.532467984	0.372364	-1.429966	0.152727	0.701776
si:ch73-147o17.1	306.8790068	0.059432057	0.130589	0.4551076	0.649032	0.960708
BX005012.4	0.674801605	0.02322667	2.4147609	0.0096186	0.992326	NA
hbba1	0	NA	NA	NA	NA	NA
si:ch73-31d8.2	48.88567644	-0.095643941	0.2783608	-0.343597	0.731149	0.971557
BX323076.1	2.198752646	0.705381762	1.5165421	0.4651251	0.641842	NA
BX120016.1	0.158795395	0.967652056	4.0804729	0.2371421	0.812547	NA
CR457447.1	0	NA	NA	NA	NA	NA
zgc:174263	382.0301075	-0.159591978	0.1172092	-1.3616	0.173324	0.732435
soga3a	0	NA	NA	NA	NA	NA
BX322555.3	115.7573208	0.17167277	0.2036489	0.8429842	0.399237	0.900683
si:dkey-117a8.2	0	NA	NA	NA	NA	NA
BX537104.1	0.48983248	-0.887840757	2.6934859	-0.329625	0.741683	NA
si:dkey-42l23.9	0.855602886	-0.591621869	2.0951335	-0.282379	0.777653	NA
CR354429.1	0	NA	NA	NA	NA	NA
yeats2	121.4186558	0.023571357	0.1858863	0.1268052	0.899095	0.990121
si:dkey-77f5.13	65.57354357	-0.155449213	0.257007	-0.604844	0.545282	0.945526
si:ch211-270g19.5	66.3508935	0.201872892	0.2457055	0.8216052	0.411302	0.903475
BX908753.1	6.174849227	-0.091928317	0.7814145	-0.117643	0.90635	NA
si:dkey-4c15.16	47.8547376	0.459503638	0.2997926	1.5327383	0.12534	0.654648
AL808129.1	270.2043669	0.203377523	0.1285345	1.5822802	0.113586	0.631853
CR925802.1	0	NA	NA	NA	NA	NA
si:ch211-40k21.5	211.495429	-0.178396867	0.145199	-1.228637	0.219208	0.785734
CR847898.2	23.23554107	0.307403253	0.4001385	0.7682422	0.442343	0.916336
AL954322.3	4.880236855	0.748213537	0.8935654	0.837335	0.402404	NA
si:ch211-266k8.6	1.456841292	-0.346576717	1.5569079	-0.222606	0.823842	NA
CU929145.2	26.44237416	0.151971181	0.380224	0.3996886	0.689386	0.966763
si:ch211-222n22.1	1.870901621	0.845910272	1.4518162	0.5826566	0.560125	NA
CR405687.1	1.35525647	1.617437907	1.7202882	0.9402134	0.347108	NA
CU571382.2	0	NA	NA	NA	NA	NA
CR848759.1	1.607627531	1.245488276	1.753893	0.7101278	0.477625	NA
CU929145.3	0.476626891	2.395187592	2.815932	0.8505843	0.395	NA
si:ch211-147g22.4	24.97718098	-0.264286389	0.4137771	-0.638717	0.523007	0.939336
CR381689.1	1.551702801	-0.222354593	1.6346833	-0.136023	0.891803	NA
CR352232.1	0.173729368	-0.955901296	4.0804729	-0.234262	0.814781	NA
CU856222.1	770.8434557	0.066600528	0.1016647	0.6550996	0.512404	0.936253
zgc:174574	110.0867051	-0.141616921	0.1927341	-0.734779	0.462474	0.922698
BX119902.3	0.63196552	0.01215153	2.613686	0.0046492	0.99629	NA
si:ch211-261d7.3	498.0506785	-0.078279751	0.115072	-0.680268	0.496335	0.932415
ANPEP	183.7631319	-0.730149974	0.1517108	-4.812775	1.49E-06	0.000139
FP102892.1	0	NA	NA	NA	NA	NA
BX323076.2	11.40698677	0.898418085	0.574336	1.5642725	0.117754	0.641446
BX629344.1	113.6882938	0.042919251	0.1878508	0.2284752	0.819277	0.981594
CU855917.1	0.680773643	-2.882718692	2.4687055	-1.167705	0.242926	NA
slc26a6	16.83594978	0.335163754	0.4716124	0.7106763	0.477285	0.926857
CU855758.1	0.513487682	-0.887846295	2.6557956	-0.334305	0.738149	NA
BX571762.1	0.158795395	0.967652056	4.0804729	0.2371421	0.812547	NA

FP016018.3	0	NA	NA	NA	NA	NA
CR792422.2	4.038546139	-0.707933183	0.9582437	-0.738782	0.460039	NA
flrt2	244.4152706	-0.019897686	0.1365865	-0.145678	0.884175	0.988784
BX530017.1	0	NA	NA	NA	NA	NA
CR925755.1	0	NA	NA	NA	NA	NA
tbpl2	0.158795395	0.967652056	4.0804729	0.2371421	0.812547	NA
BX000358.1	0	NA	NA	NA	NA	NA
si:dkey-238d18.10	0.325573202	0.005883621	3.3172783	0.0017736	0.998585	NA
BX901883.1	0	NA	NA	NA	NA	NA
zgc:193812	47.97401404	-0.317130423	0.299112	-1.06024	0.289036	0.845979
CT573316.1	0	NA	NA	NA	NA	NA
CR812470.2	2.136154996	-0.279267899	1.4098922	-0.198077	0.842984	NA
si:zfos-1714f5.3	2.827899952	-1.709122766	1.2214816	-1.399221	0.161747	NA
BX927078.1	0	NA	NA	NA	NA	NA
CT027805.1	0.966459371	0.961723876	2.0054341	0.479559	0.631541	NA
si:dkey-276j7.3	7.892235628	0.101739213	0.6834315	0.1488653	0.88166	NA
BX649242.1	1.197089583	0.415558675	1.7998043	0.230891	0.817399	NA
CR848791.3	0.339758314	0.005883863	3.2682461	0.0018003	0.998564	NA
BX005183.1	0	NA	NA	NA	NA	NA
si:dkey-81l17.6	187.350222	-0.23765477	0.1569426	-1.514279	0.129955	0.662595
si:ch211-14i3.2	19.12315223	-0.302570172	0.4370063	-0.69237	0.488705	0.931926
BX546473.1	0	NA	NA	NA	NA	NA
BX957270.1	0.182570949	0.967652056	4.0804729	0.2371421	0.812547	NA
BX005060.1	0	NA	NA	NA	NA	NA
CR759826.1	0	NA	NA	NA	NA	NA
si:dkey-170g13.2	0.158915748	0.967652056	4.0804729	0.2371421	0.812547	NA
CU571161.1	0	NA	NA	NA	NA	NA
CT573342.1	5.519902796	0.46241121	0.8095749	0.5711778	0.567879	NA
CT573316.2	0.173729368	-0.955901296	4.0804729	-0.234262	0.814781	NA
CR759841.1	3.041937827	0.664810714	1.0965847	0.6062557	0.544345	NA
CT583687.1	0.340386822	-1.882368278	3.2159502	-0.585323	0.558331	NA
BX000434.1	0	NA	NA	NA	NA	NA
slc9a1b	48.88911376	0.505743746	0.3471873	1.4566886	0.145202	0.690034
si:ch211-33e4.2	5.718702763	-0.663820507	0.8233197	-0.806273	0.420085	NA
CR847994.1	16.5742137	0.014013269	0.4644058	0.0301746	0.975928	0.996762
AL845550.1	0.638219549	-2.807461932	2.539214	-1.105642	0.268881	NA
AL772300.1	10.98429696	-1.420828401	0.6039759	-2.352459	0.01865	NA
CR387932.1	0.347458735	-1.908042286	3.4571486	-0.551912	0.581009	NA
AL935186.2	0.341366344	1.891903201	3.2128156	0.5888614	0.555954	NA
ten1	51.14878385	0.126483738	0.275052	0.459854	0.645621	0.96029
CR854965.2	0.182570949	0.967652056	4.0804729	0.2371421	0.812547	NA
BX649628.1	0	NA	NA	NA	NA	NA
si:ch211-147g22.7	8.044396228	-0.781054512	0.6792357	-1.149902	0.250184	NA
BX324164.3	43.20202888	-0.167326545	0.3019965	-0.554068	0.579532	0.951257
CT027551.1	0	NA	NA	NA	NA	NA
BX901883.2	121.5870552	0.069345145	0.1936111	0.3581671	0.720218	0.969318
CU638693.3	0.173729368	-0.955901296	4.0804729	-0.234262	0.814781	NA
BX279523.3	1.960192347	2.322231426	1.5804913	1.4693098	0.141749	NA
CR790363.1	0.690594747	1.536595024	2.3851901	0.6442233	0.519431	NA
CU655820.1	6.572316727	0.049818768	0.8183131	0.0608798	0.951455	NA
CU896541.1	16.29187582	-0.192060946	0.483512	-0.397221	0.691205	0.967295
si:dkey-244a7.1	53.19323965	0.190392108	0.2635978	0.7222826	0.470121	0.924787
FP017274.1	133.7321142	0.281295834	0.1792218	1.5695402	0.116522	0.639148
mcf2b	35.78604007	0.090673808	0.3336363	0.2717744	0.785795	0.97627

si:ch211-139d20.3	27.77300961	0.05739004	0.3606299	0.1591383	0.87356	0.987858
ftr90	16.63096384	0.114846659	0.5123931	0.2241378	0.82265	0.982189
si:dkey-108k21.7	68.62442564	0.259281764	0.2443764	1.0609934	0.288693	0.845979
BX248418.2	0	NA	NA	NA	NA	NA
CR790366.3	0	NA	NA	NA	NA	NA
CR387933.1	0.491320158	0.8528132	2.8656354	0.2976	0.766008	NA
CR936416.1	0.648748228	-0.034664259	2.4451882	-0.014177	0.988689	NA
CR812472.2	0	NA	NA	NA	NA	NA
CR382363.1	1.217246733	-1.273475927	1.9578775	-0.650437	0.51541	NA
BX005183.2	0.341486697	1.892317624	3.2124068	0.5890654	0.555817	NA
BX465853.1	0	NA	NA	NA	NA	NA
AL929182.1	0	NA	NA	NA	NA	NA
si:ch211-15d5.12	107.0048005	-0.204967644	0.2018565	-1.015413	0.309909	0.861992
CR854907.1	3.326134883	0.022489837	1.0222522	0.0220003	0.982448	NA
FP101990.1	0	NA	NA	NA	NA	NA
BX571853.1	4.39326942	0.68580426	0.9365575	0.7322607	0.464009	NA
BX530407.4	5.104964052	1.76863102	0.9437094	1.8741268	0.060913	NA
si:dkey-33c14.7	5.016822446	0.200051291	0.8525089	0.2346618	0.814471	NA
BX465208.1	0	NA	NA	NA	NA	NA
BX897720.1	0	NA	NA	NA	NA	NA
si:dkey-77f5.6	52.5545694	-0.074146764	0.2683928	-0.276262	0.782347	0.97627
BX005459.1	0	NA	NA	NA	NA	NA
BX539313.1	4.686342584	-0.419930032	0.8711711	-0.482029	0.629785	NA
ugt5a4	0.807663976	0.546685639	2.2534268	0.2426019	0.808314	NA
si:ch73-288o11.4	1.151428495	1.30584825	1.8815101	0.6940426	0.487655	NA
FP340271.1	0.157187365	-0.955901296	4.0804729	-0.234262	0.814781	NA
BX470254.1	0.157187365	-0.955901296	4.0804729	-0.234262	0.814781	NA
BX511308.1	1.686533266	-1.934326135	1.6695123	-1.158617	0.246612	NA
si:dkey-273g18.1	5.796305574	0.741436564	0.8000319	0.9267587	0.354052	NA
si:dkey-81e3.2	20.70724128	0.363199873	0.4292852	0.8460573	0.397521	0.899672
BX547992.2	0	NA	NA	NA	NA	NA
BX548045.1	0	NA	NA	NA	NA	NA
CU571319.1	1.622870238	0.01605098	1.4877883	0.0107885	0.991392	NA
AL928870.1	0.157187365	-0.955901296	4.0804729	-0.234262	0.814781	NA
si:ch211-106e7.2	26.35385037	-0.337552933	0.3951181	-0.854309	0.392934	0.897836
lrrc53	1.812471815	0.241486745	1.4258259	0.1693662	0.865509	NA
si:ch73-306e8.2	10.98411511	0.562515779	0.5898357	0.9536821	0.340245	NA
CR847793.1	1.340710299	1.573968029	1.8034464	0.8727556	0.382796	NA
si:ch211-63b16.3	7.180081778	0.791589682	0.7564258	1.0464869	0.295336	NA
CR786571.4	0.31437473	-1.788572922	3.5930657	-0.497785	0.618636	NA
si:ch73-250a16.5	76.29955184	-0.028251456	0.2315903	-0.121989	0.902908	0.990121
si:dkeyp-50b9.1	5.702888291	0.175323886	0.8963739	0.1955924	0.844929	NA
CU929360.1	9.680643111	0.833305129	0.6353457	1.3115775	0.189663	NA
CU638870.1	38.01053975	-0.237476022	0.3073743	-0.772596	0.439762	0.916082
BX569790.2	0	NA	NA	NA	NA	NA
BX470242.2	0	NA	NA	NA	NA	NA
BX005461.3	2.675108832	0.038232043	1.152332	0.033178	0.973533	NA
CR392003.1	4.685875714	-0.181728785	0.8657474	-0.20991	0.833738	NA
CT027718.1	0.633934609	1.436045836	2.6103598	0.5501333	0.582228	NA
BX649398.2	4.91104545	0.790386396	0.8756804	0.9025969	0.36674	NA
CU928222.1	0	NA	NA	NA	NA	NA
AL928908.1	0	NA	NA	NA	NA	NA
BX510360.1	0	NA	NA	NA	NA	NA
CR788230.2	0.31437473	-1.788572922	3.5930657	-0.497785	0.618636	NA

CU855930.1	24.39740769	0.078879168	0.4280408	0.1842795	0.853794	0.985174
si:dkey-77f5.15	6.289842561	-0.774742143	0.7514455	-1.031002	0.30254	NA
si:dkey-195m11.8	268.149357	0.091491249	0.1324561	0.6907287	0.489736	0.931926
si:ch211-66i15.5	0	NA	NA	NA	NA	NA
BX890600.1	0.157187365	-0.955901296	4.0804729	-0.234262	0.814781	NA
CR925728.3	1.18140675	2.512918391	1.9105304	1.3152988	0.188409	NA
CR381647.2	0.158795395	0.967652056	4.0804729	0.2371421	0.812547	NA
chmp6a	9.394222941	-0.434401994	0.6673994	-0.650888	0.515119	NA
CU929070.1	92.21993194	0.123469067	0.2055253	0.6007487	0.548007	0.945768
si:ch211-225b7.5	34.06108377	-0.235810461	0.3281564	-0.718592	0.472393	0.924838
BX539313.2	3.643811529	-1.80152991	1.1058623	-1.629073	0.103298	NA
CR931782.1	0	NA	NA	NA	NA	NA
coa3b	86.30464274	0.04249941	0.2457703	0.1729233	0.862712	0.985841
BX119963.2	3.955911939	-1.876942077	1.0796772	-1.738429	0.082135	NA
si:dkey-208m12.3	0	NA	NA	NA	NA	NA
si:dkey-125e8.3	12.56134134	-0.4941014	0.5768	-0.856625	0.391652	0.897688
CU929339.2	0	NA	NA	NA	NA	NA
BX957326.3	1.973785738	0.45896702	1.3297967	0.3451407	0.729989	NA
CR788249.2	0.31437473	-1.788572922	3.5930657	-0.497785	0.618636	NA
trmt61b	150.2828484	-0.085547126	0.1734383	-0.493242	0.621841	0.957354
CU207343.2	0.474778155	0.852397662	2.898154	0.2941175	0.768668	NA
CU693502.1	221.3101446	-0.102204529	0.1427623	-0.715907	0.474049	0.924993
si:ch73-1f23.1	2.144686848	-1.208846332	1.3383997	-0.903203	0.366418	NA
BX927362.1	2.141422715	1.160869486	1.3025367	0.8912375	0.372802	NA
FP015862.2	0.831827332	-0.648936467	2.1146571	-0.306876	0.758938	NA
BX004999.2	0	NA	NA	NA	NA	NA
zgc:113340	69.95923107	0.119198891	0.2375988	0.5016814	0.615892	0.95688
CR396585.1	0.323844819	-1.825095364	3.274639	-0.557342	0.577293	NA
si:dkey-4c2.11	9.977864257	-0.571357388	0.6132901	-0.931627	0.351529	NA
qkia	2881.289586	-0.03699402	0.070206	-0.526935	0.598238	0.955488
si:dkey-263j23.1	4.469711363	-0.11796696	0.9177674	-0.128537	0.897724	NA
si:dkey-77f5.11	31.34882788	0.51313601	0.3710536	1.382916	0.166691	0.723339
si:ch211-213d14.2	152.9345436	-0.08004157	0.1698433	-0.471267	0.63745	0.958872
cfap99	13.52303194	-0.207871856	0.5165143	-0.402451	0.687352	0.966376
si:ch211-175f11.5	0.822518881	-0.527767586	2.1171028	-0.249288	0.803138	NA
si:ch73-390p7.2	118.448011	0.104316082	0.1941628	0.5372609	0.591087	0.954193
AL928908.2	0	NA	NA	NA	NA	NA
ugt1b1	227.950371	0.110168217	0.1568173	0.7025261	0.482351	0.928726
AL772314.1	424.2219685	0.040637168	0.148904	0.2729085	0.784924	0.97627
si:dkey-148h10.5	17.52369308	-0.575696083	0.4741307	-1.214214	0.224666	0.791954
CR382327.2	24.8271213	0.278309056	0.3917065	0.7105041	0.477392	0.926857
CU467639.1	0.682973393	2.892694129	2.4661364	1.172966	0.240809	NA
si:dkey-6e2.2	309.3147879	-0.047234418	0.1319895	-0.357865	0.720444	0.969318
BX511253.1	0.500282092	2.45023829	2.5970012	0.9434876	0.345432	NA
CR450813.2	0	NA	NA	NA	NA	NA
BX511311.1	0	NA	NA	NA	NA	NA
si:dkey-111k8.3	166.2946529	-0.190983902	0.1581818	-1.207369	0.22729	0.794617
CU694807.1	0	NA	NA	NA	NA	NA
si:ch73-288o11.5	126.2594875	-0.057901207	0.1859325	-0.31141	0.755489	0.975318
CR626907.3	234.4595434	0.161769066	0.3884104	0.41649	0.677051	0.965257
DNAL1 (1 of many)	0	NA	NA	NA	NA	NA
BX571780.1	1.11645447	0.405375708	1.833693	0.2210707	0.825037	NA
BX324164.4	30.16960328	-0.611569131	0.3634936	-1.682476	0.092477	0.579378
CR848836.1	0	NA	NA	NA	NA	NA

si:dkey-245f22.3	19.12723936	0.766112449	0.4418851	1.7337367	0.082965	0.555552
BX890629.1	2.233132691	-0.235299971	1.4332413	-0.164173	0.869595	NA
BX649529.1	84.25139889	0.01649667	0.2221376	0.0742633	0.940801	0.992857
CT573383.1	62.19163897	0.215808073	0.264438	0.8161007	0.414443	0.906237
CR854989.1	3.658151491	0.292741938	0.9784745	0.299182	0.764801	NA
CR751227.1	1.626327003	1.212512227	1.5424246	0.7861079	0.431804	NA
BX323016.1	2.83873836	0.138056734	1.1655859	0.1184441	0.905716	NA
BX537110.1	24.72615587	0.611474063	0.3997772	1.5295369	0.126131	0.654806
AL935300.2	0.960634429	2.169987751	2.2906136	0.9473391	0.343466	NA
si:dkey-111k8.5	53.22991432	0.173154407	0.2659985	0.6509602	0.515072	0.937646
si:ch73-364h19.1	51.48557676	0.108049174	0.2779881	0.3886827	0.697511	0.9679
BX005129.2	3.284247079	-0.313094724	1.0504113	-0.298069	0.765651	NA
AL928724.1	0.31437473	-1.788572922	3.5930657	-0.497785	0.618636	NA
CU466287.2	13.11159212	-0.215265505	0.5445696	-0.395295	0.692625	0.96736
ms4a17a.5	6.111320098	-0.073296755	0.7543206	-0.097169	0.922592	NA
si:dkey-7j14.5	1239.217744	0.042076589	0.0895978	0.4696161	0.638629	0.959628
CR847983.1	0	NA	NA	NA	NA	NA
xkr5b	93.70253278	0.070833517	0.2069149	0.3423316	0.732101	0.971665
pimr26	0	NA	NA	NA	NA	NA
si:ch73-93k15.5	0	NA	NA	NA	NA	NA
BX571880.1	229.9505776	0.149697633	0.1852455	0.808104	0.419031	0.906406
si:ch211-252f13.5	40.82664262	0.474739684	0.3277137	1.4486418	0.147438	0.694625
CR848844.1	28.81556003	0.251471617	0.36599	0.6870996	0.49202	0.931926
wwp1	1204.204945	-0.032005617	0.0854015	-0.374766	0.707834	0.968664
CR627496.1	0.981942784	-1.029083186	1.9080416	-0.53934	0.589652	NA
si:ch211-39f2.3	10.42746954	-0.880917124	0.5993614	-1.469759	0.141627	NA
pimr62	0	NA	NA	NA	NA	NA
CU137680.1	0.157187365	-0.955901296	4.0804729	-0.234262	0.814781	NA
CU633855.1	0	NA	NA	NA	NA	NA
cbx6b	34.23560852	0.058896199	0.3338099	0.1764363	0.859951	0.985773
si:ch211-40k21.9	61.33725214	-0.554340549	0.2547479	-2.176036	0.029553	0.332786
AL954861.1	0.173729368	-0.955901296	4.0804729	-0.234262	0.814781	NA
CR450727.1	82.74240847	0.241046591	0.2340864	1.0297336	0.303135	0.85729
CT573103.1	0	NA	NA	NA	NA	NA
si:dkey-7i4.15	44.26999235	0.389340673	0.2994472	1.300198	0.193533	0.757219
AL772362.1	0.81823394	3.165585784	2.3439761	1.3505196	0.176849	NA
BX908750.1	41.17425501	0.246213923	0.3027558	0.8132427	0.416079	0.906237
cyp8b2	5.987299308	0.019910089	0.7638936	0.026064	0.979206	NA
BX511311.2	0.157187365	-0.955901296	4.0804729	-0.234262	0.814781	NA
cyp8b3	15.60974665	-0.065063711	0.4763226	-0.136596	0.89135	0.989291
BX511311.3	117.3057233	0.178817055	0.2158699	0.8283558	0.407469	0.901305
CR847503.2	0	NA	NA	NA	NA	NA
BX005083.1	0.330916733	-1.849579229	3.2489867	-0.569279	0.569167	NA
si:ch211-151m7.6	0.682853041	2.891318346	2.3286279	1.2416403	0.214369	NA
si:ch73-190f9.4	0.340386822	-1.882368278	3.2159502	-0.585323	0.558331	NA
BX072576.3	0	NA	NA	NA	NA	NA
si:ch211-256e16.10	0	NA	NA	NA	NA	NA
si:dkey-208c12.2	18.78687471	0.715100547	0.4464967	1.6015807	0.109248	0.623132
CR391977.1	0	NA	NA	NA	NA	NA
FP102921.2	0.847620474	0.550669627	2.2003584	0.2502636	0.802383	NA
si:dkey-114l24.2	11.82398567	1.674951796	0.5839487	2.8683203	0.004127	0.089082
ptprt	23.70192028	0.124673582	0.4038711	0.3086965	0.757552	0.975374
si:ch211-107e6.5	4.471949143	-0.081400179	0.8874605	-0.091723	0.926918	NA
CR847898.3	5.051188451	-1.291838366	0.8871025	-1.456245	0.145325	NA

CU914602.1	17.08399337	0.473699881	0.4631911	1.0226879	0.306455	0.860027
zgc:92912	86.41794598	0.399373115	0.2350906	1.6988049	0.089356	0.572876
BX294100.2	0.476506538	2.394892832	2.8161716	0.8504073	0.395099	NA
CT573048.1	0.647019846	-1.455099716	2.4289208	-0.599073	0.549125	NA
BX663519.1	6.591603422	-0.126700525	0.7352199	-0.17233	0.863178	NA
CR376740.2	0.863344592	-1.91853867	2.2027225	-0.870985	0.383762	NA
arfgap3	236.2125636	-0.209036898	0.1445967	-1.445654	0.148274	0.696298
si:dkey-238d18.6	0	NA	NA	NA	NA	NA
CR548634.2	1.951268442	-0.471982518	1.416302	-0.33325	0.738946	NA
BX664750.3	0	NA	NA	NA	NA	NA
si:dkey-175d9.1	0.173729368	-0.955901296	4.0804729	-0.234262	0.814781	NA
proser3	80.45932118	-0.402543955	0.2179145	-1.847256	0.06471	0.497355
si:dkey-40c23.1	5.101474207	-0.853404779	0.8763694	-0.973796	0.330158	NA
BX548024.1	0.514116189	-2.475533565	2.7461255	-0.901464	0.367342	NA
FP236741.1	5.306123322	0.375251937	0.8149602	0.4604543	0.64519	NA
BX682556.1	2.982881774	0.648103983	1.1218246	0.5777231	0.563451	NA
tac3b	60.05258724	0.07257642	0.2482995	0.2922938	0.770062	0.975687
BX088688.3	0.705528719	0.06743937	2.3808454	0.0283258	0.977402	NA
CR925804.1	1.667503729	3.073229702	1.7154383	1.7915128	0.073211	NA
si:ch211-66h3.4	0.316103113	0.005883447	3.3519121	0.0017553	0.9986	NA
CT868708.1	0.831198824	0.593716313	2.0278563	0.2927803	0.76969	NA
BX072559.1	1.164005578	0.473149093	1.8147078	0.2607302	0.794301	NA
CU571382.3	0	NA	NA	NA	NA	NA
BX465205.2	0	NA	NA	NA	NA	NA
CU137681.1	7.045272643	0.337401201	0.7266857	0.4643014	0.642432	NA
tmem82	71.79713447	-0.169528191	0.2358122	-0.718912	0.472195	0.924838
BX324166.1	0	NA	NA	NA	NA	NA
CU929312.1	1.519085666	-0.960820623	1.561202	-0.615436	0.538267	NA
BX005183.3	106.2204441	-0.029756887	0.2132781	-0.139522	0.889038	0.989291
CU896691.2	0.955930692	-0.991824331	2.0140721	-0.492447	0.622403	NA
si:dkey-24l11.2	0	NA	NA	NA	NA	NA
igfn1.3	1849.118466	0.684935461	0.0861049	7.9546665	1.80E-15	9.60E-13
si:ch211-108d22.2	117.0079712	0.318981691	0.229949	1.3871848	0.165385	0.721223
BX908796.1	0	NA	NA	NA	NA	NA
si:ch211-178n15.1	124.8612983	-0.059397147	0.2064856	-0.287658	0.773609	0.975687
FP102073.1	0.166657454	-0.955901296	4.0804729	-0.234262	0.814781	NA
BX571794.1	0.173729368	-0.955901296	4.0804729	-0.234262	0.814781	NA
si:ch211-210b2.1	170.3112529	-0.178401773	0.1805636	-0.988027	0.323139	0.870607
BX936342.1	0.341366344	1.891903201	3.2128156	0.5888614	0.555954	NA
si:dkey-42p14.6	0	NA	NA	NA	NA	NA
CU137681.2	0	NA	NA	NA	NA	NA
BX323450.1	0	NA	NA	NA	NA	NA
CR762400.1	0.837170863	-1.914744291	2.2413088	-0.854297	0.39294	NA
si:dkey-7i4.14	4.661580719	0.621544004	0.8764442	0.7091655	0.478222	NA
FP102794.2	0.182570949	0.967652056	4.0804729	0.2371421	0.812547	NA
si:ch211-282b22.1	166.6205777	0.197977429	0.177783	1.1135901	0.265455	0.831302
BX005101.1	0	NA	NA	NA	NA	NA
CU306817.1	4.140681396	-0.580134982	0.9250477	-0.627141	0.530567	NA
wu:fb18f06	516.0257822	-0.123252684	0.3886999	-0.31709	0.751176	0.97419
AL928908.3	8.127267753	1.080969928	0.7364091	1.4678932	0.142133	NA
si:ch211-246i5.5	253.2077275	-0.120891522	0.1344496	-0.899159	0.368568	0.888057
CR848661.1	0.166657454	-0.955901296	4.0804729	-0.234262	0.814781	NA
si:ch73-265h17.4	6.621521321	0.903554781	0.8599918	1.0506551	0.293417	NA
BX120006.3	0.325573202	0.005883621	3.3172783	0.0017736	0.998585	NA

CR589941.1	0.158795395	0.967652056	4.0804729	0.2371421	0.812547	NA
CU469568.3	0.166657454	-0.955901296	4.0804729	-0.234262	0.814781	NA
CU138509.1	2.08755984	-0.687114731	1.4598757	-0.470667	0.637879	NA
CU571091.1	0	NA	NA	NA	NA	NA
BX936393.1	2.456158504	0.590997614	1.296749	0.4557533	0.648567	NA
BX511021.2	6.006776622	0.357849769	0.8333441	0.4294142	0.667622	NA
si:dkey-35i13.1	1043.012251	0.057803802	0.0960238	0.6019734	0.547192	0.945526
FP243382.1	0	NA	NA	NA	NA	NA
atg2b	1.956333238	0.979788459	1.397679	0.7010111	0.483296	NA
tgm8	0.339758314	0.005883863	3.2682461	0.0018003	0.998564	NA
si:dkey-234i14.2	161.5812134	-0.203754263	0.1732602	-1.176001	0.239594	0.808091
si:dkey-23k10.3	13.59131788	-0.50396505	0.5597502	-0.900339	0.36794	0.887482
CR556709.1	0.332524763	0.005883742	3.292841	0.0017868	0.998574	NA
CR381579.2	0.31437473	-1.788572922	3.5930657	-0.497785	0.618636	NA
si:dkey-7i4.16	15.52073907	0.059145308	0.5073256	0.1165825	0.907191	0.990702
BX001030.2	0	NA	NA	NA	NA	NA
BX936406.2	0.158795395	0.967652056	4.0804729	0.2371421	0.812547	NA
trappc2	717.5011266	-0.054309982	0.1102939	-0.492412	0.622428	0.957354
BX511124.1	0	NA	NA	NA	NA	NA
tesmin	0.497574187	-2.437446738	2.6012773	-0.937019	0.348749	NA
BX324206.2	251.2353742	-0.016693373	0.1474868	-0.113186	0.909883	0.990702
si:ch211-235m3.10	97.61100882	0.139975718	0.2047979	0.6834823	0.494302	0.931926
CU076099.1	6.786828283	-0.807092876	0.7399194	-1.090785	0.275368	NA
BX569785.1	3.331127399	-1.220879481	1.059262	-1.152576	0.249085	NA
si:ch211-139l2.1	0.871516381	0.597595468	2.1830013	0.2737495	0.784277	NA
BX005048.1	2.825303351	-0.153247859	1.0990189	-0.139441	0.889102	NA
CR381579.3	0	NA	NA	NA	NA	NA
FP016235.1	1.371798472	1.585251294	1.7681373	0.8965657	0.369951	NA
CU633978.1	0.489712128	-0.887840729	2.693685	-0.329601	0.741702	NA
CU207301.2	0.476747244	2.393381959	3.0525537	0.7840589	0.433006	NA
CR450824.1	0.957659075	-0.005829811	1.9622935	-0.002971	0.99763	NA
ftr90	2.537720814	1.029316458	1.2979922	0.7930066	0.427774	NA
si:ch211-241f5.3	13.84922759	-0.348914221	0.5666871	-0.615709	0.538087	0.943336
si:ch211-244k5.1	0.678375468	-2.878459328	2.4926399	-1.154783	0.248179	NA
CR383683.1	0	NA	NA	NA	NA	NA
sptbn5	301.4082733	-0.133044559	0.128733	-1.033492	0.301374	0.856944
si:ch211-235i11.3	56.22873339	0.194131864	0.2708137	0.7168465	0.473469	0.924993
FP325112.1	1.213552517	0.460811839	1.8129902	0.2541723	0.799362	NA
CT573316.3	0	NA	NA	NA	NA	NA
si:ch73-265h17.5	0.31598276	0.005883445	3.3523626	0.001755	0.9986	NA
CT867948.1	0.666939546	1.487980453	2.2924711	0.6490727	0.516291	NA
BX629346.2	0	NA	NA	NA	NA	NA
pclaf	94.8660572	-0.087538199	0.209761	-0.417323	0.676442	0.965257
si:ch211-116o3.5	345.9632217	0.01337926	0.1190701	0.1123646	0.910534	0.990805
si:ch211-248a14.8	412.9098232	-0.810080242	0.1135077	-7.136784	9.55E-13	3.37E-10
CU137681.3	0.365141898	1.973577737	3.3927314	0.5817076	0.560764	NA
si:ch73-71c20.5	189.641317	-0.057835776	0.1598409	-0.361833	0.717477	0.96909
si:dkey-117a8.4	0.31598276	0.005883445	3.3523626	0.001755	0.9986	NA
si:dkey-23k10.2	5.820720922	0.08913485	0.7749794	0.1150158	0.908433	NA
AL954831.1	11.63417131	-2.156752745	0.6607951	-3.263875	0.001099	0.033254
si:ch1073-66l23.1	0.166657454	-0.955901296	4.0804729	-0.234262	0.814781	NA
ccdc36	0.325573202	0.005883621	3.3172783	0.0017736	0.998585	NA
birc6-as2	277.3386825	0.065289253	0.1307809	0.4992264	0.61762	0.957354
BX323807.2	0.182570949	0.967652056	4.0804729	0.2371421	0.812547	NA

CR786576.1	0.166657454	-0.955901296	4.0804729	-0.234262	0.814781	NA
BX957311.1	0.657349104	1.487939685	2.418178	0.6153144	0.538347	NA
CR848667.1	64.87118854	-0.238473278	0.2582381	-0.923463	0.355766	0.882675
BX927318.1	0	NA	NA	NA	NA	NA
FP017215.1	61.0240387	0.093037795	0.2626269	0.3542584	0.723145	0.970402
BX571794.2	0	NA	NA	NA	NA	NA
stard10	407.0578637	-0.111008579	0.1216448	-0.912563	0.361472	0.885719
CU469514.1	9.633229107	-0.217189841	0.6519728	-0.333127	0.739038	NA
BX005129.3	1.307705362	1.570322523	1.7311786	0.9070829	0.364363	NA
CU207301.3	55.05136116	-0.196518874	0.2690863	-0.730319	0.465195	0.923671
BX927282.3	0.323844819	-1.825095364	3.274639	-0.557342	0.577293	NA
BX936342.2	7.119768586	-0.51306066	0.7780991	-0.659377	0.509654	NA
si:dkey-96g2.1	81.11515261	0.224024542	0.239657	0.9347716	0.349906	0.880195
si:ch211-149e23.4	6.830865503	0.964924105	0.7301415	1.3215577	0.186315	NA
CR388132.1	0.182570949	0.967652056	4.0804729	0.2371421	0.812547	NA
CR352243.2	1.345005284	-3.868052513	1.8793794	-2.058154	0.039575	NA
CR936482.1	50.86453932	-0.054686354	0.2721382	-0.200951	0.840737	0.985092
CR932000.1	2.419057007	1.447667353	1.3204404	1.0963519	0.272925	NA
BX957346.3	0.650476611	1.436325931	2.5890223	0.5547754	0.579048	NA
CR628365.1	5.646950269	0.164583181	0.7969749	0.2065099	0.836393	NA
si:dkey-188h10.3	29.72911578	-0.105150101	0.3739276	-0.281204	0.778554	0.975942
AL929222.2	232.3066419	0.004785965	0.1459721	0.0327869	0.973845	0.996316
si:dkey-238d18.9	0	NA	NA	NA	NA	NA
elob	69.67235698	0.039713676	0.2552904	0.1555627	0.876378	0.987881
CT954240.1	11.69215885	0.509933207	0.5805413	0.8783754	0.37974	0.895192
si:dkey-276j7.2	6.240876056	0.146482846	0.7465726	0.1962071	0.844448	NA
CU861501.1	0	NA	NA	NA	NA	NA
BX323987.1	0	NA	NA	NA	NA	NA
si:rp71-77l1.1	194.8013077	-0.062351452	0.160018	-0.389653	0.696793	0.96736
CU464118.1	16.84385782	-0.343728319	0.46631	-0.737124	0.461047	0.922038
CR381621.1	0.496945679	-0.834564081	2.8549631	-0.29232	0.770042	NA
BX511195.1	0.157187365	-0.955901296	4.0804729	-0.234262	0.814781	NA
CR854827.3	6.167897666	-0.089508803	0.7668564	-0.116722	0.907081	NA
si:rp71-77l1.2	5.839687843	-0.092318545	0.8058766	-0.114557	0.908797	NA
slirp	464.7855245	0.057915276	0.1187364	0.4877635	0.625717	0.957354
CR749763.5	0	NA	NA	NA	NA	NA
CR626882.3	2.3196264	0.865094859	1.3065791	0.6621067	0.507903	NA
BX511010.1	0.158795395	0.967652056	4.0804729	0.2371421	0.812547	NA
CU929206.2	2.096119431	-0.243119099	1.3460585	-0.180616	0.856669	NA
CR628327.1	2.658446477	0.052794587	1.2320497	0.042851	0.96582	NA
BX005234.1	12.91137592	0.444713445	0.5303063	0.8385973	0.401695	0.90084
CU693494.2	7.571844566	-0.500165671	0.7311573	-0.684074	0.493928	NA
CT573392.1	7.193105622	-0.345332504	0.7160026	-0.482306	0.629588	NA
BX936364.1	1.824409104	2.133966183	1.6102851	1.3252101	0.185101	NA
BX537342.1	66.33248613	0.069146889	0.2473129	0.2795928	0.77979	0.976146
mhc1lia	4.867857203	-0.695495728	0.9608646	-0.723823	0.469174	NA
BX000438.1	13.92085297	0.925284113	0.513457	1.8020674	0.071535	0.522931
BX649397.1	0.339758314	0.005883863	3.2682461	0.0018003	0.998564	NA
si:ch211-167j9.4	163.9225224	0.016326865	0.1714781	0.0952125	0.924146	0.992676
BX571786.1	0.498674062	0.926637565	2.6784892	0.3459553	0.729376	NA
si:ch211-185a18.2	120.4820836	0.002172586	0.1949908	0.011142	0.99111	0.997679
CR391910.3	31.26832741	-0.168322895	0.3476509	-0.484172	0.628264	0.957354
CR387996.1	8.146601351	0.072717656	0.6561651	0.1108222	0.911757	NA
BX465205.3	0	NA	NA	NA	NA	NA

si:ch1073-80i24.3	1.03821512	0.108887395	2.0296353	0.0536487	0.957215	NA
BX005183.4	0	NA	NA	NA	NA	NA
CR385053.2	0	NA	NA	NA	NA	NA
BX649559.1	2.956467339	0.638032639	1.0953084	0.5825141	0.56022	NA
CU683879.2	1.817897916	-0.178150001	1.5333529	-0.116183	0.907507	NA
si:dkey-42l23.4	0.365141898	1.973577737	3.3927314	0.5817076	0.560764	NA
ccdc162	0.82223689	-0.634107248	2.2243871	-0.285071	0.77559	NA
CU467862.1	0.158795395	0.967652056	4.0804729	0.2371421	0.812547	NA
BX572077.2	0.173729368	-0.955901296	4.0804729	-0.234262	0.814781	NA
pon3.2	128.4781951	0.097044816	0.1810421	0.5360346	0.591935	0.954434
si:dkey-234i14.6	56.70970534	-0.332815008	0.2749378	-1.21051	0.226083	0.793638
FP325112.2	0.65919784	2.852228985	2.3514193	1.2129819	0.225137	NA
si:ch211-33e4.3	0.341486697	1.892317624	3.2124068	0.5890654	0.555817	NA
CR626944.1	26.42144536	-0.379804236	0.3714747	-1.022423	0.306581	0.860027
zgc:162952	101.0213417	-0.333126545	0.197469	-1.686982	0.091607	0.57849
AL845357.1	0	NA	NA	NA	NA	NA
tomm6	454.3891173	-0.096069955	0.1074166	-0.894368	0.371125	0.891368
CU929037.3	0.990234925	0.997648457	1.9937656	0.500384	0.616805	NA
zgc:194207	21.93246185	-0.329975865	0.4139957	-0.797051	0.425421	0.908944
CR788235.1	9.7309971	-0.269219972	0.6451779	-0.41728	0.676473	NA
BX470154.1	0.658957134	2.851427774	2.5143902	1.1340434	0.256776	NA
si:dkey-238d18.12	0.157187365	-0.955901296	4.0804729	-0.234262	0.814781	NA
si:ch211-275j6.5	149.6087404	-0.175761561	0.1686202	-1.042352	0.297249	0.853945
lypc	249.8912572	-0.325457356	0.1459595	-2.229778	0.025762	0.310555
CR376747.1	0.347458735	-1.908042286	3.4571486	-0.551912	0.581009	NA
BX530017.2	0.649418021	-1.460710349	2.4263694	-0.602015	0.547164	NA
si:dkey-77f5.4	87.18585644	-0.215387036	0.2146738	-1.003322	0.315705	0.866931
si:ch211-256e16.8	0.158915748	0.967652056	4.0804729	0.2371421	0.812547	NA
CR925777.1	1.84053231	-1.37010149	1.4806889	-0.925314	0.354803	NA
BX324126.1	0.158795395	0.967652056	4.0804729	0.2371421	0.812547	NA
si:ch73-144d13.5	37.44085726	0.227053115	0.3242909	0.7001526	0.483832	0.929461
BX927323.1	3.447718472	-0.16613126	1.0336897	-0.160717	0.872316	NA
CU928129.1	18.65434265	0.139546779	0.4738975	0.2944662	0.768402	0.975687
si:dkey-117a8.1	11.00960235	0.470630309	0.6157065	0.7643744	0.444644	NA
CT027582.2	0	NA	NA	NA	NA	NA
znf576.1	445.6729692	0.080945564	0.1110559	0.7288719	0.46608	0.923865
BX005129.4	2.43077492	0.191228689	1.3198632	0.1448852	0.884801	NA
CR933839.1	0	NA	NA	NA	NA	NA
CU464125.1	0	NA	NA	NA	NA	NA
smco4	174.7682576	0.059958828	0.1605675	0.3734181	0.708837	0.968664
BX465227.1	107.1683688	-0.006803363	0.2049323	-0.033198	0.973517	0.996315
si:dkey-239b22.2	827.8343215	0.07292412	0.095995	0.7596659	0.447454	0.917154
lrp1aa	2439.892972	0.078945423	0.0799365	0.987602	0.323348	0.870761
CR848825.1	0	NA	NA	NA	NA	NA
CU463231.1	1.81137194	-0.850924164	1.44611	-0.588423	0.556249	NA
CR382327.3	36.86108574	0.061235614	0.3480782	0.1759249	0.860353	0.985773
BX571712.1	0.69605863	0.094390757	2.3912825	0.0394729	0.968513	NA
CR388050.1	0.173729368	-0.955901296	4.0804729	-0.234262	0.814781	NA
pimr40	0	NA	NA	NA	NA	NA
si:dkey-42l23.1	1.005718337	-0.979164561	1.8926618	-0.517348	0.604913	NA
si:ch211-235i11.6	24.12032578	-0.614508688	0.4040379	-1.520918	0.12828	0.658954
si:dkey-42l23.6	0.157187365	-0.955901296	4.0804729	-0.234262	0.814781	NA
CR933730.1	8.095741417	0.139573932	0.679507	0.2054047	0.837256	NA
znf1134	106.9072146	-0.00841241	0.1926622	-0.043664	0.965172	0.996154

BX088540.1	1.383275679	-0.656799705	1.8044095	-0.363997	0.71586	NA
si:ch211-105j21.9	98.47180405	-0.139140114	0.2292979	-0.606809	0.543977	0.945438
si:dkey-74h17.3	0.323844819	-1.825095364	3.274639	-0.557342	0.577293	NA
si:ch211-269k10.4	3.014007976	-0.610927598	1.0969643	-0.556926	0.577578	NA
BX927395.1	0	NA	NA	NA	NA	NA
CT583687.2	0.158915748	0.967652056	4.0804729	0.2371421	0.812547	NA
CR792439.1	1.721188513	1.296357207	1.608938	0.8057223	0.420403	NA
BX323586.3	165.451494	0.058072462	0.1729969	0.335685	0.737108	0.972535
CR377210.1	4.38081309	0.128634759	0.9796286	0.1313097	0.89553	NA
BX255963.2	1.345943521	-1.646286758	1.8089979	-0.910055	0.362794	NA
CT025854.1	0.157187365	-0.955901296	4.0804729	-0.234262	0.814781	NA
BX324134.1	0	NA	NA	NA	NA	NA
CR376776.1	0.641435609	-0.01573847	2.4541227	-0.006413	0.994883	NA
CU041346.1	0	NA	NA	NA	NA	NA
si:dkeyp-84f3.9	361.4847919	-0.038771604	0.1154981	-0.33569	0.737104	0.972535
CR847983.2	0	NA	NA	NA	NA	NA
CR381579.4	0.317711143	1.810477155	3.2978238	0.5489915	0.583011	NA
BX294160.1	26.88149346	-0.169685455	0.3637669	-0.466468	0.640881	0.959994
si:dkey-1m11.6	9.44411535	0.650130297	0.6391851	1.0171237	0.309095	NA
pimr13	0	NA	NA	NA	NA	NA
si:ch1073-145m9.1	38.32885917	-0.011612691	0.318611	-0.036448	0.970925	0.996315
si:ch211-137j23.6	2.169603562	-0.184549162	1.2705658	-0.14525	0.884514	NA
BX530721.1	1.479637323	-1.831571174	1.7087478	-1.071879	0.283774	NA
BX548170.1	0	NA	NA	NA	NA	NA
CR786577.1	103.5934394	-0.030313038	0.1988818	-0.152417	0.878858	0.987881
si:dkey-40g16.5	1.821862836	0.277118821	1.3683718	0.2025172	0.839512	NA
si:ch211-167b20.8	311.7006891	0.150778341	0.1245838	1.2102562	0.226181	0.793699
CU694532.1	19.19541012	-0.227887683	0.4908593	-0.464263	0.64246	0.959994
BX323824.1	10.70795982	-0.21578648	0.5841125	-0.369426	0.71181	NA
CR936518.2	1.649579861	1.177080862	1.5643645	0.7524339	0.45179	NA
CR759927.2	13.58033287	-0.196388789	0.5152725	-0.381136	0.703103	0.968134
CU928013.1	126.7394458	-0.118007886	0.178447	-0.661305	0.508417	0.93575
CU104777.1	30.61815223	-0.78046587	0.3689007	-2.115653	0.034374	0.360397
BX293559.1	0	NA	NA	NA	NA	NA
si:ch73-265h17.2	1.664675471	0.590386896	1.4423769	0.4093153	0.682308	NA
si:ch1073-100f3.2	35.07456725	0.09260592	0.3370951	0.2747175	0.783533	0.97627
CR751227.2	4.442074287	-0.662365379	0.9584808	-0.691058	0.489529	NA
BX470264.1	246.3363215	0.070730094	0.1374265	0.5146758	0.60678	0.956718
CR352228.1	1.332030356	0.764519523	1.7290471	0.4421623	0.658372	NA
BX000451.1	0.998646424	-0.967051882	1.9878844	-0.486473	0.626632	NA
sgip1a	1125.270141	0.191988865	0.0874445	2.1955504	0.028124	0.325071
BX649266.2	0	NA	NA	NA	NA	NA
si:ch73-90p23.1	4.934307356	-0.614482725	0.8761549	-0.70134	0.483091	NA
si:ch73-204p21.2	388.4779185	-0.170628114	0.1233484	-1.383302	0.166572	0.723276
BX927184.1	193.5061304	0.024448382	0.1610901	0.1517684	0.87937	0.987881
CR387921.1	0.513487682	-0.887846295	2.6557956	-0.334305	0.738149	NA
CU928083.1	2.308396687	-0.898297445	1.409892	-0.637139	0.524034	NA
BX322555.4	3.399928744	0.950587486	1.0958152	0.8674706	0.385684	NA
si:dkey-195m11.11	28.72459228	-0.33776229	0.3641779	-0.927465	0.353685	0.881976
BX293567.1	4.783024248	-1.644244418	0.9096227	-1.807611	0.070667	NA
CU179756.1	1.702446761	-1.140108979	1.5927163	-0.715827	0.474098	NA
BX649356.1	0	NA	NA	NA	NA	NA
CT574587.1	1.643418446	0.563086621	1.5874073	0.354721	0.722799	NA
AL929309.1	0.31759079	1.807926197	3.579045	0.5051421	0.613459	NA

si:ch211-137j23.7	0	NA	NA	NA	NA	NA
AL954666.1	4.809334783	-0.331918441	0.9043803	-0.367012	0.71361	NA
tex36	1.456092431	0.343959518	1.5906412	0.2162395	0.828801	NA
BX465857.1	0.665169878	-0.08121369	2.4261845	-0.033474	0.973297	NA
CR385026.2	0	NA	NA	NA	NA	NA
BX663528.1	0	NA	NA	NA	NA	NA
CU464134.1	0.474778155	0.852397662	2.898154	0.2941175	0.768668	NA
si:ch73-40a17.4	32.29036248	-0.520445309	0.3677643	-1.41516	0.157022	0.707677
BX005392.3	5.466176727	0.792517542	0.8459409	0.9368474	0.348837	NA
BX323557.1	12.77086786	0.595985107	0.5635802	1.0574983	0.290284	0.846262
BX510649.1	3.103483828	-1.476946765	1.190938	-1.240154	0.214918	NA
CR762392.1	0	NA	NA	NA	NA	NA
si:dkey-22l14.10	29.8871531	0.295386378	0.3563554	0.8289094	0.407156	0.901305
FP102895.1	0	NA	NA	NA	NA	NA
CT955996.1	0	NA	NA	NA	NA	NA
asb5a	66.3211357	-0.285809649	0.2452683	-1.165294	0.2439	0.813336
CU467879.1	0	NA	NA	NA	NA	NA
pimr175	0.317711143	1.810477155	3.2978238	0.5489915	0.583011	NA
si:ch211-207d10.2	0	NA	NA	NA	NA	NA
CR926464.1	0.838899245	-0.667458734	2.2321093	-0.299026	0.76492	NA
BX664614.1	15.55092889	-0.091148896	0.4914172	-0.185482	0.852851	0.985174
CU137681.4	0	NA	NA	NA	NA	NA
CR354430.3	1.797777553	-1.440446692	1.5706073	-0.917127	0.359076	NA
mespbb	1.030002046	-2.236497097	2.086282	-1.072001	0.283719	NA
BX901883.3	0.791121974	0.591938164	2.3987483	0.2467696	0.805087	NA
BX663520.1	4.208877474	1.074534053	0.9885303	1.0870016	0.277036	NA
CU469568.4	1.320910952	-0.001675893	1.6383744	-0.001023	0.999184	NA
si:ch211-284e13.14	18.85396831	-0.474657591	0.4492145	-1.056639	0.290676	0.84639
BX469902.1	62.85561173	0.38259446	0.2547833	1.5016468	0.133188	0.667628
CT956066.1	1.98262732	0.994786605	1.343037	0.7406993	0.458876	NA
CR391915.1	0.158915748	0.967652056	4.0804729	0.2371421	0.812547	NA
CU137680.2	0	NA	NA	NA	NA	NA
si:dkey-248g15.3	191.121003	0.002817772	0.1772154	0.0159003	0.987314	0.997136
BX511132.1	0	NA	NA	NA	NA	NA
BX571762.2	130.1716261	0.083799569	0.1766581	0.4743603	0.635243	0.957927
CR589875.2	16.32207788	0.45625932	0.5049516	0.9035704	0.366223	0.887239
arhgef33	48.64014882	-0.300311793	0.2833073	-1.060021	0.289135	0.845979
BX936420.1	0.648627875	-0.032651702	2.3257531	-0.014039	0.988799	NA
BX950217.1	1.323549833	-0.005077815	1.6547343	-0.003069	0.997552	NA
BX942813.1	11.67413946	-0.127086185	0.5580985	-0.227713	0.81987	0.981594
FP089511.1	1.972456444	-2.287264619	1.5585685	-1.467542	0.142229	NA
BX004769.1	0.499302569	-0.916836436	2.6772503	-0.342455	0.732009	NA
BX957251.1	0.515216064	0.926382026	2.6525479	0.3492423	0.726907	NA
CT033842.1	0.182570949	0.967652056	4.0804729	0.2371421	0.812547	NA
si:ch1073-190k2.1	358.9433959	-0.091089292	0.1497559	-0.608252	0.543021	0.944974
CU855685.1	10.72470475	-0.202537037	0.5923279	-0.341934	0.732401	NA
CR812464.1	0.158795395	0.967652056	4.0804729	0.2371421	0.812547	NA
BX000364.1	0	NA	NA	NA	NA	NA
CR387984.1	0	NA	NA	NA	NA	NA
ugt1b3	3.13451943	-0.517093665	1.149453	-0.449861	0.652811	NA
BX005375.1	25.37001786	-0.055226379	0.3811113	-0.144909	0.884783	0.988838
BX957228.1	0	NA	NA	NA	NA	NA
BX323067.1	4.541776841	0.344891935	0.9083119	0.3797065	0.704163	NA
BX571712.2	14.86318731	-0.534655767	0.5435277	-0.983677	0.325274	0.871659

EIF3J (1 of many)	0.333314908	-1.858695402	3.5126701	-0.52914	0.596708	NA
CR293498.1	0	NA	NA	NA	NA	NA
BX005450.1	5.653478658	0.426888659	0.8909377	0.4791453	0.631835	NA
si:ch211-191c10.1	1.514490996	-1.013920941	1.6627523	-0.609785	0.542004	NA
FP102071.1	0	NA	NA	NA	NA	NA
BX571774.1	0.48448895	0.85344211	2.878836	0.2964539	0.766883	NA
CR848784.1	0.967129164	-0.023635743	1.9568585	-0.012078	0.990363	NA
si:ch211-272b8.7	1.008426242	2.234978276	1.9960971	1.1196742	0.262853	NA
CR376785.1	0	NA	NA	NA	NA	NA
BX640549.1	1.176929177	-1.307615778	1.8713043	-0.698772	0.484694	NA
ftr91	7.979834853	-0.731789211	0.7242042	-1.010474	0.312268	NA
BX004996.1	0.158795395	0.967652056	4.0804729	0.2371421	0.812547	NA
AL929523.1	1.682365422	-3.065563608	1.7200617	-1.78224	0.07471	NA
CU633486.1	1.862139108	0.2356309	1.4264702	0.1651846	0.868799	NA
BX000438.2	132.4173	-0.005910491	0.1943937	-0.030405	0.975744	0.996762
itgae.1	4.974666197	-0.592366691	0.8515414	-0.69564	0.486654	NA
si:ch211-130m23.5	233.3268214	0.04817647	0.1399716	0.3441875	0.730705	0.97149
CU571179.1	0	NA	NA	NA	NA	NA
CT573139.2	0.646899493	-1.455098895	2.4290529	-0.59904	0.549146	NA
BX539313.3	3.381035397	-1.21552056	1.0793432	-1.126167	0.260095	NA
pet100	186.1489145	0.061533679	0.189009	0.3255595	0.744758	0.974094
BX005129.5	1.659762023	4.182023077	1.7410023	2.4020779	0.016302	NA
si:dkey-111k8.2	48.78551658	0.168201866	0.2738274	0.6142624	0.539042	0.943733
CR936378.1	30.88982164	-0.14780929	0.3396145	-0.435227	0.663398	0.964736
AL928707.1	0.158915748	0.967652056	4.0804729	0.2371421	0.812547	NA
si:dkey-262k9.2	995.1567206	0.154634414	0.0915283	1.6894708	0.091129	0.578217
CR548622.1	3.790102121	0.575435662	1.0798266	0.5328963	0.594105	NA
si:dkey-238d18.8	0	NA	NA	NA	NA	NA
si:dkey-77f5.14	70.73560484	-0.024680714	0.2450442	-0.100719	0.919773	0.991852
BX890602.1	1.551033009	0.394774418	1.6365296	0.2412266	0.80938	NA
si:ch211-129o18.4	7.720834242	-0.215556094	0.7229517	-0.298161	0.76558	NA
BX571794.3	1.498097085	0.945410071	1.6553522	0.5711232	0.567916	NA
si:ch73-144d13.8	37.72280437	-0.250449251	0.3235075	-0.774168	0.438831	0.916019
si:dkey-7i4.8	39.18859393	-0.16454278	0.3245404	-0.507002	0.612153	0.95688
CR848840.1	0.999464308	0.976746318	1.8950081	0.5154312	0.606252	NA
si:ch211-69l10.4	32.85859801	0.432747138	0.3642163	1.1881596	0.234771	0.803509
CU927934.3	22.89521562	0.098826346	0.4008947	0.2465144	0.805284	0.979883
FP015862.3	0.166657454	-0.955901296	4.0804729	-0.234262	0.814781	NA
AL928707.2	0.158915748	0.967652056	4.0804729	0.2371421	0.812547	NA
CR391995.1	0.476386185	2.392464142	3.0533824	0.7835455	0.433307	NA
BX005332.1	0	NA	NA	NA	NA	NA
BX663503.2	172.9168667	0.153815179	0.1668246	0.9220173	0.35652	0.883422
CR751602.2	45.35494351	-0.295769686	0.2911593	-1.015835	0.309708	0.86172
FP017161.1	50.72267992	0.133122001	0.2701291	0.4928088	0.622148	0.957354
CU855758.2	0	NA	NA	NA	NA	NA
CT025912.1	0.166657454	-0.955901296	4.0804729	-0.234262	0.814781	NA
BX248121.1	645.428053	0.188712365	0.1013034	1.8628442	0.062484	0.487868
BX571712.3	0.838899245	-0.667458734	2.2321093	-0.299026	0.76492	NA
opn6b	126.8556012	0.17658931	0.179001	0.9865269	0.323875	0.870928
BX548024.2	1.796479253	-0.257679604	1.3864521	-0.185855	0.852558	NA
CT009746.1	0	NA	NA	NA	NA	NA
dscaml1	126.2938883	-0.001463809	0.1874491	-0.007809	0.993769	0.998672
fkbp3	519.7262997	-0.24586739	0.1296047	-1.897057	0.05782	0.471676
alp3	9.113249852	0.898005973	0.7091082	1.2663878	0.205374	NA

si:ch1073-205c8.5	1.634147777	0.520279905	1.6026912	0.3246289	0.745462	NA
dre-mir-365-1	0.173729368	-0.955901296	4.0804729	-0.234262	0.814781	NA
CU302323.1	1.329401519	-1.62552701	1.7303732	-0.939408	0.347521	NA
AL929457.2	3.031292051	1.40883543	1.1840831	1.1898113	0.234121	NA
CU207354.2	0	NA	NA	NA	NA	NA
CABZ01085140.1	61.22997612	-0.033605812	0.2631849	-0.127689	0.898395	0.990121
znf1049	1.169857263	-1.300491357	1.7916478	-0.725863	0.467923	NA
trip4	125.2639069	0.213317418	0.1884146	1.1321701	0.257563	0.825314
sh3bgrl3	1685.31789	-0.010650689	0.0778537	-0.136804	0.891186	0.989291
kcnq1.2	9.097185257	-0.354565764	0.6353434	-0.558069	0.576797	NA
si:dkey-238o14.3	3.641807913	-0.787910966	1.0211598	-0.771584	0.440361	NA
dre-mir-18a	0	NA	NA	NA	NA	NA
rab3gap1	698.012494	-0.023124938	0.1091781	-0.211809	0.832256	0.983557
gnl2	869.2445345	-0.095510841	0.0924737	-1.032844	0.301677	0.856944
GABRR1 (1 of many)	120.8644967	-0.106837044	0.1879576	-0.56841	0.569756	0.948983
CU459093.1	40.92957672	0.009907416	0.3087323	0.0320906	0.9744	0.996523
RF00001	0.356300316	0.005884122	3.214964	0.0018302	0.99854	NA
si:dkey-30f3.2	5.673008144	0.919904837	0.8608273	1.0686288	0.285237	NA
CU929178.1	0.173729368	-0.955901296	4.0804729	-0.234262	0.814781	NA
BX000463.1	0.663561848	-1.490280258	2.4116568	-0.617949	0.536609	NA
si:dkey-146c18.5	3.833970299	0.396630376	0.9903035	0.4005139	0.688778	NA
CT027696.1	1.891927985	-0.775950481	1.4470384	-0.536234	0.591797	NA
slc12a1	87.18982559	0.040763002	0.2455623	0.1659986	0.868158	0.987245
jac3	0.506415768	-0.866825728	2.6674086	-0.324969	0.745204	NA
RF00001	0	NA	NA	NA	NA	NA
zgc:64065	10.91911913	0.343226364	0.5695862	0.602589	0.546782	NA
BX248243.1	0.317831496	1.808837541	3.5779923	0.5055454	0.613176	NA
tefb	581.9970588	0.24389815	0.105039	2.3219765	0.020234	0.267275
tmem106c	277.4826543	-0.014984047	0.1400603	-0.106983	0.914803	0.991119
si:ch211-215e19.8	0.365141898	1.973577737	3.3927314	0.5817076	0.560764	NA
dusp2	233.5278498	0.138904846	0.1417618	0.9798468	0.327162	0.872658
CABZ01079550.1	0.496945679	-0.834564081	2.8549631	-0.29232	0.770042	NA
btc	99.43588229	-0.023568585	0.2050375	-0.114948	0.908487	0.990702
WSCD1	83.90017707	-0.288260233	0.2130744	-1.352862	0.1761	0.736505
ctsa	623.0650258	-0.001529535	0.1034028	-0.014792	0.988198	0.997369
si:dkey-178j11.5	15.55266447	-0.686098184	0.4871106	-1.408506	0.158981	0.709638
trappc10	1009.167556	-0.130090753	0.088053	-1.477415	0.139565	0.678238
CABZ01012959.1	0.657159727	-2.841717825	2.5166161	-1.129182	0.258821	NA
RF00001	0.317711143	1.810477155	3.2978238	0.5489915	0.583011	NA
FP101882.1	65.1021981	0.353084185	0.2465107	1.4323279	0.15205	0.700984
BX005085.2	10.74016116	-0.235684483	0.6424558	-0.366849	0.713731	NA
si:dkey-44g17.6	14.17468941	0.303781361	0.5653158	0.5373657	0.591015	0.954193
BX324212.1	0	NA	NA	NA	NA	NA
si:dkeyp-97a10.2	44.41811919	-0.091673951	0.2913454	-0.314657	0.753022	0.97481
bola3	124.6823516	-0.127192919	0.1873884	-0.678766	0.497286	0.93322
BX511136.1	2.456546305	0.172107456	1.1977978	0.1436866	0.885748	NA
si:ch211-281k19.2	0.158795395	0.967652056	4.0804729	0.2371421	0.812547	NA
stk40	399.235394	0.173594676	0.1283085	1.3529479	0.176072	0.736505
ankrd13d	420.713693	0.035182978	0.1121833	0.3136205	0.753809	0.975056
supt3h	178.5429782	-0.435238978	0.178916	-2.432644	0.014989	0.224348
CR391944.1	7.295004671	0.275591555	0.6887728	0.4001197	0.689068	NA
si:dkey-57l19.1	0.507044276	-2.459587493	2.7586536	-0.89159	0.372613	NA
si:dkey-285e18.6	0	NA	NA	NA	NA	NA
BX572628.1	0.983791519	-0.057426352	2.0613195	-0.027859	0.977775	NA

uts2d	7.419932704	-0.448196698	0.6978264	-0.642275	0.520694	NA
BX255896.1	9.719213419	0.118752603	0.6225485	0.1907524	0.84872	NA
CR925797.1	0.820749213	-1.887809118	2.2327507	-0.845508	0.397827	NA
lhfpl2b	243.1483451	0.102392157	0.1429995	0.7160317	0.473972	0.924993
CT583728.1	0	NA	NA	NA	NA	NA
CR792416.1	1.45775179	-1.018923527	1.7692292	-0.575914	0.564673	NA
cpxm1b	197.037273	0.135533174	0.1530873	0.8853325	0.375977	0.894587
cntn4	152.8218179	-0.128483877	0.1769948	-0.725919	0.467888	0.924328
zgc:77748	737.7895863	-1.232063517	0.1093474	-11.26742	1.90E-29	3.76E-26
traj56	0	NA	NA	NA	NA	NA
aipl2	46.9954613	-0.089833452	0.3035798	-0.295914	0.767296	0.975687
si:dkey-176f19.4	3.443952973	-0.435106774	1.0297039	-0.422555	0.67262	NA
zgc:162193	551.2710146	0.081312951	0.1083249	0.7506394	0.45287	0.919598
tradv19.0	0	NA	NA	NA	NA	NA
BX537115.1	0.332524763	0.005883742	3.292841	0.0017868	0.998574	NA
dgkab	77.26768522	-0.205764954	0.2372628	-0.867245	0.385808	0.89551
si:rp71-1h20.5	78.60608787	-0.22159652	0.2241161	-0.988758	0.322782	0.870524
si:dkey-28i19.3	5.292017277	0.34933705	0.8128001	0.4297945	0.667345	NA
or109-4	0	NA	NA	NA	NA	NA
si:dkeyp-86c4.1	2.124970071	-2.419204104	1.5265325	-1.584771	0.113018	NA
zgc:113184	160.6485615	-0.243550155	0.171379	-1.42112	0.155282	0.705118
si:dkey-27n6.1	0.166657454	-0.955901296	4.0804729	-0.234262	0.814781	NA
BX663612.1	0.996206963	-1.055667267	2.0852244	-0.506261	0.612674	NA
si:dkey-16p6.1	5.381435391	-1.208118264	0.8739192	-1.382414	0.166845	NA
si:dkey-16l2.20	178.5246919	-0.018050191	0.1578101	-0.114379	0.908937	0.990702
znf998	2.128375508	0.245522961	1.3038686	0.1883034	0.850639	NA
SLC39A11	129.5207874	-0.215158188	0.184571	-1.165721	0.243727	0.813138
znf1077	21.17379652	-0.12051169	0.4381171	-0.275067	0.783265	0.97627
CU633999.1	1.439509144	0.307590293	1.6554512	0.1858045	0.852598	NA
RF00001	1.189776963	0.42714694	1.8029205	0.2369195	0.812719	NA
BX928743.1	0.157187365	-0.955901296	4.0804729	-0.234262	0.814781	NA
mtmr3	301.6206996	0.006200062	0.1250889	0.0495652	0.960469	0.995421
AL954698.1	0	NA	NA	NA	NA	NA
APCDD1	1.320721575	-2.698764302	1.8311672	-1.473795	0.140537	NA
CU984600.2	10.99311694	-0.013814254	0.6650156	-0.020773	0.983427	NA
ptrh1	89.69912902	0.218102872	0.2182961	0.9991148	0.317739	0.867607
fn3krp	24.16548739	-0.122945396	0.4019684	-0.305858	0.759713	0.975374
si:ch211-199b20.3	56.977667	-0.301495134	0.2583697	-1.166914	0.243245	0.812077
wrn	61.05396364	0.081517238	0.2495839	0.3266125	0.743961	0.974094
BX465836.1	2.022123736	-2.267580007	1.552276	-1.46081	0.144068	NA
BICD2	56.94804649	0.259110568	0.2589587	1.0005866	0.317027	0.867335
si:ch211-215e19.3	0	NA	NA	NA	NA	NA
si:dkey-261j11.1	3.942076599	-1.013670318	0.9702486	-1.044753	0.296137	NA
si:ch211-209j12.6	2.096668871	-0.692607419	1.3431709	-0.515651	0.606098	NA
st8sia1	844.5070591	0.094174903	0.1008553	0.9337624	0.350426	0.880195
AL773558.1	5.708872458	-0.02773411	0.9247313	-0.029992	0.976074	NA
tmem56b	971.4030849	-0.038999982	0.0922145	-0.422927	0.672349	0.965257
olfcg7	0.889079191	3.262746891	2.2913259	1.4239558	0.154459	NA
smpd3	73.32021555	-0.027859836	0.22991	-0.121177	0.903551	0.990235
BX649608.1	0	NA	NA	NA	NA	NA
thrap3b	2261.342623	-0.010481351	0.0711223	-0.147371	0.882839	0.98852
BX649416.1	53.60538342	-0.045770553	0.2630893	-0.173973	0.861886	0.985841
hhex	14.68993115	0.198707968	0.4963793	0.4003148	0.688925	0.966672
tiam2b	79.67744755	-0.38814013	0.2218065	-1.749904	0.080135	0.549631

actr5	132.861162	-0.161729619	0.1790676	-0.903176	0.366432	0.887239
zgc:174698	3.239563102	0.480334624	1.1068762	0.4339552	0.664321	NA
fbn2b	1021.795639	0.160805832	0.0858477	1.8731526	0.061047	0.482482
CR626866.1	0	NA	NA	NA	NA	NA
zgc:85932	529.4942398	-0.111501416	0.1052089	-1.05981	0.289231	0.845979
meis2a	5087.14443	-0.102321621	0.066546	-1.537608	0.124145	0.652708
brcc3	324.9271196	-0.074545509	0.1323179	-0.563382	0.573175	0.949762
CR762475.1	0.339758314	0.005883863	3.2682461	0.0018003	0.998564	NA
CR388206.1	0.157187365	-0.955901296	4.0804729	-0.234262	0.814781	NA
cidea	10.02439555	-0.377589963	0.6165179	-0.612456	0.540236	NA
CU928222.2	0	NA	NA	NA	NA	NA
det1	92.33115289	-0.13038842	0.2054192	-0.634743	0.525596	0.94013
asmt	128.7957324	0.002635005	0.1785362	0.0147589	0.988224	0.997369
cpne9	15.53176486	0.057385526	0.5156125	0.1112958	0.911382	0.991066
si:ch73-329n5.6	140.6374343	0.060574453	0.4426821	0.1368351	0.891161	0.989291
pcdh15a	127.9704139	0.221233902	0.1949085	1.1350656	0.256348	0.824633
RF00001	0	NA	NA	NA	NA	NA
si:dkeyp-85h7.1	0.333314908	-1.858695402	3.5126701	-0.52914	0.596708	NA
CU929294.1	132.1715623	0.167528511	0.1917934	0.8734843	0.382399	0.895192
RF00001	0.173729368	-0.955901296	4.0804729	-0.234262	0.814781	NA
znf1053	5.449655059	-0.995702829	0.8513733	-1.169526	0.242192	NA
nipbla	1398.330357	-0.100100533	0.0865972	-1.155933	0.247708	0.81711
zbtb14	406.6613573	0.01674086	0.120984	0.1383726	0.889946	0.989291
si:dkeyp-13d12.16	0.173729368	-0.955901296	4.0804729	-0.234262	0.814781	NA
CR550307.1	0.647689638	-2.824814845	2.5064236	-1.12703	0.25973	NA
si:zfos-451d2.2	1.506000429	0.335521124	1.4976273	0.2240351	0.82273	NA
rbm5	3141.230551	-0.098481924	0.0764768	-1.287736	0.197838	0.763028
si:dkey-237m9.1	0.50625413	-0.940106488	2.8357787	-0.331516	0.740255	NA
ctxn1	6.844777673	-0.067128461	0.7284496	-0.092153	0.926577	NA
CU695232.1	165.3790571	0.057137067	0.1745061	0.3274216	0.743349	0.973765
tax1bp1a	328.7278018	0.082760432	0.1230541	0.672553	0.501232	0.934613
vstm2l	57.60034064	-0.005907236	0.264838	-0.022305	0.982205	0.996944
unc80	50.13093578	0.102834168	0.2996973	0.3431267	0.731503	0.971557
trpc3	9.872380037	-0.137263378	0.6033258	-0.227511	0.820026	NA
si:dkey-27i16.2	47.3472299	0.291764818	0.2884229	1.0115867	0.311736	0.864257
col5a3a	76.08549615	0.074463662	0.2366238	0.3146922	0.752995	0.97481
efna3b	79.04919804	-0.249548263	0.2209693	-1.129335	0.258757	0.826867
gnsb	110.6922712	-0.343687121	0.189131	-1.81719	0.069188	0.514556
CU928205.1	0	NA	NA	NA	NA	NA
taok1b	972.58687	-0.061656748	0.0900883	-0.684403	0.49372	0.931926
si:ch211-76m11.8	0.815164977	-0.617697568	2.1259573	-0.29055	0.771395	NA
CR936540.1	75.77942509	0.159731854	0.2244938	0.7115203	0.476762	0.926808
si:zfos-932h1.3	105.4930857	-0.05162169	0.2022021	-0.255297	0.798493	0.978884
scarb2a	1635.346699	0.086034055	0.087224	0.986358	0.323957	0.870928
nadka	14.55978462	0.185689948	0.5337484	0.3478979	0.727917	0.970402
cyp1a	1437.064878	-0.065502	0.1068789	-0.612862	0.539968	0.944206
BX908386.1	0.849390142	1.928131185	2.2127708	0.8713651	0.383555	NA
dync1li1	1966.442083	0.001884222	0.0826091	0.0228089	0.981803	0.996944
BX005396.1	0	NA	NA	NA	NA	NA
dre-mir-193a-1	0	NA	NA	NA	NA	NA
grm1b	6.732479047	0.754966059	0.7843974	0.962479	0.335809	NA
si:cabz01054394.6	47.54974681	-0.39470585	0.2838393	-1.390596	0.164348	0.720101
CU280645.1	0.98968449	-2.214754691	2.0224815	-1.095068	0.273487	NA
RF00001	0.182570949	0.967652056	4.0804729	0.2371421	0.812547	NA

spef2	43.97129581	0.111935603	0.2965955	0.3774016	0.705875	0.968412
exosc3	208.0118658	-0.346123413	0.1789949	-1.933705	0.053149	0.452412
BX537137.2	0.317711143	1.810477155	3.2978238	0.5489915	0.583011	NA
CR381673.1	0.182570949	0.967652056	4.0804729	0.2371421	0.812547	NA
si:ch73-340n8.4	90.28430206	-0.122035329	0.2132988	-0.572133	0.567232	0.948739
RF00001	0	NA	NA	NA	NA	NA
RF00003	0	NA	NA	NA	NA	NA
CABZ01065679.1	37.3949236	0.271830897	0.3219752	0.8442603	0.398524	0.900359
rab18b	751.0368971	0.088888791	0.0920187	0.9659863	0.334051	0.875256
mamdc2a	159.0175397	-0.202175341	0.167191	-1.209248	0.226568	0.794071
si:ch1073-181h11.4	0	NA	NA	NA	NA	NA
znf326	23.0799193	0.186773162	0.4094505	0.4561557	0.648278	0.960708
hdac4	60.76873116	0.057808833	0.2548845	0.226804	0.820576	0.981594
mxd3	401.4056701	-0.087814767	0.115698	-0.759	0.447853	0.917154
si:ch211-66c13.1	1.132247612	1.34783488	1.8934551	0.7118388	0.476565	NA
BX322574.1	4.611023263	-1.317200024	0.9017012	-1.460794	0.144072	NA
atp5pd	2396.246574	-0.043796388	0.0798598	-0.548416	0.583406	0.952214
si:ch211-229l10.4	11.05634901	0.137494197	0.5671683	0.2424222	0.808453	NA
il17rd	310.4100375	-0.138642847	0.1482044	-0.935484	0.349539	0.880195
cyp19a1b	20.56430223	0.465977237	0.4340648	1.0735199	0.283038	0.84466
nudcd3	710.6150048	-0.022124961	0.1089887	-0.203002	0.839133	0.984979
BX901957.1	33.83258878	-0.073733795	0.3328361	-0.221532	0.824678	0.982196
lrrc74b	15.98998287	0.890832543	0.4948909	1.8000584	0.071851	0.524277
srp54	1643.1918	-0.050960338	0.077205	-0.660065	0.509212	0.936123
aplp1	5761.396008	0.027493911	0.0727656	0.3778419	0.705548	0.968412
si:dkey-165o8.1	2.83080852	-0.460814609	1.1456242	-0.402239	0.687508	NA
BX276102.1	9.587787396	0.480088934	0.6252589	0.7678242	0.442592	NA
FLNB	798.4427961	-0.228644484	0.1307586	-1.7486	0.08036	0.549728
polr1a	558.3874288	-0.16633736	0.1024196	-1.624078	0.104359	0.612162
si:ch211-207l14.1	353.838779	0.312516183	0.127991	2.4417044	0.014618	0.220655
tnfrsf11b	88.63379949	-0.029799262	0.2264694	-0.131582	0.895315	0.989814
CU694197.1	273.7062234	-0.048817193	0.1577634	-0.309433	0.756992	0.975374
si:dkey-38l22.2	17.01912224	0.577863385	0.4825279	1.1975751	0.231082	0.79927
si:dkey-14o6.1	6.3247798	0.027106836	0.7544923	0.0359273	0.97134	NA
ctps1b	382.7903906	-0.029496158	0.1206516	-0.244474	0.806864	0.980254
tmem176l.3a	120.9409546	0.034402466	0.1845699	0.1863926	0.852137	0.985174
znf1015	58.0352584	-0.248946989	0.2572061	-0.967889	0.3331	0.874926
si:ch211-76m11.8	12.36151027	0.360300723	0.5945433	0.6060126	0.544506	0.945438
psmg3	241.5202147	-0.021519443	0.1507331	-0.142765	0.886476	0.988936
ly6pge	1474.030971	0.198224772	0.0958237	2.0686397	0.03858	0.385234
tradv24.0.2	0	NA	NA	NA	NA	NA
CU929451.2	69.21514171	-0.197263506	0.2433581	-0.810589	0.417602	0.906237
FP016106.1	12.90937069	-1.151361144	0.5722714	-2.011915	0.044229	0.413497
CABZ01079480.1	98.9598207	-0.174143427	0.2169581	-0.802659	0.422172	0.90651
fam136a	321.8858667	-0.179950224	0.1364088	-1.319198	0.187103	0.751002
LO017877.1	5.671247677	-0.426798239	0.855794	-0.498716	0.617979	NA
CR925723.1	16.98308779	-0.026130274	0.4578471	-0.057072	0.954488	0.994533
map3k1	243.1405284	-0.181804782	0.1485835	-1.223586	0.221108	0.787641
si:dkey-22a18.2	6.177570678	-0.065425124	0.7481135	-0.087453	0.930311	NA
BX936393.2	0.531799352	0.999478841	2.790358	0.3581902	0.720201	NA
stbd1	26.71734673	0.11154504	0.3843605	0.2902094	0.771656	0.975687
BX005376.1	10.90989328	-0.63593865	0.5958944	-1.0672	0.285881	NA
si:dkey-19b23.10	2.267882965	0.837931821	1.3107878	0.6392582	0.522655	NA
ninl	219.8682276	0.288538393	0.1604242	1.7985968	0.072082	0.525083

CT956093.1	0	NA	NA	NA	NA	NA
RF00067	0	NA	NA	NA	NA	NA
SNTA1	3.455571483	1.681513788	1.1001295	1.5284689	0.126396	NA
trgv2	0	NA	NA	NA	NA	NA
CABZ01079241.1	192.8212833	-0.228189563	0.1537424	-1.484233	0.137747	0.675209
si:dkey-56m15.8	1.16439338	-0.410527374	1.7446119	-0.235312	0.813967	NA
RF00003	0	NA	NA	NA	NA	NA
asic1c	5.624435233	-0.159965696	0.7980323	-0.20045	0.841129	NA
RF00026	0	NA	NA	NA	NA	NA
znf1078	5.839499462	0.068800878	0.7882975	0.0872778	0.930451	NA
comp	835.3434762	0.142818298	0.0930737	1.5344643	0.124915	0.654531
paf1	496.9300857	0.010702796	0.1092339	0.0979805	0.921948	0.992224
utp6	400.0694503	0.055248128	0.1103641	0.5005988	0.616653	0.95688
BX005003.1	663.7988188	0.010365567	0.108021	0.0959588	0.923553	0.992588
BX537156.1	0.340386822	-1.882368278	3.2159502	-0.585323	0.558331	NA
si:ch211-9d9.8	0.49144051	0.855669345	2.6923358	0.3178167	0.750624	NA
slc6a6b	84.57474916	0.2109597	0.2321302	0.908799	0.363456	0.886042
nr1h3	642.8861513	0.063849665	0.0997894	0.6398444	0.522274	0.939056
si:ch211-62a1.4	7.552956713	-0.370037951	0.7098191	-0.521313	0.602149	NA
nars2	75.11066598	-0.162736279	0.2377069	-0.684609	0.493591	0.931926
zfand6	423.964232	0.033299338	0.1153548	0.2886688	0.772835	0.975687
si:dkey-3h2.4	6.606781356	0.306746111	0.7918925	0.3873583	0.698491	NA
si:ch211-1o7.2	6.035469875	1.036586853	0.8262829	1.2545181	0.209654	NA
tmem56a	243.6600524	0.077831759	0.1435111	0.5423395	0.587585	0.953319
si:dkey-151g22.1	1.142466561	0.35959156	1.8215264	0.1974122	0.843505	NA
pank4	406.5982538	-0.107324429	0.1122231	-0.956349	0.338896	0.87745
BX957236.1	0	NA	NA	NA	NA	NA
ccl39.5	0	NA	NA	NA	NA	NA
si:dkey-11f4.14	11.93137573	0.428904507	0.5863508	0.731481	0.464485	0.92346
CU570782.1	1.169857263	-1.300491357	1.7916478	-0.725863	0.467923	NA
gig2g	0.489712128	-0.887840729	2.693685	-0.329601	0.741702	NA
si:ch73-366l1.5	1882.411743	0.309883419	0.1020133	3.037678	0.002384	0.06083
ube2a	1617.287571	0.037746261	0.0783489	0.4817712	0.629969	0.957354
RF00001	0	NA	NA	NA	NA	NA
arr3b	4137.706069	0.280057317	0.0930103	3.0110347	0.002604	0.064922
tec	13.74754857	-0.536332414	0.527064	-1.017585	0.308875	0.861088
FO704758.1	6.500106906	1.160543244	0.7832065	1.4817844	0.138398	NA
anxa1d	158.61189	-0.22358518	0.1608581	-1.389953	0.164543	0.720706
CR792424.1	0.982922306	1.012515237	1.9057885	0.5312842	0.595222	NA
RF00017	0.158795395	0.967652056	4.0804729	0.2371421	0.812547	NA
CU929365.1	0	NA	NA	NA	NA	NA
tollip	463.9657819	-0.013816074	0.1074165	-0.128622	0.897657	0.990121
traj32	0	NA	NA	NA	NA	NA
BX682557.1	0.316103113	0.005883447	3.3519121	0.0017553	0.9986	NA
olfcd2	0.340386822	-1.882368278	3.2159502	-0.585323	0.558331	NA
si:ch211-145h19.3	0	NA	NA	NA	NA	NA
tradv25.0.1	0	NA	NA	NA	NA	NA
hrasa	401.8865128	-0.140262998	0.1149185	-1.220543	0.222259	0.788551
si:dkey-161l11.79	0	NA	NA	NA	NA	NA
CT583728.2	0.332524763	0.005883742	3.292841	0.0017868	0.998574	NA
lyar	226.8703708	-0.03817917	0.1434715	-0.26611	0.790155	0.977338
BX005286.1	8.159675302	0.174331196	0.6584333	0.2647667	0.791189	NA
zgc:174650	3.134842705	-0.458897072	1.042204	-0.440314	0.65971	NA
ntrk2b	32.20720299	0.184625119	0.3622896	0.5096065	0.610327	0.95688

si:ch73-215f7.1	23.47302916	0.36876377	0.4068	0.9064988	0.364672	0.886637
CU855694.1	0	NA	NA	NA	NA	NA
si:ch211-59d8.3	10.16378166	-0.622580834	0.6191497	-1.005542	0.314636	NA
si:dkey-16p6.1	25.49221013	0.091270184	0.3940013	0.2316494	0.81681	0.981594
RF00001	0.498553709	0.926267934	2.6786929	0.345791	0.7295	NA
BX321870.1	0	NA	NA	NA	NA	NA
BX649431.1	0	NA	NA	NA	NA	NA
CU856358.1	0	NA	NA	NA	NA	NA
gpr75	350.978494	0.002199647	0.1298472	0.0169403	0.986484	0.996965
rtn4rl1b	213.2651203	-0.176573653	0.1516734	-1.16417	0.244355	0.813858
ccne2	282.197018	-0.031733823	0.1254604	-0.252939	0.800315	0.979377
si:dkey-106c17.2	0.476506538	2.394892832	2.8161716	0.8504073	0.395099	NA
RF00067	3.381660525	2.057543943	1.2163753	1.691537	0.090734	NA
BX004823.1	42.57074357	-0.081369024	0.315278	-0.258087	0.79634	0.978498
si:dkey-25i10.1	1.32448807	1.535255761	1.8059615	0.8501044	0.395267	NA
spata22	403.9047556	0.124028259	0.1169171	1.060822	0.288771	0.845979
zc3h7ba	137.5255511	-0.278508799	0.1872075	-1.487701	0.13683	0.673303
CR388378.2	0.846012444	-0.57358332	2.1013569	-0.272959	0.784885	NA
ncdn	261.2419366	0.012182987	0.1313551	0.0927485	0.926103	0.992702
olfcg6	2.424519895	-0.607345615	1.3474589	-0.450734	0.652181	NA
CABZ01085700.1	35.38934176	0.417671442	0.3392108	1.2313033	0.218209	0.785379
BX323564.1	5.605289719	-0.246905315	0.8349939	-0.295697	0.767461	NA
BX005082.1	0	NA	NA	NA	NA	NA
zgc:194202	3.420847211	-0.730704325	1.0710489	-0.682232	0.495092	NA
CR381534.1	0.998364433	-1.059644339	2.0638714	-0.513426	0.607654	NA
RF00001	0	NA	NA	NA	NA	NA
si:ch73-233f7.4	2.109664749	1.71033585	1.3929894	1.2278168	0.219516	NA
si:ch1073-286c18.5	0	NA	NA	NA	NA	NA
BX927336.1	8.943187208	0.014314188	0.6479758	0.0220906	0.982376	NA
si:ch211-8c17.2	7.050766526	0.605744517	0.7194384	0.8419685	0.399806	NA
FP016142.2	0	NA	NA	NA	NA	NA
sgcd	189.4169618	0.212240123	0.1574495	1.3479882	0.177662	0.738343
srsf11	3572.780912	-0.033364544	0.0696688	-0.478902	0.632008	0.957356
BX511079.1	0	NA	NA	NA	NA	NA
CU914631.1	0	NA	NA	NA	NA	NA
BX908763.1	0.166657454	-0.955901296	4.0804729	-0.234262	0.814781	NA
pdgfab	178.5086261	0.087124546	0.1582262	0.5506327	0.581885	0.951949
CR457444.2	0.688945432	-0.015421672	2.3988056	-0.006429	0.994871	NA
wbp1	434.1992781	-0.173028911	0.1170679	-1.478021	0.139402	0.678238
si:ch211-237a4.2	0.317831496	1.808837541	3.5779923	0.5055454	0.613176	NA
zgc:194887	12.93792003	-1.268981203	0.5619265	-2.258269	0.023929	0.298907
CR318595.1	0.538871265	0.999144257	2.7784799	0.359601	0.719146	NA
gchfr	120.7497264	0.014457965	0.1841064	0.0785305	0.937406	0.99279
cyb5a	902.4326026	-0.165390553	0.1077369	-1.535134	0.124751	0.654423
tubb2b	0	NA	NA	NA	NA	NA
zgc:92140	559.6069731	-0.158698921	0.1057089	-1.501282	0.133283	0.667628
FP102843.1	0	NA	NA	NA	NA	NA
hist1h2a11	2.67954512	1.611931768	1.2115434	1.330478	0.183361	NA
CU570791.1	17.46894406	0.053398912	0.4549276	0.1173789	0.90656	0.990702
RF00001	0.473290478	-0.833892061	2.901156	-0.287434	0.77378	NA
pithd1	37.66697394	0.230622263	0.330215	0.6984004	0.484927	0.93001
ora1	2.31240739	1.381217865	1.4121345	0.9781065	0.328022	NA
BX914220.1	0.698456806	0.09172817	2.5265115	0.0363063	0.971038	NA
si:dkey-14o6.4	5.109475157	0.828136354	0.924321	0.8959402	0.370285	NA

slx1b	59.42422703	-0.15385172	0.2500971	-0.615168	0.538444	0.943336
AL840638.1	300.2030541	-0.089096099	0.128295	-0.694463	0.487392	0.931584
si:dkey-4c15.8	11.21791393	-0.405563501	0.5939797	-0.68279	0.494739	0.931926
si:ch211-207e19.2	3.886328202	-1.242679728	1.1175577	-1.11196	0.266155	NA
RF00001	0	NA	NA	NA	NA	NA
chrm2a	7.636445584	-0.154084639	0.7088569	-0.217371	0.82792	NA
si:dkey-15h8.16	49.30612151	-0.283192345	0.2942314	-0.962482	0.335808	0.876088
BX901954.1	0	NA	NA	NA	NA	NA
ly6m4	0.157187365	-0.955901296	4.0804729	-0.234262	0.814781	NA
erap2	57.74011064	-0.294076039	0.2819816	-1.042891	0.296999	0.853798
si:ch211-188p14.2	47.99709062	0.110240614	0.3068129	0.3593089	0.719364	0.969183
CR759843.1	11.07293909	-0.472807103	0.5629643	-0.839853	0.400991	0.90084
ccne1	98.55878726	0.141208615	0.2050041	0.6888088	0.490944	0.931926
si:dkey-23f9.4	85.51865506	-0.233666216	0.2132648	-1.095663	0.273226	0.83816
traj54	0	NA	NA	NA	NA	NA
efna5b	6.414523943	0.02818101	0.7575298	0.0372012	0.970325	NA
nitr12	0	NA	NA	NA	NA	NA
gsptl1	3757.40464	-0.025701234	0.0754832	-0.34049	0.733488	0.971993
BX914214.1	0.317711143	1.810477155	3.2978238	0.5489915	0.583011	NA
tradv6.0.1	0	NA	NA	NA	NA	NA
RF00001	0.654133044	-1.416854166	2.5841447	-0.548287	0.583495	NA
CABZ01044277.1	60.10679619	0.304305239	0.2827951	1.0760625	0.281899	0.843666
AL929586.1	0.650115553	1.435486287	2.5894636	0.5543566	0.579335	NA
irf2bp2b	1194.815269	-0.064521664	0.0866774	-0.744389	0.456641	0.921215
pycr1b	881.5958153	-0.279129124	0.0980881	-2.845697	0.004431	0.094528
CU984600.3	42.92788039	0.180510593	0.2968063	0.6081764	0.54307	0.944974
nbeal2	78.71985441	0.108937449	0.220452	0.4941549	0.621197	0.957354
si:ch211-126j24.1	1756.782478	-0.071035668	0.0806322	-0.880984	0.378326	0.895192
rgn	237.1130003	-0.40390148	0.1366833	-2.955017	0.003127	0.073687
mthfd2	834.4704244	0.266219566	0.0887038	3.0012191	0.002689	0.066466
ankdd1a	114.3633169	0.125375789	0.1886694	0.6645264	0.506353	0.935724
iqgap3	374.5298937	-0.02728181	0.1132035	-0.240998	0.809557	0.980642
si:dkey-21e2.7	0.811320409	-1.830272858	2.4089013	-0.759796	0.447377	NA
pcdh11	459.8545392	0.212423101	0.1150529	1.8463077	0.064848	0.497901
BX957252.1	0.650235905	1.438231835	2.4251649	0.593045	0.553151	NA
FQ976914.1	5.936982178	1.210905628	0.8743087	1.3849863	0.166057	NA
KIF1C	15.91399746	-0.211098881	0.48486	-0.435381	0.663286	0.964736
CR788237.1	0	NA	NA	NA	NA	NA
CU469526.1	123.88158	0.075406108	0.1908861	0.3950319	0.692819	0.96736
AL935300.3	19.71919619	0.087739814	0.4388179	0.1999459	0.841523	0.985174
si:ch73-359m17.9	97.16854799	-0.263087172	0.2376107	-1.10722	0.268199	0.833475
fgf19	6.839045498	-0.288191774	0.7605834	-0.378909	0.704756	NA
si:dkeyp-53d3.3	27.06831652	-0.214658371	0.3990952	-0.537863	0.590672	0.954193
CT583690.1	0.809151653	1.852283317	2.2398915	0.8269523	0.408264	NA
si:dkey-93h22.8	164.456329	0.127097933	0.1665638	0.7630586	0.445428	0.917117
grin3bb	51.00932363	-0.039549605	0.2866548	-0.137969	0.890265	0.989291
BX323031.1	3.941983985	-0.271453339	0.9760577	-0.278112	0.780926	NA
exd1	6.237168294	0.920795852	0.7829033	1.1761297	0.239543	NA
map4k4	2852.563222	-0.005372783	0.0883414	-0.060818	0.951504	0.99381
prelid1a	1249.57148	-0.189079338	0.0888524	-2.128017	0.033336	0.355558
BX001023.1	57.23910727	0.144741234	0.2661439	0.5438457	0.586548	0.953319
ppfia1	387.4889802	-0.122699004	0.1270198	-0.965983	0.334052	0.875256
traj55	0	NA	NA	NA	NA	NA
CU467043.1	0	NA	NA	NA	NA	NA

zgc:174180	0.475018861	0.853207185	2.8976509	0.2944479	0.768416	NA
nup85	284.8671963	-0.024016366	0.1298668	-0.184931	0.853283	0.985174
si:zfos-2326c3.2	282.0618722	0.138523626	0.1359205	1.0191517	0.308131	0.861088
CU207221.1	448.3428586	0.10850416	0.1172489	0.9254177	0.354749	0.882105
RF00017	0	NA	NA	NA	NA	NA
zmiz2	149.5622231	-0.22635365	0.17605	-1.285735	0.198536	0.763597
CU855895.1	0.157187365	-0.955901296	4.0804729	-0.234262	0.814781	NA
tmprss2	216.6618321	-0.294022008	0.1687168	-1.742696	0.081387	0.552279
pygo1	4.160480743	-0.11077953	0.9203258	-0.12037	0.90419	NA
CABZ01101739.1	0	NA	NA	NA	NA	NA
ptpn23b	342.1052186	-0.22635034	0.1177543	-1.922225	0.054577	0.457793
CT583672.1	346.5157002	-0.158752734	0.1277183	-1.242991	0.213871	0.782251
FQ311908.1	13.64209218	-0.305831924	0.5370285	-0.569489	0.569024	0.948983
vclb	287.0600162	0.014412766	0.1341576	0.1074316	0.914447	0.991119
rela	62.4079676	0.207658617	0.2535066	0.8191449	0.412704	0.904974
si:ch211-11c3.9	0	NA	NA	NA	NA	NA
il1b	306.4322875	-0.508896421	0.1622073	-3.13732	0.001705	0.047485
zgc:165603	6.410050868	0.05972795	0.8026957	0.0744092	0.940685	NA
si:ch73-236c18.7	1.959762264	0.47107059	1.4392594	0.3273007	0.74344	NA
dre-mir-365-2	0.473290478	-0.833892061	2.901156	-0.287434	0.77378	NA
CR388231.1	1.182894427	3.688339242	2.0602285	1.7902573	0.073413	NA
CABZ01078350.1	17.60538014	-0.052328811	0.4489944	-0.116547	0.907219	0.990702
CR925761.1	8.712356334	0.04384244	0.6411351	0.0683825	0.945481	NA
parp11	3.102057144	1.463366333	1.1440555	1.2791043	0.20086	NA
CU655844.1	1.107104734	0.423303955	1.9651206	0.2154086	0.829449	NA
FP245456.1	25.16683053	-0.087657766	0.3862923	-0.226921	0.820485	0.981594
BX649331.1	2.401490789	-1.294064303	1.4085804	-0.918701	0.358252	NA
si:ch73-170d6.3	3.127678178	0.441663518	1.0960635	0.4029543	0.686982	NA
rgrb	528.0464331	-0.066522301	0.126063	-0.527691	0.597714	0.95534
rptor	507.6650939	-0.157207185	0.1077282	-1.459295	0.144484	0.688868
BX511018.1	0.330916733	-1.849579229	3.2489867	-0.569279	0.569167	NA
si:dkey-108k24.2	0	NA	NA	NA	NA	NA
en1b	305.391028	-0.143280052	0.1282421	-1.117262	0.263882	0.830245
CU929403.1	1.489338074	0.356860712	1.5490398	0.2303754	0.8178	NA
pxdn	60.35922059	0.015841172	0.2532064	0.0625623	0.950115	0.99381
ccdc14	28.48167532	0.125954154	0.3549548	0.3548457	0.722705	0.970375
si:dkey-201i2.4	1.981016035	-1.571021256	1.5219811	-1.032221	0.301968	NA
scn4aa	7.896745316	0.048573328	0.7125698	0.0681664	0.945653	NA
CABZ01046427.2	0	NA	NA	NA	NA	NA
CT737182.1	0.988894344	-1.041944082	1.9792901	-0.526423	0.598594	NA
lrtm1	29.0914112	0.205733327	0.3548793	0.5797276	0.562098	0.948728
BX548018.1	0	NA	NA	NA	NA	NA
tfec	33.49626566	-0.395506211	0.3542435	-1.116481	0.264216	0.830245
dhrs13l1	409.3305702	0.114914642	0.120537	0.9533561	0.34041	0.87805
MYH6 (1 of many)	111.0973914	0.019817969	0.1897691	0.104432	0.916827	0.991541
tbcd	267.5935663	-0.081801822	0.129466	-0.63184	0.527491	0.941391
lzts3a	28.11073296	0.088554217	0.3695161	0.2396491	0.810602	0.980958
csf3b	50.19936568	-0.737701901	0.297491	-2.479745	0.013148	0.204871
elmo1	2680.883408	0.001005374	0.0712585	0.0141088	0.988743	0.997589
si:ch211-156p11.1	0.651026051	-0.042574319	2.4423909	-0.017431	0.986092	NA
znf1125	0.860946417	-1.914579776	2.2239452	-0.860893	0.389297	NA
rgs2	43.86041134	-0.245204265	0.3016308	-0.812928	0.416259	0.906237
musk	109.5188747	0.233836275	0.1873417	1.2481804	0.211965	0.77986
BX544876.2	0	NA	NA	NA	NA	NA

pcxa	1560.026235	0.069314067	0.0819323	0.8459918	0.397557	0.899672
CABZ01007829.1	0.158915748	0.967652056	4.0804729	0.2371421	0.812547	NA
BX005124.1	0.31759079	1.807926197	3.579045	0.5051421	0.613459	NA
slitrk6	398.2123569	-0.046404694	0.1124886	-0.412528	0.679953	0.965311
cbx8b	213.7201571	0.182187303	0.1494865	1.2187545	0.222937	0.789115
gig2d	1.219995923	1.398342718	1.8849492	0.7418464	0.45818	NA
si:zfos-741a10.3	5.335374045	-0.302275223	0.9138716	-0.330763	0.740823	NA
RF00003	0	NA	NA	NA	NA	NA
BX640547.2	5.195470755	0.912320558	0.879281	1.0375756	0.299468	NA
CU693379.1	501.8505006	0.024229501	0.1169424	0.2071917	0.83586	0.984089
BX547930.4	3.576015002	0.723892456	1.0389736	0.6967381	0.485967	NA
nsmce1	45.05178135	-0.047348893	0.2950948	-0.160453	0.872524	0.987846
cratb	192.2792226	-0.241889456	0.155588	-1.55468	0.120022	0.645215
polr2b	1681.489936	-0.101731105	0.0802315	-1.26797	0.204809	0.772822
glrx3	1359.614161	-0.00493099	0.0865142	-0.056996	0.954548	0.994533
rangrf	99.3202694	0.067534011	0.2022106	0.3339785	0.738396	0.972689
cd7al	33.05653468	-0.200690885	0.3531273	-0.568325	0.569815	0.948983
pcdh2g28	4472.016408	0.100947841	0.0733842	1.3756081	0.168943	0.725919
sowahca	6.779039746	0.376200166	0.7328624	0.5133299	0.607721	NA
cdhr5b	49.96449632	-0.763550201	0.3064301	-2.49176	0.012711	0.200229
znrf2b	87.62886328	0.181219937	0.2203748	0.8223259	0.410891	0.903176
CABZ01078120.1	1.761237778	-0.220188127	1.5792016	-0.13943	0.88911	NA
traj7	0	NA	NA	NA	NA	NA
CR382300.1	107.2602411	0.089899996	0.201011	0.4472393	0.654702	0.961305
si:ch211-194m7.5	0.696687138	-1.510024977	2.3787501	-0.634798	0.52556	NA
znf1156	91.58864917	-0.240600849	0.2305156	-1.043751	0.296601	0.853709
sbno2a	24.86181743	0.617157066	0.387499	1.5926674	0.111235	0.627048
map7d1a	573.7954203	0.099501733	0.1023761	0.9719231	0.331089	0.874339
cyp2aa4	125.7680763	0.36016594	0.1849913	1.9469344	0.051543	0.445067
vwa1l	35.61519989	-0.715722004	0.3342349	-2.141374	0.032244	0.349508
RF00001	0	NA	NA	NA	NA	NA
snap91a	1894.067791	0.063131165	0.0927	0.6810267	0.495855	0.932377
VSTM2B	270.6625202	0.093108027	0.1389924	0.6698784	0.502935	0.935304
LO018021.1	56.99129899	-0.049711108	0.2663014	-0.186672	0.851918	0.985174
RF00001	0	NA	NA	NA	NA	NA
stx12	743.1352455	0.082599897	0.0992498	0.8322425	0.405272	0.901305
CU571064.1	0.706628594	2.93253102	2.4623982	1.1909248	0.233683	NA
kcnc3b	7.322184666	0.409835028	0.739849	0.5539441	0.579617	NA
CABZ01055522.1	322.3974079	0.0453186	0.1329552	0.3408561	0.733212	0.971947
si:dkey-58i8.5	1.181217373	-0.359330701	1.8241705	-0.196983	0.843841	NA
ftr67	396.4709291	-0.130636425	0.1148426	-1.137526	0.255318	0.823868
sec14l1	380.4313588	-0.084394963	0.1189644	-0.709413	0.478068	0.926857
tcerg1a	894.9858486	-0.11904693	0.0947426	-1.25653	0.208924	0.776073
CT583651.2	195.0379942	0.042391538	0.1469616	0.2884532	0.773	0.975687
cdh22	6.461447713	0.209972681	0.7603219	0.2761629	0.782423	NA
farsa	1018.876793	-0.012375301	0.1028036	-0.120378	0.904184	0.990291
plcl2	107.4039336	0.091298843	0.1995879	0.4574367	0.647357	0.960581
CT573793.1	0	NA	NA	NA	NA	NA
gcdhb	255.9526167	0.042309616	0.1479898	0.2858955	0.774958	0.975687
fam168a	107.0998129	-0.040150023	0.1909534	-0.210261	0.833464	0.983583
sox4b	2710.733948	-0.244249715	0.0744018	-3.282845	0.001028	0.031702
BX664606.1	0	NA	NA	NA	NA	NA
tgm5l	34.4199515	-0.207708702	0.3463261	-0.599749	0.548674	0.946312
sdr42e1	44.01117965	0.308146991	0.3022397	1.0195451	0.307944	0.861088

CABZ01044108.1	0.787665208	-1.829930327	2.4308619	-0.752791	0.451576	NA
BX005073.1	0	NA	NA	NA	NA	NA
FQ311879.1	0	NA	NA	NA	NA	NA
lrp12	943.8601133	0.153950691	0.0961259	1.6015532	0.109254	0.623132
SLC25A29	24.25732633	0.739340938	0.3962642	1.8657777	0.062072	0.486493
si:dkey-11d20.1	0	NA	NA	NA	NA	NA
smdt1b	781.4049987	0.033200608	0.1241096	0.2675103	0.789076	0.976965
ehd1a	808.4201482	0.022671463	0.0981495	0.2309891	0.817323	0.981594
RF00001	0	NA	NA	NA	NA	NA
oxsm	229.1286349	-0.305580226	0.1418551	-2.154171	0.031227	0.342093
RYR2 (1 of many)	750.2721649	0.056259225	0.1109853	0.506907	0.61222	0.95688
lifra	12.80252764	0.114122239	0.5634539	0.2025405	0.839494	0.984979
nop14	600.0700145	-0.122114966	0.0982572	-1.242809	0.213938	0.782251
fam57a	27.36358095	0.108417088	0.3880671	0.2793772	0.779955	0.976146
plpp4	2.147436038	0.228613569	1.259339	0.1815346	0.855948	NA
RF00001	0	NA	NA	NA	NA	NA
BX548044.7	2.427105035	0.46344952	1.3765106	0.3366843	0.736355	NA
PRDM15	36.22081447	-0.032196485	0.3255794	-0.09889	0.921226	0.992138
XPO5	178.3195967	0.127300938	0.172466	0.7381219	0.46044	0.921978
gga3b	85.66899765	-0.0444142	0.2221221	-0.199954	0.841517	0.985174
exoc6b	441.7139641	-0.076794418	0.1081171	-0.71029	0.477525	0.926857
si:dkey-199m13.5	5.183181456	-1.027821731	0.8746423	-1.175134	0.239941	NA
BX927164.1	0.158795395	0.967652056	4.0804729	0.2371421	0.812547	NA
si:dkey-4j21.2	2.453597695	-1.030026991	1.2425449	-0.828966	0.407124	NA
pclob	1222.154155	0.189927177	0.087273	2.176242	0.029537	0.332786
si:dkey-238i5.3	0.665331516	0.047715978	2.3078578	0.0206754	0.983505	NA
stac3	774.5872899	0.19897792	0.0943196	2.1096136	0.034892	0.363705
BX649355.1	0.158795395	0.967652056	4.0804729	0.2371421	0.812547	NA
dre-mir-735	0	NA	NA	NA	NA	NA
CU861459.1	0	NA	NA	NA	NA	NA
si:zfos-128g4.2	105.5601953	0.185330963	0.195938	0.9458652	0.344217	0.878434
cyp2aa9	64.25285371	-0.111729623	0.2835926	-0.393979	0.693596	0.96736
si:ch211-223a21.1	15.98118161	-0.112316432	0.4802103	-0.23389	0.81507	0.981081
BX324119.1	0.317711143	1.810477155	3.2978238	0.5489915	0.583011	NA
RF00001	0	NA	NA	NA	NA	NA
BX323544.1	0.158795395	0.967652056	4.0804729	0.2371421	0.812547	NA
pds5b	2768.360704	-0.070888703	0.0769692	-0.921001	0.35705	0.883467
si:ch211-255f4.2	47.48880026	0.193407177	0.3115185	0.620853	0.534696	0.943336
zmp:0000001082	162.1268903	-0.298224875	0.1739813	-1.71412	0.086507	0.563804
ubxn8	78.15363296	0.278106179	0.2250376	1.235821	0.216525	0.783739
tfe3a	14.79044833	0.36323802	0.4876477	0.7448779	0.456346	0.921215
pex1	176.9316525	0.271026582	0.1681947	1.6113861	0.107096	0.617015
herc7	26.56607235	-0.171995386	0.3850002	-0.446741	0.655062	0.961614
abraxas2	185.0561926	0.00224679	0.1508673	0.0148925	0.988118	0.997369
CU062501.1	65.19889207	0.225986522	0.244101	0.9257909	0.354555	0.882105
SHC3	66.73244984	0.380916449	0.2477295	1.5376306	0.124139	0.652708
zgc:153293	126.9419817	-0.238852033	0.1844661	-1.294829	0.195379	0.760412
CU633999.2	21.70432983	0.125009722	0.4093853	0.3053596	0.760092	0.975374
faslg	0.476626891	2.395187592	2.815932	0.8505843	0.395	NA
si:ch211-216p19.5	3.433455288	0.980028049	1.0442268	0.9385203	0.347977	NA
krit1	254.7331374	0.018806502	0.1363993	0.1378783	0.890337	0.989291
mcoln2	23.43760561	0.120070688	0.4178697	0.28734	0.773852	0.975687
smarcc1b	466.5887912	-0.028731967	0.1047327	-0.274336	0.783826	0.97627
zgc:173577	14.26794098	-0.056777712	0.494552	-0.114806	0.908599	0.990702

suz12b	1062.23034	-0.001148608	0.0857221	-0.013399	0.989309	0.997589
prdm1b	88.62165193	-0.157508378	0.2281822	-0.690275	0.490021	0.931926
si:ch211-196h24.1	1.659851382	-1.161637942	1.5333985	-0.757558	0.448716	NA
tex15	5.774379846	0.909870758	0.8633749	1.0538536	0.29195	NA
gigyf1a	149.6941371	-0.029245418	0.1811093	-0.161479	0.871716	0.987811
rrp7a	210.0038249	-0.261288605	0.1588517	-1.644858	0.099999	0.601211
bxdc2	524.9596208	0.136109212	0.1129905	1.2046071	0.228355	0.795541
kif5aa	5049.689071	6.94E-05	0.0724928	0.0009574	0.999236	0.999704
si:ch73-315f9.2	49.26796873	0.309194207	0.3224497	0.9588913	0.337614	0.876395
trdj1	0	NA	NA	NA	NA	NA
BX323855.1	0.475018861	0.853207185	2.8976509	0.2944479	0.768416	NA
CU856520.1	786.6676429	-0.12370277	0.0915474	-1.351243	0.176618	0.737268
rp9	202.1449903	-0.081245786	0.1691777	-0.480239	0.631057	0.957356
dre-mir-7148	0	NA	NA	NA	NA	NA
nme8	24.07013768	0.150923552	0.3918345	0.3851717	0.70011	0.967998
CT583726.1	0	NA	NA	NA	NA	NA
pald1a	442.6963556	-0.145517038	0.1087621	-1.337939	0.180916	0.741776
mslna	32.46571953	0.157201382	0.3566634	0.4407556	0.65939	0.96288
si:dkeyp-92c9.4	24.59835614	-1.039087982	0.4042065	-2.570686	0.01015	0.172128
BX957297.1	4.644996436	-0.222709595	0.9580771	-0.232455	0.816185	NA
gata4	18.96587374	-0.30650826	0.4813192	-0.636809	0.524249	0.939336
stard13b	514.5454899	-0.050956289	0.1054343	-0.483299	0.628883	0.957354
ssh2b	442.2046332	-0.0632761	0.1253753	-0.504693	0.613774	0.95688
znf1100	1.422967141	0.331098809	1.7641049	0.1876866	0.851122	NA
RF00003	0	NA	NA	NA	NA	NA
pcdh1g13	56.38889471	0.154675993	0.257483	0.6007231	0.548024	0.945768
si:ch211-9d9.7	0.988265837	0.023857189	1.9450119	0.0122658	0.990214	NA
CABZ01064972.1	9.225082698	0.394168422	0.6401605	0.6157337	0.53807	NA
rabgap1	1061.2075	0.018624493	0.0861439	0.2162021	0.82883	0.982721
RSBN1	236.5701417	-0.300193977	0.1420116	-2.11387	0.034526	0.361039
CU459186.1	9.676719148	1.163617693	0.673983	1.7264792	0.084261	NA
FP003590.1	1.468709556	-0.366786288	1.7646744	-0.207849	0.835347	NA
mrps16	316.8208371	0.002990016	0.1397579	0.0213943	0.982931	0.996944
CR759816.1	6.647152399	-0.001195502	0.7555354	-0.001582	0.998737	NA
si:ch211-76m11.15	0	NA	NA	NA	NA	NA
si:ch211-153f2.3	44.48146205	-0.080912585	0.2844388	-0.284464	0.776055	0.975826
dre-mir-126b	0	NA	NA	NA	NA	NA
CABZ01112656.1	469.8396397	0.046356306	0.1131624	0.4096442	0.682067	0.965476
pid1	44.2061838	-0.183034136	0.2863546	-0.639187	0.522701	0.939336
BX649431.2	0	NA	NA	NA	NA	NA
znf1040	53.48904879	0.139546136	0.2701863	0.5164811	0.605518	0.95651
znf1071	2.142291928	0.636310703	1.3817343	0.460516	0.645146	NA
si:ch211-193i15.1	1.297255751	-0.034484508	1.7090399	-0.020178	0.983902	NA
CU459012.1	41.02807001	0.48009166	0.3065433	1.5661461	0.117314	0.640853
zgc:162858	268.727853	0.025861641	0.1303567	0.1983914	0.842739	0.985174
znf1095	5.394696306	-0.537123051	0.8723075	-0.61575	0.53806	NA
zgc:114119	272.4184328	-0.032941306	0.1500319	-0.219562	0.826212	0.982196
CR352263.1	21.5181699	0.474499749	0.4312154	1.1003775	0.271168	0.835995
cyp2k6	9.91112297	0.475431706	0.6007469	0.791401	0.42871	NA
RF01190	0	NA	NA	NA	NA	NA
si:ch73-311h14.2	279.0415166	0.054093216	0.1282537	0.4217673	0.673195	0.965257
RF00001	0.173729368	-0.955901296	4.0804729	-0.234262	0.814781	NA
CR847947.1	1.226716822	-1.285857512	1.9544125	-0.657925	0.510586	NA
creb5a	39.19487413	-0.060995929	0.3115613	-0.195775	0.844786	0.985174

cyp2aa1	157.8551472	-0.52841137	0.162381	-3.254146	0.001137	0.034075
palm2	7.456507971	0.731710543	0.690453	1.0597543	0.289256	NA
EGFLAM	8.324457277	0.123426632	0.6458845	0.1910971	0.84845	NA
si:dkey-50i6.6	72.65467274	-0.13839676	0.2321426	-0.596171	0.551061	0.946549
fxyd1	932.4310712	0.106540959	0.1064992	1.000392	0.317121	0.867335
wacb	90.41815055	0.024717357	0.2080204	0.1188218	0.905417	0.990702
CR749168.1	0	NA	NA	NA	NA	NA
RF00001	0.157187365	-0.955901296	4.0804729	-0.234262	0.814781	NA
setdb1b	1322.860223	-0.048769512	0.0864266	-0.564288	0.572558	0.949494
faua	7273.909	-0.266488144	0.0793922	-3.356603	0.000789	0.025611
CABZ01034698.1	0	NA	NA	NA	NA	NA
si:ch211-188p14.5	0	NA	NA	NA	NA	NA
CU915778.1	14.85371436	0.692023048	0.5233783	1.3222234	0.186094	0.750443
si:dkey-185m8.2	8.490569789	0.66696346	0.6914491	0.964588	0.334751	NA
tradv25.0.2	0	NA	NA	NA	NA	NA
trdj2	0.333314908	-1.858695402	3.5126701	-0.52914	0.596708	NA
scn4ba	31.62310177	0.772851606	0.3496023	2.2106596	0.027059	0.316986
FO818704.1	1.483603486	-0.918010655	1.6988633	-0.540368	0.588944	NA
unc5a	9.713252842	-0.344927183	0.6467364	-0.533335	0.593802	NA
pcdh1g33	320.9360715	0.137318698	0.1247418	1.1008237	0.270973	0.835943
CU138542.1	0.471562095	-2.373056629	3.0645586	-0.774355	0.438721	NA
kcng1	55.41502347	0.086382386	0.280046	0.3084578	0.757734	0.975374
pcbp2	3451.783891	-0.028214195	0.069165	-0.407926	0.683328	0.965476
cfap157	9.245153283	-0.72966595	0.6659777	-1.095631	0.27324	NA
BX548000.1	2.973102951	0.995041575	1.2096859	0.8225619	0.410757	NA
cacnb2a	255.6424349	0.032567974	0.1322826	0.2461999	0.805527	0.979893
si:ch211-227p7.5	12.47398025	-0.027849693	0.5355055	-0.052006	0.958524	0.994935
BX323797.1	0.166657454	-0.955901296	4.0804729	-0.234262	0.814781	NA
znf1070	6.319239139	-1.6994311	0.8038043	-2.114235	0.034495	NA
rragcb	83.19835446	0.0256479	0.2297274	0.1116449	0.911105	0.991066
tradv2.0.3	0	NA	NA	NA	NA	NA
BX088596.1	29.66386225	0.276362456	0.3635036	0.7602744	0.447091	0.917154
CR790365.1	0	NA	NA	NA	NA	NA
CU929262.1	0.522329263	0.999930388	2.8066578	0.3562709	0.721638	NA
zgc:111868	12.62365995	-0.634782671	0.5663675	-1.120796	0.262375	0.830166
znf1154	17.71968251	0.101369884	0.4822863	0.2101861	0.833522	0.983583
tradv6.0.2	0	NA	NA	NA	NA	NA
BX323051.1	0.31437473	-1.788572922	3.5930657	-0.497785	0.618636	NA
si:dkey-161l11.67	0	NA	NA	NA	NA	NA
si:ch211-147m6.3	0	NA	NA	NA	NA	NA
bsk146	326.2903964	0.199815595	0.1291439	1.5472318	0.121807	0.648874
FP074867.1	6.956273952	-0.403105631	0.706295	-0.570733	0.568181	NA
si:zfos-741a10.4	1.957123383	0.475071774	1.3245505	0.3586664	0.719845	NA
si:ch211-106g8.54	0	NA	NA	NA	NA	NA
CABZ01032982.1	0.656369582	-1.475482481	2.3019817	-0.640962	0.521547	NA
CU467646.3	0.166657454	-0.955901296	4.0804729	-0.234262	0.814781	NA
FO905040.1	24.57415202	-0.024418099	0.3906613	-0.062505	0.950161	0.99381
aclya	2581.315186	0.008055313	0.0746656	0.1078852	0.914087	0.991119
sbno1	1399.165849	-0.043423083	0.0895748	-0.484769	0.62784	0.957354
CR376766.1	0	NA	NA	NA	NA	NA
BX572078.1	1.1953612	-0.381847657	1.7325832	-0.220392	0.825566	NA
pcif1	105.2315161	0.162727361	0.1933374	0.8416757	0.399969	0.90084
RF00001	0	NA	NA	NA	NA	NA
BX511142.1	1.518457159	-0.245850314	1.574579	-0.156137	0.875925	NA

edar	29.0118824	0.189892777	0.3738043	0.5080005	0.611453	0.95688
znf1087	7.764594022	-0.11622613	0.7011629	-0.165762	0.868344	NA
CU929418.1	0	NA	NA	NA	NA	NA
RASGRP2	15.05517927	0.17213103	0.5337243	0.3225092	0.747067	0.97419
dock1	670.0463526	-0.18328455	0.1064793	-1.721317	0.085193	0.560519
CR974475.1	0	NA	NA	NA	NA	NA
gabrb4	27.30931701	-0.424577776	0.3866483	-1.098098	0.272162	0.837242
LO017849.1	0.981393344	-0.052272524	2.0628158	-0.02534	0.979783	NA
CABZ01015815.1	58.93141907	-0.458538039	0.2919866	-1.570408	0.11632	0.638744
gch2	1224.574865	0.073567434	0.0956055	0.7694893	0.441603	0.916336
dre-mir-19a	0	NA	NA	NA	NA	NA
rpl24	14195.05076	-0.379886338	0.0800115	-4.747895	2.06E-06	0.000186
afmid	91.44296157	-0.282983778	0.2262094	-1.250982	0.210941	0.778412
FP016019.1	0.672911584	-1.510325414	2.402234	-0.628717	0.529534	NA
clic6	115.7569428	-0.005815523	0.1966647	-0.029571	0.976409	0.996762
CU929444.1	394.5200744	0.050289418	0.1215397	0.4137696	0.679043	0.965257
zgc:175280	111.3582233	0.315336173	0.2020818	1.5604379	0.118656	0.642318
si:dkey-71l4.3	7.169771436	-0.327716293	0.7146028	-0.458599	0.646522	NA
UNC13B	148.3203073	0.241906098	0.1661955	1.4555516	0.145517	0.691029
FP017279.1	0	NA	NA	NA	NA	NA
nagk	287.2698817	-0.277605655	0.1314839	-2.111329	0.034744	0.36274
si:dkey-33i11.4	642.6802927	0.222202619	0.1331352	1.669	0.095117	0.586483
si:dkey-218f9.10	165.8069126	0.158354118	0.1581654	1.0011934	0.316733	0.867129
si:dkey-6d5.1	0.317831496	1.808837541	3.5779923	0.5055454	0.613176	NA
naa38	345.2213857	-0.225996795	0.1224446	-1.845707	0.064935	0.497901
CU459186.2	6.411359828	3.098962696	0.9807952	3.159643	0.00158	NA
f3a	182.9781941	0.172999603	0.1526085	1.1336174	0.256955	0.82511
RF01848	0	NA	NA	NA	NA	NA
CABZ01055168.1	0	NA	NA	NA	NA	NA
phlda1	64.3118799	-0.109735668	0.2420799	-0.453304	0.65033	0.960708
si:ch73-72b7.1	6.627804464	-0.725680598	0.7913929	-0.916966	0.35916	NA
zgc:195356	8.008038641	1.150864211	0.6939898	1.6583301	0.097251	NA
BX663503.3	649.1456667	0.146404878	0.1049464	1.3950449	0.163002	0.717875
btr29	2.005182646	-0.002059822	1.3014479	-0.001583	0.998737	NA
fbxw5	928.8015334	0.144560936	0.0847481	1.7057717	0.088051	0.568063
tradv25.0.3	0	NA	NA	NA	NA	NA
CR762408.1	0	NA	NA	NA	NA	NA
fnta	568.4822278	-0.161692002	0.1049247	-1.541029	0.12331	0.651258
igfbp3	749.1265963	0.105275801	0.0901052	1.1683655	0.242659	0.811434
BX511066.1	0	NA	NA	NA	NA	NA
bzw1b	2296.607739	0.017438773	0.0882527	0.1976004	0.843358	0.985174
hs3st3b1a	23.22468388	-0.141927043	0.4360191	-0.325506	0.744798	0.974094
si:dkey-190j3.2	3.1004604	0.186119465	1.1084336	0.1679121	0.866652	NA
tpst2	34.22509963	0.284805214	0.3359159	0.8478467	0.396523	0.899672
katnal1	345.858906	-0.052943687	0.1216195	-0.435322	0.663329	0.964736
CR450748.1	0.173729368	-0.955901296	4.0804729	-0.234262	0.814781	NA
si:ch211-186j3.3	1.147930445	-0.458203818	1.7520625	-0.261523	0.79369	NA
CR749168.2	0.476506538	2.394892832	2.8161716	0.8504073	0.395099	NA
dnaaf4	62.39585465	0.013885945	0.2523496	0.0550266	0.956117	0.994543
RF00017	0.182570949	0.967652056	4.0804729	0.2371421	0.812547	NA
naccla	67.00743287	0.194723733	0.250129	0.7784933	0.436278	0.914408
RF00001	0	NA	NA	NA	NA	NA
cxcl8b.3	27.6686491	-0.051375908	0.3756265	-0.136774	0.891209	0.989291
si:dkey-161l11.78	0	NA	NA	NA	NA	NA

BX000701.1	25.54882446	0.090786149	0.3857409	0.2353553	0.813933	0.981081
BX530097.1	28.63386579	0.497532591	0.3598996	1.3824204	0.166843	0.723339
BX666065.1	0.949676663	1.00110294	2.1117873	0.4740548	0.635461	NA
hmgb1a	11831.28857	0.094904786	0.0697414	1.3608093	0.173574	0.732917
dok1b	54.94044735	0.108928637	0.2664224	0.4088569	0.682645	0.965476
CR927050.1	0	NA	NA	NA	NA	NA
LO018568.1	4.886277917	0.955634684	0.9316713	1.0257209	0.305023	NA
ankrd55	158.4130072	0.026597303	0.1654422	0.160765	0.872279	0.987846
fkbp10a	135.6680189	0.313287085	0.1826326	1.7153952	0.086273	0.563208
map2k6	960.7179482	0.006416299	0.0908289	0.0706416	0.943683	0.993364
chia.2	476.4418514	-0.547457952	0.141774	-3.861483	0.000113	0.005422
slc1a5	2061.180421	0.032206346	0.0719362	0.4477073	0.654364	0.961305
purg	53.794484	-0.003428656	0.2628636	-0.013043	0.989593	0.997612
CU856516.1	19.46748355	0.1783056	0.4559835	0.3910352	0.695771	0.96736
CABZ01058261.1	809.7535176	-0.121724307	0.0963266	-1.263663	0.206351	0.774011
ier2a	578.5960162	-0.003982058	0.1279248	-0.031128	0.975167	0.996762
actc1b	121343.8334	0.256736709	0.1885659	1.3615227	0.173349	0.732435
BX927287.1	8.165032132	0.161869301	0.6979331	0.2319267	0.816595	NA
itchb	23.39186765	-0.791758691	0.4011224	-1.973858	0.048398	0.433433
zgc:123103	4187.827342	0.29633398	0.1065553	2.7810346	0.005419	0.109223
BX908731.1	1.845758743	0.297900097	1.3652675	0.2181991	0.827274	NA
atp1b2a	6201.51426	0.14092081	0.0748472	1.8827812	0.05973	0.478372
CU466285.2	0	NA	NA	NA	NA	NA
CR855379.1	0.157187365	-0.955901296	4.0804729	-0.234262	0.814781	NA
si:dkey-11f4.7	6.547578104	0.586494051	0.7661767	0.7654814	0.443985	NA
BX681417.1	0	NA	NA	NA	NA	NA
si:dkey-220o5.5	1.149418122	0.346570459	1.8373228	0.188628	0.850384	NA
glyr1	1437.987874	-0.047753251	0.0764458	-0.624668	0.532189	0.942546
CR318603.1	0.157187365	-0.955901296	4.0804729	-0.234262	0.814781	NA
atp8b2	3.81066062	-0.936135673	1.0219372	-0.91604	0.359646	NA
zgc:109982	43.90276539	0.184565236	0.2923074	0.6314081	0.527774	0.941629
ghrhr2	2.096858248	0.683236533	1.3795241	0.4952697	0.62041	NA
BX571942.1	48.81365614	0.109791605	0.273683	0.4011634	0.6883	0.966672
ppp2r2bb	213.4827598	-0.059128587	0.1461025	-0.404706	0.685693	0.966307
prpsap1	88.1294897	-0.250207259	0.2236023	-1.118983	0.263147	0.830245
CABZ01076667.1	946.0835093	-0.156299491	0.1011049	-1.545915	0.122125	0.649408
galnt13	340.4177665	0.11372149	0.1254199	0.9067257	0.364552	0.886564
RF00001	0	NA	NA	NA	NA	NA
CU929119.1	7.121035593	0.914852805	0.732911	1.2482455	0.211941	NA
si:zfos-2372e4.1	8.426024301	-0.774171085	0.7311647	-1.058819	0.289682	NA
hook2	183.38059	0.091006939	0.1636744	0.5560243	0.578194	0.951024
10-Sep	1089.617294	-0.086346917	0.0946944	-0.911848	0.361849	0.885944
rasd4	644.1627495	-0.056254801	0.0990496	-0.567946	0.570072	0.948983
traj47	0	NA	NA	NA	NA	NA
acap3b	97.12589676	0.000744951	0.1980832	0.0037608	0.996999	0.99906
ppp2cb	3229.804651	-0.066682098	0.0713794	-0.934193	0.350205	0.880195
si:dkey-68o6.5	19.17726267	-0.452151334	0.4595601	-0.983879	0.325175	0.871629
CR450749.1	0	NA	NA	NA	NA	NA
CT030005.1	0	NA	NA	NA	NA	NA
ipcef1	69.87123002	0.050866203	0.2356759	0.2158312	0.829119	0.982737
CU861469.1	0.365141898	1.973577737	3.3927314	0.5817076	0.560764	NA
BX465836.2	0	NA	NA	NA	NA	NA
pum1	2896.705788	-0.018743832	0.0728329	-0.257354	0.796906	0.978569
si:ch73-119p20.1	1566.410153	0.276343947	0.0886815	3.1161398	0.001832	0.050201

vps52	458.5684559	0.046222149	0.1174992	0.3933825	0.694037	0.96736
tradv15.0	0.349228403	0.005884014	3.2372587	0.0018176	0.99855	NA
PITPNC1	15.91354488	-0.040021703	0.5158585	-0.077583	0.93816	0.99279
si:dkey-16p6.1	35.27611629	-0.319078287	0.3314616	-0.96264	0.335728	0.876088
slc7a5	34.71063869	0.340655741	0.3293108	1.0344506	0.300926	0.856434
vasna	661.8251428	0.078197773	0.0986452	0.7927178	0.427942	0.910858
si:dkeyp-13d12.11	0	NA	NA	NA	NA	NA
si:dkeyp-115e12.7	239.343443	-0.136764746	0.140144	-0.975887	0.32912	0.873436
zgc:103508	268.6404644	-0.017128839	0.1325772	-0.129199	0.8972	0.990121
atxn3	193.5225737	-0.113479004	0.1653661	-0.686229	0.492569	0.931926
ugt5b3	125.6277591	-0.523901918	0.2174344	-2.409472	0.015976	0.233052
znf1039	0.325573202	0.005883621	3.3172783	0.0017736	0.998585	NA
utp20	398.5713136	-0.134031727	0.1121758	-1.194837	0.232151	0.800166
si:dkey-112e7.2	477.6553297	-0.13743551	0.1276759	-1.07644	0.28173	0.84357
si:ch73-359m17.6	0	NA	NA	NA	NA	NA
epb41a	404.6518357	-0.198372706	0.1378772	-1.438764	0.150217	0.698425
si:dkey-228b2.6	0	NA	NA	NA	NA	NA
st3gal1l3	0.862674799	-0.605687625	2.2107371	-0.273975	0.784104	NA
RF00001	0	NA	NA	NA	NA	NA
lsr	483.036738	-0.117378623	0.1108791	-1.058618	0.289774	0.846246
CR847534.1	7.234557703	0.953295225	0.7123752	1.3381927	0.180834	NA
BX914214.2	6.726419345	-0.660832165	0.7697994	-0.858447	0.390646	NA
si:ch73-199g24.2	475.7958517	-0.169732623	0.1142291	-1.485896	0.137307	0.674358
BX511034.4	0	NA	NA	NA	NA	NA
xrcc5	478.5186347	-0.261903812	0.1049876	-2.494615	0.012609	0.19947
cdc37	1619.338543	-0.195132638	0.0899508	-2.169326	0.030058	0.335259
AL954741.1	53.18664226	0.030993751	0.2827346	0.1096214	0.91271	0.991119
RF00001	0.340386822	-1.882368278	3.2159502	-0.585323	0.558331	NA
si:ch211-51f19.1	32.07101858	0.262220564	0.3445963	0.7609499	0.446687	0.917154
RF00001	0	NA	NA	NA	NA	NA
zgc:175088	1182.952614	0.130954811	0.0975219	1.3428252	0.179329	0.740258
si:dkey-10p5.10	168.1641062	0.085200478	0.1596739	0.5335906	0.593625	0.954434
si:zfos-169g10.3	67.40276824	-0.0813297	0.2548424	-0.319137	0.749622	0.97419
jpt1b	1858.197714	0.075589036	0.1015449	0.7443906	0.45664	0.921215
naga	361.8527613	-0.339905708	0.158203	-2.148541	0.031671	0.345305
nsdhl	182.873566	-0.134358337	0.1588585	-0.845774	0.397679	0.899672
si:ch211-255f4.9	2.483816655	-0.573279681	1.1753477	-0.487753	0.625725	NA
CU651657.1	0.31437473	-1.788572922	3.5930657	-0.497785	0.618636	NA
CU138553.1	0	NA	NA	NA	NA	NA
btbd17a	282.1843005	0.141818737	0.1569176	0.9037784	0.366113	0.887239
dlg2	144.7888514	-0.126838805	0.1695905	-0.747912	0.454513	0.920666
gig2i	0.340386822	-1.882368278	3.2159502	-0.585323	0.558331	NA
zgc:174944	6.055371038	-0.223957455	0.787585	-0.28436	0.776135	NA
BX927130.1	4.247249532	-0.558419465	0.9767229	-0.571728	0.567507	NA
h2afy	308.3258181	-0.249656891	0.1264054	-1.975049	0.048263	0.432986
si:ch211-246b8.5	0	NA	NA	NA	NA	NA
CABZ01064859.1	142.7179342	-0.332225283	0.1735729	-1.914038	0.055615	0.461579
ly6m5	17.69934361	-0.13843067	0.4476909	-0.30921	0.757162	0.975374
mvda	176.088135	0.076651525	0.1520755	0.504036	0.614236	0.95688
si:ch211-209j12.2	13.17200047	-0.846668638	0.542944	-1.559403	0.118901	0.642772
pacsin3	744.2335557	0.214438578	0.0977721	2.1932484	0.028289	0.325989
RF00003	0	NA	NA	NA	NA	NA
crfb15	8.725440575	-0.616138647	0.6566336	-0.938329	0.348075	NA
zgc:92162	12.06708507	1.214920528	0.5599675	2.1696268	0.030035	0.335259

si:rp71-1d10.8	94.19793314	0.231592426	0.2089373	1.1084305	0.267676	0.833349
znf1079	7.138487098	0.215459069	0.7046981	0.3057466	0.759798	NA
si:dkey-220o21.4	0	NA	NA	NA	NA	NA
CU469503.1	0	NA	NA	NA	NA	NA
igfbp1a	1413.876652	-0.083720398	0.0982867	-0.851798	0.394326	0.898733
si:rp71-62i8.1	15.82470861	-0.591903602	0.5086297	-1.163722	0.244537	0.814189
slc25a13	9.300484764	0.408599165	0.675567	0.6048241	0.545296	NA
BX890548.1	495.6206185	0.070437673	0.1122364	0.6275832	0.530277	0.942209
hccsb	849.3500933	0.17318808	0.0870751	1.9889517	0.046707	0.424613
CR855320.1	1.717690463	0.06652635	1.4948007	0.0445052	0.964502	NA
si:dkey-30e9.6	11.6618742	0.521074356	0.5668249	0.9192862	0.357946	0.884398
CABZ01088346.1	65.19297549	0.454414593	0.2791855	1.6276439	0.1036	0.609882
lhb	2.83127539	-0.872060563	1.1245163	-0.775498	0.438045	NA
BX511034.5	8.242286414	0.840274856	0.6709519	1.2523623	0.210438	NA
evx1	0	NA	NA	NA	NA	NA
slc25a42	49.98183601	-0.147849544	0.2927179	-0.505092	0.613494	0.95688
AL954861.2	4.799179691	-1.307683096	1.0171432	-1.285643	0.198568	NA
cygb1	1018.248577	-0.013757532	0.0887583	-0.155	0.876821	0.987881
CT583728.3	0	NA	NA	NA	NA	NA
zgc:158320	52.39152625	-0.167443361	0.2783064	-0.601651	0.547406	0.945526
sp9	359.2515001	0.00323551	0.1325109	0.0244169	0.98052	0.996944
poldip3	509.756642	0.016454472	0.1051621	0.1564677	0.875664	0.987881
CABZ01050249.1	160.5216918	-0.010126998	0.1678151	-0.060346	0.95188	0.993864
si:cabz01030277.1	0.157187365	-0.955901296	4.0804729	-0.234262	0.814781	NA
ppp4r1l	99.51796872	0.102973647	0.1980476	0.519944	0.603103	0.956045
rpl13	19224.30695	-0.298393436	0.0714708	-4.175042	2.98E-05	0.001748
si:dkey-27n6.1	4.817559143	-0.478299764	0.8585534	-0.5571	0.577459	NA
si:ch211-209n20.1	3.773212606	-0.13350886	1.0172283	-0.131248	0.895579	NA
dnajb1a	122.9066103	0.303494832	0.1895599	1.6010496	0.109366	0.623408
si:dkey-238k10.1	20.38671354	-0.709000744	0.4375454	-1.620405	0.105145	0.613317
si:ch211-223l2.4	400.2724438	0.023923717	0.12532	0.190901	0.848603	0.985174
lrrtm4l2	16.07403826	0.073580354	0.4800498	0.1532765	0.87818	0.987881
jpt1a	269.6070401	-0.069952666	0.1336105	-0.523557	0.600587	0.956045
BX957256.1	0.356300316	0.005884122	3.214964	0.0018302	0.99854	NA
lama2	48.29573717	-0.045031053	0.3030867	-0.148575	0.881889	0.988344
si:dkey-161l11.76	0	NA	NA	NA	NA	NA
cables1	41.58582107	0.728152819	0.3070006	2.371829	0.0177	0.246724
rbpms	30.02789003	-0.30919787	0.3516941	-0.879167	0.379311	0.895192
cfap410	76.21022526	-0.459124291	0.2286693	-2.007809	0.044664	0.41583
ccl33.3	0.795406914	-3.1255921	2.3628864	-1.322786	0.185907	NA
CR753902.1	0.681245011	1.537021632	2.3941387	0.6419936	0.520877	NA
CU694952.1	1.222514451	1.394641811	1.9626635	0.7105863	0.477341	NA
lrit1b	485.2550907	0.383384956	0.1304477	2.9389934	0.003293	0.076602
FP085432.1	0.332524763	0.005883742	3.292841	0.0017868	0.998574	NA
BX640537.1	12.56705356	-0.853291997	0.5575675	-1.530383	0.125922	0.654803
zgc:162182	7.339482317	-0.675478786	0.7226065	-0.934781	0.349901	NA
si:dkeyp-79b7.3	1.331129901	-0.800050342	1.731093	-0.462165	0.643963	NA
zgc:158343	567.527813	0.110009074	0.1217362	0.9036679	0.366172	0.887239
bcan	553.3047495	0.210325924	0.1120247	1.8774951	0.06045	0.480058
BX914205.1	0.356300316	0.005884122	3.214964	0.0018302	0.99854	NA
CR759907.1	1.513391121	-2.914329743	1.8767386	-1.552869	0.120454	NA
ly6m3	0.530029684	-0.939524117	2.7937867	-0.336291	0.736652	NA
nme2b.2	9767.75073	-0.046751006	0.1141382	-0.4096	0.6821	0.965476
si:ch73-236c18.9	0	NA	NA	NA	NA	NA

CR391930.1	0.490502273	-2.421143305	2.7891168	-0.868068	0.385357	NA
lcat	104.0061088	0.134309038	0.2166706	0.6198766	0.535339	0.943336
CABZ01073265.1	808.1490536	-0.041515975	0.2257211	-0.183926	0.854072	0.985174
CU179702.1	0	NA	NA	NA	NA	NA
nkx1.2lb	197.223797	-0.143295001	0.159496	-0.898424	0.36896	0.888432
si:dkey-15h8.17	0.166657454	-0.955901296	4.0804729	-0.234262	0.814781	NA
atg16l1	893.9627816	-0.066217208	0.0877	-0.755042	0.450224	0.918761
med24	598.6838571	0.034506385	0.1080627	0.3193182	0.749485	0.97419
si:ch211-246b8.1	2.471988433	-0.636198482	1.2642899	-0.503206	0.614819	NA
BX927335.1	0	NA	NA	NA	NA	NA
tradv23.1.1	0	NA	NA	NA	NA	NA
AL844563.1	0	NA	NA	NA	NA	NA
si:ch1073-268j14.1	9.970721675	1.114761363	0.6146778	1.8135703	0.069744	NA
CABZ01073736.1	8.784617977	-0.164288717	0.6451832	-0.254639	0.799002	NA
serpl	891.6095337	-0.183739697	0.0875256	-2.099269	0.035793	0.368633
CR936249.1	3.702677086	0.603405183	1.0854107	0.5559234	0.578263	NA
cbx4	377.0237115	-0.069638581	0.1235241	-0.563765	0.572914	0.949762
znf1122	0.670675047	-1.455259498	2.4036813	-0.605429	0.544894	NA
cc2d1b	259.6782447	-0.229118038	0.1329035	-1.723943	0.084718	0.560519
slit1b	770.152998	0.020171934	0.1031793	0.1955037	0.844999	0.985174
pcdh1g18	61.38345388	0.079729248	0.2851639	0.2795909	0.779791	0.976146
sh3d21	624.2993817	0.18543055	0.096688	1.9178229	0.055133	0.460392
atrxl	891.7032067	0.056399801	0.0930332	0.606233	0.54436	0.945438
dre-mir-455-1	0.157187365	-0.955901296	4.0804729	-0.234262	0.814781	NA
elof1	1675.525913	-0.025769161	0.081183	-0.317421	0.750924	0.97419
tradv14.0	1.505880076	0.335389091	1.4976509	0.2239434	0.822801	NA
ogt.1	1820.240962	-0.048677588	0.0773123	-0.629623	0.528942	0.942109
BX293991.1	0.48983248	-0.887840757	2.6934859	-0.329625	0.741683	NA
ddx46	1414.25758	-0.186299438	0.0963852	-1.932864	0.053253	0.452412
asb10	36.60633581	-0.003204204	0.3187319	-0.010053	0.991979	0.997833
prlh	6.726854768	0.93497721	0.7668996	1.2191651	0.222782	NA
rnf213a	185.0859879	-0.133789411	0.162406	-0.823796	0.410055	0.902642
si:dkey-190j3.2	0.647019846	-1.455099716	2.4289208	-0.599073	0.549125	NA
RF00026	0	NA	NA	NA	NA	NA
pcdh2ab2	0	NA	NA	NA	NA	NA
BX000985.1	0	NA	NA	NA	NA	NA
muc5.3	186.9089533	-0.831015679	0.1628131	-5.104109	3.32E-07	3.89E-05
CT476811.1	12.45473035	-0.257336447	0.5386158	-0.477774	0.632811	0.957356
FQ312013.1	0.182570949	0.967652056	4.0804729	0.2371421	0.812547	NA
CR855306.1	0.173729368	-0.955901296	4.0804729	-0.234262	0.814781	NA
zgc:174863	139.3194022	0.031400238	0.1714969	0.1830951	0.854723	0.985174
hs2st1a	209.564806	0.141246766	0.1460743	0.9669516	0.333568	0.874933
SYDE2	142.3069866	-0.176392156	0.1817706	-0.970411	0.331842	0.874653
vdra	7.581226139	0.865049559	0.7247968	1.1935063	0.232671	NA
m1ap	0.507044276	-2.459587493	2.7586536	-0.89159	0.372613	NA
si:ch73-233f7.7	1.880182333	-0.761974007	1.5642365	-0.487122	0.626172	NA
tmem176l.1	302.5261865	0.16374095	0.1284128	1.275114	0.202269	0.768575
CR847906.1	3.378008714	-0.868895362	1.0549881	-0.823607	0.410163	NA
si:ch73-309g22.1	0.802200093	1.852032281	2.2653592	0.8175446	0.413617	NA
FP016247.1	0.325452849	0.005883619	3.3177086	0.0017734	0.998585	NA
ndufa4l	3780.696576	-0.028262384	0.0808669	-0.349492	0.72672	0.970402
clec3bb	32.73585744	-0.356939913	0.3842769	-0.928861	0.352961	0.881552
FQ311879.2	0.166657454	-0.955901296	4.0804729	-0.234262	0.814781	NA
traj5	0	NA	NA	NA	NA	NA

CABZ01049362.1	0	NA	NA	NA	NA	NA
btr23	28.92290011	0.162706229	0.3664295	0.4440314	0.65702	0.96215
cnn3a	1180.803754	0.091960526	0.0814411	1.129166	0.258828	0.826961
CABZ01034698.2	0.672403429	0.029250563	2.3004318	0.0127152	0.989855	NA
zgc:112234	15.96779089	0.305163475	0.4950296	0.616455	0.537594	0.943336
ephx4	215.5254552	0.036032352	0.1551833	0.2321922	0.816389	0.981594
mettl23	114.6678199	-0.237451189	0.1856175	-1.27925	0.200809	0.766855
adssl1	2356.231366	0.435886268	0.082429	5.2880179	1.24E-07	1.67E-05
si:ch211-119e14.1	41.87024966	0.103546509	0.309385	0.3346849	0.737863	0.972535
zgc:66475	586.297669	0.018934111	0.1040385	0.1819915	0.855589	0.985174
CU571163.1	0.31598276	0.005883445	3.3523626	0.001755	0.9986	NA
si:ch73-252p3.1	120.032181	0.119533561	0.1886205	0.6337252	0.52626	0.940506
gbe1b	156.1574246	0.144301855	0.1888547	0.7640891	0.444814	0.916797
si:ch1073-13h15.3	108.7582279	-0.130986022	0.214783	-0.609853	0.541959	0.944974
tradv4.0.2	0	NA	NA	NA	NA	NA
nkiras1	189.4864098	0.045876371	0.151593	0.3026286	0.762173	0.975374
dus3l	317.2121174	-0.181645854	0.1208969	-1.502485	0.132972	0.667628
BX957252.2	1.162156842	-0.331037298	1.8315853	-0.180738	0.856573	NA
pimr58	0	NA	NA	NA	NA	NA
ubl3a	1702.225887	0.032029317	0.0901646	0.3552314	0.722416	0.970375
zgc:113279	35.33950733	0.281889871	0.3525154	0.7996526	0.423912	0.908025
CABZ01066926.1	180.5706831	-0.230255912	0.162589	-1.416184	0.156722	0.707054
si:ch73-379j16.2	0.158915748	0.967652056	4.0804729	0.2371421	0.812547	NA
si:ch211-188p14.3	13.39133822	0.385968066	0.5166186	0.7471045	0.455	0.921022
dicp2.2	0.330916733	-1.849579229	3.2489867	-0.569279	0.569167	NA
FQ311924.1	6.991360453	0.724092315	0.7663631	0.9448423	0.344739	NA
CR388156.1	0	NA	NA	NA	NA	NA
si:dkey-165b20.3	2.674799102	-1.096281918	1.2186616	-0.899579	0.368345	NA
snx25	789.6391458	0.019290137	0.0907036	0.2126723	0.831583	0.983358
kynu	373.7687561	-0.305899751	0.1259347	-2.429035	0.015139	0.225353
nbeal1	183.5542334	-0.066535151	0.1557964	-0.427065	0.669332	0.965092
sec16b	134.8679911	-0.019270104	0.175525	-0.109786	0.912579	0.991119
foxo1a	555.3070014	-0.210005377	0.1085926	-1.933883	0.053128	0.452412
CU929079.1	0	NA	NA	NA	NA	NA
CT027744.1	10.47067067	-0.072941999	0.6075585	-0.120058	0.904438	NA
NPC1L1	117.5097746	-1.243417721	0.2043813	-6.083813	1.17E-09	2.42E-07
mir196a-2	0	NA	NA	NA	NA	NA
lyrm5a	190.3535567	0.185713151	0.1508206	1.2313517	0.218191	0.785379
BX088603.1	3.447021936	0.398019593	1.126451	0.3533395	0.723834	NA
lygl2	0.489712128	-0.887840729	2.693685	-0.329601	0.741702	NA
inpp5ka	354.8828119	-0.10970812	0.116275	-0.943523	0.345414	0.878434
lmbrd2a	764.9863844	-0.071518096	0.1050196	-0.680998	0.495873	0.932377
CU856423.1	19.28514996	0.15548996	0.4502241	0.3453612	0.729823	0.971143
RF00001	1.624478268	0.588556441	1.4939716	0.3939542	0.693615	NA
CU468012.2	0.182570949	0.967652056	4.0804729	0.2371421	0.812547	NA
hmgn2	14357.07147	0.05463941	0.0784898	0.6961339	0.486345	0.930833
CR759927.3	41.39801979	-0.2809935	0.3002988	-0.935713	0.349421	0.880195
CU929203.1	0	NA	NA	NA	NA	NA
RF00001	0	NA	NA	NA	NA	NA
si:dkey-182g1.10	77.54161271	-0.110791098	0.2344985	-0.47246	0.636599	0.958607
BX005124.2	0	NA	NA	NA	NA	NA
aldh18a1	1140.063993	-0.07415612	0.0861797	-0.860482	0.389523	0.896421
CR762480.1	0.476626891	2.395187592	2.815932	0.8505843	0.395	NA
CR382383.2	2.114070043	0.218508395	1.3099	0.166813	0.867517	NA

tradv23.3.8	0.356300316	0.005884122	3.214964	0.0018302	0.99854	NA
BX914205.2	0	NA	NA	NA	NA	NA
r3hcc1	136.6526376	-0.193127644	0.1716745	-1.124964	0.260604	0.828546
CU468012.3	3.550638206	-1.661963272	1.086592	-1.529519	0.126136	NA
BX088603.2	0	NA	NA	NA	NA	NA
btd	58.41587528	-0.21171544	0.2619187	-0.808325	0.418903	0.906406
si:dkey-211i20.5	3.352976145	-0.245756704	1.0634298	-0.231098	0.817239	NA
CABZ01067558.1	13.27770705	0.155024746	0.5238775	0.2959179	0.767293	0.975687
pigrl2.3	1.95189695	-1.022617052	1.416056	-0.722159	0.470197	NA
si:dkey-93h22.7	6.861220273	1.440278251	0.8156337	1.7658396	0.077423	NA
BX842700.1	1.364416829	-0.705571612	1.7443167	-0.404497	0.685847	NA
clca5.1	0	NA	NA	NA	NA	NA
si:ch73-160p18.4	0.816075475	-1.880611245	2.2559232	-0.833633	0.404488	NA
tpbga	43.98206215	0.323218084	0.3105524	1.0407843	0.297976	0.85401
RF00001	2.634753246	1.586156655	1.2604672	1.2583879	0.208252	NA
zgc:66483	5.993670435	-0.96823712	0.7946797	-1.218399	0.223072	NA
purab	388.5377394	0.271460576	0.1157198	2.3458447	0.018984	0.257116
RF00001	0	NA	NA	NA	NA	NA
FP236734.1	146.6383251	-0.11336903	0.1761359	-0.643645	0.519806	0.938945
sdr39u1	340.3083294	0.079887976	0.1171666	0.6818324	0.495345	0.931928
si:dkey-1b17.6	43.90914671	-0.005591668	0.2860721	-0.019546	0.984405	0.996944
CABZ01083166.1	179.627543	-0.06673429	0.1610337	-0.414412	0.678572	0.965257
si:ch211-69b22.5	42.27532016	0.439466309	0.3287663	1.3367134	0.181316	0.742543
chmp1b	1363.209082	-0.094043917	0.0927915	-1.013497	0.310823	0.862927
BX908721.1	2.995228194	-0.657850111	1.165773	-0.564304	0.572547	NA
CR855307.1	9.631142079	-0.279625443	0.619518	-0.45136	0.65173	NA
BX510915.1	30.80292364	-0.018584542	0.3918958	-0.047422	0.962177	0.995907
cmtm6	366.1595121	-0.205315973	0.1192426	-1.721834	0.0851	0.560519
sec14l7	197.0492339	0.569469111	0.1497938	3.8016875	0.000144	0.006578
akap8l	3088.87346	-0.085087992	0.0704403	-1.207945	0.227069	0.794587
si:ch211-11c3.11	0.341366344	1.891903201	3.2128156	0.5888614	0.555954	NA
szl	1.504941839	-0.942645884	1.6627636	-0.566915	0.570772	NA
tradv8.0	0	NA	NA	NA	NA	NA
trim35-19	0.657349104	1.487939685	2.418178	0.6153144	0.538347	NA
eed	445.7581518	-0.030201619	0.1240588	-0.243446	0.80766	0.980642
dcun1d1	342.6970018	0.095674816	0.1189818	0.804113	0.421332	0.90651
si:ch211-76m11.9	0.476747244	2.393381959	3.0525537	0.7840589	0.433006	NA
zgc:153184	226.8566821	0.206245141	0.1453614	1.4188443	0.155944	0.705963
pcdh1g3	36.50158422	0.049260196	0.3489757	0.1411565	0.887746	0.989291
LO018363.1	131.4230172	0.171551363	0.1805338	0.9502449	0.341988	0.878434
CABZ01079663.1	179.3120758	0.019870946	0.1524942	0.1303062	0.896324	0.989971
bin3	376.8460056	-0.260887073	0.1184766	-2.202013	0.027664	0.321596
xpot	1154.3208	-0.0246672	0.07977	-0.309229	0.757147	0.975374
marveld3	48.83349966	-0.234710724	0.2788057	-0.841843	0.399876	0.90084
pimr207	0.158795395	0.967652056	4.0804729	0.2371421	0.812547	NA
CR381657.1	0.785936826	-3.109243185	2.5574139	-1.215776	0.22407	NA
akt1	39.55505128	-0.139820031	0.3037023	-0.460385	0.64524	0.96029
CU915256.1	2.796063914	0.167534363	1.1001741	0.1522799	0.878966	NA
cox5ab	1470.647981	-0.02481047	0.0771448	-0.321609	0.747749	0.97419
selenof	1051.188241	-0.025807986	0.1039742	-0.248215	0.803968	0.979643
snrpg	903.8585101	-0.151150129	0.1140459	-1.325345	0.185057	0.748632
CR927050.2	0.157187365	-0.955901296	4.0804729	-0.234262	0.814781	NA
znf1074	13.29483637	-0.224602221	0.5493318	-0.408864	0.682639	0.965476
capgb	688.663817	0.077687531	0.0948609	0.8189622	0.412808	0.904974

si:dkey-203a12.5	0	NA	NA	NA	NA	NA
dicp3.1	16.89878279	0.008180872	0.4802249	0.0170355	0.986408	0.996965
kdm4b	2785.610575	-0.138284795	0.0806305	-1.715043	0.086337	0.563258
BX927335.2	0	NA	NA	NA	NA	NA
bambib	114.1406147	-0.119557193	0.1952897	-0.612204	0.540403	0.944487
BX569789.1	25.0514473	-0.029124774	0.3927715	-0.074152	0.940889	0.992857
tent2	398.7220921	-0.024759574	0.1253347	-0.197548	0.843399	0.985174
BX004980.1	0	NA	NA	NA	NA	NA
trim35-24	1.627395884	-1.946676945	1.7019099	-1.143819	0.252699	NA
gnb5a	453.1443823	0.20558749	0.107267	1.9165965	0.055289	0.46072
CT009597.1	0	NA	NA	NA	NA	NA
FP016142.3	0	NA	NA	NA	NA	NA
spink2.3	0	NA	NA	NA	NA	NA
vaspa	8.748110461	0.385984871	0.6371778	0.6057726	0.544666	NA
CR550302.1	0.182570949	0.967652056	4.0804729	0.2371421	0.812547	NA
ccnyl1	250.5359027	0.223369736	0.1356189	1.6470393	0.09955	0.600153
AL935065.1	4.479178197	-1.011012122	0.9008241	-1.122319	0.261727	NA
neurl1b	323.6507737	-0.082696256	0.1327247	-0.623066	0.533241	0.942971
si:dkey-61n16.5	331.588926	0.353175992	0.1391742	2.5376534	0.01116	0.183437
BX548059.1	4.615657976	-1.338301759	0.92645	-1.444548	0.148585	NA
BX649620.1	0.690594747	1.536595024	2.3851901	0.6442233	0.519431	NA
ahrra	52.59574207	0.378551592	0.2749894	1.3766044	0.168635	0.725919
foxred1	100.252725	0.030589039	0.2042163	0.1497875	0.880932	0.988111
CR385021.1	0.547712847	2.559541715	2.9076406	0.8802813	0.378707	NA
cacna1hb	14.49287227	0.265246616	0.5144126	0.5156301	0.606113	0.956692
BX284696.1	0	NA	NA	NA	NA	NA
si:dkey-29j8.1	4.697744131	-0.113304709	0.9439408	-0.120034	0.904456	NA
zgc:163073	145.3089833	0.245058545	0.1672105	1.4655691	0.142766	0.684998
si:dkey-152b24.7	13.23078345	-0.216209287	0.5138231	-0.420785	0.673912	0.965257
si:rp71-1h20.9	17.26017264	0.081229216	0.4608622	0.1762549	0.860094	0.985773
CU929391.3	0.853712865	-1.943683846	2.3771108	-0.817666	0.413548	NA
evlb	1193.520553	-0.115171949	0.0816411	-1.41071	0.15833	0.709224
zgc:153993	304.9499926	-0.007393738	0.1324339	-0.05583	0.955478	0.994543
relt	224.0692345	0.309356164	0.1438998	2.1498027	0.031571	0.344716
FP236161.1	0.977029335	3.42365565	2.0852004	1.6418833	0.100614	NA
ssbp2	278.4292385	0.024578083	0.1364066	0.1801825	0.857009	0.985597
zgc:56622	126.2421546	-0.435808422	0.1817915	-2.397298	0.016516	0.237008
pcdh10a	1545.581541	-0.073497523	0.0862555	-0.852091	0.394164	0.898673
pkma	7941.585755	-0.057501322	0.067448	-0.852528	0.393921	0.898496
gfra1a	320.0691379	0.280745949	0.1328106	2.1138829	0.034525	0.361039
CIR1	259.8516835	-0.16359465	0.139181	-1.175409	0.239831	0.808111
gig2f	0	NA	NA	NA	NA	NA
ccr9b	3.679219139	-0.48839622	0.9924277	-0.492123	0.622633	NA
mvb12a	227.955772	0.02873696	0.1478868	0.1943173	0.845927	0.985174
heatr1	767.0188349	-0.005509898	0.0927142	-0.059429	0.952611	0.99398
zgc:113019	202.0230697	0.082797869	0.1508259	0.5489634	0.583031	0.952214
gap43	2139.173001	0.019225713	0.0896445	0.2144661	0.830184	0.982916
baz1a	298.5114713	-0.051094153	0.1228805	-0.415803	0.677554	0.965257
rgs3a	380.4015754	-0.0263079	0.1136866	-0.231407	0.816998	0.981594
CABZ01111953.1	215.2478369	-0.089847307	0.1517639	-0.59202	0.553837	0.947449
si:ch73-46n24.1	0.31759079	1.807926197	3.579045	0.5051421	0.613459	NA
ube2d2l	617.0218176	0.150653387	0.0972145	1.5497015	0.121213	0.647458
BX682557.2	0	NA	NA	NA	NA	NA
si:dkey-250k10.4	2.131591567	1.170707889	1.3441419	0.8709704	0.38377	NA

si:dkey-19b23.7	156.2264787	-0.004262054	0.1656223	-0.025734	0.97947	0.996866
zgc:63863	124.3379288	0.007011227	0.1840643	0.0380912	0.969615	0.996315
pfk1b	780.3899054	-0.042757747	0.1036368	-0.412573	0.67992	0.965311
wdr47b	173.7349475	0.184075227	0.1769415	1.0403171	0.298193	0.85401
igf2bp2b	387.7366417	0.135267961	0.1125501	1.2018465	0.229423	0.797069
BX510342.1	0.157187365	-0.955901296	4.0804729	-0.234262	0.814781	NA
vox	3.804730862	0.641602525	0.9698754	0.6615309	0.508272	NA
CR855389.1	45.62640399	-0.570638036	0.3002381	-1.900618	0.057352	0.469987
BX323861.1	0	NA	NA	NA	NA	NA
myl12.1	6064.844339	-0.103167869	0.0769235	-1.341174	0.179864	0.741062
fhdc2	142.609791	0.075729667	0.1742408	0.4346265	0.663834	0.964736
si:ch211-272n13.3	402.6813317	0.114983138	0.1183384	0.9716467	0.331226	0.874339
tradv18.0	0	NA	NA	NA	NA	NA
CU104782.1	0	NA	NA	NA	NA	NA
gne	475.0179339	0.165408351	0.1045408	1.5822372	0.113595	0.631853
CU928046.1	123.9341753	0.107852788	0.1954931	0.5516962	0.581157	0.951731
olfcg4	4.150461214	0.118731858	0.9105978	0.1303889	0.896259	NA
cyb5b	301.4579944	0.123358698	0.1404252	0.8784657	0.379691	0.895192
glula	12029.18197	0.085545503	0.1133966	0.7543923	0.450614	0.918761
si:ch211-11n16.2	252.3814353	0.120017515	0.1423221	0.8432811	0.399071	0.90062
CT737196.1	0	NA	NA	NA	NA	NA
CR383669.2	0.672283077	0.02892376	2.3005571	0.0125725	0.989969	NA
pcdh2ac	0	NA	NA	NA	NA	NA
si:dkey-146m20.13	0	NA	NA	NA	NA	NA
actn4	7284.67497	-0.124543711	0.0681255	-1.82815	0.067527	0.508678
echdc3	47.65139508	-0.237735358	0.3210538	-0.740484	0.459006	0.921978
si:ch211-76m11.7	0	NA	NA	NA	NA	NA
pdk3b	8.718331628	0.255277613	0.6653737	0.3836605	0.70123	NA
BX908776.1	0.790881268	0.5906245	2.2495814	0.2625486	0.792899	NA
cspg5b	1014.862917	0.047137282	0.0875876	0.538173	0.590458	0.954193
si:dkey-285b23.3	0.832617477	-1.907662728	2.2444108	-0.849961	0.395347	NA
RF00001	0	NA	NA	NA	NA	NA
mon2	1349.579981	-0.086071101	0.0832782	-1.033537	0.301352	0.856944
si:dkey-11f4.16	98.45865754	0.061588494	0.2044358	0.3012608	0.763216	0.975374
topaz1	41.68294903	-0.105516006	0.3057983	-0.345051	0.730056	0.971177
pnp5b	207.9239098	0.237317314	0.1459255	1.6262907	0.103888	0.610484
pik3c2g	104.9575627	-0.1320205	0.2059779	-0.640945	0.521558	0.939056
CABZ01059119.1	87.7352532	0.539531716	0.2141558	2.519342	0.011757	0.190723
dre-mir-202	0	NA	NA	NA	NA	NA
BX572620.1	17.24894677	0.307768317	0.473166	0.6504447	0.515405	0.9377
si:dkey-203a12.7	0.182570949	0.967652056	4.0804729	0.2371421	0.812547	NA
si:dkey-31f5.11	418.1230334	0.049979523	0.1103631	0.4528644	0.650646	0.960708
FBXO46	595.5799799	-0.099490529	0.1092908	-0.910329	0.362649	0.885944
tusc2a	164.7196597	-0.23587716	0.160344	-1.47107	0.141272	0.681968
RF00001	0	NA	NA	NA	NA	NA
sb:cb1058	268.7883553	0.262476929	0.1528671	1.7170274	0.085974	0.562932
tymp	27.8912609	0.671635447	0.361172	1.8596001	0.062942	0.489621
nots	12.38700104	-0.836973318	0.5897774	-1.419134	0.15586	0.705963
zgc:174944	22.67377897	-0.203419231	0.4014704	-0.506685	0.612376	0.95688
BX927336.2	6.509954753	1.527179986	0.8028912	1.9021008	0.057158	NA
BX005396.2	2.32686674	-2.565353802	1.5613754	-1.643009	0.100381	NA
BX255908.1	0.365141898	1.973577737	3.3927314	0.5817076	0.560764	NA
fbxo7	186.9362415	-0.193779145	0.1500476	-1.291451	0.196547	0.761835
si:dkey-199m13.7	20.97030462	0.094247662	0.4151862	0.227001	0.820423	0.981594

si:ch211-9d9.1	37.14154406	-0.151363711	0.3243138	-0.46672	0.6407	0.959994
lpar1	55.49140712	0.209159923	0.2635784	0.7935396	0.427463	0.91075
prkcab	12.33841013	-0.268471598	0.5417226	-0.495589	0.620185	0.957354
CU138533.1	40.61605992	0.550518875	0.3173462	1.7347578	0.082784	0.554784
nedd4a	397.6334196	-0.327715035	0.1144402	-2.863635	0.004188	0.090115
GNAZ	123.3757602	-0.174368075	0.1901921	-0.9168	0.359248	0.884762
zgc:174275	1.157441819	-0.400541316	1.7643421	-0.22702	0.820408	NA
si:ch211-154o6.3	177.3228913	-0.181958968	0.1602432	-1.135518	0.256159	0.824633
ebf3a	301.2118018	-0.135792757	0.1254921	-1.082082	0.279216	0.842806
iqsec3a	861.7003598	0.028203337	0.095185	0.2963004	0.767001	0.975687
GPR180	39.38571579	0.244423125	0.3035155	0.8053069	0.420643	0.90651
FP103005.1	3.817387012	0.38285336	1.0044972	0.3811393	0.7031	NA
HTR7	9.483353519	-0.547160225	0.631377	-0.866614	0.386153	NA
BX677667.1	0	NA	NA	NA	NA	NA
RF01684	2.356421422	0.450380838	1.3913112	0.3237096	0.746158	NA
pkmb	4789.264641	0.139600642	0.1019224	1.3696762	0.170788	0.728307
RF00001	0	NA	NA	NA	NA	NA
si:ch73-170d6.2	10.53279627	-0.4566533	0.6109773	-0.747415	0.454813	NA
hnrnpabb	6178.411591	0.005706738	0.0732479	0.0779099	0.9379	0.99279
si:ch73-103b2.1	51.14010998	-0.383177296	0.2741884	-1.397496	0.162264	0.716781
traj4	0	NA	NA	NA	NA	NA
tnni4a	93.48181044	0.178891971	0.2157696	0.8290878	0.407055	0.901305
myo7aa	215.7863756	0.008510649	0.1425412	0.0597066	0.952389	0.993904
zgc:163083	26.46046472	-0.33594114	0.4055902	-0.828277	0.407513	0.901305
agap2	635.9680967	-0.154130693	0.099192	-1.553862	0.120217	0.645215
CT956056.1	0	NA	NA	NA	NA	NA
BX908401.1	36.767219	0.033047771	0.3297214	0.1002294	0.920162	0.992044
si:dkeyp-72e1.6	44.85087373	-0.578087548	0.2871671	-2.01307	0.044107	0.412803
sp6	40.38114839	-0.204782487	0.3184864	-0.642987	0.520233	0.939056
CU302200.1	1.860572363	-0.226435781	1.3626586	-0.166172	0.868022	NA
trim105	95.12252962	-0.259585088	0.2107181	-1.231907	0.217984	0.785379
si:ch211-25d12.7	0.473170125	-0.833888596	2.9014002	-0.287409	0.773799	NA
snn	57.58189072	0.393544675	0.2572029	1.5300941	0.125993	0.654803
CU207221.2	21.11340314	-0.03604632	0.4168272	-0.086478	0.931087	0.992702
si:ch211-57n23.1	39.5577377	0.068654908	0.3111088	0.2206781	0.825343	0.982196
CU571336.1	2.683918177	-2.803180903	1.4392211	-1.947707	0.05145	NA
BX640537.2	32.93772574	0.112729967	0.3735726	0.3017619	0.762834	0.975374
CR388184.1	0.840040406	1.927209636	2.1227864	0.9078679	0.363948	NA
si:ch211-27e6.1	7.005191142	-0.29150516	0.7366964	-0.395692	0.692332	NA
si:dkeyp-51g9.4	2.06687758	0.027890935	1.3936251	0.0200132	0.984033	NA
twnk	207.6542715	-0.300733085	0.1490554	-2.017592	0.043634	0.410116
il17rc	286.3189045	0.374507686	0.1373002	2.7276557	0.006379	0.122178
si:ch211-19312.10	23.18646194	0.244748782	0.403159	0.6070775	0.543799	0.945438
MTFP1	204.9169377	-0.087321815	0.1652696	-0.52836	0.59725	0.955244
RF00001	0.182570949	0.967652056	4.0804729	0.2371421	0.812547	NA
si:ch211-208f21.3	0.31598276	0.005883445	3.3523626	0.001755	0.9986	NA
lrrc8c	214.5225513	0.146985845	0.1412989	1.0402478	0.298225	0.85401
snrnp35	96.19861313	-0.503198848	0.2004923	-2.509817	0.012079	0.193721
BX323064.1	0	NA	NA	NA	NA	NA
p2ry2.1	7.686238723	0.905106639	0.7436065	1.217185	0.223534	NA
BX649345.1	6.928321529	-0.164650584	0.7819297	-0.21057	0.833223	NA
znf1005	48.52948737	-0.181945693	0.3108744	-0.585271	0.558366	0.948478
arl4aa	137.6379867	-0.327919682	0.1727064	-1.898712	0.057602	0.470971
CR407584.1	4.452408044	0.738780139	0.9600981	0.769484	0.441606	NA

zgc:163040	7.217046222	0.249912588	0.712674	0.3506689	0.725837	NA
tbce	235.5415712	0.267651974	0.1369528	1.9543367	0.050661	0.44139
mir222b	0	NA	NA	NA	NA	NA
zgc:154054	639.1282175	-0.165429788	0.1025221	-1.613601	0.106614	0.615567
BX072564.1	0.339758314	0.005883863	3.2682461	0.0018003	0.998564	NA
si:dkey-26m3.1	0	NA	NA	NA	NA	NA
dpf1	175.1724056	-0.062573269	0.1651671	-0.378848	0.7048	0.968134
chrm5a	7.575903096	-0.251113184	0.6779628	-0.370394	0.711089	NA
col27a1b	71.11189579	-0.122420672	0.2402746	-0.509503	0.6104	0.95688
pcdh1g26	61.00242111	-0.043384692	0.2811305	-0.154322	0.877356	0.987881
BX005245.1	0	NA	NA	NA	NA	NA
ppa1a	202.2198824	0.081115681	0.1616854	0.5016884	0.615887	0.95688
CU862015.1	0.323844819	-1.825095364	3.274639	-0.557342	0.577293	NA
nlrc3l	95.35066408	0.039657778	0.2123434	0.1867625	0.851847	0.985174
CABZ01112864.1	4.556811079	1.584511623	1.0617252	1.4923934	0.135596	NA
pigrl3.5	0.347458735	-1.908042286	3.4571486	-0.551912	0.581009	NA
mapre2	1423.226763	0.038923656	0.0770332	0.5052841	0.613359	0.95688
RF00001	0	NA	NA	NA	NA	NA
CU467646.4	46.54281291	-0.417427984	0.2809949	-1.485536	0.137402	0.674358
fam189a2	97.68265608	0.116264376	0.2037794	0.5705404	0.568311	0.948983
si:ch211-209f23.7	0	NA	NA	NA	NA	NA
FP236551.1	1.624478268	0.588556441	1.4939716	0.3939542	0.693615	NA
cldn17	9.414033575	-0.520851077	0.6507942	-0.800331	0.423519	NA
PRLHR	24.30876824	0.183873881	0.391612	0.4695308	0.63869	0.959628
plekhm1	453.179876	-0.058744852	0.1244391	-0.472077	0.636872	0.958848
CT955967.1	0.341486697	1.892317624	3.2124068	0.5890654	0.555817	NA
BX957318.1	4.736642882	0.23517262	0.8760395	0.2684498	0.788353	NA
smyhc1	8939.109754	0.257900697	0.0743849	3.4671131	0.000526	0.018573
elovl1a	304.9871278	-0.223122746	0.1449279	-1.539544	0.123672	0.652129
bnip3	190.3322986	-0.13322257	0.1578203	-0.844141	0.398591	0.900359
si:dkey-27n6.1	3.679362733	-0.85411697	1.0750053	-0.794524	0.426891	NA
BX005073.2	0	NA	NA	NA	NA	NA
FP085428.1	5.450125432	-1.177132257	0.8381374	-1.404462	0.160181	NA
polr2a	2533.116337	0.141598376	0.0869292	1.6288939	0.103335	0.609625
CU929143.1	0.158795395	0.967652056	4.0804729	0.2371421	0.812547	NA
zgc:152863	96.26207383	0.165294872	0.2079429	0.7949051	0.426669	0.910217
CR383676.1	34370.01673	0.100061954	0.0886386	1.1288753	0.25895	0.827219
nup153	1344.633717	0.098979435	0.0885547	1.1177214	0.263686	0.830245
mia2	825.3288308	0.100300011	0.1048809	0.9563229	0.338909	0.87745
ldb3b	2604.870776	0.301696413	0.0820937	3.6750267	0.000238	0.009838
tgfbr3	19.00748015	0.11796851	0.4394388	0.2684526	0.788351	0.976703
bpifc1	30.58590228	-0.046569194	0.3723347	-0.125073	0.900465	0.990121
si:ch1073-228h2.2	24.78490701	-0.130869736	0.4160346	-0.314565	0.753092	0.97481
ccl39.7	10.71462019	0.380935245	0.5883895	0.6474202	0.51736	NA
CABZ01023255.1	52.25580006	-0.322945834	0.3072165	-1.051199	0.293167	0.849017
ccnl12	217.7383995	0.143429477	0.1473691	0.973267	0.330421	0.874098
si:ch211-207i20.2	34.13036583	-0.124103375	0.3287053	-0.377552	0.705763	0.968412
FP236356.2	0.5046461	-2.45370851	2.7629627	-0.888072	0.374502	NA
RF00001	0	NA	NA	NA	NA	NA
creld1a	308.8649357	-0.021316026	0.1242642	-0.171538	0.863801	0.985952
RF00001	0	NA	NA	NA	NA	NA
apbb2b	131.251511	-0.167920278	0.1820672	-0.922299	0.356373	0.883422
rhot1a	1151.026833	0.110614563	0.0924729	1.1961834	0.231625	0.799752
cox20	183.2018039	0.059437386	0.1699023	0.3498328	0.726464	0.970402

tcf3b	1781.846181	-0.012951706	0.0773224	-0.167503	0.866975	0.986978
CR352226.5	0	NA	NA	NA	NA	NA
znf1096	9.843824584	0.420880813	0.6957888	0.6048974	0.545247	NA
ctbs	88.17584463	0.006064638	0.2174718	0.027887	0.977752	0.996762
glulb	4860.189691	0.021889041	0.0805195	0.2718477	0.785739	0.97627
si:ch211-76m11.5	9.877042991	-0.274446229	0.6275933	-0.4373	0.661894	NA
arhgap39	127.786876	0.042048365	0.1849503	0.2273496	0.820152	0.981594
RF00001	0	NA	NA	NA	NA	NA
strn	165.5128755	-0.064269826	0.1596663	-0.402526	0.687297	0.966376
CR855860.1	394.3256793	0.044174011	0.118422	0.3730221	0.709132	0.968664
dhx38	324.6350671	0.114385308	0.1197329	0.9553372	0.339407	0.87805
tradv13.0	0	NA	NA	NA	NA	NA
arhgap11a	198.7287514	-0.120445302	0.1491423	-0.807586	0.419329	0.906406
pim1	2272.965472	0.118613676	0.0769399	1.5416402	0.123161	0.651012
plaat1	312.3851483	-0.048373045	0.1348553	-0.358703	0.719817	0.969318
hist1h2a6	6.110358118	0.509229993	0.8189605	0.6218005	0.534073	NA
si:ch211-231f6.6	167.3541704	0.057080137	0.1585608	0.3599889	0.718855	0.969092
olfch1	0	NA	NA	NA	NA	NA
si:dkeyp-13d12.21	0.182570949	0.967652056	4.0804729	0.2371421	0.812547	NA
pole2	89.02379485	-0.304040591	0.2238653	-1.358141	0.174419	0.734611
TADA1	87.64226053	0.111437189	0.2221703	0.5015845	0.61596	0.95688
GABRB2 (1 of many)	7.010487842	0.576861993	0.7216973	0.799313	0.424109	NA
CU929317.1	0	NA	NA	NA	NA	NA
BX901923.1	7.395291192	0.19907077	0.6900843	0.2884731	0.772985	NA
CU571069.1	164.4888233	-0.005184673	0.1793476	-0.028909	0.976938	0.996762
CABZ01111454.1	28.69321602	0.004735797	0.3632458	0.0130374	0.989598	0.997612
prtga	0.182570949	0.967652056	4.0804729	0.2371421	0.812547	NA
pimr184	0.853245996	-0.479250517	2.1942342	-0.218414	0.827107	NA
si:dkey-206d17.15	0	NA	NA	NA	NA	NA
ergic1	340.8753067	0.124688694	0.1200049	1.0390301	0.298791	0.85478
tradv25.0.4	0	NA	NA	NA	NA	NA
BX571954.1	0	NA	NA	NA	NA	NA
spink2.4	0	NA	NA	NA	NA	NA
CABZ01044099.1	96.81499681	0.12648868	0.2166236	0.58391	0.559281	0.948478
tox2	423.200705	-0.015668457	0.1113362	-0.140731	0.888082	0.989291
CABZ01088109.1	1160.823596	-0.146414048	0.0880031	-1.663737	0.096165	0.590549
ppfibp1b	580.321423	-0.018190765	0.1016633	-0.178931	0.857992	0.985703
RF00001	0.166657454	-0.955901296	4.0804729	-0.234262	0.814781	NA
exosc7	232.1062265	-0.211511625	0.1383643	-1.528657	0.126349	0.655241
si:dkey-16p6.1	6.184096407	0.718676014	0.7989648	0.8995089	0.368382	NA
CU467837.1	10.234578	-0.281582702	0.6232063	-0.451829	0.651392	NA
RF00001	0.158795395	0.967652056	4.0804729	0.2371421	0.812547	NA
dre-mir-137-2	0	NA	NA	NA	NA	NA
mgrn1a	485.2671909	-0.034193447	0.1165604	-0.293354	0.769252	0.975687
BX908757.1	0	NA	NA	NA	NA	NA
abcg2a	751.4292625	-1.039265093	0.1058698	-9.816447	9.57E-23	1.35E-19
CU929568.1	0.31437473	-1.788572922	3.5930657	-0.497785	0.618636	NA
RF00026	0	NA	NA	NA	NA	NA
dnai1.1	1.353015435	-1.655062633	1.8376086	-0.900661	0.367769	NA
ap3b1a	367.3943564	-0.236680056	0.1192767	-1.984295	0.047223	0.426582
sugt1	491.9141165	-0.03940381	0.1069388	-0.368471	0.712522	0.969087
CABZ01084603.1	255.1741332	0.089192317	0.1435684	0.6212529	0.534433	0.943336
AL929114.1	1.005718337	-0.979164561	1.8926618	-0.517348	0.604913	NA
adipoqb	34.16082087	0.129042703	0.3511856	0.3674488	0.713284	0.96909

evx1	19.54516434	0.586445503	0.4360346	1.3449516	0.178641	0.739486
zmp:0000001114	670.1084045	-0.082168058	0.1052482	-0.780707	0.434975	0.914408
camk2b2	38.70318967	0.532630067	0.3270466	1.6286062	0.103396	0.609625
si:dkey-11d20.1	0.158915748	0.967652056	4.0804729	0.2371421	0.812547	NA
ppp2r2ca	1316.797304	-0.052371263	0.0795918	-0.657999	0.510539	0.936253
anxa1b	1072.46189	-0.144215037	0.1112382	-1.296453	0.19482	0.758937
CU855936.1	0.491320158	0.8528132	2.8656354	0.2976	0.766008	NA
mafgb	32.43365753	-0.112565801	0.3585206	-0.313973	0.753542	0.974981
CR382281.1	1.828745373	-1.411271231	1.4320856	-0.985466	0.324395	NA
si:dkey-238o14.1	2.718924439	0.785042324	1.1758509	0.6676377	0.504365	NA
RF00001	0	NA	NA	NA	NA	NA
RF00001	0	NA	NA	NA	NA	NA
zgc:101785	20.82676863	0.075058454	0.4420575	0.1697934	0.865173	0.986367
impg2b	5.734970528	0.731581719	0.8178718	0.8944944	0.371057	NA
CR385054.1	82.49920586	0.213680111	0.2303095	0.9277955	0.353514	0.881844
cul3b	971.6626299	0.101661285	0.0841181	1.2085541	0.226834	0.794528
si:ch211-237a4.2	7.615124033	-0.003840573	0.7000084	-0.005486	0.995622	NA
znf1118	14.65955055	0.120888953	0.5097825	0.2371383	0.812549	0.981081
AL935044.2	1.497509863	1.861276769	1.7055995	1.0912742	0.275152	NA
sf3a3	997.2596008	0.013628079	0.0879765	0.154906	0.876895	0.987881
dnah1	64.0122166	0.085318459	0.2432238	0.3507817	0.725752	0.970402
mrps24	306.1593443	0.014477272	0.1343987	0.1077189	0.914219	0.991119
CR925761.2	2.554623875	1.013353592	1.2467	0.8128287	0.416316	NA
caskb	1019.372097	-0.080445573	0.0893633	-0.900208	0.368009	0.887482
si:dkey-21c1.8	34.55194685	-0.170373028	0.33732	-0.505078	0.613504	0.95688
znf1178	16.70296231	0.281004253	0.481877	0.5831451	0.559796	0.948478
CU861477.1	94.84539028	-0.014512351	0.2150459	-0.067485	0.946196	0.99381
brd3b	1487.515464	-0.022445129	0.0797764	-0.28135	0.778442	0.975942
cdh29	25.65717718	1.107884363	0.4005631	2.7658175	0.005678	0.113103
CU929402.1	3.41793168	1.638142378	1.1414629	1.4351254	0.151251	NA
cnmd	3779.694411	0.104431578	0.0770193	1.3559153	0.175126	0.735545
dre-mir-7146	0.166657454	-0.955901296	4.0804729	-0.234262	0.814781	NA
dcp1a	323.4884779	0.018129126	0.1204287	0.1505383	0.88034	0.987881
il17ra2	0	NA	NA	NA	NA	NA
CR749168.3	0	NA	NA	NA	NA	NA
si:ch211-113d22.2	587.3696536	0.080626361	0.1063649	0.7580165	0.448441	0.917768
CR626874.1	1.127931677	-2.447047199	1.952687	-1.253169	0.210144	NA
znf1083	25.64486352	0.147627522	0.3847376	0.3837096	0.701194	0.968134
lrp6	414.3864585	-0.025313437	0.1269737	-0.19936	0.841981	0.985174
CR936977.1	0	NA	NA	NA	NA	NA
CABZ01092282.1	1146.524676	0.139117649	0.108015	1.2879477	0.197764	0.76301
RF00026	0	NA	NA	NA	NA	NA
znf1150	0.317831496	1.808837541	3.5779923	0.5055454	0.613176	NA
axin2	762.9209838	0.053555395	0.0923836	0.5797069	0.562112	0.948728
FQ377903.1	13.81332716	-0.986820805	0.5687519	-1.735064	0.08273	0.554784
appl2	1012.78214	-0.010051772	0.0875402	-0.114825	0.908584	0.990702
C12orf75	61.10210875	0.064904884	0.260097	0.2495411	0.802942	0.979643
FP067440.1	0	NA	NA	NA	NA	NA
si:ch211-217i17.1	3.888835444	-0.489446539	1.0697469	-0.457535	0.647287	NA
tamm41	149.0913474	0.011360604	0.177558	0.0639825	0.948984	0.99381
tradv6.0.4	0	NA	NA	NA	NA	NA
angptl1b	32.49012908	0.373080633	0.3542804	1.053066	0.292311	0.848099
si:dkey-205i10.2	0.65888811	-1.481402282	2.5795287	-0.574292	0.56577	NA
CR382337.2	1.533229493	-0.971650616	1.6564407	-0.586589	0.557479	NA

CABZ01059120.1	71.30608976	0.080059506	0.2338665	0.34233	0.732103	0.971665
tox4b	49.25922448	0.174650712	0.3164606	0.5518877	0.581025	0.951731
si:dkey-16p6.1	6.456730677	-0.3547383	0.7515022	-0.472039	0.636899	NA
CR749167.1	0	NA	NA	NA	NA	NA
si:dkeyp-9d4.3	1787.929684	0.008161104	0.0765459	0.1066172	0.915093	0.991123
CSDC2 (1 of many)	10.02367344	-0.377966933	0.5994719	-0.6305	0.528368	NA
pam	2293.968848	-0.026382608	0.0785344	-0.335937	0.736918	0.972535
CU638702.1	0	NA	NA	NA	NA	NA
CT573484.1	4.835953136	0.510857596	0.8913881	0.5731035	0.566575	NA
gb:ai877918	93.70770872	-0.192535538	0.209687	-0.918204	0.358512	0.884398
lamp1a	382.5902499	0.042108734	0.1301805	0.3234643	0.746344	0.97419
BX908782.1	17.90037117	-0.157945832	0.4664721	-0.338597	0.734914	0.972117
CU655842.1	205.7434152	0.220836991	0.1561363	1.4143856	0.157249	0.707876
si:ch211-188p14.4	147.7455926	0.311892072	0.1826224	1.7078522	0.087664	0.566637
trgv6	2.320976029	-1.326661722	1.2906399	-1.02791	0.303992	NA
znf1155	54.54246151	-0.057983317	0.271029	-0.213938	0.830596	0.982994
AL953858.1	0	NA	NA	NA	NA	NA
znf1160	25.38853007	-0.455651904	0.3871819	-1.176842	0.239259	0.807768
zgc:171929	2.933211226	-0.653480876	1.1152832	-0.585933	0.557921	NA
sncaip	158.8583979	0.274365828	0.1849982	1.483073	0.138055	0.675718
si:ch1073-185p12.2	1.353177072	-1.582120833	1.7185249	-0.920627	0.357245	NA
BX537249.1	0.671303554	-2.865684129	2.3396875	-1.224815	0.220645	NA
CABZ01056637.1	0.829470442	-0.541620008	2.1123709	-0.256404	0.797639	NA
RF00001	0.166657454	-0.955901296	4.0804729	-0.234262	0.814781	NA
klhdc4	345.2435287	-0.031832718	0.1264382	-0.251765	0.801223	0.979377
prpf39	1823.396397	-0.065908248	0.0757504	-0.870071	0.384262	0.895192
RF00001	0.158915748	0.967652056	4.0804729	0.2371421	0.812547	NA
si:dkey-247i3.6	2.963590581	-0.346567885	1.1088018	-0.312561	0.754614	NA
si:ch211-117l17.7	0	NA	NA	NA	NA	NA
AL935294.1	0	NA	NA	NA	NA	NA
si:dkey-264f17.1	27.97840008	-0.16639401	0.3638447	-0.457322	0.64744	0.960581
BX511018.2	3.32782198	0.279241825	1.0160105	0.2748415	0.783438	NA
utrn	1590.872021	-0.100990437	0.0878569	-1.149487	0.250355	0.820164
gpa33a	670.3836967	-0.04437651	0.1011532	-0.438706	0.660875	0.964022
kif16ba	237.6232604	0.044256514	0.1383706	0.3198405	0.749089	0.97419
SLC7A1 (1 of many)	395.6881252	-0.138732623	0.11781	-1.177597	0.238957	0.807768
si:ch211-238e22.5	3.989147291	0.007523638	0.9387223	0.0080148	0.993605	NA
ccdc135	34.82358301	-0.251284371	0.3300903	-0.761259	0.446502	0.917154
glis2b	21.31136374	-0.164432288	0.4265768	-0.385469	0.69989	0.967998
CU633762.1	0	NA	NA	NA	NA	NA
BX511246.1	9.835419103	0.588048129	0.6455741	0.9108918	0.362352	NA
CU929203.2	0	NA	NA	NA	NA	NA
csppla	63.93927409	-0.008683435	0.2510539	-0.034588	0.972408	0.996315
tradv23.1.2	0	NA	NA	NA	NA	NA
CT737123.1	2.316570736	-0.03491942	1.3074695	-0.026708	0.978693	NA
dph5	239.4641513	0.091006765	0.1400371	0.649876	0.515772	0.9377
ebf3a	3697.732232	-0.080948593	0.0701461	-1.154001	0.2485	0.817939
FP016234.1	101.6871095	0.673217265	0.2005863	3.356248	0.00079	0.025611
CU929199.1	13.67491115	-0.208312711	0.5089144	-0.409328	0.682299	0.965476
c1r	3.937441886	-0.990111676	0.9675049	-1.023366	0.306135	NA
si:ch211-266k2.1	20.04734258	0.143441055	0.4673143	0.3069477	0.758883	0.975374
si:ch211-152c8.2	3.266913936	-1.251468769	1.188681	-1.052821	0.292423	NA
capzb	4132.581408	0.077744851	0.0727805	1.0682093	0.285426	0.845164
CU571169.1	0	NA	NA	NA	NA	NA

si:ch73-29l19.1	218.2654268	0.114710204	0.1418301	0.808786	0.418638	0.906406
si:dkey-88e18.8	113.1021724	0.161352242	0.1945047	0.8295543	0.406791	0.901305
zgc:174314	0.996248248	-0.965226587	1.8965975	-0.508925	0.610805	NA
si:dkeyp-104b3.2	0.173729368	-0.955901296	4.0804729	-0.234262	0.814781	NA
zgc:171759	22.92131974	0.149121861	0.4335132	0.3439846	0.730858	0.971497
them6	147.2797219	0.14139785	0.1682815	0.8402458	0.400771	0.90084
si:ch1073-266p11.2	61.15527232	-0.380888789	0.2549623	-1.493903	0.135201	0.670689
BX936382.1	11.581685	-0.408241051	0.5680881	-0.718623	0.472373	0.924838
si:ch211-161c3.5	88.72091911	0.066063912	0.2068987	0.3193055	0.749495	0.97419
thumpd1	192.6842601	0.084776058	0.1616406	0.5244725	0.59995	0.956045
rhcgb	272.5906642	0.421031705	0.1445126	2.9134594	0.003574	0.080595
GRM7	381.7630063	0.007557062	0.1136505	0.0664939	0.946985	0.99381
or109-7	0	NA	NA	NA	NA	NA
FP085414.1	877.1003832	0.014832829	0.0953216	0.1556083	0.876342	0.987881
or109-3	0	NA	NA	NA	NA	NA
dre-mir-19b	0	NA	NA	NA	NA	NA
WDR7	424.2435554	0.10290882	0.1350989	0.7617297	0.446221	0.917154
CU457819.1	3.530753003	-0.085782296	1.0323748	-0.083092	0.933778	NA
znfx1	13.39876903	0.093225446	0.5244067	0.1777732	0.858901	0.985773
si:dkeyp-6a3.1	0.633934609	1.436045836	2.6103598	0.5501333	0.582228	NA
BX571843.1	0.182570949	0.967652056	4.0804729	0.2371421	0.812547	NA
dnai2a	5.988316613	0.319580571	0.7702907	0.4148831	0.678228	NA
SLA (1 of many)	1.856287422	1.405559207	1.4793983	0.9500884	0.342067	NA
impg2b	71.3552805	-0.0631177	0.2312419	-0.272951	0.784891	0.97627
fam166b	13.17395232	0.239767559	0.5686034	0.421678	0.67326	0.965257
arhgef15	11.11276253	0.091693356	0.5930325	0.1546178	0.877123	0.987881
zgc:113209	130.4433694	-0.150080054	0.1780852	-0.842743	0.399372	0.900683
ccl39.3	7.122660549	-0.938511882	0.8097241	-1.159051	0.246435	NA
pja2	288.0023274	0.015491998	0.1259472	0.1230039	0.902104	0.990121
CABZ01065328.2	0	NA	NA	NA	NA	NA
si:dkey-237g15.2	18.72540532	-0.379741482	0.4508756	-0.842231	0.399659	0.90084
CU638714.1	0	NA	NA	NA	NA	NA
chdh	37.92015805	-0.031859004	0.3140259	-0.101453	0.919191	0.991623
adamts5	34.60198641	0.210927522	0.3374371	0.625087	0.531914	0.942359
SEMA4F	75.25698508	0.024489506	0.2290929	0.1068977	0.91487	0.991119
si:dkey-165e24.1	0.522329263	0.999930388	2.8066578	0.3562709	0.721638	NA
hoxa13a	11.60214301	-0.059252558	0.580608	-0.102053	0.918715	0.991623
pip5k1cb	728.2454833	0.184682976	0.1058022	1.7455488	0.080889	0.550801
slc15a1a	42.78139219	-0.784105953	0.3106105	-2.524403	0.01159	0.188618
ahsa1b	588.0730288	0.091951577	0.0977258	0.9409136	0.346749	0.8794
RF00324	6.108135033	0.716915284	0.7815635	0.9172834	0.358994	NA
BX248331.1	0.325573202	0.005883621	3.3172783	0.0017736	0.998585	NA
fut10	25.50999217	0.11435983	0.3828603	0.2986986	0.76517	0.975374
AL627305.1	43.47699549	-0.30198357	0.3056223	-0.988094	0.323107	0.870607
FQ377896.1	2.730002558	1.164557975	1.2466845	0.934124	0.35024	NA
CR354444.1	0.814656822	0.635171646	2.1267048	0.2986647	0.765196	NA
znf1056	18.0919475	-0.021511275	0.4570967	-0.047061	0.962465	0.995907
SMIM10	227.6849449	-0.411359158	0.1417361	-2.90229	0.003704	0.082491
lat	5.323335117	0.191773592	0.8197107	0.2339528	0.815022	NA
znf1104	3.498376573	-0.388948229	0.9971241	-0.39007	0.696485	NA
pik3r6a	130.4107144	-0.035448194	0.1768388	-0.200455	0.841125	0.985174
RF00001	2.953958854	-0.371730764	1.1500285	-0.323236	0.746516	NA
BX936384.1	0.979664961	-1.029240033	2.0739984	-0.496259	0.619712	NA
arf4b	39.39482376	-0.328170406	0.3791276	-0.865593	0.386713	0.89551

si:ch211-196h24.2	2.045231758	-1.513476115	1.5521905	-0.975058	0.329531	NA
zgc:158464	298.1907307	-0.16055397	0.1294064	-1.240695	0.214718	0.782493
tradv24.0.3	0	NA	NA	NA	NA	NA
si:dkey-71l4.1	5.168555971	-0.290064932	0.8601644	-0.33722	0.735951	NA
prkacaa	341.4517694	0.152972774	0.1229257	1.2444328	0.21334	0.781631
aglb	600.8318686	0.599873831	0.1127465	5.3205546	1.03E-07	1.43E-05
si:ch1073-398f15.1	63.78795932	-0.055607614	0.2475232	-0.224656	0.822247	0.982186
AL844192.1	13.67360522	0.175879281	0.5175307	0.3398432	0.733975	0.971993
si:ch73-44m9.3	115.7118541	-0.187770395	0.1892038	-0.992424	0.320991	0.868715
CR382300.2	0	NA	NA	NA	NA	NA
AREL1	722.9580426	0.042009074	0.0962762	0.4363392	0.662591	0.964665
si:ch211-125c23.3	3.618473728	-0.751032777	1.0632385	-0.706363	0.479962	NA
znf1174	24.33863418	-0.719425702	0.3987637	-1.80414	0.071209	0.522678
dcun1d5	552.3670142	0.212974315	0.1025138	2.0775185	0.037754	0.380501
FO907110.1	1.507608459	0.992720806	1.5647909	0.6344112	0.525813	NA
si:dkey-102m7.3	116.0278933	-0.167290192	0.199459	-0.83872	0.401627	0.90084
BX276088.1	0.317831496	1.808837541	3.5779923	0.5055454	0.613176	NA
CT955963.1	8.691691029	0.379288338	0.6581911	0.5762587	0.56444	NA
eef2b	36670.44012	-0.223817843	0.2018751	-1.108694	0.267562	0.833349
alad	344.171939	0.036066689	0.1174579	0.3070605	0.758797	0.975374
si:ch211-175l6.1	0.158915748	0.967652056	4.0804729	0.2371421	0.812547	NA
txnrd3	2518.362752	0.137658006	0.0800999	1.7185787	0.085691	0.562009
timm23b	27.20703105	-0.01859262	0.3702897	-0.050211	0.959954	0.995388
amdhd2	293.969269	-0.196452095	0.1408094	-1.395164	0.162966	0.717875
CABZ01012885.1	0	NA	NA	NA	NA	NA
si:dkey-83h2.3	1.275370218	0.72451682	1.7471001	0.4146968	0.678364	NA
si:dkey-34d22.2	0.158795395	0.967652056	4.0804729	0.2371421	0.812547	NA
rock2bl	2.158246708	1.184029034	1.3774349	0.8595898	0.390015	NA
il10ra	8.545342319	0.5811507	0.6673353	0.8708526	0.383835	NA
CT990567.1	0.157187365	-0.955901296	4.0804729	-0.234262	0.814781	NA
slc25a19	84.0406713	-0.223036373	0.2316692	-0.962737	0.33568	0.876088
si:dkey-61p9.9	2.801287092	-0.168150196	1.1305696	-0.148731	0.881766	NA
si:dkey-78o7.3	0	NA	NA	NA	NA	NA
abhd5b	1.163413857	-2.485799194	1.9161281	-1.297303	0.194527	NA
nitr11a	0	NA	NA	NA	NA	NA
rps18	9979.126698	-0.409341163	0.0731741	-5.594069	2.22E-08	3.54E-06
lnpep	325.0702971	-0.037377404	0.1225936	-0.304889	0.760451	0.975374
RF00001	0	NA	NA	NA	NA	NA
pip5k1aa	128.0981508	0.171633107	0.1812325	0.9470327	0.343622	0.878434
pde6c	6011.802827	0.438323281	0.0855308	5.1247395	2.98E-07	3.57E-05
pax7a	83.5485484	-0.156000983	0.2348116	-0.664367	0.506456	0.935724
FO704610.1	326.6743038	-0.094560177	0.1247382	-0.758069	0.44841	0.917768
fgd1	38.71987558	-0.294104961	0.3263102	-0.901305	0.367426	0.887458
CT027704.1	0.323844819	-1.825095364	3.274639	-0.557342	0.577293	NA
BX957252.3	3.077193001	-0.154008103	1.0912032	-0.141136	0.887762	NA
zgc:112265	1723.495788	0.167686763	0.0974543	1.7206709	0.085311	0.560628
si:ch211-166e11.5	340.8490766	-0.069783208	0.1213695	-0.574965	0.565315	0.948739
camlg	360.4179128	-0.116430778	0.1318322	-0.883174	0.377142	0.895192
or106-5	0	NA	NA	NA	NA	NA
si:ch73-106n3.1	1.658751506	-3.048600777	1.7233157	-1.769032	0.076889	NA
si:ch211-171l17.14	2.703090012	0.370984433	1.1646911	0.318526	0.750086	NA
BX323861.2	0	NA	NA	NA	NA	NA
si:ch211-227e10.2	0	NA	NA	NA	NA	NA
ints1	775.8014877	-0.071906354	0.1024695	-0.701734	0.482845	0.929133

tpcn2	131.6225081	-0.011728757	0.1792922	-0.065417	0.947842	0.99381
znf1129	18.42736756	0.039461202	0.489447	0.0806241	0.935741	0.992702
ptpro	702.6627082	-0.019479434	0.1090399	-0.178645	0.858217	0.985773
BX323994.2	0.158795395	0.967652056	4.0804729	0.2371421	0.812547	NA
si:ch73-233f7.8	0.837961008	-3.186152199	2.1620582	-1.473666	0.140571	NA
fam167b	59.30824169	0.111294206	0.2507295	0.4438815	0.657128	0.962166
BX547934.2	168.3461972	-0.319859399	0.2088538	-1.531499	0.125646	0.654803
irgf4	0.657349104	1.487939685	2.418178	0.6153144	0.538347	NA
CU634008.1	99.37413701	-0.525882383	0.2026709	-2.59476	0.009466	0.163186
CR376738.1	2.328478025	-0.005256243	1.2225774	-0.004299	0.99657	NA
znf1091	4.879149437	-0.212744868	0.932643	-0.22811	0.819561	NA
sec22bb	668.3033109	0.001271717	0.09541	0.013329	0.989365	0.997589
cnga2b	42.12379177	-0.33449609	0.3161408	-1.058061	0.290028	0.846246
zgc:153383	174.9449925	0.099712911	0.1707906	0.5838313	0.559334	0.948478
fastkd3	170.2476391	-0.10134366	0.1568105	-0.646281	0.518097	0.938592
cfh	842.9454564	0.222621278	0.1113192	1.9998457	0.045517	0.41824
CR376766.2	148.5505613	0.211054032	0.1668853	1.2646656	0.205991	0.773798
si:dkeyp-13d12.17	0	NA	NA	NA	NA	NA
si:ch211-286o17.1	530.739335	-0.064587759	0.1127577	-0.572802	0.566779	0.948739
BX322631.1	9.197622972	0.277755221	0.6203236	0.4477586	0.654327	NA
btr21	0.496945679	-0.834564081	2.8549631	-0.29232	0.770042	NA
olfcs2	6.86352947	0.064782053	0.7391502	0.087644	0.93016	NA
nop53	1225.743428	-0.330898622	0.0946542	-3.495868	0.000473	0.017082
BX649313.1	0.173729368	-0.955901296	4.0804729	-0.234262	0.814781	NA
pnkp	209.3510501	-0.189187446	0.1507979	-1.254576	0.209633	0.776996
ptk2aa	286.7790876	0.005520691	0.1432143	0.0385485	0.96925	0.996315
si:dkey-217g21.3	1.621141855	-0.556340483	1.5189056	-0.366277	0.714158	NA
CABZ01078737.1	135.0149027	-0.001202098	0.179601	-0.006693	0.99466	0.998694
paqr7a	3.658498007	-0.008872829	0.9857908	-0.009001	0.992819	NA
BX908799.1	0	NA	NA	NA	NA	NA
si:dkey-193i10.1	7.566659171	-0.39566301	0.6966114	-0.567982	0.570047	NA
clrn3	55.32506765	-1.361005286	0.2797441	-4.86518	1.14E-06	0.000112
nrl	45.33579642	-0.147178691	0.3031034	-0.485573	0.62727	0.957354
fkrp	159.0324325	0.226233404	0.1694279	1.335278	0.181785	0.743853
znf1182	21.65750824	0.700874972	0.4182495	1.675734	0.09379	0.582661
BX004768.1	0.158915748	0.967652056	4.0804729	0.2371421	0.812547	NA
LO017721.1	4.418573093	0.111234785	0.9310672	0.1194702	0.904903	NA
fgf10b	8.710816333	-0.169329627	0.6481616	-0.261246	0.793903	NA
si:dkeyp-110c7.4	109.5821498	0.15278814	0.2194992	0.6960762	0.486381	0.930833
si:dkey-43f9.4	50.81171396	-0.36989569	0.2842908	-1.301118	0.193218	0.757173
smg6	587.299695	-0.244541672	0.1050335	-2.328225	0.0199	0.265882
pmt	310.8553678	-0.326067087	0.1254221	-2.599758	0.009329	0.162001
vent	1.815221005	2.20642324	1.6004445	1.3786315	0.168008	NA
CR450808.1	8.992305308	-0.278144435	0.6767663	-0.41099	0.68108	NA
foxo6a	25.48467138	-0.28952464	0.3894308	-0.743456	0.457206	0.921679
nploc4	816.6587721	-0.088007307	0.0966652	-0.910434	0.362594	0.885944
RF00001	0	NA	NA	NA	NA	NA
BX511111.1	3.742404164	-0.118445449	0.9878159	-0.119906	0.904557	NA
CT025669.1	2.420114602	0.545754006	1.3691689	0.3986024	0.690186	NA
pbx1a	1177.859459	-0.012723985	0.0838692	-0.151712	0.879414	0.987881
hic2	116.4224664	0.273060285	0.1967728	1.387693	0.165231	0.721223
CABZ01079251.1	64.34127833	-0.975956486	0.2508988	-3.889842	0.0001	0.004886
cpeb4b	1194.937762	0.042466884	0.0837781	0.506897	0.612227	0.95688
CR848025.1	3.177360012	0.801305058	1.0662248	0.7515348	0.452331	NA

sos1	638.1855155	-0.099929386	0.1126165	-0.887343	0.374894	0.894316
ppp1r27b	34.10031871	-0.22980837	0.3514806	-0.653829	0.513222	0.936463
ddx52	301.9045673	0.073925178	0.1410721	0.5240242	0.600262	0.956045
crb1	115.0529746	0.317188189	0.2003127	1.5834654	0.113315	0.631061
RF00001	0	NA	NA	NA	NA	NA
ubn2b	431.3470818	-0.013148209	0.1237133	-0.10628	0.91536	0.991171
si:dkey-82i20.2	0.473170125	-0.833888596	2.9014002	-0.287409	0.773799	NA
CR855379.2	1.000134101	0.001091541	2.0514706	0.0005321	0.999575	NA
rps27l	283.2342147	-0.420763106	0.1488158	-2.827408	0.004693	0.098715
cfap20	355.1004149	-0.037043686	0.1216072	-0.304618	0.760657	0.975374
dusp1	1244.198128	0.026944604	0.0984473	0.2736956	0.784319	0.97627
tradv5.0.1	0	NA	NA	NA	NA	NA
CR387919.1	33.13524704	0.263678307	0.3343181	0.7887049	0.430284	0.911945
spop	1097.077554	0.021345974	0.0890123	0.2398093	0.810478	0.980868
si:dkey-25o1.7	6.846637695	-0.330464206	0.7217227	-0.457883	0.647037	NA
abca3b	2280.575252	0.029804456	0.0733777	0.4061784	0.684612	0.966068
map1lc3c	38.99366051	-1.006405589	0.3288713	-3.060181	0.002212	0.05751
sh3bp5a	846.2109856	0.082216966	0.0898454	0.9150936	0.360142	0.885686
abhd14b	261.7585128	-0.353062691	0.1353734	-2.608066	0.009106	0.159622
CU639471.1	0.31437473	-1.788572922	3.5930657	-0.497785	0.618636	NA
si:ch1073-178p5.3	0.323844819	-1.825095364	3.274639	-0.557342	0.577293	NA
[truncated: 518,444 more chars]
