# Supplementary material for: Mycn regulates intestinal development through ribosomal biogenesis in a zebrafish model of Feingold syndrome 1
Source: PLoS Biol. 2022 Nov 1;20(11):e3001856. doi: 10.1371/journal.pbio.3001856 (PMC9624419; doi:10.1371/journal.pbio.3001856)
Supplement: Attachment — Submitted filename: Answers to Referee Comments.pdf [file pbio.3001856.s017.pdf]

Answers to Referee Comments

reviewer #1

(Evidence, reproducibility and clarity (Required))

Major
What percent of mice survive to adulthood, what is the cause of death in those that do not survive? In fertile survivors, do progeny all survive to adulthood or is there again a significant number that die prior to adulthood. What is the basis for the heterogeneity in survival?

Any insights into mTOR signaling, relative effects on S6 vs eIF4E? I think that authors did polysome profiling, not ribosome profiling, as few specific targets are discussed. If authors have knowledge of specific targets, this would improve the manuscript.

Reviewer #1 (Significance (Required)):

Careful analysis of developmental trajectory and effects of mycn knockout in fish, with single cell RNAseq data, and some analysis of translation control and the proteome.

The analysis of GI development is new, much of the effects on ribosome biogenesis is known in mammals, but not in fish, and rescues are new.

Mycn enthusiasts, medical geneticsts and fish people

I am a physician scientist interested in MYCN and cancer

We would like to thank reviewer #1 for his/her comments and very constructive suggestions about our manuscript. We are now able to provide a much stronger and more detailed manuscript. We looked at all comments in great details and did our best to address them all.

Detailed responses to reviewer #1:

What percent of mice survive to adulthood, what is the cause of death in those that do not survive?

According to the published studies of MYCN, the homologous Mycn mutants of mice cannot survive to adulthood, they die prenatally at about E11.5 of gestation. Stanton et al. (1992) showed that the cause of death in mice is hypoplasia of multi-organs including the central and peripheral nervous systems, mesonephros, lung, and gut. Besides, the study show that the homozygous mutant embryos bleed easily upon manipulation, exhibit distended aortas, and are severely anemic when examined at E12. These observations suggest a failure in the developing cardiovascular system, leading to spontaneous bleeding that would also result in embryonic death (Stanton and Perkins et al., 1992). We have added this part in the introduction.

In fertile survivors, do progeny all survive to adulthood or is there again a significant number that die prior to adulthood.

We do find lots of *mycn* mutant embryos died from 10 dpf to 15 dpf. We have added the statistical data of the survival rates in the supplement Figure S2C

What is the basis for the heterogeneity in survival?

In general, the heterogeneity of survival is due to many factors including environmental conditions and genetic background. In this particular case of *mycn* mutant zebrafish, we speculate besides factors mentioned above, competition of food and different expression level of other myc family genes in individual fish might be also the factors resulting to the survival heterogeneity.

Any insights into mTOR signaling, relative effects on S6 vs eIF4E?

We would like to thank reviewer 1 for the good question. In our RNA-seq and qPCR data, we do find the transcriptional level of *rps6*, is decreased in 3dpf *mycn* mutant, interestingly, chip-seq data shows that it is a direct target of MYCN in mouse stem cell (Main text, figure 5B, E). But we did not find eIF4E a significant change in our RNA-Seq data. Besides, we carried out Ribo-seq in WT and *mycn* mutant embryos, and found that among the genes that showed reduced translation efficiency in *mycn* mutant, mTOR pathway was enriched (Figure 7B). As the reviewer suggested, we performed Western Blot of phosphorylated S6 and eIF4EBP, and found both the phosphorylated S6 and p-eIF4EBP decreased dramatically in the *mycn* mutants, which further confirmed the downregulation of mTOR signaling (Figure 7C).

I think that authors did polysome profiling, not ribosome profiling, as few specific targets are discussed. If authors have knowledge of specific targets, this would improve the manuscript.

We carried out the Ribo-seq and calculated the translation efficiency of each gene in each replicate and further performed differential translation analysis between WT and *mycn* mutant embryos. We found more than 3,000 genes showed lower translation efficiency in mycn mutant embryos (Figure S7), and these genes were enriched for several important signaling pathways including mTOR signaling pathway which was reported to be involved in regulating ribosome biogenesis and translation (Figure 7B)

reviewer #2

(Evidence, reproducibility and clarity (Required)):

The manuscript of Li Y-F and al.; describes the role of ribosomal biogenesis in a zebrafish model of Feingold syndrome. The work is quite appreciable for the production of a new animal model to investigate this syndrome, but the conclusions seem not appropriate. The main molecular defect displayed by this model is represented by a decrease in ribosomal gene expression that the author associate with a decrease in ribosome biogenesis. However, the authors do not show any experiments about rRNA processing. Has this model a defect in 90S ribosomal formation? Moreover, establishing a direct role of ribosome biogenesis should improve ribosome biogenesis to rescue the nonfunctional myc model. By rapamycin, the authors decrease the protein synthesis to mimic similar defects observed in the zebrafish, they did not show if a decrease of ribosome gene expression occurs. Morever, they are aging on protein synthesis and do not on ribosome biogenesis.

Reviewer #2 (Significance (Required)):

The manuscript proposes a new vertebrate model to investigate the Feingold syndrome, but at molecular level is poor investigated

We would like to thank reviewer #2 for his/her comments about our manuscript and for his/her appreciating the novelty of our work. We looked at all comments and questions detailly and carried out experiments accordingly.

Detailed responses to reviewer #2:

The main molecular defect displayed by this model is represented by a decrease in ribosomal gene expression that the author associate with a decrease in ribosome biogenesis. However, the authors do not show any experiments about rRNA processing. Has this model a defect in 90S ribosomal formation?

We would like to thank the reviewer for pointing out this very important question. By DEG and GO analysis of our RNA-seq data, we found that the expression of many *rpls* and *rps* genes which involved in the rRNA processing are reduced in the *mycn* mutant, including *rpl7, rpl35, rpl7a, rps14, rps7, rpl10a, rps16, rps8a, nop53*(Supplemental Table 2). Studying in mammalian models also support that MYCN play an important role in rRNA processing (van Riggelen and Yetil et al., 2010). In our zebrafish model, we first carried out the GSEA using our RNA-Seq data of 48hpf and 72hpf in the zebrafish and found the rRNA processing were impaired in the *mycn* mutants (Figure S6D and S6E). Then, using probes for the 5'ETS, ITS1 and ITS2 regions, we performed Northern blot to evaluate the rRNA processing in the WT and *mycn* mutant. Although no obvious aberrant intermediates of the processed pre-rRNA were detected by the ITS1 and ITS2 probe, we found an extra small band using the 5'ETS-1 probe which is indicative of the impairment of rRNA processing (Figure 6A). The 90S particle is composed of approximately 70 assembly factors that form stable sub-complexes, which associate in a sequential and hierarchical manner with the nascent pre-rRNA. Within the 90S particle a series of initial cleavages occur in the 5'-ETS of the rRNA at sites A0, A1 followed by cleavage in ITS1 at site A2, yielding the 20S (precursor to 18S) and 27S pre-rRNAs (precursor to 5.8S and 25S), respectively, thereby separating the maturation pathways of the small and large subunit(Grandi and Rybin et al., 2002; Perez-Fernandez and Martin-Marcos et al., 2011; Zhang and Wu et al., 2016) . The aberrant processing of 5'ETS might imply on the impairment of the 90S pre-ribosome.

Moreover, establishing a direct role of ribosome biogenesis should improve ribosome biogenesis to rescue the nonfunctional myc model. By rapamycin, the authors decrease the protein synthesis to mimic similar defects observed in the zebrafish, they did not show if a decrease of ribosome gene expression occurs. Moreover, they are aging on protein synthesis and do not on ribosome biogenesis.

We thank the reviewer very much for this very constructive question. We do find that the expression level of *rps6*, which is a ribosome gene, decreased in *mycn* mutant by RNA-seq and qPCR (Figure 5B, E). And our sucrose density gradient centrifugation assay also showed that the amount of 40s, 60s, 80s and polyribosome are all decreased in *mycn* mutant (Figure 6B). To further address the reviewer's question, we carried out

qPCR of the ribosome genes in zebrafish embryos after treatment of rapamycin, and found many genes which decreased in the *mycn* mutant were unchanged. However, there are also some ribosome genes were decreased in the embryos treated with rapamycin, such as *rps8a, rpl24, rpl4, rpl12, rpl27l, rpl6* and *rps17*. The *rpl6, rpl24, rps3* and *rps6* which were downregulated in the *mycn* mutants were previously reported involving in the development of digestive organs (Figure S7A). We tried to rescue the intestinal defect of *mycn* mutant by injecting the mRNA of these genes, as showed in the figure S7C, E, some of those mRNA have minor rescue on the intestinal defect. The Northern blot results mentioned above also suggests impaired ribosome biogenesis in *mycn* mutant.

reviewer #3

(Evidence, reproducibility and clarity (Required)):

Summary
This study presents a loss-of-function model in zebrafish for Feingold syndrome. The phenotype is described in detail (in situ hybridization, reporter lines and histology) and by using different -omics techniques (bulk and single-cell transcriptomics, metabolomics and genomics). An impressive amount of validation (mainly using in situ hybridization was performed. Moreover, the authors uncover clues towards the pathophysiological mechanism. In addition, a putative treatment is also suggested.

Major comments
1) Quantification/statistical evaluation.
There is a consistent lack of quantification in this study, which is very problematic. Many of the phenotypic claims in the model are illustrated in a qualitative way. This is unacceptable. Especially since quantification is possible in most cases. For example:
Figure 1A Eyes are smaller at 4dpf; please quantify and statistically evaluate if this is a significant difference
Figure 1C Quantify length of intestine (GFP signal) and statistically evaluate.
Figure 4 Claims are made regarding proliferation (PCNA) and apoptosis (TUNEL). Although the figures look convincing, there is NO quantification presented although this can easily be done. The authors should provide quantifications and statistical evaluation of this data before claiming that there are phenotypic differences. The same holds true for the flow cytometry data in Figure 3D
Figure 6 Quantification and appropriate statistical evaluation is lacking again. The data in Figure 6A an B, D and F should be quantified. How can one assess reproducibility and proportion of a change if no quantification, nor statistics is provided?

It is also unclear how many biological and technical replicates of an experiment have been performed. Please provide this information.

2) More focus on skeletal abnormalities. Are there any abnormalities in the fins or axial skeleton. Especially the fins need to be studied as the hands are highly affected in Feingold syndrome. Short stature is also a common feature; why not measure length in zebrafish larvae to verify this? Why not quantify Fig1B (craniofacial skeleton)? Measuring the angles of different craniofacial elements is a common way of quantification when assessing abnormalities in the craniofacioal structures of zebrafish larvae.

3) How could it be explained that Feingold syndrome is a dominant disease whereas the model in zebrafish is a homozygous (recessive) model and still show similar features?

4) How can it be ruled out that the morphological features (short intestine, no swim bladder, smaller eyes) observed in knockout embryos are not due to a global developmental delay in the knockouts?

5) It is an important result (if supported by quantification) to see that supplementation with Leucine or Rheb-150 shown improvement of the phenotype. Does this supplementation also improve the skeletal features in zebrafish? Is there an additive/synergistic effect if one would combine both supplements?

Minor comments
1) In figure 2, a dramatic reduction of ifabp is described. Could this reduction been found back in the transcriptome data?
2) Results section; second paragraph: "some of the homozygotic mycn mutant fish..." Homozygotic should read homozygous.

Reviewer #3 (Significance (Required)):

Description of relevant disease model for Feingold syndrome. The authors use state-of-the-art techniques (single cell-sequencing) and established genomic techniques (Tol2 trans genesis, Crispr/Cas9, reporter lines, in situ hybridization).
The data presented looks convincing and seems of good quality. However, because of a lack of quantifications it is impossible to determine if the results presented are representative for the vast majority of mutant vs normal embryos.
This study provides some important insights in the pathophysiology. In addition it provides a hint towards experimental therapy.

Expertise:

Human clinical genetics/ human disease modeling in zebrafish/ zebrafish genomics/ metabolic disease/ heart and neurological disease

We would like to thank reviewer #3 for his/her very helpful comments and suggestions about our manuscript and his/her appreciation for our work. We have finished the revision and now able to provide a much stronger and more detailed manuscript.

Detailed responses to reviewer #3:

Quantification/statistical evaluation.
There is a consistent lack of quantification in this study, which is very problematic. Many of the phenotypic claims in the model are illustrated in a qualitative way. This is unacceptable. Especially since quantification is possible in most cases. For example: Figure 1A Eyes are smaller at 4dpf; please quantify and statistically evaluate if this is a significant difference.
We have measured and quantified the area of the eyes, and we added the data in the Figure S2E.

Figure 1C Quantify length of intestine (GFP signal) and statistically evaluate.
We measured and analyze the length of the intestine in both the *mycn* mutant and wild type embryo at 7dpf (Figure 2D) and found a significant shorter intestine in the *mycn* mutants.

Figure 4 Claims are made regarding proliferation (PCNA) and apoptosis (TUNEL). Although the figures look convincing, there is NO quantification presented although this can easily be done. The authors should provide quantifications and statistical evaluation of this data before claiming that there are phenotypic differences. The same holds true for the flow cytometry data in Figure 3D
We have quantified the number of proliferating cells (Figure 4A), and for the TUNEL assay, we did not find any signal in all the sections. For the cytometry data (Figure 4D), experiments were performed in triplicate.

Figure 6 Quantification and appropriate statistical evaluation is lacking again. The data in Figure 6A an B, D and F should be quantified. How can one assess reproducibility and proportion of a change if no quantification, nor statistics is provided? It is also unclear

how many biological and technical replicates of an experiment have been performed. Please provide this information.

We have supplied the quantification and biological replicates information in the new manuscript. The data in Figure 6B an C were carried out for 2-3 times. For ribosome profiling, we collect 200-300 embryos of WT and mutant each time, and the trend were the same for the replicate experiments. For the western blot in Figure 6C, we collected 30 embryos for each experiment and the results are consistent in more than three replicate experiments. As for figure 7 D, E and F, we have supplied the statistical data in the panels.

More focus on skeletal abnormalities. Are there any abnormalities in the fins or axial skeleton? Especially the fins need to be studied as the hands are highly affected in Feingold syndrome.

To address the reviewer's question. We collected the embryos of WT and *mycn* mutant at 30hpf and performed *in situ* hybridization of *shha* and *hand2.* And we found decreased expression of both markers, indicating a developmental defect of the fin bud in the *mycn* mutants (Figure 2E). Besides, we also stain the axial skeleton by Alizarin Red S in the *mycn* mutant and WT at 7dpf, but no obvious difference was observed in the *mycn* mutants (Figure S2F).

Short stature is also a common feature; why not measure length in zebrafish larvae to verify this? Why not quantify Fig1B (craniofacial skeleton)? Measuring the angles of different craniofacial elements is a common way of quantification when assessing abnormalities in the craniofacial structures of zebrafish larvae.

We have supplied this data in the Figure S2D.

How could it be explained that Feingold syndrome is a dominant disease whereas the model in zebrafish is a homozygous (recessive) model and still show similar features?

Our speculation is that, in zebrafish, the *myc* family members have multiple paralogues (*myca*, *mycb*, *mycla*, *myclb* and *mycn*) which may have redundant functions, this probably the reason that the heterozygotes of *mycn* mutant do not show obvious phenotype. We have added more discussion about this in the manuscript.

How can it be ruled out that the morphological features (short intestine, no swim bladder, smaller eyes) observed in knockout embryos are not due to a global developmental delay in the knockouts?

To ruled out the possibility of phenotypes in *mycn* mutant were due to a global developmental delay, we measured the body length at the different stages of the WT and *mycn* mutant, and we found that there is no significant difference (Figure S2C). Besides, we have stained the islet at 72 and 96hpf by *in situ* hybridization, not like other digestive organs, the size of the islet has no obvious difference (figure S3). Those data suggest that the morphological features in the *mycn* mutant are not due to the developmental delay.

It is an important result (if supported by quantification) to see that supplementation with Leucine or Rheb-150 shown improvement of the phenotype. Does this supplementation also improve the skeletal features in zebrafish? Is there an additive/synergistic effect if one would combine both supplements?

We thank reviewer 3 for this good question. We performed rescue experiments according to the reviewer's question. We didn't see obvious improvement of the malformed pharyngeal arch by supplement with Leucine. And we didn't find additive/synergistic effect of Leucine/Rheb-150 on the intestinal defect of *mycn* mutant either (Figure S7D).

Minor comments:1) In figure 2, a dramatic reduction of ifabp is described. Could this reduction be found back in the transcriptome data?

In our transcriptome data, we do find the reduction of the expression level of *ifabp* in *mycn* mutant, we also had searched our transcriptome data and found that the expression of many intestinal genes, are decreased significantly. We have added this data with a supplementary table of the bulk RNA-seq (Supplementary Table S2).

Minor comments: 2) Results section; second paragraph: "some of the homozygotic mycn mutant fish..." Homozygotic should read homozygous.

We have corrected it accordingly.

References:

Grandi, P. and V. Rybin, et al. (2002). "90S pre-ribosomes include the 35S pre-rRNA, the U3 snoRNP, and 40S subunit processing factors but predominantly lack 60S synthesis factors." Mol Cell 10 (1): 105-15.

Perez-Fernandez, J. and P. Martin-Marcos, et al. (2011). "Elucidation of the assembly events required for the recruitment of Utp20, Imp4 and Bms1 onto nascent pre-ribosomes." Nucleic Acids Res 39 (18): 8105-21.
Zhang, L. and C. Wu, et al. (2016). "Stepwise and dynamic assembly of the earliest precursors of small ribosomal subunits in yeast." Genes Dev 30 (6): 718-32.
