# Supplementary material for: Mycn regulates intestinal development through ribosomal biogenesis in a zebrafish model of Feingold syndrome 1
Source: PLoS Biol. 2022 Nov 1;20(11):e3001856. doi: 10.1371/journal.pbio.3001856 (PMC9624419; doi:10.1371/journal.pbio.3001856)
Supplement: Attachment — Submitted filename: 123654_1_rebuttal_2342358_rljlql.docx [file pbio.3001856.s015.docx]

**Manuscript number:** RC-2022-01241R

**Corresponding author(s):** Peng-Fei Xu, Xi Jin

## 1. General Statements [optional]

N/A. Please see also the cover letter.

## 2. Description of the planned revisions

Comments and suggestions from reviewers

------------------------------------------------------------------------------

Reviewer #1 (Evidence, reproducibility and clarity (Required)):

Major

What percent of mice survive to adulthood, what is the cause of death in those that do not survive? In fertile survivors, do progeny all survive to adulthood or is there again a significant number that die prior to adulthood. What is the basis for the heterogeneity in survival?

Any insights into mTOR signaling, relative effects on S6 vs eIF4E? I think that authors did polysome profiling, not ribosome profiling, as few specific targets are discussed. If authors have knowledge of specific targets, this would improve the manuscript.

Reviewer #1 (Significance (Required)):

Careful analysis of developmental trajectory and effects of mycn knockout in fish, with single cell RNAseq data, and some analysis of translation control and the proteome.

The analysis of GI development is new, much of the effects on ribosome biogenesis is known in mammals, but not in fish, and rescues are new.

Mycn enthusiasts, medical geneticsts and fish people

I am a physician scientist interested in MYCN and cancer

Reviewer #2 (Evidence, reproducibility and clarity (Required)):

The manuscript of Li Y-F and al.; describes the role of ribosomal biogenesis in a zebrafish model of Feingold syndrome. The work is quite appreciable for the production of a new animal model to investigate this syndrome, but the conclusions seem not appropriate. The main molecular defect displayed by this model is represented by a decrease in ribosomal gene expression that the author associate with a decrease in ribosome biogenesis. However, the authors do not show any experiments about rRNA processing. Has this model a defect in 90S ribosomal formation? Moreover, establishing a direct role of ribosome biogenesis should improve ribosome biogenesis to rescue the nonfunctional myc model. By rapamycin, the authors decrease the protein synthesis to mimic similar defects observed in the zebrafish, they did not show if a decrease of ribosome gene expression occurs. Morever, they are aging on protein synthesis and do not on ribosome biogenesis.

Reviewer #2 (Significance (Required)):

The manuscript proposes a new vertebrate model to investigate the Feingold syndrome, but at molecular level is poor investigated

Reviewer #3 (Evidence, reproducibility and clarity (Required)):

Summary

This study presents a loss-of-function model in zebrafish for Feingold syndrome. The phenotype is described in detail (in situ hybridization, reporter lines and histology) and by using different -omics techniques (bulk and single-cell transcriptomics, metabolomics and genomics). An impressive amount of validation (mainly using in situ hybridization was performed. Moreover, the authors uncover clues towards the pathophysiological mechanism. In addition, a putative treatment is also suggested.

Major comments

1) Quantification/statistical evaluation.

There is a consistent lack of quantification in this study, which is very problematic. Many of the phenotypic claims in the model are illustrated in a qualitative way. This is unacceptable. Especially since quantification is possible in most cases. For example:

Figure 1A Eyes are smaller at 4dpf; please quantify and statistically evaluate if this is a significant difference

Figure 1C Quantify length of intestine (GFP signal) and statistically evaluate.

Figure 4 Claims are made regarding proliferation (PCNA) and apoptosis (TUNEL). Although the figures look convincing, there is NO quantification presented although this can easily be done. The authors should provide quantifications and statistical evaluation of this data before claiming that there are phenotypic differences. The same holds true for the flow cytometry data in Figure 3D

Figure 6 Quantification and appropriate statistical evaluation is lacking again. The data in Figure 6A an B, D and F should be quantified. How can one assess reproducibility and proportion of a change if no quantification, nor statistics is provided?

It is also unclear how many biological and technical replicates of an experiment have been performed. Please provide this information.

2) More focus on skeletal abnormalities. Are there any abnormalities in the fins or axial skeleton. Especially the fins need to be studied as the hands are highly affected in Feingold syndrome. Short stature is also a common feature; why not measure length in zebrafish larvae to verify this? Why not quantify Fig1B (craniofacial skeleton)? Measuring the angles of different craniofacial elements is a common way of quantification when assessing abnormalities in the craniofacioal structures of zebrafish larvae.

3) How could it be explained that Feingold syndrome is a dominant disease whereas the model in zebrafish is a homozygous (recessive) model and still show similar features?

4) How can it be ruled out that the morphological features (short intestine, no swim bladder, smaller eyes) observed in knockout embryos are not due to a global developmental delay in the knockouts?

5) It is an important result (if supported by quantification) to see that supplementation with Leucine or Rheb-150 shown improvement of the phenotype. Does this supplementation also improve the skeletal features in zebrafish? Is there an additive/synergistic effect if one would combine both supplements?

Minor comments

1) In figure 2, a dramatic reduction of ifabp is described. Could this reduction been found back in the transcriptome data?

2) Results section; second paragraph: "some of the homozygotic mycn mutant fish..." Homozygotic should read homozygous.

Reviewer #3 (Significance (Required)):

Description of relevant disease model for Feingold syndrome. The authors use state-of-the-art techniques (single cell-sequencing) and established genomic techniques (Tol2 trans genesis, Crispr/Cas9, reporter lines, in situ hybridization).

The data presented looks convincing and seems of good quality. However, because of a lack of quantifications it is impossible to determine if the results presented are representative for the vast majority of mutant vs normal embryos.

This study provides some important insights in the pathophysiology. In addition it provides a hint towards experimental therapy.

Expertise:

Human clinical genetics/ human disease modeling in zebrafish/ zebrafish genomics/ metabolic disease/ heart and neurological disease

**Reply to reviewer #1:**

We would like to thank reviewer #1 for his/her comments and very constructive suggestions about our manuscript. We will carry out experiments and revisions accordingly:

*Question: What percent of mice survive to adulthood, what is the cause of death in those that do not survive?*

**Answer**: According to the published studies of MYCN, the homologous Mycn mutants of mice cannot survive to adulthood, they die prenatally at about E11.5 of gestation. Stanton et al. (1992) showed that the cause of death in mice is hypoplasia of multi-organs including the central and peripheral nervous systems, mesonephros, lung, and gut. Besides, the study show that the homozygous mutant embryos bleed easily upon manipulation, exhibit distended aortas, and are severely anemic when examined at E12. These observations suggest a failure in the developing cardiovascular system, leading to spontaneous bleeding that would also result in embryonic death (Stanton and Perkins et al., 1992).

We plan to add this information in the introduction of our manuscript.

*Question: In fertile survivors, do progeny all survive to adulthood or is there again a significant number that die prior to adulthood.*

**Answer**: Our understanding is that this question of reviewer 1 was asking about the survival rate of *mycn* mutant fish. We thank reviewer 1 for raising this question, interestingly, we found that most of the homozygous progenies of *mycn* heterozygotes died prior to adulthood (only 2 homozygotes survived in 200 progenies), however, the survival rate is significantly increased in the progenies of *mycn* homozygotes, and keep increasing with the increase of the generations. Our speculation is that this is due to the genetic compensation in zebrafish.

We will add the statistical data of the survival rates in the supplement.

*Question: What is the basis for the heterogeneity in survival?*

**Answer**: In general, the heterogeneity of survival is due to many factors including environmental conditions and genetic background. In this particular case of *mycn* mutant zebrafish, we speculate besides factors mentioned above, competition of food and different expression level of other myc family genes in individual fish might be also the factors resulting to the survival heterogeneity.

*Question: Any insights into mTOR signaling, relative effects on S6 vs eIF4E?*

**Answer**: In our RNA-seq and qPCR data, we do find the transcriptional level of *rps6*, is decreased in 3dpf *mycn* mutant, interestingly, chip-seq data shows that it is a direct target of MYCN in mouse stem cell (Main text, figure 5B, E). But we did not find eIF4E a significant change in our RNA-Seq data.

To further investigate the mTOR signaling pathway in *mycn* mutant fish, we plan to perform Western Blot assay of phosphorylated S6 and eIF4E which can represent their activities.

*Question: I think that authors did polysome profiling, not ribosome profiling, as few specific targets are discussed. If authors have knowledge of specific targets, this would improve the manuscript.*

**Answer**: We thank the reviewer for this very constructive suggestion. This really will improve our work and provide a more profound understanding of the molecular mechanism that result to the developmental defects in *mycn* mutant.

For the specific targets, we will try to explore it by ribo-seq, and we will carry on it following the plan below:

1. Harvest the WT and mutant zebrafish at 3dpf.
2. Ribosome extraction, RNA digestion, purifying ribosome protecting fragments, library preparation and then sequence.
3. Correlation analysis with ribo-Seq and RNA-Seq.
4. KEGG and GO analysis and find the specific targets or pathway.
5. Western Blot or ribo-qPCR verification.

**Reply to reviewer #2:**

We would like to thank reviewer #2 for his/her comments about our manuscript and for his/her appreciating the novelty of our work. We looked at all comments and questions detailly and we will carry out experiments and revisions accordingly:

*Question: The main molecular defect displayed by this model is represented by a decrease in ribosomal gene expression that the author associate with a decrease in ribosome biogenesis. However, the authors do not show any experiments about rRNA processing. Has this model a defect in 90S ribosomal formation?*

**Answer**: We thank the reviewer for this very good question. By DEG and GO analysis of our RNA-seq data we found that the expression of many *rpls* and *rps* genes involved in the rRNA processing are reduced in the *mycn* mutant, including *rpl7, rpl35, rpl7a, rps14, rps7, rpl10a, rps16, rps8a, nop53*. Studying in mammalian models also support that MYCN play an important role in rRNA processing (van Riggelen and Yetil et al., 2010).

We agree that experiments about rRNA processing will significantly improve the quality of our work. Firstly, we plan to use CX-5461 which is an inhibitor of RNA polymerase I to block the rRNA processing in zebrafish embryo, to validate if this would mimic the intestinal defects of *mycn* mutant. Secondly, we plan to use Northern Blot and Western Blot to determine the 90S pre-ribosome components and rRNA processing.

*Question: By rapamycin, the authors decrease the protein synthesis to mimic similar defects observed in the zebrafish, they did not show if a decrease of ribosome gene expression occurs. Moreover, they are aging on protein synthesis and do not on ribosome biogenesis.*

**Answer**: We thank the reviewer for his/her prudent question. We do find that the expression level of *rps6*, which is a ribosome gene, decreased in *mycn* mutant by RNA-seq and qPCR (Figure 5B, E). And our sucrose density gradient centrifugation assay also showed that the amount of 40s, 60s, 80s and polyribosome are all decreased in *mycn* mutant (Figure 6A).

To further support our conclusion, firstly, we plan to use qPCR to verify if the expression of the ribosome genes is decreased by rapamycin treatment in zebrafish embryo. Secondly, we plan to use the mRNA of several ribosome genes that decreased in *mycn* mutant to rescue the intestinal defect phenotype.

**Reply to reviewer #3:**

We would like to thank reviewer #3 for his/her very helpful comments and suggestions about our manuscript and his/her appreciation for our work. We will carry out experiments and revisions according to his/her suggestions:

*Question: Figure 1C Quantify length of intestine (GFP signal) and statistically evaluate.*

**Answer**: We thank the reviewer for his/her question. We will measure and analyze the length of the intestine in both the *mycn* mutant and wild type embryo at day 5, 6 and 7.

*Question: 2) More focus on skeletal abnormalities. Are there any abnormalities in the fins or axial skeleton? Especially the fins need to be studied as the hands are highly affected in Feingold syndrome.*

**Answer**: We thank the reviewer for these questions and suggestions.

According to the suggestion, we plan to collect the embryos of WT and *mycn* mutant at 30hpf and 5dpf to verify if there is any abnormality in the fin and axial skeleton. We plan to use *shha* (has been completed and please see part 3) and *hand2* as probes to verify the phenotype of the fin by *in situ* hybridization. Besides, we also plan to stain the axial skeleton by Alizarin Red S.

*Question: 3) How could it be explained that Feingold syndrome is a dominant disease whereas the model in zebrafish is a homozygous (recessive) model and still show similar features?*

**Answer**: Our speculation is that, in zebrafish, the myc family members have multiple paralogues (*myca*, *mycb*, *mycla*, *myclb* and *mycn*) which may have redundant functions, this probably the reason that the heterozygotes of *mycn* mutant do not show obvious phenotype. We will discuss more about this in the manuscript.

*Question: 4) How can it be ruled out that the morphological features (short intestine, no swim bladder, smaller eyes) observed in knockout embryos are not due to a global developmental delay in the knockouts?*

**Answer**: To answer this question, we measured the body length at the different stages of the WT and *mycn* mutant, and we found that there is no significant difference (please see part 3). Besides, we have stained the islet at 72 and 96hpf by *in situ* hybridization, not like other digestive organs, the size of the islet has no obvious difference (figure S3). Those data suggest that the morphological features in the *mycn* mutant are not due to the developmental delay.

*Question: 5) It is an important result (if supported by quantification) to see that supplementation with Leucine or Rheb-150 shown improvement of the phenotype. Does this supplementation also improve the skeletal features in zebrafish? Is there an additive/synergistic effect if one would combine both supplements?*

**Answer**: We thank the reviewer for these questions and suggestions.

To answer these questions, firstly we plan to supplement the *mycn* mutant embryo with Leucine and Rheb-150 to verify if the skeletal defects could be rescued. We also will supplement the mutant embryo with both Leucine and Rheb-150 at different combinations of concentrations to investigate if there is an additive/synergistic effect on both the intestinal and skeletal defects.

*Question: Minor comments: 1) In figure 2, a dramatic reduction of ifabp is described. Could this reduction be found back in the transcriptome data?*

**Answer**: Yes, in our transcriptome data, we do find the reduction of the expression level of *ifabp* in *mycn* mutant at 72hpf, we also had searched our transcriptome data and found that the expression of many intestinal genes, are decreased significantly. We added new supplementary tables of the bulk RNA-seq data.

*Question: Minor comments: 2) Results section; second paragraph: "some of the homozygotic mycn mutant fish..." Homozygotic should read homozygous.*

**Answer**: We thank the reviewer for pointing out this mistake. We have corrected it accordingly.

## 3. Description of the revisions that have already been incorporated in the transferred manuscript

**Reply to reviewer #3:**

*Question: 1) Quantification/statistical evaluation.*

*There is a consistent lack of quantification in this study, which is very problematic. Many of the phenotypic claims in the model are illustrated in a qualitative way. This is unacceptable. Especially since quantification is possible in most cases. For example:*

*Figure 1A Eyes are smaller at 4dpf; please quantify and statistically evaluate if this is a significant difference.*

**Answer**: Thanks very much for this question. We have measured and quantified the area of the eyes, as well as the body length, as shown below, the size of eyes is significantly decreased in the *mycn* mutant at 4dpf. As for body length, it showed no difference (see figures in question 2). And we will add the new data in the supplement.

*Question: Figure 4 Claims are made regarding proliferation (PCNA) and apoptosis (TUNEL). Although the figures look convincing, there is NO quantification presented although this can easily be done. The authors should provide quantifications and statistical evaluation of this data before claiming that there are phenotypic differences. The same holds true for the flow cytometry data in Figure 3D*

**Answer**: Thanks very much for the question. We have quantified the proliferating cells number, and found it significantly reduced in the intestine of *mycn* mutant. And for the TUNEL assay, we did not find any signal in all the sections, please see figure below. For the cytometry data (actually is in Figure 4D), experiments were performed in triplicate. We will add the new data in the supplement.

*Question: Figure 6 Quantification and appropriate statistical evaluation is lacking again. The data in Figure 6A and B, D and F should be quantified. How can one assess reproducibility and proportion of a change if no quantification, nor statistics is provided? It is also unclear how many biological and technical replicates of an experiment have been performed. Please provide this information.*

**Answer**: We really thank the reviewer for his/her good critic. We have supplied the quantification and biological replicates information in the new manuscript. The data in Figure 6A and B were carried out for 2-3 times. For ribosome profiling, we collect 200-300 embryos of WT and mutant each time, and the trend were the same for the replicate experiments. For the western blot in Figure 6B, we collected 30 embryos for each experiment and the results are consistent in more than three replicate experiments. As for figure 6 D, E and F, we have supplied the statistical data in the panels.

*Question: 2) More focus on skeletal abnormalities. Are there any abnormalities in the fins or axial skeleton? Especially the fins need to be studied as the hands are highly affected in Feingold syndrome.*

**Answer**: We have partially addressed this question by *in situ* hybridization of *shha* in *mycn* mutant and wild type embryos at 30hpf, and we find that the size of the fin bud in *mycn* mutant is notably decreased, as shown below:

*Question: Short stature is also a common feature; why not measure length in zebrafish larvae to verify this? Why not quantify Fig1B (craniofacial skeleton)? Measuring the angles of different craniofacial elements is a common way of quantification when assessing abnormalities in the craniofacial structures of zebrafish larvae.*

**Answer**: We have done the measurements and quantification according to the reviewer’s suggestion. There is no difference of the body length in the wild type and *mycn* mutant at 3dpf and 4dpf. Besides, we measured the angle of the M-PQ, it shows no difference, but it differs widely in the *mycn* mutant. The data are shown below, we plan to add these data in the supplement:

## 4. Description of analyses that authors prefer not to carry out

N/A.

**References**

Stanton, B. R. and A. S. Perkins, et al. (1992). "Loss of N-myc function results in embryonic lethality and failure of the epithelial component of the embryo to develop." Genes Dev **6** (12A): 2235-47.

van Riggelen, J. and A. Yetil, et al. (2010). "MYC as a regulator of ribosome biogenesis and protein synthesis." Nat Rev Cancer **10** (4): 301-9.
